# Effects of Pyroligneous Acid on Diversity and Dynamics of Antibiotic Resistance Genes in Alfalfa Silage

**DOI:** 10.1128/spectrum.01554-22

**Published:** 2022-07-11

**Authors:** Qing Zhang, Xuan Zou, Shuo Wu, Nier Wu, Xiaoyang Chen, Wei Zhou

**Affiliations:** a College of Forestry and Landscape Architecture, Guangdong Province Research Center of Woody Forage Engineering Technology, South China Agricultural University, Guangzhou, China; b College of Forestry and Landscape Architecture, Guangdong Research and Development Centre of Modern Agriculture (Woody forage) Industrial Technology, South China Agricultural University, Guangzhou, China; c College of Forestry and Landscape Architecture, Guangdong Key Laboratory for Innovative Development and Utilization of Forest Plant Germplasm, South China Agricultural University, Guangzhou, China; d College of Forestry and Landscape Architecture, State Key Laboratory for Conservation and Utilization of Subtropical Agro-bioresources, South China Agricultural University, Guangzhou, China; e Inner Mongolia Key Laboratory of Microbial Ecology of Silage, Inner Mongolia Engineering Research Center of Development and Utilization of Microbial Resources in Silage, Hohhot, China; South China Agricultural University

**Keywords:** alfalfa silage, antibiotic resistance genes, bacterial communities, pyroligneous acid

## Abstract

Antibiotic resistance genes (ARGs) are recognized as contaminants due to their potential risk for human and environment. The aim of the present study is to investigate the effects of pyroligneous acid (PA), a waste of biochar production, on fermentation characteristics, diversity, and dynamics of ARGs during ensiling of alfalfa using metagenomic analysis. The results indicated that PA decreased (*P* < 0.05) dry matter loss, pH value, gas production, coliform bacteria count, protease activity, and nonprotein-N, ammonia-N, and butyric acid contents and increased (*P* < 0.05) lactic acid content during ensiling. During fermentation, *Bacteria*, *Firmicutes*, and *Lactobacillus* were the most abundant at kingdom, phylum, and genus levels, respectively. Pyroligneous acid reduced the relative abundance of *Bacteria* and *Firmicutes* and increased that of *Lactobacillus*. The detected ARGs belonged to 36 drug classes, including mainly macrolides, tetracycline, lincosamides, and phenicol. These types of ARGs decreased during fermentation and were further reduced by PA. These types of ARGs were positively correlated (*P* < 0.05) with fermentation parameters like pH value and ammonia-N content and with bacterial communities. At the genus level, the top several drug classes, including macrolide, tetracycline, lincosamide, phenicol, oxazolidinone, streptogramin, pleuromutilin, and glycopeptide, were positively correlated with *Staphylococcus*, *Streptococcus*, *Listeria*, *Bacillus*, *Klebsiella*, *Clostridium*, and *Enterobacter*, the potential hosts of ARGs. Overall, ARGs in alfalfa silage were abundant and were influenced by the fermentation parameters and microbial community composition. Ensiling could be a feasible way to mitigate ARGs in forages. The addition of PA could not only improve fermentation quality but also reduce ARG pollution of alfalfa silage.

**IMPORTANCE** Antibiotic resistance genes (ARGs) are considered environmental pollutants posing a potential human health risk. Silage is an important and traditional feed, mainly for ruminants. ARGs in silages might influence the diversity and distribution of ARGs in animal intestinal and feces and then the manure and the manured soil. However, the diversity and dynamics of ARGs in silage during fermentation are still unknown. We ensiled alfalfa, one of the most widely used forages, with or without pyroligneous acid (PA), which was proved to have the ability to reduce ARGs in soils. The results showed that ARGs in alfalfa silage were abundant and were influenced by the fermentation parameters and microbial community. The majority of ARGs in alfalfa silage reduced during fermentation. The addition of PA could improve silage quality and reduce ARG pollution in alfalfa silage. This study can provide useful information for understanding and controlling ARG pollution in animal production.

## INTRODUCTION

The overuse and misuse of antibiotics in animal agriculture potentially contribute to the emergence and spread of antibiotic resistance genes (ARGs), which are considered environmental pollutants posing a potential human health and ecological environment risk worldwide (1). It has been estimated that antimicrobial resistance is responsible for 0.7 million annual deaths worldwide and will increase to 10 million by 2050 if no effective measure is taken now (2). Animal production, especially that of cattle and pigs, is one of the largest users of antibiotics (3). Thus, the continuous increase or high persistence of antibiotic resistance bacteria in livestock environments receives more public concern than that in other environments. Most previous studies focused on the quantitative detection and removal of ARGs in manure and relevant environments, as bacteria carrying ARGs and various pathogens could be transferred to humans from contaminated food, water, etc. (4–6). The animal intestinal microbiome is also a reservoir of ARGs, which might be affected by the microbial community and ARGs in feed. Silage, an important and traditional feed mainly for ruminant, is a product fermented by many microorganisms adhered to the surface of forages under anaerobic condition (7). It is largely produced all over the world due to the increase of consumers’ demand for meat and dairy products (8). Silage might be a potential ARG source for animals, as ARGs can be transferred from contaminated soil and water to forage plants via rhizosphere and phyllosphere microbiomes. Wu et al. (9) investigated ARGs in sweet corn kernel silage and found that ARGs associated with tetracycline antibiotic showed the largest abundance. Xu et al. (10) reported that *tetM*, *oqxB*, *lmrD*, *lnuA*, *ermB*, and *tetS* were the dominant ARGs in corn silage by analyzing silage samples from six climate zones in China. However, the diversity, dynamics, and treatments of ARGs in alfalfa silage are still unknown.

Pyroligneous acid (PA), a brown acidic liquid, is the byproduct of pyrolysis of biomass like lignin, hemicelluloses, and cellulose during biochar making. It is made up of 80 to 90% water and 10 to 20% organic chemicals, including organic acids, phenolic compounds, furan, and pyran derivatives (11). It will cause wastewater disposal problems when discharged directly (12). However, it has been proved to be beneficial in many areas of agriculture, such as insecticide, fertilizer, and smoke flavor source for food due to its biological properties, including antimicrobial, antioxidant, anti-inflammatory, and antiviral (13). Previous studies proved that PA had strong antibacterial activity on *Escherichia*, *Salmonella*, and *Pseudomonas* from animals (14, 15). Our previous study found that PA could improve fermentation quality and change bacterial community of rice straw and stylo silages (16). Moreover, Zheng et al. (6, 13) reported that the application of PA significantly reduced the absolute abundance of ARGs to more than 80% in the soil. To the best of our knowledge, the effect of PA on the diversity of ARGs in silage is unknown.

Therefore, we hypothesized that the addition of PA could reduce the relative abundance of ARGs in silage due to its antibacterial activity. The aims of this study were to (i) explore the diversity of ARGs in alfalfa silage and their dynamics during fermentation using metagenomic analysis, (ii) investigate the effect of PA on mitigation of ARGs pollution during the ensiling of alfalfa, and (iii) explore the factors influencing ARGs abundance in the alfalfa silage. This study can provide useful information for understanding and controlling ARG pollution in silage production.

## RESULTS AND DISCUSSION

### Fermentation quality and microbial diversity of alfalfa silage.

**FIG 1:**
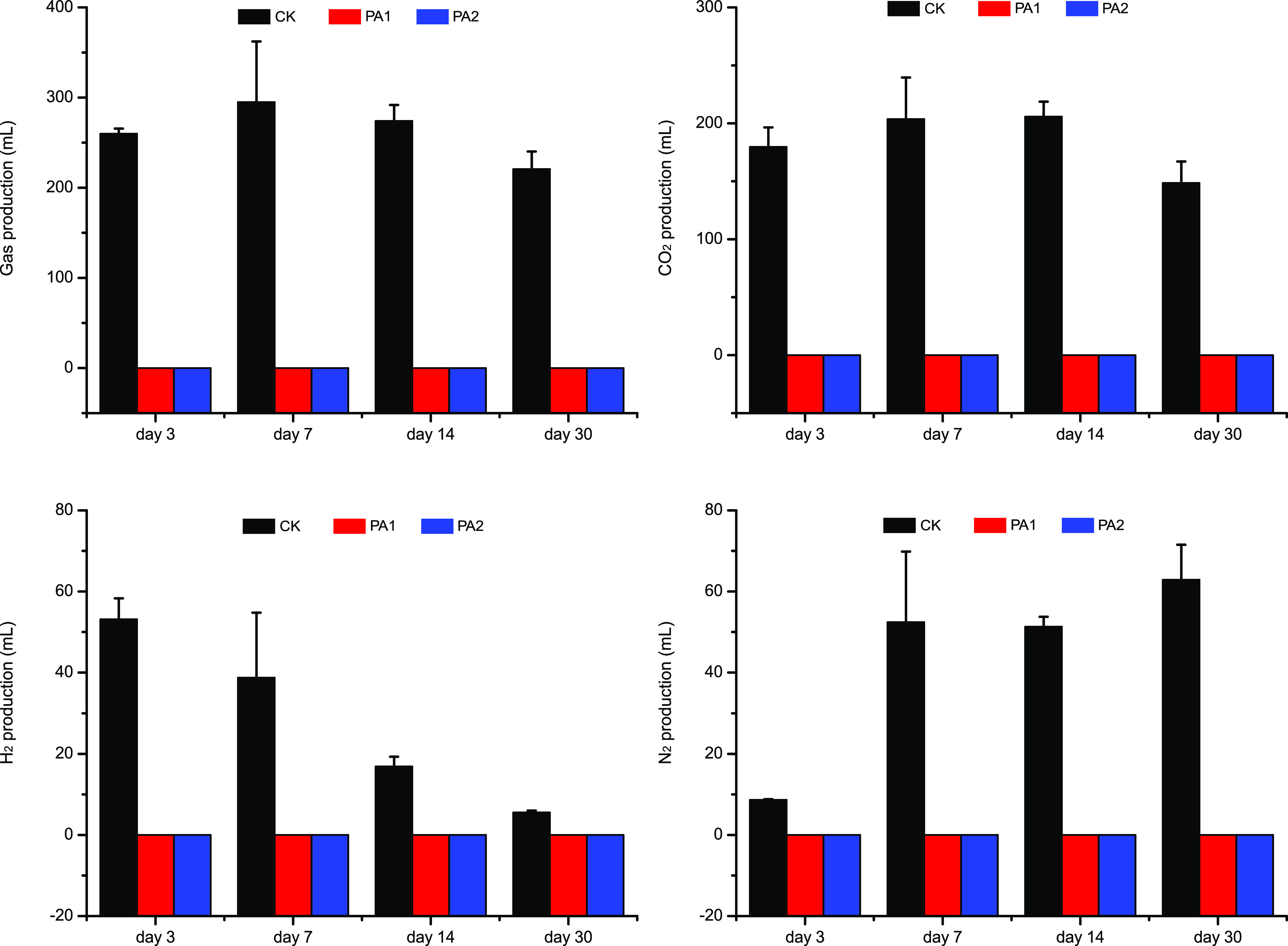
Gas production, CO_2_ production, H_2_ production, and N_2_ production of alfalfa silage after fermentation for 3, 7, 14, or 30 days with or without 1% pyroligneous acid (PA1) or 2% pyroligneous acid (PA2).

Silage, a microbial fermentation product of fresh matter like forages and agricultural byproducts, is widely used in livestock production. During fermentation, epiphytic microorganisms like lactic acid bacteria produce acids (such as lactic acid and acetic acid), and then the pH reduction inhibits microbial activities (17). Due to high buffering capacity and high number of undesirable microorganisms, alfalfa is difficult to ensile directly (7). In our study, poor fermentation of alfalfa silage indicated by high pH value and ammonia-N and butyric acid contents was observed in the control group (Table 1). Pyroligneous acid is an acidic liquid as organic acids are the main components (18). It partially explained the reduced pH (*P* < 0.05) in the PA treatments at early stage of ensiling. Moreover, lactic acid, a key metabolite of lactic acid bacteria, is the main factor for pH decline. The content of lactic acid also increased (*P* < 0.05) in the PA treatments. It indicates that the addition of PA is beneficial for lactic acid fermentation during ensiling. The rapid acidification caused by PA might limit the microbial activities during ensiling, which led to the reduction of dry matter loss and gas production (Fig. 1). Both butyric acid and ammonia-N are undesirable in silage due to their adverse effects on livestock production (19). Butyric acid content in alfalfa silage in the current study was reduced (*P* < 0.05) by PA. This is probably because the direct acidification inhibited the activities of *Clostridium* spp., the producer of butyric acid. As a key indicator of protein decomposition during ensiling, the content of ammonia-N is influenced by the activities of undesirable microorganisms. The low utilization efficiency and animal excretion of ammonia-N will bring a negative influence on the environment. In our study, PA decreased (*P* < 0.05) ammonia-N content (Table 2), which was in accord with the decline of coliform bacteria count. Similar results were found in stylo and rice straw silage in our previous study (16). The reduction of nonprotein-N content was also consistent with the decline of protease activities in PA-treated silage (Table 3). This suggests that PA application in silage production is beneficial for protein preservation during fermentation. Therefore, PA, the wastewater produced during biochar production, could be used to improve silage fermentation.

**TABLE 1:** Fermentation characteristics of alfalfa ensiled with 1% or 2% pyroligneous acid (PA)^*a*^

Fermentation characteristic	Ensiling material	Ensiling time (days)					SEM	Significance		
		3	7	14	30	Mean		T	A	T × A
DM (% FM)	CK	22.0^b^	21.4^c^	23.5^b^	24.7^a^	22.9^b^	1.13	0.01	<0.01	0.002
	1% PA	24.0^a^	24.3^a^	22.7^b^	22.5^b^	23.4^a^				
	2% PA	24.3^a^	23.7^b^	24.5^a^	22.0^b^	23.6^a^				
	Mean	23.5^AB^	23.2^BC^	23.6^A^	23.1^C^					
DM loss (%)	CK	0.49^a^	0.80^a^	1.22^a^	2.72^a^	1.31^a^	0.753	<0.01	<0.01	<0.01
	1% PA	0.23^b^	0.47^b^	0.81^b^	1.84^b^	0.84^b^				
	2% PA	0.15^c^	0.34^c^	0.77^b^	1.69^c^	0.74^c^				
	Mean	0.29^D^	0.54^C^	0.93^B^	2.08^A^					
pH	CK	5.45^a^	5.48^a^	5.46^a^	5.56^a^	5.49^a^	0.450	<0.01	<0.01	<0.01
	1% PA	4.75^b^	4.66^b^	4.53^b^	4.55^b^	4.62^b^				
	2% PA	4.70^b^	4.47^c^	4.41^c^	4.50^b^	4.52^c^				
	Mean	4.97^A^	4.80^C^	4.87^B^	4.87^B^					
Lactic acid (% DM)	CK	0.91^b^	1.16^b^	0.96^c^	0.13^b^	0.79^c^	0.914	<0.01	<0.01	<0.01
	1% PA	1.59^a^	2.12^a^	2.54^a^	3.12^a^	2.34^a^				
	2% PA	1.15^b^	2.02^a^	2.35^b^	3.11^a^	2.16^b^				
	Mean	1.21^D^	1.77^C^	1.95^B^	2.12^A^					
Acetic acid (% DM)	CK	0.90^a^	1.24^a^	1.19^a^	1.12	1.11^a^	0.302	<0.01	<0.01	<0.01
	1% PA	0.43^b^	0.71^b^	0.87^b^	1.15	0.79^b^				
	2% PA	0.35^b^	0.63^b^	0.70^c^	1.14	0.71^c^				
	Mean	0.56^D^	0.86^C^	0.92^B^	1.14^A^					
Butyric acid (% DM)	CK	ND	ND	0.36^a^	1.50^a^	0.93^a^	0.493	<0.01		
	1% PA	ND	ND	0.19^b^	0.23^b^	0.21^b^				
	2% PA	ND	ND	0.13^b^	0.23^b^	0.18^b^				
	Mean	ND	ND	0.23^B^	0.65^A^					
Lactic acid bacteria (log_10_cfu/g FM)	CK	9.07	8.53^b^	8.27	8.00	8.47	0.388	<0.01	0.364	0.254
	1% PA	8.95	8.60^ab^	8.32	8.01	8.47				
	2% PA	8.65	8.67^a^	8.26	7.93	8.38				
	Mean	8.89^A^	8.60^B^	8.28^C^	7.98^D^					
Coliform bacteria (log_10_cfu/g FM)	CK	7.88^a^	7.21^a^	6.48	4.24	6.65^a^	1.317	<0.01	<0.01	0.609
	1% PA	6.16^b^	5.09^b^	<3.00	<3.00	5.62^b^				
	2% PA	5.24^c^	4.17^b^	<3.00	<3.00	4.70^c^				
	Mean	6.43^A^	5.49^B^	6.47^A^	4.23^C^					

a DM, dry matter; FM, fresh matter; SEM, standard error of mean; ND, not detected; T, time of ensiling; A, additives; T × A, interaction of ensiling time and additives; means with different letters in the same column (a to c) or row (A to D) indicate a significant difference (*P* < 0.05).

**TABLE 2:** Protein fraction of alfalfa ensiled with 1% or 2% pyroligneous acid (PA)^*a*^

Fractionation characteristic	Ensiling material	Ensiling time (days)					SEM	Significance		
		3	7	14	30	Mean		T	A	T × A
Crude protein (% DM)	CK	15.3	15.2a	14.0	15.4	15.0	0.776	<0.01	0.272	0.177
	1% PA	14.4	14.2b	14.6	15.4	14.7				
	2% PA	14.0	14.6b	14.3	15.5	14.6				
	Mean	14.5^B^	14.7^B^	14.3^B^	15.4^A^					
True protein-N (% TN)	CK	40.6^c^	34.8^c^	32.8^b^	30.5^b^	34.7^c^	5.84	<0.01	<0.01	0.231
	1% PA	45.8^b^	41.1^b^	39.6^a^	37.0^a^	40.9^b^				
	2% PA	49.4^a^	46.8^a^	42.5^a^	36.7^a^	43.8^a^				
	Mean	45.3^A^	40.9^B^	38.3^C^	34.8^D^					
Nonprotein-N (% TN)	CK	59.4^a^	65.2^a^	67.2^a^	69.5^a^	65.3^a^	5.84	<0.01	<0.01	0.231
	1% PA	54.2^b^	58.9^b^	60.4^b^	63.0^b^	59.1^b^				
	2% PA	50.6^c^	53.2^c^	57.5^b^	63.3^b^	56.2^c^				
	Mean	54.7^D^	59.1^C^	61.7^B^	65.2^A^					
Ammonia-N (g/kg TN)	CK	9.34^a^	13.5^a^	15.4^a^	12.7^a^	12.8^a^	4.360	<0.01	<0.01	<0.01
	1% PA	2.87^b^	5.14^b^	6.62^b^	6.11^b^	5.19^b^				
	2% PA	1.71^c^	3.08^c^	7.49^b^	4.73^c^	4.26^c^				
	Mean	4.65^D^	7.24^C^	7.86^B^	9.85^A^					

a DM, dry matter; TN, total N; SEM, standard error of means; T, time of ensiling; A, additives; T × A, interaction of ensiling time and additives; means with different letters in the same column (a to c) or row (A to D) indicate a significant difference (*P* < 0.05).

**TABLE 3:** Protease activity of alfalfa ensiled with 1% or 2% pyroligneous acid (PA)^*a*^

Protease	Ensiling material	Ensiling time (days)					SEM	Significance		
		3	7	14	30	Mean		T	A	T × A
Aminopeptidase (units/h/DM)	CK	65.0^a^	21.5^a^	12.7^a^	11.5	27.7^a^	2.229	<0.01	0.272	0.177
	1% PA	22.6^b^	11.0^b^	12.1^a^	12.0	14.4^b^				
	2% PA	17.1^c^	12.1^b^	10.8^b^	12.2	13.1^c^				
	Mean	34.9^A^	14.9^B^	11.9^C^	11.9^C^					
Carboxypeptidase (units/h/DM	CK	65.8^a^	66.4^a^	65.3	61.7	64.8^a^	9.242	0.016	0.016	<0.01
	1% PA	58.2^b^	59.3^b^	61.9	67.3	61.7^b^				
	2% PA	56.8^b^	59.0^b^	62.9	66.1	61.2^b^				
	Mean	60.3^B^	61.6^B^	63.4^AB^	65^A^					
Acid protease (units/h/DM)	CK	63.7^a^	59.5^a^	54.2^a^	45.7^b^	55.8^a^	3.727	<0.01	<0.01	0.231
	1% PA	53.2^b^	48.3^b^	51.5^ab^	48.7^b^	50.4^b^				
	2% PA	54.9^b^	50.6^b^	48.3^b^	54.2^a^	52.0^b^				
	Mean	57.3^A^	52.8^B^	51.3^BC^	49.5^C^					

a DM, dry matter; SEM, standard error of means; T, time of ensiling; A, additives; T × A, interaction of ensiling time and additives; Means with different letters in the same column (a to c) or row (A to C) indicate a significant difference (*P* < 0.05).

**FIG 2:**
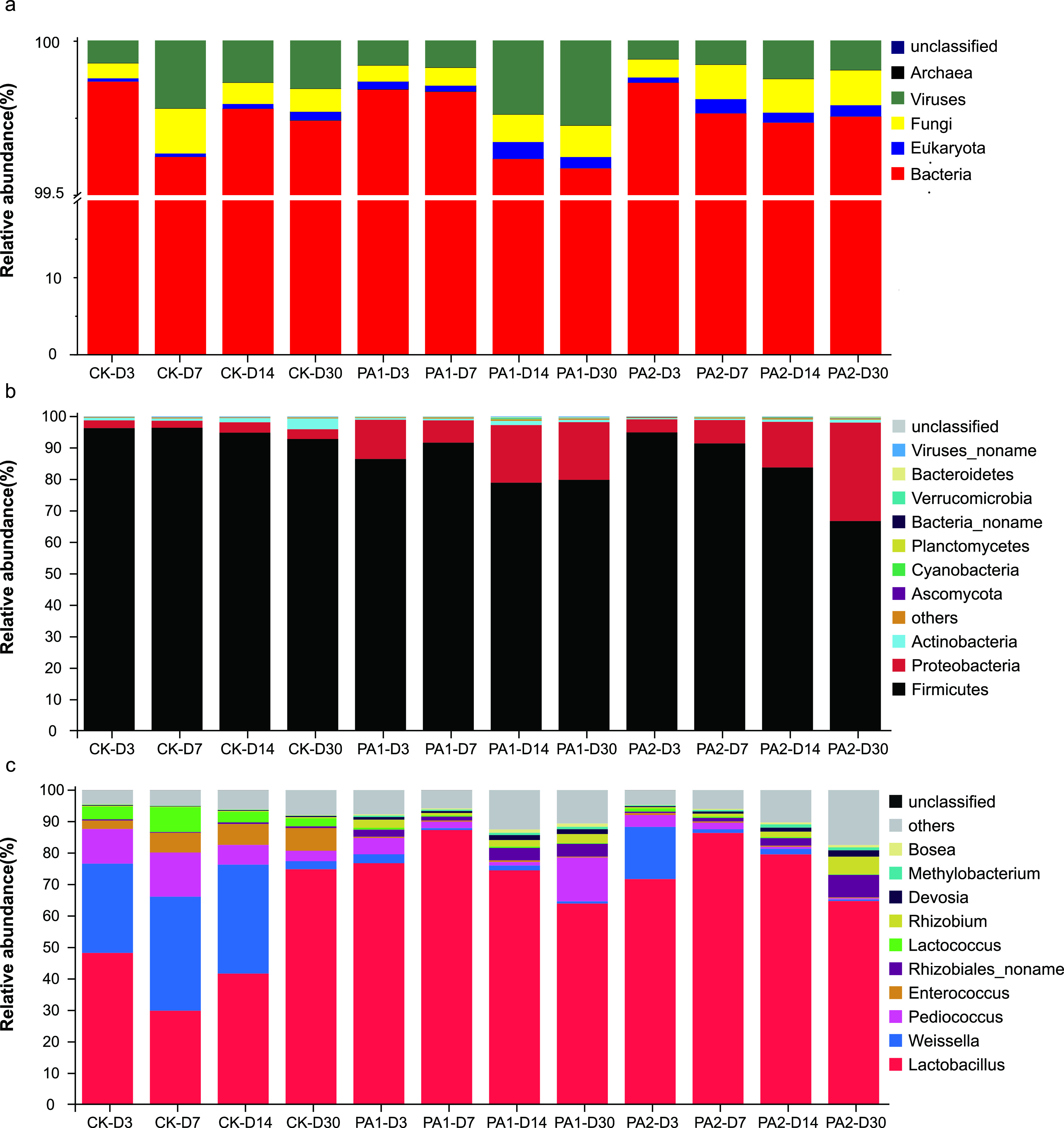
The diversity and relative abundance of microbial communities in alfalfa silage at kingdom (a), phylum (b), and genus (c) level, respectively (CK, the control; PA1, 1% pyroligneous acid; PA2, 2% pyroligneous acid; D3, D7, D14, D30, after ensiling for 3, 7, 14, or 30 days, respectively).

The improved fermentation quality was further confirmed by the microbial community. As shown in Fig. 2a, *Bacteria* was the most abundant kingdom, accounting for more than 99.5% of the total abundance in all alfalfa silage samples. Viruses, *Fungi*, *Archaea*, and *Eukaryota* showed a low abundance, which was consistent with what was found in fermented corn kernel (9). Further exploration is needed to find out their roles during ensiling. Notably, *Firmicutes* and *Proteobacteria* were the two most abundant bacterial phyla (Fig. 2b). The relative abundance of *Proteobacteria* increased and *Firmicutes* decreased during fermentation. This is in accordance with Liu et al. (7). Furthermore, the addition of PA lowered the abundance of *Firmicutes* and increased that of *Proteobacteria*. These changes are different from what we observed in our previous experiments using stylo and rice straw as silage materials (16). This might be because of different microbial communities in different silages. At the genus level, bacterial community was dominated by *Lactobacillus*, *Enterococcus*, *Pediococcus*, *Weissella*, and *Lactococcus* in the control groups (Fig. 2c). It is difficult to explain the poor fermentation quality, as these genera were considered lactic acid-producing bacteria in silage. The same results were observed by Liu et al. (7). *Lactobacillus*, which can grow rapidly and produce lactic acid as the main product, is known as the key indicator of fermentation quality (19). The abundance of *Lactobacillus* increased after the addition of PA, which was consistent with the increase of lactic acid content. The addition of PA reduced the abundance of undesirable microorganisms like *Clostridium*, *Streptococcus*, *Bacillus*, *Staphylococcus*, and *Listeria* (Table S1). This confirmed that PA might be used as a good alternative to traditional acids additives in silage production. Our previous study found that the relative abundance of *Lactococcus* was positively correlated with gas production, CO_2_ production, and H_2_ production (20). The decrease of *Lactococcus* in PA treatments might explain the reduction of these gases.

**FIG 3:**
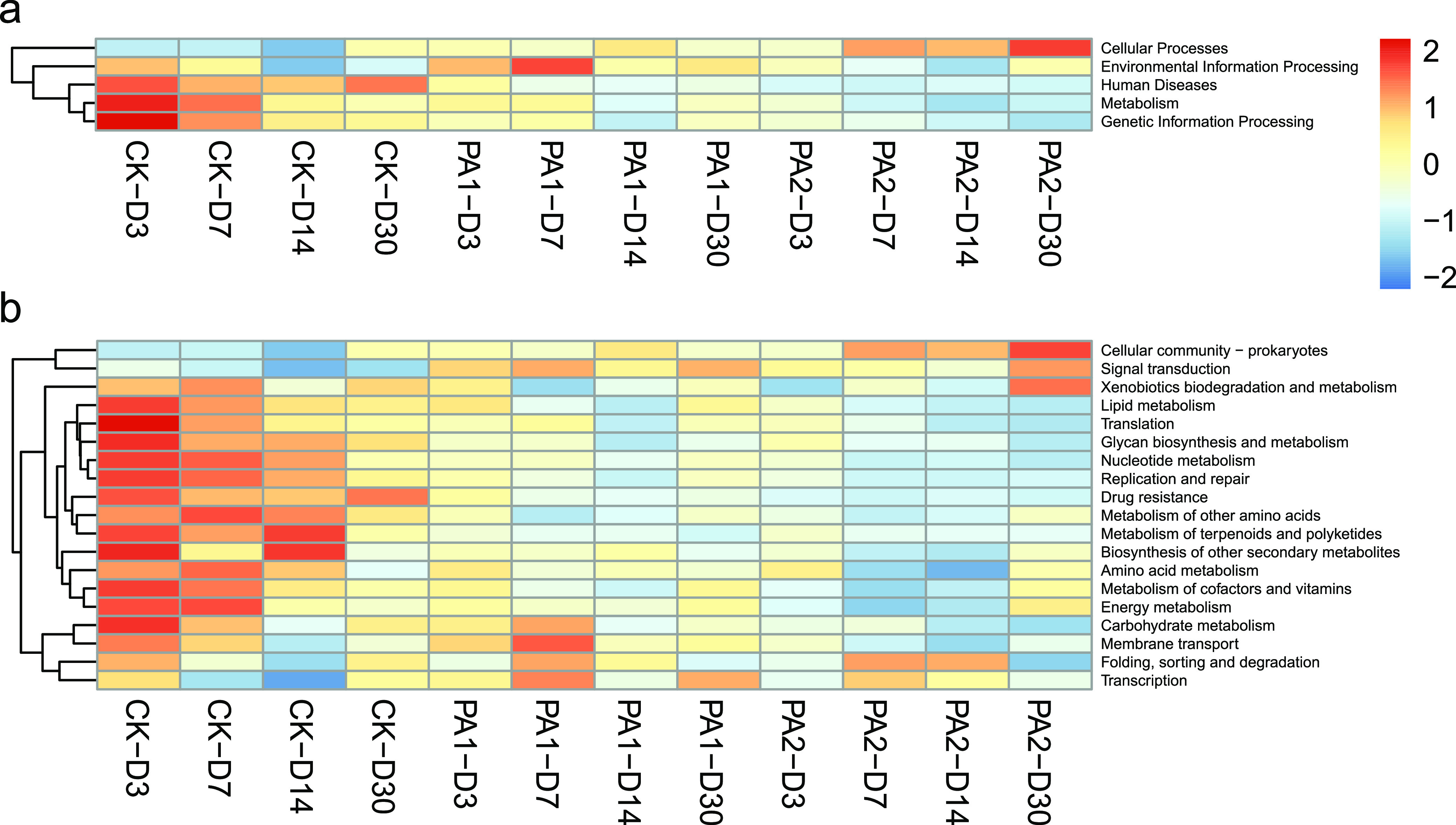
Heat map of potential metabolic pathways at level A (a) and level B (b), based on Kyoto Encyclopedia of Genes and Genomes ontology (KEGG; CK, the control; PA1, 1% pyroligneous acid; PA2, 2% pyroligneous acid; D3, D7, D14, D30, after ensiling for 3, 7, 14, or 30 days, respectively).

According to the heat map of the KEGG pathway analysis, pathways involved in the human diseases, metabolism, environmental information processing, and genetic information processing at level A and the metabolism of energy, lipid, amino acid, nucleotide and carbohydrate, cofactors and vitamins, translation, glycan biosynthesis and metabolism, replication and repair, drug resistance, and membrane transport at level B reduced during ensiling (Fig. 3). This might be because of the reduction of available resource such as oxygen, carbon source, and nitrogen source during anaerobic fermentation. The addition of PA further reduced these pathways, which could have resulted from the direct acidification inhibiting the microbial activities. Our previous study found that PA reduced these pathways in stylo silage using 16S rRNA gene-predicted function (16).

### ARGs in alfalfa silage.

**FIG 4:**
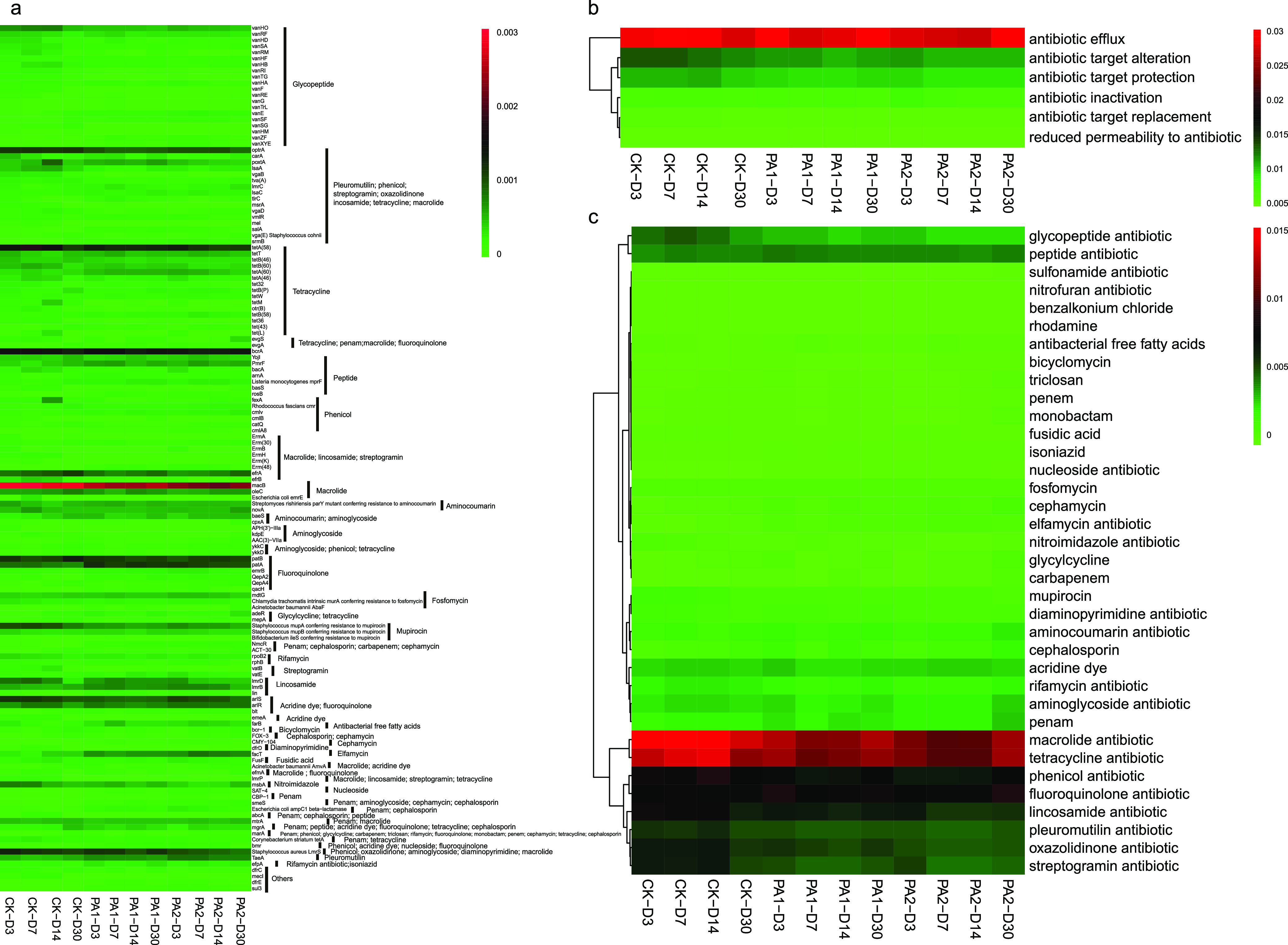
Heat map of ARGs (relative abundance of >0.01%) in alfalfa silage (a) and classification by resistance mechanism (b) and antibiotics (c) (CK, the control; PA1, 1% pyroligneous acid; PA2, 2% pyroligneous acid; D3, D7, D14, D30, after ensiling for 3, 7, 14, or 30 days, respectively).

Compared to conventional quantitative PCR, which is the method most frequently used to quantify many ARGs but is time-consuming, metagenomic analysis could possibly perform broad-spectrum scanning of ARGs that have been deposited in the Comprehensive Antibiotic Research Database (CARD) (2, 21). In the current study, more than 21,800 antibiotic resistance ontologies were detected in alfalfa silages (Fig. 4a, Table S2). It is known that the ARGs could transfer to plants via colonizing in leaf tissues from contaminated soil. The abundance of ARGs in alfalfa silage might be caused by alfalfa being contaminated with pathogens or ARGs preharvest via soil and irrigation water. Fertilization with animal manure is a traditional and common agricultural practice in China and other parts of the world (22). Manure is not only an essential source of nitrogen but also a rich source of antibiotic-resistant bacteria for agricultural soil (23). These ARGs in alfalfa might come from manured soils via root endophytes and phyllosphere. Similarly, abundant ARGs were found on the surface of vegetables like cucumbers, tomatoes, lettuce, and peppers that were fertilized with dairy or swine manure (24–26). To the best of our knowledge, this is the first report about occurrence and removal of ARGs in forage silage. The diversity of ARGs in alfalfa silage in the present study is alarming and indicates a serious issue, as any contaminated forages could be consumed by animals and the ARGs could spread to humans via dust, animal and agricultural products, etc. To improve food safety and human health, policies and management tools are needed to control or eliminate ARGs effectively in agricultural ecosystems.

The abundance and diversity of the top 142 ARGs (relative abundance of >0.01%) in alfalfa silage are shown in Fig. 4a. The results showed that *macB* was the most abundant in alfalfa silage. Hu et al. (27) reported that *macB* was widely distributed in the United States population. In addition to *macB*, resistant genes like *optrA*, *tetA*, *bcrA*, *efrA*, and *patB* were also very abundant in the detected ARGs. This might be due to their broad range of host bacteria. *tetM* and *tetW* were reported as the most prevalent tetracycline resistance gene types in the oral and fecal metagenomes of Europeans according to research performed by Seville et al. (28). *tetB* has the broadest host range in the Gram-negative microorganisms. Interestingly, all these genes became less and less abundant during fermentation. Pyroligneous acid, a byproduct of biochar production, has recently been used as a multifunctional soil amendment. This could promote plant growth by elevating nutrient availability and reducing heavy metal mobility and shift bacterial community by killing potential pathogen (18). The addition of PA decreased the abundance of these ARGs in alfalfa silage. Zheng et al. (13) also found that PA and its fractions had high removal efficiency for most ARGs in the soil. *Planctomycetes* species are considered drivers to the horizontal gene transfer of ARGs (29). Zheng et al. (13) reported that the addition of PA enhanced the abundance of phylum *Planctomycetes* and thus increased relative abundance of *tetO* and *tetW*. In the present investigation, PA increased the relative abundance of *Planctomycetes*, while it showed little effect on the relative abundance of *tetW*. The present investigation of ARG reduction in silage by PA is significant in controlling the pollution in livestock system since the silage production is a crucial part which links ARGs from soil to animals. Perhaps more efforts should be devoted to elevate the efficiency of the silage additives like PA in controlling ARG pollution in silage.

The detected ARGs in alfalfa silage in the present study belonged mainly to antibiotic efflux, cellular protection, and antibiotic inactivation (Fig. 4b, Table S3). These ARG classifications were also commonly detected in Chinese swine farms by Zhu et al. (5). Ensiling is an efficient and low-cost method to preserve high-moisture biomass (like forages and agricultural byproducts) through microbial fermentation. As shown in Fig. 4b, the majority of types of resistance mechanisms, including antibiotic target alteration and antibiotic target replacement, decreased after 30 days of ensiling. This might be because of the decline of available nutrients and competition between microorganisms. Zhang et al. (30) also considered that nutrition in swine manure could influence the microbial community structure and ARG distribution by exerting selective pressure to the microbes. Similarly, anaerobic digestion is also an effective method to remove pathogens and ARGs in sludge (31). This indicates that ensiling might be an effective method to mitigate ARG proliferation during animal production.

As shown in Fig. 4c, the resistance genes detected in alfalfa silage are potentially resistant to many classes of antibiotics, including macrolides, aminoglycosides, and tetracycline, which are critically important for human medicine. According to the FDA classes, macrolides, fluoroquinolones, and penicillins are the most widely used in human medicine worldwide (32). In the present study, resistance genes related to macrolides, fluoroquinolones, and tetracyclines were relatively abundant (Table S4). This might be because the soil was contaminated by manure, as many antimicrobials essential for human medicine had been used in food-producing animal production. For example, macrolides and lincosamides are common drugs in cattle production to treat many infections in Europe (33). Tetracyclines are one of the most extensively used antibiotics in cattle production industry to treat skin, respiratory, and gastrointestinal tract diseases. Aminoglycosides, with a broad antimicrobial spectrum, are often used as anti-infectants in synergy with other antibiotics by inhibiting protein synthesis and altering the integrity of bacterial cell membranes. These resistance genes were frequently detected in alfalfa silage in the present study. This might be a sign of danger, and measures should be taken. The relative abundance of these resistance genes in alfalfa silage decreased after 30 days of fermentation. Guo et al. (34) reported that bamboo-derived PA could remove ARGs during production of swine manure compost. In our study, the majority of ARGs, including those related to macrolides, lincosamides, aminoglycosides, and tetracycline, in alfalfa silage were reduced by the addition of PA. This might be because of the microbial community shift caused by PA. Extensive studies proved that organic acids and phenols in PA inhibited pathogens synergistically by cell membrane damage, lipid dissolution, and protein denaturation. This was further confirmed by the decrease of relevant pathways (Fig. 3). Previous studies also found that application of PA significantly reduced the number of phyla such as *Bacteroidetes*, *Firmicutes*, and *Actinobacteria*, which are always considered ARG-carrying pathogens (13, 35). The addition of PA increased the bacterial richness (Chao 1 and ACE) and decreased bacterial diversity (Shannon, Simpson) in the alfalfa silage (Table S5). These results indicated that PA could selectively inhibit specific bacteria in the alfalfa silage. Therefore, the reduction of these ARGs’ abundance might be because the addition of PA inhibited those potential ARGs-carrying pathogens. This is different from what was reported by Zheng et al. (6), who found that the application of PA decreased the bacterial community richness of the rhizosphere soil without any influence on the bacterial diversity. Some types with relative low abundance, such as cephalosporin, cephamycin, penam, carbapenem, and monobactam, increased during silage fermentation. The reason remained unclear, as the fate of these ARGs in silage was rarely explored. It might be attributed to the different reactions of microorganisms to anaerobic conditions or possible horizontal gene transfer in different bacterial communities. More efforts should be devoted to fully understanding the fate of various ARGs in silage during anaerobic fermentation process.

### Relationship between the bacterial communities and ARGs.

**FIG 5:**
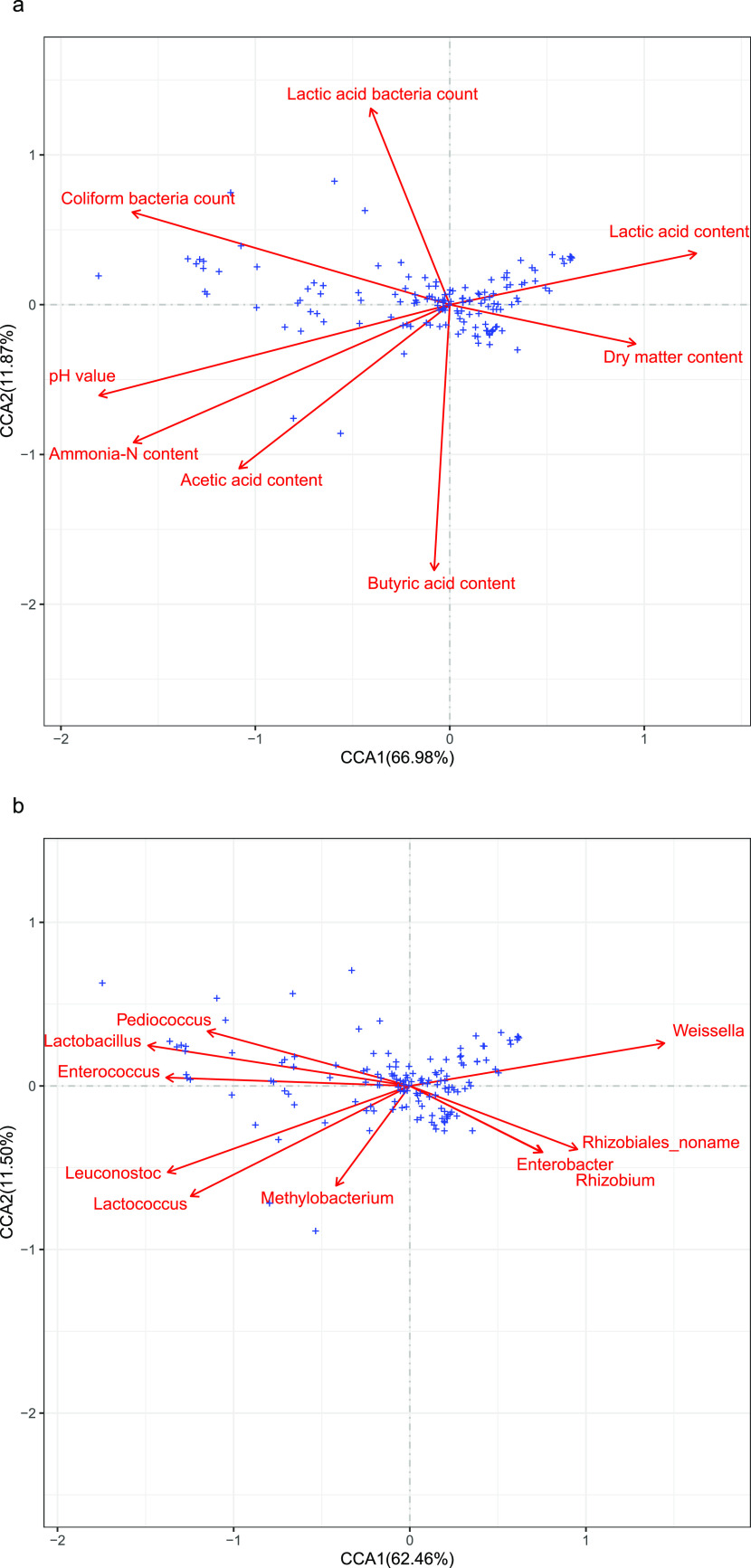
Canonical correlation analysis of ARGs (relative abundance of >0.01%) and (a) fermentation parameters and (b) bacterial communities (top 10).

**FIG 6:**
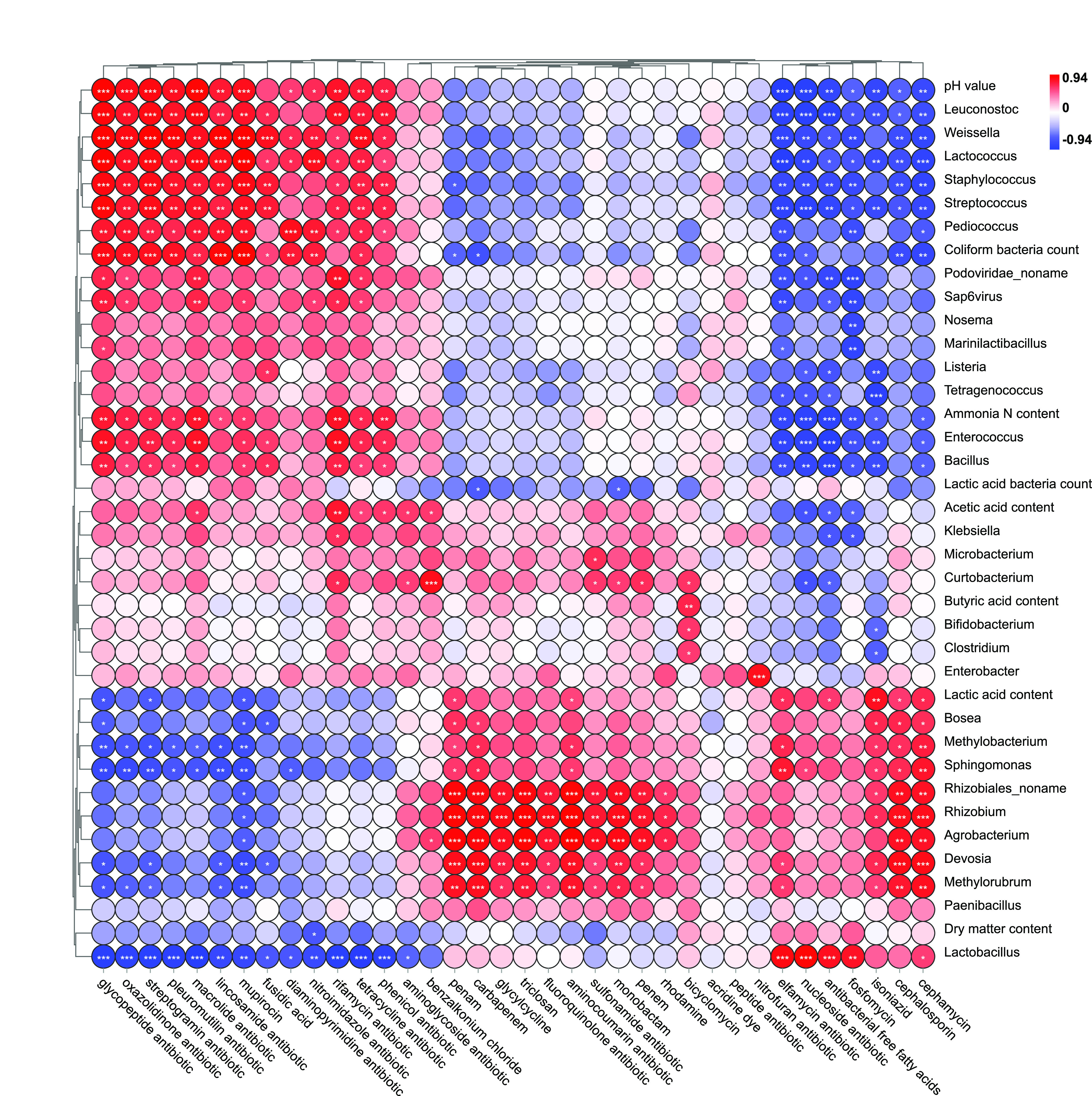
Heat map of correlations between ARGs (36 antibiotics classes) and fermentation parameters and between ARGs and bacterial communities (top 35) (*, *P* < 0.05; **, *P* < 0.01; ***, *P* < 0.001).

**FIG 7:**
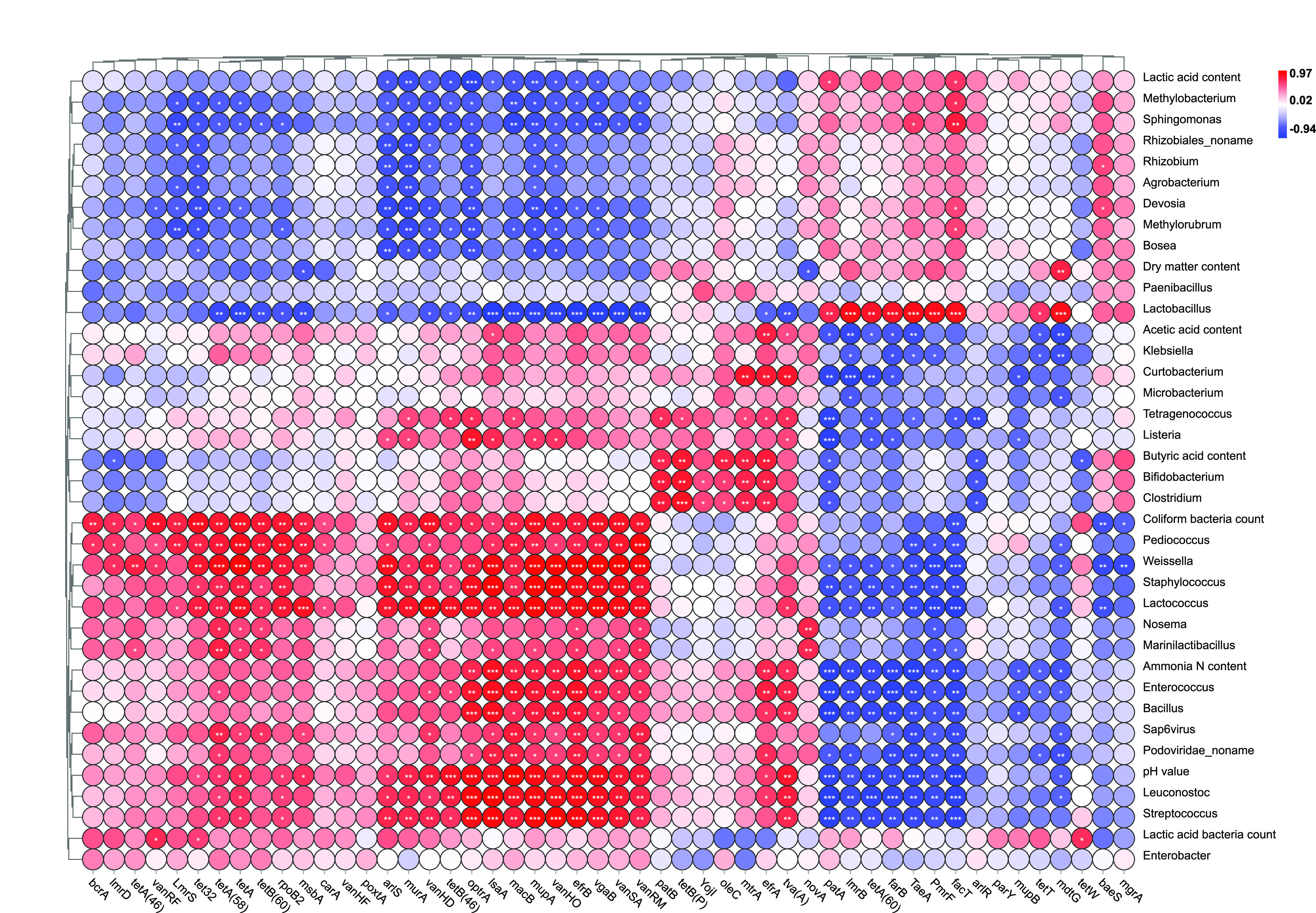
Heat map of correlation between the ARGs (top 50) and fermentation parameters and between ARGs and bacterial communities (top 35) (*, *P*< 0.05; **, *P* < 0.01; ***, *P* < 0.001).

Previous studies proved that physicochemical properties of soils like pH value, electrical conductivity, soil organic carbon, available phosphate, and ammonia-N had a great influence on ARGs diversity via shaping succession and evolution of bacterial community (6). Similarly, the silage fermentation parameters, including pH value, lactic acid content, acetic acid content, ammonia-N content, and coliform bacteria count, also influenced the abundance of ARGs in alfalfa silage in the present study (Fig. 5 to 7). For example, pH is an important parameter in silage fermentation, as relatively low pH value could inhibit the microbial activities and shift microbial community. Zheng et al. (13) reported that pH value of soil accounted for the variation in ARGs, though it was not a driving factor. In our study, the ARGs with relatively high abundance, including *macB*, *optrA*, *tetA*, *bcrA*, *efrA*, and *patB*, were positively related to pH value (Fig. 7). Positive correlation between pH value and ARGs was also reported by Zhao et al. (36) in landfills. The same correlation was also discovered between coliform bacteria count (or ammonia-N content) and the relative abundance of these ARGs.

The relative abundance of ARGs could reflect the ratio of ARG-carrying bacteria relative to total bacteria. The findings in Fig. 5 to 7 also suggested that the bacterial community in alfalfa silage had a significant influence on ARG abundance. This was consistent with Xie et al. (29) and Xu et al. (4), who reported that the microbial community was a key driving factor for the change of ARGs in animal manure and organic fertilizer by exploring the correlation between some specific ARGs and main microbial genera. The pathogens might positively correlate with the ARGs, as pathogenic bacteria are the potential hosts of ARGs. Huang et al. (37) found that some dominant genera in *Firmicutes*, including *Streptococcus* and *Clostridiaceae*, showed significant correlations with the variation of the antibiotic resistome in anaerobic digestion of swine manure using redundancy analysis. Zheng et al. (13) reported similar results in soil. In the present study, *Firmicutes* declined during ensiling, especially after the addition of PA. This may partially explain the reduction of ARG abundance during alfalfa fermentation.

At the genus level, the top several drug classes, including macrolide, tetracycline, lincosamide, phenicol, oxazolidinone, streptogramin, pleuromutilin, and glycopeptide were positively correlated with *Staphylococcus*, *Streptococcus*, *Listeria*, *Bacillus*, *Klebsiella*, *Clostridium*, and *Enterobacter*, the potential hosts of ARGs. According to previous studies, almost all these genera are pathogenic bacteria in the livestock production system (1). *Staphylococcus* is typically detected in chicken microflora. As an opportunistic pathogen, it can cause severe infections, especially bacteria that carry ARGs, though it is mostly harmless (3). *Streptococcus*, obligate or facultative aerobes, was one of the most abundant genera in slurry and swine gut microbiota (38, 39). Tian et al. (39) found that the decrease of possible hosts like *Streptococcus* by thermophilic digestion might contribute to the reduction of ARG abundance. The decrease of *Streptococcus* bacteria in our study might be because of the anaerobic condition during ensiling. *Lactobacillus*, the dominant genus in alfalfa silage, was negatively correlated with macrolide, tetracycline, lincosamide, phenicol, oxazolidinone, streptogramin, pleuromutilin, glycopeptide, mupirocin, diaminopyrimidine, nitroimidazole, diaminopyrimidine, rifamycin, and aminoglycoside, most of which were more abundant than the remaining ones. This might be because *Lactobacillus* dominated the fermentation process and inhibited the pathogenic bacteria carrying ARGs. Lactic acid bacteria inoculants containing *Lactobacillus* are commonly used in silage production. These inoculants could promote silage fermentation by changing bacterial community structure and making *Lactobacillus* dominated. The effect of lactic acid bacteria inoculants on ARGs during silage fermentation should be explored further. The relative abundance of ARGs in the present study was also influenced by other lactic acid bacteria, including *Enterococcus*, *Leuconostoc*, *Pediococcus*, *Weissella*, and *Lactococcus*. This might be because these genera are also hosts of these ARGs. For example, tigecycline and macrolide resistance has been observed in *Enterococcus* species, such as E. hirae, E. faecalis, E. faecium, and E. durans in cattle and poultry manure from Europe (3, 40). Su et al. (41) reported that *Lactococcus* in landfill leachates was significantly correlated with the ARGs. The above genera were always considered to be beneficial for silage fermentation. More studies should be conducted to verify their specific roles and mechanisms of ARG contamination during ensiling.

The three antibiotic classes, macrolides, lincosamides, and streptogramins B, have similar antibacterial action modes and comparable antibacterial spectra. Bacterial resistance to these antibiotics in staphylococci was widely reported in clinical specimens and poultry manure (42, 43). In our study, the relative abundance of ARGs belonging to these antibiotic classes and *Staphylococcus* was positively correlated (*P* < 0.01). The fluoroquinolones, which have a broad antimicrobial spectrum, showed a promising activity against both Gram-negative and Gram-positive microbes. Fluoroquinolone resistance genes were detected in *Enterobacteriaceae* in dairy calves (3). In the current study, fluoroquinolone resistance genes were positively correlated with *Enterobacter*, though the correlation was not significant. The resistant genes related to fluoroquinolones also showed a positive correlation (*P* < 0.05) with genera like *Rhizobiales_noname*, *Devosia*, *Methylorubrum*, *Rhizobium*, and *Agrobacterium*. Tetracyclines are consumed after penicillin throughout the world due to their broad antibacterial spectrum, relative safety, and low cost. In the present study, the resistant genes related to tetracyclines were positively correlated (*P* < 0.05) with *Staphylococcus*, *Streptococcus*, *Bacillus*, and *Klebsiella*. All these genera were undesirable in silage fermentation due to their competition with lactic acid bacteria. Silage dominated by these undesirable bacteria like *Bacillus*, *Klebsiella*, *Clostridium*, and *Enterobacter* was always low in fermentation quality (20). Therefore, the pathogens in the silage not only decrease the productivity of animals but also increase the risk of ARGs spreading.

The relationship between the ARGs and bacterial community was further confirmed by the significant correlations showed in Fig. 7 and Fig. S1. Brenciani et al. (44) observed *tetM* in Streptococcus pyogenes isolates in the presence of erythromycin. This may partially explain the positive correlation between *Streptococcus* and *tetM*. Additionally, ARGs like *macB*, *optrA*, *tetA*, *bcrA*, *efrA*, *arlS*, *efrA*, *msbA*, and *vanHO* were positively correlated with genera such as *Streptococcus*, *Bacillus*, *Marinilactibacillus*, *Staphylococcus*, *Klebsiella*, *Listeria*, and *Microbacterium*, suggesting that these bacteria might be the hosts of these ARGs widely detected in alfalfa silage. Resistance gene *catQ* was positively correlated with *Clostridium* and *Streptococcus*, and *fexA* was positively correlated with *Bacillus* and *Staphylococcus*. This is consistent with van Hoek et al. (45), who reviewed and found *catQ* and *fexA* in these genera, respectively.

### Conclusion.

Overall, ARGs in alfalfa silage were abundant and were influenced by the fermentation parameters and microbial community composition. Ensiling could be a feasible way to mitigate ARGs in forages, as the majority of ARGs in alfalfa silage reduced during fermentation. More studies should be conducted to provide a strong impetus to more fully understand abundance, diversity, and dynamics of ARGs during ensiling. The addition of PA could not only improve fermentation quality but also reduce ARG pollution of alfalfa silage, and 2% PA performed better than 1% PA. PA could reduce the relative abundance of ARGs by directly inhibiting potential ARG-hosting bacteria. Considering the seriousness ARGs pollution in livestock production, more efficient silage additives should be developed to reduce ARG contamination.

## MATERIALS AND METHODS

### Silage preparation.

Alfalfa was harvested at flowering stage in an experimental field of South China Agricultural University (Guangzhou, China) without application of chemical fertilizers and insecticides. The materials were immediately chopped (about 1-cm lengths), homogenized, and then treated with 1% PA and 2% PA on a fresh weight basis according to our previous study (16). Pyroligneous acid, kindly provided by Xiwei Xu (South China Agricultural University), was obtained using the method described by Xu et al. (46) and then filtered using cellulose acetate membrane (0.45 μm). The control group was treated with the same volume of sterile distilled water. After thorough mixing, about 200 g of silage materials was immediately packed and sealed using polyethylene bags (12 bags for each treatment). All these bags were kept at room temperature (27°C to 32°C). Subsequently, to determine the fermentation quality and microbial community, silages from three bags were randomly sampled after 3, 7, 14, and 30 days of ensiling.

### Gas production analyses.

As described previously in Chen et al. (20), the volume of these bags was detected in a thermostatic water bath (25°C) using a 5,000 mL beaker. The difference between before and after ensiling was calculated as gas production. A gas chromatographer (Shimadzu GC-20A) equipped with a thermal conductivity detector (TCD) was used to analyze the concentrations of CO_2_, N_2_, and H_2_ based on the ratio of peak area.

### Fermentation quality analyses.

Dynamics of fermentation parameters were analyzed according to our previous study (47). First, a 20-g silage sample was mixed with 180 mL distilled water and then stored at 4°C overnight. The pH value was measured immediately after filtration using a pH meter. The concentrations of organic acids were measured using high-pressure liquid chromatography (HPLC) method, and ammonia-N content was determined using the phenol-hypochlorite colorimetric method (20). Second, another 20 g of silage and 180 mL sterilized normal saline were homogenized and serially diluted. The lactic acid bacteria and coliform bacteria were cultured using MRS agar plate and violet red bile agar (VRBA) plate, respectively (20, 47). Last, the remained silage was dried at 65°C for 48 h to determine the dry matter content. The protease activity was measured according to our previous study (48), and 20-g samples were homogenized with 100 mL of 0.1 M sodium phosphate buffer (pH 6.0 with 5 mM hyposulfite). After filtration and centrifugation, the supernatant was used to analyze the activities of acid proteinase, carboxypeptidase, and aminopeptidase. The protein fraction was determined according to the method of Guo et al. (49), and 25% (wt/vol) trichloroacetic acid was used to precipitate the protein. Crude protein and true protein-N were analyzed using a Kjeldahl nitrogen analyzer, where nonprotein-N was calculated by their difference.

### DNA extraction and 16S rRNA gene sequencing.

Microbial DNA extraction from silage samples was performed using a HiPure bacterial DNA kit (Magen, Guangzhou, China). The V3-V4 hypervariable regions of 16S rRNA were amplified using primers (341F: CCTACGGGNGGCWGCAG; 806R: GGACTACHVGGGTATCTAAT) according to He et al. (47). A 50-μL PCR including 5 μL of 10× KOD buffer, 1.5 μL of each primer (5 μM), 1 μL of KOD polymerase, 5 μL of 2.5 mM deoxynucleoside triphosphates (dNTPs), and 100 ng of template DNA was performed. Using an Illumina Hiseq 2500PE250 sequencer, the sequencing of these PCR products was conducted immediately after purification.

### Metagenome sequencing.

Metagenomic analysis was conducted according to Li and Zhang (50) by Gene Denovo Co., Ltd. (Guangzhou, China). Briefly, NEBNext Ultra DNA library prep kit (NEB, USA) was used to construct sequencing libraries. Then Illumina HiSeq TM2500 platform (Illumina, USA) was used to sequence the library preparations after cluster generation. For each unique open reading frame, functional annotation was conducted by alignment and searching against the Kyoto Encyclopedia of Genes and Genomes and the Comprehensive Antibiotic Research Database.

### Statistical analysis.

The effects of fermentation time and PA on fermentation parameters of alfalfa silage were analyzed by two-way analysis of variance (ANOVA; IBM SPSS 20.0). Statistical significance was considered when *P* value was lower than 0.05. The correlation between ARGs and fermentation parameters and between ARGs and main bacterial communities was analyzed by canonical correlation analysis. Figures were made and processed by a free online platform of OmicShare tools (http://www.omicshare.com/tools) and the software Adobe Illustrator CS 6.0.

### Data availability.

The raw sequences of 16S rRNA gene were deposited in the NCBI database to be available to the public (PRJNA759354). The data set of metagenomic analysis was uploaded to the NCBI with the accession number SRP338492.
