# Supplementary material for: Effects of Pyroligneous Acid on Diversity and Dynamics of Antibiotic Resistance Genes in Alfalfa Silage
Source: Microbiol Spectr. 2022 Jul 11;10(4):e01554-22. doi: 10.1128/spectrum.01554-22 (PMC9430785; doi:10.1128/spectrum.01554-22)
Supplement: Supplemental file 1 — Supplemental material. Download spectrum.01554-22-s0001.pdf, PDF file, 5.1 MB [file spectrum.01554-22-s0001.pdf]

**Fig. S1** Heat map of correlation between ARGs (relative abundance > 0.01%) and fermentation parameters, bacterial communities (top 35) (*, $P< 0.05$; **, $P< 0.01$; ***, $P< 0.001$).

**Table S1** The relative abundance of microbial communities (top 30) in alfalfa silage genus level(CK, the control; PA1, 1% pyroligneous acid; PA2, 2% pyroligneous acid; D3, D7, D14, D30, after ensiling for 3, 7, 14, 30 days, respectively).

**Table S2** The relative abundance of Antibiotic Resistance Ontologies in alfalfa silage (CK, the control; PA1, 1% pyroligneous acid; PA2, 2% pyroligneous acid; D3, D7, D14, D30, after ensiling for 3, 7, 14, 30 days, respectively).

**Table S3** The relative abundance of ARGs in alfalfa silage classified by resistance mechanism (CK, the control; PA1, 1%pyroligneous acid; PA2, 2% pyroligneous acid; D3, D7, D14, D30, after ensiling for 3, 7, 14, 30 days, respectively).

**Table S4** The relative abundance of ARGs in alfalfa silage classified by antibiotics (CK, the control; PA1, 1% pyroligneous acid; PA2, 2% pyroligneous acid; D3, D7, D14, D30, after ensiling for 3, 7, 14, 30 days, respectively).

**Table S5** The microbial richness and diversity in the alfalfa silage (CK, the control; PA1, 1% pyroligneous acid; PA2, 2% pyroligneous acid; D3, D7, D14, D30, after ensiling for 3, 7, 14, 30 days, respectively).

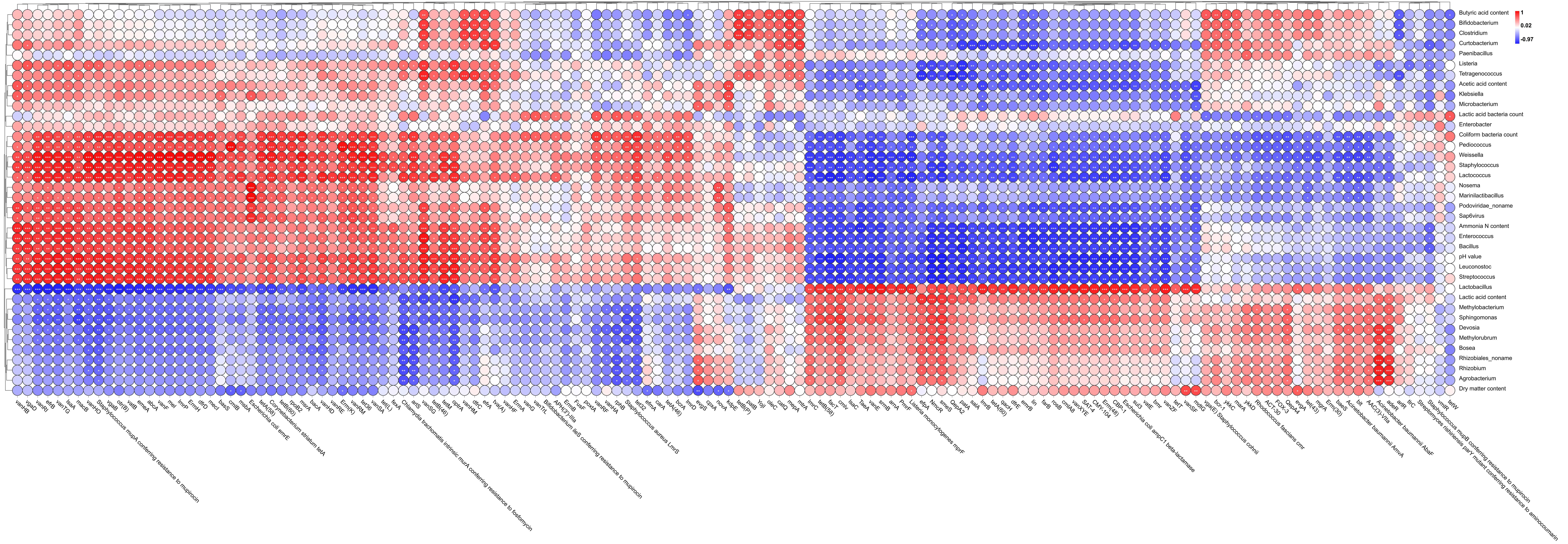
1
0.02
-0.97
Butyric acid content
Bifidobacterium
Clostridium
Curtobacterium
Paenibacillus
Listeria
Tetragenococcus
Acetic acid content
Klebsiella
Microbacterium
Lactic acid bacteria count
Enterobacter
Coliform bacteria count
Pediococcus
Weissella
Staphylococcus
Lactococcus
Nosema
Marinilactibacillus
Podoviridae_noname
Sap6virus
Ammonia N content
Enterococcus
Bacillus
pH value
Leuconostoc
Streptococcus
Lactobacillus
Lactic acid content
Methylobacterium
Sphingomonas
Devosia
Methylorubrum
Bosea
Rhizobiales_noname
Rhizobium
Agrobacterium
Dry matter content
vanHB
vgaD
vanRI
efrB
vanTG
lsaA
macB
vanHO
Staphylococcus mupA conferring resistance to mupirocin
vgaB
otr(B)
vatB
emeA
abcA
vanF
mel
lmrP
ErmH
dfrD
mecI
basS
cmlB
msbA
Escherichia coli emrE
tetA(58)
Corynebacterium striatum tetA
tetB(60)
rpoB2
msrA
bacA
vanHD
vanRE
Erm(K)
vanRM
tet36
vanSA
tet(L)
fexA
Chlamydia trachomatis intrinsic murA conferring resistance to fosfomycin
arlS
vanSG
tetB(46)
tetM
optrA
vanHM
dfrC
efrA
tva(A)
vanHF
ErmA
vanG
vanTrL
Bifidobacterium ileS conferring resistance to mupirocin
APH(3')-IIIa
ErmB
FusF
poxtA
vanRF
vanHA
rphB
Staphylococcus aureus LmrS
tet32
efmA
carA
tetA(46)
bcrA
lmrD
evgS
cpxA
novA
kdpE
tetB(P)
patB
YojI
oleC
catQ
mepA
mtrA
lmrC
tetB(58)
facT
cmlv
lsaC
TaeA
vanE
srmB
arnA
PmrF
Listeria monocytogenes mprF
efpA
NmcR
smeS
QepA2
patA
salA
lmrB
tetA(60)
qacH
dfrE
emrB
lin
farB
rosB
cmlA8
vanXYE
SAT-4
CMY-104
Erm(48)
CBP-1
Escherichia coli ampC1 beta-lactamase
sul3
vatE
bmr
vanZF
tetT
vanSF
mdtG
vga(E) Staphylococcus cohnii
bcr-1
ykkC
marA
ykkD
Rhodococcus fascians cmr
ACT-30
FOX-3
QepA4
evgA
tet(43)
mgrA
Erm(30)
baeS
Acinetobacter baumannii AmvA
blt
AAC(3)-VIIa
Acinetobacter baumannii AbaF
adeR
arlR
tlrC
Streptomyces rishiriensis parY mutant conferring resistance to aminocoumarin
Staphylococcus mupB conferring resistance to mupirocin
vmlR
tetW

| Genus | CK-D3 | CK-D7 | CK-D14 | CK-D30 | PA1-D3 | PA1-D7 | PA1-D1 | PA1-D3 | PA2-D3 | PA2-D7 | PA2-D1 | PA2-D30 |
|---|---|---|---|---|---|---|---|---|---|---|---|---|
| Weissella | 0.260 | 0.296 | 0.277 | 0.024 | 0.017 | 0.004 | 0.008 | 0.004 | 0.093 | 0.007 | 0.011 | 0.003 |
| Lactobacillus | 0.428 | 0.254 | 0.351 | 0.599 | 0.749 | 0.855 | 0.724 | 0.672 | 0.769 | 0.852 | 0.768 | 0.613 |
| Enterococcus | 0.043 | 0.120 | 0.115 | 0.124 | 0.004 | 0.003 | 0.005 | 0.002 | 0.004 | 0.003 | 0.003 | 0.003 |
| Pediococcus | 0.073 | 0.100 | 0.044 | 0.023 | 0.027 | 0.011 | 0.006 | 0.078 | 0.019 | 0.011 | 0.004 | 0.002 |
| Lactococcus | 0.092 | 0.090 | 0.045 | 0.043 | 0.006 | 0.004 | 0.007 | 0.002 | 0.016 | 0.003 | 0.005 | 0.003 |
| Leuconostoc | 0.007 | 0.007 | 0.009 | 0.007 | 0.001 | 0.001 | 0.001 | 0.002 | 0.002 | 0.002 | 0.002 | 0.002 |
| Enterobacter | 0.001 | 0.005 | 0.003 | 0.001 | 0.009 | 0.001 | 0.005 | 0.001 | 0.001 | 0.000 | 0.002 | 0.001 |
| Rhizobiales_nona | 0.002 | 0.002 | 0.003 | 0.004 | 0.011 | 0.006 | 0.019 | 0.022 | 0.002 | 0.006 | 0.013 | 0.042 |
| Rhizobium | 0.002 | 0.002 | 0.003 | 0.003 | 0.016 | 0.005 | 0.013 | 0.019 | 0.002 | 0.006 | 0.012 | 0.039 |
| Methylobacterium | 0.001 | 0.002 | 0.002 | 0.002 | 0.004 | 0.004 | 0.005 | 0.007 | 0.002 | 0.004 | 0.010 | 0.009 |
| Microbacterium | 0.002 | 0.002 | 0.002 | 0.001 | 0.001 | 0.001 | 0.003 | 0.001 | 0.001 | 0.001 | 0.001 | 0.002 |
| Streptococcus | 0.001 | 0.002 | 0.002 | 0.001 | 0.000 | 0.001 | 0.000 | 0.000 | 0.001 | 0.001 | 0.001 | 0.000 |
| Devosia | 0.002 | 0.001 | 0.002 | 0.004 | 0.007 | 0.004 | 0.012 | 0.013 | 0.002 | 0.005 | 0.011 | 0.018 |
| Bacillus | 0.001 | 0.001 | 0.001 | 0.001 | 0.000 | 0.000 | 0.001 | 0.000 | 0.001 | 0.001 | 0.001 | 0.001 |
| Klebsiella | 0.000 | 0.001 | 0.001 | 0.001 | 0.001 | 0.000 | 0.001 | 0.000 | 0.000 | 0.000 | 0.000 | 0.001 |
| Sphingomonas | 0.000 | 0.001 | 0.001 | 0.000 | 0.003 | 0.002 | 0.003 | 0.003 | 0.003 | 0.003 | 0.007 | 0.006 |
| Agrobacterium | 0.001 | 0.001 | 0.002 | 0.001 | 0.004 | 0.002 | 0.005 | 0.006 | 0.001 | 0.003 | 0.004 | 0.015 |
| Bifidobacterium | 0.001 | 0.001 | 0.007 | 0.036 | 0.000 | 0.000 | 0.000 | 0.000 | 0.000 | 0.000 | 0.000 | 0.000 |
| Podoviridae_nona | 0.000 | 0.001 | 0.000 | 0.000 | 0.000 | 0.000 | 0.000 | 0.000 | 0.000 | 0.000 | 0.000 | 0.000 |
| Methylorubrum | 0.001 | 0.001 | 0.001 | 0.001 | 0.001 | 0.001 | 0.003 | 0.002 | 0.002 | 0.001 | 0.003 | 0.005 |
| Staphylococcus | 0.001 | 0.001 | 0.001 | 0.000 | 0.000 | 0.000 | 0.000 | 0.000 | 0.000 | 0.000 | 0.000 | 0.000 |
| Curtobacterium | 0.001 | 0.001 | 0.001 | 0.001 | 0.000 | 0.000 | 0.000 | 0.000 | 0.000 | 0.000 | 0.001 | 0.001 |
| Nosema | 0.000 | 0.001 | 0.000 | 0.000 | 0.000 | 0.000 | 0.000 | 0.000 | 0.000 | 0.000 | 0.000 | 0.000 |
| Clostridium | 0.000 | 0.001 | 0.001 | 0.004 | 0.000 | 0.000 | 0.000 | 0.000 | 0.000 | 0.000 | 0.000 | 0.000 |
| Bosea | 0.002 | 0.001 | 0.001 | 0.001 | 0.003 | 0.003 | 0.008 | 0.008 | 0.001 | 0.003 | 0.005 | 0.006 |
| Listeria | 0.000 | 0.001 | 0.001 | 0.001 | 0.000 | 0.000 | 0.000 | 0.000 | 0.000 | 0.000 | 0.000 | 0.000 |
| Marinilactibacil | 0.000 | 0.001 | 0.000 | 0.000 | 0.000 | 0.000 | 0.000 | 0.000 | 0.000 | 0.000 | 0.000 | 0.000 |
| Sap6virus | 0.000 | 0.000 | 0.000 | 0.000 | 0.000 | 0.000 | 0.000 | 0.000 | 0.000 | 0.000 | 0.000 | 0.000 |
| Tetragenococcus | 0.000 | 0.000 | 0.000 | 0.001 | 0.000 | 0.000 | 0.000 | 0.000 | 0.000 | 0.000 | 0.000 | 0.000 |
| Paenibacillus | 0.000 | 0.000 | 0.000 | 0.000 | 0.000 | 0.000 | 0.001 | 0.000 | 0.000 | 0.001 | 0.000 | 0.001 |

| GeneID | ARO_Nam | CK-D3 | CK-D7 | CK-D14 | CK-D30 | PA1-D3 | PA1-D7 | PA1-D14 | PA1-D30 | PA2-D3 | PA2-D7 | PA2-D14 | PA2-D30 |
|---|---|---|---|---|---|---|---|---|---|---|---|---|---|
| Unigene140 | vanSA | 4.753E-06 | 2.560E-07 | 3.605E-06 | 6.478E-07 | 2.592E-07 | 1.261E-07 | 5.164E-07 | 0.000E+00 | 0.000E+00 | 0.000E+00 | 1.201E-06 | 0.000E+00 |
| Unigene141 | vanRF | 3.883E-06 | 0.000E+00 | 2.938E-06 | 5.197E-07 | 0.000E+00 | 0.000E+00 | 5.695E-07 | 0.000E+00 | 0.000E+00 | 0.000E+00 | 0.000E+00 | 0.000E+00 |
| Unigene152 | vanHD | 2.872E-06 | 2.934E-06 | 2.947E-06 | 1.533E-06 | 0.000E+00 | 0.000E+00 | 5.114E-07 | 3.082E-07 | 6.854E-07 | 0.000E+00 | 3.776E-08 | 0.000E+00 |
| Unigene173 | novA | 1.297E-06 | 1.107E-06 | 5.232E-06 | 7.616E-06 | 0.000E+00 | 0.000E+00 | 5.848E-07 | 0.000E+00 | 0.000E+00 | 0.000E+00 | 0.000E+00 | 1.136E-07 |
| Unigene186 | vanHO | 1.105E-04 | 1.967E-04 | 1.706E-04 | 0.000E+00 | 0.000E+00 | 0.000E+00 | 0.000E+00 | 0.000E+00 | 0.000E+00 | 0.000E+00 | 0.000E+00 | 0.000E+00 |
| Unigene191 | vanHB | 2.540E-05 | 3.736E-05 | 2.233E-05 | 0.000E+00 | 0.000E+00 | 0.000E+00 | 0.000E+00 | 0.000E+00 | 0.000E+00 | 0.000E+00 | 0.000E+00 | 0.000E+00 |
| Unigene198 | Corynebac | 2.923E-05 | 9.362E-06 | 7.832E-06 | 5.611E-06 | 3.478E-07 | 0.000E+00 | 0.000E+00 | 1.827E-07 | 9.408E-07 | 0.000E+00 | 0.000E+00 | 0.000E+00 |
| Unigene199 | patB | 2.233E-05 | 9.522E-06 | 6.093E-06 | 4.166E-06 | 3.307E-07 | 0.000E+00 | 0.000E+00 | 0.000E+00 | 9.297E-07 | 0.000E+00 | 0.000E+00 | 0.000E+00 |
| Unigene217 | tetA(58) | 9.342E-06 | 4.109E-06 | 3.389E-06 | 1.112E-05 | 6.756E-05 | 9.333E-05 | 9.620E-05 | 7.298E-05 | 1.104E-04 | 1.096E-04 | 9.310E-05 | 6.243E-05 |
| Unigene239 | tetB(58) | 1.890E-05 | 1.195E-05 | 2.381E-05 | 1.056E-04 | 1.638E-05 | 1.231E-05 | 3.058E-05 | 0.000E+00 | 0.000E+00 | 2.999E-05 | 2.547E-05 | 1.473E-05 |
| Unigene240 | tetA(58) | 5.170E-05 | 3.637E-05 | 7.529E-05 | 1.482E-04 | 8.682E-05 | 6.073E-05 | 7.912E-05 | 7.379E-05 | 0.000E+00 | 9.662E-05 | 9.492E-05 | 1.019E-04 |
| Unigene292 | lmrD | 4.919E-06 | 2.229E-05 | 3.414E-06 | 8.987E-07 | 1.294E-06 | 4.023E-07 | 3.044E-07 | 2.078E-07 | 1.291E-06 | 4.867E-07 | 2.036E-07 | 1.148E-07 |
| Unigene323 | patA | 3.985E-07 | 0.000E+00 | 1.303E-07 | 0.000E+00 | 0.000E+00 | 8.616E-08 | 0.000E+00 | 0.000E+00 | 0.000E+00 | 0.000E+00 | 0.000E+00 | 0.000E+00 |
| Unigene441 | tetT | 6.077E-06 | 2.192E-05 | 6.365E-06 | 4.040E-06 | 1.212E-06 | 4.096E-07 | 2.348E-07 | 3.361E-07 | 1.375E-06 | 7.428E-07 | 3.640E-07 | 2.329E-07 |
| Unigene449 | macB | 4.611E-06 | 2.040E-05 | 3.205E-06 | 1.067E-06 | 8.539E-07 | 4.674E-07 | 3.721E-07 | 0.000E+00 | 1.050E-06 | 8.027E-07 | 0.000E+00 | 0.000E+00 |
| Unigene458 | oleC | 2.906E-06 | 3.987E-06 | 3.259E-06 | 6.359E-06 | 4.612E-07 | 0.000E+00 | 1.915E-07 | 0.000E+00 | 7.563E-08 | 0.000E+00 | 7.915E-08 | 1.227E-07 |
| Unigene528 | novA | 1.808E-06 | 8.641E-07 | 4.864E-07 | 0.000E+00 | 0.000E+00 | 3.152E-08 | 0.000E+00 | 0.000E+00 | 0.000E+00 | 3.248E-07 | 0.000E+00 | 0.000E+00 |
| Unigene563 | poxtA | 1.927E-06 | 1.146E-06 | 1.733E-06 | 5.271E-07 | 0.000E+00 | 0.000E+00 | 0.000E+00 | 0.000E+00 | 0.000E+00 | 0.000E+00 | 0.000E+00 | 0.000E+00 |
| Unigene564 | TaeA | 2.167E-06 | 7.381E-07 | 1.613E-06 | 5.282E-07 | 0.000E+00 | 0.000E+00 | 0.000E+00 | 0.000E+00 | 0.000E+00 | 0.000E+00 | 0.000E+00 | 0.000E+00 |
| Unigene581 | macB | 2.620E-06 | 9.242E-07 | 1.194E-06 | 8.031E-07 | 0.000E+00 | 0.000E+00 | 0.000E+00 | 1.638E-07 | 0.000E+00 | 0.000E+00 | 0.000E+00 | 0.000E+00 |
| Unigene585 | vanHO | 2.674E-06 | 1.145E-06 | 1.696E-06 | 6.256E-07 | 0.000E+00 | 0.000E+00 | 0.000E+00 | 1.522E-07 | 0.000E+00 | 0.000E+00 | 0.000E+00 | 0.000E+00 |
| Unigene596 | msrC | 3.382E-06 | 1.406E-06 | 2.413E-06 | 1.087E-06 | 0.000E+00 | 0.000E+00 | 0.000E+00 | 1.196E-07 | 0.000E+00 | 0.000E+00 | 0.000E+00 | 0.000E+00 |
| Unigene633 | srmB | 1.266E-05 | 5.472E-06 | 3.308E-06 | 1.184E-05 | 8.424E-05 | 1.118E-04 | 1.165E-04 | 9.114E-05 | 1.388E-04 | 1.363E-04 | 1.094E-04 | 7.284E-05 |
| Unigene640 | macB | 1.173E-05 | 3.886E-06 | 3.429E-06 | 1.124E-05 | 7.556E-05 | 9.922E-05 | 1.083E-04 | 8.099E-05 | 1.286E-04 | 1.299E-04 | 9.920E-05 | 6.131E-05 |
| Unigene665 | baeS | 6.082E-07 | 0.000E+00 | 0.000E+00 | 0.000E+00 | 0.000E+00 | 0.000E+00 | 0.000E+00 | 0.000E+00 | 0.000E+00 | 0.000E+00 | 0.000E+00 | 8.958E-06 |
| Unigene712 | otr(B) | 2.911E-06 | 3.388E-06 | 2.185E-06 | 1.759E-06 | 2.768E-06 | 1.764E-06 | 1.611E-06 | 9.780E-07 | 1.229E-06 | 4.421E-06 | 6.078E-06 | 2.033E-06 |
| Unigene717 | bcr-1 | 3.007E-06 | 3.533E-06 | 2.374E-06 | 1.708E-06 | 2.558E-06 | 2.260E-06 | 1.768E-06 | 1.179E-06 | 1.720E-06 | 5.444E-06 | 5.832E-06 | 1.999E-06 |
| Unigene724 | bcr-1 | 3.158E-06 | 3.417E-06 | 2.745E-06 | 1.936E-06 | 3.308E-06 | 1.731E-06 | 1.548E-06 | 1.211E-06 | 1.109E-06 | 5.177E-06 | 5.083E-06 | 1.573E-06 |
| Unigene757 | tetT | 4.861E-05 | 2.805E-05 | 2.082E-05 | 2.228E-05 | 2.405E-06 | 2.234E-06 | 3.256E-06 | 8.875E-07 | 7.226E-06 | 8.470E-07 | 1.664E-06 | 1.485E-06 |
| Unigene865 | patA | 1.088E-04 | 2.018E-04 | 1.737E-04 | 8.057E-06 | 9.026E-06 | 1.978E-07 | 3.321E-06 | 1.282E-06 | 5.128E-05 | 0.000E+00 | 3.433E-06 | 1.168E-06 |
| Unigene866 | optrA | 1.074E-04 | 1.874E-04 | 1.914E-04 | 8.273E-06 | 7.573E-06 | 2.644E-07 | 3.518E-06 | 1.855E-06 | 6.275E-05 | 3.065E-07 | 2.902E-06 | 8.311E-07 |
| Unigene892 | macB | 2.506E-05 | 9.916E-06 | 7.087E-06 | 4.518E-06 | 1.624E-07 | 9.483E-08 | 0.000E+00 | 6.826E-08 | 9.587E-07 | 0.000E+00 | 0.000E+00 | 0.000E+00 |
| Unigene927 | YojI | 3.006E-05 | 9.813E-07 | 6.353E-07 | 6.227E-08 | 0.000E+00 | 9.999E-08 | 0.000E+00 | 0.000E+00 | 0.000E+00 | 0.000E+00 | 0.000E+00 | 0.000E+00 |
| Unigene974 | arlS | 2.336E-05 | 8.166E-06 | 6.053E-06 | 5.421E-06 | 4.185E-07 | 1.527E-07 | 1.042E-07 | 1.099E-07 | 9.264E-07 | 0.000E+00 | 5.387E-08 | 8.349E-08 |
| Unigene975 | mtrA | 1.985E-05 | 6.401E-06 | 5.590E-06 | 3.835E-06 | 0.000E+00 | 1.466E-07 | 0.000E+00 | 1.583E-07 | 6.424E-07 | 0.000E+00 | 0.000E+00 | 1.069E-07 |
| Unigene977 | mel | 2.442E-05 | 9.214E-06 | 6.231E-06 | 5.124E-06 | 0.000E+00 | 2.585E-07 | 2.940E-08 | 2.171E-07 | 5.808E-07 | 0.000E+00 | 0.000E+00 | 3.140E-08 |
| Unigene1035 | Staphylocc | 2.609E-06 | 3.269E-06 | 2.583E-06 | 2.087E-06 | 2.373E-06 | 1.959E-06 | 1.495E-06 | 1.105E-06 | 1.287E-06 | 5.736E-06 | 5.243E-06 | 1.719E-06 |
| Unigene1057 | arlR | 1.212E-05 | 5.518E-06 | 3.781E-06 | 1.200E-05 | 7.950E-05 | 1.098E-04 | 1.028E-04 | 9.050E-05 | 1.393E-04 | 1.372E-04 | 1.042E-04 | 7.415E-05 |
| Unigene1058 | arlS | 1.196E-05 | 3.793E-06 | 3.083E-06 | 1.213E-05 | 8.128E-05 | 1.148E-04 | 1.110E-04 | 8.691E-05 | 1.408E-04 | 1.363E-04 | 1.133E-04 | 7.478E-05 |
| Unigene1077 | facT | 6.169E-06 | 0.000E+00 | 0.000E+00 | 0.000E+00 | 3.384E-05 | 1.593E-05 | 2.130E-05 | 2.667E-05 | 3.562E-06 | 0.000E+00 | 0.000E+00 | 0.000E+00 |
| Unigene1209 | rphB | 9.677E-05 | 1.621E-04 | 1.395E-04 | 6.560E-06 | 7.934E-06 | 3.926E-07 | 3.096E-06 | 1.868E-06 | 4.143E-05 | 1.798E-07 | 2.385E-06 | 4.770E-07 |
| Unigene1257 | arlS | 7.454E-05 | 6.220E-05 | 1.231E-04 | 1.730E-04 | 1.373E-04 | 9.441E-05 | 1.249E-04 | 1.195E-04 | 9.792E-05 | 1.448E-04 | 1.579E-04 | 1.698E-04 |
| Unigene1262 | sul2 | 1.945E-07 | 1.423E-07 | 2.544E-07 | 1.309E-07 | 0.000E+00 | 0.000E+00 | 0.000E+00 | 0.000E+00 | 0.000E+00 | 0.000E+00 | 0.000E+00 | 0.000E+00 |
| Unigene1269 | Escherichia | 4.130E-05 | 2.122E-05 | 6.905E-06 | 1.273E-05 | 2.581E-06 | 1.846E-06 | 3.062E-06 | 4.785E-07 | 7.131E-06 | 0.000E+00 | 1.758E-06 | 1.696E-06 |
| Unigene1275 | vgaB | 2.545E-05 | 4.655E-06 | 4.239E-06 | 0.000E+00 | 8.726E-08 | 4.245E-08 | 0.000E+00 | 0.000E+00 | 0.000E+00 | 0.000E+00 | 0.000E+00 | 0.000E+00 |
| Unigene1317 | Klebsiella p | 6.744E-07 | 1.312E-06 | 7.219E-07 | 4.334E-07 | 7.254E-06 | 2.651E-07 | 0.000E+00 | 3.578E-07 | 0.000E+00 | 0.000E+00 | 0.000E+00 | 0.000E+00 |
| Unigene1392 | poxtA | 1.105E-05 | 2.986E-06 | 3.074E-06 | 1.174E-05 | 7.218E-05 | 1.002E-04 | 9.349E-05 | 7.853E-05 | 1.251E-04 | 1.273E-04 | 9.875E-05 | 6.476E-05 |
| Unigene1396 | Streptomy | 3.119E-06 | 0.000E+00 | 3.259E-06 | 5.039E-07 | 1.906E-07 | 6.743E-08 | 4.487E-07 | 0.000E+00 | 0.000E+00 | 0.000E+00 | 8.027E-07 | 0.000E+00 |
| Unigene1402 | vanYB | 4.413E-06 | 2.307E-07 | 2.145E-06 | 0.000E+00 | 4.670E-07 | 0.000E+00 | 9.304E-08 | 0.000E+00 | 0.000E+00 | 0.000E+00 | 6.731E-07 | 0.000E+00 |
| Unigene1421 | bcrA | 3.311E-06 | 0.000E+00 | 3.390E-06 | 3.634E-07 | 1.199E-07 | 0.000E+00 | 5.178E-07 | 0.000E+00 | 0.000E+00 | 0.000E+00 | 1.070E-06 | 8.507E-08 |
| Unigene1451 | efrA | 8.033E-07 | 5.679E-06 | 9.477E-06 | 1.553E-05 | 2.075E-08 | 1.010E-07 | 6.202E-08 | 0.000E+00 | 0.000E+00 | 0.000E+00 | 2.137E-08 | 0.000E+00 |
| Unigene1452 | efrB | 9.073E-07 | 5.915E-06 | 9.935E-06 | 1.510E-05 | 0.000E+00 | 0.000E+00 | 1.004E-07 | 0.000E+00 | 0.000E+00 | 0.000E+00 | 0.000E+00 | 0.000E+00 |
| Unigene1500 | tetA(58) | 7.390E-05 | 2.136E-04 | 6.425E-06 | 0.000E+00 | 1.853E-06 | 0.000E+00 | 0.000E+00 | 0.000E+00 | 1.276E-06 | 0.000E+00 | 1.336E-06 | 0.000E+00 |
| Unigene1512 | facT | 5.553E-05 | 5.448E-05 | 9.217E-05 | 6.369E-05 | 1.110E-04 | 7.958E-05 | 9.345E-05 | 9.638E-05 | 8.845E-05 | 1.159E-04 | 1.195E-04 | 1.209E-04 |
| Unigene1529 | salA | 1.313E-06 | 1.049E-05 | 5.333E-06 | 3.425E-06 | 0.000E+00 | 7.886E-08 | 4.037E-08 | 4.258E-08 | 0.000E+00 | 0.000E+00 | 2.086E-07 | 0.000E+00 |
| Unigene1534 | mtrA | 2.319E-06 | 1.563E-05 | 5.188E-06 | 4.309E-06 | 0.000E+00 | 0.000E+00 | 0.000E+00 | 0.000E+00 | 0.000E+00 | 0.000E+00 | 0.000E+00 | 0.000E+00 |
| Unigene1535 | vanSG | 2.007E-06 | 1.520E-05 | 5.069E-06 | 3.284E-06 | 6.194E-08 | 1.005E-07 | 0.000E+00 | 0.000E+00 | 0.000E+00 | 0.000E+00 | 0.000E+00 | 0.000E+00 |

| | | | | | | | | | | | | | |
|---|---|---|---|---|---|---|---|---|---|---|---|---|---|
| Unigene1545 | tetA(58) | 2.091E-06 | 1.406E-05 | 7.651E-06 | 4.619E-06 | 0.000E+00 | 0.000E+00 | 0.000E+00 | 0.000E+00 | 0.000E+00 | 0.000E+00 | 0.000E+00 | 0.000E+00 |
| Unigene1564 | arlS | 9.418E-05 | 1.863E-04 | 1.175E-04 | 0.000E+00 | 5.919E-06 | 1.412E-07 | 3.411E-06 | 9.145E-07 | 3.477E-05 | 0.000E+00 | 2.599E-06 | 0.000E+00 |
| Unigene1565 | arlR | 9.726E-05 | 1.755E-04 | 1.390E-04 | 0.000E+00 | 6.595E-06 | 0.000E+00 | 3.798E-06 | 0.000E+00 | 4.096E-05 | 0.000E+00 | 2.759E-06 | 0.000E+00 |
| Unigene1573 | vmlR | 8.206E-05 | 1.314E-04 | 1.191E-04 | 5.783E-06 | 7.029E-06 | 4.493E-07 | 3.782E-06 | 1.671E-06 | 5.118E-05 | 0.000E+00 | 2.694E-06 | 9.826E-07 |
| Unigene1599 | CBP-1 | 1.196E-05 | 3.028E-06 | 3.038E-06 | 1.263E-05 | 8.000E-05 | 1.053E-04 | 1.042E-04 | 8.105E-05 | 1.266E-04 | 1.335E-04 | 1.070E-04 | 7.062E-05 |
| Unigene1601 | vanE | 1.277E-05 | 5.165E-06 | 3.801E-06 | 1.287E-05 | 8.728E-05 | 1.234E-04 | 1.181E-04 | 1.015E-04 | 1.472E-04 | 1.570E-04 | 1.285E-04 | 8.632E-05 |
| Unigene1605 | poxtA | 1.292E-05 | 4.669E-06 | 3.652E-06 | 1.292E-05 | 8.681E-05 | 1.223E-04 | 1.275E-04 | 9.836E-05 | 1.489E-04 | 1.458E-04 | 1.233E-04 | 7.835E-05 |
| Unigene1611 | Chlamydia | 1.232E-05 | 4.872E-06 | 3.421E-06 | 1.218E-05 | 8.332E-05 | 1.168E-04 | 1.152E-04 | 9.055E-05 | 1.451E-04 | 1.484E-04 | 1.151E-04 | 7.118E-05 |
| Unigene1635 | Staphylocc | 9.458E-05 | 1.697E-04 | 1.732E-04 | 7.305E-06 | 9.041E-06 | 1.955E-07 | 3.302E-06 | 1.636E-06 | 5.473E-05 | 0.000E+00 | 2.663E-06 | 6.146E-07 |
| Unigene1771 | msbA | 2.521E-06 | 2.025E-06 | 3.119E-06 | 1.623E-06 | 2.030E-07 | 1.777E-07 | 5.459E-07 | 2.346E-07 | 6.389E-07 | 0.000E+00 | 1.672E-07 | 1.512E-07 |
| Unigene1896 | blt | 1.099E-06 | 8.656E-07 | 9.397E-07 | 1.479E-06 | 1.756E-05 | 5.116E-06 | 1.356E-05 | 1.526E-05 | 9.913E-06 | 1.716E-05 | 1.743E-05 | 6.393E-06 |
| Unigene1929 | mgrA | 4.261E-06 | 2.528E-05 | 3.401E-06 | 2.042E-06 | 1.123E-06 | 7.807E-07 | 3.197E-07 | 0.000E+00 | 1.815E-06 | 0.000E+00 | 0.000E+00 | 0.000E+00 |
| Unigene1946 | lmrB | 4.694E-06 | 1.837E-05 | 3.386E-06 | 8.225E-07 | 9.049E-07 | 3.244E-07 | 1.661E-07 | 0.000E+00 | 1.335E-06 | 7.880E-07 | 3.187E-07 | 0.000E+00 |
| Unigene1993 | patB | 4.798E-05 | 2.622E-05 | 1.733E-05 | 2.485E-05 | 1.472E-06 | 1.736E-06 | 3.510E-06 | 5.624E-07 | 7.987E-06 | 9.392E-07 | 2.893E-06 | 2.041E-06 |
| Unigene2042 | macB | 2.774E-06 | 3.044E-06 | 2.346E-06 | 4.057E-06 | 0.000E+00 | 1.034E-07 | 2.646E-07 | 0.000E+00 | 0.000E+00 | 0.000E+00 | 0.000E+00 | 0.000E+00 |
| Unigene2068 | tva(A) | 3.318E-06 | 1.040E-07 | 2.047E-06 | 0.000E+00 | 2.106E-07 | 1.025E-07 | 2.623E-07 | 0.000E+00 | 0.000E+00 | 0.000E+00 | 1.084E-07 | 0.000E+00 |
| Unigene2117 | AcrS | 4.129E-06 | 8.743E-06 | 9.535E-06 | 1.146E-05 | 9.267E-08 | 1.353E-07 | 4.616E-08 | 0.000E+00 | 0.000E+00 | 3.252E-07 | 0.000E+00 | 0.000E+00 |
| Unigene2128 | patB | 1.048E-06 | 1.106E-06 | 5.773E-06 | 6.959E-06 | 0.000E+00 | 0.000E+00 | 4.906E-07 | 0.000E+00 | 0.000E+00 | 0.000E+00 | 0.000E+00 | 0.000E+00 |
| Unigene2137 | abeS | 1.423E-06 | 0.000E+00 | 5.061E-06 | 8.621E-06 | 0.000E+00 | 0.000E+00 | 0.000E+00 | 0.000E+00 | 0.000E+00 | 0.000E+00 | 0.000E+00 | 0.000E+00 |
| Unigene2139 | macB | 1.871E-06 | 4.056E-06 | 7.069E-06 | 1.025E-05 | 0.000E+00 | 0.000E+00 | 5.178E-07 | 0.000E+00 | 0.000E+00 | 0.000E+00 | 0.000E+00 | 0.000E+00 |
| Unigene2159 | vga(E) Stap | 3.937E-06 | 2.103E-05 | 3.992E-06 | 8.392E-07 | 1.361E-06 | 6.620E-07 | 2.420E-07 | 0.000E+00 | 1.147E-06 | 0.000E+00 | 4.003E-07 | 0.000E+00 |
| Unigene2235 | macB | 6.075E-06 | 2.385E-05 | 3.284E-06 | 1.196E-06 | 2.002E-06 | 3.339E-07 | 1.709E-07 | 0.000E+00 | 1.182E-06 | 4.588E-07 | 0.000E+00 | 0.000E+00 |
| Unigene2237 | tetT | 4.294E-06 | 2.168E-05 | 3.955E-06 | 1.009E-06 | 1.265E-06 | 3.600E-07 | 1.746E-07 | 3.887E-07 | 1.111E-06 | 3.904E-07 | 3.207E-07 | 2.071E-07 |
| Unigene2253 | MuxC | 2.378E-07 | 0.000E+00 | 0.000E+00 | 0.000E+00 | 0.000E+00 | 0.000E+00 | 0.000E+00 | 0.000E+00 | 0.000E+00 | 0.000E+00 | 0.000E+00 | 0.000E+00 |
| Unigene2337 | macB | 2.336E-06 | 2.774E-06 | 1.954E-06 | 0.000E+00 | 0.000E+00 | 0.000E+00 | 6.357E-07 | 0.000E+00 | 5.441E-07 | 0.000E+00 | 0.000E+00 | 1.810E-07 |
| Unigene2369 | QepA2 | 4.324E-05 | 4.289E-05 | 4.708E-05 | 6.625E-06 | 1.332E-04 | 8.414E-05 | 5.015E-05 | 1.164E-04 | 9.765E-05 | 1.243E-04 | 7.780E-05 | 1.622E-04 |
| Unigene2412 | MdtK | 0.000E+00 | 0.000E+00 | 0.000E+00 | 5.935E-07 | 7.032E-06 | 0.000E+00 | 0.000E+00 | 0.000E+00 | 0.000E+00 | 0.000E+00 | 0.000E+00 | 0.000E+00 |
| Unigene2414 | bcr-1 | 3.938E-06 | 1.950E-05 | 3.220E-06 | 6.155E-07 | 9.897E-07 | 3.041E-07 | 2.076E-07 | 2.189E-07 | 1.255E-06 | 5.223E-07 | 2.950E-07 | 2.217E-07 |
| Unigene2418 | emeA | 3.115E-06 | 1.872E-05 | 3.311E-06 | 7.387E-07 | 8.913E-07 | 2.296E-07 | 2.612E-07 | 4.682E-07 | 9.800E-07 | 4.732E-07 | 5.128E-07 | 2.789E-07 |
| Unigene2495 | bcrA | 4.695E-06 | 9.190E-06 | 1.209E-05 | 1.521E-05 | 1.739E-07 | 1.269E-07 | 0.000E+00 | 4.567E-08 | 0.000E+00 | 0.000E+00 | 1.343E-07 | 0.000E+00 |
| Unigene2521 | macB | 7.421E-07 | 1.086E-06 | 3.762E-06 | 6.120E-06 | 0.000E+00 | 0.000E+00 | 5.019E-07 | 0.000E+00 | 0.000E+00 | 0.000E+00 | 0.000E+00 | 4.873E-08 |
| Unigene2528 | vanRF | 3.281E-06 | 9.793E-06 | 8.026E-06 | 1.064E-05 | 0.000E+00 | 0.000E+00 | 0.000E+00 | 0.000E+00 | 0.000E+00 | 2.436E-07 | 1.501E-07 | 0.000E+00 |
| Unigene2529 | vanSL | 3.981E-06 | 9.523E-06 | 9.734E-06 | 1.002E-05 | 6.910E-08 | 1.009E-07 | 0.000E+00 | 0.000E+00 | 0.000E+00 | 0.000E+00 | 0.000E+00 | 0.000E+00 |
| Unigene2536 | vanRF | 1.277E-05 | 4.981E-06 | 3.948E-06 | 1.488E-05 | 1.060E-04 | 1.427E-04 | 1.279E-04 | 1.213E-04 | 1.898E-04 | 1.924E-04 | 1.563E-04 | 1.011E-04 |
| Unigene2537 | vanSF | 1.318E-05 | 4.089E-06 | 2.728E-06 | 1.423E-05 | 8.933E-05 | 1.235E-04 | 1.323E-04 | 1.059E-04 | 1.572E-04 | 1.580E-04 | 1.336E-04 | 8.650E-05 |
| Unigene2619 | tet(42) | 4.566E-05 | 1.688E-05 | 6.871E-06 | 1.147E-05 | 2.102E-06 | 1.754E-06 | 3.321E-06 | 9.467E-07 | 7.505E-06 | 5.722E-07 | 1.639E-06 | 1.853E-06 |
| Unigene2621 | lmrD | 4.951E-05 | 2.858E-05 | 2.181E-05 | 2.559E-05 | 2.329E-06 | 2.083E-06 | 3.907E-06 | 7.377E-07 | 7.515E-06 | 9.788E-07 | 2.098E-06 | 1.440E-06 |
| Unigene2627 | evgS | 3.166E-05 | 1.999E-05 | 1.686E-05 | 1.829E-05 | 1.331E-06 | 1.273E-06 | 2.354E-06 | 0.000E+00 | 0.000E+00 | 3.911E-07 | 1.370E-06 | 6.835E-07 |
| Unigene2636 | bcrA | 7.711E-07 | 5.770E-07 | 1.479E-06 | 1.097E-06 | 0.000E+00 | 0.000E+00 | 0.000E+00 | 0.000E+00 | 0.000E+00 | 0.000E+00 | 0.000E+00 | 8.286E-08 |
| Unigene2694 | optrA | 2.976E-06 | 9.835E-06 | 9.552E-06 | 1.129E-05 | 0.000E+00 | 0.000E+00 | 0.000E+00 | 1.962E-08 | 0.000E+00 | 0.000E+00 | 0.000E+00 | 0.000E+00 |
| Unigene2725 | vanF | 9.695E-05 | 1.590E-04 | 1.512E-04 | 6.298E-06 | 7.366E-06 | 3.037E-07 | 2.519E-06 | 2.361E-06 | 5.819E-05 | 4.069E-07 | 3.085E-06 | 8.966E-07 |
| Unigene2736 | tetW | 1.122E-05 | 0.000E+00 | 0.000E+00 | 0.000E+00 | 1.806E-05 | 1.649E-05 | 5.847E-08 | 0.000E+00 | 1.109E-05 | 0.000E+00 | 0.000E+00 | 0.000E+00 |
| Unigene2757 | YojI | 2.605E-06 | 4.200E-06 | 3.303E-06 | 5.362E-06 | 4.007E-07 | 0.000E+00 | 3.105E-07 | 9.355E-08 | 1.533E-07 | 6.696E-08 | 0.000E+00 | 2.368E-07 |
| Unigene2764 | mdtG | 9.157E-05 | 1.462E-04 | 1.436E-04 | 7.315E-06 | 6.823E-06 | 0.000E+00 | 3.311E-06 | 2.481E-06 | 5.232E-05 | 0.000E+00 | 2.582E-06 | 5.274E-07 |
| Unigene2815 | patA | 7.111E-06 | 0.000E+00 | 0.000E+00 | 1.701E-06 | 2.983E-05 | 1.416E-05 | 2.346E-05 | 2.441E-05 | 0.000E+00 | 0.000E+00 | 0.000E+00 | 0.000E+00 |
| Unigene2829 | NmcR | 1.473E-05 | 4.958E-06 | 4.216E-06 | 1.502E-05 | 1.009E-04 | 1.422E-04 | 1.550E-04 | 1.229E-04 | 1.801E-04 | 1.845E-04 | 1.430E-04 | 9.875E-05 |
| Unigene2837 | tva(A) | 7.961E-06 | 6.485E-07 | 5.493E-07 | 0.000E+00 | 3.109E-06 | 6.724E-08 | 2.065E-07 | 0.000E+00 | 0.000E+00 | 0.000E+00 | 0.000E+00 | 0.000E+00 |
| Unigene2844 | rphB | 1.093E-05 | 0.000E+00 | 0.000E+00 | 1.315E-08 | 1.566E-05 | 1.579E-05 | 1.874E-07 | 0.000E+00 | 1.353E-06 | 0.000E+00 | 0.000E+00 | 0.000E+00 |
| Unigene2864 | abeS | 4.002E-05 | 3.863E-06 | 0.000E+00 | 0.000E+00 | 0.000E+00 | 0.000E+00 | 4.341E-06 | 0.000E+00 | 0.000E+00 | 0.000E+00 | 0.000E+00 | 0.000E+00 |
| Unigene2979 | mdtG | 1.005E-04 | 4.675E-05 | 5.369E-07 | 0.000E+00 | 2.237E-05 | 8.458E-05 | 0.000E+00 | 6.082E-08 | 0.000E+00 | 0.000E+00 | 0.000E+00 | 2.156E-07 |
| Unigene3001 | carA | 9.034E-05 | 3.827E-05 | 1.171E-07 | 0.000E+00 | 2.629E-06 | 3.238E-05 | 6.604E-08 | 0.000E+00 | 0.000E+00 | 1.108E-07 | 0.000E+00 | 0.000E+00 |
| Unigene3085 | tetA(58) | 1.084E-04 | 1.818E-04 | 1.739E-04 | 6.936E-06 | 9.110E-06 | 0.000E+00 | 3.570E-06 | 1.749E-06 | 6.110E-05 | 2.318E-07 | 3.309E-06 | 6.150E-07 |
| Unigene3094 | mdtG | 1.160E-06 | 0.000E+00 | 0.000E+00 | 2.397E-07 | 4.746E-06 | 9.494E-06 | 1.839E-06 | 7.819E-06 | 5.246E-06 | 3.584E-06 | 0.000E+00 | 0.000E+00 |
| Unigene3110 | vanSA | 9.355E-05 | 1.657E-04 | 1.647E-04 | 7.514E-06 | 7.916E-06 | 3.403E-07 | 3.326E-06 | 1.336E-06 | 5.330E-05 | 3.825E-07 | 3.077E-06 | 0.000E+00 |
| Unigene3111 | vanRF | 1.051E-04 | 1.819E-04 | 1.816E-04 | 6.992E-06 | 9.771E-06 | 0.000E+00 | 3.314E-06 | 1.038E-06 | 6.049E-05 | 0.000E+00 | 3.800E-06 | 8.848E-07 |
| Unigene3160 | efrA | 7.631E-06 | 8.626E-05 | 1.472E-04 | 7.470E-06 | 6.009E-06 | 6.638E-07 | 1.294E-07 | 1.809E-06 | 5.791E-05 | 1.303E-07 | 1.773E-06 | 6.912E-08 |
| Unigene3173 | Staphylocc | 2.956E-06 | 4.334E-06 | 3.795E-06 | 5.958E-06 | 4.746E-07 | 4.992E-08 | 4.600E-07 | 0.000E+00 | 1.640E-07 | 0.000E+00 | 1.189E-07 | 9.553E-08 |
| Unigene3176 | vanSC | 2.626E-06 | 4.667E-06 | 3.557E-06 | 5.966E-06 | 3.488E-07 | 1.543E-07 | 2.843E-07 | 0.000E+00 | 1.872E-07 | 0.000E+00 | 0.000E+00 | 2.024E-07 |

| | | | | | | | | | | | | | |
|---|---|---|---|---|---|---|---|---|---|---|---|---|---|
| Unigene3177 | vanRC | 3.247E-06 | 4.581E-06 | 4.232E-06 | 6.276E-06 | 5.668E-07 | 0.000E+00 | 4.620E-07 | 0.000E+00 | 1.521E-07 | 0.000E+00 | 0.000E+00 | 0.000E+00 |
| Unigene3178 | vanTC | 2.901E-06 | 4.178E-06 | 2.982E-06 | 6.402E-06 | 2.899E-07 | 0.000E+00 | 3.398E-07 | 0.000E+00 | 1.845E-07 | 0.000E+00 | 1.405E-07 | 2.177E-07 |
| Unigene3179 | vanXYC | 2.310E-06 | 3.833E-06 | 3.814E-06 | 5.916E-06 | 0.000E+00 | 0.000E+00 | 0.000E+00 | 0.000E+00 | 0.000E+00 | 0.000E+00 | 0.000E+00 | 0.000E+00 |
| Unigene3180 | vanC | 3.188E-06 | 4.845E-06 | 2.828E-06 | 5.727E-06 | 4.087E-07 | 0.000E+00 | 4.411E-07 | 0.000E+00 | 2.010E-07 | 0.000E+00 | 0.000E+00 | 1.087E-07 |
| Unigene3232 | macB | 1.010E-06 | 1.370E-06 | 4.901E-06 | 6.949E-06 | 0.000E+00 | 0.000E+00 | 5.881E-07 | 0.000E+00 | 0.000E+00 | 5.919E-08 | 3.039E-08 | 9.421E-08 |
| Unigene3233 | macB | 3.524E-06 | 6.085E-06 | 7.331E-06 | 9.585E-06 | 0.000E+00 | 0.000E+00 | 0.000E+00 | 0.000E+00 | 0.000E+00 | 0.000E+00 | 0.000E+00 | 0.000E+00 |
| Unigene3252 | tet(59) | 4.341E-06 | 9.413E-06 | 1.138E-05 | 9.936E-06 | 0.000E+00 | 1.138E-07 | 0.000E+00 | 0.000E+00 | 0.000E+00 | 1.758E-07 | 3.009E-08 | 0.000E+00 |
| Unigene3271 | tetA(58) | 7.387E-05 | 1.279E-04 | 8.947E-05 | 0.000E+00 | 0.000E+00 | 0.000E+00 | 0.000E+00 | 0.000E+00 | 0.000E+00 | 0.000E+00 | 0.000E+00 | 0.000E+00 |
| Unigene3325 | macB | 1.060E-04 | 2.106E-04 | 1.886E-04 | 0.000E+00 | 8.438E-06 | 3.193E-07 | 3.176E-06 | 9.851E-07 | 5.691E-05 | 2.820E-07 | 2.993E-06 | 4.988E-07 |
| Unigene3361 | tetA(58) | 2.193E-05 | 6.206E-05 | 1.084E-04 | 4.222E-06 | 5.330E-06 | 0.000E+00 | 0.000E+00 | 0.000E+00 | 3.266E-05 | 0.000E+00 | 0.000E+00 | 8.101E-08 |
| Unigene3378 | vanHF | 6.707E-05 | 5.349E-05 | 1.108E-04 | 1.554E-04 | 1.191E-04 | 8.581E-05 | 1.076E-04 | 1.039E-04 | 9.181E-05 | 1.289E-04 | 1.315E-04 | 1.348E-04 |
| Unigene3406 | vanXYE | 1.210E-05 | 3.565E-06 | 2.749E-06 | 1.365E-05 | 8.846E-05 | 1.190E-04 | 1.117E-04 | 9.659E-05 | 1.502E-04 | 1.494E-04 | 1.307E-04 | 7.914E-05 |
| Unigene3460 | vanRM | 2.620E-06 | 4.981E-06 | 4.591E-06 | 3.968E-06 | 1.039E-07 | 2.023E-07 | 0.000E+00 | 0.000E+00 | 0.000E+00 | 0.000E+00 | 0.000E+00 | 0.000E+00 |
| Unigene3461 | vanSG | 2.450E-06 | 5.340E-06 | 5.401E-06 | 5.164E-06 | 1.578E-07 | 0.000E+00 | 0.000E+00 | 0.000E+00 | 0.000E+00 | 2.769E-07 | 0.000E+00 | 0.000E+00 |
| Unigene3499 | mtrA | 4.086E-07 | 6.777E-06 | 1.007E-05 | 1.311E-05 | 5.044E-08 | 0.000E+00 | 0.000E+00 | 0.000E+00 | 0.000E+00 | 0.000E+00 | 0.000E+00 | 0.000E+00 |
| Unigene3500 | arlS | 7.997E-07 | 4.728E-06 | 8.810E-06 | 1.211E-05 | 0.000E+00 | 0.000E+00 | 4.023E-08 | 0.000E+00 | 1.987E-08 | 0.000E+00 | 0.000E+00 | 0.000E+00 |
| Unigene3616 | optrA | 4.486E-05 | 2.911E-05 | 1.613E-05 | 2.070E-05 | 1.977E-06 | 1.420E-06 | 2.813E-06 | 1.038E-06 | 1.009E-05 | 8.022E-07 | 2.229E-06 | 1.602E-06 |
| Unigene3622 | vanRF | 3.467E-05 | 1.679E-05 | 6.381E-06 | 1.176E-05 | 2.287E-06 | 1.264E-06 | 2.278E-06 | 4.915E-07 | 7.619E-06 | 8.860E-07 | 1.819E-06 | 1.548E-06 |
| Unigene3641 | lsaA | 4.174E-05 | 1.814E-05 | 6.954E-06 | 1.108E-05 | 2.016E-06 | 1.378E-06 | 2.893E-06 | 6.809E-07 | 7.463E-06 | 4.332E-07 | 2.126E-06 | 1.328E-06 |
| Unigene3658 | macB | 2.186E-06 | 1.694E-06 | 3.113E-06 | 0.000E+00 | 0.000E+00 | 3.707E-07 | 3.796E-07 | 0.000E+00 | 5.154E-07 | 0.000E+00 | 0.000E+00 | 0.000E+00 |
| Unigene3717 | patB | 2.981E-06 | 3.891E-06 | 2.717E-06 | 1.835E-06 | 2.312E-06 | 1.869E-06 | 1.816E-06 | 6.795E-07 | 1.234E-06 | 5.129E-06 | 6.255E-06 | 2.106E-06 |
| Unigene3718 | efrA | 3.420E-06 | 3.287E-06 | 2.286E-06 | 1.886E-06 | 2.567E-06 | 1.879E-06 | 2.169E-06 | 6.690E-07 | 1.394E-06 | 5.540E-06 | 5.012E-06 | 1.530E-06 |
| Unigene3725 | macB | 3.300E-06 | 4.094E-06 | 2.722E-06 | 1.642E-06 | 2.710E-06 | 2.120E-06 | 2.541E-06 | 1.675E-07 | 1.777E-06 | 7.139E-06 | 4.267E-06 | 2.261E-06 |
| Unigene3751 | mepA | 2.626E-06 | 4.348E-06 | 4.439E-06 | 7.599E-06 | 0.000E+00 | 0.000E+00 | 0.000E+00 | 0.000E+00 | 0.000E+00 | 0.000E+00 | 0.000E+00 | 0.000E+00 |
| Unigene3753 | vanSM | 2.031E-06 | 6.452E-06 | 4.510E-06 | 8.599E-06 | 0.000E+00 | 0.000E+00 | 0.000E+00 | 0.000E+00 | 0.000E+00 | 0.000E+00 | 4.076E-08 | 0.000E+00 |
| Unigene3767 | macB | 2.690E-06 | 5.792E-06 | 3.846E-06 | 6.131E-06 | 3.131E-07 | 5.539E-08 | 4.537E-07 | 0.000E+00 | 1.960E-07 | 0.000E+00 | 8.792E-08 | 2.423E-07 |
| Unigene3770 | catB3 | 4.080E-06 | 5.149E-06 | 3.953E-06 | 6.283E-06 | 6.798E-07 | 0.000E+00 | 3.386E-07 | 0.000E+00 | 0.000E+00 | 0.000E+00 | 1.750E-07 | 1.808E-07 |
| Unigene3793 | pmrA | 2.522E-06 | 6.420E-06 | 8.794E-06 | 9.972E-06 | 1.491E-07 | 5.802E-08 | 0.000E+00 | 6.264E-08 | 0.000E+00 | 2.092E-07 | 0.000E+00 | 0.000E+00 |
| Unigene3812 | Erm(49) | 3.417E-06 | 5.107E-06 | 4.031E-06 | 5.616E-06 | 0.000E+00 | 0.000E+00 | 3.219E-07 | 0.000E+00 | 3.973E-07 | 0.000E+00 | 0.000E+00 | 0.000E+00 |
| Unigene3819 | oleC | 3.081E-06 | 3.430E-06 | 2.848E-06 | 5.501E-06 | 3.472E-07 | 0.000E+00 | 1.977E-07 | 0.000E+00 | 9.760E-08 | 0.000E+00 | 1.021E-07 | 0.000E+00 |
| Unigene3853 | rpoB2 | 4.179E-06 | 3.838E-07 | 3.299E-06 | 3.531E-07 | 2.988E-07 | 4.847E-08 | 4.168E-07 | 0.000E+00 | 0.000E+00 | 0.000E+00 | 7.795E-07 | 0.000E+00 |
| Unigene3880 | tetA(60) | 1.045E-06 | 9.254E-07 | 1.655E-06 | 1.536E-06 | 1.772E-05 | 5.311E-06 | 1.386E-05 | 1.645E-05 | 9.897E-06 | 1.725E-05 | 1.673E-05 | 6.652E-06 |
| Unigene3917 | vanRM | 2.715E-06 | 5.574E-06 | 5.427E-06 | 2.843E-06 | 0.000E+00 | 5.436E-08 | 0.000E+00 | 0.000E+00 | 0.000E+00 | 2.240E-07 | 0.000E+00 | 0.000E+00 |
| Unigene3933 | tetA(58) | 4.661E-06 | 9.275E-06 | 8.720E-06 | 1.113E-05 | 3.872E-07 | 0.000E+00 | 0.000E+00 | 0.000E+00 | 0.000E+00 | 0.000E+00 | 0.000E+00 | 0.000E+00 |
| Unigene3935 | MexW | 1.639E-07 | 0.000E+00 | 0.000E+00 | 0.000E+00 | 0.000E+00 | 0.000E+00 | 0.000E+00 | 0.000E+00 | 0.000E+00 | 0.000E+00 | 0.000E+00 | 0.000E+00 |
| Unigene3952 | CMY-104 | 1.006E-05 | 3.353E-06 | 3.130E-06 | 1.272E-05 | 7.620E-05 | 1.150E-04 | 1.041E-04 | 9.032E-05 | 1.337E-04 | 1.411E-04 | 1.075E-04 | 6.810E-05 |
| Unigene3978 | tetA(46) | 1.407E-05 | 4.168E-06 | 3.197E-06 | 1.216E-05 | 8.271E-05 | 1.126E-04 | 1.211E-04 | 9.315E-05 | 1.416E-04 | 1.416E-04 | 1.154E-04 | 7.297E-05 |
| Unigene3979 | novA | 1.234E-05 | 4.567E-06 | 2.860E-06 | 1.220E-05 | 8.543E-05 | 1.180E-04 | 1.210E-04 | 9.203E-05 | 1.447E-04 | 1.452E-04 | 1.233E-04 | 7.209E-05 |
| Unigene4034 | bacA | 1.165E-05 | 0.000E+00 | 0.000E+00 | 0.000E+00 | 2.054E-05 | 1.743E-05 | 8.598E-08 | 0.000E+00 | 1.151E-05 | 0.000E+00 | 0.000E+00 | 0.000E+00 |
| Unigene4063 | Streptomy | 4.286E-05 | 2.318E-05 | 1.813E-05 | 2.048E-05 | 1.721E-06 | 1.710E-06 | 3.173E-06 | 4.231E-07 | 7.096E-06 | 9.545E-07 | 2.092E-06 | 1.636E-06 |
| Unigene4100 | Corynebac | 4.035E-07 | 0.000E+00 | 0.000E+00 | 0.000E+00 | 0.000E+00 | 0.000E+00 | 0.000E+00 | 0.000E+00 | 0.000E+00 | 0.000E+00 | 0.000E+00 | 0.000E+00 |
| Unigene4113 | mdtC | 6.280E-07 | 0.000E+00 | 0.000E+00 | 0.000E+00 | 0.000E+00 | 0.000E+00 | 8.804E-07 | 4.398E-07 | 0.000E+00 | 0.000E+00 | 0.000E+00 | 2.227E-06 |
| Unigene4140 | kdpE | 1.444E-06 | 1.585E-06 | 8.227E-07 | 0.000E+00 | 0.000E+00 | 0.000E+00 | 0.000E+00 | 0.000E+00 | 0.000E+00 | 0.000E+00 | 0.000E+00 | 0.000E+00 |
| Unigene4167 | novA | 1.736E-07 | 0.000E+00 | 0.000E+00 | 0.000E+00 | 3.213E-07 | 0.000E+00 | 0.000E+00 | 0.000E+00 | 0.000E+00 | 0.000E+00 | 0.000E+00 | 0.000E+00 |
| Unigene4182 | efrA | 2.070E-06 | 9.620E-07 | 1.885E-06 | 4.331E-07 | 0.000E+00 | 0.000E+00 | 0.000E+00 | 8.707E-08 | 0.000E+00 | 0.000E+00 | 0.000E+00 | 0.000E+00 |
| Unigene4183 | patB | 2.502E-06 | 9.217E-07 | 1.545E-06 | 5.300E-07 | 0.000E+00 | 0.000E+00 | 0.000E+00 | 1.021E-07 | 0.000E+00 | 0.000E+00 | 0.000E+00 | 0.000E+00 |
| Unigene4210 | Staphylocc | 2.176E-06 | 1.141E-06 | 1.308E-06 | 5.803E-07 | 0.000E+00 | 0.000E+00 | 0.000E+00 | 1.427E-07 | 0.000E+00 | 0.000E+00 | 0.000E+00 | 0.000E+00 |
| Unigene4217 | efrA | 2.292E-06 | 7.108E-07 | 1.173E-06 | 1.308E-06 | 0.000E+00 | 0.000E+00 | 0.000E+00 | 0.000E+00 | 0.000E+00 | 0.000E+00 | 0.000E+00 | 0.000E+00 |
| Unigene4232 | Staphylocc | 2.510E-06 | 1.008E-06 | 1.836E-06 | 5.632E-07 | 0.000E+00 | 0.000E+00 | 0.000E+00 | 0.000E+00 | 0.000E+00 | 0.000E+00 | 0.000E+00 | 0.000E+00 |
| Unigene4298 | Listeria mc | 2.547E-06 | 4.479E-06 | 3.470E-06 | 5.798E-06 | 3.379E-07 | 1.233E-07 | 4.208E-07 | 0.000E+00 | 1.385E-07 | 0.000E+00 | 1.015E-07 | 2.247E-07 |
| Unigene4301 | mgrA | 3.117E-06 | 5.050E-06 | 3.932E-06 | 5.770E-06 | 0.000E+00 | 0.000E+00 | 1.643E-07 | 0.000E+00 | 8.111E-08 | 0.000E+00 | 0.000E+00 | 0.000E+00 |
| Unigene4343 | patA | 9.472E-07 | 0.000E+00 | 0.000E+00 | 0.000E+00 | 1.343E-05 | 1.561E-05 | 0.000E+00 | 0.000E+00 | 0.000E+00 | 0.000E+00 | 0.000E+00 | 0.000E+00 |
| Unigene4344 | tetB(46) | 8.365E-07 | 0.000E+00 | 0.000E+00 | 0.000E+00 | 1.930E-05 | 1.850E-05 | 0.000E+00 | 0.000E+00 | 0.000E+00 | 0.000E+00 | 0.000E+00 | 4.494E-08 |
| Unigene4394 | Staphylocc | 3.279E-06 | 3.812E-06 | 2.336E-06 | 1.815E-06 | 2.159E-06 | 1.917E-06 | 2.070E-06 | 8.364E-07 | 1.142E-06 | 5.263E-06 | 5.849E-06 | 1.981E-06 |
| Unigene4437 | arr-2 | 4.094E-05 | 1.489E-05 | 4.667E-06 | 1.087E-05 | 1.992E-06 | 2.276E-06 | 2.934E-06 | 3.640E-07 | 0.000E+00 | 3.474E-07 | 1.249E-06 | 0.000E+00 |
| Unigene4458 | bacA | 3.804E-05 | 2.556E-05 | 1.757E-05 | 2.018E-05 | 1.750E-06 | 0.000E+00 | 3.105E-06 | 0.000E+00 | 0.000E+00 | 0.000E+00 | 1.714E-06 | 0.000E+00 |
| Unigene4495 | mecA | 2.568E-06 | 3.769E-06 | 3.123E-06 | 4.852E-06 | 2.975E-07 | 0.000E+00 | 4.010E-07 | 0.000E+00 | 2.238E-07 | 0.000E+00 | 1.802E-08 | 1.117E-07 |
| Unigene4503 | vanYM | 2.451E-06 | 4.330E-06 | 3.959E-06 | 6.127E-06 | 5.645E-07 | 0.000E+00 | 2.646E-07 | 0.000E+00 | 8.167E-08 | 0.000E+00 | 0.000E+00 | 0.000E+00 |

| | | | | | | | | | | | | | |
|---|---|---|---|---|---|---|---|---|---|---|---|---|---|
| Unigene4512 | Staphylocc | 1.184E-05 | 4.038E-06 | 3.055E-06 | 1.268E-05 | 7.805E-05 | 1.114E-04 | 1.078E-04 | 8.720E-05 | 1.339E-04 | 1.360E-04 | 1.058E-04 | 7.089E-05 |
| Unigene4541 | efrA | 1.155E-05 | 2.022E-08 | 0.000E+00 | 0.000E+00 | 1.850E-05 | 1.737E-05 | 0.000E+00 | 0.000E+00 | 1.418E-05 | 2.463E-07 | 0.000E+00 | 0.000E+00 |
| Unigene4542 | patB | 1.081E-05 | 0.000E+00 | 0.000E+00 | 0.000E+00 | 1.727E-05 | 1.553E-05 | 5.819E-08 | 0.000E+00 | 1.257E-05 | 4.490E-07 | 0.000E+00 | 0.000E+00 |
| Unigene4543 | Escherichi | 9.411E-06 | 0.000E+00 | 0.000E+00 | 0.000E+00 | 1.415E-05 | 1.530E-05 | 0.000E+00 | 0.000E+00 | 1.309E-05 | 0.000E+00 | 0.000E+00 | 3.890E-08 |
| Unigene4585 | lmrC | 1.200E-05 | 5.108E-06 | 3.771E-06 | 1.199E-05 | 8.439E-05 | 1.194E-04 | 1.195E-04 | 1.020E-04 | 1.494E-04 | 1.480E-04 | 1.180E-04 | 7.283E-05 |
| Unigene4753 | FusF | 7.114E-05 | 5.998E-05 | 1.144E-04 | 1.582E-04 | 1.355E-04 | 8.837E-05 | 1.194E-04 | 1.098E-04 | 1.008E-04 | 1.420E-04 | 1.429E-04 | 1.559E-04 |
| Unigene4771 | msbA | 3.382E-05 | 1.518E-05 | 6.989E-06 | 1.143E-05 | 1.366E-06 | 1.773E-06 | 2.665E-06 | 5.981E-07 | 6.776E-06 | 4.566E-07 | 1.582E-06 | 1.999E-06 |
| Unigene4776 | tetA(46) | 1.801E-06 | 2.816E-06 | 1.134E-06 | 6.516E-07 | 2.534E-06 | 1.898E-06 | 1.262E-06 | 1.069E-06 | 8.037E-07 | 1.705E-06 | 2.077E-06 | 1.153E-06 |
| Unigene4796 | SAT-4 | 1.326E-05 | 4.875E-06 | 0.000E+00 | 1.377E-05 | 9.403E-05 | 1.266E-04 | 1.153E-04 | 1.033E-04 | 1.484E-04 | 1.544E-04 | 1.401E-04 | 7.624E-05 |
| Unigene4855 | macB | 1.145E-06 | 3.157E-06 | 2.587E-06 | 5.971E-06 | 0.000E+00 | 0.000E+00 | 0.000E+00 | 0.000E+00 | 0.000E+00 | 0.000E+00 | 0.000E+00 | 0.000E+00 |
| Unigene4856 | macB | 1.786E-06 | 3.249E-06 | 2.628E-06 | 5.267E-06 | 0.000E+00 | 0.000E+00 | 0.000E+00 | 0.000E+00 | 0.000E+00 | 0.000E+00 | 0.000E+00 | 0.000E+00 |
| Unigene4862 | mecI | 3.328E-05 | 3.261E-05 | 1.747E-04 | 0.000E+00 | 6.808E-06 | 0.000E+00 | 0.000E+00 | 0.000E+00 | 5.989E-05 | 0.000E+00 | 0.000E+00 | 6.738E-07 |
| Unigene4878 | kdpE | 1.580E-06 | 1.420E-05 | 7.039E-06 | 3.148E-06 | 0.000E+00 | 1.385E-07 | 0.000E+00 | 1.495E-07 | 0.000E+00 | 0.000E+00 | 0.000E+00 | 0.000E+00 |
| Unigene4930 | Erm(K) | 4.815E-06 | 2.218E-05 | 3.937E-06 | 8.472E-07 | 1.216E-06 | 5.126E-07 | 2.018E-07 | 0.000E+00 | 1.754E-06 | 8.126E-07 | 0.000E+00 | 0.000E+00 |
| Unigene5029 | tet(H) | 5.070E-05 | 7.317E-05 | 3.099E-05 | 1.456E-05 | 2.111E-05 | 5.780E-06 | 4.102E-06 | 5.902E-05 | 1.404E-05 | 6.907E-06 | 1.670E-06 | 1.009E-06 |
| Unigene5043 | patA | 1.159E-05 | 4.651E-06 | 4.117E-06 | 1.405E-05 | 8.179E-05 | 1.111E-04 | 1.285E-04 | 9.650E-05 | 1.558E-04 | 1.510E-04 | 1.239E-04 | 8.068E-05 |
| Unigene5083 | tetA(58) | 4.076E-05 | 2.160E-05 | 1.474E-05 | 1.710E-05 | 1.632E-06 | 1.469E-06 | 2.439E-06 | 7.287E-07 | 7.104E-06 | 1.064E-06 | 1.680E-06 | 1.389E-06 |
| Unigene5099 | oleC | 6.567E-07 | 6.653E-07 | 4.412E-06 | 5.560E-06 | 0.000E+00 | 0.000E+00 | 3.914E-07 | 0.000E+00 | 0.000E+00 | 0.000E+00 | 0.000E+00 | 0.000E+00 |
| Unigene5108 | aadK | 2.530E-06 | 3.776E-06 | 3.575E-06 | 5.177E-06 | 2.998E-07 | 0.000E+00 | 1.867E-07 | 0.000E+00 | 1.475E-07 | 0.000E+00 | 0.000E+00 | 2.791E-07 |
| Unigene5222 | vanRB | 4.304E-07 | 1.207E-06 | 0.000E+00 | 1.304E-06 | 0.000E+00 | 0.000E+00 | 0.000E+00 | 0.000E+00 | 0.000E+00 | 0.000E+00 | 0.000E+00 | 0.000E+00 |
| Unigene5230 | macB | 1.361E-06 | 1.727E-06 | 6.260E-07 | 1.331E-06 | 5.434E-06 | 9.839E-06 | 1.732E-05 | 0.000E+00 | 1.329E-05 | 0.000E+00 | 0.000E+00 | 0.000E+00 |
| Unigene5247 | lsaC | 1.196E-06 | 3.179E-06 | 2.256E-06 | 2.879E-06 | 5.109E-08 | 9.942E-08 | 3.054E-07 | 0.000E+00 | 1.005E-07 | 0.000E+00 | 1.052E-07 | 1.087E-07 |
| Unigene5323 | pmrA | 1.549E-06 | 0.000E+00 | 0.000E+00 | 0.000E+00 | 0.000E+00 | 0.000E+00 | 0.000E+00 | 0.000E+00 | 0.000E+00 | 0.000E+00 | 0.000E+00 | 0.000E+00 |
| Unigene5330 | mel | 1.084E-06 | 0.000E+00 | 0.000E+00 | 0.000E+00 | 0.000E+00 | 0.000E+00 | 0.000E+00 | 0.000E+00 | 0.000E+00 | 0.000E+00 | 0.000E+00 | 0.000E+00 |
| Unigene5387 | Staphylocc | 5.997E-05 | 5.133E-05 | 9.843E-05 | 1.582E-04 | 1.035E-04 | 7.324E-05 | 9.436E-05 | 9.318E-05 | 0.000E+00 | 1.149E-04 | 1.197E-04 | 1.196E-04 |
| Unigene5412 | Acinetoba | 1.360E-06 | 0.000E+00 | 0.000E+00 | 0.000E+00 | 0.000E+00 | 0.000E+00 | 0.000E+00 | 0.000E+00 | 0.000E+00 | 0.000E+00 | 0.000E+00 | 0.000E+00 |
| Unigene5438 | Klebsiella p | 9.632E-07 | 2.054E-06 | 1.404E-06 | 5.002E-07 | 9.967E-06 | 6.545E-07 | 0.000E+00 | 0.000E+00 | 0.000E+00 | 2.759E-07 | 3.777E-07 | 0.000E+00 |
| Unigene5465 | vanHO | 1.076E-05 | 3.746E-06 | 2.520E-06 | 1.313E-05 | 8.339E-05 | 1.113E-04 | 1.169E-04 | 8.978E-05 | 1.427E-04 | 1.383E-04 | 1.321E-04 | 7.854E-05 |
| Unigene5503 | carA | 0.000E+00 | 0.000E+00 | 0.000E+00 | 0.000E+00 | 0.000E+00 | 0.000E+00 | 0.000E+00 | 0.000E+00 | 0.000E+00 | 0.000E+00 | 0.000E+00 | 2.557E-07 |
| Unigene5512 | PmrF | 4.547E-06 | 3.429E-07 | 3.065E-06 | 5.608E-07 | 3.471E-07 | 0.000E+00 | 1.921E-07 | 0.000E+00 | 0.000E+00 | 0.000E+00 | 1.112E-06 | 4.103E-08 |
| Unigene5514 | PmrF | 4.318E-06 | 3.702E-07 | 3.939E-06 | 6.472E-07 | 1.874E-07 | 1.094E-07 | 1.867E-07 | 0.000E+00 | 0.000E+00 | 0.000E+00 | 1.042E-06 | 0.000E+00 |
| Unigene5566 | tetA(58) | 4.483E-06 | 1.106E-05 | 1.035E-05 | 1.105E-05 | 0.000E+00 | 7.755E-08 | 0.000E+00 | 8.373E-08 | 0.000E+00 | 2.397E-07 | 0.000E+00 | 0.000E+00 |
| Unigene5569 | bcrA | 1.899E-05 | 3.567E-07 | 6.023E-07 | 0.000E+00 | 0.000E+00 | 0.000E+00 | 0.000E+00 | 4.215E-08 | 0.000E+00 | 0.000E+00 | 0.000E+00 | 0.000E+00 |
| Unigene5575 | tetA(46) | 1.044E-06 | 5.507E-06 | 5.293E-06 | 5.711E-06 | 0.000E+00 | 0.000E+00 | 0.000E+00 | 0.000E+00 | 0.000E+00 | 0.000E+00 | 0.000E+00 | 0.000E+00 |
| Unigene5629 | lnuE | 4.261E-07 | 8.185E-07 | 5.331E-06 | 5.540E-06 | 0.000E+00 | 0.000E+00 | 7.074E-07 | 0.000E+00 | 0.000E+00 | 0.000E+00 | 0.000E+00 | 0.000E+00 |
| Unigene5661 | tmrB | 1.139E-06 | 1.598E-06 | 5.403E-06 | 7.798E-06 | 0.000E+00 | 0.000E+00 | 4.904E-07 | 0.000E+00 | 0.000E+00 | 0.000E+00 | 0.000E+00 | 0.000E+00 |
| Unigene5673 | mtrA | 3.663E-06 | 2.111E-05 | 1.438E-05 | 1.133E-05 | 0.000E+00 | 0.000E+00 | 0.000E+00 | 0.000E+00 | 0.000E+00 | 0.000E+00 | 0.000E+00 | 0.000E+00 |
| Unigene5729 | Staphylocc | 2.584E-05 | 6.947E-07 | 3.327E-07 | 0.000E+00 | 0.000E+00 | 4.887E-08 | 0.000E+00 | 0.000E+00 | 0.000E+00 | 0.000E+00 | 0.000E+00 | 0.000E+00 |
| Unigene5782 | Escherichi | 1.324E-05 | 5.189E-06 | 3.039E-06 | 1.475E-05 | 9.589E-05 | 1.293E-04 | 1.394E-04 | 1.126E-04 | 1.751E-04 | 1.719E-04 | 1.365E-04 | 9.061E-05 |
| Unigene5817 | patA | 4.121E-05 | 2.184E-05 | 1.447E-05 | 1.594E-05 | 1.663E-06 | 1.372E-06 | 2.865E-06 | 6.813E-07 | 6.269E-06 | 5.937E-07 | 2.148E-06 | 1.260E-06 |
| Unigene5848 | TaeA | 4.713E-05 | 2.709E-05 | 1.511E-05 | 2.079E-05 | 1.500E-06 | 1.870E-06 | 3.409E-06 | 6.403E-07 | 7.518E-06 | 5.641E-07 | 2.124E-06 | 1.147E-06 |
| Unigene5860 | otr(B) | 4.473E-05 | 6.424E-05 | 2.900E-05 | 1.335E-05 | 1.809E-05 | 5.757E-06 | 3.629E-06 | 5.738E-05 | 1.145E-05 | 6.618E-06 | 1.988E-06 | 8.323E-07 |
| Unigene5863 | Staphylocc | 4.481E-05 | 6.204E-05 | 2.735E-05 | 1.249E-05 | 1.736E-05 | 5.988E-06 | 3.117E-06 | 5.029E-05 | 1.143E-05 | 6.067E-06 | 1.717E-06 | 1.036E-06 |
| Unigene5892 | bcrA | 4.304E-05 | 5.852E-05 | 2.648E-05 | 1.268E-05 | 1.667E-05 | 7.093E-06 | 3.098E-06 | 6.071E-05 | 1.214E-05 | 6.870E-06 | 1.301E-06 | 1.293E-06 |
| Unigene5924 | arlR | 4.351E-05 | 5.858E-05 | 2.780E-05 | 1.134E-05 | 1.603E-05 | 5.537E-06 | 3.900E-06 | 5.688E-05 | 1.109E-05 | 6.124E-06 | 1.129E-06 | 9.442E-07 |
| Unigene5925 | arlS | 4.757E-05 | 6.445E-05 | 2.744E-05 | 1.406E-05 | 1.745E-05 | 6.734E-06 | 3.520E-06 | 6.280E-05 | 1.345E-05 | 7.231E-06 | 1.993E-06 | 1.133E-06 |
| Unigene6011 | Streptomy | 3.079E-06 | 4.615E-06 | 3.639E-06 | 5.901E-06 | 5.885E-07 | 0.000E+00 | 2.748E-07 | 0.000E+00 | 2.714E-07 | 0.000E+00 | 0.000E+00 | 1.761E-07 |
| Unigene6014 | tetA(58) | 6.048E-06 | 8.568E-06 | 1.573E-05 | 1.747E-05 | 0.000E+00 | 1.140E-07 | 6.616E-07 | 0.000E+00 | 0.000E+00 | 0.000E+00 | 0.000E+00 | 4.156E-08 |
| Unigene6067 | lmrD | 3.007E-06 | 4.553E-06 | 3.590E-06 | 5.744E-06 | 4.025E-07 | 1.175E-07 | 3.007E-07 | 0.000E+00 | 2.376E-07 | 0.000E+00 | 8.288E-08 | 1.499E-07 |
| Unigene6068 | efrA | 2.948E-06 | 3.417E-06 | 3.399E-06 | 5.377E-06 | 2.661E-07 | 9.959E-08 | 8.157E-08 | 1.075E-07 | 1.812E-07 | 0.000E+00 | 1.054E-07 | 1.742E-07 |
| Unigene6078 | efrA | 3.159E-06 | 3.814E-06 | 3.228E-06 | 6.249E-06 | 3.303E-07 | 0.000E+00 | 2.879E-07 | 0.000E+00 | 1.422E-07 | 0.000E+00 | 1.275E-07 | 1.098E-07 |
| Unigene6103 | tetA(46) | 3.943E-06 | 0.000E+00 | 0.000E+00 | 0.000E+00 | 0.000E+00 | 0.000E+00 | 0.000E+00 | 0.000E+00 | 0.000E+00 | 0.000E+00 | 0.000E+00 | 0.000E+00 |
| Unigene6104 | msbA | 6.996E-06 | 0.000E+00 | 0.000E+00 | 0.000E+00 | 4.725E-08 | 0.000E+00 | 0.000E+00 | 0.000E+00 | 0.000E+00 | 0.000E+00 | 0.000E+00 | 0.000E+00 |
| Unigene6105 | tetA(46) | 6.371E-06 | 2.311E-06 | 0.000E+00 | 0.000E+00 | 1.180E-07 | 0.000E+00 | 0.000E+00 | 0.000E+00 | 2.611E-06 | 0.000E+00 | 0.000E+00 | 0.000E+00 |
| Unigene6119 | macB | 1.051E-06 | 1.015E-06 | 4.413E-06 | 5.475E-06 | 0.000E+00 | 1.363E-07 | 8.839E-07 | 0.000E+00 | 0.000E+00 | 0.000E+00 | 0.000E+00 | 0.000E+00 |
| Unigene6191 | pgpB | 4.214E-06 | 1.043E-05 | 9.590E-06 | 1.236E-05 | 4.981E-08 | 1.454E-07 | 0.000E+00 | 0.000E+00 | 0.000E+00 | 0.000E+00 | 1.026E-07 | 0.000E+00 |
| Unigene6194 | Staphylocc | 4.248E-06 | 1.129E-05 | 1.128E-05 | 1.267E-05 | 0.000E+00 | 4.662E-08 | 0.000E+00 | 0.000E+00 | 0.000E+00 | 2.402E-07 | 0.000E+00 | 0.000E+00 |
| Unigene6256 | poxtA | 5.505E-06 | 1.053E-06 | 2.455E-07 | 8.423E-08 | 0.000E+00 | 4.509E-08 | 0.000E+00 | 0.000E+00 | 0.000E+00 | 0.000E+00 | 0.000E+00 | 0.000E+00 |

| | | | | | | | | | | | | | |
|---|---|---|---|---|---|---|---|---|---|---|---|---|---|
| Unigene6263 | YojI | 1.110E-06 | 5.514E-07 | 4.519E-07 | 8.879E-07 | 3.023E-07 | 2.263E-08 | 0.000E+00 | 0.000E+00 | 0.000E+00 | 0.000E+00 | 0.000E+00 | 0.000E+00 |
| Unigene6336 | efrA | 5.212E-05 | 1.252E-04 | 1.131E-04 | 4.539E-06 | 6.961E-06 | 2.454E-07 | 3.517E-07 | 2.491E-06 | 2.878E-06 | 0.000E+00 | 2.856E-06 | 0.000E+00 |
| Unigene6385 | adeN | 4.197E-05 | 2.022E-05 | 6.357E-06 | 1.417E-05 | 2.441E-06 | 1.218E-06 | 2.930E-06 | 9.864E-07 | 6.465E-06 | 3.765E-07 | 1.547E-06 | 1.465E-06 |
| Unigene6447 | oleI | 8.412E-07 | 9.232E-07 | 4.712E-06 | 6.229E-06 | 0.000E+00 | 0.000E+00 | 5.431E-07 | 0.000E+00 | 0.000E+00 | 0.000E+00 | 8.018E-08 | 4.143E-08 |
| Unigene6498 | adeR | 1.365E-05 | 1.715E-05 | 4.507E-05 | 2.059E-06 | 1.153E-04 | 6.643E-05 | 8.273E-06 | 1.122E-04 | 4.135E-05 | 6.422E-05 | 5.254E-05 | 1.588E-04 |
| Unigene6534 | oleC | 5.337E-06 | 1.233E-05 | 1.170E-05 | 1.308E-05 | 2.391E-07 | 0.000E+00 | 1.429E-07 | 0.000E+00 | 0.000E+00 | 2.877E-07 | 0.000E+00 | 0.000E+00 |
| Unigene6543 | oleC | 3.290E-06 | 0.000E+00 | 2.443E-06 | 4.939E-07 | 3.952E-07 | 1.442E-07 | 3.445E-07 | 0.000E+00 | 0.000E+00 | 0.000E+00 | 0.000E+00 | 0.000E+00 |
| Unigene6549 | patB | 4.763E-06 | 3.267E-07 | 4.340E-06 | 6.872E-07 | 3.307E-07 | 0.000E+00 | 5.648E-07 | 0.000E+00 | 0.000E+00 | 0.000E+00 | 7.297E-07 | 0.000E+00 |
| Unigene6602 | vanSA | 2.555E-05 | 8.620E-06 | 6.804E-06 | 4.329E-06 | 2.237E-07 | 1.244E-07 | 0.000E+00 | 6.716E-08 | 7.861E-07 | 0.000E+00 | 0.000E+00 | 0.000E+00 |
| Unigene6618 | arlS | 2.983E-05 | 9.636E-06 | 6.621E-06 | 5.525E-06 | 4.052E-07 | 5.633E-08 | 0.000E+00 | 0.000E+00 | 6.265E-07 | 0.000E+00 | 0.000E+00 | 0.000E+00 |
| Unigene6619 | arlR | 2.333E-05 | 8.806E-06 | 6.098E-06 | 3.513E-06 | 5.153E-07 | 0.000E+00 | 0.000E+00 | 0.000E+00 | 1.318E-06 | 0.000E+00 | 5.305E-08 | 0.000E+00 |
| Unigene6648 | vanSA | 7.372E-06 | 0.000E+00 | 0.000E+00 | 2.162E-06 | 3.514E-05 | 1.896E-05 | 2.370E-05 | 2.563E-05 | 3.199E-06 | 0.000E+00 | 0.000E+00 | 0.000E+00 |
| Unigene6649 | vanRF | 7.275E-06 | 0.000E+00 | 0.000E+00 | 1.892E-06 | 3.397E-05 | 1.926E-05 | 2.444E-05 | 2.685E-05 | 3.523E-06 | 0.000E+00 | 0.000E+00 | 0.000E+00 |
| Unigene6671 | vanHB | 8.984E-06 | 0.000E+00 | 0.000E+00 | 2.742E-06 | 3.635E-05 | 1.634E-05 | 2.634E-05 | 2.594E-05 | 3.444E-06 | 0.000E+00 | 0.000E+00 | 0.000E+00 |
| Unigene6681 | Streptomy | 3.381E-05 | 1.384E-05 | 1.367E-05 | 5.626E-07 | 9.195E-07 | 1.721E-07 | 0.000E+00 | 0.000E+00 | 0.000E+00 | 0.000E+00 | 0.000E+00 | 0.000E+00 |
| Unigene6702 | optrA | 2.869E-05 | 0.000E+00 | 0.000E+00 | 0.000E+00 | 0.000E+00 | 2.014E-07 | 0.000E+00 | 0.000E+00 | 0.000E+00 | 0.000E+00 | 0.000E+00 | 0.000E+00 |
| Unigene6710 | lmrP | 2.809E-05 | 0.000E+00 | 0.000E+00 | 0.000E+00 | 0.000E+00 | 1.972E-07 | 0.000E+00 | 0.000E+00 | 0.000E+00 | 0.000E+00 | 0.000E+00 | 0.000E+00 |
| Unigene6712 | vanSM | 2.936E-05 | 0.000E+00 | 4.493E-07 | 0.000E+00 | 0.000E+00 | 1.547E-07 | 0.000E+00 | 0.000E+00 | 0.000E+00 | 0.000E+00 | 0.000E+00 | 0.000E+00 |
| Unigene6721 | arlS | 2.583E-05 | 7.166E-07 | 0.000E+00 | 0.000E+00 | 0.000E+00 | 5.647E-08 | 0.000E+00 | 0.000E+00 | 0.000E+00 | 0.000E+00 | 0.000E+00 | 0.000E+00 |
| Unigene6734 | novA | 2.978E-05 | 0.000E+00 | 0.000E+00 | 0.000E+00 | 0.000E+00 | 0.000E+00 | 0.000E+00 | 0.000E+00 | 0.000E+00 | 0.000E+00 | 0.000E+00 | 0.000E+00 |
| Unigene6748 | emrB | 2.976E-05 | 1.010E-06 | 0.000E+00 | 0.000E+00 | 0.000E+00 | 9.476E-08 | 0.000E+00 | 0.000E+00 | 0.000E+00 | 0.000E+00 | 0.000E+00 | 2.590E-08 |
| Unigene6801 | golS | 0.000E+00 | 4.188E-07 | 0.000E+00 | 0.000E+00 | 0.000E+00 | 0.000E+00 | 0.000E+00 | 0.000E+00 | 0.000E+00 | 0.000E+00 | 0.000E+00 | 4.240E-06 |
| Unigene6838 | patB | 3.517E-06 | 7.834E-06 | 1.103E-05 | 9.569E-06 | 1.170E-07 | 9.105E-08 | 0.000E+00 | 0.000E+00 | 0.000E+00 | 9.383E-08 | 2.409E-08 | 0.000E+00 |
| Unigene6846 | Corynebac | 1.206E-06 | 0.000E+00 | 0.000E+00 | 0.000E+00 | 0.000E+00 | 0.000E+00 | 0.000E+00 | 0.000E+00 | 0.000E+00 | 0.000E+00 | 0.000E+00 | 0.000E+00 |
| Unigene6867 | arlR | 1.190E-06 | 0.000E+00 | 0.000E+00 | 0.000E+00 | 0.000E+00 | 0.000E+00 | 1.054E-07 | 0.000E+00 | 0.000E+00 | 0.000E+00 | 0.000E+00 | 0.000E+00 |
| Unigene6869 | macB | 8.113E-07 | 0.000E+00 | 0.000E+00 | 0.000E+00 | 0.000E+00 | 0.000E+00 | 0.000E+00 | 0.000E+00 | 0.000E+00 | 0.000E+00 | 0.000E+00 | 0.000E+00 |
| Unigene6871 | efrA | 4.986E-06 | 9.978E-06 | 1.069E-05 | 1.064E-05 | 0.000E+00 | 8.106E-08 | 0.000E+00 | 0.000E+00 | 0.000E+00 | 0.000E+00 | 0.000E+00 | 0.000E+00 |
| Unigene6872 | patB | 4.551E-06 | 1.064E-05 | 1.126E-05 | 1.550E-05 | 0.000E+00 | 0.000E+00 | 0.000E+00 | 0.000E+00 | 0.000E+00 | 0.000E+00 | 0.000E+00 | 1.089E-07 |
| Unigene6900 | vanHO | 6.346E-05 | 8.597E-05 | 1.229E-04 | 7.953E-06 | 7.381E-06 | 2.453E-07 | 2.009E-06 | 1.438E-06 | 0.000E+00 | 0.000E+00 | 2.521E-06 | 6.129E-07 |
| Unigene6932 | macB | 2.842E-06 | 4.117E-06 | 2.640E-06 | 6.045E-06 | 0.000E+00 | 0.000E+00 | 2.097E-07 | 0.000E+00 | 2.071E-07 | 0.000E+00 | 0.000E+00 | 0.000E+00 |
| Unigene6933 | tetB(60) | 2.457E-06 | 3.106E-06 | 3.361E-06 | 5.489E-06 | 4.136E-07 | 0.000E+00 | 1.648E-07 | 0.000E+00 | 2.442E-07 | 0.000E+00 | 0.000E+00 | 0.000E+00 |
| Unigene6945 | tva(A) | 3.683E-06 | 4.534E-06 | 3.488E-06 | 5.724E-06 | 0.000E+00 | 0.000E+00 | 0.000E+00 | 0.000E+00 | 0.000E+00 | 0.000E+00 | 0.000E+00 | 0.000E+00 |
| Unigene6946 | carA | 2.664E-06 | 4.199E-06 | 3.452E-06 | 6.278E-06 | 2.656E-07 | 0.000E+00 | 0.000E+00 | 0.000E+00 | 0.000E+00 | 0.000E+00 | 0.000E+00 | 0.000E+00 |
| Unigene6958 | macB | 9.782E-07 | 2.863E-06 | 3.017E-06 | 4.563E-06 | 0.000E+00 | 0.000E+00 | 0.000E+00 | 0.000E+00 | 0.000E+00 | 0.000E+00 | 0.000E+00 | 0.000E+00 |
| Unigene6965 | tva(A) | 2.877E-07 | 0.000E+00 | 5.646E-08 | 0.000E+00 | 0.000E+00 | 0.000E+00 | 0.000E+00 | 0.000E+00 | 0.000E+00 | 0.000E+00 | 0.000E+00 | 0.000E+00 |
| Unigene7047 | cmlA5 | 2.868E-06 | 3.687E-06 | 2.305E-06 | 2.013E-06 | 2.579E-06 | 2.008E-06 | 1.481E-06 | 1.084E-06 | 1.015E-06 | 4.868E-06 | 5.154E-06 | 1.775E-06 |
| Unigene7063 | PmrF | 4.999E-05 | 2.687E-05 | 1.975E-05 | 2.459E-05 | 1.891E-06 | 1.988E-06 | 3.618E-06 | 9.539E-07 | 6.922E-06 | 5.310E-07 | 2.181E-06 | 2.254E-06 |
| Unigene7097 | vanXYE | 4.171E-07 | 0.000E+00 | 3.683E-07 | 5.054E-07 | 0.000E+00 | 9.829E-06 | 1.892E-06 | 0.000E+00 | 4.376E-06 | 3.159E-06 | 0.000E+00 | 0.000E+00 |
| Unigene7131 | patB | 9.377E-05 | 1.416E-04 | 1.272E-04 | 5.652E-06 | 7.354E-06 | 3.316E-07 | 3.508E-06 | 1.671E-06 | 5.162E-05 | 0.000E+00 | 2.719E-06 | 1.148E-06 |
| Unigene7154 | salA | 4.971E-05 | 6.675E-05 | 3.048E-05 | 1.513E-05 | 1.771E-05 | 5.651E-06 | 2.845E-06 | 5.527E-05 | 1.309E-05 | 7.522E-06 | 1.296E-06 | 7.209E-07 |
| Unigene7162 | Staphylocc | 4.772E-05 | 7.118E-05 | 3.161E-05 | 1.504E-05 | 1.987E-05 | 6.626E-06 | 3.291E-06 | 5.909E-05 | 1.263E-05 | 7.156E-06 | 1.922E-06 | 1.234E-06 |
| Unigene7198 | carA | 4.789E-05 | 6.702E-05 | 2.739E-05 | 1.601E-05 | 1.945E-05 | 6.975E-06 | 3.087E-06 | 5.862E-05 | 1.303E-05 | 7.095E-06 | 2.333E-06 | 1.255E-06 |
| Unigene7211 | msbA | 4.638E-05 | 6.454E-05 | 2.491E-05 | 1.419E-05 | 1.713E-05 | 7.407E-06 | 3.402E-06 | 5.429E-05 | 1.042E-05 | 6.996E-06 | 2.361E-06 | 4.153E-07 |
| Unigene7219 | lsaC | 4.526E-05 | 6.208E-05 | 2.805E-05 | 1.466E-05 | 1.863E-05 | 6.693E-06 | 3.460E-06 | 5.252E-05 | 1.199E-05 | 6.429E-06 | 1.238E-06 | 8.882E-07 |
| Unigene7322 | macB | 3.507E-06 | 5.642E-06 | 3.246E-06 | 4.387E-06 | 1.771E-07 | 1.034E-06 | 1.941E-06 | 6.512E-07 | 1.045E-06 | 0.000E+00 | 8.661E-07 | 4.711E-07 |
| Unigene7339 | lmrB | 7.053E-05 | 5.658E-05 | 1.135E-04 | 1.591E-04 | 1.263E-04 | 8.821E-05 | 1.216E-04 | 1.103E-04 | 9.406E-05 | 1.414E-04 | 1.487E-04 | 1.582E-04 |
| Unigene7354 | QepA2 | 3.923E-07 | 0.000E+00 | 0.000E+00 | 0.000E+00 | 0.000E+00 | 0.000E+00 | 0.000E+00 | 0.000E+00 | 0.000E+00 | 0.000E+00 | 0.000E+00 | 0.000E+00 |
| Unigene7380 | msbA | 8.334E-06 | 0.000E+00 | 0.000E+00 | 2.789E-06 | 3.490E-05 | 1.690E-05 | 2.762E-05 | 2.647E-05 | 3.124E-06 | 0.000E+00 | 0.000E+00 | 0.000E+00 |
| Unigene7476 | dfrC | 5.630E-05 | 4.597E-05 | 9.383E-05 | 1.345E-04 | 9.293E-05 | 7.083E-05 | 9.300E-05 | 9.270E-05 | 9.205E-05 | 1.264E-04 | 1.058E-04 | 1.109E-04 |
| Unigene7494 | Streptomy | 2.219E-06 | 1.757E-06 | 2.177E-06 | 1.536E-06 | 2.817E-07 | 0.000E+00 | 4.560E-07 | 3.330E-07 | 6.062E-07 | 0.000E+00 | 0.000E+00 | 0.000E+00 |
| Unigene7541 | Staphylocc | 2.903E-06 | 2.156E-06 | 2.656E-06 | 1.763E-06 | 2.667E-07 | 0.000E+00 | 3.865E-07 | 2.293E-07 | 6.202E-07 | 0.000E+00 | 0.000E+00 | 2.580E-08 |
| Unigene7546 | Staphylocc | 2.735E-06 | 2.607E-06 | 2.646E-06 | 2.010E-06 | 2.282E-07 | 9.717E-08 | 3.695E-07 | 1.948E-07 | 7.017E-07 | 8.582E-08 | 0.000E+00 | 1.366E-07 |
| Unigene7551 | Corynebac | 2.729E-06 | 2.689E-06 | 3.469E-06 | 1.973E-06 | 0.000E+00 | 0.000E+00 | 5.834E-07 | 2.534E-07 | 7.456E-07 | 0.000E+00 | 1.774E-07 | 1.833E-07 |
| Unigene7572 | sul4 | 3.898E-05 | 1.606E-05 | 6.134E-06 | 1.208E-05 | 1.436E-06 | 1.820E-06 | 2.428E-06 | 5.262E-07 | 6.406E-06 | 9.040E-07 | 1.238E-06 | 1.634E-06 |
| Unigene7576 | dfrK | 4.579E-05 | 2.485E-05 | 1.713E-05 | 2.059E-05 | 1.737E-06 | 2.029E-06 | 3.462E-06 | 7.302E-07 | 7.453E-06 | 7.666E-07 | 0.000E+00 | 2.218E-06 |
| Unigene7614 | macB | 4.753E-07 | 5.881E-06 | 1.030E-05 | 1.684E-05 | 0.000E+00 | 0.000E+00 | 0.000E+00 | 0.000E+00 | 0.000E+00 | 0.000E+00 | 0.000E+00 | 0.000E+00 |
| Unigene7633 | patA | 6.961E-05 | 5.761E-05 | 1.071E-04 | 1.711E-04 | 1.172E-04 | 8.576E-05 | 1.288E-04 | 1.067E-04 | 8.975E-05 | 1.296E-04 | 1.526E-04 | 1.351E-04 |
| Unigene7721 | Staphylocc | 2.597E-06 | 2.681E-06 | 3.044E-06 | 1.222E-06 | 2.933E-07 | 1.427E-07 | 3.409E-07 | 2.825E-07 | 8.418E-07 | 0.000E+00 | 0.000E+00 | 0.000E+00 |

| | | | | | | | | | | | | | |
|---|---|---|---|---|---|---|---|---|---|---|---|---|---|
| Unigene7760 | vanHA | 1.149E-05 | 0.000E+00 | 0.000E+00 | 0.000E+00 | 1.723E-05 | 1.552E-05 | 0.000E+00 | 0.000E+00 | 0.000E+00 | 0.000E+00 | 0.000E+00 | 0.000E+00 |
| Unigene7772 | arlR | 1.110E-05 | 0.000E+00 | 0.000E+00 | 0.000E+00 | 2.017E-05 | 1.558E-05 | 0.000E+00 | 0.000E+00 | 1.370E-05 | 0.000E+00 | 0.000E+00 | 0.000E+00 |
| Unigene7804 | tetB(58) | 8.113E-07 | 2.812E-07 | 1.550E-06 | 4.312E-07 | 0.000E+00 | 9.232E-08 | 0.000E+00 | 0.000E+00 | 0.000E+00 | 0.000E+00 | 4.884E-08 | 0.000E+00 |
| Unigene7829 | efrB | 1.134E-04 | 1.871E-04 | 1.787E-04 | 7.289E-06 | 9.199E-06 | 0.000E+00 | 3.087E-06 | 9.820E-07 | 5.614E-05 | 0.000E+00 | 2.988E-06 | 0.000E+00 |
| Unigene7865 | Staphylocc | 1.209E-05 | 4.978E-06 | 3.171E-06 | 1.155E-05 | 8.052E-05 | 1.145E-04 | 1.076E-04 | 8.736E-05 | 1.354E-04 | 1.352E-04 | 1.081E-04 | 6.900E-05 |
| Unigene7913 | mtrA | 1.023E-04 | 4.546E-05 | 0.000E+00 | 0.000E+00 | 2.041E-05 | 8.697E-05 | 0.000E+00 | 2.628E-07 | 0.000E+00 | 0.000E+00 | 0.000E+00 | 0.000E+00 |
| Unigene7914 | arlS | 9.736E-05 | 4.115E-05 | 2.388E-07 | 3.511E-08 | 2.196E-05 | 8.609E-05 | 0.000E+00 | 0.000E+00 | 0.000E+00 | 0.000E+00 | 0.000E+00 | 0.000E+00 |
| Unigene7925 | msrA | 9.871E-05 | 3.924E-05 | 2.315E-07 | 1.430E-07 | 2.139E-05 | 8.551E-05 | 0.000E+00 | 0.000E+00 | 0.000E+00 | 0.000E+00 | 1.620E-07 | 3.626E-07 |
| Unigene7971 | tetA(58) | 1.306E-05 | 5.409E-06 | 2.990E-06 | 1.174E-05 | 7.670E-05 | 1.031E-04 | 1.055E-04 | 8.155E-05 | 1.299E-04 | 1.306E-04 | 1.028E-04 | 7.000E-05 |
| Unigene8102 | vanHF | 8.947E-05 | 4.323E-05 | 0.000E+00 | 0.000E+00 | 2.056E-05 | 8.063E-05 | 0.000E+00 | 0.000E+00 | 0.000E+00 | 0.000E+00 | 3.834E-08 | 0.000E+00 |
| Unigene8116 | vanHD | 9.348E-05 | 3.937E-05 | 2.854E-07 | 0.000E+00 | 1.752E-05 | 8.534E-05 | 0.000E+00 | 0.000E+00 | 0.000E+00 | 0.000E+00 | 0.000E+00 | 0.000E+00 |
| Unigene8117 | vanRE | 8.171E-05 | 3.610E-05 | 0.000E+00 | 0.000E+00 | 1.858E-05 | 7.899E-05 | 0.000E+00 | 0.000E+00 | 0.000E+00 | 0.000E+00 | 0.000E+00 | 0.000E+00 |
| Unigene8118 | cpxA | 8.929E-05 | 4.054E-05 | 3.193E-07 | 8.216E-08 | 1.961E-05 | 8.015E-05 | 2.251E-08 | 0.000E+00 | 0.000E+00 | 4.532E-08 | 2.327E-08 | 0.000E+00 |
| Unigene8196 | TaeA | 2.628E-06 | 4.117E-06 | 3.461E-06 | 5.149E-06 | 0.000E+00 | 0.000E+00 | 2.486E-07 | 0.000E+00 | 2.455E-07 | 0.000E+00 | 1.028E-07 | 2.124E-07 |
| Unigene8285 | rpoB2 | 1.162E-04 | 1.482E-04 | 1.409E-04 | 0.000E+00 | 6.997E-06 | 4.315E-07 | 0.000E+00 | 1.408E-06 | 0.000E+00 | 3.656E-07 | 0.000E+00 | 0.000E+00 |
| Unigene8320 | vanL | 1.294E-06 | 2.775E-06 | 7.270E-06 | 8.045E-06 | 0.000E+00 | 0.000E+00 | 2.603E-07 | 0.000E+00 | 0.000E+00 | 0.000E+00 | 0.000E+00 | 0.000E+00 |
| Unigene8346 | macB | 2.789E-05 | 1.004E-05 | 7.727E-06 | 4.873E-06 | 2.898E-07 | 1.057E-07 | 1.624E-07 | 1.142E-07 | 8.375E-07 | 0.000E+00 | 0.000E+00 | 1.734E-07 |
| Unigene8384 | vanRM | 1.339E-06 | 1.596E-05 | 4.176E-06 | 2.656E-06 | 0.000E+00 | 1.034E-07 | 0.000E+00 | 0.000E+00 | 0.000E+00 | 0.000E+00 | 1.094E-07 | 0.000E+00 |
| Unigene8385 | basS | 1.763E-06 | 1.739E-05 | 5.338E-06 | 3.048E-06 | 9.962E-08 | 0.000E+00 | 0.000E+00 | 0.000E+00 | 0.000E+00 | 0.000E+00 | 2.051E-07 | 0.000E+00 |
| Unigene8407 | macB | 6.914E-06 | 4.121E-06 | 0.000E+00 | 4.624E-07 | 0.000E+00 | 0.000E+00 | 2.534E-07 | 0.000E+00 | 0.000E+00 | 0.000E+00 | 1.571E-07 | 0.000E+00 |
| Unigene8437 | PmrF | 2.233E-06 | 2.414E-06 | 1.859E-06 | 1.196E-06 | 9.774E-07 | 9.145E-07 | 1.498E-06 | 3.555E-07 | 4.808E-07 | 5.352E-06 | 3.638E-06 | 9.999E-07 |
| Unigene8449 | Staphylocc | 3.710E-06 | 3.667E-06 | 2.413E-06 | 2.525E-06 | 2.960E-06 | 1.950E-06 | 1.860E-06 | 8.134E-07 | 1.590E-06 | 5.731E-06 | 5.697E-06 | 1.623E-06 |
| Unigene8470 | patB | 2.864E-06 | 2.076E-06 | 3.045E-06 | 1.317E-06 | 0.000E+00 | 1.985E-07 | 3.048E-07 | 3.858E-07 | 6.623E-07 | 4.091E-08 | 0.000E+00 | 0.000E+00 |
| Unigene8471 | efrA | 2.079E-06 | 2.402E-06 | 2.684E-06 | 1.529E-06 | 1.823E-07 | 7.886E-08 | 2.018E-07 | 1.916E-07 | 7.575E-07 | 0.000E+00 | 6.258E-08 | 2.587E-07 |
| Unigene8474 | vanHO | 2.695E-05 | 0.000E+00 | 0.000E+00 | 0.000E+00 | 0.000E+00 | 1.036E-07 | 0.000E+00 | 0.000E+00 | 0.000E+00 | 0.000E+00 | 0.000E+00 | 0.000E+00 |
| Unigene8476 | vatB | 3.293E-05 | 8.902E-07 | 0.000E+00 | 0.000E+00 | 0.000E+00 | 0.000E+00 | 0.000E+00 | 0.000E+00 | 0.000E+00 | 0.000E+00 | 0.000E+00 | 0.000E+00 |
| Unigene8497 | facT | 2.546E-05 | 7.553E-06 | 6.077E-06 | 4.148E-06 | 3.012E-07 | 1.578E-07 | 2.308E-08 | 1.217E-07 | 7.293E-07 | 0.000E+00 | 0.000E+00 | 0.000E+00 |
| Unigene8501 | lmrP | 2.750E-05 | 9.846E-06 | 6.628E-06 | 4.179E-06 | 5.043E-07 | 1.155E-07 | 0.000E+00 | 9.350E-08 | 0.000E+00 | 0.000E+00 | 0.000E+00 | 0.000E+00 |
| Unigene8521 | evgS | 1.059E-04 | 1.844E-04 | 1.730E-04 | 7.999E-06 | 9.034E-06 | 4.547E-07 | 3.763E-06 | 2.005E-06 | 6.037E-05 | 0.000E+00 | 3.047E-06 | 3.729E-07 |
| Unigene8621 | lmrB | 6.616E-06 | 0.000E+00 | 0.000E+00 | 2.127E-06 | 3.413E-05 | 8.019E-06 | 2.258E-05 | 2.373E-05 | 0.000E+00 | 0.000E+00 | 0.000E+00 | 0.000E+00 |
| Unigene8626 | patA | 6.622E-06 | 0.000E+00 | 0.000E+00 | 0.000E+00 | 3.739E-05 | 1.412E-05 | 2.239E-05 | 2.582E-05 | 0.000E+00 | 0.000E+00 | 0.000E+00 | 0.000E+00 |
| Unigene8648 | pmrA | 4.214E-06 | 2.051E-05 | 4.239E-06 | 1.097E-06 | 8.539E-07 | 4.871E-07 | 1.760E-07 | 2.475E-07 | 1.101E-06 | 5.314E-07 | 1.819E-07 | 0.000E+00 |
| Unigene8655 | macB | 5.044E-06 | 2.114E-05 | 3.959E-06 | 1.407E-06 | 8.005E-07 | 5.712E-07 | 3.721E-07 | 6.168E-07 | 1.313E-06 | 7.492E-07 | 4.945E-07 | 0.000E+00 |
| Unigene8689 | evgA | 3.228E-06 | 4.218E-06 | 2.866E-06 | 5.381E-06 | 1.708E-07 | 0.000E+00 | 3.402E-07 | 0.000E+00 | 2.240E-07 | 0.000E+00 | 0.000E+00 | 4.845E-07 |
| Unigene8743 | TaeA | 2.673E-06 | 3.825E-06 | 3.735E-06 | 5.860E-06 | 3.007E-07 | 0.000E+00 | 3.745E-07 | 3.950E-08 | 1.664E-07 | 0.000E+00 | 0.000E+00 | 2.400E-07 |
| Unigene8746 | dfrE | 3.631E-06 | 4.564E-06 | 4.469E-06 | 6.799E-06 | 0.000E+00 | 0.000E+00 | 0.000E+00 | 0.000E+00 | 0.000E+00 | 0.000E+00 | 0.000E+00 | 5.462E-07 |
| Unigene8782 | tet32 | 9.432E-05 | 4.195E-05 | 3.316E-07 | 1.437E-07 | 1.988E-05 | 8.400E-05 | 7.874E-08 | 0.000E+00 | 0.000E+00 | 3.962E-08 | 1.628E-07 | 0.000E+00 |
| Unigene8798 | Staphylocc | 9.737E-05 | 4.429E-05 | 3.610E-07 | 0.000E+00 | 2.258E-05 | 8.558E-05 | 0.000E+00 | 3.220E-07 | 0.000E+00 | 0.000E+00 | 2.893E-07 | 0.000E+00 |
| Unigene8808 | TaeA | 8.478E-05 | 3.492E-05 | 5.101E-07 | 0.000E+00 | 2.991E-05 | 8.008E-05 | 0.000E+00 | 6.067E-08 | 0.000E+00 | 0.000E+00 | 0.000E+00 | 3.687E-07 |
| Unigene8812 | patA | 8.117E-05 | 3.378E-05 | 0.000E+00 | 0.000E+00 | 1.683E-05 | 7.044E-05 | 4.763E-08 | 0.000E+00 | 0.000E+00 | 0.000E+00 | 9.847E-08 | 0.000E+00 |
| Unigene8817 | optrA | 9.540E-05 | 4.012E-05 | 3.274E-07 | 0.000E+00 | 2.122E-05 | 8.079E-05 | 0.000E+00 | 0.000E+00 | 0.000E+00 | 0.000E+00 | 4.771E-08 | 0.000E+00 |
| Unigene8839 | farB | 9.684E-05 | 4.394E-05 | 2.200E-07 | 0.000E+00 | 2.102E-05 | 8.315E-05 | 0.000E+00 | 0.000E+00 | 0.000E+00 | 0.000E+00 | 0.000E+00 | 0.000E+00 |
| Unigene8852 | macB | 9.506E-05 | 4.155E-05 | 2.614E-07 | 8.279E-08 | 1.951E-05 | 7.890E-05 | 0.000E+00 | 0.000E+00 | 0.000E+00 | 0.000E+00 | 1.641E-07 | 0.000E+00 |
| Unigene8865 | Bifidobact | 9.629E-05 | 4.473E-05 | 2.563E-07 | 0.000E+00 | 1.841E-05 | 7.804E-05 | 3.614E-08 | 7.623E-08 | 0.000E+00 | 0.000E+00 | 1.868E-07 | 0.000E+00 |
| Unigene8885 | carA | 9.773E-05 | 3.868E-05 | 3.757E-07 | 0.000E+00 | 2.104E-05 | 7.554E-05 | 2.230E-08 | 0.000E+00 | 0.000E+00 | 8.979E-08 | 6.915E-08 | 7.145E-08 |
| Unigene8886 | tlrC | 9.192E-05 | 4.476E-05 | 3.193E-07 | 8.216E-08 | 2.140E-05 | 8.266E-05 | 0.000E+00 | 0.000E+00 | 0.000E+00 | 6.798E-08 | 4.653E-08 | 1.202E-07 |
| Unigene8928 | Chlamydia | 8.973E-05 | 3.987E-05 | 3.975E-07 | 0.000E+00 | 1.918E-05 | 7.787E-05 | 1.401E-07 | 1.182E-07 | 0.000E+00 | 0.000E+00 | 0.000E+00 | 0.000E+00 |
| Unigene8956 | tetA(58) | 8.189E-05 | 3.845E-05 | 3.334E-07 | 1.144E-07 | 1.946E-05 | 8.440E-05 | 0.000E+00 | 0.000E+00 | 0.000E+00 | 0.000E+00 | 0.000E+00 | 0.000E+00 |
| Unigene8982 | poxtA | 9.174E-05 | 4.069E-05 | 2.565E-07 | 0.000E+00 | 1.665E-05 | 7.191E-05 | 0.000E+00 | 5.085E-08 | 0.000E+00 | 3.882E-07 | 0.000E+00 | 0.000E+00 |
| Unigene9002 | lmrD | 9.165E-05 | 4.106E-05 | 3.828E-07 | 0.000E+00 | 1.820E-05 | 7.783E-05 | 0.000E+00 | 0.000E+00 | 0.000E+00 | 4.575E-08 | 0.000E+00 | 0.000E+00 |
| Unigene9009 | rpoB2 | 8.621E-05 | 4.069E-05 | 2.884E-07 | 8.095E-08 | 1.765E-05 | 7.417E-05 | 1.972E-08 | 3.119E-08 | 0.000E+00 | 1.290E-07 | 1.426E-07 | 7.370E-08 |
| Unigene9014 | tetT | 9.418E-05 | 4.448E-05 | 4.078E-07 | 0.000E+00 | 1.845E-05 | 8.049E-05 | 0.000E+00 | 0.000E+00 | 0.000E+00 | 1.029E-07 | 7.043E-08 | 1.820E-07 |
| Unigene9041 | patB | 8.559E-05 | 4.100E-05 | 8.179E-07 | 0.000E+00 | 1.263E-06 | 5.128E-05 | 0.000E+00 | 0.000E+00 | 0.000E+00 | 0.000E+00 | 4.334E-08 | 0.000E+00 |
| Unigene9042 | tetB(46) | 9.879E-05 | 4.410E-05 | 1.123E-07 | 0.000E+00 | 1.865E-06 | 6.394E-05 | 0.000E+00 | 0.000E+00 | 0.000E+00 | 0.000E+00 | 1.746E-07 | 0.000E+00 |
| Unigene9048 | macB | 8.957E-05 | 3.951E-05 | 2.002E-07 | 1.030E-07 | 1.872E-05 | 7.790E-05 | 0.000E+00 | 0.000E+00 | 0.000E+00 | 0.000E+00 | 0.000E+00 | 0.000E+00 |
| Unigene9049 | macB | 8.698E-05 | 4.133E-05 | 0.000E+00 | 0.000E+00 | 1.652E-05 | 7.326E-05 | 0.000E+00 | 0.000E+00 | 0.000E+00 | 1.051E-07 | 0.000E+00 | 0.000E+00 |
| Unigene9058 | PmrF | 9.247E-05 | 4.086E-05 | 1.973E-07 | 7.616E-08 | 1.813E-05 | 7.680E-05 | 0.000E+00 | 0.000E+00 | 0.000E+00 | 1.960E-07 | 0.000E+00 | 0.000E+00 |
| Unigene9092 | Streptomy | 5.777E-06 | 1.199E-05 | 1.069E-05 | 1.214E-05 | 1.655E-07 | 8.948E-08 | 0.000E+00 | 0.000E+00 | 0.000E+00 | 2.028E-07 | 0.000E+00 | 1.957E-08 |

| | | | | | | | | | | | | | |
|---|---|---|---|---|---|---|---|---|---|---|---|---|---|
| Unigene9132 | tlrC | 0.000E+00 | 0.000E+00 | 0.000E+00 | 0.000E+00 | 0.000E+00 | 1.554E-06 | 5.545E-06 | 5.705E-06 | 0.000E+00 | 0.000E+00 | 0.000E+00 | 5.146E-06 |
| Unigene9139 | tet(43) | 7.471E-06 | 0.000E+00 | 0.000E+00 | 2.226E-06 | 3.555E-05 | 1.857E-05 | 2.622E-05 | 2.822E-05 | 4.436E-06 | 0.000E+00 | 0.000E+00 | 0.000E+00 |
| Unigene9183 | optrA | 2.650E-05 | 7.256E-07 | 3.081E-07 | 0.000E+00 | 0.000E+00 | 1.072E-07 | 0.000E+00 | 0.000E+00 | 0.000E+00 | 1.657E-07 | 0.000E+00 | 0.000E+00 |
| Unigene9185 | Staphylocc | 4.236E-05 | 2.367E-05 | 1.927E-05 | 0.000E+00 | 0.000E+00 | 1.167E-07 | 0.000E+00 | 0.000E+00 | 0.000E+00 | 1.203E-07 | 0.000E+00 | 0.000E+00 |
| Unigene9195 | tetB(P) | 2.914E-05 | 0.000E+00 | 0.000E+00 | 0.000E+00 | 0.000E+00 | 0.000E+00 | 0.000E+00 | 0.000E+00 | 0.000E+00 | 2.974E-07 | 0.000E+00 | 0.000E+00 |
| Unigene9199 | patB | 6.231E-05 | 4.215E-05 | 0.000E+00 | 0.000E+00 | 0.000E+00 | 0.000E+00 | 0.000E+00 | 0.000E+00 | 0.000E+00 | 0.000E+00 | 0.000E+00 | 0.000E+00 |
| Unigene9200 | Corynebac | 2.847E-05 | 0.000E+00 | 0.000E+00 | 0.000E+00 | 0.000E+00 | 0.000E+00 | 0.000E+00 | 0.000E+00 | 0.000E+00 | 3.029E-07 | 0.000E+00 | 0.000E+00 |
| Unigene9216 | macB | 2.683E-05 | 1.417E-06 | 0.000E+00 | 0.000E+00 | 0.000E+00 | 0.000E+00 | 0.000E+00 | 0.000E+00 | 0.000E+00 | 0.000E+00 | 0.000E+00 | 0.000E+00 |
| Unigene9225 | PmrF | 4.660E-07 | 4.649E-07 | 1.663E-07 | 0.000E+00 | 1.883E-07 | 0.000E+00 | 0.000E+00 | 0.000E+00 | 0.000E+00 | 0.000E+00 | 0.000E+00 | 0.000E+00 |
| Unigene9312 | bcrA | 2.709E-05 | 0.000E+00 | 0.000E+00 | 0.000E+00 | 0.000E+00 | 2.273E-07 | 0.000E+00 | 0.000E+00 | 0.000E+00 | 0.000E+00 | 0.000E+00 | 0.000E+00 |
| Unigene9315 | tetW | 2.681E-05 | 0.000E+00 | 0.000E+00 | 0.000E+00 | 0.000E+00 | 9.566E-08 | 0.000E+00 | 0.000E+00 | 0.000E+00 | 0.000E+00 | 0.000E+00 | 0.000E+00 |
| Unigene9377 | patA | 1.199E-05 | 4.724E-06 | 3.879E-06 | 1.255E-05 | 9.329E-05 | 1.221E-04 | 1.233E-04 | 1.071E-04 | 1.549E-04 | 1.539E-04 | 1.255E-04 | 8.332E-05 |
| Unigene9401 | mel | 2.305E-05 | 0.000E+00 | 0.000E+00 | 0.000E+00 | 0.000E+00 | 0.000E+00 | 0.000E+00 | 0.000E+00 | 0.000E+00 | 0.000E+00 | 0.000E+00 | 0.000E+00 |
| Unigene9425 | Streptomy | 1.030E-04 | 4.347E-05 | 2.579E-07 | 1.327E-07 | 2.228E-05 | 8.872E-05 | 0.000E+00 | 0.000E+00 | 0.000E+00 | 1.830E-07 | 0.000E+00 | 2.330E-07 |
| Unigene9453 | msbA | 9.364E-05 | 4.181E-05 | 3.805E-07 | 1.468E-07 | 2.601E-05 | 1.005E-04 | 5.364E-08 | 2.263E-07 | 0.000E+00 | 0.000E+00 | 1.109E-07 | 0.000E+00 |
| Unigene9460 | macB | 9.741E-05 | 4.559E-05 | 5.941E-07 | 1.389E-07 | 2.301E-05 | 9.389E-05 | 9.137E-08 | 8.031E-08 | 0.000E+00 | 1.380E-07 | 9.444E-08 | 0.000E+00 |
| Unigene9487 | efrA | 5.425E-05 | 6.859E-05 | 4.542E-05 | 0.000E+00 | 0.000E+00 | 0.000E+00 | 0.000E+00 | 0.000E+00 | 0.000E+00 | 0.000E+00 | 0.000E+00 | 0.000E+00 |
| Unigene9506 | msbA | 1.038E-06 | 5.180E-07 | 9.262E-07 | 1.716E-06 | 0.000E+00 | 5.102E-08 | 0.000E+00 | 0.000E+00 | 0.000E+00 | 0.000E+00 | 0.000E+00 | 0.000E+00 |
| Unigene9543 | macB | 1.135E-06 | 4.890E-06 | 1.056E-05 | 1.312E-05 | 0.000E+00 | 0.000E+00 | 0.000E+00 | 0.000E+00 | 0.000E+00 | 0.000E+00 | 0.000E+00 | 0.000E+00 |
| Unigene9561 | arlR | 0.000E+00 | 0.000E+00 | 0.000E+00 | 0.000E+00 | 0.000E+00 | 0.000E+00 | 6.584E-06 | 3.472E-06 | 0.000E+00 | 1.657E-06 | 1.501E-06 | 4.550E-06 |
| Unigene9601 | macB | 1.093E-05 | 0.000E+00 | 0.000E+00 | 0.000E+00 | 1.672E-05 | 1.477E-05 | 0.000E+00 | 0.000E+00 | 1.231E-05 | 0.000E+00 | 0.000E+00 | 0.000E+00 |
| Unigene9716 | tet(W/N/W | 3.774E-06 | 9.844E-06 | 1.039E-05 | 1.127E-05 | 1.950E-07 | 0.000E+00 | 0.000E+00 | 0.000E+00 | 5.756E-08 | 3.129E-07 | 0.000E+00 | 0.000E+00 |
| Unigene9727 | vanTG | 3.239E-06 | 3.435E-06 | 2.346E-06 | 2.107E-06 | 2.681E-06 | 1.903E-06 | 2.021E-06 | 1.294E-06 | 9.503E-07 | 5.666E-06 | 5.769E-06 | 1.439E-06 |
| Unigene9730 | lsaA | 2.806E-06 | 4.261E-06 | 3.437E-06 | 1.683E-06 | 2.414E-06 | 1.802E-06 | 2.592E-06 | 9.112E-07 | 2.052E-06 | 6.064E-06 | 5.599E-06 | 1.895E-06 |
| Unigene9739 | arlS | 3.610E-06 | 3.448E-06 | 2.384E-06 | 1.612E-06 | 2.545E-06 | 2.326E-06 | 2.324E-06 | 6.888E-07 | 1.536E-06 | 5.935E-06 | 5.539E-06 | 2.257E-06 |
| Unigene9740 | mtrA | 2.747E-06 | 3.467E-06 | 2.291E-06 | 1.665E-06 | 2.238E-06 | 2.227E-06 | 1.976E-06 | 8.551E-07 | 1.401E-06 | 5.713E-06 | 4.347E-06 | 1.407E-06 |
| Unigene9761 | Staphylocc | 2.941E-06 | 3.506E-06 | 2.440E-06 | 1.822E-06 | 2.807E-06 | 1.922E-06 | 2.101E-06 | 7.957E-07 | 1.329E-06 | 5.777E-06 | 4.877E-06 | 1.864E-06 |
| Unigene9810 | oleC | 1.980E-06 | 1.729E-05 | 7.348E-06 | 6.259E-06 | 0.000E+00 | 3.257E-07 | 0.000E+00 | 0.000E+00 | 0.000E+00 | 0.000E+00 | 0.000E+00 | 0.000E+00 |
| Unigene9828 | mtrA | 1.147E-05 | 4.665E-06 | 3.128E-06 | 1.082E-05 | 8.122E-05 | 1.199E-04 | 1.232E-04 | 9.789E-05 | 1.443E-04 | 1.489E-04 | 1.220E-04 | 7.956E-05 |
| Unigene9829 | smeS | 1.184E-05 | 4.789E-06 | 3.596E-06 | 1.386E-05 | 8.789E-05 | 1.194E-04 | 1.225E-04 | 9.662E-05 | 1.480E-04 | 1.491E-04 | 1.264E-04 | 8.370E-05 |
| Unigene9840 | evgA | 4.512E-05 | 2.145E-05 | 1.982E-05 | 2.178E-05 | 1.635E-06 | 1.920E-06 | 3.202E-06 | 8.887E-07 | 7.544E-06 | 6.785E-07 | 3.135E-06 | 1.860E-06 |
| Unigene9849 | tetA(58) | 4.057E-06 | 7.512E-06 | 8.377E-06 | 9.833E-06 | 0.000E+00 | 1.144E-07 | 0.000E+00 | 0.000E+00 | 0.000E+00 | 0.000E+00 | 0.000E+00 | 0.000E+00 |
| Unigene9864 | basS | 1.568E-06 | 2.086E-07 | 6.759E-07 | 1.247E-06 | 1.425E-06 | 3.446E-05 | 4.495E-06 | 0.000E+00 | 1.682E-05 | 0.000E+00 | 8.151E-08 | 3.987E-06 |
| Unigene9867 | PmrF | 1.566E-06 | 2.242E-07 | 3.675E-07 | 1.100E-06 | 1.929E-06 | 8.960E-05 | 3.429E-06 | 0.000E+00 | 1.485E-05 | 5.310E-07 | 0.000E+00 | 2.737E-06 |
| Unigene9882 | bacA | 4.644E-06 | 2.064E-05 | 3.260E-06 | 7.625E-07 | 8.389E-07 | 5.714E-07 | 1.671E-07 | 0.000E+00 | 1.238E-06 | 5.047E-07 | 3.023E-07 | 2.678E-07 |
| Unigene9963 | rphB | 1.115E-05 | 3.769E-06 | 2.762E-06 | 1.089E-05 | 7.039E-05 | 9.916E-05 | 1.015E-04 | 7.973E-05 | 1.233E-04 | 1.256E-04 | 9.981E-05 | 6.913E-05 |
| Unigene9965 | tetA(58) | 1.029E-05 | 3.184E-06 | 2.648E-06 | 1.107E-05 | 6.625E-05 | 9.101E-05 | 8.926E-05 | 7.646E-05 | 1.133E-04 | 1.133E-04 | 9.322E-05 | 6.312E-05 |
| Unigene9966 | tetB(58) | 1.135E-05 | 3.400E-06 | 3.040E-06 | 1.032E-05 | 7.861E-05 | 1.083E-04 | 1.024E-04 | 8.005E-05 | 1.325E-04 | 1.321E-04 | 1.028E-04 | 6.300E-05 |
| Unigene10043 | lmrB | 3.052E-06 | 6.699E-06 | 8.806E-06 | 8.081E-06 | 0.000E+00 | 1.222E-07 | 0.000E+00 | 0.000E+00 | 0.000E+00 | 1.007E-07 | 0.000E+00 | 2.672E-08 |
| Unigene10136 | tetB(60) | 2.233E-06 | 4.092E-06 | 3.133E-06 | 5.330E-06 | 5.331E-07 | 3.990E-08 | 3.473E-07 | 0.000E+00 | 1.009E-07 | 0.000E+00 | 0.000E+00 | 2.836E-07 |
| Unigene10137 | tetA(60) | 2.538E-06 | 3.674E-06 | 3.068E-06 | 6.129E-06 | 4.904E-07 | 1.591E-07 | 2.850E-07 | 0.000E+00 | 4.021E-08 | 0.000E+00 | 0.000E+00 | 6.522E-08 |
| Unigene10177 | efrA | 2.532E-06 | 2.463E-06 | 2.597E-06 | 1.249E-06 | 0.000E+00 | 1.244E-07 | 5.095E-07 | 1.679E-07 | 7.232E-07 | 0.000E+00 | 6.582E-08 | 0.000E+00 |
| Unigene10198 | vanRF | 2.364E-06 | 3.336E-06 | 3.362E-06 | 5.798E-06 | 0.000E+00 | 1.043E-07 | 3.738E-07 | 0.000E+00 | 0.000E+00 | 0.000E+00 | 0.000E+00 | 0.000E+00 |
| Unigene10199 | baeS | 2.438E-06 | 4.125E-06 | 3.007E-06 | 5.241E-06 | 4.970E-07 | 0.000E+00 | 6.190E-07 | 7.833E-08 | 1.956E-07 | 0.000E+00 | 0.000E+00 | 0.000E+00 |
| Unigene10209 | Staphylocc | 2.663E-06 | 3.234E-06 | 2.913E-06 | 4.628E-06 | 4.607E-07 | 0.000E+00 | 2.416E-07 | 0.000E+00 | 1.431E-07 | 0.000E+00 | 9.986E-08 | 1.548E-07 |
| Unigene10215 | VatI | 3.964E-06 | 3.481E-06 | 3.001E-06 | 6.062E-06 | 0.000E+00 | 0.000E+00 | 0.000E+00 | 0.000E+00 | 0.000E+00 | 0.000E+00 | 0.000E+00 | 0.000E+00 |
| Unigene10234 | efpA | 2.510E-05 | 9.608E-06 | 7.046E-06 | 4.144E-06 | 3.292E-07 | 2.711E-07 | 0.000E+00 | 1.064E-07 | 0.000E+00 | 0.000E+00 | 0.000E+00 | 1.617E-07 |
| Unigene10239 | emrB | 2.652E-05 | 9.725E-06 | 7.453E-06 | 4.269E-06 | 4.269E-07 | 1.222E-07 | 0.000E+00 | 1.583E-07 | 9.142E-07 | 0.000E+00 | 0.000E+00 | 2.672E-08 |
| Unigene10243 | tetA(58) | 6.204E-06 | 1.300E-05 | 1.132E-05 | 1.306E-05 | 0.000E+00 | 1.407E-07 | 4.802E-08 | 0.000E+00 | 0.000E+00 | 0.000E+00 | 0.000E+00 | 5.129E-08 |
| Unigene10248 | arlS | 2.240E-05 | 8.466E-06 | 5.956E-06 | 4.337E-06 | 3.408E-07 | 3.080E-07 | 0.000E+00 | 7.674E-08 | 5.749E-07 | 0.000E+00 | 0.000E+00 | 0.000E+00 |
| Unigene10249 | arlR | 2.096E-05 | 1.022E-05 | 7.620E-06 | 4.394E-06 | 0.000E+00 | 1.011E-07 | 0.000E+00 | 0.000E+00 | 4.602E-07 | 0.000E+00 | 0.000E+00 | 0.000E+00 |
| Unigene10292 | Staphylocc | 4.869E-05 | 2.662E-05 | 1.967E-05 | 2.454E-05 | 1.959E-06 | 1.711E-06 | 2.702E-06 | 1.082E-06 | 7.807E-06 | 7.303E-07 | 2.198E-06 | 1.603E-06 |
| Unigene10300 | bcrA | 1.575E-06 | 1.589E-05 | 7.125E-06 | 3.534E-06 | 0.000E+00 | 0.000E+00 | 0.000E+00 | 0.000E+00 | 0.000E+00 | 0.000E+00 | 0.000E+00 | 0.000E+00 |
| Unigene10344 | poxtA | 6.777E-05 | 1.794E-04 | 6.959E-04 | 5.583E-05 | 5.394E-06 | 3.385E-06 | 1.974E-07 | 5.089E-07 | 1.674E-05 | 0.000E+00 | 0.000E+00 | 9.369E-08 |
| Unigene10370 | ErmD | 1.982E-06 | 0.000E+00 | 0.000E+00 | 0.000E+00 | 0.000E+00 | 0.000E+00 | 0.000E+00 | 0.000E+00 | 0.000E+00 | 0.000E+00 | 0.000E+00 | 0.000E+00 |
| Unigene10373 | tlrC | 1.465E-06 | 0.000E+00 | 0.000E+00 | 0.000E+00 | 0.000E+00 | 0.000E+00 | 0.000E+00 | 0.000E+00 | 0.000E+00 | 0.000E+00 | 0.000E+00 | 0.000E+00 |
| Unigene10463 | tva(A) | 1.331E-05 | 0.000E+00 | 0.000E+00 | 0.000E+00 | 1.948E-05 | 1.667E-05 | 0.000E+00 | 0.000E+00 | 1.722E-05 | 0.000E+00 | 0.000E+00 | 0.000E+00 |
| Unigene10477 | blaR1 | 1.542E-06 | 3.567E-06 | 3.477E-06 | 6.230E-06 | 0.000E+00 | 0.000E+00 | 0.000E+00 | 0.000E+00 | 0.000E+00 | 0.000E+00 | 0.000E+00 | 0.000E+00 |

| | | | | | | | | | | | | | |
|---|---|---|---|---|---|---|---|---|---|---|---|---|---|
| Unigene10494 | bmr | 9.888E-07 | 0.000E+00 | 3.995E-07 | 4.111E-07 | 3.845E-06 | 9.620E-06 | 1.609E-06 | 8.283E-06 | 4.418E-06 | 0.000E+00 | 0.000E+00 | 9.969E-07 |
| Unigene10586 | arlR | 1.365E-05 | 1.956E-05 | 4.060E-05 | 4.739E-06 | 1.044E-04 | 5.816E-05 | 1.096E-05 | 8.753E-05 | 3.440E-05 | 5.629E-05 | 4.332E-05 | 1.191E-04 |
| Unigene10587 | baeS | 1.367E-05 | 2.178E-05 | 4.356E-05 | 4.506E-06 | 1.128E-04 | 6.188E-05 | 1.327E-05 | 1.012E-04 | 3.927E-05 | 6.172E-05 | 4.841E-05 | 1.311E-04 |
| Unigene10588 | PmrF | 1.491E-05 | 2.163E-05 | 4.512E-05 | 4.845E-06 | 1.111E-04 | 6.172E-05 | 1.293E-05 | 9.956E-05 | 3.749E-05 | 6.432E-05 | 5.033E-05 | 1.317E-04 |
| Unigene10607 | Corynebac | 7.028E-06 | 0.000E+00 | 0.000E+00 | 2.781E-06 | 3.463E-05 | 1.795E-05 | 2.575E-05 | 2.891E-05 | 3.151E-06 | 0.000E+00 | 0.000E+00 | 0.000E+00 |
| Unigene10725 | oleC | 1.438E-06 | 7.172E-07 | 1.240E-06 | 1.188E-06 | 1.878E-05 | 5.651E-06 | 1.331E-05 | 1.754E-05 | 1.281E-05 | 1.878E-05 | 1.978E-05 | 7.878E-06 |
| Unigene10774 | efrA | 1.331E-06 | 9.876E-07 | 4.911E-06 | 6.285E-06 | 0.000E+00 | 8.106E-08 | 3.734E-07 | 0.000E+00 | 0.000E+00 | 0.000E+00 | 1.287E-07 | 2.216E-08 |
| Unigene10831 | erm(32) | 4.272E-06 | 1.811E-05 | 3.067E-06 | 5.992E-07 | 7.471E-07 | 4.704E-07 | 2.189E-07 | 0.000E+00 | 9.080E-07 | 1.102E-06 | 2.263E-07 | 0.000E+00 |
| Unigene10832 | lmrD | 3.990E-06 | 1.880E-05 | 2.791E-06 | 9.430E-07 | 8.016E-07 | 3.671E-07 | 1.879E-07 | 1.734E-07 | 8.815E-07 | 5.674E-07 | 3.884E-07 | 1.505E-07 |
| Unigene10929 | vanHO | 1.514E-06 | 1.733E-06 | 1.328E-06 | 3.156E-06 | 0.000E+00 | 0.000E+00 | 0.000E+00 | 0.000E+00 | 0.000E+00 | 0.000E+00 | 0.000E+00 | 0.000E+00 |
| Unigene11062 | baeS | 4.231E-05 | 6.615E-05 | 2.797E-05 | 1.484E-05 | 1.241E-05 | 6.760E-06 | 3.197E-06 | 5.958E-05 | 1.356E-05 | 7.885E-06 | 1.380E-06 | 8.629E-07 |
| Unigene11063 | vanRM | 4.088E-05 | 5.945E-05 | 2.491E-05 | 1.282E-05 | 1.211E-05 | 5.568E-06 | 2.850E-06 | 5.732E-05 | 1.101E-05 | 6.762E-06 | 1.473E-06 | 5.979E-07 |
| Unigene11119 | cmx | 4.910E-05 | 6.629E-05 | 2.890E-05 | 1.523E-05 | 1.853E-05 | 6.815E-06 | 3.488E-06 | 5.684E-05 | 1.423E-05 | 7.476E-06 | 1.585E-06 | 9.957E-07 |
| Unigene11167 | TaeA | 3.932E-06 | 1.773E-05 | 2.640E-06 | 6.443E-07 | 9.195E-07 | 3.169E-07 | 1.145E-07 | 0.000E+00 | 8.858E-07 | 5.186E-07 | 1.381E-07 | 1.019E-07 |
| Unigene11176 | lmrB | 4.910E-06 | 1.792E-05 | 3.435E-06 | 8.304E-07 | 9.396E-07 | 3.810E-07 | 2.600E-07 | 1.645E-07 | 7.447E-07 | 9.422E-07 | 5.644E-07 | 0.000E+00 |
| Unigene11188 | macB | 4.721E-06 | 2.293E-05 | 3.822E-06 | 8.343E-07 | 1.224E-06 | 5.742E-07 | 4.572E-07 | 8.725E-07 | 1.613E-06 | 8.546E-07 | 1.508E-06 | 6.510E-07 |
| Unigene11196 | lsaC | 6.268E-07 | 3.841E-06 | 4.100E-07 | 4.747E-07 | 3.482E-07 | 3.953E-07 | 1.156E-07 | 1.219E-07 | 7.993E-07 | 2.327E-07 | 5.377E-07 | 0.000E+00 |
| Unigene11217 | Streptomy | 1.159E-06 | 9.811E-07 | 1.494E-06 | 2.173E-06 | 1.938E-05 | 7.051E-06 | 1.610E-05 | 1.648E-05 | 1.196E-05 | 2.038E-05 | 2.149E-05 | 7.083E-06 |
| Unigene11264 | lsaA | 1.815E-06 | 1.549E-05 | 6.488E-06 | 4.800E-06 | 0.000E+00 | 0.000E+00 | 7.174E-08 | 0.000E+00 | 0.000E+00 | 0.000E+00 | 0.000E+00 | 0.000E+00 |
| Unigene11288 | efrA | 1.604E-06 | 1.703E-05 | 6.862E-06 | 3.860E-06 | 0.000E+00 | 0.000E+00 | 6.192E-08 | 0.000E+00 | 0.000E+00 | 0.000E+00 | 1.707E-07 | 0.000E+00 |
| Unigene11289 | efrB | 1.961E-06 | 1.835E-05 | 6.475E-06 | 4.165E-06 | 4.073E-08 | 9.908E-08 | 8.115E-08 | 0.000E+00 | 0.000E+00 | 8.168E-08 | 1.258E-07 | 0.000E+00 |
| Unigene11303 | baeS | 1.592E-06 | 1.101E-06 | 1.630E-06 | 1.852E-06 | 2.106E-05 | 6.284E-06 | 1.382E-05 | 1.720E-05 | 1.142E-05 | 1.850E-05 | 1.931E-05 | 6.666E-06 |
| Unigene11304 | smeR | 1.722E-06 | 8.398E-07 | 1.689E-06 | 1.980E-06 | 2.152E-05 | 5.790E-06 | 1.434E-05 | 1.742E-05 | 1.145E-05 | 2.264E-05 | 1.887E-05 | 8.083E-06 |
| Unigene11305 | macB | 1.047E-06 | 8.271E-07 | 1.931E-06 | 1.437E-06 | 1.800E-05 | 5.386E-06 | 1.196E-05 | 1.378E-05 | 8.878E-06 | 1.660E-05 | 1.494E-05 | 6.434E-06 |
| Unigene11315 | bacA | 1.118E-06 | 8.889E-07 | 1.627E-06 | 1.129E-06 | 2.074E-05 | 6.796E-06 | 1.460E-05 | 1.684E-05 | 1.197E-05 | 1.654E-05 | 1.672E-05 | 8.251E-06 |
| Unigene11338 | macB | 3.927E-05 | 2.231E-05 | 1.493E-05 | 1.621E-05 | 2.197E-06 | 1.243E-06 | 2.952E-06 | 9.661E-07 | 6.634E-06 | 0.000E+00 | 0.000E+00 | 1.087E-06 |
| Unigene11418 | tetA(58) | 1.761E-06 | 2.523E-06 | 4.895E-06 | 2.223E-06 | 0.000E+00 | 0.000E+00 | 5.413E-08 | 0.000E+00 | 0.000E+00 | 1.090E-07 | 0.000E+00 | 0.000E+00 |
| Unigene11432 | tet32 | 2.450E-05 | 9.206E-06 | 6.845E-06 | 3.293E-06 | 5.037E-07 | 0.000E+00 | 5.790E-08 | 1.221E-07 | 1.220E-06 | 0.000E+00 | 0.000E+00 | 6.184E-08 |
| Unigene11524 | lmrB | 1.051E-05 | 0.000E+00 | 0.000E+00 | 0.000E+00 | 1.745E-05 | 1.730E-05 | 0.000E+00 | 0.000E+00 | 1.069E-05 | 0.000E+00 | 0.000E+00 | 0.000E+00 |
| Unigene11525 | mdtG | 1.014E-05 | 0.000E+00 | 0.000E+00 | 0.000E+00 | 1.551E-05 | 1.638E-05 | 0.000E+00 | 0.000E+00 | 1.048E-05 | 3.764E-07 | 0.000E+00 | 0.000E+00 |
| Unigene11645 | Staphylocc | 4.687E-05 | 6.538E-05 | 2.754E-05 | 1.307E-05 | 1.596E-05 | 5.875E-06 | 3.148E-06 | 5.193E-05 | 1.029E-05 | 6.472E-06 | 1.717E-06 | 9.872E-07 |
| Unigene11661 | bcrA | 4.133E-05 | 6.346E-05 | 3.069E-05 | 1.395E-05 | 1.940E-05 | 5.957E-06 | 2.884E-06 | 6.036E-05 | 1.298E-05 | 7.632E-06 | 1.448E-06 | 1.716E-06 |
| Unigene11664 | bcrA | 4.242E-05 | 6.582E-05 | 3.026E-05 | 1.348E-05 | 1.659E-05 | 5.625E-06 | 3.384E-06 | 5.703E-05 | 1.202E-05 | 6.976E-06 | 1.614E-06 | 1.460E-06 |
| Unigene11679 | macB | 4.916E-05 | 6.637E-05 | 3.014E-05 | 1.467E-05 | 1.875E-05 | 7.208E-06 | 3.461E-06 | 6.054E-05 | 1.116E-05 | 5.891E-06 | 1.525E-06 | 1.033E-06 |
| Unigene11690 | patB | 4.654E-05 | 6.206E-05 | 2.804E-05 | 1.333E-05 | 1.741E-05 | 6.122E-06 | 3.286E-06 | 5.380E-05 | 1.086E-05 | 6.901E-06 | 1.963E-06 | 8.520E-07 |
| Unigene11691 | efrA | 4.660E-05 | 6.477E-05 | 2.982E-05 | 1.441E-05 | 1.935E-05 | 6.859E-06 | 3.655E-06 | 5.827E-05 | 1.259E-05 | 6.200E-06 | 2.271E-06 | 1.184E-06 |
| Unigene11704 | vanSE | 4.716E-05 | 7.056E-05 | 2.840E-05 | 1.350E-05 | 2.062E-05 | 6.213E-06 | 3.550E-06 | 5.973E-05 | 1.286E-05 | 7.917E-06 | 1.605E-06 | 1.053E-06 |
| Unigene11705 | vanRF | 4.690E-05 | 6.572E-05 | 2.820E-05 | 1.284E-05 | 1.644E-05 | 6.871E-06 | 3.919E-06 | 5.580E-05 | 1.166E-05 | 6.322E-06 | 2.233E-06 | 8.050E-07 |
| Unigene11731 | vanHO | 4.811E-05 | 6.970E-05 | 3.094E-05 | 1.410E-05 | 2.160E-05 | 6.700E-06 | 3.882E-06 | 5.954E-05 | 1.269E-05 | 7.700E-06 | 1.831E-06 | 7.647E-07 |
| Unigene11741 | tetB(60) | 4.471E-05 | 7.245E-05 | 2.574E-05 | 1.244E-05 | 1.801E-05 | 5.455E-06 | 3.860E-06 | 6.089E-05 | 1.184E-05 | 6.117E-06 | 1.740E-06 | 1.140E-06 |
| Unigene11742 | tetB(46) | 4.453E-05 | 6.291E-05 | 2.608E-05 | 1.377E-05 | 1.880E-05 | 5.257E-06 | 3.306E-06 | 5.609E-05 | 1.076E-05 | 5.929E-06 | 1.840E-06 | 9.958E-07 |
| Unigene11769 | tetT | 4.871E-05 | 6.553E-05 | 2.721E-05 | 1.407E-05 | 1.759E-05 | 5.683E-06 | 2.969E-06 | 5.378E-05 | 1.353E-05 | 7.075E-06 | 1.887E-06 | 1.221E-06 |
| Unigene11775 | rpoB2 | 4.721E-05 | 6.601E-05 | 2.703E-05 | 1.509E-05 | 1.829E-05 | 6.038E-06 | 2.785E-06 | 5.073E-05 | 1.210E-05 | 6.252E-06 | 1.817E-06 | 9.281E-07 |
| Unigene11793 | Staphylocc | 6.351E-05 | 5.095E-05 | 1.043E-04 | 1.526E-04 | 1.106E-04 | 7.913E-05 | 1.056E-04 | 9.803E-05 | 8.544E-05 | 1.264E-04 | 1.245E-04 | 1.238E-04 |
| Unigene11848 | tetA(58) | 4.921E-06 | 1.080E-05 | 9.729E-06 | 1.116E-05 | 1.632E-07 | 0.000E+00 | 1.219E-07 | 0.000E+00 | 0.000E+00 | 0.000E+00 | 0.000E+00 | 0.000E+00 |
| Unigene11855 | vgaB | 3.201E-05 | 0.000E+00 | 0.000E+00 | 0.000E+00 | 0.000E+00 | 0.000E+00 | 0.000E+00 | 0.000E+00 | 0.000E+00 | 0.000E+00 | 0.000E+00 | 0.000E+00 |
| Unigene11909 | optrA | 2.832E-06 | 4.880E-06 | 3.334E-06 | 5.465E-06 | 5.126E-07 | 1.134E-07 | 2.321E-07 | 1.469E-07 | 1.376E-07 | 0.000E+00 | 0.000E+00 | 0.000E+00 |
| Unigene11912 | patB | 2.876E-06 | 3.565E-06 | 2.623E-06 | 5.706E-06 | 3.759E-07 | 0.000E+00 | 1.498E-07 | 0.000E+00 | 0.000E+00 | 0.000E+00 | 0.000E+00 | 1.600E-07 |
| Unigene11935 | efrB | 0.000E+00 | 0.000E+00 | 0.000E+00 | 0.000E+00 | 6.580E-07 | 0.000E+00 | 0.000E+00 | 0.000E+00 | 0.000E+00 | 0.000E+00 | 0.000E+00 | 5.834E-07 |
| Unigene12031 | lmrD | 2.430E-05 | 8.180E-06 | 7.183E-06 | 4.052E-06 | 1.960E-07 | 0.000E+00 | 0.000E+00 | 0.000E+00 | 0.000E+00 | 0.000E+00 | 0.000E+00 | 0.000E+00 |
| Unigene12043 | vgaA | 1.571E-06 | 1.390E-05 | 5.279E-06 | 3.414E-06 | 0.000E+00 | 0.000E+00 | 0.000E+00 | 0.000E+00 | 0.000E+00 | 0.000E+00 | 0.000E+00 | 0.000E+00 |
| Unigene12094 | macB | 5.614E-07 | 7.188E-07 | 4.591E-08 | 9.921E-07 | 5.197E-08 | 0.000E+00 | 0.000E+00 | 0.000E+00 | 0.000E+00 | 0.000E+00 | 0.000E+00 | 0.000E+00 |
| Unigene12138 | patB | 9.764E-05 | 4.285E-05 | 4.799E-07 | 0.000E+00 | 2.067E-05 | 8.918E-05 | 0.000E+00 | 0.000E+00 | 0.000E+00 | 0.000E+00 | 4.662E-08 | 0.000E+00 |
| Unigene12162 | tetB(60) | 9.273E-05 | 3.669E-05 | 0.000E+00 | 0.000E+00 | 1.897E-05 | 7.548E-05 | 0.000E+00 | 0.000E+00 | 0.000E+00 | 0.000E+00 | 0.000E+00 | 0.000E+00 |
| Unigene12177 | efrA | 9.111E-05 | 4.120E-05 | 2.387E-07 | 0.000E+00 | 1.987E-05 | 8.699E-05 | 4.142E-08 | 0.000E+00 | 0.000E+00 | 8.338E-08 | 0.000E+00 | 1.327E-07 |
| Unigene12178 | patB | 9.627E-05 | 4.209E-05 | 1.523E-07 | 1.741E-08 | 1.933E-05 | 7.654E-05 | 0.000E+00 | 0.000E+00 | 0.000E+00 | 0.000E+00 | 7.890E-08 | 0.000E+00 |
| Unigene12181 | vanRF | 9.722E-05 | 4.311E-05 | 5.072E-07 | 9.490E-08 | 2.161E-05 | 8.772E-05 | 0.000E+00 | 0.000E+00 | 0.000E+00 | 1.047E-07 | 0.000E+00 | 0.000E+00 |
| Unigene12182 | vanSA | 9.245E-05 | 4.055E-05 | 2.742E-07 | 0.000E+00 | 1.953E-05 | 8.490E-05 | 6.186E-08 | 0.000E+00 | 0.000E+00 | 0.000E+00 | 0.000E+00 | 0.000E+00 |

| | | | | | | | | | | | | | |
|---|---|---|---|---|---|---|---|---|---|---|---|---|---|
| Unigene12223 | Staphylocc | 9.352E-05 | 4.176E-05 | 2.331E-07 | 4.797E-08 | 2.151E-05 | 8.212E-05 | 5.258E-08 | 0.000E+00 | 0.000E+00 | 0.000E+00 | 1.087E-07 | 0.000E+00 |
| Unigene12254 | macB | 9.488E-05 | 4.202E-05 | 7.875E-07 | 0.000E+00 | 1.928E-05 | 7.815E-05 | 0.000E+00 | 0.000E+00 | 0.000E+00 | 1.324E-07 | 0.000E+00 | 1.405E-07 |
| Unigene12284 | baeS | 6.321E-05 | 5.448E-05 | 1.021E-04 | 6.818E-05 | 1.263E-04 | 8.352E-05 | 1.045E-04 | 1.108E-04 | 1.008E-04 | 1.378E-04 | 1.410E-04 | 1.545E-04 |
| Unigene12285 | arlR | 6.281E-05 | 5.657E-05 | 1.012E-04 | 6.501E-05 | 1.223E-04 | 8.396E-05 | 1.023E-04 | 1.144E-04 | 9.434E-05 | 1.271E-04 | 1.412E-04 | 1.509E-04 |
| Unigene12310 | bcr-1 | 6.892E-07 | 9.760E-08 | 0.000E+00 | 1.122E-07 | 0.000E+00 | 2.884E-07 | 0.000E+00 | 0.000E+00 | 0.000E+00 | 0.000E+00 | 0.000E+00 | 1.051E-07 |
| Unigene12402 | efrA | 3.232E-06 | 4.572E-06 | 4.051E-06 | 5.886E-06 | 4.566E-07 | 0.000E+00 | 3.308E-07 | 6.542E-08 | 2.450E-07 | 0.000E+00 | 1.496E-07 | 1.104E-07 |
| Unigene12403 | patB | 2.677E-06 | 4.822E-06 | 4.109E-06 | 5.892E-06 | 4.194E-07 | 0.000E+00 | 3.989E-07 | 0.000E+00 | 2.438E-07 | 0.000E+00 | 1.374E-07 | 1.014E-07 |
| Unigene12473 | macB | 4.187E-06 | 5.504E-06 | 5.828E-06 | 4.277E-06 | 0.000E+00 | 1.579E-07 | 0.000E+00 | 0.000E+00 | 0.000E+00 | 2.169E-07 | 1.114E-07 | 0.000E+00 |
| Unigene12549 | FPH-1 | 4.977E-05 | 7.327E-05 | 3.267E-05 | 1.749E-05 | 2.022E-05 | 7.596E-06 | 3.342E-06 | 5.918E-05 | 1.299E-05 | 8.287E-06 | 1.382E-06 | 1.006E-06 |
| Unigene12551 | optrA | 5.636E-05 | 7.513E-05 | 3.069E-05 | 1.655E-05 | 2.376E-05 | 5.742E-06 | 4.510E-06 | 5.783E-05 | 1.346E-05 | 6.835E-06 | 1.886E-06 | 1.137E-06 |
| Unigene12555 | vatB | 4.641E-05 | 6.671E-05 | 2.494E-05 | 1.298E-05 | 1.725E-05 | 4.716E-06 | 3.283E-06 | 4.698E-05 | 1.055E-05 | 4.600E-06 | 1.730E-06 | 5.500E-07 |
| Unigene12571 | Klebsiella p | 4.555E-05 | 6.468E-05 | 2.842E-05 | 1.340E-05 | 0.000E+00 | 5.762E-06 | 3.561E-06 | 5.176E-05 | 1.319E-05 | 5.825E-06 | 0.000E+00 | 8.321E-07 |
| Unigene12585 | lsaA | 5.152E-05 | 6.575E-05 | 2.941E-05 | 1.554E-05 | 1.985E-05 | 6.180E-06 | 3.392E-06 | 5.800E-05 | 1.254E-05 | 7.071E-06 | 1.939E-06 | 1.131E-06 |
| Unigene12596 | Streptomy | 5.040E-05 | 7.138E-05 | 3.065E-05 | 1.572E-05 | 2.092E-05 | 5.986E-06 | 3.303E-06 | 5.595E-05 | 1.346E-05 | 6.723E-06 | 1.536E-06 | 1.274E-06 |
| Unigene12636 | macB | 5.196E-05 | 7.358E-05 | 3.048E-05 | 1.624E-05 | 2.141E-05 | 6.855E-06 | 3.281E-06 | 5.692E-05 | 1.370E-05 | 6.406E-06 | 2.057E-06 | 1.054E-06 |
| Unigene12646 | efpA | 4.918E-05 | 7.709E-05 | 3.326E-05 | 1.709E-05 | 2.319E-05 | 6.896E-06 | 4.316E-06 | 5.979E-05 | 1.555E-05 | 6.570E-06 | 2.313E-06 | 1.167E-06 |
| Unigene12653 | mtrA | 4.552E-05 | 6.203E-05 | 2.734E-05 | 1.305E-05 | 1.783E-05 | 6.015E-06 | 3.461E-06 | 5.528E-05 | 1.226E-05 | 5.635E-06 | 1.788E-06 | 1.141E-06 |
| Unigene12654 | arlS | 5.069E-05 | 7.396E-05 | 3.020E-05 | 1.542E-05 | 2.106E-05 | 6.269E-06 | 4.060E-06 | 5.760E-05 | 1.352E-05 | 6.791E-06 | 1.998E-06 | 1.404E-06 |
| Unigene12659 | emrB | 5.015E-05 | 7.583E-05 | 2.946E-05 | 1.560E-05 | 1.945E-05 | 6.340E-06 | 3.196E-06 | 5.714E-05 | 1.282E-05 | 5.704E-06 | 2.142E-06 | 8.799E-07 |
| Unigene12666 | RlmA(II) | 5.157E-05 | 7.212E-05 | 3.122E-05 | 1.630E-05 | 2.045E-05 | 6.979E-06 | 4.211E-06 | 6.311E-05 | 1.365E-05 | 7.492E-06 | 2.242E-06 | 1.090E-06 |
| Unigene12710 | Listeria mc | 2.961E-06 | 3.070E-06 | 2.745E-06 | 1.858E-06 | 2.100E-07 | 1.090E-07 | 3.765E-07 | 2.941E-07 | 6.472E-07 | 0.000E+00 | 0.000E+00 | 1.489E-07 |
| Unigene12780 | tetT | 1.023E-06 | 1.370E-06 | 1.847E-06 | 1.653E-06 | 2.150E-05 | 6.944E-06 | 1.501E-05 | 1.714E-05 | 1.071E-05 | 1.857E-05 | 1.938E-05 | 7.230E-06 |
| Unigene12787 | macB | 1.196E-06 | 1.078E-06 | 1.760E-06 | 1.294E-06 | 1.788E-05 | 6.555E-06 | 1.266E-05 | 1.655E-05 | 1.167E-05 | 1.555E-05 | 1.670E-05 | 7.823E-06 |
| Unigene12792 | facT | 1.347E-06 | 6.778E-07 | 1.340E-06 | 1.852E-06 | 1.873E-05 | 5.907E-06 | 1.361E-05 | 1.697E-05 | 1.141E-05 | 1.887E-05 | 1.971E-05 | 6.946E-06 |
| Unigene12805 | vatH | 2.665E-06 | 4.691E-06 | 3.898E-06 | 7.972E-06 | 0.000E+00 | 0.000E+00 | 2.226E-07 | 0.000E+00 | 0.000E+00 | 0.000E+00 | 0.000E+00 | 0.000E+00 |
| Unigene12836 | lsaA | 3.155E-06 | 4.838E-06 | 3.731E-06 | 6.087E-06 | 4.561E-07 | 0.000E+00 | 3.587E-07 | 2.522E-08 | 1.417E-07 | 0.000E+00 | 0.000E+00 | 7.662E-08 |
| Unigene12837 | tetA(46) | 3.066E-06 | 5.410E-06 | 3.156E-06 | 7.618E-06 | 5.343E-07 | 6.498E-08 | 7.651E-07 | 0.000E+00 | 3.285E-07 | 1.004E-07 | 0.000E+00 | 1.421E-07 |
| Unigene12868 | tet32 | 3.727E-06 | 3.087E-07 | 3.468E-06 | 6.214E-07 | 2.735E-07 | 7.603E-08 | 4.670E-07 | 0.000E+00 | 0.000E+00 | 0.000E+00 | 8.045E-07 | 0.000E+00 |
| Unigene12901 | tetT | 3.121E-06 | 4.102E-06 | 2.478E-06 | 1.597E-06 | 2.897E-06 | 2.175E-06 | 2.364E-06 | 1.103E-06 | 1.257E-06 | 5.650E-06 | 6.060E-06 | 1.820E-06 |
| Unigene12905 | efrB | 2.498E-06 | 4.137E-06 | 3.699E-06 | 4.957E-06 | 4.626E-07 | 2.132E-07 | 2.183E-07 | 7.674E-08 | 4.072E-07 | 1.221E-07 | 2.507E-08 | 5.181E-08 |
| Unigene12909 | vanSO | 2.714E-06 | 3.990E-06 | 3.163E-06 | 4.943E-06 | 4.308E-07 | 1.310E-07 | 5.633E-07 | 0.000E+00 | 1.060E-07 | 0.000E+00 | 8.316E-08 | 2.865E-07 |
| Unigene12910 | arlR | 3.430E-06 | 4.435E-06 | 4.103E-06 | 6.738E-06 | 0.000E+00 | 0.000E+00 | 0.000E+00 | 0.000E+00 | 0.000E+00 | 0.000E+00 | 0.000E+00 | 0.000E+00 |
| Unigene12913 | vgaALC | 2.535E-06 | 4.041E-06 | 3.191E-06 | 5.119E-06 | 5.844E-07 | 0.000E+00 | 4.763E-07 | 0.000E+00 | 0.000E+00 | 0.000E+00 | 0.000E+00 | 5.653E-08 |
| Unigene12918 | vanRA | 4.495E-06 | 4.356E-06 | 3.754E-06 | 5.602E-06 | 3.188E-07 | 3.102E-07 | 3.176E-07 | 0.000E+00 | 0.000E+00 | 0.000E+00 | 0.000E+00 | 0.000E+00 |
| Unigene12921 | patB | 3.506E-06 | 4.697E-06 | 3.694E-06 | 6.422E-06 | 4.010E-07 | 0.000E+00 | 4.375E-07 | 0.000E+00 | 1.691E-07 | 0.000E+00 | 7.865E-08 | 1.422E-07 |
| Unigene12922 | patA | 3.041E-06 | 4.838E-06 | 3.054E-06 | 5.324E-06 | 6.397E-07 | 5.836E-08 | 3.385E-07 | 6.301E-08 | 9.833E-08 | 0.000E+00 | 6.175E-08 | 1.914E-07 |
| Unigene12924 | macB | 3.969E-06 | 4.724E-06 | 3.332E-06 | 6.230E-06 | 3.719E-07 | 0.000E+00 | 6.351E-07 | 0.000E+00 | 0.000E+00 | 0.000E+00 | 0.000E+00 | 0.000E+00 |
| Unigene12937 | bcrA | 2.946E-06 | 4.499E-06 | 3.100E-06 | 7.718E-06 | 4.744E-07 | 0.000E+00 | 3.781E-07 | 0.000E+00 | 0.000E+00 | 0.000E+00 | 0.000E+00 | 0.000E+00 |
| Unigene12980 | cmrA | 3.013E-07 | 0.000E+00 | 2.534E-07 | 0.000E+00 | 0.000E+00 | 0.000E+00 | 0.000E+00 | 0.000E+00 | 0.000E+00 | 0.000E+00 | 0.000E+00 | 0.000E+00 |
| Unigene13003 | TaeA | 4.437E-05 | 2.360E-05 | 1.583E-05 | 1.884E-05 | 2.092E-06 | 1.546E-06 | 2.876E-06 | 7.939E-07 | 7.205E-06 | 9.325E-07 | 1.835E-06 | 1.402E-06 |
| Unigene13030 | bcrA | 7.765E-07 | 8.522E-07 | 3.628E-06 | 6.161E-06 | 0.000E+00 | 3.997E-08 | 2.046E-07 | 0.000E+00 | 0.000E+00 | 0.000E+00 | 4.230E-08 | 0.000E+00 |
| Unigene13058 | efpA | 2.507E-06 | 2.232E-06 | 1.555E-06 | 5.063E-06 | 3.628E-05 | 6.159E-05 | 6.682E-05 | 6.081E-05 | 3.301E-05 | 2.698E-05 | 1.523E-05 | 4.948E-06 |
| Unigene13086 | tetA(58) | 2.442E-06 | 2.524E-06 | 2.256E-06 | 2.609E-06 | 4.416E-07 | 0.000E+00 | 3.771E-07 | 0.000E+00 | 2.793E-07 | 6.325E-08 | 0.000E+00 | 2.685E-07 |
| Unigene13090 | evgA | 1.567E-06 | 2.981E-06 | 1.333E-06 | 2.532E-06 | 0.000E+00 | 0.000E+00 | 2.312E-07 | 0.000E+00 | 5.709E-08 | 0.000E+00 | 1.195E-07 | 1.235E-07 |
| Unigene13113 | msbA | 7.880E-06 | 0.000E+00 | 0.000E+00 | 2.433E-06 | 3.423E-05 | 1.606E-05 | 2.201E-05 | 2.375E-05 | 3.584E-06 | 0.000E+00 | 2.072E-08 | 0.000E+00 |
| Unigene13150 | macB | 1.869E-06 | 3.303E-06 | 4.474E-06 | 2.256E-06 | 5.065E-08 | 0.000E+00 | 0.000E+00 | 0.000E+00 | 0.000E+00 | 0.000E+00 | 0.000E+00 | 0.000E+00 |
| Unigene13151 | arlR | 2.376E-06 | 5.163E-06 | 4.936E-06 | 2.681E-06 | 1.552E-07 | 2.014E-07 | 0.000E+00 | 0.000E+00 | 0.000E+00 | 0.000E+00 | 0.000E+00 | 0.000E+00 |
| Unigene13152 | baeS | 1.853E-06 | 1.369E-06 | 2.797E-06 | 1.223E-06 | 0.000E+00 | 0.000E+00 | 0.000E+00 | 0.000E+00 | 0.000E+00 | 2.778E-07 | 0.000E+00 | 0.000E+00 |
| Unigene13158 | bcrA | 4.763E-06 | 8.775E-06 | 9.014E-06 | 1.074E-05 | 0.000E+00 | 0.000E+00 | 0.000E+00 | 0.000E+00 | 0.000E+00 | 0.000E+00 | 0.000E+00 | 0.000E+00 |
| Unigene13190 | vanYM | 2.871E-05 | 8.925E-06 | 7.778E-06 | 5.308E-06 | 5.019E-07 | 0.000E+00 | 0.000E+00 | 9.588E-08 | 0.000E+00 | 0.000E+00 | 0.000E+00 | 0.000E+00 |
| Unigene13194 | rpoB2 | 2.637E-05 | 1.109E-05 | 7.906E-06 | 4.260E-06 | 4.530E-07 | 2.970E-07 | 1.177E-07 | 6.207E-08 | 1.724E-06 | 0.000E+00 | 0.000E+00 | 7.334E-08 |
| Unigene13219 | tet32 | 1.136E-05 | 3.686E-06 | 3.082E-06 | 1.417E-05 | 8.556E-05 | 1.207E-04 | 1.192E-04 | 9.860E-05 | 1.528E-04 | 1.520E-04 | 1.263E-04 | 7.785E-05 |
| Unigene13282 | cdeA | 4.665E-06 | 9.014E-06 | 8.199E-06 | 9.912E-06 | 6.358E-08 | 6.187E-08 | 0.000E+00 | 0.000E+00 | 0.000E+00 | 0.000E+00 | 0.000E+00 | 0.000E+00 |
| Unigene13324 | vanHD | 1.185E-05 | 4.506E-06 | 3.671E-06 | 1.314E-05 | 8.647E-05 | 1.181E-04 | 1.239E-04 | 9.766E-05 | 1.493E-04 | 1.575E-04 | 1.254E-04 | 7.169E-05 |
| Unigene13344 | vmlR | 2.905E-06 | 1.823E-05 | 7.266E-06 | 5.040E-06 | 0.000E+00 | 0.000E+00 | 0.000E+00 | 0.000E+00 | 0.000E+00 | 0.000E+00 | 1.404E-07 | 0.000E+00 |
| Unigene13348 | cdeA | 2.316E-06 | 1.851E-05 | 6.679E-06 | 4.039E-06 | 0.000E+00 | 1.264E-07 | 0.000E+00 | 0.000E+00 | 0.000E+00 | 0.000E+00 | 0.000E+00 | 0.000E+00 |
| Unigene13385 | efmA | 3.113E-06 | 4.418E-06 | 6.688E-06 | 6.937E-06 | 0.000E+00 | 2.901E-08 | 8.909E-08 | 3.132E-08 | 0.000E+00 | 1.196E-07 | 0.000E+00 | 0.000E+00 |
| Unigene13420 | arlR | 5.263E-06 | 1.939E-05 | 1.913E-05 | 2.192E-05 | 0.000E+00 | 1.524E-07 | 8.321E-07 | 0.000E+00 | 0.000E+00 | 0.000E+00 | 0.000E+00 | 0.000E+00 |

| | | | | | | | | | | | | | |
|---|---|---|---|---|---|---|---|---|---|---|---|---|---|
| Unigene13421 | arlS | 1.029E-06 | 1.600E-06 | 5.258E-06 | 6.926E-06 | 0.000E+00 | 1.390E-07 | 3.796E-07 | 2.502E-08 | 0.000E+00 | 0.000E+00 | 0.000E+00 | 0.000E+00 |
| Unigene13425 | oleC | 7.507E-05 | 7.031E-05 | 1.270E-04 | 1.322E-04 | 1.414E-04 | 1.038E-04 | 1.390E-04 | 1.288E-04 | 1.173E-04 | 1.569E-04 | 1.821E-04 | 1.822E-04 |
| Unigene13473 | Staphylocc | 1.295E-05 | 4.504E-06 | 3.780E-06 | 1.209E-05 | 8.958E-05 | 1.247E-04 | 1.311E-04 | 1.032E-04 | 1.583E-04 | 1.575E-04 | 1.296E-04 | 7.664E-05 |
| Unigene13542 | patA | 0.000E+00 | 0.000E+00 | 2.308E-07 | 0.000E+00 | 0.000E+00 | 0.000E+00 | 3.254E-07 | 0.000E+00 | 0.000E+00 | 0.000E+00 | 0.000E+00 | 0.000E+00 |
| Unigene13654 | bcrA | 1.970E-06 | 4.684E-06 | 4.044E-06 | 7.293E-06 | 4.457E-07 | 3.943E-08 | 2.826E-07 | 0.000E+00 | 7.973E-08 | 0.000E+00 | 1.669E-07 | 0.000E+00 |
| Unigene13677 | vanRB | 7.826E-07 | 4.831E-06 | 4.607E-06 | 6.174E-06 | 0.000E+00 | 0.000E+00 | 0.000E+00 | 0.000E+00 | 0.000E+00 | 0.000E+00 | 0.000E+00 | 0.000E+00 |
| Unigene13678 | adeS | 1.201E-06 | 3.378E-06 | 2.578E-06 | 5.231E-06 | 0.000E+00 | 0.000E+00 | 0.000E+00 | 0.000E+00 | 0.000E+00 | 0.000E+00 | 0.000E+00 | 0.000E+00 |
| Unigene13681 | macB | 1.509E-06 | 3.741E-06 | 3.153E-06 | 6.588E-06 | 0.000E+00 | 0.000E+00 | 0.000E+00 | 0.000E+00 | 0.000E+00 | 0.000E+00 | 0.000E+00 | 0.000E+00 |
| Unigene13682 | macB | 1.864E-06 | 4.326E-06 | 3.894E-06 | 6.858E-06 | 2.959E-08 | 0.000E+00 | 0.000E+00 | 0.000E+00 | 0.000E+00 | 0.000E+00 | 0.000E+00 | 0.000E+00 |
| Unigene13740 | optrA | 2.753E-05 | 1.012E-05 | 7.705E-06 | 4.894E-06 | 5.494E-07 | 2.138E-07 | 8.756E-08 | 0.000E+00 | 1.578E-06 | 0.000E+00 | 0.000E+00 | 4.676E-08 |
| Unigene13744 | lmrD | 4.300E-05 | 2.332E-05 | 1.963E-05 | 2.143E-05 | 1.711E-06 | 2.440E-06 | 3.339E-06 | 7.142E-07 | 7.356E-06 | 9.166E-07 | 1.786E-06 | 1.945E-06 |
| Unigene13752 | macB | 1.461E-06 | 0.000E+00 | 0.000E+00 | 0.000E+00 | 0.000E+00 | 0.000E+00 | 0.000E+00 | 0.000E+00 | 0.000E+00 | 0.000E+00 | 0.000E+00 | 0.000E+00 |
| Unigene13771 | vanRF | 1.118E-06 | 0.000E+00 | 0.000E+00 | 0.000E+00 | 0.000E+00 | 0.000E+00 | 0.000E+00 | 0.000E+00 | 0.000E+00 | 0.000E+00 | 0.000E+00 | 0.000E+00 |
| Unigene13772 | vanSA | 1.546E-06 | 0.000E+00 | 0.000E+00 | 0.000E+00 | 0.000E+00 | 6.427E-08 | 0.000E+00 | 0.000E+00 | 0.000E+00 | 0.000E+00 | 0.000E+00 | 0.000E+00 |
| Unigene13777 | patB | 1.920E-06 | 0.000E+00 | 0.000E+00 | 0.000E+00 | 0.000E+00 | 0.000E+00 | 0.000E+00 | 0.000E+00 | 0.000E+00 | 0.000E+00 | 3.093E-08 | 0.000E+00 |
| Unigene13781 | tetA(58) | 1.300E-06 | 0.000E+00 | 0.000E+00 | 0.000E+00 | 0.000E+00 | 7.807E-08 | 0.000E+00 | 0.000E+00 | 0.000E+00 | 0.000E+00 | 0.000E+00 | 0.000E+00 |
| Unigene13787 | arlS | 3.071E-06 | 4.347E-06 | 3.097E-06 | 6.160E-06 | 6.824E-07 | 4.580E-08 | 4.688E-07 | 2.472E-08 | 1.389E-07 | 0.000E+00 | 1.211E-07 | 1.502E-07 |
| Unigene13794 | pmrA | 2.971E-06 | 4.025E-06 | 3.362E-06 | 5.433E-06 | 3.568E-07 | 0.000E+00 | 2.666E-07 | 0.000E+00 | 2.048E-07 | 8.945E-08 | 0.000E+00 | 6.328E-08 |
| Unigene13834 | bcrA | 6.758E-05 | 1.391E-04 | 1.851E-04 | 7.847E-06 | 8.907E-06 | 3.801E-07 | 2.024E-06 | 1.888E-06 | 6.222E-05 | 6.267E-07 | 2.695E-06 | 6.650E-07 |
| Unigene13855 | optrA | 8.826E-05 | 3.982E-05 | 2.631E-07 | 6.770E-08 | 1.843E-05 | 7.302E-05 | 0.000E+00 | 0.000E+00 | 0.000E+00 | 0.000E+00 | 9.586E-08 | 0.000E+00 |
| Unigene13879 | patB | 8.903E-05 | 3.880E-05 | 0.000E+00 | 0.000E+00 | 1.769E-05 | 7.990E-05 | 0.000E+00 | 0.000E+00 | 0.000E+00 | 0.000E+00 | 9.769E-08 | 0.000E+00 |
| Unigene13972 | vanZF | 1.636E-06 | 0.000E+00 | 0.000E+00 | 0.000E+00 | 0.000E+00 | 0.000E+00 | 0.000E+00 | 0.000E+00 | 0.000E+00 | 0.000E+00 | 0.000E+00 | 0.000E+00 |
| Unigene13977 | vanL | 1.594E-06 | 0.000E+00 | 0.000E+00 | 0.000E+00 | 0.000E+00 | 9.232E-08 | 0.000E+00 | 0.000E+00 | 0.000E+00 | 0.000E+00 | 0.000E+00 | 0.000E+00 |
| Unigene14003 | vanXYL | 1.460E-06 | 0.000E+00 | 0.000E+00 | 0.000E+00 | 0.000E+00 | 0.000E+00 | 0.000E+00 | 0.000E+00 | 0.000E+00 | 0.000E+00 | 0.000E+00 | 0.000E+00 |
| Unigene14028 | vanTG | 2.963E-06 | 3.979E-06 | 3.218E-06 | 6.304E-06 | 3.835E-07 | 3.110E-07 | 3.184E-07 | 0.000E+00 | 0.000E+00 | 0.000E+00 | 0.000E+00 | 1.360E-07 |
| Unigene14059 | optrA | 3.222E-06 | 5.654E-06 | 3.962E-06 | 6.608E-06 | 3.977E-07 | 1.075E-07 | 2.862E-07 | 0.000E+00 | 2.174E-07 | 0.000E+00 | 9.100E-08 | 1.411E-07 |
| Unigene14152 | oqxB | 4.156E-07 | 0.000E+00 | 0.000E+00 | 1.399E-07 | 0.000E+00 | 0.000E+00 | 0.000E+00 | 0.000E+00 | 0.000E+00 | 0.000E+00 | 0.000E+00 | 0.000E+00 |
| Unigene14230 | lmrB | 3.642E-06 | 2.595E-07 | 3.133E-06 | 5.970E-07 | 2.627E-07 | 7.670E-08 | 4.711E-07 | 0.000E+00 | 0.000E+00 | 0.000E+00 | 6.763E-07 | 0.000E+00 |
| Unigene14231 | mecI | 4.122E-06 | 4.601E-07 | 4.251E-06 | 0.000E+00 | 0.000E+00 | 0.000E+00 | 0.000E+00 | 0.000E+00 | 0.000E+00 | 0.000E+00 | 7.992E-07 | 0.000E+00 |
| Unigene14288 | oleC | 1.964E-06 | 2.110E-06 | 2.248E-06 | 2.024E-06 | 0.000E+00 | 0.000E+00 | 0.000E+00 | 4.776E-07 | 0.000E+00 | 0.000E+00 | 0.000E+00 | 0.000E+00 |
| Unigene14299 | GOB-5 | 9.068E-07 | 7.961E-07 | 2.195E-06 | 8.546E-07 | 0.000E+00 | 0.000E+00 | 0.000E+00 | 0.000E+00 | 0.000E+00 | 0.000E+00 | 0.000E+00 | 0.000E+00 |
| Unigene14302 | rpoB2 | 9.723E-07 | 0.000E+00 | 0.000E+00 | 0.000E+00 | 0.000E+00 | 7.848E-06 | 9.326E-07 | 4.615E-06 | 0.000E+00 | 0.000E+00 | 0.000E+00 | 0.000E+00 |
| Unigene14329 | bcrA | 2.478E-06 | 3.885E-06 | 3.022E-06 | 5.612E-06 | 5.112E-07 | 0.000E+00 | 2.742E-07 | 0.000E+00 | 2.708E-07 | 0.000E+00 | 0.000E+00 | 1.674E-07 |
| Unigene14384 | macB | 3.165E-07 | 0.000E+00 | 0.000E+00 | 0.000E+00 | 0.000E+00 | 0.000E+00 | 0.000E+00 | 0.000E+00 | 1.729E-07 | 0.000E+00 | 0.000E+00 | 0.000E+00 |
| Unigene14402 | patB | 3.530E-06 | 4.388E-06 | 2.921E-06 | 1.718E-06 | 2.268E-06 | 1.931E-06 | 2.448E-06 | 7.446E-07 | 1.999E-06 | 7.533E-06 | 6.714E-06 | 1.760E-06 |
| Unigene14403 | tetA(58) | 3.236E-06 | 4.279E-06 | 2.946E-06 | 2.008E-06 | 2.425E-06 | 1.812E-06 | 2.071E-06 | 1.092E-06 | 1.321E-06 | 5.559E-06 | 7.314E-06 | 2.074E-06 |
| Unigene14437 | PmrF | 1.047E-06 | 0.000E+00 | 0.000E+00 | 3.205E-08 | 0.000E+00 | 0.000E+00 | 0.000E+00 | 0.000E+00 | 0.000E+00 | 0.000E+00 | 0.000E+00 | 0.000E+00 |
| Unigene14444 | vgaB | 3.837E-06 | 3.166E-07 | 2.916E-06 | 5.826E-07 | 1.602E-07 | 0.000E+00 | 4.150E-07 | 1.347E-07 | 0.000E+00 | 0.000E+00 | 9.569E-07 | 3.410E-08 |
| Unigene14465 | vanTG | 3.891E-06 | 2.386E-07 | 2.901E-06 | 4.610E-07 | 1.932E-07 | 0.000E+00 | 1.925E-07 | 0.000E+00 | 0.000E+00 | 0.000E+00 | 8.703E-07 | 0.000E+00 |
| Unigene14467 | Chlamydia | 3.653E-06 | 3.818E-07 | 2.926E-06 | 4.768E-07 | 1.933E-07 | 2.686E-08 | 3.575E-07 | 0.000E+00 | 0.000E+00 | 0.000E+00 | 5.685E-07 | 0.000E+00 |
| Unigene14492 | vanHO | 1.866E-06 | 1.675E-05 | 6.407E-06 | 3.599E-06 | 0.000E+00 | 0.000E+00 | 1.475E-07 | 0.000E+00 | 0.000E+00 | 0.000E+00 | 1.905E-07 | 7.875E-08 |
| Unigene14513 | TaeA | 1.497E-05 | 5.797E-06 | 3.263E-06 | 1.319E-05 | 9.433E-05 | 1.281E-04 | 1.354E-04 | 1.082E-04 | 1.640E-04 | 1.703E-04 | 1.307E-04 | 7.782E-05 |
| Unigene14525 | macB | 4.145E-06 | 1.056E-05 | 1.048E-05 | 1.237E-05 | 0.000E+00 | 0.000E+00 | 8.821E-08 | 3.101E-08 | 0.000E+00 | 0.000E+00 | 0.000E+00 | 0.000E+00 |
| Unigene14544 | vanHD | 2.717E-06 | 8.619E-06 | 9.838E-06 | 9.878E-06 | 0.000E+00 | 0.000E+00 | 0.000E+00 | 0.000E+00 | 0.000E+00 | 0.000E+00 | 0.000E+00 | 0.000E+00 |
| Unigene14554 | lmrP | 1.116E-04 | 1.973E-04 | 1.787E-04 | 0.000E+00 | 8.249E-06 | 4.788E-07 | 4.239E-06 | 2.402E-06 | 5.672E-05 | 0.000E+00 | 3.606E-06 | 8.315E-07 |
| Unigene14585 | macB | 2.272E-06 | 4.046E-06 | 3.702E-06 | 4.199E-06 | 4.763E-07 | 1.390E-07 | 2.847E-07 | 0.000E+00 | 0.000E+00 | 0.000E+00 | 1.961E-07 | 0.000E+00 |
| Unigene14621 | lsaC | 6.358E-07 | 0.000E+00 | 0.000E+00 | 0.000E+00 | 0.000E+00 | 1.137E-05 | 0.000E+00 | 0.000E+00 | 3.314E-06 | 0.000E+00 | 0.000E+00 | 0.000E+00 |
| Unigene14721 | arlR | 5.237E-07 | 0.000E+00 | 1.495E-06 | 2.500E-06 | 0.000E+00 | 0.000E+00 | 0.000E+00 | 1.111E-06 | 0.000E+00 | 0.000E+00 | 1.634E-06 | 8.442E-07 |
| Unigene14728 | Escherichia | 0.000E+00 | 5.583E-06 | 0.000E+00 | 0.000E+00 | 0.000E+00 | 0.000E+00 | 0.000E+00 | 0.000E+00 | 0.000E+00 | 0.000E+00 | 0.000E+00 | 0.000E+00 |
| Unigene14834 | bcrA | 3.809E-06 | 1.542E-05 | 2.429E-06 | 1.442E-07 | 1.164E-06 | 5.147E-08 | 2.635E-07 | 1.111E-07 | 5.204E-07 | 4.243E-07 | 1.634E-07 | 0.000E+00 |
| Unigene14836 | vanRM | 3.712E-06 | 1.624E-05 | 3.181E-06 | 1.423E-07 | 9.918E-07 | 1.524E-07 | 3.120E-07 | 5.485E-07 | 8.217E-07 | 1.047E-07 | 0.000E+00 | 1.111E-07 |
| Unigene14840 | msbA | 3.599E-06 | 1.749E-05 | 2.705E-06 | 5.424E-07 | 5.768E-07 | 1.742E-07 | 1.189E-07 | 1.672E-07 | 8.806E-07 | 2.194E-07 | 8.192E-08 | 6.349E-08 |
| Unigene14871 | macB | 2.566E-06 | 4.200E-06 | 3.357E-06 | 6.274E-06 | 5.002E-07 | 3.407E-07 | 4.983E-07 | 0.000E+00 | 4.921E-08 | 0.000E+00 | 1.030E-07 | 2.129E-07 |
| Unigene14937 | patB | 6.807E-05 | 5.950E-05 | 1.178E-04 | 1.650E-04 | 1.323E-04 | 9.343E-05 | 1.114E-04 | 1.125E-04 | 9.674E-05 | 1.429E-04 | 1.422E-04 | 1.528E-04 |
| Unigene14990 | mdtG | 1.164E-06 | 1.306E-06 | 5.127E-06 | 6.791E-06 | 8.621E-08 | 0.000E+00 | 5.153E-07 | 0.000E+00 | 0.000E+00 | 0.000E+00 | 8.876E-08 | 0.000E+00 |
| Unigene15020 | tetA(58) | 1.937E-06 | 2.047E-06 | 2.499E-06 | 1.920E-06 | 0.000E+00 | 0.000E+00 | 6.748E-07 | 4.605E-07 | 9.016E-07 | 0.000E+00 | 8.206E-08 | 0.000E+00 |
| Unigene15040 | oleB | 1.036E-05 | 0.000E+00 | 0.000E+00 | 0.000E+00 | 1.771E-05 | 1.648E-05 | 0.000E+00 | 0.000E+00 | 1.130E-05 | 0.000E+00 | 0.000E+00 | 0.000E+00 |
| Unigene15060 | tetA(58) | 2.256E-06 | 2.095E-06 | 2.980E-06 | 0.000E+00 | 0.000E+00 | 0.000E+00 | 8.643E-07 | 4.558E-07 | 7.587E-07 | 0.000E+00 | 0.000E+00 | 0.000E+00 |

| | | | | | | | | | | | | | |
|---|---|---|---|---|---|---|---|---|---|---|---|---|---|
| Unigene15090 | msbA | 1.300E-05 | 4.937E-06 | 3.405E-06 | 1.278E-05 | 8.417E-05 | 1.211E-04 | 1.265E-04 | 1.000E-04 | 1.528E-04 | 1.510E-04 | 1.257E-04 | 7.442E-05 |
| Unigene15094 | macB | 1.085E-05 | 4.177E-06 | 2.720E-06 | 1.034E-05 | 6.656E-05 | 9.499E-05 | 9.631E-05 | 7.360E-05 | 1.148E-04 | 1.167E-04 | 9.481E-05 | 5.793E-05 |
| Unigene15102 | patA | 1.241E-05 | 3.518E-06 | 3.594E-06 | 1.447E-05 | 8.470E-05 | 1.169E-04 | 1.263E-04 | 9.193E-05 | 1.461E-04 | 1.499E-04 | 1.236E-04 | 8.264E-05 |
| Unigene15103 | oleC | 1.333E-05 | 5.269E-06 | 3.330E-06 | 1.049E-05 | 9.274E-05 | 1.294E-04 | 1.348E-04 | 1.073E-04 | 1.614E-04 | 1.495E-04 | 1.331E-04 | 6.330E-05 |
| Unigene15175 | bcrA | 3.268E-06 | 1.572E-06 | 5.549E-06 | 2.967E-06 | 0.000E+00 | 0.000E+00 | 8.129E-08 | 0.000E+00 | 0.000E+00 | 2.455E-07 | 0.000E+00 | 0.000E+00 |
| Unigene15176 | TaeA | 2.239E-06 | 2.180E-06 | 4.429E-06 | 2.735E-06 | 1.605E-07 | 0.000E+00 | 0.000E+00 | 0.000E+00 | 0.000E+00 | 2.816E-07 | 0.000E+00 | 0.000E+00 |
| Unigene15268 | macB | 2.322E-06 | 1.772E-05 | 7.591E-06 | 4.772E-06 | 0.000E+00 | 1.168E-07 | 0.000E+00 | 3.152E-08 | 0.000E+00 | 7.520E-08 | 0.000E+00 | 0.000E+00 |
| Unigene15269 | arlR | 1.961E-06 | 2.115E-05 | 7.602E-06 | 4.201E-06 | 0.000E+00 | 1.551E-07 | 0.000E+00 | 0.000E+00 | 5.227E-08 | 0.000E+00 | 0.000E+00 | 0.000E+00 |
| Unigene15270 | vanSG | 1.875E-06 | 2.001E-05 | 7.391E-06 | 4.626E-06 | 0.000E+00 | 0.000E+00 | 3.402E-08 | 0.000E+00 | 0.000E+00 | 6.849E-08 | 1.407E-07 | 3.634E-08 |
| Unigene15295 | optrA | 3.399E-06 | 4.040E-06 | 2.522E-06 | 1.775E-06 | 2.118E-06 | 2.079E-06 | 2.330E-06 | 8.515E-07 | 1.848E-06 | 5.688E-06 | 6.277E-06 | 1.999E-06 |
| Unigene15303 | tetB(46) | 7.955E-07 | 0.000E+00 | 0.000E+00 | 0.000E+00 | 0.000E+00 | 7.106E-06 | 0.000E+00 | 0.000E+00 | 3.569E-06 | 2.938E-06 | 0.000E+00 | 0.000E+00 |
| Unigene15338 | lmrD | 6.406E-06 | 6.847E-06 | 5.464E-06 | 5.200E-06 | 1.542E-04 | 2.695E-04 | 2.404E-04 | 2.623E-04 | 1.547E-04 | 4.986E-05 | 6.188E-05 | 4.157E-06 |
| Unigene15355 | arr-4 | 5.538E-06 | 1.242E-05 | 1.056E-05 | 1.366E-05 | 0.000E+00 | 0.000E+00 | 0.000E+00 | 0.000E+00 | 0.000E+00 | 0.000E+00 | 0.000E+00 | 0.000E+00 |
| Unigene15378 | vanRF | 2.690E-06 | 4.538E-06 | 4.399E-06 | 3.219E-06 | 0.000E+00 | 0.000E+00 | 0.000E+00 | 0.000E+00 | 0.000E+00 | 0.000E+00 | 0.000E+00 | 0.000E+00 |
| Unigene15379 | adeS | 1.997E-06 | 1.496E-06 | 3.297E-06 | 2.064E-06 | 1.044E-07 | 1.016E-07 | 2.600E-08 | 2.742E-08 | 0.000E+00 | 1.570E-07 | 2.687E-08 | 0.000E+00 |
| Unigene15402 | Corynebac | 2.023E-06 | 1.497E-05 | 7.475E-06 | 5.346E-06 | 0.000E+00 | 0.000E+00 | 4.763E-08 | 0.000E+00 | 0.000E+00 | 0.000E+00 | 0.000E+00 | 0.000E+00 |
| Unigene15406 | vmlR | 1.970E-06 | 1.564E-05 | 6.246E-06 | 3.367E-06 | 0.000E+00 | 1.638E-07 | 1.118E-07 | 0.000E+00 | 0.000E+00 | 0.000E+00 | 0.000E+00 | 0.000E+00 |
| Unigene15411 | efrB | 1.734E-06 | 1.522E-05 | 5.665E-06 | 3.429E-06 | 0.000E+00 | 5.797E-08 | 0.000E+00 | 0.000E+00 | 0.000E+00 | 0.000E+00 | 1.431E-07 | 4.225E-08 |
| Unigene15412 | efrA | 1.611E-06 | 1.398E-05 | 5.906E-06 | 3.067E-06 | 0.000E+00 | 0.000E+00 | 0.000E+00 | 0.000E+00 | 0.000E+00 | 0.000E+00 | 1.483E-07 | 0.000E+00 |
| Unigene15449 | macB | 1.967E-06 | 1.895E-06 | 4.846E-06 | 6.974E-06 | 0.000E+00 | 0.000E+00 | 5.777E-07 | 0.000E+00 | 0.000E+00 | 0.000E+00 | 0.000E+00 | 1.898E-07 |
| Unigene15465 | vanL | 5.529E-06 | 1.197E-05 | 9.649E-06 | 1.219E-05 | 0.000E+00 | 0.000E+00 | 0.000E+00 | 0.000E+00 | 0.000E+00 | 0.000E+00 | 0.000E+00 | 0.000E+00 |
| Unigene15472 | vanSF | 1.051E-05 | 0.000E+00 | 0.000E+00 | 0.000E+00 | 1.525E-05 | 1.588E-05 | 0.000E+00 | 0.000E+00 | 1.253E-05 | 2.517E-07 | 0.000E+00 | 0.000E+00 |
| Unigene15473 | vanRM | 1.247E-05 | 0.000E+00 | 0.000E+00 | 0.000E+00 | 2.099E-05 | 1.532E-05 | 0.000E+00 | 0.000E+00 | 0.000E+00 | 0.000E+00 | 0.000E+00 | 0.000E+00 |
| Unigene15485 | efrA | 4.224E-06 | 2.759E-07 | 4.687E-07 | 0.000E+00 | 0.000E+00 | 0.000E+00 | 0.000E+00 | 0.000E+00 | 0.000E+00 | 0.000E+00 | 0.000E+00 | 0.000E+00 |
| Unigene15502 | Staphylocc | 4.012E-06 | 1.894E-05 | 2.747E-06 | 9.072E-07 | 7.892E-07 | 3.614E-07 | 1.619E-07 | 7.316E-08 | 9.363E-07 | 6.284E-07 | 5.019E-07 | 9.879E-08 |
| Unigene15507 | Streptomy | 4.192E-06 | 2.000E-05 | 3.303E-06 | 7.666E-07 | 1.192E-06 | 4.103E-07 | 2.557E-07 | 5.008E-07 | 1.118E-06 | 6.067E-07 | 4.153E-07 | 1.561E-07 |
| Unigene15513 | lsaA | 5.024E-06 | 2.148E-05 | 3.697E-06 | 6.553E-07 | 1.256E-06 | 6.337E-07 | 2.085E-07 | 0.000E+00 | 1.373E-06 | 4.897E-07 | 3.113E-07 | 1.237E-07 |
| Unigene15570 | vgaB | 9.719E-05 | 1.703E-04 | 1.571E-04 | 7.376E-06 | 8.066E-06 | 3.783E-07 | 3.001E-06 | 1.634E-06 | 5.689E-05 | 0.000E+00 | 3.202E-06 | 0.000E+00 |
| Unigene15608 | vanL | 2.537E-06 | 2.528E-06 | 3.062E-06 | 2.002E-06 | 0.000E+00 | 9.457E-08 | 0.000E+00 | 0.000E+00 | 4.462E-07 | 2.274E-07 | 0.000E+00 | 1.034E-07 |
| Unigene15631 | efrB | 9.845E-05 | 1.775E-04 | 1.723E-04 | 0.000E+00 | 8.517E-06 | 0.000E+00 | 4.615E-06 | 1.962E-06 | 5.943E-05 | 0.000E+00 | 2.500E-06 | 0.000E+00 |
| Unigene15636 | ramA | 2.711E-06 | 1.053E-06 | 3.519E-06 | 1.432E-06 | 0.000E+00 | 4.509E-08 | 0.000E+00 | 4.868E-08 | 0.000E+00 | 3.252E-07 | 4.771E-08 | 0.000E+00 |
| Unigene15643 | tet36 | 7.384E-07 | 5.770E-06 | 9.765E-06 | 1.477E-05 | 0.000E+00 | 3.801E-08 | 0.000E+00 | 0.000E+00 | 0.000E+00 | 0.000E+00 | 4.022E-08 | 0.000E+00 |
| Unigene15723 | vanRI | 4.887E-06 | 3.967E-06 | 5.843E-06 | 4.811E-06 | 0.000E+00 | 0.000E+00 | 0.000E+00 | 0.000E+00 | 0.000E+00 | 1.830E-07 | 0.000E+00 | 0.000E+00 |
| Unigene15724 | vanSM | 3.064E-06 | 3.050E-06 | 3.846E-06 | 3.922E-06 | 1.187E-07 | 0.000E+00 | 0.000E+00 | 0.000E+00 | 0.000E+00 | 0.000E+00 | 0.000E+00 | 0.000E+00 |
| Unigene15732 | bcr-1 | 2.718E-06 | 1.067E-06 | 2.060E-06 | 6.917E-07 | 0.000E+00 | 0.000E+00 | 0.000E+00 | 1.289E-07 | 0.000E+00 | 0.000E+00 | 0.000E+00 | 0.000E+00 |
| Unigene15745 | macB | 2.719E-06 | 8.486E-07 | 1.947E-06 | 6.506E-07 | 0.000E+00 | 0.000E+00 | 0.000E+00 | 9.025E-08 | 0.000E+00 | 0.000E+00 | 0.000E+00 | 0.000E+00 |
| Unigene15849 | vanHF | 2.682E-06 | 1.099E-06 | 1.744E-06 | 7.831E-07 | 0.000E+00 | 0.000E+00 | 0.000E+00 | 1.886E-07 | 0.000E+00 | 0.000E+00 | 0.000E+00 | 0.000E+00 |
| Unigene15864 | arlS | 2.523E-06 | 1.149E-06 | 1.583E-06 | 8.492E-07 | 0.000E+00 | 0.000E+00 | 0.000E+00 | 3.205E-07 | 0.000E+00 | 0.000E+00 | 0.000E+00 | 0.000E+00 |
| Unigene15865 | mtrA | 3.723E-06 | 1.844E-06 | 1.737E-06 | 5.043E-07 | 0.000E+00 | 0.000E+00 | 0.000E+00 | 3.180E-07 | 0.000E+00 | 0.000E+00 | 0.000E+00 | 0.000E+00 |
| Unigene15912 | vanSM | 6.694E-06 | 0.000E+00 | 0.000E+00 | 2.871E-06 | 3.474E-05 | 1.595E-05 | 2.187E-05 | 2.210E-05 | 2.517E-06 | 0.000E+00 | 0.000E+00 | 0.000E+00 |
| Unigene15913 | vanRG | 7.332E-06 | 0.000E+00 | 0.000E+00 | 2.212E-06 | 3.094E-05 | 1.400E-05 | 2.340E-05 | 2.284E-05 | 2.706E-06 | 0.000E+00 | 0.000E+00 | 0.000E+00 |
| Unigene15914 | bcrA | 7.150E-06 | 0.000E+00 | 0.000E+00 | 1.858E-06 | 3.613E-05 | 1.486E-05 | 2.309E-05 | 2.471E-05 | 2.883E-06 | 0.000E+00 | 0.000E+00 | 0.000E+00 |
| Unigene15925 | evgA | 5.301E-06 | 0.000E+00 | 0.000E+00 | 2.730E-06 | 2.850E-05 | 1.129E-05 | 1.848E-05 | 2.394E-05 | 0.000E+00 | 0.000E+00 | 0.000E+00 | 0.000E+00 |
| Unigene15938 | ykkD | 6.559E-05 | 6.501E-05 | 1.212E-04 | 1.277E-04 | 1.186E-04 | 8.685E-05 | 1.209E-04 | 1.068E-04 | 8.792E-05 | 1.495E-04 | 1.564E-04 | 1.597E-04 |
| Unigene15968 | vatF | 1.582E-06 | 0.000E+00 | 0.000E+00 | 0.000E+00 | 0.000E+00 | 0.000E+00 | 0.000E+00 | 0.000E+00 | 0.000E+00 | 0.000E+00 | 0.000E+00 | 0.000E+00 |
| Unigene15972 | sul4 | 8.152E-07 | 6.576E-06 | 9.958E-06 | 1.457E-05 | 0.000E+00 | 0.000E+00 | 0.000E+00 | 0.000E+00 | 0.000E+00 | 0.000E+00 | 1.399E-07 | 0.000E+00 |
| Unigene15984 | tetT | 2.233E-06 | 3.377E-06 | 2.691E-06 | 5.167E-06 | 4.493E-07 | 7.603E-08 | 1.362E-07 | 0.000E+00 | 1.345E-07 | 5.876E-08 | 1.207E-07 | 1.039E-07 |
| Unigene16032 | arlS | 2.863E-05 | 9.402E-07 | 4.203E-07 | 0.000E+00 | 0.000E+00 | 3.704E-08 | 0.000E+00 | 0.000E+00 | 0.000E+00 | 0.000E+00 | 0.000E+00 | 0.000E+00 |
| Unigene16042 | Chlamydia | 4.391E-05 | 1.638E-05 | 0.000E+00 | 0.000E+00 | 0.000E+00 | 2.487E-07 | 0.000E+00 | 0.000E+00 | 0.000E+00 | 0.000E+00 | 0.000E+00 | 0.000E+00 |
| Unigene16044 | vanTG | 2.843E-05 | 7.226E-07 | 0.000E+00 | 0.000E+00 | 2.286E-08 | 1.334E-07 | 0.000E+00 | 0.000E+00 | 0.000E+00 | 1.146E-07 | 0.000E+00 | 2.432E-08 |
| Unigene16064 | macB | 8.513E-05 | 9.707E-05 | 9.485E-05 | 0.000E+00 | 4.507E-06 | 3.421E-07 | 0.000E+00 | 0.000E+00 | 0.000E+00 | 0.000E+00 | 0.000E+00 | 0.000E+00 |
| Unigene16065 | tetB(46) | 4.118E-05 | 1.946E-05 | 0.000E+00 | 0.000E+00 | 0.000E+00 | 1.829E-07 | 0.000E+00 | 0.000E+00 | 0.000E+00 | 0.000E+00 | 0.000E+00 | 0.000E+00 |
| Unigene16084 | Staphylocc | 4.845E-05 | 9.208E-08 | 2.058E-08 | 0.000E+00 | 0.000E+00 | 2.267E-08 | 0.000E+00 | 0.000E+00 | 0.000E+00 | 1.402E-07 | 0.000E+00 | 0.000E+00 |
| Unigene16109 | lmrD | 4.146E-05 | 2.052E-05 | 7.827E-06 | 1.301E-05 | 2.400E-06 | 1.662E-06 | 4.000E-06 | 6.304E-07 | 6.629E-06 | 5.554E-07 | 2.043E-06 | 2.062E-06 |
| Unigene16135 | oleC | 3.688E-05 | 2.158E-05 | 1.891E-05 | 2.027E-05 | 1.673E-06 | 1.362E-06 | 3.424E-06 | 8.309E-07 | 0.000E+00 | 3.660E-07 | 1.315E-06 | 0.000E+00 |
| Unigene16160 | tetA(58) | 4.617E-07 | 0.000E+00 | 2.788E-07 | 5.020E-07 | 3.077E-06 | 7.640E-06 | 1.454E-06 | 8.249E-06 | 3.338E-06 | 2.888E-06 | 0.000E+00 | 7.975E-07 |
| Unigene16241 | dfrA3 | 1.076E-06 | 2.362E-06 | 2.046E-06 | 0.000E+00 | 1.405E-05 | 0.000E+00 | 2.456E-06 | 0.000E+00 | 0.000E+00 | 0.000E+00 | 0.000E+00 | 0.000E+00 |
| Unigene16265 | vanHA | 1.880E-06 | 1.943E-06 | 2.539E-06 | 1.513E-06 | 1.513E-07 | 0.000E+00 | 3.618E-07 | 0.000E+00 | 3.573E-07 | 0.000E+00 | 0.000E+00 | 9.660E-08 |

| | | | | | | | | | | | | | |
|---|---|---|---|---|---|---|---|---|---|---|---|---|---|
| Unigene16291 | mdtG | 2.162E-05 | 6.785E-06 | 5.914E-06 | 3.422E-06 | 4.310E-07 | 0.000E+00 | 0.000E+00 | 6.038E-08 | 7.633E-07 | 0.000E+00 | 0.000E+00 | 6.115E-08 |
| Unigene16310 | cmlv | 4.588E-06 | 1.984E-05 | 3.537E-06 | 1.020E-06 | 1.335E-06 | 0.000E+00 | 3.627E-07 | 3.188E-07 | 1.642E-06 | 0.000E+00 | 0.000E+00 | 3.551E-07 |
| Unigene16315 | msbA | 5.624E-06 | 2.197E-05 | 3.780E-06 | 7.602E-07 | 1.033E-06 | 2.393E-07 | 2.450E-07 | 4.135E-07 | 1.646E-06 | 8.879E-07 | 0.000E+00 | 3.140E-07 |
| Unigene16320 | YojI | 5.016E-06 | 2.221E-05 | 4.177E-06 | 1.119E-06 | 1.555E-06 | 0.000E+00 | 2.905E-07 | 5.446E-07 | 1.402E-06 | 0.000E+00 | 4.670E-07 | 1.723E-07 |
| Unigene16337 | patA | 2.117E-06 | 2.561E-06 | 2.205E-06 | 0.000E+00 | 2.880E-07 | 1.869E-07 | 5.739E-07 | 0.000E+00 | 4.251E-07 | 0.000E+00 | 9.886E-08 | 0.000E+00 |
| Unigene16359 | FusF | 2.242E-06 | 0.000E+00 | 9.385E-06 | 1.398E-05 | 0.000E+00 | 0.000E+00 | 0.000E+00 | 0.000E+00 | 0.000E+00 | 0.000E+00 | 0.000E+00 | 0.000E+00 |
| Unigene16389 | baeS | 9.199E-06 | 2.725E-06 | 1.673E-05 | 1.051E-04 | 4.755E-06 | 4.832E-06 | 1.460E-05 | 5.521E-07 | 0.000E+00 | 1.212E-05 | 8.413E-06 | 2.879E-06 |
| Unigene16438 | kdpE | 2.483E-06 | 1.470E-05 | 8.217E-06 | 5.900E-06 | 0.000E+00 | 2.105E-07 | 0.000E+00 | 0.000E+00 | 0.000E+00 | 0.000E+00 | 0.000E+00 | 0.000E+00 |
| Unigene16443 | macB | 1.334E-06 | 1.347E-05 | 7.670E-06 | 4.133E-06 | 0.000E+00 | 0.000E+00 | 0.000E+00 | 0.000E+00 | 0.000E+00 | 0.000E+00 | 0.000E+00 | 0.000E+00 |
| Unigene16476 | macB | 2.324E-06 | 1.604E-05 | 8.996E-06 | 5.883E-06 | 0.000E+00 | 0.000E+00 | 0.000E+00 | 0.000E+00 | 0.000E+00 | 0.000E+00 | 0.000E+00 | 1.881E-08 |
| Unigene16478 | efrA | 1.827E-06 | 1.492E-05 | 8.927E-06 | 5.350E-06 | 0.000E+00 | 9.874E-08 | 0.000E+00 | 0.000E+00 | 0.000E+00 | 0.000E+00 | 0.000E+00 | 2.159E-08 |
| Unigene16517 | arlR | 1.387E-06 | 1.837E-06 | 2.299E-06 | 0.000E+00 | 0.000E+00 | 0.000E+00 | 0.000E+00 | 0.000E+00 | 6.272E-07 | 0.000E+00 | 0.000E+00 | 0.000E+00 |
| Unigene16543 | efmA | 4.307E-05 | 1.917E-05 | 1.230E-05 | 1.522E-05 | 2.282E-06 | 1.333E-06 | 2.672E-06 | 5.396E-07 | 6.877E-06 | 6.580E-07 | 2.174E-06 | 1.609E-06 |
| Unigene16566 | efrA | 1.350E-05 | 5.377E-06 | 3.491E-06 | 1.399E-05 | 9.220E-05 | 1.352E-04 | 1.312E-04 | 1.217E-04 | 1.704E-04 | 1.721E-04 | 1.360E-04 | 9.617E-05 |
| Unigene16595 | FusF | 4.678E-07 | 1.489E-06 | 1.744E-06 | 1.984E-06 | 0.000E+00 | 0.000E+00 | 0.000E+00 | 0.000E+00 | 0.000E+00 | 0.000E+00 | 0.000E+00 | 0.000E+00 |
| Unigene16624 | mepA | 2.321E-06 | 2.187E-06 | 3.404E-06 | 1.894E-06 | 0.000E+00 | 2.281E-07 | 6.486E-07 | 3.010E-07 | 6.149E-07 | 0.000E+00 | 0.000E+00 | 1.385E-07 |
| Unigene16634 | patB | 2.382E-06 | 2.791E-06 | 2.810E-06 | 1.514E-06 | 8.921E-08 | 0.000E+00 | 5.629E-07 | 3.124E-07 | 9.654E-07 | 0.000E+00 | 2.143E-07 | 0.000E+00 |
| Unigene16666 | arlR | 1.277E-05 | 3.920E-06 | 2.651E-06 | 1.211E-05 | 8.490E-05 | 1.268E-04 | 1.267E-04 | 1.021E-04 | 1.533E-04 | 1.564E-04 | 1.289E-04 | 7.350E-05 |
| Unigene16668 | facT | 1.309E-05 | 4.097E-06 | 3.030E-06 | 1.415E-05 | 9.589E-05 | 1.352E-04 | 1.344E-04 | 1.237E-04 | 1.805E-04 | 1.821E-04 | 1.426E-04 | 9.279E-05 |
| Unigene16686 | YojI | 2.282E-05 | 8.171E-06 | 6.586E-06 | 3.979E-06 | 2.717E-07 | 1.469E-07 | 1.504E-07 | 6.343E-08 | 4.455E-07 | 0.000E+00 | 0.000E+00 | 9.635E-08 |
| Unigene16708 | lmrB | 2.106E-05 | 7.643E-06 | 5.813E-06 | 4.395E-06 | 4.298E-07 | 0.000E+00 | 0.000E+00 | 2.540E-07 | 6.343E-07 | 0.000E+00 | 0.000E+00 | 5.716E-08 |
| Unigene16745 | smeS | 3.212E-06 | 2.573E-07 | 2.714E-06 | 3.077E-07 | 2.083E-07 | 1.014E-07 | 3.632E-07 | 0.000E+00 | 0.000E+00 | 0.000E+00 | 7.776E-07 | 0.000E+00 |
| Unigene16795 | novA | 1.852E-06 | 1.511E-05 | 6.777E-06 | 5.357E-06 | 0.000E+00 | 0.000E+00 | 0.000E+00 | 0.000E+00 | 0.000E+00 | 0.000E+00 | 0.000E+00 | 0.000E+00 |
| Unigene16827 | QepA2 | 3.356E-06 | 2.971E-06 | 2.634E-06 | 1.830E-06 | 2.783E-06 | 2.152E-06 | 1.832E-06 | 9.139E-07 | 1.320E-06 | 5.732E-06 | 5.911E-06 | 1.613E-06 |
| Unigene16833 | YojI | 3.558E-06 | 3.232E-06 | 2.604E-06 | 2.091E-06 | 2.138E-06 | 2.081E-06 | 2.679E-06 | 6.467E-07 | 9.880E-07 | 5.912E-06 | 4.536E-06 | 2.103E-06 |
| Unigene16835 | tetT | 2.739E-06 | 4.360E-06 | 2.972E-06 | 1.737E-06 | 2.722E-06 | 2.090E-06 | 1.887E-06 | 6.218E-07 | 1.564E-06 | 5.578E-06 | 5.153E-06 | 1.835E-06 |
| Unigene16854 | vanRA | 8.082E-06 | 0.000E+00 | 0.000E+00 | 2.507E-06 | 3.554E-05 | 1.321E-05 | 2.204E-05 | 2.495E-05 | 2.927E-06 | 0.000E+00 | 0.000E+00 | 0.000E+00 |
| Unigene16855 | bcrA | 6.741E-06 | 0.000E+00 | 0.000E+00 | 1.733E-06 | 3.079E-05 | 1.548E-05 | 2.034E-05 | 2.043E-05 | 3.157E-06 | 0.000E+00 | 0.000E+00 | 0.000E+00 |
| Unigene16872 | bcrA | 6.244E-06 | 0.000E+00 | 0.000E+00 | 1.476E-06 | 3.227E-05 | 1.422E-05 | 2.416E-05 | 2.233E-05 | 3.243E-06 | 0.000E+00 | 0.000E+00 | 0.000E+00 |
| Unigene16908 | arlR | 6.907E-06 | 0.000E+00 | 0.000E+00 | 2.989E-06 | 2.986E-05 | 1.321E-05 | 1.664E-05 | 2.002E-05 | 0.000E+00 | 0.000E+00 | 0.000E+00 | 0.000E+00 |
| Unigene16909 | arlS | 6.489E-06 | 0.000E+00 | 0.000E+00 | 2.136E-06 | 3.469E-05 | 1.662E-05 | 2.262E-05 | 2.353E-05 | 2.846E-06 | 0.000E+00 | 0.000E+00 | 0.000E+00 |
| Unigene16922 | smeR | 5.404E-06 | 0.000E+00 | 0.000E+00 | 1.497E-06 | 3.204E-05 | 1.396E-05 | 2.006E-05 | 2.445E-05 | 2.823E-06 | 0.000E+00 | 0.000E+00 | 0.000E+00 |
| Unigene16971 | YojI | 6.532E-06 | 0.000E+00 | 0.000E+00 | 2.103E-06 | 3.211E-05 | 1.395E-05 | 2.019E-05 | 2.250E-05 | 2.828E-06 | 0.000E+00 | 0.000E+00 | 0.000E+00 |
| Unigene16972 | carA | 6.091E-06 | 0.000E+00 | 0.000E+00 | 1.606E-06 | 3.007E-05 | 1.650E-05 | 2.183E-05 | 2.611E-05 | 0.000E+00 | 0.000E+00 | 0.000E+00 | 0.000E+00 |
| Unigene16990 | tetA(60) | 7.216E-06 | 0.000E+00 | 0.000E+00 | 2.114E-06 | 3.125E-05 | 1.568E-05 | 2.006E-05 | 2.235E-05 | 2.147E-06 | 0.000E+00 | 0.000E+00 | 0.000E+00 |
| Unigene16991 | tetB(60) | 6.732E-06 | 0.000E+00 | 0.000E+00 | 2.007E-06 | 3.348E-05 | 1.498E-05 | 2.275E-05 | 2.441E-05 | 2.740E-06 | 0.000E+00 | 0.000E+00 | 0.000E+00 |
| Unigene17020 | Rhodococ | 2.333E-05 | 9.942E-07 | 0.000E+00 | 0.000E+00 | 0.000E+00 | 8.902E-08 | 0.000E+00 | 0.000E+00 | 0.000E+00 | 0.000E+00 | 0.000E+00 | 0.000E+00 |
| Unigene17102 | lmrD | 3.857E-06 | 1.586E-05 | 1.554E-05 | 1.845E-05 | 0.000E+00 | 0.000E+00 | 0.000E+00 | 0.000E+00 | 0.000E+00 | 2.262E-07 | 0.000E+00 | 0.000E+00 |
| Unigene17112 | salA | 1.379E-05 | 6.295E-06 | 3.242E-06 | 1.372E-05 | 9.708E-05 | 1.305E-04 | 1.343E-04 | 1.152E-04 | 1.692E-04 | 1.641E-04 | 1.412E-04 | 8.334E-05 |
| Unigene17262 | Escherichi | 5.032E-06 | 0.000E+00 | 0.000E+00 | 2.595E-06 | 3.562E-05 | 0.000E+00 | 2.405E-05 | 2.587E-05 | 0.000E+00 | 0.000E+00 | 0.000E+00 | 0.000E+00 |
| Unigene17325 | adeR | 6.847E-06 | 0.000E+00 | 0.000E+00 | 1.552E-06 | 2.996E-05 | 1.344E-05 | 2.047E-05 | 2.159E-05 | 0.000E+00 | 0.000E+00 | 0.000E+00 | 0.000E+00 |
| Unigene17327 | macB | 5.401E-06 | 0.000E+00 | 0.000E+00 | 2.685E-06 | 3.063E-05 | 1.318E-05 | 1.784E-05 | 2.006E-05 | 2.325E-06 | 0.000E+00 | 0.000E+00 | 0.000E+00 |
| Unigene17336 | Streptomy | 6.992E-06 | 0.000E+00 | 0.000E+00 | 2.008E-06 | 3.105E-05 | 1.387E-05 | 2.021E-05 | 2.175E-05 | 2.314E-06 | 0.000E+00 | 0.000E+00 | 0.000E+00 |
| Unigene17346 | rpoB2 | 4.247E-07 | 0.000E+00 | 0.000E+00 | 0.000E+00 | 0.000E+00 | 7.653E-08 | 0.000E+00 | 0.000E+00 | 0.000E+00 | 0.000E+00 | 0.000E+00 | 0.000E+00 |
| Unigene17389 | mtrA | 3.413E-06 | 1.014E-05 | 9.023E-06 | 1.020E-05 | 0.000E+00 | 1.537E-07 | 0.000E+00 | 5.533E-08 | 0.000E+00 | 2.112E-07 | 0.000E+00 | 0.000E+00 |
| Unigene17390 | baeS | 4.891E-06 | 9.913E-06 | 1.037E-05 | 1.172E-05 | 7.245E-08 | 1.410E-07 | 0.000E+00 | 0.000E+00 | 0.000E+00 | 0.000E+00 | 1.492E-07 | 0.000E+00 |
| Unigene17391 | macB | 3.336E-06 | 8.612E-06 | 9.267E-06 | 1.068E-05 | 5.220E-08 | 0.000E+00 | 1.040E-07 | 0.000E+00 | 0.000E+00 | 0.000E+00 | 0.000E+00 | 0.000E+00 |
| Unigene17417 | msbA | 1.946E-06 | 3.829E-06 | 2.703E-06 | 3.968E-06 | 0.000E+00 | 0.000E+00 | 0.000E+00 | 0.000E+00 | 0.000E+00 | 0.000E+00 | 0.000E+00 | 1.736E-07 |
| Unigene17418 | tetA(60) | 3.261E-06 | 3.897E-06 | 3.164E-06 | 7.024E-06 | 5.635E-07 | 7.833E-08 | 2.807E-07 | 0.000E+00 | 7.920E-08 | 0.000E+00 | 0.000E+00 | 0.000E+00 |
| Unigene17440 | qacH | 0.000E+00 | 2.340E-05 | 1.835E-05 | 2.102E-05 | 0.000E+00 | 2.066E-06 | 0.000E+00 | 0.000E+00 | 0.000E+00 | 0.000E+00 | 0.000E+00 | 0.000E+00 |
| Unigene17443 | vanTC | 4.015E-05 | 2.334E-05 | 1.776E-05 | 2.131E-05 | 0.000E+00 | 1.242E-06 | 2.946E-06 | 0.000E+00 | 0.000E+00 | 7.804E-07 | 1.378E-06 | 0.000E+00 |
| Unigene17444 | TaeA | 1.251E-06 | 0.000E+00 | 4.911E-08 | 0.000E+00 | 6.116E-07 | 4.328E-07 | 0.000E+00 | 0.000E+00 | 0.000E+00 | 1.221E-05 | 0.000E+00 | 0.000E+00 |
| Unigene17498 | tsnR | 1.328E-06 | 2.551E-06 | 1.369E-06 | 2.683E-06 | 7.379E-08 | 2.872E-07 | 0.000E+00 | 0.000E+00 | 0.000E+00 | 0.000E+00 | 0.000E+00 | 2.355E-07 |
| Unigene17530 | patB | 1.093E-06 | 8.893E-07 | 3.920E-06 | 5.271E-06 | 0.000E+00 | 0.000E+00 | 3.546E-07 | 0.000E+00 | 0.000E+00 | 0.000E+00 | 4.311E-08 | 2.227E-08 |
| Unigene17551 | lsaA | 4.537E-06 | 8.418E-06 | 1.382E-05 | 1.178E-05 | 1.920E-07 | 1.635E-07 | 6.457E-07 | 1.513E-07 | 0.000E+00 | 7.221E-08 | 1.977E-07 | 7.662E-08 |
| Unigene17558 | macB | 8.596E-06 | 2.858E-06 | 2.401E-06 | 6.304E-06 | 5.430E-05 | 8.024E-05 | 1.050E-04 | 8.052E-05 | 4.968E-05 | 4.361E-05 | 2.508E-05 | 5.636E-06 |
| Unigene17563 | tetA(58) | 1.301E-05 | 4.603E-06 | 3.700E-06 | 1.476E-05 | 9.714E-05 | 1.307E-04 | 1.371E-04 | 1.153E-04 | 1.744E-04 | 1.708E-04 | 1.357E-04 | 8.487E-05 |
| Unigene17575 | evgA | 1.683E-06 | 2.183E-06 | 2.602E-06 | 1.545E-06 | 0.000E+00 | 0.000E+00 | 0.000E+00 | 0.000E+00 | 6.688E-07 | 0.000E+00 | 0.000E+00 | 0.000E+00 |

| | | | | | | | | | | | | | |
|---|---|---|---|---|---|---|---|---|---|---|---|---|---|
| Unigene17663 | YojI | 2.478E-06 | 1.754E-05 | 7.377E-06 | 4.984E-06 | 0.000E+00 | 0.000E+00 | 0.000E+00 | 0.000E+00 | 0.000E+00 | 0.000E+00 | 2.362E-07 | 0.000E+00 |
| Unigene17669 | bcrA | 2.746E-06 | 1.319E-05 | 7.391E-06 | 4.684E-06 | 0.000E+00 | 1.203E-07 | 4.106E-08 | 0.000E+00 | 0.000E+00 | 0.000E+00 | 1.698E-07 | 0.000E+00 |
| Unigene17670 | macB | 1.736E-06 | 1.972E-05 | 7.531E-06 | 3.972E-06 | 0.000E+00 | 0.000E+00 | 0.000E+00 | 0.000E+00 | 0.000E+00 | 0.000E+00 | 0.000E+00 | 9.117E-08 |
| Unigene17689 | patB | 6.091E-07 | 0.000E+00 | 0.000E+00 | 6.150E-07 | 1.489E-06 | 9.789E-06 | 2.472E-06 | 8.626E-06 | 5.858E-06 | 0.000E+00 | 0.000E+00 | 7.679E-07 |
| Unigene17721 | bcrA | 2.108E-06 | 1.749E-05 | 8.671E-06 | 4.630E-06 | 0.000E+00 | 0.000E+00 | 0.000E+00 | 0.000E+00 | 0.000E+00 | 0.000E+00 | 4.230E-08 | 0.000E+00 |
| Unigene17755 | carA | 1.262E-06 | 1.130E-06 | 1.532E-06 | 1.509E-06 | 2.195E-05 | 6.750E-06 | 1.599E-05 | 1.834E-05 | 1.285E-05 | 2.064E-05 | 2.108E-05 | 8.479E-06 |
| Unigene17756 | tva(A) | 1.814E-06 | 1.202E-06 | 1.350E-06 | 2.085E-06 | 1.925E-05 | 5.782E-06 | 1.222E-05 | 1.745E-05 | 1.217E-05 | 1.934E-05 | 1.964E-05 | 6.248E-06 |
| Unigene17908 | bcrA | 2.608E-06 | 4.836E-06 | 3.470E-06 | 6.901E-06 | 0.000E+00 | 0.000E+00 | 1.659E-07 | 6.997E-08 | 6.552E-07 | 0.000E+00 | 0.000E+00 | 3.189E-07 |
| Unigene17912 | vanRA | 3.679E-06 | 4.694E-06 | 3.655E-06 | 5.944E-06 | 8.684E-07 | 0.000E+00 | 1.527E-07 | 0.000E+00 | 4.021E-07 | 0.000E+00 | 1.578E-07 | 2.718E-07 |
| Unigene17915 | bcrA | 3.394E-06 | 4.619E-06 | 3.897E-06 | 6.787E-06 | 5.279E-07 | 0.000E+00 | 1.878E-07 | 0.000E+00 | 1.113E-07 | 0.000E+00 | 0.000E+00 | 0.000E+00 |
| Unigene17918 | macB | 1.976E-06 | 4.049E-06 | 3.534E-06 | 4.568E-06 | 6.343E-07 | 2.374E-07 | 3.889E-07 | 0.000E+00 | 4.800E-08 | 0.000E+00 | 0.000E+00 | 1.557E-07 |
| Unigene18031 | kdpE | 2.981E-06 | 3.834E-06 | 2.514E-06 | 2.916E-06 | 0.000E+00 | 0.000E+00 | 0.000E+00 | 0.000E+00 | 0.000E+00 | 0.000E+00 | 0.000E+00 | 1.652E-07 |
| Unigene18054 | facT | 2.312E-06 | 1.419E-05 | 7.919E-06 | 5.603E-06 | 2.335E-08 | 2.499E-07 | 6.978E-08 | 2.453E-08 | 9.188E-08 | 0.000E+00 | 0.000E+00 | 0.000E+00 |
| Unigene18154 | TaeA | 3.930E-06 | 2.157E-06 | 1.653E-06 | 4.299E-06 | 7.443E-05 | 9.867E-05 | 6.876E-05 | 3.080E-05 | 2.690E-04 | 2.500E-04 | 2.066E-04 | 1.781E-04 |
| Unigene18161 | TaeA | 2.307E-06 | 1.927E-06 | 2.399E-06 | 1.669E-06 | 0.000E+00 | 5.583E-08 | 6.288E-07 | 1.809E-07 | 5.081E-07 | 0.000E+00 | 0.000E+00 | 0.000E+00 |
| Unigene18162 | dfrE | 0.000E+00 | 0.000E+00 | 3.053E-06 | 0.000E+00 | 0.000E+00 | 0.000E+00 | 0.000E+00 | 0.000E+00 | 0.000E+00 | 0.000E+00 | 0.000E+00 | 0.000E+00 |
| Unigene18286 | Staphylocc | 3.860E-05 | 2.367E-05 | 2.027E-05 | 2.122E-05 | 2.161E-06 | 1.942E-06 | 2.831E-06 | 7.896E-07 | 0.000E+00 | 6.594E-07 | 1.717E-06 | 1.174E-06 |
| Unigene18349 | baeS | 3.122E-06 | 3.896E-06 | 3.045E-06 | 5.292E-06 | 5.161E-07 | 1.406E-07 | 2.057E-07 | 0.000E+00 | 2.234E-07 | 0.000E+00 | 8.503E-08 | 1.977E-07 |
| Unigene18350 | mtrA | 3.374E-06 | 3.953E-06 | 1.924E-06 | 6.354E-06 | 4.559E-07 | 4.929E-08 | 0.000E+00 | 0.000E+00 | 2.990E-07 | 0.000E+00 | 0.000E+00 | 1.078E-07 |
| Unigene18356 | YojI | 3.696E-06 | 4.459E-06 | 3.196E-06 | 6.394E-06 | 3.659E-07 | 7.913E-08 | 5.266E-07 | 4.272E-08 | 1.200E-07 | 0.000E+00 | 0.000E+00 | 2.163E-07 |
| Unigene18383 | cdeA | 1.825E-06 | 1.104E-06 | 6.266E-06 | 6.874E-06 | 0.000E+00 | 0.000E+00 | 4.401E-07 | 8.191E-08 | 0.000E+00 | 0.000E+00 | 5.351E-08 | 0.000E+00 |
| Unigene18481 | blt | 7.001E-05 | 5.744E-05 | 1.130E-04 | 1.598E-04 | 1.205E-04 | 8.664E-05 | 1.110E-04 | 1.100E-04 | 9.545E-05 | 1.404E-04 | 1.437E-04 | 1.453E-04 |
| Unigene18502 | vanHO | 1.750E-06 | 0.000E+00 | 0.000E+00 | 0.000E+00 | 0.000E+00 | 0.000E+00 | 0.000E+00 | 0.000E+00 | 0.000E+00 | 0.000E+00 | 0.000E+00 | 0.000E+00 |
| Unigene18599 | cpxA | 5.411E-06 | 1.049E-05 | 1.050E-05 | 1.105E-05 | 0.000E+00 | 2.326E-07 | 1.361E-07 | 1.435E-07 | 0.000E+00 | 3.425E-07 | 0.000E+00 | 0.000E+00 |
| Unigene18600 | arlR | 6.217E-06 | 9.763E-06 | 1.159E-05 | 1.164E-05 | 0.000E+00 | 0.000E+00 | 0.000E+00 | 0.000E+00 | 0.000E+00 | 0.000E+00 | 0.000E+00 | 0.000E+00 |
| Unigene18672 | lmrB | 8.044E-05 | 1.252E-04 | 1.204E-04 | 4.291E-06 | 6.421E-06 | 3.458E-07 | 2.048E-06 | 1.387E-06 | 5.151E-05 | 0.000E+00 | 2.247E-06 | 4.591E-07 |
| Unigene18856 | novA | 7.580E-05 | 6.034E-05 | 1.200E-04 | 1.677E-04 | 1.294E-04 | 9.051E-05 | 1.186E-04 | 1.165E-04 | 1.011E-04 | 1.445E-04 | 1.431E-04 | 1.560E-04 |
| Unigene18918 | bcrA | 1.272E-06 | 0.000E+00 | 0.000E+00 | 0.000E+00 | 0.000E+00 | 8.645E-06 | 2.643E-05 | 2.779E-05 | 3.337E-06 | 0.000E+00 | 0.000E+00 | 0.000E+00 |
| Unigene18940 | macB | 5.515E-06 | 9.989E-06 | 1.071E-05 | 1.180E-05 | 0.000E+00 | 0.000E+00 | 0.000E+00 | 1.351E-07 | 0.000E+00 | 0.000E+00 | 0.000E+00 | 0.000E+00 |
| Unigene18941 | bcrA | 3.859E-06 | 1.157E-05 | 9.903E-06 | 1.150E-05 | 0.000E+00 | 0.000E+00 | 2.053E-07 | 0.000E+00 | 0.000E+00 | 1.653E-07 | 0.000E+00 | 0.000E+00 |
| Unigene18995 | macB | 1.137E-05 | 0.000E+00 | 0.000E+00 | 0.000E+00 | 1.573E-05 | 1.516E-05 | 0.000E+00 | 0.000E+00 | 1.202E-05 | 0.000E+00 | 0.000E+00 | 0.000E+00 |
| Unigene19016 | vanHB | 1.162E-05 | 0.000E+00 | 0.000E+00 | 0.000E+00 | 2.020E-05 | 1.665E-05 | 3.805E-08 | 0.000E+00 | 1.379E-05 | 0.000E+00 | 0.000E+00 | 0.000E+00 |
| Unigene19020 | macB | 9.618E-06 | 0.000E+00 | 0.000E+00 | 0.000E+00 | 1.883E-05 | 1.572E-05 | 0.000E+00 | 0.000E+00 | 1.368E-05 | 0.000E+00 | 0.000E+00 | 0.000E+00 |
| Unigene19040 | mdtG | 2.600E-06 | 2.232E-06 | 3.082E-06 | 1.066E-06 | 2.574E-07 | 1.948E-07 | 1.424E-07 | 3.906E-07 | 8.159E-07 | 0.000E+00 | 0.000E+00 | 6.086E-08 |
| Unigene19052 | novA | 2.052E-06 | 0.000E+00 | 3.487E-06 | 0.000E+00 | 0.000E+00 | 0.000E+00 | 7.755E-07 | 0.000E+00 | 6.017E-07 | 0.000E+00 | 0.000E+00 | 1.775E-07 |
| Unigene19055 | cfrA | 2.569E-06 | 2.189E-06 | 2.699E-06 | 1.953E-06 | 0.000E+00 | 6.535E-08 | 5.018E-07 | 0.000E+00 | 4.625E-07 | 0.000E+00 | 0.000E+00 | 0.000E+00 |
| Unigene19132 | macB | 1.212E-05 | 5.259E-06 | 3.609E-06 | 1.303E-05 | 8.692E-05 | 1.178E-04 | 1.246E-04 | 9.933E-05 | 1.534E-04 | 1.539E-04 | 1.281E-04 | 7.740E-05 |
| Unigene19151 | poxtA | 2.736E-06 | 5.124E-06 | 5.225E-06 | 7.403E-06 | 8.104E-08 | 0.000E+00 | 0.000E+00 | 0.000E+00 | 0.000E+00 | 0.000E+00 | 0.000E+00 | 0.000E+00 |
| Unigene19168 | novA | 6.361E-07 | 0.000E+00 | 2.080E-07 | 1.606E-07 | 1.178E-07 | 0.000E+00 | 0.000E+00 | 1.237E-07 | 0.000E+00 | 0.000E+00 | 0.000E+00 | 0.000E+00 |
| Unigene19175 | arlS | 8.990E-05 | 1.406E-04 | 1.285E-04 | 6.770E-06 | 7.709E-06 | 4.055E-07 | 3.710E-06 | 1.669E-06 | 5.583E-05 | 1.306E-07 | 2.172E-06 | 8.867E-07 |
| Unigene19176 | vanRI | 8.487E-05 | 1.288E-04 | 1.196E-04 | 5.750E-06 | 6.525E-06 | 3.877E-07 | 0.000E+00 | 1.989E-06 | 5.082E-05 | 0.000E+00 | 2.667E-06 | 7.949E-07 |
| Unigene19177 | mel | 8.773E-05 | 1.432E-04 | 1.236E-04 | 6.440E-06 | 7.318E-06 | 1.135E-07 | 4.328E-06 | 2.267E-06 | 5.252E-05 | 4.093E-07 | 2.672E-06 | 1.148E-06 |
| Unigene19220 | macB | 3.822E-06 | 2.736E-07 | 3.750E-06 | 4.195E-07 | 4.615E-07 | 0.000E+00 | 2.299E-07 | 0.000E+00 | 0.000E+00 | 0.000E+00 | 9.504E-07 | 0.000E+00 |
| Unigene19227 | Staphylocc | 3.672E-06 | 2.806E-07 | 4.036E-06 | 3.286E-07 | 2.065E-07 | 5.025E-08 | 2.315E-07 | 0.000E+00 | 0.000E+00 | 0.000E+00 | 8.507E-07 | 0.000E+00 |
| Unigene19323 | mdtG | 6.644E-05 | 5.435E-05 | 1.067E-04 | 1.779E-04 | 1.128E-04 | 8.247E-05 | 1.066E-04 | 9.715E-05 | 8.709E-05 | 1.277E-04 | 1.368E-04 | 1.415E-04 |
| Unigene19332 | arnA | 3.321E-06 | 3.418E-07 | 2.954E-06 | 5.241E-07 | 3.844E-07 | 0.000E+00 | 1.915E-07 | 0.000E+00 | 1.891E-08 | 0.000E+00 | 9.103E-07 | 0.000E+00 |
| Unigene19386 | macB | 3.279E-06 | 4.839E-06 | 3.745E-06 | 6.525E-06 | 5.051E-07 | 1.043E-07 | 3.202E-07 | 4.824E-08 | 2.409E-07 | 0.000E+00 | 1.261E-07 | 2.443E-07 |
| Unigene19390 | vanHD | 3.133E-06 | 4.843E-06 | 3.635E-06 | 6.767E-06 | 5.145E-07 | 1.472E-07 | 2.713E-07 | 0.000E+00 | 2.084E-07 | 0.000E+00 | 0.000E+00 | 2.898E-07 |
| Unigene19443 | tetA(60) | 4.818E-05 | 7.249E-05 | 2.920E-05 | 1.415E-05 | 1.925E-05 | 7.130E-06 | 2.960E-06 | 6.546E-05 | 1.369E-05 | 6.907E-06 | 1.982E-06 | 9.701E-07 |
| Unigene19446 | PmrF | 4.934E-05 | 6.654E-05 | 2.634E-05 | 1.509E-05 | 1.973E-05 | 7.077E-06 | 3.623E-06 | 5.991E-05 | 1.968E-05 | 6.168E-06 | 1.434E-06 | 9.467E-07 |
| Unigene19508 | Erm(34) | 3.830E-06 | 9.087E-06 | 1.149E-05 | 1.385E-05 | 0.000E+00 | 0.000E+00 | 0.000E+00 | 0.000E+00 | 0.000E+00 | 0.000E+00 | 0.000E+00 | 0.000E+00 |
| Unigene19531 | efrA | 1.865E-06 | 1.573E-06 | 2.918E-06 | 2.730E-06 | 1.021E-05 | 2.473E-05 | 4.039E-06 | 6.627E-07 | 1.489E-05 | 1.283E-05 | 0.000E+00 | 1.150E-06 |
| Unigene19552 | vgaC | 2.381E-06 | 4.337E-06 | 5.980E-06 | 7.019E-06 | 0.000E+00 | 0.000E+00 | 0.000E+00 | 5.557E-08 | 0.000E+00 | 0.000E+00 | 0.000E+00 | 0.000E+00 |
| Unigene19558 | PmrF | 8.433E-07 | 0.000E+00 | 0.000E+00 | 0.000E+00 | 0.000E+00 | 3.646E-08 | 0.000E+00 | 0.000E+00 | 0.000E+00 | 0.000E+00 | 0.000E+00 | 0.000E+00 |
| Unigene19614 | ceoB | 0.000E+00 | 0.000E+00 | 0.000E+00 | 1.242E-07 | 4.098E-07 | 0.000E+00 | 0.000E+00 | 3.588E-07 | 0.000E+00 | 5.137E-07 | 3.868E-07 | 6.904E-07 |
| Unigene19654 | vanRM | 8.889E-07 | 7.188E-07 | 3.810E-06 | 5.905E-06 | 0.000E+00 | 0.000E+00 | 5.695E-07 | 0.000E+00 | 0.000E+00 | 0.000E+00 | 0.000E+00 | 0.000E+00 |
| Unigene19655 | vanSF | 7.188E-07 | 7.889E-07 | 4.702E-06 | 5.851E-06 | 0.000E+00 | 0.000E+00 | 3.375E-07 | 0.000E+00 | 0.000E+00 | 0.000E+00 | 0.000E+00 | 0.000E+00 |
| Unigene19662 | macB | 0.000E+00 | 0.000E+00 | 0.000E+00 | 0.000E+00 | 7.936E-07 | 0.000E+00 | 0.000E+00 | 0.000E+00 | 0.000E+00 | 0.000E+00 | 1.226E-06 | 3.325E-06 |

| | | | | | | | | | | | | | |
|---|---|---|---|---|---|---|---|---|---|---|---|---|---|
| Unigene19682 | carA | 7.668E-05 | 6.374E-05 | 1.131E-04 | 1.596E-04 | 1.382E-04 | 9.853E-05 | 1.286E-04 | 1.195E-04 | 1.089E-04 | 1.540E-04 | 1.382E-04 | 1.600E-04 |
| Unigene19685 | kdpE | 3.386E-06 | 5.090E-06 | 8.328E-06 | 9.554E-06 | 0.000E+00 | 0.000E+00 | 0.000E+00 | 0.000E+00 | 0.000E+00 | 0.000E+00 | 0.000E+00 | 0.000E+00 |
| Unigene19728 | arnA | 2.704E-06 | 3.391E-06 | 3.249E-06 | 4.625E-06 | 0.000E+00 | 0.000E+00 | 4.275E-07 | 0.000E+00 | 0.000E+00 | 0.000E+00 | 0.000E+00 | 1.304E-07 |
| Unigene19812 | patA | 1.316E-05 | 0.000E+00 | 0.000E+00 | 0.000E+00 | 2.157E-05 | 1.829E-05 | 0.000E+00 | 0.000E+00 | 1.373E-05 | 0.000E+00 | 0.000E+00 | 0.000E+00 |
| Unigene19819 | Staphylocc | 1.094E-05 | 0.000E+00 | 0.000E+00 | 0.000E+00 | 1.796E-05 | 1.630E-05 | 1.346E-08 | 0.000E+00 | 1.135E-05 | 2.573E-07 | 1.391E-08 | 0.000E+00 |
| Unigene19841 | optrA | 1.012E-04 | 1.796E-04 | 1.416E-04 | 0.000E+00 | 9.729E-06 | 0.000E+00 | 0.000E+00 | 0.000E+00 | 5.117E-05 | 0.000E+00 | 2.931E-06 | 1.321E-06 |
| Unigene19842 | poxtA | 1.044E-04 | 1.828E-04 | 1.701E-04 | 0.000E+00 | 8.416E-06 | 3.421E-07 | 3.743E-06 | 0.000E+00 | 5.310E-05 | 0.000E+00 | 2.828E-06 | 0.000E+00 |
| Unigene19853 | oleC | 1.115E-06 | 0.000E+00 | 0.000E+00 | 0.000E+00 | 0.000E+00 | 0.000E+00 | 0.000E+00 | 0.000E+00 | 0.000E+00 | 0.000E+00 | 0.000E+00 | 0.000E+00 |
| Unigene19869 | tet32 | 7.686E-07 | 5.483E-06 | 9.393E-06 | 1.485E-05 | 0.000E+00 | 9.442E-08 | 1.160E-07 | 0.000E+00 | 0.000E+00 | 0.000E+00 | 0.000E+00 | 0.000E+00 |
| Unigene19876 | vmlR | 1.312E-06 | 9.601E-08 | 0.000E+00 | 0.000E+00 | 0.000E+00 | 0.000E+00 | 0.000E+00 | 0.000E+00 | 0.000E+00 | 0.000E+00 | 0.000E+00 | 0.000E+00 |
| Unigene19878 | vanHD | 1.494E-06 | 0.000E+00 | 0.000E+00 | 0.000E+00 | 0.000E+00 | 0.000E+00 | 0.000E+00 | 0.000E+00 | 0.000E+00 | 0.000E+00 | 0.000E+00 | 0.000E+00 |
| Unigene19884 | Listeria mc | 1.363E-06 | 0.000E+00 | 0.000E+00 | 0.000E+00 | 0.000E+00 | 0.000E+00 | 0.000E+00 | 0.000E+00 | 0.000E+00 | 0.000E+00 | 0.000E+00 | 0.000E+00 |
| Unigene19909 | efmA | 3.602E-05 | 2.672E-05 | 0.000E+00 | 1.565E-06 | 6.737E-05 | 4.781E-05 | 4.511E-05 | 5.716E-05 | 7.903E-05 | 9.112E-05 | 4.464E-05 | 6.790E-05 |
| Unigene19918 | efrA | 7.330E-06 | 0.000E+00 | 0.000E+00 | 2.948E-06 | 3.520E-05 | 1.772E-05 | 2.490E-05 | 3.089E-05 | 0.000E+00 | 0.000E+00 | 0.000E+00 | 4.827E-08 |
| Unigene20055 | macB | 8.252E-07 | 0.000E+00 | 2.223E-07 | 4.902E-07 | 3.397E-06 | 7.382E-06 | 1.039E-06 | 7.744E-06 | 3.820E-06 | 3.154E-06 | 0.000E+00 | 8.033E-07 |
| Unigene20083 | macB | 2.391E-06 | 1.796E-05 | 6.491E-06 | 4.722E-06 | 0.000E+00 | 0.000E+00 | 0.000E+00 | 0.000E+00 | 0.000E+00 | 1.776E-07 | 3.039E-08 | 0.000E+00 |
| Unigene20085 | oleD | 2.690E-06 | 1.889E-05 | 9.481E-06 | 6.791E-06 | 0.000E+00 | 5.935E-08 | 0.000E+00 | 0.000E+00 | 0.000E+00 | 0.000E+00 | 0.000E+00 | 1.947E-07 |
| Unigene20104 | YojI | 5.225E-06 | 2.176E-05 | 1.364E-05 | 1.147E-05 | 0.000E+00 | 0.000E+00 | 2.514E-07 | 0.000E+00 | 0.000E+00 | 0.000E+00 | 1.654E-07 | 7.324E-08 |
| Unigene20150 | mepA | 2.250E-06 | 1.608E-05 | 7.775E-06 | 4.806E-06 | 0.000E+00 | 1.521E-07 | 0.000E+00 | 0.000E+00 | 0.000E+00 | 0.000E+00 | 1.877E-07 | 5.542E-08 |
| Unigene20161 | kdpE | 2.819E-07 | 2.682E-06 | 4.196E-06 | 9.727E-06 | 0.000E+00 | 0.000E+00 | 0.000E+00 | 5.485E-08 | 0.000E+00 | 0.000E+00 | 0.000E+00 | 0.000E+00 |
| Unigene20180 | vanHO | 1.304E-06 | 0.000E+00 | 0.000E+00 | 0.000E+00 | 0.000E+00 | 0.000E+00 | 0.000E+00 | 0.000E+00 | 0.000E+00 | 0.000E+00 | 0.000E+00 | 0.000E+00 |
| Unigene20187 | lmrP | 1.575E-06 | 0.000E+00 | 0.000E+00 | 0.000E+00 | 0.000E+00 | 0.000E+00 | 0.000E+00 | 0.000E+00 | 0.000E+00 | 0.000E+00 | 0.000E+00 | 0.000E+00 |
| Unigene20200 | Staphylocc | 1.482E-06 | 2.502E-08 | 0.000E+00 | 0.000E+00 | 0.000E+00 | 0.000E+00 | 0.000E+00 | 0.000E+00 | 0.000E+00 | 0.000E+00 | 0.000E+00 | 0.000E+00 |
| Unigene20215 | Staphylocc | 1.494E-06 | 0.000E+00 | 0.000E+00 | 6.468E-08 | 0.000E+00 | 0.000E+00 | 0.000E+00 | 0.000E+00 | 0.000E+00 | 0.000E+00 | 0.000E+00 | 0.000E+00 |
| Unigene20249 | carA | 2.115E-06 | 0.000E+00 | 0.000E+00 | 0.000E+00 | 0.000E+00 | 0.000E+00 | 0.000E+00 | 0.000E+00 | 0.000E+00 | 0.000E+00 | 0.000E+00 | 0.000E+00 |
| Unigene20272 | vanRI | 1.777E-06 | 7.908E-07 | 1.037E-06 | 1.552E-06 | 1.745E-05 | 5.608E-06 | 1.451E-05 | 1.878E-05 | 1.171E-05 | 1.616E-05 | 1.714E-05 | 7.211E-06 |
| Unigene20287 | Corynebac | 0.000E+00 | 1.763E-07 | 3.152E-07 | 0.000E+00 | 0.000E+00 | 0.000E+00 | 0.000E+00 | 0.000E+00 | 0.000E+00 | 0.000E+00 | 0.000E+00 | 0.000E+00 |
| Unigene20320 | vanXYN | 2.647E-06 | 4.190E-06 | 2.001E-06 | 2.497E-06 | 2.603E-06 | 1.454E-06 | 1.633E-06 | 8.609E-07 | 1.802E-06 | 4.833E-06 | 4.616E-06 | 1.487E-06 |
| Unigene20322 | Streptomy | 2.583E-06 | 3.374E-06 | 1.990E-06 | 1.584E-06 | 2.253E-06 | 1.919E-06 | 1.614E-06 | 1.184E-06 | 9.526E-07 | 5.049E-06 | 4.804E-06 | 1.517E-06 |
| Unigene20328 | FusF | 3.098E-05 | 0.000E+00 | 0.000E+00 | 0.000E+00 | 0.000E+00 | 0.000E+00 | 0.000E+00 | 0.000E+00 | 0.000E+00 | 0.000E+00 | 0.000E+00 | 0.000E+00 |
| Unigene20335 | ErmH | 3.203E-05 | 0.000E+00 | 0.000E+00 | 0.000E+00 | 0.000E+00 | 0.000E+00 | 0.000E+00 | 0.000E+00 | 0.000E+00 | 0.000E+00 | 0.000E+00 | 0.000E+00 |
| Unigene20339 | Staphylocc | 2.999E-05 | 1.043E-06 | 5.690E-07 | 0.000E+00 | 0.000E+00 | 1.045E-07 | 0.000E+00 | 0.000E+00 | 0.000E+00 | 0.000E+00 | 0.000E+00 | 0.000E+00 |
| Unigene20472 | arlS | 7.399E-06 | 0.000E+00 | 0.000E+00 | 2.371E-06 | 3.203E-05 | 1.790E-05 | 2.529E-05 | 2.630E-05 | 3.342E-06 | 0.000E+00 | 0.000E+00 | 0.000E+00 |
| Unigene20473 | vanRM | 6.843E-06 | 0.000E+00 | 0.000E+00 | 2.455E-06 | 3.246E-05 | 1.713E-05 | 2.412E-05 | 2.438E-05 | 4.625E-06 | 0.000E+00 | 0.000E+00 | 0.000E+00 |
| Unigene20507 | Streptomy | 3.559E-06 | 3.046E-07 | 3.829E-06 | 3.463E-07 | 4.898E-07 | 3.530E-08 | 2.349E-07 | 0.000E+00 | 0.000E+00 | 0.000E+00 | 1.177E-06 | 0.000E+00 |
| Unigene20579 | bcrA | 1.137E-06 | 6.721E-07 | 3.605E-06 | 5.477E-06 | 0.000E+00 | 1.891E-07 | 3.389E-07 | 1.532E-07 | 0.000E+00 | 0.000E+00 | 0.000E+00 | 0.000E+00 |
| Unigene20615 | macB | 4.211E-05 | 2.515E-05 | 2.005E-05 | 2.388E-05 | 1.849E-06 | 2.008E-06 | 2.656E-06 | 5.873E-07 | 0.000E+00 | 4.312E-07 | 1.417E-06 | 0.000E+00 |
| Unigene20621 | optrA | 5.249E-05 | 2.971E-05 | 2.234E-05 | 2.347E-05 | 2.290E-06 | 2.249E-06 | 4.167E-06 | 1.226E-06 | 8.079E-06 | 7.285E-07 | 2.312E-06 | 1.640E-06 |
| Unigene20662 | patA | 1.007E-06 | 0.000E+00 | 0.000E+00 | 4.624E-07 | 0.000E+00 | 8.019E-06 | 0.000E+00 | 0.000E+00 | 4.204E-06 | 0.000E+00 | 0.000E+00 | 0.000E+00 |
| Unigene20667 | oleC | 3.985E-07 | 0.000E+00 | 3.910E-07 | 0.000E+00 | 0.000E+00 | 0.000E+00 | 0.000E+00 | 0.000E+00 | 0.000E+00 | 0.000E+00 | 0.000E+00 | 0.000E+00 |
| Unigene20685 | vanRI | 6.927E-06 | 0.000E+00 | 0.000E+00 | 1.725E-06 | 3.340E-05 | 1.578E-05 | 2.387E-05 | 2.312E-05 | 3.331E-06 | 0.000E+00 | 0.000E+00 | 0.000E+00 |
| Unigene20688 | tetA(58) | 7.334E-06 | 0.000E+00 | 0.000E+00 | 2.794E-06 | 2.991E-05 | 1.818E-05 | 2.212E-05 | 2.374E-05 | 3.840E-06 | 0.000E+00 | 0.000E+00 | 0.000E+00 |
| Unigene20700 | Escherichia | 7.258E-06 | 0.000E+00 | 0.000E+00 | 2.733E-06 | 3.636E-05 | 1.824E-05 | 2.639E-05 | 2.835E-05 | 3.514E-06 | 0.000E+00 | 0.000E+00 | 0.000E+00 |
| Unigene20755 | PmrF | 3.715E-06 | 3.972E-07 | 3.598E-06 | 5.603E-07 | 2.412E-07 | 0.000E+00 | 3.204E-07 | 0.000E+00 | 0.000E+00 | 0.000E+00 | 9.659E-07 | 0.000E+00 |
| Unigene20786 | baeS | 9.427E-07 | 0.000E+00 | 0.000E+00 | 0.000E+00 | 4.444E-06 | 0.000E+00 | 0.000E+00 | 0.000E+00 | 0.000E+00 | 0.000E+00 | 0.000E+00 | 0.000E+00 |
| Unigene20821 | vgaB | 2.955E-05 | 9.148E-06 | 7.088E-06 | 4.540E-06 | 3.929E-07 | 0.000E+00 | 0.000E+00 | 3.538E-07 | 7.178E-07 | 0.000E+00 | 0.000E+00 | 0.000E+00 |
| Unigene20907 | tetA(58) | 2.699E-06 | 2.725E-06 | 2.119E-06 | 2.144E-06 | 2.559E-06 | 2.490E-06 | 1.513E-06 | 7.141E-07 | 1.101E-06 | 6.494E-06 | 4.240E-06 | 1.914E-06 |
| Unigene20982 | macB | 2.677E-06 | 1.989E-05 | 8.510E-06 | 5.109E-06 | 0.000E+00 | 0.000E+00 | 0.000E+00 | 0.000E+00 | 0.000E+00 | 0.000E+00 | 0.000E+00 | 0.000E+00 |
| Unigene20983 | macB | 3.285E-06 | 1.500E-05 | 6.354E-06 | 6.058E-06 | 0.000E+00 | 0.000E+00 | 0.000E+00 | 0.000E+00 | 0.000E+00 | 0.000E+00 | 0.000E+00 | 0.000E+00 |
| Unigene20993 | vatE | 2.860E-06 | 1.794E-06 | 4.878E-06 | 2.407E-06 | 0.000E+00 | 0.000E+00 | 0.000E+00 | 0.000E+00 | 0.000E+00 | 0.000E+00 | 0.000E+00 | 0.000E+00 |
| Unigene21029 | Staphylocc | 7.218E-06 | 0.000E+00 | 0.000E+00 | 1.890E-06 | 3.257E-05 | 1.768E-05 | 2.463E-05 | 2.577E-05 | 3.725E-06 | 0.000E+00 | 0.000E+00 | 0.000E+00 |
| Unigene21036 | TaeA | 7.173E-06 | 0.000E+00 | 0.000E+00 | 2.849E-06 | 3.517E-05 | 1.649E-05 | 2.562E-05 | 2.886E-05 | 0.000E+00 | 0.000E+00 | 0.000E+00 | 0.000E+00 |
| Unigene21055 | QepA4 | 7.601E-05 | 7.040E-05 | 1.252E-04 | 1.729E-04 | 1.387E-04 | 9.916E-05 | 1.281E-04 | 1.212E-04 | 1.109E-04 | 1.580E-04 | 1.720E-04 | 1.806E-04 |
| Unigene21079 | vgaE | 6.291E-06 | 3.316E-06 | 0.000E+00 | 9.194E-07 | 0.000E+00 | 2.237E-07 | 4.580E-08 | 0.000E+00 | 0.000E+00 | 0.000E+00 | 1.894E-07 | 0.000E+00 |
| Unigene21089 | optrA | 8.515E-06 | 4.570E-06 | 7.345E-07 | 1.559E-06 | 1.039E-07 | 1.011E-07 | 0.000E+00 | 0.000E+00 | 6.494E-06 | 0.000E+00 | 3.746E-07 | 0.000E+00 |
| Unigene21092 | macB | 7.206E-06 | 3.313E-06 | 6.688E-07 | 1.475E-06 | 0.000E+00 | 2.105E-07 | 1.617E-07 | 0.000E+00 | 6.173E-06 | 0.000E+00 | 1.671E-07 | 0.000E+00 |
| Unigene21093 | macB | 6.591E-06 | 4.606E-06 | 4.487E-07 | 2.309E-06 | 2.988E-07 | 8.724E-08 | 1.489E-07 | 0.000E+00 | 5.792E-06 | 0.000E+00 | 3.077E-08 | 6.359E-08 |
| Unigene21096 | bcrA | 8.401E-06 | 3.884E-06 | 2.455E-07 | 1.119E-06 | 2.780E-07 | 7.729E-08 | 1.187E-07 | 0.000E+00 | 0.000E+00 | 0.000E+00 | 0.000E+00 | 0.000E+00 |

| | | | | | | | | | | | | | |
|---|---|---|---|---|---|---|---|---|---|---|---|---|---|
| Unigene21128 | optrA | 1.258E-06 | 6.146E-06 | 1.027E-05 | 1.533E-05 | 0.000E+00 | 0.000E+00 | 0.000E+00 | 0.000E+00 | 0.000E+00 | 0.000E+00 | 0.000E+00 | 0.000E+00 |
| Unigene21155 | vanHM | 7.013E-05 | 7.131E-06 | 0.000E+00 | 0.000E+00 | 0.000E+00 | 0.000E+00 | 0.000E+00 | 0.000E+00 | 0.000E+00 | 0.000E+00 | 0.000E+00 | 0.000E+00 |
| Unigene21169 | lmrB | 1.154E-06 | 1.215E-06 | 4.875E-06 | 7.252E-06 | 2.616E-08 | 0.000E+00 | 4.951E-07 | 0.000E+00 | 0.000E+00 | 0.000E+00 | 8.080E-08 | 1.113E-07 |
| Unigene21173 | vatE | 2.108E-05 | 1.499E-06 | 0.000E+00 | 0.000E+00 | 0.000E+00 | 0.000E+00 | 0.000E+00 | 0.000E+00 | 0.000E+00 | 0.000E+00 | 0.000E+00 | 0.000E+00 |
| Unigene21191 | tetA(60) | 3.363E-07 | 0.000E+00 | 0.000E+00 | 0.000E+00 | 0.000E+00 | 2.272E-07 | 0.000E+00 | 0.000E+00 | 0.000E+00 | 0.000E+00 | 0.000E+00 | 0.000E+00 |
| Unigene21209 | macB | 3.383E-06 | 6.292E-06 | 7.054E-06 | 6.880E-06 | 0.000E+00 | 1.016E-07 | 0.000E+00 | 0.000E+00 | 0.000E+00 | 0.000E+00 | 0.000E+00 | 0.000E+00 |
| Unigene21243 | kdpE | 8.570E-07 | 0.000E+00 | 0.000E+00 | 9.616E-08 | 0.000E+00 | 5.662E-07 | 0.000E+00 | 0.000E+00 | 6.245E-07 | 0.000E+00 | 0.000E+00 | 0.000E+00 |
| Unigene21269 | bcr-1 | 6.472E-07 | 1.450E-06 | 0.000E+00 | 0.000E+00 | 6.112E-06 | 2.624E-07 | 0.000E+00 | 3.148E-07 | 8.843E-08 | 0.000E+00 | 0.000E+00 | 0.000E+00 |
| Unigene21286 | basS | 2.844E-07 | 0.000E+00 | 0.000E+00 | 0.000E+00 | 3.160E-07 | 0.000E+00 | 0.000E+00 | 0.000E+00 | 0.000E+00 | 0.000E+00 | 2.169E-07 | 3.362E-07 |
| Unigene21291 | tetB(60) | 1.041E-04 | 1.846E-04 | 1.725E-04 | 0.000E+00 | 8.870E-06 | 2.533E-07 | 0.000E+00 | 1.683E-06 | 5.372E-05 | 3.614E-07 | 0.000E+00 | 0.000E+00 |
| Unigene21305 | lfrA | 7.128E-06 | 0.000E+00 | 0.000E+00 | 2.522E-06 | 3.563E-05 | 1.779E-05 | 2.608E-05 | 2.692E-05 | 3.945E-06 | 0.000E+00 | 0.000E+00 | 0.000E+00 |
| Unigene21324 | efrB | 2.218E-06 | 1.951E-06 | 3.148E-06 | 1.555E-06 | 2.444E-07 | 1.783E-07 | 4.666E-07 | 0.000E+00 | 7.613E-07 | 0.000E+00 | 0.000E+00 | 0.000E+00 |
| Unigene21386 | arlR | 5.668E-06 | 2.351E-05 | 3.677E-06 | 1.649E-06 | 9.606E-07 | 5.193E-07 | 2.658E-07 | 0.000E+00 | 1.733E-06 | 0.000E+00 | 4.396E-07 | 5.110E-07 |
| Unigene21411 | lmrD | 5.096E-05 | 6.300E-05 | 3.051E-05 | 1.519E-05 | 2.123E-05 | 5.679E-06 | 4.360E-06 | 5.444E-05 | 1.218E-05 | 6.748E-06 | 1.841E-06 | 1.152E-06 |
| Unigene21456 | patB | 4.972E-05 | 6.792E-05 | 2.930E-05 | 1.306E-05 | 1.573E-05 | 7.172E-06 | 3.012E-06 | 5.247E-05 | 1.353E-05 | 8.291E-06 | 2.384E-06 | 5.530E-07 |
| Unigene21462 | vanRI | 3.782E-05 | 5.648E-05 | 2.399E-05 | 1.211E-05 | 1.628E-05 | 7.771E-06 | 3.294E-06 | 4.890E-05 | 1.021E-05 | 7.090E-06 | 1.624E-06 | 7.577E-07 |
| Unigene21475 | lmrB | 4.283E-05 | 5.788E-05 | 2.697E-05 | 1.301E-05 | 1.787E-05 | 6.401E-06 | 3.188E-06 | 5.129E-05 | 1.181E-05 | 6.417E-06 | 1.687E-06 | 8.988E-07 |
| Unigene21493 | msbA | 5.233E-07 | 0.000E+00 | 0.000E+00 | 0.000E+00 | 0.000E+00 | 7.071E-08 | 0.000E+00 | 0.000E+00 | 0.000E+00 | 0.000E+00 | 0.000E+00 | 0.000E+00 |
| Unigene21608 | msbA | 3.112E-06 | 3.909E-06 | 4.083E-06 | 6.417E-06 | 4.373E-07 | 6.080E-08 | 3.320E-07 | 0.000E+00 | 2.868E-07 | 0.000E+00 | 1.072E-07 | 1.551E-07 |
| Unigene21609 | novA | 3.415E-06 | 4.373E-06 | 3.315E-06 | 6.286E-06 | 3.876E-07 | 0.000E+00 | 2.235E-07 | 1.072E-07 | 2.007E-07 | 0.000E+00 | 1.470E-07 | 1.736E-07 |
| Unigene21644 | tetB(46) | 2.833E-05 | 9.235E-06 | 7.709E-06 | 4.399E-06 | 3.309E-07 | 8.050E-08 | 0.000E+00 | 8.692E-08 | 0.000E+00 | 0.000E+00 | 0.000E+00 | 0.000E+00 |
| Unigene21645 | efrA | 2.389E-05 | 8.952E-06 | 5.937E-06 | 4.872E-06 | 3.829E-07 | 5.381E-07 | 0.000E+00 | 3.129E-07 | 1.130E-06 | 0.000E+00 | 0.000E+00 | 0.000E+00 |
| Unigene21650 | oleC | 2.729E-05 | 8.161E-06 | 6.910E-06 | 4.859E-06 | 4.859E-07 | 2.364E-07 | 0.000E+00 | 0.000E+00 | 0.000E+00 | 0.000E+00 | 0.000E+00 | 0.000E+00 |
| Unigene21685 | arlR | 2.273E-06 | 4.683E-06 | 3.504E-06 | 5.246E-06 | 5.668E-07 | 0.000E+00 | 3.080E-07 | 0.000E+00 | 0.000E+00 | 0.000E+00 | 0.000E+00 | 3.838E-07 |
| Unigene21687 | macB | 3.448E-06 | 3.557E-06 | 3.057E-06 | 5.580E-06 | 2.769E-07 | 0.000E+00 | 2.299E-07 | 0.000E+00 | 1.362E-07 | 0.000E+00 | 9.504E-08 | 1.964E-07 |
| Unigene21703 | fusB | 2.402E-06 | 3.680E-06 | 2.603E-06 | 1.920E-06 | 2.613E-06 | 2.110E-06 | 1.828E-06 | 0.000E+00 | 1.422E-06 | 5.519E-06 | 5.839E-06 | 1.597E-06 |
| Unigene21717 | kdpE | 3.133E-06 | 1.859E-05 | 2.539E-06 | 1.238E-06 | 7.566E-07 | 4.908E-07 | 2.010E-07 | 3.710E-07 | 3.970E-07 | 0.000E+00 | 0.000E+00 | 0.000E+00 |
| Unigene21718 | evgS | 4.196E-06 | 1.841E-05 | 3.383E-06 | 8.177E-07 | 1.063E-06 | 4.112E-07 | 2.444E-07 | 3.724E-07 | 1.314E-06 | 7.107E-07 | 3.368E-07 | 1.160E-07 |
| Unigene21725 | Escherichia | 4.202E-06 | 9.785E-06 | 1.549E-05 | 1.149E-05 | 2.277E-07 | 4.801E-07 | 6.427E-07 | 1.994E-07 | 0.000E+00 | 0.000E+00 | 3.907E-08 | 8.075E-08 |
| Unigene21846 | oleC | 6.291E-06 | 0.000E+00 | 0.000E+00 | 1.533E-06 | 3.215E-05 | 1.646E-05 | 2.478E-05 | 2.335E-05 | 2.893E-06 | 0.000E+00 | 0.000E+00 | 0.000E+00 |
| Unigene21860 | Streptomy | 9.932E-07 | 6.432E-06 | 9.957E-06 | 1.510E-05 | 0.000E+00 | 0.000E+00 | 0.000E+00 | 1.932E-08 | 0.000E+00 | 0.000E+00 | 0.000E+00 | 3.913E-08 |
| Unigene21877 | Listeria mc | 1.436E-06 | 9.957E-07 | 4.525E-06 | 6.731E-06 | 0.000E+00 | 8.172E-08 | 2.649E-07 | 0.000E+00 | 0.000E+00 | 0.000E+00 | 2.883E-08 | 4.468E-08 |
| Unigene21881 | carA | 2.939E-06 | 3.902E-06 | 8.512E-06 | 1.115E-05 | 0.000E+00 | 0.000E+00 | 2.098E-07 | 0.000E+00 | 0.000E+00 | 0.000E+00 | 0.000E+00 | 0.000E+00 |
| Unigene21908 | carA | 3.232E-06 | 4.294E-06 | 3.213E-06 | 6.056E-06 | 6.615E-07 | 1.379E-07 | 3.766E-07 | 0.000E+00 | 2.324E-07 | 0.000E+00 | 1.216E-07 | 3.016E-07 |
| Unigene21935 | vanHD | 5.044E-07 | 0.000E+00 | 8.249E-08 | 0.000E+00 | 0.000E+00 | 4.544E-08 | 0.000E+00 | 0.000E+00 | 0.000E+00 | 0.000E+00 | 0.000E+00 | 0.000E+00 |
| Unigene21959 | vanRI | 4.847E-05 | 2.968E-05 | 2.242E-05 | 2.368E-05 | 2.861E-06 | 1.442E-06 | 3.868E-06 | 0.000E+00 | 9.298E-06 | 7.684E-07 | 2.788E-06 | 1.413E-06 |
| Unigene21989 | cmlA1 | 1.565E-06 | 1.265E-06 | 1.508E-06 | 1.635E-06 | 2.049E-05 | 5.875E-06 | 1.486E-05 | 1.781E-05 | 1.062E-05 | 1.740E-05 | 1.934E-05 | 7.365E-06 |
| Unigene22041 | emrB | 2.464E-05 | 9.577E-06 | 5.987E-06 | 4.466E-06 | 2.365E-07 | 2.812E-07 | 0.000E+00 | 1.656E-07 | 9.564E-07 | 0.000E+00 | 0.000E+00 | 0.000E+00 |
| Unigene22047 | msbA | 4.125E-07 | 0.000E+00 | 3.036E-07 | 0.000E+00 | 0.000E+00 | 5.203E-07 | 1.636E-06 | 9.229E-07 | 0.000E+00 | 0.000E+00 | 7.472E-07 | 1.300E-06 |
| Unigene22078 | ErmH | 3.526E-06 | 4.788E-07 | 3.246E-06 | 3.304E-07 | 2.423E-07 | 0.000E+00 | 2.816E-07 | 0.000E+00 | 0.000E+00 | 0.000E+00 | 9.564E-07 | 0.000E+00 |
| Unigene22083 | patA | 3.009E-05 | 8.977E-06 | 6.996E-06 | 4.638E-06 | 4.859E-07 | 0.000E+00 | 0.000E+00 | 2.553E-07 | 0.000E+00 | 0.000E+00 | 0.000E+00 | 0.000E+00 |
| Unigene22084 | ykkD | 1.763E-05 | 0.000E+00 | 5.530E-06 | 0.000E+00 | 0.000E+00 | 0.000E+00 | 0.000E+00 | 0.000E+00 | 0.000E+00 | 0.000E+00 | 0.000E+00 | 0.000E+00 |
| Unigene22085 | ykkC | 2.807E-05 | 9.662E-06 | 7.295E-06 | 4.248E-06 | 6.520E-07 | 0.000E+00 | 0.000E+00 | 0.000E+00 | 0.000E+00 | 0.000E+00 | 0.000E+00 | 0.000E+00 |
| Unigene22096 | lsaC | 6.026E-07 | 0.000E+00 | 5.068E-07 | 0.000E+00 | 0.000E+00 | 0.000E+00 | 0.000E+00 | 0.000E+00 | 0.000E+00 | 0.000E+00 | 0.000E+00 | 0.000E+00 |
| Unigene22100 | kdpE | 4.286E-06 | 1.063E-05 | 9.003E-06 | 1.073E-05 | 9.920E-08 | 1.689E-07 | 4.941E-08 | 0.000E+00 | 0.000E+00 | 1.243E-07 | 0.000E+00 | 0.000E+00 |
| Unigene22114 | efmA | 0.000E+00 | 0.000E+00 | 2.172E-05 | 0.000E+00 | 6.558E-05 | 2.725E-05 | 0.000E+00 | 4.199E-05 | 0.000E+00 | 0.000E+00 | 2.761E-05 | 7.795E-05 |
| Unigene22169 | oleC | 4.594E-05 | 2.728E-05 | 1.651E-05 | 2.115E-05 | 2.635E-06 | 1.614E-06 | 4.180E-06 | 9.740E-07 | 8.352E-06 | 5.382E-07 | 2.512E-06 | 1.713E-06 |
| Unigene22183 | oleC | 3.363E-07 | 0.000E+00 | 0.000E+00 | 0.000E+00 | 0.000E+00 | 0.000E+00 | 0.000E+00 | 0.000E+00 | 0.000E+00 | 0.000E+00 | 0.000E+00 | 0.000E+00 |
| Unigene22278 | tetB(P) | 4.129E-05 | 2.396E-05 | 2.112E-05 | 2.349E-05 | 0.000E+00 | 1.731E-06 | 3.500E-06 | 5.513E-07 | 0.000E+00 | 0.000E+00 | 0.000E+00 | 1.165E-06 |
| Unigene22293 | oleC | 5.076E-06 | 0.000E+00 | 0.000E+00 | 0.000E+00 | 0.000E+00 | 0.000E+00 | 0.000E+00 | 0.000E+00 | 0.000E+00 | 0.000E+00 | 0.000E+00 | 0.000E+00 |
| Unigene22302 | dfrE | 4.108E-06 | 1.066E-05 | 8.830E-06 | 1.199E-05 | 0.000E+00 | 0.000E+00 | 0.000E+00 | 0.000E+00 | 0.000E+00 | 0.000E+00 | 0.000E+00 | 0.000E+00 |
| Unigene22324 | Staphylocc | 2.653E-05 | 0.000E+00 | 4.703E-08 | 0.000E+00 | 0.000E+00 | 0.000E+00 | 0.000E+00 | 0.000E+00 | 0.000E+00 | 0.000E+00 | 0.000E+00 | 0.000E+00 |
| Unigene22341 | Klebsiella p | 3.903E-07 | 3.059E-07 | 0.000E+00 | 0.000E+00 | 0.000E+00 | 0.000E+00 | 0.000E+00 | 1.952E-07 | 1.219E-07 | 0.000E+00 | 0.000E+00 | 0.000E+00 |
| Unigene22368 | vmlR | 2.947E-06 | 3.528E-06 | 2.848E-06 | 4.914E-06 | 4.960E-07 | 0.000E+00 | 3.953E-07 | 0.000E+00 | 9.760E-08 | 0.000E+00 | 0.000E+00 | 2.639E-07 |
| Unigene22395 | TaeA | 9.009E-07 | 1.026E-06 | 8.507E-07 | 1.219E-06 | 1.815E-05 | 5.421E-06 | 1.441E-05 | 1.617E-05 | 1.007E-05 | 1.663E-05 | 1.664E-05 | 7.414E-06 |
| Unigene22465 | ugd | 2.035E-06 | 1.101E-06 | 2.981E-06 | 1.802E-06 | 0.000E+00 | 3.014E-08 | 3.085E-08 | 3.254E-08 | 0.000E+00 | 1.863E-07 | 1.275E-07 | 3.295E-08 |
| Unigene22495 | tet36 | 6.804E-06 | 0.000E+00 | 0.000E+00 | 2.070E-06 | 3.240E-05 | 1.425E-05 | 2.225E-05 | 2.313E-05 | 3.069E-06 | 0.000E+00 | 0.000E+00 | 0.000E+00 |
| Unigene22528 | MOX-9 | 5.975E-06 | 0.000E+00 | 0.000E+00 | 0.000E+00 | 3.352E-05 | 1.280E-05 | 1.867E-05 | 2.061E-05 | 2.659E-06 | 0.000E+00 | 0.000E+00 | 0.000E+00 |

| | | | | | | | | | | | | | |
|---|---|---|---|---|---|---|---|---|---|---|---|---|---|
| Unigene22536 | macB | 6.876E-06 | 0.000E+00 | 0.000E+00 | 2.066E-06 | 3.342E-05 | 1.624E-05 | 2.237E-05 | 2.393E-05 | 3.073E-06 | 0.000E+00 | 0.000E+00 | 0.000E+00 |
| Unigene22563 | bcrA | 7.985E-07 | 7.178E-06 | 1.127E-05 | 1.678E-05 | 0.000E+00 | 0.000E+00 | 0.000E+00 | 0.000E+00 | 0.000E+00 | 0.000E+00 | 0.000E+00 | 0.000E+00 |
| Unigene22583 | vanYF | 5.445E-06 | 2.314E-05 | 3.986E-06 | 1.222E-06 | 9.121E-07 | 4.672E-07 | 2.870E-07 | 0.000E+00 | 1.559E-06 | 6.258E-07 | 3.460E-07 | 5.619E-07 |
| Unigene22595 | tetA(58) | 3.772E-06 | 2.042E-05 | 3.912E-06 | 9.957E-07 | 6.191E-07 | 0.000E+00 | 1.423E-07 | 0.000E+00 | 1.359E-06 | 5.731E-07 | 6.865E-07 | 0.000E+00 |
| Unigene22621 | msbA | 4.158E-06 | 2.060E-05 | 3.808E-06 | 1.120E-06 | 9.625E-07 | 3.746E-07 | 1.342E-07 | 2.022E-07 | 1.231E-06 | 4.246E-07 | 2.775E-07 | 2.253E-07 |
| Unigene22622 | lmrD | 4.330E-06 | 1.994E-05 | 3.955E-06 | 1.079E-06 | 7.914E-07 | 3.850E-07 | 2.282E-07 | 3.939E-07 | 1.537E-06 | 6.056E-07 | 4.503E-07 | 8.863E-08 |
| Unigene22699 | Staphylocc | 4.699E-06 | 1.111E-05 | 1.297E-05 | 1.396E-05 | 1.071E-07 | 1.172E-07 | 2.000E-07 | 0.000E+00 | 0.000E+00 | 2.148E-07 | 0.000E+00 | 0.000E+00 |
| Unigene22703 | lmrD | 7.763E-07 | 6.437E-06 | 4.020E-07 | 3.484E-07 | 1.437E-07 | 1.865E-07 | 1.432E-07 | 2.517E-07 | 6.363E-07 | 5.044E-07 | 2.960E-07 | 3.059E-07 |
| Unigene22725 | Streptomy | 2.363E-05 | 8.530E-06 | 6.332E-06 | 4.510E-06 | 2.825E-07 | 2.234E-07 | 7.036E-08 | 1.484E-07 | 7.296E-07 | 0.000E+00 | 0.000E+00 | 0.000E+00 |
| Unigene22791 | vanTG | 7.552E-06 | 0.000E+00 | 0.000E+00 | 2.838E-06 | 3.577E-05 | 1.648E-05 | 2.611E-05 | 2.714E-05 | 3.506E-06 | 0.000E+00 | 0.000E+00 | 0.000E+00 |
| Unigene22817 | Corynebac | 3.798E-06 | 8.729E-06 | 7.372E-06 | 9.532E-06 | 0.000E+00 | 0.000E+00 | 1.523E-07 | 0.000E+00 | 0.000E+00 | 0.000E+00 | 0.000E+00 | 0.000E+00 |
| Unigene22825 | dfrA12 | 5.005E-07 | 0.000E+00 | 3.274E-07 | 0.000E+00 | 0.000E+00 | 0.000E+00 | 0.000E+00 | 0.000E+00 | 0.000E+00 | 0.000E+00 | 0.000E+00 | 0.000E+00 |
| Unigene22838 | macB | 6.911E-06 | 0.000E+00 | 0.000E+00 | 2.385E-06 | 3.610E-05 | 1.636E-05 | 2.140E-05 | 2.420E-05 | 3.107E-06 | 0.000E+00 | 0.000E+00 | 0.000E+00 |
| Unigene22850 | YojI | 6.349E-05 | 5.774E-05 | 1.049E-04 | 1.642E-04 | 1.032E-04 | 7.789E-05 | 1.056E-04 | 1.020E-04 | 0.000E+00 | 1.266E-04 | 1.295E-04 | 1.307E-04 |
| Unigene22920 | mepA | 4.608E-06 | 1.006E-05 | 1.001E-05 | 1.108E-05 | 0.000E+00 | 0.000E+00 | 0.000E+00 | 0.000E+00 | 0.000E+00 | 1.563E-07 | 0.000E+00 | 0.000E+00 |
| Unigene22944 | ykkD | 2.989E-06 | 3.280E-06 | 0.000E+00 | 2.415E-06 | 2.546E-06 | 1.292E-06 | 1.654E-06 | 0.000E+00 | 0.000E+00 | 5.549E-06 | 8.775E-06 | 2.355E-06 |
| Unigene22945 | ykkC | 3.521E-06 | 3.865E-06 | 3.551E-06 | 2.371E-06 | 1.847E-06 | 0.000E+00 | 3.248E-06 | 0.000E+00 | 2.138E-06 | 0.000E+00 | 4.476E-06 | 0.000E+00 |
| Unigene22948 | YojI | 3.668E-06 | 3.285E-06 | 2.937E-06 | 1.961E-06 | 2.187E-06 | 2.099E-06 | 1.761E-06 | 1.070E-06 | 1.356E-06 | 5.377E-06 | 6.663E-06 | 1.658E-06 |
| Unigene23051 | carA | 4.913E-07 | 8.089E-07 | 7.232E-07 | 0.000E+00 | 0.000E+00 | 0.000E+00 | 5.438E-08 | 0.000E+00 | 0.000E+00 | 0.000E+00 | 0.000E+00 | 0.000E+00 |
| Unigene23052 | efrA | 6.383E-07 | 3.002E-07 | 8.948E-07 | 1.842E-07 | 0.000E+00 | 0.000E+00 | 0.000E+00 | 0.000E+00 | 0.000E+00 | 0.000E+00 | 0.000E+00 | 0.000E+00 |
| Unigene23068 | emrB | 2.124E-06 | 1.538E-05 | 7.618E-06 | 5.886E-06 | 0.000E+00 | 5.102E-08 | 0.000E+00 | 0.000E+00 | 0.000E+00 | 0.000E+00 | 0.000E+00 | 0.000E+00 |
| Unigene23099 | dfrE | 8.661E-06 | 0.000E+00 | 0.000E+00 | 2.584E-06 | 3.863E-05 | 1.603E-05 | 2.404E-05 | 2.206E-05 | 0.000E+00 | 0.000E+00 | 0.000E+00 | 0.000E+00 |
| Unigene23101 | TaeA | 7.289E-06 | 0.000E+00 | 0.000E+00 | 2.127E-06 | 3.158E-05 | 1.410E-05 | 2.052E-05 | 2.287E-05 | 3.075E-06 | 0.000E+00 | 0.000E+00 | 0.000E+00 |
| Unigene23174 | mtrA | 6.354E-05 | 5.240E-05 | 1.006E-04 | 1.629E-04 | 1.158E-04 | 8.355E-05 | 1.139E-04 | 1.093E-04 | 9.037E-05 | 1.306E-04 | 1.334E-04 | 1.460E-04 |
| Unigene23182 | bcrA | 2.199E-06 | 2.228E-06 | 2.988E-06 | 0.000E+00 | 0.000E+00 | 0.000E+00 | 2.247E-07 | 0.000E+00 | 4.068E-07 | 0.000E+00 | 0.000E+00 | 0.000E+00 |
| Unigene23188 | mecA | 2.359E-06 | 2.642E-06 | 2.690E-06 | 1.320E-06 | 0.000E+00 | 0.000E+00 | 5.469E-07 | 3.721E-07 | 5.401E-07 | 0.000E+00 | 9.117E-08 | 0.000E+00 |
| Unigene23233 | macB | 2.427E-06 | 8.543E-07 | 1.797E-06 | 7.860E-07 | 0.000E+00 | 0.000E+00 | 0.000E+00 | 0.000E+00 | 0.000E+00 | 0.000E+00 | 0.000E+00 | 0.000E+00 |
| Unigene23250 | PmrF | 5.367E-07 | 5.358E-06 | 9.756E-06 | 1.414E-05 | 0.000E+00 | 0.000E+00 | 0.000E+00 | 0.000E+00 | 0.000E+00 | 0.000E+00 | 0.000E+00 | 0.000E+00 |
| Unigene23279 | adeL | 4.955E-07 | 0.000E+00 | 0.000E+00 | 0.000E+00 | 0.000E+00 | 1.990E-06 | 0.000E+00 | 4.586E-06 | 6.577E-07 | 0.000E+00 | 2.105E-06 | 4.770E-06 |
| Unigene23338 | efrA | 4.267E-06 | 1.508E-05 | 3.066E-06 | 6.614E-07 | 9.147E-07 | 4.046E-07 | 1.864E-07 | 1.529E-07 | 1.084E-06 | 5.837E-07 | 5.137E-07 | 1.327E-07 |
| Unigene23348 | abeS | 3.017E-06 | 0.000E+00 | 0.000E+00 | 2.133E-06 | 0.000E+00 | 0.000E+00 | 0.000E+00 | 0.000E+00 | 1.868E-06 | 4.369E-06 | 6.212E-06 | 0.000E+00 |
| Unigene23353 | vanRF | 7.581E-05 | 6.913E-05 | 1.319E-04 | 1.669E-04 | 1.523E-04 | 1.049E-04 | 1.388E-04 | 1.360E-04 | 1.097E-04 | 1.740E-04 | 1.755E-04 | 1.911E-04 |
| Unigene23354 | Staphylocc | 7.834E-05 | 4.195E-05 | 1.117E-04 | 6.059E-08 | 2.400E-06 | 1.297E-07 | 0.000E+00 | 1.401E-07 | 5.539E-05 | 1.337E-07 | 0.000E+00 | 3.783E-07 |
| Unigene23372 | TaeA | 2.054E-06 | 2.523E-06 | 3.263E-06 | 1.630E-06 | 0.000E+00 | 0.000E+00 | 3.789E-07 | 0.000E+00 | 7.484E-07 | 0.000E+00 | 0.000E+00 | 0.000E+00 |
| Unigene23394 | MdtK | 9.409E-07 | 1.271E-06 | 4.995E-06 | 5.287E-06 | 0.000E+00 | 0.000E+00 | 5.607E-07 | 0.000E+00 | 0.000E+00 | 0.000E+00 | 0.000E+00 | 0.000E+00 |
| Unigene23409 | lsaC | 1.152E-05 | 3.448E-06 | 2.905E-06 | 1.192E-05 | 7.734E-05 | 1.093E-04 | 1.030E-04 | 8.143E-05 | 1.380E-04 | 1.351E-04 | 1.086E-04 | 7.217E-05 |
| Unigene23412 | bcrA | 1.306E-05 | 3.385E-06 | 2.323E-06 | 1.239E-05 | 8.169E-05 | 1.082E-04 | 1.036E-04 | 8.947E-05 | 1.328E-04 | 1.380E-04 | 1.164E-04 | 7.228E-05 |
| Unigene23433 | mgrA | 4.942E-05 | 7.306E-05 | 3.262E-05 | 1.641E-05 | 1.484E-05 | 5.856E-06 | 4.271E-06 | 5.899E-05 | 1.492E-05 | 8.101E-06 | 1.952E-06 | 9.649E-07 |
| Unigene23543 | msbA | 8.997E-07 | 0.000E+00 | 3.964E-07 | 4.450E-07 | 0.000E+00 | 9.151E-06 | 1.240E-06 | 8.873E-06 | 5.599E-06 | 3.416E-06 | 0.000E+00 | 8.465E-07 |
| Unigene23593 | novA | 1.764E-06 | 1.016E-06 | 1.428E-06 | 1.915E-06 | 2.028E-05 | 7.723E-06 | 1.381E-05 | 1.843E-05 | 1.181E-05 | 2.029E-05 | 2.184E-05 | 6.828E-06 |
| Unigene23599 | macB | 1.381E-06 | 7.838E-07 | 1.028E-06 | 1.683E-06 | 1.767E-05 | 7.412E-06 | 1.407E-05 | 1.684E-05 | 1.114E-05 | 1.729E-05 | 1.547E-05 | 6.753E-06 |
| Unigene23600 | macB | 1.233E-06 | 1.076E-06 | 1.567E-06 | 2.122E-06 | 2.254E-05 | 7.209E-06 | 1.526E-05 | 1.711E-05 | 1.118E-05 | 1.982E-05 | 2.337E-05 | 7.750E-06 |
| Unigene23638 | Chlamydia | 7.238E-07 | 7.149E-07 | 6.865E-07 | 1.023E-06 | 1.581E-05 | 4.955E-06 | 1.017E-05 | 1.343E-05 | 8.913E-06 | 1.320E-05 | 1.515E-05 | 5.162E-06 |
| Unigene23656 | patB | 1.360E-06 | 1.323E-06 | 1.301E-06 | 1.686E-06 | 1.781E-05 | 5.732E-06 | 1.345E-05 | 1.610E-05 | 1.027E-05 | 1.567E-05 | 1.599E-05 | 6.308E-06 |
| Unigene23657 | efrA | 1.331E-06 | 6.995E-07 | 1.821E-06 | 1.458E-06 | 1.785E-05 | 5.249E-06 | 1.346E-05 | 1.459E-05 | 8.605E-06 | 1.600E-05 | 1.595E-05 | 8.043E-06 |
| Unigene23671 | lsaC | 4.874E-05 | 2.423E-05 | 1.807E-05 | 2.121E-05 | 2.479E-06 | 2.585E-06 | 3.176E-06 | 0.000E+00 | 6.577E-06 | 7.547E-07 | 1.413E-06 | 1.507E-06 |
| Unigene23723 | vanHA | 1.091E-04 | 1.950E-04 | 1.820E-04 | 0.000E+00 | 8.196E-06 | 0.000E+00 | 0.000E+00 | 1.542E-06 | 5.680E-05 | 0.000E+00 | 0.000E+00 | 0.000E+00 |
| Unigene23747 | kdpE | 4.893E-05 | 3.116E-05 | 2.071E-05 | 2.305E-05 | 2.227E-06 | 2.521E-06 | 5.117E-06 | 9.074E-07 | 1.024E-05 | 7.748E-07 | 2.761E-06 | 1.886E-06 |
| Unigene23750 | macB | 4.435E-05 | 2.555E-05 | 1.803E-05 | 2.355E-05 | 2.136E-06 | 1.504E-06 | 1.766E-06 | 8.118E-07 | 6.886E-06 | 0.000E+00 | 1.638E-06 | 1.451E-06 |
| Unigene23757 | msbA | 3.189E-05 | 1.954E-05 | 1.403E-05 | 1.877E-05 | 1.506E-06 | 1.277E-06 | 1.791E-06 | 5.106E-07 | 4.829E-06 | 6.334E-07 | 1.501E-06 | 1.034E-06 |
| Unigene23760 | efrA | 4.925E-05 | 2.919E-05 | 1.721E-05 | 1.966E-05 | 2.818E-06 | 1.797E-06 | 4.550E-06 | 3.574E-07 | 8.079E-06 | 6.334E-07 | 1.901E-06 | 1.913E-06 |
| Unigene23761 | arlS | 4.706E-05 | 2.492E-05 | 1.722E-05 | 2.079E-05 | 2.428E-06 | 1.642E-06 | 3.340E-06 | 6.559E-07 | 7.438E-06 | 5.796E-07 | 1.952E-06 | 1.796E-06 |
| Unigene23762 | arlR | 4.694E-05 | 2.718E-05 | 1.920E-05 | 2.421E-05 | 1.700E-06 | 2.206E-06 | 3.029E-06 | 7.038E-07 | 5.272E-06 | 4.133E-07 | 1.910E-06 | 2.193E-06 |
| Unigene23776 | tetA(58) | 6.391E-06 | 1.230E-05 | 1.191E-05 | 1.176E-05 | 0.000E+00 | 2.624E-07 | 0.000E+00 | 1.417E-07 | 0.000E+00 | 5.858E-07 | 0.000E+00 | 0.000E+00 |
| Unigene23777 | patB | 5.342E-06 | 1.097E-05 | 8.183E-06 | 1.056E-05 | 1.708E-07 | 0.000E+00 | 1.276E-07 | 0.000E+00 | 0.000E+00 | 0.000E+00 | 0.000E+00 | 0.000E+00 |
| Unigene23780 | bcrA | 1.566E-06 | 5.544E-07 | 9.418E-07 | 1.005E-05 | 8.418E-07 | 1.638E-07 | 1.571E-05 | 1.751E-05 | 0.000E+00 | 2.983E-06 | 0.000E+00 | 2.986E-06 |
| Unigene23829 | msrA | 2.443E-06 | 1.135E-06 | 0.000E+00 | 0.000E+00 | 0.000E+00 | 0.000E+00 | 0.000E+00 | 0.000E+00 | 0.000E+00 | 0.000E+00 | 0.000E+00 | 0.000E+00 |
| Unigene23855 | TaeA | 9.526E-05 | 1.492E-04 | 1.448E-04 | 5.930E-06 | 8.711E-06 | 1.651E-07 | 2.836E-06 | 1.803E-06 | 5.676E-05 | 0.000E+00 | 3.223E-06 | 9.228E-07 |

| | | | | | | | | | | | | | |
|---|---|---|---|---|---|---|---|---|---|---|---|---|---|
| Unigene23916 | vanHO | 3.748E-06 | 9.753E-06 | 9.100E-06 | 1.031E-05 | 1.436E-07 | 0.000E+00 | 2.503E-07 | 0.000E+00 | 0.000E+00 | 2.160E-07 | 7.392E-08 | 0.000E+00 |
| Unigene23961 | Streptomy | 1.923E-06 | 1.965E-06 | 1.220E-06 | 1.088E-06 | 1.860E-06 | 1.380E-06 | 1.138E-06 | 6.193E-07 | 9.061E-07 | 3.583E-06 | 3.262E-06 | 8.623E-07 |
| Unigene23966 | efrB | 8.249E-07 | 1.167E-06 | 4.820E-06 | 6.942E-06 | 0.000E+00 | 1.982E-08 | 7.709E-07 | 0.000E+00 | 0.000E+00 | 0.000E+00 | 6.290E-08 | 4.333E-08 |
| Unigene23967 | efrA | 8.019E-07 | 1.003E-06 | 5.233E-06 | 5.405E-06 | 6.215E-08 | 6.048E-08 | 4.128E-07 | 0.000E+00 | 0.000E+00 | 0.000E+00 | 4.266E-08 | 0.000E+00 |
| Unigene23975 | lmrD | 3.772E-06 | 1.318E-05 | 2.581E-06 | 5.767E-07 | 6.678E-07 | 1.300E-07 | 2.329E-07 | 2.631E-07 | 5.585E-07 | 1.841E-07 | 1.031E-07 | 8.882E-08 |
| Unigene23985 | optrA | 4.985E-06 | 2.216E-05 | 3.567E-06 | 1.280E-06 | 1.085E-06 | 3.368E-07 | 2.069E-07 | 1.455E-07 | 1.362E-06 | 9.256E-07 | 3.327E-07 | 2.946E-07 |
| Unigene24003 | rphB | 4.502E-06 | 1.993E-05 | 3.409E-06 | 1.151E-06 | 1.224E-06 | 2.827E-07 | 1.654E-07 | 3.053E-07 | 1.388E-06 | 6.659E-07 | 4.060E-07 | 2.208E-07 |
| Unigene24044 | Listeria mc | 3.649E-05 | 2.500E-05 | 5.805E-05 | 1.281E-04 | 5.436E-05 | 3.888E-05 | 5.819E-05 | 4.793E-05 | 3.847E-05 | 6.264E-05 | 6.854E-05 | 6.365E-05 |
| Unigene24047 | sul4 | 2.082E-05 | 7.901E-06 | 6.494E-06 | 3.948E-06 | 0.000E+00 | 0.000E+00 | 0.000E+00 | 0.000E+00 | 0.000E+00 | 0.000E+00 | 0.000E+00 | 0.000E+00 |
| Unigene24072 | ErmH | 1.171E-04 | 2.215E-04 | 1.953E-04 | 0.000E+00 | 9.275E-06 | 0.000E+00 | 4.540E-06 | 2.352E-06 | 6.270E-05 | 0.000E+00 | 3.787E-06 | 0.000E+00 |
| Unigene24095 | macB | 4.902E-06 | 9.339E-06 | 1.207E-05 | 1.532E-05 | 0.000E+00 | 0.000E+00 | 0.000E+00 | 0.000E+00 | 0.000E+00 | 0.000E+00 | 0.000E+00 | 0.000E+00 |
| Unigene24135 | baeS | 4.117E-06 | 1.114E-05 | 9.148E-06 | 1.069E-05 | 1.210E-07 | 1.177E-07 | 4.821E-08 | 1.525E-07 | 0.000E+00 | 1.456E-07 | 0.000E+00 | 2.575E-08 |
| Unigene24136 | vanRM | 3.750E-06 | 1.005E-05 | 9.945E-06 | 1.041E-05 | 0.000E+00 | 1.448E-07 | 1.482E-07 | 0.000E+00 | 0.000E+00 | 1.989E-07 | 0.000E+00 | 0.000E+00 |
| Unigene24294 | vanSM | 1.613E-06 | 4.632E-06 | 3.534E-06 | 6.537E-06 | 0.000E+00 | 0.000E+00 | 2.445E-08 | 0.000E+00 | 0.000E+00 | 0.000E+00 | 0.000E+00 | 0.000E+00 |
| Unigene24342 | arlS | 6.872E-07 | 8.620E-08 | 0.000E+00 | 2.974E-07 | 3.970E-06 | 7.472E-06 | 1.521E-06 | 7.013E-06 | 4.099E-06 | 3.565E-06 | 0.000E+00 | 6.267E-07 |
| Unigene24423 | tetA(60) | 4.494E-06 | 1.102E-05 | 1.069E-05 | 1.060E-05 | 0.000E+00 | 0.000E+00 | 0.000E+00 | 0.000E+00 | 0.000E+00 | 2.558E-07 | 1.126E-07 | 0.000E+00 |
| Unigene24428 | efrA | 4.821E-06 | 2.047E-05 | 3.651E-06 | 6.703E-07 | 1.106E-06 | 3.588E-07 | 7.733E-08 | 3.466E-07 | 1.489E-06 | 6.421E-07 | 2.398E-07 | 6.194E-08 |
| Unigene24429 | patB | 4.108E-06 | 2.211E-05 | 3.980E-06 | 9.199E-07 | 1.146E-06 | 4.088E-07 | 2.093E-07 | 5.016E-07 | 1.127E-06 | 6.127E-07 | 3.932E-07 | 0.000E+00 |
| Unigene24444 | macB | 4.064E-06 | 2.154E-05 | 3.266E-06 | 5.472E-07 | 7.310E-07 | 4.603E-07 | 3.855E-07 | 0.000E+00 | 1.396E-06 | 0.000E+00 | 3.099E-07 | 5.033E-07 |
| Unigene24445 | tetB(46) | 3.500E-06 | 1.945E-05 | 2.886E-06 | 1.128E-06 | 1.158E-06 | 4.830E-07 | 0.000E+00 | 0.000E+00 | 1.343E-06 | 1.286E-06 | 3.833E-07 | 1.760E-07 |
| Unigene24476 | arlS | 2.749E-06 | 3.429E-06 | 2.328E-06 | 2.278E-06 | 2.245E-06 | 2.006E-06 | 1.560E-06 | 1.261E-06 | 1.387E-06 | 5.234E-06 | 4.837E-06 | 1.555E-06 |
| Unigene24477 | mtrA | 2.935E-06 | 3.221E-06 | 2.400E-06 | 1.886E-06 | 1.976E-06 | 1.971E-06 | 1.427E-06 | 8.823E-07 | 1.069E-06 | 6.835E-06 | 5.290E-06 | 1.839E-06 |
| Unigene24491 | efrA | 1.014E-06 | 6.739E-06 | 1.209E-05 | 1.545E-05 | 0.000E+00 | 0.000E+00 | 0.000E+00 | 4.384E-08 | 0.000E+00 | 0.000E+00 | 0.000E+00 | 0.000E+00 |
| Unigene24492 | efrB | 6.383E-07 | 6.745E-06 | 1.006E-05 | 1.567E-05 | 0.000E+00 | 0.000E+00 | 0.000E+00 | 6.386E-08 | 0.000E+00 | 0.000E+00 | 0.000E+00 | 0.000E+00 |
| Unigene24509 | oleC | 2.426E-06 | 3.010E-06 | 2.726E-06 | 1.385E-06 | 4.297E-07 | 0.000E+00 | 7.005E-07 | 0.000E+00 | 8.071E-07 | 0.000E+00 | 0.000E+00 | 2.078E-07 |
| Unigene24522 | kdpE | 2.472E-06 | 1.755E-06 | 2.854E-06 | 1.664E-06 | 0.000E+00 | 0.000E+00 | 0.000E+00 | 0.000E+00 | 0.000E+00 | 3.240E-07 | 0.000E+00 | 0.000E+00 |
| Unigene24524 | macB | 1.996E-06 | 1.229E-06 | 2.675E-06 | 1.967E-06 | 2.164E-07 | 2.632E-07 | 0.000E+00 | 0.000E+00 | 0.000E+00 | 0.000E+00 | 0.000E+00 | 0.000E+00 |
| Unigene24612 | patA | 1.517E-06 | 1.267E-05 | 5.189E-06 | 4.597E-06 | 0.000E+00 | 0.000E+00 | 0.000E+00 | 0.000E+00 | 0.000E+00 | 0.000E+00 | 5.260E-08 | 0.000E+00 |
| Unigene24613 | tetA(58) | 2.218E-06 | 1.668E-05 | 6.972E-06 | 2.986E-06 | 0.000E+00 | 0.000E+00 | 0.000E+00 | 0.000E+00 | 0.000E+00 | 0.000E+00 | 0.000E+00 | 0.000E+00 |
| Unigene24631 | emrB | 4.735E-05 | 2.721E-05 | 1.676E-05 | 2.021E-05 | 2.086E-06 | 1.839E-06 | 3.497E-06 | 1.289E-06 | 7.196E-06 | 1.231E-06 | 2.452E-06 | 1.671E-06 |
| Unigene24662 | arlS | 4.756E-05 | 2.648E-05 | 1.809E-05 | 2.173E-05 | 2.157E-06 | 2.099E-06 | 3.818E-06 | 8.000E-07 | 8.165E-06 | 0.000E+00 | 1.882E-06 | 1.647E-06 |
| Unigene24697 | efrA | 5.252E-06 | 1.112E-05 | 9.315E-06 | 1.131E-05 | 2.448E-07 | 1.787E-07 | 0.000E+00 | 8.573E-08 | 2.007E-08 | 2.864E-07 | 0.000E+00 | 0.000E+00 |
| Unigene24700 | vanSG | 4.939E-06 | 1.145E-05 | 1.504E-05 | 1.395E-05 | 2.580E-07 | 2.511E-07 | 7.882E-07 | 1.084E-07 | 3.384E-08 | 2.070E-07 | 1.771E-07 | 1.098E-07 |
| Unigene24701 | vanRI | 3.421E-05 | 2.039E-05 | 2.026E-05 | 1.987E-05 | 1.890E-06 | 1.392E-06 | 2.646E-06 | 8.051E-07 | 0.000E+00 | 0.000E+00 | 0.000E+00 | 1.196E-06 |
| Unigene24722 | macB | 2.552E-06 | 3.828E-06 | 3.005E-06 | 4.638E-06 | 3.307E-07 | 0.000E+00 | 2.824E-07 | 4.964E-08 | 1.395E-07 | 0.000E+00 | 0.000E+00 | 1.508E-07 |
| Unigene24723 | baeS | 3.281E-06 | 4.429E-06 | 4.635E-06 | 6.460E-06 | 4.373E-07 | 3.192E-07 | 3.631E-07 | 0.000E+00 | 1.793E-07 | 0.000E+00 | 0.000E+00 | 2.714E-07 |
| Unigene24724 | vanRB | 2.655E-06 | 4.448E-06 | 3.382E-06 | 6.632E-06 | 0.000E+00 | 0.000E+00 | 0.000E+00 | 0.000E+00 | 0.000E+00 | 0.000E+00 | 0.000E+00 | 0.000E+00 |
| Unigene24739 | lsaA | 3.470E-05 | 2.819E-05 | 3.110E-05 | 1.566E-05 | 4.426E-05 | 3.767E-05 | 3.911E-05 | 5.154E-05 | 6.505E-05 | 7.289E-05 | 4.369E-05 | 5.660E-05 |
| Unigene24815 | PmrF | 1.283E-06 | 1.120E-06 | 1.711E-06 | 1.927E-06 | 2.000E-05 | 5.976E-06 | 1.300E-05 | 1.575E-05 | 1.352E-05 | 2.056E-05 | 2.213E-05 | 7.740E-06 |
| Unigene24854 | baeS | 1.265E-05 | 4.340E-06 | 3.519E-06 | 1.254E-05 | 8.843E-05 | 1.223E-04 | 1.172E-04 | 9.916E-05 | 1.510E-04 | 1.526E-04 | 1.230E-04 | 8.066E-05 |
| Unigene24858 | patB | 1.415E-05 | 5.424E-06 | 3.832E-06 | 1.376E-05 | 9.466E-05 | 1.378E-04 | 1.551E-04 | 1.077E-04 | 1.669E-04 | 1.769E-04 | 1.372E-04 | 8.988E-05 |
| Unigene24907 | Listeria mc | 1.889E-06 | 1.502E-05 | 5.377E-06 | 3.601E-06 | 0.000E+00 | 2.705E-08 | 5.539E-08 | 0.000E+00 | 0.000E+00 | 0.000E+00 | 0.000E+00 | 5.916E-08 |
| Unigene24926 | PmrF | 1.448E-06 | 1.643E-05 | 3.293E-06 | 2.060E-06 | 0.000E+00 | 3.557E-08 | 0.000E+00 | 0.000E+00 | 0.000E+00 | 0.000E+00 | 0.000E+00 | 0.000E+00 |
| Unigene24927 | PmrF | 1.589E-06 | 1.584E-05 | 3.963E-06 | 1.839E-06 | 0.000E+00 | 0.000E+00 | 0.000E+00 | 0.000E+00 | 0.000E+00 | 0.000E+00 | 7.574E-08 | 0.000E+00 |
| Unigene24961 | patB | 3.270E-06 | 4.591E-06 | 3.711E-06 | 5.903E-06 | 4.262E-07 | 3.950E-08 | 4.448E-07 | 0.000E+00 | 0.000E+00 | 0.000E+00 | 0.000E+00 | 2.159E-07 |
| Unigene24962 | efrA | 3.352E-06 | 4.167E-06 | 3.180E-06 | 5.218E-06 | 3.498E-07 | 8.008E-08 | 3.075E-07 | 0.000E+00 | 2.834E-07 | 0.000E+00 | 6.355E-08 | 1.095E-07 |
| Unigene24977 | adeR | 0.000E+00 | 5.173E-06 | 6.457E-06 | 9.429E-06 | 0.000E+00 | 0.000E+00 | 0.000E+00 | 0.000E+00 | 0.000E+00 | 0.000E+00 | 0.000E+00 | 0.000E+00 |
| Unigene24981 | adeR | 3.353E-06 | 4.766E-06 | 2.882E-06 | 6.739E-06 | 2.838E-07 | 1.036E-07 | 1.060E-07 | 0.000E+00 | 1.745E-07 | 0.000E+00 | 2.191E-07 | 0.000E+00 |
| Unigene24985 | patA | 2.769E-06 | 3.737E-06 | 2.718E-06 | 5.089E-06 | 0.000E+00 | 0.000E+00 | 8.040E-07 | 1.060E-07 | 2.481E-07 | 0.000E+00 | 0.000E+00 | 3.220E-07 |
| Unigene25028 | mefE | 2.961E-06 | 4.556E-06 | 7.108E-06 | 7.315E-06 | 0.000E+00 | 0.000E+00 | 0.000E+00 | 0.000E+00 | 0.000E+00 | 0.000E+00 | 0.000E+00 | 0.000E+00 |
| Unigene25050 | tet32 | 6.723E-06 | 0.000E+00 | 0.000E+00 | 2.021E-06 | 3.179E-05 | 1.395E-05 | 2.104E-05 | 2.297E-05 | 2.878E-06 | 0.000E+00 | 0.000E+00 | 0.000E+00 |
| Unigene25052 | patA | 6.085E-06 | 0.000E+00 | 0.000E+00 | 2.381E-06 | 3.555E-05 | 6.709E-06 | 2.132E-05 | 2.503E-05 | 0.000E+00 | 0.000E+00 | 0.000E+00 | 0.000E+00 |
| Unigene25098 | YojI | 7.948E-07 | 7.716E-07 | 7.799E-07 | 2.284E-06 | 0.000E+00 | 0.000E+00 | 0.000E+00 | 0.000E+00 | 0.000E+00 | 0.000E+00 | 0.000E+00 | 0.000E+00 |
| Unigene25144 | oleC | 3.486E-06 | 9.758E-06 | 8.228E-06 | 9.131E-06 | 1.684E-07 | 5.461E-08 | 0.000E+00 | 0.000E+00 | 0.000E+00 | 2.251E-07 | 0.000E+00 | 0.000E+00 |
| Unigene25201 | arlR | 3.040E-06 | 7.679E-06 | 7.907E-06 | 0.000E+00 | 0.000E+00 | 0.000E+00 | 0.000E+00 | 0.000E+00 | 0.000E+00 | 0.000E+00 | 0.000E+00 | 0.000E+00 |
| Unigene25457 | vanRF | 9.179E-07 | 0.000E+00 | 0.000E+00 | 0.000E+00 | 0.000E+00 | 0.000E+00 | 0.000E+00 | 0.000E+00 | 0.000E+00 | 0.000E+00 | 0.000E+00 | 0.000E+00 |
| Unigene25462 | blt | 1.103E-06 | 0.000E+00 | 0.000E+00 | 0.000E+00 | 0.000E+00 | 0.000E+00 | 0.000E+00 | 0.000E+00 | 0.000E+00 | 0.000E+00 | 0.000E+00 | 0.000E+00 |
| Unigene25509 | TaeA | 1.110E-05 | 0.000E+00 | 0.000E+00 | 0.000E+00 | 1.581E-05 | 1.503E-05 | 0.000E+00 | 0.000E+00 | 1.263E-05 | 0.000E+00 | 0.000E+00 | 0.000E+00 |

| | | | | | | | | | | | | | |
|---|---|---|---|---|---|---|---|---|---|---|---|---|---|
| Unigene25554 | bcrA | 1.410E-06 | 1.100E-06 | 5.024E-06 | 8.168E-06 | 0.000E+00 | 0.000E+00 | 5.338E-07 | 0.000E+00 | 0.000E+00 | 0.000E+00 | 0.000E+00 | 0.000E+00 |
| Unigene25609 | novA | 5.613E-06 | 4.310E-06 | 3.132E-06 | 1.264E-05 | 9.195E-05 | 1.286E-04 | 1.211E-04 | 1.170E-04 | 1.685E-04 | 1.699E-04 | 1.393E-04 | 9.531E-05 |
| Unigene25610 | patA | 6.312E-06 | 4.639E-06 | 3.097E-06 | 1.372E-05 | 9.848E-05 | 1.381E-04 | 1.352E-04 | 1.226E-04 | 1.831E-04 | 1.839E-04 | 1.472E-04 | 9.034E-05 |
| Unigene25628 | Staphylocc | 7.414E-06 | 0.000E+00 | 0.000E+00 | 2.118E-06 | 3.307E-05 | 1.513E-05 | 2.330E-05 | 2.584E-05 | 3.256E-06 | 0.000E+00 | 0.000E+00 | 0.000E+00 |
| Unigene25647 | vmlR | 8.184E-06 | 0.000E+00 | 0.000E+00 | 1.530E-06 | 3.614E-05 | 1.502E-05 | 2.549E-05 | 2.571E-05 | 2.982E-06 | 0.000E+00 | 0.000E+00 | 0.000E+00 |
| Unigene25657 | Staphylocc | 6.925E-06 | 0.000E+00 | 0.000E+00 | 1.788E-06 | 3.147E-05 | 1.517E-05 | 2.111E-05 | 2.435E-05 | 2.737E-06 | 0.000E+00 | 0.000E+00 | 0.000E+00 |
| Unigene25768 | Erm(48) | 1.061E-06 | 5.624E-07 | 2.155E-06 | 1.885E-06 | 2.204E-05 | 7.438E-06 | 1.470E-05 | 1.871E-05 | 1.228E-05 | 1.994E-05 | 1.758E-05 | 8.912E-06 |
| Unigene25810 | optrA | 1.281E-06 | 9.949E-07 | 1.064E-06 | 1.811E-06 | 1.660E-05 | 5.305E-06 | 1.392E-05 | 1.615E-05 | 9.262E-06 | 1.574E-05 | 1.603E-05 | 7.408E-06 |
| Unigene25835 | macB | 5.869E-07 | 5.905E-07 | 1.392E-06 | 2.914E-06 | 0.000E+00 | 0.000E+00 | 1.083E-07 | 1.541E-06 | 0.000E+00 | 5.993E-07 | 7.833E-07 | 8.672E-07 |
| Unigene25940 | efrA | 7.403E-06 | 0.000E+00 | 0.000E+00 | 1.977E-06 | 3.371E-05 | 1.615E-05 | 2.182E-05 | 2.386E-05 | 3.363E-06 | 0.000E+00 | 0.000E+00 | 0.000E+00 |
| Unigene25941 | patB | 6.552E-06 | 0.000E+00 | 0.000E+00 | 2.228E-06 | 3.090E-05 | 1.470E-05 | 1.935E-05 | 2.448E-05 | 3.365E-06 | 0.000E+00 | 0.000E+00 | 0.000E+00 |
| Unigene25967 | bcrA | 7.388E-06 | 0.000E+00 | 0.000E+00 | 2.260E-06 | 3.559E-05 | 1.684E-05 | 2.480E-05 | 2.437E-05 | 3.686E-06 | 0.000E+00 | 0.000E+00 | 0.000E+00 |
| Unigene25995 | patA | 7.274E-06 | 0.000E+00 | 0.000E+00 | 2.096E-06 | 3.280E-05 | 1.479E-05 | 1.969E-05 | 0.000E+00 | 0.000E+00 | 0.000E+00 | 0.000E+00 | 0.000E+00 |
| Unigene25996 | poxtA | 5.945E-06 | 0.000E+00 | 0.000E+00 | 0.000E+00 | 3.205E-05 | 1.414E-05 | 2.119E-05 | 2.359E-05 | 0.000E+00 | 0.000E+00 | 0.000E+00 | 0.000E+00 |
| Unigene26017 | mgrA | 8.716E-06 | 3.365E-06 | 2.241E-06 | 6.010E-06 | 4.381E-05 | 6.336E-05 | 7.431E-05 | 6.652E-05 | 3.568E-05 | 1.748E-05 | 1.602E-05 | 1.421E-06 |
| Unigene26038 | vanTrL | 1.249E-05 | 0.000E+00 | 0.000E+00 | 0.000E+00 | 2.068E-05 | 1.798E-05 | 0.000E+00 | 0.000E+00 | 1.167E-05 | 0.000E+00 | 0.000E+00 | 0.000E+00 |
| Unigene26046 | pmrA | 8.306E-06 | 0.000E+00 | 0.000E+00 | 0.000E+00 | 1.615E-05 | 1.520E-05 | 0.000E+00 | 0.000E+00 | 0.000E+00 | 0.000E+00 | 0.000E+00 | 0.000E+00 |
| Unigene26063 | ErmQ | 1.232E-05 | 0.000E+00 | 0.000E+00 | 0.000E+00 | 1.908E-05 | 1.580E-05 | 0.000E+00 | 0.000E+00 | 1.455E-05 | 2.550E-07 | 0.000E+00 | 0.000E+00 |
| Unigene26066 | Staphylocc | 1.112E-05 | 0.000E+00 | 0.000E+00 | 0.000E+00 | 1.789E-05 | 1.599E-05 | 0.000E+00 | 0.000E+00 | 1.201E-05 | 0.000E+00 | 0.000E+00 | 0.000E+00 |
| Unigene26068 | norA | 1.256E-05 | 0.000E+00 | 0.000E+00 | 0.000E+00 | 1.967E-05 | 1.546E-05 | 0.000E+00 | 0.000E+00 | 1.370E-05 | 2.465E-07 | 0.000E+00 | 0.000E+00 |
| Unigene26076 | tetB(46) | 1.404E-05 | 3.476E-06 | 3.108E-06 | 1.191E-05 | 8.431E-05 | 1.179E-04 | 1.202E-04 | 9.709E-05 | 1.427E-04 | 1.452E-04 | 1.185E-04 | 7.122E-05 |
| Unigene26107 | tetT | 1.356E-05 | 4.764E-06 | 3.746E-06 | 1.390E-05 | 9.348E-05 | 1.340E-04 | 1.222E-04 | 1.057E-04 | 1.556E-04 | 1.545E-04 | 1.179E-04 | 7.777E-05 |
| Unigene26110 | rpoB2 | 1.328E-05 | 5.018E-06 | 3.233E-06 | 1.317E-05 | 9.150E-05 | 1.306E-04 | 1.228E-04 | 1.083E-04 | 1.570E-04 | 1.555E-04 | 1.214E-04 | 7.773E-05 |
| Unigene26124 | vatH | 1.215E-05 | 0.000E+00 | 0.000E+00 | 0.000E+00 | 1.491E-05 | 1.889E-05 | 0.000E+00 | 0.000E+00 | 0.000E+00 | 0.000E+00 | 0.000E+00 | 0.000E+00 |
| Unigene26130 | MexL | 1.283E-05 | 0.000E+00 | 0.000E+00 | 0.000E+00 | 1.950E-05 | 1.927E-05 | 0.000E+00 | 0.000E+00 | 1.592E-05 | 0.000E+00 | 0.000E+00 | 0.000E+00 |
| Unigene26131 | rpoB2 | 1.192E-05 | 1.011E-08 | 0.000E+00 | 0.000E+00 | 1.890E-05 | 1.634E-05 | 5.098E-08 | 0.000E+00 | 1.205E-05 | 2.976E-07 | 0.000E+00 | 0.000E+00 |
| Unigene26136 | tetT | 1.146E-05 | 3.398E-08 | 0.000E+00 | 0.000E+00 | 1.880E-05 | 1.779E-05 | 1.028E-07 | 5.421E-08 | 1.249E-05 | 2.932E-07 | 0.000E+00 | 0.000E+00 |
| Unigene26163 | novA | 1.103E-05 | 0.000E+00 | 0.000E+00 | 0.000E+00 | 1.761E-05 | 1.827E-05 | 0.000E+00 | 0.000E+00 | 1.206E-05 | 4.327E-07 | 0.000E+00 | 0.000E+00 |
| Unigene26164 | tetB(46) | 1.273E-05 | 0.000E+00 | 0.000E+00 | 0.000E+00 | 1.580E-05 | 1.642E-05 | 0.000E+00 | 0.000E+00 | 8.749E-06 | 0.000E+00 | 0.000E+00 | 0.000E+00 |
| Unigene26173 | optrA | 8.305E-07 | 0.000E+00 | 0.000E+00 | 5.105E-07 | 0.000E+00 | 1.070E-05 | 8.392E-07 | 0.000E+00 | 4.025E-06 | 0.000E+00 | 0.000E+00 | 0.000E+00 |
| Unigene26178 | bcrA | 1.701E-06 | 1.564E-05 | 7.887E-06 | 5.197E-06 | 0.000E+00 | 0.000E+00 | 0.000E+00 | 0.000E+00 | 0.000E+00 | 0.000E+00 | 0.000E+00 | 0.000E+00 |
| Unigene26180 | vanRI | 2.644E-06 | 1.537E-05 | 8.055E-06 | 6.463E-06 | 0.000E+00 | 0.000E+00 | 0.000E+00 | 1.083E-07 | 0.000E+00 | 0.000E+00 | 0.000E+00 | 0.000E+00 |
| Unigene26181 | vanSM | 1.946E-06 | 1.681E-05 | 8.536E-06 | 5.965E-06 | 0.000E+00 | 1.732E-07 | 0.000E+00 | 0.000E+00 | 0.000E+00 | 0.000E+00 | 0.000E+00 | 0.000E+00 |
| Unigene26185 | otrC | 2.500E-06 | 1.529E-05 | 8.587E-06 | 7.304E-06 | 0.000E+00 | 0.000E+00 | 0.000E+00 | 0.000E+00 | 0.000E+00 | 0.000E+00 | 0.000E+00 | 0.000E+00 |
| Unigene26198 | vanRG | 8.081E-07 | 3.734E-07 | 1.628E-06 | 1.160E-06 | 0.000E+00 | 0.000E+00 | 0.000E+00 | 0.000E+00 | 0.000E+00 | 0.000E+00 | 0.000E+00 | 0.000E+00 |
| Unigene26229 | lmrD | 1.072E-05 | 0.000E+00 | 0.000E+00 | 0.000E+00 | 2.036E-05 | 1.915E-05 | 1.435E-07 | 0.000E+00 | 1.374E-05 | 0.000E+00 | 0.000E+00 | 0.000E+00 |
| Unigene26236 | vanHF | 1.183E-05 | 0.000E+00 | 0.000E+00 | 0.000E+00 | 1.917E-05 | 1.691E-05 | 1.788E-07 | 0.000E+00 | 1.240E-05 | 0.000E+00 | 3.696E-08 | 0.000E+00 |
| Unigene26260 | lsaC | 9.919E-07 | 1.089E-06 | 1.650E-06 | 1.611E-06 | 2.019E-05 | 6.131E-06 | 1.515E-05 | 1.893E-05 | 1.160E-05 | 1.871E-05 | 1.847E-05 | 8.844E-06 |
| Unigene26262 | mdtG | 1.126E-06 | 1.236E-06 | 1.207E-06 | 2.141E-06 | 2.216E-05 | 7.585E-06 | 1.304E-05 | 1.867E-05 | 1.208E-05 | 1.867E-05 | 2.117E-05 | 6.808E-06 |
| Unigene26265 | vanTG | 1.711E-06 | 7.958E-07 | 1.167E-06 | 1.552E-06 | 2.065E-05 | 6.929E-06 | 1.512E-05 | 1.750E-05 | 1.084E-05 | 1.758E-05 | 1.884E-05 | 8.125E-06 |
| Unigene26306 | optrA | 9.587E-05 | 1.599E-04 | 1.526E-04 | 6.626E-06 | 8.318E-06 | 4.467E-07 | 3.659E-06 | 1.929E-06 | 5.423E-05 | 3.682E-07 | 3.025E-06 | 4.689E-07 |
| Unigene26323 | tlrC | 3.491E-05 | 2.627E-05 | 2.155E-05 | 2.245E-05 | 0.000E+00 | 1.014E-06 | 3.558E-06 | 7.817E-07 | 0.000E+00 | 0.000E+00 | 1.481E-06 | 1.267E-06 |
| Unigene26355 | lnuA | 2.761E-05 | 0.000E+00 | 0.000E+00 | 0.000E+00 | 0.000E+00 | 0.000E+00 | 0.000E+00 | 0.000E+00 | 0.000E+00 | 0.000E+00 | 0.000E+00 | 0.000E+00 |
| Unigene26463 | bcrA | 3.516E-07 | 0.000E+00 | 0.000E+00 | 0.000E+00 | 0.000E+00 | 0.000E+00 | 0.000E+00 | 0.000E+00 | 0.000E+00 | 0.000E+00 | 0.000E+00 | 0.000E+00 |
| Unigene26479 | cmlB | 2.350E-05 | 7.622E-06 | 5.818E-06 | 3.742E-06 | 3.354E-07 | 0.000E+00 | 1.519E-07 | 0.000E+00 | 8.100E-07 | 0.000E+00 | 3.140E-08 | 6.489E-08 |
| Unigene26615 | patB | 1.068E-05 | 3.431E-06 | 3.785E-06 | 1.218E-05 | 8.165E-05 | 1.160E-04 | 1.174E-04 | 9.545E-05 | 1.447E-04 | 1.424E-04 | 1.165E-04 | 7.199E-05 |
| Unigene26617 | farB | 1.150E-05 | 4.000E-06 | 3.812E-06 | 1.362E-05 | 9.347E-05 | 1.272E-04 | 1.246E-04 | 1.107E-04 | 1.621E-04 | 1.674E-04 | 1.385E-04 | 8.684E-05 |
| Unigene26642 | efrA | 1.751E-06 | 2.914E-06 | 4.877E-06 | 7.301E-06 | 0.000E+00 | 0.000E+00 | 0.000E+00 | 0.000E+00 | 0.000E+00 | 0.000E+00 | 0.000E+00 | 0.000E+00 |
| Unigene26732 | efrB | 2.211E-06 | 2.212E-06 | 2.616E-06 | 1.313E-06 | 2.889E-07 | 1.757E-07 | 3.958E-07 | 3.415E-07 | 8.527E-07 | 0.000E+00 | 2.231E-07 | 0.000E+00 |
| Unigene26786 | NmcR | 6.350E-07 | 0.000E+00 | 0.000E+00 | 0.000E+00 | 0.000E+00 | 0.000E+00 | 0.000E+00 | 0.000E+00 | 0.000E+00 | 0.000E+00 | 0.000E+00 | 0.000E+00 |
| Unigene26914 | oleC | 2.254E-06 | 1.736E-05 | 8.472E-06 | 5.669E-06 | 0.000E+00 | 0.000E+00 | 0.000E+00 | 0.000E+00 | 0.000E+00 | 0.000E+00 | 0.000E+00 | 0.000E+00 |
| Unigene26924 | bcrA | 1.839E-06 | 1.490E-05 | 6.604E-06 | 3.240E-06 | 0.000E+00 | 0.000E+00 | 0.000E+00 | 0.000E+00 | 0.000E+00 | 0.000E+00 | 0.000E+00 | 0.000E+00 |
| Unigene26945 | macB | 9.672E-07 | 1.951E-05 | 6.604E-06 | 4.721E-06 | 0.000E+00 | 1.307E-07 | 0.000E+00 | 0.000E+00 | 0.000E+00 | 0.000E+00 | 0.000E+00 | 1.429E-07 |
| Unigene26946 | macB | 2.012E-06 | 1.600E-05 | 6.730E-06 | 3.027E-06 | 0.000E+00 | 0.000E+00 | 0.000E+00 | 0.000E+00 | 0.000E+00 | 0.000E+00 | 1.409E-07 | 0.000E+00 |
| Unigene27003 | efmA | 1.548E-06 | 1.481E-05 | 6.541E-06 | 4.240E-06 | 0.000E+00 | 1.419E-07 | 2.905E-08 | 0.000E+00 | 0.000E+00 | 0.000E+00 | 0.000E+00 | 0.000E+00 |
| Unigene27022 | Staphylocc | 8.036E-05 | 3.960E-05 | 1.920E-07 | 0.000E+00 | 1.573E-05 | 7.162E-05 | 1.353E-08 | 1.142E-07 | 0.000E+00 | 6.810E-08 | 0.000E+00 | 1.156E-07 |
| Unigene27033 | mecB | 8.396E-05 | 3.860E-05 | 3.656E-07 | 7.525E-08 | 1.649E-05 | 7.247E-05 | 0.000E+00 | 3.479E-08 | 0.000E+00 | 6.641E-08 | 5.114E-08 | 0.000E+00 |
| Unigene27044 | Staphylocc | 8.306E-05 | 4.024E-05 | 2.271E-07 | 1.052E-07 | 1.649E-05 | 7.211E-05 | 0.000E+00 | 1.891E-07 | 0.000E+00 | 1.031E-07 | 0.000E+00 | 0.000E+00 |

| | | | | | | | | | | | | | |
|---|---|---|---|---|---|---|---|---|---|---|---|---|---|
| Unigene27055 | lmrC | 9.483E-05 | 3.913E-05 | 0.000E+00 | 0.000E+00 | 1.631E-05 | 7.595E-05 | 0.000E+00 | 0.000E+00 | 0.000E+00 | 1.524E-07 | 0.000E+00 | 0.000E+00 |
| Unigene27122 | Streptomy | 8.308E-05 | 3.948E-05 | 3.072E-07 | 0.000E+00 | 1.613E-05 | 7.077E-05 | 0.000E+00 | 0.000E+00 | 0.000E+00 | 0.000E+00 | 1.319E-07 | 3.895E-08 |
| Unigene27163 | oleC | 6.969E-05 | 3.910E-05 | 3.145E-07 | 0.000E+00 | 1.847E-05 | 6.534E-05 | 0.000E+00 | 0.000E+00 | 0.000E+00 | 0.000E+00 | 0.000E+00 | 0.000E+00 |
| Unigene27165 | bcrA | 9.148E-05 | 4.347E-05 | 0.000E+00 | 0.000E+00 | 1.770E-05 | 8.425E-05 | 0.000E+00 | 0.000E+00 | 0.000E+00 | 0.000E+00 | 0.000E+00 | 0.000E+00 |
| Unigene27225 | dfrC | 9.237E-05 | 4.320E-05 | 4.446E-07 | 1.716E-07 | 1.711E-05 | 7.708E-05 | 0.000E+00 | 0.000E+00 | 0.000E+00 | 0.000E+00 | 1.296E-07 | 0.000E+00 |
| Unigene27228 | rosB | 8.316E-05 | 3.818E-05 | 1.620E-07 | 0.000E+00 | 1.752E-05 | 7.280E-05 | 0.000E+00 | 0.000E+00 | 0.000E+00 | 0.000E+00 | 2.204E-07 | 0.000E+00 |
| Unigene27259 | bcrA | 7.995E-05 | 3.444E-05 | 4.888E-08 | 0.000E+00 | 1.716E-05 | 6.538E-05 | 0.000E+00 | 0.000E+00 | 0.000E+00 | 0.000E+00 | 0.000E+00 | 0.000E+00 |
| Unigene27265 | tetW | 8.458E-05 | 3.927E-05 | 3.110E-07 | 0.000E+00 | 1.557E-05 | 7.271E-05 | 3.898E-08 | 4.111E-08 | 0.000E+00 | 3.924E-08 | 2.014E-08 | 6.245E-08 |
| Unigene27305 | tetB(60) | 8.036E-05 | 3.856E-05 | 2.288E-07 | 5.433E-08 | 1.598E-05 | 7.227E-05 | 0.000E+00 | 0.000E+00 | 0.000E+00 | 0.000E+00 | 0.000E+00 | 0.000E+00 |
| Unigene27306 | tetA(60) | 8.220E-05 | 4.013E-05 | 1.985E-07 | 0.000E+00 | 1.606E-05 | 7.371E-05 | 0.000E+00 | 0.000E+00 | 0.000E+00 | 1.229E-07 | 1.052E-07 | 0.000E+00 |
| Unigene27330 | carA | 9.034E-05 | 4.282E-05 | 9.716E-08 | 9.999E-08 | 1.408E-05 | 6.590E-05 | 0.000E+00 | 0.000E+00 | 0.000E+00 | 1.471E-07 | 0.000E+00 | 0.000E+00 |
| Unigene27331 | tva(A) | 8.567E-05 | 3.684E-05 | 2.455E-07 | 6.317E-08 | 1.984E-05 | 7.892E-05 | 0.000E+00 | 0.000E+00 | 0.000E+00 | 6.969E-08 | 0.000E+00 | 0.000E+00 |
| Unigene27344 | arnA | 8.026E-05 | 3.490E-05 | 2.017E-07 | 0.000E+00 | 1.508E-05 | 6.535E-05 | 0.000E+00 | 0.000E+00 | 0.000E+00 | 0.000E+00 | 1.960E-07 | 0.000E+00 |
| Unigene27405 | arlS | 8.645E-05 | 3.944E-05 | 1.007E-07 | 0.000E+00 | 1.604E-05 | 7.661E-05 | 0.000E+00 | 0.000E+00 | 0.000E+00 | 1.372E-07 | 0.000E+00 | 7.282E-08 |
| Unigene27406 | arlR | 7.849E-05 | 3.719E-05 | 1.752E-07 | 9.017E-08 | 1.830E-05 | 6.796E-05 | 4.941E-08 | 0.000E+00 | 0.000E+00 | 1.492E-07 | 4.086E-07 | 0.000E+00 |
| Unigene27420 | novA | 8.831E-05 | 3.956E-05 | 3.004E-07 | 0.000E+00 | 1.681E-05 | 7.230E-05 | 0.000E+00 | 0.000E+00 | 0.000E+00 | 0.000E+00 | 0.000E+00 | 0.000E+00 |
| Unigene27437 | bcrA | 7.903E-05 | 3.763E-05 | 2.865E-07 | 8.423E-08 | 1.719E-05 | 6.912E-05 | 0.000E+00 | 0.000E+00 | 0.000E+00 | 0.000E+00 | 0.000E+00 | 0.000E+00 |
| Unigene27464 | Staphylocc | 8.899E-05 | 4.087E-05 | 2.477E-07 | 0.000E+00 | 1.681E-05 | 7.327E-05 | 0.000E+00 | 0.000E+00 | 0.000E+00 | 1.036E-07 | 9.117E-08 | 3.140E-08 |
| Unigene27467 | farB | 8.633E-05 | 4.217E-05 | 3.079E-07 | 6.791E-08 | 1.691E-05 | 7.173E-05 | 0.000E+00 | 0.000E+00 | 0.000E+00 | 9.989E-08 | 0.000E+00 | 0.000E+00 |
| Unigene27468 | farB | 8.400E-05 | 4.078E-05 | 1.586E-07 | 0.000E+00 | 1.593E-05 | 7.553E-05 | 0.000E+00 | 0.000E+00 | 0.000E+00 | 0.000E+00 | 0.000E+00 | 0.000E+00 |
| Unigene27518 | lmrD | 7.135E-06 | 0.000E+00 | 0.000E+00 | 2.530E-06 | 3.493E-05 | 1.009E-05 | 2.424E-05 | 2.585E-05 | 3.008E-06 | 0.000E+00 | 0.000E+00 | 0.000E+00 |
| Unigene27536 | poxtA | 2.799E-06 | 4.657E-06 | 2.704E-06 | 5.565E-06 | 0.000E+00 | 0.000E+00 | 3.873E-07 | 0.000E+00 | 0.000E+00 | 0.000E+00 | 0.000E+00 | 1.034E-07 |
| Unigene27595 | efrA | 2.688E-05 | 9.835E-06 | 6.883E-06 | 4.506E-06 | 2.856E-07 | 1.985E-07 | 1.219E-07 | 1.072E-07 | 1.124E-06 | 0.000E+00 | 2.100E-08 | 0.000E+00 |
| Unigene27596 | tetA(46) | 2.584E-05 | 9.072E-06 | 6.466E-06 | 4.662E-06 | 6.204E-07 | 2.616E-07 | 6.181E-08 | 1.304E-07 | 6.511E-07 | 0.000E+00 | 2.129E-08 | 4.401E-08 |
| Unigene27612 | mdtG | 1.002E-06 | 3.473E-07 | 3.105E-07 | 5.326E-08 | 0.000E+00 | 8.553E-08 | 2.919E-07 | 6.157E-08 | 6.630E-07 | 0.000E+00 | 0.000E+00 | 0.000E+00 |
| Unigene27650 | evgA | 7.324E-07 | 5.998E-06 | 1.111E-05 | 1.371E-05 | 0.000E+00 | 0.000E+00 | 6.235E-08 | 0.000E+00 | 0.000E+00 | 0.000E+00 | 0.000E+00 | 0.000E+00 |
| Unigene27663 | macB | 2.714E-05 | 1.122E-05 | 6.911E-06 | 4.396E-06 | 0.000E+00 | 0.000E+00 | 0.000E+00 | 0.000E+00 | 0.000E+00 | 0.000E+00 | 0.000E+00 | 0.000E+00 |
| Unigene27683 | cmlv | 4.670E-05 | 1.867E-05 | 7.075E-06 | 1.462E-05 | 2.002E-06 | 1.712E-06 | 3.295E-06 | 4.463E-07 | 7.641E-06 | 6.693E-07 | 1.906E-06 | 1.420E-06 |
| Unigene27733 | vanHO | 1.233E-06 | 1.207E-06 | 4.511E-06 | 6.560E-06 | 0.000E+00 | 0.000E+00 | 4.056E-07 | 0.000E+00 | 0.000E+00 | 0.000E+00 | 0.000E+00 | 0.000E+00 |
| Unigene27782 | lmrD | 9.506E-06 | 0.000E+00 | 0.000E+00 | 0.000E+00 | 1.600E-05 | 1.587E-05 | 0.000E+00 | 0.000E+00 | 1.092E-05 | 0.000E+00 | 0.000E+00 | 0.000E+00 |
| Unigene27816 | patA | 1.110E-06 | 5.463E-07 | 4.509E-06 | 5.298E-06 | 0.000E+00 | 0.000E+00 | 0.000E+00 | 0.000E+00 | 0.000E+00 | 0.000E+00 | 0.000E+00 | 0.000E+00 |
| Unigene27818 | tet(38) | 1.047E-06 | 8.069E-07 | 4.918E-06 | 6.884E-06 | 0.000E+00 | 0.000E+00 | 3.945E-07 | 0.000E+00 | 0.000E+00 | 0.000E+00 | 0.000E+00 | 2.633E-08 |
| Unigene27889 | bacA | 1.283E-05 | 5.135E-06 | 3.634E-06 | 1.484E-05 | 9.936E-05 | 1.323E-04 | 1.384E-04 | 1.173E-04 | 1.721E-04 | 1.741E-04 | 1.374E-04 | 9.041E-05 |
| Unigene27951 | macB | 5.732E-06 | 9.179E-06 | 9.498E-06 | 1.120E-05 | 0.000E+00 | 5.080E-08 | 0.000E+00 | 0.000E+00 | 0.000E+00 | 0.000E+00 | 0.000E+00 | 0.000E+00 |
| Unigene28007 | Staphylocc | 2.062E-05 | 8.620E-05 | 2.557E-05 | 0.000E+00 | 5.780E-06 | 1.355E-07 | 1.966E-06 | 1.683E-06 | 0.000E+00 | 0.000E+00 | 1.888E-06 | 2.717E-07 |
| Unigene28049 | arlR | 3.863E-07 | 2.422E-07 | 0.000E+00 | 2.229E-07 | 0.000E+00 | 0.000E+00 | 0.000E+00 | 0.000E+00 | 0.000E+00 | 0.000E+00 | 0.000E+00 | 0.000E+00 |
| Unigene28060 | TaeA | 1.101E-05 | 4.408E-06 | 2.692E-06 | 1.167E-05 | 7.876E-05 | 9.980E-05 | 9.959E-05 | 8.312E-05 | 1.258E-04 | 1.277E-04 | 1.054E-04 | 6.741E-05 |
| Unigene28062 | dfrE | 1.010E-05 | 3.600E-06 | 2.318E-06 | 1.186E-05 | 6.524E-05 | 1.021E-04 | 8.902E-05 | 6.969E-05 | 1.157E-04 | 1.122E-04 | 8.969E-05 | 5.995E-05 |
| Unigene28068 | QepA2 | 1.024E-05 | 4.260E-06 | 2.740E-06 | 1.135E-05 | 6.855E-05 | 9.826E-05 | 9.783E-05 | 7.411E-05 | 1.185E-04 | 1.154E-04 | 9.335E-05 | 6.051E-05 |
| Unigene28087 | PmrF | 1.358E-06 | 1.788E-06 | 9.659E-07 | 3.770E-07 | 2.149E-06 | 1.248E-06 | 7.513E-07 | 6.339E-07 | 5.936E-07 | 1.248E-06 | 9.707E-07 | 8.827E-07 |
| Unigene28160 | arlR | 7.173E-07 | 7.978E-06 | 1.136E-05 | 1.661E-05 | 0.000E+00 | 0.000E+00 | 0.000E+00 | 0.000E+00 | 0.000E+00 | 0.000E+00 | 0.000E+00 | 0.000E+00 |
| Unigene28172 | Corynebac | 1.136E-05 | 4.574E-06 | 2.994E-06 | 1.276E-05 | 8.153E-05 | 1.162E-04 | 1.165E-04 | 9.046E-05 | 1.443E-04 | 1.458E-04 | 1.147E-04 | 7.280E-05 |
| Unigene28265 | emrY | 2.577E-07 | 7.072E-08 | 0.000E+00 | 0.000E+00 | 0.000E+00 | 0.000E+00 | 0.000E+00 | 0.000E+00 | 0.000E+00 | 0.000E+00 | 0.000E+00 | 0.000E+00 |
| Unigene28276 | tetA(58) | 1.042E-05 | 3.798E-06 | 3.147E-06 | 1.099E-05 | 6.938E-05 | 1.004E-04 | 9.532E-05 | 7.767E-05 | 1.157E-04 | 1.162E-04 | 9.089E-05 | 6.150E-05 |
| Unigene28293 | bcr-1 | 1.185E-06 | 0.000E+00 | 0.000E+00 | 0.000E+00 | 1.663E-05 | 3.125E-05 | 0.000E+00 | 2.713E-08 | 0.000E+00 | 0.000E+00 | 0.000E+00 | 0.000E+00 |
| Unigene28340 | oqxA | 0.000E+00 | 1.763E-06 | 0.000E+00 | 0.000E+00 | 8.216E-06 | 0.000E+00 | 2.214E-06 | 0.000E+00 | 0.000E+00 | 0.000E+00 | 0.000E+00 | 0.000E+00 |
| Unigene28377 | QepA2 | 5.272E-05 | 7.529E-05 | 3.018E-05 | 1.622E-05 | 2.061E-05 | 7.747E-06 | 3.406E-06 | 5.556E-05 | 1.314E-05 | 7.007E-06 | 2.184E-06 | 1.301E-06 |
| Unigene28412 | macB | 7.379E-06 | 0.000E+00 | 0.000E+00 | 1.306E-06 | 3.051E-05 | 1.443E-05 | 2.284E-05 | 2.495E-05 | 3.727E-06 | 0.000E+00 | 0.000E+00 | 0.000E+00 |
| Unigene28427 | qacH | 9.855E-06 | 0.000E+00 | 0.000E+00 | 0.000E+00 | 4.335E-05 | 0.000E+00 | 2.860E-05 | 3.169E-05 | 0.000E+00 | 0.000E+00 | 0.000E+00 | 0.000E+00 |
| Unigene28489 | pmrA | 4.410E-05 | 2.151E-05 | 1.421E-05 | 1.864E-05 | 2.255E-06 | 1.202E-06 | 2.954E-06 | 9.736E-07 | 7.172E-06 | 5.885E-07 | 1.399E-06 | 1.183E-06 |
| Unigene28604 | tetA(58) | 3.290E-06 | 4.250E-06 | 2.387E-06 | 1.661E-06 | 2.779E-06 | 2.445E-06 | 2.238E-06 | 1.320E-06 | 1.161E-06 | 6.260E-06 | 6.037E-06 | 1.256E-06 |
| Unigene28690 | patB | 6.712E-06 | 0.000E+00 | 0.000E+00 | 2.334E-06 | 3.431E-05 | 1.397E-05 | 2.300E-05 | 2.576E-05 | 2.719E-06 | 0.000E+00 | 0.000E+00 | 0.000E+00 |
| Unigene28724 | cmlv | 1.247E-05 | 4.172E-06 | 3.020E-06 | 1.306E-05 | 8.579E-05 | 1.202E-04 | 1.210E-04 | 9.592E-05 | 1.465E-04 | 1.447E-04 | 1.189E-04 | 6.951E-05 |
| Unigene28764 | bcr-1 | 2.229E-06 | 2.990E-06 | 2.997E-06 | 2.307E-06 | 3.730E-06 | 1.993E-06 | 1.980E-06 | 8.352E-07 | 1.384E-06 | 6.223E-06 | 6.894E-06 | 1.464E-06 |
| Unigene28767 | tetA(58) | 2.894E-06 | 2.973E-06 | 2.221E-06 | 1.948E-06 | 2.679E-06 | 1.564E-06 | 2.259E-06 | 0.000E+00 | 1.460E-06 | 5.663E-06 | 5.645E-06 | 1.403E-06 |
| Unigene28815 | bcrA | 8.931E-06 | 1.771E-06 | 1.126E-06 | 4.093E-06 | 2.518E-05 | 4.129E-05 | 4.724E-05 | 4.107E-05 | 2.278E-05 | 2.717E-05 | 1.707E-05 | 4.748E-06 |
| Unigene28856 | optrA | 2.001E-06 | 1.550E-05 | 6.021E-06 | 3.514E-06 | 0.000E+00 | 2.181E-07 | 7.443E-08 | 0.000E+00 | 0.000E+00 | 7.492E-08 | 2.308E-07 | 0.000E+00 |
| Unigene28878 | mdtG | 1.314E-05 | 5.451E-06 | 3.934E-06 | 1.285E-05 | 9.911E-05 | 1.394E-04 | 1.445E-04 | 1.214E-04 | 1.750E-04 | 1.757E-04 | 1.464E-04 | 9.325E-05 |

| | | | | | | | | | | | | | |
|---|---|---|---|---|---|---|---|---|---|---|---|---|---|
| Unigene28912 | Chlamydia | 7.686E-07 | 6.585E-06 | 9.390E-06 | 1.575E-05 | 0.000E+00 | 0.000E+00 | 0.000E+00 | 0.000E+00 | 0.000E+00 | 0.000E+00 | 0.000E+00 | 0.000E+00 |
| Unigene28950 | mef(B) | 1.433E-06 | 0.000E+00 | 0.000E+00 | 0.000E+00 | 4.806E-07 | 4.676E-07 | 0.000E+00 | 0.000E+00 | 2.128E-06 | 1.807E-07 | 1.051E-06 | 0.000E+00 |
| Unigene28951 | msrE | 1.129E-06 | 0.000E+00 | 2.172E-08 | 0.000E+00 | 6.641E-07 | 3.351E-07 | 0.000E+00 | 0.000E+00 | 2.347E-06 | 0.000E+00 | 1.418E-06 | 0.000E+00 |
| Unigene28984 | efmA | 7.887E-06 | 0.000E+00 | 0.000E+00 | 2.443E-06 | 3.651E-05 | 1.553E-05 | 2.493E-05 | 2.559E-05 | 2.754E-06 | 0.000E+00 | 0.000E+00 | 0.000E+00 |
| Unigene28991 | mgrA | 6.725E-06 | 0.000E+00 | 0.000E+00 | 1.859E-06 | 3.665E-05 | 1.485E-05 | 2.703E-05 | 2.776E-05 | 0.000E+00 | 0.000E+00 | 0.000E+00 | 0.000E+00 |
| Unigene29001 | macB | 6.614E-06 | 0.000E+00 | 0.000E+00 | 1.646E-06 | 2.789E-05 | 1.168E-05 | 1.897E-05 | 2.175E-05 | 1.731E-06 | 0.000E+00 | 0.000E+00 | 0.000E+00 |
| Unigene29011 | patB | 8.144E-06 | 0.000E+00 | 0.000E+00 | 2.003E-06 | 3.455E-05 | 1.601E-05 | 2.204E-05 | 2.655E-05 | 2.583E-06 | 0.000E+00 | 0.000E+00 | 0.000E+00 |
| Unigene29012 | tetA(46) | 7.331E-06 | 0.000E+00 | 0.000E+00 | 1.791E-06 | 3.450E-05 | 1.598E-05 | 2.050E-05 | 2.241E-05 | 4.136E-06 | 0.000E+00 | 0.000E+00 | 0.000E+00 |
| Unigene29016 | arlS | 7.664E-06 | 0.000E+00 | 0.000E+00 | 2.326E-06 | 3.240E-05 | 1.534E-05 | 2.191E-05 | 2.279E-05 | 3.427E-06 | 0.000E+00 | 0.000E+00 | 0.000E+00 |
| Unigene29017 | mtrA | 5.471E-06 | 0.000E+00 | 0.000E+00 | 2.578E-06 | 2.512E-05 | 1.321E-05 | 1.867E-05 | 1.799E-05 | 2.093E-06 | 0.000E+00 | 0.000E+00 | 0.000E+00 |
| Unigene29020 | macB | 7.048E-06 | 0.000E+00 | 0.000E+00 | 1.993E-06 | 2.829E-05 | 1.910E-05 | 2.231E-05 | 2.430E-05 | 3.184E-06 | 0.000E+00 | 0.000E+00 | 0.000E+00 |
| Unigene29045 | bacA | 7.686E-06 | 0.000E+00 | 0.000E+00 | 2.627E-06 | 3.380E-05 | 1.615E-05 | 2.151E-05 | 2.452E-05 | 3.016E-06 | 0.000E+00 | 0.000E+00 | 0.000E+00 |
| Unigene29066 | Staphylocc | 6.973E-06 | 0.000E+00 | 0.000E+00 | 1.933E-06 | 3.231E-05 | 1.662E-05 | 2.171E-05 | 2.471E-05 | 3.277E-06 | 0.000E+00 | 1.531E-08 | 0.000E+00 |
| Unigene29069 | Staphylocc | 6.941E-06 | 0.000E+00 | 0.000E+00 | 1.609E-06 | 3.446E-05 | 1.673E-05 | 2.261E-05 | 2.532E-05 | 3.101E-06 | 0.000E+00 | 0.000E+00 | 0.000E+00 |
| Unigene29070 | tet(38) | 7.519E-06 | 0.000E+00 | 0.000E+00 | 2.727E-06 | 3.257E-05 | 1.712E-05 | 2.255E-05 | 2.464E-05 | 3.070E-06 | 0.000E+00 | 0.000E+00 | 0.000E+00 |
| Unigene29100 | msrC | 1.863E-06 | 1.573E-05 | 6.967E-06 | 5.532E-06 | 0.000E+00 | 1.421E-07 | 0.000E+00 | 0.000E+00 | 7.186E-08 | 0.000E+00 | 0.000E+00 | 0.000E+00 |
| Unigene29130 | lmrB | 8.207E-07 | 4.253E-07 | 1.029E-06 | 5.065E-07 | 1.520E-07 | 9.858E-08 | 0.000E+00 | 1.597E-07 | 0.000E+00 | 0.000E+00 | 7.823E-08 | 0.000E+00 |
| Unigene29148 | vmlR | 1.555E-06 | 4.401E-06 | 4.135E-06 | 6.239E-06 | 0.000E+00 | 0.000E+00 | 0.000E+00 | 0.000E+00 | 0.000E+00 | 0.000E+00 | 0.000E+00 | 0.000E+00 |
| Unigene29197 | vatC | 1.110E-06 | 2.966E-06 | 1.184E-06 | 1.072E-06 | 1.715E-06 | 9.911E-07 | 1.495E-06 | 6.759E-07 | 8.438E-07 | 4.246E-06 | 3.753E-06 | 4.563E-07 |
| Unigene29215 | efrA | 1.903E-05 | 2.517E-06 | 0.000E+00 | 0.000E+00 | 5.599E-08 | 4.086E-07 | 5.578E-08 | 0.000E+00 | 0.000E+00 | 1.684E-07 | 0.000E+00 | 4.468E-07 |
| Unigene29247 | QepA2 | 1.921E-06 | 1.776E-05 | 7.187E-06 | 3.721E-06 | 0.000E+00 | 2.444E-08 | 0.000E+00 | 0.000E+00 | 0.000E+00 | 0.000E+00 | 0.000E+00 | 0.000E+00 |
| Unigene29322 | cdeA | 1.114E-06 | 6.841E-06 | 1.019E-05 | 1.630E-05 | 0.000E+00 | 0.000E+00 | 0.000E+00 | 0.000E+00 | 0.000E+00 | 0.000E+00 | 0.000E+00 | 0.000E+00 |
| Unigene29325 | mecI | 7.874E-05 | 1.663E-04 | 2.587E-05 | 0.000E+00 | 4.275E-06 | 0.000E+00 | 0.000E+00 | 1.664E-07 | 6.932E-06 | 0.000E+00 | 7.336E-07 | 0.000E+00 |
| Unigene29373 | vanHB | 2.233E-06 | 1.858E-05 | 5.386E-06 | 2.505E-06 | 0.000E+00 | 1.341E-07 | 0.000E+00 | 0.000E+00 | 0.000E+00 | 0.000E+00 | 0.000E+00 | 0.000E+00 |
| Unigene29377 | efrA | 2.548E-06 | 1.925E-05 | 9.282E-06 | 6.692E-06 | 0.000E+00 | 1.172E-07 | 4.500E-08 | 3.164E-08 | 0.000E+00 | 0.000E+00 | 0.000E+00 | 0.000E+00 |
| Unigene29382 | patB | 2.488E-06 | 1.878E-05 | 7.830E-06 | 4.870E-06 | 0.000E+00 | 9.276E-08 | 5.698E-08 | 0.000E+00 | 3.751E-08 | 0.000E+00 | 9.815E-08 | 0.000E+00 |
| Unigene29452 | msbA | 2.703E-05 | 8.571E-06 | 7.274E-06 | 4.506E-06 | 3.398E-07 | 0.000E+00 | 1.195E-07 | 1.680E-07 | 0.000E+00 | 0.000E+00 | 0.000E+00 | 4.254E-08 |
| Unigene29530 | macB | 3.117E-06 | 0.000E+00 | 2.220E-06 | 3.554E-07 | 0.000E+00 | 0.000E+00 | 0.000E+00 | 0.000E+00 | 0.000E+00 | 0.000E+00 | 1.265E-06 | 0.000E+00 |
| Unigene29540 | patA | 3.570E-06 | 4.086E-06 | 2.852E-06 | 7.107E-06 | 0.000E+00 | 1.654E-07 | 2.822E-07 | 0.000E+00 | 0.000E+00 | 0.000E+00 | 0.000E+00 | 0.000E+00 |
| Unigene29565 | tetB(46) | 1.076E-04 | 1.744E-04 | 1.631E-04 | 0.000E+00 | 8.686E-06 | 0.000E+00 | 3.090E-06 | 1.869E-06 | 4.590E-05 | 0.000E+00 | 2.470E-06 | 0.000E+00 |
| Unigene29577 | Streptomy | 2.920E-06 | 4.250E-06 | 3.473E-06 | 6.459E-06 | 5.642E-07 | 3.431E-08 | 4.215E-07 | 0.000E+00 | 1.735E-07 | 0.000E+00 | 1.089E-07 | 2.251E-07 |
| Unigene29604 | lmrB | 9.051E-05 | 4.029E-05 | 2.945E-07 | 2.332E-08 | 1.839E-05 | 7.818E-05 | 0.000E+00 | 0.000E+00 | 0.000E+00 | 0.000E+00 | 2.113E-07 | 1.365E-07 |
| Unigene29608 | bcrA | 8.451E-05 | 3.618E-05 | 6.097E-07 | 0.000E+00 | 3.161E-06 | 5.703E-05 | 0.000E+00 | 0.000E+00 | 0.000E+00 | 1.457E-07 | 7.482E-08 | 0.000E+00 |
| Unigene29615 | tetA(58) | 8.022E-05 | 3.668E-05 | 1.242E-07 | 0.000E+00 | 1.613E-05 | 7.248E-05 | 0.000E+00 | 0.000E+00 | 0.000E+00 | 0.000E+00 | 0.000E+00 | 0.000E+00 |
| Unigene29617 | macB | 7.872E-05 | 3.649E-05 | 0.000E+00 | 0.000E+00 | 1.533E-05 | 7.044E-05 | 0.000E+00 | 0.000E+00 | 0.000E+00 | 0.000E+00 | 0.000E+00 | 0.000E+00 |
| Unigene29638 | vanRF | 8.451E-05 | 3.504E-05 | 0.000E+00 | 0.000E+00 | 1.973E-05 | 7.892E-05 | 0.000E+00 | 0.000E+00 | 0.000E+00 | 0.000E+00 | 2.502E-07 | 0.000E+00 |
| Unigene29644 | lmrB | 8.599E-05 | 3.988E-05 | 1.600E-07 | 0.000E+00 | 1.809E-05 | 7.808E-05 | 2.578E-08 | 0.000E+00 | 0.000E+00 | 0.000E+00 | 7.992E-08 | 0.000E+00 |
| Unigene29779 | vanSE | 5.327E-07 | 8.575E-07 | 3.972E-06 | 5.845E-06 | 0.000E+00 | 0.000E+00 | 5.895E-07 | 0.000E+00 | 0.000E+00 | 0.000E+00 | 0.000E+00 | 1.259E-07 |
| Unigene29783 | optrA | 7.384E-05 | 6.334E-05 | 1.238E-04 | 1.726E-04 | 1.359E-04 | 9.466E-05 | 1.306E-04 | 1.236E-04 | 1.064E-04 | 1.525E-04 | 1.598E-04 | 1.620E-04 |
| Unigene29803 | tet(42) | 3.920E-06 | 9.436E-06 | 1.528E-05 | 1.171E-05 | 1.802E-07 | 3.799E-07 | 8.079E-07 | 1.578E-07 | 0.000E+00 | 1.506E-07 | 9.278E-08 | 0.000E+00 |
| Unigene29805 | tetA(60) | 5.853E-05 | 4.742E-05 | 9.897E-05 | 1.438E-04 | 1.041E-04 | 7.736E-05 | 9.711E-05 | 9.441E-05 | 8.106E-05 | 1.153E-04 | 1.169E-04 | 1.229E-04 |
| Unigene29864 | novA | 7.353E-07 | 0.000E+00 | 0.000E+00 | 4.698E-07 | 0.000E+00 | 1.123E-05 | 4.318E-07 | 3.503E-08 | 1.312E-06 | 4.698E-06 | 0.000E+00 | 0.000E+00 |
| Unigene29898 | apmA | 7.238E-06 | 0.000E+00 | 0.000E+00 | 4.185E-08 | 0.000E+00 | 0.000E+00 | 0.000E+00 | 0.000E+00 | 0.000E+00 | 0.000E+00 | 0.000E+00 | 0.000E+00 |
| Unigene29920 | vanTC | 4.094E-07 | 0.000E+00 | 5.738E-08 | 0.000E+00 | 0.000E+00 | 0.000E+00 | 0.000E+00 | 0.000E+00 | 0.000E+00 | 0.000E+00 | 0.000E+00 | 0.000E+00 |
| Unigene29929 | lmrB | 1.237E-05 | 4.820E-06 | 3.474E-06 | 1.349E-05 | 8.639E-05 | 1.186E-04 | 1.215E-04 | 9.637E-05 | 1.500E-04 | 1.451E-04 | 1.156E-04 | 7.607E-05 |
| Unigene29930 | tetA(60) | 1.146E-05 | 4.226E-06 | 3.345E-06 | 1.161E-05 | 8.220E-05 | 1.095E-04 | 1.141E-04 | 9.233E-05 | 1.386E-04 | 1.393E-04 | 1.120E-04 | 7.235E-05 |
| Unigene29931 | tetB(60) | 1.197E-05 | 4.597E-06 | 3.986E-06 | 1.280E-05 | 8.310E-05 | 1.149E-04 | 1.210E-04 | 9.167E-05 | 1.470E-04 | 1.508E-04 | 1.176E-04 | 7.409E-05 |
| Unigene29972 | optrA | 2.242E-07 | 0.000E+00 | 0.000E+00 | 0.000E+00 | 2.092E-06 | 0.000E+00 | 0.000E+00 | 0.000E+00 | 0.000E+00 | 0.000E+00 | 0.000E+00 | 0.000E+00 |
| Unigene29993 | tetA(46) | 1.491E-06 | 6.418E-07 | 1.004E-06 | 1.181E-06 | 1.861E-05 | 5.816E-06 | 1.230E-05 | 1.597E-05 | 1.013E-05 | 1.401E-05 | 1.549E-05 | 5.599E-06 |
| Unigene30075 | Staphylocc | 4.780E-05 | 6.479E-05 | 2.773E-05 | 1.588E-05 | 1.999E-05 | 7.179E-06 | 3.163E-06 | 5.671E-05 | 1.223E-05 | 6.847E-06 | 1.424E-06 | 8.869E-07 |
| Unigene30078 | Erm(K) | 4.863E-05 | 6.706E-05 | 2.853E-05 | 1.227E-05 | 1.734E-05 | 5.161E-06 | 3.549E-06 | 5.947E-05 | 1.090E-05 | 6.271E-06 | 1.630E-06 | 1.390E-06 |
| Unigene30084 | lmrB | 4.279E-05 | 5.956E-05 | 2.519E-05 | 1.367E-05 | 1.791E-05 | 5.234E-06 | 3.424E-06 | 4.974E-05 | 1.044E-05 | 6.093E-06 | 2.000E-06 | 7.154E-07 |
| Unigene30086 | msbA | 4.291E-05 | 5.583E-05 | 2.466E-05 | 1.168E-05 | 1.517E-05 | 5.287E-06 | 2.824E-06 | 4.657E-05 | 1.023E-05 | 6.870E-06 | 2.043E-06 | 1.508E-06 |
| Unigene30096 | poxtA | 5.314E-05 | 7.544E-05 | 3.195E-05 | 1.671E-05 | 2.312E-05 | 6.815E-06 | 4.354E-06 | 6.584E-05 | 1.399E-05 | 8.660E-06 | 1.518E-06 | 7.844E-07 |
| Unigene30124 | macB | 4.669E-05 | 6.828E-05 | 2.989E-05 | 1.500E-05 | 1.274E-05 | 6.950E-06 | 3.557E-06 | 6.554E-05 | 1.367E-05 | 8.144E-06 | 1.457E-06 | 1.413E-06 |
| Unigene30180 | floR | 3.532E-06 | 1.514E-07 | 3.276E-06 | 2.507E-07 | 3.678E-07 | 2.983E-08 | 3.970E-07 | 0.000E+00 | 0.000E+00 | 0.000E+00 | 9.152E-07 | 0.000E+00 |
| Unigene30215 | Staphylocc | 2.826E-05 | 9.829E-07 | 5.902E-07 | 0.000E+00 | 5.940E-08 | 5.780E-08 | 0.000E+00 | 0.000E+00 | 0.000E+00 | 0.000E+00 | 0.000E+00 | 0.000E+00 |
| Unigene30228 | lmrD | 2.683E-07 | 0.000E+00 | 0.000E+00 | 0.000E+00 | 0.000E+00 | 0.000E+00 | 5.938E-07 | 3.131E-07 | 0.000E+00 | 3.652E-07 | 0.000E+00 | 6.342E-07 |

| | | | | | | | | | | | | | |
|---|---|---|---|---|---|---|---|---|---|---|---|---|---|
| Unigene30253 | Corynebac | 2.396E-06 | 4.016E-06 | 2.607E-06 | 1.188E-06 | 2.807E-06 | 2.025E-06 | 1.446E-06 | 6.610E-07 | 9.523E-07 | 6.940E-06 | 5.531E-06 | 1.390E-06 |
| Unigene30337 | msbA | 1.098E-05 | 3.796E-06 | 3.017E-06 | 1.100E-05 | 7.571E-05 | 1.013E-04 | 9.707E-05 | 8.258E-05 | 1.243E-04 | 1.299E-04 | 1.012E-04 | 6.727E-05 |
| Unigene30396 | vanHB | 2.618E-06 | 3.154E-07 | 3.290E-06 | 0.000E+00 | 2.838E-07 | 0.000E+00 | 4.240E-07 | 0.000E+00 | 0.000E+00 | 0.000E+00 | 8.035E-07 | 0.000E+00 |
| Unigene30444 | fexA | 6.305E-05 | 1.732E-04 | 6.569E-04 | 5.474E-05 | 5.392E-06 | 2.574E-06 | 1.774E-07 | 6.681E-07 | 7.457E-06 | 0.000E+00 | 0.000E+00 | 0.000E+00 |
| Unigene30486 | mef(B) | 1.138E-06 | 1.107E-06 | 4.365E-06 | 6.295E-06 | 0.000E+00 | 8.389E-08 | 4.867E-07 | 0.000E+00 | 0.000E+00 | 0.000E+00 | 8.876E-08 | 3.057E-08 |
| Unigene30497 | tetA(46) | 2.813E-05 | 7.151E-07 | 0.000E+00 | 0.000E+00 | 0.000E+00 | 1.207E-07 | 0.000E+00 | 0.000E+00 | 0.000E+00 | 0.000E+00 | 0.000E+00 | 0.000E+00 |
| Unigene30498 | msbA | 2.651E-05 | 6.438E-07 | 4.137E-07 | 0.000E+00 | 0.000E+00 | 2.180E-07 | 0.000E+00 | 0.000E+00 | 0.000E+00 | 2.246E-07 | 0.000E+00 | 1.300E-07 |
| Unigene30522 | oleC | 3.000E-05 | 0.000E+00 | 0.000E+00 | 0.000E+00 | 0.000E+00 | 0.000E+00 | 0.000E+00 | 0.000E+00 | 0.000E+00 | 0.000E+00 | 0.000E+00 | 0.000E+00 |
| Unigene30538 | poxtA | 5.301E-07 | 4.654E-07 | 0.000E+00 | 6.958E-07 | 0.000E+00 | 0.000E+00 | 0.000E+00 | 0.000E+00 | 0.000E+00 | 0.000E+00 | 0.000E+00 | 0.000E+00 |
| Unigene30597 | tet(38) | 4.255E-06 | 6.078E-06 | 7.201E-06 | 7.524E-06 | 1.000E-07 | 9.734E-08 | 0.000E+00 | 0.000E+00 | 0.000E+00 | 2.006E-07 | 0.000E+00 | 0.000E+00 |
| Unigene30736 | lsaC | 2.074E-06 | 1.444E-05 | 5.661E-06 | 3.731E-06 | 0.000E+00 | 7.007E-08 | 0.000E+00 | 0.000E+00 | 0.000E+00 | 0.000E+00 | 1.483E-07 | 0.000E+00 |
| Unigene30755 | YojI | 1.840E-06 | 3.115E-06 | 6.733E-06 | 9.480E-06 | 1.386E-07 | 1.011E-07 | 3.452E-07 | 0.000E+00 | 3.409E-08 | 0.000E+00 | 1.070E-07 | 0.000E+00 |
| Unigene30759 | baeS | 5.027E-06 | 0.000E+00 | 3.413E-06 | 4.665E-07 | 2.415E-07 | 0.000E+00 | 0.000E+00 | 0.000E+00 | 0.000E+00 | 0.000E+00 | 9.014E-07 | 0.000E+00 |
| Unigene30760 | arlR | 3.633E-06 | 6.056E-07 | 2.978E-06 | 5.108E-07 | 1.533E-07 | 4.971E-08 | 1.120E-06 | 0.000E+00 | 0.000E+00 | 0.000E+00 | 1.052E-06 | 0.000E+00 |
| Unigene30770 | arlS | 3.627E-06 | 2.075E-07 | 3.542E-06 | 3.645E-07 | 1.337E-07 | 9.291E-08 | 2.283E-07 | 0.000E+00 | 0.000E+00 | 0.000E+00 | 6.882E-07 | 0.000E+00 |
| Unigene30799 | vanHD | 2.236E-05 | 1.494E-06 | 5.088E-07 | 0.000E+00 | 0.000E+00 | 0.000E+00 | 0.000E+00 | 0.000E+00 | 0.000E+00 | 0.000E+00 | 0.000E+00 | 0.000E+00 |
| Unigene30822 | tetB(60) | 4.064E-06 | 2.189E-05 | 3.259E-06 | 1.106E-06 | 7.066E-07 | 5.730E-07 | 3.324E-07 | 3.712E-07 | 1.448E-06 | 7.086E-07 | 4.851E-07 | 0.000E+00 |
| Unigene30866 | smeS | 2.799E-06 | 5.161E-06 | 7.640E-06 | 8.039E-06 | 1.701E-07 | 9.457E-08 | 0.000E+00 | 0.000E+00 | 0.000E+00 | 1.218E-07 | 7.505E-08 | 0.000E+00 |
| Unigene30868 | facT | 4.392E-06 | 7.842E-06 | 9.115E-06 | 1.078E-05 | 0.000E+00 | 0.000E+00 | 1.628E-07 | 2.453E-08 | 0.000E+00 | 0.000E+00 | 0.000E+00 | 0.000E+00 |
| Unigene30990 | mdtG | 3.525E-06 | 3.552E-06 | 2.581E-06 | 1.727E-06 | 2.484E-06 | 2.133E-06 | 2.126E-06 | 5.527E-07 | 1.409E-06 | 5.187E-06 | 6.410E-06 | 2.021E-06 |
| Unigene30998 | patB | 1.543E-06 | 0.000E+00 | 0.000E+00 | 0.000E+00 | 0.000E+00 | 8.134E-08 | 0.000E+00 | 0.000E+00 | 0.000E+00 | 0.000E+00 | 0.000E+00 | 0.000E+00 |
| Unigene30999 | tetB(46) | 1.931E-06 | 0.000E+00 | 0.000E+00 | 0.000E+00 | 0.000E+00 | 0.000E+00 | 0.000E+00 | 0.000E+00 | 0.000E+00 | 0.000E+00 | 0.000E+00 | 0.000E+00 |
| Unigene31021 | efrB | 2.074E-06 | 0.000E+00 | 0.000E+00 | 0.000E+00 | 9.218E-06 | 1.668E-05 | 6.935E-06 | 1.538E-05 | 1.138E-05 | 0.000E+00 | 0.000E+00 | 5.108E-06 |
| Unigene31032 | arlS | 3.579E-06 | 5.092E-07 | 2.883E-06 | 4.462E-07 | 2.209E-07 | 9.554E-08 | 3.913E-07 | 0.000E+00 | 0.000E+00 | 0.000E+00 | 6.318E-07 | 0.000E+00 |
| Unigene31033 | arlR | 2.667E-06 | 0.000E+00 | 3.443E-06 | 4.252E-07 | 1.039E-07 | 0.000E+00 | 2.589E-07 | 0.000E+00 | 0.000E+00 | 0.000E+00 | 5.887E-07 | 0.000E+00 |
| Unigene31040 | msbA | 2.913E-06 | 2.997E-07 | 3.644E-06 | 6.803E-07 | 2.225E-07 | 7.873E-08 | 5.441E-07 | 0.000E+00 | 0.000E+00 | 0.000E+00 | 8.955E-07 | 0.000E+00 |
| Unigene31047 | msbA | 2.715E-06 | 0.000E+00 | 2.220E-06 | 0.000E+00 | 0.000E+00 | 0.000E+00 | 0.000E+00 | 0.000E+00 | 0.000E+00 | 0.000E+00 | 0.000E+00 | 0.000E+00 |
| Unigene31050 | mdtG | 3.744E-06 | 2.586E-07 | 3.545E-06 | 3.701E-07 | 3.199E-07 | 2.264E-07 | 4.926E-07 | 0.000E+00 | 0.000E+00 | 0.000E+00 | 8.385E-07 | 0.000E+00 |
| Unigene31105 | vga(E) Stap | 1.118E-06 | 7.771E-06 | 1.170E-05 | 2.070E-05 | 0.000E+00 | 5.036E-08 | 0.000E+00 | 0.000E+00 | 0.000E+00 | 0.000E+00 | 1.066E-07 | 0.000E+00 |
| Unigene31109 | vmlR | 3.572E-07 | 0.000E+00 | 0.000E+00 | 0.000E+00 | 0.000E+00 | 0.000E+00 | 0.000E+00 | 0.000E+00 | 0.000E+00 | 0.000E+00 | 0.000E+00 | 0.000E+00 |
| Unigene31153 | tetT | 0.000E+00 | 0.000E+00 | 0.000E+00 | 0.000E+00 | 2.643E-06 | 0.000E+00 | 0.000E+00 | 0.000E+00 | 0.000E+00 | 0.000E+00 | 0.000E+00 | 0.000E+00 |
| Unigene31154 | macB | 4.125E-06 | 1.176E-05 | 9.678E-06 | 1.241E-05 | 2.490E-07 | 1.454E-07 | 0.000E+00 | 5.233E-08 | 0.000E+00 | 0.000E+00 | 1.026E-07 | 0.000E+00 |
| Unigene31183 | AcrS | 1.412E-06 | 1.523E-05 | 7.835E-06 | 3.982E-06 | 0.000E+00 | 2.632E-07 | 0.000E+00 | 0.000E+00 | 0.000E+00 | 0.000E+00 | 0.000E+00 | 0.000E+00 |
| Unigene31192 | macB | 1.730E-06 | 1.551E-05 | 6.459E-06 | 2.940E-06 | 0.000E+00 | 0.000E+00 | 0.000E+00 | 0.000E+00 | 0.000E+00 | 0.000E+00 | 9.654E-08 | 1.496E-07 |
| Unigene31193 | baeS | 1.761E-06 | 1.460E-05 | 7.519E-06 | 3.490E-06 | 0.000E+00 | 0.000E+00 | 0.000E+00 | 0.000E+00 | 0.000E+00 | 0.000E+00 | 0.000E+00 | 0.000E+00 |
| Unigene31194 | smeR | 1.659E-06 | 1.353E-05 | 7.256E-06 | 4.499E-06 | 0.000E+00 | 1.537E-07 | 0.000E+00 | 0.000E+00 | 0.000E+00 | 0.000E+00 | 1.627E-07 | 0.000E+00 |
| Unigene31203 | bcrA | 2.276E-06 | 1.458E-05 | 6.660E-06 | 4.848E-06 | 0.000E+00 | 8.948E-08 | 1.374E-07 | 0.000E+00 | 0.000E+00 | 0.000E+00 | 0.000E+00 | 0.000E+00 |
| Unigene31223 | vanHO | 1.322E-05 | 4.349E-06 | 3.218E-06 | 1.156E-05 | 7.969E-05 | 1.163E-04 | 1.183E-04 | 8.600E-05 | 1.397E-04 | 1.333E-04 | 1.121E-04 | 6.961E-05 |
| Unigene31373 | golS | 1.492E-06 | 0.000E+00 | 0.000E+00 | 0.000E+00 | 9.773E-06 | 0.000E+00 | 0.000E+00 | 0.000E+00 | 0.000E+00 | 0.000E+00 | 0.000E+00 | 0.000E+00 |
| Unigene31415 | mtrA | 5.080E-07 | 7.603E-07 | 1.178E-06 | 2.845E-06 | 3.078E-07 | 0.000E+00 | 4.089E-07 | 8.625E-07 | 0.000E+00 | 0.000E+00 | 8.452E-07 | 1.146E-06 |
| Unigene31424 | poxtA | 1.783E-06 | 3.712E-06 | 2.775E-06 | 4.191E-06 | 1.708E-07 | 6.647E-08 | 2.722E-07 | 0.000E+00 | 4.032E-07 | 0.000E+00 | 1.407E-07 | 2.907E-07 |
| Unigene31444 | cmrA | 3.097E-06 | 1.843E-05 | 7.807E-06 | 6.040E-06 | 0.000E+00 | 0.000E+00 | 0.000E+00 | 0.000E+00 | 0.000E+00 | 0.000E+00 | 0.000E+00 | 0.000E+00 |
| Unigene31446 | lmrB | 2.873E-06 | 1.917E-05 | 9.981E-06 | 6.015E-06 | 0.000E+00 | 1.782E-07 | 0.000E+00 | 0.000E+00 | 0.000E+00 | 0.000E+00 | 0.000E+00 | 0.000E+00 |
| Unigene31469 | Staphylocc | 1.096E-06 | 8.655E-07 | 1.141E-06 | 1.795E-06 | 1.884E-05 | 6.083E-06 | 1.315E-05 | 1.604E-05 | 1.094E-05 | 1.626E-05 | 1.734E-05 | 7.015E-06 |
| Unigene31470 | emrY | 1.225E-06 | 6.241E-07 | 9.871E-07 | 1.347E-06 | 1.839E-05 | 5.627E-06 | 1.428E-05 | 1.567E-05 | 9.872E-06 | 1.552E-05 | 1.706E-05 | 6.799E-06 |
| Unigene31476 | efrA | 2.109E-05 | 0.000E+00 | 0.000E+00 | 0.000E+00 | 0.000E+00 | 0.000E+00 | 0.000E+00 | 0.000E+00 | 6.291E-07 | 0.000E+00 | 0.000E+00 | 0.000E+00 |
| Unigene31487 | vatB | 1.695E-05 | 0.000E+00 | 0.000E+00 | 0.000E+00 | 0.000E+00 | 0.000E+00 | 0.000E+00 | 0.000E+00 | 0.000E+00 | 0.000E+00 | 0.000E+00 | 0.000E+00 |
| Unigene31496 | macB | 2.872E-06 | 4.282E-06 | 1.935E-06 | 2.121E-06 | 2.381E-06 | 1.668E-06 | 1.661E-06 | 6.005E-07 | 1.218E-06 | 5.444E-06 | 5.933E-06 | 1.824E-06 |
| Unigene31498 | kdpE | 3.179E-06 | 3.221E-06 | 2.832E-06 | 1.976E-06 | 2.717E-06 | 2.538E-06 | 2.490E-06 | 1.370E-06 | 1.069E-06 | 6.211E-06 | 6.434E-06 | 1.503E-06 |
| Unigene31536 | RSA-1 | 1.747E-05 | 2.396E-05 | 5.848E-05 | 6.461E-05 | 6.149E-06 | 1.446E-05 | 6.533E-05 | 1.633E-05 | 1.413E-05 | 2.632E-05 | 6.911E-05 | 2.122E-05 |
| Unigene31577 | YojI | 1.277E-05 | 5.207E-06 | 3.259E-06 | 1.290E-05 | 8.981E-05 | 1.299E-04 | 1.350E-04 | 1.105E-04 | 1.645E-04 | 1.620E-04 | 1.314E-04 | 8.080E-05 |
| Unigene31600 | macB | 6.029E-07 | 0.000E+00 | 0.000E+00 | 0.000E+00 | 0.000E+00 | 0.000E+00 | 0.000E+00 | 0.000E+00 | 0.000E+00 | 0.000E+00 | 0.000E+00 | 2.193E-07 |
| Unigene31622 | vanYF | 4.435E-06 | 8.880E-06 | 1.499E-05 | 1.373E-05 | 5.019E-07 | 3.108E-07 | 6.363E-07 | 1.438E-07 | 0.000E+00 | 0.000E+00 | 0.000E+00 | 0.000E+00 |
| Unigene31649 | PmrF | 1.961E-05 | 2.355E-06 | 6.348E-06 | 3.533E-06 | 1.467E-07 | 1.070E-07 | 1.461E-07 | 1.926E-07 | 0.000E+00 | 0.000E+00 | 0.000E+00 | 0.000E+00 |
| Unigene31654 | bcrA | 1.892E-05 | 3.989E-06 | 6.907E-06 | 4.340E-06 | 2.564E-07 | 0.000E+00 | 3.193E-08 | 1.347E-07 | 0.000E+00 | 0.000E+00 | 0.000E+00 | 0.000E+00 |
| Unigene31726 | Erm(34) | 3.098E-05 | 1.363E-05 | 7.274E-06 | 4.906E-06 | 1.999E-07 | 0.000E+00 | 2.390E-07 | 8.401E-08 | 1.141E-06 | 0.000E+00 | 0.000E+00 | 1.701E-07 |
| Unigene31737 | macB | 2.715E-05 | 9.933E-06 | 7.284E-06 | 4.591E-06 | 0.000E+00 | 4.509E-08 | 1.846E-07 | 0.000E+00 | 9.573E-07 | 0.000E+00 | 0.000E+00 | 0.000E+00 |
| Unigene31783 | bcrA | 9.365E-05 | 1.578E-04 | 1.455E-04 | 7.256E-06 | 8.138E-06 | 4.547E-07 | 3.569E-06 | 1.350E-06 | 5.585E-05 | 0.000E+00 | 3.688E-06 | 0.000E+00 |

| | | | | | | | | | | | | | |
|---|---|---|---|---|---|---|---|---|---|---|---|---|---|
| Unigene31843 | vanRG | 1.212E-06 | 1.117E-06 | 1.427E-06 | 1.224E-06 | 1.938E-05 | 5.135E-06 | 1.159E-05 | 1.505E-05 | 1.007E-05 | 1.550E-05 | 1.780E-05 | 9.052E-06 |
| Unigene31925 | Listeria mc | 7.270E-07 | 0.000E+00 | 2.972E-07 | 5.506E-07 | 3.998E-06 | 9.052E-06 | 2.173E-06 | 8.189E-06 | 4.768E-06 | 4.198E-06 | 0.000E+00 | 1.189E-06 |
| Unigene31951 | Staphylocc | 8.041E-07 | 8.046E-07 | 1.299E-06 | 6.925E-07 | 1.839E-07 | 1.534E-07 | 2.879E-07 | 1.104E-07 | 0.000E+00 | 0.000E+00 | 0.000E+00 | 2.795E-08 |
| Unigene32043 | srmB | 2.670E-06 | 1.820E-06 | 5.160E-06 | 2.778E-06 | 0.000E+00 | 0.000E+00 | 0.000E+00 | 4.722E-08 | 0.000E+00 | 2.253E-07 | 0.000E+00 | 0.000E+00 |
| Unigene32073 | oleB | 7.665E-07 | 0.000E+00 | 0.000E+00 | 2.977E-08 | 0.000E+00 | 2.231E-07 | 0.000E+00 | 2.409E-07 | 0.000E+00 | 0.000E+00 | 5.733E-07 | 1.132E-05 |
| Unigene32081 | lmrD | 2.874E-05 | 1.090E-05 | 6.867E-06 | 4.182E-06 | 4.018E-07 | 0.000E+00 | 1.801E-07 | 2.111E-07 | 1.107E-06 | 0.000E+00 | 0.000E+00 | 0.000E+00 |
| Unigene32095 | carA | 2.916E-06 | 5.257E-06 | 8.038E-06 | 7.957E-06 | 1.157E-07 | 0.000E+00 | 1.152E-07 | 0.000E+00 | 0.000E+00 | 0.000E+00 | 3.970E-08 | 0.000E+00 |
| Unigene32102 | adeS | 0.000E+00 | 0.000E+00 | 0.000E+00 | 0.000E+00 | 0.000E+00 | 0.000E+00 | 3.789E-07 | 0.000E+00 | 0.000E+00 | 0.000E+00 | 0.000E+00 | 5.203E-07 |
| Unigene32144 | poxtA | 1.093E-05 | 0.000E+00 | 0.000E+00 | 0.000E+00 | 1.764E-05 | 1.632E-05 | 0.000E+00 | 0.000E+00 | 1.086E-05 | 0.000E+00 | 0.000E+00 | 0.000E+00 |
| Unigene32206 | arlR | 9.840E-05 | 2.156E-04 | 1.996E-04 | 7.662E-06 | 9.195E-06 | 0.000E+00 | 0.000E+00 | 0.000E+00 | 5.820E-05 | 0.000E+00 | 3.524E-06 | 0.000E+00 |
| Unigene32228 | Staphylocc | 2.794E-06 | 2.046E-05 | 1.026E-05 | 8.806E-06 | 0.000E+00 | 3.472E-08 | 0.000E+00 | 5.624E-08 | 0.000E+00 | 0.000E+00 | 2.021E-07 | 5.695E-08 |
| Unigene32262 | Erm(30) | 1.616E-06 | 1.890E-05 | 8.612E-06 | 6.379E-06 | 0.000E+00 | 0.000E+00 | 3.252E-07 | 0.000E+00 | 0.000E+00 | 2.455E-07 | 1.260E-07 | 0.000E+00 |
| Unigene32271 | mef(B) | 2.125E-06 | 1.735E-05 | 6.335E-06 | 4.185E-06 | 0.000E+00 | 2.010E-07 | 0.000E+00 | 0.000E+00 | 0.000E+00 | 0.000E+00 | 6.078E-08 | 0.000E+00 |
| Unigene32272 | patB | 2.534E-06 | 1.800E-05 | 7.315E-06 | 4.654E-06 | 0.000E+00 | 0.000E+00 | 6.097E-08 | 0.000E+00 | 0.000E+00 | 0.000E+00 | 2.100E-07 | 4.341E-08 |
| Unigene32273 | efrA | 2.433E-06 | 1.618E-05 | 5.619E-06 | 3.912E-06 | 0.000E+00 | 1.214E-07 | 6.213E-08 | 0.000E+00 | 0.000E+00 | 8.338E-08 | 1.927E-07 | 0.000E+00 |
| Unigene32295 | novA | 4.454E-06 | 1.241E-05 | 1.036E-05 | 1.577E-05 | 0.000E+00 | 0.000E+00 | 0.000E+00 | 0.000E+00 | 0.000E+00 | 0.000E+00 | 0.000E+00 | 0.000E+00 |
| Unigene32355 | Bacillus sul | 3.925E-05 | 2.470E-05 | 2.140E-05 | 2.244E-05 | 1.431E-06 | 1.618E-06 | 3.208E-06 | 8.181E-07 | 6.626E-06 | 6.120E-07 | 1.907E-06 | 1.388E-06 |
| Unigene32394 | vanRI | 2.282E-06 | 3.668E-06 | 2.720E-06 | 1.893E-06 | 2.445E-06 | 1.762E-06 | 1.985E-06 | 2.379E-06 | 1.871E-06 | 0.000E+00 | 5.595E-06 | 1.542E-06 |
| Unigene32411 | optrA | 6.642E-07 | 1.093E-07 | 3.259E-07 | 4.024E-07 | 4.317E-06 | 9.909E-06 | 1.103E-06 | 6.667E-06 | 4.392E-06 | 4.365E-06 | 0.000E+00 | 0.000E+00 |
| Unigene32413 | rosB | 3.999E-07 | 3.192E-07 | 0.000E+00 | 0.000E+00 | 0.000E+00 | 0.000E+00 | 0.000E+00 | 0.000E+00 | 0.000E+00 | 0.000E+00 | 0.000E+00 | 0.000E+00 |
| Unigene32593 | bcrA | 2.020E-06 | 2.989E-06 | 2.586E-06 | 3.371E-06 | 5.367E-07 | 0.000E+00 | 3.889E-07 | 0.000E+00 | 2.880E-07 | 0.000E+00 | 1.005E-07 | 3.634E-07 |
| Unigene32669 | Staphylocc | 4.578E-05 | 6.259E-05 | 2.686E-05 | 1.316E-05 | 1.761E-05 | 6.170E-06 | 3.508E-06 | 5.553E-05 | 1.229E-05 | 5.936E-06 | 1.905E-06 | 1.045E-06 |
| Unigene32712 | oleC | 4.020E-05 | 5.854E-05 | 2.648E-05 | 1.189E-05 | 1.713E-05 | 6.411E-06 | 3.938E-06 | 5.657E-05 | 1.144E-05 | 6.371E-06 | 1.793E-06 | 1.452E-06 |
| Unigene32774 | TaeA | 4.660E-05 | 6.216E-05 | 2.778E-05 | 1.366E-05 | 1.856E-05 | 5.909E-06 | 3.729E-06 | 6.053E-05 | 1.306E-05 | 6.683E-06 | 1.809E-06 | 1.097E-06 |
| Unigene32776 | dfrC | 4.067E-05 | 5.979E-05 | 2.769E-05 | 1.080E-05 | 1.549E-05 | 7.096E-06 | 4.194E-06 | 5.624E-05 | 1.191E-05 | 6.408E-06 | 1.703E-06 | 1.280E-06 |
| Unigene32778 | novA | 4.077E-05 | 5.103E-05 | 2.610E-05 | 1.095E-05 | 1.448E-05 | 5.958E-06 | 2.856E-06 | 4.866E-05 | 1.042E-05 | 6.383E-06 | 1.151E-06 | 1.086E-06 |
| Unigene32825 | macB | 3.357E-06 | 1.711E-05 | 3.474E-06 | 9.287E-07 | 1.175E-06 | 3.480E-07 | 2.545E-07 | 3.220E-07 | 1.005E-06 | 4.098E-07 | 2.104E-07 | 0.000E+00 |
| Unigene32834 | vanZA | 4.926E-06 | 1.992E-05 | 2.608E-06 | 9.818E-07 | 6.481E-07 | 4.905E-07 | 1.435E-07 | 0.000E+00 | 1.417E-06 | 0.000E+00 | 0.000E+00 | 0.000E+00 |
| Unigene32875 | oleC | 5.749E-06 | 0.000E+00 | 0.000E+00 | 1.861E-06 | 3.291E-05 | 1.689E-05 | 2.390E-05 | 2.559E-05 | 3.182E-06 | 0.000E+00 | 0.000E+00 | 0.000E+00 |
| Unigene32914 | vanHD | 3.050E-06 | 3.421E-06 | 2.171E-06 | 2.099E-06 | 3.091E-06 | 2.174E-06 | 1.447E-06 | 1.056E-06 | 8.793E-07 | 4.742E-06 | 4.946E-06 | 1.585E-06 |
| Unigene32937 | tet(L) | 8.899E-05 | 1.534E-04 | 4.211E-04 | 2.077E-06 | 6.409E-07 | 2.983E-07 | 0.000E+00 | 0.000E+00 | 1.711E-04 | 0.000E+00 | 0.000E+00 | 0.000E+00 |
| Unigene32955 | Streptomy | 1.649E-06 | 1.483E-05 | 5.342E-06 | 3.622E-06 | 0.000E+00 | 0.000E+00 | 0.000E+00 | 0.000E+00 | 0.000E+00 | 0.000E+00 | 1.099E-07 | 0.000E+00 |
| Unigene32973 | dfrE | 1.956E-06 | 1.367E-05 | 6.143E-06 | 3.424E-06 | 0.000E+00 | 0.000E+00 | 0.000E+00 | 0.000E+00 | 0.000E+00 | 0.000E+00 | 0.000E+00 | 0.000E+00 |
| Unigene32974 | TaeA | 1.707E-06 | 1.611E-05 | 6.734E-06 | 4.423E-06 | 0.000E+00 | 9.321E-08 | 3.817E-08 | 0.000E+00 | 0.000E+00 | 0.000E+00 | 5.917E-08 | 6.115E-08 |
| Unigene32990 | vatE | 4.454E-06 | 4.669E-06 | 6.482E-06 | 3.811E-05 | 6.227E-06 | 3.571E-06 | 6.381E-05 | 6.210E-05 | 1.525E-04 | 9.979E-06 | 0.000E+00 | 2.958E-07 |
| Unigene33051 | tetA(58) | 7.027E-07 | 0.000E+00 | 0.000E+00 | 0.000E+00 | 0.000E+00 | 0.000E+00 | 0.000E+00 | 0.000E+00 | 0.000E+00 | 0.000E+00 | 0.000E+00 | 0.000E+00 |
| Unigene33108 | sul3 | 4.538E-06 | 2.108E-05 | 3.362E-06 | 7.284E-07 | 1.135E-06 | 3.249E-07 | 0.000E+00 | 0.000E+00 | 1.150E-06 | 4.687E-07 | 0.000E+00 | 0.000E+00 |
| Unigene33112 | dfrE | 3.772E-06 | 1.968E-05 | 2.002E-06 | 8.118E-07 | 7.557E-07 | 0.000E+00 | 2.053E-07 | 0.000E+00 | 1.217E-06 | 0.000E+00 | 0.000E+00 | 0.000E+00 |
| Unigene33139 | tetT | 2.805E-06 | 2.419E-06 | 2.812E-06 | 2.003E-06 | 1.558E-07 | 6.498E-08 | 6.209E-07 | 3.976E-07 | 4.599E-07 | 0.000E+00 | 0.000E+00 | 9.474E-08 |
| Unigene33180 | lmrD | 1.011E-04 | 1.735E-04 | 1.758E-04 | 8.003E-06 | 8.163E-06 | 3.708E-07 | 0.000E+00 | 1.960E-06 | 5.701E-05 | 3.218E-07 | 2.767E-06 | 8.749E-07 |
| Unigene33215 | tetB(60) | 5.841E-06 | 1.012E-05 | 7.843E-06 | 7.948E-06 | 0.000E+00 | 0.000E+00 | 0.000E+00 | 0.000E+00 | 0.000E+00 | 0.000E+00 | 0.000E+00 | 0.000E+00 |
| Unigene33292 | macB | 7.358E-06 | 0.000E+00 | 0.000E+00 | 2.104E-06 | 3.227E-05 | 1.490E-05 | 2.000E-05 | 2.333E-05 | 0.000E+00 | 0.000E+00 | 0.000E+00 | 0.000E+00 |
| Unigene33331 | patA | 8.248E-07 | 0.000E+00 | 0.000E+00 | 0.000E+00 | 3.582E-06 | 7.093E-06 | 1.369E-06 | 7.833E-06 | 2.991E-06 | 2.798E-06 | 0.000E+00 | 9.306E-07 |
| Unigene33385 | MdtK | 2.563E-06 | 2.680E-06 | 2.657E-06 | 1.294E-06 | 2.418E-07 | 2.614E-08 | 2.944E-07 | 1.129E-07 | 3.964E-07 | 0.000E+00 | 0.000E+00 | 0.000E+00 |
| Unigene33426 | arr-5 | 6.472E-06 | 8.472E-06 | 1.515E-05 | 1.228E-05 | 2.599E-07 | 3.372E-07 | 1.812E-06 | 2.730E-07 | 3.409E-07 | 0.000E+00 | 0.000E+00 | 9.216E-08 |
| Unigene33504 | golS | 1.311E-06 | 7.570E-07 | 6.091E-06 | 8.149E-06 | 0.000E+00 | 0.000E+00 | 7.634E-07 | 1.610E-07 | 0.000E+00 | 0.000E+00 | 0.000E+00 | 0.000E+00 |
| Unigene33558 | optrA | 1.261E-05 | 4.810E-06 | 3.302E-06 | 1.194E-05 | 8.682E-05 | 1.218E-04 | 1.228E-04 | 9.863E-05 | 1.484E-04 | 1.511E-04 | 1.166E-04 | 7.828E-05 |
| Unigene33608 | vanRE | 1.146E-06 | 2.624E-06 | 4.497E-06 | 6.087E-06 | 0.000E+00 | 0.000E+00 | 0.000E+00 | 0.000E+00 | 0.000E+00 | 0.000E+00 | 0.000E+00 | 0.000E+00 |
| Unigene33643 | vanHF | 2.152E-06 | 1.421E-05 | 7.407E-06 | 5.216E-06 | 0.000E+00 | 0.000E+00 | 1.466E-07 | 0.000E+00 | 0.000E+00 | 0.000E+00 | 3.030E-07 | 0.000E+00 |
| Unigene33657 | macB | 2.926E-06 | 5.525E-07 | 0.000E+00 | 0.000E+00 | 0.000E+00 | 0.000E+00 | 0.000E+00 | 0.000E+00 | 0.000E+00 | 0.000E+00 | 0.000E+00 | 3.347E-07 |
| Unigene33669 | arlR | 7.826E-07 | 6.066E-06 | 2.880E-07 | 0.000E+00 | 0.000E+00 | 0.000E+00 | 1.083E-07 | 0.000E+00 | 5.346E-07 | 0.000E+00 | 0.000E+00 | 0.000E+00 |
| Unigene33728 | adeR | 1.052E-06 | 7.348E-07 | 4.739E-06 | 6.230E-06 | 0.000E+00 | 5.170E-08 | 5.293E-07 | 0.000E+00 | 0.000E+00 | 0.000E+00 | 0.000E+00 | 0.000E+00 |
| Unigene33729 | vanSG | 1.537E-06 | 1.215E-06 | 4.887E-06 | 6.954E-06 | 0.000E+00 | 0.000E+00 | 9.187E-07 | 0.000E+00 | 0.000E+00 | 0.000E+00 | 0.000E+00 | 0.000E+00 |
| Unigene33735 | vanZA | 1.627E-06 | 4.806E-07 | 5.954E-06 | 6.823E-06 | 0.000E+00 | 0.000E+00 | 5.539E-07 | 0.000E+00 | 0.000E+00 | 0.000E+00 | 0.000E+00 | 0.000E+00 |
| Unigene33758 | baeS | 9.330E-07 | 5.120E-07 | 4.455E-06 | 5.590E-06 | 0.000E+00 | 0.000E+00 | 3.786E-07 | 0.000E+00 | 0.000E+00 | 0.000E+00 | 0.000E+00 | 0.000E+00 |
| Unigene33766 | lmrB | 2.772E-06 | 4.951E-06 | 3.919E-06 | 5.907E-06 | 3.393E-07 | 7.619E-08 | 3.380E-07 | 8.227E-08 | 2.825E-07 | 0.000E+00 | 1.344E-07 | 1.944E-07 |
| Unigene33809 | tetA(58) | 4.304E-07 | 8.975E-07 | 4.477E-06 | 5.694E-06 | 0.000E+00 | 0.000E+00 | 4.287E-07 | 0.000E+00 | 0.000E+00 | 0.000E+00 | 4.923E-08 | 0.000E+00 |
| Unigene33875 | macB | 3.057E-05 | 1.176E-05 | 7.302E-06 | 5.217E-06 | 0.000E+00 | 3.587E-07 | 0.000E+00 | 2.213E-07 | 0.000E+00 | 0.000E+00 | 0.000E+00 | 1.681E-07 |

| | | | | | | | | | | | | | |
|---|---|---|---|---|---|---|---|---|---|---|---|---|---|
| Unigene33891 | TaeA | 2.421E-05 | 9.064E-06 | 5.511E-06 | 4.575E-06 | 2.097E-07 | 0.000E+00 | 0.000E+00 | 0.000E+00 | 5.674E-07 | 0.000E+00 | 0.000E+00 | 0.000E+00 |
| Unigene33908 | tetA(60) | 2.242E-06 | 2.172E-06 | 2.700E-06 | 1.656E-06 | 0.000E+00 | 2.648E-07 | 2.920E-07 | 2.420E-07 | 5.149E-07 | 0.000E+00 | 0.000E+00 | 0.000E+00 |
| Unigene33909 | novA | 1.753E-06 | 2.066E-06 | 2.499E-06 | 1.416E-06 | 1.435E-07 | 0.000E+00 | 4.698E-07 | 3.878E-07 | 5.850E-07 | 0.000E+00 | 0.000E+00 | 8.726E-08 |
| Unigene34097 | vatE | 1.306E-06 | 0.000E+00 | 3.075E-07 | 3.692E-07 | 4.004E-06 | 1.073E-05 | 1.850E-06 | 0.000E+00 | 4.681E-06 | 0.000E+00 | 0.000E+00 | 0.000E+00 |
| Unigene34154 | poxtA | 5.139E-07 | 4.407E-06 | 9.424E-06 | 1.258E-05 | 0.000E+00 | 0.000E+00 | 0.000E+00 | 0.000E+00 | 0.000E+00 | 0.000E+00 | 0.000E+00 | 0.000E+00 |
| Unigene34176 | YojI | 3.525E-06 | 3.563E-06 | 2.670E-06 | 2.560E-06 | 3.023E-06 | 1.872E-06 | 2.259E-06 | 1.191E-06 | 1.048E-06 | 5.993E-06 | 4.563E-06 | 2.010E-06 |
| Unigene34197 | macB | 2.364E-06 | 3.760E-06 | 2.651E-06 | 1.803E-06 | 2.519E-06 | 2.191E-06 | 1.388E-06 | 1.295E-06 | 1.266E-06 | 5.590E-06 | 4.526E-06 | 1.426E-06 |
| Unigene34218 | tetA(60) | 1.252E-05 | 5.776E-06 | 3.635E-06 | 1.404E-05 | 9.308E-05 | 1.322E-04 | 1.373E-04 | 1.124E-04 | 1.720E-04 | 1.698E-04 | 1.312E-04 | 8.569E-05 |
| Unigene34225 | optrA | 1.408E-05 | 5.588E-06 | 3.614E-06 | 1.506E-05 | 1.001E-04 | 1.343E-04 | 1.461E-04 | 1.165E-04 | 1.793E-04 | 1.796E-04 | 1.507E-04 | 9.283E-05 |
| Unigene34243 | vanRB | 6.816E-07 | 1.443E-06 | 5.255E-07 | 0.000E+00 | 7.302E-06 | 5.263E-07 | 0.000E+00 | 5.683E-08 | 0.000E+00 | 0.000E+00 | 2.228E-07 | 0.000E+00 |
| Unigene34278 | Staphylocc | 9.663E-06 | 0.000E+00 | 0.000E+00 | 0.000E+00 | 1.468E-05 | 1.460E-05 | 0.000E+00 | 0.000E+00 | 9.935E-06 | 4.252E-07 | 0.000E+00 | 0.000E+00 |
| Unigene34308 | Staphylocc | 2.194E-06 | 1.725E-05 | 6.877E-06 | 4.265E-06 | 0.000E+00 | 0.000E+00 | 0.000E+00 | 2.754E-08 | 0.000E+00 | 0.000E+00 | 1.889E-07 | 0.000E+00 |
| Unigene34333 | tlrC | 0.000E+00 | 0.000E+00 | 0.000E+00 | 0.000E+00 | 1.528E-05 | 3.118E-05 | 0.000E+00 | 0.000E+00 | 0.000E+00 | 0.000E+00 | 1.124E-07 | 0.000E+00 |
| Unigene34338 | tcr3 | 2.258E-05 | 8.178E-06 | 6.471E-06 | 3.645E-06 | 1.542E-07 | 7.505E-08 | 0.000E+00 | 1.080E-07 | 5.564E-07 | 0.000E+00 | 0.000E+00 | 0.000E+00 |
| Unigene34350 | fusC | 2.477E-05 | 1.175E-05 | 6.924E-06 | 5.660E-06 | 0.000E+00 | 0.000E+00 | 0.000E+00 | 2.337E-07 | 9.846E-07 | 0.000E+00 | 0.000E+00 | 1.183E-07 |
| Unigene34508 | lmrB | 1.220E-05 | 3.969E-06 | 3.178E-06 | 1.241E-05 | 8.084E-05 | 1.142E-04 | 1.120E-04 | 8.753E-05 | 1.420E-04 | 1.406E-04 | 1.144E-04 | 6.989E-05 |
| Unigene34520 | macB | 9.197E-07 | 1.110E-06 | 4.196E-06 | 5.154E-06 | 0.000E+00 | 0.000E+00 | 5.598E-07 | 0.000E+00 | 0.000E+00 | 0.000E+00 | 0.000E+00 | 0.000E+00 |
| Unigene34522 | mepR | 0.000E+00 | 1.873E-06 | 0.000E+00 | 0.000E+00 | 0.000E+00 | 0.000E+00 | 0.000E+00 | 0.000E+00 | 0.000E+00 | 0.000E+00 | 0.000E+00 | 0.000E+00 |
| Unigene34566 | lsaC | 7.779E-06 | 0.000E+00 | 0.000E+00 | 1.909E-06 | 3.695E-05 | 1.816E-05 | 2.478E-05 | 2.440E-05 | 4.450E-06 | 0.000E+00 | 0.000E+00 | 0.000E+00 |
| Unigene34569 | optrA | 6.780E-06 | 0.000E+00 | 0.000E+00 | 2.233E-06 | 3.255E-05 | 1.678E-05 | 2.231E-05 | 2.476E-05 | 3.150E-06 | 0.000E+00 | 0.000E+00 | 0.000E+00 |
| Unigene34594 | macB | 7.478E-07 | 2.553E-06 | 1.427E-06 | 0.000E+00 | 0.000E+00 | 0.000E+00 | 3.035E-06 | 0.000E+00 | 0.000E+00 | 0.000E+00 | 7.128E-07 | 0.000E+00 |
| Unigene34652 | vanHO | 3.261E-07 | 8.231E-07 | 0.000E+00 | 0.000E+00 | 2.862E-06 | 2.115E-07 | 0.000E+00 | 0.000E+00 | 0.000E+00 | 0.000E+00 | 0.000E+00 | 0.000E+00 |
| Unigene34657 | tet36 | 9.803E-05 | 1.635E-04 | 1.545E-04 | 7.444E-06 | 8.441E-06 | 1.554E-07 | 4.227E-06 | 1.822E-06 | 5.499E-05 | 4.346E-07 | 2.772E-06 | 7.524E-07 |
| Unigene34679 | evgA | 7.951E-07 | 6.981E-07 | 1.144E-06 | 1.927E-06 | 1.584E-05 | 4.985E-06 | 1.273E-05 | 1.479E-05 | 9.154E-06 | 1.517E-05 | 1.552E-05 | 7.393E-06 |
| Unigene34714 | tetB(46) | 4.863E-05 | 2.593E-05 | 1.991E-05 | 2.285E-05 | 2.850E-06 | 1.697E-06 | 3.645E-06 | 1.564E-06 | 7.324E-06 | 9.384E-07 | 1.971E-06 | 1.765E-06 |
| Unigene34724 | vanSF | 5.954E-06 | 1.199E-05 | 1.053E-05 | 1.136E-05 | 0.000E+00 | 0.000E+00 | 0.000E+00 | 9.563E-08 | 0.000E+00 | 3.042E-07 | 0.000E+00 | 0.000E+00 |
| Unigene34726 | Staphylocc | 5.831E-06 | 1.191E-05 | 1.110E-05 | 1.162E-05 | 4.889E-08 | 0.000E+00 | 0.000E+00 | 0.000E+00 | 0.000E+00 | 2.451E-07 | 0.000E+00 | 2.601E-08 |
| Unigene34749 | optrA | 8.447E-07 | 8.609E-07 | 4.460E-06 | 6.723E-06 | 0.000E+00 | 0.000E+00 | 4.452E-07 | 0.000E+00 | 0.000E+00 | 0.000E+00 | 6.902E-08 | 0.000E+00 |
| Unigene34778 | cmlB | 4.905E-05 | 6.974E-05 | 2.925E-05 | 1.328E-05 | 2.025E-05 | 6.142E-06 | 2.825E-06 | 5.498E-05 | 1.245E-05 | 6.482E-06 | 2.198E-06 | 6.489E-07 |
| Unigene34804 | mecD | 3.236E-06 | 9.185E-06 | 8.056E-06 | 1.099E-05 | 1.771E-08 | 1.034E-07 | 0.000E+00 | 1.116E-07 | 0.000E+00 | 5.327E-08 | 5.470E-08 | 9.421E-08 |
| Unigene34838 | vanSF | 2.676E-06 | 2.278E-06 | 2.975E-06 | 1.875E-06 | 6.068E-08 | 0.000E+00 | 0.000E+00 | 2.550E-07 | 0.000E+00 | 0.000E+00 | 0.000E+00 | 0.000E+00 |
| Unigene34841 | vanSG | 4.575E-05 | 2.048E-05 | 7.479E-06 | 1.091E-05 | 2.010E-06 | 1.972E-06 | 3.490E-06 | 9.203E-07 | 7.942E-06 | 1.119E-06 | 1.733E-06 | 1.955E-06 |
| Unigene34843 | macB | 4.266E-05 | 2.534E-05 | 1.921E-05 | 2.288E-05 | 2.317E-06 | 1.691E-06 | 3.567E-06 | 5.533E-07 | 6.683E-06 | 7.393E-07 | 2.169E-06 | 9.525E-07 |
| Unigene35023 | FusF | 2.740E-06 | 4.647E-06 | 5.915E-06 | 5.383E-06 | 0.000E+00 | 0.000E+00 | 0.000E+00 | 2.326E-07 | 0.000E+00 | 0.000E+00 | 0.000E+00 | 0.000E+00 |
| Unigene35066 | optrA | 2.951E-06 | 3.577E-06 | 5.128E-06 | 4.036E-06 | 9.108E-08 | 0.000E+00 | 0.000E+00 | 4.785E-08 | 0.000E+00 | 4.566E-08 | 0.000E+00 | 0.000E+00 |
| Unigene35074 | optrA | 1.209E-06 | 4.499E-06 | 4.163E-06 | 6.809E-06 | 0.000E+00 | 0.000E+00 | 0.000E+00 | 0.000E+00 | 0.000E+00 | 0.000E+00 | 0.000E+00 | 0.000E+00 |
| Unigene35158 | VatI | 3.964E-06 | 1.882E-05 | 3.233E-06 | 1.040E-06 | 1.201E-06 | 7.235E-07 | 0.000E+00 | 1.803E-07 | 1.294E-06 | 6.309E-07 | 7.067E-07 | 0.000E+00 |
| Unigene35175 | tetA(58) | 4.227E-06 | 9.748E-06 | 1.081E-05 | 1.065E-05 | 0.000E+00 | 0.000E+00 | 0.000E+00 | 0.000E+00 | 0.000E+00 | 3.805E-07 | 0.000E+00 | 1.514E-07 |
| Unigene35224 | arlR | 1.363E-06 | 7.220E-07 | 1.383E-06 | 1.566E-06 | 1.571E-05 | 4.673E-06 | 1.170E-05 | 1.475E-05 | 1.120E-05 | 1.481E-05 | 1.612E-05 | 7.553E-06 |
| Unigene35225 | arlS | 1.282E-06 | 9.487E-07 | 1.375E-06 | 1.475E-06 | 1.884E-05 | 5.961E-06 | 1.296E-05 | 1.653E-05 | 1.023E-05 | 1.557E-05 | 1.606E-05 | 7.540E-06 |
| Unigene35327 | adeL | 4.797E-07 | 0.000E+00 | 7.397E-07 | 0.000E+00 | 8.756E-07 | 9.632E-07 | 3.755E-06 | 4.000E-06 | 0.000E+00 | 1.031E-06 | 1.842E-06 | 3.767E-06 |
| Unigene35405 | tetA(46) | 9.425E-07 | 8.189E-07 | 1.927E-07 | 1.983E-07 | 0.000E+00 | 0.000E+00 | 0.000E+00 | 0.000E+00 | 0.000E+00 | 0.000E+00 | 0.000E+00 | 0.000E+00 |
| Unigene35539 | Listeria mc | 2.432E-06 | 1.730E-05 | 6.973E-06 | 4.339E-06 | 0.000E+00 | 5.448E-08 | 1.255E-07 | 0.000E+00 | 0.000E+00 | 5.614E-08 | 0.000E+00 | 4.468E-08 |
| Unigene35642 | arlR | 1.263E-06 | 1.004E-06 | 1.411E-06 | 2.288E-06 | 2.091E-05 | 7.441E-06 | 1.591E-05 | 1.566E-05 | 1.147E-05 | 1.849E-05 | 2.297E-05 | 8.033E-06 |
| Unigene35742 | vanSG | 2.815E-06 | 9.854E-06 | 9.335E-06 | 1.004E-05 | 0.000E+00 | 0.000E+00 | 0.000E+00 | 0.000E+00 | 0.000E+00 | 0.000E+00 | 0.000E+00 | 0.000E+00 |
| Unigene35783 | Pseudomo | 5.626E-07 | 0.000E+00 | 1.311E-06 | 8.522E-07 | 1.164E-05 | 2.281E-07 | 0.000E+00 | 0.000E+00 | 0.000E+00 | 0.000E+00 | 0.000E+00 | 0.000E+00 |
| Unigene35792 | catQ | 4.069E-05 | 1.620E-05 | 6.924E-06 | 9.855E-06 | 1.001E-06 | 1.082E-06 | 2.216E-06 | 0.000E+00 | 7.549E-06 | 6.133E-07 | 1.660E-06 | 1.420E-06 |
| Unigene35820 | tetA(58) | 2.532E-06 | 9.807E-07 | 3.653E-07 | 0.000E+00 | 0.000E+00 | 0.000E+00 | 0.000E+00 | 0.000E+00 | 0.000E+00 | 0.000E+00 | 0.000E+00 | 0.000E+00 |
| Unigene35870 | vanTG | 0.000E+00 | 0.000E+00 | 0.000E+00 | 0.000E+00 | 0.000E+00 | 0.000E+00 | 0.000E+00 | 0.000E+00 | 0.000E+00 | 0.000E+00 | 0.000E+00 | 6.922E-07 |
| Unigene35908 | efrA | 3.706E-06 | 8.940E-06 | 9.912E-06 | 1.049E-05 | 0.000E+00 | 4.067E-08 | 0.000E+00 | 0.000E+00 | 0.000E+00 | 1.048E-07 | 0.000E+00 | 0.000E+00 |
| Unigene35911 | vanZF | 4.004E-07 | 0.000E+00 | 0.000E+00 | 0.000E+00 | 0.000E+00 | 5.951E-07 | 0.000E+00 | 0.000E+00 | 0.000E+00 | 0.000E+00 | 0.000E+00 | 0.000E+00 |
| Unigene35994 | PmrF | 2.185E-06 | 1.163E-06 | 1.592E-06 | 3.678E-07 | 0.000E+00 | 0.000E+00 | 0.000E+00 | 7.729E-08 | 0.000E+00 | 0.000E+00 | 0.000E+00 | 0.000E+00 |
| Unigene36006 | Staphylocc | 2.464E-06 | 1.135E-06 | 1.555E-06 | 7.555E-07 | 0.000E+00 | 0.000E+00 | 0.000E+00 | 0.000E+00 | 0.000E+00 | 0.000E+00 | 0.000E+00 | 0.000E+00 |
| Unigene36011 | Staphylocc | 2.626E-06 | 1.096E-06 | 1.912E-06 | 6.181E-07 | 0.000E+00 | 0.000E+00 | 0.000E+00 | 1.504E-07 | 0.000E+00 | 0.000E+00 | 0.000E+00 | 0.000E+00 |
| Unigene36014 | Erm(K) | 2.246E-06 | 9.145E-07 | 2.097E-06 | 4.756E-07 | 0.000E+00 | 0.000E+00 | 0.000E+00 | 1.269E-07 | 0.000E+00 | 0.000E+00 | 0.000E+00 | 0.000E+00 |
| Unigene36024 | Chlamydia | 2.289E-06 | 1.061E-06 | 1.498E-06 | 4.624E-07 | 0.000E+00 | 0.000E+00 | 0.000E+00 | 1.782E-07 | 0.000E+00 | 0.000E+00 | 0.000E+00 | 0.000E+00 |
| Unigene36026 | mdtG | 2.771E-06 | 8.857E-07 | 1.346E-06 | 5.433E-07 | 0.000E+00 | 0.000E+00 | 0.000E+00 | 0.000E+00 | 0.000E+00 | 0.000E+00 | 0.000E+00 | 0.000E+00 |
| Unigene36031 | vanTC | 3.208E-06 | 1.024E-06 | 1.860E-06 | 5.300E-07 | 0.000E+00 | 0.000E+00 | 0.000E+00 | 0.000E+00 | 0.000E+00 | 0.000E+00 | 0.000E+00 | 0.000E+00 |

| | | | | | | | | | | | | | |
|---|---|---|---|---|---|---|---|---|---|---|---|---|---|
| Unigene36047 | rpoB2 | 2.661E-06 | 1.080E-06 | 1.905E-06 | 5.499E-07 | 0.000E+00 | 0.000E+00 | 0.000E+00 | 7.177E-08 | 0.000E+00 | 0.000E+00 | 0.000E+00 | 0.000E+00 |
| Unigene36054 | tetT | 2.466E-06 | 8.798E-07 | 2.087E-06 | 6.382E-07 | 0.000E+00 | 0.000E+00 | 0.000E+00 | 1.799E-08 | 0.000E+00 | 0.000E+00 | 0.000E+00 | 0.000E+00 |
| Unigene36083 | carA | 2.700E-06 | 4.233E-07 | 1.400E-06 | 6.231E-07 | 0.000E+00 | 0.000E+00 | 0.000E+00 | 9.003E-08 | 0.000E+00 | 0.000E+00 | 0.000E+00 | 0.000E+00 |
| Unigene36084 | macB | 1.800E-06 | 1.111E-06 | 2.060E-06 | 4.922E-07 | 0.000E+00 | 0.000E+00 | 0.000E+00 | 1.750E-07 | 0.000E+00 | 0.000E+00 | 0.000E+00 | 0.000E+00 |
| Unigene36124 | tet(44) | 2.365E-06 | 1.132E-06 | 1.815E-06 | 8.980E-07 | 0.000E+00 | 0.000E+00 | 0.000E+00 | 1.246E-07 | 0.000E+00 | 0.000E+00 | 0.000E+00 | 0.000E+00 |
| Unigene36137 | baeR | 3.195E-06 | 9.798E-07 | 1.568E-06 | 8.066E-07 | 0.000E+00 | 0.000E+00 | 0.000E+00 | 0.000E+00 | 0.000E+00 | 0.000E+00 | 0.000E+00 | 0.000E+00 |
| Unigene36139 | macB | 2.257E-06 | 9.288E-07 | 2.056E-06 | 3.256E-07 | 0.000E+00 | 0.000E+00 | 0.000E+00 | 9.408E-08 | 0.000E+00 | 0.000E+00 | 0.000E+00 | 0.000E+00 |
| Unigene36142 | bcrA | 2.195E-06 | 9.084E-07 | 1.130E-06 | 5.814E-07 | 0.000E+00 | 0.000E+00 | 0.000E+00 | 8.401E-08 | 0.000E+00 | 0.000E+00 | 0.000E+00 | 0.000E+00 |
| Unigene36156 | macB | 2.343E-06 | 0.000E+00 | 1.126E-06 | 4.829E-07 | 0.000E+00 | 0.000E+00 | 0.000E+00 | 0.000E+00 | 0.000E+00 | 0.000E+00 | 0.000E+00 | 5.653E-08 |
| Unigene36161 | optrA | 2.548E-06 | 1.030E-06 | 1.842E-06 | 5.247E-07 | 0.000E+00 | 0.000E+00 | 0.000E+00 | 2.348E-07 | 0.000E+00 | 0.000E+00 | 0.000E+00 | 0.000E+00 |
| Unigene36199 | bmr | 1.830E-06 | 1.309E-06 | 1.605E-06 | 5.881E-07 | 0.000E+00 | 0.000E+00 | 0.000E+00 | 1.619E-07 | 0.000E+00 | 0.000E+00 | 0.000E+00 | 0.000E+00 |
| Unigene36215 | evgS | 3.157E-06 | 4.368E-06 | 3.229E-06 | 6.061E-06 | 4.693E-07 | 7.210E-08 | 2.460E-07 | 5.190E-08 | 1.458E-07 | 4.953E-08 | 1.017E-07 | 2.365E-07 |
| Unigene36238 | arlS | 3.397E-06 | 4.380E-06 | 3.473E-06 | 6.308E-06 | 5.014E-07 | 3.081E-07 | 4.995E-07 | 0.000E+00 | 7.788E-08 | 0.000E+00 | 0.000E+00 | 3.369E-07 |
| Unigene36276 | emrB | 1.540E-06 | 0.000E+00 | 0.000E+00 | 0.000E+00 | 0.000E+00 | 0.000E+00 | 0.000E+00 | 0.000E+00 | 0.000E+00 | 0.000E+00 | 2.381E-08 | 0.000E+00 |
| Unigene36293 | lmrC | 1.752E-06 | 0.000E+00 | 0.000E+00 | 0.000E+00 | 0.000E+00 | 0.000E+00 | 0.000E+00 | 0.000E+00 | 0.000E+00 | 0.000E+00 | 0.000E+00 | 0.000E+00 |
| Unigene36306 | bcrA | 1.712E-06 | 0.000E+00 | 0.000E+00 | 0.000E+00 | 0.000E+00 | 0.000E+00 | 0.000E+00 | 0.000E+00 | 0.000E+00 | 0.000E+00 | 0.000E+00 | 0.000E+00 |
| Unigene36316 | mtrA | 1.277E-06 | 0.000E+00 | 0.000E+00 | 0.000E+00 | 0.000E+00 | 0.000E+00 | 0.000E+00 | 0.000E+00 | 0.000E+00 | 0.000E+00 | 0.000E+00 | 0.000E+00 |
| Unigene36317 | arlS | 1.845E-06 | 5.678E-08 | 0.000E+00 | 0.000E+00 | 0.000E+00 | 3.728E-08 | 0.000E+00 | 0.000E+00 | 0.000E+00 | 0.000E+00 | 0.000E+00 | 0.000E+00 |
| Unigene36328 | tetA(60) | 1.764E-06 | 3.910E-08 | 0.000E+00 | 0.000E+00 | 0.000E+00 | 0.000E+00 | 0.000E+00 | 0.000E+00 | 0.000E+00 | 0.000E+00 | 0.000E+00 | 0.000E+00 |
| Unigene36329 | tetB(60) | 1.251E-06 | 0.000E+00 | 0.000E+00 | 0.000E+00 | 0.000E+00 | 0.000E+00 | 0.000E+00 | 0.000E+00 | 0.000E+00 | 0.000E+00 | 0.000E+00 | 0.000E+00 |
| Unigene36330 | macB | 2.187E-06 | 0.000E+00 | 0.000E+00 | 0.000E+00 | 0.000E+00 | 0.000E+00 | 0.000E+00 | 0.000E+00 | 0.000E+00 | 0.000E+00 | 0.000E+00 | 0.000E+00 |
| Unigene36337 | lsaC | 1.814E-06 | 0.000E+00 | 0.000E+00 | 0.000E+00 | 0.000E+00 | 0.000E+00 | 0.000E+00 | 0.000E+00 | 0.000E+00 | 0.000E+00 | 0.000E+00 | 0.000E+00 |
| Unigene36381 | vanRE | 1.733E-06 | 1.421E-05 | 6.129E-06 | 3.085E-06 | 0.000E+00 | 0.000E+00 | 0.000E+00 | 0.000E+00 | 0.000E+00 | 0.000E+00 | 5.215E-08 | 2.695E-08 |
| Unigene36382 | vanSE | 1.336E-06 | 1.602E-05 | 7.556E-06 | 4.368E-06 | 0.000E+00 | 0.000E+00 | 0.000E+00 | 0.000E+00 | 0.000E+00 | 0.000E+00 | 1.207E-07 | 2.078E-08 |
| Unigene36419 | tetA(46) | 2.064E-06 | 1.561E-05 | 5.326E-06 | 3.416E-06 | 0.000E+00 | 6.198E-08 | 1.269E-07 | 0.000E+00 | 0.000E+00 | 0.000E+00 | 8.745E-08 | 0.000E+00 |
| Unigene36423 | vanRM | 1.697E-06 | 1.568E-05 | 5.213E-06 | 4.193E-06 | 0.000E+00 | 1.931E-07 | 4.941E-08 | 0.000E+00 | 0.000E+00 | 0.000E+00 | 1.532E-07 | 0.000E+00 |
| Unigene36424 | baeS | 1.702E-06 | 1.440E-05 | 5.889E-06 | 4.121E-06 | 0.000E+00 | 7.078E-08 | 0.000E+00 | 0.000E+00 | 0.000E+00 | 4.863E-08 | 0.000E+00 | 0.000E+00 |
| Unigene36480 | vanSA | 3.444E-06 | 5.392E-06 | 3.301E-06 | 6.579E-06 | 4.746E-07 | 0.000E+00 | 5.615E-07 | 0.000E+00 | 2.626E-07 | 0.000E+00 | 9.163E-08 | 9.468E-08 |
| Unigene36481 | vanRF | 2.199E-06 | 4.981E-06 | 4.223E-06 | 6.614E-06 | 5.197E-07 | 0.000E+00 | 1.036E-07 | 5.461E-08 | 3.579E-07 | 0.000E+00 | 1.605E-07 | 3.318E-07 |
| Unigene36516 | basS | 2.591E-05 | 0.000E+00 | 0.000E+00 | 0.000E+00 | 0.000E+00 | 0.000E+00 | 0.000E+00 | 0.000E+00 | 0.000E+00 | 0.000E+00 | 0.000E+00 | 0.000E+00 |
| Unigene36600 | sdiA | 5.663E-07 | 0.000E+00 | 0.000E+00 | 0.000E+00 | 0.000E+00 | 0.000E+00 | 0.000E+00 | 0.000E+00 | 0.000E+00 | 0.000E+00 | 0.000E+00 | 0.000E+00 |
| Unigene36620 | lmrD | 3.846E-06 | 0.000E+00 | 0.000E+00 | 0.000E+00 | 0.000E+00 | 0.000E+00 | 0.000E+00 | 0.000E+00 | 0.000E+00 | 0.000E+00 | 7.296E-06 | 0.000E+00 |
| Unigene36645 | srmB | 4.872E-07 | 1.783E-07 | 9.562E-07 | 1.640E-07 | 0.000E+00 | 0.000E+00 | 0.000E+00 | 8.057E-07 | 0.000E+00 | 0.000E+00 | 0.000E+00 | 2.592E-06 |
| Unigene36669 | tetA(58) | 0.000E+00 | 0.000E+00 | 0.000E+00 | 0.000E+00 | 0.000E+00 | 0.000E+00 | 0.000E+00 | 0.000E+00 | 9.224E-07 | 0.000E+00 | 0.000E+00 | 0.000E+00 |
| Unigene36747 | dfrE | 7.778E-07 | 9.960E-07 | 5.215E-06 | 5.433E-06 | 0.000E+00 | 0.000E+00 | 1.435E-07 | 0.000E+00 | 0.000E+00 | 0.000E+00 | 0.000E+00 | 0.000E+00 |
| Unigene36748 | TaeA | 9.454E-07 | 6.980E-07 | 4.351E-06 | 6.110E-06 | 5.729E-08 | 7.433E-08 | 4.756E-07 | 0.000E+00 | 0.000E+00 | 0.000E+00 | 0.000E+00 | 0.000E+00 |
| Unigene36777 | tetB(P) | 3.195E-06 | 3.409E-07 | 3.875E-06 | 2.240E-07 | 3.697E-07 | 1.439E-07 | 3.438E-07 | 0.000E+00 | 0.000E+00 | 0.000E+00 | 6.598E-07 | 0.000E+00 |
| Unigene36790 | Staphylocc | 4.161E-06 | 3.195E-07 | 3.285E-06 | 4.655E-07 | 2.965E-07 | 2.623E-08 | 2.954E-07 | 0.000E+00 | 0.000E+00 | 0.000E+00 | 8.465E-07 | 0.000E+00 |
| Unigene36821 | macB | 5.104E-05 | 7.464E-05 | 2.925E-05 | 1.662E-05 | 1.975E-05 | 5.287E-06 | 3.107E-06 | 5.669E-05 | 1.455E-05 | 7.486E-06 | 2.627E-06 | 1.156E-06 |
| Unigene36827 | tetA(58) | 4.786E-05 | 7.444E-05 | 2.986E-05 | 1.654E-05 | 1.707E-05 | 7.011E-06 | 3.809E-06 | 5.789E-05 | 1.176E-05 | 6.942E-06 | 2.445E-06 | 9.850E-07 |
| Unigene36832 | cmlB | 5.362E-05 | 6.998E-05 | 3.277E-05 | 1.469E-05 | 1.911E-05 | 6.590E-06 | 3.314E-06 | 5.847E-05 | 1.466E-05 | 6.643E-06 | 1.607E-06 | 8.458E-07 |
| Unigene36843 | vanRM | 5.095E-05 | 7.173E-05 | 2.607E-05 | 1.535E-05 | 2.022E-05 | 6.474E-06 | 3.107E-06 | 6.001E-05 | 1.043E-05 | 7.817E-06 | 1.659E-06 | 9.954E-07 |
| Unigene36844 | vanSA | 4.946E-05 | 6.893E-05 | 2.608E-05 | 1.522E-05 | 2.038E-05 | 6.322E-06 | 3.582E-06 | 5.262E-05 | 1.201E-05 | 5.693E-06 | 1.624E-06 | 1.275E-06 |
| Unigene36898 | oleC | 2.887E-05 | 1.754E-05 | 1.978E-05 | 1.857E-05 | 1.509E-06 | 1.355E-06 | 2.389E-06 | 0.000E+00 | 0.000E+00 | 6.207E-07 | 1.195E-06 | 0.000E+00 |
| Unigene36923 | mgrA | 1.244E-06 | 1.366E-06 | 1.436E-06 | 1.922E-06 | 2.122E-05 | 6.805E-06 | 1.442E-05 | 1.769E-05 | 1.392E-05 | 2.226E-05 | 2.160E-05 | 6.749E-06 |
| Unigene36938 | tetB(46) | 1.323E-06 | 1.299E-06 | 1.128E-06 | 1.160E-06 | 1.927E-05 | 5.647E-06 | 1.164E-05 | 1.634E-05 | 1.005E-05 | 1.858E-05 | 1.765E-05 | 7.656E-06 |
| Unigene36939 | tetA(46) | 1.172E-06 | 7.444E-07 | 1.543E-06 | 2.211E-06 | 2.415E-05 | 6.633E-06 | 1.396E-05 | 1.681E-05 | 1.267E-05 | 2.164E-05 | 2.479E-05 | 6.961E-06 |
| Unigene36954 | lsaC | 1.054E-06 | 7.230E-07 | 1.120E-06 | 1.286E-06 | 1.976E-05 | 7.786E-06 | 1.434E-05 | 2.076E-05 | 1.339E-05 | 2.417E-05 | 2.160E-05 | 9.033E-06 |
| Unigene36956 | tlrC | 1.628E-06 | 1.141E-06 | 1.242E-06 | 2.283E-06 | 2.446E-05 | 8.015E-06 | 1.731E-05 | 2.063E-05 | 1.443E-05 | 2.256E-05 | 2.120E-05 | 8.176E-06 |
| Unigene36989 | macB | 2.660E-06 | 2.178E-06 | 2.623E-06 | 1.827E-06 | 1.350E-07 | 1.168E-07 | 3.885E-07 | 2.994E-07 | 4.722E-07 | 1.504E-07 | 0.000E+00 | 1.277E-07 |
| Unigene36990 | arlR | 2.248E-06 | 2.887E-06 | 3.003E-06 | 2.463E-06 | 1.594E-07 | 2.068E-07 | 4.234E-07 | 0.000E+00 | 5.227E-07 | 0.000E+00 | 0.000E+00 | 0.000E+00 |
| Unigene36991 | vanSG | 2.336E-06 | 2.362E-06 | 3.107E-06 | 1.583E-06 | 0.000E+00 | 0.000E+00 | 0.000E+00 | 3.588E-07 | 8.400E-07 | 0.000E+00 | 0.000E+00 | 0.000E+00 |
| Unigene37013 | Corynebac | 1.808E-06 | 1.890E-06 | 1.774E-06 | 1.869E-06 | 0.000E+00 | 0.000E+00 | 0.000E+00 | 0.000E+00 | 8.938E-07 | 0.000E+00 | 0.000E+00 | 0.000E+00 |
| Unigene37054 | lmrB | 3.334E-06 | 2.032E-05 | 3.202E-06 | 9.075E-07 | 6.831E-07 | 2.045E-07 | 2.356E-07 | 3.312E-07 | 1.396E-06 | 3.952E-07 | 6.492E-07 | 0.000E+00 |
| Unigene37171 | YojI | 2.576E-05 | 8.295E-06 | 5.944E-06 | 4.603E-06 | 6.730E-07 | 2.807E-07 | 0.000E+00 | 4.041E-07 | 1.261E-06 | 0.000E+00 | 0.000E+00 | 0.000E+00 |
| Unigene37173 | tetT | 2.771E-05 | 1.148E-05 | 8.417E-06 | 4.784E-06 | 5.479E-07 | 2.908E-07 | 1.489E-07 | 3.140E-07 | 2.287E-06 | 0.000E+00 | 0.000E+00 | 7.066E-08 |
| Unigene37176 | Corynebac | 2.402E-05 | 6.481E-06 | 5.972E-06 | 3.988E-06 | 3.220E-07 | 7.833E-08 | 2.005E-07 | 0.000E+00 | 0.000E+00 | 0.000E+00 | 0.000E+00 | 2.569E-07 |
| Unigene37200 | rphB | 6.266E-06 | 0.000E+00 | 0.000E+00 | 1.826E-06 | 3.314E-05 | 1.473E-05 | 2.386E-05 | 2.324E-05 | 3.095E-06 | 0.000E+00 | 0.000E+00 | 0.000E+00 |

| | | | | | | | | | | | | | |
|---|---|---|---|---|---|---|---|---|---|---|---|---|---|
| Unigene37216 | patA | 8.254E-06 | 0.000E+00 | 0.000E+00 | 2.313E-06 | 3.303E-05 | 1.504E-05 | 2.219E-05 | 2.366E-05 | 3.259E-06 | 0.000E+00 | 0.000E+00 | 0.000E+00 |
| Unigene37270 | Acinetobac | 7.495E-06 | 0.000E+00 | 0.000E+00 | 1.947E-06 | 3.116E-05 | 1.485E-05 | 2.161E-05 | 2.522E-05 | 2.731E-06 | 0.000E+00 | 0.000E+00 | 0.000E+00 |
| Unigene37293 | tetA(58) | 7.093E-05 | 2.100E-04 | 1.163E-04 | 0.000E+00 | 2.969E-06 | 0.000E+00 | 3.580E-06 | 8.209E-08 | 5.857E-05 | 3.525E-07 | 1.368E-06 | 1.247E-07 |
| Unigene37310 | bacA | 9.676E-07 | 5.947E-07 | 3.380E-06 | 6.488E-06 | 0.000E+00 | 8.368E-08 | 4.284E-07 | 0.000E+00 | 0.000E+00 | 0.000E+00 | 0.000E+00 | 0.000E+00 |
| Unigene37347 | patB | 6.194E-05 | 4.986E-05 | 1.070E-04 | 1.716E-04 | 1.087E-04 | 7.796E-05 | 1.100E-04 | 9.888E-05 | 8.288E-05 | 1.132E-04 | 1.157E-04 | 1.249E-04 |
| Unigene37348 | patB | 7.796E-05 | 6.376E-05 | 1.305E-04 | 1.534E-04 | 1.375E-04 | 9.315E-05 | 1.068E-04 | 1.277E-04 | 1.097E-04 | 1.530E-04 | 1.607E-04 | 1.652E-04 |
| Unigene37359 | dfrA22 | 2.334E-06 | 2.988E-06 | 1.717E-06 | 1.047E-06 | 1.512E-06 | 1.682E-06 | 1.363E-06 | 1.740E-06 | 1.134E-06 | 4.621E-06 | 4.004E-06 | 1.149E-06 |
| Unigene37360 | TaeA | 2.933E-06 | 3.912E-06 | 2.660E-06 | 2.066E-06 | 2.766E-06 | 1.825E-06 | 2.435E-06 | 7.962E-07 | 1.211E-06 | 5.471E-06 | 5.949E-06 | 2.378E-06 |
| Unigene37391 | Staphylocc | 2.536E-05 | 0.000E+00 | 0.000E+00 | 0.000E+00 | 0.000E+00 | 1.332E-07 | 0.000E+00 | 0.000E+00 | 0.000E+00 | 9.150E-08 | 0.000E+00 | 0.000E+00 |
| Unigene37449 | optrA | 1.439E-05 | 4.673E-06 | 3.384E-06 | 1.310E-05 | 9.079E-05 | 1.246E-04 | 1.299E-04 | 1.032E-04 | 1.582E-04 | 1.585E-04 | 1.271E-04 | 8.023E-05 |
| Unigene37462 | tetA(46) | 4.621E-06 | 2.142E-05 | 3.649E-06 | 7.555E-07 | 1.271E-06 | 4.044E-07 | 1.461E-07 | 1.027E-07 | 1.154E-06 | 7.599E-07 | 4.279E-07 | 0.000E+00 |
| Unigene37503 | lmrD | 1.315E-05 | 4.950E-06 | 3.555E-06 | 1.277E-05 | 8.889E-05 | 1.245E-04 | 1.307E-04 | 1.035E-04 | 1.558E-04 | 1.529E-04 | 1.266E-04 | 8.023E-05 |
| Unigene37504 | patA | 1.247E-05 | 5.148E-06 | 3.393E-06 | 1.226E-05 | 8.985E-05 | 1.214E-04 | 1.203E-04 | 9.736E-05 | 1.532E-04 | 1.504E-04 | 1.209E-04 | 7.359E-05 |
| Unigene37548 | mtrA | 5.410E-07 | 0.000E+00 | 0.000E+00 | 0.000E+00 | 0.000E+00 | 0.000E+00 | 0.000E+00 | 0.000E+00 | 6.570E-08 | 0.000E+00 | 0.000E+00 | 0.000E+00 |
| Unigene37561 | otr(B) | 2.634E-05 | 8.598E-06 | 7.298E-06 | 4.755E-06 | 3.178E-07 | 1.665E-07 | 0.000E+00 | 1.027E-07 | 8.177E-07 | 0.000E+00 | 0.000E+00 | 0.000E+00 |
| Unigene37655 | patA | 6.960E-07 | 6.524E-06 | 9.617E-06 | 1.552E-05 | 0.000E+00 | 0.000E+00 | 0.000E+00 | 0.000E+00 | 0.000E+00 | 0.000E+00 | 0.000E+00 | 6.671E-08 |
| Unigene37656 | lmrD | 1.131E-06 | 6.966E-06 | 1.068E-05 | 1.681E-05 | 1.216E-07 | 0.000E+00 | 0.000E+00 | 0.000E+00 | 0.000E+00 | 0.000E+00 | 0.000E+00 | 0.000E+00 |
| Unigene37673 | vanYB | 1.685E-06 | 1.503E-05 | 7.166E-06 | 4.058E-06 | 0.000E+00 | 0.000E+00 | 0.000E+00 | 0.000E+00 | 0.000E+00 | 0.000E+00 | 0.000E+00 | 0.000E+00 |
| Unigene37701 | tetT | 7.598E-06 | 0.000E+00 | 0.000E+00 | 1.969E-06 | 3.209E-05 | 1.678E-05 | 2.484E-05 | 2.619E-05 | 3.406E-06 | 0.000E+00 | 0.000E+00 | 0.000E+00 |
| Unigene37731 | tetB(46) | 7.283E-06 | 0.000E+00 | 0.000E+00 | 2.439E-06 | 3.363E-05 | 1.800E-05 | 2.637E-05 | 2.473E-05 | 3.160E-06 | 0.000E+00 | 0.000E+00 | 0.000E+00 |
| Unigene37732 | tetB(60) | 6.754E-06 | 0.000E+00 | 0.000E+00 | 2.813E-06 | 3.913E-05 | 1.642E-05 | 2.715E-05 | 2.975E-05 | 3.753E-06 | 0.000E+00 | 0.000E+00 | 0.000E+00 |
| Unigene37739 | macB | 6.856E-06 | 0.000E+00 | 0.000E+00 | 2.063E-06 | 4.050E-05 | 1.482E-05 | 2.146E-05 | 2.295E-05 | 3.126E-06 | 0.000E+00 | 0.000E+00 | 0.000E+00 |
| Unigene37746 | tetA(58) | 7.691E-06 | 0.000E+00 | 0.000E+00 | 2.500E-06 | 3.374E-05 | 1.527E-05 | 2.181E-05 | 2.504E-05 | 2.994E-06 | 0.000E+00 | 0.000E+00 | 0.000E+00 |
| Unigene37750 | poxtA | 7.219E-06 | 0.000E+00 | 0.000E+00 | 2.647E-06 | 2.447E-05 | 1.332E-05 | 2.458E-05 | 2.072E-05 | 0.000E+00 | 0.000E+00 | 0.000E+00 | 0.000E+00 |
| Unigene37777 | bcrA | 2.065E-05 | 2.637E-05 | 7.034E-05 | 7.932E-05 | 7.115E-06 | 2.144E-05 | 8.898E-05 | 2.033E-05 | 1.966E-05 | 3.134E-05 | 9.325E-05 | 2.459E-05 |
| Unigene37832 | evgS | 8.784E-08 | 0.000E+00 | 0.000E+00 | 0.000E+00 | 0.000E+00 | 0.000E+00 | 0.000E+00 | 0.000E+00 | 0.000E+00 | 0.000E+00 | 0.000E+00 | 0.000E+00 |
| Unigene37921 | YojI | 3.114E-05 | 1.287E-06 | 0.000E+00 | 0.000E+00 | 0.000E+00 | 1.585E-07 | 0.000E+00 | 0.000E+00 | 0.000E+00 | 0.000E+00 | 0.000E+00 | 0.000E+00 |
| Unigene37922 | tetT | 7.246E-05 | 7.106E-05 | 0.000E+00 | 0.000E+00 | 0.000E+00 | 3.295E-07 | 0.000E+00 | 0.000E+00 | 0.000E+00 | 0.000E+00 | 0.000E+00 | 0.000E+00 |
| Unigene37928 | evgS | 2.874E-05 | 0.000E+00 | 3.508E-08 | 0.000E+00 | 0.000E+00 | 3.864E-08 | 0.000E+00 | 0.000E+00 | 0.000E+00 | 0.000E+00 | 0.000E+00 | 0.000E+00 |
| Unigene37929 | vanRM | 2.656E-05 | 0.000E+00 | 0.000E+00 | 0.000E+00 | 0.000E+00 | 9.306E-08 | 0.000E+00 | 0.000E+00 | 0.000E+00 | 2.397E-07 | 0.000E+00 | 0.000E+00 |
| Unigene37934 | vatB | 1.162E-05 | 0.000E+00 | 0.000E+00 | 0.000E+00 | 0.000E+00 | 0.000E+00 | 0.000E+00 | 0.000E+00 | 0.000E+00 | 0.000E+00 | 0.000E+00 | 0.000E+00 |
| Unigene37940 | bacA | 3.212E-05 | 8.100E-07 | 0.000E+00 | 0.000E+00 | 0.000E+00 | 0.000E+00 | 0.000E+00 | 0.000E+00 | 0.000E+00 | 0.000E+00 | 0.000E+00 | 0.000E+00 |
| Unigene37944 | macB | 2.722E-05 | 0.000E+00 | 0.000E+00 | 0.000E+00 | 0.000E+00 | 9.269E-08 | 0.000E+00 | 0.000E+00 | 0.000E+00 | 0.000E+00 | 0.000E+00 | 0.000E+00 |
| Unigene38014 | vatE | 6.473E-05 | 6.457E-05 | 2.327E-06 | 0.000E+00 | 1.157E-06 | 0.000E+00 | 0.000E+00 | 0.000E+00 | 0.000E+00 | 0.000E+00 | 1.257E-06 | 0.000E+00 |
| Unigene38015 | NmcR | 6.165E-05 | 6.712E-05 | 2.648E-06 | 0.000E+00 | 1.736E-06 | 0.000E+00 | 0.000E+00 | 0.000E+00 | 0.000E+00 | 0.000E+00 | 1.300E-06 | 0.000E+00 |
| Unigene38045 | bcrA | 1.745E-05 | 0.000E+00 | 0.000E+00 | 0.000E+00 | 0.000E+00 | 8.309E-08 | 0.000E+00 | 0.000E+00 | 0.000E+00 | 0.000E+00 | 0.000E+00 | 0.000E+00 |
| Unigene38095 | patA | 3.933E-06 | 4.869E-06 | 4.693E-06 | 6.259E-06 | 4.544E-07 | 0.000E+00 | 5.223E-07 | 0.000E+00 | 3.439E-08 | 0.000E+00 | 2.519E-07 | 2.975E-07 |
| Unigene38118 | arlS | 2.579E-05 | 1.106E-06 | 5.751E-07 | 0.000E+00 | 7.813E-08 | 0.000E+00 | 0.000E+00 | 0.000E+00 | 0.000E+00 | 0.000E+00 | 0.000E+00 | 0.000E+00 |
| Unigene38119 | mtrA | 2.730E-05 | 4.840E-07 | 5.193E-07 | 0.000E+00 | 0.000E+00 | 0.000E+00 | 0.000E+00 | 0.000E+00 | 0.000E+00 | 0.000E+00 | 0.000E+00 | 0.000E+00 |
| Unigene38135 | tetA(58) | 9.823E-07 | 3.920E-07 | 1.490E-06 | 8.566E-07 | 0.000E+00 | 0.000E+00 | 0.000E+00 | 0.000E+00 | 0.000E+00 | 0.000E+00 | 0.000E+00 | 0.000E+00 |
| Unigene38144 | bcr-1 | 3.749E-06 | 3.896E-07 | 3.635E-06 | 2.464E-07 | 2.958E-07 | 0.000E+00 | 3.438E-07 | 0.000E+00 | 0.000E+00 | 0.000E+00 | 7.867E-07 | 0.000E+00 |
| Unigene38159 | lnuE | 0.000E+00 | 5.158E-06 | 0.000E+00 | 3.122E-06 | 0.000E+00 | 0.000E+00 | 0.000E+00 | 0.000E+00 | 0.000E+00 | 0.000E+00 | 0.000E+00 | 0.000E+00 |
| Unigene38164 | vanYB | 3.673E-06 | 9.486E-06 | 9.244E-06 | 9.993E-06 | 1.440E-07 | 9.343E-08 | 0.000E+00 | 0.000E+00 | 0.000E+00 | 3.370E-07 | 0.000E+00 | 0.000E+00 |
| Unigene38167 | lmrB | 2.189E-06 | 1.214E-06 | 1.266E-06 | 4.886E-07 | 0.000E+00 | 0.000E+00 | 0.000E+00 | 0.000E+00 | 0.000E+00 | 0.000E+00 | 0.000E+00 | 0.000E+00 |
| Unigene38216 | emrB | 1.991E-06 | 8.839E-07 | 1.295E-06 | 6.099E-07 | 0.000E+00 | 0.000E+00 | 0.000E+00 | 1.044E-07 | 0.000E+00 | 0.000E+00 | 0.000E+00 | 0.000E+00 |
| Unigene38299 | RlmA(II) | 1.170E-06 | 1.155E-06 | 1.148E-06 | 1.890E-06 | 1.966E-05 | 6.954E-06 | 1.696E-05 | 1.838E-05 | 1.287E-05 | 2.063E-05 | 1.766E-05 | 6.728E-06 |
| Unigene38309 | macB | 1.503E-06 | 7.979E-07 | 1.474E-06 | 1.566E-06 | 1.588E-05 | 5.659E-06 | 9.817E-06 | 1.386E-05 | 1.060E-05 | 1.857E-05 | 1.752E-05 | 6.417E-06 |
| Unigene38318 | farB | 1.492E-06 | 9.676E-07 | 1.641E-06 | 1.781E-06 | 2.047E-05 | 6.476E-06 | 1.419E-05 | 1.741E-05 | 1.164E-05 | 1.876E-05 | 1.885E-05 | 7.989E-06 |
| Unigene38333 | efrA | 1.098E-06 | 1.085E-06 | 2.047E-06 | 1.885E-06 | 2.110E-05 | 6.152E-06 | 1.480E-05 | 1.734E-05 | 1.206E-05 | 1.869E-05 | 2.085E-05 | 7.830E-06 |
| Unigene38334 | patB | 1.268E-06 | 9.824E-07 | 1.738E-06 | 1.525E-06 | 2.037E-05 | 6.915E-06 | 1.412E-05 | 1.665E-05 | 1.058E-05 | 1.760E-05 | 1.986E-05 | 7.847E-06 |
| Unigene38347 | efrA | 1.530E-06 | 1.129E-06 | 1.216E-06 | 2.344E-06 | 2.189E-05 | 6.586E-06 | 1.483E-05 | 1.779E-05 | 1.141E-05 | 1.951E-05 | 1.756E-05 | 8.105E-06 |
| Unigene38364 | oleC | 9.093E-07 | 8.871E-07 | 1.239E-06 | 1.785E-06 | 1.813E-05 | 6.990E-06 | 1.392E-05 | 1.580E-05 | 9.938E-06 | 1.671E-05 | 1.901E-05 | 6.210E-06 |
| Unigene38383 | Staphylocc | 1.267E-06 | 1.202E-06 | 1.307E-06 | 1.757E-06 | 2.028E-05 | 6.733E-06 | 1.493E-05 | 1.436E-05 | 1.096E-05 | 1.763E-05 | 1.860E-05 | 7.235E-06 |
| Unigene38386 | tet36 | 1.505E-06 | 9.767E-07 | 1.445E-06 | 1.650E-06 | 1.961E-05 | 6.927E-06 | 1.302E-05 | 1.709E-05 | 1.058E-05 | 1.857E-05 | 1.853E-05 | 6.656E-06 |
| Unigene38387 | oleC | 1.351E-06 | 1.208E-06 | 1.080E-06 | 1.466E-06 | 2.007E-05 | 5.410E-06 | 1.401E-05 | 1.869E-05 | 1.094E-05 | 1.678E-05 | 1.820E-05 | 8.164E-06 |
| Unigene38440 | vanTG | 2.882E-07 | 0.000E+00 | 0.000E+00 | 0.000E+00 | 0.000E+00 | 0.000E+00 | 0.000E+00 | 0.000E+00 | 0.000E+00 | 0.000E+00 | 0.000E+00 | 0.000E+00 |
| Unigene38462 | lmrD | 2.554E-05 | 9.011E-06 | 7.096E-06 | 3.810E-06 | 1.552E-07 | 0.000E+00 | 0.000E+00 | 1.223E-07 | 0.000E+00 | 0.000E+00 | 0.000E+00 | 0.000E+00 |
| Unigene38469 | ykkD | 1.704E-05 | 1.093E-05 | 4.888E-06 | 4.125E-06 | 0.000E+00 | 0.000E+00 | 0.000E+00 | 0.000E+00 | 0.000E+00 | 0.000E+00 | 0.000E+00 | 0.000E+00 |

| | | | | | | | | | | | | | |
|---|---|---|---|---|---|---|---|---|---|---|---|---|---|
| Unigene38478 | Corynebac | 2.406E-06 | 3.504E-06 | 3.391E-06 | 5.256E-06 | 3.887E-07 | 0.000E+00 | 0.000E+00 | 1.532E-07 | 0.000E+00 | 0.000E+00 | 0.000E+00 | 3.102E-07 |
| Unigene38518 | FosX | 3.770E-06 | 4.827E-06 | 3.853E-06 | 6.424E-06 | 0.000E+00 | 0.000E+00 | 0.000E+00 | 0.000E+00 | 0.000E+00 | 0.000E+00 | 0.000E+00 | 0.000E+00 |
| Unigene38595 | vanZF | 1.137E-05 | 3.774E-06 | 3.347E-06 | 1.067E-05 | 8.272E-05 | 1.199E-04 | 1.142E-04 | 8.957E-05 | 1.369E-04 | 1.402E-04 | 1.135E-04 | 7.100E-05 |
| Unigene38653 | efrA | 7.326E-07 | 4.020E-07 | 8.536E-07 | 0.000E+00 | 1.068E-06 | 1.633E-06 | 5.422E-06 | 4.436E-06 | 8.508E-07 | 1.020E-06 | 2.933E-06 | 3.572E-06 |
| Unigene38680 | vanRF | 3.368E-06 | 3.800E-06 | 2.249E-06 | 1.842E-06 | 2.079E-06 | 1.366E-06 | 1.553E-06 | 0.000E+00 | 9.715E-07 | 5.524E-06 | 5.726E-06 | 1.493E-06 |
| Unigene38695 | bcrA | 4.137E-05 | 2.426E-05 | 1.555E-05 | 2.008E-05 | 1.656E-06 | 1.533E-06 | 2.776E-06 | 8.486E-07 | 6.595E-06 | 5.264E-07 | 2.121E-06 | 1.031E-06 |
| Unigene38699 | vanZA | 3.628E-06 | 1.167E-05 | 8.962E-06 | 1.314E-05 | 1.390E-07 | 0.000E+00 | 0.000E+00 | 0.000E+00 | 0.000E+00 | 0.000E+00 | 0.000E+00 | 0.000E+00 |
| Unigene38772 | adeS | 1.582E-07 | 0.000E+00 | 0.000E+00 | 0.000E+00 | 0.000E+00 | 0.000E+00 | 0.000E+00 | 0.000E+00 | 0.000E+00 | 1.322E-07 | 2.263E-07 | 0.000E+00 |
| Unigene38800 | patA | 8.837E-06 | 0.000E+00 | 0.000E+00 | 1.942E-06 | 3.103E-05 | 1.688E-05 | 2.387E-05 | 2.416E-05 | 3.253E-06 | 0.000E+00 | 0.000E+00 | 0.000E+00 |
| Unigene38836 | macB | 1.085E-05 | 3.708E-06 | 2.732E-06 | 1.137E-05 | 7.508E-05 | 1.038E-04 | 9.660E-05 | 8.052E-05 | 1.272E-04 | 1.265E-04 | 1.045E-04 | 6.346E-05 |
| Unigene38841 | bcrA | 3.448E-06 | 1.023E-05 | 9.963E-06 | 1.067E-05 | 1.684E-07 | 8.192E-08 | 0.000E+00 | 0.000E+00 | 0.000E+00 | 1.477E-07 | 0.000E+00 | 0.000E+00 |
| Unigene38962 | vanHF | 3.095E-05 | 9.106E-06 | 6.583E-06 | 4.287E-06 | 5.041E-07 | 0.000E+00 | 0.000E+00 | 1.513E-07 | 1.275E-06 | 0.000E+00 | 0.000E+00 | 0.000E+00 |
| Unigene38968 | Staphylocc | 2.528E-05 | 1.123E-05 | 6.104E-06 | 4.933E-06 | 5.631E-07 | 3.985E-07 | 0.000E+00 | 5.379E-08 | 0.000E+00 | 0.000E+00 | 0.000E+00 | 0.000E+00 |
| Unigene39000 | Streptomy | 4.124E-05 | 5.472E-05 | 2.492E-05 | 1.000E-05 | 1.626E-05 | 5.457E-06 | 2.907E-06 | 4.817E-05 | 9.927E-06 | 5.676E-06 | 2.244E-06 | 7.669E-07 |
| Unigene39050 | tet32 | 4.463E-05 | 5.931E-05 | 2.615E-05 | 1.223E-05 | 1.587E-05 | 5.564E-06 | 3.345E-06 | 5.338E-05 | 1.133E-05 | 7.080E-06 | 1.660E-06 | 9.595E-07 |
| Unigene39072 | tet36 | 4.285E-05 | 5.747E-05 | 2.703E-05 | 1.197E-05 | 1.561E-05 | 5.696E-06 | 2.760E-06 | 5.007E-05 | 1.083E-05 | 6.391E-06 | 1.942E-06 | 1.052E-06 |
| Unigene39086 | vanHF | 4.518E-05 | 5.947E-05 | 2.738E-05 | 1.188E-05 | 1.631E-05 | 5.851E-06 | 2.905E-06 | 5.069E-05 | 1.307E-05 | 6.824E-06 | 2.224E-06 | 1.303E-06 |
| Unigene39146 | optrA | 2.884E-06 | 7.204E-07 | 2.049E-06 | 6.829E-07 | 0.000E+00 | 0.000E+00 | 0.000E+00 | 4.643E-08 | 0.000E+00 | 0.000E+00 | 0.000E+00 | 0.000E+00 |
| Unigene39150 | vanSF | 2.116E-06 | 9.288E-07 | 2.076E-06 | 3.052E-07 | 0.000E+00 | 0.000E+00 | 0.000E+00 | 3.528E-08 | 0.000E+00 | 0.000E+00 | 0.000E+00 | 0.000E+00 |
| Unigene39151 | vanRF | 2.206E-06 | 1.038E-06 | 1.900E-06 | 5.910E-07 | 0.000E+00 | 0.000E+00 | 0.000E+00 | 0.000E+00 | 0.000E+00 | 0.000E+00 | 0.000E+00 | 0.000E+00 |
| Unigene39155 | efrB | 2.490E-06 | 9.272E-07 | 1.178E-06 | 9.429E-07 | 0.000E+00 | 0.000E+00 | 0.000E+00 | 1.557E-07 | 0.000E+00 | 0.000E+00 | 0.000E+00 | 0.000E+00 |
| Unigene39156 | Staphylocc | 2.581E-06 | 1.113E-06 | 1.650E-06 | 7.445E-07 | 0.000E+00 | 0.000E+00 | 0.000E+00 | 5.379E-08 | 0.000E+00 | 0.000E+00 | 0.000E+00 | 0.000E+00 |
| Unigene39165 | lmrB | 3.246E-06 | 8.969E-07 | 2.027E-06 | 8.252E-07 | 0.000E+00 | 0.000E+00 | 0.000E+00 | 1.325E-07 | 0.000E+00 | 0.000E+00 | 0.000E+00 | 0.000E+00 |
| Unigene39188 | Staphylocc | 2.830E-06 | 7.643E-07 | 1.675E-06 | 7.713E-07 | 0.000E+00 | 0.000E+00 | 0.000E+00 | 1.049E-07 | 0.000E+00 | 0.000E+00 | 0.000E+00 | 0.000E+00 |
| Unigene39196 | macB | 2.887E-06 | 1.107E-06 | 1.694E-06 | 8.090E-07 | 0.000E+00 | 0.000E+00 | 0.000E+00 | 1.290E-07 | 0.000E+00 | 0.000E+00 | 0.000E+00 | 0.000E+00 |
| Unigene39200 | novA | 2.424E-06 | 1.003E-06 | 2.214E-06 | 5.273E-07 | 0.000E+00 | 0.000E+00 | 0.000E+00 | 1.524E-07 | 0.000E+00 | 0.000E+00 | 0.000E+00 | 0.000E+00 |
| Unigene39201 | lmrD | 2.755E-06 | 1.022E-06 | 1.936E-06 | 4.888E-07 | 0.000E+00 | 0.000E+00 | 0.000E+00 | 1.956E-07 | 0.000E+00 | 0.000E+00 | 0.000E+00 | 0.000E+00 |
| Unigene39217 | Streptomy | 2.715E-06 | 1.065E-06 | 1.437E-06 | 3.987E-07 | 0.000E+00 | 0.000E+00 | 0.000E+00 | 3.073E-07 | 0.000E+00 | 0.000E+00 | 0.000E+00 | 0.000E+00 |
| Unigene39236 | patA | 2.887E-06 | 1.200E-06 | 1.888E-06 | 6.184E-07 | 0.000E+00 | 0.000E+00 | 0.000E+00 | 0.000E+00 | 0.000E+00 | 0.000E+00 | 0.000E+00 | 0.000E+00 |
| Unigene39303 | vanRI | 0.000E+00 | 5.879E-06 | 1.092E-05 | 1.463E-05 | 0.000E+00 | 0.000E+00 | 0.000E+00 | 0.000E+00 | 0.000E+00 | 0.000E+00 | 0.000E+00 | 0.000E+00 |
| Unigene39304 | vanSG | 6.752E-07 | 7.291E-06 | 1.120E-05 | 1.669E-05 | 0.000E+00 | 0.000E+00 | 0.000E+00 | 0.000E+00 | 0.000E+00 | 0.000E+00 | 0.000E+00 | 0.000E+00 |
| Unigene39372 | macB | 4.594E-06 | 2.080E-05 | 3.370E-06 | 7.105E-07 | 8.276E-07 | 2.237E-07 | 0.000E+00 | 3.865E-07 | 1.131E-06 | 6.454E-07 | 1.894E-07 | 3.913E-07 |
| Unigene39383 | msbA | 4.165E-06 | 2.048E-05 | 3.150E-06 | 6.134E-07 | 7.230E-07 | 4.221E-07 | 9.604E-08 | 3.545E-07 | 1.138E-06 | 4.350E-07 | 5.459E-07 | 2.564E-07 |
| Unigene39437 | bcrA | 8.141E-07 | 4.273E-07 | 4.862E-07 | 3.574E-07 | 3.932E-08 | 0.000E+00 | 3.917E-08 | 1.653E-07 | 6.190E-07 | 0.000E+00 | 1.620E-07 | 0.000E+00 |
| Unigene39438 | facT | 1.744E-06 | 2.237E-06 | 2.619E-06 | 1.019E-06 | 0.000E+00 | 6.816E-08 | 6.745E-07 | 2.208E-07 | 3.675E-07 | 0.000E+00 | 0.000E+00 | 0.000E+00 |
| Unigene39547 | Staphylocc | 3.841E-06 | 2.428E-07 | 3.375E-06 | 5.281E-07 | 2.011E-07 | 0.000E+00 | 1.781E-07 | 0.000E+00 | 0.000E+00 | 0.000E+00 | 9.893E-07 | 0.000E+00 |
| Unigene39563 | vanHD | 3.252E-06 | 2.278E-07 | 3.904E-06 | 3.843E-07 | 1.922E-07 | 0.000E+00 | 3.446E-07 | 0.000E+00 | 0.000E+00 | 0.000E+00 | 1.108E-06 | 0.000E+00 |
| Unigene39577 | macB | 1.424E-05 | 5.514E-06 | 4.560E-06 | 1.547E-05 | 1.000E-04 | 1.381E-04 | 1.467E-04 | 1.207E-04 | 1.761E-04 | 1.603E-04 | 1.190E-04 | 7.329E-05 |
| Unigene39596 | novA | 7.850E-05 | 1.779E-04 | 1.437E-04 | 0.000E+00 | 5.767E-06 | 4.762E-07 | 2.995E-06 | 0.000E+00 | 5.983E-05 | 0.000E+00 | 1.620E-06 | 0.000E+00 |
| Unigene39621 | tet(42) | 2.982E-05 | 1.117E-05 | 7.411E-06 | 4.435E-06 | 3.873E-07 | 7.537E-08 | 0.000E+00 | 2.441E-07 | 9.398E-07 | 0.000E+00 | 0.000E+00 | 0.000E+00 |
| Unigene39683 | bcrA | 2.636E-05 | 1.141E-05 | 6.484E-06 | 4.631E-06 | 3.209E-07 | 0.000E+00 | 0.000E+00 | 1.686E-07 | 9.866E-07 | 0.000E+00 | 0.000E+00 | 0.000E+00 |
| Unigene39698 | msbA | 2.852E-05 | 1.027E-05 | 7.349E-06 | 4.446E-06 | 4.871E-07 | 7.900E-08 | 8.087E-08 | 1.066E-07 | 9.384E-07 | 0.000E+00 | 0.000E+00 | 0.000E+00 |
| Unigene39705 | optrA | 3.169E-05 | 1.141E-05 | 6.727E-06 | 4.264E-06 | 6.557E-07 | 0.000E+00 | 0.000E+00 | 1.060E-07 | 0.000E+00 | 0.000E+00 | 0.000E+00 | 0.000E+00 |
| Unigene39707 | Corynebac | 2.727E-05 | 7.985E-06 | 6.455E-06 | 3.519E-06 | 3.388E-07 | 0.000E+00 | 4.821E-08 | 1.525E-07 | 0.000E+00 | 0.000E+00 | 0.000E+00 | 0.000E+00 |
| Unigene39720 | srmB | 3.823E-07 | 0.000E+00 | 0.000E+00 | 5.516E-08 | 0.000E+00 | 0.000E+00 | 0.000E+00 | 0.000E+00 | 0.000E+00 | 0.000E+00 | 0.000E+00 | 0.000E+00 |
| Unigene39729 | MCR-9.1 | 5.662E-06 | 0.000E+00 | 0.000E+00 | 2.541E-06 | 3.550E-05 | 3.518E-07 | 2.173E-05 | 2.740E-05 | 0.000E+00 | 0.000E+00 | 0.000E+00 | 0.000E+00 |
| Unigene39780 | ErmE | 9.088E-07 | 6.982E-06 | 1.174E-05 | 1.766E-05 | 0.000E+00 | 0.000E+00 | 0.000E+00 | 0.000E+00 | 0.000E+00 | 0.000E+00 | 0.000E+00 | 0.000E+00 |
| Unigene39808 | optrA | 2.511E-06 | 1.922E-06 | 2.981E-06 | 1.427E-06 | 7.032E-08 | 9.123E-08 | 3.269E-07 | 1.478E-07 | 4.843E-07 | 0.000E+00 | 9.654E-08 | 0.000E+00 |
| Unigene39840 | macB | 2.769E-05 | 7.756E-07 | 4.570E-07 | 0.000E+00 | 0.000E+00 | 1.215E-07 | 0.000E+00 | 0.000E+00 | 0.000E+00 | 1.968E-07 | 0.000E+00 | 0.000E+00 |
| Unigene39880 | tet(V) | 5.794E-07 | 0.000E+00 | 1.624E-07 | 1.114E-07 | 2.329E-06 | 5.935E-06 | 1.160E-06 | 3.704E-06 | 4.553E-06 | 0.000E+00 | 0.000E+00 | 0.000E+00 |
| Unigene39956 | efrA | 3.134E-07 | 0.000E+00 | 0.000E+00 | 0.000E+00 | 0.000E+00 | 3.388E-07 | 0.000E+00 | 0.000E+00 | 0.000E+00 | 0.000E+00 | 0.000E+00 | 0.000E+00 |
| Unigene39990 | patB | 1.121E-06 | 6.535E-06 | 1.040E-05 | 1.715E-05 | 0.000E+00 | 0.000E+00 | 0.000E+00 | 6.233E-08 | 0.000E+00 | 0.000E+00 | 6.108E-08 | 0.000E+00 |
| Unigene39992 | optrA | 9.450E-07 | 6.969E-06 | 1.075E-05 | 1.668E-05 | 0.000E+00 | 0.000E+00 | 9.175E-08 | 0.000E+00 | 0.000E+00 | 0.000E+00 | 9.483E-08 | 0.000E+00 |
| Unigene40000 | bcrA | 8.804E-07 | 0.000E+00 | 0.000E+00 | 0.000E+00 | 1.364E-05 | 2.993E-05 | 0.000E+00 | 0.000E+00 | 0.000E+00 | 0.000E+00 | 0.000E+00 | 0.000E+00 |
| Unigene40060 | tetT | 3.293E-06 | 2.665E-07 | 3.336E-06 | 3.831E-07 | 3.203E-07 | 4.922E-08 | 4.703E-07 | 1.240E-07 | 0.000E+00 | 0.000E+00 | 8.854E-07 | 0.000E+00 |
| Unigene40061 | lmrC | 3.401E-06 | 2.574E-07 | 3.193E-06 | 2.961E-07 | 1.954E-07 | 1.268E-07 | 3.569E-07 | 0.000E+00 | 0.000E+00 | 0.000E+00 | 7.714E-07 | 3.466E-08 |
| Unigene40067 | emrB | 3.699E-06 | 3.466E-07 | 3.032E-06 | 6.378E-07 | 4.010E-07 | 2.439E-08 | 3.246E-07 | 0.000E+00 | 4.931E-08 | 0.000E+00 | 8.257E-07 | 0.000E+00 |
| Unigene40085 | bcrA | 6.588E-06 | 0.000E+00 | 0.000E+00 | 2.107E-06 | 3.159E-05 | 1.666E-05 | 2.402E-05 | 2.555E-05 | 3.600E-06 | 0.000E+00 | 0.000E+00 | 0.000E+00 |

| | | | | | | | | | | | | | |
|---|---|---|---|---|---|---|---|---|---|---|---|---|---|
| Unigene40097 | oleC | 2.532E-06 | 1.146E-05 | 3.017E-06 | 0.000E+00 | 1.055E-06 | 1.955E-07 | 3.002E-07 | 0.000E+00 | 5.436E-07 | 4.533E-07 | 0.000E+00 | 1.603E-07 |
| Unigene40121 | novA | 1.004E-04 | 1.786E-04 | 1.520E-04 | 0.000E+00 | 6.534E-06 | 0.000E+00 | 3.327E-06 | 0.000E+00 | 0.000E+00 | 0.000E+00 | 2.691E-06 | 6.694E-07 |
| Unigene40124 | vanRB | 3.879E-06 | 9.630E-06 | 7.386E-06 | 1.073E-05 | 0.000E+00 | 1.997E-07 | 0.000E+00 | 0.000E+00 | 0.000E+00 | 0.000E+00 | 0.000E+00 | 0.000E+00 |
| Unigene40322 | vgaB | 2.120E-07 | 0.000E+00 | 0.000E+00 | 0.000E+00 | 0.000E+00 | 0.000E+00 | 0.000E+00 | 0.000E+00 | 0.000E+00 | 0.000E+00 | 0.000E+00 | 0.000E+00 |
| Unigene40346 | bcrA | 3.029E-06 | 3.636E-06 | 2.570E-06 | 1.944E-06 | 1.963E-06 | 1.978E-06 | 1.676E-06 | 8.103E-07 | 1.655E-06 | 5.589E-06 | 5.414E-06 | 1.678E-06 |
| Unigene40476 | cmlv | 7.192E-07 | 1.032E-06 | 8.143E-07 | 1.955E-07 | 8.174E-06 | 2.392E-07 | 6.735E-07 | 3.874E-07 | 0.000E+00 | 0.000E+00 | 4.430E-07 | 0.000E+00 |
| Unigene40593 | bcrA | 4.019E-06 | 2.105E-05 | 3.369E-06 | 5.919E-07 | 3.256E-07 | 0.000E+00 | 0.000E+00 | 3.421E-07 | 9.610E-07 | 5.597E-07 | 4.310E-07 | 1.485E-07 |
| Unigene40596 | erm(32) | 4.280E-06 | 2.133E-05 | 3.738E-06 | 5.948E-07 | 1.701E-06 | 2.123E-07 | 1.738E-07 | 3.209E-07 | 1.159E-06 | 1.269E-06 | 4.043E-07 | 2.321E-07 |
| Unigene40607 | vatE | 5.018E-06 | 1.959E-05 | 2.081E-06 | 0.000E+00 | 9.195E-07 | 0.000E+00 | 0.000E+00 | 3.623E-07 | 1.131E-06 | 1.325E-06 | 0.000E+00 | 0.000E+00 |
| Unigene40608 | optrA | 4.422E-05 | 2.435E-05 | 1.642E-05 | 2.062E-05 | 2.188E-06 | 2.264E-06 | 3.947E-06 | 7.260E-07 | 7.636E-06 | 8.776E-07 | 1.826E-06 | 1.274E-06 |
| Unigene40723 | fexA | 1.112E-06 | 1.021E-06 | 4.633E-06 | 6.923E-06 | 0.000E+00 | 9.816E-08 | 4.774E-07 | 2.650E-08 | 0.000E+00 | 0.000E+00 | 1.558E-07 | 0.000E+00 |
| Unigene40763 | tetA(46) | 2.161E-06 | 4.593E-06 | 2.843E-06 | 4.922E-06 | 5.109E-07 | 0.000E+00 | 0.000E+00 | 0.000E+00 | 0.000E+00 | 0.000E+00 | 0.000E+00 | 0.000E+00 |
| Unigene40778 | carA | 6.831E-06 | 0.000E+00 | 0.000E+00 | 2.477E-06 | 3.229E-05 | 1.588E-05 | 2.440E-05 | 2.463E-05 | 3.517E-06 | 0.000E+00 | 0.000E+00 | 0.000E+00 |
| Unigene40788 | vanHO | 6.337E-06 | 0.000E+00 | 0.000E+00 | 3.233E-06 | 3.399E-05 | 1.613E-05 | 2.075E-05 | 2.516E-05 | 2.345E-06 | 0.000E+00 | 0.000E+00 | 0.000E+00 |
| Unigene40798 | tetA(58) | 7.197E-06 | 0.000E+00 | 0.000E+00 | 1.454E-06 | 3.218E-05 | 1.482E-05 | 2.123E-05 | 2.596E-05 | 2.557E-06 | 0.000E+00 | 0.000E+00 | 0.000E+00 |
| Unigene40801 | bcrA | 7.633E-06 | 0.000E+00 | 0.000E+00 | 1.955E-06 | 3.491E-05 | 1.831E-05 | 1.751E-05 | 2.151E-05 | 4.558E-06 | 0.000E+00 | 0.000E+00 | 0.000E+00 |
| Unigene40825 | novA | 2.594E-06 | 1.650E-05 | 5.515E-06 | 3.396E-06 | 0.000E+00 | 5.193E-08 | 0.000E+00 | 0.000E+00 | 0.000E+00 | 0.000E+00 | 5.495E-08 | 0.000E+00 |
| Unigene40860 | macB | 9.867E-07 | 1.547E-06 | 4.380E-06 | 5.789E-06 | 0.000E+00 | 0.000E+00 | 3.120E-07 | 0.000E+00 | 0.000E+00 | 0.000E+00 | 0.000E+00 | 0.000E+00 |
| Unigene40861 | macB | 9.684E-07 | 1.240E-06 | 5.226E-06 | 8.149E-06 | 0.000E+00 | 8.724E-08 | 6.550E-07 | 0.000E+00 | 0.000E+00 | 0.000E+00 | 0.000E+00 | 3.180E-08 |
| Unigene40930 | PmrF | 3.032E-06 | 3.546E-06 | 2.048E-06 | 1.982E-06 | 2.761E-06 | 1.988E-06 | 2.145E-06 | 8.992E-07 | 1.114E-06 | 6.118E-06 | 4.662E-06 | 1.968E-06 |
| Unigene41012 | lnuE | 1.752E-06 | 3.570E-06 | 2.455E-06 | 5.370E-06 | 0.000E+00 | 0.000E+00 | 0.000E+00 | 0.000E+00 | 0.000E+00 | 0.000E+00 | 0.000E+00 | 0.000E+00 |
| Unigene41028 | bcrA | 1.009E-06 | 4.745E-06 | 2.734E-06 | 5.821E-06 | 0.000E+00 | 0.000E+00 | 0.000E+00 | 0.000E+00 | 0.000E+00 | 0.000E+00 | 0.000E+00 | 0.000E+00 |
| Unigene41031 | smeR | 3.174E-06 | 7.617E-06 | 1.008E-05 | 1.298E-05 | 0.000E+00 | 0.000E+00 | 0.000E+00 | 0.000E+00 | 0.000E+00 | 0.000E+00 | 0.000E+00 | 0.000E+00 |
| Unigene41083 | macB | 4.170E-06 | 1.003E-05 | 1.328E-05 | 1.389E-05 | 0.000E+00 | 0.000E+00 | 0.000E+00 | 0.000E+00 | 0.000E+00 | 0.000E+00 | 0.000E+00 | 0.000E+00 |
| Unigene41087 | patB | 3.768E-07 | 8.731E-07 | 1.274E-06 | 2.325E-06 | 5.116E-07 | 2.263E-07 | 0.000E+00 | 1.124E-06 | 0.000E+00 | 4.664E-07 | 1.293E-06 | 7.423E-07 |
| Unigene41089 | msbA | 8.820E-07 | 0.000E+00 | 3.462E-07 | 0.000E+00 | 0.000E+00 | 0.000E+00 | 0.000E+00 | 0.000E+00 | 0.000E+00 | 0.000E+00 | 0.000E+00 | 0.000E+00 |
| Unigene41105 | Staphylocc | 1.146E-05 | 0.000E+00 | 0.000E+00 | 0.000E+00 | 1.968E-05 | 1.701E-05 | 0.000E+00 | 0.000E+00 | 1.188E-05 | 0.000E+00 | 0.000E+00 | 0.000E+00 |
| Unigene41112 | Staphylocc | 1.176E-05 | 0.000E+00 | 0.000E+00 | 0.000E+00 | 1.689E-05 | 1.686E-05 | 0.000E+00 | 0.000E+00 | 1.107E-05 | 0.000E+00 | 0.000E+00 | 0.000E+00 |
| Unigene41129 | sul3 | 1.212E-05 | 3.175E-08 | 0.000E+00 | 0.000E+00 | 1.620E-05 | 1.432E-05 | 0.000E+00 | 0.000E+00 | 1.084E-05 | 0.000E+00 | 0.000E+00 | 0.000E+00 |
| Unigene41139 | TaeA | 1.183E-05 | 0.000E+00 | 0.000E+00 | 0.000E+00 | 1.862E-05 | 1.653E-05 | 0.000E+00 | 0.000E+00 | 1.127E-05 | 0.000E+00 | 0.000E+00 | 0.000E+00 |
| Unigene41194 | macB | 2.144E-05 | 8.398E-06 | 6.961E-06 | 4.829E-06 | 3.099E-07 | 0.000E+00 | 0.000E+00 | 2.791E-07 | 8.276E-07 | 0.000E+00 | 0.000E+00 | 0.000E+00 |
| Unigene41329 | bcrA | 5.682E-07 | 6.938E-06 | 7.771E-06 | 1.546E-05 | 5.918E-08 | 0.000E+00 | 1.179E-07 | 0.000E+00 | 0.000E+00 | 0.000E+00 | 1.219E-07 | 0.000E+00 |
| Unigene41351 | lmrD | 9.579E-07 | 1.227E-06 | 5.169E-06 | 6.320E-06 | 0.000E+00 | 0.000E+00 | 1.767E-07 | 0.000E+00 | 0.000E+00 | 0.000E+00 | 3.652E-08 | 0.000E+00 |
| Unigene41508 | mdtG | 1.167E-05 | 3.151E-06 | 2.607E-06 | 1.143E-05 | 7.852E-05 | 1.024E-04 | 1.003E-04 | 7.905E-05 | 1.258E-04 | 1.272E-04 | 1.082E-04 | 6.597E-05 |
| Unigene41556 | Staphylocc | 4.662E-05 | 2.673E-05 | 1.949E-05 | 2.259E-05 | 2.149E-06 | 2.053E-06 | 3.346E-06 | 6.354E-07 | 7.785E-06 | 7.742E-07 | 2.014E-06 | 1.561E-06 |
| Unigene41579 | Staphylocc | 2.501E-05 | 9.003E-06 | 6.517E-06 | 4.744E-06 | 4.884E-07 | 2.376E-07 | 7.683E-08 | 1.486E-07 | 1.290E-06 | 0.000E+00 | 0.000E+00 | 0.000E+00 |
| Unigene41703 | optrA | 2.615E-05 | 9.513E-06 | 6.807E-06 | 4.326E-06 | 5.492E-07 | 2.672E-07 | 1.277E-07 | 1.346E-07 | 7.204E-07 | 0.000E+00 | 0.000E+00 | 0.000E+00 |
| Unigene41708 | vanF | 2.495E-05 | 9.959E-06 | 6.671E-06 | 4.057E-06 | 0.000E+00 | 3.037E-07 | 0.000E+00 | 2.296E-07 | 9.826E-07 | 0.000E+00 | 0.000E+00 | 0.000E+00 |
| Unigene41768 | tetA(58) | 2.488E-05 | 0.000E+00 | 0.000E+00 | 0.000E+00 | 0.000E+00 | 0.000E+00 | 0.000E+00 | 0.000E+00 | 0.000E+00 | 0.000E+00 | 0.000E+00 | 0.000E+00 |
| Unigene41775 | mdtG | 2.396E-05 | 0.000E+00 | 5.923E-07 | 0.000E+00 | 0.000E+00 | 0.000E+00 | 0.000E+00 | 0.000E+00 | 0.000E+00 | 0.000E+00 | 0.000E+00 | 9.306E-08 |
| Unigene41838 | patB | 2.484E-05 | 8.135E-07 | 4.514E-07 | 0.000E+00 | 2.839E-08 | 8.289E-08 | 0.000E+00 | 0.000E+00 | 0.000E+00 | 0.000E+00 | 0.000E+00 | 6.042E-08 |
| Unigene41854 | MexK | 0.000E+00 | 0.000E+00 | 0.000E+00 | 0.000E+00 | 4.269E-06 | 0.000E+00 | 0.000E+00 | 0.000E+00 | 0.000E+00 | 0.000E+00 | 0.000E+00 | 0.000E+00 |
| Unigene41865 | mtrA | 3.120E-06 | 4.809E-06 | 3.805E-06 | 6.881E-06 | 4.843E-07 | 4.960E-08 | 1.777E-07 | 1.071E-07 | 1.254E-07 | 0.000E+00 | 2.100E-07 | 1.627E-07 |
| Unigene41878 | arlS | 8.593E-05 | 1.366E-04 | 1.254E-04 | 5.576E-06 | 7.662E-06 | 3.775E-07 | 3.647E-06 | 2.115E-06 | 5.355E-05 | 0.000E+00 | 2.322E-06 | 7.740E-07 |
| Unigene41891 | macB | 3.689E-06 | 1.954E-05 | 3.077E-06 | 6.519E-07 | 8.880E-07 | 3.323E-07 | 2.382E-07 | 2.153E-07 | 1.546E-06 | 4.795E-07 | 4.220E-07 | 1.817E-07 |
| Unigene41892 | novA | 3.885E-06 | 2.071E-05 | 3.609E-06 | 9.026E-07 | 1.031E-06 | 0.000E+00 | 3.805E-07 | 4.013E-07 | 1.353E-06 | 6.893E-07 | 5.112E-07 | 0.000E+00 |
| Unigene41893 | tetB(P) | 5.380E-06 | 2.157E-05 | 3.848E-06 | 1.286E-06 | 1.095E-06 | 4.662E-07 | 2.500E-07 | 3.356E-07 | 1.347E-06 | 5.947E-07 | 3.758E-07 | 2.427E-07 |
| Unigene41968 | lmrD | 2.107E-05 | 9.313E-07 | 7.440E-07 | 9.116E-08 | 4.011E-08 | 5.855E-08 | 0.000E+00 | 0.000E+00 | 0.000E+00 | 1.408E-07 | 0.000E+00 | 0.000E+00 |
| Unigene41969 | oleC | 1.448E-05 | 1.392E-06 | 3.863E-07 | 0.000E+00 | 0.000E+00 | 9.457E-08 | 0.000E+00 | 0.000E+00 | 0.000E+00 | 1.462E-07 | 0.000E+00 | 0.000E+00 |
| Unigene41981 | novA | 4.678E-07 | 0.000E+00 | 0.000E+00 | 0.000E+00 | 0.000E+00 | 0.000E+00 | 0.000E+00 | 0.000E+00 | 0.000E+00 | 0.000E+00 | 0.000E+00 | 0.000E+00 |
| Unigene42011 | ugd | 1.245E-06 | 3.006E-06 | 2.741E-06 | 3.603E-06 | 3.073E-07 | 1.196E-07 | 4.592E-07 | 0.000E+00 | 3.930E-07 | 0.000E+00 | 6.328E-08 | 3.270E-08 |
| Unigene42090 | efrB | 9.946E-07 | 6.550E-06 | 1.041E-05 | 1.759E-05 | 0.000E+00 | 0.000E+00 | 0.000E+00 | 0.000E+00 | 0.000E+00 | 0.000E+00 | 0.000E+00 | 0.000E+00 |
| Unigene42110 | bcrA | 8.967E-07 | 1.160E-06 | 4.713E-06 | 5.562E-06 | 0.000E+00 | 0.000E+00 | 3.899E-07 | 0.000E+00 | 0.000E+00 | 0.000E+00 | 0.000E+00 | 0.000E+00 |
| Unigene42123 | msbA | 9.968E-05 | 1.581E-04 | 1.558E-04 | 7.275E-06 | 8.310E-06 | 3.386E-07 | 4.241E-06 | 1.807E-06 | 5.774E-05 | 1.437E-07 | 3.414E-06 | 6.969E-07 |
| Unigene42124 | tetA(46) | 9.539E-05 | 1.558E-04 | 1.455E-04 | 6.316E-06 | 7.962E-06 | 3.220E-07 | 3.255E-06 | 2.086E-06 | 5.270E-05 | 3.733E-07 | 2.683E-06 | 7.041E-07 |
| Unigene42158 | tetA(60) | 1.214E-06 | 1.608E-06 | 1.232E-06 | 4.820E-06 | 3.693E-05 | 5.943E-05 | 6.673E-05 | 5.420E-05 | 3.116E-05 | 2.187E-05 | 1.657E-05 | 4.355E-06 |
| Unigene42160 | vanTG | 3.451E-07 | 0.000E+00 | 0.000E+00 | 0.000E+00 | 1.394E-07 | 0.000E+00 | 0.000E+00 | 0.000E+00 | 0.000E+00 | 0.000E+00 | 0.000E+00 | 0.000E+00 |
| Unigene42198 | efrA | 3.185E-06 | 9.330E-06 | 8.795E-06 | 1.077E-05 | 0.000E+00 | 8.008E-08 | 8.199E-08 | 0.000E+00 | 0.000E+00 | 1.032E-07 | 2.118E-08 | 4.378E-08 |

| | | | | | | | | | | | | | |
|---|---|---|---|---|---|---|---|---|---|---|---|---|---|
| Unigene42224 | vanL | 4.072E-06 | 2.039E-05 | 2.917E-06 | 8.705E-07 | 1.255E-06 | 4.499E-07 | 2.961E-07 | 2.776E-07 | 1.429E-06 | 6.953E-07 | 4.080E-07 | 0.000E+00 |
| Unigene42228 | Staphylocc | 1.458E-06 | 9.143E-07 | 1.272E-06 | 1.753E-06 | 2.082E-05 | 6.779E-06 | 1.457E-05 | 1.845E-05 | 1.128E-05 | 1.745E-05 | 1.938E-05 | 8.042E-06 |
| Unigene42283 | farB | 6.794E-07 | 0.000E+00 | 0.000E+00 | 3.983E-07 | 0.000E+00 | 9.263E-06 | 1.577E-06 | 8.339E-06 | 4.383E-06 | 3.174E-06 | 0.000E+00 | 4.663E-07 |
| Unigene42297 | tetA(46) | 2.657E-06 | 2.496E-06 | 3.094E-06 | 1.467E-06 | 0.000E+00 | 4.132E-08 | 4.442E-07 | 2.231E-07 | 7.520E-07 | 6.387E-08 | 0.000E+00 | 6.777E-08 |
| Unigene42423 | mtrA | 8.495E-07 | 7.769E-07 | 3.380E-06 | 5.766E-06 | 0.000E+00 | 0.000E+00 | 5.745E-07 | 0.000E+00 | 0.000E+00 | 0.000E+00 | 0.000E+00 | 0.000E+00 |
| Unigene42424 | vanSF | 9.454E-07 | 9.635E-07 | 4.175E-06 | 6.751E-06 | 0.000E+00 | 0.000E+00 | 4.734E-07 | 0.000E+00 | 2.460E-08 | 0.000E+00 | 0.000E+00 | 0.000E+00 |
| Unigene42428 | abeS | 0.000E+00 | 6.813E-07 | 0.000E+00 | 1.463E-06 | 2.023E-05 | 6.263E-06 | 1.317E-05 | 1.751E-05 | 1.346E-05 | 1.717E-05 | 1.752E-05 | 9.172E-06 |
| Unigene42431 | poxtA | 1.581E-06 | 1.119E-06 | 1.551E-06 | 3.244E-06 | 2.074E-05 | 5.788E-06 | 1.592E-05 | 1.982E-05 | 1.009E-05 | 1.971E-05 | 2.018E-05 | 5.847E-06 |
| Unigene42499 | lmrB | 7.257E-06 | 0.000E+00 | 0.000E+00 | 2.113E-06 | 3.349E-05 | 1.591E-05 | 2.324E-05 | 2.387E-05 | 3.804E-06 | 0.000E+00 | 0.000E+00 | 0.000E+00 |
| Unigene42503 | efrA | 6.765E-06 | 0.000E+00 | 0.000E+00 | 2.037E-06 | 3.375E-05 | 1.634E-05 | 2.018E-05 | 2.318E-05 | 2.897E-06 | 0.000E+00 | 0.000E+00 | 0.000E+00 |
| Unigene42504 | tetB(60) | 7.303E-06 | 0.000E+00 | 0.000E+00 | 2.533E-06 | 3.279E-05 | 1.623E-05 | 2.489E-05 | 2.777E-05 | 3.656E-06 | 0.000E+00 | 0.000E+00 | 0.000E+00 |
| Unigene42515 | patA | 6.445E-06 | 0.000E+00 | 0.000E+00 | 2.264E-06 | 3.138E-05 | 1.531E-05 | 2.384E-05 | 2.588E-05 | 2.777E-06 | 0.000E+00 | 0.000E+00 | 0.000E+00 |
| Unigene42516 | lmrD | 7.278E-06 | 0.000E+00 | 0.000E+00 | 2.469E-06 | 3.471E-05 | 1.564E-05 | 2.031E-05 | 2.456E-05 | 3.482E-06 | 0.000E+00 | 0.000E+00 | 0.000E+00 |
| Unigene42543 | Staphylocc | 6.843E-06 | 0.000E+00 | 0.000E+00 | 2.325E-06 | 3.646E-05 | 1.508E-05 | 2.260E-05 | 2.738E-05 | 3.810E-06 | 0.000E+00 | 0.000E+00 | 0.000E+00 |
| Unigene42569 | tetA(58) | 7.426E-05 | 6.009E-05 | 1.274E-04 | 1.782E-04 | 1.349E-04 | 9.924E-05 | 1.208E-04 | 1.152E-04 | 9.963E-05 | 1.405E-04 | 1.396E-04 | 1.464E-04 |
| Unigene42587 | macB | 2.587E-06 | 3.508E-06 | 2.393E-06 | 2.097E-06 | 2.654E-06 | 2.084E-06 | 2.115E-06 | 1.154E-06 | 1.135E-06 | 6.076E-06 | 5.674E-06 | 2.045E-06 |
| Unigene42637 | vanHO | 1.179E-04 | 1.752E-04 | 1.638E-04 | 6.971E-06 | 9.326E-06 | 5.255E-07 | 3.300E-06 | 1.740E-06 | 6.139E-05 | 3.249E-07 | 3.522E-06 | 3.448E-07 |
| Unigene42644 | rpoB2 | 1.725E-07 | 0.000E+00 | 0.000E+00 | 0.000E+00 | 3.114E-07 | 0.000E+00 | 6.682E-07 | 0.000E+00 | 0.000E+00 | 0.000E+00 | 0.000E+00 | 0.000E+00 |
| Unigene42657 | efrA | 9.340E-07 | 5.884E-06 | 9.917E-06 | 1.390E-05 | 0.000E+00 | 0.000E+00 | 0.000E+00 | 0.000E+00 | 0.000E+00 | 0.000E+00 | 4.274E-08 | 0.000E+00 |
| Unigene42658 | patB | 6.759E-07 | 6.817E-06 | 1.007E-05 | 1.574E-05 | 0.000E+00 | 0.000E+00 | 0.000E+00 | 0.000E+00 | 0.000E+00 | 0.000E+00 | 0.000E+00 | 0.000E+00 |
| Unigene42685 | tetT | 3.804E-07 | 0.000E+00 | 3.199E-07 | 0.000E+00 | 0.000E+00 | 1.175E-07 | 0.000E+00 | 0.000E+00 | 0.000E+00 | 0.000E+00 | 0.000E+00 | 0.000E+00 |
| Unigene42718 | patA | 2.799E-06 | 4.273E-06 | 3.176E-06 | 1.855E-06 | 2.284E-06 | 2.033E-06 | 2.469E-06 | 1.327E-06 | 1.817E-06 | 5.262E-06 | 5.354E-06 | 1.810E-06 |
| Unigene42744 | YojI | 8.384E-07 | 3.425E-06 | 8.456E-06 | 1.261E-05 | 0.000E+00 | 0.000E+00 | 0.000E+00 | 0.000E+00 | 0.000E+00 | 0.000E+00 | 0.000E+00 | 0.000E+00 |
| Unigene42772 | adeS | 7.124E-06 | 0.000E+00 | 0.000E+00 | 2.742E-06 | 3.426E-05 | 1.532E-05 | 2.095E-05 | 2.538E-05 | 2.695E-06 | 0.000E+00 | 0.000E+00 | 0.000E+00 |
| Unigene42773 | arlR | 7.838E-06 | 0.000E+00 | 0.000E+00 | 1.920E-06 | 3.359E-05 | 1.815E-05 | 2.233E-05 | 2.582E-05 | 4.157E-06 | 0.000E+00 | 0.000E+00 | 0.000E+00 |
| Unigene42776 | PmrF | 7.260E-06 | 0.000E+00 | 0.000E+00 | 2.258E-06 | 3.161E-05 | 1.703E-05 | 2.403E-05 | 2.493E-05 | 3.684E-06 | 0.000E+00 | 0.000E+00 | 0.000E+00 |
| Unigene42779 | macB | 8.311E-06 | 0.000E+00 | 0.000E+00 | 2.108E-06 | 3.225E-05 | 1.341E-05 | 2.240E-05 | 2.610E-05 | 3.203E-06 | 0.000E+00 | 0.000E+00 | 0.000E+00 |
| Unigene42803 | mtrA | 2.717E-05 | 1.086E-05 | 5.634E-06 | 4.337E-06 | 2.144E-07 | 0.000E+00 | 0.000E+00 | 0.000E+00 | 1.107E-06 | 0.000E+00 | 0.000E+00 | 0.000E+00 |
| Unigene42821 | Staphylocc | 2.597E-05 | 9.961E-06 | 6.427E-06 | 4.508E-06 | 5.494E-07 | 1.589E-07 | 4.438E-08 | 1.716E-07 | 1.154E-06 | 0.000E+00 | 0.000E+00 | 0.000E+00 |
| Unigene42964 | YojI | 3.088E-06 | 3.355E-06 | 3.428E-06 | 1.764E-06 | 0.000E+00 | 1.686E-07 | 3.797E-07 | 2.184E-07 | 4.772E-07 | 0.000E+00 | 0.000E+00 | 0.000E+00 |
| Unigene42992 | bcrA | 2.624E-06 | 2.352E-06 | 2.833E-06 | 1.369E-06 | 0.000E+00 | 0.000E+00 | 0.000E+00 | 0.000E+00 | 6.215E-07 | 0.000E+00 | 0.000E+00 | 0.000E+00 |
| Unigene43008 | PmrF | 2.501E-06 | 9.029E-07 | 1.679E-06 | 1.562E-06 | 0.000E+00 | 0.000E+00 | 1.093E-07 | 0.000E+00 | 0.000E+00 | 2.566E-07 | 0.000E+00 | 0.000E+00 |
| Unigene43045 | vanRO | 4.678E-07 | 0.000E+00 | 0.000E+00 | 2.953E-07 | 0.000E+00 | 0.000E+00 | 0.000E+00 | 0.000E+00 | 0.000E+00 | 0.000E+00 | 0.000E+00 | 0.000E+00 |
| Unigene43062 | vatB | 5.569E-06 | 1.913E-06 | 3.086E-06 | 1.576E-05 | 1.052E-06 | 0.000E+00 | 9.477E-06 | 3.096E-07 | 0.000E+00 | 7.217E-06 | 8.451E-06 | 1.254E-06 |
| Unigene43063 | catB11 | 7.000E-06 | 2.247E-06 | 3.873E-06 | 1.767E-05 | 4.136E-07 | 0.000E+00 | 1.150E-05 | 0.000E+00 | 0.000E+00 | 8.751E-06 | 8.688E-06 | 1.188E-06 |
| Unigene43110 | msbA | 5.847E-06 | 0.000E+00 | 0.000E+00 | 2.336E-06 | 3.460E-05 | 8.076E-06 | 0.000E+00 | 0.000E+00 | 0.000E+00 | 0.000E+00 | 0.000E+00 | 0.000E+00 |
| Unigene43112 | bcrA | 5.701E-06 | 0.000E+00 | 0.000E+00 | 3.310E-06 | 3.657E-05 | 9.514E-06 | 0.000E+00 | 0.000E+00 | 0.000E+00 | 0.000E+00 | 0.000E+00 | 0.000E+00 |
| Unigene43122 | salA | 6.996E-06 | 0.000E+00 | 0.000E+00 | 2.080E-06 | 3.476E-05 | 1.574E-05 | 2.225E-05 | 2.765E-05 | 0.000E+00 | 0.000E+00 | 0.000E+00 | 0.000E+00 |
| Unigene43159 | MexW | 0.000E+00 | 0.000E+00 | 0.000E+00 | 0.000E+00 | 0.000E+00 | 0.000E+00 | 0.000E+00 | 1.738E-07 | 0.000E+00 | 0.000E+00 | 0.000E+00 | 8.802E-08 |
| Unigene43180 | macB | 4.394E-05 | 0.000E+00 | 0.000E+00 | 0.000E+00 | 0.000E+00 | 0.000E+00 | 0.000E+00 | 0.000E+00 | 0.000E+00 | 0.000E+00 | 0.000E+00 | 0.000E+00 |
| Unigene43181 | macB | 5.355E-05 | 0.000E+00 | 0.000E+00 | 0.000E+00 | 0.000E+00 | 0.000E+00 | 0.000E+00 | 1.278E-07 | 0.000E+00 | 0.000E+00 | 0.000E+00 | 3.236E-08 |
| Unigene43198 | macB | 1.108E-05 | 3.162E-06 | 3.137E-06 | 1.046E-05 | 6.877E-05 | 1.020E-04 | 9.497E-05 | 9.328E-05 | 1.241E-04 | 1.134E-04 | 9.636E-05 | 6.434E-05 |
| Unigene43263 | poxtA | 3.116E-06 | 5.066E-06 | 3.433E-06 | 1.018E-05 | 1.044E-04 | 1.948E-04 | 2.052E-04 | 2.319E-04 | 1.147E-04 | 4.447E-05 | 4.025E-05 | 2.398E-06 |
| Unigene43363 | msbA | 1.471E-06 | 1.444E-06 | 1.329E-06 | 1.563E-06 | 1.720E-05 | 5.691E-06 | 1.375E-05 | 1.523E-05 | 1.045E-05 | 1.604E-05 | 1.771E-05 | 6.131E-06 |
| Unigene43364 | macB | 1.510E-06 | 8.702E-07 | 1.074E-06 | 1.563E-06 | 2.299E-05 | 5.265E-06 | 1.329E-05 | 1.626E-05 | 1.127E-05 | 1.745E-05 | 1.887E-05 | 7.587E-06 |
| Unigene43389 | macB | 1.238E-06 | 8.361E-07 | 4.812E-06 | 8.702E-06 | 1.058E-07 | 0.000E+00 | 6.850E-07 | 0.000E+00 | 0.000E+00 | 0.000E+00 | 0.000E+00 | 5.628E-08 |
| Unigene43403 | vatB | 1.065E-04 | 2.031E-04 | 1.855E-04 | 9.228E-06 | 7.509E-06 | 0.000E+00 | 4.009E-06 | 1.893E-06 | 6.063E-05 | 0.000E+00 | 3.773E-06 | 0.000E+00 |
| Unigene43435 | Staphylocc | 1.110E-05 | 0.000E+00 | 0.000E+00 | 0.000E+00 | 1.804E-05 | 1.584E-05 | 0.000E+00 | 0.000E+00 | 1.276E-05 | 3.347E-07 | 1.322E-08 | 0.000E+00 |
| Unigene43468 | kdpE | 5.937E-07 | 0.000E+00 | 9.466E-07 | 0.000E+00 | 0.000E+00 | 8.022E-08 | 0.000E+00 | 0.000E+00 | 0.000E+00 | 0.000E+00 | 0.000E+00 | 0.000E+00 |
| Unigene43475 | oleC | 3.024E-06 | 2.092E-05 | 9.661E-06 | 5.930E-06 | 0.000E+00 | 0.000E+00 | 0.000E+00 | 0.000E+00 | 0.000E+00 | 0.000E+00 | 0.000E+00 | 0.000E+00 |
| Unigene43476 | NmcR | 8.389E-07 | 6.365E-06 | 1.013E-05 | 1.753E-05 | 0.000E+00 | 0.000E+00 | 0.000E+00 | 0.000E+00 | 0.000E+00 | 0.000E+00 | 0.000E+00 | 0.000E+00 |
| Unigene43521 | novA | 4.035E-06 | 9.595E-06 | 9.993E-06 | 1.154E-05 | 0.000E+00 | 0.000E+00 | 0.000E+00 | 0.000E+00 | 0.000E+00 | 0.000E+00 | 0.000E+00 | 0.000E+00 |
| Unigene43582 | Escherichia | 3.386E-06 | 5.036E-06 | 3.012E-06 | 5.017E-06 | 2.110E-07 | 6.842E-08 | 2.102E-07 | 0.000E+00 | 0.000E+00 | 0.000E+00 | 1.448E-07 | 1.122E-07 |
| Unigene43612 | arlR | 1.778E-06 | 0.000E+00 | 0.000E+00 | 0.000E+00 | 0.000E+00 | 0.000E+00 | 0.000E+00 | 0.000E+00 | 0.000E+00 | 0.000E+00 | 0.000E+00 | 0.000E+00 |
| Unigene43613 | arlS | 1.173E-06 | 1.287E-07 | 0.000E+00 | 0.000E+00 | 0.000E+00 | 0.000E+00 | 0.000E+00 | 0.000E+00 | 0.000E+00 | 0.000E+00 | 0.000E+00 | 0.000E+00 |
| Unigene43676 | tetA(58) | 2.310E-05 | 4.597E-06 | 6.184E-06 | 3.454E-06 | 1.281E-07 | 2.908E-07 | 0.000E+00 | 0.000E+00 | 4.200E-08 | 0.000E+00 | 0.000E+00 | 4.542E-08 |
| Unigene43699 | bmr | 1.294E-05 | 4.795E-06 | 3.758E-06 | 1.419E-05 | 9.126E-05 | 1.306E-04 | 1.354E-04 | 1.067E-04 | 1.661E-04 | 1.576E-04 | 1.370E-04 | 8.466E-05 |
| Unigene43778 | Staphylocc | 3.233E-06 | 5.190E-06 | 3.898E-06 | 6.269E-06 | 5.182E-07 | 1.217E-07 | 3.916E-07 | 5.632E-08 | 2.110E-07 | 7.167E-08 | 1.104E-07 | 2.662E-07 |

| | | | | | | | | | | | | | |
|---|---|---|---|---|---|---|---|---|---|---|---|---|---|
| Unigene43785 | tetA(46) | 1.203E-05 | 0.000E+00 | 0.000E+00 | 0.000E+00 | 2.030E-05 | 1.661E-05 | 0.000E+00 | 0.000E+00 | 1.364E-05 | 0.000E+00 | 0.000E+00 | 0.000E+00 |
| Unigene43786 | novA | 1.008E-05 | 0.000E+00 | 0.000E+00 | 0.000E+00 | 1.599E-05 | 1.538E-05 | 0.000E+00 | 2.151E-08 | 1.128E-05 | 2.463E-07 | 2.108E-08 | 0.000E+00 |
| Unigene43832 | optrA | 1.136E-05 | 0.000E+00 | 0.000E+00 | 0.000E+00 | 1.762E-05 | 1.542E-05 | 0.000E+00 | 0.000E+00 | 1.245E-05 | 0.000E+00 | 1.899E-08 | 1.963E-08 |
| Unigene43878 | evgA | 3.570E-06 | 4.534E-06 | 7.256E-06 | 6.849E-06 | 2.266E-07 | 0.000E+00 | 0.000E+00 | 0.000E+00 | 0.000E+00 | 0.000E+00 | 0.000E+00 | 0.000E+00 |
| Unigene43900 | dfrE | 1.102E-06 | 0.000E+00 | 0.000E+00 | 0.000E+00 | 0.000E+00 | 0.000E+00 | 0.000E+00 | 0.000E+00 | 0.000E+00 | 0.000E+00 | 0.000E+00 | 0.000E+00 |
| Unigene43910 | TaeA | 1.410E-06 | 0.000E+00 | 0.000E+00 | 0.000E+00 | 0.000E+00 | 2.540E-08 | 0.000E+00 | 0.000E+00 | 0.000E+00 | 0.000E+00 | 0.000E+00 | 0.000E+00 |
| Unigene43946 | optrA | 6.083E-07 | 0.000E+00 | 0.000E+00 | 0.000E+00 | 4.435E-06 | 9.988E-06 | 2.567E-06 | 7.678E-06 | 4.904E-06 | 3.727E-06 | 0.000E+00 | 5.393E-07 |
| Unigene43957 | vanSL | 9.081E-07 | 3.140E-07 | 2.929E-07 | 6.281E-08 | 4.146E-08 | 0.000E+00 | 1.514E-07 | 7.260E-08 | 5.710E-07 | 0.000E+00 | 0.000E+00 | 1.470E-08 |
| Unigene43958 | kdpE | 8.384E-07 | 4.090E-07 | 2.742E-07 | 0.000E+00 | 5.175E-08 | 0.000E+00 | 0.000E+00 | 0.000E+00 | 6.618E-07 | 0.000E+00 | 0.000E+00 | 0.000E+00 |
| Unigene43976 | tetB(60) | 1.334E-06 | 1.757E-06 | 5.672E-06 | 7.633E-06 | 6.586E-08 | 9.613E-08 | 3.609E-07 | 0.000E+00 | 0.000E+00 | 0.000E+00 | 0.000E+00 | 0.000E+00 |
| Unigene44040 | Staphylocc | 2.944E-05 | 0.000E+00 | 0.000E+00 | 0.000E+00 | 0.000E+00 | 0.000E+00 | 0.000E+00 | 0.000E+00 | 0.000E+00 | 0.000E+00 | 0.000E+00 | 0.000E+00 |
| Unigene44053 | msrA | 2.937E-05 | 0.000E+00 | 0.000E+00 | 0.000E+00 | 0.000E+00 | 0.000E+00 | 0.000E+00 | 0.000E+00 | 0.000E+00 | 0.000E+00 | 0.000E+00 | 0.000E+00 |
| Unigene44095 | macB | 4.457E-07 | 6.289E-07 | 1.374E-06 | 3.183E-06 | 4.244E-07 | 0.000E+00 | 2.819E-07 | 1.189E-06 | 0.000E+00 | 8.156E-07 | 1.020E-06 | 6.773E-07 |
| Unigene44115 | lin | 2.954E-06 | 4.515E-06 | 3.188E-06 | 6.392E-06 | 4.219E-07 | 6.842E-08 | 3.736E-07 | 0.000E+00 | 1.614E-07 | 0.000E+00 | 1.448E-07 | 0.000E+00 |
| Unigene44193 | lmrD | 4.919E-06 | 1.788E-05 | 3.404E-06 | 5.322E-07 | 8.782E-07 | 0.000E+00 | 0.000E+00 | 0.000E+00 | 9.120E-07 | 4.892E-07 | 0.000E+00 | 0.000E+00 |
| Unigene44196 | rpoB2 | 4.441E-06 | 2.092E-05 | 3.625E-06 | 1.126E-06 | 1.029E-06 | 4.665E-07 | 2.885E-07 | 3.672E-07 | 1.287E-06 | 8.412E-07 | 5.758E-07 | 4.463E-07 |
| Unigene44230 | optrA | 3.769E-06 | 2.551E-07 | 3.145E-06 | 5.533E-07 | 2.767E-07 | 7.180E-08 | 2.573E-07 | 0.000E+00 | 0.000E+00 | 0.000E+00 | 7.598E-07 | 0.000E+00 |
| Unigene44264 | arnA | 1.144E-05 | 0.000E+00 | 0.000E+00 | 0.000E+00 | 1.501E-05 | 1.648E-05 | 0.000E+00 | 0.000E+00 | 1.280E-05 | 3.403E-07 | 0.000E+00 | 0.000E+00 |
| Unigene44282 | mtrA | 1.223E-05 | 0.000E+00 | 0.000E+00 | 0.000E+00 | 1.847E-05 | 1.564E-05 | 0.000E+00 | 0.000E+00 | 1.211E-05 | 0.000E+00 | 0.000E+00 | 0.000E+00 |
| Unigene44286 | patB | 1.324E-05 | 0.000E+00 | 0.000E+00 | 0.000E+00 | 1.760E-05 | 1.763E-05 | 0.000E+00 | 0.000E+00 | 1.139E-05 | 0.000E+00 | 0.000E+00 | 0.000E+00 |
| Unigene44356 | optrA | 2.336E-06 | 2.203E-06 | 1.614E-06 | 1.661E-06 | 9.139E-07 | 9.960E-07 | 1.712E-06 | 3.841E-07 | 7.553E-07 | 4.619E-06 | 4.667E-06 | 1.011E-06 |
| Unigene44387 | Corynebac | 8.081E-07 | 5.741E-06 | 1.085E-05 | 1.327E-05 | 0.000E+00 | 0.000E+00 | 0.000E+00 | 0.000E+00 | 0.000E+00 | 0.000E+00 | 0.000E+00 | 0.000E+00 |
| Unigene44478 | lmrB | 3.808E-05 | 2.057E-05 | 1.382E-05 | 1.734E-05 | 2.164E-06 | 1.207E-06 | 2.603E-06 | 6.377E-07 | 6.880E-06 | 5.821E-07 | 1.467E-06 | 1.376E-06 |
| Unigene44522 | tetA(58) | 2.739E-05 | 9.891E-06 | 7.577E-06 | 4.603E-06 | 3.006E-07 | 2.700E-07 | 0.000E+00 | 1.458E-07 | 7.052E-07 | 0.000E+00 | 0.000E+00 | 0.000E+00 |
| Unigene44540 | oleC | 1.426E-06 | 9.011E-07 | 5.173E-06 | 6.982E-06 | 9.602E-08 | 0.000E+00 | 2.391E-07 | 0.000E+00 | 0.000E+00 | 0.000E+00 | 0.000E+00 | 0.000E+00 |
| Unigene44574 | bcrA | 4.478E-06 | 1.158E-05 | 1.114E-05 | 9.534E-06 | 3.856E-08 | 1.501E-07 | 7.683E-08 | 0.000E+00 | 0.000E+00 | 3.093E-07 | 0.000E+00 | 0.000E+00 |
| Unigene44594 | patA | 2.562E-07 | 0.000E+00 | 0.000E+00 | 0.000E+00 | 0.000E+00 | 0.000E+00 | 0.000E+00 | 0.000E+00 | 0.000E+00 | 0.000E+00 | 0.000E+00 | 0.000E+00 |
| Unigene44638 | ANT(6)-Ia | 9.714E-07 | 5.167E-06 | 1.100E-05 | 1.403E-05 | 0.000E+00 | 0.000E+00 | 0.000E+00 | 0.000E+00 | 0.000E+00 | 0.000E+00 | 0.000E+00 | 0.000E+00 |
| Unigene44717 | msbA | 4.138E-05 | 6.349E-08 | 0.000E+00 | 0.000E+00 | 0.000E+00 | 0.000E+00 | 0.000E+00 | 0.000E+00 | 0.000E+00 | 0.000E+00 | 1.412E-06 | 6.838E-08 |
| Unigene44747 | mdtB | 0.000E+00 | 0.000E+00 | 0.000E+00 | 0.000E+00 | 0.000E+00 | 0.000E+00 | 0.000E+00 | 0.000E+00 | 0.000E+00 | 0.000E+00 | 0.000E+00 | 1.645E-07 |
| Unigene44826 | Staphylocc | 2.920E-06 | 4.315E-06 | 4.100E-06 | 5.694E-06 | 2.246E-07 | 7.285E-08 | 1.989E-07 | 0.000E+00 | 1.473E-07 | 0.000E+00 | 0.000E+00 | 1.593E-07 |
| Unigene44848 | tet(44) | 3.686E-06 | 1.061E-05 | 7.528E-06 | 6.942E-06 | 0.000E+00 | 1.077E-07 | 0.000E+00 | 0.000E+00 | 0.000E+00 | 0.000E+00 | 0.000E+00 | 0.000E+00 |
| Unigene44978 | patA | 1.156E-06 | 1.025E-06 | 1.527E-06 | 1.661E-06 | 1.773E-05 | 6.249E-06 | 1.358E-05 | 1.432E-05 | 1.084E-05 | 1.516E-05 | 2.126E-05 | 8.987E-06 |
| Unigene44981 | baeS | 1.386E-06 | 9.540E-07 | 1.245E-06 | 2.159E-06 | 2.062E-05 | 6.349E-06 | 1.586E-05 | 1.730E-05 | 1.217E-05 | 2.078E-05 | 2.126E-05 | 6.498E-06 |
| Unigene44982 | arlR | 6.987E-07 | 1.074E-06 | 1.280E-06 | 1.929E-06 | 1.951E-05 | 5.539E-06 | 1.356E-05 | 1.669E-05 | 1.034E-05 | 1.925E-05 | 1.913E-05 | 7.763E-06 |
| Unigene44985 | emrB | 1.640E-06 | 1.257E-06 | 1.433E-06 | 1.679E-06 | 2.356E-05 | 6.241E-06 | 1.534E-05 | 1.773E-05 | 1.264E-05 | 2.049E-05 | 2.184E-05 | 7.461E-06 |
| Unigene45026 | SPG-1 | 1.559E-06 | 1.002E-06 | 1.194E-06 | 1.229E-06 | 5.069E-07 | 4.932E-07 | 5.891E-07 | 4.438E-07 | 7.896E-07 | 6.353E-07 | 9.568E-07 | 9.438E-07 |
| Unigene45063 | tlrC | 2.459E-06 | 2.952E-06 | 3.884E-06 | 5.394E-06 | 4.696E-07 | 1.246E-07 | 0.000E+00 | 0.000E+00 | 0.000E+00 | 0.000E+00 | 0.000E+00 | 0.000E+00 |
| Unigene45111 | tetA(58) | 8.469E-05 | 1.456E-04 | 1.315E-04 | 6.426E-06 | 7.466E-06 | 4.637E-07 | 3.640E-06 | 1.753E-06 | 4.997E-05 | 0.000E+00 | 2.576E-06 | 8.028E-07 |
| Unigene45116 | oleC | 1.102E-06 | 1.016E-06 | 5.625E-06 | 5.789E-06 | 0.000E+00 | 9.535E-08 | 1.952E-07 | 0.000E+00 | 0.000E+00 | 0.000E+00 | 0.000E+00 | 5.213E-08 |
| Unigene45175 | patB | 1.027E-06 | 0.000E+00 | 0.000E+00 | 0.000E+00 | 0.000E+00 | 0.000E+00 | 0.000E+00 | 0.000E+00 | 0.000E+00 | 0.000E+00 | 0.000E+00 | 0.000E+00 |
| Unigene45176 | vanHA | 1.169E-06 | 7.546E-08 | 0.000E+00 | 0.000E+00 | 0.000E+00 | 0.000E+00 | 0.000E+00 | 0.000E+00 | 0.000E+00 | 0.000E+00 | 0.000E+00 | 0.000E+00 |
| Unigene45178 | mdtG | 1.472E-06 | 0.000E+00 | 0.000E+00 | 0.000E+00 | 0.000E+00 | 0.000E+00 | 0.000E+00 | 0.000E+00 | 0.000E+00 | 0.000E+00 | 6.124E-08 | 0.000E+00 |
| Unigene45182 | lsaC | 9.584E-07 | 2.391E-07 | 0.000E+00 | 0.000E+00 | 0.000E+00 | 0.000E+00 | 0.000E+00 | 0.000E+00 | 0.000E+00 | 0.000E+00 | 0.000E+00 | 0.000E+00 |
| Unigene45198 | Staphylocc | 2.860E-06 | 4.284E-06 | 3.305E-06 | 6.438E-06 | 5.049E-07 | 1.300E-07 | 3.550E-07 | 0.000E+00 | 1.315E-07 | 2.978E-08 | 4.587E-08 | 1.896E-07 |
| Unigene45200 | pgpB | 3.669E-06 | 5.497E-06 | 4.038E-06 | 7.911E-06 | 6.610E-07 | 1.072E-07 | 2.195E-07 | 0.000E+00 | 3.794E-07 | 0.000E+00 | 0.000E+00 | 0.000E+00 |
| Unigene45204 | Acinetobac | 3.011E-06 | 4.325E-06 | 3.259E-06 | 6.372E-06 | 3.935E-07 | 0.000E+00 | 3.430E-07 | 5.169E-08 | 1.210E-07 | 0.000E+00 | 0.000E+00 | 2.094E-07 |
| Unigene45215 | vanYF | 4.193E-05 | 1.961E-05 | 6.931E-06 | 1.294E-05 | 1.643E-06 | 7.104E-07 | 2.682E-06 | 7.670E-07 | 6.284E-06 | 0.000E+00 | 1.644E-06 | 1.990E-06 |
| Unigene45236 | Erm(38) | 7.742E-06 | 0.000E+00 | 0.000E+00 | 1.470E-06 | 3.385E-05 | 1.574E-05 | 2.472E-05 | 2.313E-05 | 3.804E-06 | 0.000E+00 | 0.000E+00 | 0.000E+00 |
| Unigene45244 | tetB(60) | 6.118E-06 | 0.000E+00 | 0.000E+00 | 1.704E-06 | 3.112E-05 | 1.163E-05 | 2.247E-05 | 2.352E-05 | 1.768E-06 | 0.000E+00 | 0.000E+00 | 0.000E+00 |
| Unigene45362 | bcrA | 2.171E-06 | 1.892E-06 | 2.350E-06 | 1.515E-06 | 0.000E+00 | 0.000E+00 | 3.887E-07 | 4.100E-07 | 8.375E-07 | 0.000E+00 | 0.000E+00 | 0.000E+00 |
| Unigene45397 | emrB | 1.179E-05 | 4.384E-06 | 3.056E-06 | 1.251E-05 | 8.239E-05 | 1.140E-04 | 1.129E-04 | 8.824E-05 | 1.396E-04 | 1.391E-04 | 1.087E-04 | 7.281E-05 |
| Unigene45402 | lsaA | 3.514E-06 | 9.640E-06 | 9.438E-06 | 1.024E-05 | 0.000E+00 | 0.000E+00 | 0.000E+00 | 0.000E+00 | 0.000E+00 | 0.000E+00 | 0.000E+00 | 0.000E+00 |
| Unigene45487 | dfrE | 0.000E+00 | 0.000E+00 | 0.000E+00 | 0.000E+00 | 0.000E+00 | 8.339E-06 | 0.000E+00 | 6.961E-06 | 4.392E-06 | 0.000E+00 | 0.000E+00 | 0.000E+00 |
| Unigene45564 | patB | 5.380E-07 | 6.561E-08 | 0.000E+00 | 0.000E+00 | 0.000E+00 | 0.000E+00 | 0.000E+00 | 0.000E+00 | 0.000E+00 | 0.000E+00 | 0.000E+00 | 0.000E+00 |
| Unigene45740 | macB | 3.782E-06 | 4.497E-06 | 5.080E-06 | 5.683E-06 | 0.000E+00 | 1.460E-07 | 2.990E-07 | 0.000E+00 | 3.937E-07 | 0.000E+00 | 0.000E+00 | 0.000E+00 |
| Unigene45743 | arlR | 2.968E-06 | 3.512E-06 | 2.366E-06 | 1.499E-06 | 2.473E-06 | 2.056E-06 | 1.643E-06 | 1.245E-06 | 1.267E-06 | 5.942E-06 | 5.571E-06 | 0.000E+00 |
| Unigene45744 | baeS | 3.269E-06 | 3.980E-06 | 2.669E-06 | 2.096E-06 | 3.022E-06 | 1.883E-06 | 2.060E-06 | 1.114E-06 | 2.164E-06 | 5.634E-06 | 6.250E-06 | 1.889E-06 |

| | | | | | | | | | | | | | |
|---|---|---|---|---|---|---|---|---|---|---|---|---|---|
| Unigene45781 | evgA | 2.550E-06 | 3.470E-06 | 2.102E-06 | 2.369E-06 | 0.000E+00 | 0.000E+00 | 3.951E-07 | 0.000E+00 | 7.246E-07 | 0.000E+00 | 0.000E+00 | 0.000E+00 |
| Unigene45818 | msbA | 1.152E-06 | 1.206E-06 | 5.131E-06 | 7.339E-06 | 0.000E+00 | 7.665E-08 | 5.886E-07 | 0.000E+00 | 0.000E+00 | 0.000E+00 | 2.028E-08 | 0.000E+00 |
| Unigene45857 | basS | 4.922E-06 | 1.234E-05 | 1.356E-05 | 1.526E-05 | 1.272E-07 | 0.000E+00 | 0.000E+00 | 3.340E-08 | 0.000E+00 | 0.000E+00 | 0.000E+00 | 0.000E+00 |
| Unigene45858 | vanRE | 3.108E-06 | 7.873E-06 | 9.244E-06 | 1.352E-05 | 0.000E+00 | 0.000E+00 | 0.000E+00 | 0.000E+00 | 0.000E+00 | 0.000E+00 | 0.000E+00 | 0.000E+00 |
| Unigene45861 | vanHM | 0.000E+00 | 0.000E+00 | 0.000E+00 | 2.512E-07 | 0.000E+00 | 0.000E+00 | 0.000E+00 | 0.000E+00 | 0.000E+00 | 0.000E+00 | 0.000E+00 | 0.000E+00 |
| Unigene45906 | tetA(58) | 5.598E-07 | 0.000E+00 | 1.038E-06 | 8.793E-07 | 0.000E+00 | 0.000E+00 | 0.000E+00 | 0.000E+00 | 0.000E+00 | 0.000E+00 | 0.000E+00 | 0.000E+00 |
| Unigene45910 | tetA(58) | 1.384E-06 | 1.176E-06 | 4.644E-06 | 6.988E-06 | 0.000E+00 | 0.000E+00 | 2.471E-07 | 0.000E+00 | 0.000E+00 | 0.000E+00 | 0.000E+00 | 0.000E+00 |
| Unigene45964 | poxtA | 1.488E-07 | 0.000E+00 | 0.000E+00 | 0.000E+00 | 0.000E+00 | 0.000E+00 | 0.000E+00 | 4.052E-07 | 0.000E+00 | 0.000E+00 | 1.702E-07 | 2.344E-07 |
| Unigene46003 | ramA | 1.257E-06 | 3.450E-06 | 2.431E-06 | 6.198E-06 | 0.000E+00 | 0.000E+00 | 0.000E+00 | 8.632E-08 | 0.000E+00 | 0.000E+00 | 0.000E+00 | 0.000E+00 |
| Unigene46070 | otr(B) | 6.886E-06 | 0.000E+00 | 0.000E+00 | 2.516E-06 | 3.322E-05 | 1.366E-05 | 2.073E-05 | 2.232E-05 | 3.120E-06 | 0.000E+00 | 0.000E+00 | 0.000E+00 |
| Unigene46093 | baeS | 6.867E-06 | 0.000E+00 | 0.000E+00 | 2.010E-06 | 3.460E-05 | 1.707E-05 | 2.033E-05 | 2.382E-05 | 3.036E-06 | 0.000E+00 | 0.000E+00 | 0.000E+00 |
| Unigene46094 | vanRM | 8.325E-06 | 0.000E+00 | 0.000E+00 | 1.967E-06 | 3.016E-05 | 1.494E-05 | 2.465E-05 | 2.093E-05 | 2.565E-06 | 0.000E+00 | 0.000E+00 | 0.000E+00 |
| Unigene46106 | Streptomy | 2.900E-05 | 1.432E-05 | 9.387E-06 | 5.056E-06 | 4.421E-07 | 1.972E-07 | 1.468E-07 | 1.161E-07 | 1.558E-06 | 1.662E-07 | 0.000E+00 | 9.799E-08 |
| Unigene46182 | tetT | 1.369E-05 | 5.159E-06 | 3.357E-06 | 1.448E-05 | 9.526E-05 | 1.334E-04 | 1.406E-04 | 1.145E-04 | 1.709E-04 | 1.654E-04 | 1.445E-04 | 8.525E-05 |
| Unigene46204 | novA | 1.015E-06 | 7.465E-06 | 9.164E-06 | 1.456E-05 | 0.000E+00 | 0.000E+00 | 0.000E+00 | 0.000E+00 | 0.000E+00 | 0.000E+00 | 0.000E+00 | 0.000E+00 |
| Unigene46208 | Staphylocc | 7.485E-07 | 6.880E-06 | 1.090E-05 | 1.647E-05 | 0.000E+00 | 0.000E+00 | 0.000E+00 | 0.000E+00 | 0.000E+00 | 0.000E+00 | 0.000E+00 | 0.000E+00 |
| Unigene46266 | macB | 3.127E-05 | 2.082E-05 | 1.697E-05 | 2.081E-05 | 1.494E-06 | 1.246E-06 | 2.392E-06 | 0.000E+00 | 0.000E+00 | 6.421E-07 | 1.099E-06 | 1.931E-06 |
| Unigene46271 | mgrA | 3.733E-06 | 7.471E-06 | 1.379E-05 | 1.094E-05 | 1.626E-07 | 0.000E+00 | 7.291E-07 | 0.000E+00 | 0.000E+00 | 0.000E+00 | 0.000E+00 | 1.730E-07 |
| Unigene46290 | cmlv | 8.597E-07 | 0.000E+00 | 5.442E-08 | 4.761E-07 | 1.849E-07 | 1.268E-05 | 1.688E-06 | 0.000E+00 | 6.335E-06 | 4.603E-06 | 0.000E+00 | 0.000E+00 |
| Unigene46302 | Staphylocc | 1.163E-06 | 8.682E-07 | 1.301E-06 | 1.715E-06 | 1.796E-05 | 5.294E-06 | 1.325E-05 | 1.604E-05 | 9.993E-06 | 1.558E-05 | 1.771E-05 | 7.232E-06 |
| Unigene46341 | Staphylocc | 7.893E-07 | 6.115E-06 | 1.049E-05 | 1.462E-05 | 0.000E+00 | 0.000E+00 | 0.000E+00 | 0.000E+00 | 0.000E+00 | 0.000E+00 | 0.000E+00 | 1.372E-08 |
| Unigene46351 | lmrD | 3.225E-06 | 1.002E-05 | 8.396E-06 | 1.035E-05 | 1.774E-07 | 1.726E-07 | 0.000E+00 | 0.000E+00 | 0.000E+00 | 1.423E-07 | 1.096E-07 | 0.000E+00 |
| Unigene46398 | efpA | 6.271E-06 | 0.000E+00 | 0.000E+00 | 2.096E-06 | 3.213E-05 | 1.341E-05 | 2.319E-05 | 2.494E-05 | 2.835E-06 | 0.000E+00 | 0.000E+00 | 0.000E+00 |
| Unigene46408 | AAC(3)-VII | 7.344E-06 | 2.026E-06 | 7.553E-06 | 3.970E-05 | 1.571E-06 | 1.189E-06 | 1.065E-05 | 0.000E+00 | 0.000E+00 | 1.269E-06 | 7.187E-06 | 2.089E-06 |
| Unigene46469 | optrA | 2.913E-06 | 2.063E-07 | 2.490E-06 | 6.643E-07 | 2.610E-07 | 0.000E+00 | 3.640E-07 | 0.000E+00 | 0.000E+00 | 0.000E+00 | 9.137E-07 | 0.000E+00 |
| Unigene46470 | poxtA | 3.817E-06 | 3.256E-07 | 3.688E-06 | 4.993E-07 | 3.296E-07 | 0.000E+00 | 3.503E-07 | 0.000E+00 | 0.000E+00 | 0.000E+00 | 1.018E-06 | 0.000E+00 |
| Unigene46503 | oleC | 2.161E-06 | 1.650E-05 | 9.260E-06 | 5.344E-06 | 0.000E+00 | 0.000E+00 | 0.000E+00 | 0.000E+00 | 0.000E+00 | 0.000E+00 | 0.000E+00 | 0.000E+00 |
| Unigene46533 | tva(A) | 2.428E-06 | 1.775E-05 | 7.017E-06 | 4.273E-06 | 0.000E+00 | 0.000E+00 | 1.452E-07 | 0.000E+00 | 0.000E+00 | 0.000E+00 | 0.000E+00 | 0.000E+00 |
| Unigene46575 | macB | 6.005E-06 | 1.634E-06 | 2.875E-06 | 1.067E-06 | 0.000E+00 | 0.000E+00 | 0.000E+00 | 4.486E-07 | 0.000E+00 | 0.000E+00 | 0.000E+00 | 0.000E+00 |
| Unigene46584 | fusD | 4.918E-05 | 1.809E-05 | 7.004E-06 | 1.208E-05 | 2.099E-06 | 1.929E-06 | 3.718E-06 | 7.965E-07 | 8.089E-06 | 0.000E+00 | 1.861E-06 | 1.241E-06 |
| Unigene46618 | macB | 1.563E-06 | 1.721E-05 | 6.813E-06 | 4.411E-06 | 0.000E+00 | 0.000E+00 | 0.000E+00 | 0.000E+00 | 0.000E+00 | 0.000E+00 | 0.000E+00 | 0.000E+00 |
| Unigene46666 | patB | 2.710E-06 | 3.818E-06 | 4.009E-06 | 6.985E-06 | 0.000E+00 | 0.000E+00 | 0.000E+00 | 0.000E+00 | 0.000E+00 | 0.000E+00 | 0.000E+00 | 0.000E+00 |
| Unigene46667 | patB | 3.000E-06 | 2.766E-06 | 3.964E-06 | 5.170E-06 | 2.666E-07 | 0.000E+00 | 3.099E-07 | 0.000E+00 | 0.000E+00 | 0.000E+00 | 1.373E-07 | 0.000E+00 |
| Unigene46700 | tetA(58) | 3.552E-06 | 4.542E-06 | 3.858E-06 | 6.512E-06 | 6.130E-07 | 0.000E+00 | 0.000E+00 | 0.000E+00 | 0.000E+00 | 0.000E+00 | 0.000E+00 | 0.000E+00 |
| Unigene46702 | macB | 1.652E-06 | 4.144E-07 | 7.872E-07 | 1.287E-06 | 1.049E-07 | 0.000E+00 | 0.000E+00 | 0.000E+00 | 0.000E+00 | 0.000E+00 | 0.000E+00 | 0.000E+00 |
| Unigene46719 | Staphylocc | 4.408E-06 | 2.022E-05 | 3.498E-06 | 9.818E-07 | 8.641E-07 | 3.924E-07 | 3.443E-07 | 4.691E-07 | 1.332E-06 | 8.521E-07 | 3.856E-07 | 2.299E-07 |
| Unigene46743 | Staphylocc | 3.181E-06 | 1.689E-05 | 3.073E-06 | 4.589E-07 | 9.376E-07 | 3.333E-07 | 1.078E-07 | 1.326E-07 | 8.692E-07 | 3.073E-07 | 2.228E-07 | 2.110E-07 |
| Unigene46779 | macB | 2.560E-05 | 5.301E-07 | 4.476E-07 | 1.355E-07 | 8.943E-08 | 2.611E-07 | 0.000E+00 | 0.000E+00 | 0.000E+00 | 2.092E-07 | 0.000E+00 | 3.172E-08 |
| Unigene46781 | oleC | 2.268E-05 | 2.660E-07 | 4.756E-07 | 0.000E+00 | 0.000E+00 | 1.310E-07 | 0.000E+00 | 0.000E+00 | 0.000E+00 | 2.160E-07 | 0.000E+00 | 0.000E+00 |
| Unigene46813 | bcrA | 7.891E-07 | 0.000E+00 | 2.816E-07 | 5.071E-07 | 3.427E-06 | 9.189E-06 | 1.588E-06 | 7.620E-06 | 3.411E-06 | 2.997E-06 | 0.000E+00 | 9.327E-07 |
| Unigene46825 | Chlamydia | 4.342E-05 | 2.536E-05 | 2.040E-05 | 2.262E-05 | 2.234E-06 | 1.848E-06 | 3.255E-06 | 5.282E-07 | 7.447E-06 | 4.761E-07 | 2.157E-06 | 1.545E-06 |
| Unigene46832 | emrY | 1.514E-06 | 1.194E-06 | 1.160E-06 | 2.030E-06 | 2.154E-05 | 6.468E-06 | 1.625E-05 | 2.170E-05 | 1.437E-05 | 2.000E-05 | 2.099E-05 | 8.442E-06 |
| Unigene46891 | poxtA | 1.549E-06 | 1.228E-06 | 2.112E-06 | 1.478E-06 | 2.090E-05 | 6.561E-06 | 1.391E-05 | 1.718E-05 | 1.049E-05 | 1.956E-05 | 1.979E-05 | 8.140E-06 |
| Unigene46928 | vgaC | 7.270E-07 | 1.117E-06 | 1.807E-06 | 3.132E-06 | 1.766E-05 | 8.960E-06 | 1.888E-05 | 1.612E-05 | 1.176E-05 | 2.014E-05 | 1.957E-05 | 6.875E-06 |
| Unigene46934 | vanSF | 1.788E-06 | 9.975E-07 | 1.582E-06 | 2.369E-06 | 2.147E-05 | 7.163E-06 | 1.360E-05 | 1.776E-05 | 1.279E-05 | 1.822E-05 | 1.952E-05 | 7.971E-06 |
| Unigene46935 | vanRF | 1.598E-06 | 8.251E-07 | 1.153E-06 | 1.851E-06 | 2.020E-05 | 5.587E-06 | 1.342E-05 | 1.804E-05 | 1.120E-05 | 1.827E-05 | 1.908E-05 | 7.276E-06 |
| Unigene46945 | mecI | 1.283E-06 | 8.603E-07 | 9.090E-07 | 1.799E-06 | 2.106E-05 | 6.702E-06 | 1.278E-05 | 1.664E-05 | 1.137E-05 | 1.889E-05 | 1.981E-05 | 7.496E-06 |
| Unigene46961 | msbA | 1.564E-06 | 1.103E-06 | 1.863E-06 | 1.692E-06 | 2.401E-05 | 7.003E-06 | 1.434E-05 | 1.849E-05 | 1.227E-05 | 2.097E-05 | 2.221E-05 | 7.922E-06 |
| Unigene46962 | novA | 1.076E-06 | 1.079E-06 | 1.748E-06 | 1.724E-06 | 2.174E-05 | 6.578E-06 | 1.310E-05 | 1.860E-05 | 1.202E-05 | 2.085E-05 | 1.950E-05 | 6.908E-06 |
| Unigene46967 | PmrF | 1.494E-06 | 6.333E-07 | 1.998E-06 | 2.228E-06 | 2.036E-05 | 6.458E-06 | 1.469E-05 | 1.716E-05 | 1.291E-05 | 1.955E-05 | 2.023E-05 | 8.145E-06 |
| Unigene47080 | Staphylocc | 2.358E-05 | 8.618E-06 | 6.522E-06 | 4.489E-06 | 7.154E-07 | 2.919E-07 | 0.000E+00 | 0.000E+00 | 1.203E-06 | 0.000E+00 | 0.000E+00 | 0.000E+00 |
| Unigene47120 | oleB | 3.856E-06 | 2.281E-07 | 3.444E-06 | 1.865E-07 | 3.335E-07 | 0.000E+00 | 1.789E-07 | 0.000E+00 | 0.000E+00 | 0.000E+00 | 8.188E-07 | 0.000E+00 |
| Unigene47256 | AAC(6')-Ie | 3.828E-06 | 1.075E-05 | 1.117E-05 | 1.118E-05 | 2.299E-07 | 2.237E-07 | 0.000E+00 | 4.026E-08 | 0.000E+00 | 2.689E-07 | 1.183E-07 | 0.000E+00 |
| Unigene47296 | vanL | 3.527E-06 | 4.782E-06 | 3.548E-06 | 6.735E-06 | 5.928E-07 | 3.525E-07 | 4.593E-07 | 0.000E+00 | 2.268E-07 | 0.000E+00 | 0.000E+00 | 1.752E-07 |
| Unigene47299 | vgaALC | 2.327E-06 | 5.373E-06 | 2.949E-06 | 6.852E-06 | 9.154E-07 | 0.000E+00 | 6.437E-07 | 1.132E-07 | 0.000E+00 | 0.000E+00 | 1.109E-07 | 0.000E+00 |
| Unigene47303 | arlR | 2.200E-06 | 3.989E-06 | 3.754E-06 | 6.230E-06 | 6.907E-07 | 2.068E-07 | 0.000E+00 | 0.000E+00 | 0.000E+00 | 0.000E+00 | 0.000E+00 | 0.000E+00 |
| Unigene47304 | arlS | 2.917E-06 | 5.138E-06 | 3.872E-06 | 6.262E-06 | 0.000E+00 | 1.590E-07 | 2.713E-07 | 0.000E+00 | 1.607E-07 | 0.000E+00 | 5.607E-08 | 1.449E-07 |
| Unigene47314 | poxtA | 2.437E-06 | 3.589E-06 | 3.208E-06 | 6.183E-06 | 0.000E+00 | 0.000E+00 | 3.619E-07 | 0.000E+00 | 6.498E-08 | 0.000E+00 | 0.000E+00 | 3.513E-08 |

| | | | | | | | | | | | | | |
|---|---|---|---|---|---|---|---|---|---|---|---|---|---|
| Unigene47363 | floR | 9.832E-07 | 6.744E-06 | 9.835E-06 | 1.757E-05 | 0.000E+00 | 0.000E+00 | 0.000E+00 | 1.594E-07 | 0.000E+00 | 0.000E+00 | 0.000E+00 | 0.000E+00 |
| Unigene47369 | tetB(60) | 9.276E-07 | 5.823E-06 | 1.256E-05 | 1.885E-05 | 0.000E+00 | 0.000E+00 | 8.213E-08 | 0.000E+00 | 0.000E+00 | 0.000E+00 | 0.000E+00 | 0.000E+00 |
| Unigene47370 | macB | 8.454E-07 | 5.947E-06 | 1.109E-05 | 1.579E-05 | 0.000E+00 | 2.908E-07 | 0.000E+00 | 4.486E-08 | 0.000E+00 | 0.000E+00 | 0.000E+00 | 0.000E+00 |
| Unigene47400 | emrB | 1.198E-06 | 9.676E-07 | 4.813E-06 | 7.533E-06 | 0.000E+00 | 0.000E+00 | 2.502E-07 | 0.000E+00 | 0.000E+00 | 0.000E+00 | 0.000E+00 | 0.000E+00 |
| Unigene47419 | macB | 2.606E-06 | 1.845E-06 | 2.763E-06 | 1.698E-06 | 0.000E+00 | 0.000E+00 | 3.256E-07 | 4.416E-07 | 7.350E-07 | 0.000E+00 | 0.000E+00 | 0.000E+00 |
| Unigene47442 | oleC | 1.363E-05 | 4.415E-06 | 2.763E-06 | 1.097E-05 | 8.597E-05 | 1.228E-04 | 1.165E-04 | 9.549E-05 | 1.433E-04 | 1.534E-04 | 1.148E-04 | 7.215E-05 |
| Unigene47481 | tetT | 3.747E-06 | 5.285E-06 | 4.375E-06 | 7.848E-06 | 3.440E-07 | 1.004E-07 | 2.913E-07 | 9.036E-08 | 1.354E-07 | 5.174E-08 | 1.948E-07 | 3.660E-08 |
| Unigene47533 | mdtN | 1.430E-06 | 2.728E-06 | 1.838E-06 | 3.782E-07 | 1.226E-05 | 5.153E-07 | 2.902E-06 | 6.757E-07 | 0.000E+00 | 0.000E+00 | 6.622E-07 | 0.000E+00 |
| Unigene47639 | Streptomy | 2.779E-06 | 7.625E-07 | 1.902E-06 | 4.894E-07 | 0.000E+00 | 0.000E+00 | 0.000E+00 | 0.000E+00 | 0.000E+00 | 0.000E+00 | 0.000E+00 | 1.910E-08 |
| Unigene47641 | patA | 2.385E-06 | 8.178E-07 | 1.521E-06 | 4.515E-07 | 0.000E+00 | 0.000E+00 | 0.000E+00 | 1.392E-07 | 0.000E+00 | 0.000E+00 | 0.000E+00 | 0.000E+00 |
| Unigene47654 | dfrE | 1.452E-06 | 6.521E-07 | 7.125E-07 | 1.133E-06 | 0.000E+00 | 0.000E+00 | 0.000E+00 | 0.000E+00 | 0.000E+00 | 0.000E+00 | 0.000E+00 | 0.000E+00 |
| Unigene47655 | TaeA | 2.490E-06 | 7.486E-07 | 1.355E-06 | 3.616E-07 | 0.000E+00 | 0.000E+00 | 0.000E+00 | 9.952E-08 | 0.000E+00 | 0.000E+00 | 0.000E+00 | 2.016E-08 |
| Unigene47722 | vanHB | 1.032E-06 | 1.545E-06 | 5.832E-06 | 7.075E-06 | 0.000E+00 | 0.000E+00 | 1.731E-07 | 0.000E+00 | 0.000E+00 | 0.000E+00 | 0.000E+00 | 0.000E+00 |
| Unigene47849 | msbA | 2.244E-06 | 1.452E-05 | 5.661E-06 | 2.636E-06 | 0.000E+00 | 0.000E+00 | 0.000E+00 | 0.000E+00 | 0.000E+00 | 0.000E+00 | 0.000E+00 | 0.000E+00 |
| Unigene47886 | mdtG | 4.800E-06 | 2.084E-05 | 3.255E-06 | 8.714E-07 | 7.190E-07 | 3.790E-07 | 1.492E-07 | 3.777E-07 | 1.356E-06 | 6.008E-07 | 5.861E-07 | 2.550E-07 |
| Unigene47890 | PmrF | 2.756E-06 | 1.578E-05 | 3.194E-06 | 3.018E-07 | 5.903E-07 | 1.077E-07 | 2.573E-07 | 1.551E-07 | 8.712E-07 | 4.069E-07 | 2.659E-07 | 7.851E-08 |
| Unigene47891 | emeA | 5.130E-06 | 2.115E-05 | 3.919E-06 | 8.121E-07 | 8.318E-07 | 3.897E-07 | 0.000E+00 | 0.000E+00 | 1.455E-06 | 8.959E-07 | 6.662E-07 | 0.000E+00 |
| Unigene47892 | qacH | 5.745E-06 | 2.192E-05 | 3.847E-06 | 1.013E-06 | 1.418E-06 | 0.000E+00 | 3.028E-07 | 0.000E+00 | 8.970E-07 | 0.000E+00 | 0.000E+00 | 0.000E+00 |
| Unigene47917 | vanHB | 4.510E-07 | 6.470E-06 | 1.116E-05 | 1.532E-05 | 0.000E+00 | 0.000E+00 | 0.000E+00 | 0.000E+00 | 0.000E+00 | 0.000E+00 | 0.000E+00 | 0.000E+00 |
| Unigene47923 | srmB | 5.217E-06 | 2.013E-06 | 2.040E-06 | 6.915E-06 | 6.683E-05 | 9.270E-05 | 8.273E-05 | 7.417E-05 | 1.229E-04 | 1.214E-04 | 9.991E-05 | 6.465E-05 |
| Unigene47974 | bcrA | 1.399E-06 | 0.000E+00 | 0.000E+00 | 0.000E+00 | 0.000E+00 | 0.000E+00 | 0.000E+00 | 0.000E+00 | 0.000E+00 | 0.000E+00 | 0.000E+00 | 0.000E+00 |
| Unigene48048 | baeS | 1.138E-06 | 1.344E-06 | 1.370E-06 | 1.540E-06 | 1.823E-05 | 5.967E-06 | 1.410E-05 | 1.682E-05 | 9.507E-06 | 1.754E-05 | 1.828E-05 | 7.514E-06 |
| Unigene48055 | FusF | 1.551E-06 | 1.478E-05 | 5.647E-06 | 3.588E-06 | 0.000E+00 | 0.000E+00 | 0.000E+00 | 0.000E+00 | 0.000E+00 | 0.000E+00 | 1.145E-07 | 0.000E+00 |
| Unigene48081 | ErmA | 1.815E-04 | 2.362E-06 | 2.586E-06 | 8.435E-05 | 0.000E+00 | 2.659E-06 | 5.347E-07 | 1.456E-05 | 0.000E+00 | 2.153E-06 | 4.170E-06 | 0.000E+00 |
| Unigene48172 | Pseudomo | 1.062E-06 | 3.447E-06 | 0.000E+00 | 8.860E-07 | 1.062E-05 | 0.000E+00 | 0.000E+00 | 1.132E-06 | 0.000E+00 | 0.000E+00 | 0.000E+00 | 0.000E+00 |
| Unigene48244 | emrB | 6.978E-07 | 6.325E-06 | 9.277E-06 | 1.543E-05 | 0.000E+00 | 0.000E+00 | 0.000E+00 | 0.000E+00 | 0.000E+00 | 0.000E+00 | 0.000E+00 | 0.000E+00 |
| Unigene48285 | mgrA | 4.472E-05 | 2.113E-05 | 8.475E-06 | 1.072E-05 | 2.196E-06 | 0.000E+00 | 2.997E-06 | 7.690E-07 | 6.880E-06 | 1.142E-06 | 0.000E+00 | 2.336E-06 |
| Unigene48363 | vanSF | 1.420E-06 | 8.692E-07 | 5.199E-06 | 7.474E-06 | 0.000E+00 | 2.952E-08 | 2.116E-07 | 0.000E+00 | 0.000E+00 | 0.000E+00 | 0.000E+00 | 6.456E-08 |
| Unigene48538 | macB | 3.078E-06 | 3.731E-06 | 5.253E-06 | 5.054E-06 | 2.676E-07 | 0.000E+00 | 2.074E-07 | 0.000E+00 | 8.777E-08 | 0.000E+00 | 2.756E-07 | 1.582E-07 |
| Unigene48539 | macB | 1.961E-06 | 3.254E-06 | 3.332E-06 | 3.380E-06 | 0.000E+00 | 0.000E+00 | 5.822E-07 | 5.582E-08 | 1.568E-07 | 0.000E+00 | 2.735E-07 | 0.000E+00 |
| Unigene48541 | vanSG | 2.338E-06 | 3.617E-06 | 5.224E-06 | 4.352E-06 | 2.191E-07 | 3.045E-08 | 4.676E-07 | 0.000E+00 | 1.539E-07 | 0.000E+00 | 1.289E-07 | 1.998E-07 |
| Unigene48556 | cmrA | 5.181E-06 | 1.859E-06 | 5.670E-06 | 3.330E-05 | 1.660E-06 | 0.000E+00 | 7.719E-06 | 0.000E+00 | 0.000E+00 | 0.000E+00 | 6.382E-06 | 1.531E-06 |
| Unigene48576 | mtrA | 2.577E-06 | 4.317E-06 | 2.573E-06 | 4.885E-06 | 4.018E-07 | 4.887E-08 | 0.000E+00 | 0.000E+00 | 0.000E+00 | 0.000E+00 | 0.000E+00 | 0.000E+00 |
| Unigene48577 | arlS | 2.506E-06 | 3.981E-06 | 3.817E-06 | 6.530E-06 | 6.230E-07 | 5.683E-08 | 5.043E-07 | 0.000E+00 | 1.341E-07 | 0.000E+00 | 2.005E-08 | 2.279E-07 |
| Unigene48580 | AIM-1 | 2.520E-06 | 4.171E-06 | 3.258E-06 | 6.907E-06 | 4.888E-07 | 0.000E+00 | 0.000E+00 | 0.000E+00 | 2.623E-07 | 0.000E+00 | 0.000E+00 | 0.000E+00 |
| Unigene48658 | tetA(58) | 2.781E-06 | 2.943E-06 | 2.501E-06 | 5.149E-06 | 5.885E-07 | 0.000E+00 | 4.031E-07 | 0.000E+00 | 3.619E-08 | 0.000E+00 | 1.136E-07 | 1.174E-07 |
| Unigene48678 | lsaC | 1.255E-06 | 8.227E-06 | 5.631E-07 | 5.795E-07 | 3.188E-07 | 2.714E-07 | 3.970E-08 | 4.187E-08 | 7.056E-07 | 4.395E-07 | 2.051E-07 | 1.272E-07 |
| Unigene48691 | tetA(58) | 4.627E-06 | 2.088E-05 | 3.423E-06 | 7.046E-07 | 1.102E-06 | 3.970E-07 | 2.032E-07 | 1.715E-07 | 1.044E-06 | 2.864E-07 | 2.941E-07 | 0.000E+00 |
| Unigene48715 | vanZF | 1.041E-06 | 5.986E-07 | 1.898E-06 | 1.803E-06 | 1.851E-05 | 5.789E-06 | 1.356E-05 | 1.760E-05 | 1.252E-05 | 1.602E-05 | 1.503E-05 | 6.682E-06 |
| Unigene48734 | vmlR | 1.375E-06 | 8.657E-07 | 9.129E-07 | 1.859E-06 | 1.995E-05 | 5.969E-06 | 1.471E-05 | 1.662E-05 | 1.004E-05 | 1.620E-05 | 1.818E-05 | 8.607E-06 |
| Unigene48811 | efpA | 4.508E-06 | 0.000E+00 | 0.000E+00 | 0.000E+00 | 5.385E-08 | 0.000E+00 | 0.000E+00 | 0.000E+00 | 0.000E+00 | 0.000E+00 | 0.000E+00 | 0.000E+00 |
| Unigene48851 | tetA(58) | 3.509E-06 | 4.070E-06 | 5.978E-06 | 4.681E-06 | 3.678E-08 | 1.074E-07 | 0.000E+00 | 0.000E+00 | 0.000E+00 | 0.000E+00 | 7.574E-08 | 7.827E-08 |
| Unigene48893 | lsaA | 2.131E-05 | 3.852E-06 | 6.004E-06 | 4.416E-06 | 6.392E-08 | 1.037E-07 | 0.000E+00 | 2.239E-08 | 0.000E+00 | 0.000E+00 | 0.000E+00 | 0.000E+00 |
| Unigene48948 | vatE | 1.419E-06 | 6.813E-06 | 4.525E-06 | 8.239E-06 | 0.000E+00 | 0.000E+00 | 0.000E+00 | 0.000E+00 | 0.000E+00 | 0.000E+00 | 0.000E+00 | 0.000E+00 |
| Unigene49090 | patB | 4.697E-06 | 1.176E-05 | 1.177E-05 | 1.302E-05 | 0.000E+00 | 9.232E-08 | 0.000E+00 | 0.000E+00 | 0.000E+00 | 0.000E+00 | 9.769E-08 | 5.047E-08 |
| Unigene49096 | Chlamydia | 1.882E-06 | 2.568E-06 | 6.770E-06 | 8.503E-06 | 0.000E+00 | 0.000E+00 | 3.738E-07 | 0.000E+00 | 0.000E+00 | 0.000E+00 | 8.279E-08 | 8.555E-08 |
| Unigene49122 | Acinetoba | 6.843E-06 | 0.000E+00 | 0.000E+00 | 2.646E-06 | 3.513E-05 | 9.322E-06 | 2.399E-05 | 2.568E-05 | 3.531E-06 | 0.000E+00 | 0.000E+00 | 0.000E+00 |
| Unigene49163 | bcrA | 3.725E-06 | 9.856E-06 | 9.787E-06 | 1.070E-05 | 1.839E-07 | 0.000E+00 | 0.000E+00 | 0.000E+00 | 0.000E+00 | 0.000E+00 | 0.000E+00 | 0.000E+00 |
| Unigene49175 | vanSG | 2.519E-06 | 5.732E-06 | 7.803E-06 | 9.408E-06 | 0.000E+00 | 2.837E-08 | 0.000E+00 | 0.000E+00 | 0.000E+00 | 0.000E+00 | 0.000E+00 | 0.000E+00 |
| Unigene49196 | macB | 4.914E-06 | 1.158E-05 | 1.388E-05 | 1.591E-05 | 1.412E-07 | 0.000E+00 | 2.813E-07 | 0.000E+00 | 4.630E-08 | 3.303E-07 | 0.000E+00 | 0.000E+00 |
| Unigene49199 | cmeR | 4.187E-06 | 1.117E-05 | 1.290E-05 | 1.563E-05 | 0.000E+00 | 1.053E-07 | 0.000E+00 | 0.000E+00 | 0.000E+00 | 0.000E+00 | 1.114E-07 | 0.000E+00 |
| Unigene49250 | smeS | 6.343E-07 | 4.383E-07 | 6.455E-07 | 5.931E-07 | 8.091E-07 | 1.194E-06 | 3.744E-06 | 3.483E-06 | 4.879E-07 | 9.945E-07 | 2.177E-06 | 3.693E-06 |
| Unigene49251 | vanRO | 4.761E-07 | 5.748E-07 | 0.000E+00 | 6.731E-07 | 8.463E-07 | 1.544E-06 | 4.690E-06 | 5.113E-06 | 0.000E+00 | 2.068E-06 | 2.778E-06 | 4.784E-06 |
| Unigene49255 | mgrA | 6.950E-05 | 5.467E-05 | 1.082E-04 | 1.620E-04 | 1.222E-04 | 8.479E-05 | 1.102E-04 | 1.081E-04 | 9.815E-05 | 1.319E-04 | 1.357E-04 | 1.389E-04 |
| Unigene49278 | lsaC | 4.353E-06 | 2.080E-05 | 2.732E-06 | 2.184E-07 | 7.208E-07 | 2.046E-07 | 2.992E-08 | 2.840E-07 | 1.123E-06 | 4.819E-07 | 1.237E-07 | 1.278E-07 |
| Unigene49371 | tetA(58) | 7.085E-07 | 5.832E-07 | 1.695E-06 | 8.049E-07 | 0.000E+00 | 0.000E+00 | 0.000E+00 | 0.000E+00 | 0.000E+00 | 0.000E+00 | 0.000E+00 | 0.000E+00 |
| Unigene49376 | Agrobacte | 5.124E-07 | 0.000E+00 | 0.000E+00 | 3.449E-07 | 0.000E+00 | 9.170E-06 | 5.041E-07 | 0.000E+00 | 5.600E-07 | 4.313E-06 | 0.000E+00 | 5.384E-07 |
| Unigene49400 | Streptomy | 1.113E-05 | 4.025E-06 | 2.857E-06 | 1.052E-05 | 7.012E-05 | 1.026E-04 | 1.000E-04 | 7.855E-05 | 1.243E-04 | 1.188E-04 | 9.628E-05 | 6.707E-05 |

| | | | | | | | | | | | | | |
|---|---|---|---|---|---|---|---|---|---|---|---|---|---|
| Unigene49419 | bcrA | 4.270E-07 | 0.000E+00 | 9.636E-07 | 0.000E+00 | 6.356E-06 | 0.000E+00 | 3.308E-07 | 0.000E+00 | 0.000E+00 | 0.000E+00 | 0.000E+00 | 0.000E+00 |
| Unigene49539 | tlrC | 1.425E-06 | 0.000E+00 | 0.000E+00 | 0.000E+00 | 0.000E+00 | 0.000E+00 | 0.000E+00 | 0.000E+00 | 0.000E+00 | 0.000E+00 | 0.000E+00 | 1.359E-08 |
| Unigene49572 | efpA | 7.313E-07 | 5.733E-07 | 1.640E-06 | 1.055E-06 | 2.901E-08 | 0.000E+00 | 2.890E-08 | 0.000E+00 | 0.000E+00 | 0.000E+00 | 0.000E+00 | 0.000E+00 |
| Unigene49620 | lsaB | 9.099E-07 | 4.211E-06 | 3.494E-06 | 6.272E-06 | 0.000E+00 | 0.000E+00 | 0.000E+00 | 0.000E+00 | 0.000E+00 | 0.000E+00 | 0.000E+00 | 0.000E+00 |
| Unigene49646 | RlmA(II) | 2.612E-06 | 2.824E-06 | 4.361E-06 | 3.071E-06 | 8.662E-08 | 0.000E+00 | 4.315E-08 | 0.000E+00 | 0.000E+00 | 3.040E-07 | 0.000E+00 | 4.608E-08 |
| Unigene49650 | macB | 2.519E-06 | 4.124E-06 | 3.352E-06 | 5.648E-06 | 0.000E+00 | 0.000E+00 | 7.089E-07 | 0.000E+00 | 2.800E-07 | 0.000E+00 | 0.000E+00 | 0.000E+00 |
| Unigene49825 | msbA | 4.649E-07 | 0.000E+00 | 1.303E-07 | 0.000E+00 | 0.000E+00 | 1.436E-07 | 0.000E+00 | 0.000E+00 | 0.000E+00 | 0.000E+00 | 0.000E+00 | 0.000E+00 |
| Unigene49858 | macB | 2.371E-05 | 1.136E-06 | 6.497E-07 | 0.000E+00 | 0.000E+00 | 0.000E+00 | 0.000E+00 | 0.000E+00 | 0.000E+00 | 3.227E-07 | 0.000E+00 | 0.000E+00 |
| Unigene49960 | lmrB | 3.355E-06 | 4.825E-07 | 3.678E-06 | 5.141E-07 | 3.342E-07 | 0.000E+00 | 2.305E-07 | 0.000E+00 | 0.000E+00 | 5.155E-08 | 8.735E-07 | 0.000E+00 |
| Unigene49994 | macB | 1.410E-06 | 4.074E-06 | 3.366E-06 | 5.219E-06 | 0.000E+00 | 0.000E+00 | 0.000E+00 | 0.000E+00 | 0.000E+00 | 0.000E+00 | 0.000E+00 | 0.000E+00 |
| Unigene50014 | Staphylocc | 1.155E-05 | 0.000E+00 | 0.000E+00 | 0.000E+00 | 1.898E-05 | 1.781E-05 | 0.000E+00 | 0.000E+00 | 1.301E-05 | 2.211E-07 | 0.000E+00 | 0.000E+00 |
| Unigene50022 | Listeria mc | 1.267E-05 | 0.000E+00 | 0.000E+00 | 0.000E+00 | 1.903E-05 | 1.848E-05 | 0.000E+00 | 0.000E+00 | 1.243E-05 | 3.611E-07 | 0.000E+00 | 0.000E+00 |
| Unigene50127 | patB | 4.173E-06 | 1.096E-05 | 8.993E-06 | 1.103E-05 | 2.727E-07 | 8.846E-08 | 0.000E+00 | 1.433E-07 | 0.000E+00 | 0.000E+00 | 0.000E+00 | 0.000E+00 |
| Unigene50145 | lmrB | 1.369E-05 | 5.164E-06 | 3.578E-06 | 1.262E-05 | 8.743E-05 | 1.197E-04 | 1.286E-04 | 1.031E-04 | 1.522E-04 | 1.511E-04 | 1.253E-04 | 7.754E-05 |
| Unigene50166 | Escherichia | 1.195E-05 | 3.193E-06 | 3.192E-06 | 1.226E-05 | 7.725E-05 | 1.069E-04 | 1.084E-04 | 8.503E-05 | 1.358E-04 | 1.370E-04 | 1.117E-04 | 7.206E-05 |
| Unigene50169 | lmrD | 1.120E-05 | 5.474E-06 | 3.285E-06 | 1.247E-05 | 8.104E-05 | 1.121E-04 | 1.124E-04 | 9.165E-05 | 1.366E-04 | 1.379E-04 | 1.112E-04 | 6.795E-05 |
| Unigene50222 | Streptomy | 1.376E-05 | 5.841E-06 | 3.189E-06 | 1.353E-05 | 9.650E-05 | 1.399E-04 | 1.400E-04 | 1.213E-04 | 1.772E-04 | 1.777E-04 | 1.416E-04 | 8.962E-05 |
| Unigene50244 | mtrA | 1.190E-05 | 4.654E-06 | 3.445E-06 | 1.275E-05 | 9.188E-05 | 1.321E-04 | 1.291E-04 | 1.173E-04 | 1.663E-04 | 1.693E-04 | 1.314E-04 | 8.601E-05 |
| Unigene50245 | arlS | 1.500E-05 | 6.065E-06 | 3.919E-06 | 1.417E-05 | 1.045E-04 | 1.449E-04 | 1.489E-04 | 1.271E-04 | 1.874E-04 | 1.849E-04 | 1.506E-04 | 9.788E-05 |
| Unigene50274 | tetA(58) | 7.559E-07 | 2.001E-06 | 0.000E+00 | 0.000E+00 | 7.360E-06 | 8.171E-07 | 1.624E-06 | 0.000E+00 | 0.000E+00 | 0.000E+00 | 0.000E+00 | 0.000E+00 |
| Unigene50308 | efrB | 0.000E+00 | 1.328E-06 | 8.480E-07 | 0.000E+00 | 6.385E-06 | 0.000E+00 | 0.000E+00 | 3.531E-07 | 0.000E+00 | 0.000E+00 | 0.000E+00 | 0.000E+00 |
| Unigene50410 | bacA | 3.322E-06 | 0.000E+00 | 3.371E-06 | 6.100E-07 | 2.097E-07 | 0.000E+00 | 2.925E-07 | 0.000E+00 | 0.000E+00 | 0.000E+00 | 7.342E-07 | 0.000E+00 |
| Unigene50426 | BJP-1 | 0.000E+00 | 4.921E-07 | 4.799E-07 | 0.000E+00 | 0.000E+00 | 0.000E+00 | 3.338E-06 | 2.712E-06 | 0.000E+00 | 1.226E-06 | 9.791E-07 | 2.987E-06 |
| Unigene50469 | arnA | 2.926E-05 | 9.062E-06 | 6.730E-06 | 4.235E-06 | 3.611E-07 | 2.811E-07 | 0.000E+00 | 0.000E+00 | 1.279E-06 | 0.000E+00 | 0.000E+00 | 0.000E+00 |
| Unigene50486 | tetB(58) | 4.141E-05 | 5.762E-05 | 2.929E-05 | 1.421E-05 | 1.386E-05 | 4.979E-06 | 2.317E-06 | 4.911E-05 | 1.194E-05 | 6.017E-06 | 1.485E-06 | 1.386E-06 |
| Unigene50487 | tetA(58) | 4.180E-05 | 5.761E-05 | 2.311E-05 | 1.424E-05 | 1.448E-05 | 6.152E-06 | 3.702E-06 | 5.487E-05 | 1.293E-05 | 7.453E-06 | 1.144E-06 | 6.930E-07 |
| Unigene50494 | Staphylocc | 3.953E-05 | 5.856E-05 | 2.726E-05 | 1.232E-05 | 1.286E-05 | 6.538E-06 | 2.897E-06 | 5.082E-05 | 1.187E-05 | 7.729E-06 | 1.078E-06 | 9.284E-07 |
| Unigene50495 | Staphylocc | 2.180E-05 | 3.900E-05 | 0.000E+00 | 0.000E+00 | 0.000E+00 | 0.000E+00 | 0.000E+00 | 4.462E-05 | 0.000E+00 | 0.000E+00 | 0.000E+00 | 0.000E+00 |
| Unigene50512 | macB | 3.973E-05 | 5.874E-05 | 2.582E-05 | 1.305E-05 | 1.467E-05 | 5.995E-06 | 3.076E-06 | 5.292E-05 | 1.161E-05 | 6.675E-06 | 1.487E-06 | 6.329E-07 |
| Unigene50525 | msbA | 4.301E-06 | 8.207E-06 | 8.897E-06 | 1.049E-05 | 6.660E-08 | 1.620E-07 | 6.634E-08 | 0.000E+00 | 0.000E+00 | 1.669E-07 | 5.143E-08 | 0.000E+00 |
| Unigene50661 | vanSO | 1.921E-07 | 0.000E+00 | 0.000E+00 | 0.000E+00 | 0.000E+00 | 0.000E+00 | 0.000E+00 | 0.000E+00 | 0.000E+00 | 0.000E+00 | 0.000E+00 | 0.000E+00 |
| Unigene50676 | RlmA(II) | 1.030E-06 | 6.952E-06 | 9.398E-06 | 1.603E-05 | 0.000E+00 | 8.250E-08 | 0.000E+00 | 8.908E-08 | 0.000E+00 | 0.000E+00 | 0.000E+00 | 0.000E+00 |
| Unigene50686 | facT | 5.332E-07 | 0.000E+00 | 2.429E-07 | 4.231E-07 | 4.041E-06 | 8.009E-06 | 1.349E-06 | 6.869E-06 | 3.414E-06 | 0.000E+00 | 0.000E+00 | 8.554E-07 |
| Unigene50731 | YojI | 2.147E-05 | 8.479E-06 | 6.046E-06 | 4.318E-06 | 0.000E+00 | 0.000E+00 | 0.000E+00 | 0.000E+00 | 4.021E-07 | 0.000E+00 | 0.000E+00 | 0.000E+00 |
| Unigene50757 | vgaALC | 3.969E-06 | 7.611E-06 | 9.010E-06 | 9.707E-06 | 1.063E-07 | 0.000E+00 | 0.000E+00 | 0.000E+00 | 0.000E+00 | 0.000E+00 | 0.000E+00 | 0.000E+00 |
| Unigene50797 | MdtK | 3.402E-06 | 4.766E-06 | 5.445E-06 | 6.627E-06 | 2.680E-08 | 0.000E+00 | 0.000E+00 | 0.000E+00 | 0.000E+00 | 8.063E-08 | 0.000E+00 | 0.000E+00 |
| Unigene50827 | bcrA | 2.597E-05 | 8.693E-06 | 7.617E-06 | 4.111E-06 | 0.000E+00 | 1.706E-07 | 0.000E+00 | 7.366E-08 | 0.000E+00 | 0.000E+00 | 0.000E+00 | 0.000E+00 |
| Unigene50932 | optrA | 1.450E-06 | 1.129E-06 | 1.390E-06 | 1.703E-06 | 1.922E-05 | 6.126E-06 | 1.493E-05 | 1.756E-05 | 1.217E-05 | 1.892E-05 | 1.817E-05 | 8.831E-06 |
| Unigene50994 | macB | 3.714E-06 | 1.677E-05 | 2.569E-06 | 9.616E-07 | 6.876E-07 | 2.573E-07 | 2.108E-07 | 2.223E-07 | 1.145E-06 | 0.000E+00 | 0.000E+00 | 0.000E+00 |
| Unigene51007 | msrC | 4.221E-06 | 2.008E-05 | 2.883E-06 | 5.851E-07 | 1.287E-06 | 2.684E-07 | 3.206E-07 | 4.348E-07 | 1.312E-06 | 3.688E-07 | 1.420E-07 | 0.000E+00 |
| Unigene51021 | evgA | 5.226E-06 | 2.176E-05 | 2.866E-06 | 0.000E+00 | 8.539E-07 | 0.000E+00 | 0.000E+00 | 0.000E+00 | 1.176E-06 | 5.137E-07 | 0.000E+00 | 0.000E+00 |
| Unigene51042 | lmrD | 1.219E-05 | 4.400E-06 | 2.567E-06 | 1.270E-05 | 8.532E-05 | 1.176E-04 | 1.165E-04 | 1.013E-04 | 1.484E-04 | 1.491E-04 | 1.190E-04 | 7.626E-05 |
| Unigene51229 | macB | 0.000E+00 | 0.000E+00 | 0.000E+00 | 0.000E+00 | 0.000E+00 | 0.000E+00 | 0.000E+00 | 3.731E-07 | 0.000E+00 | 0.000E+00 | 0.000E+00 | 1.007E-06 |
| Unigene51240 | rpoB2 | 2.849E-06 | 4.179E-06 | 3.508E-06 | 2.008E-06 | 2.787E-06 | 1.976E-06 | 1.854E-06 | 1.286E-06 | 1.958E-06 | 5.719E-06 | 5.688E-06 | 1.790E-06 |
| Unigene51249 | Acinetobac | 1.836E-06 | 8.034E-06 | 4.721E-06 | 8.463E-06 | 2.874E-08 | 0.000E+00 | 0.000E+00 | 0.000E+00 | 0.000E+00 | 0.000E+00 | 0.000E+00 | 0.000E+00 |
| Unigene51265 | vgaB | 8.339E-06 | 0.000E+00 | 0.000E+00 | 2.037E-06 | 3.765E-05 | 1.827E-05 | 2.630E-05 | 2.993E-05 | 3.675E-06 | 0.000E+00 | 0.000E+00 | 0.000E+00 |
| Unigene51278 | optrA | 7.862E-06 | 0.000E+00 | 0.000E+00 | 2.232E-06 | 3.399E-05 | 1.745E-05 | 2.421E-05 | 2.639E-05 | 3.305E-06 | 0.000E+00 | 0.000E+00 | 0.000E+00 |
| Unigene51343 | pmrA | 1.076E-06 | 9.993E-07 | 4.873E-06 | 6.687E-06 | 0.000E+00 | 0.000E+00 | 0.000E+00 | 0.000E+00 | 0.000E+00 | 0.000E+00 | 9.468E-08 | 0.000E+00 |
| Unigene51355 | macB | 4.448E-06 | 1.995E-05 | 3.367E-06 | 9.839E-07 | 1.224E-06 | 6.411E-07 | 2.813E-07 | 0.000E+00 | 1.667E-06 | 3.303E-07 | 0.000E+00 | 0.000E+00 |
| Unigene51382 | sul4 | 2.993E-06 | 3.752E-06 | 2.481E-06 | 1.961E-06 | 2.831E-06 | 1.968E-06 | 2.529E-06 | 1.062E-06 | 1.415E-06 | 5.903E-06 | 5.830E-06 | 2.080E-06 |
| Unigene51491 | NmcR | 6.815E-07 | 0.000E+00 | 0.000E+00 | 0.000E+00 | 0.000E+00 | 0.000E+00 | 0.000E+00 | 0.000E+00 | 0.000E+00 | 0.000E+00 | 0.000E+00 | 0.000E+00 |
| Unigene51530 | tetT | 2.615E-06 | 3.583E-06 | 3.531E-06 | 5.123E-06 | 3.120E-07 | 3.795E-08 | 3.303E-07 | 2.049E-08 | 1.151E-07 | 0.000E+00 | 0.000E+00 | 0.000E+00 |
| Unigene51532 | arlR | 1.885E-06 | 3.482E-06 | 2.617E-06 | 2.879E-06 | 6.130E-07 | 0.000E+00 | 2.545E-07 | 0.000E+00 | 0.000E+00 | 0.000E+00 | 0.000E+00 | 1.631E-07 |
| Unigene51593 | TaeA | 2.664E-05 | 9.113E-07 | 0.000E+00 | 0.000E+00 | 0.000E+00 | 0.000E+00 | 0.000E+00 | 0.000E+00 | 0.000E+00 | 0.000E+00 | 0.000E+00 | 0.000E+00 |
| Unigene51607 | lsaC | 3.074E-07 | 0.000E+00 | 0.000E+00 | 0.000E+00 | 0.000E+00 | 0.000E+00 | 0.000E+00 | 0.000E+00 | 0.000E+00 | 0.000E+00 | 0.000E+00 | 0.000E+00 |
| Unigene51628 | tetA(46) | 2.605E-06 | 4.413E-06 | 3.760E-06 | 6.157E-06 | 4.404E-07 | 6.122E-08 | 4.178E-07 | 0.000E+00 | 6.190E-08 | 0.000E+00 | 0.000E+00 | 2.454E-07 |
| Unigene51817 | patB | 2.618E-06 | 7.758E-06 | 8.375E-06 | 1.050E-05 | 8.726E-08 | 1.415E-07 | 4.346E-07 | 0.000E+00 | 0.000E+00 | 0.000E+00 | 0.000E+00 | 0.000E+00 |
| Unigene51873 | farB | 4.575E-06 | 2.039E-05 | 3.722E-06 | 9.751E-07 | 8.429E-07 | 2.983E-07 | 2.799E-07 | 2.147E-07 | 1.483E-06 | 0.000E+00 | 3.156E-07 | 0.000E+00 |

| | | | | | | | | | | | | | |
|---|---|---|---|---|---|---|---|---|---|---|---|---|---|
| Unigene51919 | vanHO | 3.565E-06 | 3.486E-06 | 2.544E-06 | 1.931E-06 | 2.196E-06 | 1.542E-06 | 2.511E-06 | 9.836E-07 | 1.134E-06 | 6.860E-06 | 4.597E-06 | 2.299E-06 |
| Unigene51943 | Streptomy | 2.923E-05 | 2.022E-05 | 1.716E-05 | 1.954E-05 | 1.258E-06 | 1.260E-06 | 2.544E-06 | 7.194E-07 | 4.387E-06 | 0.000E+00 | 1.124E-06 | 1.043E-06 |
| Unigene51960 | vanHO | 3.096E-07 | 1.317E-06 | 1.785E-06 | 1.681E-06 | 0.000E+00 | 0.000E+00 | 0.000E+00 | 0.000E+00 | 0.000E+00 | 0.000E+00 | 0.000E+00 | 0.000E+00 |
| Unigene52028 | arlS | 3.348E-06 | 4.127E-06 | 2.368E-06 | 2.019E-06 | 2.391E-06 | 2.162E-06 | 2.141E-06 | 1.040E-06 | 1.283E-06 | 5.279E-06 | 5.769E-06 | 1.953E-06 |
| Unigene52136 | tetA(46) | 1.360E-06 | 1.285E-05 | 4.126E-06 | 2.748E-06 | 0.000E+00 | 8.914E-08 | 0.000E+00 | 0.000E+00 | 0.000E+00 | 0.000E+00 | 0.000E+00 | 0.000E+00 |
| Unigene52160 | mecA | 1.275E-06 | 1.531E-05 | 6.898E-06 | 4.733E-06 | 0.000E+00 | 0.000E+00 | 5.293E-08 | 0.000E+00 | 1.742E-08 | 0.000E+00 | 3.647E-08 | 1.884E-08 |
| Unigene52166 | bcrA | 1.455E-06 | 1.474E-05 | 8.898E-06 | 5.330E-06 | 0.000E+00 | 0.000E+00 | 0.000E+00 | 0.000E+00 | 0.000E+00 | 0.000E+00 | 0.000E+00 | 0.000E+00 |
| Unigene52176 | PEDO-2 | 1.948E-06 | 1.368E-05 | 7.118E-06 | 3.737E-06 | 0.000E+00 | 0.000E+00 | 0.000E+00 | 0.000E+00 | 0.000E+00 | 0.000E+00 | 0.000E+00 | 0.000E+00 |
| Unigene52178 | bacA | 1.780E-06 | 1.483E-05 | 8.090E-06 | 4.651E-06 | 0.000E+00 | 1.674E-07 | 0.000E+00 | 0.000E+00 | 0.000E+00 | 0.000E+00 | 1.771E-07 | 0.000E+00 |
| Unigene52220 | macB | 3.701E-06 | 2.228E-07 | 3.540E-06 | 4.573E-07 | 3.123E-07 | 0.000E+00 | 3.111E-07 | 0.000E+00 | 0.000E+00 | 0.000E+00 | 8.932E-07 | 0.000E+00 |
| Unigene52224 | patA | 8.687E-06 | 2.468E-06 | 1.843E-05 | 1.027E-04 | 5.781E-06 | 4.720E-06 | 1.249E-05 | 0.000E+00 | 9.640E-08 | 1.292E-05 | 7.466E-06 | 3.180E-06 |
| Unigene52258 | adeR | 2.731E-07 | 4.196E-07 | 1.072E-07 | 0.000E+00 | 0.000E+00 | 0.000E+00 | 0.000E+00 | 0.000E+00 | 0.000E+00 | 0.000E+00 | 0.000E+00 | 0.000E+00 |
| Unigene52334 | lsaC | 4.505E-06 | 1.026E-05 | 1.275E-05 | 1.229E-05 | 1.241E-07 | 3.622E-07 | 8.241E-07 | 1.738E-07 | 0.000E+00 | 2.489E-07 | 0.000E+00 | 8.802E-08 |
| Unigene52437 | carA | 4.672E-05 | 6.484E-05 | 2.554E-05 | 1.313E-05 | 1.886E-05 | 5.383E-06 | 3.144E-06 | 5.632E-05 | 1.249E-05 | 7.259E-06 | 1.988E-06 | 1.027E-06 |
| Unigene52472 | Staphylocc | 3.429E-06 | 3.928E-07 | 3.047E-06 | 3.498E-07 | 3.335E-07 | 7.488E-08 | 3.578E-07 | 0.000E+00 | 0.000E+00 | 0.000E+00 | 6.339E-07 | 0.000E+00 |
| Unigene52570 | PmrF | 1.432E-06 | 1.060E-06 | 1.275E-06 | 1.581E-06 | 1.910E-05 | 7.563E-06 | 1.272E-05 | 1.653E-05 | 1.030E-05 | 1.703E-05 | 1.635E-05 | 7.324E-06 |
| Unigene52621 | poxtA | 1.103E-05 | 8.511E-08 | 2.854E-07 | 0.000E+00 | 0.000E+00 | 8.384E-08 | 0.000E+00 | 0.000E+00 | 0.000E+00 | 0.000E+00 | 0.000E+00 | 0.000E+00 |
| Unigene52632 | lsaC | 2.421E-06 | 2.903E-06 | 1.980E-06 | 1.403E-06 | 2.092E-06 | 1.309E-06 | 1.340E-06 | 5.757E-07 | 7.840E-07 | 5.444E-06 | 4.308E-06 | 1.166E-06 |
| Unigene52652 | arr-4 | 0.000E+00 | 1.784E-06 | 0.000E+00 | 1.642E-06 | 0.000E+00 | 0.000E+00 | 0.000E+00 | 0.000E+00 | 0.000E+00 | 0.000E+00 | 0.000E+00 | 0.000E+00 |
| Unigene52672 | YojI | 2.183E-06 | 1.872E-05 | 7.957E-06 | 5.701E-06 | 0.000E+00 | 1.349E-07 | 0.000E+00 | 0.000E+00 | 0.000E+00 | 0.000E+00 | 0.000E+00 | 0.000E+00 |
| Unigene52806 | facT | 6.660E-06 | 0.000E+00 | 0.000E+00 | 0.000E+00 | 0.000E+00 | 0.000E+00 | 0.000E+00 | 0.000E+00 | 2.028E-08 | 0.000E+00 | 0.000E+00 | 0.000E+00 |
| Unigene52842 | mtrA | 3.775E-06 | 4.817E-06 | 7.594E-06 | 8.721E-06 | 0.000E+00 | 2.041E-07 | 0.000E+00 | 0.000E+00 | 0.000E+00 | 0.000E+00 | 0.000E+00 | 0.000E+00 |
| Unigene52843 | baeS | 3.860E-06 | 8.084E-06 | 9.905E-06 | 1.178E-05 | 0.000E+00 | 4.797E-08 | 0.000E+00 | 0.000E+00 | 0.000E+00 | 0.000E+00 | 2.538E-08 | 0.000E+00 |
| Unigene52889 | sul3 | 1.270E-05 | 6.013E-06 | 3.363E-06 | 8.029E-06 | 9.523E-05 | 1.245E-04 | 1.319E-04 | 1.053E-04 | 1.676E-04 | 1.641E-04 | 1.247E-04 | 8.229E-05 |
| Unigene52900 | evgS | 4.978E-07 | 0.000E+00 | 3.006E-07 | 0.000E+00 | 0.000E+00 | 0.000E+00 | 0.000E+00 | 0.000E+00 | 0.000E+00 | 0.000E+00 | 0.000E+00 | 0.000E+00 |
| Unigene53007 | bcrA | 8.678E-07 | 3.762E-06 | 2.682E-06 | 4.250E-06 | 0.000E+00 | 0.000E+00 | 0.000E+00 | 0.000E+00 | 0.000E+00 | 0.000E+00 | 0.000E+00 | 0.000E+00 |
| Unigene53011 | efrA | 4.442E-06 | 1.125E-05 | 9.969E-06 | 1.166E-05 | 2.427E-07 | 1.181E-07 | 6.045E-08 | 0.000E+00 | 0.000E+00 | 2.028E-07 | 0.000E+00 | 0.000E+00 |
| Unigene53018 | Staphylocc | 8.664E-07 | 6.503E-06 | 1.091E-05 | 1.603E-05 | 0.000E+00 | 7.553E-08 | 6.186E-08 | 0.000E+00 | 0.000E+00 | 0.000E+00 | 0.000E+00 | 0.000E+00 |
| Unigene53034 | bacA | 3.865E-06 | 3.826E-06 | 1.970E-06 | 1.951E-06 | 2.483E-06 | 2.212E-06 | 2.516E-06 | 0.000E+00 | 1.491E-06 | 0.000E+00 | 0.000E+00 | 1.612E-06 |
| Unigene53045 | Erm(48) | 4.174E-06 | 3.594E-06 | 2.896E-06 | 2.071E-06 | 2.519E-06 | 2.334E-06 | 2.669E-06 | 7.561E-07 | 1.652E-06 | 7.056E-06 | 6.257E-06 | 2.212E-06 |
| Unigene53059 | efmA | 2.429E-05 | 0.000E+00 | 0.000E+00 | 0.000E+00 | 0.000E+00 | 2.803E-08 | 0.000E+00 | 0.000E+00 | 0.000E+00 | 8.665E-08 | 0.000E+00 | 1.226E-07 |
| Unigene53287 | marA | 2.296E-06 | 8.533E-06 | 6.438E-06 | 9.607E-06 | 0.000E+00 | 0.000E+00 | 0.000E+00 | 0.000E+00 | 0.000E+00 | 0.000E+00 | 0.000E+00 | 0.000E+00 |
| Unigene53345 | patB | 4.024E-06 | 1.874E-05 | 3.575E-06 | 7.272E-07 | 8.471E-07 | 5.038E-07 | 0.000E+00 | 3.461E-07 | 1.204E-06 | 5.663E-07 | 6.784E-07 | 0.000E+00 |
| Unigene53346 | patB | 5.619E-06 | 2.169E-05 | 3.871E-06 | 8.451E-07 | 1.594E-06 | 1.723E-07 | 3.087E-07 | 3.256E-07 | 1.655E-06 | 8.879E-07 | 1.823E-07 | 0.000E+00 |
| Unigene53378 | vanSF | 1.085E-06 | 6.171E-06 | 3.753E-06 | 8.214E-06 | 0.000E+00 | 0.000E+00 | 3.159E-08 | 0.000E+00 | 0.000E+00 | 0.000E+00 | 0.000E+00 | 0.000E+00 |
| Unigene53449 | pmrA | 9.169E-07 | 5.683E-06 | 1.008E-05 | 1.501E-05 | 0.000E+00 | 0.000E+00 | 0.000E+00 | 0.000E+00 | 0.000E+00 | 0.000E+00 | 0.000E+00 | 0.000E+00 |
| Unigene53464 | ANT(6)-Ia | 4.857E-07 | 7.373E-05 | 9.114E-05 | 1.509E-07 | 8.716E-07 | 3.635E-07 | 0.000E+00 | 0.000E+00 | 6.954E-05 | 0.000E+00 | 0.000E+00 | 2.650E-07 |
| Unigene53477 | PmrF | 6.706E-05 | 5.297E-05 | 1.037E-04 | 1.711E-04 | 1.190E-04 | 8.539E-05 | 1.119E-04 | 1.002E-04 | 0.000E+00 | 1.261E-04 | 1.372E-04 | 1.399E-04 |
| Unigene53513 | lmrB | 5.109E-06 | 1.062E-05 | 1.017E-05 | 1.205E-05 | 2.616E-07 | 0.000E+00 | 0.000E+00 | 0.000E+00 | 0.000E+00 | 2.361E-07 | 0.000E+00 | 0.000E+00 |
| Unigene53514 | cmrA | 5.313E-06 | 9.436E-06 | 9.903E-06 | 1.068E-05 | 0.000E+00 | 0.000E+00 | 5.910E-08 | 0.000E+00 | 0.000E+00 | 0.000E+00 | 0.000E+00 | 0.000E+00 |
| Unigene53559 | macB | 8.421E-06 | 0.000E+00 | 0.000E+00 | 1.842E-06 | 3.914E-05 | 1.603E-05 | 2.144E-05 | 3.249E-05 | 2.608E-06 | 0.000E+00 | 0.000E+00 | 0.000E+00 |
| Unigene53560 | macB | 6.993E-06 | 0.000E+00 | 0.000E+00 | 1.587E-06 | 3.315E-05 | 1.872E-05 | 2.709E-05 | 2.638E-05 | 4.532E-06 | 0.000E+00 | 0.000E+00 | 0.000E+00 |
| Unigene53635 | lmrC | 9.517E-07 | 6.469E-06 | 1.050E-05 | 2.403E-05 | 3.411E-08 | 1.659E-08 | 1.699E-08 | 0.000E+00 | 1.678E-08 | 0.000E+00 | 0.000E+00 | 1.814E-08 |
| Unigene53702 | tetA(46) | 3.751E-06 | 1.076E-05 | 1.082E-05 | 1.210E-05 | 0.000E+00 | 0.000E+00 | 3.219E-08 | 0.000E+00 | 0.000E+00 | 9.719E-08 | 0.000E+00 | 0.000E+00 |
| Unigene53715 | otr(B) | 8.880E-07 | 6.965E-06 | 1.112E-05 | 1.712E-05 | 0.000E+00 | 2.823E-08 | 0.000E+00 | 0.000E+00 | 0.000E+00 | 0.000E+00 | 0.000E+00 | 0.000E+00 |
| Unigene53761 | vanHO | 3.748E-06 | 2.482E-07 | 3.773E-06 | 4.568E-07 | 0.000E+00 | 0.000E+00 | 5.007E-07 | 0.000E+00 | 0.000E+00 | 0.000E+00 | 7.023E-07 | 0.000E+00 |
| Unigene53762 | vanHO | 4.188E-06 | 6.011E-07 | 3.983E-06 | 4.880E-07 | 2.863E-07 | 0.000E+00 | 2.139E-07 | 0.000E+00 | 0.000E+00 | 0.000E+00 | 1.069E-06 | 0.000E+00 |
| Unigene53791 | vmlR | 7.173E-07 | 0.000E+00 | 1.760E-07 | 0.000E+00 | 4.064E-06 | 9.344E-06 | 1.747E-06 | 5.610E-06 | 4.391E-06 | 0.000E+00 | 0.000E+00 | 0.000E+00 |
| Unigene53815 | vmlR | 4.343E-07 | 0.000E+00 | 0.000E+00 | 5.847E-07 | 0.000E+00 | 5.738E-07 | 0.000E+00 | 3.379E-07 | 4.746E-07 | 0.000E+00 | 0.000E+00 | 0.000E+00 |
| Unigene53819 | tetA(58) | 2.818E-05 | 8.252E-06 | 7.885E-06 | 4.702E-06 | 4.215E-07 | 0.000E+00 | 0.000E+00 | 0.000E+00 | 0.000E+00 | 0.000E+00 | 0.000E+00 | 0.000E+00 |
| Unigene53820 | tetB(58) | 2.686E-05 | 9.993E-06 | 6.254E-06 | 4.639E-06 | 0.000E+00 | 0.000E+00 | 9.161E-08 | 9.661E-08 | 0.000E+00 | 0.000E+00 | 0.000E+00 | 4.892E-08 |
| Unigene53864 | optrA | 7.326E-06 | 0.000E+00 | 0.000E+00 | 2.185E-06 | 3.432E-05 | 1.691E-05 | 2.188E-05 | 2.577E-05 | 2.799E-06 | 0.000E+00 | 0.000E+00 | 0.000E+00 |
| Unigene53896 | emrY | 2.315E-06 | 1.839E-05 | 7.195E-06 | 4.745E-06 | 0.000E+00 | 7.362E-08 | 0.000E+00 | 0.000E+00 | 0.000E+00 | 0.000E+00 | 2.597E-08 | 0.000E+00 |
| Unigene53917 | vanZA | 4.716E-05 | 1.994E-05 | 8.433E-06 | 1.439E-05 | 2.251E-06 | 1.813E-06 | 2.475E-06 | 7.340E-07 | 7.866E-06 | 0.000E+00 | 2.078E-06 | 2.312E-06 |
| Unigene53918 | Escherichi | 8.777E-07 | 0.000E+00 | 0.000E+00 | 4.596E-07 | 4.189E-06 | 9.207E-06 | 0.000E+00 | 8.044E-06 | 3.624E-06 | 4.780E-06 | 0.000E+00 | 7.301E-07 |
| Unigene53946 | vanRA | 3.219E-06 | 3.243E-06 | 2.683E-06 | 2.182E-06 | 2.401E-06 | 1.812E-06 | 1.806E-06 | 1.235E-06 | 1.350E-06 | 6.042E-06 | 5.448E-06 | 2.085E-06 |
| Unigene53954 | msbA | 9.223E-07 | 0.000E+00 | 1.006E-07 | 5.692E-07 | 0.000E+00 | 9.970E-06 | 2.835E-07 | 8.553E-06 | 1.288E-06 | 0.000E+00 | 0.000E+00 | 7.873E-07 |
| Unigene53970 | mdtG | 6.939E-06 | 0.000E+00 | 0.000E+00 | 2.887E-06 | 3.263E-05 | 1.717E-05 | 2.534E-05 | 2.497E-05 | 3.522E-06 | 0.000E+00 | 0.000E+00 | 0.000E+00 |

| | | | | | | | | | | | | | |
|---|---|---|---|---|---|---|---|---|---|---|---|---|---|
| Unigene53987 | patA | 2.755E-06 | 3.187E-06 | 1.863E-06 | 1.767E-06 | 2.440E-06 | 1.731E-06 | 2.143E-06 | 8.257E-07 | 1.058E-06 | 5.765E-06 | 4.472E-06 | 1.980E-06 |
| Unigene54010 | ugd | 2.158E-06 | 0.000E+00 | 0.000E+00 | 0.000E+00 | 0.000E+00 | 0.000E+00 | 0.000E+00 | 0.000E+00 | 0.000E+00 | 0.000E+00 | 0.000E+00 | 0.000E+00 |
| Unigene54046 | tlrC | 1.933E-05 | 0.000E+00 | 0.000E+00 | 4.856E-06 | 7.083E-05 | 8.175E-05 | 5.046E-05 | 5.813E-05 | 7.865E-06 | 0.000E+00 | 0.000E+00 | 0.000E+00 |
| Unigene54089 | Staphylocc | 2.270E-06 | 2.174E-06 | 3.137E-06 | 1.637E-06 | 0.000E+00 | 1.002E-07 | 3.846E-07 | 2.298E-07 | 5.697E-07 | 0.000E+00 | 6.624E-08 | 4.107E-08 |
| Unigene54117 | YojI | 1.369E-05 | 5.580E-06 | 4.438E-06 | 1.504E-05 | 9.245E-05 | 1.324E-04 | 1.451E-04 | 1.197E-04 | 1.666E-04 | 1.681E-04 | 1.300E-04 | 8.571E-05 |
| Unigene54154 | Staphylocc | 1.090E-04 | 2.089E-04 | 1.823E-04 | 7.905E-06 | 9.592E-06 | 0.000E+00 | 4.047E-06 | 2.049E-06 | 5.882E-05 | 0.000E+00 | 2.561E-06 | 8.949E-07 |
| Unigene54226 | lmrD | 2.796E-06 | 3.856E-06 | 2.906E-06 | 5.368E-06 | 3.065E-07 | 1.392E-07 | 6.718E-07 | 2.147E-08 | 1.407E-07 | 0.000E+00 | 6.312E-08 | 1.087E-07 |
| Unigene54251 | msbA | 1.365E-06 | 3.525E-06 | 2.758E-06 | 4.075E-06 | 2.230E-07 | 8.681E-08 | 3.555E-07 | 2.343E-08 | 2.852E-07 | 8.945E-08 | 2.296E-08 | 3.559E-07 |
| Unigene54335 | abcA | 1.157E-06 | 5.001E-06 | 7.291E-06 | 1.819E-05 | 0.000E+00 | 0.000E+00 | 0.000E+00 | 0.000E+00 | 0.000E+00 | 7.809E-08 | 4.009E-08 | 0.000E+00 |
| Unigene54342 | poxtA | 2.589E-06 | 1.931E-05 | 3.810E-06 | 6.127E-07 | 9.437E-07 | 3.061E-07 | 1.343E-07 | 4.250E-07 | 1.238E-06 | 0.000E+00 | 0.000E+00 | 1.434E-07 |
| Unigene54355 | catQ | 3.353E-06 | 7.635E-06 | 1.223E-05 | 1.097E-05 | 2.780E-07 | 6.492E-07 | 2.769E-07 | 0.000E+00 | 0.000E+00 | 0.000E+00 | 0.000E+00 | 2.958E-07 |
| Unigene54432 | Streptomy | 3.576E-06 | 8.705E-06 | 9.233E-06 | 1.054E-05 | 1.413E-07 | 8.591E-08 | 7.036E-08 | 0.000E+00 | 0.000E+00 | 1.948E-07 | 1.818E-08 | 0.000E+00 |
| Unigene54450 | Streptomy | 1.261E-05 | 0.000E+00 | 0.000E+00 | 0.000E+00 | 1.907E-05 | 1.664E-05 | 7.328E-08 | 5.797E-08 | 1.248E-05 | 2.951E-07 | 9.468E-08 | 1.957E-08 |
| Unigene54458 | vanRM | 1.255E-05 | 0.000E+00 | 0.000E+00 | 0.000E+00 | 1.608E-05 | 1.447E-05 | 0.000E+00 | 0.000E+00 | 1.123E-05 | 0.000E+00 | 0.000E+00 | 0.000E+00 |
| Unigene54459 | arlS | 1.158E-05 | 0.000E+00 | 0.000E+00 | 0.000E+00 | 1.818E-05 | 1.529E-05 | 3.842E-08 | 0.000E+00 | 1.248E-05 | 0.000E+00 | 0.000E+00 | 0.000E+00 |
| Unigene54473 | vanHO | 1.190E-05 | 0.000E+00 | 0.000E+00 | 0.000E+00 | 1.878E-05 | 1.606E-05 | 0.000E+00 | 0.000E+00 | 1.201E-05 | 0.000E+00 | 0.000E+00 | 0.000E+00 |
| Unigene54484 | tetA(58) | 1.133E-05 | 0.000E+00 | 0.000E+00 | 0.000E+00 | 1.803E-05 | 1.826E-05 | 0.000E+00 | 0.000E+00 | 1.224E-05 | 0.000E+00 | 0.000E+00 | 0.000E+00 |
| Unigene54562 | vanRA | 1.978E-06 | 2.012E-06 | 2.415E-06 | 1.998E-06 | 5.361E-08 | 0.000E+00 | 0.000E+00 | 0.000E+00 | 0.000E+00 | 1.613E-07 | 0.000E+00 | 0.000E+00 |
| Unigene54564 | novA | 1.738E-06 | 9.538E-07 | 2.518E-06 | 1.463E-06 | 1.379E-07 | 2.237E-07 | 0.000E+00 | 0.000E+00 | 0.000E+00 | 3.688E-07 | 0.000E+00 | 0.000E+00 |
| Unigene54590 | vanTG | 1.496E-06 | 0.000E+00 | 0.000E+00 | 0.000E+00 | 0.000E+00 | 0.000E+00 | 0.000E+00 | 0.000E+00 | 0.000E+00 | 0.000E+00 | 0.000E+00 | 0.000E+00 |
| Unigene54592 | arlR | 1.413E-06 | 0.000E+00 | 0.000E+00 | 0.000E+00 | 0.000E+00 | 0.000E+00 | 0.000E+00 | 0.000E+00 | 0.000E+00 | 0.000E+00 | 0.000E+00 | 0.000E+00 |
| Unigene54593 | adeS | 1.466E-06 | 0.000E+00 | 0.000E+00 | 0.000E+00 | 0.000E+00 | 0.000E+00 | 0.000E+00 | 0.000E+00 | 0.000E+00 | 0.000E+00 | 0.000E+00 | 0.000E+00 |
| Unigene54726 | vanHF | 2.651E-05 | 1.056E-05 | 6.143E-06 | 4.313E-06 | 3.622E-07 | 2.820E-07 | 0.000E+00 | 3.045E-07 | 0.000E+00 | 0.000E+00 | 0.000E+00 | 0.000E+00 |
| Unigene54751 | vanHO | 2.982E-05 | 9.604E-07 | 6.678E-07 | 0.000E+00 | 0.000E+00 | 0.000E+00 | 0.000E+00 | 0.000E+00 | 0.000E+00 | 0.000E+00 | 0.000E+00 | 0.000E+00 |
| Unigene54755 | efrA | 2.148E-06 | 2.297E-06 | 2.835E-06 | 1.571E-06 | 2.469E-07 | 1.001E-07 | 5.739E-07 | 2.162E-07 | 7.489E-07 | 0.000E+00 | 6.355E-08 | 1.751E-07 |
| Unigene54756 | patB | 2.711E-06 | 2.976E-06 | 2.812E-06 | 1.438E-06 | 9.533E-08 | 1.113E-07 | 5.128E-07 | 2.404E-07 | 5.439E-07 | 0.000E+00 | 1.178E-07 | 0.000E+00 |
| Unigene54761 | efrA | 2.317E-06 | 2.157E-06 | 3.005E-06 | 1.410E-06 | 3.162E-07 | 0.000E+00 | 6.899E-07 | 3.005E-07 | 5.628E-07 | 3.019E-08 | 0.000E+00 | 1.762E-07 |
| Unigene54762 | efrB | 2.874E-06 | 3.231E-06 | 3.405E-06 | 2.283E-06 | 1.947E-07 | 9.472E-08 | 5.043E-07 | 3.273E-07 | 6.895E-07 | 0.000E+00 | 1.002E-07 | 1.243E-07 |
| Unigene54778 | clbB | 4.086E-07 | 0.000E+00 | 0.000E+00 | 0.000E+00 | 0.000E+00 | 0.000E+00 | 0.000E+00 | 0.000E+00 | 0.000E+00 | 0.000E+00 | 0.000E+00 | 0.000E+00 |
| Unigene54829 | patA | 0.000E+00 | 0.000E+00 | 1.572E-06 | 0.000E+00 | 1.704E-05 | 0.000E+00 | 1.330E-05 | 1.643E-05 | 8.508E-06 | 1.326E-05 | 1.702E-05 | 0.000E+00 |
| Unigene54830 | macB | 1.441E-06 | 9.490E-07 | 1.508E-06 | 0.000E+00 | 1.548E-05 | 6.958E-06 | 1.170E-05 | 1.357E-05 | 8.400E-06 | 1.520E-05 | 1.297E-05 | 4.883E-06 |
| Unigene54833 | efpA | 1.183E-06 | 8.899E-07 | 1.462E-06 | 1.328E-06 | 1.719E-05 | 5.070E-06 | 1.193E-05 | 1.532E-05 | 8.335E-06 | 1.675E-05 | 1.502E-05 | 5.751E-06 |
| Unigene54865 | golS | 4.463E-06 | 2.493E-05 | 3.128E-06 | 0.000E+00 | 1.328E-06 | 2.585E-07 | 0.000E+00 | 6.512E-07 | 1.655E-06 | 0.000E+00 | 0.000E+00 | 0.000E+00 |
| Unigene54867 | efpA | 4.542E-06 | 2.202E-05 | 3.504E-06 | 8.339E-07 | 1.187E-06 | 3.939E-07 | 2.419E-07 | 5.670E-07 | 1.009E-06 | 5.682E-07 | 4.168E-07 | 0.000E+00 |
| Unigene54879 | Staphylocc | 4.452E-06 | 2.100E-05 | 3.463E-06 | 1.060E-06 | 1.127E-06 | 4.488E-07 | 2.553E-07 | 3.096E-07 | 1.147E-06 | 6.167E-07 | 3.298E-07 | 1.772E-07 |
| Unigene54902 | YojI | 5.019E-06 | 1.918E-05 | 2.770E-06 | 1.014E-06 | 1.150E-06 | 0.000E+00 | 2.083E-07 | 0.000E+00 | 1.200E-06 | 5.941E-07 | 6.100E-07 | 0.000E+00 |
| Unigene54903 | tetA(60) | 4.338E-06 | 2.211E-05 | 2.805E-06 | 8.149E-07 | 7.471E-07 | 3.272E-07 | 1.489E-07 | 0.000E+00 | 1.838E-06 | 7.866E-07 | 0.000E+00 | 0.000E+00 |
| Unigene54991 | macB | 1.128E-04 | 1.780E-04 | 1.813E-04 | 7.473E-06 | 7.681E-06 | 5.790E-07 | 3.718E-06 | 2.160E-06 | 6.673E-05 | 0.000E+00 | 3.620E-06 | 7.482E-07 |
| Unigene54997 | QepA2 | 2.847E-06 | 4.478E-06 | 3.387E-06 | 6.316E-06 | 3.985E-07 | 0.000E+00 | 4.962E-07 | 5.233E-08 | 2.940E-07 | 0.000E+00 | 1.795E-07 | 1.855E-07 |
| Unigene55012 | arlS | 2.699E-05 | 9.898E-06 | 6.670E-06 | 4.622E-06 | 3.795E-07 | 1.108E-07 | 2.079E-07 | 0.000E+00 | 9.894E-07 | 0.000E+00 | 0.000E+00 | 0.000E+00 |
| Unigene55044 | vanSF | 1.038E-06 | 7.314E-06 | 1.125E-05 | 1.677E-05 | 0.000E+00 | 0.000E+00 | 0.000E+00 | 0.000E+00 | 0.000E+00 | 0.000E+00 | 0.000E+00 | 0.000E+00 |
| Unigene55045 | vanRF | 9.824E-07 | 7.394E-06 | 9.411E-06 | 1.724E-05 | 0.000E+00 | 0.000E+00 | 0.000E+00 | 0.000E+00 | 0.000E+00 | 0.000E+00 | 0.000E+00 | 0.000E+00 |
| Unigene55054 | Bifidobacte | 8.872E-06 | 0.000E+00 | 0.000E+00 | 2.860E-06 | 3.539E-05 | 1.901E-05 | 2.475E-05 | 2.618E-05 | 4.092E-06 | 5.440E-08 | 0.000E+00 | 3.848E-08 |
| Unigene55085 | TaeA | 3.502E-06 | 1.857E-07 | 3.337E-06 | 3.929E-07 | 1.692E-07 | 9.145E-08 | 2.247E-07 | 1.382E-07 | 0.000E+00 | 0.000E+00 | 9.096E-07 | 0.000E+00 |
| Unigene55089 | vatC | 3.736E-06 | 0.000E+00 | 1.796E-06 | 7.546E-08 | 1.660E-07 | 1.212E-07 | 2.481E-07 | 0.000E+00 | 0.000E+00 | 0.000E+00 | 2.992E-07 | 0.000E+00 |
| Unigene55117 | baeS | 1.065E-06 | 3.859E-06 | 3.032E-06 | 6.455E-06 | 0.000E+00 | 0.000E+00 | 0.000E+00 | 8.290E-08 | 0.000E+00 | 0.000E+00 | 0.000E+00 | 0.000E+00 |
| Unigene55123 | patA | 3.662E-06 | 5.345E-06 | 3.242E-06 | 6.539E-06 | 0.000E+00 | 1.020E-07 | 5.850E-07 | 0.000E+00 | 2.063E-07 | 0.000E+00 | 0.000E+00 | 0.000E+00 |
| Unigene55150 | tetW | 1.109E-06 | 0.000E+00 | 0.000E+00 | 0.000E+00 | 0.000E+00 | 0.000E+00 | 0.000E+00 | 0.000E+00 | 0.000E+00 | 0.000E+00 | 0.000E+00 | 0.000E+00 |
| Unigene55156 | poxtA | 1.182E-06 | 0.000E+00 | 0.000E+00 | 0.000E+00 | 0.000E+00 | 0.000E+00 | 2.181E-08 | 0.000E+00 | 0.000E+00 | 0.000E+00 | 0.000E+00 | 0.000E+00 |
| Unigene55197 | evgS | 7.288E-07 | 1.341E-06 | 9.465E-07 | 2.229E-06 | 3.572E-07 | 2.317E-08 | 0.000E+00 | 0.000E+00 | 0.000E+00 | 0.000E+00 | 0.000E+00 | 0.000E+00 |
| Unigene55225 | baeS | 2.740E-05 | 8.753E-06 | 7.279E-06 | 4.589E-06 | 2.475E-07 | 3.131E-07 | 0.000E+00 | 1.560E-07 | 1.096E-06 | 0.000E+00 | 2.548E-08 | 0.000E+00 |
| Unigene55226 | mtrA | 2.649E-05 | 9.975E-06 | 7.950E-06 | 5.151E-06 | 6.191E-07 | 9.269E-08 | 0.000E+00 | 2.002E-07 | 1.406E-06 | 0.000E+00 | 0.000E+00 | 0.000E+00 |
| Unigene55264 | vatE | 1.034E-05 | 5.503E-06 | 3.075E-06 | 1.366E-05 | 8.855E-05 | 1.162E-04 | 1.222E-04 | 1.020E-04 | 1.503E-04 | 1.450E-04 | 1.166E-04 | 7.427E-05 |
| Unigene55269 | rosB | 1.348E-05 | 5.387E-06 | 3.572E-06 | 1.245E-05 | 8.278E-05 | 1.193E-04 | 1.236E-04 | 9.966E-05 | 1.521E-04 | 1.471E-04 | 1.317E-04 | 7.877E-05 |
| Unigene55282 | macB | 3.057E-06 | 4.205E-06 | 3.519E-06 | 6.503E-06 | 4.981E-07 | 0.000E+00 | 0.000E+00 | 0.000E+00 | 0.000E+00 | 0.000E+00 | 1.865E-07 | 3.372E-07 |
| Unigene55290 | optrA | 1.349E-06 | 1.697E-06 | 1.033E-06 | 4.320E-07 | 1.462E-06 | 1.138E-06 | 8.012E-07 | 6.145E-07 | 6.474E-07 | 7.331E-07 | 1.317E-06 | 9.724E-07 |
| Unigene55361 | mtrA | 2.458E-06 | 2.647E-06 | 2.321E-06 | 1.311E-06 | 0.000E+00 | 0.000E+00 | 0.000E+00 | 2.707E-07 | 8.111E-07 | 0.000E+00 | 0.000E+00 | 0.000E+00 |
| Unigene55362 | arlS | 2.044E-06 | 2.223E-06 | 2.571E-06 | 1.576E-06 | 0.000E+00 | 0.000E+00 | 3.497E-07 | 3.688E-07 | 5.890E-07 | 0.000E+00 | 6.377E-08 | 1.538E-07 |

| | | | | | | | | | | | | | |
|---|---|---|---|---|---|---|---|---|---|---|---|---|---|
| Unigene55398 | macB | 2.880E-05 | 0.000E+00 | 0.000E+00 | 0.000E+00 | 0.000E+00 | 0.000E+00 | 0.000E+00 | 0.000E+00 | 0.000E+00 | 0.000E+00 | 0.000E+00 | 0.000E+00 |
| Unigene55399 | tetB(46) | 2.543E-05 | 0.000E+00 | 0.000E+00 | 0.000E+00 | 0.000E+00 | 0.000E+00 | 0.000E+00 | 0.000E+00 | 0.000E+00 | 0.000E+00 | 0.000E+00 | 0.000E+00 |
| Unigene55443 | bcrA | 2.964E-06 | 0.000E+00 | 3.793E-07 | 5.400E-06 | 7.022E-05 | 8.247E-05 | 7.110E-05 | 4.685E-05 | 1.794E-04 | 2.485E-04 | 2.291E-04 | 1.902E-04 |
| Unigene55450 | Escherichia | 8.372E-07 | 0.000E+00 | 3.347E-07 | 3.758E-07 | 0.000E+00 | 8.783E-06 | 1.098E-06 | 7.854E-06 | 3.321E-06 | 0.000E+00 | 0.000E+00 | 0.000E+00 |
| Unigene55500 | tet(41) | 1.450E-06 | 0.000E+00 | 0.000E+00 | 0.000E+00 | 0.000E+00 | 0.000E+00 | 0.000E+00 | 0.000E+00 | 0.000E+00 | 0.000E+00 | 0.000E+00 | 0.000E+00 |
| Unigene55505 | optrA | 6.922E-07 | 0.000E+00 | 0.000E+00 | 0.000E+00 | 0.000E+00 | 0.000E+00 | 0.000E+00 | 0.000E+00 | 0.000E+00 | 0.000E+00 | 0.000E+00 | 0.000E+00 |
| Unigene55573 | vanRB | 1.930E-06 | 8.473E-07 | 2.699E-06 | 1.559E-06 | 0.000E+00 | 5.216E-08 | 0.000E+00 | 0.000E+00 | 0.000E+00 | 0.000E+00 | 0.000E+00 | 0.000E+00 |
| Unigene55581 | vanL | 2.557E-06 | 1.746E-06 | 3.305E-06 | 1.858E-06 | 1.039E-07 | 6.743E-08 | 0.000E+00 | 0.000E+00 | 0.000E+00 | 0.000E+00 | 0.000E+00 | 0.000E+00 |
| Unigene55597 | macB | 6.438E-06 | 0.000E+00 | 0.000E+00 | 1.532E-06 | 3.300E-05 | 1.720E-05 | 2.392E-05 | 2.356E-05 | 3.166E-06 | 0.000E+00 | 0.000E+00 | 0.000E+00 |
| Unigene55618 | tetB(46) | 6.229E-05 | 5.184E-05 | 1.026E-04 | 1.447E-04 | 1.266E-04 | 8.737E-05 | 1.004E-04 | 1.087E-04 | 8.392E-05 | 1.432E-04 | 1.227E-04 | 1.449E-04 |
| Unigene55619 | tetB(46) | 7.385E-05 | 5.824E-05 | 1.134E-04 | 1.766E-04 | 1.055E-04 | 7.321E-05 | 1.164E-04 | 9.182E-05 | 9.854E-05 | 1.082E-04 | 1.439E-04 | 1.162E-04 |
| Unigene55715 | vatB | 2.323E-06 | 5.827E-06 | 3.907E-06 | 8.712E-06 | 0.000E+00 | 0.000E+00 | 0.000E+00 | 0.000E+00 | 0.000E+00 | 0.000E+00 | 0.000E+00 | 0.000E+00 |
| Unigene55745 | vanE | 1.156E-05 | 0.000E+00 | 0.000E+00 | 0.000E+00 | 2.027E-05 | 1.649E-05 | 0.000E+00 | 0.000E+00 | 1.341E-05 | 0.000E+00 | 0.000E+00 | 0.000E+00 |
| Unigene55798 | TaeA | 4.402E-06 | 9.287E-06 | 1.013E-05 | 1.151E-05 | 0.000E+00 | 0.000E+00 | 0.000E+00 | 0.000E+00 | 0.000E+00 | 0.000E+00 | 0.000E+00 | 0.000E+00 |
| Unigene55839 | facT | 1.085E-05 | 3.722E-06 | 2.974E-06 | 1.207E-05 | 7.673E-05 | 1.061E-04 | 1.052E-04 | 8.208E-05 | 1.279E-04 | 1.332E-04 | 1.079E-04 | 6.865E-05 |
| Unigene55860 | efrA | 2.320E-05 | 7.974E-06 | 6.513E-06 | 4.033E-06 | 4.108E-07 | 1.399E-07 | 0.000E+00 | 1.079E-07 | 0.000E+00 | 0.000E+00 | 0.000E+00 | 0.000E+00 |
| Unigene55861 | patB | 2.305E-05 | 9.374E-06 | 6.446E-06 | 4.268E-06 | 3.913E-07 | 7.615E-08 | 0.000E+00 | 1.233E-07 | 7.507E-07 | 0.000E+00 | 0.000E+00 | 1.041E-07 |
| Unigene55872 | macB | 1.129E-06 | 0.000E+00 | 0.000E+00 | 3.801E-07 | 4.914E-06 | 8.750E-06 | 0.000E+00 | 1.029E-05 | 4.663E-06 | 0.000E+00 | 0.000E+00 | 1.409E-06 |
| Unigene55958 | optrA | 1.266E-06 | 2.246E-06 | 5.569E-06 | 7.159E-06 | 0.000E+00 | 2.281E-08 | 1.868E-07 | 0.000E+00 | 0.000E+00 | 0.000E+00 | 9.654E-08 | 0.000E+00 |
| Unigene55961 | mdtG | 4.491E-06 | 9.418E-06 | 8.813E-06 | 9.653E-06 | 0.000E+00 | 0.000E+00 | 3.445E-07 | 0.000E+00 | 0.000E+00 | 0.000E+00 | 0.000E+00 | 0.000E+00 |
| Unigene56043 | lmrB | 7.189E-06 | 0.000E+00 | 0.000E+00 | 2.515E-06 | 3.390E-05 | 1.526E-05 | 2.509E-05 | 2.550E-05 | 3.672E-06 | 0.000E+00 | 0.000E+00 | 0.000E+00 |
| Unigene56044 | vgaB | 7.336E-06 | 0.000E+00 | 0.000E+00 | 2.599E-06 | 3.505E-05 | 1.926E-05 | 2.640E-05 | 2.786E-05 | 4.385E-06 | 0.000E+00 | 0.000E+00 | 0.000E+00 |
| Unigene56073 | marA | 4.547E-07 | 0.000E+00 | 2.231E-07 | 0.000E+00 | 8.418E-08 | 0.000E+00 | 0.000E+00 | 0.000E+00 | 0.000E+00 | 0.000E+00 | 0.000E+00 | 0.000E+00 |
| Unigene56150 | kdpE | 2.236E-06 | 1.672E-05 | 8.639E-06 | 5.645E-06 | 0.000E+00 | 0.000E+00 | 0.000E+00 | 0.000E+00 | 1.018E-07 | 0.000E+00 | 0.000E+00 | 5.506E-08 |
| Unigene56217 | macB | 2.455E-06 | 3.727E-06 | 2.850E-06 | 6.693E-06 | 5.454E-07 | 3.538E-07 | 3.622E-07 | 4.776E-08 | 4.025E-07 | 0.000E+00 | 1.404E-07 | 0.000E+00 |
| Unigene56345 | Streptomy | 1.112E-05 | 0.000E+00 | 0.000E+00 | 0.000E+00 | 1.737E-05 | 1.628E-05 | 7.152E-08 | 5.658E-08 | 1.206E-05 | 3.060E-07 | 0.000E+00 | 0.000E+00 |
| Unigene56356 | catB3 | 1.127E-05 | 0.000E+00 | 0.000E+00 | 0.000E+00 | 1.710E-05 | 1.241E-05 | 0.000E+00 | 0.000E+00 | 1.221E-05 | 3.441E-07 | 0.000E+00 | 0.000E+00 |
| Unigene56357 | vanHA | 1.202E-05 | 0.000E+00 | 0.000E+00 | 0.000E+00 | 1.880E-05 | 1.737E-05 | 0.000E+00 | 0.000E+00 | 1.104E-05 | 0.000E+00 | 0.000E+00 | 0.000E+00 |
| Unigene56360 | dfrI | 1.102E-05 | 0.000E+00 | 0.000E+00 | 0.000E+00 | 1.160E-05 | 1.731E-05 | 0.000E+00 | 0.000E+00 | 1.449E-05 | 0.000E+00 | 0.000E+00 | 0.000E+00 |
| Unigene56362 | TaeA | 1.158E-05 | 0.000E+00 | 0.000E+00 | 0.000E+00 | 1.767E-05 | 1.781E-05 | 1.704E-07 | 0.000E+00 | 1.262E-05 | 1.525E-07 | 1.957E-08 | 0.000E+00 |
| Unigene56398 | fexA | 1.088E-05 | 0.000E+00 | 0.000E+00 | 0.000E+00 | 1.646E-05 | 1.589E-05 | 0.000E+00 | 0.000E+00 | 1.310E-06 | 0.000E+00 | 0.000E+00 | 0.000E+00 |
| Unigene56446 | PEDO-2 | 1.133E-06 | 5.650E-07 | 3.991E-06 | 5.355E-06 | 0.000E+00 | 0.000E+00 | 6.838E-07 | 0.000E+00 | 0.000E+00 | 0.000E+00 | 0.000E+00 | 0.000E+00 |
| Unigene56462 | tet(59) | 3.191E-06 | 1.705E-05 | 1.048E-05 | 9.987E-06 | 0.000E+00 | 5.702E-08 | 4.962E-07 | 0.000E+00 | 0.000E+00 | 0.000E+00 | 9.050E-08 | 6.235E-08 |
| Unigene56694 | PmrF | 1.396E-06 | 1.614E-05 | 3.107E-06 | 1.822E-06 | 0.000E+00 | 1.472E-07 | 0.000E+00 | 0.000E+00 | 0.000E+00 | 0.000E+00 | 1.169E-07 | 0.000E+00 |
| Unigene56697 | oleC | 2.323E-06 | 1.726E-05 | 6.733E-06 | 6.768E-06 | 0.000E+00 | 5.773E-08 | 0.000E+00 | 0.000E+00 | 2.918E-08 | 0.000E+00 | 9.163E-08 | 0.000E+00 |
| Unigene56721 | Streptomy | 5.044E-07 | 2.768E-07 | 6.874E-07 | 3.396E-07 | 3.113E-08 | 0.000E+00 | 0.000E+00 | 0.000E+00 | 0.000E+00 | 0.000E+00 | 0.000E+00 | 0.000E+00 |
| Unigene56756 | lin | 3.619E-06 | 2.367E-07 | 2.892E-06 | 4.840E-07 | 2.929E-07 | 7.772E-08 | 3.448E-07 | 0.000E+00 | 0.000E+00 | 0.000E+00 | 4.386E-07 | 0.000E+00 |
| Unigene56811 | bcrA | 5.527E-07 | 0.000E+00 | 1.338E-06 | 1.712E-06 | 0.000E+00 | 0.000E+00 | 0.000E+00 | 0.000E+00 | 0.000E+00 | 0.000E+00 | 0.000E+00 | 0.000E+00 |
| Unigene56886 | vanHA | 1.506E-06 | 9.015E-08 | 0.000E+00 | 0.000E+00 | 0.000E+00 | 0.000E+00 | 0.000E+00 | 0.000E+00 | 0.000E+00 | 0.000E+00 | 0.000E+00 | 0.000E+00 |
| Unigene57071 | vanRM | 1.387E-06 | 1.218E-05 | 5.819E-06 | 4.008E-06 | 5.313E-08 | 1.034E-07 | 1.588E-07 | 0.000E+00 | 0.000E+00 | 0.000E+00 | 0.000E+00 | 5.653E-08 |
| Unigene57077 | Staphylocc | 1.439E-06 | 0.000E+00 | 0.000E+00 | 0.000E+00 | 0.000E+00 | 0.000E+00 | 0.000E+00 | 0.000E+00 | 0.000E+00 | 0.000E+00 | 0.000E+00 | 0.000E+00 |
| Unigene57098 | Staphylocc | 1.623E-06 | 0.000E+00 | 0.000E+00 | 0.000E+00 | 0.000E+00 | 0.000E+00 | 0.000E+00 | 0.000E+00 | 1.314E-08 | 0.000E+00 | 0.000E+00 | 0.000E+00 |
| Unigene57115 | msbA | 3.843E-07 | 0.000E+00 | 0.000E+00 | 0.000E+00 | 0.000E+00 | 0.000E+00 | 0.000E+00 | 0.000E+00 | 0.000E+00 | 0.000E+00 | 0.000E+00 | 0.000E+00 |
| Unigene57155 | Streptomy | 6.943E-06 | 0.000E+00 | 0.000E+00 | 2.392E-06 | 3.407E-05 | 1.880E-05 | 2.415E-05 | 2.595E-05 | 3.183E-06 | 0.000E+00 | 0.000E+00 | 0.000E+00 |
| Unigene57200 | tetB(60) | 2.290E-06 | 3.391E-06 | 3.585E-06 | 6.130E-06 | 3.635E-07 | 5.895E-08 | 2.414E-07 | 0.000E+00 | 1.788E-07 | 0.000E+00 | 1.455E-07 | 2.148E-07 |
| Unigene57252 | vanL | 1.298E-06 | 1.657E-05 | 4.316E-06 | 2.109E-06 | 0.000E+00 | 0.000E+00 | 0.000E+00 | 3.694E-08 | 0.000E+00 | 0.000E+00 | 0.000E+00 | 0.000E+00 |
| Unigene57258 | catB3 | 7.625E-07 | 1.660E-05 | 4.198E-06 | 2.738E-06 | 0.000E+00 | 0.000E+00 | 0.000E+00 | 9.890E-08 | 0.000E+00 | 0.000E+00 | 1.938E-07 | 0.000E+00 |
| Unigene57310 | Staphylocc | 1.708E-07 | 0.000E+00 | 0.000E+00 | 4.024E-07 | 0.000E+00 | 0.000E+00 | 6.301E-08 | 0.000E+00 | 0.000E+00 | 0.000E+00 | 0.000E+00 | 0.000E+00 |
| Unigene57322 | oleC | 2.168E-06 | 1.909E-06 | 1.497E-06 | 8.649E-07 | 0.000E+00 | 1.447E-07 | 5.629E-07 | 1.250E-07 | 6.729E-07 | 0.000E+00 | 0.000E+00 | 0.000E+00 |
| Unigene57339 | lmrD | 1.021E-06 | 0.000E+00 | 0.000E+00 | 6.081E-07 | 0.000E+00 | 8.660E-06 | 1.478E-06 | 8.587E-06 | 3.977E-06 | 4.345E-06 | 0.000E+00 | 8.665E-07 |
| Unigene57364 | vanRF | 3.967E-06 | 0.000E+00 | 0.000E+00 | 8.012E-07 | 2.002E-05 | 2.761E-06 | 7.985E-06 | 8.855E-06 | 1.355E-07 | 0.000E+00 | 2.836E-08 | 0.000E+00 |
| Unigene57366 | PmrF | 7.368E-06 | 0.000E+00 | 0.000E+00 | 2.249E-06 | 3.422E-05 | 1.478E-05 | 2.799E-05 | 2.960E-05 | 3.670E-06 | 0.000E+00 | 0.000E+00 | 0.000E+00 |
| Unigene57400 | macB | 2.401E-06 | 1.967E-05 | 8.413E-06 | 5.498E-06 | 0.000E+00 | 0.000E+00 | 0.000E+00 | 0.000E+00 | 0.000E+00 | 0.000E+00 | 0.000E+00 | 0.000E+00 |
| Unigene57402 | optrA | 1.933E-06 | 1.582E-05 | 8.661E-06 | 5.305E-06 | 0.000E+00 | 0.000E+00 | 0.000E+00 | 0.000E+00 | 0.000E+00 | 0.000E+00 | 6.701E-08 | 0.000E+00 |
| Unigene57440 | tetB(P) | 1.687E-06 | 9.373E-08 | 4.190E-08 | 0.000E+00 | 0.000E+00 | 2.308E-08 | 0.000E+00 | 0.000E+00 | 0.000E+00 | 0.000E+00 | 0.000E+00 | 0.000E+00 |
| Unigene57441 | lmrD | 1.814E-06 | 0.000E+00 | 0.000E+00 | 0.000E+00 | 0.000E+00 | 8.914E-08 | 0.000E+00 | 0.000E+00 | 0.000E+00 | 0.000E+00 | 0.000E+00 | 0.000E+00 |
| Unigene57447 | bacA | 1.107E-06 | 0.000E+00 | 0.000E+00 | 0.000E+00 | 0.000E+00 | 0.000E+00 | 0.000E+00 | 0.000E+00 | 0.000E+00 | 0.000E+00 | 0.000E+00 | 0.000E+00 |
| Unigene57451 | cmlA8 | 1.575E-06 | 9.740E-08 | 6.531E-08 | 0.000E+00 | 0.000E+00 | 0.000E+00 | 0.000E+00 | 0.000E+00 | 0.000E+00 | 0.000E+00 | 0.000E+00 | 0.000E+00 |

| | | | | | | | | | | | | | |
|---|---|---|---|---|---|---|---|---|---|---|---|---|---|
| Unigene57464 | PmrF | 1.542E-06 | 0.000E+00 | 0.000E+00 | 0.000E+00 | 0.000E+00 | 0.000E+00 | 0.000E+00 | 0.000E+00 | 0.000E+00 | 0.000E+00 | 0.000E+00 | 0.000E+00 |
| Unigene57467 | TaeA | 1.449E-06 | 0.000E+00 | 0.000E+00 | 0.000E+00 | 0.000E+00 | 0.000E+00 | 0.000E+00 | 0.000E+00 | 0.000E+00 | 0.000E+00 | 0.000E+00 | 0.000E+00 |
| Unigene57478 | msbA | 1.623E-06 | 4.948E-08 | 0.000E+00 | 0.000E+00 | 0.000E+00 | 4.874E-08 | 0.000E+00 | 0.000E+00 | 0.000E+00 | 0.000E+00 | 0.000E+00 | 0.000E+00 |
| Unigene57486 | poxtA | 1.409E-06 | 0.000E+00 | 0.000E+00 | 0.000E+00 | 0.000E+00 | 4.616E-08 | 0.000E+00 | 0.000E+00 | 0.000E+00 | 0.000E+00 | 0.000E+00 | 0.000E+00 |
| Unigene57490 | YojI | 1.555E-06 | 4.551E-08 | 0.000E+00 | 0.000E+00 | 0.000E+00 | 0.000E+00 | 4.589E-08 | 0.000E+00 | 0.000E+00 | 0.000E+00 | 2.372E-08 | 0.000E+00 |
| Unigene57508 | bcrA | 1.710E-06 | 0.000E+00 | 0.000E+00 | 0.000E+00 | 0.000E+00 | 0.000E+00 | 0.000E+00 | 0.000E+00 | 0.000E+00 | 0.000E+00 | 0.000E+00 | 0.000E+00 |
| Unigene57510 | bcrA | 1.781E-06 | 0.000E+00 | 0.000E+00 | 0.000E+00 | 0.000E+00 | 0.000E+00 | 0.000E+00 | 0.000E+00 | 0.000E+00 | 0.000E+00 | 0.000E+00 | 0.000E+00 |
| Unigene57537 | patA | 3.759E-06 | 3.610E-06 | 2.121E-06 | 1.898E-06 | 1.879E-06 | 1.727E-06 | 1.976E-06 | 9.872E-07 | 1.541E-06 | 5.234E-06 | 5.482E-06 | 2.444E-06 |
| Unigene57648 | TaeA | 2.159E-07 | 0.000E+00 | 0.000E+00 | 0.000E+00 | 0.000E+00 | 5.446E-07 | 9.559E-07 | 0.000E+00 | 0.000E+00 | 0.000E+00 | 0.000E+00 | 0.000E+00 |
| Unigene57708 | lmrB | 1.278E-06 | 0.000E+00 | 0.000E+00 | 0.000E+00 | 0.000E+00 | 0.000E+00 | 0.000E+00 | 0.000E+00 | 2.540E-08 | 0.000E+00 | 0.000E+00 | 0.000E+00 |
| Unigene57734 | oleC | 1.027E-06 | 0.000E+00 | 0.000E+00 | 0.000E+00 | 0.000E+00 | 0.000E+00 | 0.000E+00 | 0.000E+00 | 0.000E+00 | 0.000E+00 | 0.000E+00 | 0.000E+00 |
| Unigene57735 | oleC | 1.628E-06 | 0.000E+00 | 0.000E+00 | 0.000E+00 | 0.000E+00 | 0.000E+00 | 0.000E+00 | 0.000E+00 | 0.000E+00 | 0.000E+00 | 0.000E+00 | 0.000E+00 |
| Unigene57769 | efmA | 2.045E-05 | 1.087E-06 | 7.721E-06 | 1.052E-07 | 6.078E-07 | 0.000E+00 | 0.000E+00 | 0.000E+00 | 6.834E-07 | 1.451E-07 | 0.000E+00 | 0.000E+00 |
| Unigene57794 | Staphylocc | 4.434E-06 | 1.996E-05 | 3.499E-06 | 9.127E-07 | 9.769E-07 | 4.357E-07 | 3.244E-07 | 2.424E-07 | 1.241E-06 | 8.163E-07 | 5.588E-07 | 1.444E-07 |
| Unigene57797 | fusC | 4.864E-06 | 2.227E-05 | 3.616E-06 | 7.442E-07 | 1.310E-06 | 0.000E+00 | 1.631E-07 | 0.000E+00 | 1.235E-06 | 0.000E+00 | 5.620E-07 | 0.000E+00 |
| Unigene57819 | lmrD | 3.472E-06 | 3.493E-06 | 2.538E-06 | 1.644E-06 | 2.531E-06 | 2.268E-06 | 2.342E-06 | 1.013E-06 | 1.799E-06 | 6.467E-06 | 5.440E-06 | 1.988E-06 |
| Unigene57820 | patA | 3.617E-06 | 3.495E-06 | 2.666E-06 | 1.981E-06 | 2.659E-06 | 1.342E-06 | 2.031E-06 | 8.401E-07 | 1.475E-06 | 5.532E-06 | 4.693E-06 | 1.872E-06 |
| Unigene57833 | optrA | 3.165E-06 | 3.734E-06 | 2.620E-06 | 1.917E-06 | 2.110E-06 | 2.074E-06 | 2.211E-06 | 8.542E-07 | 1.557E-06 | 6.214E-06 | 5.656E-06 | 2.315E-06 |
| Unigene57834 | lmrB | 2.801E-06 | 4.329E-06 | 3.229E-06 | 1.721E-06 | 3.060E-06 | 1.716E-06 | 2.247E-06 | 9.263E-07 | 1.480E-06 | 6.578E-06 | 5.820E-06 | 1.904E-06 |
| Unigene57836 | tlrC | 3.879E-06 | 5.068E-06 | 2.538E-06 | 3.451E-06 | 1.642E-06 | 2.247E-06 | 2.658E-06 | 8.625E-07 | 1.615E-06 | 6.379E-06 | 6.445E-06 | 2.620E-06 |
| Unigene57889 | vanRO | 1.296E-07 | 0.000E+00 | 0.000E+00 | 1.964E-07 | 3.601E-08 | 0.000E+00 | 0.000E+00 | 0.000E+00 | 0.000E+00 | 0.000E+00 | 0.000E+00 | 0.000E+00 |
| Unigene57897 | efrA | 4.732E-06 | 1.121E-05 | 1.407E-05 | 1.450E-05 | 8.118E-08 | 9.874E-08 | 0.000E+00 | 0.000E+00 | 0.000E+00 | 2.442E-07 | 4.179E-08 | 0.000E+00 |
| Unigene57898 | patB | 5.624E-06 | 1.096E-05 | 1.370E-05 | 1.572E-05 | 1.005E-07 | 0.000E+00 | 0.000E+00 | 4.222E-08 | 0.000E+00 | 2.619E-07 | 4.137E-08 | 0.000E+00 |
| Unigene57925 | PmrF | 7.378E-06 | 8.623E-07 | 7.374E-07 | 1.587E-06 | 3.575E-05 | 4.036E-05 | 2.646E-05 | 1.471E-05 | 1.023E-04 | 1.009E-04 | 9.323E-05 | 7.131E-05 |
| Unigene57927 | arlR | 7.965E-06 | 7.669E-07 | 3.657E-07 | 1.693E-06 | 3.095E-05 | 3.676E-05 | 2.572E-05 | 1.539E-05 | 9.439E-05 | 8.806E-05 | 8.126E-05 | 6.998E-05 |
| Unigene57928 | arlS | 9.533E-06 | 3.885E-07 | 3.936E-07 | 2.311E-06 | 3.791E-05 | 4.007E-05 | 2.648E-05 | 1.694E-05 | 1.091E-04 | 9.891E-05 | 9.175E-05 | 7.324E-05 |
| Unigene57942 | tetB(60) | 3.765E-06 | 5.173E-06 | 3.868E-06 | 7.274E-06 | 5.269E-07 | 1.602E-07 | 5.905E-07 | 0.000E+00 | 2.916E-07 | 0.000E+00 | 6.781E-08 | 2.102E-07 |
| Unigene57969 | vanRG | 3.849E-06 | 3.552E-06 | 3.391E-06 | 7.288E-06 | 6.803E-07 | 1.419E-07 | 5.809E-07 | 0.000E+00 | 1.434E-07 | 0.000E+00 | 2.001E-07 | 2.068E-07 |
| Unigene57978 | macB | 5.945E-06 | 9.236E-06 | 1.064E-05 | 1.311E-05 | 0.000E+00 | 0.000E+00 | 0.000E+00 | 0.000E+00 | 0.000E+00 | 0.000E+00 | 0.000E+00 | 0.000E+00 |
| Unigene58006 | optrA | 1.385E-06 | 7.424E-06 | 1.283E-05 | 1.736E-05 | 6.592E-08 | 0.000E+00 | 4.378E-08 | 6.926E-08 | 0.000E+00 | 0.000E+00 | 0.000E+00 | 0.000E+00 |
| Unigene58025 | tetA(58) | 4.972E-07 | 7.795E-07 | 5.018E-06 | 7.136E-06 | 0.000E+00 | 0.000E+00 | 5.109E-07 | 0.000E+00 | 0.000E+00 | 0.000E+00 | 4.062E-08 | 0.000E+00 |
| Unigene58029 | vanSM | 2.613E-06 | 3.645E-06 | 4.388E-06 | 3.465E-06 | 0.000E+00 | 7.180E-08 | 2.450E-08 | 0.000E+00 | 0.000E+00 | 1.233E-07 | 0.000E+00 | 0.000E+00 |
| Unigene58030 | vanRI | 3.462E-06 | 8.883E-06 | 9.043E-06 | 9.779E-06 | 0.000E+00 | 0.000E+00 | 0.000E+00 | 0.000E+00 | 0.000E+00 | 1.042E-07 | 0.000E+00 | 0.000E+00 |
| Unigene58032 | bcrA | 4.540E-06 | 9.916E-06 | 1.056E-05 | 1.261E-05 | 0.000E+00 | 9.816E-08 | 1.005E-07 | 1.590E-07 | 0.000E+00 | 0.000E+00 | 1.558E-07 | 0.000E+00 |
| Unigene58067 | optrA | 5.118E-06 | 2.202E-05 | 1.238E-05 | 1.147E-05 | 1.938E-07 | 1.886E-07 | 3.862E-07 | 0.000E+00 | 0.000E+00 | 8.639E-08 | 2.883E-07 | 1.146E-07 |
| Unigene58107 | oleC | 8.439E-07 | 1.184E-06 | 2.300E-07 | 2.131E-07 | 2.344E-07 | 1.014E-07 | 7.783E-08 | 1.368E-07 | 6.662E-07 | 3.395E-07 | 1.073E-07 | 0.000E+00 |
| Unigene58146 | salA | 2.170E-06 | 4.502E-06 | 2.960E-06 | 5.126E-06 | 3.269E-07 | 1.392E-07 | 4.275E-07 | 0.000E+00 | 3.820E-07 | 0.000E+00 | 1.683E-07 | 1.739E-07 |
| Unigene58166 | lmrC | 1.427E-06 | 3.132E-08 | 2.801E-08 | 0.000E+00 | 0.000E+00 | 0.000E+00 | 0.000E+00 | 0.000E+00 | 0.000E+00 | 0.000E+00 | 0.000E+00 | 0.000E+00 |
| Unigene58168 | tetT | 2.323E-06 | 0.000E+00 | 5.099E-07 | 0.000E+00 | 0.000E+00 | 0.000E+00 | 0.000E+00 | 0.000E+00 | 0.000E+00 | 0.000E+00 | 0.000E+00 | 0.000E+00 |
| Unigene58223 | Staphylocc | 3.869E-05 | 2.352E-05 | 1.971E-05 | 2.131E-05 | 1.850E-06 | 1.378E-06 | 2.635E-06 | 7.442E-07 | 6.442E-06 | 4.783E-07 | 1.979E-06 | 1.230E-06 |
| Unigene58238 | tetB(60) | 1.133E-04 | 2.183E-04 | 1.986E-04 | 7.596E-06 | 9.906E-06 | 3.231E-07 | 0.000E+00 | 1.803E-06 | 6.294E-05 | 0.000E+00 | 4.160E-06 | 8.832E-07 |
| Unigene58245 | TaeA | 1.928E-07 | 0.000E+00 | 0.000E+00 | 1.558E-07 | 0.000E+00 | 4.169E-08 | 0.000E+00 | 0.000E+00 | 0.000E+00 | 0.000E+00 | 3.088E-07 | 0.000E+00 |
| Unigene58262 | bcr-1 | 2.332E-05 | 7.586E-06 | 6.195E-06 | 4.374E-06 | 2.048E-07 | 0.000E+00 | 0.000E+00 | 0.000E+00 | 0.000E+00 | 0.000E+00 | 0.000E+00 | 0.000E+00 |
| Unigene58280 | evgS | 0.000E+00 | 0.000E+00 | 0.000E+00 | 0.000E+00 | 0.000E+00 | 0.000E+00 | 9.893E-06 | 6.087E-06 | 0.000E+00 | 0.000E+00 | 0.000E+00 | 5.087E-06 |
| Unigene58465 | Agrobacte | 6.687E-07 | 2.469E-06 | 6.562E-07 | 1.842E-07 | 1.959E-06 | 1.249E-06 | 1.547E-06 | 6.386E-07 | 7.309E-07 | 1.422E-06 | 1.530E-06 | 1.150E-06 |
| Unigene58532 | mecA | 8.203E-07 | 5.825E-07 | 4.419E-07 | 2.046E-06 | 1.072E-07 | 0.000E+00 | 0.000E+00 | 0.000E+00 | 0.000E+00 | 0.000E+00 | 0.000E+00 | 0.000E+00 |
| Unigene58655 | patA | 1.055E-05 | 4.213E-06 | 2.462E-06 | 1.129E-05 | 7.622E-05 | 1.061E-04 | 1.033E-04 | 8.030E-05 | 1.308E-04 | 1.283E-04 | 1.053E-04 | 7.052E-05 |
| Unigene58823 | APH(6)-Id | 2.857E-07 | 0.000E+00 | 1.869E-07 | 0.000E+00 | 0.000E+00 | 1.029E-07 | 3.162E-07 | 0.000E+00 | 0.000E+00 | 0.000E+00 | 3.268E-07 | 2.814E-07 |
| Unigene58924 | Staphylocc | 1.021E-06 | 0.000E+00 | 0.000E+00 | 0.000E+00 | 0.000E+00 | 0.000E+00 | 1.592E-08 | 0.000E+00 | 0.000E+00 | 0.000E+00 | 0.000E+00 | 0.000E+00 |
| Unigene58937 | oleI | 2.561E-06 | 3.050E-06 | 3.127E-06 | 1.678E-06 | 2.724E-07 | 0.000E+00 | 6.633E-07 | 0.000E+00 | 4.466E-07 | 0.000E+00 | 0.000E+00 | 0.000E+00 |
| Unigene59030 | bcrA | 2.910E-06 | 7.873E-06 | 8.367E-06 | 8.884E-06 | 0.000E+00 | 0.000E+00 | 0.000E+00 | 0.000E+00 | 0.000E+00 | 2.639E-07 | 0.000E+00 | 0.000E+00 |
| Unigene59045 | facT | 2.424E-06 | 2.339E-06 | 2.337E-06 | 1.118E-06 | 2.321E-07 | 0.000E+00 | 3.237E-07 | 1.951E-07 | 5.481E-07 | 0.000E+00 | 0.000E+00 | 7.409E-08 |
| Unigene59061 | tetA(60) | 1.345E-05 | 4.233E-06 | 3.593E-06 | 1.455E-05 | 9.155E-05 | 1.342E-04 | 1.337E-04 | 1.140E-04 | 1.690E-04 | 1.766E-04 | 1.405E-04 | 9.118E-05 |
| Unigene59143 | Listeria mc | 7.522E-07 | 6.241E-06 | 1.030E-05 | 1.607E-05 | 0.000E+00 | 0.000E+00 | 0.000E+00 | 2.976E-08 | 0.000E+00 | 0.000E+00 | 0.000E+00 | 0.000E+00 |
| Unigene59197 | abcA | 9.536E-05 | 1.832E-04 | 1.726E-04 | 7.678E-06 | 8.872E-06 | 0.000E+00 | 4.287E-06 | 0.000E+00 | 5.368E-05 | 0.000E+00 | 3.829E-06 | 0.000E+00 |
| Unigene59198 | YojI | 3.765E-05 | 2.301E-05 | 1.956E-05 | 2.109E-05 | 1.527E-06 | 1.486E-06 | 2.633E-06 | 9.566E-07 | 0.000E+00 | 0.000E+00 | 1.784E-06 | 0.000E+00 |
| Unigene59233 | arnA | 1.527E-06 | 8.196E-07 | 9.326E-07 | 1.474E-06 | 1.501E-05 | 5.834E-06 | 1.304E-05 | 1.577E-05 | 9.720E-06 | 1.558E-05 | 1.437E-05 | 6.460E-06 |
| Unigene59270 | bacA | 0.000E+00 | 0.000E+00 | 0.000E+00 | 0.000E+00 | 0.000E+00 | 0.000E+00 | 0.000E+00 | 0.000E+00 | 0.000E+00 | 0.000E+00 | 0.000E+00 | 3.419E-07 |

| | | | | | | | | | | | | | |
|---|---|---|---|---|---|---|---|---|---|---|---|---|---|
| Unigene59325 | Listeria mc | 2.290E-06 | 2.334E-06 | 2.959E-06 | 1.390E-06 | 0.000E+00 | 1.217E-07 | 6.231E-07 | 2.921E-07 | 5.880E-07 | 9.756E-08 | 1.002E-07 | 8.874E-08 |
| Unigene59347 | efrA | 2.116E-06 | 7.412E-07 | 4.418E-07 | 0.000E+00 | 0.000E+00 | 0.000E+00 | 0.000E+00 | 0.000E+00 | 0.000E+00 | 0.000E+00 | 0.000E+00 | 0.000E+00 |
| Unigene59355 | Corynebac | 7.145E-07 | 0.000E+00 | 0.000E+00 | 0.000E+00 | 7.611E-06 | 5.453E-07 | 1.535E-06 | 3.925E-07 | 0.000E+00 | 0.000E+00 | 4.808E-07 | 0.000E+00 |
| Unigene59412 | FosA2 | 0.000E+00 | 0.000E+00 | 0.000E+00 | 4.481E-07 | 0.000E+00 | 0.000E+00 | 0.000E+00 | 0.000E+00 | 0.000E+00 | 0.000E+00 | 0.000E+00 | 0.000E+00 |
| Unigene59464 | vanHB | 0.000E+00 | 0.000E+00 | 0.000E+00 | 0.000E+00 | 0.000E+00 | 3.651E-07 | 0.000E+00 | 0.000E+00 | 0.000E+00 | 0.000E+00 | 0.000E+00 | 0.000E+00 |
| Unigene59524 | vanYB | 7.346E-07 | 8.537E-07 | 4.028E-06 | 5.411E-06 | 0.000E+00 | 9.343E-08 | 5.739E-07 | 0.000E+00 | 0.000E+00 | 0.000E+00 | 0.000E+00 | 0.000E+00 |
| Unigene59558 | vatB | 1.962E-06 | 5.546E-06 | 8.692E-06 | 7.144E-06 | 0.000E+00 | 0.000E+00 | 0.000E+00 | 0.000E+00 | 0.000E+00 | 1.987E-07 | 0.000E+00 | 2.108E-07 |
| Unigene59595 | vanTG | 3.304E-06 | 1.925E-05 | 3.328E-06 | 9.448E-07 | 1.332E-06 | 4.425E-07 | 1.942E-07 | 4.778E-07 | 1.119E-06 | 4.886E-07 | 6.689E-07 | 3.456E-07 |
| Unigene59598 | lmrD | 4.114E-06 | 1.760E-05 | 3.071E-06 | 7.457E-07 | 9.376E-07 | 3.801E-07 | 0.000E+00 | 0.000E+00 | 9.993E-07 | 5.484E-07 | 4.425E-07 | 3.325E-07 |
| Unigene59621 | Staphylocc | 2.503E-06 | 4.621E-06 | 2.685E-06 | 6.189E-06 | 1.237E-04 | 2.331E-04 | 2.184E-04 | 2.423E-04 | 1.315E-04 | 9.318E-05 | 4.669E-05 | 2.184E-06 |
| Unigene59629 | arlR | 5.076E-07 | 0.000E+00 | 0.000E+00 | 5.125E-07 | 0.000E+00 | 0.000E+00 | 0.000E+00 | 0.000E+00 | 0.000E+00 | 0.000E+00 | 0.000E+00 | 0.000E+00 |
| Unigene59630 | macB | 3.146E-07 | 0.000E+00 | 0.000E+00 | 0.000E+00 | 0.000E+00 | 0.000E+00 | 0.000E+00 | 0.000E+00 | 0.000E+00 | 0.000E+00 | 0.000E+00 | 0.000E+00 |
| Unigene59654 | bcrA | 5.594E-07 | 2.745E-06 | 3.342E-05 | 3.323E-07 | 2.961E-06 | 2.846E-07 | 0.000E+00 | 0.000E+00 | 1.737E-04 | 7.698E-07 | 0.000E+00 | 3.890E-08 |
| Unigene59658 | MexW | 2.783E-07 | 0.000E+00 | 0.000E+00 | 0.000E+00 | 0.000E+00 | 0.000E+00 | 2.053E-07 | 1.083E-07 | 0.000E+00 | 0.000E+00 | 1.061E-07 | 1.645E-07 |
| Unigene59682 | ugd | 2.102E-06 | 1.712E-05 | 4.723E-06 | 2.682E-06 | 0.000E+00 | 0.000E+00 | 0.000E+00 | 3.229E-08 | 0.000E+00 | 0.000E+00 | 0.000E+00 | 0.000E+00 |
| Unigene59739 | abeS | 3.620E-06 | 3.973E-06 | 0.000E+00 | 2.843E-06 | 0.000E+00 | 0.000E+00 | 0.000E+00 | 0.000E+00 | 8.243E-07 | 0.000E+00 | 0.000E+00 | 0.000E+00 |
| Unigene59799 | LRA-10 | 9.782E-08 | 0.000E+00 | 0.000E+00 | 0.000E+00 | 0.000E+00 | 0.000E+00 | 0.000E+00 | 0.000E+00 | 0.000E+00 | 0.000E+00 | 0.000E+00 | 0.000E+00 |
| Unigene59820 | PmrF | 2.109E-06 | 1.668E-06 | 1.460E-06 | 6.889E-07 | 3.445E-08 | 0.000E+00 | 3.775E-07 | 1.448E-07 | 0.000E+00 | 0.000E+00 | 0.000E+00 | 1.100E-07 |
| Unigene59840 | vatB | 1.537E-07 | 0.000E+00 | 0.000E+00 | 0.000E+00 | 0.000E+00 | 0.000E+00 | 0.000E+00 | 0.000E+00 | 0.000E+00 | 0.000E+00 | 0.000E+00 | 0.000E+00 |
| Unigene59869 | Acinetoba | 1.921E-07 | 0.000E+00 | 6.285E-08 | 0.000E+00 | 0.000E+00 | 0.000E+00 | 0.000E+00 | 0.000E+00 | 0.000E+00 | 0.000E+00 | 0.000E+00 | 0.000E+00 |
| Unigene59887 | macB | 1.626E-06 | 2.362E-06 | 1.971E-06 | 6.761E-07 | 0.000E+00 | 0.000E+00 | 5.293E-07 | 2.233E-07 | 4.182E-07 | 0.000E+00 | 0.000E+00 | 0.000E+00 |
| Unigene59898 | kdpE | 7.111E-06 | 0.000E+00 | 0.000E+00 | 2.173E-06 | 3.373E-05 | 1.037E-05 | 2.294E-05 | 2.632E-05 | 0.000E+00 | 0.000E+00 | 0.000E+00 | 0.000E+00 |
| Unigene59899 | pmrA | 7.334E-06 | 0.000E+00 | 0.000E+00 | 2.784E-06 | 3.301E-05 | 1.632E-05 | 2.293E-05 | 2.578E-05 | 3.189E-06 | 0.000E+00 | 0.000E+00 | 0.000E+00 |
| Unigene59906 | Acinetoba | 5.486E-07 | 0.000E+00 | 0.000E+00 | 0.000E+00 | 4.688E-07 | 0.000E+00 | 7.939E-07 | 9.851E-07 | 0.000E+00 | 5.641E-07 | 7.723E-07 | 1.596E-06 |
| Unigene60001 | bcrA | 1.260E-06 | 9.874E-07 | 1.342E-06 | 1.563E-06 | 2.075E-05 | 6.964E-06 | 1.585E-05 | 1.857E-05 | 1.298E-05 | 1.884E-05 | 1.931E-05 | 7.997E-06 |
| Unigene60007 | poxtA | 1.100E-06 | 1.338E-06 | 1.575E-06 | 1.711E-06 | 1.820E-05 | 6.234E-06 | 1.540E-05 | 1.721E-05 | 1.030E-05 | 1.877E-05 | 1.775E-05 | 7.659E-06 |
| Unigene60015 | macB | 1.058E-06 | 1.009E-06 | 1.624E-06 | 1.486E-06 | 2.232E-05 | 7.456E-06 | 1.496E-05 | 1.846E-05 | 1.277E-05 | 2.013E-05 | 2.125E-05 | 6.685E-06 |
| Unigene60030 | macB | 1.494E-06 | 6.092E-07 | 9.217E-07 | 1.509E-06 | 1.860E-05 | 6.601E-06 | 1.215E-05 | 1.575E-05 | 1.092E-05 | 1.731E-05 | 1.998E-05 | 7.318E-06 |
| Unigene60033 | cmlv | 1.762E-06 | 9.377E-07 | 1.703E-06 | 1.618E-06 | 2.168E-05 | 6.927E-06 | 1.415E-05 | 2.004E-05 | 1.097E-05 | 2.094E-05 | 2.089E-05 | 7.417E-06 |
| Unigene60073 | bcrA | 8.582E-07 | 1.449E-06 | 5.830E-07 | 0.000E+00 | 2.200E-06 | 2.569E-06 | 9.425E-06 | 0.000E+00 | 3.463E-06 | 0.000E+00 | 0.000E+00 | 0.000E+00 |
| Unigene60123 | abeS | 2.665E-06 | 1.528E-05 | 6.199E-06 | 4.386E-06 | 0.000E+00 | 0.000E+00 | 0.000E+00 | 0.000E+00 | 0.000E+00 | 0.000E+00 | 3.388E-07 | 0.000E+00 |
| Unigene60161 | bacA | 1.001E-04 | 1.733E-04 | 1.727E-04 | 7.728E-06 | 9.580E-06 | 0.000E+00 | 3.697E-06 | 1.496E-06 | 5.693E-05 | 4.760E-07 | 2.488E-06 | 5.051E-07 |
| Unigene60165 | macB | 9.298E-05 | 1.481E-04 | 1.498E-04 | 6.580E-06 | 8.668E-06 | 1.854E-07 | 3.511E-06 | 2.052E-06 | 5.042E-05 | 0.000E+00 | 2.060E-06 | 6.081E-07 |
| Unigene60200 | mdtC | 6.241E-07 | 0.000E+00 | 0.000E+00 | 0.000E+00 | 0.000E+00 | 0.000E+00 | 4.145E-07 | 5.245E-07 | 0.000E+00 | 0.000E+00 | 2.285E-07 | 0.000E+00 |
| Unigene60232 | vanHO | 3.127E-05 | 2.280E-05 | 1.472E-06 | 5.202E-06 | 9.020E-06 | 1.276E-05 | 3.800E-05 | 5.747E-06 | 5.071E-05 | 5.637E-05 | 1.895E-05 | 1.387E-06 |
| Unigene60267 | vanE | 3.294E-06 | 4.082E-07 | 3.702E-06 | 7.244E-07 | 1.476E-07 | 1.436E-07 | 3.529E-07 | 0.000E+00 | 0.000E+00 | 0.000E+00 | 7.902E-07 | 0.000E+00 |
| Unigene60328 | bacA | 3.793E-06 | 3.611E-06 | 2.545E-06 | 5.980E-06 | 5.590E-07 | 0.000E+00 | 4.284E-07 | 0.000E+00 | 0.000E+00 | 0.000E+00 | 0.000E+00 | 1.373E-07 |
| Unigene60367 | patA | 6.927E-06 | 0.000E+00 | 0.000E+00 | 2.200E-06 | 3.528E-05 | 1.790E-05 | 2.237E-05 | 2.593E-05 | 4.047E-06 | 0.000E+00 | 0.000E+00 | 0.000E+00 |
| Unigene60377 | tetA(46) | 7.217E-06 | 0.000E+00 | 0.000E+00 | 2.487E-06 | 3.226E-05 | 1.752E-05 | 2.157E-05 | 2.598E-05 | 3.920E-06 | 0.000E+00 | 0.000E+00 | 0.000E+00 |
| Unigene60393 | fusB | 4.881E-06 | 9.678E-06 | 1.581E-05 | 1.240E-05 | 0.000E+00 | 2.270E-07 | 6.971E-07 | 3.063E-07 | 0.000E+00 | 0.000E+00 | 1.201E-07 | 0.000E+00 |
| Unigene60407 | efrA | 9.731E-07 | 1.089E-06 | 4.554E-06 | 5.877E-06 | 0.000E+00 | 4.046E-08 | 3.521E-07 | 0.000E+00 | 0.000E+00 | 0.000E+00 | 0.000E+00 | 6.636E-08 |
| Unigene60449 | lmrD | 3.266E-06 | 3.676E-06 | 2.177E-06 | 2.368E-06 | 2.047E-06 | 1.720E-06 | 1.622E-06 | 8.797E-07 | 1.968E-06 | 4.944E-06 | 4.119E-06 | 1.287E-06 |
| Unigene60472 | Staphylocc | 2.702E-06 | 8.435E-07 | 5.485E-07 | 9.408E-08 | 0.000E+00 | 7.553E-08 | 0.000E+00 | 0.000E+00 | 0.000E+00 | 0.000E+00 | 0.000E+00 | 0.000E+00 |
| Unigene60484 | Staphylocc | 3.180E-06 | 3.208E-06 | 4.397E-06 | 2.757E-06 | 0.000E+00 | 1.215E-07 | 5.155E-07 | 3.374E-07 | 7.899E-07 | 0.000E+00 | 0.000E+00 | 9.492E-08 |
| Unigene60487 | PmrF | 6.168E-07 | 0.000E+00 | 0.000E+00 | 4.153E-07 | 3.350E-06 | 8.150E-06 | 0.000E+00 | 8.880E-06 | 4.420E-06 | 0.000E+00 | 0.000E+00 | 1.094E-06 |
| Unigene60639 | Streptomy | 1.079E-06 | 9.016E-07 | 1.217E-06 | 1.692E-06 | 1.652E-05 | 6.182E-06 | 1.161E-05 | 1.504E-05 | 8.327E-06 | 1.425E-05 | 1.640E-05 | 6.321E-06 |
| Unigene60711 | NmcR | 8.157E-07 | 5.542E-06 | 1.052E-05 | 1.534E-05 | 0.000E+00 | 4.199E-08 | 0.000E+00 | 0.000E+00 | 0.000E+00 | 0.000E+00 | 0.000E+00 | 0.000E+00 |
| Unigene60773 | tetA(58) | 7.041E-06 | 0.000E+00 | 0.000E+00 | 2.005E-06 | 3.550E-05 | 1.667E-05 | 2.418E-05 | 2.638E-05 | 2.920E-06 | 0.000E+00 | 0.000E+00 | 0.000E+00 |
| Unigene60781 | lsaA | 7.101E-06 | 0.000E+00 | 0.000E+00 | 2.112E-06 | 3.124E-05 | 1.627E-05 | 2.300E-05 | 2.400E-05 | 2.547E-06 | 0.000E+00 | 0.000E+00 | 0.000E+00 |
| Unigene60791 | lmrD | 8.079E-06 | 0.000E+00 | 0.000E+00 | 2.232E-06 | 3.435E-05 | 1.751E-05 | 2.269E-05 | 2.639E-05 | 3.405E-06 | 0.000E+00 | 0.000E+00 | 0.000E+00 |
| Unigene60792 | patA | 7.436E-06 | 0.000E+00 | 0.000E+00 | 2.541E-06 | 3.378E-05 | 1.535E-05 | 2.521E-05 | 2.628E-05 | 3.571E-06 | 0.000E+00 | 0.000E+00 | 2.220E-08 |
| Unigene60797 | bcrA | 6.740E-06 | 0.000E+00 | 0.000E+00 | 1.968E-06 | 3.212E-05 | 1.734E-05 | 2.319E-05 | 2.460E-05 | 4.482E-06 | 0.000E+00 | 0.000E+00 | 0.000E+00 |
| Unigene60838 | oleC | 5.899E-06 | 0.000E+00 | 0.000E+00 | 1.765E-06 | 2.822E-05 | 1.181E-06 | 2.515E-05 | 2.659E-05 | 0.000E+00 | 0.000E+00 | 0.000E+00 | 0.000E+00 |
| Unigene60890 | YojI | 1.230E-05 | 5.032E-06 | 2.846E-06 | 1.058E-05 | 7.027E-05 | 1.017E-04 | 1.014E-04 | 7.563E-05 | 1.166E-04 | 1.256E-04 | 1.017E-04 | 6.536E-05 |
| Unigene60949 | novA | 9.308E-07 | 6.416E-06 | 1.107E-05 | 1.408E-05 | 0.000E+00 | 0.000E+00 | 0.000E+00 | 0.000E+00 | 0.000E+00 | 0.000E+00 | 0.000E+00 | 0.000E+00 |
| Unigene61011 | mgrA | 1.302E-06 | 0.000E+00 | 0.000E+00 | 0.000E+00 | 0.000E+00 | 0.000E+00 | 0.000E+00 | 0.000E+00 | 0.000E+00 | 0.000E+00 | 0.000E+00 | 0.000E+00 |
| Unigene61096 | vanRE | 3.405E-06 | 1.834E-05 | 3.653E-06 | 8.711E-07 | 5.044E-07 | 9.816E-08 | 0.000E+00 | 3.180E-07 | 0.000E+00 | 5.563E-07 | 0.000E+00 | 1.073E-07 |
| Unigene61097 | evgS | 4.508E-06 | 2.024E-05 | 3.485E-06 | 8.391E-07 | 1.500E-06 | 5.389E-07 | 2.069E-07 | 3.152E-07 | 1.294E-06 | 6.479E-07 | 4.039E-07 | 0.000E+00 |

| | | | | | | | | | | | | | |
|---|---|---|---|---|---|---|---|---|---|---|---|---|---|
| Unigene61160 | NmcR | 7.197E-07 | 0.000E+00 | 0.000E+00 | 0.000E+00 | 3.478E-06 | 7.936E-06 | 1.434E-06 | 6.721E-06 | 3.383E-06 | 0.000E+00 | 0.000E+00 | 4.254E-07 |
| Unigene61184 | baeS | 8.804E-07 | 5.368E-07 | 6.079E-06 | 6.651E-06 | 0.000E+00 | 0.000E+00 | 3.970E-07 | 0.000E+00 | 0.000E+00 | 0.000E+00 | 3.730E-08 | 0.000E+00 |
| Unigene61185 | mtrA | 5.688E-07 | 1.769E-06 | 3.256E-06 | 5.792E-06 | 0.000E+00 | 0.000E+00 | 2.098E-07 | 0.000E+00 | 0.000E+00 | 0.000E+00 | 0.000E+00 | 0.000E+00 |
| Unigene61292 | patA | 6.565E-07 | 0.000E+00 | 0.000E+00 | 0.000E+00 | 0.000E+00 | 0.000E+00 | 0.000E+00 | 0.000E+00 | 0.000E+00 | 0.000E+00 | 0.000E+00 | 0.000E+00 |
| Unigene61305 | Chlamydia | 2.679E-05 | 8.780E-06 | 6.395E-06 | 4.310E-06 | 5.111E-07 | 1.658E-07 | 5.657E-08 | 1.492E-07 | 7.822E-07 | 0.000E+00 | 0.000E+00 | 0.000E+00 |
| Unigene61307 | vanTG | 2.637E-05 | 9.042E-06 | 6.420E-06 | 4.807E-06 | 5.796E-07 | 1.645E-07 | 0.000E+00 | 0.000E+00 | 7.603E-07 | 0.000E+00 | 0.000E+00 | 0.000E+00 |
| Unigene61322 | tetA(46) | 4.145E-06 | 1.333E-06 | 1.479E-06 | 8.118E-06 | 3.954E-05 | 4.331E-05 | 3.591E-05 | 2.619E-05 | 1.095E-04 | 1.141E-04 | 1.021E-04 | 7.814E-05 |
| Unigene61331 | RlmA(II) | 7.797E-07 | 8.986E-07 | 5.088E-06 | 7.047E-06 | 0.000E+00 | 0.000E+00 | 3.020E-07 | 0.000E+00 | 0.000E+00 | 0.000E+00 | 0.000E+00 | 0.000E+00 |
| Unigene61347 | RlmA(II) | 1.793E-06 | 8.173E-06 | 5.279E-06 | 8.267E-06 | 1.299E-07 | 0.000E+00 | 0.000E+00 | 0.000E+00 | 0.000E+00 | 0.000E+00 | 0.000E+00 | 0.000E+00 |
| Unigene61351 | APH(3')-III | 1.561E-04 | 1.127E-05 | 8.207E-06 | 3.280E-07 | 1.056E-05 | 6.057E-06 | 0.000E+00 | 1.943E-06 | 0.000E+00 | 0.000E+00 | 0.000E+00 | 0.000E+00 |
| Unigene61353 | ErmB | 1.583E-04 | 1.709E-05 | 1.041E-04 | 7.067E-07 | 1.769E-05 | 6.667E-06 | 2.130E-06 | 1.685E-06 | 2.063E-04 | 1.316E-06 | 0.000E+00 | 1.654E-06 |
| Unigene61428 | bcrA | 6.208E-07 | 1.022E-06 | 5.076E-06 | 6.304E-06 | 0.000E+00 | 0.000E+00 | 3.817E-07 | 0.000E+00 | 0.000E+00 | 0.000E+00 | 0.000E+00 | 0.000E+00 |
| Unigene61521 | tetA(58) | 3.345E-06 | 3.785E-07 | 2.673E-06 | 1.741E-07 | 3.065E-07 | 0.000E+00 | 3.817E-07 | 0.000E+00 | 0.000E+00 | 0.000E+00 | 1.578E-07 | 0.000E+00 |
| Unigene61535 | bcrA | 3.738E-06 | 5.843E-06 | 7.039E-06 | 7.015E-06 | 8.389E-08 | 0.000E+00 | 0.000E+00 | 0.000E+00 | 0.000E+00 | 0.000E+00 | 0.000E+00 | 0.000E+00 |
| Unigene61546 | lmrD | 2.712E-05 | 0.000E+00 | 0.000E+00 | 0.000E+00 | 0.000E+00 | 0.000E+00 | 0.000E+00 | 0.000E+00 | 0.000E+00 | 0.000E+00 | 0.000E+00 | 0.000E+00 |
| Unigene61548 | lmrD | 3.062E-05 | 0.000E+00 | 0.000E+00 | 0.000E+00 | 0.000E+00 | 0.000E+00 | 0.000E+00 | 0.000E+00 | 0.000E+00 | 0.000E+00 | 0.000E+00 | 0.000E+00 |
| Unigene61553 | Staphylocc | 2.804E-05 | 0.000E+00 | 0.000E+00 | 0.000E+00 | 0.000E+00 | 0.000E+00 | 0.000E+00 | 0.000E+00 | 0.000E+00 | 0.000E+00 | 0.000E+00 | 0.000E+00 |
| Unigene61557 | mecI | 2.808E-05 | 0.000E+00 | 0.000E+00 | 0.000E+00 | 0.000E+00 | 0.000E+00 | 0.000E+00 | 0.000E+00 | 0.000E+00 | 0.000E+00 | 0.000E+00 | 0.000E+00 |
| Unigene61565 | patA | 2.935E-05 | 0.000E+00 | 4.766E-07 | 0.000E+00 | 0.000E+00 | 0.000E+00 | 0.000E+00 | 0.000E+00 | 0.000E+00 | 0.000E+00 | 0.000E+00 | 0.000E+00 |
| Unigene61596 | ceoB | 0.000E+00 | 0.000E+00 | 0.000E+00 | 0.000E+00 | 0.000E+00 | 3.752E-07 | 0.000E+00 | 0.000E+00 | 0.000E+00 | 0.000E+00 | 0.000E+00 | 0.000E+00 |
| Unigene61613 | iri | 3.668E-07 | 6.710E-08 | 0.000E+00 | 0.000E+00 | 0.000E+00 | 0.000E+00 | 0.000E+00 | 0.000E+00 | 0.000E+00 | 0.000E+00 | 0.000E+00 | 0.000E+00 |
| Unigene61656 | tetT | 1.168E-06 | 7.378E-06 | 1.094E-05 | 1.712E-05 | 0.000E+00 | 0.000E+00 | 0.000E+00 | 2.392E-08 | 0.000E+00 | 0.000E+00 | 0.000E+00 | 0.000E+00 |
| Unigene61669 | fexA | 9.921E-05 | 2.283E-06 | 4.878E-05 | 9.131E-08 | 0.000E+00 | 6.598E-07 | 0.000E+00 | 3.501E-05 | 2.322E-06 | 8.562E-07 | 9.298E-05 | 0.000E+00 |
| Unigene61685 | patB | 2.846E-06 | 3.023E-06 | 2.973E-06 | 1.799E-06 | 2.652E-07 | 1.787E-07 | 5.487E-07 | 5.358E-07 | 1.044E-06 | 0.000E+00 | 1.260E-07 | 1.736E-07 |
| Unigene61686 | efrA | 2.770E-06 | 2.506E-06 | 2.791E-06 | 1.361E-06 | 0.000E+00 | 0.000E+00 | 3.521E-07 | 3.276E-07 | 6.136E-07 | 0.000E+00 | 0.000E+00 | 0.000E+00 |
| Unigene61701 | tetB(58) | 3.092E-06 | 3.439E-06 | 2.468E-06 | 2.123E-06 | 2.794E-06 | 2.941E-06 | 1.734E-06 | 9.143E-07 | 1.397E-06 | 5.373E-06 | 6.272E-06 | 1.754E-06 |
| Unigene61758 | optrA | 5.733E-07 | 5.566E-06 | 8.957E-06 | 1.519E-05 | 0.000E+00 | 0.000E+00 | 0.000E+00 | 0.000E+00 | 0.000E+00 | 0.000E+00 | 0.000E+00 | 0.000E+00 |
| Unigene61775 | Bifidobacte | 7.239E-07 | 0.000E+00 | 0.000E+00 | 0.000E+00 | 0.000E+00 | 0.000E+00 | 0.000E+00 | 0.000E+00 | 0.000E+00 | 0.000E+00 | 0.000E+00 | 2.334E-07 |
| Unigene61781 | optrA | 1.818E-05 | 1.298E-05 | 1.475E-05 | 1.588E-05 | 7.154E-07 | 8.793E-07 | 1.782E-06 | 2.769E-07 | 2.463E-06 | 0.000E+00 | 7.947E-07 | 7.611E-07 |
| Unigene61830 | patA | 2.989E-06 | 2.322E-06 | 3.249E-06 | 1.579E-06 | 1.533E-07 | 0.000E+00 | 4.580E-07 | 2.684E-07 | 6.534E-07 | 0.000E+00 | 0.000E+00 | 0.000E+00 |
| Unigene61831 | tetA(58) | 2.451E-06 | 2.234E-06 | 2.527E-06 | 1.049E-06 | 0.000E+00 | 1.796E-07 | 2.759E-07 | 2.182E-07 | 7.719E-07 | 0.000E+00 | 0.000E+00 | 2.946E-07 |
| Unigene61835 | Chlamydia | 1.983E-06 | 3.320E-06 | 2.968E-06 | 1.377E-06 | 2.479E-07 | 0.000E+00 | 4.665E-07 | 0.000E+00 | 9.213E-07 | 0.000E+00 | 0.000E+00 | 0.000E+00 |
| Unigene61840 | oleB | 2.029E-06 | 1.865E-06 | 2.917E-06 | 0.000E+00 | 2.621E-07 | 0.000E+00 | 7.312E-07 | 2.203E-07 | 7.737E-07 | 0.000E+00 | 0.000E+00 | 0.000E+00 |
| Unigene62026 | lsaC | 1.289E-06 | 8.132E-07 | 4.837E-06 | 5.661E-06 | 0.000E+00 | 6.965E-08 | 2.139E-07 | 0.000E+00 | 0.000E+00 | 0.000E+00 | 0.000E+00 | 0.000E+00 |
| Unigene62099 | rphB | 1.369E-05 | 2.155E-05 | 1.081E-06 | 5.357E-06 | 8.218E-06 | 1.456E-05 | 1.857E-05 | 6.978E-06 | 5.319E-05 | 5.483E-05 | 1.201E-05 | 1.634E-06 |
| Unigene62115 | oleC | 4.046E-06 | 1.053E-05 | 1.060E-05 | 1.043E-05 | 0.000E+00 | 0.000E+00 | 4.763E-08 | 0.000E+00 | 0.000E+00 | 0.000E+00 | 0.000E+00 | 0.000E+00 |
| Unigene62131 | macB | 6.329E-07 | 5.905E-06 | 4.835E-06 | 1.137E-05 | 0.000E+00 | 1.955E-07 | 1.001E-07 | 0.000E+00 | 0.000E+00 | 0.000E+00 | 0.000E+00 | 0.000E+00 |
| Unigene62220 | arlR | 1.545E-05 | 1.464E-06 | 0.000E+00 | 0.000E+00 | 0.000E+00 | 0.000E+00 | 0.000E+00 | 0.000E+00 | 0.000E+00 | 0.000E+00 | 0.000E+00 | 0.000E+00 |
| Unigene62302 | tetT | 1.722E-06 | 1.367E-06 | 2.142E-06 | 2.173E-06 | 2.075E-05 | 6.796E-06 | 1.385E-05 | 1.629E-05 | 1.042E-05 | 1.747E-05 | 1.878E-05 | 7.922E-06 |
| Unigene62435 | smeR | 3.745E-06 | 8.220E-06 | 8.186E-06 | 8.999E-06 | 0.000E+00 | 0.000E+00 | 0.000E+00 | 0.000E+00 | 0.000E+00 | 0.000E+00 | 0.000E+00 | 0.000E+00 |
| Unigene62504 | Acinetobac | 1.442E-06 | 1.551E-05 | 8.058E-06 | 5.043E-06 | 0.000E+00 | 0.000E+00 | 0.000E+00 | 0.000E+00 | 0.000E+00 | 0.000E+00 | 0.000E+00 | 0.000E+00 |
| Unigene62509 | vanHO | 2.233E-06 | 1.742E-05 | 7.072E-06 | 5.604E-06 | 0.000E+00 | 0.000E+00 | 0.000E+00 | 0.000E+00 | 0.000E+00 | 0.000E+00 | 1.161E-07 | 0.000E+00 |
| Unigene62569 | vanHA | 9.415E-07 | 9.595E-07 | 1.188E-06 | 1.562E-06 | 2.040E-05 | 6.725E-06 | 1.303E-05 | 1.621E-05 | 1.003E-05 | 1.498E-05 | 1.762E-05 | 7.115E-06 |
| Unigene62573 | Staphylocc | 9.457E-07 | 8.696E-07 | 1.204E-06 | 1.781E-06 | 1.823E-05 | 5.996E-06 | 1.355E-05 | 1.629E-05 | 1.120E-05 | 1.543E-05 | 1.652E-05 | 7.311E-06 |
| Unigene62585 | marA | 1.187E-06 | 8.826E-07 | 8.266E-07 | 1.972E-06 | 1.885E-05 | 7.079E-06 | 1.670E-05 | 1.859E-05 | 1.050E-05 | 1.881E-05 | 1.441E-05 | 9.596E-06 |
| Unigene62590 | rphB | 1.104E-06 | 9.164E-07 | 1.216E-06 | 1.523E-06 | 1.863E-05 | 4.935E-06 | 1.298E-05 | 1.548E-05 | 9.847E-06 | 1.502E-05 | 1.608E-05 | 7.195E-06 |
| Unigene62591 | patA | 1.050E-06 | 9.670E-07 | 1.030E-06 | 1.439E-06 | 1.760E-05 | 5.998E-06 | 1.384E-05 | 1.600E-05 | 9.486E-06 | 1.437E-05 | 1.690E-05 | 6.647E-06 |
| Unigene62592 | lmrD | 1.250E-06 | 1.014E-06 | 8.710E-07 | 1.427E-06 | 1.743E-05 | 5.072E-06 | 1.219E-05 | 1.446E-05 | 9.642E-06 | 1.532E-05 | 1.608E-05 | 6.274E-06 |
| Unigene62598 | bcrA | 1.220E-06 | 7.305E-07 | 1.342E-06 | 1.307E-06 | 1.869E-05 | 6.196E-06 | 1.625E-05 | 1.886E-05 | 9.053E-06 | 1.755E-05 | 1.586E-05 | 8.304E-06 |
| Unigene62600 | norB | 9.611E-07 | 9.307E-07 | 1.313E-06 | 1.351E-06 | 1.752E-05 | 6.478E-06 | 1.343E-05 | 1.546E-05 | 9.536E-06 | 1.535E-05 | 1.597E-05 | 7.484E-06 |
| Unigene62638 | patA | 5.923E-06 | 0.000E+00 | 0.000E+00 | 2.054E-06 | 3.415E-05 | 1.598E-05 | 2.247E-05 | 2.475E-05 | 2.915E-06 | 0.000E+00 | 0.000E+00 | 0.000E+00 |
| Unigene62639 | oleC | 7.516E-06 | 0.000E+00 | 0.000E+00 | 2.571E-06 | 3.463E-05 | 1.869E-05 | 2.659E-05 | 2.857E-05 | 4.085E-06 | 0.000E+00 | 0.000E+00 | 0.000E+00 |
| Unigene62869 | bmr | 5.347E-05 | 7.113E-05 | 2.823E-05 | 1.588E-05 | 2.080E-05 | 6.231E-06 | 3.676E-06 | 6.088E-05 | 1.434E-05 | 7.920E-06 | 2.072E-06 | 9.734E-07 |
| Unigene62882 | optrA | 5.012E-05 | 7.359E-05 | 3.029E-05 | 1.669E-05 | 2.172E-05 | 6.235E-06 | 3.192E-06 | 5.811E-05 | 1.326E-05 | 7.688E-06 | 1.706E-06 | 1.246E-06 |
| Unigene62883 | macB | 4.815E-05 | 6.859E-05 | 2.963E-05 | 1.509E-05 | 2.021E-05 | 6.554E-06 | 3.503E-06 | 5.535E-05 | 1.180E-05 | 6.754E-06 | 1.791E-06 | 8.269E-07 |
| Unigene62888 | Corynebac | 5.156E-05 | 7.471E-05 | 3.045E-05 | 1.570E-05 | 2.355E-05 | 6.547E-06 | 3.620E-06 | 5.598E-05 | 1.460E-05 | 7.077E-06 | 2.103E-06 | 8.628E-07 |
| Unigene62891 | efrA | 5.037E-05 | 6.711E-05 | 2.925E-05 | 1.431E-05 | 2.008E-05 | 5.983E-06 | 3.956E-06 | 5.839E-05 | 1.230E-05 | 7.681E-06 | 2.117E-06 | 8.794E-07 |
| Unigene63098 | PmrF | 1.109E-06 | 4.044E-06 | 3.423E-06 | 5.235E-06 | 0.000E+00 | 0.000E+00 | 0.000E+00 | 0.000E+00 | 0.000E+00 | 7.265E-08 | 0.000E+00 | 0.000E+00 |

| | | | | | | | | | | | | | |
|---|---|---|---|---|---|---|---|---|---|---|---|---|---|
| Unigene63149 | aadS | 2.152E-06 | 1.782E-06 | 4.927E-06 | 2.783E-06 | 1.258E-07 | 0.000E+00 | 0.000E+00 | 0.000E+00 | 0.000E+00 | 1.682E-07 | 0.000E+00 | 0.000E+00 |
| Unigene63172 | kdpE | 2.637E-07 | 1.737E-07 | 0.000E+00 | 0.000E+00 | 0.000E+00 | 0.000E+00 | 0.000E+00 | 0.000E+00 | 0.000E+00 | 0.000E+00 | 0.000E+00 | 0.000E+00 |
| Unigene63182 | ugd | 4.368E-07 | 1.307E-07 | 0.000E+00 | 0.000E+00 | 2.206E-07 | 0.000E+00 | 0.000E+00 | 0.000E+00 | 0.000E+00 | 0.000E+00 | 0.000E+00 | 0.000E+00 |
| Unigene63225 | vanRM | 2.500E-06 | 1.960E-06 | 3.286E-06 | 1.668E-06 | 9.920E-08 | 0.000E+00 | 7.906E-07 | 0.000E+00 | 5.856E-07 | 0.000E+00 | 0.000E+00 | 0.000E+00 |
| Unigene63226 | baeS | 1.867E-06 | 2.145E-06 | 3.060E-06 | 1.441E-06 | 2.440E-07 | 0.000E+00 | 5.104E-07 | 2.820E-07 | 6.240E-07 | 2.202E-07 | 0.000E+00 | 0.000E+00 |
| Unigene63250 | baeS | 9.842E-07 | 1.176E-06 | 4.764E-06 | 6.449E-06 | 0.000E+00 | 0.000E+00 | 2.663E-07 | 0.000E+00 | 0.000E+00 | 0.000E+00 | 1.501E-07 | 5.170E-08 |
| Unigene63363 | patB | 2.745E-06 | 1.612E-05 | 8.316E-06 | 5.358E-06 | 0.000E+00 | 0.000E+00 | 0.000E+00 | 0.000E+00 | 0.000E+00 | 0.000E+00 | 0.000E+00 | 0.000E+00 |
| Unigene63393 | efrA | 0.000E+00 | 0.000E+00 | 0.000E+00 | 0.000E+00 | 0.000E+00 | 2.693E-07 | 1.957E-06 | 0.000E+00 | 0.000E+00 | 0.000E+00 | 0.000E+00 | 0.000E+00 |
| Unigene63411 | efrA | 1.462E-06 | 8.641E-07 | 1.196E-06 | 6.058E-07 | 2.083E-07 | 1.216E-07 | 1.245E-07 | 1.532E-07 | 0.000E+00 | 4.176E-08 | 0.000E+00 | 1.551E-07 |
| Unigene63414 | macB | 2.023E-06 | 1.817E-06 | 4.061E-06 | 2.786E-06 | 0.000E+00 | 0.000E+00 | 0.000E+00 | 0.000E+00 | 4.021E-07 | 0.000E+00 | 1.578E-07 | 0.000E+00 |
| Unigene63427 | vmlR | 6.781E-06 | 0.000E+00 | 1.474E-06 | 5.289E-06 | 4.358E-05 | 6.725E-05 | 6.067E-05 | 5.905E-05 | 7.083E-05 | 7.721E-05 | 0.000E+00 | 3.739E-05 |
| Unigene63475 | evgS | 2.288E-06 | 7.847E-07 | 8.418E-07 | 1.805E-07 | 3.971E-08 | 0.000E+00 | 0.000E+00 | 0.000E+00 | 0.000E+00 | 0.000E+00 | 0.000E+00 | 0.000E+00 |
| Unigene63480 | bcrA | 7.463E-06 | 0.000E+00 | 0.000E+00 | 2.664E-06 | 3.605E-05 | 1.722E-05 | 2.516E-05 | 2.658E-05 | 4.515E-06 | 0.000E+00 | 0.000E+00 | 0.000E+00 |
| Unigene63493 | macB | 1.655E-06 | 0.000E+00 | 0.000E+00 | 0.000E+00 | 0.000E+00 | 0.000E+00 | 0.000E+00 | 0.000E+00 | 0.000E+00 | 0.000E+00 | 0.000E+00 | 0.000E+00 |
| Unigene63520 | patB | 1.382E-06 | 0.000E+00 | 0.000E+00 | 0.000E+00 | 0.000E+00 | 0.000E+00 | 0.000E+00 | 0.000E+00 | 0.000E+00 | 0.000E+00 | 0.000E+00 | 0.000E+00 |
| Unigene63521 | tetA(58) | 1.187E-06 | 4.203E-08 | 0.000E+00 | 0.000E+00 | 0.000E+00 | 0.000E+00 | 0.000E+00 | 0.000E+00 | 0.000E+00 | 0.000E+00 | 0.000E+00 | 0.000E+00 |
| Unigene63525 | baeS | 1.870E-06 | 0.000E+00 | 0.000E+00 | 0.000E+00 | 0.000E+00 | 0.000E+00 | 0.000E+00 | 0.000E+00 | 0.000E+00 | 0.000E+00 | 0.000E+00 | 0.000E+00 |
| Unigene63527 | mtrA | 1.603E-06 | 0.000E+00 | 0.000E+00 | 0.000E+00 | 0.000E+00 | 0.000E+00 | 0.000E+00 | 0.000E+00 | 0.000E+00 | 0.000E+00 | 0.000E+00 | 0.000E+00 |
| Unigene63535 | vmlR | 8.083E-07 | 2.772E-08 | 0.000E+00 | 0.000E+00 | 0.000E+00 | 0.000E+00 | 1.118E-07 | 0.000E+00 | 0.000E+00 | 0.000E+00 | 0.000E+00 | 0.000E+00 |
| Unigene63549 | msbA | 1.291E-06 | 6.161E-08 | 0.000E+00 | 0.000E+00 | 0.000E+00 | 0.000E+00 | 2.071E-08 | 0.000E+00 | 0.000E+00 | 0.000E+00 | 0.000E+00 | 0.000E+00 |
| Unigene63550 | patB | 1.403E-06 | 9.744E-08 | 0.000E+00 | 0.000E+00 | 0.000E+00 | 3.839E-08 | 0.000E+00 | 0.000E+00 | 0.000E+00 | 0.000E+00 | 0.000E+00 | 0.000E+00 |
| Unigene63566 | Staphylocc | 1.725E-06 | 0.000E+00 | 0.000E+00 | 0.000E+00 | 0.000E+00 | 2.591E-08 | 0.000E+00 | 0.000E+00 | 0.000E+00 | 0.000E+00 | 0.000E+00 | 0.000E+00 |
| Unigene63591 | MdtK | 2.988E-05 | 2.104E-05 | 1.740E-05 | 1.969E-05 | 1.431E-06 | 1.256E-06 | 2.097E-06 | 7.076E-07 | 0.000E+00 | 7.597E-07 | 1.040E-06 | 9.255E-07 |
| Unigene63610 | macB | 4.420E-06 | 9.065E-06 | 1.003E-05 | 1.298E-05 | 1.488E-07 | 0.000E+00 | 0.000E+00 | 0.000E+00 | 0.000E+00 | 0.000E+00 | 0.000E+00 | 0.000E+00 |
| Unigene63719 | carA | 2.844E-06 | 3.278E-06 | 4.047E-06 | 0.000E+00 | 1.580E-07 | 0.000E+00 | 1.574E-07 | 0.000E+00 | 5.699E-07 | 0.000E+00 | 0.000E+00 | 5.603E-08 |
| Unigene63728 | macB | 8.691E-07 | 9.993E-07 | 4.914E-06 | 6.143E-06 | 9.195E-08 | 0.000E+00 | 5.038E-07 | 0.000E+00 | 0.000E+00 | 0.000E+00 | 0.000E+00 | 0.000E+00 |
| Unigene63732 | MuxC | 3.183E-07 | 0.000E+00 | 0.000E+00 | 0.000E+00 | 0.000E+00 | 1.377E-07 | 0.000E+00 | 0.000E+00 | 6.959E-08 | 0.000E+00 | 1.457E-07 | 0.000E+00 |
| Unigene63851 | Escherichia | 5.805E-06 | 1.082E-05 | 1.117E-05 | 1.212E-05 | 2.202E-07 | 0.000E+00 | 0.000E+00 | 0.000E+00 | 0.000E+00 | 9.463E-08 | 1.296E-07 | 3.347E-08 |
| Unigene63854 | macB | 2.805E-06 | 2.624E-06 | 2.978E-06 | 1.439E-06 | 2.043E-07 | 0.000E+00 | 3.054E-07 | 3.220E-07 | 3.016E-07 | 0.000E+00 | 0.000E+00 | 0.000E+00 |
| Unigene63962 | tva(A) | 1.831E-06 | 3.846E-06 | 2.462E-06 | 3.538E-06 | 5.525E-07 | 9.775E-08 | 3.503E-07 | 7.916E-08 | 1.729E-07 | 0.000E+00 | 7.757E-08 | 5.611E-07 |
| Unigene64036 | TaeA | 5.970E-06 | 0.000E+00 | 0.000E+00 | 2.642E-06 | 3.512E-05 | 8.485E-06 | 0.000E+00 | 0.000E+00 | 0.000E+00 | 0.000E+00 | 0.000E+00 | 0.000E+00 |
| Unigene64110 | Staphylocc | 1.233E-05 | 4.869E-06 | 3.380E-06 | 1.298E-05 | 9.523E-05 | 1.350E-04 | 1.425E-04 | 1.164E-04 | 1.707E-04 | 1.679E-04 | 1.354E-04 | 8.940E-05 |
| Unigene64175 | tet(44) | 4.135E-06 | 1.168E-05 | 1.192E-05 | 1.413E-05 | 1.130E-07 | 0.000E+00 | 0.000E+00 | 0.000E+00 | 0.000E+00 | 1.510E-07 | 0.000E+00 | 1.202E-07 |
| Unigene64190 | tetB(60) | 2.830E-06 | 3.433E-06 | 2.375E-06 | 1.316E-06 | 1.944E-06 | 2.254E-06 | 1.978E-06 | 1.217E-06 | 1.017E-06 | 5.143E-06 | 5.281E-06 | 2.641E-06 |
| Unigene64243 | bacA | 2.129E-06 | 1.997E-06 | 2.279E-06 | 1.368E-06 | 0.000E+00 | 0.000E+00 | 2.999E-07 | 0.000E+00 | 5.500E-07 | 1.725E-07 | 0.000E+00 | 0.000E+00 |
| Unigene64245 | PEDO-2 | 2.574E-06 | 2.260E-06 | 2.223E-06 | 1.612E-06 | 0.000E+00 | 0.000E+00 | 0.000E+00 | 0.000E+00 | 8.441E-07 | 0.000E+00 | 0.000E+00 | 0.000E+00 |
| Unigene64282 | patB | 7.718E-07 | 2.491E-08 | 0.000E+00 | 3.439E-07 | 3.884E-06 | 9.669E-06 | 1.708E-06 | 7.499E-06 | 4.962E-06 | 3.439E-06 | 0.000E+00 | 9.123E-07 |
| Unigene64306 | tetA(58) | 2.981E-05 | 1.276E-06 | 0.000E+00 | 0.000E+00 | 0.000E+00 | 9.667E-08 | 0.000E+00 | 0.000E+00 | 0.000E+00 | 0.000E+00 | 0.000E+00 | 0.000E+00 |
| Unigene64309 | Streptomy | 6.542E-05 | 6.156E-05 | 0.000E+00 | 0.000E+00 | 0.000E+00 | 2.509E-07 | 0.000E+00 | 0.000E+00 | 0.000E+00 | 2.586E-07 | 0.000E+00 | 0.000E+00 |
| Unigene64355 | vanRG | 8.037E-07 | 7.840E-06 | 1.016E-05 | 1.515E-05 | 0.000E+00 | 0.000E+00 | 0.000E+00 | 0.000E+00 | 0.000E+00 | 0.000E+00 | 0.000E+00 | 0.000E+00 |
| Unigene64360 | vatE | 1.124E-06 | 0.000E+00 | 0.000E+00 | 0.000E+00 | 5.769E-06 | 8.912E-06 | 0.000E+00 | 0.000E+00 | 0.000E+00 | 4.174E-06 | 0.000E+00 | 0.000E+00 |
| Unigene64400 | vanSA | 3.200E-06 | 3.429E-06 | 2.621E-06 | 2.010E-06 | 2.324E-06 | 1.743E-06 | 2.315E-06 | 8.530E-07 | 1.019E-06 | 5.530E-06 | 5.390E-06 | 1.370E-06 |
| Unigene64401 | arlR | 3.247E-06 | 2.749E-06 | 2.366E-06 | 1.920E-06 | 2.782E-06 | 1.604E-06 | 1.951E-06 | 1.029E-06 | 9.631E-07 | 5.890E-06 | 6.207E-06 | 1.316E-06 |
| Unigene64443 | macB | 3.027E-06 | 8.553E-07 | 2.088E-06 | 4.540E-07 | 0.000E+00 | 0.000E+00 | 0.000E+00 | 3.149E-07 | 0.000E+00 | 0.000E+00 | 0.000E+00 | 0.000E+00 |
| Unigene64451 | patA | 2.449E-06 | 1.440E-06 | 1.588E-06 | 5.300E-07 | 0.000E+00 | 0.000E+00 | 0.000E+00 | 2.042E-07 | 0.000E+00 | 0.000E+00 | 0.000E+00 | 0.000E+00 |
| Unigene64459 | evgA | 3.851E-06 | 2.175E-06 | 1.445E-06 | 6.863E-07 | 0.000E+00 | 0.000E+00 | 0.000E+00 | 0.000E+00 | 0.000E+00 | 0.000E+00 | 0.000E+00 | 0.000E+00 |
| Unigene64498 | bcrA | 2.317E-06 | 8.620E-07 | 1.541E-06 | 7.138E-07 | 0.000E+00 | 0.000E+00 | 0.000E+00 | 4.584E-08 | 0.000E+00 | 0.000E+00 | 0.000E+00 | 0.000E+00 |
| Unigene64514 | tlrC | 2.170E-06 | 1.786E-06 | 3.017E-06 | 7.305E-07 | 0.000E+00 | 0.000E+00 | 0.000E+00 | 1.583E-07 | 0.000E+00 | 0.000E+00 | 0.000E+00 | 0.000E+00 |
| Unigene64554 | tetA(58) | 2.983E-06 | 0.000E+00 | 2.038E-06 | 6.481E-07 | 0.000E+00 | 0.000E+00 | 0.000E+00 | 3.966E-07 | 0.000E+00 | 0.000E+00 | 0.000E+00 | 0.000E+00 |
| Unigene64571 | lmrC | 1.849E-06 | 0.000E+00 | 1.608E-06 | 6.791E-07 | 0.000E+00 | 0.000E+00 | 0.000E+00 | 0.000E+00 | 0.000E+00 | 0.000E+00 | 0.000E+00 | 0.000E+00 |
| Unigene64577 | patA | 1.779E-06 | 1.255E-06 | 1.247E-06 | 2.995E-07 | 0.000E+00 | 0.000E+00 | 0.000E+00 | 1.978E-07 | 0.000E+00 | 0.000E+00 | 0.000E+00 | 0.000E+00 |
| Unigene64586 | lmrB | 2.009E-06 | 1.191E-06 | 2.220E-06 | 6.529E-07 | 0.000E+00 | 0.000E+00 | 0.000E+00 | 1.617E-07 | 0.000E+00 | 0.000E+00 | 0.000E+00 | 0.000E+00 |
| Unigene64614 | vanHF | 1.278E-06 | 0.000E+00 | 0.000E+00 | 0.000E+00 | 0.000E+00 | 0.000E+00 | 0.000E+00 | 0.000E+00 | 0.000E+00 | 0.000E+00 | 0.000E+00 | 0.000E+00 |
| Unigene64621 | fexA | 1.196E-06 | 0.000E+00 | 0.000E+00 | 0.000E+00 | 0.000E+00 | 0.000E+00 | 0.000E+00 | 0.000E+00 | 0.000E+00 | 0.000E+00 | 0.000E+00 | 0.000E+00 |
| Unigene64627 | baeS | 1.360E-06 | 0.000E+00 | 0.000E+00 | 0.000E+00 | 0.000E+00 | 0.000E+00 | 0.000E+00 | 0.000E+00 | 0.000E+00 | 0.000E+00 | 0.000E+00 | 0.000E+00 |
| Unigene64628 | vanRF | 8.166E-07 | 0.000E+00 | 0.000E+00 | 0.000E+00 | 0.000E+00 | 1.039E-07 | 0.000E+00 | 0.000E+00 | 0.000E+00 | 0.000E+00 | 0.000E+00 | 0.000E+00 |
| Unigene64724 | tlrC | 2.780E-05 | 8.347E-06 | 2.826E-06 | 5.588E-06 | 1.312E-06 | 8.571E-07 | 1.325E-06 | 1.889E-07 | 5.978E-06 | 3.064E-07 | 2.961E-07 | 8.415E-07 |
| Unigene64787 | macB | 4.598E-07 | 4.694E-06 | 9.926E-06 | 1.305E-05 | 0.000E+00 | 0.000E+00 | 0.000E+00 | 0.000E+00 | 0.000E+00 | 0.000E+00 | 0.000E+00 | 0.000E+00 |

| | | | | | | | | | | | | | |
|---|---|---|---|---|---|---|---|---|---|---|---|---|---|
| Unigene64798 | msbA | 3.982E-06 | 0.000E+00 | 3.344E-06 | 5.414E-07 | 1.702E-07 | 2.070E-07 | 4.662E-07 | 0.000E+00 | 0.000E+00 | 0.000E+00 | 8.322E-07 | 0.000E+00 |
| Unigene64799 | tetB(46) | 3.026E-06 | 2.050E-07 | 3.446E-06 | 6.414E-07 | 4.981E-07 | 0.000E+00 | 1.654E-07 | 0.000E+00 | 0.000E+00 | 0.000E+00 | 7.693E-07 | 0.000E+00 |
| Unigene64818 | arlS | 8.985E-07 | 6.738E-06 | 1.068E-05 | 1.553E-05 | 0.000E+00 | 9.250E-08 | 0.000E+00 | 0.000E+00 | 0.000E+00 | 0.000E+00 | 0.000E+00 | 0.000E+00 |
| Unigene64859 | srmB | 3.801E-06 | 4.710E-07 | 3.279E-06 | 9.287E-08 | 5.109E-07 | 0.000E+00 | 1.696E-07 | 0.000E+00 | 0.000E+00 | 0.000E+00 | 9.468E-07 | 0.000E+00 |
| Unigene64879 | OXA-18 | 0.000E+00 | 0.000E+00 | 0.000E+00 | 0.000E+00 | 6.592E-07 | 0.000E+00 | 0.000E+00 | 0.000E+00 | 0.000E+00 | 0.000E+00 | 0.000E+00 | 0.000E+00 |
| Unigene64891 | msbA | 1.466E-06 | 8.775E-07 | 1.275E-06 | 3.028E-06 | 4.071E-07 | 0.000E+00 | 0.000E+00 | 0.000E+00 | 0.000E+00 | 0.000E+00 | 0.000E+00 | 0.000E+00 |
| Unigene64938 | macB | 5.937E-07 | 8.144E-08 | 3.641E-08 | 0.000E+00 | 3.298E-07 | 0.000E+00 | 0.000E+00 | 0.000E+00 | 0.000E+00 | 0.000E+00 | 0.000E+00 | 0.000E+00 |
| Unigene64949 | vanHM | 2.884E-06 | 1.838E-06 | 2.647E-06 | 1.941E-06 | 6.890E-08 | 2.347E-07 | 6.864E-08 | 0.000E+00 | 0.000E+00 | 2.763E-07 | 0.000E+00 | 0.000E+00 |
| Unigene65006 | patA | 3.662E-06 | 1.836E-05 | 3.347E-06 | 7.345E-07 | 8.638E-07 | 2.440E-07 | 0.000E+00 | 3.513E-07 | 1.261E-06 | 0.000E+00 | 1.148E-07 | 8.894E-08 |
| Unigene65031 | macB | 3.336E-06 | 4.383E-06 | 3.965E-06 | 5.646E-06 | 0.000E+00 | 0.000E+00 | 5.200E-07 | 0.000E+00 | 3.081E-07 | 0.000E+00 | 2.687E-07 | 2.222E-07 |
| Unigene65050 | tetA(58) | 3.219E-06 | 4.794E-06 | 4.016E-06 | 7.197E-06 | 4.087E-07 | 0.000E+00 | 0.000E+00 | 5.367E-08 | 0.000E+00 | 0.000E+00 | 1.052E-07 | 2.718E-07 |
| Unigene65051 | oleC | 2.700E-06 | 3.192E-06 | 2.691E-06 | 5.118E-06 | 0.000E+00 | 4.491E-08 | 4.138E-07 | 0.000E+00 | 0.000E+00 | 0.000E+00 | 0.000E+00 | 1.473E-07 |
| Unigene65059 | Chlamydia | 3.196E-06 | 4.220E-06 | 3.062E-06 | 5.521E-06 | 7.489E-07 | 0.000E+00 | 3.868E-07 | 5.828E-08 | 1.091E-07 | 0.000E+00 | 1.428E-07 | 5.902E-08 |
| Unigene65172 | bmr | 1.431E-06 | 1.163E-07 | 1.014E-06 | 2.596E-06 | 0.000E+00 | 0.000E+00 | 3.813E-07 | 1.145E-06 | 0.000E+00 | 4.724E-07 | 6.063E-07 | 2.193E-07 |
| Unigene65209 | lsaC | 1.577E-06 | 3.292E-06 | 2.154E-06 | 3.528E-06 | 4.122E-07 | 5.014E-08 | 4.278E-07 | 0.000E+00 | 2.535E-07 | 0.000E+00 | 7.074E-08 | 1.827E-07 |
| Unigene65253 | rphB | 2.686E-05 | 0.000E+00 | 0.000E+00 | 0.000E+00 | 0.000E+00 | 1.309E-07 | 0.000E+00 | 0.000E+00 | 0.000E+00 | 0.000E+00 | 0.000E+00 | 0.000E+00 |
| Unigene65254 | lmrB | 6.873E-07 | 7.075E-06 | 1.002E-05 | 1.501E-05 | 0.000E+00 | 0.000E+00 | 0.000E+00 | 0.000E+00 | 0.000E+00 | 0.000E+00 | 0.000E+00 | 8.404E-08 |
| Unigene65352 | arlR | 4.591E-06 | 1.023E-05 | 1.215E-05 | 1.169E-05 | 0.000E+00 | 0.000E+00 | 0.000E+00 | 0.000E+00 | 0.000E+00 | 0.000E+00 | 1.094E-07 | 0.000E+00 |
| Unigene65357 | emrB | 2.787E-06 | 1.020E-06 | 1.717E-06 | 5.018E-07 | 0.000E+00 | 0.000E+00 | 0.000E+00 | 5.044E-08 | 0.000E+00 | 0.000E+00 | 0.000E+00 | 0.000E+00 |
| Unigene65360 | Staphylocc | 2.124E-06 | 9.747E-07 | 1.844E-06 | 6.499E-07 | 0.000E+00 | 0.000E+00 | 0.000E+00 | 1.502E-08 | 0.000E+00 | 0.000E+00 | 0.000E+00 | 0.000E+00 |
| Unigene65371 | bcrA | 2.790E-06 | 1.166E-06 | 1.651E-06 | 7.602E-07 | 0.000E+00 | 0.000E+00 | 0.000E+00 | 3.618E-07 | 0.000E+00 | 0.000E+00 | 0.000E+00 | 0.000E+00 |
| Unigene65379 | tetA(58) | 2.387E-06 | 4.798E-07 | 2.046E-06 | 5.433E-07 | 0.000E+00 | 0.000E+00 | 0.000E+00 | 1.177E-07 | 0.000E+00 | 0.000E+00 | 0.000E+00 | 0.000E+00 |
| Unigene65464 | vanTC | 4.072E-06 | 1.067E-05 | 8.896E-06 | 9.515E-06 | 0.000E+00 | 1.285E-07 | 0.000E+00 | 0.000E+00 | 0.000E+00 | 0.000E+00 | 1.700E-07 | 0.000E+00 |
| Unigene65482 | oleC | 6.329E-07 | 7.267E-06 | 1.127E-05 | 1.583E-05 | 2.705E-08 | 0.000E+00 | 0.000E+00 | 0.000E+00 | 0.000E+00 | 0.000E+00 | 2.228E-07 | 0.000E+00 |
| Unigene65502 | bcrA | 1.076E-06 | 5.905E-07 | 1.248E-06 | 2.025E-06 | 2.103E-05 | 5.975E-06 | 1.429E-05 | 1.815E-05 | 1.278E-05 | 2.087E-05 | 2.299E-05 | 8.672E-06 |
| Unigene65511 | tetA(60) | 1.285E-06 | 1.115E-06 | 1.276E-06 | 1.841E-06 | 2.112E-05 | 7.302E-06 | 1.416E-05 | 1.622E-05 | 1.138E-05 | 2.074E-05 | 1.911E-05 | 7.278E-06 |
| Unigene65543 | vanHF | 1.441E-06 | 6.327E-07 | 1.257E-06 | 1.585E-06 | 2.128E-05 | 5.643E-06 | 1.609E-05 | 1.712E-05 | 1.141E-05 | 2.048E-05 | 1.905E-05 | 8.025E-06 |
| Unigene65544 | vanHF | 1.253E-06 | 7.756E-07 | 1.229E-06 | 2.108E-06 | 1.988E-05 | 6.250E-06 | 1.326E-05 | 1.653E-05 | 1.271E-05 | 1.689E-05 | 1.951E-05 | 6.720E-06 |
| Unigene65549 | Staphylocc | 1.339E-06 | 6.446E-07 | 1.844E-06 | 1.898E-06 | 2.044E-05 | 5.740E-06 | 1.414E-05 | 1.752E-05 | 1.230E-05 | 1.942E-05 | 1.967E-05 | 8.220E-06 |
| Unigene65552 | lmrB | 1.455E-06 | 1.065E-06 | 1.363E-06 | 1.625E-06 | 2.180E-05 | 5.911E-06 | 1.437E-05 | 1.737E-05 | 1.145E-05 | 1.980E-05 | 2.008E-05 | 7.532E-06 |
| Unigene65566 | arlS | 1.624E-06 | 1.021E-06 | 1.511E-06 | 1.931E-06 | 2.011E-05 | 6.182E-06 | 1.318E-05 | 1.683E-05 | 1.220E-05 | 1.772E-05 | 1.883E-05 | 7.199E-06 |
| Unigene65718 | macB | 1.009E-06 | 1.213E-06 | 1.838E-06 | 1.698E-06 | 2.241E-05 | 7.478E-06 | 1.733E-05 | 1.828E-05 | 1.439E-05 | 2.087E-05 | 2.275E-05 | 8.744E-06 |
| Unigene65725 | Streptococ | 1.051E-06 | 1.099E-06 | 1.179E-06 | 2.325E-06 | 2.224E-05 | 7.142E-06 | 1.568E-05 | 2.062E-05 | 1.329E-05 | 2.213E-05 | 1.924E-05 | 6.507E-06 |
| Unigene65726 | QepA2 | 1.362E-06 | 8.850E-07 | 1.064E-06 | 2.274E-06 | 2.341E-05 | 7.244E-06 | 1.517E-05 | 1.808E-05 | 1.194E-05 | 2.001E-05 | 2.064E-05 | 7.460E-06 |
| Unigene65752 | ugd | 1.414E-06 | 1.437E-06 | 1.310E-06 | 1.533E-06 | 2.097E-05 | 7.330E-06 | 1.484E-05 | 1.870E-05 | 1.325E-05 | 2.039E-05 | 2.003E-05 | 8.881E-06 |
| Unigene65767 | tetA(58) | 2.260E-06 | 4.212E-06 | 2.886E-06 | 5.216E-06 | 3.985E-07 | 3.102E-07 | 3.176E-07 | 0.000E+00 | 0.000E+00 | 0.000E+00 | 0.000E+00 | 0.000E+00 |
| Unigene65882 | vanSB | 2.838E-06 | 5.113E-06 | 4.014E-06 | 5.851E-06 | 7.094E-07 | 1.023E-07 | 2.356E-07 | 2.760E-08 | 1.034E-07 | 0.000E+00 | 1.082E-07 | 1.677E-07 |
| Unigene65883 | vanRB | 2.727E-06 | 3.374E-06 | 3.065E-06 | 5.758E-06 | 0.000E+00 | 0.000E+00 | 4.939E-07 | 0.000E+00 | 3.252E-07 | 0.000E+00 | 0.000E+00 | 1.758E-07 |
| Unigene65884 | macB | 3.273E-06 | 4.511E-06 | 3.648E-06 | 5.499E-06 | 3.199E-07 | 1.698E-07 | 2.318E-07 | 3.056E-08 | 5.723E-08 | 0.000E+00 | 2.995E-08 | 3.095E-07 |
| Unigene65885 | macB | 2.571E-06 | 4.128E-06 | 3.551E-06 | 4.616E-06 | 3.174E-07 | 0.000E+00 | 0.000E+00 | 0.000E+00 | 2.082E-07 | 0.000E+00 | 0.000E+00 | 1.688E-07 |
| Unigene65985 | evgA | 1.040E-06 | 7.588E-06 | 1.132E-05 | 1.512E-05 | 0.000E+00 | 0.000E+00 | 0.000E+00 | 0.000E+00 | 0.000E+00 | 0.000E+00 | 0.000E+00 | 0.000E+00 |
| Unigene65988 | RlmA(II) | 2.144E-06 | 2.824E-06 | 2.831E-06 | 1.732E-06 | 0.000E+00 | 0.000E+00 | 0.000E+00 | 2.730E-07 | 1.150E-06 | 1.303E-07 | 0.000E+00 | 0.000E+00 |
| Unigene66032 | vmlR | 6.796E-07 | 4.807E-06 | 1.045E-05 | 1.468E-05 | 0.000E+00 | 0.000E+00 | 0.000E+00 | 0.000E+00 | 0.000E+00 | 0.000E+00 | 0.000E+00 | 0.000E+00 |
| Unigene66105 | mecC | 3.205E-06 | 2.475E-07 | 3.019E-06 | 4.392E-07 | 1.968E-07 | 1.741E-08 | 3.031E-07 | 0.000E+00 | 0.000E+00 | 3.589E-08 | 8.660E-07 | 3.808E-08 |
| Unigene66139 | adeR | 3.262E-06 | 3.929E-06 | 3.367E-06 | 5.989E-06 | 5.648E-07 | 0.000E+00 | 0.000E+00 | 0.000E+00 | 0.000E+00 | 0.000E+00 | 0.000E+00 | 1.001E-07 |
| Unigene66252 | vanSG | 7.805E-07 | 8.770E-07 | 4.304E-06 | 6.681E-06 | 0.000E+00 | 4.018E-08 | 7.404E-07 | 0.000E+00 | 0.000E+00 | 0.000E+00 | 0.000E+00 | 2.197E-08 |
| Unigene66274 | ugd | 1.245E-06 | 1.154E-06 | 9.771E-07 | 0.000E+00 | 0.000E+00 | 5.981E-08 | 1.837E-07 | 3.229E-08 | 5.744E-07 | 6.163E-08 | 0.000E+00 | 0.000E+00 |
| Unigene66353 | patB | 3.610E-05 | 2.270E-05 | 1.938E-05 | 1.981E-05 | 1.786E-06 | 1.363E-06 | 2.911E-06 | 7.677E-07 | 5.591E-06 | 5.088E-07 | 1.902E-06 | 1.166E-06 |
| Unigene66367 | vanHB | 1.990E-06 | 8.330E-06 | 5.130E-06 | 8.005E-06 | 0.000E+00 | 0.000E+00 | 0.000E+00 | 0.000E+00 | 0.000E+00 | 0.000E+00 | 0.000E+00 | 0.000E+00 |
| Unigene66451 | vanYB | 2.590E-06 | 2.581E-06 | 3.128E-06 | 1.569E-06 | 2.214E-07 | 4.308E-08 | 2.205E-07 | 0.000E+00 | 7.405E-07 | 1.776E-07 | 0.000E+00 | 0.000E+00 |
| Unigene66497 | tetA(60) | 6.111E-06 | 0.000E+00 | 0.000E+00 | 0.000E+00 | 0.000E+00 | 0.000E+00 | 0.000E+00 | 0.000E+00 | 1.917E-07 | 0.000E+00 | 0.000E+00 | 0.000E+00 |
| Unigene66563 | YojI | 1.102E-06 | 0.000E+00 | 3.462E-07 | 0.000E+00 | 4.311E-06 | 1.096E-05 | 1.611E-06 | 8.905E-06 | 4.964E-06 | 2.947E-06 | 0.000E+00 | 0.000E+00 |
| Unigene66567 | rpoB2 | 3.213E-06 | 4.816E-06 | 4.314E-06 | 6.372E-06 | 4.054E-07 | 1.347E-07 | 3.743E-07 | 1.039E-08 | 2.432E-07 | 0.000E+00 | 1.120E-07 | 2.104E-07 |
| Unigene66682 | AcrS | 1.037E-06 | 0.000E+00 | 9.202E-07 | 0.000E+00 | 0.000E+00 | 0.000E+00 | 0.000E+00 | 0.000E+00 | 0.000E+00 | 0.000E+00 | 5.081E-07 | 0.000E+00 |
| Unigene66783 | vgaD | 2.690E-06 | 4.609E-06 | 3.959E-06 | 5.516E-06 | 4.574E-07 | 0.000E+00 | 3.038E-07 | 0.000E+00 | 3.600E-07 | 6.116E-08 | 9.420E-08 | 0.000E+00 |
| Unigene66794 | patB | 9.410E-07 | 1.123E-06 | 4.416E-06 | 4.173E-06 | 0.000E+00 | 0.000E+00 | 5.434E-07 | 0.000E+00 | 0.000E+00 | 0.000E+00 | 0.000E+00 | 0.000E+00 |
| Unigene66806 | bacA | 3.638E-06 | 8.793E-06 | 8.355E-06 | 1.008E-05 | 8.600E-08 | 4.184E-08 | 0.000E+00 | 0.000E+00 | 0.000E+00 | 4.312E-07 | 0.000E+00 | 0.000E+00 |
| Unigene66832 | cmrA | 2.044E-06 | 2.539E-06 | 2.666E-06 | 1.222E-06 | 0.000E+00 | 0.000E+00 | 4.466E-07 | 2.198E-07 | 5.880E-07 | 0.000E+00 | 1.846E-07 | 0.000E+00 |

| | | | | | | | | | | | | | |
|---|---|---|---|---|---|---|---|---|---|---|---|---|---|
| Unigene66833 | lmrB | 2.519E-06 | 2.688E-06 | 2.495E-06 | 1.569E-06 | 0.000E+00 | 0.000E+00 | 4.951E-07 | 5.771E-07 | 8.492E-07 | 0.000E+00 | 0.000E+00 | 3.061E-07 |
| Unigene66952 | patB | 1.021E-06 | 1.774E-06 | 1.168E-06 | 1.288E-06 | 1.994E-05 | 6.805E-06 | 1.713E-05 | 1.911E-05 | 1.144E-05 | 1.687E-05 | 1.936E-05 | 6.083E-06 |
| Unigene67081 | bcrA | 2.956E-06 | 4.283E-06 | 2.707E-06 | 4.975E-06 | 4.817E-07 | 8.522E-08 | 5.235E-07 | 0.000E+00 | 1.292E-07 | 8.781E-08 | 0.000E+00 | 3.261E-07 |
| Unigene67122 | macB | 1.087E-06 | 3.490E-07 | 1.170E-06 | 9.367E-07 | 0.000E+00 | 0.000E+00 | 0.000E+00 | 0.000E+00 | 0.000E+00 | 0.000E+00 | 0.000E+00 | 0.000E+00 |
| Unigene67129 | baeR | 0.000E+00 | 0.000E+00 | 0.000E+00 | 0.000E+00 | 0.000E+00 | 0.000E+00 | 0.000E+00 | 0.000E+00 | 0.000E+00 | 0.000E+00 | 0.000E+00 | 4.454E-06 |
| Unigene67227 | bcrA | 7.995E-05 | 4.020E-05 | 1.797E-07 | 0.000E+00 | 1.668E-05 | 7.009E-05 | 0.000E+00 | 0.000E+00 | 0.000E+00 | 0.000E+00 | 1.048E-07 | 1.624E-07 |
| Unigene67262 | efrA | 8.657E-05 | 3.799E-05 | 2.693E-07 | 0.000E+00 | 1.817E-05 | 7.407E-05 | 1.215E-07 | 0.000E+00 | 0.000E+00 | 0.000E+00 | 1.256E-07 | 0.000E+00 |
| Unigene67286 | tetT | 8.608E-05 | 3.888E-05 | 2.216E-07 | 0.000E+00 | 1.795E-05 | 7.463E-05 | 6.818E-08 | 0.000E+00 | 0.000E+00 | 0.000E+00 | 9.396E-08 | 1.456E-07 |
| Unigene67318 | vanHD | 8.824E-05 | 4.391E-05 | 3.161E-07 | 0.000E+00 | 1.915E-05 | 7.892E-05 | 0.000E+00 | 1.128E-07 | 0.000E+00 | 0.000E+00 | 1.474E-07 | 0.000E+00 |
| Unigene67355 | msbA | 9.109E-05 | 4.643E-05 | 1.790E-06 | 1.302E-07 | 1.852E-05 | 8.481E-05 | 0.000E+00 | 0.000E+00 | 0.000E+00 | 1.847E-07 | 1.686E-07 | 1.525E-07 |
| Unigene67363 | tetA(58) | 8.015E-05 | 3.883E-05 | 1.303E-06 | 0.000E+00 | 1.761E-05 | 7.166E-05 | 0.000E+00 | 0.000E+00 | 0.000E+00 | 0.000E+00 | 0.000E+00 | 0.000E+00 |
| Unigene67378 | msbA | 8.561E-05 | 3.791E-05 | 1.257E-07 | 0.000E+00 | 1.750E-05 | 7.699E-05 | 9.451E-08 | 0.000E+00 | 0.000E+00 | 0.000E+00 | 0.000E+00 | 0.000E+00 |
| Unigene67449 | baeS | 4.743E-07 | 0.000E+00 | 2.036E-07 | 0.000E+00 | 0.000E+00 | 1.282E-07 | 0.000E+00 | 0.000E+00 | 0.000E+00 | 0.000E+00 | 0.000E+00 | 0.000E+00 |
| Unigene67572 | lmrC | 0.000E+00 | 0.000E+00 | 0.000E+00 | 4.762E-07 | 0.000E+00 | 0.000E+00 | 0.000E+00 | 4.453E-06 | 0.000E+00 | 0.000E+00 | 2.060E-06 | 3.750E-06 |
| Unigene67590 | macB | 2.270E-06 | 0.000E+00 | 0.000E+00 | 2.547E-07 | 0.000E+00 | 4.544E-08 | 0.000E+00 | 0.000E+00 | 0.000E+00 | 0.000E+00 | 0.000E+00 | 0.000E+00 |
| Unigene67600 | vanRO | 1.237E-07 | 0.000E+00 | 0.000E+00 | 0.000E+00 | 0.000E+00 | 0.000E+00 | 0.000E+00 | 0.000E+00 | 0.000E+00 | 0.000E+00 | 0.000E+00 | 0.000E+00 |
| Unigene67654 | Corynebac | 0.000E+00 | 0.000E+00 | 0.000E+00 | 0.000E+00 | 0.000E+00 | 0.000E+00 | 0.000E+00 | 0.000E+00 | 0.000E+00 | 0.000E+00 | 0.000E+00 | 2.770E-06 |
| Unigene67678 | oleC | 4.233E-06 | 9.438E-06 | 1.294E-05 | 1.251E-05 | 1.470E-07 | 0.000E+00 | 9.761E-08 | 0.000E+00 | 0.000E+00 | 0.000E+00 | 0.000E+00 | 0.000E+00 |
| Unigene67714 | PEDO-2 | 1.133E-06 | 5.650E-06 | 9.295E-06 | 1.456E-05 | 0.000E+00 | 0.000E+00 | 0.000E+00 | 0.000E+00 | 0.000E+00 | 0.000E+00 | 5.889E-08 | 0.000E+00 |
| Unigene67728 | abeS | 1.609E-06 | 0.000E+00 | 0.000E+00 | 0.000E+00 | 0.000E+00 | 0.000E+00 | 0.000E+00 | 0.000E+00 | 0.000E+00 | 0.000E+00 | 0.000E+00 | 0.000E+00 |
| Unigene67878 | tva(A) | 1.102E-04 | 1.349E-04 | 1.258E-04 | 5.944E-06 | 7.719E-06 | 2.608E-07 | 3.738E-07 | 3.041E-06 | 5.675E-05 | 3.225E-07 | 2.870E-06 | 5.133E-07 |
| Unigene67981 | macB | 2.556E-06 | 1.202E-06 | 3.878E-06 | 2.603E-06 | 4.772E-08 | 2.322E-08 | 7.131E-08 | 0.000E+00 | 0.000E+00 | 7.178E-08 | 2.211E-07 | 0.000E+00 |
| Unigene67994 | Staphylocc | 4.019E-06 | 1.009E-05 | 9.160E-06 | 1.060E-05 | 2.187E-07 | 1.002E-07 | 3.846E-08 | 2.704E-08 | 0.000E+00 | 9.032E-08 | 7.949E-08 | 5.476E-08 |
| Unigene68004 | mefC | 2.464E-07 | 0.000E+00 | 0.000E+00 | 0.000E+00 | 0.000E+00 | 8.879E-08 | 0.000E+00 | 0.000E+00 | 0.000E+00 | 0.000E+00 | 0.000E+00 | 1.748E-06 |
| Unigene68053 | efrB | 2.948E-06 | 4.188E-06 | 3.513E-06 | 6.446E-06 | 3.817E-07 | 0.000E+00 | 2.602E-07 | 4.222E-08 | 1.384E-07 | 0.000E+00 | 0.000E+00 | 1.283E-07 |
| Unigene68054 | efrA | 2.505E-06 | 5.009E-06 | 3.349E-06 | 5.939E-06 | 3.298E-07 | 1.203E-07 | 2.464E-07 | 0.000E+00 | 1.622E-07 | 0.000E+00 | 0.000E+00 | 8.772E-08 |
| Unigene68057 | tva(A) | 6.822E-07 | 5.275E-06 | 8.641E-06 | 1.487E-05 | 0.000E+00 | 0.000E+00 | 0.000E+00 | 0.000E+00 | 0.000E+00 | 0.000E+00 | 0.000E+00 | 0.000E+00 |
| Unigene68107 | Corynebac | 1.026E-05 | 0.000E+00 | 0.000E+00 | 0.000E+00 | 1.771E-05 | 1.500E-05 | 0.000E+00 | 0.000E+00 | 1.011E-05 | 0.000E+00 | 0.000E+00 | 0.000E+00 |
| Unigene68173 | lmrD | 1.555E-06 | 3.183E-06 | 0.000E+00 | 0.000E+00 | 1.088E-05 | 5.907E-07 | 0.000E+00 | 0.000E+00 | 0.000E+00 | 0.000E+00 | 0.000E+00 | 0.000E+00 |
| Unigene68178 | macB | 1.044E-06 | 8.421E-06 | 1.132E-05 | 1.967E-05 | 0.000E+00 | 0.000E+00 | 0.000E+00 | 0.000E+00 | 0.000E+00 | 0.000E+00 | 0.000E+00 | 0.000E+00 |
| Unigene68179 | macB | 9.973E-07 | 6.423E-06 | 9.786E-06 | 1.519E-05 | 0.000E+00 | 0.000E+00 | 0.000E+00 | 0.000E+00 | 0.000E+00 | 0.000E+00 | 3.002E-08 | 0.000E+00 |
| Unigene68197 | oleC | 3.185E-06 | 2.362E-06 | 3.294E-06 | 1.304E-06 | 0.000E+00 | 0.000E+00 | 0.000E+00 | 3.014E-07 | 9.879E-07 | 0.000E+00 | 0.000E+00 | 5.087E-08 |
| Unigene68220 | oleC | 8.590E-07 | 5.557E-06 | 4.436E-07 | 0.000E+00 | 3.014E-07 | 9.775E-08 | 0.000E+00 | 4.749E-07 | 0.000E+00 | 0.000E+00 | 1.551E-07 | 0.000E+00 |
| Unigene68238 | efpA | 2.547E-07 | 0.000E+00 | 0.000E+00 | 0.000E+00 | 0.000E+00 | 0.000E+00 | 0.000E+00 | 0.000E+00 | 0.000E+00 | 0.000E+00 | 0.000E+00 | 0.000E+00 |
| Unigene68246 | optrA | 4.060E-06 | 1.892E-05 | 2.988E-06 | 8.201E-07 | 1.147E-06 | 2.926E-07 | 2.809E-07 | 2.172E-07 | 1.036E-06 | 5.277E-07 | 3.677E-07 | 2.400E-07 |
| Unigene68260 | arlS | 4.455E-06 | 2.049E-05 | 4.013E-06 | 6.883E-07 | 1.109E-06 | 3.421E-07 | 1.347E-07 | 4.262E-07 | 1.703E-06 | 5.424E-07 | 3.342E-07 | 1.439E-07 |
| Unigene68262 | arlR | 3.853E-06 | 2.125E-05 | 4.380E-06 | 3.796E-07 | 9.918E-07 | 4.064E-07 | 2.600E-07 | 0.000E+00 | 6.676E-07 | 7.328E-07 | 1.612E-07 | 3.332E-07 |
| Unigene68263 | PmrF | 4.081E-06 | 1.825E-05 | 2.946E-06 | 7.153E-07 | 1.536E-06 | 6.199E-07 | 2.613E-07 | 1.969E-07 | 1.143E-06 | 0.000E+00 | 1.543E-07 | 2.791E-07 |
| Unigene68339 | vatE | 1.358E-06 | 0.000E+00 | 3.454E-07 | 4.570E-06 | 0.000E+00 | 0.000E+00 | 1.369E-05 | 1.608E-05 | 0.000E+00 | 0.000E+00 | 0.000E+00 | 0.000E+00 |
| Unigene68387 | vanSG | 8.277E-07 | 3.461E-07 | 1.508E-06 | 7.164E-07 | 0.000E+00 | 0.000E+00 | 0.000E+00 | 0.000E+00 | 0.000E+00 | 1.317E-07 | 0.000E+00 | 0.000E+00 |
| Unigene68413 | YojI | 4.183E-06 | 1.851E-05 | 3.278E-06 | 1.036E-06 | 1.374E-06 | 3.413E-07 | 0.000E+00 | 0.000E+00 | 9.776E-07 | 6.741E-07 | 3.912E-07 | 0.000E+00 |
| Unigene68420 | vatH | 3.485E-06 | 2.185E-06 | 3.194E-06 | 1.469E-06 | 0.000E+00 | 0.000E+00 | 3.814E-07 | 0.000E+00 | 0.000E+00 | 0.000E+00 | 0.000E+00 | 0.000E+00 |
| Unigene68491 | bcrA | 1.279E-05 | 0.000E+00 | 0.000E+00 | 0.000E+00 | 1.902E-05 | 1.718E-05 | 0.000E+00 | 0.000E+00 | 1.180E-05 | 0.000E+00 | 0.000E+00 | 0.000E+00 |
| Unigene68497 | tetB(P) | 1.125E-05 | 2.241E-08 | 0.000E+00 | 0.000E+00 | 1.926E-05 | 1.664E-05 | 0.000E+00 | 0.000E+00 | 1.241E-05 | 0.000E+00 | 0.000E+00 | 0.000E+00 |
| Unigene68498 | PmrF | 1.108E-05 | 0.000E+00 | 0.000E+00 | 0.000E+00 | 1.545E-05 | 1.601E-05 | 0.000E+00 | 0.000E+00 | 1.243E-05 | 0.000E+00 | 0.000E+00 | 0.000E+00 |
| Unigene68500 | PmrF | 1.232E-05 | 0.000E+00 | 0.000E+00 | 0.000E+00 | 1.432E-05 | 1.540E-05 | 0.000E+00 | 0.000E+00 | 1.270E-05 | 0.000E+00 | 0.000E+00 | 0.000E+00 |
| Unigene68504 | msbA | 3.964E-07 | 0.000E+00 | 0.000E+00 | 0.000E+00 | 0.000E+00 | 0.000E+00 | 0.000E+00 | 0.000E+00 | 0.000E+00 | 0.000E+00 | 0.000E+00 | 0.000E+00 |
| Unigene68520 | TaeA | 8.764E-07 | 0.000E+00 | 1.911E-07 | 1.475E-07 | 0.000E+00 | 1.295E-05 | 3.772E-07 | 0.000E+00 | 1.596E-06 | 0.000E+00 | 0.000E+00 | 5.180E-07 |
| Unigene68541 | vanTE | 4.993E-05 | 7.168E-05 | 3.120E-05 | 1.539E-05 | 1.986E-05 | 5.987E-06 | 3.493E-06 | 5.828E-05 | 1.345E-05 | 6.936E-06 | 2.101E-06 | 1.017E-06 |
| Unigene68551 | Chlamydia | 4.761E-05 | 6.314E-05 | 2.692E-05 | 1.417E-05 | 1.768E-05 | 4.664E-06 | 3.174E-06 | 5.184E-05 | 1.187E-05 | 6.728E-06 | 1.742E-06 | 1.020E-06 |
| Unigene68575 | Staphylocc | 5.030E-05 | 6.743E-05 | 2.950E-05 | 1.606E-05 | 1.866E-05 | 6.044E-06 | 2.802E-06 | 5.411E-05 | 1.139E-05 | 5.594E-06 | 1.689E-06 | 9.975E-07 |
| Unigene68651 | lmrB | 3.665E-07 | 2.011E-07 | 1.284E-07 | 3.966E-07 | 2.036E-07 | 2.830E-08 | 0.000E+00 | 0.000E+00 | 0.000E+00 | 0.000E+00 | 0.000E+00 | 0.000E+00 |
| Unigene68676 | oleC | 1.972E-05 | 2.078E-05 | 6.361E-05 | 7.038E-05 | 6.489E-06 | 1.704E-05 | 6.382E-05 | 1.871E-05 | 1.286E-05 | 2.745E-05 | 7.425E-05 | 2.461E-05 |
| Unigene68690 | lmrD | 4.196E-06 | 9.438E-06 | 1.313E-05 | 1.175E-05 | 1.385E-07 | 3.593E-07 | 5.518E-07 | 3.879E-07 | 9.081E-08 | 2.777E-07 | 3.802E-07 | 0.000E+00 |
| Unigene68730 | tetA(58) | 1.524E-06 | 2.903E-06 | 2.332E-06 | 3.758E-06 | 2.490E-07 | 1.454E-07 | 3.473E-07 | 0.000E+00 | 3.920E-07 | 0.000E+00 | 5.128E-08 | 1.590E-07 |
| Unigene68863 | vanRL | 4.353E-05 | 2.031E-05 | 1.152E-05 | 1.655E-05 | 2.536E-06 | 1.939E-06 | 4.421E-06 | 0.000E+00 | 6.415E-06 | 0.000E+00 | 1.212E-06 | 2.120E-06 |
| Unigene68885 | facT | 2.772E-06 | 0.000E+00 | 0.000E+00 | 0.000E+00 | 6.787E-06 | 1.005E-06 | 0.000E+00 | 0.000E+00 | 3.807E-06 | 1.310E-05 | 5.466E-06 | 1.401E-06 |
| Unigene68938 | efmA | 2.519E-06 | 2.880E-08 | 5.150E-08 | 5.300E-08 | 5.831E-08 | 0.000E+00 | 0.000E+00 | 0.000E+00 | 0.000E+00 | 0.000E+00 | 0.000E+00 | 2.358E-06 |

| | | | | | | | | | | | | | |
|---|---|---|---|---|---|---|---|---|---|---|---|---|---|
| Unigene68943 | Staphylocc | 8.623E-07 | 3.909E-06 | 5.150E-07 | 2.650E-07 | 1.666E-07 | 1.621E-07 | 2.490E-07 | 8.752E-08 | 3.278E-07 | 3.341E-07 | 1.715E-07 | 0.000E+00 |
| Unigene69018 | vanHF | 9.631E-07 | 7.654E-07 | 4.823E-06 | 6.305E-06 | 0.000E+00 | 0.000E+00 | 4.043E-07 | 0.000E+00 | 0.000E+00 | 0.000E+00 | 0.000E+00 | 0.000E+00 |
| Unigene69054 | mgrA | 9.474E-07 | 0.000E+00 | 0.000E+00 | 5.467E-07 | 0.000E+00 | 9.072E-06 | 0.000E+00 | 0.000E+00 | 5.695E-06 | 0.000E+00 | 0.000E+00 | 0.000E+00 |
| Unigene69058 | adeR | 1.281E-06 | 1.293E-06 | 1.408E-06 | 2.380E-06 | 2.129E-05 | 8.142E-06 | 1.412E-05 | 1.944E-05 | 1.053E-05 | 1.758E-05 | 1.764E-05 | 7.813E-06 |
| Unigene69059 | macB | 1.713E-06 | 1.095E-06 | 1.469E-06 | 1.998E-06 | 2.130E-05 | 6.310E-06 | 1.333E-05 | 1.737E-05 | 1.125E-05 | 1.834E-05 | 1.851E-05 | 6.939E-06 |
| Unigene69089 | oleB | 9.282E-07 | 0.000E+00 | 2.024E-07 | 3.819E-07 | 3.208E-06 | 7.247E-06 | 1.294E-06 | 5.457E-06 | 3.870E-06 | 2.527E-06 | 0.000E+00 | 5.689E-07 |
| Unigene69123 | Rhodococ | 6.459E-05 | 5.849E-05 | 1.143E-04 | 1.332E-04 | 1.223E-04 | 8.559E-05 | 1.100E-04 | 1.142E-04 | 8.981E-05 | 1.291E-04 | 1.407E-04 | 1.493E-04 |
| Unigene69137 | vanZA | 4.402E-06 | 9.509E-06 | 1.577E-05 | 1.115E-05 | 3.881E-07 | 3.021E-07 | 5.413E-07 | 4.078E-07 | 0.000E+00 | 0.000E+00 | 0.000E+00 | 0.000E+00 |
| Unigene69149 | vga(E) Stap | 3.365E-06 | 4.578E-06 | 3.674E-06 | 5.792E-06 | 6.319E-07 | 0.000E+00 | 5.246E-07 | 0.000E+00 | 5.181E-08 | 0.000E+00 | 0.000E+00 | 0.000E+00 |
| Unigene69168 | optrA | 1.167E-05 | 0.000E+00 | 0.000E+00 | 0.000E+00 | 1.753E-05 | 1.597E-05 | 4.394E-08 | 0.000E+00 | 1.248E-05 | 4.202E-07 | 0.000E+00 | 0.000E+00 |
| Unigene69222 | lmrD | 3.025E-06 | 9.433E-07 | 1.754E-06 | 7.984E-07 | 0.000E+00 | 0.000E+00 | 0.000E+00 | 0.000E+00 | 0.000E+00 | 0.000E+00 | 0.000E+00 | 0.000E+00 |
| Unigene69244 | tetT | 2.537E-06 | 8.532E-07 | 1.606E-06 | 6.197E-07 | 0.000E+00 | 0.000E+00 | 0.000E+00 | 2.388E-08 | 0.000E+00 | 0.000E+00 | 0.000E+00 | 0.000E+00 |
| Unigene69248 | bcrA | 2.123E-06 | 1.165E-06 | 1.077E-06 | 5.174E-07 | 0.000E+00 | 0.000E+00 | 0.000E+00 | 0.000E+00 | 0.000E+00 | 0.000E+00 | 0.000E+00 | 0.000E+00 |
| Unigene69269 | bacA | 3.041E-06 | 9.413E-07 | 1.339E-06 | 3.543E-07 | 0.000E+00 | 0.000E+00 | 0.000E+00 | 0.000E+00 | 0.000E+00 | 0.000E+00 | 0.000E+00 | 4.608E-08 |
| Unigene69301 | Corynebac | 2.539E-06 | 6.635E-07 | 1.265E-06 | 8.139E-07 | 0.000E+00 | 0.000E+00 | 0.000E+00 | 4.704E-08 | 0.000E+00 | 0.000E+00 | 0.000E+00 | 0.000E+00 |
| Unigene69302 | patB | 2.807E-06 | 8.869E-07 | 1.711E-06 | 4.724E-07 | 0.000E+00 | 0.000E+00 | 0.000E+00 | 1.986E-07 | 0.000E+00 | 0.000E+00 | 0.000E+00 | 0.000E+00 |
| Unigene69363 | vanRM | 1.734E-06 | 1.415E-06 | 1.702E-06 | 2.694E-07 | 0.000E+00 | 0.000E+00 | 0.000E+00 | 0.000E+00 | 0.000E+00 | 0.000E+00 | 0.000E+00 | 0.000E+00 |
| Unigene69364 | baeS | 2.739E-06 | 8.659E-07 | 1.957E-06 | 3.541E-07 | 0.000E+00 | 0.000E+00 | 0.000E+00 | 0.000E+00 | 0.000E+00 | 0.000E+00 | 0.000E+00 | 0.000E+00 |
| Unigene69366 | vanZF | 1.739E-06 | 9.543E-07 | 1.706E-06 | 4.390E-07 | 0.000E+00 | 0.000E+00 | 0.000E+00 | 0.000E+00 | 0.000E+00 | 0.000E+00 | 0.000E+00 | 0.000E+00 |
| Unigene69368 | PmrF | 2.491E-06 | 9.493E-07 | 1.901E-06 | 4.542E-07 | 0.000E+00 | 0.000E+00 | 0.000E+00 | 4.038E-08 | 0.000E+00 | 0.000E+00 | 0.000E+00 | 0.000E+00 |
| Unigene69412 | tetA(46) | 4.103E-07 | 1.001E-07 | 5.816E-07 | 0.000E+00 | 8.611E-07 | 4.436E-07 | 1.564E-06 | 0.000E+00 | 3.488E-07 | 0.000E+00 | 0.000E+00 | 3.180E-06 |
| Unigene69433 | vanHO | 1.020E-04 | 4.510E-05 | 8.146E-07 | 0.000E+00 | 2.256E-05 | 9.126E-05 | 0.000E+00 | 0.000E+00 | 0.000E+00 | 2.845E-07 | 3.652E-08 | 0.000E+00 |
| Unigene69458 | bcrA | 8.995E-05 | 3.781E-05 | 5.329E-07 | 2.057E-07 | 3.017E-06 | 7.724E-05 | 0.000E+00 | 0.000E+00 | 0.000E+00 | 0.000E+00 | 0.000E+00 | 0.000E+00 |
| Unigene69509 | patA | 8.767E-05 | 4.081E-05 | 1.336E-07 | 0.000E+00 | 2.048E-05 | 8.329E-05 | 0.000E+00 | 0.000E+00 | 0.000E+00 | 1.517E-07 | 0.000E+00 | 0.000E+00 |
| Unigene69510 | efrB | 9.857E-05 | 3.805E-05 | 2.832E-07 | 0.000E+00 | 2.024E-05 | 7.871E-05 | 0.000E+00 | 0.000E+00 | 0.000E+00 | 0.000E+00 | 0.000E+00 | 0.000E+00 |
| Unigene69516 | macB | 9.305E-05 | 4.266E-05 | 3.379E-07 | 0.000E+00 | 1.975E-05 | 7.682E-05 | 0.000E+00 | 0.000E+00 | 0.000E+00 | 0.000E+00 | 0.000E+00 | 0.000E+00 |
| Unigene69530 | vanG | 1.050E-04 | 4.535E-05 | 3.500E-07 | 9.005E-08 | 2.028E-05 | 9.219E-05 | 0.000E+00 | 0.000E+00 | 0.000E+00 | 0.000E+00 | 0.000E+00 | 1.054E-07 |
| Unigene69562 | lmrD | 9.112E-05 | 4.044E-05 | 5.068E-07 | 8.693E-08 | 1.750E-05 | 7.659E-05 | 0.000E+00 | 0.000E+00 | 0.000E+00 | 1.918E-07 | 0.000E+00 | 5.087E-08 |
| Unigene69683 | bcrA | 9.478E-05 | 4.656E-05 | 8.858E-07 | 0.000E+00 | 1.929E-05 | 5.149E-05 | 0.000E+00 | 0.000E+00 | 0.000E+00 | 0.000E+00 | 0.000E+00 | 0.000E+00 |
| Unigene69699 | bcrA | 9.060E-05 | 4.150E-05 | 0.000E+00 | 0.000E+00 | 2.363E-05 | 8.291E-05 | 5.617E-08 | 0.000E+00 | 0.000E+00 | 0.000E+00 | 5.806E-08 | 1.200E-07 |
| Unigene69704 | macB | 9.217E-05 | 4.271E-05 | 0.000E+00 | 0.000E+00 | 2.312E-05 | 7.444E-05 | 0.000E+00 | 0.000E+00 | 0.000E+00 | 0.000E+00 | 9.541E-08 | 0.000E+00 |
| Unigene69709 | Staphylocc | 9.794E-05 | 4.052E-05 | 2.530E-07 | 7.102E-08 | 1.971E-05 | 8.089E-05 | 5.189E-08 | 0.000E+00 | 0.000E+00 | 0.000E+00 | 3.486E-07 | 0.000E+00 |
| Unigene69822 | lin | 9.096E-05 | 3.981E-05 | 3.836E-07 | 6.233E-08 | 1.861E-05 | 7.916E-05 | 0.000E+00 | 2.401E-08 | 0.000E+00 | 0.000E+00 | 0.000E+00 | 0.000E+00 |
| Unigene69847 | vatB | 1.289E-06 | 8.611E-07 | 2.969E-06 | 5.263E-06 | 0.000E+00 | 0.000E+00 | 0.000E+00 | 0.000E+00 | 0.000E+00 | 0.000E+00 | 0.000E+00 | 6.624E-08 |
| Unigene69848 | vanRB | 1.196E-06 | 0.000E+00 | 3.128E-06 | 5.936E-06 | 0.000E+00 | 0.000E+00 | 0.000E+00 | 0.000E+00 | 0.000E+00 | 0.000E+00 | 0.000E+00 | 0.000E+00 |
| Unigene69852 | ugd | 8.277E-07 | 0.000E+00 | 0.000E+00 | 0.000E+00 | 0.000E+00 | 0.000E+00 | 0.000E+00 | 0.000E+00 | 0.000E+00 | 0.000E+00 | 0.000E+00 | 0.000E+00 |
| Unigene69900 | Corynebac | 9.462E-07 | 7.181E-06 | 1.032E-05 | 1.338E-05 | 6.007E-08 | 1.169E-07 | 8.976E-08 | 0.000E+00 | 0.000E+00 | 0.000E+00 | 0.000E+00 | 3.196E-08 |
| Unigene69915 | tlrC | 5.557E-07 | 7.208E-07 | 1.537E-06 | 3.316E-06 | 0.000E+00 | 0.000E+00 | 0.000E+00 | 1.710E-06 | 1.104E-07 | 1.069E-06 | 1.271E-06 | 9.554E-07 |
| Unigene69932 | efrA | 0.000E+00 | 0.000E+00 | 0.000E+00 | 0.000E+00 | 0.000E+00 | 0.000E+00 | 0.000E+00 | 4.584E-07 | 0.000E+00 | 0.000E+00 | 1.797E-07 | 0.000E+00 |
| Unigene69982 | lmrD | 2.127E-06 | 1.614E-05 | 7.335E-06 | 3.632E-06 | 0.000E+00 | 2.367E-07 | 0.000E+00 | 0.000E+00 | 0.000E+00 | 0.000E+00 | 1.073E-07 | 0.000E+00 |
| Unigene69989 | macB | 2.228E-06 | 1.499E-05 | 5.856E-06 | 4.117E-06 | 0.000E+00 | 1.363E-07 | 0.000E+00 | 0.000E+00 | 0.000E+00 | 0.000E+00 | 0.000E+00 | 0.000E+00 |
| Unigene70133 | vanHD | 2.196E-07 | 0.000E+00 | 0.000E+00 | 0.000E+00 | 0.000E+00 | 0.000E+00 | 0.000E+00 | 0.000E+00 | 0.000E+00 | 0.000E+00 | 0.000E+00 | 2.225E-07 |
| Unigene70149 | Agrobacte | 1.145E-06 | 6.449E-06 | 9.435E-06 | 1.807E-05 | 0.000E+00 | 0.000E+00 | 0.000E+00 | 0.000E+00 | 0.000E+00 | 0.000E+00 | 0.000E+00 | 0.000E+00 |
| Unigene70171 | tetA(58) | 2.985E-05 | 2.231E-06 | 5.373E-05 | 6.318E-06 | 4.427E-06 | 8.616E-08 | 7.498E-07 | 1.861E-06 | 0.000E+00 | 0.000E+00 | 1.048E-06 | 0.000E+00 |
| Unigene70190 | vgaB | 1.720E-06 | 0.000E+00 | 0.000E+00 | 0.000E+00 | 0.000E+00 | 0.000E+00 | 0.000E+00 | 0.000E+00 | 0.000E+00 | 0.000E+00 | 0.000E+00 | 0.000E+00 |
| Unigene70222 | vgaE | 1.330E-06 | 0.000E+00 | 0.000E+00 | 8.139E-08 | 0.000E+00 | 0.000E+00 | 0.000E+00 | 0.000E+00 | 0.000E+00 | 0.000E+00 | 0.000E+00 | 0.000E+00 |
| Unigene70227 | vanHO | 9.456E-07 | 0.000E+00 | 0.000E+00 | 0.000E+00 | 0.000E+00 | 1.057E-07 | 0.000E+00 | 0.000E+00 | 0.000E+00 | 0.000E+00 | 0.000E+00 | 0.000E+00 |
| Unigene70234 | macB | 1.693E-06 | 0.000E+00 | 2.251E-06 | 0.000E+00 | 1.123E-06 | 2.952E-08 | 0.000E+00 | 3.188E-07 | 0.000E+00 | 0.000E+00 | 0.000E+00 | 0.000E+00 |
| Unigene70235 | macB | 1.357E-06 | 0.000E+00 | 2.387E-06 | 0.000E+00 | 1.923E-06 | 0.000E+00 | 0.000E+00 | 5.461E-08 | 0.000E+00 | 0.000E+00 | 0.000E+00 | 0.000E+00 |
| Unigene70261 | Streptomy | 1.503E-06 | 0.000E+00 | 1.569E-08 | 0.000E+00 | 0.000E+00 | 0.000E+00 | 1.769E-08 | 0.000E+00 | 1.747E-08 | 0.000E+00 | 0.000E+00 | 0.000E+00 |
| Unigene70294 | YojI | 3.389E-06 | 2.952E-07 | 2.666E-06 | 3.260E-07 | 5.080E-07 | 0.000E+00 | 3.275E-07 | 0.000E+00 | 0.000E+00 | 0.000E+00 | 1.139E-06 | 0.000E+00 |
| Unigene70396 | baeS | 1.113E-05 | 0.000E+00 | 0.000E+00 | 0.000E+00 | 1.766E-05 | 1.651E-05 | 0.000E+00 | 0.000E+00 | 1.324E-05 | 0.000E+00 | 0.000E+00 | 0.000E+00 |
| Unigene70397 | vanRM | 1.235E-05 | 0.000E+00 | 0.000E+00 | 0.000E+00 | 1.657E-05 | 1.469E-05 | 0.000E+00 | 0.000E+00 | 1.289E-05 | 0.000E+00 | 0.000E+00 | 0.000E+00 |
| Unigene70491 | msbA | 2.001E-06 | 2.879E-06 | 3.447E-06 | 5.029E-06 | 0.000E+00 | 0.000E+00 | 0.000E+00 | 0.000E+00 | 0.000E+00 | 0.000E+00 | 0.000E+00 | 0.000E+00 |
| Unigene70494 | tetB(P) | 2.279E-06 | 4.415E-06 | 2.897E-06 | 6.083E-06 | 3.522E-07 | 4.284E-08 | 1.755E-07 | 0.000E+00 | 2.382E-07 | 0.000E+00 | 6.800E-08 | 0.000E+00 |
| Unigene70496 | tetO | 2.832E-07 | 0.000E+00 | 0.000E+00 | 0.000E+00 | 0.000E+00 | 3.061E-07 | 0.000E+00 | 6.941E-07 | 0.000E+00 | 0.000E+00 | 0.000E+00 | 1.807E-06 |
| Unigene70500 | Staphylocc | 2.120E-06 | 1.653E-05 | 7.751E-06 | 5.614E-06 | 0.000E+00 | 2.504E-08 | 1.282E-08 | 0.000E+00 | 0.000E+00 | 0.000E+00 | 6.624E-08 | 0.000E+00 |
| Unigene70517 | mepA | 3.930E-06 | 2.136E-05 | 4.522E-06 | 1.512E-06 | 1.143E-06 | 3.034E-07 | 1.553E-07 | 3.549E-07 | 8.948E-07 | 6.775E-07 | 0.000E+00 | 1.659E-07 |

| | | | | | | | | | | | | | |
|---|---|---|---|---|---|---|---|---|---|---|---|---|---|
| Unigene70604 | novA | 2.270E-06 | 8.528E-06 | 6.103E-06 | 9.319E-06 | 0.000E+00 | 0.000E+00 | 0.000E+00 | 2.154E-08 | 0.000E+00 | 0.000E+00 | 0.000E+00 | 0.000E+00 |
| Unigene70639 | ugd | 5.256E-07 | 5.768E-07 | 0.000E+00 | 4.190E-07 | 6.146E-08 | 0.000E+00 | 0.000E+00 | 0.000E+00 | 0.000E+00 | 0.000E+00 | 0.000E+00 | 0.000E+00 |
| Unigene70648 | vanSN | 4.139E-06 | 2.018E-05 | 3.095E-06 | 8.132E-07 | 1.292E-06 | 2.176E-07 | 2.971E-07 | 1.306E-07 | 1.736E-06 | 6.728E-07 | 0.000E+00 | 2.909E-07 |
| Unigene70649 | mtrA | 3.716E-06 | 1.888E-05 | 3.689E-06 | 8.291E-07 | 1.008E-06 | 0.000E+00 | 0.000E+00 | 0.000E+00 | 1.181E-06 | 5.777E-07 | 0.000E+00 | 4.086E-07 |
| Unigene70733 | macB | 3.517E-06 | 7.260E-06 | 8.175E-06 | 9.386E-06 | 0.000E+00 | 0.000E+00 | 0.000E+00 | 2.444E-07 | 0.000E+00 | 0.000E+00 | 0.000E+00 | 4.949E-08 |
| Unigene70734 | vanRM | 1.180E-06 | 4.385E-06 | 3.742E-06 | 5.685E-06 | 0.000E+00 | 0.000E+00 | 0.000E+00 | 0.000E+00 | 0.000E+00 | 0.000E+00 | 0.000E+00 | 0.000E+00 |
| Unigene70735 | vanSG | 1.385E-06 | 3.352E-06 | 3.136E-06 | 6.096E-06 | 0.000E+00 | 0.000E+00 | 0.000E+00 | 0.000E+00 | 0.000E+00 | 0.000E+00 | 0.000E+00 | 0.000E+00 |
| Unigene70739 | catB2 | 0.000E+00 | 0.000E+00 | 0.000E+00 | 0.000E+00 | 0.000E+00 | 0.000E+00 | 0.000E+00 | 0.000E+00 | 0.000E+00 | 0.000E+00 | 0.000E+00 | 3.951E-06 |
| Unigene70740 | macB | 0.000E+00 | 0.000E+00 | 0.000E+00 | 0.000E+00 | 0.000E+00 | 1.554E-06 | 4.445E-06 | 0.000E+00 | 0.000E+00 | 0.000E+00 | 0.000E+00 | 0.000E+00 |
| Unigene70807 | optrA | 1.692E-06 | 8.714E-07 | 1.456E-06 | 1.709E-06 | 2.200E-05 | 7.544E-06 | 1.367E-05 | 1.690E-05 | 1.283E-05 | 1.939E-05 | 2.067E-05 | 7.310E-06 |
| Unigene70814 | vatB | 7.851E-07 | 0.000E+00 | 0.000E+00 | 0.000E+00 | 0.000E+00 | 0.000E+00 | 1.871E-05 | 2.194E-05 | 0.000E+00 | 0.000E+00 | 0.000E+00 | 0.000E+00 |
| Unigene70825 | macB | 8.792E-07 | 0.000E+00 | 2.054E-07 | 5.496E-07 | 3.814E-06 | 9.957E-06 | 1.761E-06 | 8.406E-06 | 3.341E-06 | 3.824E-06 | 0.000E+00 | 9.403E-07 |
| Unigene70859 | ugd | 9.857E-07 | 1.022E-06 | 4.137E-06 | 6.608E-06 | 0.000E+00 | 0.000E+00 | 4.848E-07 | 0.000E+00 | 0.000E+00 | 0.000E+00 | 9.396E-08 | 0.000E+00 |
| Unigene70909 | ugd | 4.690E-07 | 4.542E-07 | 1.245E-06 | 1.198E-06 | 0.000E+00 | 5.965E-08 | 1.221E-07 | 0.000E+00 | 0.000E+00 | 0.000E+00 | 0.000E+00 | 6.522E-08 |
| Unigene70983 | efrA | 1.084E-06 | 9.370E-07 | 5.013E-06 | 6.719E-06 | 0.000E+00 | 5.860E-08 | 4.050E-07 | 0.000E+00 | 0.000E+00 | 0.000E+00 | 6.201E-08 | 6.407E-08 |
| Unigene71005 | tetT | 1.068E-05 | 0.000E+00 | 0.000E+00 | 0.000E+00 | 1.716E-05 | 1.470E-05 | 0.000E+00 | 0.000E+00 | 1.087E-05 | 0.000E+00 | 0.000E+00 | 0.000E+00 |
| Unigene71015 | tet(42) | 3.657E-06 | 5.146E-07 | 2.806E-06 | 4.735E-07 | 3.386E-07 | 0.000E+00 | 5.189E-07 | 0.000E+00 | 0.000E+00 | 0.000E+00 | 6.972E-07 | 0.000E+00 |
| Unigene71033 | arlS | 2.331E-05 | 5.510E-07 | 5.354E-07 | 4.408E-08 | 0.000E+00 | 0.000E+00 | 0.000E+00 | 0.000E+00 | 0.000E+00 | 1.216E-07 | 0.000E+00 | 0.000E+00 |
| Unigene71050 | vanHF | 1.112E-06 | 0.000E+00 | 2.888E-07 | 5.284E-07 | 4.360E-06 | 8.945E-06 | 1.882E-06 | 0.000E+00 | 4.290E-06 | 3.607E-06 | 0.000E+00 | 6.959E-07 |
| Unigene71128 | cdeA | 1.191E-05 | 0.000E+00 | 0.000E+00 | 0.000E+00 | 1.601E-05 | 1.672E-05 | 0.000E+00 | 0.000E+00 | 1.034E-05 | 0.000E+00 | 0.000E+00 | 0.000E+00 |
| Unigene71129 | arlS | 1.219E-05 | 0.000E+00 | 0.000E+00 | 0.000E+00 | 1.865E-05 | 1.728E-05 | 0.000E+00 | 0.000E+00 | 1.083E-05 | 2.136E-07 | 0.000E+00 | 0.000E+00 |
| Unigene71130 | arlR | 1.203E-05 | 0.000E+00 | 0.000E+00 | 0.000E+00 | 1.728E-05 | 1.641E-05 | 0.000E+00 | 0.000E+00 | 1.381E-05 | 0.000E+00 | 0.000E+00 | 0.000E+00 |
| Unigene71311 | arlR | 3.726E-06 | 2.004E-05 | 3.519E-06 | 1.035E-06 | 5.175E-07 | 0.000E+00 | 0.000E+00 | 4.350E-07 | 1.324E-06 | 5.189E-07 | 4.795E-07 | 1.652E-07 |
| Unigene71312 | arlS | 4.287E-06 | 2.163E-05 | 3.090E-06 | 9.497E-07 | 7.289E-07 | 3.783E-07 | 1.452E-07 | 3.063E-07 | 1.195E-06 | 5.847E-07 | 4.003E-07 | 2.068E-07 |
| Unigene71348 | oleC | 4.598E-07 | 2.019E-07 | 2.707E-07 | 3.096E-07 | 1.022E-07 | 0.000E+00 | 0.000E+00 | 0.000E+00 | 0.000E+00 | 0.000E+00 | 7.013E-08 | 0.000E+00 |
| Unigene71423 | msrC | 3.052E-06 | 4.169E-06 | 3.749E-06 | 5.100E-06 | 5.611E-07 | 1.662E-07 | 1.944E-07 | 5.126E-08 | 7.200E-08 | 0.000E+00 | 2.010E-07 | 7.787E-08 |
| Unigene71465 | Staphylocc | 1.290E-06 | 2.920E-08 | 0.000E+00 | 0.000E+00 | 0.000E+00 | 4.314E-08 | 4.416E-08 | 0.000E+00 | 0.000E+00 | 0.000E+00 | 0.000E+00 | 0.000E+00 |
| Unigene71471 | efrB | 1.537E-06 | 0.000E+00 | 0.000E+00 | 0.000E+00 | 0.000E+00 | 0.000E+00 | 0.000E+00 | 0.000E+00 | 0.000E+00 | 0.000E+00 | 0.000E+00 | 0.000E+00 |
| Unigene71576 | Escherichia | 4.304E-06 | 1.848E-06 | 0.000E+00 | 2.173E-06 | 0.000E+00 | 0.000E+00 | 0.000E+00 | 0.000E+00 | 0.000E+00 | 0.000E+00 | 5.351E-07 | 5.530E-07 |
| Unigene71589 | patA | 4.876E-05 | 5.821E-05 | 2.764E-05 | 1.483E-05 | 1.865E-05 | 6.709E-06 | 4.744E-06 | 6.217E-05 | 1.191E-05 | 6.764E-06 | 1.634E-06 | 7.916E-07 |
| Unigene71592 | bcrA | 4.153E-05 | 6.592E-05 | 2.666E-05 | 1.483E-05 | 1.287E-05 | 5.213E-06 | 3.903E-06 | 6.137E-05 | 1.259E-05 | 7.256E-06 | 1.153E-06 | 1.404E-06 |
| Unigene71608 | tet(44) | 6.194E-07 | 1.593E-06 | 7.814E-07 | 5.004E-07 | 5.702E-06 | 3.635E-07 | 1.841E-06 | 3.099E-07 | 0.000E+00 | 0.000E+00 | 5.061E-07 | 2.719E-07 |
| Unigene71658 | adeS | 2.142E-06 | 2.405E-06 | 2.628E-06 | 1.155E-06 | 0.000E+00 | 1.842E-07 | 5.658E-07 | 3.126E-07 | 7.716E-07 | 0.000E+00 | 0.000E+00 | 1.151E-07 |
| Unigene71700 | Streptomy | 6.117E-07 | 5.423E-06 | 8.511E-06 | 1.367E-05 | 0.000E+00 | 5.087E-08 | 1.736E-08 | 0.000E+00 | 0.000E+00 | 0.000E+00 | 0.000E+00 | 0.000E+00 |
| Unigene71819 | msbA | 5.739E-06 | 3.824E-06 | 2.514E-07 | 5.174E-07 | 5.123E-07 | 0.000E+00 | 0.000E+00 | 0.000E+00 | 0.000E+00 | 0.000E+00 | 0.000E+00 | 0.000E+00 |
| Unigene71845 | bcrA | 5.794E-07 | 7.267E-07 | 4.304E-06 | 6.436E-06 | 0.000E+00 | 0.000E+00 | 2.748E-07 | 0.000E+00 | 0.000E+00 | 0.000E+00 | 0.000E+00 | 0.000E+00 |
| Unigene71848 | FusF | 3.369E-06 | 5.022E-06 | 3.010E-06 | 6.042E-06 | 5.027E-07 | 0.000E+00 | 2.782E-07 | 0.000E+00 | 1.649E-07 | 0.000E+00 | 5.752E-08 | 3.566E-07 |
| Unigene71859 | bcrA | 1.857E-06 | 1.389E-06 | 2.526E-06 | 1.364E-06 | 1.406E-07 | 0.000E+00 | 4.670E-07 | 0.000E+00 | 5.996E-07 | 0.000E+00 | 0.000E+00 | 0.000E+00 |
| Unigene71999 | mtrA | 5.688E-07 | 0.000E+00 | 0.000E+00 | 0.000E+00 | 0.000E+00 | 7.686E-07 | 3.043E-06 | 0.000E+00 | 4.145E-07 | 0.000E+00 | 0.000E+00 | 0.000E+00 |
| Unigene72036 | lsaA | 2.217E-06 | 2.008E-06 | 2.365E-06 | 1.717E-06 | 1.674E-07 | 0.000E+00 | 4.287E-07 | 7.536E-08 | 5.880E-07 | 0.000E+00 | 4.923E-08 | 0.000E+00 |
| Unigene72082 | smeR | 1.090E-06 | 5.723E-07 | 3.442E-06 | 5.026E-06 | 1.053E-07 | 0.000E+00 | 0.000E+00 | 0.000E+00 | 0.000E+00 | 0.000E+00 | 0.000E+00 | 0.000E+00 |
| Unigene72117 | golS | 2.869E-06 | 5.590E-06 | 4.153E-06 | 7.171E-06 | 0.000E+00 | 0.000E+00 | 0.000E+00 | 0.000E+00 | 0.000E+00 | 0.000E+00 | 0.000E+00 | 0.000E+00 |
| Unigene72129 | efrA | 6.791E-06 | 0.000E+00 | 0.000E+00 | 2.553E-06 | 3.282E-05 | 1.535E-05 | 2.395E-05 | 2.611E-05 | 3.438E-06 | 0.000E+00 | 0.000E+00 | 0.000E+00 |
| Unigene72135 | tetA(58) | 7.557E-06 | 0.000E+00 | 0.000E+00 | 2.326E-06 | 3.182E-05 | 1.626E-05 | 2.003E-05 | 2.504E-05 | 3.029E-06 | 0.000E+00 | 0.000E+00 | 0.000E+00 |
| Unigene72142 | vgaB | 7.080E-06 | 0.000E+00 | 0.000E+00 | 2.079E-06 | 3.202E-05 | 1.585E-05 | 2.106E-05 | 2.330E-05 | 3.750E-06 | 0.000E+00 | 0.000E+00 | 0.000E+00 |
| Unigene72160 | vanSM | 1.069E-06 | 7.821E-07 | 6.643E-07 | 1.439E-07 | 0.000E+00 | 0.000E+00 | 1.577E-07 | 1.248E-07 | 6.231E-07 | 0.000E+00 | 0.000E+00 | 0.000E+00 |
| Unigene72193 | vmlR | 5.509E-07 | 0.000E+00 | 3.603E-07 | 0.000E+00 | 4.121E-06 | 9.607E-06 | 1.544E-06 | 8.402E-06 | 4.937E-06 | 3.027E-06 | 0.000E+00 | 0.000E+00 |
| Unigene72250 | YojI | 2.669E-05 | 1.083E-05 | 7.300E-06 | 4.564E-06 | 5.123E-07 | 9.970E-08 | 6.805E-08 | 0.000E+00 | 9.744E-07 | 0.000E+00 | 0.000E+00 | 0.000E+00 |
| Unigene72274 | vatE | 1.056E-05 | 0.000E+00 | 0.000E+00 | 0.000E+00 | 0.000E+00 | 0.000E+00 | 0.000E+00 | 5.735E-08 | 0.000E+00 | 0.000E+00 | 0.000E+00 | 0.000E+00 |
| Unigene72410 | poxtA | 9.782E-07 | 5.205E-06 | 9.366E-06 | 1.512E-05 | 0.000E+00 | 0.000E+00 | 0.000E+00 | 0.000E+00 | 0.000E+00 | 0.000E+00 | 0.000E+00 | 0.000E+00 |
| Unigene72419 | PmrF | 2.369E-05 | 4.023E-06 | 2.198E-06 | 2.708E-06 | 1.094E-06 | 5.137E-07 | 1.428E-06 | 2.377E-07 | 0.000E+00 | 3.781E-07 | 1.553E-07 | 3.210E-07 |
| Unigene72474 | efrB | 3.085E-06 | 3.910E-06 | 2.793E-06 | 6.267E-06 | 4.284E-07 | 5.955E-08 | 2.032E-07 | 6.430E-08 | 1.204E-07 | 0.000E+00 | 0.000E+00 | 1.302E-07 |
| Unigene72535 | vanHB | 1.203E-06 | 0.000E+00 | 1.276E-07 | 0.000E+00 | 4.514E-06 | 7.977E-06 | 0.000E+00 | 7.361E-06 | 4.015E-06 | 0.000E+00 | 0.000E+00 | 6.148E-07 |
| Unigene72537 | vanRE | 2.518E-06 | 4.875E-06 | 3.100E-06 | 1.433E-06 | 2.747E-06 | 2.970E-06 | 3.497E-06 | 8.551E-07 | 8.508E-07 | 1.137E-05 | 1.241E-05 | 2.111E-06 |
| Unigene72538 | baeS | 3.819E-06 | 5.256E-06 | 2.558E-06 | 2.571E-06 | 2.728E-06 | 2.785E-06 | 3.690E-06 | 1.238E-06 | 1.325E-06 | 1.050E-05 | 7.766E-06 | 2.508E-06 |
| Unigene72570 | pgpB | 1.223E-06 | 1.181E-06 | 5.279E-06 | 6.124E-06 | 0.000E+00 | 0.000E+00 | 3.789E-07 | 0.000E+00 | 0.000E+00 | 0.000E+00 | 0.000E+00 | 0.000E+00 |
| Unigene72573 | otr(B) | 9.148E-07 | 9.084E-07 | 4.788E-06 | 6.401E-06 | 0.000E+00 | 0.000E+00 | 1.929E-07 | 0.000E+00 | 0.000E+00 | 0.000E+00 | 0.000E+00 | 0.000E+00 |
| Unigene72632 | lmrD | 4.928E-06 | 5.991E-06 | 7.283E-06 | 9.260E-06 | 0.000E+00 | 0.000E+00 | 0.000E+00 | 0.000E+00 | 0.000E+00 | 1.750E-07 | 4.492E-08 | 0.000E+00 |

| | | | | | | | | | | | | | |
|---|---|---|---|---|---|---|---|---|---|---|---|---|---|
| Unigene72633 | efrA | 5.203E-06 | 7.593E-06 | 7.905E-06 | 9.190E-06 | 6.215E-08 | 1.210E-07 | 0.000E+00 | 0.000E+00 | 0.000E+00 | 2.077E-07 | 4.266E-08 | 8.817E-08 |
| Unigene72667 | vanSG | 1.764E-06 | 2.152E-06 | 2.223E-06 | 1.408E-06 | 2.420E-07 | 0.000E+00 | 2.893E-07 | 3.305E-07 | 6.190E-07 | 0.000E+00 | 2.492E-08 | 1.030E-07 |
| Unigene72742 | lmrD | 2.131E-06 | 1.845E-06 | 2.562E-06 | 1.970E-06 | 4.501E-07 | 9.085E-07 | 1.229E-06 | 3.503E-07 | 7.053E-07 | 1.087E-06 | 1.133E-06 | 1.330E-06 |
| Unigene72869 | sul4 | 4.148E-06 | 8.313E-06 | 1.165E-05 | 9.743E-06 | 2.003E-07 | 2.599E-07 | 4.657E-07 | 2.807E-07 | 0.000E+00 | 0.000E+00 | 2.407E-07 | 1.066E-07 |
| Unigene72884 | vanUG | 0.000E+00 | 0.000E+00 | 0.000E+00 | 0.000E+00 | 0.000E+00 | 0.000E+00 | 5.604E-06 | 7.942E-06 | 0.000E+00 | 0.000E+00 | 0.000E+00 | 0.000E+00 |
| Unigene73158 | macB | 1.609E-06 | 7.066E-07 | 9.926E-07 | 1.718E-06 | 1.660E-05 | 6.263E-06 | 1.456E-05 | 1.438E-05 | 1.005E-05 | 1.588E-05 | 1.567E-05 | 7.175E-06 |
| Unigene73162 | tlrC | 1.598E-06 | 0.000E+00 | 0.000E+00 | 0.000E+00 | 0.000E+00 | 8.462E-06 | 0.000E+00 | 0.000E+00 | 3.559E-06 | 0.000E+00 | 0.000E+00 | 0.000E+00 |
| Unigene73177 | vanHD | 6.642E-07 | 0.000E+00 | 1.955E-07 | 1.677E-07 | 4.870E-06 | 9.119E-06 | 1.580E-06 | 8.141E-06 | 5.263E-06 | 3.145E-06 | 0.000E+00 | 0.000E+00 |
| Unigene73202 | tetB(60) | 3.132E-06 | 3.963E-06 | 2.676E-06 | 5.712E-06 | 3.684E-07 | 7.967E-08 | 0.000E+00 | 8.603E-08 | 1.812E-07 | 0.000E+00 | 1.054E-07 | 6.534E-08 |
| Unigene73254 | mdtG | 1.495E-06 | 0.000E+00 | 0.000E+00 | 0.000E+00 | 0.000E+00 | 0.000E+00 | 0.000E+00 | 0.000E+00 | 0.000E+00 | 0.000E+00 | 0.000E+00 | 0.000E+00 |
| Unigene73258 | Streptomy | 1.314E-06 | 0.000E+00 | 0.000E+00 | 0.000E+00 | 1.737E-08 | 0.000E+00 | 0.000E+00 | 0.000E+00 | 0.000E+00 | 0.000E+00 | 3.578E-08 | 0.000E+00 |
| Unigene73370 | baeR | 2.071E-06 | 1.892E-05 | 7.068E-06 | 4.865E-06 | 7.502E-08 | 0.000E+00 | 0.000E+00 | 0.000E+00 | 0.000E+00 | 0.000E+00 | 0.000E+00 | 0.000E+00 |
| Unigene73411 | vanHA | 5.789E-06 | 1.064E-05 | 9.786E-06 | 1.134E-05 | 0.000E+00 | 0.000E+00 | 3.482E-08 | 0.000E+00 | 0.000E+00 | 3.855E-07 | 0.000E+00 | 1.488E-07 |
| Unigene73418 | tet(43) | 2.458E-05 | 6.510E-06 | 8.378E-06 | 3.189E-05 | 9.641E-06 | 9.041E-06 | 2.227E-05 | 1.307E-06 | 1.432E-05 | 4.350E-05 | 2.272E-05 | 6.184E-06 |
| Unigene73425 | adeR | 2.804E-06 | 4.414E-06 | 3.327E-06 | 5.861E-06 | 4.105E-07 | 2.350E-08 | 3.368E-07 | 0.000E+00 | 4.752E-08 | 0.000E+00 | 0.000E+00 | 7.708E-08 |
| Unigene73478 | vanL | 7.003E-06 | 0.000E+00 | 0.000E+00 | 2.458E-06 | 3.252E-05 | 1.693E-05 | 2.468E-05 | 2.677E-05 | 3.515E-06 | 0.000E+00 | 0.000E+00 | 0.000E+00 |
| Unigene73480 | vanHB | 7.313E-06 | 0.000E+00 | 0.000E+00 | 2.147E-06 | 3.439E-05 | 1.943E-05 | 2.606E-05 | 2.873E-05 | 3.732E-06 | 0.000E+00 | 0.000E+00 | 0.000E+00 |
| Unigene73489 | lin | 7.818E-06 | 0.000E+00 | 0.000E+00 | 2.617E-06 | 3.025E-05 | 9.401E-06 | 2.100E-05 | 2.625E-05 | 0.000E+00 | 0.000E+00 | 0.000E+00 | 0.000E+00 |
| Unigene73532 | novA | 4.899E-06 | 1.280E-07 | 2.861E-08 | 0.000E+00 | 0.000E+00 | 6.305E-08 | 0.000E+00 | 0.000E+00 | 0.000E+00 | 0.000E+00 | 0.000E+00 | 0.000E+00 |
| Unigene73533 | bcr-1 | 4.764E-06 | 2.601E-08 | 0.000E+00 | 0.000E+00 | 0.000E+00 | 2.562E-08 | 0.000E+00 | 0.000E+00 | 0.000E+00 | 0.000E+00 | 0.000E+00 | 0.000E+00 |
| Unigene73576 | vanHB | 1.305E-06 | 1.467E-06 | 1.125E-06 | 1.607E-07 | 1.592E-06 | 7.227E-07 | 3.171E-07 | 5.202E-07 | 6.959E-07 | 1.419E-06 | 1.165E-06 | 6.773E-07 |
| Unigene73580 | bcrA | 5.399E-07 | 0.000E+00 | 0.000E+00 | 2.337E-07 | 0.000E+00 | 2.001E-06 | 1.238E-06 | 8.103E-06 | 4.679E-06 | 0.000E+00 | 0.000E+00 | 2.279E-07 |
| Unigene73635 | facT | 8.656E-06 | 2.627E-06 | 1.969E-06 | 4.151E-06 | 8.827E-05 | 1.169E-04 | 1.041E-04 | 9.734E-05 | 1.488E-04 | 1.444E-04 | 1.219E-04 | 8.100E-05 |
| Unigene73654 | Staphylocc | 2.115E-06 | 7.318E-07 | 7.670E-07 | 4.643E-08 | 0.000E+00 | 0.000E+00 | 0.000E+00 | 0.000E+00 | 0.000E+00 | 0.000E+00 | 0.000E+00 | 0.000E+00 |
| Unigene73664 | Listeria mc | 4.079E-06 | 1.019E-05 | 9.269E-06 | 1.113E-05 | 1.529E-07 | 0.000E+00 | 0.000E+00 | 0.000E+00 | 0.000E+00 | 9.756E-08 | 0.000E+00 | 4.437E-08 |
| Unigene73751 | efrA | 1.938E-05 | 9.574E-06 | 6.868E-06 | 5.327E-06 | 6.383E-07 | 1.412E-07 | 0.000E+00 | 6.097E-08 | 0.000E+00 | 0.000E+00 | 0.000E+00 | 0.000E+00 |
| Unigene73792 | macB | 1.196E-06 | 6.876E-06 | 1.098E-05 | 1.637E-05 | 0.000E+00 | 0.000E+00 | 0.000E+00 | 0.000E+00 | 0.000E+00 | 0.000E+00 | 0.000E+00 | 0.000E+00 |
| Unigene73808 | kdpE | 9.522E-08 | 0.000E+00 | 0.000E+00 | 0.000E+00 | 0.000E+00 | 0.000E+00 | 0.000E+00 | 2.223E-07 | 0.000E+00 | 0.000E+00 | 0.000E+00 | 0.000E+00 |
| Unigene73845 | cmlv | 3.361E-06 | 3.272E-07 | 3.005E-06 | 5.748E-07 | 1.807E-07 | 1.172E-07 | 2.700E-07 | 0.000E+00 | 0.000E+00 | 0.000E+00 | 5.271E-07 | 0.000E+00 |
| Unigene73847 | bcrA | 3.867E-06 | 4.429E-07 | 4.817E-06 | 4.754E-07 | 2.241E-07 | 0.000E+00 | 1.861E-07 | 0.000E+00 | 3.675E-08 | 0.000E+00 | 9.616E-07 | 0.000E+00 |
| Unigene73870 | vanTE | 6.010E-07 | 0.000E+00 | 2.808E-07 | 4.046E-07 | 4.324E-06 | 8.198E-06 | 1.489E-06 | 7.249E-06 | 4.129E-06 | 3.379E-06 | 0.000E+00 | 9.133E-07 |
| Unigene73884 | smeS | 2.436E-06 | 1.428E-05 | 6.312E-06 | 4.466E-06 | 1.051E-07 | 1.023E-07 | 1.309E-07 | 0.000E+00 | 0.000E+00 | 0.000E+00 | 0.000E+00 | 0.000E+00 |
| Unigene73885 | vanRB | 2.124E-06 | 1.373E-05 | 5.881E-06 | 3.765E-06 | 0.000E+00 | 0.000E+00 | 0.000E+00 | 0.000E+00 | 0.000E+00 | 0.000E+00 | 1.620E-07 | 0.000E+00 |
| Unigene73943 | srmB | 4.346E-06 | 5.664E-06 | 3.608E-06 | 4.663E-06 | 1.857E-07 | 6.550E-07 | 1.318E-06 | 6.585E-07 | 6.851E-07 | 3.491E-07 | 1.147E-06 | 9.385E-07 |
| Unigene73969 | vanL | 1.256E-06 | 8.458E-07 | 1.792E-06 | 1.182E-06 | 2.071E-05 | 6.294E-06 | 1.510E-05 | 1.752E-05 | 1.123E-05 | 1.844E-05 | 1.858E-05 | 7.287E-06 |
| Unigene73996 | lsaA | 1.208E-06 | 5.585E-06 | 9.416E-06 | 1.500E-05 | 0.000E+00 | 9.324E-08 | 2.387E-08 | 0.000E+00 | 0.000E+00 | 0.000E+00 | 0.000E+00 | 0.000E+00 |
| Unigene74142 | CRP | 3.571E-06 | 4.078E-06 | 5.113E-06 | 6.237E-06 | 0.000E+00 | 0.000E+00 | 1.068E-07 | 0.000E+00 | 0.000E+00 | 0.000E+00 | 0.000E+00 | 0.000E+00 |
| Unigene74193 | tetA(46) | 2.552E-06 | 3.407E-06 | 2.462E-06 | 4.681E-06 | 3.780E-07 | 4.598E-08 | 1.883E-07 | 0.000E+00 | 2.324E-07 | 0.000E+00 | 0.000E+00 | 2.011E-07 |
| Unigene74210 | vanRE | 8.554E-07 | 4.546E-06 | 2.783E-06 | 0.000E+00 | 1.155E-05 | 8.761E-07 | 4.484E-06 | 2.207E-06 | 0.000E+00 | 0.000E+00 | 1.287E-06 | 1.118E-06 |
| Unigene74263 | emrB | 2.052E-06 | 1.936E-05 | 8.224E-06 | 5.246E-06 | 0.000E+00 | 1.180E-07 | 0.000E+00 | 0.000E+00 | 0.000E+00 | 0.000E+00 | 0.000E+00 | 0.000E+00 |
| Unigene74267 | pgpB | 3.179E-06 | 1.702E-05 | 8.111E-06 | 4.593E-06 | 0.000E+00 | 0.000E+00 | 0.000E+00 | 0.000E+00 | 0.000E+00 | 0.000E+00 | 0.000E+00 | 0.000E+00 |
| Unigene74268 | Staphylocc | 3.213E-06 | 2.121E-05 | 1.045E-05 | 8.601E-06 | 5.599E-08 | 1.498E-07 | 0.000E+00 | 7.353E-08 | 0.000E+00 | 0.000E+00 | 1.153E-07 | 0.000E+00 |
| Unigene74274 | Escherichia | 3.474E-06 | 1.937E-05 | 1.020E-05 | 1.127E-05 | 0.000E+00 | 0.000E+00 | 5.939E-07 | 0.000E+00 | 0.000E+00 | 0.000E+00 | 0.000E+00 | 1.001E-07 |
| Unigene74277 | macB | 2.793E-06 | 1.553E-05 | 7.189E-06 | 4.716E-06 | 0.000E+00 | 0.000E+00 | 0.000E+00 | 0.000E+00 | 0.000E+00 | 1.020E-07 | 0.000E+00 | 0.000E+00 |
| Unigene74300 | tetA(58) | 6.957E-06 | 0.000E+00 | 0.000E+00 | 2.575E-06 | 3.874E-05 | 1.168E-06 | 2.697E-05 | 2.799E-05 | 0.000E+00 | 0.000E+00 | 0.000E+00 | 0.000E+00 |
| Unigene74336 | Corynebac | 1.554E-06 | 3.280E-08 | 0.000E+00 | 0.000E+00 | 0.000E+00 | 0.000E+00 | 0.000E+00 | 0.000E+00 | 0.000E+00 | 0.000E+00 | 0.000E+00 | 0.000E+00 |
| Unigene74341 | vanTG | 1.237E-06 | 0.000E+00 | 0.000E+00 | 0.000E+00 | 0.000E+00 | 0.000E+00 | 0.000E+00 | 0.000E+00 | 0.000E+00 | 0.000E+00 | 0.000E+00 | 0.000E+00 |
| Unigene74344 | qacA | 1.390E-06 | 0.000E+00 | 0.000E+00 | 0.000E+00 | 0.000E+00 | 0.000E+00 | 2.230E-08 | 0.000E+00 | 0.000E+00 | 0.000E+00 | 0.000E+00 | 0.000E+00 |
| Unigene74349 | msbA | 9.672E-07 | 0.000E+00 | 0.000E+00 | 0.000E+00 | 0.000E+00 | 0.000E+00 | 0.000E+00 | 0.000E+00 | 0.000E+00 | 0.000E+00 | 0.000E+00 | 0.000E+00 |
| Unigene74374 | lmrD | 1.830E-06 | 0.000E+00 | 0.000E+00 | 0.000E+00 | 0.000E+00 | 0.000E+00 | 6.015E-08 | 0.000E+00 | 0.000E+00 | 0.000E+00 | 0.000E+00 | 2.141E-08 |
| Unigene74375 | patA | 1.336E-06 | 0.000E+00 | 0.000E+00 | 0.000E+00 | 0.000E+00 | 0.000E+00 | 0.000E+00 | 0.000E+00 | 0.000E+00 | 0.000E+00 | 0.000E+00 | 0.000E+00 |
| Unigene74386 | oleC | 2.242E-05 | 7.936E-06 | 6.082E-06 | 3.999E-06 | 4.782E-07 | 1.861E-07 | 4.763E-08 | 0.000E+00 | 0.000E+00 | 0.000E+00 | 0.000E+00 | 0.000E+00 |
| Unigene74412 | Bifidobacte | 0.000E+00 | 0.000E+00 | 0.000E+00 | 0.000E+00 | 0.000E+00 | 0.000E+00 | 0.000E+00 | 4.717E-07 | 0.000E+00 | 0.000E+00 | 0.000E+00 | 0.000E+00 |
| Unigene74450 | MexL | 1.314E-05 | 0.000E+00 | 0.000E+00 | 0.000E+00 | 2.139E-05 | 1.959E-05 | 0.000E+00 | 0.000E+00 | 1.343E-05 | 0.000E+00 | 0.000E+00 | 0.000E+00 |
| Unigene74451 | carA | 1.240E-05 | 0.000E+00 | 0.000E+00 | 0.000E+00 | 2.037E-05 | 1.801E-05 | 0.000E+00 | 0.000E+00 | 1.361E-05 | 3.440E-07 | 0.000E+00 | 0.000E+00 |
| Unigene74457 | vatH | 1.161E-05 | 0.000E+00 | 0.000E+00 | 0.000E+00 | 2.107E-05 | 2.024E-05 | 0.000E+00 | 0.000E+00 | 1.116E-05 | 0.000E+00 | 0.000E+00 | 0.000E+00 |
| Unigene74464 | Streptomy | 1.272E-05 | 0.000E+00 | 0.000E+00 | 0.000E+00 | 1.781E-05 | 1.661E-05 | 0.000E+00 | 3.220E-08 | 1.264E-05 | 0.000E+00 | 3.156E-08 | 0.000E+00 |
| Unigene74482 | Staphylocc | 1.239E-05 | 0.000E+00 | 0.000E+00 | 0.000E+00 | 1.698E-05 | 1.676E-05 | 0.000E+00 | 0.000E+00 | 0.000E+00 | 0.000E+00 | 0.000E+00 | 0.000E+00 |

| | | | | | | | | | | | | | |
|---|---|---|---|---|---|---|---|---|---|---|---|---|---|
| Unigene74547 | vanHF | 2.421E-06 | 4.539E-06 | 3.464E-06 | 5.806E-06 | 0.000E+00 | 0.000E+00 | 0.000E+00 | 1.177E-07 | 0.000E+00 | 0.000E+00 | 0.000E+00 | 0.000E+00 |
| Unigene74610 | vanZF | 9.984E-07 | 0.000E+00 | 0.000E+00 | 2.240E-07 | 3.913E-06 | 8.454E-06 | 1.013E-06 | 0.000E+00 | 4.243E-06 | 3.769E-06 | 0.000E+00 | 0.000E+00 |
| Unigene74637 | golS | 3.263E-06 | 6.389E-06 | 6.318E-06 | 4.364E-06 | 0.000E+00 | 0.000E+00 | 0.000E+00 | 0.000E+00 | 0.000E+00 | 0.000E+00 | 0.000E+00 | 0.000E+00 |
| Unigene74730 | tetA(58) | 3.602E-06 | 9.370E-06 | 1.187E-05 | 1.199E-05 | 0.000E+00 | 3.845E-07 | 1.132E-06 | 2.076E-07 | 0.000E+00 | 1.981E-07 | 0.000E+00 | 0.000E+00 |
| Unigene74785 | vmlR | 3.138E-05 | 2.252E-05 | 1.922E-05 | 1.970E-05 | 2.032E-06 | 1.551E-06 | 2.898E-06 | 0.000E+00 | 0.000E+00 | 9.989E-07 | 1.149E-06 | 0.000E+00 |
| Unigene74826 | tetA(60) | 0.000E+00 | 0.000E+00 | 0.000E+00 | 0.000E+00 | 0.000E+00 | 8.522E-08 | 0.000E+00 | 0.000E+00 | 0.000E+00 | 0.000E+00 | 0.000E+00 | 0.000E+00 |
| Unigene74858 | efrB | 6.937E-06 | 0.000E+00 | 0.000E+00 | 2.407E-06 | 3.395E-05 | 1.748E-05 | 2.246E-05 | 2.642E-05 | 3.615E-06 | 0.000E+00 | 0.000E+00 | 0.000E+00 |
| Unigene74863 | mecC | 7.454E-06 | 0.000E+00 | 0.000E+00 | 2.160E-06 | 3.417E-05 | 1.715E-05 | 2.498E-05 | 2.779E-05 | 3.153E-06 | 0.000E+00 | 0.000E+00 | 0.000E+00 |
| Unigene74864 | emrB | 7.632E-06 | 0.000E+00 | 0.000E+00 | 2.051E-06 | 3.519E-05 | 1.703E-05 | 2.586E-05 | 2.624E-05 | 3.342E-06 | 0.000E+00 | 0.000E+00 | 0.000E+00 |
| Unigene75007 | vanHO | 1.145E-05 | 0.000E+00 | 0.000E+00 | 0.000E+00 | 1.950E-05 | 1.667E-05 | 0.000E+00 | 0.000E+00 | 1.255E-05 | 0.000E+00 | 0.000E+00 | 0.000E+00 |
| Unigene75158 | pmrA | 1.049E-06 | 1.122E-06 | 1.689E-06 | 1.739E-06 | 2.041E-05 | 6.514E-06 | 1.521E-05 | 1.648E-05 | 1.155E-05 | 1.741E-05 | 1.880E-05 | 6.487E-06 |
| Unigene75198 | vanZF | 8.523E-07 | 9.354E-07 | 4.025E-06 | 5.325E-06 | 0.000E+00 | 5.758E-08 | 3.537E-07 | 0.000E+00 | 0.000E+00 | 0.000E+00 | 0.000E+00 | 0.000E+00 |
| Unigene75238 | marA | 0.000E+00 | 0.000E+00 | 0.000E+00 | 0.000E+00 | 7.599E-06 | 0.000E+00 | 0.000E+00 | 0.000E+00 | 0.000E+00 | 0.000E+00 | 0.000E+00 | 0.000E+00 |
| Unigene75274 | bcrA | 5.279E-06 | 0.000E+00 | 0.000E+00 | 1.281E-06 | 4.127E-05 | 3.841E-07 | 2.685E-05 | 3.430E-05 | 0.000E+00 | 0.000E+00 | 0.000E+00 | 0.000E+00 |
| Unigene75340 | arlR | 4.185E-05 | 2.827E-05 | 2.172E-05 | 2.212E-05 | 0.000E+00 | 0.000E+00 | 3.583E-06 | 8.336E-07 | 0.000E+00 | 0.000E+00 | 0.000E+00 | 1.407E-06 |
| Unigene75350 | vgaC | 5.028E-08 | 0.000E+00 | 0.000E+00 | 0.000E+00 | 0.000E+00 | 0.000E+00 | 0.000E+00 | 0.000E+00 | 0.000E+00 | 0.000E+00 | 0.000E+00 | 2.377E-07 |
| Unigene75354 | msbA | 4.314E-06 | 1.493E-05 | 3.835E-06 | 0.000E+00 | 7.894E-07 | 1.646E-07 | 3.932E-07 | 4.147E-07 | 1.109E-06 | 7.350E-07 | 0.000E+00 | 1.200E-07 |
| Unigene75411 | patA | 9.911E-07 | 7.769E-07 | 7.145E-07 | 1.082E-06 | 1.798E-07 | 0.000E+00 | 0.000E+00 | 0.000E+00 | 0.000E+00 | 0.000E+00 | 0.000E+00 | 0.000E+00 |
| Unigene75590 | mepA | 1.383E-06 | 1.158E-06 | 5.314E-06 | 7.836E-06 | 0.000E+00 | 0.000E+00 | 4.930E-07 | 0.000E+00 | 0.000E+00 | 0.000E+00 | 0.000E+00 | 0.000E+00 |
| Unigene75596 | oleC | 1.403E-06 | 1.074E-06 | 6.343E-06 | 8.117E-06 | 0.000E+00 | 0.000E+00 | 1.412E-07 | 0.000E+00 | 0.000E+00 | 0.000E+00 | 0.000E+00 | 0.000E+00 |
| Unigene75875 | tetA(58) | 8.257E-06 | 0.000E+00 | 0.000E+00 | 1.597E-06 | 4.167E-05 | 1.614E-05 | 2.576E-05 | 2.886E-05 | 0.000E+00 | 0.000E+00 | 0.000E+00 | 0.000E+00 |
| Unigene75908 | tetA(46) | 5.907E-06 | 0.000E+00 | 0.000E+00 | 2.166E-06 | 2.867E-05 | 1.570E-05 | 2.165E-05 | 2.191E-05 | 2.755E-06 | 0.000E+00 | 0.000E+00 | 0.000E+00 |
| Unigene75909 | oleC | 7.499E-06 | 0.000E+00 | 0.000E+00 | 2.455E-06 | 3.527E-05 | 1.545E-05 | 2.283E-05 | 2.432E-05 | 2.883E-06 | 0.000E+00 | 0.000E+00 | 0.000E+00 |
| Unigene75933 | lsaC | 2.660E-06 | 1.783E-07 | 1.514E-06 | 2.870E-07 | 2.255E-07 | 4.389E-08 | 3.370E-07 | 0.000E+00 | 0.000E+00 | 0.000E+00 | 1.626E-07 | 0.000E+00 |
| Unigene76040 | mgrA | 0.000E+00 | 2.084E-06 | 0.000E+00 | 0.000E+00 | 0.000E+00 | 0.000E+00 | 0.000E+00 | 0.000E+00 | 0.000E+00 | 0.000E+00 | 0.000E+00 | 0.000E+00 |
| Unigene76196 | carA | 4.288E-06 | 1.790E-05 | 2.905E-06 | 9.804E-07 | 7.011E-07 | 2.799E-07 | 8.954E-08 | 2.833E-07 | 1.114E-06 | 5.407E-07 | 2.406E-07 | 1.530E-07 |
| Unigene76316 | macB | 2.483E-06 | 4.138E-06 | 2.707E-06 | 5.619E-06 | 4.598E-07 | 0.000E+00 | 3.054E-07 | 0.000E+00 | 0.000E+00 | 0.000E+00 | 5.260E-08 | 0.000E+00 |
| Unigene76322 | carA | 2.764E-06 | 4.004E-06 | 2.531E-06 | 4.800E-06 | 6.141E-07 | 0.000E+00 | 4.078E-07 | 0.000E+00 | 1.208E-07 | 0.000E+00 | 0.000E+00 | 3.049E-07 |
| Unigene76325 | Staphyloco | 2.757E-05 | 1.014E-05 | 6.888E-06 | 4.065E-06 | 3.521E-07 | 3.084E-07 | 1.052E-07 | 1.110E-07 | 1.576E-06 | 0.000E+00 | 0.000E+00 | 0.000E+00 |
| Unigene76370 | vanRM | 3.494E-07 | 1.534E-06 | 2.194E-06 | 0.000E+00 | 0.000E+00 | 0.000E+00 | 0.000E+00 | 0.000E+00 | 0.000E+00 | 0.000E+00 | 0.000E+00 | 0.000E+00 |
| Unigene76407 | vanRM | 1.801E-06 | 3.599E-06 | 3.036E-06 | 2.052E-06 | 2.309E-06 | 1.947E-06 | 2.709E-06 | 1.186E-06 | 1.363E-06 | 5.350E-06 | 5.758E-06 | 1.365E-06 |
| Unigene76409 | tva(A) | 7.909E-06 | 1.413E-06 | 1.593E-05 | 9.626E-05 | 4.087E-06 | 4.076E-06 | 1.323E-05 | 8.051E-07 | 0.000E+00 | 1.137E-05 | 6.312E-06 | 2.228E-06 |
| Unigene76416 | efpA | 2.059E-07 | 0.000E+00 | 0.000E+00 | 0.000E+00 | 0.000E+00 | 0.000E+00 | 0.000E+00 | 0.000E+00 | 0.000E+00 | 0.000E+00 | 0.000E+00 | 0.000E+00 |
| Unigene76444 | tetA(60) | 1.854E-06 | 6.338E-07 | 0.000E+00 | 0.000E+00 | 0.000E+00 | 0.000E+00 | 3.364E-08 | 0.000E+00 | 0.000E+00 | 0.000E+00 | 0.000E+00 | 0.000E+00 |
| Unigene76450 | baeR | 0.000E+00 | 0.000E+00 | 0.000E+00 | 0.000E+00 | 0.000E+00 | 0.000E+00 | 0.000E+00 | 0.000E+00 | 0.000E+00 | 0.000E+00 | 0.000E+00 | 2.719E-06 |
| Unigene76492 | patB | 1.201E-06 | 8.852E-07 | 5.557E-06 | 7.694E-06 | 0.000E+00 | 3.710E-08 | 5.888E-07 | 0.000E+00 | 0.000E+00 | 5.735E-08 | 0.000E+00 | 2.029E-08 |
| Unigene76532 | NmcR | 0.000E+00 | 0.000E+00 | 0.000E+00 | 0.000E+00 | 1.002E-05 | 0.000E+00 | 2.235E-06 | 0.000E+00 | 0.000E+00 | 0.000E+00 | 0.000E+00 | 0.000E+00 |
| Unigene76563 | bcrA | 2.399E-05 | 9.458E-06 | 6.353E-06 | 3.810E-06 | 0.000E+00 | 2.014E-07 | 1.547E-07 | 4.350E-07 | 0.000E+00 | 0.000E+00 | 0.000E+00 | 0.000E+00 |
| Unigene76737 | vatH | 1.427E-06 | 4.218E-07 | 1.616E-07 | 0.000E+00 | 0.000E+00 | 0.000E+00 | 0.000E+00 | 0.000E+00 | 0.000E+00 | 0.000E+00 | 0.000E+00 | 2.596E-07 |
| Unigene76755 | tva(A) | 9.456E-07 | 1.396E-06 | 4.607E-06 | 6.651E-06 | 0.000E+00 | 7.050E-08 | 6.135E-07 | 0.000E+00 | 0.000E+00 | 0.000E+00 | 0.000E+00 | 3.854E-08 |
| Unigene76790 | msbA | 4.732E-05 | 2.674E-05 | 1.827E-05 | 2.132E-05 | 2.305E-06 | 2.104E-06 | 3.739E-06 | 1.115E-06 | 8.028E-06 | 6.955E-07 | 1.554E-06 | 1.389E-06 |
| Unigene76871 | efrA | 2.043E-06 | 1.705E-05 | 8.215E-06 | 4.939E-06 | 0.000E+00 | 1.244E-07 | 0.000E+00 | 0.000E+00 | 0.000E+00 | 0.000E+00 | 0.000E+00 | 0.000E+00 |
| Unigene76890 | vanHB | 6.705E-05 | 1.181E-04 | 1.170E-04 | 0.000E+00 | 0.000E+00 | 0.000E+00 | 0.000E+00 | 0.000E+00 | 0.000E+00 | 0.000E+00 | 0.000E+00 | 9.936E-07 |
| Unigene76945 | msbA | 4.099E-06 | 8.773E-06 | 1.498E-05 | 1.382E-05 | 0.000E+00 | 3.877E-07 | 7.372E-07 | 5.383E-07 | 0.000E+00 | 0.000E+00 | 4.689E-07 | 0.000E+00 |
| Unigene77104 | arlR | 7.784E-07 | 0.000E+00 | 0.000E+00 | 2.774E-07 | 3.510E-06 | 1.054E-05 | 1.216E-06 | 0.000E+00 | 4.454E-06 | 3.621E-06 | 0.000E+00 | 0.000E+00 |
| Unigene77197 | bcrA | 1.662E-06 | 9.601E-07 | 2.275E-06 | 4.859E-07 | 0.000E+00 | 0.000E+00 | 0.000E+00 | 1.021E-07 | 0.000E+00 | 0.000E+00 | 0.000E+00 | 0.000E+00 |
| Unigene77223 | arlR | 2.573E-06 | 8.215E-07 | 1.377E-06 | 4.252E-07 | 0.000E+00 | 0.000E+00 | 0.000E+00 | 5.461E-08 | 0.000E+00 | 0.000E+00 | 0.000E+00 | 0.000E+00 |
| Unigene77224 | arlS | 2.952E-06 | 8.922E-07 | 1.763E-06 | 5.400E-07 | 0.000E+00 | 0.000E+00 | 0.000E+00 | 1.748E-07 | 0.000E+00 | 0.000E+00 | 0.000E+00 | 0.000E+00 |
| Unigene77288 | tetA(60) | 2.290E-06 | 9.603E-07 | 1.333E-06 | 4.512E-07 | 0.000E+00 | 0.000E+00 | 0.000E+00 | 0.000E+00 | 0.000E+00 | 0.000E+00 | 0.000E+00 | 0.000E+00 |
| Unigene77289 | tetB(60) | 2.522E-06 | 8.364E-07 | 1.424E-06 | 5.863E-07 | 0.000E+00 | 0.000E+00 | 0.000E+00 | 6.354E-08 | 0.000E+00 | 0.000E+00 | 0.000E+00 | 0.000E+00 |
| Unigene77314 | tetW | 2.475E-06 | 9.247E-07 | 1.722E-06 | 4.786E-07 | 0.000E+00 | 0.000E+00 | 0.000E+00 | 1.434E-07 | 0.000E+00 | 0.000E+00 | 0.000E+00 | 0.000E+00 |
| Unigene77362 | bcrA | 3.206E-06 | 3.953E-06 | 3.361E-06 | 5.100E-06 | 4.879E-07 | 0.000E+00 | 0.000E+00 | 0.000E+00 | 3.360E-07 | 0.000E+00 | 1.005E-07 | 3.634E-07 |
| Unigene77403 | PmrF | 5.442E-05 | 3.989E-05 | 8.400E-05 | 1.381E-04 | 9.635E-05 | 7.005E-05 | 7.898E-05 | 8.525E-05 | 0.000E+00 | 1.068E-04 | 9.753E-05 | 1.068E-04 |
| Unigene77424 | tmrB | 2.358E-06 | 2.024E-05 | 7.533E-06 | 4.822E-06 | 0.000E+00 | 1.307E-07 | 0.000E+00 | 0.000E+00 | 0.000E+00 | 0.000E+00 | 0.000E+00 | 0.000E+00 |
| Unigene77687 | tetA(58) | 1.142E-06 | 6.835E-07 | 4.753E-07 | 1.502E-06 | 7.687E-08 | 0.000E+00 | 0.000E+00 | 0.000E+00 | 0.000E+00 | 0.000E+00 | 0.000E+00 | 0.000E+00 |
| Unigene77766 | Staphyloco | 3.765E-06 | 3.516E-07 | 3.432E-06 | 4.044E-07 | 3.115E-07 | 8.659E-08 | 3.546E-07 | 0.000E+00 | 0.000E+00 | 0.000E+00 | 7.330E-07 | 0.000E+00 |
| Unigene77881 | vgaD | 2.989E-07 | 0.000E+00 | 0.000E+00 | 0.000E+00 | 0.000E+00 | 0.000E+00 | 0.000E+00 | 0.000E+00 | 0.000E+00 | 0.000E+00 | 0.000E+00 | 0.000E+00 |
| Unigene77904 | oleC | 3.504E-06 | 1.010E-05 | 9.447E-06 | 9.579E-06 | 0.000E+00 | 1.913E-07 | 0.000E+00 | 1.239E-07 | 0.000E+00 | 0.000E+00 | 0.000E+00 | 0.000E+00 |

| | | | | | | | | | | | | | |
|---|---|---|---|---|---|---|---|---|---|---|---|---|---|
| Unigene77940 | bcrA | 9.494E-07 | 4.631E-07 | 4.831E-07 | 0.000E+00 | 0.000E+00 | 7.603E-08 | 2.724E-07 | 0.000E+00 | 5.765E-07 | 0.000E+00 | 0.000E+00 | 0.000E+00 |
| Unigene77953 | mepA | 1.460E-05 | 0.000E+00 | 0.000E+00 | 0.000E+00 | 2.130E-05 | 1.827E-05 | 0.000E+00 | 0.000E+00 | 1.240E-05 | 4.359E-07 | 0.000E+00 | 0.000E+00 |
| Unigene77955 | poxtA | 1.364E-05 | 0.000E+00 | 0.000E+00 | 0.000E+00 | 1.657E-05 | 1.589E-05 | 0.000E+00 | 0.000E+00 | 1.074E-05 | 0.000E+00 | 0.000E+00 | 0.000E+00 |
| Unigene77958 | optrA | 1.295E-05 | 0.000E+00 | 0.000E+00 | 0.000E+00 | 2.084E-05 | 1.692E-05 | 9.249E-08 | 0.000E+00 | 1.242E-05 | 2.095E-07 | 0.000E+00 | 0.000E+00 |
| Unigene78106 | arnA | 5.606E-05 | 4.184E-05 | 8.467E-05 | 1.529E-04 | 9.036E-05 | 6.710E-05 | 8.559E-05 | 8.066E-05 | 0.000E+00 | 1.035E-04 | 1.033E-04 | 1.024E-04 |
| Unigene78192 | macB | 3.304E-06 | 9.206E-06 | 9.727E-06 | 1.010E-05 | 0.000E+00 | 0.000E+00 | 0.000E+00 | 0.000E+00 | 0.000E+00 | 0.000E+00 | 0.000E+00 | 0.000E+00 |
| Unigene78244 | vmlR | 8.748E-07 | 6.577E-06 | 1.039E-05 | 1.564E-05 | 2.430E-08 | 9.457E-08 | 0.000E+00 | 0.000E+00 | 0.000E+00 | 0.000E+00 | 0.000E+00 | 0.000E+00 |
| Unigene78427 | vanHA | 1.260E-06 | 9.221E-07 | 1.522E-06 | 1.729E-06 | 2.046E-05 | 6.323E-06 | 1.359E-05 | 1.878E-05 | 1.060E-05 | 1.577E-05 | 1.556E-05 | 7.066E-06 |
| Unigene78446 | NmcR | 8.814E-07 | 0.000E+00 | 3.603E-07 | 0.000E+00 | 1.714E-06 | 9.885E-06 | 1.829E-06 | 1.076E-05 | 5.780E-06 | 4.623E-06 | 0.000E+00 | 0.000E+00 |
| Unigene78459 | macB | 2.772E-06 | 1.599E-06 | 2.674E-06 | 1.898E-06 | 0.000E+00 | 2.540E-07 | 2.080E-07 | 0.000E+00 | 4.622E-07 | 0.000E+00 | 0.000E+00 | 0.000E+00 |
| Unigene78460 | macB | 2.556E-06 | 2.510E-06 | 2.560E-06 | 1.222E-06 | 3.287E-07 | 1.454E-07 | 5.061E-07 | 0.000E+00 | 7.056E-07 | 0.000E+00 | 0.000E+00 | 1.908E-07 |
| Unigene78463 | baeS | 2.872E-06 | 3.833E-06 | 4.366E-06 | 2.112E-06 | 0.000E+00 | 9.582E-08 | 2.747E-07 | 2.276E-07 | 6.587E-07 | 0.000E+00 | 0.000E+00 | 3.353E-07 |
| Unigene78552 | efrB | 1.491E-06 | 7.519E-07 | 1.503E-06 | 1.180E-06 | 1.536E-05 | 5.446E-06 | 1.231E-05 | 1.515E-05 | 9.382E-06 | 1.666E-05 | 1.558E-05 | 6.050E-06 |
| Unigene78555 | vmlR | 1.360E-06 | 1.038E-06 | 1.479E-06 | 1.642E-06 | 1.652E-05 | 5.592E-06 | 1.649E-05 | 1.729E-05 | 1.069E-05 | 1.686E-05 | 1.589E-05 | 7.198E-06 |
| Unigene78572 | tetA(58) | 9.614E-07 | 1.015E-06 | 1.451E-06 | 1.344E-06 | 1.475E-05 | 7.075E-06 | 1.248E-05 | 1.623E-05 | 8.689E-06 | 1.516E-05 | 1.573E-05 | 7.605E-06 |
| Unigene78687 | vanRM | 3.120E-06 | 4.962E-06 | 4.303E-06 | 6.072E-06 | 4.520E-07 | 1.466E-07 | 2.502E-07 | 0.000E+00 | 0.000E+00 | 0.000E+00 | 0.000E+00 | 1.069E-07 |
| Unigene78688 | arlS | 2.961E-06 | 5.165E-06 | 3.209E-06 | 6.850E-06 | 3.682E-07 | 2.389E-08 | 3.913E-07 | 0.000E+00 | 1.449E-07 | 0.000E+00 | 0.000E+00 | 2.089E-07 |
| Unigene78757 | patB | 4.508E-07 | 0.000E+00 | 0.000E+00 | 2.732E-07 | 0.000E+00 | 0.000E+00 | 0.000E+00 | 0.000E+00 | 0.000E+00 | 0.000E+00 | 0.000E+00 | 0.000E+00 |
| Unigene78821 | macB | 3.719E-06 | 2.301E-06 | 6.172E-06 | 6.791E-06 | 1.758E-07 | 0.000E+00 | 0.000E+00 | 0.000E+00 | 0.000E+00 | 3.525E-07 | 0.000E+00 | 0.000E+00 |
| Unigene78845 | novA | 1.067E-06 | 7.967E-07 | 1.383E-06 | 1.595E-06 | 1.546E-05 | 7.339E-06 | 1.139E-05 | 1.665E-05 | 1.101E-05 | 1.865E-05 | 1.690E-05 | 7.773E-06 |
| Unigene78946 | mgrA | 3.160E-06 | 3.469E-06 | 2.437E-06 | 2.128E-06 | 3.511E-06 | 1.871E-06 | 2.915E-06 | 7.026E-07 | 1.809E-06 | 4.694E-06 | 4.906E-06 | 1.512E-06 |
| Unigene78980 | tmrB | 1.189E-06 | 5.836E-06 | 9.699E-06 | 1.731E-05 | 0.000E+00 | 0.000E+00 | 0.000E+00 | 0.000E+00 | 0.000E+00 | 0.000E+00 | 0.000E+00 | 0.000E+00 |
| Unigene79005 | vanHD | 0.000E+00 | 0.000E+00 | 0.000E+00 | 0.000E+00 | 0.000E+00 | 3.292E-07 | 0.000E+00 | 0.000E+00 | 5.547E-08 | 0.000E+00 | 0.000E+00 | 0.000E+00 |
| Unigene79056 | ARL-1 | 1.984E-06 | 4.175E-06 | 5.332E-06 | 8.341E-06 | 1.509E-08 | 0.000E+00 | 0.000E+00 | 0.000E+00 | 0.000E+00 | 0.000E+00 | 0.000E+00 | 0.000E+00 |
| Unigene79088 | mecA | 8.927E-07 | 6.648E-07 | 4.693E-06 | 5.972E-06 | 0.000E+00 | 0.000E+00 | 3.705E-07 | 0.000E+00 | 0.000E+00 | 0.000E+00 | 5.470E-08 | 1.884E-08 |
| Unigene79276 | arlS | 1.162E-05 | 2.130E-06 | 1.597E-05 | 1.017E-04 | 5.351E-06 | 4.430E-06 | 1.400E-05 | 7.553E-07 | 0.000E+00 | 1.105E-05 | 1.464E-05 | 2.748E-06 |
| Unigene79325 | macB | 5.124E-07 | 6.135E-07 | 1.371E-06 | 6.115E-07 | 0.000E+00 | 0.000E+00 | 0.000E+00 | 0.000E+00 | 0.000E+00 | 0.000E+00 | 0.000E+00 | 0.000E+00 |
| Unigene79332 | macB | 8.481E-07 | 4.072E-07 | 1.638E-06 | 1.258E-06 | 0.000E+00 | 0.000E+00 | 0.000E+00 | 0.000E+00 | 0.000E+00 | 0.000E+00 | 0.000E+00 | 0.000E+00 |
| Unigene79380 | vanUG | 1.968E-06 | 0.000E+00 | 0.000E+00 | 0.000E+00 | 0.000E+00 | 0.000E+00 | 0.000E+00 | 0.000E+00 | 0.000E+00 | 0.000E+00 | 0.000E+00 | 0.000E+00 |
| Unigene79426 | tetA(58) | 7.587E-07 | 6.397E-06 | 8.595E-06 | 1.518E-05 | 0.000E+00 | 0.000E+00 | 0.000E+00 | 0.000E+00 | 0.000E+00 | 0.000E+00 | 0.000E+00 | 0.000E+00 |
| Unigene79436 | vanSF | 2.048E-06 | 1.867E-05 | 8.200E-06 | 4.109E-06 | 9.102E-08 | 2.952E-08 | 0.000E+00 | 0.000E+00 | 0.000E+00 | 0.000E+00 | 1.874E-07 | 0.000E+00 |
| Unigene79506 | arlR | 4.618E-06 | 2.137E-05 | 3.224E-06 | 8.173E-07 | 1.481E-06 | 4.118E-07 | 1.054E-07 | 2.223E-07 | 1.665E-06 | 5.834E-07 | 4.902E-07 | 0.000E+00 |
| Unigene79588 | evgS | 0.000E+00 | 0.000E+00 | 0.000E+00 | 0.000E+00 | 4.627E-07 | 3.752E-07 | 0.000E+00 | 0.000E+00 | 2.276E-07 | 0.000E+00 | 4.765E-07 | 0.000E+00 |
| Unigene79592 | bcrA | 6.380E-07 | 7.935E-07 | 4.131E-06 | 6.872E-06 | 0.000E+00 | 9.195E-08 | 3.295E-07 | 0.000E+00 | 0.000E+00 | 0.000E+00 | 0.000E+00 | 0.000E+00 |
| Unigene79652 | arlR | 0.000E+00 | 0.000E+00 | 0.000E+00 | 0.000E+00 | 0.000E+00 | 0.000E+00 | 0.000E+00 | 0.000E+00 | 0.000E+00 | 3.654E-07 | 0.000E+00 | 0.000E+00 |
| Unigene79732 | lmrD | 1.100E-06 | 1.056E-06 | 1.484E-06 | 1.701E-06 | 1.749E-05 | 5.612E-06 | 1.472E-05 | 1.529E-05 | 1.007E-05 | 1.739E-05 | 1.561E-05 | 6.949E-06 |
| Unigene79736 | vatE | 1.507E-06 | 1.084E-06 | 1.377E-06 | 1.312E-06 | 1.571E-05 | 5.788E-06 | 1.087E-05 | 1.462E-05 | 9.488E-06 | 1.598E-05 | 1.653E-05 | 7.496E-06 |
| Unigene79808 | qacH | 0.000E+00 | 0.000E+00 | 0.000E+00 | 0.000E+00 | 4.285E-06 | 6.584E-07 | 0.000E+00 | 0.000E+00 | 0.000E+00 | 0.000E+00 | 0.000E+00 | 0.000E+00 |
| Unigene79864 | lmrB | 6.479E-07 | 2.540E-08 | 0.000E+00 | 0.000E+00 | 1.910E-05 | 2.276E-06 | 0.000E+00 | 0.000E+00 | 0.000E+00 | 0.000E+00 | 0.000E+00 | 0.000E+00 |
| Unigene79865 | bcrA | 8.661E-07 | 0.000E+00 | 0.000E+00 | 0.000E+00 | 1.691E-05 | 2.557E-06 | 0.000E+00 | 0.000E+00 | 0.000E+00 | 0.000E+00 | 0.000E+00 | 0.000E+00 |
| Unigene79872 | macB | 1.268E-06 | 0.000E+00 | 0.000E+00 | 0.000E+00 | 1.136E-05 | 0.000E+00 | 0.000E+00 | 0.000E+00 | 0.000E+00 | 0.000E+00 | 0.000E+00 | 0.000E+00 |
| Unigene79988 | vanZF | 8.124E-06 | 2.952E-07 | 6.863E-07 | 5.650E-06 | 3.586E-07 | 0.000E+00 | 5.240E-06 | 1.231E-05 | 0.000E+00 | 1.360E-05 | 0.000E+00 | 7.186E-06 |
| Unigene80097 | vgaD | 2.365E-07 | 2.163E-07 | 1.934E-07 | 2.388E-07 | 0.000E+00 | 0.000E+00 | 0.000E+00 | 0.000E+00 | 4.308E-08 | 0.000E+00 | 4.509E-08 | 4.659E-08 |
| Unigene80172 | bacA | 3.857E-07 | 0.000E+00 | 0.000E+00 | 0.000E+00 | 0.000E+00 | 0.000E+00 | 5.079E-06 | 0.000E+00 | 0.000E+00 | 0.000E+00 | 0.000E+00 | 4.969E-06 |
| Unigene80187 | kdpE | 1.071E-06 | 6.947E-07 | 3.679E-06 | 5.507E-06 | 0.000E+00 | 0.000E+00 | 5.389E-07 | 0.000E+00 | 0.000E+00 | 0.000E+00 | 0.000E+00 | 0.000E+00 |
| Unigene80188 | vanSA | 1.134E-06 | 1.468E-06 | 4.109E-06 | 5.550E-06 | 0.000E+00 | 0.000E+00 | 6.437E-07 | 0.000E+00 | 0.000E+00 | 0.000E+00 | 6.653E-08 | 8.594E-08 |
| Unigene80210 | msrA | 3.398E-07 | 0.000E+00 | 0.000E+00 | 0.000E+00 | 0.000E+00 | 0.000E+00 | 0.000E+00 | 0.000E+00 | 0.000E+00 | 0.000E+00 | 0.000E+00 | 0.000E+00 |
| Unigene80256 | macB | 2.098E-06 | 1.491E-05 | 6.704E-06 | 3.749E-06 | 5.977E-08 | 0.000E+00 | 0.000E+00 | 6.280E-08 | 0.000E+00 | 0.000E+00 | 0.000E+00 | 0.000E+00 |
| Unigene80257 | macB | 1.598E-06 | 1.759E-05 | 4.841E-06 | 4.128E-06 | 0.000E+00 | 1.524E-07 | 1.040E-07 | 0.000E+00 | 0.000E+00 | 0.000E+00 | 0.000E+00 | 0.000E+00 |
| Unigene80300 | vanSG | 1.041E-06 | 6.892E-06 | 1.042E-05 | 1.468E-05 | 0.000E+00 | 0.000E+00 | 0.000E+00 | 0.000E+00 | 0.000E+00 | 0.000E+00 | 0.000E+00 | 0.000E+00 |
| Unigene80301 | mtrA | 7.085E-07 | 7.533E-06 | 9.689E-06 | 1.529E-05 | 0.000E+00 | 9.574E-08 | 0.000E+00 | 0.000E+00 | 0.000E+00 | 0.000E+00 | 0.000E+00 | 0.000E+00 |
| Unigene80307 | macB | 7.144E-07 | 7.105E-06 | 1.279E-05 | 1.754E-05 | 0.000E+00 | 2.896E-07 | 0.000E+00 | 0.000E+00 | 0.000E+00 | 0.000E+00 | 0.000E+00 | 0.000E+00 |
| Unigene80398 | pmrA | 2.308E-06 | 2.974E-06 | 3.054E-06 | 1.545E-06 | 0.000E+00 | 0.000E+00 | 2.970E-07 | 0.000E+00 | 5.866E-07 | 0.000E+00 | 0.000E+00 | 2.537E-07 |
| Unigene80423 | lmrD | 2.679E-06 | 1.813E-06 | 3.111E-06 | 0.000E+00 | 0.000E+00 | 0.000E+00 | 5.930E-07 | 3.127E-07 | 4.880E-07 | 0.000E+00 | 5.107E-08 | 0.000E+00 |
| Unigene80467 | tetA(46) | 2.016E-06 | 1.528E-05 | 8.432E-06 | 5.576E-06 | 0.000E+00 | 0.000E+00 | 0.000E+00 | 2.200E-08 | 0.000E+00 | 0.000E+00 | 0.000E+00 | 0.000E+00 |
| Unigene80468 | tetA(46) | 1.901E-06 | 1.539E-05 | 7.461E-06 | 5.517E-06 | 0.000E+00 | 1.397E-07 | 0.000E+00 | 0.000E+00 | 0.000E+00 | 0.000E+00 | 0.000E+00 | 0.000E+00 |
| Unigene80498 | novA | 0.000E+00 | 0.000E+00 | 0.000E+00 | 0.000E+00 | 0.000E+00 | 0.000E+00 | 0.000E+00 | 0.000E+00 | 0.000E+00 | 0.000E+00 | 0.000E+00 | 1.926E-06 |
| Unigene80539 | patA | 1.623E-06 | 1.123E-06 | 1.385E-06 | 9.619E-07 | 2.073E-05 | 7.056E-06 | 1.386E-05 | 1.870E-05 | 1.134E-05 | 1.737E-05 | 1.574E-05 | 7.381E-06 |

| | | | | | | | | | | | | | |
|---|---|---|---|---|---|---|---|---|---|---|---|---|---|
| Unigene80560 | Corynebac | 1.768E-06 | 1.137E-06 | 1.256E-06 | 2.432E-06 | 1.869E-05 | 6.689E-06 | 1.471E-05 | 1.868E-05 | 1.116E-05 | 1.728E-05 | 1.900E-05 | 9.080E-06 |
| Unigene80600 | bmr | 7.821E-07 | 1.273E-06 | 1.111E-06 | 1.770E-06 | 1.968E-05 | 6.035E-06 | 1.295E-05 | 1.637E-05 | 1.032E-05 | 1.688E-05 | 1.820E-05 | 7.459E-06 |
| Unigene80601 | qacH | 0.000E+00 | 1.435E-06 | 1.480E-06 | 0.000E+00 | 1.508E-05 | 6.523E-06 | 1.135E-05 | 1.279E-05 | 9.123E-06 | 1.311E-05 | 1.231E-05 | 4.279E-06 |
| Unigene80767 | lsaC | 3.835E-05 | 1.978E-05 | 7.124E-06 | 1.158E-05 | 2.234E-06 | 1.650E-06 | 3.214E-06 | 6.084E-07 | 8.424E-06 | 5.807E-07 | 2.129E-06 | 1.320E-06 |
| Unigene80784 | tetA(58) | 5.804E-07 | 4.900E-08 | 1.314E-07 | 0.000E+00 | 0.000E+00 | 0.000E+00 | 0.000E+00 | 0.000E+00 | 0.000E+00 | 0.000E+00 | 1.532E-07 | 0.000E+00 |
| Unigene80785 | tetB(58) | 3.098E-06 | 3.217E-07 | 2.835E-06 | 0.000E+00 | 0.000E+00 | 0.000E+00 | 1.853E-07 | 0.000E+00 | 0.000E+00 | 0.000E+00 | 0.000E+00 | 0.000E+00 |
| Unigene80841 | macB | 2.354E-06 | 7.381E-07 | 1.650E-06 | 5.093E-07 | 0.000E+00 | 0.000E+00 | 0.000E+00 | 4.906E-08 | 0.000E+00 | 0.000E+00 | 0.000E+00 | 0.000E+00 |
| Unigene80937 | tetA(60) | 1.068E-04 | 1.916E-04 | 1.835E-04 | 7.799E-06 | 8.683E-06 | 5.005E-07 | 4.448E-06 | 1.794E-06 | 6.317E-05 | 0.000E+00 | 3.093E-06 | 1.029E-06 |
| Unigene81008 | vanHB | 9.293E-07 | 1.020E-06 | 1.056E-06 | 4.149E-06 | 0.000E+00 | 2.485E-06 | 2.165E-07 | 5.709E-06 | 1.283E-06 | 0.000E+00 | 0.000E+00 | 4.047E-07 |
| Unigene81034 | tetA(58) | 3.435E-06 | 4.042E-06 | 3.289E-06 | 5.767E-06 | 1.839E-07 | 0.000E+00 | 4.122E-07 | 1.932E-07 | 2.714E-07 | 0.000E+00 | 0.000E+00 | 0.000E+00 |
| Unigene81084 | Klebsiella p | 0.000E+00 | 6.846E-07 | 2.040E-07 | 2.625E-07 | 0.000E+00 | 0.000E+00 | 0.000E+00 | 0.000E+00 | 0.000E+00 | 0.000E+00 | 0.000E+00 | 0.000E+00 |
| Unigene81163 | macB | 0.000E+00 | 6.617E-07 | 0.000E+00 | 5.620E-07 | 0.000E+00 | 0.000E+00 | 0.000E+00 | 0.000E+00 | 0.000E+00 | 0.000E+00 | 0.000E+00 | 1.096E-07 |
| Unigene81210 | macB | 3.452E-07 | 1.326E-06 | 0.000E+00 | 0.000E+00 | 0.000E+00 | 0.000E+00 | 0.000E+00 | 0.000E+00 | 0.000E+00 | 0.000E+00 | 0.000E+00 | 0.000E+00 |
| Unigene81392 | tet(59) | 2.362E-07 | 1.267E-06 | 1.674E-06 | 1.749E-06 | 0.000E+00 | 0.000E+00 | 0.000E+00 | 0.000E+00 | 0.000E+00 | 0.000E+00 | 0.000E+00 | 0.000E+00 |
| Unigene81418 | lmrD | 1.860E-07 | 6.707E-07 | 1.303E-07 | 0.000E+00 | 0.000E+00 | 0.000E+00 | 0.000E+00 | 0.000E+00 | 0.000E+00 | 0.000E+00 | 0.000E+00 | 0.000E+00 |
| Unigene81517 | arr-1 | 0.000E+00 | 6.182E-06 | 0.000E+00 | 0.000E+00 | 0.000E+00 | 0.000E+00 | 0.000E+00 | 0.000E+00 | 3.157E-07 | 0.000E+00 | 0.000E+00 | 0.000E+00 |
| Unigene81551 | mdtN | 0.000E+00 | 7.575E-07 | 4.002E-07 | 0.000E+00 | 0.000E+00 | 0.000E+00 | 0.000E+00 | 0.000E+00 | 0.000E+00 | 0.000E+00 | 0.000E+00 | 0.000E+00 |
| Unigene81612 | marA | 5.433E-05 | 4.581E-05 | 8.743E-05 | 1.598E-04 | 9.263E-05 | 6.380E-05 | 8.710E-05 | 8.941E-05 | 6.979E-05 | 1.063E-04 | 1.077E-04 | 1.178E-04 |
| Unigene81616 | macB | 2.425E-05 | 1.980E-05 | 1.683E-05 | 1.794E-05 | 0.000E+00 | 1.727E-06 | 0.000E+00 | 0.000E+00 | 2.722E-06 | 6.281E-07 | 0.000E+00 | 1.833E-06 |
| Unigene81627 | oleC | 0.000E+00 | 4.175E-07 | 0.000E+00 | 0.000E+00 | 0.000E+00 | 0.000E+00 | 0.000E+00 | 0.000E+00 | 0.000E+00 | 0.000E+00 | 0.000E+00 | 0.000E+00 |
| Unigene81824 | facT | 6.996E-05 | 6.360E-05 | 1.182E-04 | 1.602E-04 | 1.353E-04 | 9.380E-05 | 1.230E-04 | 1.226E-04 | 1.032E-04 | 1.549E-04 | 1.592E-04 | 1.687E-04 |
| Unigene81902 | patA | 4.374E-07 | 5.281E-07 | 1.245E-06 | 2.385E-06 | 1.458E-07 | 0.000E+00 | 0.000E+00 | 9.190E-07 | 0.000E+00 | 4.873E-07 | 1.351E-06 | 4.653E-07 |
| Unigene81907 | TaeA | 4.214E-06 | 1.437E-06 | 1.606E-07 | 0.000E+00 | 0.000E+00 | 0.000E+00 | 0.000E+00 | 0.000E+00 | 0.000E+00 | 0.000E+00 | 0.000E+00 | 0.000E+00 |
| Unigene81976 | efmA | 0.000E+00 | 2.504E-06 | 0.000E+00 | 0.000E+00 | 0.000E+00 | 0.000E+00 | 1.435E-07 | 0.000E+00 | 0.000E+00 | 0.000E+00 | 0.000E+00 | 4.597E-07 |
| Unigene81978 | arlS | 1.176E-05 | 5.072E-06 | 3.435E-06 | 1.233E-05 | 8.962E-05 | 1.277E-04 | 1.304E-04 | 1.025E-04 | 1.528E-04 | 1.551E-04 | 1.269E-04 | 7.656E-05 |
| Unigene82005 | mdtG | 2.276E-06 | 1.590E-05 | 6.903E-06 | 4.728E-06 | 0.000E+00 | 0.000E+00 | 0.000E+00 | 0.000E+00 | 1.131E-07 | 0.000E+00 | 0.000E+00 | 0.000E+00 |
| Unigene82038 | lmrP | 3.929E-05 | 2.240E-05 | 1.711E-05 | 1.857E-05 | 1.897E-06 | 1.538E-06 | 3.263E-06 | 4.529E-07 | 7.520E-06 | 9.220E-07 | 2.012E-06 | 1.376E-06 |
| Unigene82128 | arlR | 0.000E+00 | 5.723E-07 | 0.000E+00 | 0.000E+00 | 0.000E+00 | 0.000E+00 | 0.000E+00 | 0.000E+00 | 0.000E+00 | 0.000E+00 | 0.000E+00 | 0.000E+00 |
| Unigene82155 | lsaA | 1.084E-04 | 1.932E-04 | 1.772E-04 | 0.000E+00 | 9.363E-06 | 0.000E+00 | 3.150E-06 | 0.000E+00 | 5.768E-05 | 0.000E+00 | 3.256E-06 | 5.643E-07 |
| Unigene82202 | evgA | 0.000E+00 | 1.780E-06 | 0.000E+00 | 0.000E+00 | 0.000E+00 | 0.000E+00 | 1.414E-06 | 0.000E+00 | 3.222E-07 | 0.000E+00 | 1.686E-07 | 0.000E+00 |
| Unigene82205 | sdiA | 0.000E+00 | 0.000E+00 | 0.000E+00 | 0.000E+00 | 4.308E-07 | 0.000E+00 | 0.000E+00 | 0.000E+00 | 0.000E+00 | 0.000E+00 | 0.000E+00 | 0.000E+00 |
| Unigene82251 | evgS | 0.000E+00 | 9.914E-07 | 0.000E+00 | 0.000E+00 | 5.962E-06 | 0.000E+00 | 0.000E+00 | 0.000E+00 | 0.000E+00 | 0.000E+00 | 0.000E+00 | 0.000E+00 |
| Unigene82257 | Acinetobac | 0.000E+00 | 2.279E-06 | 0.000E+00 | 0.000E+00 | 8.691E-06 | 0.000E+00 | 0.000E+00 | 0.000E+00 | 0.000E+00 | 0.000E+00 | 0.000E+00 | 0.000E+00 |
| Unigene82267 | vanSF | 2.913E-06 | 2.309E-06 | 3.678E-06 | 2.696E-06 | 8.988E-08 | 1.749E-07 | 5.969E-08 | 0.000E+00 | 0.000E+00 | 0.000E+00 | 3.085E-08 | 0.000E+00 |
| Unigene82364 | adeL | 0.000E+00 | 1.134E-06 | 0.000E+00 | 0.000E+00 | 0.000E+00 | 0.000E+00 | 1.183E-06 | 0.000E+00 | 0.000E+00 | 0.000E+00 | 0.000E+00 | 0.000E+00 |
| Unigene82367 | adeL | 0.000E+00 | 9.490E-07 | 0.000E+00 | 2.910E-07 | 0.000E+00 | 0.000E+00 | 0.000E+00 | 0.000E+00 | 0.000E+00 | 0.000E+00 | 0.000E+00 | 0.000E+00 |
| Unigene82615 | macB | 0.000E+00 | 1.444E-06 | 0.000E+00 | 0.000E+00 | 0.000E+00 | 0.000E+00 | 0.000E+00 | 0.000E+00 | 0.000E+00 | 0.000E+00 | 0.000E+00 | 0.000E+00 |
| Unigene82625 | efrB | 4.188E-06 | 8.875E-06 | 9.041E-06 | 1.093E-05 | 0.000E+00 | 0.000E+00 | 0.000E+00 | 0.000E+00 | 0.000E+00 | 0.000E+00 | 0.000E+00 | 0.000E+00 |
| Unigene82633 | ugd | 1.106E-07 | 5.465E-07 | 0.000E+00 | 1.955E-07 | 3.073E-08 | 0.000E+00 | 0.000E+00 | 1.937E-07 | 3.023E-07 | 0.000E+00 | 9.492E-08 | 6.539E-08 |
| Unigene82650 | rphB | 4.919E-05 | 2.581E-05 | 2.167E-05 | 2.341E-05 | 2.324E-06 | 2.282E-06 | 4.424E-06 | 8.068E-07 | 6.432E-06 | 7.700E-07 | 2.201E-06 | 2.054E-06 |
| Unigene82653 | optrA | 7.279E-05 | 6.452E-05 | 1.213E-04 | 1.794E-04 | 1.352E-04 | 9.385E-05 | 1.303E-04 | 1.210E-04 | 1.020E-04 | 1.570E-04 | 1.572E-04 | 1.635E-04 |
| Unigene82720 | cpxA | 0.000E+00 | 7.436E-07 | 0.000E+00 | 0.000E+00 | 0.000E+00 | 0.000E+00 | 4.852E-07 | 9.303E-08 | 0.000E+00 | 0.000E+00 | 0.000E+00 | 0.000E+00 |
| Unigene82748 | macB | 1.681E-07 | 1.061E-06 | 3.217E-06 | 3.650E-06 | 0.000E+00 | 0.000E+00 | 0.000E+00 | 0.000E+00 | 0.000E+00 | 0.000E+00 | 0.000E+00 | 0.000E+00 |
| Unigene82761 | optrA | 7.841E-05 | 5.925E-05 | 1.203E-04 | 1.657E-04 | 1.403E-04 | 1.035E-04 | 1.265E-04 | 1.259E-04 | 1.093E-04 | 1.580E-04 | 1.622E-04 | 1.600E-04 |
| Unigene82808 | vanSF | 0.000E+00 | 5.272E-07 | 0.000E+00 | 0.000E+00 | 0.000E+00 | 0.000E+00 | 0.000E+00 | 0.000E+00 | 0.000E+00 | 0.000E+00 | 0.000E+00 | 0.000E+00 |
| Unigene82838 | tetW | 1.111E-05 | 4.207E-06 | 3.036E-06 | 1.223E-05 | 8.223E-05 | 1.097E-04 | 1.035E-04 | 8.677E-05 | 1.355E-04 | 1.355E-04 | 1.058E-04 | 7.515E-05 |
| Unigene82851 | optrA | 3.857E-07 | 1.016E-06 | 6.812E-07 | 0.000E+00 | 4.842E-06 | 2.502E-07 | 0.000E+00 | 4.052E-07 | 0.000E+00 | 0.000E+00 | 0.000E+00 | 0.000E+00 |
| Unigene82871 | vanTN | 0.000E+00 | 1.257E-06 | 1.006E-06 | 0.000E+00 | 0.000E+00 | 0.000E+00 | 1.234E-06 | 0.000E+00 | 0.000E+00 | 0.000E+00 | 0.000E+00 | 1.425E-07 |
| Unigene82878 | vgaE | 9.150E-07 | 6.829E-07 | 1.508E-06 | 0.000E+00 | 4.473E-07 | 2.374E-07 | 0.000E+00 | 1.623E-06 | 0.000E+00 | 0.000E+00 | 1.172E-06 | 6.489E-07 |
| Unigene82891 | AcrS | 0.000E+00 | 4.677E-07 | 3.136E-07 | 2.152E-07 | 0.000E+00 | 0.000E+00 | 0.000E+00 | 0.000E+00 | 0.000E+00 | 0.000E+00 | 0.000E+00 | 0.000E+00 |
| Unigene82892 | AcrE | 0.000E+00 | 7.459E-07 | 2.778E-07 | 1.716E-07 | 0.000E+00 | 0.000E+00 | 0.000E+00 | 0.000E+00 | 0.000E+00 | 0.000E+00 | 0.000E+00 | 0.000E+00 |
| Unigene83015 | Streptomy | 8.967E-08 | 4.921E-07 | 2.772E-06 | 4.935E-06 | 4.981E-08 | 0.000E+00 | 0.000E+00 | 0.000E+00 | 0.000E+00 | 0.000E+00 | 0.000E+00 | 0.000E+00 |
| Unigene83260 | rosB | 3.159E-07 | 9.586E-07 | 5.653E-07 | 3.003E-07 | 5.574E-07 | 0.000E+00 | 1.111E-06 | 0.000E+00 | 0.000E+00 | 0.000E+00 | 2.976E-07 | 0.000E+00 |
| Unigene83295 | MexK | 0.000E+00 | 6.005E-07 | 0.000E+00 | 9.208E-08 | 0.000E+00 | 0.000E+00 | 0.000E+00 | 0.000E+00 | 0.000E+00 | 0.000E+00 | 0.000E+00 | 0.000E+00 |
| Unigene83306 | Chlamydia | 6.728E-05 | 5.582E-05 | 1.175E-04 | 1.451E-04 | 1.269E-04 | 8.887E-05 | 1.190E-04 | 1.112E-04 | 1.014E-04 | 1.362E-04 | 1.412E-04 | 1.474E-04 |
| Unigene83326 | pmrA | 8.639E-07 | 6.925E-06 | 3.828E-06 | 2.247E-06 | 0.000E+00 | 0.000E+00 | 0.000E+00 | 0.000E+00 | 0.000E+00 | 0.000E+00 | 0.000E+00 | 0.000E+00 |
| Unigene83397 | macB | 0.000E+00 | 1.401E-06 | 9.842E-07 | 0.000E+00 | 8.611E-07 | 0.000E+00 | 1.564E-06 | 0.000E+00 | 0.000E+00 | 0.000E+00 | 0.000E+00 | 0.000E+00 |
| Unigene83497 | vanTmL | 7.775E-07 | 4.813E-06 | 4.394E-06 | 1.046E-05 | 0.000E+00 | 0.000E+00 | 1.033E-07 | 0.000E+00 | 0.000E+00 | 0.000E+00 | 0.000E+00 | 0.000E+00 |

| | | | | | | | | | | | | | |
|---|---|---|---|---|---|---|---|---|---|---|---|---|---|
| Unigene83498 | oleC | 5.036E-07 | 4.887E-06 | 9.752E-06 | 1.440E-05 | 0.000E+00 | 0.000E+00 | 0.000E+00 | 0.000E+00 | 0.000E+00 | 0.000E+00 | 0.000E+00 | 0.000E+00 |
| Unigene83616 | AAC(6')-Ie | 0.000E+00 | 2.898E-07 | 9.068E-07 | 0.000E+00 | 0.000E+00 | 0.000E+00 | 0.000E+00 | 3.853E-07 | 0.000E+00 | 0.000E+00 | 7.551E-08 | 0.000E+00 |
| Unigene83659 | cdeA | 3.699E-07 | 5.499E-06 | 2.376E-06 | 2.037E-06 | 0.000E+00 | 0.000E+00 | 0.000E+00 | 0.000E+00 | 0.000E+00 | 0.000E+00 | 0.000E+00 | 0.000E+00 |
| Unigene83711 | bcrA | 0.000E+00 | 8.089E-07 | 0.000E+00 | 0.000E+00 | 3.275E-07 | 0.000E+00 | 0.000E+00 | 0.000E+00 | 0.000E+00 | 0.000E+00 | 0.000E+00 | 0.000E+00 |
| Unigene83734 | Acinetoba | 0.000E+00 | 1.886E-06 | 6.599E-07 | 0.000E+00 | 0.000E+00 | 0.000E+00 | 1.626E-06 | 3.780E-07 | 0.000E+00 | 0.000E+00 | 0.000E+00 | 0.000E+00 |
| Unigene83758 | Corynebac | 0.000E+00 | 6.179E-07 | 0.000E+00 | 0.000E+00 | 0.000E+00 | 0.000E+00 | 0.000E+00 | 0.000E+00 | 0.000E+00 | 0.000E+00 | 0.000E+00 | 0.000E+00 |
| Unigene83759 | optrA | 4.343E-06 | 2.173E-05 | 3.748E-06 | 8.223E-07 | 9.046E-07 | 2.934E-07 | 1.502E-07 | 4.526E-07 | 1.293E-06 | 6.479E-07 | 4.657E-07 | 2.292E-07 |
| Unigene83768 | kdpE | 0.000E+00 | 2.362E-06 | 1.220E-06 | 0.000E+00 | 0.000E+00 | 0.000E+00 | 2.223E-06 | 0.000E+00 | 0.000E+00 | 0.000E+00 | 0.000E+00 | 0.000E+00 |
| Unigene83771 | arlS | 1.080E-04 | 1.920E-04 | 1.835E-04 | 7.613E-06 | 9.936E-06 | 3.149E-07 | 3.831E-06 | 2.120E-06 | 5.993E-05 | 4.199E-07 | 3.783E-06 | 6.278E-07 |
| Unigene83785 | efrB | 3.116E-07 | 1.388E-06 | 1.799E-06 | 1.462E-06 | 0.000E+00 | 0.000E+00 | 0.000E+00 | 0.000E+00 | 0.000E+00 | 0.000E+00 | 0.000E+00 | 0.000E+00 |
| Unigene83786 | efrA | 2.611E-07 | 1.371E-06 | 1.738E-06 | 1.638E-06 | 0.000E+00 | 0.000E+00 | 0.000E+00 | 0.000E+00 | 0.000E+00 | 0.000E+00 | 0.000E+00 | 2.204E-08 |
| Unigene83861 | vanHD | 4.797E-06 | 1.075E-05 | 9.414E-06 | 1.208E-05 | 0.000E+00 | 0.000E+00 | 0.000E+00 | 0.000E+00 | 0.000E+00 | 0.000E+00 | 0.000E+00 | 0.000E+00 |
| Unigene83908 | mefE | 2.880E-07 | 3.850E-06 | 2.184E-06 | 2.353E-06 | 0.000E+00 | 0.000E+00 | 0.000E+00 | 0.000E+00 | 0.000E+00 | 0.000E+00 | 0.000E+00 | 0.000E+00 |
| Unigene83924 | rphB | 0.000E+00 | 1.870E-06 | 1.869E-06 | 1.328E-06 | 0.000E+00 | 0.000E+00 | 0.000E+00 | 0.000E+00 | 0.000E+00 | 0.000E+00 | 0.000E+00 | 0.000E+00 |
| Unigene83931 | efrA | 6.800E-05 | 5.438E-05 | 1.097E-04 | 1.581E-04 | 1.138E-04 | 8.345E-05 | 1.088E-04 | 1.033E-04 | 8.850E-05 | 1.322E-04 | 1.333E-04 | 1.335E-04 |
| Unigene83932 | patB | 6.776E-05 | 5.889E-05 | 1.140E-04 | 1.630E-04 | 1.199E-04 | 8.044E-05 | 1.153E-04 | 1.095E-04 | 9.220E-05 | 1.340E-04 | 1.408E-04 | 1.374E-04 |
| Unigene83975 | mdtH | 9.236E-08 | 6.082E-07 | 0.000E+00 | 4.663E-08 | 0.000E+00 | 0.000E+00 | 0.000E+00 | 0.000E+00 | 0.000E+00 | 0.000E+00 | 0.000E+00 | 0.000E+00 |
| Unigene83988 | YojI | 3.398E-06 | 3.285E-06 | 3.930E-06 | 5.841E-06 | 3.595E-07 | 8.746E-08 | 4.477E-07 | 0.000E+00 | 1.769E-07 | 0.000E+00 | 0.000E+00 | 4.781E-08 |
| Unigene84086 | vanSA | 3.509E-06 | 3.533E-06 | 2.391E-06 | 1.874E-06 | 2.094E-06 | 2.038E-06 | 2.022E-06 | 1.016E-06 | 1.363E-06 | 5.654E-06 | 6.303E-06 | 2.023E-06 |
| Unigene84096 | lmrD | 1.450E-07 | 8.913E-07 | 4.269E-07 | 1.757E-07 | 0.000E+00 | 0.000E+00 | 9.630E-08 | 0.000E+00 | 0.000E+00 | 0.000E+00 | 0.000E+00 | 0.000E+00 |
| Unigene84180 | vanTE | 0.000E+00 | 1.542E-06 | 1.085E-06 | 3.622E-07 | 8.368E-06 | 0.000E+00 | 0.000E+00 | 0.000E+00 | 0.000E+00 | 0.000E+00 | 0.000E+00 | 0.000E+00 |
| Unigene84226 | vanRA | 5.214E-06 | 1.145E-05 | 1.153E-05 | 1.240E-05 | 0.000E+00 | 0.000E+00 | 0.000E+00 | 0.000E+00 | 0.000E+00 | 0.000E+00 | 0.000E+00 | 0.000E+00 |
| Unigene84227 | AAC(6')-Ib | 3.605E-06 | 1.108E-05 | 1.176E-05 | 9.522E-06 | 0.000E+00 | 0.000E+00 | 0.000E+00 | 0.000E+00 | 0.000E+00 | 0.000E+00 | 0.000E+00 | 0.000E+00 |
| Unigene84273 | ACT-29 | 0.000E+00 | 8.347E-07 | 5.251E-07 | 0.000E+00 | 0.000E+00 | 0.000E+00 | 0.000E+00 | 0.000E+00 | 0.000E+00 | 0.000E+00 | 0.000E+00 | 0.000E+00 |
| Unigene84335 | msbA | 0.000E+00 | 5.571E-07 | 9.961E-08 | 2.306E-07 | 0.000E+00 | 0.000E+00 | 0.000E+00 | 0.000E+00 | 0.000E+00 | 0.000E+00 | 0.000E+00 | 0.000E+00 |
| Unigene84449 | tetA(58) | 0.000E+00 | 6.179E-07 | 0.000E+00 | 0.000E+00 | 0.000E+00 | 0.000E+00 | 0.000E+00 | 0.000E+00 | 0.000E+00 | 0.000E+00 | 0.000E+00 | 0.000E+00 |
| Unigene84482 | carA | 0.000E+00 | 5.530E-07 | 0.000E+00 | 0.000E+00 | 0.000E+00 | 0.000E+00 | 0.000E+00 | 0.000E+00 | 0.000E+00 | 0.000E+00 | 0.000E+00 | 0.000E+00 |
| Unigene84511 | cmlv | 7.521E-05 | 6.105E-05 | 1.206E-04 | 1.699E-04 | 1.299E-04 | 9.393E-05 | 1.181E-04 | 1.197E-04 | 1.010E-04 | 1.474E-04 | 1.529E-04 | 1.556E-04 |
| Unigene84512 | macB | 7.213E-05 | 6.082E-05 | 1.166E-04 | 1.604E-04 | 1.254E-04 | 8.726E-05 | 1.244E-04 | 1.130E-04 | 9.845E-05 | 1.395E-04 | 1.425E-04 | 1.496E-04 |
| Unigene84542 | poxtA | 0.000E+00 | 1.142E-06 | 0.000E+00 | 8.943E-08 | 0.000E+00 | 0.000E+00 | 0.000E+00 | 0.000E+00 | 0.000E+00 | 0.000E+00 | 0.000E+00 | 0.000E+00 |
| Unigene84561 | Klebsiella p | 0.000E+00 | 5.288E-07 | 3.940E-07 | 0.000E+00 | 0.000E+00 | 0.000E+00 | 0.000E+00 | 0.000E+00 | 0.000E+00 | 0.000E+00 | 0.000E+00 | 0.000E+00 |
| Unigene84562 | Klebsiella p | 0.000E+00 | 7.242E-07 | 3.486E-07 | 0.000E+00 | 0.000E+00 | 0.000E+00 | 0.000E+00 | 0.000E+00 | 0.000E+00 | 0.000E+00 | 0.000E+00 | 0.000E+00 |
| Unigene84607 | lin | 1.299E-05 | 4.775E-06 | 3.020E-06 | 1.256E-05 | 9.023E-05 | 1.189E-04 | 1.282E-04 | 1.028E-04 | 1.516E-04 | 1.630E-04 | 1.303E-04 | 8.113E-05 |
| Unigene84669 | patB | 7.126E-05 | 5.812E-05 | 1.170E-04 | 1.643E-04 | 1.313E-04 | 9.112E-05 | 1.195E-04 | 1.113E-04 | 1.028E-04 | 1.434E-04 | 1.542E-04 | 1.686E-04 |
| Unigene84752 | tetA(58) | 0.000E+00 | 7.842E-07 | 0.000E+00 | 0.000E+00 | 4.203E-07 | 0.000E+00 | 0.000E+00 | 0.000E+00 | 0.000E+00 | 0.000E+00 | 0.000E+00 | 0.000E+00 |
| Unigene84756 | arnA | 0.000E+00 | 1.196E-06 | 5.680E-07 | 0.000E+00 | 5.296E-07 | 0.000E+00 | 1.997E-06 | 0.000E+00 | 0.000E+00 | 0.000E+00 | 0.000E+00 | 0.000E+00 |
| Unigene84762 | optrA | 6.689E-05 | 5.836E-05 | 1.155E-04 | 1.636E-04 | 1.217E-04 | 8.491E-05 | 1.173E-04 | 1.090E-04 | 9.414E-05 | 1.360E-04 | 1.457E-04 | 1.465E-04 |
| Unigene84830 | oleC | 0.000E+00 | 2.298E-06 | 0.000E+00 | 0.000E+00 | 0.000E+00 | 0.000E+00 | 0.000E+00 | 0.000E+00 | 0.000E+00 | 0.000E+00 | 0.000E+00 | 0.000E+00 |
| Unigene84853 | Klebsiella p | 0.000E+00 | 6.675E-07 | 2.754E-07 | 0.000E+00 | 0.000E+00 | 0.000E+00 | 0.000E+00 | 0.000E+00 | 0.000E+00 | 0.000E+00 | 0.000E+00 | 0.000E+00 |
| Unigene84893 | basS | 0.000E+00 | 4.044E-07 | 3.616E-07 | 0.000E+00 | 0.000E+00 | 0.000E+00 | 0.000E+00 | 0.000E+00 | 0.000E+00 | 0.000E+00 | 0.000E+00 | 0.000E+00 |
| Unigene84902 | macB | 7.518E-07 | 7.065E-06 | 4.150E-06 | 3.369E-06 | 0.000E+00 | 0.000E+00 | 0.000E+00 | 0.000E+00 | 0.000E+00 | 0.000E+00 | 0.000E+00 | 0.000E+00 |
| Unigene84903 | macB | 3.720E-07 | 1.166E-06 | 1.616E-06 | 2.173E-06 | 0.000E+00 | 0.000E+00 | 0.000E+00 | 0.000E+00 | 0.000E+00 | 0.000E+00 | 0.000E+00 | 0.000E+00 |
| Unigene85001 | macB | 4.424E-06 | 2.283E-05 | 3.832E-06 | 9.256E-07 | 1.063E-06 | 7.755E-07 | 4.411E-08 | 4.652E-07 | 1.612E-06 | 5.327E-07 | 4.103E-07 | 3.297E-07 |
| Unigene85002 | macB | 2.483E-06 | 7.054E-06 | 6.115E-06 | 7.621E-06 | 0.000E+00 | 0.000E+00 | 0.000E+00 | 0.000E+00 | 0.000E+00 | 0.000E+00 | 0.000E+00 | 0.000E+00 |
| Unigene85008 | vanHD | 1.999E-07 | 1.060E-06 | 7.191E-07 | 1.312E-06 | 0.000E+00 | 0.000E+00 | 0.000E+00 | 0.000E+00 | 0.000E+00 | 0.000E+00 | 0.000E+00 | 0.000E+00 |
| Unigene85014 | vanHD | 0.000E+00 | 1.018E-06 | 1.123E-06 | 1.093E-06 | 0.000E+00 | 0.000E+00 | 0.000E+00 | 0.000E+00 | 0.000E+00 | 0.000E+00 | 0.000E+00 | 0.000E+00 |
| Unigene85023 | macB | 0.000E+00 | 5.551E-07 | 2.482E-06 | 3.204E-06 | 0.000E+00 | 0.000E+00 | 0.000E+00 | 0.000E+00 | 0.000E+00 | 0.000E+00 | 0.000E+00 | 0.000E+00 |
| Unigene85094 | adeL | 0.000E+00 | 8.689E-07 | 1.766E-07 | 0.000E+00 | 0.000E+00 | 0.000E+00 | 0.000E+00 | 0.000E+00 | 0.000E+00 | 0.000E+00 | 0.000E+00 | 0.000E+00 |
| Unigene85095 | otr(B) | 0.000E+00 | 8.283E-07 | 1.693E-07 | 1.742E-07 | 0.000E+00 | 0.000E+00 | 0.000E+00 | 0.000E+00 | 0.000E+00 | 0.000E+00 | 0.000E+00 | 0.000E+00 |
| Unigene85107 | msbA | 1.989E-07 | 1.945E-06 | 3.744E-06 | 5.223E-06 | 0.000E+00 | 0.000E+00 | 0.000E+00 | 0.000E+00 | 0.000E+00 | 0.000E+00 | 0.000E+00 | 0.000E+00 |
| Unigene85110 | AcrF | 0.000E+00 | 5.733E-07 | 0.000E+00 | 2.110E-07 | 0.000E+00 | 0.000E+00 | 0.000E+00 | 0.000E+00 | 0.000E+00 | 0.000E+00 | 0.000E+00 | 0.000E+00 |
| Unigene85116 | kdpE | 0.000E+00 | 6.695E-06 | 5.819E-06 | 8.941E-06 | 0.000E+00 | 0.000E+00 | 0.000E+00 | 0.000E+00 | 0.000E+00 | 0.000E+00 | 4.846E-08 | 0.000E+00 |
| Unigene85128 | vatB | 0.000E+00 | 1.937E-06 | 9.497E-07 | 0.000E+00 | 6.641E-06 | 0.000E+00 | 0.000E+00 | 0.000E+00 | 0.000E+00 | 0.000E+00 | 0.000E+00 | 0.000E+00 |
| Unigene85130 | Acinetoba | 0.000E+00 | 2.721E-07 | 0.000E+00 | 1.502E-07 | 0.000E+00 | 0.000E+00 | 5.488E-08 | 1.158E-07 | 0.000E+00 | 0.000E+00 | 0.000E+00 | 0.000E+00 |
| Unigene85174 | efrA | 9.148E-07 | 6.741E-06 | 1.116E-05 | 1.544E-05 | 0.000E+00 | 0.000E+00 | 0.000E+00 | 0.000E+00 | 0.000E+00 | 0.000E+00 | 0.000E+00 | 0.000E+00 |
| Unigene85362 | mtrA | 0.000E+00 | 0.000E+00 | 1.104E-06 | 0.000E+00 | 6.652E-06 | 0.000E+00 | 0.000E+00 | 0.000E+00 | 0.000E+00 | 0.000E+00 | 0.000E+00 | 0.000E+00 |
| Unigene85418 | macB | 5.260E-05 | 4.171E-05 | 7.957E-05 | 1.189E-04 | 9.389E-05 | 6.360E-05 | 8.454E-05 | 7.795E-05 | 7.531E-05 | 1.089E-04 | 1.024E-04 | 1.023E-04 |
| Unigene85434 | Staphyloco | 2.242E-07 | 3.075E-07 | 0.000E+00 | 5.659E-08 | 0.000E+00 | 0.000E+00 | 0.000E+00 | 6.542E-08 | 0.000E+00 | 0.000E+00 | 0.000E+00 | 0.000E+00 |

| | | | | | | | | | | | | | |
|---|---|---|---|---|---|---|---|---|---|---|---|---|---|
| Unigene85464 | efrA | 2.868E-06 | 1.763E-05 | 8.093E-06 | 5.206E-06 | 2.083E-08 | 8.106E-08 | 6.224E-08 | 0.000E+00 | 0.000E+00 | 0.000E+00 | 0.000E+00 | 0.000E+00 |
| Unigene85472 | arlR | 0.000E+00 | 6.823E-07 | 0.000E+00 | 0.000E+00 | 0.000E+00 | 0.000E+00 | 0.000E+00 | 0.000E+00 | 0.000E+00 | 0.000E+00 | 0.000E+00 | 1.131E-07 |
| Unigene85526 | vanHO | 1.002E-04 | 1.735E-04 | 1.720E-04 | 7.761E-06 | 8.614E-06 | 5.170E-07 | 2.873E-06 | 1.675E-06 | 6.041E-05 | 3.425E-07 | 2.305E-06 | 4.038E-08 |
| Unigene85600 | tetB(60) | 6.045E-08 | 5.308E-07 | 0.000E+00 | 6.104E-08 | 0.000E+00 | 0.000E+00 | 1.338E-07 | 0.000E+00 | 0.000E+00 | 0.000E+00 | 0.000E+00 | 0.000E+00 |
| Unigene85626 | adeR | 0.000E+00 | 7.316E-07 | 1.635E-06 | 2.308E-06 | 0.000E+00 | 2.059E-07 | 0.000E+00 | 0.000E+00 | 0.000E+00 | 0.000E+00 | 1.198E-06 | 9.004E-07 |
| Unigene85642 | evgS | 0.000E+00 | 0.000E+00 | 0.000E+00 | 0.000E+00 | 0.000E+00 | 0.000E+00 | 0.000E+00 | 0.000E+00 | 0.000E+00 | 0.000E+00 | 0.000E+00 | 3.617E-07 |
| Unigene85768 | emeA | 3.906E-05 | 2.465E-05 | 1.870E-05 | 2.117E-05 | 1.629E-06 | 1.555E-06 | 3.551E-06 | 7.749E-07 | 0.000E+00 | 0.000E+00 | 1.582E-06 | 1.439E-06 |
| Unigene85771 | Staphylocc | 1.063E-04 | 2.042E-04 | 1.812E-04 | 0.000E+00 | 7.633E-06 | 0.000E+00 | 3.995E-06 | 0.000E+00 | 5.374E-05 | 0.000E+00 | 2.824E-06 | 0.000E+00 |
| Unigene85815 | novA | 0.000E+00 | 7.794E-07 | 3.787E-07 | 2.962E-07 | 3.773E-07 | 1.335E-07 | 2.905E-07 | 0.000E+00 | 0.000E+00 | 1.032E-07 | 0.000E+00 | 0.000E+00 |
| Unigene85891 | arlR | 1.770E-06 | 3.937E-06 | 4.845E-06 | 7.526E-06 | 0.000E+00 | 0.000E+00 | 0.000E+00 | 0.000E+00 | 0.000E+00 | 0.000E+00 | 0.000E+00 | 0.000E+00 |
| Unigene85985 | APH(3')-IIa | 4.091E-07 | 7.364E-06 | 6.423E-07 | 1.239E-07 | 2.727E-07 | 0.000E+00 | 0.000E+00 | 2.865E-07 | 6.708E-07 | 4.102E-07 | 0.000E+00 | 9.672E-08 |
| Unigene85990 | macB | 0.000E+00 | 5.671E-06 | 1.158E-05 | 8.233E-06 | 2.317E-06 | 8.199E-07 | 1.731E-05 | 1.439E-06 | 2.694E-06 | 2.957E-06 | 0.000E+00 | 0.000E+00 |
| Unigene85993 | macB | 0.000E+00 | 1.377E-06 | 5.208E-07 | 4.385E-07 | 0.000E+00 | 0.000E+00 | 1.228E-06 | 0.000E+00 | 0.000E+00 | 0.000E+00 | 0.000E+00 | 3.992E-07 |
| Unigene85996 | farB | 6.522E-05 | 5.578E-05 | 1.147E-04 | 1.654E-04 | 1.204E-04 | 9.015E-05 | 1.080E-04 | 1.086E-04 | 9.590E-05 | 1.359E-04 | 1.289E-04 | 1.379E-04 |
| Unigene86045 | clbB | 0.000E+00 | 2.763E-06 | 1.710E-06 | 5.866E-07 | 0.000E+00 | 5.382E-07 | 2.143E-06 | 0.000E+00 | 0.000E+00 | 0.000E+00 | 0.000E+00 | 0.000E+00 |
| Unigene86069 | vmlR | 8.024E-07 | 8.407E-07 | 4.653E-06 | 6.998E-06 | 0.000E+00 | 0.000E+00 | 3.633E-07 | 0.000E+00 | 0.000E+00 | 0.000E+00 | 0.000E+00 | 0.000E+00 |
| Unigene86080 | PmrF | 0.000E+00 | 6.433E-07 | 0.000E+00 | 0.000E+00 | 0.000E+00 | 0.000E+00 | 0.000E+00 | 0.000E+00 | 0.000E+00 | 0.000E+00 | 0.000E+00 | 0.000E+00 |
| Unigene86086 | tetB(46) | 4.579E-05 | 2.688E-05 | 2.159E-05 | 2.559E-05 | 2.885E-06 | 1.604E-06 | 3.244E-06 | 9.961E-07 | 7.543E-06 | 1.033E-06 | 1.995E-06 | 1.053E-06 |
| Unigene86173 | vanHD | 0.000E+00 | 3.473E-07 | 0.000E+00 | 0.000E+00 | 0.000E+00 | 0.000E+00 | 0.000E+00 | 0.000E+00 | 0.000E+00 | 0.000E+00 | 0.000E+00 | 0.000E+00 |
| Unigene86174 | tetA(58) | 2.869E-07 | 4.724E-07 | 1.056E-06 | 2.354E-07 | 0.000E+00 | 0.000E+00 | 0.000E+00 | 0.000E+00 | 0.000E+00 | 0.000E+00 | 0.000E+00 | 0.000E+00 |
| Unigene86194 | vanYM | 0.000E+00 | 4.898E-07 | 0.000E+00 | 2.904E-06 | 2.754E-07 | 5.360E-08 | 0.000E+00 | 8.682E-07 | 0.000E+00 | 0.000E+00 | 6.239E-07 | 5.275E-07 |
| Unigene86220 | MexB | 0.000E+00 | 3.881E-07 | 1.487E-07 | 0.000E+00 | 0.000E+00 | 0.000E+00 | 0.000E+00 | 0.000E+00 | 0.000E+00 | 0.000E+00 | 0.000E+00 | 0.000E+00 |
| Unigene86249 | APH(2'')-II | 0.000E+00 | 3.595E-04 | 0.000E+00 | 0.000E+00 | 0.000E+00 | 0.000E+00 | 0.000E+00 | 1.703E-07 | 0.000E+00 | 0.000E+00 | 0.000E+00 | 0.000E+00 |
| Unigene86258 | Escherichia | 0.000E+00 | 3.682E-04 | 0.000E+00 | 0.000E+00 | 0.000E+00 | 0.000E+00 | 0.000E+00 | 0.000E+00 | 0.000E+00 | 0.000E+00 | 0.000E+00 | 0.000E+00 |
| Unigene86299 | TaeA | 0.000E+00 | 2.007E-06 | 0.000E+00 | 0.000E+00 | 7.687E-06 | 0.000E+00 | 0.000E+00 | 0.000E+00 | 0.000E+00 | 0.000E+00 | 0.000E+00 | 0.000E+00 |
| Unigene86330 | Acinetobac | 0.000E+00 | 4.649E-07 | 0.000E+00 | 0.000E+00 | 0.000E+00 | 0.000E+00 | 0.000E+00 | 0.000E+00 | 0.000E+00 | 0.000E+00 | 0.000E+00 | 0.000E+00 |
| Unigene86349 | Corynebac | 0.000E+00 | 2.715E-07 | 6.068E-08 | 0.000E+00 | 0.000E+00 | 0.000E+00 | 0.000E+00 | 0.000E+00 | 0.000E+00 | 0.000E+00 | 0.000E+00 | 0.000E+00 |
| Unigene86358 | lmrB | 0.000E+00 | 4.418E-07 | 0.000E+00 | 1.355E-07 | 4.472E-07 | 0.000E+00 | 0.000E+00 | 0.000E+00 | 0.000E+00 | 0.000E+00 | 0.000E+00 | 0.000E+00 |
| Unigene86365 | Staphylocc | 5.562E-05 | 6.090E-05 | 1.046E-04 | 6.947E-05 | 1.264E-04 | 8.933E-05 | 1.052E-04 | 1.189E-04 | 9.391E-05 | 1.297E-04 | 1.355E-04 | 1.643E-04 |
| Unigene86415 | arlR | 6.744E-05 | 6.089E-05 | 1.108E-04 | 1.343E-04 | 1.391E-04 | 9.129E-05 | 1.120E-04 | 1.081E-04 | 9.113E-05 | 1.438E-04 | 1.475E-04 | 1.497E-04 |
| Unigene86479 | macB | 2.522E-07 | 1.292E-06 | 1.279E-06 | 1.401E-06 | 0.000E+00 | 0.000E+00 | 0.000E+00 | 0.000E+00 | 0.000E+00 | 0.000E+00 | 0.000E+00 | 0.000E+00 |
| Unigene86497 | kdpE | 0.000E+00 | 7.126E-07 | 3.413E-06 | 3.185E-06 | 0.000E+00 | 0.000E+00 | 0.000E+00 | 0.000E+00 | 0.000E+00 | 0.000E+00 | 0.000E+00 | 0.000E+00 |
| Unigene86579 | cmx | 0.000E+00 | 1.318E-06 | 0.000E+00 | 3.964E-07 | 4.079E-06 | 2.746E-07 | 0.000E+00 | 0.000E+00 | 0.000E+00 | 0.000E+00 | 0.000E+00 | 0.000E+00 |
| Unigene86602 | efrA | 1.016E-06 | 5.863E-06 | 1.030E-05 | 1.497E-05 | 0.000E+00 | 0.000E+00 | 1.041E-07 | 0.000E+00 | 0.000E+00 | 0.000E+00 | 0.000E+00 | 0.000E+00 |
| Unigene86625 | Klebsiella p | 0.000E+00 | 7.914E-07 | 2.721E-07 | 0.000E+00 | 4.775E-06 | 0.000E+00 | 0.000E+00 | 0.000E+00 | 0.000E+00 | 0.000E+00 | 0.000E+00 | 0.000E+00 |
| Unigene86681 | FOX-3 | 7.099E-05 | 5.866E-05 | 1.119E-04 | 1.702E-04 | 1.250E-04 | 8.746E-05 | 1.152E-04 | 1.096E-04 | 9.609E-05 | 1.407E-04 | 1.445E-04 | 1.496E-04 |
| Unigene86717 | bcrA | 8.337E-05 | 6.511E-05 | 1.231E-04 | 1.848E-04 | 1.444E-04 | 1.025E-04 | 1.361E-04 | 1.163E-04 | 1.111E-04 | 1.496E-04 | 1.640E-04 | 1.678E-04 |
| Unigene86829 | vanRG | 1.191E-06 | 5.905E-06 | 3.828E-06 | 4.438E-06 | 0.000E+00 | 0.000E+00 | 0.000E+00 | 0.000E+00 | 0.000E+00 | 0.000E+00 | 0.000E+00 | 0.000E+00 |
| Unigene86837 | vanZF | 3.303E-06 | 5.729E-06 | 6.220E-06 | 8.284E-06 | 0.000E+00 | 0.000E+00 | 0.000E+00 | 0.000E+00 | 0.000E+00 | 0.000E+00 | 0.000E+00 | 0.000E+00 |
| Unigene86929 | tetA(58) | 9.320E-07 | 1.023E-06 | 8.729E-07 | 1.412E-06 | 1.530E-05 | 4.763E-06 | 1.191E-05 | 1.459E-05 | 8.381E-06 | 1.199E-05 | 1.425E-05 | 6.409E-06 |
| Unigene87018 | farB | 1.109E-05 | 2.965E-06 | 2.390E-06 | 8.965E-06 | 9.214E-05 | 1.277E-04 | 1.023E-04 | 1.024E-04 | 1.648E-04 | 1.537E-04 | 1.264E-04 | 7.945E-05 |
| Unigene87025 | adeL | 0.000E+00 | 3.794E-07 | 0.000E+00 | 2.182E-07 | 0.000E+00 | 0.000E+00 | 0.000E+00 | 0.000E+00 | 0.000E+00 | 0.000E+00 | 0.000E+00 | 0.000E+00 |
| Unigene87082 | vanSA | 1.699E-07 | 4.351E-07 | 2.223E-07 | 1.144E-07 | 0.000E+00 | 0.000E+00 | 0.000E+00 | 0.000E+00 | 0.000E+00 | 0.000E+00 | 0.000E+00 | 0.000E+00 |
| Unigene87185 | vanTG | 1.029E-04 | 1.811E-04 | 1.664E-04 | 7.334E-06 | 8.228E-06 | 3.781E-07 | 3.324E-06 | 1.321E-06 | 5.820E-05 | 3.896E-07 | 2.848E-06 | 4.621E-07 |
| Unigene87257 | vanHO | 0.000E+00 | 1.938E-06 | 1.569E-06 | 1.480E-06 | 0.000E+00 | 0.000E+00 | 0.000E+00 | 0.000E+00 | 0.000E+00 | 0.000E+00 | 0.000E+00 | 3.938E-08 |
| Unigene87265 | macB | 0.000E+00 | 1.588E-06 | 1.908E-06 | 1.826E-06 | 0.000E+00 | 0.000E+00 | 0.000E+00 | 0.000E+00 | 0.000E+00 | 0.000E+00 | 0.000E+00 | 0.000E+00 |
| Unigene87300 | arnA | 0.000E+00 | 7.474E-07 | 0.000E+00 | 0.000E+00 | 0.000E+00 | 0.000E+00 | 0.000E+00 | 0.000E+00 | 0.000E+00 | 0.000E+00 | 0.000E+00 | 0.000E+00 |
| Unigene87332 | vanRM | 0.000E+00 | 7.141E-07 | 0.000E+00 | 2.022E-06 | 0.000E+00 | 0.000E+00 | 0.000E+00 | 0.000E+00 | 0.000E+00 | 0.000E+00 | 0.000E+00 | 0.000E+00 |
| Unigene87333 | baeS | 3.681E-07 | 1.521E-06 | 1.381E-06 | 1.181E-06 | 0.000E+00 | 2.340E-08 | 0.000E+00 | 0.000E+00 | 0.000E+00 | 0.000E+00 | 0.000E+00 | 0.000E+00 |
| Unigene87353 | TolC | 0.000E+00 | 4.724E-07 | 1.689E-07 | 0.000E+00 | 0.000E+00 | 0.000E+00 | 0.000E+00 | 0.000E+00 | 0.000E+00 | 0.000E+00 | 0.000E+00 | 0.000E+00 |
| Unigene87380 | rosB | 1.681E-07 | 6.766E-07 | 3.299E-07 | 0.000E+00 | 0.000E+00 | 0.000E+00 | 0.000E+00 | 0.000E+00 | 0.000E+00 | 0.000E+00 | 0.000E+00 | 0.000E+00 |
| Unigene87563 | novA | 0.000E+00 | 6.241E-07 | 0.000E+00 | 0.000E+00 | 0.000E+00 | 0.000E+00 | 0.000E+00 | 0.000E+00 | 0.000E+00 | 0.000E+00 | 0.000E+00 | 0.000E+00 |
| Unigene87568 | MexK | 2.050E-07 | 4.499E-07 | 2.514E-07 | 0.000E+00 | 0.000E+00 | 0.000E+00 | 1.701E-07 | 0.000E+00 | 5.600E-08 | 0.000E+00 | 4.689E-07 | 0.000E+00 |
| Unigene87590 | Pseudomo | 0.000E+00 | 3.192E-07 | 3.567E-07 | 0.000E+00 | 0.000E+00 | 7.860E-08 | 4.023E-07 | 0.000E+00 | 0.000E+00 | 0.000E+00 | 1.663E-07 | 0.000E+00 |
| Unigene87674 | bcrA | 2.733E-07 | 2.999E-06 | 2.748E-06 | 4.036E-06 | 0.000E+00 | 0.000E+00 | 0.000E+00 | 0.000E+00 | 0.000E+00 | 0.000E+00 | 0.000E+00 | 0.000E+00 |
| Unigene87675 | tetA(58) | 2.261E-07 | 2.928E-06 | 1.908E-06 | 3.561E-06 | 0.000E+00 | 2.444E-07 | 0.000E+00 | 0.000E+00 | 0.000E+00 | 0.000E+00 | 0.000E+00 | 0.000E+00 |
| Unigene87687 | vatE | 6.761E-05 | 6.300E-05 | 1.152E-04 | 1.688E-04 | 1.403E-04 | 9.130E-05 | 1.244E-04 | 1.181E-04 | 1.006E-04 | 1.467E-04 | 1.466E-04 | 1.642E-04 |
| Unigene87779 | efrA | 0.000E+00 | 0.000E+00 | 0.000E+00 | 0.000E+00 | 0.000E+00 | 0.000E+00 | 9.132E-07 | 0.000E+00 | 0.000E+00 | 0.000E+00 | 0.000E+00 | 0.000E+00 |

| | | | | | | | | | | | | | |
|---|---|---|---|---|---|---|---|---|---|---|---|---|---|
| Unigene87811 | macB | 4.579E-08 | 9.548E-07 | 3.549E-06 | 2.913E-06 | 0.000E+00 | 0.000E+00 | 0.000E+00 | 0.000E+00 | 0.000E+00 | 0.000E+00 | 0.000E+00 | 0.000E+00 |
| Unigene87828 | mdtC | 0.000E+00 | 5.265E-07 | 0.000E+00 | 0.000E+00 | 0.000E+00 | 0.000E+00 | 0.000E+00 | 0.000E+00 | 0.000E+00 | 0.000E+00 | 0.000E+00 | 0.000E+00 |
| Unigene87852 | macB | 0.000E+00 | 0.000E+00 | 5.801E-08 | 1.194E-07 | 0.000E+00 | 0.000E+00 | 0.000E+00 | 0.000E+00 | 0.000E+00 | 0.000E+00 | 0.000E+00 | 0.000E+00 |
| Unigene87858 | tlrC | 1.025E-07 | 5.998E-07 | 2.011E-07 | 0.000E+00 | 0.000E+00 | 0.000E+00 | 0.000E+00 | 0.000E+00 | 0.000E+00 | 0.000E+00 | 0.000E+00 | 0.000E+00 |
| Unigene87872 | tetB(46) | 0.000E+00 | 5.025E-07 | 0.000E+00 | 0.000E+00 | 0.000E+00 | 4.950E-08 | 0.000E+00 | 0.000E+00 | 0.000E+00 | 0.000E+00 | 0.000E+00 | 0.000E+00 |
| Unigene87904 | kdpE | 0.000E+00 | 1.202E-06 | 8.409E-07 | 0.000E+00 | 0.000E+00 | 0.000E+00 | 0.000E+00 | 0.000E+00 | 0.000E+00 | 0.000E+00 | 0.000E+00 | 0.000E+00 |
| Unigene87915 | mdtP | 0.000E+00 | 7.310E-07 | 2.614E-07 | 1.644E-07 | 0.000E+00 | 0.000E+00 | 0.000E+00 | 0.000E+00 | 0.000E+00 | 0.000E+00 | 0.000E+00 | 0.000E+00 |
| Unigene87961 | Klebsiella p | 4.165E-07 | 6.095E-07 | 0.000E+00 | 0.000E+00 | 0.000E+00 | 0.000E+00 | 0.000E+00 | 0.000E+00 | 0.000E+00 | 0.000E+00 | 0.000E+00 | 0.000E+00 |
| Unigene87979 | optrA | 3.531E-07 | 1.439E-06 | 1.320E-06 | 1.647E-06 | 0.000E+00 | 0.000E+00 | 0.000E+00 | 0.000E+00 | 0.000E+00 | 0.000E+00 | 0.000E+00 | 0.000E+00 |
| Unigene88072 | Klebsiella p | 0.000E+00 | 9.049E-07 | 3.236E-07 | 0.000E+00 | 0.000E+00 | 0.000E+00 | 0.000E+00 | 0.000E+00 | 0.000E+00 | 0.000E+00 | 0.000E+00 | 1.949E-07 |
| Unigene88154 | msbA | 4.133E-06 | 2.196E-05 | 3.037E-06 | 1.030E-06 | 9.911E-07 | 5.905E-07 | 1.411E-07 | 3.188E-07 | 1.015E-06 | 9.330E-07 | 3.540E-07 | 2.367E-07 |
| Unigene88173 | patB | 5.736E-06 | 1.318E-05 | 1.086E-05 | 1.428E-05 | 0.000E+00 | 0.000E+00 | 0.000E+00 | 0.000E+00 | 0.000E+00 | 0.000E+00 | 0.000E+00 | 0.000E+00 |
| Unigene88198 | Staphylocc | 9.968E-05 | 1.565E-04 | 1.499E-04 | 6.641E-06 | 8.693E-06 | 4.748E-07 | 3.466E-06 | 2.010E-06 | 5.605E-05 | 3.347E-07 | 2.988E-06 | 8.197E-07 |
| Unigene88226 | YojI | 0.000E+00 | 1.167E-06 | 3.051E-06 | 0.000E+00 | 0.000E+00 | 0.000E+00 | 0.000E+00 | 0.000E+00 | 0.000E+00 | 0.000E+00 | 0.000E+00 | 0.000E+00 |
| Unigene88244 | dfrC | 0.000E+00 | 1.957E-06 | 3.061E-06 | 0.000E+00 | 0.000E+00 | 0.000E+00 | 0.000E+00 | 0.000E+00 | 0.000E+00 | 0.000E+00 | 0.000E+00 | 0.000E+00 |
| Unigene88246 | oleC | 0.000E+00 | 1.977E-06 | 0.000E+00 | 0.000E+00 | 0.000E+00 | 0.000E+00 | 0.000E+00 | 0.000E+00 | 0.000E+00 | 0.000E+00 | 0.000E+00 | 0.000E+00 |
| Unigene88385 | YojI | 2.964E-07 | 8.459E-07 | 1.105E-06 | 2.963E-06 | 3.293E-07 | 1.602E-07 | 0.000E+00 | 1.349E-06 | 2.916E-07 | 0.000E+00 | 1.628E-06 | 1.366E-06 |
| Unigene88472 | Listeria mc | 1.877E-07 | 1.305E-06 | 1.093E-06 | 1.377E-06 | 0.000E+00 | 2.705E-08 | 0.000E+00 | 0.000E+00 | 0.000E+00 | 0.000E+00 | 4.294E-08 | 0.000E+00 |
| Unigene88689 | vanHO | 7.480E-07 | 8.841E-07 | 4.630E-06 | 6.421E-06 | 0.000E+00 | 3.110E-08 | 3.184E-07 | 0.000E+00 | 0.000E+00 | 0.000E+00 | 0.000E+00 | 0.000E+00 |
| Unigene88735 | tetA(58) | 6.725E-08 | 5.905E-07 | 3.299E-07 | 0.000E+00 | 7.471E-08 | 0.000E+00 | 0.000E+00 | 0.000E+00 | 0.000E+00 | 0.000E+00 | 0.000E+00 | 0.000E+00 |
| Unigene88737 | macB | 6.622E-07 | 6.904E-06 | 1.364E-05 | 1.768E-05 | 0.000E+00 | 4.474E-08 | 0.000E+00 | 0.000E+00 | 0.000E+00 | 0.000E+00 | 0.000E+00 | 0.000E+00 |
| Unigene88859 | tetB(60) | 7.053E-07 | 5.974E-06 | 1.010E-05 | 1.505E-05 | 0.000E+00 | 0.000E+00 | 6.004E-08 | 0.000E+00 | 0.000E+00 | 8.058E-08 | 0.000E+00 | 0.000E+00 |
| Unigene88860 | tetA(60) | 3.869E-07 | 5.763E-06 | 8.678E-06 | 1.302E-05 | 0.000E+00 | 0.000E+00 | 0.000E+00 | 0.000E+00 | 0.000E+00 | 0.000E+00 | 0.000E+00 | 0.000E+00 |
| Unigene88872 | macB | 5.380E-07 | 1.299E-06 | 1.478E-06 | 1.902E-06 | 0.000E+00 | 0.000E+00 | 0.000E+00 | 0.000E+00 | 0.000E+00 | 0.000E+00 | 0.000E+00 | 0.000E+00 |
| Unigene88937 | mepA | 0.000E+00 | 1.027E-06 | 0.000E+00 | 1.606E-06 | 0.000E+00 | 0.000E+00 | 0.000E+00 | 0.000E+00 | 0.000E+00 | 0.000E+00 | 0.000E+00 | 0.000E+00 |
| Unigene88962 | tet32 | 9.981E-05 | 1.647E-04 | 1.563E-04 | 6.934E-06 | 8.730E-06 | 4.018E-07 | 3.800E-06 | 1.508E-06 | 5.915E-05 | 2.169E-07 | 2.976E-06 | 6.485E-07 |
| Unigene88963 | efrA | 7.686E-05 | 6.325E-05 | 1.244E-04 | 1.829E-04 | 1.361E-04 | 9.497E-05 | 1.228E-04 | 1.064E-04 | 1.078E-04 | 1.520E-04 | 1.451E-04 | 1.488E-04 |
| Unigene88973 | vmlR | 0.000E+00 | 7.751E-07 | 0.000E+00 | 0.000E+00 | 0.000E+00 | 0.000E+00 | 0.000E+00 | 0.000E+00 | 0.000E+00 | 0.000E+00 | 0.000E+00 | 0.000E+00 |
| Unigene89003 | tet(43) | 0.000E+00 | 1.795E-06 | 2.105E-06 | 4.971E-07 | 0.000E+00 | 0.000E+00 | 0.000E+00 | 0.000E+00 | 0.000E+00 | 0.000E+00 | 0.000E+00 | 0.000E+00 |
| Unigene89065 | Staphylocc | 9.046E-07 | 6.723E-06 | 1.018E-05 | 1.540E-05 | 0.000E+00 | 8.578E-08 | 8.782E-08 | 0.000E+00 | 0.000E+00 | 0.000E+00 | 0.000E+00 | 5.628E-08 |
| Unigene89076 | poxtA | 6.251E-07 | 7.595E-06 | 9.638E-06 | 1.294E-05 | 0.000E+00 | 4.827E-08 | 9.883E-08 | 0.000E+00 | 0.000E+00 | 0.000E+00 | 0.000E+00 | 0.000E+00 |
| Unigene89118 | vanHB | 3.830E-06 | 1.301E-06 | 0.000E+00 | 0.000E+00 | 0.000E+00 | 0.000E+00 | 0.000E+00 | 0.000E+00 | 0.000E+00 | 0.000E+00 | 0.000E+00 | 0.000E+00 |
| Unigene89223 | Listeria mc | 1.009E-06 | 6.684E-06 | 1.188E-05 | 1.695E-05 | 0.000E+00 | 0.000E+00 | 1.378E-08 | 0.000E+00 | 0.000E+00 | 0.000E+00 | 0.000E+00 | 0.000E+00 |
| Unigene89317 | poxtA | 2.649E-05 | 1.075E-05 | 8.167E-06 | 4.829E-06 | 2.554E-07 | 0.000E+00 | 0.000E+00 | 0.000E+00 | 1.206E-06 | 0.000E+00 | 0.000E+00 | 0.000E+00 |
| Unigene89383 | tetA(46) | 7.986E-07 | 5.536E-06 | 9.981E-06 | 1.732E-05 | 9.339E-08 | 4.544E-08 | 0.000E+00 | 0.000E+00 | 0.000E+00 | 0.000E+00 | 0.000E+00 | 0.000E+00 |
| Unigene89490 | mdtB | 0.000E+00 | 6.539E-07 | 2.407E-07 | 0.000E+00 | 0.000E+00 | 0.000E+00 | 0.000E+00 | 0.000E+00 | 0.000E+00 | 0.000E+00 | 0.000E+00 | 0.000E+00 |
| Unigene89507 | emeA | 1.090E-06 | 5.830E-06 | 1.107E-05 | 1.634E-05 | 0.000E+00 | 0.000E+00 | 9.045E-08 | 0.000E+00 | 0.000E+00 | 0.000E+00 | 1.558E-07 | 0.000E+00 |
| Unigene89524 | otr(A) | 0.000E+00 | 7.479E-07 | 0.000E+00 | 6.157E-07 | 0.000E+00 | 0.000E+00 | 0.000E+00 | 0.000E+00 | 0.000E+00 | 0.000E+00 | 0.000E+00 | 0.000E+00 |
| Unigene89548 | mdtB | 1.888E-07 | 0.000E+00 | 0.000E+00 | 0.000E+00 | 0.000E+00 | 0.000E+00 | 0.000E+00 | 0.000E+00 | 0.000E+00 | 0.000E+00 | 0.000E+00 | 0.000E+00 |
| Unigene89568 | lmrD | 0.000E+00 | 5.624E-07 | 0.000E+00 | 0.000E+00 | 8.132E-08 | 0.000E+00 | 0.000E+00 | 0.000E+00 | 0.000E+00 | 0.000E+00 | 0.000E+00 | 0.000E+00 |
| Unigene89620 | efmA | 2.501E-06 | 6.691E-06 | 8.078E-06 | 9.892E-06 | 0.000E+00 | 0.000E+00 | 5.767E-08 | 0.000E+00 | 0.000E+00 | 0.000E+00 | 0.000E+00 | 0.000E+00 |
| Unigene89766 | msrC | 7.317E-07 | 5.102E-06 | 7.517E-06 | 1.869E-05 | 0.000E+00 | 9.306E-08 | 7.145E-08 | 2.512E-08 | 0.000E+00 | 0.000E+00 | 2.462E-08 | 0.000E+00 |
| Unigene89785 | tetA(58) | 5.401E-07 | 3.967E-06 | 7.460E-06 | 1.821E-05 | 0.000E+00 | 0.000E+00 | 0.000E+00 | 0.000E+00 | 0.000E+00 | 0.000E+00 | 1.426E-07 | 0.000E+00 |
| Unigene89786 | patA | 4.139E-07 | 5.501E-06 | 6.272E-06 | 1.518E-05 | 0.000E+00 | 9.942E-08 | 0.000E+00 | 0.000E+00 | 0.000E+00 | 0.000E+00 | 0.000E+00 | 0.000E+00 |
| Unigene89801 | vanRL | 0.000E+00 | 1.197E-06 | 8.837E-07 | 1.819E-06 | 0.000E+00 | 0.000E+00 | 0.000E+00 | 0.000E+00 | 0.000E+00 | 0.000E+00 | 0.000E+00 | 0.000E+00 |
| Unigene89881 | efrA | 2.620E-07 | 1.273E-06 | 1.542E-06 | 1.739E-06 | 2.079E-08 | 0.000E+00 | 0.000E+00 | 0.000E+00 | 0.000E+00 | 0.000E+00 | 0.000E+00 | 0.000E+00 |
| Unigene89889 | mef(B) | 5.112E-07 | 1.318E-06 | 1.429E-06 | 1.549E-06 | 0.000E+00 | 0.000E+00 | 0.000E+00 | 0.000E+00 | 0.000E+00 | 0.000E+00 | 0.000E+00 | 0.000E+00 |
| Unigene89957 | basS | 0.000E+00 | 2.928E-07 | 4.363E-08 | 0.000E+00 | 0.000E+00 | 0.000E+00 | 0.000E+00 | 0.000E+00 | 0.000E+00 | 0.000E+00 | 0.000E+00 | 0.000E+00 |
| Unigene89985 | macB | 5.422E-07 | 6.729E-06 | 9.494E-06 | 1.491E-05 | 0.000E+00 | 0.000E+00 | 0.000E+00 | 0.000E+00 | 0.000E+00 | 0.000E+00 | 0.000E+00 | 0.000E+00 |
| Unigene89998 | tetB(46) | 0.000E+00 | 6.056E-07 | 0.000E+00 | 0.000E+00 | 0.000E+00 | 0.000E+00 | 0.000E+00 | 0.000E+00 | 0.000E+00 | 0.000E+00 | 0.000E+00 | 0.000E+00 |
| Unigene90048 | adeL | 0.000E+00 | 8.998E-07 | 0.000E+00 | 0.000E+00 | 5.692E-08 | 0.000E+00 | 0.000E+00 | 0.000E+00 | 0.000E+00 | 0.000E+00 | 0.000E+00 | 0.000E+00 |
| Unigene90062 | TriB | 0.000E+00 | 9.490E-07 | 4.713E-07 | 2.425E-07 | 0.000E+00 | 0.000E+00 | 0.000E+00 | 2.804E-07 | 0.000E+00 | 0.000E+00 | 0.000E+00 | 0.000E+00 |
| Unigene90089 | emrB | 2.629E-06 | 2.449E-06 | 2.753E-06 | 1.116E-06 | 0.000E+00 | 0.000E+00 | 4.646E-07 | 4.126E-07 | 7.003E-07 | 7.384E-08 | 0.000E+00 | 0.000E+00 |
| Unigene90125 | vanRL | 1.896E-06 | 6.763E-07 | 1.581E-06 | 6.223E-07 | 0.000E+00 | 0.000E+00 | 0.000E+00 | 0.000E+00 | 0.000E+00 | 0.000E+00 | 0.000E+00 | 0.000E+00 |
| Unigene90343 | salA | 6.329E-07 | 2.214E-06 | 2.872E-06 | 2.037E-06 | 0.000E+00 | 0.000E+00 | 0.000E+00 | 0.000E+00 | 0.000E+00 | 0.000E+00 | 0.000E+00 | 0.000E+00 |
| Unigene90382 | TaeA | 0.000E+00 | 1.207E-06 | 2.158E-06 | 1.606E-06 | 0.000E+00 | 0.000E+00 | 0.000E+00 | 0.000E+00 | 0.000E+00 | 0.000E+00 | 0.000E+00 | 0.000E+00 |
| Unigene90481 | PmrF | 1.282E-05 | 4.927E-06 | 2.993E-06 | 1.332E-05 | 8.177E-05 | 1.175E-04 | 1.087E-04 | 8.748E-05 | 1.368E-04 | 1.386E-04 | 1.077E-04 | 7.400E-05 |
| Unigene90528 | vanRI | 3.998E-06 | 9.101E-06 | 1.395E-05 | 1.510E-05 | 0.000E+00 | 0.000E+00 | 0.000E+00 | 0.000E+00 | 0.000E+00 | 0.000E+00 | 0.000E+00 | 0.000E+00 |

| | | | | | | | | | | | | | |
|---|---|---|---|---|---|---|---|---|---|---|---|---|---|
| Unigene90529 | ugd | 0.000E+00 | 6.005E-07 | 0.000E+00 | 0.000E+00 | 0.000E+00 | 0.000E+00 | 0.000E+00 | 0.000E+00 | 0.000E+00 | 0.000E+00 | 0.000E+00 | 0.000E+00 |
| Unigene90547 | salA | 0.000E+00 | 4.294E-07 | 0.000E+00 | 0.000E+00 | 0.000E+00 | 0.000E+00 | 0.000E+00 | 0.000E+00 | 0.000E+00 | 0.000E+00 | 0.000E+00 | 0.000E+00 |
| Unigene90587 | marA | 0.000E+00 | 9.601E-07 | 0.000E+00 | 0.000E+00 | 0.000E+00 | 0.000E+00 | 0.000E+00 | 0.000E+00 | 0.000E+00 | 0.000E+00 | 0.000E+00 | 0.000E+00 |
| Unigene90610 | mphB | 7.421E-08 | 4.072E-07 | 0.000E+00 | 0.000E+00 | 0.000E+00 | 0.000E+00 | 0.000E+00 | 0.000E+00 | 0.000E+00 | 0.000E+00 | 0.000E+00 | 0.000E+00 |
| Unigene90674 | vanRE | 0.000E+00 | 4.621E-07 | 1.607E-06 | 1.276E-06 | 0.000E+00 | 0.000E+00 | 0.000E+00 | 0.000E+00 | 0.000E+00 | 0.000E+00 | 0.000E+00 | 0.000E+00 |
| Unigene90675 | vanSE | 0.000E+00 | 1.910E-06 | 1.150E-06 | 1.972E-06 | 0.000E+00 | 0.000E+00 | 0.000E+00 | 0.000E+00 | 0.000E+00 | 0.000E+00 | 0.000E+00 | 0.000E+00 |
| Unigene90828 | Escherichia | 0.000E+00 | 4.144E-07 | 0.000E+00 | 0.000E+00 | 0.000E+00 | 0.000E+00 | 0.000E+00 | 0.000E+00 | 0.000E+00 | 0.000E+00 | 0.000E+00 | 0.000E+00 |
| Unigene90831 | oleC | 0.000E+00 | 1.079E-06 | 0.000E+00 | 8.273E-08 | 0.000E+00 | 0.000E+00 | 0.000E+00 | 0.000E+00 | 0.000E+00 | 0.000E+00 | 0.000E+00 | 0.000E+00 |
| Unigene90886 | cpxA | 0.000E+00 | 2.969E-06 | 1.780E-06 | 7.140E-07 | 6.694E-06 | 0.000E+00 | 0.000E+00 | 0.000E+00 | 9.072E-07 | 0.000E+00 | 0.000E+00 | 1.817E-07 |
| Unigene90908 | baeS | 2.850E-07 | 4.419E-06 | 1.329E-06 | 1.979E-06 | 0.000E+00 | 0.000E+00 | 0.000E+00 | 0.000E+00 | 0.000E+00 | 0.000E+00 | 0.000E+00 | 0.000E+00 |
| Unigene90915 | macB | 0.000E+00 | 8.630E-07 | 3.655E-06 | 4.054E-06 | 0.000E+00 | 0.000E+00 | 0.000E+00 | 0.000E+00 | 0.000E+00 | 0.000E+00 | 0.000E+00 | 0.000E+00 |
| Unigene91010 | vanRM | 1.373E-06 | 5.735E-06 | 6.735E-06 | 6.752E-06 | 0.000E+00 | 0.000E+00 | 0.000E+00 | 0.000E+00 | 0.000E+00 | 0.000E+00 | 0.000E+00 | 0.000E+00 |
| Unigene91011 | vanSF | 5.735E-07 | 1.589E-06 | 1.795E-06 | 2.068E-06 | 0.000E+00 | 2.952E-08 | 0.000E+00 | 0.000E+00 | 0.000E+00 | 0.000E+00 | 0.000E+00 | 0.000E+00 |
| Unigene91043 | oleC | 0.000E+00 | 7.143E-07 | 1.703E-07 | 0.000E+00 | 0.000E+00 | 0.000E+00 | 0.000E+00 | 0.000E+00 | 0.000E+00 | 0.000E+00 | 0.000E+00 | 0.000E+00 |
| Unigene91095 | tlrC | 4.168E-05 | 2.421E-05 | 1.724E-05 | 2.047E-05 | 1.770E-06 | 1.581E-06 | 2.896E-06 | 6.830E-07 | 6.253E-06 | 6.699E-07 | 1.636E-06 | 1.499E-06 |
| Unigene91120 | macB | 7.812E-05 | 6.221E-05 | 1.271E-04 | 1.661E-04 | 1.420E-04 | 1.001E-04 | 1.281E-04 | 1.275E-04 | 1.042E-04 | 1.529E-04 | 1.585E-04 | 1.661E-04 |
| Unigene91149 | tetB(P) | 0.000E+00 | 2.526E-07 | 0.000E+00 | 0.000E+00 | 0.000E+00 | 0.000E+00 | 0.000E+00 | 0.000E+00 | 0.000E+00 | 0.000E+00 | 0.000E+00 | 0.000E+00 |
| Unigene91173 | chrB | 0.000E+00 | 8.249E-07 | 6.599E-07 | 0.000E+00 | 5.406E-06 | 0.000E+00 | 4.816E-07 | 0.000E+00 | 0.000E+00 | 0.000E+00 | 0.000E+00 | 0.000E+00 |
| Unigene91195 | rosB | 3.843E-08 | 5.061E-07 | 0.000E+00 | 0.000E+00 | 0.000E+00 | 0.000E+00 | 0.000E+00 | 0.000E+00 | 0.000E+00 | 0.000E+00 | 0.000E+00 | 0.000E+00 |
| Unigene91244 | mdsC | 6.942E-08 | 5.714E-07 | 1.022E-07 | 2.454E-07 | 0.000E+00 | 0.000E+00 | 0.000E+00 | 0.000E+00 | 0.000E+00 | 0.000E+00 | 0.000E+00 | 0.000E+00 |
| Unigene91434 | baeR | 2.546E-06 | 2.900E-06 | 2.451E-06 | 2.086E-06 | 0.000E+00 | 2.077E-07 | 6.380E-07 | 2.804E-07 | 6.825E-07 | 0.000E+00 | 2.198E-07 | 1.136E-07 |
| Unigene91471 | Listeria mc | 5.206E-05 | 4.960E-05 | 9.235E-05 | 6.203E-05 | 1.104E-04 | 7.918E-05 | 9.641E-05 | 1.072E-04 | 8.927E-05 | 1.246E-04 | 1.235E-04 | 1.368E-04 |
| Unigene91481 | ykkC | 7.992E-05 | 6.291E-05 | 1.306E-04 | 1.963E-04 | 1.561E-04 | 1.035E-04 | 1.338E-04 | 1.152E-04 | 1.173E-04 | 1.679E-04 | 1.709E-04 | 1.769E-04 |
| Unigene91578 | gadX | 0.000E+00 | 6.034E-07 | 0.000E+00 | 0.000E+00 | 0.000E+00 | 0.000E+00 | 0.000E+00 | 0.000E+00 | 0.000E+00 | 0.000E+00 | 0.000E+00 | 0.000E+00 |
| Unigene91614 | msbA | 3.130E-05 | 2.067E-05 | 1.700E-05 | 2.009E-05 | 1.597E-06 | 1.533E-06 | 2.365E-06 | 3.311E-07 | 0.000E+00 | 6.109E-07 | 1.320E-06 | 1.051E-06 |
| Unigene91627 | vgaC | 0.000E+00 | 2.277E-06 | 0.000E+00 | 0.000E+00 | 7.009E-06 | 0.000E+00 | 0.000E+00 | 0.000E+00 | 0.000E+00 | 0.000E+00 | 0.000E+00 | 0.000E+00 |
| Unigene91686 | abeS | 0.000E+00 | 0.000E+00 | 2.270E-06 | 4.265E-06 | 0.000E+00 | 0.000E+00 | 0.000E+00 | 0.000E+00 | 0.000E+00 | 0.000E+00 | 0.000E+00 | 0.000E+00 |
| Unigene91690 | AcrS | 7.608E-07 | 1.014E-06 | 3.999E-06 | 5.598E-06 | 0.000E+00 | 0.000E+00 | 3.007E-07 | 0.000E+00 | 0.000E+00 | 0.000E+00 | 0.000E+00 | 0.000E+00 |
| Unigene91839 | macB | 0.000E+00 | 4.853E-07 | 2.893E-07 | 1.488E-07 | 0.000E+00 | 0.000E+00 | 0.000E+00 | 0.000E+00 | 0.000E+00 | 0.000E+00 | 0.000E+00 | 0.000E+00 |
| Unigene91882 | vanHA | 0.000E+00 | 6.947E-07 | 0.000E+00 | 0.000E+00 | 0.000E+00 | 0.000E+00 | 0.000E+00 | 0.000E+00 | 0.000E+00 | 0.000E+00 | 0.000E+00 | 0.000E+00 |
| Unigene92132 | macB | 5.630E-07 | 6.351E-06 | 8.993E-06 | 1.532E-05 | 0.000E+00 | 0.000E+00 | 0.000E+00 | 0.000E+00 | 0.000E+00 | 0.000E+00 | 0.000E+00 | 0.000E+00 |
| Unigene92163 | lmrD | 0.000E+00 | 9.462E-07 | 1.504E-06 | 1.644E-06 | 0.000E+00 | 0.000E+00 | 0.000E+00 | 0.000E+00 | 0.000E+00 | 0.000E+00 | 0.000E+00 | 0.000E+00 |
| Unigene92185 | vgaE | 6.068E-08 | 9.101E-07 | 4.505E-06 | 1.532E-06 | 0.000E+00 | 0.000E+00 | 4.477E-08 | 0.000E+00 | 0.000E+00 | 0.000E+00 | 0.000E+00 | 0.000E+00 |
| Unigene92207 | emeA | 0.000E+00 | 1.968E-06 | 2.148E-06 | 0.000E+00 | 0.000E+00 | 0.000E+00 | 0.000E+00 | 0.000E+00 | 0.000E+00 | 0.000E+00 | 0.000E+00 | 0.000E+00 |
| Unigene92217 | Streptomy | 0.000E+00 | 1.899E-06 | 2.490E-06 | 3.311E-06 | 0.000E+00 | 0.000E+00 | 0.000E+00 | 0.000E+00 | 0.000E+00 | 0.000E+00 | 0.000E+00 | 0.000E+00 |
| Unigene92229 | macB | 0.000E+00 | 1.884E-06 | 2.342E-06 | 1.564E-06 | 0.000E+00 | 0.000E+00 | 0.000E+00 | 0.000E+00 | 0.000E+00 | 0.000E+00 | 0.000E+00 | 0.000E+00 |
| Unigene92232 | vmlR | 0.000E+00 | 2.499E-06 | 2.991E-06 | 1.854E-06 | 0.000E+00 | 0.000E+00 | 0.000E+00 | 0.000E+00 | 0.000E+00 | 0.000E+00 | 0.000E+00 | 0.000E+00 |
| Unigene92264 | qacH | 0.000E+00 | 0.000E+00 | 3.984E-07 | 0.000E+00 | 0.000E+00 | 0.000E+00 | 0.000E+00 | 0.000E+00 | 0.000E+00 | 0.000E+00 | 0.000E+00 | 0.000E+00 |
| Unigene92320 | bcrA | 0.000E+00 | 5.272E-07 | 0.000E+00 | 0.000E+00 | 0.000E+00 | 0.000E+00 | 0.000E+00 | 0.000E+00 | 0.000E+00 | 0.000E+00 | 0.000E+00 | 0.000E+00 |
| Unigene92360 | arlR | 5.347E-05 | 4.693E-05 | 9.788E-05 | 1.420E-04 | 9.699E-05 | 6.644E-05 | 9.667E-05 | 8.720E-05 | 7.590E-05 | 1.090E-04 | 1.174E-04 | 1.122E-04 |
| Unigene92401 | macB | 0.000E+00 | 1.645E-06 | 2.534E-06 | 1.824E-06 | 0.000E+00 | 0.000E+00 | 0.000E+00 | 0.000E+00 | 0.000E+00 | 0.000E+00 | 0.000E+00 | 0.000E+00 |
| Unigene92441 | dfrA3 | 0.000E+00 | 4.049E-07 | 0.000E+00 | 0.000E+00 | 0.000E+00 | 0.000E+00 | 0.000E+00 | 0.000E+00 | 0.000E+00 | 0.000E+00 | 0.000E+00 | 0.000E+00 |
| Unigene92479 | cfrA | 3.806E-06 | 1.030E-05 | 1.162E-05 | 1.129E-05 | 0.000E+00 | 0.000E+00 | 3.317E-08 | 0.000E+00 | 0.000E+00 | 0.000E+00 | 0.000E+00 | 0.000E+00 |
| Unigene92644 | macB | 2.268E-07 | 4.812E-06 | 1.632E-06 | 1.909E-06 | 0.000E+00 | 0.000E+00 | 0.000E+00 | 2.941E-08 | 0.000E+00 | 0.000E+00 | 0.000E+00 | 0.000E+00 |
| Unigene92738 | PmrF | 0.000E+00 | 4.233E-07 | 7.569E-08 | 1.947E-07 | 0.000E+00 | 0.000E+00 | 0.000E+00 | 0.000E+00 | 0.000E+00 | 0.000E+00 | 0.000E+00 | 0.000E+00 |
| Unigene92747 | tetA(58) | 0.000E+00 | 3.848E-06 | 0.000E+00 | 0.000E+00 | 0.000E+00 | 0.000E+00 | 0.000E+00 | 0.000E+00 | 1.138E-06 | 0.000E+00 | 0.000E+00 | 6.564E-07 |
| Unigene92753 | Klebsiella p | 0.000E+00 | 1.312E-06 | 5.866E-07 | 2.012E-07 | 0.000E+00 | 0.000E+00 | 0.000E+00 | 0.000E+00 | 0.000E+00 | 0.000E+00 | 0.000E+00 | 0.000E+00 |
| Unigene92760 | bcrA | 0.000E+00 | 1.962E-06 | 0.000E+00 | 0.000E+00 | 0.000E+00 | 0.000E+00 | 0.000E+00 | 0.000E+00 | 0.000E+00 | 0.000E+00 | 0.000E+00 | 0.000E+00 |
| Unigene92771 | macB | 0.000E+00 | 1.191E-06 | 0.000E+00 | 0.000E+00 | 0.000E+00 | 0.000E+00 | 0.000E+00 | 0.000E+00 | 0.000E+00 | 0.000E+00 | 0.000E+00 | 0.000E+00 |
| Unigene92783 | tetB(46) | 0.000E+00 | 1.138E-06 | 0.000E+00 | 0.000E+00 | 0.000E+00 | 0.000E+00 | 0.000E+00 | 0.000E+00 | 0.000E+00 | 0.000E+00 | 0.000E+00 | 0.000E+00 |
| Unigene92784 | tetA(60) | 0.000E+00 | 8.763E-07 | 0.000E+00 | 0.000E+00 | 0.000E+00 | 0.000E+00 | 0.000E+00 | 0.000E+00 | 0.000E+00 | 0.000E+00 | 0.000E+00 | 0.000E+00 |
| Unigene92788 | vanHA | 0.000E+00 | 1.300E-06 | 0.000E+00 | 0.000E+00 | 0.000E+00 | 0.000E+00 | 0.000E+00 | 0.000E+00 | 0.000E+00 | 0.000E+00 | 0.000E+00 | 0.000E+00 |
| Unigene92807 | bcrA | 0.000E+00 | 1.136E-06 | 0.000E+00 | 1.881E-06 | 0.000E+00 | 0.000E+00 | 0.000E+00 | 0.000E+00 | 0.000E+00 | 0.000E+00 | 0.000E+00 | 0.000E+00 |
| Unigene92850 | basS | 0.000E+00 | 9.954E-07 | 0.000E+00 | 8.326E-08 | 0.000E+00 | 0.000E+00 | 0.000E+00 | 0.000E+00 | 0.000E+00 | 0.000E+00 | 0.000E+00 | 0.000E+00 |
| Unigene92877 | lsaC | 6.965E-05 | 6.198E-05 | 1.105E-04 | 1.648E-04 | 1.273E-04 | 8.935E-05 | 1.221E-04 | 1.236E-04 | 1.011E-04 | 1.457E-04 | 1.452E-04 | 1.564E-04 |
| Unigene92990 | dfrA20 | 0.000E+00 | 7.826E-07 | 0.000E+00 | 0.000E+00 | 0.000E+00 | 0.000E+00 | 0.000E+00 | 0.000E+00 | 0.000E+00 | 0.000E+00 | 0.000E+00 | 0.000E+00 |
| Unigene93052 | vanHF | 1.157E-07 | 6.984E-07 | 0.000E+00 | 0.000E+00 | 0.000E+00 | 0.000E+00 | 0.000E+00 | 0.000E+00 | 0.000E+00 | 0.000E+00 | 0.000E+00 | 0.000E+00 |
| Unigene93062 | otr(B) | 0.000E+00 | 6.721E-07 | 1.717E-07 | 4.417E-08 | 0.000E+00 | 0.000E+00 | 0.000E+00 | 0.000E+00 | 0.000E+00 | 0.000E+00 | 0.000E+00 | 0.000E+00 |

| | | | | | | | | | | | | | |
|---|---|---|---|---|---|---|---|---|---|---|---|---|---|
| Unigene93138 | tetB(60) | 8.724E-06 | 5.386E-06 | 3.032E-06 | 1.182E-05 | 7.754E-05 | 9.714E-05 | 9.797E-05 | 9.102E-05 | 1.239E-04 | 1.149E-04 | 9.497E-05 | 5.908E-05 |
| Unigene93154 | vanHB | 0.000E+00 | 8.329E-07 | 8.510E-07 | 0.000E+00 | 5.420E-07 | 0.000E+00 | 0.000E+00 | 0.000E+00 | 0.000E+00 | 0.000E+00 | 1.023E-06 | 6.407E-07 |
| Unigene93176 | YojI | 2.779E-07 | 4.794E-06 | 2.805E-06 | 2.706E-06 | 0.000E+00 | 0.000E+00 | 0.000E+00 | 0.000E+00 | 0.000E+00 | 0.000E+00 | 0.000E+00 | 0.000E+00 |
| Unigene93183 | lmrD | 0.000E+00 | 0.000E+00 | 0.000E+00 | 0.000E+00 | 0.000E+00 | 0.000E+00 | 0.000E+00 | 0.000E+00 | 0.000E+00 | 0.000E+00 | 5.884E-07 | 0.000E+00 |
| Unigene93278 | Erm(34) | 3.534E-07 | 9.482E-07 | 4.239E-07 | 0.000E+00 | 0.000E+00 | 0.000E+00 | 0.000E+00 | 0.000E+00 | 0.000E+00 | 0.000E+00 | 0.000E+00 | 0.000E+00 |
| Unigene93296 | RlmA(II) | 0.000E+00 | 4.423E-07 | 2.373E-07 | 0.000E+00 | 0.000E+00 | 0.000E+00 | 0.000E+00 | 0.000E+00 | 0.000E+00 | 0.000E+00 | 0.000E+00 | 0.000E+00 |
| Unigene93352 | smeS | 0.000E+00 | 2.350E-07 | 0.000E+00 | 0.000E+00 | 0.000E+00 | 0.000E+00 | 5.925E-08 | 0.000E+00 | 0.000E+00 | 0.000E+00 | 0.000E+00 | 0.000E+00 |
| Unigene93358 | baeR | 0.000E+00 | 1.031E-06 | 3.227E-07 | 2.372E-07 | 0.000E+00 | 0.000E+00 | 0.000E+00 | 0.000E+00 | 0.000E+00 | 0.000E+00 | 0.000E+00 | 0.000E+00 |
| Unigene93424 | efrA | 0.000E+00 | 6.787E-07 | 0.000E+00 | 0.000E+00 | 0.000E+00 | 0.000E+00 | 0.000E+00 | 0.000E+00 | 0.000E+00 | 0.000E+00 | 0.000E+00 | 0.000E+00 |
| Unigene93432 | lsaA | 2.653E-05 | 9.728E-07 | 2.629E-07 | 2.082E-08 | 4.580E-08 | 0.000E+00 | 0.000E+00 | 0.000E+00 | 0.000E+00 | 0.000E+00 | 0.000E+00 | 0.000E+00 |
| Unigene93449 | macB | 0.000E+00 | 1.186E-06 | 1.383E-06 | 2.040E-06 | 0.000E+00 | 0.000E+00 | 0.000E+00 | 0.000E+00 | 0.000E+00 | 0.000E+00 | 0.000E+00 | 0.000E+00 |
| Unigene93480 | NmcR | 0.000E+00 | 8.000E-07 | 2.384E-07 | 0.000E+00 | 0.000E+00 | 0.000E+00 | 0.000E+00 | 0.000E+00 | 0.000E+00 | 0.000E+00 | 0.000E+00 | 0.000E+00 |
| Unigene93602 | vanSG | 2.532E-06 | 7.619E-06 | 7.072E-06 | 7.278E-06 | 0.000E+00 | 0.000E+00 | 0.000E+00 | 0.000E+00 | 0.000E+00 | 0.000E+00 | 2.336E-08 | 0.000E+00 |
| Unigene93607 | tetA(60) | 2.092E-06 | 7.568E-06 | 6.012E-06 | 6.683E-06 | 4.744E-08 | 0.000E+00 | 0.000E+00 | 0.000E+00 | 0.000E+00 | 0.000E+00 | 0.000E+00 | 0.000E+00 |
| Unigene93637 | PmrF | 8.650E-07 | 1.405E-06 | 8.827E-07 | 5.241E-07 | 1.883E-06 | 1.197E-06 | 7.275E-07 | 6.865E-07 | 7.941E-07 | 2.235E-06 | 1.781E-06 | 1.022E-06 |
| Unigene93683 | macB | 5.058E-05 | 2.749E-05 | 2.106E-05 | 2.362E-05 | 2.478E-06 | 1.792E-06 | 4.036E-06 | 5.981E-07 | 6.621E-06 | 5.372E-07 | 2.172E-06 | 1.639E-06 |
| Unigene93981 | dfrK | 5.284E-07 | 1.882E-05 | 1.414E-06 | 3.396E-07 | 0.000E+00 | 0.000E+00 | 0.000E+00 | 0.000E+00 | 0.000E+00 | 0.000E+00 | 0.000E+00 | 0.000E+00 |
| Unigene94048 | Staphylocc | 4.190E-05 | 2.495E-05 | 2.045E-05 | 2.201E-05 | 2.001E-06 | 1.930E-06 | 3.341E-06 | 5.494E-07 | 6.333E-06 | 6.147E-07 | 2.228E-06 | 1.266E-06 |
| Unigene94145 | efrA | 0.000E+00 | 1.869E-06 | 0.000E+00 | 0.000E+00 | 0.000E+00 | 0.000E+00 | 0.000E+00 | 0.000E+00 | 0.000E+00 | 0.000E+00 | 0.000E+00 | 0.000E+00 |
| Unigene94150 | mgrA | 0.000E+00 | 1.848E-06 | 0.000E+00 | 0.000E+00 | 0.000E+00 | 0.000E+00 | 0.000E+00 | 0.000E+00 | 0.000E+00 | 0.000E+00 | 0.000E+00 | 0.000E+00 |
| Unigene94153 | tetA(58) | 0.000E+00 | 2.452E-06 | 0.000E+00 | 0.000E+00 | 0.000E+00 | 0.000E+00 | 0.000E+00 | 0.000E+00 | 0.000E+00 | 0.000E+00 | 0.000E+00 | 0.000E+00 |
| Unigene94168 | lsaA | 0.000E+00 | 1.744E-06 | 0.000E+00 | 0.000E+00 | 0.000E+00 | 0.000E+00 | 0.000E+00 | 0.000E+00 | 0.000E+00 | 0.000E+00 | 0.000E+00 | 0.000E+00 |
| Unigene94173 | tetA(58) | 0.000E+00 | 1.992E-06 | 0.000E+00 | 0.000E+00 | 0.000E+00 | 0.000E+00 | 0.000E+00 | 0.000E+00 | 0.000E+00 | 0.000E+00 | 0.000E+00 | 0.000E+00 |
| Unigene94189 | bcrA | 0.000E+00 | 2.031E-06 | 0.000E+00 | 0.000E+00 | 0.000E+00 | 0.000E+00 | 0.000E+00 | 0.000E+00 | 0.000E+00 | 0.000E+00 | 0.000E+00 | 0.000E+00 |
| Unigene94263 | efrB | 0.000E+00 | 4.168E-07 | 1.863E-07 | 0.000E+00 | 0.000E+00 | 0.000E+00 | 0.000E+00 | 0.000E+00 | 0.000E+00 | 0.000E+00 | 0.000E+00 | 0.000E+00 |
| Unigene94285 | lmrC | 3.387E-07 | 1.006E-06 | 5.670E-07 | 0.000E+00 | 6.043E-06 | 0.000E+00 | 0.000E+00 | 0.000E+00 | 0.000E+00 | 0.000E+00 | 0.000E+00 | 0.000E+00 |
| Unigene94327 | bcrA | 6.854E-05 | 5.531E-05 | 1.078E-04 | 1.570E-04 | 1.267E-04 | 8.736E-05 | 1.118E-04 | 1.117E-04 | 9.475E-05 | 1.344E-04 | 1.381E-04 | 1.524E-04 |
| Unigene94436 | arlS | 6.807E-05 | 5.728E-05 | 1.143E-04 | 1.674E-04 | 1.283E-04 | 8.766E-05 | 1.128E-04 | 1.056E-04 | 9.603E-05 | 1.392E-04 | 1.389E-04 | 1.443E-04 |
| Unigene94437 | mtrA | 6.146E-05 | 4.939E-05 | 1.013E-04 | 1.460E-04 | 1.052E-04 | 7.955E-05 | 9.431E-05 | 9.478E-05 | 8.282E-05 | 1.194E-04 | 1.195E-04 | 1.254E-04 |
| Unigene94510 | tetM | 0.000E+00 | 2.486E-07 | 0.000E+00 | 0.000E+00 | 0.000E+00 | 0.000E+00 | 0.000E+00 | 0.000E+00 | 0.000E+00 | 0.000E+00 | 0.000E+00 | 0.000E+00 |
| Unigene94553 | vanSF | 0.000E+00 | 1.019E-06 | 4.314E-06 | 4.137E-06 | 0.000E+00 | 0.000E+00 | 1.511E-07 | 0.000E+00 | 0.000E+00 | 0.000E+00 | 0.000E+00 | 0.000E+00 |
| Unigene94590 | vanHD | 4.813E-07 | 2.226E-06 | 9.782E-07 | 4.166E-07 | 8.631E-06 | 8.919E-07 | 2.055E-06 | 0.000E+00 | 0.000E+00 | 0.000E+00 | 0.000E+00 | 0.000E+00 |
| Unigene94695 | patA | 0.000E+00 | 2.853E-07 | 0.000E+00 | 0.000E+00 | 0.000E+00 | 0.000E+00 | 0.000E+00 | 0.000E+00 | 0.000E+00 | 0.000E+00 | 0.000E+00 | 0.000E+00 |
| Unigene94746 | macB | 0.000E+00 | 6.635E-07 | 0.000E+00 | 0.000E+00 | 0.000E+00 | 0.000E+00 | 0.000E+00 | 0.000E+00 | 0.000E+00 | 0.000E+00 | 0.000E+00 | 0.000E+00 |
| Unigene94803 | norB | 0.000E+00 | 7.114E-07 | 0.000E+00 | 3.927E-07 | 0.000E+00 | 0.000E+00 | 0.000E+00 | 0.000E+00 | 0.000E+00 | 0.000E+00 | 0.000E+00 | 0.000E+00 |
| Unigene94871 | AcrS | 4.936E-07 | 1.517E-06 | 3.390E-07 | 0.000E+00 | 0.000E+00 | 1.601E-07 | 1.147E-06 | 0.000E+00 | 0.000E+00 | 0.000E+00 | 3.388E-07 | 0.000E+00 |
| Unigene94874 | opmE | 0.000E+00 | 4.853E-07 | 0.000E+00 | 0.000E+00 | 0.000E+00 | 0.000E+00 | 0.000E+00 | 0.000E+00 | 0.000E+00 | 0.000E+00 | 0.000E+00 | 0.000E+00 |
| Unigene94875 | emrA | 0.000E+00 | 6.408E-07 | 0.000E+00 | 0.000E+00 | 0.000E+00 | 0.000E+00 | 0.000E+00 | 0.000E+00 | 0.000E+00 | 0.000E+00 | 0.000E+00 | 0.000E+00 |
| Unigene94894 | efrB | 1.833E-07 | 1.086E-06 | 4.083E-06 | 3.369E-06 | 0.000E+00 | 0.000E+00 | 0.000E+00 | 0.000E+00 | 0.000E+00 | 0.000E+00 | 0.000E+00 | 0.000E+00 |
| Unigene94912 | farB | 0.000E+00 | 5.779E-07 | 1.101E-06 | 3.514E-06 | 3.052E-07 | 0.000E+00 | 0.000E+00 | 1.897E-06 | 0.000E+00 | 6.886E-07 | 9.951E-07 | 5.683E-07 |
| Unigene94932 | YojI | 0.000E+00 | 3.552E-07 | 0.000E+00 | 0.000E+00 | 0.000E+00 | 0.000E+00 | 0.000E+00 | 0.000E+00 | 0.000E+00 | 0.000E+00 | 0.000E+00 | 0.000E+00 |
| Unigene95203 | patB | 8.758E-07 | 9.220E-07 | 3.964E-06 | 5.541E-06 | 0.000E+00 | 1.159E-07 | 4.352E-07 | 0.000E+00 | 0.000E+00 | 0.000E+00 | 0.000E+00 | 0.000E+00 |
| Unigene95204 | efrA | 7.778E-07 | 9.553E-07 | 3.998E-06 | 5.910E-06 | 0.000E+00 | 0.000E+00 | 5.944E-07 | 0.000E+00 | 0.000E+00 | 0.000E+00 | 4.237E-08 | 0.000E+00 |
| Unigene95223 | YojI | 4.482E-05 | 3.032E-05 | 2.446E-05 | 2.568E-05 | 2.141E-06 | 1.634E-06 | 3.871E-06 | 1.107E-06 | 0.000E+00 | 9.576E-07 | 2.374E-06 | 7.708E-07 |
| Unigene95245 | ykkD | 0.000E+00 | 5.110E-06 | 9.340E-06 | 1.651E-05 | 0.000E+00 | 0.000E+00 | 0.000E+00 | 0.000E+00 | 0.000E+00 | 0.000E+00 | 0.000E+00 | 0.000E+00 |
| Unigene95275 | RlmA(II) | 0.000E+00 | 7.330E-07 | 0.000E+00 | 0.000E+00 | 0.000E+00 | 0.000E+00 | 0.000E+00 | 0.000E+00 | 0.000E+00 | 0.000E+00 | 0.000E+00 | 0.000E+00 |
| Unigene95304 | bcrA | 0.000E+00 | 7.498E-07 | 0.000E+00 | 0.000E+00 | 0.000E+00 | 0.000E+00 | 0.000E+00 | 0.000E+00 | 0.000E+00 | 0.000E+00 | 0.000E+00 | 0.000E+00 |
| Unigene95318 | bcrA | 0.000E+00 | 5.481E-07 | 0.000E+00 | 0.000E+00 | 0.000E+00 | 0.000E+00 | 0.000E+00 | 0.000E+00 | 0.000E+00 | 0.000E+00 | 0.000E+00 | 0.000E+00 |
| Unigene95382 | Staphylocc | 0.000E+00 | 8.144E-07 | 3.608E-06 | 2.861E-06 | 0.000E+00 | 0.000E+00 | 0.000E+00 | 0.000E+00 | 0.000E+00 | 0.000E+00 | 0.000E+00 | 0.000E+00 |
| Unigene95419 | Klebsiella p | 0.000E+00 | 8.932E-07 | 0.000E+00 | 0.000E+00 | 0.000E+00 | 0.000E+00 | 0.000E+00 | 0.000E+00 | 0.000E+00 | 0.000E+00 | 0.000E+00 | 0.000E+00 |
| Unigene95517 | aadS | 3.020E-07 | 4.185E-06 | 2.334E-06 | 1.983E-06 | 0.000E+00 | 0.000E+00 | 0.000E+00 | 0.000E+00 | 0.000E+00 | 0.000E+00 | 0.000E+00 | 0.000E+00 |
| Unigene95518 | arlS | 3.317E-06 | 6.984E-06 | 6.282E-06 | 8.179E-06 | 0.000E+00 | 0.000E+00 | 0.000E+00 | 0.000E+00 | 0.000E+00 | 0.000E+00 | 0.000E+00 | 0.000E+00 |
| Unigene95542 | poxtA | 0.000E+00 | 5.041E-07 | 4.507E-07 | 0.000E+00 | 0.000E+00 | 0.000E+00 | 0.000E+00 | 0.000E+00 | 0.000E+00 | 0.000E+00 | 0.000E+00 | 0.000E+00 |
| Unigene95546 | bcrA | 1.025E-07 | 1.087E-06 | 2.883E-06 | 3.829E-06 | 0.000E+00 | 0.000E+00 | 0.000E+00 | 0.000E+00 | 0.000E+00 | 0.000E+00 | 0.000E+00 | 0.000E+00 |
| Unigene95643 | ugd | 2.908E-08 | 8.299E-07 | 0.000E+00 | 0.000E+00 | 3.231E-08 | 0.000E+00 | 0.000E+00 | 0.000E+00 | 0.000E+00 | 0.000E+00 | 0.000E+00 | 0.000E+00 |
| Unigene95731 | patB | 1.882E-06 | 1.347E-05 | 7.628E-06 | 4.793E-06 | 0.000E+00 | 1.327E-07 | 0.000E+00 | 4.776E-08 | 0.000E+00 | 0.000E+00 | 0.000E+00 | 0.000E+00 |
| Unigene95755 | rpoB2 | 0.000E+00 | 0.000E+00 | 0.000E+00 | 1.175E-07 | 0.000E+00 | 0.000E+00 | 0.000E+00 | 0.000E+00 | 0.000E+00 | 0.000E+00 | 0.000E+00 | 0.000E+00 |
| Unigene95833 | vanRB | 1.832E-07 | 9.548E-07 | 2.920E-07 | 2.081E-07 | 0.000E+00 | 0.000E+00 | 0.000E+00 | 1.336E-07 | 0.000E+00 | 0.000E+00 | 0.000E+00 | 0.000E+00 |

| | | | | | | | | | | | | | |
|---|---|---|---|---|---|---|---|---|---|---|---|---|---|
| Unigene95858 | tetB(46) | 5.728E-07 | 1.212E-06 | 1.204E-06 | 0.000E+00 | 5.591E-06 | 0.000E+00 | 2.762E-06 | 0.000E+00 | 0.000E+00 | 0.000E+00 | 0.000E+00 | 0.000E+00 |
| Unigene95930 | bcrA | 3.647E-08 | 5.204E-07 | 2.756E-06 | 3.168E-06 | 0.000E+00 | 0.000E+00 | 0.000E+00 | 0.000E+00 | 0.000E+00 | 0.000E+00 | 0.000E+00 | 0.000E+00 |
| Unigene95937 | adeR | 6.969E-05 | 6.054E-05 | 1.130E-04 | 1.680E-04 | 1.489E-04 | 1.057E-04 | 1.159E-04 | 1.395E-04 | 1.026E-04 | 1.516E-04 | 1.489E-04 | 1.899E-04 |
| Unigene95984 | AcrS | 1.071E-07 | 5.288E-07 | 2.626E-07 | 0.000E+00 | 0.000E+00 | 0.000E+00 | 3.555E-07 | 2.499E-07 | 0.000E+00 | 0.000E+00 | 0.000E+00 | 0.000E+00 |
| Unigene96004 | ramA | 3.289E-06 | 3.688E-06 | 2.526E-06 | 4.296E-06 | 3.177E-07 | 7.729E-08 | 2.769E-07 | 0.000E+00 | 0.000E+00 | 0.000E+00 | 0.000E+00 | 2.113E-07 |
| Unigene96011 | oleC | 1.701E-07 | 1.214E-06 | 3.005E-06 | 2.362E-06 | 0.000E+00 | 0.000E+00 | 0.000E+00 | 0.000E+00 | 0.000E+00 | 0.000E+00 | 4.865E-08 | 0.000E+00 |
| Unigene96163 | efrA | 7.375E-07 | 6.268E-06 | 1.156E-05 | 1.659E-05 | 0.000E+00 | 0.000E+00 | 0.000E+00 | 0.000E+00 | 0.000E+00 | 0.000E+00 | 4.326E-08 | 0.000E+00 |
| Unigene96164 | patB | 6.563E-07 | 5.782E-06 | 9.524E-06 | 1.486E-05 | 0.000E+00 | 0.000E+00 | 0.000E+00 | 0.000E+00 | 0.000E+00 | 0.000E+00 | 0.000E+00 | 6.125E-08 |
| Unigene96182 | novA | 8.150E-05 | 1.867E-04 | 0.000E+00 | 0.000E+00 | 0.000E+00 | 0.000E+00 | 0.000E+00 | 1.634E-06 | 5.444E-05 | 0.000E+00 | 0.000E+00 | 8.962E-07 |
| Unigene96185 | Erm(K) | 4.444E-05 | 2.500E-05 | 1.834E-05 | 1.936E-05 | 2.648E-06 | 1.472E-06 | 3.467E-06 | 0.000E+00 | 6.178E-06 | 7.966E-07 | 1.558E-06 | 1.771E-06 |
| Unigene96267 | vanRB | 2.895E-07 | 1.059E-06 | 4.735E-08 | 8.283E-07 | 0.000E+00 | 0.000E+00 | 0.000E+00 | 0.000E+00 | 0.000E+00 | 0.000E+00 | 0.000E+00 | 0.000E+00 |
| Unigene96345 | bcrA | 4.465E-07 | 8.330E-07 | 1.402E-06 | 1.713E-06 | 0.000E+00 | 0.000E+00 | 0.000E+00 | 0.000E+00 | 0.000E+00 | 0.000E+00 | 0.000E+00 | 0.000E+00 |
| Unigene96348 | vanRI | 2.908E-07 | 9.575E-07 | 1.902E-06 | 2.888E-06 | 0.000E+00 | 0.000E+00 | 0.000E+00 | 0.000E+00 | 0.000E+00 | 0.000E+00 | 0.000E+00 | 0.000E+00 |
| Unigene96349 | baeS | 4.483E-07 | 1.062E-06 | 1.482E-06 | 1.573E-06 | 0.000E+00 | 0.000E+00 | 0.000E+00 | 0.000E+00 | 0.000E+00 | 0.000E+00 | 0.000E+00 | 0.000E+00 |
| Unigene96370 | vgaD | 0.000E+00 | 6.099E-07 | 0.000E+00 | 0.000E+00 | 0.000E+00 | 0.000E+00 | 0.000E+00 | 0.000E+00 | 0.000E+00 | 0.000E+00 | 0.000E+00 | 0.000E+00 |
| Unigene96389 | abeS | 0.000E+00 | 4.313E-06 | 3.397E-06 | 3.212E-06 | 0.000E+00 | 0.000E+00 | 0.000E+00 | 0.000E+00 | 0.000E+00 | 0.000E+00 | 0.000E+00 | 0.000E+00 |
| Unigene96444 | emrB | 8.104E-07 | 1.779E-06 | 1.281E-06 | 4.774E-07 | 7.227E-06 | 0.000E+00 | 1.470E-06 | 0.000E+00 | 0.000E+00 | 0.000E+00 | 7.467E-07 | 0.000E+00 |
| Unigene96460 | bcrA | 0.000E+00 | 9.336E-07 | 2.754E-06 | 3.951E-06 | 0.000E+00 | 0.000E+00 | 0.000E+00 | 0.000E+00 | 0.000E+00 | 0.000E+00 | 0.000E+00 | 0.000E+00 |
| Unigene96495 | ACC-4 | 0.000E+00 | 6.594E-07 | 1.340E-07 | 0.000E+00 | 0.000E+00 | 0.000E+00 | 0.000E+00 | 0.000E+00 | 0.000E+00 | 0.000E+00 | 0.000E+00 | 0.000E+00 |
| Unigene96518 | vanL | 4.988E-05 | 2.655E-05 | 1.736E-05 | 2.296E-05 | 2.094E-06 | 1.972E-06 | 3.768E-06 | 1.064E-06 | 6.678E-06 | 1.016E-06 | 1.843E-06 | 1.114E-06 |
| Unigene96536 | vanRM | 1.047E-04 | 1.665E-04 | 1.524E-04 | 7.031E-06 | 8.702E-06 | 2.994E-07 | 3.327E-06 | 1.939E-06 | 5.668E-05 | 0.000E+00 | 2.715E-06 | 1.029E-06 |
| Unigene96585 | msbA | 1.505E-08 | 1.800E-06 | 4.282E-07 | 4.255E-07 | 0.000E+00 | 0.000E+00 | 0.000E+00 | 0.000E+00 | 0.000E+00 | 0.000E+00 | 0.000E+00 | 0.000E+00 |
| Unigene96643 | efrA | 0.000E+00 | 6.119E-07 | 0.000E+00 | 0.000E+00 | 0.000E+00 | 0.000E+00 | 0.000E+00 | 0.000E+00 | 0.000E+00 | 0.000E+00 | 0.000E+00 | 0.000E+00 |
| Unigene96677 | macB | 3.738E-07 | 6.095E-06 | 9.039E-06 | 1.467E-05 | 0.000E+00 | 8.659E-08 | 0.000E+00 | 0.000E+00 | 0.000E+00 | 0.000E+00 | 0.000E+00 | 0.000E+00 |
| Unigene96697 | Acinetoba | 0.000E+00 | 9.288E-07 | 6.643E-07 | 0.000E+00 | 6.178E-07 | 0.000E+00 | 1.739E-06 | 0.000E+00 | 0.000E+00 | 0.000E+00 | 0.000E+00 | 0.000E+00 |
| Unigene96700 | vanHD | 0.000E+00 | 1.151E-06 | 0.000E+00 | 0.000E+00 | 6.766E-07 | 0.000E+00 | 1.535E-06 | 0.000E+00 | 0.000E+00 | 0.000E+00 | 0.000E+00 | 0.000E+00 |
| Unigene96810 | Streptomy | 9.546E-08 | 8.681E-07 | 4.015E-07 | 2.479E-07 | 1.212E-07 | 0.000E+00 | 3.924E-07 | 0.000E+00 | 0.000E+00 | 0.000E+00 | 0.000E+00 | 0.000E+00 |
| Unigene96822 | AAC(6')-In | 9.014E-07 | 1.051E-06 | 6.523E-06 | 7.680E-06 | 0.000E+00 | 0.000E+00 | 4.988E-07 | 0.000E+00 | 0.000E+00 | 0.000E+00 | 0.000E+00 | 0.000E+00 |
| Unigene96834 | tetA(60) | 0.000E+00 | 1.907E-05 | 0.000E+00 | 0.000E+00 | 0.000E+00 | 0.000E+00 | 0.000E+00 | 0.000E+00 | 0.000E+00 | 0.000E+00 | 0.000E+00 | 0.000E+00 |
| Unigene96886 | TaeA | 4.338E-06 | 9.807E-06 | 9.376E-06 | 1.210E-05 | 0.000E+00 | 0.000E+00 | 0.000E+00 | 1.005E-07 | 0.000E+00 | 1.534E-07 | 0.000E+00 | 0.000E+00 |
| Unigene96890 | efrA | 0.000E+00 | 3.527E-06 | 2.306E-06 | 2.281E-06 | 0.000E+00 | 0.000E+00 | 0.000E+00 | 0.000E+00 | 0.000E+00 | 0.000E+00 | 0.000E+00 | 0.000E+00 |
| Unigene96891 | MexK | 0.000E+00 | 7.310E-07 | 2.042E-07 | 0.000E+00 | 0.000E+00 | 0.000E+00 | 0.000E+00 | 0.000E+00 | 0.000E+00 | 0.000E+00 | 0.000E+00 | 0.000E+00 |
| Unigene97046 | basS | 0.000E+00 | 1.369E-06 | 0.000E+00 | 0.000E+00 | 0.000E+00 | 0.000E+00 | 0.000E+00 | 1.820E-07 | 1.364E-07 | 0.000E+00 | 0.000E+00 | 0.000E+00 |
| Unigene97126 | TaeA | 1.956E-07 | 6.442E-07 | 0.000E+00 | 0.000E+00 | 0.000E+00 | 0.000E+00 | 0.000E+00 | 0.000E+00 | 0.000E+00 | 0.000E+00 | 0.000E+00 | 0.000E+00 |
| Unigene97214 | smeC | 3.596E-07 | 6.982E-07 | 4.343E-07 | 3.073E-07 | 0.000E+00 | 0.000E+00 | 0.000E+00 | 0.000E+00 | 0.000E+00 | 0.000E+00 | 0.000E+00 | 0.000E+00 |
| Unigene97221 | vanF | 2.652E-05 | 1.141E-06 | 3.032E-07 | 0.000E+00 | 0.000E+00 | 0.000E+00 | 0.000E+00 | 0.000E+00 | 0.000E+00 | 0.000E+00 | 0.000E+00 | 0.000E+00 |
| Unigene97324 | tetB(P) | 1.301E-05 | 4.221E-06 | 3.111E-06 | 1.208E-05 | 8.763E-05 | 1.181E-04 | 1.176E-04 | 9.460E-05 | 1.477E-04 | 1.489E-04 | 1.175E-04 | 7.566E-05 |
| Unigene97334 | ANT(4')-Ib | 0.000E+00 | 3.762E-06 | 9.366E-07 | 2.629E-07 | 0.000E+00 | 0.000E+00 | 0.000E+00 | 0.000E+00 | 0.000E+00 | 0.000E+00 | 0.000E+00 | 7.180E-07 |
| Unigene97359 | TaeA | 2.410E-07 | 1.455E-06 | 1.250E-06 | 1.269E-06 | 0.000E+00 | 5.583E-08 | 0.000E+00 | 0.000E+00 | 0.000E+00 | 0.000E+00 | 0.000E+00 | 0.000E+00 |
| Unigene97360 | dfrE | 0.000E+00 | 1.145E-06 | 1.984E-06 | 1.515E-06 | 0.000E+00 | 0.000E+00 | 0.000E+00 | 0.000E+00 | 0.000E+00 | 0.000E+00 | 0.000E+00 | 0.000E+00 |
| Unigene97380 | tetA(58) | 6.388E-05 | 5.097E-05 | 9.694E-05 | 1.321E-04 | 1.062E-04 | 7.071E-05 | 9.946E-05 | 8.782E-05 | 7.991E-05 | 1.154E-04 | 1.157E-04 | 1.182E-04 |
| Unigene97384 | arlR | 1.356E-07 | 1.240E-06 | 0.000E+00 | 0.000E+00 | 4.872E-06 | 0.000E+00 | 0.000E+00 | 0.000E+00 | 0.000E+00 | 0.000E+00 | 0.000E+00 | 0.000E+00 |
| Unigene97417 | emrB | 0.000E+00 | 8.902E-07 | 6.101E-07 | 0.000E+00 | 0.000E+00 | 0.000E+00 | 0.000E+00 | 0.000E+00 | 0.000E+00 | 0.000E+00 | 0.000E+00 | 0.000E+00 |
| Unigene97427 | macB | 0.000E+00 | 3.668E-06 | 2.696E-07 | 0.000E+00 | 0.000E+00 | 0.000E+00 | 0.000E+00 | 4.276E-07 | 1.001E-07 | 0.000E+00 | 0.000E+00 | 0.000E+00 |
| Unigene97438 | efrA | 2.685E-06 | 3.889E-06 | 2.928E-06 | 6.083E-06 | 2.486E-07 | 4.032E-08 | 4.747E-07 | 0.000E+00 | 2.038E-07 | 0.000E+00 | 0.000E+00 | 2.204E-07 |
| Unigene97500 | vatA | 0.000E+00 | 6.471E-07 | 1.302E-06 | 9.923E-08 | 5.458E-08 | 0.000E+00 | 0.000E+00 | 5.735E-08 | 1.612E-04 | 0.000E+00 | 0.000E+00 | 0.000E+00 |
| Unigene97519 | farB | 8.053E-07 | 1.080E-06 | 4.061E-06 | 6.032E-06 | 0.000E+00 | 0.000E+00 | 5.199E-07 | 0.000E+00 | 0.000E+00 | 0.000E+00 | 0.000E+00 | 0.000E+00 |
| Unigene97627 | patA | 0.000E+00 | 1.354E-06 | 1.252E-07 | 0.000E+00 | 2.362E-07 | 0.000E+00 | 0.000E+00 | 0.000E+00 | 0.000E+00 | 0.000E+00 | 0.000E+00 | 0.000E+00 |
| Unigene97704 | patA | 1.105E-04 | 1.964E-04 | 1.686E-04 | 0.000E+00 | 9.526E-06 | 3.404E-07 | 4.345E-06 | 1.816E-06 | 5.330E-05 | 0.000E+00 | 3.368E-06 | 7.443E-07 |
| Unigene97756 | tva(A) | 6.936E-05 | 6.143E-05 | 1.172E-04 | 1.614E-04 | 1.360E-04 | 9.739E-05 | 1.164E-04 | 1.169E-04 | 9.582E-05 | 1.496E-04 | 1.458E-04 | 1.601E-04 |
| Unigene97877 | mdtC | 0.000E+00 | 3.059E-07 | 0.000E+00 | 0.000E+00 | 0.000E+00 | 0.000E+00 | 0.000E+00 | 0.000E+00 | 0.000E+00 | 0.000E+00 | 0.000E+00 | 0.000E+00 |
| Unigene97933 | TolC | 0.000E+00 | 0.000E+00 | 0.000E+00 | 0.000E+00 | 2.255E-07 | 0.000E+00 | 0.000E+00 | 0.000E+00 | 0.000E+00 | 0.000E+00 | 0.000E+00 | 0.000E+00 |
| Unigene97997 | tetA(60) | 0.000E+00 | 4.853E-07 | 0.000E+00 | 0.000E+00 | 0.000E+00 | 0.000E+00 | 0.000E+00 | 0.000E+00 | 0.000E+00 | 0.000E+00 | 0.000E+00 | 0.000E+00 |
| Unigene98122 | bcrA | 0.000E+00 | 6.418E-07 | 0.000E+00 | 0.000E+00 | 0.000E+00 | 0.000E+00 | 0.000E+00 | 0.000E+00 | 0.000E+00 | 0.000E+00 | 0.000E+00 | 0.000E+00 |
| Unigene98138 | novA | 0.000E+00 | 1.387E-06 | 0.000E+00 | 1.271E-05 | 0.000E+00 | 0.000E+00 | 0.000E+00 | 0.000E+00 | 0.000E+00 | 0.000E+00 | 0.000E+00 | 0.000E+00 |
| Unigene98146 | macB | 0.000E+00 | 1.089E-06 | 2.160E-06 | 1.879E-06 | 0.000E+00 | 0.000E+00 | 0.000E+00 | 0.000E+00 | 0.000E+00 | 0.000E+00 | 0.000E+00 | 0.000E+00 |
| Unigene98252 | carA | 4.383E-07 | 1.227E-06 | 5.376E-07 | 2.877E-07 | 5.721E-06 | 0.000E+00 | 0.000E+00 | 1.535E-07 | 0.000E+00 | 0.000E+00 | 0.000E+00 | 0.000E+00 |
| Unigene98362 | arlR | 0.000E+00 | 1.074E-06 | 2.514E-06 | 4.516E-06 | 0.000E+00 | 0.000E+00 | 0.000E+00 | 0.000E+00 | 0.000E+00 | 0.000E+00 | 0.000E+00 | 0.000E+00 |

| | | | | | | | | | | | | | |
|---|---|---|---|---|---|---|---|---|---|---|---|---|---|
| Unigene98394 | otr(B) | 4.728E-06 | 1.911E-05 | 3.469E-06 | 7.586E-07 | 1.006E-06 | 5.255E-07 | 4.891E-08 | 4.642E-07 | 1.980E-06 | 4.184E-07 | 3.033E-07 | 3.134E-07 |
| Unigene98476 | kdpE | 2.857E-07 | 7.838E-07 | 1.869E-07 | 0.000E+00 | 0.000E+00 | 0.000E+00 | 0.000E+00 | 0.000E+00 | 0.000E+00 | 0.000E+00 | 0.000E+00 | 0.000E+00 |
| Unigene98599 | Staphylocc | 2.490E-05 | 1.080E-04 | 3.343E-05 | 1.447E-05 | 1.014E-04 | 2.022E-05 | 2.283E-05 | 2.177E-05 | 6.024E-05 | 0.000E+00 | 1.411E-05 | 3.360E-06 |
| Unigene98624 | bacA | 0.000E+00 | 1.580E-04 | 0.000E+00 | 2.746E-07 | 0.000E+00 | 0.000E+00 | 1.462E-06 | 0.000E+00 | 1.911E-06 | 0.000E+00 | 0.000E+00 | 0.000E+00 |
| Unigene98625 | bcrA | 0.000E+00 | 1.492E-04 | 8.306E-05 | 0.000E+00 | 0.000E+00 | 0.000E+00 | 7.027E-07 | 0.000E+00 | 2.666E-06 | 0.000E+00 | 0.000E+00 | 0.000E+00 |
| Unigene98641 | lmrB | 7.064E-08 | 1.266E-06 | 3.581E-06 | 4.304E-06 | 0.000E+00 | 0.000E+00 | 0.000E+00 | 0.000E+00 | 0.000E+00 | 0.000E+00 | 0.000E+00 | 0.000E+00 |
| Unigene98740 | Pseudomo | 0.000E+00 | 3.859E-07 | 0.000E+00 | 0.000E+00 | 1.563E-07 | 2.281E-07 | 0.000E+00 | 0.000E+00 | 0.000E+00 | 0.000E+00 | 0.000E+00 | 0.000E+00 |
| Unigene98747 | Staphylocc | 7.054E-05 | 6.321E-05 | 1.188E-04 | 1.702E-04 | 1.346E-04 | 9.110E-05 | 1.210E-04 | 1.160E-04 | 1.018E-04 | 1.482E-04 | 1.490E-04 | 1.602E-04 |
| Unigene98784 | msrE | 1.685E-07 | 7.164E-07 | 4.752E-07 | 1.488E-07 | 0.000E+00 | 0.000E+00 | 0.000E+00 | 0.000E+00 | 0.000E+00 | 0.000E+00 | 0.000E+00 | 0.000E+00 |
| Unigene98813 | GOB-6 | 0.000E+00 | 6.854E-07 | 1.885E-07 | 0.000E+00 | 0.000E+00 | 0.000E+00 | 0.000E+00 | 0.000E+00 | 0.000E+00 | 0.000E+00 | 0.000E+00 | 0.000E+00 |
| Unigene98840 | cmrA | 1.335E-07 | 7.912E-07 | 4.192E-06 | 4.233E-06 | 0.000E+00 | 0.000E+00 | 0.000E+00 | 0.000E+00 | 0.000E+00 | 0.000E+00 | 0.000E+00 | 0.000E+00 |
| Unigene98871 | Klebsiella p | 1.464E-07 | 4.820E-07 | 0.000E+00 | 0.000E+00 | 0.000E+00 | 0.000E+00 | 0.000E+00 | 0.000E+00 | 0.000E+00 | 0.000E+00 | 0.000E+00 | 0.000E+00 |
| Unigene98875 | vanN | 1.824E-07 | 5.671E-07 | 1.790E-07 | 6.753E-07 | 0.000E+00 | 0.000E+00 | 0.000E+00 | 0.000E+00 | 0.000E+00 | 0.000E+00 | 0.000E+00 | 0.000E+00 |
| Unigene98954 | efrA | 5.231E-06 | 1.066E-05 | 1.098E-05 | 1.081E-05 | 0.000E+00 | 4.395E-08 | 0.000E+00 | 0.000E+00 | 0.000E+00 | 3.019E-07 | 0.000E+00 | 0.000E+00 |
| Unigene99059 | basS | 0.000E+00 | 8.950E-07 | 0.000E+00 | 0.000E+00 | 0.000E+00 | 0.000E+00 | 1.504E-06 | 2.380E-07 | 0.000E+00 | 0.000E+00 | 0.000E+00 | 0.000E+00 |
| Unigene99068 | patA | 0.000E+00 | 1.376E-06 | 0.000E+00 | 0.000E+00 | 0.000E+00 | 0.000E+00 | 0.000E+00 | 0.000E+00 | 0.000E+00 | 0.000E+00 | 0.000E+00 | 0.000E+00 |
| Unigene99110 | AcrS | 0.000E+00 | 7.481E-07 | 3.201E-06 | 4.228E-06 | 0.000E+00 | 0.000E+00 | 0.000E+00 | 0.000E+00 | 0.000E+00 | 0.000E+00 | 0.000E+00 | 0.000E+00 |
| Unigene99121 | arlR | 4.331E-06 | 8.457E-06 | 1.002E-05 | 1.142E-05 | 0.000E+00 | 0.000E+00 | 0.000E+00 | 0.000E+00 | 0.000E+00 | 0.000E+00 | 0.000E+00 | 0.000E+00 |
| Unigene99128 | lin | 1.532E-07 | 7.565E-07 | 1.879E-07 | 3.480E-07 | 0.000E+00 | 4.140E-08 | 0.000E+00 | 0.000E+00 | 0.000E+00 | 0.000E+00 | 0.000E+00 | 0.000E+00 |
| Unigene99139 | Acinetobac | 5.799E-07 | 2.687E-06 | 1.370E-06 | 8.892E-07 | 0.000E+00 | 0.000E+00 | 1.830E-06 | 5.265E-07 | 0.000E+00 | 0.000E+00 | 7.125E-07 | 0.000E+00 |
| Unigene99219 | rosB | 0.000E+00 | 1.359E-06 | 0.000E+00 | 0.000E+00 | 0.000E+00 | 0.000E+00 | 0.000E+00 | 0.000E+00 | 0.000E+00 | 0.000E+00 | 0.000E+00 | 0.000E+00 |
| Unigene99224 | tetA(58) | 4.422E-06 | 1.127E-05 | 1.223E-05 | 1.417E-05 | 0.000E+00 | 0.000E+00 | 0.000E+00 | 0.000E+00 | 0.000E+00 | 0.000E+00 | 0.000E+00 | 0.000E+00 |
| Unigene99296 | tetA(58) | 5.153E-05 | 4.553E-05 | 9.328E-05 | 1.362E-04 | 1.097E-04 | 6.926E-05 | 9.399E-05 | 8.403E-05 | 7.737E-05 | 1.105E-04 | 1.107E-04 | 1.042E-04 |
| Unigene99300 | mdtC | 0.000E+00 | 5.368E-07 | 0.000E+00 | 0.000E+00 | 0.000E+00 | 0.000E+00 | 0.000E+00 | 0.000E+00 | 0.000E+00 | 0.000E+00 | 0.000E+00 | 0.000E+00 |
| Unigene99387 | golS | 0.000E+00 | 7.991E-07 | 0.000E+00 | 8.170E-08 | 0.000E+00 | 0.000E+00 | 0.000E+00 | 0.000E+00 | 0.000E+00 | 0.000E+00 | 0.000E+00 | 0.000E+00 |
| Unigene99395 | evgA | 1.177E-05 | 4.081E-06 | 2.377E-06 | 1.166E-05 | 7.053E-05 | 9.982E-05 | 9.995E-05 | 7.371E-05 | 1.154E-04 | 1.121E-04 | 9.544E-05 | 5.807E-05 |
| Unigene99407 | vanRM | 6.055E-07 | 4.652E-06 | 3.154E-06 | 2.070E-06 | 0.000E+00 | 0.000E+00 | 0.000E+00 | 0.000E+00 | 0.000E+00 | 0.000E+00 | 0.000E+00 | 0.000E+00 |
| Unigene99409 | macB | 3.843E-07 | 5.108E-06 | 2.137E-06 | 2.630E-06 | 0.000E+00 | 0.000E+00 | 0.000E+00 | 0.000E+00 | 0.000E+00 | 0.000E+00 | 0.000E+00 | 0.000E+00 |
| Unigene99467 | tetB(46) | 0.000E+00 | 6.131E-07 | 3.462E-07 | 1.188E-07 | 0.000E+00 | 0.000E+00 | 1.301E-07 | 0.000E+00 | 0.000E+00 | 0.000E+00 | 0.000E+00 | 0.000E+00 |
| Unigene99506 | efrA | 4.922E-06 | 9.776E-06 | 9.658E-06 | 1.132E-05 | 0.000E+00 | 0.000E+00 | 4.142E-08 | 0.000E+00 | 0.000E+00 | 0.000E+00 | 0.000E+00 | 0.000E+00 |
| Unigene99567 | bcrA | 8.713E-08 | 4.781E-07 | 0.000E+00 | 0.000E+00 | 4.840E-08 | 0.000E+00 | 0.000E+00 | 0.000E+00 | 0.000E+00 | 0.000E+00 | 0.000E+00 | 0.000E+00 |
| Unigene99683 | lmrC | 0.000E+00 | 6.216E-07 | 0.000E+00 | 3.959E-07 | 0.000E+00 | 0.000E+00 | 0.000E+00 | 0.000E+00 | 0.000E+00 | 0.000E+00 | 0.000E+00 | 0.000E+00 |
| Unigene99758 | FusF | 1.233E-05 | 5.720E-06 | 3.900E-06 | 1.253E-05 | 9.971E-05 | 1.353E-04 | 1.393E-04 | 1.131E-04 | 1.803E-04 | 1.749E-04 | 1.374E-04 | 8.868E-05 |
| Unigene99926 | Staphylocc | 4.239E-05 | 2.528E-05 | 2.052E-05 | 2.113E-05 | 2.171E-06 | 1.822E-06 | 3.609E-06 | 5.417E-07 | 0.000E+00 | 7.891E-07 | 1.746E-06 | 1.227E-06 |
| Unigene99931 | tetB(46) | 0.000E+00 | 6.776E-07 | 0.000E+00 | 0.000E+00 | 0.000E+00 | 0.000E+00 | 0.000E+00 | 0.000E+00 | 0.000E+00 | 0.000E+00 | 0.000E+00 | 0.000E+00 |
| Unigene99977 | facT | 0.000E+00 | 8.304E-07 | 3.238E-06 | 3.799E-06 | 0.000E+00 | 0.000E+00 | 0.000E+00 | 0.000E+00 | 0.000E+00 | 0.000E+00 | 0.000E+00 | 0.000E+00 |
| Unigene99996 | Listeria mc | 7.079E-08 | 6.216E-07 | 3.126E-07 | 1.787E-07 | 0.000E+00 | 0.000E+00 | 1.959E-07 | 0.000E+00 | 0.000E+00 | 0.000E+00 | 0.000E+00 | 0.000E+00 |
| Unigene100045 | patA | 0.000E+00 | 3.085E-07 | 0.000E+00 | 0.000E+00 | 0.000E+00 | 0.000E+00 | 0.000E+00 | 0.000E+00 | 0.000E+00 | 0.000E+00 | 0.000E+00 | 0.000E+00 |
| Unigene100054 | lsaC | 0.000E+00 | 1.079E-06 | 0.000E+00 | 0.000E+00 | 0.000E+00 | 0.000E+00 | 0.000E+00 | 0.000E+00 | 0.000E+00 | 0.000E+00 | 0.000E+00 | 0.000E+00 |
| Unigene100078 | vanSL | 1.758E-07 | 3.514E-06 | 1.737E-06 | 1.610E-06 | 0.000E+00 | 0.000E+00 | 1.390E-08 | 0.000E+00 | 0.000E+00 | 0.000E+00 | 4.309E-08 | 0.000E+00 |
| Unigene100079 | kdpE | 0.000E+00 | 3.207E-06 | 1.729E-06 | 1.311E-06 | 0.000E+00 | 0.000E+00 | 0.000E+00 | 0.000E+00 | 0.000E+00 | 1.550E-07 | 0.000E+00 | 0.000E+00 |
| Unigene100081 | mtrA | 3.304E-07 | 3.937E-06 | 2.315E-06 | 2.097E-06 | 0.000E+00 | 0.000E+00 | 0.000E+00 | 0.000E+00 | 0.000E+00 | 0.000E+00 | 0.000E+00 | 0.000E+00 |
| Unigene100269 | mdtN | 0.000E+00 | 5.135E-07 | 3.884E-07 | 0.000E+00 | 0.000E+00 | 0.000E+00 | 0.000E+00 | 0.000E+00 | 0.000E+00 | 0.000E+00 | 0.000E+00 | 0.000E+00 |
| Unigene100276 | patB | 0.000E+00 | 6.101E-07 | 0.000E+00 | 0.000E+00 | 0.000E+00 | 0.000E+00 | 0.000E+00 | 0.000E+00 | 0.000E+00 | 0.000E+00 | 0.000E+00 | 0.000E+00 |
| Unigene100305 | macB | 0.000E+00 | 4.980E-07 | 1.908E-07 | 0.000E+00 | 0.000E+00 | 0.000E+00 | 0.000E+00 | 0.000E+00 | 0.000E+00 | 0.000E+00 | 0.000E+00 | 0.000E+00 |
| Unigene100355 | Erm(48) | 1.475E-05 | 5.169E-06 | 4.479E-06 | 1.573E-05 | 9.998E-05 | 1.374E-04 | 1.498E-04 | 1.198E-04 | 1.708E-04 | 1.826E-04 | 1.472E-04 | 8.629E-05 |
| Unigene100393 | salA | 1.289E-07 | 1.320E-06 | 2.445E-06 | 2.928E-06 | 0.000E+00 | 2.322E-08 | 0.000E+00 | 0.000E+00 | 0.000E+00 | 0.000E+00 | 0.000E+00 | 0.000E+00 |
| Unigene100395 | srmB | 9.223E-07 | 7.086E-06 | 1.140E-05 | 1.576E-05 | 0.000E+00 | 0.000E+00 | 0.000E+00 | 3.987E-08 | 0.000E+00 | 0.000E+00 | 7.815E-08 | 0.000E+00 |
| Unigene100402 | vanRL | 0.000E+00 | 3.459E-07 | 1.767E-07 | 1.364E-07 | 0.000E+00 | 0.000E+00 | 0.000E+00 | 1.051E-07 | 0.000E+00 | 0.000E+00 | 0.000E+00 | 0.000E+00 |
| Unigene100461 | Streptomy | 2.388E-07 | 1.083E-06 | 1.374E-06 | 1.993E-06 | 0.000E+00 | 0.000E+00 | 0.000E+00 | 0.000E+00 | 0.000E+00 | 0.000E+00 | 0.000E+00 | 0.000E+00 |
| Unigene100494 | vanG | 0.000E+00 | 8.045E-07 | 8.055E-07 | 2.072E-07 | 0.000E+00 | 1.585E-07 | 1.136E-06 | 0.000E+00 | 0.000E+00 | 0.000E+00 | 0.000E+00 | 0.000E+00 |
| Unigene100496 | mdtG | 0.000E+00 | 1.322E-06 | 6.422E-07 | 2.379E-07 | 5.526E-07 | 0.000E+00 | 9.562E-07 | 0.000E+00 | 0.000E+00 | 0.000E+00 | 0.000E+00 | 0.000E+00 |
| Unigene100528 | mefE | 4.165E-07 | 5.714E-07 | 1.703E-07 | 0.000E+00 | 0.000E+00 | 1.501E-07 | 0.000E+00 | 0.000E+00 | 0.000E+00 | 0.000E+00 | 0.000E+00 | 0.000E+00 |
| Unigene100529 | PEDO-2 | 0.000E+00 | 9.606E-07 | 3.385E-06 | 3.015E-06 | 0.000E+00 | 0.000E+00 | 0.000E+00 | 0.000E+00 | 0.000E+00 | 0.000E+00 | 0.000E+00 | 0.000E+00 |
| Unigene100555 | rosA | 2.997E-08 | 7.895E-07 | 3.235E-07 | 1.513E-07 | 0.000E+00 | 0.000E+00 | 0.000E+00 | 0.000E+00 | 0.000E+00 | 0.000E+00 | 0.000E+00 | 0.000E+00 |
| Unigene100627 | optrA | 0.000E+00 | 1.697E-06 | 2.337E-06 | 1.456E-06 | 0.000E+00 | 0.000E+00 | 0.000E+00 | 0.000E+00 | 0.000E+00 | 0.000E+00 | 0.000E+00 | 0.000E+00 |
| Unigene100635 | YojI | 0.000E+00 | 1.357E-06 | 0.000E+00 | 0.000E+00 | 0.000E+00 | 0.000E+00 | 0.000E+00 | 0.000E+00 | 0.000E+00 | 0.000E+00 | 0.000E+00 | 0.000E+00 |
| Unigene100642 | bacA | 0.000E+00 | 1.588E-06 | 2.077E-06 | 4.809E-06 | 0.000E+00 | 0.000E+00 | 0.000E+00 | 0.000E+00 | 0.000E+00 | 0.000E+00 | 0.000E+00 | 0.000E+00 |

| | | | | | | | | | | | | | |
|---|---|---|---|---|---|---|---|---|---|---|---|---|---|
| Unigene100678 | CRP | 0.000E+00 | 7.414E-07 | 3.740E-06 | 4.531E-06 | 0.000E+00 | 0.000E+00 | 5.340E-08 | 0.000E+00 | 0.000E+00 | 0.000E+00 | 0.000E+00 | 0.000E+00 |
| Unigene100707 | cpxA | 3.689E-07 | 1.653E-06 | 1.508E-06 | 1.739E-06 | 0.000E+00 | 0.000E+00 | 0.000E+00 | 0.000E+00 | 0.000E+00 | 0.000E+00 | 0.000E+00 | 0.000E+00 |
| Unigene100805 | rosB | 0.000E+00 | 3.645E-07 | 0.000E+00 | 6.707E-08 | 0.000E+00 | 0.000E+00 | 0.000E+00 | 0.000E+00 | 0.000E+00 | 0.000E+00 | 1.520E-07 | 0.000E+00 |
| Unigene100871 | msrA | 1.086E-04 | 1.975E-04 | 1.728E-04 | 0.000E+00 | 7.882E-06 | 0.000E+00 | 3.796E-06 | 0.000E+00 | 5.362E-05 | 2.617E-07 | 3.225E-06 | 0.000E+00 |
| Unigene100882 | bcrA | 0.000E+00 | 6.384E-07 | 0.000E+00 | 0.000E+00 | 0.000E+00 | 0.000E+00 | 0.000E+00 | 0.000E+00 | 0.000E+00 | 0.000E+00 | 0.000E+00 | 0.000E+00 |
| Unigene100971 | vanSL | 0.000E+00 | 1.120E-06 | 2.162E-06 | 1.616E-06 | 0.000E+00 | 0.000E+00 | 0.000E+00 | 0.000E+00 | 0.000E+00 | 0.000E+00 | 0.000E+00 | 0.000E+00 |
| Unigene101063 | efrB | 0.000E+00 | 1.450E-06 | 2.593E-06 | 1.334E-06 | 0.000E+00 | 0.000E+00 | 0.000E+00 | 0.000E+00 | 0.000E+00 | 0.000E+00 | 0.000E+00 | 0.000E+00 |
| Unigene101075 | optrA | 0.000E+00 | 1.143E-06 | 1.865E-06 | 1.235E-06 | 0.000E+00 | 0.000E+00 | 0.000E+00 | 0.000E+00 | 0.000E+00 | 0.000E+00 | 0.000E+00 | 0.000E+00 |
| Unigene101107 | macB | 3.658E-06 | 9.654E-06 | 8.420E-06 | 8.448E-06 | 0.000E+00 | 0.000E+00 | 0.000E+00 | 3.140E-08 | 0.000E+00 | 0.000E+00 | 0.000E+00 | 0.000E+00 |
| Unigene101184 | patA | 0.000E+00 | 6.594E-07 | 0.000E+00 | 0.000E+00 | 0.000E+00 | 0.000E+00 | 0.000E+00 | 0.000E+00 | 0.000E+00 | 0.000E+00 | 0.000E+00 | 0.000E+00 |
| Unigene101271 | Corynebac | 0.000E+00 | 0.000E+00 | 0.000E+00 | 0.000E+00 | 3.321E-07 | 0.000E+00 | 0.000E+00 | 0.000E+00 | 0.000E+00 | 0.000E+00 | 0.000E+00 | 0.000E+00 |
| Unigene101360 | oleC | 6.616E-07 | 2.102E-06 | 1.025E-06 | 0.000E+00 | 0.000E+00 | 0.000E+00 | 0.000E+00 | 0.000E+00 | 0.000E+00 | 0.000E+00 | 0.000E+00 | 0.000E+00 |
| Unigene101389 | sul4 | 0.000E+00 | 6.259E-07 | 0.000E+00 | 0.000E+00 | 0.000E+00 | 0.000E+00 | 1.978E-06 | 0.000E+00 | 6.233E-07 | 0.000E+00 | 0.000E+00 | 0.000E+00 |
| Unigene101472 | macB | 0.000E+00 | 1.692E-06 | 0.000E+00 | 1.700E-06 | 0.000E+00 | 0.000E+00 | 0.000E+00 | 0.000E+00 | 0.000E+00 | 0.000E+00 | 0.000E+00 | 0.000E+00 |
| Unigene101550 | efmA | 0.000E+00 | 4.086E-07 | 0.000E+00 | 0.000E+00 | 0.000E+00 | 0.000E+00 | 0.000E+00 | 0.000E+00 | 0.000E+00 | 0.000E+00 | 0.000E+00 | 0.000E+00 |
| Unigene101591 | arnA | 0.000E+00 | 5.399E-07 | 0.000E+00 | 0.000E+00 | 0.000E+00 | 0.000E+00 | 0.000E+00 | 0.000E+00 | 0.000E+00 | 0.000E+00 | 0.000E+00 | 0.000E+00 |
| Unigene101665 | vanTG | 7.231E-07 | 6.190E-06 | 1.005E-05 | 1.487E-05 | 0.000E+00 | 0.000E+00 | 0.000E+00 | 0.000E+00 | 0.000E+00 | 0.000E+00 | 0.000E+00 | 0.000E+00 |
| Unigene101696 | macB | 5.332E-07 | 3.990E-06 | 5.089E-06 | 1.415E-05 | 0.000E+00 | 0.000E+00 | 0.000E+00 | 0.000E+00 | 0.000E+00 | 0.000E+00 | 0.000E+00 | 0.000E+00 |
| Unigene101700 | mtrA | 4.932E-07 | 5.314E-06 | 8.447E-06 | 1.340E-05 | 0.000E+00 | 0.000E+00 | 0.000E+00 | 0.000E+00 | 0.000E+00 | 0.000E+00 | 0.000E+00 | 0.000E+00 |
| Unigene101757 | arlS | 1.972E-07 | 3.920E-06 | 1.957E-06 | 1.881E-06 | 0.000E+00 | 2.369E-08 | 0.000E+00 | 0.000E+00 | 0.000E+00 | 0.000E+00 | 0.000E+00 | 0.000E+00 |
| Unigene101780 | rpoB2 | 0.000E+00 | 3.889E-07 | 0.000E+00 | 0.000E+00 | 0.000E+00 | 0.000E+00 | 4.992E-07 | 4.136E-07 | 0.000E+00 | 0.000E+00 | 4.791E-07 | 3.046E-07 |
| Unigene101840 | macB | 0.000E+00 | 0.000E+00 | 3.118E-06 | 3.850E-06 | 0.000E+00 | 0.000E+00 | 0.000E+00 | 0.000E+00 | 0.000E+00 | 0.000E+00 | 0.000E+00 | 0.000E+00 |
| Unigene101859 | macB | 0.000E+00 | 3.062E-07 | 2.346E-07 | 0.000E+00 | 0.000E+00 | 0.000E+00 | 0.000E+00 | 0.000E+00 | 0.000E+00 | 0.000E+00 | 0.000E+00 | 0.000E+00 |
| Unigene101965 | bcrA | 2.952E-07 | 8.835E-07 | 1.580E-07 | 1.355E-07 | 0.000E+00 | 0.000E+00 | 0.000E+00 | 0.000E+00 | 0.000E+00 | 0.000E+00 | 0.000E+00 | 0.000E+00 |
| Unigene102012 | efrB | 3.420E-06 | 8.764E-06 | 8.784E-06 | 9.998E-06 | 0.000E+00 | 1.935E-07 | 7.926E-08 | 6.269E-08 | 0.000E+00 | 1.596E-07 | 0.000E+00 | 0.000E+00 |
| Unigene102015 | emeA | 2.701E-05 | 1.034E-06 | 3.928E-07 | 2.378E-08 | 2.616E-08 | 0.000E+00 | 0.000E+00 | 0.000E+00 | 0.000E+00 | 0.000E+00 | 0.000E+00 | 0.000E+00 |
| Unigene102091 | rosA | 0.000E+00 | 1.323E-06 | 0.000E+00 | 0.000E+00 | 8.296E-06 | 0.000E+00 | 0.000E+00 | 0.000E+00 | 0.000E+00 | 0.000E+00 | 0.000E+00 | 0.000E+00 |
| Unigene102204 | mdtM | 0.000E+00 | 7.531E-07 | 0.000E+00 | 3.464E-07 | 0.000E+00 | 0.000E+00 | 0.000E+00 | 0.000E+00 | 0.000E+00 | 0.000E+00 | 0.000E+00 | 0.000E+00 |
| Unigene102232 | vanTC | 9.088E-06 | 3.243E-06 | 2.134E-06 | 7.600E-06 | 5.810E-05 | 7.844E-05 | 8.493E-05 | 6.563E-05 | 9.765E-05 | 0.000E+00 | 0.000E+00 | 4.340E-05 |
| Unigene102287 | macB | 0.000E+00 | 1.485E-06 | 8.361E-07 | 0.000E+00 | 5.569E-07 | 0.000E+00 | 1.350E-06 | 0.000E+00 | 0.000E+00 | 0.000E+00 | 0.000E+00 | 0.000E+00 |
| Unigene102290 | PmrF | 5.642E-07 | 5.308E-06 | 5.141E-07 | 0.000E+00 | 0.000E+00 | 0.000E+00 | 0.000E+00 | 0.000E+00 | 3.524E-07 | 0.000E+00 | 0.000E+00 | 0.000E+00 |
| Unigene102332 | fusD | 3.487E-07 | 6.014E-06 | 7.772E-06 | 1.474E-05 | 0.000E+00 | 0.000E+00 | 0.000E+00 | 0.000E+00 | 0.000E+00 | 0.000E+00 | 0.000E+00 | 0.000E+00 |
| Unigene102402 | facT | 3.427E-07 | 7.522E-07 | 2.914E-07 | 5.075E-07 | 0.000E+00 | 0.000E+00 | 0.000E+00 | 0.000E+00 | 0.000E+00 | 0.000E+00 | 0.000E+00 | 8.101E-08 |
| Unigene102431 | acrB | 4.740E-08 | 3.642E-07 | 0.000E+00 | 0.000E+00 | 0.000E+00 | 0.000E+00 | 0.000E+00 | 0.000E+00 | 0.000E+00 | 0.000E+00 | 0.000E+00 | 0.000E+00 |
| Unigene102534 | vanG | 0.000E+00 | 7.006E-07 | 2.237E-07 | 0.000E+00 | 0.000E+00 | 0.000E+00 | 0.000E+00 | 0.000E+00 | 0.000E+00 | 0.000E+00 | 0.000E+00 | 0.000E+00 |
| Unigene102543 | lmrD | 0.000E+00 | 9.924E-07 | 0.000E+00 | 0.000E+00 | 5.960E-06 | 0.000E+00 | 0.000E+00 | 0.000E+00 | 0.000E+00 | 0.000E+00 | 0.000E+00 | 0.000E+00 |
| Unigene102579 | NmcR | 1.048E-06 | 2.799E-06 | 1.817E-06 | 6.703E-07 | 1.075E-05 | 0.000E+00 | 2.243E-06 | 5.301E-07 | 0.000E+00 | 0.000E+00 | 8.792E-07 | 0.000E+00 |
| Unigene102613 | patB | 5.162E-07 | 2.571E-06 | 1.636E-06 | 0.000E+00 | 9.131E-06 | 8.585E-07 | 0.000E+00 | 0.000E+00 | 0.000E+00 | 0.000E+00 | 1.181E-06 | 0.000E+00 |
| Unigene102622 | msbA | 2.768E-07 | 4.558E-06 | 2.372E-06 | 3.094E-06 | 0.000E+00 | 0.000E+00 | 0.000E+00 | 2.154E-08 | 0.000E+00 | 0.000E+00 | 0.000E+00 | 0.000E+00 |
| Unigene102623 | patB | 4.267E-07 | 4.642E-06 | 2.130E-06 | 2.997E-06 | 0.000E+00 | 0.000E+00 | 0.000E+00 | 0.000E+00 | 0.000E+00 | 0.000E+00 | 2.122E-08 | 0.000E+00 |
| Unigene102626 | optrA | 3.795E-07 | 4.851E-06 | 2.453E-06 | 2.570E-06 | 0.000E+00 | 0.000E+00 | 4.941E-08 | 0.000E+00 | 0.000E+00 | 0.000E+00 | 0.000E+00 | 0.000E+00 |
| Unigene102629 | evgA | 7.139E-07 | 4.701E-06 | 2.902E-06 | 2.523E-06 | 0.000E+00 | 0.000E+00 | 0.000E+00 | 0.000E+00 | 0.000E+00 | 0.000E+00 | 0.000E+00 | 0.000E+00 |
| Unigene102843 | msbA | 1.524E-07 | 7.025E-07 | 2.692E-07 | 6.156E-08 | 0.000E+00 | 0.000E+00 | 0.000E+00 | 0.000E+00 | 0.000E+00 | 0.000E+00 | 0.000E+00 | 0.000E+00 |
| Unigene102844 | macB | 1.050E-07 | 9.217E-07 | 3.811E-06 | 3.816E-06 | 0.000E+00 | 0.000E+00 | 0.000E+00 | 0.000E+00 | 0.000E+00 | 0.000E+00 | 0.000E+00 | 0.000E+00 |
| Unigene102868 | rosB | 6.574E-07 | 1.785E-06 | 9.166E-07 | 3.494E-07 | 7.956E-06 | 2.992E-07 | 1.647E-06 | 3.231E-07 | 0.000E+00 | 0.000E+00 | 0.000E+00 | 0.000E+00 |
| Unigene102877 | tetB(60) | 0.000E+00 | 1.724E-06 | 2.303E-06 | 1.732E-06 | 0.000E+00 | 0.000E+00 | 0.000E+00 | 0.000E+00 | 0.000E+00 | 0.000E+00 | 0.000E+00 | 0.000E+00 |
| Unigene102878 | tetA(60) | 0.000E+00 | 1.901E-06 | 2.368E-06 | 1.786E-06 | 0.000E+00 | 0.000E+00 | 0.000E+00 | 0.000E+00 | 0.000E+00 | 0.000E+00 | 0.000E+00 | 0.000E+00 |
| Unigene102883 | efrB | 0.000E+00 | 1.567E-06 | 0.000E+00 | 1.744E-06 | 0.000E+00 | 0.000E+00 | 0.000E+00 | 0.000E+00 | 0.000E+00 | 0.000E+00 | 0.000E+00 | 0.000E+00 |
| Unigene102926 | lmrB | 0.000E+00 | 1.910E-06 | 0.000E+00 | 0.000E+00 | 0.000E+00 | 0.000E+00 | 0.000E+00 | 0.000E+00 | 0.000E+00 | 0.000E+00 | 0.000E+00 | 0.000E+00 |
| Unigene102940 | efrA | 1.928E-07 | 8.042E-07 | 1.892E-07 | 1.558E-07 | 0.000E+00 | 0.000E+00 | 0.000E+00 | 0.000E+00 | 0.000E+00 | 0.000E+00 | 0.000E+00 | 0.000E+00 |
| Unigene103127 | cpxA | 0.000E+00 | 1.125E-06 | 6.575E-07 | 9.950E-08 | 0.000E+00 | 0.000E+00 | 1.592E-06 | 0.000E+00 | 0.000E+00 | 0.000E+00 | 0.000E+00 | 0.000E+00 |
| Unigene103156 | vgaB | 1.018E-06 | 4.756E-06 | 7.904E-06 | 6.901E-06 | 0.000E+00 | 0.000E+00 | 0.000E+00 | 0.000E+00 | 0.000E+00 | 6.479E-08 | 9.980E-08 | 1.031E-07 |
| Unigene103190 | patA | 0.000E+00 | 1.153E-06 | 4.627E-07 | 4.545E-07 | 0.000E+00 | 0.000E+00 | 0.000E+00 | 0.000E+00 | 0.000E+00 | 0.000E+00 | 0.000E+00 | 0.000E+00 |
| Unigene103193 | lmrB | 7.575E-05 | 6.152E-05 | 1.188E-04 | 1.603E-04 | 1.374E-04 | 9.222E-05 | 1.293E-04 | 1.209E-04 | 1.048E-04 | 1.549E-04 | 1.605E-04 | 1.743E-04 |
| Unigene103215 | bcr-1 | 0.000E+00 | 3.710E-07 | 0.000E+00 | 0.000E+00 | 0.000E+00 | 0.000E+00 | 0.000E+00 | 0.000E+00 | 0.000E+00 | 0.000E+00 | 0.000E+00 | 0.000E+00 |
| Unigene103218 | bcrA | 0.000E+00 | 5.905E-07 | 0.000E+00 | 0.000E+00 | 0.000E+00 | 0.000E+00 | 0.000E+00 | 0.000E+00 | 0.000E+00 | 0.000E+00 | 0.000E+00 | 0.000E+00 |
| Unigene103231 | vanKI | 8.833E-07 | 6.815E-06 | 1.003E-05 | 1.500E-05 | 0.000E+00 | 1.157E-07 | 0.000E+00 | 0.000E+00 | 0.000E+00 | 0.000E+00 | 0.000E+00 | 0.000E+00 |
| Unigene103360 | catQ | 6.417E-05 | 5.841E-05 | 1.084E-04 | 1.378E-04 | 1.211E-04 | 7.903E-05 | 1.072E-04 | 1.067E-04 | 9.292E-05 | 1.354E-04 | 1.341E-04 | 1.421E-04 |

| | | | | | | | | | | | | | |
|---|---|---|---|---|---|---|---|---|---|---|---|---|---|
| Unigene103463 | baeS | 0.000E+00 | 7.515E-07 | 1.344E-06 | 1.087E-06 | 0.000E+00 | 0.000E+00 | 0.000E+00 | 0.000E+00 | 0.000E+00 | 0.000E+00 | 0.000E+00 | 0.000E+00 |
| Unigene103464 | macB | 0.000E+00 | 8.324E-07 | 1.302E-06 | 2.106E-06 | 0.000E+00 | 0.000E+00 | 0.000E+00 | 0.000E+00 | 0.000E+00 | 0.000E+00 | 0.000E+00 | 0.000E+00 |
| Unigene103499 | qacA | 6.217E-07 | 5.721E-06 | 6.452E-06 | 1.347E-05 | 0.000E+00 | 1.034E-07 | 0.000E+00 | 0.000E+00 | 0.000E+00 | 0.000E+00 | 0.000E+00 | 0.000E+00 |
| Unigene103523 | smeC | 0.000E+00 | 2.301E-07 | 0.000E+00 | 0.000E+00 | 7.762E-08 | 0.000E+00 | 1.005E-06 | 0.000E+00 | 0.000E+00 | 0.000E+00 | 0.000E+00 | 0.000E+00 |
| Unigene103574 | macB | 0.000E+00 | 4.523E-07 | 2.516E-06 | 1.480E-05 | 0.000E+00 | 0.000E+00 | 0.000E+00 | 0.000E+00 | 0.000E+00 | 0.000E+00 | 0.000E+00 | 0.000E+00 |
| Unigene103596 | efrA | 3.144E-06 | 4.518E-06 | 4.168E-06 | 6.028E-06 | 2.703E-07 | 1.011E-07 | 4.142E-07 | 0.000E+00 | 1.432E-07 | 4.169E-08 | 1.070E-07 | 1.548E-07 |
| Unigene103619 | Acinetoba | 0.000E+00 | 9.394E-07 | 1.680E-07 | 3.210E-07 | 0.000E+00 | 0.000E+00 | 0.000E+00 | 0.000E+00 | 0.000E+00 | 0.000E+00 | 0.000E+00 | 0.000E+00 |
| Unigene103622 | patB | 0.000E+00 | 5.696E-07 | 0.000E+00 | 0.000E+00 | 0.000E+00 | 0.000E+00 | 0.000E+00 | 0.000E+00 | 0.000E+00 | 0.000E+00 | 0.000E+00 | 0.000E+00 |
| Unigene103654 | tetB(P) | 0.000E+00 | 9.430E-07 | 1.907E-06 | 2.024E-06 | 0.000E+00 | 0.000E+00 | 0.000E+00 | 0.000E+00 | 0.000E+00 | 0.000E+00 | 0.000E+00 | 0.000E+00 |
| Unigene103680 | facT | 7.096E-05 | 5.833E-05 | 1.113E-04 | 1.404E-04 | 1.350E-04 | 8.898E-05 | 1.163E-04 | 1.153E-04 | 1.009E-04 | 1.390E-04 | 1.499E-04 | 1.555E-04 |
| Unigene103683 | Rhodococ | 6.979E-05 | 5.959E-05 | 1.191E-04 | 1.731E-04 | 1.349E-04 | 9.809E-05 | 1.247E-04 | 1.136E-04 | 1.064E-04 | 1.518E-04 | 1.632E-04 | 1.644E-04 |
| Unigene103701 | baeR | 3.816E-07 | 5.863E-07 | 2.246E-07 | 4.624E-07 | 1.272E-07 | 1.072E-06 | 1.900E-06 | 0.000E+00 | 4.587E-07 | 0.000E+00 | 0.000E+00 | 9.020E-07 |
| Unigene103739 | AAC(6')-Iz | 0.000E+00 | 5.781E-07 | 0.000E+00 | 0.000E+00 | 0.000E+00 | 0.000E+00 | 0.000E+00 | 0.000E+00 | 0.000E+00 | 0.000E+00 | 0.000E+00 | 0.000E+00 |
| Unigene103841 | evgS | 4.497E-07 | 1.833E-06 | 8.510E-07 | 5.838E-07 | 6.048E-06 | 0.000E+00 | 1.013E-06 | 3.749E-07 | 0.000E+00 | 0.000E+00 | 0.000E+00 | 0.000E+00 |
| Unigene103945 | oleB | 0.000E+00 | 1.270E-07 | 0.000E+00 | 0.000E+00 | 0.000E+00 | 0.000E+00 | 0.000E+00 | 4.052E-07 | 0.000E+00 | 0.000E+00 | 0.000E+00 | 2.735E-07 |
| Unigene104028 | tetT | 0.000E+00 | 4.499E-07 | 0.000E+00 | 0.000E+00 | 1.992E-06 | 0.000E+00 | 0.000E+00 | 0.000E+00 | 0.000E+00 | 0.000E+00 | 0.000E+00 | 0.000E+00 |
| Unigene104068 | efrA | 0.000E+00 | 6.489E-06 | 1.110E-05 | 1.481E-05 | 0.000E+00 | 0.000E+00 | 0.000E+00 | 0.000E+00 | 0.000E+00 | 0.000E+00 | 9.017E-08 | 0.000E+00 |
| Unigene104101 | lmrC | 5.485E-06 | 1.470E-06 | 9.860E-06 | 6.215E-05 | 2.512E-06 | 3.078E-06 | 6.256E-06 | 0.000E+00 | 0.000E+00 | 1.912E-06 | 7.471E-06 | 1.980E-06 |
| Unigene104126 | OXY-2-7 | 0.000E+00 | 3.280E-07 | 1.955E-07 | 0.000E+00 | 0.000E+00 | 0.000E+00 | 0.000E+00 | 0.000E+00 | 0.000E+00 | 0.000E+00 | 0.000E+00 | 0.000E+00 |
| Unigene104172 | vanRN | 0.000E+00 | 4.686E-07 | 0.000E+00 | 0.000E+00 | 0.000E+00 | 0.000E+00 | 0.000E+00 | 0.000E+00 | 0.000E+00 | 0.000E+00 | 0.000E+00 | 6.561E-07 |
| Unigene104199 | arnA | 0.000E+00 | 9.508E-07 | 0.000E+00 | 0.000E+00 | 1.013E-07 | 0.000E+00 | 0.000E+00 | 0.000E+00 | 0.000E+00 | 0.000E+00 | 0.000E+00 | 5.389E-08 |
| Unigene104272 | Acinetoba | 2.542E-07 | 1.054E-06 | 3.325E-07 | 1.711E-07 | 0.000E+00 | 0.000E+00 | 0.000E+00 | 0.000E+00 | 0.000E+00 | 0.000E+00 | 0.000E+00 | 0.000E+00 |
| Unigene104336 | cpxA | 0.000E+00 | 3.590E-07 | 2.246E-07 | 1.982E-07 | 0.000E+00 | 0.000E+00 | 1.810E-07 | 1.527E-07 | 0.000E+00 | 0.000E+00 | 0.000E+00 | 0.000E+00 |
| Unigene104390 | msbA | 2.338E-06 | 1.766E-05 | 8.156E-06 | 5.737E-06 | 0.000E+00 | 1.975E-08 | 1.011E-07 | 0.000E+00 | 0.000E+00 | 0.000E+00 | 1.672E-07 | 0.000E+00 |
| Unigene104472 | tetM | 2.515E-04 | 2.536E-04 | 5.284E-04 | 2.128E-04 | 2.246E-05 | 1.069E-05 | 9.211E-05 | 3.375E-05 | 1.759E-04 | 7.489E-06 | 4.271E-05 | 1.416E-05 |
| Unigene104488 | macB | 0.000E+00 | 5.157E-07 | 3.227E-06 | 4.603E-06 | 0.000E+00 | 0.000E+00 | 0.000E+00 | 0.000E+00 | 0.000E+00 | 0.000E+00 | 0.000E+00 | 0.000E+00 |
| Unigene104500 | TriB | 2.303E-07 | 7.945E-07 | 9.364E-07 | 0.000E+00 | 7.129E-06 | 0.000E+00 | 0.000E+00 | 1.536E-07 | 0.000E+00 | 0.000E+00 | 0.000E+00 | 0.000E+00 |
| Unigene104506 | bcrA | 2.226E-07 | 4.887E-07 | 3.422E-06 | 3.597E-06 | 0.000E+00 | 0.000E+00 | 0.000E+00 | 0.000E+00 | 0.000E+00 | 0.000E+00 | 0.000E+00 | 0.000E+00 |
| Unigene104748 | mtrA | 0.000E+00 | 3.235E-07 | 7.232E-08 | 0.000E+00 | 0.000E+00 | 0.000E+00 | 0.000E+00 | 0.000E+00 | 0.000E+00 | 0.000E+00 | 0.000E+00 | 0.000E+00 |
| Unigene104783 | TolC | 8.844E-08 | 5.824E-07 | 1.446E-07 | 0.000E+00 | 0.000E+00 | 0.000E+00 | 0.000E+00 | 0.000E+00 | 0.000E+00 | 0.000E+00 | 0.000E+00 | 0.000E+00 |
| Unigene105053 | MCR-4.4 | 3.053E-07 | 1.277E-06 | 6.739E-07 | 2.890E-07 | 7.630E-06 | 3.300E-07 | 0.000E+00 | 2.895E-07 | 0.000E+00 | 1.063E-07 | 0.000E+00 | 0.000E+00 |
| Unigene105102 | Staphyloc | 0.000E+00 | 4.972E-07 | 0.000E+00 | 0.000E+00 | 0.000E+00 | 0.000E+00 | 0.000E+00 | 0.000E+00 | 0.000E+00 | 0.000E+00 | 0.000E+00 | 0.000E+00 |
| Unigene105231 | macB | 6.063E-05 | 4.853E-05 | 9.629E-05 | 1.653E-04 | 1.090E-04 | 7.699E-05 | 9.459E-05 | 9.442E-05 | 7.354E-05 | 1.153E-04 | 1.133E-04 | 1.277E-04 |
| Unigene105247 | TaeA | 2.477E-07 | 8.457E-07 | 2.970E-07 | 7.503E-07 | 0.000E+00 | 0.000E+00 | 0.000E+00 | 6.424E-08 | 6.016E-08 | 0.000E+00 | 0.000E+00 | 0.000E+00 |
| Unigene105279 | pmrA | 1.878E-07 | 6.185E-07 | 2.870E-06 | 2.872E-06 | 2.981E-08 | 0.000E+00 | 0.000E+00 | 0.000E+00 | 0.000E+00 | 0.000E+00 | 0.000E+00 | 0.000E+00 |
| Unigene105306 | arlR | 0.000E+00 | 7.157E-07 | 0.000E+00 | 0.000E+00 | 0.000E+00 | 0.000E+00 | 0.000E+00 | 0.000E+00 | 0.000E+00 | 0.000E+00 | 0.000E+00 | 0.000E+00 |
| Unigene105461 | rpoB2 | 1.131E-06 | 6.658E-06 | 1.102E-05 | 1.657E-05 | 0.000E+00 | 1.926E-08 | 5.915E-08 | 2.079E-08 | 0.000E+00 | 0.000E+00 | 2.038E-08 | 0.000E+00 |
| Unigene105532 | macB | 1.324E-06 | 5.296E-06 | 4.776E-06 | 4.614E-06 | 0.000E+00 | 0.000E+00 | 0.000E+00 | 0.000E+00 | 0.000E+00 | 0.000E+00 | 0.000E+00 | 0.000E+00 |
| Unigene105540 | efrA | 7.244E-05 | 6.153E-05 | 1.239E-04 | 1.735E-04 | 1.346E-04 | 9.685E-05 | 1.247E-04 | 1.247E-04 | 1.087E-04 | 1.494E-04 | 1.575E-04 | 1.669E-04 |
| Unigene105567 | vanZF | 2.131E-07 | 5.846E-06 | 2.509E-06 | 2.743E-06 | 0.000E+00 | 0.000E+00 | 0.000E+00 | 6.218E-08 | 0.000E+00 | 0.000E+00 | 0.000E+00 | 0.000E+00 |
| Unigene105614 | lmrC | 5.124E-08 | 2.812E-07 | 2.011E-07 | 3.105E-07 | 0.000E+00 | 0.000E+00 | 0.000E+00 | 0.000E+00 | 0.000E+00 | 0.000E+00 | 0.000E+00 | 0.000E+00 |
| Unigene105637 | vanTG | 0.000E+00 | 9.763E-07 | 2.083E-06 | 2.144E-06 | 0.000E+00 | 0.000E+00 | 0.000E+00 | 0.000E+00 | 0.000E+00 | 0.000E+00 | 0.000E+00 | 0.000E+00 |
| Unigene105652 | poxtA | 3.647E-07 | 4.884E-06 | 2.434E-06 | 1.473E-06 | 0.000E+00 | 0.000E+00 | 0.000E+00 | 0.000E+00 | 0.000E+00 | 0.000E+00 | 0.000E+00 | 0.000E+00 |
| Unigene105705 | mecB | 0.000E+00 | 9.259E-07 | 2.811E-06 | 2.990E-06 | 0.000E+00 | 0.000E+00 | 0.000E+00 | 0.000E+00 | 0.000E+00 | 0.000E+00 | 0.000E+00 | 0.000E+00 |
| Unigene105833 | kdpE | 0.000E+00 | 6.594E-07 | 0.000E+00 | 2.206E-07 | 0.000E+00 | 0.000E+00 | 2.418E-07 | 0.000E+00 | 0.000E+00 | 0.000E+00 | 0.000E+00 | 0.000E+00 |
| Unigene105925 | msbA | 0.000E+00 | 5.781E-07 | 0.000E+00 | 0.000E+00 | 0.000E+00 | 0.000E+00 | 0.000E+00 | 0.000E+00 | 0.000E+00 | 0.000E+00 | 0.000E+00 | 0.000E+00 |
| Unigene105952 | efrA | 1.305E-07 | 1.146E-06 | 4.245E-06 | 3.164E-06 | 0.000E+00 | 0.000E+00 | 0.000E+00 | 0.000E+00 | 0.000E+00 | 0.000E+00 | 0.000E+00 | 0.000E+00 |
| Unigene105953 | patB | 1.956E-08 | 9.448E-07 | 3.148E-06 | 3.378E-06 | 0.000E+00 | 0.000E+00 | 0.000E+00 | 0.000E+00 | 0.000E+00 | 0.000E+00 | 0.000E+00 | 0.000E+00 |
| Unigene106157 | baeR | 0.000E+00 | 0.000E+00 | 1.051E-06 | 0.000E+00 | 0.000E+00 | 0.000E+00 | 0.000E+00 | 0.000E+00 | 0.000E+00 | 0.000E+00 | 0.000E+00 | 0.000E+00 |
| Unigene106204 | tet(59) | 0.000E+00 | 7.657E-07 | 3.370E-06 | 3.766E-06 | 0.000E+00 | 0.000E+00 | 0.000E+00 | 0.000E+00 | 0.000E+00 | 0.000E+00 | 0.000E+00 | 0.000E+00 |
| Unigene106237 | catA4 | 0.000E+00 | 1.359E-06 | 1.028E-06 | 0.000E+00 | 0.000E+00 | 6.176E-07 | 0.000E+00 | 0.000E+00 | 0.000E+00 | 0.000E+00 | 0.000E+00 | 0.000E+00 |
| Unigene106259 | msrC | 3.566E-07 | 7.367E-07 | 1.852E-06 | 1.207E-06 | 0.000E+00 | 0.000E+00 | 4.643E-08 | 0.000E+00 | 0.000E+00 | 0.000E+00 | 0.000E+00 | 9.917E-08 |
| Unigene106358 | poxtA | 0.000E+00 | 5.368E-07 | 0.000E+00 | 0.000E+00 | 0.000E+00 | 0.000E+00 | 0.000E+00 | 0.000E+00 | 0.000E+00 | 0.000E+00 | 0.000E+00 | 0.000E+00 |
| Unigene106489 | vanRE | 0.000E+00 | 1.181E-06 | 0.000E+00 | 0.000E+00 | 0.000E+00 | 0.000E+00 | 1.531E-06 | 0.000E+00 | 0.000E+00 | 0.000E+00 | 0.000E+00 | 0.000E+00 |
| Unigene106539 | vanRF | 6.550E-07 | 5.648E-07 | 4.131E-06 | 7.748E-06 | 0.000E+00 | 0.000E+00 | 8.802E-07 | 0.000E+00 | 0.000E+00 | 0.000E+00 | 1.605E-07 | 0.000E+00 |
| Unigene106687 | macB | 1.964E-06 | 3.702E-06 | 4.776E-06 | 6.037E-06 | 0.000E+00 | 0.000E+00 | 0.000E+00 | 0.000E+00 | 0.000E+00 | 0.000E+00 | 0.000E+00 | 0.000E+00 |
| Unigene106745 | bcrC | 0.000E+00 | 8.726E-07 | 0.000E+00 | 0.000E+00 | 0.000E+00 | 0.000E+00 | 1.349E-06 | 0.000E+00 | 0.000E+00 | 0.000E+00 | 0.000E+00 | 0.000E+00 |
| Unigene106776 | patA | 8.145E-07 | 1.082E-06 | 1.010E-06 | 1.645E-06 | 2.167E-05 | 6.303E-06 | 1.295E-05 | 1.871E-05 | 1.115E-05 | 1.972E-05 | 1.917E-05 | 9.222E-06 |

| | | | | | | | | | | | | | |
|---|---|---|---|---|---|---|---|---|---|---|---|---|---|
| Unigene106778 | vanSM | 2.044E-06 | 8.169E-06 | 1.109E-05 | 1.219E-05 | 0.000E+00 | 0.000E+00 | 8.537E-08 | 0.000E+00 | 0.000E+00 | 0.000E+00 | 0.000E+00 | 0.000E+00 |
| Unigene106784 | tmrB | 0.000E+00 | 1.042E-06 | 0.000E+00 | 0.000E+00 | 0.000E+00 | 0.000E+00 | 0.000E+00 | 0.000E+00 | 0.000E+00 | 0.000E+00 | 0.000E+00 | 0.000E+00 |
| Unigene106869 | eptA | 0.000E+00 | 5.066E-07 | 2.548E-07 | 5.826E-08 | 0.000E+00 | 0.000E+00 | 0.000E+00 | 0.000E+00 | 0.000E+00 | 0.000E+00 | 0.000E+00 | 0.000E+00 |
| Unigene106940 | vanSL | 2.485E-08 | 2.073E-06 | 4.389E-07 | 3.262E-07 | 0.000E+00 | 0.000E+00 | 0.000E+00 | 0.000E+00 | 0.000E+00 | 0.000E+00 | 0.000E+00 | 0.000E+00 |
| Unigene106941 | kdpE | 0.000E+00 | 2.311E-06 | 3.672E-07 | 2.362E-07 | 0.000E+00 | 0.000E+00 | 0.000E+00 | 0.000E+00 | 0.000E+00 | 0.000E+00 | 0.000E+00 | 0.000E+00 |
| Unigene106951 | macB | 0.000E+00 | 1.554E-06 | 1.852E-07 | 4.289E-07 | 0.000E+00 | 0.000E+00 | 0.000E+00 | 0.000E+00 | 0.000E+00 | 0.000E+00 | 0.000E+00 | 0.000E+00 |
| Unigene106952 | macB | 2.690E-08 | 1.860E-06 | 2.904E-07 | 7.606E-07 | 0.000E+00 | 0.000E+00 | 0.000E+00 | 0.000E+00 | 0.000E+00 | 0.000E+00 | 0.000E+00 | 0.000E+00 |
| Unigene106999 | vanHD | 0.000E+00 | 3.338E-07 | 0.000E+00 | 0.000E+00 | 0.000E+00 | 0.000E+00 | 0.000E+00 | 0.000E+00 | 0.000E+00 | 0.000E+00 | 0.000E+00 | 0.000E+00 |
| Unigene107031 | oleC | 7.859E-05 | 6.737E-05 | 1.377E-04 | 1.872E-04 | 1.539E-04 | 1.063E-04 | 1.432E-04 | 1.345E-04 | 1.164E-04 | 1.753E-04 | 1.791E-04 | 1.850E-04 |
| Unigene107104 | bcrA | 0.000E+00 | 4.374E-07 | 1.466E-07 | 1.509E-07 | 0.000E+00 | 0.000E+00 | 0.000E+00 | 0.000E+00 | 0.000E+00 | 0.000E+00 | 0.000E+00 | 0.000E+00 |
| Unigene107111 | vgaB | 2.170E-05 | 7.267E-06 | 6.949E-06 | 4.133E-06 | 4.598E-07 | 0.000E+00 | 0.000E+00 | 2.684E-07 | 0.000E+00 | 0.000E+00 | 0.000E+00 | 0.000E+00 |
| Unigene107410 | vanHB | 0.000E+00 | 2.346E-07 | 0.000E+00 | 0.000E+00 | 0.000E+00 | 0.000E+00 | 0.000E+00 | 0.000E+00 | 0.000E+00 | 0.000E+00 | 0.000E+00 | 0.000E+00 |
| Unigene107445 | Escherichia | 0.000E+00 | 6.157E-07 | 0.000E+00 | 0.000E+00 | 0.000E+00 | 0.000E+00 | 0.000E+00 | 0.000E+00 | 0.000E+00 | 0.000E+00 | 0.000E+00 | 0.000E+00 |
| Unigene107591 | golS | 0.000E+00 | 1.331E-06 | 0.000E+00 | 0.000E+00 | 0.000E+00 | 0.000E+00 | 0.000E+00 | 0.000E+00 | 0.000E+00 | 0.000E+00 | 0.000E+00 | 0.000E+00 |
| Unigene107672 | vgaB | 0.000E+00 | 1.449E-06 | 0.000E+00 | 0.000E+00 | 0.000E+00 | 0.000E+00 | 0.000E+00 | 0.000E+00 | 0.000E+00 | 0.000E+00 | 0.000E+00 | 0.000E+00 |
| Unigene107745 | Corynebac | 0.000E+00 | 3.473E-07 | 0.000E+00 | 0.000E+00 | 0.000E+00 | 0.000E+00 | 0.000E+00 | 0.000E+00 | 0.000E+00 | 0.000E+00 | 0.000E+00 | 0.000E+00 |
| Unigene107885 | tetB(P) | 0.000E+00 | 6.795E-07 | 1.341E-06 | 2.867E-06 | 3.321E-07 | 1.616E-07 | 2.127E-07 | 1.246E-06 | 1.167E-07 | 0.000E+00 | 1.270E-06 | 9.842E-07 |
| Unigene107902 | vanHO | 0.000E+00 | 1.927E-06 | 2.392E-06 | 1.937E-06 | 0.000E+00 | 0.000E+00 | 0.000E+00 | 0.000E+00 | 0.000E+00 | 0.000E+00 | 0.000E+00 | 0.000E+00 |
| Unigene107919 | msrC | 7.922E-07 | 5.603E-06 | 1.049E-05 | 1.467E-05 | 0.000E+00 | 9.515E-08 | 0.000E+00 | 5.137E-08 | 0.000E+00 | 0.000E+00 | 0.000E+00 | 0.000E+00 |
| Unigene107927 | vmlR | 0.000E+00 | 6.947E-07 | 4.347E-07 | 0.000E+00 | 0.000E+00 | 0.000E+00 | 0.000E+00 | 0.000E+00 | 0.000E+00 | 0.000E+00 | 0.000E+00 | 0.000E+00 |
| Unigene107971 | bcrA | 9.080E-07 | 1.096E-06 | 3.742E-06 | 7.060E-06 | 0.000E+00 | 0.000E+00 | 3.517E-07 | 0.000E+00 | 0.000E+00 | 0.000E+00 | 0.000E+00 | 0.000E+00 |
| Unigene107987 | cmlv | 2.663E-06 | 3.161E-06 | 2.400E-06 | 1.811E-06 | 1.932E-06 | 1.762E-06 | 1.714E-06 | 8.246E-07 | 1.069E-06 | 5.600E-06 | 5.284E-06 | 1.734E-06 |
| Unigene107999 | Streptomy | 7.826E-05 | 6.530E-05 | 1.316E-04 | 1.764E-04 | 1.380E-04 | 1.003E-04 | 1.331E-04 | 1.260E-04 | 1.074E-04 | 1.567E-04 | 1.677E-04 | 1.752E-04 |
| Unigene108004 | MdtK | 0.000E+00 | 1.275E-06 | 6.335E-07 | 0.000E+00 | 0.000E+00 | 0.000E+00 | 1.239E-06 | 0.000E+00 | 0.000E+00 | 0.000E+00 | 0.000E+00 | 0.000E+00 |
| Unigene108067 | vanHF | 0.000E+00 | 2.445E-06 | 3.253E-06 | 2.854E-06 | 0.000E+00 | 0.000E+00 | 0.000E+00 | 0.000E+00 | 0.000E+00 | 0.000E+00 | 0.000E+00 | 0.000E+00 |
| Unigene108179 | oleC | 5.327E-08 | 7.308E-07 | 0.000E+00 | 1.614E-07 | 1.775E-07 | 0.000E+00 | 5.895E-08 | 0.000E+00 | 2.911E-07 | 8.901E-08 | 2.742E-07 | 1.259E-07 |
| Unigene108220 | tetB(58) | 4.737E-07 | 6.685E-07 | 1.394E-06 | 1.025E-06 | 0.000E+00 | 0.000E+00 | 0.000E+00 | 0.000E+00 | 0.000E+00 | 0.000E+00 | 0.000E+00 | 0.000E+00 |
| Unigene108295 | arlR | 0.000E+00 | 1.430E-06 | 6.155E-07 | 1.072E-06 | 0.000E+00 | 0.000E+00 | 0.000E+00 | 0.000E+00 | 0.000E+00 | 0.000E+00 | 0.000E+00 | 0.000E+00 |
| Unigene108332 | baeS | 0.000E+00 | 1.649E-06 | 0.000E+00 | 0.000E+00 | 0.000E+00 | 0.000E+00 | 0.000E+00 | 0.000E+00 | 0.000E+00 | 0.000E+00 | 0.000E+00 | 0.000E+00 |
| Unigene108458 | vanRG | 0.000E+00 | 5.954E-07 | 0.000E+00 | 0.000E+00 | 0.000E+00 | 0.000E+00 | 0.000E+00 | 0.000E+00 | 0.000E+00 | 0.000E+00 | 0.000E+00 | 0.000E+00 |
| Unigene108554 | Acinetoba | 6.959E-05 | 5.836E-05 | 1.129E-04 | 1.597E-04 | 1.257E-04 | 9.052E-05 | 1.185E-04 | 1.137E-04 | 9.800E-05 | 1.445E-04 | 1.492E-04 | 1.549E-04 |
| Unigene108589 | Staphyloc | 1.489E-05 | 5.021E-06 | 3.887E-06 | 1.484E-05 | 9.038E-05 | 1.265E-04 | 1.305E-04 | 1.004E-04 | 1.599E-04 | 1.529E-04 | 1.286E-04 | 7.927E-05 |
| Unigene108662 | macB | 0.000E+00 | 2.170E-06 | 2.752E-06 | 0.000E+00 | 0.000E+00 | 0.000E+00 | 0.000E+00 | 0.000E+00 | 0.000E+00 | 0.000E+00 | 0.000E+00 | 0.000E+00 |
| Unigene108721 | efpA | 1.443E-06 | 2.094E-06 | 1.440E-06 | 1.358E-06 | 1.060E-06 | 1.216E-06 | 1.110E-06 | 3.996E-07 | 4.544E-07 | 3.896E-06 | 3.804E-06 | 1.098E-06 |
| Unigene108888 | YojI | 1.137E-07 | 7.485E-07 | 1.487E-07 | 0.000E+00 | 0.000E+00 | 0.000E+00 | 0.000E+00 | 0.000E+00 | 0.000E+00 | 0.000E+00 | 0.000E+00 | 0.000E+00 |
| Unigene108945 | bcrA | 2.614E-06 | 1.673E-05 | 6.928E-06 | 5.484E-06 | 0.000E+00 | 0.000E+00 | 0.000E+00 | 1.189E-07 | 0.000E+00 | 0.000E+00 | 0.000E+00 | 0.000E+00 |
| Unigene109196 | bacA | 0.000E+00 | 1.509E-06 | 0.000E+00 | 0.000E+00 | 2.225E-06 | 0.000E+00 | 0.000E+00 | 0.000E+00 | 0.000E+00 | 0.000E+00 | 0.000E+00 | 0.000E+00 |
| Unigene109279 | arlR | 0.000E+00 | 3.737E-07 | 0.000E+00 | 2.063E-07 | 0.000E+00 | 0.000E+00 | 0.000E+00 | 0.000E+00 | 0.000E+00 | 0.000E+00 | 0.000E+00 | 0.000E+00 |
| Unigene109351 | vanHD | 1.359E-07 | 8.052E-07 | 3.733E-06 | 3.018E-06 | 0.000E+00 | 0.000E+00 | 0.000E+00 | 0.000E+00 | 0.000E+00 | 0.000E+00 | 0.000E+00 | 0.000E+00 |
| Unigene109359 | Staphyloc | 0.000E+00 | 1.683E-06 | 1.297E-06 | 1.525E-06 | 0.000E+00 | 0.000E+00 | 0.000E+00 | 0.000E+00 | 0.000E+00 | 0.000E+00 | 0.000E+00 | 0.000E+00 |
| Unigene109364 | oleC | 2.425E-06 | 2.468E-06 | 2.380E-06 | 1.603E-06 | 2.450E-07 | 0.000E+00 | 4.881E-07 | 0.000E+00 | 7.230E-07 | 0.000E+00 | 1.009E-07 | 0.000E+00 |
| Unigene109419 | bcrA | 0.000E+00 | 5.493E-07 | 0.000E+00 | 0.000E+00 | 9.267E-08 | 0.000E+00 | 0.000E+00 | 0.000E+00 | 0.000E+00 | 0.000E+00 | 0.000E+00 | 0.000E+00 |
| Unigene109515 | rpoB2 | 0.000E+00 | 1.420E-06 | 2.459E-06 | 2.781E-05 | 0.000E+00 | 0.000E+00 | 0.000E+00 | 0.000E+00 | 0.000E+00 | 0.000E+00 | 0.000E+00 | 0.000E+00 |
| Unigene109580 | bcr-1 | 0.000E+00 | 1.329E-06 | 0.000E+00 | 4.890E-07 | 6.694E-06 | 0.000E+00 | 0.000E+00 | 1.570E-07 | 0.000E+00 | 0.000E+00 | 4.616E-07 | 0.000E+00 |
| Unigene109656 | YojI | 0.000E+00 | 5.326E-06 | 1.014E-05 | 1.525E-05 | 0.000E+00 | 0.000E+00 | 0.000E+00 | 0.000E+00 | 0.000E+00 | 0.000E+00 | 0.000E+00 | 0.000E+00 |
| Unigene109721 | tetA(58) | 0.000E+00 | 7.873E-07 | 0.000E+00 | 0.000E+00 | 0.000E+00 | 0.000E+00 | 0.000E+00 | 0.000E+00 | 0.000E+00 | 0.000E+00 | 0.000E+00 | 0.000E+00 |
| Unigene109742 | arlS | 0.000E+00 | 5.962E-06 | 0.000E+00 | 0.000E+00 | 0.000E+00 | 0.000E+00 | 0.000E+00 | 0.000E+00 | 0.000E+00 | 0.000E+00 | 0.000E+00 | 0.000E+00 |
| Unigene109768 | arlR | 0.000E+00 | 4.724E-07 | 3.472E-06 | 2.994E-06 | 0.000E+00 | 0.000E+00 | 0.000E+00 | 0.000E+00 | 0.000E+00 | 0.000E+00 | 0.000E+00 | 0.000E+00 |
| Unigene109794 | RlmA(II) | 0.000E+00 | 3.388E-07 | 2.164E-07 | 0.000E+00 | 9.798E-07 | 0.000E+00 | 9.761E-08 | 5.147E-08 | 0.000E+00 | 0.000E+00 | 0.000E+00 | 0.000E+00 |
| Unigene109943 | arlS | 1.675E-05 | 1.859E-05 | 0.000E+00 | 0.000E+00 | 0.000E+00 | 0.000E+00 | 0.000E+00 | 0.000E+00 | 0.000E+00 | 0.000E+00 | 0.000E+00 | 0.000E+00 |
| Unigene109958 | oleC | 8.722E-05 | 1.471E-04 | 1.564E-04 | 7.385E-06 | 7.518E-06 | 4.089E-07 | 0.000E+00 | 2.012E-06 | 5.697E-05 | 2.809E-07 | 2.933E-06 | 1.143E-06 |
| Unigene110089 | adeL | 0.000E+00 | 2.134E-07 | 0.000E+00 | 1.964E-07 | 0.000E+00 | 0.000E+00 | 0.000E+00 | 0.000E+00 | 0.000E+00 | 0.000E+00 | 0.000E+00 | 0.000E+00 |
| Unigene110153 | macB | 6.871E-08 | 1.297E-06 | 4.113E-06 | 3.691E-06 | 0.000E+00 | 0.000E+00 | 0.000E+00 | 0.000E+00 | 0.000E+00 | 0.000E+00 | 0.000E+00 | 0.000E+00 |
| Unigene110154 | cpxA | 1.537E-07 | 1.012E-06 | 2.866E-06 | 4.408E-06 | 0.000E+00 | 0.000E+00 | 0.000E+00 | 0.000E+00 | 0.000E+00 | 0.000E+00 | 0.000E+00 | 0.000E+00 |
| Unigene110182 | tetA(58) | 9.480E-08 | 5.723E-07 | 4.651E-07 | 0.000E+00 | 0.000E+00 | 0.000E+00 | 0.000E+00 | 0.000E+00 | 0.000E+00 | 0.000E+00 | 0.000E+00 | 0.000E+00 |
| Unigene110187 | efrA | 1.179E-07 | 6.471E-07 | 1.446E-07 | 8.931E-08 | 0.000E+00 | 0.000E+00 | 0.000E+00 | 0.000E+00 | 0.000E+00 | 0.000E+00 | 0.000E+00 | 0.000E+00 |
| Unigene110227 | tetA(46) | 8.510E-07 | 5.832E-06 | 1.045E-05 | 1.438E-05 | 0.000E+00 | 0.000E+00 | 0.000E+00 | 0.000E+00 | 0.000E+00 | 0.000E+00 | 0.000E+00 | 0.000E+00 |
| Unigene110314 | tetA(58) | 1.276E-07 | 4.201E-07 | 4.173E-08 | 0.000E+00 | 0.000E+00 | 0.000E+00 | 0.000E+00 | 0.000E+00 | 2.789E-07 | 0.000E+00 | 0.000E+00 | 0.000E+00 |

| | | | | | | | | | | | | | |
|---|---|---|---|---|---|---|---|---|---|---|---|---|---|
| Unigene110374 | tva(A) | 2.952E-07 | 1.296E-06 | 1.770E-06 | 1.888E-06 | 0.000E+00 | 0.000E+00 | 0.000E+00 | 0.000E+00 | 0.000E+00 | 0.000E+00 | 0.000E+00 | 0.000E+00 |
| Unigene110573 | lmrB | 1.275E-06 | 8.102E-07 | 1.273E-06 | 1.559E-06 | 1.904E-05 | 4.812E-06 | 1.382E-05 | 1.666E-05 | 1.081E-05 | 1.645E-05 | 1.635E-05 | 8.303E-06 |
| Unigene110586 | erm(32) | 1.805E-06 | 1.629E-05 | 5.846E-06 | 3.099E-06 | 0.000E+00 | 0.000E+00 | 0.000E+00 | 0.000E+00 | 0.000E+00 | 0.000E+00 | 2.065E-07 | 8.536E-08 |
| Unigene110598 | poxtA | 0.000E+00 | 4.072E-07 | 0.000E+00 | 0.000E+00 | 0.000E+00 | 0.000E+00 | 0.000E+00 | 0.000E+00 | 0.000E+00 | 0.000E+00 | 0.000E+00 | 0.000E+00 |
| Unigene110687 | tetB(60) | 2.756E-05 | 1.015E-05 | 6.420E-06 | 4.004E-06 | 2.002E-07 | 0.000E+00 | 0.000E+00 | 1.473E-07 | 0.000E+00 | 0.000E+00 | 0.000E+00 | 0.000E+00 |
| Unigene110699 | Streptomy | 0.000E+00 | 1.204E-06 | 1.840E-06 | 1.637E-06 | 0.000E+00 | 0.000E+00 | 0.000E+00 | 0.000E+00 | 0.000E+00 | 0.000E+00 | 0.000E+00 | 0.000E+00 |
| Unigene110725 | bcrA | 0.000E+00 | 1.011E-06 | 4.108E-07 | 0.000E+00 | 4.651E-06 | 0.000E+00 | 0.000E+00 | 4.398E-07 | 0.000E+00 | 0.000E+00 | 0.000E+00 | 0.000E+00 |
| Unigene110761 | tetA(58) | 1.080E-06 | 9.575E-07 | 6.563E-06 | 6.209E-06 | 0.000E+00 | 0.000E+00 | 4.598E-07 | 0.000E+00 | 0.000E+00 | 0.000E+00 | 0.000E+00 | 0.000E+00 |
| Unigene110769 | efrB | 3.241E-08 | 1.316E-06 | 2.894E-06 | 3.142E-06 | 0.000E+00 | 0.000E+00 | 0.000E+00 | 0.000E+00 | 0.000E+00 | 0.000E+00 | 0.000E+00 | 0.000E+00 |
| Unigene110852 | adeG | 5.198E-08 | 3.994E-07 | 0.000E+00 | 0.000E+00 | 0.000E+00 | 0.000E+00 | 0.000E+00 | 0.000E+00 | 0.000E+00 | 0.000E+00 | 0.000E+00 | 0.000E+00 |
| Unigene110939 | poxtA | 0.000E+00 | 0.000E+00 | 4.512E-07 | 0.000E+00 | 0.000E+00 | 0.000E+00 | 0.000E+00 | 0.000E+00 | 0.000E+00 | 0.000E+00 | 0.000E+00 | 0.000E+00 |
| Unigene110987 | bmr | 2.560E-05 | 8.246E-07 | 3.686E-07 | 0.000E+00 | 1.192E-07 | 0.000E+00 | 0.000E+00 | 0.000E+00 | 0.000E+00 | 0.000E+00 | 0.000E+00 | 0.000E+00 |
| Unigene111008 | NmcR | 0.000E+00 | 1.625E-06 | 0.000E+00 | 0.000E+00 | 0.000E+00 | 0.000E+00 | 2.598E-06 | 0.000E+00 | 0.000E+00 | 0.000E+00 | 0.000E+00 | 0.000E+00 |
| Unigene111041 | vanHD | 2.386E-05 | 1.340E-06 | 0.000E+00 | 0.000E+00 | 0.000E+00 | 0.000E+00 | 0.000E+00 | 0.000E+00 | 0.000E+00 | 0.000E+00 | 0.000E+00 | 0.000E+00 |
| Unigene111050 | macB | 7.259E-05 | 6.072E-05 | 1.186E-04 | 1.713E-04 | 1.339E-04 | 9.376E-05 | 1.168E-04 | 1.200E-04 | 9.976E-05 | 1.503E-04 | 1.560E-04 | 1.656E-04 |
| Unigene111077 | Klebsiella p | 0.000E+00 | 1.389E-06 | 0.000E+00 | 3.196E-07 | 0.000E+00 | 0.000E+00 | 0.000E+00 | 0.000E+00 | 0.000E+00 | 0.000E+00 | 0.000E+00 | 0.000E+00 |
| Unigene111223 | srmB | 8.792E-07 | 2.091E-06 | 1.150E-06 | 5.074E-07 | 7.093E-06 | 3.395E-07 | 1.529E-06 | 4.398E-07 | 0.000E+00 | 0.000E+00 | 5.987E-07 | 0.000E+00 |
| Unigene111238 | oqxA | 0.000E+00 | 1.536E-06 | 0.000E+00 | 0.000E+00 | 4.391E-06 | 0.000E+00 | 0.000E+00 | 0.000E+00 | 0.000E+00 | 0.000E+00 | 0.000E+00 | 1.947E-07 |
| Unigene111246 | bcrA | 0.000E+00 | 4.468E-07 | 2.854E-07 | 0.000E+00 | 0.000E+00 | 0.000E+00 | 0.000E+00 | 0.000E+00 | 0.000E+00 | 0.000E+00 | 0.000E+00 | 0.000E+00 |
| Unigene111363 | vmlR | 1.681E-07 | 4.152E-07 | 4.949E-07 | 1.698E-07 | 0.000E+00 | 0.000E+00 | 0.000E+00 | 0.000E+00 | 0.000E+00 | 0.000E+00 | 0.000E+00 | 0.000E+00 |
| Unigene111613 | lmrC | 0.000E+00 | 0.000E+00 | 0.000E+00 | 0.000E+00 | 0.000E+00 | 1.886E-07 | 0.000E+00 | 2.716E-07 | 0.000E+00 | 0.000E+00 | 0.000E+00 | 4.125E-07 |
| Unigene111627 | mdtG | 0.000E+00 | 6.538E-07 | 2.192E-07 | 7.520E-08 | 0.000E+00 | 0.000E+00 | 0.000E+00 | 0.000E+00 | 0.000E+00 | 0.000E+00 | 0.000E+00 | 0.000E+00 |
| Unigene111643 | adeR | 0.000E+00 | 9.155E-07 | 0.000E+00 | 5.054E-07 | 0.000E+00 | 0.000E+00 | 0.000E+00 | 0.000E+00 | 0.000E+00 | 0.000E+00 | 0.000E+00 | 0.000E+00 |
| Unigene111676 | qacH | 0.000E+00 | 7.515E-07 | 0.000E+00 | 0.000E+00 | 0.000E+00 | 0.000E+00 | 0.000E+00 | 0.000E+00 | 0.000E+00 | 0.000E+00 | 0.000E+00 | 0.000E+00 |
| Unigene111701 | vanSM | 0.000E+00 | 5.061E-07 | 0.000E+00 | 0.000E+00 | 0.000E+00 | 0.000E+00 | 0.000E+00 | 0.000E+00 | 0.000E+00 | 0.000E+00 | 0.000E+00 | 0.000E+00 |
| Unigene111718 | bcrA | 0.000E+00 | 6.589E-07 | 4.985E-07 | 0.000E+00 | 0.000E+00 | 0.000E+00 | 1.329E-06 | 0.000E+00 | 0.000E+00 | 0.000E+00 | 0.000E+00 | 0.000E+00 |
| Unigene111753 | bcrA | 2.594E-06 | 8.078E-06 | 8.632E-06 | 9.167E-06 | 0.000E+00 | 0.000E+00 | 0.000E+00 | 0.000E+00 | 0.000E+00 | 0.000E+00 | 0.000E+00 | 0.000E+00 |
| Unigene111795 | bcrA | 4.103E-06 | 2.026E-05 | 3.456E-06 | 0.000E+00 | 0.000E+00 | 0.000E+00 | 0.000E+00 | 0.000E+00 | 1.565E-06 | 0.000E+00 | 0.000E+00 | 3.203E-07 |
| Unigene111871 | Pseudomo | 0.000E+00 | 8.491E-07 | 4.140E-07 | 0.000E+00 | 0.000E+00 | 0.000E+00 | 0.000E+00 | 0.000E+00 | 0.000E+00 | 0.000E+00 | 0.000E+00 | 0.000E+00 |
| Unigene111982 | rosA | 0.000E+00 | 8.222E-07 | 0.000E+00 | 0.000E+00 | 0.000E+00 | 0.000E+00 | 0.000E+00 | 0.000E+00 | 0.000E+00 | 0.000E+00 | 0.000E+00 | 0.000E+00 |
| Unigene112003 | golS | 0.000E+00 | 4.120E-07 | 2.455E-07 | 1.895E-07 | 0.000E+00 | 0.000E+00 | 0.000E+00 | 0.000E+00 | 0.000E+00 | 0.000E+00 | 0.000E+00 | 0.000E+00 |
| Unigene112023 | cmx | 0.000E+00 | 1.423E-06 | 0.000E+00 | 0.000E+00 | 0.000E+00 | 0.000E+00 | 0.000E+00 | 0.000E+00 | 0.000E+00 | 0.000E+00 | 0.000E+00 | 0.000E+00 |
| Unigene112055 | H-NS | 0.000E+00 | 1.137E-06 | 0.000E+00 | 2.415E-07 | 9.386E-06 | 0.000E+00 | 0.000E+00 | 0.000E+00 | 0.000E+00 | 0.000E+00 | 0.000E+00 | 0.000E+00 |
| Unigene112278 | mgrA | 5.985E-06 | 2.388E-06 | 3.144E-06 | 7.753E-06 | 4.446E-05 | 4.078E-05 | 3.171E-05 | 1.933E-05 | 9.534E-05 | 1.273E-04 | 8.498E-05 | 6.709E-05 |
| Unigene112402 | msrE | 4.304E-07 | 5.621E-06 | 8.911E-06 | 1.165E-05 | 0.000E+00 | 0.000E+00 | 0.000E+00 | 0.000E+00 | 0.000E+00 | 0.000E+00 | 0.000E+00 | 0.000E+00 |
| Unigene112405 | mdtG | 4.950E-07 | 5.090E-06 | 1.005E-05 | 1.526E-05 | 2.894E-08 | 1.127E-07 | 2.883E-08 | 0.000E+00 | 0.000E+00 | 0.000E+00 | 0.000E+00 | 0.000E+00 |
| Unigene112408 | msbA | 6.394E-07 | 5.113E-06 | 8.981E-06 | 1.448E-05 | 0.000E+00 | 5.925E-08 | 0.000E+00 | 0.000E+00 | 0.000E+00 | 0.000E+00 | 0.000E+00 | 0.000E+00 |
| Unigene112413 | rpoB2 | 9.109E-06 | 2.974E-05 | 2.444E-05 | 2.353E-05 | 0.000E+00 | 1.824E-07 | 7.074E-07 | 1.762E-07 | 0.000E+00 | 2.472E-07 | 2.539E-07 | 0.000E+00 |
| Unigene112526 | otr(B) | 7.481E-07 | 5.289E-06 | 9.630E-06 | 1.535E-05 | 0.000E+00 | 0.000E+00 | 0.000E+00 | 0.000E+00 | 0.000E+00 | 0.000E+00 | 0.000E+00 | 0.000E+00 |
| Unigene112592 | tlrC | 0.000E+00 | 2.938E-07 | 1.051E-07 | 3.244E-07 | 0.000E+00 | 5.787E-08 | 0.000E+00 | 0.000E+00 | 0.000E+00 | 0.000E+00 | 0.000E+00 | 0.000E+00 |
| Unigene112659 | efrA | 2.201E-06 | 2.395E-06 | 2.983E-06 | 1.883E-06 | 2.693E-07 | 0.000E+00 | 4.747E-07 | 2.394E-07 | 7.338E-07 | 1.870E-07 | 0.000E+00 | 1.543E-07 |
| Unigene112781 | patA | 0.000E+00 | 1.205E-06 | 1.939E-06 | 1.375E-06 | 0.000E+00 | 0.000E+00 | 0.000E+00 | 0.000E+00 | 0.000E+00 | 0.000E+00 | 0.000E+00 | 0.000E+00 |
| Unigene112849 | bcrA | 4.804E-07 | 1.265E-06 | 4.006E-06 | 4.802E-06 | 0.000E+00 | 0.000E+00 | 1.063E-07 | 0.000E+00 | 0.000E+00 | 0.000E+00 | 0.000E+00 | 0.000E+00 |
| Unigene112931 | Chlamydia | 3.929E-06 | 2.076E-05 | 3.167E-06 | 1.188E-06 | 7.228E-07 | 0.000E+00 | 2.216E-07 | 4.089E-07 | 1.176E-06 | 0.000E+00 | 3.435E-07 | 3.254E-07 |
| Unigene112963 | arlR | 1.120E-05 | 3.840E-06 | 2.961E-06 | 1.201E-05 | 7.338E-05 | 1.136E-04 | 1.164E-04 | 8.884E-05 | 1.364E-04 | 1.365E-04 | 1.015E-04 | 6.447E-05 |
| Unigene113086 | Erm(34) | 9.032E-07 | 2.155E-06 | 2.042E-06 | 7.931E-07 | 9.380E-06 | 0.000E+00 | 2.043E-06 | 4.584E-07 | 0.000E+00 | 3.500E-07 | 8.086E-07 | 3.249E-07 |
| Unigene113141 | vatE | 0.000E+00 | 5.761E-07 | 0.000E+00 | 0.000E+00 | 0.000E+00 | 0.000E+00 | 0.000E+00 | 0.000E+00 | 0.000E+00 | 0.000E+00 | 0.000E+00 | 0.000E+00 |
| Unigene113353 | arnA | 0.000E+00 | 1.943E-06 | 0.000E+00 | 0.000E+00 | 9.987E-06 | 7.730E-07 | 0.000E+00 | 5.564E-07 | 0.000E+00 | 0.000E+00 | 0.000E+00 | 0.000E+00 |
| Unigene113362 | smeS | 9.042E-08 | 4.962E-07 | 2.218E-07 | 3.196E-07 | 0.000E+00 | 0.000E+00 | 5.004E-08 | 0.000E+00 | 0.000E+00 | 0.000E+00 | 0.000E+00 | 0.000E+00 |
| Unigene113414 | cmlA8 | 1.188E-05 | 2.838E-06 | 1.978E-06 | 1.118E-05 | 7.547E-05 | 1.002E-04 | 9.825E-05 | 7.451E-05 | 1.289E-04 | 1.178E-04 | 1.067E-04 | 6.621E-05 |
| Unigene113441 | aadA24 | 0.000E+00 | 1.855E-06 | 0.000E+00 | 0.000E+00 | 0.000E+00 | 0.000E+00 | 0.000E+00 | 0.000E+00 | 0.000E+00 | 0.000E+00 | 0.000E+00 | 0.000E+00 |
| Unigene113476 | optrA | 0.000E+00 | 1.404E-06 | 0.000E+00 | 5.466E-07 | 0.000E+00 | 0.000E+00 | 0.000E+00 | 0.000E+00 | 0.000E+00 | 0.000E+00 | 0.000E+00 | 0.000E+00 |
| Unigene113521 | macB | 0.000E+00 | 1.673E-06 | 1.584E-06 | 2.173E-06 | 0.000E+00 | 0.000E+00 | 0.000E+00 | 0.000E+00 | 0.000E+00 | 0.000E+00 | 0.000E+00 | 0.000E+00 |
| Unigene113532 | hp1181 | 0.000E+00 | 6.998E-07 | 1.955E-07 | 0.000E+00 | 0.000E+00 | 0.000E+00 | 0.000E+00 | 0.000E+00 | 0.000E+00 | 0.000E+00 | 0.000E+00 | 0.000E+00 |
| Unigene113611 | vanRO | 0.000E+00 | 9.669E-07 | 0.000E+00 | 0.000E+00 | 0.000E+00 | 0.000E+00 | 0.000E+00 | 0.000E+00 | 0.000E+00 | 0.000E+00 | 0.000E+00 | 0.000E+00 |
| Unigene113617 | macB | 0.000E+00 | 3.528E-06 | 1.513E-06 | 6.294E-07 | 1.068E-05 | 4.256E-07 | 2.832E-06 | 7.275E-07 | 0.000E+00 | 3.289E-07 | 0.000E+00 | 0.000E+00 |
| Unigene113816 | mdtG | 0.000E+00 | 1.017E-06 | 0.000E+00 | 0.000E+00 | 0.000E+00 | 0.000E+00 | 1.104E-06 | 0.000E+00 | 0.000E+00 | 0.000E+00 | 0.000E+00 | 0.000E+00 |
| Unigene113868 | arlS | 2.618E-06 | 1.553E-05 | 1.030E-05 | 9.006E-06 | 0.000E+00 | 1.679E-07 | 4.420E-07 | 0.000E+00 | 0.000E+00 | 0.000E+00 | 7.613E-08 | 5.245E-08 |

| | | | | | | | | | | | | | |
|---|---|---|---|---|---|---|---|---|---|---|---|---|---|
| Unigene113938 | baeS | 5.240E-08 | 1.074E-06 | 3.154E-06 | 3.740E-06 | 0.000E+00 | 0.000E+00 | 0.000E+00 | 0.000E+00 | 0.000E+00 | 0.000E+00 | 0.000E+00 | 0.000E+00 |
| Unigene113939 | lsaB | 0.000E+00 | 8.435E-07 | 3.056E-06 | 3.064E-06 | 0.000E+00 | 0.000E+00 | 0.000E+00 | 0.000E+00 | 0.000E+00 | 0.000E+00 | 0.000E+00 | 0.000E+00 |
| Unigene113968 | vgaALC | 0.000E+00 | 7.029E-07 | 0.000E+00 | 0.000E+00 | 0.000E+00 | 0.000E+00 | 0.000E+00 | 0.000E+00 | 0.000E+00 | 0.000E+00 | 0.000E+00 | 0.000E+00 |
| Unigene114008 | cpxA | 4.467E-05 | 2.380E-05 | 1.601E-05 | 2.007E-05 | 2.065E-06 | 1.834E-06 | 3.318E-06 | 5.968E-07 | 6.274E-06 | 5.178E-07 | 2.206E-06 | 1.346E-06 |
| Unigene114013 | vatB | 5.546E-07 | 5.844E-06 | 9.361E-06 | 1.703E-05 | 0.000E+00 | 5.996E-08 | 0.000E+00 | 0.000E+00 | 0.000E+00 | 0.000E+00 | 0.000E+00 | 0.000E+00 |
| Unigene114040 | vanYB | 0.000E+00 | 1.267E-06 | 7.686E-07 | 0.000E+00 | 0.000E+00 | 0.000E+00 | 0.000E+00 | 0.000E+00 | 0.000E+00 | 0.000E+00 | 0.000E+00 | 0.000E+00 |
| Unigene114122 | Acinetoba | 1.231E-07 | 6.216E-07 | 4.591E-07 | 1.989E-07 | 0.000E+00 | 0.000E+00 | 0.000E+00 | 0.000E+00 | 0.000E+00 | 0.000E+00 | 0.000E+00 | 0.000E+00 |
| Unigene114213 | ramA | 0.000E+00 | 0.000E+00 | 0.000E+00 | 1.695E-06 | 0.000E+00 | 0.000E+00 | 0.000E+00 | 1.383E-06 | 0.000E+00 | 0.000E+00 | 0.000E+00 | 0.000E+00 |
| Unigene114241 | vanHF | 0.000E+00 | 5.107E-07 | 1.712E-07 | 0.000E+00 | 0.000E+00 | 0.000E+00 | 0.000E+00 | 0.000E+00 | 0.000E+00 | 0.000E+00 | 0.000E+00 | 0.000E+00 |
| Unigene114437 | msbA | 0.000E+00 | 1.510E-06 | 2.459E-06 | 0.000E+00 | 0.000E+00 | 0.000E+00 | 0.000E+00 | 0.000E+00 | 0.000E+00 | 0.000E+00 | 0.000E+00 | 0.000E+00 |
| Unigene114443 | arlS | 1.758E-07 | 8.298E-07 | 4.123E-06 | 4.705E-06 | 0.000E+00 | 0.000E+00 | 0.000E+00 | 0.000E+00 | 0.000E+00 | 0.000E+00 | 0.000E+00 | 0.000E+00 |
| Unigene114555 | mepR | 0.000E+00 | 7.381E-07 | 0.000E+00 | 0.000E+00 | 0.000E+00 | 0.000E+00 | 0.000E+00 | 0.000E+00 | 0.000E+00 | 0.000E+00 | 0.000E+00 | 0.000E+00 |
| Unigene114733 | efrB | 3.222E-08 | 6.718E-07 | 3.161E-07 | 1.301E-07 | 0.000E+00 | 0.000E+00 | 0.000E+00 | 0.000E+00 | 0.000E+00 | 0.000E+00 | 0.000E+00 | 0.000E+00 |
| Unigene114734 | bcr-1 | 0.000E+00 | 7.815E-07 | 0.000E+00 | 8.788E-07 | 0.000E+00 | 0.000E+00 | 0.000E+00 | 0.000E+00 | 0.000E+00 | 0.000E+00 | 0.000E+00 | 0.000E+00 |
| Unigene114880 | evgS | 0.000E+00 | 8.904E-07 | 8.380E-08 | 0.000E+00 | 0.000E+00 | 0.000E+00 | 0.000E+00 | 0.000E+00 | 0.000E+00 | 0.000E+00 | 0.000E+00 | 0.000E+00 |
| Unigene114910 | vanRG | 0.000E+00 | 8.729E-07 | 3.443E-06 | 3.024E-06 | 0.000E+00 | 0.000E+00 | 0.000E+00 | 0.000E+00 | 0.000E+00 | 0.000E+00 | 0.000E+00 | 0.000E+00 |
| Unigene114911 | vanSE | 0.000E+00 | 7.016E-07 | 2.962E-06 | 3.371E-06 | 0.000E+00 | 0.000E+00 | 0.000E+00 | 0.000E+00 | 0.000E+00 | 0.000E+00 | 0.000E+00 | 0.000E+00 |
| Unigene114931 | efrA | 5.352E-07 | 6.300E-06 | 9.309E-06 | 1.402E-05 | 8.202E-08 | 0.000E+00 | 0.000E+00 | 0.000E+00 | 0.000E+00 | 0.000E+00 | 0.000E+00 | 0.000E+00 |
| Unigene114932 | tetB(60) | 7.802E-07 | 5.218E-06 | 8.244E-06 | 1.325E-05 | 0.000E+00 | 7.846E-08 | 0.000E+00 | 0.000E+00 | 0.000E+00 | 0.000E+00 | 0.000E+00 | 4.290E-08 |
| Unigene115161 | Staphyloco | 6.423E-05 | 5.472E-05 | 1.080E-04 | 1.605E-04 | 1.219E-04 | 8.666E-05 | 1.053E-04 | 1.031E-04 | 8.976E-05 | 1.316E-04 | 1.293E-04 | 1.333E-04 |
| Unigene115163 | Klebsiella p | 0.000E+00 | 6.442E-07 | 0.000E+00 | 0.000E+00 | 0.000E+00 | 0.000E+00 | 0.000E+00 | 0.000E+00 | 0.000E+00 | 0.000E+00 | 0.000E+00 | 0.000E+00 |
| Unigene115174 | qacB | 2.246E-08 | 1.948E-06 | 2.645E-07 | 0.000E+00 | 0.000E+00 | 0.000E+00 | 0.000E+00 | 0.000E+00 | 0.000E+00 | 0.000E+00 | 0.000E+00 | 3.452E-07 |
| Unigene115183 | msbA | 0.000E+00 | 7.646E-07 | 0.000E+00 | 0.000E+00 | 0.000E+00 | 0.000E+00 | 0.000E+00 | 0.000E+00 | 0.000E+00 | 0.000E+00 | 0.000E+00 | 0.000E+00 |
| Unigene115250 | tcr3 | 1.645E-07 | 7.220E-07 | 5.302E-07 | 0.000E+00 | 3.132E-07 | 0.000E+00 | 0.000E+00 | 1.371E-07 | 0.000E+00 | 0.000E+00 | 0.000E+00 | 0.000E+00 |
| Unigene115261 | Bifidobact | 0.000E+00 | 5.905E-07 | 0.000E+00 | 0.000E+00 | 0.000E+00 | 0.000E+00 | 0.000E+00 | 0.000E+00 | 0.000E+00 | 0.000E+00 | 0.000E+00 | 0.000E+00 |
| Unigene115307 | vanSA | 0.000E+00 | 6.672E-07 | 0.000E+00 | 0.000E+00 | 0.000E+00 | 0.000E+00 | 0.000E+00 | 0.000E+00 | 0.000E+00 | 0.000E+00 | 0.000E+00 | 0.000E+00 |
| Unigene115327 | Listeria mc | 5.292E-06 | 1.070E-05 | 1.060E-05 | 1.178E-05 | 8.399E-08 | 1.634E-07 | 6.972E-08 | 1.471E-08 | 0.000E+00 | 1.825E-07 | 7.206E-08 | 0.000E+00 |
| Unigene115352 | vanL | 0.000E+00 | 5.467E-07 | 0.000E+00 | 0.000E+00 | 0.000E+00 | 0.000E+00 | 0.000E+00 | 0.000E+00 | 0.000E+00 | 0.000E+00 | 0.000E+00 | 0.000E+00 |
| Unigene115392 | mecD | 1.446E-06 | 4.727E-06 | 3.677E-06 | 7.270E-06 | 0.000E+00 | 0.000E+00 | 0.000E+00 | 3.630E-08 | 0.000E+00 | 0.000E+00 | 0.000E+00 | 0.000E+00 |
| Unigene115501 | qacH | 0.000E+00 | 7.619E-07 | 0.000E+00 | 0.000E+00 | 0.000E+00 | 0.000E+00 | 5.378E-06 | 0.000E+00 | 0.000E+00 | 0.000E+00 | 2.581E-06 | 4.411E-06 |
| Unigene115504 | optrA | 3.587E-06 | 4.608E-06 | 3.419E-06 | 6.400E-06 | 3.849E-07 | 1.102E-07 | 1.128E-07 | 0.000E+00 | 1.559E-07 | 0.000E+00 | 1.166E-07 | 1.927E-07 |
| Unigene115590 | patA | 0.000E+00 | 5.288E-07 | 0.000E+00 | 0.000E+00 | 0.000E+00 | 0.000E+00 | 0.000E+00 | 0.000E+00 | 0.000E+00 | 0.000E+00 | 0.000E+00 | 0.000E+00 |
| Unigene115739 | patA | 0.000E+00 | 1.911E-06 | 0.000E+00 | 0.000E+00 | 0.000E+00 | 0.000E+00 | 0.000E+00 | 0.000E+00 | 0.000E+00 | 0.000E+00 | 0.000E+00 | 0.000E+00 |
| Unigene115754 | cmlv | 0.000E+00 | 5.808E-07 | 0.000E+00 | 0.000E+00 | 0.000E+00 | 0.000E+00 | 0.000E+00 | 0.000E+00 | 0.000E+00 | 0.000E+00 | 0.000E+00 | 0.000E+00 |
| Unigene115843 | srmB | 0.000E+00 | 8.069E-07 | 6.558E-08 | 0.000E+00 | 0.000E+00 | 0.000E+00 | 0.000E+00 | 0.000E+00 | 0.000E+00 | 0.000E+00 | 0.000E+00 | 0.000E+00 |
| Unigene115969 | adeL | 0.000E+00 | 1.066E-06 | 0.000E+00 | 0.000E+00 | 0.000E+00 | 0.000E+00 | 0.000E+00 | 0.000E+00 | 0.000E+00 | 0.000E+00 | 0.000E+00 | 0.000E+00 |
| Unigene115970 | BJP-1 | 9.158E-08 | 0.000E+00 | 2.246E-06 | 1.896E-06 | 0.000E+00 | 0.000E+00 | 0.000E+00 | 0.000E+00 | 0.000E+00 | 0.000E+00 | 0.000E+00 | 0.000E+00 |
| Unigene116027 | arlS | 0.000E+00 | 1.142E-06 | 2.083E-06 | 1.723E-06 | 0.000E+00 | 0.000E+00 | 0.000E+00 | 2.429E-08 | 0.000E+00 | 0.000E+00 | 0.000E+00 | 0.000E+00 |
| Unigene116028 | arlR | 0.000E+00 | 1.031E-06 | 1.890E-06 | 4.318E-06 | 0.000E+00 | 0.000E+00 | 0.000E+00 | 0.000E+00 | 0.000E+00 | 0.000E+00 | 0.000E+00 | 0.000E+00 |
| Unigene116188 | bcrA | 3.563E-07 | 7.821E-07 | 1.294E-06 | 3.310E-06 | 8.312E-07 | 3.081E-07 | 3.155E-07 | 9.981E-07 | 0.000E+00 | 7.541E-07 | 1.182E-06 | 6.738E-07 |
| Unigene116342 | emrB | 1.155E-07 | 7.856E-07 | 3.172E-07 | 1.632E-07 | 0.000E+00 | 0.000E+00 | 0.000E+00 | 0.000E+00 | 0.000E+00 | 0.000E+00 | 0.000E+00 | 0.000E+00 |
| Unigene116416 | novA | 0.000E+00 | 5.265E-07 | 0.000E+00 | 0.000E+00 | 0.000E+00 | 0.000E+00 | 0.000E+00 | 0.000E+00 | 0.000E+00 | 0.000E+00 | 0.000E+00 | 0.000E+00 |
| Unigene116481 | Streptoco | 0.000E+00 | 6.516E-07 | 0.000E+00 | 0.000E+00 | 0.000E+00 | 0.000E+00 | 0.000E+00 | 8.662E-08 | 0.000E+00 | 0.000E+00 | 0.000E+00 | 0.000E+00 |
| Unigene116706 | vanRL | 4.598E-06 | 1.368E-05 | 1.502E-05 | 1.728E-05 | 0.000E+00 | 0.000E+00 | 0.000E+00 | 0.000E+00 | 0.000E+00 | 0.000E+00 | 0.000E+00 | 0.000E+00 |
| Unigene116735 | optrA | 2.957E-06 | 4.346E-06 | 3.918E-06 | 5.787E-06 | 3.898E-07 | 7.225E-08 | 4.623E-07 | 0.000E+00 | 2.009E-07 | 0.000E+00 | 9.556E-08 | 1.777E-07 |
| Unigene116887 | vanHA | 0.000E+00 | 3.981E-07 | 0.000E+00 | 0.000E+00 | 0.000E+00 | 0.000E+00 | 0.000E+00 | 0.000E+00 | 0.000E+00 | 0.000E+00 | 0.000E+00 | 2.144E-07 |
| Unigene116896 | Klebsiella p | 0.000E+00 | 1.048E-06 | 0.000E+00 | 1.286E-07 | 0.000E+00 | 0.000E+00 | 0.000E+00 | 0.000E+00 | 0.000E+00 | 0.000E+00 | 0.000E+00 | 0.000E+00 |
| Unigene116923 | mdtA | 6.079E-07 | 8.340E-07 | 9.246E-07 | 3.683E-07 | 6.720E-06 | 0.000E+00 | 7.401E-07 | 4.612E-07 | 0.000E+00 | 0.000E+00 | 0.000E+00 | 0.000E+00 |
| Unigene116951 | lmrD | 3.271E-07 | 7.538E-07 | 3.273E-06 | 3.534E-06 | 0.000E+00 | 0.000E+00 | 0.000E+00 | 0.000E+00 | 0.000E+00 | 0.000E+00 | 0.000E+00 | 0.000E+00 |
| Unigene116958 | patA | 5.425E-07 | 2.630E-06 | 1.198E-06 | 0.000E+00 | 1.025E-05 | 0.000E+00 | 1.401E-06 | 0.000E+00 | 0.000E+00 | 5.540E-07 | 0.000E+00 | 0.000E+00 |
| Unigene116988 | macB | 1.330E-06 | 6.022E-06 | 1.072E-05 | 1.660E-05 | 0.000E+00 | 0.000E+00 | 0.000E+00 | 0.000E+00 | 0.000E+00 | 0.000E+00 | 0.000E+00 | 0.000E+00 |
| Unigene116990 | Rhodococ | 8.944E-07 | 6.723E-06 | 9.840E-06 | 1.514E-05 | 0.000E+00 | 0.000E+00 | 0.000E+00 | 0.000E+00 | 0.000E+00 | 0.000E+00 | 0.000E+00 | 0.000E+00 |
| Unigene117060 | efrA | 5.076E-08 | 4.456E-07 | 1.494E-07 | 0.000E+00 | 0.000E+00 | 0.000E+00 | 0.000E+00 | 0.000E+00 | 0.000E+00 | 0.000E+00 | 0.000E+00 | 0.000E+00 |
| Unigene117119 | otr(B) | 0.000E+00 | 1.955E-07 | 0.000E+00 | 0.000E+00 | 0.000E+00 | 0.000E+00 | 0.000E+00 | 8.318E-08 | 0.000E+00 | 0.000E+00 | 0.000E+00 | 0.000E+00 |
| Unigene117200 | ceoB | 0.000E+00 | 0.000E+00 | 0.000E+00 | 0.000E+00 | 0.000E+00 | 0.000E+00 | 0.000E+00 | 0.000E+00 | 0.000E+00 | 0.000E+00 | 2.917E-07 | 0.000E+00 |
| Unigene117224 | cpxA | 4.197E-07 | 7.538E-07 | 3.744E-07 | 3.853E-07 | 0.000E+00 | 0.000E+00 | 0.000E+00 | 0.000E+00 | 0.000E+00 | 0.000E+00 | 0.000E+00 | 0.000E+00 |
| Unigene117232 | Klebsiella p | 0.000E+00 | 4.158E-07 | 0.000E+00 | 0.000E+00 | 0.000E+00 | 0.000E+00 | 0.000E+00 | 0.000E+00 | 0.000E+00 | 0.000E+00 | 0.000E+00 | 0.000E+00 |
| Unigene117266 | vanHO | 0.000E+00 | 9.248E-07 | 1.304E-06 | 2.095E-06 | 0.000E+00 | 0.000E+00 | 0.000E+00 | 0.000E+00 | 0.000E+00 | 0.000E+00 | 0.000E+00 | 0.000E+00 |

| | | | | | | | | | | | | | |
|---|---|---|---|---|---|---|---|---|---|---|---|---|---|
| Unigene117323 | patB | 0.000E+00 | 8.251E-07 | 1.844E-07 | 0.000E+00 | 0.000E+00 | 0.000E+00 | 0.000E+00 | 0.000E+00 | 0.000E+00 | 0.000E+00 | 0.000E+00 | 0.000E+00 |
| Unigene117331 | efrB | 0.000E+00 | 1.847E-06 | 1.024E-06 | 6.240E-07 | 3.787E-06 | 0.000E+00 | 1.391E-06 | 0.000E+00 | 0.000E+00 | 0.000E+00 | 0.000E+00 | 0.000E+00 |
| Unigene117451 | tetA(58) | 0.000E+00 | 8.435E-07 | 0.000E+00 | 0.000E+00 | 0.000E+00 | 0.000E+00 | 0.000E+00 | 0.000E+00 | 0.000E+00 | 0.000E+00 | 0.000E+00 | 0.000E+00 |
| Unigene117481 | PmrF | 0.000E+00 | 7.335E-07 | 7.020E-05 | 0.000E+00 | 4.455E-07 | 0.000E+00 | 0.000E+00 | 0.000E+00 | 5.566E-05 | 0.000E+00 | 0.000E+00 | 0.000E+00 |
| Unigene117490 | emrB | 1.377E-05 | 4.876E-06 | 3.154E-06 | 1.355E-05 | 9.419E-05 | 1.296E-04 | 1.362E-04 | 1.127E-04 | 1.713E-04 | 1.709E-04 | 1.382E-04 | 8.444E-05 |
| Unigene117522 | vanYB | 0.000E+00 | 0.000E+00 | 3.180E-06 | 3.142E-06 | 0.000E+00 | 0.000E+00 | 0.000E+00 | 0.000E+00 | 0.000E+00 | 0.000E+00 | 0.000E+00 | 0.000E+00 |
| Unigene117682 | oleC | 0.000E+00 | 4.542E-07 | 0.000E+00 | 0.000E+00 | 9.195E-07 | 3.132E-07 | 0.000E+00 | 0.000E+00 | 0.000E+00 | 0.000E+00 | 0.000E+00 | 2.495E-06 |
| Unigene117692 | bacA | 0.000E+00 | 1.189E-06 | 2.583E-06 | 2.501E-06 | 0.000E+00 | 0.000E+00 | 0.000E+00 | 0.000E+00 | 0.000E+00 | 0.000E+00 | 0.000E+00 | 0.000E+00 |
| Unigene117710 | lsaA | 0.000E+00 | 1.333E-06 | 2.013E-06 | 1.818E-06 | 0.000E+00 | 0.000E+00 | 0.000E+00 | 0.000E+00 | 0.000E+00 | 0.000E+00 | 0.000E+00 | 0.000E+00 |
| Unigene117720 | AcrS | 6.277E-07 | 1.083E-06 | 0.000E+00 | 0.000E+00 | 7.172E-06 | 5.816E-07 | 0.000E+00 | 4.187E-07 | 0.000E+00 | 0.000E+00 | 0.000E+00 | 3.180E-07 |
| Unigene117726 | arlR | 0.000E+00 | 1.359E-06 | 0.000E+00 | 0.000E+00 | 6.400E-06 | 0.000E+00 | 0.000E+00 | 1.667E-07 | 0.000E+00 | 0.000E+00 | 0.000E+00 | 0.000E+00 |
| Unigene117759 | dfrA3 | 0.000E+00 | 3.227E-07 | 0.000E+00 | 0.000E+00 | 0.000E+00 | 0.000E+00 | 0.000E+00 | 0.000E+00 | 0.000E+00 | 0.000E+00 | 1.345E-06 | 2.433E-06 |
| Unigene117790 | tetA(58) | 3.172E-05 | 6.691E-06 | 5.804E-06 | 2.854E-07 | 0.000E+00 | 1.091E-07 | 0.000E+00 | 0.000E+00 | 0.000E+00 | 0.000E+00 | 0.000E+00 | 0.000E+00 |
| Unigene117898 | vanHB | 0.000E+00 | 1.928E-06 | 4.402E-06 | 4.815E-06 | 0.000E+00 | 0.000E+00 | 0.000E+00 | 0.000E+00 | 0.000E+00 | 0.000E+00 | 0.000E+00 | 0.000E+00 |
| Unigene117956 | vgaALC | 0.000E+00 | 6.216E-07 | 3.751E-06 | 2.812E-06 | 0.000E+00 | 0.000E+00 | 0.000E+00 | 0.000E+00 | 0.000E+00 | 0.000E+00 | 0.000E+00 | 0.000E+00 |
| Unigene117998 | Klebsiella p | 0.000E+00 | 5.624E-07 | 4.609E-07 | 3.881E-07 | 0.000E+00 | 0.000E+00 | 0.000E+00 | 0.000E+00 | 0.000E+00 | 0.000E+00 | 0.000E+00 | 0.000E+00 |
| Unigene118005 | baeS | 0.000E+00 | 6.653E-07 | 3.357E-06 | 3.782E-06 | 0.000E+00 | 0.000E+00 | 0.000E+00 | 0.000E+00 | 0.000E+00 | 0.000E+00 | 0.000E+00 | 0.000E+00 |
| Unigene118026 | vanHF | 0.000E+00 | 3.535E-06 | 1.210E-06 | 8.305E-07 | 0.000E+00 | 0.000E+00 | 0.000E+00 | 0.000E+00 | 0.000E+00 | 0.000E+00 | 0.000E+00 | 0.000E+00 |
| Unigene118028 | tlrC | 8.701E-08 | 5.730E-07 | 3.984E-07 | 2.929E-08 | 0.000E+00 | 0.000E+00 | 0.000E+00 | 0.000E+00 | 0.000E+00 | 0.000E+00 | 0.000E+00 | 0.000E+00 |
| Unigene118029 | MexB | 0.000E+00 | 6.947E-07 | 0.000E+00 | 0.000E+00 | 0.000E+00 | 0.000E+00 | 0.000E+00 | 0.000E+00 | 0.000E+00 | 0.000E+00 | 0.000E+00 | 0.000E+00 |
| Unigene118083 | vmlR | 0.000E+00 | 7.381E-07 | 4.242E-07 | 2.183E-06 | 0.000E+00 | 0.000E+00 | 0.000E+00 | 0.000E+00 | 0.000E+00 | 0.000E+00 | 0.000E+00 | 0.000E+00 |
| Unigene118128 | TaeA | 0.000E+00 | 1.754E-07 | 4.704E-07 | 0.000E+00 | 0.000E+00 | 0.000E+00 | 0.000E+00 | 0.000E+00 | 0.000E+00 | 0.000E+00 | 0.000E+00 | 0.000E+00 |
| Unigene118167 | oleC | 7.955E-07 | 1.333E-06 | 4.519E-07 | 0.000E+00 | 1.628E-06 | 2.399E-06 | 1.344E-05 | 0.000E+00 | 4.347E-06 | 0.000E+00 | 0.000E+00 | 5.939E-07 |
| Unigene118172 | H-NS | 0.000E+00 | 3.535E-06 | 0.000E+00 | 1.038E-06 | 0.000E+00 | 0.000E+00 | 0.000E+00 | 0.000E+00 | 0.000E+00 | 0.000E+00 | 1.333E-06 | 2.430E-07 |
| Unigene118222 | Acinetoba | 0.000E+00 | 3.955E-07 | 0.000E+00 | 0.000E+00 | 0.000E+00 | 0.000E+00 | 5.698E-08 | 0.000E+00 | 0.000E+00 | 0.000E+00 | 0.000E+00 | 0.000E+00 |
| Unigene118227 | Klebsiella p | 0.000E+00 | 2.228E-07 | 0.000E+00 | 0.000E+00 | 0.000E+00 | 0.000E+00 | 0.000E+00 | 0.000E+00 | 0.000E+00 | 0.000E+00 | 0.000E+00 | 0.000E+00 |
| Unigene118240 | vatE | 0.000E+00 | 7.833E-07 | 3.286E-06 | 3.548E-06 | 0.000E+00 | 0.000E+00 | 0.000E+00 | 0.000E+00 | 0.000E+00 | 0.000E+00 | 0.000E+00 | 0.000E+00 |
| Unigene118250 | patB | 2.271E-07 | 6.544E-07 | 1.281E-06 | 3.297E-06 | 5.362E-07 | 3.990E-07 | 0.000E+00 | 1.127E-06 | 0.000E+00 | 7.274E-07 | 8.444E-07 | 7.047E-07 |
| Unigene118260 | vanSC | 0.000E+00 | 0.000E+00 | 0.000E+00 | 3.829E-07 | 0.000E+00 | 0.000E+00 | 0.000E+00 | 0.000E+00 | 0.000E+00 | 0.000E+00 | 0.000E+00 | 0.000E+00 |
| Unigene118341 | tetA(60) | 3.977E-06 | 1.871E-05 | 2.805E-06 | 8.248E-07 | 8.877E-07 | 2.303E-07 | 1.179E-07 | 2.694E-07 | 1.009E-06 | 6.329E-07 | 3.656E-07 | 0.000E+00 |
| Unigene118354 | tetA(58) | 4.894E-06 | 3.690E-06 | 1.870E-06 | 1.162E-05 | 1.378E-05 | 1.155E-05 | 3.874E-05 | 3.297E-05 | 1.527E-05 | 1.095E-05 | 0.000E+00 | 3.180E-06 |
| Unigene118355 | oleC | 0.000E+00 | 3.533E-07 | 1.805E-07 | 0.000E+00 | 0.000E+00 | 0.000E+00 | 0.000E+00 | 5.367E-08 | 0.000E+00 | 0.000E+00 | 0.000E+00 | 0.000E+00 |
| Unigene118521 | Klebsiella p | 0.000E+00 | 7.095E-07 | 2.660E-07 | 4.001E-07 | 0.000E+00 | 0.000E+00 | 0.000E+00 | 0.000E+00 | 0.000E+00 | 0.000E+00 | 0.000E+00 | 0.000E+00 |
| Unigene118589 | vanRG | 0.000E+00 | 1.558E-06 | 2.471E-06 | 1.942E-06 | 0.000E+00 | 0.000E+00 | 0.000E+00 | 0.000E+00 | 0.000E+00 | 0.000E+00 | 0.000E+00 | 0.000E+00 |
| Unigene118590 | vanSG | 1.450E-07 | 1.241E-06 | 1.964E-06 | 1.669E-06 | 0.000E+00 | 0.000E+00 | 0.000E+00 | 0.000E+00 | 0.000E+00 | 0.000E+00 | 0.000E+00 | 0.000E+00 |
| Unigene118591 | vanYB | 2.270E-07 | 1.395E-06 | 1.693E-06 | 1.742E-06 | 0.000E+00 | 0.000E+00 | 0.000E+00 | 0.000E+00 | 0.000E+00 | 0.000E+00 | 0.000E+00 | 0.000E+00 |
| Unigene118635 | smeR | 0.000E+00 | 1.074E-06 | 1.008E-06 | 0.000E+00 | 0.000E+00 | 0.000E+00 | 2.219E-06 | 0.000E+00 | 0.000E+00 | 0.000E+00 | 0.000E+00 | 0.000E+00 |
| Unigene118784 | vanZA | 0.000E+00 | 4.120E-07 | 2.640E-06 | 3.475E-06 | 0.000E+00 | 0.000E+00 | 0.000E+00 | 0.000E+00 | 0.000E+00 | 0.000E+00 | 0.000E+00 | 0.000E+00 |
| Unigene118896 | qnrE1 | 0.000E+00 | 1.044E-06 | 6.384E-07 | 0.000E+00 | 5.560E-07 | 0.000E+00 | 7.755E-07 | 0.000E+00 | 0.000E+00 | 0.000E+00 | 0.000E+00 | 0.000E+00 |
| Unigene119020 | mexQ | 0.000E+00 | 3.690E-07 | 0.000E+00 | 2.264E-07 | 0.000E+00 | 0.000E+00 | 0.000E+00 | 0.000E+00 | 0.000E+00 | 0.000E+00 | 0.000E+00 | 0.000E+00 |
| Unigene119112 | vmlR | 1.038E-06 | 1.554E-06 | 1.065E-06 | 1.811E-06 | 2.407E-05 | 8.469E-06 | 2.027E-05 | 2.055E-05 | 1.599E-05 | 2.560E-05 | 2.370E-05 | 1.015E-05 |
| Unigene119118 | msrC | 0.000E+00 | 7.576E-07 | 0.000E+00 | 0.000E+00 | 0.000E+00 | 0.000E+00 | 0.000E+00 | 0.000E+00 | 0.000E+00 | 0.000E+00 | 0.000E+00 | 0.000E+00 |
| Unigene119149 | macB | 0.000E+00 | 1.566E-06 | 1.779E-06 | 1.675E-06 | 0.000E+00 | 0.000E+00 | 0.000E+00 | 0.000E+00 | 0.000E+00 | 0.000E+00 | 0.000E+00 | 0.000E+00 |
| Unigene119186 | tetA(58) | 2.500E-06 | 3.528E-06 | 2.015E-06 | 1.623E-06 | 2.877E-06 | 2.075E-06 | 2.026E-06 | 8.338E-07 | 8.784E-07 | 3.929E-06 | 5.720E-06 | 1.478E-06 |
| Unigene119214 | OpmD | 0.000E+00 | 3.488E-07 | 0.000E+00 | 0.000E+00 | 0.000E+00 | 0.000E+00 | 0.000E+00 | 5.299E-08 | 0.000E+00 | 0.000E+00 | 0.000E+00 | 0.000E+00 |
| Unigene119221 | vatB | 4.428E-07 | 8.748E-07 | 9.993E-07 | 2.549E-06 | 0.000E+00 | 0.000E+00 | 0.000E+00 | 0.000E+00 | 0.000E+00 | 0.000E+00 | 0.000E+00 | 0.000E+00 |
| Unigene119296 | ugd | 2.248E-07 | 4.830E-06 | 1.923E-06 | 1.719E-06 | 0.000E+00 | 0.000E+00 | 0.000E+00 | 0.000E+00 | 0.000E+00 | 0.000E+00 | 0.000E+00 | 0.000E+00 |
| Unigene119302 | Streptomy | 0.000E+00 | 2.430E-07 | 0.000E+00 | 0.000E+00 | 0.000E+00 | 0.000E+00 | 0.000E+00 | 1.551E-07 | 9.679E-08 | 0.000E+00 | 0.000E+00 | 3.140E-07 |
| Unigene119321 | golS | 0.000E+00 | 7.199E-06 | 1.056E-05 | 1.719E-05 | 0.000E+00 | 0.000E+00 | 1.423E-07 | 0.000E+00 | 0.000E+00 | 0.000E+00 | 0.000E+00 | 0.000E+00 |
| Unigene119330 | macB | 3.318E-07 | 4.838E-06 | 5.488E-06 | 1.115E-05 | 0.000E+00 | 0.000E+00 | 0.000E+00 | 0.000E+00 | 0.000E+00 | 0.000E+00 | 0.000E+00 | 0.000E+00 |
| Unigene119535 | arlS | 3.874E-05 | 2.614E-05 | 2.087E-05 | 2.270E-05 | 1.668E-06 | 1.967E-06 | 3.424E-06 | 5.311E-07 | 0.000E+00 | 5.829E-07 | 1.743E-06 | 1.560E-06 |
| Unigene119567 | vatH | 5.170E-07 | 1.915E-06 | 3.171E-07 | 3.263E-07 | 3.590E-07 | 1.048E-07 | 2.146E-07 | 0.000E+00 | 7.417E-07 | 2.520E-07 | 1.848E-07 | 0.000E+00 |
| Unigene119581 | bcr-1 | 0.000E+00 | 7.201E-07 | 2.575E-07 | 0.000E+00 | 0.000E+00 | 0.000E+00 | 0.000E+00 | 0.000E+00 | 0.000E+00 | 0.000E+00 | 0.000E+00 | 0.000E+00 |
| Unigene119651 | tetB(46) | 0.000E+00 | 1.139E-06 | 0.000E+00 | 0.000E+00 | 0.000E+00 | 0.000E+00 | 0.000E+00 | 0.000E+00 | 0.000E+00 | 0.000E+00 | 0.000E+00 | 0.000E+00 |
| Unigene119652 | tetB(46) | 0.000E+00 | 1.416E-06 | 0.000E+00 | 1.600E-06 | 0.000E+00 | 0.000E+00 | 0.000E+00 | 0.000E+00 | 0.000E+00 | 0.000E+00 | 0.000E+00 | 0.000E+00 |
| Unigene119697 | macB | 0.000E+00 | 7.316E-07 | 3.083E-06 | 3.798E-06 | 0.000E+00 | 0.000E+00 | 0.000E+00 | 0.000E+00 | 0.000E+00 | 0.000E+00 | 0.000E+00 | 0.000E+00 |
| Unigene119715 | rpoB2 | 0.000E+00 | 3.845E-07 | 3.438E-07 | 0.000E+00 | 0.000E+00 | 0.000E+00 | 2.216E-07 | 0.000E+00 | 0.000E+00 | 0.000E+00 | 0.000E+00 | 0.000E+00 |
| Unigene119902 | vanHO | 0.000E+00 | 5.808E-07 | 3.462E-07 | 2.375E-07 | 0.000E+00 | 0.000E+00 | 0.000E+00 | 0.000E+00 | 0.000E+00 | 0.000E+00 | 0.000E+00 | 0.000E+00 |

| | | | | | | | | | | | | | |
|---|---|---|---|---|---|---|---|---|---|---|---|---|---|
| Unigene119910 | bcrA | 7.466E-07 | 1.109E-06 | 1.465E-06 | 1.419E-06 | 1.664E-05 | 6.504E-06 | 1.191E-05 | 1.528E-05 | 8.400E-06 | 1.624E-05 | 1.512E-05 | 7.527E-06 |
| Unigene120033 | vanSG | 0.000E+00 | 1.203E-06 | 1.532E-06 | 1.295E-06 | 0.000E+00 | 0.000E+00 | 0.000E+00 | 0.000E+00 | 0.000E+00 | 0.000E+00 | 0.000E+00 | 0.000E+00 |
| Unigene120062 | tlrC | 0.000E+00 | 4.682E-07 | 3.721E-07 | 0.000E+00 | 0.000E+00 | 0.000E+00 | 0.000E+00 | 0.000E+00 | 0.000E+00 | 0.000E+00 | 0.000E+00 | 0.000E+00 |
| Unigene120134 | AcrF | 0.000E+00 | 7.109E-07 | 0.000E+00 | 0.000E+00 | 0.000E+00 | 0.000E+00 | 0.000E+00 | 0.000E+00 | 0.000E+00 | 0.000E+00 | 1.647E-07 | 0.000E+00 |
| Unigene120142 | tetB(60) | 2.680E-06 | 3.665E-06 | 2.994E-06 | 4.538E-06 | 3.206E-07 | 0.000E+00 | 5.019E-07 | 0.000E+00 | 3.154E-07 | 0.000E+00 | 0.000E+00 | 1.462E-07 |
| Unigene120204 | oleC | 4.146E-06 | 5.115E-06 | 3.867E-06 | 8.063E-06 | 4.568E-07 | 1.852E-07 | 3.034E-07 | 0.000E+00 | 7.491E-08 | 0.000E+00 | 0.000E+00 | 1.215E-07 |
| Unigene120273 | golS | 0.000E+00 | 1.053E-06 | 0.000E+00 | 0.000E+00 | 0.000E+00 | 0.000E+00 | 0.000E+00 | 0.000E+00 | 0.000E+00 | 0.000E+00 | 0.000E+00 | 0.000E+00 |
| Unigene120320 | vanSA | 1.527E-06 | 3.146E-06 | 2.667E-06 | 3.609E-06 | 2.482E-07 | 4.025E-08 | 2.472E-07 | 0.000E+00 | 0.000E+00 | 0.000E+00 | 1.278E-07 | 8.802E-08 |
| Unigene120321 | macB | 3.025E-06 | 3.995E-06 | 2.968E-06 | 5.487E-06 | 2.974E-07 | 0.000E+00 | 2.962E-07 | 0.000E+00 | 0.000E+00 | 8.945E-08 | 1.225E-07 | 1.266E-07 |
| Unigene120484 | macB | 0.000E+00 | 6.561E-07 | 0.000E+00 | 0.000E+00 | 0.000E+00 | 0.000E+00 | 0.000E+00 | 0.000E+00 | 0.000E+00 | 0.000E+00 | 0.000E+00 | 0.000E+00 |
| Unigene120522 | emrB | 0.000E+00 | 1.255E-06 | 1.584E-06 | 1.630E-06 | 0.000E+00 | 0.000E+00 | 0.000E+00 | 0.000E+00 | 0.000E+00 | 0.000E+00 | 0.000E+00 | 0.000E+00 |
| Unigene120550 | bcrC | 0.000E+00 | 1.163E-06 | 0.000E+00 | 0.000E+00 | 5.476E-06 | 5.157E-07 | 0.000E+00 | 0.000E+00 | 0.000E+00 | 0.000E+00 | 0.000E+00 | 0.000E+00 |
| Unigene120652 | abeS | 4.719E-07 | 1.036E-06 | 0.000E+00 | 0.000E+00 | 0.000E+00 | 0.000E+00 | 0.000E+00 | 0.000E+00 | 0.000E+00 | 0.000E+00 | 0.000E+00 | 0.000E+00 |
| Unigene120708 | vanRM | 3.408E-07 | 2.458E-06 | 1.051E-06 | 2.065E-06 | 0.000E+00 | 0.000E+00 | 0.000E+00 | 0.000E+00 | 0.000E+00 | 0.000E+00 | 0.000E+00 | 0.000E+00 |
| Unigene120711 | bcrA | 2.232E-07 | 4.704E-06 | 1.227E-06 | 1.623E-06 | 0.000E+00 | 0.000E+00 | 0.000E+00 | 0.000E+00 | 0.000E+00 | 0.000E+00 | 0.000E+00 | 0.000E+00 |
| Unigene120853 | AcrE | 2.438E-07 | 9.555E-07 | 2.734E-07 | 0.000E+00 | 0.000E+00 | 0.000E+00 | 0.000E+00 | 0.000E+00 | 0.000E+00 | 0.000E+00 | 0.000E+00 | 0.000E+00 |
| Unigene120904 | vanHF | 0.000E+00 | 8.508E-07 | 3.590E-06 | 4.165E-06 | 0.000E+00 | 0.000E+00 | 0.000E+00 | 0.000E+00 | 0.000E+00 | 0.000E+00 | 0.000E+00 | 0.000E+00 |
| Unigene120919 | kdpE | 0.000E+00 | 8.883E-07 | 1.402E-07 | 0.000E+00 | 0.000E+00 | 0.000E+00 | 0.000E+00 | 0.000E+00 | 0.000E+00 | 0.000E+00 | 0.000E+00 | 0.000E+00 |
| Unigene120945 | ramA | 0.000E+00 | 5.180E-07 | 0.000E+00 | 0.000E+00 | 0.000E+00 | 0.000E+00 | 0.000E+00 | 0.000E+00 | 0.000E+00 | 0.000E+00 | 0.000E+00 | 0.000E+00 |
| Unigene120952 | mtrA | 0.000E+00 | 4.072E-07 | 0.000E+00 | 0.000E+00 | 0.000E+00 | 0.000E+00 | 0.000E+00 | 0.000E+00 | 0.000E+00 | 0.000E+00 | 0.000E+00 | 0.000E+00 |
| Unigene121016 | tva(A) | 2.409E-07 | 4.847E-07 | 3.034E-06 | 2.879E-06 | 0.000E+00 | 0.000E+00 | 0.000E+00 | 0.000E+00 | 0.000E+00 | 0.000E+00 | 0.000E+00 | 0.000E+00 |
| Unigene121240 | srmB | 0.000E+00 | 3.394E-07 | 0.000E+00 | 0.000E+00 | 0.000E+00 | 0.000E+00 | 0.000E+00 | 0.000E+00 | 0.000E+00 | 0.000E+00 | 0.000E+00 | 0.000E+00 |
| Unigene121442 | optrA | 0.000E+00 | 3.355E-07 | 0.000E+00 | 0.000E+00 | 0.000E+00 | 0.000E+00 | 0.000E+00 | 0.000E+00 | 0.000E+00 | 0.000E+00 | 0.000E+00 | 0.000E+00 |
| Unigene121496 | msbA | 0.000E+00 | 3.028E-07 | 0.000E+00 | 0.000E+00 | 0.000E+00 | 0.000E+00 | 0.000E+00 | 0.000E+00 | 0.000E+00 | 0.000E+00 | 0.000E+00 | 0.000E+00 |
| Unigene121607 | tetA(58) | 0.000E+00 | 4.662E-07 | 2.315E-07 | 0.000E+00 | 0.000E+00 | 0.000E+00 | 0.000E+00 | 0.000E+00 | 0.000E+00 | 0.000E+00 | 0.000E+00 | 0.000E+00 |
| Unigene121633 | mgrA | 0.000E+00 | 1.156E-06 | 0.000E+00 | 1.520E-07 | 0.000E+00 | 3.254E-07 | 5.829E-07 | 0.000E+00 | 0.000E+00 | 4.610E-06 | 4.562E-06 | 0.000E+00 |
| Unigene121655 | Enterobact | 0.000E+00 | 7.188E-07 | 1.836E-07 | 4.252E-07 | 0.000E+00 | 0.000E+00 | 0.000E+00 | 0.000E+00 | 0.000E+00 | 0.000E+00 | 0.000E+00 | 0.000E+00 |
| Unigene121656 | AcrS | 0.000E+00 | 8.163E-07 | 4.379E-07 | 0.000E+00 | 0.000E+00 | 0.000E+00 | 0.000E+00 | 0.000E+00 | 0.000E+00 | 0.000E+00 | 0.000E+00 | 0.000E+00 |
| Unigene121658 | macB | 5.308E-07 | 5.984E-06 | 8.333E-06 | 1.359E-05 | 0.000E+00 | 0.000E+00 | 1.068E-07 | 0.000E+00 | 0.000E+00 | 0.000E+00 | 0.000E+00 | 0.000E+00 |
| Unigene121774 | LlmA 23S r | 1.296E-07 | 9.248E-07 | 0.000E+00 | 1.309E-07 | 7.201E-08 | 0.000E+00 | 0.000E+00 | 0.000E+00 | 0.000E+00 | 0.000E+00 | 0.000E+00 | 0.000E+00 |
| Unigene121860 | tetA(60) | 0.000E+00 | 8.748E-07 | 3.147E-06 | 3.603E-06 | 0.000E+00 | 0.000E+00 | 0.000E+00 | 0.000E+00 | 0.000E+00 | 0.000E+00 | 0.000E+00 | 0.000E+00 |
| Unigene121896 | vgaB | 5.723E-07 | 1.005E-06 | 4.006E-06 | 6.204E-06 | 0.000E+00 | 0.000E+00 | 4.645E-07 | 0.000E+00 | 0.000E+00 | 0.000E+00 | 0.000E+00 | 4.510E-08 |
| Unigene121910 | efrA | 0.000E+00 | 1.382E-06 | 1.011E-06 | 0.000E+00 | 0.000E+00 | 0.000E+00 | 0.000E+00 | 0.000E+00 | 0.000E+00 | 0.000E+00 | 0.000E+00 | 0.000E+00 |
| Unigene121943 | tetA(60) | 2.786E-06 | 4.507E-06 | 3.453E-06 | 5.757E-06 | 3.869E-07 | 0.000E+00 | 1.826E-07 | 2.140E-08 | 4.007E-08 | 0.000E+00 | 4.194E-08 | 2.167E-07 |
| Unigene121944 | bcrA | 2.379E-06 | 4.287E-06 | 3.694E-06 | 5.809E-06 | 3.945E-07 | 0.000E+00 | 3.144E-07 | 0.000E+00 | 1.941E-07 | 0.000E+00 | 0.000E+00 | 1.679E-07 |
| Unigene122001 | evgS | 0.000E+00 | 4.656E-07 | 0.000E+00 | 0.000E+00 | 0.000E+00 | 0.000E+00 | 0.000E+00 | 2.251E-07 | 0.000E+00 | 0.000E+00 | 0.000E+00 | 0.000E+00 |
| Unigene122152 | Staphylocc | 0.000E+00 | 0.000E+00 | 1.160E-07 | 0.000E+00 | 0.000E+00 | 0.000E+00 | 0.000E+00 | 0.000E+00 | 0.000E+00 | 0.000E+00 | 0.000E+00 | 0.000E+00 |
| Unigene122153 | tetA(46) | 5.525E-07 | 1.085E-06 | 4.223E-06 | 5.022E-06 | 0.000E+00 | 0.000E+00 | 3.540E-07 | 1.018E-07 | 0.000E+00 | 0.000E+00 | 0.000E+00 | 0.000E+00 |
| Unigene122196 | Staphylocc | 9.729E-07 | 9.407E-07 | 4.353E-06 | 5.977E-06 | 2.574E-08 | 6.261E-08 | 3.717E-07 | 2.704E-08 | 1.266E-08 | 0.000E+00 | 0.000E+00 | 0.000E+00 |
| Unigene122271 | hmrM | 0.000E+00 | 4.641E-07 | 1.614E-07 | 1.186E-07 | 0.000E+00 | 0.000E+00 | 0.000E+00 | 0.000E+00 | 0.000E+00 | 0.000E+00 | 0.000E+00 | 0.000E+00 |
| Unigene122276 | PmrF | 0.000E+00 | 3.302E-06 | 3.191E-06 | 5.063E-06 | 0.000E+00 | 0.000E+00 | 0.000E+00 | 0.000E+00 | 0.000E+00 | 0.000E+00 | 0.000E+00 | 0.000E+00 |
| Unigene122338 | acrB | 8.942E-08 | 8.505E-07 | 3.217E-07 | 3.010E-07 | 0.000E+00 | 0.000E+00 | 0.000E+00 | 0.000E+00 | 0.000E+00 | 0.000E+00 | 0.000E+00 | 0.000E+00 |
| Unigene122475 | tetA(60) | 0.000E+00 | 1.036E-06 | 6.756E-07 | 0.000E+00 | 0.000E+00 | 0.000E+00 | 1.154E-06 | 0.000E+00 | 0.000E+00 | 0.000E+00 | 0.000E+00 | 0.000E+00 |
| Unigene122540 | lmrD | 3.927E-07 | 4.504E-06 | 3.044E-06 | 3.272E-06 | 0.000E+00 | 0.000E+00 | 2.173E-08 | 0.000E+00 | 0.000E+00 | 0.000E+00 | 0.000E+00 | 0.000E+00 |
| Unigene122570 | msbA | 0.000E+00 | 3.059E-07 | 2.188E-07 | 0.000E+00 | 0.000E+00 | 0.000E+00 | 0.000E+00 | 0.000E+00 | 0.000E+00 | 0.000E+00 | 0.000E+00 | 0.000E+00 |
| Unigene122604 | arlR | 7.453E-07 | 1.585E-06 | 8.684E-07 | 1.364E-06 | 1.832E-05 | 4.230E-06 | 1.433E-05 | 1.734E-05 | 1.161E-05 | 1.640E-05 | 1.790E-05 | 7.378E-06 |
| Unigene122686 | tetA(58) | 0.000E+00 | 7.357E-07 | 1.039E-06 | 2.708E-06 | 4.703E-07 | 0.000E+00 | 3.514E-07 | 9.471E-07 | 1.928E-07 | 7.860E-07 | 8.071E-07 | 3.753E-07 |
| Unigene122823 | poxtA | 0.000E+00 | 7.669E-07 | 0.000E+00 | 1.976E-07 | 0.000E+00 | 0.000E+00 | 0.000E+00 | 0.000E+00 | 0.000E+00 | 0.000E+00 | 0.000E+00 | 0.000E+00 |
| Unigene122845 | kdpE | 0.000E+00 | 6.748E-07 | 4.223E-07 | 0.000E+00 | 0.000E+00 | 0.000E+00 | 0.000E+00 | 0.000E+00 | 0.000E+00 | 0.000E+00 | 0.000E+00 | 0.000E+00 |
| Unigene122938 | macB | 0.000E+00 | 7.485E-07 | 4.610E-06 | 5.662E-06 | 0.000E+00 | 0.000E+00 | 0.000E+00 | 0.000E+00 | 0.000E+00 | 0.000E+00 | 0.000E+00 | 0.000E+00 |
| Unigene123034 | chrB | 0.000E+00 | 1.216E-06 | 0.000E+00 | 0.000E+00 | 0.000E+00 | 0.000E+00 | 0.000E+00 | 0.000E+00 | 0.000E+00 | 0.000E+00 | 0.000E+00 | 0.000E+00 |
| Unigene123035 | mecD | 0.000E+00 | 1.077E-06 | 6.360E-07 | 0.000E+00 | 0.000E+00 | 0.000E+00 | 1.127E-06 | 0.000E+00 | 0.000E+00 | 0.000E+00 | 0.000E+00 | 0.000E+00 |
| Unigene123043 | YojI | 2.696E-06 | 8.934E-07 | 2.446E-06 | 7.449E-07 | 0.000E+00 | 0.000E+00 | 0.000E+00 | 1.485E-07 | 0.000E+00 | 0.000E+00 | 0.000E+00 | 0.000E+00 |
| Unigene123295 | Corynebac | 0.000E+00 | 8.606E-07 | 2.137E-07 | 0.000E+00 | 0.000E+00 | 0.000E+00 | 0.000E+00 | 0.000E+00 | 0.000E+00 | 0.000E+00 | 0.000E+00 | 0.000E+00 |
| Unigene123318 | acrD | 1.594E-07 | 2.624E-07 | 0.000E+00 | 0.000E+00 | 0.000E+00 | 0.000E+00 | 0.000E+00 | 0.000E+00 | 0.000E+00 | 0.000E+00 | 0.000E+00 | 0.000E+00 |
| Unigene123365 | TaeA | 2.232E-05 | 8.478E-06 | 5.605E-06 | 3.444E-06 | 4.736E-07 | 0.000E+00 | 7.549E-08 | 0.000E+00 | 1.044E-06 | 0.000E+00 | 0.000E+00 | 0.000E+00 |
| Unigene123380 | smeF | 0.000E+00 | 7.103E-07 | 0.000E+00 | 0.000E+00 | 0.000E+00 | 0.000E+00 | 4.029E-07 | 0.000E+00 | 0.000E+00 | 0.000E+00 | 9.254E-08 | 0.000E+00 |
| Unigene123394 | vanTN | 9.869E-07 | 1.328E-06 | 0.000E+00 | 1.832E-06 | 0.000E+00 | 0.000E+00 | 0.000E+00 | 0.000E+00 | 0.000E+00 | 0.000E+00 | 0.000E+00 | 0.000E+00 |

| | | | | | | | | | | | | | |
|---|---|---|---|---|---|---|---|---|---|---|---|---|---|
| Unigene123417 | msbA | 6.079E-08 | 7.506E-07 | 2.386E-07 | 1.535E-07 | 0.000E+00 | 0.000E+00 | 0.000E+00 | 0.000E+00 | 0.000E+00 | 0.000E+00 | 0.000E+00 | 0.000E+00 |
| Unigene123434 | macB | 0.000E+00 | 3.473E-07 | 0.000E+00 | 0.000E+00 | 0.000E+00 | 0.000E+00 | 0.000E+00 | 4.433E-07 | 0.000E+00 | 0.000E+00 | 0.000E+00 | 0.000E+00 |
| Unigene123519 | optrA | 0.000E+00 | 5.037E-07 | 1.551E-06 | 0.000E+00 | 4.532E-07 | 0.000E+00 | 0.000E+00 | 2.024E-06 | 0.000E+00 | 0.000E+00 | 0.000E+00 | 8.439E-07 |
| Unigene123660 | lsaC | 3.951E-07 | 8.006E-07 | 0.000E+00 | 0.000E+00 | 1.688E-07 | 0.000E+00 | 1.009E-07 | 0.000E+00 | 0.000E+00 | 7.111E-07 | 1.008E-06 | 5.389E-07 |
| Unigene123758 | smeD | 0.000E+00 | 4.113E-07 | 0.000E+00 | 1.081E-07 | 0.000E+00 | 0.000E+00 | 0.000E+00 | 0.000E+00 | 0.000E+00 | 0.000E+00 | 0.000E+00 | 0.000E+00 |
| Unigene123858 | TaeA | 7.737E-05 | 6.721E-05 | 1.217E-04 | 1.779E-04 | 1.322E-04 | 9.137E-05 | 1.391E-04 | 1.220E-04 | 1.036E-04 | 1.569E-04 | 1.495E-04 | 1.727E-04 |
| Unigene123900 | Listeria mc | 2.738E-05 | 9.623E-06 | 7.039E-06 | 4.102E-06 | 3.713E-07 | 0.000E+00 | 5.691E-08 | 2.101E-07 | 9.274E-07 | 0.000E+00 | 0.000E+00 | 0.000E+00 |
| Unigene123999 | arnA | 0.000E+00 | 9.625E-07 | 1.754E-06 | 1.397E-06 | 0.000E+00 | 0.000E+00 | 0.000E+00 | 7.874E-08 | 0.000E+00 | 0.000E+00 | 0.000E+00 | 0.000E+00 |
| Unigene124008 | mdtN | 0.000E+00 | 1.620E-06 | 8.079E-07 | 0.000E+00 | 0.000E+00 | 0.000E+00 | 2.294E-06 | 0.000E+00 | 9.309E-08 | 0.000E+00 | 0.000E+00 | 0.000E+00 |
| Unigene124180 | lin | 0.000E+00 | 5.451E-07 | 0.000E+00 | 0.000E+00 | 0.000E+00 | 0.000E+00 | 0.000E+00 | 0.000E+00 | 0.000E+00 | 0.000E+00 | 0.000E+00 | 0.000E+00 |
| Unigene124235 | vanSM | 0.000E+00 | 5.507E-07 | 2.188E-07 | 5.630E-08 | 0.000E+00 | 0.000E+00 | 0.000E+00 | 0.000E+00 | 0.000E+00 | 0.000E+00 | 0.000E+00 | 0.000E+00 |
| Unigene124320 | Acinetobac | 7.238E-08 | 6.884E-07 | 3.788E-07 | 0.000E+00 | 0.000E+00 | 0.000E+00 | 0.000E+00 | 0.000E+00 | 0.000E+00 | 0.000E+00 | 0.000E+00 | 0.000E+00 |
| Unigene124337 | patB | 3.754E-07 | 1.202E-06 | 0.000E+00 | 0.000E+00 | 0.000E+00 | 0.000E+00 | 9.693E-07 | 1.095E-07 | 0.000E+00 | 0.000E+00 | 0.000E+00 | 0.000E+00 |
| Unigene124378 | adeL | 0.000E+00 | 7.522E-07 | 0.000E+00 | 0.000E+00 | 0.000E+00 | 0.000E+00 | 0.000E+00 | 0.000E+00 | 0.000E+00 | 0.000E+00 | 0.000E+00 | 0.000E+00 |
| Unigene124530 | otrC | 3.743E-07 | 8.215E-07 | 0.000E+00 | 1.575E-07 | 0.000E+00 | 0.000E+00 | 0.000E+00 | 0.000E+00 | 0.000E+00 | 0.000E+00 | 0.000E+00 | 0.000E+00 |
| Unigene124535 | Staphylocc | 0.000E+00 | 1.115E-06 | 2.085E-06 | 1.957E-06 | 0.000E+00 | 0.000E+00 | 0.000E+00 | 0.000E+00 | 0.000E+00 | 0.000E+00 | 0.000E+00 | 0.000E+00 |
| Unigene124549 | otr(B) | 0.000E+00 | 2.241E-06 | 2.640E-06 | 0.000E+00 | 0.000E+00 | 0.000E+00 | 0.000E+00 | 0.000E+00 | 0.000E+00 | 0.000E+00 | 0.000E+00 | 0.000E+00 |
| Unigene124577 | mtrA | 0.000E+00 | 2.940E-07 | 2.935E-06 | 3.607E-06 | 0.000E+00 | 0.000E+00 | 0.000E+00 | 0.000E+00 | 0.000E+00 | 0.000E+00 | 0.000E+00 | 0.000E+00 |
| Unigene124876 | efrB | 0.000E+00 | 7.086E-07 | 0.000E+00 | 1.087E-07 | 0.000E+00 | 0.000E+00 | 0.000E+00 | 0.000E+00 | 0.000E+00 | 0.000E+00 | 0.000E+00 | 0.000E+00 |
| Unigene124950 | Acinetobac | 7.387E-08 | 7.296E-07 | 2.416E-07 | 1.492E-07 | 0.000E+00 | 0.000E+00 | 0.000E+00 | 0.000E+00 | 0.000E+00 | 0.000E+00 | 0.000E+00 | 0.000E+00 |
| Unigene125036 | mdtG | 0.000E+00 | 1.487E-06 | 1.825E-06 | 0.000E+00 | 0.000E+00 | 0.000E+00 | 0.000E+00 | 0.000E+00 | 0.000E+00 | 0.000E+00 | 0.000E+00 | 0.000E+00 |
| Unigene125041 | macB | 0.000E+00 | 1.111E-06 | 1.838E-06 | 1.483E-06 | 0.000E+00 | 0.000E+00 | 0.000E+00 | 0.000E+00 | 0.000E+00 | 0.000E+00 | 0.000E+00 | 0.000E+00 |
| Unigene125047 | vanRF | 0.000E+00 | 8.729E-07 | 2.249E-06 | 0.000E+00 | 0.000E+00 | 0.000E+00 | 0.000E+00 | 0.000E+00 | 0.000E+00 | 0.000E+00 | 0.000E+00 | 0.000E+00 |
| Unigene125121 | macB | 0.000E+00 | 1.590E-06 | 1.340E-06 | 1.546E-06 | 0.000E+00 | 0.000E+00 | 0.000E+00 | 0.000E+00 | 0.000E+00 | 0.000E+00 | 0.000E+00 | 0.000E+00 |
| Unigene125165 | efrB | 0.000E+00 | 6.871E-07 | 7.679E-08 | 0.000E+00 | 0.000E+00 | 0.000E+00 | 0.000E+00 | 0.000E+00 | 0.000E+00 | 0.000E+00 | 0.000E+00 | 0.000E+00 |
| Unigene125357 | Bifidobacte | 1.127E-07 | 3.091E-07 | 0.000E+00 | 0.000E+00 | 0.000E+00 | 0.000E+00 | 0.000E+00 | 0.000E+00 | 0.000E+00 | 0.000E+00 | 0.000E+00 | 6.659E-08 |
| Unigene125396 | AAC(6')-Ib | 0.000E+00 | 6.364E-07 | 0.000E+00 | 3.253E-07 | 0.000E+00 | 0.000E+00 | 0.000E+00 | 0.000E+00 | 0.000E+00 | 0.000E+00 | 0.000E+00 | 0.000E+00 |
| Unigene125469 | bcrA | 0.000E+00 | 5.597E-07 | 2.002E-07 | 5.150E-08 | 0.000E+00 | 0.000E+00 | 0.000E+00 | 0.000E+00 | 0.000E+00 | 0.000E+00 | 0.000E+00 | 0.000E+00 |
| Unigene125740 | msbA | 0.000E+00 | 4.279E-07 | 0.000E+00 | 0.000E+00 | 0.000E+00 | 0.000E+00 | 0.000E+00 | 0.000E+00 | 0.000E+00 | 0.000E+00 | 0.000E+00 | 0.000E+00 |
| Unigene125741 | lsaA | 3.818E-06 | 8.680E-06 | 8.588E-06 | 1.016E-05 | 0.000E+00 | 2.680E-08 | 0.000E+00 | 0.000E+00 | 0.000E+00 | 0.000E+00 | 0.000E+00 | 0.000E+00 |
| Unigene125752 | arlS | 2.401E-06 | 2.352E-06 | 2.608E-06 | 1.472E-06 | 0.000E+00 | 0.000E+00 | 0.000E+00 | 2.002E-07 | 7.262E-07 | 0.000E+00 | 0.000E+00 | 0.000E+00 |
| Unigene125798 | Corynebac | 0.000E+00 | 5.761E-07 | 0.000E+00 | 0.000E+00 | 0.000E+00 | 0.000E+00 | 0.000E+00 | 0.000E+00 | 0.000E+00 | 0.000E+00 | 0.000E+00 | 0.000E+00 |
| Unigene125869 | Corynebac | 0.000E+00 | 9.185E-07 | 0.000E+00 | 0.000E+00 | 0.000E+00 | 0.000E+00 | 0.000E+00 | 0.000E+00 | 0.000E+00 | 0.000E+00 | 0.000E+00 | 0.000E+00 |
| Unigene125900 | macB | 0.000E+00 | 6.763E-07 | 0.000E+00 | 0.000E+00 | 0.000E+00 | 0.000E+00 | 0.000E+00 | 0.000E+00 | 0.000E+00 | 0.000E+00 | 0.000E+00 | 0.000E+00 |
| Unigene125920 | PmrF | 8.086E-07 | 1.638E-06 | 1.526E-06 | 4.083E-07 | 7.083E-06 | 0.000E+00 | 1.136E-06 | 3.630E-07 | 0.000E+00 | 2.079E-07 | 6.759E-07 | 0.000E+00 |
| Unigene126009 | Klebsiella p | 0.000E+00 | 2.386E-07 | 1.600E-07 | 0.000E+00 | 0.000E+00 | 0.000E+00 | 0.000E+00 | 0.000E+00 | 0.000E+00 | 0.000E+00 | 0.000E+00 | 0.000E+00 |
| Unigene126010 | emrR | 6.079E-08 | 6.672E-07 | 1.790E-07 | 1.228E-07 | 0.000E+00 | 0.000E+00 | 0.000E+00 | 0.000E+00 | 0.000E+00 | 0.000E+00 | 0.000E+00 | 0.000E+00 |
| Unigene126066 | lsaC | 1.614E-07 | 5.314E-07 | 1.082E-06 | 7.063E-07 | 0.000E+00 | 0.000E+00 | 0.000E+00 | 0.000E+00 | 0.000E+00 | 0.000E+00 | 0.000E+00 | 0.000E+00 |
| Unigene126104 | arnA | 8.277E-08 | 6.359E-07 | 2.030E-07 | 2.507E-07 | 4.598E-08 | 0.000E+00 | 3.206E-07 | 0.000E+00 | 0.000E+00 | 0.000E+00 | 4.734E-08 | 0.000E+00 |
| Unigene126105 | PmrF | 2.931E-07 | 6.433E-07 | 0.000E+00 | 0.000E+00 | 0.000E+00 | 0.000E+00 | 0.000E+00 | 0.000E+00 | 0.000E+00 | 0.000E+00 | 0.000E+00 | 0.000E+00 |
| Unigene126110 | Chlamydia | 0.000E+00 | 5.799E-07 | 0.000E+00 | 0.000E+00 | 0.000E+00 | 0.000E+00 | 0.000E+00 | 0.000E+00 | 0.000E+00 | 0.000E+00 | 0.000E+00 | 0.000E+00 |
| Unigene126166 | bcrA | 0.000E+00 | 3.094E-07 | 0.000E+00 | 0.000E+00 | 0.000E+00 | 0.000E+00 | 0.000E+00 | 0.000E+00 | 0.000E+00 | 0.000E+00 | 4.837E-07 | 0.000E+00 |
| Unigene126235 | FosA5 | 0.000E+00 | 1.037E-06 | 0.000E+00 | 0.000E+00 | 0.000E+00 | 0.000E+00 | 0.000E+00 | 0.000E+00 | 0.000E+00 | 0.000E+00 | 0.000E+00 | 0.000E+00 |
| Unigene126264 | SRT-2 | 0.000E+00 | 5.025E-07 | 0.000E+00 | 0.000E+00 | 6.994E-07 | 0.000E+00 | 0.000E+00 | 0.000E+00 | 0.000E+00 | 0.000E+00 | 0.000E+00 | 0.000E+00 |
| Unigene126347 | vanHF | 2.320E-06 | 2.214E-06 | 3.299E-06 | 0.000E+00 | 0.000E+00 | 0.000E+00 | 0.000E+00 | 0.000E+00 | 7.350E-07 | 0.000E+00 | 0.000E+00 | 0.000E+00 |
| Unigene126453 | bcrA | 1.301E-07 | 7.527E-07 | 2.785E-07 | 1.910E-07 | 0.000E+00 | 0.000E+00 | 0.000E+00 | 0.000E+00 | 0.000E+00 | 0.000E+00 | 0.000E+00 | 0.000E+00 |
| Unigene126533 | tetA(58) | 4.579E-07 | 5.025E-07 | 0.000E+00 | 0.000E+00 | 1.272E-07 | 0.000E+00 | 0.000E+00 | 0.000E+00 | 0.000E+00 | 0.000E+00 | 0.000E+00 | 0.000E+00 |
| Unigene126664 | otr(B) | 0.000E+00 | 1.150E-06 | 3.534E-06 | 3.438E-06 | 0.000E+00 | 0.000E+00 | 0.000E+00 | 0.000E+00 | 0.000E+00 | 0.000E+00 | 0.000E+00 | 0.000E+00 |
| Unigene126692 | vanSL | 2.706E-06 | 4.108E-06 | 6.122E-06 | 6.726E-06 | 0.000E+00 | 0.000E+00 | 0.000E+00 | 0.000E+00 | 0.000E+00 | 0.000E+00 | 0.000E+00 | 5.746E-08 |
| Unigene126701 | mepR | 0.000E+00 | 1.983E-06 | 0.000E+00 | 1.507E-06 | 6.108E-07 | 4.245E-07 | 0.000E+00 | 2.384E-06 | 0.000E+00 | 0.000E+00 | 1.114E-05 | 0.000E+00 |
| Unigene126723 | patB | 6.407E-07 | 4.261E-06 | 2.718E-06 | 2.017E-06 | 0.000E+00 | 0.000E+00 | 0.000E+00 | 0.000E+00 | 0.000E+00 | 0.000E+00 | 0.000E+00 | 0.000E+00 |
| Unigene126771 | bacA | 9.308E-07 | 6.865E-06 | 9.535E-06 | 1.763E-05 | 0.000E+00 | 1.207E-07 | 0.000E+00 | 0.000E+00 | 0.000E+00 | 0.000E+00 | 0.000E+00 | 0.000E+00 |
| Unigene126877 | MCR-4.4 | 0.000E+00 | 1.243E-06 | 7.473E-07 | 0.000E+00 | 0.000E+00 | 0.000E+00 | 1.470E-06 | 0.000E+00 | 0.000E+00 | 0.000E+00 | 3.351E-07 | 0.000E+00 |
| Unigene126919 | macB | 0.000E+00 | 9.323E-07 | 3.175E-07 | 2.042E-07 | 0.000E+00 | 0.000E+00 | 0.000E+00 | 0.000E+00 | 0.000E+00 | 0.000E+00 | 0.000E+00 | 0.000E+00 |
| Unigene126942 | bmr | 0.000E+00 | 1.767E-06 | 2.291E-06 | 0.000E+00 | 0.000E+00 | 0.000E+00 | 0.000E+00 | 0.000E+00 | 0.000E+00 | 0.000E+00 | 0.000E+00 | 0.000E+00 |
| Unigene127011 | oleC | 0.000E+00 | 8.385E-07 | 3.124E-07 | 1.286E-07 | 0.000E+00 | 0.000E+00 | 0.000E+00 | 0.000E+00 | 0.000E+00 | 0.000E+00 | 0.000E+00 | 0.000E+00 |
| Unigene127184 | vanHD | 0.000E+00 | 6.373E-07 | 1.106E-06 | 2.380E-06 | 3.795E-07 | 0.000E+00 | 0.000E+00 | 9.569E-07 | 7.467E-08 | 0.000E+00 | 1.563E-06 | 7.268E-07 |
| Unigene127206 | efrA | 1.113E-07 | 1.201E-06 | 3.732E-06 | 4.140E-06 | 0.000E+00 | 0.000E+00 | 0.000E+00 | 0.000E+00 | 0.000E+00 | 0.000E+00 | 0.000E+00 | 0.000E+00 |

| | | | | | | | | | | | | | |
|---|---|---|---|---|---|---|---|---|---|---|---|---|---|
| Unigene127238 | tetT | 8.856E-06 | 2.841E-05 | 2.139E-05 | 1.995E-05 | 2.236E-07 | 1.339E-07 | 5.826E-07 | 0.000E+00 | 0.000E+00 | 3.449E-07 | 0.000E+00 | 0.000E+00 |
| Unigene127503 | patB | 1.083E-06 | 5.942E-06 | 1.086E-05 | 1.808E-05 | 0.000E+00 | 0.000E+00 | 0.000E+00 | 0.000E+00 | 0.000E+00 | 0.000E+00 | 0.000E+00 | 4.000E-08 |
| Unigene127527 | lmrD | 0.000E+00 | 2.879E-06 | 1.309E-06 | 4.939E-07 | 7.706E-06 | 0.000E+00 | 0.000E+00 | 4.152E-07 | 0.000E+00 | 0.000E+00 | 0.000E+00 | 0.000E+00 |
| Unigene127641 | ykkC | 0.000E+00 | 0.000E+00 | 0.000E+00 | 0.000E+00 | 0.000E+00 | 4.389E-07 | 0.000E+00 | 0.000E+00 | 0.000E+00 | 0.000E+00 | 0.000E+00 | 0.000E+00 |
| Unigene127662 | bcrA | 0.000E+00 | 4.705E-07 | 2.524E-07 | 1.342E-06 | 0.000E+00 | 0.000E+00 | 0.000E+00 | 0.000E+00 | 0.000E+00 | 0.000E+00 | 0.000E+00 | 0.000E+00 |
| Unigene127672 | vanG | 1.402E-07 | 1.077E-06 | 3.439E-07 | 1.416E-07 | 0.000E+00 | 0.000E+00 | 0.000E+00 | 0.000E+00 | 0.000E+00 | 0.000E+00 | 0.000E+00 | 0.000E+00 |
| Unigene127877 | mepA | 1.196E-06 | 1.023E-06 | 1.877E-06 | 0.000E+00 | 0.000E+00 | 0.000E+00 | 0.000E+00 | 1.116E-07 | 0.000E+00 | 0.000E+00 | 0.000E+00 | 0.000E+00 |
| Unigene127886 | tetA(58) | 0.000E+00 | 3.403E-06 | 0.000E+00 | 0.000E+00 | 0.000E+00 | 0.000E+00 | 0.000E+00 | 2.838E-07 | 0.000E+00 | 0.000E+00 | 0.000E+00 | 0.000E+00 |
| Unigene127895 | macB | 3.858E-07 | 0.000E+00 | 1.514E-06 | 1.688E-06 | 0.000E+00 | 0.000E+00 | 0.000E+00 | 0.000E+00 | 0.000E+00 | 0.000E+00 | 0.000E+00 | 0.000E+00 |
| Unigene127913 | poxtA | 0.000E+00 | 6.988E-07 | 0.000E+00 | 2.572E-07 | 1.415E-07 | 0.000E+00 | 0.000E+00 | 0.000E+00 | 0.000E+00 | 0.000E+00 | 0.000E+00 | 0.000E+00 |
| Unigene127921 | lmrC | 2.632E-06 | 3.396E-06 | 3.172E-06 | 2.565E-06 | 4.053E-06 | 2.346E-06 | 2.658E-06 | 5.390E-07 | 2.221E-06 | 6.688E-06 | 7.026E-06 | 2.183E-06 |
| Unigene127935 | TaeA | 0.000E+00 | 6.585E-06 | 1.095E-05 | 1.684E-05 | 0.000E+00 | 0.000E+00 | 0.000E+00 | 0.000E+00 | 0.000E+00 | 0.000E+00 | 0.000E+00 | 0.000E+00 |
| Unigene127937 | tetT | 1.132E-06 | 6.296E-06 | 1.127E-05 | 1.583E-05 | 0.000E+00 | 5.028E-08 | 6.864E-08 | 0.000E+00 | 0.000E+00 | 0.000E+00 | 5.321E-08 | 0.000E+00 |
| Unigene127966 | mepR | 4.579E-07 | 6.700E-07 | 4.643E-06 | 6.165E-06 | 0.000E+00 | 0.000E+00 | 0.000E+00 | 0.000E+00 | 0.000E+00 | 0.000E+00 | 0.000E+00 | 0.000E+00 |
| Unigene127976 | optrA | 0.000E+00 | 1.734E-06 | 1.211E-06 | 0.000E+00 | 1.053E-05 | 3.202E-07 | 0.000E+00 | 0.000E+00 | 0.000E+00 | 0.000E+00 | 0.000E+00 | 0.000E+00 |
| Unigene128067 | arnA | 9.636E-08 | 1.093E-06 | 2.206E-07 | 0.000E+00 | 6.031E-06 | 0.000E+00 | 0.000E+00 | 2.999E-07 | 0.000E+00 | 0.000E+00 | 2.572E-07 | 0.000E+00 |
| Unigene128071 | macB | 2.038E-07 | 8.052E-07 | 1.200E-06 | 7.820E-07 | 0.000E+00 | 0.000E+00 | 0.000E+00 | 0.000E+00 | 0.000E+00 | 0.000E+00 | 0.000E+00 | 0.000E+00 |
| Unigene128077 | optrA | 2.640E-06 | 1.956E-06 | 2.475E-06 | 1.613E-06 | 1.307E-07 | 0.000E+00 | 5.210E-07 | 3.532E-07 | 8.820E-07 | 3.746E-08 | 0.000E+00 | 2.782E-07 |
| Unigene128121 | macB | 0.000E+00 | 5.662E-07 | 0.000E+00 | 0.000E+00 | 0.000E+00 | 0.000E+00 | 0.000E+00 | 0.000E+00 | 0.000E+00 | 0.000E+00 | 0.000E+00 | 0.000E+00 |
| Unigene128123 | CRP | 1.062E-06 | 4.607E-06 | 3.362E-06 | 3.265E-06 | 0.000E+00 | 0.000E+00 | 0.000E+00 | 0.000E+00 | 0.000E+00 | 0.000E+00 | 0.000E+00 | 0.000E+00 |
| Unigene128133 | macB | 3.830E-06 | 9.007E-06 | 8.769E-06 | 9.899E-06 | 0.000E+00 | 0.000E+00 | 0.000E+00 | 0.000E+00 | 0.000E+00 | 0.000E+00 | 0.000E+00 | 0.000E+00 |
| Unigene128240 | TaeA | 6.640E-07 | 5.512E-06 | 9.155E-06 | 1.386E-05 | 0.000E+00 | 1.841E-08 | 0.000E+00 | 0.000E+00 | 0.000E+00 | 0.000E+00 | 0.000E+00 | 0.000E+00 |
| Unigene128242 | dfrE | 0.000E+00 | 5.010E-06 | 8.127E-06 | 1.218E-05 | 0.000E+00 | 0.000E+00 | 0.000E+00 | 0.000E+00 | 0.000E+00 | 0.000E+00 | 0.000E+00 | 0.000E+00 |
| Unigene128405 | cfrC | 0.000E+00 | 2.795E-07 | 1.249E-07 | 1.929E-07 | 0.000E+00 | 0.000E+00 | 7.047E-08 | 0.000E+00 | 0.000E+00 | 0.000E+00 | 0.000E+00 | 0.000E+00 |
| Unigene128567 | catI | 0.000E+00 | 1.568E-06 | 3.737E-07 | 0.000E+00 | 0.000E+00 | 0.000E+00 | 0.000E+00 | 0.000E+00 | 0.000E+00 | 0.000E+00 | 0.000E+00 | 0.000E+00 |
| Unigene128724 | tetA(58) | 0.000E+00 | 3.645E-07 | 0.000E+00 | 0.000E+00 | 0.000E+00 | 0.000E+00 | 0.000E+00 | 0.000E+00 | 0.000E+00 | 0.000E+00 | 0.000E+00 | 0.000E+00 |
| Unigene128739 | MdtK | 8.444E-07 | 4.422E-06 | 3.362E-06 | 2.290E-06 | 0.000E+00 | 0.000E+00 | 0.000E+00 | 0.000E+00 | 0.000E+00 | 0.000E+00 | 0.000E+00 | 0.000E+00 |
| Unigene128801 | tetA(58) | 0.000E+00 | 6.509E-07 | 4.157E-07 | 0.000E+00 | 0.000E+00 | 0.000E+00 | 9.846E-07 | 0.000E+00 | 0.000E+00 | 0.000E+00 | 3.392E-07 | 0.000E+00 |
| Unigene128848 | vanN | 3.482E-06 | 1.096E-05 | 1.120E-05 | 1.164E-05 | 0.000E+00 | 0.000E+00 | 0.000E+00 | 0.000E+00 | 0.000E+00 | 0.000E+00 | 0.000E+00 | 0.000E+00 |
| Unigene128936 | PmpM | 3.926E-06 | 6.171E-06 | 7.990E-06 | 9.422E-06 | 0.000E+00 | 0.000E+00 | 0.000E+00 | 0.000E+00 | 0.000E+00 | 0.000E+00 | 0.000E+00 | 0.000E+00 |
| Unigene128958 | vanRO | 0.000E+00 | 7.141E-07 | 2.946E-07 | 0.000E+00 | 0.000E+00 | 0.000E+00 | 0.000E+00 | 0.000E+00 | 0.000E+00 | 0.000E+00 | 0.000E+00 | 0.000E+00 |
| Unigene128971 | TaeA | 0.000E+00 | 6.793E-07 | 2.336E-07 | 0.000E+00 | 0.000E+00 | 0.000E+00 | 0.000E+00 | 0.000E+00 | 0.000E+00 | 0.000E+00 | 0.000E+00 | 0.000E+00 |
| Unigene128990 | tlrC | 0.000E+00 | 1.687E-06 | 0.000E+00 | 2.218E-06 | 0.000E+00 | 0.000E+00 | 0.000E+00 | 0.000E+00 | 0.000E+00 | 0.000E+00 | 0.000E+00 | 0.000E+00 |
| Unigene128993 | macB | 0.000E+00 | 1.151E-06 | 2.099E-06 | 2.076E-06 | 0.000E+00 | 0.000E+00 | 0.000E+00 | 0.000E+00 | 0.000E+00 | 0.000E+00 | 0.000E+00 | 0.000E+00 |
| Unigene129002 | Staphylocc | 0.000E+00 | 1.528E-06 | 2.233E-06 | 1.294E-06 | 0.000E+00 | 0.000E+00 | 0.000E+00 | 0.000E+00 | 0.000E+00 | 0.000E+00 | 0.000E+00 | 0.000E+00 |
| Unigene129084 | oleC | 5.186E-07 | 8.537E-07 | 2.120E-06 | 1.396E-06 | 0.000E+00 | 0.000E+00 | 0.000E+00 | 0.000E+00 | 0.000E+00 | 0.000E+00 | 0.000E+00 | 0.000E+00 |
| Unigene129087 | mepA | 6.040E-07 | 2.504E-06 | 2.327E-06 | 2.146E-06 | 0.000E+00 | 0.000E+00 | 0.000E+00 | 0.000E+00 | 0.000E+00 | 0.000E+00 | 0.000E+00 | 0.000E+00 |
| Unigene129115 | novA | 0.000E+00 | 1.423E-06 | 1.320E-06 | 1.358E-06 | 0.000E+00 | 0.000E+00 | 0.000E+00 | 0.000E+00 | 0.000E+00 | 0.000E+00 | 0.000E+00 | 0.000E+00 |
| Unigene129219 | emrB | 0.000E+00 | 5.061E-07 | 0.000E+00 | 0.000E+00 | 0.000E+00 | 0.000E+00 | 0.000E+00 | 0.000E+00 | 0.000E+00 | 0.000E+00 | 0.000E+00 | 0.000E+00 |
| Unigene129237 | srmB | 0.000E+00 | 4.397E-07 | 0.000E+00 | 0.000E+00 | 0.000E+00 | 0.000E+00 | 0.000E+00 | 0.000E+00 | 6.256E-08 | 0.000E+00 | 0.000E+00 | 0.000E+00 |
| Unigene129336 | vanRF | 7.741E-08 | 5.947E-07 | 0.000E+00 | 0.000E+00 | 0.000E+00 | 0.000E+00 | 0.000E+00 | 0.000E+00 | 0.000E+00 | 0.000E+00 | 0.000E+00 | 0.000E+00 |
| Unigene129440 | basS | 0.000E+00 | 8.894E-07 | 6.626E-07 | 0.000E+00 | 1.951E-06 | 0.000E+00 | 4.484E-07 | 0.000E+00 | 0.000E+00 | 0.000E+00 | 3.605E-07 | 0.000E+00 |
| Unigene129472 | macA | 0.000E+00 | 7.447E-07 | 0.000E+00 | 0.000E+00 | 0.000E+00 | 0.000E+00 | 0.000E+00 | 0.000E+00 | 0.000E+00 | 0.000E+00 | 0.000E+00 | 0.000E+00 |
| Unigene129601 | macB | 2.438E-07 | 1.070E-06 | 3.759E-07 | 0.000E+00 | 0.000E+00 | 0.000E+00 | 0.000E+00 | 0.000E+00 | 0.000E+00 | 0.000E+00 | 0.000E+00 | 0.000E+00 |
| Unigene129608 | tetA(58) | 0.000E+00 | 1.640E-06 | 0.000E+00 | 0.000E+00 | 5.028E-06 | 0.000E+00 | 0.000E+00 | 0.000E+00 | 0.000E+00 | 0.000E+00 | 0.000E+00 | 0.000E+00 |
| Unigene129664 | Corynebac | 0.000E+00 | 3.642E-07 | 4.651E-08 | 0.000E+00 | 6.846E-07 | 0.000E+00 | 0.000E+00 | 0.000E+00 | 0.000E+00 | 0.000E+00 | 2.169E-07 | 0.000E+00 |
| Unigene129680 | vanWG | 0.000E+00 | 4.294E-07 | 0.000E+00 | 1.185E-07 | 0.000E+00 | 0.000E+00 | 0.000E+00 | 0.000E+00 | 0.000E+00 | 0.000E+00 | 0.000E+00 | 0.000E+00 |
| Unigene129722 | macB | 0.000E+00 | 6.830E-07 | 3.409E-06 | 3.639E-06 | 0.000E+00 | 0.000E+00 | 0.000E+00 | 0.000E+00 | 0.000E+00 | 0.000E+00 | 0.000E+00 | 0.000E+00 |
| Unigene129755 | lnuE | 0.000E+00 | 6.826E-07 | 1.526E-06 | 3.203E-06 | 0.000E+00 | 0.000E+00 | 0.000E+00 | 0.000E+00 | 0.000E+00 | 0.000E+00 | 0.000E+00 | 0.000E+00 |
| Unigene129759 | PmrF | 0.000E+00 | 1.943E-06 | 6.756E-05 | 0.000E+00 | 0.000E+00 | 0.000E+00 | 0.000E+00 | 0.000E+00 | 0.000E+00 | 0.000E+00 | 0.000E+00 | 0.000E+00 |
| Unigene129771 | adeJ | 0.000E+00 | 3.634E-07 | 2.707E-07 | 2.229E-07 | 0.000E+00 | 0.000E+00 | 0.000E+00 | 0.000E+00 | 0.000E+00 | 0.000E+00 | 0.000E+00 | 0.000E+00 |
| Unigene129774 | arlS | 1.154E-06 | 8.803E-07 | 9.790E-07 | 1.482E-06 | 1.895E-05 | 5.837E-06 | 1.507E-05 | 1.827E-05 | 1.144E-05 | 1.905E-05 | 1.833E-05 | 8.348E-06 |
| Unigene129874 | msbA | 0.000E+00 | 0.000E+00 | 0.000E+00 | 0.000E+00 | 2.740E-07 | 0.000E+00 | 0.000E+00 | 0.000E+00 | 0.000E+00 | 0.000E+00 | 0.000E+00 | 0.000E+00 |
| Unigene129953 | H-NS | 0.000E+00 | 8.748E-07 | 0.000E+00 | 0.000E+00 | 0.000E+00 | 0.000E+00 | 0.000E+00 | 0.000E+00 | 0.000E+00 | 0.000E+00 | 0.000E+00 | 0.000E+00 |
| Unigene130092 | catA8 | 0.000E+00 | 1.804E-06 | 0.000E+00 | 1.107E-06 | 0.000E+00 | 0.000E+00 | 0.000E+00 | 0.000E+00 | 4.356E-07 | 0.000E+00 | 0.000E+00 | 5.299E-07 |
| Unigene130140 | lnuA | 1.129E-06 | 5.832E-07 | 3.910E-07 | 0.000E+00 | 9.593E-07 | 2.334E-05 | 0.000E+00 | 0.000E+00 | 0.000E+00 | 0.000E+00 | 0.000E+00 | 0.000E+00 |
| Unigene130213 | vanRM | 0.000E+00 | 1.097E-06 | 0.000E+00 | 1.394E-06 | 0.000E+00 | 0.000E+00 | 0.000E+00 | 0.000E+00 | 0.000E+00 | 0.000E+00 | 0.000E+00 | 0.000E+00 |
| Unigene130217 | efrA | 6.754E-07 | 7.413E-07 | 0.000E+00 | 0.000E+00 | 4.890E-06 | 0.000E+00 | 0.000E+00 | 0.000E+00 | 0.000E+00 | 0.000E+00 | 0.000E+00 | 0.000E+00 |

| | | | | | | | | | | | | | |
|---|---|---|---|---|---|---|---|---|---|---|---|---|---|
| Unigene130269 | catB8 | 0.000E+00 | 9.770E-07 | 0.000E+00 | 0.000E+00 | 1.075E-06 | 0.000E+00 | 0.000E+00 | 0.000E+00 | 0.000E+00 | 0.000E+00 | 0.000E+00 | 0.000E+00 |
| Unigene130323 | MexB | 0.000E+00 | 3.710E-07 | 0.000E+00 | 0.000E+00 | 0.000E+00 | 0.000E+00 | 0.000E+00 | 0.000E+00 | 0.000E+00 | 0.000E+00 | 0.000E+00 | 0.000E+00 |
| Unigene130502 | tet(45) | 9.397E-08 | 6.704E-07 | 2.305E-08 | 3.321E-07 | 0.000E+00 | 0.000E+00 | 0.000E+00 | 0.000E+00 | 0.000E+00 | 0.000E+00 | 0.000E+00 | 0.000E+00 |
| Unigene130539 | patB | 8.814E-07 | 1.229E-06 | 5.567E-06 | 6.972E-06 | 8.160E-08 | 0.000E+00 | 3.455E-07 | 0.000E+00 | 0.000E+00 | 0.000E+00 | 8.402E-08 | 0.000E+00 |
| Unigene130600 | bcrA | 0.000E+00 | 1.021E-06 | 3.161E-06 | 3.622E-06 | 0.000E+00 | 0.000E+00 | 0.000E+00 | 0.000E+00 | 0.000E+00 | 0.000E+00 | 0.000E+00 | 0.000E+00 |
| Unigene130623 | aadA27 | 0.000E+00 | 2.344E-06 | 3.224E-06 | 2.198E-06 | 0.000E+00 | 0.000E+00 | 0.000E+00 | 0.000E+00 | 4.489E-08 | 0.000E+00 | 0.000E+00 | 0.000E+00 |
| Unigene130640 | pmrA | 0.000E+00 | 5.665E-07 | 0.000E+00 | 0.000E+00 | 0.000E+00 | 8.585E-08 | 0.000E+00 | 0.000E+00 | 0.000E+00 | 0.000E+00 | 0.000E+00 | 0.000E+00 |
| Unigene130745 | vanRM | 2.402E-06 | 5.957E-06 | 7.259E-06 | 9.459E-06 | 0.000E+00 | 0.000E+00 | 0.000E+00 | 0.000E+00 | 0.000E+00 | 0.000E+00 | 0.000E+00 | 0.000E+00 |
| Unigene130764 | macB | 0.000E+00 | 9.346E-07 | 3.912E-06 | 3.244E-06 | 0.000E+00 | 0.000E+00 | 0.000E+00 | 0.000E+00 | 0.000E+00 | 0.000E+00 | 0.000E+00 | 0.000E+00 |
| Unigene130812 | baeS | 0.000E+00 | 1.336E-06 | 1.813E-06 | 1.624E-06 | 0.000E+00 | 0.000E+00 | 0.000E+00 | 0.000E+00 | 0.000E+00 | 0.000E+00 | 0.000E+00 | 0.000E+00 |
| Unigene130828 | kdpE | 6.561E-08 | 6.481E-07 | 0.000E+00 | 0.000E+00 | 0.000E+00 | 0.000E+00 | 0.000E+00 | 0.000E+00 | 0.000E+00 | 0.000E+00 | 0.000E+00 | 0.000E+00 |
| Unigene130926 | emrY | 0.000E+00 | 4.581E-07 | 0.000E+00 | 0.000E+00 | 0.000E+00 | 0.000E+00 | 0.000E+00 | 0.000E+00 | 0.000E+00 | 0.000E+00 | 0.000E+00 | 0.000E+00 |
| Unigene130999 | PmrF | 3.653E-07 | 7.290E-07 | 1.629E-07 | 0.000E+00 | 7.379E-08 | 7.180E-08 | 0.000E+00 | 0.000E+00 | 1.815E-07 | 2.220E-07 | 0.000E+00 | 0.000E+00 |
| Unigene131004 | YojI | 0.000E+00 | 5.135E-07 | 1.148E-07 | 0.000E+00 | 0.000E+00 | 0.000E+00 | 0.000E+00 | 0.000E+00 | 0.000E+00 | 0.000E+00 | 0.000E+00 | 0.000E+00 |
| Unigene131084 | mdtP | 2.819E-07 | 9.540E-07 | 4.841E-07 | 0.000E+00 | 0.000E+00 | 0.000E+00 | 9.621E-07 | 0.000E+00 | 0.000E+00 | 0.000E+00 | 0.000E+00 | 0.000E+00 |
| Unigene131103 | OprN | 0.000E+00 | 6.716E-07 | 2.502E-07 | 0.000E+00 | 1.303E-06 | 0.000E+00 | 0.000E+00 | 0.000E+00 | 0.000E+00 | 0.000E+00 | 0.000E+00 | 0.000E+00 |
| Unigene131120 | tetA(58) | 0.000E+00 | 1.291E-06 | 2.137E-06 | 1.452E-06 | 0.000E+00 | 0.000E+00 | 0.000E+00 | 0.000E+00 | 0.000E+00 | 0.000E+00 | 0.000E+00 | 0.000E+00 |
| Unigene131179 | adeB | 0.000E+00 | 0.000E+00 | 0.000E+00 | 1.109E-07 | 0.000E+00 | 0.000E+00 | 0.000E+00 | 0.000E+00 | 0.000E+00 | 0.000E+00 | 0.000E+00 | 0.000E+00 |
| Unigene131184 | bcrA | 6.767E-07 | 1.300E-06 | 5.246E-06 | 5.433E-06 | 0.000E+00 | 0.000E+00 | 5.617E-07 | 0.000E+00 | 0.000E+00 | 0.000E+00 | 0.000E+00 | 2.000E-08 |
| Unigene131229 | gimA | 1.143E-06 | 1.163E-06 | 4.650E-06 | 6.643E-06 | 0.000E+00 | 0.000E+00 | 3.394E-07 | 0.000E+00 | 0.000E+00 | 0.000E+00 | 0.000E+00 | 0.000E+00 |
| Unigene131239 | tetB(P) | 2.276E-05 | 8.307E-06 | 6.470E-06 | 3.763E-06 | 1.911E-07 | 0.000E+00 | 0.000E+00 | 0.000E+00 | 1.149E-06 | 0.000E+00 | 0.000E+00 | 0.000E+00 |
| Unigene131286 | adeL | 0.000E+00 | 1.181E-06 | 6.687E-07 | 0.000E+00 | 0.000E+00 | 0.000E+00 | 0.000E+00 | 8.373E-08 | 0.000E+00 | 0.000E+00 | 0.000E+00 | 0.000E+00 |
| Unigene131360 | vanRF | 0.000E+00 | 0.000E+00 | 3.565E-06 | 3.998E-06 | 0.000E+00 | 0.000E+00 | 0.000E+00 | 0.000E+00 | 0.000E+00 | 0.000E+00 | 0.000E+00 | 0.000E+00 |
| Unigene131376 | SRT-2 | 2.284E-07 | 5.571E-07 | 2.241E-07 | 0.000E+00 | 9.022E-07 | 0.000E+00 | 2.809E-07 | 0.000E+00 | 0.000E+00 | 0.000E+00 | 2.322E-07 | 0.000E+00 |
| Unigene131403 | tet32 | 0.000E+00 | 1.181E-06 | 1.973E-06 | 1.585E-06 | 0.000E+00 | 0.000E+00 | 0.000E+00 | 0.000E+00 | 0.000E+00 | 0.000E+00 | 0.000E+00 | 0.000E+00 |
| Unigene131428 | macB | 0.000E+00 | 5.288E-07 | 2.626E-07 | 0.000E+00 | 0.000E+00 | 0.000E+00 | 0.000E+00 | 0.000E+00 | 5.851E-08 | 0.000E+00 | 0.000E+00 | 0.000E+00 |
| Unigene131485 | poxtA | 4.147E-07 | 5.488E-06 | 4.974E-07 | 1.629E-07 | 2.560E-07 | 7.472E-08 | 0.000E+00 | 2.421E-07 | 2.266E-07 | 0.000E+00 | 7.907E-08 | 0.000E+00 |
| Unigene131553 | novA | 0.000E+00 | 5.579E-07 | 4.988E-07 | 2.139E-07 | 0.000E+00 | 0.000E+00 | 0.000E+00 | 0.000E+00 | 0.000E+00 | 0.000E+00 | 0.000E+00 | 0.000E+00 |
| Unigene131575 | tetA(46) | 9.938E-07 | 1.363E-06 | 4.857E-06 | 6.755E-06 | 0.000E+00 | 6.198E-08 | 7.615E-07 | 0.000E+00 | 2.089E-08 | 0.000E+00 | 0.000E+00 | 4.518E-08 |
| Unigene131594 | tet32 | 3.341E-05 | 2.135E-05 | 1.704E-05 | 2.023E-05 | 1.767E-06 | 1.547E-06 | 2.738E-06 | 5.568E-07 | 0.000E+00 | 4.133E-07 | 1.233E-06 | 1.107E-06 |
| Unigene131687 | tetA(58) | 0.000E+00 | 2.353E-06 | 0.000E+00 | 0.000E+00 | 0.000E+00 | 0.000E+00 | 0.000E+00 | 0.000E+00 | 0.000E+00 | 0.000E+00 | 0.000E+00 | 0.000E+00 |
| Unigene131728 | oleB | 9.236E-08 | 9.123E-07 | 1.813E-07 | 2.332E-07 | 0.000E+00 | 0.000E+00 | 0.000E+00 | 0.000E+00 | 0.000E+00 | 0.000E+00 | 0.000E+00 | 0.000E+00 |
| Unigene131852 | AcrS | 2.788E-07 | 1.469E-06 | 1.641E-06 | 2.027E-06 | 0.000E+00 | 0.000E+00 | 0.000E+00 | 0.000E+00 | 0.000E+00 | 0.000E+00 | 0.000E+00 | 0.000E+00 |
| Unigene131983 | efrA | 1.205E-05 | 3.930E-06 | 2.934E-06 | 1.243E-05 | 8.023E-05 | 1.155E-04 | 1.107E-04 | 9.205E-05 | 1.379E-04 | 1.339E-04 | 1.128E-04 | 7.101E-05 |
| Unigene131989 | TaeA | 0.000E+00 | 0.000E+00 | 0.000E+00 | 0.000E+00 | 0.000E+00 | 0.000E+00 | 0.000E+00 | 0.000E+00 | 0.000E+00 | 0.000E+00 | 0.000E+00 | 5.106E-06 |
| Unigene132009 | vanHD | 0.000E+00 | 4.168E-07 | 0.000E+00 | 0.000E+00 | 0.000E+00 | 0.000E+00 | 0.000E+00 | 0.000E+00 | 0.000E+00 | 0.000E+00 | 0.000E+00 | 0.000E+00 |
| Unigene132011 | tetA(58) | 1.681E-07 | 5.536E-07 | 1.650E-07 | 1.698E-07 | 2.335E-08 | 4.544E-08 | 0.000E+00 | 0.000E+00 | 0.000E+00 | 0.000E+00 | 0.000E+00 | 9.936E-08 |
| Unigene132033 | Agrobacte | 0.000E+00 | 5.288E-07 | 2.101E-07 | 2.162E-07 | 0.000E+00 | 0.000E+00 | 0.000E+00 | 0.000E+00 | 0.000E+00 | 0.000E+00 | 0.000E+00 | 0.000E+00 |
| Unigene132035 | acrB | 1.383E-07 | 8.804E-07 | 4.614E-07 | 1.955E-07 | 0.000E+00 | 0.000E+00 | 3.674E-07 | 0.000E+00 | 0.000E+00 | 0.000E+00 | 0.000E+00 | 0.000E+00 |
| Unigene132158 | evgS | 2.867E-06 | 4.522E-06 | 3.434E-06 | 5.778E-06 | 4.274E-07 | 6.708E-08 | 3.434E-07 | 0.000E+00 | 5.426E-08 | 0.000E+00 | 0.000E+00 | 8.802E-08 |
| Unigene132191 | adeS | 0.000E+00 | 8.435E-07 | 0.000E+00 | 0.000E+00 | 0.000E+00 | 0.000E+00 | 0.000E+00 | 0.000E+00 | 0.000E+00 | 0.000E+00 | 0.000E+00 | 0.000E+00 |
| Unigene132223 | kdpE | 6.521E-07 | 1.278E-06 | 3.839E-06 | 5.362E-06 | 0.000E+00 | 0.000E+00 | 0.000E+00 | 0.000E+00 | 0.000E+00 | 0.000E+00 | 0.000E+00 | 0.000E+00 |
| Unigene132336 | Acinetoba | 0.000E+00 | 1.139E-06 | 7.339E-07 | 0.000E+00 | 2.359E-06 | 0.000E+00 | 0.000E+00 | 1.690E-07 | 0.000E+00 | 0.000E+00 | 0.000E+00 | 0.000E+00 |
| Unigene132347 | patA | 0.000E+00 | 3.865E-07 | 1.536E-07 | 1.185E-07 | 0.000E+00 | 0.000E+00 | 0.000E+00 | 0.000E+00 | 0.000E+00 | 0.000E+00 | 0.000E+00 | 0.000E+00 |
| Unigene132425 | lmrB | 0.000E+00 | 1.426E-06 | 2.483E-06 | 0.000E+00 | 0.000E+00 | 0.000E+00 | 0.000E+00 | 0.000E+00 | 0.000E+00 | 0.000E+00 | 0.000E+00 | 0.000E+00 |
| Unigene132446 | poxtA | 0.000E+00 | 1.419E-06 | 0.000E+00 | 1.600E-06 | 0.000E+00 | 0.000E+00 | 0.000E+00 | 0.000E+00 | 0.000E+00 | 0.000E+00 | 0.000E+00 | 0.000E+00 |
| Unigene132450 | vanHA | 0.000E+00 | 2.355E-06 | 0.000E+00 | 0.000E+00 | 0.000E+00 | 0.000E+00 | 0.000E+00 | 0.000E+00 | 0.000E+00 | 0.000E+00 | 0.000E+00 | 0.000E+00 |
| Unigene132561 | marA | 0.000E+00 | 0.000E+00 | 1.553E-07 | 0.000E+00 | 0.000E+00 | 0.000E+00 | 0.000E+00 | 0.000E+00 | 0.000E+00 | 0.000E+00 | 0.000E+00 | 0.000E+00 |
| Unigene132609 | AcrS | 0.000E+00 | 1.278E-06 | 0.000E+00 | 0.000E+00 | 0.000E+00 | 0.000E+00 | 0.000E+00 | 0.000E+00 | 0.000E+00 | 0.000E+00 | 0.000E+00 | 0.000E+00 |
| Unigene132610 | AcrE | 2.265E-07 | 9.323E-07 | 0.000E+00 | 2.573E-07 | 4.719E-07 | 0.000E+00 | 1.661E-06 | 0.000E+00 | 0.000E+00 | 0.000E+00 | 2.267E-07 | 0.000E+00 |
| Unigene132674 | tetA(58) | 2.030E-07 | 0.000E+00 | 2.490E-07 | 0.000E+00 | 0.000E+00 | 0.000E+00 | 0.000E+00 | 0.000E+00 | 0.000E+00 | 0.000E+00 | 0.000E+00 | 0.000E+00 |
| Unigene132763 | cmlB1 | 0.000E+00 | 4.218E-07 | 0.000E+00 | 0.000E+00 | 0.000E+00 | 0.000E+00 | 0.000E+00 | 0.000E+00 | 0.000E+00 | 0.000E+00 | 0.000E+00 | 0.000E+00 |
| Unigene132765 | vanZF | 0.000E+00 | 9.354E-07 | 2.248E-06 | 2.636E-06 | 0.000E+00 | 0.000E+00 | 0.000E+00 | 0.000E+00 | 0.000E+00 | 0.000E+00 | 0.000E+00 | 0.000E+00 |
| Unigene132863 | macB | 0.000E+00 | 8.697E-07 | 3.519E-06 | 1.642E-06 | 0.000E+00 | 0.000E+00 | 0.000E+00 | 0.000E+00 | 0.000E+00 | 0.000E+00 | 0.000E+00 | 0.000E+00 |
| Unigene132935 | Listeria mc | 0.000E+00 | 1.335E-06 | 2.547E-06 | 0.000E+00 | 0.000E+00 | 0.000E+00 | 0.000E+00 | 0.000E+00 | 0.000E+00 | 0.000E+00 | 0.000E+00 | 0.000E+00 |
| Unigene132950 | basS | 1.494E-07 | 4.101E-07 | 2.933E-07 | 0.000E+00 | 1.204E-06 | 4.039E-08 | 0.000E+00 | 0.000E+00 | 0.000E+00 | 0.000E+00 | 2.992E-07 | 0.000E+00 |
| Unigene132963 | ugd | 0.000E+00 | 1.032E-06 | 3.528E-07 | 0.000E+00 | 0.000E+00 | 0.000E+00 | 0.000E+00 | 0.000E+00 | 0.000E+00 | 0.000E+00 | 0.000E+00 | 0.000E+00 |
| Unigene132975 | vanZF | 0.000E+00 | 4.967E-07 | 1.480E-07 | 0.000E+00 | 7.820E-07 | 1.087E-07 | 0.000E+00 | 0.000E+00 | 0.000E+00 | 0.000E+00 | 5.176E-07 | 6.538E-07 |

| | | | | | | | | | | | | | |
|---|---|---|---|---|---|---|---|---|---|---|---|---|---|
| Unigene132978 | tetA(60) | 0.000E+00 | 3.690E-07 | 0.000E+00 | 0.000E+00 | 0.000E+00 | 0.000E+00 | 0.000E+00 | 0.000E+00 | 0.000E+00 | 0.000E+00 | 0.000E+00 | 0.000E+00 |
| Unigene132999 | Acinetoba | 3.668E-07 | 1.959E-06 | 1.248E-06 | 5.186E-07 | 1.111E-05 | 5.023E-07 | 1.489E-06 | 5.994E-07 | 0.000E+00 | 3.269E-07 | 0.000E+00 | 0.000E+00 |
| Unigene133190 | arlS | 5.555E-07 | 6.739E-07 | 0.000E+00 | 3.189E-06 | 6.172E-07 | 0.000E+00 | 2.265E-07 | 0.000E+00 | 0.000E+00 | 0.000E+00 | 1.572E-06 | 8.986E-07 |
| Unigene133237 | TriC | 0.000E+00 | 3.108E-07 | 0.000E+00 | 0.000E+00 | 0.000E+00 | 0.000E+00 | 0.000E+00 | 0.000E+00 | 0.000E+00 | 0.000E+00 | 0.000E+00 | 0.000E+00 |
| Unigene133310 | vatE | 0.000E+00 | 6.652E-06 | 0.000E+00 | 0.000E+00 | 0.000E+00 | 0.000E+00 | 0.000E+00 | 0.000E+00 | 0.000E+00 | 0.000E+00 | 0.000E+00 | 0.000E+00 |
| Unigene133345 | adeL | 0.000E+00 | 2.480E-06 | 1.021E-06 | 5.071E-07 | 1.068E-05 | 0.000E+00 | 0.000E+00 | 0.000E+00 | 0.000E+00 | 0.000E+00 | 0.000E+00 | 0.000E+00 |
| Unigene133361 | macB | 0.000E+00 | 1.447E-06 | 0.000E+00 | 1.755E-06 | 0.000E+00 | 0.000E+00 | 0.000E+00 | 0.000E+00 | 0.000E+00 | 0.000E+00 | 0.000E+00 | 0.000E+00 |
| Unigene133382 | msbA | 0.000E+00 | 1.167E-06 | 1.044E-06 | 0.000E+00 | 0.000E+00 | 0.000E+00 | 0.000E+00 | 0.000E+00 | 0.000E+00 | 0.000E+00 | 0.000E+00 | 0.000E+00 |
| Unigene133403 | vanHO | 0.000E+00 | 5.135E-07 | 0.000E+00 | 0.000E+00 | 0.000E+00 | 0.000E+00 | 0.000E+00 | 0.000E+00 | 0.000E+00 | 0.000E+00 | 0.000E+00 | 0.000E+00 |
| Unigene133445 | tetA(46) | 0.000E+00 | 3.075E-07 | 0.000E+00 | 0.000E+00 | 0.000E+00 | 0.000E+00 | 0.000E+00 | 0.000E+00 | 0.000E+00 | 0.000E+00 | 0.000E+00 | 6.624E-07 |
| Unigene133486 | mecA | 7.110E-07 | 7.596E-06 | 1.060E-05 | 1.514E-05 | 0.000E+00 | 0.000E+00 | 0.000E+00 | 0.000E+00 | 0.000E+00 | 0.000E+00 | 0.000E+00 | 0.000E+00 |
| Unigene133549 | efmA | 0.000E+00 | 5.442E-07 | 6.812E-07 | 0.000E+00 | 0.000E+00 | 0.000E+00 | 0.000E+00 | 0.000E+00 | 0.000E+00 | 0.000E+00 | 0.000E+00 | 0.000E+00 |
| Unigene133711 | oleC | 0.000E+00 | 4.740E-07 | 0.000E+00 | 0.000E+00 | 0.000E+00 | 0.000E+00 | 0.000E+00 | 0.000E+00 | 0.000E+00 | 0.000E+00 | 0.000E+00 | 0.000E+00 |
| Unigene133759 | mdtH | 4.230E-07 | 1.161E-06 | 4.151E-07 | 3.204E-07 | 8.841E-06 | 2.858E-07 | 1.551E-06 | 2.777E-07 | 0.000E+00 | 2.062E-07 | 1.512E-07 | 0.000E+00 |
| Unigene133760 | tetA(58) | 0.000E+00 | 1.348E-06 | 0.000E+00 | 0.000E+00 | 6.589E-06 | 0.000E+00 | 0.000E+00 | 0.000E+00 | 0.000E+00 | 0.000E+00 | 0.000E+00 | 0.000E+00 |
| Unigene133766 | Pseudomo | 0.000E+00 | 1.712E-06 | 0.000E+00 | 0.000E+00 | 0.000E+00 | 0.000E+00 | 0.000E+00 | 0.000E+00 | 0.000E+00 | 0.000E+00 | 0.000E+00 | 0.000E+00 |
| Unigene133768 | rpoB2 | 0.000E+00 | 7.042E-07 | 3.390E-07 | 0.000E+00 | 0.000E+00 | 1.067E-07 | 0.000E+00 | 0.000E+00 | 0.000E+00 | 0.000E+00 | 0.000E+00 | 0.000E+00 |
| Unigene133900 | macB | 0.000E+00 | 3.634E-07 | 2.437E-07 | 0.000E+00 | 0.000E+00 | 0.000E+00 | 0.000E+00 | 0.000E+00 | 0.000E+00 | 0.000E+00 | 0.000E+00 | 0.000E+00 |
| Unigene133993 | mdtH | 0.000E+00 | 0.000E+00 | 0.000E+00 | 0.000E+00 | 2.427E-06 | 0.000E+00 | 0.000E+00 | 0.000E+00 | 0.000E+00 | 0.000E+00 | 0.000E+00 | 0.000E+00 |
| Unigene134040 | macB | 0.000E+00 | 1.069E-06 | 1.247E-06 | 1.283E-06 | 0.000E+00 | 0.000E+00 | 0.000E+00 | 0.000E+00 | 0.000E+00 | 0.000E+00 | 0.000E+00 | 0.000E+00 |
| Unigene134057 | efrB | 1.248E-06 | 2.238E-06 | 5.624E-06 | 7.333E-06 | 0.000E+00 | 3.801E-08 | 4.670E-07 | 0.000E+00 | 0.000E+00 | 0.000E+00 | 1.006E-07 | 0.000E+00 |
| Unigene134074 | marA | 0.000E+00 | 0.000E+00 | 3.299E-07 | 8.489E-08 | 0.000E+00 | 0.000E+00 | 9.304E-08 | 9.812E-08 | 0.000E+00 | 0.000E+00 | 0.000E+00 | 0.000E+00 |
| Unigene134111 | facT | 0.000E+00 | 6.411E-07 | 0.000E+00 | 0.000E+00 | 0.000E+00 | 0.000E+00 | 0.000E+00 | 0.000E+00 | 0.000E+00 | 0.000E+00 | 0.000E+00 | 0.000E+00 |
| Unigene134232 | mdtG | 0.000E+00 | 1.528E-06 | 2.863E-06 | 1.784E-06 | 0.000E+00 | 0.000E+00 | 0.000E+00 | 0.000E+00 | 0.000E+00 | 0.000E+00 | 0.000E+00 | 0.000E+00 |
| Unigene134236 | vanHB | 0.000E+00 | 1.614E-06 | 0.000E+00 | 0.000E+00 | 0.000E+00 | 0.000E+00 | 0.000E+00 | 0.000E+00 | 0.000E+00 | 0.000E+00 | 0.000E+00 | 0.000E+00 |
| Unigene134246 | efrA | 0.000E+00 | 1.632E-06 | 2.152E-06 | 1.576E-06 | 0.000E+00 | 0.000E+00 | 0.000E+00 | 0.000E+00 | 0.000E+00 | 0.000E+00 | 0.000E+00 | 0.000E+00 |
| Unigene134247 | patB | 0.000E+00 | 1.544E-06 | 2.433E-06 | 1.953E-06 | 0.000E+00 | 0.000E+00 | 0.000E+00 | 0.000E+00 | 0.000E+00 | 0.000E+00 | 0.000E+00 | 0.000E+00 |
| Unigene134249 | Staphyloc | 0.000E+00 | 1.211E-06 | 2.695E-06 | 1.591E-06 | 0.000E+00 | 0.000E+00 | 0.000E+00 | 0.000E+00 | 0.000E+00 | 0.000E+00 | 0.000E+00 | 0.000E+00 |
| Unigene134320 | cmx | 2.083E-07 | 9.397E-07 | 2.725E-07 | 1.869E-07 | 0.000E+00 | 0.000E+00 | 3.073E-07 | 0.000E+00 | 0.000E+00 | 0.000E+00 | 0.000E+00 | 0.000E+00 |
| Unigene134357 | arlR | 1.930E-07 | 5.296E-07 | 3.882E-06 | 3.849E-06 | 0.000E+00 | 0.000E+00 | 0.000E+00 | 0.000E+00 | 0.000E+00 | 0.000E+00 | 0.000E+00 | 0.000E+00 |
| Unigene134529 | MexD | 0.000E+00 | 0.000E+00 | 0.000E+00 | 0.000E+00 | 0.000E+00 | 0.000E+00 | 3.285E-07 | 0.000E+00 | 0.000E+00 | 0.000E+00 | 0.000E+00 | 0.000E+00 |
| Unigene134568 | adeR | 0.000E+00 | 2.410E-07 | 0.000E+00 | 3.105E-07 | 0.000E+00 | 0.000E+00 | 0.000E+00 | 0.000E+00 | 0.000E+00 | 0.000E+00 | 5.024E-08 | 0.000E+00 |
| Unigene134646 | macB | 3.125E-07 | 5.756E-06 | 9.665E-06 | 1.447E-05 | 0.000E+00 | 0.000E+00 | 0.000E+00 | 0.000E+00 | 0.000E+00 | 0.000E+00 | 1.554E-08 | 4.818E-08 |
| Unigene134647 | carA | 0.000E+00 | 1.767E-06 | 1.024E-06 | 0.000E+00 | 0.000E+00 | 0.000E+00 | 0.000E+00 | 0.000E+00 | 0.000E+00 | 0.000E+00 | 0.000E+00 | 0.000E+00 |
| Unigene134842 | arlR | 0.000E+00 | 1.042E-06 | 4.436E-07 | 0.000E+00 | 0.000E+00 | 0.000E+00 | 0.000E+00 | 0.000E+00 | 0.000E+00 | 0.000E+00 | 0.000E+00 | 1.603E-07 |
| Unigene134866 | lnuD | 0.000E+00 | 1.864E-05 | 8.818E-05 | 0.000E+00 | 5.815E-07 | 5.030E-07 | 0.000E+00 | 0.000E+00 | 5.702E-05 | 0.000E+00 | 0.000E+00 | 0.000E+00 |
| Unigene134867 | AAC(6')-Ia | 0.000E+00 | 1.871E-05 | 9.247E-05 | 0.000E+00 | 0.000E+00 | 0.000E+00 | 0.000E+00 | 0.000E+00 | 6.591E-05 | 0.000E+00 | 0.000E+00 | 0.000E+00 |
| Unigene134868 | aadA13 | 1.227E-07 | 2.146E-05 | 9.310E-05 | 0.000E+00 | 1.318E-06 | 1.769E-07 | 0.000E+00 | 0.000E+00 | 5.818E-05 | 0.000E+00 | 0.000E+00 | 0.000E+00 |
| Unigene134914 | macB | 6.666E-07 | 6.793E-07 | 1.495E-06 | 8.654E-07 | 0.000E+00 | 1.029E-07 | 0.000E+00 | 0.000E+00 | 0.000E+00 | 0.000E+00 | 0.000E+00 | 0.000E+00 |
| Unigene134938 | adeL | 0.000E+00 | 1.278E-06 | 9.347E-07 | 0.000E+00 | 0.000E+00 | 0.000E+00 | 0.000E+00 | 0.000E+00 | 0.000E+00 | 0.000E+00 | 0.000E+00 | 0.000E+00 |
| Unigene135028 | optrA | 0.000E+00 | 7.538E-07 | 0.000E+00 | 0.000E+00 | 0.000E+00 | 0.000E+00 | 0.000E+00 | 0.000E+00 | 0.000E+00 | 0.000E+00 | 0.000E+00 | 0.000E+00 |
| Unigene135144 | vanL | 0.000E+00 | 7.989E-07 | 3.540E-06 | 3.739E-06 | 0.000E+00 | 0.000E+00 | 0.000E+00 | 0.000E+00 | 0.000E+00 | 0.000E+00 | 0.000E+00 | 0.000E+00 |
| Unigene135198 | tetA(58) | 0.000E+00 | 1.066E-06 | 3.890E-06 | 4.003E-06 | 0.000E+00 | 0.000E+00 | 0.000E+00 | 0.000E+00 | 0.000E+00 | 0.000E+00 | 0.000E+00 | 0.000E+00 |
| Unigene135405 | Klebsiella p | 0.000E+00 | 6.580E-07 | 2.167E-07 | 0.000E+00 | 0.000E+00 | 0.000E+00 | 0.000E+00 | 0.000E+00 | 0.000E+00 | 0.000E+00 | 0.000E+00 | 0.000E+00 |
| Unigene135466 | efrA | 0.000E+00 | 7.498E-07 | 0.000E+00 | 6.037E-07 | 0.000E+00 | 0.000E+00 | 0.000E+00 | 0.000E+00 | 0.000E+00 | 0.000E+00 | 0.000E+00 | 0.000E+00 |
| Unigene135683 | Staphyloc | 0.000E+00 | 7.439E-07 | 0.000E+00 | 0.000E+00 | 0.000E+00 | 0.000E+00 | 0.000E+00 | 0.000E+00 | 0.000E+00 | 0.000E+00 | 0.000E+00 | 0.000E+00 |
| Unigene135761 | patA | 2.538E-07 | 7.242E-07 | 1.494E-07 | 0.000E+00 | 0.000E+00 | 0.000E+00 | 0.000E+00 | 0.000E+00 | 0.000E+00 | 0.000E+00 | 0.000E+00 | 0.000E+00 |
| Unigene135766 | evgS | 0.000E+00 | 5.272E-07 | 0.000E+00 | 0.000E+00 | 0.000E+00 | 0.000E+00 | 0.000E+00 | 0.000E+00 | 0.000E+00 | 0.000E+00 | 0.000E+00 | 0.000E+00 |
| Unigene135787 | dfrG | 0.000E+00 | 5.691E-07 | 1.145E-06 | 1.309E-06 | 0.000E+00 | 0.000E+00 | 0.000E+00 | 0.000E+00 | 0.000E+00 | 0.000E+00 | 0.000E+00 | 0.000E+00 |
| Unigene135800 | bacA | 3.108E-07 | 4.135E-06 | 9.502E-05 | 4.315E-07 | 2.287E-06 | 5.039E-07 | 0.000E+00 | 0.000E+00 | 1.699E-04 | 4.760E-07 | 0.000E+00 | 0.000E+00 |
| Unigene135820 | tlrB confer | 4.595E-06 | 9.439E-06 | 1.410E-05 | 1.091E-05 | 3.490E-07 | 4.245E-07 | 3.912E-07 | 0.000E+00 | 4.292E-08 | 0.000E+00 | 1.348E-07 | 0.000E+00 |
| Unigene136045 | marA | 8.163E-07 | 2.973E-06 | 1.893E-06 | 4.871E-07 | 1.171E-05 | 4.813E-07 | 3.532E-06 | 0.000E+00 | 0.000E+00 | 0.000E+00 | 8.913E-07 | 0.000E+00 |
| Unigene136055 | lmrB | 0.000E+00 | 1.897E-06 | 0.000E+00 | 0.000E+00 | 0.000E+00 | 0.000E+00 | 0.000E+00 | 0.000E+00 | 0.000E+00 | 0.000E+00 | 0.000E+00 | 0.000E+00 |
| Unigene136079 | vanHD | 0.000E+00 | 1.718E-06 | 2.877E-06 | 0.000E+00 | 0.000E+00 | 0.000E+00 | 0.000E+00 | 0.000E+00 | 0.000E+00 | 0.000E+00 | 0.000E+00 | 0.000E+00 |
| Unigene136165 | lsaA | 0.000E+00 | 1.207E-06 | 3.110E-06 | 3.941E-06 | 0.000E+00 | 0.000E+00 | 0.000E+00 | 0.000E+00 | 0.000E+00 | 0.000E+00 | 0.000E+00 | 0.000E+00 |
| Unigene136185 | vanWB | 0.000E+00 | 6.465E-07 | 2.697E-07 | 0.000E+00 | 6.544E-07 | 3.821E-07 | 0.000E+00 | 0.000E+00 | 1.288E-07 | 0.000E+00 | 3.594E-07 | 1.485E-06 |
| Unigene136200 | mdsB | 0.000E+00 | 0.000E+00 | 0.000E+00 | 2.188E-07 | 0.000E+00 | 0.000E+00 | 0.000E+00 | 0.000E+00 | 0.000E+00 | 0.000E+00 | 0.000E+00 | 0.000E+00 |
| Unigene136228 | baeS | 5.103E-07 | 6.112E-06 | 3.461E-06 | 3.293E-06 | 0.000E+00 | 0.000E+00 | 0.000E+00 | 2.590E-08 | 0.000E+00 | 0.000E+00 | 0.000E+00 | 0.000E+00 |

| | | | | | | | | | | | | | |
|---|---|---|---|---|---|---|---|---|---|---|---|---|---|
| Unigene136256 | poxtA | 3.132E-07 | 5.019E-06 | 3.811E-06 | 3.796E-06 | 0.000E+00 | 0.000E+00 | 0.000E+00 | 3.656E-08 | 0.000E+00 | 0.000E+00 | 7.166E-08 | 0.000E+00 |
| Unigene136258 | mecR1 | 4.719E-07 | 5.663E-06 | 3.612E-06 | 3.749E-06 | 0.000E+00 | 0.000E+00 | 0.000E+00 | 0.000E+00 | 0.000E+00 | 0.000E+00 | 0.000E+00 | 0.000E+00 |
| Unigene136287 | Staphylocc | 0.000E+00 | 9.852E-07 | 2.404E-06 | 1.715E-06 | 0.000E+00 | 0.000E+00 | 0.000E+00 | 1.416E-08 | 0.000E+00 | 0.000E+00 | 0.000E+00 | 0.000E+00 |
| Unigene136344 | eptA | 0.000E+00 | 6.427E-07 | 2.873E-07 | 1.848E-07 | 0.000E+00 | 0.000E+00 | 0.000E+00 | 0.000E+00 | 0.000E+00 | 0.000E+00 | 0.000E+00 | 0.000E+00 |
| Unigene136567 | msbA | 1.026E-06 | 2.833E-06 | 2.777E-06 | 5.841E-06 | 0.000E+00 | 0.000E+00 | 0.000E+00 | 0.000E+00 | 0.000E+00 | 0.000E+00 | 0.000E+00 | 0.000E+00 |
| Unigene136617 | dfrA3 | 0.000E+00 | 6.797E-07 | 0.000E+00 | 0.000E+00 | 0.000E+00 | 0.000E+00 | 0.000E+00 | 0.000E+00 | 0.000E+00 | 0.000E+00 | 0.000E+00 | 0.000E+00 |
| Unigene136684 | Corynebac | 0.000E+00 | 7.553E-07 | 0.000E+00 | 2.527E-07 | 0.000E+00 | 0.000E+00 | 0.000E+00 | 0.000E+00 | 0.000E+00 | 0.000E+00 | 0.000E+00 | 0.000E+00 |
| Unigene136685 | vanRG | 0.000E+00 | 1.933E-06 | 8.759E-07 | 0.000E+00 | 8.845E-06 | 4.173E-07 | 2.430E-06 | 0.000E+00 | 0.000E+00 | 0.000E+00 | 0.000E+00 | 0.000E+00 |
| Unigene136741 | oqxB | 4.295E-08 | 6.836E-07 | 3.161E-07 | 2.169E-07 | 0.000E+00 | 0.000E+00 | 0.000E+00 | 0.000E+00 | 0.000E+00 | 0.000E+00 | 0.000E+00 | 0.000E+00 |
| Unigene136802 | Acinetobac | 0.000E+00 | 6.638E-07 | 9.371E-08 | 2.250E-07 | 0.000E+00 | 0.000E+00 | 0.000E+00 | 0.000E+00 | 0.000E+00 | 0.000E+00 | 0.000E+00 | 0.000E+00 |
| Unigene136920 | msbA | 4.060E-07 | 3.038E-07 | 6.520E-07 | 7.082E-07 | 7.792E-07 | 1.636E-06 | 4.371E-06 | 3.598E-06 | 3.631E-07 | 1.049E-06 | 2.132E-06 | 3.665E-06 |
| Unigene136944 | novA | 1.999E-07 | 1.775E-06 | 4.280E-06 | 4.203E-06 | 0.000E+00 | 0.000E+00 | 0.000E+00 | 0.000E+00 | 0.000E+00 | 0.000E+00 | 0.000E+00 | 0.000E+00 |
| Unigene136945 | novA | 3.192E-07 | 1.772E-06 | 4.699E-06 | 4.286E-06 | 0.000E+00 | 0.000E+00 | 0.000E+00 | 0.000E+00 | 0.000E+00 | 0.000E+00 | 0.000E+00 | 0.000E+00 |
| Unigene137117 | ugd | 3.843E-07 | 3.826E-06 | 1.347E-06 | 1.441E-06 | 0.000E+00 | 0.000E+00 | 0.000E+00 | 0.000E+00 | 0.000E+00 | 6.116E-08 | 0.000E+00 | 0.000E+00 |
| Unigene137176 | RlmA(II) | 0.000E+00 | 1.605E-06 | 1.950E-06 | 1.628E-06 | 0.000E+00 | 0.000E+00 | 0.000E+00 | 0.000E+00 | 0.000E+00 | 0.000E+00 | 0.000E+00 | 0.000E+00 |
| Unigene137209 | patB | 5.943E-07 | 1.305E-06 | 1.013E-06 | 2.843E-07 | 6.498E-06 | 0.000E+00 | 7.962E-07 | 4.381E-07 | 0.000E+00 | 0.000E+00 | 0.000E+00 | 1.109E-07 |
| Unigene137314 | efrA | 1.491E-07 | 1.190E-06 | 3.271E-06 | 3.695E-06 | 0.000E+00 | 0.000E+00 | 0.000E+00 | 0.000E+00 | 0.000E+00 | 0.000E+00 | 0.000E+00 | 0.000E+00 |
| Unigene137487 | Erm(47) | 0.000E+00 | 9.385E-07 | 1.783E-06 | 0.000E+00 | 0.000E+00 | 0.000E+00 | 0.000E+00 | 0.000E+00 | 0.000E+00 | 0.000E+00 | 0.000E+00 | 0.000E+00 |
| Unigene137638 | adeR | 0.000E+00 | 2.138E-06 | 0.000E+00 | 0.000E+00 | 0.000E+00 | 0.000E+00 | 0.000E+00 | 0.000E+00 | 0.000E+00 | 0.000E+00 | 0.000E+00 | 0.000E+00 |
| Unigene137683 | arlS | 0.000E+00 | 1.751E-06 | 2.818E-06 | 1.611E-06 | 0.000E+00 | 0.000E+00 | 0.000E+00 | 0.000E+00 | 0.000E+00 | 0.000E+00 | 0.000E+00 | 0.000E+00 |
| Unigene137684 | mtrA | 0.000E+00 | 1.737E-06 | 2.262E-06 | 7.396E-06 | 0.000E+00 | 0.000E+00 | 0.000E+00 | 0.000E+00 | 0.000E+00 | 0.000E+00 | 0.000E+00 | 0.000E+00 |
| Unigene137751 | Escherichia | 0.000E+00 | 5.863E-07 | 0.000E+00 | 0.000E+00 | 0.000E+00 | 0.000E+00 | 0.000E+00 | 0.000E+00 | 0.000E+00 | 0.000E+00 | 0.000E+00 | 0.000E+00 |
| Unigene137866 | Pseudomo | 0.000E+00 | 6.239E-07 | 4.383E-07 | 0.000E+00 | 0.000E+00 | 0.000E+00 | 0.000E+00 | 0.000E+00 | 0.000E+00 | 0.000E+00 | 0.000E+00 | 0.000E+00 |
| Unigene137911 | vatB | 0.000E+00 | 6.653E-07 | 6.940E-07 | 3.316E-06 | 0.000E+00 | 0.000E+00 | 0.000E+00 | 0.000E+00 | 0.000E+00 | 0.000E+00 | 0.000E+00 | 0.000E+00 |
| Unigene137994 | tet(43) | 0.000E+00 | 5.180E-07 | 0.000E+00 | 0.000E+00 | 0.000E+00 | 0.000E+00 | 4.701E-06 | 0.000E+00 | 0.000E+00 | 0.000E+00 | 0.000E+00 | 0.000E+00 |
| Unigene138006 | tetA(58) | 0.000E+00 | 1.082E-06 | 0.000E+00 | 0.000E+00 | 0.000E+00 | 0.000E+00 | 0.000E+00 | 0.000E+00 | 0.000E+00 | 0.000E+00 | 0.000E+00 | 0.000E+00 |
| Unigene138010 | clbB | 0.000E+00 | 7.220E-07 | 0.000E+00 | 0.000E+00 | 0.000E+00 | 0.000E+00 | 0.000E+00 | 0.000E+00 | 0.000E+00 | 0.000E+00 | 0.000E+00 | 0.000E+00 |
| Unigene138037 | Erm(34) | 9.119E-07 | 1.401E-06 | 4.724E-06 | 7.698E-06 | 0.000E+00 | 0.000E+00 | 4.441E-07 | 0.000E+00 | 0.000E+00 | 0.000E+00 | 4.172E-08 | 0.000E+00 |
| Unigene138092 | macB | 1.978E-07 | 9.986E-07 | 4.813E-06 | 3.595E-06 | 0.000E+00 | 0.000E+00 | 0.000E+00 | 0.000E+00 | 0.000E+00 | 0.000E+00 | 0.000E+00 | 0.000E+00 |
| Unigene138143 | bcrA | 0.000E+00 | 9.463E-07 | 3.452E-06 | 3.935E-06 | 0.000E+00 | 0.000E+00 | 0.000E+00 | 0.000E+00 | 0.000E+00 | 0.000E+00 | 0.000E+00 | 4.077E-08 |
| Unigene138233 | vanHO | 0.000E+00 | 5.484E-07 | 3.498E-06 | 3.936E-06 | 0.000E+00 | 0.000E+00 | 0.000E+00 | 0.000E+00 | 0.000E+00 | 0.000E+00 | 0.000E+00 | 0.000E+00 |
| Unigene138252 | Pseudomo | 0.000E+00 | 1.113E-06 | 0.000E+00 | 0.000E+00 | 8.182E-06 | 0.000E+00 | 0.000E+00 | 0.000E+00 | 0.000E+00 | 0.000E+00 | 0.000E+00 | 0.000E+00 |
| Unigene138257 | Listeria mc | 3.906E-07 | 1.729E-06 | 1.867E-06 | 1.769E-06 | 0.000E+00 | 0.000E+00 | 0.000E+00 | 0.000E+00 | 0.000E+00 | 0.000E+00 | 0.000E+00 | 0.000E+00 |
| Unigene138270 | lmrD | 0.000E+00 | 9.023E-07 | 5.557E-07 | 0.000E+00 | 0.000E+00 | 0.000E+00 | 1.072E-06 | 0.000E+00 | 0.000E+00 | 0.000E+00 | 3.135E-07 | 8.637E-08 |
| Unigene138285 | vanTG | 0.000E+00 | 5.509E-07 | 2.770E-07 | 3.801E-07 | 0.000E+00 | 0.000E+00 | 0.000E+00 | 0.000E+00 | 0.000E+00 | 0.000E+00 | 0.000E+00 | 0.000E+00 |
| Unigene138390 | tetB(46) | 0.000E+00 | 7.023E-07 | 0.000E+00 | 0.000E+00 | 0.000E+00 | 0.000E+00 | 0.000E+00 | 0.000E+00 | 0.000E+00 | 0.000E+00 | 0.000E+00 | 0.000E+00 |
| Unigene138438 | macB | 1.142E-07 | 3.132E-07 | 1.680E-07 | 2.882E-07 | 0.000E+00 | 0.000E+00 | 0.000E+00 | 0.000E+00 | 0.000E+00 | 0.000E+00 | 0.000E+00 | 0.000E+00 |
| Unigene138526 | mdtN | 0.000E+00 | 1.271E-06 | 0.000E+00 | 0.000E+00 | 0.000E+00 | 0.000E+00 | 0.000E+00 | 0.000E+00 | 0.000E+00 | 0.000E+00 | 0.000E+00 | 0.000E+00 |
| Unigene138530 | macB | 0.000E+00 | 0.000E+00 | 2.869E-07 | 0.000E+00 | 0.000E+00 | 0.000E+00 | 0.000E+00 | 0.000E+00 | 0.000E+00 | 0.000E+00 | 0.000E+00 | 4.839E-07 |
| Unigene138534 | Pseudomo | 0.000E+00 | 1.355E-06 | 0.000E+00 | 0.000E+00 | 0.000E+00 | 0.000E+00 | 2.733E-06 | 0.000E+00 | 0.000E+00 | 0.000E+00 | 0.000E+00 | 0.000E+00 |
| Unigene138586 | Acinetobac | 8.406E-08 | 5.074E-07 | 0.000E+00 | 1.273E-07 | 4.670E-08 | 0.000E+00 | 3.721E-07 | 0.000E+00 | 0.000E+00 | 0.000E+00 | 0.000E+00 | 0.000E+00 |
| Unigene138619 | bcrA | 0.000E+00 | 5.249E-07 | 0.000E+00 | 0.000E+00 | 0.000E+00 | 0.000E+00 | 0.000E+00 | 0.000E+00 | 0.000E+00 | 0.000E+00 | 0.000E+00 | 0.000E+00 |
| Unigene138651 | macB | 5.223E-08 | 8.599E-07 | 1.691E-06 | 1.266E-06 | 0.000E+00 | 0.000E+00 | 0.000E+00 | 0.000E+00 | 0.000E+00 | 0.000E+00 | 0.000E+00 | 0.000E+00 |
| Unigene138696 | PmrF | 0.000E+00 | 2.581E-06 | 0.000E+00 | 2.375E-06 | 0.000E+00 | 0.000E+00 | 0.000E+00 | 0.000E+00 | 0.000E+00 | 0.000E+00 | 0.000E+00 | 0.000E+00 |
| Unigene138780 | mef(B) | 5.342E-06 | 1.145E-05 | 1.110E-05 | 1.127E-05 | 0.000E+00 | 0.000E+00 | 0.000E+00 | 0.000E+00 | 0.000E+00 | 1.676E-07 | 2.869E-08 | 0.000E+00 |
| Unigene138784 | msbA | 0.000E+00 | 5.952E-07 | 3.081E-07 | 2.018E-07 | 0.000E+00 | 0.000E+00 | 0.000E+00 | 0.000E+00 | 0.000E+00 | 0.000E+00 | 0.000E+00 | 0.000E+00 |
| Unigene138790 | bcrA | 3.685E-07 | 0.000E+00 | 6.870E-07 | 0.000E+00 | 3.889E-06 | 2.789E-07 | 0.000E+00 | 0.000E+00 | 0.000E+00 | 0.000E+00 | 0.000E+00 | 0.000E+00 |
| Unigene138886 | macB | 2.301E-05 | 8.288E-06 | 7.193E-06 | 4.441E-06 | 6.146E-07 | 0.000E+00 | 0.000E+00 | 2.260E-07 | 8.163E-07 | 0.000E+00 | 0.000E+00 | 0.000E+00 |
| Unigene138910 | arnA | 0.000E+00 | 7.474E-07 | 8.910E-08 | 4.585E-08 | 3.026E-07 | 9.816E-08 | 1.005E-07 | 0.000E+00 | 0.000E+00 | 0.000E+00 | 1.558E-07 | 0.000E+00 |
| Unigene138912 | Staphylocc | 0.000E+00 | 4.360E-07 | 0.000E+00 | 0.000E+00 | 0.000E+00 | 0.000E+00 | 0.000E+00 | 0.000E+00 | 0.000E+00 | 0.000E+00 | 7.574E-08 | 0.000E+00 |
| Unigene138954 | vanRM | 0.000E+00 | 2.909E-07 | 0.000E+00 | 5.353E-08 | 0.000E+00 | 0.000E+00 | 0.000E+00 | 0.000E+00 | 0.000E+00 | 0.000E+00 | 0.000E+00 | 0.000E+00 |
| Unigene138962 | Pseudomo | 0.000E+00 | 5.199E-07 | 0.000E+00 | 0.000E+00 | 0.000E+00 | 0.000E+00 | 0.000E+00 | 0.000E+00 | 0.000E+00 | 0.000E+00 | 0.000E+00 | 0.000E+00 |
| Unigene139032 | RSA-1 | 3.416E-07 | 9.373E-07 | 5.363E-07 | 3.449E-07 | 1.746E-06 | 0.000E+00 | 8.582E-06 | 3.987E-08 | 3.323E-06 | 0.000E+00 | 0.000E+00 | 0.000E+00 |
| Unigene139048 | mdtC | 0.000E+00 | 7.873E-07 | 0.000E+00 | 5.174E-08 | 0.000E+00 | 0.000E+00 | 0.000E+00 | 0.000E+00 | 0.000E+00 | 0.000E+00 | 0.000E+00 | 0.000E+00 |
| Unigene139049 | emrB | 1.140E-07 | 8.006E-07 | 1.118E-07 | 1.151E-07 | 0.000E+00 | 0.000E+00 | 0.000E+00 | 0.000E+00 | 0.000E+00 | 0.000E+00 | 0.000E+00 | 0.000E+00 |
| Unigene139050 | baeS | 0.000E+00 | 6.252E-07 | 0.000E+00 | 0.000E+00 | 0.000E+00 | 0.000E+00 | 0.000E+00 | 0.000E+00 | 0.000E+00 | 0.000E+00 | 0.000E+00 | 0.000E+00 |
| Unigene139118 | novA | 6.829E-07 | 7.090E-07 | 4.202E-06 | 5.014E-06 | 4.101E-08 | 0.000E+00 | 3.064E-07 | 0.000E+00 | 0.000E+00 | 0.000E+00 | 0.000E+00 | 0.000E+00 |
| Unigene139167 | Staphylocc | 0.000E+00 | 0.000E+00 | 1.265E-06 | 0.000E+00 | 0.000E+00 | 0.000E+00 | 0.000E+00 | 0.000E+00 | 0.000E+00 | 0.000E+00 | 0.000E+00 | 0.000E+00 |

| | | | | | | | | | | | | | |
|---|---|---|---|---|---|---|---|---|---|---|---|---|---|
| Unigene139256 | baeS | 0.000E+00 | 0.000E+00 | 7.366E-07 | 0.000E+00 | 0.000E+00 | 1.205E-06 | 3.978E-06 | 3.771E-06 | 0.000E+00 | 1.597E-06 | 1.769E-06 | 4.195E-06 |
| Unigene139257 | arlR | 0.000E+00 | 0.000E+00 | 6.371E-07 | 5.152E-07 | 0.000E+00 | 1.504E-06 | 5.236E-06 | 2.220E-06 | 9.631E-07 | 1.085E-06 | 0.000E+00 | 4.166E-06 |
| Unigene139269 | Escherichia | 0.000E+00 | 1.108E-06 | 2.190E-06 | 1.744E-06 | 0.000E+00 | 0.000E+00 | 0.000E+00 | 0.000E+00 | 0.000E+00 | 0.000E+00 | 0.000E+00 | 0.000E+00 |
| Unigene139278 | lsaA | 2.047E-05 | 2.863E-05 | 6.768E-05 | 5.459E-05 | 6.368E-05 | 4.659E-05 | 5.716E-05 | 6.794E-05 | 3.345E-05 | 5.210E-05 | 8.099E-05 | 8.692E-05 |
| Unigene139300 | YojI | 0.000E+00 | 0.000E+00 | 8.060E-07 | 8.158E-05 | 0.000E+00 | 0.000E+00 | 0.000E+00 | 9.588E-08 | 0.000E+00 | 0.000E+00 | 0.000E+00 | 0.000E+00 |
| Unigene139334 | Staphylocc | 0.000E+00 | 0.000E+00 | 8.238E-07 | 7.660E-05 | 0.000E+00 | 0.000E+00 | 0.000E+00 | 2.579E-08 | 0.000E+00 | 0.000E+00 | 0.000E+00 | 0.000E+00 |
| Unigene139453 | tetB(60) | 6.280E-05 | 5.097E-05 | 1.049E-04 | 1.545E-04 | 1.113E-04 | 7.995E-05 | 1.076E-04 | 9.907E-05 | 8.571E-05 | 1.275E-04 | 1.285E-04 | 1.279E-04 |
| Unigene139725 | VatI | 0.000E+00 | 0.000E+00 | 1.185E-06 | 0.000E+00 | 0.000E+00 | 0.000E+00 | 0.000E+00 | 0.000E+00 | 0.000E+00 | 0.000E+00 | 0.000E+00 | 0.000E+00 |
| Unigene139843 | MdtK | 2.165E-08 | 4.752E-08 | 1.190E-06 | 0.000E+00 | 4.810E-08 | 0.000E+00 | 4.792E-08 | 5.054E-08 | 1.420E-07 | 1.206E-07 | 0.000E+00 | 1.100E-06 |
| Unigene139917 | tetB(46) | 0.000E+00 | 0.000E+00 | 1.285E-06 | 0.000E+00 | 4.545E-08 | 0.000E+00 | 4.528E-08 | 0.000E+00 | 0.000E+00 | 0.000E+00 | 0.000E+00 | 9.188E-07 |
| Unigene139945 | lmrB | 9.487E-06 | 4.190E-06 | 3.133E-06 | 1.201E-05 | 7.401E-05 | 1.018E-04 | 9.911E-05 | 7.855E-05 | 1.182E-04 | 1.183E-04 | 9.653E-05 | 6.247E-05 |
| Unigene139954 | macB | 0.000E+00 | 2.758E-06 | 1.897E-06 | 3.448E-06 | 0.000E+00 | 0.000E+00 | 0.000E+00 | 0.000E+00 | 0.000E+00 | 0.000E+00 | 0.000E+00 | 0.000E+00 |
| Unigene140027 | Streptomy | 7.889E-08 | 1.732E-07 | 1.208E-06 | 1.593E-07 | 0.000E+00 | 0.000E+00 | 0.000E+00 | 0.000E+00 | 0.000E+00 | 0.000E+00 | 0.000E+00 | 0.000E+00 |
| Unigene140057 | tetA(60) | 0.000E+00 | 2.942E-07 | 2.630E-07 | 0.000E+00 | 0.000E+00 | 0.000E+00 | 0.000E+00 | 0.000E+00 | 0.000E+00 | 0.000E+00 | 0.000E+00 | 0.000E+00 |
| Unigene140100 | Staphylocc | 2.483E-05 | 8.937E-06 | 5.838E-06 | 4.491E-06 | 3.635E-07 | 3.275E-07 | 1.475E-07 | 1.132E-07 | 1.165E-06 | 0.000E+00 | 0.000E+00 | 1.146E-07 |
| Unigene140112 | mdtN | 0.000E+00 | 3.797E-08 | 1.460E-06 | 6.988E-08 | 0.000E+00 | 0.000E+00 | 0.000E+00 | 0.000E+00 | 0.000E+00 | 7.708E-08 | 0.000E+00 | 8.997E-07 |
| Unigene140134 | BahA | 1.836E-07 | 3.627E-07 | 9.009E-07 | 6.675E-07 | 0.000E+00 | 0.000E+00 | 0.000E+00 | 0.000E+00 | 0.000E+00 | 0.000E+00 | 0.000E+00 | 0.000E+00 |
| Unigene140180 | lmrD | 3.009E-06 | 4.043E-06 | 3.490E-06 | 6.704E-06 | 4.255E-07 | 1.972E-07 | 4.037E-07 | 0.000E+00 | 2.193E-07 | 0.000E+00 | 0.000E+00 | 1.725E-07 |
| Unigene140250 | msrC | 1.006E-07 | 0.000E+00 | 6.414E-07 | 8.642E-05 | 0.000E+00 | 0.000E+00 | 0.000E+00 | 0.000E+00 | 0.000E+00 | 0.000E+00 | 0.000E+00 | 0.000E+00 |
| Unigene140299 | bacA | 0.000E+00 | 0.000E+00 | 1.025E-06 | 0.000E+00 | 0.000E+00 | 0.000E+00 | 0.000E+00 | 0.000E+00 | 0.000E+00 | 0.000E+00 | 0.000E+00 | 0.000E+00 |
| Unigene140321 | vanSG | 0.000E+00 | 0.000E+00 | 1.047E-06 | 6.409E-05 | 0.000E+00 | 0.000E+00 | 0.000E+00 | 2.165E-07 | 0.000E+00 | 0.000E+00 | 0.000E+00 | 0.000E+00 |
| Unigene140322 | mtrA | 0.000E+00 | 0.000E+00 | 8.324E-07 | 5.978E-05 | 0.000E+00 | 0.000E+00 | 0.000E+00 | 5.211E-08 | 0.000E+00 | 0.000E+00 | 0.000E+00 | 0.000E+00 |
| Unigene140344 | macB | 0.000E+00 | 6.549E-08 | 1.073E-06 | 0.000E+00 | 8.838E-08 | 2.150E-08 | 6.604E-08 | 2.322E-08 | 1.522E-07 | 6.647E-08 | 4.550E-08 | 8.934E-07 |
| Unigene140399 | evgS | 0.000E+00 | 0.000E+00 | 2.357E-07 | 0.000E+00 | 0.000E+00 | 0.000E+00 | 0.000E+00 | 0.000E+00 | 5.250E-08 | 0.000E+00 | 1.648E-07 | 0.000E+00 |
| Unigene140401 | macB | 1.137E-07 | 0.000E+00 | 4.461E-07 | 4.209E-07 | 0.000E+00 | 0.000E+00 | 0.000E+00 | 0.000E+00 | 0.000E+00 | 0.000E+00 | 0.000E+00 | 0.000E+00 |
| Unigene140406 | tetB(46) | 1.724E-07 | 4.542E-07 | 1.455E-06 | 3.100E-06 | 0.000E+00 | 1.491E-07 | 3.435E-07 | 1.369E-06 | 0.000E+00 | 2.689E-07 | 1.144E-06 | 1.223E-06 |
| Unigene140409 | macB | 3.937E-07 | 0.000E+00 | 1.846E-06 | 3.313E-06 | 0.000E+00 | 0.000E+00 | 0.000E+00 | 1.532E-06 | 0.000E+00 | 8.283E-07 | 9.006E-07 | 1.086E-06 |
| Unigene140410 | tetB(60) | 3.223E-07 | 5.109E-07 | 1.230E-06 | 3.074E-06 | 5.967E-07 | 2.710E-07 | 1.189E-07 | 1.003E-06 | 1.370E-07 | 6.382E-07 | 1.085E-06 | 7.619E-07 |
| Unigene140411 | tetA(60) | 3.817E-07 | 8.578E-07 | 1.641E-06 | 2.790E-06 | 4.442E-07 | 1.572E-07 | 2.615E-07 | 1.528E-06 | 5.960E-08 | 8.909E-07 | 8.940E-07 | 8.164E-07 |
| Unigene140439 | adeL | 0.000E+00 | 0.000E+00 | 0.000E+00 | 0.000E+00 | 0.000E+00 | 0.000E+00 | 2.842E-06 | 0.000E+00 | 0.000E+00 | 0.000E+00 | 0.000E+00 | 2.983E-06 |
| Unigene140586 | oleB | 0.000E+00 | 4.434E-07 | 7.928E-07 | 1.113E-07 | 0.000E+00 | 0.000E+00 | 0.000E+00 | 0.000E+00 | 0.000E+00 | 0.000E+00 | 0.000E+00 | 0.000E+00 |
| Unigene140648 | tetB(P) | 1.596E-07 | 0.000E+00 | 2.908E-07 | 1.842E-07 | 0.000E+00 | 0.000E+00 | 0.000E+00 | 0.000E+00 | 0.000E+00 | 0.000E+00 | 0.000E+00 | 0.000E+00 |
| Unigene140653 | msbA | 5.531E-07 | 3.311E-07 | 4.934E-07 | 3.046E-07 | 0.000E+00 | 0.000E+00 | 0.000E+00 | 0.000E+00 | 0.000E+00 | 0.000E+00 | 0.000E+00 | 0.000E+00 |
| Unigene140674 | vanHB | 2.385E-07 | 3.271E-07 | 7.897E-07 | 8.127E-07 | 0.000E+00 | 0.000E+00 | 0.000E+00 | 0.000E+00 | 0.000E+00 | 0.000E+00 | 0.000E+00 | 0.000E+00 |
| Unigene140721 | otr(B) | 9.651E-05 | 1.849E-04 | 1.732E-04 | 7.104E-06 | 8.959E-06 | 1.895E-07 | 3.711E-06 | 1.791E-06 | 5.777E-05 | 1.953E-07 | 3.409E-06 | 7.771E-07 |
| Unigene141004 | tetA(58) | 0.000E+00 | 0.000E+00 | 1.031E-06 | 0.000E+00 | 0.000E+00 | 0.000E+00 | 0.000E+00 | 9.812E-08 | 0.000E+00 | 0.000E+00 | 0.000E+00 | 0.000E+00 |
| Unigene141021 | msrE | 0.000E+00 | 0.000E+00 | 7.542E-07 | 0.000E+00 | 0.000E+00 | 0.000E+00 | 0.000E+00 | 0.000E+00 | 0.000E+00 | 0.000E+00 | 0.000E+00 | 0.000E+00 |
| Unigene141087 | mdtG | 0.000E+00 | 0.000E+00 | 1.113E-05 | 0.000E+00 | 0.000E+00 | 9.693E-07 | 0.000E+00 | 0.000E+00 | 0.000E+00 | 0.000E+00 | 0.000E+00 | 0.000E+00 |
| Unigene141112 | oleC | 0.000E+00 | 0.000E+00 | 1.446E-05 | 0.000E+00 | 0.000E+00 | 0.000E+00 | 0.000E+00 | 0.000E+00 | 0.000E+00 | 0.000E+00 | 0.000E+00 | 0.000E+00 |
| Unigene141163 | Staphylocc | 2.304E-08 | 0.000E+00 | 1.280E-05 | 0.000E+00 | 3.840E-08 | 7.722E-07 | 2.805E-07 | 0.000E+00 | 0.000E+00 | 0.000E+00 | 0.000E+00 | 0.000E+00 |
| Unigene141316 | Staphylocc | 3.816E-07 | 4.690E-07 | 1.333E-06 | 2.959E-06 | 4.069E-07 | 3.465E-07 | 3.041E-07 | 1.372E-06 | 6.673E-08 | 8.501E-07 | 1.030E-06 | 5.953E-07 |
| Unigene141378 | rpoB2 | 5.268E-07 | 3.724E-07 | 1.165E-06 | 2.381E-06 | 2.381E-07 | 1.158E-07 | 3.064E-07 | 1.011E-06 | 7.808E-08 | 4.476E-07 | 9.704E-07 | 4.222E-07 |
| Unigene141387 | msbA | 0.000E+00 | 0.000E+00 | 8.237E-07 | 0.000E+00 | 0.000E+00 | 0.000E+00 | 0.000E+00 | 0.000E+00 | 0.000E+00 | 0.000E+00 | 0.000E+00 | 0.000E+00 |
| Unigene141408 | msbA | 0.000E+00 | 0.000E+00 | 1.246E-06 | 5.030E-08 | 1.107E-07 | 2.693E-08 | 0.000E+00 | 0.000E+00 | 0.000E+00 | 0.000E+00 | 0.000E+00 | 1.295E-06 |
| Unigene141422 | vatE | 0.000E+00 | 5.565E-07 | 3.151E-06 | 2.105E-06 | 0.000E+00 | 0.000E+00 | 0.000E+00 | 0.000E+00 | 0.000E+00 | 0.000E+00 | 0.000E+00 | 0.000E+00 |
| Unigene141435 | lmrC | 0.000E+00 | 1.027E-07 | 1.836E-07 | 1.890E-07 | 2.599E-07 | 1.517E-07 | 6.213E-07 | 3.823E-07 | 0.000E+00 | 0.000E+00 | 3.746E-07 | 9.954E-07 |
| Unigene141445 | arlR | 1.397E-07 | 0.000E+00 | 5.942E-07 | 2.822E-07 | 0.000E+00 | 0.000E+00 | 0.000E+00 | 0.000E+00 | 0.000E+00 | 0.000E+00 | 5.328E-08 | 0.000E+00 |
| Unigene141467 | TaeA | 4.464E-07 | 2.321E-07 | 1.176E-06 | 2.942E-06 | 2.871E-07 | 0.000E+00 | 2.600E-07 | 1.152E-06 | 1.797E-07 | 6.805E-07 | 1.048E-06 | 3.332E-07 |
| Unigene141471 | dfrE | 3.241E-07 | 0.000E+00 | 1.272E-06 | 2.880E-06 | 0.000E+00 | 0.000E+00 | 2.152E-07 | 6.053E-07 | 0.000E+00 | 0.000E+00 | 6.673E-07 | 5.363E-07 |
| Unigene141495 | dfrA3 | 0.000E+00 | 0.000E+00 | 9.598E-07 | 3.704E-07 | 0.000E+00 | 4.626E-07 | 2.030E-06 | 0.000E+00 | 0.000E+00 | 0.000E+00 | 1.818E-06 | 3.107E-06 |
| Unigene141548 | tetA(60) | 7.953E-07 | 1.403E-06 | 1.775E-06 | 1.921E-06 | 0.000E+00 | 0.000E+00 | 0.000E+00 | 3.640E-08 | 0.000E+00 | 0.000E+00 | 0.000E+00 | 0.000E+00 |
| Unigene141598 | lmrC | 5.425E-07 | 0.000E+00 | 7.098E-07 | 3.652E-07 | 0.000E+00 | 1.173E-06 | 4.703E-06 | 0.000E+00 | 0.000E+00 | 0.000E+00 | 0.000E+00 | 3.812E-06 |
| Unigene141607 | baeS | 2.922E-06 | 3.854E-06 | 3.001E-06 | 1.922E-06 | 2.844E-06 | 2.106E-06 | 2.231E-06 | 5.553E-07 | 1.659E-06 | 6.889E-06 | 5.416E-06 | 2.356E-06 |
| Unigene141618 | vgaB | 0.000E+00 | 0.000E+00 | 7.078E-07 | 0.000E+00 | 0.000E+00 | 0.000E+00 | 2.395E-06 | 2.175E-06 | 5.585E-07 | 7.366E-07 | 0.000E+00 | 2.629E-06 |
| Unigene141648 | Erm(30) | 7.894E-05 | 6.835E-05 | 1.303E-04 | 1.717E-04 | 1.499E-04 | 1.032E-04 | 1.432E-04 | 1.349E-04 | 1.180E-04 | 1.691E-04 | 1.777E-04 | 1.848E-04 |
| Unigene141695 | smeR | 3.471E-07 | 0.000E+00 | 1.306E-06 | 0.000E+00 | 0.000E+00 | 0.000E+00 | 0.000E+00 | 0.000E+00 | 0.000E+00 | 0.000E+00 | 0.000E+00 | 0.000E+00 |
| Unigene141810 | tetA(60) | 0.000E+00 | 1.757E-07 | 1.775E-06 | 1.410E-05 | 0.000E+00 | 0.000E+00 | 0.000E+00 | 0.000E+00 | 0.000E+00 | 0.000E+00 | 0.000E+00 | 0.000E+00 |
| Unigene141824 | vanRF | 0.000E+00 | 1.610E-07 | 1.392E-06 | 1.338E-05 | 0.000E+00 | 0.000E+00 | 0.000E+00 | 0.000E+00 | 0.000E+00 | 0.000E+00 | 0.000E+00 | 0.000E+00 |

| | | | | | | | | | | | | | |
|---|---|---|---|---|---|---|---|---|---|---|---|---|---|
| Unigene141827 | bcrA | 0.000E+00 | 0.000E+00 | 2.082E-06 | 1.219E-05 | 0.000E+00 | 0.000E+00 | 0.000E+00 | 0.000E+00 | 0.000E+00 | 0.000E+00 | 0.000E+00 | 0.000E+00 |
| Unigene141852 | macB | 9.480E-08 | 1.561E-07 | 2.186E-06 | 1.527E-05 | 0.000E+00 | 0.000E+00 | 0.000E+00 | 0.000E+00 | 0.000E+00 | 0.000E+00 | 0.000E+00 | 0.000E+00 |
| Unigene141917 | poxtA | 0.000E+00 | 0.000E+00 | 1.215E-05 | 0.000E+00 | 0.000E+00 | 0.000E+00 | 0.000E+00 | 0.000E+00 | 0.000E+00 | 0.000E+00 | 0.000E+00 | 0.000E+00 |
| Unigene141928 | macB | 0.000E+00 | 0.000E+00 | 1.263E-05 | 0.000E+00 | 0.000E+00 | 0.000E+00 | 0.000E+00 | 0.000E+00 | 0.000E+00 | 0.000E+00 | 0.000E+00 | 0.000E+00 |
| Unigene141938 | tetA(58) | 0.000E+00 | 0.000E+00 | 1.244E-05 | 0.000E+00 | 0.000E+00 | 0.000E+00 | 0.000E+00 | 0.000E+00 | 0.000E+00 | 0.000E+00 | 0.000E+00 | 0.000E+00 |
| Unigene141944 | vanHO | 0.000E+00 | 0.000E+00 | 1.294E-05 | 0.000E+00 | 0.000E+00 | 0.000E+00 | 0.000E+00 | 0.000E+00 | 0.000E+00 | 0.000E+00 | 0.000E+00 | 0.000E+00 |
| Unigene141958 | cmlA8 | 0.000E+00 | 0.000E+00 | 1.212E-05 | 0.000E+00 | 0.000E+00 | 0.000E+00 | 0.000E+00 | 0.000E+00 | 0.000E+00 | 0.000E+00 | 0.000E+00 | 0.000E+00 |
| Unigene141959 | bacA | 0.000E+00 | 0.000E+00 | 1.297E-05 | 0.000E+00 | 0.000E+00 | 1.061E-06 | 0.000E+00 | 0.000E+00 | 0.000E+00 | 0.000E+00 | 0.000E+00 | 0.000E+00 |
| Unigene141963 | bcrA | 0.000E+00 | 0.000E+00 | 1.168E-05 | 0.000E+00 | 0.000E+00 | 0.000E+00 | 0.000E+00 | 0.000E+00 | 0.000E+00 | 0.000E+00 | 0.000E+00 | 0.000E+00 |
| Unigene141991 | patA | 0.000E+00 | 0.000E+00 | 1.094E-05 | 0.000E+00 | 0.000E+00 | 8.840E-07 | 3.497E-07 | 0.000E+00 | 0.000E+00 | 0.000E+00 | 0.000E+00 | 0.000E+00 |
| Unigene141992 | lmrD | 0.000E+00 | 0.000E+00 | 1.290E-05 | 0.000E+00 | 0.000E+00 | 1.148E-06 | 5.281E-07 | 0.000E+00 | 0.000E+00 | 0.000E+00 | 0.000E+00 | 0.000E+00 |
| Unigene142014 | patB | 1.352E-07 | 1.780E-07 | 1.061E-06 | 1.911E-07 | 0.000E+00 | 0.000E+00 | 0.000E+00 | 0.000E+00 | 0.000E+00 | 0.000E+00 | 0.000E+00 | 0.000E+00 |
| Unigene142090 | Bifidobacte | 2.198E-07 | 2.851E-07 | 1.500E-06 | 6.457E-06 | 0.000E+00 | 0.000E+00 | 0.000E+00 | 0.000E+00 | 0.000E+00 | 0.000E+00 | 0.000E+00 | 0.000E+00 |
| Unigene142127 | patB | 6.883E-08 | 1.007E-07 | 4.953E-07 | 2.317E-07 | 0.000E+00 | 0.000E+00 | 0.000E+00 | 0.000E+00 | 0.000E+00 | 0.000E+00 | 0.000E+00 | 0.000E+00 |
| Unigene142184 | mdtH | 0.000E+00 | 0.000E+00 | 1.389E-06 | 5.392E-08 | 2.076E-07 | 0.000E+00 | 0.000E+00 | 0.000E+00 | 2.918E-08 | 0.000E+00 | 6.108E-08 | 1.105E-06 |
| Unigene142271 | mtrA | 0.000E+00 | 0.000E+00 | 5.528E-07 | 1.479E-06 | 0.000E+00 | 0.000E+00 | 0.000E+00 | 0.000E+00 | 0.000E+00 | 0.000E+00 | 0.000E+00 | 0.000E+00 |
| Unigene142340 | YojI | 2.562E-07 | 3.528E-06 | 3.497E-06 | 5.080E-06 | 0.000E+00 | 0.000E+00 | 0.000E+00 | 0.000E+00 | 0.000E+00 | 0.000E+00 | 0.000E+00 | 0.000E+00 |
| Unigene142373 | Listeria mc | 2.054E-05 | 5.075E-06 | 4.087E-06 | 1.393E-05 | 9.285E-05 | 1.276E-04 | 1.327E-04 | 1.108E-04 | 1.611E-04 | 1.640E-04 | 1.373E-04 | 8.688E-05 |
| Unigene142426 | rphB | 1.348E-08 | 0.000E+00 | 8.468E-07 | 7.892E-05 | 0.000E+00 | 0.000E+00 | 0.000E+00 | 1.102E-07 | 0.000E+00 | 0.000E+00 | 0.000E+00 | 0.000E+00 |
| Unigene142484 | tetB(60) | 3.279E-07 | 2.617E-07 | 6.434E-07 | 0.000E+00 | 0.000E+00 | 0.000E+00 | 1.089E-06 | 1.879E-06 | 0.000E+00 | 0.000E+00 | 9.547E-07 | 2.537E-06 |
| Unigene142497 | lmrC | 0.000E+00 | 9.680E-08 | 3.245E-07 | 0.000E+00 | 0.000E+00 | 3.575E-07 | 0.000E+00 | 9.008E-07 | 0.000E+00 | 5.158E-07 | 0.000E+00 | 0.000E+00 |
| Unigene142498 | baeS | 0.000E+00 | 4.972E-07 | 5.557E-07 | 0.000E+00 | 0.000E+00 | 0.000E+00 | 0.000E+00 | 0.000E+00 | 0.000E+00 | 0.000E+00 | 0.000E+00 | 0.000E+00 |
| Unigene142618 | efrA | 1.125E-07 | 8.024E-07 | 3.127E-06 | 4.165E-06 | 0.000E+00 | 0.000E+00 | 0.000E+00 | 0.000E+00 | 0.000E+00 | 0.000E+00 | 0.000E+00 | 0.000E+00 |
| Unigene142622 | vanTC | 0.000E+00 | 0.000E+00 | 1.062E-06 | 0.000E+00 | 0.000E+00 | 0.000E+00 | 0.000E+00 | 0.000E+00 | 0.000E+00 | 0.000E+00 | 0.000E+00 | 0.000E+00 |
| Unigene142632 | bcrA | 2.850E-07 | 0.000E+00 | 9.789E-07 | 2.339E-06 | 0.000E+00 | 0.000E+00 | 1.577E-07 | 1.248E-06 | 0.000E+00 | 3.969E-07 | 8.151E-07 | 8.423E-08 |
| Unigene142775 | macB | 0.000E+00 | 3.690E-07 | 9.898E-07 | 1.273E-07 | 0.000E+00 | 0.000E+00 | 0.000E+00 | 0.000E+00 | 0.000E+00 | 0.000E+00 | 0.000E+00 | 0.000E+00 |
| Unigene142822 | baeS | 5.249E-07 | 5.113E-06 | 8.562E-06 | 1.352E-05 | 0.000E+00 | 0.000E+00 | 3.631E-08 | 0.000E+00 | 0.000E+00 | 0.000E+00 | 0.000E+00 | 0.000E+00 |
| Unigene142836 | MOX-2 | 0.000E+00 | 0.000E+00 | 1.053E-05 | 0.000E+00 | 0.000E+00 | 0.000E+00 | 0.000E+00 | 0.000E+00 | 0.000E+00 | 0.000E+00 | 0.000E+00 | 0.000E+00 |
| Unigene142842 | arlR | 0.000E+00 | 0.000E+00 | 1.405E-05 | 0.000E+00 | 0.000E+00 | 0.000E+00 | 0.000E+00 | 0.000E+00 | 0.000E+00 | 0.000E+00 | 0.000E+00 | 0.000E+00 |
| Unigene142972 | mdtM | 0.000E+00 | 1.635E-06 | 0.000E+00 | 0.000E+00 | 9.257E-06 | 3.579E-07 | 0.000E+00 | 0.000E+00 | 0.000E+00 | 0.000E+00 | 0.000E+00 | 0.000E+00 |
| Unigene142985 | arlS | 3.404E-06 | 7.979E-06 | 8.435E-06 | 1.028E-05 | 0.000E+00 | 0.000E+00 | 0.000E+00 | 0.000E+00 | 0.000E+00 | 0.000E+00 | 0.000E+00 | 0.000E+00 |
| Unigene142992 | bcrA | 0.000E+00 | 0.000E+00 | 6.923E-07 | 0.000E+00 | 0.000E+00 | 0.000E+00 | 0.000E+00 | 0.000E+00 | 0.000E+00 | 0.000E+00 | 0.000E+00 | 0.000E+00 |
| Unigene143053 | msrC | 0.000E+00 | 0.000E+00 | 1.040E-06 | 0.000E+00 | 0.000E+00 | 0.000E+00 | 0.000E+00 | 0.000E+00 | 0.000E+00 | 0.000E+00 | 0.000E+00 | 0.000E+00 |
| Unigene143140 | vanRG | 0.000E+00 | 0.000E+00 | 6.628E-07 | 0.000E+00 | 0.000E+00 | 0.000E+00 | 0.000E+00 | 0.000E+00 | 0.000E+00 | 0.000E+00 | 0.000E+00 | 0.000E+00 |
| Unigene143141 | vanSE | 0.000E+00 | 0.000E+00 | 8.654E-07 | 0.000E+00 | 0.000E+00 | 0.000E+00 | 0.000E+00 | 0.000E+00 | 0.000E+00 | 0.000E+00 | 0.000E+00 | 0.000E+00 |
| Unigene143333 | LlmA 23S r | 2.562E-07 | 0.000E+00 | 6.424E-07 | 0.000E+00 | 9.804E-07 | 6.155E-07 | 1.764E-06 | 1.030E-06 | 2.489E-07 | 8.562E-07 | 1.009E-06 | 4.206E-06 |
| Unigene143403 | tetA(58) | 3.416E-07 | 6.092E-07 | 3.268E-06 | 2.932E-06 | 0.000E+00 | 0.000E+00 | 0.000E+00 | 0.000E+00 | 0.000E+00 | 0.000E+00 | 0.000E+00 | 0.000E+00 |
| Unigene143407 | vanRL | 9.480E-08 | 7.283E-07 | 3.070E-06 | 4.164E-06 | 0.000E+00 | 0.000E+00 | 0.000E+00 | 0.000E+00 | 0.000E+00 | 0.000E+00 | 0.000E+00 | 0.000E+00 |
| Unigene143408 | vanSG | 1.915E-07 | 1.219E-06 | 3.494E-06 | 3.596E-06 | 0.000E+00 | 0.000E+00 | 0.000E+00 | 0.000E+00 | 0.000E+00 | 0.000E+00 | 0.000E+00 | 0.000E+00 |
| Unigene143409 | macB | 9.316E-08 | 4.601E-07 | 2.971E-06 | 3.622E-06 | 0.000E+00 | 0.000E+00 | 0.000E+00 | 0.000E+00 | 0.000E+00 | 0.000E+00 | 0.000E+00 | 0.000E+00 |
| Unigene143516 | Corynebac | 0.000E+00 | 0.000E+00 | 3.641E-07 | 0.000E+00 | 0.000E+00 | 0.000E+00 | 3.658E-06 | 2.480E-06 | 0.000E+00 | 0.000E+00 | 0.000E+00 | 3.030E-06 |
| Unigene143528 | cmx | 0.000E+00 | 0.000E+00 | 4.044E-07 | 0.000E+00 | 0.000E+00 | 0.000E+00 | 0.000E+00 | 0.000E+00 | 0.000E+00 | 0.000E+00 | 0.000E+00 | 0.000E+00 |
| Unigene143766 | vanRG | 0.000E+00 | 0.000E+00 | 4.768E-07 | 0.000E+00 | 0.000E+00 | 0.000E+00 | 0.000E+00 | 0.000E+00 | 0.000E+00 | 0.000E+00 | 0.000E+00 | 0.000E+00 |
| Unigene143795 | tva(A) | 0.000E+00 | 0.000E+00 | 1.586E-06 | 5.055E-06 | 0.000E+00 | 0.000E+00 | 0.000E+00 | 0.000E+00 | 0.000E+00 | 0.000E+00 | 0.000E+00 | 0.000E+00 |
| Unigene143796 | lmrD | 0.000E+00 | 2.086E-07 | 1.082E-06 | 6.143E-06 | 0.000E+00 | 0.000E+00 | 0.000E+00 | 0.000E+00 | 0.000E+00 | 0.000E+00 | 0.000E+00 | 0.000E+00 |
| Unigene143848 | adeS | 2.206E-07 | 7.532E-07 | 4.185E-06 | 3.737E-06 | 0.000E+00 | 0.000E+00 | 0.000E+00 | 0.000E+00 | 0.000E+00 | 0.000E+00 | 0.000E+00 | 0.000E+00 |
| Unigene143879 | lmrC | 0.000E+00 | 0.000E+00 | 7.929E-07 | 5.154E-07 | 0.000E+00 | 0.000E+00 | 0.000E+00 | 0.000E+00 | 0.000E+00 | 0.000E+00 | 0.000E+00 | 0.000E+00 |
| Unigene143926 | tetA(58) | 1.798E-07 | 0.000E+00 | 8.823E-07 | 0.000E+00 | 0.000E+00 | 0.000E+00 | 0.000E+00 | 0.000E+00 | 0.000E+00 | 0.000E+00 | 0.000E+00 | 0.000E+00 |
| Unigene143933 | msbA | 0.000E+00 | 0.000E+00 | 1.104E-06 | 7.120E-05 | 0.000E+00 | 0.000E+00 | 0.000E+00 | 0.000E+00 | 0.000E+00 | 0.000E+00 | 0.000E+00 | 0.000E+00 |
| Unigene143938 | tetB(P) | 0.000E+00 | 0.000E+00 | 8.382E-07 | 1.319E-05 | 0.000E+00 | 0.000E+00 | 0.000E+00 | 0.000E+00 | 0.000E+00 | 0.000E+00 | 0.000E+00 | 0.000E+00 |
| Unigene144038 | hmrM | 0.000E+00 | 2.578E-08 | 1.222E-06 | 0.000E+00 | 0.000E+00 | 0.000E+00 | 5.200E-08 | 0.000E+00 | 0.000E+00 | 0.000E+00 | 0.000E+00 | 8.609E-07 |
| Unigene144060 | PmrF | 1.239E-05 | 4.717E-06 | 2.924E-06 | 1.150E-05 | 8.284E-05 | 1.139E-04 | 1.099E-04 | 9.462E-05 | 1.470E-04 | 1.388E-04 | 1.115E-04 | 7.312E-05 |
| Unigene144176 | vanRN | 0.000E+00 | 0.000E+00 | 1.225E-05 | 0.000E+00 | 0.000E+00 | 1.031E-06 | 0.000E+00 | 0.000E+00 | 0.000E+00 | 0.000E+00 | 0.000E+00 | 0.000E+00 |
| Unigene144181 | tetB(46) | 8.121E-08 | 5.348E-07 | 1.167E-05 | 0.000E+00 | 0.000E+00 | 0.000E+00 | 0.000E+00 | 0.000E+00 | 0.000E+00 | 0.000E+00 | 0.000E+00 | 9.119E-07 |
| Unigene144182 | patB | 0.000E+00 | 0.000E+00 | 1.211E-05 | 0.000E+00 | 0.000E+00 | 1.016E-06 | 5.671E-07 | 1.495E-07 | 0.000E+00 | 0.000E+00 | 0.000E+00 | 0.000E+00 |
| Unigene144196 | Staphylocc | 0.000E+00 | 0.000E+00 | 1.232E-05 | 0.000E+00 | 0.000E+00 | 8.937E-07 | 5.608E-07 | 0.000E+00 | 0.000E+00 | 0.000E+00 | 0.000E+00 | 0.000E+00 |
| Unigene144231 | sul4 | 0.000E+00 | 0.000E+00 | 1.279E-05 | 0.000E+00 | 0.000E+00 | 1.105E-06 | 5.342E-07 | 0.000E+00 | 0.000E+00 | 0.000E+00 | 0.000E+00 | 0.000E+00 |
| Unigene144244 | vanYB | 0.000E+00 | 0.000E+00 | 1.232E-05 | 0.000E+00 | 0.000E+00 | 0.000E+00 | 0.000E+00 | 0.000E+00 | 0.000E+00 | 0.000E+00 | 0.000E+00 | 0.000E+00 |

| | | | | | | | | | | | | | |
|---|---|---|---|---|---|---|---|---|---|---|---|---|---|
| Unigene144270 | Staphylocc | 0.000E+00 | 0.000E+00 | 1.362E-05 | 0.000E+00 | 0.000E+00 | 7.974E-07 | 0.000E+00 | 0.000E+00 | 0.000E+00 | 0.000E+00 | 0.000E+00 | 0.000E+00 |
| Unigene144277 | mepA | 0.000E+00 | 0.000E+00 | 1.267E-05 | 0.000E+00 | 0.000E+00 | 0.000E+00 | 0.000E+00 | 0.000E+00 | 0.000E+00 | 0.000E+00 | 0.000E+00 | 0.000E+00 |
| Unigene144280 | lmrB | 0.000E+00 | 0.000E+00 | 1.203E-05 | 0.000E+00 | 0.000E+00 | 7.853E-07 | 5.276E-07 | 0.000E+00 | 0.000E+00 | 0.000E+00 | 0.000E+00 | 0.000E+00 |
| Unigene144282 | facT | 0.000E+00 | 0.000E+00 | 1.139E-05 | 0.000E+00 | 0.000E+00 | 8.084E-07 | 5.518E-07 | 0.000E+00 | 0.000E+00 | 0.000E+00 | 0.000E+00 | 0.000E+00 |
| Unigene144314 | vanHA | 0.000E+00 | 0.000E+00 | 1.357E-05 | 0.000E+00 | 0.000E+00 | 0.000E+00 | 3.620E-07 | 0.000E+00 | 0.000E+00 | 0.000E+00 | 0.000E+00 | 0.000E+00 |
| Unigene144332 | novA | 0.000E+00 | 0.000E+00 | 1.309E-05 | 0.000E+00 | 4.859E-08 | 6.147E-07 | 4.357E-07 | 0.000E+00 | 0.000E+00 | 0.000E+00 | 0.000E+00 | 0.000E+00 |
| Unigene144344 | YojI | 0.000E+00 | 0.000E+00 | 1.364E-05 | 0.000E+00 | 0.000E+00 | 0.000E+00 | 0.000E+00 | 0.000E+00 | 0.000E+00 | 0.000E+00 | 0.000E+00 | 0.000E+00 |
| Unigene144351 | optrA | 0.000E+00 | 0.000E+00 | 1.383E-05 | 0.000E+00 | 0.000E+00 | 0.000E+00 | 0.000E+00 | 0.000E+00 | 0.000E+00 | 0.000E+00 | 0.000E+00 | 0.000E+00 |
| Unigene144353 | mdtG | 0.000E+00 | 0.000E+00 | 1.335E-05 | 0.000E+00 | 0.000E+00 | 0.000E+00 | 0.000E+00 | 0.000E+00 | 0.000E+00 | 0.000E+00 | 0.000E+00 | 0.000E+00 |
| Unigene144379 | poxtA | 0.000E+00 | 0.000E+00 | 1.056E-05 | 0.000E+00 | 0.000E+00 | 1.551E-06 | 0.000E+00 | 0.000E+00 | 0.000E+00 | 0.000E+00 | 0.000E+00 | 0.000E+00 |
| Unigene144396 | vatE | 0.000E+00 | 0.000E+00 | 1.362E-05 | 0.000E+00 | 0.000E+00 | 0.000E+00 | 0.000E+00 | 0.000E+00 | 0.000E+00 | 0.000E+00 | 0.000E+00 | 0.000E+00 |
| Unigene144402 | lin | 0.000E+00 | 0.000E+00 | 1.174E-05 | 0.000E+00 | 0.000E+00 | 7.521E-07 | 0.000E+00 | 0.000E+00 | 0.000E+00 | 0.000E+00 | 0.000E+00 | 0.000E+00 |
| Unigene144408 | patB | 0.000E+00 | 0.000E+00 | 1.284E-05 | 0.000E+00 | 0.000E+00 | 0.000E+00 | 0.000E+00 | 0.000E+00 | 0.000E+00 | 0.000E+00 | 0.000E+00 | 0.000E+00 |
| Unigene144413 | golS | 0.000E+00 | 0.000E+00 | 1.419E-05 | 0.000E+00 | 0.000E+00 | 0.000E+00 | 0.000E+00 | 4.019E-07 | 0.000E+00 | 0.000E+00 | 0.000E+00 | 0.000E+00 |
| Unigene144428 | tetA(58) | 0.000E+00 | 0.000E+00 | 1.283E-06 | 0.000E+00 | 0.000E+00 | 0.000E+00 | 0.000E+00 | 0.000E+00 | 0.000E+00 | 0.000E+00 | 0.000E+00 | 0.000E+00 |
| Unigene144521 | PmrF | 0.000E+00 | 0.000E+00 | 1.271E-06 | 0.000E+00 | 0.000E+00 | 0.000E+00 | 0.000E+00 | 0.000E+00 | 0.000E+00 | 0.000E+00 | 0.000E+00 | 0.000E+00 |
| Unigene144549 | Staphylocc | 0.000E+00 | 1.124E-06 | 2.344E-06 | 1.240E-06 | 0.000E+00 | 0.000E+00 | 0.000E+00 | 3.983E-08 | 0.000E+00 | 0.000E+00 | 0.000E+00 | 0.000E+00 |
| Unigene144674 | arlS | 3.460E-07 | 4.747E-07 | 1.426E-06 | 3.459E-06 | 5.958E-07 | 1.309E-07 | 2.106E-07 | 1.111E-06 | 0.000E+00 | 5.396E-07 | 1.088E-06 | 4.294E-07 |
| Unigene144675 | lmrP | 0.000E+00 | 7.183E-07 | 1.721E-06 | 3.093E-06 | 2.036E-07 | 0.000E+00 | 2.028E-07 | 1.620E-06 | 0.000E+00 | 7.291E-07 | 1.078E-06 | 8.355E-07 |
| Unigene144681 | vatH | 6.974E-07 | 5.959E-06 | 1.007E-05 | 1.595E-05 | 0.000E+00 | 0.000E+00 | 5.513E-08 | 0.000E+00 | 0.000E+00 | 0.000E+00 | 0.000E+00 | 0.000E+00 |
| Unigene144722 | PmrF | 0.000E+00 | 0.000E+00 | 3.046E-07 | 2.090E-07 | 0.000E+00 | 0.000E+00 | 0.000E+00 | 0.000E+00 | 0.000E+00 | 0.000E+00 | 0.000E+00 | 0.000E+00 |
| Unigene144731 | lmrD | 0.000E+00 | 0.000E+00 | 1.001E-06 | 0.000E+00 | 0.000E+00 | 0.000E+00 | 0.000E+00 | 0.000E+00 | 0.000E+00 | 0.000E+00 | 0.000E+00 | 0.000E+00 |
| Unigene144732 | lmrD | 0.000E+00 | 0.000E+00 | 6.701E-07 | 0.000E+00 | 0.000E+00 | 0.000E+00 | 0.000E+00 | 0.000E+00 | 0.000E+00 | 0.000E+00 | 0.000E+00 | 0.000E+00 |
| Unigene144812 | baeS | 0.000E+00 | 0.000E+00 | 2.919E-07 | 0.000E+00 | 0.000E+00 | 0.000E+00 | 0.000E+00 | 0.000E+00 | 0.000E+00 | 0.000E+00 | 0.000E+00 | 0.000E+00 |
| Unigene144841 | oleC | 0.000E+00 | 0.000E+00 | 1.105E-06 | 4.982E-06 | 0.000E+00 | 0.000E+00 | 0.000E+00 | 0.000E+00 | 0.000E+00 | 0.000E+00 | 0.000E+00 | 0.000E+00 |
| Unigene144874 | lmrD | 1.027E-07 | 7.888E-08 | 1.128E-06 | 2.903E-07 | 0.000E+00 | 0.000E+00 | 0.000E+00 | 0.000E+00 | 0.000E+00 | 0.000E+00 | 0.000E+00 | 0.000E+00 |
| Unigene144919 | TaeA | 0.000E+00 | 2.113E-07 | 2.531E-06 | 1.390E-05 | 0.000E+00 | 0.000E+00 | 0.000E+00 | 0.000E+00 | 0.000E+00 | 0.000E+00 | 0.000E+00 | 0.000E+00 |
| Unigene144942 | tetB(P) | 1.596E-08 | 8.761E-08 | 2.036E-06 | 1.088E-05 | 0.000E+00 | 0.000E+00 | 0.000E+00 | 0.000E+00 | 0.000E+00 | 0.000E+00 | 0.000E+00 | 0.000E+00 |
| Unigene144945 | dfrA26 | 9.522E-08 | 1.568E-07 | 1.682E-06 | 1.500E-05 | 0.000E+00 | 0.000E+00 | 0.000E+00 | 0.000E+00 | 0.000E+00 | 0.000E+00 | 0.000E+00 | 0.000E+00 |
| Unigene144966 | evgA | 0.000E+00 | 0.000E+00 | 2.583E-06 | 1.618E-05 | 0.000E+00 | 0.000E+00 | 0.000E+00 | 0.000E+00 | 0.000E+00 | 0.000E+00 | 0.000E+00 | 0.000E+00 |
| Unigene144977 | Staphylocc | 0.000E+00 | 9.862E-08 | 2.050E-06 | 1.615E-05 | 0.000E+00 | 0.000E+00 | 0.000E+00 | 0.000E+00 | 0.000E+00 | 0.000E+00 | 0.000E+00 | 0.000E+00 |
| Unigene144994 | lmrD | 0.000E+00 | 1.373E-07 | 2.169E-06 | 1.179E-05 | 0.000E+00 | 0.000E+00 | 0.000E+00 | 0.000E+00 | 0.000E+00 | 0.000E+00 | 0.000E+00 | 0.000E+00 |
| Unigene145007 | lmrB | 4.618E-06 | 1.865E-05 | 3.285E-06 | 8.627E-07 | 6.157E-07 | 4.493E-07 | 3.067E-07 | 5.121E-07 | 1.388E-06 | 7.459E-07 | 7.396E-07 | 3.002E-07 |
| Unigene145040 | macB | 0.000E+00 | 7.086E-07 | 2.772E-06 | 3.830E-06 | 0.000E+00 | 0.000E+00 | 0.000E+00 | 0.000E+00 | 0.000E+00 | 0.000E+00 | 0.000E+00 | 0.000E+00 |
| Unigene145053 | APH(6)-Id | 3.053E-07 | 0.000E+00 | 6.365E-07 | 0.000E+00 | 9.750E-07 | 9.487E-07 | 9.713E-07 | 1.470E-06 | 0.000E+00 | 0.000E+00 | 1.135E-06 | 2.977E-06 |
| Unigene145108 | MexD | 8.967E-08 | 0.000E+00 | 2.053E-07 | 0.000E+00 | 2.324E-07 | 0.000E+00 | 0.000E+00 | 4.884E-07 | 0.000E+00 | 0.000E+00 | 4.445E-07 | 8.479E-07 |
| Unigene145152 | NmcR | 0.000E+00 | 0.000E+00 | 7.366E-07 | 0.000E+00 | 0.000E+00 | 0.000E+00 | 0.000E+00 | 0.000E+00 | 0.000E+00 | 0.000E+00 | 0.000E+00 | 0.000E+00 |
| Unigene145191 | vanHB | 0.000E+00 | 0.000E+00 | 1.326E-05 | 0.000E+00 | 0.000E+00 | 0.000E+00 | 1.150E-06 | 0.000E+00 | 0.000E+00 | 0.000E+00 | 0.000E+00 | 0.000E+00 |
| Unigene145201 | tetT | 0.000E+00 | 1.754E-06 | 2.195E-06 | 0.000E+00 | 0.000E+00 | 0.000E+00 | 0.000E+00 | 0.000E+00 | 0.000E+00 | 0.000E+00 | 0.000E+00 | 0.000E+00 |
| Unigene145217 | evgA | 9.827E-08 | 1.618E-07 | 1.012E-06 | 0.000E+00 | 0.000E+00 | 0.000E+00 | 0.000E+00 | 0.000E+00 | 0.000E+00 | 0.000E+00 | 0.000E+00 | 1.394E-06 |
| Unigene145219 | lmrD | 0.000E+00 | 4.705E-08 | 1.683E-06 | 0.000E+00 | 0.000E+00 | 0.000E+00 | 0.000E+00 | 0.000E+00 | 0.000E+00 | 0.000E+00 | 0.000E+00 | 1.013E-06 |
| Unigene145247 | patB | 1.109E-05 | 3.113E-06 | 3.007E-06 | 1.146E-05 | 7.165E-05 | 1.041E-04 | 1.007E-04 | 8.586E-05 | 1.167E-04 | 1.159E-04 | 1.003E-04 | 6.163E-05 |
| Unigene145346 | macB | 0.000E+00 | 0.000E+00 | 6.812E-07 | 0.000E+00 | 0.000E+00 | 0.000E+00 | 5.378E-06 | 0.000E+00 | 0.000E+00 | 0.000E+00 | 2.046E-06 | 3.756E-06 |
| Unigene145402 | vanHD | 8.869E-05 | 1.368E-04 | 1.359E-04 | 6.533E-06 | 9.057E-06 | 3.211E-07 | 4.091E-06 | 1.772E-06 | 5.437E-05 | 5.148E-07 | 3.058E-06 | 0.000E+00 |
| Unigene145498 | vanSM | 2.381E-06 | 2.247E-06 | 2.569E-06 | 2.260E-06 | 0.000E+00 | 0.000E+00 | 0.000E+00 | 0.000E+00 | 0.000E+00 | 0.000E+00 | 0.000E+00 | 0.000E+00 |
| Unigene145552 | golS | 0.000E+00 | 0.000E+00 | 8.421E-07 | 0.000E+00 | 0.000E+00 | 0.000E+00 | 0.000E+00 | 0.000E+00 | 0.000E+00 | 0.000E+00 | 0.000E+00 | 0.000E+00 |
| Unigene145570 | bcrA | 4.139E-08 | 9.538E-07 | 2.924E-06 | 3.385E-06 | 0.000E+00 | 0.000E+00 | 0.000E+00 | 0.000E+00 | 0.000E+00 | 0.000E+00 | 0.000E+00 | 0.000E+00 |
| Unigene145575 | smeR | 0.000E+00 | 6.243E-07 | 3.070E-06 | 3.255E-06 | 0.000E+00 | 0.000E+00 | 0.000E+00 | 0.000E+00 | 0.000E+00 | 0.000E+00 | 0.000E+00 | 0.000E+00 |
| Unigene145585 | hmrM | 2.995E-07 | 0.000E+00 | 1.696E-06 | 6.841E-06 | 0.000E+00 | 0.000E+00 | 0.000E+00 | 0.000E+00 | 0.000E+00 | 0.000E+00 | 0.000E+00 | 0.000E+00 |
| Unigene145638 | vmlR | 0.000E+00 | 7.201E-08 | 6.438E-07 | 0.000E+00 | 0.000E+00 | 0.000E+00 | 0.000E+00 | 0.000E+00 | 0.000E+00 | 0.000E+00 | 0.000E+00 | 0.000E+00 |
| Unigene145681 | vatH | 0.000E+00 | 0.000E+00 | 4.446E-07 | 8.064E-05 | 0.000E+00 | 0.000E+00 | 0.000E+00 | 0.000E+00 | 0.000E+00 | 0.000E+00 | 0.000E+00 | 0.000E+00 |
| Unigene145797 | efrA | 5.754E-08 | 9.157E-07 | 3.049E-06 | 3.051E-06 | 0.000E+00 | 0.000E+00 | 0.000E+00 | 0.000E+00 | 0.000E+00 | 0.000E+00 | 6.582E-08 | 0.000E+00 |
| Unigene145809 | oleC | 0.000E+00 | 3.254E-06 | 2.334E-06 | 1.848E-06 | 0.000E+00 | 0.000E+00 | 0.000E+00 | 0.000E+00 | 0.000E+00 | 0.000E+00 | 0.000E+00 | 0.000E+00 |
| Unigene145952 | cmlv | 0.000E+00 | 5.905E-08 | 9.238E-07 | 8.149E-08 | 1.793E-07 | 2.908E-08 | 0.000E+00 | 0.000E+00 | 1.470E-07 | 0.000E+00 | 1.231E-07 | 1.335E-06 |
| Unigene145953 | adeL | 0.000E+00 | 7.847E-08 | 1.263E-06 | 1.444E-07 | 1.191E-07 | 0.000E+00 | 0.000E+00 | 4.173E-08 | 7.814E-08 | 0.000E+00 | 1.636E-07 | 1.732E-06 |
| Unigene145960 | bcrA | 0.000E+00 | 0.000E+00 | 8.027E-07 | 0.000E+00 | 0.000E+00 | 0.000E+00 | 0.000E+00 | 0.000E+00 | 0.000E+00 | 0.000E+00 | 0.000E+00 | 0.000E+00 |
| Unigene146075 | evgA | 0.000E+00 | 0.000E+00 | 4.895E-07 | 0.000E+00 | 0.000E+00 | 0.000E+00 | 0.000E+00 | 0.000E+00 | 0.000E+00 | 0.000E+00 | 0.000E+00 | 0.000E+00 |
| Unigene146084 | vanHO | 7.423E-05 | 5.873E-05 | 1.139E-04 | 1.844E-04 | 1.261E-04 | 9.281E-05 | 1.217E-04 | 1.120E-04 | 9.824E-05 | 1.403E-04 | 1.478E-04 | 1.507E-04 |

| | | | | | | | | | | | | | |
|---|---|---|---|---|---|---|---|---|---|---|---|---|---|
| Unigene146096 | macB | 0.000E+00 | 0.000E+00 | 3.232E-07 | 0.000E+00 | 0.000E+00 | 0.000E+00 | 0.000E+00 | 0.000E+00 | 0.000E+00 | 0.000E+00 | 0.000E+00 | 0.000E+00 |
| Unigene146104 | patA | 4.428E-08 | 0.000E+00 | 1.608E-06 | 0.000E+00 | 9.839E-08 | 0.000E+00 | 0.000E+00 | 0.000E+00 | 9.679E-08 | 0.000E+00 | 0.000E+00 | 3.140E-07 |
| Unigene146150 | tetA(58) | 1.976E-06 | 1.274E-05 | 6.093E-06 | 3.848E-06 | 0.000E+00 | 0.000E+00 | 0.000E+00 | 0.000E+00 | 0.000E+00 | 0.000E+00 | 2.018E-07 | 0.000E+00 |
| Unigene146163 | tetW | 0.000E+00 | 2.830E-07 | 1.923E-06 | 1.402E-05 | 0.000E+00 | 0.000E+00 | 0.000E+00 | 0.000E+00 | 0.000E+00 | 0.000E+00 | 0.000E+00 | 0.000E+00 |
| Unigene146170 | tetA(58) | 0.000E+00 | 0.000E+00 | 1.894E-06 | 1.328E-05 | 0.000E+00 | 0.000E+00 | 0.000E+00 | 0.000E+00 | 0.000E+00 | 0.000E+00 | 0.000E+00 | 0.000E+00 |
| Unigene146171 | efrA | 0.000E+00 | 2.209E-07 | 1.634E-06 | 1.234E-05 | 0.000E+00 | 0.000E+00 | 0.000E+00 | 0.000E+00 | 0.000E+00 | 0.000E+00 | 0.000E+00 | 0.000E+00 |
| Unigene146172 | patB | 0.000E+00 | 2.902E-07 | 1.541E-06 | 1.307E-05 | 0.000E+00 | 0.000E+00 | 0.000E+00 | 0.000E+00 | 0.000E+00 | 0.000E+00 | 0.000E+00 | 0.000E+00 |
| Unigene146177 | macB | 0.000E+00 | 0.000E+00 | 2.127E-06 | 1.455E-05 | 0.000E+00 | 0.000E+00 | 0.000E+00 | 0.000E+00 | 0.000E+00 | 0.000E+00 | 0.000E+00 | 0.000E+00 |
| Unigene146193 | patB | 2.960E-06 | 0.000E+00 | 1.061E-05 | 9.773E-06 | 0.000E+00 | 0.000E+00 | 0.000E+00 | 0.000E+00 | 0.000E+00 | 0.000E+00 | 0.000E+00 | 0.000E+00 |
| Unigene146222 | evgS | 0.000E+00 | 0.000E+00 | 4.693E-07 | 0.000E+00 | 0.000E+00 | 0.000E+00 | 0.000E+00 | 0.000E+00 | 0.000E+00 | 0.000E+00 | 0.000E+00 | 0.000E+00 |
| Unigene146314 | macB | 0.000E+00 | 0.000E+00 | 3.672E-07 | 0.000E+00 | 0.000E+00 | 0.000E+00 | 0.000E+00 | 0.000E+00 | 0.000E+00 | 0.000E+00 | 0.000E+00 | 0.000E+00 |
| Unigene146325 | tet32 | 3.973E-07 | 6.541E-07 | 1.121E-06 | 2.541E-06 | 2.575E-07 | 8.948E-08 | 2.565E-07 | 1.237E-06 | 1.086E-07 | 5.532E-07 | 9.657E-07 | 8.023E-07 |
| Unigene146329 | poxtA | 1.352E-07 | 6.785E-07 | 1.346E-06 | 2.282E-06 | 0.000E+00 | 2.506E-07 | 1.924E-07 | 1.601E-06 | 0.000E+00 | 6.671E-07 | 1.083E-06 | 7.992E-07 |
| Unigene146375 | oleB | 0.000E+00 | 0.000E+00 | 0.000E+00 | 0.000E+00 | 0.000E+00 | 0.000E+00 | 0.000E+00 | 0.000E+00 | 0.000E+00 | 0.000E+00 | 0.000E+00 | 3.287E-06 |
| Unigene146422 | vatH | 0.000E+00 | 0.000E+00 | 8.848E-07 | 0.000E+00 | 0.000E+00 | 0.000E+00 | 0.000E+00 | 0.000E+00 | 0.000E+00 | 0.000E+00 | 0.000E+00 | 0.000E+00 |
| Unigene146442 | tetA(58) | 5.021E-07 | 1.142E-06 | 1.443E-06 | 1.304E-06 | 0.000E+00 | 0.000E+00 | 0.000E+00 | 0.000E+00 | 0.000E+00 | 0.000E+00 | 0.000E+00 | 0.000E+00 |
| Unigene146447 | marA | 4.356E-08 | 0.000E+00 | 1.069E-06 | 1.232E-06 | 0.000E+00 | 0.000E+00 | 0.000E+00 | 0.000E+00 | 0.000E+00 | 0.000E+00 | 0.000E+00 | 0.000E+00 |
| Unigene146516 | vgaALC | 9.565E-08 | 9.448E-07 | 9.385E-07 | 7.244E-07 | 0.000E+00 | 0.000E+00 | 0.000E+00 | 0.000E+00 | 0.000E+00 | 0.000E+00 | 0.000E+00 | 0.000E+00 |
| Unigene146580 | adeL | 0.000E+00 | 1.763E-07 | 6.303E-07 | 0.000E+00 | 0.000E+00 | 0.000E+00 | 0.000E+00 | 0.000E+00 | 0.000E+00 | 0.000E+00 | 0.000E+00 | 0.000E+00 |
| Unigene146902 | hp1181 | 0.000E+00 | 0.000E+00 | 1.391E-06 | 7.040E-08 | 0.000E+00 | 0.000E+00 | 0.000E+00 | 0.000E+00 | 1.270E-07 | 0.000E+00 | 1.063E-07 | 9.340E-07 |
| Unigene146927 | Escherichia | 0.000E+00 | 0.000E+00 | 7.542E-07 | 0.000E+00 | 0.000E+00 | 0.000E+00 | 0.000E+00 | 0.000E+00 | 0.000E+00 | 0.000E+00 | 0.000E+00 | 0.000E+00 |
| Unigene146959 | tetA(58) | 0.000E+00 | 0.000E+00 | 0.000E+00 | 0.000E+00 | 0.000E+00 | 0.000E+00 | 0.000E+00 | 0.000E+00 | 0.000E+00 | 0.000E+00 | 0.000E+00 | 4.279E-06 |
| Unigene147066 | vgaB | 0.000E+00 | 0.000E+00 | 8.159E-07 | 6.989E-05 | 0.000E+00 | 0.000E+00 | 0.000E+00 | 0.000E+00 | 0.000E+00 | 0.000E+00 | 0.000E+00 | 0.000E+00 |
| Unigene147120 | mecA | 3.110E-07 | 6.485E-07 | 1.449E-06 | 1.774E-06 | 0.000E+00 | 0.000E+00 | 0.000E+00 | 0.000E+00 | 0.000E+00 | 0.000E+00 | 0.000E+00 | 0.000E+00 |
| Unigene147139 | vanSF | 7.276E-07 | 5.884E-07 | 7.890E-07 | 1.121E-06 | 1.676E-05 | 6.085E-06 | 1.221E-05 | 1.748E-05 | 9.584E-06 | 1.706E-05 | 1.362E-05 | 7.966E-06 |
| Unigene147140 | bcrA | 6.725E-07 | 3.885E-07 | 1.528E-06 | 1.430E-06 | 1.840E-05 | 7.040E-06 | 1.207E-05 | 1.710E-05 | 9.169E-06 | 1.621E-05 | 1.668E-05 | 8.326E-06 |
| Unigene147169 | oleC | 1.655E-07 | 0.000E+00 | 0.000E+00 | 0.000E+00 | 0.000E+00 | 6.263E-07 | 1.374E-06 | 2.898E-06 | 1.809E-07 | 0.000E+00 | 1.326E-06 | 2.837E-06 |
| Unigene147178 | tetB(P) | 1.633E-07 | 8.964E-08 | 1.122E-06 | 2.474E-07 | 0.000E+00 | 2.207E-08 | 0.000E+00 | 0.000E+00 | 0.000E+00 | 0.000E+00 | 0.000E+00 | 0.000E+00 |
| Unigene147204 | patA | 0.000E+00 | 0.000E+00 | 5.557E-07 | 0.000E+00 | 0.000E+00 | 0.000E+00 | 0.000E+00 | 0.000E+00 | 0.000E+00 | 0.000E+00 | 0.000E+00 | 0.000E+00 |
| Unigene147222 | macB | 0.000E+00 | 0.000E+00 | 5.364E-07 | 0.000E+00 | 4.794E-07 | 0.000E+00 | 0.000E+00 | 0.000E+00 | 0.000E+00 | 0.000E+00 | 0.000E+00 | 0.000E+00 |
| Unigene147226 | vga(E) Stap | 6.756E-05 | 5.699E-05 | 1.140E-04 | 1.531E-04 | 1.283E-04 | 8.484E-05 | 1.168E-04 | 1.102E-04 | 9.684E-05 | 1.390E-04 | 1.444E-04 | 1.472E-04 |
| Unigene147343 | macB | 0.000E+00 | 0.000E+00 | 1.187E-06 | 7.362E-05 | 0.000E+00 | 0.000E+00 | 0.000E+00 | 3.620E-08 | 0.000E+00 | 0.000E+00 | 0.000E+00 | 0.000E+00 |
| Unigene147359 | lmrB | 0.000E+00 | 0.000E+00 | 1.249E-06 | 0.000E+00 | 0.000E+00 | 0.000E+00 | 0.000E+00 | 0.000E+00 | 0.000E+00 | 0.000E+00 | 0.000E+00 | 0.000E+00 |
| Unigene147429 | Listeria mc | 1.238E-07 | 9.513E-08 | 1.191E-06 | 1.625E-07 | 0.000E+00 | 0.000E+00 | 0.000E+00 | 0.000E+00 | 0.000E+00 | 0.000E+00 | 0.000E+00 | 0.000E+00 |
| Unigene147482 | efrA | 0.000E+00 | 0.000E+00 | 4.474E-07 | 4.144E-07 | 0.000E+00 | 0.000E+00 | 0.000E+00 | 0.000E+00 | 0.000E+00 | 0.000E+00 | 0.000E+00 | 0.000E+00 |
| Unigene147483 | vgaB | 0.000E+00 | 0.000E+00 | 7.039E-07 | 0.000E+00 | 0.000E+00 | 0.000E+00 | 0.000E+00 | 4.652E-08 | 0.000E+00 | 0.000E+00 | 1.368E-07 | 0.000E+00 |
| Unigene147510 | bcrA | 1.469E-07 | 8.061E-08 | 1.117E-06 | 1.483E-07 | 1.224E-07 | 0.000E+00 | 0.000E+00 | 2.143E-07 | 0.000E+00 | 0.000E+00 | 3.781E-07 | 0.000E+00 |
| Unigene147522 | Corynebac | 0.000E+00 | 0.000E+00 | 4.061E-07 | 0.000E+00 | 0.000E+00 | 0.000E+00 | 0.000E+00 | 0.000E+00 | 0.000E+00 | 0.000E+00 | 0.000E+00 | 0.000E+00 |
| Unigene147536 | msbA | 3.238E-07 | 0.000E+00 | 6.354E-07 | 2.692E-07 | 4.020E-07 | 4.529E-07 | 3.309E-06 | 2.934E-06 | 4.579E-07 | 7.425E-07 | 1.808E-06 | 1.823E-06 |
| Unigene147580 | TaeA | 0.000E+00 | 0.000E+00 | 6.121E-07 | 3.674E-07 | 0.000E+00 | 0.000E+00 | 0.000E+00 | 0.000E+00 | 0.000E+00 | 0.000E+00 | 0.000E+00 | 0.000E+00 |
| Unigene147593 | vatH | 0.000E+00 | 0.000E+00 | 8.421E-07 | 7.999E-07 | 0.000E+00 | 0.000E+00 | 0.000E+00 | 0.000E+00 | 0.000E+00 | 0.000E+00 | 0.000E+00 | 0.000E+00 |
| Unigene147614 | vgaALC | 0.000E+00 | 5.249E-07 | 2.909E-06 | 3.477E-06 | 0.000E+00 | 0.000E+00 | 0.000E+00 | 0.000E+00 | 0.000E+00 | 0.000E+00 | 0.000E+00 | 0.000E+00 |
| Unigene147647 | smeS | 0.000E+00 | 0.000E+00 | 1.261E-06 | 5.089E-08 | 0.000E+00 | 0.000E+00 | 0.000E+00 | 0.000E+00 | 1.928E-07 | 0.000E+00 | 2.883E-08 | 9.532E-07 |
| Unigene147648 | mtrA | 0.000E+00 | 0.000E+00 | 1.012E-06 | 4.527E-08 | 0.000E+00 | 0.000E+00 | 0.000E+00 | 0.000E+00 | 0.000E+00 | 0.000E+00 | 0.000E+00 | 1.060E-06 |
| Unigene147673 | mef(B) | 1.806E-07 | 2.549E-07 | 9.368E-07 | 1.563E-07 | 0.000E+00 | 0.000E+00 | 0.000E+00 | 0.000E+00 | 0.000E+00 | 0.000E+00 | 0.000E+00 | 0.000E+00 |
| Unigene147796 | macB | 0.000E+00 | 0.000E+00 | 5.642E-07 | 0.000E+00 | 0.000E+00 | 0.000E+00 | 0.000E+00 | 4.602E-06 | 4.489E-07 | 0.000E+00 | 1.644E-06 | 4.029E-06 |
| Unigene147819 | vanTC | 0.000E+00 | 3.536E-07 | 4.426E-07 | 4.555E-07 | 0.000E+00 | 0.000E+00 | 0.000E+00 | 0.000E+00 | 0.000E+00 | 0.000E+00 | 0.000E+00 | 0.000E+00 |
| Unigene147820 | vanXYN | 0.000E+00 | 0.000E+00 | 4.631E-07 | 0.000E+00 | 0.000E+00 | 0.000E+00 | 0.000E+00 | 0.000E+00 | 0.000E+00 | 0.000E+00 | 0.000E+00 | 0.000E+00 |
| Unigene147859 | PmrF | 1.337E-07 | 5.135E-07 | 8.853E-07 | 2.463E-06 | 0.000E+00 | 0.000E+00 | 0.000E+00 | 0.000E+00 | 0.000E+00 | 0.000E+00 | 0.000E+00 | 0.000E+00 |
| Unigene147909 | tetB(P) | 1.518E-07 | 1.999E-07 | 3.589E-06 | 1.922E-05 | 0.000E+00 | 0.000E+00 | 0.000E+00 | 0.000E+00 | 0.000E+00 | 0.000E+00 | 0.000E+00 | 0.000E+00 |
| Unigene147941 | arlR | 0.000E+00 | 0.000E+00 | 2.011E-06 | 1.268E-05 | 0.000E+00 | 0.000E+00 | 0.000E+00 | 0.000E+00 | 0.000E+00 | 0.000E+00 | 0.000E+00 | 0.000E+00 |
| Unigene147942 | cpxA | 1.578E-08 | 8.658E-08 | 2.105E-06 | 1.388E-05 | 0.000E+00 | 0.000E+00 | 0.000E+00 | 0.000E+00 | 0.000E+00 | 0.000E+00 | 0.000E+00 | 0.000E+00 |
| Unigene147946 | msbA | 0.000E+00 | 1.732E-07 | 1.807E-06 | 1.185E-05 | 0.000E+00 | 0.000E+00 | 0.000E+00 | 0.000E+00 | 0.000E+00 | 0.000E+00 | 0.000E+00 | 0.000E+00 |
| Unigene147950 | tetA(58) | 0.000E+00 | 8.317E-08 | 1.822E-06 | 1.370E-05 | 0.000E+00 | 0.000E+00 | 0.000E+00 | 0.000E+00 | 0.000E+00 | 0.000E+00 | 0.000E+00 | 0.000E+00 |
| Unigene147952 | macB | 0.000E+00 | 2.684E-07 | 2.000E-06 | 1.329E-05 | 0.000E+00 | 0.000E+00 | 0.000E+00 | 0.000E+00 | 0.000E+00 | 0.000E+00 | 0.000E+00 | 0.000E+00 |
| Unigene147966 | tetA(46) | 0.000E+00 | 0.000E+00 | 1.245E-06 | 8.968E-06 | 0.000E+00 | 0.000E+00 | 0.000E+00 | 0.000E+00 | 0.000E+00 | 0.000E+00 | 0.000E+00 | 0.000E+00 |
| Unigene147985 | tetB(58) | 7.027E-07 | 3.374E-07 | 1.595E-06 | 7.540E-07 | 0.000E+00 | 0.000E+00 | 0.000E+00 | 0.000E+00 | 0.000E+00 | 0.000E+00 | 0.000E+00 | 0.000E+00 |
| Unigene147988 | bcrA | 4.344E-07 | 6.968E-07 | 1.541E-06 | 7.761E-07 | 3.712E-08 | 0.000E+00 | 0.000E+00 | 0.000E+00 | 0.000E+00 | 0.000E+00 | 0.000E+00 | 0.000E+00 |

| | | | | | | | | | | | | | |
|---|---|---|---|---|---|---|---|---|---|---|---|---|---|
| Unigene148107 | vanHO | 0.000E+00 | 0.000E+00 | 1.754E-06 | 5.515E-06 | 0.000E+00 | 0.000E+00 | 0.000E+00 | 0.000E+00 | 0.000E+00 | 0.000E+00 | 0.000E+00 | 0.000E+00 |
| Unigene148111 | mtrA | 0.000E+00 | 0.000E+00 | 2.809E-06 | 6.330E-06 | 0.000E+00 | 0.000E+00 | 0.000E+00 | 0.000E+00 | 0.000E+00 | 0.000E+00 | 0.000E+00 | 0.000E+00 |
| Unigene148112 | tetA(58) | 0.000E+00 | 0.000E+00 | 1.820E-06 | 5.654E-06 | 0.000E+00 | 0.000E+00 | 0.000E+00 | 0.000E+00 | 0.000E+00 | 0.000E+00 | 0.000E+00 | 0.000E+00 |
| Unigene148133 | vgaC | 0.000E+00 | 0.000E+00 | 1.421E-06 | 6.102E-06 | 0.000E+00 | 0.000E+00 | 0.000E+00 | 0.000E+00 | 0.000E+00 | 0.000E+00 | 0.000E+00 | 0.000E+00 |
| Unigene148177 | patB | 6.697E-08 | 1.470E-07 | 1.051E-06 | 1.127E-07 | 0.000E+00 | 0.000E+00 | 0.000E+00 | 0.000E+00 | 0.000E+00 | 0.000E+00 | 0.000E+00 | 0.000E+00 |
| Unigene148178 | efrA | 3.762E-08 | 1.445E-07 | 1.403E-06 | 2.849E-07 | 0.000E+00 | 0.000E+00 | 0.000E+00 | 0.000E+00 | 0.000E+00 | 0.000E+00 | 0.000E+00 | 0.000E+00 |
| Unigene148239 | efrA | 0.000E+00 | 0.000E+00 | 7.391E-07 | 0.000E+00 | 0.000E+00 | 0.000E+00 | 0.000E+00 | 0.000E+00 | 0.000E+00 | 0.000E+00 | 0.000E+00 | 0.000E+00 |
| Unigene148267 | Listeria mc | 2.828E-06 | 3.328E-06 | 2.297E-06 | 1.809E-06 | 2.473E-06 | 1.964E-06 | 2.067E-06 | 1.001E-06 | 1.552E-06 | 5.715E-06 | 5.576E-06 | 1.679E-06 |
| Unigene148283 | bacA | 0.000E+00 | 6.896E-07 | 4.624E-07 | 0.000E+00 | 0.000E+00 | 0.000E+00 | 0.000E+00 | 0.000E+00 | 0.000E+00 | 0.000E+00 | 0.000E+00 | 0.000E+00 |
| Unigene148288 | macB | 0.000E+00 | 2.977E-07 | 3.061E-06 | 2.073E-05 | 0.000E+00 | 0.000E+00 | 0.000E+00 | 0.000E+00 | 0.000E+00 | 0.000E+00 | 0.000E+00 | 0.000E+00 |
| Unigene148313 | tetT | 0.000E+00 | 0.000E+00 | 6.896E-07 | 0.000E+00 | 0.000E+00 | 0.000E+00 | 0.000E+00 | 0.000E+00 | 0.000E+00 | 0.000E+00 | 0.000E+00 | 0.000E+00 |
| Unigene148314 | optrA | 0.000E+00 | 0.000E+00 | 8.895E-07 | 0.000E+00 | 0.000E+00 | 0.000E+00 | 0.000E+00 | 0.000E+00 | 0.000E+00 | 0.000E+00 | 0.000E+00 | 0.000E+00 |
| Unigene148350 | lmrB | 1.177E-07 | 1.292E-07 | 1.248E-06 | 2.853E-07 | 0.000E+00 | 0.000E+00 | 0.000E+00 | 0.000E+00 | 0.000E+00 | 0.000E+00 | 0.000E+00 | 0.000E+00 |
| Unigene148355 | vanRA | 1.334E-07 | 1.952E-07 | 1.309E-06 | 2.694E-07 | 0.000E+00 | 0.000E+00 | 0.000E+00 | 0.000E+00 | 0.000E+00 | 0.000E+00 | 0.000E+00 | 0.000E+00 |
| Unigene148356 | vanSM | 0.000E+00 | 1.727E-07 | 1.389E-06 | 6.354E-08 | 0.000E+00 | 0.000E+00 | 0.000E+00 | 0.000E+00 | 0.000E+00 | 0.000E+00 | 0.000E+00 | 0.000E+00 |
| Unigene148373 | efmA | 7.613E-08 | 0.000E+00 | 1.071E-06 | 3.588E-07 | 0.000E+00 | 0.000E+00 | 0.000E+00 | 0.000E+00 | 0.000E+00 | 0.000E+00 | 0.000E+00 | 0.000E+00 |
| Unigene148378 | mdtG | 2.556E-08 | 0.000E+00 | 1.028E-06 | 4.388E-07 | 0.000E+00 | 0.000E+00 | 0.000E+00 | 0.000E+00 | 0.000E+00 | 0.000E+00 | 0.000E+00 | 0.000E+00 |
| Unigene148384 | msbA | 0.000E+00 | 1.114E-07 | 1.394E-06 | 2.050E-07 | 0.000E+00 | 0.000E+00 | 0.000E+00 | 0.000E+00 | 0.000E+00 | 0.000E+00 | 0.000E+00 | 0.000E+00 |
| Unigene148389 | cfrA | 6.011E-08 | 1.649E-07 | 8.848E-07 | 2.732E-07 | 0.000E+00 | 0.000E+00 | 0.000E+00 | 0.000E+00 | 0.000E+00 | 0.000E+00 | 0.000E+00 | 0.000E+00 |
| Unigene148390 | AAC(6')-lic | 0.000E+00 | 1.305E-07 | 1.517E-06 | 0.000E+00 | 0.000E+00 | 0.000E+00 | 0.000E+00 | 0.000E+00 | 0.000E+00 | 0.000E+00 | 0.000E+00 | 0.000E+00 |
| Unigene148395 | macB | 5.961E-08 | 0.000E+00 | 1.082E-06 | 3.913E-07 | 0.000E+00 | 0.000E+00 | 0.000E+00 | 0.000E+00 | 0.000E+00 | 0.000E+00 | 0.000E+00 | 0.000E+00 |
| Unigene148408 | lmrD | 2.917E-07 | 1.245E-06 | 1.845E-06 | 1.407E-06 | 0.000E+00 | 0.000E+00 | 0.000E+00 | 0.000E+00 | 0.000E+00 | 0.000E+00 | 0.000E+00 | 0.000E+00 |
| Unigene148476 | macB | 7.470E-05 | 6.534E-05 | 1.169E-04 | 1.550E-04 | 1.332E-04 | 9.265E-05 | 1.139E-04 | 1.176E-04 | 1.022E-04 | 1.479E-04 | 1.557E-04 | 1.633E-04 |
| Unigene148516 | vanHM | 0.000E+00 | 0.000E+00 | 8.239E-07 | 6.595E-07 | 0.000E+00 | 0.000E+00 | 0.000E+00 | 0.000E+00 | 0.000E+00 | 0.000E+00 | 0.000E+00 | 0.000E+00 |
| Unigene148534 | msbA | 0.000E+00 | 0.000E+00 | 9.217E-07 | 0.000E+00 | 0.000E+00 | 0.000E+00 | 0.000E+00 | 0.000E+00 | 0.000E+00 | 0.000E+00 | 0.000E+00 | 0.000E+00 |
| Unigene148548 | macB | 0.000E+00 | 2.783E-07 | 2.702E-06 | 1.482E-05 | 0.000E+00 | 0.000E+00 | 0.000E+00 | 0.000E+00 | 0.000E+00 | 0.000E+00 | 0.000E+00 | 0.000E+00 |
| Unigene148566 | Pseudomo | 0.000E+00 | 2.013E-07 | 8.399E-07 | 5.556E-07 | 0.000E+00 | 0.000E+00 | 0.000E+00 | 0.000E+00 | 1.002E-07 | 0.000E+00 | 0.000E+00 | 0.000E+00 |
| Unigene148609 | evgA | 0.000E+00 | 0.000E+00 | 1.332E-06 | 0.000E+00 | 0.000E+00 | 0.000E+00 | 0.000E+00 | 0.000E+00 | 0.000E+00 | 0.000E+00 | 0.000E+00 | 2.080E-06 |
| Unigene148611 | efrA | 0.000E+00 | 0.000E+00 | 7.456E-07 | 0.000E+00 | 7.091E-07 | 0.000E+00 | 0.000E+00 | 0.000E+00 | 0.000E+00 | 0.000E+00 | 0.000E+00 | 3.449E-06 |
| Unigene148664 | GOB-6 | 0.000E+00 | 0.000E+00 | 1.369E-06 | 1.006E-07 | 0.000E+00 | 0.000E+00 | 0.000E+00 | 0.000E+00 | 0.000E+00 | 0.000E+00 | 1.140E-07 | 1.354E-06 |
| Unigene148666 | Klebsiella p | 0.000E+00 | 9.422E-08 | 1.460E-06 | 2.890E-08 | 9.538E-08 | 0.000E+00 | 0.000E+00 | 0.000E+00 | 1.564E-07 | 0.000E+00 | 9.820E-08 | 8.457E-07 |
| Unigene148868 | poxtA | 0.000E+00 | 6.411E-07 | 1.026E-06 | 6.830E-07 | 0.000E+00 | 1.196E-06 | 0.000E+00 | 3.768E-06 | 0.000E+00 | 0.000E+00 | 2.286E-06 | 4.942E-06 |
| Unigene148975 | arnA | 1.081E-07 | 0.000E+00 | 9.444E-06 | 0.000E+00 | 0.000E+00 | 5.845E-07 | 8.976E-07 | 6.311E-08 | 0.000E+00 | 0.000E+00 | 0.000E+00 | 0.000E+00 |
| Unigene148994 | bcrA | 0.000E+00 | 0.000E+00 | 1.244E-05 | 0.000E+00 | 0.000E+00 | 0.000E+00 | 0.000E+00 | 0.000E+00 | 0.000E+00 | 0.000E+00 | 0.000E+00 | 0.000E+00 |
| Unigene149009 | Staphylocc | 0.000E+00 | 0.000E+00 | 1.277E-05 | 0.000E+00 | 0.000E+00 | 8.138E-07 | 0.000E+00 | 7.753E-08 | 0.000E+00 | 0.000E+00 | 0.000E+00 | 0.000E+00 |
| Unigene149018 | emeA | 0.000E+00 | 0.000E+00 | 1.195E-05 | 0.000E+00 | 0.000E+00 | 0.000E+00 | 0.000E+00 | 0.000E+00 | 0.000E+00 | 0.000E+00 | 0.000E+00 | 0.000E+00 |
| Unigene149030 | msbA | 7.137E-07 | 6.507E-06 | 1.023E-06 | 0.000E+00 | 0.000E+00 | 0.000E+00 | 0.000E+00 | 0.000E+00 | 0.000E+00 | 0.000E+00 | 0.000E+00 | 0.000E+00 |
| Unigene149057 | bcr-1 | 0.000E+00 | 0.000E+00 | 9.141E-07 | 8.142E-05 | 0.000E+00 | 0.000E+00 | 0.000E+00 | 0.000E+00 | 0.000E+00 | 0.000E+00 | 0.000E+00 | 0.000E+00 |
| Unigene149077 | efrA | 2.824E-07 | 0.000E+00 | 8.591E-07 | 0.000E+00 | 9.726E-07 | 0.000E+00 | 1.469E-06 | 8.241E-07 | 0.000E+00 | 0.000E+00 | 9.692E-07 | 2.971E-06 |
| Unigene149080 | vgaB | 1.029E-04 | 1.570E-04 | 1.539E-04 | 8.012E-06 | 9.916E-06 | 3.216E-07 | 4.061E-06 | 1.968E-06 | 6.059E-05 | 0.000E+00 | 2.836E-06 | 0.000E+00 |
| Unigene149081 | msbA | 0.000E+00 | 2.525E-07 | 6.599E-07 | 2.502E-07 | 4.719E-07 | 6.505E-07 | 3.330E-06 | 2.396E-06 | 0.000E+00 | 0.000E+00 | 0.000E+00 | 1.966E-06 |
| Unigene149086 | bcrA | 0.000E+00 | 3.863E-07 | 0.000E+00 | 7.108E-07 | 0.000E+00 | 0.000E+00 | 0.000E+00 | 0.000E+00 | 0.000E+00 | 0.000E+00 | 0.000E+00 | 0.000E+00 |
| Unigene149148 | tet(43) | 0.000E+00 | 1.002E-06 | 3.839E-07 | 0.000E+00 | 0.000E+00 | 0.000E+00 | 0.000E+00 | 0.000E+00 | 0.000E+00 | 0.000E+00 | 0.000E+00 | 0.000E+00 |
| Unigene149185 | ceoB | 0.000E+00 | 0.000E+00 | 3.406E-07 | 0.000E+00 | 0.000E+00 | 0.000E+00 | 4.610E-07 | 0.000E+00 | 0.000E+00 | 0.000E+00 | 0.000E+00 | 0.000E+00 |
| Unigene149292 | tetA(46) | 0.000E+00 | 0.000E+00 | 1.297E-06 | 0.000E+00 | 0.000E+00 | 0.000E+00 | 0.000E+00 | 0.000E+00 | 0.000E+00 | 0.000E+00 | 0.000E+00 | 0.000E+00 |
| Unigene149293 | tetA(58) | 0.000E+00 | 0.000E+00 | 0.000E+00 | 0.000E+00 | 0.000E+00 | 0.000E+00 | 3.219E-06 | 0.000E+00 | 0.000E+00 | 0.000E+00 | 1.283E-06 | 1.670E-06 |
| Unigene149315 | tetA(46) | 0.000E+00 | 0.000E+00 | 7.282E-07 | 0.000E+00 | 0.000E+00 | 0.000E+00 | 0.000E+00 | 0.000E+00 | 0.000E+00 | 0.000E+00 | 0.000E+00 | 0.000E+00 |
| Unigene149427 | YojI | 0.000E+00 | 0.000E+00 | 3.896E-07 | 2.406E-07 | 0.000E+00 | 0.000E+00 | 3.955E-07 | 0.000E+00 | 0.000E+00 | 0.000E+00 | 0.000E+00 | 7.040E-07 |
| Unigene149432 | vanRB | 0.000E+00 | 0.000E+00 | 9.555E-07 | 3.933E-07 | 0.000E+00 | 0.000E+00 | 0.000E+00 | 0.000E+00 | 0.000E+00 | 0.000E+00 | 0.000E+00 | 0.000E+00 |
| Unigene149454 | novA | 0.000E+00 | 0.000E+00 | 2.673E-07 | 0.000E+00 | 0.000E+00 | 0.000E+00 | 0.000E+00 | 0.000E+00 | 0.000E+00 | 0.000E+00 | 0.000E+00 | 0.000E+00 |
| Unigene149832 | macB | 6.134E-05 | 1.289E-04 | 7.114E-05 | 2.049E-05 | 1.042E-04 | 1.379E-04 | 1.472E-04 | 1.225E-04 | 1.747E-04 | 1.686E-04 | 1.427E-04 | 8.585E-05 |
| Unigene149856 | tetB(P) | 4.045E-05 | 2.461E-05 | 1.877E-05 | 2.076E-05 | 1.781E-06 | 1.810E-06 | 2.511E-06 | 6.100E-07 | 6.049E-06 | 4.366E-07 | 1.617E-06 | 1.446E-06 |
| Unigene149866 | msbA | 0.000E+00 | 0.000E+00 | 1.227E-06 | 0.000E+00 | 0.000E+00 | 0.000E+00 | 0.000E+00 | 0.000E+00 | 0.000E+00 | 0.000E+00 | 0.000E+00 | 0.000E+00 |
| Unigene149960 | poxtA | 6.452E-05 | 5.340E-05 | 1.068E-04 | 1.508E-04 | 1.309E-04 | 8.391E-05 | 1.062E-04 | 1.150E-04 | 8.484E-05 | 1.288E-04 | 1.253E-04 | 1.558E-04 |
| Unigene150079 | vanRG | 0.000E+00 | 0.000E+00 | 2.155E-07 | 0.000E+00 | 0.000E+00 | 0.000E+00 | 2.430E-07 | 0.000E+00 | 0.000E+00 | 0.000E+00 | 0.000E+00 | 0.000E+00 |
| Unigene150102 | vanRB | 3.726E-06 | 7.157E-06 | 6.856E-06 | 7.479E-06 | 0.000E+00 | 1.511E-07 | 0.000E+00 | 0.000E+00 | 0.000E+00 | 0.000E+00 | 0.000E+00 | 0.000E+00 |
| Unigene150135 | lmrD | 9.119E-08 | 2.002E-08 | 1.127E-06 | 1.842E-07 | 0.000E+00 | 0.000E+00 | 0.000E+00 | 0.000E+00 | 0.000E+00 | 0.000E+00 | 0.000E+00 | 0.000E+00 |
| Unigene150136 | patA | 7.538E-08 | 4.136E-08 | 1.091E-06 | 2.284E-07 | 0.000E+00 | 0.000E+00 | 0.000E+00 | 0.000E+00 | 0.000E+00 | 0.000E+00 | 0.000E+00 | 0.000E+00 |

| | | | | | | | | | | | | | |
|---|---|---|---|---|---|---|---|---|---|---|---|---|---|
| Unigene150139 | otr(B) | 1.964E-07 | 1.677E-07 | 1.114E-06 | 2.204E-07 | 0.000E+00 | 0.000E+00 | 0.000E+00 | 0.000E+00 | 0.000E+00 | 0.000E+00 | 0.000E+00 | 0.000E+00 |
| Unigene150188 | baeS | 9.418E-08 | 1.550E-07 | 1.201E-06 | 1.427E-07 | 0.000E+00 | 0.000E+00 | 0.000E+00 | 0.000E+00 | 0.000E+00 | 0.000E+00 | 0.000E+00 | 0.000E+00 |
| Unigene150212 | bcrA | 5.223E-08 | 0.000E+00 | 4.613E-07 | 2.110E-07 | 0.000E+00 | 0.000E+00 | 0.000E+00 | 0.000E+00 | 0.000E+00 | 0.000E+00 | 0.000E+00 | 0.000E+00 |
| Unigene150308 | macB | 9.937E-05 | 1.779E-04 | 1.742E-04 | 7.293E-06 | 8.763E-06 | 2.807E-07 | 3.934E-06 | 1.705E-06 | 5.690E-05 | 2.169E-07 | 3.119E-06 | 6.331E-07 |
| Unigene150324 | patB | 0.000E+00 | 1.005E-07 | 6.290E-07 | 0.000E+00 | 0.000E+00 | 0.000E+00 | 0.000E+00 | 0.000E+00 | 0.000E+00 | 0.000E+00 | 0.000E+00 | 0.000E+00 |
| Unigene150375 | cmeR | 1.501E-07 | 1.648E-07 | 1.129E-06 | 0.000E+00 | 0.000E+00 | 0.000E+00 | 0.000E+00 | 0.000E+00 | 0.000E+00 | 0.000E+00 | 0.000E+00 | 0.000E+00 |
| Unigene150390 | bcr-1 | 5.434E-08 | 2.982E-08 | 1.360E-06 | 2.744E-08 | 1.509E-07 | 0.000E+00 | 0.000E+00 | 0.000E+00 | 2.079E-07 | 0.000E+00 | 9.324E-08 | 1.317E-06 |
| Unigene150394 | cmlv | 0.000E+00 | 0.000E+00 | 1.249E-06 | 5.587E-08 | 0.000E+00 | 0.000E+00 | 0.000E+00 | 0.000E+00 | 2.419E-07 | 0.000E+00 | 6.328E-08 | 9.482E-07 |
| Unigene150405 | mdtN | 0.000E+00 | 0.000E+00 | 1.004E-06 | 3.826E-08 | 8.418E-08 | 0.000E+00 | 0.000E+00 | 0.000E+00 | 0.000E+00 | 0.000E+00 | 1.734E-07 | 8.957E-07 |
| Unigene150427 | tetA(46) | 0.000E+00 | 1.429E-07 | 1.149E-06 | 0.000E+00 | 0.000E+00 | 0.000E+00 | 0.000E+00 | 0.000E+00 | 0.000E+00 | 0.000E+00 | 0.000E+00 | 0.000E+00 |
| Unigene150440 | optrA | 7.956E-08 | 2.619E-07 | 1.034E-06 | 2.812E-07 | 0.000E+00 | 0.000E+00 | 0.000E+00 | 0.000E+00 | 0.000E+00 | 0.000E+00 | 0.000E+00 | 0.000E+00 |
| Unigene150502 | bcrA | 5.231E-05 | 5.691E-05 | 9.153E-05 | 6.682E-05 | 1.308E-04 | 7.905E-05 | 1.027E-04 | 1.110E-04 | 9.069E-05 | 1.231E-04 | 1.397E-04 | 1.538E-04 |
| Unigene150504 | fexA | 5.903E-05 | 6.098E-05 | 1.064E-04 | 7.343E-05 | 1.301E-04 | 8.720E-05 | 1.059E-04 | 1.107E-04 | 1.057E-04 | 1.343E-04 | 1.442E-04 | 1.571E-04 |
| Unigene150668 | vatB | 0.000E+00 | 0.000E+00 | 1.424E-06 | 0.000E+00 | 0.000E+00 | 0.000E+00 | 0.000E+00 | 0.000E+00 | 0.000E+00 | 0.000E+00 | 0.000E+00 | 0.000E+00 |
| Unigene150706 | Chlamydia | 0.000E+00 | 1.862E-07 | 2.592E-06 | 1.395E-05 | 0.000E+00 | 0.000E+00 | 0.000E+00 | 0.000E+00 | 0.000E+00 | 0.000E+00 | 0.000E+00 | 0.000E+00 |
| Unigene150709 | vanO | 0.000E+00 | 9.226E-08 | 2.062E-06 | 1.132E-05 | 0.000E+00 | 0.000E+00 | 0.000E+00 | 0.000E+00 | 0.000E+00 | 0.000E+00 | 0.000E+00 | 0.000E+00 |
| Unigene150734 | blaR1 | 2.387E-07 | 5.240E-07 | 1.099E-06 | 1.224E-06 | 0.000E+00 | 0.000E+00 | 0.000E+00 | 0.000E+00 | 0.000E+00 | 0.000E+00 | 0.000E+00 | 0.000E+00 |
| Unigene150739 | smeR | 4.270E-07 | 3.280E-07 | 1.466E-06 | 1.121E-06 | 2.846E-07 | 0.000E+00 | 4.726E-08 | 0.000E+00 | 0.000E+00 | 0.000E+00 | 0.000E+00 | 0.000E+00 |
| Unigene150929 | vanRF | 0.000E+00 | 0.000E+00 | 8.409E-07 | 1.923E-07 | 0.000E+00 | 0.000E+00 | 0.000E+00 | 0.000E+00 | 0.000E+00 | 0.000E+00 | 0.000E+00 | 0.000E+00 |
| Unigene150944 | bcrA | 0.000E+00 | 0.000E+00 | 0.000E+00 | 0.000E+00 | 6.905E-07 | 0.000E+00 | 0.000E+00 | 0.000E+00 | 0.000E+00 | 0.000E+00 | 0.000E+00 | 3.490E-06 |
| Unigene150983 | vmlR | 0.000E+00 | 3.536E-07 | 5.058E-07 | 0.000E+00 | 0.000E+00 | 0.000E+00 | 0.000E+00 | 0.000E+00 | 0.000E+00 | 0.000E+00 | 0.000E+00 | 0.000E+00 |
| Unigene151009 | macB | 0.000E+00 | 0.000E+00 | 8.619E-07 | 0.000E+00 | 0.000E+00 | 0.000E+00 | 0.000E+00 | 0.000E+00 | 0.000E+00 | 0.000E+00 | 0.000E+00 | 0.000E+00 |
| Unigene151036 | oleC | 0.000E+00 | 3.202E-07 | 1.903E-06 | 7.001E-06 | 0.000E+00 | 0.000E+00 | 0.000E+00 | 0.000E+00 | 0.000E+00 | 0.000E+00 | 0.000E+00 | 0.000E+00 |
| Unigene151038 | tetA(58) | 0.000E+00 | 0.000E+00 | 9.282E-07 | 3.224E-06 | 0.000E+00 | 0.000E+00 | 0.000E+00 | 0.000E+00 | 0.000E+00 | 0.000E+00 | 0.000E+00 | 0.000E+00 |
| Unigene151068 | efpA | 2.459E-07 | 0.000E+00 | 0.000E+00 | 0.000E+00 | 0.000E+00 | 0.000E+00 | 0.000E+00 | 0.000E+00 | 0.000E+00 | 0.000E+00 | 0.000E+00 | 0.000E+00 |
| Unigene151081 | emrA | 0.000E+00 | 1.014E-06 | 1.082E-06 | 0.000E+00 | 0.000E+00 | 0.000E+00 | 0.000E+00 | 0.000E+00 | 0.000E+00 | 0.000E+00 | 0.000E+00 | 0.000E+00 |
| Unigene151102 | macB | 1.448E-07 | 2.118E-07 | 1.373E-06 | 1.462E-07 | 0.000E+00 | 0.000E+00 | 0.000E+00 | 0.000E+00 | 0.000E+00 | 0.000E+00 | 0.000E+00 | 0.000E+00 |
| Unigene151188 | FusF | 9.439E-08 | 5.180E-08 | 8.798E-07 | 5.242E-07 | 0.000E+00 | 0.000E+00 | 0.000E+00 | 0.000E+00 | 0.000E+00 | 0.000E+00 | 0.000E+00 | 0.000E+00 |
| Unigene151216 | Acinetoba | 3.081E-07 | 0.000E+00 | 2.488E-06 | 7.874E-06 | 0.000E+00 | 0.000E+00 | 0.000E+00 | 0.000E+00 | 0.000E+00 | 0.000E+00 | 0.000E+00 | 0.000E+00 |
| Unigene151221 | tet(38) | 2.759E-07 | 1.009E-07 | 1.782E-06 | 6.872E-06 | 0.000E+00 | 0.000E+00 | 0.000E+00 | 0.000E+00 | 0.000E+00 | 0.000E+00 | 0.000E+00 | 0.000E+00 |
| Unigene151225 | efrB | 1.083E-07 | 1.387E-07 | 1.293E-06 | 2.644E-07 | 0.000E+00 | 0.000E+00 | 0.000E+00 | 0.000E+00 | 0.000E+00 | 0.000E+00 | 0.000E+00 | 0.000E+00 |
| Unigene151226 | efrA | 9.228E-08 | 4.051E-08 | 8.874E-07 | 3.262E-07 | 0.000E+00 | 0.000E+00 | 0.000E+00 | 0.000E+00 | 0.000E+00 | 0.000E+00 | 0.000E+00 | 0.000E+00 |
| Unigene151234 | vanRF | 6.456E-08 | 7.086E-08 | 1.140E-06 | 6.519E-08 | 0.000E+00 | 0.000E+00 | 0.000E+00 | 0.000E+00 | 0.000E+00 | 0.000E+00 | 0.000E+00 | 0.000E+00 |
| Unigene151263 | efpA | 0.000E+00 | 3.253E-07 | 4.363E-07 | 0.000E+00 | 0.000E+00 | 0.000E+00 | 0.000E+00 | 0.000E+00 | 0.000E+00 | 0.000E+00 | 0.000E+00 | 0.000E+00 |
| Unigene151275 | marA | 3.710E-08 | 0.000E+00 | 1.238E-06 | 0.000E+00 | 1.237E-07 | 0.000E+00 | 1.232E-07 | 4.331E-08 | 1.622E-07 | 0.000E+00 | 0.000E+00 | 1.140E-06 |
| Unigene151455 | oleC | 0.000E+00 | 0.000E+00 | 8.678E-07 | 8.291E-05 | 0.000E+00 | 0.000E+00 | 0.000E+00 | 0.000E+00 | 0.000E+00 | 0.000E+00 | 0.000E+00 | 0.000E+00 |
| Unigene151463 | TaeA | 2.265E-08 | 0.000E+00 | 1.067E-06 | 7.521E-05 | 0.000E+00 | 0.000E+00 | 0.000E+00 | 0.000E+00 | 0.000E+00 | 0.000E+00 | 0.000E+00 | 0.000E+00 |
| Unigene151466 | vanHM | 0.000E+00 | 0.000E+00 | 7.869E-07 | 8.045E-05 | 0.000E+00 | 0.000E+00 | 0.000E+00 | 0.000E+00 | 0.000E+00 | 0.000E+00 | 0.000E+00 | 0.000E+00 |
| Unigene151509 | bcrA | 0.000E+00 | 0.000E+00 | 1.618E-06 | 0.000E+00 | 0.000E+00 | 0.000E+00 | 0.000E+00 | 0.000E+00 | 0.000E+00 | 0.000E+00 | 0.000E+00 | 0.000E+00 |
| Unigene151570 | cmlB | 5.448E-08 | 0.000E+00 | 1.149E-06 | 0.000E+00 | 3.026E-08 | 0.000E+00 | 0.000E+00 | 0.000E+00 | 0.000E+00 | 1.517E-07 | 0.000E+00 | 8.050E-07 |
| Unigene151731 | Staphylocc | 3.340E-07 | 4.451E-07 | 1.217E-06 | 2.482E-06 | 3.711E-07 | 0.000E+00 | 0.000E+00 | 1.225E-06 | 0.000E+00 | 3.189E-07 | 6.823E-07 | 7.332E-07 |
| Unigene151749 | emrY | 2.618E-06 | 7.761E-07 | 1.369E-06 | 6.562E-07 | 0.000E+00 | 0.000E+00 | 0.000E+00 | 0.000E+00 | 0.000E+00 | 0.000E+00 | 0.000E+00 | 0.000E+00 |
| Unigene151778 | Staphylocc | 0.000E+00 | 0.000E+00 | 6.643E-07 | 0.000E+00 | 0.000E+00 | 0.000E+00 | 0.000E+00 | 1.976E-07 | 0.000E+00 | 0.000E+00 | 0.000E+00 | 0.000E+00 |
| Unigene151833 | oleB | 6.045E-08 | 1.106E-07 | 1.285E-06 | 5.982E-06 | 0.000E+00 | 0.000E+00 | 0.000E+00 | 0.000E+00 | 0.000E+00 | 0.000E+00 | 0.000E+00 | 0.000E+00 |
| Unigene151865 | macB | 0.000E+00 | 1.589E-07 | 1.420E-07 | 1.462E-07 | 0.000E+00 | 0.000E+00 | 0.000E+00 | 0.000E+00 | 0.000E+00 | 0.000E+00 | 0.000E+00 | 0.000E+00 |
| Unigene151910 | lsaA | 3.534E-06 | 8.193E-06 | 8.337E-06 | 7.859E-06 | 0.000E+00 | 0.000E+00 | 0.000E+00 | 0.000E+00 | 0.000E+00 | 0.000E+00 | 0.000E+00 | 0.000E+00 |
| Unigene151929 | mefC | 3.729E-07 | 3.508E-07 | 1.071E-06 | 2.690E-06 | 3.551E-07 | 2.303E-07 | 2.948E-07 | 1.275E-06 | 2.038E-07 | 2.967E-07 | 9.445E-07 | 7.241E-07 |
| Unigene151945 | macB | 1.170E-07 | 4.172E-07 | 1.148E-06 | 2.835E-06 | 1.949E-07 | 1.897E-07 | 3.883E-07 | 1.331E-06 | 6.392E-08 | 7.166E-07 | 1.271E-06 | 6.221E-07 |
| Unigene151967 | macB | 1.281E-07 | 2.812E-07 | 1.550E-06 | 2.932E-06 | 2.846E-07 | 0.000E+00 | 4.726E-07 | 1.196E-06 | 0.000E+00 | 5.232E-07 | 8.792E-07 | 0.000E+00 |
| Unigene152130 | marA | 0.000E+00 | 0.000E+00 | 0.000E+00 | 0.000E+00 | 1.077E-07 | 0.000E+00 | 0.000E+00 | 0.000E+00 | 1.060E-07 | 0.000E+00 | 0.000E+00 | 3.437E-07 |
| Unigene152135 | bcrA | 9.236E-08 | 0.000E+00 | 1.042E-06 | 0.000E+00 | 5.130E-08 | 0.000E+00 | 0.000E+00 | 0.000E+00 | 0.000E+00 | 0.000E+00 | 0.000E+00 | 9.826E-07 |
| Unigene152259 | poxtA | 0.000E+00 | 2.801E-07 | 7.512E-07 | 3.865E-07 | 0.000E+00 | 0.000E+00 | 0.000E+00 | 0.000E+00 | 0.000E+00 | 0.000E+00 | 0.000E+00 | 0.000E+00 |
| Unigene152310 | mtrA | 4.579E-08 | 0.000E+00 | 1.078E-06 | 1.849E-07 | 0.000E+00 | 0.000E+00 | 0.000E+00 | 0.000E+00 | 0.000E+00 | 0.000E+00 | 0.000E+00 | 0.000E+00 |
| Unigene152311 | arlS | 0.000E+00 | 7.693E-08 | 1.187E-06 | 2.478E-07 | 0.000E+00 | 0.000E+00 | 0.000E+00 | 0.000E+00 | 0.000E+00 | 0.000E+00 | 0.000E+00 | 0.000E+00 |
| Unigene152430 | Streptomy | 4.454E-06 | 2.001E-05 | 3.274E-06 | 9.771E-07 | 8.711E-07 | 2.344E-07 | 2.769E-07 | 2.921E-07 | 1.368E-06 | 5.018E-07 | 2.862E-07 | 1.183E-07 |
| Unigene152455 | macB | 0.000E+00 | 0.000E+00 | 9.926E-07 | 5.572E-07 | 0.000E+00 | 0.000E+00 | 0.000E+00 | 0.000E+00 | 0.000E+00 | 0.000E+00 | 0.000E+00 | 0.000E+00 |
| Unigene152464 | macB | 0.000E+00 | 0.000E+00 | 8.579E-07 | 2.716E-07 | 0.000E+00 | 0.000E+00 | 0.000E+00 | 0.000E+00 | 0.000E+00 | 0.000E+00 | 0.000E+00 | 0.000E+00 |
| Unigene152519 | arlS | 0.000E+00 | 0.000E+00 | 9.131E-07 | 0.000E+00 | 0.000E+00 | 0.000E+00 | 0.000E+00 | 0.000E+00 | 0.000E+00 | 0.000E+00 | 0.000E+00 | 0.000E+00 |

| | | | | | | | | | | | | | |
|---|---|---|---|---|---|---|---|---|---|---|---|---|---|
| Unigene152539 | macB | 0.000E+00 | 5.748E-06 | 1.635E-06 | 2.019E-06 | 0.000E+00 | 0.000E+00 | 0.000E+00 | 0.000E+00 | 0.000E+00 | 0.000E+00 | 0.000E+00 | 0.000E+00 |
| Unigene152608 | tlrC | 0.000E+00 | 0.000E+00 | 1.257E-06 | 1.294E-07 | 0.000E+00 | 0.000E+00 | 0.000E+00 | 0.000E+00 | 0.000E+00 | 0.000E+00 | 0.000E+00 | 0.000E+00 |
| Unigene152695 | catB2 | 0.000E+00 | 0.000E+00 | 7.506E-07 | 1.030E-07 | 1.416E-06 | 0.000E+00 | 9.595E-07 | 1.131E-06 | 0.000E+00 | 0.000E+00 | 1.283E-06 | 3.798E-06 |
| Unigene152732 | vga(E) Sta | 0.000E+00 | 1.803E-07 | 9.269E-07 | 0.000E+00 | 0.000E+00 | 0.000E+00 | 2.273E-07 | 0.000E+00 | 1.347E-07 | 9.150E-08 | 0.000E+00 | 8.253E-07 |
| Unigene152781 | lsaC | 0.000E+00 | 0.000E+00 | 3.352E-07 | 0.000E+00 | 0.000E+00 | 0.000E+00 | 0.000E+00 | 0.000E+00 | 0.000E+00 | 0.000E+00 | 0.000E+00 | 0.000E+00 |
| Unigene152911 | arlR | 0.000E+00 | 0.000E+00 | 8.255E-07 | 0.000E+00 | 0.000E+00 | 0.000E+00 | 0.000E+00 | 0.000E+00 | 0.000E+00 | 0.000E+00 | 0.000E+00 | 0.000E+00 |
| Unigene152912 | arlS | 9.543E-08 | 1.833E-07 | 1.124E-06 | 1.446E-07 | 0.000E+00 | 0.000E+00 | 0.000E+00 | 0.000E+00 | 0.000E+00 | 0.000E+00 | 0.000E+00 | 0.000E+00 |
| Unigene152950 | macB | 0.000E+00 | 2.109E-07 | 1.169E-06 | 1.552E-07 | 0.000E+00 | 0.000E+00 | 0.000E+00 | 0.000E+00 | 0.000E+00 | 0.000E+00 | 0.000E+00 | 0.000E+00 |
| Unigene152951 | tetB(46) | 1.113E-07 | 0.000E+00 | 1.347E-06 | 1.499E-07 | 0.000E+00 | 0.000E+00 | 0.000E+00 | 0.000E+00 | 0.000E+00 | 0.000E+00 | 0.000E+00 | 0.000E+00 |
| Unigene152962 | tva(A) | 9.017E-08 | 1.649E-07 | 1.150E-06 | 2.732E-07 | 0.000E+00 | 0.000E+00 | 0.000E+00 | 0.000E+00 | 0.000E+00 | 0.000E+00 | 0.000E+00 | 0.000E+00 |
| Unigene152963 | carA | 0.000E+00 | 1.125E-07 | 1.575E-06 | 1.725E-07 | 0.000E+00 | 0.000E+00 | 0.000E+00 | 0.000E+00 | 0.000E+00 | 0.000E+00 | 0.000E+00 | 0.000E+00 |
| Unigene152979 | Streptomy | 0.000E+00 | 8.857E-08 | 1.887E-06 | 1.396E-05 | 0.000E+00 | 0.000E+00 | 0.000E+00 | 0.000E+00 | 0.000E+00 | 0.000E+00 | 0.000E+00 | 0.000E+00 |
| Unigene152990 | patA | 0.000E+00 | 2.440E-07 | 2.225E-06 | 1.257E-05 | 0.000E+00 | 0.000E+00 | 0.000E+00 | 0.000E+00 | 0.000E+00 | 0.000E+00 | 0.000E+00 | 0.000E+00 |
| Unigene153020 | Streptomy | 4.516E-07 | 7.691E-07 | 1.222E-06 | 2.422E-06 | 0.000E+00 | 1.515E-07 | 1.206E-07 | 1.236E-06 | 3.404E-08 | 4.857E-07 | 7.125E-07 | 3.865E-07 |
| Unigene153024 | mdtG | 4.199E-07 | 5.761E-07 | 1.185E-06 | 2.730E-06 | 0.000E+00 | 1.135E-07 | 2.614E-07 | 1.409E-06 | 0.000E+00 | 5.555E-07 | 1.081E-06 | 8.686E-07 |
| Unigene153036 | poxtA | 0.000E+00 | 0.000E+00 | 6.043E-07 | 0.000E+00 | 0.000E+00 | 0.000E+00 | 0.000E+00 | 0.000E+00 | 0.000E+00 | 0.000E+00 | 0.000E+00 | 0.000E+00 |
| Unigene153112 | kdpE | 0.000E+00 | 6.846E-07 | 4.080E-07 | 0.000E+00 | 0.000E+00 | 0.000E+00 | 0.000E+00 | 0.000E+00 | 0.000E+00 | 0.000E+00 | 0.000E+00 | 0.000E+00 |
| Unigene153137 | bcrA | 2.675E-07 | 1.581E-07 | 1.332E-06 | 6.503E-06 | 0.000E+00 | 0.000E+00 | 0.000E+00 | 0.000E+00 | 0.000E+00 | 0.000E+00 | 0.000E+00 | 0.000E+00 |
| Unigene153182 | macB | 0.000E+00 | 6.561E-07 | 3.158E-06 | 3.343E-06 | 0.000E+00 | 0.000E+00 | 0.000E+00 | 0.000E+00 | 0.000E+00 | 0.000E+00 | 0.000E+00 | 0.000E+00 |
| Unigene153231 | pmrA | 2.697E-08 | 1.184E-07 | 1.297E-06 | 2.996E-07 | 0.000E+00 | 0.000E+00 | 0.000E+00 | 0.000E+00 | 0.000E+00 | 0.000E+00 | 0.000E+00 | 0.000E+00 |
| Unigene153244 | bacA | 0.000E+00 | 0.000E+00 | 1.022E-06 | 7.298E-05 | 0.000E+00 | 0.000E+00 | 0.000E+00 | 4.502E-08 | 0.000E+00 | 0.000E+00 | 0.000E+00 | 0.000E+00 |
| Unigene153250 | YojI | 0.000E+00 | 0.000E+00 | 7.888E-07 | 7.319E-05 | 0.000E+00 | 0.000E+00 | 0.000E+00 | 7.218E-08 | 0.000E+00 | 0.000E+00 | 0.000E+00 | 0.000E+00 |
| Unigene153287 | vmlR | 2.187E-07 | 0.000E+00 | 1.116E-06 | 2.650E-07 | 0.000E+00 | 0.000E+00 | 0.000E+00 | 0.000E+00 | 0.000E+00 | 0.000E+00 | 0.000E+00 | 0.000E+00 |
| Unigene153328 | smeS | 5.319E-07 | 4.939E-07 | 1.365E-06 | 0.000E+00 | 4.545E-08 | 0.000E+00 | 0.000E+00 | 2.388E-07 | 0.000E+00 | 0.000E+00 | 6.552E-07 | 0.000E+00 |
| Unigene153371 | bcrA | 2.083E-07 | 3.810E-07 | 3.406E-07 | 0.000E+00 | 0.000E+00 | 0.000E+00 | 0.000E+00 | 0.000E+00 | 0.000E+00 | 0.000E+00 | 0.000E+00 | 0.000E+00 |
| Unigene153384 | cpxA | 0.000E+00 | 1.140E-06 | 5.582E-07 | 0.000E+00 | 0.000E+00 | 0.000E+00 | 0.000E+00 | 0.000E+00 | 0.000E+00 | 0.000E+00 | 0.000E+00 | 0.000E+00 |
| Unigene153394 | vanSL | 2.920E-07 | 0.000E+00 | 8.452E-07 | 2.949E-07 | 1.281E-06 | 5.051E-07 | 1.487E-06 | 1.414E-06 | 2.234E-07 | 5.204E-07 | 1.052E-06 | 3.710E-06 |
| Unigene153395 | adeR | 0.000E+00 | 0.000E+00 | 0.000E+00 | 0.000E+00 | 1.573E-07 | 0.000E+00 | 0.000E+00 | 2.479E-07 | 0.000E+00 | 0.000E+00 | 0.000E+00 | 0.000E+00 |
| Unigene153499 | arlS | 0.000E+00 | 0.000E+00 | 1.150E-06 | 0.000E+00 | 0.000E+00 | 0.000E+00 | 0.000E+00 | 0.000E+00 | 0.000E+00 | 0.000E+00 | 0.000E+00 | 0.000E+00 |
| Unigene153535 | efrB | 0.000E+00 | 0.000E+00 | 6.235E-06 | 6.932E-06 | 0.000E+00 | 0.000E+00 | 0.000E+00 | 0.000E+00 | 0.000E+00 | 0.000E+00 | 0.000E+00 | 0.000E+00 |
| Unigene153572 | vanHO | 0.000E+00 | 0.000E+00 | 1.292E-06 | 0.000E+00 | 0.000E+00 | 0.000E+00 | 0.000E+00 | 0.000E+00 | 0.000E+00 | 0.000E+00 | 0.000E+00 | 0.000E+00 |
| Unigene153730 | lmrD | 3.677E-06 | 2.187E-07 | 3.182E-06 | 3.841E-07 | 2.817E-07 | 9.791E-08 | 4.411E-07 | 0.000E+00 | 0.000E+00 | 0.000E+00 | 8.081E-07 | 0.000E+00 |
| Unigene153739 | macB | 0.000E+00 | 0.000E+00 | 1.598E-06 | 0.000E+00 | 0.000E+00 | 0.000E+00 | 0.000E+00 | 0.000E+00 | 0.000E+00 | 0.000E+00 | 0.000E+00 | 0.000E+00 |
| Unigene153746 | tetT | 0.000E+00 | 0.000E+00 | 7.226E-07 | 0.000E+00 | 0.000E+00 | 0.000E+00 | 0.000E+00 | 0.000E+00 | 0.000E+00 | 0.000E+00 | 0.000E+00 | 0.000E+00 |
| Unigene153768 | mecI | 0.000E+00 | 0.000E+00 | 1.291E-06 | 0.000E+00 | 0.000E+00 | 0.000E+00 | 0.000E+00 | 0.000E+00 | 0.000E+00 | 0.000E+00 | 0.000E+00 | 0.000E+00 |
| Unigene153770 | baeS | 0.000E+00 | 0.000E+00 | 1.208E-06 | 0.000E+00 | 0.000E+00 | 0.000E+00 | 0.000E+00 | 0.000E+00 | 0.000E+00 | 0.000E+00 | 0.000E+00 | 0.000E+00 |
| Unigene153784 | tetA(46) | 1.163E-07 | 1.596E-07 | 6.563E-07 | 2.056E-07 | 0.000E+00 | 0.000E+00 | 0.000E+00 | 0.000E+00 | 0.000E+00 | 0.000E+00 | 0.000E+00 | 0.000E+00 |
| Unigene153811 | vanRF | 3.233E-07 | 1.064E-06 | 4.305E-06 | 6.062E-06 | 0.000E+00 | 0.000E+00 | 0.000E+00 | 0.000E+00 | 0.000E+00 | 0.000E+00 | 0.000E+00 | 0.000E+00 |
| Unigene153822 | macB | 0.000E+00 | 9.419E-06 | 0.000E+00 | 0.000E+00 | 0.000E+00 | 0.000E+00 | 0.000E+00 | 0.000E+00 | 0.000E+00 | 0.000E+00 | 0.000E+00 | 0.000E+00 |
| Unigene153868 | vanHO | 0.000E+00 | 0.000E+00 | 6.103E-07 | 8.611E-05 | 0.000E+00 | 0.000E+00 | 0.000E+00 | 0.000E+00 | 0.000E+00 | 0.000E+00 | 0.000E+00 | 0.000E+00 |
| Unigene154156 | macB | 0.000E+00 | 6.889E-07 | 2.596E-06 | 2.852E-06 | 0.000E+00 | 0.000E+00 | 0.000E+00 | 0.000E+00 | 0.000E+00 | 0.000E+00 | 0.000E+00 | 0.000E+00 |
| Unigene154226 | patB | 5.653E-08 | 9.100E-07 | 3.828E-06 | 3.045E-06 | 0.000E+00 | 0.000E+00 | 0.000E+00 | 0.000E+00 | 0.000E+00 | 0.000E+00 | 0.000E+00 | 0.000E+00 |
| Unigene154227 | novA | 1.474E-07 | 4.853E-07 | 3.471E-06 | 3.126E-06 | 0.000E+00 | 0.000E+00 | 0.000E+00 | 0.000E+00 | 0.000E+00 | 0.000E+00 | 0.000E+00 | 0.000E+00 |
| Unigene154235 | macB | 1.276E-07 | 6.535E-07 | 2.754E-06 | 3.393E-06 | 0.000E+00 | 0.000E+00 | 0.000E+00 | 0.000E+00 | 0.000E+00 | 0.000E+00 | 0.000E+00 | 0.000E+00 |
| Unigene154240 | tetA(58) | 6.380E-07 | 1.400E-06 | 8.346E-07 | 0.000E+00 | 7.891E-06 | 0.000E+00 | 0.000E+00 | 0.000E+00 | 9.297E-08 | 0.000E+00 | 0.000E+00 | 0.000E+00 |
| Unigene154421 | blt | 0.000E+00 | 0.000E+00 | 9.088E-07 | 0.000E+00 | 0.000E+00 | 0.000E+00 | 0.000E+00 | 0.000E+00 | 0.000E+00 | 0.000E+00 | 0.000E+00 | 0.000E+00 |
| Unigene154459 | poxtA | 0.000E+00 | 6.616E-08 | 1.213E-06 | 0.000E+00 | 0.000E+00 | 0.000E+00 | 0.000E+00 | 0.000E+00 | 1.318E-07 | 0.000E+00 | 0.000E+00 | 9.975E-07 |
| Unigene154494 | lmrD | 3.514E-07 | 3.856E-07 | 1.164E-06 | 2.927E-06 | 0.000E+00 | 0.000E+00 | 3.402E-07 | 1.538E-06 | 0.000E+00 | 4.892E-07 | 0.000E+00 | 0.000E+00 |
| Unigene154496 | Staphyloc | 2.394E-07 | 4.379E-07 | 1.227E-06 | 2.713E-06 | 4.137E-07 | 2.301E-07 | 2.355E-07 | 1.242E-06 | 0.000E+00 | 4.445E-07 | 1.187E-06 | 9.905E-07 |
| Unigene154524 | evgS | 1.748E-07 | 2.740E-07 | 7.104E-07 | 0.000E+00 | 7.766E-07 | 3.239E-07 | 1.160E-06 | 1.107E-06 | 0.000E+00 | 8.065E-07 | 8.853E-07 | 2.597E-06 |
| Unigene154546 | lmrD | 0.000E+00 | 0.000E+00 | 9.570E-07 | 0.000E+00 | 6.991E-07 | 1.633E-06 | 4.039E-06 | 4.260E-06 | 0.000E+00 | 1.542E-06 | 2.087E-06 | 4.091E-06 |
| Unigene154591 | lmrD | 0.000E+00 | 0.000E+00 | 6.713E-07 | 2.826E-07 | 0.000E+00 | 1.109E-06 | 4.474E-06 | 3.049E-06 | 0.000E+00 | 7.621E-07 | 1.316E-06 | 2.389E-06 |
| Unigene154672 | rpoB2 | 0.000E+00 | 0.000E+00 | 5.839E-07 | 0.000E+00 | 0.000E+00 | 0.000E+00 | 0.000E+00 | 0.000E+00 | 0.000E+00 | 0.000E+00 | 0.000E+00 | 0.000E+00 |
| Unigene154748 | efrA | 0.000E+00 | 1.957E-07 | 6.708E-07 | 6.003E-08 | 7.595E-07 | 0.000E+00 | 9.540E-07 | 8.674E-07 | 9.746E-08 | 0.000E+00 | 0.000E+00 | 2.284E-06 |
| Unigene154805 | vanTG | 0.000E+00 | 0.000E+00 | 8.798E-07 | 0.000E+00 | 0.000E+00 | 0.000E+00 | 0.000E+00 | 0.000E+00 | 0.000E+00 | 0.000E+00 | 0.000E+00 | 0.000E+00 |
| Unigene154809 | mdtG | 0.000E+00 | 0.000E+00 | 7.486E-07 | 0.000E+00 | 0.000E+00 | 0.000E+00 | 0.000E+00 | 0.000E+00 | 0.000E+00 | 0.000E+00 | 0.000E+00 | 0.000E+00 |
| Unigene154810 | lsaA | 0.000E+00 | 0.000E+00 | 9.502E-07 | 0.000E+00 | 0.000E+00 | 0.000E+00 | 0.000E+00 | 0.000E+00 | 0.000E+00 | 0.000E+00 | 0.000E+00 | 0.000E+00 |
| Unigene154887 | oleB | 0.000E+00 | 0.000E+00 | 7.175E-07 | 0.000E+00 | 0.000E+00 | 0.000E+00 | 0.000E+00 | 0.000E+00 | 0.000E+00 | 0.000E+00 | 0.000E+00 | 0.000E+00 |

| | | | | | | | | | | | | | |
|---|---|---|---|---|---|---|---|---|---|---|---|---|---|
| Unigene154889 | macB | 0.000E+00 | 0.000E+00 | 1.229E-06 | 0.000E+00 | 0.000E+00 | 0.000E+00 | 0.000E+00 | 0.000E+00 | 0.000E+00 | 0.000E+00 | 0.000E+00 | 0.000E+00 |
| Unigene154890 | patB | 0.000E+00 | 0.000E+00 | 1.056E-06 | 0.000E+00 | 0.000E+00 | 0.000E+00 | 0.000E+00 | 0.000E+00 | 0.000E+00 | 0.000E+00 | 0.000E+00 | 0.000E+00 |
| Unigene154967 | vanG | 1.878E-07 | 2.945E-07 | 1.843E-06 | 5.284E-06 | 0.000E+00 | 0.000E+00 | 0.000E+00 | 0.000E+00 | 0.000E+00 | 0.000E+00 | 0.000E+00 | 0.000E+00 |
| Unigene154978 | Streptomy | 2.257E-07 | 1.817E-07 | 2.274E-06 | 7.340E-06 | 0.000E+00 | 0.000E+00 | 0.000E+00 | 0.000E+00 | 0.000E+00 | 0.000E+00 | 0.000E+00 | 0.000E+00 |
| Unigene155070 | OprN | 2.118E-08 | 0.000E+00 | 8.937E-07 | 8.556E-08 | 1.177E-07 | 0.000E+00 | 0.000E+00 | 0.000E+00 | 1.621E-07 | 0.000E+00 | 0.000E+00 | 6.009E-07 |
| Unigene155071 | Klebsiella p | 0.000E+00 | 2.254E-07 | 1.612E-06 | 6.221E-08 | 0.000E+00 | 2.220E-08 | 4.545E-08 | 7.191E-08 | 1.347E-07 | 0.000E+00 | 9.396E-08 | 1.335E-06 |
| Unigene155072 | farA | 0.000E+00 | 6.453E-08 | 1.327E-06 | 0.000E+00 | 0.000E+00 | 0.000E+00 | 0.000E+00 | 0.000E+00 | 9.640E-08 | 0.000E+00 | 1.009E-07 | 8.340E-07 |
| Unigene155124 | abeS | 0.000E+00 | 0.000E+00 | 1.558E-06 | 0.000E+00 | 0.000E+00 | 0.000E+00 | 0.000E+00 | 0.000E+00 | 0.000E+00 | 0.000E+00 | 0.000E+00 | 9.383E-07 |
| Unigene155173 | vanRA | 0.000E+00 | 0.000E+00 | 0.000E+00 | 0.000E+00 | 0.000E+00 | 0.000E+00 | 8.574E-07 | 1.507E-06 | 5.645E-07 | 0.000E+00 | 0.000E+00 | 4.986E-06 |
| Unigene155220 | bcrA | 0.000E+00 | 4.072E-07 | 2.002E-06 | 6.651E-06 | 0.000E+00 | 0.000E+00 | 0.000E+00 | 0.000E+00 | 0.000E+00 | 0.000E+00 | 0.000E+00 | 0.000E+00 |
| Unigene155227 | macB | 0.000E+00 | 0.000E+00 | 1.846E-06 | 5.847E-06 | 0.000E+00 | 0.000E+00 | 0.000E+00 | 0.000E+00 | 0.000E+00 | 0.000E+00 | 0.000E+00 | 0.000E+00 |
| Unigene155231 | adeJ | 0.000E+00 | 4.154E-07 | 3.714E-07 | 0.000E+00 | 0.000E+00 | 0.000E+00 | 0.000E+00 | 0.000E+00 | 0.000E+00 | 0.000E+00 | 0.000E+00 | 0.000E+00 |
| Unigene155287 | Klebsiella p | 0.000E+00 | 0.000E+00 | 3.579E-06 | 0.000E+00 | 0.000E+00 | 0.000E+00 | 0.000E+00 | 3.193E-07 | 0.000E+00 | 0.000E+00 | 0.000E+00 | 0.000E+00 |
| Unigene155299 | evgA | 0.000E+00 | 0.000E+00 | 1.028E-06 | 0.000E+00 | 0.000E+00 | 0.000E+00 | 0.000E+00 | 0.000E+00 | 0.000E+00 | 0.000E+00 | 0.000E+00 | 0.000E+00 |
| Unigene155337 | vanSM | 0.000E+00 | 5.688E-07 | 1.162E-06 | 0.000E+00 | 1.124E-06 | 0.000E+00 | 5.162E-06 | 4.667E-06 | 0.000E+00 | 1.622E-06 | 2.936E-06 | 4.055E-06 |
| Unigene155364 | vanHB | 0.000E+00 | 0.000E+00 | 0.000E+00 | 0.000E+00 | 0.000E+00 | 0.000E+00 | 1.484E-06 | 1.006E-06 | 0.000E+00 | 0.000E+00 | 0.000E+00 | 0.000E+00 |
| Unigene155372 | vanL | 6.329E-07 | 7.244E-06 | 9.493E-06 | 1.695E-05 | 0.000E+00 | 0.000E+00 | 0.000E+00 | 0.000E+00 | 0.000E+00 | 0.000E+00 | 0.000E+00 | 0.000E+00 |
| Unigene155488 | mtrA | 3.454E-06 | 4.860E-07 | 3.302E-06 | 4.024E-07 | 1.476E-07 | 0.000E+00 | 2.940E-07 | 0.000E+00 | 0.000E+00 | 0.000E+00 | 5.065E-07 | 0.000E+00 |
| Unigene155511 | Corynebac | 0.000E+00 | 0.000E+00 | 1.241E-06 | 0.000E+00 | 0.000E+00 | 0.000E+00 | 0.000E+00 | 0.000E+00 | 0.000E+00 | 0.000E+00 | 0.000E+00 | 0.000E+00 |
| Unigene155517 | poxtA | 0.000E+00 | 0.000E+00 | 8.774E-07 | 0.000E+00 | 0.000E+00 | 0.000E+00 | 0.000E+00 | 0.000E+00 | 0.000E+00 | 0.000E+00 | 0.000E+00 | 0.000E+00 |
| Unigene155700 | efpA | 0.000E+00 | 0.000E+00 | 3.116E-07 | 2.850E-07 | 7.839E-08 | 0.000E+00 | 0.000E+00 | 0.000E+00 | 0.000E+00 | 0.000E+00 | 0.000E+00 | 0.000E+00 |
| Unigene155721 | dfrC | 0.000E+00 | 0.000E+00 | 1.374E-06 | 9.078E-05 | 0.000E+00 | 0.000E+00 | 0.000E+00 | 0.000E+00 | 0.000E+00 | 0.000E+00 | 0.000E+00 | 0.000E+00 |
| Unigene155734 | efmA | 5.353E-08 | 0.000E+00 | 9.455E-07 | 6.217E-07 | 0.000E+00 | 5.787E-08 | 0.000E+00 | 0.000E+00 | 0.000E+00 | 0.000E+00 | 0.000E+00 | 0.000E+00 |
| Unigene155761 | vanRE | 7.912E-08 | 0.000E+00 | 1.553E-06 | 7.990E-08 | 8.790E-08 | 0.000E+00 | 0.000E+00 | 0.000E+00 | 5.765E-08 | 0.000E+00 | 1.508E-07 | 1.309E-06 |
| Unigene155768 | evgS | 2.391E-08 | 0.000E+00 | 1.126E-06 | 1.207E-07 | 7.969E-08 | 2.585E-08 | 2.646E-08 | 1.116E-07 | 1.437E-07 | 3.995E-08 | 4.103E-08 | 1.046E-06 |
| Unigene155777 | cmlv | 8.050E-08 | 5.890E-08 | 1.316E-06 | 0.000E+00 | 3.279E-07 | 2.901E-08 | 0.000E+00 | 0.000E+00 | 2.053E-07 | 0.000E+00 | 0.000E+00 | 1.364E-06 |
| Unigene155789 | kdpE | 0.000E+00 | 0.000E+00 | 1.173E-06 | 4.829E-08 | 0.000E+00 | 0.000E+00 | 0.000E+00 | 5.582E-08 | 4.182E-07 | 0.000E+00 | 5.470E-08 | 7.349E-07 |
| Unigene155790 | smeS | 0.000E+00 | 0.000E+00 | 1.471E-06 | 0.000E+00 | 1.225E-07 | 0.000E+00 | 0.000E+00 | 0.000E+00 | 7.230E-08 | 0.000E+00 | 0.000E+00 | 1.095E-06 |
| Unigene155815 | lmrB | 2.537E-05 | 9.043E-06 | 6.527E-06 | 4.620E-06 | 0.000E+00 | 1.009E-07 | 1.033E-07 | 1.090E-07 | 7.143E-07 | 0.000E+00 | 0.000E+00 | 0.000E+00 |
| Unigene155886 | baeR | 0.000E+00 | 9.226E-08 | 2.145E-06 | 9.295E-06 | 0.000E+00 | 0.000E+00 | 0.000E+00 | 0.000E+00 | 0.000E+00 | 0.000E+00 | 0.000E+00 | 0.000E+00 |
| Unigene155939 | tetA(58) | 0.000E+00 | 2.591E-07 | 2.085E-06 | 1.298E-05 | 0.000E+00 | 0.000E+00 | 0.000E+00 | 0.000E+00 | 0.000E+00 | 0.000E+00 | 0.000E+00 | 0.000E+00 |
| Unigene155941 | mtrA | 0.000E+00 | 0.000E+00 | 1.988E-06 | 1.441E-05 | 0.000E+00 | 0.000E+00 | 0.000E+00 | 0.000E+00 | 0.000E+00 | 0.000E+00 | 0.000E+00 | 0.000E+00 |
| Unigene155985 | vanRF | 0.000E+00 | 0.000E+00 | 1.351E-05 | 0.000E+00 | 0.000E+00 | 1.219E-06 | 0.000E+00 | 0.000E+00 | 0.000E+00 | 0.000E+00 | 0.000E+00 | 0.000E+00 |
| Unigene155986 | vanSF | 0.000E+00 | 0.000E+00 | 1.230E-05 | 0.000E+00 | 0.000E+00 | 0.000E+00 | 0.000E+00 | 0.000E+00 | 0.000E+00 | 0.000E+00 | 0.000E+00 | 0.000E+00 |
| Unigene156063 | lmrB | 0.000E+00 | 0.000E+00 | 9.940E-07 | 0.000E+00 | 0.000E+00 | 0.000E+00 | 0.000E+00 | 0.000E+00 | 0.000E+00 | 0.000E+00 | 0.000E+00 | 0.000E+00 |
| Unigene156118 | baeS | 0.000E+00 | 0.000E+00 | 1.039E-06 | 7.929E-05 | 0.000E+00 | 0.000E+00 | 0.000E+00 | 1.124E-07 | 0.000E+00 | 0.000E+00 | 0.000E+00 | 0.000E+00 |
| Unigene156119 | arlR | 0.000E+00 | 0.000E+00 | 9.181E-07 | 7.398E-05 | 0.000E+00 | 0.000E+00 | 0.000E+00 | 0.000E+00 | 0.000E+00 | 0.000E+00 | 0.000E+00 | 0.000E+00 |
| Unigene156122 | PmrF | 0.000E+00 | 0.000E+00 | 9.929E-07 | 7.453E-05 | 0.000E+00 | 0.000E+00 | 0.000E+00 | 0.000E+00 | 0.000E+00 | 0.000E+00 | 0.000E+00 | 0.000E+00 |
| Unigene156228 | optrA | 1.427E-07 | 2.237E-08 | 1.220E-06 | 4.116E-08 | 0.000E+00 | 0.000E+00 | 0.000E+00 | 0.000E+00 | 0.000E+00 | 0.000E+00 | 2.331E-08 | 0.000E+00 |
| Unigene156229 | bacA | 7.741E-08 | 1.274E-07 | 1.557E-06 | 1.954E-07 | 0.000E+00 | 0.000E+00 | 0.000E+00 | 0.000E+00 | 0.000E+00 | 0.000E+00 | 0.000E+00 | 0.000E+00 |
| Unigene156400 | Corynebac | 0.000E+00 | 0.000E+00 | 4.763E-07 | 0.000E+00 | 0.000E+00 | 0.000E+00 | 0.000E+00 | 0.000E+00 | 0.000E+00 | 0.000E+00 | 0.000E+00 | 0.000E+00 |
| Unigene156475 | mexM | 0.000E+00 | 0.000E+00 | 1.219E-06 | 0.000E+00 | 0.000E+00 | 1.038E-06 | 3.813E-06 | 0.000E+00 | 0.000E+00 | 0.000E+00 | 1.389E-06 | 3.806E-06 |
| Unigene156574 | tetB(58) | 2.085E-07 | 2.289E-07 | 1.391E-06 | 1.811E-06 | 2.317E-07 | 0.000E+00 | 1.385E-07 | 1.071E-06 | 0.000E+00 | 6.969E-07 | 1.050E-06 | 2.465E-07 |
| Unigene156575 | tetA(58) | 3.427E-07 | 2.633E-07 | 1.009E-06 | 2.941E-06 | 2.665E-07 | 0.000E+00 | 2.655E-07 | 1.440E-06 | 1.498E-07 | 6.871E-07 | 7.840E-07 | 5.266E-07 |
| Unigene156584 | PmrF | 0.000E+00 | 0.000E+00 | 9.845E-07 | 0.000E+00 | 0.000E+00 | 0.000E+00 | 0.000E+00 | 0.000E+00 | 0.000E+00 | 0.000E+00 | 0.000E+00 | 0.000E+00 |
| Unigene156592 | rphB | 0.000E+00 | 0.000E+00 | 1.254E-06 | 0.000E+00 | 0.000E+00 | 0.000E+00 | 0.000E+00 | 0.000E+00 | 0.000E+00 | 0.000E+00 | 0.000E+00 | 0.000E+00 |
| Unigene156666 | efrB | 1.655E-07 | 0.000E+00 | 1.421E-06 | 2.507E-07 | 0.000E+00 | 0.000E+00 | 0.000E+00 | 0.000E+00 | 0.000E+00 | 0.000E+00 | 0.000E+00 | 0.000E+00 |
| Unigene156708 | vatE | 0.000E+00 | 1.147E-07 | 1.230E-06 | 2.110E-07 | 0.000E+00 | 0.000E+00 | 0.000E+00 | 0.000E+00 | 0.000E+00 | 0.000E+00 | 0.000E+00 | 0.000E+00 |
| Unigene156760 | facT | 2.675E-07 | 5.419E-07 | 1.393E-06 | 3.012E-06 | 0.000E+00 | 2.002E-07 | 2.732E-07 | 1.057E-06 | 0.000E+00 | 2.521E-07 | 1.271E-06 | 8.512E-07 |
| Unigene156805 | emeA | 0.000E+00 | 0.000E+00 | 5.369E-07 | 0.000E+00 | 0.000E+00 | 0.000E+00 | 0.000E+00 | 0.000E+00 | 0.000E+00 | 0.000E+00 | 0.000E+00 | 0.000E+00 |
| Unigene156913 | vanRI | 1.334E-07 | 0.000E+00 | 7.417E-07 | 0.000E+00 | 0.000E+00 | 0.000E+00 | 0.000E+00 | 0.000E+00 | 0.000E+00 | 0.000E+00 | 0.000E+00 | 0.000E+00 |
| Unigene156955 | macB | 0.000E+00 | 0.000E+00 | 1.048E-06 | 0.000E+00 | 0.000E+00 | 0.000E+00 | 0.000E+00 | 0.000E+00 | 0.000E+00 | 0.000E+00 | 0.000E+00 | 0.000E+00 |
| Unigene157001 | macB | 0.000E+00 | 5.880E-07 | 2.760E-06 | 2.886E-06 | 0.000E+00 | 0.000E+00 | 0.000E+00 | 0.000E+00 | 0.000E+00 | 0.000E+00 | 0.000E+00 | 0.000E+00 |
| Unigene157004 | vanHB | 0.000E+00 | 5.072E-07 | 3.142E-06 | 2.600E-06 | 0.000E+00 | 0.000E+00 | 0.000E+00 | 0.000E+00 | 0.000E+00 | 0.000E+00 | 0.000E+00 | 0.000E+00 |
| Unigene157018 | macB | 9.357E-08 | 0.000E+00 | 1.591E-06 | 0.000E+00 | 3.465E-08 | 3.372E-08 | 6.904E-08 | 0.000E+00 | 1.023E-07 | 0.000E+00 | 2.497E-07 | 1.511E-06 |
| Unigene157057 | tetB(P) | 0.000E+00 | 8.981E-08 | 8.631E-07 | 6.625E-05 | 0.000E+00 | 2.211E-08 | 0.000E+00 | 7.163E-08 | 0.000E+00 | 0.000E+00 | 0.000E+00 | 0.000E+00 |
| Unigene157067 | rpoB2 | 0.000E+00 | 0.000E+00 | 0.000E+00 | 2.527E-07 | 0.000E+00 | 4.058E-07 | 1.662E-06 | 0.000E+00 | 1.368E-07 | 0.000E+00 | 0.000E+00 | 2.810E-06 |
| Unigene157118 | macB | 0.000E+00 | 0.000E+00 | 9.282E-07 | 0.000E+00 | 0.000E+00 | 1.236E-06 | 0.000E+00 | 0.000E+00 | 0.000E+00 | 0.000E+00 | 0.000E+00 | 3.774E-06 |

| | | | | | | | | | | | | | |
|---|---|---|---|---|---|---|---|---|---|---|---|---|---|
| Unigene157135 | Bifidobact | 1.810E-07 | 2.781E-07 | 2.993E-06 | 1.649E-05 | 0.000E+00 | 0.000E+00 | 0.000E+00 | 0.000E+00 | 0.000E+00 | 0.000E+00 | 0.000E+00 | 0.000E+00 |
| Unigene157270 | tetA(58) | 1.752E-07 | 0.000E+00 | 4.127E-07 | 0.000E+00 | 0.000E+00 | 0.000E+00 | 0.000E+00 | 0.000E+00 | 0.000E+00 | 0.000E+00 | 0.000E+00 | 0.000E+00 |
| Unigene157331 | macB | 0.000E+00 | 0.000E+00 | 6.423E-07 | 0.000E+00 | 0.000E+00 | 5.307E-07 | 0.000E+00 | 0.000E+00 | 0.000E+00 | 0.000E+00 | 0.000E+00 | 0.000E+00 |
| Unigene157334 | vanSE | 9.384E-08 | 0.000E+00 | 4.297E-07 | 3.159E-07 | 0.000E+00 | 0.000E+00 | 0.000E+00 | 0.000E+00 | 0.000E+00 | 0.000E+00 | 0.000E+00 | 0.000E+00 |
| Unigene157363 | bcrA | 0.000E+00 | 9.760E-08 | 6.981E-07 | 5.478E-05 | 0.000E+00 | 0.000E+00 | 0.000E+00 | 0.000E+00 | 0.000E+00 | 0.000E+00 | 0.000E+00 | 0.000E+00 |
| Unigene157375 | optrA | 0.000E+00 | 0.000E+00 | 9.262E-07 | 7.481E-05 | 0.000E+00 | 0.000E+00 | 0.000E+00 | 0.000E+00 | 0.000E+00 | 0.000E+00 | 0.000E+00 | 0.000E+00 |
| Unigene157512 | oleC | 3.865E-07 | 1.339E-07 | 7.584E-07 | 4.108E-07 | 8.135E-07 | 9.015E-07 | 2.679E-06 | 2.184E-06 | 3.335E-07 | 1.042E-06 | 1.373E-06 | 3.342E-06 |
| Unigene157537 | arnA | 0.000E+00 | 7.404E-08 | 1.059E-06 | 4.816E-05 | 0.000E+00 | 0.000E+00 | 0.000E+00 | 7.874E-08 | 0.000E+00 | 0.000E+00 | 0.000E+00 | 0.000E+00 |
| Unigene157582 | optrA | 0.000E+00 | 0.000E+00 | 0.000E+00 | 0.000E+00 | 0.000E+00 | 0.000E+00 | 0.000E+00 | 0.000E+00 | 0.000E+00 | 1.712E-07 | 0.000E+00 | 0.000E+00 |
| Unigene157694 | macB | 0.000E+00 | 0.000E+00 | 8.977E-07 | 0.000E+00 | 0.000E+00 | 0.000E+00 | 0.000E+00 | 0.000E+00 | 0.000E+00 | 0.000E+00 | 0.000E+00 | 2.523E-06 |
| Unigene157701 | cmlv | 0.000E+00 | 0.000E+00 | 4.512E-07 | 0.000E+00 | 0.000E+00 | 0.000E+00 | 0.000E+00 | 2.505E-07 | 0.000E+00 | 0.000E+00 | 0.000E+00 | 0.000E+00 |
| Unigene157721 | tetB(60) | 5.425E-08 | 0.000E+00 | 7.985E-07 | 7.953E-05 | 0.000E+00 | 0.000E+00 | 0.000E+00 | 1.478E-07 | 0.000E+00 | 0.000E+00 | 0.000E+00 | 0.000E+00 |
| Unigene157722 | tetA(60) | 3.672E-08 | 0.000E+00 | 9.189E-07 | 7.810E-05 | 0.000E+00 | 0.000E+00 | 0.000E+00 | 8.573E-08 | 0.000E+00 | 0.000E+00 | 0.000E+00 | 0.000E+00 |
| Unigene157766 | PmrF | 0.000E+00 | 0.000E+00 | 2.706E-06 | 0.000E+00 | 0.000E+00 | 0.000E+00 | 0.000E+00 | 0.000E+00 | 0.000E+00 | 0.000E+00 | 0.000E+00 | 0.000E+00 |
| Unigene157767 | PmrF | 0.000E+00 | 0.000E+00 | 3.135E-06 | 0.000E+00 | 0.000E+00 | 0.000E+00 | 0.000E+00 | 0.000E+00 | 0.000E+00 | 0.000E+00 | 0.000E+00 | 0.000E+00 |
| Unigene157838 | aadK | 0.000E+00 | 0.000E+00 | 4.703E-05 | 1.335E-06 | 0.000E+00 | 0.000E+00 | 0.000E+00 | 4.287E-08 | 1.606E-07 | 0.000E+00 | 0.000E+00 | 0.000E+00 |
| Unigene157841 | Pseudomo | 0.000E+00 | 0.000E+00 | 6.839E-07 | 0.000E+00 | 0.000E+00 | 0.000E+00 | 0.000E+00 | 0.000E+00 | 0.000E+00 | 0.000E+00 | 0.000E+00 | 0.000E+00 |
| Unigene157842 | lmrC | 1.204E-07 | 0.000E+00 | 7.091E-07 | 0.000E+00 | 1.204E-06 | 5.859E-07 | 1.178E-06 | 1.148E-06 | 0.000E+00 | 6.038E-07 | 8.726E-07 | 3.725E-06 |
| Unigene157949 | TaeA | 0.000E+00 | 4.157E-07 | 3.632E-06 | 3.355E-06 | 0.000E+00 | 0.000E+00 | 0.000E+00 | 0.000E+00 | 0.000E+00 | 0.000E+00 | 0.000E+00 | 0.000E+00 |
| Unigene157950 | dfrE | 0.000E+00 | 5.368E-07 | 3.119E-06 | 3.272E-06 | 0.000E+00 | 0.000E+00 | 0.000E+00 | 0.000E+00 | 0.000E+00 | 0.000E+00 | 0.000E+00 | 0.000E+00 |
| Unigene157955 | vanHF | 6.581E-08 | 4.334E-07 | 2.486E-06 | 2.559E-06 | 0.000E+00 | 0.000E+00 | 0.000E+00 | 0.000E+00 | 0.000E+00 | 0.000E+00 | 0.000E+00 | 0.000E+00 |
| Unigene158065 | vanSA | 0.000E+00 | 0.000E+00 | 5.965E-07 | 0.000E+00 | 0.000E+00 | 0.000E+00 | 0.000E+00 | 0.000E+00 | 0.000E+00 | 0.000E+00 | 0.000E+00 | 0.000E+00 |
| Unigene158084 | OXY-2-1 | 0.000E+00 | 2.435E-07 | 1.007E-06 | 9.242E-07 | 0.000E+00 | 0.000E+00 | 0.000E+00 | 0.000E+00 | 0.000E+00 | 0.000E+00 | 0.000E+00 | 0.000E+00 |
| Unigene158107 | Erm(34) | 1.571E-07 | 0.000E+00 | 1.695E-06 | 0.000E+00 | 2.618E-07 | 0.000E+00 | 0.000E+00 | 0.000E+00 | 0.000E+00 | 0.000E+00 | 8.984E-08 | 1.485E-06 |
| Unigene158132 | Acinetoba | 0.000E+00 | 1.086E-06 | 7.339E-07 | 0.000E+00 | 7.558E-06 | 2.869E-07 | 9.612E-07 | 5.351E-07 | 1.318E-07 | 2.688E-07 | 0.000E+00 | 0.000E+00 |
| Unigene158159 | lmrD | 0.000E+00 | 0.000E+00 | 4.161E-07 | 0.000E+00 | 1.767E-07 | 0.000E+00 | 0.000E+00 | 0.000E+00 | 0.000E+00 | 0.000E+00 | 0.000E+00 | 8.772E-07 |
| Unigene158403 | tet32 | 3.169E-06 | 3.306E-06 | 2.170E-06 | 2.426E-06 | 2.147E-06 | 1.769E-06 | 2.004E-06 | 7.520E-07 | 1.237E-06 | 6.362E-06 | 5.517E-06 | 1.770E-06 |
| Unigene158408 | macB | 2.117E-07 | 1.936E-08 | 1.731E-06 | 4.649E-06 | 0.000E+00 | 0.000E+00 | 0.000E+00 | 0.000E+00 | 0.000E+00 | 0.000E+00 | 0.000E+00 | 0.000E+00 |
| Unigene158413 | msbA | 7.941E-08 | 1.743E-07 | 2.026E-06 | 5.854E-06 | 0.000E+00 | 0.000E+00 | 0.000E+00 | 0.000E+00 | 0.000E+00 | 0.000E+00 | 0.000E+00 | 0.000E+00 |
| Unigene158433 | patA | 0.000E+00 | 1.417E-07 | 1.267E-06 | 4.346E-08 | 0.000E+00 | 0.000E+00 | 4.763E-08 | 1.005E-07 | 1.411E-07 | 0.000E+00 | 4.923E-08 | 1.526E-06 |
| Unigene158438 | AcrF | 5.203E-08 | 5.711E-08 | 1.072E-06 | 7.356E-08 | 5.780E-08 | 2.250E-08 | 2.303E-08 | 0.000E+00 | 1.706E-07 | 3.478E-08 | 1.428E-07 | 1.230E-06 |
| Unigene158439 | AcrE | 0.000E+00 | 0.000E+00 | 9.898E-07 | 2.830E-08 | 6.226E-08 | 0.000E+00 | 1.240E-07 | 2.290E-07 | 6.125E-08 | 1.249E-07 | 0.000E+00 | 9.274E-07 |
| Unigene158440 | AcrS | 0.000E+00 | 0.000E+00 | 1.051E-06 | 5.150E-08 | 0.000E+00 | 0.000E+00 | 0.000E+00 | 0.000E+00 | 5.574E-08 | 0.000E+00 | 0.000E+00 | 1.869E-06 |
| Unigene158547 | Chlamydia | 0.000E+00 | 0.000E+00 | 1.220E-05 | 0.000E+00 | 0.000E+00 | 7.985E-07 | 0.000E+00 | 0.000E+00 | 0.000E+00 | 0.000E+00 | 0.000E+00 | 0.000E+00 |
| Unigene158552 | YojI | 0.000E+00 | 0.000E+00 | 1.195E-05 | 0.000E+00 | 0.000E+00 | 1.070E-06 | 3.080E-07 | 0.000E+00 | 0.000E+00 | 0.000E+00 | 0.000E+00 | 0.000E+00 |
| Unigene158556 | patA | 0.000E+00 | 0.000E+00 | 1.203E-05 | 0.000E+00 | 0.000E+00 | 0.000E+00 | 0.000E+00 | 0.000E+00 | 0.000E+00 | 0.000E+00 | 0.000E+00 | 0.000E+00 |
| Unigene158559 | bcrA | 0.000E+00 | 0.000E+00 | 1.403E-05 | 0.000E+00 | 0.000E+00 | 9.959E-07 | 0.000E+00 | 0.000E+00 | 0.000E+00 | 0.000E+00 | 0.000E+00 | 0.000E+00 |
| Unigene158562 | vanL | 0.000E+00 | 0.000E+00 | 1.239E-05 | 0.000E+00 | 0.000E+00 | 8.549E-07 | 0.000E+00 | 0.000E+00 | 0.000E+00 | 0.000E+00 | 0.000E+00 | 0.000E+00 |
| Unigene158575 | Staphyloc | 0.000E+00 | 0.000E+00 | 1.217E-05 | 0.000E+00 | 0.000E+00 | 7.483E-07 | 4.704E-07 | 0.000E+00 | 0.000E+00 | 0.000E+00 | 0.000E+00 | 0.000E+00 |
| Unigene158582 | patA | 0.000E+00 | 0.000E+00 | 1.348E-05 | 0.000E+00 | 0.000E+00 | 8.041E-07 | 0.000E+00 | 0.000E+00 | 0.000E+00 | 0.000E+00 | 0.000E+00 | 0.000E+00 |
| Unigene158718 | smeR | 0.000E+00 | 0.000E+00 | 0.000E+00 | 0.000E+00 | 0.000E+00 | 0.000E+00 | 9.640E-07 | 0.000E+00 | 0.000E+00 | 0.000E+00 | 0.000E+00 | 0.000E+00 |
| Unigene158738 | tetW | 8.553E-05 | 1.391E-04 | 1.374E-04 | 0.000E+00 | 7.199E-06 | 3.396E-07 | 0.000E+00 | 0.000E+00 | 4.502E-05 | 0.000E+00 | 2.650E-06 | 0.000E+00 |
| Unigene158759 | Streptomy | 0.000E+00 | 0.000E+00 | 6.713E-07 | 0.000E+00 | 0.000E+00 | 0.000E+00 | 0.000E+00 | 0.000E+00 | 0.000E+00 | 0.000E+00 | 0.000E+00 | 0.000E+00 |
| Unigene158776 | tet(K) | 0.000E+00 | 4.952E-08 | 1.970E-06 | 1.292E-05 | 0.000E+00 | 0.000E+00 | 0.000E+00 | 0.000E+00 | 0.000E+00 | 0.000E+00 | 0.000E+00 | 0.000E+00 |
| Unigene158803 | AAC(6')-Ib | 0.000E+00 | 0.000E+00 | 1.265E-06 | 9.621E-07 | 0.000E+00 | 0.000E+00 | 0.000E+00 | 0.000E+00 | 0.000E+00 | 0.000E+00 | 0.000E+00 | 0.000E+00 |
| Unigene158876 | Corynebac | 3.174E-08 | 0.000E+00 | 1.339E-06 | 0.000E+00 | 1.058E-07 | 1.029E-07 | 0.000E+00 | 7.410E-08 | 1.041E-07 | 0.000E+00 | 0.000E+00 | 5.628E-07 |
| Unigene158908 | efrA | 4.961E-07 | 4.084E-07 | 1.521E-06 | 2.599E-06 | 0.000E+00 | 0.000E+00 | 0.000E+00 | 1.194E-06 | 0.000E+00 | 5.527E-07 | 8.158E-07 | 9.896E-07 |
| Unigene158911 | Staphyloc | 2.299E-07 | 4.037E-07 | 1.196E-06 | 2.577E-06 | 2.810E-07 | 0.000E+00 | 3.817E-07 | 1.583E-06 | 0.000E+00 | 5.379E-07 | 1.105E-06 | 9.240E-07 |
| Unigene158916 | macB | 2.593E-07 | 0.000E+00 | 1.060E-06 | 1.833E-06 | 0.000E+00 | 0.000E+00 | 0.000E+00 | 1.261E-06 | 1.417E-07 | 0.000E+00 | 0.000E+00 | 0.000E+00 |
| Unigene158939 | emrR | 0.000E+00 | 0.000E+00 | 1.173E-06 | 0.000E+00 | 5.579E-06 | 0.000E+00 | 1.323E-06 | 0.000E+00 | 0.000E+00 | 0.000E+00 | 6.838E-07 | 2.120E-07 |
| Unigene158971 | tet32 | 0.000E+00 | 3.878E-08 | 9.015E-07 | 5.185E-05 | 0.000E+00 | 1.910E-08 | 0.000E+00 | 8.249E-08 | 0.000E+00 | 0.000E+00 | 0.000E+00 | 0.000E+00 |
| Unigene158989 | otr(B) | 0.000E+00 | 0.000E+00 | 8.302E-07 | 0.000E+00 | 0.000E+00 | 0.000E+00 | 0.000E+00 | 0.000E+00 | 0.000E+00 | 0.000E+00 | 0.000E+00 | 0.000E+00 |
| Unigene158991 | Staphyloc | 0.000E+00 | 0.000E+00 | 9.825E-07 | 0.000E+00 | 0.000E+00 | 0.000E+00 | 0.000E+00 | 0.000E+00 | 0.000E+00 | 0.000E+00 | 0.000E+00 | 0.000E+00 |
| Unigene159019 | sul4 | 0.000E+00 | 0.000E+00 | 1.009E-06 | 0.000E+00 | 0.000E+00 | 0.000E+00 | 0.000E+00 | 0.000E+00 | 0.000E+00 | 0.000E+00 | 2.715E-07 | 0.000E+00 |
| Unigene159031 | evgS | 5.835E-08 | 6.404E-08 | 1.271E-06 | 5.892E-08 | 1.297E-07 | 0.000E+00 | 0.000E+00 | 6.811E-08 | 1.020E-07 | 0.000E+00 | 1.068E-07 | 1.379E-06 |
| Unigene159103 | Streptomy | 8.020E-08 | 4.401E-08 | 1.325E-06 | 6.749E-08 | 8.910E-08 | 1.300E-07 | 0.000E+00 | 7.801E-08 | 2.045E-07 | 1.191E-07 | 1.070E-07 | 1.059E-06 |
| Unigene159128 | vanHD | 0.000E+00 | 0.000E+00 | 3.249E-07 | 0.000E+00 | 0.000E+00 | 0.000E+00 | 0.000E+00 | 0.000E+00 | 0.000E+00 | 0.000E+00 | 0.000E+00 | 0.000E+00 |
| Unigene159152 | tetA(58) | 0.000E+00 | 0.000E+00 | 1.075E-06 | 0.000E+00 | 0.000E+00 | 0.000E+00 | 0.000E+00 | 0.000E+00 | 0.000E+00 | 0.000E+00 | 0.000E+00 | 0.000E+00 |

| | | | | | | | | | | | | | |
|---|---|---|---|---|---|---|---|---|---|---|---|---|---|
| Unigene159161 | ErmX | 0.000E+00 | 0.000E+00 | 1.417E-06 | 0.000E+00 | 0.000E+00 | 0.000E+00 | 0.000E+00 | 0.000E+00 | 0.000E+00 | 0.000E+00 | 0.000E+00 | 0.000E+00 |
| Unigene159396 | oleB | 0.000E+00 | 4.618E-07 | 0.000E+00 | 0.000E+00 | 0.000E+00 | 0.000E+00 | 0.000E+00 | 0.000E+00 | 0.000E+00 | 0.000E+00 | 0.000E+00 | 0.000E+00 |
| Unigene159583 | arr-1 | 0.000E+00 | 7.758E-07 | 3.699E-06 | 2.935E-06 | 0.000E+00 | 0.000E+00 | 0.000E+00 | 0.000E+00 | 0.000E+00 | 0.000E+00 | 0.000E+00 | 0.000E+00 |
| Unigene159686 | novA | 0.000E+00 | 2.255E-07 | 2.914E-06 | 1.413E-05 | 0.000E+00 | 0.000E+00 | 0.000E+00 | 0.000E+00 | 0.000E+00 | 0.000E+00 | 0.000E+00 | 0.000E+00 |
| Unigene159687 | msbA | 1.017E-07 | 2.046E-07 | 2.444E-06 | 1.316E-05 | 0.000E+00 | 0.000E+00 | 0.000E+00 | 0.000E+00 | 0.000E+00 | 0.000E+00 | 0.000E+00 | 0.000E+00 |
| Unigene159690 | vgaB | 0.000E+00 | 0.000E+00 | 2.421E-06 | 1.446E-05 | 0.000E+00 | 0.000E+00 | 0.000E+00 | 0.000E+00 | 0.000E+00 | 0.000E+00 | 0.000E+00 | 0.000E+00 |
| Unigene159691 | macB | 0.000E+00 | 0.000E+00 | 2.381E-06 | 1.535E-05 | 0.000E+00 | 0.000E+00 | 0.000E+00 | 0.000E+00 | 0.000E+00 | 0.000E+00 | 0.000E+00 | 0.000E+00 |
| Unigene159696 | oleC | 1.695E-08 | 2.046E-07 | 2.344E-06 | 1.365E-05 | 0.000E+00 | 0.000E+00 | 0.000E+00 | 0.000E+00 | 0.000E+00 | 0.000E+00 | 0.000E+00 | 0.000E+00 |
| Unigene159801 | otr(B) | 7.892E-08 | 2.310E-07 | 3.614E-07 | 6.376E-07 | 2.923E-08 | 0.000E+00 | 0.000E+00 | 0.000E+00 | 0.000E+00 | 0.000E+00 | 9.028E-08 | 9.329E-08 |
| Unigene159971 | vanSA | 0.000E+00 | 1.398E-06 | 0.000E+00 | 0.000E+00 | 0.000E+00 | 0.000E+00 | 0.000E+00 | 0.000E+00 | 0.000E+00 | 0.000E+00 | 0.000E+00 | 0.000E+00 |
| Unigene159990 | oleI | 8.384E-08 | 9.202E-07 | 2.825E-06 | 2.427E-06 | 0.000E+00 | 0.000E+00 | 0.000E+00 | 0.000E+00 | 0.000E+00 | 0.000E+00 | 0.000E+00 | 0.000E+00 |
| Unigene160069 | macB | 0.000E+00 | 0.000E+00 | 1.221E-06 | 7.833E-05 | 0.000E+00 | 0.000E+00 | 0.000E+00 | 9.373E-08 | 0.000E+00 | 0.000E+00 | 0.000E+00 | 0.000E+00 |
| Unigene160103 | novA | 5.100E-08 | 0.000E+00 | 7.005E-07 | 0.000E+00 | 0.000E+00 | 0.000E+00 | 0.000E+00 | 0.000E+00 | 0.000E+00 | 0.000E+00 | 0.000E+00 | 0.000E+00 |
| Unigene160146 | macB | 0.000E+00 | 0.000E+00 | 1.139E-06 | 6.947E-05 | 0.000E+00 | 0.000E+00 | 0.000E+00 | 3.012E-08 | 0.000E+00 | 0.000E+00 | 0.000E+00 | 0.000E+00 |
| Unigene160202 | Escherichia | 2.202E-07 | 1.027E-06 | 7.561E-07 | 1.945E-07 | 5.809E-07 | 0.000E+00 | 1.157E-06 | 3.533E-07 | 0.000E+00 | 0.000E+00 | 3.777E-07 | 0.000E+00 |
| Unigene160253 | facT | 0.000E+00 | 0.000E+00 | 4.399E-07 | 0.000E+00 | 0.000E+00 | 0.000E+00 | 0.000E+00 | 0.000E+00 | 0.000E+00 | 0.000E+00 | 0.000E+00 | 0.000E+00 |
| Unigene160390 | lmrC | 0.000E+00 | 0.000E+00 | 1.302E-06 | 4.800E-06 | 0.000E+00 | 0.000E+00 | 0.000E+00 | 0.000E+00 | 0.000E+00 | 0.000E+00 | 0.000E+00 | 0.000E+00 |
| Unigene160469 | vanZF | 0.000E+00 | 0.000E+00 | 1.101E-06 | 0.000E+00 | 0.000E+00 | 0.000E+00 | 0.000E+00 | 0.000E+00 | 0.000E+00 | 0.000E+00 | 0.000E+00 | 0.000E+00 |
| Unigene160497 | Erm(33) | 4.233E-06 | 0.000E+00 | 1.372E-05 | 1.229E-05 | 0.000E+00 | 7.103E-06 | 0.000E+00 | 2.059E-07 | 0.000E+00 | 0.000E+00 | 0.000E+00 | 0.000E+00 |
| Unigene160571 | efrA | 0.000E+00 | 0.000E+00 | 5.427E-07 | 0.000E+00 | 0.000E+00 | 0.000E+00 | 0.000E+00 | 0.000E+00 | 0.000E+00 | 0.000E+00 | 0.000E+00 | 0.000E+00 |
| Unigene160574 | msbA | 0.000E+00 | 9.388E-08 | 5.036E-07 | 3.628E-07 | 1.121E-06 | 3.514E-07 | 1.041E-06 | 8.386E-07 | 1.122E-07 | 6.289E-07 | 1.037E-06 | 2.790E-06 |
| Unigene160592 | lmrD | 0.000E+00 | 0.000E+00 | 7.345E-07 | 0.000E+00 | 0.000E+00 | 0.000E+00 | 0.000E+00 | 0.000E+00 | 0.000E+00 | 0.000E+00 | 0.000E+00 | 0.000E+00 |
| Unigene160726 | tcr3 | 0.000E+00 | 5.272E-08 | 8.013E-07 | 8.295E-05 | 0.000E+00 | 0.000E+00 | 0.000E+00 | 8.411E-08 | 0.000E+00 | 0.000E+00 | 0.000E+00 | 0.000E+00 |
| Unigene160860 | macB | 0.000E+00 | 0.000E+00 | 1.023E-06 | 0.000E+00 | 2.317E-07 | 0.000E+00 | 0.000E+00 | 0.000E+00 | 9.117E-08 | 0.000E+00 | 4.771E-08 | 9.859E-07 |
| Unigene160861 | lin | 0.000E+00 | 0.000E+00 | 1.122E-06 | 3.980E-08 | 8.757E-08 | 0.000E+00 | 0.000E+00 | 0.000E+00 | 0.000E+00 | 0.000E+00 | 0.000E+00 | 8.386E-07 |
| Unigene160902 | macB | 0.000E+00 | 0.000E+00 | 1.286E-06 | 6.506E-08 | 1.193E-07 | 9.287E-08 | 0.000E+00 | 0.000E+00 | 2.113E-07 | 2.153E-07 | 7.370E-08 | 1.345E-06 |
| Unigene160944 | Staphylocc | 3.391E-08 | 1.489E-07 | 1.386E-06 | 9.131E-08 | 8.790E-08 | 0.000E+00 | 0.000E+00 | 0.000E+00 | 1.482E-07 | 1.007E-07 | 1.293E-07 | 1.109E-06 |
| Unigene161030 | vanSA | 0.000E+00 | 0.000E+00 | 9.040E-07 | 1.116E-07 | 0.000E+00 | 0.000E+00 | 0.000E+00 | 0.000E+00 | 0.000E+00 | 0.000E+00 | 0.000E+00 | 0.000E+00 |
| Unigene161031 | vanRI | 1.428E-07 | 0.000E+00 | 8.409E-07 | 1.442E-07 | 0.000E+00 | 0.000E+00 | 0.000E+00 | 0.000E+00 | 0.000E+00 | 0.000E+00 | 0.000E+00 | 0.000E+00 |
| Unigene161067 | evgA | 2.624E-07 | 1.728E-07 | 3.605E-07 | 0.000E+00 | 0.000E+00 | 0.000E+00 | 1.162E-07 | 0.000E+00 | 0.000E+00 | 0.000E+00 | 0.000E+00 | 0.000E+00 |
| Unigene161074 | bcrB | 3.402E-07 | 3.267E-06 | 1.322E-04 | 0.000E+00 | 3.685E-06 | 3.678E-07 | 0.000E+00 | 0.000E+00 | 1.296E-04 | 2.843E-07 | 0.000E+00 | 0.000E+00 |
| Unigene161082 | efpA | 0.000E+00 | 0.000E+00 | 5.253E-07 | 0.000E+00 | 0.000E+00 | 0.000E+00 | 0.000E+00 | 0.000E+00 | 0.000E+00 | 0.000E+00 | 0.000E+00 | 0.000E+00 |
| Unigene161121 | Acinetoba | 0.000E+00 | 0.000E+00 | 6.759E-07 | 0.000E+00 | 0.000E+00 | 0.000E+00 | 0.000E+00 | 0.000E+00 | 0.000E+00 | 0.000E+00 | 0.000E+00 | 0.000E+00 |
| Unigene161171 | patA | 0.000E+00 | 0.000E+00 | 4.520E-07 | 0.000E+00 | 0.000E+00 | 0.000E+00 | 0.000E+00 | 0.000E+00 | 0.000E+00 | 0.000E+00 | 1.882E-07 | 0.000E+00 |
| Unigene161250 | optrA | 0.000E+00 | 6.397E-07 | 6.159E-07 | 0.000E+00 | 4.284E-06 | 0.000E+00 | 0.000E+00 | 0.000E+00 | 0.000E+00 | 0.000E+00 | 0.000E+00 | 0.000E+00 |
| Unigene161313 | msbA | 2.656E-06 | 1.163E-06 | 1.259E-06 | 6.028E-07 | 0.000E+00 | 0.000E+00 | 0.000E+00 | 1.219E-07 | 0.000E+00 | 0.000E+00 | 0.000E+00 | 1.764E-08 |
| Unigene161323 | baeS | 7.328E-05 | 6.192E-05 | 1.185E-04 | 1.713E-04 | 1.302E-04 | 9.332E-05 | 1.254E-04 | 1.172E-04 | 1.046E-04 | 1.465E-04 | 1.459E-04 | 1.539E-04 |
| Unigene161357 | tet(43) | 6.711E-05 | 4.994E-05 | 1.060E-04 | 1.618E-04 | 1.154E-04 | 8.300E-05 | 1.021E-04 | 1.010E-04 | 8.866E-05 | 1.243E-04 | 1.285E-04 | 1.302E-04 |
| Unigene161397 | macB | 0.000E+00 | 0.000E+00 | 9.254E-07 | 0.000E+00 | 0.000E+00 | 0.000E+00 | 0.000E+00 | 0.000E+00 | 0.000E+00 | 0.000E+00 | 0.000E+00 | 0.000E+00 |
| Unigene161411 | rphA | 1.328E-07 | 5.832E-07 | 6.952E-07 | 4.472E-07 | 0.000E+00 | 0.000E+00 | 0.000E+00 | 0.000E+00 | 0.000E+00 | 0.000E+00 | 0.000E+00 | 0.000E+00 |
| Unigene161417 | macB | 5.779E-07 | 5.686E-07 | 9.581E-07 | 7.043E-07 | 5.756E-07 | 1.443E-06 | 4.675E-06 | 3.675E-06 | 4.791E-07 | 1.598E-06 | 1.915E-06 | 4.334E-06 |
| Unigene161432 | srmB | 0.000E+00 | 1.954E-07 | 1.048E-06 | 3.339E-07 | 0.000E+00 | 0.000E+00 | 0.000E+00 | 0.000E+00 | 0.000E+00 | 0.000E+00 | 0.000E+00 | 0.000E+00 |
| Unigene161456 | arlR | 0.000E+00 | 0.000E+00 | 9.385E-07 | 0.000E+00 | 0.000E+00 | 0.000E+00 | 1.059E-07 | 1.116E-07 | 0.000E+00 | 0.000E+00 | 0.000E+00 | 1.357E-06 |
| Unigene161457 | basS | 0.000E+00 | 0.000E+00 | 1.163E-06 | 0.000E+00 | 2.026E-07 | 0.000E+00 | 0.000E+00 | 0.000E+00 | 2.492E-07 | 0.000E+00 | 2.608E-08 | 1.051E-06 |
| Unigene161467 | evgS | 3.398E-08 | 0.000E+00 | 1.478E-06 | 8.006E-08 | 8.808E-08 | 3.673E-08 | 1.379E-07 | 0.000E+00 | 3.714E-08 | 5.047E-08 | 2.591E-08 | 1.245E-06 |
| Unigene161469 | Klebsiella p | 0.000E+00 | 0.000E+00 | 1.250E-06 | 1.144E-07 | 3.146E-08 | 0.000E+00 | 0.000E+00 | 0.000E+00 | 2.785E-07 | 0.000E+00 | 9.717E-08 | 7.363E-07 |
| Unigene161473 | YojI | 0.000E+00 | 8.604E-08 | 1.096E-06 | 0.000E+00 | 2.177E-08 | 0.000E+00 | 4.338E-08 | 0.000E+00 | 4.284E-08 | 0.000E+00 | 6.726E-08 | 1.436E-06 |
| Unigene161513 | Staphylocc | 3.245E-06 | 3.452E-07 | 2.761E-06 | 3.845E-07 | 3.126E-07 | 3.579E-08 | 3.481E-07 | 0.000E+00 | 0.000E+00 | 0.000E+00 | 5.491E-07 | 0.000E+00 |
| Unigene161613 | efmA | 1.541E-07 | 9.301E-07 | 1.260E-06 | 1.608E-06 | 0.000E+00 | 0.000E+00 | 0.000E+00 | 0.000E+00 | 0.000E+00 | 0.000E+00 | 0.000E+00 | 0.000E+00 |
| Unigene161651 | mepA | 2.261E-07 | 9.924E-07 | 4.481E-06 | 3.835E-06 | 0.000E+00 | 0.000E+00 | 0.000E+00 | 0.000E+00 | 0.000E+00 | 0.000E+00 | 0.000E+00 | 0.000E+00 |
| Unigene161790 | ugd | 0.000E+00 | 0.000E+00 | 1.629E-06 | 0.000E+00 | 0.000E+00 | 0.000E+00 | 0.000E+00 | 0.000E+00 | 0.000E+00 | 0.000E+00 | 0.000E+00 | 0.000E+00 |
| Unigene161793 | macB | 0.000E+00 | 0.000E+00 | 5.404E-07 | 5.989E-07 | 0.000E+00 | 0.000E+00 | 4.688E-08 | 0.000E+00 | 0.000E+00 | 0.000E+00 | 0.000E+00 | 0.000E+00 |
| Unigene161904 | lmrD | 0.000E+00 | 0.000E+00 | 9.133E-07 | 0.000E+00 | 6.080E-06 | 0.000E+00 | 0.000E+00 | 0.000E+00 | 0.000E+00 | 0.000E+00 | 0.000E+00 | 0.000E+00 |
| Unigene161914 | Escherichia | 0.000E+00 | 0.000E+00 | 9.417E-07 | 0.000E+00 | 0.000E+00 | 0.000E+00 | 0.000E+00 | 0.000E+00 | 0.000E+00 | 0.000E+00 | 0.000E+00 | 0.000E+00 |
| Unigene161922 | efpA | 0.000E+00 | 1.990E-07 | 2.373E-07 | 6.104E-08 | 0.000E+00 | 0.000E+00 | 0.000E+00 | 0.000E+00 | 0.000E+00 | 0.000E+00 | 0.000E+00 | 0.000E+00 |
| Unigene161958 | acrD | 0.000E+00 | 2.273E-08 | 1.352E-06 | 1.046E-08 | 3.452E-08 | 2.239E-08 | 0.000E+00 | 0.000E+00 | 1.698E-07 | 0.000E+00 | 1.658E-07 | 1.053E-06 |
| Unigene162016 | PmrF | 0.000E+00 | 7.223E-08 | 4.520E-07 | 1.362E-06 | 0.000E+00 | 0.000E+00 | 0.000E+00 | 0.000E+00 | 0.000E+00 | 0.000E+00 | 0.000E+00 | 0.000E+00 |
| Unigene162017 | PmrF | 0.000E+00 | 2.536E-07 | 1.004E-06 | 2.333E-06 | 0.000E+00 | 0.000E+00 | 0.000E+00 | 0.000E+00 | 0.000E+00 | 0.000E+00 | 0.000E+00 | 0.000E+00 |

| | | | | | | | | | | | | | |
|---|---|---|---|---|---|---|---|---|---|---|---|---|---|
| Unigene162047 | Corynebac | 0.000E+00 | 3.091E-08 | 1.354E-06 | 0.000E+00 | 9.388E-08 | 3.045E-08 | 0.000E+00 | 0.000E+00 | 1.539E-07 | 1.255E-07 | 3.222E-08 | 1.299E-06 |
| Unigene162067 | Klebsiella p | 0.000E+00 | 0.000E+00 | 0.000E+00 | 0.000E+00 | 0.000E+00 | 0.000E+00 | 0.000E+00 | 0.000E+00 | 0.000E+00 | 3.269E-07 | 0.000E+00 | 0.000E+00 |
| Unigene162068 | Klebsiella p | 0.000E+00 | 0.000E+00 | 1.408E-06 | 0.000E+00 | 0.000E+00 | 0.000E+00 | 0.000E+00 | 0.000E+00 | 0.000E+00 | 0.000E+00 | 0.000E+00 | 1.166E-06 |
| Unigene162091 | adeL | 6.386E-08 | 0.000E+00 | 1.159E-06 | 6.449E-08 | 1.064E-07 | 3.452E-08 | 0.000E+00 | 0.000E+00 | 1.047E-07 | 0.000E+00 | 1.096E-07 | 5.661E-07 |
| Unigene162104 | vgaD | 1.708E-05 | 7.848E-06 | 2.244E-05 | 9.934E-05 | 6.691E-06 | 7.901E-06 | 2.596E-05 | 3.660E-06 | 9.813E-06 | 2.088E-05 | 1.964E-05 | 6.239E-06 |
| Unigene162155 | Staphylocc | 1.145E-06 | 9.763E-07 | 1.196E-06 | 1.600E-06 | 1.791E-05 | 5.994E-06 | 1.378E-05 | 1.519E-05 | 9.843E-06 | 1.550E-05 | 1.546E-05 | 6.410E-06 |
| Unigene162168 | macB | 0.000E+00 | 0.000E+00 | 7.602E-07 | 0.000E+00 | 0.000E+00 | 0.000E+00 | 2.144E-06 | 2.060E-06 | 0.000E+00 | 0.000E+00 | 0.000E+00 | 5.189E-06 |
| Unigene162224 | vgaB | 0.000E+00 | 0.000E+00 | 7.114E-07 | 2.697E-07 | 0.000E+00 | 0.000E+00 | 0.000E+00 | 0.000E+00 | 0.000E+00 | 0.000E+00 | 0.000E+00 | 0.000E+00 |
| Unigene162289 | optrA | 0.000E+00 | 3.117E-07 | 1.269E-06 | 7.010E-07 | 0.000E+00 | 0.000E+00 | 0.000E+00 | 0.000E+00 | 0.000E+00 | 0.000E+00 | 0.000E+00 | 0.000E+00 |
| Unigene162301 | emrB | 2.669E-07 | 3.831E-07 | 1.914E-06 | 6.407E-06 | 0.000E+00 | 0.000E+00 | 0.000E+00 | 0.000E+00 | 0.000E+00 | 0.000E+00 | 0.000E+00 | 0.000E+00 |
| Unigene162359 | emeA | 9.971E-05 | 1.795E-04 | 1.765E-04 | 7.537E-06 | 8.815E-06 | 3.563E-07 | 2.554E-06 | 1.841E-06 | 5.322E-05 | 2.885E-07 | 2.586E-06 | 7.514E-07 |
| Unigene162388 | tetA(58) | 0.000E+00 | 0.000E+00 | 5.939E-07 | 4.754E-07 | 0.000E+00 | 0.000E+00 | 0.000E+00 | 0.000E+00 | 2.940E-07 | 0.000E+00 | 0.000E+00 | 0.000E+00 |
| Unigene162490 | macB | 0.000E+00 | 2.672E-07 | 1.672E-06 | 2.458E-07 | 0.000E+00 | 0.000E+00 | 0.000E+00 | 0.000E+00 | 0.000E+00 | 0.000E+00 | 0.000E+00 | 0.000E+00 |
| Unigene162542 | tetA(58) | 0.000E+00 | 0.000E+00 | 7.993E-07 | 0.000E+00 | 6.524E-07 | 1.393E-06 | 3.879E-06 | 3.604E-06 | 0.000E+00 | 1.583E-06 | 1.907E-06 | 3.986E-06 |
| Unigene162565 | bcrA | 0.000E+00 | 1.097E-07 | 1.831E-06 | 1.393E-05 | 0.000E+00 | 0.000E+00 | 0.000E+00 | 0.000E+00 | 0.000E+00 | 0.000E+00 | 0.000E+00 | 0.000E+00 |
| Unigene162567 | lmrB | 1.284E-07 | 2.113E-07 | 2.519E-06 | 1.491E-05 | 0.000E+00 | 0.000E+00 | 0.000E+00 | 0.000E+00 | 0.000E+00 | 0.000E+00 | 0.000E+00 | 0.000E+00 |
| Unigene162597 | tetA(46) | 0.000E+00 | 0.000E+00 | 5.323E-07 | 0.000E+00 | 9.710E-07 | 3.258E-07 | 8.673E-07 | 8.092E-07 | 1.977E-07 | 6.044E-07 | 4.827E-07 | 1.568E-06 |
| Unigene162678 | patB | 3.438E-08 | 9.433E-08 | 1.214E-06 | 2.430E-07 | 0.000E+00 | 0.000E+00 | 0.000E+00 | 0.000E+00 | 0.000E+00 | 0.000E+00 | 0.000E+00 | 0.000E+00 |
| Unigene162679 | efrA | 7.472E-08 | 1.435E-07 | 1.540E-06 | 2.641E-07 | 0.000E+00 | 0.000E+00 | 0.000E+00 | 0.000E+00 | 0.000E+00 | 0.000E+00 | 0.000E+00 | 0.000E+00 |
| Unigene162825 | bcrA | 0.000E+00 | 3.108E-07 | 6.946E-07 | 7.863E-07 | 0.000E+00 | 0.000E+00 | 0.000E+00 | 0.000E+00 | 0.000E+00 | 0.000E+00 | 0.000E+00 | 0.000E+00 |
| Unigene162841 | mdtG | 0.000E+00 | 0.000E+00 | 1.208E-06 | 8.109E-08 | 5.947E-08 | 2.894E-08 | 0.000E+00 | 3.124E-08 | 5.851E-08 | 1.193E-07 | 1.225E-07 | 1.171E-06 |
| Unigene162858 | baeS | 0.000E+00 | 0.000E+00 | 1.208E-06 | 9.946E-08 | 0.000E+00 | 5.324E-08 | 0.000E+00 | 0.000E+00 | 1.076E-07 | 5.486E-08 | 5.633E-08 | 9.313E-07 |
| Unigene162938 | cdeA | 0.000E+00 | 8.825E-07 | 3.481E-06 | 2.937E-06 | 0.000E+00 | 0.000E+00 | 0.000E+00 | 0.000E+00 | 0.000E+00 | 0.000E+00 | 0.000E+00 | 0.000E+00 |
| Unigene162992 | Listeria mc | 3.758E-08 | 1.787E-07 | 9.833E-07 | 3.036E-07 | 0.000E+00 | 0.000E+00 | 0.000E+00 | 0.000E+00 | 0.000E+00 | 0.000E+00 | 0.000E+00 | 0.000E+00 |
| Unigene162995 | carA | 0.000E+00 | 1.040E-07 | 1.116E-06 | 1.915E-07 | 0.000E+00 | 0.000E+00 | 0.000E+00 | 0.000E+00 | 0.000E+00 | 0.000E+00 | 0.000E+00 | 0.000E+00 |
| Unigene163162 | cfrC | 2.717E-08 | 0.000E+00 | 2.293E-06 | 1.509E-05 | 0.000E+00 | 0.000E+00 | 0.000E+00 | 0.000E+00 | 0.000E+00 | 0.000E+00 | 0.000E+00 | 0.000E+00 |
| Unigene163178 | RbpA | 0.000E+00 | 0.000E+00 | 2.002E-06 | 1.574E-05 | 0.000E+00 | 0.000E+00 | 0.000E+00 | 0.000E+00 | 0.000E+00 | 0.000E+00 | 0.000E+00 | 0.000E+00 |
| Unigene163185 | macB | 0.000E+00 | 0.000E+00 | 1.581E-06 | 2.872E-06 | 0.000E+00 | 0.000E+00 | 0.000E+00 | 0.000E+00 | 0.000E+00 | 0.000E+00 | 0.000E+00 | 0.000E+00 |
| Unigene163215 | bacA | 0.000E+00 | 1.618E-07 | 1.953E-06 | 1.477E-05 | 0.000E+00 | 0.000E+00 | 0.000E+00 | 0.000E+00 | 0.000E+00 | 0.000E+00 | 0.000E+00 | 0.000E+00 |
| Unigene163233 | oleB | 0.000E+00 | 0.000E+00 | 1.740E-06 | 1.215E-05 | 0.000E+00 | 0.000E+00 | 0.000E+00 | 0.000E+00 | 0.000E+00 | 0.000E+00 | 0.000E+00 | 0.000E+00 |
| Unigene163250 | lmrD | 2.110E-08 | 1.158E-07 | 1.635E-06 | 9.161E-06 | 0.000E+00 | 0.000E+00 | 0.000E+00 | 0.000E+00 | 0.000E+00 | 0.000E+00 | 0.000E+00 | 0.000E+00 |
| Unigene163354 | macB | 8.678E-08 | 0.000E+00 | 1.107E-06 | 3.067E-07 | 0.000E+00 | 0.000E+00 | 0.000E+00 | 0.000E+00 | 0.000E+00 | 0.000E+00 | 0.000E+00 | 0.000E+00 |
| Unigene163430 | pmrA | 0.000E+00 | 2.624E-07 | 3.910E-07 | 1.207E-07 | 0.000E+00 | 0.000E+00 | 0.000E+00 | 0.000E+00 | 0.000E+00 | 0.000E+00 | 0.000E+00 | 0.000E+00 |
| Unigene163485 | mdtG | 0.000E+00 | 0.000E+00 | 4.083E-07 | 0.000E+00 | 0.000E+00 | 1.928E-07 | 0.000E+00 | 0.000E+00 | 0.000E+00 | 0.000E+00 | 0.000E+00 | 0.000E+00 |
| Unigene163507 | TaeA | 8.553E-08 | 7.510E-08 | 1.057E-06 | 1.727E-07 | 0.000E+00 | 0.000E+00 | 0.000E+00 | 0.000E+00 | 0.000E+00 | 0.000E+00 | 0.000E+00 | 0.000E+00 |
| Unigene163509 | dfrE | 0.000E+00 | 4.144E-07 | 1.605E-06 | 2.542E-07 | 0.000E+00 | 0.000E+00 | 0.000E+00 | 0.000E+00 | 0.000E+00 | 0.000E+00 | 0.000E+00 | 0.000E+00 |
| Unigene163510 | mdtA | 0.000E+00 | 0.000E+00 | 2.707E-07 | 0.000E+00 | 0.000E+00 | 0.000E+00 | 0.000E+00 | 0.000E+00 | 0.000E+00 | 0.000E+00 | 0.000E+00 | 0.000E+00 |
| Unigene163531 | carA | 0.000E+00 | 1.562E-07 | 1.788E-06 | 5.749E-08 | 0.000E+00 | 0.000E+00 | 0.000E+00 | 0.000E+00 | 0.000E+00 | 3.488E-07 | 1.302E-07 | 1.312E-06 |
| Unigene163562 | emrB | 7.263E-07 | 2.098E-07 | 1.707E-06 | 1.351E-07 | 1.062E-07 | 0.000E+00 | 0.000E+00 | 1.339E-07 | 0.000E+00 | 0.000E+00 | 4.372E-07 | 0.000E+00 |
| Unigene163743 | lsaC | 0.000E+00 | 0.000E+00 | 9.691E-07 | 0.000E+00 | 0.000E+00 | 0.000E+00 | 0.000E+00 | 0.000E+00 | 1.704E-07 | 0.000E+00 | 1.189E-07 | 9.831E-07 |
| Unigene163757 | vanHB | 0.000E+00 | 0.000E+00 | 8.875E-07 | 0.000E+00 | 6.930E-08 | 0.000E+00 | 0.000E+00 | 3.640E-08 | 1.364E-07 | 1.042E-07 | 7.135E-08 | 1.106E-06 |
| Unigene163758 | Acinetoba | 0.000E+00 | 1.936E-07 | 1.360E-06 | 0.000E+00 | 0.000E+00 | 0.000E+00 | 0.000E+00 | 0.000E+00 | 1.102E-07 | 0.000E+00 | 0.000E+00 | 1.132E-06 |
| Unigene163770 | eptA | 5.785E-08 | 0.000E+00 | 8.515E-07 | 3.895E-08 | 8.569E-08 | 0.000E+00 | 2.134E-08 | 0.000E+00 | 8.430E-08 | 6.444E-08 | 8.823E-08 | 1.527E-06 |
| Unigene163774 | macB | 0.000E+00 | 0.000E+00 | 1.212E-06 | 0.000E+00 | 0.000E+00 | 7.028E-08 | 0.000E+00 | 1.138E-07 | 1.777E-07 | 0.000E+00 | 1.116E-07 | 9.222E-07 |
| Unigene163808 | tetB(58) | 0.000E+00 | 5.094E-07 | 6.625E-07 | 2.983E-07 | 0.000E+00 | 0.000E+00 | 4.670E-08 | 0.000E+00 | 1.384E-07 | 0.000E+00 | 0.000E+00 | 0.000E+00 |
| Unigene163884 | bcrA | 8.910E-07 | 5.265E-07 | 5.044E-07 | 4.741E-06 | 0.000E+00 | 2.149E-06 | 8.609E-06 | 5.520E-06 | 1.161E-06 | 2.405E-06 | 6.037E-06 | 3.240E-07 |
| Unigene163899 | macB | 1.494E-07 | 2.343E-07 | 1.760E-06 | 1.561E-05 | 0.000E+00 | 0.000E+00 | 0.000E+00 | 0.000E+00 | 0.000E+00 | 0.000E+00 | 0.000E+00 | 0.000E+00 |
| Unigene163905 | patB | 3.802E-08 | 1.669E-07 | 2.127E-06 | 1.298E-05 | 0.000E+00 | 0.000E+00 | 0.000E+00 | 0.000E+00 | 0.000E+00 | 0.000E+00 | 0.000E+00 | 0.000E+00 |
| Unigene163912 | oleB | 0.000E+00 | 1.239E-07 | 2.326E-06 | 1.550E-05 | 0.000E+00 | 0.000E+00 | 0.000E+00 | 0.000E+00 | 0.000E+00 | 0.000E+00 | 0.000E+00 | 0.000E+00 |
| Unigene163913 | tlrC | 0.000E+00 | 2.435E-07 | 2.649E-06 | 1.195E-05 | 0.000E+00 | 0.000E+00 | 0.000E+00 | 0.000E+00 | 0.000E+00 | 0.000E+00 | 0.000E+00 | 0.000E+00 |
| Unigene163918 | vanTG | 0.000E+00 | 1.303E-07 | 2.121E-06 | 1.547E-05 | 0.000E+00 | 0.000E+00 | 0.000E+00 | 0.000E+00 | 0.000E+00 | 0.000E+00 | 0.000E+00 | 0.000E+00 |
| Unigene164013 | arlS | 8.237E-07 | 5.537E-06 | 5.557E-07 | 7.798E-08 | 4.290E-07 | 1.948E-07 | 5.698E-08 | 2.103E-07 | 3.376E-07 | 2.007E-07 | 0.000E+00 | 1.826E-07 |
| Unigene164041 | golS | 0.000E+00 | 0.000E+00 | 1.338E-06 | 0.000E+00 | 5.051E-07 | 0.000E+00 | 0.000E+00 | 0.000E+00 | 8.282E-08 | 0.000E+00 | 0.000E+00 | 1.523E-06 |
| Unigene164044 | arnA | 0.000E+00 | 2.242E-07 | 1.069E-06 | 0.000E+00 | 2.270E-07 | 7.362E-08 | 0.000E+00 | 3.975E-08 | 1.489E-07 | 0.000E+00 | 7.790E-08 | 1.409E-06 |
| Unigene164065 | evgS | 0.000E+00 | 6.344E-07 | 3.927E-07 | 8.980E-08 | 0.000E+00 | 0.000E+00 | 0.000E+00 | 0.000E+00 | 0.000E+00 | 0.000E+00 | 0.000E+00 | 0.000E+00 |
| Unigene164078 | vanHB | 6.622E-07 | 7.267E-08 | 9.746E-07 | 2.273E-06 | 1.471E-07 | 0.000E+00 | 4.763E-07 | 1.198E-06 | 0.000E+00 | 5.163E-07 | 3.408E-07 | 0.000E+00 |
| Unigene164094 | vanG | 0.000E+00 | 0.000E+00 | 1.180E-06 | 0.000E+00 | 1.303E-07 | 0.000E+00 | 0.000E+00 | 0.000E+00 | 1.602E-07 | 0.000E+00 | 0.000E+00 | 1.352E-06 |
| Unigene164123 | Staphylocc | 3.126E-07 | 5.806E-07 | 1.085E-06 | 2.902E-06 | 4.808E-07 | 2.859E-07 | 2.528E-07 | 1.137E-06 | 9.198E-08 | 8.571E-07 | 9.902E-07 | 5.542E-07 |

| | | | | | | | | | | | | | |
|---|---|---|---|---|---|---|---|---|---|---|---|---|---|
| Unigene164131 | msbA | 3.413E-07 | 5.323E-07 | 1.322E-06 | 2.467E-06 | 4.590E-07 | 0.000E+00 | 2.783E-07 | 1.405E-06 | 9.817E-08 | 5.603E-07 | 1.089E-06 | 7.432E-07 |
| Unigene164132 | macB | 2.759E-07 | 7.125E-07 | 1.210E-06 | 3.311E-06 | 3.065E-07 | 2.983E-07 | 1.257E-07 | 1.250E-06 | 0.000E+00 | 9.763E-07 | 8.911E-07 | 1.113E-06 |
| Unigene164143 | vanSA | 3.480E-07 | 6.048E-07 | 1.167E-06 | 2.284E-06 | 9.022E-07 | 1.254E-07 | 4.815E-07 | 9.479E-07 | 0.000E+00 | 3.877E-07 | 1.062E-06 | 8.913E-07 |
| Unigene164152 | efrA | 3.775E-07 | 5.920E-07 | 7.145E-07 | 2.505E-06 | 3.895E-07 | 4.373E-07 | 0.000E+00 | 1.354E-06 | 0.000E+00 | 6.609E-07 | 6.170E-07 | 6.375E-07 |
| Unigene164162 | Listeria mc | 2.905E-07 | 7.069E-07 | 1.462E-06 | 2.895E-06 | 4.630E-07 | 1.638E-07 | 2.935E-07 | 1.268E-06 | 1.518E-07 | 7.878E-07 | 1.141E-06 | 8.210E-07 |
| Unigene164166 | patA | 2.812E-07 | 8.230E-07 | 1.048E-06 | 2.669E-06 | 4.165E-07 | 0.000E+00 | 3.734E-07 | 1.182E-06 | 0.000E+00 | 3.968E-07 | 9.649E-07 | 9.306E-07 |
| Unigene164167 | lmrD | 2.536E-07 | 4.175E-07 | 1.742E-06 | 2.927E-06 | 2.616E-07 | 2.154E-07 | 2.606E-07 | 1.776E-06 | 5.940E-08 | 5.448E-07 | 1.533E-06 | 7.922E-07 |
| Unigene164227 | macB | 0.000E+00 | 0.000E+00 | 6.520E-07 | 0.000E+00 | 4.763E-08 | 2.317E-08 | 2.372E-08 | 0.000E+00 | 7.028E-08 | 9.551E-08 | 9.807E-08 | 7.094E-07 |
| Unigene164232 | emrB | 0.000E+00 | 0.000E+00 | 1.037E-06 | 0.000E+00 | 7.830E-08 | 0.000E+00 | 2.600E-08 | 0.000E+00 | 1.541E-07 | 0.000E+00 | 6.719E-08 | 1.166E-06 |
| Unigene164312 | macB | 0.000E+00 | 0.000E+00 | 8.019E-07 | 0.000E+00 | 0.000E+00 | 0.000E+00 | 0.000E+00 | 1.272E-06 | 0.000E+00 | 0.000E+00 | 0.000E+00 | 3.596E-06 |
| Unigene164329 | vgaD | 4.493E-07 | 6.229E-07 | 1.091E-06 | 3.152E-06 | 0.000E+00 | 1.790E-07 | 3.402E-07 | 1.905E-06 | 7.754E-08 | 6.323E-07 | 1.326E-06 | 4.752E-07 |
| Unigene164363 | vanTE | 0.000E+00 | 0.000E+00 | 6.992E-07 | 5.709E-06 | 0.000E+00 | 0.000E+00 | 0.000E+00 | 0.000E+00 | 0.000E+00 | 0.000E+00 | 0.000E+00 | 0.000E+00 |
| Unigene164374 | vanSA | 0.000E+00 | 0.000E+00 | 2.262E-06 | 0.000E+00 | 0.000E+00 | 0.000E+00 | 0.000E+00 | 0.000E+00 | 0.000E+00 | 0.000E+00 | 0.000E+00 | 0.000E+00 |
| Unigene164456 | macA | 0.000E+00 | 0.000E+00 | 1.306E-06 | 1.168E-07 | 0.000E+00 | 3.127E-08 | 0.000E+00 | 0.000E+00 | 1.265E-07 | 9.667E-08 | 0.000E+00 | 9.231E-07 |
| Unigene164457 | macB | 4.974E-08 | 1.092E-07 | 1.025E-06 | 8.371E-08 | 9.210E-08 | 0.000E+00 | 0.000E+00 | 0.000E+00 | 1.268E-07 | 0.000E+00 | 0.000E+00 | 9.603E-07 |
| Unigene164462 | msbA | 0.000E+00 | 0.000E+00 | 9.197E-07 | 0.000E+00 | 4.165E-08 | 0.000E+00 | 0.000E+00 | 2.188E-08 | 1.434E-07 | 0.000E+00 | 6.433E-08 | 1.174E-06 |
| Unigene164463 | msbA | 3.654E-08 | 0.000E+00 | 1.004E-06 | 3.690E-08 | 1.015E-07 | 0.000E+00 | 4.044E-08 | 6.397E-08 | 1.198E-07 | 6.105E-08 | 8.359E-08 | 1.663E-06 |
| Unigene164638 | tlrC | 0.000E+00 | 0.000E+00 | 3.564E-07 | 6.419E-07 | 5.296E-06 | 1.001E-05 | 0.000E+00 | 8.214E-06 | 0.000E+00 | 3.995E-06 | 0.000E+00 | 0.000E+00 |
| Unigene164815 | tetB(58) | 0.000E+00 | 0.000E+00 | 7.246E-07 | 0.000E+00 | 0.000E+00 | 0.000E+00 | 0.000E+00 | 0.000E+00 | 0.000E+00 | 0.000E+00 | 0.000E+00 | 0.000E+00 |
| Unigene164945 | mecC | 0.000E+00 | 0.000E+00 | 1.666E-06 | 6.390E-06 | 0.000E+00 | 0.000E+00 | 0.000E+00 | 0.000E+00 | 0.000E+00 | 0.000E+00 | 0.000E+00 | 0.000E+00 |
| Unigene164988 | Staphylocc | 2.543E-07 | 4.947E-07 | 1.157E-06 | 2.649E-06 | 6.548E-07 | 0.000E+00 | 1.663E-07 | 1.133E-06 | 1.137E-07 | 7.081E-07 | 8.990E-07 | 8.470E-07 |
| Unigene165099 | Klebsiella p | 0.000E+00 | 0.000E+00 | 3.486E-07 | 0.000E+00 | 0.000E+00 | 0.000E+00 | 0.000E+00 | 0.000E+00 | 0.000E+00 | 0.000E+00 | 0.000E+00 | 0.000E+00 |
| Unigene165166 | arlS | 8.805E-07 | 3.093E-06 | 3.059E-06 | 3.112E-06 | 0.000E+00 | 0.000E+00 | 0.000E+00 | 0.000E+00 | 0.000E+00 | 0.000E+00 | 0.000E+00 | 0.000E+00 |
| Unigene165286 | vanRO | 6.987E-08 | 6.646E-07 | 3.839E-06 | 3.575E-06 | 0.000E+00 | 0.000E+00 | 0.000E+00 | 0.000E+00 | 0.000E+00 | 0.000E+00 | 0.000E+00 | 0.000E+00 |
| Unigene165296 | Klebsiella p | 0.000E+00 | 0.000E+00 | 3.199E-07 | 0.000E+00 | 0.000E+00 | 0.000E+00 | 7.217E-07 | 0.000E+00 | 0.000E+00 | 0.000E+00 | 0.000E+00 | 1.606E-06 |
| Unigene165414 | vanF | 0.000E+00 | 0.000E+00 | 6.162E-07 | 0.000E+00 | 0.000E+00 | 0.000E+00 | 0.000E+00 | 0.000E+00 | 0.000E+00 | 0.000E+00 | 0.000E+00 | 0.000E+00 |
| Unigene165524 | bacA | 0.000E+00 | 4.758E-07 | 1.547E-06 | 0.000E+00 | 0.000E+00 | 0.000E+00 | 0.000E+00 | 0.000E+00 | 0.000E+00 | 0.000E+00 | 0.000E+00 | 1.118E-06 |
| Unigene165537 | TolC | 6.508E-08 | 0.000E+00 | 1.128E-06 | 4.381E-08 | 2.410E-08 | 0.000E+00 | 9.604E-08 | 1.266E-07 | 0.000E+00 | 0.000E+00 | 4.963E-08 | 1.154E-06 |
| Unigene165672 | carA | 0.000E+00 | 0.000E+00 | 1.614E-06 | 0.000E+00 | 0.000E+00 | 0.000E+00 | 0.000E+00 | 0.000E+00 | 0.000E+00 | 0.000E+00 | 0.000E+00 | 0.000E+00 |
| Unigene165678 | Acinetoba | 0.000E+00 | 0.000E+00 | 5.346E-07 | 0.000E+00 | 0.000E+00 | 0.000E+00 | 0.000E+00 | 0.000E+00 | 0.000E+00 | 0.000E+00 | 0.000E+00 | 0.000E+00 |
| Unigene165769 | adeL | 0.000E+00 | 0.000E+00 | 8.798E-07 | 0.000E+00 | 1.116E-06 | 2.326E-07 | 1.985E-06 | 9.629E-07 | 3.136E-07 | 0.000E+00 | 0.000E+00 | 4.409E-06 |
| Unigene165770 | adeL | 0.000E+00 | 0.000E+00 | 8.095E-07 | 0.000E+00 | 0.000E+00 | 0.000E+00 | 0.000E+00 | 0.000E+00 | 0.000E+00 | 0.000E+00 | 1.231E-06 | 0.000E+00 |
| Unigene165845 | vanE | 9.466E-08 | 6.926E-08 | 6.192E-07 | 2.708E-07 | 0.000E+00 | 0.000E+00 | 6.985E-08 | 0.000E+00 | 0.000E+00 | 0.000E+00 | 0.000E+00 | 0.000E+00 |
| Unigene165877 | oleC | 0.000E+00 | 0.000E+00 | 0.000E+00 | 0.000E+00 | 0.000E+00 | 0.000E+00 | 4.281E-06 | 0.000E+00 | 0.000E+00 | 0.000E+00 | 1.327E-06 | 0.000E+00 |
| Unigene165969 | MexD | 0.000E+00 | 0.000E+00 | 1.940E-07 | 0.000E+00 | 0.000E+00 | 2.442E-07 | 5.939E-07 | 3.297E-07 | 0.000E+00 | 5.978E-07 | 0.000E+00 | 1.102E-06 |
| Unigene165970 | AcrS | 4.139E-07 | 0.000E+00 | 0.000E+00 | 0.000E+00 | 1.609E-06 | 0.000E+00 | 0.000E+00 | 1.268E-06 | 4.523E-07 | 6.915E-07 | 1.243E-06 | 2.874E-06 |
| Unigene165971 | macB | 3.813E-07 | 0.000E+00 | 8.106E-07 | 4.920E-07 | 1.200E-06 | 4.122E-07 | 1.102E-06 | 1.187E-06 | 3.704E-07 | 1.038E-06 | 9.692E-07 | 3.655E-06 |
| Unigene166091 | msbA | 9.557E-05 | 1.744E-04 | 1.890E-04 | 8.321E-06 | 9.813E-06 | 2.884E-07 | 0.000E+00 | 1.834E-06 | 5.715E-05 | 0.000E+00 | 2.679E-06 | 0.000E+00 |
| Unigene166278 | tetA(58) | 6.622E-08 | 6.177E-07 | 3.574E-06 | 4.447E-06 | 0.000E+00 | 0.000E+00 | 0.000E+00 | 0.000E+00 | 0.000E+00 | 0.000E+00 | 0.000E+00 | 0.000E+00 |
| Unigene166293 | vanZF | 5.076E-08 | 0.000E+00 | 8.964E-07 | 4.100E-07 | 0.000E+00 | 0.000E+00 | 0.000E+00 | 0.000E+00 | 0.000E+00 | 0.000E+00 | 0.000E+00 | 0.000E+00 |
| Unigene166299 | YojI | 0.000E+00 | 8.527E-08 | 1.448E-06 | 2.746E-07 | 0.000E+00 | 0.000E+00 | 0.000E+00 | 0.000E+00 | 0.000E+00 | 0.000E+00 | 0.000E+00 | 0.000E+00 |
| Unigene166341 | macB | 1.803E-07 | 1.319E-07 | 7.668E-07 | 2.428E-07 | 0.000E+00 | 0.000E+00 | 0.000E+00 | 1.403E-07 | 0.000E+00 | 0.000E+00 | 0.000E+00 | 0.000E+00 |
| Unigene166350 | lsaA | 4.261E-08 | 1.871E-07 | 1.192E-06 | 1.291E-07 | 0.000E+00 | 0.000E+00 | 0.000E+00 | 0.000E+00 | 0.000E+00 | 0.000E+00 | 0.000E+00 | 0.000E+00 |
| Unigene166523 | msbA | 0.000E+00 | 0.000E+00 | 3.979E-07 | 3.549E-07 | 4.505E-07 | 5.845E-07 | 0.000E+00 | 1.988E-06 | 0.000E+00 | 0.000E+00 | 0.000E+00 | 2.109E-06 |
| Unigene166577 | Klebsiella p | 0.000E+00 | 0.000E+00 | 2.640E-06 | 0.000E+00 | 1.067E-07 | 0.000E+00 | 0.000E+00 | 0.000E+00 | 0.000E+00 | 0.000E+00 | 0.000E+00 | 2.385E-06 |
| Unigene166682 | novA | 0.000E+00 | 0.000E+00 | 1.207E-07 | 0.000E+00 | 0.000E+00 | 0.000E+00 | 0.000E+00 | 0.000E+00 | 0.000E+00 | 0.000E+00 | 0.000E+00 | 0.000E+00 |
| Unigene166754 | macB | 7.924E-07 | 8.239E-07 | 1.678E-06 | 5.475E-07 | 0.000E+00 | 0.000E+00 | 0.000E+00 | 0.000E+00 | 0.000E+00 | 0.000E+00 | 0.000E+00 | 0.000E+00 |
| Unigene166756 | tetA(58) | 2.786E-07 | 3.440E-07 | 1.333E-06 | 7.033E-07 | 0.000E+00 | 0.000E+00 | 0.000E+00 | 0.000E+00 | 0.000E+00 | 0.000E+00 | 0.000E+00 | 0.000E+00 |
| Unigene166794 | vanYB | 0.000E+00 | 1.327E-07 | 1.463E-06 | 3.663E-07 | 0.000E+00 | 0.000E+00 | 0.000E+00 | 0.000E+00 | 0.000E+00 | 0.000E+00 | 0.000E+00 | 0.000E+00 |
| Unigene166809 | arnA | 0.000E+00 | 6.119E-08 | 6.018E-07 | 1.689E-07 | 0.000E+00 | 0.000E+00 | 0.000E+00 | 0.000E+00 | 0.000E+00 | 0.000E+00 | 0.000E+00 | 0.000E+00 |
| Unigene166854 | msbA | 0.000E+00 | 0.000E+00 | 0.000E+00 | 4.318E-07 | 0.000E+00 | 0.000E+00 | 0.000E+00 | 0.000E+00 | 0.000E+00 | 0.000E+00 | 0.000E+00 | 0.000E+00 |
| Unigene166866 | adeL | 0.000E+00 | 0.000E+00 | 1.307E-06 | 0.000E+00 | 0.000E+00 | 0.000E+00 | 0.000E+00 | 0.000E+00 | 0.000E+00 | 0.000E+00 | 0.000E+00 | 0.000E+00 |
| Unigene166931 | macB | 5.475E-06 | 1.108E-05 | 9.530E-06 | 1.077E-05 | 0.000E+00 | 0.000E+00 | 0.000E+00 | 0.000E+00 | 0.000E+00 | 0.000E+00 | 0.000E+00 | 0.000E+00 |
| Unigene166956 | bcrA | 0.000E+00 | 0.000E+00 | 8.584E-07 | 2.208E-07 | 0.000E+00 | 0.000E+00 | 0.000E+00 | 0.000E+00 | 0.000E+00 | 0.000E+00 | 0.000E+00 | 0.000E+00 |
| Unigene167099 | bcrA | 0.000E+00 | 3.398E-07 | 1.443E-06 | 3.987E-06 | 0.000E+00 | 0.000E+00 | 0.000E+00 | 0.000E+00 | 0.000E+00 | 0.000E+00 | 0.000E+00 | 0.000E+00 |
| Unigene167122 | Staphylocc | 1.601E-08 | 0.000E+00 | 9.584E-07 | 6.754E-05 | 0.000E+00 | 5.193E-08 | 0.000E+00 | 1.308E-07 | 0.000E+00 | 0.000E+00 | 0.000E+00 | 0.000E+00 |
| Unigene167129 | mtrA | 3.251E-06 | 0.000E+00 | 2.965E-06 | 4.161E-07 | 3.052E-07 | 1.980E-07 | 0.000E+00 | 1.603E-07 | 0.000E+00 | 0.000E+00 | 1.152E-06 | 0.000E+00 |
| Unigene167167 | patA | 2.558E-05 | 9.343E-06 | 7.242E-06 | 4.409E-06 | 2.232E-07 | 1.382E-07 | 1.011E-07 | 1.493E-07 | 7.787E-07 | 0.000E+00 | 0.000E+00 | 0.000E+00 |

| | | | | | | | | | | | | | |
|---|---|---|---|---|---|---|---|---|---|---|---|---|---|
| Unigene167184 | Streptomy | 0.000E+00 | 0.000E+00 | 1.006E-06 | 0.000E+00 | 0.000E+00 | 0.000E+00 | 0.000E+00 | 0.000E+00 | 0.000E+00 | 0.000E+00 | 0.000E+00 | 0.000E+00 |
| Unigene167199 | optrA | 0.000E+00 | 0.000E+00 | 1.068E-06 | 0.000E+00 | 0.000E+00 | 0.000E+00 | 0.000E+00 | 0.000E+00 | 0.000E+00 | 0.000E+00 | 0.000E+00 | 0.000E+00 |
| Unigene167289 | bcrA | 0.000E+00 | 0.000E+00 | 5.467E-07 | 1.758E-07 | 0.000E+00 | 0.000E+00 | 0.000E+00 | 0.000E+00 | 0.000E+00 | 0.000E+00 | 1.593E-07 | 0.000E+00 |
| Unigene167303 | ugd | 1.910E-07 | 0.000E+00 | 4.061E-07 | 0.000E+00 | 3.537E-08 | 0.000E+00 | 0.000E+00 | 0.000E+00 | 0.000E+00 | 0.000E+00 | 0.000E+00 | 0.000E+00 |
| Unigene167309 | vanHA | 6.920E-08 | 4.936E-07 | 5.092E-07 | 6.988E-08 | 0.000E+00 | 0.000E+00 | 7.658E-08 | 0.000E+00 | 0.000E+00 | 0.000E+00 | 0.000E+00 | 0.000E+00 |
| Unigene167375 | vanL | 0.000E+00 | 7.009E-08 | 2.068E-06 | 6.577E-06 | 0.000E+00 | 0.000E+00 | 0.000E+00 | 0.000E+00 | 0.000E+00 | 0.000E+00 | 0.000E+00 | 0.000E+00 |
| Unigene167392 | vanSL | 6.300E-08 | 8.574E-07 | 3.140E-06 | 3.753E-06 | 0.000E+00 | 0.000E+00 | 0.000E+00 | 0.000E+00 | 0.000E+00 | 0.000E+00 | 0.000E+00 | 0.000E+00 |
| Unigene167394 | baeS | 0.000E+00 | 8.279E-07 | 2.656E-06 | 3.226E-06 | 0.000E+00 | 0.000E+00 | 0.000E+00 | 0.000E+00 | 0.000E+00 | 0.000E+00 | 0.000E+00 | 0.000E+00 |
| Unigene167470 | tetA(58) | 1.145E-07 | 0.000E+00 | 1.984E-06 | 7.090E-06 | 0.000E+00 | 0.000E+00 | 0.000E+00 | 0.000E+00 | 0.000E+00 | 0.000E+00 | 0.000E+00 | 0.000E+00 |
| Unigene167476 | tetB(P) | 0.000E+00 | 2.453E-07 | 1.379E-06 | 7.029E-06 | 0.000E+00 | 0.000E+00 | 0.000E+00 | 0.000E+00 | 0.000E+00 | 0.000E+00 | 0.000E+00 | 0.000E+00 |
| Unigene167480 | dfrA3 | 4.825E-08 | 0.000E+00 | 2.746E-06 | 7.552E-06 | 0.000E+00 | 0.000E+00 | 0.000E+00 | 0.000E+00 | 0.000E+00 | 0.000E+00 | 0.000E+00 | 0.000E+00 |
| Unigene167522 | oleC | 1.729E-07 | 5.691E-07 | 1.145E-06 | 3.229E-06 | 0.000E+00 | 0.000E+00 | 5.261E-07 | 1.614E-06 | 4.723E-08 | 6.739E-07 | 1.384E-06 | 1.022E-06 |
| Unigene167612 | lmrD | 1.145E-07 | 0.000E+00 | 3.679E-06 | 1.743E-05 | 0.000E+00 | 0.000E+00 | 0.000E+00 | 0.000E+00 | 0.000E+00 | 0.000E+00 | 0.000E+00 | 0.000E+00 |
| Unigene167623 | optrA | 1.437E-07 | 7.888E-07 | 3.204E-06 | 3.898E-06 | 0.000E+00 | 0.000E+00 | 0.000E+00 | 0.000E+00 | 0.000E+00 | 0.000E+00 | 0.000E+00 | 0.000E+00 |
| Unigene167702 | TaeA | 0.000E+00 | 1.638E-07 | 1.611E-06 | 6.764E-06 | 0.000E+00 | 0.000E+00 | 0.000E+00 | 0.000E+00 | 0.000E+00 | 0.000E+00 | 0.000E+00 | 0.000E+00 |
| Unigene167719 | lmrB | 2.299E-07 | 1.514E-07 | 1.421E-06 | 6.617E-06 | 0.000E+00 | 0.000E+00 | 0.000E+00 | 0.000E+00 | 0.000E+00 | 0.000E+00 | 0.000E+00 | 0.000E+00 |
| Unigene167757 | Rhodococ | 0.000E+00 | 0.000E+00 | 7.799E-07 | 0.000E+00 | 0.000E+00 | 0.000E+00 | 0.000E+00 | 0.000E+00 | 0.000E+00 | 0.000E+00 | 0.000E+00 | 0.000E+00 |
| Unigene167781 | sul4 | 0.000E+00 | 0.000E+00 | 1.428E-06 | 0.000E+00 | 0.000E+00 | 0.000E+00 | 0.000E+00 | 0.000E+00 | 0.000E+00 | 0.000E+00 | 0.000E+00 | 0.000E+00 |
| Unigene167807 | patB | 1.907E-06 | 4.858E-06 | 6.901E-06 | 8.392E-06 | 0.000E+00 | 0.000E+00 | 0.000E+00 | 0.000E+00 | 0.000E+00 | 0.000E+00 | 0.000E+00 | 0.000E+00 |
| Unigene167884 | Escherichia | 0.000E+00 | 0.000E+00 | 8.255E-06 | 0.000E+00 | 0.000E+00 | 0.000E+00 | 0.000E+00 | 0.000E+00 | 0.000E+00 | 0.000E+00 | 0.000E+00 | 0.000E+00 |
| Unigene168027 | smeR | 0.000E+00 | 1.181E-06 | 0.000E+00 | 0.000E+00 | 7.744E-06 | 5.563E-07 | 0.000E+00 | 0.000E+00 | 0.000E+00 | 0.000E+00 | 0.000E+00 | 0.000E+00 |
| Unigene168037 | rosB | 0.000E+00 | 0.000E+00 | 9.822E-07 | 3.610E-08 | 5.957E-08 | 0.000E+00 | 0.000E+00 | 1.043E-07 | 1.954E-08 | 5.973E-08 | 4.089E-08 | 1.352E-06 |
| Unigene168039 | optrA | 1.864E-07 | 5.579E-08 | 1.463E-06 | 1.369E-07 | 9.413E-08 | 0.000E+00 | 0.000E+00 | 0.000E+00 | 1.111E-07 | 0.000E+00 | 7.753E-08 | 9.614E-07 |
| Unigene168090 | vanRF | 1.839E-07 | 0.000E+00 | 4.512E-07 | 0.000E+00 | 0.000E+00 | 0.000E+00 | 0.000E+00 | 0.000E+00 | 0.000E+00 | 0.000E+00 | 0.000E+00 | 0.000E+00 |
| Unigene168093 | adeN | 0.000E+00 | 7.690E-07 | 3.143E-06 | 3.639E-06 | 0.000E+00 | 0.000E+00 | 0.000E+00 | 0.000E+00 | 0.000E+00 | 0.000E+00 | 0.000E+00 | 0.000E+00 |
| Unigene168139 | Corynebac | 0.000E+00 | 1.776E-07 | 1.720E-06 | 3.268E-07 | 0.000E+00 | 0.000E+00 | 0.000E+00 | 0.000E+00 | 0.000E+00 | 0.000E+00 | 0.000E+00 | 0.000E+00 |
| Unigene168148 | vanTG | 8.654E-08 | 3.166E-08 | 1.217E-06 | 1.457E-07 | 0.000E+00 | 0.000E+00 | 3.193E-08 | 0.000E+00 | 0.000E+00 | 0.000E+00 | 0.000E+00 | 0.000E+00 |
| Unigene168153 | vanL | 6.622E-08 | 0.000E+00 | 1.494E-06 | 1.337E-07 | 0.000E+00 | 0.000E+00 | 0.000E+00 | 0.000E+00 | 0.000E+00 | 0.000E+00 | 0.000E+00 | 0.000E+00 |
| Unigene168193 | arlS | 9.093E-08 | 1.996E-07 | 9.517E-07 | 1.530E-07 | 0.000E+00 | 0.000E+00 | 0.000E+00 | 0.000E+00 | 0.000E+00 | 0.000E+00 | 0.000E+00 | 0.000E+00 |
| Unigene168194 | mtrA | 0.000E+00 | 0.000E+00 | 7.070E-07 | 1.455E-07 | 0.000E+00 | 0.000E+00 | 0.000E+00 | 0.000E+00 | 0.000E+00 | 0.000E+00 | 0.000E+00 | 0.000E+00 |
| Unigene168195 | macB | 1.508E-07 | 1.204E-07 | 1.036E-06 | 2.215E-07 | 0.000E+00 | 0.000E+00 | 0.000E+00 | 0.000E+00 | 0.000E+00 | 0.000E+00 | 0.000E+00 | 0.000E+00 |
| Unigene168200 | Staphylocc | 1.315E-07 | 1.443E-07 | 9.246E-07 | 2.213E-07 | 0.000E+00 | 0.000E+00 | 0.000E+00 | 0.000E+00 | 0.000E+00 | 0.000E+00 | 0.000E+00 | 0.000E+00 |
| Unigene168229 | MexW | 0.000E+00 | 0.000E+00 | 3.500E-07 | 0.000E+00 | 0.000E+00 | 0.000E+00 | 0.000E+00 | 0.000E+00 | 0.000E+00 | 0.000E+00 | 0.000E+00 | 0.000E+00 |
| Unigene168308 | basS | 6.548E-08 | 1.198E-07 | 1.885E-06 | 6.612E-08 | 1.212E-07 | 2.359E-08 | 0.000E+00 | 2.548E-08 | 1.908E-07 | 0.000E+00 | 4.993E-08 | 1.109E-06 |
| Unigene168309 | arlR | 0.000E+00 | 0.000E+00 | 2.279E-06 | 4.787E-08 | 0.000E+00 | 0.000E+00 | 0.000E+00 | 0.000E+00 | 1.554E-07 | 1.056E-07 | 2.169E-07 | 1.625E-06 |
| Unigene168310 | OprA | 0.000E+00 | 2.045E-07 | 1.874E-06 | 2.352E-08 | 1.294E-07 | 0.000E+00 | 0.000E+00 | 1.359E-07 | 2.800E-07 | 1.297E-07 | 2.664E-08 | 1.734E-06 |
| Unigene168322 | arlR | 0.000E+00 | 1.040E-07 | 1.302E-06 | 0.000E+00 | 1.580E-07 | 0.000E+00 | 0.000E+00 | 0.000E+00 | 1.036E-07 | 0.000E+00 | 5.422E-08 | 1.401E-06 |
| Unigene168323 | vanSC | 4.608E-08 | 2.529E-08 | 1.515E-06 | 4.653E-08 | 0.000E+00 | 0.000E+00 | 2.550E-08 | 2.689E-08 | 1.511E-07 | 0.000E+00 | 2.372E-07 | 1.389E-06 |
| Unigene168328 | aadA2 | 0.000E+00 | 0.000E+00 | 2.000E-06 | 7.625E-08 | 1.258E-07 | 0.000E+00 | 0.000E+00 | 0.000E+00 | 1.651E-07 | 4.626E-07 | 2.591E-07 | 5.400E-06 |
| Unigene168329 | sul1 | 1.921E-07 | 0.000E+00 | 2.338E-06 | 0.000E+00 | 8.539E-08 | 4.154E-08 | 0.000E+00 | 8.971E-08 | 2.100E-07 | 2.140E-07 | 4.835E-07 | 5.269E-06 |
| Unigene168332 | lfrA | 2.348E-07 | 1.406E-07 | 1.802E-06 | 1.940E-07 | 3.558E-07 | 3.924E-07 | 6.616E-07 | 5.981E-07 | 2.800E-07 | 7.135E-07 | 1.099E-06 | 3.407E-06 |
| Unigene168487 | evgA | 0.000E+00 | 0.000E+00 | 2.186E-06 | 1.460E-05 | 0.000E+00 | 0.000E+00 | 0.000E+00 | 0.000E+00 | 0.000E+00 | 0.000E+00 | 0.000E+00 | 0.000E+00 |
| Unigene168493 | bcrA | 0.000E+00 | 2.187E-07 | 2.118E-06 | 1.234E-05 | 0.000E+00 | 0.000E+00 | 0.000E+00 | 0.000E+00 | 0.000E+00 | 0.000E+00 | 0.000E+00 | 0.000E+00 |
| Unigene168512 | APH(6)-Id | 0.000E+00 | 0.000E+00 | 2.237E-07 | 0.000E+00 | 0.000E+00 | 0.000E+00 | 0.000E+00 | 0.000E+00 | 0.000E+00 | 0.000E+00 | 0.000E+00 | 2.156E-07 |
| Unigene168727 | PmrF | 0.000E+00 | 1.737E-07 | 0.000E+00 | 0.000E+00 | 0.000E+00 | 0.000E+00 | 0.000E+00 | 0.000E+00 | 0.000E+00 | 0.000E+00 | 0.000E+00 | 0.000E+00 |
| Unigene168813 | macB | 0.000E+00 | 3.533E-07 | 6.768E-07 | 9.751E-07 | 0.000E+00 | 0.000E+00 | 0.000E+00 | 0.000E+00 | 0.000E+00 | 0.000E+00 | 0.000E+00 | 0.000E+00 |
| Unigene168852 | LRA-13 | 0.000E+00 | 0.000E+00 | 3.199E-07 | 0.000E+00 | 1.811E-07 | 0.000E+00 | 9.022E-07 | 0.000E+00 | 0.000E+00 | 0.000E+00 | 6.216E-08 | 4.497E-07 |
| Unigene168933 | bcrA | 0.000E+00 | 0.000E+00 | 5.960E-07 | 3.067E-07 | 0.000E+00 | 0.000E+00 | 0.000E+00 | 0.000E+00 | 0.000E+00 | 0.000E+00 | 0.000E+00 | 0.000E+00 |
| Unigene168958 | tetA(60) | 0.000E+00 | 2.228E-08 | 1.335E-06 | 1.230E-07 | 9.022E-08 | 4.389E-08 | 6.741E-08 | 0.000E+00 | 4.438E-08 | 2.262E-07 | 9.289E-08 | 1.200E-06 |
| Unigene169038 | Staphylocc | 6.161E-07 | 6.617E-06 | 6.175E-06 | 6.222E-06 | 0.000E+00 | 0.000E+00 | 0.000E+00 | 0.000E+00 | 0.000E+00 | 0.000E+00 | 0.000E+00 | 0.000E+00 |
| Unigene169043 | tetA(58) | 0.000E+00 | 0.000E+00 | 4.975E-07 | 1.707E-07 | 0.000E+00 | 0.000E+00 | 0.000E+00 | 0.000E+00 | 0.000E+00 | 0.000E+00 | 0.000E+00 | 0.000E+00 |
| Unigene169127 | tetB(P) | 7.053E-05 | 5.750E-05 | 1.147E-04 | 1.627E-04 | 1.244E-04 | 8.764E-05 | 1.151E-04 | 1.142E-04 | 9.625E-05 | 1.389E-04 | 1.401E-04 | 1.439E-04 |
| Unigene169224 | smeS | 2.321E-08 | 0.000E+00 | 1.122E-06 | 2.930E-08 | 9.027E-08 | 0.000E+00 | 1.927E-08 | 4.742E-08 | 1.078E-07 | 2.586E-08 | 4.647E-08 | 9.741E-07 |
| Unigene169256 | VatI | 0.000E+00 | 0.000E+00 | 1.402E-06 | 1.779E-06 | 0.000E+00 | 0.000E+00 | 0.000E+00 | 0.000E+00 | 0.000E+00 | 0.000E+00 | 0.000E+00 | 0.000E+00 |
| Unigene169409 | Klebsiella p | 0.000E+00 | 0.000E+00 | 1.253E-05 | 0.000E+00 | 0.000E+00 | 0.000E+00 | 0.000E+00 | 0.000E+00 | 0.000E+00 | 0.000E+00 | 0.000E+00 | 0.000E+00 |
| Unigene169417 | Escherichia | 0.000E+00 | 0.000E+00 | 1.492E-05 | 0.000E+00 | 0.000E+00 | 0.000E+00 | 0.000E+00 | 0.000E+00 | 0.000E+00 | 0.000E+00 | 0.000E+00 | 0.000E+00 |
| Unigene169432 | arlR | 0.000E+00 | 0.000E+00 | 1.330E-05 | 0.000E+00 | 0.000E+00 | 0.000E+00 | 0.000E+00 | 0.000E+00 | 0.000E+00 | 0.000E+00 | 0.000E+00 | 0.000E+00 |
| Unigene169433 | basS | 0.000E+00 | 0.000E+00 | 1.252E-05 | 0.000E+00 | 0.000E+00 | 8.400E-07 | 0.000E+00 | 0.000E+00 | 0.000E+00 | 0.000E+00 | 0.000E+00 | 0.000E+00 |

| | | | | | | | | | | | | | |
|---|---|---|---|---|---|---|---|---|---|---|---|---|---|
| Unigene169501 | ugd | 1.106E-07 | 1.822E-07 | 1.194E-06 | 3.073E-07 | 0.000E+00 | 0.000E+00 | 0.000E+00 | 0.000E+00 | 0.000E+00 | 0.000E+00 | 0.000E+00 | 0.000E+00 |
| Unigene169635 | vanZF | 0.000E+00 | 3.863E-07 | 1.233E-06 | 2.386E-06 | 1.117E-07 | 0.000E+00 | 0.000E+00 | 1.878E-06 | 0.000E+00 | 7.282E-07 | 9.778E-07 | 0.000E+00 |
| Unigene169639 | vanL | 4.270E-07 | 8.435E-07 | 1.229E-06 | 2.875E-06 | 2.846E-07 | 3.385E-07 | 1.575E-07 | 1.661E-06 | 1.867E-07 | 3.805E-07 | 1.107E-06 | 6.729E-07 |
| Unigene169662 | RlmA(II) | 0.000E+00 | 0.000E+00 | 9.499E-07 | 6.606E-05 | 0.000E+00 | 0.000E+00 | 0.000E+00 | 0.000E+00 | 0.000E+00 | 0.000E+00 | 0.000E+00 | 0.000E+00 |
| Unigene169851 | mel | 0.000E+00 | 0.000E+00 | 1.303E-07 | 0.000E+00 | 0.000E+00 | 0.000E+00 | 0.000E+00 | 0.000E+00 | 0.000E+00 | 0.000E+00 | 0.000E+00 | 0.000E+00 |
| Unigene169869 | vanSC | 1.266E-07 | 0.000E+00 | 3.105E-07 | 0.000E+00 | 0.000E+00 | 0.000E+00 | 0.000E+00 | 0.000E+00 | 0.000E+00 | 0.000E+00 | 0.000E+00 | 0.000E+00 |
| Unigene169903 | msbA | 0.000E+00 | 2.118E-07 | 3.409E-06 | 1.657E-05 | 0.000E+00 | 0.000E+00 | 0.000E+00 | 0.000E+00 | 0.000E+00 | 0.000E+00 | 0.000E+00 | 0.000E+00 |
| Unigene169916 | macB | 0.000E+00 | 0.000E+00 | 7.356E-07 | 5.255E-06 | 0.000E+00 | 0.000E+00 | 0.000E+00 | 0.000E+00 | 0.000E+00 | 0.000E+00 | 0.000E+00 | 0.000E+00 |
| Unigene170022 | Bifidobacte | 0.000E+00 | 0.000E+00 | 0.000E+00 | 0.000E+00 | 0.000E+00 | 0.000E+00 | 0.000E+00 | 0.000E+00 | 0.000E+00 | 0.000E+00 | 1.331E-07 | 0.000E+00 |
| Unigene170092 | bcrA | 0.000E+00 | 0.000E+00 | 4.479E-07 | 1.054E-06 | 0.000E+00 | 0.000E+00 | 0.000E+00 | 0.000E+00 | 0.000E+00 | 0.000E+00 | 0.000E+00 | 0.000E+00 |
| Unigene170169 | Streptomy | 0.000E+00 | 3.013E-07 | 2.693E-07 | 0.000E+00 | 0.000E+00 | 0.000E+00 | 0.000E+00 | 0.000E+00 | 0.000E+00 | 0.000E+00 | 0.000E+00 | 0.000E+00 |
| Unigene170269 | otr(B) | 0.000E+00 | 0.000E+00 | 3.559E-07 | 4.273E-07 | 0.000E+00 | 0.000E+00 | 0.000E+00 | 0.000E+00 | 0.000E+00 | 0.000E+00 | 0.000E+00 | 0.000E+00 |
| Unigene170325 | vanA | 0.000E+00 | 0.000E+00 | 6.178E-07 | 0.000E+00 | 0.000E+00 | 0.000E+00 | 0.000E+00 | 0.000E+00 | 0.000E+00 | 0.000E+00 | 0.000E+00 | 0.000E+00 |
| Unigene170450 | macB | 0.000E+00 | 0.000E+00 | 1.150E-06 | 0.000E+00 | 0.000E+00 | 0.000E+00 | 0.000E+00 | 0.000E+00 | 0.000E+00 | 0.000E+00 | 0.000E+00 | 0.000E+00 |
| Unigene170548 | vanSF | 0.000E+00 | 0.000E+00 | 8.798E-07 | 6.083E-05 | 0.000E+00 | 0.000E+00 | 0.000E+00 | 9.515E-08 | 0.000E+00 | 0.000E+00 | 0.000E+00 | 0.000E+00 |
| Unigene170570 | basS | 0.000E+00 | 0.000E+00 | 5.590E-07 | 0.000E+00 | 5.860E-07 | 8.667E-07 | 3.573E-06 | 2.807E-06 | 5.996E-07 | 9.166E-07 | 1.786E-06 | 2.968E-06 |
| Unigene170595 | rosB | 0.000E+00 | 0.000E+00 | 1.290E-06 | 5.241E-08 | 3.844E-08 | 1.870E-08 | 7.658E-08 | 6.058E-08 | 1.134E-07 | 1.156E-07 | 7.915E-08 | 1.186E-06 |
| Unigene170596 | dfrA3 | 0.000E+00 | 0.000E+00 | 1.386E-06 | 0.000E+00 | 0.000E+00 | 0.000E+00 | 0.000E+00 | 0.000E+00 | 0.000E+00 | 0.000E+00 | 0.000E+00 | 1.590E-06 |
| Unigene170625 | qacH | 0.000E+00 | 0.000E+00 | 0.000E+00 | 0.000E+00 | 1.314E-05 | 2.706E-05 | 0.000E+00 | 8.705E-07 | 2.329E-06 | 3.038E-05 | 0.000E+00 | 0.000E+00 |
| Unigene170695 | golS | 0.000E+00 | 0.000E+00 | 0.000E+00 | 0.000E+00 | 0.000E+00 | 0.000E+00 | 3.797E-06 | 0.000E+00 | 0.000E+00 | 0.000E+00 | 1.427E-06 | 0.000E+00 |
| Unigene170707 | bacA | 5.286E-07 | 7.044E-07 | 1.556E-06 | 2.402E-06 | 2.936E-07 | 0.000E+00 | 2.925E-07 | 1.454E-06 | 0.000E+00 | 4.626E-07 | 7.342E-07 | 8.033E-07 |
| Unigene170713 | poxtA | 2.989E-07 | 2.343E-07 | 1.089E-06 | 2.070E-06 | 0.000E+00 | 0.000E+00 | 4.253E-07 | 1.595E-06 | 0.000E+00 | 9.037E-07 | 7.815E-07 | 0.000E+00 |
| Unigene170715 | YojI | 3.110E-07 | 6.371E-07 | 1.587E-06 | 2.743E-06 | 0.000E+00 | 2.241E-07 | 3.671E-07 | 1.525E-06 | 0.000E+00 | 3.926E-07 | 1.043E-06 | 8.087E-07 |
| Unigene170747 | vanRI | 0.000E+00 | 5.135E-08 | 9.181E-07 | 8.031E-07 | 0.000E+00 | 0.000E+00 | 0.000E+00 | 0.000E+00 | 0.000E+00 | 0.000E+00 | 0.000E+00 | 0.000E+00 |
| Unigene170748 | baeS | 0.000E+00 | 1.707E-07 | 7.629E-07 | 6.281E-07 | 0.000E+00 | 0.000E+00 | 0.000E+00 | 0.000E+00 | 0.000E+00 | 0.000E+00 | 0.000E+00 | 0.000E+00 |
| Unigene170801 | patB | 0.000E+00 | 0.000E+00 | 1.358E-06 | 3.177E-08 | 3.495E-08 | 0.000E+00 | 1.393E-07 | 0.000E+00 | 0.000E+00 | 1.752E-07 | 1.440E-07 | 7.810E-07 |
| Unigene170839 | emrY | 0.000E+00 | 3.948E-07 | 4.172E-07 | 9.908E-08 | 0.000E+00 | 0.000E+00 | 0.000E+00 | 0.000E+00 | 0.000E+00 | 0.000E+00 | 0.000E+00 | 0.000E+00 |
| Unigene170888 | oleB | 0.000E+00 | 0.000E+00 | 8.764E-07 | 0.000E+00 | 2.362E-07 | 9.195E-08 | 0.000E+00 | 4.964E-08 | 9.297E-08 | 0.000E+00 | 0.000E+00 | 1.005E-06 |
| Unigene170938 | vanHO | 9.607E-08 | 0.000E+00 | 9.113E-07 | 7.862E-05 | 0.000E+00 | 0.000E+00 | 0.000E+00 | 1.869E-07 | 0.000E+00 | 0.000E+00 | 0.000E+00 | 0.000E+00 |
| Unigene170944 | optrA | 0.000E+00 | 0.000E+00 | 1.123E-06 | 8.533E-05 | 0.000E+00 | 0.000E+00 | 0.000E+00 | 2.672E-08 | 0.000E+00 | 0.000E+00 | 0.000E+00 | 0.000E+00 |
| Unigene170965 | vanHO | 0.000E+00 | 0.000E+00 | 1.131E-06 | 0.000E+00 | 0.000E+00 | 0.000E+00 | 0.000E+00 | 0.000E+00 | 0.000E+00 | 0.000E+00 | 0.000E+00 | 0.000E+00 |
| Unigene171059 | patB | 0.000E+00 | 0.000E+00 | 3.991E-07 | 0.000E+00 | 0.000E+00 | 0.000E+00 | 3.274E-06 | 2.892E-06 | 2.425E-07 | 1.359E-06 | 1.903E-06 | 2.054E-06 |
| Unigene171135 | cmx | 0.000E+00 | 2.829E-07 | 5.374E-07 | 1.301E-07 | 0.000E+00 | 0.000E+00 | 0.000E+00 | 3.760E-08 | 0.000E+00 | 0.000E+00 | 0.000E+00 | 0.000E+00 |
| Unigene171163 | macB | 0.000E+00 | 1.078E-06 | 3.226E-06 | 3.493E-06 | 0.000E+00 | 0.000E+00 | 0.000E+00 | 0.000E+00 | 0.000E+00 | 0.000E+00 | 0.000E+00 | 0.000E+00 |
| Unigene171401 | optrA | 0.000E+00 | 0.000E+00 | 7.039E-07 | 0.000E+00 | 0.000E+00 | 0.000E+00 | 0.000E+00 | 0.000E+00 | 0.000E+00 | 0.000E+00 | 0.000E+00 | 0.000E+00 |
| Unigene171415 | macB | 1.410E-07 | 1.031E-07 | 8.760E-07 | 0.000E+00 | 0.000E+00 | 0.000E+00 | 0.000E+00 | 0.000E+00 | 0.000E+00 | 0.000E+00 | 0.000E+00 | 0.000E+00 |
| Unigene171432 | tva(A) | 0.000E+00 | 0.000E+00 | 7.430E-07 | 0.000E+00 | 1.151E-06 | 0.000E+00 | 1.852E-06 | 0.000E+00 | 0.000E+00 | 0.000E+00 | 0.000E+00 | 2.779E-06 |
| Unigene171464 | oleB | 0.000E+00 | 8.543E-07 | 1.078E-06 | 8.785E-07 | 3.662E-06 | 9.900E-08 | 5.068E-07 | 0.000E+00 | 0.000E+00 | 0.000E+00 | 0.000E+00 | 1.624E-07 |
| Unigene171665 | arlR | 0.000E+00 | 2.443E-07 | 3.641E-07 | 0.000E+00 | 0.000E+00 | 0.000E+00 | 0.000E+00 | 0.000E+00 | 0.000E+00 | 0.000E+00 | 0.000E+00 | 0.000E+00 |
| Unigene171750 | tetB(P) | 0.000E+00 | 0.000E+00 | 1.206E-05 | 0.000E+00 | 0.000E+00 | 9.491E-07 | 0.000E+00 | 1.192E-07 | 0.000E+00 | 0.000E+00 | 0.000E+00 | 0.000E+00 |
| Unigene171767 | emrA | 3.363E-07 | 4.921E-07 | 4.766E-07 | 0.000E+00 | 0.000E+00 | 0.000E+00 | 0.000E+00 | 0.000E+00 | 0.000E+00 | 0.000E+00 | 0.000E+00 | 0.000E+00 |
| Unigene171795 | Corynebac | 0.000E+00 | 0.000E+00 | 7.943E-07 | 0.000E+00 | 0.000E+00 | 0.000E+00 | 7.466E-08 | 1.181E-07 | 2.581E-07 | 0.000E+00 | 0.000E+00 | 1.196E-06 |
| Unigene171882 | vanO | 0.000E+00 | 0.000E+00 | 3.239E-07 | 0.000E+00 | 0.000E+00 | 0.000E+00 | 0.000E+00 | 1.541E-07 | 0.000E+00 | 0.000E+00 | 0.000E+00 | 0.000E+00 |
| Unigene171895 | Acinetoba | 3.400E-07 | 2.133E-07 | 6.435E-07 | 2.698E-07 | 1.187E-06 | 4.201E-07 | 2.016E-06 | 1.276E-06 | 0.000E+00 | 6.764E-07 | 1.000E-06 | 3.962E-06 |
| Unigene172004 | macB | 0.000E+00 | 0.000E+00 | 0.000E+00 | 0.000E+00 | 0.000E+00 | 0.000E+00 | 6.301E-08 | 6.645E-08 | 0.000E+00 | 0.000E+00 | 1.302E-07 | 0.000E+00 |
| Unigene172033 | vanRI | 7.142E-07 | 1.254E-06 | 6.213E-06 | 6.202E-06 | 0.000E+00 | 0.000E+00 | 4.215E-07 | 0.000E+00 | 0.000E+00 | 0.000E+00 | 0.000E+00 | 0.000E+00 |
| Unigene172071 | baeS | 2.407E-07 | 6.076E-07 | 1.299E-06 | 3.063E-06 | 6.953E-07 | 1.041E-07 | 2.398E-07 | 1.573E-06 | 2.368E-07 | 9.385E-07 | 1.212E-06 | 6.829E-07 |
| Unigene172131 | golS | 2.115E-06 | 0.000E+00 | 4.455E-06 | 5.841E-06 | 0.000E+00 | 0.000E+00 | 0.000E+00 | 0.000E+00 | 0.000E+00 | 0.000E+00 | 0.000E+00 | 0.000E+00 |
| Unigene172248 | sul4 | 0.000E+00 | 0.000E+00 | 1.343E-06 | 0.000E+00 | 1.690E-07 | 4.110E-08 | 0.000E+00 | 0.000E+00 | 0.000E+00 | 0.000E+00 | 0.000E+00 | 1.483E-06 |
| Unigene172264 | patB | 0.000E+00 | 0.000E+00 | 1.786E-06 | 0.000E+00 | 0.000E+00 | 8.553E-08 | 0.000E+00 | 0.000E+00 | 3.027E-07 | 0.000E+00 | 0.000E+00 | 1.216E-06 |
| Unigene172270 | tetA(58) | 0.000E+00 | 0.000E+00 | 1.571E-06 | 0.000E+00 | 9.879E-08 | 0.000E+00 | 4.921E-08 | 5.190E-08 | 0.000E+00 | 9.906E-08 | 1.017E-07 | 9.986E-07 |
| Unigene172278 | evgS | 2.670E-08 | 5.861E-08 | 1.362E-06 | 9.437E-08 | 1.928E-07 | 2.886E-08 | 0.000E+00 | 1.558E-08 | 1.605E-07 | 4.461E-08 | 6.108E-08 | 1.452E-06 |
| Unigene172302 | acrB | 0.000E+00 | 0.000E+00 | 4.325E-07 | 0.000E+00 | 0.000E+00 | 0.000E+00 | 0.000E+00 | 0.000E+00 | 0.000E+00 | 0.000E+00 | 0.000E+00 | 0.000E+00 |
| Unigene172333 | Streptomy | 0.000E+00 | 0.000E+00 | 3.655E-07 | 2.925E-07 | 0.000E+00 | 0.000E+00 | 0.000E+00 | 0.000E+00 | 0.000E+00 | 0.000E+00 | 0.000E+00 | 0.000E+00 |
| Unigene172348 | tetA(58) | 0.000E+00 | 0.000E+00 | 1.504E-06 | 1.245E-06 | 0.000E+00 | 0.000E+00 | 0.000E+00 | 0.000E+00 | 0.000E+00 | 0.000E+00 | 0.000E+00 | 0.000E+00 |
| Unigene172453 | tetB(46) | 0.000E+00 | 0.000E+00 | 3.950E-07 | 0.000E+00 | 0.000E+00 | 0.000E+00 | 0.000E+00 | 0.000E+00 | 0.000E+00 | 0.000E+00 | 0.000E+00 | 0.000E+00 |
| Unigene172454 | YojI | 0.000E+00 | 0.000E+00 | 8.148E-07 | 6.750E-05 | 0.000E+00 | 0.000E+00 | 0.000E+00 | 0.000E+00 | 0.000E+00 | 0.000E+00 | 0.000E+00 | 0.000E+00 |
| Unigene172489 | efpA | 0.000E+00 | 0.000E+00 | 9.754E-07 | 4.768E-07 | 0.000E+00 | 0.000E+00 | 0.000E+00 | 0.000E+00 | 0.000E+00 | 0.000E+00 | 0.000E+00 | 0.000E+00 |

| | | | | | | | | | | | | | |
|---|---|---|---|---|---|---|---|---|---|---|---|---|---|
| Unigene172519 | tet(41) | 2.334E-07 | 1.025E-07 | 1.145E-06 | 2.357E-06 | 0.000E+00 | 0.000E+00 | 0.000E+00 | 1.471E-06 | 0.000E+00 | 6.240E-07 | 8.811E-07 | 0.000E+00 |
| Unigene172523 | vatE | 0.000E+00 | 0.000E+00 | 1.092E-06 | 2.890E-06 | 0.000E+00 | 0.000E+00 | 1.173E-07 | 2.042E-06 | 0.000E+00 | 1.181E-06 | 9.095E-07 | 0.000E+00 |
| Unigene172630 | basS | 2.441E-06 | 4.292E-06 | 2.674E-06 | 2.417E-06 | 2.449E-06 | 1.896E-06 | 2.439E-06 | 8.299E-07 | 1.243E-06 | 6.072E-06 | 5.287E-06 | 1.877E-06 |
| Unigene172746 | vanRM | 0.000E+00 | 1.602E-07 | 5.934E-07 | 8.423E-08 | 8.572E-07 | 3.832E-07 | 9.924E-07 | 5.598E-07 | 1.595E-07 | 3.949E-07 | 8.110E-07 | 2.465E-06 |
| Unigene172841 | macB | 1.701E-07 | 0.000E+00 | 0.000E+00 | 6.013E-07 | 8.977E-07 | 1.655E-06 | 4.848E-06 | 5.064E-06 | 9.297E-07 | 1.706E-06 | 2.141E-06 | 3.620E-06 |
| Unigene172909 | lsaB | 0.000E+00 | 8.726E-08 | 7.542E-07 | 6.958E-07 | 0.000E+00 | 0.000E+00 | 0.000E+00 | 0.000E+00 | 0.000E+00 | 0.000E+00 | 0.000E+00 | 0.000E+00 |
| Unigene172936 | efrA | 0.000E+00 | 0.000E+00 | 8.093E-07 | 0.000E+00 | 0.000E+00 | 0.000E+00 | 0.000E+00 | 0.000E+00 | 0.000E+00 | 0.000E+00 | 0.000E+00 | 0.000E+00 |
| Unigene173009 | macB | 0.000E+00 | 0.000E+00 | 8.528E-07 | 7.536E-05 | 0.000E+00 | 1.491E-08 | 0.000E+00 | 6.441E-08 | 0.000E+00 | 0.000E+00 | 0.000E+00 | 0.000E+00 |
| Unigene173028 | vanSA | 1.163E-07 | 5.107E-07 | 1.940E-06 | 2.056E-06 | 0.000E+00 | 0.000E+00 | 0.000E+00 | 0.000E+00 | 0.000E+00 | 0.000E+00 | 0.000E+00 | 0.000E+00 |
| Unigene173031 | oleC | 0.000E+00 | 0.000E+00 | 3.695E-07 | 0.000E+00 | 1.195E-07 | 0.000E+00 | 7.145E-07 | 0.000E+00 | 0.000E+00 | 0.000E+00 | 0.000E+00 | 0.000E+00 |
| Unigene173182 | Erm(30) | 0.000E+00 | 6.384E-07 | 9.274E-07 | 2.790E-06 | 0.000E+00 | 0.000E+00 | 4.828E-07 | 1.570E-06 | 0.000E+00 | 0.000E+00 | 0.000E+00 | 0.000E+00 |
| Unigene173184 | vgaE | 2.747E-07 | 3.015E-07 | 1.572E-06 | 2.312E-06 | 0.000E+00 | 0.000E+00 | 1.520E-07 | 1.657E-06 | 0.000E+00 | 4.081E-07 | 1.519E-06 | 0.000E+00 |
| Unigene173213 | oleC | 2.580E-08 | 1.416E-07 | 2.279E-06 | 9.850E-06 | 0.000E+00 | 0.000E+00 | 0.000E+00 | 0.000E+00 | 0.000E+00 | 0.000E+00 | 0.000E+00 | 0.000E+00 |
| Unigene173239 | msbA | 1.201E-07 | 2.449E-07 | 1.684E-06 | 1.199E-05 | 0.000E+00 | 0.000E+00 | 0.000E+00 | 0.000E+00 | 0.000E+00 | 0.000E+00 | 0.000E+00 | 0.000E+00 |
| Unigene173240 | msbA | 0.000E+00 | 0.000E+00 | 1.971E-06 | 1.445E-05 | 0.000E+00 | 0.000E+00 | 0.000E+00 | 0.000E+00 | 0.000E+00 | 0.000E+00 | 0.000E+00 | 0.000E+00 |
| Unigene173273 | baeS | 2.548E-07 | 0.000E+00 | 1.417E-06 | 5.461E-06 | 0.000E+00 | 0.000E+00 | 0.000E+00 | 0.000E+00 | 0.000E+00 | 0.000E+00 | 0.000E+00 | 0.000E+00 |
| Unigene173274 | mtrA | 3.499E-07 | 0.000E+00 | 1.631E-06 | 5.963E-06 | 0.000E+00 | 0.000E+00 | 0.000E+00 | 0.000E+00 | 0.000E+00 | 0.000E+00 | 0.000E+00 | 0.000E+00 |
| Unigene173374 | smeA | 0.000E+00 | 0.000E+00 | 1.081E-06 | 0.000E+00 | 0.000E+00 | 0.000E+00 | 0.000E+00 | 0.000E+00 | 0.000E+00 | 0.000E+00 | 0.000E+00 | 0.000E+00 |
| Unigene173427 | efrA | 0.000E+00 | 0.000E+00 | 5.738E-07 | 6.112E-05 | 0.000E+00 | 0.000E+00 | 0.000E+00 | 0.000E+00 | 0.000E+00 | 0.000E+00 | 0.000E+00 | 0.000E+00 |
| Unigene173432 | mgrA | 0.000E+00 | 0.000E+00 | 1.006E-06 | 9.772E-05 | 0.000E+00 | 0.000E+00 | 0.000E+00 | 0.000E+00 | 0.000E+00 | 0.000E+00 | 0.000E+00 | 0.000E+00 |
| Unigene173434 | tetA(58) | 0.000E+00 | 0.000E+00 | 1.056E-06 | 8.708E-05 | 0.000E+00 | 0.000E+00 | 0.000E+00 | 4.331E-08 | 0.000E+00 | 0.000E+00 | 0.000E+00 | 0.000E+00 |
| Unigene173505 | iri | 1.341E-07 | 2.207E-07 | 3.947E-07 | 0.000E+00 | 0.000E+00 | 0.000E+00 | 0.000E+00 | 3.913E-08 | 0.000E+00 | 0.000E+00 | 0.000E+00 | 0.000E+00 |
| Unigene173579 | patB | 0.000E+00 | 4.218E-07 | 3.469E-06 | 4.113E-06 | 0.000E+00 | 0.000E+00 | 0.000E+00 | 0.000E+00 | 0.000E+00 | 0.000E+00 | 0.000E+00 | 0.000E+00 |
| Unigene173584 | bcrA | 7.033E-08 | 9.648E-07 | 3.968E-06 | 3.551E-06 | 0.000E+00 | 0.000E+00 | 0.000E+00 | 0.000E+00 | 0.000E+00 | 0.000E+00 | 0.000E+00 | 0.000E+00 |
| Unigene173587 | arlS | 1.231E-07 | 7.026E-07 | 2.585E-06 | 3.456E-06 | 0.000E+00 | 0.000E+00 | 0.000E+00 | 0.000E+00 | 0.000E+00 | 0.000E+00 | 0.000E+00 | 0.000E+00 |
| Unigene173601 | novA | 0.000E+00 | 3.546E-07 | 1.569E-06 | 6.020E-06 | 0.000E+00 | 0.000E+00 | 3.576E-08 | 0.000E+00 | 0.000E+00 | 0.000E+00 | 0.000E+00 | 0.000E+00 |
| Unigene173712 | arlS | 1.149E-06 | 7.247E-07 | 9.358E-07 | 1.827E-06 | 2.081E-05 | 6.477E-06 | 1.440E-05 | 1.924E-05 | 1.045E-05 | 1.855E-05 | 1.673E-05 | 8.180E-06 |
| Unigene173719 | msbA | 0.000E+00 | 9.084E-08 | 1.917E-06 | 7.038E-06 | 0.000E+00 | 0.000E+00 | 0.000E+00 | 0.000E+00 | 0.000E+00 | 0.000E+00 | 0.000E+00 | 0.000E+00 |
| Unigene173737 | tetA(58) | 0.000E+00 | 3.009E-07 | 1.849E-06 | 1.602E-05 | 0.000E+00 | 0.000E+00 | 0.000E+00 | 0.000E+00 | 0.000E+00 | 0.000E+00 | 0.000E+00 | 0.000E+00 |
| Unigene173746 | mgtA | 0.000E+00 | 0.000E+00 | 3.101E-06 | 2.051E-05 | 0.000E+00 | 0.000E+00 | 0.000E+00 | 0.000E+00 | 0.000E+00 | 0.000E+00 | 0.000E+00 | 0.000E+00 |
| Unigene173797 | macB | 8.903E-05 | 1.431E-04 | 1.563E-04 | 7.579E-06 | 6.977E-06 | 0.000E+00 | 0.000E+00 | 0.000E+00 | 5.826E-05 | 0.000E+00 | 2.716E-06 | 0.000E+00 |
| Unigene173798 | NmcR | 0.000E+00 | 0.000E+00 | 8.884E-07 | 0.000E+00 | 9.671E-07 | 1.280E-06 | 2.968E-06 | 3.455E-06 | 0.000E+00 | 1.668E-06 | 1.992E-06 | 4.322E-06 |
| Unigene173811 | vanHM | 4.159E-06 | 1.061E-05 | 9.360E-06 | 1.060E-05 | 0.000E+00 | 0.000E+00 | 0.000E+00 | 0.000E+00 | 0.000E+00 | 0.000E+00 | 0.000E+00 | 0.000E+00 |
| Unigene173815 | aadS | 0.000E+00 | 0.000E+00 | 3.666E-07 | 7.546E-08 | 0.000E+00 | 0.000E+00 | 0.000E+00 | 0.000E+00 | 0.000E+00 | 0.000E+00 | 0.000E+00 | 0.000E+00 |
| Unigene173854 | vanHO | 0.000E+00 | 7.179E-08 | 1.572E-06 | 1.321E-07 | 2.180E-07 | 0.000E+00 | 0.000E+00 | 0.000E+00 | 0.000E+00 | 7.287E-08 | 7.482E-08 | 9.665E-07 |
| Unigene173862 | adeH | 0.000E+00 | 2.578E-08 | 8.530E-07 | 4.745E-08 | 5.220E-08 | 0.000E+00 | 0.000E+00 | 0.000E+00 | 2.568E-08 | 5.234E-08 | 0.000E+00 | 6.109E-07 |
| Unigene173863 | emrA | 2.981E-08 | 1.309E-07 | 9.359E-07 | 0.000E+00 | 0.000E+00 | 0.000E+00 | 0.000E+00 | 0.000E+00 | 1.303E-07 | 0.000E+00 | 6.819E-08 | 1.304E-06 |
| Unigene173865 | mepR | 0.000E+00 | 0.000E+00 | 1.466E-06 | 0.000E+00 | 0.000E+00 | 0.000E+00 | 1.654E-07 | 0.000E+00 | 0.000E+00 | 0.000E+00 | 0.000E+00 | 2.031E-06 |
| Unigene173867 | FosA5 | 0.000E+00 | 0.000E+00 | 1.683E-06 | 7.874E-08 | 0.000E+00 | 0.000E+00 | 0.000E+00 | 0.000E+00 | 5.113E-07 | 0.000E+00 | 0.000E+00 | 1.567E-06 |
| Unigene173870 | Escherichia | 0.000E+00 | 0.000E+00 | 0.000E+00 | 0.000E+00 | 1.067E-07 | 0.000E+00 | 0.000E+00 | 0.000E+00 | 0.000E+00 | 2.140E-07 | 0.000E+00 | 1.703E-06 |
| Unigene173872 | Acinetobac | 4.668E-08 | 7.685E-08 | 1.351E-06 | 0.000E+00 | 0.000E+00 | 0.000E+00 | 0.000E+00 | 2.724E-08 | 1.020E-07 | 0.000E+00 | 8.010E-08 | 1.269E-06 |
| Unigene173938 | vanHA | 1.258E-07 | 5.525E-07 | 3.643E-06 | 3.685E-06 | 0.000E+00 | 0.000E+00 | 0.000E+00 | 0.000E+00 | 0.000E+00 | 0.000E+00 | 0.000E+00 | 0.000E+00 |
| Unigene174006 | bcrA | 0.000E+00 | 7.060E-07 | 4.591E-07 | 0.000E+00 | 0.000E+00 | 0.000E+00 | 7.767E-07 | 0.000E+00 | 0.000E+00 | 0.000E+00 | 0.000E+00 | 0.000E+00 |
| Unigene174153 | smeC | 0.000E+00 | 0.000E+00 | 1.439E-06 | 0.000E+00 | 0.000E+00 | 0.000E+00 | 0.000E+00 | 0.000E+00 | 0.000E+00 | 0.000E+00 | 0.000E+00 | 0.000E+00 |
| Unigene174160 | macB | 0.000E+00 | 3.374E-07 | 7.757E-07 | 0.000E+00 | 0.000E+00 | 1.567E-06 | 3.111E-06 | 4.101E-06 | 5.760E-07 | 0.000E+00 | 2.060E-06 | 3.374E-06 |
| Unigene174168 | AAC(6')-Ie | 0.000E+00 | 7.949E-07 | 3.689E-06 | 3.726E-06 | 0.000E+00 | 0.000E+00 | 0.000E+00 | 0.000E+00 | 0.000E+00 | 0.000E+00 | 0.000E+00 | 0.000E+00 |
| Unigene174192 | Streptomy | 2.339E-07 | 0.000E+00 | 4.131E-07 | 6.142E-07 | 0.000E+00 | 0.000E+00 | 0.000E+00 | 0.000E+00 | 0.000E+00 | 0.000E+00 | 0.000E+00 | 0.000E+00 |
| Unigene174196 | vanRN | 0.000E+00 | 0.000E+00 | 1.554E-06 | 7.244E-06 | 0.000E+00 | 0.000E+00 | 0.000E+00 | 0.000E+00 | 0.000E+00 | 0.000E+00 | 0.000E+00 | 0.000E+00 |
| Unigene174197 | bcrA | 1.730E-07 | 0.000E+00 | 1.901E-06 | 6.149E-06 | 0.000E+00 | 0.000E+00 | 0.000E+00 | 0.000E+00 | 0.000E+00 | 0.000E+00 | 0.000E+00 | 0.000E+00 |
| Unigene174219 | smeF | 0.000E+00 | 0.000E+00 | 1.877E-07 | 0.000E+00 | 0.000E+00 | 0.000E+00 | 0.000E+00 | 0.000E+00 | 0.000E+00 | 0.000E+00 | 0.000E+00 | 5.653E-07 |
| Unigene174565 | Staphyloco | 2.307E-08 | 0.000E+00 | 9.279E-07 | 5.583E-05 | 0.000E+00 | 0.000E+00 | 0.000E+00 | 9.423E-08 | 0.000E+00 | 0.000E+00 | 0.000E+00 | 0.000E+00 |
| Unigene174613 | novA | 5.816E-08 | 2.234E-07 | 1.313E-06 | 5.873E-08 | 3.554E-07 | 9.431E-08 | 0.000E+00 | 0.000E+00 | 0.000E+00 | 0.000E+00 | 0.000E+00 | 1.031E-06 |
| Unigene174630 | vanTN | 0.000E+00 | 0.000E+00 | 1.320E-06 | 0.000E+00 | 6.641E-08 | 0.000E+00 | 0.000E+00 | 1.047E-07 | 3.267E-08 | 0.000E+00 | 0.000E+00 | 1.343E-06 |
| Unigene174637 | ramA | 0.000E+00 | 0.000E+00 | 1.271E-06 | 0.000E+00 | 0.000E+00 | 0.000E+00 | 0.000E+00 | 0.000E+00 | 0.000E+00 | 0.000E+00 | 0.000E+00 | 0.000E+00 |
| Unigene174638 | Pseudomo | 0.000E+00 | 0.000E+00 | 1.320E-06 | 0.000E+00 | 0.000E+00 | 0.000E+00 | 0.000E+00 | 0.000E+00 | 0.000E+00 | 0.000E+00 | 0.000E+00 | 1.004E-06 |
| Unigene174703 | optrA | 1.471E-07 | 4.573E-07 | 3.848E-07 | 1.980E-07 | 0.000E+00 | 0.000E+00 | 0.000E+00 | 0.000E+00 | 0.000E+00 | 0.000E+00 | 0.000E+00 | 0.000E+00 |
| Unigene174751 | FOX-9 | 2.729E-05 | 8.519E-06 | 6.131E-06 | 5.093E-06 | 3.113E-07 | 4.241E-07 | 3.101E-08 | 1.308E-07 | 1.041E-06 | 0.000E+00 | 0.000E+00 | 0.000E+00 |
| Unigene174800 | Streptomy | 5.604E-08 | 1.230E-07 | 3.299E-07 | 1.698E-07 | 1.245E-07 | 0.000E+00 | 0.000E+00 | 0.000E+00 | 0.000E+00 | 0.000E+00 | 0.000E+00 | 0.000E+00 |

| | | | | | | | | | | | | | |
|---|---|---|---|---|---|---|---|---|---|---|---|---|---|
| Unigene175204 | oleC | 4.778E-06 | 8.407E-06 | 9.842E-06 | 1.083E-05 | 0.000E+00 | 0.000E+00 | 0.000E+00 | 4.258E-08 | 0.000E+00 | 0.000E+00 | 0.000E+00 | 0.000E+00 |
| Unigene175216 | vanRI | 1.494E-07 | 1.148E-06 | 2.982E-06 | 0.000E+00 | 0.000E+00 | 0.000E+00 | 0.000E+00 | 0.000E+00 | 0.000E+00 | 0.000E+00 | 0.000E+00 | 0.000E+00 |
| Unigene175225 | adeR | 0.000E+00 | 0.000E+00 | 4.873E-07 | 0.000E+00 | 0.000E+00 | 0.000E+00 | 0.000E+00 | 0.000E+00 | 0.000E+00 | 0.000E+00 | 0.000E+00 | 0.000E+00 |
| Unigene175313 | bcrA | 0.000E+00 | 1.147E-07 | 1.435E-06 | 7.912E-06 | 0.000E+00 | 0.000E+00 | 0.000E+00 | 0.000E+00 | 0.000E+00 | 0.000E+00 | 0.000E+00 | 0.000E+00 |
| Unigene175390 | PmrF | 1.695E-07 | 0.000E+00 | 5.819E-07 | 4.278E-07 | 0.000E+00 | 0.000E+00 | 0.000E+00 | 0.000E+00 | 0.000E+00 | 0.000E+00 | 0.000E+00 | 2.003E-07 |
| Unigene175402 | tetA(60) | 0.000E+00 | 7.201E-08 | 1.223E-06 | 0.000E+00 | 0.000E+00 | 0.000E+00 | 0.000E+00 | 0.000E+00 | 1.434E-07 | 1.096E-07 | 7.505E-08 | 6.204E-07 |
| Unigene175404 | TaeA | 0.000E+00 | 0.000E+00 | 1.560E-06 | 4.116E-08 | 9.056E-08 | 0.000E+00 | 0.000E+00 | 0.000E+00 | 8.909E-08 | 9.081E-08 | 0.000E+00 | 9.635E-07 |
| Unigene175413 | bcrA | 0.000E+00 | 0.000E+00 | 7.151E-07 | 0.000E+00 | 0.000E+00 | 0.000E+00 | 4.745E-08 | 0.000E+00 | 9.371E-08 | 0.000E+00 | 0.000E+00 | 7.094E-07 |
| Unigene175417 | srmB | 0.000E+00 | 0.000E+00 | 1.478E-06 | 1.739E-07 | 4.782E-08 | 9.306E-08 | 0.000E+00 | 0.000E+00 | 0.000E+00 | 0.000E+00 | 4.923E-08 | 1.017E-06 |
| Unigene175425 | novA | 4.598E-08 | 0.000E+00 | 1.173E-06 | 0.000E+00 | 1.022E-07 | 0.000E+00 | 0.000E+00 | 0.000E+00 | 0.000E+00 | 0.000E+00 | 0.000E+00 | 1.196E-06 |
| Unigene175503 | Streptomy | 0.000E+00 | 2.184E-07 | 1.236E-06 | 2.679E-07 | 0.000E+00 | 0.000E+00 | 0.000E+00 | 0.000E+00 | 0.000E+00 | 0.000E+00 | 0.000E+00 | 0.000E+00 |
| Unigene175798 | clbB | 0.000E+00 | 0.000E+00 | 7.674E-07 | 6.536E-07 | 8.389E-07 | 0.000E+00 | 0.000E+00 | 0.000E+00 | 0.000E+00 | 0.000E+00 | 2.283E-06 | 4.367E-06 |
| Unigene175842 | vanRD | 0.000E+00 | 0.000E+00 | 6.599E-07 | 0.000E+00 | 0.000E+00 | 0.000E+00 | 4.035E-06 | 0.000E+00 | 0.000E+00 | 0.000E+00 | 0.000E+00 | 4.016E-06 |
| Unigene175884 | novA | 0.000E+00 | 1.750E-07 | 2.346E-07 | 0.000E+00 | 0.000E+00 | 0.000E+00 | 0.000E+00 | 0.000E+00 | 0.000E+00 | 8.879E-08 | 4.103E-07 | 4.240E-07 |
| Unigene175947 | mtrA | 0.000E+00 | 7.350E-07 | 2.891E-06 | 1.262E-06 | 0.000E+00 | 0.000E+00 | 0.000E+00 | 0.000E+00 | 0.000E+00 | 0.000E+00 | 0.000E+00 | 0.000E+00 |
| Unigene176051 | patA | 0.000E+00 | 0.000E+00 | 5.984E-07 | 8.587E-05 | 0.000E+00 | 0.000E+00 | 0.000E+00 | 3.051E-07 | 0.000E+00 | 0.000E+00 | 0.000E+00 | 0.000E+00 |
| Unigene176052 | vanHD | 0.000E+00 | 0.000E+00 | 3.152E-07 | 0.000E+00 | 0.000E+00 | 0.000E+00 | 1.185E-07 | 0.000E+00 | 0.000E+00 | 0.000E+00 | 0.000E+00 | 0.000E+00 |
| Unigene176096 | lnuA | 4.700E-05 | 2.297E-05 | 3.125E-05 | 8.390E-05 | 1.558E-05 | 1.262E-05 | 1.209E-05 | 0.000E+00 | 4.213E-06 | 0.000E+00 | 0.000E+00 | 6.328E-07 |
| Unigene176228 | mtrA | 0.000E+00 | 0.000E+00 | 3.591E-07 | 0.000E+00 | 0.000E+00 | 0.000E+00 | 0.000E+00 | 0.000E+00 | 0.000E+00 | 0.000E+00 | 0.000E+00 | 0.000E+00 |
| Unigene176254 | acrB | 1.448E-07 | 4.766E-07 | 5.682E-07 | 0.000E+00 | 0.000E+00 | 0.000E+00 | 0.000E+00 | 0.000E+00 | 0.000E+00 | 0.000E+00 | 0.000E+00 | 0.000E+00 |
| Unigene176313 | oleC | 0.000E+00 | 0.000E+00 | 4.685E-07 | 1.854E-07 | 0.000E+00 | 0.000E+00 | 0.000E+00 | 0.000E+00 | 0.000E+00 | 0.000E+00 | 0.000E+00 | 0.000E+00 |
| Unigene176329 | patB | 0.000E+00 | 0.000E+00 | 0.000E+00 | 0.000E+00 | 0.000E+00 | 1.283E-07 | 0.000E+00 | 0.000E+00 | 0.000E+00 | 0.000E+00 | 0.000E+00 | 0.000E+00 |
| Unigene176335 | lfrA | 0.000E+00 | 2.139E-08 | 8.225E-07 | 4.134E-07 | 0.000E+00 | 0.000E+00 | 0.000E+00 | 0.000E+00 | 0.000E+00 | 0.000E+00 | 0.000E+00 | 0.000E+00 |
| Unigene176337 | evgA | 0.000E+00 | 0.000E+00 | 9.137E-07 | 7.836E-07 | 0.000E+00 | 0.000E+00 | 0.000E+00 | 0.000E+00 | 0.000E+00 | 0.000E+00 | 0.000E+00 | 0.000E+00 |
| Unigene176387 | PmrF | 0.000E+00 | 0.000E+00 | 1.268E-06 | 7.056E-08 | 7.762E-08 | 0.000E+00 | 0.000E+00 | 0.000E+00 | 1.146E-07 | 0.000E+00 | 0.000E+00 | 1.074E-06 |
| Unigene176394 | bcrA | 0.000E+00 | 0.000E+00 | 1.639E-06 | 3.568E-07 | 1.035E-06 | 3.125E-07 | 0.000E+00 | 0.000E+00 | 0.000E+00 | 2.862E-07 | 6.613E-07 | 1.746E-06 |
| Unigene176400 | H-NS | 0.000E+00 | 3.423E-07 | 1.377E-06 | 0.000E+00 | 0.000E+00 | 0.000E+00 | 0.000E+00 | 2.730E-07 | 0.000E+00 | 0.000E+00 | 0.000E+00 | 1.751E-06 |
| Unigene176411 | tetA(58) | 0.000E+00 | 0.000E+00 | 6.290E-07 | 4.300E-06 | 0.000E+00 | 0.000E+00 | 0.000E+00 | 0.000E+00 | 0.000E+00 | 0.000E+00 | 0.000E+00 | 0.000E+00 |
| Unigene176430 | macB | 3.010E-07 | 0.000E+00 | 1.587E-06 | 2.925E-06 | 0.000E+00 | 0.000E+00 | 2.915E-07 | 1.274E-06 | 0.000E+00 | 9.639E-07 | 8.177E-07 | 7.560E-07 |
| Unigene176431 | macB | 3.153E-07 | 4.326E-07 | 1.199E-06 | 2.786E-06 | 7.006E-07 | 2.983E-07 | 2.617E-07 | 8.281E-07 | 0.000E+00 | 6.147E-07 | 1.127E-06 | 3.727E-07 |
| Unigene176486 | macB | 0.000E+00 | 0.000E+00 | 0.000E+00 | 2.921E-07 | 0.000E+00 | 0.000E+00 | 0.000E+00 | 0.000E+00 | 0.000E+00 | 0.000E+00 | 0.000E+00 | 0.000E+00 |
| Unigene176575 | otr(B) | 0.000E+00 | 0.000E+00 | 1.077E-07 | 1.109E-07 | 0.000E+00 | 0.000E+00 | 0.000E+00 | 0.000E+00 | 0.000E+00 | 0.000E+00 | 0.000E+00 | 0.000E+00 |
| Unigene176598 | bcrA | 0.000E+00 | 0.000E+00 | 0.000E+00 | 0.000E+00 | 0.000E+00 | 0.000E+00 | 0.000E+00 | 0.000E+00 | 0.000E+00 | 0.000E+00 | 0.000E+00 | 3.261E-07 |
| Unigene176683 | PmrF | 3.128E-08 | 0.000E+00 | 2.026E-06 | 3.159E-08 | 3.475E-08 | 0.000E+00 | 0.000E+00 | 7.302E-08 | 0.000E+00 | 0.000E+00 | 0.000E+00 | 1.405E-06 |
| Unigene176730 | tet(44) | 8.849E-08 | 1.942E-07 | 1.268E-06 | 1.072E-07 | 3.932E-08 | 1.531E-07 | 2.350E-07 | 2.272E-07 | 1.354E-07 | 1.774E-07 | 1.012E-07 | 7.322E-07 |
| Unigene176731 | cpxA | 0.000E+00 | 0.000E+00 | 1.297E-06 | 6.209E-08 | 1.366E-07 | 0.000E+00 | 6.805E-08 | 0.000E+00 | 1.344E-07 | 0.000E+00 | 0.000E+00 | 1.127E-06 |
| Unigene176753 | macB | 0.000E+00 | 0.000E+00 | 3.594E-07 | 0.000E+00 | 0.000E+00 | 0.000E+00 | 0.000E+00 | 0.000E+00 | 0.000E+00 | 0.000E+00 | 0.000E+00 | 0.000E+00 |
| Unigene176817 | bcrA | 0.000E+00 | 0.000E+00 | 7.250E-07 | 0.000E+00 | 4.156E-06 | 0.000E+00 | 0.000E+00 | 0.000E+00 | 0.000E+00 | 0.000E+00 | 0.000E+00 | 0.000E+00 |
| Unigene176846 | Staphylocc | 0.000E+00 | 4.941E-08 | 1.127E-06 | 8.252E-05 | 0.000E+00 | 0.000E+00 | 0.000E+00 | 0.000E+00 | 0.000E+00 | 0.000E+00 | 0.000E+00 | 0.000E+00 |
| Unigene176848 | patB | 5.275E-08 | 3.859E-08 | 1.018E-06 | 8.524E-05 | 0.000E+00 | 1.901E-08 | 0.000E+00 | 1.026E-07 | 0.000E+00 | 0.000E+00 | 0.000E+00 | 0.000E+00 |
| Unigene176849 | efrA | 0.000E+00 | 0.000E+00 | 7.841E-07 | 7.846E-05 | 0.000E+00 | 2.009E-08 | 0.000E+00 | 0.000E+00 | 0.000E+00 | 0.000E+00 | 0.000E+00 | 0.000E+00 |
| Unigene176860 | vanHA | 0.000E+00 | 0.000E+00 | 1.053E-06 | 7.517E-05 | 0.000E+00 | 0.000E+00 | 0.000E+00 | 2.277E-07 | 0.000E+00 | 0.000E+00 | 0.000E+00 | 0.000E+00 |
| Unigene176887 | kdpE | 0.000E+00 | 1.022E-07 | 2.377E-06 | 1.359E-05 | 0.000E+00 | 0.000E+00 | 0.000E+00 | 0.000E+00 | 0.000E+00 | 0.000E+00 | 0.000E+00 | 0.000E+00 |
| Unigene176888 | bcrA | 0.000E+00 | 0.000E+00 | 2.787E-06 | 1.542E-05 | 0.000E+00 | 0.000E+00 | 0.000E+00 | 0.000E+00 | 0.000E+00 | 0.000E+00 | 0.000E+00 | 0.000E+00 |
| Unigene176893 | poxtA | 0.000E+00 | 0.000E+00 | 2.224E-06 | 1.349E-05 | 0.000E+00 | 0.000E+00 | 0.000E+00 | 0.000E+00 | 0.000E+00 | 0.000E+00 | 0.000E+00 | 0.000E+00 |
| Unigene176904 | vanD | 0.000E+00 | 0.000E+00 | 2.063E-06 | 1.393E-05 | 0.000E+00 | 0.000E+00 | 0.000E+00 | 0.000E+00 | 0.000E+00 | 0.000E+00 | 0.000E+00 | 0.000E+00 |
| Unigene177025 | lmrD | 3.449E-08 | 1.136E-07 | 3.722E-07 | 3.134E-07 | 1.533E-07 | 0.000E+00 | 0.000E+00 | 0.000E+00 | 0.000E+00 | 0.000E+00 | 0.000E+00 | 0.000E+00 |
| Unigene177030 | mdtG | 0.000E+00 | 4.269E-07 | 5.088E-07 | 1.309E-07 | 0.000E+00 | 0.000E+00 | 0.000E+00 | 0.000E+00 | 0.000E+00 | 0.000E+00 | 0.000E+00 | 0.000E+00 |
| Unigene177048 | tet(W/N/W | 0.000E+00 | 0.000E+00 | 3.726E-07 | 0.000E+00 | 0.000E+00 | 0.000E+00 | 0.000E+00 | 0.000E+00 | 0.000E+00 | 0.000E+00 | 0.000E+00 | 0.000E+00 |
| Unigene177056 | efrA | 2.997E-07 | 3.619E-07 | 7.647E-07 | 5.448E-07 | 6.327E-07 | 8.424E-07 | 3.881E-06 | 3.499E-06 | 4.259E-07 | 1.369E-06 | 1.509E-06 | 3.295E-06 |
| Unigene177115 | efrA | 0.000E+00 | 7.805E-07 | 3.250E-06 | 3.912E-06 | 0.000E+00 | 0.000E+00 | 0.000E+00 | 0.000E+00 | 0.000E+00 | 0.000E+00 | 0.000E+00 | 0.000E+00 |
| Unigene177124 | baeS | 0.000E+00 | 0.000E+00 | 0.000E+00 | 0.000E+00 | 0.000E+00 | 0.000E+00 | 4.007E-06 | 3.724E-06 | 0.000E+00 | 0.000E+00 | 0.000E+00 | 0.000E+00 |
| Unigene177166 | acrB | 0.000E+00 | 3.192E-07 | 0.000E+00 | 0.000E+00 | 0.000E+00 | 0.000E+00 | 0.000E+00 | 0.000E+00 | 0.000E+00 | 0.000E+00 | 0.000E+00 | 0.000E+00 |
| Unigene177284 | rpoB2 | 0.000E+00 | 0.000E+00 | 5.086E-07 | 0.000E+00 | 0.000E+00 | 0.000E+00 | 0.000E+00 | 0.000E+00 | 0.000E+00 | 0.000E+00 | 0.000E+00 | 0.000E+00 |
| Unigene177354 | vanHA | 4.722E-07 | 1.851E-07 | 1.225E-06 | 1.703E-07 | 0.000E+00 | 0.000E+00 | 7.466E-08 | 0.000E+00 | 0.000E+00 | 0.000E+00 | 6.173E-07 | 0.000E+00 |
| Unigene177365 | MexW | 0.000E+00 | 2.866E-07 | 3.075E-07 | 0.000E+00 | 0.000E+00 | 0.000E+00 | 0.000E+00 | 0.000E+00 | 0.000E+00 | 0.000E+00 | 2.987E-07 | 0.000E+00 |
| Unigene177377 | lfrA | 5.124E-08 | 4.499E-07 | 4.022E-07 | 3.105E-07 | 0.000E+00 | 0.000E+00 | 0.000E+00 | 5.981E-08 | 0.000E+00 | 0.000E+00 | 0.000E+00 | 0.000E+00 |
| Unigene177402 | adeS | 2.370E-08 | 5.202E-08 | 1.372E-06 | 0.000E+00 | 7.899E-08 | 7.686E-08 | 2.623E-08 | 0.000E+00 | 7.771E-08 | 0.000E+00 | 2.711E-08 | 1.457E-06 |

| | | | | | | | | | | | | | |
|---|---|---|---|---|---|---|---|---|---|---|---|---|---|
| Unigene177403 | arlR | 0.000E+00 | 0.000E+00 | 1.555E-06 | 0.000E+00 | 1.067E-07 | 0.000E+00 | 0.000E+00 | 0.000E+00 | 0.000E+00 | 0.000E+00 | 0.000E+00 | 1.192E-06 |
| Unigene177499 | efrB | 0.000E+00 | 0.000E+00 | 9.826E-07 | 0.000E+00 | 9.468E-08 | 0.000E+00 | 0.000E+00 | 2.487E-08 | 9.315E-08 | 0.000E+00 | 2.437E-08 | 1.360E-06 |
| Unigene177505 | farB | 2.265E-08 | 0.000E+00 | 1.445E-06 | 2.288E-08 | 0.000E+00 | 0.000E+00 | 7.521E-08 | 0.000E+00 | 7.428E-08 | 1.009E-07 | 5.182E-08 | 1.151E-06 |
| Unigene177518 | vmlR | 4.446E-08 | 6.344E-07 | 2.487E-06 | 2.963E-06 | 0.000E+00 | 0.000E+00 | 0.000E+00 | 0.000E+00 | 0.000E+00 | 0.000E+00 | 0.000E+00 | 0.000E+00 |
| Unigene177530 | tlrC | 0.000E+00 | 3.317E-08 | 1.216E-06 | 4.578E-08 | 1.679E-07 | 0.000E+00 | 0.000E+00 | 8.820E-08 | 3.304E-08 | 0.000E+00 | 5.186E-08 | 9.646E-07 |
| Unigene177601 | rpoB2 | 3.590E-08 | 0.000E+00 | 1.209E-05 | 0.000E+00 | 4.985E-08 | 9.799E-07 | 4.072E-07 | 1.257E-07 | 0.000E+00 | 0.000E+00 | 0.000E+00 | 0.000E+00 |
| Unigene177605 | tetT | 0.000E+00 | 0.000E+00 | 1.279E-05 | 0.000E+00 | 0.000E+00 | 9.140E-07 | 3.402E-07 | 1.077E-07 | 0.000E+00 | 0.000E+00 | 0.000E+00 | 0.000E+00 |
| Unigene177634 | efrA | 0.000E+00 | 0.000E+00 | 1.063E-05 | 0.000E+00 | 0.000E+00 | 7.478E-07 | 0.000E+00 | 0.000E+00 | 0.000E+00 | 0.000E+00 | 0.000E+00 | 0.000E+00 |
| Unigene177635 | tetB(60) | 0.000E+00 | 0.000E+00 | 1.248E-05 | 0.000E+00 | 0.000E+00 | 0.000E+00 | 0.000E+00 | 0.000E+00 | 0.000E+00 | 0.000E+00 | 0.000E+00 | 0.000E+00 |
| Unigene177680 | lfrA | 0.000E+00 | 0.000E+00 | 5.557E-07 | 0.000E+00 | 0.000E+00 | 0.000E+00 | 0.000E+00 | 0.000E+00 | 0.000E+00 | 0.000E+00 | 0.000E+00 | 0.000E+00 |
| Unigene177767 | evgA | 0.000E+00 | 0.000E+00 | 1.940E-06 | 6.408E-06 | 0.000E+00 | 0.000E+00 | 0.000E+00 | 0.000E+00 | 0.000E+00 | 0.000E+00 | 0.000E+00 | 0.000E+00 |
| Unigene177922 | evgA | 0.000E+00 | 3.761E-08 | 3.362E-07 | 3.460E-07 | 0.000E+00 | 0.000E+00 | 0.000E+00 | 0.000E+00 | 0.000E+00 | 0.000E+00 | 0.000E+00 | 0.000E+00 |
| Unigene177934 | macB | 0.000E+00 | 9.373E-08 | 4.190E-07 | 2.587E-07 | 3.795E-07 | 7.847E-07 | 1.654E-06 | 6.978E-07 | 0.000E+00 | 7.610E-07 | 9.769E-07 | 2.725E-06 |
| Unigene177972 | emrB | 0.000E+00 | 0.000E+00 | 9.395E-07 | 6.906E-08 | 2.533E-08 | 0.000E+00 | 0.000E+00 | 0.000E+00 | 9.967E-08 | 0.000E+00 | 1.043E-07 | 9.162E-07 |
| Unigene177973 | mdtC | 3.134E-08 | 3.440E-08 | 1.056E-06 | 0.000E+00 | 0.000E+00 | 0.000E+00 | 8.093E-08 | 6.097E-08 | 1.941E-07 | 4.655E-08 | 7.170E-08 | 1.050E-06 |
| Unigene177974 | mdtB | 2.067E-08 | 7.941E-08 | 1.227E-06 | 7.306E-08 | 9.187E-08 | 0.000E+00 | 0.000E+00 | 0.000E+00 | 1.130E-07 | 4.606E-08 | 3.547E-08 | 8.552E-07 |
| Unigene177975 | mdtA | 5.223E-08 | 0.000E+00 | 1.025E-06 | 0.000E+00 | 0.000E+00 | 1.412E-07 | 0.000E+00 | 3.048E-08 | 1.142E-07 | 0.000E+00 | 8.962E-08 | 1.327E-06 |
| Unigene178036 | vanSO | 3.736E-08 | 4.101E-08 | 2.200E-07 | 1.509E-07 | 0.000E+00 | 0.000E+00 | 0.000E+00 | 4.361E-08 | 0.000E+00 | 0.000E+00 | 0.000E+00 | 0.000E+00 |
| Unigene178120 | vmlR | 5.998E-06 | 4.069E-06 | 1.712E-06 | 5.176E-06 | 4.790E-05 | 5.659E-05 | 7.242E-05 | 4.922E-05 | 6.838E-05 | 0.000E+00 | 5.352E-05 | 0.000E+00 |
| Unigene178190 | rosB | 0.000E+00 | 0.000E+00 | 7.130E-07 | 7.558E-05 | 0.000E+00 | 0.000E+00 | 0.000E+00 | 1.305E-07 | 0.000E+00 | 0.000E+00 | 0.000E+00 | 0.000E+00 |
| Unigene178323 | macB | 0.000E+00 | 0.000E+00 | 1.181E-05 | 0.000E+00 | 0.000E+00 | 1.234E-06 | 9.235E-07 | 0.000E+00 | 0.000E+00 | 0.000E+00 | 0.000E+00 | 0.000E+00 |
| Unigene178365 | cmrA | 0.000E+00 | 0.000E+00 | 1.162E-05 | 0.000E+00 | 0.000E+00 | 0.000E+00 | 6.268E-07 | 0.000E+00 | 0.000E+00 | 0.000E+00 | 0.000E+00 | 0.000E+00 |
| Unigene178366 | Rhodococ | 0.000E+00 | 0.000E+00 | 1.250E-05 | 0.000E+00 | 0.000E+00 | 0.000E+00 | 0.000E+00 | 0.000E+00 | 0.000E+00 | 0.000E+00 | 0.000E+00 | 0.000E+00 |
| Unigene178375 | qacA | 0.000E+00 | 0.000E+00 | 1.289E-05 | 0.000E+00 | 0.000E+00 | 1.023E-06 | 0.000E+00 | 0.000E+00 | 0.000E+00 | 0.000E+00 | 0.000E+00 | 0.000E+00 |
| Unigene178395 | vatE | 0.000E+00 | 0.000E+00 | 1.302E-05 | 0.000E+00 | 0.000E+00 | 0.000E+00 | 0.000E+00 | 0.000E+00 | 0.000E+00 | 0.000E+00 | 0.000E+00 | 0.000E+00 |
| Unigene178396 | lmrB | 0.000E+00 | 0.000E+00 | 1.140E-05 | 0.000E+00 | 2.522E-08 | 0.000E+00 | 5.527E-07 | 0.000E+00 | 0.000E+00 | 0.000E+00 | 0.000E+00 | 0.000E+00 |
| Unigene178398 | bcrA | 0.000E+00 | 0.000E+00 | 1.114E-05 | 0.000E+00 | 0.000E+00 | 9.472E-07 | 0.000E+00 | 0.000E+00 | 0.000E+00 | 0.000E+00 | 0.000E+00 | 0.000E+00 |
| Unigene178406 | bmr | 0.000E+00 | 0.000E+00 | 1.231E-05 | 0.000E+00 | 0.000E+00 | 0.000E+00 | 0.000E+00 | 0.000E+00 | 0.000E+00 | 0.000E+00 | 0.000E+00 | 0.000E+00 |
| Unigene178425 | emrB | 0.000E+00 | 0.000E+00 | 1.248E-05 | 0.000E+00 | 0.000E+00 | 0.000E+00 | 0.000E+00 | 0.000E+00 | 0.000E+00 | 0.000E+00 | 0.000E+00 | 0.000E+00 |
| Unigene178445 | lmrB | 0.000E+00 | 0.000E+00 | 1.366E-05 | 0.000E+00 | 0.000E+00 | 1.123E-06 | 0.000E+00 | 0.000E+00 | 0.000E+00 | 0.000E+00 | 0.000E+00 | 0.000E+00 |
| Unigene178456 | macB | 0.000E+00 | 0.000E+00 | 1.266E-05 | 0.000E+00 | 0.000E+00 | 1.230E-06 | 0.000E+00 | 0.000E+00 | 0.000E+00 | 0.000E+00 | 0.000E+00 | 0.000E+00 |
| Unigene178464 | lmrB | 0.000E+00 | 0.000E+00 | 1.316E-05 | 0.000E+00 | 0.000E+00 | 0.000E+00 | 6.394E-07 | 0.000E+00 | 0.000E+00 | 0.000E+00 | 0.000E+00 | 0.000E+00 |
| Unigene178472 | lmrD | 0.000E+00 | 0.000E+00 | 1.374E-05 | 0.000E+00 | 0.000E+00 | 0.000E+00 | 0.000E+00 | 0.000E+00 | 0.000E+00 | 0.000E+00 | 0.000E+00 | 0.000E+00 |
| Unigene178475 | macB | 0.000E+00 | 0.000E+00 | 1.236E-05 | 0.000E+00 | 0.000E+00 | 0.000E+00 | 0.000E+00 | 0.000E+00 | 0.000E+00 | 0.000E+00 | 0.000E+00 | 0.000E+00 |
| Unigene178488 | poxtA | 0.000E+00 | 0.000E+00 | 1.327E-05 | 0.000E+00 | 0.000E+00 | 1.048E-06 | 4.692E-07 | 0.000E+00 | 0.000E+00 | 0.000E+00 | 0.000E+00 | 0.000E+00 |
| Unigene178525 | tetA(58) | 0.000E+00 | 0.000E+00 | 1.078E-05 | 0.000E+00 | 0.000E+00 | 7.723E-07 | 5.930E-07 | 0.000E+00 | 0.000E+00 | 0.000E+00 | 0.000E+00 | 0.000E+00 |
| Unigene178528 | Staphyloco | 0.000E+00 | 0.000E+00 | 1.274E-05 | 0.000E+00 | 0.000E+00 | 6.197E-07 | 6.589E-07 | 0.000E+00 | 0.000E+00 | 0.000E+00 | 0.000E+00 | 0.000E+00 |
| Unigene178541 | facT | 0.000E+00 | 0.000E+00 | 1.154E-05 | 0.000E+00 | 0.000E+00 | 7.875E-07 | 3.455E-07 | 0.000E+00 | 0.000E+00 | 0.000E+00 | 0.000E+00 | 0.000E+00 |
| Unigene178546 | tetT | 0.000E+00 | 0.000E+00 | 1.277E-05 | 0.000E+00 | 0.000E+00 | 1.218E-06 | 4.156E-07 | 0.000E+00 | 0.000E+00 | 0.000E+00 | 0.000E+00 | 0.000E+00 |
| Unigene178547 | Vibrio ang | 0.000E+00 | 0.000E+00 | 1.355E-05 | 0.000E+00 | 0.000E+00 | 0.000E+00 | 0.000E+00 | 0.000E+00 | 0.000E+00 | 0.000E+00 | 0.000E+00 | 0.000E+00 |
| Unigene178565 | Agrobacte | 0.000E+00 | 0.000E+00 | 1.255E-05 | 0.000E+00 | 0.000E+00 | 0.000E+00 | 0.000E+00 | 0.000E+00 | 0.000E+00 | 0.000E+00 | 0.000E+00 | 0.000E+00 |
| Unigene178572 | mgrA | 0.000E+00 | 0.000E+00 | 1.307E-05 | 0.000E+00 | 0.000E+00 | 0.000E+00 | 0.000E+00 | 0.000E+00 | 0.000E+00 | 0.000E+00 | 0.000E+00 | 0.000E+00 |
| Unigene178574 | lmrB | 0.000E+00 | 0.000E+00 | 1.341E-05 | 0.000E+00 | 0.000E+00 | 0.000E+00 | 0.000E+00 | 0.000E+00 | 0.000E+00 | 0.000E+00 | 0.000E+00 | 0.000E+00 |
| Unigene178593 | arlS | 0.000E+00 | 0.000E+00 | 1.200E-05 | 0.000E+00 | 0.000E+00 | 1.011E-06 | 4.986E-07 | 0.000E+00 | 0.000E+00 | 0.000E+00 | 0.000E+00 | 0.000E+00 |
| Unigene178594 | mtrA | 0.000E+00 | 0.000E+00 | 1.295E-05 | 0.000E+00 | 0.000E+00 | 9.445E-07 | 0.000E+00 | 0.000E+00 | 0.000E+00 | 0.000E+00 | 0.000E+00 | 0.000E+00 |
| Unigene178604 | Streptomy | 0.000E+00 | 0.000E+00 | 1.246E-05 | 0.000E+00 | 0.000E+00 | 1.257E-06 | 4.962E-07 | 0.000E+00 | 0.000E+00 | 0.000E+00 | 0.000E+00 | 0.000E+00 |
| Unigene178639 | vanHA | 0.000E+00 | 0.000E+00 | 1.357E-05 | 0.000E+00 | 0.000E+00 | 0.000E+00 | 0.000E+00 | 0.000E+00 | 0.000E+00 | 0.000E+00 | 0.000E+00 | 0.000E+00 |
| Unigene178693 | arlS | 4.502E-08 | 1.482E-07 | 3.092E-07 | 3.182E-07 | 0.000E+00 | 0.000E+00 | 0.000E+00 | 0.000E+00 | 0.000E+00 | 0.000E+00 | 0.000E+00 | 0.000E+00 |
| Unigene178694 | evgS | 2.117E-07 | 5.034E-07 | 4.500E-07 | 1.425E-07 | 0.000E+00 | 0.000E+00 | 0.000E+00 | 0.000E+00 | 0.000E+00 | 0.000E+00 | 0.000E+00 | 0.000E+00 |
| Unigene178793 | vanHA | 0.000E+00 | 3.623E-07 | 1.101E-06 | 6.333E-07 | 0.000E+00 | 0.000E+00 | 0.000E+00 | 0.000E+00 | 0.000E+00 | 0.000E+00 | 0.000E+00 | 0.000E+00 |
| Unigene178839 | lmrC | 0.000E+00 | 0.000E+00 | 6.878E-07 | 0.000E+00 | 0.000E+00 | 5.305E-07 | 0.000E+00 | 1.023E-06 | 0.000E+00 | 0.000E+00 | 0.000E+00 | 3.231E-06 |
| Unigene178861 | vanXYL | 2.502E-07 | 4.577E-07 | 1.391E-06 | 2.737E-06 | 0.000E+00 | 0.000E+00 | 6.001E-07 | 1.314E-06 | 0.000E+00 | 7.898E-07 | 1.240E-06 | 0.000E+00 |
| Unigene178908 | lsaB | 0.000E+00 | 0.000E+00 | 2.781E-06 | 6.260E-07 | 0.000E+00 | 0.000E+00 | 0.000E+00 | 0.000E+00 | 0.000E+00 | 0.000E+00 | 0.000E+00 | 0.000E+00 |
| Unigene178959 | mgrA | 0.000E+00 | 0.000E+00 | 1.988E-06 | 7.945E-05 | 0.000E+00 | 0.000E+00 | 0.000E+00 | 0.000E+00 | 0.000E+00 | 0.000E+00 | 0.000E+00 | 0.000E+00 |
| Unigene179090 | patB | 0.000E+00 | 2.271E-07 | 2.558E-06 | 1.387E-05 | 0.000E+00 | 0.000E+00 | 0.000E+00 | 0.000E+00 | 0.000E+00 | 0.000E+00 | 0.000E+00 | 0.000E+00 |
| Unigene179094 | Staphyloco | 0.000E+00 | 1.079E-07 | 2.085E-06 | 1.470E-05 | 0.000E+00 | 0.000E+00 | 0.000E+00 | 0.000E+00 | 0.000E+00 | 0.000E+00 | 0.000E+00 | 0.000E+00 |
| Unigene179122 | TaeA | 0.000E+00 | 1.694E-07 | 2.136E-06 | 1.271E-05 | 0.000E+00 | 0.000E+00 | 0.000E+00 | 0.000E+00 | 0.000E+00 | 0.000E+00 | 0.000E+00 | 0.000E+00 |
| Unigene179133 | Staphyloco | 0.000E+00 | 0.000E+00 | 4.150E-07 | 0.000E+00 | 0.000E+00 | 0.000E+00 | 1.040E-07 | 0.000E+00 | 0.000E+00 | 0.000E+00 | 0.000E+00 | 0.000E+00 |

| | | | | | | | | | | | | | |
|---|---|---|---|---|---|---|---|---|---|---|---|---|---|
| Unigene179183 | adeL | 0.000E+00 | 0.000E+00 | 6.354E-07 | 2.515E-07 | 0.000E+00 | 0.000E+00 | 3.308E-07 | 2.326E-07 | 0.000E+00 | 0.000E+00 | 0.000E+00 | 0.000E+00 |
| Unigene179263 | Bifidobacte | 1.683E-07 | 7.123E-07 | 1.753E-06 | 3.317E-07 | 1.041E-06 | 1.646E-07 | 1.259E-06 | 6.079E-07 | 2.977E-07 | 1.696E-07 | 6.232E-07 | 3.277E-06 |
| Unigene179313 | tetB(46) | 1.805E-08 | 0.000E+00 | 1.134E-06 | 1.641E-07 | 8.023E-08 | 0.000E+00 | 3.996E-08 | 0.000E+00 | 1.776E-07 | 0.000E+00 | 4.130E-08 | 9.816E-07 |
| Unigene179314 | tetA(60) | 1.808E-08 | 9.924E-08 | 1.153E-06 | 1.826E-08 | 1.205E-07 | 0.000E+00 | 6.004E-08 | 6.333E-08 | 5.930E-08 | 1.410E-07 | 1.448E-07 | 1.090E-06 |
| Unigene179335 | evgS | 0.000E+00 | 0.000E+00 | 4.327E-07 | 0.000E+00 | 0.000E+00 | 0.000E+00 | 0.000E+00 | 0.000E+00 | 0.000E+00 | 0.000E+00 | 0.000E+00 | 9.383E-07 |
| Unigene179340 | msbA | 0.000E+00 | 0.000E+00 | 1.026E-06 | 6.348E-05 | 0.000E+00 | 0.000E+00 | 0.000E+00 | 5.085E-08 | 0.000E+00 | 0.000E+00 | 0.000E+00 | 0.000E+00 |
| Unigene179450 | basS | 0.000E+00 | 0.000E+00 | 4.779E-07 | 0.000E+00 | 0.000E+00 | 0.000E+00 | 9.311E-07 | 0.000E+00 | 0.000E+00 | 0.000E+00 | 0.000E+00 | 0.000E+00 |
| Unigene179466 | arlS | 5.556E-08 | 1.016E-07 | 1.199E-06 | 2.244E-07 | 0.000E+00 | 0.000E+00 | 0.000E+00 | 0.000E+00 | 0.000E+00 | 0.000E+00 | 0.000E+00 | 0.000E+00 |
| Unigene179467 | mtrA | 0.000E+00 | 0.000E+00 | 1.168E-06 | 2.774E-07 | 0.000E+00 | 0.000E+00 | 0.000E+00 | 0.000E+00 | 0.000E+00 | 0.000E+00 | 0.000E+00 | 0.000E+00 |
| Unigene179607 | dfrA12 | 0.000E+00 | 0.000E+00 | 9.316E-07 | 0.000E+00 | 7.032E-08 | 0.000E+00 | 0.000E+00 | 0.000E+00 | 1.384E-07 | 0.000E+00 | 7.240E-08 | 1.272E-06 |
| Unigene179803 | Escherichia | 0.000E+00 | 0.000E+00 | 0.000E+00 | 4.865E-07 | 0.000E+00 | 0.000E+00 | 0.000E+00 | 0.000E+00 | 0.000E+00 | 0.000E+00 | 0.000E+00 | 0.000E+00 |
| Unigene179866 | bcrA | 0.000E+00 | 0.000E+00 | 2.208E-06 | 1.198E-06 | 0.000E+00 | 0.000E+00 | 0.000E+00 | 0.000E+00 | 0.000E+00 | 0.000E+00 | 0.000E+00 | 0.000E+00 |
| Unigene179990 | lnuA | 0.000E+00 | 0.000E+00 | 1.108E-06 | 0.000E+00 | 0.000E+00 | 0.000E+00 | 0.000E+00 | 0.000E+00 | 0.000E+00 | 0.000E+00 | 0.000E+00 | 0.000E+00 |
| Unigene180051 | Streptomy | 5.116E-08 | 0.000E+00 | 1.054E-06 | 1.550E-07 | 2.652E-07 | 7.374E-08 | 0.000E+00 | 0.000E+00 | 1.677E-07 | 1.330E-07 | 0.000E+00 | 9.272E-07 |
| Unigene180070 | tetA(58) | 3.717E-08 | 0.000E+00 | 1.204E-06 | 1.877E-08 | 0.000E+00 | 2.009E-08 | 0.000E+00 | 2.169E-08 | 1.828E-07 | 4.140E-08 | 0.000E+00 | 1.186E-06 |
| Unigene180190 | tetA(58) | 3.988E-06 | 8.713E-06 | 8.232E-06 | 1.117E-05 | 0.000E+00 | 0.000E+00 | 0.000E+00 | 2.196E-07 | 0.000E+00 | 0.000E+00 | 4.304E-08 | 4.447E-08 |
| Unigene180220 | Agrobacte | 3.553E-07 | 0.000E+00 | 5.478E-07 | 0.000E+00 | 7.894E-07 | 0.000E+00 | 7.303E-07 | 8.294E-07 | 2.219E-07 | 6.219E-07 | 0.000E+00 | 1.920E-06 |
| Unigene180339 | adeS | 4.658E-08 | 6.646E-07 | 8.456E-07 | 8.467E-07 | 0.000E+00 | 0.000E+00 | 0.000E+00 | 0.000E+00 | 0.000E+00 | 0.000E+00 | 0.000E+00 | 0.000E+00 |
| Unigene180347 | patB | 0.000E+00 | 0.000E+00 | 0.000E+00 | 0.000E+00 | 0.000E+00 | 0.000E+00 | 0.000E+00 | 0.000E+00 | 0.000E+00 | 0.000E+00 | 0.000E+00 | 4.022E-06 |
| Unigene180354 | patA | 3.741E-06 | 4.269E-07 | 2.871E-06 | 0.000E+00 | 3.086E-07 | 0.000E+00 | 5.124E-07 | 0.000E+00 | 0.000E+00 | 0.000E+00 | 9.745E-07 | 0.000E+00 |
| Unigene180418 | cmlB | 0.000E+00 | 0.000E+00 | 4.525E-07 | 0.000E+00 | 0.000E+00 | 0.000E+00 | 0.000E+00 | 0.000E+00 | 0.000E+00 | 0.000E+00 | 0.000E+00 | 0.000E+00 |
| Unigene180499 | bcrA | 3.087E-07 | 7.260E-07 | 3.894E-06 | 3.563E-06 | 0.000E+00 | 0.000E+00 | 0.000E+00 | 0.000E+00 | 0.000E+00 | 0.000E+00 | 0.000E+00 | 0.000E+00 |
| Unigene180508 | macB | 0.000E+00 | 0.000E+00 | 6.870E-07 | 0.000E+00 | 8.597E-07 | 1.115E-06 | 4.201E-06 | 3.075E-06 | 5.034E-07 | 8.826E-07 | 0.000E+00 | 4.007E-06 |
| Unigene180571 | cmlv | 0.000E+00 | 0.000E+00 | 6.599E-07 | 0.000E+00 | 0.000E+00 | 0.000E+00 | 2.634E-06 | 0.000E+00 | 0.000E+00 | 0.000E+00 | 0.000E+00 | 2.201E-06 |
| Unigene180624 | FusF | 5.052E-08 | 0.000E+00 | 1.274E-05 | 0.000E+00 | 0.000E+00 | 0.000E+00 | 0.000E+00 | 0.000E+00 | 0.000E+00 | 0.000E+00 | 0.000E+00 | 0.000E+00 |
| Unigene180706 | tcr3 | 0.000E+00 | 0.000E+00 | 1.156E-05 | 0.000E+00 | 0.000E+00 | 8.014E-07 | 2.813E-07 | 0.000E+00 | 0.000E+00 | 0.000E+00 | 0.000E+00 | 0.000E+00 |
| Unigene180713 | macB | 0.000E+00 | 0.000E+00 | 1.316E-05 | 0.000E+00 | 0.000E+00 | 0.000E+00 | 0.000E+00 | 0.000E+00 | 0.000E+00 | 0.000E+00 | 0.000E+00 | 0.000E+00 |
| Unigene180721 | blt | 0.000E+00 | 0.000E+00 | 1.400E-05 | 0.000E+00 | 0.000E+00 | 5.423E-07 | 3.886E-07 | 0.000E+00 | 0.000E+00 | 0.000E+00 | 0.000E+00 | 0.000E+00 |
| Unigene180725 | bcrA | 0.000E+00 | 0.000E+00 | 1.316E-05 | 0.000E+00 | 0.000E+00 | 0.000E+00 | 0.000E+00 | 0.000E+00 | 0.000E+00 | 0.000E+00 | 0.000E+00 | 0.000E+00 |
| Unigene180728 | vanRL | 0.000E+00 | 0.000E+00 | 1.208E-05 | 0.000E+00 | 0.000E+00 | 1.467E-06 | 2.146E-07 | 0.000E+00 | 0.000E+00 | 0.000E+00 | 0.000E+00 | 0.000E+00 |
| Unigene180766 | patA | 4.287E-08 | 0.000E+00 | 1.161E-05 | 0.000E+00 | 0.000E+00 | 0.000E+00 | 0.000E+00 | 0.000E+00 | 0.000E+00 | 0.000E+00 | 0.000E+00 | 0.000E+00 |
| Unigene180794 | arlR | 0.000E+00 | 0.000E+00 | 1.134E-05 | 0.000E+00 | 0.000E+00 | 8.635E-07 | 0.000E+00 | 0.000E+00 | 0.000E+00 | 0.000E+00 | 0.000E+00 | 0.000E+00 |
| Unigene180795 | arlS | 0.000E+00 | 0.000E+00 | 1.338E-05 | 0.000E+00 | 0.000E+00 | 7.180E-07 | 5.146E-07 | 0.000E+00 | 0.000E+00 | 0.000E+00 | 0.000E+00 | 0.000E+00 |
| Unigene180901 | efpA | 0.000E+00 | 0.000E+00 | 1.166E-05 | 0.000E+00 | 0.000E+00 | 5.428E-07 | 3.176E-07 | 0.000E+00 | 0.000E+00 | 0.000E+00 | 0.000E+00 | 0.000E+00 |
| Unigene180906 | PmrF | 0.000E+00 | 0.000E+00 | 1.103E-05 | 0.000E+00 | 3.795E-08 | 0.000E+00 | 2.646E-07 | 0.000E+00 | 0.000E+00 | 0.000E+00 | 0.000E+00 | 0.000E+00 |
| Unigene180917 | Streptomy | 0.000E+00 | 0.000E+00 | 1.158E-05 | 0.000E+00 | 1.732E-08 | 1.045E-06 | 6.041E-07 | 0.000E+00 | 0.000E+00 | 0.000E+00 | 0.000E+00 | 0.000E+00 |
| Unigene180943 | dfrC | 0.000E+00 | 0.000E+00 | 9.850E-06 | 0.000E+00 | 0.000E+00 | 8.511E-07 | 0.000E+00 | 0.000E+00 | 0.000E+00 | 0.000E+00 | 0.000E+00 | 0.000E+00 |
| Unigene180945 | TaeA | 0.000E+00 | 0.000E+00 | 1.086E-05 | 0.000E+00 | 0.000E+00 | 8.835E-07 | 4.711E-07 | 0.000E+00 | 0.000E+00 | 0.000E+00 | 0.000E+00 | 0.000E+00 |
| Unigene180993 | tetW | 0.000E+00 | 0.000E+00 | 1.280E-05 | 0.000E+00 | 0.000E+00 | 1.006E-06 | 2.914E-07 | 0.000E+00 | 0.000E+00 | 0.000E+00 | 0.000E+00 | 0.000E+00 |
| Unigene181015 | vanHO | 0.000E+00 | 0.000E+00 | 1.364E-05 | 0.000E+00 | 0.000E+00 | 0.000E+00 | 0.000E+00 | 0.000E+00 | 0.000E+00 | 0.000E+00 | 0.000E+00 | 0.000E+00 |
| Unigene181165 | MdtK | 0.000E+00 | 0.000E+00 | 0.000E+00 | 1.156E-07 | 0.000E+00 | 6.187E-08 | 0.000E+00 | 0.000E+00 | 0.000E+00 | 0.000E+00 | 0.000E+00 | 0.000E+00 |
| Unigene181201 | lsaC | 0.000E+00 | 0.000E+00 | 1.508E-06 | 0.000E+00 | 0.000E+00 | 0.000E+00 | 0.000E+00 | 0.000E+00 | 0.000E+00 | 0.000E+00 | 1.465E-07 | 0.000E+00 |
| Unigene181278 | rpoB2 | 0.000E+00 | 2.475E-07 | 3.477E-07 | 0.000E+00 | 0.000E+00 | 0.000E+00 | 0.000E+00 | 0.000E+00 | 0.000E+00 | 0.000E+00 | 0.000E+00 | 3.427E-07 |
| Unigene181300 | Streptomy | 0.000E+00 | 0.000E+00 | 4.446E-07 | 0.000E+00 | 0.000E+00 | 0.000E+00 | 0.000E+00 | 0.000E+00 | 0.000E+00 | 0.000E+00 | 0.000E+00 | 0.000E+00 |
| Unigene181310 | bcrA | 0.000E+00 | 7.157E-07 | 9.954E-07 | 7.683E-07 | 0.000E+00 | 0.000E+00 | 0.000E+00 | 0.000E+00 | 0.000E+00 | 0.000E+00 | 0.000E+00 | 0.000E+00 |
| Unigene181322 | TaeA | 0.000E+00 | 0.000E+00 | 1.152E-05 | 0.000E+00 | 0.000E+00 | 0.000E+00 | 0.000E+00 | 0.000E+00 | 0.000E+00 | 0.000E+00 | 0.000E+00 | 0.000E+00 |
| Unigene181362 | vanSF | 2.683E-08 | 1.178E-07 | 1.211E-06 | 4.877E-07 | 0.000E+00 | 0.000E+00 | 0.000E+00 | 0.000E+00 | 0.000E+00 | 0.000E+00 | 0.000E+00 | 0.000E+00 |
| Unigene181363 | vanRF | 0.000E+00 | 3.081E-07 | 1.515E-06 | 2.362E-07 | 0.000E+00 | 0.000E+00 | 0.000E+00 | 0.000E+00 | 0.000E+00 | 0.000E+00 | 0.000E+00 | 0.000E+00 |
| Unigene181375 | macB | 3.654E-07 | 0.000E+00 | 6.773E-07 | 0.000E+00 | 1.173E-06 | 4.828E-07 | 1.483E-06 | 1.232E-06 | 0.000E+00 | 0.000E+00 | 0.000E+00 | 3.984E-06 |
| Unigene181413 | bcrA | 0.000E+00 | 0.000E+00 | 1.916E-06 | 1.210E-05 | 0.000E+00 | 0.000E+00 | 0.000E+00 | 0.000E+00 | 0.000E+00 | 0.000E+00 | 0.000E+00 | 0.000E+00 |
| Unigene181423 | lfrA | 0.000E+00 | 8.636E-08 | 1.718E-06 | 1.359E-05 | 0.000E+00 | 0.000E+00 | 0.000E+00 | 0.000E+00 | 0.000E+00 | 0.000E+00 | 0.000E+00 | 0.000E+00 |
| Unigene181436 | tetA(58) | 0.000E+00 | 0.000E+00 | 2.566E-06 | 1.438E-05 | 0.000E+00 | 0.000E+00 | 0.000E+00 | 0.000E+00 | 0.000E+00 | 0.000E+00 | 0.000E+00 | 0.000E+00 |
| Unigene181483 | vmlR | 2.919E-07 | 2.746E-07 | 9.003E-07 | 1.011E-06 | 0.000E+00 | 0.000E+00 | 0.000E+00 | 0.000E+00 | 0.000E+00 | 0.000E+00 | 0.000E+00 | 1.479E-07 |
| Unigene181498 | oleC | 0.000E+00 | 0.000E+00 | 8.798E-07 | 7.602E-05 | 0.000E+00 | 0.000E+00 | 0.000E+00 | 2.093E-07 | 0.000E+00 | 0.000E+00 | 0.000E+00 | 0.000E+00 |
| Unigene181655 | Staphylocc | 0.000E+00 | 0.000E+00 | 6.838E-07 | 0.000E+00 | 0.000E+00 | 0.000E+00 | 0.000E+00 | 0.000E+00 | 0.000E+00 | 0.000E+00 | 0.000E+00 | 0.000E+00 |
| Unigene181659 | efpA | 0.000E+00 | 0.000E+00 | 4.493E-07 | 0.000E+00 | 0.000E+00 | 0.000E+00 | 0.000E+00 | 0.000E+00 | 0.000E+00 | 0.000E+00 | 0.000E+00 | 0.000E+00 |
| Unigene181693 | mtrA | 1.159E-04 | 1.933E-04 | 1.843E-04 | 8.341E-06 | 9.771E-06 | 4.827E-07 | 3.805E-06 | 0.000E+00 | 6.115E-05 | 0.000E+00 | 4.239E-06 | 0.000E+00 |
| Unigene181748 | basS | 1.548E-07 | 0.000E+00 | 5.317E-07 | 0.000E+00 | 0.000E+00 | 0.000E+00 | 0.000E+00 | 0.000E+00 | 0.000E+00 | 0.000E+00 | 0.000E+00 | 0.000E+00 |

| | | | | | | | | | | | | | |
|---|---|---|---|---|---|---|---|---|---|---|---|---|---|
| Unigene182003 | Staphylocc | 2.053E-07 | 1.578E-07 | 1.733E-06 | 7.237E-06 | 0.000E+00 | 0.000E+00 | 0.000E+00 | 0.000E+00 | 0.000E+00 | 0.000E+00 | 0.000E+00 | 0.000E+00 |
| Unigene182081 | vanHO | 0.000E+00 | 7.050E-08 | 5.988E-07 | 0.000E+00 | 1.249E-06 | 6.945E-07 | 1.813E-06 | 1.050E-06 | 0.000E+00 | 6.083E-07 | 9.920E-07 | 2.772E-06 |
| Unigene182085 | patA | 0.000E+00 | 0.000E+00 | 7.078E-07 | 0.000E+00 | 9.349E-07 | 5.199E-07 | 0.000E+00 | 0.000E+00 | 0.000E+00 | 0.000E+00 | 8.595E-07 | 1.883E-06 |
| Unigene182091 | patA | 3.409E-07 | 5.846E-07 | 3.136E-06 | 2.711E-06 | 0.000E+00 | 0.000E+00 | 0.000E+00 | 0.000E+00 | 0.000E+00 | 0.000E+00 | 0.000E+00 | 0.000E+00 |
| Unigene182102 | tetA(46) | 0.000E+00 | 0.000E+00 | 6.142E-07 | 0.000E+00 | 0.000E+00 | 0.000E+00 | 0.000E+00 | 3.688E-06 | 0.000E+00 | 0.000E+00 | 0.000E+00 | 4.756E-06 |
| Unigene182128 | efrA | 1.633E-07 | 5.974E-08 | 1.282E-06 | 5.186E-06 | 0.000E+00 | 0.000E+00 | 0.000E+00 | 0.000E+00 | 0.000E+00 | 0.000E+00 | 0.000E+00 | 0.000E+00 |
| Unigene182129 | tetA(58) | 0.000E+00 | 1.265E-07 | 1.094E-06 | 4.230E-06 | 0.000E+00 | 0.000E+00 | 0.000E+00 | 0.000E+00 | 0.000E+00 | 0.000E+00 | 0.000E+00 | 0.000E+00 |
| Unigene182134 | tetW | 1.375E-07 | 3.018E-07 | 1.670E-06 | 5.971E-06 | 0.000E+00 | 0.000E+00 | 0.000E+00 | 0.000E+00 | 0.000E+00 | 0.000E+00 | 0.000E+00 | 0.000E+00 |
| Unigene182171 | dfrB6 | 2.178E-07 | 0.000E+00 | 5.557E-07 | 0.000E+00 | 0.000E+00 | 0.000E+00 | 6.268E-07 | 0.000E+00 | 4.761E-08 | 0.000E+00 | 4.983E-08 | 0.000E+00 |
| Unigene182248 | efrB | 0.000E+00 | 0.000E+00 | 1.221E-07 | 1.256E-07 | 2.073E-07 | 0.000E+00 | 0.000E+00 | 3.630E-07 | 0.000E+00 | 0.000E+00 | 1.423E-07 | 3.676E-07 |
| Unigene182252 | baeR | 0.000E+00 | 0.000E+00 | 5.936E-07 | 6.109E-07 | 5.761E-07 | 1.261E-06 | 4.926E-06 | 5.094E-06 | 8.029E-07 | 0.000E+00 | 2.274E-06 | 5.874E-06 |
| Unigene182282 | Acinetoba | 5.147E-07 | 1.480E-06 | 1.347E-06 | 2.970E-07 | 4.003E-06 | 0.000E+00 | 0.000E+00 | 2.861E-07 | 0.000E+00 | 0.000E+00 | 0.000E+00 | 0.000E+00 |
| Unigene182606 | facT | 0.000E+00 | 0.000E+00 | 1.173E-06 | 0.000E+00 | 0.000E+00 | 0.000E+00 | 2.594E-08 | 0.000E+00 | 0.000E+00 | 1.828E-07 | 2.682E-08 | 1.081E-06 |
| Unigene182620 | bcrA | 0.000E+00 | 1.158E-07 | 6.469E-07 | 2.663E-07 | 0.000E+00 | 0.000E+00 | 0.000E+00 | 0.000E+00 | 0.000E+00 | 0.000E+00 | 0.000E+00 | 0.000E+00 |
| Unigene182733 | novA | 1.500E-07 | 5.761E-07 | 8.461E-07 | 2.461E-06 | 3.332E-07 | 0.000E+00 | 2.075E-07 | 1.050E-06 | 1.434E-07 | 6.265E-07 | 1.179E-06 | 8.420E-07 |
| Unigene182753 | carA | 0.000E+00 | 9.712E-08 | 2.032E-06 | 1.374E-05 | 0.000E+00 | 0.000E+00 | 0.000E+00 | 0.000E+00 | 0.000E+00 | 0.000E+00 | 0.000E+00 | 0.000E+00 |
| Unigene182760 | bcrA | 0.000E+00 | 7.973E-07 | 4.811E-06 | 3.209E-06 | 0.000E+00 | 0.000E+00 | 0.000E+00 | 0.000E+00 | 0.000E+00 | 0.000E+00 | 0.000E+00 | 0.000E+00 |
| Unigene182762 | vanRM | 0.000E+00 | 7.702E-07 | 3.030E-06 | 3.307E-06 | 0.000E+00 | 0.000E+00 | 0.000E+00 | 0.000E+00 | 0.000E+00 | 0.000E+00 | 0.000E+00 | 0.000E+00 |
| Unigene182763 | vanSF | 6.628E-08 | 7.760E-07 | 3.317E-06 | 3.302E-06 | 0.000E+00 | 0.000E+00 | 0.000E+00 | 0.000E+00 | 0.000E+00 | 0.000E+00 | 0.000E+00 | 0.000E+00 |
| Unigene182796 | Chlamydia | 4.947E-08 | 0.000E+00 | 6.311E-07 | 6.070E-05 | 0.000E+00 | 0.000E+00 | 0.000E+00 | 5.775E-08 | 0.000E+00 | 0.000E+00 | 0.000E+00 | 0.000E+00 |
| Unigene182838 | MexD | 0.000E+00 | 0.000E+00 | 0.000E+00 | 0.000E+00 | 0.000E+00 | 3.213E-07 | 0.000E+00 | 0.000E+00 | 0.000E+00 | 0.000E+00 | 0.000E+00 | 0.000E+00 |
| Unigene182886 | vanHO | 5.543E-07 | 7.873E-07 | 1.696E-06 | 2.700E-06 | 0.000E+00 | 0.000E+00 | 1.443E-07 | 1.180E-06 | 1.426E-07 | 5.448E-07 | 8.952E-07 | 4.240E-07 |
| Unigene183002 | vanTG | 0.000E+00 | 0.000E+00 | 1.070E-06 | 7.102E-05 | 0.000E+00 | 0.000E+00 | 0.000E+00 | 6.699E-08 | 0.000E+00 | 0.000E+00 | 0.000E+00 | 0.000E+00 |
| Unigene183071 | arlS | 5.198E-08 | 1.141E-07 | 7.141E-07 | 1.575E-07 | 0.000E+00 | 0.000E+00 | 0.000E+00 | 0.000E+00 | 0.000E+00 | 0.000E+00 | 0.000E+00 | 0.000E+00 |
| Unigene183093 | vanHB | 0.000E+00 | 0.000E+00 | 6.540E-07 | 0.000E+00 | 0.000E+00 | 0.000E+00 | 2.810E-06 | 0.000E+00 | 0.000E+00 | 0.000E+00 | 1.343E-06 | 0.000E+00 |
| Unigene183122 | optrA | 5.191E-07 | 4.144E-07 | 1.713E-06 | 1.763E-06 | 0.000E+00 | 0.000E+00 | 4.701E-07 | 1.708E-06 | 2.579E-07 | 8.937E-07 | 1.673E-06 | 8.368E-07 |
| Unigene183138 | TaeA | 0.000E+00 | 0.000E+00 | 6.748E-07 | 1.225E-07 | 5.842E-07 | 1.312E-07 | 0.000E+00 | 0.000E+00 | 0.000E+00 | 0.000E+00 | 0.000E+00 | 0.000E+00 |
| Unigene183174 | Acinetoba | 0.000E+00 | 5.726E-07 | 6.399E-07 | 0.000E+00 | 0.000E+00 | 0.000E+00 | 0.000E+00 | 0.000E+00 | 9.503E-08 | 0.000E+00 | 0.000E+00 | 0.000E+00 |
| Unigene183192 | smeS | 2.296E-06 | 1.046E-06 | 1.623E-06 | 4.716E-07 | 0.000E+00 | 0.000E+00 | 0.000E+00 | 1.136E-07 | 0.000E+00 | 0.000E+00 | 0.000E+00 | 0.000E+00 |
| Unigene183210 | arlR | 0.000E+00 | 0.000E+00 | 8.174E-07 | 2.804E-07 | 0.000E+00 | 0.000E+00 | 0.000E+00 | 0.000E+00 | 0.000E+00 | 0.000E+00 | 0.000E+00 | 0.000E+00 |
| Unigene183299 | srmB | 0.000E+00 | 0.000E+00 | 9.961E-07 | 6.560E-07 | 1.353E-06 | 1.668E-06 | 6.336E-06 | 2.796E-06 | 7.101E-07 | 1.764E-06 | 2.647E-06 | 3.648E-06 |
| Unigene183317 | tet(38) | 1.627E-07 | 0.000E+00 | 1.824E-06 | 6.477E-06 | 0.000E+00 | 0.000E+00 | 0.000E+00 | 0.000E+00 | 0.000E+00 | 0.000E+00 | 0.000E+00 | 0.000E+00 |
| Unigene183426 | novA | 0.000E+00 | 0.000E+00 | 4.566E-07 | 0.000E+00 | 0.000E+00 | 0.000E+00 | 3.219E-07 | 0.000E+00 | 1.271E-07 | 3.888E-07 | 0.000E+00 | 4.812E-07 |
| Unigene183459 | macB | 0.000E+00 | 0.000E+00 | 9.968E-07 | 5.433E-06 | 0.000E+00 | 0.000E+00 | 0.000E+00 | 0.000E+00 | 0.000E+00 | 0.000E+00 | 0.000E+00 | 0.000E+00 |
| Unigene183465 | tetB(46) | 0.000E+00 | 2.105E-07 | 1.338E-06 | 6.197E-06 | 0.000E+00 | 0.000E+00 | 0.000E+00 | 0.000E+00 | 0.000E+00 | 0.000E+00 | 0.000E+00 | 0.000E+00 |
| Unigene183469 | Chlamydia | 1.454E-07 | 0.000E+00 | 2.093E-06 | 7.170E-06 | 0.000E+00 | 0.000E+00 | 0.000E+00 | 0.000E+00 | 0.000E+00 | 0.000E+00 | 0.000E+00 | 0.000E+00 |
| Unigene183479 | msbA | 3.029E-07 | 0.000E+00 | 1.643E-06 | 7.034E-06 | 0.000E+00 | 0.000E+00 | 0.000E+00 | 0.000E+00 | 0.000E+00 | 0.000E+00 | 0.000E+00 | 0.000E+00 |
| Unigene183480 | novA | 0.000E+00 | 2.220E-07 | 9.129E-07 | 5.760E-06 | 0.000E+00 | 0.000E+00 | 0.000E+00 | 0.000E+00 | 0.000E+00 | 0.000E+00 | 0.000E+00 | 0.000E+00 |
| Unigene183567 | vanTrL | 0.000E+00 | 0.000E+00 | 1.124E-06 | 0.000E+00 | 0.000E+00 | 9.775E-08 | 0.000E+00 | 7.036E-08 | 6.589E-08 | 0.000E+00 | 3.448E-08 | 1.069E-06 |
| Unigene183639 | NmcR | 0.000E+00 | 2.504E-07 | 5.596E-07 | 0.000E+00 | 0.000E+00 | 0.000E+00 | 0.000E+00 | 0.000E+00 | 0.000E+00 | 0.000E+00 | 0.000E+00 | 0.000E+00 |
| Unigene183640 | fexA | 0.000E+00 | 0.000E+00 | 2.112E-07 | 0.000E+00 | 1.913E-07 | 0.000E+00 | 0.000E+00 | 0.000E+00 | 0.000E+00 | 0.000E+00 | 0.000E+00 | 0.000E+00 |
| Unigene183702 | PmrF | 2.690E-07 | 0.000E+00 | 7.542E-07 | 0.000E+00 | 1.409E-06 | 2.908E-07 | 1.404E-06 | 8.074E-07 | 0.000E+00 | 0.000E+00 | 0.000E+00 | 2.953E-06 |
| Unigene183829 | emrB | 4.024E-06 | 1.008E-05 | 9.075E-06 | 1.057E-05 | 1.482E-07 | 9.613E-08 | 0.000E+00 | 0.000E+00 | 0.000E+00 | 0.000E+00 | 2.543E-08 | 0.000E+00 |
| Unigene183841 | patB | 0.000E+00 | 0.000E+00 | 9.060E-07 | 0.000E+00 | 0.000E+00 | 0.000E+00 | 0.000E+00 | 0.000E+00 | 0.000E+00 | 0.000E+00 | 0.000E+00 | 0.000E+00 |
| Unigene183958 | arlS | 1.993E-07 | 3.554E-07 | 1.100E-06 | 2.566E-06 | 1.384E-07 | 2.154E-07 | 2.481E-07 | 1.337E-06 | 2.722E-08 | 6.659E-07 | 9.117E-07 | 6.183E-07 |
| Unigene183959 | arlR | 2.319E-07 | 0.000E+00 | 9.102E-07 | 2.904E-06 | 7.214E-07 | 0.000E+00 | 2.053E-07 | 1.191E-06 | 0.000E+00 | 0.000E+00 | 1.804E-06 | 4.934E-07 |
| Unigene183961 | vanTG | 2.014E-07 | 5.999E-07 | 1.129E-06 | 2.499E-06 | 5.434E-07 | 1.866E-07 | 9.552E-08 | 8.731E-07 | 2.516E-07 | 6.410E-07 | 8.886E-07 | 8.502E-07 |
| Unigene184087 | mdtG | 8.030E-08 | 0.000E+00 | 8.142E-07 | 7.603E-05 | 0.000E+00 | 0.000E+00 | 0.000E+00 | 1.875E-07 | 0.000E+00 | 0.000E+00 | 0.000E+00 | 0.000E+00 |
| Unigene184111 | TolC | 0.000E+00 | 1.303E-07 | 3.494E-07 | 0.000E+00 | 0.000E+00 | 0.000E+00 | 1.751E-07 | 0.000E+00 | 0.000E+00 | 0.000E+00 | 0.000E+00 | 0.000E+00 |
| Unigene184114 | Staphylocc | 4.304E-08 | 0.000E+00 | 8.658E-07 | 8.284E-05 | 0.000E+00 | 0.000E+00 | 0.000E+00 | 2.512E-08 | 0.000E+00 | 0.000E+00 | 0.000E+00 | 0.000E+00 |
| Unigene184117 | tetT | 0.000E+00 | 0.000E+00 | 3.443E-07 | 0.000E+00 | 0.000E+00 | 0.000E+00 | 0.000E+00 | 0.000E+00 | 0.000E+00 | 0.000E+00 | 0.000E+00 | 0.000E+00 |
| Unigene184132 | poxtA | 0.000E+00 | 0.000E+00 | 1.394E-06 | 0.000E+00 | 0.000E+00 | 0.000E+00 | 0.000E+00 | 0.000E+00 | 0.000E+00 | 0.000E+00 | 0.000E+00 | 0.000E+00 |
| Unigene184207 | Acinetoba | 2.314E-08 | 0.000E+00 | 1.340E-06 | 0.000E+00 | 7.712E-08 | 0.000E+00 | 0.000E+00 | 0.000E+00 | 1.517E-07 | 7.733E-08 | 1.059E-07 | 1.313E-06 |
| Unigene184244 | efrB | 0.000E+00 | 0.000E+00 | 6.285E-07 | 0.000E+00 | 0.000E+00 | 0.000E+00 | 0.000E+00 | 0.000E+00 | 0.000E+00 | 0.000E+00 | 0.000E+00 | 0.000E+00 |
| Unigene184245 | bcrA | 0.000E+00 | 0.000E+00 | 4.738E-07 | 0.000E+00 | 0.000E+00 | 0.000E+00 | 0.000E+00 | 0.000E+00 | 0.000E+00 | 0.000E+00 | 0.000E+00 | 0.000E+00 |
| Unigene184263 | lmrD | 0.000E+00 | 0.000E+00 | 4.140E-07 | 0.000E+00 | 6.563E-07 | 0.000E+00 | 0.000E+00 | 7.388E-07 | 0.000E+00 | 7.521E-07 | 0.000E+00 | 9.975E-07 |
| Unigene184310 | adeS | 0.000E+00 | 1.836E-07 | 5.197E-07 | 0.000E+00 | 0.000E+00 | 0.000E+00 | 0.000E+00 | 0.000E+00 | 0.000E+00 | 0.000E+00 | 0.000E+00 | 0.000E+00 |
| Unigene184403 | msbA | 0.000E+00 | 0.000E+00 | 1.000E-06 | 0.000E+00 | 0.000E+00 | 0.000E+00 | 0.000E+00 | 0.000E+00 | 0.000E+00 | 0.000E+00 | 0.000E+00 | 0.000E+00 |

| | | | | | | | | | | | | | |
|---|---|---|---|---|---|---|---|---|---|---|---|---|---|
| Unigene184404 | carA | 0.000E+00 | 0.000E+00 | 8.093E-07 | 0.000E+00 | 0.000E+00 | 0.000E+00 | 0.000E+00 | 0.000E+00 | 0.000E+00 | 0.000E+00 | 0.000E+00 | 0.000E+00 |
| Unigene184416 | vgaD | 0.000E+00 | 0.000E+00 | 1.265E-05 | 0.000E+00 | 0.000E+00 | 9.674E-07 | 0.000E+00 | 0.000E+00 | 0.000E+00 | 0.000E+00 | 0.000E+00 | 0.000E+00 |
| Unigene184417 | Staphylocc | 0.000E+00 | 0.000E+00 | 1.229E-05 | 0.000E+00 | 0.000E+00 | 1.116E-06 | 0.000E+00 | 8.034E-08 | 0.000E+00 | 0.000E+00 | 0.000E+00 | 0.000E+00 |
| Unigene184422 | emrB | 0.000E+00 | 0.000E+00 | 1.369E-05 | 0.000E+00 | 0.000E+00 | 0.000E+00 | 0.000E+00 | 0.000E+00 | 0.000E+00 | 0.000E+00 | 0.000E+00 | 0.000E+00 |
| Unigene184477 | optrA | 3.321E-08 | 0.000E+00 | 1.255E-05 | 0.000E+00 | 5.534E-08 | 6.642E-07 | 7.167E-07 | 0.000E+00 | 0.000E+00 | 0.000E+00 | 0.000E+00 | 0.000E+00 |
| Unigene184552 | vanRM | 0.000E+00 | 0.000E+00 | 1.118E-05 | 0.000E+00 | 0.000E+00 | 0.000E+00 | 0.000E+00 | 0.000E+00 | 0.000E+00 | 0.000E+00 | 0.000E+00 | 0.000E+00 |
| Unigene184553 | baeS | 0.000E+00 | 0.000E+00 | 1.268E-05 | 0.000E+00 | 0.000E+00 | 7.878E-07 | 0.000E+00 | 0.000E+00 | 0.000E+00 | 0.000E+00 | 0.000E+00 | 0.000E+00 |
| Unigene184560 | Acinetobac | 0.000E+00 | 0.000E+00 | 1.164E-05 | 0.000E+00 | 0.000E+00 | 1.134E-06 | 0.000E+00 | 0.000E+00 | 0.000E+00 | 0.000E+00 | 0.000E+00 | 0.000E+00 |
| Unigene184565 | efrA | 0.000E+00 | 0.000E+00 | 1.297E-05 | 0.000E+00 | 0.000E+00 | 1.251E-06 | 4.268E-07 | 0.000E+00 | 0.000E+00 | 0.000E+00 | 0.000E+00 | 0.000E+00 |
| Unigene184668 | vanTG | 0.000E+00 | 0.000E+00 | 1.338E-05 | 0.000E+00 | 0.000E+00 | 0.000E+00 | 7.725E-07 | 0.000E+00 | 0.000E+00 | 0.000E+00 | 0.000E+00 | 0.000E+00 |
| Unigene184683 | mdtG | 0.000E+00 | 0.000E+00 | 1.157E-05 | 0.000E+00 | 1.172E-07 | 8.553E-07 | 7.297E-07 | 1.539E-07 | 0.000E+00 | 0.000E+00 | 0.000E+00 | 0.000E+00 |
| Unigene184698 | optrA | 0.000E+00 | 0.000E+00 | 9.898E-06 | 0.000E+00 | 0.000E+00 | 0.000E+00 | 6.911E-07 | 0.000E+00 | 0.000E+00 | 0.000E+00 | 0.000E+00 | 0.000E+00 |
| Unigene184707 | Erm(48) | 0.000E+00 | 0.000E+00 | 1.187E-05 | 0.000E+00 | 0.000E+00 | 0.000E+00 | 5.195E-07 | 1.264E-07 | 0.000E+00 | 0.000E+00 | 0.000E+00 | 0.000E+00 |
| Unigene184710 | Staphylocc | 0.000E+00 | 0.000E+00 | 1.363E-05 | 0.000E+00 | 0.000E+00 | 8.935E-07 | 2.990E-07 | 9.276E-08 | 0.000E+00 | 0.000E+00 | 0.000E+00 | 0.000E+00 |
| Unigene184756 | patA | 0.000E+00 | 0.000E+00 | 1.863E-06 | 0.000E+00 | 3.516E-07 | 0.000E+00 | 0.000E+00 | 0.000E+00 | 0.000E+00 | 0.000E+00 | 0.000E+00 | 1.389E-06 |
| Unigene184757 | lmrD | 0.000E+00 | 0.000E+00 | 1.691E-06 | 1.273E-07 | 0.000E+00 | 0.000E+00 | 0.000E+00 | 4.906E-08 | 2.297E-07 | 0.000E+00 | 0.000E+00 | 6.956E-07 |
| Unigene184904 | rpoB2 | 0.000E+00 | 0.000E+00 | 9.598E-07 | 0.000E+00 | 0.000E+00 | 0.000E+00 | 0.000E+00 | 0.000E+00 | 0.000E+00 | 0.000E+00 | 0.000E+00 | 0.000E+00 |
| Unigene184920 | adeF | 0.000E+00 | 0.000E+00 | 1.288E-07 | 0.000E+00 | 0.000E+00 | 0.000E+00 | 0.000E+00 | 0.000E+00 | 0.000E+00 | 0.000E+00 | 0.000E+00 | 0.000E+00 |
| Unigene184968 | arnA | 1.093E-05 | 3.804E-06 | 3.757E-06 | 1.333E-05 | 6.886E-05 | 1.072E-04 | 1.033E-04 | 8.191E-05 | 1.355E-04 | 1.351E-04 | 1.045E-04 | 7.093E-05 |
| Unigene184991 | vanHO | 0.000E+00 | 1.206E-06 | 2.315E-06 | 3.785E-06 | 0.000E+00 | 0.000E+00 | 0.000E+00 | 0.000E+00 | 0.000E+00 | 0.000E+00 | 0.000E+00 | 0.000E+00 |
| Unigene184997 | vanSG | 0.000E+00 | 2.963E-07 | 5.298E-07 | 0.000E+00 | 0.000E+00 | 0.000E+00 | 0.000E+00 | 2.701E-07 | 0.000E+00 | 0.000E+00 | 0.000E+00 | 0.000E+00 |
| Unigene185010 | vanRG | 0.000E+00 | 1.489E-07 | 1.198E-06 | 1.370E-06 | 0.000E+00 | 0.000E+00 | 0.000E+00 | 0.000E+00 | 0.000E+00 | 0.000E+00 | 0.000E+00 | 0.000E+00 |
| Unigene185011 | vanSF | 5.279E-07 | 4.679E-07 | 1.136E-06 | 1.333E-06 | 0.000E+00 | 0.000E+00 | 0.000E+00 | 0.000E+00 | 0.000E+00 | 0.000E+00 | 0.000E+00 | 9.599E-08 |
| Unigene185091 | smeS | 0.000E+00 | 0.000E+00 | 4.106E-07 | 0.000E+00 | 0.000E+00 | 0.000E+00 | 0.000E+00 | 0.000E+00 | 0.000E+00 | 0.000E+00 | 0.000E+00 | 1.131E-06 |
| Unigene185122 | mexM | 0.000E+00 | 0.000E+00 | 6.599E-07 | 0.000E+00 | 0.000E+00 | 0.000E+00 | 0.000E+00 | 0.000E+00 | 0.000E+00 | 0.000E+00 | 0.000E+00 | 0.000E+00 |
| Unigene185145 | tlrC | 0.000E+00 | 6.807E-08 | 3.043E-07 | 2.192E-07 | 0.000E+00 | 0.000E+00 | 0.000E+00 | 0.000E+00 | 0.000E+00 | 0.000E+00 | 0.000E+00 | 0.000E+00 |
| Unigene185166 | adeL | 0.000E+00 | 0.000E+00 | 3.810E-07 | 0.000E+00 | 0.000E+00 | 0.000E+00 | 0.000E+00 | 0.000E+00 | 0.000E+00 | 0.000E+00 | 0.000E+00 | 0.000E+00 |
| Unigene185356 | optrA | 0.000E+00 | 0.000E+00 | 6.464E-07 | 8.626E-05 | 0.000E+00 | 0.000E+00 | 0.000E+00 | 0.000E+00 | 0.000E+00 | 0.000E+00 | 0.000E+00 | 0.000E+00 |
| Unigene185377 | tetA(58) | 0.000E+00 | 0.000E+00 | 0.000E+00 | 0.000E+00 | 0.000E+00 | 1.122E-06 | 5.487E-06 | 4.216E-06 | 0.000E+00 | 0.000E+00 | 2.374E-06 | 4.906E-06 |
| Unigene185426 | evgS | 0.000E+00 | 1.810E-07 | 6.068E-07 | 0.000E+00 | 0.000E+00 | 0.000E+00 | 0.000E+00 | 0.000E+00 | 0.000E+00 | 0.000E+00 | 0.000E+00 | 0.000E+00 |
| Unigene185513 | bcrA | 0.000E+00 | 0.000E+00 | 0.000E+00 | 0.000E+00 | 0.000E+00 | 0.000E+00 | 5.133E-06 | 0.000E+00 | 0.000E+00 | 0.000E+00 | 0.000E+00 | 0.000E+00 |
| Unigene185519 | vgaB | 3.550E-06 | 1.043E-05 | 1.067E-05 | 1.124E-05 | 0.000E+00 | 7.995E-08 | 0.000E+00 | 0.000E+00 | 0.000E+00 | 0.000E+00 | 0.000E+00 | 0.000E+00 |
| Unigene185563 | tlrC | 4.339E-08 | 0.000E+00 | 1.064E-06 | 0.000E+00 | 2.410E-07 | 0.000E+00 | 0.000E+00 | 0.000E+00 | 1.186E-07 | 0.000E+00 | 0.000E+00 | 5.641E-07 |
| Unigene185628 | lsaC | 4.052E-07 | 1.976E-07 | 1.502E-06 | 2.046E-07 | 1.500E-07 | 0.000E+00 | 0.000E+00 | 1.051E-07 | 0.000E+00 | 0.000E+00 | 3.605E-07 | 0.000E+00 |
| Unigene185709 | Corynebac | 0.000E+00 | 0.000E+00 | 4.288E-07 | 4.412E-07 | 0.000E+00 | 0.000E+00 | 0.000E+00 | 0.000E+00 | 0.000E+00 | 0.000E+00 | 0.000E+00 | 0.000E+00 |
| Unigene185821 | baeS | 0.000E+00 | 4.330E-07 | 1.584E-06 | 1.485E-06 | 0.000E+00 | 0.000E+00 | 0.000E+00 | 0.000E+00 | 0.000E+00 | 0.000E+00 | 0.000E+00 | 0.000E+00 |
| Unigene185846 | Listeria mc | 6.608E-07 | 9.032E-07 | 4.612E-06 | 6.018E-06 | 0.000E+00 | 0.000E+00 | 3.174E-07 | 0.000E+00 | 0.000E+00 | 0.000E+00 | 2.852E-08 | 0.000E+00 |
| Unigene185925 | novA | 0.000E+00 | 4.781E-08 | 1.325E-06 | 1.036E-04 | 0.000E+00 | 0.000E+00 | 0.000E+00 | 5.085E-08 | 0.000E+00 | 0.000E+00 | 0.000E+00 | 0.000E+00 |
| Unigene185954 | macB | 0.000E+00 | 4.703E-07 | 2.756E-06 | 2.596E-06 | 0.000E+00 | 0.000E+00 | 0.000E+00 | 0.000E+00 | 0.000E+00 | 0.000E+00 | 0.000E+00 | 0.000E+00 |
| Unigene185956 | tetA(58) | 1.076E-07 | 4.330E-07 | 2.710E-06 | 2.137E-06 | 0.000E+00 | 0.000E+00 | 0.000E+00 | 0.000E+00 | 0.000E+00 | 0.000E+00 | 0.000E+00 | 0.000E+00 |
| Unigene186023 | ugd | 5.301E-08 | 0.000E+00 | 0.000E+00 | 0.000E+00 | 1.884E-06 | 0.000E+00 | 7.040E-07 | 0.000E+00 | 0.000E+00 | 0.000E+00 | 6.063E-07 | 0.000E+00 |
| Unigene186063 | Bifidobacte | 0.000E+00 | 0.000E+00 | 3.890E-07 | 0.000E+00 | 0.000E+00 | 0.000E+00 | 0.000E+00 | 0.000E+00 | 0.000E+00 | 0.000E+00 | 0.000E+00 | 0.000E+00 |
| Unigene186105 | vanHB | 0.000E+00 | 0.000E+00 | 1.062E-06 | 4.514E-05 | 0.000E+00 | 0.000E+00 | 0.000E+00 | 0.000E+00 | 0.000E+00 | 0.000E+00 | 0.000E+00 | 0.000E+00 |
| Unigene186109 | tetA(60) | 0.000E+00 | 0.000E+00 | 9.096E-07 | 6.690E-05 | 0.000E+00 | 0.000E+00 | 0.000E+00 | 1.273E-07 | 0.000E+00 | 0.000E+00 | 0.000E+00 | 0.000E+00 |
| Unigene186110 | tetB(46) | 0.000E+00 | 0.000E+00 | 7.026E-07 | 6.620E-05 | 0.000E+00 | 0.000E+00 | 0.000E+00 | 0.000E+00 | 0.000E+00 | 0.000E+00 | 0.000E+00 | 0.000E+00 |
| Unigene186134 | lmrD | 2.202E-07 | 3.108E-07 | 7.718E-07 | 0.000E+00 | 8.389E-07 | 2.721E-07 | 1.219E-06 | 8.447E-07 | 4.127E-07 | 9.113E-07 | 8.637E-07 | 4.500E-06 |
| Unigene186135 | vanTC | 7.119E-07 | 4.507E-07 | 3.304E-06 | 5.474E-06 | 0.000E+00 | 0.000E+00 | 2.727E-07 | 0.000E+00 | 0.000E+00 | 0.000E+00 | 0.000E+00 | 3.236E-08 |
| Unigene186263 | msbA | 1.187E-07 | 1.955E-07 | 1.427E-06 | 3.162E-06 | 0.000E+00 | 0.000E+00 | 0.000E+00 | 0.000E+00 | 0.000E+00 | 0.000E+00 | 0.000E+00 | 0.000E+00 |
| Unigene186300 | lsaC | 0.000E+00 | 0.000E+00 | 1.008E-06 | 0.000E+00 | 0.000E+00 | 0.000E+00 | 0.000E+00 | 0.000E+00 | 0.000E+00 | 0.000E+00 | 0.000E+00 | 0.000E+00 |
| Unigene186306 | vanSA | 0.000E+00 | 0.000E+00 | 9.776E-07 | 0.000E+00 | 0.000E+00 | 0.000E+00 | 0.000E+00 | 0.000E+00 | 0.000E+00 | 0.000E+00 | 0.000E+00 | 0.000E+00 |
| Unigene186307 | vanRF | 0.000E+00 | 0.000E+00 | 1.245E-06 | 0.000E+00 | 0.000E+00 | 0.000E+00 | 0.000E+00 | 0.000E+00 | 0.000E+00 | 0.000E+00 | 0.000E+00 | 0.000E+00 |
| Unigene186365 | novA | 1.689E-07 | 3.522E-07 | 1.575E-06 | 6.772E-06 | 0.000E+00 | 0.000E+00 | 0.000E+00 | 0.000E+00 | 0.000E+00 | 0.000E+00 | 0.000E+00 | 0.000E+00 |
| Unigene186366 | novA | 1.404E-07 | 2.697E-07 | 1.946E-06 | 6.222E-06 | 0.000E+00 | 0.000E+00 | 0.000E+00 | 0.000E+00 | 0.000E+00 | 0.000E+00 | 0.000E+00 | 0.000E+00 |
| Unigene186386 | PmrF | 0.000E+00 | 0.000E+00 | 7.943E-07 | 4.428E-07 | 0.000E+00 | 0.000E+00 | 0.000E+00 | 0.000E+00 | 0.000E+00 | 0.000E+00 | 0.000E+00 | 0.000E+00 |
| Unigene186536 | bcrA | 0.000E+00 | 8.346E-08 | 1.455E-06 | 9.176E-06 | 0.000E+00 | 0.000E+00 | 0.000E+00 | 0.000E+00 | 0.000E+00 | 0.000E+00 | 0.000E+00 | 0.000E+00 |
| Unigene186537 | tva(A) | 0.000E+00 | 1.718E-07 | 2.188E-06 | 9.285E-06 | 0.000E+00 | 0.000E+00 | 0.000E+00 | 0.000E+00 | 0.000E+00 | 0.000E+00 | 0.000E+00 | 0.000E+00 |
| Unigene186547 | macB | 0.000E+00 | 1.222E-07 | 1.675E-06 | 6.032E-06 | 0.000E+00 | 0.000E+00 | 0.000E+00 | 0.000E+00 | 0.000E+00 | 0.000E+00 | 0.000E+00 | 0.000E+00 |
| Unigene186554 | Acinetobac | 2.391E-07 | 2.221E-07 | 1.913E-06 | 7.541E-06 | 0.000E+00 | 0.000E+00 | 0.000E+00 | 0.000E+00 | 0.000E+00 | 0.000E+00 | 0.000E+00 | 0.000E+00 |

| | | | | | | | | | | | | | |
|---|---|---|---|---|---|---|---|---|---|---|---|---|---|
| Unigene186567 | Staphylocc | 0.000E+00 | 0.000E+00 | 1.003E-06 | 0.000E+00 | 0.000E+00 | 0.000E+00 | 0.000E+00 | 0.000E+00 | 0.000E+00 | 0.000E+00 | 0.000E+00 | 0.000E+00 |
| Unigene186587 | patB | 7.150E-08 | 5.885E-07 | 2.701E-06 | 3.195E-06 | 0.000E+00 | 0.000E+00 | 0.000E+00 | 0.000E+00 | 0.000E+00 | 0.000E+00 | 0.000E+00 | 0.000E+00 |
| Unigene186588 | efrA | 2.037E-07 | 7.317E-07 | 3.471E-06 | 3.460E-06 | 0.000E+00 | 0.000E+00 | 0.000E+00 | 0.000E+00 | 0.000E+00 | 0.000E+00 | 0.000E+00 | 0.000E+00 |
| Unigene186636 | macB | 5.801E-08 | 1.273E-07 | 5.123E-07 | 6.443E-07 | 0.000E+00 | 0.000E+00 | 0.000E+00 | 0.000E+00 | 0.000E+00 | 0.000E+00 | 0.000E+00 | 6.856E-08 |
| Unigene186639 | ramA | 0.000E+00 | 4.656E-07 | 6.812E-07 | 9.347E-07 | 0.000E+00 | 0.000E+00 | 0.000E+00 | 0.000E+00 | 0.000E+00 | 0.000E+00 | 0.000E+00 | 0.000E+00 |
| Unigene186674 | lsaC | 8.473E-08 | 0.000E+00 | 1.538E-06 | 2.781E-06 | 5.648E-07 | 0.000E+00 | 2.344E-07 | 7.912E-07 | 2.778E-07 | 6.607E-07 | 6.300E-07 | 1.001E-06 |
| Unigene186696 | tlrC | 1.087E-07 | 1.909E-06 | 1.120E-06 | 1.811E-06 | 0.000E+00 | 0.000E+00 | 0.000E+00 | 0.000E+00 | 0.000E+00 | 0.000E+00 | 0.000E+00 | 0.000E+00 |
| Unigene186706 | patB | 0.000E+00 | 3.592E-07 | 2.770E-06 | 3.718E-06 | 0.000E+00 | 0.000E+00 | 0.000E+00 | 0.000E+00 | 0.000E+00 | 0.000E+00 | 0.000E+00 | 0.000E+00 |
| Unigene186711 | AAC(6')-33 | 0.000E+00 | 5.808E-07 | 2.942E-06 | 3.147E-06 | 0.000E+00 | 0.000E+00 | 0.000E+00 | 0.000E+00 | 0.000E+00 | 0.000E+00 | 0.000E+00 | 0.000E+00 |
| Unigene186729 | patB | 0.000E+00 | 1.282E-06 | 2.046E-06 | 0.000E+00 | 3.660E-06 | 0.000E+00 | 0.000E+00 | 0.000E+00 | 5.014E-07 | 0.000E+00 | 5.248E-07 | 2.169E-06 |
| Unigene186762 | macB | 0.000E+00 | 2.132E-06 | 9.983E-07 | 0.000E+00 | 0.000E+00 | 0.000E+00 | 1.501E-06 | 0.000E+00 | 0.000E+00 | 0.000E+00 | 6.348E-07 | 0.000E+00 |
| Unigene186779 | AcrF | 3.003E-07 | 1.373E-06 | 9.330E-07 | 0.000E+00 | 3.058E-06 | 0.000E+00 | 1.219E-06 | 0.000E+00 | 0.000E+00 | 1.115E-07 | 8.015E-07 | 0.000E+00 |
| Unigene186801 | vanHD | 9.842E-08 | 6.121E-07 | 2.607E-06 | 3.048E-06 | 0.000E+00 | 0.000E+00 | 0.000E+00 | 0.000E+00 | 0.000E+00 | 0.000E+00 | 0.000E+00 | 0.000E+00 |
| Unigene186836 | Klebsiella p | 0.000E+00 | 0.000E+00 | 1.602E-06 | 9.157E-08 | 6.716E-08 | 0.000E+00 | 1.004E-07 | 7.056E-08 | 1.652E-07 | 0.000E+00 | 0.000E+00 | 1.000E-06 |
| Unigene186881 | vanRD | 0.000E+00 | 0.000E+00 | 0.000E+00 | 0.000E+00 | 0.000E+00 | 0.000E+00 | 0.000E+00 | 0.000E+00 | 0.000E+00 | 0.000E+00 | 0.000E+00 | 3.980E-06 |
| Unigene186913 | Streptococ | 0.000E+00 | 1.088E-07 | 1.946E-06 | 5.007E-07 | 0.000E+00 | 0.000E+00 | 0.000E+00 | 0.000E+00 | 0.000E+00 | 0.000E+00 | 0.000E+00 | 0.000E+00 |
| Unigene186983 | sul3 | 7.866E-07 | 3.083E-07 | 1.103E-06 | 4.709E-06 | 6.242E-08 | 3.098E-06 | 6.343E-06 | 7.838E-06 | 9.826E-07 | 1.596E-06 | 7.616E-06 | 3.653E-07 |
| Unigene186993 | msbA | 7.631E-08 | 0.000E+00 | 7.114E-07 | 2.312E-07 | 0.000E+00 | 0.000E+00 | 1.774E-06 | 7.571E-07 | 0.000E+00 | 5.526E-07 | 0.000E+00 | 1.759E-06 |
| Unigene187093 | rpoB2 | 0.000E+00 | 0.000E+00 | 3.536E-07 | 0.000E+00 | 0.000E+00 | 2.226E-07 | 0.000E+00 | 0.000E+00 | 0.000E+00 | 0.000E+00 | 0.000E+00 | 0.000E+00 |
| Unigene187249 | vgbA | 0.000E+00 | 3.149E-07 | 2.499E-06 | 2.173E-07 | 7.969E-08 | 0.000E+00 | 0.000E+00 | 0.000E+00 | 1.869E-04 | 0.000E+00 | 0.000E+00 | 0.000E+00 |
| Unigene187372 | arlR | 0.000E+00 | 0.000E+00 | 6.946E-07 | 4.098E-06 | 0.000E+00 | 0.000E+00 | 0.000E+00 | 0.000E+00 | 0.000E+00 | 0.000E+00 | 0.000E+00 | 0.000E+00 |
| Unigene187376 | bcrA | 3.893E-07 | 4.662E-07 | 1.667E-06 | 8.578E-07 | 0.000E+00 | 0.000E+00 | 0.000E+00 | 0.000E+00 | 0.000E+00 | 0.000E+00 | 0.000E+00 | 0.000E+00 |
| Unigene187392 | vanRB | 4.847E-08 | 5.320E-07 | 3.044E-06 | 3.524E-06 | 0.000E+00 | 0.000E+00 | 0.000E+00 | 0.000E+00 | 0.000E+00 | 0.000E+00 | 0.000E+00 | 0.000E+00 |
| Unigene187418 | emeA | 0.000E+00 | 0.000E+00 | 1.042E-06 | 0.000E+00 | 0.000E+00 | 0.000E+00 | 0.000E+00 | 0.000E+00 | 0.000E+00 | 0.000E+00 | 0.000E+00 | 0.000E+00 |
| Unigene187419 | abeS | 0.000E+00 | 0.000E+00 | 8.964E-07 | 0.000E+00 | 0.000E+00 | 0.000E+00 | 0.000E+00 | 0.000E+00 | 0.000E+00 | 0.000E+00 | 0.000E+00 | 0.000E+00 |
| Unigene187434 | APH(3'')-Ib | 0.000E+00 | 0.000E+00 | 1.566E-06 | 0.000E+00 | 0.000E+00 | 6.391E-08 | 0.000E+00 | 6.901E-08 | 0.000E+00 | 0.000E+00 | 2.029E-07 | 1.048E-06 |
| Unigene187435 | APH(6)-Id | 3.857E-08 | 1.693E-07 | 2.384E-06 | 0.000E+00 | 1.714E-07 | 4.169E-08 | 1.707E-07 | 1.351E-07 | 3.372E-07 | 0.000E+00 | 0.000E+00 | 2.097E-06 |
| Unigene187455 | oleC | 0.000E+00 | 0.000E+00 | 9.269E-07 | 0.000E+00 | 0.000E+00 | 0.000E+00 | 0.000E+00 | 0.000E+00 | 0.000E+00 | 0.000E+00 | 0.000E+00 | 0.000E+00 |
| Unigene187531 | bcrA | 0.000E+00 | 0.000E+00 | 2.994E-06 | 1.494E-05 | 0.000E+00 | 0.000E+00 | 0.000E+00 | 0.000E+00 | 0.000E+00 | 0.000E+00 | 0.000E+00 | 0.000E+00 |
| Unigene187546 | optrA | 0.000E+00 | 0.000E+00 | 5.193E-07 | 3.563E-07 | 0.000E+00 | 0.000E+00 | 0.000E+00 | 0.000E+00 | 0.000E+00 | 0.000E+00 | 0.000E+00 | 0.000E+00 |
| Unigene187553 | bmr | 0.000E+00 | 3.754E-07 | 5.493E-07 | 3.768E-07 | 0.000E+00 | 0.000E+00 | 0.000E+00 | 0.000E+00 | 0.000E+00 | 0.000E+00 | 0.000E+00 | 0.000E+00 |
| Unigene187640 | SRT-2 | 0.000E+00 | 0.000E+00 | 1.180E-06 | 4.858E-08 | 7.126E-08 | 0.000E+00 | 5.324E-08 | 3.744E-08 | 7.011E-08 | 5.359E-08 | 1.101E-07 | 9.857E-07 |
| Unigene187674 | Enterococ | 9.735E-07 | 1.462E-06 | 4.625E-06 | 5.226E-06 | 0.000E+00 | 0.000E+00 | 3.970E-07 | 0.000E+00 | 0.000E+00 | 0.000E+00 | 0.000E+00 | 0.000E+00 |
| Unigene187703 | TaeA | 1.181E-07 | 0.000E+00 | 1.125E-06 | 1.192E-07 | 5.621E-08 | 1.823E-08 | 0.000E+00 | 0.000E+00 | 9.217E-08 | 0.000E+00 | 1.736E-07 | 1.635E-06 |
| Unigene187704 | LlmA 23S r | 0.000E+00 | 2.952E-08 | 9.766E-07 | 5.433E-08 | 5.977E-08 | 0.000E+00 | 0.000E+00 | 6.280E-08 | 1.470E-07 | 0.000E+00 | 0.000E+00 | 6.677E-07 |
| Unigene187717 | msrA | 0.000E+00 | 7.632E-07 | 4.309E-07 | 1.848E-07 | 0.000E+00 | 0.000E+00 | 0.000E+00 | 0.000E+00 | 0.000E+00 | 0.000E+00 | 0.000E+00 | 0.000E+00 |
| Unigene187730 | vanTG | 1.345E-06 | 7.381E-07 | 3.079E-07 | 9.055E-08 | 0.000E+00 | 0.000E+00 | 0.000E+00 | 0.000E+00 | 0.000E+00 | 0.000E+00 | 0.000E+00 | 0.000E+00 |
| Unigene187860 | macB | 0.000E+00 | 0.000E+00 | 1.004E-06 | 0.000E+00 | 0.000E+00 | 0.000E+00 | 3.944E-06 | 3.871E-06 | 0.000E+00 | 0.000E+00 | 0.000E+00 | 3.419E-06 |
| Unigene187897 | efrA | 1.082E-06 | 1.023E-06 | 1.268E-06 | 1.548E-06 | 2.247E-05 | 4.874E-06 | 1.480E-05 | 1.491E-05 | 9.428E-06 | 1.915E-05 | 1.967E-05 | 8.242E-06 |
| Unigene187900 | bcrA | 0.000E+00 | 0.000E+00 | 3.570E-07 | 0.000E+00 | 0.000E+00 | 0.000E+00 | 0.000E+00 | 0.000E+00 | 0.000E+00 | 0.000E+00 | 0.000E+00 | 0.000E+00 |
| Unigene187956 | facT | 0.000E+00 | 0.000E+00 | 9.470E-07 | 0.000E+00 | 0.000E+00 | 0.000E+00 | 0.000E+00 | 0.000E+00 | 0.000E+00 | 0.000E+00 | 0.000E+00 | 0.000E+00 |
| Unigene187988 | tet(43) | 1.006E-07 | 0.000E+00 | 0.000E+00 | 6.601E-07 | 0.000E+00 | 0.000E+00 | 0.000E+00 | 2.348E-07 | 0.000E+00 | 0.000E+00 | 0.000E+00 | 0.000E+00 |
| Unigene188070 | macB | 2.349E-07 | 3.610E-07 | 1.337E-06 | 3.796E-07 | 0.000E+00 | 0.000E+00 | 0.000E+00 | 0.000E+00 | 0.000E+00 | 0.000E+00 | 0.000E+00 | 0.000E+00 |
| Unigene188073 | vanSA | 9.376E-07 | 6.860E-07 | 2.278E-06 | 8.116E-07 | 0.000E+00 | 0.000E+00 | 0.000E+00 | 0.000E+00 | 0.000E+00 | 0.000E+00 | 0.000E+00 | 0.000E+00 |
| Unigene188074 | Escherichia | 7.475E-07 | 3.053E-07 | 1.518E-06 | 1.246E-06 | 0.000E+00 | 0.000E+00 | 0.000E+00 | 0.000E+00 | 0.000E+00 | 0.000E+00 | 0.000E+00 | 0.000E+00 |
| Unigene188075 | tetA(58) | 5.863E-07 | 2.271E-07 | 1.117E-06 | 8.707E-07 | 0.000E+00 | 0.000E+00 | 0.000E+00 | 0.000E+00 | 0.000E+00 | 0.000E+00 | 0.000E+00 | 0.000E+00 |
| Unigene188120 | macB | 0.000E+00 | 0.000E+00 | 7.929E-07 | 1.718E-07 | 0.000E+00 | 0.000E+00 | 0.000E+00 | 0.000E+00 | 0.000E+00 | 0.000E+00 | 0.000E+00 | 0.000E+00 |
| Unigene188202 | mtrA | 0.000E+00 | 0.000E+00 | 0.000E+00 | 6.809E-06 | 0.000E+00 | 0.000E+00 | 0.000E+00 | 0.000E+00 | 0.000E+00 | 0.000E+00 | 0.000E+00 | 0.000E+00 |
| Unigene188260 | poxtA | 2.875E-07 | 0.000E+00 | 1.169E-06 | 3.733E-07 | 0.000E+00 | 9.323E-07 | 3.227E-06 | 0.000E+00 | 0.000E+00 | 6.405E-07 | 1.879E-06 | 2.524E-06 |
| Unigene188282 | carA | 3.317E-07 | 6.599E-07 | 1.444E-06 | 2.973E-06 | 5.528E-07 | 0.000E+00 | 1.836E-07 | 1.210E-06 | 2.266E-07 | 5.312E-07 | 9.960E-07 | 6.617E-07 |
| Unigene188287 | carA | 3.913E-07 | 6.084E-07 | 1.376E-06 | 2.832E-06 | 4.709E-07 | 0.000E+00 | 1.083E-07 | 1.637E-06 | 0.000E+00 | 6.901E-07 | 8.952E-07 | 7.708E-07 |
| Unigene188395 | vmlR | 0.000E+00 | 0.000E+00 | 9.401E-07 | 0.000E+00 | 0.000E+00 | 0.000E+00 | 0.000E+00 | 0.000E+00 | 0.000E+00 | 0.000E+00 | 0.000E+00 | 0.000E+00 |
| Unigene188398 | macB | 0.000E+00 | 0.000E+00 | 1.191E-06 | 7.843E-05 | 0.000E+00 | 0.000E+00 | 0.000E+00 | 0.000E+00 | 0.000E+00 | 0.000E+00 | 0.000E+00 | 0.000E+00 |
| Unigene188513 | tet(38) | 0.000E+00 | 2.497E-07 | 2.076E-06 | 1.564E-05 | 0.000E+00 | 0.000E+00 | 0.000E+00 | 0.000E+00 | 0.000E+00 | 0.000E+00 | 0.000E+00 | 0.000E+00 |
| Unigene188630 | blt | 1.835E-07 | 0.000E+00 | 2.004E-06 | 7.043E-06 | 0.000E+00 | 0.000E+00 | 0.000E+00 | 0.000E+00 | 0.000E+00 | 0.000E+00 | 0.000E+00 | 0.000E+00 |
| Unigene188657 | QnrS8 | 0.000E+00 | 0.000E+00 | 1.061E-06 | 0.000E+00 | 0.000E+00 | 0.000E+00 | 0.000E+00 | 0.000E+00 | 0.000E+00 | 0.000E+00 | 0.000E+00 | 1.045E-06 |
| Unigene188661 | bcrA | 3.104E-07 | 9.841E-07 | 1.354E-06 | 1.741E-06 | 0.000E+00 | 0.000E+00 | 0.000E+00 | 0.000E+00 | 0.000E+00 | 0.000E+00 | 0.000E+00 | 0.000E+00 |
| Unigene188748 | tetA(58) | 0.000E+00 | 6.236E-07 | 3.066E-06 | 3.837E-06 | 0.000E+00 | 0.000E+00 | 0.000E+00 | 0.000E+00 | 0.000E+00 | 0.000E+00 | 0.000E+00 | 0.000E+00 |

| | | | | | | | | | | | | | |
|---|---|---|---|---|---|---|---|---|---|---|---|---|---|
| Unigene188819 | macB | 4.321E-08 | 0.000E+00 | 1.187E-06 | 1.309E-07 | 0.000E+00 | 0.000E+00 | 0.000E+00 | 0.000E+00 | 0.000E+00 | 0.000E+00 | 0.000E+00 | 0.000E+00 |
| Unigene188821 | vanSB | 9.459E-08 | 0.000E+00 | 1.439E-06 | 3.105E-07 | 0.000E+00 | 0.000E+00 | 0.000E+00 | 0.000E+00 | 0.000E+00 | 0.000E+00 | 0.000E+00 | 0.000E+00 |
| Unigene188822 | vanRB | 0.000E+00 | 2.696E-07 | 1.109E-06 | 2.481E-07 | 0.000E+00 | 0.000E+00 | 0.000E+00 | 0.000E+00 | 0.000E+00 | 0.000E+00 | 0.000E+00 | 0.000E+00 |
| Unigene188823 | macB | 1.047E-07 | 2.011E-07 | 1.028E-06 | 4.230E-07 | 0.000E+00 | 0.000E+00 | 0.000E+00 | 0.000E+00 | 0.000E+00 | 0.000E+00 | 0.000E+00 | 0.000E+00 |
| Unigene188885 | Acinetoba | 4.788E-06 | 3.450E-07 | 5.220E-06 | 3.370E-06 | 6.716E-06 | 3.657E-05 | 1.389E-05 | 1.524E-06 | 1.583E-05 | 4.768E-06 | 3.319E-07 | 4.687E-06 |
| Unigene188895 | bcrA | 1.034E-05 | 4.177E-06 | 2.886E-06 | 1.066E-05 | 7.922E-05 | 1.060E-04 | 1.205E-04 | 1.002E-04 | 1.375E-04 | 1.471E-04 | 1.201E-04 | 7.648E-05 |
| Unigene188976 | aadA9 | 0.000E+00 | 0.000E+00 | 1.011E-06 | 0.000E+00 | 0.000E+00 | 0.000E+00 | 0.000E+00 | 2.406E-07 | 0.000E+00 | 0.000E+00 | 0.000E+00 | 0.000E+00 |
| Unigene189054 | vanZF | 0.000E+00 | 1.298E-07 | 3.307E-06 | 1.579E-05 | 0.000E+00 | 0.000E+00 | 0.000E+00 | 0.000E+00 | 0.000E+00 | 0.000E+00 | 0.000E+00 | 0.000E+00 |
| Unigene189057 | Streptomy | 3.083E-08 | 3.384E-07 | 2.617E-06 | 1.571E-05 | 0.000E+00 | 0.000E+00 | 0.000E+00 | 0.000E+00 | 0.000E+00 | 0.000E+00 | 0.000E+00 | 0.000E+00 |
| Unigene189069 | vanG | 0.000E+00 | 0.000E+00 | 2.518E-06 | 1.309E-05 | 0.000E+00 | 0.000E+00 | 0.000E+00 | 0.000E+00 | 0.000E+00 | 0.000E+00 | 0.000E+00 | 0.000E+00 |
| Unigene189077 | ErmR | 3.505E-08 | 0.000E+00 | 2.167E-06 | 1.561E-05 | 0.000E+00 | 0.000E+00 | 0.000E+00 | 0.000E+00 | 0.000E+00 | 0.000E+00 | 0.000E+00 | 0.000E+00 |
| Unigene189102 | macB | 4.483E-07 | 0.000E+00 | 1.265E-06 | 1.981E-06 | 0.000E+00 | 0.000E+00 | 0.000E+00 | 0.000E+00 | 0.000E+00 | 0.000E+00 | 0.000E+00 | 0.000E+00 |
| Unigene189190 | evgA | 1.020E-07 | 1.679E-07 | 1.251E-06 | 1.030E-07 | 0.000E+00 | 0.000E+00 | 0.000E+00 | 0.000E+00 | 0.000E+00 | 0.000E+00 | 0.000E+00 | 0.000E+00 |
| Unigene189209 | oleC | 2.644E-08 | 5.803E-08 | 9.598E-07 | 1.869E-07 | 0.000E+00 | 0.000E+00 | 0.000E+00 | 0.000E+00 | 0.000E+00 | 0.000E+00 | 0.000E+00 | 0.000E+00 |
| Unigene189259 | efrB | 0.000E+00 | 1.000E-06 | 1.491E-06 | 1.606E-06 | 0.000E+00 | 0.000E+00 | 0.000E+00 | 0.000E+00 | 0.000E+00 | 0.000E+00 | 0.000E+00 | 0.000E+00 |
| Unigene189260 | efrA | 2.778E-07 | 9.147E-07 | 1.127E-06 | 1.683E-06 | 0.000E+00 | 0.000E+00 | 0.000E+00 | 0.000E+00 | 0.000E+00 | 0.000E+00 | 0.000E+00 | 0.000E+00 |
| Unigene189301 | bcrA | 0.000E+00 | 0.000E+00 | 1.115E-06 | 0.000E+00 | 1.753E-07 | 3.411E-08 | 0.000E+00 | 7.366E-08 | 1.380E-07 | 7.030E-08 | 7.219E-08 | 5.968E-07 |
| Unigene189419 | mecA | 0.000E+00 | 6.967E-08 | 1.137E-06 | 1.122E-07 | 0.000E+00 | 0.000E+00 | 0.000E+00 | 3.705E-08 | 0.000E+00 | 0.000E+00 | 0.000E+00 | 0.000E+00 |
| Unigene189462 | Staphyloc | 3.587E-06 | 1.694E-05 | 1.325E-05 | 1.124E-05 | 0.000E+00 | 3.709E-07 | 4.142E-07 | 0.000E+00 | 0.000E+00 | 0.000E+00 | 0.000E+00 | 3.687E-08 |
| Unigene189487 | patA | 0.000E+00 | 1.993E-07 | 1.247E-06 | 0.000E+00 | 1.009E-07 | 0.000E+00 | 0.000E+00 | 0.000E+00 | 1.985E-07 | 0.000E+00 | 0.000E+00 | 6.440E-07 |
| Unigene189498 | TaeA | 0.000E+00 | 0.000E+00 | 1.525E-06 | 9.234E-08 | 0.000E+00 | 0.000E+00 | 3.374E-08 | 1.067E-07 | 3.665E-07 | 0.000E+00 | 0.000E+00 | 9.728E-07 |
| Unigene189503 | oleC | 4.236E-08 | 6.974E-08 | 1.102E-06 | 4.278E-08 | 0.000E+00 | 4.580E-08 | 0.000E+00 | 0.000E+00 | 6.945E-08 | 1.888E-07 | 1.211E-07 | 9.013E-07 |
| Unigene189553 | mecC | 0.000E+00 | 1.210E-07 | 2.704E-06 | 1.590E-05 | 0.000E+00 | 0.000E+00 | 0.000E+00 | 0.000E+00 | 0.000E+00 | 0.000E+00 | 0.000E+00 | 0.000E+00 |
| Unigene189666 | efrB | 1.283E-07 | 2.012E-07 | 1.205E-06 | 1.666E-07 | 0.000E+00 | 0.000E+00 | 0.000E+00 | 0.000E+00 | 0.000E+00 | 0.000E+00 | 0.000E+00 | 0.000E+00 |
| Unigene189667 | efrA | 5.624E-08 | 2.057E-08 | 1.159E-06 | 2.839E-07 | 0.000E+00 | 0.000E+00 | 0.000E+00 | 2.188E-08 | 0.000E+00 | 0.000E+00 | 0.000E+00 | 0.000E+00 |
| Unigene189672 | Staphyloc | 7.320E-08 | 1.071E-07 | 1.113E-06 | 1.725E-07 | 0.000E+00 | 0.000E+00 | 0.000E+00 | 0.000E+00 | 0.000E+00 | 0.000E+00 | 0.000E+00 | 0.000E+00 |
| Unigene189764 | efrA | 0.000E+00 | 2.624E-07 | 3.519E-07 | 0.000E+00 | 0.000E+00 | 0.000E+00 | 0.000E+00 | 0.000E+00 | 0.000E+00 | 0.000E+00 | 0.000E+00 | 0.000E+00 |
| Unigene189801 | facT | 8.196E-07 | 8.304E-07 | 4.660E-06 | 6.367E-06 | 0.000E+00 | 0.000E+00 | 1.861E-07 | 0.000E+00 | 0.000E+00 | 0.000E+00 | 0.000E+00 | 0.000E+00 |
| Unigene189877 | apmA | 2.700E-08 | 7.409E-08 | 9.406E-07 | 8.316E-07 | 0.000E+00 | 0.000E+00 | 0.000E+00 | 0.000E+00 | 0.000E+00 | 0.000E+00 | 0.000E+00 | 0.000E+00 |
| Unigene189903 | bcr-1 | 0.000E+00 | 0.000E+00 | 7.655E-07 | 2.988E-07 | 6.874E-07 | 0.000E+00 | 0.000E+00 | 8.164E-07 | 0.000E+00 | 0.000E+00 | 1.015E-06 | 0.000E+00 |
| Unigene190013 | rosB | 0.000E+00 | 2.029E-08 | 1.270E-06 | 0.000E+00 | 1.027E-07 | 0.000E+00 | 0.000E+00 | 0.000E+00 | 1.414E-07 | 4.119E-08 | 0.000E+00 | 1.071E-06 |
| Unigene190031 | patA | 4.457E-07 | 1.922E-06 | 1.562E-06 | 5.144E-07 | 0.000E+00 | 0.000E+00 | 0.000E+00 | 3.716E-07 | 0.000E+00 | 0.000E+00 | 0.000E+00 | 0.000E+00 |
| Unigene190068 | Acinetoba | 2.434E-08 | 0.000E+00 | 1.314E-06 | 1.229E-07 | 0.000E+00 | 5.263E-08 | 0.000E+00 | 0.000E+00 | 2.395E-07 | 0.000E+00 | 1.392E-07 | 1.468E-06 |
| Unigene190070 | adeL | 3.575E-08 | 0.000E+00 | 1.614E-06 | 0.000E+00 | 0.000E+00 | 0.000E+00 | 0.000E+00 | 0.000E+00 | 0.000E+00 | 0.000E+00 | 1.227E-07 | 1.099E-06 |
| Unigene190098 | ramA | 0.000E+00 | 0.000E+00 | 8.122E-07 | 0.000E+00 | 0.000E+00 | 0.000E+00 | 0.000E+00 | 0.000E+00 | 0.000E+00 | 0.000E+00 | 0.000E+00 | 9.784E-07 |
| Unigene190155 | cmeR | 0.000E+00 | 0.000E+00 | 1.564E-06 | 1.444E-05 | 0.000E+00 | 0.000E+00 | 0.000E+00 | 0.000E+00 | 0.000E+00 | 0.000E+00 | 0.000E+00 | 0.000E+00 |
| Unigene190162 | vanHO | 3.301E-08 | 2.536E-07 | 2.105E-06 | 1.373E-05 | 0.000E+00 | 0.000E+00 | 0.000E+00 | 0.000E+00 | 0.000E+00 | 0.000E+00 | 0.000E+00 | 0.000E+00 |
| Unigene190178 | vanRF | 0.000E+00 | 0.000E+00 | 2.991E-06 | 1.576E-05 | 0.000E+00 | 0.000E+00 | 0.000E+00 | 0.000E+00 | 0.000E+00 | 0.000E+00 | 0.000E+00 | 0.000E+00 |
| Unigene190184 | mtrA | 0.000E+00 | 2.293E-07 | 2.255E-06 | 1.234E-05 | 0.000E+00 | 0.000E+00 | 0.000E+00 | 0.000E+00 | 0.000E+00 | 0.000E+00 | 0.000E+00 | 0.000E+00 |
| Unigene190191 | lmrC | 0.000E+00 | 0.000E+00 | 2.701E-06 | 1.191E-05 | 0.000E+00 | 0.000E+00 | 0.000E+00 | 0.000E+00 | 0.000E+00 | 0.000E+00 | 0.000E+00 | 0.000E+00 |
| Unigene190195 | mtrA | 0.000E+00 | 0.000E+00 | 1.731E-06 | 1.318E-05 | 0.000E+00 | 0.000E+00 | 0.000E+00 | 0.000E+00 | 0.000E+00 | 0.000E+00 | 0.000E+00 | 0.000E+00 |
| Unigene190196 | baeS | 0.000E+00 | 6.315E-08 | 2.597E-06 | 1.275E-05 | 0.000E+00 | 0.000E+00 | 0.000E+00 | 0.000E+00 | 0.000E+00 | 0.000E+00 | 0.000E+00 | 0.000E+00 |
| Unigene190221 | bcrA | 0.000E+00 | 1.003E-07 | 1.816E-06 | 1.273E-05 | 0.000E+00 | 0.000E+00 | 0.000E+00 | 0.000E+00 | 0.000E+00 | 0.000E+00 | 0.000E+00 | 0.000E+00 |
| Unigene190232 | evgA | 0.000E+00 | 2.624E-07 | 1.916E-06 | 1.409E-05 | 0.000E+00 | 0.000E+00 | 0.000E+00 | 0.000E+00 | 0.000E+00 | 0.000E+00 | 0.000E+00 | 0.000E+00 |
| Unigene190236 | macB | 0.000E+00 | 7.953E-08 | 1.884E-06 | 9.146E-06 | 0.000E+00 | 0.000E+00 | 0.000E+00 | 0.000E+00 | 0.000E+00 | 0.000E+00 | 0.000E+00 | 0.000E+00 |
| Unigene190365 | Streptococ | 0.000E+00 | 0.000E+00 | 2.797E-07 | 0.000E+00 | 0.000E+00 | 0.000E+00 | 0.000E+00 | 0.000E+00 | 0.000E+00 | 0.000E+00 | 0.000E+00 | 0.000E+00 |
| Unigene190390 | macB | 1.089E-05 | 0.000E+00 | 1.632E-05 | 0.000E+00 | 0.000E+00 | 0.000E+00 | 0.000E+00 | 0.000E+00 | 0.000E+00 | 0.000E+00 | 0.000E+00 | 0.000E+00 |
| Unigene190411 | tlrC | 1.701E-07 | 1.400E-07 | 6.677E-07 | 6.872E-07 | 0.000E+00 | 0.000E+00 | 0.000E+00 | 0.000E+00 | 0.000E+00 | 0.000E+00 | 0.000E+00 | 0.000E+00 |
| Unigene190414 | otr(B) | 0.000E+00 | 0.000E+00 | 0.000E+00 | 0.000E+00 | 7.969E-07 | 0.000E+00 | 9.107E-07 | 0.000E+00 | 0.000E+00 | 5.406E-07 | 7.964E-07 | 2.294E-06 |
| Unigene190422 | Bifidobact | 0.000E+00 | 0.000E+00 | 7.296E-07 | 0.000E+00 | 0.000E+00 | 0.000E+00 | 0.000E+00 | 0.000E+00 | 0.000E+00 | 0.000E+00 | 0.000E+00 | 0.000E+00 |
| Unigene190467 | carA | 0.000E+00 | 0.000E+00 | 1.827E-07 | 0.000E+00 | 0.000E+00 | 2.817E-07 | 6.593E-07 | 6.953E-07 | 0.000E+00 | 0.000E+00 | 2.981E-07 | 4.841E-07 |
| Unigene190481 | vgaB | 2.740E-05 | 1.096E-06 | 6.531E-07 | 0.000E+00 | 0.000E+00 | 0.000E+00 | 0.000E+00 | 0.000E+00 | 0.000E+00 | 0.000E+00 | 0.000E+00 | 0.000E+00 |
| Unigene190526 | adeL | 0.000E+00 | 1.177E-07 | 1.298E-06 | 1.083E-07 | 0.000E+00 | 3.864E-08 | 0.000E+00 | 0.000E+00 | 2.344E-07 | 0.000E+00 | 0.000E+00 | 1.225E-06 |
| Unigene190642 | vatB | 0.000E+00 | 0.000E+00 | 1.403E-04 | 0.000E+00 | 0.000E+00 | 0.000E+00 | 0.000E+00 | 0.000E+00 | 0.000E+00 | 0.000E+00 | 0.000E+00 | 0.000E+00 |
| Unigene190684 | vmlR | 0.000E+00 | 6.700E-07 | 5.616E-07 | 0.000E+00 | 0.000E+00 | 0.000E+00 | 0.000E+00 | 0.000E+00 | 0.000E+00 | 0.000E+00 | 0.000E+00 | 0.000E+00 |
| Unigene190755 | tetB(46) | 0.000E+00 | 3.302E-07 | 5.904E-07 | 4.907E-07 | 5.656E-07 | 0.000E+00 | 2.484E-06 | 1.675E-06 | 0.000E+00 | 0.000E+00 | 1.191E-06 | 1.586E-06 |
| Unigene190758 | Acinetoba | 0.000E+00 | 0.000E+00 | 6.599E-07 | 3.396E-07 | 0.000E+00 | 0.000E+00 | 0.000E+00 | 0.000E+00 | 0.000E+00 | 0.000E+00 | 0.000E+00 | 0.000E+00 |
| Unigene190870 | tetA(58) | 1.894E-07 | 4.990E-07 | 5.948E-07 | 8.417E-07 | 0.000E+00 | 0.000E+00 | 0.000E+00 | 8.845E-08 | 0.000E+00 | 0.000E+00 | 4.334E-08 | 0.000E+00 |

| | | | | | | | | | | | | | |
|---|---|---|---|---|---|---|---|---|---|---|---|---|---|
| Unigene190892 | vanRF | 1.112E-06 | 3.172E-06 | 1.745E-06 | 0.000E+00 | 0.000E+00 | 0.000E+00 | 0.000E+00 | 0.000E+00 | 0.000E+00 | 0.000E+00 | 0.000E+00 | 0.000E+00 |
| Unigene190926 | qacB | 0.000E+00 | 0.000E+00 | 3.359E-07 | 0.000E+00 | 0.000E+00 | 0.000E+00 | 0.000E+00 | 0.000E+00 | 0.000E+00 | 0.000E+00 | 0.000E+00 | 0.000E+00 |
| Unigene190982 | arlS | 0.000E+00 | 1.887E-08 | 7.927E-07 | 7.590E-05 | 0.000E+00 | 0.000E+00 | 0.000E+00 | 1.204E-07 | 0.000E+00 | 0.000E+00 | 0.000E+00 | 0.000E+00 |
| Unigene191040 | Chlamydia | 0.000E+00 | 0.000E+00 | 9.469E-07 | 0.000E+00 | 0.000E+00 | 0.000E+00 | 0.000E+00 | 0.000E+00 | 0.000E+00 | 0.000E+00 | 0.000E+00 | 0.000E+00 |
| Unigene191156 | evgS | 0.000E+00 | 0.000E+00 | 4.124E-07 | 0.000E+00 | 1.121E-06 | 0.000E+00 | 0.000E+00 | 0.000E+00 | 0.000E+00 | 0.000E+00 | 0.000E+00 | 1.490E-06 |
| Unigene191171 | emrB | 6.782E-08 | 7.939E-07 | 3.438E-06 | 3.150E-06 | 0.000E+00 | 0.000E+00 | 0.000E+00 | 0.000E+00 | 0.000E+00 | 0.000E+00 | 0.000E+00 | 0.000E+00 |
| Unigene191311 | oleC | 8.643E-08 | 9.486E-07 | 3.138E-06 | 4.975E-06 | 0.000E+00 | 0.000E+00 | 0.000E+00 | 0.000E+00 | 0.000E+00 | 0.000E+00 | 0.000E+00 | 0.000E+00 |
| Unigene191323 | SRT-2 | 3.479E-07 | 3.546E-07 | 7.803E-07 | 0.000E+00 | 7.123E-06 | 2.418E-07 | 0.000E+00 | 2.321E-07 | 0.000E+00 | 0.000E+00 | 8.528E-08 | 0.000E+00 |
| Unigene191349 | OXY-2-6 | 0.000E+00 | 1.130E-07 | 2.526E-07 | 0.000E+00 | 0.000E+00 | 0.000E+00 | 0.000E+00 | 0.000E+00 | 0.000E+00 | 0.000E+00 | 0.000E+00 | 0.000E+00 |
| Unigene191410 | AcrS | 0.000E+00 | 0.000E+00 | 3.519E-07 | 0.000E+00 | 0.000E+00 | 0.000E+00 | 0.000E+00 | 0.000E+00 | 0.000E+00 | 0.000E+00 | 0.000E+00 | 0.000E+00 |
| Unigene191454 | TaeA | 6.010E-07 | 2.199E-07 | 1.039E-06 | 4.335E-07 | 1.145E-06 | 9.281E-07 | 4.687E-06 | 2.405E-06 | 6.568E-07 | 1.562E-06 | 1.997E-06 | 3.586E-06 |
| Unigene191609 | Acinetobac | 0.000E+00 | 0.000E+00 | 1.187E-06 | 7.476E-08 | 8.225E-08 | 0.000E+00 | 0.000E+00 | 0.000E+00 | 2.428E-07 | 0.000E+00 | 0.000E+00 | 9.335E-07 |
| Unigene191630 | mgrA | 0.000E+00 | 0.000E+00 | 2.624E-06 | 9.878E-07 | 0.000E+00 | 0.000E+00 | 0.000E+00 | 0.000E+00 | 0.000E+00 | 0.000E+00 | 0.000E+00 | 0.000E+00 |
| Unigene191704 | YojI | 0.000E+00 | 9.373E-08 | 1.299E-06 | 8.624E-08 | 0.000E+00 | 0.000E+00 | 7.089E-08 | 2.492E-08 | 4.667E-08 | 0.000E+00 | 0.000E+00 | 1.287E-06 |
| Unigene191715 | bcrA | 0.000E+00 | 8.835E-08 | 1.343E-06 | 0.000E+00 | 5.962E-08 | 0.000E+00 | 0.000E+00 | 0.000E+00 | 1.760E-07 | 1.495E-07 | 1.228E-07 | 1.522E-06 |
| Unigene191720 | emrR | 0.000E+00 | 0.000E+00 | 8.897E-07 | 0.000E+00 | 0.000E+00 | 0.000E+00 | 0.000E+00 | 0.000E+00 | 0.000E+00 | 0.000E+00 | 0.000E+00 | 1.215E-06 |
| Unigene191721 | Klebsiella p | 0.000E+00 | 0.000E+00 | 1.336E-06 | 1.100E-07 | 0.000E+00 | 0.000E+00 | 3.015E-08 | 0.000E+00 | 2.977E-08 | 0.000E+00 | 1.870E-07 | 9.338E-07 |
| Unigene191722 | emrB | 0.000E+00 | 0.000E+00 | 8.816E-07 | 2.110E-08 | 1.161E-07 | 0.000E+00 | 0.000E+00 | 0.000E+00 | 1.370E-07 | 6.982E-08 | 2.390E-08 | 1.358E-06 |
| Unigene191779 | PmrF | 5.754E-08 | 0.000E+00 | 5.646E-07 | 0.000E+00 | 0.000E+00 | 0.000E+00 | 0.000E+00 | 0.000E+00 | 0.000E+00 | 0.000E+00 | 0.000E+00 | 0.000E+00 |
| Unigene191797 | basS | 2.788E-07 | 0.000E+00 | 2.735E-07 | 0.000E+00 | 0.000E+00 | 0.000E+00 | 0.000E+00 | 0.000E+00 | 0.000E+00 | 0.000E+00 | 0.000E+00 | 0.000E+00 |
| Unigene191806 | vanTC | 2.885E-08 | 3.166E-08 | 4.529E-07 | 4.078E-07 | 0.000E+00 | 0.000E+00 | 0.000E+00 | 0.000E+00 | 0.000E+00 | 0.000E+00 | 3.300E-08 | 0.000E+00 |
| Unigene191839 | efmA | 0.000E+00 | 0.000E+00 | 9.137E-07 | 0.000E+00 | 0.000E+00 | 0.000E+00 | 0.000E+00 | 0.000E+00 | 0.000E+00 | 0.000E+00 | 0.000E+00 | 0.000E+00 |
| Unigene191850 | macB | 0.000E+00 | 0.000E+00 | 7.039E-07 | 0.000E+00 | 0.000E+00 | 0.000E+00 | 0.000E+00 | 0.000E+00 | 0.000E+00 | 0.000E+00 | 0.000E+00 | 0.000E+00 |
| Unigene191857 | vanHA | 1.137E-05 | 3.617E-06 | 3.424E-06 | 1.168E-05 | 7.912E-05 | 1.153E-04 | 1.061E-04 | 9.037E-05 | 1.375E-04 | 1.285E-04 | 1.015E-04 | 6.814E-05 |
| Unigene191879 | vanHA | 0.000E+00 | 0.000E+00 | 1.666E-05 | 1.508E-05 | 1.976E-05 | 0.000E+00 | 4.875E-06 | 1.754E-05 | 1.290E-05 | 0.000E+00 | 0.000E+00 | 0.000E+00 |
| Unigene191962 | macB | 1.338E-06 | 1.263E-06 | 4.964E-06 | 7.352E-06 | 8.921E-08 | 0.000E+00 | 1.481E-07 | 0.000E+00 | 0.000E+00 | 0.000E+00 | 3.062E-08 | 0.000E+00 |
| Unigene192049 | Bifidobacte | 0.000E+00 | 5.486E-08 | 1.189E-06 | 6.310E-08 | 1.805E-07 | 0.000E+00 | 0.000E+00 | 0.000E+00 | 2.185E-07 | 0.000E+00 | 0.000E+00 | 7.386E-07 |
| Unigene192054 | mecC | 1.697E-08 | 9.314E-08 | 1.099E-06 | 5.142E-08 | 2.263E-07 | 0.000E+00 | 0.000E+00 | 9.905E-08 | 9.275E-08 | 0.000E+00 | 1.165E-07 | 1.244E-06 |
| Unigene192070 | lmrC | 0.000E+00 | 3.149E-08 | 1.295E-06 | 0.000E+00 | 3.188E-08 | 0.000E+00 | 0.000E+00 | 0.000E+00 | 1.254E-07 | 6.393E-08 | 6.564E-08 | 8.818E-07 |
| Unigene192199 | patA | 1.318E-07 | 4.820E-08 | 9.481E-07 | 5.712E-05 | 0.000E+00 | 0.000E+00 | 0.000E+00 | 0.000E+00 | 0.000E+00 | 0.000E+00 | 0.000E+00 | 0.000E+00 |
| Unigene192259 | macB | 1.176E-07 | 3.227E-07 | 2.308E-07 | 0.000E+00 | 0.000E+00 | 0.000E+00 | 0.000E+00 | 0.000E+00 | 0.000E+00 | 0.000E+00 | 0.000E+00 | 0.000E+00 |
| Unigene192288 | Staphylocc | 4.697E-07 | 5.624E-07 | 1.362E-06 | 3.169E-06 | 0.000E+00 | 0.000E+00 | 3.072E-07 | 1.121E-06 | 4.667E-08 | 7.135E-07 | 9.769E-07 | 4.542E-07 |
| Unigene192370 | macB | 2.089E-07 | 0.000E+00 | 0.000E+00 | 0.000E+00 | 0.000E+00 | 0.000E+00 | 0.000E+00 | 0.000E+00 | 0.000E+00 | 0.000E+00 | 0.000E+00 | 0.000E+00 |
| Unigene192372 | lmrD | 5.896E-08 | 9.706E-08 | 1.157E-06 | 0.000E+00 | 1.310E-07 | 0.000E+00 | 0.000E+00 | 0.000E+00 | 1.933E-07 | 0.000E+00 | 0.000E+00 | 1.150E-06 |
| Unigene192618 | eptA | 1.974E-08 | 4.334E-08 | 1.066E-06 | 0.000E+00 | 1.097E-07 | 2.134E-08 | 4.370E-08 | 0.000E+00 | 1.726E-07 | 8.797E-08 | 2.258E-08 | 1.027E-06 |
| Unigene192619 | arlR | 4.804E-08 | 0.000E+00 | 9.898E-07 | 9.702E-08 | 2.135E-07 | 0.000E+00 | 5.316E-08 | 0.000E+00 | 0.000E+00 | 0.000E+00 | 0.000E+00 | 1.192E-06 |
| Unigene192623 | Acinetobac | 1.072E-07 | 0.000E+00 | 1.325E-06 | 6.493E-08 | 4.763E-08 | 4.634E-08 | 0.000E+00 | 0.000E+00 | 1.640E-07 | 1.671E-07 | 4.904E-08 | 9.628E-07 |
| Unigene192646 | PmrF | 0.000E+00 | 0.000E+00 | 1.336E-05 | 0.000E+00 | 0.000E+00 | 0.000E+00 | 0.000E+00 | 0.000E+00 | 0.000E+00 | 0.000E+00 | 0.000E+00 | 0.000E+00 |
| Unigene192744 | msrE | 0.000E+00 | 9.323E-08 | 1.528E-06 | 2.288E-07 | 0.000E+00 | 0.000E+00 | 0.000E+00 | 0.000E+00 | 0.000E+00 | 0.000E+00 | 0.000E+00 | 0.000E+00 |
| Unigene192745 | msrC | 0.000E+00 | 3.374E-07 | 1.433E-06 | 0.000E+00 | 0.000E+00 | 0.000E+00 | 0.000E+00 | 0.000E+00 | 0.000E+00 | 0.000E+00 | 0.000E+00 | 0.000E+00 |
| Unigene192776 | bcrA | 9.303E-08 | 0.000E+00 | 1.339E-06 | 3.131E-07 | 0.000E+00 | 0.000E+00 | 0.000E+00 | 0.000E+00 | 0.000E+00 | 0.000E+00 | 0.000E+00 | 0.000E+00 |
| Unigene192785 | RlmA(II) | 3.509E-07 | 1.112E-06 | 1.530E-06 | 2.008E-06 | 0.000E+00 | 0.000E+00 | 0.000E+00 | 0.000E+00 | 0.000E+00 | 0.000E+00 | 0.000E+00 | 0.000E+00 |
| Unigene193027 | fexA | 2.289E-07 | 2.513E-07 | 0.000E+00 | 0.000E+00 | 0.000E+00 | 0.000E+00 | 0.000E+00 | 1.336E-07 | 0.000E+00 | 0.000E+00 | 0.000E+00 | 0.000E+00 |
| Unigene193043 | sul2 | 1.273E-07 | 1.398E-07 | 5.623E-07 | 0.000E+00 | 0.000E+00 | 0.000E+00 | 0.000E+00 | 7.432E-08 | 0.000E+00 | 0.000E+00 | 0.000E+00 | 0.000E+00 |
| Unigene193059 | mtrA | 0.000E+00 | 1.093E-06 | 1.023E-06 | 1.627E-06 | 0.000E+00 | 0.000E+00 | 0.000E+00 | 0.000E+00 | 0.000E+00 | 0.000E+00 | 0.000E+00 | 0.000E+00 |
| Unigene193100 | macB | 2.866E-06 | 9.386E-06 | 6.824E-06 | 6.216E-06 | 0.000E+00 | 0.000E+00 | 0.000E+00 | 0.000E+00 | 0.000E+00 | 0.000E+00 | 0.000E+00 | 0.000E+00 |
| Unigene193121 | APH(3'')-Ib | 0.000E+00 | 0.000E+00 | 3.959E-06 | 0.000E+00 | 3.558E-07 | 0.000E+00 | 0.000E+00 | 2.243E-07 | 4.900E-07 | 0.000E+00 | 0.000E+00 | 5.754E-06 |
| Unigene193175 | ErmE | 1.459E-07 | 0.000E+00 | 8.232E-07 | 4.052E-07 | 0.000E+00 | 0.000E+00 | 0.000E+00 | 0.000E+00 | 0.000E+00 | 0.000E+00 | 0.000E+00 | 0.000E+00 |
| Unigene193191 | poxtA | 0.000E+00 | 0.000E+00 | 6.748E-07 | 0.000E+00 | 0.000E+00 | 0.000E+00 | 3.268E-06 | 0.000E+00 | 0.000E+00 | 0.000E+00 | 1.596E-06 | 3.443E-06 |
| Unigene193225 | tva(A) | 1.303E-07 | 1.226E-07 | 5.115E-07 | 3.384E-07 | 8.066E-07 | 4.427E-07 | 9.684E-07 | 1.043E-06 | 3.052E-07 | 5.807E-07 | 8.944E-07 | 3.609E-06 |
| Unigene193266 | YojI | 8.060E-08 | 0.000E+00 | 1.107E-06 | 1.628E-07 | 0.000E+00 | 0.000E+00 | 0.000E+00 | 0.000E+00 | 0.000E+00 | 0.000E+00 | 0.000E+00 | 0.000E+00 |
| Unigene193268 | kdpE | 1.888E-07 | 0.000E+00 | 1.343E-06 | 2.859E-07 | 0.000E+00 | 0.000E+00 | 0.000E+00 | 0.000E+00 | 0.000E+00 | 0.000E+00 | 0.000E+00 | 0.000E+00 |
| Unigene193343 | lmrB | 0.000E+00 | 0.000E+00 | 7.304E-07 | 8.002E-05 | 0.000E+00 | 0.000E+00 | 0.000E+00 | 7.899E-08 | 0.000E+00 | 0.000E+00 | 0.000E+00 | 0.000E+00 |
| Unigene193378 | macB | 0.000E+00 | 0.000E+00 | 2.816E-06 | 1.357E-05 | 0.000E+00 | 0.000E+00 | 0.000E+00 | 0.000E+00 | 0.000E+00 | 0.000E+00 | 0.000E+00 | 0.000E+00 |
| Unigene193407 | Corynebac | 0.000E+00 | 0.000E+00 | 6.631E-07 | 0.000E+00 | 5.775E-06 | 0.000E+00 | 0.000E+00 | 0.000E+00 | 0.000E+00 | 0.000E+00 | 0.000E+00 | 0.000E+00 |
| Unigene193410 | kdpE | 0.000E+00 | 9.524E-07 | 9.650E-07 | 1.694E-06 | 0.000E+00 | 0.000E+00 | 0.000E+00 | 0.000E+00 | 0.000E+00 | 0.000E+00 | 0.000E+00 | 0.000E+00 |
| Unigene193459 | dfrA20 | 0.000E+00 | 0.000E+00 | 1.075E-06 | 0.000E+00 | 0.000E+00 | 0.000E+00 | 0.000E+00 | 0.000E+00 | 0.000E+00 | 0.000E+00 | 0.000E+00 | 0.000E+00 |
| Unigene193498 | Listeria mc | 7.507E-08 | 8.651E-07 | 3.290E-06 | 3.247E-06 | 0.000E+00 | 0.000E+00 | 0.000E+00 | 0.000E+00 | 0.000E+00 | 0.000E+00 | 0.000E+00 | 0.000E+00 |

| | | | | | | | | | | | | | |
|---|---|---|---|---|---|---|---|---|---|---|---|---|---|
| Unigene193673 | patA | 4.719E-08 | 2.072E-07 | 4.168E-07 | 4.766E-07 | 0.000E+00 | 0.000E+00 | 5.223E-08 | 0.000E+00 | 0.000E+00 | 0.000E+00 | 0.000E+00 | 0.000E+00 |
| Unigene193689 | poxtA | 1.526E-07 | 5.863E-07 | 3.744E-06 | 3.506E-06 | 0.000E+00 | 0.000E+00 | 0.000E+00 | 0.000E+00 | 0.000E+00 | 0.000E+00 | 0.000E+00 | 0.000E+00 |
| Unigene193706 | evgA | 0.000E+00 | 0.000E+00 | 0.000E+00 | 0.000E+00 | 0.000E+00 | 0.000E+00 | 0.000E+00 | 0.000E+00 | 0.000E+00 | 0.000E+00 | 0.000E+00 | 9.645E-07 |
| Unigene193726 | patA | 0.000E+00 | 0.000E+00 | 3.274E-07 | 0.000E+00 | 0.000E+00 | 0.000E+00 | 0.000E+00 | 0.000E+00 | 0.000E+00 | 0.000E+00 | 0.000E+00 | 0.000E+00 |
| Unigene193832 | patB | 3.772E-05 | 2.349E-05 | 2.029E-05 | 2.100E-05 | 0.000E+00 | 1.614E-06 | 2.755E-06 | 4.470E-07 | 7.031E-06 | 8.958E-07 | 0.000E+00 | 0.000E+00 |
| Unigene193849 | cpxA | 0.000E+00 | 1.849E-07 | 1.701E-06 | 7.292E-08 | 1.872E-07 | 1.301E-07 | 7.992E-08 | 0.000E+00 | 1.052E-07 | 0.000E+00 | 2.203E-07 | 1.024E-06 |
| Unigene193911 | PmrF | 2.181E-07 | 0.000E+00 | 1.177E-06 | 2.203E-07 | 0.000E+00 | 0.000E+00 | 0.000E+00 | 0.000E+00 | 0.000E+00 | 0.000E+00 | 0.000E+00 | 0.000E+00 |
| Unigene193914 | efrA | 4.173E-07 | 3.598E-07 | 4.972E-07 | 5.117E-07 | 5.960E-07 | 9.989E-07 | 3.563E-06 | 3.410E-06 | 1.629E-07 | 1.395E-06 | 1.602E-06 | 3.417E-06 |
| Unigene194005 | abcA | 0.000E+00 | 0.000E+00 | 5.129E-07 | 0.000E+00 | 0.000E+00 | 0.000E+00 | 0.000E+00 | 0.000E+00 | 0.000E+00 | 0.000E+00 | 0.000E+00 | 0.000E+00 |
| Unigene194124 | macB | 0.000E+00 | 0.000E+00 | 4.461E-07 | 0.000E+00 | 0.000E+00 | 0.000E+00 | 0.000E+00 | 0.000E+00 | 0.000E+00 | 0.000E+00 | 0.000E+00 | 0.000E+00 |
| Unigene194201 | vanSG | 1.783E-07 | 7.503E-07 | 1.108E-06 | 3.302E-07 | 0.000E+00 | 0.000E+00 | 0.000E+00 | 0.000E+00 | 0.000E+00 | 0.000E+00 | 0.000E+00 | 0.000E+00 |
| Unigene194352 | tetA(58) | 0.000E+00 | 0.000E+00 | 5.425E-07 | 0.000E+00 | 0.000E+00 | 0.000E+00 | 0.000E+00 | 1.092E-06 | 0.000E+00 | 0.000E+00 | 0.000E+00 | 0.000E+00 |
| Unigene194355 | carA | 0.000E+00 | 9.119E-08 | 1.549E-06 | 0.000E+00 | 0.000E+00 | 0.000E+00 | 0.000E+00 | 4.849E-08 | 1.816E-07 | 0.000E+00 | 0.000E+00 | 5.402E-07 |
| Unigene194363 | rphB | 0.000E+00 | 5.957E-08 | 9.853E-07 | 4.111E-08 | 1.206E-07 | 1.467E-08 | 1.051E-07 | 0.000E+00 | 1.186E-07 | 6.046E-08 | 0.000E+00 | 1.283E-06 |
| Unigene194375 | tetA(58) | 0.000E+00 | 0.000E+00 | 4.061E-07 | 0.000E+00 | 8.867E-07 | 0.000E+00 | 2.519E-06 | 3.278E-06 | 0.000E+00 | 0.000E+00 | 2.029E-06 | 4.088E-06 |
| Unigene194408 | vatB | 0.000E+00 | 6.248E-08 | 0.000E+00 | 0.000E+00 | 0.000E+00 | 0.000E+00 | 5.041E-07 | 0.000E+00 | 0.000E+00 | 0.000E+00 | 1.302E-07 | 2.019E-07 |
| Unigene194464 | macB | 0.000E+00 | 0.000E+00 | 4.168E-07 | 0.000E+00 | 0.000E+00 | 0.000E+00 | 1.567E-07 | 0.000E+00 | 0.000E+00 | 0.000E+00 | 0.000E+00 | 0.000E+00 |
| Unigene194654 | baeR | 0.000E+00 | 9.800E-08 | 1.095E-06 | 0.000E+00 | 0.000E+00 | 4.827E-08 | 2.471E-07 | 0.000E+00 | 2.928E-07 | 2.984E-07 | 1.021E-07 | 1.108E-06 |
| Unigene194693 | CRP | 0.000E+00 | 0.000E+00 | 1.468E-06 | 1.608E-06 | 0.000E+00 | 0.000E+00 | 0.000E+00 | 0.000E+00 | 0.000E+00 | 0.000E+00 | 0.000E+00 | 0.000E+00 |
| Unigene194764 | arlS | 2.429E-08 | 5.332E-08 | 1.239E-06 | 2.453E-07 | 0.000E+00 | 0.000E+00 | 0.000E+00 | 0.000E+00 | 0.000E+00 | 0.000E+00 | 0.000E+00 | 0.000E+00 |
| Unigene194766 | Staphylocc | 1.121E-07 | 0.000E+00 | 5.499E-07 | 0.000E+00 | 0.000E+00 | 0.000E+00 | 0.000E+00 | 0.000E+00 | 0.000E+00 | 0.000E+00 | 0.000E+00 | 0.000E+00 |
| Unigene194780 | tetA(58) | 0.000E+00 | 0.000E+00 | 1.545E-07 | 0.000E+00 | 0.000E+00 | 0.000E+00 | 0.000E+00 | 0.000E+00 | 0.000E+00 | 0.000E+00 | 0.000E+00 | 0.000E+00 |
| Unigene194833 | tetA(58) | 0.000E+00 | 1.504E-07 | 9.415E-07 | 1.730E-07 | 0.000E+00 | 0.000E+00 | 0.000E+00 | 0.000E+00 | 0.000E+00 | 0.000E+00 | 0.000E+00 | 0.000E+00 |
| Unigene194956 | tetA(58) | 0.000E+00 | 0.000E+00 | 2.876E-07 | 0.000E+00 | 0.000E+00 | 0.000E+00 | 0.000E+00 | 0.000E+00 | 0.000E+00 | 0.000E+00 | 0.000E+00 | 0.000E+00 |
| Unigene195073 | vanG | 0.000E+00 | 1.154E-07 | 8.254E-07 | 0.000E+00 | 0.000E+00 | 2.273E-07 | 0.000E+00 | 0.000E+00 | 1.532E-07 | 0.000E+00 | 0.000E+00 | 1.119E-06 |
| Unigene195100 | macB | 1.334E-07 | 2.440E-07 | 1.309E-06 | 6.106E-06 | 0.000E+00 | 0.000E+00 | 0.000E+00 | 0.000E+00 | 0.000E+00 | 0.000E+00 | 0.000E+00 | 0.000E+00 |
| Unigene195115 | Chlamydia | 0.000E+00 | 9.230E-07 | 2.039E-06 | 1.749E-06 | 0.000E+00 | 0.000E+00 | 0.000E+00 | 0.000E+00 | 0.000E+00 | 0.000E+00 | 0.000E+00 | 0.000E+00 |
| Unigene195139 | tetB(46) | 0.000E+00 | 0.000E+00 | 1.343E-06 | 4.876E-06 | 0.000E+00 | 0.000E+00 | 0.000E+00 | 0.000E+00 | 0.000E+00 | 0.000E+00 | 0.000E+00 | 0.000E+00 |
| Unigene195140 | optrA | 0.000E+00 | 0.000E+00 | 1.634E-06 | 5.986E-06 | 0.000E+00 | 0.000E+00 | 0.000E+00 | 0.000E+00 | 0.000E+00 | 0.000E+00 | 0.000E+00 | 0.000E+00 |
| Unigene195250 | Staphylocc | 9.357E-08 | 1.027E-07 | 9.443E-07 | 3.509E-07 | 0.000E+00 | 0.000E+00 | 0.000E+00 | 0.000E+00 | 0.000E+00 | 0.000E+00 | 3.058E-08 | 0.000E+00 |
| Unigene195324 | macB | 0.000E+00 | 9.155E-08 | 1.187E-06 | 4.212E-08 | 2.317E-07 | 4.509E-08 | 0.000E+00 | 9.736E-08 | 0.000E+00 | 0.000E+00 | 9.541E-08 | 7.888E-07 |
| Unigene195338 | vanHO | 8.152E-08 | 0.000E+00 | 1.173E-06 | 0.000E+00 | 0.000E+00 | 0.000E+00 | 0.000E+00 | 0.000E+00 | 2.079E-07 | 0.000E+00 | 0.000E+00 | 8.351E-07 |
| Unigene195395 | oleC | 2.110E-07 | 0.000E+00 | 1.035E-06 | 7.031E-06 | 0.000E+00 | 0.000E+00 | 0.000E+00 | 0.000E+00 | 0.000E+00 | 0.000E+00 | 0.000E+00 | 0.000E+00 |
| Unigene195403 | macB | 0.000E+00 | 0.000E+00 | 1.009E-06 | 4.434E-06 | 0.000E+00 | 0.000E+00 | 0.000E+00 | 0.000E+00 | 0.000E+00 | 0.000E+00 | 0.000E+00 | 0.000E+00 |
| Unigene195407 | evgA | 0.000E+00 | 0.000E+00 | 1.350E-06 | 5.728E-06 | 0.000E+00 | 0.000E+00 | 0.000E+00 | 0.000E+00 | 0.000E+00 | 0.000E+00 | 0.000E+00 | 0.000E+00 |
| Unigene195527 | vanHA | 0.000E+00 | 0.000E+00 | 1.049E-06 | 0.000E+00 | 0.000E+00 | 0.000E+00 | 0.000E+00 | 0.000E+00 | 0.000E+00 | 0.000E+00 | 0.000E+00 | 0.000E+00 |
| Unigene195591 | efrA | 0.000E+00 | 0.000E+00 | 4.667E-07 | 0.000E+00 | 0.000E+00 | 0.000E+00 | 0.000E+00 | 0.000E+00 | 0.000E+00 | 0.000E+00 | 0.000E+00 | 9.135E-07 |
| Unigene195769 | vanHD | 6.348E-08 | 6.967E-08 | 9.655E-07 | 1.282E-07 | 0.000E+00 | 0.000E+00 | 0.000E+00 | 0.000E+00 | 0.000E+00 | 0.000E+00 | 0.000E+00 | 0.000E+00 |
| Unigene195781 | basS | 0.000E+00 | 0.000E+00 | 4.350E-07 | 0.000E+00 | 0.000E+00 | 0.000E+00 | 0.000E+00 | 0.000E+00 | 0.000E+00 | 0.000E+00 | 0.000E+00 | 0.000E+00 |
| Unigene195790 | acrD | 1.459E-07 | 1.601E-07 | 5.011E-07 | 2.210E-07 | 0.000E+00 | 0.000E+00 | 0.000E+00 | 2.980E-07 | 0.000E+00 | 0.000E+00 | 0.000E+00 | 0.000E+00 |
| Unigene195816 | Staphylocc | 0.000E+00 | 0.000E+00 | 4.297E-07 | 0.000E+00 | 2.085E-07 | 0.000E+00 | 0.000E+00 | 0.000E+00 | 2.051E-07 | 0.000E+00 | 0.000E+00 | 0.000E+00 |
| Unigene195828 | vanRM | 0.000E+00 | 4.900E-08 | 8.762E-07 | 0.000E+00 | 0.000E+00 | 0.000E+00 | 0.000E+00 | 0.000E+00 | 0.000E+00 | 0.000E+00 | 0.000E+00 | 0.000E+00 |
| Unigene195838 | optrA | 0.000E+00 | 0.000E+00 | 3.259E-07 | 0.000E+00 | 0.000E+00 | 0.000E+00 | 0.000E+00 | 0.000E+00 | 0.000E+00 | 0.000E+00 | 0.000E+00 | 0.000E+00 |
| Unigene195849 | macB | 1.913E-07 | 7.348E-07 | 1.736E-06 | 3.042E-06 | 4.250E-07 | 0.000E+00 | 4.763E-07 | 1.563E-06 | 1.568E-07 | 6.926E-07 | 1.039E-06 | 7.914E-07 |
| Unigene195851 | emrB | 2.801E-07 | 1.537E-07 | 1.076E-06 | 1.815E-06 | 0.000E+00 | 0.000E+00 | 0.000E+00 | 7.628E-07 | 5.102E-08 | 4.680E-07 | 7.209E-07 | 2.483E-07 |
| Unigene195894 | tetA(58) | 0.000E+00 | 0.000E+00 | 7.341E-07 | 5.333E-07 | 0.000E+00 | 0.000E+00 | 0.000E+00 | 0.000E+00 | 0.000E+00 | 0.000E+00 | 0.000E+00 | 0.000E+00 |
| Unigene195898 | ramA | 1.668E-07 | 5.035E-07 | 1.391E-06 | 1.306E-06 | 0.000E+00 | 0.000E+00 | 0.000E+00 | 0.000E+00 | 0.000E+00 | 0.000E+00 | 0.000E+00 | 0.000E+00 |
| Unigene195911 | ErmA | 0.000E+00 | 0.000E+00 | 9.598E-07 | 0.000E+00 | 0.000E+00 | 0.000E+00 | 0.000E+00 | 0.000E+00 | 0.000E+00 | 0.000E+00 | 0.000E+00 | 0.000E+00 |
| Unigene195915 | mdtF | 0.000E+00 | 0.000E+00 | 0.000E+00 | 0.000E+00 | 0.000E+00 | 0.000E+00 | 0.000E+00 | 0.000E+00 | 0.000E+00 | 0.000E+00 | 2.849E-07 | 0.000E+00 |
| Unigene195946 | tetB(60) | 1.291E-07 | 7.873E-07 | 9.010E-07 | 2.173E-07 | 4.941E-07 | 9.306E-08 | 1.270E-06 | 8.373E-08 | 0.000E+00 | 0.000E+00 | 1.969E-07 | 0.000E+00 |
| Unigene195987 | smeR | 0.000E+00 | 0.000E+00 | 0.000E+00 | 0.000E+00 | 0.000E+00 | 1.029E-06 | 1.782E-06 | 0.000E+00 | 3.200E-07 | 0.000E+00 | 0.000E+00 | 0.000E+00 |
| Unigene196028 | vanHO | 0.000E+00 | 0.000E+00 | 9.628E-07 | 0.000E+00 | 0.000E+00 | 1.096E-06 | 1.774E-06 | 2.023E-06 | 0.000E+00 | 9.473E-07 | 0.000E+00 | 3.170E-06 |
| Unigene196043 | bmr | 0.000E+00 | 0.000E+00 | 2.607E-07 | 0.000E+00 | 0.000E+00 | 0.000E+00 | 0.000E+00 | 0.000E+00 | 0.000E+00 | 0.000E+00 | 0.000E+00 | 0.000E+00 |
| Unigene196101 | clbB | 0.000E+00 | 9.108E-08 | 1.167E-06 | 0.000E+00 | 0.000E+00 | 1.495E-07 | 0.000E+00 | 0.000E+00 | 0.000E+00 | 2.157E-07 | 0.000E+00 | 1.079E-06 |
| Unigene196233 | patB | 0.000E+00 | 0.000E+00 | 1.368E-06 | 0.000E+00 | 0.000E+00 | 0.000E+00 | 0.000E+00 | 0.000E+00 | 0.000E+00 | 0.000E+00 | 0.000E+00 | 0.000E+00 |
| Unigene196258 | vgaB | 0.000E+00 | 0.000E+00 | 1.370E-06 | 6.754E-06 | 0.000E+00 | 0.000E+00 | 0.000E+00 | 0.000E+00 | 0.000E+00 | 0.000E+00 | 0.000E+00 | 0.000E+00 |
| Unigene196335 | optrA | 0.000E+00 | 0.000E+00 | 8.908E-07 | 0.000E+00 | 0.000E+00 | 0.000E+00 | 0.000E+00 | 0.000E+00 | 0.000E+00 | 0.000E+00 | 0.000E+00 | 0.000E+00 |
| Unigene196376 | patB | 4.235E-07 | 7.394E-07 | 1.209E-06 | 3.324E-06 | 4.705E-07 | 1.249E-07 | 1.704E-07 | 1.326E-06 | 0.000E+00 | 5.361E-07 | 1.013E-06 | 7.963E-07 |

| | | | | | | | | | | | | | |
|---|---|---|---|---|---|---|---|---|---|---|---|---|---|
| Unigene196593 | tetB(60) | 1.270E-07 | 5.974E-08 | 1.193E-06 | 9.162E-08 | 0.000E+00 | 0.000E+00 | 0.000E+00 | 0.000E+00 | 0.000E+00 | 0.000E+00 | 0.000E+00 | 0.000E+00 |
| Unigene196594 | tetA(60) | 7.234E-08 | 1.985E-07 | 1.153E-06 | 2.739E-07 | 0.000E+00 | 0.000E+00 | 0.000E+00 | 0.000E+00 | 0.000E+00 | 0.000E+00 | 0.000E+00 | 0.000E+00 |
| Unigene196619 | patB | 8.506E-08 | 4.668E-08 | 1.043E-06 | 1.718E-07 | 0.000E+00 | 0.000E+00 | 0.000E+00 | 0.000E+00 | 0.000E+00 | 0.000E+00 | 0.000E+00 | 0.000E+00 |
| Unigene196620 | patB | 0.000E+00 | 0.000E+00 | 1.413E-06 | 3.635E-07 | 0.000E+00 | 0.000E+00 | 0.000E+00 | 0.000E+00 | 0.000E+00 | 0.000E+00 | 0.000E+00 | 0.000E+00 |
| Unigene196626 | macB | 0.000E+00 | 2.578E-07 | 8.299E-07 | 2.847E-07 | 0.000E+00 | 0.000E+00 | 0.000E+00 | 0.000E+00 | 0.000E+00 | 0.000E+00 | 0.000E+00 | 0.000E+00 |
| Unigene196667 | vanHO | 0.000E+00 | 0.000E+00 | 2.866E-07 | 0.000E+00 | 0.000E+00 | 0.000E+00 | 0.000E+00 | 0.000E+00 | 0.000E+00 | 0.000E+00 | 0.000E+00 | 0.000E+00 |
| Unigene196687 | lsaA | 0.000E+00 | 0.000E+00 | 2.497E-06 | 2.199E-06 | 0.000E+00 | 0.000E+00 | 0.000E+00 | 0.000E+00 | 0.000E+00 | 0.000E+00 | 0.000E+00 | 0.000E+00 |
| Unigene196704 | lmrD | 0.000E+00 | 2.271E-07 | 3.046E-07 | 0.000E+00 | 0.000E+00 | 0.000E+00 | 0.000E+00 | 0.000E+00 | 0.000E+00 | 0.000E+00 | 0.000E+00 | 0.000E+00 |
| Unigene196707 | novA | 1.849E-07 | 1.015E-07 | 2.159E-06 | 6.945E-06 | 0.000E+00 | 0.000E+00 | 0.000E+00 | 0.000E+00 | 0.000E+00 | 0.000E+00 | 0.000E+00 | 0.000E+00 |
| Unigene196710 | patB | 1.641E-07 | 1.801E-07 | 1.679E-06 | 6.321E-06 | 0.000E+00 | 0.000E+00 | 0.000E+00 | 0.000E+00 | 0.000E+00 | 0.000E+00 | 0.000E+00 | 0.000E+00 |
| Unigene196711 | macB | 0.000E+00 | 0.000E+00 | 2.157E-06 | 7.167E-06 | 0.000E+00 | 0.000E+00 | 0.000E+00 | 0.000E+00 | 0.000E+00 | 0.000E+00 | 0.000E+00 | 0.000E+00 |
| Unigene196713 | macB | 1.599E-07 | 2.883E-07 | 1.748E-06 | 7.394E-06 | 0.000E+00 | 0.000E+00 | 0.000E+00 | 0.000E+00 | 0.000E+00 | 0.000E+00 | 0.000E+00 | 0.000E+00 |
| Unigene196813 | TaeA | 0.000E+00 | 0.000E+00 | 8.592E-07 | 5.123E-05 | 0.000E+00 | 0.000E+00 | 0.000E+00 | 1.376E-07 | 0.000E+00 | 0.000E+00 | 0.000E+00 | 0.000E+00 |
| Unigene196954 | Staphylocc | 2.483E-05 | 1.028E-06 | 4.035E-07 | 2.307E-08 | 2.538E-08 | 0.000E+00 | 0.000E+00 | 0.000E+00 | 0.000E+00 | 0.000E+00 | 0.000E+00 | 0.000E+00 |
| Unigene196965 | acrB | 0.000E+00 | 1.027E-06 | 4.284E-07 | 6.299E-08 | 0.000E+00 | 0.000E+00 | 0.000E+00 | 0.000E+00 | 0.000E+00 | 0.000E+00 | 0.000E+00 | 0.000E+00 |
| Unigene197029 | msbA | 0.000E+00 | 1.141E-07 | 1.785E-06 | 0.000E+00 | 0.000E+00 | 0.000E+00 | 0.000E+00 | 6.067E-08 | 0.000E+00 | 2.316E-07 | 0.000E+00 | 1.229E-06 |
| Unigene197068 | evgS | 1.129E-07 | 0.000E+00 | 7.383E-07 | 1.900E-07 | 0.000E+00 | 0.000E+00 | 0.000E+00 | 0.000E+00 | 0.000E+00 | 0.000E+00 | 4.304E-08 | 0.000E+00 |
| Unigene197107 | farA | 0.000E+00 | 0.000E+00 | 1.885E-07 | 0.000E+00 | 0.000E+00 | 0.000E+00 | 0.000E+00 | 0.000E+00 | 0.000E+00 | 0.000E+00 | 0.000E+00 | 0.000E+00 |
| Unigene197109 | patB | 4.565E-07 | 7.873E-07 | 7.999E-07 | 4.610E-07 | 2.173E-06 | 2.503E-06 | 8.913E-06 | 0.000E+00 | 3.920E-06 | 0.000E+00 | 0.000E+00 | 1.966E-06 |
| Unigene197208 | tetA(58) | 0.000E+00 | 0.000E+00 | 7.857E-07 | 2.022E-07 | 0.000E+00 | 0.000E+00 | 0.000E+00 | 0.000E+00 | 0.000E+00 | 0.000E+00 | 0.000E+00 | 0.000E+00 |
| Unigene197277 | Bifidobacte | 0.000E+00 | 7.421E-08 | 2.019E-06 | 1.266E-05 | 0.000E+00 | 0.000E+00 | 0.000E+00 | 0.000E+00 | 0.000E+00 | 0.000E+00 | 0.000E+00 | 0.000E+00 |
| Unigene197291 | emrY | 0.000E+00 | 1.621E-07 | 1.946E-06 | 1.438E-05 | 0.000E+00 | 0.000E+00 | 0.000E+00 | 0.000E+00 | 0.000E+00 | 0.000E+00 | 0.000E+00 | 0.000E+00 |
| Unigene197342 | rosB | 0.000E+00 | 6.984E-07 | 2.271E-07 | 0.000E+00 | 0.000E+00 | 0.000E+00 | 0.000E+00 | 0.000E+00 | 0.000E+00 | 0.000E+00 | 0.000E+00 | 0.000E+00 |
| Unigene197367 | tetA(58) | 8.423E-08 | 6.933E-08 | 1.012E-06 | 1.063E-07 | 0.000E+00 | 0.000E+00 | 0.000E+00 | 0.000E+00 | 0.000E+00 | 0.000E+00 | 2.409E-08 | 0.000E+00 |
| Unigene197441 | lsaA | 2.322E-07 | 2.549E-07 | 1.405E-06 | 2.541E-06 | 0.000E+00 | 0.000E+00 | 2.570E-07 | 1.581E-06 | 0.000E+00 | 3.449E-07 | 9.298E-07 | 1.373E-07 |
| Unigene197444 | bcrA | 1.787E-07 | 6.277E-07 | 1.649E-06 | 3.790E-06 | 5.560E-07 | 0.000E+00 | 3.561E-07 | 1.919E-06 | 0.000E+00 | 9.159E-07 | 1.717E-06 | 7.183E-07 |
| Unigene197445 | mecI | 0.000E+00 | 0.000E+00 | 7.847E-07 | 3.230E-06 | 0.000E+00 | 0.000E+00 | 0.000E+00 | 1.612E-06 | 0.000E+00 | 0.000E+00 | 0.000E+00 | 0.000E+00 |
| Unigene197508 | PmrF | 0.000E+00 | 0.000E+00 | 4.854E-07 | 9.367E-07 | 0.000E+00 | 0.000E+00 | 0.000E+00 | 0.000E+00 | 0.000E+00 | 0.000E+00 | 0.000E+00 | 0.000E+00 |
| Unigene197516 | Streptomy | 0.000E+00 | 0.000E+00 | 5.999E-07 | 5.396E-05 | 0.000E+00 | 0.000E+00 | 0.000E+00 | 1.427E-07 | 0.000E+00 | 0.000E+00 | 0.000E+00 | 0.000E+00 |
| Unigene197527 | tetA(60) | 0.000E+00 | 7.719E-08 | 4.140E-07 | 0.000E+00 | 0.000E+00 | 0.000E+00 | 0.000E+00 | 0.000E+00 | 0.000E+00 | 0.000E+00 | 0.000E+00 | 0.000E+00 |
| Unigene197707 | adeS | 0.000E+00 | 0.000E+00 | 4.272E-07 | 0.000E+00 | 0.000E+00 | 0.000E+00 | 0.000E+00 | 0.000E+00 | 0.000E+00 | 0.000E+00 | 0.000E+00 | 0.000E+00 |
| Unigene198032 | blt | 3.738E-07 | 4.396E-07 | 1.598E-06 | 2.831E-06 | 5.932E-07 | 2.598E-07 | 2.955E-07 | 9.350E-07 | 3.210E-07 | 5.056E-07 | 1.558E-06 | 9.468E-07 |
| Unigene198047 | farB | 2.280E-07 | 1.251E-07 | 8.500E-07 | 2.325E-06 | 1.520E-07 | 9.858E-08 | 2.775E-07 | 1.277E-06 | 7.475E-08 | 3.555E-07 | 5.998E-07 | 3.503E-07 |
| Unigene198074 | patB | 0.000E+00 | 1.495E-07 | 8.019E-07 | 1.719E-07 | 0.000E+00 | 0.000E+00 | 0.000E+00 | 0.000E+00 | 0.000E+00 | 0.000E+00 | 0.000E+00 | 0.000E+00 |
| Unigene198115 | tetB(60) | 0.000E+00 | 1.305E-07 | 1.342E-06 | 4.803E-07 | 0.000E+00 | 0.000E+00 | 0.000E+00 | 0.000E+00 | 0.000E+00 | 0.000E+00 | 0.000E+00 | 0.000E+00 |
| Unigene198124 | optrA | 8.315E-08 | 5.476E-08 | 1.485E-06 | 1.176E-07 | 0.000E+00 | 0.000E+00 | 0.000E+00 | 0.000E+00 | 0.000E+00 | 0.000E+00 | 0.000E+00 | 0.000E+00 |
| Unigene198150 | mtrA | 4.502E-08 | 1.976E-07 | 1.458E-06 | 4.092E-07 | 0.000E+00 | 0.000E+00 | 0.000E+00 | 0.000E+00 | 0.000E+00 | 0.000E+00 | 0.000E+00 | 0.000E+00 |
| Unigene198167 | macB | 0.000E+00 | 0.000E+00 | 4.186E-07 | 9.573E-08 | 0.000E+00 | 0.000E+00 | 0.000E+00 | 0.000E+00 | 0.000E+00 | 0.000E+00 | 0.000E+00 | 0.000E+00 |
| Unigene198241 | cfrC | 2.174E-07 | 0.000E+00 | 1.600E-06 | 4.582E-06 | 0.000E+00 | 0.000E+00 | 0.000E+00 | 0.000E+00 | 0.000E+00 | 0.000E+00 | 0.000E+00 | 0.000E+00 |
| Unigene198256 | adeR | 6.698E-07 | 7.351E-07 | 3.668E-06 | 5.127E-06 | 9.602E-08 | 0.000E+00 | 3.348E-07 | 0.000E+00 | 0.000E+00 | 0.000E+00 | 0.000E+00 | 0.000E+00 |
| Unigene198281 | Acinetoba | 0.000E+00 | 0.000E+00 | 2.596E-07 | 0.000E+00 | 0.000E+00 | 0.000E+00 | 0.000E+00 | 0.000E+00 | 0.000E+00 | 0.000E+00 | 0.000E+00 | 0.000E+00 |
| Unigene198304 | Staphylocc | 0.000E+00 | 0.000E+00 | 6.378E-07 | 0.000E+00 | 0.000E+00 | 0.000E+00 | 0.000E+00 | 0.000E+00 | 0.000E+00 | 0.000E+00 | 0.000E+00 | 0.000E+00 |
| Unigene198364 | patB | 0.000E+00 | 5.229E-07 | 4.675E-07 | 1.203E-07 | 0.000E+00 | 0.000E+00 | 0.000E+00 | 0.000E+00 | 0.000E+00 | 0.000E+00 | 0.000E+00 | 0.000E+00 |
| Unigene198419 | kdpE | 0.000E+00 | 3.937E-08 | 4.223E-07 | 2.173E-07 | 0.000E+00 | 0.000E+00 | 0.000E+00 | 0.000E+00 | 0.000E+00 | 0.000E+00 | 0.000E+00 | 0.000E+00 |
| Unigene198502 | cdeA | 1.637E-07 | 9.499E-07 | 3.604E-06 | 3.543E-06 | 0.000E+00 | 0.000E+00 | 0.000E+00 | 0.000E+00 | 0.000E+00 | 0.000E+00 | 0.000E+00 | 0.000E+00 |
| Unigene198622 | Listeria mc | 1.638E-07 | 8.850E-07 | 3.647E-06 | 3.741E-06 | 0.000E+00 | 0.000E+00 | 0.000E+00 | 0.000E+00 | 0.000E+00 | 0.000E+00 | 0.000E+00 | 0.000E+00 |
| Unigene198670 | tetA(46) | 9.340E-08 | 4.101E-08 | 1.155E-06 | 5.659E-08 | 2.075E-08 | 0.000E+00 | 0.000E+00 | 4.361E-08 | 1.838E-07 | 0.000E+00 | 0.000E+00 | 7.508E-07 |
| Unigene198700 | lmrD | 0.000E+00 | 0.000E+00 | 1.082E-06 | 5.830E-07 | 0.000E+00 | 0.000E+00 | 0.000E+00 | 0.000E+00 | 0.000E+00 | 0.000E+00 | 0.000E+00 | 0.000E+00 |
| Unigene198738 | macB | 0.000E+00 | 0.000E+00 | 2.526E-07 | 2.080E-07 | 0.000E+00 | 0.000E+00 | 0.000E+00 | 0.000E+00 | 0.000E+00 | 0.000E+00 | 0.000E+00 | 0.000E+00 |
| Unigene198856 | arlR | 0.000E+00 | 0.000E+00 | 7.091E-07 | 0.000E+00 | 0.000E+00 | 0.000E+00 | 1.200E-06 | 0.000E+00 | 0.000E+00 | 0.000E+00 | 1.056E-06 | 1.851E-06 |
| Unigene198952 | baeS | 2.502E-07 | 4.120E-07 | 1.154E-06 | 2.754E-06 | 0.000E+00 | 0.000E+00 | 1.385E-07 | 1.577E-06 | 0.000E+00 | 7.805E-07 | 9.160E-07 | 4.733E-07 |
| Unigene198956 | bcrA | 0.000E+00 | 0.000E+00 | 3.219E-07 | 0.000E+00 | 0.000E+00 | 0.000E+00 | 0.000E+00 | 0.000E+00 | 0.000E+00 | 0.000E+00 | 0.000E+00 | 0.000E+00 |
| Unigene198973 | YojI | 4.236E-08 | 3.720E-07 | 2.910E-07 | 0.000E+00 | 0.000E+00 | 0.000E+00 | 0.000E+00 | 0.000E+00 | 0.000E+00 | 0.000E+00 | 0.000E+00 | 0.000E+00 |
| Unigene198987 | Staphylocc | 4.060E-08 | 0.000E+00 | 5.976E-07 | 0.000E+00 | 0.000E+00 | 0.000E+00 | 0.000E+00 | 0.000E+00 | 0.000E+00 | 0.000E+00 | 0.000E+00 | 0.000E+00 |
| Unigene199059 | msbA | 0.000E+00 | 0.000E+00 | 1.249E-07 | 0.000E+00 | 0.000E+00 | 0.000E+00 | 0.000E+00 | 0.000E+00 | 0.000E+00 | 0.000E+00 | 0.000E+00 | 0.000E+00 |
| Unigene199181 | efrA | 2.145E-07 | 1.766E-07 | 2.105E-06 | 5.036E-06 | 0.000E+00 | 0.000E+00 | 0.000E+00 | 0.000E+00 | 0.000E+00 | 0.000E+00 | 0.000E+00 | 0.000E+00 |
| Unigene199182 | lmrD | 2.566E-07 | 0.000E+00 | 2.895E-06 | 1.082E-05 | 0.000E+00 | 0.000E+00 | 0.000E+00 | 0.000E+00 | 0.000E+00 | 0.000E+00 | 0.000E+00 | 0.000E+00 |
| Unigene199200 | novA | 1.681E-07 | 0.000E+00 | 1.815E-06 | 8.192E-06 | 0.000E+00 | 0.000E+00 | 0.000E+00 | 0.000E+00 | 0.000E+00 | 0.000E+00 | 0.000E+00 | 0.000E+00 |

| | | | | | | | | | | | | | |
|---|---|---|---|---|---|---|---|---|---|---|---|---|---|
| Unigene199210 | cpxA | 0.000E+00 | 0.000E+00 | 2.995E-07 | 0.000E+00 | 0.000E+00 | 0.000E+00 | 0.000E+00 | 0.000E+00 | 0.000E+00 | 0.000E+00 | 0.000E+00 | 0.000E+00 |
| Unigene199337 | efrA | 0.000E+00 | 0.000E+00 | 3.519E-07 | 0.000E+00 | 3.985E-08 | 0.000E+00 | 0.000E+00 | 0.000E+00 | 0.000E+00 | 0.000E+00 | 0.000E+00 | 0.000E+00 |
| Unigene199345 | arlS | 0.000E+00 | 0.000E+00 | 6.211E-07 | 0.000E+00 | 0.000E+00 | 0.000E+00 | 0.000E+00 | 0.000E+00 | 0.000E+00 | 0.000E+00 | 0.000E+00 | 0.000E+00 |
| Unigene199349 | LRA-12 | 3.372E-06 | 1.029E-05 | 7.104E-06 | 1.011E-05 | 1.653E-07 | 2.680E-07 | 0.000E+00 | 0.000E+00 | 0.000E+00 | 0.000E+00 | 0.000E+00 | 0.000E+00 |
| Unigene199361 | msrA | 0.000E+00 | 0.000E+00 | 0.000E+00 | 0.000E+00 | 0.000E+00 | 0.000E+00 | 0.000E+00 | 0.000E+00 | 0.000E+00 | 0.000E+00 | 0.000E+00 | 2.020E-06 |
| Unigene199427 | Agrobacte | 1.397E-07 | 7.669E-08 | 4.114E-07 | 9.172E-07 | 0.000E+00 | 0.000E+00 | 0.000E+00 | 0.000E+00 | 0.000E+00 | 1.557E-07 | 0.000E+00 | 0.000E+00 |
| Unigene199485 | bcrA | 0.000E+00 | 0.000E+00 | 1.460E-06 | 4.442E-06 | 0.000E+00 | 0.000E+00 | 0.000E+00 | 0.000E+00 | 0.000E+00 | 0.000E+00 | 0.000E+00 | 0.000E+00 |
| Unigene199548 | macB | 0.000E+00 | 0.000E+00 | 6.020E-07 | 0.000E+00 | 0.000E+00 | 0.000E+00 | 0.000E+00 | 0.000E+00 | 0.000E+00 | 0.000E+00 | 0.000E+00 | 0.000E+00 |
| Unigene199549 | macB | 0.000E+00 | 0.000E+00 | 9.373E-07 | 0.000E+00 | 0.000E+00 | 0.000E+00 | 0.000E+00 | 0.000E+00 | 0.000E+00 | 0.000E+00 | 0.000E+00 | 0.000E+00 |
| Unigene199570 | tet(V) | 0.000E+00 | 0.000E+00 | 1.333E-06 | 2.637E-08 | 0.000E+00 | 2.823E-08 | 0.000E+00 | 0.000E+00 | 8.563E-08 | 0.000E+00 | 0.000E+00 | 1.050E-06 |
| Unigene199625 | Enterococ | 0.000E+00 | 1.293E-06 | 3.419E-06 | 2.846E-06 | 0.000E+00 | 0.000E+00 | 0.000E+00 | 0.000E+00 | 0.000E+00 | 0.000E+00 | 0.000E+00 | 0.000E+00 |
| Unigene199644 | efrA | 0.000E+00 | 5.770E-08 | 2.321E-06 | 1.276E-05 | 0.000E+00 | 0.000E+00 | 0.000E+00 | 0.000E+00 | 0.000E+00 | 0.000E+00 | 0.000E+00 | 0.000E+00 |
| Unigene199650 | Staphyloc | 0.000E+00 | 2.081E-07 | 2.023E-06 | 1.383E-05 | 0.000E+00 | 0.000E+00 | 0.000E+00 | 0.000E+00 | 0.000E+00 | 0.000E+00 | 0.000E+00 | 0.000E+00 |
| Unigene199700 | bcrA | 0.000E+00 | 0.000E+00 | 1.206E-06 | 0.000E+00 | 0.000E+00 | 0.000E+00 | 0.000E+00 | 0.000E+00 | 0.000E+00 | 0.000E+00 | 0.000E+00 | 0.000E+00 |
| Unigene199727 | bcr-1 | 1.645E-07 | 6.962E-07 | 1.383E-06 | 2.681E-06 | 2.871E-07 | 1.778E-07 | 1.040E-07 | 1.042E-06 | 0.000E+00 | 6.019E-07 | 7.256E-07 | 5.276E-07 |
| Unigene199773 | lmrD | 8.825E-05 | 1.452E-04 | 1.259E-04 | 0.000E+00 | 0.000E+00 | 0.000E+00 | 0.000E+00 | 0.000E+00 | 0.000E+00 | 0.000E+00 | 0.000E+00 | 0.000E+00 |
| Unigene199785 | vanHA | 4.719E-07 | 0.000E+00 | 7.718E-07 | 1.906E-07 | 0.000E+00 | 0.000E+00 | 1.393E-07 | 0.000E+00 | 0.000E+00 | 0.000E+00 | 0.000E+00 | 0.000E+00 |
| Unigene199893 | Staphyloc | 0.000E+00 | 7.786E-08 | 1.671E-06 | 5.827E-06 | 0.000E+00 | 0.000E+00 | 0.000E+00 | 0.000E+00 | 0.000E+00 | 0.000E+00 | 0.000E+00 | 0.000E+00 |
| Unigene199920 | TriC | 0.000E+00 | 0.000E+00 | 3.519E-07 | 0.000E+00 | 0.000E+00 | 0.000E+00 | 0.000E+00 | 0.000E+00 | 0.000E+00 | 0.000E+00 | 0.000E+00 | 0.000E+00 |
| Unigene200003 | tet32 | 1.996E-08 | 6.573E-08 | 8.619E-07 | 1.210E-07 | 0.000E+00 | 0.000E+00 | 0.000E+00 | 0.000E+00 | 0.000E+00 | 0.000E+00 | 0.000E+00 | 0.000E+00 |
| Unigene200024 | msbA | 0.000E+00 | 5.864E-07 | 4.050E-06 | 3.889E-06 | 0.000E+00 | 1.992E-08 | 0.000E+00 | 0.000E+00 | 0.000E+00 | 0.000E+00 | 0.000E+00 | 0.000E+00 |
| Unigene200072 | tetA(60) | 2.765E-05 | 9.008E-06 | 6.757E-06 | 3.894E-06 | 3.060E-07 | 1.588E-07 | 0.000E+00 | 0.000E+00 | 0.000E+00 | 0.000E+00 | 6.301E-08 | 0.000E+00 |
| Unigene200097 | Klebsiella p | 0.000E+00 | 1.043E-06 | 5.715E-07 | 2.477E-07 | 0.000E+00 | 0.000E+00 | 0.000E+00 | 0.000E+00 | 5.026E-07 | 0.000E+00 | 0.000E+00 | 0.000E+00 |
| Unigene200163 | cpxA | 2.383E-07 | 1.207E-07 | 1.349E-06 | 6.590E-06 | 0.000E+00 | 0.000E+00 | 0.000E+00 | 0.000E+00 | 0.000E+00 | 0.000E+00 | 0.000E+00 | 0.000E+00 |
| Unigene200175 | Streptomy | 5.131E-07 | 6.688E-07 | 1.369E-06 | 2.559E-06 | 4.810E-07 | 1.560E-07 | 3.195E-07 | 1.610E-06 | 1.227E-07 | 6.074E-07 | 1.046E-06 | 6.824E-07 |
| Unigene200195 | mgrA | 0.000E+00 | 0.000E+00 | 7.039E-07 | 0.000E+00 | 0.000E+00 | 0.000E+00 | 0.000E+00 | 0.000E+00 | 0.000E+00 | 0.000E+00 | 0.000E+00 | 0.000E+00 |
| Unigene200356 | optrA | 1.989E-07 | 1.528E-07 | 8.977E-07 | 3.214E-07 | 7.955E-07 | 4.945E-07 | 1.431E-06 | 1.416E-06 | 1.739E-07 | 5.539E-07 | 1.069E-06 | 3.503E-06 |
| Unigene200416 | lmrB | 2.353E-07 | 1.174E-07 | 1.532E-06 | 6.265E-06 | 0.000E+00 | 0.000E+00 | 0.000E+00 | 0.000E+00 | 0.000E+00 | 0.000E+00 | 0.000E+00 | 0.000E+00 |
| Unigene200418 | bcrA | 0.000E+00 | 1.402E-07 | 1.222E-06 | 5.223E-06 | 0.000E+00 | 0.000E+00 | 0.000E+00 | 0.000E+00 | 0.000E+00 | 0.000E+00 | 0.000E+00 | 0.000E+00 |
| Unigene200472 | tetB(60) | 0.000E+00 | 1.625E-07 | 1.453E-07 | 0.000E+00 | 1.152E-06 | 0.000E+00 | 0.000E+00 | 0.000E+00 | 0.000E+00 | 0.000E+00 | 0.000E+00 | 0.000E+00 |
| Unigene200567 | mgrA | 0.000E+00 | 0.000E+00 | 1.598E-06 | 0.000E+00 | 1.573E-07 | 0.000E+00 | 0.000E+00 | 0.000E+00 | 0.000E+00 | 0.000E+00 | 8.098E-08 | 0.000E+00 |
| Unigene200580 | tetO | 8.121E-08 | 0.000E+00 | 9.363E-07 | 8.201E-08 | 4.511E-07 | 0.000E+00 | 2.472E-07 | 0.000E+00 | 1.997E-07 | 2.035E-07 | 0.000E+00 | 6.959E-07 |
| Unigene200637 | macB | 0.000E+00 | 0.000E+00 | 1.179E-05 | 0.000E+00 | 0.000E+00 | 1.006E-06 | 0.000E+00 | 0.000E+00 | 0.000E+00 | 0.000E+00 | 0.000E+00 | 0.000E+00 |
| Unigene200638 | macB | 0.000E+00 | 0.000E+00 | 1.033E-05 | 0.000E+00 | 0.000E+00 | 6.095E-07 | 0.000E+00 | 0.000E+00 | 0.000E+00 | 0.000E+00 | 0.000E+00 | 0.000E+00 |
| Unigene200642 | msbA | 1.818E-08 | 0.000E+00 | 1.273E-05 | 0.000E+00 | 0.000E+00 | 9.628E-07 | 0.000E+00 | 8.486E-08 | 0.000E+00 | 0.000E+00 | 0.000E+00 | 0.000E+00 |
| Unigene200645 | pmrA | 0.000E+00 | 0.000E+00 | 1.188E-05 | 0.000E+00 | 0.000E+00 | 8.032E-07 | 3.959E-07 | 6.424E-08 | 0.000E+00 | 0.000E+00 | 0.000E+00 | 0.000E+00 |
| Unigene200670 | kdpE | 0.000E+00 | 0.000E+00 | 8.876E-07 | 0.000E+00 | 0.000E+00 | 0.000E+00 | 0.000E+00 | 0.000E+00 | 1.561E-07 | 0.000E+00 | 0.000E+00 | 1.407E-06 |
| Unigene200671 | vanSG | 6.005E-08 | 1.318E-08 | 1.355E-06 | 3.638E-08 | 6.671E-08 | 1.298E-08 | 3.987E-08 | 7.009E-08 | 5.250E-08 | 1.070E-07 | 4.121E-08 | 8.375E-07 |
| Unigene200787 | YojI | 6.215E-07 | 3.837E-07 | 4.955E-07 | 9.022E-07 | 7.768E-07 | 1.386E-06 | 4.471E-06 | 4.580E-06 | 4.246E-07 | 1.385E-06 | 2.488E-06 | 4.683E-06 |
| Unigene200847 | efrB | 1.008E-05 | 9.734E-06 | 4.024E-06 | 5.634E-06 | 4.061E-07 | 8.075E-07 | 1.601E-06 | 4.824E-07 | 6.949E-07 | 1.416E-07 | 1.473E-06 | 4.133E-07 |
| Unigene200873 | efrA | 0.000E+00 | 0.000E+00 | 1.488E-06 | 1.156E-07 | 1.590E-07 | 0.000E+00 | 9.502E-08 | 1.670E-07 | 6.256E-08 | 9.564E-08 | 9.820E-08 | 1.387E-06 |
| Unigene200943 | Streptomy | 0.000E+00 | 0.000E+00 | 5.338E-07 | 0.000E+00 | 0.000E+00 | 0.000E+00 | 0.000E+00 | 0.000E+00 | 0.000E+00 | 0.000E+00 | 0.000E+00 | 0.000E+00 |
| Unigene200966 | tetQ | 0.000E+00 | 2.380E-06 | 2.160E-06 | 1.443E-06 | 0.000E+00 | 0.000E+00 | 0.000E+00 | 3.835E-08 | 0.000E+00 | 0.000E+00 | 0.000E+00 | 0.000E+00 |
| Unigene200972 | vanTG | 2.014E-07 | 4.105E-07 | 9.881E-07 | 2.499E-06 | 0.000E+00 | 0.000E+00 | 3.184E-07 | 1.209E-06 | 0.000E+00 | 0.000E+00 | 5.924E-07 | 6.461E-07 |
| Unigene200994 | poxtA | 1.559E-07 | 1.027E-07 | 1.163E-06 | 9.448E-08 | 0.000E+00 | 0.000E+00 | 3.452E-08 | 0.000E+00 | 0.000E+00 | 0.000E+00 | 0.000E+00 | 0.000E+00 |
| Unigene201002 | PmrF | 6.622E-08 | 0.000E+00 | 1.267E-06 | 2.675E-07 | 0.000E+00 | 0.000E+00 | 0.000E+00 | 0.000E+00 | 0.000E+00 | 0.000E+00 | 0.000E+00 | 0.000E+00 |
| Unigene201084 | macB | 9.650E-08 | 0.000E+00 | 1.231E-06 | 0.000E+00 | 0.000E+00 | 1.043E-07 | 0.000E+00 | 0.000E+00 | 2.637E-07 | 0.000E+00 | 1.380E-07 | 8.555E-07 |
| Unigene201142 | vanHA | 0.000E+00 | 0.000E+00 | 1.369E-06 | 0.000E+00 | 0.000E+00 | 0.000E+00 | 0.000E+00 | 0.000E+00 | 0.000E+00 | 0.000E+00 | 0.000E+00 | 0.000E+00 |
| Unigene201148 | tetA(46) | 0.000E+00 | 0.000E+00 | 1.599E-06 | 0.000E+00 | 0.000E+00 | 0.000E+00 | 0.000E+00 | 0.000E+00 | 0.000E+00 | 0.000E+00 | 0.000E+00 | 0.000E+00 |
| Unigene201150 | tetA(46) | 0.000E+00 | 0.000E+00 | 2.575E-07 | 0.000E+00 | 6.803E-07 | 0.000E+00 | 0.000E+00 | 0.000E+00 | 0.000E+00 | 0.000E+00 | 6.004E-07 | 0.000E+00 |
| Unigene201251 | vanSL | 0.000E+00 | 0.000E+00 | 1.721E-07 | 0.000E+00 | 0.000E+00 | 0.000E+00 | 0.000E+00 | 0.000E+00 | 0.000E+00 | 0.000E+00 | 0.000E+00 | 0.000E+00 |
| Unigene201287 | MdtK | 0.000E+00 | 2.952E-07 | 3.695E-07 | 1.087E-07 | 0.000E+00 | 0.000E+00 | 0.000E+00 | 0.000E+00 | 0.000E+00 | 0.000E+00 | 0.000E+00 | 0.000E+00 |
| Unigene201339 | vanSL | 6.403E-07 | 8.268E-07 | 4.103E-06 | 5.439E-06 | 0.000E+00 | 0.000E+00 | 4.308E-07 | 0.000E+00 | 4.117E-08 | 0.000E+00 | 4.309E-08 | 1.484E-08 |
| Unigene201454 | macB | 3.261E-06 | 7.311E-06 | 6.947E-06 | 7.291E-06 | 1.035E-07 | 0.000E+00 | 5.155E-08 | 0.000E+00 | 0.000E+00 | 3.632E-07 | 0.000E+00 | 0.000E+00 |
| Unigene201541 | Staphyloc | 4.042E-06 | 9.700E-06 | 1.293E-05 | 1.525E-05 | 0.000E+00 | 0.000E+00 | 0.000E+00 | 0.000E+00 | 0.000E+00 | 0.000E+00 | 0.000E+00 | 0.000E+00 |
| Unigene201635 | baeR | 4.142E-06 | 1.976E-07 | 3.181E-06 | 5.456E-07 | 0.000E+00 | 0.000E+00 | 6.478E-07 | 1.577E-07 | 0.000E+00 | 0.000E+00 | 8.755E-07 | 0.000E+00 |
| Unigene201654 | bacA | 0.000E+00 | 0.000E+00 | 1.953E-06 | 7.145E-06 | 0.000E+00 | 0.000E+00 | 0.000E+00 | 0.000E+00 | 0.000E+00 | 0.000E+00 | 0.000E+00 | 0.000E+00 |
| Unigene201665 | tetA(46) | 0.000E+00 | 7.761E-07 | 1.200E-06 | 1.544E-06 | 0.000E+00 | 0.000E+00 | 0.000E+00 | 0.000E+00 | 0.000E+00 | 0.000E+00 | 0.000E+00 | 0.000E+00 |

| | | | | | | | | | | | | | |
|---|---|---|---|---|---|---|---|---|---|---|---|---|---|
| Unigene201688 | lmrD | 0.000E+00 | 0.000E+00 | 8.480E-07 | 8.728E-08 | 9.602E-08 | 0.000E+00 | 0.000E+00 | 0.000E+00 | 1.889E-07 | 0.000E+00 | 0.000E+00 | 9.194E-07 |
| Unigene201734 | smeS | 2.425E-07 | 6.050E-07 | 6.058E-07 | 0.000E+00 | 3.184E-07 | 0.000E+00 | 1.147E-06 | 0.000E+00 | 0.000E+00 | 0.000E+00 | 0.000E+00 | 0.000E+00 |
| Unigene201804 | marA | 0.000E+00 | 1.058E-06 | 0.000E+00 | 0.000E+00 | 0.000E+00 | 0.000E+00 | 0.000E+00 | 0.000E+00 | 0.000E+00 | 0.000E+00 | 0.000E+00 | 0.000E+00 |
| Unigene201839 | vanHD | 0.000E+00 | 0.000E+00 | 1.046E-06 | 8.474E-05 | 0.000E+00 | 3.601E-08 | 0.000E+00 | 0.000E+00 | 0.000E+00 | 0.000E+00 | 0.000E+00 | 0.000E+00 |
| Unigene202001 | patA | 0.000E+00 | 0.000E+00 | 2.960E-07 | 0.000E+00 | 0.000E+00 | 0.000E+00 | 0.000E+00 | 0.000E+00 | 0.000E+00 | 0.000E+00 | 0.000E+00 | 0.000E+00 |
| Unigene202035 | farB | 4.628E-08 | 0.000E+00 | 1.044E-06 | 0.000E+00 | 0.000E+00 | 7.505E-08 | 0.000E+00 | 5.402E-08 | 1.517E-07 | 0.000E+00 | 1.853E-07 | 1.149E-06 |
| Unigene202050 | oleC | 0.000E+00 | 5.669E-07 | 2.407E-06 | 2.130E-06 | 0.000E+00 | 0.000E+00 | 0.000E+00 | 0.000E+00 | 0.000E+00 | 0.000E+00 | 0.000E+00 | 0.000E+00 |
| Unigene202215 | vanHD | 0.000E+00 | 1.561E-07 | 1.326E-06 | 0.000E+00 | 1.317E-07 | 0.000E+00 | 0.000E+00 | 0.000E+00 | 1.554E-07 | 0.000E+00 | 8.133E-08 | 1.373E-06 |
| Unigene202364 | vgaE | 2.463E-05 | 8.425E-06 | 7.510E-06 | 4.956E-06 | 4.001E-07 | 1.217E-07 | 9.965E-08 | 1.577E-07 | 0.000E+00 | 0.000E+00 | 0.000E+00 | 0.000E+00 |
| Unigene202416 | baeS | 0.000E+00 | 0.000E+00 | 1.600E-07 | 0.000E+00 | 0.000E+00 | 0.000E+00 | 0.000E+00 | 0.000E+00 | 0.000E+00 | 0.000E+00 | 0.000E+00 | 0.000E+00 |
| Unigene202447 | macB | 0.000E+00 | 0.000E+00 | 0.000E+00 | 0.000E+00 | 0.000E+00 | 0.000E+00 | 0.000E+00 | 0.000E+00 | 1.316E-07 | 0.000E+00 | 0.000E+00 | 0.000E+00 |
| Unigene202455 | golS | 0.000E+00 | 0.000E+00 | 8.477E-07 | 1.586E-07 | 0.000E+00 | 0.000E+00 | 0.000E+00 | 0.000E+00 | 0.000E+00 | 4.375E-07 | 0.000E+00 | 1.393E-06 |
| Unigene202463 | macB | 0.000E+00 | 0.000E+00 | 1.337E-06 | 0.000E+00 | 1.044E-07 | 0.000E+00 | 0.000E+00 | 0.000E+00 | 0.000E+00 | 2.094E-07 | 0.000E+00 | 6.109E-07 |
| Unigene202474 | Acinetoba | 0.000E+00 | 1.437E-07 | 2.634E-06 | 1.391E-05 | 0.000E+00 | 0.000E+00 | 0.000E+00 | 0.000E+00 | 0.000E+00 | 0.000E+00 | 0.000E+00 | 0.000E+00 |
| Unigene202493 | rpoB2 | 0.000E+00 | 0.000E+00 | 2.919E-07 | 0.000E+00 | 0.000E+00 | 0.000E+00 | 0.000E+00 | 0.000E+00 | 0.000E+00 | 0.000E+00 | 0.000E+00 | 0.000E+00 |
| Unigene202571 | vanZF | 0.000E+00 | 0.000E+00 | 1.289E-06 | 0.000E+00 | 0.000E+00 | 0.000E+00 | 0.000E+00 | 0.000E+00 | 0.000E+00 | 0.000E+00 | 0.000E+00 | 0.000E+00 |
| Unigene202573 | baeS | 0.000E+00 | 0.000E+00 | 7.617E-07 | 0.000E+00 | 0.000E+00 | 0.000E+00 | 0.000E+00 | 0.000E+00 | 0.000E+00 | 0.000E+00 | 0.000E+00 | 0.000E+00 |
| Unigene202574 | vanRM | 0.000E+00 | 0.000E+00 | 9.486E-07 | 0.000E+00 | 0.000E+00 | 0.000E+00 | 0.000E+00 | 0.000E+00 | 0.000E+00 | 0.000E+00 | 0.000E+00 | 0.000E+00 |
| Unigene202581 | carA | 0.000E+00 | 0.000E+00 | 7.976E-07 | 0.000E+00 | 0.000E+00 | 0.000E+00 | 0.000E+00 | 0.000E+00 | 0.000E+00 | 0.000E+00 | 0.000E+00 | 0.000E+00 |
| Unigene202697 | PmrF | 0.000E+00 | 0.000E+00 | 7.832E-07 | 0.000E+00 | 0.000E+00 | 0.000E+00 | 0.000E+00 | 0.000E+00 | 0.000E+00 | 0.000E+00 | 0.000E+00 | 0.000E+00 |
| Unigene202744 | lmrB | 0.000E+00 | 0.000E+00 | 7.968E-07 | 0.000E+00 | 0.000E+00 | 0.000E+00 | 0.000E+00 | 0.000E+00 | 0.000E+00 | 0.000E+00 | 0.000E+00 | 0.000E+00 |
| Unigene202745 | lmrB | 0.000E+00 | 0.000E+00 | 1.433E-06 | 0.000E+00 | 0.000E+00 | 0.000E+00 | 0.000E+00 | 0.000E+00 | 0.000E+00 | 0.000E+00 | 0.000E+00 | 0.000E+00 |
| Unigene202756 | vanRM | 0.000E+00 | 0.000E+00 | 1.660E-06 | 0.000E+00 | 0.000E+00 | 0.000E+00 | 0.000E+00 | 0.000E+00 | 0.000E+00 | 0.000E+00 | 0.000E+00 | 0.000E+00 |
| Unigene202819 | lfrA | 0.000E+00 | 0.000E+00 | 1.271E-06 | 5.058E-06 | 0.000E+00 | 0.000E+00 | 0.000E+00 | 0.000E+00 | 0.000E+00 | 0.000E+00 | 0.000E+00 | 0.000E+00 |
| Unigene202932 | cmlv | 0.000E+00 | 0.000E+00 | 9.840E-07 | 0.000E+00 | 0.000E+00 | 0.000E+00 | 0.000E+00 | 0.000E+00 | 0.000E+00 | 0.000E+00 | 0.000E+00 | 0.000E+00 |
| Unigene202935 | rpoB2 | 6.311E-08 | 0.000E+00 | 1.053E-06 | 1.912E-07 | 0.000E+00 | 0.000E+00 | 0.000E+00 | 0.000E+00 | 0.000E+00 | 0.000E+00 | 0.000E+00 | 0.000E+00 |
| Unigene202984 | lmrB | 4.584E-07 | 6.355E-07 | 1.184E-06 | 2.729E-06 | 1.608E-07 | 0.000E+00 | 2.136E-07 | 1.859E-06 | 1.318E-07 | 4.031E-07 | 1.187E-06 | 8.555E-07 |
| Unigene202995 | mdtA | 0.000E+00 | 1.031E-06 | 7.636E-07 | 0.000E+00 | 5.962E-06 | 0.000E+00 | 0.000E+00 | 0.000E+00 | 0.000E+00 | 0.000E+00 | 0.000E+00 | 0.000E+00 |
| Unigene203033 | abcA | 0.000E+00 | 0.000E+00 | 1.197E-05 | 0.000E+00 | 0.000E+00 | 0.000E+00 | 0.000E+00 | 0.000E+00 | 0.000E+00 | 0.000E+00 | 0.000E+00 | 0.000E+00 |
| Unigene203100 | adeR | 0.000E+00 | 0.000E+00 | 1.269E-05 | 0.000E+00 | 0.000E+00 | 0.000E+00 | 0.000E+00 | 0.000E+00 | 0.000E+00 | 0.000E+00 | 0.000E+00 | 0.000E+00 |
| Unigene203187 | fexA | 1.582E-07 | 1.985E-07 | 8.872E-07 | 5.661E-06 | 0.000E+00 | 0.000E+00 | 0.000E+00 | 0.000E+00 | 0.000E+00 | 0.000E+00 | 0.000E+00 | 0.000E+00 |
| Unigene203312 | tetB(60) | 0.000E+00 | 0.000E+00 | 1.351E-06 | 0.000E+00 | 0.000E+00 | 0.000E+00 | 0.000E+00 | 0.000E+00 | 0.000E+00 | 0.000E+00 | 0.000E+00 | 0.000E+00 |
| Unigene203421 | arnA | 0.000E+00 | 0.000E+00 | 1.029E-06 | 0.000E+00 | 0.000E+00 | 0.000E+00 | 0.000E+00 | 0.000E+00 | 0.000E+00 | 0.000E+00 | 0.000E+00 | 0.000E+00 |
| Unigene203513 | QepA2 | 0.000E+00 | 0.000E+00 | 1.109E-06 | 0.000E+00 | 0.000E+00 | 0.000E+00 | 0.000E+00 | 0.000E+00 | 0.000E+00 | 0.000E+00 | 0.000E+00 | 0.000E+00 |
| Unigene203522 | tetB(58) | 1.699E-07 | 0.000E+00 | 0.000E+00 | 0.000E+00 | 0.000E+00 | 0.000E+00 | 0.000E+00 | 0.000E+00 | 0.000E+00 | 0.000E+00 | 0.000E+00 | 0.000E+00 |
| Unigene203523 | YojI | 0.000E+00 | 0.000E+00 | 5.512E-07 | 0.000E+00 | 0.000E+00 | 0.000E+00 | 0.000E+00 | 0.000E+00 | 0.000E+00 | 0.000E+00 | 0.000E+00 | 0.000E+00 |
| Unigene203600 | novA | 0.000E+00 | 0.000E+00 | 6.196E-07 | 0.000E+00 | 0.000E+00 | 0.000E+00 | 0.000E+00 | 0.000E+00 | 0.000E+00 | 0.000E+00 | 0.000E+00 | 0.000E+00 |
| Unigene203633 | tetA(58) | 0.000E+00 | 0.000E+00 | 1.264E-06 | 0.000E+00 | 1.547E-07 | 7.529E-08 | 0.000E+00 | 0.000E+00 | 0.000E+00 | 0.000E+00 | 0.000E+00 | 8.644E-07 |
| Unigene203649 | bcrA | 1.842E-07 | 4.909E-07 | 3.227E-06 | 4.330E-06 | 0.000E+00 | 0.000E+00 | 0.000E+00 | 0.000E+00 | 0.000E+00 | 0.000E+00 | 0.000E+00 | 0.000E+00 |
| Unigene203740 | Acinetoba | 2.832E-07 | 0.000E+00 | 1.412E-06 | 5.457E-06 | 0.000E+00 | 0.000E+00 | 0.000E+00 | 0.000E+00 | 0.000E+00 | 0.000E+00 | 0.000E+00 | 0.000E+00 |
| Unigene203749 | vatE | 0.000E+00 | 0.000E+00 | 1.508E-06 | 0.000E+00 | 0.000E+00 | 0.000E+00 | 0.000E+00 | 0.000E+00 | 0.000E+00 | 0.000E+00 | 0.000E+00 | 0.000E+00 |
| Unigene203777 | Staphyloco | 0.000E+00 | 0.000E+00 | 2.697E-07 | 0.000E+00 | 2.909E-07 | 1.274E-07 | 3.042E-07 | 1.528E-07 | 0.000E+00 | 2.041E-07 | 1.497E-07 | 6.963E-07 |
| Unigene203903 | tetA(58) | 2.117E-05 | 8.964E-06 | 5.122E-06 | 4.016E-06 | 1.184E-07 | 1.152E-07 | 0.000E+00 | 4.145E-08 | 8.151E-07 | 0.000E+00 | 0.000E+00 | 0.000E+00 |
| Unigene203973 | Streptomy | 0.000E+00 | 0.000E+00 | 1.272E-07 | 0.000E+00 | 0.000E+00 | 0.000E+00 | 3.587E-07 | 3.783E-07 | 0.000E+00 | 0.000E+00 | 2.224E-07 | 9.194E-07 |
| Unigene204026 | efrA | 0.000E+00 | 0.000E+00 | 4.114E-07 | 0.000E+00 | 0.000E+00 | 0.000E+00 | 0.000E+00 | 0.000E+00 | 0.000E+00 | 0.000E+00 | 0.000E+00 | 0.000E+00 |
| Unigene204099 | efrA | 2.704E-08 | 0.000E+00 | 1.724E-06 | 1.423E-04 | 0.000E+00 | 0.000E+00 | 0.000E+00 | 0.000E+00 | 0.000E+00 | 0.000E+00 | 0.000E+00 | 0.000E+00 |
| Unigene204134 | smeE | 1.157E-07 | 0.000E+00 | 2.271E-07 | 0.000E+00 | 0.000E+00 | 0.000E+00 | 0.000E+00 | 0.000E+00 | 0.000E+00 | 0.000E+00 | 0.000E+00 | 0.000E+00 |
| Unigene204181 | tet32 | 0.000E+00 | 0.000E+00 | 8.741E-07 | 0.000E+00 | 0.000E+00 | 0.000E+00 | 0.000E+00 | 0.000E+00 | 0.000E+00 | 0.000E+00 | 0.000E+00 | 0.000E+00 |
| Unigene204228 | arnA | 0.000E+00 | 6.345E-07 | 8.825E-07 | 0.000E+00 | 0.000E+00 | 0.000E+00 | 0.000E+00 | 0.000E+00 | 0.000E+00 | 0.000E+00 | 0.000E+00 | 0.000E+00 |
| Unigene204335 | acrB | 2.050E-08 | 0.000E+00 | 1.448E-06 | 5.174E-08 | 1.025E-07 | 0.000E+00 | 1.474E-07 | 5.981E-08 | 4.480E-08 | 6.849E-08 | 4.689E-08 | 1.284E-06 |
| Unigene204336 | Enterobact | 0.000E+00 | 0.000E+00 | 1.433E-06 | 8.190E-08 | 1.802E-07 | 2.923E-08 | 0.000E+00 | 9.467E-08 | 0.000E+00 | 0.000E+00 | 6.185E-08 | 1.342E-06 |
| Unigene204337 | AcrS | 9.694E-08 | 0.000E+00 | 1.141E-06 | 1.468E-07 | 1.077E-07 | 0.000E+00 | 0.000E+00 | 0.000E+00 | 3.179E-07 | 0.000E+00 | 0.000E+00 | 1.490E-06 |
| Unigene204362 | adeR | 0.000E+00 | 1.948E-07 | 8.708E-07 | 2.240E-07 | 0.000E+00 | 0.000E+00 | 0.000E+00 | 0.000E+00 | 0.000E+00 | 0.000E+00 | 0.000E+00 | 0.000E+00 |
| Unigene204422 | patA | 0.000E+00 | 3.598E-07 | 9.359E-07 | 0.000E+00 | 7.616E-07 | 0.000E+00 | 0.000E+00 | 3.201E-06 | 0.000E+00 | 1.361E-06 | 1.568E-06 | 3.594E-06 |
| Unigene204463 | SRT-2 | 6.292E-08 | 0.000E+00 | 3.087E-07 | 2.542E-07 | 0.000E+00 | 0.000E+00 | 0.000E+00 | 0.000E+00 | 0.000E+00 | 0.000E+00 | 0.000E+00 | 0.000E+00 |
| Unigene204539 | mdsC | 0.000E+00 | 0.000E+00 | 8.779E-07 | 0.000E+00 | 0.000E+00 | 0.000E+00 | 0.000E+00 | 0.000E+00 | 0.000E+00 | 0.000E+00 | 0.000E+00 | 0.000E+00 |
| Unigene204549 | TaeA | 3.953E-07 | 4.716E-07 | 1.788E-06 | 3.107E-06 | 0.000E+00 | 2.416E-07 | 3.614E-07 | 1.344E-06 | 0.000E+00 | 7.276E-07 | 9.044E-07 | 7.314E-07 |
| Unigene204624 | macB | 0.000E+00 | 1.164E-06 | 8.427E-07 | 2.041E-07 | 1.852E-06 | 0.000E+00 | 0.000E+00 | 0.000E+00 | 0.000E+00 | 0.000E+00 | 0.000E+00 | 0.000E+00 |

| | | | | | | | | | | | | | |
|---|---|---|---|---|---|---|---|---|---|---|---|---|---|
| Unigene204684 | PmrF | 0.000E+00 | 0.000E+00 | 1.111E-06 | 0.000E+00 | 0.000E+00 | 0.000E+00 | 0.000E+00 | 0.000E+00 | 0.000E+00 | 0.000E+00 | 0.000E+00 | 0.000E+00 |
| Unigene204687 | vanSA | 0.000E+00 | 0.000E+00 | 1.209E-06 | 0.000E+00 | 0.000E+00 | 0.000E+00 | 0.000E+00 | 0.000E+00 | 0.000E+00 | 0.000E+00 | 0.000E+00 | 0.000E+00 |
| Unigene204746 | dfrA20 | 0.000E+00 | 0.000E+00 | 5.097E-07 | 0.000E+00 | 0.000E+00 | 0.000E+00 | 0.000E+00 | 0.000E+00 | 0.000E+00 | 0.000E+00 | 0.000E+00 | 0.000E+00 |
| Unigene204766 | mdtB | 0.000E+00 | 0.000E+00 | 1.588E-07 | 0.000E+00 | 0.000E+00 | 0.000E+00 | 1.791E-07 | 0.000E+00 | 0.000E+00 | 0.000E+00 | 0.000E+00 | 0.000E+00 |
| Unigene204783 | lsaA | 8.643E-08 | 6.877E-07 | 3.329E-06 | 4.342E-06 | 0.000E+00 | 0.000E+00 | 0.000E+00 | 0.000E+00 | 0.000E+00 | 0.000E+00 | 0.000E+00 | 0.000E+00 |
| Unigene204803 | srmB | 0.000E+00 | 0.000E+00 | 1.036E-06 | 8.826E-05 | 0.000E+00 | 0.000E+00 | 0.000E+00 | 0.000E+00 | 0.000E+00 | 0.000E+00 | 0.000E+00 | 0.000E+00 |
| Unigene204809 | lsaA | 0.000E+00 | 2.367E-08 | 6.348E-07 | 8.379E-05 | 0.000E+00 | 2.331E-08 | 0.000E+00 | 1.258E-07 | 0.000E+00 | 0.000E+00 | 0.000E+00 | 0.000E+00 |
| Unigene204831 | oleB | 0.000E+00 | 0.000E+00 | 5.356E-07 | 1.575E-07 | 9.529E-07 | 2.107E-07 | 8.630E-07 | 6.371E-07 | 0.000E+00 | 0.000E+00 | 4.014E-07 | 2.212E-06 |
| Unigene204895 | NmcR | 0.000E+00 | 3.429E-07 | 8.174E-07 | 0.000E+00 | 1.157E-06 | 5.253E-07 | 1.613E-06 | 1.013E-06 | 4.552E-07 | 0.000E+00 | 9.926E-07 | 3.446E-06 |
| Unigene204958 | optrA | 3.620E-07 | 7.945E-07 | 1.550E-06 | 3.024E-06 | 2.925E-07 | 0.000E+00 | 1.093E-07 | 1.728E-06 | 0.000E+00 | 5.498E-07 | 9.410E-07 | 4.278E-07 |
| Unigene205039 | oleB | 0.000E+00 | 0.000E+00 | 7.690E-07 | 0.000E+00 | 8.236E-07 | 3.435E-07 | 8.439E-07 | 6.923E-07 | 0.000E+00 | 6.135E-07 | 8.722E-07 | 2.303E-06 |
| Unigene205125 | tetA(58) | 2.606E-07 | 3.678E-07 | 7.307E-07 | 2.406E-06 | 5.377E-07 | 2.415E-07 | 2.884E-07 | 1.825E-06 | 0.000E+00 | 5.392E-07 | 1.065E-06 | 2.641E-07 |
| Unigene205126 | baeS | 5.945E-08 | 8.482E-07 | 1.283E-06 | 2.641E-06 | 6.274E-07 | 2.249E-07 | 3.290E-07 | 1.076E-06 | 0.000E+00 | 7.285E-07 | 1.360E-06 | 7.730E-07 |
| Unigene205170 | emeA | 0.000E+00 | 0.000E+00 | 5.835E-07 | 0.000E+00 | 0.000E+00 | 0.000E+00 | 0.000E+00 | 0.000E+00 | 0.000E+00 | 0.000E+00 | 0.000E+00 | 0.000E+00 |
| Unigene205308 | Staphylocc | 2.508E-05 | 8.742E-06 | 6.765E-06 | 5.174E-06 | 1.552E-07 | 2.770E-07 | 0.000E+00 | 1.087E-07 | 0.000E+00 | 0.000E+00 | 0.000E+00 | 1.101E-07 |
| Unigene205356 | macB | 0.000E+00 | 0.000E+00 | 8.573E-07 | 7.996E-05 | 0.000E+00 | 0.000E+00 | 0.000E+00 | 0.000E+00 | 0.000E+00 | 0.000E+00 | 0.000E+00 | 0.000E+00 |
| Unigene205400 | oleC | 0.000E+00 | 0.000E+00 | 6.768E-07 | 1.672E-07 | 7.356E-07 | 3.579E-07 | 8.855E-07 | 7.407E-07 | 0.000E+00 | 3.073E-07 | 3.472E-07 | 1.957E-06 |
| Unigene205403 | lmrC | 1.942E-07 | 1.563E-07 | 1.575E-06 | 5.609E-06 | 0.000E+00 | 0.000E+00 | 0.000E+00 | 0.000E+00 | 0.000E+00 | 0.000E+00 | 0.000E+00 | 0.000E+00 |
| Unigene205430 | LRA-13 | 0.000E+00 | 0.000E+00 | 5.744E-07 | 0.000E+00 | 7.433E-07 | 3.616E-07 | 1.080E-06 | 5.206E-07 | 0.000E+00 | 4.347E-07 | 0.000E+00 | 2.801E-06 |
| Unigene205431 | NmcR | 2.196E-07 | 0.000E+00 | 0.000E+00 | 0.000E+00 | 0.000E+00 | 0.000E+00 | 1.337E-06 | 1.581E-06 | 0.000E+00 | 0.000E+00 | 9.629E-07 | 5.062E-06 |
| Unigene205472 | cpxA | 0.000E+00 | 5.157E-08 | 1.360E-06 | 9.490E-08 | 7.830E-08 | 0.000E+00 | 1.040E-07 | 0.000E+00 | 5.136E-08 | 0.000E+00 | 8.062E-08 | 1.027E-06 |
| Unigene205535 | tetA(58) | 0.000E+00 | 0.000E+00 | 7.782E-07 | 2.381E-07 | 6.191E-07 | 8.110E-07 | 1.779E-06 | 1.026E-06 | 0.000E+00 | 5.731E-07 | 1.201E-06 | 3.572E-06 |
| Unigene205667 | tetA(58) | 3.641E-07 | 8.879E-07 | 9.923E-07 | 8.987E-07 | 0.000E+00 | 0.000E+00 | 0.000E+00 | 0.000E+00 | 0.000E+00 | 0.000E+00 | 0.000E+00 | 0.000E+00 |
| Unigene205668 | ramA | 0.000E+00 | 1.003E-06 | 1.346E-06 | 0.000E+00 | 0.000E+00 | 0.000E+00 | 0.000E+00 | 0.000E+00 | 0.000E+00 | 0.000E+00 | 0.000E+00 | 0.000E+00 |
| Unigene205694 | vanHF | 5.782E-07 | 7.403E-07 | 5.043E-07 | 4.444E-06 | 4.389E-06 | 6.806E-06 | 1.497E-05 | 8.811E-06 | 8.074E-07 | 9.840E-06 | 2.058E-06 | 2.810E-06 |
| Unigene205740 | optrA | 0.000E+00 | 0.000E+00 | 9.096E-07 | 6.362E-05 | 0.000E+00 | 0.000E+00 | 0.000E+00 | 9.661E-08 | 0.000E+00 | 0.000E+00 | 0.000E+00 | 0.000E+00 |
| Unigene205903 | evgS | 3.871E-07 | 2.549E-07 | 5.697E-07 | 1.954E-07 | 6.020E-07 | 2.092E-07 | 1.199E-06 | 6.777E-07 | 0.000E+00 | 3.881E-07 | 4.427E-07 | 2.105E-06 |
| Unigene205948 | gimA | 1.725E-06 | 5.229E-06 | 4.594E-06 | 3.428E-06 | 0.000E+00 | 0.000E+00 | 0.000E+00 | 0.000E+00 | 0.000E+00 | 0.000E+00 | 0.000E+00 | 0.000E+00 |
| Unigene205993 | MexD | 0.000E+00 | 0.000E+00 | 3.121E-07 | 0.000E+00 | 0.000E+00 | 0.000E+00 | 0.000E+00 | 0.000E+00 | 0.000E+00 | 0.000E+00 | 0.000E+00 | 0.000E+00 |
| Unigene206050 | tetA(58) | 4.559E-06 | 1.001E-05 | 9.228E-06 | 1.153E-05 | 0.000E+00 | 0.000E+00 | 0.000E+00 | 0.000E+00 | 0.000E+00 | 0.000E+00 | 0.000E+00 | 0.000E+00 |
| Unigene206185 | evgS | 0.000E+00 | 0.000E+00 | 7.542E-07 | 0.000E+00 | 0.000E+00 | 0.000E+00 | 0.000E+00 | 2.736E-06 | 0.000E+00 | 0.000E+00 | 0.000E+00 | 2.271E-06 |
| Unigene206202 | patA | 0.000E+00 | 0.000E+00 | 0.000E+00 | 0.000E+00 | 2.288E-07 | 0.000E+00 | 0.000E+00 | 0.000E+00 | 0.000E+00 | 0.000E+00 | 0.000E+00 | 0.000E+00 |
| Unigene206288 | bcrA | 0.000E+00 | 0.000E+00 | 2.020E-06 | 0.000E+00 | 0.000E+00 | 0.000E+00 | 0.000E+00 | 0.000E+00 | 0.000E+00 | 0.000E+00 | 0.000E+00 | 0.000E+00 |
| Unigene206382 | vgaA | 4.304E-07 | 7.873E-07 | 1.220E-06 | 4.346E-07 | 0.000E+00 | 0.000E+00 | 0.000E+00 | 0.000E+00 | 0.000E+00 | 0.000E+00 | 0.000E+00 | 0.000E+00 |
| Unigene206399 | evgS | 1.775E-07 | 1.217E-07 | 7.184E-07 | 3.137E-07 | 7.641E-07 | 5.037E-07 | 8.839E-07 | 1.139E-06 | 3.152E-07 | 7.909E-07 | 1.066E-06 | 3.566E-06 |
| Unigene206460 | carA | 5.407E-06 | 7.697E-06 | 8.562E-06 | 8.866E-06 | 0.000E+00 | 0.000E+00 | 0.000E+00 | 0.000E+00 | 0.000E+00 | 0.000E+00 | 0.000E+00 | 0.000E+00 |
| Unigene206464 | Corynebac | 2.738E-06 | 1.867E-05 | 8.314E-06 | 6.311E-06 | 0.000E+00 | 0.000E+00 | 0.000E+00 | 0.000E+00 | 0.000E+00 | 0.000E+00 | 2.948E-07 | 0.000E+00 |
| Unigene206567 | lmrD | 0.000E+00 | 1.583E-07 | 4.812E-07 | 0.000E+00 | 0.000E+00 | 2.183E-07 | 1.277E-06 | 0.000E+00 | 0.000E+00 | 0.000E+00 | 0.000E+00 | 1.978E-06 |
| Unigene206587 | bcrA | 1.414E-05 | 0.000E+00 | 5.663E-07 | 2.082E-07 | 0.000E+00 | 0.000E+00 | 0.000E+00 | 0.000E+00 | 0.000E+00 | 0.000E+00 | 0.000E+00 | 0.000E+00 |
| Unigene206668 | tetT | 2.973E-06 | 5.284E-06 | 7.990E-06 | 1.034E-05 | 0.000E+00 | 3.493E-08 | 0.000E+00 | 0.000E+00 | 0.000E+00 | 0.000E+00 | 0.000E+00 | 0.000E+00 |
| Unigene206680 | Acinetoba | 6.292E-08 | 2.072E-07 | 3.087E-07 | 0.000E+00 | 0.000E+00 | 0.000E+00 | 0.000E+00 | 0.000E+00 | 0.000E+00 | 0.000E+00 | 0.000E+00 | 0.000E+00 |
| Unigene206761 | vanZF | 0.000E+00 | 0.000E+00 | 1.094E-06 | 0.000E+00 | 0.000E+00 | 0.000E+00 | 0.000E+00 | 0.000E+00 | 0.000E+00 | 0.000E+00 | 0.000E+00 | 0.000E+00 |
| Unigene206859 | bcrA | 0.000E+00 | 3.121E-07 | 1.674E-06 | 5.600E-06 | 0.000E+00 | 0.000E+00 | 0.000E+00 | 0.000E+00 | 0.000E+00 | 0.000E+00 | 0.000E+00 | 0.000E+00 |
| Unigene206874 | tlrC | 0.000E+00 | 1.534E-07 | 4.114E-07 | 2.352E-07 | 0.000E+00 | 0.000E+00 | 0.000E+00 | 0.000E+00 | 0.000E+00 | 0.000E+00 | 0.000E+00 | 0.000E+00 |
| Unigene206908 | Staphylocc | 0.000E+00 | 1.248E-06 | 2.074E-06 | 1.407E-06 | 0.000E+00 | 0.000E+00 | 0.000E+00 | 0.000E+00 | 0.000E+00 | 0.000E+00 | 0.000E+00 | 0.000E+00 |
| Unigene207001 | Bifidobacte | 8.341E-08 | 0.000E+00 | 4.092E-07 | 1.053E-07 | 6.023E-07 | 3.832E-07 | 5.539E-07 | 8.032E-07 | 1.140E-07 | 3.484E-07 | 5.486E-07 | 1.454E-06 |
| Unigene207103 | tetA(58) | 2.572E-07 | 3.058E-07 | 7.992E-07 | 4.978E-07 | 5.001E-07 | 9.500E-07 | 4.009E-06 | 2.502E-06 | 2.108E-07 | 5.731E-07 | 1.201E-06 | 2.635E-06 |
| Unigene207230 | Staphylocc | 2.884E-06 | 4.841E-06 | 3.467E-06 | 5.974E-06 | 3.672E-07 | 1.355E-07 | 3.406E-07 | 5.322E-08 | 2.118E-07 | 7.619E-08 | 1.173E-07 | 2.829E-07 |
| Unigene207232 | efrA | 1.639E-07 | 0.000E+00 | 5.359E-07 | 0.000E+00 | 0.000E+00 | 0.000E+00 | 0.000E+00 | 0.000E+00 | 0.000E+00 | 0.000E+00 | 0.000E+00 | 0.000E+00 |
| Unigene207285 | msbA | 2.335E-07 | 8.083E-07 | 1.216E-06 | 2.957E-06 | 0.000E+00 | 1.165E-07 | 2.783E-07 | 9.645E-07 | 0.000E+00 | 6.604E-07 | 1.623E-06 | 7.856E-07 |
| Unigene207308 | basS | 3.860E-07 | 0.000E+00 | 1.018E-06 | 0.000E+00 | 1.367E-06 | 6.259E-07 | 1.629E-06 | 8.448E-07 | 2.637E-07 | 9.406E-07 | 7.451E-07 | 3.508E-06 |
| Unigene207320 | vmlR | 0.000E+00 | 2.002E-07 | 1.110E-06 | 5.893E-07 | 0.000E+00 | 0.000E+00 | 0.000E+00 | 0.000E+00 | 0.000E+00 | 0.000E+00 | 0.000E+00 | 0.000E+00 |
| Unigene207359 | oleC | 1.204E-07 | 1.322E-07 | 5.909E-07 | 0.000E+00 | 0.000E+00 | 4.774E-07 | 2.333E-06 | 1.711E-06 | 0.000E+00 | 0.000E+00 | 0.000E+00 | 2.468E-06 |
| Unigene207368 | tetA(58) | 8.784E-08 | 9.640E-08 | 5.171E-07 | 4.435E-07 | 0.000E+00 | 0.000E+00 | 0.000E+00 | 0.000E+00 | 0.000E+00 | 0.000E+00 | 0.000E+00 | 0.000E+00 |
| Unigene207496 | msbA | 0.000E+00 | 0.000E+00 | 8.629E-07 | 0.000E+00 | 0.000E+00 | 0.000E+00 | 0.000E+00 | 0.000E+00 | 0.000E+00 | 0.000E+00 | 0.000E+00 | 0.000E+00 |
| Unigene207540 | mdtH | 0.000E+00 | 0.000E+00 | 0.000E+00 | 0.000E+00 | 6.793E-06 | 0.000E+00 | 0.000E+00 | 0.000E+00 | 0.000E+00 | 0.000E+00 | 0.000E+00 | 0.000E+00 |
| Unigene207707 | blt | 0.000E+00 | 0.000E+00 | 0.000E+00 | 7.819E-07 | 0.000E+00 | 0.000E+00 | 2.955E-08 | 0.000E+00 | 0.000E+00 | 0.000E+00 | 0.000E+00 | 0.000E+00 |
| Unigene207776 | mecI | 0.000E+00 | 0.000E+00 | 0.000E+00 | 1.079E-06 | 0.000E+00 | 0.000E+00 | 0.000E+00 | 0.000E+00 | 7.010E-07 | 0.000E+00 | 0.000E+00 | 0.000E+00 |

| | | | | | | | | | | | | | |
|---|---|---|---|---|---|---|---|---|---|---|---|---|---|
| Unigene207859 | evgS | 0.000E+00 | 2.068E-07 | 0.000E+00 | 7.231E-07 | 0.000E+00 | 0.000E+00 | 0.000E+00 | 0.000E+00 | 0.000E+00 | 6.927E-07 | 0.000E+00 | 0.000E+00 |
| Unigene207860 | mtrA | 0.000E+00 | 1.001E-07 | 0.000E+00 | 1.243E-06 | 0.000E+00 | 0.000E+00 | 0.000E+00 | 0.000E+00 | 0.000E+00 | 1.067E-06 | 0.000E+00 | 0.000E+00 |
| Unigene207985 | evgA | 1.006E-07 | 0.000E+00 | 1.973E-07 | 3.554E-07 | 0.000E+00 | 0.000E+00 | 0.000E+00 | 0.000E+00 | 1.649E-07 | 0.000E+00 | 0.000E+00 | 1.783E-07 |
| Unigene208005 | lsaC | 0.000E+00 | 0.000E+00 | 0.000E+00 | 6.439E-07 | 0.000E+00 | 0.000E+00 | 0.000E+00 | 0.000E+00 | 0.000E+00 | 0.000E+00 | 0.000E+00 | 0.000E+00 |
| Unigene208104 | bcrA | 0.000E+00 | 0.000E+00 | 0.000E+00 | 3.602E-07 | 0.000E+00 | 0.000E+00 | 0.000E+00 | 0.000E+00 | 0.000E+00 | 0.000E+00 | 0.000E+00 | 0.000E+00 |
| Unigene208284 | lmrB | 0.000E+00 | 0.000E+00 | 5.676E-07 | 7.735E-05 | 0.000E+00 | 2.502E-08 | 0.000E+00 | 8.103E-08 | 0.000E+00 | 0.000E+00 | 0.000E+00 | 0.000E+00 |
| Unigene208290 | poxtA | 4.171E-08 | 0.000E+00 | 7.366E-07 | 7.429E-05 | 0.000E+00 | 0.000E+00 | 0.000E+00 | 0.000E+00 | 0.000E+00 | 0.000E+00 | 0.000E+00 | 0.000E+00 |
| Unigene208396 | vanRE | 0.000E+00 | 0.000E+00 | 0.000E+00 | 2.397E-07 | 0.000E+00 | 0.000E+00 | 0.000E+00 | 0.000E+00 | 0.000E+00 | 0.000E+00 | 0.000E+00 | 0.000E+00 |
| Unigene208420 | tetT | 0.000E+00 | 1.176E-07 | 4.656E-07 | 4.915E-06 | 0.000E+00 | 6.619E-08 | 0.000E+00 | 0.000E+00 | 0.000E+00 | 0.000E+00 | 0.000E+00 | 0.000E+00 |
| Unigene208456 | msbA | 0.000E+00 | 1.090E-07 | 2.274E-07 | 6.921E-06 | 0.000E+00 | 0.000E+00 | 0.000E+00 | 0.000E+00 | 0.000E+00 | 0.000E+00 | 0.000E+00 | 0.000E+00 |
| Unigene208494 | facT | 0.000E+00 | 7.436E-07 | 2.737E-07 | 6.841E-07 | 0.000E+00 | 0.000E+00 | 3.970E-07 | 1.861E-07 | 0.000E+00 | 0.000E+00 | 0.000E+00 | 0.000E+00 |
| Unigene208546 | msbA | 1.324E-07 | 1.307E-07 | 1.480E-06 | 5.961E-06 | 0.000E+00 | 0.000E+00 | 0.000E+00 | 0.000E+00 | 0.000E+00 | 0.000E+00 | 0.000E+00 | 0.000E+00 |
| Unigene208614 | catB2 | 0.000E+00 | 0.000E+00 | 0.000E+00 | 3.112E-06 | 0.000E+00 | 0.000E+00 | 0.000E+00 | 0.000E+00 | 0.000E+00 | 0.000E+00 | 0.000E+00 | 0.000E+00 |
| Unigene208729 | Staphylocc | 0.000E+00 | 1.047E-07 | 0.000E+00 | 9.309E-07 | 0.000E+00 | 0.000E+00 | 0.000E+00 | 0.000E+00 | 0.000E+00 | 8.853E-07 | 0.000E+00 | 2.818E-07 |
| Unigene208732 | Erm(48) | 0.000E+00 | 0.000E+00 | 0.000E+00 | 1.317E-06 | 0.000E+00 | 0.000E+00 | 0.000E+00 | 0.000E+00 | 0.000E+00 | 0.000E+00 | 0.000E+00 | 0.000E+00 |
| Unigene208781 | tetA(58) | 1.494E-07 | 7.654E-07 | 2.688E-06 | 2.666E-06 | 0.000E+00 | 0.000E+00 | 0.000E+00 | 0.000E+00 | 0.000E+00 | 0.000E+00 | 0.000E+00 | 0.000E+00 |
| Unigene208852 | patB | 1.148E-06 | 6.787E-07 | 1.248E-06 | 6.066E-07 | 3.926E-08 | 0.000E+00 | 7.822E-08 | 2.887E-07 | 0.000E+00 | 0.000E+00 | 6.063E-08 | 4.177E-08 |
| Unigene208882 | oleC | 0.000E+00 | 0.000E+00 | 0.000E+00 | 8.167E-07 | 0.000E+00 | 0.000E+00 | 0.000E+00 | 0.000E+00 | 0.000E+00 | 0.000E+00 | 0.000E+00 | 0.000E+00 |
| Unigene208913 | bcrA | 0.000E+00 | 0.000E+00 | 0.000E+00 | 1.050E-06 | 0.000E+00 | 0.000E+00 | 0.000E+00 | 0.000E+00 | 0.000E+00 | 0.000E+00 | 0.000E+00 | 0.000E+00 |
| Unigene208934 | bcrA | 0.000E+00 | 0.000E+00 | 0.000E+00 | 2.661E-07 | 0.000E+00 | 0.000E+00 | 0.000E+00 | 0.000E+00 | 0.000E+00 | 0.000E+00 | 0.000E+00 | 0.000E+00 |
| Unigene208980 | evgA | 0.000E+00 | 0.000E+00 | 2.666E-07 | 8.780E-07 | 0.000E+00 | 0.000E+00 | 0.000E+00 | 0.000E+00 | 0.000E+00 | 0.000E+00 | 0.000E+00 | 0.000E+00 |
| Unigene209008 | vmlR | 0.000E+00 | 0.000E+00 | 0.000E+00 | 9.300E-07 | 0.000E+00 | 5.764E-07 | 0.000E+00 | 2.829E-07 | 0.000E+00 | 0.000E+00 | 0.000E+00 | 0.000E+00 |
| Unigene209117 | baeS | 0.000E+00 | 6.293E-07 | 3.508E-06 | 4.053E-06 | 0.000E+00 | 0.000E+00 | 0.000E+00 | 0.000E+00 | 0.000E+00 | 0.000E+00 | 0.000E+00 | 0.000E+00 |
| Unigene209165 | tetA(58) | 0.000E+00 | 0.000E+00 | 1.331E-07 | 5.935E-07 | 0.000E+00 | 0.000E+00 | 0.000E+00 | 0.000E+00 | 0.000E+00 | 0.000E+00 | 0.000E+00 | 0.000E+00 |
| Unigene209225 | vanHD | 0.000E+00 | 0.000E+00 | 0.000E+00 | 9.478E-07 | 0.000E+00 | 0.000E+00 | 0.000E+00 | 0.000E+00 | 0.000E+00 | 0.000E+00 | 0.000E+00 | 0.000E+00 |
| Unigene209240 | macB | 2.282E-06 | 0.000E+00 | 0.000E+00 | 0.000E+00 | 0.000E+00 | 0.000E+00 | 0.000E+00 | 0.000E+00 | 0.000E+00 | 0.000E+00 | 0.000E+00 | 0.000E+00 |
| Unigene209276 | YojI | 8.847E-07 | 7.271E-06 | 1.157E-05 | 1.554E-05 | 0.000E+00 | 8.896E-08 | 9.108E-08 | 0.000E+00 | 0.000E+00 | 0.000E+00 | 2.353E-08 | 0.000E+00 |
| Unigene209387 | patB | 0.000E+00 | 0.000E+00 | 3.417E-07 | 4.850E-06 | 0.000E+00 | 3.963E-08 | 0.000E+00 | 0.000E+00 | 0.000E+00 | 0.000E+00 | 0.000E+00 | 0.000E+00 |
| Unigene209431 | efrA | 1.312E-07 | 8.641E-07 | 3.366E-06 | 3.578E-06 | 0.000E+00 | 0.000E+00 | 0.000E+00 | 0.000E+00 | 0.000E+00 | 0.000E+00 | 0.000E+00 | 0.000E+00 |
| Unigene209448 | vanHO | 1.353E-07 | 0.000E+00 | 0.000E+00 | 9.226E-07 | 0.000E+00 | 0.000E+00 | 0.000E+00 | 0.000E+00 | 0.000E+00 | 0.000E+00 | 0.000E+00 | 0.000E+00 |
| Unigene209489 | tetA(58) | 2.005E-07 | 2.567E-07 | 1.639E-07 | 8.099E-07 | 1.114E-07 | 0.000E+00 | 3.698E-08 | 0.000E+00 | 1.461E-07 | 0.000E+00 | 0.000E+00 | 0.000E+00 |
| Unigene209512 | vanSF | 0.000E+00 | 0.000E+00 | 9.943E-07 | 5.360E-07 | 0.000E+00 | 0.000E+00 | 0.000E+00 | 0.000E+00 | 0.000E+00 | 0.000E+00 | 0.000E+00 | 0.000E+00 |
| Unigene209514 | patA | 0.000E+00 | 0.000E+00 | 0.000E+00 | 7.367E-07 | 0.000E+00 | 0.000E+00 | 0.000E+00 | 0.000E+00 | 0.000E+00 | 0.000E+00 | 0.000E+00 | 0.000E+00 |
| Unigene209517 | bcrA | 0.000E+00 | 0.000E+00 | 0.000E+00 | 5.970E-07 | 0.000E+00 | 0.000E+00 | 0.000E+00 | 0.000E+00 | 0.000E+00 | 0.000E+00 | 0.000E+00 | 0.000E+00 |
| Unigene209543 | msbA | 0.000E+00 | 0.000E+00 | 0.000E+00 | 3.803E-07 | 0.000E+00 | 0.000E+00 | 0.000E+00 | 0.000E+00 | 0.000E+00 | 0.000E+00 | 0.000E+00 | 0.000E+00 |
| Unigene209548 | ugd | 0.000E+00 | 0.000E+00 | 0.000E+00 | 7.260E-07 | 0.000E+00 | 1.036E-07 | 1.061E-07 | 0.000E+00 | 0.000E+00 | 0.000E+00 | 0.000E+00 | 0.000E+00 |
| Unigene209634 | oleB | 0.000E+00 | 0.000E+00 | 0.000E+00 | 4.690E-07 | 0.000E+00 | 0.000E+00 | 0.000E+00 | 0.000E+00 | 0.000E+00 | 0.000E+00 | 0.000E+00 | 0.000E+00 |
| Unigene209648 | vanSC | 1.100E-07 | 2.174E-07 | 2.159E-07 | 7.111E-07 | 2.445E-08 | 2.379E-08 | 2.435E-08 | 0.000E+00 | 7.215E-08 | 0.000E+00 | 0.000E+00 | 0.000E+00 |
| Unigene209710 | tet(30) | 0.000E+00 | 0.000E+00 | 0.000E+00 | 3.196E-07 | 0.000E+00 | 0.000E+00 | 0.000E+00 | 0.000E+00 | 0.000E+00 | 0.000E+00 | 0.000E+00 | 0.000E+00 |
| Unigene209776 | tetB(P) | 0.000E+00 | 0.000E+00 | 7.953E-08 | 1.944E-06 | 0.000E+00 | 0.000E+00 | 0.000E+00 | 0.000E+00 | 0.000E+00 | 0.000E+00 | 0.000E+00 | 0.000E+00 |
| Unigene209809 | adeN | 0.000E+00 | 0.000E+00 | 0.000E+00 | 2.421E-06 | 0.000E+00 | 0.000E+00 | 0.000E+00 | 0.000E+00 | 0.000E+00 | 0.000E+00 | 0.000E+00 | 0.000E+00 |
| Unigene209811 | rphB | 0.000E+00 | 0.000E+00 | 1.320E-08 | 1.508E-06 | 0.000E+00 | 0.000E+00 | 0.000E+00 | 0.000E+00 | 0.000E+00 | 0.000E+00 | 0.000E+00 | 0.000E+00 |
| Unigene209815 | bcrA | 0.000E+00 | 0.000E+00 | 6.946E-08 | 1.180E-06 | 0.000E+00 | 0.000E+00 | 0.000E+00 | 0.000E+00 | 0.000E+00 | 0.000E+00 | 0.000E+00 | 0.000E+00 |
| Unigene209816 | vanSM | 0.000E+00 | 0.000E+00 | 0.000E+00 | 1.297E-06 | 0.000E+00 | 0.000E+00 | 0.000E+00 | 0.000E+00 | 0.000E+00 | 0.000E+00 | 0.000E+00 | 0.000E+00 |
| Unigene209817 | vanRE | 0.000E+00 | 0.000E+00 | 9.141E-08 | 1.223E-06 | 0.000E+00 | 0.000E+00 | 0.000E+00 | 0.000E+00 | 0.000E+00 | 0.000E+00 | 0.000E+00 | 0.000E+00 |
| Unigene209845 | poxtA | 0.000E+00 | 0.000E+00 | 0.000E+00 | 3.865E-07 | 0.000E+00 | 0.000E+00 | 0.000E+00 | 0.000E+00 | 0.000E+00 | 0.000E+00 | 0.000E+00 | 0.000E+00 |
| Unigene209873 | arlS | 4.733E-06 | 1.964E-05 | 3.327E-06 | 8.084E-07 | 7.063E-07 | 4.327E-07 | 2.606E-07 | 0.000E+00 | 1.338E-06 | 5.246E-07 | 0.000E+00 | 0.000E+00 |
| Unigene209905 | Staphylocc | 0.000E+00 | 0.000E+00 | 0.000E+00 | 6.037E-07 | 0.000E+00 | 0.000E+00 | 0.000E+00 | 0.000E+00 | 0.000E+00 | 0.000E+00 | 0.000E+00 | 0.000E+00 |
| Unigene209946 | efrB | 1.690E-07 | 0.000E+00 | 0.000E+00 | 7.396E-07 | 0.000E+00 | 3.045E-07 | 0.000E+00 | 0.000E+00 | 0.000E+00 | 0.000E+00 | 0.000E+00 | 0.000E+00 |
| Unigene209997 | vmlR | 0.000E+00 | 1.824E-07 | 3.261E-07 | 4.950E-06 | 0.000E+00 | 0.000E+00 | 0.000E+00 | 0.000E+00 | 0.000E+00 | 0.000E+00 | 0.000E+00 | 0.000E+00 |
| Unigene210059 | tet32 | 0.000E+00 | 0.000E+00 | 1.387E-07 | 4.443E-06 | 0.000E+00 | 0.000E+00 | 0.000E+00 | 0.000E+00 | 0.000E+00 | 0.000E+00 | 0.000E+00 | 0.000E+00 |
| Unigene210065 | poxtA | 0.000E+00 | 0.000E+00 | 2.732E-07 | 4.137E-06 | 0.000E+00 | 0.000E+00 | 0.000E+00 | 0.000E+00 | 0.000E+00 | 0.000E+00 | 0.000E+00 | 0.000E+00 |
| Unigene210120 | MexW | 0.000E+00 | 1.763E-07 | 0.000E+00 | 5.676E-07 | 0.000E+00 | 0.000E+00 | 0.000E+00 | 0.000E+00 | 0.000E+00 | 0.000E+00 | 0.000E+00 | 0.000E+00 |
| Unigene210198 | vmlR | 0.000E+00 | 0.000E+00 | 0.000E+00 | 4.509E-07 | 0.000E+00 | 0.000E+00 | 0.000E+00 | 0.000E+00 | 0.000E+00 | 0.000E+00 | 0.000E+00 | 0.000E+00 |
| Unigene210214 | mdtG | 0.000E+00 | 0.000E+00 | 0.000E+00 | 7.630E-07 | 1.042E-06 | 4.225E-07 | 0.000E+00 | 0.000E+00 | 5.126E-07 | 7.836E-07 | 0.000E+00 | 0.000E+00 |
| Unigene210215 | lsaA | 3.019E-07 | 0.000E+00 | 0.000E+00 | 7.404E-07 | 0.000E+00 | 0.000E+00 | 0.000E+00 | 0.000E+00 | 3.535E-07 | 0.000E+00 | 0.000E+00 | 0.000E+00 |
| Unigene210324 | vanWI | 0.000E+00 | 0.000E+00 | 5.306E-08 | 1.529E-06 | 0.000E+00 | 0.000E+00 | 0.000E+00 | 0.000E+00 | 0.000E+00 | 0.000E+00 | 0.000E+00 | 0.000E+00 |
| Unigene210347 | Staphylocc | 0.000E+00 | 0.000E+00 | 0.000E+00 | 1.720E-06 | 0.000E+00 | 0.000E+00 | 0.000E+00 | 0.000E+00 | 0.000E+00 | 0.000E+00 | 0.000E+00 | 0.000E+00 |

| | | | | | | | | | | | | | |
|---|---|---|---|---|---|---|---|---|---|---|---|---|---|
| Unigene210348 | emrA | 0.000E+00 | 0.000E+00 | 0.000E+00 | 2.293E-06 | 0.000E+00 | 0.000E+00 | 0.000E+00 | 0.000E+00 | 0.000E+00 | 0.000E+00 | 0.000E+00 | 0.000E+00 |
| Unigene210349 | emrK | 0.000E+00 | 0.000E+00 | 3.249E-08 | 1.672E-06 | 0.000E+00 | 0.000E+00 | 0.000E+00 | 0.000E+00 | 0.000E+00 | 0.000E+00 | 0.000E+00 | 0.000E+00 |
| Unigene210420 | tetA(58) | 0.000E+00 | 0.000E+00 | 2.046E-07 | 4.801E-06 | 0.000E+00 | 0.000E+00 | 0.000E+00 | 0.000E+00 | 0.000E+00 | 0.000E+00 | 0.000E+00 | 0.000E+00 |
| Unigene210538 | macB | 2.129E-07 | 0.000E+00 | 8.735E-07 | 5.433E-06 | 0.000E+00 | 0.000E+00 | 0.000E+00 | 0.000E+00 | 0.000E+00 | 0.000E+00 | 0.000E+00 | 0.000E+00 |
| Unigene210658 | Staphylocc | 0.000E+00 | 2.036E-07 | 0.000E+00 | 1.194E-06 | 0.000E+00 | 0.000E+00 | 0.000E+00 | 0.000E+00 | 0.000E+00 | 0.000E+00 | 0.000E+00 | 0.000E+00 |
| Unigene210680 | tetA(58) | 0.000E+00 | 0.000E+00 | 0.000E+00 | 5.500E-06 | 0.000E+00 | 0.000E+00 | 0.000E+00 | 0.000E+00 | 0.000E+00 | 0.000E+00 | 0.000E+00 | 0.000E+00 |
| Unigene210701 | vanHB | 7.392E-05 | 5.930E-05 | 1.166E-04 | 1.864E-04 | 1.315E-04 | 9.471E-05 | 1.345E-04 | 1.201E-04 | 1.008E-04 | 1.471E-04 | 1.596E-04 | 1.658E-04 |
| Unigene210793 | rpoB2 | 0.000E+00 | 0.000E+00 | 0.000E+00 | 5.041E-07 | 0.000E+00 | 0.000E+00 | 0.000E+00 | 0.000E+00 | 0.000E+00 | 0.000E+00 | 0.000E+00 | 0.000E+00 |
| Unigene210818 | macB | 0.000E+00 | 0.000E+00 | 0.000E+00 | 6.965E-07 | 0.000E+00 | 0.000E+00 | 0.000E+00 | 0.000E+00 | 0.000E+00 | 0.000E+00 | 0.000E+00 | 0.000E+00 |
| Unigene210830 | RlmA(II) | 0.000E+00 | 0.000E+00 | 0.000E+00 | 5.863E-07 | 0.000E+00 | 0.000E+00 | 0.000E+00 | 0.000E+00 | 0.000E+00 | 0.000E+00 | 0.000E+00 | 0.000E+00 |
| Unigene210849 | bacA | 0.000E+00 | 0.000E+00 | 1.503E-07 | 5.955E-06 | 0.000E+00 | 0.000E+00 | 0.000E+00 | 0.000E+00 | 0.000E+00 | 0.000E+00 | 0.000E+00 | 0.000E+00 |
| Unigene210870 | evgA | 3.904E-06 | 8.184E-06 | 9.380E-06 | 1.122E-05 | 1.112E-07 | 3.246E-07 | 0.000E+00 | 0.000E+00 | 0.000E+00 | 2.788E-07 | 0.000E+00 | 0.000E+00 |
| Unigene210936 | farA | 0.000E+00 | 0.000E+00 | 0.000E+00 | 2.739E-07 | 0.000E+00 | 0.000E+00 | 0.000E+00 | 0.000E+00 | 0.000E+00 | 0.000E+00 | 0.000E+00 | 0.000E+00 |
| Unigene211014 | lin | 0.000E+00 | 0.000E+00 | 7.821E-07 | 5.030E-07 | 8.818E-06 | 3.949E-07 | 8.454E-07 | 0.000E+00 | 0.000E+00 | 0.000E+00 | 0.000E+00 | 0.000E+00 |
| Unigene211040 | tetA(46) | 4.537E-07 | 0.000E+00 | 0.000E+00 | 5.237E-07 | 0.000E+00 | 0.000E+00 | 0.000E+00 | 0.000E+00 | 0.000E+00 | 0.000E+00 | 0.000E+00 | 0.000E+00 |
| Unigene211211 | salA | 0.000E+00 | 0.000E+00 | 0.000E+00 | 5.960E-07 | 0.000E+00 | 0.000E+00 | 0.000E+00 | 0.000E+00 | 0.000E+00 | 0.000E+00 | 0.000E+00 | 0.000E+00 |
| Unigene211246 | OprA | 1.251E-07 | 2.060E-07 | 0.000E+00 | 4.422E-07 | 0.000E+00 | 0.000E+00 | 0.000E+00 | 0.000E+00 | 0.000E+00 | 0.000E+00 | 0.000E+00 | 0.000E+00 |
| Unigene211259 | tetA(58) | 2.191E-06 | 1.683E-05 | 8.061E-06 | 5.238E-06 | 0.000E+00 | 1.045E-07 | 0.000E+00 | 0.000E+00 | 0.000E+00 | 0.000E+00 | 0.000E+00 | 0.000E+00 |
| Unigene211296 | GOB-6 | 0.000E+00 | 1.833E-06 | 7.232E-07 | 4.465E-07 | 6.550E-06 | 0.000E+00 | 0.000E+00 | 0.000E+00 | 0.000E+00 | 0.000E+00 | 0.000E+00 | 0.000E+00 |
| Unigene211319 | bcrA | 1.257E-05 | 0.000E+00 | 0.000E+00 | 2.602E-06 | 3.261E-05 | 7.190E-06 | 2.262E-05 | 2.256E-05 | 0.000E+00 | 0.000E+00 | 0.000E+00 | 3.005E-06 |
| Unigene211321 | macB | 6.748E-06 | 0.000E+00 | 0.000E+00 | 2.210E-06 | 2.766E-05 | 1.390E-05 | 2.240E-05 | 2.469E-05 | 2.741E-06 | 0.000E+00 | 0.000E+00 | 0.000E+00 |
| Unigene211339 | vmlR | 0.000E+00 | 0.000E+00 | 0.000E+00 | 3.563E-07 | 0.000E+00 | 0.000E+00 | 0.000E+00 | 0.000E+00 | 0.000E+00 | 0.000E+00 | 0.000E+00 | 0.000E+00 |
| Unigene211341 | carA | 0.000E+00 | 0.000E+00 | 3.688E-07 | 7.117E-07 | 0.000E+00 | 5.080E-08 | 0.000E+00 | 0.000E+00 | 0.000E+00 | 0.000E+00 | 0.000E+00 | 0.000E+00 |
| Unigene211351 | Agrobacte | 5.169E-07 | 1.031E-07 | 3.227E-07 | 9.964E-07 | 0.000E+00 | 0.000E+00 | 0.000E+00 | 0.000E+00 | 5.136E-08 | 0.000E+00 | 0.000E+00 | 0.000E+00 |
| Unigene211407 | vanSN | 0.000E+00 | 0.000E+00 | 0.000E+00 | 6.112E-07 | 0.000E+00 | 0.000E+00 | 0.000E+00 | 0.000E+00 | 0.000E+00 | 0.000E+00 | 0.000E+00 | 0.000E+00 |
| Unigene211456 | MexW | 0.000E+00 | 0.000E+00 | 0.000E+00 | 6.827E-07 | 0.000E+00 | 0.000E+00 | 0.000E+00 | 0.000E+00 | 0.000E+00 | 0.000E+00 | 0.000E+00 | 0.000E+00 |
| Unigene211514 | efpA | 4.365E-08 | 2.395E-07 | 1.713E-07 | 7.053E-07 | 0.000E+00 | 0.000E+00 | 0.000E+00 | 0.000E+00 | 0.000E+00 | 0.000E+00 | 0.000E+00 | 0.000E+00 |
| Unigene211627 | msbA | 0.000E+00 | 0.000E+00 | 0.000E+00 | 4.226E-07 | 0.000E+00 | 0.000E+00 | 0.000E+00 | 0.000E+00 | 0.000E+00 | 0.000E+00 | 0.000E+00 | 0.000E+00 |
| Unigene211646 | Corynebac | 2.134E-07 | 0.000E+00 | 0.000E+00 | 7.080E-07 | 0.000E+00 | 1.977E-07 | 0.000E+00 | 1.423E-07 | 3.332E-07 | 0.000E+00 | 0.000E+00 | 0.000E+00 |
| Unigene211676 | Escherichia | 2.454E-06 | 5.180E-06 | 2.933E-06 | 6.513E-06 | 0.000E+00 | 6.802E-08 | 0.000E+00 | 0.000E+00 | 6.878E-08 | 0.000E+00 | 7.198E-08 | 1.859E-07 |
| Unigene211685 | vmlR | 0.000E+00 | 0.000E+00 | 0.000E+00 | 3.790E-07 | 0.000E+00 | 0.000E+00 | 0.000E+00 | 0.000E+00 | 0.000E+00 | 0.000E+00 | 0.000E+00 | 0.000E+00 |
| Unigene211715 | vanHF | 0.000E+00 | 0.000E+00 | 2.871E-07 | 5.351E-06 | 0.000E+00 | 0.000E+00 | 0.000E+00 | 0.000E+00 | 0.000E+00 | 0.000E+00 | 0.000E+00 | 0.000E+00 |
| Unigene211773 | msbA | 0.000E+00 | 0.000E+00 | 0.000E+00 | 3.927E-07 | 0.000E+00 | 0.000E+00 | 0.000E+00 | 0.000E+00 | 0.000E+00 | 0.000E+00 | 0.000E+00 | 0.000E+00 |
| Unigene211852 | mtrA | 0.000E+00 | 0.000E+00 | 0.000E+00 | 4.643E-07 | 0.000E+00 | 0.000E+00 | 3.562E-07 | 0.000E+00 | 0.000E+00 | 0.000E+00 | 0.000E+00 | 0.000E+00 |
| Unigene212098 | baeR | 0.000E+00 | 0.000E+00 | 0.000E+00 | 7.154E-07 | 0.000E+00 | 0.000E+00 | 0.000E+00 | 0.000E+00 | 0.000E+00 | 0.000E+00 | 0.000E+00 | 3.350E-06 |
| Unigene212137 | efpA | 8.678E-08 | 1.190E-07 | 4.896E-07 | 5.477E-07 | 0.000E+00 | 2.345E-08 | 0.000E+00 | 0.000E+00 | 0.000E+00 | 0.000E+00 | 7.445E-08 | 0.000E+00 |
| Unigene212139 | cmlv | 0.000E+00 | 2.574E-07 | 2.812E-07 | 6.840E-07 | 0.000E+00 | 5.633E-08 | 0.000E+00 | 3.041E-08 | 8.543E-08 | 0.000E+00 | 5.960E-08 | 6.159E-08 |
| Unigene212296 | Staphylocc | 0.000E+00 | 0.000E+00 | 0.000E+00 | 4.409E-07 | 0.000E+00 | 0.000E+00 | 0.000E+00 | 0.000E+00 | 0.000E+00 | 0.000E+00 | 0.000E+00 | 0.000E+00 |
| Unigene212298 | Staphylocc | 0.000E+00 | 3.994E-08 | 7.975E-07 | 5.853E-05 | 0.000E+00 | 0.000E+00 | 0.000E+00 | 8.496E-08 | 0.000E+00 | 0.000E+00 | 0.000E+00 | 0.000E+00 |
| Unigene212372 | fexA | 0.000E+00 | 0.000E+00 | 3.138E-07 | 1.130E-06 | 0.000E+00 | 0.000E+00 | 0.000E+00 | 0.000E+00 | 0.000E+00 | 0.000E+00 | 0.000E+00 | 0.000E+00 |
| Unigene212380 | patB | 3.730E-08 | 1.842E-07 | 0.000E+00 | 1.055E-06 | 0.000E+00 | 0.000E+00 | 0.000E+00 | 0.000E+00 | 0.000E+00 | 0.000E+00 | 0.000E+00 | 3.527E-07 |
| Unigene212381 | tetA(46) | 0.000E+00 | 1.433E-07 | 0.000E+00 | 9.039E-07 | 0.000E+00 | 0.000E+00 | 0.000E+00 | 0.000E+00 | 0.000E+00 | 6.855E-07 | 0.000E+00 | 0.000E+00 |
| Unigene212430 | poxtA | 0.000E+00 | 0.000E+00 | 0.000E+00 | 4.724E-07 | 0.000E+00 | 0.000E+00 | 0.000E+00 | 0.000E+00 | 0.000E+00 | 0.000E+00 | 0.000E+00 | 0.000E+00 |
| Unigene212551 | macB | 0.000E+00 | 0.000E+00 | 0.000E+00 | 6.585E-07 | 0.000E+00 | 0.000E+00 | 0.000E+00 | 0.000E+00 | 0.000E+00 | 0.000E+00 | 0.000E+00 | 0.000E+00 |
| Unigene212574 | oleC | 0.000E+00 | 0.000E+00 | 0.000E+00 | 6.791E-07 | 0.000E+00 | 0.000E+00 | 0.000E+00 | 0.000E+00 | 0.000E+00 | 0.000E+00 | 0.000E+00 | 0.000E+00 |
| Unigene212674 | macB | 0.000E+00 | 0.000E+00 | 0.000E+00 | 5.071E-06 | 0.000E+00 | 0.000E+00 | 0.000E+00 | 0.000E+00 | 0.000E+00 | 0.000E+00 | 0.000E+00 | 0.000E+00 |
| Unigene212775 | vatH | 0.000E+00 | 0.000E+00 | 3.265E-07 | 5.638E-06 | 0.000E+00 | 0.000E+00 | 0.000E+00 | 0.000E+00 | 0.000E+00 | 0.000E+00 | 0.000E+00 | 0.000E+00 |
| Unigene212799 | Staphylocc | 0.000E+00 | 0.000E+00 | 2.355E-07 | 4.039E-06 | 0.000E+00 | 5.766E-08 | 0.000E+00 | 0.000E+00 | 0.000E+00 | 0.000E+00 | 0.000E+00 | 0.000E+00 |
| Unigene212834 | mecA | 0.000E+00 | 0.000E+00 | 0.000E+00 | 2.570E-07 | 0.000E+00 | 0.000E+00 | 0.000E+00 | 0.000E+00 | 0.000E+00 | 0.000E+00 | 0.000E+00 | 0.000E+00 |
| Unigene212846 | arlR | 1.121E-07 | 0.000E+00 | 5.499E-08 | 2.830E-07 | 0.000E+00 | 0.000E+00 | 0.000E+00 | 0.000E+00 | 0.000E+00 | 0.000E+00 | 0.000E+00 | 0.000E+00 |
| Unigene212930 | vanSA | 0.000E+00 | 1.884E-07 | 2.246E-07 | 5.780E-07 | 0.000E+00 | 0.000E+00 | 0.000E+00 | 0.000E+00 | 0.000E+00 | 0.000E+00 | 0.000E+00 | 0.000E+00 |
| Unigene212938 | msbA | 0.000E+00 | 0.000E+00 | 3.078E-07 | 4.562E-06 | 0.000E+00 | 3.391E-08 | 0.000E+00 | 0.000E+00 | 0.000E+00 | 0.000E+00 | 0.000E+00 | 0.000E+00 |
| Unigene213068 | Staphylocc | 0.000E+00 | 0.000E+00 | 0.000E+00 | 9.490E-07 | 0.000E+00 | 0.000E+00 | 0.000E+00 | 0.000E+00 | 4.622E-07 | 0.000E+00 | 0.000E+00 | 0.000E+00 |
| Unigene213074 | vanTG | 0.000E+00 | 0.000E+00 | 0.000E+00 | 4.477E-07 | 0.000E+00 | 0.000E+00 | 0.000E+00 | 0.000E+00 | 0.000E+00 | 0.000E+00 | 4.610E-08 | 0.000E+00 |
| Unigene213101 | tetB(P) | 8.075E-08 | 1.108E-07 | 0.000E+00 | 1.142E-06 | 0.000E+00 | 2.182E-08 | 0.000E+00 | 0.000E+00 | 0.000E+00 | 8.321E-07 | 0.000E+00 | 1.909E-07 |
| Unigene213187 | mtrA | 0.000E+00 | 2.601E-07 | 1.860E-07 | 5.265E-07 | 0.000E+00 | 0.000E+00 | 0.000E+00 | 0.000E+00 | 0.000E+00 | 0.000E+00 | 5.422E-08 | 0.000E+00 |
| Unigene213188 | smeS | 1.270E-07 | 0.000E+00 | 1.557E-07 | 1.218E-06 | 7.052E-08 | 0.000E+00 | 0.000E+00 | 3.705E-08 | 0.000E+00 | 0.000E+00 | 0.000E+00 | 0.000E+00 |
| Unigene213294 | vanI | 0.000E+00 | 1.875E-07 | 5.586E-08 | 2.875E-07 | 0.000E+00 | 0.000E+00 | 0.000E+00 | 0.000E+00 | 0.000E+00 | 0.000E+00 | 0.000E+00 | 0.000E+00 |

| | | | | | | | | | | | | | |
|---|---|---|---|---|---|---|---|---|---|---|---|---|---|
| Unigene213346 | arnA | 0.000E+00 | 0.000E+00 | 0.000E+00 | 3.575E-06 | 0.000E+00 | 0.000E+00 | 0.000E+00 | 0.000E+00 | 0.000E+00 | 0.000E+00 | 0.000E+00 | 0.000E+00 |
| Unigene213487 | sdiA | 0.000E+00 | 0.000E+00 | 2.231E-07 | 0.000E+00 | 0.000E+00 | 0.000E+00 | 0.000E+00 | 0.000E+00 | 0.000E+00 | 0.000E+00 | 0.000E+00 | 0.000E+00 |
| Unigene213553 | TaeA | 0.000E+00 | 1.780E-06 | 1.482E-06 | 8.745E-07 | 3.644E-06 | 0.000E+00 | 1.652E-06 | 0.000E+00 | 0.000E+00 | 0.000E+00 | 0.000E+00 | 0.000E+00 |
| Unigene213622 | macB | 0.000E+00 | 0.000E+00 | 0.000E+00 | 1.158E-06 | 0.000E+00 | 0.000E+00 | 0.000E+00 | 0.000E+00 | 0.000E+00 | 1.081E-06 | 0.000E+00 | 0.000E+00 |
| Unigene213638 | macB | 8.942E-08 | 1.636E-07 | 2.632E-07 | 7.224E-07 | 0.000E+00 | 6.444E-08 | 0.000E+00 | 0.000E+00 | 0.000E+00 | 6.641E-08 | 0.000E+00 | 0.000E+00 |
| Unigene213660 | Staphylocc | 0.000E+00 | 0.000E+00 | 6.689E-07 | 5.809E-05 | 0.000E+00 | 0.000E+00 | 0.000E+00 | 1.092E-07 | 0.000E+00 | 0.000E+00 | 0.000E+00 | 0.000E+00 |
| Unigene213685 | macB | 6.511E-08 | 0.000E+00 | 0.000E+00 | 1.003E-06 | 0.000E+00 | 0.000E+00 | 0.000E+00 | 0.000E+00 | 0.000E+00 | 7.435E-07 | 0.000E+00 | 2.501E-07 |
| Unigene213691 | macB | 0.000E+00 | 0.000E+00 | 1.922E-07 | 4.493E-06 | 0.000E+00 | 1.513E-08 | 0.000E+00 | 0.000E+00 | 0.000E+00 | 0.000E+00 | 0.000E+00 | 0.000E+00 |
| Unigene213693 | lsaC | 0.000E+00 | 0.000E+00 | 4.493E-07 | 4.161E-06 | 0.000E+00 | 4.950E-08 | 0.000E+00 | 0.000E+00 | 0.000E+00 | 0.000E+00 | 0.000E+00 | 0.000E+00 |
| Unigene213701 | TaeA | 3.459E-07 | 1.687E-07 | 1.885E-07 | 7.761E-07 | 4.269E-08 | 0.000E+00 | 8.506E-08 | 0.000E+00 | 0.000E+00 | 0.000E+00 | 0.000E+00 | 0.000E+00 |
| Unigene213703 | macB | 0.000E+00 | 2.214E-07 | 2.574E-06 | 1.542E-05 | 0.000E+00 | 0.000E+00 | 0.000E+00 | 0.000E+00 | 0.000E+00 | 0.000E+00 | 0.000E+00 | 0.000E+00 |
| Unigene213730 | adeL | 0.000E+00 | 0.000E+00 | 0.000E+00 | 4.555E-07 | 0.000E+00 | 0.000E+00 | 0.000E+00 | 0.000E+00 | 0.000E+00 | 0.000E+00 | 0.000E+00 | 0.000E+00 |
| Unigene213845 | macB | 0.000E+00 | 0.000E+00 | 0.000E+00 | 4.046E-07 | 1.272E-07 | 0.000E+00 | 1.267E-07 | 3.340E-07 | 0.000E+00 | 0.000E+00 | 0.000E+00 | 0.000E+00 |
| Unigene213850 | bcrA | 0.000E+00 | 0.000E+00 | 0.000E+00 | 3.602E-07 | 0.000E+00 | 0.000E+00 | 0.000E+00 | 0.000E+00 | 6.498E-08 | 0.000E+00 | 0.000E+00 | 0.000E+00 |
| Unigene213866 | mef(B) | 0.000E+00 | 0.000E+00 | 0.000E+00 | 6.894E-07 | 0.000E+00 | 0.000E+00 | 1.079E-07 | 0.000E+00 | 0.000E+00 | 0.000E+00 | 0.000E+00 | 0.000E+00 |
| Unigene213992 | vanRB | 0.000E+00 | 0.000E+00 | 0.000E+00 | 7.200E-07 | 0.000E+00 | 0.000E+00 | 0.000E+00 | 0.000E+00 | 0.000E+00 | 0.000E+00 | 0.000E+00 | 0.000E+00 |
| Unigene214059 | msbA | 0.000E+00 | 0.000E+00 | 2.411E-07 | 5.210E-06 | 0.000E+00 | 0.000E+00 | 0.000E+00 | 0.000E+00 | 0.000E+00 | 0.000E+00 | 0.000E+00 | 0.000E+00 |
| Unigene214137 | evgA | 2.538E-07 | 5.571E-08 | 3.486E-07 | 4.613E-07 | 0.000E+00 | 0.000E+00 | 0.000E+00 | 0.000E+00 | 5.547E-08 | 0.000E+00 | 0.000E+00 | 0.000E+00 |
| Unigene214138 | evgS | 1.147E-07 | 2.140E-07 | 2.364E-07 | 6.371E-07 | 7.646E-08 | 0.000E+00 | 3.809E-08 | 1.339E-08 | 2.508E-08 | 0.000E+00 | 5.249E-08 | 9.492E-08 |
| Unigene214184 | oleC | 0.000E+00 | 0.000E+00 | 5.518E-07 | 1.666E-06 | 0.000E+00 | 0.000E+00 | 0.000E+00 | 0.000E+00 | 0.000E+00 | 0.000E+00 | 0.000E+00 | 0.000E+00 |
| Unigene214202 | Klebsiella p | 0.000E+00 | 0.000E+00 | 0.000E+00 | 2.937E-07 | 0.000E+00 | 0.000E+00 | 0.000E+00 | 0.000E+00 | 0.000E+00 | 0.000E+00 | 0.000E+00 | 0.000E+00 |
| Unigene214311 | Staphylocc | 7.100E-05 | 6.023E-05 | 1.156E-04 | 1.686E-04 | 1.240E-04 | 9.093E-05 | 1.144E-04 | 1.072E-04 | 9.818E-05 | 1.423E-04 | 1.443E-04 | 1.467E-04 |
| Unigene214378 | msbA | 1.104E-07 | 0.000E+00 | 0.000E+00 | 1.022E-06 | 0.000E+00 | 0.000E+00 | 0.000E+00 | 1.932E-07 | 3.820E-07 | 0.000E+00 | 0.000E+00 | 0.000E+00 |
| Unigene214435 | macB | 0.000E+00 | 0.000E+00 | 2.554E-07 | 1.630E-06 | 0.000E+00 | 0.000E+00 | 0.000E+00 | 0.000E+00 | 0.000E+00 | 0.000E+00 | 0.000E+00 | 0.000E+00 |
| Unigene214440 | arlR | 0.000E+00 | 0.000E+00 | 2.869E-07 | 5.315E-07 | 0.000E+00 | 0.000E+00 | 0.000E+00 | 0.000E+00 | 0.000E+00 | 0.000E+00 | 0.000E+00 | 0.000E+00 |
| Unigene214467 | vanRB | 3.471E-07 | 8.163E-07 | 2.919E-07 | 1.052E-06 | 0.000E+00 | 0.000E+00 | 0.000E+00 | 0.000E+00 | 0.000E+00 | 0.000E+00 | 0.000E+00 | 0.000E+00 |
| Unigene214481 | mdtA | 8.060E-08 | 0.000E+00 | 0.000E+00 | 4.477E-07 | 0.000E+00 | 0.000E+00 | 0.000E+00 | 0.000E+00 | 0.000E+00 | 0.000E+00 | 0.000E+00 | 0.000E+00 |
| Unigene214533 | efpA | 0.000E+00 | 4.283E-07 | 0.000E+00 | 0.000E+00 | 0.000E+00 | 0.000E+00 | 0.000E+00 | 0.000E+00 | 0.000E+00 | 0.000E+00 | 0.000E+00 | 0.000E+00 |
| Unigene214576 | emrB | 0.000E+00 | 0.000E+00 | 0.000E+00 | 3.747E-07 | 0.000E+00 | 0.000E+00 | 0.000E+00 | 0.000E+00 | 0.000E+00 | 0.000E+00 | 0.000E+00 | 0.000E+00 |
| Unigene214797 | arlS | 5.028E-07 | 0.000E+00 | 7.401E-07 | 2.031E-07 | 0.000E+00 | 0.000E+00 | 0.000E+00 | 0.000E+00 | 0.000E+00 | 0.000E+00 | 0.000E+00 | 0.000E+00 |
| Unigene214884 | novA | 1.133E-07 | 2.175E-07 | 2.778E-07 | 9.150E-07 | 6.292E-08 | 0.000E+00 | 0.000E+00 | 0.000E+00 | 0.000E+00 | 0.000E+00 | 6.478E-08 | 1.674E-07 |
| Unigene214930 | patA | 0.000E+00 | 0.000E+00 | 0.000E+00 | 5.310E-07 | 0.000E+00 | 0.000E+00 | 8.954E-08 | 0.000E+00 | 0.000E+00 | 0.000E+00 | 4.627E-08 | 0.000E+00 |
| Unigene214995 | lsaC | 1.523E-07 | 0.000E+00 | 2.739E-07 | 4.228E-06 | 0.000E+00 | 0.000E+00 | 0.000E+00 | 0.000E+00 | 0.000E+00 | 0.000E+00 | 0.000E+00 | 0.000E+00 |
| Unigene215008 | msbA | 0.000E+00 | 0.000E+00 | 1.897E-07 | 3.904E-07 | 0.000E+00 | 0.000E+00 | 0.000E+00 | 0.000E+00 | 0.000E+00 | 0.000E+00 | 0.000E+00 | 0.000E+00 |
| Unigene215010 | evgA | 0.000E+00 | 5.846E-08 | 0.000E+00 | 7.531E-07 | 0.000E+00 | 0.000E+00 | 0.000E+00 | 0.000E+00 | 0.000E+00 | 0.000E+00 | 0.000E+00 | 0.000E+00 |
| Unigene215188 | tetA(58) | 3.961E-07 | 0.000E+00 | 0.000E+00 | 4.000E-07 | 0.000E+00 | 0.000E+00 | 0.000E+00 | 0.000E+00 | 0.000E+00 | 0.000E+00 | 0.000E+00 | 0.000E+00 |
| Unigene215301 | vgaB | 0.000E+00 | 0.000E+00 | 0.000E+00 | 4.794E-07 | 0.000E+00 | 0.000E+00 | 0.000E+00 | 0.000E+00 | 0.000E+00 | 0.000E+00 | 0.000E+00 | 0.000E+00 |
| Unigene215403 | arlS | 0.000E+00 | 0.000E+00 | 0.000E+00 | 5.537E-05 | 0.000E+00 | 2.285E-08 | 0.000E+00 | 0.000E+00 | 0.000E+00 | 0.000E+00 | 0.000E+00 | 0.000E+00 |
| Unigene215492 | mdtG | 0.000E+00 | 0.000E+00 | 0.000E+00 | 4.907E-07 | 0.000E+00 | 0.000E+00 | 0.000E+00 | 0.000E+00 | 0.000E+00 | 0.000E+00 | 0.000E+00 | 0.000E+00 |
| Unigene215547 | AxyY | 0.000E+00 | 3.652E-07 | 0.000E+00 | 3.921E-07 | 0.000E+00 | 0.000E+00 | 0.000E+00 | 1.295E-07 | 0.000E+00 | 0.000E+00 | 0.000E+00 | 0.000E+00 |
| Unigene215637 | arlR | 3.639E-07 | 0.000E+00 | 0.000E+00 | 3.149E-07 | 0.000E+00 | 0.000E+00 | 5.753E-08 | 0.000E+00 | 1.136E-07 | 0.000E+00 | 0.000E+00 | 0.000E+00 |
| Unigene215871 | mdtG | 0.000E+00 | 0.000E+00 | 0.000E+00 | 4.953E-07 | 0.000E+00 | 0.000E+00 | 0.000E+00 | 0.000E+00 | 0.000E+00 | 0.000E+00 | 0.000E+00 | 0.000E+00 |
| Unigene215888 | vanG | 0.000E+00 | 8.748E-08 | 4.171E-07 | 1.717E-06 | 0.000E+00 | 0.000E+00 | 0.000E+00 | 0.000E+00 | 0.000E+00 | 0.000E+00 | 0.000E+00 | 0.000E+00 |
| Unigene216025 | Streptomy | 0.000E+00 | 0.000E+00 | 3.535E-07 | 5.855E-06 | 0.000E+00 | 1.770E-08 | 0.000E+00 | 0.000E+00 | 0.000E+00 | 0.000E+00 | 0.000E+00 | 0.000E+00 |
| Unigene216118 | Acinetoba | 0.000E+00 | 2.491E-08 | 3.118E-07 | 1.169E-06 | 0.000E+00 | 0.000E+00 | 0.000E+00 | 0.000E+00 | 0.000E+00 | 0.000E+00 | 0.000E+00 | 0.000E+00 |
| Unigene216204 | efrA | 0.000E+00 | 0.000E+00 | 7.232E-08 | 6.698E-07 | 0.000E+00 | 0.000E+00 | 0.000E+00 | 0.000E+00 | 0.000E+00 | 0.000E+00 | 0.000E+00 | 0.000E+00 |
| Unigene216252 | msbA | 0.000E+00 | 0.000E+00 | 0.000E+00 | 4.107E-07 | 0.000E+00 | 0.000E+00 | 0.000E+00 | 0.000E+00 | 0.000E+00 | 0.000E+00 | 0.000E+00 | 0.000E+00 |
| Unigene216259 | msbA | 0.000E+00 | 0.000E+00 | 0.000E+00 | 4.559E-07 | 0.000E+00 | 0.000E+00 | 0.000E+00 | 0.000E+00 | 0.000E+00 | 0.000E+00 | 0.000E+00 | 0.000E+00 |
| Unigene216340 | Agrobacte | 7.421E-07 | 3.394E-07 | 0.000E+00 | 9.367E-07 | 0.000E+00 | 0.000E+00 | 0.000E+00 | 0.000E+00 | 0.000E+00 | 0.000E+00 | 0.000E+00 | 0.000E+00 |
| Unigene216438 | msbA | 2.562E-07 | 0.000E+00 | 0.000E+00 | 6.037E-07 | 0.000E+00 | 0.000E+00 | 0.000E+00 | 0.000E+00 | 6.067E-07 | 0.000E+00 | 0.000E+00 | 0.000E+00 |
| Unigene216605 | cpxA | 0.000E+00 | 0.000E+00 | 1.997E-07 | 4.470E-06 | 0.000E+00 | 0.000E+00 | 0.000E+00 | 0.000E+00 | 0.000E+00 | 0.000E+00 | 0.000E+00 | 0.000E+00 |
| Unigene216607 | tetA(46) | 0.000E+00 | 0.000E+00 | 0.000E+00 | 1.417E-07 | 0.000E+00 | 5.057E-08 | 0.000E+00 | 0.000E+00 | 0.000E+00 | 0.000E+00 | 0.000E+00 | 0.000E+00 |
| Unigene216628 | bcrA | 0.000E+00 | 0.000E+00 | 0.000E+00 | 2.957E-07 | 0.000E+00 | 0.000E+00 | 0.000E+00 | 0.000E+00 | 0.000E+00 | 0.000E+00 | 0.000E+00 | 0.000E+00 |
| Unigene216630 | efrB | 0.000E+00 | 0.000E+00 | 0.000E+00 | 3.473E-07 | 0.000E+00 | 0.000E+00 | 0.000E+00 | 0.000E+00 | 0.000E+00 | 0.000E+00 | 0.000E+00 | 0.000E+00 |
| Unigene216682 | vanTrL | 0.000E+00 | 4.168E-07 | 0.000E+00 | 3.835E-07 | 0.000E+00 | 0.000E+00 | 0.000E+00 | 0.000E+00 | 0.000E+00 | 0.000E+00 | 0.000E+00 | 0.000E+00 |
| Unigene216691 | novA | 5.586E-07 | 3.872E-07 | 2.741E-07 | 3.652E-06 | 7.512E-07 | 8.422E-07 | 1.277E-05 | 1.328E-05 | 0.000E+00 | 1.899E-06 | 0.000E+00 | 8.688E-08 |
| Unigene216698 | efrB | 0.000E+00 | 0.000E+00 | 0.000E+00 | 3.582E-07 | 0.000E+00 | 0.000E+00 | 0.000E+00 | 0.000E+00 | 0.000E+00 | 0.000E+00 | 0.000E+00 | 0.000E+00 |
| Unigene216769 | Corynebac | 0.000E+00 | 0.000E+00 | 4.693E-07 | 5.920E-05 | 0.000E+00 | 0.000E+00 | 0.000E+00 | 0.000E+00 | 0.000E+00 | 0.000E+00 | 0.000E+00 | 0.000E+00 |

| | | | | | | | | | | | | | |
|---|---|---|---|---|---|---|---|---|---|---|---|---|---|
| Unigene216779 | Streptomy | 0.000E+00 | 6.401E-08 | 0.000E+00 | 5.006E-07 | 0.000E+00 | 0.000E+00 | 0.000E+00 | 0.000E+00 | 0.000E+00 | 0.000E+00 | 0.000E+00 | 0.000E+00 |
| Unigene216878 | FosA6 | 0.000E+00 | 5.905E-07 | 0.000E+00 | 7.761E-07 | 0.000E+00 | 0.000E+00 | 0.000E+00 | 0.000E+00 | 0.000E+00 | 0.000E+00 | 0.000E+00 | 0.000E+00 |
| Unigene216967 | macB | 0.000E+00 | 0.000E+00 | 1.624E-07 | 4.305E-06 | 0.000E+00 | 0.000E+00 | 0.000E+00 | 0.000E+00 | 0.000E+00 | 0.000E+00 | 0.000E+00 | 0.000E+00 |
| Unigene216971 | msbA | 0.000E+00 | 0.000E+00 | 3.596E-07 | 4.609E-06 | 0.000E+00 | 0.000E+00 | 0.000E+00 | 0.000E+00 | 0.000E+00 | 0.000E+00 | 0.000E+00 | 0.000E+00 |
| Unigene216986 | oleC | 2.624E-07 | 0.000E+00 | 0.000E+00 | 5.742E-07 | 0.000E+00 | 0.000E+00 | 0.000E+00 | 0.000E+00 | 1.434E-07 | 0.000E+00 | 0.000E+00 | 0.000E+00 |
| Unigene217174 | Corynebac | 0.000E+00 | 0.000E+00 | 0.000E+00 | 4.702E-07 | 1.149E-07 | 0.000E+00 | 0.000E+00 | 0.000E+00 | 0.000E+00 | 0.000E+00 | 0.000E+00 | 0.000E+00 |
| Unigene217211 | baeS | 0.000E+00 | 0.000E+00 | 0.000E+00 | 5.719E-07 | 0.000E+00 | 0.000E+00 | 0.000E+00 | 0.000E+00 | 0.000E+00 | 0.000E+00 | 0.000E+00 | 0.000E+00 |
| Unigene217240 | bacA | 0.000E+00 | 0.000E+00 | 0.000E+00 | 4.346E-07 | 0.000E+00 | 0.000E+00 | 0.000E+00 | 0.000E+00 | 0.000E+00 | 0.000E+00 | 0.000E+00 | 0.000E+00 |
| Unigene217263 | mef(B) | 1.736E-07 | 8.707E-07 | 3.138E-06 | 2.829E-06 | 0.000E+00 | 0.000E+00 | 0.000E+00 | 0.000E+00 | 0.000E+00 | 0.000E+00 | 0.000E+00 | 0.000E+00 |
| Unigene217271 | golS | 0.000E+00 | 7.133E-07 | 2.693E-06 | 5.178E-06 | 0.000E+00 | 0.000E+00 | 0.000E+00 | 0.000E+00 | 0.000E+00 | 0.000E+00 | 0.000E+00 | 0.000E+00 |
| Unigene217294 | MexD | 0.000E+00 | 0.000E+00 | 0.000E+00 | 0.000E+00 | 0.000E+00 | 0.000E+00 | 0.000E+00 | 0.000E+00 | 0.000E+00 | 0.000E+00 | 7.202E-07 | 0.000E+00 |
| Unigene217308 | efrB | 0.000E+00 | 0.000E+00 | 0.000E+00 | 3.969E-07 | 0.000E+00 | 0.000E+00 | 0.000E+00 | 0.000E+00 | 1.611E-07 | 0.000E+00 | 0.000E+00 | 0.000E+00 |
| Unigene217332 | tetB(46) | 0.000E+00 | 8.453E-08 | 5.182E-07 | 6.422E-05 | 0.000E+00 | 2.379E-08 | 0.000E+00 | 0.000E+00 | 0.000E+00 | 0.000E+00 | 0.000E+00 | 0.000E+00 |
| Unigene217682 | rpoB2 | 0.000E+00 | 0.000E+00 | 0.000E+00 | 1.772E-07 | 0.000E+00 | 0.000E+00 | 0.000E+00 | 0.000E+00 | 0.000E+00 | 0.000E+00 | 0.000E+00 | 0.000E+00 |
| Unigene217691 | patB | 0.000E+00 | 2.082E-07 | 0.000E+00 | 7.836E-07 | 0.000E+00 | 0.000E+00 | 0.000E+00 | 0.000E+00 | 0.000E+00 | 7.107E-07 | 0.000E+00 | 1.223E-07 |
| Unigene217715 | novA | 0.000E+00 | 0.000E+00 | 0.000E+00 | 4.527E-07 | 0.000E+00 | 0.000E+00 | 0.000E+00 | 0.000E+00 | 9.800E-08 | 0.000E+00 | 0.000E+00 | 0.000E+00 |
| Unigene217935 | Staphylocc | 0.000E+00 | 0.000E+00 | 2.400E-07 | 3.704E-06 | 0.000E+00 | 3.525E-08 | 0.000E+00 | 0.000E+00 | 0.000E+00 | 0.000E+00 | 0.000E+00 | 0.000E+00 |
| Unigene217998 | tetA(58) | 0.000E+00 | 2.221E-07 | 0.000E+00 | 8.856E-07 | 0.000E+00 | 0.000E+00 | 0.000E+00 | 0.000E+00 | 0.000E+00 | 1.165E-06 | 0.000E+00 | 0.000E+00 |
| Unigene218026 | Acinetoba | 4.277E-07 | 0.000E+00 | 0.000E+00 | 0.000E+00 | 0.000E+00 | 8.507E-07 | 2.632E-06 | 3.055E-06 | 0.000E+00 | 0.000E+00 | 1.174E-06 | 2.426E-06 |
| Unigene218146 | evgA | 0.000E+00 | 2.203E-07 | 1.970E-07 | 6.892E-07 | 0.000E+00 | 0.000E+00 | 0.000E+00 | 0.000E+00 | 0.000E+00 | 0.000E+00 | 0.000E+00 | 9.492E-08 |
| Unigene218243 | TolC | 0.000E+00 | 4.955E-07 | 0.000E+00 | 5.319E-07 | 0.000E+00 | 0.000E+00 | 0.000E+00 | 0.000E+00 | 0.000E+00 | 0.000E+00 | 0.000E+00 | 0.000E+00 |
| Unigene218272 | tetA(60) | 0.000E+00 | 0.000E+00 | 0.000E+00 | 3.835E-07 | 0.000E+00 | 0.000E+00 | 0.000E+00 | 0.000E+00 | 0.000E+00 | 0.000E+00 | 0.000E+00 | 0.000E+00 |
| Unigene218283 | arlS | 0.000E+00 | 8.287E-07 | 0.000E+00 | 0.000E+00 | 0.000E+00 | 0.000E+00 | 0.000E+00 | 0.000E+00 | 0.000E+00 | 0.000E+00 | 0.000E+00 | 0.000E+00 |
| Unigene218324 | macB | 0.000E+00 | 0.000E+00 | 0.000E+00 | 0.000E+00 | 0.000E+00 | 0.000E+00 | 0.000E+00 | 5.887E-07 | 0.000E+00 | 0.000E+00 | 0.000E+00 | 0.000E+00 |
| Unigene218332 | Streptomy | 0.000E+00 | 0.000E+00 | 0.000E+00 | 5.020E-07 | 0.000E+00 | 0.000E+00 | 0.000E+00 | 0.000E+00 | 0.000E+00 | 0.000E+00 | 0.000E+00 | 0.000E+00 |
| Unigene218420 | mgrA | 0.000E+00 | 0.000E+00 | 0.000E+00 | 4.876E-07 | 3.372E-06 | 7.978E-06 | 0.000E+00 | 6.521E-06 | 4.222E-06 | 0.000E+00 | 0.000E+00 | 0.000E+00 |
| Unigene218447 | poxtA | 0.000E+00 | 0.000E+00 | 0.000E+00 | 7.342E-07 | 0.000E+00 | 0.000E+00 | 0.000E+00 | 0.000E+00 | 0.000E+00 | 0.000E+00 | 0.000E+00 | 0.000E+00 |
| Unigene218524 | patA | 0.000E+00 | 0.000E+00 | 0.000E+00 | 2.914E-07 | 0.000E+00 | 0.000E+00 | 0.000E+00 | 0.000E+00 | 0.000E+00 | 0.000E+00 | 0.000E+00 | 0.000E+00 |
| Unigene218558 | oleC | 0.000E+00 | 0.000E+00 | 0.000E+00 | 9.361E-07 | 0.000E+00 | 0.000E+00 | 0.000E+00 | 0.000E+00 | 0.000E+00 | 0.000E+00 | 0.000E+00 | 0.000E+00 |
| Unigene218579 | cmx | 0.000E+00 | 0.000E+00 | 0.000E+00 | 3.463E-05 | 0.000E+00 | 0.000E+00 | 0.000E+00 | 0.000E+00 | 0.000E+00 | 0.000E+00 | 0.000E+00 | 0.000E+00 |
| Unigene218594 | bcrA | 0.000E+00 | 0.000E+00 | 3.446E-07 | 1.096E-06 | 0.000E+00 | 0.000E+00 | 0.000E+00 | 0.000E+00 | 0.000E+00 | 0.000E+00 | 0.000E+00 | 0.000E+00 |
| Unigene218628 | msbA | 3.283E-07 | 5.404E-07 | 1.110E-06 | 2.560E-06 | 6.889E-07 | 1.577E-07 | 4.239E-07 | 1.384E-06 | 3.987E-08 | 1.077E-06 | 1.106E-06 | 6.467E-07 |
| Unigene218820 | arlS | 0.000E+00 | 9.924E-08 | 3.771E-07 | 4.748E-06 | 0.000E+00 | 0.000E+00 | 0.000E+00 | 0.000E+00 | 0.000E+00 | 0.000E+00 | 0.000E+00 | 0.000E+00 |
| Unigene218821 | arlR | 0.000E+00 | 0.000E+00 | 0.000E+00 | 5.457E-06 | 0.000E+00 | 0.000E+00 | 0.000E+00 | 0.000E+00 | 0.000E+00 | 0.000E+00 | 0.000E+00 | 0.000E+00 |
| Unigene218837 | RlmA(II) | 0.000E+00 | 1.610E-07 | 0.000E+00 | 5.927E-07 | 0.000E+00 | 0.000E+00 | 0.000E+00 | 0.000E+00 | 0.000E+00 | 0.000E+00 | 0.000E+00 | 1.156E-07 |
| Unigene218853 | efpA | 0.000E+00 | 1.648E-07 | 0.000E+00 | 4.043E-07 | 0.000E+00 | 5.410E-08 | 0.000E+00 | 0.000E+00 | 0.000E+00 | 0.000E+00 | 0.000E+00 | 0.000E+00 |
| Unigene218965 | tetA(46) | 7.281E-07 | 6.593E-06 | 1.064E-05 | 1.638E-05 | 0.000E+00 | 0.000E+00 | 0.000E+00 | 0.000E+00 | 0.000E+00 | 0.000E+00 | 0.000E+00 | 2.391E-08 |
| Unigene218995 | Staphylocc | 0.000E+00 | 0.000E+00 | 0.000E+00 | 5.583E-07 | 0.000E+00 | 0.000E+00 | 0.000E+00 | 0.000E+00 | 0.000E+00 | 0.000E+00 | 0.000E+00 | 0.000E+00 |
| Unigene219095 | tlrC | 0.000E+00 | 0.000E+00 | 3.051E-07 | 1.005E-06 | 0.000E+00 | 0.000E+00 | 0.000E+00 | 0.000E+00 | 0.000E+00 | 0.000E+00 | 0.000E+00 | 0.000E+00 |
| Unigene219188 | sul4 | 8.030E-08 | 8.813E-08 | 2.758E-07 | 1.176E-06 | 0.000E+00 | 0.000E+00 | 4.444E-08 | 0.000E+00 | 0.000E+00 | 0.000E+00 | 0.000E+00 | 0.000E+00 |
| Unigene219250 | Chlamydia | 0.000E+00 | 0.000E+00 | 0.000E+00 | 5.315E-07 | 0.000E+00 | 0.000E+00 | 0.000E+00 | 0.000E+00 | 0.000E+00 | 0.000E+00 | 0.000E+00 | 0.000E+00 |
| Unigene219310 | efpA | 1.471E-07 | 2.152E-07 | 2.165E-07 | 6.435E-07 | 5.446E-08 | 5.299E-08 | 0.000E+00 | 0.000E+00 | 2.679E-08 | 0.000E+00 | 1.121E-07 | 0.000E+00 |
| Unigene219341 | AcrS | 0.000E+00 | 1.534E-07 | 4.114E-07 | 7.526E-07 | 0.000E+00 | 0.000E+00 | 0.000E+00 | 0.000E+00 | 1.018E-07 | 0.000E+00 | 0.000E+00 | 0.000E+00 |
| Unigene219407 | cpxA | 0.000E+00 | 0.000E+00 | 2.136E-07 | 2.826E-07 | 0.000E+00 | 0.000E+00 | 4.474E-07 | 0.000E+00 | 0.000E+00 | 0.000E+00 | 0.000E+00 | 0.000E+00 |
| Unigene219446 | bcrA | 0.000E+00 | 0.000E+00 | 0.000E+00 | 4.695E-07 | 0.000E+00 | 0.000E+00 | 0.000E+00 | 0.000E+00 | 0.000E+00 | 0.000E+00 | 0.000E+00 | 0.000E+00 |
| Unigene219500 | vanHF | 1.997E-06 | 1.542E-06 | 2.540E-06 | 1.382E-06 | 0.000E+00 | 0.000E+00 | 0.000E+00 | 0.000E+00 | 0.000E+00 | 0.000E+00 | 0.000E+00 | 0.000E+00 |
| Unigene219671 | arlR | 0.000E+00 | 0.000E+00 | 1.641E-07 | 2.027E-06 | 0.000E+00 | 0.000E+00 | 0.000E+00 | 0.000E+00 | 0.000E+00 | 0.000E+00 | 0.000E+00 | 0.000E+00 |
| Unigene219726 | patA | 0.000E+00 | 0.000E+00 | 2.455E-07 | 0.000E+00 | 0.000E+00 | 0.000E+00 | 0.000E+00 | 0.000E+00 | 0.000E+00 | 0.000E+00 | 0.000E+00 | 0.000E+00 |
| Unigene219881 | efpA | 0.000E+00 | 1.646E-07 | 2.943E-07 | 6.815E-07 | 0.000E+00 | 0.000E+00 | 0.000E+00 | 0.000E+00 | 0.000E+00 | 0.000E+00 | 0.000E+00 | 0.000E+00 |
| Unigene219911 | lmrC | 0.000E+00 | 0.000E+00 | 0.000E+00 | 2.173E-07 | 0.000E+00 | 0.000E+00 | 0.000E+00 | 0.000E+00 | 0.000E+00 | 0.000E+00 | 0.000E+00 | 0.000E+00 |
| Unigene219916 | catQ | 4.561E-06 | 1.079E-05 | 1.012E-05 | 1.386E-05 | 0.000E+00 | 0.000E+00 | 0.000E+00 | 0.000E+00 | 0.000E+00 | 0.000E+00 | 0.000E+00 | 0.000E+00 |
| Unigene220006 | arnA | 0.000E+00 | 0.000E+00 | 0.000E+00 | 4.873E-07 | 0.000E+00 | 0.000E+00 | 0.000E+00 | 0.000E+00 | 0.000E+00 | 0.000E+00 | 0.000E+00 | 0.000E+00 |
| Unigene220050 | vanTC | 2.087E-06 | 1.507E-05 | 6.907E-06 | 4.747E-06 | 0.000E+00 | 5.436E-08 | 0.000E+00 | 0.000E+00 | 0.000E+00 | 0.000E+00 | 8.627E-08 | 0.000E+00 |
| Unigene220056 | tetA(46) | 1.196E-06 | 0.000E+00 | 0.000E+00 | 4.996E-07 | 0.000E+00 | 9.894E-06 | 0.000E+00 | 1.039E-05 | 4.100E-06 | 0.000E+00 | 0.000E+00 | 0.000E+00 |
| Unigene220158 | tetA(58) | 0.000E+00 | 0.000E+00 | 2.155E-07 | 2.218E-07 | 0.000E+00 | 0.000E+00 | 0.000E+00 | 0.000E+00 | 0.000E+00 | 0.000E+00 | 0.000E+00 | 0.000E+00 |
| Unigene220245 | sul4 | 0.000E+00 | 0.000E+00 | 0.000E+00 | 5.787E-07 | 0.000E+00 | 0.000E+00 | 0.000E+00 | 0.000E+00 | 0.000E+00 | 0.000E+00 | 0.000E+00 | 0.000E+00 |
| Unigene220278 | Streptomy | 0.000E+00 | 0.000E+00 | 0.000E+00 | 2.415E-07 | 0.000E+00 | 0.000E+00 | 0.000E+00 | 0.000E+00 | 0.000E+00 | 0.000E+00 | 0.000E+00 | 0.000E+00 |
| Unigene220360 | sul3 | 1.726E-07 | 1.263E-07 | 0.000E+00 | 3.486E-07 | 0.000E+00 | 0.000E+00 | 4.458E-07 | 5.373E-07 | 0.000E+00 | 0.000E+00 | 3.291E-07 | 0.000E+00 |

| | | | | | | | | | | | | | |
|---|---|---|---|---|---|---|---|---|---|---|---|---|---|
| Unigene220367 | efrA | 0.000E+00 | 0.000E+00 | 0.000E+00 | 5.133E-07 | 0.000E+00 | 0.000E+00 | 9.377E-08 | 0.000E+00 | 0.000E+00 | 0.000E+00 | 4.846E-08 | 0.000E+00 |
| Unigene220388 | novA | 0.000E+00 | 0.000E+00 | 0.000E+00 | 5.433E-07 | 0.000E+00 | 0.000E+00 | 0.000E+00 | 0.000E+00 | 0.000E+00 | 0.000E+00 | 5.128E-07 | 0.000E+00 |
| Unigene220466 | efrA | 3.216E-07 | 3.530E-07 | 8.607E-07 | 1.890E-06 | 5.522E-07 | 3.161E-07 | 0.000E+00 | 1.468E-06 | 9.587E-08 | 6.514E-07 | 8.696E-07 | 6.567E-07 |
| Unigene220513 | mtrA | 4.638E-06 | 8.348E-06 | 1.220E-05 | 1.433E-05 | 0.000E+00 | 0.000E+00 | 0.000E+00 | 0.000E+00 | 0.000E+00 | 0.000E+00 | 0.000E+00 | 0.000E+00 |
| Unigene220575 | basS | 6.769E-06 | 0.000E+00 | 0.000E+00 | 1.967E-06 | 3.065E-05 | 7.628E-06 | 2.346E-05 | 2.609E-05 | 0.000E+00 | 0.000E+00 | 0.000E+00 | 0.000E+00 |
| Unigene220685 | vanRI | 4.761E-06 | 7.368E-06 | 8.830E-06 | 1.005E-05 | 5.818E-07 | 0.000E+00 | 7.377E-07 | 0.000E+00 | 0.000E+00 | 0.000E+00 | 0.000E+00 | 3.939E-07 |
| Unigene220729 | tetT | 0.000E+00 | 1.687E-08 | 9.955E-07 | 6.616E-05 | 0.000E+00 | 3.323E-08 | 0.000E+00 | 2.153E-07 | 0.000E+00 | 0.000E+00 | 0.000E+00 | 0.000E+00 |
| Unigene220822 | basS | 0.000E+00 | 0.000E+00 | 0.000E+00 | 5.995E-07 | 0.000E+00 | 0.000E+00 | 1.643E-07 | 0.000E+00 | 0.000E+00 | 0.000E+00 | 0.000E+00 | 0.000E+00 |
| Unigene220881 | tetA(58) | 0.000E+00 | 0.000E+00 | 0.000E+00 | 0.000E+00 | 0.000E+00 | 0.000E+00 | 0.000E+00 | 4.141E-07 | 0.000E+00 | 0.000E+00 | 0.000E+00 | 0.000E+00 |
| Unigene220957 | patB | 0.000E+00 | 0.000E+00 | 0.000E+00 | 7.172E-07 | 0.000E+00 | 0.000E+00 | 0.000E+00 | 0.000E+00 | 5.822E-07 | 0.000E+00 | 0.000E+00 | 0.000E+00 |
| Unigene220970 | oleC | 0.000E+00 | 0.000E+00 | 0.000E+00 | 9.194E-07 | 0.000E+00 | 0.000E+00 | 4.397E-06 | 4.444E-06 | 0.000E+00 | 0.000E+00 | 0.000E+00 | 0.000E+00 |
| Unigene221062 | tet(38) | 0.000E+00 | 0.000E+00 | 5.346E-07 | 5.043E-06 | 0.000E+00 | 0.000E+00 | 0.000E+00 | 0.000E+00 | 0.000E+00 | 0.000E+00 | 0.000E+00 | 0.000E+00 |
| Unigene221080 | tet37 | 0.000E+00 | 0.000E+00 | 0.000E+00 | 1.545E-06 | 0.000E+00 | 0.000E+00 | 0.000E+00 | 0.000E+00 | 0.000E+00 | 0.000E+00 | 0.000E+00 | 0.000E+00 |
| Unigene221096 | msbA | 0.000E+00 | 0.000E+00 | 3.641E-08 | 1.948E-06 | 0.000E+00 | 0.000E+00 | 0.000E+00 | 0.000E+00 | 0.000E+00 | 0.000E+00 | 0.000E+00 | 0.000E+00 |
| Unigene221097 | novA | 0.000E+00 | 0.000E+00 | 7.219E-08 | 1.876E-06 | 0.000E+00 | 0.000E+00 | 0.000E+00 | 0.000E+00 | 0.000E+00 | 0.000E+00 | 0.000E+00 | 0.000E+00 |
| Unigene221128 | smeR | 0.000E+00 | 0.000E+00 | 0.000E+00 | 1.698E-06 | 0.000E+00 | 0.000E+00 | 0.000E+00 | 0.000E+00 | 0.000E+00 | 0.000E+00 | 0.000E+00 | 0.000E+00 |
| Unigene221130 | macB | 0.000E+00 | 0.000E+00 | 0.000E+00 | 1.868E-06 | 0.000E+00 | 0.000E+00 | 0.000E+00 | 0.000E+00 | 0.000E+00 | 0.000E+00 | 0.000E+00 | 0.000E+00 |
| Unigene221141 | macB | 0.000E+00 | 0.000E+00 | 0.000E+00 | 2.278E-06 | 0.000E+00 | 0.000E+00 | 0.000E+00 | 0.000E+00 | 0.000E+00 | 0.000E+00 | 0.000E+00 | 0.000E+00 |
| Unigene221144 | vanTG | 0.000E+00 | 0.000E+00 | 5.442E-08 | 2.156E-06 | 0.000E+00 | 0.000E+00 | 0.000E+00 | 0.000E+00 | 0.000E+00 | 0.000E+00 | 0.000E+00 | 0.000E+00 |
| Unigene221150 | smeR | 3.165E-07 | 3.039E-07 | 0.000E+00 | 7.990E-07 | 4.395E-08 | 0.000E+00 | 0.000E+00 | 0.000E+00 | 0.000E+00 | 0.000E+00 | 4.525E-08 | 0.000E+00 |
| Unigene221212 | lmrB | 0.000E+00 | 0.000E+00 | 0.000E+00 | 5.544E-07 | 0.000E+00 | 0.000E+00 | 0.000E+00 | 0.000E+00 | 0.000E+00 | 0.000E+00 | 0.000E+00 | 0.000E+00 |
| Unigene221323 | macB | 9.119E-08 | 0.000E+00 | 0.000E+00 | 9.208E-07 | 0.000E+00 | 0.000E+00 | 0.000E+00 | 0.000E+00 | 0.000E+00 | 1.084E-06 | 0.000E+00 | 4.311E-07 |
| Unigene221341 | vanSF | 0.000E+00 | 0.000E+00 | 0.000E+00 | 1.226E-06 | 0.000E+00 | 0.000E+00 | 0.000E+00 | 0.000E+00 | 0.000E+00 | 0.000E+00 | 0.000E+00 | 0.000E+00 |
| Unigene221342 | vanRF | 0.000E+00 | 0.000E+00 | 0.000E+00 | 8.504E-07 | 0.000E+00 | 0.000E+00 | 0.000E+00 | 0.000E+00 | 0.000E+00 | 0.000E+00 | 0.000E+00 | 0.000E+00 |
| Unigene221376 | vanHA | 9.752E-08 | 7.136E-08 | 0.000E+00 | 8.535E-07 | 0.000E+00 | 0.000E+00 | 0.000E+00 | 0.000E+00 | 0.000E+00 | 9.777E-07 | 0.000E+00 | 1.921E-07 |
| Unigene221404 | Acinetobac | 0.000E+00 | 0.000E+00 | 0.000E+00 | 1.012E-06 | 0.000E+00 | 0.000E+00 | 0.000E+00 | 0.000E+00 | 0.000E+00 | 1.090E-06 | 0.000E+00 | 0.000E+00 |
| Unigene221412 | efrA | 0.000E+00 | 0.000E+00 | 8.948E-08 | 6.446E-07 | 0.000E+00 | 0.000E+00 | 2.018E-07 | 8.160E-07 | 0.000E+00 | 0.000E+00 | 0.000E+00 | 7.186E-08 |
| Unigene221416 | poxtA | 0.000E+00 | 0.000E+00 | 0.000E+00 | 0.000E+00 | 0.000E+00 | 0.000E+00 | 0.000E+00 | 0.000E+00 | 0.000E+00 | 5.162E-07 | 0.000E+00 | 0.000E+00 |
| Unigene221445 | golS | 1.182E-07 | 2.595E-07 | 3.867E-07 | 3.980E-07 | 0.000E+00 | 0.000E+00 | 0.000E+00 | 0.000E+00 | 0.000E+00 | 0.000E+00 | 0.000E+00 | 0.000E+00 |
| Unigene221501 | patB | 0.000E+00 | 0.000E+00 | 0.000E+00 | 7.761E-07 | 0.000E+00 | 0.000E+00 | 0.000E+00 | 0.000E+00 | 0.000E+00 | 0.000E+00 | 0.000E+00 | 0.000E+00 |
| Unigene221573 | vanSA | 0.000E+00 | 0.000E+00 | 0.000E+00 | 6.104E-07 | 0.000E+00 | 0.000E+00 | 0.000E+00 | 0.000E+00 | 0.000E+00 | 0.000E+00 | 6.915E-08 | 0.000E+00 |
| Unigene221650 | mepA | 0.000E+00 | 0.000E+00 | 0.000E+00 | 6.737E-06 | 0.000E+00 | 0.000E+00 | 0.000E+00 | 0.000E+00 | 0.000E+00 | 0.000E+00 | 0.000E+00 | 0.000E+00 |
| Unigene221728 | efrA | 0.000E+00 | 0.000E+00 | 1.660E-07 | 3.998E-06 | 0.000E+00 | 0.000E+00 | 0.000E+00 | 0.000E+00 | 0.000E+00 | 0.000E+00 | 0.000E+00 | 0.000E+00 |
| Unigene221759 | basS | 0.000E+00 | 3.623E-08 | 3.563E-07 | 6.666E-07 | 3.667E-08 | 7.136E-08 | 0.000E+00 | 0.000E+00 | 0.000E+00 | 0.000E+00 | 3.776E-08 | 2.341E-07 |
| Unigene221833 | PmrF | 0.000E+00 | 4.150E-07 | 1.147E-06 | 1.875E-06 | 0.000E+00 | 0.000E+00 | 0.000E+00 | 0.000E+00 | 0.000E+00 | 0.000E+00 | 0.000E+00 | 0.000E+00 |
| Unigene221844 | Bifidobacte | 0.000E+00 | 0.000E+00 | 0.000E+00 | 6.350E-07 | 0.000E+00 | 0.000E+00 | 0.000E+00 | 0.000E+00 | 0.000E+00 | 0.000E+00 | 0.000E+00 | 0.000E+00 |
| Unigene222020 | vanSO | 1.830E-07 | 2.410E-07 | 2.155E-07 | 3.326E-07 | 2.033E-08 | 0.000E+00 | 0.000E+00 | 0.000E+00 | 0.000E+00 | 0.000E+00 | 4.187E-08 | 0.000E+00 |
| Unigene222102 | tetA(58) | 0.000E+00 | 4.880E-07 | 5.235E-07 | 7.633E-07 | 4.940E-08 | 0.000E+00 | 0.000E+00 | 0.000E+00 | 0.000E+00 | 0.000E+00 | 0.000E+00 | 1.577E-07 |
| Unigene222177 | baeS | 2.532E-07 | 8.336E-08 | 3.975E-07 | 5.880E-07 | 0.000E+00 | 0.000E+00 | 0.000E+00 | 0.000E+00 | 0.000E+00 | 0.000E+00 | 0.000E+00 | 0.000E+00 |
| Unigene222179 | mtrA | 0.000E+00 | 0.000E+00 | 0.000E+00 | 5.433E-07 | 0.000E+00 | 0.000E+00 | 0.000E+00 | 0.000E+00 | 0.000E+00 | 0.000E+00 | 0.000E+00 | 0.000E+00 |
| Unigene222301 | bcrA | 1.494E-07 | 0.000E+00 | 5.866E-07 | 8.552E-07 | 0.000E+00 | 0.000E+00 | 0.000E+00 | 0.000E+00 | 0.000E+00 | 0.000E+00 | 0.000E+00 | 2.355E-07 |
| Unigene222353 | tetA(58) | 7.787E-07 | 5.167E-06 | 8.717E-06 | 1.244E-05 | 0.000E+00 | 1.531E-07 | 0.000E+00 | 0.000E+00 | 0.000E+00 | 0.000E+00 | 0.000E+00 | 0.000E+00 |
| Unigene222393 | tetA(46) | 0.000E+00 | 0.000E+00 | 0.000E+00 | 4.041E-07 | 0.000E+00 | 0.000E+00 | 0.000E+00 | 0.000E+00 | 0.000E+00 | 0.000E+00 | 0.000E+00 | 3.153E-07 |
| Unigene222417 | YojI | 0.000E+00 | 0.000E+00 | 4.766E-07 | 1.245E-06 | 0.000E+00 | 0.000E+00 | 0.000E+00 | 0.000E+00 | 0.000E+00 | 0.000E+00 | 0.000E+00 | 0.000E+00 |
| Unigene222439 | tetA(60) | 0.000E+00 | 0.000E+00 | 0.000E+00 | 5.940E-07 | 0.000E+00 | 0.000E+00 | 0.000E+00 | 0.000E+00 | 0.000E+00 | 0.000E+00 | 0.000E+00 | 0.000E+00 |
| Unigene222518 | arlS | 0.000E+00 | 0.000E+00 | 0.000E+00 | 5.108E-07 | 0.000E+00 | 0.000E+00 | 0.000E+00 | 0.000E+00 | 0.000E+00 | 0.000E+00 | 0.000E+00 | 0.000E+00 |
| Unigene222709 | Staphylocc | 0.000E+00 | 1.203E-07 | 0.000E+00 | 7.303E-07 | 0.000E+00 | 0.000E+00 | 0.000E+00 | 0.000E+00 | 0.000E+00 | 0.000E+00 | 0.000E+00 | 0.000E+00 |
| Unigene222739 | pmrA | 0.000E+00 | 0.000E+00 | 0.000E+00 | 4.229E-07 | 0.000E+00 | 0.000E+00 | 0.000E+00 | 0.000E+00 | 0.000E+00 | 0.000E+00 | 0.000E+00 | 0.000E+00 |
| Unigene222817 | novA | 1.073E-07 | 8.835E-08 | 1.764E-06 | 6.503E-06 | 0.000E+00 | 0.000E+00 | 0.000E+00 | 0.000E+00 | 0.000E+00 | 0.000E+00 | 0.000E+00 | 0.000E+00 |
| Unigene222886 | optrA | 0.000E+00 | 0.000E+00 | 2.448E-07 | 5.173E-06 | 0.000E+00 | 1.798E-08 | 0.000E+00 | 0.000E+00 | 0.000E+00 | 0.000E+00 | 0.000E+00 | 0.000E+00 |
| Unigene222905 | Agrobacte | 2.773E-07 | 0.000E+00 | 0.000E+00 | 1.008E-06 | 0.000E+00 | 0.000E+00 | 0.000E+00 | 0.000E+00 | 6.668E-07 | 0.000E+00 | 0.000E+00 | 0.000E+00 |
| Unigene222989 | efpA | 8.557E-08 | 1.174E-07 | 1.889E-07 | 9.073E-07 | 0.000E+00 | 6.938E-08 | 4.735E-08 | 4.994E-08 | 4.676E-08 | 0.000E+00 | 0.000E+00 | 2.023E-07 |
| Unigene223037 | msbA | 0.000E+00 | 0.000E+00 | 0.000E+00 | 6.478E-07 | 6.479E-08 | 0.000E+00 | 6.455E-08 | 0.000E+00 | 0.000E+00 | 0.000E+00 | 0.000E+00 | 0.000E+00 |
| Unigene223087 | baeS | 0.000E+00 | 0.000E+00 | 0.000E+00 | 1.241E-06 | 0.000E+00 | 0.000E+00 | 0.000E+00 | 0.000E+00 | 3.604E-07 | 0.000E+00 | 0.000E+00 | 0.000E+00 |
| Unigene223172 | poxtA | 0.000E+00 | 0.000E+00 | 0.000E+00 | 4.882E-05 | 0.000E+00 | 0.000E+00 | 0.000E+00 | 0.000E+00 | 0.000E+00 | 0.000E+00 | 0.000E+00 | 0.000E+00 |
| Unigene223175 | apmA | 0.000E+00 | 0.000E+00 | 0.000E+00 | 4.663E-05 | 0.000E+00 | 0.000E+00 | 0.000E+00 | 0.000E+00 | 0.000E+00 | 0.000E+00 | 0.000E+00 | 0.000E+00 |
| Unigene223187 | marA | 0.000E+00 | 0.000E+00 | 2.575E-07 | 4.960E-06 | 0.000E+00 | 0.000E+00 | 0.000E+00 | 0.000E+00 | 0.000E+00 | 0.000E+00 | 0.000E+00 | 0.000E+00 |
| Unigene223188 | vanSG | 0.000E+00 | 0.000E+00 | 3.748E-07 | 3.986E-06 | 0.000E+00 | 0.000E+00 | 0.000E+00 | 0.000E+00 | 0.000E+00 | 0.000E+00 | 0.000E+00 | 0.000E+00 |

| | | | | | | | | | | | | | |
|---|---|---|---|---|---|---|---|---|---|---|---|---|---|
| Unigene223189 | smeR | 0.000E+00 | 0.000E+00 | 2.346E-07 | 4.105E-06 | 0.000E+00 | 0.000E+00 | 0.000E+00 | 0.000E+00 | 0.000E+00 | 0.000E+00 | 0.000E+00 | 0.000E+00 |
| Unigene223277 | lmrC | 0.000E+00 | 0.000E+00 | 0.000E+00 | 3.881E-07 | 0.000E+00 | 0.000E+00 | 0.000E+00 | 0.000E+00 | 0.000E+00 | 0.000E+00 | 0.000E+00 | 0.000E+00 |
| Unigene223296 | mdtG | 0.000E+00 | 0.000E+00 | 0.000E+00 | 5.340E-06 | 0.000E+00 | 0.000E+00 | 0.000E+00 | 0.000E+00 | 0.000E+00 | 0.000E+00 | 0.000E+00 | 0.000E+00 |
| Unigene223298 | lsaA | 0.000E+00 | 9.299E-08 | 4.157E-07 | 4.748E-06 | 0.000E+00 | 2.290E-08 | 0.000E+00 | 0.000E+00 | 0.000E+00 | 0.000E+00 | 0.000E+00 | 0.000E+00 |
| Unigene223300 | golS | 0.000E+00 | 0.000E+00 | 4.270E-07 | 4.754E-06 | 0.000E+00 | 0.000E+00 | 0.000E+00 | 0.000E+00 | 0.000E+00 | 0.000E+00 | 0.000E+00 | 0.000E+00 |
| Unigene223433 | efrA | 0.000E+00 | 0.000E+00 | 0.000E+00 | 5.673E-07 | 1.440E-07 | 0.000E+00 | 1.435E-07 | 0.000E+00 | 0.000E+00 | 0.000E+00 | 0.000E+00 | 0.000E+00 |
| Unigene223532 | bcrA | 0.000E+00 | 0.000E+00 | 0.000E+00 | 1.510E-05 | 0.000E+00 | 0.000E+00 | 0.000E+00 | 0.000E+00 | 0.000E+00 | 0.000E+00 | 0.000E+00 | 0.000E+00 |
| Unigene223533 | bcrA | 0.000E+00 | 0.000E+00 | 0.000E+00 | 1.553E-05 | 0.000E+00 | 0.000E+00 | 0.000E+00 | 0.000E+00 | 0.000E+00 | 0.000E+00 | 0.000E+00 | 0.000E+00 |
| Unigene223575 | bacA | 2.205E-07 | 1.452E-07 | 3.029E-07 | 8.906E-07 | 0.000E+00 | 1.430E-07 | 0.000E+00 | 1.029E-07 | 0.000E+00 | 0.000E+00 | 1.513E-07 | 0.000E+00 |
| Unigene223612 | oleB | 0.000E+00 | 0.000E+00 | 0.000E+00 | 3.921E-07 | 0.000E+00 | 5.996E-08 | 0.000E+00 | 0.000E+00 | 0.000E+00 | 0.000E+00 | 0.000E+00 | 0.000E+00 |
| Unigene223613 | tet(38) | 0.000E+00 | 0.000E+00 | 0.000E+00 | 6.874E-07 | 0.000E+00 | 0.000E+00 | 0.000E+00 | 0.000E+00 | 0.000E+00 | 0.000E+00 | 0.000E+00 | 0.000E+00 |
| Unigene223614 | macB | 0.000E+00 | 0.000E+00 | 0.000E+00 | 7.217E-07 | 0.000E+00 | 0.000E+00 | 0.000E+00 | 0.000E+00 | 0.000E+00 | 0.000E+00 | 0.000E+00 | 0.000E+00 |
| Unigene223756 | vanHB | 0.000E+00 | 0.000E+00 | 0.000E+00 | 1.945E-06 | 0.000E+00 | 0.000E+00 | 0.000E+00 | 0.000E+00 | 0.000E+00 | 0.000E+00 | 0.000E+00 | 0.000E+00 |
| Unigene223757 | Acinetobac | 0.000E+00 | 0.000E+00 | 0.000E+00 | 1.737E-06 | 0.000E+00 | 0.000E+00 | 0.000E+00 | 0.000E+00 | 0.000E+00 | 0.000E+00 | 0.000E+00 | 0.000E+00 |
| Unigene223793 | cfr(B) | 0.000E+00 | 0.000E+00 | 3.017E-08 | 1.925E-06 | 0.000E+00 | 0.000E+00 | 0.000E+00 | 0.000E+00 | 0.000E+00 | 0.000E+00 | 0.000E+00 | 0.000E+00 |
| Unigene223796 | arnA | 0.000E+00 | 0.000E+00 | 3.395E-08 | 1.887E-06 | 0.000E+00 | 0.000E+00 | 0.000E+00 | 0.000E+00 | 0.000E+00 | 0.000E+00 | 0.000E+00 | 0.000E+00 |
| Unigene223811 | patB | 0.000E+00 | 0.000E+00 | 8.447E-08 | 2.347E-06 | 0.000E+00 | 0.000E+00 | 0.000E+00 | 0.000E+00 | 0.000E+00 | 0.000E+00 | 0.000E+00 | 0.000E+00 |
| Unigene223921 | bacA | 0.000E+00 | 2.019E-07 | 3.158E-07 | 5.572E-07 | 5.109E-08 | 0.000E+00 | 0.000E+00 | 0.000E+00 | 0.000E+00 | 0.000E+00 | 0.000E+00 | 1.631E-07 |
| Unigene223931 | baeS | 0.000E+00 | 0.000E+00 | 0.000E+00 | 5.377E-06 | 0.000E+00 | 0.000E+00 | 0.000E+00 | 0.000E+00 | 0.000E+00 | 0.000E+00 | 0.000E+00 | 0.000E+00 |
| Unigene223933 | vanZF | 0.000E+00 | 5.733E-08 | 3.075E-07 | 5.697E-06 | 0.000E+00 | 0.000E+00 | 0.000E+00 | 0.000E+00 | 0.000E+00 | 0.000E+00 | 0.000E+00 | 0.000E+00 |
| Unigene223981 | baeS | 2.280E-07 | 0.000E+00 | 3.579E-07 | 7.827E-07 | 0.000E+00 | 0.000E+00 | 0.000E+00 | 0.000E+00 | 0.000E+00 | 0.000E+00 | 0.000E+00 | 1.617E-07 |
| Unigene224008 | mdtG | 0.000E+00 | 0.000E+00 | 0.000E+00 | 1.100E-06 | 0.000E+00 | 0.000E+00 | 0.000E+00 | 0.000E+00 | 0.000E+00 | 0.000E+00 | 0.000E+00 | 0.000E+00 |
| Unigene224059 | bcrA | 3.414E-06 | 9.803E-06 | 9.678E-06 | 1.202E-05 | 0.000E+00 | 0.000E+00 | 0.000E+00 | 0.000E+00 | 0.000E+00 | 3.073E-07 | 1.183E-07 | 0.000E+00 |
| Unigene224224 | Corynebac | 0.000E+00 | 0.000E+00 | 2.037E-07 | 6.289E-07 | 0.000E+00 | 0.000E+00 | 0.000E+00 | 0.000E+00 | 0.000E+00 | 0.000E+00 | 0.000E+00 | 0.000E+00 |
| Unigene224236 | novA | 0.000E+00 | 4.168E-07 | 0.000E+00 | 5.113E-07 | 7.032E-07 | 8.993E-07 | 3.443E-06 | 2.807E-06 | 2.965E-07 | 1.108E-06 | 1.179E-06 | 2.116E-06 |
| Unigene224275 | cipA | 0.000E+00 | 1.316E-07 | 0.000E+00 | 6.356E-07 | 0.000E+00 | 0.000E+00 | 0.000E+00 | 0.000E+00 | 0.000E+00 | 0.000E+00 | 0.000E+00 | 4.251E-07 |
| Unigene224365 | tetA(58) | 0.000E+00 | 0.000E+00 | 0.000E+00 | 6.493E-07 | 9.525E-08 | 4.634E-08 | 1.898E-07 | 0.000E+00 | 0.000E+00 | 0.000E+00 | 4.904E-08 | 0.000E+00 |
| Unigene224419 | tetA(60) | 3.175E-06 | 4.240E-06 | 7.204E-06 | 8.108E-06 | 8.988E-08 | 0.000E+00 | 0.000E+00 | 0.000E+00 | 0.000E+00 | 0.000E+00 | 0.000E+00 | 0.000E+00 |
| Unigene224421 | poxtA | 0.000E+00 | 0.000E+00 | 0.000E+00 | 8.106E-07 | 0.000E+00 | 2.345E-07 | 0.000E+00 | 2.279E-07 | 2.371E-07 | 0.000E+00 | 0.000E+00 | 0.000E+00 |
| Unigene224458 | tap | 0.000E+00 | 0.000E+00 | 0.000E+00 | 7.342E-07 | 2.692E-08 | 0.000E+00 | 0.000E+00 | 1.697E-07 | 0.000E+00 | 0.000E+00 | 0.000E+00 | 0.000E+00 |
| Unigene224501 | patA | 0.000E+00 | 2.660E-07 | 0.000E+00 | 6.852E-07 | 0.000E+00 | 0.000E+00 | 0.000E+00 | 0.000E+00 | 0.000E+00 | 0.000E+00 | 0.000E+00 | 0.000E+00 |
| Unigene224554 | dfrE | 0.000E+00 | 0.000E+00 | 0.000E+00 | 8.099E-07 | 0.000E+00 | 0.000E+00 | 0.000E+00 | 0.000E+00 | 0.000E+00 | 0.000E+00 | 0.000E+00 | 0.000E+00 |
| Unigene224559 | vatH | 0.000E+00 | 5.463E-07 | 7.515E-07 | 8.894E-07 | 0.000E+00 | 0.000E+00 | 0.000E+00 | 0.000E+00 | 0.000E+00 | 0.000E+00 | 0.000E+00 | 0.000E+00 |
| Unigene224686 | carA | 0.000E+00 | 0.000E+00 | 0.000E+00 | 5.908E-07 | 0.000E+00 | 0.000E+00 | 0.000E+00 | 0.000E+00 | 0.000E+00 | 0.000E+00 | 0.000E+00 | 0.000E+00 |
| Unigene224713 | facT | 0.000E+00 | 1.429E-07 | 3.619E-07 | 7.667E-07 | 0.000E+00 | 0.000E+00 | 0.000E+00 | 0.000E+00 | 0.000E+00 | 0.000E+00 | 0.000E+00 | 0.000E+00 |
| Unigene224718 | bcrA | 0.000E+00 | 0.000E+00 | 0.000E+00 | 3.219E-07 | 0.000E+00 | 0.000E+00 | 0.000E+00 | 0.000E+00 | 0.000E+00 | 0.000E+00 | 0.000E+00 | 0.000E+00 |
| Unigene224732 | bcrA | 2.046E-07 | 0.000E+00 | 0.000E+00 | 0.000E+00 | 5.454E-07 | 0.000E+00 | 1.404E-06 | 1.576E-06 | 0.000E+00 | 0.000E+00 | 9.828E-07 | 1.814E-06 |
| Unigene224780 | tetA(58) | 0.000E+00 | 5.175E-07 | 2.103E-07 | 1.126E-06 | 0.000E+00 | 0.000E+00 | 0.000E+00 | 0.000E+00 | 0.000E+00 | 0.000E+00 | 0.000E+00 | 0.000E+00 |
| Unigene224789 | oleC | 0.000E+00 | 0.000E+00 | 0.000E+00 | 9.692E-07 | 0.000E+00 | 0.000E+00 | 0.000E+00 | 0.000E+00 | 0.000E+00 | 0.000E+00 | 0.000E+00 | 0.000E+00 |
| Unigene224794 | tetB(60) | 0.000E+00 | 0.000E+00 | 0.000E+00 | 4.142E-07 | 0.000E+00 | 0.000E+00 | 0.000E+00 | 0.000E+00 | 0.000E+00 | 0.000E+00 | 0.000E+00 | 0.000E+00 |
| Unigene224829 | Staphylocc | 0.000E+00 | 0.000E+00 | 1.963E-07 | 5.657E-06 | 0.000E+00 | 0.000E+00 | 0.000E+00 | 0.000E+00 | 0.000E+00 | 0.000E+00 | 0.000E+00 | 0.000E+00 |
| Unigene224832 | Corynebac | 0.000E+00 | 0.000E+00 | 2.919E-07 | 4.907E-06 | 0.000E+00 | 0.000E+00 | 0.000E+00 | 0.000E+00 | 0.000E+00 | 0.000E+00 | 0.000E+00 | 0.000E+00 |
| Unigene224844 | vanSO | 0.000E+00 | 0.000E+00 | 0.000E+00 | 4.134E-07 | 0.000E+00 | 0.000E+00 | 0.000E+00 | 0.000E+00 | 0.000E+00 | 0.000E+00 | 0.000E+00 | 0.000E+00 |
| Unigene224887 | bacA | 0.000E+00 | 0.000E+00 | 0.000E+00 | 8.232E-07 | 0.000E+00 | 0.000E+00 | 0.000E+00 | 0.000E+00 | 0.000E+00 | 0.000E+00 | 0.000E+00 | 0.000E+00 |
| Unigene225225 | vanHO | 1.639E-07 | 0.000E+00 | 2.144E-07 | 4.412E-07 | 0.000E+00 | 0.000E+00 | 1.209E-07 | 0.000E+00 | 0.000E+00 | 0.000E+00 | 1.874E-07 | 0.000E+00 |
| Unigene225252 | bcrA | 0.000E+00 | 0.000E+00 | 0.000E+00 | 5.488E-07 | 0.000E+00 | 3.447E-06 | 7.618E-06 | 0.000E+00 | 3.524E-06 | 2.422E-06 | 5.926E-06 | 0.000E+00 |
| Unigene225282 | tva(A) | 0.000E+00 | 0.000E+00 | 0.000E+00 | 7.719E-07 | 0.000E+00 | 0.000E+00 | 0.000E+00 | 0.000E+00 | 0.000E+00 | 0.000E+00 | 0.000E+00 | 0.000E+00 |
| Unigene225298 | smeR | 1.237E-07 | 0.000E+00 | 0.000E+00 | 6.869E-07 | 0.000E+00 | 0.000E+00 | 0.000E+00 | 0.000E+00 | 0.000E+00 | 0.000E+00 | 0.000E+00 | 0.000E+00 |
| Unigene225370 | TaeA | 1.218E-07 | 2.674E-07 | 3.187E-07 | 6.355E-07 | 0.000E+00 | 1.097E-07 | 0.000E+00 | 0.000E+00 | 0.000E+00 | 6.785E-08 | 4.645E-08 | 1.200E-07 |
| Unigene225414 | bcrA | 0.000E+00 | 0.000E+00 | 0.000E+00 | 1.937E-06 | 0.000E+00 | 0.000E+00 | 0.000E+00 | 0.000E+00 | 0.000E+00 | 0.000E+00 | 0.000E+00 | 0.000E+00 |
| Unigene225421 | vanHM | 0.000E+00 | 0.000E+00 | 6.085E-08 | 1.910E-06 | 0.000E+00 | 0.000E+00 | 0.000E+00 | 0.000E+00 | 0.000E+00 | 0.000E+00 | 0.000E+00 | 0.000E+00 |
| Unigene225432 | vanHM | 0.000E+00 | 0.000E+00 | 0.000E+00 | 1.708E-06 | 0.000E+00 | 0.000E+00 | 0.000E+00 | 0.000E+00 | 0.000E+00 | 0.000E+00 | 0.000E+00 | 0.000E+00 |
| Unigene225443 | catV | 0.000E+00 | 0.000E+00 | 4.821E-08 | 2.133E-06 | 0.000E+00 | 0.000E+00 | 0.000E+00 | 0.000E+00 | 0.000E+00 | 0.000E+00 | 0.000E+00 | 0.000E+00 |
| Unigene225444 | macB | 0.000E+00 | 0.000E+00 | 0.000E+00 | 1.327E-06 | 0.000E+00 | 0.000E+00 | 0.000E+00 | 0.000E+00 | 0.000E+00 | 0.000E+00 | 0.000E+00 | 0.000E+00 |
| Unigene225446 | macB | 0.000E+00 | 0.000E+00 | 4.945E-08 | 2.138E-06 | 0.000E+00 | 0.000E+00 | 0.000E+00 | 0.000E+00 | 0.000E+00 | 0.000E+00 | 0.000E+00 | 0.000E+00 |
| Unigene225455 | bcrA | 0.000E+00 | 0.000E+00 | 0.000E+00 | 1.864E-06 | 0.000E+00 | 0.000E+00 | 0.000E+00 | 0.000E+00 | 0.000E+00 | 0.000E+00 | 0.000E+00 | 0.000E+00 |
| Unigene225457 | vanRF | 0.000E+00 | 0.000E+00 | 0.000E+00 | 1.417E-06 | 0.000E+00 | 0.000E+00 | 0.000E+00 | 0.000E+00 | 0.000E+00 | 0.000E+00 | 0.000E+00 | 0.000E+00 |
| Unigene225458 | vanSF | 0.000E+00 | 0.000E+00 | 2.204E-08 | 1.701E-06 | 0.000E+00 | 0.000E+00 | 0.000E+00 | 0.000E+00 | 0.000E+00 | 0.000E+00 | 0.000E+00 | 0.000E+00 |

| | | | | | | | | | | | | | |
|---|---|---|---|---|---|---|---|---|---|---|---|---|---|
| Unigene225466 | ramA | 0.000E+00 | 0.000E+00 | 3.666E-08 | 1.698E-06 | 0.000E+00 | 0.000E+00 | 0.000E+00 | 0.000E+00 | 0.000E+00 | 0.000E+00 | 0.000E+00 | 0.000E+00 |
| Unigene225472 | bcr-1 | 0.000E+00 | 0.000E+00 | 1.402E-07 | 1.803E-06 | 0.000E+00 | 0.000E+00 | 0.000E+00 | 0.000E+00 | 0.000E+00 | 0.000E+00 | 0.000E+00 | 0.000E+00 |
| Unigene225476 | vanHO | 0.000E+00 | 0.000E+00 | 6.360E-08 | 1.833E-06 | 0.000E+00 | 0.000E+00 | 0.000E+00 | 0.000E+00 | 0.000E+00 | 0.000E+00 | 0.000E+00 | 0.000E+00 |
| Unigene225477 | oleD | 0.000E+00 | 0.000E+00 | 3.199E-08 | 9.549E-07 | 0.000E+00 | 0.000E+00 | 0.000E+00 | 0.000E+00 | 0.000E+00 | 0.000E+00 | 0.000E+00 | 0.000E+00 |
| Unigene225481 | TaeA | 0.000E+00 | 0.000E+00 | 1.609E-08 | 1.673E-06 | 0.000E+00 | 0.000E+00 | 0.000E+00 | 0.000E+00 | 0.000E+00 | 0.000E+00 | 0.000E+00 | 0.000E+00 |
| Unigene225489 | tetA(58) | 0.000E+00 | 0.000E+00 | 0.000E+00 | 1.832E-06 | 0.000E+00 | 0.000E+00 | 0.000E+00 | 0.000E+00 | 0.000E+00 | 0.000E+00 | 0.000E+00 | 0.000E+00 |
| Unigene225495 | tetB(P) | 0.000E+00 | 0.000E+00 | 4.873E-08 | 1.789E-06 | 0.000E+00 | 0.000E+00 | 0.000E+00 | 0.000E+00 | 0.000E+00 | 0.000E+00 | 0.000E+00 | 0.000E+00 |
| Unigene225496 | dfrG | 0.000E+00 | 0.000E+00 | 1.955E-07 | 1.543E-06 | 0.000E+00 | 0.000E+00 | 0.000E+00 | 0.000E+00 | 0.000E+00 | 0.000E+00 | 0.000E+00 | 0.000E+00 |
| Unigene225501 | baeS | 0.000E+00 | 0.000E+00 | 2.375E-08 | 1.589E-06 | 0.000E+00 | 0.000E+00 | 0.000E+00 | 0.000E+00 | 0.000E+00 | 0.000E+00 | 0.000E+00 | 0.000E+00 |
| Unigene225502 | kdpE | 0.000E+00 | 0.000E+00 | 0.000E+00 | 1.586E-06 | 0.000E+00 | 0.000E+00 | 0.000E+00 | 0.000E+00 | 0.000E+00 | 0.000E+00 | 0.000E+00 | 0.000E+00 |
| Unigene225619 | poxtA | 0.000E+00 | 0.000E+00 | 0.000E+00 | 3.574E-07 | 0.000E+00 | 0.000E+00 | 0.000E+00 | 0.000E+00 | 0.000E+00 | 0.000E+00 | 0.000E+00 | 0.000E+00 |
| Unigene225657 | tetA(58) | 0.000E+00 | 0.000E+00 | 0.000E+00 | 5.326E-06 | 0.000E+00 | 0.000E+00 | 0.000E+00 | 0.000E+00 | 0.000E+00 | 0.000E+00 | 0.000E+00 | 0.000E+00 |
| Unigene225694 | efrA | 0.000E+00 | 0.000E+00 | 0.000E+00 | 4.527E-07 | 0.000E+00 | 0.000E+00 | 0.000E+00 | 0.000E+00 | 0.000E+00 | 7.492E-07 | 0.000E+00 | 3.180E-07 |
| Unigene225697 | patB | 1.695E-07 | 0.000E+00 | 0.000E+00 | 1.241E-06 | 0.000E+00 | 0.000E+00 | 0.000E+00 | 0.000E+00 | 0.000E+00 | 1.133E-06 | 0.000E+00 | 3.505E-07 |
| Unigene225766 | patB | 0.000E+00 | 0.000E+00 | 0.000E+00 | 3.958E-06 | 0.000E+00 | 0.000E+00 | 0.000E+00 | 0.000E+00 | 0.000E+00 | 0.000E+00 | 0.000E+00 | 0.000E+00 |
| Unigene225767 | carA | 0.000E+00 | 0.000E+00 | 1.498E-07 | 4.123E-06 | 0.000E+00 | 0.000E+00 | 0.000E+00 | 0.000E+00 | 0.000E+00 | 0.000E+00 | 0.000E+00 | 0.000E+00 |
| Unigene225778 | emrY | 0.000E+00 | 1.271E-06 | 2.005E-06 | 1.490E-06 | 0.000E+00 | 0.000E+00 | 0.000E+00 | 0.000E+00 | 0.000E+00 | 0.000E+00 | 5.193E-08 | 0.000E+00 |
| Unigene225837 | tetB(P) | 0.000E+00 | 0.000E+00 | 0.000E+00 | 5.682E-05 | 0.000E+00 | 3.552E-08 | 0.000E+00 | 0.000E+00 | 0.000E+00 | 0.000E+00 | 0.000E+00 | 0.000E+00 |
| Unigene225965 | Staphylocc | 0.000E+00 | 0.000E+00 | 0.000E+00 | 7.478E-07 | 0.000E+00 | 0.000E+00 | 0.000E+00 | 0.000E+00 | 3.035E-07 | 0.000E+00 | 0.000E+00 | 0.000E+00 |
| Unigene226054 | tva(A) | 0.000E+00 | 0.000E+00 | 0.000E+00 | 7.652E-07 | 0.000E+00 | 0.000E+00 | 4.990E-06 | 0.000E+00 | 0.000E+00 | 0.000E+00 | 0.000E+00 | 3.628E-06 |
| Unigene226117 | arlS | 0.000E+00 | 0.000E+00 | 1.527E-07 | 4.692E-06 | 0.000E+00 | 0.000E+00 | 0.000E+00 | 0.000E+00 | 0.000E+00 | 0.000E+00 | 0.000E+00 | 0.000E+00 |
| Unigene226129 | vanG | 0.000E+00 | 0.000E+00 | 0.000E+00 | 1.019E-06 | 0.000E+00 | 0.000E+00 | 0.000E+00 | 0.000E+00 | 3.675E-07 | 0.000E+00 | 0.000E+00 | 0.000E+00 |
| Unigene226385 | tet(43) | 0.000E+00 | 0.000E+00 | 0.000E+00 | 5.025E-07 | 0.000E+00 | 0.000E+00 | 0.000E+00 | 0.000E+00 | 0.000E+00 | 0.000E+00 | 0.000E+00 | 0.000E+00 |
| Unigene226395 | gimA | 1.478E-06 | 2.494E-06 | 4.460E-06 | 4.977E-06 | 0.000E+00 | 0.000E+00 | 0.000E+00 | 0.000E+00 | 0.000E+00 | 0.000E+00 | 0.000E+00 | 0.000E+00 |
| Unigene226476 | patB | 0.000E+00 | 0.000E+00 | 2.640E-07 | 5.976E-07 | 0.000E+00 | 0.000E+00 | 0.000E+00 | 0.000E+00 | 0.000E+00 | 0.000E+00 | 0.000E+00 | 0.000E+00 |
| Unigene226504 | lmrB | 1.620E-07 | 0.000E+00 | 0.000E+00 | 8.880E-07 | 0.000E+00 | 5.003E-08 | 0.000E+00 | 1.621E-07 | 4.047E-07 | 0.000E+00 | 0.000E+00 | 0.000E+00 |
| Unigene226552 | vanRI | 0.000E+00 | 0.000E+00 | 0.000E+00 | 4.673E-07 | 0.000E+00 | 0.000E+00 | 0.000E+00 | 0.000E+00 | 0.000E+00 | 0.000E+00 | 0.000E+00 | 0.000E+00 |
| Unigene226603 | optrA | 0.000E+00 | 5.025E-08 | 4.493E-08 | 5.086E-07 | 0.000E+00 | 0.000E+00 | 0.000E+00 | 0.000E+00 | 0.000E+00 | 0.000E+00 | 5.238E-08 | 0.000E+00 |
| Unigene226660 | lmrB | 0.000E+00 | 0.000E+00 | 0.000E+00 | 4.643E-07 | 0.000E+00 | 0.000E+00 | 0.000E+00 | 0.000E+00 | 0.000E+00 | 0.000E+00 | 0.000E+00 | 0.000E+00 |
| Unigene226920 | lmrD | 0.000E+00 | 0.000E+00 | 1.343E-06 | 5.862E-06 | 0.000E+00 | 0.000E+00 | 0.000E+00 | 0.000E+00 | 0.000E+00 | 0.000E+00 | 0.000E+00 | 0.000E+00 |
| Unigene226966 | vanG | 0.000E+00 | 0.000E+00 | 0.000E+00 | 4.920E-07 | 4.511E-08 | 0.000E+00 | 0.000E+00 | 0.000E+00 | 0.000E+00 | 0.000E+00 | 0.000E+00 | 0.000E+00 |
| Unigene226967 | vanRF | 0.000E+00 | 0.000E+00 | 0.000E+00 | 4.179E-07 | 0.000E+00 | 0.000E+00 | 7.125E-07 | 2.040E-06 | 0.000E+00 | 0.000E+00 | 8.416E-07 | 0.000E+00 |
| Unigene226981 | Acinetoba | 0.000E+00 | 1.669E-07 | 0.000E+00 | 5.759E-07 | 0.000E+00 | 0.000E+00 | 0.000E+00 | 0.000E+00 | 0.000E+00 | 0.000E+00 | 0.000E+00 | 0.000E+00 |
| Unigene226985 | OprA | 8.374E-08 | 0.000E+00 | 8.216E-08 | 4.651E-07 | 0.000E+00 | 0.000E+00 | 0.000E+00 | 2.444E-07 | 0.000E+00 | 0.000E+00 | 0.000E+00 | 0.000E+00 |
| Unigene226995 | mdtC | 1.237E-07 | 0.000E+00 | 0.000E+00 | 4.996E-07 | 0.000E+00 | 0.000E+00 | 0.000E+00 | 0.000E+00 | 0.000E+00 | 0.000E+00 | 0.000E+00 | 0.000E+00 |
| Unigene227041 | MexF | 0.000E+00 | 0.000E+00 | 0.000E+00 | 4.254E-07 | 5.850E-08 | 0.000E+00 | 0.000E+00 | 4.917E-07 | 0.000E+00 | 0.000E+00 | 0.000E+00 | 8.299E-08 |
| Unigene227065 | bmr | 0.000E+00 | 4.144E-08 | 0.000E+00 | 3.431E-07 | 0.000E+00 | 0.000E+00 | 0.000E+00 | 0.000E+00 | 0.000E+00 | 0.000E+00 | 0.000E+00 | 0.000E+00 |
| Unigene227101 | emrB | 0.000E+00 | 0.000E+00 | 0.000E+00 | 3.159E-07 | 0.000E+00 | 0.000E+00 | 4.154E-07 | 0.000E+00 | 0.000E+00 | 0.000E+00 | 0.000E+00 | 0.000E+00 |
| Unigene227106 | bcrA | 0.000E+00 | 0.000E+00 | 0.000E+00 | 1.393E-07 | 4.598E-07 | 0.000E+00 | 0.000E+00 | 0.000E+00 | 0.000E+00 | 0.000E+00 | 2.104E-07 | 0.000E+00 |
| Unigene227131 | Pseudomo | 0.000E+00 | 0.000E+00 | 5.797E-07 | 6.392E-07 | 8.438E-07 | 7.299E-07 | 1.775E-06 | 7.881E-07 | 2.767E-07 | 4.231E-07 | 1.207E-06 | 4.838E-06 |
| Unigene227209 | efrA | 0.000E+00 | 0.000E+00 | 0.000E+00 | 3.992E-07 | 0.000E+00 | 0.000E+00 | 2.916E-07 | 0.000E+00 | 0.000E+00 | 0.000E+00 | 3.517E-07 | 0.000E+00 |
| Unigene227294 | Staphylocc | 7.826E-08 | 0.000E+00 | 0.000E+00 | 1.087E-06 | 8.694E-07 | 1.480E-07 | 0.000E+00 | 2.969E-07 | 5.132E-07 | 5.448E-07 | 0.000E+00 | 0.000E+00 |
| Unigene227360 | APH(6)-Ic | 0.000E+00 | 0.000E+00 | 0.000E+00 | 4.289E-07 | 0.000E+00 | 0.000E+00 | 0.000E+00 | 0.000E+00 | 0.000E+00 | 0.000E+00 | 0.000E+00 | 0.000E+00 |
| Unigene227550 | bcrA | 0.000E+00 | 4.880E-08 | 1.309E-07 | 1.616E-06 | 0.000E+00 | 0.000E+00 | 0.000E+00 | 0.000E+00 | 0.000E+00 | 0.000E+00 | 0.000E+00 | 0.000E+00 |
| Unigene227567 | lsaC | 0.000E+00 | 0.000E+00 | 0.000E+00 | 1.827E-06 | 0.000E+00 | 0.000E+00 | 0.000E+00 | 0.000E+00 | 0.000E+00 | 0.000E+00 | 0.000E+00 | 0.000E+00 |
| Unigene227627 | farB | 0.000E+00 | 2.157E-07 | 0.000E+00 | 5.706E-07 | 0.000E+00 | 0.000E+00 | 0.000E+00 | 0.000E+00 | 0.000E+00 | 1.122E-06 | 0.000E+00 | 4.356E-07 |
| Unigene227666 | ErmD | 0.000E+00 | 0.000E+00 | 5.983E-07 | 7.280E-05 | 0.000E+00 | 0.000E+00 | 0.000E+00 | 0.000E+00 | 0.000E+00 | 0.000E+00 | 0.000E+00 | 0.000E+00 |
| Unigene227671 | Agrobacte | 0.000E+00 | 0.000E+00 | 0.000E+00 | 8.218E-07 | 0.000E+00 | 0.000E+00 | 0.000E+00 | 0.000E+00 | 0.000E+00 | 0.000E+00 | 0.000E+00 | 0.000E+00 |
| Unigene227733 | abcA | 0.000E+00 | 0.000E+00 | 1.037E-06 | 5.490E-06 | 0.000E+00 | 0.000E+00 | 0.000E+00 | 0.000E+00 | 0.000E+00 | 0.000E+00 | 0.000E+00 | 0.000E+00 |
| Unigene227755 | macB | 0.000E+00 | 0.000E+00 | 5.468E-07 | 1.602E-06 | 0.000E+00 | 0.000E+00 | 0.000E+00 | 0.000E+00 | 0.000E+00 | 0.000E+00 | 0.000E+00 | 0.000E+00 |
| Unigene227757 | tetA(58) | 0.000E+00 | 0.000E+00 | 3.812E-07 | 1.334E-06 | 0.000E+00 | 0.000E+00 | 0.000E+00 | 0.000E+00 | 0.000E+00 | 0.000E+00 | 0.000E+00 | 0.000E+00 |
| Unigene227804 | vanC | 1.289E-07 | 1.768E-07 | 2.213E-07 | 7.482E-07 | 0.000E+00 | 0.000E+00 | 0.000E+00 | 0.000E+00 | 7.042E-08 | 0.000E+00 | 3.685E-08 | 7.616E-08 |
| Unigene227873 | arlR | 0.000E+00 | 0.000E+00 | 0.000E+00 | 3.396E-07 | 0.000E+00 | 0.000E+00 | 0.000E+00 | 0.000E+00 | 0.000E+00 | 0.000E+00 | 0.000E+00 | 0.000E+00 |
| Unigene228087 | ugd | 0.000E+00 | 0.000E+00 | 0.000E+00 | 2.797E-05 | 0.000E+00 | 0.000E+00 | 0.000E+00 | 0.000E+00 | 0.000E+00 | 0.000E+00 | 0.000E+00 | 0.000E+00 |
| Unigene228151 | oleB | 0.000E+00 | 0.000E+00 | 0.000E+00 | 5.583E-07 | 0.000E+00 | 0.000E+00 | 2.354E-07 | 0.000E+00 | 0.000E+00 | 0.000E+00 | 0.000E+00 | 0.000E+00 |
| Unigene228179 | mgrA | 1.454E-07 | 0.000E+00 | 0.000E+00 | 1.248E-06 | 1.131E-06 | 1.179E-06 | 0.000E+00 | 0.000E+00 | 0.000E+00 | 0.000E+00 | 0.000E+00 | 0.000E+00 |
| Unigene228191 | Staphylocc | 0.000E+00 | 1.471E-07 | 1.879E-07 | 3.789E-06 | 0.000E+00 | 0.000E+00 | 0.000E+00 | 0.000E+00 | 0.000E+00 | 0.000E+00 | 0.000E+00 | 0.000E+00 |
| Unigene228295 | macB | 0.000E+00 | 0.000E+00 | 0.000E+00 | 5.719E-07 | 0.000E+00 | 0.000E+00 | 0.000E+00 | 0.000E+00 | 0.000E+00 | 0.000E+00 | 0.000E+00 | 0.000E+00 |

| | | | | | | | | | | | | | |
|---|---|---|---|---|---|---|---|---|---|---|---|---|---|
| Unigene228296 | tetB(60) | 1.464E-07 | 0.000E+00 | 0.000E+00 | 1.072E-06 | 0.000E+00 | 0.000E+00 | 0.000E+00 | 0.000E+00 | 0.000E+00 | 0.000E+00 | 0.000E+00 | 2.163E-07 |
| Unigene228335 | fexA | 0.000E+00 | 0.000E+00 | 3.213E-07 | 1.653E-06 | 0.000E+00 | 0.000E+00 | 0.000E+00 | 0.000E+00 | 0.000E+00 | 0.000E+00 | 0.000E+00 | 0.000E+00 |
| Unigene228420 | vanHO | 0.000E+00 | 0.000E+00 | 0.000E+00 | 5.673E-07 | 0.000E+00 | 0.000E+00 | 0.000E+00 | 0.000E+00 | 0.000E+00 | 0.000E+00 | 0.000E+00 | 0.000E+00 |
| Unigene228535 | basS | 7.222E-08 | 5.944E-08 | 1.772E-07 | 6.563E-07 | 0.000E+00 | 0.000E+00 | 0.000E+00 | 0.000E+00 | 7.893E-08 | 0.000E+00 | 0.000E+00 | 2.134E-08 |
| Unigene228536 | mtrA | 0.000E+00 | 1.521E-07 | 2.719E-07 | 4.663E-07 | 0.000E+00 | 0.000E+00 | 0.000E+00 | 0.000E+00 | 0.000E+00 | 0.000E+00 | 1.057E-07 | 1.638E-07 |
| Unigene228704 | evgS | 5.148E-08 | 0.000E+00 | 0.000E+00 | 3.639E-07 | 0.000E+00 | 0.000E+00 | 0.000E+00 | 0.000E+00 | 0.000E+00 | 0.000E+00 | 0.000E+00 | 3.651E-07 |
| Unigene228711 | macB | 0.000E+00 | 0.000E+00 | 3.219E-08 | 5.300E-07 | 0.000E+00 | 0.000E+00 | 0.000E+00 | 0.000E+00 | 0.000E+00 | 0.000E+00 | 0.000E+00 | 0.000E+00 |
| Unigene228733 | tetA(46) | 0.000E+00 | 9.627E-08 | 0.000E+00 | 1.211E-06 | 0.000E+00 | 0.000E+00 | 0.000E+00 | 0.000E+00 | 0.000E+00 | 5.863E-07 | 0.000E+00 | 0.000E+00 |
| Unigene228799 | norB | 0.000E+00 | 0.000E+00 | 0.000E+00 | 3.796E-07 | 0.000E+00 | 0.000E+00 | 0.000E+00 | 1.097E-07 | 0.000E+00 | 0.000E+00 | 0.000E+00 | 0.000E+00 |
| Unigene228838 | macB | 4.913E-08 | 2.966E-07 | 1.446E-07 | 4.465E-07 | 0.000E+00 | 0.000E+00 | 0.000E+00 | 0.000E+00 | 0.000E+00 | 0.000E+00 | 0.000E+00 | 0.000E+00 |
| Unigene228898 | oleB | 0.000E+00 | 0.000E+00 | 0.000E+00 | 4.808E-07 | 0.000E+00 | 0.000E+00 | 4.215E-07 | 0.000E+00 | 0.000E+00 | 0.000E+00 | 0.000E+00 | 0.000E+00 |
| Unigene228903 | Acinetobac | 0.000E+00 | 0.000E+00 | 0.000E+00 | 3.042E-07 | 0.000E+00 | 0.000E+00 | 0.000E+00 | 1.005E-07 | 0.000E+00 | 0.000E+00 | 0.000E+00 | 0.000E+00 |
| Unigene228915 | Klebsiella p | 0.000E+00 | 1.059E-06 | 0.000E+00 | 5.995E-07 | 0.000E+00 | 0.000E+00 | 0.000E+00 | 0.000E+00 | 0.000E+00 | 0.000E+00 | 0.000E+00 | 0.000E+00 |
| Unigene228944 | evgA | 0.000E+00 | 2.880E-07 | 1.545E-07 | 4.240E-07 | 0.000E+00 | 0.000E+00 | 0.000E+00 | 4.289E-07 | 0.000E+00 | 0.000E+00 | 0.000E+00 | 0.000E+00 |
| Unigene229159 | salA | 0.000E+00 | 0.000E+00 | 0.000E+00 | 4.368E-07 | 0.000E+00 | 0.000E+00 | 0.000E+00 | 0.000E+00 | 0.000E+00 | 0.000E+00 | 0.000E+00 | 0.000E+00 |
| Unigene229331 | adeR | 0.000E+00 | 0.000E+00 | 0.000E+00 | 0.000E+00 | 5.101E-06 | 0.000E+00 | 0.000E+00 | 0.000E+00 | 0.000E+00 | 0.000E+00 | 0.000E+00 | 0.000E+00 |
| Unigene229506 | lmrD | 0.000E+00 | 8.173E-08 | 3.836E-07 | 4.850E-06 | 0.000E+00 | 0.000E+00 | 0.000E+00 | 0.000E+00 | 0.000E+00 | 0.000E+00 | 0.000E+00 | 0.000E+00 |
| Unigene229539 | novA | 3.941E-08 | 8.652E-08 | 1.992E-06 | 1.268E-05 | 0.000E+00 | 0.000E+00 | 0.000E+00 | 0.000E+00 | 0.000E+00 | 0.000E+00 | 0.000E+00 | 0.000E+00 |
| Unigene229746 | macB | 0.000E+00 | 0.000E+00 | 2.129E-07 | 5.696E-06 | 0.000E+00 | 0.000E+00 | 0.000E+00 | 0.000E+00 | 0.000E+00 | 0.000E+00 | 0.000E+00 | 0.000E+00 |
| Unigene229752 | efrB | 0.000E+00 | 0.000E+00 | 4.497E-07 | 5.179E-06 | 0.000E+00 | 0.000E+00 | 0.000E+00 | 0.000E+00 | 0.000E+00 | 0.000E+00 | 0.000E+00 | 0.000E+00 |
| Unigene229807 | macB | 4.203E-07 | 0.000E+00 | 0.000E+00 | 9.338E-07 | 1.401E-06 | 1.515E-07 | 0.000E+00 | 0.000E+00 | 5.513E-07 | 1.061E-06 | 0.000E+00 | 0.000E+00 |
| Unigene229814 | macB | 0.000E+00 | 0.000E+00 | 0.000E+00 | 7.814E-07 | 0.000E+00 | 0.000E+00 | 0.000E+00 | 0.000E+00 | 0.000E+00 | 0.000E+00 | 0.000E+00 | 0.000E+00 |
| Unigene229848 | rpoB2 | 0.000E+00 | 0.000E+00 | 2.962E-07 | 5.092E-06 | 0.000E+00 | 0.000E+00 | 3.930E-08 | 0.000E+00 | 0.000E+00 | 0.000E+00 | 0.000E+00 | 0.000E+00 |
| Unigene229938 | lmrD | 0.000E+00 | 0.000E+00 | 0.000E+00 | 1.119E-06 | 0.000E+00 | 0.000E+00 | 0.000E+00 | 0.000E+00 | 0.000E+00 | 1.097E-06 | 0.000E+00 | 1.638E-07 |
| Unigene229939 | patA | 0.000E+00 | 1.428E-07 | 0.000E+00 | 1.051E-06 | 0.000E+00 | 0.000E+00 | 0.000E+00 | 0.000E+00 | 0.000E+00 | 8.902E-07 | 0.000E+00 | 1.757E-07 |
| Unigene229949 | novA | 0.000E+00 | 2.617E-07 | 3.802E-07 | 3.612E-07 | 4.967E-07 | 0.000E+00 | 3.167E-06 | 7.306E-07 | 2.280E-07 | 6.641E-07 | 1.125E-06 | 1.762E-06 |
| Unigene229963 | oleC | 4.598E-07 | 8.075E-07 | 1.805E-06 | 1.022E-06 | 0.000E+00 | 0.000E+00 | 0.000E+00 | 0.000E+00 | 0.000E+00 | 0.000E+00 | 0.000E+00 | 0.000E+00 |
| Unigene229969 | ugd | 0.000E+00 | 0.000E+00 | 0.000E+00 | 6.439E-07 | 0.000E+00 | 0.000E+00 | 0.000E+00 | 0.000E+00 | 0.000E+00 | 0.000E+00 | 0.000E+00 | 0.000E+00 |
| Unigene229989 | bcrA | 3.587E-06 | 0.000E+00 | 0.000E+00 | 0.000E+00 | 0.000E+00 | 0.000E+00 | 0.000E+00 | 0.000E+00 | 0.000E+00 | 0.000E+00 | 0.000E+00 | 0.000E+00 |
| Unigene230046 | arlR | 1.701E-07 | 4.668E-08 | 1.461E-06 | 6.056E-06 | 0.000E+00 | 0.000E+00 | 0.000E+00 | 0.000E+00 | 0.000E+00 | 0.000E+00 | 0.000E+00 | 0.000E+00 |
| Unigene230115 | vanRE | 0.000E+00 | 0.000E+00 | 0.000E+00 | 6.459E-07 | 2.090E-07 | 0.000E+00 | 0.000E+00 | 0.000E+00 | 0.000E+00 | 0.000E+00 | 1.435E-07 | 0.000E+00 |
| Unigene230224 | vanTG | 0.000E+00 | 0.000E+00 | 0.000E+00 | 5.795E-07 | 0.000E+00 | 0.000E+00 | 0.000E+00 | 0.000E+00 | 0.000E+00 | 0.000E+00 | 0.000E+00 | 0.000E+00 |
| Unigene230282 | YojI | 0.000E+00 | 0.000E+00 | 0.000E+00 | 1.852E-07 | 0.000E+00 | 0.000E+00 | 0.000E+00 | 0.000E+00 | 0.000E+00 | 0.000E+00 | 0.000E+00 | 0.000E+00 |
| Unigene230342 | tetA(58) | 0.000E+00 | 0.000E+00 | 1.955E-07 | 9.512E-07 | 0.000E+00 | 0.000E+00 | 0.000E+00 | 0.000E+00 | 0.000E+00 | 0.000E+00 | 0.000E+00 | 0.000E+00 |
| Unigene230417 | cipA | 2.255E-07 | 2.475E-07 | 3.793E-07 | 7.157E-07 | 0.000E+00 | 1.393E-07 | 0.000E+00 | 0.000E+00 | 0.000E+00 | 0.000E+00 | 0.000E+00 | 0.000E+00 |
| Unigene230494 | Staphylocc | 0.000E+00 | 0.000E+00 | 0.000E+00 | 3.972E-06 | 0.000E+00 | 0.000E+00 | 0.000E+00 | 0.000E+00 | 0.000E+00 | 0.000E+00 | 0.000E+00 | 0.000E+00 |
| Unigene230531 | mgrA | 0.000E+00 | 0.000E+00 | 0.000E+00 | 7.102E-07 | 3.125E-07 | 0.000E+00 | 0.000E+00 | 0.000E+00 | 0.000E+00 | 0.000E+00 | 0.000E+00 | 0.000E+00 |
| Unigene230599 | msbA | 0.000E+00 | 0.000E+00 | 0.000E+00 | 6.107E-07 | 0.000E+00 | 0.000E+00 | 0.000E+00 | 0.000E+00 | 0.000E+00 | 0.000E+00 | 0.000E+00 | 0.000E+00 |
| Unigene230626 | adeS | 8.909E-06 | 0.000E+00 | 0.000E+00 | 3.476E-06 | 3.644E-05 | 1.729E-05 | 2.411E-05 | 2.947E-05 | 3.794E-06 | 0.000E+00 | 0.000E+00 | 0.000E+00 |
| Unigene230698 | bcrA | 0.000E+00 | 0.000E+00 | 3.771E-07 | 4.366E-07 | 0.000E+00 | 0.000E+00 | 0.000E+00 | 0.000E+00 | 0.000E+00 | 0.000E+00 | 0.000E+00 | 0.000E+00 |
| Unigene230820 | tet(38) | 3.930E-05 | 2.462E-05 | 1.844E-05 | 2.030E-05 | 1.550E-06 | 1.867E-06 | 3.210E-06 | 6.461E-07 | 0.000E+00 | 5.426E-07 | 1.266E-06 | 1.151E-06 |
| Unigene230891 | tet(30) | 6.367E-08 | 9.317E-08 | 3.332E-07 | 6.644E-07 | 0.000E+00 | 0.000E+00 | 0.000E+00 | 0.000E+00 | 4.639E-08 | 0.000E+00 | 0.000E+00 | 1.756E-07 |
| Unigene230893 | bcrA | 0.000E+00 | 0.000E+00 | 0.000E+00 | 6.392E-07 | 0.000E+00 | 0.000E+00 | 0.000E+00 | 0.000E+00 | 0.000E+00 | 0.000E+00 | 0.000E+00 | 0.000E+00 |
| Unigene231037 | mdtM | 0.000E+00 | 0.000E+00 | 8.908E-07 | 0.000E+00 | 5.193E-06 | 0.000E+00 | 1.005E-06 | 0.000E+00 | 0.000E+00 | 0.000E+00 | 0.000E+00 | 0.000E+00 |
| Unigene231056 | lsaA | 9.291E-07 | 1.020E-06 | 4.177E-06 | 5.891E-06 | 0.000E+00 | 0.000E+00 | 3.587E-07 | 0.000E+00 | 0.000E+00 | 0.000E+00 | 0.000E+00 | 2.554E-08 |
| Unigene231139 | bcrA | 4.067E-07 | 0.000E+00 | 3.628E-07 | 7.468E-07 | 0.000E+00 | 0.000E+00 | 0.000E+00 | 0.000E+00 | 0.000E+00 | 0.000E+00 | 0.000E+00 | 0.000E+00 |
| Unigene231299 | bcrA | 0.000E+00 | 0.000E+00 | 1.772E-07 | 4.047E-06 | 0.000E+00 | 3.903E-08 | 0.000E+00 | 0.000E+00 | 0.000E+00 | 0.000E+00 | 0.000E+00 | 0.000E+00 |
| Unigene231300 | MexL | 0.000E+00 | 0.000E+00 | 2.823E-07 | 6.043E-06 | 0.000E+00 | 0.000E+00 | 0.000E+00 | 0.000E+00 | 0.000E+00 | 0.000E+00 | 0.000E+00 | 0.000E+00 |
| Unigene231360 | Listeria mc | 0.000E+00 | 0.000E+00 | 0.000E+00 | 4.417E-07 | 0.000E+00 | 0.000E+00 | 0.000E+00 | 0.000E+00 | 0.000E+00 | 0.000E+00 | 0.000E+00 | 0.000E+00 |
| Unigene231424 | vanRF | 0.000E+00 | 0.000E+00 | 5.533E-07 | 5.219E-06 | 0.000E+00 | 0.000E+00 | 0.000E+00 | 0.000E+00 | 0.000E+00 | 0.000E+00 | 0.000E+00 | 0.000E+00 |
| Unigene231441 | tetB(60) | 0.000E+00 | 0.000E+00 | 0.000E+00 | 4.704E-07 | 0.000E+00 | 0.000E+00 | 1.547E-07 | 0.000E+00 | 5.091E-08 | 0.000E+00 | 0.000E+00 | 0.000E+00 |
| Unigene231490 | vanTG | 0.000E+00 | 0.000E+00 | 0.000E+00 | 9.958E-07 | 0.000E+00 | 0.000E+00 | 0.000E+00 | 0.000E+00 | 6.657E-07 | 1.583E-06 | 0.000E+00 | 0.000E+00 |
| Unigene231541 | msbA | 0.000E+00 | 2.094E-07 | 4.493E-07 | 4.277E-06 | 0.000E+00 | 0.000E+00 | 0.000E+00 | 0.000E+00 | 0.000E+00 | 0.000E+00 | 0.000E+00 | 0.000E+00 |
| Unigene231542 | Corynebac | 0.000E+00 | 1.575E-07 | 5.631E-07 | 4.093E-06 | 0.000E+00 | 0.000E+00 | 0.000E+00 | 0.000E+00 | 0.000E+00 | 0.000E+00 | 0.000E+00 | 0.000E+00 |
| Unigene231556 | patB | 0.000E+00 | 0.000E+00 | 0.000E+00 | 5.628E-07 | 0.000E+00 | 0.000E+00 | 0.000E+00 | 0.000E+00 | 0.000E+00 | 0.000E+00 | 0.000E+00 | 0.000E+00 |
| Unigene231581 | vanE | 0.000E+00 | 1.266E-07 | 0.000E+00 | 5.826E-07 | 0.000E+00 | 0.000E+00 | 0.000E+00 | 0.000E+00 | 0.000E+00 | 0.000E+00 | 0.000E+00 | 0.000E+00 |
| Unigene231693 | cmlv | 0.000E+00 | 0.000E+00 | 0.000E+00 | 5.777E-07 | 0.000E+00 | 5.890E-08 | 1.809E-07 | 0.000E+00 | 0.000E+00 | 0.000E+00 | 0.000E+00 | 0.000E+00 |
| Unigene231712 | rpoB2 | 0.000E+00 | 0.000E+00 | 6.180E-07 | 0.000E+00 | 0.000E+00 | 0.000E+00 | 0.000E+00 | 0.000E+00 | 0.000E+00 | 0.000E+00 | 0.000E+00 | 0.000E+00 |

| | | | | | | | | | | | | | |
|---|---|---|---|---|---|---|---|---|---|---|---|---|---|
| Unigene231855 | optrA | 0.000E+00 | 0.000E+00 | 0.000E+00 | 5.512E-07 | 0.000E+00 | 0.000E+00 | 0.000E+00 | 5.916E-07 | 0.000E+00 | 0.000E+00 | 0.000E+00 | 0.000E+00 |
| Unigene231943 | arlS | 0.000E+00 | 0.000E+00 | 2.673E-07 | 5.227E-06 | 0.000E+00 | 0.000E+00 | 0.000E+00 | 0.000E+00 | 0.000E+00 | 0.000E+00 | 0.000E+00 | 0.000E+00 |
| Unigene231955 | Corynebac | 0.000E+00 | 0.000E+00 | 0.000E+00 | 9.558E-07 | 0.000E+00 | 0.000E+00 | 0.000E+00 | 0.000E+00 | 0.000E+00 | 0.000E+00 | 0.000E+00 | 0.000E+00 |
| Unigene231984 | iri | 0.000E+00 | 2.045E-07 | 0.000E+00 | 2.352E-07 | 0.000E+00 | 0.000E+00 | 0.000E+00 | 0.000E+00 | 0.000E+00 | 0.000E+00 | 0.000E+00 | 0.000E+00 |
| Unigene232036 | otr(B) | 0.000E+00 | 0.000E+00 | 0.000E+00 | 6.104E-07 | 0.000E+00 | 0.000E+00 | 0.000E+00 | 0.000E+00 | 0.000E+00 | 0.000E+00 | 0.000E+00 | 9.051E-07 |
| Unigene232134 | bcrA | 1.699E-07 | 2.486E-07 | 0.000E+00 | 5.147E-07 | 0.000E+00 | 0.000E+00 | 0.000E+00 | 0.000E+00 | 0.000E+00 | 0.000E+00 | 0.000E+00 | 0.000E+00 |
| Unigene232144 | poxtA | 0.000E+00 | 7.381E-08 | 0.000E+00 | 4.075E-07 | 0.000E+00 | 0.000E+00 | 0.000E+00 | 0.000E+00 | 0.000E+00 | 0.000E+00 | 0.000E+00 | 0.000E+00 |
| Unigene232289 | emrB | 0.000E+00 | 0.000E+00 | 1.339E-07 | 4.525E-06 | 0.000E+00 | 2.459E-08 | 0.000E+00 | 0.000E+00 | 0.000E+00 | 0.000E+00 | 0.000E+00 | 0.000E+00 |
| Unigene232337 | poxtA | 0.000E+00 | 0.000E+00 | 1.312E-06 | 5.558E-06 | 0.000E+00 | 0.000E+00 | 0.000E+00 | 0.000E+00 | 0.000E+00 | 0.000E+00 | 0.000E+00 | 0.000E+00 |
| Unigene232380 | lmrC | 0.000E+00 | 1.669E-07 | 1.405E-06 | 1.327E-05 | 0.000E+00 | 0.000E+00 | 0.000E+00 | 0.000E+00 | 0.000E+00 | 0.000E+00 | 0.000E+00 | 0.000E+00 |
| Unigene232516 | PmrF | 6.581E-08 | 0.000E+00 | 0.000E+00 | 5.981E-07 | 0.000E+00 | 0.000E+00 | 5.827E-07 | 1.536E-07 | 5.035E-07 | 9.164E-07 | 0.000E+00 | 0.000E+00 |
| Unigene232540 | vatB | 0.000E+00 | 0.000E+00 | 0.000E+00 | 7.442E-07 | 0.000E+00 | 0.000E+00 | 0.000E+00 | 0.000E+00 | 0.000E+00 | 0.000E+00 | 0.000E+00 | 0.000E+00 |
| Unigene232659 | patB | 0.000E+00 | 0.000E+00 | 0.000E+00 | 5.970E-07 | 0.000E+00 | 0.000E+00 | 0.000E+00 | 0.000E+00 | 0.000E+00 | 0.000E+00 | 0.000E+00 | 0.000E+00 |
| Unigene232714 | Rhodococ | 0.000E+00 | 0.000E+00 | 8.080E-08 | 9.424E-07 | 0.000E+00 | 0.000E+00 | 3.038E-08 | 0.000E+00 | 0.000E+00 | 0.000E+00 | 0.000E+00 | 0.000E+00 |
| Unigene232725 | tetB(P) | 0.000E+00 | 0.000E+00 | 0.000E+00 | 4.126E-07 | 0.000E+00 | 0.000E+00 | 0.000E+00 | 0.000E+00 | 0.000E+00 | 0.000E+00 | 0.000E+00 | 0.000E+00 |
| Unigene232831 | macB | 1.380E-07 | 0.000E+00 | 0.000E+00 | 1.114E-06 | 0.000E+00 | 1.491E-07 | 0.000E+00 | 0.000E+00 | 4.523E-07 | 0.000E+00 | 0.000E+00 | 0.000E+00 |
| Unigene232835 | poxtA | 1.783E-07 | 0.000E+00 | 0.000E+00 | 1.051E-06 | 0.000E+00 | 1.928E-07 | 3.948E-07 | 0.000E+00 | 5.848E-07 | 0.000E+00 | 0.000E+00 | 0.000E+00 |
| Unigene232912 | abeS | 0.000E+00 | 0.000E+00 | 0.000E+00 | 8.961E-07 | 0.000E+00 | 0.000E+00 | 0.000E+00 | 0.000E+00 | 0.000E+00 | 0.000E+00 | 0.000E+00 | 0.000E+00 |
| Unigene233037 | Klebsiella p | 0.000E+00 | 0.000E+00 | 1.001E-07 | 2.575E-07 | 0.000E+00 | 0.000E+00 | 0.000E+00 | 0.000E+00 | 0.000E+00 | 0.000E+00 | 0.000E+00 | 0.000E+00 |
| Unigene233054 | evgS | 0.000E+00 | 2.661E-07 | 1.487E-07 | 4.285E-07 | 1.010E-07 | 0.000E+00 | 1.677E-07 | 1.415E-07 | 0.000E+00 | 1.013E-07 | 0.000E+00 | 0.000E+00 |
| Unigene233090 | bcrA | 0.000E+00 | 0.000E+00 | 3.641E-07 | 5.287E-06 | 0.000E+00 | 0.000E+00 | 0.000E+00 | 0.000E+00 | 0.000E+00 | 0.000E+00 | 0.000E+00 | 0.000E+00 |
| Unigene233098 | vanRF | 0.000E+00 | 0.000E+00 | 0.000E+00 | 5.856E-07 | 0.000E+00 | 0.000E+00 | 0.000E+00 | 0.000E+00 | 0.000E+00 | 0.000E+00 | 0.000E+00 | 0.000E+00 |
| Unigene233145 | cfrC | 0.000E+00 | 0.000E+00 | 2.400E-07 | 1.152E-06 | 0.000E+00 | 0.000E+00 | 0.000E+00 | 0.000E+00 | 0.000E+00 | 0.000E+00 | 0.000E+00 | 0.000E+00 |
| Unigene233163 | efrA | 0.000E+00 | 0.000E+00 | 0.000E+00 | 3.122E-07 | 0.000E+00 | 0.000E+00 | 0.000E+00 | 0.000E+00 | 0.000E+00 | 0.000E+00 | 0.000E+00 | 0.000E+00 |
| Unigene233174 | efrB | 0.000E+00 | 0.000E+00 | 1.412E-07 | 3.634E-07 | 0.000E+00 | 0.000E+00 | 0.000E+00 | 0.000E+00 | 0.000E+00 | 0.000E+00 | 0.000E+00 | 0.000E+00 |
| Unigene233258 | bcrA | 0.000E+00 | 0.000E+00 | 0.000E+00 | 6.434E-07 | 0.000E+00 | 0.000E+00 | 0.000E+00 | 0.000E+00 | 0.000E+00 | 0.000E+00 | 0.000E+00 | 0.000E+00 |
| Unigene233259 | Corynebac | 0.000E+00 | 0.000E+00 | 4.327E-08 | 6.680E-07 | 0.000E+00 | 0.000E+00 | 0.000E+00 | 0.000E+00 | 0.000E+00 | 0.000E+00 | 0.000E+00 | 0.000E+00 |
| Unigene233294 | macB | 0.000E+00 | 8.271E-07 | 0.000E+00 | 1.903E-06 | 0.000E+00 | 0.000E+00 | 0.000E+00 | 0.000E+00 | 0.000E+00 | 0.000E+00 | 0.000E+00 | 0.000E+00 |
| Unigene233405 | arlR | 0.000E+00 | 1.618E-07 | 0.000E+00 | 3.721E-06 | 0.000E+00 | 0.000E+00 | 0.000E+00 | 0.000E+00 | 0.000E+00 | 0.000E+00 | 0.000E+00 | 0.000E+00 |
| Unigene233435 | vanO | 0.000E+00 | 0.000E+00 | 1.567E-06 | 5.914E-06 | 0.000E+00 | 0.000E+00 | 0.000E+00 | 0.000E+00 | 0.000E+00 | 0.000E+00 | 0.000E+00 | 0.000E+00 |
| Unigene233576 | poxtA | 0.000E+00 | 0.000E+00 | 0.000E+00 | 2.739E-07 | 3.616E-06 | 0.000E+00 | 0.000E+00 | 6.438E-06 | 0.000E+00 | 0.000E+00 | 0.000E+00 | 0.000E+00 |
| Unigene233637 | lmrB | 0.000E+00 | 0.000E+00 | 0.000E+00 | 1.027E-06 | 0.000E+00 | 0.000E+00 | 0.000E+00 | 0.000E+00 | 0.000E+00 | 0.000E+00 | 0.000E+00 | 0.000E+00 |
| Unigene233760 | mecB | 2.285E-07 | 0.000E+00 | 1.793E-07 | 8.997E-07 | 7.614E-08 | 0.000E+00 | 0.000E+00 | 0.000E+00 | 4.994E-08 | 0.000E+00 | 7.840E-08 | 0.000E+00 |
| Unigene233837 | vanHA | 0.000E+00 | 0.000E+00 | 0.000E+00 | 4.008E-07 | 0.000E+00 | 0.000E+00 | 0.000E+00 | 0.000E+00 | 0.000E+00 | 0.000E+00 | 0.000E+00 | 0.000E+00 |
| Unigene233853 | vanD | 0.000E+00 | 0.000E+00 | 0.000E+00 | 1.740E-06 | 0.000E+00 | 0.000E+00 | 0.000E+00 | 0.000E+00 | 0.000E+00 | 0.000E+00 | 0.000E+00 | 0.000E+00 |
| Unigene234035 | bcrA | 0.000E+00 | 0.000E+00 | 0.000E+00 | 8.823E-07 | 0.000E+00 | 0.000E+00 | 0.000E+00 | 0.000E+00 | 1.005E-07 | 0.000E+00 | 0.000E+00 | 0.000E+00 |
| Unigene234094 | tva(A) | 0.000E+00 | 0.000E+00 | 0.000E+00 | 5.113E-07 | 0.000E+00 | 0.000E+00 | 0.000E+00 | 0.000E+00 | 0.000E+00 | 0.000E+00 | 0.000E+00 | 0.000E+00 |
| Unigene234235 | golS | 0.000E+00 | 0.000E+00 | 0.000E+00 | 4.527E-07 | 0.000E+00 | 0.000E+00 | 0.000E+00 | 0.000E+00 | 0.000E+00 | 0.000E+00 | 0.000E+00 | 0.000E+00 |
| Unigene234250 | tetA(58) | 0.000E+00 | 9.916E-07 | 1.290E-06 | 2.074E-06 | 0.000E+00 | 0.000E+00 | 0.000E+00 | 0.000E+00 | 0.000E+00 | 0.000E+00 | 0.000E+00 | 0.000E+00 |
| Unigene234273 | macB | 0.000E+00 | 0.000E+00 | 3.937E-07 | 1.142E-06 | 0.000E+00 | 0.000E+00 | 0.000E+00 | 0.000E+00 | 0.000E+00 | 0.000E+00 | 0.000E+00 | 0.000E+00 |
| Unigene234276 | macB | 0.000E+00 | 3.976E-08 | 2.844E-07 | 9.512E-07 | 0.000E+00 | 0.000E+00 | 0.000E+00 | 0.000E+00 | 0.000E+00 | 0.000E+00 | 0.000E+00 | 0.000E+00 |
| Unigene234329 | Bifidobact | 0.000E+00 | 0.000E+00 | 0.000E+00 | 1.058E-06 | 0.000E+00 | 0.000E+00 | 0.000E+00 | 0.000E+00 | 0.000E+00 | 0.000E+00 | 0.000E+00 | 0.000E+00 |
| Unigene234382 | carA | 0.000E+00 | 1.051E-07 | 0.000E+00 | 8.061E-07 | 0.000E+00 | 0.000E+00 | 0.000E+00 | 0.000E+00 | 0.000E+00 | 4.980E-07 | 0.000E+00 | 3.019E-07 |
| Unigene234383 | msbA | 3.666E-08 | 1.408E-07 | 0.000E+00 | 9.996E-07 | 0.000E+00 | 0.000E+00 | 0.000E+00 | 0.000E+00 | 0.000E+00 | 3.676E-07 | 0.000E+00 | 2.383E-07 |
| Unigene234402 | oleC | 0.000E+00 | 0.000E+00 | 0.000E+00 | 4.808E-07 | 0.000E+00 | 0.000E+00 | 0.000E+00 | 0.000E+00 | 1.041E-07 | 0.000E+00 | 0.000E+00 | 0.000E+00 |
| Unigene234429 | oleB | 0.000E+00 | 0.000E+00 | 3.092E-07 | 2.728E-07 | 2.501E-07 | 3.894E-07 | 1.046E-06 | 1.051E-06 | 3.937E-07 | 8.526E-07 | 1.442E-06 | 2.129E-06 |
| Unigene234454 | macB | 5.064E-07 | 9.262E-07 | 0.000E+00 | 4.261E-07 | 8.672E-06 | 0.000E+00 | 0.000E+00 | 0.000E+00 | 0.000E+00 | 0.000E+00 | 0.000E+00 | 0.000E+00 |
| Unigene234472 | tetA(58) | 0.000E+00 | 0.000E+00 | 0.000E+00 | 3.105E-07 | 0.000E+00 | 0.000E+00 | 0.000E+00 | 0.000E+00 | 0.000E+00 | 0.000E+00 | 0.000E+00 | 0.000E+00 |
| Unigene234534 | kdpE | 0.000E+00 | 0.000E+00 | 0.000E+00 | 7.902E-07 | 0.000E+00 | 0.000E+00 | 0.000E+00 | 0.000E+00 | 0.000E+00 | 0.000E+00 | 0.000E+00 | 0.000E+00 |
| Unigene234678 | poxtA | 0.000E+00 | 0.000E+00 | 0.000E+00 | 4.329E-06 | 0.000E+00 | 0.000E+00 | 0.000E+00 | 0.000E+00 | 0.000E+00 | 0.000E+00 | 0.000E+00 | 0.000E+00 |
| Unigene234703 | patB | 0.000E+00 | 0.000E+00 | 0.000E+00 | 5.583E-07 | 0.000E+00 | 0.000E+00 | 0.000E+00 | 0.000E+00 | 0.000E+00 | 0.000E+00 | 0.000E+00 | 0.000E+00 |
| Unigene234718 | Staphyloc | 0.000E+00 | 0.000E+00 | 0.000E+00 | 4.707E-07 | 0.000E+00 | 0.000E+00 | 0.000E+00 | 0.000E+00 | 0.000E+00 | 0.000E+00 | 0.000E+00 | 0.000E+00 |
| Unigene234806 | poxtA | 7.631E-06 | 0.000E+00 | 0.000E+00 | 2.338E-06 | 2.815E-05 | 1.423E-05 | 2.405E-05 | 2.482E-05 | 2.343E-06 | 0.000E+00 | 0.000E+00 | 0.000E+00 |
| Unigene234869 | PmrF | 0.000E+00 | 0.000E+00 | 0.000E+00 | 4.774E-05 | 0.000E+00 | 0.000E+00 | 0.000E+00 | 0.000E+00 | 0.000E+00 | 0.000E+00 | 0.000E+00 | 0.000E+00 |
| Unigene234917 | macB | 0.000E+00 | 0.000E+00 | 0.000E+00 | 7.193E-07 | 0.000E+00 | 5.674E-07 | 0.000E+00 | 0.000E+00 | 5.737E-07 | 1.002E-06 | 0.000E+00 | 0.000E+00 |
| Unigene234943 | Streptomy | 0.000E+00 | 0.000E+00 | 0.000E+00 | 1.811E-07 | 0.000E+00 | 0.000E+00 | 0.000E+00 | 0.000E+00 | 0.000E+00 | 0.000E+00 | 0.000E+00 | 0.000E+00 |
| Unigene234996 | Bifidobact | 0.000E+00 | 0.000E+00 | 6.509E-08 | 2.103E-06 | 0.000E+00 | 0.000E+00 | 0.000E+00 | 0.000E+00 | 0.000E+00 | 0.000E+00 | 0.000E+00 | 0.000E+00 |
| Unigene235022 | Chlamydia | 0.000E+00 | 0.000E+00 | 0.000E+00 | 1.426E-06 | 0.000E+00 | 0.000E+00 | 0.000E+00 | 0.000E+00 | 0.000E+00 | 0.000E+00 | 0.000E+00 | 0.000E+00 |

| | | | | | | | | | | | | | |
|---|---|---|---|---|---|---|---|---|---|---|---|---|---|
| Unigene235080 | Staphylocc | 0.000E+00 | 0.000E+00 | 5.091E-08 | 1.970E-06 | 0.000E+00 | 0.000E+00 | 0.000E+00 | 0.000E+00 | 0.000E+00 | 0.000E+00 | 0.000E+00 | 0.000E+00 |
| Unigene235092 | ErmY | 0.000E+00 | 0.000E+00 | 0.000E+00 | 1.707E-06 | 0.000E+00 | 0.000E+00 | 0.000E+00 | 0.000E+00 | 0.000E+00 | 0.000E+00 | 0.000E+00 | 0.000E+00 |
| Unigene235096 | Staphylocc | 0.000E+00 | 0.000E+00 | 1.637E-08 | 1.769E-06 | 0.000E+00 | 0.000E+00 | 0.000E+00 | 0.000E+00 | 0.000E+00 | 0.000E+00 | 0.000E+00 | 0.000E+00 |
| Unigene235129 | Corynebac | 3.804E-07 | 0.000E+00 | 3.199E-07 | 0.000E+00 | 0.000E+00 | 0.000E+00 | 0.000E+00 | 0.000E+00 | 0.000E+00 | 0.000E+00 | 0.000E+00 | 0.000E+00 |
| Unigene235168 | lsaC | 0.000E+00 | 0.000E+00 | 6.640E-08 | 5.467E-07 | 0.000E+00 | 0.000E+00 | 0.000E+00 | 0.000E+00 | 0.000E+00 | 0.000E+00 | 0.000E+00 | 0.000E+00 |
| Unigene235216 | macB | 0.000E+00 | 0.000E+00 | 0.000E+00 | 2.603E-07 | 0.000E+00 | 0.000E+00 | 0.000E+00 | 0.000E+00 | 0.000E+00 | 0.000E+00 | 0.000E+00 | 0.000E+00 |
| Unigene235263 | poxtA | 0.000E+00 | 0.000E+00 | 6.247E-07 | 1.286E-06 | 0.000E+00 | 0.000E+00 | 0.000E+00 | 0.000E+00 | 0.000E+00 | 0.000E+00 | 0.000E+00 | 0.000E+00 |
| Unigene235324 | Acinetoba | 3.829E-08 | 2.732E-07 | 3.344E-06 | 1.715E-05 | 0.000E+00 | 0.000E+00 | 0.000E+00 | 0.000E+00 | 0.000E+00 | 0.000E+00 | 0.000E+00 | 0.000E+00 |
| Unigene235357 | tva(A) | 0.000E+00 | 0.000E+00 | 0.000E+00 | 6.536E-07 | 0.000E+00 | 0.000E+00 | 0.000E+00 | 0.000E+00 | 0.000E+00 | 0.000E+00 | 0.000E+00 | 0.000E+00 |
| Unigene235401 | bcrA | 1.575E-06 | 5.802E-06 | 6.033E-06 | 9.351E-06 | 0.000E+00 | 0.000E+00 | 0.000E+00 | 0.000E+00 | 0.000E+00 | 0.000E+00 | 0.000E+00 | 0.000E+00 |
| Unigene235444 | tetB(P) | 2.075E-07 | 2.580E-07 | 2.714E-07 | 7.262E-07 | 0.000E+00 | 0.000E+00 | 0.000E+00 | 1.614E-08 | 0.000E+00 | 0.000E+00 | 4.746E-08 | 4.904E-08 |
| Unigene235470 | vanTE | 0.000E+00 | 2.506E-07 | 0.000E+00 | 1.095E-06 | 0.000E+00 | 0.000E+00 | 0.000E+00 | 0.000E+00 | 0.000E+00 | 0.000E+00 | 0.000E+00 | 3.036E-07 |
| Unigene235491 | msbA | 2.538E-08 | 0.000E+00 | 0.000E+00 | 7.688E-07 | 0.000E+00 | 0.000E+00 | 0.000E+00 | 0.000E+00 | 0.000E+00 | 7.916E-07 | 0.000E+00 | 2.400E-07 |
| Unigene235494 | bcrA | 0.000E+00 | 0.000E+00 | 0.000E+00 | 7.144E-07 | 0.000E+00 | 0.000E+00 | 0.000E+00 | 0.000E+00 | 0.000E+00 | 0.000E+00 | 0.000E+00 | 0.000E+00 |
| Unigene235512 | efrA | 0.000E+00 | 1.128E-06 | 5.044E-07 | 3.114E-07 | 0.000E+00 | 0.000E+00 | 0.000E+00 | 0.000E+00 | 0.000E+00 | 0.000E+00 | 0.000E+00 | 0.000E+00 |
| Unigene235519 | efrB | 0.000E+00 | 0.000E+00 | 0.000E+00 | 7.204E-07 | 0.000E+00 | 0.000E+00 | 0.000E+00 | 0.000E+00 | 0.000E+00 | 0.000E+00 | 0.000E+00 | 0.000E+00 |
| Unigene235567 | ykkC | 0.000E+00 | 0.000E+00 | 0.000E+00 | 6.296E-06 | 0.000E+00 | 0.000E+00 | 0.000E+00 | 0.000E+00 | 0.000E+00 | 0.000E+00 | 0.000E+00 | 0.000E+00 |
| Unigene235572 | tetB(60) | 5.962E-07 | 6.886E-06 | 1.109E-05 | 1.568E-05 | 0.000E+00 | 3.069E-08 | 0.000E+00 | 1.326E-07 | 0.000E+00 | 0.000E+00 | 3.248E-08 | 0.000E+00 |
| Unigene235773 | baeS | 9.565E-08 | 5.774E-07 | 0.000E+00 | 2.898E-07 | 0.000E+00 | 0.000E+00 | 0.000E+00 | 0.000E+00 | 0.000E+00 | 0.000E+00 | 0.000E+00 | 0.000E+00 |
| Unigene235837 | tlrC | 0.000E+00 | 0.000E+00 | 0.000E+00 | 3.276E-07 | 0.000E+00 | 0.000E+00 | 0.000E+00 | 0.000E+00 | 0.000E+00 | 0.000E+00 | 0.000E+00 | 0.000E+00 |
| Unigene235861 | tlrC | 0.000E+00 | 0.000E+00 | 0.000E+00 | 9.131E-07 | 0.000E+00 | 0.000E+00 | 0.000E+00 | 0.000E+00 | 4.447E-07 | 0.000E+00 | 0.000E+00 | 0.000E+00 |
| Unigene235862 | bcrA | 2.593E-07 | 0.000E+00 | 0.000E+00 | 1.396E-06 | 0.000E+00 | 0.000E+00 | 0.000E+00 | 1.009E-07 | 5.195E-07 | 0.000E+00 | 0.000E+00 | 0.000E+00 |
| Unigene235876 | carA | 0.000E+00 | 0.000E+00 | 0.000E+00 | 3.710E-07 | 0.000E+00 | 0.000E+00 | 0.000E+00 | 0.000E+00 | 0.000E+00 | 0.000E+00 | 0.000E+00 | 0.000E+00 |
| Unigene235888 | rpoB2 | 0.000E+00 | 0.000E+00 | 3.603E-07 | 4.506E-06 | 0.000E+00 | 0.000E+00 | 0.000E+00 | 0.000E+00 | 0.000E+00 | 0.000E+00 | 0.000E+00 | 0.000E+00 |
| Unigene236038 | efpA | 1.355E-07 | 2.380E-07 | 4.521E-07 | 5.748E-07 | 6.022E-08 | 0.000E+00 | 8.999E-08 | 0.000E+00 | 0.000E+00 | 0.000E+00 | 0.000E+00 | 1.922E-07 |
| Unigene236184 | tetA(58) | 0.000E+00 | 0.000E+00 | 0.000E+00 | 1.227E-06 | 0.000E+00 | 0.000E+00 | 0.000E+00 | 0.000E+00 | 0.000E+00 | 0.000E+00 | 0.000E+00 | 0.000E+00 |
| Unigene236250 | vgaALC | 0.000E+00 | 0.000E+00 | 0.000E+00 | 7.572E-07 | 0.000E+00 | 0.000E+00 | 0.000E+00 | 0.000E+00 | 0.000E+00 | 0.000E+00 | 0.000E+00 | 0.000E+00 |
| Unigene236429 | tva(A) | 0.000E+00 | 0.000E+00 | 0.000E+00 | 3.763E-07 | 0.000E+00 | 0.000E+00 | 0.000E+00 | 0.000E+00 | 0.000E+00 | 0.000E+00 | 0.000E+00 | 5.506E-08 |
| Unigene236438 | efmA | 0.000E+00 | 0.000E+00 | 2.707E-07 | 4.711E-06 | 0.000E+00 | 0.000E+00 | 0.000E+00 | 0.000E+00 | 0.000E+00 | 0.000E+00 | 0.000E+00 | 0.000E+00 |
| Unigene236448 | macB | 0.000E+00 | 0.000E+00 | 1.433E-07 | 4.720E-06 | 0.000E+00 | 0.000E+00 | 0.000E+00 | 0.000E+00 | 0.000E+00 | 0.000E+00 | 0.000E+00 | 0.000E+00 |
| Unigene236478 | ErmC | 0.000E+00 | 0.000E+00 | 2.169E-07 | 5.321E-06 | 0.000E+00 | 0.000E+00 | 0.000E+00 | 0.000E+00 | 0.000E+00 | 0.000E+00 | 0.000E+00 | 0.000E+00 |
| Unigene236522 | lmrD | 0.000E+00 | 2.595E-08 | 0.000E+00 | 5.254E-07 | 0.000E+00 | 0.000E+00 | 0.000E+00 | 0.000E+00 | 0.000E+00 | 0.000E+00 | 0.000E+00 | 0.000E+00 |
| Unigene236532 | mtrA | 2.430E-07 | 1.143E-07 | 2.214E-07 | 5.783E-07 | 3.856E-08 | 0.000E+00 | 0.000E+00 | 0.000E+00 | 0.000E+00 | 0.000E+00 | 0.000E+00 | 1.026E-07 |
| Unigene236564 | rpoB2 | 1.278E-07 | 1.871E-07 | 0.000E+00 | 1.054E-06 | 0.000E+00 | 0.000E+00 | 0.000E+00 | 0.000E+00 | 0.000E+00 | 1.163E-06 | 0.000E+00 | 1.007E-07 |
| Unigene236573 | vanSF | 0.000E+00 | 0.000E+00 | 0.000E+00 | 5.927E-07 | 0.000E+00 | 0.000E+00 | 0.000E+00 | 0.000E+00 | 0.000E+00 | 0.000E+00 | 0.000E+00 | 0.000E+00 |
| Unigene236634 | oleC | 0.000E+00 | 0.000E+00 | 0.000E+00 | 0.000E+00 | 4.763E-07 | 2.781E-07 | 9.489E-07 | 4.503E-07 | 0.000E+00 | 0.000E+00 | 0.000E+00 | 7.601E-07 |
| Unigene236705 | macB | 0.000E+00 | 0.000E+00 | 3.339E-07 | 5.497E-06 | 0.000E+00 | 0.000E+00 | 0.000E+00 | 0.000E+00 | 0.000E+00 | 0.000E+00 | 0.000E+00 | 0.000E+00 |
| Unigene236706 | ACC-3 | 0.000E+00 | 0.000E+00 | 1.711E-07 | 4.930E-06 | 0.000E+00 | 2.693E-08 | 0.000E+00 | 0.000E+00 | 0.000E+00 | 0.000E+00 | 0.000E+00 | 0.000E+00 |
| Unigene236731 | novA | 0.000E+00 | 0.000E+00 | 0.000E+00 | 2.362E-07 | 0.000E+00 | 4.215E-08 | 2.589E-07 | 0.000E+00 | 0.000E+00 | 0.000E+00 | 1.784E-07 | 0.000E+00 |
| Unigene236780 | patA | 0.000E+00 | 0.000E+00 | 0.000E+00 | 8.029E-07 | 0.000E+00 | 0.000E+00 | 0.000E+00 | 0.000E+00 | 0.000E+00 | 0.000E+00 | 0.000E+00 | 0.000E+00 |
| Unigene236788 | Acinetoba | 0.000E+00 | 2.731E-07 | 1.221E-07 | 7.537E-07 | 0.000E+00 | 0.000E+00 | 0.000E+00 | 0.000E+00 | 0.000E+00 | 0.000E+00 | 0.000E+00 | 0.000E+00 |
| Unigene236790 | ErmR | 0.000E+00 | 0.000E+00 | 3.591E-07 | 1.146E-06 | 0.000E+00 | 0.000E+00 | 0.000E+00 | 0.000E+00 | 0.000E+00 | 0.000E+00 | 0.000E+00 | 0.000E+00 |
| Unigene236793 | baeS | 0.000E+00 | 0.000E+00 | 0.000E+00 | 7.507E-07 | 0.000E+00 | 0.000E+00 | 0.000E+00 | 0.000E+00 | 0.000E+00 | 0.000E+00 | 0.000E+00 | 0.000E+00 |
| Unigene236794 | smeR | 0.000E+00 | 0.000E+00 | 0.000E+00 | 7.244E-07 | 0.000E+00 | 0.000E+00 | 0.000E+00 | 0.000E+00 | 0.000E+00 | 0.000E+00 | 0.000E+00 | 0.000E+00 |
| Unigene236803 | rpoB2 | 0.000E+00 | 0.000E+00 | 0.000E+00 | 1.605E-06 | 0.000E+00 | 0.000E+00 | 0.000E+00 | 0.000E+00 | 0.000E+00 | 0.000E+00 | 0.000E+00 | 0.000E+00 |
| Unigene236936 | vanRF | 0.000E+00 | 0.000E+00 | 0.000E+00 | 6.924E-07 | 0.000E+00 | 0.000E+00 | 0.000E+00 | 0.000E+00 | 0.000E+00 | 0.000E+00 | 0.000E+00 | 0.000E+00 |
| Unigene236992 | baeS | 0.000E+00 | 0.000E+00 | 0.000E+00 | 8.890E-07 | 0.000E+00 | 0.000E+00 | 0.000E+00 | 0.000E+00 | 0.000E+00 | 9.262E-07 | 0.000E+00 | 2.023E-07 |
| Unigene236993 | arlR | 0.000E+00 | 1.547E-07 | 0.000E+00 | 7.117E-07 | 0.000E+00 | 0.000E+00 | 0.000E+00 | 0.000E+00 | 0.000E+00 | 0.000E+00 | 0.000E+00 | 3.332E-07 |
| Unigene237033 | mtrA | 0.000E+00 | 0.000E+00 | 3.105E-07 | 2.009E-06 | 0.000E+00 | 0.000E+00 | 0.000E+00 | 0.000E+00 | 0.000E+00 | 0.000E+00 | 0.000E+00 | 0.000E+00 |
| Unigene237034 | cpxA | 0.000E+00 | 0.000E+00 | 2.876E-07 | 1.607E-06 | 0.000E+00 | 0.000E+00 | 0.000E+00 | 0.000E+00 | 0.000E+00 | 0.000E+00 | 0.000E+00 | 0.000E+00 |
| Unigene237038 | tlrC | 0.000E+00 | 2.275E-08 | 1.831E-07 | 1.424E-06 | 0.000E+00 | 0.000E+00 | 0.000E+00 | 0.000E+00 | 0.000E+00 | 0.000E+00 | 0.000E+00 | 0.000E+00 |
| Unigene237059 | vanRI | 0.000E+00 | 0.000E+00 | 0.000E+00 | 3.018E-07 | 0.000E+00 | 0.000E+00 | 0.000E+00 | 0.000E+00 | 0.000E+00 | 0.000E+00 | 0.000E+00 | 0.000E+00 |
| Unigene237090 | novA | 0.000E+00 | 0.000E+00 | 0.000E+00 | 4.673E-07 | 3.428E-07 | 0.000E+00 | 0.000E+00 | 0.000E+00 | 0.000E+00 | 0.000E+00 | 0.000E+00 | 0.000E+00 |
| Unigene237129 | oleD | 0.000E+00 | 2.923E-07 | 3.136E-07 | 8.069E-07 | 0.000E+00 | 0.000E+00 | 1.769E-07 | 0.000E+00 | 0.000E+00 | 0.000E+00 | 0.000E+00 | 9.445E-08 |
| Unigene237164 | PmrF | 0.000E+00 | 0.000E+00 | 0.000E+00 | 6.000E-07 | 0.000E+00 | 0.000E+00 | 0.000E+00 | 0.000E+00 | 0.000E+00 | 0.000E+00 | 0.000E+00 | 0.000E+00 |
| Unigene237181 | vanHO | 0.000E+00 | 0.000E+00 | 0.000E+00 | 5.821E-07 | 0.000E+00 | 0.000E+00 | 0.000E+00 | 0.000E+00 | 0.000E+00 | 0.000E+00 | 0.000E+00 | 0.000E+00 |
| Unigene237203 | patB | 0.000E+00 | 0.000E+00 | 3.943E-07 | 4.959E-07 | 0.000E+00 | 0.000E+00 | 0.000E+00 | 0.000E+00 | 0.000E+00 | 0.000E+00 | 0.000E+00 | 0.000E+00 |
| Unigene237219 | arlR | 0.000E+00 | 6.151E-08 | 0.000E+00 | 3.396E-07 | 0.000E+00 | 0.000E+00 | 0.000E+00 | 0.000E+00 | 0.000E+00 | 0.000E+00 | 0.000E+00 | 0.000E+00 |

| | | | | | | | | | | | | | |
|---|---|---|---|---|---|---|---|---|---|---|---|---|---|
| Unigene237234 | tetA(60) | 3.885E-08 | 0.000E+00 | 0.000E+00 | 3.923E-07 | 0.000E+00 | 0.000E+00 | 2.580E-07 | 0.000E+00 | 0.000E+00 | 0.000E+00 | 2.222E-07 | 1.837E-07 |
| Unigene237275 | optrA | 0.000E+00 | 2.843E-07 | 0.000E+00 | 8.049E-07 | 0.000E+00 | 0.000E+00 | 0.000E+00 | 0.000E+00 | 0.000E+00 | 1.154E-06 | 0.000E+00 | 0.000E+00 |
| Unigene237346 | bcrA | 0.000E+00 | 0.000E+00 | 1.820E-07 | 3.747E-07 | 0.000E+00 | 0.000E+00 | 0.000E+00 | 0.000E+00 | 0.000E+00 | 0.000E+00 | 0.000E+00 | 0.000E+00 |
| Unigene237418 | tetA(58) | 0.000E+00 | 0.000E+00 | 5.656E-07 | 1.358E-06 | 0.000E+00 | 0.000E+00 | 0.000E+00 | 0.000E+00 | 0.000E+00 | 0.000E+00 | 0.000E+00 | 0.000E+00 |
| Unigene237445 | vanTG | 6.642E-08 | 0.000E+00 | 1.955E-07 | 6.037E-07 | 0.000E+00 | 7.180E-08 | 0.000E+00 | 0.000E+00 | 0.000E+00 | 0.000E+00 | 1.520E-07 | 0.000E+00 |
| Unigene237458 | Listeria mc | 1.243E-08 | 0.000E+00 | 0.000E+00 | 9.536E-07 | 0.000E+00 | 0.000E+00 | 0.000E+00 | 0.000E+00 | 0.000E+00 | 8.305E-07 | 0.000E+00 | 3.378E-07 |
| Unigene237461 | PmrF | 0.000E+00 | 0.000E+00 | 0.000E+00 | 7.336E-07 | 0.000E+00 | 0.000E+00 | 0.000E+00 | 0.000E+00 | 0.000E+00 | 7.586E-07 | 0.000E+00 | 3.220E-07 |
| Unigene237541 | vanRG | 0.000E+00 | 0.000E+00 | 0.000E+00 | 3.773E-07 | 0.000E+00 | 0.000E+00 | 0.000E+00 | 0.000E+00 | 0.000E+00 | 0.000E+00 | 0.000E+00 | 0.000E+00 |
| Unigene237584 | tlrC | 0.000E+00 | 0.000E+00 | 0.000E+00 | 5.604E-07 | 0.000E+00 | 0.000E+00 | 0.000E+00 | 3.599E-07 | 0.000E+00 | 0.000E+00 | 0.000E+00 | 0.000E+00 |
| Unigene237627 | msbA | 0.000E+00 | 0.000E+00 | 0.000E+00 | 8.906E-07 | 0.000E+00 | 0.000E+00 | 0.000E+00 | 0.000E+00 | 0.000E+00 | 0.000E+00 | 0.000E+00 | 0.000E+00 |
| Unigene237744 | vatB | 0.000E+00 | 0.000E+00 | 0.000E+00 | 7.451E-07 | 0.000E+00 | 0.000E+00 | 0.000E+00 | 0.000E+00 | 0.000E+00 | 0.000E+00 | 0.000E+00 | 0.000E+00 |
| Unigene237809 | rpoB2 | 0.000E+00 | 0.000E+00 | 0.000E+00 | 5.719E-07 | 0.000E+00 | 8.746E-08 | 0.000E+00 | 4.722E-07 | 0.000E+00 | 0.000E+00 | 0.000E+00 | 0.000E+00 |
| Unigene237874 | AAC(6')-Isa | 0.000E+00 | 2.024E-07 | 1.810E-07 | 6.830E-07 | 0.000E+00 | 0.000E+00 | 0.000E+00 | 0.000E+00 | 6.720E-08 | 0.000E+00 | 7.033E-08 | 0.000E+00 |
| Unigene237911 | novA | 0.000E+00 | 1.927E-08 | 2.584E-07 | 2.996E-06 | 0.000E+00 | 0.000E+00 | 0.000E+00 | 0.000E+00 | 0.000E+00 | 0.000E+00 | 0.000E+00 | 0.000E+00 |
| Unigene237912 | novA | 3.465E-08 | 0.000E+00 | 5.101E-07 | 4.374E-06 | 0.000E+00 | 0.000E+00 | 0.000E+00 | 0.000E+00 | 0.000E+00 | 0.000E+00 | 0.000E+00 | 0.000E+00 |
| Unigene238102 | patA | 1.109E-07 | 0.000E+00 | 1.814E-07 | 5.228E-07 | 0.000E+00 | 0.000E+00 | 0.000E+00 | 0.000E+00 | 0.000E+00 | 0.000E+00 | 0.000E+00 | 0.000E+00 |
| Unigene238133 | vanHF | 0.000E+00 | 0.000E+00 | 0.000E+00 | 1.482E-06 | 0.000E+00 | 0.000E+00 | 0.000E+00 | 0.000E+00 | 0.000E+00 | 0.000E+00 | 0.000E+00 | 0.000E+00 |
| Unigene238348 | bcrA | 0.000E+00 | 0.000E+00 | 0.000E+00 | 2.905E-06 | 0.000E+00 | 0.000E+00 | 0.000E+00 | 0.000E+00 | 0.000E+00 | 0.000E+00 | 0.000E+00 | 0.000E+00 |
| Unigene238360 | macB | 0.000E+00 | 0.000E+00 | 3.110E-07 | 5.643E-06 | 0.000E+00 | 0.000E+00 | 0.000E+00 | 0.000E+00 | 0.000E+00 | 0.000E+00 | 0.000E+00 | 0.000E+00 |
| Unigene238383 | vanZF | 2.091E-06 | 1.660E-05 | 6.456E-06 | 3.601E-06 | 0.000E+00 | 6.647E-08 | 0.000E+00 | 0.000E+00 | 0.000E+00 | 0.000E+00 | 0.000E+00 | 0.000E+00 |
| Unigene238433 | Staphylocc | 0.000E+00 | 0.000E+00 | 0.000E+00 | 3.987E-07 | 0.000E+00 | 5.336E-08 | 0.000E+00 | 0.000E+00 | 0.000E+00 | 0.000E+00 | 0.000E+00 | 0.000E+00 |
| Unigene238445 | tetA(58) | 7.186E-06 | 5.258E-07 | 5.062E-07 | 2.754E-06 | 2.800E-05 | 2.972E-05 | 2.170E-05 | 1.230E-05 | 7.846E-05 | 8.378E-05 | 6.934E-05 | 5.610E-05 |
| Unigene238526 | dfrG | 0.000E+00 | 0.000E+00 | 0.000E+00 | 5.300E-07 | 0.000E+00 | 0.000E+00 | 0.000E+00 | 0.000E+00 | 5.737E-07 | 0.000E+00 | 0.000E+00 | 0.000E+00 |
| Unigene238571 | oleC | 0.000E+00 | 8.230E-08 | 1.545E-06 | 1.314E-05 | 0.000E+00 | 0.000E+00 | 0.000E+00 | 0.000E+00 | 0.000E+00 | 0.000E+00 | 0.000E+00 | 0.000E+00 |
| Unigene238666 | Escherichia | 0.000E+00 | 4.144E-07 | 0.000E+00 | 4.448E-07 | 0.000E+00 | 0.000E+00 | 0.000E+00 | 0.000E+00 | 0.000E+00 | 0.000E+00 | 0.000E+00 | 0.000E+00 |
| Unigene238667 | efrA | 0.000E+00 | 0.000E+00 | 0.000E+00 | 4.515E-07 | 0.000E+00 | 3.222E-08 | 6.598E-08 | 0.000E+00 | 0.000E+00 | 0.000E+00 | 0.000E+00 | 0.000E+00 |
| Unigene238937 | vgaB | 0.000E+00 | 0.000E+00 | 0.000E+00 | 5.025E-07 | 0.000E+00 | 0.000E+00 | 1.377E-07 | 0.000E+00 | 0.000E+00 | 0.000E+00 | 0.000E+00 | 0.000E+00 |
| Unigene239026 | bcrA | 0.000E+00 | 0.000E+00 | 0.000E+00 | 6.707E-07 | 0.000E+00 | 0.000E+00 | 0.000E+00 | 0.000E+00 | 0.000E+00 | 0.000E+00 | 0.000E+00 | 0.000E+00 |
| Unigene239028 | efpA | 1.145E-07 | 0.000E+00 | 0.000E+00 | 4.046E-07 | 1.272E-07 | 0.000E+00 | 0.000E+00 | 0.000E+00 | 0.000E+00 | 0.000E+00 | 0.000E+00 | 0.000E+00 |
| Unigene239153 | kdpE | 0.000E+00 | 0.000E+00 | 4.332E-07 | 4.458E-07 | 0.000E+00 | 0.000E+00 | 0.000E+00 | 0.000E+00 | 0.000E+00 | 0.000E+00 | 0.000E+00 | 0.000E+00 |
| Unigene239213 | mdtC | 0.000E+00 | 3.494E-07 | 0.000E+00 | 5.787E-07 | 0.000E+00 | 0.000E+00 | 2.114E-07 | 0.000E+00 | 0.000E+00 | 0.000E+00 | 0.000E+00 | 0.000E+00 |
| Unigene239269 | mecC | 0.000E+00 | 0.000E+00 | 0.000E+00 | 4.315E-07 | 0.000E+00 | 0.000E+00 | 0.000E+00 | 0.000E+00 | 0.000E+00 | 0.000E+00 | 0.000E+00 | 0.000E+00 |
| Unigene239322 | patA | 0.000E+00 | 0.000E+00 | 0.000E+00 | 1.863E-07 | 0.000E+00 | 0.000E+00 | 0.000E+00 | 0.000E+00 | 0.000E+00 | 0.000E+00 | 0.000E+00 | 0.000E+00 |
| Unigene239395 | bcrA | 2.354E-06 | 3.567E-06 | 5.572E-06 | 4.603E-06 | 4.151E-08 | 1.212E-07 | 0.000E+00 | 0.000E+00 | 0.000E+00 | 0.000E+00 | 8.547E-08 | 0.000E+00 |
| Unigene239397 | golS | 0.000E+00 | 0.000E+00 | 0.000E+00 | 7.330E-07 | 0.000E+00 | 0.000E+00 | 0.000E+00 | 0.000E+00 | 0.000E+00 | 0.000E+00 | 0.000E+00 | 0.000E+00 |
| Unigene239430 | facT | 1.705E-07 | 7.016E-08 | 2.718E-07 | 7.961E-07 | 0.000E+00 | 0.000E+00 | 0.000E+00 | 1.244E-07 | 4.658E-08 | 0.000E+00 | 0.000E+00 | 0.000E+00 |
| Unigene239579 | arnA | 2.399E-07 | 1.128E-07 | 4.371E-07 | 8.651E-07 | 0.000E+00 | 0.000E+00 | 0.000E+00 | 0.000E+00 | 0.000E+00 | 0.000E+00 | 3.920E-08 | 0.000E+00 |
| Unigene239657 | VatI | 0.000E+00 | 0.000E+00 | 0.000E+00 | 2.890E-07 | 0.000E+00 | 0.000E+00 | 0.000E+00 | 0.000E+00 | 0.000E+00 | 0.000E+00 | 0.000E+00 | 0.000E+00 |
| Unigene239843 | dfrI | 0.000E+00 | 0.000E+00 | 0.000E+00 | 1.140E-06 | 0.000E+00 | 0.000E+00 | 0.000E+00 | 0.000E+00 | 0.000E+00 | 0.000E+00 | 0.000E+00 | 0.000E+00 |
| Unigene239850 | vanHD | 0.000E+00 | 1.107E-07 | 3.629E-07 | 8.489E-07 | 0.000E+00 | 3.635E-08 | 0.000E+00 | 0.000E+00 | 0.000E+00 | 0.000E+00 | 0.000E+00 | 0.000E+00 |
| Unigene239864 | bacA | 1.137E-06 | 3.077E-06 | 7.435E-07 | 0.000E+00 | 5.893E-07 | 0.000E+00 | 8.386E-08 | 0.000E+00 | 0.000E+00 | 0.000E+00 | 0.000E+00 | 3.583E-07 |
| Unigene239974 | bacA | 0.000E+00 | 0.000E+00 | 0.000E+00 | 7.789E-07 | 0.000E+00 | 0.000E+00 | 0.000E+00 | 0.000E+00 | 3.794E-07 | 0.000E+00 | 0.000E+00 | 0.000E+00 |
| Unigene239989 | lsaA | 4.295E-08 | 9.429E-08 | 0.000E+00 | 9.760E-07 | 0.000E+00 | 0.000E+00 | 0.000E+00 | 0.000E+00 | 0.000E+00 | 8.374E-07 | 0.000E+00 | 2.539E-07 |
| Unigene239992 | vanHB | 6.424E-08 | 0.000E+00 | 0.000E+00 | 8.109E-07 | 0.000E+00 | 2.778E-07 | 0.000E+00 | 1.500E-07 | 3.160E-07 | 0.000E+00 | 0.000E+00 | 0.000E+00 |
| Unigene239993 | vanHO | 0.000E+00 | 0.000E+00 | 0.000E+00 | 7.114E-07 | 0.000E+00 | 0.000E+00 | 0.000E+00 | 1.495E-07 | 5.600E-07 | 7.135E-07 | 0.000E+00 | 0.000E+00 |
| Unigene240054 | patB | 7.374E-06 | 0.000E+00 | 0.000E+00 | 1.930E-06 | 3.260E-05 | 1.705E-05 | 2.464E-05 | 2.523E-05 | 3.472E-06 | 0.000E+00 | 0.000E+00 | 0.000E+00 |
| Unigene240090 | macB | 0.000E+00 | 0.000E+00 | 0.000E+00 | 9.216E-07 | 0.000E+00 | 0.000E+00 | 0.000E+00 | 0.000E+00 | 0.000E+00 | 0.000E+00 | 0.000E+00 | 0.000E+00 |
| Unigene240158 | vgaA | 0.000E+00 | 0.000E+00 | 0.000E+00 | 6.998E-07 | 8.104E-08 | 0.000E+00 | 0.000E+00 | 0.000E+00 | 0.000E+00 | 0.000E+00 | 1.252E-07 | 0.000E+00 |
| Unigene240202 | sul4 | 0.000E+00 | 0.000E+00 | 0.000E+00 | 5.174E-07 | 0.000E+00 | 0.000E+00 | 0.000E+00 | 0.000E+00 | 0.000E+00 | 0.000E+00 | 0.000E+00 | 0.000E+00 |
| Unigene240258 | patB | 1.582E-07 | 1.930E-07 | 1.173E-06 | 4.740E-06 | 0.000E+00 | 0.000E+00 | 0.000E+00 | 0.000E+00 | 0.000E+00 | 0.000E+00 | 0.000E+00 | 0.000E+00 |
| Unigene240385 | Staphylocc | 6.588E-07 | 4.378E-06 | 7.182E-07 | 3.696E-07 | 2.846E-07 | 0.000E+00 | 0.000E+00 | 8.544E-08 | 8.000E-07 | 1.631E-07 | 0.000E+00 | 0.000E+00 |
| Unigene240402 | mtrA | 2.801E-06 | 0.000E+00 | 0.000E+00 | 1.343E-06 | 1.182E-05 | 2.776E-06 | 1.408E-05 | 1.501E-05 | 3.061E-07 | 0.000E+00 | 0.000E+00 | 0.000E+00 |
| Unigene240416 | efrA | 0.000E+00 | 2.376E-06 | 2.103E-06 | 1.629E-06 | 0.000E+00 | 0.000E+00 | 0.000E+00 | 0.000E+00 | 0.000E+00 | 0.000E+00 | 0.000E+00 | 0.000E+00 |
| Unigene240437 | evgA | 0.000E+00 | 3.508E-07 | 0.000E+00 | 7.531E-07 | 0.000E+00 | 0.000E+00 | 0.000E+00 | 0.000E+00 | 1.747E-07 | 0.000E+00 | 0.000E+00 | 0.000E+00 |
| Unigene240561 | SPG-1 | 0.000E+00 | 0.000E+00 | 0.000E+00 | 5.884E-07 | 0.000E+00 | 0.000E+00 | 0.000E+00 | 0.000E+00 | 2.123E-07 | 0.000E+00 | 0.000E+00 | 0.000E+00 |
| Unigene240576 | ugd | 0.000E+00 | 0.000E+00 | 0.000E+00 | 4.125E-06 | 0.000E+00 | 0.000E+00 | 0.000E+00 | 0.000E+00 | 0.000E+00 | 0.000E+00 | 0.000E+00 | 0.000E+00 |
| Unigene240670 | bcrA | 0.000E+00 | 0.000E+00 | 0.000E+00 | 1.215E-06 | 0.000E+00 | 0.000E+00 | 0.000E+00 | 0.000E+00 | 0.000E+00 | 0.000E+00 | 0.000E+00 | 0.000E+00 |
| Unigene240671 | vanRI | 0.000E+00 | 0.000E+00 | 0.000E+00 | 1.324E-06 | 0.000E+00 | 0.000E+00 | 0.000E+00 | 0.000E+00 | 0.000E+00 | 0.000E+00 | 0.000E+00 | 0.000E+00 |

| | | | | | | | | | | | | | |
|---|---|---|---|---|---|---|---|---|---|---|---|---|---|
| Unigene240672 | vanSG | 0.000E+00 | 0.000E+00 | 0.000E+00 | 1.493E-06 | 0.000E+00 | 0.000E+00 | 0.000E+00 | 0.000E+00 | 0.000E+00 | 0.000E+00 | 0.000E+00 | 0.000E+00 |
| Unigene240674 | Acinetoba | 0.000E+00 | 0.000E+00 | 4.799E-08 | 1.852E-06 | 0.000E+00 | 0.000E+00 | 0.000E+00 | 0.000E+00 | 0.000E+00 | 0.000E+00 | 0.000E+00 | 0.000E+00 |
| Unigene240705 | lmrD | 0.000E+00 | 3.158E-08 | 8.469E-08 | 1.598E-06 | 0.000E+00 | 0.000E+00 | 0.000E+00 | 0.000E+00 | 0.000E+00 | 0.000E+00 | 0.000E+00 | 0.000E+00 |
| Unigene240795 | efpA | 0.000E+00 | 0.000E+00 | 0.000E+00 | 5.601E-07 | 0.000E+00 | 0.000E+00 | 0.000E+00 | 0.000E+00 | 0.000E+00 | 0.000E+00 | 0.000E+00 | 0.000E+00 |
| Unigene240866 | tetB(58) | 0.000E+00 | 0.000E+00 | 0.000E+00 | 1.801E-07 | 0.000E+00 | 0.000E+00 | 0.000E+00 | 0.000E+00 | 0.000E+00 | 0.000E+00 | 0.000E+00 | 0.000E+00 |
| Unigene240971 | bcrA | 0.000E+00 | 0.000E+00 | 0.000E+00 | 1.033E-06 | 0.000E+00 | 0.000E+00 | 0.000E+00 | 0.000E+00 | 0.000E+00 | 0.000E+00 | 0.000E+00 | 2.102E-07 |
| Unigene241033 | pmrA | 0.000E+00 | 0.000E+00 | 2.896E-07 | 7.858E-07 | 3.935E-06 | 7.484E-06 | 1.218E-06 | 7.047E-06 | 3.901E-06 | 2.302E-06 | 0.000E+00 | 6.026E-07 |
| Unigene241051 | tetA(58) | 8.820E-08 | 0.000E+00 | 4.760E-07 | 4.453E-07 | 4.899E-08 | 0.000E+00 | 0.000E+00 | 0.000E+00 | 0.000E+00 | 0.000E+00 | 0.000E+00 | 1.043E-07 |
| Unigene241082 | macB | 0.000E+00 | 0.000E+00 | 0.000E+00 | 8.763E-07 | 0.000E+00 | 0.000E+00 | 0.000E+00 | 0.000E+00 | 0.000E+00 | 0.000E+00 | 0.000E+00 | 0.000E+00 |
| Unigene241107 | emeA | 0.000E+00 | 0.000E+00 | 3.484E-07 | 4.026E-06 | 0.000E+00 | 0.000E+00 | 0.000E+00 | 0.000E+00 | 0.000E+00 | 0.000E+00 | 0.000E+00 | 0.000E+00 |
| Unigene241138 | lmrD | 0.000E+00 | 0.000E+00 | 0.000E+00 | 7.349E-07 | 0.000E+00 | 0.000E+00 | 0.000E+00 | 0.000E+00 | 0.000E+00 | 0.000E+00 | 0.000E+00 | 0.000E+00 |
| Unigene241177 | tetQ | 0.000E+00 | 0.000E+00 | 1.500E-07 | 3.532E-06 | 0.000E+00 | 0.000E+00 | 0.000E+00 | 0.000E+00 | 0.000E+00 | 0.000E+00 | 0.000E+00 | 0.000E+00 |
| Unigene241264 | poxtA | 6.137E-07 | 0.000E+00 | 4.416E-07 | 4.545E-07 | 4.545E-06 | 9.376E-06 | 1.087E-06 | 6.972E-06 | 3.980E-06 | 3.418E-06 | 0.000E+00 | 0.000E+00 |
| Unigene241285 | efmA | 8.967E-07 | 1.068E-06 | 4.098E-06 | 6.545E-06 | 0.000E+00 | 0.000E+00 | 3.119E-07 | 0.000E+00 | 0.000E+00 | 0.000E+00 | 0.000E+00 | 0.000E+00 |
| Unigene241393 | tetA(46) | 1.542E-07 | 0.000E+00 | 0.000E+00 | 1.121E-06 | 7.878E-07 | 6.333E-07 | 0.000E+00 | 0.000E+00 | 4.381E-07 | 8.586E-07 | 0.000E+00 | 0.000E+00 |
| Unigene241394 | tetB(46) | 0.000E+00 | 0.000E+00 | 0.000E+00 | 1.020E-06 | 1.431E-06 | 1.016E-06 | 0.000E+00 | 0.000E+00 | 4.187E-07 | 6.207E-07 | 0.000E+00 | 0.000E+00 |
| Unigene241445 | Streptomy | 0.000E+00 | 0.000E+00 | 0.000E+00 | 8.016E-07 | 0.000E+00 | 0.000E+00 | 0.000E+00 | 0.000E+00 | 0.000E+00 | 0.000E+00 | 0.000E+00 | 0.000E+00 |
| Unigene241527 | tetT | 0.000E+00 | 0.000E+00 | 0.000E+00 | 6.455E-07 | 0.000E+00 | 0.000E+00 | 0.000E+00 | 0.000E+00 | 0.000E+00 | 0.000E+00 | 0.000E+00 | 0.000E+00 |
| Unigene241538 | vatB | 0.000E+00 | 5.229E-07 | 2.493E-06 | 1.965E-06 | 0.000E+00 | 0.000E+00 | 0.000E+00 | 0.000E+00 | 0.000E+00 | 0.000E+00 | 0.000E+00 | 0.000E+00 |
| Unigene241566 | vanXYL | 0.000E+00 | 0.000E+00 | 0.000E+00 | 3.468E-07 | 0.000E+00 | 0.000E+00 | 0.000E+00 | 0.000E+00 | 0.000E+00 | 0.000E+00 | 0.000E+00 | 0.000E+00 |
| Unigene241616 | Corynebac | 0.000E+00 | 0.000E+00 | 0.000E+00 | 6.150E-07 | 0.000E+00 | 0.000E+00 | 0.000E+00 | 0.000E+00 | 0.000E+00 | 0.000E+00 | 0.000E+00 | 0.000E+00 |
| Unigene241663 | arlS | 0.000E+00 | 1.682E-07 | 0.000E+00 | 1.083E-06 | 0.000E+00 | 0.000E+00 | 0.000E+00 | 0.000E+00 | 0.000E+00 | 9.483E-07 | 0.000E+00 | 2.415E-07 |
| Unigene241804 | tetA(58) | 0.000E+00 | 0.000E+00 | 0.000E+00 | 4.811E-07 | 0.000E+00 | 0.000E+00 | 0.000E+00 | 0.000E+00 | 0.000E+00 | 0.000E+00 | 0.000E+00 | 0.000E+00 |
| Unigene241805 | tetB(58) | 0.000E+00 | 0.000E+00 | 0.000E+00 | 9.230E-07 | 0.000E+00 | 0.000E+00 | 0.000E+00 | 0.000E+00 | 0.000E+00 | 0.000E+00 | 0.000E+00 | 0.000E+00 |
| Unigene241827 | bcrA | 0.000E+00 | 0.000E+00 | 0.000E+00 | 5.525E-07 | 0.000E+00 | 0.000E+00 | 0.000E+00 | 0.000E+00 | 0.000E+00 | 0.000E+00 | 0.000E+00 | 0.000E+00 |
| Unigene241828 | patB | 0.000E+00 | 3.192E-07 | 1.679E-07 | 8.292E-07 | 0.000E+00 | 0.000E+00 | 0.000E+00 | 0.000E+00 | 0.000E+00 | 0.000E+00 | 0.000E+00 | 0.000E+00 |
| Unigene241853 | farB | 4.304E-08 | 1.181E-07 | 0.000E+00 | 8.910E-07 | 0.000E+00 | 0.000E+00 | 0.000E+00 | 0.000E+00 | 0.000E+00 | 1.199E-06 | 0.000E+00 | 4.579E-07 |
| Unigene241923 | fexA | 1.563E-07 | 1.470E-07 | 1.051E-06 | 4.418E-06 | 0.000E+00 | 0.000E+00 | 0.000E+00 | 0.000E+00 | 0.000E+00 | 0.000E+00 | 0.000E+00 | 0.000E+00 |
| Unigene241970 | Acinetoba | 1.104E-07 | 0.000E+00 | 0.000E+00 | 1.672E-07 | 0.000E+00 | 0.000E+00 | 1.832E-07 | 1.932E-07 | 0.000E+00 | 0.000E+00 | 0.000E+00 | 0.000E+00 |
| Unigene241998 | novA | 0.000E+00 | 0.000E+00 | 1.657E-07 | 8.040E-07 | 0.000E+00 | 0.000E+00 | 0.000E+00 | 0.000E+00 | 0.000E+00 | 0.000E+00 | 0.000E+00 | 0.000E+00 |
| Unigene242026 | Streptomy | 0.000E+00 | 3.702E-08 | 4.965E-08 | 1.856E-06 | 0.000E+00 | 0.000E+00 | 0.000E+00 | 0.000E+00 | 0.000E+00 | 0.000E+00 | 0.000E+00 | 0.000E+00 |
| Unigene242166 | tetA(58) | 0.000E+00 | 0.000E+00 | 2.906E-07 | 0.000E+00 | 0.000E+00 | 1.067E-07 | 0.000E+00 | 0.000E+00 | 0.000E+00 | 0.000E+00 | 0.000E+00 | 0.000E+00 |
| Unigene242237 | AAC(3)-VII | 0.000E+00 | 0.000E+00 | 0.000E+00 | 6.841E-07 | 0.000E+00 | 0.000E+00 | 8.821E-08 | 0.000E+00 | 0.000E+00 | 0.000E+00 | 0.000E+00 | 4.711E-08 |
| Unigene242265 | tetA(46) | 0.000E+00 | 1.663E-07 | 5.056E-07 | 9.641E-07 | 0.000E+00 | 0.000E+00 | 0.000E+00 | 0.000E+00 | 0.000E+00 | 0.000E+00 | 0.000E+00 | 0.000E+00 |
| Unigene242276 | TaeA | 1.360E-05 | 4.868E-06 | 3.786E-06 | 1.524E-05 | 9.104E-05 | 1.256E-04 | 1.386E-04 | 1.085E-04 | 1.627E-04 | 1.643E-04 | 1.301E-04 | 8.652E-05 |
| Unigene242300 | evgS | 6.362E-07 | 2.505E-06 | 1.276E-06 | 6.564E-07 | 1.019E-05 | 0.000E+00 | 1.791E-06 | 0.000E+00 | 0.000E+00 | 0.000E+00 | 0.000E+00 | 0.000E+00 |
| Unigene242310 | tetA(58) | 6.045E-08 | 0.000E+00 | 2.076E-07 | 2.747E-07 | 3.358E-08 | 0.000E+00 | 0.000E+00 | 0.000E+00 | 0.000E+00 | 0.000E+00 | 3.457E-08 | 0.000E+00 |
| Unigene242496 | patA | 0.000E+00 | 0.000E+00 | 2.103E-07 | 5.368E-06 | 0.000E+00 | 0.000E+00 | 0.000E+00 | 0.000E+00 | 0.000E+00 | 0.000E+00 | 0.000E+00 | 0.000E+00 |
| Unigene242544 | tlrC | 0.000E+00 | 0.000E+00 | 0.000E+00 | 5.900E-07 | 0.000E+00 | 0.000E+00 | 0.000E+00 | 0.000E+00 | 0.000E+00 | 0.000E+00 | 0.000E+00 | 0.000E+00 |
| Unigene242682 | vanSM | 1.216E-07 | 2.002E-07 | 0.000E+00 | 6.753E-07 | 0.000E+00 | 0.000E+00 | 0.000E+00 | 0.000E+00 | 0.000E+00 | 0.000E+00 | 0.000E+00 | 2.874E-07 |
| Unigene242725 | mepA | 6.588E-08 | 2.651E-07 | 4.309E-07 | 7.761E-07 | 0.000E+00 | 0.000E+00 | 0.000E+00 | 0.000E+00 | 0.000E+00 | 0.000E+00 | 0.000E+00 | 0.000E+00 |
| Unigene242743 | lmrD | 0.000E+00 | 0.000E+00 | 1.689E-07 | 0.000E+00 | 0.000E+00 | 0.000E+00 | 0.000E+00 | 0.000E+00 | 0.000E+00 | 0.000E+00 | 0.000E+00 | 0.000E+00 |
| Unigene242779 | lmrB | 2.056E-07 | 0.000E+00 | 0.000E+00 | 4.499E-07 | 0.000E+00 | 2.223E-07 | 5.310E-07 | 0.000E+00 | 2.622E-07 | 2.672E-07 | 0.000E+00 | 0.000E+00 |
| Unigene242977 | tet(44) | 0.000E+00 | 0.000E+00 | 0.000E+00 | 5.981E-07 | 0.000E+00 | 0.000E+00 | 0.000E+00 | 0.000E+00 | 0.000E+00 | 0.000E+00 | 0.000E+00 | 0.000E+00 |
| Unigene243035 | novA | 0.000E+00 | 0.000E+00 | 3.312E-07 | 5.794E-06 | 0.000E+00 | 0.000E+00 | 0.000E+00 | 0.000E+00 | 0.000E+00 | 0.000E+00 | 0.000E+00 | 0.000E+00 |
| Unigene243042 | adeN | 0.000E+00 | 0.000E+00 | 2.218E-07 | 1.415E-06 | 0.000E+00 | 0.000E+00 | 0.000E+00 | 0.000E+00 | 0.000E+00 | 0.000E+00 | 0.000E+00 | 0.000E+00 |
| Unigene243044 | bcrA | 0.000E+00 | 0.000E+00 | 0.000E+00 | 2.147E-06 | 0.000E+00 | 0.000E+00 | 0.000E+00 | 0.000E+00 | 0.000E+00 | 0.000E+00 | 0.000E+00 | 0.000E+00 |
| Unigene243077 | Staphyloc | 2.582E-07 | 0.000E+00 | 2.956E-07 | 6.085E-07 | 0.000E+00 | 0.000E+00 | 0.000E+00 | 5.024E-08 | 0.000E+00 | 0.000E+00 | 0.000E+00 | 0.000E+00 |
| Unigene243150 | patB | 0.000E+00 | 0.000E+00 | 0.000E+00 | 6.245E-07 | 0.000E+00 | 0.000E+00 | 0.000E+00 | 0.000E+00 | 0.000E+00 | 0.000E+00 | 0.000E+00 | 0.000E+00 |
| Unigene243151 | efrA | 0.000E+00 | 0.000E+00 | 0.000E+00 | 6.000E-07 | 0.000E+00 | 0.000E+00 | 0.000E+00 | 0.000E+00 | 0.000E+00 | 0.000E+00 | 0.000E+00 | 0.000E+00 |
| Unigene243191 | macB | 0.000E+00 | 0.000E+00 | 0.000E+00 | 4.759E-07 | 0.000E+00 | 0.000E+00 | 0.000E+00 | 0.000E+00 | 0.000E+00 | 0.000E+00 | 0.000E+00 | 0.000E+00 |
| Unigene243281 | Acinetoba | 0.000E+00 | 0.000E+00 | 6.322E-08 | 3.253E-07 | 0.000E+00 | 0.000E+00 | 0.000E+00 | 0.000E+00 | 0.000E+00 | 0.000E+00 | 0.000E+00 | 0.000E+00 |
| Unigene243291 | novA | 1.855E-07 | 0.000E+00 | 0.000E+00 | 9.367E-07 | 9.687E-07 | 2.006E-07 | 2.875E-07 | 1.299E-07 | 4.055E-07 | 9.713E-07 | 0.000E+00 | 0.000E+00 |
| Unigene243292 | tetA(60) | 1.862E-07 | 0.000E+00 | 0.000E+00 | 7.708E-07 | 1.158E-06 | 3.824E-07 | 4.121E-07 | 2.390E-07 | 4.680E-07 | 1.306E-06 | 0.000E+00 | 0.000E+00 |
| Unigene243327 | efrA | 8.325E-08 | 2.513E-07 | 3.063E-07 | 7.146E-07 | 0.000E+00 | 0.000E+00 | 0.000E+00 | 0.000E+00 | 0.000E+00 | 0.000E+00 | 0.000E+00 | 0.000E+00 |
| Unigene243374 | mtrA | 0.000E+00 | 0.000E+00 | 0.000E+00 | 1.364E-06 | 0.000E+00 | 0.000E+00 | 0.000E+00 | 0.000E+00 | 0.000E+00 | 0.000E+00 | 0.000E+00 | 0.000E+00 |
| Unigene243375 | adeS | 0.000E+00 | 0.000E+00 | 0.000E+00 | 1.130E-06 | 0.000E+00 | 0.000E+00 | 0.000E+00 | 0.000E+00 | 0.000E+00 | 0.000E+00 | 0.000E+00 | 0.000E+00 |
| Unigene243415 | tlrC | 0.000E+00 | 0.000E+00 | 0.000E+00 | 2.442E-07 | 0.000E+00 | 0.000E+00 | 0.000E+00 | 0.000E+00 | 0.000E+00 | 0.000E+00 | 0.000E+00 | 0.000E+00 |

| | | | | | | | | | | | | | |
|---|---|---|---|---|---|---|---|---|---|---|---|---|---|
| Unigene243466 | bcrA | 0.000E+00 | 0.000E+00 | 0.000E+00 | 6.791E-07 | 0.000E+00 | 0.000E+00 | 0.000E+00 | 0.000E+00 | 0.000E+00 | 0.000E+00 | 0.000E+00 | 0.000E+00 |
| Unigene243467 | bcrA | 0.000E+00 | 0.000E+00 | 2.354E-07 | 8.997E-07 | 0.000E+00 | 0.000E+00 | 0.000E+00 | 0.000E+00 | 0.000E+00 | 0.000E+00 | 0.000E+00 | 0.000E+00 |
| Unigene243493 | lmrB | 0.000E+00 | 0.000E+00 | 0.000E+00 | 4.233E-07 | 0.000E+00 | 0.000E+00 | 0.000E+00 | 0.000E+00 | 0.000E+00 | 0.000E+00 | 0.000E+00 | 0.000E+00 |
| Unigene243494 | facT | 0.000E+00 | 0.000E+00 | 0.000E+00 | 5.537E-07 | 0.000E+00 | 0.000E+00 | 0.000E+00 | 0.000E+00 | 0.000E+00 | 0.000E+00 | 0.000E+00 | 0.000E+00 |
| Unigene243683 | vanE | 0.000E+00 | 0.000E+00 | 0.000E+00 | 4.043E-07 | 0.000E+00 | 0.000E+00 | 1.662E-07 | 0.000E+00 | 0.000E+00 | 0.000E+00 | 0.000E+00 | 0.000E+00 |
| Unigene243828 | arlR | 0.000E+00 | 0.000E+00 | 0.000E+00 | 1.005E-06 | 0.000E+00 | 1.691E-06 | 5.404E-06 | 0.000E+00 | 0.000E+00 | 0.000E+00 | 0.000E+00 | 0.000E+00 |
| Unigene243935 | oleC | 0.000E+00 | 0.000E+00 | 0.000E+00 | 9.097E-07 | 0.000E+00 | 0.000E+00 | 0.000E+00 | 0.000E+00 | 7.658E-07 | 0.000E+00 | 0.000E+00 | 0.000E+00 |
| Unigene243984 | Bifidobacte | 0.000E+00 | 0.000E+00 | 0.000E+00 | 5.433E-07 | 0.000E+00 | 0.000E+00 | 0.000E+00 | 0.000E+00 | 0.000E+00 | 0.000E+00 | 0.000E+00 | 0.000E+00 |
| Unigene244006 | tet(V) | 4.970E-08 | 1.364E-07 | 2.682E-07 | 6.023E-07 | 0.000E+00 | 0.000E+00 | 2.750E-08 | 0.000E+00 | 2.716E-08 | 0.000E+00 | 0.000E+00 | 0.000E+00 |
| Unigene244066 | Corynebac | 0.000E+00 | 0.000E+00 | 0.000E+00 | 6.970E-07 | 4.511E-08 | 0.000E+00 | 0.000E+00 | 0.000E+00 | 0.000E+00 | 0.000E+00 | 2.322E-07 | 0.000E+00 |
| Unigene244102 | arnA | 0.000E+00 | 0.000E+00 | 0.000E+00 | 7.361E-07 | 0.000E+00 | 0.000E+00 | 0.000E+00 | 0.000E+00 | 0.000E+00 | 7.733E-07 | 0.000E+00 | 0.000E+00 |
| Unigene244121 | macB | 0.000E+00 | 0.000E+00 | 0.000E+00 | 3.721E-07 | 0.000E+00 | 0.000E+00 | 0.000E+00 | 0.000E+00 | 0.000E+00 | 0.000E+00 | 0.000E+00 | 8.711E-08 |
| Unigene244183 | Streptomy | 0.000E+00 | 0.000E+00 | 0.000E+00 | 3.357E-07 | 6.716E-08 | 3.267E-08 | 6.690E-08 | 0.000E+00 | 0.000E+00 | 0.000E+00 | 1.037E-07 | 0.000E+00 |
| Unigene244185 | vanRO | 0.000E+00 | 3.658E-07 | 1.869E-07 | 3.846E-07 | 0.000E+00 | 3.088E-07 | 6.850E-07 | 0.000E+00 | 0.000E+00 | 3.182E-07 | 5.991E-07 | 1.294E-06 |
| Unigene244189 | CblA-1 | 0.000E+00 | 0.000E+00 | 0.000E+00 | 5.655E-07 | 0.000E+00 | 0.000E+00 | 1.328E-07 | 0.000E+00 | 0.000E+00 | 0.000E+00 | 0.000E+00 | 0.000E+00 |
| Unigene244208 | vanYB | 0.000E+00 | 0.000E+00 | 0.000E+00 | 3.650E-06 | 0.000E+00 | 0.000E+00 | 0.000E+00 | 0.000E+00 | 0.000E+00 | 0.000E+00 | 0.000E+00 | 0.000E+00 |
| Unigene244209 | ACC-3 | 0.000E+00 | 0.000E+00 | 9.776E-08 | 4.527E-06 | 0.000E+00 | 0.000E+00 | 0.000E+00 | 0.000E+00 | 0.000E+00 | 0.000E+00 | 0.000E+00 | 0.000E+00 |
| Unigene244308 | mtrA | 4.952E-07 | 1.976E-07 | 8.394E-07 | 2.228E-06 | 5.502E-07 | 1.947E-07 | 2.990E-07 | 8.934E-07 | 0.000E+00 | 7.523E-07 | 1.081E-06 | 0.000E+00 |
| Unigene244358 | emrB | 0.000E+00 | 2.648E-07 | 0.000E+00 | 3.898E-07 | 0.000E+00 | 0.000E+00 | 1.602E-07 | 1.690E-07 | 0.000E+00 | 0.000E+00 | 0.000E+00 | 0.000E+00 |
| Unigene244549 | kdpE | 0.000E+00 | 0.000E+00 | 0.000E+00 | 3.543E-07 | 0.000E+00 | 0.000E+00 | 0.000E+00 | 0.000E+00 | 0.000E+00 | 0.000E+00 | 0.000E+00 | 0.000E+00 |
| Unigene244624 | tlrC | 0.000E+00 | 0.000E+00 | 0.000E+00 | 5.358E-07 | 0.000E+00 | 0.000E+00 | 0.000E+00 | 0.000E+00 | 0.000E+00 | 0.000E+00 | 0.000E+00 | 0.000E+00 |
| Unigene244639 | lfrA | 1.287E-07 | 0.000E+00 | 1.516E-07 | 4.419E-07 | 0.000E+00 | 0.000E+00 | 0.000E+00 | 0.000E+00 | 0.000E+00 | 0.000E+00 | 0.000E+00 | 0.000E+00 |
| Unigene244783 | efpA | 0.000E+00 | 0.000E+00 | 0.000E+00 | 2.183E-06 | 0.000E+00 | 0.000E+00 | 0.000E+00 | 0.000E+00 | 0.000E+00 | 0.000E+00 | 0.000E+00 | 0.000E+00 |
| Unigene245201 | bacA | 0.000E+00 | 0.000E+00 | 0.000E+00 | 1.503E-06 | 0.000E+00 | 0.000E+00 | 0.000E+00 | 0.000E+00 | 0.000E+00 | 0.000E+00 | 0.000E+00 | 0.000E+00 |
| Unigene245227 | optrA | 0.000E+00 | 0.000E+00 | 4.941E-08 | 1.644E-06 | 0.000E+00 | 0.000E+00 | 0.000E+00 | 0.000E+00 | 0.000E+00 | 0.000E+00 | 0.000E+00 | 0.000E+00 |
| Unigene245307 | macB | 3.387E-07 | 7.435E-07 | 1.278E-07 | 6.577E-07 | 2.894E-08 | 0.000E+00 | 2.883E-08 | 0.000E+00 | 0.000E+00 | 0.000E+00 | 0.000E+00 | 0.000E+00 |
| Unigene245501 | optrA | 0.000E+00 | 0.000E+00 | 0.000E+00 | 5.192E-07 | 0.000E+00 | 0.000E+00 | 0.000E+00 | 0.000E+00 | 0.000E+00 | 0.000E+00 | 0.000E+00 | 0.000E+00 |
| Unigene245575 | cpxA | 1.376E-07 | 1.511E-07 | 2.701E-07 | 6.949E-07 | 4.170E-08 | 0.000E+00 | 0.000E+00 | 2.921E-08 | 4.103E-08 | 0.000E+00 | 1.431E-08 | 1.479E-07 |
| Unigene245576 | kdpE | 4.699E-08 | 0.000E+00 | 1.844E-07 | 4.745E-07 | 0.000E+00 | 5.080E-08 | 0.000E+00 | 0.000E+00 | 0.000E+00 | 0.000E+00 | 0.000E+00 | 0.000E+00 |
| Unigene245702 | macB | 0.000E+00 | 0.000E+00 | 0.000E+00 | 6.501E-07 | 0.000E+00 | 0.000E+00 | 0.000E+00 | 0.000E+00 | 0.000E+00 | 0.000E+00 | 0.000E+00 | 0.000E+00 |
| Unigene245830 | basS | 0.000E+00 | 0.000E+00 | 4.710E-07 | 6.867E-07 | 0.000E+00 | 0.000E+00 | 0.000E+00 | 0.000E+00 | 0.000E+00 | 0.000E+00 | 0.000E+00 | 0.000E+00 |
| Unigene245844 | oleC | 2.526E-07 | 0.000E+00 | 0.000E+00 | 6.122E-07 | 0.000E+00 | 2.731E-07 | 0.000E+00 | 0.000E+00 | 5.521E-07 | 0.000E+00 | 0.000E+00 | 0.000E+00 |
| Unigene246021 | Corynebac | 0.000E+00 | 0.000E+00 | 0.000E+00 | 4.969E-07 | 0.000E+00 | 0.000E+00 | 0.000E+00 | 0.000E+00 | 0.000E+00 | 0.000E+00 | 0.000E+00 | 0.000E+00 |
| Unigene246050 | arlS | 0.000E+00 | 0.000E+00 | 0.000E+00 | 8.168E-07 | 0.000E+00 | 1.325E-07 | 0.000E+00 | 0.000E+00 | 3.751E-07 | 0.000E+00 | 0.000E+00 | 0.000E+00 |
| Unigene246238 | macB | 0.000E+00 | 0.000E+00 | 1.683E-07 | 6.061E-07 | 0.000E+00 | 0.000E+00 | 0.000E+00 | 0.000E+00 | 0.000E+00 | 0.000E+00 | 0.000E+00 | 0.000E+00 |
| Unigene246323 | vgaD | 0.000E+00 | 0.000E+00 | 2.618E-07 | 2.694E-07 | 0.000E+00 | 0.000E+00 | 0.000E+00 | 0.000E+00 | 0.000E+00 | 0.000E+00 | 0.000E+00 | 0.000E+00 |
| Unigene246377 | tet32 | 0.000E+00 | 0.000E+00 | 0.000E+00 | 3.642E-07 | 0.000E+00 | 0.000E+00 | 0.000E+00 | 0.000E+00 | 0.000E+00 | 0.000E+00 | 0.000E+00 | 0.000E+00 |
| Unigene246390 | bcrA | 0.000E+00 | 0.000E+00 | 3.798E-07 | 6.254E-07 | 0.000E+00 | 0.000E+00 | 0.000E+00 | 0.000E+00 | 0.000E+00 | 0.000E+00 | 0.000E+00 | 0.000E+00 |
| Unigene246463 | APH(2'')-If | 0.000E+00 | 0.000E+00 | 0.000E+00 | 3.652E-07 | 0.000E+00 | 0.000E+00 | 0.000E+00 | 0.000E+00 | 0.000E+00 | 0.000E+00 | 0.000E+00 | 0.000E+00 |
| Unigene246517 | sul3 | 0.000E+00 | 1.539E-07 | 2.407E-07 | 5.663E-07 | 0.000E+00 | 0.000E+00 | 0.000E+00 | 0.000E+00 | 0.000E+00 | 0.000E+00 | 0.000E+00 | 1.657E-07 |
| Unigene246682 | cmlB1 | 0.000E+00 | 0.000E+00 | 0.000E+00 | 7.858E-07 | 0.000E+00 | 0.000E+00 | 8.909E-08 | 4.072E-07 | 0.000E+00 | 0.000E+00 | 9.208E-08 | 0.000E+00 |
| Unigene246717 | vanHA | 0.000E+00 | 1.410E-07 | 0.000E+00 | 6.811E-07 | 0.000E+00 | 0.000E+00 | 0.000E+00 | 0.000E+00 | 0.000E+00 | 9.661E-07 | 0.000E+00 | 0.000E+00 |
| Unigene246816 | bcrA | 0.000E+00 | 2.262E-07 | 0.000E+00 | 1.624E-06 | 0.000E+00 | 0.000E+00 | 0.000E+00 | 0.000E+00 | 0.000E+00 | 1.194E-06 | 0.000E+00 | 6.335E-07 |
| Unigene246896 | Streptomy | 0.000E+00 | 0.000E+00 | 0.000E+00 | 5.387E-07 | 0.000E+00 | 0.000E+00 | 2.530E-07 | 0.000E+00 | 3.332E-08 | 0.000E+00 | 2.789E-07 | 0.000E+00 |
| Unigene246996 | Corynebac | 0.000E+00 | 0.000E+00 | 0.000E+00 | 8.085E-07 | 0.000E+00 | 0.000E+00 | 0.000E+00 | 0.000E+00 | 0.000E+00 | 0.000E+00 | 0.000E+00 | 0.000E+00 |
| Unigene246998 | cfrC | 0.000E+00 | 0.000E+00 | 0.000E+00 | 0.000E+00 | 0.000E+00 | 7.976E-07 | 0.000E+00 | 2.368E-06 | 0.000E+00 | 0.000E+00 | 0.000E+00 | 2.326E-06 |
| Unigene247065 | tlrC | 0.000E+00 | 0.000E+00 | 1.085E-07 | 4.465E-07 | 0.000E+00 | 0.000E+00 | 0.000E+00 | 8.603E-08 | 0.000E+00 | 0.000E+00 | 0.000E+00 | 0.000E+00 |
| Unigene247094 | optrA | 4.154E-08 | 2.052E-07 | 0.000E+00 | 8.600E-07 | 0.000E+00 | 0.000E+00 | 0.000E+00 | 0.000E+00 | 0.000E+00 | 1.157E-06 | 0.000E+00 | 3.437E-07 |
| Unigene247103 | arlR | 0.000E+00 | 4.055E-07 | 0.000E+00 | 6.062E-07 | 0.000E+00 | 0.000E+00 | 0.000E+00 | 0.000E+00 | 0.000E+00 | 0.000E+00 | 0.000E+00 | 0.000E+00 |
| Unigene247122 | Acinetoba | 0.000E+00 | 0.000E+00 | 5.893E-07 | 1.137E-06 | 0.000E+00 | 0.000E+00 | 0.000E+00 | 0.000E+00 | 0.000E+00 | 0.000E+00 | 0.000E+00 | 0.000E+00 |
| Unigene247131 | Staphyloc | 0.000E+00 | 0.000E+00 | 0.000E+00 | 6.753E-07 | 0.000E+00 | 0.000E+00 | 0.000E+00 | 0.000E+00 | 0.000E+00 | 0.000E+00 | 0.000E+00 | 0.000E+00 |
| Unigene247132 | Streptomy | 0.000E+00 | 0.000E+00 | 2.266E-07 | 5.596E-07 | 0.000E+00 | 0.000E+00 | 0.000E+00 | 5.390E-08 | 0.000E+00 | 0.000E+00 | 0.000E+00 | 0.000E+00 |
| Unigene247430 | macB | 0.000E+00 | 1.961E-06 | 2.844E-06 | 2.245E-06 | 0.000E+00 | 0.000E+00 | 0.000E+00 | 0.000E+00 | 0.000E+00 | 0.000E+00 | 0.000E+00 | 0.000E+00 |
| Unigene247473 | tetB(60) | 2.727E-06 | 3.526E-06 | 2.713E-06 | 2.151E-06 | 1.826E-06 | 1.818E-06 | 1.282E-06 | 1.047E-06 | 1.143E-06 | 4.287E-06 | 5.983E-06 | 1.811E-06 |
| Unigene247587 | adeR | 0.000E+00 | 0.000E+00 | 0.000E+00 | 1.797E-06 | 0.000E+00 | 0.000E+00 | 0.000E+00 | 0.000E+00 | 0.000E+00 | 0.000E+00 | 0.000E+00 | 0.000E+00 |
| Unigene247591 | vanRM | 0.000E+00 | 0.000E+00 | 0.000E+00 | 1.543E-06 | 0.000E+00 | 0.000E+00 | 0.000E+00 | 3.794E-08 | 0.000E+00 | 0.000E+00 | 0.000E+00 | 0.000E+00 |
| Unigene247595 | macB | 0.000E+00 | 0.000E+00 | 0.000E+00 | 1.427E-06 | 0.000E+00 | 0.000E+00 | 0.000E+00 | 0.000E+00 | 0.000E+00 | 0.000E+00 | 0.000E+00 | 0.000E+00 |
| Unigene247800 | Staphyloc | 0.000E+00 | 0.000E+00 | 0.000E+00 | 3.486E-07 | 0.000E+00 | 0.000E+00 | 1.592E-07 | 0.000E+00 | 0.000E+00 | 0.000E+00 | 0.000E+00 | 0.000E+00 |

| | | | | | | | | | | | | | |
|---|---|---|---|---|---|---|---|---|---|---|---|---|---|
| Unigene247831 | patB | 9.316E-08 | 0.000E+00 | 0.000E+00 | 5.645E-07 | 0.000E+00 | 0.000E+00 | 0.000E+00 | 0.000E+00 | 0.000E+00 | 0.000E+00 | 0.000E+00 | 0.000E+00 |
| Unigene247890 | patA | 0.000E+00 | 0.000E+00 | 0.000E+00 | 1.286E-07 | 0.000E+00 | 0.000E+00 | 0.000E+00 | 0.000E+00 | 0.000E+00 | 0.000E+00 | 0.000E+00 | 0.000E+00 |
| Unigene247964 | Corynebac | 0.000E+00 | 0.000E+00 | 0.000E+00 | 9.478E-07 | 0.000E+00 | 0.000E+00 | 0.000E+00 | 3.913E-08 | 0.000E+00 | 0.000E+00 | 0.000E+00 | 0.000E+00 |
| Unigene247993 | rosA | 0.000E+00 | 0.000E+00 | 0.000E+00 | 4.075E-07 | 0.000E+00 | 0.000E+00 | 0.000E+00 | 0.000E+00 | 0.000E+00 | 1.319E-06 | 0.000E+00 | 3.243E-06 |
| Unigene247996 | TaeA | 0.000E+00 | 0.000E+00 | 0.000E+00 | 2.379E-07 | 0.000E+00 | 0.000E+00 | 4.346E-08 | 0.000E+00 | 0.000E+00 | 0.000E+00 | 1.797E-07 | 0.000E+00 |
| Unigene248078 | patA | 0.000E+00 | 9.941E-08 | 3.733E-07 | 5.378E-06 | 0.000E+00 | 0.000E+00 | 0.000E+00 | 0.000E+00 | 0.000E+00 | 0.000E+00 | 0.000E+00 | 0.000E+00 |
| Unigene248211 | cmlv | 0.000E+00 | 0.000E+00 | 0.000E+00 | 1.122E-06 | 0.000E+00 | 0.000E+00 | 0.000E+00 | 0.000E+00 | 0.000E+00 | 0.000E+00 | 0.000E+00 | 1.922E-07 |
| Unigene248235 | eptA | 0.000E+00 | 0.000E+00 | 0.000E+00 | 3.018E-07 | 0.000E+00 | 0.000E+00 | 0.000E+00 | 0.000E+00 | 0.000E+00 | 0.000E+00 | 0.000E+00 | 0.000E+00 |
| Unigene248551 | macB | 2.093E-07 | 0.000E+00 | 0.000E+00 | 7.610E-07 | 0.000E+00 | 0.000E+00 | 0.000E+00 | 0.000E+00 | 5.034E-07 | 0.000E+00 | 0.000E+00 | 0.000E+00 |
| Unigene248579 | macB | 0.000E+00 | 0.000E+00 | 0.000E+00 | 5.613E-07 | 0.000E+00 | 0.000E+00 | 0.000E+00 | 0.000E+00 | 0.000E+00 | 0.000E+00 | 0.000E+00 | 0.000E+00 |
| Unigene248624 | rpoB2 | 0.000E+00 | 0.000E+00 | 5.088E-07 | 4.800E-07 | 0.000E+00 | 0.000E+00 | 1.937E-06 | 2.018E-06 | 0.000E+00 | 0.000E+00 | 9.392E-07 | 2.452E-06 |
| Unigene248733 | evgS | 0.000E+00 | 0.000E+00 | 5.586E-08 | 4.024E-07 | 0.000E+00 | 0.000E+00 | 0.000E+00 | 0.000E+00 | 0.000E+00 | 0.000E+00 | 0.000E+00 | 0.000E+00 |
| Unigene248782 | poxtA | 0.000E+00 | 0.000E+00 | 0.000E+00 | 2.835E-07 | 0.000E+00 | 0.000E+00 | 0.000E+00 | 0.000E+00 | 0.000E+00 | 0.000E+00 | 0.000E+00 | 0.000E+00 |
| Unigene248878 | adeF | 0.000E+00 | 0.000E+00 | 3.536E-07 | 2.599E-07 | 0.000E+00 | 0.000E+00 | 0.000E+00 | 0.000E+00 | 0.000E+00 | 0.000E+00 | 0.000E+00 | 0.000E+00 |
| Unigene249018 | PmrF | 0.000E+00 | 0.000E+00 | 0.000E+00 | 7.657E-07 | 0.000E+00 | 0.000E+00 | 0.000E+00 | 0.000E+00 | 0.000E+00 | 0.000E+00 | 0.000E+00 | 0.000E+00 |
| Unigene249025 | tet(59) | 0.000E+00 | 0.000E+00 | 0.000E+00 | 3.813E-07 | 0.000E+00 | 0.000E+00 | 0.000E+00 | 0.000E+00 | 0.000E+00 | 0.000E+00 | 0.000E+00 | 0.000E+00 |
| Unigene249077 | dfrI | 6.561E-07 | 8.641E-07 | 8.369E-07 | 6.625E-07 | 1.516E-05 | 4.610E-06 | 9.730E-06 | 1.310E-05 | 8.534E-06 | 2.127E-05 | 2.184E-05 | 6.204E-06 |
| Unigene249088 | vanHD | 1.941E-07 | 1.826E-07 | 6.259E-07 | 6.441E-07 | 9.243E-08 | 0.000E+00 | 0.000E+00 | 0.000E+00 | 3.031E-08 | 0.000E+00 | 0.000E+00 | 1.639E-07 |
| Unigene249170 | patB | 0.000E+00 | 0.000E+00 | 1.913E-07 | 5.118E-07 | 0.000E+00 | 0.000E+00 | 0.000E+00 | 0.000E+00 | 0.000E+00 | 0.000E+00 | 0.000E+00 | 0.000E+00 |
| Unigene249198 | vanSA | 0.000E+00 | 0.000E+00 | 3.347E-07 | 5.323E-06 | 0.000E+00 | 0.000E+00 | 0.000E+00 | 0.000E+00 | 0.000E+00 | 0.000E+00 | 0.000E+00 | 0.000E+00 |
| Unigene249227 | lmrD | 0.000E+00 | 0.000E+00 | 1.126E-06 | 5.752E-06 | 0.000E+00 | 0.000E+00 | 0.000E+00 | 0.000E+00 | 0.000E+00 | 0.000E+00 | 0.000E+00 | 0.000E+00 |
| Unigene249243 | YojI | 0.000E+00 | 3.204E-07 | 3.274E-07 | 1.095E-06 | 0.000E+00 | 0.000E+00 | 0.000E+00 | 0.000E+00 | 0.000E+00 | 0.000E+00 | 0.000E+00 | 0.000E+00 |
| Unigene249244 | bcrA | 1.855E-07 | 4.581E-07 | 4.551E-07 | 7.025E-07 | 0.000E+00 | 0.000E+00 | 0.000E+00 | 0.000E+00 | 3.042E-07 | 0.000E+00 | 0.000E+00 | 0.000E+00 |
| Unigene249404 | arnA | 0.000E+00 | 0.000E+00 | 0.000E+00 | 5.351E-07 | 0.000E+00 | 0.000E+00 | 9.022E-08 | 1.427E-07 | 0.000E+00 | 0.000E+00 | 0.000E+00 | 0.000E+00 |
| Unigene249424 | sul4 | 0.000E+00 | 2.277E-07 | 0.000E+00 | 7.184E-07 | 0.000E+00 | 0.000E+00 | 0.000E+00 | 0.000E+00 | 0.000E+00 | 0.000E+00 | 0.000E+00 | 4.205E-07 |
| Unigene249522 | vmlR | 0.000E+00 | 7.744E-07 | 3.877E-06 | 3.634E-06 | 0.000E+00 | 0.000E+00 | 0.000E+00 | 0.000E+00 | 0.000E+00 | 0.000E+00 | 0.000E+00 | 0.000E+00 |
| Unigene249561 | novA | 0.000E+00 | 1.263E-07 | 0.000E+00 | 4.648E-07 | 0.000E+00 | 0.000E+00 | 0.000E+00 | 0.000E+00 | 0.000E+00 | 0.000E+00 | 0.000E+00 | 0.000E+00 |
| Unigene249571 | bacA | 0.000E+00 | 0.000E+00 | 0.000E+00 | 6.014E-07 | 1.764E-07 | 0.000E+00 | 0.000E+00 | 0.000E+00 | 0.000E+00 | 0.000E+00 | 9.084E-08 | 0.000E+00 |
| Unigene249609 | mdtG | 9.004E-08 | 1.878E-06 | 5.301E-07 | 7.274E-07 | 0.000E+00 | 0.000E+00 | 0.000E+00 | 0.000E+00 | 0.000E+00 | 0.000E+00 | 0.000E+00 | 0.000E+00 |
| Unigene249739 | lmrB | 4.658E-08 | 1.789E-07 | 0.000E+00 | 7.761E-07 | 0.000E+00 | 0.000E+00 | 0.000E+00 | 0.000E+00 | 0.000E+00 | 0.000E+00 | 0.000E+00 | 3.028E-07 |
| Unigene249753 | oleC | 0.000E+00 | 0.000E+00 | 0.000E+00 | 3.865E-07 | 0.000E+00 | 0.000E+00 | 0.000E+00 | 0.000E+00 | 0.000E+00 | 0.000E+00 | 0.000E+00 | 0.000E+00 |
| Unigene249785 | patB | 0.000E+00 | 0.000E+00 | 0.000E+00 | 5.740E-07 | 0.000E+00 | 0.000E+00 | 0.000E+00 | 0.000E+00 | 0.000E+00 | 0.000E+00 | 0.000E+00 | 0.000E+00 |
| Unigene249802 | vanSG | 0.000E+00 | 0.000E+00 | 0.000E+00 | 4.484E-07 | 0.000E+00 | 0.000E+00 | 1.134E-07 | 3.190E-07 | 7.467E-08 | 0.000E+00 | 1.954E-07 | 0.000E+00 |
| Unigene249807 | Streptomy | 1.012E-07 | 7.937E-08 | 1.362E-06 | 4.703E-06 | 0.000E+00 | 0.000E+00 | 0.000E+00 | 0.000E+00 | 0.000E+00 | 0.000E+00 | 0.000E+00 | 0.000E+00 |
| Unigene249896 | vanHO | 3.913E-07 | 4.652E-07 | 9.598E-07 | 3.787E-06 | 5.796E-07 | 0.000E+00 | 3.609E-07 | 1.370E-06 | 1.069E-07 | 4.359E-07 | 1.343E-06 | 0.000E+00 |
| Unigene250105 | efrB | 0.000E+00 | 1.104E-07 | 0.000E+00 | 9.139E-07 | 0.000E+00 | 0.000E+00 | 0.000E+00 | 0.000E+00 | 0.000E+00 | 6.721E-07 | 0.000E+00 | 2.972E-07 |
| Unigene250144 | Staphylocc | 0.000E+00 | 0.000E+00 | 0.000E+00 | 5.137E-07 | 0.000E+00 | 0.000E+00 | 0.000E+00 | 0.000E+00 | 0.000E+00 | 0.000E+00 | 0.000E+00 | 0.000E+00 |
| Unigene250148 | ugd | 0.000E+00 | 1.510E-07 | 1.350E-07 | 4.891E-06 | 0.000E+00 | 0.000E+00 | 0.000E+00 | 0.000E+00 | 0.000E+00 | 0.000E+00 | 0.000E+00 | 0.000E+00 |
| Unigene250231 | baeS | 0.000E+00 | 2.721E-08 | 2.433E-08 | 4.757E-07 | 0.000E+00 | 0.000E+00 | 0.000E+00 | 0.000E+00 | 0.000E+00 | 0.000E+00 | 0.000E+00 | 0.000E+00 |
| Unigene250397 | facT | 1.945E-07 | 0.000E+00 | 6.996E-07 | 0.000E+00 | 0.000E+00 | 0.000E+00 | 0.000E+00 | 0.000E+00 | 1.417E-07 | 0.000E+00 | 0.000E+00 | 0.000E+00 |
| Unigene250511 | PmrF | 0.000E+00 | 0.000E+00 | 0.000E+00 | 1.083E-06 | 1.384E-06 | 0.000E+00 | 0.000E+00 | 2.423E-07 | 3.403E-07 | 8.094E-07 | 0.000E+00 | 0.000E+00 |
| Unigene250601 | ykkC | 0.000E+00 | 6.252E-06 | 1.153E-05 | 1.771E-05 | 0.000E+00 | 0.000E+00 | 0.000E+00 | 0.000E+00 | 0.000E+00 | 0.000E+00 | 0.000E+00 | 0.000E+00 |
| Unigene250630 | Streptomy | 0.000E+00 | 0.000E+00 | 0.000E+00 | 4.486E-07 | 0.000E+00 | 0.000E+00 | 0.000E+00 | 0.000E+00 | 0.000E+00 | 0.000E+00 | 0.000E+00 | 0.000E+00 |
| Unigene250663 | efrA | 0.000E+00 | 4.714E-08 | 1.264E-07 | 4.771E-07 | 0.000E+00 | 0.000E+00 | 0.000E+00 | 0.000E+00 | 0.000E+00 | 0.000E+00 | 0.000E+00 | 0.000E+00 |
| Unigene250674 | sul4 | 0.000E+00 | 0.000E+00 | 3.849E-07 | 0.000E+00 | 0.000E+00 | 0.000E+00 | 0.000E+00 | 0.000E+00 | 0.000E+00 | 0.000E+00 | 0.000E+00 | 0.000E+00 |
| Unigene250688 | rphA | 0.000E+00 | 1.474E-08 | 0.000E+00 | 7.573E-05 | 0.000E+00 | 0.000E+00 | 0.000E+00 | 0.000E+00 | 0.000E+00 | 0.000E+00 | 0.000E+00 | 0.000E+00 |
| Unigene250766 | efrB | 0.000E+00 | 0.000E+00 | 0.000E+00 | 4.481E-07 | 0.000E+00 | 0.000E+00 | 0.000E+00 | 0.000E+00 | 0.000E+00 | 0.000E+00 | 0.000E+00 | 3.278E-07 |
| Unigene250769 | arlS | 0.000E+00 | 0.000E+00 | 0.000E+00 | 6.476E-07 | 1.583E-07 | 0.000E+00 | 0.000E+00 | 0.000E+00 | 0.000E+00 | 0.000E+00 | 0.000E+00 | 0.000E+00 |
| Unigene250784 | oleC | 0.000E+00 | 0.000E+00 | 0.000E+00 | 6.093E-07 | 0.000E+00 | 0.000E+00 | 0.000E+00 | 0.000E+00 | 0.000E+00 | 0.000E+00 | 0.000E+00 | 0.000E+00 |
| Unigene250851 | macB | 0.000E+00 | 2.352E-07 | 3.786E-07 | 5.628E-07 | 0.000E+00 | 0.000E+00 | 0.000E+00 | 5.004E-08 | 0.000E+00 | 0.000E+00 | 4.904E-08 | 0.000E+00 |
| Unigene250858 | lmrD | 0.000E+00 | 0.000E+00 | 0.000E+00 | 5.065E-07 | 0.000E+00 | 0.000E+00 | 0.000E+00 | 0.000E+00 | 0.000E+00 | 0.000E+00 | 0.000E+00 | 0.000E+00 |
| Unigene251040 | Klebsiella p | 0.000E+00 | 0.000E+00 | 0.000E+00 | 5.749E-07 | 6.325E-08 | 0.000E+00 | 3.150E-07 | 0.000E+00 | 0.000E+00 | 0.000E+00 | 0.000E+00 | 0.000E+00 |
| Unigene251089 | mtrA | 0.000E+00 | 0.000E+00 | 0.000E+00 | 3.622E-07 | 0.000E+00 | 0.000E+00 | 0.000E+00 | 0.000E+00 | 0.000E+00 | 0.000E+00 | 0.000E+00 | 0.000E+00 |
| Unigene251104 | Staphylocc | 0.000E+00 | 0.000E+00 | 0.000E+00 | 5.388E-07 | 0.000E+00 | 0.000E+00 | 0.000E+00 | 0.000E+00 | 0.000E+00 | 0.000E+00 | 0.000E+00 | 0.000E+00 |
| Unigene251245 | optrA | 0.000E+00 | 0.000E+00 | 0.000E+00 | 6.159E-07 | 0.000E+00 | 0.000E+00 | 0.000E+00 | 0.000E+00 | 0.000E+00 | 0.000E+00 | 0.000E+00 | 0.000E+00 |
| Unigene251280 | vanSM | 0.000E+00 | 0.000E+00 | 0.000E+00 | 3.196E-07 | 0.000E+00 | 0.000E+00 | 0.000E+00 | 0.000E+00 | 0.000E+00 | 0.000E+00 | 0.000E+00 | 0.000E+00 |
| Unigene251369 | vanZF | 0.000E+00 | 0.000E+00 | 0.000E+00 | 0.000E+00 | 0.000E+00 | 0.000E+00 | 0.000E+00 | 0.000E+00 | 0.000E+00 | 6.309E-07 | 0.000E+00 | 0.000E+00 |
| Unigene251424 | basS | 0.000E+00 | 0.000E+00 | 0.000E+00 | 5.193E-07 | 0.000E+00 | 0.000E+00 | 0.000E+00 | 5.079E-07 | 0.000E+00 | 0.000E+00 | 0.000E+00 | 0.000E+00 |

| | | | | | | | | | | | | | |
|---|---|---|---|---|---|---|---|---|---|---|---|---|---|
| Unigene251629 | MexI | 0.000E+00 | 0.000E+00 | 0.000E+00 | 2.690E-07 | 0.000E+00 | 0.000E+00 | 0.000E+00 | 0.000E+00 | 0.000E+00 | 5.934E-07 | 0.000E+00 | 0.000E+00 |
| Unigene251682 | macB | 2.552E-07 | 0.000E+00 | 1.419E-06 | 6.700E-06 | 0.000E+00 | 0.000E+00 | 0.000E+00 | 0.000E+00 | 0.000E+00 | 0.000E+00 | 0.000E+00 | 0.000E+00 |
| Unigene251708 | optrA | 0.000E+00 | 0.000E+00 | 0.000E+00 | 4.787E-07 | 0.000E+00 | 0.000E+00 | 0.000E+00 | 0.000E+00 | 0.000E+00 | 0.000E+00 | 0.000E+00 | 0.000E+00 |
| Unigene251802 | msbA | 0.000E+00 | 0.000E+00 | 3.744E-08 | 1.580E-06 | 0.000E+00 | 0.000E+00 | 0.000E+00 | 0.000E+00 | 0.000E+00 | 0.000E+00 | 0.000E+00 | 0.000E+00 |
| Unigene251803 | tetB(60) | 0.000E+00 | 0.000E+00 | 0.000E+00 | 1.760E-06 | 0.000E+00 | 0.000E+00 | 0.000E+00 | 0.000E+00 | 0.000E+00 | 0.000E+00 | 0.000E+00 | 0.000E+00 |
| Unigene251831 | bcrC | 0.000E+00 | 0.000E+00 | 5.707E-08 | 1.116E-06 | 0.000E+00 | 0.000E+00 | 0.000E+00 | 0.000E+00 | 0.000E+00 | 0.000E+00 | 0.000E+00 | 0.000E+00 |
| Unigene251870 | PmrF | 0.000E+00 | 0.000E+00 | 0.000E+00 | 1.867E-06 | 0.000E+00 | 0.000E+00 | 0.000E+00 | 0.000E+00 | 0.000E+00 | 0.000E+00 | 0.000E+00 | 0.000E+00 |
| Unigene251871 | arlR | 0.000E+00 | 0.000E+00 | 4.672E-08 | 1.731E-06 | 0.000E+00 | 0.000E+00 | 0.000E+00 | 0.000E+00 | 0.000E+00 | 0.000E+00 | 0.000E+00 | 0.000E+00 |
| Unigene251872 | smeS | 0.000E+00 | 0.000E+00 | 0.000E+00 | 1.680E-06 | 0.000E+00 | 0.000E+00 | 0.000E+00 | 0.000E+00 | 0.000E+00 | 0.000E+00 | 0.000E+00 | 0.000E+00 |
| Unigene251881 | vanHM | 0.000E+00 | 0.000E+00 | 8.091E-08 | 2.290E-06 | 0.000E+00 | 0.000E+00 | 0.000E+00 | 0.000E+00 | 0.000E+00 | 0.000E+00 | 0.000E+00 | 0.000E+00 |
| Unigene252045 | smeS | 0.000E+00 | 0.000E+00 | 0.000E+00 | 3.790E-07 | 0.000E+00 | 0.000E+00 | 0.000E+00 | 0.000E+00 | 0.000E+00 | 0.000E+00 | 0.000E+00 | 0.000E+00 |
| Unigene252079 | otr(B) | 0.000E+00 | 1.789E-07 | 7.999E-07 | 8.232E-07 | 0.000E+00 | 0.000E+00 | 0.000E+00 | 0.000E+00 | 0.000E+00 | 0.000E+00 | 0.000E+00 | 0.000E+00 |
| Unigene252103 | Acinetoba | 0.000E+00 | 0.000E+00 | 0.000E+00 | 3.426E-07 | 4.846E-07 | 0.000E+00 | 2.146E-07 | 0.000E+00 | 5.298E-08 | 0.000E+00 | 5.544E-08 | 2.292E-07 |
| Unigene252106 | oleI | 1.108E-06 | 2.144E-06 | 2.432E-06 | 4.240E-06 | 0.000E+00 | 0.000E+00 | 0.000E+00 | 0.000E+00 | 0.000E+00 | 0.000E+00 | 0.000E+00 | 0.000E+00 |
| Unigene252119 | tet(43) | 2.196E-07 | 1.096E-07 | 4.505E-07 | 6.451E-07 | 2.218E-08 | 6.474E-08 | 0.000E+00 | 0.000E+00 | 2.182E-08 | 0.000E+00 | 2.284E-08 | 1.180E-07 |
| Unigene252162 | oleB | 2.948E-07 | 0.000E+00 | 0.000E+00 | 9.923E-07 | 0.000E+00 | 0.000E+00 | 0.000E+00 | 0.000E+00 | 0.000E+00 | 0.000E+00 | 0.000E+00 | 4.065E-07 |
| Unigene252211 | lmrD | 8.942E-08 | 9.814E-08 | 6.434E-07 | 6.923E-07 | 0.000E+00 | 9.667E-08 | 0.000E+00 | 0.000E+00 | 0.000E+00 | 0.000E+00 | 0.000E+00 | 0.000E+00 |
| Unigene252243 | fusH | 0.000E+00 | 0.000E+00 | 0.000E+00 | 3.987E-07 | 0.000E+00 | 0.000E+00 | 0.000E+00 | 5.761E-08 | 0.000E+00 | 0.000E+00 | 0.000E+00 | 0.000E+00 |
| Unigene252261 | acrB | 0.000E+00 | 0.000E+00 | 3.519E-07 | 5.886E-07 | 0.000E+00 | 0.000E+00 | 4.466E-07 | 2.617E-07 | 0.000E+00 | 0.000E+00 | 0.000E+00 | 1.590E-07 |
| Unigene252434 | arlR | 2.893E-06 | 4.190E-06 | 0.000E+00 | 5.959E-06 | 0.000E+00 | 0.000E+00 | 0.000E+00 | 0.000E+00 | 0.000E+00 | 0.000E+00 | 0.000E+00 | 0.000E+00 |
| Unigene252447 | cpxA | 0.000E+00 | 2.846E-07 | 3.816E-07 | 0.000E+00 | 0.000E+00 | 0.000E+00 | 0.000E+00 | 0.000E+00 | 0.000E+00 | 0.000E+00 | 0.000E+00 | 0.000E+00 |
| Unigene252556 | efrA | 0.000E+00 | 0.000E+00 | 0.000E+00 | 5.481E-07 | 0.000E+00 | 0.000E+00 | 0.000E+00 | 0.000E+00 | 0.000E+00 | 0.000E+00 | 0.000E+00 | 1.132E-07 |
| Unigene252694 | novA | 1.495E-06 | 8.710E-07 | 3.387E-06 | 2.255E-06 | 0.000E+00 | 0.000E+00 | 0.000E+00 | 0.000E+00 | 0.000E+00 | 0.000E+00 | 0.000E+00 | 0.000E+00 |
| Unigene252758 | vgaB | 5.380E-08 | 1.181E-07 | 1.056E-07 | 4.890E-07 | 0.000E+00 | 0.000E+00 | 0.000E+00 | 0.000E+00 | 0.000E+00 | 0.000E+00 | 0.000E+00 | 0.000E+00 |
| Unigene252770 | novA | 0.000E+00 | 0.000E+00 | 0.000E+00 | 3.874E-07 | 0.000E+00 | 0.000E+00 | 1.061E-07 | 0.000E+00 | 0.000E+00 | 0.000E+00 | 0.000E+00 | 2.267E-08 |
| Unigene252813 | acrB | 0.000E+00 | 7.873E-07 | 0.000E+00 | 5.886E-07 | 0.000E+00 | 0.000E+00 | 0.000E+00 | 0.000E+00 | 0.000E+00 | 0.000E+00 | 0.000E+00 | 0.000E+00 |
| Unigene252830 | tet(30) | 0.000E+00 | 0.000E+00 | 0.000E+00 | 5.447E-07 | 0.000E+00 | 0.000E+00 | 0.000E+00 | 0.000E+00 | 0.000E+00 | 0.000E+00 | 0.000E+00 | 0.000E+00 |
| Unigene252839 | vanTE | 0.000E+00 | 0.000E+00 | 0.000E+00 | 1.127E-06 | 0.000E+00 | 0.000E+00 | 0.000E+00 | 0.000E+00 | 0.000E+00 | 0.000E+00 | 0.000E+00 | 0.000E+00 |
| Unigene252857 | vanRA | 0.000E+00 | 0.000E+00 | 0.000E+00 | 2.977E-07 | 0.000E+00 | 0.000E+00 | 0.000E+00 | 0.000E+00 | 0.000E+00 | 0.000E+00 | 0.000E+00 | 3.833E-06 |
| Unigene252888 | oleC | 0.000E+00 | 0.000E+00 | 1.325E-07 | 8.638E-07 | 0.000E+00 | 0.000E+00 | 0.000E+00 | 0.000E+00 | 0.000E+00 | 1.505E-07 | 0.000E+00 | 5.322E-08 |
| Unigene252902 | tetA(58) | 0.000E+00 | 0.000E+00 | 1.077E-07 | 0.000E+00 | 0.000E+00 | 0.000E+00 | 0.000E+00 | 0.000E+00 | 0.000E+00 | 0.000E+00 | 0.000E+00 | 0.000E+00 |
| Unigene252942 | tetB(58) | 0.000E+00 | 1.384E-07 | 0.000E+00 | 7.216E-07 | 0.000E+00 | 0.000E+00 | 0.000E+00 | 0.000E+00 | 0.000E+00 | 9.364E-07 | 0.000E+00 | 3.478E-07 |
| Unigene252943 | tetA(58) | 0.000E+00 | 0.000E+00 | 0.000E+00 | 8.358E-07 | 0.000E+00 | 0.000E+00 | 0.000E+00 | 0.000E+00 | 0.000E+00 | 8.511E-07 | 0.000E+00 | 1.505E-07 |
| Unigene252953 | mecD | 0.000E+00 | 0.000E+00 | 0.000E+00 | 3.881E-07 | 0.000E+00 | 0.000E+00 | 0.000E+00 | 0.000E+00 | 0.000E+00 | 0.000E+00 | 0.000E+00 | 0.000E+00 |
| Unigene252970 | oleB | 0.000E+00 | 0.000E+00 | 0.000E+00 | 4.582E-07 | 0.000E+00 | 0.000E+00 | 0.000E+00 | 0.000E+00 | 0.000E+00 | 0.000E+00 | 0.000E+00 | 0.000E+00 |
| Unigene252974 | Klebsiella p | 0.000E+00 | 1.050E-07 | 0.000E+00 | 4.346E-07 | 0.000E+00 | 0.000E+00 | 0.000E+00 | 0.000E+00 | 0.000E+00 | 0.000E+00 | 0.000E+00 | 0.000E+00 |
| Unigene253198 | Streptomy | 0.000E+00 | 0.000E+00 | 2.292E-07 | 4.293E-06 | 0.000E+00 | 1.683E-08 | 0.000E+00 | 0.000E+00 | 0.000E+00 | 0.000E+00 | 0.000E+00 | 0.000E+00 |
| Unigene253217 | farA | 0.000E+00 | 0.000E+00 | 3.312E-07 | 3.409E-07 | 0.000E+00 | 0.000E+00 | 0.000E+00 | 0.000E+00 | 0.000E+00 | 0.000E+00 | 0.000E+00 | 0.000E+00 |
| Unigene253229 | bcrA | 1.594E-07 | 0.000E+00 | 0.000E+00 | 8.451E-07 | 0.000E+00 | 0.000E+00 | 0.000E+00 | 0.000E+00 | 0.000E+00 | 7.103E-07 | 0.000E+00 | 7.066E-07 |
| Unigene253338 | Staphylocc | 9.144E-07 | 6.255E-06 | 9.870E-06 | 1.619E-05 | 2.362E-08 | 6.897E-08 | 0.000E+00 | 0.000E+00 | 0.000E+00 | 0.000E+00 | 9.730E-08 | 7.541E-08 |
| Unigene253360 | novA | 0.000E+00 | 0.000E+00 | 0.000E+00 | 3.784E-07 | 0.000E+00 | 0.000E+00 | 0.000E+00 | 0.000E+00 | 1.755E-07 | 0.000E+00 | 0.000E+00 | 0.000E+00 |
| Unigene253398 | mdtG | 0.000E+00 | 2.350E-07 | 3.677E-07 | 3.244E-07 | 0.000E+00 | 0.000E+00 | 0.000E+00 | 2.499E-07 | 0.000E+00 | 0.000E+00 | 0.000E+00 | 0.000E+00 |
| Unigene253450 | Acinetoba | 0.000E+00 | 0.000E+00 | 9.076E-08 | 6.538E-07 | 0.000E+00 | 0.000E+00 | 0.000E+00 | 0.000E+00 | 0.000E+00 | 0.000E+00 | 0.000E+00 | 0.000E+00 |
| Unigene253601 | bcrA | 0.000E+00 | 0.000E+00 | 0.000E+00 | 5.286E-07 | 0.000E+00 | 0.000E+00 | 6.437E-08 | 0.000E+00 | 0.000E+00 | 0.000E+00 | 0.000E+00 | 0.000E+00 |
| Unigene253621 | aadK | 0.000E+00 | 0.000E+00 | 0.000E+00 | 1.644E-06 | 0.000E+00 | 0.000E+00 | 0.000E+00 | 0.000E+00 | 0.000E+00 | 0.000E+00 | 0.000E+00 | 0.000E+00 |
| Unigene253622 | Staphylocc | 0.000E+00 | 2.486E-07 | 0.000E+00 | 9.608E-07 | 0.000E+00 | 0.000E+00 | 0.000E+00 | 0.000E+00 | 0.000E+00 | 7.318E-07 | 0.000E+00 | 2.678E-07 |
| Unigene253649 | macB | 0.000E+00 | 0.000E+00 | 0.000E+00 | 1.073E-06 | 0.000E+00 | 0.000E+00 | 0.000E+00 | 0.000E+00 | 0.000E+00 | 0.000E+00 | 0.000E+00 | 0.000E+00 |
| Unigene253683 | novA | 0.000E+00 | 0.000E+00 | 0.000E+00 | 6.506E-07 | 0.000E+00 | 0.000E+00 | 0.000E+00 | 0.000E+00 | 0.000E+00 | 0.000E+00 | 1.106E-07 | 0.000E+00 |
| Unigene253746 | bcrA | 0.000E+00 | 4.705E-08 | 2.945E-07 | 9.091E-07 | 0.000E+00 | 0.000E+00 | 0.000E+00 | 0.000E+00 | 0.000E+00 | 0.000E+00 | 0.000E+00 | 0.000E+00 |
| Unigene253805 | qacH | 0.000E+00 | 0.000E+00 | 0.000E+00 | 7.108E-07 | 0.000E+00 | 0.000E+00 | 0.000E+00 | 0.000E+00 | 0.000E+00 | 0.000E+00 | 0.000E+00 | 0.000E+00 |
| Unigene253812 | mtrA | 0.000E+00 | 2.435E-07 | 0.000E+00 | 6.721E-07 | 0.000E+00 | 0.000E+00 | 0.000E+00 | 0.000E+00 | 0.000E+00 | 0.000E+00 | 0.000E+00 | 0.000E+00 |
| Unigene253923 | evgS | 0.000E+00 | 0.000E+00 | 0.000E+00 | 4.269E-07 | 0.000E+00 | 0.000E+00 | 0.000E+00 | 0.000E+00 | 0.000E+00 | 0.000E+00 | 0.000E+00 | 0.000E+00 |
| Unigene253988 | novA | 7.741E-08 | 0.000E+00 | 0.000E+00 | 2.345E-07 | 0.000E+00 | 0.000E+00 | 0.000E+00 | 0.000E+00 | 0.000E+00 | 0.000E+00 | 0.000E+00 | 0.000E+00 |
| Unigene254106 | arlS | 0.000E+00 | 0.000E+00 | 0.000E+00 | 5.099E-07 | 0.000E+00 | 0.000E+00 | 0.000E+00 | 0.000E+00 | 0.000E+00 | 0.000E+00 | 0.000E+00 | 0.000E+00 |
| Unigene254112 | vanRM | 0.000E+00 | 0.000E+00 | 0.000E+00 | 5.197E-07 | 0.000E+00 | 0.000E+00 | 0.000E+00 | 0.000E+00 | 0.000E+00 | 0.000E+00 | 0.000E+00 | 0.000E+00 |
| Unigene254184 | lmrD | 0.000E+00 | 0.000E+00 | 5.414E-08 | 4.458E-07 | 0.000E+00 | 0.000E+00 | 0.000E+00 | 0.000E+00 | 0.000E+00 | 0.000E+00 | 0.000E+00 | 0.000E+00 |
| Unigene254285 | tetB(60) | 0.000E+00 | 0.000E+00 | 3.132E-07 | 3.223E-07 | 0.000E+00 | 0.000E+00 | 0.000E+00 | 0.000E+00 | 0.000E+00 | 0.000E+00 | 0.000E+00 | 0.000E+00 |
| Unigene254290 | Streptomy | 0.000E+00 | 0.000E+00 | 0.000E+00 | 7.594E-07 | 0.000E+00 | 0.000E+00 | 0.000E+00 | 0.000E+00 | 0.000E+00 | 0.000E+00 | 0.000E+00 | 0.000E+00 |

| | | | | | | | | | | | | | |
|---|---|---|---|---|---|---|---|---|---|---|---|---|---|
| Unigene254292 | Corynebac | 1.198E-07 | 2.104E-07 | 1.411E-07 | 8.712E-07 | 7.987E-08 | 0.000E+00 | 0.000E+00 | 0.000E+00 | 0.000E+00 | 0.000E+00 | 0.000E+00 | 0.000E+00 |
| Unigene254369 | sul4 | 6.356E-07 | 0.000E+00 | 0.000E+00 | 5.502E-07 | 3.077E-06 | 8.785E-06 | 1.558E-06 | 0.000E+00 | 5.210E-06 | 3.338E-06 | 0.000E+00 | 1.127E-06 |
| Unigene254377 | kdpE | 0.000E+00 | 0.000E+00 | 0.000E+00 | 6.640E-07 | 0.000E+00 | 0.000E+00 | 0.000E+00 | 1.047E-06 | 0.000E+00 | 0.000E+00 | 0.000E+00 | 0.000E+00 |
| Unigene254382 | Chlamydia | 0.000E+00 | 0.000E+00 | 7.166E-08 | 4.450E-06 | 0.000E+00 | 0.000E+00 | 0.000E+00 | 0.000E+00 | 0.000E+00 | 0.000E+00 | 0.000E+00 | 0.000E+00 |
| Unigene254423 | vanHO | 0.000E+00 | 0.000E+00 | 0.000E+00 | 5.942E-07 | 0.000E+00 | 0.000E+00 | 0.000E+00 | 0.000E+00 | 0.000E+00 | 0.000E+00 | 0.000E+00 | 0.000E+00 |
| Unigene254547 | msbA | 0.000E+00 | 0.000E+00 | 0.000E+00 | 6.531E-07 | 0.000E+00 | 0.000E+00 | 0.000E+00 | 0.000E+00 | 0.000E+00 | 0.000E+00 | 0.000E+00 | 0.000E+00 |
| Unigene254557 | otr(A) | 0.000E+00 | 0.000E+00 | 0.000E+00 | 1.013E-06 | 0.000E+00 | 0.000E+00 | 0.000E+00 | 0.000E+00 | 0.000E+00 | 0.000E+00 | 0.000E+00 | 0.000E+00 |
| Unigene254565 | TaeA | 0.000E+00 | 5.650E-08 | 0.000E+00 | 2.599E-07 | 0.000E+00 | 0.000E+00 | 0.000E+00 | 0.000E+00 | 0.000E+00 | 0.000E+00 | 0.000E+00 | 0.000E+00 |
| Unigene254705 | efrA | 0.000E+00 | 0.000E+00 | 0.000E+00 | 3.747E-07 | 0.000E+00 | 0.000E+00 | 0.000E+00 | 0.000E+00 | 0.000E+00 | 0.000E+00 | 0.000E+00 | 0.000E+00 |
| Unigene254735 | hp1181 | 0.000E+00 | 2.675E-07 | 0.000E+00 | 4.220E-07 | 3.869E-08 | 0.000E+00 | 4.239E-07 | 8.129E-08 | 0.000E+00 | 0.000E+00 | 0.000E+00 | 0.000E+00 |
| Unigene254816 | arnA | 2.943E-07 | 0.000E+00 | 0.000E+00 | 6.936E-07 | 5.813E-07 | 0.000E+00 | 2.172E-07 | 0.000E+00 | 3.575E-07 | 0.000E+00 | 0.000E+00 | 0.000E+00 |
| Unigene254842 | efrA | 0.000E+00 | 0.000E+00 | 0.000E+00 | 6.225E-07 | 0.000E+00 | 0.000E+00 | 0.000E+00 | 0.000E+00 | 0.000E+00 | 0.000E+00 | 0.000E+00 | 0.000E+00 |
| Unigene254989 | oleB | 0.000E+00 | 0.000E+00 | 0.000E+00 | 0.000E+00 | 0.000E+00 | 0.000E+00 | 1.809E-06 | 0.000E+00 | 0.000E+00 | 0.000E+00 | 0.000E+00 | 0.000E+00 |
| Unigene255031 | efrB | 0.000E+00 | 0.000E+00 | 0.000E+00 | 6.452E-07 | 1.868E-07 | 0.000E+00 | 7.443E-08 | 0.000E+00 | 0.000E+00 | 0.000E+00 | 1.154E-07 | 0.000E+00 |
| Unigene255197 | tet32 | 0.000E+00 | 9.493E-08 | 0.000E+00 | 6.813E-07 | 0.000E+00 | 0.000E+00 | 0.000E+00 | 0.000E+00 | 0.000E+00 | 9.250E-07 | 0.000E+00 | 3.067E-07 |
| Unigene255256 | Staphylocc | 0.000E+00 | 0.000E+00 | 0.000E+00 | 4.571E-07 | 0.000E+00 | 0.000E+00 | 0.000E+00 | 0.000E+00 | 0.000E+00 | 0.000E+00 | 0.000E+00 | 0.000E+00 |
| Unigene255310 | patA | 0.000E+00 | 7.973E-07 | 0.000E+00 | 8.252E-07 | 0.000E+00 | 4.908E-08 | 0.000E+00 | 0.000E+00 | 0.000E+00 | 0.000E+00 | 0.000E+00 | 0.000E+00 |
| Unigene255311 | tetA(58) | 0.000E+00 | 0.000E+00 | 0.000E+00 | 9.230E-07 | 0.000E+00 | 0.000E+00 | 0.000E+00 | 0.000E+00 | 0.000E+00 | 7.405E-07 | 0.000E+00 | 3.437E-07 |
| Unigene255429 | evgS | 4.171E-08 | 1.373E-07 | 8.185E-08 | 3.369E-07 | 0.000E+00 | 0.000E+00 | 0.000E+00 | 0.000E+00 | 0.000E+00 | 0.000E+00 | 0.000E+00 | 0.000E+00 |
| Unigene255495 | vanHA | 0.000E+00 | 0.000E+00 | 0.000E+00 | 3.822E-07 | 0.000E+00 | 0.000E+00 | 0.000E+00 | 1.893E-07 | 0.000E+00 | 0.000E+00 | 0.000E+00 | 0.000E+00 |
| Unigene255512 | vanI | 2.461E-07 | 3.473E-07 | 5.176E-07 | 7.102E-07 | 0.000E+00 | 0.000E+00 | 0.000E+00 | 0.000E+00 | 0.000E+00 | 0.000E+00 | 0.000E+00 | 0.000E+00 |
| Unigene255517 | lsaA | 0.000E+00 | 0.000E+00 | 0.000E+00 | 5.415E-07 | 0.000E+00 | 0.000E+00 | 0.000E+00 | 0.000E+00 | 0.000E+00 | 0.000E+00 | 0.000E+00 | 0.000E+00 |
| Unigene255575 | poxtA | 0.000E+00 | 0.000E+00 | 0.000E+00 | 3.881E-07 | 0.000E+00 | 0.000E+00 | 0.000E+00 | 0.000E+00 | 0.000E+00 | 0.000E+00 | 2.198E-07 | 0.000E+00 |
| Unigene255578 | poxtA | 0.000E+00 | 0.000E+00 | 0.000E+00 | 8.915E-07 | 0.000E+00 | 0.000E+00 | 0.000E+00 | 0.000E+00 | 0.000E+00 | 0.000E+00 | 0.000E+00 | 0.000E+00 |
| Unigene255602 | vanRI | 0.000E+00 | 0.000E+00 | 0.000E+00 | 3.768E-07 | 0.000E+00 | 0.000E+00 | 0.000E+00 | 0.000E+00 | 0.000E+00 | 0.000E+00 | 0.000E+00 | 0.000E+00 |
| Unigene255632 | tetA(46) | 0.000E+00 | 0.000E+00 | 0.000E+00 | 6.291E-07 | 0.000E+00 | 0.000E+00 | 1.254E-07 | 0.000E+00 | 0.000E+00 | 0.000E+00 | 1.620E-07 | 0.000E+00 |
| Unigene255667 | tetB(60) | 0.000E+00 | 0.000E+00 | 0.000E+00 | 6.936E-07 | 0.000E+00 | 0.000E+00 | 0.000E+00 | 0.000E+00 | 0.000E+00 | 0.000E+00 | 0.000E+00 | 0.000E+00 |
| Unigene255713 | tet(42) | 4.295E-06 | 1.982E-05 | 2.719E-06 | 9.094E-07 | 7.952E-07 | 3.994E-07 | 2.556E-07 | 4.582E-07 | 1.388E-06 | 6.173E-07 | 0.000E+00 | 0.000E+00 |
| Unigene255731 | otr(B) | 0.000E+00 | 0.000E+00 | 0.000E+00 | 4.476E-06 | 0.000E+00 | 0.000E+00 | 0.000E+00 | 0.000E+00 | 0.000E+00 | 0.000E+00 | 0.000E+00 | 0.000E+00 |
| Unigene255819 | novA | 1.272E-07 | 1.396E-07 | 0.000E+00 | 5.137E-07 | 0.000E+00 | 0.000E+00 | 2.815E-08 | 2.969E-08 | 0.000E+00 | 0.000E+00 | 0.000E+00 | 6.014E-08 |
| Unigene255822 | adeL | 0.000E+00 | 2.790E-07 | 3.325E-07 | 3.850E-07 | 0.000E+00 | 0.000E+00 | 4.688E-08 | 0.000E+00 | 0.000E+00 | 0.000E+00 | 0.000E+00 | 0.000E+00 |
| Unigene255837 | vanSF | 0.000E+00 | 0.000E+00 | 0.000E+00 | 1.627E-06 | 0.000E+00 | 0.000E+00 | 0.000E+00 | 0.000E+00 | 0.000E+00 | 0.000E+00 | 0.000E+00 | 0.000E+00 |
| Unigene255871 | msbA | 0.000E+00 | 0.000E+00 | 0.000E+00 | 2.937E-07 | 0.000E+00 | 0.000E+00 | 0.000E+00 | 0.000E+00 | 0.000E+00 | 0.000E+00 | 0.000E+00 | 0.000E+00 |
| Unigene255875 | facT | 0.000E+00 | 0.000E+00 | 0.000E+00 | 0.000E+00 | 0.000E+00 | 0.000E+00 | 0.000E+00 | 1.419E-07 | 0.000E+00 | 0.000E+00 | 0.000E+00 | 0.000E+00 |
| Unigene256003 | oleC | 0.000E+00 | 0.000E+00 | 0.000E+00 | 0.000E+00 | 0.000E+00 | 2.270E-07 | 0.000E+00 | 0.000E+00 | 0.000E+00 | 0.000E+00 | 0.000E+00 | 1.241E-06 |
| Unigene256049 | oleC | 0.000E+00 | 8.317E-07 | 4.065E-06 | 3.265E-06 | 0.000E+00 | 0.000E+00 | 0.000E+00 | 0.000E+00 | 0.000E+00 | 0.000E+00 | 0.000E+00 | 0.000E+00 |
| Unigene256090 | Chlamydia | 0.000E+00 | 0.000E+00 | 0.000E+00 | 4.390E-07 | 0.000E+00 | 0.000E+00 | 0.000E+00 | 0.000E+00 | 0.000E+00 | 0.000E+00 | 0.000E+00 | 0.000E+00 |
| Unigene256100 | smeS | 0.000E+00 | 0.000E+00 | 0.000E+00 | 4.695E-07 | 0.000E+00 | 0.000E+00 | 0.000E+00 | 0.000E+00 | 0.000E+00 | 0.000E+00 | 0.000E+00 | 0.000E+00 |
| Unigene256226 | arnA | 0.000E+00 | 0.000E+00 | 0.000E+00 | 4.695E-07 | 0.000E+00 | 0.000E+00 | 0.000E+00 | 0.000E+00 | 0.000E+00 | 0.000E+00 | 0.000E+00 | 0.000E+00 |
| Unigene256261 | lmrC | 0.000E+00 | 0.000E+00 | 4.661E-08 | 7.436E-07 | 0.000E+00 | 0.000E+00 | 0.000E+00 | 0.000E+00 | 0.000E+00 | 0.000E+00 | 0.000E+00 | 0.000E+00 |
| Unigene256413 | baeS | 2.479E-08 | 0.000E+00 | 9.731E-08 | 6.009E-07 | 0.000E+00 | 0.000E+00 | 0.000E+00 | 0.000E+00 | 0.000E+00 | 0.000E+00 | 0.000E+00 | 0.000E+00 |
| Unigene256414 | arlR | 9.316E-08 | 0.000E+00 | 0.000E+00 | 0.000E+00 | 0.000E+00 | 0.000E+00 | 0.000E+00 | 0.000E+00 | 0.000E+00 | 0.000E+00 | 0.000E+00 | 0.000E+00 |
| Unigene256541 | mtrA | 2.261E-07 | 0.000E+00 | 5.767E-07 | 3.652E-07 | 0.000E+00 | 0.000E+00 | 2.102E-06 | 1.267E-06 | 0.000E+00 | 0.000E+00 | 0.000E+00 | 5.718E-06 |
| Unigene256704 | lmrB | 0.000E+00 | 0.000E+00 | 0.000E+00 | 5.842E-07 | 0.000E+00 | 0.000E+00 | 0.000E+00 | 0.000E+00 | 0.000E+00 | 0.000E+00 | 0.000E+00 | 0.000E+00 |
| Unigene256779 | MuxC | 5.624E-08 | 0.000E+00 | 0.000E+00 | 4.543E-07 | 1.041E-07 | 0.000E+00 | 2.282E-07 | 0.000E+00 | 0.000E+00 | 0.000E+00 | 2.359E-07 | 0.000E+00 |
| Unigene256829 | bcrA | 0.000E+00 | 0.000E+00 | 0.000E+00 | 0.000E+00 | 0.000E+00 | 0.000E+00 | 0.000E+00 | 3.293E-07 | 0.000E+00 | 0.000E+00 | 0.000E+00 | 0.000E+00 |
| Unigene256921 | baeS | 0.000E+00 | 4.164E-07 | 0.000E+00 | 5.224E-07 | 0.000E+00 | 0.000E+00 | 0.000E+00 | 0.000E+00 | 0.000E+00 | 0.000E+00 | 0.000E+00 | 0.000E+00 |
| Unigene256922 | mtrA | 0.000E+00 | 0.000E+00 | 0.000E+00 | 4.147E-07 | 0.000E+00 | 0.000E+00 | 0.000E+00 | 0.000E+00 | 0.000E+00 | 0.000E+00 | 0.000E+00 | 0.000E+00 |
| Unigene257069 | TaeA | 0.000E+00 | 0.000E+00 | 0.000E+00 | 8.112E-07 | 0.000E+00 | 0.000E+00 | 0.000E+00 | 0.000E+00 | 0.000E+00 | 0.000E+00 | 0.000E+00 | 0.000E+00 |
| Unigene257072 | macB | 0.000E+00 | 0.000E+00 | 0.000E+00 | 0.000E+00 | 0.000E+00 | 0.000E+00 | 0.000E+00 | 0.000E+00 | 0.000E+00 | 0.000E+00 | 0.000E+00 | 4.276E-06 |
| Unigene257190 | smeS | 0.000E+00 | 0.000E+00 | 1.118E-07 | 1.888E-06 | 0.000E+00 | 0.000E+00 | 0.000E+00 | 0.000E+00 | 0.000E+00 | 0.000E+00 | 0.000E+00 | 0.000E+00 |
| Unigene257191 | mtrA | 0.000E+00 | 0.000E+00 | 0.000E+00 | 1.851E-06 | 0.000E+00 | 0.000E+00 | 0.000E+00 | 0.000E+00 | 0.000E+00 | 0.000E+00 | 0.000E+00 | 0.000E+00 |
| Unigene257326 | arlS | 0.000E+00 | 0.000E+00 | 0.000E+00 | 6.519E-07 | 0.000E+00 | 0.000E+00 | 0.000E+00 | 0.000E+00 | 0.000E+00 | 0.000E+00 | 0.000E+00 | 0.000E+00 |
| Unigene257364 | otr(B) | 2.864E-07 | 1.123E-07 | 1.606E-07 | 6.817E-07 | 4.545E-08 | 2.211E-08 | 2.264E-08 | 0.000E+00 | 0.000E+00 | 0.000E+00 | 4.680E-08 | 7.254E-08 |
| Unigene257435 | mel | 0.000E+00 | 7.854E-07 | 2.965E-06 | 2.703E-06 | 0.000E+00 | 0.000E+00 | 0.000E+00 | 0.000E+00 | 0.000E+00 | 0.000E+00 | 0.000E+00 | 0.000E+00 |
| Unigene257436 | efmA | 9.963E-08 | 8.201E-07 | 3.519E-06 | 3.270E-06 | 0.000E+00 | 0.000E+00 | 0.000E+00 | 0.000E+00 | 0.000E+00 | 0.000E+00 | 0.000E+00 | 0.000E+00 |
| Unigene257442 | mtrA | 0.000E+00 | 0.000E+00 | 0.000E+00 | 4.591E-07 | 0.000E+00 | 0.000E+00 | 0.000E+00 | 0.000E+00 | 0.000E+00 | 0.000E+00 | 0.000E+00 | 0.000E+00 |
| Unigene257503 | abcA | 0.000E+00 | 0.000E+00 | 0.000E+00 | 5.054E-07 | 0.000E+00 | 0.000E+00 | 0.000E+00 | 0.000E+00 | 0.000E+00 | 0.000E+00 | 0.000E+00 | 0.000E+00 |

| | | | | | | | | | | | | | |
|---|---|---|---|---|---|---|---|---|---|---|---|---|---|
| Unigene257505 | msrA | 0.000E+00 | 0.000E+00 | 0.000E+00 | 6.139E-07 | 0.000E+00 | 0.000E+00 | 0.000E+00 | 0.000E+00 | 0.000E+00 | 0.000E+00 | 0.000E+00 | 0.000E+00 |
| Unigene257544 | tetT | 0.000E+00 | 0.000E+00 | 3.218E-07 | 4.512E-06 | 0.000E+00 | 0.000E+00 | 0.000E+00 | 0.000E+00 | 0.000E+00 | 0.000E+00 | 0.000E+00 | 0.000E+00 |
| Unigene257638 | macB | 0.000E+00 | 0.000E+00 | 0.000E+00 | 4.527E-07 | 0.000E+00 | 0.000E+00 | 0.000E+00 | 0.000E+00 | 0.000E+00 | 0.000E+00 | 0.000E+00 | 0.000E+00 |
| Unigene257653 | msbA | 2.915E-06 | 3.782E-06 | 2.914E-06 | 1.703E-06 | 2.383E-06 | 1.684E-06 | 2.495E-06 | 7.489E-07 | 1.503E-06 | 6.269E-06 | 6.081E-06 | 2.535E-06 |
| Unigene257705 | macB | 0.000E+00 | 5.013E-07 | 0.000E+00 | 8.713E-07 | 0.000E+00 | 0.000E+00 | 0.000E+00 | 0.000E+00 | 0.000E+00 | 0.000E+00 | 1.161E-07 | 0.000E+00 |
| Unigene257742 | msbA | 0.000E+00 | 0.000E+00 | 1.257E-07 | 3.881E-07 | 0.000E+00 | 0.000E+00 | 0.000E+00 | 2.990E-07 | 0.000E+00 | 0.000E+00 | 0.000E+00 | 0.000E+00 |
| Unigene257750 | bcrA | 0.000E+00 | 0.000E+00 | 0.000E+00 | 7.983E-07 | 0.000E+00 | 0.000E+00 | 0.000E+00 | 0.000E+00 | 0.000E+00 | 0.000E+00 | 0.000E+00 | 0.000E+00 |
| Unigene258028 | mdtG | 6.963E-05 | 6.121E-05 | 1.133E-04 | 1.652E-04 | 1.218E-04 | 8.940E-05 | 1.178E-04 | 1.137E-04 | 9.460E-05 | 1.408E-04 | 1.400E-04 | 1.471E-04 |
| Unigene258052 | cmlv | 0.000E+00 | 0.000E+00 | 0.000E+00 | 5.525E-07 | 0.000E+00 | 0.000E+00 | 0.000E+00 | 0.000E+00 | 0.000E+00 | 0.000E+00 | 0.000E+00 | 1.617E-07 |
| Unigene258074 | vanHD | 0.000E+00 | 0.000E+00 | 0.000E+00 | 7.221E-07 | 0.000E+00 | 0.000E+00 | 0.000E+00 | 0.000E+00 | 0.000E+00 | 0.000E+00 | 0.000E+00 | 0.000E+00 |
| Unigene258082 | macB | 0.000E+00 | 0.000E+00 | 0.000E+00 | 4.179E-07 | 0.000E+00 | 0.000E+00 | 0.000E+00 | 0.000E+00 | 0.000E+00 | 0.000E+00 | 0.000E+00 | 0.000E+00 |
| Unigene258100 | novA | 0.000E+00 | 0.000E+00 | 0.000E+00 | 3.939E-07 | 0.000E+00 | 0.000E+00 | 0.000E+00 | 0.000E+00 | 0.000E+00 | 0.000E+00 | 0.000E+00 | 0.000E+00 |
| Unigene258110 | novA | 0.000E+00 | 0.000E+00 | 0.000E+00 | 5.275E-07 | 0.000E+00 | 0.000E+00 | 0.000E+00 | 0.000E+00 | 0.000E+00 | 1.164E-07 | 2.390E-07 | 0.000E+00 |
| Unigene258135 | bcrA | 1.741E-07 | 0.000E+00 | 6.150E-07 | 8.791E-07 | 0.000E+00 | 0.000E+00 | 0.000E+00 | 0.000E+00 | 0.000E+00 | 0.000E+00 | 0.000E+00 | 0.000E+00 |
| Unigene258137 | evgA | 1.461E-07 | 2.137E-07 | 1.911E-07 | 1.131E-06 | 0.000E+00 | 2.105E-07 | 0.000E+00 | 0.000E+00 | 0.000E+00 | 0.000E+00 | 0.000E+00 | 1.151E-07 |
| Unigene258156 | lmrD | 0.000E+00 | 0.000E+00 | 0.000E+00 | 7.761E-07 | 0.000E+00 | 0.000E+00 | 0.000E+00 | 0.000E+00 | 0.000E+00 | 0.000E+00 | 0.000E+00 | 0.000E+00 |
| Unigene258203 | QepA2 | 0.000E+00 | 0.000E+00 | 0.000E+00 | 5.671E-07 | 0.000E+00 | 0.000E+00 | 0.000E+00 | 0.000E+00 | 0.000E+00 | 0.000E+00 | 0.000E+00 | 0.000E+00 |
| Unigene258207 | lmrB | 0.000E+00 | 3.645E-08 | 0.000E+00 | 8.719E-07 | 0.000E+00 | 0.000E+00 | 0.000E+00 | 0.000E+00 | 0.000E+00 | 0.000E+00 | 0.000E+00 | 0.000E+00 |
| Unigene258222 | PmrF | 0.000E+00 | 0.000E+00 | 0.000E+00 | 5.663E-07 | 0.000E+00 | 0.000E+00 | 0.000E+00 | 0.000E+00 | 0.000E+00 | 0.000E+00 | 0.000E+00 | 0.000E+00 |
| Unigene258273 | mdtP | 0.000E+00 | 5.344E-08 | 1.433E-07 | 3.933E-07 | 0.000E+00 | 0.000E+00 | 0.000E+00 | 0.000E+00 | 0.000E+00 | 0.000E+00 | 0.000E+00 | 0.000E+00 |
| Unigene258281 | NmcR | 0.000E+00 | 0.000E+00 | 0.000E+00 | 8.103E-07 | 0.000E+00 | 0.000E+00 | 0.000E+00 | 0.000E+00 | 0.000E+00 | 0.000E+00 | 0.000E+00 | 0.000E+00 |
| Unigene258302 | Chlamydia | 4.730E-08 | 0.000E+00 | 0.000E+00 | 9.552E-07 | 0.000E+00 | 0.000E+00 | 0.000E+00 | 0.000E+00 | 0.000E+00 | 7.376E-07 | 0.000E+00 | 3.354E-07 |
| Unigene258375 | TaeA | 0.000E+00 | 8.336E-07 | 0.000E+00 | 4.794E-07 | 0.000E+00 | 2.395E-07 | 0.000E+00 | 0.000E+00 | 0.000E+00 | 0.000E+00 | 0.000E+00 | 0.000E+00 |
| Unigene258491 | vgaD | 0.000E+00 | 0.000E+00 | 0.000E+00 | 7.373E-07 | 0.000E+00 | 0.000E+00 | 0.000E+00 | 0.000E+00 | 0.000E+00 | 0.000E+00 | 0.000E+00 | 0.000E+00 |
| Unigene258492 | bcrA | 0.000E+00 | 0.000E+00 | 0.000E+00 | 5.821E-07 | 0.000E+00 | 0.000E+00 | 0.000E+00 | 0.000E+00 | 0.000E+00 | 0.000E+00 | 0.000E+00 | 0.000E+00 |
| Unigene258559 | PmrF | 0.000E+00 | 1.687E-07 | 0.000E+00 | 3.105E-07 | 0.000E+00 | 0.000E+00 | 0.000E+00 | 0.000E+00 | 0.000E+00 | 0.000E+00 | 0.000E+00 | 0.000E+00 |
| Unigene258659 | vanTG | 0.000E+00 | 0.000E+00 | 0.000E+00 | 2.991E-07 | 0.000E+00 | 0.000E+00 | 0.000E+00 | 0.000E+00 | 0.000E+00 | 0.000E+00 | 0.000E+00 | 0.000E+00 |
| Unigene258667 | OXA-156 | 1.713E-07 | 3.761E-08 | 3.699E-07 | 1.177E-06 | 0.000E+00 | 0.000E+00 | 0.000E+00 | 0.000E+00 | 0.000E+00 | 0.000E+00 | 0.000E+00 | 1.215E-07 |
| Unigene258809 | efrA | 1.618E-07 | 0.000E+00 | 7.938E-08 | 8.987E-07 | 4.224E-06 | 1.341E-05 | 1.352E-05 | 4.423E-05 | 4.392E-06 | 0.000E+00 | 2.465E-05 | 1.179E-06 |
| Unigene258817 | ugd | 0.000E+00 | 0.000E+00 | 0.000E+00 | 5.530E-05 | 0.000E+00 | 0.000E+00 | 0.000E+00 | 0.000E+00 | 0.000E+00 | 0.000E+00 | 0.000E+00 | 0.000E+00 |
| Unigene258889 | bcrA | 0.000E+00 | 5.761E-08 | 0.000E+00 | 3.710E-07 | 0.000E+00 | 0.000E+00 | 0.000E+00 | 0.000E+00 | 0.000E+00 | 0.000E+00 | 0.000E+00 | 0.000E+00 |
| Unigene258984 | vanTE | 0.000E+00 | 0.000E+00 | 0.000E+00 | 3.951E-07 | 0.000E+00 | 0.000E+00 | 0.000E+00 | 5.138E-07 | 0.000E+00 | 0.000E+00 | 5.595E-07 | 0.000E+00 |
| Unigene259006 | ugd | 0.000E+00 | 0.000E+00 | 0.000E+00 | 1.560E-05 | 0.000E+00 | 0.000E+00 | 0.000E+00 | 0.000E+00 | 0.000E+00 | 0.000E+00 | 0.000E+00 | 0.000E+00 |
| Unigene259015 | patB | 0.000E+00 | 0.000E+00 | 3.087E-07 | 0.000E+00 | 0.000E+00 | 0.000E+00 | 0.000E+00 | 0.000E+00 | 0.000E+00 | 0.000E+00 | 0.000E+00 | 0.000E+00 |
| Unigene259110 | YojI | 2.614E-07 | 1.434E-07 | 0.000E+00 | 3.959E-07 | 0.000E+00 | 0.000E+00 | 0.000E+00 | 0.000E+00 | 0.000E+00 | 0.000E+00 | 0.000E+00 | 5.149E-08 |
| Unigene259117 | tetA(58) | 5.207E-07 | 0.000E+00 | 0.000E+00 | 4.381E-07 | 4.242E-06 | 8.396E-06 | 0.000E+00 | 7.191E-06 | 3.746E-06 | 0.000E+00 | 0.000E+00 | 8.206E-07 |
| Unigene259199 | mtrA | 4.559E-08 | 2.502E-07 | 4.026E-07 | 7.827E-07 | 0.000E+00 | 0.000E+00 | 0.000E+00 | 0.000E+00 | 0.000E+00 | 0.000E+00 | 0.000E+00 | 2.156E-07 |
| Unigene259224 | bcrA | 0.000E+00 | 2.827E-06 | 2.080E-06 | 9.484E-07 | 1.252E-05 | 0.000E+00 | 0.000E+00 | 0.000E+00 | 0.000E+00 | 0.000E+00 | 0.000E+00 | 0.000E+00 |
| Unigene259528 | efmA | 0.000E+00 | 0.000E+00 | 8.260E-06 | 0.000E+00 | 0.000E+00 | 0.000E+00 | 0.000E+00 | 0.000E+00 | 0.000E+00 | 0.000E+00 | 0.000E+00 | 0.000E+00 |
| Unigene259552 | poxtA | 0.000E+00 | 0.000E+00 | 0.000E+00 | 2.233E-06 | 0.000E+00 | 0.000E+00 | 0.000E+00 | 0.000E+00 | 0.000E+00 | 0.000E+00 | 0.000E+00 | 0.000E+00 |
| Unigene259590 | vmlR | 0.000E+00 | 0.000E+00 | 0.000E+00 | 5.075E-07 | 0.000E+00 | 0.000E+00 | 0.000E+00 | 0.000E+00 | 0.000E+00 | 0.000E+00 | 0.000E+00 | 0.000E+00 |
| Unigene259638 | Erm(43) | 0.000E+00 | 0.000E+00 | 3.555E-08 | 4.390E-07 | 0.000E+00 | 0.000E+00 | 0.000E+00 | 0.000E+00 | 0.000E+00 | 0.000E+00 | 0.000E+00 | 0.000E+00 |
| Unigene259722 | macB | 0.000E+00 | 0.000E+00 | 0.000E+00 | 4.003E-07 | 0.000E+00 | 0.000E+00 | 0.000E+00 | 0.000E+00 | 0.000E+00 | 0.000E+00 | 0.000E+00 | 0.000E+00 |
| Unigene259742 | bcrA | 0.000E+00 | 0.000E+00 | 9.512E-08 | 4.258E-06 | 0.000E+00 | 0.000E+00 | 0.000E+00 | 0.000E+00 | 0.000E+00 | 0.000E+00 | 0.000E+00 | 0.000E+00 |
| Unigene259744 | optrA | 0.000E+00 | 0.000E+00 | 2.876E-07 | 5.602E-06 | 0.000E+00 | 0.000E+00 | 0.000E+00 | 0.000E+00 | 0.000E+00 | 0.000E+00 | 0.000E+00 | 0.000E+00 |
| Unigene259770 | efmA | 2.064E-06 | 5.825E-05 | 4.195E-05 | 5.837E-06 | 2.179E-06 | 3.292E-06 | 0.000E+00 | 1.205E-07 | 2.510E-06 | 0.000E+00 | 1.358E-06 | 5.734E-06 |
| Unigene259786 | TaeA | 0.000E+00 | 0.000E+00 | 0.000E+00 | 5.288E-07 | 0.000E+00 | 0.000E+00 | 0.000E+00 | 0.000E+00 | 0.000E+00 | 0.000E+00 | 0.000E+00 | 0.000E+00 |
| Unigene259832 | salA | 1.375E-06 | 1.240E-05 | 3.754E-06 | 3.380E-06 | 0.000E+00 | 0.000E+00 | 0.000E+00 | 0.000E+00 | 0.000E+00 | 0.000E+00 | 0.000E+00 | 0.000E+00 |
| Unigene259905 | oleC | 0.000E+00 | 0.000E+00 | 0.000E+00 | 8.329E-07 | 0.000E+00 | 0.000E+00 | 0.000E+00 | 0.000E+00 | 0.000E+00 | 0.000E+00 | 0.000E+00 | 0.000E+00 |
| Unigene259957 | bacA | 0.000E+00 | 0.000E+00 | 0.000E+00 | 2.957E-07 | 0.000E+00 | 0.000E+00 | 0.000E+00 | 0.000E+00 | 0.000E+00 | 0.000E+00 | 0.000E+00 | 0.000E+00 |
| Unigene260038 | tetA(58) | 7.190E-06 | 0.000E+00 | 0.000E+00 | 2.303E-06 | 2.920E-05 | 1.571E-05 | 2.031E-05 | 2.379E-05 | 2.818E-06 | 0.000E+00 | 0.000E+00 | 0.000E+00 |
| Unigene260039 | msbA | 7.746E-06 | 0.000E+00 | 0.000E+00 | 2.551E-06 | 3.354E-05 | 1.780E-05 | 2.531E-05 | 2.600E-05 | 3.570E-06 | 0.000E+00 | 0.000E+00 | 0.000E+00 |
| Unigene260064 | msbA | 0.000E+00 | 0.000E+00 | 0.000E+00 | 4.303E-07 | 0.000E+00 | 0.000E+00 | 0.000E+00 | 0.000E+00 | 0.000E+00 | 0.000E+00 | 0.000E+00 | 0.000E+00 |
| Unigene260071 | mecB | 0.000E+00 | 9.800E-08 | 1.898E-07 | 4.749E-06 | 0.000E+00 | 0.000E+00 | 0.000E+00 | 0.000E+00 | 0.000E+00 | 0.000E+00 | 0.000E+00 | 0.000E+00 |
| Unigene260106 | vgaB | 0.000E+00 | 0.000E+00 | 1.564E-07 | 5.795E-06 | 0.000E+00 | 0.000E+00 | 0.000E+00 | 0.000E+00 | 0.000E+00 | 0.000E+00 | 0.000E+00 | 0.000E+00 |
| Unigene260121 | patB | 1.552E-07 | 1.703E-07 | 2.538E-07 | 5.746E-07 | 0.000E+00 | 0.000E+00 | 0.000E+00 | 0.000E+00 | 0.000E+00 | 0.000E+00 | 0.000E+00 | 0.000E+00 |
| Unigene260149 | Staphylocc | 0.000E+00 | 0.000E+00 | 0.000E+00 | 8.693E-07 | 0.000E+00 | 0.000E+00 | 0.000E+00 | 0.000E+00 | 0.000E+00 | 0.000E+00 | 0.000E+00 | 0.000E+00 |
| Unigene260160 | tetA(60) | 3.483E-07 | 0.000E+00 | 0.000E+00 | 7.589E-07 | 0.000E+00 | 0.000E+00 | 0.000E+00 | 8.559E-08 | 3.005E-07 | 0.000E+00 | 0.000E+00 | 0.000E+00 |

| | | | | | | | | | | | | | |
|---|---|---|---|---|---|---|---|---|---|---|---|---|---|
| Unigene260195 | Pseudomo | 0.000E+00 | 0.000E+00 | 0.000E+00 | 3.826E-07 | 2.525E-07 | 0.000E+00 | 0.000E+00 | 0.000E+00 | 0.000E+00 | 0.000E+00 | 0.000E+00 | 0.000E+00 |
| Unigene260230 | Streptomy | 0.000E+00 | 0.000E+00 | 0.000E+00 | 4.220E-07 | 0.000E+00 | 0.000E+00 | 0.000E+00 | 0.000E+00 | 0.000E+00 | 0.000E+00 | 0.000E+00 | 0.000E+00 |
| Unigene260344 | MdtK | 0.000E+00 | 0.000E+00 | 0.000E+00 | 2.484E-07 | 0.000E+00 | 0.000E+00 | 0.000E+00 | 0.000E+00 | 0.000E+00 | 0.000E+00 | 0.000E+00 | 0.000E+00 |
| Unigene260406 | facT | 0.000E+00 | 0.000E+00 | 0.000E+00 | 4.939E-07 | 0.000E+00 | 0.000E+00 | 0.000E+00 | 0.000E+00 | 0.000E+00 | 0.000E+00 | 0.000E+00 | 7.227E-08 |
| Unigene260418 | efpA | 0.000E+00 | 0.000E+00 | 0.000E+00 | 4.633E-07 | 0.000E+00 | 0.000E+00 | 0.000E+00 | 0.000E+00 | 0.000E+00 | 0.000E+00 | 0.000E+00 | 0.000E+00 |
| Unigene260461 | QepA4 | 0.000E+00 | 5.684E-07 | 3.388E-07 | 9.297E-07 | 0.000E+00 | 0.000E+00 | 2.547E-07 | 0.000E+00 | 0.000E+00 | 1.282E-07 | 0.000E+00 | 0.000E+00 |
| Unigene260548 | oleC | 0.000E+00 | 0.000E+00 | 0.000E+00 | 5.054E-07 | 0.000E+00 | 0.000E+00 | 0.000E+00 | 0.000E+00 | 0.000E+00 | 0.000E+00 | 0.000E+00 | 0.000E+00 |
| Unigene260563 | macB | 0.000E+00 | 0.000E+00 | 0.000E+00 | 8.412E-07 | 0.000E+00 | 0.000E+00 | 0.000E+00 | 0.000E+00 | 0.000E+00 | 0.000E+00 | 0.000E+00 | 0.000E+00 |
| Unigene260564 | efpA | 1.368E-07 | 4.003E-07 | 2.237E-07 | 1.243E-06 | 0.000E+00 | 0.000E+00 | 5.046E-08 | 0.000E+00 | 9.967E-08 | 0.000E+00 | 0.000E+00 | 0.000E+00 |
| Unigene260809 | vmlR | 0.000E+00 | 0.000E+00 | 0.000E+00 | 6.392E-07 | 0.000E+00 | 0.000E+00 | 0.000E+00 | 0.000E+00 | 0.000E+00 | 0.000E+00 | 0.000E+00 | 0.000E+00 |
| Unigene260886 | tetA(58) | 0.000E+00 | 0.000E+00 | 0.000E+00 | 5.650E-07 | 0.000E+00 | 0.000E+00 | 0.000E+00 | 0.000E+00 | 0.000E+00 | 0.000E+00 | 0.000E+00 | 0.000E+00 |
| Unigene260900 | vanSF | 2.458E-07 | 1.499E-07 | 4.824E-07 | 1.020E-06 | 0.000E+00 | 0.000E+00 | 0.000E+00 | 0.000E+00 | 0.000E+00 | 0.000E+00 | 3.124E-08 | 0.000E+00 |
| Unigene261266 | srmB | 0.000E+00 | 0.000E+00 | 0.000E+00 | 5.075E-07 | 0.000E+00 | 0.000E+00 | 0.000E+00 | 0.000E+00 | 0.000E+00 | 0.000E+00 | 0.000E+00 | 0.000E+00 |
| Unigene261285 | vanSG | 0.000E+00 | 0.000E+00 | 0.000E+00 | 1.214E-06 | 0.000E+00 | 0.000E+00 | 0.000E+00 | 0.000E+00 | 0.000E+00 | 0.000E+00 | 0.000E+00 | 0.000E+00 |
| Unigene261331 | vanHA | 0.000E+00 | 0.000E+00 | 0.000E+00 | 4.794E-07 | 0.000E+00 | 0.000E+00 | 0.000E+00 | 0.000E+00 | 0.000E+00 | 0.000E+00 | 0.000E+00 | 0.000E+00 |
| Unigene261440 | kdpE | 0.000E+00 | 0.000E+00 | 0.000E+00 | 5.197E-07 | 0.000E+00 | 0.000E+00 | 5.178E-08 | 0.000E+00 | 0.000E+00 | 0.000E+00 | 0.000E+00 | 0.000E+00 |
| Unigene261475 | Corynebac | 0.000E+00 | 1.869E-07 | 1.671E-07 | 5.158E-07 | 0.000E+00 | 0.000E+00 | 2.638E-07 | 1.192E-07 | 0.000E+00 | 0.000E+00 | 0.000E+00 | 0.000E+00 |
| Unigene261484 | bcrA | 0.000E+00 | 0.000E+00 | 0.000E+00 | 5.219E-07 | 0.000E+00 | 0.000E+00 | 0.000E+00 | 0.000E+00 | 0.000E+00 | 0.000E+00 | 0.000E+00 | 0.000E+00 |
| Unigene261488 | tet(K) | 0.000E+00 | 0.000E+00 | 1.774E-07 | 7.533E-07 | 0.000E+00 | 0.000E+00 | 0.000E+00 | 0.000E+00 | 0.000E+00 | 0.000E+00 | 0.000E+00 | 0.000E+00 |
| Unigene261642 | tetA(58) | 0.000E+00 | 9.226E-08 | 4.124E-08 | 5.942E-07 | 0.000E+00 | 0.000E+00 | 0.000E+00 | 0.000E+00 | 0.000E+00 | 0.000E+00 | 0.000E+00 | 0.000E+00 |
| Unigene261781 | tet(A) | 0.000E+00 | 0.000E+00 | 0.000E+00 | 5.694E-07 | 0.000E+00 | 0.000E+00 | 5.200E-08 | 0.000E+00 | 0.000E+00 | 0.000E+00 | 0.000E+00 | 0.000E+00 |
| Unigene261789 | vanSF | 0.000E+00 | 7.619E-08 | 1.703E-07 | 4.907E-07 | 0.000E+00 | 0.000E+00 | 0.000E+00 | 0.000E+00 | 0.000E+00 | 0.000E+00 | 0.000E+00 | 0.000E+00 |
| Unigene261790 | vanRF | 0.000E+00 | 2.513E-07 | 2.696E-07 | 6.936E-07 | 0.000E+00 | 0.000E+00 | 0.000E+00 | 0.000E+00 | 0.000E+00 | 0.000E+00 | 0.000E+00 | 0.000E+00 |
| Unigene261822 | vanHB | 0.000E+00 | 0.000E+00 | 0.000E+00 | 5.279E-07 | 4.840E-08 | 0.000E+00 | 9.643E-08 | 0.000E+00 | 0.000E+00 | 0.000E+00 | 0.000E+00 | 0.000E+00 |
| Unigene261826 | vanHD | 0.000E+00 | 0.000E+00 | 0.000E+00 | 7.897E-07 | 0.000E+00 | 0.000E+00 | 0.000E+00 | 0.000E+00 | 0.000E+00 | 9.914E-07 | 0.000E+00 | 1.913E-07 |
| Unigene261866 | bcrA | 0.000E+00 | 1.473E-07 | 1.580E-07 | 5.148E-07 | 0.000E+00 | 0.000E+00 | 2.376E-07 | 0.000E+00 | 0.000E+00 | 5.978E-08 | 9.208E-08 | 0.000E+00 |
| Unigene261891 | novA | 0.000E+00 | 0.000E+00 | 0.000E+00 | 5.286E-07 | 0.000E+00 | 0.000E+00 | 0.000E+00 | 0.000E+00 | 0.000E+00 | 0.000E+00 | 0.000E+00 | 0.000E+00 |
| Unigene261997 | acrB | 0.000E+00 | 0.000E+00 | 0.000E+00 | 3.995E-07 | 0.000E+00 | 0.000E+00 | 0.000E+00 | 9.235E-08 | 0.000E+00 | 0.000E+00 | 0.000E+00 | 0.000E+00 |
| Unigene262032 | tetA(58) | 0.000E+00 | 0.000E+00 | 0.000E+00 | 6.258E-07 | 0.000E+00 | 0.000E+00 | 0.000E+00 | 0.000E+00 | 0.000E+00 | 0.000E+00 | 0.000E+00 | 0.000E+00 |
| Unigene262034 | efpA | 0.000E+00 | 0.000E+00 | 0.000E+00 | 3.196E-07 | 0.000E+00 | 0.000E+00 | 0.000E+00 | 0.000E+00 | 0.000E+00 | 0.000E+00 | 0.000E+00 | 0.000E+00 |
| Unigene262077 | emrB | 0.000E+00 | 0.000E+00 | 0.000E+00 | 6.468E-07 | 0.000E+00 | 0.000E+00 | 0.000E+00 | 0.000E+00 | 0.000E+00 | 0.000E+00 | 0.000E+00 | 0.000E+00 |
| Unigene262109 | macB | 0.000E+00 | 0.000E+00 | 0.000E+00 | 7.951E-07 | 0.000E+00 | 0.000E+00 | 0.000E+00 | 2.553E-07 | 0.000E+00 | 0.000E+00 | 0.000E+00 | 0.000E+00 |
| Unigene262129 | tetA(58) | 0.000E+00 | 0.000E+00 | 7.542E-08 | 6.597E-07 | 0.000E+00 | 0.000E+00 | 0.000E+00 | 0.000E+00 | 0.000E+00 | 0.000E+00 | 0.000E+00 | 0.000E+00 |
| Unigene262196 | arlS | 0.000E+00 | 0.000E+00 | 0.000E+00 | 5.154E-07 | 0.000E+00 | 0.000E+00 | 0.000E+00 | 0.000E+00 | 0.000E+00 | 0.000E+00 | 0.000E+00 | 0.000E+00 |
| Unigene262257 | tva(A) | 0.000E+00 | 0.000E+00 | 0.000E+00 | 2.390E-06 | 0.000E+00 | 0.000E+00 | 0.000E+00 | 0.000E+00 | 0.000E+00 | 0.000E+00 | 0.000E+00 | 0.000E+00 |
| Unigene262378 | patA | 0.000E+00 | 0.000E+00 | 4.641E-07 | 0.000E+00 | 0.000E+00 | 0.000E+00 | 0.000E+00 | 0.000E+00 | 0.000E+00 | 0.000E+00 | 0.000E+00 | 0.000E+00 |
| Unigene262385 | oleC | 0.000E+00 | 0.000E+00 | 0.000E+00 | 7.448E-07 | 0.000E+00 | 0.000E+00 | 0.000E+00 | 0.000E+00 | 0.000E+00 | 0.000E+00 | 0.000E+00 | 0.000E+00 |
| Unigene262528 | patA | 0.000E+00 | 0.000E+00 | 7.899E-08 | 6.503E-07 | 0.000E+00 | 0.000E+00 | 0.000E+00 | 0.000E+00 | 0.000E+00 | 0.000E+00 | 0.000E+00 | 0.000E+00 |
| Unigene262645 | patB | 0.000E+00 | 0.000E+00 | 0.000E+00 | 1.111E-06 | 0.000E+00 | 0.000E+00 | 0.000E+00 | 0.000E+00 | 0.000E+00 | 0.000E+00 | 0.000E+00 | 0.000E+00 |
| Unigene262649 | Staphyloc | 4.392E-08 | 2.410E-07 | 0.000E+00 | 1.353E-06 | 0.000E+00 | 0.000E+00 | 0.000E+00 | 0.000E+00 | 0.000E+00 | 7.828E-07 | 0.000E+00 | 3.374E-07 |
| Unigene262654 | bacA | 0.000E+00 | 0.000E+00 | 0.000E+00 | 1.740E-06 | 0.000E+00 | 0.000E+00 | 0.000E+00 | 0.000E+00 | 0.000E+00 | 0.000E+00 | 0.000E+00 | 0.000E+00 |
| Unigene262809 | patA | 0.000E+00 | 0.000E+00 | 0.000E+00 | 9.411E-07 | 0.000E+00 | 0.000E+00 | 0.000E+00 | 0.000E+00 | 0.000E+00 | 0.000E+00 | 0.000E+00 | 0.000E+00 |
| Unigene262834 | patA | 0.000E+00 | 0.000E+00 | 5.515E-07 | 4.595E-06 | 0.000E+00 | 0.000E+00 | 0.000E+00 | 0.000E+00 | 0.000E+00 | 0.000E+00 | 0.000E+00 | 0.000E+00 |
| Unigene262837 | efrB | 0.000E+00 | 8.947E-08 | 4.799E-07 | 4.898E-06 | 0.000E+00 | 0.000E+00 | 0.000E+00 | 0.000E+00 | 0.000E+00 | 0.000E+00 | 0.000E+00 | 0.000E+00 |
| Unigene262838 | patB | 0.000E+00 | 0.000E+00 | 4.867E-07 | 4.701E-06 | 0.000E+00 | 0.000E+00 | 0.000E+00 | 0.000E+00 | 0.000E+00 | 0.000E+00 | 0.000E+00 | 0.000E+00 |
| Unigene262926 | Erm(34) | 0.000E+00 | 0.000E+00 | 0.000E+00 | 0.000E+00 | 0.000E+00 | 0.000E+00 | 6.496E-07 | 0.000E+00 | 0.000E+00 | 0.000E+00 | 0.000E+00 | 0.000E+00 |
| Unigene262970 | vanSF | 0.000E+00 | 0.000E+00 | 0.000E+00 | 3.069E-07 | 0.000E+00 | 0.000E+00 | 0.000E+00 | 0.000E+00 | 0.000E+00 | 0.000E+00 | 0.000E+00 | 0.000E+00 |
| Unigene262999 | lmrC | 0.000E+00 | 0.000E+00 | 0.000E+00 | 4.617E-07 | 0.000E+00 | 0.000E+00 | 1.012E-07 | 0.000E+00 | 0.000E+00 | 0.000E+00 | 1.743E-07 | 0.000E+00 |
| Unigene263013 | msbA | 1.006E-07 | 0.000E+00 | 0.000E+00 | 7.616E-07 | 0.000E+00 | 0.000E+00 | 0.000E+00 | 0.000E+00 | 0.000E+00 | 0.000E+00 | 0.000E+00 | 2.377E-07 |
| Unigene263140 | mtrA | 0.000E+00 | 0.000E+00 | 0.000E+00 | 8.252E-07 | 0.000E+00 | 0.000E+00 | 0.000E+00 | 0.000E+00 | 0.000E+00 | 0.000E+00 | 0.000E+00 | 0.000E+00 |
| Unigene263213 | bcrA | 0.000E+00 | 0.000E+00 | 0.000E+00 | 6.604E-07 | 0.000E+00 | 0.000E+00 | 0.000E+00 | 0.000E+00 | 0.000E+00 | 0.000E+00 | 0.000E+00 | 0.000E+00 |
| Unigene263216 | patB | 1.004E-07 | 1.322E-07 | 2.364E-07 | 7.298E-07 | 6.691E-08 | 4.340E-08 | 0.000E+00 | 0.000E+00 | 8.777E-08 | 0.000E+00 | 0.000E+00 | 1.898E-07 |
| Unigene263237 | optrA | 0.000E+00 | 0.000E+00 | 0.000E+00 | 9.361E-07 | 0.000E+00 | 0.000E+00 | 0.000E+00 | 0.000E+00 | 0.000E+00 | 0.000E+00 | 0.000E+00 | 0.000E+00 |
| Unigene263348 | lnuE | 0.000E+00 | 0.000E+00 | 6.675E-07 | 8.743E-07 | 0.000E+00 | 0.000E+00 | 0.000E+00 | 0.000E+00 | 0.000E+00 | 0.000E+00 | 0.000E+00 | 0.000E+00 |
| Unigene263474 | patB | 0.000E+00 | 0.000E+00 | 3.731E-08 | 1.497E-06 | 0.000E+00 | 0.000E+00 | 0.000E+00 | 0.000E+00 | 0.000E+00 | 0.000E+00 | 0.000E+00 | 0.000E+00 |
| Unigene263478 | vanSM | 0.000E+00 | 0.000E+00 | 5.817E-08 | 1.886E-06 | 0.000E+00 | 0.000E+00 | 0.000E+00 | 0.000E+00 | 0.000E+00 | 0.000E+00 | 0.000E+00 | 0.000E+00 |
| Unigene263479 | vanRG | 0.000E+00 | 0.000E+00 | 4.512E-08 | 2.229E-06 | 0.000E+00 | 0.000E+00 | 0.000E+00 | 0.000E+00 | 0.000E+00 | 0.000E+00 | 0.000E+00 | 0.000E+00 |
| Unigene263536 | TaeA | 0.000E+00 | 0.000E+00 | 4.223E-08 | 1.739E-06 | 0.000E+00 | 0.000E+00 | 0.000E+00 | 0.000E+00 | 0.000E+00 | 0.000E+00 | 0.000E+00 | 0.000E+00 |

| | | | | | | | | | | | | | |
|---|---|---|---|---|---|---|---|---|---|---|---|---|---|
| Unigene263547 | bcrA | 0.000E+00 | 0.000E+00 | 0.000E+00 | 1.611E-06 | 0.000E+00 | 0.000E+00 | 0.000E+00 | 0.000E+00 | 0.000E+00 | 0.000E+00 | 0.000E+00 | 0.000E+00 |
| Unigene263551 | sul4 | 0.000E+00 | 0.000E+00 | 0.000E+00 | 2.294E-06 | 0.000E+00 | 0.000E+00 | 0.000E+00 | 0.000E+00 | 0.000E+00 | 0.000E+00 | 0.000E+00 | 0.000E+00 |
| Unigene263559 | lmrB | 0.000E+00 | 0.000E+00 | 0.000E+00 | 2.020E-06 | 0.000E+00 | 0.000E+00 | 0.000E+00 | 0.000E+00 | 0.000E+00 | 0.000E+00 | 0.000E+00 | 0.000E+00 |
| Unigene263578 | Staphylocc | 0.000E+00 | 0.000E+00 | 4.455E-08 | 1.559E-06 | 0.000E+00 | 0.000E+00 | 0.000E+00 | 0.000E+00 | 0.000E+00 | 0.000E+00 | 0.000E+00 | 0.000E+00 |
| Unigene263596 | bcrA | 0.000E+00 | 0.000E+00 | 0.000E+00 | 1.657E-06 | 0.000E+00 | 0.000E+00 | 0.000E+00 | 0.000E+00 | 0.000E+00 | 0.000E+00 | 0.000E+00 | 0.000E+00 |
| Unigene263599 | vanRA | 0.000E+00 | 0.000E+00 | 0.000E+00 | 1.630E-06 | 0.000E+00 | 0.000E+00 | 0.000E+00 | 0.000E+00 | 0.000E+00 | 0.000E+00 | 0.000E+00 | 0.000E+00 |
| Unigene263752 | macB | 0.000E+00 | 8.748E-08 | 4.693E-07 | 1.529E-06 | 0.000E+00 | 0.000E+00 | 0.000E+00 | 0.000E+00 | 0.000E+00 | 0.000E+00 | 0.000E+00 | 0.000E+00 |
| Unigene263755 | vanD | 0.000E+00 | 0.000E+00 | 3.017E-07 | 8.796E-07 | 0.000E+00 | 0.000E+00 | 0.000E+00 | 0.000E+00 | 0.000E+00 | 0.000E+00 | 0.000E+00 | 0.000E+00 |
| Unigene263810 | tetW | 0.000E+00 | 0.000E+00 | 3.473E-08 | 2.198E-06 | 0.000E+00 | 0.000E+00 | 0.000E+00 | 0.000E+00 | 0.000E+00 | 0.000E+00 | 0.000E+00 | 0.000E+00 |
| Unigene263845 | mecB | 0.000E+00 | 0.000E+00 | 0.000E+00 | 1.688E-06 | 0.000E+00 | 0.000E+00 | 0.000E+00 | 0.000E+00 | 0.000E+00 | 0.000E+00 | 0.000E+00 | 0.000E+00 |
| Unigene263880 | patA | 0.000E+00 | 0.000E+00 | 0.000E+00 | 1.786E-06 | 0.000E+00 | 0.000E+00 | 0.000E+00 | 0.000E+00 | 0.000E+00 | 0.000E+00 | 0.000E+00 | 0.000E+00 |
| Unigene263951 | emrR | 0.000E+00 | 0.000E+00 | 0.000E+00 | 2.101E-06 | 0.000E+00 | 0.000E+00 | 0.000E+00 | 0.000E+00 | 0.000E+00 | 0.000E+00 | 0.000E+00 | 0.000E+00 |
| Unigene263952 | lmrB | 0.000E+00 | 0.000E+00 | 0.000E+00 | 1.540E-06 | 0.000E+00 | 0.000E+00 | 0.000E+00 | 0.000E+00 | 0.000E+00 | 0.000E+00 | 0.000E+00 | 0.000E+00 |
| Unigene263956 | msbA | 0.000E+00 | 2.054E-08 | 3.672E-08 | 1.776E-06 | 0.000E+00 | 0.000E+00 | 0.000E+00 | 0.000E+00 | 0.000E+00 | 0.000E+00 | 0.000E+00 | 0.000E+00 |
| Unigene263961 | bcrA | 0.000E+00 | 0.000E+00 | 3.428E-08 | 2.399E-06 | 0.000E+00 | 0.000E+00 | 0.000E+00 | 0.000E+00 | 0.000E+00 | 0.000E+00 | 0.000E+00 | 0.000E+00 |
| Unigene263962 | vanSL | 0.000E+00 | 0.000E+00 | 0.000E+00 | 1.729E-06 | 0.000E+00 | 0.000E+00 | 0.000E+00 | 0.000E+00 | 0.000E+00 | 0.000E+00 | 0.000E+00 | 0.000E+00 |
| Unigene263963 | vanRF | 0.000E+00 | 0.000E+00 | 0.000E+00 | 2.681E-06 | 0.000E+00 | 0.000E+00 | 0.000E+00 | 0.000E+00 | 0.000E+00 | 0.000E+00 | 0.000E+00 | 0.000E+00 |
| Unigene263967 | patA | 0.000E+00 | 0.000E+00 | 0.000E+00 | 2.031E-06 | 0.000E+00 | 0.000E+00 | 0.000E+00 | 0.000E+00 | 0.000E+00 | 0.000E+00 | 0.000E+00 | 0.000E+00 |
| Unigene263980 | vanHM | 0.000E+00 | 0.000E+00 | 0.000E+00 | 2.187E-06 | 0.000E+00 | 0.000E+00 | 0.000E+00 | 0.000E+00 | 0.000E+00 | 0.000E+00 | 0.000E+00 | 0.000E+00 |
| Unigene264094 | facT | 1.471E-07 | 9.226E-08 | 2.887E-07 | 8.064E-07 | 0.000E+00 | 0.000E+00 | 0.000E+00 | 0.000E+00 | 4.594E-08 | 0.000E+00 | 0.000E+00 | 0.000E+00 |
| Unigene264105 | arlS | 0.000E+00 | 0.000E+00 | 0.000E+00 | 7.025E-07 | 0.000E+00 | 0.000E+00 | 0.000E+00 | 0.000E+00 | 0.000E+00 | 0.000E+00 | 0.000E+00 | 0.000E+00 |
| Unigene264135 | arnA | 2.418E-06 | 0.000E+00 | 2.205E-06 | 0.000E+00 | 0.000E+00 | 0.000E+00 | 0.000E+00 | 0.000E+00 | 0.000E+00 | 0.000E+00 | 0.000E+00 | 0.000E+00 |
| Unigene264153 | vanZF | 0.000E+00 | 4.031E-08 | 0.000E+00 | 4.450E-07 | 0.000E+00 | 0.000E+00 | 0.000E+00 | 0.000E+00 | 0.000E+00 | 0.000E+00 | 0.000E+00 | 0.000E+00 |
| Unigene264319 | fexA | 2.766E-08 | 0.000E+00 | 0.000E+00 | 8.938E-07 | 0.000E+00 | 0.000E+00 | 0.000E+00 | 0.000E+00 | 0.000E+00 | 0.000E+00 | 0.000E+00 | 3.270E-07 |
| Unigene264360 | Streptomy | 1.585E-07 | 3.697E-07 | 4.861E-07 | 7.004E-07 | 0.000E+00 | 0.000E+00 | 0.000E+00 | 2.313E-08 | 6.498E-08 | 0.000E+00 | 0.000E+00 | 0.000E+00 |
| Unigene264367 | rpoB2 | 1.374E-07 | 2.564E-07 | 4.315E-07 | 8.187E-07 | 0.000E+00 | 0.000E+00 | 6.084E-08 | 4.812E-08 | 0.000E+00 | 0.000E+00 | 7.860E-08 | 0.000E+00 |
| Unigene264497 | lsaA | 0.000E+00 | 0.000E+00 | 0.000E+00 | 3.234E-07 | 0.000E+00 | 0.000E+00 | 0.000E+00 | 0.000E+00 | 0.000E+00 | 0.000E+00 | 0.000E+00 | 0.000E+00 |
| Unigene264650 | tetA(58) | 2.203E-05 | 6.255E-06 | 3.255E-06 | 1.924E-05 | 1.210E-04 | 1.510E-04 | 1.428E-04 | 1.614E-04 | 1.534E-04 | 1.386E-04 | 1.307E-04 | 7.455E-05 |
| Unigene264692 | YojI | 0.000E+00 | 8.683E-08 | 0.000E+00 | 8.389E-07 | 0.000E+00 | 0.000E+00 | 0.000E+00 | 0.000E+00 | 0.000E+00 | 1.102E-06 | 0.000E+00 | 0.000E+00 |
| Unigene264706 | Bifidobacte | 0.000E+00 | 0.000E+00 | 0.000E+00 | 2.716E-07 | 0.000E+00 | 0.000E+00 | 0.000E+00 | 0.000E+00 | 0.000E+00 | 0.000E+00 | 0.000E+00 | 0.000E+00 |
| Unigene264903 | MexJ | 0.000E+00 | 0.000E+00 | 0.000E+00 | 4.673E-07 | 0.000E+00 | 0.000E+00 | 0.000E+00 | 0.000E+00 | 0.000E+00 | 0.000E+00 | 0.000E+00 | 0.000E+00 |
| Unigene265099 | tetT | 0.000E+00 | 0.000E+00 | 0.000E+00 | 5.539E-07 | 0.000E+00 | 0.000E+00 | 0.000E+00 | 0.000E+00 | 0.000E+00 | 0.000E+00 | 0.000E+00 | 0.000E+00 |
| Unigene265128 | tetB(60) | 0.000E+00 | 0.000E+00 | 0.000E+00 | 2.481E-07 | 0.000E+00 | 0.000E+00 | 3.806E-07 | 0.000E+00 | 0.000E+00 | 0.000E+00 | 0.000E+00 | 0.000E+00 |
| Unigene265185 | oleC | 0.000E+00 | 0.000E+00 | 0.000E+00 | 5.147E-07 | 0.000E+00 | 0.000E+00 | 0.000E+00 | 0.000E+00 | 0.000E+00 | 0.000E+00 | 0.000E+00 | 0.000E+00 |
| Unigene265243 | PmrF | 0.000E+00 | 0.000E+00 | 2.253E-07 | 5.400E-06 | 0.000E+00 | 0.000E+00 | 0.000E+00 | 0.000E+00 | 0.000E+00 | 0.000E+00 | 0.000E+00 | 0.000E+00 |
| Unigene265247 | oleC | 0.000E+00 | 4.631E-08 | 3.726E-07 | 5.539E-06 | 0.000E+00 | 0.000E+00 | 0.000E+00 | 0.000E+00 | 0.000E+00 | 0.000E+00 | 0.000E+00 | 0.000E+00 |
| Unigene265320 | facT | 0.000E+00 | 0.000E+00 | 3.270E-07 | 4.327E-07 | 0.000E+00 | 0.000E+00 | 0.000E+00 | 0.000E+00 | 0.000E+00 | 0.000E+00 | 0.000E+00 | 0.000E+00 |
| Unigene265321 | Acinetobac | 0.000E+00 | 2.624E-07 | 1.341E-07 | 5.519E-07 | 0.000E+00 | 3.693E-08 | 3.024E-07 | 1.595E-07 | 0.000E+00 | 0.000E+00 | 0.000E+00 | 0.000E+00 |
| Unigene265462 | lfrA | 0.000E+00 | 0.000E+00 | 0.000E+00 | 7.808E-07 | 0.000E+00 | 0.000E+00 | 0.000E+00 | 0.000E+00 | 0.000E+00 | 0.000E+00 | 0.000E+00 | 0.000E+00 |
| Unigene265479 | oleC | 0.000E+00 | 0.000E+00 | 0.000E+00 | 7.874E-07 | 0.000E+00 | 0.000E+00 | 0.000E+00 | 0.000E+00 | 0.000E+00 | 0.000E+00 | 0.000E+00 | 0.000E+00 |
| Unigene265486 | lmrD | 1.993E-07 | 0.000E+00 | 0.000E+00 | 8.597E-07 | 0.000E+00 | 0.000E+00 | 0.000E+00 | 1.480E-07 | 3.366E-07 | 0.000E+00 | 0.000E+00 | 0.000E+00 |
| Unigene265567 | evgA | 0.000E+00 | 0.000E+00 | 0.000E+00 | 4.841E-07 | 0.000E+00 | 0.000E+00 | 0.000E+00 | 0.000E+00 | 2.911E-07 | 0.000E+00 | 0.000E+00 | 0.000E+00 |
| Unigene265582 | lmrD | 0.000E+00 | 0.000E+00 | 0.000E+00 | 1.033E-06 | 0.000E+00 | 0.000E+00 | 0.000E+00 | 0.000E+00 | 0.000E+00 | 0.000E+00 | 0.000E+00 | 0.000E+00 |
| Unigene265704 | bcrA | 0.000E+00 | 4.860E-08 | 4.345E-08 | 7.602E-07 | 0.000E+00 | 0.000E+00 | 0.000E+00 | 0.000E+00 | 0.000E+00 | 0.000E+00 | 0.000E+00 | 0.000E+00 |
| Unigene265713 | oleC | 0.000E+00 | 0.000E+00 | 0.000E+00 | 1.056E-06 | 0.000E+00 | 0.000E+00 | 0.000E+00 | 0.000E+00 | 5.714E-07 | 0.000E+00 | 0.000E+00 | 0.000E+00 |
| Unigene265763 | oleC | 0.000E+00 | 0.000E+00 | 0.000E+00 | 3.796E-07 | 0.000E+00 | 0.000E+00 | 0.000E+00 | 0.000E+00 | 0.000E+00 | 0.000E+00 | 0.000E+00 | 0.000E+00 |
| Unigene265785 | Streptomy | 3.698E-07 | 6.493E-07 | 3.991E-07 | 1.083E-06 | 0.000E+00 | 0.000E+00 | 2.046E-07 | 1.295E-07 | 0.000E+00 | 1.648E-07 | 0.000E+00 | 0.000E+00 |
| Unigene265823 | tcr3 | 0.000E+00 | 0.000E+00 | 0.000E+00 | 6.338E-07 | 0.000E+00 | 0.000E+00 | 0.000E+00 | 0.000E+00 | 0.000E+00 | 0.000E+00 | 0.000E+00 | 0.000E+00 |
| Unigene265853 | vanHD | 0.000E+00 | 0.000E+00 | 0.000E+00 | 5.065E-07 | 0.000E+00 | 0.000E+00 | 0.000E+00 | 0.000E+00 | 0.000E+00 | 0.000E+00 | 0.000E+00 | 0.000E+00 |
| Unigene265905 | macB | 0.000E+00 | 0.000E+00 | 0.000E+00 | 3.813E-07 | 0.000E+00 | 0.000E+00 | 0.000E+00 | 0.000E+00 | 0.000E+00 | 0.000E+00 | 0.000E+00 | 0.000E+00 |
| Unigene266065 | macB | 0.000E+00 | 0.000E+00 | 0.000E+00 | 2.384E-06 | 3.038E-05 | 1.491E-05 | 2.093E-05 | 2.161E-05 | 0.000E+00 | 0.000E+00 | 0.000E+00 | 0.000E+00 |
| Unigene266224 | tetT | 0.000E+00 | 0.000E+00 | 5.940E-08 | 1.880E-06 | 0.000E+00 | 0.000E+00 | 0.000E+00 | 0.000E+00 | 0.000E+00 | 0.000E+00 | 0.000E+00 | 0.000E+00 |
| Unigene266229 | rpoB2 | 0.000E+00 | 0.000E+00 | 6.817E-08 | 1.868E-06 | 0.000E+00 | 0.000E+00 | 0.000E+00 | 0.000E+00 | 0.000E+00 | 0.000E+00 | 0.000E+00 | 0.000E+00 |
| Unigene266317 | Agrobacte | 0.000E+00 | 0.000E+00 | 0.000E+00 | 3.969E-07 | 0.000E+00 | 0.000E+00 | 0.000E+00 | 0.000E+00 | 0.000E+00 | 1.040E-06 | 0.000E+00 | 0.000E+00 |
| Unigene266365 | PmrF | 0.000E+00 | 0.000E+00 | 0.000E+00 | 5.139E-07 | 0.000E+00 | 0.000E+00 | 0.000E+00 | 0.000E+00 | 0.000E+00 | 0.000E+00 | 0.000E+00 | 0.000E+00 |
| Unigene266388 | optrA | 0.000E+00 | 0.000E+00 | 0.000E+00 | 4.823E-07 | 0.000E+00 | 0.000E+00 | 0.000E+00 | 0.000E+00 | 0.000E+00 | 0.000E+00 | 0.000E+00 | 0.000E+00 |
| Unigene266491 | macB | 3.698E-08 | 2.029E-07 | 1.814E-07 | 6.161E-06 | 0.000E+00 | 0.000E+00 | 0.000E+00 | 0.000E+00 | 0.000E+00 | 0.000E+00 | 0.000E+00 | 0.000E+00 |
| Unigene266591 | marA | 0.000E+00 | 0.000E+00 | 0.000E+00 | 6.487E-07 | 0.000E+00 | 0.000E+00 | 0.000E+00 | 0.000E+00 | 0.000E+00 | 0.000E+00 | 0.000E+00 | 0.000E+00 |

| | | | | | | | | | | | | | |
|---|---|---|---|---|---|---|---|---|---|---|---|---|---|
| Unigene266683 | poxtA | 0.000E+00 | 0.000E+00 | 0.000E+00 | 2.519E-05 | 0.000E+00 | 0.000E+00 | 0.000E+00 | 0.000E+00 | 0.000E+00 | 0.000E+00 | 0.000E+00 | 0.000E+00 |
| Unigene266687 | PmrF | 0.000E+00 | 0.000E+00 | 0.000E+00 | 2.467E-05 | 0.000E+00 | 0.000E+00 | 0.000E+00 | 0.000E+00 | 0.000E+00 | 0.000E+00 | 0.000E+00 | 0.000E+00 |
| Unigene266709 | vanHD | 0.000E+00 | 0.000E+00 | 2.854E-07 | 5.188E-06 | 0.000E+00 | 0.000E+00 | 0.000E+00 | 0.000E+00 | 0.000E+00 | 0.000E+00 | 0.000E+00 | 0.000E+00 |
| Unigene266911 | vanHD | 0.000E+00 | 0.000E+00 | 6.923E-07 | 8.633E-05 | 0.000E+00 | 0.000E+00 | 0.000E+00 | 0.000E+00 | 0.000E+00 | 0.000E+00 | 0.000E+00 | 0.000E+00 |
| Unigene267033 | lsaC | 0.000E+00 | 0.000E+00 | 0.000E+00 | 4.989E-07 | 0.000E+00 | 0.000E+00 | 0.000E+00 | 0.000E+00 | 0.000E+00 | 0.000E+00 | 0.000E+00 | 0.000E+00 |
| Unigene267041 | emrY | 0.000E+00 | 0.000E+00 | 0.000E+00 | 4.955E-06 | 0.000E+00 | 0.000E+00 | 0.000E+00 | 0.000E+00 | 0.000E+00 | 0.000E+00 | 0.000E+00 | 0.000E+00 |
| Unigene267072 | evgS | 0.000E+00 | 0.000E+00 | 0.000E+00 | 5.030E-07 | 2.214E-07 | 0.000E+00 | 0.000E+00 | 0.000E+00 | 1.089E-07 | 0.000E+00 | 2.659E-07 | 0.000E+00 |
| Unigene267109 | TaeA | 0.000E+00 | 0.000E+00 | 1.553E-07 | 7.790E-07 | 0.000E+00 | 2.566E-07 | 4.597E-07 | 1.316E-06 | 0.000E+00 | 0.000E+00 | 7.014E-07 | 0.000E+00 |
| Unigene267226 | srmB | 0.000E+00 | 0.000E+00 | 0.000E+00 | 7.160E-07 | 0.000E+00 | 0.000E+00 | 0.000E+00 | 0.000E+00 | 0.000E+00 | 0.000E+00 | 0.000E+00 | 0.000E+00 |
| Unigene267240 | tetA(58) | 0.000E+00 | 0.000E+00 | 0.000E+00 | 1.767E-06 | 0.000E+00 | 0.000E+00 | 0.000E+00 | 0.000E+00 | 0.000E+00 | 0.000E+00 | 0.000E+00 | 0.000E+00 |
| Unigene267268 | bmr | 0.000E+00 | 8.835E-08 | 7.109E-07 | 6.891E-05 | 0.000E+00 | 0.000E+00 | 0.000E+00 | 0.000E+00 | 0.000E+00 | 0.000E+00 | 0.000E+00 | 0.000E+00 |
| Unigene267289 | optrA | 0.000E+00 | 0.000E+00 | 0.000E+00 | 4.876E-07 | 0.000E+00 | 0.000E+00 | 0.000E+00 | 0.000E+00 | 0.000E+00 | 0.000E+00 | 0.000E+00 | 0.000E+00 |
| Unigene267309 | qacB | 0.000E+00 | 0.000E+00 | 0.000E+00 | 5.744E-07 | 0.000E+00 | 0.000E+00 | 0.000E+00 | 0.000E+00 | 0.000E+00 | 0.000E+00 | 0.000E+00 | 0.000E+00 |
| Unigene267327 | TaeA | 0.000E+00 | 0.000E+00 | 0.000E+00 | 6.791E-07 | 0.000E+00 | 0.000E+00 | 0.000E+00 | 0.000E+00 | 0.000E+00 | 0.000E+00 | 0.000E+00 | 0.000E+00 |
| Unigene267382 | arlS | 6.071E-05 | 5.672E-05 | 1.017E-04 | 7.203E-05 | 1.180E-04 | 8.829E-05 | 1.141E-04 | 1.164E-04 | 1.005E-04 | 1.413E-04 | 1.388E-04 | 1.600E-04 |
| Unigene267396 | bcrA | 0.000E+00 | 0.000E+00 | 1.314E-07 | 1.217E-06 | 0.000E+00 | 0.000E+00 | 0.000E+00 | 0.000E+00 | 0.000E+00 | 0.000E+00 | 0.000E+00 | 0.000E+00 |
| Unigene267586 | tetA(58) | 0.000E+00 | 0.000E+00 | 0.000E+00 | 1.215E-06 | 0.000E+00 | 0.000E+00 | 0.000E+00 | 0.000E+00 | 0.000E+00 | 0.000E+00 | 0.000E+00 | 0.000E+00 |
| Unigene267597 | macB | 8.574E-08 | 0.000E+00 | 0.000E+00 | 7.359E-07 | 1.810E-06 | 0.000E+00 | 0.000E+00 | 2.502E-07 | 3.748E-07 | 0.000E+00 | 0.000E+00 | 0.000E+00 |
| Unigene267599 | msbA | 0.000E+00 | 0.000E+00 | 0.000E+00 | 1.031E-06 | 1.323E-06 | 0.000E+00 | 0.000E+00 | 0.000E+00 | 2.789E-07 | 0.000E+00 | 0.000E+00 | 0.000E+00 |
| Unigene267646 | patB | 1.862E-07 | 2.043E-07 | 1.096E-07 | 9.023E-07 | 0.000E+00 | 0.000E+00 | 0.000E+00 | 0.000E+00 | 0.000E+00 | 0.000E+00 | 8.518E-08 | 0.000E+00 |
| Unigene267730 | vanHF | 3.055E-06 | 9.185E-06 | 9.678E-06 | 1.264E-05 | 0.000E+00 | 0.000E+00 | 0.000E+00 | 0.000E+00 | 0.000E+00 | 0.000E+00 | 0.000E+00 | 0.000E+00 |
| Unigene267752 | Erm(K) | 0.000E+00 | 0.000E+00 | 0.000E+00 | 5.105E-07 | 0.000E+00 | 0.000E+00 | 0.000E+00 | 0.000E+00 | 0.000E+00 | 0.000E+00 | 0.000E+00 | 0.000E+00 |
| Unigene267797 | lmrB | 0.000E+00 | 0.000E+00 | 0.000E+00 | 6.174E-07 | 0.000E+00 | 0.000E+00 | 0.000E+00 | 0.000E+00 | 0.000E+00 | 0.000E+00 | 0.000E+00 | 0.000E+00 |
| Unigene267874 | tetA(58) | 5.674E-06 | 2.339E-05 | 3.588E-06 | 1.486E-06 | 1.121E-06 | 3.635E-07 | 0.000E+00 | 0.000E+00 | 1.286E-06 | 0.000E+00 | 0.000E+00 | 0.000E+00 |
| Unigene267892 | smeS | 0.000E+00 | 0.000E+00 | 0.000E+00 | 0.000E+00 | 0.000E+00 | 1.723E-07 | 0.000E+00 | 0.000E+00 | 0.000E+00 | 0.000E+00 | 0.000E+00 | 0.000E+00 |
| Unigene267934 | baeS | 8.748E-08 | 2.640E-07 | 0.000E+00 | 6.846E-07 | 0.000E+00 | 0.000E+00 | 0.000E+00 | 0.000E+00 | 0.000E+00 | 1.023E-06 | 0.000E+00 | 4.912E-07 |
| Unigene267935 | vanRM | 0.000E+00 | 1.317E-07 | 0.000E+00 | 1.091E-06 | 0.000E+00 | 0.000E+00 | 0.000E+00 | 0.000E+00 | 0.000E+00 | 9.803E-07 | 0.000E+00 | 1.418E-07 |
| Unigene268062 | dfrC | 0.000E+00 | 0.000E+00 | 0.000E+00 | 4.346E-06 | 0.000E+00 | 0.000E+00 | 0.000E+00 | 0.000E+00 | 0.000E+00 | 0.000E+00 | 0.000E+00 | 0.000E+00 |
| Unigene268064 | TaeA | 0.000E+00 | 0.000E+00 | 2.694E-07 | 3.882E-06 | 0.000E+00 | 3.710E-08 | 0.000E+00 | 0.000E+00 | 0.000E+00 | 0.000E+00 | 0.000E+00 | 0.000E+00 |
| Unigene268093 | bcrA | 0.000E+00 | 0.000E+00 | 4.548E-07 | 1.237E-06 | 0.000E+00 | 0.000E+00 | 0.000E+00 | 0.000E+00 | 0.000E+00 | 0.000E+00 | 0.000E+00 | 0.000E+00 |
| Unigene268107 | mgrA | 0.000E+00 | 0.000E+00 | 0.000E+00 | 4.116E-07 | 0.000E+00 | 0.000E+00 | 0.000E+00 | 0.000E+00 | 0.000E+00 | 0.000E+00 | 0.000E+00 | 0.000E+00 |
| Unigene268123 | smeS | 0.000E+00 | 0.000E+00 | 7.821E-08 | 5.232E-07 | 0.000E+00 | 0.000E+00 | 0.000E+00 | 0.000E+00 | 0.000E+00 | 0.000E+00 | 4.559E-08 | 0.000E+00 |
| Unigene268142 | PmrF | 0.000E+00 | 0.000E+00 | 0.000E+00 | 6.414E-07 | 0.000E+00 | 0.000E+00 | 0.000E+00 | 0.000E+00 | 0.000E+00 | 0.000E+00 | 0.000E+00 | 0.000E+00 |
| Unigene268153 | macB | 0.000E+00 | 0.000E+00 | 0.000E+00 | 1.099E-06 | 0.000E+00 | 0.000E+00 | 0.000E+00 | 0.000E+00 | 0.000E+00 | 0.000E+00 | 0.000E+00 | 0.000E+00 |
| Unigene268211 | tetB(58) | 0.000E+00 | 0.000E+00 | 0.000E+00 | 8.666E-07 | 0.000E+00 | 0.000E+00 | 0.000E+00 | 0.000E+00 | 0.000E+00 | 0.000E+00 | 0.000E+00 | 0.000E+00 |
| Unigene268243 | arlS | 0.000E+00 | 0.000E+00 | 0.000E+00 | 6.928E-07 | 0.000E+00 | 0.000E+00 | 0.000E+00 | 0.000E+00 | 0.000E+00 | 0.000E+00 | 0.000E+00 | 0.000E+00 |
| Unigene268418 | macB | 0.000E+00 | 0.000E+00 | 2.053E-07 | 4.226E-06 | 0.000E+00 | 6.462E-08 | 0.000E+00 | 0.000E+00 | 0.000E+00 | 0.000E+00 | 0.000E+00 | 0.000E+00 |
| Unigene268534 | PmrF | 0.000E+00 | 0.000E+00 | 0.000E+00 | 9.658E-07 | 0.000E+00 | 0.000E+00 | 0.000E+00 | 0.000E+00 | 0.000E+00 | 0.000E+00 | 0.000E+00 | 0.000E+00 |
| Unigene268541 | qacH | 9.576E-06 | 0.000E+00 | 0.000E+00 | 6.878E-06 | 5.330E-05 | 6.712E-05 | 7.397E-05 | 7.536E-05 | 1.195E-04 | 1.143E-04 | 8.898E-05 | 4.562E-05 |
| Unigene268575 | macB | 0.000E+00 | 0.000E+00 | 0.000E+00 | 5.995E-07 | 0.000E+00 | 0.000E+00 | 0.000E+00 | 0.000E+00 | 0.000E+00 | 0.000E+00 | 1.273E-07 | 0.000E+00 |
| Unigene268673 | erm(32) | 0.000E+00 | 0.000E+00 | 1.796E-07 | 4.176E-06 | 0.000E+00 | 0.000E+00 | 0.000E+00 | 0.000E+00 | 0.000E+00 | 0.000E+00 | 0.000E+00 | 0.000E+00 |
| Unigene268703 | lsaC | 0.000E+00 | 0.000E+00 | 0.000E+00 | 3.927E-07 | 0.000E+00 | 0.000E+00 | 0.000E+00 | 0.000E+00 | 0.000E+00 | 0.000E+00 | 0.000E+00 | 0.000E+00 |
| Unigene268705 | mdtG | 0.000E+00 | 0.000E+00 | 0.000E+00 | 5.262E-07 | 0.000E+00 | 0.000E+00 | 0.000E+00 | 0.000E+00 | 0.000E+00 | 0.000E+00 | 0.000E+00 | 0.000E+00 |
| Unigene268742 | vmlR | 0.000E+00 | 0.000E+00 | 0.000E+00 | 8.396E-07 | 0.000E+00 | 0.000E+00 | 0.000E+00 | 0.000E+00 | 0.000E+00 | 0.000E+00 | 0.000E+00 | 0.000E+00 |
| Unigene268759 | mdtC | 0.000E+00 | 0.000E+00 | 1.518E-07 | 1.927E-06 | 0.000E+00 | 0.000E+00 | 0.000E+00 | 0.000E+00 | 0.000E+00 | 0.000E+00 | 0.000E+00 | 0.000E+00 |
| Unigene268767 | vanRG | 0.000E+00 | 0.000E+00 | 6.834E-08 | 1.301E-06 | 0.000E+00 | 0.000E+00 | 0.000E+00 | 0.000E+00 | 0.000E+00 | 0.000E+00 | 0.000E+00 | 0.000E+00 |
| Unigene268826 | poxtA | 0.000E+00 | 0.000E+00 | 0.000E+00 | 2.052E-06 | 0.000E+00 | 0.000E+00 | 0.000E+00 | 0.000E+00 | 0.000E+00 | 0.000E+00 | 0.000E+00 | 0.000E+00 |
| Unigene268895 | Streptomy | 0.000E+00 | 0.000E+00 | 0.000E+00 | 3.253E-07 | 0.000E+00 | 0.000E+00 | 0.000E+00 | 0.000E+00 | 0.000E+00 | 0.000E+00 | 0.000E+00 | 0.000E+00 |
| Unigene268922 | efmA | 0.000E+00 | 0.000E+00 | 0.000E+00 | 4.435E-07 | 0.000E+00 | 0.000E+00 | 0.000E+00 | 0.000E+00 | 0.000E+00 | 0.000E+00 | 0.000E+00 | 0.000E+00 |
| Unigene268942 | Streptococ | 1.391E-07 | 0.000E+00 | 0.000E+00 | 1.124E-06 | 1.288E-06 | 3.510E-07 | 4.620E-07 | 2.165E-07 | 5.576E-07 | 7.750E-07 | 0.000E+00 | 0.000E+00 |
| Unigene269358 | facT | 1.362E-07 | 3.737E-07 | 2.673E-07 | 5.502E-07 | 0.000E+00 | 0.000E+00 | 0.000E+00 | 0.000E+00 | 0.000E+00 | 0.000E+00 | 0.000E+00 | 0.000E+00 |
| Unigene269392 | Staphyloco | 1.488E-07 | 0.000E+00 | 0.000E+00 | 9.013E-07 | 0.000E+00 | 0.000E+00 | 0.000E+00 | 0.000E+00 | 0.000E+00 | 6.076E-07 | 0.000E+00 | 3.517E-07 |
| Unigene269463 | Klebsiella p | 0.000E+00 | 0.000E+00 | 0.000E+00 | 0.000E+00 | 0.000E+00 | 0.000E+00 | 0.000E+00 | 0.000E+00 | 4.055E-07 | 0.000E+00 | 0.000E+00 | 0.000E+00 |
| Unigene269571 | tetA(58) | 1.371E-07 | 0.000E+00 | 2.017E-07 | 3.806E-07 | 0.000E+00 | 0.000E+00 | 0.000E+00 | 0.000E+00 | 0.000E+00 | 0.000E+00 | 0.000E+00 | 0.000E+00 |
| Unigene269572 | facT | 0.000E+00 | 0.000E+00 | 0.000E+00 | 3.356E-07 | 0.000E+00 | 0.000E+00 | 0.000E+00 | 0.000E+00 | 0.000E+00 | 0.000E+00 | 0.000E+00 | 9.821E-08 |
| Unigene269677 | evgS | 0.000E+00 | 0.000E+00 | 0.000E+00 | 5.848E-07 | 0.000E+00 | 0.000E+00 | 0.000E+00 | 3.914E-07 | 0.000E+00 | 0.000E+00 | 2.092E-07 | 0.000E+00 |
| Unigene269761 | otrC | 0.000E+00 | 0.000E+00 | 0.000E+00 | 6.450E-07 | 0.000E+00 | 0.000E+00 | 0.000E+00 | 0.000E+00 | 0.000E+00 | 0.000E+00 | 0.000E+00 | 0.000E+00 |
| Unigene269770 | ugd | 0.000E+00 | 0.000E+00 | 0.000E+00 | 5.300E-07 | 0.000E+00 | 0.000E+00 | 0.000E+00 | 0.000E+00 | 0.000E+00 | 0.000E+00 | 0.000E+00 | 0.000E+00 |

| | | | | | | | | | | | | | |
|---|---|---|---|---|---|---|---|---|---|---|---|---|---|
| Unigene269828 | AcrF | 0.000E+00 | 0.000E+00 | 3.931E-07 | 5.780E-07 | 0.000E+00 | 0.000E+00 | 0.000E+00 | 0.000E+00 | 0.000E+00 | 0.000E+00 | 0.000E+00 | 0.000E+00 |
| Unigene269829 | AcrE | 0.000E+00 | 4.644E-07 | 0.000E+00 | 6.715E-07 | 0.000E+00 | 0.000E+00 | 0.000E+00 | 0.000E+00 | 0.000E+00 | 0.000E+00 | 0.000E+00 | 0.000E+00 |
| Unigene269833 | rphA | 0.000E+00 | 0.000E+00 | 0.000E+00 | 7.569E-07 | 0.000E+00 | 0.000E+00 | 0.000E+00 | 0.000E+00 | 0.000E+00 | 0.000E+00 | 0.000E+00 | 0.000E+00 |
| Unigene269856 | tet(43) | 9.061E-08 | 2.983E-07 | 5.779E-07 | 1.007E-06 | 0.000E+00 | 0.000E+00 | 0.000E+00 | 0.000E+00 | 0.000E+00 | 0.000E+00 | 2.591E-08 | 1.339E-07 |
| Unigene269866 | oleC | 0.000E+00 | 0.000E+00 | 0.000E+00 | 2.484E-07 | 0.000E+00 | 0.000E+00 | 0.000E+00 | 0.000E+00 | 0.000E+00 | 0.000E+00 | 0.000E+00 | 0.000E+00 |
| Unigene269868 | lmrB | 1.098E-07 | 0.000E+00 | 3.232E-07 | 5.544E-07 | 0.000E+00 | 0.000E+00 | 0.000E+00 | 0.000E+00 | 0.000E+00 | 0.000E+00 | 0.000E+00 | 0.000E+00 |
| Unigene269892 | efrA | 4.670E-07 | 4.716E-07 | 2.383E-07 | 1.811E-06 | 6.226E-08 | 0.000E+00 | 0.000E+00 | 0.000E+00 | 0.000E+00 | 0.000E+00 | 0.000E+00 | 0.000E+00 |
| Unigene269893 | efrB | 2.822E-07 | 3.872E-07 | 3.808E-07 | 1.514E-06 | 0.000E+00 | 0.000E+00 | 0.000E+00 | 0.000E+00 | 0.000E+00 | 0.000E+00 | 2.018E-08 | 0.000E+00 |
| Unigene269899 | tlrC | 0.000E+00 | 0.000E+00 | 0.000E+00 | 4.624E-07 | 0.000E+00 | 0.000E+00 | 0.000E+00 | 0.000E+00 | 0.000E+00 | 0.000E+00 | 0.000E+00 | 0.000E+00 |
| Unigene270063 | Corynebac | 0.000E+00 | 0.000E+00 | 0.000E+00 | 1.923E-07 | 0.000E+00 | 1.029E-07 | 0.000E+00 | 0.000E+00 | 0.000E+00 | 0.000E+00 | 0.000E+00 | 0.000E+00 |
| Unigene270086 | lmrB | 0.000E+00 | 0.000E+00 | 0.000E+00 | 7.598E-07 | 0.000E+00 | 0.000E+00 | 0.000E+00 | 0.000E+00 | 0.000E+00 | 0.000E+00 | 0.000E+00 | 0.000E+00 |
| Unigene270141 | novA | 0.000E+00 | 0.000E+00 | 0.000E+00 | 0.000E+00 | 0.000E+00 | 0.000E+00 | 0.000E+00 | 0.000E+00 | 3.016E-07 | 0.000E+00 | 0.000E+00 | 0.000E+00 |
| Unigene270160 | carA | 0.000E+00 | 0.000E+00 | 0.000E+00 | 5.433E-07 | 0.000E+00 | 0.000E+00 | 0.000E+00 | 0.000E+00 | 0.000E+00 | 0.000E+00 | 0.000E+00 | 0.000E+00 |
| Unigene270185 | PmrF | 0.000E+00 | 0.000E+00 | 0.000E+00 | 1.397E-05 | 0.000E+00 | 0.000E+00 | 0.000E+00 | 0.000E+00 | 0.000E+00 | 0.000E+00 | 0.000E+00 | 0.000E+00 |
| Unigene270198 | oleC | 0.000E+00 | 0.000E+00 | 0.000E+00 | 5.195E-07 | 0.000E+00 | 0.000E+00 | 0.000E+00 | 0.000E+00 | 0.000E+00 | 0.000E+00 | 0.000E+00 | 0.000E+00 |
| Unigene270214 | catB9 | 0.000E+00 | 6.216E-08 | 0.000E+00 | 4.575E-07 | 0.000E+00 | 0.000E+00 | 0.000E+00 | 0.000E+00 | 0.000E+00 | 0.000E+00 | 0.000E+00 | 0.000E+00 |
| Unigene270222 | bmr | 0.000E+00 | 0.000E+00 | 0.000E+00 | 1.416E-06 | 0.000E+00 | 0.000E+00 | 0.000E+00 | 0.000E+00 | 0.000E+00 | 1.112E-06 | 0.000E+00 | 4.463E-07 |
| Unigene270305 | farB | 0.000E+00 | 1.228E-07 | 0.000E+00 | 8.132E-07 | 0.000E+00 | 0.000E+00 | 0.000E+00 | 0.000E+00 | 0.000E+00 | 7.725E-07 | 0.000E+00 | 4.760E-07 |
| Unigene270307 | Staphylocc | 8.073E-08 | 8.860E-08 | 0.000E+00 | 1.013E-06 | 0.000E+00 | 0.000E+00 | 0.000E+00 | 0.000E+00 | 0.000E+00 | 9.764E-07 | 0.000E+00 | 3.135E-07 |
| Unigene270383 | tetB(46) | 0.000E+00 | 0.000E+00 | 0.000E+00 | 4.829E-07 | 0.000E+00 | 0.000E+00 | 0.000E+00 | 0.000E+00 | 0.000E+00 | 0.000E+00 | 0.000E+00 | 0.000E+00 |
| Unigene270505 | vanRM | 0.000E+00 | 4.108E-07 | 6.427E-07 | 6.142E-07 | 0.000E+00 | 0.000E+00 | 0.000E+00 | 0.000E+00 | 0.000E+00 | 0.000E+00 | 0.000E+00 | 0.000E+00 |
| Unigene270513 | efrA | 0.000E+00 | 0.000E+00 | 0.000E+00 | 6.822E-07 | 0.000E+00 | 0.000E+00 | 0.000E+00 | 0.000E+00 | 0.000E+00 | 0.000E+00 | 0.000E+00 | 0.000E+00 |
| Unigene270518 | Acinetobac | 0.000E+00 | 6.306E-07 | 3.588E-07 | 4.483E-07 | 0.000E+00 | 0.000E+00 | 0.000E+00 | 0.000E+00 | 0.000E+00 | 0.000E+00 | 0.000E+00 | 0.000E+00 |
| Unigene270539 | efrB | 0.000E+00 | 0.000E+00 | 1.100E-06 | 5.446E-05 | 0.000E+00 | 0.000E+00 | 0.000E+00 | 0.000E+00 | 0.000E+00 | 0.000E+00 | 0.000E+00 | 0.000E+00 |
| Unigene270556 | bcrA | 0.000E+00 | 0.000E+00 | 1.781E-07 | 1.152E-06 | 0.000E+00 | 0.000E+00 | 0.000E+00 | 0.000E+00 | 0.000E+00 | 0.000E+00 | 0.000E+00 | 0.000E+00 |
| Unigene270632 | tetA(58) | 4.598E-07 | 2.523E-07 | 0.000E+00 | 3.715E-07 | 0.000E+00 | 0.000E+00 | 1.527E-07 | 0.000E+00 | 0.000E+00 | 0.000E+00 | 0.000E+00 | 0.000E+00 |
| Unigene270670 | tetA(58) | 0.000E+00 | 0.000E+00 | 0.000E+00 | 5.202E-07 | 0.000E+00 | 0.000E+00 | 0.000E+00 | 0.000E+00 | 0.000E+00 | 0.000E+00 | 0.000E+00 | 0.000E+00 |
| Unigene270810 | msbA | 0.000E+00 | 0.000E+00 | 0.000E+00 | 3.622E-07 | 0.000E+00 | 0.000E+00 | 0.000E+00 | 0.000E+00 | 0.000E+00 | 0.000E+00 | 0.000E+00 | 0.000E+00 |
| Unigene270848 | evgA | 0.000E+00 | 1.470E-07 | 2.629E-07 | 9.919E-07 | 0.000E+00 | 0.000E+00 | 0.000E+00 | 0.000E+00 | 0.000E+00 | 0.000E+00 | 0.000E+00 | 1.055E-07 |
| Unigene270902 | baeR | 0.000E+00 | 0.000E+00 | 4.345E-08 | 1.252E-06 | 0.000E+00 | 0.000E+00 | 0.000E+00 | 0.000E+00 | 0.000E+00 | 0.000E+00 | 0.000E+00 | 0.000E+00 |
| Unigene270904 | macB | 0.000E+00 | 0.000E+00 | 0.000E+00 | 1.960E-06 | 0.000E+00 | 0.000E+00 | 0.000E+00 | 0.000E+00 | 0.000E+00 | 0.000E+00 | 0.000E+00 | 0.000E+00 |
| Unigene270919 | patA | 0.000E+00 | 0.000E+00 | 0.000E+00 | 9.500E-07 | 1.960E-07 | 0.000E+00 | 0.000E+00 | 0.000E+00 | 6.427E-08 | 0.000E+00 | 2.690E-07 | 0.000E+00 |
| Unigene270933 | arlS | 0.000E+00 | 0.000E+00 | 0.000E+00 | 8.449E-07 | 0.000E+00 | 0.000E+00 | 0.000E+00 | 0.000E+00 | 3.302E-07 | 0.000E+00 | 0.000E+00 | 0.000E+00 |
| Unigene271015 | msbA | 0.000E+00 | 0.000E+00 | 0.000E+00 | 5.077E-07 | 0.000E+00 | 0.000E+00 | 0.000E+00 | 0.000E+00 | 0.000E+00 | 0.000E+00 | 0.000E+00 | 0.000E+00 |
| Unigene271162 | novA | 0.000E+00 | 0.000E+00 | 0.000E+00 | 4.610E-07 | 0.000E+00 | 0.000E+00 | 0.000E+00 | 0.000E+00 | 0.000E+00 | 0.000E+00 | 0.000E+00 | 0.000E+00 |
| Unigene271168 | oleC | 2.138E-07 | 2.346E-07 | 0.000E+00 | 2.878E-07 | 0.000E+00 | 0.000E+00 | 0.000E+00 | 0.000E+00 | 0.000E+00 | 0.000E+00 | 0.000E+00 | 0.000E+00 |
| Unigene271239 | bcrA | 0.000E+00 | 0.000E+00 | 0.000E+00 | 7.172E-07 | 0.000E+00 | 0.000E+00 | 0.000E+00 | 0.000E+00 | 0.000E+00 | 0.000E+00 | 0.000E+00 | 0.000E+00 |
| Unigene271283 | msbA | 0.000E+00 | 0.000E+00 | 0.000E+00 | 4.405E-07 | 0.000E+00 | 0.000E+00 | 0.000E+00 | 0.000E+00 | 0.000E+00 | 0.000E+00 | 0.000E+00 | 0.000E+00 |
| Unigene271300 | optrA | 0.000E+00 | 1.465E-07 | 1.048E-07 | 4.853E-07 | 0.000E+00 | 0.000E+00 | 0.000E+00 | 0.000E+00 | 0.000E+00 | 0.000E+00 | 0.000E+00 | 0.000E+00 |
| Unigene271337 | efrA | 0.000E+00 | 0.000E+00 | 0.000E+00 | 5.821E-07 | 0.000E+00 | 0.000E+00 | 4.253E-08 | 0.000E+00 | 0.000E+00 | 0.000E+00 | 0.000E+00 | 0.000E+00 |
| Unigene271338 | emrB | 0.000E+00 | 0.000E+00 | 0.000E+00 | 6.663E-07 | 5.639E-08 | 0.000E+00 | 0.000E+00 | 0.000E+00 | 0.000E+00 | 0.000E+00 | 0.000E+00 | 0.000E+00 |
| Unigene271408 | oleB | 0.000E+00 | 0.000E+00 | 2.209E-07 | 3.637E-07 | 0.000E+00 | 0.000E+00 | 0.000E+00 | 0.000E+00 | 0.000E+00 | 0.000E+00 | 0.000E+00 | 0.000E+00 |
| Unigene271615 | smeR | 0.000E+00 | 0.000E+00 | 0.000E+00 | 4.435E-07 | 0.000E+00 | 0.000E+00 | 0.000E+00 | 0.000E+00 | 0.000E+00 | 0.000E+00 | 0.000E+00 | 0.000E+00 |
| Unigene271817 | efrA | 0.000E+00 | 0.000E+00 | 4.436E-08 | 1.933E-06 | 0.000E+00 | 0.000E+00 | 0.000E+00 | 0.000E+00 | 0.000E+00 | 0.000E+00 | 0.000E+00 | 0.000E+00 |
| Unigene271818 | patB | 0.000E+00 | 0.000E+00 | 3.462E-08 | 1.817E-06 | 0.000E+00 | 0.000E+00 | 0.000E+00 | 0.000E+00 | 0.000E+00 | 0.000E+00 | 0.000E+00 | 0.000E+00 |
| Unigene271820 | vanRI | 0.000E+00 | 0.000E+00 | 0.000E+00 | 1.748E-06 | 0.000E+00 | 0.000E+00 | 0.000E+00 | 0.000E+00 | 0.000E+00 | 0.000E+00 | 0.000E+00 | 0.000E+00 |
| Unigene271821 | baeS | 0.000E+00 | 0.000E+00 | 2.271E-08 | 1.776E-06 | 0.000E+00 | 0.000E+00 | 0.000E+00 | 0.000E+00 | 0.000E+00 | 0.000E+00 | 0.000E+00 | 0.000E+00 |
| Unigene271826 | vanHD | 0.000E+00 | 0.000E+00 | 6.640E-08 | 1.913E-06 | 0.000E+00 | 0.000E+00 | 0.000E+00 | 0.000E+00 | 0.000E+00 | 0.000E+00 | 0.000E+00 | 0.000E+00 |
| Unigene271858 | TaeA | 0.000E+00 | 0.000E+00 | 1.872E-07 | 1.464E-06 | 0.000E+00 | 0.000E+00 | 0.000E+00 | 0.000E+00 | 0.000E+00 | 0.000E+00 | 0.000E+00 | 0.000E+00 |
| Unigene271862 | fexA | 0.000E+00 | 0.000E+00 | 0.000E+00 | 1.533E-06 | 0.000E+00 | 0.000E+00 | 0.000E+00 | 0.000E+00 | 0.000E+00 | 0.000E+00 | 0.000E+00 | 0.000E+00 |
| Unigene272151 | evgA | 5.076E-08 | 2.228E-07 | 0.000E+00 | 9.738E-07 | 0.000E+00 | 0.000E+00 | 0.000E+00 | 0.000E+00 | 0.000E+00 | 6.219E-07 | 0.000E+00 | 5.999E-08 |
| Unigene272200 | TaeA | 0.000E+00 | 0.000E+00 | 2.009E-07 | 3.676E-07 | 0.000E+00 | 0.000E+00 | 2.518E-08 | 0.000E+00 | 4.973E-08 | 0.000E+00 | 0.000E+00 | 8.067E-08 |
| Unigene272212 | arlR | 0.000E+00 | 0.000E+00 | 0.000E+00 | 7.461E-07 | 0.000E+00 | 0.000E+00 | 0.000E+00 | 0.000E+00 | 0.000E+00 | 0.000E+00 | 0.000E+00 | 0.000E+00 |
| Unigene272215 | macB | 0.000E+00 | 0.000E+00 | 0.000E+00 | 5.237E-07 | 0.000E+00 | 0.000E+00 | 0.000E+00 | 0.000E+00 | 0.000E+00 | 0.000E+00 | 0.000E+00 | 0.000E+00 |
| Unigene272348 | vanSM | 0.000E+00 | 2.147E-07 | 6.719E-07 | 6.915E-07 | 0.000E+00 | 0.000E+00 | 0.000E+00 | 0.000E+00 | 0.000E+00 | 0.000E+00 | 0.000E+00 | 0.000E+00 |
| Unigene272357 | Staphylocc | 1.054E-04 | 1.957E-04 | 1.850E-04 | 7.127E-06 | 9.236E-06 | 4.190E-07 | 4.009E-06 | 1.716E-06 | 6.012E-05 | 1.787E-07 | 3.119E-06 | 9.638E-07 |
| Unigene272412 | bcrA | 0.000E+00 | 0.000E+00 | 4.213E-07 | 5.175E-06 | 0.000E+00 | 0.000E+00 | 0.000E+00 | 0.000E+00 | 0.000E+00 | 0.000E+00 | 0.000E+00 | 0.000E+00 |
| Unigene272444 | vanTN | 0.000E+00 | 0.000E+00 | 4.578E-07 | 4.535E-06 | 0.000E+00 | 0.000E+00 | 0.000E+00 | 0.000E+00 | 0.000E+00 | 0.000E+00 | 0.000E+00 | 0.000E+00 |

| | | | | | | | | | | | | | |
|---|---|---|---|---|---|---|---|---|---|---|---|---|---|
| Unigene272476 | arlS | 0.000E+00 | 0.000E+00 | 0.000E+00 | 3.652E-07 | 0.000E+00 | 0.000E+00 | 0.000E+00 | 0.000E+00 | 0.000E+00 | 0.000E+00 | 0.000E+00 | 0.000E+00 |
| Unigene272478 | oleB | 0.000E+00 | 0.000E+00 | 3.845E-07 | 4.261E-07 | 0.000E+00 | 0.000E+00 | 0.000E+00 | 0.000E+00 | 0.000E+00 | 0.000E+00 | 0.000E+00 | 0.000E+00 |
| Unigene272750 | kdpE | 0.000E+00 | 3.413E-07 | 0.000E+00 | 3.768E-07 | 0.000E+00 | 0.000E+00 | 0.000E+00 | 0.000E+00 | 0.000E+00 | 0.000E+00 | 0.000E+00 | 0.000E+00 |
| Unigene272807 | mdtB | 0.000E+00 | 0.000E+00 | 0.000E+00 | 2.603E-07 | 0.000E+00 | 0.000E+00 | 0.000E+00 | 0.000E+00 | 0.000E+00 | 0.000E+00 | 2.211E-07 | 0.000E+00 |
| Unigene272828 | rpoB2 | 0.000E+00 | 1.561E-07 | 0.000E+00 | 1.085E-06 | 0.000E+00 | 0.000E+00 | 0.000E+00 | 0.000E+00 | 0.000E+00 | 6.688E-07 | 0.000E+00 | 3.175E-07 |
| Unigene272861 | bcrC | 0.000E+00 | 0.000E+00 | 0.000E+00 | 7.568E-07 | 0.000E+00 | 0.000E+00 | 0.000E+00 | 0.000E+00 | 0.000E+00 | 0.000E+00 | 0.000E+00 | 0.000E+00 |
| Unigene272898 | macB | 0.000E+00 | 0.000E+00 | 0.000E+00 | 1.611E-06 | 0.000E+00 | 0.000E+00 | 0.000E+00 | 0.000E+00 | 0.000E+00 | 0.000E+00 | 0.000E+00 | 0.000E+00 |
| Unigene272899 | macB | 0.000E+00 | 0.000E+00 | 1.383E-07 | 1.661E-06 | 0.000E+00 | 0.000E+00 | 0.000E+00 | 0.000E+00 | 0.000E+00 | 0.000E+00 | 0.000E+00 | 0.000E+00 |
| Unigene272905 | bcrA | 0.000E+00 | 0.000E+00 | 0.000E+00 | 2.145E-06 | 0.000E+00 | 0.000E+00 | 0.000E+00 | 0.000E+00 | 0.000E+00 | 0.000E+00 | 0.000E+00 | 0.000E+00 |
| Unigene272911 | patB | 0.000E+00 | 0.000E+00 | 7.345E-08 | 1.625E-06 | 0.000E+00 | 0.000E+00 | 0.000E+00 | 0.000E+00 | 0.000E+00 | 0.000E+00 | 0.000E+00 | 0.000E+00 |
| Unigene272912 | novA | 0.000E+00 | 0.000E+00 | 1.777E-08 | 1.134E-06 | 0.000E+00 | 0.000E+00 | 0.000E+00 | 0.000E+00 | 0.000E+00 | 0.000E+00 | 0.000E+00 | 0.000E+00 |
| Unigene272929 | vanRI | 0.000E+00 | 0.000E+00 | 3.124E-08 | 1.447E-06 | 0.000E+00 | 0.000E+00 | 0.000E+00 | 0.000E+00 | 0.000E+00 | 0.000E+00 | 0.000E+00 | 0.000E+00 |
| Unigene272946 | carA | 0.000E+00 | 0.000E+00 | 0.000E+00 | 1.616E-06 | 0.000E+00 | 0.000E+00 | 0.000E+00 | 0.000E+00 | 0.000E+00 | 0.000E+00 | 0.000E+00 | 0.000E+00 |
| Unigene272958 | tva(A) | 0.000E+00 | 0.000E+00 | 0.000E+00 | 1.278E-06 | 0.000E+00 | 0.000E+00 | 0.000E+00 | 0.000E+00 | 0.000E+00 | 0.000E+00 | 0.000E+00 | 0.000E+00 |
| Unigene272960 | vanZF | 0.000E+00 | 0.000E+00 | 0.000E+00 | 1.943E-06 | 0.000E+00 | 0.000E+00 | 0.000E+00 | 0.000E+00 | 0.000E+00 | 0.000E+00 | 0.000E+00 | 0.000E+00 |
| Unigene272989 | oleC | 0.000E+00 | 0.000E+00 | 4.531E-08 | 2.145E-06 | 0.000E+00 | 0.000E+00 | 0.000E+00 | 0.000E+00 | 0.000E+00 | 0.000E+00 | 0.000E+00 | 0.000E+00 |
| Unigene272996 | bcrA | 0.000E+00 | 0.000E+00 | 0.000E+00 | 1.647E-06 | 0.000E+00 | 0.000E+00 | 0.000E+00 | 0.000E+00 | 0.000E+00 | 0.000E+00 | 0.000E+00 | 0.000E+00 |
| Unigene272997 | baeS | 0.000E+00 | 0.000E+00 | 1.242E-07 | 1.598E-06 | 0.000E+00 | 0.000E+00 | 0.000E+00 | 0.000E+00 | 0.000E+00 | 0.000E+00 | 0.000E+00 | 0.000E+00 |
| Unigene272998 | vanRI | 0.000E+00 | 0.000E+00 | 9.302E-08 | 1.580E-06 | 0.000E+00 | 0.000E+00 | 0.000E+00 | 0.000E+00 | 0.000E+00 | 0.000E+00 | 0.000E+00 | 0.000E+00 |
| Unigene273023 | vmlR | 0.000E+00 | 0.000E+00 | 0.000E+00 | 8.149E-07 | 0.000E+00 | 0.000E+00 | 0.000E+00 | 0.000E+00 | 0.000E+00 | 0.000E+00 | 0.000E+00 | 0.000E+00 |
| Unigene273046 | TaeA | 0.000E+00 | 0.000E+00 | 0.000E+00 | 1.749E-06 | 0.000E+00 | 0.000E+00 | 0.000E+00 | 0.000E+00 | 0.000E+00 | 0.000E+00 | 0.000E+00 | 0.000E+00 |
| Unigene273067 | tcr3 | 0.000E+00 | 0.000E+00 | 0.000E+00 | 1.510E-06 | 0.000E+00 | 0.000E+00 | 0.000E+00 | 0.000E+00 | 0.000E+00 | 0.000E+00 | 0.000E+00 | 0.000E+00 |
| Unigene273196 | arlR | 0.000E+00 | 0.000E+00 | 0.000E+00 | 3.692E-07 | 0.000E+00 | 0.000E+00 | 0.000E+00 | 0.000E+00 | 0.000E+00 | 0.000E+00 | 0.000E+00 | 0.000E+00 |
| Unigene273259 | optrA | 4.502E-08 | 5.435E-07 | 0.000E+00 | 6.365E-07 | 0.000E+00 | 0.000E+00 | 0.000E+00 | 0.000E+00 | 0.000E+00 | 0.000E+00 | 0.000E+00 | 1.064E-07 |
| Unigene273285 | mgrA | 0.000E+00 | 0.000E+00 | 0.000E+00 | 9.232E-07 | 0.000E+00 | 0.000E+00 | 0.000E+00 | 0.000E+00 | 0.000E+00 | 0.000E+00 | 0.000E+00 | 0.000E+00 |
| Unigene273309 | lin | 0.000E+00 | 0.000E+00 | 0.000E+00 | 5.289E-07 | 3.544E-06 | 0.000E+00 | 0.000E+00 | 6.558E-06 | 3.643E-06 | 2.864E-06 | 0.000E+00 | 0.000E+00 |
| Unigene273391 | efpA | 0.000E+00 | 0.000E+00 | 1.852E-07 | 7.149E-07 | 0.000E+00 | 0.000E+00 | 0.000E+00 | 0.000E+00 | 0.000E+00 | 0.000E+00 | 1.080E-07 | 0.000E+00 |
| Unigene273410 | lmrC | 0.000E+00 | 0.000E+00 | 0.000E+00 | 3.477E-07 | 0.000E+00 | 0.000E+00 | 0.000E+00 | 0.000E+00 | 0.000E+00 | 0.000E+00 | 0.000E+00 | 0.000E+00 |
| Unigene273423 | msbA | 0.000E+00 | 0.000E+00 | 3.778E-08 | 1.711E-06 | 0.000E+00 | 0.000E+00 | 0.000E+00 | 0.000E+00 | 0.000E+00 | 0.000E+00 | 0.000E+00 | 0.000E+00 |
| Unigene273431 | lmrD | 0.000E+00 | 0.000E+00 | 0.000E+00 | 1.797E-06 | 0.000E+00 | 0.000E+00 | 0.000E+00 | 0.000E+00 | 0.000E+00 | 0.000E+00 | 0.000E+00 | 0.000E+00 |
| Unigene273466 | tet(45) | 0.000E+00 | 0.000E+00 | 0.000E+00 | 1.834E-06 | 0.000E+00 | 0.000E+00 | 0.000E+00 | 0.000E+00 | 0.000E+00 | 0.000E+00 | 0.000E+00 | 0.000E+00 |
| Unigene273528 | efpA | 2.178E-08 | 1.673E-07 | 4.275E-07 | 8.138E-07 | 0.000E+00 | 0.000E+00 | 2.411E-08 | 0.000E+00 | 7.142E-08 | 0.000E+00 | 4.983E-08 | 1.545E-07 |
| Unigene273609 | Acinetobac | 0.000E+00 | 0.000E+00 | 4.945E-08 | 1.807E-06 | 0.000E+00 | 0.000E+00 | 0.000E+00 | 0.000E+00 | 0.000E+00 | 0.000E+00 | 0.000E+00 | 0.000E+00 |
| Unigene273614 | emrY | 0.000E+00 | 0.000E+00 | 2.087E-08 | 1.933E-06 | 0.000E+00 | 0.000E+00 | 0.000E+00 | 0.000E+00 | 0.000E+00 | 0.000E+00 | 0.000E+00 | 0.000E+00 |
| Unigene273621 | BJP-1 | 0.000E+00 | 0.000E+00 | 0.000E+00 | 2.334E-06 | 0.000E+00 | 0.000E+00 | 0.000E+00 | 0.000E+00 | 0.000E+00 | 0.000E+00 | 0.000E+00 | 0.000E+00 |
| Unigene273628 | vanRN | 0.000E+00 | 0.000E+00 | 0.000E+00 | 1.388E-06 | 0.000E+00 | 0.000E+00 | 0.000E+00 | 0.000E+00 | 0.000E+00 | 0.000E+00 | 0.000E+00 | 0.000E+00 |
| Unigene273629 | bcrA | 0.000E+00 | 0.000E+00 | 0.000E+00 | 1.172E-06 | 0.000E+00 | 0.000E+00 | 0.000E+00 | 0.000E+00 | 0.000E+00 | 0.000E+00 | 0.000E+00 | 0.000E+00 |
| Unigene273631 | vanSG | 0.000E+00 | 0.000E+00 | 0.000E+00 | 1.863E-06 | 0.000E+00 | 0.000E+00 | 0.000E+00 | 0.000E+00 | 0.000E+00 | 0.000E+00 | 0.000E+00 | 0.000E+00 |
| Unigene273666 | Corynebac | 0.000E+00 | 0.000E+00 | 0.000E+00 | 1.879E-06 | 0.000E+00 | 0.000E+00 | 0.000E+00 | 0.000E+00 | 0.000E+00 | 0.000E+00 | 0.000E+00 | 0.000E+00 |
| Unigene273669 | Staphyloco | 0.000E+00 | 0.000E+00 | 2.389E-08 | 1.561E-06 | 0.000E+00 | 0.000E+00 | 0.000E+00 | 0.000E+00 | 0.000E+00 | 0.000E+00 | 0.000E+00 | 0.000E+00 |
| Unigene273696 | bcrA | 0.000E+00 | 0.000E+00 | 0.000E+00 | 1.546E-06 | 0.000E+00 | 0.000E+00 | 0.000E+00 | 0.000E+00 | 0.000E+00 | 0.000E+00 | 0.000E+00 | 0.000E+00 |
| Unigene273701 | macB | 0.000E+00 | 0.000E+00 | 4.980E-08 | 1.333E-06 | 0.000E+00 | 0.000E+00 | 0.000E+00 | 0.000E+00 | 0.000E+00 | 0.000E+00 | 0.000E+00 | 0.000E+00 |
| Unigene273709 | msbA | 0.000E+00 | 0.000E+00 | 0.000E+00 | 2.011E-06 | 0.000E+00 | 0.000E+00 | 0.000E+00 | 0.000E+00 | 0.000E+00 | 0.000E+00 | 0.000E+00 | 0.000E+00 |
| Unigene273710 | novA | 0.000E+00 | 0.000E+00 | 1.477E-07 | 2.431E-06 | 0.000E+00 | 0.000E+00 | 0.000E+00 | 0.000E+00 | 0.000E+00 | 0.000E+00 | 0.000E+00 | 0.000E+00 |
| Unigene273719 | tetA(58) | 0.000E+00 | 0.000E+00 | 0.000E+00 | 1.677E-06 | 0.000E+00 | 0.000E+00 | 0.000E+00 | 0.000E+00 | 0.000E+00 | 0.000E+00 | 0.000E+00 | 0.000E+00 |
| Unigene273721 | evgA | 0.000E+00 | 0.000E+00 | 0.000E+00 | 1.932E-06 | 0.000E+00 | 0.000E+00 | 0.000E+00 | 0.000E+00 | 0.000E+00 | 0.000E+00 | 0.000E+00 | 0.000E+00 |
| Unigene273727 | bcrA | 0.000E+00 | 0.000E+00 | 0.000E+00 | 2.264E-06 | 0.000E+00 | 0.000E+00 | 0.000E+00 | 0.000E+00 | 0.000E+00 | 0.000E+00 | 0.000E+00 | 0.000E+00 |
| Unigene273750 | optrA | 0.000E+00 | 0.000E+00 | 1.461E-07 | 1.138E-06 | 0.000E+00 | 0.000E+00 | 0.000E+00 | 0.000E+00 | 0.000E+00 | 0.000E+00 | 0.000E+00 | 0.000E+00 |
| Unigene273782 | tetA(58) | 0.000E+00 | 0.000E+00 | 0.000E+00 | 6.575E-07 | 0.000E+00 | 3.704E-08 | 0.000E+00 | 4.000E-07 | 0.000E+00 | 0.000E+00 | 0.000E+00 | 0.000E+00 |
| Unigene273908 | basS | 3.146E-07 | 0.000E+00 | 6.174E-08 | 3.177E-07 | 0.000E+00 | 0.000E+00 | 0.000E+00 | 0.000E+00 | 0.000E+00 | 0.000E+00 | 0.000E+00 | 0.000E+00 |
| Unigene273914 | msbA | 0.000E+00 | 0.000E+00 | 0.000E+00 | 4.591E-07 | 0.000E+00 | 0.000E+00 | 0.000E+00 | 0.000E+00 | 0.000E+00 | 0.000E+00 | 0.000E+00 | 0.000E+00 |
| Unigene274034 | macB | 0.000E+00 | 0.000E+00 | 0.000E+00 | 1.161E-06 | 0.000E+00 | 0.000E+00 | 0.000E+00 | 0.000E+00 | 0.000E+00 | 0.000E+00 | 0.000E+00 | 0.000E+00 |
| Unigene274056 | macB | 0.000E+00 | 0.000E+00 | 0.000E+00 | 1.031E-06 | 0.000E+00 | 0.000E+00 | 0.000E+00 | 0.000E+00 | 0.000E+00 | 0.000E+00 | 0.000E+00 | 0.000E+00 |
| Unigene274187 | bcr-1 | 0.000E+00 | 1.431E-07 | 0.000E+00 | 4.610E-07 | 0.000E+00 | 0.000E+00 | 0.000E+00 | 0.000E+00 | 0.000E+00 | 0.000E+00 | 0.000E+00 | 0.000E+00 |
| Unigene274262 | otr(A) | 0.000E+00 | 0.000E+00 | 0.000E+00 | 3.582E-07 | 0.000E+00 | 0.000E+00 | 0.000E+00 | 0.000E+00 | 0.000E+00 | 0.000E+00 | 0.000E+00 | 2.096E-07 |
| Unigene274307 | optrA | 6.463E-08 | 1.773E-07 | 3.171E-07 | 8.321E-07 | 5.385E-08 | 6.986E-08 | 5.364E-08 | 0.000E+00 | 3.532E-08 | 0.000E+00 | 0.000E+00 | 9.549E-08 |
| Unigene274372 | macB | 0.000E+00 | 0.000E+00 | 0.000E+00 | 3.622E-07 | 0.000E+00 | 0.000E+00 | 0.000E+00 | 0.000E+00 | 0.000E+00 | 0.000E+00 | 0.000E+00 | 0.000E+00 |
| Unigene274375 | macB | 0.000E+00 | 0.000E+00 | 0.000E+00 | 3.343E-07 | 0.000E+00 | 0.000E+00 | 0.000E+00 | 0.000E+00 | 6.514E-07 | 0.000E+00 | 0.000E+00 | 1.644E-06 |

| | | | | | | | | | | | | | |
|---|---|---|---|---|---|---|---|---|---|---|---|---|---|
| Unigene274407 | bcrA | 0.000E+00 | 2.837E-07 | 3.171E-07 | 3.916E-07 | 0.000E+00 | 3.493E-08 | 0.000E+00 | 2.263E-07 | 0.000E+00 | 0.000E+00 | 0.000E+00 | 0.000E+00 |
| Unigene274578 | efpA | 0.000E+00 | 0.000E+00 | 5.028E-07 | 0.000E+00 | 0.000E+00 | 0.000E+00 | 0.000E+00 | 0.000E+00 | 0.000E+00 | 0.000E+00 | 0.000E+00 | 0.000E+00 |
| Unigene274617 | evgS | 1.234E-07 | 1.625E-07 | 2.664E-07 | 6.480E-07 | 5.483E-08 | 0.000E+00 | 0.000E+00 | 2.881E-08 | 0.000E+00 | 0.000E+00 | 5.646E-08 | 0.000E+00 |
| Unigene274684 | oleB | 0.000E+00 | 0.000E+00 | 5.317E-07 | 1.133E-06 | 0.000E+00 | 0.000E+00 | 0.000E+00 | 0.000E+00 | 0.000E+00 | 0.000E+00 | 0.000E+00 | 0.000E+00 |
| Unigene274685 | optrA | 0.000E+00 | 1.783E-07 | 5.179E-07 | 1.722E-06 | 0.000E+00 | 0.000E+00 | 0.000E+00 | 0.000E+00 | 0.000E+00 | 0.000E+00 | 0.000E+00 | 0.000E+00 |
| Unigene274724 | Staphylocc | 2.294E-08 | 7.554E-08 | 8.780E-07 | 7.518E-05 | 0.000E+00 | 0.000E+00 | 0.000E+00 | 1.339E-07 | 0.000E+00 | 0.000E+00 | 0.000E+00 | 0.000E+00 |
| Unigene274877 | arlS | 2.972E-08 | 1.957E-07 | 0.000E+00 | 1.051E-06 | 0.000E+00 | 0.000E+00 | 0.000E+00 | 0.000E+00 | 0.000E+00 | 1.093E-06 | 0.000E+00 | 5.622E-07 |
| Unigene274879 | arlR | 0.000E+00 | 1.993E-07 | 0.000E+00 | 1.192E-06 | 0.000E+00 | 0.000E+00 | 0.000E+00 | 0.000E+00 | 0.000E+00 | 1.062E-06 | 0.000E+00 | 2.683E-07 |
| Unigene274892 | lsaA | 0.000E+00 | 0.000E+00 | 0.000E+00 | 6.830E-07 | 0.000E+00 | 0.000E+00 | 0.000E+00 | 0.000E+00 | 0.000E+00 | 0.000E+00 | 0.000E+00 | 0.000E+00 |
| Unigene275021 | bcrA | 0.000E+00 | 0.000E+00 | 0.000E+00 | 8.427E-07 | 0.000E+00 | 0.000E+00 | 0.000E+00 | 0.000E+00 | 7.680E-07 | 0.000E+00 | 0.000E+00 | 0.000E+00 |
| Unigene275226 | tetA(58) | 3.710E-07 | 0.000E+00 | 0.000E+00 | 5.995E-07 | 0.000E+00 | 0.000E+00 | 8.213E-08 | 0.000E+00 | 0.000E+00 | 3.307E-07 | 0.000E+00 | 0.000E+00 |
| Unigene275236 | Staphylocc | 0.000E+00 | 0.000E+00 | 0.000E+00 | 4.272E-07 | 0.000E+00 | 0.000E+00 | 0.000E+00 | 0.000E+00 | 0.000E+00 | 0.000E+00 | 0.000E+00 | 0.000E+00 |
| Unigene275353 | macB | 4.154E-08 | 7.599E-08 | 0.000E+00 | 8.530E-07 | 0.000E+00 | 0.000E+00 | 0.000E+00 | 0.000E+00 | 0.000E+00 | 8.947E-07 | 0.000E+00 | 4.256E-07 |
| Unigene275371 | vanSF | 7.135E-07 | 2.757E-06 | 5.181E-06 | 4.611E-06 | 0.000E+00 | 0.000E+00 | 0.000E+00 | 0.000E+00 | 0.000E+00 | 0.000E+00 | 0.000E+00 | 0.000E+00 |
| Unigene275379 | Streptomy | 0.000E+00 | 2.266E-07 | 1.519E-07 | 6.254E-07 | 0.000E+00 | 0.000E+00 | 5.712E-08 | 0.000E+00 | 0.000E+00 | 0.000E+00 | 0.000E+00 | 0.000E+00 |
| Unigene275529 | vanHD | 0.000E+00 | 0.000E+00 | 0.000E+00 | 1.788E-06 | 0.000E+00 | 0.000E+00 | 0.000E+00 | 0.000E+00 | 0.000E+00 | 0.000E+00 | 0.000E+00 | 0.000E+00 |
| Unigene275539 | macB | 0.000E+00 | 0.000E+00 | 4.124E-08 | 2.122E-06 | 0.000E+00 | 0.000E+00 | 0.000E+00 | 0.000E+00 | 0.000E+00 | 0.000E+00 | 0.000E+00 | 0.000E+00 |
| Unigene275541 | MdtK | 0.000E+00 | 0.000E+00 | 4.611E-08 | 2.444E-06 | 0.000E+00 | 0.000E+00 | 0.000E+00 | 0.000E+00 | 0.000E+00 | 0.000E+00 | 0.000E+00 | 0.000E+00 |
| Unigene275546 | facT | 0.000E+00 | 0.000E+00 | 4.436E-08 | 1.575E-06 | 0.000E+00 | 0.000E+00 | 0.000E+00 | 0.000E+00 | 0.000E+00 | 0.000E+00 | 0.000E+00 | 0.000E+00 |
| Unigene275557 | mdtB | 0.000E+00 | 0.000E+00 | 5.201E-08 | 1.895E-06 | 0.000E+00 | 0.000E+00 | 0.000E+00 | 0.000E+00 | 0.000E+00 | 0.000E+00 | 0.000E+00 | 0.000E+00 |
| Unigene275606 | bacA | 0.000E+00 | 0.000E+00 | 0.000E+00 | 2.025E-06 | 0.000E+00 | 0.000E+00 | 0.000E+00 | 0.000E+00 | 0.000E+00 | 0.000E+00 | 0.000E+00 | 0.000E+00 |
| Unigene275817 | evgA | 0.000E+00 | 0.000E+00 | 0.000E+00 | 2.694E-07 | 0.000E+00 | 0.000E+00 | 0.000E+00 | 0.000E+00 | 0.000E+00 | 0.000E+00 | 0.000E+00 | 0.000E+00 |
| Unigene276014 | blt | 0.000E+00 | 0.000E+00 | 0.000E+00 | 4.346E-07 | 0.000E+00 | 0.000E+00 | 0.000E+00 | 0.000E+00 | 0.000E+00 | 0.000E+00 | 0.000E+00 | 0.000E+00 |
| Unigene276015 | Staphylocc | 0.000E+00 | 4.390E-08 | 0.000E+00 | 4.039E-07 | 0.000E+00 | 0.000E+00 | 0.000E+00 | 0.000E+00 | 0.000E+00 | 0.000E+00 | 0.000E+00 | 0.000E+00 |
| Unigene276077 | arlR | 0.000E+00 | 3.307E-07 | 1.689E-07 | 6.954E-07 | 0.000E+00 | 0.000E+00 | 0.000E+00 | 0.000E+00 | 0.000E+00 | 0.000E+00 | 0.000E+00 | 0.000E+00 |
| Unigene276090 | otr(B) | 7.197E-08 | 0.000E+00 | 1.412E-07 | 4.361E-07 | 0.000E+00 | 0.000E+00 | 0.000E+00 | 0.000E+00 | 0.000E+00 | 0.000E+00 | 8.233E-08 | 8.507E-08 |
| Unigene276221 | oleB | 0.000E+00 | 0.000E+00 | 0.000E+00 | 4.120E-07 | 0.000E+00 | 0.000E+00 | 0.000E+00 | 0.000E+00 | 0.000E+00 | 0.000E+00 | 0.000E+00 | 0.000E+00 |
| Unigene276252 | arlS | 2.263E-07 | 0.000E+00 | 0.000E+00 | 9.141E-07 | 0.000E+00 | 0.000E+00 | 0.000E+00 | 0.000E+00 | 0.000E+00 | 0.000E+00 | 0.000E+00 | 2.432E-07 |
| Unigene276302 | rphA | 1.253E-07 | 2.612E-07 | 2.704E-07 | 5.692E-07 | 0.000E+00 | 0.000E+00 | 0.000E+00 | 0.000E+00 | 5.476E-08 | 0.000E+00 | 1.433E-08 | 1.333E-07 |
| Unigene276321 | AAC(6')-Ib | 0.000E+00 | 0.000E+00 | 0.000E+00 | 4.829E-07 | 0.000E+00 | 0.000E+00 | 0.000E+00 | 0.000E+00 | 0.000E+00 | 0.000E+00 | 0.000E+00 | 0.000E+00 |
| Unigene276456 | PmrF | 0.000E+00 | 0.000E+00 | 3.922E-07 | 8.134E-06 | 0.000E+00 | 0.000E+00 | 0.000E+00 | 0.000E+00 | 0.000E+00 | 0.000E+00 | 0.000E+00 | 0.000E+00 |
| Unigene276465 | Staphylocc | 0.000E+00 | 0.000E+00 | 0.000E+00 | 5.645E-07 | 0.000E+00 | 0.000E+00 | 0.000E+00 | 0.000E+00 | 0.000E+00 | 0.000E+00 | 0.000E+00 | 0.000E+00 |
| Unigene276544 | arlR | 0.000E+00 | 0.000E+00 | 3.771E-07 | 0.000E+00 | 0.000E+00 | 0.000E+00 | 0.000E+00 | 0.000E+00 | 0.000E+00 | 0.000E+00 | 0.000E+00 | 0.000E+00 |
| Unigene276545 | baeS | 1.001E-07 | 8.239E-08 | 1.719E-07 | 3.032E-07 | 0.000E+00 | 2.705E-08 | 0.000E+00 | 0.000E+00 | 0.000E+00 | 0.000E+00 | 2.862E-08 | 0.000E+00 |
| Unigene276639 | tetA(58) | 1.022E-07 | 0.000E+00 | 4.009E-07 | 7.221E-07 | 0.000E+00 | 7.362E-08 | 0.000E+00 | 0.000E+00 | 0.000E+00 | 0.000E+00 | 0.000E+00 | 0.000E+00 |
| Unigene276801 | vanZA | 0.000E+00 | 0.000E+00 | 0.000E+00 | 8.793E-07 | 0.000E+00 | 0.000E+00 | 0.000E+00 | 0.000E+00 | 0.000E+00 | 0.000E+00 | 0.000E+00 | 0.000E+00 |
| Unigene276832 | efrA | 0.000E+00 | 0.000E+00 | 0.000E+00 | 6.258E-07 | 0.000E+00 | 0.000E+00 | 0.000E+00 | 0.000E+00 | 0.000E+00 | 0.000E+00 | 0.000E+00 | 0.000E+00 |
| Unigene276934 | otr(B) | 0.000E+00 | 0.000E+00 | 0.000E+00 | 4.199E-07 | 0.000E+00 | 0.000E+00 | 0.000E+00 | 0.000E+00 | 0.000E+00 | 0.000E+00 | 0.000E+00 | 0.000E+00 |
| Unigene276947 | gadX | 0.000E+00 | 0.000E+00 | 3.128E-07 | 1.127E-06 | 0.000E+00 | 0.000E+00 | 0.000E+00 | 0.000E+00 | 0.000E+00 | 0.000E+00 | 0.000E+00 | 9.421E-08 |
| Unigene276994 | mdtM | 0.000E+00 | 0.000E+00 | 0.000E+00 | 4.567E-07 | 0.000E+00 | 0.000E+00 | 0.000E+00 | 0.000E+00 | 0.000E+00 | 0.000E+00 | 0.000E+00 | 0.000E+00 |
| Unigene277014 | msbA | 0.000E+00 | 0.000E+00 | 0.000E+00 | 0.000E+00 | 0.000E+00 | 0.000E+00 | 4.652E-06 | 0.000E+00 | 0.000E+00 | 0.000E+00 | 0.000E+00 | 0.000E+00 |
| Unigene277027 | bcrA | 0.000E+00 | 4.199E-07 | 3.285E-07 | 4.829E-07 | 0.000E+00 | 0.000E+00 | 0.000E+00 | 0.000E+00 | 0.000E+00 | 0.000E+00 | 0.000E+00 | 0.000E+00 |
| Unigene277100 | TaeA | 0.000E+00 | 0.000E+00 | 0.000E+00 | 7.244E-07 | 0.000E+00 | 0.000E+00 | 0.000E+00 | 0.000E+00 | 0.000E+00 | 0.000E+00 | 0.000E+00 | 0.000E+00 |
| Unigene277198 | lsaC | 0.000E+00 | 0.000E+00 | 5.180E-07 | 1.269E-06 | 0.000E+00 | 0.000E+00 | 0.000E+00 | 0.000E+00 | 0.000E+00 | 0.000E+00 | 0.000E+00 | 0.000E+00 |
| Unigene277200 | ugd | 0.000E+00 | 0.000E+00 | 4.277E-07 | 1.403E-06 | 0.000E+00 | 0.000E+00 | 0.000E+00 | 0.000E+00 | 0.000E+00 | 0.000E+00 | 0.000E+00 | 0.000E+00 |
| Unigene277233 | patA | 0.000E+00 | 0.000E+00 | 0.000E+00 | 0.000E+00 | 0.000E+00 | 0.000E+00 | 0.000E+00 | 4.484E-06 | 0.000E+00 | 0.000E+00 | 0.000E+00 | 0.000E+00 |
| Unigene277245 | tetB(P) | 0.000E+00 | 0.000E+00 | 0.000E+00 | 5.268E-07 | 0.000E+00 | 0.000E+00 | 0.000E+00 | 0.000E+00 | 0.000E+00 | 0.000E+00 | 0.000E+00 | 0.000E+00 |
| Unigene277326 | baeS | 1.300E-07 | 0.000E+00 | 0.000E+00 | 1.182E-06 | 0.000E+00 | 3.514E-07 | 0.000E+00 | 0.000E+00 | 3.908E-07 | 0.000E+00 | 0.000E+00 | 0.000E+00 |
| Unigene277337 | tlrC | 0.000E+00 | 0.000E+00 | 0.000E+00 | 4.959E-07 | 0.000E+00 | 0.000E+00 | 0.000E+00 | 0.000E+00 | 0.000E+00 | 0.000E+00 | 0.000E+00 | 0.000E+00 |
| Unigene277361 | vanRA | 0.000E+00 | 0.000E+00 | 0.000E+00 | 3.881E-07 | 0.000E+00 | 0.000E+00 | 0.000E+00 | 0.000E+00 | 5.134E-07 | 0.000E+00 | 0.000E+00 | 0.000E+00 |
| Unigene277459 | optrA | 4.658E-06 | 2.025E-05 | 3.047E-06 | 8.486E-07 | 1.177E-06 | 3.357E-07 | 0.000E+00 | 2.559E-07 | 1.298E-06 | 0.000E+00 | 5.015E-07 | 1.727E-07 |
| Unigene277492 | optrA | 0.000E+00 | 1.074E-07 | 1.440E-07 | 2.469E-07 | 0.000E+00 | 0.000E+00 | 0.000E+00 | 0.000E+00 | 0.000E+00 | 0.000E+00 | 0.000E+00 | 0.000E+00 |
| Unigene277676 | iri | 0.000E+00 | 2.869E-07 | 1.710E-07 | 3.519E-07 | 0.000E+00 | 0.000E+00 | 0.000E+00 | 0.000E+00 | 0.000E+00 | 0.000E+00 | 0.000E+00 | 0.000E+00 |
| Unigene277728 | efrB | 0.000E+00 | 0.000E+00 | 0.000E+00 | 2.433E-07 | 0.000E+00 | 0.000E+00 | 0.000E+00 | 0.000E+00 | 0.000E+00 | 0.000E+00 | 0.000E+00 | 0.000E+00 |
| Unigene277796 | evgS | 0.000E+00 | 0.000E+00 | 0.000E+00 | 0.000E+00 | 0.000E+00 | 4.898E-07 | 0.000E+00 | 0.000E+00 | 0.000E+00 | 0.000E+00 | 0.000E+00 | 0.000E+00 |
| Unigene277817 | tetB(60) | 0.000E+00 | 3.192E-08 | 0.000E+00 | 3.818E-07 | 0.000E+00 | 3.144E-08 | 1.287E-07 | 0.000E+00 | 0.000E+00 | 0.000E+00 | 0.000E+00 | 0.000E+00 |
| Unigene277932 | Acinetobac | 0.000E+00 | 0.000E+00 | 0.000E+00 | 2.198E-06 | 0.000E+00 | 0.000E+00 | 0.000E+00 | 0.000E+00 | 0.000E+00 | 0.000E+00 | 0.000E+00 | 0.000E+00 |
| Unigene277934 | macB | 0.000E+00 | 0.000E+00 | 4.551E-08 | 1.780E-06 | 0.000E+00 | 0.000E+00 | 0.000E+00 | 0.000E+00 | 0.000E+00 | 0.000E+00 | 0.000E+00 | 0.000E+00 |

| | | | | | | | | | | | | | |
|---|---|---|---|---|---|---|---|---|---|---|---|---|---|
| Unigene277935 | vanSB | 0.000E+00 | 0.000E+00 | 0.000E+00 | 1.567E-06 | 0.000E+00 | 0.000E+00 | 0.000E+00 | 0.000E+00 | 0.000E+00 | 0.000E+00 | 0.000E+00 | 0.000E+00 |
| Unigene277936 | kdpE | 0.000E+00 | 0.000E+00 | 9.024E-08 | 1.207E-06 | 0.000E+00 | 0.000E+00 | 0.000E+00 | 0.000E+00 | 0.000E+00 | 0.000E+00 | 0.000E+00 | 0.000E+00 |
| Unigene278003 | vanHA | 0.000E+00 | 0.000E+00 | 9.992E-08 | 1.851E-06 | 0.000E+00 | 0.000E+00 | 0.000E+00 | 0.000E+00 | 0.000E+00 | 0.000E+00 | 0.000E+00 | 0.000E+00 |
| Unigene278013 | mtrA | 0.000E+00 | 0.000E+00 | 0.000E+00 | 2.420E-06 | 0.000E+00 | 0.000E+00 | 0.000E+00 | 0.000E+00 | 0.000E+00 | 0.000E+00 | 0.000E+00 | 0.000E+00 |
| Unigene278014 | vanSA | 0.000E+00 | 0.000E+00 | 0.000E+00 | 2.007E-06 | 0.000E+00 | 0.000E+00 | 0.000E+00 | 0.000E+00 | 0.000E+00 | 0.000E+00 | 0.000E+00 | 0.000E+00 |
| Unigene278015 | macB | 0.000E+00 | 0.000E+00 | 0.000E+00 | 1.525E-06 | 0.000E+00 | 0.000E+00 | 0.000E+00 | 0.000E+00 | 0.000E+00 | 0.000E+00 | 0.000E+00 | 0.000E+00 |
| Unigene278020 | Staphylocc | 0.000E+00 | 0.000E+00 | 4.957E-08 | 1.683E-06 | 0.000E+00 | 0.000E+00 | 0.000E+00 | 0.000E+00 | 0.000E+00 | 0.000E+00 | 0.000E+00 | 0.000E+00 |
| Unigene278022 | otr(B) | 0.000E+00 | 0.000E+00 | 2.007E-08 | 1.281E-06 | 0.000E+00 | 0.000E+00 | 0.000E+00 | 0.000E+00 | 0.000E+00 | 0.000E+00 | 0.000E+00 | 0.000E+00 |
| Unigene278023 | Staphylocc | 0.000E+00 | 0.000E+00 | 2.213E-08 | 1.481E-06 | 0.000E+00 | 0.000E+00 | 0.000E+00 | 0.000E+00 | 0.000E+00 | 0.000E+00 | 0.000E+00 | 0.000E+00 |
| Unigene278028 | ramA | 0.000E+00 | 0.000E+00 | 0.000E+00 | 1.836E-06 | 0.000E+00 | 0.000E+00 | 0.000E+00 | 0.000E+00 | 0.000E+00 | 0.000E+00 | 0.000E+00 | 0.000E+00 |
| Unigene278034 | bcrA | 0.000E+00 | 0.000E+00 | 0.000E+00 | 1.751E-06 | 0.000E+00 | 0.000E+00 | 0.000E+00 | 0.000E+00 | 0.000E+00 | 0.000E+00 | 0.000E+00 | 0.000E+00 |
| Unigene278037 | vanRA | 0.000E+00 | 0.000E+00 | 0.000E+00 | 1.780E-06 | 0.000E+00 | 0.000E+00 | 0.000E+00 | 0.000E+00 | 0.000E+00 | 0.000E+00 | 0.000E+00 | 0.000E+00 |
| Unigene278038 | vanSA | 0.000E+00 | 0.000E+00 | 0.000E+00 | 1.477E-06 | 0.000E+00 | 0.000E+00 | 0.000E+00 | 0.000E+00 | 0.000E+00 | 0.000E+00 | 0.000E+00 | 0.000E+00 |
| Unigene278039 | patA | 0.000E+00 | 0.000E+00 | 0.000E+00 | 1.982E-06 | 0.000E+00 | 0.000E+00 | 0.000E+00 | 0.000E+00 | 0.000E+00 | 0.000E+00 | 0.000E+00 | 0.000E+00 |
| Unigene278052 | vanRG | 0.000E+00 | 0.000E+00 | 8.948E-08 | 1.473E-06 | 0.000E+00 | 0.000E+00 | 0.000E+00 | 0.000E+00 | 0.000E+00 | 0.000E+00 | 0.000E+00 | 0.000E+00 |
| Unigene278053 | vanSE | 0.000E+00 | 0.000E+00 | 8.702E-08 | 2.090E-06 | 0.000E+00 | 0.000E+00 | 0.000E+00 | 0.000E+00 | 0.000E+00 | 0.000E+00 | 0.000E+00 | 0.000E+00 |
| Unigene278054 | bcrA | 0.000E+00 | 0.000E+00 | 0.000E+00 | 2.116E-06 | 0.000E+00 | 0.000E+00 | 0.000E+00 | 0.000E+00 | 0.000E+00 | 0.000E+00 | 0.000E+00 | 0.000E+00 |
| Unigene278070 | novA | 0.000E+00 | 0.000E+00 | 5.452E-08 | 1.739E-06 | 0.000E+00 | 0.000E+00 | 0.000E+00 | 0.000E+00 | 0.000E+00 | 0.000E+00 | 0.000E+00 | 0.000E+00 |
| Unigene278071 | msbA | 0.000E+00 | 0.000E+00 | 1.814E-08 | 1.886E-06 | 0.000E+00 | 0.000E+00 | 0.000E+00 | 0.000E+00 | 0.000E+00 | 0.000E+00 | 0.000E+00 | 0.000E+00 |
| Unigene278084 | vanHF | 0.000E+00 | 0.000E+00 | 1.006E-07 | 1.552E-06 | 0.000E+00 | 0.000E+00 | 0.000E+00 | 0.000E+00 | 0.000E+00 | 0.000E+00 | 0.000E+00 | 0.000E+00 |
| Unigene278096 | macB | 0.000E+00 | 0.000E+00 | 4.173E-08 | 1.374E-06 | 0.000E+00 | 0.000E+00 | 0.000E+00 | 0.000E+00 | 0.000E+00 | 0.000E+00 | 0.000E+00 | 0.000E+00 |
| Unigene278121 | Streptomy | 0.000E+00 | 0.000E+00 | 4.873E-08 | 1.672E-06 | 0.000E+00 | 0.000E+00 | 0.000E+00 | 0.000E+00 | 0.000E+00 | 0.000E+00 | 0.000E+00 | 0.000E+00 |
| Unigene278141 | adeR | 0.000E+00 | 0.000E+00 | 0.000E+00 | 1.449E-06 | 0.000E+00 | 0.000E+00 | 0.000E+00 | 0.000E+00 | 0.000E+00 | 0.000E+00 | 0.000E+00 | 0.000E+00 |
| Unigene278164 | arnA | 0.000E+00 | 0.000E+00 | 0.000E+00 | 1.412E-06 | 0.000E+00 | 0.000E+00 | 0.000E+00 | 0.000E+00 | 0.000E+00 | 0.000E+00 | 0.000E+00 | 0.000E+00 |
| Unigene278178 | vanRF | 0.000E+00 | 0.000E+00 | 0.000E+00 | 1.748E-06 | 0.000E+00 | 0.000E+00 | 0.000E+00 | 0.000E+00 | 0.000E+00 | 0.000E+00 | 0.000E+00 | 0.000E+00 |
| Unigene278179 | baeS | 0.000E+00 | 2.460E-08 | 0.000E+00 | 1.924E-06 | 0.000E+00 | 0.000E+00 | 0.000E+00 | 0.000E+00 | 0.000E+00 | 0.000E+00 | 0.000E+00 | 0.000E+00 |
| Unigene278181 | bcrA | 0.000E+00 | 0.000E+00 | 0.000E+00 | 1.934E-06 | 0.000E+00 | 0.000E+00 | 0.000E+00 | 0.000E+00 | 0.000E+00 | 0.000E+00 | 0.000E+00 | 0.000E+00 |
| Unigene278231 | mecC | 0.000E+00 | 0.000E+00 | 1.446E-08 | 1.905E-06 | 0.000E+00 | 0.000E+00 | 0.000E+00 | 0.000E+00 | 0.000E+00 | 0.000E+00 | 0.000E+00 | 0.000E+00 |
| Unigene278286 | vanRE | 0.000E+00 | 0.000E+00 | 7.707E-08 | 2.022E-06 | 0.000E+00 | 0.000E+00 | 0.000E+00 | 0.000E+00 | 0.000E+00 | 0.000E+00 | 0.000E+00 | 0.000E+00 |
| Unigene278332 | tetQ | 0.000E+00 | 0.000E+00 | 5.210E-08 | 2.127E-06 | 0.000E+00 | 0.000E+00 | 0.000E+00 | 0.000E+00 | 0.000E+00 | 0.000E+00 | 0.000E+00 | 0.000E+00 |
| Unigene278355 | mtrA | 0.000E+00 | 0.000E+00 | 0.000E+00 | 1.873E-06 | 0.000E+00 | 0.000E+00 | 0.000E+00 | 0.000E+00 | 0.000E+00 | 0.000E+00 | 0.000E+00 | 0.000E+00 |
| Unigene278356 | adeS | 0.000E+00 | 0.000E+00 | 0.000E+00 | 2.087E-06 | 0.000E+00 | 0.000E+00 | 0.000E+00 | 0.000E+00 | 0.000E+00 | 0.000E+00 | 0.000E+00 | 0.000E+00 |
| Unigene278387 | PmrF | 0.000E+00 | 0.000E+00 | 1.020E-07 | 5.249E-07 | 0.000E+00 | 0.000E+00 | 0.000E+00 | 0.000E+00 | 0.000E+00 | 0.000E+00 | 0.000E+00 | 0.000E+00 |
| Unigene278431 | mgrA | 0.000E+00 | 2.427E-07 | 0.000E+00 | 9.675E-07 | 0.000E+00 | 0.000E+00 | 0.000E+00 | 0.000E+00 | 0.000E+00 | 0.000E+00 | 0.000E+00 | 3.485E-07 |
| Unigene278523 | tetB(P) | 0.000E+00 | 0.000E+00 | 2.929E-08 | 1.763E-06 | 0.000E+00 | 0.000E+00 | 0.000E+00 | 0.000E+00 | 0.000E+00 | 0.000E+00 | 0.000E+00 | 0.000E+00 |
| Unigene278576 | TaeA | 0.000E+00 | 0.000E+00 | 0.000E+00 | 3.343E-07 | 0.000E+00 | 0.000E+00 | 0.000E+00 | 0.000E+00 | 0.000E+00 | 0.000E+00 | 0.000E+00 | 0.000E+00 |
| Unigene278692 | mdtM | 0.000E+00 | 0.000E+00 | 0.000E+00 | 1.016E-06 | 0.000E+00 | 0.000E+00 | 0.000E+00 | 0.000E+00 | 0.000E+00 | 0.000E+00 | 0.000E+00 | 0.000E+00 |
| Unigene278749 | mgrA | 0.000E+00 | 0.000E+00 | 0.000E+00 | 0.000E+00 | 6.320E-06 | 0.000E+00 | 0.000E+00 | 0.000E+00 | 0.000E+00 | 0.000E+00 | 0.000E+00 | 0.000E+00 |
| Unigene278799 | vanRG | 0.000E+00 | 0.000E+00 | 0.000E+00 | 3.598E-07 | 0.000E+00 | 0.000E+00 | 0.000E+00 | 0.000E+00 | 0.000E+00 | 0.000E+00 | 0.000E+00 | 0.000E+00 |
| Unigene278942 | PmrF | 0.000E+00 | 0.000E+00 | 0.000E+00 | 5.300E-07 | 0.000E+00 | 0.000E+00 | 0.000E+00 | 0.000E+00 | 0.000E+00 | 0.000E+00 | 0.000E+00 | 0.000E+00 |
| Unigene279055 | bcrA | 0.000E+00 | 0.000E+00 | 0.000E+00 | 5.397E-07 | 0.000E+00 | 0.000E+00 | 0.000E+00 | 8.318E-08 | 5.062E-07 | 0.000E+00 | 0.000E+00 | 0.000E+00 |
| Unigene279109 | vanSM | 0.000E+00 | 0.000E+00 | 1.748E-06 | 6.764E-06 | 0.000E+00 | 0.000E+00 | 0.000E+00 | 0.000E+00 | 0.000E+00 | 0.000E+00 | 0.000E+00 | 0.000E+00 |
| Unigene279346 | tetA(46) | 0.000E+00 | 8.047E-08 | 0.000E+00 | 5.923E-07 | 0.000E+00 | 0.000E+00 | 0.000E+00 | 0.000E+00 | 0.000E+00 | 0.000E+00 | 0.000E+00 | 2.167E-08 |
| Unigene279407 | carA | 0.000E+00 | 0.000E+00 | 0.000E+00 | 6.065E-07 | 0.000E+00 | 0.000E+00 | 0.000E+00 | 0.000E+00 | 0.000E+00 | 0.000E+00 | 0.000E+00 | 0.000E+00 |
| Unigene279579 | MuxB | 0.000E+00 | 0.000E+00 | 0.000E+00 | 3.704E-07 | 0.000E+00 | 0.000E+00 | 0.000E+00 | 0.000E+00 | 0.000E+00 | 0.000E+00 | 0.000E+00 | 0.000E+00 |
| Unigene279695 | tetB(P) | 0.000E+00 | 0.000E+00 | 0.000E+00 | 7.494E-07 | 0.000E+00 | 0.000E+00 | 0.000E+00 | 0.000E+00 | 0.000E+00 | 0.000E+00 | 0.000E+00 | 0.000E+00 |
| Unigene279705 | YojI | 9.925E-05 | 1.688E-04 | 1.658E-04 | 7.454E-06 | 8.926E-06 | 1.535E-07 | 3.551E-06 | 2.220E-06 | 5.806E-05 | 0.000E+00 | 3.280E-06 | 0.000E+00 |
| Unigene279846 | efrA | 0.000E+00 | 0.000E+00 | 2.563E-07 | 3.692E-07 | 0.000E+00 | 0.000E+00 | 0.000E+00 | 0.000E+00 | 0.000E+00 | 0.000E+00 | 5.975E-08 | 0.000E+00 |
| Unigene279955 | Staphylocc | 0.000E+00 | 0.000E+00 | 0.000E+00 | 7.700E-07 | 0.000E+00 | 0.000E+00 | 0.000E+00 | 0.000E+00 | 0.000E+00 | 0.000E+00 | 0.000E+00 | 0.000E+00 |
| Unigene280109 | QepA4 | 0.000E+00 | 0.000E+00 | 0.000E+00 | 0.000E+00 | 1.162E-06 | 0.000E+00 | 0.000E+00 | 0.000E+00 | 0.000E+00 | 0.000E+00 | 0.000E+00 | 0.000E+00 |
| Unigene280195 | bacA | 0.000E+00 | 0.000E+00 | 0.000E+00 | 0.000E+00 | 7.999E-07 | 0.000E+00 | 0.000E+00 | 0.000E+00 | 0.000E+00 | 0.000E+00 | 0.000E+00 | 8.983E-07 |
| Unigene280249 | tetB(46) | 0.000E+00 | 0.000E+00 | 2.995E-07 | 0.000E+00 | 3.434E-06 | 7.177E-06 | 1.731E-06 | 6.636E-06 | 4.254E-06 | 0.000E+00 | 0.000E+00 | 0.000E+00 |
| Unigene280374 | tcr3 | 0.000E+00 | 0.000E+00 | 1.129E-07 | 0.000E+00 | 6.392E-07 | 1.866E-07 | 1.910E-07 | 0.000E+00 | 2.516E-07 | 1.282E-07 | 3.291E-07 | 8.842E-07 |
| Unigene280461 | patA | 0.000E+00 | 0.000E+00 | 0.000E+00 | 0.000E+00 | 9.424E-07 | 0.000E+00 | 9.389E-07 | 0.000E+00 | 0.000E+00 | 0.000E+00 | 0.000E+00 | 0.000E+00 |
| Unigene280477 | tetA(46) | 0.000E+00 | 0.000E+00 | 1.643E-07 | 0.000E+00 | 1.163E-06 | 0.000E+00 | 0.000E+00 | 0.000E+00 | 1.098E-06 | 0.000E+00 | 0.000E+00 | 0.000E+00 |
| Unigene280567 | rpoB2 | 4.407E-05 | 2.460E-05 | 1.867E-05 | 2.160E-05 | 2.437E-06 | 1.645E-06 | 3.289E-06 | 7.732E-07 | 6.868E-06 | 6.482E-07 | 1.618E-06 | 1.238E-06 |
| Unigene280572 | efrA | 0.000E+00 | 9.549E-08 | 7.399E-07 | 4.100E-07 | 8.700E-07 | 6.271E-07 | 1.509E-06 | 1.083E-06 | 1.585E-07 | 0.000E+00 | 1.194E-06 | 3.497E-06 |
| Unigene280695 | mdtC | 0.000E+00 | 0.000E+00 | 0.000E+00 | 0.000E+00 | 6.994E-07 | 9.590E-07 | 2.882E-06 | 1.737E-06 | 0.000E+00 | 8.607E-07 | 6.220E-07 | 2.537E-06 |

| | | | | | | | | | | | | | |
|---|---|---|---|---|---|---|---|---|---|---|---|---|---|
| Unigene280753 | otr(A) | 0.000E+00 | 4.631E-08 | 8.281E-08 | 0.000E+00 | 4.219E-07 | 0.000E+00 | 0.000E+00 | 9.851E-08 | 1.384E-07 | 0.000E+00 | 2.896E-07 | 6.484E-07 |
| Unigene280801 | macB | 0.000E+00 | 3.546E-07 | 0.000E+00 | 0.000E+00 | 6.462E-07 | 0.000E+00 | 2.181E-06 | 2.112E-06 | 0.000E+00 | 0.000E+00 | 1.700E-06 | 2.826E-06 |
| Unigene280828 | lsaC | 0.000E+00 | 0.000E+00 | 0.000E+00 | 3.646E-07 | 4.172E-06 | 5.582E-06 | 1.638E-06 | 0.000E+00 | 3.828E-06 | 2.494E-06 | 0.000E+00 | 0.000E+00 |
| Unigene280856 | adeL | 0.000E+00 | 0.000E+00 | 0.000E+00 | 0.000E+00 | 1.737E-06 | 0.000E+00 | 0.000E+00 | 0.000E+00 | 0.000E+00 | 0.000E+00 | 0.000E+00 | 0.000E+00 |
| Unigene280857 | catA4 | 0.000E+00 | 0.000E+00 | 0.000E+00 | 0.000E+00 | 8.615E-07 | 0.000E+00 | 2.146E-07 | 0.000E+00 | 0.000E+00 | 0.000E+00 | 0.000E+00 | 0.000E+00 |
| Unigene280878 | PmrF | 8.346E-07 | 0.000E+00 | 4.095E-07 | 3.612E-07 | 5.331E-06 | 1.134E-05 | 1.715E-06 | 8.628E-06 | 4.268E-06 | 4.748E-06 | 0.000E+00 | 0.000E+00 |
| Unigene280881 | tetA(58) | 7.625E-07 | 0.000E+00 | 3.325E-07 | 3.850E-07 | 4.612E-06 | 1.040E-05 | 1.969E-06 | 9.494E-06 | 5.001E-06 | 3.870E-06 | 0.000E+00 | 7.010E-07 |
| Unigene280885 | tet32 | 0.000E+00 | 0.000E+00 | 2.657E-07 | 4.193E-07 | 4.152E-06 | 1.042E-05 | 1.698E-06 | 8.535E-06 | 4.795E-06 | 4.304E-06 | 6.195E-08 | 8.963E-07 |
| Unigene280963 | tetA(58) | 0.000E+00 | 0.000E+00 | 0.000E+00 | 0.000E+00 | 6.478E-07 | 0.000E+00 | 0.000E+00 | 0.000E+00 | 0.000E+00 | 0.000E+00 | 0.000E+00 | 0.000E+00 |
| Unigene281010 | tetB(60) | 0.000E+00 | 0.000E+00 | 0.000E+00 | 0.000E+00 | 3.620E-06 | 0.000E+00 | 0.000E+00 | 0.000E+00 | 0.000E+00 | 0.000E+00 | 3.923E-07 | 0.000E+00 |
| Unigene281016 | evgS | 0.000E+00 | 0.000E+00 | 0.000E+00 | 0.000E+00 | 3.919E-06 | 0.000E+00 | 0.000E+00 | 0.000E+00 | 9.640E-08 | 0.000E+00 | 0.000E+00 | 0.000E+00 |
| Unigene281021 | evgS | 0.000E+00 | 0.000E+00 | 0.000E+00 | 0.000E+00 | 3.218E-06 | 0.000E+00 | 0.000E+00 | 0.000E+00 | 0.000E+00 | 0.000E+00 | 0.000E+00 | 3.718E-06 |
| Unigene281136 | mef(B) | 2.152E-07 | 0.000E+00 | 0.000E+00 | 3.556E-07 | 9.781E-07 | 0.000E+00 | 4.980E-06 | 3.631E-06 | 8.981E-07 | 1.504E-06 | 1.925E-06 | 3.677E-06 |
| Unigene281257 | evgS | 3.742E-07 | 1.058E-06 | 6.675E-07 | 4.122E-07 | 7.772E-06 | 4.780E-07 | 1.104E-06 | 3.838E-07 | 0.000E+00 | 0.000E+00 | 0.000E+00 | 0.000E+00 |
| Unigene281278 | msbA | 0.000E+00 | 0.000E+00 | 0.000E+00 | 0.000E+00 | 8.501E-07 | 0.000E+00 | 0.000E+00 | 0.000E+00 | 0.000E+00 | 0.000E+00 | 0.000E+00 | 0.000E+00 |
| Unigene281330 | macB | 1.836E-07 | 0.000E+00 | 0.000E+00 | 7.417E-08 | 8.160E-07 | 0.000E+00 | 0.000E+00 | 9.002E-07 | 0.000E+00 | 0.000E+00 | 0.000E+00 | 1.953E-06 |
| Unigene281396 | novA | 0.000E+00 | 1.526E-07 | 0.000E+00 | 0.000E+00 | 8.031E-07 | 0.000E+00 | 0.000E+00 | 0.000E+00 | 6.382E-07 | 0.000E+00 | 0.000E+00 | 0.000E+00 |
| Unigene281407 | emrY | 0.000E+00 | 3.041E-07 | 4.758E-07 | 1.865E-07 | 1.590E-06 | 5.991E-07 | 1.533E-06 | 1.186E-06 | 0.000E+00 | 9.260E-07 | 9.773E-07 | 3.603E-06 |
| Unigene281432 | rpoB2 | 0.000E+00 | 0.000E+00 | 0.000E+00 | 0.000E+00 | 0.000E+00 | 0.000E+00 | 0.000E+00 | 0.000E+00 | 0.000E+00 | 0.000E+00 | 0.000E+00 | 5.781E-07 |
| Unigene281453 | Enterobact | 2.877E-07 | 1.768E-06 | 0.000E+00 | 0.000E+00 | 5.018E-06 | 0.000E+00 | 1.401E-06 | 0.000E+00 | 1.226E-06 | 0.000E+00 | 0.000E+00 | 0.000E+00 |
| Unigene281465 | TriC | 0.000E+00 | 0.000E+00 | 0.000E+00 | 0.000E+00 | 4.098E-07 | 0.000E+00 | 2.722E-07 | 3.588E-07 | 0.000E+00 | 0.000E+00 | 0.000E+00 | 2.180E-07 |
| Unigene281482 | MCR-4.2 | 0.000E+00 | 0.000E+00 | 0.000E+00 | 0.000E+00 | 3.825E-07 | 0.000E+00 | 0.000E+00 | 0.000E+00 | 0.000E+00 | 0.000E+00 | 0.000E+00 | 0.000E+00 |
| Unigene281498 | TriC | 0.000E+00 | 0.000E+00 | 0.000E+00 | 0.000E+00 | 3.919E-07 | 0.000E+00 | 0.000E+00 | 0.000E+00 | 0.000E+00 | 0.000E+00 | 0.000E+00 | 1.390E-07 |
| Unigene281558 | AcrS | 0.000E+00 | 0.000E+00 | 0.000E+00 | 0.000E+00 | 9.408E-07 | 0.000E+00 | 0.000E+00 | 0.000E+00 | 9.800E-07 | 0.000E+00 | 0.000E+00 | 5.888E-08 |
| Unigene281559 | AcrE | 0.000E+00 | 0.000E+00 | 0.000E+00 | 1.430E-07 | 1.447E-06 | 0.000E+00 | 0.000E+00 | 0.000E+00 | 1.207E-06 | 0.000E+00 | 0.000E+00 | 0.000E+00 |
| Unigene281560 | AcrF | 0.000E+00 | 1.934E-07 | 0.000E+00 | 1.466E-07 | 2.061E-06 | 0.000E+00 | 1.950E-07 | 0.000E+00 | 6.571E-07 | 0.000E+00 | 0.000E+00 | 0.000E+00 |
| Unigene281593 | tetW | 6.430E-05 | 5.400E-05 | 1.054E-04 | 1.503E-04 | 1.128E-04 | 7.876E-05 | 1.039E-04 | 1.033E-04 | 8.677E-05 | 1.285E-04 | 1.258E-04 | 1.317E-04 |
| Unigene281623 | patA | 6.326E-05 | 5.170E-05 | 1.007E-04 | 1.493E-04 | 1.108E-04 | 7.820E-05 | 1.005E-04 | 9.991E-05 | 8.958E-05 | 1.284E-04 | 1.297E-04 | 1.337E-04 |
| Unigene281624 | patB | 7.100E-05 | 5.404E-05 | 1.087E-04 | 1.629E-04 | 1.268E-04 | 8.709E-05 | 1.116E-04 | 1.124E-04 | 9.091E-05 | 1.304E-04 | 1.399E-04 | 1.416E-04 |
| Unigene281654 | arlR | 0.000E+00 | 0.000E+00 | 0.000E+00 | 0.000E+00 | 8.611E-07 | 0.000E+00 | 0.000E+00 | 0.000E+00 | 0.000E+00 | 0.000E+00 | 0.000E+00 | 0.000E+00 |
| Unigene281655 | arlS | 0.000E+00 | 0.000E+00 | 0.000E+00 | 0.000E+00 | 7.847E-07 | 0.000E+00 | 0.000E+00 | 0.000E+00 | 0.000E+00 | 0.000E+00 | 0.000E+00 | 0.000E+00 |
| Unigene281667 | basS | 0.000E+00 | 0.000E+00 | 0.000E+00 | 0.000E+00 | 7.809E-07 | 0.000E+00 | 0.000E+00 | 0.000E+00 | 0.000E+00 | 0.000E+00 | 0.000E+00 | 0.000E+00 |
| Unigene281686 | arlR | 0.000E+00 | 0.000E+00 | 0.000E+00 | 0.000E+00 | 1.764E-06 | 0.000E+00 | 0.000E+00 | 0.000E+00 | 0.000E+00 | 0.000E+00 | 0.000E+00 | 0.000E+00 |
| Unigene281756 | rosB | 6.352E-07 | 1.838E-06 | 1.001E-06 | 4.276E-07 | 8.340E-06 | 3.537E-07 | 2.066E-06 | 0.000E+00 | 0.000E+00 | 0.000E+00 | 8.587E-07 | 0.000E+00 |
| Unigene281777 | tetB(46) | 3.804E-07 | 1.034E-06 | 8.710E-07 | 2.927E-07 | 7.527E-06 | 3.133E-07 | 9.824E-07 | 5.286E-07 | 0.000E+00 | 0.000E+00 | 5.387E-07 | 0.000E+00 |
| Unigene281778 | tetA(60) | 3.823E-07 | 9.991E-07 | 3.394E-07 | 2.390E-07 | 7.787E-06 | 3.543E-07 | 6.650E-07 | 2.338E-07 | 0.000E+00 | 0.000E+00 | 1.666E-07 | 0.000E+00 |
| Unigene281820 | eptA | 3.416E-08 | 0.000E+00 | 1.006E-07 | 0.000E+00 | 3.415E-07 | 0.000E+00 | 4.915E-07 | 4.386E-07 | 0.000E+00 | 0.000E+00 | 2.344E-07 | 7.268E-07 |
| Unigene281851 | novA | 0.000E+00 | 0.000E+00 | 0.000E+00 | 0.000E+00 | 8.094E-07 | 1.232E-06 | 3.515E-06 | 2.399E-06 | 0.000E+00 | 0.000E+00 | 1.816E-06 | 3.621E-06 |
| Unigene281867 | tetA(46) | 3.406E-07 | 2.639E-07 | 4.129E-07 | 4.654E-07 | 9.572E-07 | 6.498E-07 | 1.353E-06 | 1.193E-06 | 6.570E-08 | 7.812E-07 | 1.009E-06 | 3.268E-06 |
| Unigene281905 | patB | 0.000E+00 | 0.000E+00 | 0.000E+00 | 0.000E+00 | 8.429E-07 | 0.000E+00 | 0.000E+00 | 5.233E-07 | 0.000E+00 | 1.153E-07 | 0.000E+00 | 5.299E-07 |
| Unigene282014 | Streptomy | 4.775E-07 | 1.741E-06 | 1.238E-06 | 4.133E-07 | 6.062E-06 | 2.765E-07 | 1.736E-06 | 0.000E+00 | 0.000E+00 | 0.000E+00 | 4.877E-07 | 6.047E-08 |
| Unigene282022 | vanSF | 0.000E+00 | 0.000E+00 | 0.000E+00 | 0.000E+00 | 1.722E-06 | 0.000E+00 | 0.000E+00 | 0.000E+00 | 0.000E+00 | 0.000E+00 | 0.000E+00 | 0.000E+00 |
| Unigene282032 | CMY-105 | 7.570E-07 | 2.967E-08 | 3.449E-07 | 4.095E-07 | 4.085E-06 | 7.657E-06 | 1.197E-06 | 6.154E-06 | 4.403E-06 | 2.831E-06 | 0.000E+00 | 9.267E-07 |
| Unigene282130 | LlmA 23S r | 0.000E+00 | 0.000E+00 | 0.000E+00 | 0.000E+00 | 3.717E-06 | 0.000E+00 | 0.000E+00 | 0.000E+00 | 0.000E+00 | 0.000E+00 | 0.000E+00 | 0.000E+00 |
| Unigene282155 | tetT | 0.000E+00 | 0.000E+00 | 0.000E+00 | 0.000E+00 | 3.758E-06 | 0.000E+00 | 0.000E+00 | 0.000E+00 | 6.682E-08 | 3.405E-07 | 7.460E-07 | 0.000E+00 |
| Unigene282176 | bacA | 0.000E+00 | 0.000E+00 | 0.000E+00 | 0.000E+00 | 3.555E-06 | 0.000E+00 | 0.000E+00 | 0.000E+00 | 0.000E+00 | 0.000E+00 | 5.491E-07 | 0.000E+00 |
| Unigene282225 | novA | 0.000E+00 | 0.000E+00 | 0.000E+00 | 0.000E+00 | 3.353E-06 | 0.000E+00 | 0.000E+00 | 0.000E+00 | 0.000E+00 | 0.000E+00 | 6.703E-07 | 1.406E-06 |
| Unigene282247 | tetT | 0.000E+00 | 0.000E+00 | 0.000E+00 | 0.000E+00 | 4.129E-06 | 0.000E+00 | 8.226E-07 | 0.000E+00 | 0.000E+00 | 0.000E+00 | 7.895E-07 | 0.000E+00 |
| Unigene282277 | tetA(60) | 0.000E+00 | 0.000E+00 | 0.000E+00 | 0.000E+00 | 2.735E-06 | 0.000E+00 | 0.000E+00 | 0.000E+00 | 0.000E+00 | 0.000E+00 | 0.000E+00 | 0.000E+00 |
| Unigene282278 | oleC | 0.000E+00 | 0.000E+00 | 0.000E+00 | 0.000E+00 | 3.121E-06 | 0.000E+00 | 0.000E+00 | 0.000E+00 | 0.000E+00 | 0.000E+00 | 6.920E-07 | 0.000E+00 |
| Unigene282306 | vanHF | 0.000E+00 | 0.000E+00 | 0.000E+00 | 0.000E+00 | 3.798E-06 | 0.000E+00 | 0.000E+00 | 0.000E+00 | 0.000E+00 | 0.000E+00 | 4.218E-07 | 0.000E+00 |
| Unigene282307 | tetA(60) | 0.000E+00 | 0.000E+00 | 0.000E+00 | 0.000E+00 | 4.271E-06 | 0.000E+00 | 0.000E+00 | 0.000E+00 | 1.300E-07 | 0.000E+00 | 2.493E-07 | 0.000E+00 |
| Unigene282315 | msbA | 0.000E+00 | 0.000E+00 | 0.000E+00 | 0.000E+00 | 3.286E-06 | 0.000E+00 | 0.000E+00 | 0.000E+00 | 0.000E+00 | 0.000E+00 | 4.981E-07 | 1.609E-06 |
| Unigene282323 | macB | 0.000E+00 | 0.000E+00 | 0.000E+00 | 0.000E+00 | 3.508E-06 | 0.000E+00 | 0.000E+00 | 0.000E+00 | 0.000E+00 | 3.702E-07 | 0.000E+00 | 0.000E+00 |
| Unigene282325 | catB10 | 0.000E+00 | 0.000E+00 | 0.000E+00 | 0.000E+00 | 2.650E-06 | 0.000E+00 | 0.000E+00 | 1.361E-06 | 0.000E+00 | 0.000E+00 | 0.000E+00 | 0.000E+00 |
| Unigene282326 | macB | 0.000E+00 | 0.000E+00 | 0.000E+00 | 0.000E+00 | 4.013E-06 | 0.000E+00 | 0.000E+00 | 0.000E+00 | 0.000E+00 | 0.000E+00 | 0.000E+00 | 0.000E+00 |
| Unigene282347 | msbA | 0.000E+00 | 8.232E-07 | 0.000E+00 | 4.952E-07 | 6.410E-07 | 1.466E-06 | 2.043E-06 | 3.940E-06 | 0.000E+00 | 1.060E-06 | 1.749E-06 | 3.001E-06 |
| Unigene282354 | carA | 7.750E-05 | 3.490E-05 | 0.000E+00 | 3.494E-08 | 1.757E-05 | 6.190E-05 | 0.000E+00 | 0.000E+00 | 0.000E+00 | 0.000E+00 | 0.000E+00 | 0.000E+00 |

| | | | | | | | | | | | | | |
|---|---|---|---|---|---|---|---|---|---|---|---|---|---|
| Unigene282387 | OXA-18 | 0.000E+00 | 0.000E+00 | 0.000E+00 | 0.000E+00 | 3.213E-07 | 0.000E+00 | 1.281E-07 | 4.052E-07 | 0.000E+00 | 0.000E+00 | 1.323E-07 | 4.787E-07 |
| Unigene282407 | arlR | 0.000E+00 | 0.000E+00 | 0.000E+00 | 0.000E+00 | 1.056E-06 | 0.000E+00 | 0.000E+00 | 0.000E+00 | 0.000E+00 | 0.000E+00 | 0.000E+00 | 0.000E+00 |
| Unigene282446 | tetA(60) | 0.000E+00 | 0.000E+00 | 1.105E-06 | 5.077E-07 | 1.586E-06 | 9.784E-07 | 2.693E-06 | 1.714E-06 | 2.638E-07 | 1.232E-06 | 1.334E-06 | 5.539E-06 |
| Unigene282577 | macB | 0.000E+00 | 0.000E+00 | 0.000E+00 | 0.000E+00 | 0.000E+00 | 0.000E+00 | 1.300E-06 | 1.335E-06 | 0.000E+00 | 0.000E+00 | 6.013E-07 | 0.000E+00 |
| Unigene282594 | carA | 4.197E-07 | 0.000E+00 | 8.798E-07 | 5.972E-07 | 1.505E-06 | 6.394E-07 | 1.351E-06 | 1.225E-06 | 0.000E+00 | 0.000E+00 | 9.602E-07 | 3.450E-06 |
| Unigene282595 | vgaD | 3.413E-07 | 3.154E-07 | 5.640E-07 | 0.000E+00 | 1.297E-06 | 4.855E-07 | 9.742E-07 | 8.597E-07 | 2.749E-07 | 7.404E-07 | 7.808E-07 | 2.909E-06 |
| Unigene282684 | macB | 0.000E+00 | 0.000E+00 | 0.000E+00 | 0.000E+00 | 3.764E-06 | 0.000E+00 | 0.000E+00 | 0.000E+00 | 0.000E+00 | 9.494E-08 | 1.219E-07 | 0.000E+00 |
| Unigene282710 | acrB | 0.000E+00 | 0.000E+00 | 0.000E+00 | 0.000E+00 | 0.000E+00 | 0.000E+00 | 0.000E+00 | 0.000E+00 | 0.000E+00 | 0.000E+00 | 0.000E+00 | 4.646E-07 |
| Unigene282757 | mefC | 0.000E+00 | 0.000E+00 | 0.000E+00 | 0.000E+00 | 4.997E-07 | 0.000E+00 | 0.000E+00 | 0.000E+00 | 0.000E+00 | 0.000E+00 | 0.000E+00 | 0.000E+00 |
| Unigene282769 | iri | 0.000E+00 | 0.000E+00 | 0.000E+00 | 0.000E+00 | 1.278E-07 | 0.000E+00 | 1.274E-07 | 5.373E-07 | 0.000E+00 | 0.000E+00 | 0.000E+00 | 3.401E-07 |
| Unigene282852 | macB | 0.000E+00 | 0.000E+00 | 0.000E+00 | 0.000E+00 | 8.329E-07 | 0.000E+00 | 7.321E-07 | 0.000E+00 | 1.928E-07 | 0.000E+00 | 0.000E+00 | 5.734E-07 |
| Unigene282886 | vanE | 7.110E-05 | 6.254E-05 | 1.209E-04 | 1.718E-04 | 1.303E-04 | 8.874E-05 | 1.164E-04 | 1.134E-04 | 9.967E-05 | 1.439E-04 | 1.419E-04 | 1.545E-04 |
| Unigene282887 | Staphylocc | 6.789E-05 | 5.807E-05 | 1.123E-04 | 1.583E-04 | 1.232E-04 | 8.585E-05 | 1.124E-04 | 1.084E-04 | 9.560E-05 | 1.366E-04 | 1.380E-04 | 1.392E-04 |
| Unigene282992 | efrA | 0.000E+00 | 0.000E+00 | 0.000E+00 | 0.000E+00 | 5.114E-07 | 0.000E+00 | 0.000E+00 | 0.000E+00 | 0.000E+00 | 0.000E+00 | 0.000E+00 | 0.000E+00 |
| Unigene283075 | vanSA | 7.093E-05 | 6.539E-05 | 1.160E-04 | 1.359E-04 | 1.319E-04 | 8.952E-05 | 1.188E-04 | 1.267E-04 | 1.005E-04 | 1.508E-04 | 1.520E-04 | 1.667E-04 |
| Unigene283104 | bcrA | 0.000E+00 | 3.038E-07 | 0.000E+00 | 2.096E-07 | 7.303E-07 | 2.244E-07 | 1.417E-06 | 8.481E-07 | 0.000E+00 | 1.079E-06 | 1.108E-06 | 8.997E-07 |
| Unigene283107 | novA | 0.000E+00 | 0.000E+00 | 0.000E+00 | 4.604E-08 | 5.572E-07 | 0.000E+00 | 6.560E-07 | 0.000E+00 | 0.000E+00 | 0.000E+00 | 0.000E+00 | 6.467E-07 |
| Unigene283159 | macB | 6.652E-07 | 9.018E-07 | 1.421E-06 | 0.000E+00 | 8.303E-06 | 6.345E-07 | 9.960E-07 | 3.197E-07 | 0.000E+00 | 0.000E+00 | 4.923E-07 | 0.000E+00 |
| Unigene283172 | bcr-1 | 0.000E+00 | 0.000E+00 | 0.000E+00 | 0.000E+00 | 5.300E-07 | 1.719E-07 | 1.173E-07 | 6.806E-07 | 0.000E+00 | 0.000E+00 | 1.819E-07 | 6.892E-07 |
| Unigene283224 | AAC(3)-VII | 5.817E-05 | 4.932E-05 | 9.286E-05 | 9.046E-05 | 1.349E-04 | 8.212E-05 | 9.540E-05 | 1.044E-04 | 9.109E-05 | 1.378E-04 | 1.239E-04 | 1.491E-04 |
| Unigene283227 | efpA | 5.589E-05 | 5.934E-05 | 1.001E-04 | 6.409E-05 | 1.312E-04 | 8.641E-05 | 1.016E-04 | 1.190E-04 | 9.819E-05 | 1.344E-04 | 1.334E-04 | 1.747E-04 |
| Unigene283228 | vgaD | 5.663E-05 | 4.864E-05 | 9.928E-05 | 7.577E-05 | 1.255E-04 | 8.074E-05 | 1.009E-04 | 1.066E-04 | 9.094E-05 | 1.235E-04 | 1.291E-04 | 1.487E-04 |
| Unigene283338 | novA | 1.675E-07 | 2.452E-07 | 0.000E+00 | 1.692E-07 | 7.652E-07 | 0.000E+00 | 1.607E-06 | 1.065E-06 | 0.000E+00 | 6.636E-07 | 0.000E+00 | 3.015E-06 |
| Unigene283342 | novA | 0.000E+00 | 0.000E+00 | 0.000E+00 | 0.000E+00 | 9.413E-07 | 0.000E+00 | 0.000E+00 | 7.912E-07 | 0.000E+00 | 0.000E+00 | 0.000E+00 | 0.000E+00 |
| Unigene283598 | Corynebac | 0.000E+00 | 0.000E+00 | 7.778E-08 | 0.000E+00 | 9.246E-07 | 1.071E-07 | 0.000E+00 | 6.939E-07 | 2.166E-07 | 0.000E+00 | 3.627E-07 | 6.090E-07 |
| Unigene283626 | vanG | 0.000E+00 | 7.381E-07 | 6.025E-07 | 0.000E+00 | 2.274E-06 | 0.000E+00 | 0.000E+00 | 0.000E+00 | 0.000E+00 | 0.000E+00 | 0.000E+00 | 0.000E+00 |
| Unigene283637 | Enterobact | 0.000E+00 | 0.000E+00 | 0.000E+00 | 0.000E+00 | 5.977E-07 | 0.000E+00 | 0.000E+00 | 0.000E+00 | 0.000E+00 | 0.000E+00 | 0.000E+00 | 0.000E+00 |
| Unigene283776 | TaeA | 0.000E+00 | 0.000E+00 | 0.000E+00 | 0.000E+00 | 4.706E-07 | 0.000E+00 | 0.000E+00 | 0.000E+00 | 0.000E+00 | 0.000E+00 | 0.000E+00 | 0.000E+00 |
| Unigene283878 | arnA | 4.232E-07 | 1.090E-06 | 7.827E-07 | 2.795E-07 | 9.060E-06 | 3.696E-07 | 1.009E-06 | 2.470E-07 | 1.957E-07 | 0.000E+00 | 2.048E-07 | 0.000E+00 |
| Unigene283925 | poxtA | 0.000E+00 | 0.000E+00 | 0.000E+00 | 0.000E+00 | 2.926E-07 | 0.000E+00 | 0.000E+00 | 0.000E+00 | 0.000E+00 | 0.000E+00 | 0.000E+00 | 0.000E+00 |
| Unigene283952 | sul4 | 0.000E+00 | 0.000E+00 | 0.000E+00 | 0.000E+00 | 4.226E-07 | 0.000E+00 | 0.000E+00 | 0.000E+00 | 0.000E+00 | 0.000E+00 | 0.000E+00 | 0.000E+00 |
| Unigene283986 | vanSN | 0.000E+00 | 0.000E+00 | 0.000E+00 | 0.000E+00 | 6.694E-07 | 0.000E+00 | 0.000E+00 | 0.000E+00 | 0.000E+00 | 0.000E+00 | 0.000E+00 | 0.000E+00 |
| Unigene284004 | bcrA | 0.000E+00 | 0.000E+00 | 4.163E-07 | 0.000E+00 | 7.284E-07 | 0.000E+00 | 1.494E-06 | 0.000E+00 | 0.000E+00 | 0.000E+00 | 0.000E+00 | 3.237E-06 |
| Unigene284066 | Acinetoba | 0.000E+00 | 0.000E+00 | 0.000E+00 | 0.000E+00 | 5.936E-06 | 0.000E+00 | 0.000E+00 | 0.000E+00 | 1.040E-06 | 0.000E+00 | 0.000E+00 | 0.000E+00 |
| Unigene284068 | adeL | 0.000E+00 | 0.000E+00 | 0.000E+00 | 0.000E+00 | 1.395E-06 | 0.000E+00 | 0.000E+00 | 0.000E+00 | 7.448E-07 | 0.000E+00 | 0.000E+00 | 0.000E+00 |
| Unigene284125 | vanHA | 5.092E-07 | 0.000E+00 | 4.996E-07 | 3.428E-07 | 3.771E-06 | 7.082E-06 | 0.000E+00 | 6.894E-06 | 3.821E-06 | 3.479E-06 | 0.000E+00 | 0.000E+00 |
| Unigene284152 | patB | 0.000E+00 | 0.000E+00 | 0.000E+00 | 0.000E+00 | 3.617E-06 | 0.000E+00 | 0.000E+00 | 0.000E+00 | 0.000E+00 | 0.000E+00 | 3.136E-07 | 0.000E+00 |
| Unigene284191 | efrA | 0.000E+00 | 0.000E+00 | 0.000E+00 | 0.000E+00 | 3.319E-06 | 0.000E+00 | 0.000E+00 | 0.000E+00 | 0.000E+00 | 0.000E+00 | 1.395E-07 | 0.000E+00 |
| Unigene284202 | macB | 0.000E+00 | 0.000E+00 | 0.000E+00 | 0.000E+00 | 3.117E-06 | 0.000E+00 | 0.000E+00 | 0.000E+00 | 0.000E+00 | 0.000E+00 | 7.473E-07 | 0.000E+00 |
| Unigene284333 | vanHO | 0.000E+00 | 0.000E+00 | 1.025E-07 | 0.000E+00 | 4.642E-07 | 0.000E+00 | 4.239E-07 | 0.000E+00 | 0.000E+00 | 0.000E+00 | 5.577E-07 | 6.586E-07 |
| Unigene284352 | smeS | 0.000E+00 | 0.000E+00 | 0.000E+00 | 0.000E+00 | 8.145E-07 | 0.000E+00 | 0.000E+00 | 0.000E+00 | 0.000E+00 | 0.000E+00 | 0.000E+00 | 0.000E+00 |
| Unigene284371 | mefC | 0.000E+00 | 0.000E+00 | 0.000E+00 | 1.473E-07 | 8.510E-07 | 0.000E+00 | 1.211E-07 | 5.535E-07 | 0.000E+00 | 1.219E-07 | 2.921E-07 | 1.725E-07 |
| Unigene284406 | tet(W/N/W | 0.000E+00 | 0.000E+00 | 1.701E-06 | 0.000E+00 | 8.022E-06 | 0.000E+00 | 0.000E+00 | 9.597E-07 | 1.016E-06 | 0.000E+00 | 0.000E+00 | 0.000E+00 |
| Unigene284407 | patA | 2.970E-07 | 2.301E-07 | 6.170E-07 | 0.000E+00 | 8.733E-07 | 1.511E-06 | 3.113E-06 | 2.793E-06 | 4.391E-07 | 1.109E-06 | 1.638E-06 | 2.953E-06 |
| Unigene284451 | lmrD | 3.538E-07 | 1.521E-06 | 9.546E-07 | 4.168E-07 | 7.205E-06 | 3.506E-07 | 0.000E+00 | 3.785E-07 | 0.000E+00 | 0.000E+00 | 5.733E-07 | 1.045E-07 |
| Unigene284460 | sul4 | 0.000E+00 | 0.000E+00 | 1.641E-07 | 0.000E+00 | 4.336E-07 | 0.000E+00 | 0.000E+00 | 0.000E+00 | 0.000E+00 | 0.000E+00 | 1.913E-07 | 0.000E+00 |
| Unigene284503 | efrA | 1.105E-07 | 0.000E+00 | 1.085E-07 | 1.116E-07 | 3.275E-07 | 0.000E+00 | 4.486E-07 | 3.871E-07 | 0.000E+00 | 1.642E-07 | 8.430E-08 | 6.969E-07 |
| Unigene284526 | tetT | 0.000E+00 | 0.000E+00 | 0.000E+00 | 8.522E-08 | 7.032E-07 | 4.562E-08 | 0.000E+00 | 0.000E+00 | 0.000E+00 | 2.350E-07 | 9.654E-08 | 4.988E-07 |
| Unigene284587 | Acinetoba | 0.000E+00 | 0.000E+00 | 0.000E+00 | 0.000E+00 | 6.595E-07 | 0.000E+00 | 2.300E-06 | 1.473E-06 | 0.000E+00 | 7.715E-07 | 9.903E-07 | 2.865E-06 |
| Unigene284626 | NmcR | 2.478E-07 | 1.010E-06 | 4.515E-07 | 2.502E-07 | 8.022E-06 | 2.678E-07 | 6.268E-07 | 0.000E+00 | 0.000E+00 | 0.000E+00 | 2.024E-07 | 0.000E+00 |
| Unigene284627 | adeL | 1.375E-07 | 6.037E-07 | 2.024E-07 | 0.000E+00 | 4.659E-06 | 0.000E+00 | 4.185E-07 | 2.408E-07 | 0.000E+00 | 0.000E+00 | 0.000E+00 | 0.000E+00 |
| Unigene284663 | tetA(46) | 0.000E+00 | 6.672E-08 | 0.000E+00 | 0.000E+00 | 7.767E-07 | 0.000E+00 | 9.756E-07 | 9.934E-07 | 1.661E-07 | 2.709E-07 | 1.043E-06 | 1.078E-06 |
| Unigene284823 | Staphylocc | 5.342E-05 | 4.867E-05 | 9.175E-05 | 5.861E-05 | 1.188E-04 | 8.111E-05 | 9.470E-05 | 1.059E-04 | 9.406E-05 | 1.255E-04 | 1.127E-04 | 1.322E-04 |
| Unigene284870 | YojI | 5.124E-07 | 1.354E-06 | 7.262E-07 | 0.000E+00 | 3.985E-06 | 0.000E+00 | 1.092E-06 | 0.000E+00 | 0.000E+00 | 0.000E+00 | 5.861E-07 | 8.973E-08 |
| Unigene284872 | Klebsiella p | 0.000E+00 | 0.000E+00 | 2.070E-07 | 0.000E+00 | 1.373E-06 | 0.000E+00 | 1.668E-07 | 0.000E+00 | 0.000E+00 | 0.000E+00 | 0.000E+00 | 0.000E+00 |
| Unigene284901 | Staphylocc | 0.000E+00 | 0.000E+00 | 0.000E+00 | 0.000E+00 | 6.934E-07 | 0.000E+00 | 0.000E+00 | 0.000E+00 | 0.000E+00 | 0.000E+00 | 0.000E+00 | 0.000E+00 |
| Unigene284909 | vanSN | 0.000E+00 | 0.000E+00 | 0.000E+00 | 0.000E+00 | 4.539E-07 | 0.000E+00 | 0.000E+00 | 0.000E+00 | 0.000E+00 | 0.000E+00 | 0.000E+00 | 0.000E+00 |
| Unigene285088 | RlmA(II) | 0.000E+00 | 0.000E+00 | 0.000E+00 | 0.000E+00 | 1.067E-06 | 0.000E+00 | 0.000E+00 | 0.000E+00 | 0.000E+00 | 0.000E+00 | 0.000E+00 | 0.000E+00 |

| | | | | | | | | | | | | | |
|---|---|---|---|---|---|---|---|---|---|---|---|---|---|
| Unigene285118 | oleB | 0.000E+00 | 0.000E+00 | 8.325E-07 | 0.000E+00 | 1.157E-06 | 0.000E+00 | 1.110E-06 | 1.080E-06 | 8.430E-08 | 0.000E+00 | 1.412E-06 | 3.419E-06 |
| Unigene285119 | macB | 3.402E-07 | 2.801E-07 | 7.512E-07 | 3.006E-07 | 1.370E-06 | 0.000E+00 | 1.836E-06 | 0.000E+00 | 0.000E+00 | 5.212E-07 | 1.459E-06 | 3.620E-06 |
| Unigene285211 | evgS | 0.000E+00 | 0.000E+00 | 0.000E+00 | 0.000E+00 | 4.045E-07 | 8.746E-08 | 2.686E-07 | 0.000E+00 | 0.000E+00 | 0.000E+00 | 0.000E+00 | 0.000E+00 |
| Unigene285215 | tetB(60) | 8.030E-08 | 4.407E-08 | 3.152E-07 | 1.622E-07 | 6.245E-07 | 1.736E-07 | 3.555E-07 | 9.373E-07 | 1.755E-07 | 0.000E+00 | 5.511E-07 | 9.017E-07 |
| Unigene285269 | marA | 0.000E+00 | 0.000E+00 | 4.316E-07 | 0.000E+00 | 2.443E-06 | 0.000E+00 | 4.119E-07 | 0.000E+00 | 0.000E+00 | 0.000E+00 | 0.000E+00 | 0.000E+00 |
| Unigene285299 | MexF | 0.000E+00 | 4.261E-07 | 1.633E-07 | 0.000E+00 | 3.697E-07 | 0.000E+00 | 0.000E+00 | 0.000E+00 | 0.000E+00 | 0.000E+00 | 0.000E+00 | 0.000E+00 |
| Unigene285324 | mepA | 6.936E-05 | 5.649E-05 | 1.107E-04 | 1.570E-04 | 1.214E-04 | 8.845E-05 | 1.156E-04 | 1.120E-04 | 9.400E-05 | 1.361E-04 | 1.406E-04 | 1.433E-04 |
| Unigene285325 | tetT | 7.280E-05 | 5.920E-05 | 1.130E-04 | 1.670E-04 | 1.244E-04 | 9.171E-05 | 1.130E-04 | 1.120E-04 | 9.491E-05 | 1.428E-04 | 1.442E-04 | 1.468E-04 |
| Unigene285326 | rpoB2 | 7.187E-05 | 5.892E-05 | 1.138E-04 | 1.626E-04 | 1.284E-04 | 9.104E-05 | 1.139E-04 | 1.165E-04 | 9.522E-05 | 1.402E-04 | 1.401E-04 | 1.469E-04 |
| Unigene285422 | lin | 7.941E-08 | 1.743E-07 | 2.338E-07 | 0.000E+00 | 1.500E-06 | 0.000E+00 | 1.758E-07 | 0.000E+00 | 6.510E-07 | 0.000E+00 | 9.084E-08 | 0.000E+00 |
| Unigene285546 | Acinetoba | 0.000E+00 | 0.000E+00 | 0.000E+00 | 0.000E+00 | 4.919E-07 | 0.000E+00 | 0.000E+00 | 0.000E+00 | 0.000E+00 | 0.000E+00 | 0.000E+00 | 0.000E+00 |
| Unigene285559 | MuxC | 1.439E-07 | 6.768E-08 | 4.336E-07 | 2.802E-07 | 9.819E-07 | 4.000E-07 | 9.441E-07 | 8.997E-07 | 0.000E+00 | 5.953E-07 | 8.229E-07 | 3.049E-06 |
| Unigene285664 | efrA | 0.000E+00 | 0.000E+00 | 0.000E+00 | 0.000E+00 | 7.431E-07 | 0.000E+00 | 0.000E+00 | 0.000E+00 | 0.000E+00 | 0.000E+00 | 0.000E+00 | 1.031E-06 |
| Unigene285696 | patA | 8.390E-08 | 0.000E+00 | 2.470E-07 | 1.483E-07 | 5.359E-07 | 2.041E-07 | 6.732E-07 | 8.324E-07 | 6.878E-08 | 2.103E-07 | 2.879E-07 | 9.173E-07 |
| Unigene285702 | dfrD | 9.093E-05 | 1.477E-04 | 1.386E-04 | 6.418E-06 | 7.829E-06 | 0.000E+00 | 2.855E-06 | 2.057E-06 | 5.323E-05 | 0.000E+00 | 0.000E+00 | 0.000E+00 |
| Unigene285801 | norB | 2.540E-07 | 1.064E-06 | 5.438E-07 | 1.399E-07 | 8.260E-06 | 7.488E-08 | 2.044E-07 | 2.695E-07 | 0.000E+00 | 1.543E-07 | 1.321E-07 | 0.000E+00 |
| Unigene285878 | Tet(X4) | 0.000E+00 | 0.000E+00 | 0.000E+00 | 0.000E+00 | 1.127E-06 | 0.000E+00 | 0.000E+00 | 0.000E+00 | 0.000E+00 | 0.000E+00 | 0.000E+00 | 0.000E+00 |
| Unigene285889 | evgS | 1.354E-07 | 0.000E+00 | 1.772E-07 | 1.823E-07 | 8.725E-07 | 4.099E-07 | 8.492E-07 | 9.272E-07 | 2.072E-07 | 3.419E-07 | 7.641E-07 | 8.856E-07 |
| Unigene285904 | golS | 0.000E+00 | 0.000E+00 | 0.000E+00 | 0.000E+00 | 1.073E-06 | 0.000E+00 | 0.000E+00 | 0.000E+00 | 0.000E+00 | 0.000E+00 | 0.000E+00 | 9.784E-07 |
| Unigene285950 | vanI | 0.000E+00 | 0.000E+00 | 0.000E+00 | 0.000E+00 | 2.957E-07 | 0.000E+00 | 0.000E+00 | 0.000E+00 | 0.000E+00 | 0.000E+00 | 0.000E+00 | 1.798E-07 |
| Unigene285982 | NmcR | 0.000E+00 | 0.000E+00 | 6.878E-07 | 0.000E+00 | 1.285E-06 | 0.000E+00 | 1.319E-06 | 8.182E-07 | 0.000E+00 | 9.761E-07 | 8.820E-07 | 2.776E-06 |
| Unigene285996 | emrB | 0.000E+00 | 1.139E-06 | 0.000E+00 | 0.000E+00 | 4.034E-06 | 0.000E+00 | 0.000E+00 | 0.000E+00 | 6.300E-07 | 2.354E-07 | 0.000E+00 | 0.000E+00 |
| Unigene286031 | macB | 1.307E-07 | 0.000E+00 | 0.000E+00 | 3.079E-07 | 1.065E-06 | 4.238E-07 | 1.254E-06 | 1.170E-06 | 3.809E-07 | 3.397E-07 | 9.966E-07 | 1.287E-06 |
| Unigene286060 | bcrA | 0.000E+00 | 0.000E+00 | 0.000E+00 | 0.000E+00 | 4.122E-07 | 0.000E+00 | 0.000E+00 | 0.000E+00 | 0.000E+00 | 0.000E+00 | 0.000E+00 | 5.012E-07 |
| Unigene286080 | Burkholde | 0.000E+00 | 0.000E+00 | 0.000E+00 | 0.000E+00 | 3.736E-07 | 0.000E+00 | 0.000E+00 | 0.000E+00 | 0.000E+00 | 0.000E+00 | 0.000E+00 | 0.000E+00 |
| Unigene286105 | tetA(46) | 0.000E+00 | 0.000E+00 | 0.000E+00 | 0.000E+00 | 6.404E-07 | 2.493E-07 | 0.000E+00 | 5.831E-07 | 0.000E+00 | 5.565E-07 | 6.594E-07 | 9.539E-07 |
| Unigene286266 | facT | 0.000E+00 | 0.000E+00 | 0.000E+00 | 0.000E+00 | 1.677E-06 | 0.000E+00 | 0.000E+00 | 0.000E+00 | 0.000E+00 | 0.000E+00 | 0.000E+00 | 0.000E+00 |
| Unigene286297 | TaeA | 2.399E-07 | 0.000E+00 | 4.203E-07 | 2.768E-07 | 9.898E-07 | 2.223E-07 | 1.176E-06 | 7.400E-07 | 0.000E+00 | 5.917E-07 | 5.488E-07 | 3.058E-06 |
| Unigene286378 | MexK | 4.483E-08 | 9.841E-08 | 0.000E+00 | 4.527E-08 | 3.985E-07 | 1.454E-07 | 0.000E+00 | 1.047E-07 | 4.900E-08 | 0.000E+00 | 0.000E+00 | 2.120E-07 |
| Unigene286380 | oqxA | 0.000E+00 | 3.916E-07 | 2.963E-07 | 0.000E+00 | 1.281E-06 | 0.000E+00 | 0.000E+00 | 0.000E+00 | 0.000E+00 | 0.000E+00 | 2.512E-07 | 9.734E-08 |
| Unigene286435 | patA | 0.000E+00 | 0.000E+00 | 0.000E+00 | 0.000E+00 | 5.235E-07 | 0.000E+00 | 0.000E+00 | 0.000E+00 | 9.014E-07 | 6.999E-07 | 1.348E-07 | 9.284E-08 |
| Unigene286442 | Corynebac | 0.000E+00 | 0.000E+00 | 0.000E+00 | 0.000E+00 | 4.091E-07 | 0.000E+00 | 0.000E+00 | 0.000E+00 | 0.000E+00 | 0.000E+00 | 4.680E-07 | 0.000E+00 |
| Unigene286579 | kdpE | 0.000E+00 | 0.000E+00 | 0.000E+00 | 0.000E+00 | 6.010E-07 | 0.000E+00 | 0.000E+00 | 0.000E+00 | 0.000E+00 | 0.000E+00 | 0.000E+00 | 0.000E+00 |
| Unigene286637 | carA | 0.000E+00 | 9.084E-08 | 2.707E-07 | 0.000E+00 | 7.356E-07 | 0.000E+00 | 6.412E-07 | 1.031E-06 | 0.000E+00 | 0.000E+00 | 2.209E-07 | 9.458E-07 |
| Unigene286748 | macB | 0.000E+00 | 0.000E+00 | 0.000E+00 | 0.000E+00 | 5.077E-07 | 0.000E+00 | 0.000E+00 | 0.000E+00 | 0.000E+00 | 0.000E+00 | 0.000E+00 | 0.000E+00 |
| Unigene286753 | sdiA | 4.392E-08 | 0.000E+00 | 0.000E+00 | 0.000E+00 | 7.807E-07 | 0.000E+00 | 1.458E-07 | 6.152E-07 | 0.000E+00 | 0.000E+00 | 3.517E-07 | 4.672E-07 |
| Unigene286788 | TaeA | 0.000E+00 | 0.000E+00 | 0.000E+00 | 0.000E+00 | 1.459E-06 | 0.000E+00 | 0.000E+00 | 0.000E+00 | 0.000E+00 | 0.000E+00 | 0.000E+00 | 0.000E+00 |
| Unigene286791 | tetA(58) | 0.000E+00 | 0.000E+00 | 0.000E+00 | 0.000E+00 | 0.000E+00 | 0.000E+00 | 0.000E+00 | 1.709E-07 | 0.000E+00 | 0.000E+00 | 0.000E+00 | 0.000E+00 |
| Unigene286825 | rosB | 0.000E+00 | 0.000E+00 | 0.000E+00 | 0.000E+00 | 8.184E-07 | 0.000E+00 | 0.000E+00 | 7.063E-07 | 0.000E+00 | 0.000E+00 | 0.000E+00 | 7.152E-07 |
| Unigene286852 | acrB | 0.000E+00 | 0.000E+00 | 0.000E+00 | 0.000E+00 | 6.778E-07 | 0.000E+00 | 0.000E+00 | 0.000E+00 | 0.000E+00 | 0.000E+00 | 0.000E+00 | 0.000E+00 |
| Unigene286854 | bcr-1 | 0.000E+00 | 0.000E+00 | 0.000E+00 | 0.000E+00 | 1.016E-06 | 0.000E+00 | 1.191E-07 | 0.000E+00 | 4.410E-07 | 0.000E+00 | 0.000E+00 | 0.000E+00 |
| Unigene286873 | basS | 0.000E+00 | 0.000E+00 | 0.000E+00 | 0.000E+00 | 3.664E-07 | 0.000E+00 | 0.000E+00 | 0.000E+00 | 0.000E+00 | 0.000E+00 | 0.000E+00 | 0.000E+00 |
| Unigene286984 | MexW | 0.000E+00 | 0.000E+00 | 1.916E-08 | 0.000E+00 | 4.556E-07 | 1.267E-07 | 0.000E+00 | 3.191E-07 | 0.000E+00 | 0.000E+00 | 2.457E-07 | 3.924E-07 |
| Unigene287006 | macA | 1.332E-07 | 1.169E-07 | 5.227E-08 | 1.883E-07 | 1.006E-06 | 8.638E-08 | 1.091E-06 | 4.663E-07 | 0.000E+00 | 0.000E+00 | 3.961E-07 | 7.556E-07 |
| Unigene287026 | vanRE | 4.410E-05 | 2.408E-05 | 2.053E-05 | 2.145E-05 | 2.617E-06 | 2.047E-06 | 4.242E-06 | 7.547E-07 | 7.874E-06 | 6.173E-07 | 2.483E-06 | 1.201E-06 |
| Unigene287075 | novA | 0.000E+00 | 6.046E-08 | 0.000E+00 | 0.000E+00 | 5.508E-07 | 0.000E+00 | 3.048E-07 | 3.001E-07 | 1.606E-07 | 0.000E+00 | 0.000E+00 | 3.907E-07 |
| Unigene287106 | tetA(58) | 8.214E-08 | 0.000E+00 | 0.000E+00 | 0.000E+00 | 7.300E-07 | 0.000E+00 | 0.000E+00 | 0.000E+00 | 0.000E+00 | 0.000E+00 | 0.000E+00 | 0.000E+00 |
| Unigene287133 | bcrA | 0.000E+00 | 0.000E+00 | 0.000E+00 | 0.000E+00 | 4.407E-07 | 0.000E+00 | 0.000E+00 | 0.000E+00 | 0.000E+00 | 0.000E+00 | 0.000E+00 | 0.000E+00 |
| Unigene287148 | msbA | 5.721E-07 | 1.783E-06 | 1.123E-06 | 3.541E-07 | 7.833E-06 | 4.789E-07 | 1.062E-06 | 4.955E-07 | 0.000E+00 | 2.262E-07 | 6.122E-07 | 1.963E-07 |
| Unigene287174 | efrA | 0.000E+00 | 0.000E+00 | 0.000E+00 | 0.000E+00 | 7.431E-07 | 0.000E+00 | 0.000E+00 | 0.000E+00 | 0.000E+00 | 0.000E+00 | 0.000E+00 | 0.000E+00 |
| Unigene287176 | otr(B) | 0.000E+00 | 0.000E+00 | 0.000E+00 | 0.000E+00 | 3.452E-07 | 0.000E+00 | 0.000E+00 | 9.068E-08 | 0.000E+00 | 0.000E+00 | 0.000E+00 | 2.755E-07 |
| Unigene287194 | lmrC | 0.000E+00 | 6.561E-07 | 3.910E-07 | 8.049E-08 | 1.815E-06 | 0.000E+00 | 1.544E-07 | 0.000E+00 | 0.000E+00 | 0.000E+00 | 0.000E+00 | 2.355E-08 |
| Unigene287284 | emrB | 0.000E+00 | 0.000E+00 | 0.000E+00 | 0.000E+00 | 3.516E-07 | 0.000E+00 | 0.000E+00 | 0.000E+00 | 0.000E+00 | 0.000E+00 | 0.000E+00 | 0.000E+00 |
| Unigene287381 | srmB | 0.000E+00 | 0.000E+00 | 0.000E+00 | 0.000E+00 | 9.022E-07 | 0.000E+00 | 0.000E+00 | 0.000E+00 | 0.000E+00 | 0.000E+00 | 0.000E+00 | 0.000E+00 |
| Unigene287400 | evgS | 0.000E+00 | 0.000E+00 | 0.000E+00 | 0.000E+00 | 0.000E+00 | 0.000E+00 | 0.000E+00 | 8.169E-07 | 3.825E-07 | 0.000E+00 | 5.003E-07 | 8.272E-07 |
| Unigene287446 | vanHA | 1.405E-05 | 0.000E+00 | 0.000E+00 | 0.000E+00 | 2.166E-05 | 1.802E-05 | 0.000E+00 | 0.000E+00 | 1.557E-05 | 1.850E-07 | 0.000E+00 | 0.000E+00 |
| Unigene287451 | rpoB2 | 0.000E+00 | 0.000E+00 | 0.000E+00 | 0.000E+00 | 1.714E-07 | 0.000E+00 | 0.000E+00 | 0.000E+00 | 0.000E+00 | 0.000E+00 | 0.000E+00 | 0.000E+00 |
| Unigene287508 | carA | 0.000E+00 | 0.000E+00 | 0.000E+00 | 0.000E+00 | 9.030E-07 | 0.000E+00 | 0.000E+00 | 0.000E+00 | 0.000E+00 | 0.000E+00 | 0.000E+00 | 0.000E+00 |

| | | | | | | | | | | | | | |
|---|---|---|---|---|---|---|---|---|---|---|---|---|---|
| Unigene287610 | tetB(60) | 0.000E+00 | 0.000E+00 | 0.000E+00 | 0.000E+00 | 0.000E+00 | 0.000E+00 | 0.000E+00 | 0.000E+00 | 0.000E+00 | 0.000E+00 | 1.851E-07 | 1.052E-06 |
| Unigene287616 | Klebsiella p | 0.000E+00 | 0.000E+00 | 0.000E+00 | 1.231E-07 | 1.321E-06 | 0.000E+00 | 2.362E-07 | 0.000E+00 | 6.996E-07 | 0.000E+00 | 0.000E+00 | 0.000E+00 |
| Unigene287646 | vanHA | 0.000E+00 | 0.000E+00 | 0.000E+00 | 0.000E+00 | 7.158E-07 | 3.134E-07 | 0.000E+00 | 0.000E+00 | 0.000E+00 | 0.000E+00 | 0.000E+00 | 0.000E+00 |
| Unigene287767 | PmrF | 2.908E-07 | 0.000E+00 | 0.000E+00 | 1.305E-07 | 7.898E-07 | 4.890E-07 | 8.583E-07 | 7.921E-07 | 2.472E-07 | 2.880E-07 | 9.240E-07 | 1.222E-06 |
| Unigene287961 | MexD | 0.000E+00 | 0.000E+00 | 0.000E+00 | 0.000E+00 | 3.604E-07 | 0.000E+00 | 5.984E-08 | 0.000E+00 | 0.000E+00 | 0.000E+00 | 0.000E+00 | 0.000E+00 |
| Unigene287982 | oqxA | 1.647E-07 | 0.000E+00 | 0.000E+00 | 1.663E-07 | 1.830E-06 | 0.000E+00 | 0.000E+00 | 0.000E+00 | 6.300E-07 | 0.000E+00 | 0.000E+00 | 0.000E+00 |
| Unigene288010 | kdpE | 3.570E-07 | 0.000E+00 | 0.000E+00 | 0.000E+00 | 4.532E-07 | 3.308E-07 | 6.773E-07 | 0.000E+00 | 1.115E-07 | 0.000E+00 | 4.083E-07 | 5.425E-07 |
| Unigene288040 | lmrC | 6.264E-05 | 0.000E+00 | 0.000E+00 | 1.214E-07 | 2.277E-05 | 7.896E-05 | 0.000E+00 | 0.000E+00 | 0.000E+00 | 0.000E+00 | 0.000E+00 | 9.474E-08 |
| Unigene288146 | oleC | 0.000E+00 | 0.000E+00 | 0.000E+00 | 0.000E+00 | 2.780E-06 | 0.000E+00 | 0.000E+00 | 1.947E-07 | 0.000E+00 | 0.000E+00 | 0.000E+00 | 0.000E+00 |
| Unigene288153 | tetA(58) | 0.000E+00 | 0.000E+00 | 0.000E+00 | 0.000E+00 | 2.762E-06 | 0.000E+00 | 0.000E+00 | 0.000E+00 | 0.000E+00 | 0.000E+00 | 4.196E-07 | 0.000E+00 |
| Unigene288174 | macB | 0.000E+00 | 0.000E+00 | 0.000E+00 | 0.000E+00 | 2.868E-06 | 0.000E+00 | 0.000E+00 | 0.000E+00 | 0.000E+00 | 0.000E+00 | 1.991E-07 | 0.000E+00 |
| Unigene288178 | efrA | 0.000E+00 | 0.000E+00 | 0.000E+00 | 0.000E+00 | 4.515E-06 | 0.000E+00 | 0.000E+00 | 0.000E+00 | 0.000E+00 | 0.000E+00 | 2.667E-07 | 0.000E+00 |
| Unigene288179 | macB | 0.000E+00 | 0.000E+00 | 0.000E+00 | 0.000E+00 | 2.988E-06 | 0.000E+00 | 0.000E+00 | 0.000E+00 | 0.000E+00 | 0.000E+00 | 1.899E-07 | 0.000E+00 |
| Unigene288188 | oleC | 0.000E+00 | 0.000E+00 | 0.000E+00 | 0.000E+00 | 4.223E-06 | 0.000E+00 | 0.000E+00 | 0.000E+00 | 0.000E+00 | 1.077E-07 | 2.211E-07 | 0.000E+00 |
| Unigene288192 | carA | 0.000E+00 | 0.000E+00 | 0.000E+00 | 0.000E+00 | 3.214E-06 | 0.000E+00 | 0.000E+00 | 0.000E+00 | 0.000E+00 | 0.000E+00 | 3.809E-07 | 0.000E+00 |
| Unigene288230 | bcrA | 0.000E+00 | 0.000E+00 | 2.811E-07 | 3.375E-07 | 4.138E-06 | 9.860E-06 | 1.691E-06 | 8.026E-06 | 5.202E-06 | 3.883E-06 | 0.000E+00 | 8.843E-07 |
| Unigene288331 | bcrA | 6.570E-05 | 5.925E-05 | 1.113E-04 | 1.451E-04 | 1.211E-04 | 8.845E-05 | 1.101E-04 | 1.076E-04 | 9.428E-05 | 1.336E-04 | 1.379E-04 | 1.413E-04 |
| Unigene288439 | Klebsiella p | 0.000E+00 | 0.000E+00 | 5.157E-07 | 0.000E+00 | 1.475E-06 | 0.000E+00 | 3.061E-07 | 0.000E+00 | 0.000E+00 | 0.000E+00 | 0.000E+00 | 0.000E+00 |
| Unigene288507 | patA | 0.000E+00 | 0.000E+00 | 0.000E+00 | 0.000E+00 | 2.730E-06 | 0.000E+00 | 0.000E+00 | 0.000E+00 | 0.000E+00 | 0.000E+00 | 0.000E+00 | 0.000E+00 |
| Unigene288521 | lmrC | 0.000E+00 | 0.000E+00 | 0.000E+00 | 0.000E+00 | 3.321E-06 | 0.000E+00 | 0.000E+00 | 0.000E+00 | 0.000E+00 | 0.000E+00 | 5.373E-07 | 0.000E+00 |
| Unigene288530 | patA | 0.000E+00 | 0.000E+00 | 0.000E+00 | 0.000E+00 | 2.779E-06 | 0.000E+00 | 0.000E+00 | 0.000E+00 | 0.000E+00 | 0.000E+00 | 0.000E+00 | 0.000E+00 |
| Unigene288554 | lmrD | 0.000E+00 | 0.000E+00 | 0.000E+00 | 0.000E+00 | 2.200E-06 | 0.000E+00 | 0.000E+00 | 0.000E+00 | 0.000E+00 | 0.000E+00 | 0.000E+00 | 0.000E+00 |
| Unigene288555 | bcrA | 0.000E+00 | 0.000E+00 | 0.000E+00 | 0.000E+00 | 3.198E-06 | 0.000E+00 | 0.000E+00 | 0.000E+00 | 0.000E+00 | 0.000E+00 | 0.000E+00 | 0.000E+00 |
| Unigene288562 | lin | 0.000E+00 | 0.000E+00 | 0.000E+00 | 0.000E+00 | 2.725E-06 | 0.000E+00 | 0.000E+00 | 0.000E+00 | 0.000E+00 | 0.000E+00 | 4.978E-07 | 3.741E-07 |
| Unigene288575 | novA | 0.000E+00 | 0.000E+00 | 0.000E+00 | 0.000E+00 | 3.109E-06 | 0.000E+00 | 0.000E+00 | 1.561E-06 | 0.000E+00 | 0.000E+00 | 0.000E+00 | 0.000E+00 |
| Unigene288590 | tetA(58) | 0.000E+00 | 0.000E+00 | 0.000E+00 | 0.000E+00 | 2.866E-06 | 0.000E+00 | 0.000E+00 | 0.000E+00 | 0.000E+00 | 0.000E+00 | 0.000E+00 | 0.000E+00 |
| Unigene288596 | vanSO | 0.000E+00 | 0.000E+00 | 0.000E+00 | 0.000E+00 | 2.784E-06 | 0.000E+00 | 0.000E+00 | 0.000E+00 | 0.000E+00 | 0.000E+00 | 0.000E+00 | 0.000E+00 |
| Unigene288597 | arlR | 0.000E+00 | 0.000E+00 | 0.000E+00 | 0.000E+00 | 3.002E-06 | 0.000E+00 | 0.000E+00 | 0.000E+00 | 0.000E+00 | 0.000E+00 | 0.000E+00 | 0.000E+00 |
| Unigene288608 | oleC | 0.000E+00 | 0.000E+00 | 0.000E+00 | 0.000E+00 | 3.723E-06 | 0.000E+00 | 0.000E+00 | 0.000E+00 | 0.000E+00 | 0.000E+00 | 0.000E+00 | 0.000E+00 |
| Unigene288609 | TaeA | 0.000E+00 | 0.000E+00 | 0.000E+00 | 0.000E+00 | 2.542E-06 | 0.000E+00 | 0.000E+00 | 0.000E+00 | 0.000E+00 | 0.000E+00 | 0.000E+00 | 0.000E+00 |
| Unigene288612 | oleC | 0.000E+00 | 0.000E+00 | 0.000E+00 | 0.000E+00 | 2.291E-06 | 0.000E+00 | 0.000E+00 | 0.000E+00 | 0.000E+00 | 0.000E+00 | 2.051E-07 | 0.000E+00 |
| Unigene288613 | tetA(58) | 0.000E+00 | 5.974E-08 | 0.000E+00 | 0.000E+00 | 2.983E-06 | 0.000E+00 | 0.000E+00 | 0.000E+00 | 0.000E+00 | 0.000E+00 | 0.000E+00 | 1.030E-06 |
| Unigene288629 | tetA(46) | 0.000E+00 | 0.000E+00 | 0.000E+00 | 0.000E+00 | 2.384E-06 | 0.000E+00 | 0.000E+00 | 0.000E+00 | 0.000E+00 | 0.000E+00 | 2.046E-07 | 0.000E+00 |
| Unigene288662 | oleC | 0.000E+00 | 0.000E+00 | 3.969E-07 | 0.000E+00 | 2.696E-06 | 0.000E+00 | 0.000E+00 | 0.000E+00 | 0.000E+00 | 0.000E+00 | 0.000E+00 | 0.000E+00 |
| Unigene288689 | oleB | 8.701E-08 | 9.549E-08 | 0.000E+00 | 8.786E-08 | 9.022E-07 | 0.000E+00 | 3.852E-07 | 6.094E-07 | 0.000E+00 | 9.693E-08 | 1.327E-07 | 4.457E-07 |
| Unigene288712 | macB | 0.000E+00 | 0.000E+00 | 0.000E+00 | 0.000E+00 | 4.657E-07 | 0.000E+00 | 0.000E+00 | 0.000E+00 | 0.000E+00 | 0.000E+00 | 0.000E+00 | 0.000E+00 |
| Unigene288831 | macB | 0.000E+00 | 0.000E+00 | 0.000E+00 | 0.000E+00 | 1.155E-06 | 0.000E+00 | 8.458E-07 | 0.000E+00 | 0.000E+00 | 1.703E-07 | 0.000E+00 | 0.000E+00 |
| Unigene288874 | rpoB2 | 1.985E-08 | 0.000E+00 | 1.169E-07 | 1.604E-07 | 6.396E-07 | 8.585E-08 | 5.273E-07 | 4.635E-07 | 0.000E+00 | 2.433E-07 | 3.179E-07 | 8.683E-07 |
| Unigene288942 | pmrA | 0.000E+00 | 0.000E+00 | 0.000E+00 | 0.000E+00 | 1.213E-06 | 0.000E+00 | 2.653E-07 | 0.000E+00 | 0.000E+00 | 0.000E+00 | 0.000E+00 | 0.000E+00 |
| Unigene288958 | tetA(46) | 2.489E-07 | 1.214E-07 | 0.000E+00 | 0.000E+00 | 5.531E-07 | 3.289E-07 | 7.041E-07 | 1.356E-06 | 2.721E-07 | 3.698E-07 | 6.961E-07 | 1.046E-06 |
| Unigene289019 | macB | 0.000E+00 | 0.000E+00 | 0.000E+00 | 0.000E+00 | 6.497E-07 | 0.000E+00 | 0.000E+00 | 0.000E+00 | 0.000E+00 | 0.000E+00 | 0.000E+00 | 8.756E-07 |
| Unigene289051 | patA | 0.000E+00 | 0.000E+00 | 0.000E+00 | 0.000E+00 | 7.928E-07 | 0.000E+00 | 0.000E+00 | 0.000E+00 | 0.000E+00 | 0.000E+00 | 0.000E+00 | 0.000E+00 |
| Unigene289107 | macB | 0.000E+00 | 0.000E+00 | 0.000E+00 | 0.000E+00 | 4.367E-07 | 0.000E+00 | 0.000E+00 | 0.000E+00 | 0.000E+00 | 0.000E+00 | 0.000E+00 | 0.000E+00 |
| Unigene289158 | lmrD | 0.000E+00 | 0.000E+00 | 2.910E-07 | 0.000E+00 | 8.471E-07 | 0.000E+00 | 1.360E-06 | 0.000E+00 | 0.000E+00 | 0.000E+00 | 0.000E+00 | 0.000E+00 |
| Unigene289215 | kdpE | 0.000E+00 | 1.022E-07 | 3.657E-07 | 0.000E+00 | 4.657E-07 | 2.518E-07 | 8.764E-07 | 9.243E-07 | 2.036E-07 | 0.000E+00 | 4.795E-07 | 2.312E-06 |
| Unigene289360 | smeR | 0.000E+00 | 0.000E+00 | 0.000E+00 | 0.000E+00 | 2.575E-06 | 0.000E+00 | 0.000E+00 | 0.000E+00 | 0.000E+00 | 0.000E+00 | 5.003E-07 | 0.000E+00 |
| Unigene289371 | smeR | 0.000E+00 | 0.000E+00 | 0.000E+00 | 0.000E+00 | 3.274E-06 | 0.000E+00 | 0.000E+00 | 0.000E+00 | 0.000E+00 | 0.000E+00 | 0.000E+00 | 6.861E-07 |
| Unigene289375 | Acinetoba | 0.000E+00 | 0.000E+00 | 0.000E+00 | 0.000E+00 | 3.347E-06 | 0.000E+00 | 0.000E+00 | 0.000E+00 | 0.000E+00 | 0.000E+00 | 5.213E-07 | 3.591E-07 |
| Unigene289414 | adeH | 0.000E+00 | 6.675E-07 | 0.000E+00 | 0.000E+00 | 9.355E-07 | 0.000E+00 | 3.107E-07 | 0.000E+00 | 0.000E+00 | 0.000E+00 | 0.000E+00 | 0.000E+00 |
| Unigene289527 | poxtA | 0.000E+00 | 0.000E+00 | 0.000E+00 | 0.000E+00 | 3.412E-06 | 0.000E+00 | 0.000E+00 | 0.000E+00 | 0.000E+00 | 0.000E+00 | 4.780E-07 | 0.000E+00 |
| Unigene289551 | tetB(46) | 0.000E+00 | 0.000E+00 | 0.000E+00 | 0.000E+00 | 3.363E-06 | 0.000E+00 | 0.000E+00 | 1.191E-06 | 0.000E+00 | 0.000E+00 | 3.562E-07 | 0.000E+00 |
| Unigene289560 | MexL | 0.000E+00 | 0.000E+00 | 0.000E+00 | 0.000E+00 | 3.260E-06 | 0.000E+00 | 0.000E+00 | 0.000E+00 | 0.000E+00 | 0.000E+00 | 0.000E+00 | 0.000E+00 |
| Unigene289561 | Klebsiella p | 0.000E+00 | 0.000E+00 | 0.000E+00 | 0.000E+00 | 3.311E-06 | 0.000E+00 | 0.000E+00 | 0.000E+00 | 0.000E+00 | 0.000E+00 | 0.000E+00 | 0.000E+00 |
| Unigene289562 | farB | 0.000E+00 | 0.000E+00 | 0.000E+00 | 0.000E+00 | 2.887E-06 | 0.000E+00 | 0.000E+00 | 0.000E+00 | 0.000E+00 | 0.000E+00 | 0.000E+00 | 0.000E+00 |
| Unigene289569 | carA | 0.000E+00 | 0.000E+00 | 0.000E+00 | 0.000E+00 | 3.452E-06 | 0.000E+00 | 0.000E+00 | 0.000E+00 | 0.000E+00 | 0.000E+00 | 0.000E+00 | 0.000E+00 |
| Unigene289580 | macB | 0.000E+00 | 0.000E+00 | 0.000E+00 | 0.000E+00 | 2.831E-06 | 0.000E+00 | 3.917E-07 | 0.000E+00 | 0.000E+00 | 0.000E+00 | 0.000E+00 | 0.000E+00 |
| Unigene289602 | rosB | 1.030E-07 | 5.650E-08 | 5.052E-08 | 0.000E+00 | 5.148E-07 | 0.000E+00 | 1.709E-07 | 3.606E-07 | 0.000E+00 | 2.868E-07 | 0.000E+00 | 3.651E-07 |
| Unigene289628 | tetA(58) | 0.000E+00 | 0.000E+00 | 0.000E+00 | 0.000E+00 | 3.854E-06 | 0.000E+00 | 0.000E+00 | 0.000E+00 | 0.000E+00 | 0.000E+00 | 0.000E+00 | 0.000E+00 |

| | | | | | | | | | | | | | |
|---|---|---|---|---|---|---|---|---|---|---|---|---|---|
| Unigene289633 | Acinetobac | 0.000E+00 | 0.000E+00 | 0.000E+00 | 0.000E+00 | 2.988E-06 | 0.000E+00 | 0.000E+00 | 0.000E+00 | 0.000E+00 | 1.145E-07 | 3.724E-07 | 0.000E+00 |
| Unigene289649 | Staphylocc | 0.000E+00 | 0.000E+00 | 0.000E+00 | 2.974E-07 | 3.694E-06 | 1.592E-07 | 0.000E+00 | 1.633E-06 | 0.000E+00 | 3.280E-07 | 3.368E-07 | 1.465E-06 |
| Unigene289670 | Streptomy | 0.000E+00 | 7.272E-08 | 0.000E+00 | 0.000E+00 | 4.078E-06 | 0.000E+00 | 0.000E+00 | 0.000E+00 | 0.000E+00 | 4.281E-07 | 4.396E-07 | 0.000E+00 |
| Unigene289684 | evgS | 0.000E+00 | 0.000E+00 | 0.000E+00 | 0.000E+00 | 2.758E-06 | 0.000E+00 | 0.000E+00 | 0.000E+00 | 0.000E+00 | 0.000E+00 | 6.814E-07 | 0.000E+00 |
| Unigene289785 | arlR | 0.000E+00 | 0.000E+00 | 0.000E+00 | 0.000E+00 | 5.434E-07 | 0.000E+00 | 0.000E+00 | 0.000E+00 | 0.000E+00 | 0.000E+00 | 0.000E+00 | 0.000E+00 |
| Unigene289809 | macB | 1.701E-07 | 4.201E-07 | 0.000E+00 | 0.000E+00 | 3.166E-06 | 0.000E+00 | 5.648E-07 | 0.000E+00 | 7.902E-07 | 1.421E-07 | 0.000E+00 | 0.000E+00 |
| Unigene289851 | rosB | 0.000E+00 | 1.785E-06 | 1.140E-06 | 5.234E-07 | 7.764E-06 | 0.000E+00 | 1.464E-06 | 0.000E+00 | 0.000E+00 | 0.000E+00 | 7.156E-07 | 0.000E+00 |
| Unigene289874 | rosA | 0.000E+00 | 0.000E+00 | 4.929E-07 | 0.000E+00 | 2.408E-06 | 0.000E+00 | 0.000E+00 | 0.000E+00 | 7.802E-07 | 0.000E+00 | 0.000E+00 | 0.000E+00 |
| Unigene290017 | Corynebac | 6.301E-07 | 7.979E-07 | 9.987E-07 | 1.175E-06 | 0.000E+00 | 0.000E+00 | 1.073E-07 | 0.000E+00 | 5.298E-08 | 0.000E+00 | 0.000E+00 | 5.729E-08 |
| Unigene290018 | efrA | 0.000E+00 | 0.000E+00 | 0.000E+00 | 0.000E+00 | 4.316E-07 | 0.000E+00 | 0.000E+00 | 0.000E+00 | 0.000E+00 | 0.000E+00 | 0.000E+00 | 8.265E-07 |
| Unigene290054 | vanHO | 6.492E-05 | 5.071E-05 | 1.073E-04 | 1.529E-04 | 1.161E-04 | 7.891E-05 | 1.021E-04 | 9.712E-05 | 8.875E-05 | 1.230E-04 | 1.282E-04 | 1.294E-04 |
| Unigene290096 | abeM | 0.000E+00 | 0.000E+00 | 1.616E-07 | 0.000E+00 | 6.709E-07 | 0.000E+00 | 2.430E-07 | 2.563E-07 | 0.000E+00 | 0.000E+00 | 0.000E+00 | 5.840E-07 |
| Unigene290105 | patA | 0.000E+00 | 1.974E-07 | 0.000E+00 | 2.119E-07 | 1.232E-06 | 2.916E-07 | 1.592E-06 | 9.796E-07 | 0.000E+00 | 0.000E+00 | 1.440E-06 | 3.295E-06 |
| Unigene290133 | farB | 0.000E+00 | 3.735E-07 | 0.000E+00 | 5.552E-07 | 6.690E-07 | 0.000E+00 | 2.695E-06 | 1.467E-06 | 0.000E+00 | 7.583E-07 | 1.138E-06 | 1.640E-06 |
| Unigene290298 | mtrA | 0.000E+00 | 0.000E+00 | 0.000E+00 | 0.000E+00 | 2.946E-06 | 0.000E+00 | 0.000E+00 | 0.000E+00 | 0.000E+00 | 0.000E+00 | 2.384E-07 | 0.000E+00 |
| Unigene290306 | rosB | 0.000E+00 | 0.000E+00 | 0.000E+00 | 0.000E+00 | 3.242E-06 | 0.000E+00 | 0.000E+00 | 0.000E+00 | 0.000E+00 | 0.000E+00 | 0.000E+00 | 0.000E+00 |
| Unigene290314 | Staphylocc | 0.000E+00 | 0.000E+00 | 0.000E+00 | 1.769E-07 | 3.235E-06 | 0.000E+00 | 0.000E+00 | 0.000E+00 | 0.000E+00 | 0.000E+00 | 3.756E-07 | 0.000E+00 |
| Unigene290426 | Klebsiella p | 3.048E-07 | 4.015E-07 | 1.495E-07 | 2.462E-07 | 4.504E-06 | 2.307E-07 | 0.000E+00 | 4.625E-07 | 0.000E+00 | 1.698E-07 | 0.000E+00 | 0.000E+00 |
| Unigene290507 | optrA | 0.000E+00 | 0.000E+00 | 0.000E+00 | 0.000E+00 | 1.161E-06 | 2.710E-07 | 0.000E+00 | 0.000E+00 | 0.000E+00 | 9.543E-07 | 0.000E+00 | 0.000E+00 |
| Unigene290600 | vanI | 0.000E+00 | 0.000E+00 | 6.834E-07 | 0.000E+00 | 1.702E-06 | 7.152E-07 | 1.657E-06 | 1.829E-06 | 0.000E+00 | 0.000E+00 | 9.560E-07 | 3.951E-06 |
| Unigene290685 | facT | 6.413E-05 | 6.303E-05 | 1.066E-04 | 7.122E-05 | 1.304E-04 | 9.136E-05 | 1.124E-04 | 1.212E-04 | 1.073E-04 | 1.418E-04 | 1.515E-04 | 1.672E-04 |
| Unigene290694 | NmcR | 0.000E+00 | 3.484E-08 | 0.000E+00 | 2.564E-07 | 5.289E-07 | 0.000E+00 | 9.134E-07 | 5.928E-07 | 0.000E+00 | 0.000E+00 | 4.720E-07 | 7.879E-07 |
| Unigene290751 | evgS | 0.000E+00 | 0.000E+00 | 0.000E+00 | 0.000E+00 | 3.361E-07 | 0.000E+00 | 0.000E+00 | 0.000E+00 | 0.000E+00 | 0.000E+00 | 0.000E+00 | 0.000E+00 |
| Unigene290764 | MIR-23 | 3.662E-07 | 1.391E-06 | 1.133E-06 | 3.413E-07 | 9.012E-06 | 6.090E-07 | 9.041E-07 | 2.630E-07 | 0.000E+00 | 4.393E-07 | 2.578E-07 | 0.000E+00 |
| Unigene290794 | patA | 0.000E+00 | 0.000E+00 | 0.000E+00 | 0.000E+00 | 9.595E-07 | 0.000E+00 | 0.000E+00 | 0.000E+00 | 0.000E+00 | 8.018E-08 | 0.000E+00 | 8.507E-08 |
| Unigene290827 | adeL | 0.000E+00 | 0.000E+00 | 0.000E+00 | 0.000E+00 | 4.798E-07 | 0.000E+00 | 0.000E+00 | 1.680E-07 | 0.000E+00 | 0.000E+00 | 4.528E-07 | 5.104E-07 |
| Unigene290975 | msbA | 4.209E-07 | 0.000E+00 | 2.155E-07 | 3.881E-07 | 4.168E-06 | 7.240E-06 | 1.094E-06 | 6.280E-06 | 3.980E-06 | 0.000E+00 | 4.187E-08 | 0.000E+00 |
| Unigene290983 | optrA | 0.000E+00 | 0.000E+00 | 0.000E+00 | 0.000E+00 | 5.434E-07 | 0.000E+00 | 4.962E-07 | 6.185E-07 | 0.000E+00 | 0.000E+00 | 0.000E+00 | 0.000E+00 |
| Unigene291138 | OprN | 0.000E+00 | 0.000E+00 | 0.000E+00 | 0.000E+00 | 0.000E+00 | 0.000E+00 | 0.000E+00 | 0.000E+00 | 0.000E+00 | 0.000E+00 | 0.000E+00 | 1.211E-07 |
| Unigene291181 | baeR | 0.000E+00 | 0.000E+00 | 0.000E+00 | 0.000E+00 | 1.749E-07 | 0.000E+00 | 0.000E+00 | 0.000E+00 | 0.000E+00 | 0.000E+00 | 0.000E+00 | 0.000E+00 |
| Unigene291251 | adeG | 0.000E+00 | 0.000E+00 | 0.000E+00 | 0.000E+00 | 3.156E-07 | 0.000E+00 | 0.000E+00 | 0.000E+00 | 0.000E+00 | 0.000E+00 | 0.000E+00 | 2.519E-07 |
| Unigene291271 | PmrF | 0.000E+00 | 0.000E+00 | 1.332E-07 | 0.000E+00 | 6.788E-07 | 7.339E-08 | 9.767E-07 | 7.528E-07 | 0.000E+00 | 0.000E+00 | 6.601E-07 | 9.629E-07 |
| Unigene291293 | AcrF | 0.000E+00 | 0.000E+00 | 0.000E+00 | 0.000E+00 | 3.039E-07 | 0.000E+00 | 0.000E+00 | 0.000E+00 | 0.000E+00 | 0.000E+00 | 0.000E+00 | 0.000E+00 |
| Unigene291444 | efrB | 0.000E+00 | 0.000E+00 | 0.000E+00 | 0.000E+00 | 2.769E-07 | 0.000E+00 | 1.839E-07 | 5.334E-07 | 4.541E-08 | 0.000E+00 | 9.504E-08 | 1.473E-07 |
| Unigene291478 | evgS | 8.608E-08 | 0.000E+00 | 0.000E+00 | 0.000E+00 | 2.869E-07 | 0.000E+00 | 0.000E+00 | 3.014E-07 | 0.000E+00 | 0.000E+00 | 0.000E+00 | 0.000E+00 |
| Unigene291531 | arlR | 0.000E+00 | 0.000E+00 | 0.000E+00 | 0.000E+00 | 9.692E-07 | 0.000E+00 | 0.000E+00 | 0.000E+00 | 0.000E+00 | 0.000E+00 | 0.000E+00 | 0.000E+00 |
| Unigene291543 | vanZF | 0.000E+00 | 0.000E+00 | 0.000E+00 | 0.000E+00 | 0.000E+00 | 4.594E-06 | 0.000E+00 | 0.000E+00 | 0.000E+00 | 0.000E+00 | 0.000E+00 | 0.000E+00 |
| Unigene291572 | lmrC | 1.501E-07 | 0.000E+00 | 9.822E-08 | 0.000E+00 | 8.340E-07 | 0.000E+00 | 0.000E+00 | 7.010E-07 | 2.188E-07 | 0.000E+00 | 2.290E-07 | 0.000E+00 |
| Unigene291597 | efrA | 0.000E+00 | 0.000E+00 | 0.000E+00 | 0.000E+00 | 0.000E+00 | 0.000E+00 | 0.000E+00 | 0.000E+00 | 0.000E+00 | 0.000E+00 | 0.000E+00 | 3.367E-07 |
| Unigene291673 | basS | 0.000E+00 | 0.000E+00 | 0.000E+00 | 0.000E+00 | 3.455E-07 | 0.000E+00 | 0.000E+00 | 0.000E+00 | 0.000E+00 | 0.000E+00 | 7.115E-08 | 0.000E+00 |
| Unigene291674 | vanRI | 0.000E+00 | 0.000E+00 | 0.000E+00 | 0.000E+00 | 6.305E-07 | 0.000E+00 | 4.449E-07 | 5.245E-07 | 7.754E-08 | 0.000E+00 | 0.000E+00 | 4.752E-07 |
| Unigene291740 | emrB | 0.000E+00 | 0.000E+00 | 0.000E+00 | 0.000E+00 | 9.178E-07 | 0.000E+00 | 0.000E+00 | 0.000E+00 | 0.000E+00 | 0.000E+00 | 0.000E+00 | 0.000E+00 |
| Unigene291763 | efrB | 0.000E+00 | 3.737E-08 | 0.000E+00 | 0.000E+00 | 4.918E-07 | 0.000E+00 | 1.131E-07 | 2.782E-07 | 0.000E+00 | 0.000E+00 | 2.727E-07 | 2.817E-07 |
| Unigene291772 | kdpE | 9.093E-08 | 6.653E-08 | 1.487E-07 | 9.182E-08 | 1.111E-06 | 2.949E-07 | 9.057E-07 | 8.491E-07 | 1.656E-07 | 2.364E-07 | 9.361E-07 | 1.075E-06 |
| Unigene291811 | tetA(58) | 4.807E-05 | 7.855E-05 | 1.851E-04 | 7.726E-06 | 6.690E-06 | 0.000E+00 | 0.000E+00 | 2.189E-06 | 5.799E-05 | 0.000E+00 | 2.315E-06 | 5.834E-07 |
| Unigene291827 | poxtA | 8.906E-07 | 9.974E-08 | 1.605E-07 | 0.000E+00 | 1.809E-05 | 2.554E-06 | 0.000E+00 | 0.000E+00 | 0.000E+00 | 0.000E+00 | 0.000E+00 | 1.934E-07 |
| Unigene291841 | YojI | 9.849E-08 | 0.000E+00 | 9.664E-08 | 2.486E-08 | 1.915E-07 | 0.000E+00 | 1.908E-07 | 3.449E-07 | 5.382E-08 | 8.229E-08 | 8.450E-08 | 4.075E-07 |
| Unigene292031 | optrA | 0.000E+00 | 0.000E+00 | 0.000E+00 | 0.000E+00 | 7.804E-07 | 0.000E+00 | 0.000E+00 | 0.000E+00 | 0.000E+00 | 0.000E+00 | 0.000E+00 | 9.058E-07 |
| Unigene292039 | lmrD | 0.000E+00 | 0.000E+00 | 0.000E+00 | 0.000E+00 | 7.756E-07 | 8.879E-08 | 0.000E+00 | 1.103E-06 | 0.000E+00 | 0.000E+00 | 5.637E-07 | 1.117E-06 |
| Unigene292151 | ceoB | 0.000E+00 | 0.000E+00 | 0.000E+00 | 0.000E+00 | 5.467E-07 | 0.000E+00 | 2.905E-07 | 0.000E+00 | 0.000E+00 | 0.000E+00 | 0.000E+00 | 0.000E+00 |
| Unigene292265 | MexF | 7.057E-07 | 1.931E-06 | 1.505E-06 | 0.000E+00 | 8.625E-06 | 5.750E-07 | 1.823E-06 | 4.417E-07 | 4.583E-07 | 0.000E+00 | 5.499E-07 | 0.000E+00 |
| Unigene292268 | facT | 1.797E-07 | 9.369E-07 | 5.511E-07 | 0.000E+00 | 8.011E-06 | 2.186E-07 | 1.442E-06 | 2.884E-07 | 4.910E-08 | 0.000E+00 | 1.542E-07 | 0.000E+00 |
| Unigene292283 | Staphylocc | 0.000E+00 | 0.000E+00 | 0.000E+00 | 0.000E+00 | 1.343E-07 | 1.307E-07 | 6.690E-08 | 7.056E-08 | 0.000E+00 | 0.000E+00 | 0.000E+00 | 2.144E-07 |
| Unigene292419 | MuxC | 1.035E-07 | 0.000E+00 | 0.000E+00 | 0.000E+00 | 5.364E-07 | 0.000E+00 | 0.000E+00 | 2.013E-07 | 1.131E-07 | 0.000E+00 | 0.000E+00 | 8.153E-08 |
| Unigene292640 | tet(33) | 0.000E+00 | 0.000E+00 | 5.661E-07 | 4.052E-07 | 1.115E-06 | 5.423E-07 | 1.555E-06 | 0.000E+00 | 0.000E+00 | 1.062E-06 | 1.549E-06 | 4.595E-06 |
| Unigene292644 | Chlamydia | 0.000E+00 | 0.000E+00 | 0.000E+00 | 8.771E-07 | 7.237E-07 | 0.000E+00 | 0.000E+00 | 0.000E+00 | 0.000E+00 | 0.000E+00 | 0.000E+00 | 0.000E+00 |
| Unigene292698 | tetT | 7.133E-05 | 6.003E-05 | 1.216E-04 | 1.697E-04 | 1.345E-04 | 9.326E-05 | 1.178E-04 | 1.089E-04 | 9.939E-05 | 1.483E-04 | 1.503E-04 | 1.551E-04 |
| Unigene292711 | vanSG | 0.000E+00 | 0.000E+00 | 0.000E+00 | 0.000E+00 | 3.985E-07 | 0.000E+00 | 0.000E+00 | 0.000E+00 | 0.000E+00 | 0.000E+00 | 0.000E+00 | 0.000E+00 |

| | | | | | | | | | | | | | |
|---|---|---|---|---|---|---|---|---|---|---|---|---|---|
| Unigene292732 | tetM | 0.000E+00 | 0.000E+00 | 0.000E+00 | 0.000E+00 | 3.448E-07 | 0.000E+00 | 0.000E+00 | 7.850E-07 | 0.000E+00 | 0.000E+00 | 0.000E+00 | 1.834E-06 |
| Unigene292773 | tetT | 8.089E-07 | 3.032E-06 | 1.692E-06 | 6.473E-07 | 9.495E-06 | 6.105E-07 | 3.750E-06 | 6.770E-07 | 6.339E-07 | 4.591E-07 | 1.082E-06 | 2.345E-07 |
| Unigene292899 | vmlR | 0.000E+00 | 0.000E+00 | 0.000E+00 | 0.000E+00 | 6.066E-07 | 0.000E+00 | 4.266E-07 | 7.123E-07 | 0.000E+00 | 2.862E-07 | 6.246E-07 | 4.936E-07 |
| Unigene292928 | Staphylocc | 1.828E-07 | 3.761E-07 | 2.690E-07 | 4.153E-07 | 1.827E-06 | 4.075E-06 | 5.866E-06 | 8.000E-07 | 1.348E-06 | 1.191E-05 | 1.126E-05 | 3.510E-07 |
| Unigene292970 | lsaB | 0.000E+00 | 0.000E+00 | 0.000E+00 | 0.000E+00 | 5.692E-07 | 0.000E+00 | 0.000E+00 | 0.000E+00 | 0.000E+00 | 0.000E+00 | 0.000E+00 | 0.000E+00 |
| Unigene292971 | lsaE | 0.000E+00 | 0.000E+00 | 3.940E-08 | 1.216E-07 | 4.460E-07 | 0.000E+00 | 8.887E-08 | 0.000E+00 | 5.266E-07 | 0.000E+00 | 0.000E+00 | 0.000E+00 |
| Unigene293022 | MexW | 0.000E+00 | 0.000E+00 | 0.000E+00 | 0.000E+00 | 4.623E-07 | 0.000E+00 | 0.000E+00 | 0.000E+00 | 0.000E+00 | 0.000E+00 | 0.000E+00 | 2.108E-07 |
| Unigene293039 | bcrA | 0.000E+00 | 0.000E+00 | 0.000E+00 | 0.000E+00 | 6.245E-07 | 0.000E+00 | 0.000E+00 | 0.000E+00 | 0.000E+00 | 0.000E+00 | 0.000E+00 | 0.000E+00 |
| Unigene293145 | oleB | 0.000E+00 | 0.000E+00 | 0.000E+00 | 0.000E+00 | 3.132E-07 | 0.000E+00 | 0.000E+00 | 0.000E+00 | 0.000E+00 | 0.000E+00 | 0.000E+00 | 0.000E+00 |
| Unigene293237 | smeS | 0.000E+00 | 0.000E+00 | 0.000E+00 | 0.000E+00 | 2.618E-06 | 0.000E+00 | 0.000E+00 | 0.000E+00 | 0.000E+00 | 0.000E+00 | 1.634E-07 | 0.000E+00 |
| Unigene293238 | smeR | 0.000E+00 | 0.000E+00 | 0.000E+00 | 0.000E+00 | 3.919E-06 | 0.000E+00 | 0.000E+00 | 0.000E+00 | 0.000E+00 | 0.000E+00 | 0.000E+00 | 0.000E+00 |
| Unigene293242 | lmrD | 0.000E+00 | 0.000E+00 | 0.000E+00 | 0.000E+00 | 3.789E-06 | 0.000E+00 | 0.000E+00 | 0.000E+00 | 0.000E+00 | 0.000E+00 | 2.483E-07 | 0.000E+00 |
| Unigene293258 | TriA | 0.000E+00 | 0.000E+00 | 0.000E+00 | 1.739E-07 | 3.379E-06 | 0.000E+00 | 0.000E+00 | 0.000E+00 | 0.000E+00 | 0.000E+00 | 3.610E-07 | 0.000E+00 |
| Unigene293259 | MexK | 0.000E+00 | 0.000E+00 | 0.000E+00 | 0.000E+00 | 3.562E-06 | 0.000E+00 | 0.000E+00 | 0.000E+00 | 0.000E+00 | 3.289E-07 | 5.939E-07 | 1.853E-06 |
| Unigene293265 | efrA | 0.000E+00 | 0.000E+00 | 0.000E+00 | 3.612E-07 | 4.073E-06 | 0.000E+00 | 0.000E+00 | 2.087E-06 | 0.000E+00 | 0.000E+00 | 2.728E-07 | 0.000E+00 |
| Unigene293406 | arnA | 0.000E+00 | 0.000E+00 | 0.000E+00 | 0.000E+00 | 2.025E-06 | 0.000E+00 | 0.000E+00 | 0.000E+00 | 1.032E-06 | 0.000E+00 | 0.000E+00 | 0.000E+00 |
| Unigene293446 | sul4 | 0.000E+00 | 0.000E+00 | 8.463E-07 | 0.000E+00 | 1.734E-06 | 0.000E+00 | 0.000E+00 | 0.000E+00 | 0.000E+00 | 0.000E+00 | 9.396E-07 | 0.000E+00 |
| Unigene293453 | catB2 | 0.000E+00 | 0.000E+00 | 0.000E+00 | 0.000E+00 | 9.243E-07 | 0.000E+00 | 0.000E+00 | 0.000E+00 | 0.000E+00 | 0.000E+00 | 0.000E+00 | 0.000E+00 |
| Unigene293459 | efrA | 0.000E+00 | 0.000E+00 | 0.000E+00 | 0.000E+00 | 7.409E-07 | 0.000E+00 | 0.000E+00 | 0.000E+00 | 0.000E+00 | 0.000E+00 | 4.577E-07 | 0.000E+00 |
| Unigene293579 | evgA | 0.000E+00 | 0.000E+00 | 0.000E+00 | 0.000E+00 | 8.654E-07 | 3.158E-07 | 4.850E-07 | 7.956E-07 | 0.000E+00 | 0.000E+00 | 0.000E+00 | 0.000E+00 |
| Unigene293685 | farA | 8.254E-07 | 1.391E-06 | 1.012E-06 | 0.000E+00 | 7.696E-06 | 4.780E-07 | 0.000E+00 | 2.409E-07 | 0.000E+00 | 0.000E+00 | 6.407E-07 | 0.000E+00 |
| Unigene293686 | Klebsiella p | 8.419E-07 | 2.434E-06 | 1.310E-06 | 5.806E-07 | 8.988E-06 | 5.994E-07 | 1.318E-06 | 4.554E-07 | 0.000E+00 | 2.287E-07 | 1.081E-06 | 0.000E+00 |
| Unigene293687 | OprN | 4.547E-07 | 1.758E-06 | 9.985E-07 | 4.810E-07 | 6.278E-06 | 4.447E-07 | 1.486E-06 | 2.022E-07 | 0.000E+00 | 0.000E+00 | 0.000E+00 | 0.000E+00 |
| Unigene293748 | sdiA | 0.000E+00 | 3.131E-07 | 0.000E+00 | 0.000E+00 | 9.056E-07 | 0.000E+00 | 0.000E+00 | 0.000E+00 | 5.791E-07 | 0.000E+00 | 0.000E+00 | 0.000E+00 |
| Unigene293756 | Streptomy | 0.000E+00 | 0.000E+00 | 0.000E+00 | 0.000E+00 | 8.194E-07 | 0.000E+00 | 0.000E+00 | 0.000E+00 | 0.000E+00 | 0.000E+00 | 0.000E+00 | 4.616E-07 |
| Unigene293790 | NmcR | 0.000E+00 | 0.000E+00 | 0.000E+00 | 0.000E+00 | 0.000E+00 | 0.000E+00 | 0.000E+00 | 9.003E-08 | 0.000E+00 | 0.000E+00 | 0.000E+00 | 2.735E-07 |
| Unigene293876 | oleC | 0.000E+00 | 0.000E+00 | 0.000E+00 | 0.000E+00 | 2.286E-06 | 0.000E+00 | 0.000E+00 | 0.000E+00 | 0.000E+00 | 0.000E+00 | 0.000E+00 | 0.000E+00 |
| Unigene293887 | dfrA3 | 0.000E+00 | 0.000E+00 | 0.000E+00 | 0.000E+00 | 3.317E-06 | 0.000E+00 | 0.000E+00 | 0.000E+00 | 0.000E+00 | 0.000E+00 | 0.000E+00 | 0.000E+00 |
| Unigene293902 | MexW | 0.000E+00 | 0.000E+00 | 0.000E+00 | 0.000E+00 | 2.697E-06 | 0.000E+00 | 0.000E+00 | 0.000E+00 | 0.000E+00 | 0.000E+00 | 5.600E-07 | 9.162E-07 |
| Unigene293903 | MuxA | 0.000E+00 | 0.000E+00 | 0.000E+00 | 0.000E+00 | 2.950E-06 | 0.000E+00 | 0.000E+00 | 0.000E+00 | 0.000E+00 | 0.000E+00 | 2.819E-07 | 0.000E+00 |
| Unigene293936 | vanI | 0.000E+00 | 0.000E+00 | 0.000E+00 | 0.000E+00 | 2.863E-06 | 0.000E+00 | 0.000E+00 | 1.585E-06 | 0.000E+00 | 0.000E+00 | 0.000E+00 | 0.000E+00 |
| Unigene293937 | Acinetoba | 0.000E+00 | 0.000E+00 | 0.000E+00 | 0.000E+00 | 2.923E-06 | 0.000E+00 | 0.000E+00 | 0.000E+00 | 0.000E+00 | 0.000E+00 | 0.000E+00 | 7.844E-07 |
| Unigene293972 | oleB | 0.000E+00 | 5.344E-08 | 0.000E+00 | 0.000E+00 | 5.409E-07 | 0.000E+00 | 0.000E+00 | 2.273E-07 | 2.661E-08 | 0.000E+00 | 8.354E-08 | 2.590E-07 |
| Unigene293979 | bcrA | 0.000E+00 | 0.000E+00 | 0.000E+00 | 0.000E+00 | 3.501E-07 | 0.000E+00 | 2.990E-07 | 0.000E+00 | 0.000E+00 | 0.000E+00 | 0.000E+00 | 0.000E+00 |
| Unigene293988 | mdtN | 3.374E-07 | 7.407E-07 | 4.047E-07 | 1.514E-07 | 6.331E-06 | 2.837E-07 | 4.979E-07 | 3.501E-07 | 0.000E+00 | 0.000E+00 | 1.715E-07 | 0.000E+00 |
| Unigene293990 | eptA | 6.551E-07 | 1.482E-06 | 1.071E-06 | 0.000E+00 | 7.234E-06 | 3.648E-07 | 1.033E-06 | 3.708E-07 | 0.000E+00 | 0.000E+00 | 2.725E-07 | 1.643E-07 |
| Unigene293994 | hmrM | 6.399E-07 | 0.000E+00 | 8.140E-07 | 3.351E-07 | 6.056E-06 | 4.612E-07 | 0.000E+00 | 0.000E+00 | 0.000E+00 | 1.056E-07 | 4.609E-07 | 0.000E+00 |
| Unigene294102 | smeF | 2.096E-07 | 4.601E-07 | 1.828E-07 | 0.000E+00 | 6.029E-06 | 2.770E-07 | 0.000E+00 | 2.175E-07 | 0.000E+00 | 0.000E+00 | 0.000E+00 | 1.101E-07 |
| Unigene294103 | smeS | 5.546E-07 | 1.023E-06 | 5.225E-07 | 0.000E+00 | 6.605E-06 | 3.118E-07 | 8.839E-07 | 3.108E-07 | 0.000E+00 | 0.000E+00 | 2.538E-07 | 0.000E+00 |
| Unigene294166 | golS | 0.000E+00 | 0.000E+00 | 0.000E+00 | 1.563E-07 | 5.160E-07 | 0.000E+00 | 2.570E-07 | 0.000E+00 | 0.000E+00 | 0.000E+00 | 0.000E+00 | 0.000E+00 |
| Unigene294176 | CRP | 0.000E+00 | 0.000E+00 | 2.440E-06 | 0.000E+00 | 1.184E-05 | 0.000E+00 | 0.000E+00 | 0.000E+00 | 0.000E+00 | 0.000E+00 | 0.000E+00 | 0.000E+00 |
| Unigene294329 | FusF | 1.064E-04 | 2.277E-04 | 1.847E-04 | 0.000E+00 | 9.230E-06 | 7.574E-07 | 2.880E-06 | 1.694E-06 | 6.230E-05 | 0.000E+00 | 2.633E-06 | 0.000E+00 |
| Unigene294336 | efrA | 0.000E+00 | 0.000E+00 | 3.978E-07 | 0.000E+00 | 6.237E-07 | 0.000E+00 | 7.249E-07 | 1.019E-06 | 0.000E+00 | 0.000E+00 | 5.351E-07 | 3.392E-06 |
| Unigene294392 | cmlv | 0.000E+00 | 0.000E+00 | 0.000E+00 | 0.000E+00 | 1.938E-07 | 1.886E-07 | 0.000E+00 | 0.000E+00 | 1.271E-07 | 0.000E+00 | 2.661E-07 | 0.000E+00 |
| Unigene294396 | Acinetoba | 0.000E+00 | 0.000E+00 | 0.000E+00 | 0.000E+00 | 4.052E-07 | 0.000E+00 | 0.000E+00 | 0.000E+00 | 3.322E-07 | 0.000E+00 | 0.000E+00 | 0.000E+00 |
| Unigene294444 | patA | 0.000E+00 | 0.000E+00 | 0.000E+00 | 5.905E-08 | 2.599E-07 | 0.000E+00 | 2.589E-07 | 1.365E-07 | 0.000E+00 | 0.000E+00 | 0.000E+00 | 4.147E-07 |
| Unigene294488 | tetA(58) | 3.373E-07 | 4.813E-07 | 2.317E-07 | 2.384E-07 | 1.237E-06 | 2.188E-06 | 6.085E-06 | 0.000E+00 | 1.401E-06 | 0.000E+00 | 0.000E+00 | 0.000E+00 |
| Unigene294583 | adeL | 0.000E+00 | 0.000E+00 | 0.000E+00 | 0.000E+00 | 6.921E-07 | 0.000E+00 | 6.268E-07 | 4.627E-07 | 0.000E+00 | 0.000E+00 | 1.296E-07 | 4.686E-07 |
| Unigene294590 | basS | 4.638E-07 | 9.841E-07 | 8.798E-07 | 5.308E-07 | 9.275E-06 | 4.680E-07 | 0.000E+00 | 2.526E-07 | 0.000E+00 | 0.000E+00 | 0.000E+00 | 0.000E+00 |
| Unigene294615 | cpxA | 8.223E-07 | 2.656E-06 | 1.729E-06 | 0.000E+00 | 1.065E-05 | 0.000E+00 | 2.964E-06 | 0.000E+00 | 0.000E+00 | 0.000E+00 | 0.000E+00 | 0.000E+00 |
| Unigene294653 | adeG | 0.000E+00 | 9.015E-08 | 0.000E+00 | 0.000E+00 | 1.825E-07 | 0.000E+00 | 3.636E-07 | 0.000E+00 | 0.000E+00 | 0.000E+00 | 0.000E+00 | 0.000E+00 |
| Unigene294749 | MexW | 1.755E-07 | 0.000E+00 | 8.612E-08 | 0.000E+00 | 6.630E-07 | 1.898E-07 | 5.440E-07 | 3.893E-07 | 0.000E+00 | 0.000E+00 | 0.000E+00 | 5.395E-07 |
| Unigene294765 | MexD | 0.000E+00 | 0.000E+00 | 0.000E+00 | 0.000E+00 | 0.000E+00 | 0.000E+00 | 0.000E+00 | 2.269E-06 | 0.000E+00 | 0.000E+00 | 0.000E+00 | 3.741E-06 |
| Unigene294841 | ceoB | 0.000E+00 | 0.000E+00 | 0.000E+00 | 0.000E+00 | 3.804E-07 | 0.000E+00 | 0.000E+00 | 0.000E+00 | 0.000E+00 | 0.000E+00 | 0.000E+00 | 0.000E+00 |
| Unigene294846 | vmlR | 0.000E+00 | 0.000E+00 | 0.000E+00 | 0.000E+00 | 7.059E-07 | 0.000E+00 | 1.125E-06 | 4.945E-07 | 0.000E+00 | 0.000E+00 | 4.361E-07 | 9.514E-07 |
| Unigene294958 | Streptomy | 6.258E-05 | 5.107E-05 | 9.996E-05 | 1.457E-04 | 1.119E-04 | 7.963E-05 | 9.876E-05 | 9.998E-05 | 8.369E-05 | 1.199E-04 | 1.227E-04 | 1.220E-04 |
| Unigene294970 | patB | 0.000E+00 | 0.000E+00 | 0.000E+00 | 3.995E-08 | 4.395E-07 | 0.000E+00 | 0.000E+00 | 0.000E+00 | 0.000E+00 | 0.000E+00 | 5.430E-07 | 0.000E+00 |
| Unigene294983 | catB2 | 0.000E+00 | 0.000E+00 | 0.000E+00 | 0.000E+00 | 6.963E-07 | 2.259E-07 | 2.312E-07 | 3.048E-07 | 0.000E+00 | 4.655E-07 | 0.000E+00 | 4.322E-07 |

| | | | | | | | | | | | | | |
|---|---|---|---|---|---|---|---|---|---|---|---|---|---|
| Unigene294984 | macB | 0.000E+00 | 0.000E+00 | 0.000E+00 | 3.747E-08 | 8.244E-07 | 4.011E-08 | 8.213E-08 | 3.465E-07 | 1.217E-07 | 1.653E-07 | 0.000E+00 | 4.824E-07 |
| Unigene295041 | TriC | 0.000E+00 | 0.000E+00 | 0.000E+00 | 0.000E+00 | 0.000E+00 | 0.000E+00 | 0.000E+00 | 0.000E+00 | 0.000E+00 | 0.000E+00 | 0.000E+00 | 2.958E-07 |
| Unigene295102 | cmlv | 0.000E+00 | 0.000E+00 | 0.000E+00 | 0.000E+00 | 4.670E-07 | 0.000E+00 | 0.000E+00 | 0.000E+00 | 0.000E+00 | 0.000E+00 | 0.000E+00 | 0.000E+00 |
| Unigene295281 | macB | 0.000E+00 | 5.125E-07 | 0.000E+00 | 1.230E-07 | 1.872E-06 | 0.000E+00 | 0.000E+00 | 0.000E+00 | 6.657E-08 | 0.000E+00 | 0.000E+00 | 0.000E+00 |
| Unigene295391 | OXA-18 | 0.000E+00 | 0.000E+00 | 0.000E+00 | 0.000E+00 | 5.148E-07 | 0.000E+00 | 0.000E+00 | 0.000E+00 | 0.000E+00 | 0.000E+00 | 5.889E-08 | 0.000E+00 |
| Unigene295415 | LpeB | 0.000E+00 | 0.000E+00 | 0.000E+00 | 0.000E+00 | 5.434E-07 | 0.000E+00 | 0.000E+00 | 0.000E+00 | 0.000E+00 | 0.000E+00 | 0.000E+00 | 0.000E+00 |
| Unigene295425 | patB | 0.000E+00 | 0.000E+00 | 0.000E+00 | 0.000E+00 | 0.000E+00 | 0.000E+00 | 0.000E+00 | 0.000E+00 | 0.000E+00 | 0.000E+00 | 0.000E+00 | 3.893E-07 |
| Unigene295479 | TriA | 0.000E+00 | 0.000E+00 | 0.000E+00 | 0.000E+00 | 7.677E-07 | 0.000E+00 | 0.000E+00 | 0.000E+00 | 0.000E+00 | 0.000E+00 | 0.000E+00 | 0.000E+00 |
| Unigene295579 | baeS | 0.000E+00 | 0.000E+00 | 0.000E+00 | 0.000E+00 | 0.000E+00 | 0.000E+00 | 0.000E+00 | 0.000E+00 | 0.000E+00 | 0.000E+00 | 0.000E+00 | 6.896E-07 |
| Unigene295709 | adeL | 0.000E+00 | 0.000E+00 | 0.000E+00 | 0.000E+00 | 3.509E-06 | 0.000E+00 | 0.000E+00 | 0.000E+00 | 0.000E+00 | 0.000E+00 | 0.000E+00 | 0.000E+00 |
| Unigene295738 | efrA | 0.000E+00 | 0.000E+00 | 0.000E+00 | 0.000E+00 | 3.605E-06 | 0.000E+00 | 0.000E+00 | 2.127E-06 | 0.000E+00 | 0.000E+00 | 6.838E-07 | 0.000E+00 |
| Unigene295754 | Corynebac | 0.000E+00 | 0.000E+00 | 0.000E+00 | 3.183E-07 | 3.432E-06 | 6.816E-08 | 0.000E+00 | 2.134E-06 | 0.000E+00 | 3.512E-07 | 6.010E-07 | 1.962E-06 |
| Unigene295762 | patA | 0.000E+00 | 0.000E+00 | 0.000E+00 | 0.000E+00 | 3.749E-06 | 0.000E+00 | 0.000E+00 | 0.000E+00 | 0.000E+00 | 0.000E+00 | 6.555E-07 | 0.000E+00 |
| Unigene295767 | efrA | 0.000E+00 | 0.000E+00 | 0.000E+00 | 0.000E+00 | 3.406E-06 | 0.000E+00 | 0.000E+00 | 0.000E+00 | 0.000E+00 | 0.000E+00 | 4.721E-07 | 0.000E+00 |
| Unigene295801 | vanRL | 5.649E-07 | 3.248E-07 | 5.807E-07 | 6.519E-07 | 4.483E-07 | 1.570E-06 | 3.483E-06 | 3.956E-06 | 4.116E-07 | 1.289E-06 | 1.600E-06 | 3.784E-06 |
| Unigene295816 | facT | 0.000E+00 | 0.000E+00 | 0.000E+00 | 0.000E+00 | 1.497E-06 | 0.000E+00 | 1.492E-07 | 0.000E+00 | 6.875E-07 | 0.000E+00 | 0.000E+00 | 0.000E+00 |
| Unigene295824 | Klebsiella p | 0.000E+00 | 0.000E+00 | 0.000E+00 | 0.000E+00 | 9.624E-07 | 0.000E+00 | 0.000E+00 | 0.000E+00 | 0.000E+00 | 0.000E+00 | 0.000E+00 | 0.000E+00 |
| Unigene295858 | oleB | 3.013E-07 | 0.000E+00 | 0.000E+00 | 3.477E-07 | 4.256E-06 | 0.000E+00 | 0.000E+00 | 3.014E-07 | 1.129E-06 | 0.000E+00 | 4.431E-07 | 0.000E+00 |
| Unigene296060 | Staphylocc | 0.000E+00 | 0.000E+00 | 0.000E+00 | 0.000E+00 | 9.141E-07 | 3.079E-07 | 7.005E-07 | 4.063E-07 | 0.000E+00 | 0.000E+00 | 0.000E+00 | 1.534E-06 |
| Unigene296075 | rpoB2 | 0.000E+00 | 0.000E+00 | 0.000E+00 | 0.000E+00 | 3.835E-07 | 0.000E+00 | 0.000E+00 | 4.030E-07 | 0.000E+00 | 0.000E+00 | 0.000E+00 | 2.040E-07 |
| Unigene296149 | lmrD | 9.236E-08 | 0.000E+00 | 0.000E+00 | 0.000E+00 | 1.539E-07 | 1.997E-07 | 0.000E+00 | 3.234E-07 | 4.543E-07 | 0.000E+00 | 0.000E+00 | 0.000E+00 |
| Unigene296241 | patA | 0.000E+00 | 2.501E-07 | 0.000E+00 | 0.000E+00 | 7.776E-07 | 0.000E+00 | 7.387E-07 | 0.000E+00 | 1.601E-07 | 0.000E+00 | 0.000E+00 | 0.000E+00 |
| Unigene296257 | evgS | 0.000E+00 | 0.000E+00 | 0.000E+00 | 0.000E+00 | 1.095E-06 | 0.000E+00 | 9.091E-07 | 4.794E-07 | 0.000E+00 | 0.000E+00 | 0.000E+00 | 9.709E-07 |
| Unigene296265 | efrA | 3.575E-08 | 3.923E-08 | 0.000E+00 | 0.000E+00 | 7.546E-07 | 0.000E+00 | 2.374E-07 | 2.921E-07 | 7.814E-08 | 0.000E+00 | 2.045E-07 | 4.648E-07 |
| Unigene296358 | oleB | 0.000E+00 | 0.000E+00 | 0.000E+00 | 0.000E+00 | 4.782E-07 | 0.000E+00 | 0.000E+00 | 0.000E+00 | 0.000E+00 | 0.000E+00 | 0.000E+00 | 0.000E+00 |
| Unigene296436 | macB | 6.320E-07 | 1.935E-06 | 1.485E-06 | 3.863E-07 | 7.668E-06 | 2.697E-07 | 0.000E+00 | 3.494E-07 | 3.817E-07 | 0.000E+00 | 7.990E-07 | 2.752E-07 |
| Unigene296483 | mdtC | 0.000E+00 | 0.000E+00 | 0.000E+00 | 0.000E+00 | 4.754E-07 | 0.000E+00 | 0.000E+00 | 0.000E+00 | 0.000E+00 | 0.000E+00 | 0.000E+00 | 0.000E+00 |
| Unigene296523 | ceoB | 0.000E+00 | 0.000E+00 | 0.000E+00 | 0.000E+00 | 4.899E-07 | 0.000E+00 | 0.000E+00 | 0.000E+00 | 0.000E+00 | 0.000E+00 | 0.000E+00 | 0.000E+00 |
| Unigene296524 | adeG | 0.000E+00 | 0.000E+00 | 0.000E+00 | 0.000E+00 | 6.237E-07 | 0.000E+00 | 0.000E+00 | 0.000E+00 | 0.000E+00 | 0.000E+00 | 0.000E+00 | 0.000E+00 |
| Unigene296637 | bcrA | 0.000E+00 | 0.000E+00 | 1.650E-07 | 0.000E+00 | 6.537E-07 | 0.000E+00 | 1.163E-06 | 1.325E-06 | 0.000E+00 | 3.746E-07 | 1.298E-06 | 3.826E-06 |
| Unigene296662 | bcr-1 | 0.000E+00 | 0.000E+00 | 9.920E-07 | 0.000E+00 | 6.552E-06 | 0.000E+00 | 0.000E+00 | 0.000E+00 | 0.000E+00 | 0.000E+00 | 0.000E+00 | 0.000E+00 |
| Unigene296671 | vanHO | 0.000E+00 | 1.045E-07 | 1.246E-07 | 0.000E+00 | 4.231E-07 | 3.088E-07 | 5.621E-07 | 5.557E-07 | 2.428E-07 | 0.000E+00 | 5.446E-07 | 7.504E-07 |
| Unigene296829 | patA | 0.000E+00 | 0.000E+00 | 0.000E+00 | 1.672E-07 | 0.000E+00 | 8.053E-07 | 2.382E-06 | 1.546E-06 | 0.000E+00 | 8.759E-07 | 0.000E+00 | 3.375E-06 |
| Unigene296863 | bcrA | 0.000E+00 | 0.000E+00 | 0.000E+00 | 0.000E+00 | 3.290E-07 | 0.000E+00 | 0.000E+00 | 4.033E-07 | 0.000E+00 | 0.000E+00 | 0.000E+00 | 0.000E+00 |
| Unigene296905 | vatB | 0.000E+00 | 0.000E+00 | 0.000E+00 | 0.000E+00 | 4.062E-07 | 0.000E+00 | 0.000E+00 | 0.000E+00 | 0.000E+00 | 0.000E+00 | 0.000E+00 | 0.000E+00 |
| Unigene296931 | macB | 8.121E-08 | 0.000E+00 | 0.000E+00 | 0.000E+00 | 9.473E-07 | 0.000E+00 | 0.000E+00 | 0.000E+00 | 5.769E-07 | 0.000E+00 | 0.000E+00 | 0.000E+00 |
| Unigene296949 | vanHF | 0.000E+00 | 0.000E+00 | 0.000E+00 | 0.000E+00 | 9.663E-07 | 0.000E+00 | 0.000E+00 | 0.000E+00 | 0.000E+00 | 0.000E+00 | 0.000E+00 | 0.000E+00 |
| Unigene296992 | TriC | 0.000E+00 | 0.000E+00 | 0.000E+00 | 0.000E+00 | 5.546E-07 | 0.000E+00 | 0.000E+00 | 0.000E+00 | 0.000E+00 | 0.000E+00 | 0.000E+00 | 0.000E+00 |
| Unigene297013 | bcrA | 0.000E+00 | 0.000E+00 | 0.000E+00 | 0.000E+00 | 3.321E-07 | 0.000E+00 | 9.451E-08 | 4.486E-07 | 0.000E+00 | 9.513E-08 | 0.000E+00 | 3.028E-07 |
| Unigene297068 | efrA | 1.784E-08 | 5.875E-08 | 0.000E+00 | 0.000E+00 | 6.542E-07 | 5.787E-08 | 9.875E-08 | 3.124E-07 | 1.170E-07 | 1.590E-07 | 1.633E-07 | 4.429E-07 |
| Unigene297072 | mdtN | 0.000E+00 | 4.526E-07 | 0.000E+00 | 0.000E+00 | 1.208E-06 | 0.000E+00 | 2.905E-07 | 0.000E+00 | 0.000E+00 | 0.000E+00 | 0.000E+00 | 0.000E+00 |
| Unigene297139 | oleC | 2.771E-07 | 9.630E-07 | 3.172E-07 | 0.000E+00 | 8.414E-06 | 4.992E-07 | 0.000E+00 | 3.773E-07 | 0.000E+00 | 3.087E-07 | 2.641E-07 | 0.000E+00 |
| Unigene297215 | msbA | 0.000E+00 | 0.000E+00 | 0.000E+00 | 0.000E+00 | 4.576E-07 | 0.000E+00 | 0.000E+00 | 0.000E+00 | 0.000E+00 | 0.000E+00 | 0.000E+00 | 0.000E+00 |
| Unigene297258 | basS | 0.000E+00 | 0.000E+00 | 0.000E+00 | 7.931E-08 | 6.980E-07 | 0.000E+00 | 0.000E+00 | 0.000E+00 | 0.000E+00 | 0.000E+00 | 0.000E+00 | 0.000E+00 |
| Unigene297358 | baeR | 0.000E+00 | 0.000E+00 | 0.000E+00 | 0.000E+00 | 1.000E-06 | 0.000E+00 | 0.000E+00 | 0.000E+00 | 0.000E+00 | 0.000E+00 | 9.785E-07 | 1.118E-06 |
| Unigene297496 | tetA(60) | 0.000E+00 | 0.000E+00 | 0.000E+00 | 0.000E+00 | 6.395E-07 | 0.000E+00 | 0.000E+00 | 0.000E+00 | 0.000E+00 | 0.000E+00 | 0.000E+00 | 0.000E+00 |
| Unigene297522 | efrA | 0.000E+00 | 0.000E+00 | 0.000E+00 | 0.000E+00 | 6.935E-07 | 0.000E+00 | 0.000E+00 | 6.939E-07 | 0.000E+00 | 0.000E+00 | 6.800E-07 | 1.054E-06 |
| Unigene297573 | Acinetobac | 0.000E+00 | 0.000E+00 | 0.000E+00 | 0.000E+00 | 4.295E-07 | 0.000E+00 | 0.000E+00 | 1.504E-07 | 0.000E+00 | 0.000E+00 | 0.000E+00 | 0.000E+00 |
| Unigene297596 | macB | 0.000E+00 | 0.000E+00 | 0.000E+00 | 0.000E+00 | 1.328E-06 | 0.000E+00 | 0.000E+00 | 0.000E+00 | 0.000E+00 | 0.000E+00 | 0.000E+00 | 0.000E+00 |
| Unigene297602 | lmrD | 0.000E+00 | 0.000E+00 | 0.000E+00 | 0.000E+00 | 1.791E-06 | 0.000E+00 | 0.000E+00 | 0.000E+00 | 1.405E-05 | 0.000E+00 | 0.000E+00 | 0.000E+00 |
| Unigene297615 | macB | 0.000E+00 | 0.000E+00 | 0.000E+00 | 0.000E+00 | 1.312E-06 | 0.000E+00 | 2.615E-06 | 1.078E-06 | 4.695E-07 | 0.000E+00 | 0.000E+00 | 0.000E+00 |
| Unigene297669 | bcrC | 0.000E+00 | 0.000E+00 | 0.000E+00 | 0.000E+00 | 7.655E-07 | 0.000E+00 | 0.000E+00 | 0.000E+00 | 0.000E+00 | 0.000E+00 | 7.276E-07 | 0.000E+00 |
| Unigene297686 | macB | 7.295E-08 | 0.000E+00 | 0.000E+00 | 0.000E+00 | 4.457E-07 | 2.366E-07 | 4.441E-07 | 2.555E-07 | 0.000E+00 | 0.000E+00 | 2.921E-07 | 6.036E-07 |
| Unigene297714 | Rhodobac | 0.000E+00 | 0.000E+00 | 0.000E+00 | 0.000E+00 | 6.437E-07 | 0.000E+00 | 0.000E+00 | 0.000E+00 | 0.000E+00 | 0.000E+00 | 0.000E+00 | 2.935E-07 |
| Unigene297722 | Staphylocc | 1.244E-05 | 4.737E-06 | 3.500E-06 | 1.305E-05 | 8.142E-05 | 1.145E-04 | 1.163E-04 | 8.868E-05 | 1.456E-04 | 1.413E-04 | 1.150E-04 | 7.474E-05 |
| Unigene297784 | efrA | 0.000E+00 | 0.000E+00 | 0.000E+00 | 0.000E+00 | 4.820E-07 | 0.000E+00 | 0.000E+00 | 0.000E+00 | 0.000E+00 | 0.000E+00 | 0.000E+00 | 0.000E+00 |
| Unigene297957 | cmlv | 0.000E+00 | 0.000E+00 | 0.000E+00 | 0.000E+00 | 1.100E-06 | 0.000E+00 | 0.000E+00 | 0.000E+00 | 0.000E+00 | 0.000E+00 | 0.000E+00 | 0.000E+00 |
| Unigene298063 | patB | 0.000E+00 | 6.056E-07 | 0.000E+00 | 0.000E+00 | 1.226E-06 | 0.000E+00 | 0.000E+00 | 0.000E+00 | 0.000E+00 | 0.000E+00 | 0.000E+00 | 0.000E+00 |

| | | | | | | | | | | | | | |
|---|---|---|---|---|---|---|---|---|---|---|---|---|---|
| Unigene298187 | MexH | 1.462E-07 | 0.000E+00 | 1.721E-07 | 5.905E-08 | 7.146E-07 | 0.000E+00 | 7.767E-07 | 7.509E-07 | 0.000E+00 | 0.000E+00 | 0.000E+00 | 0.000E+00 |
| Unigene298297 | efrA | 0.000E+00 | 0.000E+00 | 0.000E+00 | 0.000E+00 | 1.082E-06 | 0.000E+00 | 0.000E+00 | 0.000E+00 | 0.000E+00 | 0.000E+00 | 0.000E+00 | 1.371E-06 |
| Unigene298313 | tlrC | 0.000E+00 | 1.824E-07 | 3.261E-07 | 4.195E-08 | 8.308E-07 | 2.246E-07 | 5.518E-07 | 1.358E-06 | 0.000E+00 | 3.702E-07 | 3.802E-07 | 1.719E-06 |
| Unigene298367 | Corynebac | 0.000E+00 | 0.000E+00 | 1.500E-07 | 9.261E-08 | 3.056E-07 | 0.000E+00 | 5.751E-07 | 3.211E-07 | 0.000E+00 | 1.362E-07 | 2.098E-07 | 4.697E-07 |
| Unigene298371 | patA | 0.000E+00 | 0.000E+00 | 0.000E+00 | 0.000E+00 | 5.337E-07 | 0.000E+00 | 0.000E+00 | 0.000E+00 | 0.000E+00 | 0.000E+00 | 3.297E-07 | 6.586E-07 |
| Unigene298504 | baeS | 0.000E+00 | 0.000E+00 | 0.000E+00 | 0.000E+00 | 2.804E-06 | 0.000E+00 | 9.181E-08 | 0.000E+00 | 0.000E+00 | 0.000E+00 | 3.389E-07 | 0.000E+00 |
| Unigene298505 | kdpE | 0.000E+00 | 0.000E+00 | 0.000E+00 | 0.000E+00 | 3.157E-06 | 0.000E+00 | 0.000E+00 | 0.000E+00 | 0.000E+00 | 0.000E+00 | 0.000E+00 | 0.000E+00 |
| Unigene298526 | tlrC | 0.000E+00 | 0.000E+00 | 0.000E+00 | 0.000E+00 | 3.065E-06 | 0.000E+00 | 0.000E+00 | 0.000E+00 | 0.000E+00 | 0.000E+00 | 0.000E+00 | 0.000E+00 |
| Unigene298527 | oleC | 0.000E+00 | 0.000E+00 | 0.000E+00 | 0.000E+00 | 3.797E-06 | 0.000E+00 | 0.000E+00 | 0.000E+00 | 0.000E+00 | 0.000E+00 | 2.896E-07 | 0.000E+00 |
| Unigene298529 | YojI | 1.543E-07 | 0.000E+00 | 0.000E+00 | 0.000E+00 | 1.885E-06 | 0.000E+00 | 0.000E+00 | 0.000E+00 | 0.000E+00 | 0.000E+00 | 0.000E+00 | 8.206E-07 |
| Unigene298576 | golS | 0.000E+00 | 0.000E+00 | 0.000E+00 | 0.000E+00 | 0.000E+00 | 0.000E+00 | 1.420E-06 | 0.000E+00 | 0.000E+00 | 0.000E+00 | 0.000E+00 | 1.600E-06 |
| Unigene298683 | lmrB | 4.839E-07 | 0.000E+00 | 3.391E-07 | 2.094E-07 | 3.507E-06 | 5.754E-06 | 9.945E-07 | 6.428E-06 | 2.871E-06 | 2.233E-06 | 0.000E+00 | 5.447E-07 |
| Unigene298684 | lmrD | 4.413E-07 | 3.725E-08 | 5.662E-07 | 3.428E-07 | 3.318E-06 | 6.825E-06 | 1.089E-06 | 6.260E-06 | 3.858E-06 | 2.836E-06 | 0.000E+00 | 0.000E+00 |
| Unigene298694 | tetA(58) | 8.198E-07 | 0.000E+00 | 4.022E-07 | 3.794E-07 | 3.529E-06 | 6.241E-06 | 1.323E-06 | 6.619E-06 | 3.285E-06 | 0.000E+00 | 0.000E+00 | 0.000E+00 |
| Unigene298695 | tetB(58) | 7.008E-07 | 0.000E+00 | 2.023E-07 | 4.996E-07 | 2.794E-06 | 4.947E-06 | 0.000E+00 | 0.000E+00 | 2.749E-06 | 0.000E+00 | 0.000E+00 | 0.000E+00 |
| Unigene298702 | evgS | 2.932E-08 | 0.000E+00 | 2.877E-08 | 0.000E+00 | 2.280E-07 | 1.585E-07 | 1.298E-07 | 3.422E-07 | 6.409E-08 | 0.000E+00 | 0.000E+00 | 2.426E-07 |
| Unigene298742 | cmlv | 0.000E+00 | 0.000E+00 | 0.000E+00 | 0.000E+00 | 5.784E-07 | 0.000E+00 | 0.000E+00 | 0.000E+00 | 0.000E+00 | 0.000E+00 | 0.000E+00 | 0.000E+00 |
| Unigene298781 | dfrA26 | 0.000E+00 | 0.000E+00 | 0.000E+00 | 0.000E+00 | 7.429E-07 | 0.000E+00 | 0.000E+00 | 0.000E+00 | 0.000E+00 | 0.000E+00 | 0.000E+00 | 8.623E-07 |
| Unigene298804 | bcrA | 0.000E+00 | 6.384E-08 | 0.000E+00 | 0.000E+00 | 3.877E-07 | 0.000E+00 | 0.000E+00 | 4.073E-07 | 0.000E+00 | 0.000E+00 | 3.992E-07 | 4.812E-07 |
| Unigene298852 | arlR | 0.000E+00 | 0.000E+00 | 0.000E+00 | 0.000E+00 | 1.449E-06 | 0.000E+00 | 0.000E+00 | 0.000E+00 | 0.000E+00 | 0.000E+00 | 0.000E+00 | 0.000E+00 |
| Unigene298929 | bcrA | 7.532E-07 | 1.378E-06 | 3.871E-07 | 1.811E-07 | 2.072E-06 | 2.288E-06 | 9.964E-06 | 4.187E-07 | 3.058E-06 | 0.000E+00 | 0.000E+00 | 5.935E-07 |
| Unigene298962 | basS | 2.630E-07 | 6.561E-07 | 4.458E-07 | 3.139E-07 | 7.146E-06 | 3.360E-07 | 1.217E-06 | 1.396E-07 | 7.840E-08 | 0.000E+00 | 1.368E-07 | 0.000E+00 |
| Unigene298968 | vanSB | 0.000E+00 | 0.000E+00 | 0.000E+00 | 0.000E+00 | 2.391E-07 | 0.000E+00 | 1.429E-07 | 3.517E-07 | 0.000E+00 | 0.000E+00 | 1.969E-07 | 0.000E+00 |
| Unigene298972 | adeL | 0.000E+00 | 0.000E+00 | 0.000E+00 | 0.000E+00 | 1.235E-06 | 0.000E+00 | 0.000E+00 | 0.000E+00 | 0.000E+00 | 0.000E+00 | 0.000E+00 | 0.000E+00 |
| Unigene299110 | vgaE | 0.000E+00 | 0.000E+00 | 0.000E+00 | 0.000E+00 | 3.622E-07 | 0.000E+00 | 0.000E+00 | 0.000E+00 | 1.188E-07 | 0.000E+00 | 0.000E+00 | 0.000E+00 |
| Unigene299193 | otr(B) | 0.000E+00 | 0.000E+00 | 8.363E-08 | 8.607E-08 | 5.918E-07 | 0.000E+00 | 3.066E-07 | 0.000E+00 | 0.000E+00 | 1.899E-07 | 1.462E-07 | 4.533E-07 |
| Unigene299526 | efrA | 0.000E+00 | 0.000E+00 | 0.000E+00 | 0.000E+00 | 0.000E+00 | 0.000E+00 | 0.000E+00 | 2.842E-07 | 0.000E+00 | 0.000E+00 | 0.000E+00 | 0.000E+00 |
| Unigene299552 | tetT | 0.000E+00 | 0.000E+00 | 0.000E+00 | 0.000E+00 | 1.398E-06 | 0.000E+00 | 0.000E+00 | 0.000E+00 | 4.362E-07 | 0.000E+00 | 0.000E+00 | 0.000E+00 |
| Unigene299602 | farA | 6.045E-08 | 0.000E+00 | 2.076E-07 | 3.968E-07 | 7.387E-07 | 4.901E-07 | 1.572E-06 | 1.270E-06 | 0.000E+00 | 1.683E-07 | 0.000E+00 | 1.358E-06 |
| Unigene299647 | adeL | 0.000E+00 | 0.000E+00 | 7.015E-08 | 0.000E+00 | 1.311E-06 | 0.000E+00 | 0.000E+00 | 0.000E+00 | 0.000E+00 | 0.000E+00 | 0.000E+00 | 0.000E+00 |
| Unigene299661 | tlrC | 0.000E+00 | 0.000E+00 | 0.000E+00 | 1.156E-07 | 0.000E+00 | 0.000E+00 | 0.000E+00 | 0.000E+00 | 0.000E+00 | 0.000E+00 | 0.000E+00 | 0.000E+00 |
| Unigene299689 | PmrF | 0.000E+00 | 0.000E+00 | 0.000E+00 | 0.000E+00 | 6.440E-07 | 0.000E+00 | 7.217E-07 | 0.000E+00 | 0.000E+00 | 0.000E+00 | 0.000E+00 | 9.421E-07 |
| Unigene299774 | emrK | 0.000E+00 | 0.000E+00 | 0.000E+00 | 0.000E+00 | 6.542E-07 | 0.000E+00 | 9.183E-07 | 0.000E+00 | 0.000E+00 | 0.000E+00 | 0.000E+00 | 0.000E+00 |
| Unigene299939 | kdpE | 2.839E-08 | 1.558E-07 | 0.000E+00 | 0.000E+00 | 4.100E-07 | 0.000E+00 | 9.426E-08 | 0.000E+00 | 3.103E-08 | 0.000E+00 | 0.000E+00 | 2.014E-07 |
| Unigene299990 | vanG | 0.000E+00 | 0.000E+00 | 0.000E+00 | 0.000E+00 | 1.096E-06 | 0.000E+00 | 2.407E-06 | 0.000E+00 | 0.000E+00 | 0.000E+00 | 0.000E+00 | 0.000E+00 |
| Unigene300001 | arnA | 4.060E-07 | 1.114E-07 | 2.324E-07 | 6.492E-07 | 4.210E-06 | 6.109E-06 | 1.011E-06 | 8.689E-06 | 4.512E-06 | 2.412E-06 | 0.000E+00 | 7.999E-07 |
| Unigene300019 | oleB | 0.000E+00 | 1.307E-07 | 5.844E-08 | 1.203E-07 | 6.617E-07 | 0.000E+00 | 0.000E+00 | 2.781E-07 | 4.340E-08 | 1.327E-07 | 1.363E-07 | 4.693E-07 |
| Unigene300103 | tetB(46) | 0.000E+00 | 0.000E+00 | 0.000E+00 | 0.000E+00 | 0.000E+00 | 0.000E+00 | 0.000E+00 | 0.000E+00 | 0.000E+00 | 0.000E+00 | 6.078E-07 | 0.000E+00 |
| Unigene300105 | Acinetoba | 2.440E-07 | 1.526E-06 | 1.029E-06 | 0.000E+00 | 9.514E-06 | 6.067E-07 | 1.053E-06 | 4.557E-07 | 0.000E+00 | 0.000E+00 | 4.187E-07 | 0.000E+00 |
| Unigene300157 | TaeA | 3.069E-07 | 1.254E-06 | 6.693E-07 | 0.000E+00 | 7.313E-06 | 3.871E-07 | 1.208E-06 | 2.389E-07 | 1.118E-07 | 2.469E-07 | 0.000E+00 | 1.814E-07 |
| Unigene300177 | AAC(6')-IIb | 0.000E+00 | 0.000E+00 | 0.000E+00 | 1.852E-07 | 4.075E-07 | 3.965E-07 | 7.443E-07 | 9.991E-07 | 0.000E+00 | 0.000E+00 | 1.469E-06 | 6.504E-07 |
| Unigene300218 | tetB(60) | 7.922E-07 | 9.419E-07 | 7.125E-07 | 5.666E-07 | 7.627E-06 | 4.995E-07 | 1.205E-06 | 3.853E-07 | 0.000E+00 | 0.000E+00 | 4.908E-07 | 0.000E+00 |
| Unigene300299 | cmlv | 0.000E+00 | 0.000E+00 | 4.140E-07 | 0.000E+00 | 1.055E-06 | 0.000E+00 | 5.254E-07 | 0.000E+00 | 8.647E-08 | 0.000E+00 | 0.000E+00 | 0.000E+00 |
| Unigene300397 | Staphyloco | 0.000E+00 | 0.000E+00 | 0.000E+00 | 0.000E+00 | 9.563E-07 | 0.000E+00 | 0.000E+00 | 0.000E+00 | 0.000E+00 | 0.000E+00 | 0.000E+00 | 0.000E+00 |
| Unigene300438 | rphB | 0.000E+00 | 0.000E+00 | 0.000E+00 | 0.000E+00 | 1.026E-06 | 0.000E+00 | 5.760E-07 | 0.000E+00 | 0.000E+00 | 7.247E-07 | 0.000E+00 | 0.000E+00 |
| Unigene300447 | bcrA | 0.000E+00 | 0.000E+00 | 0.000E+00 | 0.000E+00 | 9.625E-07 | 0.000E+00 | 0.000E+00 | 0.000E+00 | 0.000E+00 | 0.000E+00 | 0.000E+00 | 0.000E+00 |
| Unigene300565 | adeL | 0.000E+00 | 0.000E+00 | 0.000E+00 | 0.000E+00 | 8.976E-07 | 0.000E+00 | 8.535E-07 | 8.573E-07 | 0.000E+00 | 0.000E+00 | 0.000E+00 | 8.682E-07 |
| Unigene300649 | evgS | 2.542E-07 | 5.446E-07 | 4.394E-07 | 1.711E-07 | 4.666E-06 | 1.832E-07 | 2.545E-07 | 1.978E-07 | 0.000E+00 | 0.000E+00 | 2.907E-07 | 0.000E+00 |
| Unigene300772 | Pseudomo | 0.000E+00 | 0.000E+00 | 0.000E+00 | 0.000E+00 | 1.016E-06 | 0.000E+00 | 0.000E+00 | 0.000E+00 | 0.000E+00 | 0.000E+00 | 0.000E+00 | 0.000E+00 |
| Unigene300773 | cpxA | 0.000E+00 | 0.000E+00 | 0.000E+00 | 0.000E+00 | 1.162E-06 | 0.000E+00 | 0.000E+00 | 0.000E+00 | 0.000E+00 | 0.000E+00 | 0.000E+00 | 0.000E+00 |
| Unigene300855 | farB | 0.000E+00 | 0.000E+00 | 0.000E+00 | 0.000E+00 | 1.241E-06 | 0.000E+00 | 0.000E+00 | 0.000E+00 | 0.000E+00 | 0.000E+00 | 0.000E+00 | 0.000E+00 |
| Unigene300880 | MexI | 0.000E+00 | 0.000E+00 | 0.000E+00 | 0.000E+00 | 3.435E-07 | 0.000E+00 | 0.000E+00 | 0.000E+00 | 0.000E+00 | 0.000E+00 | 0.000E+00 | 0.000E+00 |
| Unigene300897 | emrY | 2.771E-07 | 0.000E+00 | 9.289E-07 | 5.130E-07 | 9.235E-07 | 0.000E+00 | 4.728E-06 | 4.178E-06 | 4.038E-07 | 1.466E-06 | 1.875E-06 | 4.940E-06 |
| Unigene300928 | YojI | 0.000E+00 | 0.000E+00 | 0.000E+00 | 0.000E+00 | 1.790E-06 | 0.000E+00 | 0.000E+00 | 0.000E+00 | 6.103E-07 | 0.000E+00 | 0.000E+00 | 0.000E+00 |
| Unigene300956 | mgrA | 0.000E+00 | 0.000E+00 | 0.000E+00 | 0.000E+00 | 1.425E-06 | 0.000E+00 | 0.000E+00 | 0.000E+00 | 0.000E+00 | 0.000E+00 | 0.000E+00 | 0.000E+00 |
| Unigene301037 | MexD | 0.000E+00 | 0.000E+00 | 0.000E+00 | 0.000E+00 | 4.001E-07 | 0.000E+00 | 0.000E+00 | 0.000E+00 | 0.000E+00 | 0.000E+00 | 0.000E+00 | 0.000E+00 |
| Unigene301088 | efrA | 0.000E+00 | 0.000E+00 | 0.000E+00 | 0.000E+00 | 1.013E-06 | 0.000E+00 | 1.480E-06 | 1.029E-06 | 0.000E+00 | 0.000E+00 | 0.000E+00 | 0.000E+00 |
| Unigene301160 | macB | 0.000E+00 | 0.000E+00 | 0.000E+00 | 0.000E+00 | 3.136E-06 | 0.000E+00 | 0.000E+00 | 0.000E+00 | 0.000E+00 | 0.000E+00 | 0.000E+00 | 0.000E+00 |

| | | | | | | | | | | | | | |
|---|---|---|---|---|---|---|---|---|---|---|---|---|---|
| Unigene301180 | OprN | 0.000E+00 | 0.000E+00 | 0.000E+00 | 4.928E-08 | 3.686E-06 | 0.000E+00 | 0.000E+00 | 0.000E+00 | 0.000E+00 | 0.000E+00 | 2.233E-07 | 0.000E+00 |
| Unigene301379 | tetA(58) | 0.000E+00 | 0.000E+00 | 8.549E-08 | 4.399E-08 | 9.195E-07 | 0.000E+00 | 4.821E-08 | 0.000E+00 | 9.523E-08 | 0.000E+00 | 1.495E-07 | 4.634E-07 |
| Unigene301426 | macB | 0.000E+00 | 4.525E-08 | 0.000E+00 | 0.000E+00 | 4.580E-07 | 1.783E-07 | 0.000E+00 | 1.925E-07 | 0.000E+00 | 0.000E+00 | 0.000E+00 | 7.310E-07 |
| Unigene301432 | tetA(58) | 1.403E-07 | 0.000E+00 | 0.000E+00 | 0.000E+00 | 4.158E-07 | 0.000E+00 | 0.000E+00 | 3.823E-07 | 0.000E+00 | 0.000E+00 | 2.141E-07 | 6.083E-07 |
| Unigene301471 | lin | 0.000E+00 | 0.000E+00 | 0.000E+00 | 0.000E+00 | 2.214E-07 | 0.000E+00 | 0.000E+00 | 0.000E+00 | 0.000E+00 | 0.000E+00 | 0.000E+00 | 0.000E+00 |
| Unigene301493 | mtrA | 0.000E+00 | 0.000E+00 | 0.000E+00 | 0.000E+00 | 7.864E-07 | 0.000E+00 | 0.000E+00 | 4.958E-07 | 0.000E+00 | 0.000E+00 | 5.938E-07 | 0.000E+00 |
| Unigene301574 | adeL | 4.321E-08 | 4.743E-08 | 0.000E+00 | 0.000E+00 | 8.161E-07 | 0.000E+00 | 3.348E-07 | 2.018E-07 | 0.000E+00 | 9.628E-08 | 9.886E-08 | 5.108E-07 |
| Unigene301602 | msbA | 0.000E+00 | 1.747E-06 | 7.497E-07 | 0.000E+00 | 5.187E-06 | 3.212E-07 | 0.000E+00 | 0.000E+00 | 3.479E-07 | 0.000E+00 | 5.098E-07 | 1.254E-07 |
| Unigene301857 | evgS | 2.366E-07 | 1.154E-07 | 3.525E-07 | 2.831E-07 | 8.956E-07 | 2.747E-07 | 1.067E-06 | 8.796E-07 | 8.619E-08 | 4.588E-07 | 6.715E-07 | 2.786E-06 |
| Unigene301861 | tetB(46) | 0.000E+00 | 0.000E+00 | 0.000E+00 | 0.000E+00 | 2.748E-07 | 0.000E+00 | 0.000E+00 | 0.000E+00 | 0.000E+00 | 0.000E+00 | 0.000E+00 | 0.000E+00 |
| Unigene302034 | bacA | 2.671E-05 | 1.050E-05 | 6.871E-06 | 3.932E-06 | 6.072E-07 | 2.216E-07 | 0.000E+00 | 3.987E-07 | 1.269E-06 | 0.000E+00 | 0.000E+00 | 8.075E-08 |
| Unigene302044 | novA | 0.000E+00 | 0.000E+00 | 0.000E+00 | 0.000E+00 | 5.715E-07 | 0.000E+00 | 0.000E+00 | 0.000E+00 | 0.000E+00 | 0.000E+00 | 0.000E+00 | 0.000E+00 |
| Unigene302211 | patA | 0.000E+00 | 2.054E-08 | 1.469E-07 | 2.646E-07 | 3.347E-06 | 0.000E+00 | 0.000E+00 | 0.000E+00 | 0.000E+00 | 0.000E+00 | 0.000E+00 | 0.000E+00 |
| Unigene302270 | MexF | 0.000E+00 | 0.000E+00 | 0.000E+00 | 0.000E+00 | 0.000E+00 | 0.000E+00 | 0.000E+00 | 3.094E-07 | 0.000E+00 | 0.000E+00 | 0.000E+00 | 0.000E+00 |
| Unigene302292 | mdtB | 0.000E+00 | 0.000E+00 | 0.000E+00 | 0.000E+00 | 7.245E-07 | 0.000E+00 | 0.000E+00 | 0.000E+00 | 0.000E+00 | 0.000E+00 | 0.000E+00 | 0.000E+00 |
| Unigene302306 | oleB | 0.000E+00 | 0.000E+00 | 2.338E-07 | 1.203E-07 | 3.970E-07 | 0.000E+00 | 6.152E-07 | 6.488E-07 | 8.679E-08 | 3.538E-07 | 6.359E-07 | 5.163E-07 |
| Unigene302317 | mdtG | 1.312E-07 | 0.000E+00 | 6.953E-07 | 0.000E+00 | 3.265E-06 | 0.000E+00 | 6.100E-07 | 0.000E+00 | 0.000E+00 | 0.000E+00 | 2.402E-07 | 0.000E+00 |
| Unigene302329 | macB | 3.910E-07 | 6.179E-07 | 3.223E-07 | 4.580E-07 | 1.720E-06 | 1.758E-06 | 6.751E-06 | 0.000E+00 | 2.273E-06 | 0.000E+00 | 0.000E+00 | 4.252E-07 |
| Unigene302332 | bcr-1 | 0.000E+00 | 0.000E+00 | 0.000E+00 | 0.000E+00 | 4.248E-07 | 0.000E+00 | 0.000E+00 | 6.375E-07 | 0.000E+00 | 0.000E+00 | 0.000E+00 | 0.000E+00 |
| Unigene302337 | lmrB | 0.000E+00 | 0.000E+00 | 0.000E+00 | 0.000E+00 | 1.323E-06 | 0.000E+00 | 0.000E+00 | 0.000E+00 | 0.000E+00 | 0.000E+00 | 0.000E+00 | 0.000E+00 |
| Unigene302365 | efrA | 0.000E+00 | 0.000E+00 | 0.000E+00 | 0.000E+00 | 7.502E-07 | 0.000E+00 | 0.000E+00 | 0.000E+00 | 0.000E+00 | 0.000E+00 | 0.000E+00 | 0.000E+00 |
| Unigene302376 | optrA | 0.000E+00 | 0.000E+00 | 0.000E+00 | 0.000E+00 | 7.154E-06 | 0.000E+00 | 0.000E+00 | 0.000E+00 | 1.371E-06 | 0.000E+00 | 0.000E+00 | 0.000E+00 |
| Unigene302388 | TolC | 0.000E+00 | 1.909E-07 | 0.000E+00 | 0.000E+00 | 1.449E-06 | 0.000E+00 | 0.000E+00 | 0.000E+00 | 9.741E-07 | 2.422E-07 | 0.000E+00 | 0.000E+00 |
| Unigene302477 | tetA(58) | 1.345E-07 | 0.000E+00 | 0.000E+00 | 0.000E+00 | 8.218E-07 | 0.000E+00 | 4.466E-07 | 0.000E+00 | 7.350E-08 | 4.495E-07 | 1.539E-07 | 0.000E+00 |
| Unigene302491 | QepA2 | 0.000E+00 | 0.000E+00 | 0.000E+00 | 0.000E+00 | 0.000E+00 | 0.000E+00 | 0.000E+00 | 0.000E+00 | 0.000E+00 | 0.000E+00 | 0.000E+00 | 7.033E-07 |
| Unigene302512 | APH(6)-Ic | 0.000E+00 | 0.000E+00 | 0.000E+00 | 0.000E+00 | 5.073E-06 | 0.000E+00 | 0.000E+00 | 0.000E+00 | 0.000E+00 | 0.000E+00 | 2.271E-07 | 0.000E+00 |
| Unigene302518 | smeR | 0.000E+00 | 0.000E+00 | 0.000E+00 | 0.000E+00 | 2.338E-06 | 0.000E+00 | 1.059E-07 | 0.000E+00 | 0.000E+00 | 0.000E+00 | 3.829E-07 | 0.000E+00 |
| Unigene302523 | macB | 0.000E+00 | 0.000E+00 | 0.000E+00 | 0.000E+00 | 3.207E-06 | 0.000E+00 | 0.000E+00 | 0.000E+00 | 0.000E+00 | 0.000E+00 | 3.669E-07 | 1.137E-06 |
| Unigene302575 | msbA | 0.000E+00 | 0.000E+00 | 0.000E+00 | 0.000E+00 | 4.027E-06 | 0.000E+00 | 0.000E+00 | 0.000E+00 | 0.000E+00 | 0.000E+00 | 9.285E-07 | 0.000E+00 |
| Unigene302580 | tetA(58) | 0.000E+00 | 0.000E+00 | 0.000E+00 | 0.000E+00 | 3.866E-06 | 0.000E+00 | 0.000E+00 | 2.101E-06 | 0.000E+00 | 0.000E+00 | 0.000E+00 | 0.000E+00 |
| Unigene302593 | oleC | 0.000E+00 | 0.000E+00 | 0.000E+00 | 0.000E+00 | 3.907E-06 | 0.000E+00 | 0.000E+00 | 0.000E+00 | 0.000E+00 | 0.000E+00 | 3.352E-07 | 0.000E+00 |
| Unigene302618 | efrB | 0.000E+00 | 0.000E+00 | 0.000E+00 | 0.000E+00 | 3.409E-06 | 0.000E+00 | 0.000E+00 | 0.000E+00 | 0.000E+00 | 0.000E+00 | 2.966E-07 | 0.000E+00 |
| Unigene302647 | patB | 0.000E+00 | 0.000E+00 | 0.000E+00 | 0.000E+00 | 3.544E-06 | 0.000E+00 | 0.000E+00 | 0.000E+00 | 0.000E+00 | 0.000E+00 | 1.459E-07 | 0.000E+00 |
| Unigene302651 | cpxA | 0.000E+00 | 2.696E-08 | 0.000E+00 | 0.000E+00 | 3.166E-06 | 0.000E+00 | 0.000E+00 | 0.000E+00 | 0.000E+00 | 0.000E+00 | 0.000E+00 | 0.000E+00 |
| Unigene302652 | baeR | 0.000E+00 | 0.000E+00 | 0.000E+00 | 0.000E+00 | 2.976E-06 | 0.000E+00 | 0.000E+00 | 9.381E-07 | 0.000E+00 | 0.000E+00 | 0.000E+00 | 7.388E-07 |
| Unigene302653 | mdtB | 0.000E+00 | 2.313E-08 | 0.000E+00 | 0.000E+00 | 2.857E-06 | 5.696E-08 | 1.283E-07 | 0.000E+00 | 0.000E+00 | 0.000E+00 | 2.049E-07 | 3.613E-07 |
| Unigene302655 | cpxA | 0.000E+00 | 0.000E+00 | 0.000E+00 | 0.000E+00 | 2.648E-06 | 0.000E+00 | 0.000E+00 | 8.373E-07 | 0.000E+00 | 0.000E+00 | 3.441E-07 | 0.000E+00 |
| Unigene302656 | mtrA | 0.000E+00 | 0.000E+00 | 0.000E+00 | 0.000E+00 | 2.624E-06 | 0.000E+00 | 0.000E+00 | 5.616E-07 | 0.000E+00 | 0.000E+00 | 4.003E-07 | 0.000E+00 |
| Unigene302662 | MuxC | 0.000E+00 | 1.151E-08 | 0.000E+00 | 0.000E+00 | 3.507E-06 | 0.000E+00 | 1.277E-07 | 0.000E+00 | 0.000E+00 | 0.000E+00 | 1.799E-07 | 0.000E+00 |
| Unigene302680 | oleB | 0.000E+00 | 6.298E-08 | 0.000E+00 | 0.000E+00 | 2.678E-06 | 0.000E+00 | 0.000E+00 | 0.000E+00 | 0.000E+00 | 9.589E-08 | 1.313E-07 | 0.000E+00 |
| Unigene302688 | oleC | 0.000E+00 | 0.000E+00 | 0.000E+00 | 0.000E+00 | 2.341E-06 | 0.000E+00 | 0.000E+00 | 1.256E-06 | 0.000E+00 | 0.000E+00 | 0.000E+00 | 0.000E+00 |
| Unigene302689 | oleC | 0.000E+00 | 0.000E+00 | 0.000E+00 | 0.000E+00 | 3.331E-06 | 0.000E+00 | 0.000E+00 | 0.000E+00 | 0.000E+00 | 1.965E-07 | 0.000E+00 | 0.000E+00 |
| Unigene302699 | carA | 0.000E+00 | 0.000E+00 | 0.000E+00 | 0.000E+00 | 2.820E-06 | 0.000E+00 | 0.000E+00 | 0.000E+00 | 0.000E+00 | 0.000E+00 | 6.067E-07 | 8.957E-07 |
| Unigene302700 | tva(A) | 4.287E-08 | 0.000E+00 | 8.413E-08 | 0.000E+00 | 3.762E-06 | 0.000E+00 | 0.000E+00 | 0.000E+00 | 0.000E+00 | 0.000E+00 | 0.000E+00 | 0.000E+00 |
| Unigene302743 | emrB | 0.000E+00 | 0.000E+00 | 0.000E+00 | 1.012E-07 | 7.346E-07 | 0.000E+00 | 7.540E-07 | 8.888E-07 | 0.000E+00 | 3.125E-07 | 5.043E-07 | 7.579E-07 |
| Unigene302771 | Corynebac | 0.000E+00 | 0.000E+00 | 0.000E+00 | 0.000E+00 | 6.907E-07 | 0.000E+00 | 0.000E+00 | 0.000E+00 | 0.000E+00 | 0.000E+00 | 5.470E-07 | 0.000E+00 |
| Unigene302824 | lmrD | 0.000E+00 | 0.000E+00 | 0.000E+00 | 0.000E+00 | 4.566E-07 | 8.078E-08 | 4.135E-08 | 4.361E-07 | 0.000E+00 | 1.249E-07 | 4.274E-07 | 3.533E-07 |
| Unigene302825 | novA | 1.727E-08 | 9.478E-08 | 1.695E-08 | 5.232E-08 | 5.948E-07 | 0.000E+00 | 2.485E-07 | 6.250E-07 | 0.000E+00 | 2.309E-07 | 1.185E-07 | 5.920E-07 |
| Unigene302843 | NmcR | 0.000E+00 | 0.000E+00 | 0.000E+00 | 0.000E+00 | 7.775E-07 | 0.000E+00 | 0.000E+00 | 0.000E+00 | 0.000E+00 | 0.000E+00 | 0.000E+00 | 0.000E+00 |
| Unigene302950 | tetA(58) | 0.000E+00 | 0.000E+00 | 0.000E+00 | 0.000E+00 | 5.087E-07 | 0.000E+00 | 0.000E+00 | 0.000E+00 | 0.000E+00 | 0.000E+00 | 0.000E+00 | 0.000E+00 |
| Unigene302984 | acrD | 4.354E-07 | 1.354E-06 | 8.748E-07 | 1.989E-07 | 5.113E-06 | 0.000E+00 | 8.031E-07 | 0.000E+00 | 0.000E+00 | 0.000E+00 | 3.676E-07 | 0.000E+00 |
| Unigene302990 | acrB | 0.000E+00 | 0.000E+00 | 0.000E+00 | 0.000E+00 | 5.461E-07 | 0.000E+00 | 0.000E+00 | 0.000E+00 | 0.000E+00 | 0.000E+00 | 0.000E+00 | 8.393E-07 |
| Unigene303017 | basS | 0.000E+00 | 0.000E+00 | 0.000E+00 | 0.000E+00 | 1.254E-06 | 0.000E+00 | 8.474E-07 | 0.000E+00 | 0.000E+00 | 0.000E+00 | 0.000E+00 | 0.000E+00 |
| Unigene303089 | vanHO | 0.000E+00 | 0.000E+00 | 0.000E+00 | 0.000E+00 | 2.855E-07 | 0.000E+00 | 3.910E-07 | 0.000E+00 | 0.000E+00 | 0.000E+00 | 0.000E+00 | 3.797E-07 |
| Unigene303144 | TaeA | 2.142E-06 | 5.850E-06 | 8.798E-07 | 3.521E-07 | 2.767E-07 | 0.000E+00 | 0.000E+00 | 0.000E+00 | 5.445E-07 | 0.000E+00 | 1.140E-07 | 0.000E+00 |
| Unigene303271 | vanI | 0.000E+00 | 0.000E+00 | 0.000E+00 | 9.969E-08 | 5.849E-07 | 0.000E+00 | 1.457E-07 | 1.536E-07 | 0.000E+00 | 0.000E+00 | 0.000E+00 | 3.890E-07 |
| Unigene303290 | H-NS | 0.000E+00 | 7.201E-07 | 0.000E+00 | 0.000E+00 | 3.936E-06 | 0.000E+00 | 9.440E-07 | 0.000E+00 | 0.000E+00 | 0.000E+00 | 1.051E-06 | 0.000E+00 |
| Unigene303335 | vanRE | 0.000E+00 | 0.000E+00 | 0.000E+00 | 0.000E+00 | 3.471E-07 | 0.000E+00 | 0.000E+00 | 0.000E+00 | 0.000E+00 | 0.000E+00 | 0.000E+00 | 0.000E+00 |

| | | | | | | | | | | | | | |
|---|---|---|---|---|---|---|---|---|---|---|---|---|---|
| Unigene303385 | patA | 0.000E+00 | 0.000E+00 | 0.000E+00 | 0.000E+00 | 4.698E-07 | 0.000E+00 | 3.120E-07 | 0.000E+00 | 0.000E+00 | 0.000E+00 | 0.000E+00 | 0.000E+00 |
| Unigene303392 | TriC | 0.000E+00 | 0.000E+00 | 0.000E+00 | 0.000E+00 | 3.472E-07 | 0.000E+00 | 1.977E-07 | 5.211E-07 | 0.000E+00 | 0.000E+00 | 0.000E+00 | 7.388E-07 |
| Unigene303483 | Tet(X3) | 0.000E+00 | 1.414E-06 | 0.000E+00 | 0.000E+00 | 5.127E-06 | 0.000E+00 | 1.871E-06 | 0.000E+00 | 0.000E+00 | 0.000E+00 | 0.000E+00 | 0.000E+00 |
| Unigene303567 | baeS | 0.000E+00 | 0.000E+00 | 0.000E+00 | 0.000E+00 | 0.000E+00 | 0.000E+00 | 0.000E+00 | 0.000E+00 | 0.000E+00 | 0.000E+00 | 2.715E-07 | 0.000E+00 |
| Unigene303593 | vanRI | 0.000E+00 | 0.000E+00 | 0.000E+00 | 0.000E+00 | 5.102E-07 | 0.000E+00 | 0.000E+00 | 0.000E+00 | 0.000E+00 | 0.000E+00 | 0.000E+00 | 0.000E+00 |
| Unigene303623 | bcrA | 0.000E+00 | 0.000E+00 | 0.000E+00 | 0.000E+00 | 3.795E-07 | 1.846E-07 | 0.000E+00 | 0.000E+00 | 0.000E+00 | 1.903E-07 | 0.000E+00 | 6.729E-07 |
| Unigene303650 | efrA | 0.000E+00 | 0.000E+00 | 0.000E+00 | 0.000E+00 | 5.574E-07 | 0.000E+00 | 0.000E+00 | 0.000E+00 | 0.000E+00 | 0.000E+00 | 0.000E+00 | 0.000E+00 |
| Unigene303787 | novA | 0.000E+00 | 0.000E+00 | 2.653E-07 | 4.186E-07 | 5.086E-06 | 1.130E-05 | 0.000E+00 | 0.000E+00 | 0.000E+00 | 0.000E+00 | 0.000E+00 | 0.000E+00 |
| Unigene303788 | efrA | 0.000E+00 | 0.000E+00 | 2.522E-07 | 5.006E-07 | 4.835E-06 | 0.000E+00 | 0.000E+00 | 0.000E+00 | 0.000E+00 | 0.000E+00 | 2.100E-08 | 0.000E+00 |
| Unigene303829 | Staphylocc | 3.331E-08 | 2.194E-07 | 0.000E+00 | 0.000E+00 | 7.772E-07 | 0.000E+00 | 2.212E-07 | 3.888E-07 | 1.821E-07 | 2.598E-07 | 1.143E-07 | 3.150E-07 |
| Unigene303880 | tetA(60) | 0.000E+00 | 0.000E+00 | 3.073E-07 | 2.977E-07 | 4.094E-06 | 8.047E-06 | 0.000E+00 | 0.000E+00 | 0.000E+00 | 3.736E-06 | 0.000E+00 | 0.000E+00 |
| Unigene303944 | tetA(46) | 0.000E+00 | 0.000E+00 | 2.338E-07 | 2.005E-07 | 1.279E-06 | 3.863E-07 | 1.670E-06 | 1.344E-06 | 3.472E-07 | 3.096E-07 | 9.538E-07 | 0.000E+00 |
| Unigene303958 | patA | 0.000E+00 | 0.000E+00 | 0.000E+00 | 0.000E+00 | 2.686E-07 | 0.000E+00 | 0.000E+00 | 0.000E+00 | 0.000E+00 | 0.000E+00 | 0.000E+00 | 0.000E+00 |
| Unigene304099 | patA | 0.000E+00 | 0.000E+00 | 0.000E+00 | 0.000E+00 | 1.006E-06 | 0.000E+00 | 0.000E+00 | 0.000E+00 | 5.328E-07 | 1.552E-07 | 0.000E+00 | 0.000E+00 |
| Unigene304100 | tetA(60) | 0.000E+00 | 0.000E+00 | 0.000E+00 | 0.000E+00 | 5.958E-07 | 0.000E+00 | 0.000E+00 | 0.000E+00 | 0.000E+00 | 0.000E+00 | 0.000E+00 | 0.000E+00 |
| Unigene304120 | efrA | 0.000E+00 | 0.000E+00 | 0.000E+00 | 0.000E+00 | 8.014E-07 | 0.000E+00 | 0.000E+00 | 0.000E+00 | 0.000E+00 | 0.000E+00 | 0.000E+00 | 0.000E+00 |
| Unigene304147 | vanSM | 2.997E-08 | 0.000E+00 | 1.029E-07 | 0.000E+00 | 4.162E-07 | 2.430E-07 | 6.966E-07 | 5.598E-07 | 3.276E-08 | 2.003E-07 | 5.486E-07 | 3.366E-07 |
| Unigene304215 | evgS | 0.000E+00 | 0.000E+00 | 0.000E+00 | 0.000E+00 | 7.925E-07 | 0.000E+00 | 0.000E+00 | 0.000E+00 | 6.498E-08 | 0.000E+00 | 0.000E+00 | 0.000E+00 |
| Unigene304256 | sdiA | 8.893E-08 | 0.000E+00 | 0.000E+00 | 0.000E+00 | 7.903E-07 | 0.000E+00 | 8.366E-07 | 6.747E-07 | 0.000E+00 | 0.000E+00 | 0.000E+00 | 7.883E-07 |
| Unigene304271 | bmr | 0.000E+00 | 0.000E+00 | 0.000E+00 | 0.000E+00 | 8.264E-07 | 0.000E+00 | 0.000E+00 | 0.000E+00 | 0.000E+00 | 0.000E+00 | 0.000E+00 | 0.000E+00 |
| Unigene304301 | Acinetobac | 0.000E+00 | 0.000E+00 | 1.960E-07 | 0.000E+00 | 7.211E-07 | 3.778E-07 | 5.526E-07 | 7.577E-07 | 0.000E+00 | 4.728E-07 | 7.425E-07 | 7.082E-07 |
| Unigene304328 | iri | 0.000E+00 | 0.000E+00 | 0.000E+00 | 0.000E+00 | 4.503E-07 | 0.000E+00 | 0.000E+00 | 3.871E-07 | 0.000E+00 | 0.000E+00 | 2.108E-07 | 3.485E-07 |
| Unigene304404 | iri | 0.000E+00 | 0.000E+00 | 0.000E+00 | 0.000E+00 | 5.224E-07 | 5.981E-08 | 0.000E+00 | 2.260E-07 | 0.000E+00 | 0.000E+00 | 0.000E+00 | 0.000E+00 |
| Unigene304432 | novA | 0.000E+00 | 0.000E+00 | 2.041E-07 | 9.548E-08 | 6.303E-07 | 2.249E-07 | 9.209E-07 | 9.271E-07 | 0.000E+00 | 0.000E+00 | 2.812E-07 | 1.073E-06 |
| Unigene304435 | emrY | 0.000E+00 | 0.000E+00 | 0.000E+00 | 5.419E-08 | 1.461E-06 | 0.000E+00 | 0.000E+00 | 0.000E+00 | 0.000E+00 | 0.000E+00 | 0.000E+00 | 0.000E+00 |
| Unigene304466 | cpxA | 0.000E+00 | 0.000E+00 | 0.000E+00 | 0.000E+00 | 6.798E-07 | 0.000E+00 | 0.000E+00 | 0.000E+00 | 0.000E+00 | 2.840E-07 | 0.000E+00 | 0.000E+00 |
| Unigene304492 | macB | 3.190E-07 | 9.231E-07 | 8.253E-07 | 2.929E-07 | 5.929E-06 | 2.822E-07 | 1.412E-06 | 4.740E-07 | 0.000E+00 | 0.000E+00 | 3.649E-07 | 0.000E+00 |
| Unigene304493 | smeS | 2.866E-07 | 1.355E-06 | 6.707E-07 | 3.785E-07 | 5.830E-06 | 3.337E-07 | 9.273E-07 | 2.316E-07 | 1.205E-07 | 1.228E-07 | 5.044E-07 | 0.000E+00 |
| Unigene304535 | smeS | 0.000E+00 | 0.000E+00 | 0.000E+00 | 1.150E-07 | 3.162E-07 | 0.000E+00 | 0.000E+00 | 0.000E+00 | 0.000E+00 | 0.000E+00 | 0.000E+00 | 1.346E-07 |
| Unigene304553 | tetA(58) | 0.000E+00 | 0.000E+00 | 0.000E+00 | 0.000E+00 | 9.578E-07 | 0.000E+00 | 0.000E+00 | 2.415E-07 | 0.000E+00 | 0.000E+00 | 0.000E+00 | 0.000E+00 |
| Unigene304554 | tetB(58) | 0.000E+00 | 0.000E+00 | 0.000E+00 | 2.098E-07 | 1.015E-06 | 0.000E+00 | 0.000E+00 | 0.000E+00 | 0.000E+00 | 0.000E+00 | 2.376E-07 | 0.000E+00 |
| Unigene304561 | mgrA | 0.000E+00 | 0.000E+00 | 0.000E+00 | 2.203E-07 | 9.692E-07 | 0.000E+00 | 5.633E-07 | 8.486E-07 | 0.000E+00 | 0.000E+00 | 0.000E+00 | 0.000E+00 |
| Unigene304668 | efrA | 0.000E+00 | 0.000E+00 | 0.000E+00 | 0.000E+00 | 4.092E-07 | 0.000E+00 | 0.000E+00 | 0.000E+00 | 0.000E+00 | 0.000E+00 | 0.000E+00 | 0.000E+00 |
| Unigene304671 | kdpE | 0.000E+00 | 0.000E+00 | 0.000E+00 | 0.000E+00 | 4.741E-07 | 0.000E+00 | 1.687E-07 | 0.000E+00 | 0.000E+00 | 0.000E+00 | 0.000E+00 | 0.000E+00 |
| Unigene304710 | novA | 0.000E+00 | 0.000E+00 | 0.000E+00 | 5.659E-08 | 3.736E-07 | 0.000E+00 | 0.000E+00 | 0.000E+00 | 1.225E-07 | 0.000E+00 | 3.205E-07 | 0.000E+00 |
| Unigene304797 | NmcR | 0.000E+00 | 0.000E+00 | 0.000E+00 | 0.000E+00 | 3.609E-06 | 0.000E+00 | 0.000E+00 | 0.000E+00 | 0.000E+00 | 0.000E+00 | 3.597E-07 | 0.000E+00 |
| Unigene304840 | msbA | 0.000E+00 | 0.000E+00 | 0.000E+00 | 0.000E+00 | 4.130E-06 | 0.000E+00 | 0.000E+00 | 0.000E+00 | 0.000E+00 | 0.000E+00 | 0.000E+00 | 0.000E+00 |
| Unigene304887 | vanRE | 1.824E-07 | 3.670E-07 | 0.000E+00 | 2.456E-07 | 8.104E-07 | 3.286E-07 | 1.043E-06 | 1.313E-06 | 0.000E+00 | 0.000E+00 | 8.345E-07 | 7.725E-06 |
| Unigene305054 | lmrD | 0.000E+00 | 0.000E+00 | 0.000E+00 | 0.000E+00 | 6.568E-07 | 0.000E+00 | 2.617E-07 | 2.415E-07 | 9.693E-08 | 0.000E+00 | 4.734E-07 | 0.000E+00 |
| Unigene305075 | tlrC | 0.000E+00 | 0.000E+00 | 0.000E+00 | 0.000E+00 | 5.138E-07 | 0.000E+00 | 0.000E+00 | 0.000E+00 | 0.000E+00 | 0.000E+00 | 0.000E+00 | 0.000E+00 |
| Unigene305094 | OXA-59 | 0.000E+00 | 0.000E+00 | 0.000E+00 | 0.000E+00 | 3.516E-07 | 0.000E+00 | 0.000E+00 | 7.388E-08 | 0.000E+00 | 0.000E+00 | 0.000E+00 | 2.244E-07 |
| Unigene305116 | TaeA | 0.000E+00 | 0.000E+00 | 0.000E+00 | 0.000E+00 | 5.373E-07 | 0.000E+00 | 6.690E-08 | 0.000E+00 | 0.000E+00 | 0.000E+00 | 0.000E+00 | 0.000E+00 |
| Unigene305232 | basS | 0.000E+00 | 0.000E+00 | 0.000E+00 | 0.000E+00 | 1.256E-06 | 0.000E+00 | 2.502E-07 | 0.000E+00 | 0.000E+00 | 0.000E+00 | 0.000E+00 | 0.000E+00 |
| Unigene305254 | tetB(60) | 9.119E-08 | 1.001E-07 | 0.000E+00 | 0.000E+00 | 3.039E-07 | 0.000E+00 | 1.009E-07 | 1.597E-07 | 0.000E+00 | 1.016E-07 | 1.043E-07 | 3.234E-07 |
| Unigene305337 | Staphylocc | 0.000E+00 | 5.846E-08 | 0.000E+00 | 1.883E-07 | 7.397E-07 | 0.000E+00 | 2.653E-07 | 9.948E-07 | 2.038E-07 | 1.780E-07 | 3.047E-07 | 8.815E-07 |
| Unigene305401 | optrA | 5.685E-07 | 1.337E-07 | 3.187E-07 | 0.000E+00 | 1.150E-06 | 3.731E-07 | 7.640E-07 | 2.370E-08 | 0.000E+00 | 1.061E-05 | 0.000E+00 | 0.000E+00 |
| Unigene305432 | rosB | 0.000E+00 | 0.000E+00 | 0.000E+00 | 0.000E+00 | 5.822E-07 | 0.000E+00 | 0.000E+00 | 4.078E-07 | 0.000E+00 | 0.000E+00 | 0.000E+00 | 6.194E-07 |
| Unigene305443 | farB | 0.000E+00 | 0.000E+00 | 0.000E+00 | 0.000E+00 | 3.475E-07 | 0.000E+00 | 0.000E+00 | 0.000E+00 | 0.000E+00 | 0.000E+00 | 0.000E+00 | 0.000E+00 |
| Unigene305473 | catA4 | 0.000E+00 | 0.000E+00 | 0.000E+00 | 0.000E+00 | 7.581E-07 | 0.000E+00 | 0.000E+00 | 0.000E+00 | 0.000E+00 | 0.000E+00 | 0.000E+00 | 0.000E+00 |
| Unigene305506 | adeS | 0.000E+00 | 5.997E-07 | 2.062E-07 | 0.000E+00 | 1.307E-06 | 0.000E+00 | 0.000E+00 | 4.906E-08 | 0.000E+00 | 0.000E+00 | 2.885E-07 | 4.968E-08 |
| Unigene305563 | arlR | 0.000E+00 | 0.000E+00 | 0.000E+00 | 0.000E+00 | 0.000E+00 | 0.000E+00 | 0.000E+00 | 6.759E-07 | 0.000E+00 | 0.000E+00 | 0.000E+00 | 0.000E+00 |
| Unigene305597 | MexK | 5.595E-07 | 1.984E-06 | 8.587E-07 | 3.187E-07 | 7.220E-06 | 2.482E-07 | 0.000E+00 | 2.679E-07 | 0.000E+00 | 1.918E-07 | 5.087E-07 | 0.000E+00 |
| Unigene305667 | tetA(58) | 0.000E+00 | 0.000E+00 | 1.514E-07 | 0.000E+00 | 1.071E-06 | 0.000E+00 | 0.000E+00 | 0.000E+00 | 3.794E-07 | 0.000E+00 | 0.000E+00 | 0.000E+00 |
| Unigene305741 | carA | 0.000E+00 | 0.000E+00 | 0.000E+00 | 0.000E+00 | 4.163E-07 | 1.736E-07 | 0.000E+00 | 0.000E+00 | 8.777E-08 | 0.000E+00 | 0.000E+00 | 3.797E-07 |
| Unigene305762 | patA | 0.000E+00 | 0.000E+00 | 0.000E+00 | 0.000E+00 | 0.000E+00 | 0.000E+00 | 0.000E+00 | 0.000E+00 | 0.000E+00 | 0.000E+00 | 0.000E+00 | 2.864E-06 |
| Unigene305813 | YojI | 0.000E+00 | 0.000E+00 | 1.173E-06 | 0.000E+00 | 1.377E-06 | 6.702E-07 | 0.000E+00 | 0.000E+00 | 0.000E+00 | 0.000E+00 | 0.000E+00 | 2.512E-06 |
| Unigene305814 | patA | 0.000E+00 | 0.000E+00 | 0.000E+00 | 0.000E+00 | 7.440E-07 | 0.000E+00 | 0.000E+00 | 0.000E+00 | 0.000E+00 | 0.000E+00 | 0.000E+00 | 0.000E+00 |
| Unigene305839 | cpxA | 0.000E+00 | 0.000E+00 | 0.000E+00 | 0.000E+00 | 3.912E-07 | 0.000E+00 | 0.000E+00 | 0.000E+00 | 0.000E+00 | 0.000E+00 | 0.000E+00 | 0.000E+00 |

| | | | | | | | | | | | | | |
|---|---|---|---|---|---|---|---|---|---|---|---|---|---|
| Unigene305882 | Acinetobac | 0.000E+00 | 4.842E-07 | 0.000E+00 | 0.000E+00 | 1.171E-06 | 0.000E+00 | 0.000E+00 | 0.000E+00 | 4.822E-07 | 0.000E+00 | 0.000E+00 | 7.822E-07 |
| Unigene305984 | carA | 0.000E+00 | 0.000E+00 | 0.000E+00 | 0.000E+00 | 6.831E-07 | 0.000E+00 | 0.000E+00 | 0.000E+00 | 0.000E+00 | 0.000E+00 | 0.000E+00 | 0.000E+00 |
| Unigene306042 | acrB | 9.129E-07 | 2.522E-06 | 1.399E-06 | 5.386E-07 | 8.683E-06 | 4.657E-07 | 2.123E-06 | 5.388E-07 | 0.000E+00 | 1.485E-07 | 6.101E-07 | 0.000E+00 |
| Unigene306057 | dfrA3 | 0.000E+00 | 0.000E+00 | 1.760E-07 | 0.000E+00 | 0.000E+00 | 0.000E+00 | 0.000E+00 | 6.978E-07 | 0.000E+00 | 0.000E+00 | 6.154E-07 | 0.000E+00 |
| Unigene306096 | blt | 8.406E-08 | 0.000E+00 | 1.375E-07 | 1.415E-07 | 3.300E-06 | 9.905E-06 | 5.427E-06 | 7.850E-07 | 2.052E-06 | 8.147E-06 | 1.276E-05 | 3.975E-07 |
| Unigene306133 | tva(A) | 0.000E+00 | 0.000E+00 | 0.000E+00 | 1.509E-07 | 1.204E-06 | 0.000E+00 | 8.270E-07 | 1.221E-06 | 0.000E+00 | 0.000E+00 | 0.000E+00 | 4.328E-06 |
| Unigene306189 | basS | 1.663E-07 | 2.346E-07 | 3.962E-07 | 2.638E-07 | 9.764E-07 | 4.879E-07 | 1.446E-06 | 1.109E-06 | 1.817E-07 | 3.969E-07 | 1.114E-06 | 2.976E-06 |
| Unigene306190 | vanRO | 2.935E-07 | 3.221E-07 | 4.319E-07 | 2.469E-07 | 1.630E-06 | 0.000E+00 | 1.840E-06 | 9.134E-07 | 2.673E-07 | 8.717E-07 | 1.511E-06 | 2.891E-06 |
| Unigene306284 | macB | 0.000E+00 | 0.000E+00 | 0.000E+00 | 0.000E+00 | 3.178E-06 | 0.000E+00 | 0.000E+00 | 0.000E+00 | 7.867E-08 | 2.205E-07 | 3.499E-07 | 0.000E+00 |
| Unigene306292 | PEDO-1 | 0.000E+00 | 0.000E+00 | 0.000E+00 | 2.570E-07 | 3.231E-06 | 0.000E+00 | 0.000E+00 | 0.000E+00 | 0.000E+00 | 0.000E+00 | 4.574E-07 | 0.000E+00 |
| Unigene306315 | msbA | 0.000E+00 | 0.000E+00 | 0.000E+00 | 0.000E+00 | 3.016E-06 | 0.000E+00 | 0.000E+00 | 0.000E+00 | 0.000E+00 | 0.000E+00 | 2.969E-07 | 0.000E+00 |
| Unigene306316 | lmrD | 0.000E+00 | 0.000E+00 | 0.000E+00 | 0.000E+00 | 2.911E-06 | 9.999E-08 | 0.000E+00 | 0.000E+00 | 0.000E+00 | 0.000E+00 | 0.000E+00 | 0.000E+00 |
| Unigene306348 | macB | 0.000E+00 | 0.000E+00 | 0.000E+00 | 0.000E+00 | 2.262E-06 | 0.000E+00 | 0.000E+00 | 1.396E-06 | 0.000E+00 | 0.000E+00 | 0.000E+00 | 0.000E+00 |
| Unigene306349 | lmrC | 0.000E+00 | 0.000E+00 | 0.000E+00 | 0.000E+00 | 3.319E-06 | 0.000E+00 | 0.000E+00 | 0.000E+00 | 0.000E+00 | 0.000E+00 | 4.776E-07 | 0.000E+00 |
| Unigene306352 | lmrC | 0.000E+00 | 0.000E+00 | 0.000E+00 | 0.000E+00 | 3.128E-06 | 0.000E+00 | 0.000E+00 | 0.000E+00 | 0.000E+00 | 0.000E+00 | 2.301E-07 | 0.000E+00 |
| Unigene306363 | TaeA | 0.000E+00 | 0.000E+00 | 0.000E+00 | 0.000E+00 | 3.985E-06 | 0.000E+00 | 0.000E+00 | 0.000E+00 | 0.000E+00 | 0.000E+00 | 7.074E-07 | 0.000E+00 |
| Unigene306366 | optrA | 0.000E+00 | 0.000E+00 | 0.000E+00 | 0.000E+00 | 3.292E-06 | 0.000E+00 | 0.000E+00 | 0.000E+00 | 0.000E+00 | 0.000E+00 | 6.143E-07 | 1.034E-06 |
| Unigene306380 | efrA | 0.000E+00 | 0.000E+00 | 0.000E+00 | 0.000E+00 | 3.169E-06 | 0.000E+00 | 0.000E+00 | 0.000E+00 | 0.000E+00 | 0.000E+00 | 0.000E+00 | 0.000E+00 |
| Unigene306392 | patA | 0.000E+00 | 0.000E+00 | 0.000E+00 | 0.000E+00 | 2.511E-06 | 0.000E+00 | 0.000E+00 | 5.277E-08 | 0.000E+00 | 0.000E+00 | 1.551E-07 | 0.000E+00 |
| Unigene306393 | YojI | 0.000E+00 | 0.000E+00 | 0.000E+00 | 0.000E+00 | 2.656E-06 | 0.000E+00 | 0.000E+00 | 0.000E+00 | 0.000E+00 | 0.000E+00 | 1.013E-07 | 0.000E+00 |
| Unigene306401 | lmrC | 0.000E+00 | 0.000E+00 | 0.000E+00 | 0.000E+00 | 2.632E-06 | 0.000E+00 | 0.000E+00 | 0.000E+00 | 0.000E+00 | 0.000E+00 | 2.666E-07 | 0.000E+00 |
| Unigene306426 | Corynebac | 0.000E+00 | 0.000E+00 | 0.000E+00 | 0.000E+00 | 2.396E-06 | 0.000E+00 | 0.000E+00 | 1.812E-06 | 0.000E+00 | 0.000E+00 | 4.193E-07 | 0.000E+00 |
| Unigene306432 | tva(A) | 0.000E+00 | 0.000E+00 | 0.000E+00 | 0.000E+00 | 3.586E-06 | 0.000E+00 | 0.000E+00 | 0.000E+00 | 0.000E+00 | 0.000E+00 | 0.000E+00 | 0.000E+00 |
| Unigene306444 | efrA | 0.000E+00 | 0.000E+00 | 0.000E+00 | 0.000E+00 | 3.510E-06 | 0.000E+00 | 0.000E+00 | 0.000E+00 | 0.000E+00 | 0.000E+00 | 3.750E-07 | 0.000E+00 |
| Unigene306457 | vmlR | 0.000E+00 | 0.000E+00 | 0.000E+00 | 0.000E+00 | 2.662E-06 | 0.000E+00 | 0.000E+00 | 0.000E+00 | 0.000E+00 | 0.000E+00 | 0.000E+00 | 0.000E+00 |
| Unigene306474 | tetB(60) | 0.000E+00 | 0.000E+00 | 0.000E+00 | 0.000E+00 | 2.052E-06 | 0.000E+00 | 0.000E+00 | 0.000E+00 | 0.000E+00 | 0.000E+00 | 0.000E+00 | 0.000E+00 |
| Unigene306480 | bcrA | 0.000E+00 | 0.000E+00 | 0.000E+00 | 0.000E+00 | 3.130E-06 | 0.000E+00 | 0.000E+00 | 0.000E+00 | 0.000E+00 | 0.000E+00 | 1.343E-07 | 0.000E+00 |
| Unigene306494 | optrA | 0.000E+00 | 0.000E+00 | 0.000E+00 | 0.000E+00 | 2.497E-06 | 0.000E+00 | 0.000E+00 | 0.000E+00 | 0.000E+00 | 0.000E+00 | 1.714E-07 | 0.000E+00 |
| Unigene306503 | emrY | 0.000E+00 | 0.000E+00 | 0.000E+00 | 0.000E+00 | 2.211E-06 | 0.000E+00 | 0.000E+00 | 0.000E+00 | 0.000E+00 | 0.000E+00 | 1.588E-07 | 0.000E+00 |
| Unigene306513 | patA | 0.000E+00 | 0.000E+00 | 0.000E+00 | 0.000E+00 | 2.358E-06 | 0.000E+00 | 0.000E+00 | 0.000E+00 | 0.000E+00 | 0.000E+00 | 1.349E-07 | 0.000E+00 |
| Unigene306518 | Acinetobac | 0.000E+00 | 0.000E+00 | 0.000E+00 | 0.000E+00 | 3.126E-06 | 0.000E+00 | 0.000E+00 | 0.000E+00 | 0.000E+00 | 0.000E+00 | 4.235E-07 | 1.196E-06 |
| Unigene306523 | bcrA | 0.000E+00 | 0.000E+00 | 0.000E+00 | 0.000E+00 | 2.842E-06 | 0.000E+00 | 0.000E+00 | 0.000E+00 | 0.000E+00 | 0.000E+00 | 2.418E-07 | 0.000E+00 |
| Unigene306530 | efrA | 0.000E+00 | 0.000E+00 | 0.000E+00 | 0.000E+00 | 2.634E-06 | 0.000E+00 | 0.000E+00 | 0.000E+00 | 0.000E+00 | 0.000E+00 | 4.868E-07 | 7.545E-07 |
| Unigene306538 | msrA | 0.000E+00 | 0.000E+00 | 0.000E+00 | 0.000E+00 | 2.162E-06 | 0.000E+00 | 0.000E+00 | 0.000E+00 | 0.000E+00 | 0.000E+00 | 2.182E-07 | 0.000E+00 |
| Unigene306552 | patA | 0.000E+00 | 0.000E+00 | 0.000E+00 | 0.000E+00 | 2.753E-06 | 0.000E+00 | 0.000E+00 | 0.000E+00 | 0.000E+00 | 0.000E+00 | 4.149E-07 | 0.000E+00 |
| Unigene306575 | patA | 0.000E+00 | 0.000E+00 | 0.000E+00 | 0.000E+00 | 2.686E-06 | 0.000E+00 | 0.000E+00 | 0.000E+00 | 0.000E+00 | 0.000E+00 | 0.000E+00 | 0.000E+00 |
| Unigene306578 | macB | 0.000E+00 | 0.000E+00 | 0.000E+00 | 0.000E+00 | 2.930E-06 | 3.168E-07 | 0.000E+00 | 0.000E+00 | 0.000E+00 | 0.000E+00 | 4.789E-07 | 2.277E-06 |
| Unigene306607 | bcrA | 0.000E+00 | 0.000E+00 | 0.000E+00 | 0.000E+00 | 2.967E-06 | 0.000E+00 | 0.000E+00 | 0.000E+00 | 0.000E+00 | 0.000E+00 | 5.989E-07 | 0.000E+00 |
| Unigene306612 | macB | 0.000E+00 | 0.000E+00 | 0.000E+00 | 0.000E+00 | 3.245E-06 | 0.000E+00 | 0.000E+00 | 0.000E+00 | 0.000E+00 | 0.000E+00 | 3.517E-07 | 0.000E+00 |
| Unigene306613 | msbA | 0.000E+00 | 0.000E+00 | 0.000E+00 | 0.000E+00 | 4.110E-06 | 0.000E+00 | 0.000E+00 | 0.000E+00 | 0.000E+00 | 0.000E+00 | 8.206E-07 | 0.000E+00 |
| Unigene306614 | MexH | 0.000E+00 | 0.000E+00 | 0.000E+00 | 0.000E+00 | 2.915E-06 | 0.000E+00 | 0.000E+00 | 0.000E+00 | 0.000E+00 | 0.000E+00 | 6.744E-08 | 2.439E-07 |
| Unigene306615 | mdtC | 0.000E+00 | 0.000E+00 | 0.000E+00 | 0.000E+00 | 3.092E-06 | 0.000E+00 | 4.563E-08 | 1.564E-07 | 2.253E-08 | 2.296E-08 | 1.768E-07 | 1.340E-07 |
| Unigene306620 | oleB | 0.000E+00 | 0.000E+00 | 0.000E+00 | 0.000E+00 | 4.085E-06 | 0.000E+00 | 0.000E+00 | 0.000E+00 | 0.000E+00 | 0.000E+00 | 6.198E-07 | 1.327E-06 |
| Unigene306621 | macB | 0.000E+00 | 0.000E+00 | 0.000E+00 | 0.000E+00 | 2.672E-06 | 0.000E+00 | 0.000E+00 | 0.000E+00 | 0.000E+00 | 0.000E+00 | 0.000E+00 | 0.000E+00 |
| Unigene306649 | NmcR | 0.000E+00 | 0.000E+00 | 0.000E+00 | 0.000E+00 | 3.985E-06 | 0.000E+00 | 0.000E+00 | 0.000E+00 | 0.000E+00 | 0.000E+00 | 1.727E-07 | 0.000E+00 |
| Unigene306752 | Escherichia | 7.592E-07 | 1.437E-06 | 1.336E-06 | 6.609E-07 | 6.806E-06 | 3.679E-07 | 0.000E+00 | 5.195E-07 | 0.000E+00 | 0.000E+00 | 0.000E+00 | 0.000E+00 |
| Unigene306812 | adeL | 0.000E+00 | 0.000E+00 | 0.000E+00 | 0.000E+00 | 2.181E-06 | 0.000E+00 | 0.000E+00 | 0.000E+00 | 8.344E-07 | 0.000E+00 | 0.000E+00 | 8.594E-08 |
| Unigene306819 | mtrA | 0.000E+00 | 0.000E+00 | 0.000E+00 | 0.000E+00 | 3.881E-07 | 0.000E+00 | 0.000E+00 | 0.000E+00 | 0.000E+00 | 0.000E+00 | 0.000E+00 | 0.000E+00 |
| Unigene306849 | oleB | 0.000E+00 | 0.000E+00 | 0.000E+00 | 1.656E-07 | 5.465E-07 | 0.000E+00 | 9.981E-07 | 8.852E-07 | 8.960E-08 | 2.055E-07 | 6.799E-07 | 7.510E-07 |
| Unigene306858 | msbA | 0.000E+00 | 0.000E+00 | 0.000E+00 | 0.000E+00 | 3.802E-07 | 0.000E+00 | 0.000E+00 | 0.000E+00 | 0.000E+00 | 0.000E+00 | 0.000E+00 | 0.000E+00 |
| Unigene306922 | srmB | 0.000E+00 | 0.000E+00 | 0.000E+00 | 0.000E+00 | 8.571E-07 | 0.000E+00 | 0.000E+00 | 1.706E-06 | 0.000E+00 | 0.000E+00 | 0.000E+00 | 1.200E-06 |
| Unigene306982 | MexB | 8.245E-08 | 0.000E+00 | 0.000E+00 | 0.000E+00 | 8.244E-07 | 0.000E+00 | 0.000E+00 | 1.444E-07 | 0.000E+00 | 0.000E+00 | 9.432E-08 | 3.898E-07 |
| Unigene307011 | macB | 0.000E+00 | 0.000E+00 | 0.000E+00 | 0.000E+00 | 1.103E-06 | 0.000E+00 | 0.000E+00 | 0.000E+00 | 0.000E+00 | 0.000E+00 | 0.000E+00 | 0.000E+00 |
| Unigene307026 | vanHB | 0.000E+00 | 0.000E+00 | 0.000E+00 | 0.000E+00 | 6.936E-07 | 0.000E+00 | 0.000E+00 | 0.000E+00 | 0.000E+00 | 0.000E+00 | 0.000E+00 | 0.000E+00 |
| Unigene307198 | Corynebac | 1.697E-07 | 5.961E-07 | 6.661E-07 | 0.000E+00 | 7.580E-06 | 3.669E-07 | 1.127E-06 | 2.377E-07 | 0.000E+00 | 0.000E+00 | 4.659E-07 | 0.000E+00 |
| Unigene307241 | adeS | 0.000E+00 | 0.000E+00 | 0.000E+00 | 0.000E+00 | 1.051E-06 | 0.000E+00 | 0.000E+00 | 0.000E+00 | 0.000E+00 | 0.000E+00 | 0.000E+00 | 0.000E+00 |
| Unigene307416 | Staphylocc | 0.000E+00 | 0.000E+00 | 0.000E+00 | 9.923E-08 | 4.913E-07 | 0.000E+00 | 0.000E+00 | 2.294E-07 | 1.611E-07 | 0.000E+00 | 0.000E+00 | 0.000E+00 |
| Unigene307483 | salA | 0.000E+00 | 0.000E+00 | 0.000E+00 | 0.000E+00 | 8.487E-07 | 0.000E+00 | 0.000E+00 | 2.802E-07 | 0.000E+00 | 0.000E+00 | 3.995E-07 | 0.000E+00 |

| | | | | | | | | | | | | | |
|---|---|---|---|---|---|---|---|---|---|---|---|---|---|
| Unigene307515 | rpoB2 | 1.767E-06 | 1.387E-06 | 1.760E-06 | 2.516E-06 | 1.991E-05 | 7.592E-06 | 1.397E-05 | 1.671E-05 | 1.129E-05 | 1.720E-05 | 1.888E-05 | 7.393E-06 |
| Unigene307537 | lsaC | 0.000E+00 | 0.000E+00 | 0.000E+00 | 0.000E+00 | 5.560E-07 | 0.000E+00 | 0.000E+00 | 0.000E+00 | 0.000E+00 | 0.000E+00 | 0.000E+00 | 0.000E+00 |
| Unigene307548 | sdiA | 4.465E-08 | 4.900E-07 | 0.000E+00 | 0.000E+00 | 1.141E-06 | 0.000E+00 | 0.000E+00 | 2.085E-07 | 0.000E+00 | 0.000E+00 | 0.000E+00 | 0.000E+00 |
| Unigene307557 | farA | 0.000E+00 | 1.273E-07 | 5.123E-07 | 1.172E-07 | 9.022E-07 | 3.449E-07 | 1.123E-06 | 1.049E-06 | 0.000E+00 | 9.046E-07 | 8.958E-07 | 3.325E-06 |
| Unigene307575 | macB | 0.000E+00 | 1.373E-07 | 0.000E+00 | 2.948E-07 | 4.633E-07 | 2.705E-07 | 0.000E+00 | 4.381E-07 | 3.647E-07 | 0.000E+00 | 8.110E-07 | 8.874E-07 |
| Unigene307581 | Corynebac | 4.719E-08 | 0.000E+00 | 0.000E+00 | 0.000E+00 | 7.340E-07 | 0.000E+00 | 5.745E-07 | 6.060E-07 | 0.000E+00 | 0.000E+00 | 5.398E-07 | 5.578E-07 |
| Unigene307614 | adeL | 0.000E+00 | 0.000E+00 | 2.514E-07 | 0.000E+00 | 4.269E-07 | 0.000E+00 | 0.000E+00 | 0.000E+00 | 0.000E+00 | 0.000E+00 | 0.000E+00 | 3.785E-08 |
| Unigene307677 | Staphyloco | 0.000E+00 | 0.000E+00 | 0.000E+00 | 0.000E+00 | 2.952E-06 | 0.000E+00 | 0.000E+00 | 1.634E-06 | 0.000E+00 | 3.316E-07 | 4.918E-07 | 1.082E-06 |
| Unigene307719 | tetA(58) | 0.000E+00 | 0.000E+00 | 0.000E+00 | 8.472E-08 | 3.262E-06 | 0.000E+00 | 4.643E-07 | 0.000E+00 | 0.000E+00 | 0.000E+00 | 9.837E-07 | 0.000E+00 |
| Unigene307733 | macB | 0.000E+00 | 0.000E+00 | 0.000E+00 | 0.000E+00 | 4.263E-06 | 4.509E-07 | 0.000E+00 | 0.000E+00 | 0.000E+00 | 0.000E+00 | 1.479E-06 | 0.000E+00 |
| Unigene307780 | tetB(P) | 0.000E+00 | 0.000E+00 | 0.000E+00 | 0.000E+00 | 8.656E-07 | 0.000E+00 | 0.000E+00 | 0.000E+00 | 0.000E+00 | 0.000E+00 | 0.000E+00 | 1.140E-06 |
| Unigene307832 | vanSA | 0.000E+00 | 3.615E-07 | 0.000E+00 | 0.000E+00 | 5.855E-07 | 0.000E+00 | 0.000E+00 | 2.717E-06 | 0.000E+00 | 1.321E-06 | 1.658E-06 | 2.751E-06 |
| Unigene307836 | lmrD | 0.000E+00 | 2.590E-07 | 0.000E+00 | 0.000E+00 | 7.864E-07 | 4.592E-07 | 1.149E-06 | 3.305E-07 | 0.000E+00 | 0.000E+00 | 9.717E-07 | 2.566E-06 |
| Unigene307990 | novA | 0.000E+00 | 0.000E+00 | 0.000E+00 | 0.000E+00 | 3.306E-06 | 0.000E+00 | 0.000E+00 | 0.000E+00 | 0.000E+00 | 0.000E+00 | 4.353E-07 | 1.043E-06 |
| Unigene307994 | novA | 0.000E+00 | 0.000E+00 | 0.000E+00 | 0.000E+00 | 3.253E-06 | 0.000E+00 | 0.000E+00 | 0.000E+00 | 0.000E+00 | 0.000E+00 | 0.000E+00 | 0.000E+00 |
| Unigene308073 | msbA | 0.000E+00 | 0.000E+00 | 0.000E+00 | 0.000E+00 | 6.557E-07 | 0.000E+00 | 0.000E+00 | 0.000E+00 | 1.985E-07 | 0.000E+00 | 0.000E+00 | 0.000E+00 |
| Unigene308123 | efrA | 0.000E+00 | 6.410E-08 | 8.596E-08 | 0.000E+00 | 2.076E-06 | 0.000E+00 | 1.777E-07 | 0.000E+00 | 6.064E-07 | 0.000E+00 | 5.010E-08 | 0.000E+00 |
| Unigene308125 | macA | 2.788E-08 | 0.000E+00 | 2.735E-08 | 0.000E+00 | 1.765E-06 | 0.000E+00 | 3.085E-08 | 0.000E+00 | 4.570E-07 | 0.000E+00 | 6.377E-08 | 1.318E-07 |
| Unigene308126 | macB | 1.330E-07 | 7.301E-08 | 1.469E-07 | 5.038E-08 | 2.180E-06 | 0.000E+00 | 1.104E-07 | 0.000E+00 | 6.907E-07 | 1.482E-07 | 0.000E+00 | 7.863E-08 |
| Unigene308353 | tetB(P) | 4.031E-06 | 1.930E-05 | 3.564E-06 | 8.739E-07 | 9.993E-07 | 3.693E-07 | 1.512E-07 | 3.057E-07 | 1.182E-06 | 5.327E-07 | 3.647E-07 | 0.000E+00 |
| Unigene308483 | macB | 9.080E-08 | 0.000E+00 | 0.000E+00 | 5.502E-07 | 9.079E-07 | 0.000E+00 | 1.005E-07 | 0.000E+00 | 9.925E-08 | 0.000E+00 | 2.077E-07 | 2.147E-07 |
| Unigene308499 | MexJ | 0.000E+00 | 0.000E+00 | 0.000E+00 | 0.000E+00 | 4.981E-07 | 0.000E+00 | 0.000E+00 | 0.000E+00 | 0.000E+00 | 0.000E+00 | 0.000E+00 | 0.000E+00 |
| Unigene308515 | MuxB | 0.000E+00 | 0.000E+00 | 0.000E+00 | 0.000E+00 | 2.846E-07 | 0.000E+00 | 0.000E+00 | 1.495E-07 | 0.000E+00 | 0.000E+00 | 0.000E+00 | 0.000E+00 |
| Unigene308579 | kdpE | 0.000E+00 | 0.000E+00 | 0.000E+00 | 0.000E+00 | 1.449E-06 | 0.000E+00 | 0.000E+00 | 0.000E+00 | 0.000E+00 | 0.000E+00 | 0.000E+00 | 0.000E+00 |
| Unigene308774 | patA | 0.000E+00 | 0.000E+00 | 1.268E-07 | 9.789E-08 | 6.821E-07 | 0.000E+00 | 1.180E-06 | 7.921E-07 | 0.000E+00 | 1.800E-07 | 4.066E-07 | 1.413E-06 |
| Unigene308807 | adeS | 0.000E+00 | 0.000E+00 | 0.000E+00 | 0.000E+00 | 5.263E-07 | 0.000E+00 | 0.000E+00 | 0.000E+00 | 0.000E+00 | 0.000E+00 | 0.000E+00 | 0.000E+00 |
| Unigene308822 | Pseudomo | 0.000E+00 | 0.000E+00 | 0.000E+00 | 0.000E+00 | 1.659E-06 | 0.000E+00 | 3.889E-07 | 0.000E+00 | 0.000E+00 | 0.000E+00 | 0.000E+00 | 0.000E+00 |
| Unigene308867 | Clostridiun | 0.000E+00 | 0.000E+00 | 0.000E+00 | 0.000E+00 | 6.342E-07 | 0.000E+00 | 0.000E+00 | 0.000E+00 | 0.000E+00 | 0.000E+00 | 0.000E+00 | 0.000E+00 |
| Unigene308939 | efrA | 0.000E+00 | 0.000E+00 | 0.000E+00 | 0.000E+00 | 4.872E-07 | 0.000E+00 | 0.000E+00 | 0.000E+00 | 0.000E+00 | 0.000E+00 | 0.000E+00 | 0.000E+00 |
| Unigene308988 | oleC | 0.000E+00 | 6.653E-07 | 0.000E+00 | 0.000E+00 | 2.790E-06 | 0.000E+00 | 0.000E+00 | 0.000E+00 | 0.000E+00 | 0.000E+00 | 0.000E+00 | 0.000E+00 |
| Unigene309093 | emrY | 0.000E+00 | 0.000E+00 | 4.354E-07 | 0.000E+00 | 1.356E-06 | 0.000E+00 | 0.000E+00 | 0.000E+00 | 0.000E+00 | 0.000E+00 | 0.000E+00 | 0.000E+00 |
| Unigene309123 | bcrA | 0.000E+00 | 0.000E+00 | 1.009E-07 | 6.921E-08 | 4.568E-06 | 1.334E-06 | 1.214E-06 | 0.000E+00 | 3.858E-06 | 4.581E-06 | 0.000E+00 | 0.000E+00 |
| Unigene309128 | tetA(46) | 1.501E-08 | 3.294E-08 | 2.798E-07 | 2.576E-07 | 5.202E-06 | 0.000E+00 | 0.000E+00 | 0.000E+00 | 0.000E+00 | 0.000E+00 | 0.000E+00 | 0.000E+00 |
| Unigene309144 | YojI | 1.064E-06 | 0.000E+00 | 2.611E-07 | 3.881E-07 | 4.302E-06 | 9.843E-06 | 1.669E-06 | 9.247E-06 | 5.105E-06 | 4.083E-06 | 0.000E+00 | 6.289E-07 |
| Unigene309174 | bcrA | 0.000E+00 | 0.000E+00 | 0.000E+00 | 0.000E+00 | 6.262E-07 | 0.000E+00 | 3.970E-07 | 4.187E-07 | 3.360E-07 | 3.425E-07 | 4.103E-07 | 5.451E-07 |
| Unigene309207 | vanHF | 0.000E+00 | 1.402E-07 | 3.133E-07 | 2.902E-07 | 1.242E-06 | 0.000E+00 | 1.520E-06 | 1.193E-06 | 0.000E+00 | 0.000E+00 | 1.096E-06 | 3.585E-06 |
| Unigene309212 | tetA(46) | 0.000E+00 | 0.000E+00 | 0.000E+00 | 0.000E+00 | 1.832E-07 | 0.000E+00 | 0.000E+00 | 0.000E+00 | 0.000E+00 | 0.000E+00 | 0.000E+00 | 0.000E+00 |
| Unigene309224 | arlR | 0.000E+00 | 0.000E+00 | 8.718E-07 | 0.000E+00 | 1.755E-06 | 0.000E+00 | 1.366E-06 | 0.000E+00 | 0.000E+00 | 8.248E-07 | 0.000E+00 | 4.142E-06 |
| Unigene309225 | Acinetoba | 4.205E-07 | 0.000E+00 | 0.000E+00 | 0.000E+00 | 9.618E-07 | 3.744E-07 | 0.000E+00 | 1.039E-06 | 0.000E+00 | 0.000E+00 | 6.791E-07 | 3.158E-06 |
| Unigene309287 | msrE | 0.000E+00 | 0.000E+00 | 0.000E+00 | 0.000E+00 | 4.870E-07 | 8.616E-08 | 8.821E-08 | 5.117E-07 | 0.000E+00 | 0.000E+00 | 0.000E+00 | 5.182E-07 |
| Unigene309357 | vanSC | 0.000E+00 | 0.000E+00 | 0.000E+00 | 0.000E+00 | 3.026E-07 | 0.000E+00 | 0.000E+00 | 2.650E-07 | 0.000E+00 | 0.000E+00 | 0.000E+00 | 1.073E-07 |
| Unigene309481 | arlR | 0.000E+00 | 0.000E+00 | 0.000E+00 | 2.872E-07 | 9.479E-07 | 0.000E+00 | 0.000E+00 | 1.051E-06 | 3.109E-07 | 0.000E+00 | 1.681E-06 | 2.745E-06 |
| Unigene309482 | smeS | 2.314E-07 | 0.000E+00 | 0.000E+00 | 1.869E-07 | 8.998E-07 | 0.000E+00 | 1.383E-06 | 7.563E-07 | 1.012E-07 | 0.000E+00 | 7.941E-07 | 2.216E-06 |
| Unigene309492 | lmrC | 0.000E+00 | 0.000E+00 | 0.000E+00 | 0.000E+00 | 3.162E-07 | 0.000E+00 | 0.000E+00 | 0.000E+00 | 0.000E+00 | 0.000E+00 | 0.000E+00 | 0.000E+00 |
| Unigene309831 | efpA | 9.276E-08 | 0.000E+00 | 1.365E-07 | 1.171E-07 | 3.684E-06 | 6.192E-06 | 1.566E-06 | 2.707E-08 | 3.422E-06 | 0.000E+00 | 5.305E-08 | 0.000E+00 |
| Unigene309844 | patB | 0.000E+00 | 0.000E+00 | 0.000E+00 | 0.000E+00 | 2.381E-06 | 0.000E+00 | 2.419E-06 | 1.962E-06 | 0.000E+00 | 0.000E+00 | 0.000E+00 | 1.292E-06 |
| Unigene309916 | MuxB | 0.000E+00 | 7.619E-08 | 0.000E+00 | 0.000E+00 | 3.856E-07 | 2.627E-07 | 1.152E-07 | 0.000E+00 | 0.000E+00 | 0.000E+00 | 5.559E-07 | 2.462E-07 |
| Unigene309924 | MuxB | 0.000E+00 | 0.000E+00 | 0.000E+00 | 0.000E+00 | 6.512E-07 | 0.000E+00 | 0.000E+00 | 0.000E+00 | 0.000E+00 | 0.000E+00 | 0.000E+00 | 0.000E+00 |
| Unigene309954 | macB | 1.699E-07 | 7.459E-07 | 5.001E-07 | 0.000E+00 | 1.950E-06 | 0.000E+00 | 0.000E+00 | 0.000E+00 | 0.000E+00 | 0.000E+00 | 4.535E-07 | 6.694E-08 |
| Unigene309965 | arlR | 0.000E+00 | 0.000E+00 | 0.000E+00 | 0.000E+00 | 1.087E-06 | 0.000E+00 | 0.000E+00 | 0.000E+00 | 0.000E+00 | 0.000E+00 | 0.000E+00 | 0.000E+00 |
| Unigene309994 | smeD | 0.000E+00 | 0.000E+00 | 0.000E+00 | 0.000E+00 | 6.339E-07 | 0.000E+00 | 0.000E+00 | 0.000E+00 | 0.000E+00 | 0.000E+00 | 3.108E-07 | 0.000E+00 |
| Unigene310043 | tlrC | 0.000E+00 | 3.536E-08 | 3.161E-08 | 0.000E+00 | 6.442E-07 | 0.000E+00 | 1.783E-07 | 0.000E+00 | 0.000E+00 | 2.871E-07 | 2.948E-07 | 3.427E-07 |
| Unigene310118 | tetA(60) | 2.616E-07 | 0.000E+00 | 0.000E+00 | 0.000E+00 | 1.126E-06 | 0.000E+00 | 0.000E+00 | 0.000E+00 | 0.000E+00 | 0.000E+00 | 0.000E+00 | 0.000E+00 |
| Unigene310120 | adeL | 0.000E+00 | 0.000E+00 | 0.000E+00 | 0.000E+00 | 1.504E-06 | 0.000E+00 | 0.000E+00 | 0.000E+00 | 0.000E+00 | 0.000E+00 | 0.000E+00 | 0.000E+00 |
| Unigene310167 | Agrobacte | 0.000E+00 | 0.000E+00 | 0.000E+00 | 0.000E+00 | 1.387E-06 | 0.000E+00 | 0.000E+00 | 0.000E+00 | 0.000E+00 | 0.000E+00 | 0.000E+00 | 0.000E+00 |
| Unigene310269 | lin | 0.000E+00 | 3.703E-07 | 5.150E-07 | 0.000E+00 | 1.458E-06 | 8.106E-08 | 0.000E+00 | 0.000E+00 | 0.000E+00 | 0.000E+00 | 0.000E+00 | 0.000E+00 |
| Unigene310453 | tetA(58) | 3.762E-07 | 0.000E+00 | 4.061E-07 | 2.659E-07 | 3.427E-06 | 5.572E-06 | 1.624E-06 | 0.000E+00 | 3.166E-06 | 3.143E-06 | 8.607E-08 | 0.000E+00 |
| Unigene310468 | lsaC | 4.726E-07 | 0.000E+00 | 3.555E-07 | 4.295E-07 | 4.166E-06 | 7.936E-06 | 1.482E-06 | 7.061E-06 | 4.701E-06 | 2.948E-06 | 0.000E+00 | 6.890E-07 |

| | | | | | | | | | | | | | |
|---|---|---|---|---|---|---|---|---|---|---|---|---|---|
| Unigene310542 | vanHB | 0.000E+00 | 0.000E+00 | 0.000E+00 | 0.000E+00 | 7.869E-07 | 7.293E-08 | 0.000E+00 | 1.142E-06 | 1.475E-07 | 1.503E-07 | 4.630E-07 | 5.183E-07 |
| Unigene310657 | oleC | 0.000E+00 | 0.000E+00 | 0.000E+00 | 0.000E+00 | 1.200E-06 | 0.000E+00 | 0.000E+00 | 0.000E+00 | 0.000E+00 | 0.000E+00 | 0.000E+00 | 0.000E+00 |
| Unigene310658 | bcrA | 0.000E+00 | 0.000E+00 | 0.000E+00 | 0.000E+00 | 6.550E-07 | 0.000E+00 | 0.000E+00 | 0.000E+00 | 6.444E-07 | 0.000E+00 | 0.000E+00 | 0.000E+00 |
| Unigene310693 | baeS | 0.000E+00 | 4.265E-07 | 2.640E-07 | 0.000E+00 | 1.561E-06 | 0.000E+00 | 1.323E-07 | 0.000E+00 | 0.000E+00 | 0.000E+00 | 0.000E+00 | 0.000E+00 |
| Unigene310727 | tetA(58) | 0.000E+00 | 0.000E+00 | 0.000E+00 | 0.000E+00 | 8.702E-07 | 1.783E-07 | 6.388E-07 | 0.000E+00 | 0.000E+00 | 0.000E+00 | 4.244E-07 | 6.335E-07 |
| Unigene310791 | MexK | 5.542E-07 | 8.011E-07 | 6.632E-07 | 2.594E-07 | 5.887E-06 | 3.799E-07 | 7.480E-07 | 2.367E-07 | 0.000E+00 | 0.000E+00 | 2.010E-07 | 0.000E+00 |
| Unigene310874 | vanSM | 0.000E+00 | 1.350E-06 | 9.481E-07 | 0.000E+00 | 2.952E-06 | 0.000E+00 | 6.076E-07 | 0.000E+00 | 0.000E+00 | 0.000E+00 | 0.000E+00 | 0.000E+00 |
| Unigene311007 | sul4 | 0.000E+00 | 8.879E-08 | 1.191E-07 | 0.000E+00 | 6.741E-07 | 1.749E-07 | 3.134E-07 | 6.138E-07 | 0.000E+00 | 0.000E+00 | 1.851E-07 | 4.781E-07 |
| Unigene311030 | NmcR | 0.000E+00 | 0.000E+00 | 0.000E+00 | 2.288E-07 | 1.216E-06 | 0.000E+00 | 8.775E-07 | 0.000E+00 | 0.000E+00 | 0.000E+00 | 0.000E+00 | 1.160E-06 |
| Unigene311041 | vanSA | 9.543E-07 | 1.878E-06 | 1.970E-06 | 3.555E-06 | 2.925E-07 | 7.114E-08 | 1.093E-07 | 0.000E+00 | 0.000E+00 | 0.000E+00 | 0.000E+00 | 1.945E-07 |
| Unigene311059 | macB | 3.352E-08 | 1.104E-07 | 9.867E-08 | 3.385E-08 | 4.841E-07 | 2.174E-07 | 0.000E+00 | 5.478E-07 | 0.000E+00 | 1.867E-07 | 1.150E-07 | 5.151E-07 |
| Unigene311125 | baeR | 1.915E-07 | 0.000E+00 | 0.000E+00 | 2.320E-07 | 1.106E-06 | 5.381E-07 | 1.399E-06 | 1.207E-06 | 0.000E+00 | 0.000E+00 | 8.322E-07 | 1.177E-06 |
| Unigene311236 | novA | 2.386E-08 | 0.000E+00 | 0.000E+00 | 0.000E+00 | 5.036E-07 | 0.000E+00 | 0.000E+00 | 3.620E-07 | 7.823E-08 | 0.000E+00 | 3.275E-07 | 0.000E+00 |
| Unigene311266 | mdtE | 0.000E+00 | 0.000E+00 | 0.000E+00 | 0.000E+00 | 3.867E-07 | 0.000E+00 | 0.000E+00 | 0.000E+00 | 0.000E+00 | 0.000E+00 | 0.000E+00 | 0.000E+00 |
| Unigene311294 | Escherichia | 4.066E-07 | 1.305E-06 | 3.683E-07 | 5.054E-07 | 1.564E-06 | 1.961E-06 | 1.104E-05 | 1.095E-07 | 3.761E-06 | 0.000E+00 | 0.000E+00 | 0.000E+00 |
| Unigene311498 | ugd | 3.319E-07 | 8.197E-07 | 4.343E-07 | 0.000E+00 | 5.747E-06 | 3.588E-07 | 1.531E-07 | 1.614E-07 | 0.000E+00 | 0.000E+00 | 0.000E+00 | 0.000E+00 |
| Unigene311504 | macB | 1.572E-07 | 3.698E-07 | 2.425E-07 | 0.000E+00 | 6.389E-06 | 2.428E-07 | 2.486E-08 | 3.146E-07 | 0.000E+00 | 0.000E+00 | 1.028E-07 | 0.000E+00 |
| Unigene311514 | oleC | 0.000E+00 | 0.000E+00 | 0.000E+00 | 3.610E-07 | 1.112E-06 | 4.637E-07 | 0.000E+00 | 1.043E-06 | 0.000E+00 | 0.000E+00 | 6.543E-07 | 2.916E-06 |
| Unigene311581 | bcrA | 0.000E+00 | 0.000E+00 | 0.000E+00 | 0.000E+00 | 0.000E+00 | 0.000E+00 | 1.140E-07 | 0.000E+00 | 0.000E+00 | 0.000E+00 | 0.000E+00 | 0.000E+00 |
| Unigene311589 | Acinetoba | 0.000E+00 | 0.000E+00 | 0.000E+00 | 0.000E+00 | 7.541E-07 | 0.000E+00 | 0.000E+00 | 0.000E+00 | 0.000E+00 | 0.000E+00 | 0.000E+00 | 0.000E+00 |
| Unigene311615 | macB | 8.045E-08 | 1.324E-07 | 1.184E-07 | 0.000E+00 | 5.139E-07 | 1.087E-07 | 3.339E-07 | 1.643E-07 | 1.978E-07 | 1.344E-07 | 3.221E-07 | 3.328E-07 |
| Unigene311616 | macA | 0.000E+00 | 1.081E-07 | 0.000E+00 | 1.989E-07 | 6.292E-07 | 2.662E-07 | 8.448E-07 | 8.622E-07 | 1.615E-07 | 3.566E-07 | 3.943E-07 | 7.567E-07 |
| Unigene311653 | Corynebac | 0.000E+00 | 0.000E+00 | 0.000E+00 | 0.000E+00 | 2.759E-06 | 0.000E+00 | 2.920E-06 | 0.000E+00 | 2.262E-07 | 1.383E-06 | 0.000E+00 | 0.000E+00 |
| Unigene311766 | evgS | 0.000E+00 | 0.000E+00 | 0.000E+00 | 1.494E-07 | 5.636E-07 | 0.000E+00 | 0.000E+00 | 2.961E-07 | 0.000E+00 | 0.000E+00 | 0.000E+00 | 0.000E+00 |
| Unigene311891 | poxtA | 0.000E+00 | 0.000E+00 | 0.000E+00 | 0.000E+00 | 3.110E-06 | 0.000E+00 | 1.452E-07 | 0.000E+00 | 1.434E-07 | 0.000E+00 | 0.000E+00 | 0.000E+00 |
| Unigene311892 | bcrA | 0.000E+00 | 0.000E+00 | 0.000E+00 | 0.000E+00 | 4.352E-06 | 5.102E-08 | 0.000E+00 | 0.000E+00 | 0.000E+00 | 0.000E+00 | 2.159E-07 | 0.000E+00 |
| Unigene311947 | Staphylocc | 0.000E+00 | 0.000E+00 | 4.078E-07 | 4.663E-07 | 4.335E-06 | 7.913E-06 | 1.891E-06 | 0.000E+00 | 4.366E-06 | 3.858E-06 | 0.000E+00 | 0.000E+00 |
| Unigene312032 | vanHB | 0.000E+00 | 0.000E+00 | 0.000E+00 | 0.000E+00 | 7.796E-07 | 0.000E+00 | 9.838E-07 | 0.000E+00 | 0.000E+00 | 0.000E+00 | 0.000E+00 | 0.000E+00 |
| Unigene312072 | sdiA | 3.125E-07 | 1.127E-06 | 7.886E-07 | 4.058E-07 | 6.845E-06 | 2.413E-07 | 0.000E+00 | 2.085E-07 | 0.000E+00 | 2.487E-07 | 0.000E+00 | 0.000E+00 |
| Unigene312090 | tetA(58) | 0.000E+00 | 0.000E+00 | 0.000E+00 | 0.000E+00 | 0.000E+00 | 0.000E+00 | 2.089E-07 | 0.000E+00 | 0.000E+00 | 0.000E+00 | 0.000E+00 | 4.463E-07 |
| Unigene312146 | bcrA | 0.000E+00 | 0.000E+00 | 0.000E+00 | 0.000E+00 | 1.122E-06 | 0.000E+00 | 0.000E+00 | 8.615E-07 | 0.000E+00 | 0.000E+00 | 0.000E+00 | 1.745E-06 |
| Unigene312151 | facT | 0.000E+00 | 4.438E-07 | 4.629E-07 | 0.000E+00 | 1.123E-06 | 0.000E+00 | 3.232E-07 | 0.000E+00 | 0.000E+00 | 0.000E+00 | 0.000E+00 | 2.655E-08 |
| Unigene312158 | Escherichia | 4.356E-08 | 0.000E+00 | 0.000E+00 | 0.000E+00 | 1.404E-06 | 0.000E+00 | 0.000E+00 | 0.000E+00 | 0.000E+00 | 0.000E+00 | 0.000E+00 | 0.000E+00 |
| Unigene312175 | efrA | 0.000E+00 | 0.000E+00 | 0.000E+00 | 0.000E+00 | 1.172E-06 | 4.847E-07 | 0.000E+00 | 0.000E+00 | 0.000E+00 | 8.226E-07 | 0.000E+00 | 0.000E+00 |
| Unigene312336 | macB | 2.706E-06 | 2.067E-06 | 8.229E-07 | 2.860E-06 | 1.788E-05 | 2.499E-05 | 3.126E-05 | 1.915E-05 | 3.312E-05 | 0.000E+00 | 0.000E+00 | 1.595E-05 |
| Unigene312351 | Streptomy | 3.804E-07 | 2.147E-06 | 0.000E+00 | 4.390E-07 | 7.003E-06 | 4.700E-07 | 0.000E+00 | 0.000E+00 | 2.376E-07 | 0.000E+00 | 0.000E+00 | 0.000E+00 |
| Unigene312393 | Acinetoba | 0.000E+00 | 0.000E+00 | 0.000E+00 | 0.000E+00 | 7.607E-07 | 0.000E+00 | 0.000E+00 | 1.713E-07 | 0.000E+00 | 0.000E+00 | 0.000E+00 | 0.000E+00 |
| Unigene312413 | adeS | 2.381E-07 | 2.874E-07 | 4.438E-07 | 3.366E-07 | 1.137E-06 | 3.860E-07 | 1.001E-06 | 1.306E-06 | 7.806E-08 | 9.812E-07 | 1.116E-06 | 3.405E-06 |
| Unigene312417 | adeB | 3.545E-07 | 1.453E-06 | 8.901E-07 | 3.475E-07 | 5.560E-06 | 4.283E-07 | 1.592E-06 | 3.286E-07 | 0.000E+00 | 0.000E+00 | 3.101E-07 | 0.000E+00 |
| Unigene312418 | adeS | 3.334E-07 | 1.397E-06 | 6.246E-07 | 2.449E-07 | 6.634E-06 | 6.553E-07 | 0.000E+00 | 1.061E-07 | 0.000E+00 | 0.000E+00 | 2.774E-07 | 0.000E+00 |
| Unigene312420 | bcrA | 0.000E+00 | 1.449E-06 | 1.099E-06 | 0.000E+00 | 7.021E-06 | 6.919E-07 | 0.000E+00 | 4.202E-07 | 0.000E+00 | 2.228E-07 | 0.000E+00 | 0.000E+00 |
| Unigene312439 | Klebsiella p | 2.265E-07 | 1.088E-06 | 4.446E-07 | 4.575E-07 | 4.750E-06 | 1.531E-07 | 4.074E-07 | 1.983E-07 | 0.000E+00 | 1.577E-07 | 0.000E+00 | 0.000E+00 |
| Unigene312443 | otrC | 2.865E-07 | 1.188E-06 | 4.373E-07 | 3.215E-07 | 4.739E-06 | 5.850E-07 | 1.127E-06 | 3.716E-07 | 0.000E+00 | 0.000E+00 | 2.549E-07 | 0.000E+00 |
| Unigene312460 | patA | 0.000E+00 | 0.000E+00 | 7.366E-07 | 0.000E+00 | 8.016E-06 | 4.959E-07 | 0.000E+00 | 3.894E-07 | 0.000E+00 | 0.000E+00 | 4.294E-07 | 0.000E+00 |
| Unigene312478 | patA | 0.000E+00 | 0.000E+00 | 0.000E+00 | 0.000E+00 | 6.078E-07 | 0.000E+00 | 8.410E-07 | 0.000E+00 | 0.000E+00 | 5.418E-07 | 8.345E-07 | 7.186E-07 |
| Unigene312523 | macB | 0.000E+00 | 0.000E+00 | 0.000E+00 | 0.000E+00 | 1.028E-06 | 0.000E+00 | 0.000E+00 | 0.000E+00 | 0.000E+00 | 0.000E+00 | 0.000E+00 | 3.644E-06 |
| Unigene312558 | patA | 0.000E+00 | 0.000E+00 | 2.800E-07 | 0.000E+00 | 1.223E-06 | 1.762E-07 | 8.571E-07 | 1.332E-06 | 0.000E+00 | 4.086E-07 | 2.331E-07 | 2.553E-06 |
| Unigene312740 | vmlR | 0.000E+00 | 0.000E+00 | 6.051E-08 | 0.000E+00 | 5.823E-07 | 1.666E-07 | 0.000E+00 | 5.038E-07 | 0.000E+00 | 0.000E+00 | 2.116E-07 | 5.831E-07 |
| Unigene312818 | novA | 1.111E-06 | 0.000E+00 | 0.000E+00 | 0.000E+00 | 2.245E-07 | 0.000E+00 | 0.000E+00 | 0.000E+00 | 0.000E+00 | 0.000E+00 | 0.000E+00 | 0.000E+00 |
| Unigene312849 | adeN | 0.000E+00 | 0.000E+00 | 0.000E+00 | 0.000E+00 | 4.347E-07 | 0.000E+00 | 7.578E-07 | 0.000E+00 | 0.000E+00 | 0.000E+00 | 0.000E+00 | 0.000E+00 |
| Unigene312963 | MexH | 0.000E+00 | 0.000E+00 | 0.000E+00 | 0.000E+00 | 6.444E-07 | 4.703E-07 | 0.000E+00 | 6.432E-07 | 0.000E+00 | 0.000E+00 | 0.000E+00 | 1.028E-06 |
| Unigene312979 | LlmA 23S r | 0.000E+00 | 0.000E+00 | 0.000E+00 | 0.000E+00 | 4.336E-07 | 0.000E+00 | 0.000E+00 | 1.269E-06 | 1.523E-07 | 0.000E+00 | 2.551E-07 | 7.579E-07 |
| Unigene312995 | vanRF | 0.000E+00 | 0.000E+00 | 0.000E+00 | 0.000E+00 | 8.648E-07 | 0.000E+00 | 0.000E+00 | 0.000E+00 | 0.000E+00 | 0.000E+00 | 0.000E+00 | 0.000E+00 |
| Unigene313084 | macB | 1.133E-05 | 3.398E-06 | 3.421E-06 | 1.278E-05 | 8.418E-05 | 1.173E-04 | 1.106E-04 | 9.434E-05 | 1.477E-04 | 1.453E-04 | 1.186E-04 | 7.631E-05 |
| Unigene313092 | Bifidobacte | 9.158E-08 | 0.000E+00 | 0.000E+00 | 0.000E+00 | 3.561E-07 | 0.000E+00 | 1.520E-07 | 0.000E+00 | 0.000E+00 | 0.000E+00 | 0.000E+00 | 0.000E+00 |
| Unigene313128 | baeR | 0.000E+00 | 0.000E+00 | 0.000E+00 | 0.000E+00 | 9.261E-06 | 0.000E+00 | 0.000E+00 | 0.000E+00 | 0.000E+00 | 0.000E+00 | 0.000E+00 | 0.000E+00 |
| Unigene313132 | tetB(60) | 0.000E+00 | 0.000E+00 | 0.000E+00 | 0.000E+00 | 7.032E-07 | 0.000E+00 | 0.000E+00 | 4.104E-07 | 0.000E+00 | 0.000E+00 | 8.045E-07 | 0.000E+00 |
| Unigene313137 | cfrC | 0.000E+00 | 1.009E-07 | 0.000E+00 | 0.000E+00 | 6.130E-07 | 0.000E+00 | 2.714E-07 | 4.294E-07 | 0.000E+00 | 1.024E-07 | 0.000E+00 | 3.986E-07 |

| | | | | | | | | | | | | | |
|---|---|---|---|---|---|---|---|---|---|---|---|---|---|
| Unigene313253 | msbA | 0.000E+00 | 0.000E+00 | 0.000E+00 | 3.569E-07 | 1.440E-06 | 8.491E-07 | 0.000E+00 | 1.696E-06 | 0.000E+00 | 0.000E+00 | 8.984E-07 | 3.342E-06 |
| Unigene313408 | efrA | 0.000E+00 | 0.000E+00 | 0.000E+00 | 0.000E+00 | 4.568E-07 | 0.000E+00 | 0.000E+00 | 0.000E+00 | 0.000E+00 | 0.000E+00 | 0.000E+00 | 0.000E+00 |
| Unigene313499 | Acinetoba | 0.000E+00 | 0.000E+00 | 1.955E-07 | 0.000E+00 | 9.171E-07 | 0.000E+00 | 0.000E+00 | 0.000E+00 | 0.000E+00 | 0.000E+00 | 0.000E+00 | 0.000E+00 |
| Unigene313533 | iri | 0.000E+00 | 0.000E+00 | 0.000E+00 | 0.000E+00 | 4.295E-07 | 0.000E+00 | 9.270E-07 | 0.000E+00 | 0.000E+00 | 0.000E+00 | 0.000E+00 | 0.000E+00 |
| Unigene313542 | msrC | 0.000E+00 | 0.000E+00 | 0.000E+00 | 0.000E+00 | 1.374E-06 | 0.000E+00 | 0.000E+00 | 0.000E+00 | 0.000E+00 | 0.000E+00 | 4.716E-07 | 2.095E-06 |
| Unigene313613 | patB | 0.000E+00 | 0.000E+00 | 0.000E+00 | 0.000E+00 | 5.087E-07 | 0.000E+00 | 0.000E+00 | 0.000E+00 | 0.000E+00 | 0.000E+00 | 0.000E+00 | 0.000E+00 |
| Unigene313770 | marA | 0.000E+00 | 0.000E+00 | 0.000E+00 | 0.000E+00 | 0.000E+00 | 0.000E+00 | 0.000E+00 | 0.000E+00 | 0.000E+00 | 0.000E+00 | 6.577E-07 | 0.000E+00 |
| Unigene313848 | Acinetoba | 0.000E+00 | 6.915E-07 | 6.896E-07 | 0.000E+00 | 1.454E-06 | 0.000E+00 | 3.487E-07 | 0.000E+00 | 0.000E+00 | 1.350E-07 | 3.881E-07 | 0.000E+00 |
| Unigene313860 | vgaALC | 3.416E-08 | 4.499E-07 | 3.017E-07 | 0.000E+00 | 7.590E-07 | 0.000E+00 | 0.000E+00 | 0.000E+00 | 0.000E+00 | 0.000E+00 | 0.000E+00 | 0.000E+00 |
| Unigene313875 | patB | 0.000E+00 | 0.000E+00 | 0.000E+00 | 0.000E+00 | 8.539E-07 | 0.000E+00 | 8.034E-07 | 3.987E-07 | 3.734E-07 | 0.000E+00 | 0.000E+00 | 6.057E-07 |
| Unigene313882 | macB | 0.000E+00 | 0.000E+00 | 0.000E+00 | 0.000E+00 | 5.197E-07 | 0.000E+00 | 0.000E+00 | 0.000E+00 | 0.000E+00 | 0.000E+00 | 0.000E+00 | 0.000E+00 |
| Unigene313942 | oleC | 0.000E+00 | 6.203E-07 | 2.986E-07 | 0.000E+00 | 1.690E-06 | 0.000E+00 | 2.646E-07 | 0.000E+00 | 0.000E+00 | 0.000E+00 | 0.000E+00 | 0.000E+00 |
| Unigene313995 | mdtB | 6.925E-07 | 1.237E-06 | 1.035E-06 | 4.175E-07 | 8.360E-06 | 4.581E-07 | 1.350E-06 | 3.981E-07 | 1.017E-07 | 3.685E-07 | 4.020E-07 | 1.710E-07 |
| Unigene313996 | mdtC | 7.761E-07 | 1.865E-06 | 1.122E-06 | 4.766E-07 | 8.109E-06 | 3.855E-07 | 1.938E-06 | 7.100E-07 | 0.000E+00 | 0.000E+00 | 7.198E-07 | 0.000E+00 |
| Unigene313997 | emrB | 3.192E-07 | 1.126E-06 | 5.816E-07 | 0.000E+00 | 6.484E-06 | 3.204E-07 | 9.588E-07 | 3.459E-07 | 0.000E+00 | 1.778E-07 | 3.912E-07 | 0.000E+00 |
| Unigene314073 | bcrA | 0.000E+00 | 3.634E-07 | 0.000E+00 | 0.000E+00 | 1.103E-06 | 3.579E-07 | 0.000E+00 | 0.000E+00 | 0.000E+00 | 2.305E-07 | 4.261E-07 | 1.957E-07 |
| Unigene314100 | evgS | 0.000E+00 | 1.177E-07 | 7.889E-08 | 0.000E+00 | 1.533E-06 | 0.000E+00 | 1.186E-07 | 0.000E+00 | 4.101E-07 | 8.956E-08 | 0.000E+00 | 0.000E+00 |
| Unigene314138 | AxyY | 0.000E+00 | 0.000E+00 | 0.000E+00 | 0.000E+00 | 3.388E-06 | 0.000E+00 | 0.000E+00 | 0.000E+00 | 0.000E+00 | 0.000E+00 | 0.000E+00 | 0.000E+00 |
| Unigene314167 | abeS | 0.000E+00 | 0.000E+00 | 9.181E-08 | 0.000E+00 | 4.158E-07 | 0.000E+00 | 0.000E+00 | 0.000E+00 | 0.000E+00 | 0.000E+00 | 0.000E+00 | 0.000E+00 |
| Unigene314218 | facT | 0.000E+00 | 0.000E+00 | 0.000E+00 | 0.000E+00 | 4.563E-07 | 0.000E+00 | 0.000E+00 | 0.000E+00 | 0.000E+00 | 0.000E+00 | 0.000E+00 | 0.000E+00 |
| Unigene314396 | mdtB | 0.000E+00 | 0.000E+00 | 0.000E+00 | 0.000E+00 | 3.985E-07 | 0.000E+00 | 0.000E+00 | 1.794E-07 | 0.000E+00 | 1.712E-07 | 0.000E+00 | 1.211E-07 |
| Unigene314430 | lmrB | 5.869E-07 | 2.684E-08 | 1.920E-07 | 0.000E+00 | 3.396E-06 | 9.147E-06 | 1.191E-06 | 9.106E-06 | 4.063E-06 | 2.888E-06 | 0.000E+00 | 9.539E-07 |
| Unigene314432 | tetA(58) | 0.000E+00 | 0.000E+00 | 4.140E-07 | 3.835E-07 | 3.328E-06 | 7.390E-06 | 0.000E+00 | 6.009E-06 | 4.197E-06 | 3.666E-06 | 0.000E+00 | 0.000E+00 |
| Unigene314466 | Pseudomo | 0.000E+00 | 5.191E-08 | 0.000E+00 | 1.433E-07 | 8.144E-07 | 0.000E+00 | 5.496E-07 | 0.000E+00 | 0.000E+00 | 2.108E-07 | 6.222E-07 | 7.268E-07 |
| Unigene314583 | bcrA | 0.000E+00 | 1.104E-07 | 0.000E+00 | 0.000E+00 | 3.575E-07 | 2.826E-07 | 0.000E+00 | 9.390E-08 | 0.000E+00 | 0.000E+00 | 0.000E+00 | 4.517E-07 |
| Unigene314603 | msbA | 0.000E+00 | 0.000E+00 | 0.000E+00 | 0.000E+00 | 9.825E-07 | 0.000E+00 | 0.000E+00 | 0.000E+00 | 4.833E-07 | 0.000E+00 | 0.000E+00 | 0.000E+00 |
| Unigene314604 | patA | 0.000E+00 | 1.440E-07 | 7.358E-08 | 0.000E+00 | 1.166E-06 | 0.000E+00 | 0.000E+00 | 0.000E+00 | 6.352E-07 | 0.000E+00 | 0.000E+00 | 0.000E+00 |
| Unigene314702 | evgS | 0.000E+00 | 0.000E+00 | 0.000E+00 | 0.000E+00 | 1.019E-06 | 0.000E+00 | 0.000E+00 | 0.000E+00 | 3.647E-07 | 0.000E+00 | 0.000E+00 | 0.000E+00 |
| Unigene314734 | oleB | 0.000E+00 | 0.000E+00 | 0.000E+00 | 0.000E+00 | 3.579E-06 | 0.000E+00 | 2.852E-07 | 0.000E+00 | 0.000E+00 | 0.000E+00 | 3.685E-07 | 0.000E+00 |
| Unigene314741 | efrB | 0.000E+00 | 0.000E+00 | 0.000E+00 | 0.000E+00 | 3.155E-06 | 0.000E+00 | 0.000E+00 | 0.000E+00 | 0.000E+00 | 0.000E+00 | 5.929E-07 | 0.000E+00 |
| Unigene314748 | macB | 0.000E+00 | 0.000E+00 | 0.000E+00 | 0.000E+00 | 4.181E-06 | 0.000E+00 | 0.000E+00 | 0.000E+00 | 0.000E+00 | 0.000E+00 | 3.363E-07 | 0.000E+00 |
| Unigene314769 | patA | 0.000E+00 | 0.000E+00 | 0.000E+00 | 0.000E+00 | 6.108E-07 | 0.000E+00 | 0.000E+00 | 0.000E+00 | 0.000E+00 | 0.000E+00 | 0.000E+00 | 0.000E+00 |
| Unigene314795 | macB | 0.000E+00 | 0.000E+00 | 0.000E+00 | 0.000E+00 | 6.104E-07 | 0.000E+00 | 0.000E+00 | 2.138E-07 | 0.000E+00 | 0.000E+00 | 0.000E+00 | 0.000E+00 |
| Unigene314829 | bcrA | 0.000E+00 | 0.000E+00 | 0.000E+00 | 0.000E+00 | 1.169E-06 | 0.000E+00 | 0.000E+00 | 9.905E-07 | 0.000E+00 | 0.000E+00 | 0.000E+00 | 0.000E+00 |
| Unigene314915 | Brucella su | 0.000E+00 | 0.000E+00 | 0.000E+00 | 0.000E+00 | 1.494E-07 | 0.000E+00 | 1.489E-07 | 2.355E-07 | 0.000E+00 | 0.000E+00 | 0.000E+00 | 3.975E-07 |
| Unigene315059 | lmrD | 3.101E-07 | 4.424E-07 | 3.043E-07 | 6.576E-07 | 7.923E-07 | 1.341E-06 | 2.334E-06 | 1.412E-06 | 3.050E-07 | 1.002E-06 | 1.844E-06 | 3.445E-06 |
| Unigene315074 | patA | 0.000E+00 | 0.000E+00 | 0.000E+00 | 2.744E-07 | 6.641E-07 | 0.000E+00 | 0.000E+00 | 0.000E+00 | 0.000E+00 | 0.000E+00 | 0.000E+00 | 0.000E+00 |
| Unigene315092 | bcrA | 0.000E+00 | 0.000E+00 | 0.000E+00 | 0.000E+00 | 1.045E-06 | 0.000E+00 | 0.000E+00 | 0.000E+00 | 2.571E-07 | 0.000E+00 | 0.000E+00 | 0.000E+00 |
| Unigene315189 | acrD | 0.000E+00 | 0.000E+00 | 0.000E+00 | 0.000E+00 | 4.122E-07 | 0.000E+00 | 0.000E+00 | 0.000E+00 | 0.000E+00 | 0.000E+00 | 0.000E+00 | 0.000E+00 |
| Unigene315255 | bcrA | 0.000E+00 | 3.785E-07 | 7.783E-07 | 0.000E+00 | 9.962E-07 | 7.084E-07 | 2.557E-06 | 1.087E-06 | 0.000E+00 | 7.684E-07 | 8.284E-07 | 2.120E-06 |
| Unigene315500 | patA | 0.000E+00 | 0.000E+00 | 0.000E+00 | 0.000E+00 | 9.806E-07 | 0.000E+00 | 0.000E+00 | 0.000E+00 | 0.000E+00 | 0.000E+00 | 0.000E+00 | 8.943E-07 |
| Unigene315550 | efrA | 2.069E-07 | 0.000E+00 | 0.000E+00 | 4.179E-07 | 8.210E-07 | 1.917E-07 | 4.580E-07 | 6.556E-07 | 3.877E-07 | 6.257E-07 | 8.454E-07 | 1.642E-06 |
| Unigene315600 | rpoB2 | 3.871E-08 | 0.000E+00 | 0.000E+00 | 0.000E+00 | 9.460E-07 | 0.000E+00 | 1.199E-06 | 0.000E+00 | 0.000E+00 | 0.000E+00 | 0.000E+00 | 0.000E+00 |
| Unigene315601 | blt | 0.000E+00 | 2.644E-07 | 2.364E-07 | 0.000E+00 | 5.353E-07 | 0.000E+00 | 0.000E+00 | 0.000E+00 | 0.000E+00 | 0.000E+00 | 1.837E-07 | 9.492E-08 |
| Unigene315603 | AcrS | 0.000E+00 | 0.000E+00 | 1.545E-07 | 0.000E+00 | 6.997E-07 | 4.539E-07 | 6.390E-07 | 0.000E+00 | 0.000E+00 | 5.847E-07 | 6.604E-07 | 0.000E+00 |
| Unigene315805 | macB | 1.035E-07 | 1.514E-07 | 0.000E+00 | 0.000E+00 | 8.238E-07 | 0.000E+00 | 7.634E-08 | 0.000E+00 | 3.204E-07 | 0.000E+00 | 0.000E+00 | 2.038E-08 |
| Unigene315898 | rosA | 0.000E+00 | 0.000E+00 | 0.000E+00 | 2.330E-07 | 9.294E-07 | 0.000E+00 | 1.724E-06 | 8.418E-07 | 0.000E+00 | 0.000E+00 | 0.000E+00 | 2.728E-06 |
| Unigene315928 | Acinetoba | 0.000E+00 | 0.000E+00 | 0.000E+00 | 0.000E+00 | 2.841E-06 | 8.211E-08 | 0.000E+00 | 0.000E+00 | 0.000E+00 | 0.000E+00 | 0.000E+00 | 5.985E-08 |
| Unigene315941 | novA | 0.000E+00 | 0.000E+00 | 0.000E+00 | 0.000E+00 | 3.321E-07 | 0.000E+00 | 0.000E+00 | 0.000E+00 | 0.000E+00 | 0.000E+00 | 0.000E+00 | 0.000E+00 |
| Unigene315946 | mgrA | 1.281E-07 | 2.109E-07 | 0.000E+00 | 0.000E+00 | 7.827E-07 | 0.000E+00 | 0.000E+00 | 0.000E+00 | 2.100E-07 | 0.000E+00 | 0.000E+00 | 4.542E-07 |
| Unigene315987 | salA | 1.948E-07 | 2.137E-07 | 0.000E+00 | 0.000E+00 | 9.195E-07 | 0.000E+00 | 0.000E+00 | 0.000E+00 | 5.322E-07 | 0.000E+00 | 0.000E+00 | 2.360E-06 |
| Unigene316000 | mdtC | 0.000E+00 | 1.196E-07 | 0.000E+00 | 1.375E-07 | 3.632E-07 | 0.000E+00 | 1.507E-07 | 3.498E-07 | 2.084E-07 | 6.069E-08 | 0.000E+00 | 5.474E-07 |
| Unigene316010 | Staphyloc | 7.242E-05 | 6.250E-05 | 1.194E-04 | 1.747E-04 | 1.347E-04 | 9.235E-05 | 1.187E-04 | 1.169E-04 | 9.963E-05 | 1.450E-04 | 1.525E-04 | 1.577E-04 |
| Unigene316118 | bcrA | 0.000E+00 | 0.000E+00 | 0.000E+00 | 0.000E+00 | 7.550E-07 | 0.000E+00 | 3.343E-07 | 0.000E+00 | 0.000E+00 | 0.000E+00 | 0.000E+00 | 0.000E+00 |
| Unigene316327 | lmrC | 0.000E+00 | 0.000E+00 | 0.000E+00 | 0.000E+00 | 3.919E-07 | 0.000E+00 | 0.000E+00 | 0.000E+00 | 0.000E+00 | 0.000E+00 | 0.000E+00 | 5.213E-08 |
| Unigene316351 | iri | 1.376E-07 | 0.000E+00 | 3.780E-07 | 2.223E-07 | 6.726E-07 | 1.785E-07 | 1.675E-06 | 9.315E-07 | 0.000E+00 | 2.759E-07 | 6.296E-07 | 1.236E-06 |
| Unigene316366 | baeS | 0.000E+00 | 0.000E+00 | 0.000E+00 | 0.000E+00 | 1.343E-06 | 0.000E+00 | 0.000E+00 | 0.000E+00 | 6.096E-07 | 0.000E+00 | 0.000E+00 | 0.000E+00 |
| Unigene316367 | arlR | 0.000E+00 | 0.000E+00 | 0.000E+00 | 0.000E+00 | 1.375E-06 | 0.000E+00 | 0.000E+00 | 0.000E+00 | 7.285E-07 | 0.000E+00 | 0.000E+00 | 0.000E+00 |

| | | | | | | | | | | | | | |
|---|---|---|---|---|---|---|---|---|---|---|---|---|---|
| Unigene316453 | patB | 0.000E+00 | 0.000E+00 | 0.000E+00 | 0.000E+00 | 6.245E-07 | 0.000E+00 | 0.000E+00 | 0.000E+00 | 0.000E+00 | 0.000E+00 | 0.000E+00 | 2.847E-07 |
| Unigene316454 | cpxA | 0.000E+00 | 0.000E+00 | 0.000E+00 | 0.000E+00 | 6.065E-07 | 0.000E+00 | 4.280E-07 | 3.186E-07 | 1.492E-07 | 0.000E+00 | 3.383E-07 | 3.496E-07 |
| Unigene316467 | cpxA | 0.000E+00 | 0.000E+00 | 0.000E+00 | 0.000E+00 | 1.436E-06 | 0.000E+00 | 3.770E-06 | 0.000E+00 | 0.000E+00 | 0.000E+00 | 8.868E-07 | 9.997E-07 |
| Unigene316485 | macB | 0.000E+00 | 0.000E+00 | 0.000E+00 | 0.000E+00 | 4.203E-07 | 0.000E+00 | 2.326E-07 | 5.397E-07 | 4.594E-08 | 0.000E+00 | 0.000E+00 | 5.962E-07 |
| Unigene316696 | tetA(58) | 0.000E+00 | 0.000E+00 | 0.000E+00 | 0.000E+00 | 1.090E-06 | 0.000E+00 | 0.000E+00 | 0.000E+00 | 0.000E+00 | 0.000E+00 | 0.000E+00 | 0.000E+00 |
| Unigene316730 | macB | 0.000E+00 | 0.000E+00 | 0.000E+00 | 0.000E+00 | 1.434E-06 | 0.000E+00 | 0.000E+00 | 0.000E+00 | 0.000E+00 | 0.000E+00 | 0.000E+00 | 9.157E-07 |
| Unigene316760 | arlR | 0.000E+00 | 0.000E+00 | 0.000E+00 | 0.000E+00 | 3.579E-07 | 0.000E+00 | 0.000E+00 | 0.000E+00 | 0.000E+00 | 0.000E+00 | 0.000E+00 | 0.000E+00 |
| Unigene316769 | bcr-1 | 7.432E-05 | 5.899E-05 | 1.206E-04 | 1.638E-04 | 1.301E-04 | 9.133E-05 | 1.248E-04 | 1.100E-04 | 9.858E-05 | 1.447E-04 | 1.486E-04 | 1.647E-04 |
| Unigene316858 | poxtA | 3.200E-07 | 0.000E+00 | 0.000E+00 | 0.000E+00 | 2.800E-06 | 0.000E+00 | 0.000E+00 | 0.000E+00 | 5.246E-07 | 0.000E+00 | 0.000E+00 | 0.000E+00 |
| Unigene316893 | novA | 0.000E+00 | 0.000E+00 | 0.000E+00 | 0.000E+00 | 3.054E-07 | 0.000E+00 | 3.477E-07 | 0.000E+00 | 0.000E+00 | 0.000E+00 | 0.000E+00 | 0.000E+00 |
| Unigene316942 | optrA | 0.000E+00 | 0.000E+00 | 0.000E+00 | 0.000E+00 | 4.317E-07 | 0.000E+00 | 0.000E+00 | 9.420E-07 | 0.000E+00 | 0.000E+00 | 1.368E-07 | 6.359E-07 |
| Unigene317057 | adeR | 0.000E+00 | 0.000E+00 | 0.000E+00 | 0.000E+00 | 2.846E-07 | 0.000E+00 | 0.000E+00 | 0.000E+00 | 0.000E+00 | 0.000E+00 | 0.000E+00 | 2.271E-07 |
| Unigene317063 | ANT(3'')-II | 0.000E+00 | 0.000E+00 | 0.000E+00 | 0.000E+00 | 6.113E-07 | 0.000E+00 | 2.707E-07 | 0.000E+00 | 0.000E+00 | 0.000E+00 | 0.000E+00 | 0.000E+00 |
| Unigene317099 | patB | 0.000E+00 | 0.000E+00 | 0.000E+00 | 0.000E+00 | 2.071E-06 | 6.411E-07 | 0.000E+00 | 0.000E+00 | 9.584E-06 | 0.000E+00 | 0.000E+00 | 0.000E+00 |
| Unigene317128 | lmrD | 0.000E+00 | 0.000E+00 | 0.000E+00 | 2.205E-07 | 3.118E-07 | 0.000E+00 | 0.000E+00 | 9.829E-07 | 0.000E+00 | 4.517E-07 | 6.065E-07 | 1.069E-06 |
| Unigene317161 | cpxA | 0.000E+00 | 4.909E-07 | 2.438E-07 | 1.506E-07 | 1.132E-06 | 1.075E-07 | 0.000E+00 | 0.000E+00 | 0.000E+00 | 0.000E+00 | 0.000E+00 | 0.000E+00 |
| Unigene317205 | bcrA | 0.000E+00 | 0.000E+00 | 0.000E+00 | 0.000E+00 | 5.000E-07 | 0.000E+00 | 0.000E+00 | 5.253E-07 | 1.342E-07 | 0.000E+00 | 0.000E+00 | 3.869E-07 |
| Unigene317308 | evgS | 0.000E+00 | 0.000E+00 | 0.000E+00 | 0.000E+00 | 3.039E-07 | 0.000E+00 | 3.700E-07 | 0.000E+00 | 0.000E+00 | 0.000E+00 | 0.000E+00 | 0.000E+00 |
| Unigene317322 | novA | 0.000E+00 | 0.000E+00 | 0.000E+00 | 0.000E+00 | 3.705E-06 | 0.000E+00 | 0.000E+00 | 0.000E+00 | 0.000E+00 | 0.000E+00 | 0.000E+00 | 0.000E+00 |
| Unigene317383 | basS | 0.000E+00 | 0.000E+00 | 0.000E+00 | 0.000E+00 | 4.475E-07 | 0.000E+00 | 0.000E+00 | 6.716E-08 | 0.000E+00 | 0.000E+00 | 0.000E+00 | 0.000E+00 |
| Unigene317393 | efrA | 0.000E+00 | 0.000E+00 | 0.000E+00 | 0.000E+00 | 3.302E-07 | 0.000E+00 | 4.606E-07 | 0.000E+00 | 0.000E+00 | 0.000E+00 | 0.000E+00 | 0.000E+00 |
| Unigene317469 | vanTG | 0.000E+00 | 0.000E+00 | 0.000E+00 | 0.000E+00 | 5.197E-07 | 0.000E+00 | 4.264E-07 | 0.000E+00 | 0.000E+00 | 0.000E+00 | 0.000E+00 | 0.000E+00 |
| Unigene317548 | efrB | 5.476E-07 | 1.884E-06 | 1.452E-06 | 5.082E-07 | 5.475E-06 | 2.560E-07 | 1.966E-06 | 2.764E-07 | 0.000E+00 | 0.000E+00 | 3.217E-07 | 0.000E+00 |
| Unigene317553 | OprA | 4.912E-07 | 7.959E-07 | 5.050E-07 | 0.000E+00 | 5.587E-06 | 1.264E-07 | 0.000E+00 | 8.191E-08 | 0.000E+00 | 0.000E+00 | 0.000E+00 | 0.000E+00 |
| Unigene317582 | evgS | 1.842E-08 | 6.067E-08 | 0.000E+00 | 1.861E-08 | 5.117E-07 | 7.967E-08 | 1.427E-07 | 1.721E-07 | 6.041E-08 | 1.847E-07 | 0.000E+00 | 3.702E-07 |
| Unigene317598 | adeG | 0.000E+00 | 0.000E+00 | 0.000E+00 | 0.000E+00 | 1.039E-06 | 0.000E+00 | 0.000E+00 | 0.000E+00 | 0.000E+00 | 0.000E+00 | 0.000E+00 | 3.687E-07 |
| Unigene317625 | evgS | 0.000E+00 | 0.000E+00 | 1.135E-07 | 3.505E-07 | 7.070E-07 | 2.502E-07 | 8.003E-07 | 5.908E-07 | 1.423E-07 | 3.705E-07 | 4.632E-07 | 8.718E-07 |
| Unigene317656 | sdiA | 0.000E+00 | 0.000E+00 | 0.000E+00 | 1.495E-07 | 7.129E-07 | 0.000E+00 | 2.185E-07 | 1.728E-07 | 0.000E+00 | 2.199E-07 | 0.000E+00 | 5.251E-07 |
| Unigene317690 | tetB(60) | 0.000E+00 | 0.000E+00 | 0.000E+00 | 0.000E+00 | 0.000E+00 | 2.920E-07 | 0.000E+00 | 0.000E+00 | 0.000E+00 | 9.027E-07 | 1.133E-06 | 0.000E+00 |
| Unigene317733 | efrA | 0.000E+00 | 0.000E+00 | 0.000E+00 | 0.000E+00 | 7.514E-07 | 0.000E+00 | 0.000E+00 | 1.005E-06 | 0.000E+00 | 0.000E+00 | 8.440E-07 | 1.599E-06 |
| Unigene317841 | baeS | 0.000E+00 | 0.000E+00 | 0.000E+00 | 0.000E+00 | 4.598E-07 | 0.000E+00 | 0.000E+00 | 4.831E-08 | 0.000E+00 | 0.000E+00 | 0.000E+00 | 0.000E+00 |
| Unigene317862 | lmrC | 0.000E+00 | 0.000E+00 | 0.000E+00 | 0.000E+00 | 3.475E-07 | 0.000E+00 | 0.000E+00 | 0.000E+00 | 0.000E+00 | 0.000E+00 | 0.000E+00 | 0.000E+00 |
| Unigene317885 | hmrM | 0.000E+00 | 0.000E+00 | 0.000E+00 | 0.000E+00 | 1.180E-06 | 0.000E+00 | 1.993E-06 | 1.375E-06 | 0.000E+00 | 0.000E+00 | 0.000E+00 | 3.111E-06 |
| Unigene317943 | farA | 0.000E+00 | 1.915E-07 | 0.000E+00 | 0.000E+00 | 8.400E-07 | 0.000E+00 | 0.000E+00 | 0.000E+00 | 0.000E+00 | 0.000E+00 | 0.000E+00 | 0.000E+00 |
| Unigene317993 | ErmX | 0.000E+00 | 0.000E+00 | 0.000E+00 | 0.000E+00 | 1.708E-06 | 2.770E-07 | 0.000E+00 | 0.000E+00 | 0.000E+00 | 0.000E+00 | 0.000E+00 | 0.000E+00 |
| Unigene318007 | Agrobacte | 0.000E+00 | 0.000E+00 | 0.000E+00 | 0.000E+00 | 5.625E-07 | 0.000E+00 | 0.000E+00 | 0.000E+00 | 0.000E+00 | 0.000E+00 | 0.000E+00 | 0.000E+00 |
| Unigene318035 | MexK | 9.827E-08 | 0.000E+00 | 1.768E-07 | 1.323E-07 | 8.915E-07 | 0.000E+00 | 6.163E-07 | 4.970E-07 | 0.000E+00 | 0.000E+00 | 3.559E-07 | 6.195E-07 |
| Unigene318110 | smeR | 0.000E+00 | 0.000E+00 | 0.000E+00 | 0.000E+00 | 4.617E-07 | 0.000E+00 | 0.000E+00 | 0.000E+00 | 1.009E-07 | 0.000E+00 | 0.000E+00 | 0.000E+00 |
| Unigene318245 | oleB | 0.000E+00 | 0.000E+00 | 0.000E+00 | 1.114E-07 | 0.000E+00 | 0.000E+00 | 0.000E+00 | 3.220E-07 | 0.000E+00 | 0.000E+00 | 0.000E+00 | 1.304E-06 |
| Unigene318278 | Acinetoba | 0.000E+00 | 2.228E-07 | 0.000E+00 | 0.000E+00 | 8.120E-07 | 0.000E+00 | 8.988E-08 | 4.266E-07 | 4.438E-08 | 2.714E-07 | 0.000E+00 | 2.880E-07 |
| Unigene318298 | msbA | 0.000E+00 | 9.640E-08 | 0.000E+00 | 1.331E-07 | 6.343E-07 | 0.000E+00 | 1.021E-06 | 3.588E-07 | 4.800E-08 | 3.914E-07 | 0.000E+00 | 3.271E-06 |
| Unigene318382 | mtrA | 0.000E+00 | 0.000E+00 | 0.000E+00 | 0.000E+00 | 5.852E-07 | 0.000E+00 | 0.000E+00 | 0.000E+00 | 0.000E+00 | 0.000E+00 | 0.000E+00 | 0.000E+00 |
| Unigene318435 | ACT-17 | 0.000E+00 | 0.000E+00 | 0.000E+00 | 0.000E+00 | 8.136E-07 | 0.000E+00 | 0.000E+00 | 0.000E+00 | 8.620E-07 | 0.000E+00 | 0.000E+00 | 0.000E+00 |
| Unigene318477 | tetW | 0.000E+00 | 0.000E+00 | 0.000E+00 | 0.000E+00 | 2.525E-07 | 0.000E+00 | 0.000E+00 | 0.000E+00 | 0.000E+00 | 0.000E+00 | 0.000E+00 | 0.000E+00 |
| Unigene318537 | patA | 0.000E+00 | 0.000E+00 | 0.000E+00 | 0.000E+00 | 0.000E+00 | 0.000E+00 | 0.000E+00 | 0.000E+00 | 0.000E+00 | 0.000E+00 | 0.000E+00 | 1.137E-06 |
| Unigene318583 | arlS | 8.064E-07 | 7.586E-08 | 2.487E-07 | 5.817E-07 | 3.840E-06 | 9.241E-06 | 9.690E-07 | 7.208E-06 | 4.206E-06 | 4.055E-06 | 0.000E+00 | 0.000E+00 |
| Unigene318628 | oleC | 2.205E-07 | 0.000E+00 | 2.596E-07 | 3.563E-07 | 9.798E-07 | 3.337E-07 | 1.513E-06 | 8.751E-07 | 0.000E+00 | 0.000E+00 | 0.000E+00 | 1.512E-06 |
| Unigene318642 | macB | 0.000E+00 | 0.000E+00 | 0.000E+00 | 0.000E+00 | 2.284E-07 | 0.000E+00 | 0.000E+00 | 0.000E+00 | 0.000E+00 | 0.000E+00 | 0.000E+00 | 0.000E+00 |
| Unigene318771 | mgrA | 0.000E+00 | 0.000E+00 | 0.000E+00 | 0.000E+00 | 9.719E-07 | 0.000E+00 | 0.000E+00 | 0.000E+00 | 0.000E+00 | 0.000E+00 | 0.000E+00 | 0.000E+00 |
| Unigene318793 | adeS | 0.000E+00 | 0.000E+00 | 4.745E-07 | 0.000E+00 | 6.447E-07 | 0.000E+00 | 0.000E+00 | 0.000E+00 | 0.000E+00 | 0.000E+00 | 9.128E-07 | 0.000E+00 |
| Unigene318794 | vanRO | 0.000E+00 | 0.000E+00 | 0.000E+00 | 0.000E+00 | 1.169E-06 | 0.000E+00 | 1.376E-06 | 1.340E-06 | 0.000E+00 | 7.991E-07 | 0.000E+00 | 4.296E-06 |
| Unigene318796 | mdtB | 0.000E+00 | 1.464E-07 | 5.536E-07 | 2.382E-07 | 8.889E-07 | 0.000E+00 | 9.309E-07 | 0.000E+00 | 0.000E+00 | 0.000E+00 | 0.000E+00 | 2.437E-06 |
| Unigene318865 | mgrA | 0.000E+00 | 0.000E+00 | 0.000E+00 | 0.000E+00 | 8.730E-07 | 0.000E+00 | 0.000E+00 | 7.762E-07 | 0.000E+00 | 0.000E+00 | 8.298E-07 | 7.145E-07 |
| Unigene318900 | carA | 0.000E+00 | 0.000E+00 | 0.000E+00 | 0.000E+00 | 5.831E-07 | 0.000E+00 | 0.000E+00 | 0.000E+00 | 0.000E+00 | 0.000E+00 | 0.000E+00 | 0.000E+00 |
| Unigene318939 | msbA | 0.000E+00 | 0.000E+00 | 0.000E+00 | 0.000E+00 | 1.533E-06 | 0.000E+00 | 0.000E+00 | 0.000E+00 | 0.000E+00 | 0.000E+00 | 0.000E+00 | 0.000E+00 |
| Unigene318969 | novA | 0.000E+00 | 0.000E+00 | 0.000E+00 | 3.105E-07 | 3.260E-06 | 0.000E+00 | 4.640E-07 | 0.000E+00 | 0.000E+00 | 2.491E-07 | 6.714E-07 | 7.929E-07 |
| Unigene318974 | evgS | 0.000E+00 | 0.000E+00 | 0.000E+00 | 0.000E+00 | 2.495E-06 | 2.630E-08 | 0.000E+00 | 7.666E-07 | 2.659E-08 | 1.174E-07 | 2.968E-07 | 0.000E+00 |
| Unigene318975 | msbA | 0.000E+00 | 0.000E+00 | 0.000E+00 | 0.000E+00 | 2.324E-06 | 0.000E+00 | 0.000E+00 | 0.000E+00 | 0.000E+00 | 0.000E+00 | 2.351E-07 | 0.000E+00 |

| | | | | | | | | | | | | | |
|---|---|---|---|---|---|---|---|---|---|---|---|---|---|
| Unigene319006 | novA | 0.000E+00 | 0.000E+00 | 0.000E+00 | 0.000E+00 | 2.940E-06 | 0.000E+00 | 0.000E+00 | 0.000E+00 | 0.000E+00 | 0.000E+00 | 4.749E-07 | 0.000E+00 |
| Unigene319053 | farA | 0.000E+00 | 0.000E+00 | 0.000E+00 | 0.000E+00 | 5.403E-07 | 0.000E+00 | 0.000E+00 | 1.419E-07 | 6.644E-08 | 6.772E-08 | 0.000E+00 | 2.515E-07 |
| Unigene319091 | basS | 4.531E-07 | 9.945E-07 | 5.335E-07 | 2.288E-07 | 8.053E-06 | 5.143E-07 | 5.014E-07 | 2.380E-07 | 0.000E+00 | 1.262E-07 | 2.073E-07 | 0.000E+00 |
| Unigene319133 | Streptomy | 6.622E-07 | 1.983E-06 | 9.475E-07 | 4.736E-07 | 9.119E-06 | 4.474E-07 | 2.000E-06 | 2.576E-07 | 0.000E+00 | 2.305E-07 | 4.103E-07 | 2.283E-07 |
| Unigene319499 | cpxA | 0.000E+00 | 1.716E-07 | 0.000E+00 | 2.527E-07 | 5.560E-07 | 0.000E+00 | 1.731E-07 | 2.556E-07 | 2.051E-07 | 2.439E-07 | 1.789E-07 | 8.874E-07 |
| Unigene319508 | adeS | 7.010E-08 | 1.539E-07 | 8.598E-08 | 5.309E-08 | 6.035E-07 | 1.894E-08 | 1.746E-07 | 6.750E-07 | 3.831E-08 | 2.147E-07 | 8.018E-08 | 5.593E-07 |
| Unigene319543 | mtrA | 0.000E+00 | 0.000E+00 | 0.000E+00 | 0.000E+00 | 2.964E-07 | 0.000E+00 | 0.000E+00 | 2.595E-07 | 0.000E+00 | 0.000E+00 | 0.000E+00 | 0.000E+00 |
| Unigene319627 | lmrC | 0.000E+00 | 0.000E+00 | 8.216E-08 | 0.000E+00 | 1.070E-06 | 0.000E+00 | 3.707E-07 | 7.331E-07 | 0.000E+00 | 3.731E-07 | 5.268E-07 | 1.287E-06 |
| Unigene319660 | MexW | 0.000E+00 | 0.000E+00 | 0.000E+00 | 0.000E+00 | 6.203E-07 | 0.000E+00 | 0.000E+00 | 0.000E+00 | 0.000E+00 | 0.000E+00 | 4.354E-07 | 0.000E+00 |
| Unigene319791 | mtrA | 0.000E+00 | 0.000E+00 | 0.000E+00 | 0.000E+00 | 1.189E-07 | 0.000E+00 | 0.000E+00 | 0.000E+00 | 0.000E+00 | 0.000E+00 | 0.000E+00 | 0.000E+00 |
| Unigene319827 | msbA | 0.000E+00 | 0.000E+00 | 0.000E+00 | 0.000E+00 | 4.879E-07 | 0.000E+00 | 4.051E-07 | 0.000E+00 | 0.000E+00 | 0.000E+00 | 0.000E+00 | 4.759E-07 |
| Unigene319969 | fexA | 0.000E+00 | 0.000E+00 | 0.000E+00 | 0.000E+00 | 2.846E-07 | 0.000E+00 | 0.000E+00 | 0.000E+00 | 0.000E+00 | 0.000E+00 | 0.000E+00 | 0.000E+00 |
| Unigene319999 | hmrM | 4.740E-08 | 0.000E+00 | 0.000E+00 | 0.000E+00 | 1.080E-06 | 0.000E+00 | 0.000E+00 | 0.000E+00 | 6.217E-07 | 2.112E-07 | 0.000E+00 | 0.000E+00 |
| Unigene320052 | msbA | 0.000E+00 | 0.000E+00 | 1.077E-07 | 0.000E+00 | 3.659E-07 | 1.187E-07 | 0.000E+00 | 0.000E+00 | 1.800E-07 | 0.000E+00 | 0.000E+00 | 7.787E-07 |
| Unigene320103 | macB | 0.000E+00 | 0.000E+00 | 0.000E+00 | 0.000E+00 | 5.220E-07 | 0.000E+00 | 0.000E+00 | 0.000E+00 | 0.000E+00 | 0.000E+00 | 0.000E+00 | 0.000E+00 |
| Unigene320150 | kdpE | 0.000E+00 | 0.000E+00 | 0.000E+00 | 6.965E-08 | 3.831E-07 | 7.456E-08 | 3.054E-07 | 0.000E+00 | 3.769E-08 | 0.000E+00 | 2.761E-07 | 4.484E-07 |
| Unigene320226 | macB | 0.000E+00 | 0.000E+00 | 0.000E+00 | 0.000E+00 | 3.532E-07 | 0.000E+00 | 0.000E+00 | 0.000E+00 | 0.000E+00 | 0.000E+00 | 0.000E+00 | 0.000E+00 |
| Unigene320237 | tetW | 3.357E-07 | 0.000E+00 | 5.895E-07 | 2.676E-07 | 1.099E-06 | 3.629E-07 | 1.525E-06 | 1.072E-06 | 1.352E-07 | 6.101E-07 | 8.691E-07 | 3.571E-06 |
| Unigene320240 | ACT-29 | 0.000E+00 | 4.637E-07 | 3.317E-07 | 0.000E+00 | 2.003E-06 | 0.000E+00 | 2.182E-07 | 0.000E+00 | 0.000E+00 | 0.000E+00 | 0.000E+00 | 0.000E+00 |
| Unigene320296 | evgA | 0.000E+00 | 0.000E+00 | 4.503E-07 | 0.000E+00 | 1.133E-06 | 4.410E-07 | 5.080E-07 | 7.143E-07 | 2.229E-07 | 0.000E+00 | 1.225E-06 | 2.411E-06 |
| Unigene320333 | bcrA | 0.000E+00 | 1.189E-07 | 0.000E+00 | 3.646E-08 | 6.017E-07 | 0.000E+00 | 1.998E-07 | 4.215E-07 | 0.000E+00 | 0.000E+00 | 4.543E-07 | 1.707E-07 |
| Unigene320433 | bcrA | 0.000E+00 | 0.000E+00 | 0.000E+00 | 0.000E+00 | 4.184E-07 | 0.000E+00 | 0.000E+00 | 0.000E+00 | 0.000E+00 | 0.000E+00 | 0.000E+00 | 0.000E+00 |
| Unigene320460 | Klebsiella p | 0.000E+00 | 0.000E+00 | 0.000E+00 | 0.000E+00 | 1.679E-06 | 0.000E+00 | 0.000E+00 | 0.000E+00 | 1.069E-06 | 0.000E+00 | 0.000E+00 | 0.000E+00 |
| Unigene320471 | evgS | 0.000E+00 | 0.000E+00 | 0.000E+00 | 0.000E+00 | 4.960E-07 | 0.000E+00 | 0.000E+00 | 0.000E+00 | 0.000E+00 | 0.000E+00 | 0.000E+00 | 0.000E+00 |
| Unigene320586 | evgS | 0.000E+00 | 0.000E+00 | 0.000E+00 | 0.000E+00 | 3.081E-07 | 0.000E+00 | 0.000E+00 | 0.000E+00 | 0.000E+00 | 0.000E+00 | 2.538E-07 | 1.311E-07 |
| Unigene320738 | evgS | 8.183E-08 | 4.490E-08 | 0.000E+00 | 0.000E+00 | 5.909E-07 | 0.000E+00 | 3.170E-07 | 0.000E+00 | 8.943E-08 | 0.000E+00 | 0.000E+00 | 2.902E-07 |
| Unigene320842 | PmrF | 0.000E+00 | 0.000E+00 | 0.000E+00 | 0.000E+00 | 5.560E-07 | 0.000E+00 | 0.000E+00 | 0.000E+00 | 0.000E+00 | 0.000E+00 | 0.000E+00 | 0.000E+00 |
| Unigene320874 | adeL | 0.000E+00 | 1.321E-06 | 0.000E+00 | 2.947E-07 | 6.889E-06 | 2.760E-07 | 0.000E+00 | 1.277E-07 | 0.000E+00 | 0.000E+00 | 0.000E+00 | 0.000E+00 |
| Unigene320877 | TriC | 3.880E-07 | 1.197E-06 | 7.306E-07 | 0.000E+00 | 5.837E-06 | 3.174E-07 | 9.982E-07 | 4.285E-07 | 0.000E+00 | 0.000E+00 | 2.519E-07 | 0.000E+00 |
| Unigene320878 | TriA | 3.933E-07 | 6.784E-07 | 3.308E-07 | 2.553E-07 | 5.181E-06 | 0.000E+00 | 6.841E-07 | 2.623E-07 | 0.000E+00 | 0.000E+00 | 0.000E+00 | 0.000E+00 |
| Unigene320898 | acrB | 4.579E-08 | 2.513E-08 | 1.348E-07 | 1.387E-07 | 6.867E-07 | 1.856E-07 | 6.841E-07 | 5.745E-07 | 0.000E+00 | 1.148E-07 | 0.000E+00 | 8.930E-07 |
| Unigene320899 | OprN | 6.697E-08 | 0.000E+00 | 8.762E-08 | 0.000E+00 | 1.339E-06 | 1.931E-07 | 9.142E-07 | 9.120E-07 | 1.464E-07 | 0.000E+00 | 0.000E+00 | 1.055E-06 |
| Unigene320920 | adeL | 0.000E+00 | 0.000E+00 | 0.000E+00 | 0.000E+00 | 1.195E-06 | 0.000E+00 | 0.000E+00 | 0.000E+00 | 0.000E+00 | 0.000E+00 | 0.000E+00 | 0.000E+00 |
| Unigene320976 | bcrA | 4.618E-07 | 0.000E+00 | 0.000E+00 | 5.596E-07 | 4.823E-06 | 1.018E-05 | 1.227E-06 | 7.978E-06 | 4.896E-06 | 5.247E-06 | 0.000E+00 | 0.000E+00 |
| Unigene321082 | cpxA | 1.377E-07 | 0.000E+00 | 5.178E-07 | 0.000E+00 | 1.300E-06 | 0.000E+00 | 1.193E-06 | 0.000E+00 | 2.006E-07 | 0.000E+00 | 9.448E-07 | 3.363E-06 |
| Unigene321083 | Pseudomo | 0.000E+00 | 0.000E+00 | 5.129E-07 | 3.519E-07 | 9.679E-07 | 5.651E-07 | 1.832E-06 | 9.153E-07 | 0.000E+00 | 0.000E+00 | 0.000E+00 | 3.862E-06 |
| Unigene321122 | emrB | 0.000E+00 | 0.000E+00 | 7.313E-07 | 3.998E-07 | 5.175E-07 | 5.539E-07 | 1.779E-06 | 3.126E-06 | 4.582E-07 | 1.297E-06 | 1.838E-06 | 2.836E-06 |
| Unigene321165 | tetB(60) | 5.436E-07 | 1.522E-06 | 7.358E-07 | 2.650E-07 | 8.164E-06 | 2.634E-07 | 8.299E-07 | 2.407E-07 | 0.000E+00 | 0.000E+00 | 4.932E-07 | 0.000E+00 |
| Unigene321228 | acrD | 0.000E+00 | 0.000E+00 | 0.000E+00 | 0.000E+00 | 1.129E-06 | 0.000E+00 | 0.000E+00 | 0.000E+00 | 6.044E-07 | 0.000E+00 | 0.000E+00 | 0.000E+00 |
| Unigene321378 | MexD | 0.000E+00 | 0.000E+00 | 0.000E+00 | 0.000E+00 | 8.372E-07 | 0.000E+00 | 9.731E-07 | 6.353E-07 | 0.000E+00 | 0.000E+00 | 0.000E+00 | 1.584E-06 |
| Unigene321381 | mdtE | 0.000E+00 | 0.000E+00 | 0.000E+00 | 0.000E+00 | 3.052E-07 | 4.950E-08 | 0.000E+00 | 3.741E-07 | 0.000E+00 | 0.000E+00 | 2.619E-07 | 1.624E-07 |
| Unigene321400 | acrB | 0.000E+00 | 2.518E-08 | 4.502E-08 | 2.317E-08 | 6.627E-07 | 0.000E+00 | 7.618E-08 | 3.214E-07 | 1.003E-07 | 1.278E-07 | 1.575E-07 | 6.237E-07 |
| Unigene321413 | efpA | 6.051E-05 | 6.045E-05 | 1.093E-04 | 7.012E-05 | 1.330E-04 | 9.201E-05 | 1.085E-04 | 1.206E-04 | 1.080E-04 | 1.487E-04 | 1.487E-04 | 1.681E-04 |
| Unigene321414 | lsaA | 7.137E-05 | 6.199E-05 | 1.238E-04 | 1.670E-04 | 1.381E-04 | 9.154E-05 | 1.207E-04 | 1.153E-04 | 1.015E-04 | 1.506E-04 | 1.606E-04 | 1.690E-04 |
| Unigene321503 | patA | 1.596E-07 | 0.000E+00 | 0.000E+00 | 0.000E+00 | 9.577E-07 | 0.000E+00 | 1.131E-06 | 8.572E-07 | 0.000E+00 | 4.624E-07 | 1.278E-06 | 3.887E-06 |
| Unigene321615 | adeL | 1.824E-07 | 8.006E-08 | 1.432E-07 | 1.105E-07 | 7.699E-07 | 1.577E-07 | 3.633E-07 | 3.832E-07 | 0.000E+00 | 0.000E+00 | 3.338E-07 | 6.036E-07 |
| Unigene321646 | tlrC | 4.102E-07 | 1.074E-06 | 0.000E+00 | 5.736E-07 | 6.275E-06 | 4.093E-07 | 1.013E-06 | 7.366E-08 | 0.000E+00 | 0.000E+00 | 3.249E-07 | 0.000E+00 |
| Unigene321666 | bcrA | 0.000E+00 | 0.000E+00 | 0.000E+00 | 0.000E+00 | 5.612E-07 | 0.000E+00 | 0.000E+00 | 0.000E+00 | 0.000E+00 | 0.000E+00 | 0.000E+00 | 0.000E+00 |
| Unigene321752 | Brucella su | 4.754E-08 | 6.957E-08 | 0.000E+00 | 3.201E-08 | 5.106E-07 | 5.139E-08 | 2.280E-07 | 4.624E-07 | 5.196E-08 | 1.765E-07 | 3.807E-07 | 5.807E-07 |
| Unigene321788 | efrA | 0.000E+00 | 0.000E+00 | 4.345E-08 | 4.472E-08 | 2.460E-07 | 0.000E+00 | 0.000E+00 | 0.000E+00 | 0.000E+00 | 0.000E+00 | 1.520E-07 | 5.234E-07 |
| Unigene321802 | evgS | 0.000E+00 | 0.000E+00 | 0.000E+00 | 0.000E+00 | 1.104E-06 | 0.000E+00 | 0.000E+00 | 0.000E+00 | 0.000E+00 | 0.000E+00 | 0.000E+00 | 1.251E-06 |
| Unigene321814 | lmrD | 0.000E+00 | 0.000E+00 | 0.000E+00 | 0.000E+00 | 3.634E-06 | 0.000E+00 | 1.963E-07 | 0.000E+00 | 0.000E+00 | 3.952E-07 | 3.832E-07 | 1.211E-06 |
| Unigene321845 | mgrA | 0.000E+00 | 0.000E+00 | 0.000E+00 | 0.000E+00 | 6.766E-07 | 0.000E+00 | 0.000E+00 | 0.000E+00 | 0.000E+00 | 0.000E+00 | 0.000E+00 | 0.000E+00 |
| Unigene321989 | iri | 0.000E+00 | 0.000E+00 | 0.000E+00 | 0.000E+00 | 3.013E-06 | 0.000E+00 | 0.000E+00 | 0.000E+00 | 0.000E+00 | 0.000E+00 | 1.026E-07 | 0.000E+00 |
| Unigene322236 | cmlv | 2.470E-07 | 1.175E-06 | 6.734E-07 | 0.000E+00 | 6.068E-06 | 2.077E-07 | 9.418E-07 | 4.806E-07 | 0.000E+00 | 0.000E+00 | 0.000E+00 | 2.596E-07 |
| Unigene322267 | farA | 0.000E+00 | 1.354E-07 | 0.000E+00 | 2.491E-07 | 8.220E-07 | 0.000E+00 | 0.000E+00 | 0.000E+00 | 0.000E+00 | 0.000E+00 | 0.000E+00 | 1.020E-06 |
| Unigene322269 | bcrA | 0.000E+00 | 8.578E-08 | 1.278E-07 | 5.262E-08 | 5.499E-07 | 0.000E+00 | 3.460E-07 | 5.170E-07 | 1.424E-07 | 2.902E-07 | 0.000E+00 | 6.467E-07 |
| Unigene322325 | sdiA | 0.000E+00 | 0.000E+00 | 0.000E+00 | 0.000E+00 | 4.483E-07 | 0.000E+00 | 0.000E+00 | 0.000E+00 | 0.000E+00 | 0.000E+00 | 0.000E+00 | 0.000E+00 |

| | | | | | | | | | | | | | |
|---|---|---|---|---|---|---|---|---|---|---|---|---|---|
| Unigene322381 | oleC | 0.000E+00 | 0.000E+00 | 1.669E-07 | 0.000E+00 | 4.725E-07 | 0.000E+00 | 9.414E-08 | 2.482E-07 | 0.000E+00 | 0.000E+00 | 2.432E-07 | 3.519E-07 |
| Unigene322443 | macB | 0.000E+00 | 0.000E+00 | 0.000E+00 | 0.000E+00 | 3.707E-06 | 0.000E+00 | 0.000E+00 | 0.000E+00 | 0.000E+00 | 0.000E+00 | 3.924E-07 | 0.000E+00 |
| Unigene322451 | carA | 0.000E+00 | 0.000E+00 | 0.000E+00 | 0.000E+00 | 3.521E-06 | 0.000E+00 | 0.000E+00 | 1.679E-06 | 0.000E+00 | 0.000E+00 | 4.771E-07 | 0.000E+00 |
| Unigene322517 | emrK | 3.146E-08 | 3.453E-08 | 3.087E-08 | 0.000E+00 | 4.194E-07 | 3.401E-08 | 1.393E-07 | 4.040E-07 | 1.376E-07 | 7.010E-08 | 1.080E-07 | 3.347E-07 |
| Unigene322518 | MexL | 0.000E+00 | 0.000E+00 | 9.469E-08 | 0.000E+00 | 4.288E-07 | 0.000E+00 | 1.068E-07 | 3.943E-07 | 0.000E+00 | 0.000E+00 | 0.000E+00 | 3.422E-07 |
| Unigene322575 | Staphylocc | 0.000E+00 | 0.000E+00 | 0.000E+00 | 0.000E+00 | 4.303E-07 | 0.000E+00 | 0.000E+00 | 3.014E-07 | 0.000E+00 | 0.000E+00 | 0.000E+00 | 0.000E+00 |
| Unigene322582 | novA | 0.000E+00 | 0.000E+00 | 4.124E-07 | 0.000E+00 | 1.167E-06 | 6.361E-07 | 1.396E-06 | 0.000E+00 | 0.000E+00 | 0.000E+00 | 1.202E-06 | 3.478E-06 |
| Unigene322663 | rosA | 0.000E+00 | 0.000E+00 | 0.000E+00 | 0.000E+00 | 5.661E-07 | 1.944E-07 | 6.966E-07 | 5.598E-07 | 0.000E+00 | 0.000E+00 | 0.000E+00 | 0.000E+00 |
| Unigene322679 | basS | 0.000E+00 | 0.000E+00 | 0.000E+00 | 0.000E+00 | 3.701E-07 | 0.000E+00 | 0.000E+00 | 0.000E+00 | 0.000E+00 | 0.000E+00 | 0.000E+00 | 0.000E+00 |
| Unigene322693 | tva(A) | 2.606E-07 | 2.452E-07 | 5.480E-07 | 0.000E+00 | 1.282E-06 | 1.892E-06 | 6.758E-06 | 5.128E-06 | 0.000E+00 | 1.369E-06 | 2.939E-06 | 5.457E-06 |
| Unigene322829 | vanRC | 0.000E+00 | 0.000E+00 | 0.000E+00 | 0.000E+00 | 1.715E-06 | 0.000E+00 | 0.000E+00 | 0.000E+00 | 4.338E-07 | 0.000E+00 | 0.000E+00 | 0.000E+00 |
| Unigene322864 | bcrA | 8.172E-07 | 0.000E+00 | 0.000E+00 | 0.000E+00 | 1.857E-05 | 2.098E-06 | 0.000E+00 | 0.000E+00 | 0.000E+00 | 0.000E+00 | 0.000E+00 | 0.000E+00 |
| Unigene322894 | lmrD | 0.000E+00 | 7.018E-07 | 0.000E+00 | 0.000E+00 | 3.105E-06 | 0.000E+00 | 5.257E-07 | 1.066E-07 | 0.000E+00 | 0.000E+00 | 4.179E-07 | 0.000E+00 |
| Unigene323021 | efpA | 0.000E+00 | 0.000E+00 | 3.314E-07 | 5.603E-07 | 4.556E-06 | 1.030E-05 | 0.000E+00 | 8.730E-06 | 6.434E-06 | 0.000E+00 | 0.000E+00 | 9.696E-07 |
| Unigene323069 | tetA(58) | 0.000E+00 | 0.000E+00 | 0.000E+00 | 0.000E+00 | 7.214E-07 | 0.000E+00 | 0.000E+00 | 0.000E+00 | 0.000E+00 | 0.000E+00 | 0.000E+00 | 0.000E+00 |
| Unigene323200 | cpxA | 2.765E-07 | 0.000E+00 | 7.235E-07 | 2.327E-07 | 9.215E-07 | 3.487E-07 | 1.428E-06 | 7.262E-07 | 1.007E-07 | 0.000E+00 | 9.225E-07 | 3.377E-06 |
| Unigene323300 | basS | 0.000E+00 | 1.575E-07 | 0.000E+00 | 0.000E+00 | 1.833E-06 | 0.000E+00 | 0.000E+00 | 0.000E+00 | 6.534E-07 | 0.000E+00 | 0.000E+00 | 0.000E+00 |
| Unigene323346 | Acinetobac | 0.000E+00 | 1.968E-06 | 0.000E+00 | 0.000E+00 | 6.812E-06 | 0.000E+00 | 0.000E+00 | 0.000E+00 | 8.211E-07 | 0.000E+00 | 0.000E+00 | 0.000E+00 |
| Unigene323408 | efrA | 0.000E+00 | 0.000E+00 | 0.000E+00 | 0.000E+00 | 3.443E-06 | 0.000E+00 | 0.000E+00 | 0.000E+00 | 0.000E+00 | 0.000E+00 | 3.282E-07 | 1.424E-06 |
| Unigene323438 | macB | 0.000E+00 | 0.000E+00 | 0.000E+00 | 0.000E+00 | 2.269E-06 | 0.000E+00 | 0.000E+00 | 0.000E+00 | 0.000E+00 | 0.000E+00 | 0.000E+00 | 0.000E+00 |
| Unigene323439 | tetA(60) | 0.000E+00 | 0.000E+00 | 0.000E+00 | 0.000E+00 | 2.614E-06 | 0.000E+00 | 0.000E+00 | 0.000E+00 | 0.000E+00 | 0.000E+00 | 0.000E+00 | 0.000E+00 |
| Unigene323461 | acrB | 0.000E+00 | 0.000E+00 | 0.000E+00 | 0.000E+00 | 2.886E-06 | 0.000E+00 | 0.000E+00 | 0.000E+00 | 0.000E+00 | 0.000E+00 | 2.340E-07 | 6.408E-07 |
| Unigene323462 | Enterobact | 0.000E+00 | 0.000E+00 | 0.000E+00 | 0.000E+00 | 2.256E-06 | 0.000E+00 | 0.000E+00 | 0.000E+00 | 0.000E+00 | 0.000E+00 | 9.050E-08 | 0.000E+00 |
| Unigene323518 | tetA(58) | 0.000E+00 | 0.000E+00 | 0.000E+00 | 0.000E+00 | 3.997E-06 | 0.000E+00 | 0.000E+00 | 0.000E+00 | 0.000E+00 | 0.000E+00 | 2.619E-07 | 0.000E+00 |
| Unigene323529 | patA | 0.000E+00 | 0.000E+00 | 0.000E+00 | 0.000E+00 | 3.810E-06 | 0.000E+00 | 0.000E+00 | 0.000E+00 | 0.000E+00 | 0.000E+00 | 2.367E-07 | 0.000E+00 |
| Unigene323599 | oleC | 0.000E+00 | 0.000E+00 | 1.383E-07 | 0.000E+00 | 4.698E-07 | 1.016E-07 | 0.000E+00 | 1.152E-06 | 0.000E+00 | 0.000E+00 | 2.150E-07 | 3.888E-07 |
| Unigene323611 | MexD | 9.080E-08 | 0.000E+00 | 8.910E-08 | 4.585E-08 | 2.018E-07 | 4.908E-08 | 4.020E-07 | 1.060E-07 | 0.000E+00 | 0.000E+00 | 0.000E+00 | 1.073E-07 |
| Unigene323612 | mdtE | 0.000E+00 | 0.000E+00 | 0.000E+00 | 0.000E+00 | 2.999E-07 | 0.000E+00 | 0.000E+00 | 3.151E-07 | 0.000E+00 | 0.000E+00 | 4.412E-08 | 3.647E-07 |
| Unigene323626 | tetB(60) | 0.000E+00 | 0.000E+00 | 0.000E+00 | 0.000E+00 | 1.152E-06 | 0.000E+00 | 0.000E+00 | 0.000E+00 | 7.553E-07 | 0.000E+00 | 0.000E+00 | 0.000E+00 |
| Unigene323685 | mdtG | 8.428E-08 | 1.233E-07 | 2.757E-08 | 0.000E+00 | 9.988E-07 | 0.000E+00 | 9.328E-08 | 0.000E+00 | 3.071E-07 | 0.000E+00 | 0.000E+00 | 0.000E+00 |
| Unigene323719 | Streptomy | 0.000E+00 | 8.652E-08 | 7.735E-08 | 0.000E+00 | 9.633E-07 | 8.522E-08 | 8.724E-08 | 3.220E-07 | 8.616E-08 | 0.000E+00 | 1.353E-07 | 2.795E-07 |
| Unigene323772 | vatC | 0.000E+00 | 0.000E+00 | 3.608E-07 | 2.150E-07 | 9.890E-07 | 5.230E-07 | 1.114E-06 | 6.777E-07 | 0.000E+00 | 5.821E-07 | 6.641E-07 | 2.928E-06 |
| Unigene323806 | basS | 0.000E+00 | 0.000E+00 | 0.000E+00 | 0.000E+00 | 5.513E-07 | 0.000E+00 | 0.000E+00 | 0.000E+00 | 0.000E+00 | 0.000E+00 | 0.000E+00 | 0.000E+00 |
| Unigene323823 | emrY | 0.000E+00 | 0.000E+00 | 0.000E+00 | 0.000E+00 | 8.680E-07 | 0.000E+00 | 0.000E+00 | 0.000E+00 | 0.000E+00 | 0.000E+00 | 0.000E+00 | 0.000E+00 |
| Unigene323866 | Staphylocc | 0.000E+00 | 0.000E+00 | 0.000E+00 | 0.000E+00 | 3.659E-07 | 0.000E+00 | 0.000E+00 | 3.845E-07 | 0.000E+00 | 0.000E+00 | 1.256E-07 | 1.298E-06 |
| Unigene323899 | lmrC | 0.000E+00 | 0.000E+00 | 0.000E+00 | 0.000E+00 | 5.148E-07 | 1.670E-07 | 3.989E-07 | 0.000E+00 | 0.000E+00 | 0.000E+00 | 0.000E+00 | 4.260E-07 |
| Unigene323900 | mdtA | 0.000E+00 | 5.905E-08 | 0.000E+00 | 0.000E+00 | 0.000E+00 | 0.000E+00 | 5.954E-08 | 0.000E+00 | 5.880E-08 | 1.199E-07 | 0.000E+00 | 6.359E-08 |
| Unigene323917 | smeS | 7.395E-08 | 0.000E+00 | 3.084E-07 | 0.000E+00 | 5.751E-07 | 9.993E-08 | 7.571E-07 | 6.474E-07 | 8.083E-08 | 0.000E+00 | 2.749E-07 | 6.775E-07 |
| Unigene323983 | tetT | 9.885E-07 | 0.000E+00 | 1.749E-07 | 4.909E-07 | 5.149E-06 | 8.777E-06 | 1.614E-06 | 8.228E-06 | 4.924E-06 | 3.628E-06 | 0.000E+00 | 7.662E-07 |
| Unigene324006 | mdtG | 0.000E+00 | 0.000E+00 | 2.805E-07 | 5.249E-07 | 4.129E-06 | 0.000E+00 | 0.000E+00 | 0.000E+00 | 0.000E+00 | 0.000E+00 | 0.000E+00 | 0.000E+00 |
| Unigene324013 | YojI | 0.000E+00 | 0.000E+00 | 2.974E-07 | 4.017E-07 | 4.546E-06 | 0.000E+00 | 0.000E+00 | 0.000E+00 | 0.000E+00 | 0.000E+00 | 0.000E+00 | 0.000E+00 |
| Unigene324015 | tetA(58) | 0.000E+00 | 0.000E+00 | 1.015E-07 | 1.741E-07 | 4.789E-06 | 0.000E+00 | 0.000E+00 | 8.735E-06 | 0.000E+00 | 0.000E+00 | 0.000E+00 | 0.000E+00 |
| Unigene324019 | arlR | 0.000E+00 | 9.640E-08 | 3.017E-07 | 0.000E+00 | 5.855E-07 | 3.798E-07 | 9.721E-07 | 1.230E-06 | 0.000E+00 | 0.000E+00 | 6.029E-07 | 2.855E-06 |
| Unigene324036 | msbA | 0.000E+00 | 0.000E+00 | 0.000E+00 | 0.000E+00 | 1.507E-07 | 4.887E-08 | 0.000E+00 | 0.000E+00 | 0.000E+00 | 1.007E-07 | 0.000E+00 | 2.672E-07 |
| Unigene324044 | carA | 0.000E+00 | 1.406E-07 | 1.676E-07 | 0.000E+00 | 4.744E-07 | 2.308E-07 | 1.134E-06 | 0.000E+00 | 1.400E-07 | 0.000E+00 | 0.000E+00 | 1.413E-06 |
| Unigene324166 | vanHD | 5.299E-05 | 1.457E-04 | 7.231E-05 | 0.000E+00 | 3.618E-06 | 0.000E+00 | 0.000E+00 | 0.000E+00 | 0.000E+00 | 0.000E+00 | 1.397E-06 | 0.000E+00 |
| Unigene324281 | patA | 5.518E-07 | 1.022E-06 | 6.768E-07 | 2.786E-07 | 7.126E-06 | 2.983E-07 | 0.000E+00 | 3.019E-07 | 0.000E+00 | 2.113E-07 | 4.537E-07 | 0.000E+00 |
| Unigene324303 | YojI | 5.416E-08 | 1.169E-06 | 3.189E-07 | 1.823E-08 | 2.708E-06 | 1.952E-07 | 0.000E+00 | 0.000E+00 | 0.000E+00 | 0.000E+00 | 0.000E+00 | 0.000E+00 |
| Unigene324362 | MexD | 0.000E+00 | 1.810E-07 | 0.000E+00 | 0.000E+00 | 5.496E-07 | 0.000E+00 | 0.000E+00 | 0.000E+00 | 9.012E-08 | 0.000E+00 | 9.432E-08 | 0.000E+00 |
| Unigene324364 | MSI-1 | 0.000E+00 | 0.000E+00 | 0.000E+00 | 0.000E+00 | 6.084E-07 | 0.000E+00 | 2.496E-07 | 4.136E-07 | 1.056E-07 | 0.000E+00 | 1.843E-07 | 4.570E-07 |
| Unigene324482 | poxtA | 0.000E+00 | 0.000E+00 | 0.000E+00 | 0.000E+00 | 3.415E-06 | 0.000E+00 | 0.000E+00 | 0.000E+00 | 0.000E+00 | 4.186E-07 | 4.689E-07 | 0.000E+00 |
| Unigene324504 | emrA | 0.000E+00 | 0.000E+00 | 0.000E+00 | 0.000E+00 | 2.821E-06 | 0.000E+00 | 0.000E+00 | 0.000E+00 | 0.000E+00 | 0.000E+00 | 3.457E-07 | 0.000E+00 |
| Unigene324532 | cmlv | 0.000E+00 | 0.000E+00 | 0.000E+00 | 0.000E+00 | 3.464E-06 | 0.000E+00 | 0.000E+00 | 0.000E+00 | 0.000E+00 | 0.000E+00 | 3.156E-07 | 0.000E+00 |
| Unigene324536 | novA | 0.000E+00 | 0.000E+00 | 0.000E+00 | 0.000E+00 | 3.193E-06 | 0.000E+00 | 0.000E+00 | 0.000E+00 | 0.000E+00 | 0.000E+00 | 6.849E-07 | 1.597E-06 |
| Unigene324586 | mtrA | 0.000E+00 | 3.192E-07 | 2.854E-07 | 0.000E+00 | 1.185E-06 | 1.048E-07 | 1.395E-06 | 1.018E-06 | 0.000E+00 | 0.000E+00 | 0.000E+00 | 8.594E-07 |
| Unigene324638 | carA | 0.000E+00 | 1.675E-07 | 2.621E-07 | 0.000E+00 | 8.054E-07 | 0.000E+00 | 7.179E-07 | 6.235E-07 | 4.170E-08 | 0.000E+00 | 0.000E+00 | 1.398E-06 |
| Unigene324674 | basS | 1.145E-07 | 0.000E+00 | 0.000E+00 | 0.000E+00 | 1.933E-06 | 0.000E+00 | 0.000E+00 | 0.000E+00 | 0.000E+00 | 0.000E+00 | 0.000E+00 | 0.000E+00 |
| Unigene324725 | emrB | 0.000E+00 | 0.000E+00 | 0.000E+00 | 0.000E+00 | 6.930E-07 | 0.000E+00 | 0.000E+00 | 0.000E+00 | 0.000E+00 | 0.000E+00 | 0.000E+00 | 0.000E+00 |

| | | | | | | | | | | | | | |
|---|---|---|---|---|---|---|---|---|---|---|---|---|---|
| Unigene324765 | adeS | 0.000E+00 | 0.000E+00 | 0.000E+00 | 0.000E+00 | 6.520E-07 | 0.000E+00 | 0.000E+00 | 5.328E-07 | 0.000E+00 | 0.000E+00 | 0.000E+00 | 0.000E+00 |
| Unigene324778 | smeS | 1.148E-07 | 0.000E+00 | 5.631E-08 | 2.898E-08 | 4.463E-07 | 1.551E-07 | 4.128E-07 | 4.019E-07 | 0.000E+00 | 1.279E-07 | 3.282E-07 | 5.766E-07 |
| Unigene324782 | tcr3 | 3.289E-07 | 5.673E-07 | 2.766E-07 | 0.000E+00 | 7.151E-06 | 3.810E-07 | 0.000E+00 | 5.210E-07 | 0.000E+00 | 0.000E+00 | 2.956E-07 | 0.000E+00 |
| Unigene324843 | sdiA | 0.000E+00 | 0.000E+00 | 0.000E+00 | 0.000E+00 | 6.870E-07 | 0.000E+00 | 0.000E+00 | 0.000E+00 | 0.000E+00 | 0.000E+00 | 0.000E+00 | 0.000E+00 |
| Unigene324849 | rosB | 0.000E+00 | 0.000E+00 | 0.000E+00 | 0.000E+00 | 0.000E+00 | 0.000E+00 | 0.000E+00 | 4.579E-07 | 0.000E+00 | 0.000E+00 | 1.282E-07 | 3.312E-07 |
| Unigene324852 | patB | 0.000E+00 | 0.000E+00 | 0.000E+00 | 0.000E+00 | 8.822E-07 | 0.000E+00 | 0.000E+00 | 0.000E+00 | 0.000E+00 | 0.000E+00 | 9.538E-07 | 0.000E+00 |
| Unigene324916 | bcrA | 0.000E+00 | 0.000E+00 | 0.000E+00 | 0.000E+00 | 0.000E+00 | 1.565E-07 | 0.000E+00 | 0.000E+00 | 0.000E+00 | 0.000E+00 | 0.000E+00 | 0.000E+00 |
| Unigene325012 | oleB | 0.000E+00 | 0.000E+00 | 0.000E+00 | 3.853E-07 | 1.229E-06 | 4.125E-07 | 0.000E+00 | 1.381E-06 | 0.000E+00 | 0.000E+00 | 0.000E+00 | 2.526E-06 |
| Unigene325013 | macB | 0.000E+00 | 0.000E+00 | 0.000E+00 | 0.000E+00 | 1.458E-06 | 0.000E+00 | 0.000E+00 | 9.701E-07 | 0.000E+00 | 0.000E+00 | 0.000E+00 | 1.448E-06 |
| Unigene325155 | patA | 1.865E-08 | 1.433E-07 | 0.000E+00 | 0.000E+00 | 8.080E-07 | 0.000E+00 | 1.651E-07 | 5.442E-07 | 1.223E-07 | 2.077E-07 | 0.000E+00 | 5.952E-07 |
| Unigene325240 | mdtG | 0.000E+00 | 0.000E+00 | 0.000E+00 | 0.000E+00 | 2.255E-06 | 0.000E+00 | 0.000E+00 | 0.000E+00 | 1.450E-05 | 0.000E+00 | 0.000E+00 | 0.000E+00 |
| Unigene325274 | efrA | 0.000E+00 | 0.000E+00 | 1.203E-07 | 0.000E+00 | 9.876E-07 | 0.000E+00 | 1.154E-06 | 7.872E-07 | 0.000E+00 | 0.000E+00 | 3.857E-07 | 9.784E-07 |
| Unigene325281 | MuxB | 0.000E+00 | 0.000E+00 | 0.000E+00 | 0.000E+00 | 2.415E-07 | 0.000E+00 | 3.308E-07 | 2.537E-07 | 5.940E-08 | 0.000E+00 | 0.000E+00 | 6.102E-07 |
| Unigene325334 | rpoB2 | 0.000E+00 | 0.000E+00 | 0.000E+00 | 0.000E+00 | 8.584E-07 | 0.000E+00 | 7.918E-07 | 6.681E-07 | 0.000E+00 | 3.188E-07 | 0.000E+00 | 7.780E-07 |
| Unigene325361 | cmlA6 | 0.000E+00 | 2.202E-07 | 5.168E-07 | 1.773E-07 | 1.170E-06 | 3.525E-07 | 1.721E-06 | 1.376E-06 | 0.000E+00 | 1.118E-06 | 9.468E-07 | 4.210E-06 |
| Unigene325387 | vanRE | 0.000E+00 | 0.000E+00 | 0.000E+00 | 0.000E+00 | 3.146E-07 | 0.000E+00 | 0.000E+00 | 0.000E+00 | 0.000E+00 | 0.000E+00 | 0.000E+00 | 0.000E+00 |
| Unigene325406 | tetB(60) | 2.788E-08 | 6.119E-08 | 0.000E+00 | 1.126E-07 | 4.955E-07 | 0.000E+00 | 2.468E-07 | 4.555E-07 | 9.140E-08 | 1.242E-07 | 0.000E+00 | 2.966E-07 |
| Unigene325424 | TriC | 0.000E+00 | 0.000E+00 | 3.199E-08 | 6.585E-08 | 4.166E-07 | 0.000E+00 | 1.624E-07 | 3.425E-07 | 0.000E+00 | 9.081E-08 | 1.305E-07 | 2.891E-07 |
| Unigene325471 | vanG | 0.000E+00 | 1.308E-06 | 8.254E-07 | 0.000E+00 | 1.830E-06 | 0.000E+00 | 1.009E-06 | 0.000E+00 | 8.811E-07 | 1.562E-07 | 5.212E-07 | 0.000E+00 |
| Unigene325510 | golS | 0.000E+00 | 0.000E+00 | 4.430E-07 | 0.000E+00 | 8.359E-07 | 0.000E+00 | 1.832E-06 | 3.074E-06 | 0.000E+00 | 1.425E-06 | 1.980E-06 | 3.469E-06 |
| Unigene325532 | msbA | 0.000E+00 | 0.000E+00 | 0.000E+00 | 0.000E+00 | 6.849E-07 | 0.000E+00 | 0.000E+00 | 0.000E+00 | 0.000E+00 | 0.000E+00 | 0.000E+00 | 0.000E+00 |
| Unigene325583 | efpA | 0.000E+00 | 0.000E+00 | 0.000E+00 | 0.000E+00 | 1.775E-06 | 0.000E+00 | 0.000E+00 | 0.000E+00 | 0.000E+00 | 0.000E+00 | 0.000E+00 | 0.000E+00 |
| Unigene325599 | tet(49) | 2.869E-08 | 9.448E-08 | 1.126E-07 | 0.000E+00 | 4.782E-07 | 0.000E+00 | 1.905E-07 | 2.010E-07 | 1.254E-07 | 0.000E+00 | 9.847E-08 | 2.035E-07 |
| Unigene325774 | emrB | 0.000E+00 | 0.000E+00 | 0.000E+00 | 0.000E+00 | 7.289E-07 | 0.000E+00 | 0.000E+00 | 0.000E+00 | 0.000E+00 | 0.000E+00 | 0.000E+00 | 0.000E+00 |
| Unigene325775 | Staphylocc | 0.000E+00 | 0.000E+00 | 0.000E+00 | 5.515E-07 | 8.133E-07 | 0.000E+00 | 0.000E+00 | 0.000E+00 | 3.937E-07 | 0.000E+00 | 0.000E+00 | 0.000E+00 |
| Unigene325810 | patA | 8.374E-08 | 1.379E-07 | 0.000E+00 | 4.228E-08 | 5.582E-07 | 4.526E-08 | 4.170E-07 | 2.932E-07 | 4.576E-08 | 0.000E+00 | 2.395E-07 | 3.959E-07 |
| Unigene325871 | efrA | 0.000E+00 | 0.000E+00 | 0.000E+00 | 0.000E+00 | 6.292E-07 | 0.000E+00 | 0.000E+00 | 0.000E+00 | 0.000E+00 | 0.000E+00 | 0.000E+00 | 8.607E-07 |
| Unigene325902 | tetA(60) | 0.000E+00 | 0.000E+00 | 0.000E+00 | 0.000E+00 | 3.783E-06 | 0.000E+00 | 0.000E+00 | 0.000E+00 | 0.000E+00 | 0.000E+00 | 0.000E+00 | 0.000E+00 |
| Unigene325945 | macB | 0.000E+00 | 0.000E+00 | 0.000E+00 | 0.000E+00 | 3.501E-06 | 0.000E+00 | 0.000E+00 | 0.000E+00 | 0.000E+00 | 0.000E+00 | 0.000E+00 | 0.000E+00 |
| Unigene325951 | clbB | 0.000E+00 | 0.000E+00 | 0.000E+00 | 0.000E+00 | 3.190E-06 | 0.000E+00 | 0.000E+00 | 1.721E-06 | 0.000E+00 | 0.000E+00 | 8.876E-07 | 1.896E-06 |
| Unigene325965 | mefC | 0.000E+00 | 0.000E+00 | 0.000E+00 | 0.000E+00 | 3.383E-06 | 0.000E+00 | 0.000E+00 | 0.000E+00 | 0.000E+00 | 0.000E+00 | 0.000E+00 | 0.000E+00 |
| Unigene326007 | oleB | 0.000E+00 | 0.000E+00 | 0.000E+00 | 0.000E+00 | 0.000E+00 | 0.000E+00 | 0.000E+00 | 1.705E-07 | 0.000E+00 | 0.000E+00 | 0.000E+00 | 0.000E+00 |
| Unigene326018 | TaeA | 7.067E-08 | 7.757E-08 | 5.201E-08 | 5.353E-08 | 8.048E-07 | 0.000E+00 | 6.257E-07 | 2.681E-07 | 1.545E-07 | 2.952E-07 | 2.223E-07 | 8.145E-07 |
| Unigene326047 | mdtA | 0.000E+00 | 6.947E-07 | 0.000E+00 | 0.000E+00 | 1.450E-06 | 0.000E+00 | 0.000E+00 | 0.000E+00 | 0.000E+00 | 0.000E+00 | 0.000E+00 | 0.000E+00 |
| Unigene326083 | Staphylocc | 7.266E-07 | 3.797E-08 | 3.282E-07 | 4.309E-07 | 4.023E-06 | 8.328E-06 | 1.391E-06 | 7.889E-06 | 3.668E-06 | 3.327E-06 | 0.000E+00 | 7.089E-07 |
| Unigene326117 | adeB | 9.384E-08 | 3.204E-07 | 3.069E-07 | 5.264E-08 | 1.031E-06 | 0.000E+00 | 1.385E-07 | 7.302E-08 | 0.000E+00 | 0.000E+00 | 3.339E-07 | 0.000E+00 |
| Unigene326305 | sul4 | 8.579E-07 | 2.668E-06 | 1.649E-06 | 0.000E+00 | 1.084E-05 | 7.729E-07 | 2.572E-06 | 4.590E-07 | 2.735E-07 | 0.000E+00 | 9.814E-07 | 0.000E+00 |
| Unigene326375 | mtrA | 0.000E+00 | 1.036E-07 | 0.000E+00 | 0.000E+00 | 8.389E-07 | 0.000E+00 | 4.701E-07 | 4.958E-07 | 2.579E-07 | 2.629E-07 | 0.000E+00 | 1.618E-06 |
| Unigene326482 | Staphylocc | 0.000E+00 | 0.000E+00 | 0.000E+00 | 0.000E+00 | 1.192E-06 | 0.000E+00 | 0.000E+00 | 3.132E-08 | 0.000E+00 | 0.000E+00 | 0.000E+00 | 6.343E-08 |
| Unigene326524 | mexN | 0.000E+00 | 6.561E-08 | 0.000E+00 | 0.000E+00 | 7.969E-07 | 0.000E+00 | 3.308E-07 | 7.675E-07 | 6.534E-08 | 0.000E+00 | 0.000E+00 | 4.240E-07 |
| Unigene326539 | macB | 0.000E+00 | 5.761E-08 | 0.000E+00 | 0.000E+00 | 6.997E-07 | 0.000E+00 | 5.228E-07 | 0.000E+00 | 0.000E+00 | 0.000E+00 | 6.004E-08 | 2.482E-07 |
| Unigene326572 | tetA(58) | 0.000E+00 | 0.000E+00 | 0.000E+00 | 0.000E+00 | 5.479E-07 | 1.454E-07 | 0.000E+00 | 0.000E+00 | 0.000E+00 | 0.000E+00 | 0.000E+00 | 0.000E+00 |
| Unigene326745 | vanRI | 0.000E+00 | 3.623E-08 | 0.000E+00 | 3.333E-07 | 5.134E-07 | 0.000E+00 | 0.000E+00 | 0.000E+00 | 0.000E+00 | 0.000E+00 | 0.000E+00 | 3.901E-07 |
| Unigene326752 | emrY | 0.000E+00 | 0.000E+00 | 0.000E+00 | 0.000E+00 | 8.602E-07 | 0.000E+00 | 0.000E+00 | 0.000E+00 | 0.000E+00 | 0.000E+00 | 0.000E+00 | 0.000E+00 |
| Unigene326753 | baeS | 0.000E+00 | 0.000E+00 | 0.000E+00 | 0.000E+00 | 1.022E-06 | 0.000E+00 | 0.000E+00 | 0.000E+00 | 8.293E-07 | 0.000E+00 | 0.000E+00 | 0.000E+00 |
| Unigene326802 | rosB | 0.000E+00 | 0.000E+00 | 0.000E+00 | 0.000E+00 | 0.000E+00 | 0.000E+00 | 0.000E+00 | 0.000E+00 | 2.125E-07 | 0.000E+00 | 0.000E+00 | 0.000E+00 |
| Unigene326921 | vanHO | 0.000E+00 | 0.000E+00 | 0.000E+00 | 1.612E-07 | 6.740E-07 | 2.416E-07 | 4.947E-07 | 2.982E-07 | 0.000E+00 | 0.000E+00 | 0.000E+00 | 1.057E-06 |
| Unigene327024 | novA | 0.000E+00 | 0.000E+00 | 0.000E+00 | 0.000E+00 | 1.641E-06 | 5.424E-07 | 1.882E-06 | 1.627E-06 | 0.000E+00 | 0.000E+00 | 1.180E-06 | 5.239E-06 |
| Unigene327113 | MexI | 0.000E+00 | 0.000E+00 | 0.000E+00 | 0.000E+00 | 0.000E+00 | 0.000E+00 | 0.000E+00 | 0.000E+00 | 0.000E+00 | 0.000E+00 | 0.000E+00 | 5.133E-07 |
| Unigene327132 | efrA | 0.000E+00 | 0.000E+00 | 3.388E-07 | 0.000E+00 | 6.392E-07 | 2.488E-07 | 9.871E-07 | 4.030E-07 | 9.434E-08 | 2.243E-07 | 6.911E-07 | 8.502E-07 |
| Unigene327182 | tlrC | 1.559E-07 | 8.558E-08 | 0.000E+00 | 0.000E+00 | 1.732E-07 | 4.215E-08 | 1.726E-07 | 2.275E-07 | 4.261E-08 | 0.000E+00 | 0.000E+00 | 4.608E-07 |
| Unigene327209 | NmcR | 0.000E+00 | 0.000E+00 | 0.000E+00 | 0.000E+00 | 1.135E-06 | 0.000E+00 | 0.000E+00 | 0.000E+00 | 5.980E-07 | 0.000E+00 | 0.000E+00 | 0.000E+00 |
| Unigene327213 | L1 beta-la | 0.000E+00 | 0.000E+00 | 0.000E+00 | 6.666E-08 | 8.800E-07 | 0.000E+00 | 0.000E+00 | 0.000E+00 | 0.000E+00 | 0.000E+00 | 0.000E+00 | 0.000E+00 |
| Unigene327224 | TriC | 0.000E+00 | 5.705E-08 | 0.000E+00 | 0.000E+00 | 3.465E-07 | 1.124E-07 | 0.000E+00 | 1.213E-07 | 0.000E+00 | 0.000E+00 | 2.973E-07 | 4.915E-07 |
| Unigene327264 | YojI | 0.000E+00 | 0.000E+00 | 0.000E+00 | 0.000E+00 | 4.706E-07 | 4.580E-07 | 8.439E-07 | 0.000E+00 | 2.778E-07 | 0.000E+00 | 0.000E+00 | 6.510E-07 |
| Unigene327389 | oleC | 0.000E+00 | 0.000E+00 | 0.000E+00 | 0.000E+00 | 2.448E-06 | 1.401E-07 | 0.000E+00 | 1.463E-06 | 0.000E+00 | 0.000E+00 | 6.920E-07 | 0.000E+00 |
| Unigene327390 | TaeA | 0.000E+00 | 0.000E+00 | 0.000E+00 | 0.000E+00 | 2.328E-06 | 0.000E+00 | 0.000E+00 | 1.364E-06 | 0.000E+00 | 0.000E+00 | 0.000E+00 | 0.000E+00 |
| Unigene327393 | iri | 0.000E+00 | 0.000E+00 | 0.000E+00 | 0.000E+00 | 3.241E-06 | 0.000E+00 | 0.000E+00 | 0.000E+00 | 0.000E+00 | 0.000E+00 | 4.286E-07 | 0.000E+00 |

| | | | | | | | | | | | | | |
|---|---|---|---|---|---|---|---|---|---|---|---|---|---|
| Unigene327449 | vanRI | 0.000E+00 | 0.000E+00 | 0.000E+00 | 0.000E+00 | 2.323E-06 | 0.000E+00 | 0.000E+00 | 0.000E+00 | 0.000E+00 | 0.000E+00 | 1.344E-07 | 0.000E+00 |
| Unigene327457 | lmrC | 0.000E+00 | 0.000E+00 | 0.000E+00 | 0.000E+00 | 2.807E-06 | 0.000E+00 | 0.000E+00 | 0.000E+00 | 0.000E+00 | 0.000E+00 | 0.000E+00 | 0.000E+00 |
| Unigene327486 | bcrA | 0.000E+00 | 0.000E+00 | 0.000E+00 | 0.000E+00 | 1.843E-06 | 0.000E+00 | 0.000E+00 | 0.000E+00 | 0.000E+00 | 0.000E+00 | 0.000E+00 | 0.000E+00 |
| Unigene327488 | vanRF | 0.000E+00 | 0.000E+00 | 0.000E+00 | 0.000E+00 | 1.931E-06 | 0.000E+00 | 0.000E+00 | 0.000E+00 | 0.000E+00 | 0.000E+00 | 0.000E+00 | 0.000E+00 |
| Unigene327489 | cmlA6 | 1.180E-06 | 2.616E-06 | 1.975E-06 | 6.075E-07 | 1.319E-05 | 6.754E-07 | 2.510E-06 | 5.402E-07 | 0.000E+00 | 3.867E-07 | 1.244E-06 | 0.000E+00 |
| Unigene327526 | ykkC | 0.000E+00 | 0.000E+00 | 0.000E+00 | 0.000E+00 | 0.000E+00 | 0.000E+00 | 0.000E+00 | 4.785E-07 | 0.000E+00 | 0.000E+00 | 0.000E+00 | 0.000E+00 |
| Unigene327564 | bmr | 0.000E+00 | 0.000E+00 | 0.000E+00 | 0.000E+00 | 3.903E-07 | 0.000E+00 | 0.000E+00 | 0.000E+00 | 0.000E+00 | 0.000E+00 | 0.000E+00 | 0.000E+00 |
| Unigene327615 | arlR | 0.000E+00 | 0.000E+00 | 0.000E+00 | 0.000E+00 | 0.000E+00 | 0.000E+00 | 0.000E+00 | 2.444E-06 | 0.000E+00 | 5.424E-07 | 0.000E+00 | 0.000E+00 |
| Unigene327683 | cfrC | 0.000E+00 | 0.000E+00 | 0.000E+00 | 0.000E+00 | 4.673E-07 | 0.000E+00 | 0.000E+00 | 5.728E-07 | 0.000E+00 | 0.000E+00 | 0.000E+00 | 0.000E+00 |
| Unigene327724 | Klebsiella p | 0.000E+00 | 0.000E+00 | 2.603E-07 | 0.000E+00 | 1.245E-06 | 0.000E+00 | 0.000E+00 | 0.000E+00 | 4.833E-07 | 0.000E+00 | 0.000E+00 | 0.000E+00 |
| Unigene327833 | bcrA | 0.000E+00 | 0.000E+00 | 0.000E+00 | 0.000E+00 | 5.539E-07 | 0.000E+00 | 0.000E+00 | 0.000E+00 | 0.000E+00 | 0.000E+00 | 0.000E+00 | 2.946E-07 |
| Unigene327857 | oleC | 0.000E+00 | 0.000E+00 | 0.000E+00 | 0.000E+00 | 6.842E-07 | 0.000E+00 | 0.000E+00 | 0.000E+00 | 0.000E+00 | 0.000E+00 | 0.000E+00 | 0.000E+00 |
| Unigene327998 | YojI | 2.348E-07 | 2.147E-07 | 0.000E+00 | 2.371E-07 | 6.086E-07 | 3.807E-07 | 5.630E-07 | 6.394E-07 | 1.711E-07 | 2.615E-07 | 7.161E-07 | 1.619E-06 |
| Unigene328059 | tlrC | 0.000E+00 | 0.000E+00 | 0.000E+00 | 0.000E+00 | 1.597E-06 | 0.000E+00 | 2.682E-06 | 0.000E+00 | 2.693E-07 | 0.000E+00 | 8.926E-07 | 5.146E-06 |
| Unigene328131 | vga(E) Stap | 0.000E+00 | 0.000E+00 | 2.883E-07 | 2.967E-07 | 1.102E-06 | 1.191E-07 | 7.722E-07 | 6.430E-07 | 0.000E+00 | 2.864E-07 | 1.680E-07 | 1.519E-06 |
| Unigene328142 | bcrA | 0.000E+00 | 0.000E+00 | 0.000E+00 | 0.000E+00 | 0.000E+00 | 8.553E-08 | 0.000E+00 | 0.000E+00 | 0.000E+00 | 0.000E+00 | 0.000E+00 | 3.741E-07 |
| Unigene328210 | Acinetobac | 0.000E+00 | 0.000E+00 | 0.000E+00 | 0.000E+00 | 3.344E-07 | 0.000E+00 | 0.000E+00 | 0.000E+00 | 0.000E+00 | 0.000E+00 | 0.000E+00 | 0.000E+00 |
| Unigene328254 | MexK | 0.000E+00 | 6.967E-08 | 0.000E+00 | 0.000E+00 | 7.052E-07 | 0.000E+00 | 0.000E+00 | 0.000E+00 | 0.000E+00 | 0.000E+00 | 0.000E+00 | 0.000E+00 |
| Unigene328304 | bacA | 0.000E+00 | 0.000E+00 | 0.000E+00 | 0.000E+00 | 5.753E-07 | 0.000E+00 | 0.000E+00 | 0.000E+00 | 0.000E+00 | 0.000E+00 | 1.975E-07 | 0.000E+00 |
| Unigene328368 | evgS | 0.000E+00 | 0.000E+00 | 0.000E+00 | 0.000E+00 | 0.000E+00 | 0.000E+00 | 0.000E+00 | 0.000E+00 | 0.000E+00 | 0.000E+00 | 0.000E+00 | 1.060E-06 |
| Unigene328427 | vanSL | 0.000E+00 | 0.000E+00 | 0.000E+00 | 0.000E+00 | 4.404E-07 | 0.000E+00 | 0.000E+00 | 0.000E+00 | 0.000E+00 | 0.000E+00 | 0.000E+00 | 0.000E+00 |
| Unigene328533 | patA | 0.000E+00 | 0.000E+00 | 0.000E+00 | 0.000E+00 | 7.265E-07 | 0.000E+00 | 0.000E+00 | 0.000E+00 | 0.000E+00 | 0.000E+00 | 0.000E+00 | 0.000E+00 |
| Unigene328632 | facT | 0.000E+00 | 0.000E+00 | 0.000E+00 | 0.000E+00 | 1.148E-06 | 0.000E+00 | 0.000E+00 | 0.000E+00 | 0.000E+00 | 0.000E+00 | 0.000E+00 | 0.000E+00 |
| Unigene328654 | Pseudomo | 0.000E+00 | 0.000E+00 | 2.690E-07 | 0.000E+00 | 9.137E-07 | 0.000E+00 | 0.000E+00 | 0.000E+00 | 0.000E+00 | 0.000E+00 | 3.920E-07 | 0.000E+00 |
| Unigene328706 | kdpE | 0.000E+00 | 0.000E+00 | 0.000E+00 | 0.000E+00 | 6.897E-07 | 0.000E+00 | 0.000E+00 | 0.000E+00 | 0.000E+00 | 0.000E+00 | 0.000E+00 | 0.000E+00 |
| Unigene328753 | patA | 8.506E-08 | 0.000E+00 | 0.000E+00 | 3.436E-07 | 4.252E-07 | 9.195E-08 | 2.824E-07 | 0.000E+00 | 0.000E+00 | 0.000E+00 | 3.405E-07 | 2.011E-07 |
| Unigene328767 | evgS | 1.259E-07 | 3.083E-07 | 3.516E-07 | 0.000E+00 | 1.356E-06 | 1.047E-07 | 2.358E-07 | 0.000E+00 | 0.000E+00 | 0.000E+00 | 3.656E-07 | 5.724E-08 |
| Unigene329075 | MexD | 0.000E+00 | 0.000E+00 | 0.000E+00 | 7.056E-08 | 3.881E-07 | 0.000E+00 | 1.933E-07 | 3.670E-07 | 0.000E+00 | 0.000E+00 | 1.998E-07 | 3.717E-07 |
| Unigene329104 | efrA | 0.000E+00 | 0.000E+00 | 0.000E+00 | 0.000E+00 | 5.778E-07 | 0.000E+00 | 2.159E-07 | 2.277E-07 | 7.106E-08 | 0.000E+00 | 0.000E+00 | 0.000E+00 |
| Unigene329209 | Staphylocc | 0.000E+00 | 0.000E+00 | 0.000E+00 | 0.000E+00 | 3.347E-07 | 0.000E+00 | 0.000E+00 | 0.000E+00 | 0.000E+00 | 0.000E+00 | 0.000E+00 | 0.000E+00 |
| Unigene329234 | arlR | 9.197E-08 | 0.000E+00 | 0.000E+00 | 0.000E+00 | 8.684E-07 | 0.000E+00 | 0.000E+00 | 6.441E-07 | 4.021E-07 | 0.000E+00 | 0.000E+00 | 1.685E-06 |
| Unigene329244 | Pseudomo | 0.000E+00 | 0.000E+00 | 0.000E+00 | 0.000E+00 | 2.110E-06 | 0.000E+00 | 0.000E+00 | 0.000E+00 | 0.000E+00 | 0.000E+00 | 8.045E-08 | 0.000E+00 |
| Unigene329258 | cmlv | 0.000E+00 | 0.000E+00 | 0.000E+00 | 0.000E+00 | 2.518E-06 | 0.000E+00 | 0.000E+00 | 0.000E+00 | 0.000E+00 | 0.000E+00 | 5.066E-07 | 0.000E+00 |
| Unigene329270 | OXA-18 | 0.000E+00 | 0.000E+00 | 0.000E+00 | 0.000E+00 | 2.627E-06 | 0.000E+00 | 0.000E+00 | 0.000E+00 | 0.000E+00 | 0.000E+00 | 0.000E+00 | 0.000E+00 |
| Unigene329283 | evgS | 0.000E+00 | 0.000E+00 | 0.000E+00 | 0.000E+00 | 2.623E-06 | 0.000E+00 | 1.215E-07 | 0.000E+00 | 0.000E+00 | 0.000E+00 | 2.041E-07 | 2.596E-07 |
| Unigene329292 | cpxA | 0.000E+00 | 0.000E+00 | 0.000E+00 | 0.000E+00 | 2.899E-06 | 2.496E-08 | 0.000E+00 | 0.000E+00 | 0.000E+00 | 0.000E+00 | 3.698E-07 | 0.000E+00 |
| Unigene329293 | arlR | 0.000E+00 | 0.000E+00 | 0.000E+00 | 0.000E+00 | 3.095E-06 | 0.000E+00 | 0.000E+00 | 0.000E+00 | 0.000E+00 | 0.000E+00 | 4.396E-07 | 0.000E+00 |
| Unigene329302 | golS | 0.000E+00 | 0.000E+00 | 0.000E+00 | 0.000E+00 | 3.665E-06 | 0.000E+00 | 0.000E+00 | 0.000E+00 | 0.000E+00 | 0.000E+00 | 0.000E+00 | 0.000E+00 |
| Unigene329336 | smeS | 0.000E+00 | 0.000E+00 | 0.000E+00 | 0.000E+00 | 2.713E-06 | 2.237E-08 | 0.000E+00 | 0.000E+00 | 0.000E+00 | 0.000E+00 | 1.420E-07 | 0.000E+00 |
| Unigene329395 | cpxA | 0.000E+00 | 0.000E+00 | 0.000E+00 | 0.000E+00 | 1.663E-06 | 0.000E+00 | 0.000E+00 | 0.000E+00 | 1.125E-05 | 0.000E+00 | 0.000E+00 | 0.000E+00 |
| Unigene329453 | mtrA | 4.392E-08 | 0.000E+00 | 0.000E+00 | 0.000E+00 | 9.270E-07 | 2.374E-07 | 2.916E-07 | 8.202E-07 | 4.800E-08 | 2.446E-07 | 3.014E-07 | 7.787E-07 |
| Unigene329493 | cpxA | 0.000E+00 | 0.000E+00 | 0.000E+00 | 0.000E+00 | 3.941E-07 | 0.000E+00 | 0.000E+00 | 0.000E+00 | 0.000E+00 | 0.000E+00 | 0.000E+00 | 0.000E+00 |
| Unigene329510 | basS | 0.000E+00 | 3.015E-07 | 0.000E+00 | 0.000E+00 | 1.730E-06 | 0.000E+00 | 2.787E-07 | 0.000E+00 | 0.000E+00 | 0.000E+00 | 3.928E-07 | 5.412E-08 |
| Unigene329550 | efrA | 0.000E+00 | 0.000E+00 | 0.000E+00 | 2.796E-07 | 5.713E-07 | 0.000E+00 | 0.000E+00 | 6.465E-07 | 0.000E+00 | 0.000E+00 | 0.000E+00 | 6.546E-07 |
| Unigene329646 | vanRF | 0.000E+00 | 0.000E+00 | 0.000E+00 | 0.000E+00 | 2.026E-06 | 0.000E+00 | 0.000E+00 | 0.000E+00 | 0.000E+00 | 0.000E+00 | 0.000E+00 | 0.000E+00 |
| Unigene329831 | Streptomy | 0.000E+00 | 0.000E+00 | 0.000E+00 | 0.000E+00 | 6.966E-07 | 0.000E+00 | 0.000E+00 | 0.000E+00 | 0.000E+00 | 0.000E+00 | 0.000E+00 | 0.000E+00 |
| Unigene329931 | TaeA | 0.000E+00 | 0.000E+00 | 0.000E+00 | 0.000E+00 | 1.343E-07 | 0.000E+00 | 0.000E+00 | 0.000E+00 | 0.000E+00 | 0.000E+00 | 0.000E+00 | 0.000E+00 |
| Unigene329972 | lmrB | 9.680E-07 | 0.000E+00 | 8.835E-08 | 3.637E-07 | 5.827E-06 | 9.710E-06 | 1.271E-06 | 7.410E-06 | 4.429E-06 | 4.238E-06 | 0.000E+00 | 7.450E-07 |
| Unigene329988 | Staphylocc | 0.000E+00 | 0.000E+00 | 0.000E+00 | 0.000E+00 | 0.000E+00 | 1.020E-07 | 0.000E+00 | 0.000E+00 | 0.000E+00 | 0.000E+00 | 0.000E+00 | 0.000E+00 |
| Unigene330001 | patB | 0.000E+00 | 0.000E+00 | 0.000E+00 | 1.430E-07 | 6.292E-07 | 0.000E+00 | 0.000E+00 | 1.653E-07 | 0.000E+00 | 0.000E+00 | 0.000E+00 | 4.184E-07 |
| Unigene330012 | AAC(6')-If | 0.000E+00 | 0.000E+00 | 0.000E+00 | 0.000E+00 | 2.375E-07 | 2.311E-07 | 0.000E+00 | 0.000E+00 | 0.000E+00 | 0.000E+00 | 8.151E-07 | 9.265E-07 |
| Unigene330068 | vanHF | 0.000E+00 | 0.000E+00 | 0.000E+00 | 0.000E+00 | 4.139E-06 | 0.000E+00 | 0.000E+00 | 0.000E+00 | 0.000E+00 | 0.000E+00 | 0.000E+00 | 0.000E+00 |
| Unigene330084 | mtrA | 0.000E+00 | 0.000E+00 | 0.000E+00 | 0.000E+00 | 3.804E-07 | 0.000E+00 | 0.000E+00 | 0.000E+00 | 0.000E+00 | 0.000E+00 | 4.476E-07 | 0.000E+00 |
| Unigene330181 | evgS | 0.000E+00 | 0.000E+00 | 0.000E+00 | 0.000E+00 | 4.013E-06 | 0.000E+00 | 3.748E-07 | 1.888E-06 | 0.000E+00 | 3.353E-07 | 8.930E-07 | 1.768E-06 |
| Unigene330184 | evgS | 0.000E+00 | 6.893E-08 | 0.000E+00 | 0.000E+00 | 4.000E-06 | 0.000E+00 | 0.000E+00 | 0.000E+00 | 9.152E-08 | 2.798E-07 | 7.663E-07 | 1.683E-06 |
| Unigene330198 | sdiA | 0.000E+00 | 0.000E+00 | 0.000E+00 | 0.000E+00 | 4.494E-07 | 0.000E+00 | 0.000E+00 | 0.000E+00 | 0.000E+00 | 0.000E+00 | 0.000E+00 | 8.128E-07 |
| Unigene330217 | TriB | 0.000E+00 | 3.096E-07 | 0.000E+00 | 0.000E+00 | 0.000E+00 | 0.000E+00 | 0.000E+00 | 0.000E+00 | 0.000E+00 | 0.000E+00 | 0.000E+00 | 0.000E+00 |
| Unigene330234 | catB10 | 0.000E+00 | 0.000E+00 | 0.000E+00 | 0.000E+00 | 3.985E-07 | 0.000E+00 | 0.000E+00 | 0.000E+00 | 0.000E+00 | 0.000E+00 | 0.000E+00 | 0.000E+00 |

| | | | | | | | | | | | | | |
|---|---|---|---|---|---|---|---|---|---|---|---|---|---|
| Unigene330312 | Brucella su | 0.000E+00 | 0.000E+00 | 0.000E+00 | 0.000E+00 | 6.292E-07 | 2.721E-07 | 0.000E+00 | 0.000E+00 | 0.000E+00 | 0.000E+00 | 0.000E+00 | 0.000E+00 |
| Unigene330347 | vanG | 0.000E+00 | 0.000E+00 | 1.576E-07 | 0.000E+00 | 8.921E-07 | 0.000E+00 | 7.998E-07 | 9.060E-07 | 0.000E+00 | 0.000E+00 | 2.449E-07 | 0.000E+00 |
| Unigene330429 | oleC | 0.000E+00 | 0.000E+00 | 0.000E+00 | 0.000E+00 | 5.434E-07 | 0.000E+00 | 2.953E-07 | 0.000E+00 | 0.000E+00 | 0.000E+00 | 0.000E+00 | 0.000E+00 |
| Unigene330456 | poxtA | 0.000E+00 | 2.892E-07 | 0.000E+00 | 0.000E+00 | 7.319E-07 | 0.000E+00 | 0.000E+00 | 3.588E-07 | 0.000E+00 | 1.468E-07 | 5.024E-08 | 0.000E+00 |
| Unigene330696 | Acinetobac | 0.000E+00 | 0.000E+00 | 1.627E-07 | 3.163E-07 | 7.574E-07 | 0.000E+00 | 1.264E-06 | 9.463E-07 | 3.423E-07 | 7.184E-07 | 1.349E-06 | 1.721E-06 |
| Unigene330756 | efpA | 0.000E+00 | 0.000E+00 | 0.000E+00 | 0.000E+00 | 9.910E-07 | 0.000E+00 | 0.000E+00 | 0.000E+00 | 0.000E+00 | 0.000E+00 | 0.000E+00 | 0.000E+00 |
| Unigene330790 | Klebsiella p | 0.000E+00 | 0.000E+00 | 0.000E+00 | 0.000E+00 | 6.884E-07 | 0.000E+00 | 0.000E+00 | 0.000E+00 | 0.000E+00 | 0.000E+00 | 0.000E+00 | 0.000E+00 |
| Unigene330827 | tetA(58) | 0.000E+00 | 0.000E+00 | 0.000E+00 | 0.000E+00 | 5.676E-07 | 0.000E+00 | 0.000E+00 | 0.000E+00 | 0.000E+00 | 0.000E+00 | 0.000E+00 | 0.000E+00 |
| Unigene330876 | msbA | 1.827E-07 | 1.458E-07 | 9.287E-07 | 3.186E-07 | 1.236E-06 | 6.821E-07 | 1.875E-06 | 1.512E-06 | 1.633E-07 | 7.584E-07 | 1.463E-06 | 3.965E-06 |
| Unigene330877 | novA | 2.442E-07 | 2.488E-07 | 4.620E-07 | 3.170E-07 | 9.300E-07 | 3.016E-07 | 8.878E-07 | 8.957E-07 | 1.525E-07 | 5.440E-07 | 7.979E-07 | 1.979E-06 |
| Unigene331029 | mecD | 3.148E-07 | 7.927E-07 | 6.179E-07 | 0.000E+00 | 7.242E-06 | 3.003E-07 | 7.994E-07 | 3.026E-07 | 2.024E-08 | 0.000E+00 | 0.000E+00 | 0.000E+00 |
| Unigene331038 | novA | 1.232E-07 | 2.254E-07 | 0.000E+00 | 0.000E+00 | 7.300E-07 | 8.879E-08 | 0.000E+00 | 2.876E-07 | 1.347E-07 | 2.745E-07 | 9.396E-08 | 3.884E-07 |
| Unigene331069 | evgS | 7.704E-08 | 0.000E+00 | 1.134E-07 | 1.167E-07 | 4.993E-07 | 2.776E-07 | 8.953E-07 | 4.646E-07 | 1.684E-07 | 0.000E+00 | 4.553E-07 | 1.108E-06 |
| Unigene331095 | evgS | 0.000E+00 | 0.000E+00 | 0.000E+00 | 0.000E+00 | 6.798E-07 | 0.000E+00 | 0.000E+00 | 0.000E+00 | 0.000E+00 | 0.000E+00 | 0.000E+00 | 0.000E+00 |
| Unigene331104 | farB | 0.000E+00 | 0.000E+00 | 0.000E+00 | 0.000E+00 | 6.520E-07 | 0.000E+00 | 0.000E+00 | 5.709E-07 | 0.000E+00 | 0.000E+00 | 0.000E+00 | 1.002E-06 |
| Unigene331135 | sul4 | 0.000E+00 | 0.000E+00 | 0.000E+00 | 1.242E-07 | 4.098E-07 | 0.000E+00 | 1.361E-07 | 5.742E-07 | 0.000E+00 | 0.000E+00 | 0.000E+00 | 7.268E-07 |
| Unigene331145 | lin | 0.000E+00 | 0.000E+00 | 0.000E+00 | 0.000E+00 | 6.325E-07 | 0.000E+00 | 0.000E+00 | 0.000E+00 | 0.000E+00 | 0.000E+00 | 5.210E-07 | 0.000E+00 |
| Unigene331339 | acrB | 0.000E+00 | 0.000E+00 | 0.000E+00 | 0.000E+00 | 1.784E-07 | 0.000E+00 | 0.000E+00 | 0.000E+00 | 0.000E+00 | 0.000E+00 | 0.000E+00 | 3.797E-07 |
| Unigene331456 | Staphylocc | 0.000E+00 | 5.272E-08 | 9.427E-08 | 0.000E+00 | 0.000E+00 | 0.000E+00 | 1.595E-07 | 0.000E+00 | 0.000E+00 | 0.000E+00 | 0.000E+00 | 1.136E-07 |
| Unigene331586 | norB | 0.000E+00 | 0.000E+00 | 0.000E+00 | 0.000E+00 | 1.385E-06 | 0.000E+00 | 0.000E+00 | 0.000E+00 | 6.309E-07 | 0.000E+00 | 0.000E+00 | 0.000E+00 |
| Unigene331654 | oleB | 0.000E+00 | 0.000E+00 | 0.000E+00 | 0.000E+00 | 4.367E-07 | 0.000E+00 | 0.000E+00 | 0.000E+00 | 0.000E+00 | 0.000E+00 | 0.000E+00 | 0.000E+00 |
| Unigene331714 | AAC(6')-Ic | 0.000E+00 | 0.000E+00 | 0.000E+00 | 0.000E+00 | 1.594E-06 | 0.000E+00 | 0.000E+00 | 0.000E+00 | 0.000E+00 | 0.000E+00 | 0.000E+00 | 0.000E+00 |
| Unigene331786 | efrA | 0.000E+00 | 0.000E+00 | 0.000E+00 | 0.000E+00 | 1.049E-06 | 0.000E+00 | 0.000E+00 | 0.000E+00 | 0.000E+00 | 0.000E+00 | 0.000E+00 | 0.000E+00 |
| Unigene331876 | evgS | 0.000E+00 | 0.000E+00 | 0.000E+00 | 0.000E+00 | 6.032E-07 | 0.000E+00 | 4.916E-07 | 5.185E-07 | 5.395E-08 | 0.000E+00 | 0.000E+00 | 0.000E+00 |
| Unigene332122 | baeS | 0.000E+00 | 2.187E-07 | 0.000E+00 | 0.000E+00 | 1.107E-06 | 0.000E+00 | 0.000E+00 | 0.000E+00 | 8.167E-07 | 8.324E-08 | 0.000E+00 | 0.000E+00 |
| Unigene332181 | MuxC | 0.000E+00 | 0.000E+00 | 4.591E-08 | 0.000E+00 | 7.276E-07 | 0.000E+00 | 3.107E-07 | 0.000E+00 | 0.000E+00 | 0.000E+00 | 0.000E+00 | 7.742E-07 |
| Unigene332266 | adeR | 0.000E+00 | 0.000E+00 | 0.000E+00 | 0.000E+00 | 7.032E-07 | 0.000E+00 | 6.421E-07 | 7.388E-07 | 1.153E-07 | 2.350E-07 | 3.620E-07 | 6.858E-07 |
| Unigene332279 | TaeA | 0.000E+00 | 0.000E+00 | 0.000E+00 | 0.000E+00 | 1.112E-06 | 0.000E+00 | 0.000E+00 | 0.000E+00 | 0.000E+00 | 0.000E+00 | 0.000E+00 | 0.000E+00 |
| Unigene332305 | baeS | 5.480E-07 | 1.968E-06 | 9.532E-07 | 5.282E-07 | 8.108E-06 | 5.655E-07 | 1.626E-06 | 2.907E-07 | 0.000E+00 | 0.000E+00 | 6.553E-07 | 0.000E+00 |
| Unigene332333 | MexK | 0.000E+00 | 0.000E+00 | 0.000E+00 | 0.000E+00 | 1.122E-07 | 0.000E+00 | 0.000E+00 | 0.000E+00 | 0.000E+00 | 0.000E+00 | 0.000E+00 | 5.971E-08 |
| Unigene332474 | Acinetobac | 0.000E+00 | 0.000E+00 | 0.000E+00 | 0.000E+00 | 1.034E-06 | 0.000E+00 | 0.000E+00 | 0.000E+00 | 2.603E-07 | 0.000E+00 | 0.000E+00 | 0.000E+00 |
| Unigene332548 | novA | 0.000E+00 | 0.000E+00 | 0.000E+00 | 0.000E+00 | 6.766E-07 | 0.000E+00 | 0.000E+00 | 0.000E+00 | 1.109E-07 | 0.000E+00 | 0.000E+00 | 0.000E+00 |
| Unigene332573 | novA | 3.971E-08 | 1.743E-07 | 2.338E-07 | 3.208E-07 | 4.411E-07 | 0.000E+00 | 1.758E-07 | 6.025E-07 | 0.000E+00 | 0.000E+00 | 2.725E-07 | 7.979E-07 |
| Unigene332676 | TriB | 0.000E+00 | 0.000E+00 | 0.000E+00 | 0.000E+00 | 4.341E-07 | 0.000E+00 | 0.000E+00 | 5.613E-07 | 0.000E+00 | 0.000E+00 | 0.000E+00 | 0.000E+00 |
| Unigene332738 | efrA | 0.000E+00 | 0.000E+00 | 0.000E+00 | 0.000E+00 | 2.660E-06 | 0.000E+00 | 0.000E+00 | 0.000E+00 | 0.000E+00 | 0.000E+00 | 3.720E-07 | 0.000E+00 |
| Unigene332787 | vmlR | 0.000E+00 | 0.000E+00 | 0.000E+00 | 0.000E+00 | 2.732E-06 | 0.000E+00 | 0.000E+00 | 0.000E+00 | 0.000E+00 | 0.000E+00 | 0.000E+00 | 0.000E+00 |
| Unigene332809 | novA | 0.000E+00 | 0.000E+00 | 0.000E+00 | 0.000E+00 | 3.558E-06 | 0.000E+00 | 0.000E+00 | 0.000E+00 | 0.000E+00 | 0.000E+00 | 9.280E-07 | 0.000E+00 |
| Unigene332882 | tetA(58) | 0.000E+00 | 0.000E+00 | 0.000E+00 | 0.000E+00 | 2.189E-06 | 0.000E+00 | 0.000E+00 | 0.000E+00 | 0.000E+00 | 0.000E+00 | 1.353E-07 | 1.444E-06 |
| Unigene332910 | tet(41) | 0.000E+00 | 0.000E+00 | 0.000E+00 | 0.000E+00 | 2.783E-06 | 0.000E+00 | 0.000E+00 | 0.000E+00 | 0.000E+00 | 0.000E+00 | 0.000E+00 | 0.000E+00 |
| Unigene332921 | evgS | 0.000E+00 | 0.000E+00 | 0.000E+00 | 0.000E+00 | 3.076E-06 | 0.000E+00 | 0.000E+00 | 0.000E+00 | 0.000E+00 | 2.570E-07 | 4.079E-07 | 0.000E+00 |
| Unigene332928 | tetA(58) | 0.000E+00 | 0.000E+00 | 0.000E+00 | 0.000E+00 | 2.824E-06 | 0.000E+00 | 0.000E+00 | 0.000E+00 | 7.612E-08 | 0.000E+00 | 0.000E+00 | 0.000E+00 |
| Unigene332929 | vanRM | 0.000E+00 | 0.000E+00 | 0.000E+00 | 0.000E+00 | 3.358E-06 | 2.178E-07 | 0.000E+00 | 0.000E+00 | 0.000E+00 | 0.000E+00 | 0.000E+00 | 0.000E+00 |
| Unigene332934 | tetA(58) | 0.000E+00 | 0.000E+00 | 0.000E+00 | 0.000E+00 | 2.418E-06 | 0.000E+00 | 0.000E+00 | 0.000E+00 | 0.000E+00 | 0.000E+00 | 0.000E+00 | 0.000E+00 |
| Unigene332942 | patA | 0.000E+00 | 0.000E+00 | 0.000E+00 | 0.000E+00 | 2.552E-06 | 0.000E+00 | 0.000E+00 | 0.000E+00 | 0.000E+00 | 0.000E+00 | 2.879E-07 | 0.000E+00 |
| Unigene332961 | vanG | 3.124E-07 | 8.381E-07 | 9.877E-07 | 4.206E-07 | 8.522E-06 | 4.503E-07 | 0.000E+00 | 6.077E-07 | 0.000E+00 | 0.000E+00 | 0.000E+00 | 0.000E+00 |
| Unigene332967 | MexF | 0.000E+00 | 0.000E+00 | 0.000E+00 | 0.000E+00 | 4.427E-07 | 0.000E+00 | 0.000E+00 | 0.000E+00 | 0.000E+00 | 3.552E-07 | 0.000E+00 | 0.000E+00 |
| Unigene333254 | cmlv | 0.000E+00 | 0.000E+00 | 0.000E+00 | 5.237E-08 | 8.641E-07 | 0.000E+00 | 1.320E-06 | 1.543E-06 | 0.000E+00 | 5.199E-07 | 1.008E-06 | 2.912E-06 |
| Unigene333258 | lsaC | 0.000E+00 | 0.000E+00 | 0.000E+00 | 1.132E-07 | 4.566E-07 | 0.000E+00 | 2.481E-07 | 3.489E-07 | 0.000E+00 | 2.913E-07 | 1.709E-07 | 7.508E-07 |
| Unigene333273 | tetA(60) | 0.000E+00 | 0.000E+00 | 0.000E+00 | 2.005E-07 | 5.734E-07 | 0.000E+00 | 3.076E-07 | 5.098E-07 | 0.000E+00 | 0.000E+00 | 0.000E+00 | 8.448E-07 |
| Unigene333334 | PmrF | 6.550E-07 | 0.000E+00 | 3.366E-07 | 2.205E-07 | 2.737E-06 | 7.620E-06 | 1.415E-06 | 7.354E-06 | 3.852E-06 | 0.000E+00 | 0.000E+00 | 0.000E+00 |
| Unigene333434 | optrA | 0.000E+00 | 0.000E+00 | 0.000E+00 | 0.000E+00 | 9.414E-07 | 0.000E+00 | 0.000E+00 | 0.000E+00 | 0.000E+00 | 0.000E+00 | 0.000E+00 | 0.000E+00 |
| Unigene333452 | MexF | 0.000E+00 | 0.000E+00 | 0.000E+00 | 0.000E+00 | 5.069E-07 | 0.000E+00 | 3.787E-07 | 0.000E+00 | 0.000E+00 | 0.000E+00 | 0.000E+00 | 3.146E-07 |
| Unigene333485 | FosA7 | 0.000E+00 | 0.000E+00 | 0.000E+00 | 0.000E+00 | 1.187E-06 | 0.000E+00 | 0.000E+00 | 0.000E+00 | 0.000E+00 | 0.000E+00 | 0.000E+00 | 0.000E+00 |
| Unigene333488 | oleB | 0.000E+00 | 0.000E+00 | 0.000E+00 | 1.387E-07 | 2.136E-06 | 0.000E+00 | 0.000E+00 | 0.000E+00 | 0.000E+00 | 0.000E+00 | 0.000E+00 | 0.000E+00 |
| Unigene333792 | LpeB | 0.000E+00 | 0.000E+00 | 0.000E+00 | 1.917E-07 | 6.329E-07 | 0.000E+00 | 0.000E+00 | 2.216E-07 | 0.000E+00 | 0.000E+00 | 1.448E-07 | 0.000E+00 |
| Unigene333818 | novA | 1.334E-07 | 1.789E-07 | 2.763E-07 | 5.987E-08 | 1.907E-05 | 3.533E-05 | 2.116E-06 | 1.228E-06 | 2.835E-06 | 3.882E-05 | 1.458E-05 | 1.437E-06 |
| Unigene333822 | adeL | 1.065E-07 | 1.169E-07 | 3.485E-07 | 2.152E-07 | 7.496E-07 | 1.152E-07 | 3.144E-07 | 7.047E-07 | 1.164E-07 | 3.956E-07 | 7.312E-07 | 7.556E-07 |
| Unigene333856 | smeR | 0.000E+00 | 0.000E+00 | 0.000E+00 | 0.000E+00 | 1.700E-06 | 0.000E+00 | 0.000E+00 | 0.000E+00 | 0.000E+00 | 0.000E+00 | 0.000E+00 | 0.000E+00 |

| | | | | | | | | | | | | | |
|---|---|---|---|---|---|---|---|---|---|---|---|---|---|
| Unigene333889 | macB | 0.000E+00 | 0.000E+00 | 0.000E+00 | 0.000E+00 | 2.898E-07 | 0.000E+00 | 0.000E+00 | 0.000E+00 | 0.000E+00 | 0.000E+00 | 0.000E+00 | 0.000E+00 |
| Unigene333913 | bcrA | 0.000E+00 | 0.000E+00 | 0.000E+00 | 0.000E+00 | 4.297E-07 | 0.000E+00 | 0.000E+00 | 0.000E+00 | 0.000E+00 | 0.000E+00 | 2.011E-07 | 3.325E-07 |
| Unigene333949 | mexY | 0.000E+00 | 0.000E+00 | 0.000E+00 | 0.000E+00 | 0.000E+00 | 0.000E+00 | 0.000E+00 | 0.000E+00 | 0.000E+00 | 0.000E+00 | 0.000E+00 | 6.759E-07 |
| Unigene333981 | oleC | 0.000E+00 | 2.664E-07 | 1.191E-07 | 0.000E+00 | 7.190E-07 | 0.000E+00 | 8.954E-08 | 0.000E+00 | 7.074E-07 | 3.154E-07 | 4.627E-08 | 0.000E+00 |
| Unigene333994 | Klebsiella p | 3.261E-07 | 8.459E-07 | 0.000E+00 | 0.000E+00 | 7.113E-06 | 0.000E+00 | 1.017E-06 | 3.114E-07 | 0.000E+00 | 0.000E+00 | 0.000E+00 | 0.000E+00 |
| Unigene334027 | Staphylocc | 0.000E+00 | 0.000E+00 | 0.000E+00 | 0.000E+00 | 0.000E+00 | 0.000E+00 | 0.000E+00 | 1.427E-07 | 0.000E+00 | 0.000E+00 | 0.000E+00 | 0.000E+00 |
| Unigene334070 | tetT | 4.607E-05 | 2.494E-05 | 1.750E-05 | 1.974E-05 | 2.075E-06 | 1.619E-06 | 3.127E-06 | 7.568E-07 | 6.581E-06 | 7.223E-07 | 2.225E-06 | 1.423E-06 |
| Unigene334092 | novA | 0.000E+00 | 0.000E+00 | 0.000E+00 | 0.000E+00 | 5.931E-07 | 0.000E+00 | 0.000E+00 | 0.000E+00 | 0.000E+00 | 0.000E+00 | 0.000E+00 | 0.000E+00 |
| Unigene334144 | patA | 0.000E+00 | 4.983E-08 | 0.000E+00 | 0.000E+00 | 8.070E-07 | 0.000E+00 | 3.517E-07 | 2.650E-07 | 0.000E+00 | 0.000E+00 | 1.039E-07 | 7.513E-07 |
| Unigene334150 | arnA | 0.000E+00 | 0.000E+00 | 0.000E+00 | 0.000E+00 | 9.635E-07 | 0.000E+00 | 0.000E+00 | 0.000E+00 | 0.000E+00 | 0.000E+00 | 0.000E+00 | 0.000E+00 |
| Unigene334168 | rpoB2 | 0.000E+00 | 0.000E+00 | 0.000E+00 | 0.000E+00 | 4.966E-07 | 0.000E+00 | 0.000E+00 | 0.000E+00 | 0.000E+00 | 0.000E+00 | 0.000E+00 | 0.000E+00 |
| Unigene334169 | Bifidobacte | 0.000E+00 | 0.000E+00 | 0.000E+00 | 0.000E+00 | 4.578E-07 | 0.000E+00 | 0.000E+00 | 0.000E+00 | 0.000E+00 | 0.000E+00 | 0.000E+00 | 0.000E+00 |
| Unigene334175 | VatI | 0.000E+00 | 0.000E+00 | 0.000E+00 | 0.000E+00 | 7.137E-07 | 5.787E-08 | 1.007E-06 | 1.125E-06 | 0.000E+00 | 0.000E+00 | 4.286E-07 | 1.202E-06 |
| Unigene334191 | PmrF | 0.000E+00 | 0.000E+00 | 0.000E+00 | 0.000E+00 | 4.062E-07 | 0.000E+00 | 0.000E+00 | 0.000E+00 | 0.000E+00 | 0.000E+00 | 0.000E+00 | 0.000E+00 |
| Unigene334270 | cpxA | 1.983E-07 | 5.986E-07 | 2.433E-07 | 0.000E+00 | 5.784E-06 | 2.948E-07 | 0.000E+00 | 8.682E-08 | 0.000E+00 | 0.000E+00 | 8.508E-08 | 0.000E+00 |
| Unigene334271 | Pseudomo | 3.273E-07 | 8.532E-07 | 7.226E-07 | 4.545E-07 | 7.045E-06 | 8.846E-08 | 0.000E+00 | 4.776E-07 | 0.000E+00 | 0.000E+00 | 3.744E-07 | 0.000E+00 |
| Unigene334311 | macB | 0.000E+00 | 0.000E+00 | 0.000E+00 | 0.000E+00 | 1.217E-06 | 0.000E+00 | 0.000E+00 | 0.000E+00 | 0.000E+00 | 0.000E+00 | 0.000E+00 | 0.000E+00 |
| Unigene334360 | Corynebac | 0.000E+00 | 5.859E-07 | 0.000E+00 | 0.000E+00 | 3.277E-06 | 0.000E+00 | 6.841E-07 | 0.000E+00 | 0.000E+00 | 0.000E+00 | 0.000E+00 | 2.657E-07 |
| Unigene334377 | TaeA | 0.000E+00 | 0.000E+00 | 0.000E+00 | 0.000E+00 | 0.000E+00 | 0.000E+00 | 5.141E-06 | 3.873E-06 | 0.000E+00 | 0.000E+00 | 0.000E+00 | 4.034E-06 |
| Unigene334401 | TriA | 4.653E-07 | 3.192E-07 | 1.141E-06 | 6.461E-07 | 1.163E-06 | 9.117E-07 | 2.092E-06 | 2.274E-06 | 3.179E-07 | 1.134E-06 | 1.563E-06 | 4.709E-06 |
| Unigene334402 | MexK | 2.592E-07 | 2.048E-07 | 5.900E-07 | 3.978E-07 | 9.098E-07 | 4.707E-07 | 1.469E-06 | 1.307E-06 | 2.493E-07 | 5.889E-07 | 9.960E-07 | 3.406E-06 |
| Unigene334404 | tetB(46) | 0.000E+00 | 2.452E-07 | 9.316E-07 | 3.948E-07 | 1.717E-06 | 8.251E-07 | 1.896E-06 | 1.173E-06 | 0.000E+00 | 1.037E-06 | 1.001E-06 | 4.269E-06 |
| Unigene334533 | evgS | 0.000E+00 | 0.000E+00 | 3.940E-08 | 6.082E-08 | 3.568E-07 | 1.953E-07 | 4.221E-07 | 3.749E-07 | 4.388E-08 | 0.000E+00 | 3.674E-07 | 5.932E-07 |
| Unigene334740 | macB | 0.000E+00 | 0.000E+00 | 0.000E+00 | 0.000E+00 | 5.172E-07 | 0.000E+00 | 4.580E-07 | 0.000E+00 | 0.000E+00 | 0.000E+00 | 2.959E-07 | 2.446E-07 |
| Unigene334881 | msbA | 0.000E+00 | 0.000E+00 | 0.000E+00 | 0.000E+00 | 3.248E-07 | 0.000E+00 | 0.000E+00 | 0.000E+00 | 0.000E+00 | 0.000E+00 | 0.000E+00 | 0.000E+00 |
| Unigene334888 | Corynebac | 1.464E-07 | 2.812E-07 | 0.000E+00 | 0.000E+00 | 1.138E-06 | 0.000E+00 | 0.000E+00 | 0.000E+00 | 4.400E-07 | 0.000E+00 | 0.000E+00 | 0.000E+00 |
| Unigene334985 | lmrD | 9.752E-08 | 0.000E+00 | 0.000E+00 | 0.000E+00 | 7.584E-07 | 0.000E+00 | 5.756E-07 | 0.000E+00 | 0.000E+00 | 0.000E+00 | 0.000E+00 | 0.000E+00 |
| Unigene335122 | Bifidobacte | 1.189E-07 | 0.000E+00 | 0.000E+00 | 0.000E+00 | 3.236E-06 | 0.000E+00 | 5.044E-07 | 1.573E-06 | 0.000E+00 | 0.000E+00 | 7.707E-07 | 1.569E-06 |
| Unigene335133 | adeL | 0.000E+00 | 0.000E+00 | 0.000E+00 | 0.000E+00 | 2.701E-06 | 0.000E+00 | 0.000E+00 | 0.000E+00 | 0.000E+00 | 0.000E+00 | 0.000E+00 | 0.000E+00 |
| Unigene335177 | macB | 8.359E-07 | 2.517E-06 | 1.725E-06 | 6.277E-07 | 1.219E-05 | 5.793E-07 | 3.345E-06 | 5.004E-07 | 0.000E+00 | 3.820E-07 | 9.807E-07 | 0.000E+00 |
| Unigene335237 | bcrA | 3.913E-08 | 1.288E-07 | 0.000E+00 | 3.951E-08 | 5.216E-07 | 0.000E+00 | 0.000E+00 | 2.284E-07 | 1.711E-07 | 1.743E-07 | 0.000E+00 | 4.162E-07 |
| Unigene335337 | mgrA | 0.000E+00 | 0.000E+00 | 0.000E+00 | 0.000E+00 | 3.026E-06 | 0.000E+00 | 0.000E+00 | 0.000E+00 | 0.000E+00 | 0.000E+00 | 0.000E+00 | 0.000E+00 |
| Unigene335345 | otr(B) | 0.000E+00 | 0.000E+00 | 0.000E+00 | 0.000E+00 | 2.814E-06 | 0.000E+00 | 0.000E+00 | 0.000E+00 | 0.000E+00 | 0.000E+00 | 2.229E-07 | 7.677E-08 |
| Unigene335376 | carA | 0.000E+00 | 0.000E+00 | 0.000E+00 | 0.000E+00 | 2.668E-06 | 0.000E+00 | 0.000E+00 | 0.000E+00 | 1.023E-07 | 0.000E+00 | 2.497E-07 | 0.000E+00 |
| Unigene335389 | tetB(46) | 0.000E+00 | 0.000E+00 | 0.000E+00 | 0.000E+00 | 3.487E-06 | 0.000E+00 | 0.000E+00 | 0.000E+00 | 0.000E+00 | 0.000E+00 | 7.679E-07 | 0.000E+00 |
| Unigene335476 | efpA | 0.000E+00 | 0.000E+00 | 0.000E+00 | 0.000E+00 | 2.768E-06 | 0.000E+00 | 0.000E+00 | 0.000E+00 | 0.000E+00 | 0.000E+00 | 2.073E-07 | 0.000E+00 |
| Unigene335494 | tlrC | 0.000E+00 | 0.000E+00 | 0.000E+00 | 0.000E+00 | 3.265E-06 | 0.000E+00 | 2.391E-07 | 0.000E+00 | 0.000E+00 | 0.000E+00 | 2.966E-07 | 0.000E+00 |
| Unigene335498 | APH(2'')-II | 0.000E+00 | 0.000E+00 | 0.000E+00 | 0.000E+00 | 2.072E-06 | 0.000E+00 | 0.000E+00 | 0.000E+00 | 0.000E+00 | 0.000E+00 | 2.051E-07 | 0.000E+00 |
| Unigene335521 | msbA | 0.000E+00 | 6.401E-08 | 0.000E+00 | 0.000E+00 | 2.268E-06 | 0.000E+00 | 0.000E+00 | 0.000E+00 | 0.000E+00 | 0.000E+00 | 2.668E-07 | 0.000E+00 |
| Unigene335760 | efrA | 2.200E-07 | 0.000E+00 | 0.000E+00 | 0.000E+00 | 7.777E-07 | 2.162E-07 | 7.083E-07 | 8.871E-07 | 1.093E-07 | 0.000E+00 | 9.151E-07 | 1.087E-06 |
| Unigene335905 | lmrC | 0.000E+00 | 0.000E+00 | 0.000E+00 | 0.000E+00 | 5.639E-07 | 0.000E+00 | 0.000E+00 | 2.370E-07 | 0.000E+00 | 0.000E+00 | 0.000E+00 | 0.000E+00 |
| Unigene335947 | mexY | 0.000E+00 | 0.000E+00 | 0.000E+00 | 0.000E+00 | 4.029E-07 | 0.000E+00 | 0.000E+00 | 0.000E+00 | 0.000E+00 | 0.000E+00 | 0.000E+00 | 0.000E+00 |
| Unigene336001 | msbA | 0.000E+00 | 0.000E+00 | 0.000E+00 | 0.000E+00 | 3.516E-07 | 0.000E+00 | 0.000E+00 | 0.000E+00 | 0.000E+00 | 0.000E+00 | 0.000E+00 | 0.000E+00 |
| Unigene336002 | lmrC | 0.000E+00 | 0.000E+00 | 4.624E-07 | 3.172E-07 | 7.853E-07 | 0.000E+00 | 1.434E-06 | 6.417E-07 | 0.000E+00 | 9.187E-07 | 0.000E+00 | 3.110E-06 |
| Unigene336051 | carA | 0.000E+00 | 0.000E+00 | 0.000E+00 | 0.000E+00 | 9.995E-07 | 0.000E+00 | 0.000E+00 | 0.000E+00 | 0.000E+00 | 0.000E+00 | 0.000E+00 | 0.000E+00 |
| Unigene336238 | Brucella su | 1.113E-07 | 4.072E-08 | 1.578E-07 | 0.000E+00 | 5.084E-07 | 1.738E-07 | 7.392E-07 | 4.331E-07 | 0.000E+00 | 1.516E-07 | 6.932E-07 | 5.555E-07 |
| Unigene336296 | macB | 0.000E+00 | 0.000E+00 | 0.000E+00 | 0.000E+00 | 5.197E-07 | 2.529E-07 | 1.553E-07 | 2.730E-07 | 1.023E-07 | 2.085E-07 | 0.000E+00 | 0.000E+00 |
| Unigene336340 | tetA(58) | 5.380E-07 | 1.323E-06 | 7.099E-07 | 4.684E-07 | 7.564E-06 | 2.607E-07 | 1.047E-06 | 3.681E-07 | 0.000E+00 | 0.000E+00 | 2.547E-07 | 1.535E-07 |
| Unigene336597 | tetA(58) | 0.000E+00 | 0.000E+00 | 0.000E+00 | 0.000E+00 | 1.287E-06 | 0.000E+00 | 0.000E+00 | 0.000E+00 | 0.000E+00 | 0.000E+00 | 0.000E+00 | 0.000E+00 |
| Unigene336606 | macB | 0.000E+00 | 0.000E+00 | 0.000E+00 | 0.000E+00 | 0.000E+00 | 0.000E+00 | 5.386E-07 | 0.000E+00 | 0.000E+00 | 0.000E+00 | 0.000E+00 | 0.000E+00 |
| Unigene336614 | efrA | 0.000E+00 | 0.000E+00 | 8.834E-07 | 0.000E+00 | 1.048E-06 | 6.951E-07 | 0.000E+00 | 0.000E+00 | 0.000E+00 | 9.073E-07 | 0.000E+00 | 3.091E-06 |
| Unigene336747 | otr(B) | 0.000E+00 | 0.000E+00 | 0.000E+00 | 0.000E+00 | 4.219E-07 | 0.000E+00 | 0.000E+00 | 0.000E+00 | 0.000E+00 | 0.000E+00 | 7.240E-08 | 0.000E+00 |
| Unigene336767 | otrC | 3.502E-07 | 0.000E+00 | 0.000E+00 | 0.000E+00 | 8.842E-07 | 0.000E+00 | 0.000E+00 | 0.000E+00 | 0.000E+00 | 0.000E+00 | 0.000E+00 | 0.000E+00 |
| Unigene336778 | rosB | 0.000E+00 | 0.000E+00 | 0.000E+00 | 0.000E+00 | 5.033E-07 | 0.000E+00 | 0.000E+00 | 0.000E+00 | 0.000E+00 | 0.000E+00 | 0.000E+00 | 0.000E+00 |
| Unigene336854 | CatU | 0.000E+00 | 0.000E+00 | 0.000E+00 | 0.000E+00 | 5.169E-07 | 0.000E+00 | 0.000E+00 | 0.000E+00 | 0.000E+00 | 0.000E+00 | 0.000E+00 | 0.000E+00 |
| Unigene336978 | arnA | 0.000E+00 | 1.121E-07 | 0.000E+00 | 3.439E-08 | 2.118E-06 | 0.000E+00 | 0.000E+00 | 0.000E+00 | 8.560E-07 | 0.000E+00 | 0.000E+00 | 4.025E-08 |
| Unigene337039 | cpxA | 5.380E-07 | 1.540E-07 | 7.345E-07 | 3.779E-07 | 1.715E-06 | 5.057E-07 | 2.201E-06 | 1.392E-06 | 4.858E-07 | 1.068E-06 | 1.284E-06 | 4.894E-06 |
| Unigene337104 | patA | 0.000E+00 | 0.000E+00 | 0.000E+00 | 0.000E+00 | 4.539E-07 | 0.000E+00 | 0.000E+00 | 0.000E+00 | 0.000E+00 | 0.000E+00 | 0.000E+00 | 0.000E+00 |

| | | | | | | | | | | | | | |
|---|---|---|---|---|---|---|---|---|---|---|---|---|---|
| Unigene337140 | vanHA | 0.000E+00 | 0.000E+00 | 0.000E+00 | 0.000E+00 | 8.207E-07 | 3.472E-07 | 0.000E+00 | 0.000E+00 | 0.000E+00 | 0.000E+00 | 0.000E+00 | 0.000E+00 |
| Unigene337156 | mgtA | 0.000E+00 | 0.000E+00 | 0.000E+00 | 0.000E+00 | 3.023E-07 | 0.000E+00 | 0.000E+00 | 0.000E+00 | 0.000E+00 | 0.000E+00 | 3.395E-07 | 4.093E-07 |
| Unigene337227 | ceoB | 0.000E+00 | 0.000E+00 | 0.000E+00 | 0.000E+00 | 4.879E-07 | 0.000E+00 | 0.000E+00 | 6.835E-07 | 0.000E+00 | 0.000E+00 | 1.675E-07 | 3.893E-07 |
| Unigene337241 | baeR | 0.000E+00 | 0.000E+00 | 0.000E+00 | 0.000E+00 | 4.409E-07 | 0.000E+00 | 0.000E+00 | 7.721E-07 | 0.000E+00 | 0.000E+00 | 0.000E+00 | 6.776E-07 |
| Unigene337290 | MexW | 6.711E-08 | 0.000E+00 | 0.000E+00 | 1.129E-07 | 5.716E-07 | 0.000E+00 | 2.476E-07 | 3.133E-07 | 1.467E-07 | 0.000E+00 | 1.535E-07 | 2.115E-07 |
| Unigene337301 | adeL | 0.000E+00 | 1.539E-07 | 0.000E+00 | 0.000E+00 | 2.414E-06 | 0.000E+00 | 0.000E+00 | 1.268E-06 | 0.000E+00 | 0.000E+00 | 0.000E+00 | 0.000E+00 |
| Unigene337303 | rosA | 0.000E+00 | 0.000E+00 | 0.000E+00 | 0.000E+00 | 2.643E-06 | 0.000E+00 | 0.000E+00 | 0.000E+00 | 0.000E+00 | 0.000E+00 | 0.000E+00 | 0.000E+00 |
| Unigene337324 | emrY | 0.000E+00 | 0.000E+00 | 0.000E+00 | 0.000E+00 | 3.110E-06 | 0.000E+00 | 0.000E+00 | 0.000E+00 | 0.000E+00 | 0.000E+00 | 1.837E-07 | 0.000E+00 |
| Unigene337329 | AcrS | 0.000E+00 | 0.000E+00 | 0.000E+00 | 0.000E+00 | 1.808E-06 | 0.000E+00 | 0.000E+00 | 0.000E+00 | 0.000E+00 | 0.000E+00 | 0.000E+00 | 0.000E+00 |
| Unigene337330 | MexD | 0.000E+00 | 0.000E+00 | 0.000E+00 | 0.000E+00 | 2.700E-06 | 6.679E-08 | 0.000E+00 | 0.000E+00 | 0.000E+00 | 0.000E+00 | 3.533E-07 | 0.000E+00 |
| Unigene337331 | Escherichia | 0.000E+00 | 0.000E+00 | 0.000E+00 | 0.000E+00 | 2.322E-06 | 0.000E+00 | 0.000E+00 | 0.000E+00 | 0.000E+00 | 0.000E+00 | 2.584E-07 | 0.000E+00 |
| Unigene337340 | cmx | 0.000E+00 | 0.000E+00 | 0.000E+00 | 0.000E+00 | 2.699E-06 | 0.000E+00 | 0.000E+00 | 0.000E+00 | 0.000E+00 | 0.000E+00 | 4.887E-07 | 0.000E+00 |
| Unigene337345 | patA | 0.000E+00 | 0.000E+00 | 1.736E-07 | 0.000E+00 | 2.718E-06 | 0.000E+00 | 0.000E+00 | 0.000E+00 | 0.000E+00 | 0.000E+00 | 5.058E-07 | 0.000E+00 |
| Unigene337406 | tetA(58) | 0.000E+00 | 0.000E+00 | 0.000E+00 | 0.000E+00 | 5.784E-07 | 4.221E-07 | 0.000E+00 | 0.000E+00 | 0.000E+00 | 1.933E-07 | 0.000E+00 | 0.000E+00 |
| Unigene337427 | efrB | 0.000E+00 | 0.000E+00 | 0.000E+00 | 0.000E+00 | 1.672E-06 | 0.000E+00 | 0.000E+00 | 0.000E+00 | 0.000E+00 | 0.000E+00 | 0.000E+00 | 0.000E+00 |
| Unigene337432 | lmrD | 0.000E+00 | 3.855E-07 | 0.000E+00 | 0.000E+00 | 8.868E-07 | 0.000E+00 | 0.000E+00 | 0.000E+00 | 0.000E+00 | 0.000E+00 | 0.000E+00 | 0.000E+00 |
| Unigene337514 | smeS | 7.625E-07 | 0.000E+00 | 4.780E-07 | 2.995E-07 | 5.106E-06 | 8.243E-06 | 1.641E-06 | 8.530E-06 | 3.936E-06 | 3.799E-06 | 0.000E+00 | 0.000E+00 |
| Unigene337551 | basS | 7.353E-08 | 5.380E-08 | 9.620E-08 | 0.000E+00 | 4.357E-07 | 7.949E-08 | 5.968E-07 | 7.439E-07 | 0.000E+00 | 3.003E-07 | 4.206E-07 | 1.478E-06 |
| Unigene337552 | vanRO | 1.461E-07 | 0.000E+00 | 0.000E+00 | 0.000E+00 | 7.032E-07 | 2.632E-07 | 1.293E-06 | 1.421E-06 | 0.000E+00 | 0.000E+00 | 8.354E-07 | 3.395E-06 |
| Unigene337622 | optrA | 3.015E-07 | 7.032E-07 | 5.547E-07 | 2.474E-07 | 5.736E-06 | 2.648E-07 | 6.257E-07 | 3.079E-07 | 0.000E+00 | 0.000E+00 | 1.293E-07 | 4.455E-08 |
| Unigene337625 | adeL | 2.928E-07 | 1.285E-06 | 6.105E-07 | 0.000E+00 | 8.010E-06 | 0.000E+00 | 0.000E+00 | 8.544E-08 | 0.000E+00 | 0.000E+00 | 0.000E+00 | 0.000E+00 |
| Unigene337626 | adeL | 4.632E-07 | 1.056E-06 | 4.895E-07 | 3.238E-07 | 6.610E-06 | 5.778E-07 | 0.000E+00 | 1.248E-07 | 0.000E+00 | 0.000E+00 | 0.000E+00 | 0.000E+00 |
| Unigene337627 | tetA(60) | 2.943E-07 | 1.077E-06 | 6.418E-07 | 0.000E+00 | 5.559E-06 | 0.000E+00 | 0.000E+00 | 4.199E-07 | 0.000E+00 | 0.000E+00 | 0.000E+00 | 1.160E-07 |
| Unigene337705 | mdtN | 3.647E-08 | 2.802E-07 | 0.000E+00 | 0.000E+00 | 1.580E-06 | 0.000E+00 | 0.000E+00 | 0.000E+00 | 0.000E+00 | 0.000E+00 | 0.000E+00 | 0.000E+00 |
| Unigene337722 | cfrC | 0.000E+00 | 0.000E+00 | 0.000E+00 | 0.000E+00 | 1.377E-06 | 0.000E+00 | 0.000E+00 | 8.877E-07 | 0.000E+00 | 0.000E+00 | 7.089E-07 | 0.000E+00 |
| Unigene337821 | vanG | 0.000E+00 | 0.000E+00 | 0.000E+00 | 0.000E+00 | 1.128E-07 | 0.000E+00 | 0.000E+00 | 0.000E+00 | 0.000E+00 | 0.000E+00 | 0.000E+00 | 0.000E+00 |
| Unigene337858 | Staphylocc | 0.000E+00 | 0.000E+00 | 0.000E+00 | 5.275E-08 | 2.321E-07 | 0.000E+00 | 0.000E+00 | 0.000E+00 | 0.000E+00 | 0.000E+00 | 0.000E+00 | 0.000E+00 |
| Unigene337865 | msbA | 0.000E+00 | 0.000E+00 | 0.000E+00 | 0.000E+00 | 9.011E-07 | 0.000E+00 | 9.974E-08 | 3.997E-07 | 9.850E-08 | 1.807E-07 | 1.443E-07 | 6.178E-07 |
| Unigene338005 | bcrA | 0.000E+00 | 0.000E+00 | 0.000E+00 | 0.000E+00 | 1.365E-06 | 0.000E+00 | 0.000E+00 | 0.000E+00 | 0.000E+00 | 0.000E+00 | 0.000E+00 | 0.000E+00 |
| Unigene338007 | evgA | 0.000E+00 | 0.000E+00 | 0.000E+00 | 0.000E+00 | 1.665E-06 | 0.000E+00 | 0.000E+00 | 0.000E+00 | 1.340E-05 | 0.000E+00 | 1.170E-05 | 0.000E+00 |
| Unigene338033 | tetA(60) | 1.369E-07 | 1.074E-07 | 3.263E-07 | 2.963E-07 | 7.172E-07 | 4.018E-07 | 9.527E-07 | 6.622E-07 | 0.000E+00 | 3.923E-07 | 7.609E-07 | 1.850E-06 |
| Unigene338160 | macA | 0.000E+00 | 0.000E+00 | 0.000E+00 | 0.000E+00 | 5.747E-07 | 0.000E+00 | 0.000E+00 | 4.227E-07 | 0.000E+00 | 0.000E+00 | 0.000E+00 | 3.669E-07 |
| Unigene338177 | bcrA | 0.000E+00 | 0.000E+00 | 0.000E+00 | 0.000E+00 | 9.545E-07 | 0.000E+00 | 0.000E+00 | 0.000E+00 | 0.000E+00 | 0.000E+00 | 0.000E+00 | 0.000E+00 |
| Unigene338195 | patB | 0.000E+00 | 0.000E+00 | 0.000E+00 | 0.000E+00 | 3.355E-07 | 0.000E+00 | 4.178E-08 | 0.000E+00 | 0.000E+00 | 0.000E+00 | 4.319E-08 | 1.339E-07 |
| Unigene338231 | YojI | 0.000E+00 | 0.000E+00 | 6.295E-07 | 4.123E-07 | 1.004E-06 | 0.000E+00 | 1.969E-06 | 7.829E-07 | 0.000E+00 | 0.000E+00 | 0.000E+00 | 3.619E-06 |
| Unigene338301 | patA | 0.000E+00 | 0.000E+00 | 0.000E+00 | 0.000E+00 | 0.000E+00 | 0.000E+00 | 0.000E+00 | 1.621E-07 | 0.000E+00 | 0.000E+00 | 0.000E+00 | 0.000E+00 |
| Unigene338469 | kdpE | 0.000E+00 | 2.054E-07 | 0.000E+00 | 0.000E+00 | 5.197E-07 | 0.000E+00 | 9.320E-07 | 4.915E-07 | 0.000E+00 | 0.000E+00 | 4.281E-07 | 0.000E+00 |
| Unigene338490 | macB | 0.000E+00 | 0.000E+00 | 0.000E+00 | 0.000E+00 | 7.781E-07 | 0.000E+00 | 0.000E+00 | 0.000E+00 | 0.000E+00 | 0.000E+00 | 0.000E+00 | 0.000E+00 |
| Unigene338563 | cpxA | 0.000E+00 | 0.000E+00 | 0.000E+00 | 0.000E+00 | 2.872E-06 | 0.000E+00 | 0.000E+00 | 0.000E+00 | 0.000E+00 | 0.000E+00 | 2.931E-07 | 0.000E+00 |
| Unigene338564 | mtrA | 0.000E+00 | 0.000E+00 | 0.000E+00 | 0.000E+00 | 3.423E-06 | 0.000E+00 | 0.000E+00 | 0.000E+00 | 0.000E+00 | 0.000E+00 | 4.734E-07 | 0.000E+00 |
| Unigene338582 | QepA4 | 0.000E+00 | 0.000E+00 | 0.000E+00 | 0.000E+00 | 2.446E-06 | 0.000E+00 | 0.000E+00 | 5.577E-07 | 0.000E+00 | 0.000E+00 | 3.327E-07 | 0.000E+00 |
| Unigene338589 | lmrC | 0.000E+00 | 0.000E+00 | 0.000E+00 | 0.000E+00 | 3.513E-06 | 0.000E+00 | 0.000E+00 | 0.000E+00 | 0.000E+00 | 0.000E+00 | 2.699E-07 | 0.000E+00 |
| Unigene338600 | abeM | 0.000E+00 | 0.000E+00 | 0.000E+00 | 0.000E+00 | 3.027E-06 | 0.000E+00 | 1.789E-07 | 0.000E+00 | 0.000E+00 | 0.000E+00 | 4.754E-07 | 0.000E+00 |
| Unigene338638 | tetA(60) | 0.000E+00 | 0.000E+00 | 0.000E+00 | 9.234E-08 | 3.082E-06 | 0.000E+00 | 0.000E+00 | 0.000E+00 | 0.000E+00 | 6.112E-07 | 0.000E+00 | 0.000E+00 |
| Unigene338679 | adeJ | 0.000E+00 | 0.000E+00 | 0.000E+00 | 0.000E+00 | 0.000E+00 | 0.000E+00 | 6.143E-07 | 9.968E-07 | 0.000E+00 | 0.000E+00 | 0.000E+00 | 0.000E+00 |
| Unigene338709 | adeL | 0.000E+00 | 0.000E+00 | 0.000E+00 | 0.000E+00 | 4.228E-07 | 0.000E+00 | 0.000E+00 | 0.000E+00 | 0.000E+00 | 0.000E+00 | 0.000E+00 | 0.000E+00 |
| Unigene338763 | MexK | 0.000E+00 | 0.000E+00 | 0.000E+00 | 0.000E+00 | 7.986E-07 | 0.000E+00 | 0.000E+00 | 5.684E-07 | 0.000E+00 | 0.000E+00 | 0.000E+00 | 0.000E+00 |
| Unigene338798 | arlS | 0.000E+00 | 0.000E+00 | 3.435E-07 | 2.593E-07 | 5.005E-06 | 7.923E-06 | 1.679E-06 | 7.901E-06 | 4.822E-06 | 3.354E-06 | 0.000E+00 | 1.269E-06 |
| Unigene338841 | eptA | 1.216E-07 | 0.000E+00 | 1.193E-07 | 0.000E+00 | 1.249E-06 | 0.000E+00 | 0.000E+00 | 0.000E+00 | 4.983E-07 | 0.000E+00 | 3.477E-08 | 0.000E+00 |
| Unigene338878 | tetA(46) | 0.000E+00 | 0.000E+00 | 0.000E+00 | 0.000E+00 | 0.000E+00 | 0.000E+00 | 0.000E+00 | 1.137E-07 | 0.000E+00 | 0.000E+00 | 0.000E+00 | 0.000E+00 |
| Unigene338883 | tetA(46) | 0.000E+00 | 0.000E+00 | 0.000E+00 | 0.000E+00 | 7.642E-07 | 0.000E+00 | 0.000E+00 | 0.000E+00 | 0.000E+00 | 0.000E+00 | 0.000E+00 | 0.000E+00 |
| Unigene338957 | MexD | 6.178E-08 | 6.781E-08 | 2.425E-07 | 2.080E-07 | 8.694E-07 | 2.671E-07 | 1.014E-06 | 5.769E-07 | 1.576E-07 | 4.473E-07 | 7.303E-07 | 1.826E-06 |
| Unigene338958 | Enterobact | 0.000E+00 | 0.000E+00 | 0.000E+00 | 2.908E-07 | 8.435E-07 | 0.000E+00 | 0.000E+00 | 0.000E+00 | 0.000E+00 | 0.000E+00 | 8.086E-07 | 1.919E-06 |
| Unigene338988 | smeS | 0.000E+00 | 0.000E+00 | 9.754E-08 | 0.000E+00 | 1.104E-06 | 0.000E+00 | 0.000E+00 | 0.000E+00 | 6.790E-07 | 0.000E+00 | 0.000E+00 | 0.000E+00 |
| Unigene339325 | msbA | 4.007E-08 | 0.000E+00 | 3.932E-08 | 0.000E+00 | 1.937E-06 | 0.000E+00 | 1.552E-07 | 0.000E+00 | 0.000E+00 | 0.000E+00 | 0.000E+00 | 0.000E+00 |
| Unigene339382 | bacA | 4.000E-08 | 4.390E-08 | 0.000E+00 | 8.079E-08 | 7.555E-07 | 8.648E-08 | 1.328E-07 | 0.000E+00 | 0.000E+00 | 4.902E-07 | 1.830E-07 | 5.201E-07 |
| Unigene339388 | Brucella su | 0.000E+00 | 0.000E+00 | 0.000E+00 | 0.000E+00 | 5.692E-07 | 0.000E+00 | 0.000E+00 | 0.000E+00 | 0.000E+00 | 0.000E+00 | 0.000E+00 | 0.000E+00 |
| Unigene339421 | Staphylocc | 1.840E-07 | 6.733E-08 | 4.936E-07 | 2.850E-07 | 8.315E-07 | 1.459E-07 | 8.555E-07 | 6.588E-07 | 1.743E-07 | 4.647E-07 | 7.017E-07 | 2.204E-06 |

| | | | | | | | | | | | | | |
|---|---|---|---|---|---|---|---|---|---|---|---|---|---|
| Unigene339441 | Acinetoba | 4.947E-08 | 8.144E-08 | 4.854E-08 | 0.000E+00 | 7.695E-07 | 0.000E+00 | 1.369E-07 | 4.042E-07 | 1.081E-07 | 1.102E-07 | 2.264E-07 | 3.509E-07 |
| Unigene339465 | vanRL | 0.000E+00 | 1.845E-07 | 1.375E-08 | 0.000E+00 | 5.603E-07 | 0.000E+00 | 1.861E-07 | 8.177E-07 | 6.125E-08 | 1.561E-07 | 1.923E-07 | 3.975E-07 |
| Unigene339520 | baeR | 0.000E+00 | 9.601E-08 | 0.000E+00 | 0.000E+00 | 7.775E-07 | 7.566E-07 | 1.307E-06 | 1.532E-06 | 2.868E-07 | 6.822E-07 | 6.504E-07 | 3.878E-06 |
| Unigene339596 | evgA | 0.000E+00 | 0.000E+00 | 0.000E+00 | 0.000E+00 | 7.262E-07 | 0.000E+00 | 0.000E+00 | 0.000E+00 | 0.000E+00 | 0.000E+00 | 0.000E+00 | 0.000E+00 |
| Unigene339615 | macB | 0.000E+00 | 0.000E+00 | 0.000E+00 | 0.000E+00 | 1.032E-06 | 2.456E-07 | 1.234E-06 | 9.643E-07 | 0.000E+00 | 5.522E-07 | 8.269E-07 | 1.245E-06 |
| Unigene339647 | vanHF | 0.000E+00 | 0.000E+00 | 0.000E+00 | 0.000E+00 | 1.795E-06 | 0.000E+00 | 0.000E+00 | 0.000E+00 | 0.000E+00 | 0.000E+00 | 0.000E+00 | 0.000E+00 |
| Unigene339679 | evgA | 0.000E+00 | 0.000E+00 | 0.000E+00 | 0.000E+00 | 4.623E-07 | 0.000E+00 | 0.000E+00 | 0.000E+00 | 0.000E+00 | 0.000E+00 | 0.000E+00 | 0.000E+00 |
| Unigene339837 | TaeA | 0.000E+00 | 0.000E+00 | 0.000E+00 | 0.000E+00 | 1.388E-06 | 0.000E+00 | 0.000E+00 | 0.000E+00 | 0.000E+00 | 0.000E+00 | 0.000E+00 | 0.000E+00 |
| Unigene339842 | carA | 0.000E+00 | 0.000E+00 | 0.000E+00 | 0.000E+00 | 1.266E-06 | 0.000E+00 | 0.000E+00 | 0.000E+00 | 0.000E+00 | 0.000E+00 | 0.000E+00 | 0.000E+00 |
| Unigene339866 | smeR | 0.000E+00 | 0.000E+00 | 0.000E+00 | 0.000E+00 | 9.870E-07 | 0.000E+00 | 0.000E+00 | 0.000E+00 | 0.000E+00 | 0.000E+00 | 0.000E+00 | 0.000E+00 |
| Unigene339924 | chrB | 0.000E+00 | 0.000E+00 | 0.000E+00 | 0.000E+00 | 1.318E-06 | 0.000E+00 | 0.000E+00 | 0.000E+00 | 0.000E+00 | 0.000E+00 | 0.000E+00 | 0.000E+00 |
| Unigene339925 | mecD | 0.000E+00 | 0.000E+00 | 9.071E-08 | 7.468E-08 | 1.232E-06 | 0.000E+00 | 0.000E+00 | 0.000E+00 | 4.850E-07 | 8.238E-08 | 2.115E-08 | 0.000E+00 |
| Unigene339947 | bcrA | 1.408E-06 | 0.000E+00 | 0.000E+00 | 0.000E+00 | 1.741E-05 | 2.771E-06 | 0.000E+00 | 0.000E+00 | 0.000E+00 | 0.000E+00 | 0.000E+00 | 1.280E-07 |
| Unigene339983 | acrB | 0.000E+00 | 0.000E+00 | 0.000E+00 | 0.000E+00 | 6.798E-07 | 0.000E+00 | 0.000E+00 | 0.000E+00 | 0.000E+00 | 0.000E+00 | 0.000E+00 | 0.000E+00 |
| Unigene340040 | macB | 0.000E+00 | 0.000E+00 | 0.000E+00 | 0.000E+00 | 1.103E-06 | 0.000E+00 | 0.000E+00 | 0.000E+00 | 0.000E+00 | 0.000E+00 | 0.000E+00 | 0.000E+00 |
| Unigene340145 | msbA | 0.000E+00 | 0.000E+00 | 0.000E+00 | 0.000E+00 | 3.550E-06 | 0.000E+00 | 0.000E+00 | 1.572E-06 | 0.000E+00 | 0.000E+00 | 2.963E-07 | 0.000E+00 |
| Unigene340194 | vanHD | 0.000E+00 | 0.000E+00 | 0.000E+00 | 2.451E-07 | 3.370E-06 | 0.000E+00 | 0.000E+00 | 0.000E+00 | 6.632E-08 | 4.957E-07 | 7.866E-07 | 0.000E+00 |
| Unigene340205 | novA | 0.000E+00 | 0.000E+00 | 0.000E+00 | 0.000E+00 | 2.980E-06 | 1.397E-07 | 0.000E+00 | 0.000E+00 | 0.000E+00 | 0.000E+00 | 2.587E-07 | 0.000E+00 |
| Unigene340221 | patA | 0.000E+00 | 0.000E+00 | 0.000E+00 | 0.000E+00 | 2.762E-06 | 0.000E+00 | 0.000E+00 | 0.000E+00 | 0.000E+00 | 2.164E-07 | 1.777E-07 | 0.000E+00 |
| Unigene340238 | sdiA | 0.000E+00 | 0.000E+00 | 0.000E+00 | 0.000E+00 | 2.455E-06 | 0.000E+00 | 0.000E+00 | 0.000E+00 | 0.000E+00 | 0.000E+00 | 0.000E+00 | 0.000E+00 |
| Unigene340250 | tva(A) | 0.000E+00 | 0.000E+00 | 0.000E+00 | 0.000E+00 | 2.711E-06 | 0.000E+00 | 0.000E+00 | 0.000E+00 | 0.000E+00 | 0.000E+00 | 8.036E-07 | 1.705E-06 |
| Unigene340251 | macB | 0.000E+00 | 0.000E+00 | 0.000E+00 | 0.000E+00 | 3.788E-06 | 0.000E+00 | 0.000E+00 | 0.000E+00 | 0.000E+00 | 0.000E+00 | 0.000E+00 | 0.000E+00 |
| Unigene340254 | vanRG | 0.000E+00 | 0.000E+00 | 0.000E+00 | 0.000E+00 | 2.949E-06 | 0.000E+00 | 0.000E+00 | 0.000E+00 | 0.000E+00 | 0.000E+00 | 6.478E-07 | 0.000E+00 |
| Unigene340255 | smeS | 0.000E+00 | 0.000E+00 | 0.000E+00 | 0.000E+00 | 3.222E-06 | 0.000E+00 | 0.000E+00 | 1.013E-06 | 0.000E+00 | 0.000E+00 | 5.892E-07 | 1.252E-06 |
| Unigene340337 | AcrE | 0.000E+00 | 0.000E+00 | 0.000E+00 | 0.000E+00 | 4.231E-07 | 1.373E-07 | 1.405E-07 | 0.000E+00 | 0.000E+00 | 0.000E+00 | 4.357E-07 | 6.003E-07 |
| Unigene340356 | efrB | 9.439E-08 | 1.036E-07 | 2.315E-08 | 4.766E-08 | 3.932E-07 | 0.000E+00 | 3.395E-07 | 2.754E-07 | 7.737E-08 | 1.840E-07 | 1.350E-07 | 4.463E-07 |
| Unigene340373 | tetB(46) | 0.000E+00 | 2.187E-07 | 0.000E+00 | 0.000E+00 | 1.348E-06 | 0.000E+00 | 0.000E+00 | 0.000E+00 | 9.701E-07 | 0.000E+00 | 0.000E+00 | 0.000E+00 |
| Unigene340374 | tetA(60) | 0.000E+00 | 8.075E-08 | 1.083E-07 | 0.000E+00 | 1.492E-06 | 0.000E+00 | 0.000E+00 | 0.000E+00 | 6.835E-07 | 0.000E+00 | 0.000E+00 | 0.000E+00 |
| Unigene340416 | macB | 0.000E+00 | 0.000E+00 | 2.011E-07 | 0.000E+00 | 5.123E-07 | 0.000E+00 | 0.000E+00 | 0.000E+00 | 0.000E+00 | 0.000E+00 | 0.000E+00 | 0.000E+00 |
| Unigene340529 | macB | 0.000E+00 | 0.000E+00 | 0.000E+00 | 0.000E+00 | 2.759E-07 | 0.000E+00 | 0.000E+00 | 0.000E+00 | 0.000E+00 | 0.000E+00 | 0.000E+00 | 0.000E+00 |
| Unigene340561 | msbA | 0.000E+00 | 0.000E+00 | 0.000E+00 | 9.658E-08 | 5.313E-07 | 2.068E-07 | 0.000E+00 | 0.000E+00 | 0.000E+00 | 0.000E+00 | 0.000E+00 | 0.000E+00 |
| Unigene340623 | patA | 0.000E+00 | 0.000E+00 | 0.000E+00 | 0.000E+00 | 9.326E-07 | 0.000E+00 | 0.000E+00 | 0.000E+00 | 0.000E+00 | 0.000E+00 | 0.000E+00 | 0.000E+00 |
| Unigene340624 | MexK | 0.000E+00 | 0.000E+00 | 1.424E-07 | 7.325E-08 | 3.761E-07 | 2.614E-07 | 2.944E-07 | 2.822E-07 | 0.000E+00 | 0.000E+00 | 8.298E-08 | 4.001E-07 |
| Unigene340638 | farA | 0.000E+00 | 0.000E+00 | 0.000E+00 | 0.000E+00 | 1.666E-07 | 8.106E-08 | 0.000E+00 | 0.000E+00 | 0.000E+00 | 8.353E-08 | 8.577E-08 | 2.216E-07 |
| Unigene340651 | vatF | 4.701E-07 | 2.293E-07 | 6.150E-07 | 0.000E+00 | 9.285E-07 | 4.517E-07 | 1.676E-06 | 1.219E-06 | 0.000E+00 | 8.728E-07 | 8.365E-07 | 4.569E-06 |
| Unigene340697 | AcrE | 4.531E-07 | 0.000E+00 | 0.000E+00 | 0.000E+00 | 4.813E-06 | 0.000E+00 | 9.088E-07 | 1.653E-07 | 0.000E+00 | 0.000E+00 | 0.000E+00 | 1.004E-07 |
| Unigene340741 | VatI | 0.000E+00 | 0.000E+00 | 0.000E+00 | 0.000E+00 | 4.244E-07 | 0.000E+00 | 0.000E+00 | 0.000E+00 | 0.000E+00 | 0.000E+00 | 0.000E+00 | 0.000E+00 |
| Unigene340746 | adeJ | 0.000E+00 | 0.000E+00 | 0.000E+00 | 0.000E+00 | 7.796E-07 | 0.000E+00 | 2.589E-07 | 6.485E-07 | 9.587E-08 | 1.303E-07 | 2.007E-07 | 7.258E-07 |
| Unigene340821 | tcr3 | 0.000E+00 | 0.000E+00 | 0.000E+00 | 0.000E+00 | 1.096E-06 | 0.000E+00 | 2.600E-08 | 0.000E+00 | 5.649E-07 | 1.047E-07 | 8.062E-08 | 0.000E+00 |
| Unigene340882 | novA | 8.249E-07 | 0.000E+00 | 5.036E-07 | 4.443E-07 | 3.849E-06 | 8.026E-06 | 1.846E-06 | 8.944E-06 | 4.648E-06 | 4.064E-06 | 0.000E+00 | 8.234E-07 |
| Unigene340883 | tetA(46) | 0.000E+00 | 2.040E-08 | 1.824E-07 | 6.005E-07 | 4.356E-06 | 8.578E-06 | 1.419E-06 | 6.920E-06 | 4.144E-06 | 2.878E-06 | 0.000E+00 | 5.711E-07 |
| Unigene340898 | Streptomy | 0.000E+00 | 0.000E+00 | 0.000E+00 | 0.000E+00 | 9.358E-07 | 0.000E+00 | 0.000E+00 | 0.000E+00 | 0.000E+00 | 0.000E+00 | 0.000E+00 | 0.000E+00 |
| Unigene340899 | Acinetoba | 0.000E+00 | 0.000E+00 | 0.000E+00 | 6.163E-07 | 9.794E-07 | 0.000E+00 | 0.000E+00 | 0.000E+00 | 1.482E-07 | 5.540E-07 | 0.000E+00 | 0.000E+00 |
| Unigene341015 | oleC | 0.000E+00 | 0.000E+00 | 0.000E+00 | 0.000E+00 | 4.080E-07 | 0.000E+00 | 0.000E+00 | 2.572E-07 | 0.000E+00 | 0.000E+00 | 0.000E+00 | 0.000E+00 |
| Unigene341048 | aadA13 | 0.000E+00 | 0.000E+00 | 0.000E+00 | 0.000E+00 | 8.405E-07 | 0.000E+00 | 0.000E+00 | 0.000E+00 | 1.838E-07 | 0.000E+00 | 0.000E+00 | 0.000E+00 |
| Unigene341050 | srmB | 0.000E+00 | 0.000E+00 | 0.000E+00 | 0.000E+00 | 4.427E-07 | 0.000E+00 | 0.000E+00 | 3.101E-07 | 0.000E+00 | 0.000E+00 | 0.000E+00 | 0.000E+00 |
| Unigene341065 | facT | 0.000E+00 | 0.000E+00 | 0.000E+00 | 0.000E+00 | 2.732E-07 | 0.000E+00 | 0.000E+00 | 0.000E+00 | 0.000E+00 | 0.000E+00 | 0.000E+00 | 0.000E+00 |
| Unigene341103 | lmrD | 1.562E-04 | 2.838E-04 | 2.268E-04 | 0.000E+00 | 1.363E-05 | 0.000E+00 | 5.489E-06 | 0.000E+00 | 7.300E-05 | 0.000E+00 | 4.039E-06 | 0.000E+00 |
| Unigene341155 | tlrC | 0.000E+00 | 0.000E+00 | 2.676E-07 | 0.000E+00 | 4.132E-07 | 2.144E-07 | 1.098E-06 | 0.000E+00 | 0.000E+00 | 0.000E+00 | 5.956E-07 | 8.499E-07 |
| Unigene341291 | MexW | 2.459E-07 | 0.000E+00 | 6.435E-07 | 3.622E-07 | 9.791E-07 | 3.877E-07 | 1.542E-06 | 8.254E-07 | 0.000E+00 | 6.393E-07 | 1.160E-06 | 3.174E-06 |
| Unigene341312 | Enterobact | 2.219E-07 | 0.000E+00 | 2.177E-07 | 0.000E+00 | 8.627E-07 | 0.000E+00 | 1.903E-06 | 1.036E-06 | 1.212E-07 | 0.000E+00 | 1.332E-06 | 2.491E-06 |
| Unigene341313 | acrB | 2.716E-07 | 2.178E-07 | 6.663E-07 | 4.009E-07 | 9.169E-07 | 4.179E-07 | 1.341E-06 | 1.024E-06 | 2.169E-07 | 7.797E-07 | 8.723E-07 | 3.285E-06 |
| Unigene341319 | Escherichia | 3.615E-07 | 1.160E-06 | 7.639E-07 | 3.650E-07 | 5.992E-06 | 0.000E+00 | 1.077E-06 | 3.245E-07 | 0.000E+00 | 0.000E+00 | 2.862E-07 | 0.000E+00 |
| Unigene341396 | QepA1 | 0.000E+00 | 0.000E+00 | 0.000E+00 | 0.000E+00 | 1.992E-07 | 0.000E+00 | 0.000E+00 | 0.000E+00 | 0.000E+00 | 0.000E+00 | 0.000E+00 | 0.000E+00 |
| Unigene341409 | smeR | 0.000E+00 | 0.000E+00 | 0.000E+00 | 0.000E+00 | 5.087E-07 | 0.000E+00 | 0.000E+00 | 0.000E+00 | 0.000E+00 | 0.000E+00 | 0.000E+00 | 0.000E+00 |
| Unigene341469 | macB | 0.000E+00 | 0.000E+00 | 0.000E+00 | 0.000E+00 | 3.088E-06 | 0.000E+00 | 0.000E+00 | 0.000E+00 | 0.000E+00 | 0.000E+00 | 5.450E-07 | 1.197E-06 |
| Unigene341478 | baeS | 0.000E+00 | 0.000E+00 | 0.000E+00 | 0.000E+00 | 2.903E-06 | 0.000E+00 | 0.000E+00 | 0.000E+00 | 0.000E+00 | 0.000E+00 | 2.637E-07 | 0.000E+00 |
| Unigene341495 | kdpE | 0.000E+00 | 0.000E+00 | 0.000E+00 | 0.000E+00 | 3.721E-06 | 0.000E+00 | 0.000E+00 | 0.000E+00 | 0.000E+00 | 0.000E+00 | 4.408E-07 | 0.000E+00 |

| | | | | | | | | | | | | | |
|---|---|---|---|---|---|---|---|---|---|---|---|---|---|
| Unigene341503 | vanSA | 0.000E+00 | 0.000E+00 | 0.000E+00 | 0.000E+00 | 3.841E-06 | 0.000E+00 | 2.870E-07 | 1.059E-06 | 9.446E-08 | 0.000E+00 | 3.213E-07 | 1.047E-06 |
| Unigene341524 | tetA(46) | 3.188E-07 | 0.000E+00 | 6.257E-07 | 0.000E+00 | 1.063E-06 | 0.000E+00 | 2.073E-06 | 7.443E-07 | 1.307E-07 | 7.991E-07 | 1.276E-06 | 3.015E-06 |
| Unigene341547 | Staphylocc | 7.404E-08 | 8.126E-08 | 0.000E+00 | 7.476E-08 | 7.403E-07 | 8.004E-08 | 2.185E-07 | 2.016E-07 | 1.079E-07 | 4.124E-07 | 1.129E-07 | 4.376E-07 |
| Unigene341556 | rosB | 0.000E+00 | 7.595E-08 | 0.000E+00 | 0.000E+00 | 1.422E-06 | 0.000E+00 | 0.000E+00 | 0.000E+00 | 7.752E-07 | 7.708E-08 | 0.000E+00 | 0.000E+00 |
| Unigene341567 | vanTG | 0.000E+00 | 0.000E+00 | 0.000E+00 | 0.000E+00 | 3.795E-07 | 0.000E+00 | 0.000E+00 | 0.000E+00 | 0.000E+00 | 0.000E+00 | 0.000E+00 | 0.000E+00 |
| Unigene341594 | patA | 0.000E+00 | 0.000E+00 | 0.000E+00 | 0.000E+00 | 8.434E-07 | 1.427E-07 | 4.018E-07 | 3.467E-07 | 1.082E-07 | 0.000E+00 | 6.796E-07 | 3.901E-07 |
| Unigene341602 | bcrA | 4.312E-07 | 0.000E+00 | 8.270E-07 | 2.969E-07 | 1.132E-06 | 5.085E-07 | 1.540E-06 | 1.121E-06 | 0.000E+00 | 6.768E-07 | 1.681E-06 | 2.896E-06 |
| Unigene341682 | tetA(58) | 0.000E+00 | 0.000E+00 | 0.000E+00 | 0.000E+00 | 1.814E-06 | 0.000E+00 | 4.253E-07 | 3.925E-07 | 0.000E+00 | 0.000E+00 | 0.000E+00 | 0.000E+00 |
| Unigene341726 | optrA | 0.000E+00 | 0.000E+00 | 0.000E+00 | 0.000E+00 | 8.812E-07 | 1.491E-07 | 0.000E+00 | 0.000E+00 | 0.000E+00 | 0.000E+00 | 4.339E-07 | 4.484E-07 |
| Unigene341839 | oleC | 0.000E+00 | 0.000E+00 | 0.000E+00 | 4.295E-08 | 9.450E-08 | 0.000E+00 | 9.414E-08 | 0.000E+00 | 0.000E+00 | 0.000E+00 | 0.000E+00 | 0.000E+00 |
| Unigene341850 | rosA | 0.000E+00 | 0.000E+00 | 0.000E+00 | 0.000E+00 | 6.741E-07 | 8.746E-08 | 4.477E-08 | 0.000E+00 | 0.000E+00 | 0.000E+00 | 3.239E-07 | 9.563E-08 |
| Unigene341975 | lmrD | 0.000E+00 | 0.000E+00 | 0.000E+00 | 0.000E+00 | 4.269E-07 | 0.000E+00 | 0.000E+00 | 0.000E+00 | 0.000E+00 | 0.000E+00 | 0.000E+00 | 0.000E+00 |
| Unigene342162 | tet(30) | 0.000E+00 | 0.000E+00 | 0.000E+00 | 0.000E+00 | 4.744E-07 | 0.000E+00 | 0.000E+00 | 0.000E+00 | 0.000E+00 | 0.000E+00 | 0.000E+00 | 0.000E+00 |
| Unigene342264 | PmrF | 0.000E+00 | 0.000E+00 | 0.000E+00 | 0.000E+00 | 1.094E-06 | 0.000E+00 | 0.000E+00 | 0.000E+00 | 0.000E+00 | 0.000E+00 | 0.000E+00 | 0.000E+00 |
| Unigene342344 | Klebsiella p | 0.000E+00 | 0.000E+00 | 0.000E+00 | 0.000E+00 | 1.192E-06 | 0.000E+00 | 0.000E+00 | 0.000E+00 | 0.000E+00 | 0.000E+00 | 4.092E-07 | 0.000E+00 |
| Unigene342359 | Acinetobac | 0.000E+00 | 2.495E-07 | 4.709E-07 | 3.316E-07 | 6.735E-07 | 0.000E+00 | 1.174E-06 | 6.781E-07 | 0.000E+00 | 3.939E-07 | 6.067E-07 | 2.209E-06 |
| Unigene342457 | lmrB | 3.757E-07 | 6.774E-07 | 4.739E-07 | 2.439E-07 | 8.287E-06 | 3.481E-07 | 1.307E-06 | 1.566E-07 | 0.000E+00 | 0.000E+00 | 0.000E+00 | 0.000E+00 |
| Unigene342458 | adeL | 3.301E-07 | 9.056E-07 | 5.830E-07 | 0.000E+00 | 6.344E-06 | 2.141E-07 | 6.941E-07 | 1.926E-07 | 0.000E+00 | 0.000E+00 | 0.000E+00 | 0.000E+00 |
| Unigene342518 | arlS | 0.000E+00 | 0.000E+00 | 0.000E+00 | 0.000E+00 | 4.534E-07 | 0.000E+00 | 0.000E+00 | 0.000E+00 | 0.000E+00 | 0.000E+00 | 0.000E+00 | 0.000E+00 |
| Unigene342602 | macB | 0.000E+00 | 1.352E-07 | 1.612E-07 | 2.074E-07 | 8.669E-07 | 0.000E+00 | 0.000E+00 | 6.232E-07 | 0.000E+00 | 0.000E+00 | 5.168E-07 | 9.709E-07 |
| Unigene342619 | OXA-18 | 0.000E+00 | 0.000E+00 | 0.000E+00 | 0.000E+00 | 5.505E-07 | 0.000E+00 | 0.000E+00 | 0.000E+00 | 1.547E-07 | 0.000E+00 | 0.000E+00 | 0.000E+00 |
| Unigene342644 | Acinetobac | 0.000E+00 | 0.000E+00 | 0.000E+00 | 0.000E+00 | 0.000E+00 | 0.000E+00 | 0.000E+00 | 0.000E+00 | 0.000E+00 | 0.000E+00 | 2.474E-07 | 0.000E+00 |
| Unigene342741 | MexW | 0.000E+00 | 0.000E+00 | 0.000E+00 | 0.000E+00 | 5.248E-07 | 0.000E+00 | 0.000E+00 | 0.000E+00 | 0.000E+00 | 0.000E+00 | 0.000E+00 | 0.000E+00 |
| Unigene342748 | vanHA | 0.000E+00 | 0.000E+00 | 0.000E+00 | 0.000E+00 | 8.432E-07 | 0.000E+00 | 0.000E+00 | 0.000E+00 | 0.000E+00 | 0.000E+00 | 0.000E+00 | 6.333E-07 |
| Unigene342762 | SAT-3 | 0.000E+00 | 0.000E+00 | 0.000E+00 | 0.000E+00 | 1.020E-06 | 0.000E+00 | 0.000E+00 | 0.000E+00 | 0.000E+00 | 0.000E+00 | 0.000E+00 | 1.474E-06 |
| Unigene342871 | vanHF | 0.000E+00 | 0.000E+00 | 0.000E+00 | 0.000E+00 | 9.195E-07 | 0.000E+00 | 0.000E+00 | 0.000E+00 | 0.000E+00 | 0.000E+00 | 1.065E-06 | 3.710E-06 |
| Unigene342956 | vanHD | 7.592E-07 | 2.327E-06 | 1.387E-06 | 7.138E-07 | 1.169E-05 | 2.547E-07 | 2.289E-06 | 4.889E-07 | 0.000E+00 | 0.000E+00 | 5.690E-07 | 0.000E+00 |
| Unigene343015 | oqxB | 5.178E-08 | 3.410E-08 | 0.000E+00 | 0.000E+00 | 1.070E-06 | 0.000E+00 | 1.032E-07 | 0.000E+00 | 5.886E-07 | 9.229E-08 | 2.369E-08 | 0.000E+00 |
| Unigene343021 | emrB | 0.000E+00 | 0.000E+00 | 0.000E+00 | 0.000E+00 | 5.837E-07 | 0.000E+00 | 0.000E+00 | 0.000E+00 | 0.000E+00 | 0.000E+00 | 0.000E+00 | 0.000E+00 |
| Unigene343029 | mgrA | 0.000E+00 | 0.000E+00 | 0.000E+00 | 0.000E+00 | 0.000E+00 | 0.000E+00 | 0.000E+00 | 5.636E-07 | 0.000E+00 | 0.000E+00 | 0.000E+00 | 1.141E-06 |
| Unigene343045 | AcrS | 0.000E+00 | 0.000E+00 | 9.686E-08 | 0.000E+00 | 1.316E-06 | 0.000E+00 | 0.000E+00 | 0.000E+00 | 9.711E-07 | 0.000E+00 | 0.000E+00 | 0.000E+00 |
| Unigene343112 | YojI | 0.000E+00 | 0.000E+00 | 0.000E+00 | 0.000E+00 | 6.766E-07 | 0.000E+00 | 0.000E+00 | 0.000E+00 | 0.000E+00 | 0.000E+00 | 0.000E+00 | 2.016E-06 |
| Unigene343178 | lmrD | 0.000E+00 | 0.000E+00 | 0.000E+00 | 0.000E+00 | 5.738E-07 | 0.000E+00 | 5.240E-07 | 0.000E+00 | 0.000E+00 | 0.000E+00 | 0.000E+00 | 0.000E+00 |
| Unigene343215 | optrA | 0.000E+00 | 0.000E+00 | 0.000E+00 | 0.000E+00 | 7.682E-07 | 0.000E+00 | 0.000E+00 | 0.000E+00 | 0.000E+00 | 0.000E+00 | 0.000E+00 | 0.000E+00 |
| Unigene343280 | vanRD | 1.045E-07 | 0.000E+00 | 0.000E+00 | 0.000E+00 | 9.285E-07 | 0.000E+00 | 0.000E+00 | 0.000E+00 | 0.000E+00 | 0.000E+00 | 0.000E+00 | 0.000E+00 |
| Unigene343291 | baeS | 2.538E-07 | 0.000E+00 | 3.984E-07 | 0.000E+00 | 8.740E-07 | 3.841E-07 | 6.741E-07 | 5.924E-07 | 3.328E-07 | 6.502E-07 | 5.516E-07 | 2.580E-06 |
| Unigene343310 | iri | 0.000E+00 | 0.000E+00 | 0.000E+00 | 0.000E+00 | 5.403E-07 | 0.000E+00 | 0.000E+00 | 0.000E+00 | 0.000E+00 | 0.000E+00 | 0.000E+00 | 0.000E+00 |
| Unigene343326 | efrA | 0.000E+00 | 0.000E+00 | 0.000E+00 | 0.000E+00 | 0.000E+00 | 0.000E+00 | 0.000E+00 | 0.000E+00 | 0.000E+00 | 0.000E+00 | 0.000E+00 | 3.140E-07 |
| Unigene343330 | oqxA | 0.000E+00 | 5.399E-07 | 0.000E+00 | 0.000E+00 | 1.025E-06 | 0.000E+00 | 0.000E+00 | 0.000E+00 | 0.000E+00 | 0.000E+00 | 0.000E+00 | 0.000E+00 |
| Unigene343442 | tap | 0.000E+00 | 0.000E+00 | 0.000E+00 | 0.000E+00 | 6.573E-07 | 0.000E+00 | 0.000E+00 | 0.000E+00 | 0.000E+00 | 0.000E+00 | 0.000E+00 | 0.000E+00 |
| Unigene343499 | efrA | 1.832E-07 | 0.000E+00 | 4.493E-08 | 0.000E+00 | 4.069E-07 | 2.475E-07 | 5.068E-07 | 0.000E+00 | 0.000E+00 | 4.591E-07 | 0.000E+00 | 5.953E-07 |
| Unigene343518 | efrB | 0.000E+00 | 0.000E+00 | 0.000E+00 | 0.000E+00 | 4.464E-07 | 0.000E+00 | 0.000E+00 | 0.000E+00 | 0.000E+00 | 0.000E+00 | 0.000E+00 | 0.000E+00 |
| Unigene343587 | adeS | 0.000E+00 | 0.000E+00 | 8.014E-08 | 0.000E+00 | 5.671E-07 | 0.000E+00 | 4.519E-07 | 0.000E+00 | 0.000E+00 | 0.000E+00 | 0.000E+00 | 0.000E+00 |
| Unigene343690 | basS | 0.000E+00 | 0.000E+00 | 0.000E+00 | 0.000E+00 | 1.490E-06 | 0.000E+00 | 0.000E+00 | 0.000E+00 | 1.023E-06 | 0.000E+00 | 0.000E+00 | 0.000E+00 |
| Unigene343790 | adeH | 0.000E+00 | 0.000E+00 | 0.000E+00 | 0.000E+00 | 6.757E-07 | 0.000E+00 | 0.000E+00 | 0.000E+00 | 4.346E-07 | 0.000E+00 | 0.000E+00 | 0.000E+00 |
| Unigene343809 | TriC | 0.000E+00 | 0.000E+00 | 0.000E+00 | 0.000E+00 | 0.000E+00 | 0.000E+00 | 2.018E-07 | 0.000E+00 | 0.000E+00 | 0.000E+00 | 0.000E+00 | 0.000E+00 |
| Unigene343868 | TaeA | 0.000E+00 | 0.000E+00 | 1.634E-07 | 1.682E-07 | 9.252E-07 | 0.000E+00 | 7.374E-07 | 0.000E+00 | 0.000E+00 | 0.000E+00 | 0.000E+00 | 9.844E-07 |
| Unigene343916 | efrA | 0.000E+00 | 0.000E+00 | 1.015E-07 | 0.000E+00 | 7.280E-07 | 2.237E-07 | 4.199E-07 | 9.259E-07 | 1.508E-07 | 0.000E+00 | 7.890E-07 | 8.153E-07 |
| Unigene343986 | TriB | 0.000E+00 | 0.000E+00 | 0.000E+00 | 0.000E+00 | 1.222E-06 | 0.000E+00 | 0.000E+00 | 0.000E+00 | 0.000E+00 | 0.000E+00 | 0.000E+00 | 3.865E-06 |
| Unigene343987 | TriC | 2.627E-07 | 1.384E-07 | 5.362E-07 | 2.865E-07 | 8.872E-07 | 3.862E-07 | 1.209E-06 | 7.850E-07 | 2.067E-07 | 5.619E-07 | 7.573E-07 | 2.559E-06 |
| Unigene344027 | qacH | 0.000E+00 | 0.000E+00 | 1.025E-07 | 0.000E+00 | 2.901E-06 | 0.000E+00 | 0.000E+00 | 8.048E-06 | 4.339E-06 | 0.000E+00 | 0.000E+00 | 0.000E+00 |
| Unigene344112 | PmrF | 0.000E+00 | 0.000E+00 | 0.000E+00 | 0.000E+00 | 7.416E-07 | 0.000E+00 | 0.000E+00 | 0.000E+00 | 0.000E+00 | 0.000E+00 | 0.000E+00 | 0.000E+00 |
| Unigene344203 | patB | 1.401E-07 | 1.538E-07 | 0.000E+00 | 0.000E+00 | 6.226E-07 | 0.000E+00 | 2.791E-07 | 1.962E-07 | 3.063E-08 | 1.561E-07 | 0.000E+00 | 5.631E-07 |
| Unigene344324 | macB | 0.000E+00 | 0.000E+00 | 5.477E-07 | 0.000E+00 | 7.923E-07 | 0.000E+00 | 0.000E+00 | 6.153E-07 | 0.000E+00 | 0.000E+00 | 0.000E+00 | 1.869E-06 |
| Unigene344490 | qacH | 0.000E+00 | 0.000E+00 | 0.000E+00 | 0.000E+00 | 3.463E-06 | 0.000E+00 | 0.000E+00 | 0.000E+00 | 0.000E+00 | 0.000E+00 | 0.000E+00 | 0.000E+00 |
| Unigene344493 | Staphylocc | 0.000E+00 | 4.560E-08 | 0.000E+00 | 0.000E+00 | 2.654E-06 | 0.000E+00 | 0.000E+00 | 6.547E-07 | 0.000E+00 | 0.000E+00 | 0.000E+00 | 1.473E-06 |
| Unigene344587 | golS | 0.000E+00 | 0.000E+00 | 0.000E+00 | 0.000E+00 | 8.790E-07 | 0.000E+00 | 0.000E+00 | 0.000E+00 | 0.000E+00 | 0.000E+00 | 1.810E-07 | 5.611E-07 |
| Unigene344607 | MuxC | 0.000E+00 | 0.000E+00 | 0.000E+00 | 0.000E+00 | 4.865E-07 | 0.000E+00 | 0.000E+00 | 0.000E+00 | 0.000E+00 | 0.000E+00 | 0.000E+00 | 0.000E+00 |

| | | | | | | | | | | | | | |
|---|---|---|---|---|---|---|---|---|---|---|---|---|---|
| Unigene344610 | lmrB | 2.534E-07 | 0.000E+00 | 0.000E+00 | 2.327E-07 | 2.150E-06 | 5.555E-06 | 0.000E+00 | 0.000E+00 | 3.123E-06 | 0.000E+00 | 0.000E+00 | 0.000E+00 |
| Unigene344714 | lmrC | 0.000E+00 | 0.000E+00 | 0.000E+00 | 0.000E+00 | 1.616E-06 | 0.000E+00 | 0.000E+00 | 0.000E+00 | 1.176E-06 | 0.000E+00 | 0.000E+00 | 0.000E+00 |
| Unigene344784 | ceoB | 0.000E+00 | 8.652E-08 | 0.000E+00 | 0.000E+00 | 4.379E-07 | 0.000E+00 | 8.724E-08 | 0.000E+00 | 0.000E+00 | 0.000E+00 | 0.000E+00 | 2.795E-07 |
| Unigene344791 | evgS | 0.000E+00 | 0.000E+00 | 0.000E+00 | 0.000E+00 | 6.803E-07 | 0.000E+00 | 0.000E+00 | 9.190E-07 | 0.000E+00 | 4.873E-07 | 0.000E+00 | 0.000E+00 |
| Unigene344829 | rphB | 5.748E-07 | 1.702E-06 | 9.312E-07 | 5.129E-07 | 6.816E-06 | 2.890E-07 | 1.627E-06 | 5.305E-07 | 0.000E+00 | 0.000E+00 | 0.000E+00 | 0.000E+00 |
| Unigene344922 | otr(B) | 0.000E+00 | 0.000E+00 | 2.367E-07 | 0.000E+00 | 6.433E-07 | 0.000E+00 | 3.204E-07 | 0.000E+00 | 0.000E+00 | 0.000E+00 | 0.000E+00 | 0.000E+00 |
| Unigene344925 | lmrB | 0.000E+00 | 0.000E+00 | 0.000E+00 | 0.000E+00 | 7.777E-07 | 0.000E+00 | 0.000E+00 | 0.000E+00 | 0.000E+00 | 0.000E+00 | 0.000E+00 | 0.000E+00 |
| Unigene344933 | PmrF | 0.000E+00 | 0.000E+00 | 0.000E+00 | 0.000E+00 | 1.729E-06 | 0.000E+00 | 0.000E+00 | 1.159E-07 | 0.000E+00 | 0.000E+00 | 0.000E+00 | 0.000E+00 |
| Unigene344934 | PmrF | 0.000E+00 | 0.000E+00 | 0.000E+00 | 0.000E+00 | 1.883E-06 | 0.000E+00 | 0.000E+00 | 0.000E+00 | 0.000E+00 | 0.000E+00 | 0.000E+00 | 0.000E+00 |
| Unigene345053 | vanHO | 1.362E-07 | 7.474E-08 | 1.336E-07 | 0.000E+00 | 4.918E-07 | 0.000E+00 | 4.145E-07 | 7.949E-07 | 0.000E+00 | 0.000E+00 | 7.401E-07 | 9.660E-07 |
| Unigene345076 | mtrA | 0.000E+00 | 0.000E+00 | 0.000E+00 | 0.000E+00 | 3.014E-06 | 0.000E+00 | 0.000E+00 | 0.000E+00 | 0.000E+00 | 0.000E+00 | 0.000E+00 | 0.000E+00 |
| Unigene345152 | novA | 1.570E-07 | 0.000E+00 | 1.027E-07 | 1.585E-07 | 6.587E-07 | 2.451E-07 | 7.334E-07 | 6.107E-07 | 1.906E-08 | 0.000E+00 | 0.000E+00 | 1.237E-06 |
| Unigene345332 | lmrC | 0.000E+00 | 0.000E+00 | 0.000E+00 | 0.000E+00 | 6.846E-07 | 0.000E+00 | 0.000E+00 | 0.000E+00 | 0.000E+00 | 0.000E+00 | 0.000E+00 | 1.009E-06 |
| Unigene345378 | macB | 0.000E+00 | 0.000E+00 | 0.000E+00 | 0.000E+00 | 1.397E-06 | 0.000E+00 | 0.000E+00 | 0.000E+00 | 1.095E-05 | 0.000E+00 | 1.241E-05 | 0.000E+00 |
| Unigene345488 | Bifidobact | 0.000E+00 | 0.000E+00 | 0.000E+00 | 0.000E+00 | 8.657E-07 | 0.000E+00 | 8.956E-07 | 9.096E-07 | 0.000E+00 | 0.000E+00 | 8.914E-07 | 6.023E-07 |
| Unigene345532 | tet(44) | 0.000E+00 | 0.000E+00 | 0.000E+00 | 0.000E+00 | 7.816E-07 | 0.000E+00 | 0.000E+00 | 0.000E+00 | 0.000E+00 | 0.000E+00 | 0.000E+00 | 0.000E+00 |
| Unigene345626 | Klebsiella p | 0.000E+00 | 0.000E+00 | 2.778E-07 | 0.000E+00 | 1.038E-06 | 0.000E+00 | 0.000E+00 | 0.000E+00 | 0.000E+00 | 0.000E+00 | 0.000E+00 | 0.000E+00 |
| Unigene345650 | patA | 0.000E+00 | 0.000E+00 | 0.000E+00 | 0.000E+00 | 3.307E-07 | 0.000E+00 | 0.000E+00 | 0.000E+00 | 0.000E+00 | 0.000E+00 | 0.000E+00 | 0.000E+00 |
| Unigene345756 | TaeA | 4.236E-08 | 0.000E+00 | 0.000E+00 | 8.556E-08 | 9.883E-07 | 9.159E-08 | 3.282E-07 | 4.945E-07 | 0.000E+00 | 3.775E-07 | 3.392E-07 | 3.505E-07 |
| Unigene345807 | MexJ | 0.000E+00 | 1.596E-07 | 0.000E+00 | 0.000E+00 | 9.692E-07 | 0.000E+00 | 3.862E-07 | 1.358E-07 | 0.000E+00 | 2.592E-07 | 0.000E+00 | 2.062E-07 |
| Unigene345882 | efrA | 0.000E+00 | 0.000E+00 | 0.000E+00 | 0.000E+00 | 7.727E-07 | 0.000E+00 | 0.000E+00 | 1.433E-06 | 0.000E+00 | 1.185E-06 | 0.000E+00 | 2.225E-06 |
| Unigene345950 | MexF | 0.000E+00 | 0.000E+00 | 0.000E+00 | 0.000E+00 | 0.000E+00 | 0.000E+00 | 0.000E+00 | 0.000E+00 | 0.000E+00 | 0.000E+00 | 0.000E+00 | 3.028E-07 |
| Unigene346023 | lmrD | 0.000E+00 | 6.846E-08 | 2.142E-07 | 0.000E+00 | 8.662E-07 | 6.743E-08 | 7.939E-07 | 8.737E-07 | 0.000E+00 | 0.000E+00 | 0.000E+00 | 1.032E-06 |
| Unigene346031 | kdpE | 0.000E+00 | 0.000E+00 | 0.000E+00 | 0.000E+00 | 3.097E-07 | 0.000E+00 | 0.000E+00 | 0.000E+00 | 0.000E+00 | 0.000E+00 | 5.740E-07 | 0.000E+00 |
| Unigene346086 | YojI | 0.000E+00 | 0.000E+00 | 0.000E+00 | 0.000E+00 | 5.940E-07 | 0.000E+00 | 0.000E+00 | 0.000E+00 | 0.000E+00 | 0.000E+00 | 0.000E+00 | 0.000E+00 |
| Unigene346220 | vanRE | 0.000E+00 | 0.000E+00 | 0.000E+00 | 0.000E+00 | 3.088E-07 | 0.000E+00 | 0.000E+00 | 0.000E+00 | 0.000E+00 | 0.000E+00 | 0.000E+00 | 0.000E+00 |
| Unigene346233 | vanRO | 0.000E+00 | 0.000E+00 | 0.000E+00 | 0.000E+00 | 2.440E-07 | 0.000E+00 | 0.000E+00 | 0.000E+00 | 0.000E+00 | 0.000E+00 | 0.000E+00 | 0.000E+00 |
| Unigene346295 | rosB | 0.000E+00 | 0.000E+00 | 0.000E+00 | 0.000E+00 | 0.000E+00 | 1.723E-07 | 5.293E-07 | 0.000E+00 | 0.000E+00 | 0.000E+00 | 0.000E+00 | 9.421E-07 |
| Unigene346318 | Staphyloc | 2.788E-06 | 0.000E+00 | 1.218E-06 | 2.947E-06 | 1.987E-05 | 2.913E-05 | 2.312E-05 | 2.776E-05 | 3.021E-05 | 2.701E-05 | 0.000E+00 | 1.205E-05 |
| Unigene346322 | vanHF | 3.631E-07 | 0.000E+00 | 2.915E-07 | 2.333E-07 | 5.317E-06 | 8.064E-06 | 1.900E-06 | 9.362E-06 | 3.644E-06 | 0.000E+00 | 0.000E+00 | 3.511E-07 |
| Unigene346424 | Enterobact | 6.051E-07 | 2.772E-06 | 1.910E-06 | 5.845E-07 | 8.856E-06 | 0.000E+00 | 1.776E-06 | 0.000E+00 | 4.601E-07 | 0.000E+00 | 8.727E-07 | 0.000E+00 |
| Unigene346442 | Acinetoba | 0.000E+00 | 3.200E-08 | 2.861E-08 | 2.945E-08 | 5.507E-07 | 0.000E+00 | 2.259E-07 | 2.723E-07 | 9.561E-08 | 0.000E+00 | 1.668E-07 | 2.413E-07 |
| Unigene346484 | evgS | 0.000E+00 | 0.000E+00 | 0.000E+00 | 1.549E-07 | 3.219E-06 | 0.000E+00 | 0.000E+00 | 7.632E-07 | 0.000E+00 | 1.349E-07 | 2.678E-07 | 6.679E-07 |
| Unigene346489 | arlR | 0.000E+00 | 0.000E+00 | 0.000E+00 | 0.000E+00 | 3.231E-06 | 0.000E+00 | 0.000E+00 | 0.000E+00 | 0.000E+00 | 0.000E+00 | 0.000E+00 | 0.000E+00 |
| Unigene346490 | cpxA | 0.000E+00 | 0.000E+00 | 0.000E+00 | 0.000E+00 | 2.582E-06 | 0.000E+00 | 0.000E+00 | 0.000E+00 | 0.000E+00 | 0.000E+00 | 3.290E-07 | 0.000E+00 |
| Unigene346518 | floR | 0.000E+00 | 0.000E+00 | 0.000E+00 | 0.000E+00 | 2.922E-06 | 0.000E+00 | 0.000E+00 | 0.000E+00 | 0.000E+00 | 0.000E+00 | 0.000E+00 | 0.000E+00 |
| Unigene346562 | macB | 0.000E+00 | 0.000E+00 | 0.000E+00 | 0.000E+00 | 2.173E-06 | 0.000E+00 | 0.000E+00 | 0.000E+00 | 0.000E+00 | 0.000E+00 | 9.324E-07 | 0.000E+00 |
| Unigene346569 | mdtB | 0.000E+00 | 0.000E+00 | 0.000E+00 | 0.000E+00 | 2.994E-06 | 0.000E+00 | 0.000E+00 | 0.000E+00 | 0.000E+00 | 0.000E+00 | 2.226E-07 | 0.000E+00 |
| Unigene346585 | msrA | 0.000E+00 | 0.000E+00 | 0.000E+00 | 0.000E+00 | 2.848E-06 | 0.000E+00 | 0.000E+00 | 0.000E+00 | 0.000E+00 | 0.000E+00 | 8.654E-07 | 0.000E+00 |
| Unigene346661 | otr(B) | 0.000E+00 | 0.000E+00 | 0.000E+00 | 0.000E+00 | 1.466E-06 | 0.000E+00 | 0.000E+00 | 0.000E+00 | 3.978E-07 | 0.000E+00 | 0.000E+00 | 0.000E+00 |
| Unigene346692 | arlS | 5.998E-08 | 1.053E-07 | 1.177E-08 | 6.057E-08 | 6.130E-07 | 5.187E-08 | 1.460E-07 | 3.921E-07 | 3.933E-08 | 1.871E-07 | 1.372E-07 | 6.381E-07 |
| Unigene346693 | kdpE | 0.000E+00 | 5.135E-08 | 0.000E+00 | 0.000E+00 | 6.237E-07 | 0.000E+00 | 1.036E-07 | 8.191E-07 | 0.000E+00 | 0.000E+00 | 3.211E-07 | 3.871E-07 |
| Unigene346711 | vanRB | 6.168E-07 | 2.733E-06 | 1.524E-06 | 5.998E-07 | 1.132E-05 | 5.433E-07 | 2.124E-06 | 4.000E-07 | 0.000E+00 | 0.000E+00 | 1.098E-06 | 2.430E-07 |
| Unigene346758 | oleB | 0.000E+00 | 0.000E+00 | 0.000E+00 | 0.000E+00 | 5.197E-07 | 0.000E+00 | 0.000E+00 | 0.000E+00 | 0.000E+00 | 0.000E+00 | 0.000E+00 | 0.000E+00 |
| Unigene346802 | adeR | 0.000E+00 | 0.000E+00 | 0.000E+00 | 2.324E-07 | 6.392E-07 | 9.331E-08 | 0.000E+00 | 5.037E-07 | 0.000E+00 | 9.615E-08 | 0.000E+00 | 5.781E-07 |
| Unigene346909 | smeS | 0.000E+00 | 0.000E+00 | 0.000E+00 | 0.000E+00 | 4.896E-07 | 7.940E-08 | 1.626E-07 | 3.858E-07 | 2.007E-08 | 0.000E+00 | 0.000E+00 | 2.170E-07 |
| Unigene347014 | rosB | 0.000E+00 | 0.000E+00 | 0.000E+00 | 0.000E+00 | 3.358E-07 | 0.000E+00 | 0.000E+00 | 0.000E+00 | 0.000E+00 | 0.000E+00 | 0.000E+00 | 0.000E+00 |
| Unigene347044 | rosB | 7.892E-08 | 0.000E+00 | 1.162E-07 | 0.000E+00 | 7.453E-07 | 1.138E-07 | 8.298E-07 | 3.839E-07 | 4.313E-08 | 0.000E+00 | 3.912E-07 | 4.043E-07 |
| Unigene347104 | evgS | 1.637E-07 | 0.000E+00 | 0.000E+00 | 1.299E-07 | 2.144E-06 | 0.000E+00 | 0.000E+00 | 0.000E+00 | 9.843E-07 | 0.000E+00 | 0.000E+00 | 0.000E+00 |
| Unigene347256 | tetB(60) | 1.586E-07 | 0.000E+00 | 5.188E-08 | 5.339E-08 | 6.462E-07 | 2.858E-07 | 7.022E-07 | 8.949E-07 | 3.467E-07 | 3.240E-07 | 5.443E-07 | 9.375E-07 |
| Unigene347264 | vanTG | 0.000E+00 | 0.000E+00 | 0.000E+00 | 0.000E+00 | 3.475E-07 | 0.000E+00 | 0.000E+00 | 0.000E+00 | 0.000E+00 | 0.000E+00 | 0.000E+00 | 0.000E+00 |
| Unigene347308 | TriA | 0.000E+00 | 0.000E+00 | 0.000E+00 | 0.000E+00 | 4.391E-07 | 0.000E+00 | 0.000E+00 | 1.538E-07 | 0.000E+00 | 0.000E+00 | 4.019E-07 | 3.634E-07 |
| Unigene347330 | efrA | 2.014E-07 | 0.000E+00 | 4.235E-07 | 1.453E-07 | 9.589E-07 | 1.866E-07 | 8.916E-07 | 5.037E-07 | 0.000E+00 | 0.000E+00 | 2.962E-07 | 1.972E-06 |
| Unigene347487 | bcr-1 | 0.000E+00 | 0.000E+00 | 0.000E+00 | 0.000E+00 | 0.000E+00 | 0.000E+00 | 3.032E-07 | 0.000E+00 | 0.000E+00 | 0.000E+00 | 0.000E+00 | 6.477E-07 |
| Unigene347512 | macB | 0.000E+00 | 0.000E+00 | 0.000E+00 | 0.000E+00 | 9.730E-07 | 0.000E+00 | 0.000E+00 | 0.000E+00 | 5.926E-07 | 0.000E+00 | 0.000E+00 | 0.000E+00 |
| Unigene347543 | TaeA | 2.402E-07 | 0.000E+00 | 2.357E-07 | 9.702E-08 | 1.067E-06 | 0.000E+00 | 8.506E-07 | 5.607E-07 | 5.250E-08 | 0.000E+00 | 0.000E+00 | 2.385E-06 |
| Unigene347569 | msbA | 1.251E-07 | 0.000E+00 | 0.000E+00 | 0.000E+00 | 4.170E-07 | 0.000E+00 | 0.000E+00 | 0.000E+00 | 0.000E+00 | 0.000E+00 | 0.000E+00 | 0.000E+00 |
| Unigene347614 | Staphyloc | 0.000E+00 | 6.886E-08 | 0.000E+00 | 0.000E+00 | 3.137E-07 | 2.374E-07 | 2.083E-07 | 0.000E+00 | 1.714E-07 | 0.000E+00 | 0.000E+00 | 0.000E+00 |

| | | | | | | | | | | | | | |
|---|---|---|---|---|---|---|---|---|---|---|---|---|---|
| Unigene347619 | YojI | 1.471E-07 | 0.000E+00 | 4.124E-08 | 0.000E+00 | 5.603E-07 | 0.000E+00 | 4.884E-07 | 5.642E-07 | 4.594E-08 | 1.405E-07 | 2.644E-07 | 3.726E-07 |
| Unigene347635 | lmrD | 0.000E+00 | 0.000E+00 | 0.000E+00 | 0.000E+00 | 0.000E+00 | 0.000E+00 | 3.520E-07 | 0.000E+00 | 0.000E+00 | 0.000E+00 | 0.000E+00 | 0.000E+00 |
| Unigene347745 | rosB | 0.000E+00 | 0.000E+00 | 0.000E+00 | 0.000E+00 | 7.405E-07 | 0.000E+00 | 0.000E+00 | 0.000E+00 | 0.000E+00 | 0.000E+00 | 0.000E+00 | 0.000E+00 |
| Unigene347746 | oleC | 0.000E+00 | 0.000E+00 | 0.000E+00 | 0.000E+00 | 3.842E-07 | 2.493E-07 | 0.000E+00 | 0.000E+00 | 0.000E+00 | 0.000E+00 | 0.000E+00 | 7.722E-07 |
| Unigene347853 | patB | 0.000E+00 | 8.683E-08 | 1.165E-07 | 0.000E+00 | 5.713E-07 | 0.000E+00 | 2.627E-07 | 3.232E-07 | 8.647E-08 | 0.000E+00 | 0.000E+00 | 6.079E-07 |
| Unigene348010 | vanXI | 0.000E+00 | 0.000E+00 | 0.000E+00 | 0.000E+00 | 3.198E-06 | 0.000E+00 | 0.000E+00 | 0.000E+00 | 0.000E+00 | 0.000E+00 | 0.000E+00 | 0.000E+00 |
| Unigene348012 | Streptomy | 0.000E+00 | 0.000E+00 | 0.000E+00 | 0.000E+00 | 3.693E-06 | 0.000E+00 | 0.000E+00 | 0.000E+00 | 0.000E+00 | 0.000E+00 | 7.781E-07 | 0.000E+00 |
| Unigene348018 | msbA | 0.000E+00 | 0.000E+00 | 0.000E+00 | 0.000E+00 | 2.848E-06 | 0.000E+00 | 0.000E+00 | 0.000E+00 | 0.000E+00 | 0.000E+00 | 4.067E-07 | 0.000E+00 |
| Unigene348042 | bcrA | 0.000E+00 | 0.000E+00 | 0.000E+00 | 0.000E+00 | 2.665E-06 | 0.000E+00 | 0.000E+00 | 0.000E+00 | 0.000E+00 | 0.000E+00 | 8.071E-08 | 0.000E+00 |
| Unigene348063 | baeR | 0.000E+00 | 0.000E+00 | 0.000E+00 | 0.000E+00 | 1.166E-06 | 0.000E+00 | 1.259E-06 | 8.679E-07 | 0.000E+00 | 3.411E-07 | 5.003E-07 | 0.000E+00 |
| Unigene348240 | TriB | 0.000E+00 | 0.000E+00 | 0.000E+00 | 0.000E+00 | 2.732E-07 | 0.000E+00 | 0.000E+00 | 0.000E+00 | 0.000E+00 | 0.000E+00 | 2.110E-07 | 0.000E+00 |
| Unigene348296 | tetA(58) | 0.000E+00 | 4.649E-08 | 0.000E+00 | 0.000E+00 | 3.153E-06 | 0.000E+00 | 0.000E+00 | 0.000E+00 | 0.000E+00 | 0.000E+00 | 2.423E-07 | 0.000E+00 |
| Unigene348327 | srmB | 0.000E+00 | 0.000E+00 | 0.000E+00 | 0.000E+00 | 3.196E-07 | 0.000E+00 | 0.000E+00 | 0.000E+00 | 0.000E+00 | 0.000E+00 | 0.000E+00 | 0.000E+00 |
| Unigene348421 | efrB | 0.000E+00 | 0.000E+00 | 0.000E+00 | 0.000E+00 | 6.352E-07 | 0.000E+00 | 0.000E+00 | 0.000E+00 | 3.409E-07 | 0.000E+00 | 0.000E+00 | 0.000E+00 |
| Unigene348807 | tetA(46) | 0.000E+00 | 0.000E+00 | 0.000E+00 | 0.000E+00 | 1.017E-06 | 0.000E+00 | 4.603E-06 | 4.543E-06 | 0.000E+00 | 0.000E+00 | 0.000E+00 | 3.608E-06 |
| Unigene348990 | vanRF | 0.000E+00 | 4.328E-07 | 9.950E-07 | 3.982E-06 | 6.259E-07 | 1.827E-07 | 2.494E-07 | 1.118E-06 | 0.000E+00 | 1.318E-06 | 0.000E+00 | 9.323E-07 |
| Unigene349112 | tcr3 | 0.000E+00 | 5.597E-07 | 0.000E+00 | 0.000E+00 | 1.501E-06 | 0.000E+00 | 0.000E+00 | 0.000E+00 | 0.000E+00 | 0.000E+00 | 0.000E+00 | 0.000E+00 |
| Unigene349220 | farA | 0.000E+00 | 0.000E+00 | 0.000E+00 | 0.000E+00 | 0.000E+00 | 0.000E+00 | 0.000E+00 | 9.774E-08 | 0.000E+00 | 0.000E+00 | 0.000E+00 | 4.949E-07 |
| Unigene349231 | patA | 0.000E+00 | 0.000E+00 | 0.000E+00 | 0.000E+00 | 1.294E-06 | 0.000E+00 | 0.000E+00 | 0.000E+00 | 0.000E+00 | 0.000E+00 | 0.000E+00 | 0.000E+00 |
| Unigene349378 | tetA(46) | 0.000E+00 | 0.000E+00 | 0.000E+00 | 0.000E+00 | 1.179E-06 | 0.000E+00 | 0.000E+00 | 0.000E+00 | 0.000E+00 | 0.000E+00 | 0.000E+00 | 0.000E+00 |
| Unigene349400 | rgt1438 | 0.000E+00 | 0.000E+00 | 0.000E+00 | 0.000E+00 | 6.145E-07 | 0.000E+00 | 0.000E+00 | 0.000E+00 | 0.000E+00 | 0.000E+00 | 0.000E+00 | 0.000E+00 |
| Unigene349436 | NmcR | 0.000E+00 | 0.000E+00 | 0.000E+00 | 2.570E-07 | 7.269E-07 | 0.000E+00 | 1.046E-06 | 7.213E-07 | 0.000E+00 | 0.000E+00 | 5.406E-07 | 1.203E-06 |
| Unigene349576 | oleB | 0.000E+00 | 0.000E+00 | 0.000E+00 | 0.000E+00 | 7.084E-07 | 0.000E+00 | 0.000E+00 | 0.000E+00 | 2.613E-07 | 0.000E+00 | 0.000E+00 | 0.000E+00 |
| Unigene349622 | vanG | 0.000E+00 | 0.000E+00 | 0.000E+00 | 0.000E+00 | 1.339E-06 | 0.000E+00 | 0.000E+00 | 0.000E+00 | 0.000E+00 | 0.000E+00 | 0.000E+00 | 0.000E+00 |
| Unigene349884 | msbA | 4.991E-07 | 1.063E-06 | 8.786E-07 | 1.927E-07 | 7.665E-06 | 3.491E-07 | 9.261E-07 | 3.598E-07 | 0.000E+00 | 0.000E+00 | 1.847E-07 | 1.215E-07 |
| Unigene349924 | YojI | 0.000E+00 | 0.000E+00 | 0.000E+00 | 0.000E+00 | 3.415E-07 | 0.000E+00 | 0.000E+00 | 0.000E+00 | 0.000E+00 | 0.000E+00 | 0.000E+00 | 2.180E-07 |
| Unigene350035 | Klebsiella p | 1.503E-07 | 0.000E+00 | 6.193E-07 | 0.000E+00 | 1.069E-06 | 0.000E+00 | 0.000E+00 | 0.000E+00 | 0.000E+00 | 0.000E+00 | 5.157E-07 | 0.000E+00 |
| Unigene350201 | MexL | 0.000E+00 | 0.000E+00 | 0.000E+00 | 0.000E+00 | 7.809E-07 | 0.000E+00 | 0.000E+00 | 2.272E-06 | 0.000E+00 | 0.000E+00 | 4.330E-07 | 5.752E-07 |
| Unigene350204 | vanSM | 4.276E-07 | 0.000E+00 | 1.049E-07 | 3.598E-07 | 1.425E-06 | 3.351E-06 | 7.689E-06 | 0.000E+00 | 4.478E-06 | 1.707E-06 | 6.766E-06 | 2.106E-07 |
| Unigene350261 | Staphylocc | 3.178E-07 | 7.474E-07 | 6.126E-07 | 3.209E-07 | 6.671E-06 | 4.049E-07 | 7.160E-07 | 1.060E-07 | 1.365E-07 | 2.529E-07 | 2.597E-07 | 2.683E-08 |
| Unigene350303 | lsaA | 0.000E+00 | 0.000E+00 | 0.000E+00 | 0.000E+00 | 1.437E-06 | 0.000E+00 | 0.000E+00 | 0.000E+00 | 0.000E+00 | 0.000E+00 | 0.000E+00 | 0.000E+00 |
| Unigene350307 | Staphylocc | 0.000E+00 | 0.000E+00 | 0.000E+00 | 0.000E+00 | 7.779E-07 | 0.000E+00 | 0.000E+00 | 0.000E+00 | 0.000E+00 | 0.000E+00 | 0.000E+00 | 0.000E+00 |
| Unigene350323 | APH(6)-Id | 0.000E+00 | 0.000E+00 | 0.000E+00 | 0.000E+00 | 2.589E-07 | 0.000E+00 | 0.000E+00 | 0.000E+00 | 0.000E+00 | 0.000E+00 | 0.000E+00 | 0.000E+00 |
| Unigene350328 | basS | 1.812E-07 | 3.729E-07 | 1.778E-07 | 0.000E+00 | 1.661E-06 | 2.939E-07 | 2.758E-07 | 0.000E+00 | 0.000E+00 | 0.000E+00 | 5.701E-07 | 0.000E+00 |
| Unigene350348 | msbA | 0.000E+00 | 0.000E+00 | 0.000E+00 | 0.000E+00 | 6.232E-07 | 0.000E+00 | 0.000E+00 | 0.000E+00 | 0.000E+00 | 0.000E+00 | 0.000E+00 | 0.000E+00 |
| Unigene350372 | acrB | 0.000E+00 | 0.000E+00 | 5.306E-08 | 0.000E+00 | 5.406E-07 | 0.000E+00 | 0.000E+00 | 0.000E+00 | 0.000E+00 | 0.000E+00 | 0.000E+00 | 0.000E+00 |
| Unigene350406 | bcr-1 | 0.000E+00 | 0.000E+00 | 0.000E+00 | 0.000E+00 | 1.651E-06 | 2.975E-07 | 0.000E+00 | 0.000E+00 | 0.000E+00 | 0.000E+00 | 0.000E+00 | 0.000E+00 |
| Unigene350506 | Acinetobac | 2.299E-07 | 8.580E-07 | 4.963E-07 | 0.000E+00 | 4.291E-06 | 3.231E-07 | 7.125E-07 | 3.757E-07 | 5.026E-08 | 0.000E+00 | 2.367E-07 | 0.000E+00 |
| Unigene350515 | baeS | 3.718E-07 | 1.020E-06 | 0.000E+00 | 0.000E+00 | 6.171E-06 | 1.507E-07 | 7.459E-07 | 1.085E-07 | 5.080E-08 | 0.000E+00 | 0.000E+00 | 0.000E+00 |
| Unigene350516 | oqxB | 7.057E-07 | 1.908E-06 | 1.144E-06 | 8.986E-07 | 8.034E-06 | 4.202E-07 | 1.789E-06 | 2.865E-07 | 0.000E+00 | 2.621E-07 | 0.000E+00 | 0.000E+00 |
| Unigene350662 | patA | 8.724E-07 | 6.575E-06 | 9.702E-07 | 0.000E+00 | 3.231E-07 | 1.886E-07 | 0.000E+00 | 0.000E+00 | 4.450E-07 | 0.000E+00 | 6.653E-08 | 0.000E+00 |
| Unigene350734 | kdpE | 9.694E-08 | 0.000E+00 | 2.616E-07 | 0.000E+00 | 1.158E-06 | 0.000E+00 | 1.153E-06 | 1.216E-06 | 0.000E+00 | 0.000E+00 | 3.604E-07 | 0.000E+00 |
| Unigene350762 | oleC | 0.000E+00 | 0.000E+00 | 0.000E+00 | 0.000E+00 | 0.000E+00 | 0.000E+00 | 1.361E-07 | 0.000E+00 | 1.344E-07 | 0.000E+00 | 0.000E+00 | 0.000E+00 |
| Unigene350789 | tetA(46) | 0.000E+00 | 1.696E-07 | 4.549E-07 | 0.000E+00 | 8.370E-07 | 2.924E-07 | 1.240E-06 | 5.863E-07 | 1.689E-07 | 0.000E+00 | 5.082E-07 | 2.854E-06 |
| Unigene350928 | TaeA | 4.708E-07 | 5.536E-08 | 1.815E-07 | 3.905E-07 | 3.680E-06 | 6.834E-06 | 1.079E-06 | 7.634E-06 | 3.859E-06 | 2.903E-06 | 0.000E+00 | 4.770E-07 |
| Unigene350931 | QepA2 | 5.610E-07 | 0.000E+00 | 2.163E-07 | 4.654E-07 | 3.094E-06 | 6.260E-06 | 1.441E-06 | 6.081E-06 | 3.986E-06 | 2.589E-06 | 0.000E+00 | 6.869E-07 |
| Unigene350949 | bcrA | 5.157E-07 | 0.000E+00 | 0.000E+00 | 2.083E-07 | 3.819E-06 | 5.946E-06 | 1.065E-06 | 7.183E-06 | 4.359E-06 | 3.255E-06 | 0.000E+00 | 0.000E+00 |
| Unigene350973 | macB | 0.000E+00 | 0.000E+00 | 0.000E+00 | 0.000E+00 | 3.783E-07 | 0.000E+00 | 0.000E+00 | 0.000E+00 | 0.000E+00 | 0.000E+00 | 0.000E+00 | 0.000E+00 |
| Unigene350976 | vanHD | 0.000E+00 | 0.000E+00 | 0.000E+00 | 0.000E+00 | 3.300E-07 | 0.000E+00 | 7.306E-08 | 3.082E-07 | 0.000E+00 | 0.000E+00 | 3.776E-08 | 0.000E+00 |
| Unigene350986 | rosA | 0.000E+00 | 0.000E+00 | 0.000E+00 | 1.828E-07 | 8.621E-07 | 0.000E+00 | 9.733E-07 | 1.479E-06 | 0.000E+00 | 0.000E+00 | 1.243E-06 | 1.468E-06 |
| Unigene350997 | tetA(58) | 0.000E+00 | 0.000E+00 | 0.000E+00 | 0.000E+00 | 1.699E-06 | 0.000E+00 | 0.000E+00 | 0.000E+00 | 0.000E+00 | 0.000E+00 | 0.000E+00 | 0.000E+00 |
| Unigene350998 | vmlR | 0.000E+00 | 0.000E+00 | 0.000E+00 | 0.000E+00 | 1.169E-06 | 0.000E+00 | 0.000E+00 | 0.000E+00 | 0.000E+00 | 0.000E+00 | 0.000E+00 | 0.000E+00 |
| Unigene351104 | MexW | 0.000E+00 | 0.000E+00 | 0.000E+00 | 0.000E+00 | 3.245E-07 | 0.000E+00 | 0.000E+00 | 0.000E+00 | 0.000E+00 | 0.000E+00 | 0.000E+00 | 0.000E+00 |
| Unigene351205 | acrB | 0.000E+00 | 0.000E+00 | 8.197E-08 | 0.000E+00 | 7.796E-07 | 2.709E-07 | 5.548E-07 | 8.386E-07 | 2.191E-07 | 4.095E-07 | 5.734E-07 | 6.517E-07 |
| Unigene351342 | cpxA | 0.000E+00 | 0.000E+00 | 0.000E+00 | 0.000E+00 | 2.381E-06 | 3.069E-08 | 3.142E-08 | 0.000E+00 | 0.000E+00 | 0.000E+00 | 3.572E-07 | 3.524E-07 |
| Unigene351343 | Pseudomo | 0.000E+00 | 0.000E+00 | 0.000E+00 | 3.343E-07 | 3.284E-06 | 0.000E+00 | 0.000E+00 | 0.000E+00 | 0.000E+00 | 0.000E+00 | 0.000E+00 | 0.000E+00 |
| Unigene351358 | macB | 0.000E+00 | 0.000E+00 | 0.000E+00 | 0.000E+00 | 2.557E-06 | 0.000E+00 | 0.000E+00 | 0.000E+00 | 0.000E+00 | 0.000E+00 | 5.511E-07 | 7.910E-07 |
| Unigene351359 | macB | 0.000E+00 | 0.000E+00 | 0.000E+00 | 0.000E+00 | 2.801E-06 | 0.000E+00 | 0.000E+00 | 0.000E+00 | 0.000E+00 | 0.000E+00 | 5.665E-07 | 0.000E+00 |

| | | | | | | | | | | | | | |
|---|---|---|---|---|---|---|---|---|---|---|---|---|---|
| Unigene351362 | Tet(47) | 0.000E+00 | 0.000E+00 | 0.000E+00 | 0.000E+00 | 1.897E-06 | 0.000E+00 | 0.000E+00 | 0.000E+00 | 0.000E+00 | 0.000E+00 | 1.954E-07 | 0.000E+00 |
| Unigene351368 | tlrC | 0.000E+00 | 0.000E+00 | 0.000E+00 | 0.000E+00 | 3.124E-06 | 0.000E+00 | 3.042E-07 | 0.000E+00 | 0.000E+00 | 0.000E+00 | 4.836E-07 | 0.000E+00 |
| Unigene351402 | Staphylocc | 0.000E+00 | 0.000E+00 | 1.450E-07 | 0.000E+00 | 4.269E-07 | 0.000E+00 | 1.014E-06 | 7.936E-07 | 0.000E+00 | 0.000E+00 | 4.396E-07 | 0.000E+00 |
| Unigene351511 | adeL | 0.000E+00 | 0.000E+00 | 0.000E+00 | 0.000E+00 | 0.000E+00 | 0.000E+00 | 0.000E+00 | 9.661E-07 | 0.000E+00 | 0.000E+00 | 0.000E+00 | 0.000E+00 |
| Unigene351572 | efrB | 0.000E+00 | 0.000E+00 | 0.000E+00 | 0.000E+00 | 6.157E-07 | 0.000E+00 | 9.200E-07 | 0.000E+00 | 0.000E+00 | 0.000E+00 | 0.000E+00 | 1.037E-06 |
| Unigene351742 | cpxA | 0.000E+00 | 0.000E+00 | 0.000E+00 | 0.000E+00 | 2.173E-07 | 0.000E+00 | 0.000E+00 | 0.000E+00 | 0.000E+00 | 0.000E+00 | 0.000E+00 | 0.000E+00 |
| Unigene351750 | patA | 0.000E+00 | 0.000E+00 | 0.000E+00 | 0.000E+00 | 7.142E-07 | 0.000E+00 | 5.243E-07 | 3.555E-07 | 0.000E+00 | 0.000E+00 | 1.935E-07 | 3.600E-07 |
| Unigene351756 | emrR | 0.000E+00 | 0.000E+00 | 0.000E+00 | 0.000E+00 | 1.395E-06 | 0.000E+00 | 0.000E+00 | 0.000E+00 | 0.000E+00 | 0.000E+00 | 0.000E+00 | 0.000E+00 |
| Unigene351757 | Corynebac | 0.000E+00 | 0.000E+00 | 0.000E+00 | 0.000E+00 | 4.719E-07 | 0.000E+00 | 3.917E-07 | 8.263E-08 | 0.000E+00 | 0.000E+00 | 0.000E+00 | 3.347E-07 |
| Unigene351880 | rosB | 0.000E+00 | 1.955E-07 | 0.000E+00 | 3.418E-07 | 1.306E-06 | 4.815E-07 | 1.498E-06 | 7.694E-07 | 0.000E+00 | 4.961E-07 | 7.540E-07 | 2.801E-06 |
| Unigene351966 | patA | 0.000E+00 | 0.000E+00 | 0.000E+00 | 0.000E+00 | 4.138E-07 | 0.000E+00 | 5.496E-07 | 5.314E-07 | 2.714E-07 | 0.000E+00 | 0.000E+00 | 0.000E+00 |
| Unigene351981 | evgS | 0.000E+00 | 0.000E+00 | 0.000E+00 | 0.000E+00 | 1.684E-06 | 0.000E+00 | 4.613E-06 | 0.000E+00 | 0.000E+00 | 0.000E+00 | 0.000E+00 | 0.000E+00 |
| Unigene352081 | Corynebac | 0.000E+00 | 0.000E+00 | 0.000E+00 | 0.000E+00 | 1.155E-06 | 0.000E+00 | 0.000E+00 | 0.000E+00 | 0.000E+00 | 0.000E+00 | 0.000E+00 | 0.000E+00 |
| Unigene352120 | MexA | 0.000E+00 | 0.000E+00 | 0.000E+00 | 0.000E+00 | 5.134E-07 | 0.000E+00 | 0.000E+00 | 0.000E+00 | 0.000E+00 | 0.000E+00 | 0.000E+00 | 0.000E+00 |
| Unigene352306 | bcrA | 0.000E+00 | 0.000E+00 | 0.000E+00 | 0.000E+00 | 1.522E-06 | 0.000E+00 | 0.000E+00 | 0.000E+00 | 0.000E+00 | 0.000E+00 | 0.000E+00 | 0.000E+00 |
| Unigene352352 | NmcR | 0.000E+00 | 0.000E+00 | 0.000E+00 | 0.000E+00 | 9.766E-07 | 0.000E+00 | 3.503E-07 | 7.388E-07 | 1.153E-07 | 3.525E-07 | 2.816E-07 | 4.988E-07 |
| Unigene352381 | Klebsiella p | 0.000E+00 | 6.081E-07 | 4.578E-07 | 0.000E+00 | 1.717E-06 | 0.000E+00 | 0.000E+00 | 1.361E-07 | 0.000E+00 | 0.000E+00 | 0.000E+00 | 0.000E+00 |
| Unigene352412 | farA | 0.000E+00 | 0.000E+00 | 0.000E+00 | 0.000E+00 | 2.970E-07 | 0.000E+00 | 0.000E+00 | 0.000E+00 | 1.461E-07 | 0.000E+00 | 0.000E+00 | 0.000E+00 |
| Unigene352432 | tva(A) | 6.683E-08 | 0.000E+00 | 0.000E+00 | 2.025E-07 | 8.167E-07 | 0.000E+00 | 0.000E+00 | 4.681E-07 | 7.305E-08 | 1.489E-07 | 0.000E+00 | 0.000E+00 |
| Unigene352440 | dfrA26 | 1.845E-07 | 0.000E+00 | 0.000E+00 | 0.000E+00 | 3.415E-07 | 0.000E+00 | 0.000E+00 | 0.000E+00 | 0.000E+00 | 0.000E+00 | 0.000E+00 | 0.000E+00 |
| Unigene352461 | cpxA | 0.000E+00 | 0.000E+00 | 1.628E-07 | 1.197E-07 | 6.056E-07 | 0.000E+00 | 4.197E-07 | 3.320E-07 | 0.000E+00 | 1.584E-07 | 3.524E-07 | 7.004E-07 |
| Unigene352532 | oleC | 2.183E-08 | 2.156E-07 | 0.000E+00 | 0.000E+00 | 1.503E-06 | 0.000E+00 | 0.000E+00 | 0.000E+00 | 4.055E-07 | 0.000E+00 | 2.497E-08 | 0.000E+00 |
| Unigene352551 | patB | 0.000E+00 | 0.000E+00 | 0.000E+00 | 0.000E+00 | 7.911E-07 | 2.566E-07 | 1.138E-06 | 0.000E+00 | 0.000E+00 | 0.000E+00 | 7.693E-07 | 1.356E-06 |
| Unigene352597 | patA | 0.000E+00 | 0.000E+00 | 0.000E+00 | 0.000E+00 | 1.025E-06 | 0.000E+00 | 0.000E+00 | 0.000E+00 | 0.000E+00 | 0.000E+00 | 0.000E+00 | 0.000E+00 |
| Unigene352697 | mtrA | 0.000E+00 | 0.000E+00 | 0.000E+00 | 0.000E+00 | 5.456E-07 | 0.000E+00 | 0.000E+00 | 0.000E+00 | 0.000E+00 | 0.000E+00 | 0.000E+00 | 0.000E+00 |
| Unigene352705 | MuxB | 0.000E+00 | 0.000E+00 | 1.245E-07 | 5.125E-08 | 6.625E-07 | 1.097E-07 | 4.915E-07 | 2.962E-07 | 0.000E+00 | 0.000E+00 | 2.177E-07 | 4.650E-07 |
| Unigene352818 | vgaA | 0.000E+00 | 0.000E+00 | 0.000E+00 | 0.000E+00 | 6.711E-07 | 0.000E+00 | 0.000E+00 | 0.000E+00 | 0.000E+00 | 0.000E+00 | 0.000E+00 | 0.000E+00 |
| Unigene352904 | evgS | 0.000E+00 | 0.000E+00 | 0.000E+00 | 0.000E+00 | 0.000E+00 | 0.000E+00 | 5.716E-07 | 0.000E+00 | 0.000E+00 | 0.000E+00 | 0.000E+00 | 0.000E+00 |
| Unigene352940 | carA | 0.000E+00 | 0.000E+00 | 0.000E+00 | 0.000E+00 | 0.000E+00 | 5.147E-08 | 0.000E+00 | 3.890E-07 | 0.000E+00 | 0.000E+00 | 0.000E+00 | 0.000E+00 |
| Unigene353073 | basS | 0.000E+00 | 0.000E+00 | 0.000E+00 | 0.000E+00 | 4.336E-07 | 0.000E+00 | 0.000E+00 | 1.952E-07 | 0.000E+00 | 0.000E+00 | 0.000E+00 | 6.590E-07 |
| Unigene353144 | mdtA | 0.000E+00 | 0.000E+00 | 0.000E+00 | 0.000E+00 | 1.312E-06 | 0.000E+00 | 0.000E+00 | 0.000E+00 | 0.000E+00 | 0.000E+00 | 0.000E+00 | 0.000E+00 |
| Unigene353152 | lmrD | 0.000E+00 | 0.000E+00 | 0.000E+00 | 0.000E+00 | 0.000E+00 | 0.000E+00 | 0.000E+00 | 0.000E+00 | 0.000E+00 | 0.000E+00 | 1.259E-06 | 0.000E+00 |
| Unigene353210 | adeR | 0.000E+00 | 0.000E+00 | 0.000E+00 | 3.992E-07 | 1.171E-06 | 0.000E+00 | 0.000E+00 | 1.025E-06 | 0.000E+00 | 0.000E+00 | 0.000E+00 | 2.907E-06 |
| Unigene353430 | MexB | 0.000E+00 | 0.000E+00 | 0.000E+00 | 0.000E+00 | 5.300E-07 | 0.000E+00 | 0.000E+00 | 0.000E+00 | 0.000E+00 | 0.000E+00 | 0.000E+00 | 0.000E+00 |
| Unigene353524 | novA | 0.000E+00 | 0.000E+00 | 0.000E+00 | 0.000E+00 | 6.520E-07 | 0.000E+00 | 4.021E-07 | 4.241E-07 | 0.000E+00 | 0.000E+00 | 0.000E+00 | 0.000E+00 |
| Unigene353550 | LlmA 23S r | 5.678E-08 | 3.116E-08 | 0.000E+00 | 0.000E+00 | 5.677E-07 | 1.535E-07 | 3.142E-07 | 6.628E-07 | 0.000E+00 | 1.581E-07 | 2.923E-07 | 6.041E-07 |
| Unigene353604 | Brucella su | 3.103E-07 | 0.000E+00 | 8.403E-07 | 3.634E-07 | 1.007E-06 | 5.769E-07 | 1.799E-06 | 1.420E-06 | 1.763E-07 | 1.065E-06 | 1.164E-06 | 3.726E-06 |
| Unigene353736 | lmrD | 7.428E-07 | 3.284E-06 | 1.333E-06 | 6.429E-07 | 9.337E-06 | 8.030E-07 | 2.419E-06 | 9.414E-07 | 0.000E+00 | 4.019E-07 | 0.000E+00 | 3.261E-07 |
| Unigene353742 | mdtP | 3.412E-07 | 1.223E-06 | 6.027E-07 | 4.365E-07 | 7.177E-06 | 6.394E-07 | 8.812E-07 | 5.576E-07 | 0.000E+00 | 1.520E-07 | 5.465E-07 | 0.000E+00 |
| Unigene353850 | bcrA | 3.528E-07 | 6.453E-08 | 5.481E-07 | 2.375E-07 | 1.306E-06 | 1.907E-07 | 1.041E-06 | 8.236E-07 | 3.856E-07 | 7.205E-07 | 9.752E-07 | 3.545E-06 |
| Unigene354056 | tetA(58) | 0.000E+00 | 0.000E+00 | 0.000E+00 | 0.000E+00 | 3.529E-06 | 0.000E+00 | 0.000E+00 | 0.000E+00 | 0.000E+00 | 0.000E+00 | 4.820E-07 | 0.000E+00 |
| Unigene354064 | bcrA | 0.000E+00 | 0.000E+00 | 0.000E+00 | 0.000E+00 | 3.302E-06 | 6.427E-08 | 0.000E+00 | 0.000E+00 | 0.000E+00 | 0.000E+00 | 4.420E-07 | 0.000E+00 |
| Unigene354077 | oleC | 0.000E+00 | 0.000E+00 | 0.000E+00 | 0.000E+00 | 2.630E-06 | 0.000E+00 | 0.000E+00 | 0.000E+00 | 0.000E+00 | 0.000E+00 | 1.407E-07 | 0.000E+00 |
| Unigene354089 | oleB | 0.000E+00 | 0.000E+00 | 0.000E+00 | 0.000E+00 | 2.869E-06 | 0.000E+00 | 0.000E+00 | 0.000E+00 | 0.000E+00 | 0.000E+00 | 1.894E-07 | 0.000E+00 |
| Unigene354090 | vgaE | 0.000E+00 | 0.000E+00 | 0.000E+00 | 0.000E+00 | 2.552E-06 | 0.000E+00 | 0.000E+00 | 0.000E+00 | 0.000E+00 | 0.000E+00 | 1.314E-07 | 0.000E+00 |
| Unigene354101 | carA | 0.000E+00 | 0.000E+00 | 0.000E+00 | 0.000E+00 | 2.835E-06 | 0.000E+00 | 1.130E-07 | 0.000E+00 | 0.000E+00 | 0.000E+00 | 3.737E-07 | 0.000E+00 |
| Unigene354123 | lmrD | 0.000E+00 | 0.000E+00 | 0.000E+00 | 1.778E-08 | 2.974E-06 | 0.000E+00 | 0.000E+00 | 2.056E-08 | 0.000E+00 | 7.847E-08 | 1.410E-07 | 0.000E+00 |
| Unigene354140 | tetA(58) | 0.000E+00 | 0.000E+00 | 0.000E+00 | 0.000E+00 | 3.409E-06 | 0.000E+00 | 0.000E+00 | 0.000E+00 | 0.000E+00 | 0.000E+00 | 0.000E+00 | 0.000E+00 |
| Unigene354141 | tetB(60) | 0.000E+00 | 0.000E+00 | 0.000E+00 | 0.000E+00 | 2.842E-06 | 0.000E+00 | 0.000E+00 | 0.000E+00 | 0.000E+00 | 0.000E+00 | 4.540E-07 | 0.000E+00 |
| Unigene354144 | tva(A) | 0.000E+00 | 0.000E+00 | 0.000E+00 | 0.000E+00 | 2.848E-06 | 0.000E+00 | 0.000E+00 | 0.000E+00 | 0.000E+00 | 0.000E+00 | 5.430E-07 | 0.000E+00 |
| Unigene354182 | patA | 0.000E+00 | 0.000E+00 | 0.000E+00 | 0.000E+00 | 3.383E-06 | 0.000E+00 | 0.000E+00 | 9.479E-07 | 0.000E+00 | 0.000E+00 | 0.000E+00 | 0.000E+00 |
| Unigene354192 | mtrA | 0.000E+00 | 0.000E+00 | 0.000E+00 | 0.000E+00 | 2.698E-06 | 5.147E-08 | 0.000E+00 | 5.557E-08 | 0.000E+00 | 0.000E+00 | 3.268E-07 | 0.000E+00 |
| Unigene354193 | smeS | 0.000E+00 | 0.000E+00 | 0.000E+00 | 0.000E+00 | 2.011E-06 | 0.000E+00 | 2.744E-08 | 0.000E+00 | 0.000E+00 | 1.657E-07 | 1.134E-07 | 5.861E-08 |
| Unigene354206 | efrA | 0.000E+00 | 0.000E+00 | 0.000E+00 | 0.000E+00 | 2.831E-06 | 0.000E+00 | 0.000E+00 | 0.000E+00 | 0.000E+00 | 4.506E-07 | 5.553E-07 | 0.000E+00 |
| Unigene354243 | poxtA | 0.000E+00 | 0.000E+00 | 0.000E+00 | 0.000E+00 | 2.977E-06 | 0.000E+00 | 0.000E+00 | 0.000E+00 | 0.000E+00 | 0.000E+00 | 0.000E+00 | 0.000E+00 |
| Unigene354250 | Corynebac | 0.000E+00 | 0.000E+00 | 0.000E+00 | 0.000E+00 | 2.536E-06 | 0.000E+00 | 0.000E+00 | 0.000E+00 | 0.000E+00 | 0.000E+00 | 0.000E+00 | 0.000E+00 |
| Unigene354258 | efrA | 1.904E-07 | 0.000E+00 | 0.000E+00 | 0.000E+00 | 2.391E-06 | 0.000E+00 | 2.108E-07 | 0.000E+00 | 0.000E+00 | 0.000E+00 | 3.921E-07 | 1.351E-06 |
| Unigene354259 | poxtA | 0.000E+00 | 0.000E+00 | 0.000E+00 | 0.000E+00 | 3.241E-06 | 0.000E+00 | 0.000E+00 | 0.000E+00 | 0.000E+00 | 0.000E+00 | 4.626E-07 | 1.588E-06 |

| | | | | | | | | | | | | | |
|---|---|---|---|---|---|---|---|---|---|---|---|---|---|
| Unigene354262 | evgS | 0.000E+00 | 0.000E+00 | 0.000E+00 | 0.000E+00 | 2.938E-06 | 2.072E-08 | 6.363E-08 | 0.000E+00 | 0.000E+00 | 5.337E-08 | 9.864E-08 | 0.000E+00 |
| Unigene354266 | carA | 0.000E+00 | 0.000E+00 | 0.000E+00 | 0.000E+00 | 1.881E-06 | 0.000E+00 | 0.000E+00 | 0.000E+00 | 0.000E+00 | 0.000E+00 | 0.000E+00 | 0.000E+00 |
| Unigene354276 | oleC | 0.000E+00 | 0.000E+00 | 0.000E+00 | 0.000E+00 | 2.712E-06 | 0.000E+00 | 0.000E+00 | 0.000E+00 | 0.000E+00 | 0.000E+00 | 2.792E-07 | 1.998E-07 |
| Unigene354281 | vanHO | 0.000E+00 | 0.000E+00 | 0.000E+00 | 0.000E+00 | 2.518E-06 | 0.000E+00 | 3.534E-08 | 0.000E+00 | 0.000E+00 | 0.000E+00 | 2.922E-07 | 0.000E+00 |
| Unigene354298 | efrA | 0.000E+00 | 0.000E+00 | 0.000E+00 | 0.000E+00 | 3.888E-06 | 0.000E+00 | 0.000E+00 | 0.000E+00 | 1.265E-07 | 0.000E+00 | 9.595E-07 | 0.000E+00 |
| Unigene354337 | tetA(46) | 0.000E+00 | 0.000E+00 | 0.000E+00 | 0.000E+00 | 2.715E-06 | 0.000E+00 | 0.000E+00 | 0.000E+00 | 0.000E+00 | 0.000E+00 | 1.705E-07 | 0.000E+00 |
| Unigene354338 | oleC | 0.000E+00 | 0.000E+00 | 0.000E+00 | 0.000E+00 | 2.939E-06 | 0.000E+00 | 0.000E+00 | 0.000E+00 | 0.000E+00 | 0.000E+00 | 3.027E-07 | 0.000E+00 |
| Unigene354352 | carA | 0.000E+00 | 0.000E+00 | 0.000E+00 | 0.000E+00 | 3.164E-06 | 0.000E+00 | 1.001E-07 | 0.000E+00 | 0.000E+00 | 0.000E+00 | 0.000E+00 | 0.000E+00 |
| Unigene354353 | patB | 0.000E+00 | 0.000E+00 | 0.000E+00 | 0.000E+00 | 3.464E-06 | 0.000E+00 | 0.000E+00 | 0.000E+00 | 0.000E+00 | 0.000E+00 | 0.000E+00 | 0.000E+00 |
| Unigene354364 | efrA | 0.000E+00 | 0.000E+00 | 0.000E+00 | 0.000E+00 | 3.765E-06 | 0.000E+00 | 0.000E+00 | 0.000E+00 | 0.000E+00 | 0.000E+00 | 1.798E-07 | 0.000E+00 |
| Unigene354366 | lmrC | 0.000E+00 | 0.000E+00 | 0.000E+00 | 0.000E+00 | 3.334E-06 | 3.277E-08 | 0.000E+00 | 0.000E+00 | 0.000E+00 | 0.000E+00 | 1.387E-07 | 0.000E+00 |
| Unigene354376 | bcrA | 0.000E+00 | 0.000E+00 | 0.000E+00 | 0.000E+00 | 3.287E-06 | 0.000E+00 | 0.000E+00 | 0.000E+00 | 0.000E+00 | 0.000E+00 | 2.986E-07 | 0.000E+00 |
| Unigene354503 | SRT-2 | 0.000E+00 | 0.000E+00 | 0.000E+00 | 0.000E+00 | 1.301E-06 | 0.000E+00 | 0.000E+00 | 0.000E+00 | 5.445E-07 | 0.000E+00 | 0.000E+00 | 0.000E+00 |
| Unigene354504 | tet(39) | 0.000E+00 | 8.163E-08 | 0.000E+00 | 0.000E+00 | 1.074E-06 | 0.000E+00 | 0.000E+00 | 0.000E+00 | 9.484E-07 | 0.000E+00 | 0.000E+00 | 0.000E+00 |
| Unigene355004 | novA | 0.000E+00 | 0.000E+00 | 0.000E+00 | 0.000E+00 | 4.381E-07 | 0.000E+00 | 0.000E+00 | 0.000E+00 | 6.157E-08 | 0.000E+00 | 1.289E-07 | 1.998E-07 |
| Unigene355037 | cpxA | 8.574E-08 | 0.000E+00 | 0.000E+00 | 8.658E-08 | 4.286E-07 | 0.000E+00 | 0.000E+00 | 0.000E+00 | 0.000E+00 | 0.000E+00 | 0.000E+00 | 1.013E-07 |
| Unigene355068 | patA | 0.000E+00 | 0.000E+00 | 0.000E+00 | 0.000E+00 | 4.146E-07 | 0.000E+00 | 0.000E+00 | 5.082E-07 | 0.000E+00 | 0.000E+00 | 0.000E+00 | 0.000E+00 |
| Unigene355076 | MuxB | 0.000E+00 | 8.620E-08 | 0.000E+00 | 0.000E+00 | 3.490E-07 | 0.000E+00 | 2.173E-07 | 0.000E+00 | 0.000E+00 | 0.000E+00 | 2.695E-07 | 5.106E-07 |
| Unigene355094 | tetA(60) | 0.000E+00 | 0.000E+00 | 0.000E+00 | 0.000E+00 | 1.138E-06 | 0.000E+00 | 0.000E+00 | 0.000E+00 | 0.000E+00 | 0.000E+00 | 0.000E+00 | 0.000E+00 |
| Unigene355275 | vmlR | 0.000E+00 | 0.000E+00 | 0.000E+00 | 0.000E+00 | 5.197E-07 | 0.000E+00 | 0.000E+00 | 0.000E+00 | 0.000E+00 | 0.000E+00 | 0.000E+00 | 0.000E+00 |
| Unigene355316 | evgS | 0.000E+00 | 0.000E+00 | 0.000E+00 | 0.000E+00 | 9.719E-07 | 0.000E+00 | 0.000E+00 | 0.000E+00 | 0.000E+00 | 0.000E+00 | 5.003E-07 | 0.000E+00 |
| Unigene355414 | evgS | 1.931E-07 | 0.000E+00 | 2.067E-07 | 1.773E-07 | 2.925E-07 | 4.554E-07 | 1.360E-06 | 8.196E-07 | 0.000E+00 | 5.280E-07 | 7.429E-07 | 2.033E-06 |
| Unigene355436 | Corynebac | 0.000E+00 | 0.000E+00 | 3.105E-07 | 0.000E+00 | 1.371E-06 | 0.000E+00 | 0.000E+00 | 0.000E+00 | 0.000E+00 | 0.000E+00 | 0.000E+00 | 0.000E+00 |
| Unigene355440 | patA | 3.311E-08 | 0.000E+00 | 3.249E-08 | 0.000E+00 | 7.724E-07 | 0.000E+00 | 3.298E-07 | 0.000E+00 | 0.000E+00 | 7.376E-08 | 1.894E-07 | 4.696E-07 |
| Unigene355478 | tetB(60) | 0.000E+00 | 0.000E+00 | 0.000E+00 | 0.000E+00 | 1.016E-06 | 0.000E+00 | 0.000E+00 | 0.000E+00 | 0.000E+00 | 0.000E+00 | 0.000E+00 | 0.000E+00 |
| Unigene355515 | ceoB | 0.000E+00 | 0.000E+00 | 0.000E+00 | 0.000E+00 | 0.000E+00 | 0.000E+00 | 0.000E+00 | 2.037E-07 | 0.000E+00 | 0.000E+00 | 0.000E+00 | 0.000E+00 |
| Unigene355684 | arlS | 0.000E+00 | 0.000E+00 | 0.000E+00 | 0.000E+00 | 6.991E-07 | 6.802E-08 | 6.964E-08 | 0.000E+00 | 0.000E+00 | 0.000E+00 | 0.000E+00 | 1.488E-07 |
| Unigene355814 | novA | 0.000E+00 | 0.000E+00 | 0.000E+00 | 0.000E+00 | 4.754E-07 | 0.000E+00 | 0.000E+00 | 0.000E+00 | 0.000E+00 | 0.000E+00 | 0.000E+00 | 0.000E+00 |
| Unigene355922 | macB | 0.000E+00 | 0.000E+00 | 2.256E-07 | 3.715E-07 | 4.393E-06 | 1.009E-05 | 0.000E+00 | 7.139E-06 | 4.574E-06 | 3.586E-06 | 0.000E+00 | 0.000E+00 |
| Unigene355950 | TriA | 0.000E+00 | 0.000E+00 | 0.000E+00 | 0.000E+00 | 7.336E-07 | 0.000E+00 | 0.000E+00 | 0.000E+00 | 0.000E+00 | 0.000E+00 | 0.000E+00 | 0.000E+00 |
| Unigene355975 | smeR | 0.000E+00 | 0.000E+00 | 0.000E+00 | 0.000E+00 | 7.882E-07 | 0.000E+00 | 0.000E+00 | 0.000E+00 | 0.000E+00 | 0.000E+00 | 0.000E+00 | 0.000E+00 |
| Unigene356023 | evgS | 0.000E+00 | 0.000E+00 | 0.000E+00 | 0.000E+00 | 8.117E-07 | 0.000E+00 | 1.470E-07 | 0.000E+00 | 0.000E+00 | 0.000E+00 | 0.000E+00 | 0.000E+00 |
| Unigene356353 | TriC | 0.000E+00 | 0.000E+00 | 5.898E-08 | 0.000E+00 | 4.007E-07 | 3.249E-07 | 0.000E+00 | 0.000E+00 | 0.000E+00 | 2.679E-07 | 0.000E+00 | 1.421E-07 |
| Unigene356402 | cpxA | 0.000E+00 | 0.000E+00 | 0.000E+00 | 0.000E+00 | 4.360E-07 | 0.000E+00 | 0.000E+00 | 0.000E+00 | 0.000E+00 | 0.000E+00 | 0.000E+00 | 0.000E+00 |
| Unigene356462 | msrA | 0.000E+00 | 0.000E+00 | 0.000E+00 | 0.000E+00 | 2.802E-06 | 0.000E+00 | 0.000E+00 | 0.000E+00 | 0.000E+00 | 0.000E+00 | 1.803E-07 | 0.000E+00 |
| Unigene356466 | arlR | 0.000E+00 | 0.000E+00 | 0.000E+00 | 0.000E+00 | 2.380E-06 | 0.000E+00 | 0.000E+00 | 0.000E+00 | 0.000E+00 | 0.000E+00 | 2.178E-07 | 0.000E+00 |
| Unigene356467 | basS | 0.000E+00 | 0.000E+00 | 0.000E+00 | 0.000E+00 | 2.808E-06 | 0.000E+00 | 0.000E+00 | 0.000E+00 | 0.000E+00 | 0.000E+00 | 1.024E-07 | 0.000E+00 |
| Unigene356475 | adeL | 0.000E+00 | 0.000E+00 | 0.000E+00 | 0.000E+00 | 4.132E-06 | 0.000E+00 | 0.000E+00 | 0.000E+00 | 0.000E+00 | 0.000E+00 | 2.065E-07 | 0.000E+00 |
| Unigene356476 | efpA | 0.000E+00 | 0.000E+00 | 0.000E+00 | 0.000E+00 | 2.788E-06 | 0.000E+00 | 0.000E+00 | 0.000E+00 | 0.000E+00 | 0.000E+00 | 2.658E-07 | 0.000E+00 |
| Unigene356497 | oleB | 0.000E+00 | 0.000E+00 | 0.000E+00 | 0.000E+00 | 3.257E-06 | 0.000E+00 | 3.165E-07 | 0.000E+00 | 0.000E+00 | 0.000E+00 | 3.271E-07 | 0.000E+00 |
| Unigene356498 | msbA | 0.000E+00 | 0.000E+00 | 0.000E+00 | 0.000E+00 | 3.769E-06 | 0.000E+00 | 1.073E-07 | 0.000E+00 | 0.000E+00 | 0.000E+00 | 5.914E-07 | 0.000E+00 |
| Unigene356517 | lmrD | 0.000E+00 | 0.000E+00 | 0.000E+00 | 0.000E+00 | 4.590E-06 | 0.000E+00 | 0.000E+00 | 2.411E-06 | 0.000E+00 | 0.000E+00 | 1.772E-06 | 0.000E+00 |
| Unigene356522 | Corynebac | 0.000E+00 | 0.000E+00 | 0.000E+00 | 0.000E+00 | 2.815E-06 | 0.000E+00 | 0.000E+00 | 1.406E-06 | 0.000E+00 | 0.000E+00 | 6.653E-07 | 0.000E+00 |
| Unigene356541 | lin | 0.000E+00 | 0.000E+00 | 0.000E+00 | 0.000E+00 | 2.770E-06 | 0.000E+00 | 4.716E-08 | 0.000E+00 | 0.000E+00 | 0.000E+00 | 2.194E-07 | 0.000E+00 |
| Unigene356547 | tetB(60) | 0.000E+00 | 0.000E+00 | 0.000E+00 | 0.000E+00 | 3.031E-06 | 0.000E+00 | 0.000E+00 | 0.000E+00 | 0.000E+00 | 0.000E+00 | 2.774E-07 | 9.315E-07 |
| Unigene356580 | novA | 0.000E+00 | 0.000E+00 | 0.000E+00 | 0.000E+00 | 3.428E-06 | 0.000E+00 | 0.000E+00 | 0.000E+00 | 0.000E+00 | 0.000E+00 | 0.000E+00 | 0.000E+00 |
| Unigene356584 | macB | 0.000E+00 | 0.000E+00 | 0.000E+00 | 0.000E+00 | 4.077E-06 | 0.000E+00 | 0.000E+00 | 0.000E+00 | 0.000E+00 | 0.000E+00 | 5.248E-07 | 0.000E+00 |
| Unigene356593 | tet(52) | 0.000E+00 | 0.000E+00 | 0.000E+00 | 0.000E+00 | 2.981E-06 | 0.000E+00 | 0.000E+00 | 0.000E+00 | 0.000E+00 | 1.208E-07 | 1.550E-07 | 0.000E+00 |
| Unigene356597 | tetA(46) | 0.000E+00 | 0.000E+00 | 0.000E+00 | 0.000E+00 | 2.599E-06 | 0.000E+00 | 0.000E+00 | 0.000E+00 | 0.000E+00 | 0.000E+00 | 5.797E-07 | 0.000E+00 |
| Unigene356606 | efrA | 0.000E+00 | 0.000E+00 | 0.000E+00 | 0.000E+00 | 2.955E-06 | 0.000E+00 | 0.000E+00 | 0.000E+00 | 0.000E+00 | 0.000E+00 | 0.000E+00 | 0.000E+00 |
| Unigene356618 | adeL | 0.000E+00 | 0.000E+00 | 0.000E+00 | 0.000E+00 | 3.703E-06 | 0.000E+00 | 0.000E+00 | 0.000E+00 | 0.000E+00 | 0.000E+00 | 0.000E+00 | 0.000E+00 |
| Unigene356627 | patA | 0.000E+00 | 0.000E+00 | 0.000E+00 | 0.000E+00 | 3.766E-06 | 0.000E+00 | 2.936E-07 | 0.000E+00 | 0.000E+00 | 0.000E+00 | 0.000E+00 | 0.000E+00 |
| Unigene356640 | tetA(58) | 0.000E+00 | 0.000E+00 | 0.000E+00 | 0.000E+00 | 3.013E-06 | 0.000E+00 | 0.000E+00 | 1.159E-06 | 0.000E+00 | 0.000E+00 | 2.476E-07 | 0.000E+00 |
| Unigene356656 | patB | 0.000E+00 | 0.000E+00 | 0.000E+00 | 0.000E+00 | 3.140E-06 | 0.000E+00 | 0.000E+00 | 0.000E+00 | 0.000E+00 | 0.000E+00 | 1.391E-07 | 0.000E+00 |
| Unigene356666 | Acinetoba | 0.000E+00 | 0.000E+00 | 0.000E+00 | 0.000E+00 | 3.583E-06 | 0.000E+00 | 0.000E+00 | 0.000E+00 | 0.000E+00 | 0.000E+00 | 3.459E-07 | 0.000E+00 |
| Unigene356670 | vanHO | 0.000E+00 | 0.000E+00 | 0.000E+00 | 0.000E+00 | 2.913E-06 | 0.000E+00 | 0.000E+00 | 0.000E+00 | 0.000E+00 | 0.000E+00 | 3.506E-07 | 0.000E+00 |
| Unigene356674 | novA | 0.000E+00 | 0.000E+00 | 0.000E+00 | 0.000E+00 | 2.531E-06 | 0.000E+00 | 0.000E+00 | 0.000E+00 | 0.000E+00 | 0.000E+00 | 0.000E+00 | 0.000E+00 |
| Unigene356690 | macB | 0.000E+00 | 0.000E+00 | 0.000E+00 | 0.000E+00 | 3.287E-06 | 0.000E+00 | 2.481E-07 | 0.000E+00 | 0.000E+00 | 0.000E+00 | 0.000E+00 | 0.000E+00 |

| | | | | | | | | | | | | | |
|---|---|---|---|---|---|---|---|---|---|---|---|---|---|
| Unigene356695 | efrA | 0.000E+00 | 0.000E+00 | 0.000E+00 | 0.000E+00 | 2.503E-06 | 0.000E+00 | 0.000E+00 | 0.000E+00 | 0.000E+00 | 0.000E+00 | 0.000E+00 | 1.402E-07 |
| Unigene356703 | efrA | 0.000E+00 | 0.000E+00 | 0.000E+00 | 0.000E+00 | 3.308E-06 | 0.000E+00 | 0.000E+00 | 0.000E+00 | 0.000E+00 | 0.000E+00 | 0.000E+00 | 0.000E+00 |
| Unigene356708 | efrA | 0.000E+00 | 0.000E+00 | 0.000E+00 | 0.000E+00 | 3.472E-06 | 0.000E+00 | 9.788E-08 | 0.000E+00 | 0.000E+00 | 0.000E+00 | 0.000E+00 | 8.363E-07 |
| Unigene356726 | efrA | 0.000E+00 | 0.000E+00 | 0.000E+00 | 0.000E+00 | 3.455E-06 | 2.353E-07 | 0.000E+00 | 0.000E+00 | 0.000E+00 | 0.000E+00 | 0.000E+00 | 0.000E+00 |
| Unigene356749 | vgaB | 0.000E+00 | 0.000E+00 | 0.000E+00 | 0.000E+00 | 3.700E-06 | 0.000E+00 | 1.418E-07 | 0.000E+00 | 0.000E+00 | 0.000E+00 | 0.000E+00 | 0.000E+00 |
| Unigene357010 | Streptomy | 0.000E+00 | 0.000E+00 | 0.000E+00 | 0.000E+00 | 5.289E-07 | 0.000E+00 | 0.000E+00 | 0.000E+00 | 0.000E+00 | 0.000E+00 | 0.000E+00 | 0.000E+00 |
| Unigene357043 | macB | 5.799E-07 | 0.000E+00 | 3.003E-07 | 6.506E-07 | 3.973E-06 | 8.463E-06 | 1.498E-06 | 7.521E-06 | 4.102E-06 | 3.248E-06 | 0.000E+00 | 9.710E-07 |
| Unigene357046 | oleC | 0.000E+00 | 0.000E+00 | 8.122E-08 | 0.000E+00 | 4.828E-06 | 6.711E-06 | 1.832E-06 | 7.584E-06 | 5.292E-06 | 0.000E+00 | 0.000E+00 | 0.000E+00 |
| Unigene357049 | macB | 0.000E+00 | 0.000E+00 | 3.256E-07 | 8.137E-07 | 3.423E-06 | 6.405E-06 | 1.259E-06 | 6.529E-06 | 3.730E-06 | 0.000E+00 | 0.000E+00 | 6.163E-07 |
| Unigene357322 | otr(A) | 0.000E+00 | 1.247E-07 | 0.000E+00 | 0.000E+00 | 6.735E-07 | 0.000E+00 | 0.000E+00 | 0.000E+00 | 0.000E+00 | 0.000E+00 | 0.000E+00 | 0.000E+00 |
| Unigene357331 | macB | 0.000E+00 | 0.000E+00 | 0.000E+00 | 0.000E+00 | 0.000E+00 | 0.000E+00 | 0.000E+00 | 3.884E-07 | 0.000E+00 | 0.000E+00 | 0.000E+00 | 0.000E+00 |
| Unigene357346 | macB | 0.000E+00 | 0.000E+00 | 0.000E+00 | 0.000E+00 | 1.376E-06 | 0.000E+00 | 0.000E+00 | 0.000E+00 | 0.000E+00 | 0.000E+00 | 0.000E+00 | 0.000E+00 |
| Unigene357582 | lmrD | 0.000E+00 | 0.000E+00 | 0.000E+00 | 0.000E+00 | 1.258E-06 | 0.000E+00 | 0.000E+00 | 0.000E+00 | 0.000E+00 | 0.000E+00 | 0.000E+00 | 0.000E+00 |
| Unigene357625 | vanSM | 0.000E+00 | 0.000E+00 | 0.000E+00 | 0.000E+00 | 1.537E-06 | 0.000E+00 | 7.291E-08 | 0.000E+00 | 5.280E-07 | 0.000E+00 | 0.000E+00 | 0.000E+00 |
| Unigene357626 | vanRF | 0.000E+00 | 0.000E+00 | 0.000E+00 | 0.000E+00 | 1.482E-06 | 0.000E+00 | 0.000E+00 | 0.000E+00 | 7.290E-07 | 0.000E+00 | 0.000E+00 | 0.000E+00 |
| Unigene357629 | evgS | 0.000E+00 | 0.000E+00 | 0.000E+00 | 0.000E+00 | 0.000E+00 | 0.000E+00 | 0.000E+00 | 0.000E+00 | 0.000E+00 | 0.000E+00 | 0.000E+00 | 1.076E-06 |
| Unigene357633 | acrB | 0.000E+00 | 4.880E-07 | 8.726E-08 | 0.000E+00 | 8.891E-07 | 1.923E-07 | 0.000E+00 | 9.688E-07 | 0.000E+00 | 0.000E+00 | 5.425E-07 | 5.956E-07 |
| Unigene357681 | smeR | 0.000E+00 | 0.000E+00 | 0.000E+00 | 0.000E+00 | 1.091E-06 | 0.000E+00 | 0.000E+00 | 0.000E+00 | 0.000E+00 | 0.000E+00 | 0.000E+00 | 0.000E+00 |
| Unigene357683 | marA | 0.000E+00 | 0.000E+00 | 1.891E-07 | 0.000E+00 | 9.991E-07 | 0.000E+00 | 2.062E-06 | 0.000E+00 | 2.808E-07 | 0.000E+00 | 8.450E-07 | 7.593E-07 |
| Unigene357692 | msbA | 0.000E+00 | 0.000E+00 | 0.000E+00 | 0.000E+00 | 9.545E-07 | 0.000E+00 | 1.358E-07 | 5.253E-07 | 0.000E+00 | 9.115E-08 | 2.808E-07 | 5.803E-07 |
| Unigene357903 | Acinetoba | 0.000E+00 | 1.278E-07 | 0.000E+00 | 9.408E-08 | 1.087E-06 | 0.000E+00 | 0.000E+00 | 0.000E+00 | 2.800E-07 | 0.000E+00 | 0.000E+00 | 0.000E+00 |
| Unigene357931 | emrB | 0.000E+00 | 2.284E-08 | 2.246E-07 | 6.725E-07 | 3.769E-06 | 8.347E-06 | 0.000E+00 | 6.195E-06 | 3.458E-06 | 3.037E-06 | 0.000E+00 | 6.888E-07 |
| Unigene357993 | lmrD | 0.000E+00 | 0.000E+00 | 0.000E+00 | 0.000E+00 | 0.000E+00 | 0.000E+00 | 9.616E-07 | 0.000E+00 | 0.000E+00 | 0.000E+00 | 0.000E+00 | 0.000E+00 |
| Unigene358045 | Acinetoba | 5.369E-07 | 9.429E-07 | 5.690E-07 | 0.000E+00 | 6.275E-06 | 3.018E-07 | 0.000E+00 | 2.507E-07 | 0.000E+00 | 0.000E+00 | 0.000E+00 | 0.000E+00 |
| Unigene358049 | tetA(58) | 5.884E-07 | 1.476E-06 | 8.249E-07 | 3.820E-07 | 7.191E-06 | 5.453E-07 | 9.304E-07 | 2.453E-07 | 0.000E+00 | 0.000E+00 | 0.000E+00 | 0.000E+00 |
| Unigene358173 | evgS | 1.868E-07 | 2.523E-07 | 7.612E-07 | 2.756E-07 | 1.005E-06 | 4.193E-07 | 1.065E-06 | 1.090E-06 | 1.727E-07 | 5.441E-07 | 9.860E-07 | 2.921E-06 |
| Unigene358316 | basS | 2.232E-07 | 0.000E+00 | 0.000E+00 | 0.000E+00 | 7.440E-07 | 3.861E-07 | 7.906E-07 | 1.016E-06 | 0.000E+00 | 3.730E-07 | 6.384E-07 | 6.333E-07 |
| Unigene358317 | arlR | 0.000E+00 | 0.000E+00 | 0.000E+00 | 0.000E+00 | 1.031E-06 | 0.000E+00 | 0.000E+00 | 9.745E-07 | 0.000E+00 | 0.000E+00 | 1.008E-06 | 9.320E-07 |
| Unigene358334 | vanHD | 0.000E+00 | 0.000E+00 | 0.000E+00 | 0.000E+00 | 5.413E-07 | 3.073E-07 | 0.000E+00 | 0.000E+00 | 0.000E+00 | 0.000E+00 | 0.000E+00 | 0.000E+00 |
| Unigene358456 | poxtA | 0.000E+00 | 0.000E+00 | 0.000E+00 | 8.294E-08 | 3.878E-07 | 0.000E+00 | 0.000E+00 | 2.157E-07 | 0.000E+00 | 0.000E+00 | 5.872E-07 | 0.000E+00 |
| Unigene358556 | Staphylocc | 0.000E+00 | 0.000E+00 | 0.000E+00 | 0.000E+00 | 0.000E+00 | 1.276E-06 | 0.000E+00 | 0.000E+00 | 0.000E+00 | 0.000E+00 | 0.000E+00 | 0.000E+00 |
| Unigene358566 | msbA | 0.000E+00 | 0.000E+00 | 0.000E+00 | 0.000E+00 | 0.000E+00 | 1.604E-07 | 0.000E+00 | 0.000E+00 | 0.000E+00 | 0.000E+00 | 0.000E+00 | 0.000E+00 |
| Unigene358600 | cmrA | 0.000E+00 | 0.000E+00 | 0.000E+00 | 0.000E+00 | 0.000E+00 | 6.356E-07 | 0.000E+00 | 0.000E+00 | 0.000E+00 | 0.000E+00 | 0.000E+00 | 0.000E+00 |
| Unigene358724 | kdpE | 0.000E+00 | 2.350E-07 | 3.677E-07 | 0.000E+00 | 1.189E-07 | 9.838E-07 | 1.540E-06 | 2.687E-06 | 0.000E+00 | 0.000E+00 | 5.511E-07 | 1.266E-06 |
| Unigene358807 | farB | 0.000E+00 | 0.000E+00 | 0.000E+00 | 0.000E+00 | 0.000E+00 | 3.525E-07 | 0.000E+00 | 0.000E+00 | 0.000E+00 | 0.000E+00 | 0.000E+00 | 0.000E+00 |
| Unigene358850 | msbA | 0.000E+00 | 0.000E+00 | 0.000E+00 | 0.000E+00 | 0.000E+00 | 3.381E-07 | 0.000E+00 | 5.112E-07 | 0.000E+00 | 0.000E+00 | 0.000E+00 | 0.000E+00 |
| Unigene358991 | bacA | 0.000E+00 | 0.000E+00 | 0.000E+00 | 0.000E+00 | 0.000E+00 | 9.624E-07 | 0.000E+00 | 0.000E+00 | 0.000E+00 | 0.000E+00 | 0.000E+00 | 0.000E+00 |
| Unigene359155 | evgS | 0.000E+00 | 0.000E+00 | 0.000E+00 | 0.000E+00 | 0.000E+00 | 1.756E-07 | 0.000E+00 | 0.000E+00 | 0.000E+00 | 0.000E+00 | 0.000E+00 | 0.000E+00 |
| Unigene359258 | emrB | 0.000E+00 | 0.000E+00 | 0.000E+00 | 0.000E+00 | 0.000E+00 | 6.037E-07 | 0.000E+00 | 0.000E+00 | 0.000E+00 | 0.000E+00 | 0.000E+00 | 0.000E+00 |
| Unigene359322 | mtrA | 0.000E+00 | 0.000E+00 | 0.000E+00 | 0.000E+00 | 0.000E+00 | 5.539E-07 | 1.804E-06 | 0.000E+00 | 0.000E+00 | 0.000E+00 | 0.000E+00 | 0.000E+00 |
| Unigene359327 | Erm(30) | 8.636E-05 | 3.837E-05 | 0.000E+00 | 0.000E+00 | 2.058E-05 | 8.345E-05 | 0.000E+00 | 0.000E+00 | 0.000E+00 | 0.000E+00 | 0.000E+00 | 0.000E+00 |
| Unigene359340 | farA | 0.000E+00 | 0.000E+00 | 0.000E+00 | 0.000E+00 | 7.620E-07 | 1.460E-06 | 3.962E-06 | 4.078E-06 | 5.623E-07 | 1.051E-06 | 2.011E-06 | 2.990E-06 |
| Unigene359588 | Pseudomo | 0.000E+00 | 0.000E+00 | 3.969E-08 | 0.000E+00 | 1.348E-07 | 7.434E-07 | 0.000E+00 | 9.443E-08 | 0.000E+00 | 0.000E+00 | 1.388E-07 | 0.000E+00 |
| Unigene359637 | mgrA | 0.000E+00 | 0.000E+00 | 7.659E-07 | 0.000E+00 | 0.000E+00 | 0.000E+00 | 5.430E-06 | 5.271E-06 | 0.000E+00 | 0.000E+00 | 0.000E+00 | 4.349E-06 |
| Unigene359666 | novA | 0.000E+00 | 0.000E+00 | 0.000E+00 | 0.000E+00 | 0.000E+00 | 1.508E-06 | 0.000E+00 | 0.000E+00 | 0.000E+00 | 0.000E+00 | 0.000E+00 | 0.000E+00 |
| Unigene359667 | msbA | 0.000E+00 | 0.000E+00 | 0.000E+00 | 0.000E+00 | 0.000E+00 | 1.514E-06 | 0.000E+00 | 0.000E+00 | 0.000E+00 | 0.000E+00 | 0.000E+00 | 0.000E+00 |
| Unigene359669 | msbA | 0.000E+00 | 0.000E+00 | 0.000E+00 | 0.000E+00 | 0.000E+00 | 1.620E-06 | 0.000E+00 | 0.000E+00 | 0.000E+00 | 0.000E+00 | 0.000E+00 | 0.000E+00 |
| Unigene359693 | tetT | 0.000E+00 | 0.000E+00 | 0.000E+00 | 0.000E+00 | 0.000E+00 | 1.626E-06 | 0.000E+00 | 0.000E+00 | 0.000E+00 | 0.000E+00 | 0.000E+00 | 0.000E+00 |
| Unigene359699 | vanL | 0.000E+00 | 0.000E+00 | 0.000E+00 | 0.000E+00 | 0.000E+00 | 1.173E-06 | 0.000E+00 | 0.000E+00 | 0.000E+00 | 0.000E+00 | 0.000E+00 | 0.000E+00 |
| Unigene359720 | Staphylocc | 0.000E+00 | 0.000E+00 | 0.000E+00 | 0.000E+00 | 0.000E+00 | 1.688E-06 | 0.000E+00 | 0.000E+00 | 0.000E+00 | 0.000E+00 | 0.000E+00 | 0.000E+00 |
| Unigene359785 | vanHF | 0.000E+00 | 0.000E+00 | 0.000E+00 | 0.000E+00 | 0.000E+00 | 2.059E-07 | 0.000E+00 | 2.593E-07 | 0.000E+00 | 0.000E+00 | 0.000E+00 | 2.626E-07 |
| Unigene359844 | vatE | 0.000E+00 | 0.000E+00 | 0.000E+00 | 0.000E+00 | 0.000E+00 | 7.867E-07 | 0.000E+00 | 0.000E+00 | 0.000E+00 | 0.000E+00 | 0.000E+00 | 0.000E+00 |
| Unigene360005 | lmrB | 0.000E+00 | 0.000E+00 | 0.000E+00 | 0.000E+00 | 0.000E+00 | 7.425E-07 | 0.000E+00 | 4.516E-06 | 0.000E+00 | 0.000E+00 | 0.000E+00 | 0.000E+00 |
| Unigene360013 | Staphylocc | 0.000E+00 | 0.000E+00 | 0.000E+00 | 0.000E+00 | 0.000E+00 | 1.423E-06 | 0.000E+00 | 0.000E+00 | 0.000E+00 | 0.000E+00 | 0.000E+00 | 0.000E+00 |
| Unigene360050 | PmrF | 0.000E+00 | 0.000E+00 | 0.000E+00 | 0.000E+00 | 0.000E+00 | 1.651E-06 | 0.000E+00 | 0.000E+00 | 0.000E+00 | 0.000E+00 | 0.000E+00 | 0.000E+00 |
| Unigene360055 | oleC | 0.000E+00 | 0.000E+00 | 0.000E+00 | 0.000E+00 | 0.000E+00 | 2.086E-06 | 0.000E+00 | 0.000E+00 | 0.000E+00 | 0.000E+00 | 0.000E+00 | 0.000E+00 |
| Unigene360081 | kdpE | 0.000E+00 | 0.000E+00 | 0.000E+00 | 0.000E+00 | 0.000E+00 | 4.653E-07 | 0.000E+00 | 0.000E+00 | 0.000E+00 | 0.000E+00 | 0.000E+00 | 0.000E+00 |
| Unigene360441 | vanHD | 0.000E+00 | 0.000E+00 | 0.000E+00 | 0.000E+00 | 0.000E+00 | 7.152E-07 | 0.000E+00 | 0.000E+00 | 0.000E+00 | 0.000E+00 | 0.000E+00 | 0.000E+00 |

| | | | | | | | | | | | | | |
|---|---|---|---|---|---|---|---|---|---|---|---|---|---|
| Unigene360509 | patA | 0.000E+00 | 3.383E-07 | 4.674E-07 | 3.961E-07 | 8.094E-07 | 1.121E-06 | 3.070E-06 | 2.715E-06 | 0.000E+00 | 0.000E+00 | 1.250E-06 | 3.014E-06 |
| Unigene360646 | cfrC | 4.203E-08 | 0.000E+00 | 0.000E+00 | 0.000E+00 | 1.868E-07 | 0.000E+00 | 3.721E-07 | 3.434E-07 | 0.000E+00 | 0.000E+00 | 1.442E-07 | 6.459E-07 |
| Unigene360873 | arlR | 0.000E+00 | 0.000E+00 | 0.000E+00 | 0.000E+00 | 0.000E+00 | 6.004E-07 | 0.000E+00 | 5.672E-07 | 0.000E+00 | 0.000E+00 | 0.000E+00 | 4.923E-07 |
| Unigene361152 | msbA | 0.000E+00 | 0.000E+00 | 0.000E+00 | 0.000E+00 | 0.000E+00 | 8.366E-07 | 0.000E+00 | 8.151E-06 | 0.000E+00 | 0.000E+00 | 1.368E-05 | 0.000E+00 |
| Unigene361222 | otr(B) | 0.000E+00 | 0.000E+00 | 0.000E+00 | 0.000E+00 | 0.000E+00 | 1.747E-06 | 0.000E+00 | 0.000E+00 | 0.000E+00 | 0.000E+00 | 0.000E+00 | 0.000E+00 |
| Unigene361323 | msbA | 9.755E-07 | 0.000E+00 | 0.000E+00 | 0.000E+00 | 0.000E+00 | 2.693E-06 | 0.000E+00 | 0.000E+00 | 0.000E+00 | 0.000E+00 | 0.000E+00 | 0.000E+00 |
| Unigene361374 | patB | 0.000E+00 | 0.000E+00 | 0.000E+00 | 0.000E+00 | 0.000E+00 | 8.272E-07 | 0.000E+00 | 0.000E+00 | 0.000E+00 | 0.000E+00 | 0.000E+00 | 0.000E+00 |
| Unigene361375 | macB | 0.000E+00 | 0.000E+00 | 0.000E+00 | 0.000E+00 | 0.000E+00 | 1.291E-06 | 0.000E+00 | 0.000E+00 | 0.000E+00 | 0.000E+00 | 0.000E+00 | 0.000E+00 |
| Unigene361384 | evgA | 4.261E-07 | 0.000E+00 | 0.000E+00 | 0.000E+00 | 0.000E+00 | 7.083E-06 | 0.000E+00 | 0.000E+00 | 3.202E-06 | 0.000E+00 | 0.000E+00 | 1.322E-06 |
| Unigene361470 | tetB(46) | 0.000E+00 | 0.000E+00 | 0.000E+00 | 0.000E+00 | 0.000E+00 | 1.231E-06 | 0.000E+00 | 0.000E+00 | 0.000E+00 | 0.000E+00 | 0.000E+00 | 0.000E+00 |
| Unigene361471 | tetB(46) | 0.000E+00 | 0.000E+00 | 0.000E+00 | 0.000E+00 | 0.000E+00 | 9.970E-07 | 0.000E+00 | 0.000E+00 | 0.000E+00 | 0.000E+00 | 0.000E+00 | 0.000E+00 |
| Unigene361582 | tetA(58) | 0.000E+00 | 0.000E+00 | 0.000E+00 | 0.000E+00 | 0.000E+00 | 9.448E-06 | 0.000E+00 | 0.000E+00 | 4.263E-06 | 0.000E+00 | 0.000E+00 | 0.000E+00 |
| Unigene361610 | arlS | 0.000E+00 | 0.000E+00 | 0.000E+00 | 0.000E+00 | 0.000E+00 | 8.750E-07 | 0.000E+00 | 0.000E+00 | 0.000E+00 | 0.000E+00 | 0.000E+00 | 0.000E+00 |
| Unigene361616 | kdpE | 0.000E+00 | 2.944E-07 | 1.024E-06 | 5.418E-07 | 0.000E+00 | 6.444E-07 | 0.000E+00 | 0.000E+00 | 3.909E-07 | 0.000E+00 | 7.842E-07 | 3.206E-06 |
| Unigene361841 | vanRO | 0.000E+00 | 0.000E+00 | 0.000E+00 | 0.000E+00 | 0.000E+00 | 8.907E-07 | 0.000E+00 | 0.000E+00 | 0.000E+00 | 0.000E+00 | 0.000E+00 | 0.000E+00 |
| Unigene361865 | Streptomy | 0.000E+00 | 3.119E-06 | 0.000E+00 | 0.000E+00 | 0.000E+00 | 0.000E+00 | 0.000E+00 | 0.000E+00 | 0.000E+00 | 0.000E+00 | 0.000E+00 | 0.000E+00 |
| Unigene362009 | vanSF | 0.000E+00 | 0.000E+00 | 0.000E+00 | 0.000E+00 | 0.000E+00 | 5.410E-07 | 0.000E+00 | 0.000E+00 | 0.000E+00 | 0.000E+00 | 0.000E+00 | 0.000E+00 |
| Unigene362073 | Staphylocc | 0.000E+00 | 0.000E+00 | 0.000E+00 | 0.000E+00 | 6.243E-07 | 1.260E-06 | 3.524E-06 | 4.106E-06 | 0.000E+00 | 1.136E-06 | 2.095E-06 | 3.936E-06 |
| Unigene362082 | macB | 0.000E+00 | 0.000E+00 | 0.000E+00 | 0.000E+00 | 0.000E+00 | 0.000E+00 | 0.000E+00 | 0.000E+00 | 0.000E+00 | 0.000E+00 | 0.000E+00 | 3.917E-06 |
| Unigene362112 | Staphylocc | 0.000E+00 | 0.000E+00 | 0.000E+00 | 0.000E+00 | 0.000E+00 | 9.595E-07 | 0.000E+00 | 0.000E+00 | 0.000E+00 | 0.000E+00 | 0.000E+00 | 0.000E+00 |
| Unigene362131 | TaeA | 0.000E+00 | 0.000E+00 | 0.000E+00 | 0.000E+00 | 0.000E+00 | 5.768E-07 | 0.000E+00 | 0.000E+00 | 0.000E+00 | 0.000E+00 | 0.000E+00 | 0.000E+00 |
| Unigene362246 | efmA | 0.000E+00 | 0.000E+00 | 0.000E+00 | 0.000E+00 | 0.000E+00 | 9.771E-07 | 0.000E+00 | 0.000E+00 | 0.000E+00 | 0.000E+00 | 0.000E+00 | 0.000E+00 |
| Unigene362281 | lmrB | 0.000E+00 | 0.000E+00 | 0.000E+00 | 0.000E+00 | 0.000E+00 | 1.111E-06 | 0.000E+00 | 0.000E+00 | 0.000E+00 | 0.000E+00 | 0.000E+00 | 0.000E+00 |
| Unigene362359 | vanHD | 0.000E+00 | 0.000E+00 | 0.000E+00 | 0.000E+00 | 0.000E+00 | 1.256E-06 | 0.000E+00 | 0.000E+00 | 0.000E+00 | 0.000E+00 | 0.000E+00 | 0.000E+00 |
| Unigene362389 | tetA(60) | 0.000E+00 | 0.000E+00 | 0.000E+00 | 0.000E+00 | 0.000E+00 | 1.823E-06 | 0.000E+00 | 0.000E+00 | 0.000E+00 | 0.000E+00 | 0.000E+00 | 0.000E+00 |
| Unigene362396 | bcrA | 0.000E+00 | 0.000E+00 | 0.000E+00 | 0.000E+00 | 0.000E+00 | 1.564E-06 | 5.004E-08 | 0.000E+00 | 0.000E+00 | 0.000E+00 | 0.000E+00 | 0.000E+00 |
| Unigene362413 | poxtA | 0.000E+00 | 0.000E+00 | 0.000E+00 | 0.000E+00 | 0.000E+00 | 1.172E-06 | 0.000E+00 | 0.000E+00 | 0.000E+00 | 0.000E+00 | 0.000E+00 | 0.000E+00 |
| Unigene362422 | optrA | 0.000E+00 | 0.000E+00 | 0.000E+00 | 0.000E+00 | 0.000E+00 | 1.285E-06 | 2.349E-08 | 0.000E+00 | 0.000E+00 | 0.000E+00 | 0.000E+00 | 0.000E+00 |
| Unigene362464 | Listeria mc | 0.000E+00 | 0.000E+00 | 0.000E+00 | 0.000E+00 | 0.000E+00 | 1.341E-06 | 0.000E+00 | 0.000E+00 | 0.000E+00 | 0.000E+00 | 0.000E+00 | 0.000E+00 |
| Unigene362465 | bcr-1 | 0.000E+00 | 0.000E+00 | 0.000E+00 | 0.000E+00 | 0.000E+00 | 1.122E-06 | 0.000E+00 | 0.000E+00 | 0.000E+00 | 0.000E+00 | 0.000E+00 | 0.000E+00 |
| Unigene362688 | smeS | 0.000E+00 | 0.000E+00 | 0.000E+00 | 0.000E+00 | 0.000E+00 | 1.026E-06 | 0.000E+00 | 0.000E+00 | 0.000E+00 | 0.000E+00 | 0.000E+00 | 0.000E+00 |
| Unigene362691 | lmrB | 0.000E+00 | 0.000E+00 | 0.000E+00 | 0.000E+00 | 0.000E+00 | 1.494E-06 | 0.000E+00 | 0.000E+00 | 0.000E+00 | 0.000E+00 | 0.000E+00 | 0.000E+00 |
| Unigene362701 | TaeA | 0.000E+00 | 0.000E+00 | 0.000E+00 | 0.000E+00 | 0.000E+00 | 6.781E-07 | 0.000E+00 | 0.000E+00 | 0.000E+00 | 0.000E+00 | 0.000E+00 | 0.000E+00 |
| Unigene362816 | vanF | 0.000E+00 | 0.000E+00 | 0.000E+00 | 0.000E+00 | 0.000E+00 | 8.022E-07 | 0.000E+00 | 0.000E+00 | 0.000E+00 | 0.000E+00 | 0.000E+00 | 0.000E+00 |
| Unigene362821 | ugd | 0.000E+00 | 0.000E+00 | 0.000E+00 | 0.000E+00 | 0.000E+00 | 2.261E-05 | 0.000E+00 | 0.000E+00 | 0.000E+00 | 0.000E+00 | 0.000E+00 | 0.000E+00 |
| Unigene362921 | tetA(58) | 0.000E+00 | 0.000E+00 | 0.000E+00 | 0.000E+00 | 0.000E+00 | 1.298E-06 | 0.000E+00 | 0.000E+00 | 0.000E+00 | 0.000E+00 | 0.000E+00 | 0.000E+00 |
| Unigene362941 | mtrA | 0.000E+00 | 0.000E+00 | 0.000E+00 | 0.000E+00 | 0.000E+00 | 1.092E-06 | 0.000E+00 | 0.000E+00 | 0.000E+00 | 0.000E+00 | 0.000E+00 | 0.000E+00 |
| Unigene362942 | baeS | 0.000E+00 | 0.000E+00 | 0.000E+00 | 0.000E+00 | 0.000E+00 | 1.079E-06 | 0.000E+00 | 0.000E+00 | 0.000E+00 | 0.000E+00 | 0.000E+00 | 0.000E+00 |
| Unigene363008 | Staphylocc | 0.000E+00 | 0.000E+00 | 0.000E+00 | 0.000E+00 | 0.000E+00 | 2.223E-07 | 0.000E+00 | 0.000E+00 | 0.000E+00 | 0.000E+00 | 0.000E+00 | 6.481E-07 |
| Unigene363056 | kdpE | 0.000E+00 | 0.000E+00 | 3.318E-07 | 5.899E-07 | 0.000E+00 | 8.641E-07 | 3.573E-06 | 2.548E-06 | 3.360E-07 | 8.219E-07 | 0.000E+00 | 3.670E-06 |
| Unigene363141 | lfrA | 3.746E-07 | 0.000E+00 | 0.000E+00 | 6.936E-07 | 9.480E-07 | 1.327E-06 | 2.879E-06 | 2.575E-06 | 3.867E-07 | 9.738E-07 | 1.881E-06 | 4.551E-06 |
| Unigene363167 | lmrB | 0.000E+00 | 0.000E+00 | 0.000E+00 | 0.000E+00 | 0.000E+00 | 4.754E-07 | 0.000E+00 | 0.000E+00 | 0.000E+00 | 0.000E+00 | 0.000E+00 | 0.000E+00 |
| Unigene363300 | vanHO | 0.000E+00 | 0.000E+00 | 0.000E+00 | 0.000E+00 | 0.000E+00 | 3.300E-07 | 0.000E+00 | 0.000E+00 | 0.000E+00 | 0.000E+00 | 0.000E+00 | 0.000E+00 |
| Unigene363302 | tet32 | 0.000E+00 | 0.000E+00 | 0.000E+00 | 0.000E+00 | 1.210E-06 | 4.709E-07 | 0.000E+00 | 9.661E-07 | 0.000E+00 | 7.765E-07 | 0.000E+00 | 3.296E-06 |
| Unigene363376 | Streptomy | 0.000E+00 | 0.000E+00 | 0.000E+00 | 0.000E+00 | 0.000E+00 | 5.436E-07 | 0.000E+00 | 0.000E+00 | 0.000E+00 | 0.000E+00 | 0.000E+00 | 0.000E+00 |
| Unigene363428 | mdtC | 0.000E+00 | 0.000E+00 | 0.000E+00 | 0.000E+00 | 0.000E+00 | 4.600E-07 | 0.000E+00 | 0.000E+00 | 0.000E+00 | 0.000E+00 | 0.000E+00 | 0.000E+00 |
| Unigene363489 | Corynebac | 0.000E+00 | 0.000E+00 | 0.000E+00 | 0.000E+00 | 0.000E+00 | 8.369E-06 | 0.000E+00 | 0.000E+00 | 2.646E-06 | 0.000E+00 | 0.000E+00 | 0.000E+00 |
| Unigene363619 | vanRG | 0.000E+00 | 0.000E+00 | 0.000E+00 | 0.000E+00 | 0.000E+00 | 5.764E-07 | 0.000E+00 | 0.000E+00 | 0.000E+00 | 0.000E+00 | 0.000E+00 | 4.583E-07 |
| Unigene363633 | mecI | 0.000E+00 | 0.000E+00 | 0.000E+00 | 0.000E+00 | 0.000E+00 | 6.887E-07 | 0.000E+00 | 0.000E+00 | 0.000E+00 | 0.000E+00 | 0.000E+00 | 0.000E+00 |
| Unigene363675 | vgaB | 0.000E+00 | 1.437E-07 | 4.069E-07 | 1.322E-07 | 8.002E-07 | 9.438E-07 | 3.068E-06 | 1.809E-06 | 2.624E-07 | 8.023E-07 | 1.248E-06 | 2.270E-06 |
| Unigene363792 | mdtA | 0.000E+00 | 0.000E+00 | 0.000E+00 | 0.000E+00 | 0.000E+00 | 5.474E-07 | 0.000E+00 | 0.000E+00 | 0.000E+00 | 0.000E+00 | 0.000E+00 | 0.000E+00 |
| Unigene363928 | PmrF | 0.000E+00 | 0.000E+00 | 0.000E+00 | 0.000E+00 | 0.000E+00 | 6.158E-07 | 0.000E+00 | 0.000E+00 | 0.000E+00 | 0.000E+00 | 2.896E-07 | 1.496E-07 |
| Unigene363965 | novA | 0.000E+00 | 0.000E+00 | 0.000E+00 | 0.000E+00 | 0.000E+00 | 1.177E-06 | 0.000E+00 | 0.000E+00 | 0.000E+00 | 0.000E+00 | 0.000E+00 | 0.000E+00 |
| Unigene363966 | msbA | 0.000E+00 | 0.000E+00 | 0.000E+00 | 0.000E+00 | 0.000E+00 | 1.143E-06 | 0.000E+00 | 0.000E+00 | 0.000E+00 | 0.000E+00 | 0.000E+00 | 0.000E+00 |
| Unigene363988 | novA | 0.000E+00 | 0.000E+00 | 0.000E+00 | 0.000E+00 | 0.000E+00 | 3.339E-07 | 0.000E+00 | 0.000E+00 | 0.000E+00 | 0.000E+00 | 0.000E+00 | 0.000E+00 |
| Unigene364086 | TaeA | 0.000E+00 | 0.000E+00 | 0.000E+00 | 0.000E+00 | 0.000E+00 | 1.556E-06 | 0.000E+00 | 0.000E+00 | 0.000E+00 | 0.000E+00 | 0.000E+00 | 0.000E+00 |
| Unigene364088 | dfrG | 0.000E+00 | 0.000E+00 | 0.000E+00 | 0.000E+00 | 0.000E+00 | 9.751E-07 | 0.000E+00 | 0.000E+00 | 0.000E+00 | 0.000E+00 | 0.000E+00 | 0.000E+00 |
| Unigene364120 | macB | 0.000E+00 | 0.000E+00 | 5.676E-07 | 0.000E+00 | 8.998E-07 | 6.671E-07 | 7.683E-07 | 6.302E-07 | 0.000E+00 | 4.726E-07 | 4.853E-07 | 2.872E-06 |

| | | | | | | | | | | | | | |
|---|---|---|---|---|---|---|---|---|---|---|---|---|---|
| Unigene364205 | adeR | 0.000E+00 | 0.000E+00 | 0.000E+00 | 0.000E+00 | 0.000E+00 | 4.039E-07 | 0.000E+00 | 0.000E+00 | 0.000E+00 | 0.000E+00 | 0.000E+00 | 0.000E+00 |
| Unigene364259 | PmrF | 0.000E+00 | 0.000E+00 | 0.000E+00 | 0.000E+00 | 0.000E+00 | 4.748E-07 | 0.000E+00 | 0.000E+00 | 0.000E+00 | 0.000E+00 | 0.000E+00 | 0.000E+00 |
| Unigene364316 | PmrF | 0.000E+00 | 0.000E+00 | 0.000E+00 | 0.000E+00 | 0.000E+00 | 8.779E-07 | 0.000E+00 | 0.000E+00 | 0.000E+00 | 0.000E+00 | 0.000E+00 | 0.000E+00 |
| Unigene364351 | TriC | 0.000E+00 | 0.000E+00 | 0.000E+00 | 0.000E+00 | 0.000E+00 | 3.300E-07 | 0.000E+00 | 0.000E+00 | 0.000E+00 | 0.000E+00 | 0.000E+00 | 0.000E+00 |
| Unigene364371 | macB | 0.000E+00 | 0.000E+00 | 0.000E+00 | 0.000E+00 | 0.000E+00 | 7.755E-07 | 0.000E+00 | 0.000E+00 | 0.000E+00 | 0.000E+00 | 0.000E+00 | 0.000E+00 |
| Unigene364402 | amrB | 0.000E+00 | 0.000E+00 | 0.000E+00 | 1.411E-07 | 0.000E+00 | 7.050E-07 | 0.000E+00 | 0.000E+00 | 0.000E+00 | 0.000E+00 | 2.131E-07 | 5.506E-07 |
| Unigene364412 | tetA(60) | 0.000E+00 | 0.000E+00 | 0.000E+00 | 0.000E+00 | 0.000E+00 | 4.263E-07 | 0.000E+00 | 0.000E+00 | 0.000E+00 | 0.000E+00 | 0.000E+00 | 0.000E+00 |
| Unigene364484 | msbA | 0.000E+00 | 2.904E-07 | 0.000E+00 | 0.000E+00 | 0.000E+00 | 7.628E-07 | 0.000E+00 | 0.000E+00 | 0.000E+00 | 0.000E+00 | 0.000E+00 | 3.545E-06 |
| Unigene364512 | adeL | 0.000E+00 | 0.000E+00 | 0.000E+00 | 0.000E+00 | 0.000E+00 | 5.601E-07 | 7.057E-07 | 0.000E+00 | 0.000E+00 | 0.000E+00 | 0.000E+00 | 0.000E+00 |
| Unigene364533 | smeF | 0.000E+00 | 0.000E+00 | 0.000E+00 | 0.000E+00 | 0.000E+00 | 6.763E-07 | 0.000E+00 | 0.000E+00 | 0.000E+00 | 0.000E+00 | 0.000E+00 | 0.000E+00 |
| Unigene364650 | evgS | 0.000E+00 | 0.000E+00 | 0.000E+00 | 0.000E+00 | 0.000E+00 | 4.373E-07 | 0.000E+00 | 1.889E-07 | 0.000E+00 | 0.000E+00 | 0.000E+00 | 8.607E-06 |
| Unigene364657 | mepA | 0.000E+00 | 0.000E+00 | 0.000E+00 | 0.000E+00 | 0.000E+00 | 7.122E-07 | 0.000E+00 | 0.000E+00 | 0.000E+00 | 0.000E+00 | 0.000E+00 | 0.000E+00 |
| Unigene364690 | macB | 0.000E+00 | 0.000E+00 | 0.000E+00 | 0.000E+00 | 0.000E+00 | 1.081E-06 | 0.000E+00 | 0.000E+00 | 0.000E+00 | 0.000E+00 | 0.000E+00 | 0.000E+00 |
| Unigene364696 | bmr | 0.000E+00 | 0.000E+00 | 0.000E+00 | 0.000E+00 | 0.000E+00 | 1.113E-06 | 0.000E+00 | 0.000E+00 | 0.000E+00 | 0.000E+00 | 0.000E+00 | 0.000E+00 |
| Unigene364715 | vanTC | 0.000E+00 | 0.000E+00 | 0.000E+00 | 0.000E+00 | 0.000E+00 | 9.275E-07 | 0.000E+00 | 0.000E+00 | 0.000E+00 | 0.000E+00 | 0.000E+00 | 0.000E+00 |
| Unigene364725 | bcrA | 1.325E-05 | 5.041E-06 | 4.292E-06 | 1.458E-05 | 9.952E-05 | 1.327E-04 | 1.416E-04 | 1.146E-04 | 1.711E-04 | 1.727E-04 | 1.416E-04 | 9.001E-05 |
| Unigene364818 | TaeA | 0.000E+00 | 0.000E+00 | 0.000E+00 | 0.000E+00 | 0.000E+00 | 4.496E-07 | 0.000E+00 | 0.000E+00 | 0.000E+00 | 0.000E+00 | 0.000E+00 | 0.000E+00 |
| Unigene364920 | vanG | 0.000E+00 | 0.000E+00 | 0.000E+00 | 0.000E+00 | 0.000E+00 | 5.339E-07 | 7.809E-07 | 9.059E-07 | 0.000E+00 | 0.000E+00 | 0.000E+00 | 5.421E-07 |
| Unigene365020 | optrA | 0.000E+00 | 0.000E+00 | 0.000E+00 | 0.000E+00 | 0.000E+00 | 9.818E-07 | 0.000E+00 | 0.000E+00 | 0.000E+00 | 0.000E+00 | 0.000E+00 | 0.000E+00 |
| Unigene365024 | poxtA | 0.000E+00 | 0.000E+00 | 0.000E+00 | 0.000E+00 | 0.000E+00 | 1.232E-06 | 0.000E+00 | 0.000E+00 | 0.000E+00 | 0.000E+00 | 0.000E+00 | 0.000E+00 |
| Unigene365026 | mepA | 0.000E+00 | 0.000E+00 | 0.000E+00 | 0.000E+00 | 0.000E+00 | 1.474E-06 | 0.000E+00 | 0.000E+00 | 0.000E+00 | 0.000E+00 | 0.000E+00 | 0.000E+00 |
| Unigene365105 | efmA | 0.000E+00 | 0.000E+00 | 0.000E+00 | 0.000E+00 | 0.000E+00 | 5.487E-07 | 0.000E+00 | 0.000E+00 | 0.000E+00 | 0.000E+00 | 0.000E+00 | 0.000E+00 |
| Unigene365119 | Staphylocc | 0.000E+00 | 0.000E+00 | 0.000E+00 | 0.000E+00 | 0.000E+00 | 9.177E-07 | 0.000E+00 | 0.000E+00 | 0.000E+00 | 0.000E+00 | 0.000E+00 | 0.000E+00 |
| Unigene365186 | lmrC | 0.000E+00 | 0.000E+00 | 0.000E+00 | 0.000E+00 | 9.962E-08 | 3.877E-07 | 4.466E-07 | 0.000E+00 | 0.000E+00 | 0.000E+00 | 2.564E-07 | 6.730E-06 |
| Unigene365260 | msbA | 0.000E+00 | 0.000E+00 | 0.000E+00 | 0.000E+00 | 0.000E+00 | 2.066E-06 | 0.000E+00 | 0.000E+00 | 0.000E+00 | 0.000E+00 | 0.000E+00 | 0.000E+00 |
| Unigene365288 | salA | 0.000E+00 | 0.000E+00 | 0.000E+00 | 0.000E+00 | 0.000E+00 | 1.414E-06 | 0.000E+00 | 0.000E+00 | 0.000E+00 | 0.000E+00 | 0.000E+00 | 0.000E+00 |
| Unigene365313 | lmrB | 0.000E+00 | 0.000E+00 | 0.000E+00 | 0.000E+00 | 0.000E+00 | 6.054E-07 | 0.000E+00 | 0.000E+00 | 0.000E+00 | 0.000E+00 | 0.000E+00 | 0.000E+00 |
| Unigene365346 | Chlamydia | 0.000E+00 | 0.000E+00 | 0.000E+00 | 0.000E+00 | 0.000E+00 | 3.351E-07 | 0.000E+00 | 0.000E+00 | 0.000E+00 | 0.000E+00 | 0.000E+00 | 0.000E+00 |
| Unigene365350 | vanRE | 0.000E+00 | 0.000E+00 | 0.000E+00 | 0.000E+00 | 0.000E+00 | 0.000E+00 | 0.000E+00 | 0.000E+00 | 0.000E+00 | 0.000E+00 | 0.000E+00 | 1.913E-07 |
| Unigene365365 | mtrA | 0.000E+00 | 0.000E+00 | 0.000E+00 | 0.000E+00 | 0.000E+00 | 1.250E-06 | 0.000E+00 | 0.000E+00 | 0.000E+00 | 0.000E+00 | 0.000E+00 | 5.256E-07 |
| Unigene365473 | mgrA | 0.000E+00 | 3.619E-06 | 0.000E+00 | 1.022E-05 | 6.992E-05 | 9.706E-05 | 9.380E-05 | 0.000E+00 | 0.000E+00 | 0.000E+00 | 0.000E+00 | 0.000E+00 |
| Unigene365499 | MexF | 0.000E+00 | 0.000E+00 | 0.000E+00 | 0.000E+00 | 0.000E+00 | 2.496E-07 | 0.000E+00 | 0.000E+00 | 0.000E+00 | 0.000E+00 | 0.000E+00 | 3.275E-07 |
| Unigene365603 | efrA | 0.000E+00 | 0.000E+00 | 0.000E+00 | 0.000E+00 | 0.000E+00 | 1.067E-06 | 0.000E+00 | 0.000E+00 | 0.000E+00 | 0.000E+00 | 0.000E+00 | 0.000E+00 |
| Unigene365604 | lmrD | 0.000E+00 | 0.000E+00 | 0.000E+00 | 0.000E+00 | 0.000E+00 | 9.662E-07 | 0.000E+00 | 0.000E+00 | 0.000E+00 | 0.000E+00 | 0.000E+00 | 0.000E+00 |
| Unigene365758 | vatE | 9.963E-08 | 0.000E+00 | 0.000E+00 | 1.509E-07 | 3.874E-07 | 6.462E-07 | 9.373E-07 | 9.303E-07 | 0.000E+00 | 0.000E+00 | 9.117E-07 | 2.885E-06 |
| Unigene365808 | rgt1438 | 0.000E+00 | 0.000E+00 | 0.000E+00 | 0.000E+00 | 0.000E+00 | 4.046E-07 | 0.000E+00 | 0.000E+00 | 0.000E+00 | 0.000E+00 | 0.000E+00 | 0.000E+00 |
| Unigene365820 | mecI | 0.000E+00 | 0.000E+00 | 0.000E+00 | 0.000E+00 | 0.000E+00 | 1.433E-06 | 0.000E+00 | 0.000E+00 | 0.000E+00 | 0.000E+00 | 0.000E+00 | 0.000E+00 |
| Unigene365847 | Staphylocc | 0.000E+00 | 0.000E+00 | 0.000E+00 | 0.000E+00 | 0.000E+00 | 1.482E-06 | 0.000E+00 | 0.000E+00 | 0.000E+00 | 0.000E+00 | 0.000E+00 | 0.000E+00 |
| Unigene365903 | tetA(58) | 0.000E+00 | 0.000E+00 | 0.000E+00 | 0.000E+00 | 0.000E+00 | 1.001E-06 | 0.000E+00 | 0.000E+00 | 0.000E+00 | 0.000E+00 | 0.000E+00 | 0.000E+00 |
| Unigene365905 | adeN | 0.000E+00 | 0.000E+00 | 0.000E+00 | 0.000E+00 | 0.000E+00 | 1.599E-06 | 0.000E+00 | 0.000E+00 | 0.000E+00 | 0.000E+00 | 0.000E+00 | 0.000E+00 |
| Unigene365953 | tetA(58) | 0.000E+00 | 0.000E+00 | 0.000E+00 | 0.000E+00 | 0.000E+00 | 9.123E-07 | 0.000E+00 | 0.000E+00 | 0.000E+00 | 0.000E+00 | 0.000E+00 | 0.000E+00 |
| Unigene366000 | efrA | 0.000E+00 | 0.000E+00 | 0.000E+00 | 0.000E+00 | 0.000E+00 | 9.852E-07 | 0.000E+00 | 9.506E-06 | 0.000E+00 | 0.000E+00 | 1.695E-05 | 0.000E+00 |
| Unigene366128 | cpxA | 0.000E+00 | 0.000E+00 | 0.000E+00 | 0.000E+00 | 0.000E+00 | 1.082E-07 | 6.093E-07 | 0.000E+00 | 0.000E+00 | 0.000E+00 | 0.000E+00 | 3.549E-07 |
| Unigene366166 | efrA | 0.000E+00 | 0.000E+00 | 0.000E+00 | 0.000E+00 | 0.000E+00 | 5.113E-07 | 1.745E-07 | 0.000E+00 | 0.000E+00 | 0.000E+00 | 0.000E+00 | 3.960E-06 |
| Unigene366178 | vanG | 9.565E-08 | 1.155E-06 | 6.100E-07 | 0.000E+00 | 5.419E-06 | 3.619E-07 | 0.000E+00 | 5.024E-07 | 0.000E+00 | 0.000E+00 | 0.000E+00 | 0.000E+00 |
| Unigene366224 | cpxA | 0.000E+00 | 0.000E+00 | 0.000E+00 | 4.188E-07 | 4.608E-07 | 9.216E-07 | 3.341E-06 | 2.421E-06 | 0.000E+00 | 0.000E+00 | 0.000E+00 | 2.206E-06 |
| Unigene366282 | optrA | 0.000E+00 | 0.000E+00 | 0.000E+00 | 0.000E+00 | 0.000E+00 | 1.296E-06 | 1.843E-08 | 0.000E+00 | 0.000E+00 | 0.000E+00 | 0.000E+00 | 0.000E+00 |
| Unigene366381 | mefE | 0.000E+00 | 0.000E+00 | 0.000E+00 | 0.000E+00 | 0.000E+00 | 1.929E-06 | 0.000E+00 | 0.000E+00 | 0.000E+00 | 0.000E+00 | 0.000E+00 | 0.000E+00 |
| Unigene366411 | patB | 0.000E+00 | 0.000E+00 | 0.000E+00 | 0.000E+00 | 0.000E+00 | 7.413E-07 | 0.000E+00 | 0.000E+00 | 0.000E+00 | 0.000E+00 | 0.000E+00 | 0.000E+00 |
| Unigene366412 | efrA | 0.000E+00 | 0.000E+00 | 0.000E+00 | 0.000E+00 | 0.000E+00 | 1.288E-06 | 0.000E+00 | 0.000E+00 | 0.000E+00 | 0.000E+00 | 0.000E+00 | 0.000E+00 |
| Unigene366492 | cmlB1 | 0.000E+00 | 0.000E+00 | 0.000E+00 | 0.000E+00 | 0.000E+00 | 8.812E-07 | 0.000E+00 | 0.000E+00 | 0.000E+00 | 0.000E+00 | 0.000E+00 | 0.000E+00 |
| Unigene366511 | poxtA | 0.000E+00 | 0.000E+00 | 0.000E+00 | 0.000E+00 | 0.000E+00 | 1.313E-06 | 0.000E+00 | 0.000E+00 | 0.000E+00 | 0.000E+00 | 0.000E+00 | 0.000E+00 |
| Unigene366525 | tetA(46) | 0.000E+00 | 0.000E+00 | 0.000E+00 | 0.000E+00 | 0.000E+00 | 7.954E-06 | 0.000E+00 | 0.000E+00 | 3.114E-06 | 0.000E+00 | 0.000E+00 | 0.000E+00 |
| Unigene366529 | macA | 0.000E+00 | 0.000E+00 | 0.000E+00 | 0.000E+00 | 0.000E+00 | 5.036E-07 | 0.000E+00 | 0.000E+00 | 0.000E+00 | 0.000E+00 | 0.000E+00 | 0.000E+00 |
| Unigene366549 | Staphylocc | 0.000E+00 | 0.000E+00 | 0.000E+00 | 0.000E+00 | 0.000E+00 | 6.269E-07 | 0.000E+00 | 0.000E+00 | 0.000E+00 | 0.000E+00 | 0.000E+00 | 1.637E-06 |
| Unigene366653 | baeS | 0.000E+00 | 0.000E+00 | 0.000E+00 | 0.000E+00 | 0.000E+00 | 5.729E-07 | 0.000E+00 | 0.000E+00 | 0.000E+00 | 0.000E+00 | 0.000E+00 | 0.000E+00 |
| Unigene366709 | lmrC | 0.000E+00 | 0.000E+00 | 0.000E+00 | 0.000E+00 | 0.000E+00 | 5.881E-07 | 0.000E+00 | 0.000E+00 | 0.000E+00 | 0.000E+00 | 0.000E+00 | 0.000E+00 |
| Unigene366815 | lmrD | 0.000E+00 | 0.000E+00 | 4.611E-08 | 0.000E+00 | 0.000E+00 | 6.095E-07 | 2.080E-07 | 2.742E-07 | 0.000E+00 | 0.000E+00 | 0.000E+00 | 5.054E-06 |

| | | | | | | | | | | | | | |
|---|---|---|---|---|---|---|---|---|---|---|---|---|---|
| Unigene366902 | vanHD | 0.000E+00 | 0.000E+00 | 0.000E+00 | 0.000E+00 | 0.000E+00 | 1.630E-06 | 0.000E+00 | 0.000E+00 | 0.000E+00 | 0.000E+00 | 0.000E+00 | 0.000E+00 |
| Unigene366983 | efrB | 0.000E+00 | 0.000E+00 | 0.000E+00 | 0.000E+00 | 0.000E+00 | 2.737E-07 | 0.000E+00 | 0.000E+00 | 0.000E+00 | 0.000E+00 | 0.000E+00 | 0.000E+00 |
| Unigene367028 | tetA(58) | 0.000E+00 | 0.000E+00 | 0.000E+00 | 0.000E+00 | 0.000E+00 | 4.449E-07 | 0.000E+00 | 0.000E+00 | 0.000E+00 | 0.000E+00 | 0.000E+00 | 0.000E+00 |
| Unigene367060 | cpxA | 0.000E+00 | 0.000E+00 | 0.000E+00 | 0.000E+00 | 0.000E+00 | 0.000E+00 | 0.000E+00 | 0.000E+00 | 0.000E+00 | 0.000E+00 | 5.912E-07 | 0.000E+00 |
| Unigene367178 | evgS | 0.000E+00 | 0.000E+00 | 0.000E+00 | 0.000E+00 | 0.000E+00 | 6.498E-07 | 0.000E+00 | 0.000E+00 | 0.000E+00 | 0.000E+00 | 0.000E+00 | 0.000E+00 |
| Unigene367266 | patB | 0.000E+00 | 0.000E+00 | 0.000E+00 | 0.000E+00 | 0.000E+00 | 9.306E-07 | 0.000E+00 | 5.415E-06 | 0.000E+00 | 0.000E+00 | 0.000E+00 | 0.000E+00 |
| Unigene367296 | macB | 0.000E+00 | 0.000E+00 | 0.000E+00 | 0.000E+00 | 0.000E+00 | 5.845E-07 | 0.000E+00 | 0.000E+00 | 0.000E+00 | 0.000E+00 | 0.000E+00 | 0.000E+00 |
| Unigene367333 | golS | 0.000E+00 | 0.000E+00 | 0.000E+00 | 0.000E+00 | 0.000E+00 | 6.411E-07 | 0.000E+00 | 0.000E+00 | 0.000E+00 | 0.000E+00 | 0.000E+00 | 0.000E+00 |
| Unigene367386 | vanTrL | 9.542E-05 | 3.985E-05 | 5.729E-07 | 8.423E-08 | 2.125E-05 | 7.764E-05 | 0.000E+00 | 0.000E+00 | 0.000E+00 | 1.858E-07 | 0.000E+00 | 0.000E+00 |
| Unigene367393 | bcr-1 | 1.530E-07 | 0.000E+00 | 0.000E+00 | 1.030E-07 | 0.000E+00 | 3.308E-07 | 0.000E+00 | 0.000E+00 | 0.000E+00 | 0.000E+00 | 1.750E-07 | 1.206E-07 |
| Unigene367467 | farA | 0.000E+00 | 0.000E+00 | 0.000E+00 | 0.000E+00 | 0.000E+00 | 4.081E-07 | 0.000E+00 | 0.000E+00 | 0.000E+00 | 0.000E+00 | 0.000E+00 | 0.000E+00 |
| Unigene367509 | arlR | 5.728E-07 | 0.000E+00 | 0.000E+00 | 8.263E-08 | 0.000E+00 | 1.513E-05 | 1.811E-07 | 1.910E-07 | 2.817E-06 | 0.000E+00 | 0.000E+00 | 0.000E+00 |
| Unigene367510 | basS | 1.357E-06 | 0.000E+00 | 0.000E+00 | 1.958E-07 | 2.962E-06 | 1.428E-05 | 2.012E-06 | 2.235E-06 | 2.808E-06 | 0.000E+00 | 0.000E+00 | 0.000E+00 |
| Unigene367526 | mtrA | 0.000E+00 | 0.000E+00 | 0.000E+00 | 0.000E+00 | 0.000E+00 | 4.564E-06 | 0.000E+00 | 0.000E+00 | 0.000E+00 | 0.000E+00 | 0.000E+00 | 4.151E-06 |
| Unigene367586 | lmrB | 6.711E-07 | 0.000E+00 | 3.633E-07 | 4.673E-07 | 0.000E+00 | 9.631E-06 | 1.357E-06 | 7.995E-06 | 5.134E-06 | 0.000E+00 | 0.000E+00 | 8.206E-07 |
| Unigene367591 | PmrF | 0.000E+00 | 0.000E+00 | 0.000E+00 | 0.000E+00 | 0.000E+00 | 5.979E-07 | 0.000E+00 | 0.000E+00 | 0.000E+00 | 0.000E+00 | 0.000E+00 | 0.000E+00 |
| Unigene367617 | YojI | 0.000E+00 | 0.000E+00 | 0.000E+00 | 0.000E+00 | 0.000E+00 | 8.676E-07 | 0.000E+00 | 0.000E+00 | 0.000E+00 | 0.000E+00 | 0.000E+00 | 0.000E+00 |
| Unigene367706 | novA | 0.000E+00 | 0.000E+00 | 0.000E+00 | 0.000E+00 | 0.000E+00 | 8.309E-07 | 0.000E+00 | 0.000E+00 | 0.000E+00 | 0.000E+00 | 0.000E+00 | 0.000E+00 |
| Unigene367772 | macB | 0.000E+00 | 0.000E+00 | 0.000E+00 | 0.000E+00 | 0.000E+00 | 3.381E-07 | 0.000E+00 | 0.000E+00 | 0.000E+00 | 0.000E+00 | 0.000E+00 | 0.000E+00 |
| Unigene367846 | vanSM | 0.000E+00 | 0.000E+00 | 0.000E+00 | 0.000E+00 | 0.000E+00 | 1.600E-06 | 0.000E+00 | 0.000E+00 | 0.000E+00 | 0.000E+00 | 0.000E+00 | 0.000E+00 |
| Unigene367849 | mtrA | 0.000E+00 | 0.000E+00 | 0.000E+00 | 0.000E+00 | 0.000E+00 | 1.689E-06 | 0.000E+00 | 0.000E+00 | 0.000E+00 | 0.000E+00 | 0.000E+00 | 0.000E+00 |
| Unigene367901 | Rhodococ | 0.000E+00 | 0.000E+00 | 0.000E+00 | 0.000E+00 | 0.000E+00 | 1.098E-06 | 0.000E+00 | 7.593E-06 | 0.000E+00 | 0.000E+00 | 0.000E+00 | 0.000E+00 |
| Unigene367937 | Acinetoba | 0.000E+00 | 0.000E+00 | 0.000E+00 | 0.000E+00 | 0.000E+00 | 5.915E-07 | 0.000E+00 | 0.000E+00 | 0.000E+00 | 0.000E+00 | 0.000E+00 | 0.000E+00 |
| Unigene367986 | bcrA | 0.000E+00 | 0.000E+00 | 0.000E+00 | 0.000E+00 | 0.000E+00 | 4.592E-07 | 0.000E+00 | 0.000E+00 | 0.000E+00 | 0.000E+00 | 0.000E+00 | 4.351E-06 |
| Unigene367987 | patA | 1.470E-06 | 0.000E+00 | 1.803E-07 | 2.062E-08 | 1.626E-05 | 3.697E-05 | 0.000E+00 | 9.533E-08 | 0.000E+00 | 0.000E+00 | 9.342E-08 | 0.000E+00 |
| Unigene367993 | bcrA | 0.000E+00 | 0.000E+00 | 0.000E+00 | 0.000E+00 | 0.000E+00 | 6.789E-07 | 0.000E+00 | 9.872E-06 | 0.000E+00 | 0.000E+00 | 1.729E-05 | 0.000E+00 |
| Unigene368125 | efrA | 0.000E+00 | 0.000E+00 | 0.000E+00 | 0.000E+00 | 0.000E+00 | 0.000E+00 | 0.000E+00 | 1.151E-06 | 0.000E+00 | 0.000E+00 | 0.000E+00 | 0.000E+00 |
| Unigene368145 | vatE | 0.000E+00 | 0.000E+00 | 0.000E+00 | 0.000E+00 | 0.000E+00 | 1.382E-06 | 0.000E+00 | 0.000E+00 | 0.000E+00 | 0.000E+00 | 0.000E+00 | 0.000E+00 |
| Unigene368158 | efpA | 0.000E+00 | 0.000E+00 | 0.000E+00 | 0.000E+00 | 0.000E+00 | 1.171E-06 | 0.000E+00 | 0.000E+00 | 0.000E+00 | 0.000E+00 | 0.000E+00 | 0.000E+00 |
| Unigene368209 | vanHB | 4.331E-05 | 1.188E-04 | 1.117E-04 | 5.669E-06 | 6.237E-06 | 5.780E-07 | 2.515E-06 | 0.000E+00 | 3.762E-05 | 0.000E+00 | 1.605E-06 | 0.000E+00 |
| Unigene368301 | evgS | 0.000E+00 | 0.000E+00 | 0.000E+00 | 0.000E+00 | 0.000E+00 | 0.000E+00 | 0.000E+00 | 1.441E-06 | 0.000E+00 | 0.000E+00 | 0.000E+00 | 0.000E+00 |
| Unigene368504 | acrB | 0.000E+00 | 0.000E+00 | 0.000E+00 | 0.000E+00 | 2.391E-07 | 9.306E-08 | 0.000E+00 | 0.000E+00 | 0.000E+00 | 0.000E+00 | 0.000E+00 | 1.526E-07 |
| Unigene368513 | tetB(46) | 0.000E+00 | 0.000E+00 | 0.000E+00 | 0.000E+00 | 0.000E+00 | 1.319E-06 | 4.113E-06 | 4.305E-06 | 0.000E+00 | 0.000E+00 | 2.157E-06 | 4.229E-06 |
| Unigene368828 | ykkD | 0.000E+00 | 0.000E+00 | 0.000E+00 | 0.000E+00 | 0.000E+00 | 1.551E-06 | 0.000E+00 | 0.000E+00 | 0.000E+00 | 0.000E+00 | 0.000E+00 | 0.000E+00 |
| Unigene368975 | tetB(60) | 0.000E+00 | 0.000E+00 | 0.000E+00 | 0.000E+00 | 0.000E+00 | 1.131E-06 | 0.000E+00 | 0.000E+00 | 0.000E+00 | 0.000E+00 | 0.000E+00 | 0.000E+00 |
| Unigene369074 | farA | 0.000E+00 | 1.423E-07 | 0.000E+00 | 0.000E+00 | 0.000E+00 | 5.139E-07 | 0.000E+00 | 0.000E+00 | 0.000E+00 | 0.000E+00 | 0.000E+00 | 0.000E+00 |
| Unigene369202 | otr(B) | 0.000E+00 | 0.000E+00 | 0.000E+00 | 0.000E+00 | 0.000E+00 | 1.026E-06 | 0.000E+00 | 0.000E+00 | 0.000E+00 | 0.000E+00 | 0.000E+00 | 0.000E+00 |
| Unigene369206 | Listeria mc | 0.000E+00 | 0.000E+00 | 0.000E+00 | 0.000E+00 | 0.000E+00 | 1.324E-06 | 0.000E+00 | 0.000E+00 | 0.000E+00 | 0.000E+00 | 0.000E+00 | 0.000E+00 |
| Unigene369363 | optrA | 0.000E+00 | 0.000E+00 | 0.000E+00 | 0.000E+00 | 0.000E+00 | 5.655E-07 | 0.000E+00 | 0.000E+00 | 0.000E+00 | 0.000E+00 | 0.000E+00 | 0.000E+00 |
| Unigene369450 | vanHB | 0.000E+00 | 0.000E+00 | 0.000E+00 | 0.000E+00 | 0.000E+00 | 9.392E-07 | 0.000E+00 | 0.000E+00 | 0.000E+00 | 0.000E+00 | 0.000E+00 | 0.000E+00 |
| Unigene369453 | efrA | 2.391E-07 | 0.000E+00 | 0.000E+00 | 0.000E+00 | 0.000E+00 | 4.524E-07 | 1.588E-06 | 1.535E-06 | 0.000E+00 | 0.000E+00 | 0.000E+00 | 1.449E-06 |
| Unigene369512 | patB | 0.000E+00 | 0.000E+00 | 8.770E-07 | 0.000E+00 | 0.000E+00 | 7.433E-07 | 1.750E-06 | 0.000E+00 | 0.000E+00 | 8.808E-07 | 0.000E+00 | 3.373E-06 |
| Unigene369640 | Staphyloc | 0.000E+00 | 0.000E+00 | 0.000E+00 | 0.000E+00 | 0.000E+00 | 6.036E-07 | 0.000E+00 | 0.000E+00 | 0.000E+00 | 0.000E+00 | 0.000E+00 | 0.000E+00 |
| Unigene369669 | lsaA | 0.000E+00 | 0.000E+00 | 0.000E+00 | 0.000E+00 | 0.000E+00 | 5.945E-07 | 0.000E+00 | 5.108E-06 | 0.000E+00 | 0.000E+00 | 0.000E+00 | 0.000E+00 |
| Unigene369680 | oqxB | 0.000E+00 | 0.000E+00 | 0.000E+00 | 0.000E+00 | 0.000E+00 | 3.712E-07 | 0.000E+00 | 0.000E+00 | 0.000E+00 | 0.000E+00 | 0.000E+00 | 0.000E+00 |
| Unigene369753 | tetA(60) | 0.000E+00 | 0.000E+00 | 0.000E+00 | 0.000E+00 | 0.000E+00 | 1.129E-06 | 0.000E+00 | 0.000E+00 | 0.000E+00 | 0.000E+00 | 0.000E+00 | 0.000E+00 |
| Unigene369755 | oleB | 0.000E+00 | 0.000E+00 | 0.000E+00 | 0.000E+00 | 0.000E+00 | 7.781E-07 | 0.000E+00 | 0.000E+00 | 0.000E+00 | 0.000E+00 | 0.000E+00 | 0.000E+00 |
| Unigene369807 | TaeA | 0.000E+00 | 0.000E+00 | 0.000E+00 | 0.000E+00 | 0.000E+00 | 3.029E-07 | 0.000E+00 | 0.000E+00 | 0.000E+00 | 0.000E+00 | 0.000E+00 | 2.650E-07 |
| Unigene369828 | bcrA | 0.000E+00 | 0.000E+00 | 0.000E+00 | 0.000E+00 | 0.000E+00 | 9.496E-07 | 0.000E+00 | 0.000E+00 | 0.000E+00 | 0.000E+00 | 0.000E+00 | 0.000E+00 |
| Unigene369874 | PmrF | 0.000E+00 | 0.000E+00 | 0.000E+00 | 0.000E+00 | 0.000E+00 | 9.754E-07 | 0.000E+00 | 0.000E+00 | 0.000E+00 | 0.000E+00 | 0.000E+00 | 0.000E+00 |
| Unigene370164 | msbA | 0.000E+00 | 0.000E+00 | 0.000E+00 | 0.000E+00 | 0.000E+00 | 7.837E-07 | 0.000E+00 | 0.000E+00 | 0.000E+00 | 0.000E+00 | 0.000E+00 | 0.000E+00 |
| Unigene370165 | msbA | 0.000E+00 | 0.000E+00 | 0.000E+00 | 0.000E+00 | 0.000E+00 | 1.337E-06 | 0.000E+00 | 0.000E+00 | 0.000E+00 | 0.000E+00 | 0.000E+00 | 0.000E+00 |
| Unigene370200 | bcrA | 0.000E+00 | 0.000E+00 | 0.000E+00 | 0.000E+00 | 0.000E+00 | 4.230E-07 | 0.000E+00 | 0.000E+00 | 0.000E+00 | 0.000E+00 | 0.000E+00 | 0.000E+00 |
| Unigene370231 | patB | 0.000E+00 | 0.000E+00 | 0.000E+00 | 0.000E+00 | 0.000E+00 | 4.762E-07 | 0.000E+00 | 0.000E+00 | 0.000E+00 | 0.000E+00 | 6.478E-07 | 0.000E+00 |
| Unigene370317 | vanD | 0.000E+00 | 0.000E+00 | 0.000E+00 | 0.000E+00 | 0.000E+00 | 7.405E-07 | 0.000E+00 | 0.000E+00 | 0.000E+00 | 0.000E+00 | 0.000E+00 | 0.000E+00 |
| Unigene370323 | Chlamydia | 0.000E+00 | 0.000E+00 | 0.000E+00 | 0.000E+00 | 0.000E+00 | 1.026E-06 | 0.000E+00 | 0.000E+00 | 0.000E+00 | 0.000E+00 | 0.000E+00 | 0.000E+00 |
| Unigene370363 | optrA | 0.000E+00 | 0.000E+00 | 0.000E+00 | 0.000E+00 | 3.995E-07 | 5.382E-07 | 0.000E+00 | 0.000E+00 | 3.628E-07 | 0.000E+00 | 0.000E+00 | 2.027E-06 |
| Unigene370405 | bcr-1 | 0.000E+00 | 0.000E+00 | 0.000E+00 | 0.000E+00 | 0.000E+00 | 6.655E-07 | 0.000E+00 | 0.000E+00 | 0.000E+00 | 0.000E+00 | 0.000E+00 | 0.000E+00 |

| | | | | | | | | | | | | | |
|---|---|---|---|---|---|---|---|---|---|---|---|---|---|
| Unigene370427 | vanHA | 0.000E+00 | 0.000E+00 | 0.000E+00 | 0.000E+00 | 0.000E+00 | 1.086E-06 | 0.000E+00 | 1.052E-05 | 0.000E+00 | 0.000E+00 | 0.000E+00 | 0.000E+00 |
| Unigene370428 | vanHB | 0.000E+00 | 0.000E+00 | 0.000E+00 | 0.000E+00 | 0.000E+00 | 1.118E-06 | 0.000E+00 | 1.094E-05 | 0.000E+00 | 0.000E+00 | 0.000E+00 | 0.000E+00 |
| Unigene370543 | rpoB2 | 0.000E+00 | 0.000E+00 | 0.000E+00 | 0.000E+00 | 0.000E+00 | 0.000E+00 | 0.000E+00 | 0.000E+00 | 0.000E+00 | 0.000E+00 | 0.000E+00 | 3.437E-06 |
| Unigene370670 | cpxA | 0.000E+00 | 0.000E+00 | 0.000E+00 | 2.126E-07 | 0.000E+00 | 8.598E-07 | 2.537E-06 | 2.375E-06 | 0.000E+00 | 0.000E+00 | 0.000E+00 | 2.544E-06 |
| Unigene370731 | vanHM | 3.699E-07 | 0.000E+00 | 0.000E+00 | 0.000E+00 | 0.000E+00 | 3.090E-06 | 0.000E+00 | 0.000E+00 | 0.000E+00 | 0.000E+00 | 0.000E+00 | 0.000E+00 |
| Unigene370877 | kdpE | 0.000E+00 | 0.000E+00 | 1.308E-06 | 0.000E+00 | 8.251E-06 | 0.000E+00 | 0.000E+00 | 0.000E+00 | 0.000E+00 | 0.000E+00 | 0.000E+00 | 0.000E+00 |
| Unigene370879 | NmcR | 0.000E+00 | 0.000E+00 | 0.000E+00 | 0.000E+00 | 0.000E+00 | 4.324E-07 | 0.000E+00 | 0.000E+00 | 0.000E+00 | 0.000E+00 | 0.000E+00 | 0.000E+00 |
| Unigene370900 | oleC | 0.000E+00 | 0.000E+00 | 0.000E+00 | 0.000E+00 | 0.000E+00 | 3.598E-07 | 0.000E+00 | 0.000E+00 | 0.000E+00 | 0.000E+00 | 0.000E+00 | 0.000E+00 |
| Unigene370914 | rosB | 0.000E+00 | 0.000E+00 | 0.000E+00 | 0.000E+00 | 0.000E+00 | 1.175E-07 | 0.000E+00 | 0.000E+00 | 0.000E+00 | 0.000E+00 | 0.000E+00 | 1.285E-07 |
| Unigene370970 | bcrA | 4.974E-07 | 4.023E-07 | 7.964E-07 | 2.644E-07 | 6.399E-07 | 1.500E-06 | 5.013E-06 | 3.789E-06 | 4.578E-07 | 1.312E-06 | 2.156E-06 | 5.013E-06 |
| Unigene371013 | efrA | 0.000E+00 | 0.000E+00 | 0.000E+00 | 0.000E+00 | 0.000E+00 | 5.951E-07 | 0.000E+00 | 0.000E+00 | 0.000E+00 | 0.000E+00 | 0.000E+00 | 0.000E+00 |
| Unigene371066 | oleC | 0.000E+00 | 0.000E+00 | 0.000E+00 | 0.000E+00 | 0.000E+00 | 6.051E-07 | 0.000E+00 | 4.356E-07 | 0.000E+00 | 0.000E+00 | 0.000E+00 | 0.000E+00 |
| Unigene371124 | arlS | 6.554E-05 | 5.362E-05 | 1.082E-04 | 1.416E-04 | 1.184E-04 | 8.595E-05 | 1.084E-04 | 1.050E-04 | 9.003E-05 | 1.327E-04 | 1.329E-04 | 1.392E-04 |
| Unigene371285 | adeL | 0.000E+00 | 0.000E+00 | 0.000E+00 | 0.000E+00 | 0.000E+00 | 4.308E-07 | 0.000E+00 | 0.000E+00 | 0.000E+00 | 0.000E+00 | 0.000E+00 | 0.000E+00 |
| Unigene371330 | tetA(58) | 2.869E-07 | 2.699E-07 | 8.447E-07 | 5.381E-07 | 5.920E-07 | 1.196E-06 | 4.559E-06 | 1.507E-06 | 3.808E-07 | 6.621E-07 | 1.407E-06 | 3.125E-06 |
| Unigene371364 | Bifidobacte | 0.000E+00 | 0.000E+00 | 0.000E+00 | 0.000E+00 | 0.000E+00 | 3.466E-07 | 0.000E+00 | 2.573E-07 | 0.000E+00 | 0.000E+00 | 0.000E+00 | 0.000E+00 |
| Unigene371424 | Acinetobac | 0.000E+00 | 0.000E+00 | 0.000E+00 | 0.000E+00 | 4.084E-07 | 1.669E-06 | 3.879E-06 | 3.977E-06 | 4.554E-07 | 1.502E-06 | 2.019E-06 | 3.998E-06 |
| Unigene371439 | facT | 0.000E+00 | 0.000E+00 | 0.000E+00 | 0.000E+00 | 9.267E-08 | 0.000E+00 | 0.000E+00 | 0.000E+00 | 0.000E+00 | 0.000E+00 | 0.000E+00 | 1.479E-06 |
| Unigene371448 | Staphylocc | 0.000E+00 | 0.000E+00 | 0.000E+00 | 0.000E+00 | 0.000E+00 | 8.200E-07 | 0.000E+00 | 0.000E+00 | 0.000E+00 | 0.000E+00 | 0.000E+00 | 0.000E+00 |
| Unigene371472 | dfrA3 | 0.000E+00 | 0.000E+00 | 0.000E+00 | 0.000E+00 | 0.000E+00 | 1.142E-06 | 0.000E+00 | 0.000E+00 | 0.000E+00 | 0.000E+00 | 0.000E+00 | 0.000E+00 |
| Unigene371474 | TaeA | 0.000E+00 | 0.000E+00 | 0.000E+00 | 0.000E+00 | 0.000E+00 | 1.009E-06 | 0.000E+00 | 0.000E+00 | 0.000E+00 | 0.000E+00 | 0.000E+00 | 0.000E+00 |
| Unigene371493 | lmrD | 0.000E+00 | 0.000E+00 | 0.000E+00 | 0.000E+00 | 0.000E+00 | 7.372E-07 | 0.000E+00 | 0.000E+00 | 0.000E+00 | 0.000E+00 | 0.000E+00 | 0.000E+00 |
| Unigene371525 | TaeA | 1.606E-07 | 0.000E+00 | 3.152E-07 | 0.000E+00 | 2.855E-07 | 6.250E-07 | 9.243E-07 | 7.873E-07 | 1.053E-07 | 4.651E-07 | 4.409E-07 | 2.506E-06 |
| Unigene371564 | OpmB | 0.000E+00 | 0.000E+00 | 0.000E+00 | 0.000E+00 | 0.000E+00 | 4.308E-07 | 0.000E+00 | 0.000E+00 | 0.000E+00 | 0.000E+00 | 0.000E+00 | 0.000E+00 |
| Unigene371583 | Staphylocc | 0.000E+00 | 0.000E+00 | 0.000E+00 | 0.000E+00 | 0.000E+00 | 5.030E-07 | 0.000E+00 | 0.000E+00 | 0.000E+00 | 0.000E+00 | 7.318E-07 | 3.437E-07 |
| Unigene371619 | evgS | 0.000E+00 | 0.000E+00 | 0.000E+00 | 0.000E+00 | 0.000E+00 | 2.770E-07 | 0.000E+00 | 5.981E-07 | 0.000E+00 | 0.000E+00 | 0.000E+00 | 0.000E+00 |
| Unigene371671 | patB | 0.000E+00 | 0.000E+00 | 0.000E+00 | 0.000E+00 | 0.000E+00 | 1.241E-06 | 0.000E+00 | 0.000E+00 | 0.000E+00 | 0.000E+00 | 0.000E+00 | 0.000E+00 |
| Unigene371746 | bcrA | 0.000E+00 | 0.000E+00 | 0.000E+00 | 0.000E+00 | 0.000E+00 | 9.729E-07 | 0.000E+00 | 5.937E-06 | 0.000E+00 | 0.000E+00 | 0.000E+00 | 0.000E+00 |
| Unigene371752 | Staphylocc | 0.000E+00 | 0.000E+00 | 0.000E+00 | 0.000E+00 | 0.000E+00 | 7.050E-07 | 0.000E+00 | 5.247E-06 | 0.000E+00 | 0.000E+00 | 0.000E+00 | 0.000E+00 |
| Unigene371854 | mtrA | 0.000E+00 | 0.000E+00 | 2.651E-07 | 0.000E+00 | 0.000E+00 | 5.354E-07 | 4.484E-07 | 4.204E-07 | 0.000E+00 | 0.000E+00 | 0.000E+00 | 0.000E+00 |
| Unigene372007 | mdtC | 0.000E+00 | 0.000E+00 | 0.000E+00 | 5.125E-08 | 0.000E+00 | 3.841E-07 | 0.000E+00 | 0.000E+00 | 0.000E+00 | 0.000E+00 | 3.483E-07 | 3.000E-07 |
| Unigene372038 | mef(B) | 0.000E+00 | 0.000E+00 | 0.000E+00 | 0.000E+00 | 0.000E+00 | 5.410E-07 | 0.000E+00 | 0.000E+00 | 0.000E+00 | 0.000E+00 | 0.000E+00 | 0.000E+00 |
| Unigene372085 | Brucella su | 0.000E+00 | 0.000E+00 | 0.000E+00 | 0.000E+00 | 0.000E+00 | 5.236E-07 | 0.000E+00 | 1.212E-07 | 0.000E+00 | 0.000E+00 | 0.000E+00 | 0.000E+00 |
| Unigene372243 | lmrD | 0.000E+00 | 0.000E+00 | 0.000E+00 | 0.000E+00 | 0.000E+00 | 3.752E-07 | 0.000E+00 | 0.000E+00 | 0.000E+00 | 0.000E+00 | 5.459E-07 | 0.000E+00 |
| Unigene372289 | patA | 0.000E+00 | 0.000E+00 | 0.000E+00 | 0.000E+00 | 0.000E+00 | 3.635E-07 | 0.000E+00 | 0.000E+00 | 0.000E+00 | 0.000E+00 | 0.000E+00 | 0.000E+00 |
| Unigene372317 | efrA | 0.000E+00 | 0.000E+00 | 0.000E+00 | 0.000E+00 | 0.000E+00 | 5.263E-07 | 0.000E+00 | 0.000E+00 | 0.000E+00 | 0.000E+00 | 0.000E+00 | 0.000E+00 |
| Unigene372337 | acrB | 0.000E+00 | 0.000E+00 | 0.000E+00 | 0.000E+00 | 0.000E+00 | 3.525E-07 | 0.000E+00 | 4.757E-07 | 0.000E+00 | 0.000E+00 | 0.000E+00 | 0.000E+00 |
| Unigene372389 | baeS | 0.000E+00 | 0.000E+00 | 0.000E+00 | 0.000E+00 | 0.000E+00 | 7.755E-07 | 0.000E+00 | 0.000E+00 | 0.000E+00 | 0.000E+00 | 0.000E+00 | 0.000E+00 |
| Unigene372390 | arlR | 0.000E+00 | 0.000E+00 | 0.000E+00 | 0.000E+00 | 0.000E+00 | 1.889E-06 | 0.000E+00 | 0.000E+00 | 0.000E+00 | 0.000E+00 | 0.000E+00 | 0.000E+00 |
| Unigene372417 | Streptomy | 0.000E+00 | 0.000E+00 | 0.000E+00 | 0.000E+00 | 0.000E+00 | 1.318E-06 | 0.000E+00 | 0.000E+00 | 0.000E+00 | 0.000E+00 | 0.000E+00 | 0.000E+00 |
| Unigene372471 | lmrB | 0.000E+00 | 0.000E+00 | 0.000E+00 | 0.000E+00 | 0.000E+00 | 1.161E-06 | 0.000E+00 | 0.000E+00 | 0.000E+00 | 0.000E+00 | 0.000E+00 | 0.000E+00 |
| Unigene372689 | RSA-1 | 5.518E-07 | 0.000E+00 | 1.969E-07 | 1.520E-07 | 0.000E+00 | 8.758E-06 | 5.274E-07 | 0.000E+00 | 7.676E-07 | 0.000E+00 | 0.000E+00 | 0.000E+00 |
| Unigene372711 | dfrG | 0.000E+00 | 0.000E+00 | 0.000E+00 | 0.000E+00 | 0.000E+00 | 1.206E-06 | 0.000E+00 | 0.000E+00 | 0.000E+00 | 0.000E+00 | 0.000E+00 | 0.000E+00 |
| Unigene372764 | msbA | 0.000E+00 | 0.000E+00 | 0.000E+00 | 0.000E+00 | 0.000E+00 | 1.489E-06 | 0.000E+00 | 0.000E+00 | 0.000E+00 | 0.000E+00 | 0.000E+00 | 0.000E+00 |
| Unigene372816 | Staphylocc | 0.000E+00 | 0.000E+00 | 0.000E+00 | 0.000E+00 | 0.000E+00 | 1.812E-06 | 0.000E+00 | 0.000E+00 | 0.000E+00 | 0.000E+00 | 0.000E+00 | 0.000E+00 |
| Unigene372818 | bmr | 0.000E+00 | 0.000E+00 | 0.000E+00 | 0.000E+00 | 0.000E+00 | 1.595E-06 | 0.000E+00 | 0.000E+00 | 0.000E+00 | 0.000E+00 | 0.000E+00 | 0.000E+00 |
| Unigene372819 | tetA(58) | 0.000E+00 | 0.000E+00 | 0.000E+00 | 0.000E+00 | 0.000E+00 | 1.254E-06 | 0.000E+00 | 0.000E+00 | 0.000E+00 | 0.000E+00 | 0.000E+00 | 0.000E+00 |
| Unigene372834 | lsaA | 0.000E+00 | 0.000E+00 | 0.000E+00 | 0.000E+00 | 0.000E+00 | 1.520E-06 | 0.000E+00 | 0.000E+00 | 0.000E+00 | 0.000E+00 | 0.000E+00 | 0.000E+00 |
| Unigene372970 | macB | 0.000E+00 | 0.000E+00 | 0.000E+00 | 0.000E+00 | 0.000E+00 | 0.000E+00 | 0.000E+00 | 4.486E-07 | 0.000E+00 | 0.000E+00 | 0.000E+00 | 0.000E+00 |
| Unigene373048 | MexW | 0.000E+00 | 0.000E+00 | 0.000E+00 | 0.000E+00 | 0.000E+00 | 2.475E-07 | 0.000E+00 | 0.000E+00 | 0.000E+00 | 0.000E+00 | 0.000E+00 | 0.000E+00 |
| Unigene373072 | Chlamydia | 0.000E+00 | 0.000E+00 | 0.000E+00 | 0.000E+00 | 0.000E+00 | 4.784E-07 | 0.000E+00 | 0.000E+00 | 0.000E+00 | 0.000E+00 | 0.000E+00 | 3.597E-07 |
| Unigene373142 | Streptomy | 0.000E+00 | 0.000E+00 | 0.000E+00 | 0.000E+00 | 0.000E+00 | 8.787E-06 | 0.000E+00 | 0.000E+00 | 0.000E+00 | 0.000E+00 | 0.000E+00 | 0.000E+00 |
| Unigene373332 | efpA | 4.653E-07 | 0.000E+00 | 0.000E+00 | 0.000E+00 | 0.000E+00 | 4.653E-06 | 0.000E+00 | 0.000E+00 | 0.000E+00 | 0.000E+00 | 0.000E+00 | 0.000E+00 |
| Unigene373413 | Streptomy | 0.000E+00 | 0.000E+00 | 0.000E+00 | 0.000E+00 | 0.000E+00 | 9.002E-07 | 0.000E+00 | 0.000E+00 | 0.000E+00 | 0.000E+00 | 0.000E+00 | 0.000E+00 |
| Unigene373482 | Staphylocc | 0.000E+00 | 0.000E+00 | 0.000E+00 | 0.000E+00 | 0.000E+00 | 4.211E-07 | 0.000E+00 | 0.000E+00 | 0.000E+00 | 0.000E+00 | 0.000E+00 | 0.000E+00 |
| Unigene373501 | optrA | 0.000E+00 | 0.000E+00 | 0.000E+00 | 0.000E+00 | 0.000E+00 | 9.211E-07 | 0.000E+00 | 0.000E+00 | 0.000E+00 | 0.000E+00 | 0.000E+00 | 0.000E+00 |
| Unigene373567 | mgrA | 0.000E+00 | 0.000E+00 | 0.000E+00 | 0.000E+00 | 0.000E+00 | 7.347E-07 | 3.761E-07 | 0.000E+00 | 0.000E+00 | 0.000E+00 | 2.591E-07 | 3.481E-06 |
| Unigene373618 | macB | 0.000E+00 | 0.000E+00 | 0.000E+00 | 0.000E+00 | 1.278E-07 | 0.000E+00 | 0.000E+00 | 6.045E-07 | 0.000E+00 | 0.000E+00 | 1.316E-07 | 0.000E+00 |

| | | | | | | | | | | | | | |
|---|---|---|---|---|---|---|---|---|---|---|---|---|---|
| Unigene373640 | optrA | 0.000E+00 | 0.000E+00 | 0.000E+00 | 0.000E+00 | 0.000E+00 | 5.628E-07 | 0.000E+00 | 0.000E+00 | 0.000E+00 | 0.000E+00 | 0.000E+00 | 0.000E+00 |
| Unigene373681 | vanSE | 1.679E-07 | 0.000E+00 | 0.000E+00 | 2.261E-07 | 3.731E-07 | 5.597E-07 | 1.301E-06 | 1.503E-06 | 3.059E-07 | 5.300E-07 | 4.802E-07 | 1.604E-06 |
| Unigene373741 | vanRF | 0.000E+00 | 0.000E+00 | 0.000E+00 | 0.000E+00 | 0.000E+00 | 1.011E-06 | 0.000E+00 | 9.720E-06 | 0.000E+00 | 0.000E+00 | 1.589E-05 | 0.000E+00 |
| Unigene373926 | vanHD | 0.000E+00 | 0.000E+00 | 0.000E+00 | 0.000E+00 | 0.000E+00 | 9.840E-07 | 0.000E+00 | 9.942E-06 | 0.000E+00 | 0.000E+00 | 0.000E+00 | 0.000E+00 |
| Unigene373996 | otr(B) | 0.000E+00 | 0.000E+00 | 0.000E+00 | 0.000E+00 | 0.000E+00 | 6.147E-07 | 0.000E+00 | 0.000E+00 | 0.000E+00 | 0.000E+00 | 0.000E+00 | 3.619E-07 |
| Unigene374013 | NmcR | 0.000E+00 | 0.000E+00 | 0.000E+00 | 0.000E+00 | 0.000E+00 | 3.506E-06 | 0.000E+00 | 0.000E+00 | 0.000E+00 | 0.000E+00 | 0.000E+00 | 0.000E+00 |
| Unigene374041 | Staphylocc | 0.000E+00 | 0.000E+00 | 0.000E+00 | 0.000E+00 | 0.000E+00 | 0.000E+00 | 3.120E-07 | 0.000E+00 | 0.000E+00 | 0.000E+00 | 0.000E+00 | 0.000E+00 |
| Unigene374069 | tetA(46) | 0.000E+00 | 0.000E+00 | 0.000E+00 | 0.000E+00 | 0.000E+00 | 6.500E-07 | 0.000E+00 | 0.000E+00 | 0.000E+00 | 0.000E+00 | 0.000E+00 | 4.115E-07 |
| Unigene374191 | vanHB | 2.539E-07 | 0.000E+00 | 9.344E-07 | 0.000E+00 | 7.758E-07 | 9.951E-07 | 1.054E-06 | 1.297E-06 | 0.000E+00 | 0.000E+00 | 1.271E-06 | 3.001E-06 |
| Unigene374272 | Staphylocc | 0.000E+00 | 0.000E+00 | 0.000E+00 | 0.000E+00 | 0.000E+00 | 1.616E-06 | 0.000E+00 | 0.000E+00 | 0.000E+00 | 0.000E+00 | 0.000E+00 | 0.000E+00 |
| Unigene374275 | Erm(48) | 0.000E+00 | 0.000E+00 | 0.000E+00 | 0.000E+00 | 0.000E+00 | 1.097E-06 | 0.000E+00 | 0.000E+00 | 0.000E+00 | 0.000E+00 | 0.000E+00 | 0.000E+00 |
| Unigene374300 | macB | 0.000E+00 | 0.000E+00 | 0.000E+00 | 0.000E+00 | 0.000E+00 | 5.453E-07 | 0.000E+00 | 0.000E+00 | 0.000E+00 | 0.000E+00 | 0.000E+00 | 4.968E-08 |
| Unigene374390 | catQ | 0.000E+00 | 0.000E+00 | 0.000E+00 | 0.000E+00 | 0.000E+00 | 9.867E-07 | 0.000E+00 | 0.000E+00 | 0.000E+00 | 0.000E+00 | 0.000E+00 | 0.000E+00 |
| Unigene374631 | bcrA | 2.114E-07 | 0.000E+00 | 0.000E+00 | 8.539E-08 | 0.000E+00 | 6.856E-07 | 1.334E-06 | 4.442E-07 | 1.155E-07 | 1.648E-07 | 9.673E-08 | 1.499E-06 |
| Unigene374658 | efrB | 0.000E+00 | 0.000E+00 | 0.000E+00 | 0.000E+00 | 0.000E+00 | 1.423E-06 | 0.000E+00 | 9.286E-06 | 0.000E+00 | 0.000E+00 | 1.656E-05 | 0.000E+00 |
| Unigene375029 | YojI | 0.000E+00 | 0.000E+00 | 0.000E+00 | 0.000E+00 | 0.000E+00 | 4.081E-07 | 0.000E+00 | 0.000E+00 | 0.000E+00 | 0.000E+00 | 0.000E+00 | 0.000E+00 |
| Unigene375294 | tetA(58) | 0.000E+00 | 0.000E+00 | 0.000E+00 | 0.000E+00 | 0.000E+00 | 8.724E-07 | 0.000E+00 | 0.000E+00 | 0.000E+00 | 0.000E+00 | 0.000E+00 | 0.000E+00 |
| Unigene375415 | TaeA | 0.000E+00 | 0.000E+00 | 0.000E+00 | 0.000E+00 | 0.000E+00 | 7.566E-07 | 0.000E+00 | 8.833E-06 | 0.000E+00 | 0.000E+00 | 0.000E+00 | 0.000E+00 |
| Unigene375492 | vanSN | 0.000E+00 | 0.000E+00 | 0.000E+00 | 0.000E+00 | 0.000E+00 | 3.231E-07 | 0.000E+00 | 0.000E+00 | 0.000E+00 | 0.000E+00 | 0.000E+00 | 0.000E+00 |
| Unigene375677 | emrB | 0.000E+00 | 0.000E+00 | 0.000E+00 | 0.000E+00 | 0.000E+00 | 7.694E-07 | 0.000E+00 | 0.000E+00 | 0.000E+00 | 0.000E+00 | 0.000E+00 | 0.000E+00 |
| Unigene375694 | mdtG | 0.000E+00 | 0.000E+00 | 0.000E+00 | 0.000E+00 | 0.000E+00 | 4.680E-07 | 0.000E+00 | 0.000E+00 | 0.000E+00 | 0.000E+00 | 0.000E+00 | 1.462E-07 |
| Unigene375699 | sul4 | 0.000E+00 | 0.000E+00 | 0.000E+00 | 0.000E+00 | 0.000E+00 | 6.647E-07 | 0.000E+00 | 0.000E+00 | 0.000E+00 | 0.000E+00 | 3.907E-07 | 0.000E+00 |
| Unigene375727 | vanHO | 0.000E+00 | 0.000E+00 | 0.000E+00 | 0.000E+00 | 0.000E+00 | 9.431E-07 | 0.000E+00 | 0.000E+00 | 0.000E+00 | 0.000E+00 | 0.000E+00 | 0.000E+00 |
| Unigene375768 | poxtA | 0.000E+00 | 0.000E+00 | 0.000E+00 | 0.000E+00 | 0.000E+00 | 1.032E-06 | 0.000E+00 | 6.482E-06 | 0.000E+00 | 0.000E+00 | 0.000E+00 | 0.000E+00 |
| Unigene375779 | macB | 0.000E+00 | 0.000E+00 | 0.000E+00 | 0.000E+00 | 0.000E+00 | 1.323E-06 | 0.000E+00 | 7.388E-06 | 0.000E+00 | 0.000E+00 | 0.000E+00 | 0.000E+00 |
| Unigene375782 | lmrB | 0.000E+00 | 0.000E+00 | 0.000E+00 | 0.000E+00 | 0.000E+00 | 4.043E-06 | 0.000E+00 | 0.000E+00 | 0.000E+00 | 0.000E+00 | 0.000E+00 | 0.000E+00 |
| Unigene375830 | macB | 0.000E+00 | 0.000E+00 | 0.000E+00 | 0.000E+00 | 0.000E+00 | 1.241E-06 | 0.000E+00 | 0.000E+00 | 0.000E+00 | 0.000E+00 | 0.000E+00 | 0.000E+00 |
| Unigene375849 | vanSA | 0.000E+00 | 0.000E+00 | 0.000E+00 | 0.000E+00 | 0.000E+00 | 2.983E-07 | 0.000E+00 | 0.000E+00 | 0.000E+00 | 0.000E+00 | 0.000E+00 | 0.000E+00 |
| Unigene375954 | vanHB | 0.000E+00 | 0.000E+00 | 0.000E+00 | 0.000E+00 | 0.000E+00 | 1.553E-06 | 0.000E+00 | 0.000E+00 | 0.000E+00 | 0.000E+00 | 0.000E+00 | 0.000E+00 |
| Unigene375957 | TaeA | 0.000E+00 | 0.000E+00 | 0.000E+00 | 0.000E+00 | 0.000E+00 | 1.121E-06 | 0.000E+00 | 0.000E+00 | 0.000E+00 | 0.000E+00 | 0.000E+00 | 0.000E+00 |
| Unigene376079 | rpoB2 | 0.000E+00 | 0.000E+00 | 0.000E+00 | 0.000E+00 | 0.000E+00 | 5.057E-07 | 0.000E+00 | 0.000E+00 | 0.000E+00 | 0.000E+00 | 0.000E+00 | 0.000E+00 |
| Unigene376090 | macB | 0.000E+00 | 0.000E+00 | 0.000E+00 | 0.000E+00 | 0.000E+00 | 6.087E-07 | 0.000E+00 | 0.000E+00 | 0.000E+00 | 0.000E+00 | 0.000E+00 | 0.000E+00 |
| Unigene376192 | otr(B) | 0.000E+00 | 0.000E+00 | 0.000E+00 | 0.000E+00 | 0.000E+00 | 3.972E-07 | 0.000E+00 | 0.000E+00 | 0.000E+00 | 0.000E+00 | 0.000E+00 | 0.000E+00 |
| Unigene376237 | mtrA | 0.000E+00 | 0.000E+00 | 0.000E+00 | 0.000E+00 | 0.000E+00 | 0.000E+00 | 0.000E+00 | 0.000E+00 | 0.000E+00 | 0.000E+00 | 0.000E+00 | 3.401E-07 |
| Unigene376247 | cmlv | 0.000E+00 | 0.000E+00 | 0.000E+00 | 0.000E+00 | 0.000E+00 | 3.910E-07 | 0.000E+00 | 0.000E+00 | 0.000E+00 | 0.000E+00 | 0.000E+00 | 7.482E-07 |
| Unigene376261 | TaeA | 0.000E+00 | 0.000E+00 | 0.000E+00 | 0.000E+00 | 0.000E+00 | 8.086E-07 | 0.000E+00 | 9.167E-06 | 0.000E+00 | 0.000E+00 | 1.400E-05 | 0.000E+00 |
| Unigene376286 | mtrA | 0.000E+00 | 0.000E+00 | 0.000E+00 | 0.000E+00 | 0.000E+00 | 1.947E-06 | 0.000E+00 | 0.000E+00 | 0.000E+00 | 0.000E+00 | 0.000E+00 | 0.000E+00 |
| Unigene376294 | tetB(58) | 0.000E+00 | 0.000E+00 | 0.000E+00 | 0.000E+00 | 0.000E+00 | 1.258E-06 | 0.000E+00 | 0.000E+00 | 0.000E+00 | 0.000E+00 | 0.000E+00 | 0.000E+00 |
| Unigene376295 | tetA(58) | 0.000E+00 | 0.000E+00 | 0.000E+00 | 0.000E+00 | 0.000E+00 | 1.976E-06 | 0.000E+00 | 0.000E+00 | 0.000E+00 | 0.000E+00 | 0.000E+00 | 0.000E+00 |
| Unigene376301 | efrA | 0.000E+00 | 0.000E+00 | 0.000E+00 | 0.000E+00 | 0.000E+00 | 8.779E-07 | 0.000E+00 | 0.000E+00 | 0.000E+00 | 0.000E+00 | 0.000E+00 | 0.000E+00 |
| Unigene376316 | Staphylocc | 0.000E+00 | 0.000E+00 | 0.000E+00 | 0.000E+00 | 0.000E+00 | 1.309E-06 | 0.000E+00 | 0.000E+00 | 0.000E+00 | 0.000E+00 | 0.000E+00 | 0.000E+00 |
| Unigene376319 | vanHA | 0.000E+00 | 0.000E+00 | 0.000E+00 | 0.000E+00 | 0.000E+00 | 1.933E-06 | 3.733E-08 | 0.000E+00 | 0.000E+00 | 0.000E+00 | 0.000E+00 | 0.000E+00 |
| Unigene376405 | efrB | 0.000E+00 | 0.000E+00 | 0.000E+00 | 0.000E+00 | 0.000E+00 | 8.485E-06 | 0.000E+00 | 0.000E+00 | 2.421E-06 | 0.000E+00 | 0.000E+00 | 0.000E+00 |
| Unigene376436 | novA | 0.000E+00 | 0.000E+00 | 0.000E+00 | 0.000E+00 | 0.000E+00 | 4.179E-07 | 0.000E+00 | 0.000E+00 | 0.000E+00 | 0.000E+00 | 0.000E+00 | 0.000E+00 |
| Unigene376473 | oleC | 0.000E+00 | 0.000E+00 | 0.000E+00 | 0.000E+00 | 0.000E+00 | 9.779E-07 | 0.000E+00 | 0.000E+00 | 0.000E+00 | 0.000E+00 | 0.000E+00 | 0.000E+00 |
| Unigene376501 | PmrF | 0.000E+00 | 0.000E+00 | 0.000E+00 | 0.000E+00 | 0.000E+00 | 1.316E-06 | 0.000E+00 | 0.000E+00 | 0.000E+00 | 0.000E+00 | 0.000E+00 | 0.000E+00 |
| Unigene376545 | msbA | 0.000E+00 | 0.000E+00 | 0.000E+00 | 0.000E+00 | 0.000E+00 | 1.315E-06 | 8.157E-08 | 0.000E+00 | 0.000E+00 | 0.000E+00 | 0.000E+00 | 0.000E+00 |
| Unigene376645 | TaeA | 0.000E+00 | 0.000E+00 | 0.000E+00 | 0.000E+00 | 0.000E+00 | 9.431E-07 | 0.000E+00 | 0.000E+00 | 0.000E+00 | 0.000E+00 | 0.000E+00 | 0.000E+00 |
| Unigene376723 | efmA | 0.000E+00 | 0.000E+00 | 0.000E+00 | 0.000E+00 | 0.000E+00 | 8.313E-06 | 0.000E+00 | 0.000E+00 | 0.000E+00 | 0.000E+00 | 0.000E+00 | 0.000E+00 |
| Unigene376795 | tetA(60) | 0.000E+00 | 5.705E-07 | 8.161E-07 | 9.448E-07 | 4.562E-06 | 0.000E+00 | 4.602E-07 | 0.000E+00 | 0.000E+00 | 0.000E+00 | 0.000E+00 | 0.000E+00 |
| Unigene376821 | oqxA | 0.000E+00 | 0.000E+00 | 4.077E-08 | 0.000E+00 | 0.000E+00 | 0.000E+00 | 0.000E+00 | 0.000E+00 | 0.000E+00 | 3.702E-07 | 2.376E-07 | 1.964E-07 |
| Unigene376825 | MexB | 0.000E+00 | 0.000E+00 | 0.000E+00 | 0.000E+00 | 0.000E+00 | 4.445E-07 | 0.000E+00 | 0.000E+00 | 0.000E+00 | 0.000E+00 | 0.000E+00 | 0.000E+00 |
| Unigene376840 | carA | 0.000E+00 | 0.000E+00 | 0.000E+00 | 0.000E+00 | 0.000E+00 | 0.000E+00 | 0.000E+00 | 0.000E+00 | 0.000E+00 | 2.305E-07 | 0.000E+00 | 0.000E+00 |
| Unigene376872 | basS | 0.000E+00 | 0.000E+00 | 0.000E+00 | 0.000E+00 | 0.000E+00 | 1.070E-06 | 0.000E+00 | 0.000E+00 | 0.000E+00 | 0.000E+00 | 0.000E+00 | 9.746E-08 |
| Unigene376921 | PmrF | 0.000E+00 | 0.000E+00 | 0.000E+00 | 0.000E+00 | 0.000E+00 | 1.305E-06 | 0.000E+00 | 0.000E+00 | 0.000E+00 | 0.000E+00 | 0.000E+00 | 0.000E+00 |
| Unigene376969 | smeS | 0.000E+00 | 0.000E+00 | 0.000E+00 | 0.000E+00 | 0.000E+00 | 3.664E-07 | 0.000E+00 | 0.000E+00 | 0.000E+00 | 0.000E+00 | 0.000E+00 | 0.000E+00 |
| Unigene377036 | tetA(60) | 0.000E+00 | 0.000E+00 | 2.919E-07 | 1.502E-07 | 5.509E-07 | 4.288E-07 | 0.000E+00 | 0.000E+00 | 1.084E-07 | 0.000E+00 | 0.000E+00 | 0.000E+00 |
| Unigene377072 | evgA | 0.000E+00 | 0.000E+00 | 0.000E+00 | 0.000E+00 | 0.000E+00 | 3.216E-07 | 0.000E+00 | 0.000E+00 | 0.000E+00 | 0.000E+00 | 0.000E+00 | 0.000E+00 |

| | | | | | | | | | | | | | |
|---|---|---|---|---|---|---|---|---|---|---|---|---|---|
| Unigene377201 | evgS | 0.000E+00 | 0.000E+00 | 0.000E+00 | 0.000E+00 | 0.000E+00 | 2.614E-07 | 0.000E+00 | 0.000E+00 | 0.000E+00 | 0.000E+00 | 0.000E+00 | 0.000E+00 |
| Unigene377475 | vanHB | 0.000E+00 | 0.000E+00 | 0.000E+00 | 0.000E+00 | 0.000E+00 | 9.840E-07 | 0.000E+00 | 0.000E+00 | 0.000E+00 | 0.000E+00 | 0.000E+00 | 0.000E+00 |
| Unigene377490 | arlS | 0.000E+00 | 0.000E+00 | 0.000E+00 | 0.000E+00 | 0.000E+00 | 9.880E-07 | 0.000E+00 | 0.000E+00 | 0.000E+00 | 0.000E+00 | 0.000E+00 | 0.000E+00 |
| Unigene377491 | vanRM | 0.000E+00 | 0.000E+00 | 0.000E+00 | 0.000E+00 | 0.000E+00 | 8.872E-07 | 0.000E+00 | 0.000E+00 | 0.000E+00 | 0.000E+00 | 0.000E+00 | 0.000E+00 |
| Unigene377653 | sul3 | 0.000E+00 | 0.000E+00 | 0.000E+00 | 0.000E+00 | 0.000E+00 | 1.512E-06 | 0.000E+00 | 5.118E-06 | 0.000E+00 | 0.000E+00 | 0.000E+00 | 0.000E+00 |
| Unigene377720 | vanHO | 0.000E+00 | 0.000E+00 | 0.000E+00 | 0.000E+00 | 0.000E+00 | 1.523E-06 | 0.000E+00 | 0.000E+00 | 0.000E+00 | 0.000E+00 | 0.000E+00 | 0.000E+00 |
| Unigene377725 | Staphylocc | 0.000E+00 | 0.000E+00 | 0.000E+00 | 0.000E+00 | 0.000E+00 | 1.100E-06 | 0.000E+00 | 0.000E+00 | 0.000E+00 | 0.000E+00 | 0.000E+00 | 0.000E+00 |
| Unigene377728 | Erm(K) | 0.000E+00 | 0.000E+00 | 0.000E+00 | 0.000E+00 | 0.000E+00 | 1.732E-06 | 0.000E+00 | 0.000E+00 | 0.000E+00 | 0.000E+00 | 0.000E+00 | 0.000E+00 |
| Unigene377784 | vanHD | 0.000E+00 | 0.000E+00 | 0.000E+00 | 0.000E+00 | 0.000E+00 | 1.145E-06 | 0.000E+00 | 0.000E+00 | 0.000E+00 | 0.000E+00 | 0.000E+00 | 0.000E+00 |
| Unigene377786 | macB | 0.000E+00 | 0.000E+00 | 0.000E+00 | 0.000E+00 | 0.000E+00 | 1.489E-06 | 0.000E+00 | 0.000E+00 | 0.000E+00 | 0.000E+00 | 0.000E+00 | 0.000E+00 |
| Unigene377790 | cmlv | 0.000E+00 | 0.000E+00 | 0.000E+00 | 0.000E+00 | 0.000E+00 | 1.919E-06 | 2.977E-08 | 0.000E+00 | 0.000E+00 | 0.000E+00 | 0.000E+00 | 0.000E+00 |
| Unigene377885 | arlS | 0.000E+00 | 0.000E+00 | 0.000E+00 | 0.000E+00 | 0.000E+00 | 6.204E-07 | 0.000E+00 | 0.000E+00 | 0.000E+00 | 0.000E+00 | 0.000E+00 | 0.000E+00 |
| Unigene377968 | TaeA | 0.000E+00 | 0.000E+00 | 0.000E+00 | 0.000E+00 | 0.000E+00 | 4.722E-07 | 0.000E+00 | 0.000E+00 | 0.000E+00 | 0.000E+00 | 0.000E+00 | 0.000E+00 |
| Unigene378074 | Acinetobac | 0.000E+00 | 0.000E+00 | 0.000E+00 | 0.000E+00 | 0.000E+00 | 2.488E-07 | 0.000E+00 | 0.000E+00 | 0.000E+00 | 0.000E+00 | 0.000E+00 | 0.000E+00 |
| Unigene378078 | carA | 0.000E+00 | 0.000E+00 | 0.000E+00 | 0.000E+00 | 0.000E+00 | 4.092E-07 | 0.000E+00 | 0.000E+00 | 0.000E+00 | 0.000E+00 | 0.000E+00 | 0.000E+00 |
| Unigene378120 | efmA | 0.000E+00 | 0.000E+00 | 0.000E+00 | 0.000E+00 | 0.000E+00 | 1.512E-06 | 0.000E+00 | 0.000E+00 | 0.000E+00 | 0.000E+00 | 0.000E+00 | 0.000E+00 |
| Unigene378190 | tetA(58) | 0.000E+00 | 0.000E+00 | 0.000E+00 | 0.000E+00 | 0.000E+00 | 5.419E-07 | 0.000E+00 | 0.000E+00 | 0.000E+00 | 0.000E+00 | 6.498E-07 | 2.765E-07 |
| Unigene378214 | tetQ | 0.000E+00 | 0.000E+00 | 0.000E+00 | 0.000E+00 | 0.000E+00 | 1.495E-06 | 0.000E+00 | 0.000E+00 | 0.000E+00 | 0.000E+00 | 0.000E+00 | 0.000E+00 |
| Unigene378234 | oleC | 0.000E+00 | 0.000E+00 | 0.000E+00 | 0.000E+00 | 0.000E+00 | 1.901E-06 | 0.000E+00 | 0.000E+00 | 0.000E+00 | 0.000E+00 | 0.000E+00 | 0.000E+00 |
| Unigene378245 | YojI | 0.000E+00 | 0.000E+00 | 0.000E+00 | 0.000E+00 | 0.000E+00 | 1.599E-06 | 0.000E+00 | 0.000E+00 | 0.000E+00 | 0.000E+00 | 0.000E+00 | 0.000E+00 |
| Unigene378276 | macB | 0.000E+00 | 0.000E+00 | 0.000E+00 | 0.000E+00 | 0.000E+00 | 6.703E-07 | 0.000E+00 | 0.000E+00 | 0.000E+00 | 0.000E+00 | 0.000E+00 | 0.000E+00 |
| Unigene378287 | lsaA | 0.000E+00 | 0.000E+00 | 0.000E+00 | 0.000E+00 | 0.000E+00 | 8.125E-07 | 0.000E+00 | 1.039E-05 | 0.000E+00 | 0.000E+00 | 1.448E-05 | 0.000E+00 |
| Unigene378310 | patA | 0.000E+00 | 0.000E+00 | 0.000E+00 | 0.000E+00 | 0.000E+00 | 7.007E-07 | 0.000E+00 | 0.000E+00 | 0.000E+00 | 0.000E+00 | 0.000E+00 | 0.000E+00 |
| Unigene378507 | Escherichia | 0.000E+00 | 0.000E+00 | 0.000E+00 | 0.000E+00 | 0.000E+00 | 1.185E-06 | 0.000E+00 | 0.000E+00 | 0.000E+00 | 0.000E+00 | 0.000E+00 | 0.000E+00 |
| Unigene378509 | tet(38) | 0.000E+00 | 0.000E+00 | 0.000E+00 | 0.000E+00 | 0.000E+00 | 1.352E-06 | 0.000E+00 | 0.000E+00 | 0.000E+00 | 0.000E+00 | 0.000E+00 | 0.000E+00 |
| Unigene378546 | golS | 0.000E+00 | 0.000E+00 | 0.000E+00 | 0.000E+00 | 0.000E+00 | 0.000E+00 | 0.000E+00 | 0.000E+00 | 0.000E+00 | 0.000E+00 | 0.000E+00 | 2.135E-06 |
| Unigene378741 | ceoB | 0.000E+00 | 0.000E+00 | 0.000E+00 | 0.000E+00 | 0.000E+00 | 2.146E-07 | 0.000E+00 | 0.000E+00 | 0.000E+00 | 0.000E+00 | 0.000E+00 | 0.000E+00 |
| Unigene378852 | tlrC | 0.000E+00 | 0.000E+00 | 0.000E+00 | 0.000E+00 | 0.000E+00 | 2.457E-07 | 0.000E+00 | 0.000E+00 | 0.000E+00 | 0.000E+00 | 0.000E+00 | 0.000E+00 |
| Unigene378856 | msbA | 0.000E+00 | 0.000E+00 | 0.000E+00 | 0.000E+00 | 1.573E-07 | 1.531E-07 | 0.000E+00 | 0.000E+00 | 0.000E+00 | 0.000E+00 | 0.000E+00 | 0.000E+00 |
| Unigene379005 | arlS | 0.000E+00 | 0.000E+00 | 0.000E+00 | 0.000E+00 | 1.440E-07 | 0.000E+00 | 0.000E+00 | 0.000E+00 | 0.000E+00 | 0.000E+00 | 0.000E+00 | 0.000E+00 |
| Unigene379012 | bcrA | 0.000E+00 | 0.000E+00 | 6.025E-07 | 0.000E+00 | 0.000E+00 | 6.954E-07 | 1.359E-06 | 1.468E-06 | 0.000E+00 | 9.446E-07 | 1.371E-06 | 3.491E-06 |
| Unigene379080 | mtrA | 0.000E+00 | 0.000E+00 | 0.000E+00 | 0.000E+00 | 0.000E+00 | 3.002E-07 | 0.000E+00 | 0.000E+00 | 0.000E+00 | 0.000E+00 | 0.000E+00 | 0.000E+00 |
| Unigene379144 | carA | 4.317E-05 | 2.202E-05 | 1.725E-05 | 1.892E-05 | 2.167E-06 | 1.721E-06 | 2.779E-06 | 6.803E-07 | 7.277E-06 | 6.742E-07 | 2.462E-06 | 1.616E-06 |
| Unigene379186 | TaeA | 0.000E+00 | 0.000E+00 | 0.000E+00 | 0.000E+00 | 0.000E+00 | 1.039E-06 | 0.000E+00 | 0.000E+00 | 0.000E+00 | 0.000E+00 | 0.000E+00 | 0.000E+00 |
| Unigene379200 | tva(A) | 0.000E+00 | 0.000E+00 | 0.000E+00 | 0.000E+00 | 0.000E+00 | 0.000E+00 | 0.000E+00 | 0.000E+00 | 0.000E+00 | 0.000E+00 | 3.546E-07 | 0.000E+00 |
| Unigene379430 | tet(30) | 0.000E+00 | 0.000E+00 | 7.299E-07 | 0.000E+00 | 0.000E+00 | 1.809E-06 | 4.117E-06 | 2.853E-06 | 0.000E+00 | 0.000E+00 | 1.884E-06 | 3.643E-06 |
| Unigene379455 | Bifidobacte | 0.000E+00 | 0.000E+00 | 0.000E+00 | 0.000E+00 | 0.000E+00 | 0.000E+00 | 0.000E+00 | 4.958E-07 | 0.000E+00 | 0.000E+00 | 0.000E+00 | 0.000E+00 |
| Unigene379491 | mtrA | 0.000E+00 | 0.000E+00 | 0.000E+00 | 0.000E+00 | 0.000E+00 | 5.965E-07 | 0.000E+00 | 0.000E+00 | 0.000E+00 | 0.000E+00 | 0.000E+00 | 0.000E+00 |
| Unigene379512 | emeA | 0.000E+00 | 0.000E+00 | 0.000E+00 | 0.000E+00 | 0.000E+00 | 1.122E-06 | 0.000E+00 | 0.000E+00 | 0.000E+00 | 0.000E+00 | 0.000E+00 | 0.000E+00 |
| Unigene379530 | Rhodococc | 0.000E+00 | 0.000E+00 | 0.000E+00 | 0.000E+00 | 0.000E+00 | 6.319E-07 | 0.000E+00 | 0.000E+00 | 0.000E+00 | 0.000E+00 | 0.000E+00 | 0.000E+00 |
| Unigene379562 | tetA(58) | 0.000E+00 | 0.000E+00 | 0.000E+00 | 0.000E+00 | 0.000E+00 | 8.021E-06 | 0.000E+00 | 0.000E+00 | 3.036E-06 | 0.000E+00 | 0.000E+00 | 0.000E+00 |
| Unigene379607 | oleC | 0.000E+00 | 0.000E+00 | 0.000E+00 | 0.000E+00 | 0.000E+00 | 8.741E-07 | 0.000E+00 | 0.000E+00 | 0.000E+00 | 0.000E+00 | 0.000E+00 | 0.000E+00 |
| Unigene379628 | tetA(58) | 6.048E-06 | 0.000E+00 | 0.000E+00 | 2.415E-06 | 3.660E-05 | 7.375E-06 | 1.825E-05 | 2.356E-05 | 0.000E+00 | 0.000E+00 | 0.000E+00 | 0.000E+00 |
| Unigene379718 | carA | 0.000E+00 | 0.000E+00 | 0.000E+00 | 0.000E+00 | 0.000E+00 | 0.000E+00 | 0.000E+00 | 0.000E+00 | 0.000E+00 | 0.000E+00 | 0.000E+00 | 3.904E-06 |
| Unigene379809 | Staphylocc | 0.000E+00 | 0.000E+00 | 0.000E+00 | 0.000E+00 | 0.000E+00 | 1.111E-06 | 0.000E+00 | 0.000E+00 | 0.000E+00 | 0.000E+00 | 0.000E+00 | 0.000E+00 |
| Unigene379934 | Staphylocc | 0.000E+00 | 0.000E+00 | 0.000E+00 | 0.000E+00 | 0.000E+00 | 4.164E-07 | 0.000E+00 | 0.000E+00 | 0.000E+00 | 0.000E+00 | 7.050E-07 | 1.214E-07 |
| Unigene379975 | cdeA | 0.000E+00 | 0.000E+00 | 0.000E+00 | 0.000E+00 | 0.000E+00 | 2.690E-07 | 0.000E+00 | 0.000E+00 | 0.000E+00 | 0.000E+00 | 0.000E+00 | 0.000E+00 |
| Unigene380123 | macB | 0.000E+00 | 0.000E+00 | 0.000E+00 | 0.000E+00 | 1.009E-07 | 6.871E-07 | 0.000E+00 | 0.000E+00 | 0.000E+00 | 0.000E+00 | 0.000E+00 | 1.073E-07 |
| Unigene380160 | lmrC | 0.000E+00 | 0.000E+00 | 0.000E+00 | 4.676E-07 | 0.000E+00 | 1.645E-06 | 0.000E+00 | 3.783E-06 | 0.000E+00 | 0.000E+00 | 0.000E+00 | 3.883E-06 |
| Unigene380171 | macB | 0.000E+00 | 0.000E+00 | 0.000E+00 | 0.000E+00 | 0.000E+00 | 2.195E-07 | 0.000E+00 | 0.000E+00 | 0.000E+00 | 0.000E+00 | 0.000E+00 | 0.000E+00 |
| Unigene380179 | adeA | 0.000E+00 | 0.000E+00 | 0.000E+00 | 0.000E+00 | 0.000E+00 | 6.374E-07 | 0.000E+00 | 0.000E+00 | 0.000E+00 | 0.000E+00 | 0.000E+00 | 0.000E+00 |
| Unigene380252 | vanRO | 0.000E+00 | 0.000E+00 | 0.000E+00 | 0.000E+00 | 0.000E+00 | 1.674E-07 | 0.000E+00 | 0.000E+00 | 0.000E+00 | 0.000E+00 | 0.000E+00 | 1.830E-07 |
| Unigene380264 | mdtG | 0.000E+00 | 0.000E+00 | 0.000E+00 | 0.000E+00 | 0.000E+00 | 1.296E-06 | 0.000E+00 | 0.000E+00 | 0.000E+00 | 0.000E+00 | 0.000E+00 | 0.000E+00 |
| Unigene380270 | Acinetobac | 0.000E+00 | 0.000E+00 | 0.000E+00 | 0.000E+00 | 0.000E+00 | 5.856E-07 | 0.000E+00 | 0.000E+00 | 0.000E+00 | 0.000E+00 | 0.000E+00 | 5.079E-07 |
| Unigene380271 | ACT-30 | 6.834E-05 | 5.696E-05 | 1.177E-04 | 1.636E-04 | 1.230E-04 | 8.624E-05 | 1.131E-04 | 1.132E-04 | 9.778E-05 | 1.421E-04 | 1.406E-04 | 1.485E-04 |
| Unigene380296 | pmrA | 0.000E+00 | 0.000E+00 | 0.000E+00 | 0.000E+00 | 0.000E+00 | 7.436E-07 | 0.000E+00 | 0.000E+00 | 0.000E+00 | 0.000E+00 | 0.000E+00 | 0.000E+00 |
| Unigene380299 | evgS | 0.000E+00 | 0.000E+00 | 0.000E+00 | 0.000E+00 | 0.000E+00 | 6.589E-07 | 1.093E-06 | 0.000E+00 | 0.000E+00 | 0.000E+00 | 0.000E+00 | 1.565E-06 |
| Unigene380448 | adeR | 0.000E+00 | 0.000E+00 | 0.000E+00 | 0.000E+00 | 0.000E+00 | 1.014E-06 | 0.000E+00 | 0.000E+00 | 0.000E+00 | 0.000E+00 | 0.000E+00 | 0.000E+00 |

| | | | | | | | | | | | | | |
|---|---|---|---|---|---|---|---|---|---|---|---|---|---|
| Unigene380568 | mecI | 0.000E+00 | 0.000E+00 | 0.000E+00 | 0.000E+00 | 0.000E+00 | 7.755E-07 | 0.000E+00 | 0.000E+00 | 0.000E+00 | 0.000E+00 | 0.000E+00 | 0.000E+00 |
| Unigene380585 | oleB | 0.000E+00 | 0.000E+00 | 0.000E+00 | 0.000E+00 | 0.000E+00 | 3.943E-07 | 0.000E+00 | 0.000E+00 | 0.000E+00 | 0.000E+00 | 0.000E+00 | 0.000E+00 |
| Unigene380681 | tetA(60) | 0.000E+00 | 0.000E+00 | 0.000E+00 | 0.000E+00 | 0.000E+00 | 9.155E-07 | 0.000E+00 | 0.000E+00 | 0.000E+00 | 0.000E+00 | 0.000E+00 | 0.000E+00 |
| Unigene380776 | bcr-1 | 0.000E+00 | 0.000E+00 | 0.000E+00 | 0.000E+00 | 0.000E+00 | 3.921E-07 | 0.000E+00 | 0.000E+00 | 0.000E+00 | 0.000E+00 | 0.000E+00 | 0.000E+00 |
| Unigene380858 | vatB | 0.000E+00 | 0.000E+00 | 0.000E+00 | 0.000E+00 | 0.000E+00 | 8.491E-07 | 0.000E+00 | 0.000E+00 | 0.000E+00 | 0.000E+00 | 0.000E+00 | 0.000E+00 |
| Unigene380976 | vanZF | 0.000E+00 | 0.000E+00 | 0.000E+00 | 0.000E+00 | 0.000E+00 | 1.231E-06 | 0.000E+00 | 0.000E+00 | 0.000E+00 | 0.000E+00 | 0.000E+00 | 0.000E+00 |
| Unigene380979 | Staphylocc | 0.000E+00 | 0.000E+00 | 0.000E+00 | 0.000E+00 | 0.000E+00 | 1.314E-06 | 0.000E+00 | 0.000E+00 | 0.000E+00 | 0.000E+00 | 0.000E+00 | 0.000E+00 |
| Unigene380996 | Klebsiella p | 0.000E+00 | 5.442E-08 | 0.000E+00 | 0.000E+00 | 0.000E+00 | 2.680E-07 | 5.488E-08 | 0.000E+00 | 0.000E+00 | 0.000E+00 | 0.000E+00 | 0.000E+00 |
| Unigene381044 | tetA(60) | 3.400E-07 | 0.000E+00 | 0.000E+00 | 0.000E+00 | 7.110E-07 | 1.405E-06 | 4.117E-06 | 3.222E-06 | 0.000E+00 | 1.716E-06 | 0.000E+00 | 3.688E-06 |
| Unigene381122 | tva(A) | 0.000E+00 | 0.000E+00 | 0.000E+00 | 0.000E+00 | 0.000E+00 | 5.468E-07 | 0.000E+00 | 0.000E+00 | 0.000E+00 | 0.000E+00 | 0.000E+00 | 0.000E+00 |
| Unigene381234 | tetA(46) | 0.000E+00 | 0.000E+00 | 0.000E+00 | 0.000E+00 | 0.000E+00 | 1.448E-06 | 0.000E+00 | 0.000E+00 | 0.000E+00 | 0.000E+00 | 0.000E+00 | 0.000E+00 |
| Unigene381438 | vanUG | 0.000E+00 | 0.000E+00 | 0.000E+00 | 0.000E+00 | 0.000E+00 | 1.662E-05 | 0.000E+00 | 0.000E+00 | 0.000E+00 | 0.000E+00 | 0.000E+00 | 0.000E+00 |
| Unigene381480 | novA | 0.000E+00 | 0.000E+00 | 0.000E+00 | 0.000E+00 | 0.000E+00 | 9.640E-07 | 0.000E+00 | 0.000E+00 | 0.000E+00 | 0.000E+00 | 0.000E+00 | 0.000E+00 |
| Unigene381571 | patB | 0.000E+00 | 0.000E+00 | 0.000E+00 | 0.000E+00 | 0.000E+00 | 5.712E-07 | 0.000E+00 | 0.000E+00 | 0.000E+00 | 0.000E+00 | 0.000E+00 | 0.000E+00 |
| Unigene381794 | bcrA | 0.000E+00 | 0.000E+00 | 0.000E+00 | 0.000E+00 | 0.000E+00 | 2.776E-06 | 0.000E+00 | 0.000E+00 | 3.475E-06 | 0.000E+00 | 0.000E+00 | 0.000E+00 |
| Unigene381870 | msbA | 0.000E+00 | 0.000E+00 | 0.000E+00 | 0.000E+00 | 0.000E+00 | 1.380E-06 | 0.000E+00 | 0.000E+00 | 0.000E+00 | 0.000E+00 | 0.000E+00 | 0.000E+00 |
| Unigene381872 | tlrC | 0.000E+00 | 0.000E+00 | 0.000E+00 | 0.000E+00 | 0.000E+00 | 1.617E-06 | 0.000E+00 | 0.000E+00 | 0.000E+00 | 0.000E+00 | 0.000E+00 | 0.000E+00 |
| Unigene381875 | tetA(58) | 0.000E+00 | 0.000E+00 | 0.000E+00 | 0.000E+00 | 0.000E+00 | 1.314E-06 | 0.000E+00 | 0.000E+00 | 0.000E+00 | 0.000E+00 | 0.000E+00 | 0.000E+00 |
| Unigene381883 | lmrB | 0.000E+00 | 0.000E+00 | 0.000E+00 | 0.000E+00 | 0.000E+00 | 1.568E-06 | 1.036E-07 | 0.000E+00 | 0.000E+00 | 0.000E+00 | 0.000E+00 | 0.000E+00 |
| Unigene381886 | otr(B) | 0.000E+00 | 0.000E+00 | 0.000E+00 | 0.000E+00 | 0.000E+00 | 1.638E-06 | 0.000E+00 | 0.000E+00 | 0.000E+00 | 0.000E+00 | 0.000E+00 | 0.000E+00 |
| Unigene381914 | Staphylocc | 0.000E+00 | 0.000E+00 | 0.000E+00 | 0.000E+00 | 0.000E+00 | 1.323E-06 | 0.000E+00 | 0.000E+00 | 0.000E+00 | 0.000E+00 | 0.000E+00 | 0.000E+00 |
| Unigene381936 | Enterobact | 0.000E+00 | 0.000E+00 | 6.435E-07 | 0.000E+00 | 7.286E-07 | 1.549E-06 | 4.032E-06 | 2.920E-06 | 0.000E+00 | 1.001E-06 | 0.000E+00 | 3.330E-06 |
| Unigene381952 | lmrB | 0.000E+00 | 0.000E+00 | 0.000E+00 | 0.000E+00 | 0.000E+00 | 7.920E-07 | 0.000E+00 | 0.000E+00 | 0.000E+00 | 0.000E+00 | 0.000E+00 | 0.000E+00 |
| Unigene381956 | tlrC | 0.000E+00 | 0.000E+00 | 0.000E+00 | 0.000E+00 | 0.000E+00 | 5.170E-07 | 0.000E+00 | 0.000E+00 | 0.000E+00 | 0.000E+00 | 0.000E+00 | 0.000E+00 |
| Unigene382043 | fusD | 0.000E+00 | 0.000E+00 | 0.000E+00 | 0.000E+00 | 0.000E+00 | 1.185E-06 | 0.000E+00 | 0.000E+00 | 0.000E+00 | 0.000E+00 | 0.000E+00 | 0.000E+00 |
| Unigene382045 | catB3 | 0.000E+00 | 0.000E+00 | 0.000E+00 | 0.000E+00 | 0.000E+00 | 1.072E-06 | 0.000E+00 | 0.000E+00 | 0.000E+00 | 0.000E+00 | 0.000E+00 | 0.000E+00 |
| Unigene382046 | vatB | 0.000E+00 | 0.000E+00 | 0.000E+00 | 0.000E+00 | 0.000E+00 | 6.876E-07 | 0.000E+00 | 0.000E+00 | 0.000E+00 | 0.000E+00 | 0.000E+00 | 0.000E+00 |
| Unigene382081 | vanRE | 6.695E-07 | 0.000E+00 | 0.000E+00 | 3.380E-07 | 1.647E-06 | 3.515E-06 | 6.351E-06 | 2.233E-07 | 3.502E-06 | 2.024E-06 | 8.534E-06 | 2.261E-07 |
| Unigene382349 | patA | 0.000E+00 | 0.000E+00 | 0.000E+00 | 0.000E+00 | 0.000E+00 | 7.710E-07 | 0.000E+00 | 0.000E+00 | 0.000E+00 | 0.000E+00 | 0.000E+00 | 0.000E+00 |
| Unigene382688 | arnA | 7.163E-06 | 0.000E+00 | 0.000E+00 | 1.990E-06 | 2.829E-05 | 1.288E-05 | 1.883E-05 | 2.183E-05 | 3.829E-06 | 0.000E+00 | 0.000E+00 | 0.000E+00 |
| Unigene382755 | emrB | 0.000E+00 | 0.000E+00 | 0.000E+00 | 0.000E+00 | 0.000E+00 | 9.874E-07 | 0.000E+00 | 0.000E+00 | 0.000E+00 | 0.000E+00 | 0.000E+00 | 0.000E+00 |
| Unigene382779 | emrK | 0.000E+00 | 3.067E-07 | 0.000E+00 | 0.000E+00 | 0.000E+00 | 1.843E-06 | 4.269E-06 | 3.719E-06 | 7.942E-07 | 0.000E+00 | 0.000E+00 | 4.988E-06 |
| Unigene383030 | srmB | 0.000E+00 | 0.000E+00 | 0.000E+00 | 0.000E+00 | 0.000E+00 | 9.730E-07 | 0.000E+00 | 0.000E+00 | 0.000E+00 | 0.000E+00 | 0.000E+00 | 0.000E+00 |
| Unigene383080 | poxtA | 0.000E+00 | 0.000E+00 | 0.000E+00 | 0.000E+00 | 0.000E+00 | 6.514E-07 | 0.000E+00 | 0.000E+00 | 0.000E+00 | 0.000E+00 | 0.000E+00 | 0.000E+00 |
| Unigene383226 | arlS | 0.000E+00 | 0.000E+00 | 0.000E+00 | 0.000E+00 | 0.000E+00 | 4.474E-07 | 0.000E+00 | 0.000E+00 | 0.000E+00 | 0.000E+00 | 0.000E+00 | 0.000E+00 |
| Unigene383255 | vanHF | 0.000E+00 | 0.000E+00 | 0.000E+00 | 0.000E+00 | 0.000E+00 | 4.154E-07 | 0.000E+00 | 0.000E+00 | 0.000E+00 | 0.000E+00 | 0.000E+00 | 0.000E+00 |
| Unigene383429 | lsaC | 0.000E+00 | 0.000E+00 | 0.000E+00 | 0.000E+00 | 0.000E+00 | 6.391E-07 | 0.000E+00 | 0.000E+00 | 0.000E+00 | 0.000E+00 | 0.000E+00 | 6.289E-07 |
| Unigene383457 | Chlamydia | 0.000E+00 | 0.000E+00 | 0.000E+00 | 0.000E+00 | 0.000E+00 | 1.014E-06 | 0.000E+00 | 0.000E+00 | 0.000E+00 | 0.000E+00 | 0.000E+00 | 0.000E+00 |
| Unigene383629 | patB | 0.000E+00 | 0.000E+00 | 1.175E-05 | 0.000E+00 | 0.000E+00 | 9.535E-07 | 0.000E+00 | 0.000E+00 | 0.000E+00 | 0.000E+00 | 0.000E+00 | 0.000E+00 |
| Unigene383669 | macB | 0.000E+00 | 0.000E+00 | 0.000E+00 | 0.000E+00 | 0.000E+00 | 4.011E-07 | 0.000E+00 | 0.000E+00 | 0.000E+00 | 0.000E+00 | 0.000E+00 | 0.000E+00 |
| Unigene383880 | carA | 0.000E+00 | 0.000E+00 | 0.000E+00 | 0.000E+00 | 0.000E+00 | 5.839E-07 | 0.000E+00 | 0.000E+00 | 0.000E+00 | 0.000E+00 | 0.000E+00 | 0.000E+00 |
| Unigene383977 | oleC | 0.000E+00 | 0.000E+00 | 0.000E+00 | 0.000E+00 | 0.000E+00 | 8.577E-07 | 0.000E+00 | 0.000E+00 | 0.000E+00 | 0.000E+00 | 0.000E+00 | 0.000E+00 |
| Unigene383987 | tva(A) | 0.000E+00 | 0.000E+00 | 0.000E+00 | 0.000E+00 | 0.000E+00 | 5.137E-07 | 0.000E+00 | 0.000E+00 | 0.000E+00 | 0.000E+00 | 0.000E+00 | 0.000E+00 |
| Unigene384123 | Rhodococc | 0.000E+00 | 0.000E+00 | 0.000E+00 | 0.000E+00 | 0.000E+00 | 1.232E-06 | 0.000E+00 | 0.000E+00 | 0.000E+00 | 0.000E+00 | 0.000E+00 | 0.000E+00 |
| Unigene384186 | erm(45) | 0.000E+00 | 0.000E+00 | 0.000E+00 | 0.000E+00 | 0.000E+00 | 9.496E-07 | 0.000E+00 | 0.000E+00 | 0.000E+00 | 0.000E+00 | 0.000E+00 | 0.000E+00 |
| Unigene384224 | bcrA | 0.000E+00 | 0.000E+00 | 0.000E+00 | 0.000E+00 | 0.000E+00 | 1.513E-06 | 0.000E+00 | 0.000E+00 | 0.000E+00 | 0.000E+00 | 0.000E+00 | 0.000E+00 |
| Unigene384251 | arlR | 0.000E+00 | 0.000E+00 | 0.000E+00 | 0.000E+00 | 0.000E+00 | 1.460E-06 | 0.000E+00 | 0.000E+00 | 0.000E+00 | 0.000E+00 | 0.000E+00 | 0.000E+00 |
| Unigene384252 | arlS | 0.000E+00 | 0.000E+00 | 0.000E+00 | 0.000E+00 | 0.000E+00 | 1.484E-06 | 0.000E+00 | 0.000E+00 | 0.000E+00 | 0.000E+00 | 0.000E+00 | 0.000E+00 |
| Unigene384269 | tetA(58) | 0.000E+00 | 0.000E+00 | 0.000E+00 | 1.518E-07 | 0.000E+00 | 9.098E-07 | 2.329E-06 | 1.614E-06 | 0.000E+00 | 0.000E+00 | 0.000E+00 | 0.000E+00 |
| Unigene384338 | smeR | 0.000E+00 | 0.000E+00 | 0.000E+00 | 0.000E+00 | 0.000E+00 | 8.243E-07 | 0.000E+00 | 0.000E+00 | 0.000E+00 | 0.000E+00 | 0.000E+00 | 0.000E+00 |
| Unigene384368 | optrA | 0.000E+00 | 0.000E+00 | 0.000E+00 | 0.000E+00 | 0.000E+00 | 5.840E-07 | 0.000E+00 | 0.000E+00 | 0.000E+00 | 0.000E+00 | 0.000E+00 | 4.736E-06 |
| Unigene384585 | arlR | 0.000E+00 | 0.000E+00 | 0.000E+00 | 0.000E+00 | 0.000E+00 | 5.080E-07 | 0.000E+00 | 0.000E+00 | 0.000E+00 | 0.000E+00 | 0.000E+00 | 0.000E+00 |
| Unigene384609 | macB | 0.000E+00 | 0.000E+00 | 0.000E+00 | 0.000E+00 | 0.000E+00 | 6.647E-07 | 0.000E+00 | 0.000E+00 | 0.000E+00 | 0.000E+00 | 0.000E+00 | 0.000E+00 |
| Unigene384638 | vanRE | 0.000E+00 | 0.000E+00 | 0.000E+00 | 0.000E+00 | 0.000E+00 | 1.368E-06 | 0.000E+00 | 0.000E+00 | 0.000E+00 | 0.000E+00 | 0.000E+00 | 0.000E+00 |
| Unigene384639 | evgS | 0.000E+00 | 0.000E+00 | 0.000E+00 | 0.000E+00 | 0.000E+00 | 1.547E-06 | 0.000E+00 | 0.000E+00 | 0.000E+00 | 0.000E+00 | 0.000E+00 | 0.000E+00 |
| Unigene384712 | YojI | 0.000E+00 | 0.000E+00 | 0.000E+00 | 0.000E+00 | 0.000E+00 | 2.595E-07 | 0.000E+00 | 0.000E+00 | 0.000E+00 | 0.000E+00 | 0.000E+00 | 0.000E+00 |
| Unigene384828 | tetA(58) | 0.000E+00 | 0.000E+00 | 0.000E+00 | 0.000E+00 | 0.000E+00 | 5.510E-07 | 0.000E+00 | 0.000E+00 | 0.000E+00 | 0.000E+00 | 0.000E+00 | 0.000E+00 |
| Unigene384861 | evgS | 0.000E+00 | 0.000E+00 | 0.000E+00 | 0.000E+00 | 0.000E+00 | 4.197E-07 | 0.000E+00 | 0.000E+00 | 0.000E+00 | 0.000E+00 | 0.000E+00 | 0.000E+00 |

| | | | | | | | | | | | | | |
|---|---|---|---|---|---|---|---|---|---|---|---|---|---|
| Unigene384924 | PmrF | 0.000E+00 | 0.000E+00 | 0.000E+00 | 0.000E+00 | 0.000E+00 | 1.999E-06 | 0.000E+00 | 0.000E+00 | 0.000E+00 | 0.000E+00 | 0.000E+00 | 0.000E+00 |
| Unigene384939 | farA | 0.000E+00 | 0.000E+00 | 0.000E+00 | 0.000E+00 | 7.387E-07 | 1.176E-06 | 3.880E-06 | 2.646E-06 | 0.000E+00 | 0.000E+00 | 2.144E-06 | 3.287E-06 |
| Unigene385010 | smeC | 0.000E+00 | 0.000E+00 | 0.000E+00 | 0.000E+00 | 0.000E+00 | 2.890E-07 | 0.000E+00 | 0.000E+00 | 0.000E+00 | 0.000E+00 | 0.000E+00 | 0.000E+00 |
| Unigene385055 | arnA | 0.000E+00 | 0.000E+00 | 0.000E+00 | 0.000E+00 | 0.000E+00 | 9.693E-07 | 0.000E+00 | 0.000E+00 | 0.000E+00 | 0.000E+00 | 0.000E+00 | 0.000E+00 |
| Unigene385147 | vanHF | 0.000E+00 | 0.000E+00 | 0.000E+00 | 0.000E+00 | 0.000E+00 | 1.498E-06 | 0.000E+00 | 0.000E+00 | 0.000E+00 | 0.000E+00 | 0.000E+00 | 0.000E+00 |
| Unigene385218 | tetA(58) | 0.000E+00 | 0.000E+00 | 0.000E+00 | 0.000E+00 | 0.000E+00 | 1.097E-06 | 0.000E+00 | 0.000E+00 | 0.000E+00 | 0.000E+00 | 0.000E+00 | 0.000E+00 |
| Unigene385226 | Staphylocc | 0.000E+00 | 0.000E+00 | 0.000E+00 | 0.000E+00 | 0.000E+00 | 1.282E-06 | 0.000E+00 | 0.000E+00 | 0.000E+00 | 0.000E+00 | 0.000E+00 | 0.000E+00 |
| Unigene385312 | tetA(60) | 4.304E-06 | 0.000E+00 | 0.000E+00 | 0.000E+00 | 1.261E-06 | 3.003E-06 | 0.000E+00 | 0.000E+00 | 0.000E+00 | 0.000E+00 | 0.000E+00 | 0.000E+00 |
| Unigene385456 | TaeA | 0.000E+00 | 0.000E+00 | 0.000E+00 | 0.000E+00 | 0.000E+00 | 1.655E-06 | 0.000E+00 | 0.000E+00 | 0.000E+00 | 0.000E+00 | 0.000E+00 | 0.000E+00 |
| Unigene385637 | macB | 0.000E+00 | 0.000E+00 | 0.000E+00 | 0.000E+00 | 0.000E+00 | 4.950E-07 | 0.000E+00 | 0.000E+00 | 0.000E+00 | 0.000E+00 | 0.000E+00 | 0.000E+00 |
| Unigene385768 | Acinetobac | 0.000E+00 | 0.000E+00 | 0.000E+00 | 0.000E+00 | 0.000E+00 | 2.970E-07 | 0.000E+00 | 0.000E+00 | 0.000E+00 | 0.000E+00 | 0.000E+00 | 0.000E+00 |
| Unigene385863 | msbA | 0.000E+00 | 0.000E+00 | 0.000E+00 | 0.000E+00 | 0.000E+00 | 1.687E-06 | 5.365E-06 | 3.215E-06 | 0.000E+00 | 0.000E+00 | 0.000E+00 | 4.406E-06 |
| Unigene385864 | bcrA | 0.000E+00 | 0.000E+00 | 0.000E+00 | 0.000E+00 | 0.000E+00 | 6.258E-07 | 0.000E+00 | 0.000E+00 | 0.000E+00 | 0.000E+00 | 0.000E+00 | 0.000E+00 |
| Unigene385967 | vanTG | 0.000E+00 | 0.000E+00 | 0.000E+00 | 0.000E+00 | 0.000E+00 | 8.065E-07 | 0.000E+00 | 8.842E-06 | 0.000E+00 | 0.000E+00 | 0.000E+00 | 0.000E+00 |
| Unigene386007 | Chlamydia | 0.000E+00 | 0.000E+00 | 0.000E+00 | 0.000E+00 | 0.000E+00 | 8.685E-07 | 0.000E+00 | 7.104E-06 | 0.000E+00 | 0.000E+00 | 1.401E-05 | 0.000E+00 |
| Unigene386046 | patA | 0.000E+00 | 0.000E+00 | 0.000E+00 | 0.000E+00 | 0.000E+00 | 8.138E-07 | 0.000E+00 | 0.000E+00 | 0.000E+00 | 0.000E+00 | 0.000E+00 | 0.000E+00 |
| Unigene386125 | cdeA | 0.000E+00 | 0.000E+00 | 0.000E+00 | 0.000E+00 | 0.000E+00 | 1.029E-06 | 0.000E+00 | 0.000E+00 | 0.000E+00 | 0.000E+00 | 0.000E+00 | 0.000E+00 |
| Unigene386137 | macB | 0.000E+00 | 0.000E+00 | 0.000E+00 | 0.000E+00 | 0.000E+00 | 1.333E-06 | 0.000E+00 | 0.000E+00 | 0.000E+00 | 0.000E+00 | 0.000E+00 | 0.000E+00 |
| Unigene386138 | macB | 0.000E+00 | 0.000E+00 | 0.000E+00 | 0.000E+00 | 0.000E+00 | 1.809E-06 | 0.000E+00 | 0.000E+00 | 0.000E+00 | 0.000E+00 | 0.000E+00 | 0.000E+00 |
| Unigene386143 | vgaB | 0.000E+00 | 0.000E+00 | 0.000E+00 | 0.000E+00 | 0.000E+00 | 1.893E-06 | 0.000E+00 | 0.000E+00 | 0.000E+00 | 0.000E+00 | 0.000E+00 | 0.000E+00 |
| Unigene386165 | macB | 0.000E+00 | 5.650E-08 | 0.000E+00 | 1.560E-07 | 0.000E+00 | 0.000E+00 | 0.000E+00 | 0.000E+00 | 1.125E-07 | 0.000E+00 | 2.945E-07 | 6.086E-08 |
| Unigene386223 | baeS | 3.766E-07 | 0.000E+00 | 2.112E-07 | 0.000E+00 | 0.000E+00 | 1.745E-07 | 0.000E+00 | 0.000E+00 | 0.000E+00 | 0.000E+00 | 0.000E+00 | 0.000E+00 |
| Unigene386289 | efmA | 0.000E+00 | 0.000E+00 | 0.000E+00 | 0.000E+00 | 0.000E+00 | 9.930E-07 | 1.365E-06 | 1.069E-05 | 3.499E-06 | 0.000E+00 | 0.000E+00 | 5.584E-07 |
| Unigene386375 | carA | 0.000E+00 | 0.000E+00 | 0.000E+00 | 0.000E+00 | 0.000E+00 | 8.008E-07 | 0.000E+00 | 0.000E+00 | 0.000E+00 | 0.000E+00 | 0.000E+00 | 0.000E+00 |
| Unigene386398 | adeN | 0.000E+00 | 0.000E+00 | 0.000E+00 | 0.000E+00 | 0.000E+00 | 8.438E-07 | 0.000E+00 | 0.000E+00 | 0.000E+00 | 0.000E+00 | 0.000E+00 | 0.000E+00 |
| Unigene386401 | lsaC | 0.000E+00 | 0.000E+00 | 0.000E+00 | 0.000E+00 | 0.000E+00 | 2.781E-07 | 9.015E-07 | 5.004E-08 | 0.000E+00 | 2.388E-07 | 4.904E-07 | 5.574E-07 |
| Unigene386514 | arlR | 4.658E-08 | 0.000E+00 | 0.000E+00 | 0.000E+00 | 0.000E+00 | 6.546E-07 | 0.000E+00 | 0.000E+00 | 0.000E+00 | 0.000E+00 | 0.000E+00 | 1.101E-07 |
| Unigene386521 | vanRI | 0.000E+00 | 0.000E+00 | 8.104E-07 | 0.000E+00 | 0.000E+00 | 1.046E-06 | 3.238E-06 | 0.000E+00 | 0.000E+00 | 0.000E+00 | 0.000E+00 | 4.240E-06 |
| Unigene386544 | vmlR | 0.000E+00 | 0.000E+00 | 0.000E+00 | 0.000E+00 | 0.000E+00 | 5.764E-07 | 0.000E+00 | 0.000E+00 | 0.000E+00 | 0.000E+00 | 0.000E+00 | 4.583E-07 |
| Unigene386582 | macB | 0.000E+00 | 0.000E+00 | 0.000E+00 | 0.000E+00 | 0.000E+00 | 5.428E-07 | 0.000E+00 | 0.000E+00 | 0.000E+00 | 0.000E+00 | 0.000E+00 | 0.000E+00 |
| Unigene386664 | bmr | 0.000E+00 | 0.000E+00 | 0.000E+00 | 0.000E+00 | 0.000E+00 | 8.438E-07 | 0.000E+00 | 0.000E+00 | 0.000E+00 | 0.000E+00 | 0.000E+00 | 0.000E+00 |
| Unigene386676 | carA | 0.000E+00 | 0.000E+00 | 0.000E+00 | 0.000E+00 | 0.000E+00 | 7.327E-07 | 0.000E+00 | 0.000E+00 | 0.000E+00 | 0.000E+00 | 0.000E+00 | 0.000E+00 |
| Unigene386740 | patB | 0.000E+00 | 0.000E+00 | 0.000E+00 | 0.000E+00 | 0.000E+00 | 1.517E-06 | 0.000E+00 | 0.000E+00 | 0.000E+00 | 0.000E+00 | 0.000E+00 | 0.000E+00 |
| Unigene386741 | patB | 0.000E+00 | 0.000E+00 | 0.000E+00 | 0.000E+00 | 0.000E+00 | 1.224E-06 | 0.000E+00 | 0.000E+00 | 0.000E+00 | 0.000E+00 | 0.000E+00 | 0.000E+00 |
| Unigene386746 | vanRM | 0.000E+00 | 0.000E+00 | 0.000E+00 | 0.000E+00 | 0.000E+00 | 1.354E-06 | 0.000E+00 | 0.000E+00 | 0.000E+00 | 0.000E+00 | 0.000E+00 | 0.000E+00 |
| Unigene386769 | patB | 0.000E+00 | 0.000E+00 | 0.000E+00 | 0.000E+00 | 0.000E+00 | 9.363E-07 | 0.000E+00 | 0.000E+00 | 0.000E+00 | 0.000E+00 | 0.000E+00 | 0.000E+00 |
| Unigene386774 | cpxA | 0.000E+00 | 0.000E+00 | 0.000E+00 | 0.000E+00 | 0.000E+00 | 3.681E-07 | 3.015E-07 | 0.000E+00 | 0.000E+00 | 0.000E+00 | 0.000E+00 | 0.000E+00 |
| Unigene386785 | patA | 0.000E+00 | 0.000E+00 | 0.000E+00 | 0.000E+00 | 0.000E+00 | 4.503E-07 | 0.000E+00 | 0.000E+00 | 0.000E+00 | 0.000E+00 | 0.000E+00 | 0.000E+00 |
| Unigene386793 | bcrA | 0.000E+00 | 0.000E+00 | 0.000E+00 | 3.159E-07 | 0.000E+00 | 2.705E-07 | 0.000E+00 | 0.000E+00 | 0.000E+00 | 0.000E+00 | 0.000E+00 | 0.000E+00 |
| Unigene387242 | efrA | 0.000E+00 | 0.000E+00 | 0.000E+00 | 0.000E+00 | 0.000E+00 | 0.000E+00 | 0.000E+00 | 1.064E-06 | 0.000E+00 | 0.000E+00 | 0.000E+00 | 0.000E+00 |
| Unigene387357 | macB | 0.000E+00 | 0.000E+00 | 0.000E+00 | 0.000E+00 | 0.000E+00 | 1.654E-06 | 0.000E+00 | 0.000E+00 | 0.000E+00 | 0.000E+00 | 0.000E+00 | 0.000E+00 |
| Unigene387419 | bcr-1 | 0.000E+00 | 0.000E+00 | 0.000E+00 | 0.000E+00 | 0.000E+00 | 8.412E-07 | 0.000E+00 | 0.000E+00 | 0.000E+00 | 0.000E+00 | 0.000E+00 | 0.000E+00 |
| Unigene387423 | tetA(58) | 0.000E+00 | 0.000E+00 | 0.000E+00 | 0.000E+00 | 0.000E+00 | 9.850E-07 | 0.000E+00 | 0.000E+00 | 0.000E+00 | 0.000E+00 | 0.000E+00 | 0.000E+00 |
| Unigene387622 | ugd | 0.000E+00 | 0.000E+00 | 0.000E+00 | 0.000E+00 | 0.000E+00 | 6.878E-07 | 0.000E+00 | 1.614E-07 | 0.000E+00 | 9.244E-08 | 0.000E+00 | 1.308E-07 |
| Unigene387655 | efrA | 0.000E+00 | 0.000E+00 | 0.000E+00 | 0.000E+00 | 0.000E+00 | 0.000E+00 | 6.507E-08 | 0.000E+00 | 0.000E+00 | 0.000E+00 | 0.000E+00 | 3.475E-07 |
| Unigene387748 | rphB | 0.000E+00 | 0.000E+00 | 0.000E+00 | 0.000E+00 | 0.000E+00 | 1.568E-06 | 0.000E+00 | 0.000E+00 | 0.000E+00 | 0.000E+00 | 0.000E+00 | 0.000E+00 |
| Unigene387856 | SPG-1 | 0.000E+00 | 0.000E+00 | 0.000E+00 | 0.000E+00 | 0.000E+00 | 3.984E-07 | 0.000E+00 | 0.000E+00 | 0.000E+00 | 0.000E+00 | 0.000E+00 | 0.000E+00 |
| Unigene387903 | lmrC | 0.000E+00 | 0.000E+00 | 0.000E+00 | 4.453E-08 | 0.000E+00 | 7.628E-07 | 0.000E+00 | 0.000E+00 | 0.000E+00 | 0.000E+00 | 0.000E+00 | 0.000E+00 |
| Unigene387968 | macB | 0.000E+00 | 0.000E+00 | 0.000E+00 | 0.000E+00 | 0.000E+00 | 2.366E-06 | 0.000E+00 | 0.000E+00 | 0.000E+00 | 0.000E+00 | 0.000E+00 | 0.000E+00 |
| Unigene387978 | Bifidobacte | 0.000E+00 | 0.000E+00 | 0.000E+00 | 0.000E+00 | 0.000E+00 | 1.456E-06 | 0.000E+00 | 0.000E+00 | 0.000E+00 | 0.000E+00 | 0.000E+00 | 0.000E+00 |
| Unigene388003 | Staphylocc | 0.000E+00 | 0.000E+00 | 0.000E+00 | 0.000E+00 | 0.000E+00 | 9.970E-07 | 0.000E+00 | 0.000E+00 | 0.000E+00 | 0.000E+00 | 0.000E+00 | 0.000E+00 |
| Unigene388004 | mdtG | 0.000E+00 | 0.000E+00 | 0.000E+00 | 0.000E+00 | 0.000E+00 | 8.168E-07 | 0.000E+00 | 0.000E+00 | 0.000E+00 | 0.000E+00 | 0.000E+00 | 0.000E+00 |
| Unigene388018 | adeL | 0.000E+00 | 0.000E+00 | 3.299E-07 | 0.000E+00 | 0.000E+00 | 5.816E-07 | 0.000E+00 | 0.000E+00 | 2.205E-07 | 0.000E+00 | 0.000E+00 | 7.949E-08 |
| Unigene388448 | qacA | 0.000E+00 | 0.000E+00 | 0.000E+00 | 0.000E+00 | 0.000E+00 | 2.779E-07 | 1.219E-07 | 8.573E-08 | 0.000E+00 | 1.227E-07 | 0.000E+00 | 1.302E-07 |
| Unigene388523 | TaeA | 0.000E+00 | 0.000E+00 | 0.000E+00 | 0.000E+00 | 0.000E+00 | 7.217E-07 | 0.000E+00 | 0.000E+00 | 0.000E+00 | 0.000E+00 | 0.000E+00 | 0.000E+00 |
| Unigene388582 | srmB | 0.000E+00 | 0.000E+00 | 5.623E-07 | 0.000E+00 | 6.130E-07 | 5.965E-07 | 0.000E+00 | 8.670E-07 | 2.552E-07 | 7.093E-07 | 7.526E-07 | 2.734E-06 |
| Unigene388605 | tetT | 0.000E+00 | 0.000E+00 | 0.000E+00 | 0.000E+00 | 0.000E+00 | 1.386E-06 | 0.000E+00 | 0.000E+00 | 0.000E+00 | 0.000E+00 | 0.000E+00 | 0.000E+00 |
| Unigene388635 | vanHD | 0.000E+00 | 0.000E+00 | 0.000E+00 | 0.000E+00 | 0.000E+00 | 1.146E-06 | 0.000E+00 | 0.000E+00 | 0.000E+00 | 0.000E+00 | 0.000E+00 | 0.000E+00 |

| | | | | | | | | | | | | | |
|---|---|---|---|---|---|---|---|---|---|---|---|---|---|
| Unigene388651 | Corynebac | 0.000E+00 | 0.000E+00 | 0.000E+00 | 0.000E+00 | 0.000E+00 | 5.792E-07 | 0.000E+00 | 0.000E+00 | 0.000E+00 | 0.000E+00 | 0.000E+00 | 0.000E+00 |
| Unigene388671 | Corynebac | 0.000E+00 | 0.000E+00 | 0.000E+00 | 0.000E+00 | 0.000E+00 | 1.449E-06 | 0.000E+00 | 0.000E+00 | 0.000E+00 | 0.000E+00 | 0.000E+00 | 0.000E+00 |
| Unigene388738 | Erm(30) | 0.000E+00 | 0.000E+00 | 0.000E+00 | 0.000E+00 | 0.000E+00 | 1.398E-06 | 0.000E+00 | 0.000E+00 | 0.000E+00 | 0.000E+00 | 0.000E+00 | 0.000E+00 |
| Unigene388832 | vanHB | 0.000E+00 | 0.000E+00 | 0.000E+00 | 0.000E+00 | 0.000E+00 | 6.729E-07 | 0.000E+00 | 1.405E-05 | 0.000E+00 | 0.000E+00 | 2.370E-05 | 0.000E+00 |
| Unigene388835 | vanHO | 0.000E+00 | 0.000E+00 | 0.000E+00 | 0.000E+00 | 0.000E+00 | 1.258E-06 | 0.000E+00 | 1.222E-05 | 0.000E+00 | 0.000E+00 | 0.000E+00 | 0.000E+00 |
| Unigene388857 | arnA | 0.000E+00 | 0.000E+00 | 0.000E+00 | 0.000E+00 | 0.000E+00 | 4.929E-07 | 0.000E+00 | 0.000E+00 | 0.000E+00 | 0.000E+00 | 0.000E+00 | 0.000E+00 |
| Unigene388866 | vanHD | 0.000E+00 | 0.000E+00 | 0.000E+00 | 0.000E+00 | 0.000E+00 | 5.303E-07 | 0.000E+00 | 0.000E+00 | 0.000E+00 | 0.000E+00 | 0.000E+00 | 0.000E+00 |
| Unigene388890 | Staphylocc | 0.000E+00 | 0.000E+00 | 0.000E+00 | 0.000E+00 | 0.000E+00 | 1.027E-06 | 0.000E+00 | 0.000E+00 | 0.000E+00 | 0.000E+00 | 0.000E+00 | 0.000E+00 |
| Unigene388921 | efrA | 0.000E+00 | 0.000E+00 | 0.000E+00 | 0.000E+00 | 0.000E+00 | 4.130E-07 | 0.000E+00 | 0.000E+00 | 0.000E+00 | 0.000E+00 | 0.000E+00 | 0.000E+00 |
| Unigene389083 | optrA | 0.000E+00 | 0.000E+00 | 0.000E+00 | 0.000E+00 | 0.000E+00 | 6.451E-07 | 0.000E+00 | 0.000E+00 | 0.000E+00 | 0.000E+00 | 0.000E+00 | 3.868E-07 |
| Unigene389128 | Staphylocc | 0.000E+00 | 0.000E+00 | 0.000E+00 | 0.000E+00 | 0.000E+00 | 1.612E-06 | 0.000E+00 | 0.000E+00 | 0.000E+00 | 0.000E+00 | 0.000E+00 | 0.000E+00 |
| Unigene389130 | Klebsiella p | 0.000E+00 | 0.000E+00 | 0.000E+00 | 0.000E+00 | 0.000E+00 | 1.587E-06 | 0.000E+00 | 0.000E+00 | 0.000E+00 | 0.000E+00 | 0.000E+00 | 0.000E+00 |
| Unigene389201 | evgS | 0.000E+00 | 0.000E+00 | 0.000E+00 | 0.000E+00 | 0.000E+00 | 6.763E-07 | 0.000E+00 | 0.000E+00 | 0.000E+00 | 0.000E+00 | 0.000E+00 | 0.000E+00 |
| Unigene389218 | adeG | 4.879E-07 | 0.000E+00 | 7.181E-07 | 3.832E-07 | 0.000E+00 | 1.113E-06 | 4.410E-06 | 3.227E-06 | 4.740E-07 | 1.117E-06 | 2.232E-06 | 3.588E-06 |
| Unigene389219 | ceoB | 4.652E-07 | 4.662E-07 | 6.648E-07 | 2.962E-07 | 7.752E-07 | 9.730E-07 | 3.548E-06 | 2.845E-06 | 0.000E+00 | 1.250E-06 | 1.400E-06 | 3.359E-06 |
| Unigene389300 | tetA(60) | 0.000E+00 | 0.000E+00 | 0.000E+00 | 0.000E+00 | 0.000E+00 | 2.796E-05 | 0.000E+00 | 0.000E+00 | 0.000E+00 | 0.000E+00 | 0.000E+00 | 0.000E+00 |
| Unigene389320 | MexW | 0.000E+00 | 0.000E+00 | 8.185E-08 | 1.263E-07 | 0.000E+00 | 3.156E-07 | 0.000E+00 | 0.000E+00 | 0.000E+00 | 0.000E+00 | 0.000E+00 | 6.902E-07 |
| Unigene389367 | vanSF | 4.414E-05 | 1.927E-05 | 6.844E-06 | 1.195E-05 | 1.662E-06 | 1.475E-06 | 3.020E-06 | 6.678E-07 | 8.057E-06 | 4.167E-07 | 2.668E-06 | 1.535E-06 |
| Unigene389533 | vatH | 0.000E+00 | 0.000E+00 | 0.000E+00 | 0.000E+00 | 0.000E+00 | 6.724E-07 | 0.000E+00 | 5.808E-06 | 0.000E+00 | 0.000E+00 | 0.000E+00 | 0.000E+00 |
| Unigene389603 | bcrA | 0.000E+00 | 0.000E+00 | 0.000E+00 | 0.000E+00 | 0.000E+00 | 7.621E-07 | 0.000E+00 | 9.961E-06 | 0.000E+00 | 0.000E+00 | 0.000E+00 | 0.000E+00 |
| Unigene389700 | macB | 0.000E+00 | 0.000E+00 | 0.000E+00 | 0.000E+00 | 0.000E+00 | 9.095E-07 | 0.000E+00 | 1.396E-06 | 0.000E+00 | 0.000E+00 | 0.000E+00 | 5.548E-06 |
| Unigene389992 | vanTC | 0.000E+00 | 0.000E+00 | 0.000E+00 | 0.000E+00 | 0.000E+00 | 5.057E-07 | 0.000E+00 | 0.000E+00 | 0.000E+00 | 0.000E+00 | 0.000E+00 | 0.000E+00 |
| Unigene390011 | rphA | 0.000E+00 | 0.000E+00 | 0.000E+00 | 0.000E+00 | 0.000E+00 | 6.374E-07 | 0.000E+00 | 0.000E+00 | 0.000E+00 | 0.000E+00 | 0.000E+00 | 0.000E+00 |
| Unigene390170 | macB | 0.000E+00 | 0.000E+00 | 0.000E+00 | 0.000E+00 | 0.000E+00 | 9.761E-07 | 0.000E+00 | 0.000E+00 | 0.000E+00 | 0.000E+00 | 0.000E+00 | 0.000E+00 |
| Unigene390310 | adeL | 0.000E+00 | 0.000E+00 | 0.000E+00 | 0.000E+00 | 0.000E+00 | 1.054E-06 | 1.758E-06 | 0.000E+00 | 0.000E+00 | 0.000E+00 | 2.891E-07 | 0.000E+00 |
| Unigene390444 | lmrD | 0.000E+00 | 0.000E+00 | 0.000E+00 | 0.000E+00 | 2.440E-07 | 0.000E+00 | 0.000E+00 | 3.418E-07 | 0.000E+00 | 0.000E+00 | 4.187E-07 | 1.471E-06 |
| Unigene390538 | Acinetobac | 2.902E-07 | 0.000E+00 | 0.000E+00 | 0.000E+00 | 8.327E-07 | 1.438E-06 | 3.532E-06 | 0.000E+00 | 0.000E+00 | 0.000E+00 | 1.272E-06 | 3.544E-06 |
| Unigene390637 | Corynebac | 0.000E+00 | 0.000E+00 | 0.000E+00 | 0.000E+00 | 0.000E+00 | 1.626E-06 | 0.000E+00 | 0.000E+00 | 0.000E+00 | 0.000E+00 | 0.000E+00 | 0.000E+00 |
| Unigene390671 | Staphylocc | 0.000E+00 | 0.000E+00 | 0.000E+00 | 0.000E+00 | 0.000E+00 | 3.994E-07 | 0.000E+00 | 0.000E+00 | 0.000E+00 | 0.000E+00 | 0.000E+00 | 0.000E+00 |
| Unigene390698 | baeS | 0.000E+00 | 0.000E+00 | 0.000E+00 | 0.000E+00 | 0.000E+00 | 6.677E-07 | 0.000E+00 | 0.000E+00 | 0.000E+00 | 0.000E+00 | 0.000E+00 | 0.000E+00 |
| Unigene390699 | smeR | 0.000E+00 | 0.000E+00 | 0.000E+00 | 0.000E+00 | 0.000E+00 | 6.721E-07 | 0.000E+00 | 0.000E+00 | 0.000E+00 | 0.000E+00 | 0.000E+00 | 0.000E+00 |
| Unigene390840 | Staphylocc | 0.000E+00 | 0.000E+00 | 0.000E+00 | 0.000E+00 | 0.000E+00 | 9.365E-07 | 0.000E+00 | 9.207E-06 | 0.000E+00 | 0.000E+00 | 0.000E+00 | 0.000E+00 |
| Unigene390953 | optrA | 0.000E+00 | 0.000E+00 | 0.000E+00 | 0.000E+00 | 0.000E+00 | 6.122E-07 | 0.000E+00 | 0.000E+00 | 0.000E+00 | 0.000E+00 | 0.000E+00 | 2.975E-07 |
| Unigene390967 | msbA | 0.000E+00 | 0.000E+00 | 7.991E-07 | 0.000E+00 | 0.000E+00 | 9.928E-07 | 3.337E-06 | 3.458E-06 | 0.000E+00 | 0.000E+00 | 1.546E-06 | 3.850E-06 |
| Unigene391011 | tetB(60) | 0.000E+00 | 0.000E+00 | 0.000E+00 | 0.000E+00 | 0.000E+00 | 1.965E-06 | 0.000E+00 | 0.000E+00 | 0.000E+00 | 0.000E+00 | 0.000E+00 | 0.000E+00 |
| Unigene391087 | Agrobacte | 0.000E+00 | 0.000E+00 | 1.560E-07 | 0.000E+00 | 0.000E+00 | 0.000E+00 | 0.000E+00 | 0.000E+00 | 0.000E+00 | 0.000E+00 | 0.000E+00 | 0.000E+00 |
| Unigene391107 | MexC | 0.000E+00 | 0.000E+00 | 0.000E+00 | 0.000E+00 | 0.000E+00 | 2.205E-07 | 5.644E-08 | 0.000E+00 | 0.000E+00 | 0.000E+00 | 0.000E+00 | 0.000E+00 |
| Unigene391110 | bcrA | 3.057E-07 | 2.460E-07 | 5.799E-07 | 0.000E+00 | 5.434E-07 | 1.366E-06 | 2.955E-06 | 1.927E-06 | 2.450E-07 | 6.356E-07 | 1.888E-06 | 3.951E-06 |
| Unigene391174 | Erm(K) | 0.000E+00 | 0.000E+00 | 0.000E+00 | 0.000E+00 | 0.000E+00 | 8.994E-07 | 0.000E+00 | 0.000E+00 | 0.000E+00 | 0.000E+00 | 0.000E+00 | 0.000E+00 |
| Unigene391220 | tlrC | 0.000E+00 | 0.000E+00 | 0.000E+00 | 0.000E+00 | 0.000E+00 | 1.423E-06 | 0.000E+00 | 0.000E+00 | 0.000E+00 | 0.000E+00 | 0.000E+00 | 0.000E+00 |
| Unigene391227 | Corynebac | 0.000E+00 | 0.000E+00 | 0.000E+00 | 0.000E+00 | 0.000E+00 | 8.183E-07 | 0.000E+00 | 0.000E+00 | 0.000E+00 | 0.000E+00 | 0.000E+00 | 0.000E+00 |
| Unigene391339 | adeR | 0.000E+00 | 0.000E+00 | 0.000E+00 | 0.000E+00 | 0.000E+00 | 7.961E-07 | 0.000E+00 | 0.000E+00 | 0.000E+00 | 0.000E+00 | 0.000E+00 | 0.000E+00 |
| Unigene391457 | rpoB2 | 9.292E-08 | 0.000E+00 | 2.735E-07 | 2.064E-07 | 0.000E+00 | 6.831E-07 | 9.255E-07 | 9.761E-07 | 0.000E+00 | 7.867E-07 | 8.716E-07 | 1.823E-06 |
| Unigene391478 | patB | 0.000E+00 | 0.000E+00 | 0.000E+00 | 0.000E+00 | 0.000E+00 | 6.979E-07 | 0.000E+00 | 0.000E+00 | 0.000E+00 | 0.000E+00 | 0.000E+00 | 0.000E+00 |
| Unigene391482 | vanRL | 0.000E+00 | 0.000E+00 | 0.000E+00 | 0.000E+00 | 0.000E+00 | 1.075E-06 | 0.000E+00 | 0.000E+00 | 0.000E+00 | 0.000E+00 | 0.000E+00 | 0.000E+00 |
| Unigene391539 | poxtA | 0.000E+00 | 0.000E+00 | 0.000E+00 | 0.000E+00 | 0.000E+00 | 5.170E-07 | 0.000E+00 | 1.094E-05 | 0.000E+00 | 0.000E+00 | 0.000E+00 | 0.000E+00 |
| Unigene391560 | Staphylocc | 0.000E+00 | 0.000E+00 | 0.000E+00 | 0.000E+00 | 0.000E+00 | 9.651E-07 | 0.000E+00 | 0.000E+00 | 0.000E+00 | 0.000E+00 | 0.000E+00 | 0.000E+00 |
| Unigene391619 | MexF | 0.000E+00 | 0.000E+00 | 0.000E+00 | 0.000E+00 | 0.000E+00 | 2.290E-07 | 0.000E+00 | 3.956E-07 | 0.000E+00 | 0.000E+00 | 0.000E+00 | 0.000E+00 |
| Unigene391681 | smeD | 0.000E+00 | 0.000E+00 | 0.000E+00 | 0.000E+00 | 0.000E+00 | 3.684E-07 | 0.000E+00 | 0.000E+00 | 0.000E+00 | 0.000E+00 | 0.000E+00 | 0.000E+00 |
| Unigene391882 | tlrC | 7.263E-06 | 3.100E-06 | 2.904E-06 | 0.000E+00 | 3.960E-05 | 5.898E-05 | 5.915E-05 | 0.000E+00 | 6.968E-05 | 0.000E+00 | 5.539E-05 | 0.000E+00 |
| Unigene391936 | NmcR | 0.000E+00 | 0.000E+00 | 0.000E+00 | 0.000E+00 | 0.000E+00 | 4.580E-07 | 0.000E+00 | 0.000E+00 | 0.000E+00 | 0.000E+00 | 0.000E+00 | 0.000E+00 |
| Unigene391995 | mtrA | 0.000E+00 | 0.000E+00 | 0.000E+00 | 0.000E+00 | 0.000E+00 | 1.803E-06 | 0.000E+00 | 0.000E+00 | 0.000E+00 | 0.000E+00 | 0.000E+00 | 0.000E+00 |
| Unigene392000 | efrA | 0.000E+00 | 0.000E+00 | 0.000E+00 | 0.000E+00 | 0.000E+00 | 1.786E-06 | 0.000E+00 | 0.000E+00 | 0.000E+00 | 0.000E+00 | 0.000E+00 | 0.000E+00 |
| Unigene392094 | bcrA | 0.000E+00 | 0.000E+00 | 0.000E+00 | 0.000E+00 | 0.000E+00 | 1.086E-06 | 0.000E+00 | 0.000E+00 | 0.000E+00 | 0.000E+00 | 0.000E+00 | 0.000E+00 |
| Unigene392100 | iri | 0.000E+00 | 0.000E+00 | 0.000E+00 | 0.000E+00 | 0.000E+00 | 7.299E-07 | 0.000E+00 | 2.463E-07 | 0.000E+00 | 0.000E+00 | 0.000E+00 | 0.000E+00 |
| Unigene392161 | msbA | 0.000E+00 | 0.000E+00 | 0.000E+00 | 0.000E+00 | 0.000E+00 | 5.900E-07 | 0.000E+00 | 7.463E-06 | 0.000E+00 | 0.000E+00 | 0.000E+00 | 0.000E+00 |
| Unigene392162 | msbA | 0.000E+00 | 0.000E+00 | 0.000E+00 | 0.000E+00 | 0.000E+00 | 8.482E-07 | 0.000E+00 | 0.000E+00 | 0.000E+00 | 0.000E+00 | 0.000E+00 | 0.000E+00 |
| Unigene392226 | Listeria mc | 0.000E+00 | 0.000E+00 | 0.000E+00 | 0.000E+00 | 0.000E+00 | 9.263E-06 | 2.633E-05 | 2.832E-05 | 3.678E-06 | 0.000E+00 | 0.000E+00 | 0.000E+00 |

| | | | | | | | | | | | | | |
|---|---|---|---|---|---|---|---|---|---|---|---|---|---|
| Unigene392404 | Staphylocc | 0.000E+00 | 0.000E+00 | 0.000E+00 | 0.000E+00 | 0.000E+00 | 4.759E-07 | 0.000E+00 | 0.000E+00 | 0.000E+00 | 0.000E+00 | 0.000E+00 | 0.000E+00 |
| Unigene392461 | vanRE | 0.000E+00 | 0.000E+00 | 0.000E+00 | 0.000E+00 | 0.000E+00 | 1.637E-06 | 0.000E+00 | 0.000E+00 | 0.000E+00 | 0.000E+00 | 0.000E+00 | 0.000E+00 |
| Unigene392473 | vanSM | 0.000E+00 | 0.000E+00 | 0.000E+00 | 0.000E+00 | 0.000E+00 | 1.127E-06 | 0.000E+00 | 0.000E+00 | 0.000E+00 | 0.000E+00 | 0.000E+00 | 0.000E+00 |
| Unigene392474 | vanRF | 0.000E+00 | 0.000E+00 | 0.000E+00 | 0.000E+00 | 0.000E+00 | 1.418E-06 | 0.000E+00 | 0.000E+00 | 0.000E+00 | 0.000E+00 | 0.000E+00 | 0.000E+00 |
| Unigene392708 | tetW | 0.000E+00 | 0.000E+00 | 0.000E+00 | 0.000E+00 | 0.000E+00 | 1.651E-06 | 0.000E+00 | 0.000E+00 | 0.000E+00 | 0.000E+00 | 0.000E+00 | 0.000E+00 |
| Unigene392860 | tetA(60) | 2.121E-07 | 0.000E+00 | 5.298E-07 | 3.310E-07 | 0.000E+00 | 1.167E-06 | 3.137E-06 | 3.264E-06 | 3.794E-07 | 1.267E-06 | 0.000E+00 | 3.214E-06 |
| Unigene393031 | mecC | 0.000E+00 | 0.000E+00 | 1.286E-07 | 0.000E+00 | 0.000E+00 | 1.257E-06 | 3.625E-08 | 2.103E-07 | 0.000E+00 | 0.000E+00 | 0.000E+00 | 0.000E+00 |
| Unigene393111 | macB | 0.000E+00 | 0.000E+00 | 0.000E+00 | 0.000E+00 | 0.000E+00 | 9.613E-07 | 0.000E+00 | 0.000E+00 | 0.000E+00 | 0.000E+00 | 0.000E+00 | 0.000E+00 |
| Unigene393126 | CMY-84 | 0.000E+00 | 0.000E+00 | 0.000E+00 | 0.000E+00 | 0.000E+00 | 4.524E-07 | 0.000E+00 | 0.000E+00 | 0.000E+00 | 0.000E+00 | 0.000E+00 | 0.000E+00 |
| Unigene393141 | vanD | 0.000E+00 | 0.000E+00 | 0.000E+00 | 0.000E+00 | 0.000E+00 | 8.460E-07 | 0.000E+00 | 0.000E+00 | 0.000E+00 | 0.000E+00 | 0.000E+00 | 0.000E+00 |
| Unigene393252 | oleC | 0.000E+00 | 0.000E+00 | 0.000E+00 | 0.000E+00 | 0.000E+00 | 0.000E+00 | 0.000E+00 | 0.000E+00 | 0.000E+00 | 0.000E+00 | 0.000E+00 | 1.640E-06 |
| Unigene393291 | arlR | 0.000E+00 | 0.000E+00 | 0.000E+00 | 0.000E+00 | 0.000E+00 | 1.048E-06 | 0.000E+00 | 0.000E+00 | 0.000E+00 | 0.000E+00 | 0.000E+00 | 0.000E+00 |
| Unigene393331 | vanSF | 0.000E+00 | 0.000E+00 | 0.000E+00 | 0.000E+00 | 0.000E+00 | 1.355E-06 | 0.000E+00 | 0.000E+00 | 0.000E+00 | 0.000E+00 | 0.000E+00 | 0.000E+00 |
| Unigene393334 | Agrobacte | 0.000E+00 | 0.000E+00 | 0.000E+00 | 0.000E+00 | 0.000E+00 | 9.113E-07 | 0.000E+00 | 0.000E+00 | 0.000E+00 | 0.000E+00 | 0.000E+00 | 0.000E+00 |
| Unigene393400 | TriA | 0.000E+00 | 0.000E+00 | 0.000E+00 | 0.000E+00 | 0.000E+00 | 0.000E+00 | 2.355E-06 | 0.000E+00 | 0.000E+00 | 0.000E+00 | 0.000E+00 | 0.000E+00 |
| Unigene393506 | vanHB | 0.000E+00 | 0.000E+00 | 3.731E-08 | 0.000E+00 | 4.224E-08 | 3.699E-07 | 2.525E-07 | 0.000E+00 | 0.000E+00 | 0.000E+00 | 4.349E-08 | 0.000E+00 |
| Unigene393515 | TaeA | 0.000E+00 | 0.000E+00 | 0.000E+00 | 0.000E+00 | 0.000E+00 | 7.813E-07 | 0.000E+00 | 0.000E+00 | 0.000E+00 | 0.000E+00 | 0.000E+00 | 0.000E+00 |
| Unigene393547 | patA | 4.501E-05 | 2.543E-05 | 1.963E-05 | 2.042E-05 | 2.015E-06 | 1.384E-06 | 3.858E-06 | 8.096E-07 | 6.823E-06 | 6.934E-07 | 1.933E-06 | 1.282E-06 |
| Unigene393639 | patA | 0.000E+00 | 0.000E+00 | 0.000E+00 | 0.000E+00 | 0.000E+00 | 1.454E-07 | 0.000E+00 | 2.355E-07 | 0.000E+00 | 0.000E+00 | 3.077E-07 | 1.272E-06 |
| Unigene393648 | arlR | 0.000E+00 | 0.000E+00 | 0.000E+00 | 0.000E+00 | 0.000E+00 | 2.336E-07 | 6.217E-07 | 0.000E+00 | 0.000E+00 | 0.000E+00 | 4.943E-08 | 2.043E-07 |
| Unigene393717 | evgS | 0.000E+00 | 0.000E+00 | 0.000E+00 | 0.000E+00 | 0.000E+00 | 3.943E-07 | 0.000E+00 | 0.000E+00 | 0.000E+00 | 0.000E+00 | 0.000E+00 | 0.000E+00 |
| Unigene393821 | evgS | 4.559E-08 | 0.000E+00 | 0.000E+00 | 0.000E+00 | 0.000E+00 | 1.972E-07 | 0.000E+00 | 0.000E+00 | 0.000E+00 | 0.000E+00 | 0.000E+00 | 1.617E-07 |
| Unigene393822 | mdtN | 0.000E+00 | 0.000E+00 | 0.000E+00 | 0.000E+00 | 0.000E+00 | 5.240E-07 | 0.000E+00 | 0.000E+00 | 0.000E+00 | 0.000E+00 | 0.000E+00 | 0.000E+00 |
| Unigene393856 | patA | 0.000E+00 | 0.000E+00 | 0.000E+00 | 4.666E-07 | 7.700E-07 | 1.285E-06 | 3.836E-06 | 4.161E-06 | 4.690E-07 | 0.000E+00 | 1.699E-06 | 4.682E-06 |
| Unigene393910 | Staphylocc | 0.000E+00 | 0.000E+00 | 0.000E+00 | 0.000E+00 | 0.000E+00 | 5.849E-07 | 0.000E+00 | 0.000E+00 | 0.000E+00 | 0.000E+00 | 0.000E+00 | 5.684E-07 |
| Unigene394036 | vanSF | 0.000E+00 | 0.000E+00 | 0.000E+00 | 0.000E+00 | 0.000E+00 | 6.374E-07 | 0.000E+00 | 0.000E+00 | 0.000E+00 | 0.000E+00 | 0.000E+00 | 0.000E+00 |
| Unigene394299 | NmcR | 0.000E+00 | 0.000E+00 | 0.000E+00 | 0.000E+00 | 0.000E+00 | 8.394E-07 | 0.000E+00 | 0.000E+00 | 0.000E+00 | 0.000E+00 | 0.000E+00 | 0.000E+00 |
| Unigene394334 | TaeA | 0.000E+00 | 0.000E+00 | 0.000E+00 | 0.000E+00 | 0.000E+00 | 2.266E-07 | 0.000E+00 | 0.000E+00 | 0.000E+00 | 0.000E+00 | 0.000E+00 | 0.000E+00 |
| Unigene394437 | facT | 0.000E+00 | 0.000E+00 | 0.000E+00 | 0.000E+00 | 0.000E+00 | 8.697E-07 | 0.000E+00 | 0.000E+00 | 0.000E+00 | 0.000E+00 | 0.000E+00 | 0.000E+00 |
| Unigene394442 | bcrA | 0.000E+00 | 0.000E+00 | 0.000E+00 | 0.000E+00 | 0.000E+00 | 9.008E-07 | 0.000E+00 | 0.000E+00 | 0.000E+00 | 0.000E+00 | 0.000E+00 | 0.000E+00 |
| Unigene394481 | patB | 0.000E+00 | 0.000E+00 | 0.000E+00 | 0.000E+00 | 0.000E+00 | 8.037E-07 | 0.000E+00 | 0.000E+00 | 0.000E+00 | 0.000E+00 | 0.000E+00 | 0.000E+00 |
| Unigene394505 | qacH | 0.000E+00 | 0.000E+00 | 0.000E+00 | 0.000E+00 | 0.000E+00 | 6.231E-07 | 0.000E+00 | 0.000E+00 | 0.000E+00 | 0.000E+00 | 0.000E+00 | 0.000E+00 |
| Unigene394520 | vanHB | 0.000E+00 | 0.000E+00 | 0.000E+00 | 0.000E+00 | 0.000E+00 | 4.436E-07 | 0.000E+00 | 0.000E+00 | 0.000E+00 | 0.000E+00 | 0.000E+00 | 0.000E+00 |
| Unigene394569 | Streptomy | 0.000E+00 | 0.000E+00 | 0.000E+00 | 0.000E+00 | 0.000E+00 | 1.344E-06 | 0.000E+00 | 0.000E+00 | 0.000E+00 | 0.000E+00 | 0.000E+00 | 0.000E+00 |
| Unigene394669 | Brucella su | 0.000E+00 | 0.000E+00 | 0.000E+00 | 0.000E+00 | 0.000E+00 | 0.000E+00 | 0.000E+00 | 0.000E+00 | 0.000E+00 | 0.000E+00 | 0.000E+00 | 1.514E-06 |
| Unigene394692 | mdtC | 0.000E+00 | 0.000E+00 | 0.000E+00 | 0.000E+00 | 3.129E-07 | 0.000E+00 | 0.000E+00 | 0.000E+00 | 0.000E+00 | 0.000E+00 | 0.000E+00 | 0.000E+00 |
| Unigene394766 | poxtA | 0.000E+00 | 0.000E+00 | 0.000E+00 | 0.000E+00 | 0.000E+00 | 4.474E-07 | 0.000E+00 | 0.000E+00 | 0.000E+00 | 0.000E+00 | 0.000E+00 | 0.000E+00 |
| Unigene394784 | mdtN | 2.952E-07 | 0.000E+00 | 0.000E+00 | 6.957E-07 | 9.257E-06 | 7.802E-07 | 1.706E-06 | 0.000E+00 | 0.000E+00 | 2.193E-07 | 0.000E+00 | 0.000E+00 |
| Unigene394872 | acrB | 0.000E+00 | 0.000E+00 | 0.000E+00 | 0.000E+00 | 2.255E-07 | 0.000E+00 | 0.000E+00 | 5.924E-07 | 0.000E+00 | 0.000E+00 | 2.322E-07 | 0.000E+00 |
| Unigene394909 | adeI | 0.000E+00 | 0.000E+00 | 0.000E+00 | 0.000E+00 | 0.000E+00 | 3.805E-07 | 1.002E-06 | 2.935E-07 | 0.000E+00 | 0.000E+00 | 0.000E+00 | 0.000E+00 |
| Unigene394962 | adeI | 0.000E+00 | 0.000E+00 | 0.000E+00 | 0.000E+00 | 0.000E+00 | 4.081E-07 | 0.000E+00 | 0.000E+00 | 0.000E+00 | 0.000E+00 | 2.159E-07 | 0.000E+00 |
| Unigene395083 | mepA | 0.000E+00 | 0.000E+00 | 0.000E+00 | 0.000E+00 | 0.000E+00 | 6.594E-07 | 0.000E+00 | 0.000E+00 | 0.000E+00 | 0.000E+00 | 0.000E+00 | 0.000E+00 |
| Unigene395166 | oleC | 0.000E+00 | 0.000E+00 | 0.000E+00 | 0.000E+00 | 0.000E+00 | 3.801E-07 | 0.000E+00 | 0.000E+00 | 0.000E+00 | 0.000E+00 | 0.000E+00 | 0.000E+00 |
| Unigene395231 | Streptomy | 0.000E+00 | 0.000E+00 | 0.000E+00 | 0.000E+00 | 0.000E+00 | 6.037E-07 | 0.000E+00 | 9.040E-06 | 0.000E+00 | 0.000E+00 | 0.000E+00 | 0.000E+00 |
| Unigene395415 | Streptomy | 0.000E+00 | 0.000E+00 | 0.000E+00 | 0.000E+00 | 0.000E+00 | 1.273E-06 | 0.000E+00 | 0.000E+00 | 0.000E+00 | 0.000E+00 | 0.000E+00 | 0.000E+00 |
| Unigene395430 | lmrC | 0.000E+00 | 0.000E+00 | 0.000E+00 | 0.000E+00 | 0.000E+00 | 5.662E-07 | 0.000E+00 | 0.000E+00 | 0.000E+00 | 0.000E+00 | 0.000E+00 | 0.000E+00 |
| Unigene395581 | Streptomy | 0.000E+00 | 0.000E+00 | 0.000E+00 | 0.000E+00 | 0.000E+00 | 1.474E-06 | 0.000E+00 | 0.000E+00 | 0.000E+00 | 0.000E+00 | 0.000E+00 | 0.000E+00 |
| Unigene395591 | Staphylocc | 0.000E+00 | 0.000E+00 | 9.914E-08 | 0.000E+00 | 0.000E+00 | 3.277E-07 | 0.000E+00 | 0.000E+00 | 0.000E+00 | 0.000E+00 | 0.000E+00 | 0.000E+00 |
| Unigene395827 | lmrB | 0.000E+00 | 0.000E+00 | 0.000E+00 | 0.000E+00 | 0.000E+00 | 1.181E-06 | 0.000E+00 | 0.000E+00 | 0.000E+00 | 0.000E+00 | 0.000E+00 | 0.000E+00 |
| Unigene395839 | baeS | 0.000E+00 | 0.000E+00 | 0.000E+00 | 0.000E+00 | 0.000E+00 | 1.775E-06 | 0.000E+00 | 0.000E+00 | 0.000E+00 | 0.000E+00 | 0.000E+00 | 0.000E+00 |
| Unigene395840 | vanRM | 0.000E+00 | 0.000E+00 | 0.000E+00 | 0.000E+00 | 0.000E+00 | 1.557E-06 | 0.000E+00 | 0.000E+00 | 0.000E+00 | 0.000E+00 | 0.000E+00 | 0.000E+00 |
| Unigene395902 | PmrF | 0.000E+00 | 0.000E+00 | 0.000E+00 | 0.000E+00 | 0.000E+00 | 5.628E-07 | 0.000E+00 | 0.000E+00 | 0.000E+00 | 0.000E+00 | 0.000E+00 | 0.000E+00 |
| Unigene395918 | Bifidobacte | 0.000E+00 | 0.000E+00 | 0.000E+00 | 0.000E+00 | 0.000E+00 | 2.614E-07 | 0.000E+00 | 0.000E+00 | 0.000E+00 | 0.000E+00 | 0.000E+00 | 0.000E+00 |
| Unigene396043 | Corynebac | 0.000E+00 | 0.000E+00 | 0.000E+00 | 0.000E+00 | 0.000E+00 | 1.163E-07 | 0.000E+00 | 0.000E+00 | 0.000E+00 | 0.000E+00 | 0.000E+00 | 0.000E+00 |
| Unigene396410 | Rhodococc | 0.000E+00 | 0.000E+00 | 0.000E+00 | 0.000E+00 | 3.073E-08 | 7.476E-07 | 0.000E+00 | 0.000E+00 | 0.000E+00 | 0.000E+00 | 0.000E+00 | 0.000E+00 |
| Unigene396460 | mphO | 2.289E-07 | 0.000E+00 | 0.000E+00 | 0.000E+00 | 0.000E+00 | 4.331E-07 | 0.000E+00 | 0.000E+00 | 0.000E+00 | 0.000E+00 | 0.000E+00 | 1.421E-06 |
| Unigene396493 | CMY-98 | 0.000E+00 | 0.000E+00 | 0.000E+00 | 0.000E+00 | 0.000E+00 | 5.587E-07 | 0.000E+00 | 0.000E+00 | 0.000E+00 | 0.000E+00 | 0.000E+00 | 0.000E+00 |
| Unigene396508 | tet(30) | 0.000E+00 | 0.000E+00 | 2.329E-07 | 0.000E+00 | 0.000E+00 | 5.987E-07 | 8.756E-07 | 0.000E+00 | 0.000E+00 | 4.407E-07 | 0.000E+00 | 5.611E-07 |

| | | | | | | | | | | | | | |
|---|---|---|---|---|---|---|---|---|---|---|---|---|---|
| Unigene396528 | vanRI | 0.000E+00 | 0.000E+00 | 0.000E+00 | 0.000E+00 | 0.000E+00 | 5.965E-07 | 0.000E+00 | 0.000E+00 | 0.000E+00 | 0.000E+00 | 1.052E-07 | 0.000E+00 |
| Unigene396536 | tetA(58) | 0.000E+00 | 0.000E+00 | 0.000E+00 | 0.000E+00 | 0.000E+00 | 7.494E-06 | 0.000E+00 | 0.000E+00 | 0.000E+00 | 0.000E+00 | 0.000E+00 | 0.000E+00 |
| Unigene396709 | tetA(60) | 0.000E+00 | 0.000E+00 | 0.000E+00 | 0.000E+00 | 0.000E+00 | 3.270E-07 | 0.000E+00 | 0.000E+00 | 0.000E+00 | 0.000E+00 | 0.000E+00 | 0.000E+00 |
| Unigene396711 | macB | 0.000E+00 | 0.000E+00 | 0.000E+00 | 0.000E+00 | 0.000E+00 | 6.263E-07 | 0.000E+00 | 0.000E+00 | 0.000E+00 | 0.000E+00 | 0.000E+00 | 0.000E+00 |
| Unigene396767 | Staphylocc | 0.000E+00 | 0.000E+00 | 0.000E+00 | 0.000E+00 | 0.000E+00 | 5.539E-07 | 0.000E+00 | 0.000E+00 | 0.000E+00 | 0.000E+00 | 0.000E+00 | 0.000E+00 |
| Unigene396768 | Listeria mc | 0.000E+00 | 0.000E+00 | 0.000E+00 | 0.000E+00 | 0.000E+00 | 4.653E-07 | 0.000E+00 | 0.000E+00 | 0.000E+00 | 0.000E+00 | 0.000E+00 | 0.000E+00 |
| Unigene396868 | YojI | 0.000E+00 | 0.000E+00 | 0.000E+00 | 0.000E+00 | 7.762E-07 | 0.000E+00 | 0.000E+00 | 7.340E-07 | 0.000E+00 | 0.000E+00 | 1.598E-07 | 0.000E+00 |
| Unigene396922 | lmrD | 0.000E+00 | 0.000E+00 | 0.000E+00 | 0.000E+00 | 0.000E+00 | 0.000E+00 | 0.000E+00 | 0.000E+00 | 0.000E+00 | 0.000E+00 | 0.000E+00 | 1.137E-06 |
| Unigene396951 | fexA | 0.000E+00 | 0.000E+00 | 0.000E+00 | 0.000E+00 | 0.000E+00 | 1.741E-06 | 0.000E+00 | 0.000E+00 | 0.000E+00 | 0.000E+00 | 0.000E+00 | 0.000E+00 |
| Unigene396989 | arlR | 0.000E+00 | 0.000E+00 | 0.000E+00 | 0.000E+00 | 0.000E+00 | 1.740E-06 | 0.000E+00 | 0.000E+00 | 0.000E+00 | 0.000E+00 | 0.000E+00 | 0.000E+00 |
| Unigene396990 | arlS | 0.000E+00 | 0.000E+00 | 0.000E+00 | 0.000E+00 | 0.000E+00 | 1.743E-06 | 0.000E+00 | 0.000E+00 | 0.000E+00 | 0.000E+00 | 0.000E+00 | 0.000E+00 |
| Unigene396994 | bcr-1 | 0.000E+00 | 0.000E+00 | 0.000E+00 | 0.000E+00 | 0.000E+00 | 2.129E-06 | 0.000E+00 | 0.000E+00 | 0.000E+00 | 0.000E+00 | 0.000E+00 | 0.000E+00 |
| Unigene397234 | emrB | 0.000E+00 | 0.000E+00 | 0.000E+00 | 0.000E+00 | 0.000E+00 | 5.033E-07 | 0.000E+00 | 0.000E+00 | 0.000E+00 | 0.000E+00 | 1.775E-07 | 0.000E+00 |
| Unigene397246 | mtrA | 0.000E+00 | 0.000E+00 | 0.000E+00 | 0.000E+00 | 0.000E+00 | 9.365E-07 | 0.000E+00 | 0.000E+00 | 0.000E+00 | 0.000E+00 | 0.000E+00 | 0.000E+00 |
| Unigene397247 | vanSB | 0.000E+00 | 0.000E+00 | 0.000E+00 | 0.000E+00 | 0.000E+00 | 8.199E-07 | 0.000E+00 | 0.000E+00 | 0.000E+00 | 0.000E+00 | 0.000E+00 | 0.000E+00 |
| Unigene397308 | tetB(P) | 0.000E+00 | 0.000E+00 | 0.000E+00 | 0.000E+00 | 0.000E+00 | 1.396E-06 | 0.000E+00 | 0.000E+00 | 0.000E+00 | 0.000E+00 | 0.000E+00 | 0.000E+00 |
| Unigene397411 | Bifidobacte | 0.000E+00 | 0.000E+00 | 0.000E+00 | 0.000E+00 | 0.000E+00 | 3.693E-07 | 0.000E+00 | 0.000E+00 | 0.000E+00 | 0.000E+00 | 0.000E+00 | 0.000E+00 |
| Unigene397499 | vgaE | 0.000E+00 | 0.000E+00 | 0.000E+00 | 0.000E+00 | 0.000E+00 | 3.335E-07 | 0.000E+00 | 9.003E-08 | 0.000E+00 | 0.000E+00 | 0.000E+00 | 0.000E+00 |
| Unigene397604 | cmlv | 3.004E-07 | 0.000E+00 | 0.000E+00 | 0.000E+00 | 0.000E+00 | 1.122E-06 | 4.020E-06 | 3.602E-06 | 0.000E+00 | 0.000E+00 | 1.468E-06 | 3.777E-06 |
| Unigene397605 | adeL | 3.945E-07 | 0.000E+00 | 0.000E+00 | 0.000E+00 | 0.000E+00 | 1.202E-06 | 4.009E-06 | 3.852E-06 | 0.000E+00 | 0.000E+00 | 2.010E-06 | 3.095E-06 |
| Unigene397749 | vanHD | 0.000E+00 | 0.000E+00 | 0.000E+00 | 0.000E+00 | 0.000E+00 | 6.907E-07 | 0.000E+00 | 0.000E+00 | 0.000E+00 | 0.000E+00 | 0.000E+00 | 0.000E+00 |
| Unigene397819 | tetB(46) | 0.000E+00 | 0.000E+00 | 0.000E+00 | 0.000E+00 | 0.000E+00 | 9.095E-07 | 0.000E+00 | 0.000E+00 | 0.000E+00 | 0.000E+00 | 0.000E+00 | 0.000E+00 |
| Unigene397969 | lmrD | 0.000E+00 | 0.000E+00 | 0.000E+00 | 0.000E+00 | 0.000E+00 | 7.327E-07 | 0.000E+00 | 0.000E+00 | 0.000E+00 | 0.000E+00 | 0.000E+00 | 0.000E+00 |
| Unigene398026 | vanHO | 3.860E-07 | 0.000E+00 | 0.000E+00 | 0.000E+00 | 0.000E+00 | 4.695E-07 | 0.000E+00 | 0.000E+00 | 0.000E+00 | 0.000E+00 | 0.000E+00 | 0.000E+00 |
| Unigene398032 | tet(A) | 0.000E+00 | 0.000E+00 | 0.000E+00 | 0.000E+00 | 0.000E+00 | 3.732E-07 | 0.000E+00 | 0.000E+00 | 0.000E+00 | 0.000E+00 | 0.000E+00 | 0.000E+00 |
| Unigene398043 | macB | 0.000E+00 | 0.000E+00 | 0.000E+00 | 0.000E+00 | 0.000E+00 | 1.851E-06 | 4.821E-06 | 3.955E-06 | 0.000E+00 | 0.000E+00 | 0.000E+00 | 3.565E-06 |
| Unigene398248 | Streptomy | 0.000E+00 | 0.000E+00 | 0.000E+00 | 0.000E+00 | 0.000E+00 | 1.339E-06 | 0.000E+00 | 0.000E+00 | 0.000E+00 | 0.000E+00 | 0.000E+00 | 0.000E+00 |
| Unigene398304 | efrA | 0.000E+00 | 0.000E+00 | 6.476E-07 | 0.000E+00 | 6.694E-07 | 1.303E-06 | 2.763E-06 | 3.684E-06 | 5.645E-07 | 9.909E-07 | 2.002E-06 | 3.595E-06 |
| Unigene398342 | adeL | 0.000E+00 | 0.000E+00 | 0.000E+00 | 0.000E+00 | 0.000E+00 | 3.021E-07 | 0.000E+00 | 0.000E+00 | 0.000E+00 | 0.000E+00 | 0.000E+00 | 0.000E+00 |
| Unigene398441 | patA | 1.072E-06 | 1.078E-06 | 8.762E-07 | 5.861E-07 | 7.837E-06 | 3.379E-07 | 0.000E+00 | 0.000E+00 | 0.000E+00 | 0.000E+00 | 0.000E+00 | 0.000E+00 |
| Unigene398679 | mgrA | 0.000E+00 | 0.000E+00 | 0.000E+00 | 0.000E+00 | 0.000E+00 | 1.023E-06 | 0.000E+00 | 0.000E+00 | 0.000E+00 | 0.000E+00 | 0.000E+00 | 0.000E+00 |
| Unigene398851 | tetA(60) | 1.571E-07 | 1.078E-07 | 3.853E-08 | 0.000E+00 | 6.544E-08 | 9.361E-06 | 1.260E-06 | 1.068E-05 | 4.035E-06 | 0.000E+00 | 0.000E+00 | 6.267E-07 |
| Unigene399052 | macB | 0.000E+00 | 0.000E+00 | 0.000E+00 | 0.000E+00 | 0.000E+00 | 9.942E-07 | 0.000E+00 | 0.000E+00 | 0.000E+00 | 0.000E+00 | 0.000E+00 | 0.000E+00 |
| Unigene399109 | Staphylocc | 0.000E+00 | 0.000E+00 | 0.000E+00 | 0.000E+00 | 0.000E+00 | 1.121E-06 | 0.000E+00 | 0.000E+00 | 0.000E+00 | 0.000E+00 | 0.000E+00 | 0.000E+00 |
| Unigene399324 | Rhodococ | 0.000E+00 | 0.000E+00 | 0.000E+00 | 0.000E+00 | 0.000E+00 | 9.970E-07 | 0.000E+00 | 9.819E-06 | 0.000E+00 | 0.000E+00 | 1.701E-05 | 0.000E+00 |
| Unigene399341 | macB | 0.000E+00 | 0.000E+00 | 0.000E+00 | 0.000E+00 | 0.000E+00 | 8.691E-07 | 0.000E+00 | 0.000E+00 | 0.000E+00 | 0.000E+00 | 0.000E+00 | 0.000E+00 |
| Unigene399464 | tetA(60) | 0.000E+00 | 0.000E+00 | 0.000E+00 | 0.000E+00 | 0.000E+00 | 9.115E-07 | 0.000E+00 | 0.000E+00 | 0.000E+00 | 0.000E+00 | 0.000E+00 | 0.000E+00 |
| Unigene399486 | vmlR | 0.000E+00 | 0.000E+00 | 0.000E+00 | 0.000E+00 | 0.000E+00 | 2.620E-07 | 0.000E+00 | 0.000E+00 | 0.000E+00 | 0.000E+00 | 0.000E+00 | 0.000E+00 |
| Unigene399523 | vanHB | 0.000E+00 | 0.000E+00 | 0.000E+00 | 0.000E+00 | 0.000E+00 | 1.323E-06 | 0.000E+00 | 9.627E-06 | 0.000E+00 | 0.000E+00 | 0.000E+00 | 0.000E+00 |
| Unigene399584 | Staphylocc | 0.000E+00 | 0.000E+00 | 0.000E+00 | 0.000E+00 | 0.000E+00 | 5.948E-07 | 0.000E+00 | 0.000E+00 | 0.000E+00 | 0.000E+00 | 0.000E+00 | 0.000E+00 |
| Unigene399699 | Staphylocc | 0.000E+00 | 0.000E+00 | 0.000E+00 | 0.000E+00 | 0.000E+00 | 1.035E-06 | 0.000E+00 | 0.000E+00 | 0.000E+00 | 0.000E+00 | 0.000E+00 | 0.000E+00 |
| Unigene399700 | Rhodococ | 0.000E+00 | 0.000E+00 | 0.000E+00 | 0.000E+00 | 0.000E+00 | 1.142E-06 | 0.000E+00 | 0.000E+00 | 0.000E+00 | 0.000E+00 | 0.000E+00 | 0.000E+00 |
| Unigene399752 | lmrB | 0.000E+00 | 0.000E+00 | 0.000E+00 | 0.000E+00 | 0.000E+00 | 1.065E-06 | 0.000E+00 | 0.000E+00 | 0.000E+00 | 0.000E+00 | 0.000E+00 | 0.000E+00 |
| Unigene399799 | poxtA | 0.000E+00 | 0.000E+00 | 0.000E+00 | 0.000E+00 | 0.000E+00 | 1.298E-06 | 0.000E+00 | 0.000E+00 | 0.000E+00 | 0.000E+00 | 0.000E+00 | 0.000E+00 |
| Unigene399818 | rpoB2 | 0.000E+00 | 0.000E+00 | 0.000E+00 | 0.000E+00 | 0.000E+00 | 1.448E-06 | 0.000E+00 | 0.000E+00 | 0.000E+00 | 0.000E+00 | 0.000E+00 | 0.000E+00 |
| Unigene399888 | adeR | 0.000E+00 | 0.000E+00 | 0.000E+00 | 2.280E-07 | 0.000E+00 | 0.000E+00 | 0.000E+00 | 0.000E+00 | 0.000E+00 | 0.000E+00 | 0.000E+00 | 0.000E+00 |
| Unigene400174 | bcrA | 0.000E+00 | 0.000E+00 | 0.000E+00 | 0.000E+00 | 0.000E+00 | 1.444E-06 | 0.000E+00 | 0.000E+00 | 0.000E+00 | 0.000E+00 | 0.000E+00 | 0.000E+00 |
| Unigene400256 | Staphylocc | 0.000E+00 | 0.000E+00 | 0.000E+00 | 0.000E+00 | 0.000E+00 | 9.175E-07 | 0.000E+00 | 0.000E+00 | 0.000E+00 | 0.000E+00 | 0.000E+00 | 0.000E+00 |
| Unigene400471 | cmrA | 0.000E+00 | 0.000E+00 | 0.000E+00 | 0.000E+00 | 0.000E+00 | 1.214E-06 | 0.000E+00 | 0.000E+00 | 0.000E+00 | 0.000E+00 | 0.000E+00 | 0.000E+00 |
| Unigene400474 | vanHA | 0.000E+00 | 0.000E+00 | 0.000E+00 | 0.000E+00 | 0.000E+00 | 1.308E-06 | 0.000E+00 | 0.000E+00 | 0.000E+00 | 0.000E+00 | 0.000E+00 | 0.000E+00 |
| Unigene400601 | Acinetoba | 0.000E+00 | 0.000E+00 | 0.000E+00 | 0.000E+00 | 0.000E+00 | 4.572E-07 | 0.000E+00 | 0.000E+00 | 0.000E+00 | 0.000E+00 | 0.000E+00 | 0.000E+00 |
| Unigene400717 | PmrF | 0.000E+00 | 0.000E+00 | 0.000E+00 | 0.000E+00 | 0.000E+00 | 1.238E-06 | 0.000E+00 | 0.000E+00 | 0.000E+00 | 0.000E+00 | 0.000E+00 | 0.000E+00 |
| Unigene400743 | vanHM | 0.000E+00 | 0.000E+00 | 0.000E+00 | 0.000E+00 | 0.000E+00 | 1.612E-06 | 0.000E+00 | 0.000E+00 | 0.000E+00 | 0.000E+00 | 0.000E+00 | 0.000E+00 |
| Unigene400947 | NmcR | 0.000E+00 | 0.000E+00 | 0.000E+00 | 0.000E+00 | 0.000E+00 | 9.693E-07 | 0.000E+00 | 0.000E+00 | 0.000E+00 | 0.000E+00 | 0.000E+00 | 0.000E+00 |
| Unigene400966 | YojI | 0.000E+00 | 0.000E+00 | 0.000E+00 | 0.000E+00 | 0.000E+00 | 1.228E-06 | 0.000E+00 | 0.000E+00 | 0.000E+00 | 0.000E+00 | 0.000E+00 | 0.000E+00 |
| Unigene401022 | macB | 0.000E+00 | 0.000E+00 | 0.000E+00 | 0.000E+00 | 0.000E+00 | 1.108E-06 | 0.000E+00 | 0.000E+00 | 0.000E+00 | 0.000E+00 | 0.000E+00 | 0.000E+00 |
| Unigene401030 | lmrD | 0.000E+00 | 0.000E+00 | 0.000E+00 | 0.000E+00 | 0.000E+00 | 9.601E-07 | 0.000E+00 | 0.000E+00 | 0.000E+00 | 0.000E+00 | 0.000E+00 | 0.000E+00 |
| Unigene401110 | lmrD | 0.000E+00 | 0.000E+00 | 0.000E+00 | 0.000E+00 | 0.000E+00 | 8.096E-07 | 0.000E+00 | 0.000E+00 | 0.000E+00 | 0.000E+00 | 0.000E+00 | 4.426E-07 |

| | | | | | | | | | | | | | |
|---|---|---|---|---|---|---|---|---|---|---|---|---|---|
| Unigene401205 | vanHF | 0.000E+00 | 0.000E+00 | 0.000E+00 | 0.000E+00 | 0.000E+00 | 4.596E-07 | 0.000E+00 | 0.000E+00 | 0.000E+00 | 0.000E+00 | 0.000E+00 | 0.000E+00 |
| Unigene401215 | arlS | 0.000E+00 | 0.000E+00 | 1.098E-05 | 0.000E+00 | 0.000E+00 | 1.055E-06 | 0.000E+00 | 0.000E+00 | 0.000E+00 | 0.000E+00 | 0.000E+00 | 0.000E+00 |
| Unigene401297 | Staphylocc | 4.171E-08 | 0.000E+00 | 4.092E-08 | 0.000E+00 | 0.000E+00 | 1.803E-07 | 1.385E-07 | 0.000E+00 | 0.000E+00 | 2.323E-07 | 2.385E-07 | 1.183E-06 |
| Unigene401302 | efmA | 0.000E+00 | 0.000E+00 | 0.000E+00 | 0.000E+00 | 0.000E+00 | 4.413E-07 | 0.000E+00 | 0.000E+00 | 0.000E+00 | 0.000E+00 | 0.000E+00 | 3.791E-07 |
| Unigene401392 | Bifidobacte | 0.000E+00 | 0.000E+00 | 0.000E+00 | 0.000E+00 | 0.000E+00 | 6.776E-07 | 0.000E+00 | 0.000E+00 | 0.000E+00 | 0.000E+00 | 0.000E+00 | 0.000E+00 |
| Unigene401398 | kdpE | 0.000E+00 | 0.000E+00 | 0.000E+00 | 0.000E+00 | 0.000E+00 | 9.517E-07 | 0.000E+00 | 9.991E-06 | 0.000E+00 | 0.000E+00 | 0.000E+00 | 0.000E+00 |
| Unigene401408 | smeR | 0.000E+00 | 0.000E+00 | 0.000E+00 | 0.000E+00 | 1.913E-07 | 0.000E+00 | 3.811E-07 | 0.000E+00 | 0.000E+00 | 0.000E+00 | 1.280E-06 | 0.000E+00 |
| Unigene401418 | efrA | 0.000E+00 | 0.000E+00 | 0.000E+00 | 0.000E+00 | 0.000E+00 | 1.366E-06 | 0.000E+00 | 0.000E+00 | 0.000E+00 | 0.000E+00 | 0.000E+00 | 0.000E+00 |
| Unigene401419 | lmrD | 0.000E+00 | 0.000E+00 | 0.000E+00 | 0.000E+00 | 0.000E+00 | 1.700E-06 | 0.000E+00 | 0.000E+00 | 0.000E+00 | 0.000E+00 | 0.000E+00 | 0.000E+00 |
| Unigene401420 | CMY-90 | 0.000E+00 | 0.000E+00 | 0.000E+00 | 0.000E+00 | 0.000E+00 | 1.798E-06 | 0.000E+00 | 0.000E+00 | 0.000E+00 | 0.000E+00 | 0.000E+00 | 0.000E+00 |
| Unigene401535 | tetB(46) | 0.000E+00 | 0.000E+00 | 0.000E+00 | 0.000E+00 | 0.000E+00 | 2.195E-07 | 0.000E+00 | 0.000E+00 | 0.000E+00 | 0.000E+00 | 0.000E+00 | 0.000E+00 |
| Unigene401554 | vanRM | 0.000E+00 | 0.000E+00 | 0.000E+00 | 0.000E+00 | 0.000E+00 | 1.344E-06 | 1.803E-06 | 0.000E+00 | 0.000E+00 | 0.000E+00 | 0.000E+00 | 3.040E-06 |
| Unigene401616 | mgrA | 0.000E+00 | 0.000E+00 | 0.000E+00 | 0.000E+00 | 0.000E+00 | 7.074E-07 | 0.000E+00 | 0.000E+00 | 0.000E+00 | 0.000E+00 | 4.990E-07 | 7.562E-06 |
| Unigene401718 | vgaD | 0.000E+00 | 0.000E+00 | 0.000E+00 | 0.000E+00 | 0.000E+00 | 1.246E-06 | 0.000E+00 | 0.000E+00 | 0.000E+00 | 0.000E+00 | 0.000E+00 | 0.000E+00 |
| Unigene401743 | bcrA | 0.000E+00 | 0.000E+00 | 3.392E-07 | 0.000E+00 | 0.000E+00 | 0.000E+00 | 0.000E+00 | 1.917E-06 | 0.000E+00 | 0.000E+00 | 0.000E+00 | 1.124E-06 |
| Unigene401783 | lin | 0.000E+00 | 0.000E+00 | 0.000E+00 | 0.000E+00 | 0.000E+00 | 8.983E-07 | 0.000E+00 | 0.000E+00 | 0.000E+00 | 0.000E+00 | 0.000E+00 | 0.000E+00 |
| Unigene401845 | baeS | 0.000E+00 | 0.000E+00 | 0.000E+00 | 0.000E+00 | 0.000E+00 | 1.135E-06 | 0.000E+00 | 0.000E+00 | 0.000E+00 | 0.000E+00 | 0.000E+00 | 0.000E+00 |
| Unigene401846 | vanRM | 0.000E+00 | 0.000E+00 | 0.000E+00 | 0.000E+00 | 0.000E+00 | 9.693E-07 | 0.000E+00 | 0.000E+00 | 0.000E+00 | 0.000E+00 | 0.000E+00 | 0.000E+00 |
| Unigene401930 | vanSF | 0.000E+00 | 0.000E+00 | 0.000E+00 | 0.000E+00 | 0.000E+00 | 6.138E-07 | 0.000E+00 | 0.000E+00 | 0.000E+00 | 0.000E+00 | 0.000E+00 | 0.000E+00 |
| Unigene401956 | lmrB | 0.000E+00 | 0.000E+00 | 0.000E+00 | 0.000E+00 | 0.000E+00 | 1.153E-06 | 0.000E+00 | 0.000E+00 | 0.000E+00 | 0.000E+00 | 0.000E+00 | 0.000E+00 |
| Unigene401964 | tetB(60) | 0.000E+00 | 0.000E+00 | 0.000E+00 | 0.000E+00 | 0.000E+00 | 3.063E-05 | 0.000E+00 | 0.000E+00 | 0.000E+00 | 0.000E+00 | 0.000E+00 | 0.000E+00 |
| Unigene402011 | lsaA | 0.000E+00 | 0.000E+00 | 0.000E+00 | 0.000E+00 | 0.000E+00 | 7.942E-07 | 0.000E+00 | 0.000E+00 | 0.000E+00 | 0.000E+00 | 0.000E+00 | 0.000E+00 |
| Unigene402025 | vanHF | 0.000E+00 | 0.000E+00 | 0.000E+00 | 0.000E+00 | 0.000E+00 | 9.605E-07 | 0.000E+00 | 0.000E+00 | 0.000E+00 | 0.000E+00 | 0.000E+00 | 0.000E+00 |
| Unigene402054 | bcrA | 0.000E+00 | 0.000E+00 | 0.000E+00 | 0.000E+00 | 0.000E+00 | 4.154E-07 | 0.000E+00 | 0.000E+00 | 0.000E+00 | 0.000E+00 | 0.000E+00 | 0.000E+00 |
| Unigene402150 | AcrF | 0.000E+00 | 0.000E+00 | 0.000E+00 | 0.000E+00 | 0.000E+00 | 1.994E-07 | 0.000E+00 | 0.000E+00 | 0.000E+00 | 0.000E+00 | 0.000E+00 | 0.000E+00 |
| Unigene402285 | vanHD | 0.000E+00 | 0.000E+00 | 0.000E+00 | 0.000E+00 | 0.000E+00 | 0.000E+00 | 0.000E+00 | 0.000E+00 | 0.000E+00 | 0.000E+00 | 0.000E+00 | 1.201E-06 |
| Unigene402303 | lmrC | 0.000E+00 | 0.000E+00 | 0.000E+00 | 0.000E+00 | 0.000E+00 | 9.627E-07 | 0.000E+00 | 0.000E+00 | 0.000E+00 | 0.000E+00 | 0.000E+00 | 0.000E+00 |
| Unigene402331 | patB | 0.000E+00 | 0.000E+00 | 0.000E+00 | 0.000E+00 | 0.000E+00 | 6.228E-07 | 0.000E+00 | 0.000E+00 | 0.000E+00 | 0.000E+00 | 0.000E+00 | 0.000E+00 |
| Unigene402332 | patA | 0.000E+00 | 0.000E+00 | 0.000E+00 | 0.000E+00 | 0.000E+00 | 9.844E-07 | 0.000E+00 | 0.000E+00 | 0.000E+00 | 0.000E+00 | 0.000E+00 | 0.000E+00 |
| Unigene402390 | bmr | 0.000E+00 | 0.000E+00 | 0.000E+00 | 0.000E+00 | 0.000E+00 | 6.423E-07 | 0.000E+00 | 0.000E+00 | 0.000E+00 | 0.000E+00 | 0.000E+00 | 0.000E+00 |
| Unigene402428 | lmrC | 0.000E+00 | 0.000E+00 | 0.000E+00 | 0.000E+00 | 6.991E-08 | 3.401E-07 | 0.000E+00 | 0.000E+00 | 0.000E+00 | 1.402E-07 | 0.000E+00 | 0.000E+00 |
| Unigene402441 | tetT | 0.000E+00 | 0.000E+00 | 0.000E+00 | 0.000E+00 | 0.000E+00 | 1.267E-06 | 0.000E+00 | 0.000E+00 | 0.000E+00 | 0.000E+00 | 0.000E+00 | 0.000E+00 |
| Unigene402496 | lsaC | 0.000E+00 | 0.000E+00 | 0.000E+00 | 0.000E+00 | 0.000E+00 | 1.176E-06 | 0.000E+00 | 0.000E+00 | 0.000E+00 | 0.000E+00 | 0.000E+00 | 0.000E+00 |
| Unigene402507 | FosX | 0.000E+00 | 0.000E+00 | 0.000E+00 | 0.000E+00 | 0.000E+00 | 2.195E-07 | 0.000E+00 | 0.000E+00 | 0.000E+00 | 0.000E+00 | 0.000E+00 | 4.200E-07 |
| Unigene402511 | macB | 0.000E+00 | 0.000E+00 | 0.000E+00 | 0.000E+00 | 0.000E+00 | 0.000E+00 | 0.000E+00 | 0.000E+00 | 1.558E-07 | 0.000E+00 | 0.000E+00 | 0.000E+00 |
| Unigene402530 | Streptomy | 0.000E+00 | 0.000E+00 | 0.000E+00 | 0.000E+00 | 0.000E+00 | 3.899E-07 | 0.000E+00 | 0.000E+00 | 0.000E+00 | 0.000E+00 | 0.000E+00 | 0.000E+00 |
| Unigene402543 | msbA | 0.000E+00 | 0.000E+00 | 0.000E+00 | 0.000E+00 | 0.000E+00 | 9.914E-07 | 0.000E+00 | 0.000E+00 | 0.000E+00 | 0.000E+00 | 0.000E+00 | 0.000E+00 |
| Unigene402570 | arlR | 0.000E+00 | 0.000E+00 | 0.000E+00 | 0.000E+00 | 0.000E+00 | 5.694E-07 | 0.000E+00 | 0.000E+00 | 0.000E+00 | 0.000E+00 | 0.000E+00 | 0.000E+00 |
| Unigene402615 | mdtB | 0.000E+00 | 0.000E+00 | 0.000E+00 | 0.000E+00 | 0.000E+00 | 5.539E-07 | 0.000E+00 | 0.000E+00 | 0.000E+00 | 0.000E+00 | 0.000E+00 | 0.000E+00 |
| Unigene402655 | vatB | 0.000E+00 | 0.000E+00 | 0.000E+00 | 0.000E+00 | 0.000E+00 | 8.389E-07 | 0.000E+00 | 0.000E+00 | 0.000E+00 | 0.000E+00 | 0.000E+00 | 0.000E+00 |
| Unigene402777 | tetB(60) | 0.000E+00 | 0.000E+00 | 0.000E+00 | 0.000E+00 | 0.000E+00 | 1.057E-06 | 0.000E+00 | 0.000E+00 | 0.000E+00 | 0.000E+00 | 0.000E+00 | 0.000E+00 |
| Unigene402883 | adeR | 0.000E+00 | 0.000E+00 | 0.000E+00 | 0.000E+00 | 0.000E+00 | 5.948E-07 | 0.000E+00 | 0.000E+00 | 0.000E+00 | 0.000E+00 | 0.000E+00 | 0.000E+00 |
| Unigene402901 | smeS | 0.000E+00 | 0.000E+00 | 0.000E+00 | 0.000E+00 | 0.000E+00 | 3.308E-07 | 0.000E+00 | 0.000E+00 | 0.000E+00 | 0.000E+00 | 0.000E+00 | 0.000E+00 |
| Unigene403073 | patA | 0.000E+00 | 0.000E+00 | 0.000E+00 | 0.000E+00 | 0.000E+00 | 5.132E-07 | 0.000E+00 | 0.000E+00 | 0.000E+00 | 0.000E+00 | 0.000E+00 | 0.000E+00 |
| Unigene403164 | vanHO | 0.000E+00 | 0.000E+00 | 0.000E+00 | 0.000E+00 | 0.000E+00 | 1.132E-06 | 0.000E+00 | 6.721E-06 | 0.000E+00 | 0.000E+00 | 0.000E+00 | 0.000E+00 |
| Unigene403215 | Staphylocc | 7.159E-07 | 0.000E+00 | 2.980E-07 | 3.505E-07 | 4.073E-06 | 9.568E-06 | 1.417E-06 | 8.027E-06 | 4.552E-06 | 3.625E-06 | 0.000E+00 | 8.975E-07 |
| Unigene403226 | tetA(58) | 0.000E+00 | 0.000E+00 | 0.000E+00 | 0.000E+00 | 0.000E+00 | 9.431E-07 | 0.000E+00 | 3.928E-06 | 0.000E+00 | 0.000E+00 | 0.000E+00 | 0.000E+00 |
| Unigene403254 | efrA | 0.000E+00 | 0.000E+00 | 0.000E+00 | 0.000E+00 | 0.000E+00 | 4.900E-07 | 0.000E+00 | 0.000E+00 | 0.000E+00 | 0.000E+00 | 0.000E+00 | 0.000E+00 |
| Unigene403287 | arlS | 0.000E+00 | 0.000E+00 | 0.000E+00 | 0.000E+00 | 0.000E+00 | 7.886E-07 | 0.000E+00 | 0.000E+00 | 0.000E+00 | 0.000E+00 | 0.000E+00 | 0.000E+00 |
| Unigene403340 | patB | 0.000E+00 | 0.000E+00 | 0.000E+00 | 0.000E+00 | 0.000E+00 | 1.425E-06 | 0.000E+00 | 5.163E-06 | 0.000E+00 | 0.000E+00 | 0.000E+00 | 0.000E+00 |
| Unigene403343 | vanRN | 0.000E+00 | 0.000E+00 | 0.000E+00 | 0.000E+00 | 0.000E+00 | 1.132E-06 | 0.000E+00 | 4.613E-06 | 0.000E+00 | 0.000E+00 | 0.000E+00 | 0.000E+00 |
| Unigene403344 | arlS | 0.000E+00 | 0.000E+00 | 0.000E+00 | 0.000E+00 | 0.000E+00 | 1.031E-06 | 0.000E+00 | 5.201E-06 | 0.000E+00 | 0.000E+00 | 0.000E+00 | 0.000E+00 |
| Unigene403368 | efrA | 0.000E+00 | 0.000E+00 | 0.000E+00 | 0.000E+00 | 0.000E+00 | 1.377E-07 | 0.000E+00 | 0.000E+00 | 0.000E+00 | 0.000E+00 | 0.000E+00 | 0.000E+00 |
| Unigene403373 | mtrA | 0.000E+00 | 0.000E+00 | 0.000E+00 | 0.000E+00 | 0.000E+00 | 2.983E-07 | 0.000E+00 | 0.000E+00 | 0.000E+00 | 0.000E+00 | 0.000E+00 | 0.000E+00 |
| Unigene403431 | rosB | 5.933E-07 | 0.000E+00 | 0.000E+00 | 0.000E+00 | 4.284E-06 | 1.108E-05 | 1.403E-06 | 7.807E-06 | 4.038E-06 | 0.000E+00 | 0.000E+00 | 0.000E+00 |
| Unigene403449 | vanRM | 0.000E+00 | 0.000E+00 | 0.000E+00 | 0.000E+00 | 0.000E+00 | 8.415E-07 | 0.000E+00 | 8.391E-06 | 0.000E+00 | 0.000E+00 | 0.000E+00 | 0.000E+00 |
| Unigene403582 | tetA(46) | 0.000E+00 | 0.000E+00 | 0.000E+00 | 0.000E+00 | 0.000E+00 | 4.709E-07 | 0.000E+00 | 0.000E+00 | 0.000E+00 | 0.000E+00 | 0.000E+00 | 0.000E+00 |
| Unigene403594 | Streptomy | 0.000E+00 | 0.000E+00 | 0.000E+00 | 0.000E+00 | 0.000E+00 | 5.714E-07 | 2.925E-07 | 0.000E+00 | 0.000E+00 | 0.000E+00 | 0.000E+00 | 0.000E+00 |

| | | | | | | | | | | | | | |
|---|---|---|---|---|---|---|---|---|---|---|---|---|---|
| Unigene403672 | tetA(58) | 0.000E+00 | 0.000E+00 | 0.000E+00 | 0.000E+00 | 0.000E+00 | 4.598E-07 | 0.000E+00 | 0.000E+00 | 0.000E+00 | 0.000E+00 | 0.000E+00 | 0.000E+00 |
| Unigene403787 | rpoB2 | 0.000E+00 | 0.000E+00 | 0.000E+00 | 2.867E-07 | 3.785E-07 | 1.013E-06 | 1.822E-06 | 1.690E-06 | 0.000E+00 | 4.744E-07 | 8.768E-07 | 1.342E-06 |
| Unigene404047 | adeB | 0.000E+00 | 0.000E+00 | 0.000E+00 | 0.000E+00 | 0.000E+00 | 2.326E-07 | 0.000E+00 | 0.000E+00 | 0.000E+00 | 0.000E+00 | 0.000E+00 | 0.000E+00 |
| Unigene404093 | vanSN | 0.000E+00 | 0.000E+00 | 0.000E+00 | 0.000E+00 | 0.000E+00 | 2.737E-07 | 0.000E+00 | 0.000E+00 | 0.000E+00 | 0.000E+00 | 0.000E+00 | 0.000E+00 |
| Unigene404114 | macB | 0.000E+00 | 0.000E+00 | 0.000E+00 | 0.000E+00 | 0.000E+00 | 1.471E-06 | 0.000E+00 | 0.000E+00 | 0.000E+00 | 0.000E+00 | 0.000E+00 | 0.000E+00 |
| Unigene404169 | vanHA | 0.000E+00 | 0.000E+00 | 0.000E+00 | 0.000E+00 | 0.000E+00 | 7.380E-07 | 0.000E+00 | 0.000E+00 | 0.000E+00 | 0.000E+00 | 0.000E+00 | 0.000E+00 |
| Unigene404222 | cdeA | 0.000E+00 | 0.000E+00 | 0.000E+00 | 0.000E+00 | 0.000E+00 | 7.487E-07 | 0.000E+00 | 9.268E-06 | 0.000E+00 | 0.000E+00 | 1.485E-05 | 0.000E+00 |
| Unigene404223 | arlS | 0.000E+00 | 0.000E+00 | 0.000E+00 | 0.000E+00 | 0.000E+00 | 9.950E-07 | 0.000E+00 | 1.005E-05 | 0.000E+00 | 0.000E+00 | 1.572E-05 | 0.000E+00 |
| Unigene404236 | vanHB | 0.000E+00 | 0.000E+00 | 0.000E+00 | 0.000E+00 | 0.000E+00 | 7.030E-07 | 0.000E+00 | 0.000E+00 | 0.000E+00 | 0.000E+00 | 0.000E+00 | 0.000E+00 |
| Unigene404277 | mdtG | 0.000E+00 | 0.000E+00 | 0.000E+00 | 0.000E+00 | 0.000E+00 | 1.115E-06 | 0.000E+00 | 0.000E+00 | 0.000E+00 | 0.000E+00 | 0.000E+00 | 0.000E+00 |
| Unigene404354 | ErmE | 0.000E+00 | 0.000E+00 | 0.000E+00 | 0.000E+00 | 0.000E+00 | 1.621E-06 | 0.000E+00 | 0.000E+00 | 0.000E+00 | 0.000E+00 | 0.000E+00 | 0.000E+00 |
| Unigene404432 | Chlamydia | 4.040E-05 | 7.697E-05 | 8.661E-05 | 3.913E-06 | 3.664E-06 | 5.348E-07 | 2.373E-06 | 5.775E-07 | 3.028E-05 | 0.000E+00 | 0.000E+00 | 0.000E+00 |
| Unigene404454 | farB | 0.000E+00 | 0.000E+00 | 0.000E+00 | 0.000E+00 | 0.000E+00 | 9.183E-07 | 0.000E+00 | 0.000E+00 | 0.000E+00 | 0.000E+00 | 0.000E+00 | 0.000E+00 |
| Unigene404457 | vanTC | 0.000E+00 | 0.000E+00 | 0.000E+00 | 0.000E+00 | 0.000E+00 | 8.353E-07 | 0.000E+00 | 0.000E+00 | 0.000E+00 | 0.000E+00 | 0.000E+00 | 0.000E+00 |
| Unigene404564 | facT | 0.000E+00 | 0.000E+00 | 0.000E+00 | 0.000E+00 | 0.000E+00 | 1.929E-06 | 0.000E+00 | 0.000E+00 | 0.000E+00 | 0.000E+00 | 0.000E+00 | 0.000E+00 |
| Unigene404657 | lsaC | 0.000E+00 | 9.924E-08 | 0.000E+00 | 0.000E+00 | 0.000E+00 | 3.910E-07 | 0.000E+00 | 0.000E+00 | 0.000E+00 | 0.000E+00 | 0.000E+00 | 0.000E+00 |
| Unigene404881 | oqxB | 0.000E+00 | 0.000E+00 | 0.000E+00 | 0.000E+00 | 0.000E+00 | 3.965E-07 | 0.000E+00 | 0.000E+00 | 0.000E+00 | 0.000E+00 | 0.000E+00 | 0.000E+00 |
| Unigene404931 | efrA | 0.000E+00 | 0.000E+00 | 0.000E+00 | 0.000E+00 | 0.000E+00 | 1.666E-06 | 0.000E+00 | 0.000E+00 | 0.000E+00 | 0.000E+00 | 0.000E+00 | 0.000E+00 |
| Unigene404935 | vmlR | 0.000E+00 | 0.000E+00 | 0.000E+00 | 0.000E+00 | 0.000E+00 | 1.906E-06 | 0.000E+00 | 0.000E+00 | 0.000E+00 | 0.000E+00 | 0.000E+00 | 0.000E+00 |
| Unigene404938 | vanD | 0.000E+00 | 0.000E+00 | 0.000E+00 | 0.000E+00 | 0.000E+00 | 1.551E-06 | 0.000E+00 | 0.000E+00 | 0.000E+00 | 0.000E+00 | 0.000E+00 | 0.000E+00 |
| Unigene405085 | lsaC | 0.000E+00 | 0.000E+00 | 0.000E+00 | 0.000E+00 | 0.000E+00 | 1.093E-06 | 0.000E+00 | 0.000E+00 | 0.000E+00 | 0.000E+00 | 0.000E+00 | 0.000E+00 |
| Unigene405212 | tcr3 | 0.000E+00 | 0.000E+00 | 0.000E+00 | 0.000E+00 | 0.000E+00 | 0.000E+00 | 5.988E-07 | 0.000E+00 | 0.000E+00 | 0.000E+00 | 3.782E-07 | 1.101E-06 |
| Unigene405314 | macB | 0.000E+00 | 0.000E+00 | 0.000E+00 | 0.000E+00 | 0.000E+00 | 1.884E-06 | 0.000E+00 | 0.000E+00 | 0.000E+00 | 0.000E+00 | 0.000E+00 | 0.000E+00 |
| Unigene405360 | mefE | 6.527E-06 | 0.000E+00 | 0.000E+00 | 0.000E+00 | 0.000E+00 | 7.381E-07 | 6.045E-08 | 1.594E-07 | 0.000E+00 | 1.469E-05 | 0.000E+00 | 2.905E-07 |
| Unigene405369 | arlS | 0.000E+00 | 0.000E+00 | 0.000E+00 | 0.000E+00 | 0.000E+00 | 1.015E-06 | 0.000E+00 | 0.000E+00 | 0.000E+00 | 0.000E+00 | 0.000E+00 | 0.000E+00 |
| Unigene405461 | macB | 0.000E+00 | 0.000E+00 | 0.000E+00 | 0.000E+00 | 5.023E-08 | 6.842E-07 | 0.000E+00 | 0.000E+00 | 0.000E+00 | 0.000E+00 | 0.000E+00 | 0.000E+00 |
| Unigene405508 | arlR | 0.000E+00 | 0.000E+00 | 0.000E+00 | 0.000E+00 | 0.000E+00 | 5.563E-07 | 0.000E+00 | 0.000E+00 | 0.000E+00 | 0.000E+00 | 8.562E-07 | 0.000E+00 |
| Unigene405789 | tetA(58) | 0.000E+00 | 0.000E+00 | 0.000E+00 | 0.000E+00 | 0.000E+00 | 1.509E-06 | 0.000E+00 | 0.000E+00 | 0.000E+00 | 0.000E+00 | 0.000E+00 | 0.000E+00 |
| Unigene405801 | mdtG | 0.000E+00 | 0.000E+00 | 0.000E+00 | 0.000E+00 | 0.000E+00 | 1.772E-06 | 0.000E+00 | 0.000E+00 | 0.000E+00 | 0.000E+00 | 0.000E+00 | 0.000E+00 |
| Unigene405823 | carA | 0.000E+00 | 0.000E+00 | 9.674E-06 | 0.000E+00 | 0.000E+00 | 1.148E-06 | 0.000E+00 | 0.000E+00 | 0.000E+00 | 0.000E+00 | 0.000E+00 | 0.000E+00 |
| Unigene405848 | Streptomy | 0.000E+00 | 0.000E+00 | 0.000E+00 | 0.000E+00 | 0.000E+00 | 1.181E-06 | 0.000E+00 | 1.030E-05 | 0.000E+00 | 0.000E+00 | 0.000E+00 | 0.000E+00 |
| Unigene405869 | bcrA | 0.000E+00 | 0.000E+00 | 0.000E+00 | 0.000E+00 | 0.000E+00 | 6.327E-07 | 0.000E+00 | 0.000E+00 | 0.000E+00 | 0.000E+00 | 0.000E+00 | 0.000E+00 |
| Unigene405879 | vanHF | 0.000E+00 | 0.000E+00 | 0.000E+00 | 0.000E+00 | 0.000E+00 | 8.038E-07 | 0.000E+00 | 0.000E+00 | 0.000E+00 | 0.000E+00 | 0.000E+00 | 0.000E+00 |
| Unigene405931 | bcrA | 0.000E+00 | 0.000E+00 | 0.000E+00 | 0.000E+00 | 0.000E+00 | 1.219E-06 | 8.163E-07 | 7.597E-08 | 0.000E+00 | 0.000E+00 | 0.000E+00 | 0.000E+00 |
| Unigene405936 | patA | 0.000E+00 | 0.000E+00 | 0.000E+00 | 0.000E+00 | 9.719E-08 | 1.277E-06 | 7.746E-07 | 5.106E-08 | 0.000E+00 | 0.000E+00 | 0.000E+00 | 0.000E+00 |
| Unigene405947 | facT | 0.000E+00 | 0.000E+00 | 0.000E+00 | 0.000E+00 | 1.910E-06 | 7.605E-07 | 1.499E-06 | 1.460E-06 | 0.000E+00 | 1.161E-06 | 0.000E+00 | 4.558E-06 |
| Unigene405964 | RlmA(II) | 0.000E+00 | 0.000E+00 | 0.000E+00 | 0.000E+00 | 0.000E+00 | 4.967E-07 | 0.000E+00 | 0.000E+00 | 0.000E+00 | 0.000E+00 | 0.000E+00 | 0.000E+00 |
| Unigene405970 | macB | 0.000E+00 | 0.000E+00 | 0.000E+00 | 0.000E+00 | 0.000E+00 | 4.653E-07 | 0.000E+00 | 0.000E+00 | 0.000E+00 | 0.000E+00 | 0.000E+00 | 0.000E+00 |
| Unigene405987 | msrE | 0.000E+00 | 0.000E+00 | 0.000E+00 | 0.000E+00 | 0.000E+00 | 2.543E-07 | 0.000E+00 | 0.000E+00 | 0.000E+00 | 0.000E+00 | 0.000E+00 | 0.000E+00 |
| Unigene406045 | PmrF | 0.000E+00 | 0.000E+00 | 0.000E+00 | 0.000E+00 | 0.000E+00 | 1.008E-06 | 0.000E+00 | 0.000E+00 | 0.000E+00 | 0.000E+00 | 0.000E+00 | 0.000E+00 |
| Unigene406187 | farB | 0.000E+00 | 0.000E+00 | 0.000E+00 | 0.000E+00 | 1.868E-07 | 6.134E-07 | 0.000E+00 | 4.170E-07 | 0.000E+00 | 7.023E-08 | 0.000E+00 | 6.956E-07 |
| Unigene406189 | bmr | 0.000E+00 | 0.000E+00 | 0.000E+00 | 0.000E+00 | 0.000E+00 | 5.628E-07 | 0.000E+00 | 0.000E+00 | 0.000E+00 | 0.000E+00 | 0.000E+00 | 0.000E+00 |
| Unigene406246 | optrA | 0.000E+00 | 0.000E+00 | 0.000E+00 | 0.000E+00 | 0.000E+00 | 8.257E-07 | 0.000E+00 | 0.000E+00 | 0.000E+00 | 0.000E+00 | 0.000E+00 | 0.000E+00 |
| Unigene406409 | vanSA | 0.000E+00 | 0.000E+00 | 0.000E+00 | 0.000E+00 | 0.000E+00 | 1.077E-06 | 0.000E+00 | 0.000E+00 | 0.000E+00 | 0.000E+00 | 0.000E+00 | 0.000E+00 |
| Unigene406561 | tet32 | 0.000E+00 | 0.000E+00 | 0.000E+00 | 0.000E+00 | 0.000E+00 | 1.009E-06 | 0.000E+00 | 0.000E+00 | 0.000E+00 | 0.000E+00 | 0.000E+00 | 0.000E+00 |
| Unigene406590 | tetA(60) | 3.963E-07 | 0.000E+00 | 0.000E+00 | 0.000E+00 | 0.000E+00 | 6.920E-07 | 0.000E+00 | 2.348E-06 | 0.000E+00 | 0.000E+00 | 0.000E+00 | 2.810E-06 |
| Unigene406626 | AcrF | 0.000E+00 | 1.575E-06 | 9.205E-07 | 3.343E-07 | 1.005E-05 | 4.176E-07 | 0.000E+00 | 0.000E+00 | 0.000E+00 | 0.000E+00 | 0.000E+00 | 0.000E+00 |
| Unigene406684 | lmrB | 0.000E+00 | 0.000E+00 | 0.000E+00 | 0.000E+00 | 0.000E+00 | 4.378E-07 | 0.000E+00 | 0.000E+00 | 0.000E+00 | 0.000E+00 | 0.000E+00 | 0.000E+00 |
| Unigene406685 | Agrobacte | 0.000E+00 | 0.000E+00 | 0.000E+00 | 0.000E+00 | 4.014E-07 | 5.208E-07 | 0.000E+00 | 0.000E+00 | 0.000E+00 | 0.000E+00 | 0.000E+00 | 0.000E+00 |
| Unigene406714 | lsaC | 0.000E+00 | 0.000E+00 | 0.000E+00 | 0.000E+00 | 0.000E+00 | 7.896E-07 | 0.000E+00 | 0.000E+00 | 0.000E+00 | 0.000E+00 | 0.000E+00 | 0.000E+00 |
| Unigene406738 | vanHO | 0.000E+00 | 0.000E+00 | 0.000E+00 | 0.000E+00 | 0.000E+00 | 8.332E-07 | 0.000E+00 | 0.000E+00 | 0.000E+00 | 0.000E+00 | 0.000E+00 | 0.000E+00 |
| Unigene406754 | efrA | 0.000E+00 | 0.000E+00 | 0.000E+00 | 0.000E+00 | 0.000E+00 | 7.714E-07 | 3.633E-06 | 0.000E+00 | 0.000E+00 | 0.000E+00 | 0.000E+00 | 2.834E-06 |
| Unigene406782 | basS | 0.000E+00 | 0.000E+00 | 6.103E-08 | 0.000E+00 | 0.000E+00 | 6.724E-08 | 1.377E-07 | 0.000E+00 | 0.000E+00 | 0.000E+00 | 0.000E+00 | 1.176E-06 |
| Unigene406796 | mecD | 0.000E+00 | 0.000E+00 | 0.000E+00 | 0.000E+00 | 0.000E+00 | 3.421E-07 | 0.000E+00 | 5.277E-07 | 0.000E+00 | 0.000E+00 | 5.172E-07 | 0.000E+00 |
| Unigene406948 | vanN | 0.000E+00 | 0.000E+00 | 9.567E-07 | 0.000E+00 | 0.000E+00 | 1.430E-06 | 5.511E-06 | 3.821E-06 | 6.090E-07 | 1.280E-06 | 2.031E-06 | 4.816E-06 |
| Unigene406969 | mgrA | 0.000E+00 | 0.000E+00 | 1.215E-05 | 0.000E+00 | 0.000E+00 | 1.064E-06 | 0.000E+00 | 0.000E+00 | 0.000E+00 | 0.000E+00 | 0.000E+00 | 0.000E+00 |
| Unigene407014 | arlS | 0.000E+00 | 0.000E+00 | 0.000E+00 | 0.000E+00 | 0.000E+00 | 1.245E-06 | 0.000E+00 | 0.000E+00 | 0.000E+00 | 0.000E+00 | 0.000E+00 | 0.000E+00 |
| Unigene407015 | arlR | 0.000E+00 | 0.000E+00 | 0.000E+00 | 0.000E+00 | 0.000E+00 | 1.122E-06 | 0.000E+00 | 0.000E+00 | 0.000E+00 | 0.000E+00 | 0.000E+00 | 0.000E+00 |

| | | | | | | | | | | | | | |
|---|---|---|---|---|---|---|---|---|---|---|---|---|---|
| Unigene407111 | abeS | 0.000E+00 | 0.000E+00 | 0.000E+00 | 0.000E+00 | 0.000E+00 | 2.884E-07 | 0.000E+00 | 0.000E+00 | 0.000E+00 | 0.000E+00 | 0.000E+00 | 0.000E+00 |
| Unigene407412 | macB | 7.826E-08 | 0.000E+00 | 0.000E+00 | 0.000E+00 | 6.520E-07 | 4.230E-07 | 4.330E-07 | 0.000E+00 | 0.000E+00 | 0.000E+00 | 0.000E+00 | 0.000E+00 |
| Unigene407529 | evgS | 0.000E+00 | 0.000E+00 | 0.000E+00 | 0.000E+00 | 0.000E+00 | 2.571E-07 | 0.000E+00 | 0.000E+00 | 0.000E+00 | 0.000E+00 | 0.000E+00 | 0.000E+00 |
| Unigene407541 | arlS | 0.000E+00 | 0.000E+00 | 0.000E+00 | 0.000E+00 | 0.000E+00 | 1.111E-06 | 0.000E+00 | 0.000E+00 | 0.000E+00 | 0.000E+00 | 0.000E+00 | 0.000E+00 |
| Unigene407746 | msbA | 0.000E+00 | 0.000E+00 | 0.000E+00 | 0.000E+00 | 0.000E+00 | 6.036E-07 | 0.000E+00 | 0.000E+00 | 0.000E+00 | 0.000E+00 | 0.000E+00 | 0.000E+00 |
| Unigene407790 | MexF | 0.000E+00 | 0.000E+00 | 0.000E+00 | 0.000E+00 | 0.000E+00 | 1.579E-07 | 0.000E+00 | 0.000E+00 | 0.000E+00 | 0.000E+00 | 0.000E+00 | 0.000E+00 |
| Unigene407796 | efrA | 0.000E+00 | 0.000E+00 | 0.000E+00 | 0.000E+00 | 0.000E+00 | 5.881E-07 | 0.000E+00 | 0.000E+00 | 0.000E+00 | 0.000E+00 | 0.000E+00 | 0.000E+00 |
| Unigene407840 | Pseudomo | 0.000E+00 | 0.000E+00 | 0.000E+00 | 0.000E+00 | 0.000E+00 | 2.115E-07 | 0.000E+00 | 0.000E+00 | 0.000E+00 | 0.000E+00 | 0.000E+00 | 0.000E+00 |
| Unigene407892 | bmr | 0.000E+00 | 0.000E+00 | 0.000E+00 | 0.000E+00 | 0.000E+00 | 1.026E-06 | 0.000E+00 | 8.858E-06 | 0.000E+00 | 0.000E+00 | 1.742E-05 | 0.000E+00 |
| Unigene407894 | vanHO | 0.000E+00 | 0.000E+00 | 0.000E+00 | 0.000E+00 | 0.000E+00 | 6.907E-07 | 0.000E+00 | 9.263E-06 | 0.000E+00 | 0.000E+00 | 1.708E-05 | 0.000E+00 |
| Unigene407968 | adeL | 0.000E+00 | 0.000E+00 | 0.000E+00 | 0.000E+00 | 0.000E+00 | 0.000E+00 | 0.000E+00 | 3.340E-07 | 0.000E+00 | 0.000E+00 | 0.000E+00 | 2.706E-07 |
| Unigene408102 | bcrA | 0.000E+00 | 0.000E+00 | 0.000E+00 | 0.000E+00 | 0.000E+00 | 2.541E-06 | 0.000E+00 | 0.000E+00 | 3.261E-06 | 0.000E+00 | 0.000E+00 | 0.000E+00 |
| Unigene408276 | rphA | 0.000E+00 | 0.000E+00 | 0.000E+00 | 0.000E+00 | 0.000E+00 | 5.875E-07 | 0.000E+00 | 0.000E+00 | 0.000E+00 | 0.000E+00 | 0.000E+00 | 0.000E+00 |
| Unigene408284 | novA | 0.000E+00 | 0.000E+00 | 3.009E-07 | 1.407E-07 | 0.000E+00 | 1.055E-06 | 0.000E+00 | 2.505E-06 | 0.000E+00 | 0.000E+00 | 0.000E+00 | 3.262E-06 |
| Unigene408369 | farB | 2.469E-07 | 4.742E-07 | 6.662E-07 | 0.000E+00 | 1.257E-06 | 9.119E-07 | 2.049E-06 | 1.585E-06 | 0.000E+00 | 4.813E-07 | 1.647E-06 | 3.988E-06 |
| Unigene408379 | patA | 0.000E+00 | 0.000E+00 | 0.000E+00 | 0.000E+00 | 0.000E+00 | 1.827E-06 | 0.000E+00 | 0.000E+00 | 0.000E+00 | 0.000E+00 | 0.000E+00 | 3.574E-06 |
| Unigene408466 | poxtA | 0.000E+00 | 0.000E+00 | 0.000E+00 | 0.000E+00 | 0.000E+00 | 1.467E-06 | 0.000E+00 | 0.000E+00 | 0.000E+00 | 0.000E+00 | 0.000E+00 | 0.000E+00 |
| Unigene408829 | tetB(58) | 0.000E+00 | 0.000E+00 | 0.000E+00 | 0.000E+00 | 0.000E+00 | 1.592E-06 | 4.664E-06 | 0.000E+00 | 1.118E-06 | 0.000E+00 | 0.000E+00 | 2.902E-07 |
| Unigene408850 | arnA | 0.000E+00 | 0.000E+00 | 0.000E+00 | 0.000E+00 | 0.000E+00 | 6.950E-07 | 0.000E+00 | 0.000E+00 | 0.000E+00 | 0.000E+00 | 0.000E+00 | 0.000E+00 |
| Unigene409023 | smeF | 0.000E+00 | 0.000E+00 | 1.111E-07 | 1.144E-07 | 0.000E+00 | 4.898E-07 | 0.000E+00 | 1.322E-07 | 0.000E+00 | 0.000E+00 | 0.000E+00 | 0.000E+00 |
| Unigene409095 | emrY | 0.000E+00 | 0.000E+00 | 0.000E+00 | 0.000E+00 | 0.000E+00 | 3.231E-07 | 4.631E-07 | 0.000E+00 | 0.000E+00 | 0.000E+00 | 0.000E+00 | 0.000E+00 |
| Unigene409119 | tva(A) | 0.000E+00 | 0.000E+00 | 0.000E+00 | 0.000E+00 | 0.000E+00 | 7.915E-07 | 0.000E+00 | 0.000E+00 | 0.000E+00 | 0.000E+00 | 0.000E+00 | 0.000E+00 |
| Unigene409251 | Corynebac | 0.000E+00 | 7.381E-08 | 0.000E+00 | 0.000E+00 | 0.000E+00 | 3.635E-07 | 0.000E+00 | 0.000E+00 | 0.000E+00 | 0.000E+00 | 0.000E+00 | 0.000E+00 |
| Unigene409283 | vanHD | 1.214E-07 | 0.000E+00 | 0.000E+00 | 0.000E+00 | 0.000E+00 | 9.183E-07 | 0.000E+00 | 0.000E+00 | 0.000E+00 | 0.000E+00 | 0.000E+00 | 2.271E-06 |
| Unigene409370 | lmrD | 0.000E+00 | 0.000E+00 | 0.000E+00 | 0.000E+00 | 0.000E+00 | 6.391E-07 | 0.000E+00 | 0.000E+00 | 0.000E+00 | 0.000E+00 | 0.000E+00 | 0.000E+00 |
| Unigene409486 | arlR | 0.000E+00 | 0.000E+00 | 0.000E+00 | 0.000E+00 | 0.000E+00 | 1.655E-06 | 0.000E+00 | 0.000E+00 | 0.000E+00 | 0.000E+00 | 0.000E+00 | 0.000E+00 |
| Unigene409603 | lmrD | 0.000E+00 | 0.000E+00 | 0.000E+00 | 0.000E+00 | 0.000E+00 | 3.343E-07 | 0.000E+00 | 0.000E+00 | 0.000E+00 | 0.000E+00 | 0.000E+00 | 0.000E+00 |
| Unigene409620 | acrB | 0.000E+00 | 0.000E+00 | 0.000E+00 | 0.000E+00 | 0.000E+00 | 4.965E-07 | 0.000E+00 | 0.000E+00 | 0.000E+00 | 0.000E+00 | 0.000E+00 | 0.000E+00 |
| Unigene409769 | poxtA | 0.000E+00 | 0.000E+00 | 0.000E+00 | 0.000E+00 | 1.072E-07 | 5.216E-07 | 0.000E+00 | 2.253E-07 | 0.000E+00 | 1.613E-07 | 0.000E+00 | 0.000E+00 |
| Unigene409815 | macB | 0.000E+00 | 0.000E+00 | 0.000E+00 | 0.000E+00 | 0.000E+00 | 5.592E-07 | 0.000E+00 | 0.000E+00 | 0.000E+00 | 0.000E+00 | 0.000E+00 | 0.000E+00 |
| Unigene409857 | vgaD | 0.000E+00 | 0.000E+00 | 0.000E+00 | 0.000E+00 | 0.000E+00 | 4.455E-07 | 0.000E+00 | 0.000E+00 | 0.000E+00 | 0.000E+00 | 0.000E+00 | 0.000E+00 |
| Unigene409897 | Pseudomo | 0.000E+00 | 0.000E+00 | 0.000E+00 | 0.000E+00 | 0.000E+00 | 5.465E-07 | 0.000E+00 | 0.000E+00 | 0.000E+00 | 0.000E+00 | 0.000E+00 | 0.000E+00 |
| Unigene409919 | efrA | 0.000E+00 | 0.000E+00 | 0.000E+00 | 0.000E+00 | 0.000E+00 | 1.331E-06 | 0.000E+00 | 0.000E+00 | 0.000E+00 | 0.000E+00 | 0.000E+00 | 0.000E+00 |
| Unigene409920 | patB | 0.000E+00 | 0.000E+00 | 0.000E+00 | 0.000E+00 | 0.000E+00 | 1.740E-06 | 0.000E+00 | 0.000E+00 | 0.000E+00 | 0.000E+00 | 0.000E+00 | 0.000E+00 |
| Unigene409940 | TaeA | 0.000E+00 | 0.000E+00 | 0.000E+00 | 0.000E+00 | 0.000E+00 | 1.264E-06 | 0.000E+00 | 0.000E+00 | 0.000E+00 | 0.000E+00 | 0.000E+00 | 0.000E+00 |
| Unigene409993 | ykkC | 0.000E+00 | 0.000E+00 | 0.000E+00 | 0.000E+00 | 0.000E+00 | 5.287E-07 | 0.000E+00 | 0.000E+00 | 0.000E+00 | 0.000E+00 | 0.000E+00 | 0.000E+00 |
| Unigene410147 | tetB(46) | 0.000E+00 | 0.000E+00 | 0.000E+00 | 0.000E+00 | 0.000E+00 | 0.000E+00 | 0.000E+00 | 0.000E+00 | 0.000E+00 | 0.000E+00 | 0.000E+00 | 2.891E-07 |
| Unigene410168 | qacA | 0.000E+00 | 0.000E+00 | 0.000E+00 | 0.000E+00 | 0.000E+00 | 9.554E-07 | 0.000E+00 | 0.000E+00 | 0.000E+00 | 0.000E+00 | 0.000E+00 | 0.000E+00 |
| Unigene410416 | rpoB2 | 0.000E+00 | 0.000E+00 | 0.000E+00 | 0.000E+00 | 0.000E+00 | 1.404E-06 | 0.000E+00 | 0.000E+00 | 0.000E+00 | 0.000E+00 | 0.000E+00 | 0.000E+00 |
| Unigene410422 | tetT | 0.000E+00 | 0.000E+00 | 0.000E+00 | 0.000E+00 | 0.000E+00 | 1.167E-06 | 0.000E+00 | 0.000E+00 | 0.000E+00 | 0.000E+00 | 0.000E+00 | 0.000E+00 |
| Unigene410504 | basS | 0.000E+00 | 0.000E+00 | 0.000E+00 | 0.000E+00 | 0.000E+00 | 9.123E-07 | 0.000E+00 | 0.000E+00 | 0.000E+00 | 0.000E+00 | 0.000E+00 | 0.000E+00 |
| Unigene410505 | Listeria mc | 0.000E+00 | 0.000E+00 | 0.000E+00 | 0.000E+00 | 0.000E+00 | 1.072E-06 | 0.000E+00 | 0.000E+00 | 0.000E+00 | 0.000E+00 | 0.000E+00 | 0.000E+00 |
| Unigene410515 | arnA | 0.000E+00 | 0.000E+00 | 0.000E+00 | 0.000E+00 | 0.000E+00 | 6.972E-07 | 0.000E+00 | 0.000E+00 | 0.000E+00 | 0.000E+00 | 0.000E+00 | 0.000E+00 |
| Unigene410530 | PmrF | 0.000E+00 | 0.000E+00 | 0.000E+00 | 0.000E+00 | 0.000E+00 | 3.231E-07 | 0.000E+00 | 0.000E+00 | 0.000E+00 | 0.000E+00 | 0.000E+00 | 0.000E+00 |
| Unigene410689 | rpoB2 | 0.000E+00 | 0.000E+00 | 0.000E+00 | 0.000E+00 | 0.000E+00 | 2.551E-07 | 0.000E+00 | 0.000E+00 | 2.063E-07 | 0.000E+00 | 0.000E+00 | 0.000E+00 |
| Unigene410736 | novA | 0.000E+00 | 0.000E+00 | 6.823E-07 | 4.213E-07 | 0.000E+00 | 1.297E-06 | 3.694E-06 | 2.759E-06 | 0.000E+00 | 9.101E-07 | 0.000E+00 | 2.774E-06 |
| Unigene410759 | mdsB | 0.000E+00 | 0.000E+00 | 0.000E+00 | 0.000E+00 | 0.000E+00 | 0.000E+00 | 0.000E+00 | 5.842E-07 | 0.000E+00 | 2.788E-07 | 6.440E-07 | 0.000E+00 |
| Unigene410846 | Acinetoba | 0.000E+00 | 0.000E+00 | 0.000E+00 | 0.000E+00 | 1.082E-07 | 6.842E-07 | 0.000E+00 | 5.115E-07 | 0.000E+00 | 0.000E+00 | 0.000E+00 | 6.992E-06 |
| Unigene411016 | lmrB | 0.000E+00 | 0.000E+00 | 0.000E+00 | 0.000E+00 | 0.000E+00 | 9.555E-07 | 0.000E+00 | 0.000E+00 | 0.000E+00 | 0.000E+00 | 0.000E+00 | 0.000E+00 |
| Unigene411017 | tetB(60) | 0.000E+00 | 0.000E+00 | 0.000E+00 | 0.000E+00 | 0.000E+00 | 6.726E-07 | 0.000E+00 | 9.035E-06 | 0.000E+00 | 0.000E+00 | 1.168E-05 | 0.000E+00 |
| Unigene411037 | efrB | 0.000E+00 | 0.000E+00 | 0.000E+00 | 0.000E+00 | 0.000E+00 | 3.187E-07 | 0.000E+00 | 0.000E+00 | 0.000E+00 | 0.000E+00 | 0.000E+00 | 0.000E+00 |
| Unigene411069 | arlS | 0.000E+00 | 0.000E+00 | 0.000E+00 | 0.000E+00 | 0.000E+00 | 1.501E-06 | 0.000E+00 | 0.000E+00 | 0.000E+00 | 0.000E+00 | 0.000E+00 | 0.000E+00 |
| Unigene411070 | arlR | 0.000E+00 | 0.000E+00 | 0.000E+00 | 0.000E+00 | 0.000E+00 | 1.647E-06 | 0.000E+00 | 0.000E+00 | 0.000E+00 | 0.000E+00 | 0.000E+00 | 0.000E+00 |
| Unigene411074 | bcrA | 0.000E+00 | 0.000E+00 | 0.000E+00 | 0.000E+00 | 0.000E+00 | 1.319E-06 | 0.000E+00 | 0.000E+00 | 0.000E+00 | 0.000E+00 | 0.000E+00 | 0.000E+00 |
| Unigene411158 | mexN | 0.000E+00 | 0.000E+00 | 0.000E+00 | 0.000E+00 | 0.000E+00 | 5.652E-07 | 0.000E+00 | 0.000E+00 | 0.000E+00 | 0.000E+00 | 0.000E+00 | 0.000E+00 |
| Unigene411255 | ykkC | 0.000E+00 | 0.000E+00 | 0.000E+00 | 0.000E+00 | 0.000E+00 | 3.261E-07 | 0.000E+00 | 0.000E+00 | 0.000E+00 | 0.000E+00 | 0.000E+00 | 0.000E+00 |
| Unigene411308 | MuxA | 0.000E+00 | 0.000E+00 | 0.000E+00 | 0.000E+00 | 0.000E+00 | 3.877E-07 | 0.000E+00 | 0.000E+00 | 0.000E+00 | 0.000E+00 | 0.000E+00 | 0.000E+00 |
| Unigene411384 | NmcR | 0.000E+00 | 0.000E+00 | 0.000E+00 | 0.000E+00 | 0.000E+00 | 6.703E-07 | 0.000E+00 | 2.384E-06 | 0.000E+00 | 7.314E-07 | 0.000E+00 | 0.000E+00 |

| | | | | | | | | | | | | | |
|---|---|---|---|---|---|---|---|---|---|---|---|---|---|
| Unigene411414 | novA | 0.000E+00 | 0.000E+00 | 0.000E+00 | 0.000E+00 | 0.000E+00 | 6.567E-07 | 0.000E+00 | 0.000E+00 | 0.000E+00 | 0.000E+00 | 1.673E-05 | 0.000E+00 |
| Unigene411428 | tetB(60) | 0.000E+00 | 0.000E+00 | 0.000E+00 | 0.000E+00 | 0.000E+00 | 1.028E-06 | 0.000E+00 | 0.000E+00 | 0.000E+00 | 0.000E+00 | 0.000E+00 | 0.000E+00 |
| Unigene411441 | AcrF | 0.000E+00 | 0.000E+00 | 0.000E+00 | 0.000E+00 | 0.000E+00 | 1.728E-07 | 0.000E+00 | 0.000E+00 | 0.000E+00 | 0.000E+00 | 0.000E+00 | 0.000E+00 |
| Unigene411524 | Erm(42) | 0.000E+00 | 0.000E+00 | 0.000E+00 | 0.000E+00 | 0.000E+00 | 1.054E-06 | 0.000E+00 | 0.000E+00 | 0.000E+00 | 0.000E+00 | 0.000E+00 | 0.000E+00 |
| Unigene411527 | tetA(58) | 0.000E+00 | 0.000E+00 | 0.000E+00 | 0.000E+00 | 0.000E+00 | 1.669E-06 | 0.000E+00 | 0.000E+00 | 0.000E+00 | 0.000E+00 | 0.000E+00 | 0.000E+00 |
| Unigene411540 | mdtG | 0.000E+00 | 0.000E+00 | 0.000E+00 | 0.000E+00 | 0.000E+00 | 1.633E-06 | 0.000E+00 | 0.000E+00 | 0.000E+00 | 0.000E+00 | 0.000E+00 | 0.000E+00 |
| Unigene411580 | CRP | 0.000E+00 | 0.000E+00 | 0.000E+00 | 0.000E+00 | 0.000E+00 | 2.073E-06 | 0.000E+00 | 0.000E+00 | 0.000E+00 | 0.000E+00 | 0.000E+00 | 0.000E+00 |
| Unigene411641 | Streptomy | 0.000E+00 | 0.000E+00 | 0.000E+00 | 0.000E+00 | 0.000E+00 | 0.000E+00 | 0.000E+00 | 0.000E+00 | 0.000E+00 | 0.000E+00 | 0.000E+00 | 2.808E-06 |
| Unigene411710 | macB | 0.000E+00 | 0.000E+00 | 0.000E+00 | 0.000E+00 | 0.000E+00 | 9.391E-07 | 0.000E+00 | 0.000E+00 | 0.000E+00 | 0.000E+00 | 0.000E+00 | 0.000E+00 |
| Unigene411711 | macB | 0.000E+00 | 0.000E+00 | 0.000E+00 | 0.000E+00 | 0.000E+00 | 1.050E-06 | 0.000E+00 | 0.000E+00 | 0.000E+00 | 0.000E+00 | 0.000E+00 | 0.000E+00 |
| Unigene411760 | tetA(58) | 0.000E+00 | 0.000E+00 | 0.000E+00 | 0.000E+00 | 0.000E+00 | 6.842E-07 | 0.000E+00 | 0.000E+00 | 0.000E+00 | 0.000E+00 | 0.000E+00 | 0.000E+00 |
| Unigene411842 | tetA(58) | 0.000E+00 | 0.000E+00 | 0.000E+00 | 0.000E+00 | 0.000E+00 | 1.695E-06 | 0.000E+00 | 0.000E+00 | 0.000E+00 | 0.000E+00 | 0.000E+00 | 0.000E+00 |
| Unigene411855 | tet(W/N/W | 0.000E+00 | 0.000E+00 | 0.000E+00 | 0.000E+00 | 0.000E+00 | 1.471E-06 | 0.000E+00 | 0.000E+00 | 0.000E+00 | 0.000E+00 | 0.000E+00 | 0.000E+00 |
| Unigene411859 | tetA(58) | 0.000E+00 | 0.000E+00 | 0.000E+00 | 0.000E+00 | 0.000E+00 | 1.682E-06 | 0.000E+00 | 0.000E+00 | 0.000E+00 | 0.000E+00 | 0.000E+00 | 0.000E+00 |
| Unigene411860 | tetB(58) | 0.000E+00 | 0.000E+00 | 0.000E+00 | 0.000E+00 | 0.000E+00 | 1.181E-06 | 0.000E+00 | 0.000E+00 | 0.000E+00 | 0.000E+00 | 0.000E+00 | 0.000E+00 |
| Unigene412069 | TaeA | 0.000E+00 | 0.000E+00 | 0.000E+00 | 0.000E+00 | 0.000E+00 | 6.204E-07 | 0.000E+00 | 0.000E+00 | 0.000E+00 | 0.000E+00 | 0.000E+00 | 0.000E+00 |
| Unigene412171 | efrA | 0.000E+00 | 0.000E+00 | 0.000E+00 | 0.000E+00 | 0.000E+00 | 3.972E-07 | 0.000E+00 | 6.127E-08 | 0.000E+00 | 0.000E+00 | 0.000E+00 | 0.000E+00 |
| Unigene412196 | PmrF | 0.000E+00 | 0.000E+00 | 0.000E+00 | 0.000E+00 | 0.000E+00 | 1.192E-06 | 0.000E+00 | 0.000E+00 | 0.000E+00 | 0.000E+00 | 0.000E+00 | 0.000E+00 |
| Unigene412258 | Staphyloc | 0.000E+00 | 0.000E+00 | 0.000E+00 | 0.000E+00 | 0.000E+00 | 9.535E-07 | 0.000E+00 | 7.695E-06 | 0.000E+00 | 0.000E+00 | 0.000E+00 | 0.000E+00 |
| Unigene412328 | bcrA | 0.000E+00 | 0.000E+00 | 0.000E+00 | 0.000E+00 | 0.000E+00 | 6.695E-07 | 0.000E+00 | 0.000E+00 | 0.000E+00 | 0.000E+00 | 0.000E+00 | 0.000E+00 |
| Unigene412375 | arlR | 0.000E+00 | 0.000E+00 | 0.000E+00 | 0.000E+00 | 0.000E+00 | 0.000E+00 | 0.000E+00 | 5.638E-06 | 0.000E+00 | 0.000E+00 | 0.000E+00 | 0.000E+00 |
| Unigene412567 | dfrA3 | 0.000E+00 | 0.000E+00 | 0.000E+00 | 0.000E+00 | 0.000E+00 | 1.213E-06 | 0.000E+00 | 0.000E+00 | 0.000E+00 | 0.000E+00 | 0.000E+00 | 0.000E+00 |
| Unigene412591 | cpxA | 0.000E+00 | 0.000E+00 | 0.000E+00 | 0.000E+00 | 0.000E+00 | 1.468E-06 | 0.000E+00 | 0.000E+00 | 0.000E+00 | 0.000E+00 | 0.000E+00 | 0.000E+00 |
| Unigene412728 | adeL | 0.000E+00 | 0.000E+00 | 2.400E-07 | 0.000E+00 | 0.000E+00 | 0.000E+00 | 1.005E-06 | 2.039E-07 | 0.000E+00 | 0.000E+00 | 0.000E+00 | 0.000E+00 |
| Unigene412745 | LRA-8 | 0.000E+00 | 0.000E+00 | 0.000E+00 | 0.000E+00 | 0.000E+00 | 0.000E+00 | 1.344E-06 | 0.000E+00 | 0.000E+00 | 0.000E+00 | 0.000E+00 | 0.000E+00 |
| Unigene412797 | mdtC | 0.000E+00 | 0.000E+00 | 0.000E+00 | 0.000E+00 | 0.000E+00 | 0.000E+00 | 9.045E-07 | 0.000E+00 | 0.000E+00 | 0.000E+00 | 0.000E+00 | 0.000E+00 |
| Unigene412844 | Staphyloc | 0.000E+00 | 0.000E+00 | 0.000E+00 | 0.000E+00 | 2.711E-08 | 2.638E-07 | 1.080E-06 | 1.709E-07 | 0.000E+00 | 8.154E-08 | 1.954E-07 | 4.038E-07 |
| Unigene412855 | mtrA | 0.000E+00 | 0.000E+00 | 0.000E+00 | 0.000E+00 | 0.000E+00 | 0.000E+00 | 8.887E-07 | 0.000E+00 | 0.000E+00 | 0.000E+00 | 0.000E+00 | 0.000E+00 |
| Unigene412881 | lmrC | 0.000E+00 | 0.000E+00 | 0.000E+00 | 0.000E+00 | 1.207E-07 | 0.000E+00 | 4.210E-07 | 3.806E-07 | 0.000E+00 | 0.000E+00 | 3.419E-07 | 2.248E-07 |
| Unigene413041 | tetT | 0.000E+00 | 0.000E+00 | 0.000E+00 | 0.000E+00 | 0.000E+00 | 0.000E+00 | 8.289E-07 | 0.000E+00 | 0.000E+00 | 0.000E+00 | 3.497E-08 | 0.000E+00 |
| Unigene413098 | lmrB | 0.000E+00 | 0.000E+00 | 0.000E+00 | 0.000E+00 | 0.000E+00 | 0.000E+00 | 3.529E-07 | 0.000E+00 | 0.000E+00 | 0.000E+00 | 0.000E+00 | 0.000E+00 |
| Unigene413109 | tetB(60) | 0.000E+00 | 0.000E+00 | 0.000E+00 | 0.000E+00 | 0.000E+00 | 1.795E-07 | 7.902E-07 | 3.295E-07 | 0.000E+00 | 0.000E+00 | 0.000E+00 | 4.514E-07 |
| Unigene413162 | adeL | 0.000E+00 | 0.000E+00 | 1.297E-07 | 0.000E+00 | 0.000E+00 | 0.000E+00 | 7.315E-07 | 8.641E-07 | 4.045E-07 | 2.651E-07 | 3.629E-07 | 1.375E-06 |
| Unigene413197 | CKO-1 | 0.000E+00 | 0.000E+00 | 0.000E+00 | 0.000E+00 | 0.000E+00 | 0.000E+00 | 2.135E-06 | 0.000E+00 | 0.000E+00 | 0.000E+00 | 0.000E+00 | 0.000E+00 |
| Unigene413250 | optrA | 0.000E+00 | 0.000E+00 | 0.000E+00 | 0.000E+00 | 0.000E+00 | 0.000E+00 | 1.092E-06 | 0.000E+00 | 0.000E+00 | 0.000E+00 | 0.000E+00 | 0.000E+00 |
| Unigene413410 | basS | 0.000E+00 | 0.000E+00 | 0.000E+00 | 0.000E+00 | 1.868E-07 | 0.000E+00 | 8.772E-07 | 0.000E+00 | 0.000E+00 | 0.000E+00 | 2.473E-07 | 0.000E+00 |
| Unigene413414 | efrA | 0.000E+00 | 0.000E+00 | 0.000E+00 | 0.000E+00 | 6.678E-07 | 0.000E+00 | 1.064E-06 | 0.000E+00 | 0.000E+00 | 0.000E+00 | 4.813E-07 | 0.000E+00 |
| Unigene413441 | TaeA | 0.000E+00 | 0.000E+00 | 0.000E+00 | 5.938E-08 | 2.286E-07 | 1.589E-07 | 8.785E-07 | 3.088E-07 | 0.000E+00 | 0.000E+00 | 0.000E+00 | 0.000E+00 |
| Unigene413477 | acrB | 0.000E+00 | 0.000E+00 | 0.000E+00 | 0.000E+00 | 0.000E+00 | 0.000E+00 | 6.583E-07 | 0.000E+00 | 0.000E+00 | 0.000E+00 | 1.237E-07 | 0.000E+00 |
| Unigene413590 | adeJ | 0.000E+00 | 0.000E+00 | 0.000E+00 | 0.000E+00 | 0.000E+00 | 0.000E+00 | 8.241E-07 | 0.000E+00 | 0.000E+00 | 0.000E+00 | 0.000E+00 | 0.000E+00 |
| Unigene413702 | rpoB2 | 0.000E+00 | 0.000E+00 | 0.000E+00 | 0.000E+00 | 0.000E+00 | 0.000E+00 | 4.710E-07 | 0.000E+00 | 0.000E+00 | 0.000E+00 | 0.000E+00 | 0.000E+00 |
| Unigene413758 | lmrC | 0.000E+00 | 0.000E+00 | 8.654E-08 | 0.000E+00 | 3.184E-07 | 0.000E+00 | 1.196E-06 | 0.000E+00 | 0.000E+00 | 0.000E+00 | 4.036E-07 | 0.000E+00 |
| Unigene413933 | tetA(60) | 0.000E+00 | 0.000E+00 | 0.000E+00 | 0.000E+00 | 0.000E+00 | 0.000E+00 | 1.884E-06 | 0.000E+00 | 0.000E+00 | 0.000E+00 | 0.000E+00 | 0.000E+00 |
| Unigene413934 | tetB(60) | 0.000E+00 | 0.000E+00 | 0.000E+00 | 0.000E+00 | 0.000E+00 | 0.000E+00 | 2.189E-06 | 0.000E+00 | 0.000E+00 | 0.000E+00 | 0.000E+00 | 0.000E+00 |
| Unigene414316 | vanHF | 0.000E+00 | 0.000E+00 | 1.261E-07 | 1.946E-07 | 4.639E-07 | 1.042E-07 | 1.422E-06 | 2.062E-06 | 0.000E+00 | 0.000E+00 | 5.511E-07 | 1.063E-06 |
| Unigene414413 | novA | 0.000E+00 | 0.000E+00 | 0.000E+00 | 0.000E+00 | 0.000E+00 | 0.000E+00 | 9.324E-07 | 6.258E-07 | 0.000E+00 | 0.000E+00 | 0.000E+00 | 0.000E+00 |
| Unigene414430 | vanHB | 0.000E+00 | 0.000E+00 | 0.000E+00 | 1.389E-07 | 0.000E+00 | 0.000E+00 | 2.131E-06 | 0.000E+00 | 0.000E+00 | 0.000E+00 | 0.000E+00 | 0.000E+00 |
| Unigene414441 | mdtN | 0.000E+00 | 0.000E+00 | 0.000E+00 | 0.000E+00 | 0.000E+00 | 0.000E+00 | 2.569E-06 | 0.000E+00 | 0.000E+00 | 0.000E+00 | 0.000E+00 | 0.000E+00 |
| Unigene414524 | evgS | 0.000E+00 | 0.000E+00 | 0.000E+00 | 0.000E+00 | 0.000E+00 | 0.000E+00 | 8.006E-07 | 7.916E-08 | 0.000E+00 | 0.000E+00 | 2.327E-07 | 1.336E-07 |
| Unigene414579 | bcrA | 0.000E+00 | 1.524E-07 | 0.000E+00 | 0.000E+00 | 0.000E+00 | 2.251E-07 | 4.226E-07 | 0.000E+00 | 0.000E+00 | 0.000E+00 | 0.000E+00 | 6.564E-07 |
| Unigene414676 | tetT | 0.000E+00 | 0.000E+00 | 3.299E-07 | 0.000E+00 | 0.000E+00 | 0.000E+00 | 1.351E-06 | 5.991E-07 | 0.000E+00 | 0.000E+00 | 0.000E+00 | 7.322E-07 |
| Unigene414724 | iri | 0.000E+00 | 0.000E+00 | 0.000E+00 | 0.000E+00 | 0.000E+00 | 0.000E+00 | 7.328E-07 | 0.000E+00 | 0.000E+00 | 0.000E+00 | 2.525E-07 | 0.000E+00 |
| Unigene414810 | adeL | 0.000E+00 | 0.000E+00 | 0.000E+00 | 0.000E+00 | 3.075E-07 | 0.000E+00 | 9.573E-07 | 0.000E+00 | 0.000E+00 | 0.000E+00 | 0.000E+00 | 0.000E+00 |
| Unigene414864 | lmrB | 0.000E+00 | 0.000E+00 | 0.000E+00 | 0.000E+00 | 0.000E+00 | 0.000E+00 | 7.098E-07 | 0.000E+00 | 0.000E+00 | 0.000E+00 | 0.000E+00 | 0.000E+00 |
| Unigene414941 | mtrA | 0.000E+00 | 0.000E+00 | 0.000E+00 | 8.980E-08 | 0.000E+00 | 3.365E-07 | 5.905E-07 | 2.595E-07 | 0.000E+00 | 0.000E+00 | 2.543E-07 | 0.000E+00 |
| Unigene415083 | MexD | 0.000E+00 | 0.000E+00 | 0.000E+00 | 0.000E+00 | 0.000E+00 | 0.000E+00 | 1.043E-06 | 0.000E+00 | 0.000E+00 | 0.000E+00 | 0.000E+00 | 0.000E+00 |
| Unigene415113 | lmrB | 0.000E+00 | 0.000E+00 | 0.000E+00 | 0.000E+00 | 0.000E+00 | 0.000E+00 | 1.063E-06 | 0.000E+00 | 0.000E+00 | 0.000E+00 | 0.000E+00 | 0.000E+00 |
| Unigene415213 | tetB(60) | 0.000E+00 | 0.000E+00 | 0.000E+00 | 0.000E+00 | 0.000E+00 | 0.000E+00 | 4.342E-07 | 0.000E+00 | 0.000E+00 | 0.000E+00 | 0.000E+00 | 0.000E+00 |

| | | | | | | | | | | | | | |
|---|---|---|---|---|---|---|---|---|---|---|---|---|---|
| Unigene415223 | vanRF | 0.000E+00 | 0.000E+00 | 0.000E+00 | 0.000E+00 | 0.000E+00 | 0.000E+00 | 5.982E-07 | 0.000E+00 | 0.000E+00 | 0.000E+00 | 0.000E+00 | 4.065E-07 |
| Unigene415236 | kdpE | 0.000E+00 | 0.000E+00 | 0.000E+00 | 0.000E+00 | 0.000E+00 | 0.000E+00 | 9.194E-07 | 0.000E+00 | 0.000E+00 | 0.000E+00 | 0.000E+00 | 0.000E+00 |
| Unigene415254 | kdpE | 0.000E+00 | 0.000E+00 | 0.000E+00 | 0.000E+00 | 2.610E-07 | 0.000E+00 | 8.321E-07 | 1.645E-07 | 0.000E+00 | 0.000E+00 | 0.000E+00 | 3.332E-07 |
| Unigene415288 | Staphylocc | 3.190E-07 | 0.000E+00 | 0.000E+00 | 2.050E-07 | 0.000E+00 | 0.000E+00 | 9.951E-07 | 8.125E-07 | 0.000E+00 | 0.000E+00 | 5.972E-07 | 8.228E-07 |
| Unigene415308 | efrA | 1.625E-07 | 0.000E+00 | 0.000E+00 | 0.000E+00 | 0.000E+00 | 2.460E-07 | 1.007E-06 | 9.107E-07 | 0.000E+00 | 0.000E+00 | 7.437E-07 | 7.685E-07 |
| Unigene415319 | cmx | 0.000E+00 | 1.798E-07 | 8.039E-08 | 0.000E+00 | 0.000E+00 | 0.000E+00 | 5.743E-07 | 1.052E-06 | 0.000E+00 | 0.000E+00 | 0.000E+00 | 9.361E-07 |
| Unigene415334 | MexD | 1.023E-07 | 6.735E-08 | 2.308E-07 | 9.296E-08 | 0.000E+00 | 5.197E-07 | 1.438E-06 | 5.014E-07 | 0.000E+00 | 3.190E-07 | 0.000E+00 | 6.166E-07 |
| Unigene415335 | adeA | 0.000E+00 | 0.000E+00 | 0.000E+00 | 0.000E+00 | 0.000E+00 | 0.000E+00 | 5.997E-07 | 0.000E+00 | 0.000E+00 | 0.000E+00 | 0.000E+00 | 0.000E+00 |
| Unigene415370 | oleC | 3.892E-08 | 0.000E+00 | 2.100E-07 | 0.000E+00 | 2.594E-07 | 5.890E-07 | 2.003E-06 | 8.631E-07 | 0.000E+00 | 5.419E-07 | 5.787E-07 | 1.012E-06 |
| Unigene415435 | NmcR | 0.000E+00 | 0.000E+00 | 0.000E+00 | 0.000E+00 | 0.000E+00 | 0.000E+00 | 8.704E-07 | 4.590E-07 | 0.000E+00 | 0.000E+00 | 0.000E+00 | 1.141E-06 |
| Unigene415441 | TaeA | 0.000E+00 | 0.000E+00 | 0.000E+00 | 0.000E+00 | 0.000E+00 | 0.000E+00 | 0.000E+00 | 2.102E-07 | 0.000E+00 | 0.000E+00 | 0.000E+00 | 0.000E+00 |
| Unigene415496 | macB | 0.000E+00 | 0.000E+00 | 0.000E+00 | 0.000E+00 | 0.000E+00 | 0.000E+00 | 6.702E-07 | 3.806E-07 | 0.000E+00 | 0.000E+00 | 0.000E+00 | 0.000E+00 |
| Unigene415509 | bcrA | 0.000E+00 | 0.000E+00 | 0.000E+00 | 0.000E+00 | 0.000E+00 | 0.000E+00 | 1.636E-06 | 0.000E+00 | 0.000E+00 | 0.000E+00 | 0.000E+00 | 0.000E+00 |
| Unigene415513 | lsaC | 0.000E+00 | 0.000E+00 | 0.000E+00 | 0.000E+00 | 0.000E+00 | 1.776E-07 | 1.091E-06 | 1.726E-06 | 0.000E+00 | 2.287E-07 | 0.000E+00 | 1.117E-06 |
| Unigene415551 | vanRD | 0.000E+00 | 0.000E+00 | 1.738E-07 | 0.000E+00 | 0.000E+00 | 0.000E+00 | 1.225E-06 | 0.000E+00 | 0.000E+00 | 0.000E+00 | 4.559E-07 | 0.000E+00 |
| Unigene415640 | Pseudomo | 0.000E+00 | 0.000E+00 | 0.000E+00 | 0.000E+00 | 0.000E+00 | 0.000E+00 | 1.708E-06 | 0.000E+00 | 0.000E+00 | 0.000E+00 | 0.000E+00 | 0.000E+00 |
| Unigene415662 | rosB | 0.000E+00 | 0.000E+00 | 0.000E+00 | 0.000E+00 | 0.000E+00 | 0.000E+00 | 6.301E-07 | 0.000E+00 | 0.000E+00 | 0.000E+00 | 0.000E+00 | 0.000E+00 |
| Unigene415666 | OprN | 0.000E+00 | 2.345E-07 | 8.767E-07 | 4.707E-07 | 1.165E-06 | 1.029E-06 | 1.956E-06 | 1.383E-06 | 2.335E-07 | 7.789E-07 | 1.111E-06 | 4.109E-06 |
| Unigene415668 | bcrA | 8.423E-08 | 0.000E+00 | 1.033E-07 | 1.276E-07 | 9.123E-07 | 3.415E-07 | 5.360E-07 | 8.848E-07 | 1.841E-07 | 0.000E+00 | 7.708E-07 | 7.716E-07 |
| Unigene415676 | tlrC | 0.000E+00 | 0.000E+00 | 0.000E+00 | 0.000E+00 | 0.000E+00 | 0.000E+00 | 7.939E-07 | 0.000E+00 | 0.000E+00 | 0.000E+00 | 0.000E+00 | 0.000E+00 |
| Unigene415789 | bcr-1 | 0.000E+00 | 0.000E+00 | 0.000E+00 | 0.000E+00 | 0.000E+00 | 0.000E+00 | 9.017E-07 | 0.000E+00 | 0.000E+00 | 0.000E+00 | 0.000E+00 | 0.000E+00 |
| Unigene415809 | patA | 0.000E+00 | 0.000E+00 | 0.000E+00 | 0.000E+00 | 0.000E+00 | 2.865E-07 | 1.349E-06 | 0.000E+00 | 0.000E+00 | 0.000E+00 | 0.000E+00 | 0.000E+00 |
| Unigene415824 | macB | 0.000E+00 | 0.000E+00 | 8.258E-07 | 0.000E+00 | 0.000E+00 | 0.000E+00 | 1.497E-06 | 0.000E+00 | 0.000E+00 | 0.000E+00 | 1.272E-06 | 3.091E-06 |
| Unigene415946 | Acinetoba | 0.000E+00 | 0.000E+00 | 0.000E+00 | 0.000E+00 | 0.000E+00 | 0.000E+00 | 0.000E+00 | 0.000E+00 | 0.000E+00 | 0.000E+00 | 0.000E+00 | 2.678E-07 |
| Unigene415976 | baeS | 0.000E+00 | 0.000E+00 | 0.000E+00 | 0.000E+00 | 0.000E+00 | 0.000E+00 | 7.972E-07 | 0.000E+00 | 0.000E+00 | 0.000E+00 | 0.000E+00 | 0.000E+00 |
| Unigene415998 | macA | 0.000E+00 | 1.074E-06 | 0.000E+00 | 0.000E+00 | 0.000E+00 | 0.000E+00 | 1.783E-06 | 0.000E+00 | 0.000E+00 | 0.000E+00 | 0.000E+00 | 0.000E+00 |
| Unigene416005 | LlmA 23S r | 0.000E+00 | 8.879E-08 | 0.000E+00 | 0.000E+00 | 0.000E+00 | 0.000E+00 | 3.582E-07 | 2.361E-07 | 0.000E+00 | 0.000E+00 | 2.776E-07 | 4.781E-07 |
| Unigene416058 | vatB | 0.000E+00 | 0.000E+00 | 0.000E+00 | 0.000E+00 | 0.000E+00 | 0.000E+00 | 9.030E-07 | 0.000E+00 | 0.000E+00 | 0.000E+00 | 0.000E+00 | 0.000E+00 |
| Unigene416067 | facT | 0.000E+00 | 0.000E+00 | 0.000E+00 | 0.000E+00 | 0.000E+00 | 0.000E+00 | 9.161E-07 | 6.038E-08 | 0.000E+00 | 0.000E+00 | 0.000E+00 | 3.669E-07 |
| Unigene416096 | adeL | 0.000E+00 | 0.000E+00 | 0.000E+00 | 0.000E+00 | 0.000E+00 | 0.000E+00 | 2.310E-06 | 0.000E+00 | 0.000E+00 | 0.000E+00 | 0.000E+00 | 0.000E+00 |
| Unigene416170 | smeS | 0.000E+00 | 0.000E+00 | 0.000E+00 | 0.000E+00 | 0.000E+00 | 0.000E+00 | 5.817E-07 | 0.000E+00 | 0.000E+00 | 0.000E+00 | 0.000E+00 | 0.000E+00 |
| Unigene416179 | TriA | 0.000E+00 | 0.000E+00 | 2.289E-07 | 1.178E-07 | 0.000E+00 | 1.891E-07 | 1.065E-06 | 0.000E+00 | 6.374E-08 | 0.000E+00 | 0.000E+00 | 0.000E+00 |
| Unigene416180 | TriB | 0.000E+00 | 1.305E-07 | 0.000E+00 | 2.701E-07 | 1.981E-07 | 9.640E-08 | 1.184E-06 | 2.776E-07 | 0.000E+00 | 0.000E+00 | 4.080E-07 | 8.081E-07 |
| Unigene416204 | adeG | 0.000E+00 | 0.000E+00 | 0.000E+00 | 0.000E+00 | 0.000E+00 | 0.000E+00 | 3.348E-07 | 2.522E-07 | 0.000E+00 | 0.000E+00 | 2.966E-07 | 0.000E+00 |
| Unigene416265 | macB | 1.991E-05 | 1.641E-05 | 4.495E-05 | 5.504E-05 | 3.493E-05 | 2.779E-05 | 3.451E-05 | 3.723E-05 | 2.493E-05 | 3.732E-05 | 5.167E-05 | 4.889E-05 |
| Unigene416314 | optrA | 2.127E-07 | 0.000E+00 | 0.000E+00 | 0.000E+00 | 0.000E+00 | 1.839E-07 | 1.600E-06 | 0.000E+00 | 0.000E+00 | 0.000E+00 | 0.000E+00 | 4.022E-07 |
| Unigene416320 | Streptomy | 0.000E+00 | 0.000E+00 | 3.901E-07 | 3.881E-07 | 7.802E-07 | 7.019E-07 | 3.564E-06 | 2.042E-06 | 2.173E-07 | 7.528E-07 | 1.258E-06 | 2.224E-06 |
| Unigene416582 | OXA-45 | 0.000E+00 | 0.000E+00 | 0.000E+00 | 0.000E+00 | 0.000E+00 | 0.000E+00 | 0.000E+00 | 2.672E-07 | 0.000E+00 | 0.000E+00 | 0.000E+00 | 0.000E+00 |
| Unigene416709 | patA | 0.000E+00 | 0.000E+00 | 0.000E+00 | 0.000E+00 | 0.000E+00 | 0.000E+00 | 1.254E-06 | 0.000E+00 | 0.000E+00 | 0.000E+00 | 0.000E+00 | 0.000E+00 |
| Unigene416726 | Pseudomo | 0.000E+00 | 3.087E-07 | 0.000E+00 | 0.000E+00 | 0.000E+00 | 0.000E+00 | 3.658E-06 | 1.313E-06 | 0.000E+00 | 0.000E+00 | 0.000E+00 | 0.000E+00 |
| Unigene416786 | ErmW | 0.000E+00 | 0.000E+00 | 0.000E+00 | 0.000E+00 | 0.000E+00 | 0.000E+00 | 8.571E-07 | 0.000E+00 | 0.000E+00 | 0.000E+00 | 6.061E-07 | 0.000E+00 |
| Unigene416793 | Burkholder | 0.000E+00 | 0.000E+00 | 0.000E+00 | 0.000E+00 | 0.000E+00 | 0.000E+00 | 9.161E-07 | 0.000E+00 | 0.000E+00 | 0.000E+00 | 0.000E+00 | 0.000E+00 |
| Unigene416813 | tlrC | 0.000E+00 | 0.000E+00 | 0.000E+00 | 0.000E+00 | 0.000E+00 | 0.000E+00 | 3.948E-07 | 0.000E+00 | 0.000E+00 | 0.000E+00 | 0.000E+00 | 0.000E+00 |
| Unigene416831 | novA | 0.000E+00 | 0.000E+00 | 0.000E+00 | 0.000E+00 | 0.000E+00 | 0.000E+00 | 1.240E-07 | 1.962E-07 | 0.000E+00 | 0.000E+00 | 0.000E+00 | 0.000E+00 |
| Unigene416892 | mtrA | 0.000E+00 | 0.000E+00 | 0.000E+00 | 0.000E+00 | 0.000E+00 | 0.000E+00 | 3.520E-07 | 0.000E+00 | 0.000E+00 | 0.000E+00 | 0.000E+00 | 0.000E+00 |
| Unigene416941 | vanG | 0.000E+00 | 0.000E+00 | 0.000E+00 | 0.000E+00 | 0.000E+00 | 0.000E+00 | 1.823E-06 | 0.000E+00 | 0.000E+00 | 0.000E+00 | 0.000E+00 | 1.657E-06 |
| Unigene416948 | mecA | 0.000E+00 | 3.409E-07 | 0.000E+00 | 0.000E+00 | 0.000E+00 | 0.000E+00 | 2.204E-06 | 1.301E-06 | 0.000E+00 | 0.000E+00 | 0.000E+00 | 0.000E+00 |
| Unigene416992 | gadW | 0.000E+00 | 0.000E+00 | 0.000E+00 | 0.000E+00 | 0.000E+00 | 4.308E-07 | 5.513E-07 | 1.008E-06 | 1.452E-07 | 2.590E-07 | 7.598E-07 | 1.217E-06 |
| Unigene417046 | efrA | 0.000E+00 | 0.000E+00 | 0.000E+00 | 0.000E+00 | 1.027E-06 | 4.089E-07 | 6.978E-07 | 9.812E-07 | 4.594E-08 | 0.000E+00 | 7.212E-07 | 1.341E-06 |
| Unigene417055 | sdiA | 0.000E+00 | 0.000E+00 | 0.000E+00 | 1.796E-07 | 0.000E+00 | 0.000E+00 | 8.366E-07 | 0.000E+00 | 0.000E+00 | 1.981E-07 | 0.000E+00 | 2.628E-07 |
| Unigene417086 | otr(B) | 0.000E+00 | 0.000E+00 | 0.000E+00 | 0.000E+00 | 0.000E+00 | 0.000E+00 | 1.985E-06 | 0.000E+00 | 0.000E+00 | 0.000E+00 | 0.000E+00 | 0.000E+00 |
| Unigene417114 | macB | 9.800E-08 | 6.453E-08 | 9.616E-08 | 0.000E+00 | 7.621E-07 | 8.475E-08 | 8.894E-07 | 5.491E-07 | 1.714E-07 | 2.183E-07 | 6.950E-07 | 6.023E-07 |
| Unigene417143 | TaeA | 0.000E+00 | 0.000E+00 | 0.000E+00 | 0.000E+00 | 0.000E+00 | 0.000E+00 | 7.906E-07 | 0.000E+00 | 0.000E+00 | 0.000E+00 | 5.107E-08 | 0.000E+00 |
| Unigene417295 | MexD | 0.000E+00 | 0.000E+00 | 0.000E+00 | 0.000E+00 | 1.336E-07 | 0.000E+00 | 0.000E+00 | 0.000E+00 | 0.000E+00 | 0.000E+00 | 0.000E+00 | 0.000E+00 |
| Unigene417312 | macB | 0.000E+00 | 0.000E+00 | 0.000E+00 | 0.000E+00 | 0.000E+00 | 0.000E+00 | 9.127E-07 | 0.000E+00 | 0.000E+00 | 0.000E+00 | 0.000E+00 | 0.000E+00 |
| Unigene417315 | tlrC | 0.000E+00 | 0.000E+00 | 0.000E+00 | 0.000E+00 | 0.000E+00 | 0.000E+00 | 2.707E-07 | 5.709E-07 | 0.000E+00 | 0.000E+00 | 0.000E+00 | 4.336E-07 |
| Unigene417383 | adeL | 0.000E+00 | 0.000E+00 | 0.000E+00 | 0.000E+00 | 0.000E+00 | 0.000E+00 | 1.159E-06 | 0.000E+00 | 0.000E+00 | 0.000E+00 | 0.000E+00 | 0.000E+00 |
| Unigene417448 | mexN | 0.000E+00 | 0.000E+00 | 0.000E+00 | 0.000E+00 | 0.000E+00 | 0.000E+00 | 1.041E-06 | 0.000E+00 | 0.000E+00 | 0.000E+00 | 0.000E+00 | 0.000E+00 |

| | | | | | | | | | | | | | |
|---|---|---|---|---|---|---|---|---|---|---|---|---|---|
| Unigene417459 | MexF | 0.000E+00 | 0.000E+00 | 0.000E+00 | 0.000E+00 | 0.000E+00 | 0.000E+00 | 0.000E+00 | 4.652E-07 | 0.000E+00 | 0.000E+00 | 0.000E+00 | 0.000E+00 |
| Unigene417483 | macB | 0.000E+00 | 0.000E+00 | 0.000E+00 | 0.000E+00 | 0.000E+00 | 0.000E+00 | 7.351E-07 | 0.000E+00 | 0.000E+00 | 0.000E+00 | 0.000E+00 | 0.000E+00 |
| Unigene417685 | bcrA | 0.000E+00 | 0.000E+00 | 0.000E+00 | 0.000E+00 | 0.000E+00 | 0.000E+00 | 0.000E+00 | 4.003E-07 | 0.000E+00 | 0.000E+00 | 3.433E-07 | 1.115E-06 |
| Unigene417687 | novA | 0.000E+00 | 1.669E-07 | 4.641E-07 | 1.876E-07 | 3.378E-07 | 1.826E-07 | 1.327E-06 | 3.155E-07 | 0.000E+00 | 0.000E+00 | 3.478E-07 | 1.278E-06 |
| Unigene417777 | patB | 2.374E-07 | 0.000E+00 | 0.000E+00 | 0.000E+00 | 0.000E+00 | 0.000E+00 | 1.357E-06 | 0.000E+00 | 0.000E+00 | 0.000E+00 | 0.000E+00 | 0.000E+00 |
| Unigene417874 | baeS | 0.000E+00 | 0.000E+00 | 0.000E+00 | 0.000E+00 | 0.000E+00 | 0.000E+00 | 3.609E-07 | 0.000E+00 | 0.000E+00 | 0.000E+00 | 2.797E-07 | 5.781E-07 |
| Unigene417935 | oleB | 0.000E+00 | 0.000E+00 | 1.277E-07 | 0.000E+00 | 0.000E+00 | 0.000E+00 | 1.056E-06 | 0.000E+00 | 0.000E+00 | 0.000E+00 | 1.985E-07 | 1.539E-07 |
| Unigene417969 | NmcR | 0.000E+00 | 0.000E+00 | 0.000E+00 | 0.000E+00 | 0.000E+00 | 0.000E+00 | 5.014E-07 | 0.000E+00 | 0.000E+00 | 0.000E+00 | 0.000E+00 | 1.785E-07 |
| Unigene418055 | efpA | 0.000E+00 | 0.000E+00 | 0.000E+00 | 0.000E+00 | 0.000E+00 | 0.000E+00 | 6.004E-07 | 0.000E+00 | 0.000E+00 | 0.000E+00 | 0.000E+00 | 0.000E+00 |
| Unigene418058 | vanHD | 0.000E+00 | 0.000E+00 | 0.000E+00 | 0.000E+00 | 1.952E-07 | 0.000E+00 | 5.833E-07 | 0.000E+00 | 0.000E+00 | 0.000E+00 | 0.000E+00 | 0.000E+00 |
| Unigene418182 | Burkholder | 0.000E+00 | 0.000E+00 | 0.000E+00 | 0.000E+00 | 0.000E+00 | 0.000E+00 | 9.036E-07 | 0.000E+00 | 0.000E+00 | 0.000E+00 | 6.671E-08 | 0.000E+00 |
| Unigene418254 | bcrA | 0.000E+00 | 4.533E-08 | 0.000E+00 | 0.000E+00 | 6.883E-08 | 0.000E+00 | 8.686E-07 | 1.205E-07 | 0.000E+00 | 0.000E+00 | 7.087E-08 | 0.000E+00 |
| Unigene418328 | vanHM | 1.708E-07 | 0.000E+00 | 0.000E+00 | 0.000E+00 | 0.000E+00 | 0.000E+00 | 1.512E-06 | 0.000E+00 | 0.000E+00 | 0.000E+00 | 2.344E-07 | 0.000E+00 |
| Unigene418333 | TolC | 0.000E+00 | 4.410E-07 | 0.000E+00 | 0.000E+00 | 1.166E-06 | 0.000E+00 | 2.397E-06 | 1.277E-06 | 0.000E+00 | 0.000E+00 | 5.873E-07 | 8.444E-07 |
| Unigene418339 | basS | 0.000E+00 | 0.000E+00 | 0.000E+00 | 0.000E+00 | 0.000E+00 | 0.000E+00 | 2.233E-06 | 0.000E+00 | 0.000E+00 | 0.000E+00 | 0.000E+00 | 0.000E+00 |
| Unigene418417 | Klebsiella p | 1.051E-07 | 2.693E-07 | 1.720E-07 | 0.000E+00 | 0.000E+00 | 0.000E+00 | 6.207E-07 | 0.000E+00 | 0.000E+00 | 0.000E+00 | 0.000E+00 | 0.000E+00 |
| Unigene418471 | tetA(58) | 0.000E+00 | 0.000E+00 | 0.000E+00 | 0.000E+00 | 0.000E+00 | 0.000E+00 | 4.936E-07 | 0.000E+00 | 0.000E+00 | 0.000E+00 | 0.000E+00 | 0.000E+00 |
| Unigene418555 | tetB(46) | 0.000E+00 | 0.000E+00 | 0.000E+00 | 0.000E+00 | 0.000E+00 | 0.000E+00 | 6.195E-07 | 7.260E-08 | 0.000E+00 | 0.000E+00 | 0.000E+00 | 0.000E+00 |
| Unigene418582 | emrB | 3.420E-07 | 8.006E-07 | 7.382E-07 | 1.842E-07 | 0.000E+00 | 0.000E+00 | 1.009E-06 | 0.000E+00 | 0.000E+00 | 0.000E+00 | 0.000E+00 | 0.000E+00 |
| Unigene418612 | evgS | 0.000E+00 | 0.000E+00 | 0.000E+00 | 0.000E+00 | 0.000E+00 | 0.000E+00 | 4.616E-07 | 3.408E-07 | 0.000E+00 | 0.000E+00 | 0.000E+00 | 2.465E-07 |
| Unigene418626 | msbA | 1.783E-07 | 3.262E-08 | 1.896E-07 | 1.801E-07 | 3.302E-07 | 6.266E-07 | 1.053E-06 | 7.980E-07 | 1.949E-07 | 8.940E-07 | 1.020E-06 | 2.249E-06 |
| Unigene418786 | bcr-1 | 0.000E+00 | 0.000E+00 | 0.000E+00 | 0.000E+00 | 0.000E+00 | 0.000E+00 | 1.414E-06 | 8.948E-07 | 0.000E+00 | 0.000E+00 | 0.000E+00 | 0.000E+00 |
| Unigene418788 | efrA | 0.000E+00 | 1.783E-07 | 0.000E+00 | 0.000E+00 | 0.000E+00 | 0.000E+00 | 2.202E-06 | 0.000E+00 | 0.000E+00 | 0.000E+00 | 0.000E+00 | 0.000E+00 |
| Unigene418870 | macB | 0.000E+00 | 0.000E+00 | 0.000E+00 | 0.000E+00 | 0.000E+00 | 0.000E+00 | 1.453E-06 | 0.000E+00 | 0.000E+00 | 0.000E+00 | 0.000E+00 | 0.000E+00 |
| Unigene418936 | tetA(46) | 0.000E+00 | 1.476E-07 | 0.000E+00 | 0.000E+00 | 0.000E+00 | 0.000E+00 | 5.210E-07 | 0.000E+00 | 0.000E+00 | 0.000E+00 | 0.000E+00 | 1.590E-07 |
| Unigene419049 | tlrC | 0.000E+00 | 0.000E+00 | 0.000E+00 | 2.643E-07 | 0.000E+00 | 4.087E-07 | 3.380E-06 | 2.648E-06 | 0.000E+00 | 0.000E+00 | 0.000E+00 | 1.066E-06 |
| Unigene419140 | emrR | 0.000E+00 | 0.000E+00 | 0.000E+00 | 0.000E+00 | 0.000E+00 | 0.000E+00 | 2.288E-06 | 0.000E+00 | 0.000E+00 | 0.000E+00 | 0.000E+00 | 0.000E+00 |
| Unigene419141 | emrB | 0.000E+00 | 4.136E-07 | 0.000E+00 | 0.000E+00 | 8.838E-07 | 0.000E+00 | 2.549E-06 | 1.320E-06 | 0.000E+00 | 0.000E+00 | 5.508E-07 | 4.701E-07 |
| Unigene419182 | novA | 0.000E+00 | 0.000E+00 | 0.000E+00 | 0.000E+00 | 0.000E+00 | 0.000E+00 | 9.161E-07 | 0.000E+00 | 0.000E+00 | 0.000E+00 | 0.000E+00 | 0.000E+00 |
| Unigene419220 | mexN | 0.000E+00 | 0.000E+00 | 3.373E-08 | 6.943E-08 | 0.000E+00 | 2.230E-07 | 8.751E-07 | 0.000E+00 | 0.000E+00 | 1.532E-07 | 1.966E-07 | 1.625E-07 |
| Unigene419363 | tetA(58) | 0.000E+00 | 0.000E+00 | 0.000E+00 | 0.000E+00 | 0.000E+00 | 0.000E+00 | 7.531E-07 | 0.000E+00 | 0.000E+00 | 0.000E+00 | 0.000E+00 | 0.000E+00 |
| Unigene419379 | msbA | 0.000E+00 | 0.000E+00 | 0.000E+00 | 0.000E+00 | 0.000E+00 | 0.000E+00 | 9.244E-07 | 0.000E+00 | 0.000E+00 | 0.000E+00 | 0.000E+00 | 0.000E+00 |
| Unigene419458 | abcA | 0.000E+00 | 0.000E+00 | 0.000E+00 | 0.000E+00 | 0.000E+00 | 1.879E-06 | 2.931E-06 | 3.188E-06 | 0.000E+00 | 0.000E+00 | 0.000E+00 | 0.000E+00 |
| Unigene419516 | Acinetobac | 0.000E+00 | 3.749E-08 | 6.704E-08 | 3.449E-08 | 0.000E+00 | 0.000E+00 | 5.671E-07 | 3.588E-07 | 0.000E+00 | 0.000E+00 | 0.000E+00 | 0.000E+00 |
| Unigene419544 | YojI | 0.000E+00 | 0.000E+00 | 0.000E+00 | 0.000E+00 | 0.000E+00 | 0.000E+00 | 2.517E-06 | 0.000E+00 | 0.000E+00 | 0.000E+00 | 0.000E+00 | 1.379E-06 |
| Unigene419556 | adeH | 0.000E+00 | 5.648E-07 | 0.000E+00 | 0.000E+00 | 0.000E+00 | 0.000E+00 | 1.087E-06 | 0.000E+00 | 0.000E+00 | 0.000E+00 | 0.000E+00 | 0.000E+00 |
| Unigene419562 | smeS | 0.000E+00 | 0.000E+00 | 0.000E+00 | 0.000E+00 | 0.000E+00 | 0.000E+00 | 0.000E+00 | 1.152E-07 | 0.000E+00 | 0.000E+00 | 0.000E+00 | 0.000E+00 |
| Unigene419597 | farB | 0.000E+00 | 0.000E+00 | 0.000E+00 | 0.000E+00 | 0.000E+00 | 0.000E+00 | 2.100E-06 | 0.000E+00 | 0.000E+00 | 0.000E+00 | 0.000E+00 | 7.385E-07 |
| Unigene419604 | iri | 0.000E+00 | 0.000E+00 | 0.000E+00 | 0.000E+00 | 0.000E+00 | 0.000E+00 | 8.133E-07 | 0.000E+00 | 0.000E+00 | 0.000E+00 | 0.000E+00 | 1.448E-07 |
| Unigene419747 | vanSG | 0.000E+00 | 0.000E+00 | 0.000E+00 | 0.000E+00 | 0.000E+00 | 0.000E+00 | 1.163E-06 | 0.000E+00 | 0.000E+00 | 0.000E+00 | 0.000E+00 | 7.310E-07 |
| Unigene419765 | patB | 0.000E+00 | 0.000E+00 | 0.000E+00 | 0.000E+00 | 0.000E+00 | 0.000E+00 | 0.000E+00 | 1.204E-07 | 0.000E+00 | 0.000E+00 | 0.000E+00 | 4.063E-07 |
| Unigene419820 | lsaC | 0.000E+00 | 0.000E+00 | 0.000E+00 | 0.000E+00 | 0.000E+00 | 0.000E+00 | 8.046E-07 | 0.000E+00 | 0.000E+00 | 0.000E+00 | 0.000E+00 | 0.000E+00 |
| Unigene419868 | optrA | 0.000E+00 | 4.358E-08 | 3.896E-08 | 4.010E-08 | 4.852E-07 | 1.717E-07 | 1.274E-06 | 7.415E-07 | 0.000E+00 | 0.000E+00 | 4.542E-07 | 1.408E-06 |
| Unigene419896 | msbA | 0.000E+00 | 0.000E+00 | 0.000E+00 | 0.000E+00 | 0.000E+00 | 0.000E+00 | 4.434E-07 | 0.000E+00 | 0.000E+00 | 0.000E+00 | 0.000E+00 | 0.000E+00 |
| Unigene419968 | optrA | 0.000E+00 | 0.000E+00 | 0.000E+00 | 0.000E+00 | 0.000E+00 | 0.000E+00 | 1.152E-06 | 0.000E+00 | 0.000E+00 | 0.000E+00 | 0.000E+00 | 0.000E+00 |
| Unigene419996 | ceoB | 0.000E+00 | 0.000E+00 | 0.000E+00 | 0.000E+00 | 0.000E+00 | 0.000E+00 | 8.487E-07 | 0.000E+00 | 0.000E+00 | 1.869E-07 | 0.000E+00 | 4.816E-07 |
| Unigene420045 | Corynebac | 0.000E+00 | 0.000E+00 | 1.930E-07 | 0.000E+00 | 0.000E+00 | 1.519E-07 | 1.648E-06 | 9.838E-07 | 0.000E+00 | 0.000E+00 | 0.000E+00 | 0.000E+00 |
| Unigene420055 | Staphylocc | 0.000E+00 | 5.825E-08 | 1.041E-07 | 0.000E+00 | 3.980E-07 | 2.151E-07 | 6.608E-07 | 3.252E-07 | 5.801E-08 | 2.660E-07 | 5.615E-07 | 5.489E-07 |
| Unigene420064 | efrA | 0.000E+00 | 0.000E+00 | 1.415E-07 | 0.000E+00 | 2.243E-07 | 0.000E+00 | 7.662E-07 | 9.765E-07 | 6.306E-08 | 1.607E-07 | 5.280E-07 | 2.012E-06 |
| Unigene420096 | tetB(60) | 0.000E+00 | 0.000E+00 | 0.000E+00 | 0.000E+00 | 0.000E+00 | 0.000E+00 | 5.475E-07 | 4.331E-07 | 0.000E+00 | 0.000E+00 | 0.000E+00 | 0.000E+00 |
| Unigene420150 | Klebsiella p | 0.000E+00 | 0.000E+00 | 0.000E+00 | 0.000E+00 | 0.000E+00 | 0.000E+00 | 3.842E-07 | 0.000E+00 | 0.000E+00 | 0.000E+00 | 0.000E+00 | 0.000E+00 |
| Unigene420197 | vmlR | 0.000E+00 | 0.000E+00 | 0.000E+00 | 0.000E+00 | 0.000E+00 | 0.000E+00 | 7.316E-07 | 4.287E-07 | 0.000E+00 | 0.000E+00 | 2.520E-07 | 1.259E-06 |
| Unigene420272 | adeN | 0.000E+00 | 0.000E+00 | 0.000E+00 | 0.000E+00 | 0.000E+00 | 0.000E+00 | 6.424E-07 | 4.169E-07 | 0.000E+00 | 0.000E+00 | 0.000E+00 | 2.111E-07 |
| Unigene420333 | vanRA | 1.989E-06 | 3.126E-06 | 2.174E-06 | 1.096E-06 | 0.000E+00 | 0.000E+00 | 6.004E-07 | 4.749E-07 | 3.459E-07 | 0.000E+00 | 0.000E+00 | 0.000E+00 |
| Unigene420369 | Klebsiella p | 0.000E+00 | 0.000E+00 | 0.000E+00 | 0.000E+00 | 0.000E+00 | 0.000E+00 | 4.106E-07 | 0.000E+00 | 0.000E+00 | 0.000E+00 | 0.000E+00 | 0.000E+00 |
| Unigene420434 | macA | 0.000E+00 | 0.000E+00 | 0.000E+00 | 0.000E+00 | 0.000E+00 | 0.000E+00 | 1.591E-06 | 0.000E+00 | 0.000E+00 | 0.000E+00 | 0.000E+00 | 0.000E+00 |
| Unigene420495 | bcrA | 0.000E+00 | 0.000E+00 | 2.155E-07 | 0.000E+00 | 0.000E+00 | 0.000E+00 | 1.896E-06 | 0.000E+00 | 0.000E+00 | 0.000E+00 | 0.000E+00 | 0.000E+00 |
| Unigene420496 | arlR | 0.000E+00 | 0.000E+00 | 0.000E+00 | 0.000E+00 | 0.000E+00 | 0.000E+00 | 2.842E-06 | 0.000E+00 | 0.000E+00 | 0.000E+00 | 0.000E+00 | 0.000E+00 |

| | | | | | | | | | | | | | |
|---|---|---|---|---|---|---|---|---|---|---|---|---|---|
| Unigene420590 | vanSA | 0.000E+00 | 0.000E+00 | 0.000E+00 | 0.000E+00 | 0.000E+00 | 0.000E+00 | 9.682E-07 | 0.000E+00 | 0.000E+00 | 0.000E+00 | 0.000E+00 | 0.000E+00 |
| Unigene420644 | TriA | 0.000E+00 | 0.000E+00 | 0.000E+00 | 1.148E-07 | 0.000E+00 | 4.096E-07 | 9.225E-07 | 1.769E-07 | 0.000E+00 | 0.000E+00 | 0.000E+00 | 2.687E-07 |
| Unigene420657 | msbA | 0.000E+00 | 0.000E+00 | 1.394E-07 | 0.000E+00 | 2.762E-07 | 1.919E-07 | 1.061E-06 | 1.078E-06 | 7.763E-08 | 0.000E+00 | 6.906E-07 | 2.644E-06 |
| Unigene420836 | PER-7 | 0.000E+00 | 0.000E+00 | 0.000E+00 | 0.000E+00 | 0.000E+00 | 0.000E+00 | 6.918E-07 | 0.000E+00 | 0.000E+00 | 2.984E-07 | 1.532E-07 | 2.111E-07 |
| Unigene420925 | msrA | 0.000E+00 | 0.000E+00 | 0.000E+00 | 0.000E+00 | 0.000E+00 | 0.000E+00 | 5.228E-07 | 0.000E+00 | 0.000E+00 | 0.000E+00 | 0.000E+00 | 0.000E+00 |
| Unigene420984 | cmx | 1.199E-06 | 1.491E-06 | 5.514E-06 | 6.831E-06 | 0.000E+00 | 0.000E+00 | 5.306E-07 | 0.000E+00 | 0.000E+00 | 0.000E+00 | 0.000E+00 | 3.148E-08 |
| Unigene421042 | SRT-2 | 0.000E+00 | 0.000E+00 | 0.000E+00 | 0.000E+00 | 0.000E+00 | 7.553E-08 | 7.346E-07 | 4.078E-08 | 0.000E+00 | 0.000E+00 | 0.000E+00 | 0.000E+00 |
| Unigene421102 | sul4 | 0.000E+00 | 0.000E+00 | 0.000E+00 | 0.000E+00 | 0.000E+00 | 0.000E+00 | 0.000E+00 | 0.000E+00 | 0.000E+00 | 0.000E+00 | 0.000E+00 | 1.766E-07 |
| Unigene421127 | patA | 0.000E+00 | 0.000E+00 | 0.000E+00 | 0.000E+00 | 2.679E-07 | 0.000E+00 | 6.338E-07 | 0.000E+00 | 0.000E+00 | 0.000E+00 | 2.758E-07 | 9.263E-07 |
| Unigene421180 | patB | 0.000E+00 | 0.000E+00 | 0.000E+00 | 0.000E+00 | 0.000E+00 | 0.000E+00 | 7.414E-07 | 0.000E+00 | 0.000E+00 | 0.000E+00 | 0.000E+00 | 0.000E+00 |
| Unigene421201 | sul4 | 0.000E+00 | 0.000E+00 | 0.000E+00 | 0.000E+00 | 0.000E+00 | 0.000E+00 | 3.278E-07 | 0.000E+00 | 0.000E+00 | 0.000E+00 | 0.000E+00 | 0.000E+00 |
| Unigene421233 | MuxB | 0.000E+00 | 0.000E+00 | 0.000E+00 | 0.000E+00 | 0.000E+00 | 0.000E+00 | 7.857E-07 | 0.000E+00 | 0.000E+00 | 0.000E+00 | 0.000E+00 | 0.000E+00 |
| Unigene421259 | arlR | 0.000E+00 | 0.000E+00 | 0.000E+00 | 0.000E+00 | 0.000E+00 | 0.000E+00 | 7.777E-07 | 0.000E+00 | 0.000E+00 | 0.000E+00 | 0.000E+00 | 0.000E+00 |
| Unigene421473 | lmrB | 0.000E+00 | 8.205E-07 | 6.550E-07 | 0.000E+00 | 0.000E+00 | 0.000E+00 | 1.803E-06 | 0.000E+00 | 0.000E+00 | 0.000E+00 | 0.000E+00 | 0.000E+00 |
| Unigene421497 | Acinetobac | 0.000E+00 | 0.000E+00 | 0.000E+00 | 0.000E+00 | 0.000E+00 | 0.000E+00 | 5.973E-07 | 0.000E+00 | 0.000E+00 | 0.000E+00 | 0.000E+00 | 0.000E+00 |
| Unigene421548 | msbA | 0.000E+00 | 0.000E+00 | 0.000E+00 | 0.000E+00 | 0.000E+00 | 0.000E+00 | 1.239E-06 | 0.000E+00 | 0.000E+00 | 0.000E+00 | 0.000E+00 | 2.187E-06 |
| Unigene421576 | Acinetobac | 0.000E+00 | 0.000E+00 | 0.000E+00 | 0.000E+00 | 0.000E+00 | 0.000E+00 | 3.934E-06 | 3.722E-06 | 0.000E+00 | 0.000E+00 | 2.061E-06 | 3.770E-06 |
| Unigene421586 | Bifidobacte | 0.000E+00 | 0.000E+00 | 0.000E+00 | 0.000E+00 | 0.000E+00 | 0.000E+00 | 0.000E+00 | 0.000E+00 | 0.000E+00 | 0.000E+00 | 0.000E+00 | 4.038E-07 |
| Unigene421685 | eptA | 0.000E+00 | 0.000E+00 | 0.000E+00 | 0.000E+00 | 0.000E+00 | 0.000E+00 | 6.166E-07 | 0.000E+00 | 0.000E+00 | 0.000E+00 | 0.000E+00 | 0.000E+00 |
| Unigene421809 | NmcR | 0.000E+00 | 0.000E+00 | 0.000E+00 | 0.000E+00 | 0.000E+00 | 0.000E+00 | 3.664E-07 | 6.441E-08 | 0.000E+00 | 0.000E+00 | 0.000E+00 | 0.000E+00 |
| Unigene421820 | MexK | 0.000E+00 | 0.000E+00 | 3.068E-07 | 2.972E-07 | 3.515E-06 | 0.000E+00 | 1.079E-06 | 0.000E+00 | 0.000E+00 | 0.000E+00 | 0.000E+00 | 0.000E+00 |
| Unigene421922 | arlR | 0.000E+00 | 0.000E+00 | 0.000E+00 | 0.000E+00 | 0.000E+00 | 0.000E+00 | 0.000E+00 | 0.000E+00 | 0.000E+00 | 0.000E+00 | 0.000E+00 | 1.223E-06 |
| Unigene421949 | efrA | 0.000E+00 | 0.000E+00 | 0.000E+00 | 0.000E+00 | 0.000E+00 | 0.000E+00 | 7.337E-07 | 0.000E+00 | 0.000E+00 | 0.000E+00 | 0.000E+00 | 0.000E+00 |
| Unigene421961 | patB | 0.000E+00 | 0.000E+00 | 0.000E+00 | 0.000E+00 | 4.725E-08 | 0.000E+00 | 8.943E-07 | 0.000E+00 | 0.000E+00 | 0.000E+00 | 0.000E+00 | 0.000E+00 |
| Unigene421976 | QepA2 | 0.000E+00 | 0.000E+00 | 0.000E+00 | 0.000E+00 | 1.252E-07 | 0.000E+00 | 5.611E-07 | 0.000E+00 | 0.000E+00 | 0.000E+00 | 0.000E+00 | 1.998E-07 |
| Unigene422011 | evgS | 0.000E+00 | 0.000E+00 | 0.000E+00 | 0.000E+00 | 2.907E-07 | 0.000E+00 | 0.000E+00 | 0.000E+00 | 0.000E+00 | 0.000E+00 | 0.000E+00 | 0.000E+00 |
| Unigene422019 | oleC | 0.000E+00 | 0.000E+00 | 0.000E+00 | 0.000E+00 | 0.000E+00 | 0.000E+00 | 1.096E-06 | 0.000E+00 | 0.000E+00 | 0.000E+00 | 0.000E+00 | 0.000E+00 |
| Unigene422020 | efrB | 0.000E+00 | 0.000E+00 | 0.000E+00 | 0.000E+00 | 0.000E+00 | 0.000E+00 | 7.825E-07 | 0.000E+00 | 0.000E+00 | 0.000E+00 | 0.000E+00 | 0.000E+00 |
| Unigene422066 | novA | 1.808E-07 | 0.000E+00 | 0.000E+00 | 0.000E+00 | 4.688E-07 | 0.000E+00 | 7.672E-07 | 8.092E-07 | 9.883E-08 | 0.000E+00 | 9.309E-07 | 6.057E-07 |
| Unigene422269 | novA | 1.161E-07 | 0.000E+00 | 0.000E+00 | 0.000E+00 | 0.000E+00 | 0.000E+00 | 9.638E-07 | 1.807E-07 | 0.000E+00 | 0.000E+00 | 0.000E+00 | 2.745E-07 |
| Unigene422283 | hp1181 | 0.000E+00 | 0.000E+00 | 0.000E+00 | 0.000E+00 | 0.000E+00 | 0.000E+00 | 5.235E-07 | 0.000E+00 | 0.000E+00 | 0.000E+00 | 0.000E+00 | 0.000E+00 |
| Unigene422464 | adeN | 0.000E+00 | 0.000E+00 | 0.000E+00 | 0.000E+00 | 0.000E+00 | 0.000E+00 | 0.000E+00 | 1.132E-07 | 0.000E+00 | 0.000E+00 | 0.000E+00 | 0.000E+00 |
| Unigene422591 | patA | 0.000E+00 | 0.000E+00 | 0.000E+00 | 0.000E+00 | 0.000E+00 | 0.000E+00 | 4.785E-07 | 0.000E+00 | 0.000E+00 | 0.000E+00 | 0.000E+00 | 0.000E+00 |
| Unigene422687 | tlrC | 0.000E+00 | 0.000E+00 | 0.000E+00 | 0.000E+00 | 0.000E+00 | 0.000E+00 | 5.254E-07 | 0.000E+00 | 0.000E+00 | 0.000E+00 | 0.000E+00 | 0.000E+00 |
| Unigene422714 | bcrA | 0.000E+00 | 0.000E+00 | 0.000E+00 | 0.000E+00 | 0.000E+00 | 0.000E+00 | 6.573E-07 | 0.000E+00 | 0.000E+00 | 0.000E+00 | 0.000E+00 | 0.000E+00 |
| Unigene422763 | Enterobact | 0.000E+00 | 0.000E+00 | 1.066E-07 | 0.000E+00 | 6.943E-07 | 0.000E+00 | 9.924E-07 | 6.978E-07 | 0.000E+00 | 0.000E+00 | 0.000E+00 | 1.959E-06 |
| Unigene422850 | lin | 0.000E+00 | 0.000E+00 | 0.000E+00 | 0.000E+00 | 0.000E+00 | 0.000E+00 | 1.052E-06 | 0.000E+00 | 0.000E+00 | 0.000E+00 | 0.000E+00 | 0.000E+00 |
| Unigene422969 | vanHD | 0.000E+00 | 0.000E+00 | 0.000E+00 | 0.000E+00 | 0.000E+00 | 0.000E+00 | 5.140E-07 | 0.000E+00 | 0.000E+00 | 0.000E+00 | 0.000E+00 | 0.000E+00 |
| Unigene423020 | oleB | 0.000E+00 | 4.051E-08 | 0.000E+00 | 0.000E+00 | 3.486E-07 | 0.000E+00 | 7.354E-07 | 7.971E-07 | 0.000E+00 | 0.000E+00 | 0.000E+00 | 1.200E-06 |
| Unigene423080 | mdtN | 0.000E+00 | 0.000E+00 | 0.000E+00 | 0.000E+00 | 0.000E+00 | 0.000E+00 | 6.616E-07 | 0.000E+00 | 0.000E+00 | 0.000E+00 | 0.000E+00 | 0.000E+00 |
| Unigene423152 | patB | 1.281E-07 | 0.000E+00 | 1.257E-07 | 0.000E+00 | 2.372E-07 | 0.000E+00 | 1.229E-06 | 8.473E-07 | 0.000E+00 | 0.000E+00 | 2.003E-06 | 3.180E-06 |
| Unigene423169 | OprM | 0.000E+00 | 0.000E+00 | 0.000E+00 | 0.000E+00 | 0.000E+00 | 0.000E+00 | 8.120E-07 | 0.000E+00 | 0.000E+00 | 0.000E+00 | 0.000E+00 | 0.000E+00 |
| Unigene423170 | MexD | 0.000E+00 | 0.000E+00 | 0.000E+00 | 0.000E+00 | 0.000E+00 | 0.000E+00 | 9.143E-07 | 0.000E+00 | 0.000E+00 | 0.000E+00 | 0.000E+00 | 0.000E+00 |
| Unigene423281 | macB | 0.000E+00 | 3.999E-07 | 0.000E+00 | 0.000E+00 | 0.000E+00 | 0.000E+00 | 3.321E-06 | 0.000E+00 | 0.000E+00 | 0.000E+00 | 0.000E+00 | 0.000E+00 |
| Unigene423334 | cmlv | 0.000E+00 | 0.000E+00 | 0.000E+00 | 0.000E+00 | 0.000E+00 | 0.000E+00 | 2.632E-07 | 0.000E+00 | 0.000E+00 | 0.000E+00 | 0.000E+00 | 0.000E+00 |
| Unigene423427 | lmrC | 4.287E-08 | 0.000E+00 | 1.262E-07 | 0.000E+00 | 0.000E+00 | 3.707E-07 | 1.186E-06 | 1.301E-06 | 0.000E+00 | 0.000E+00 | 1.422E-06 | 3.598E-06 |
| Unigene423459 | tet(43) | 0.000E+00 | 0.000E+00 | 0.000E+00 | 0.000E+00 | 0.000E+00 | 0.000E+00 | 5.595E-07 | 0.000E+00 | 0.000E+00 | 0.000E+00 | 0.000E+00 | 0.000E+00 |
| Unigene423555 | efrA | 0.000E+00 | 0.000E+00 | 0.000E+00 | 0.000E+00 | 0.000E+00 | 0.000E+00 | 7.510E-07 | 0.000E+00 | 0.000E+00 | 0.000E+00 | 0.000E+00 | 0.000E+00 |
| Unigene423560 | tetA(46) | 0.000E+00 | 0.000E+00 | 0.000E+00 | 0.000E+00 | 4.598E-07 | 0.000E+00 | 7.328E-07 | 0.000E+00 | 0.000E+00 | 0.000E+00 | 9.468E-08 | 8.316E-07 |
| Unigene423575 | acrB | 0.000E+00 | 0.000E+00 | 0.000E+00 | 0.000E+00 | 0.000E+00 | 0.000E+00 | 4.288E-07 | 1.392E-07 | 0.000E+00 | 0.000E+00 | 0.000E+00 | 1.585E-06 |
| Unigene423582 | tetA(46) | 0.000E+00 | 0.000E+00 | 8.774E-08 | 0.000E+00 | 0.000E+00 | 1.289E-07 | 8.247E-07 | 4.175E-07 | 0.000E+00 | 9.961E-08 | 3.410E-08 | 1.409E-07 |
| Unigene423598 | smeR | 0.000E+00 | 0.000E+00 | 0.000E+00 | 0.000E+00 | 0.000E+00 | 0.000E+00 | 0.000E+00 | 0.000E+00 | 0.000E+00 | 0.000E+00 | 1.985E-07 | 0.000E+00 |
| Unigene423634 | tetB(46) | 0.000E+00 | 0.000E+00 | 0.000E+00 | 0.000E+00 | 0.000E+00 | 0.000E+00 | 6.709E-07 | 0.000E+00 | 0.000E+00 | 0.000E+00 | 0.000E+00 | 0.000E+00 |
| Unigene423871 | cmeB | 0.000E+00 | 1.004E-07 | 0.000E+00 | 1.231E-07 | 0.000E+00 | 0.000E+00 | 3.711E-07 | 0.000E+00 | 0.000E+00 | 0.000E+00 | 0.000E+00 | 9.368E-07 |
| Unigene424078 | vanI | 0.000E+00 | 0.000E+00 | 0.000E+00 | 0.000E+00 | 0.000E+00 | 0.000E+00 | 8.479E-07 | 1.138E-06 | 0.000E+00 | 0.000E+00 | 0.000E+00 | 5.763E-07 |
| Unigene424083 | oleC | 0.000E+00 | 0.000E+00 | 0.000E+00 | 0.000E+00 | 0.000E+00 | 0.000E+00 | 4.660E-07 | 0.000E+00 | 0.000E+00 | 0.000E+00 | 0.000E+00 | 0.000E+00 |
| Unigene424114 | TaeA | 0.000E+00 | 0.000E+00 | 0.000E+00 | 0.000E+00 | 0.000E+00 | 0.000E+00 | 7.767E-07 | 0.000E+00 | 0.000E+00 | 0.000E+00 | 0.000E+00 | 0.000E+00 |
| Unigene424115 | msbA | 0.000E+00 | 0.000E+00 | 0.000E+00 | 0.000E+00 | 0.000E+00 | 0.000E+00 | 5.644E-07 | 0.000E+00 | 0.000E+00 | 0.000E+00 | 0.000E+00 | 0.000E+00 |

| | | | | | | | | | | | | | |
|---|---|---|---|---|---|---|---|---|---|---|---|---|---|
| Unigene424148 | tet(A) | 0.000E+00 | 0.000E+00 | 0.000E+00 | 0.000E+00 | 0.000E+00 | 0.000E+00 | 5.768E-07 | 5.725E-07 | 3.351E-07 | 0.000E+00 | 0.000E+00 | 7.972E-07 |
| Unigene424167 | mdtB | 0.000E+00 | 0.000E+00 | 0.000E+00 | 0.000E+00 | 0.000E+00 | 0.000E+00 | 9.888E-07 | 0.000E+00 | 0.000E+00 | 0.000E+00 | 0.000E+00 | 0.000E+00 |
| Unigene424186 | mtrA | 0.000E+00 | 0.000E+00 | 0.000E+00 | 0.000E+00 | 0.000E+00 | 0.000E+00 | 4.763E-07 | 0.000E+00 | 0.000E+00 | 0.000E+00 | 0.000E+00 | 0.000E+00 |
| Unigene424216 | efrB | 0.000E+00 | 0.000E+00 | 0.000E+00 | 0.000E+00 | 0.000E+00 | 0.000E+00 | 6.345E-07 | 0.000E+00 | 0.000E+00 | 0.000E+00 | 0.000E+00 | 4.691E-07 |
| Unigene424301 | bcrA | 0.000E+00 | 0.000E+00 | 0.000E+00 | 0.000E+00 | 0.000E+00 | 0.000E+00 | 7.797E-07 | 0.000E+00 | 0.000E+00 | 0.000E+00 | 0.000E+00 | 0.000E+00 |
| Unigene424428 | tetA(58) | 0.000E+00 | 0.000E+00 | 0.000E+00 | 0.000E+00 | 0.000E+00 | 0.000E+00 | 0.000E+00 | 0.000E+00 | 0.000E+00 | 0.000E+00 | 0.000E+00 | 4.386E-07 |
| Unigene424517 | msbA | 4.699E-08 | 0.000E+00 | 2.075E-07 | 1.423E-07 | 2.349E-07 | 0.000E+00 | 4.420E-07 | 3.565E-07 | 5.136E-08 | 1.570E-07 | 2.419E-07 | 4.999E-07 |
| Unigene424562 | patA | 0.000E+00 | 0.000E+00 | 0.000E+00 | 0.000E+00 | 1.011E-06 | 0.000E+00 | 1.007E-06 | 6.957E-07 | 0.000E+00 | 0.000E+00 | 8.612E-07 | 2.559E-06 |
| Unigene424652 | AcrF | 0.000E+00 | 0.000E+00 | 0.000E+00 | 0.000E+00 | 0.000E+00 | 0.000E+00 | 1.931E-07 | 2.716E-07 | 0.000E+00 | 0.000E+00 | 0.000E+00 | 0.000E+00 |
| Unigene424659 | mtrA | 0.000E+00 | 0.000E+00 | 0.000E+00 | 2.456E-07 | 0.000E+00 | 0.000E+00 | 8.074E-07 | 0.000E+00 | 0.000E+00 | 0.000E+00 | 0.000E+00 | 0.000E+00 |
| Unigene424914 | tetA(58) | 0.000E+00 | 0.000E+00 | 0.000E+00 | 6.227E-08 | 0.000E+00 | 9.999E-08 | 6.142E-07 | 5.398E-07 | 0.000E+00 | 0.000E+00 | 4.585E-07 | 1.348E-06 |
| Unigene424997 | tetA(58) | 0.000E+00 | 0.000E+00 | 0.000E+00 | 0.000E+00 | 0.000E+00 | 0.000E+00 | 7.835E-07 | 0.000E+00 | 0.000E+00 | 0.000E+00 | 0.000E+00 | 0.000E+00 |
| Unigene425065 | vanSC | 0.000E+00 | 0.000E+00 | 0.000E+00 | 6.693E-08 | 0.000E+00 | 0.000E+00 | 3.913E-07 | 0.000E+00 | 0.000E+00 | 0.000E+00 | 0.000E+00 | 5.484E-07 |
| Unigene425076 | oleC | 2.669E-07 | 3.381E-07 | 5.239E-07 | 1.866E-07 | 1.095E-06 | 0.000E+00 | 1.568E-06 | 7.910E-07 | 0.000E+00 | 0.000E+00 | 8.456E-07 | 2.767E-06 |
| Unigene425148 | vanHB | 0.000E+00 | 0.000E+00 | 0.000E+00 | 0.000E+00 | 0.000E+00 | 0.000E+00 | 7.674E-07 | 0.000E+00 | 0.000E+00 | 0.000E+00 | 0.000E+00 | 0.000E+00 |
| Unigene425155 | cmlv | 0.000E+00 | 0.000E+00 | 0.000E+00 | 0.000E+00 | 0.000E+00 | 0.000E+00 | 1.378E-06 | 3.229E-07 | 1.209E-07 | 0.000E+00 | 3.481E-07 | 1.373E-06 |
| Unigene425215 | Corynebac | 0.000E+00 | 0.000E+00 | 0.000E+00 | 0.000E+00 | 0.000E+00 | 0.000E+00 | 1.088E-06 | 0.000E+00 | 0.000E+00 | 0.000E+00 | 0.000E+00 | 0.000E+00 |
| Unigene425250 | lmrB | 0.000E+00 | 0.000E+00 | 0.000E+00 | 0.000E+00 | 0.000E+00 | 0.000E+00 | 4.763E-07 | 0.000E+00 | 0.000E+00 | 0.000E+00 | 0.000E+00 | 2.544E-07 |
| Unigene425278 | evgS | 0.000E+00 | 0.000E+00 | 0.000E+00 | 0.000E+00 | 0.000E+00 | 0.000E+00 | 2.382E-06 | 0.000E+00 | 0.000E+00 | 0.000E+00 | 0.000E+00 | 0.000E+00 |
| Unigene425420 | vanSC | 3.295E-06 | 4.346E-06 | 2.817E-06 | 1.862E-06 | 2.854E-06 | 1.830E-06 | 1.940E-06 | 1.058E-06 | 1.553E-06 | 5.488E-06 | 5.912E-06 | 1.572E-06 |
| Unigene425610 | msbA | 1.065E-07 | 0.000E+00 | 6.969E-08 | 1.076E-07 | 1.973E-07 | 7.678E-08 | 6.681E-07 | 5.803E-07 | 7.763E-08 | 0.000E+00 | 3.250E-07 | 8.395E-07 |
| Unigene425764 | lsaC | 0.000E+00 | 3.446E-07 | 8.216E-08 | 0.000E+00 | 1.233E-06 | 3.168E-07 | 3.220E-06 | 1.148E-06 | 0.000E+00 | 0.000E+00 | 0.000E+00 | 0.000E+00 |
| Unigene425972 | lmrC | 0.000E+00 | 0.000E+00 | 8.834E-07 | 0.000E+00 | 1.572E-06 | 0.000E+00 | 1.898E-06 | 0.000E+00 | 0.000E+00 | 0.000E+00 | 1.471E-06 | 3.496E-06 |
| Unigene425979 | evgS | 0.000E+00 | 0.000E+00 | 0.000E+00 | 0.000E+00 | 6.178E-07 | 0.000E+00 | 1.954E-06 | 0.000E+00 | 0.000E+00 | 0.000E+00 | 0.000E+00 | 0.000E+00 |
| Unigene426009 | oleB | 0.000E+00 | 0.000E+00 | 1.477E-07 | 7.598E-08 | 2.926E-07 | 2.847E-07 | 1.416E-06 | 6.587E-07 | 1.234E-07 | 2.096E-07 | 5.595E-07 | 2.535E-06 |
| Unigene426010 | macB | 2.127E-07 | 0.000E+00 | 0.000E+00 | 0.000E+00 | 2.835E-07 | 0.000E+00 | 8.943E-07 | 9.929E-07 | 0.000E+00 | 3.316E-07 | 8.757E-07 | 1.659E-06 |
| Unigene426091 | carA | 6.521E-08 | 7.157E-08 | 3.199E-08 | 1.976E-07 | 5.071E-07 | 2.820E-07 | 5.774E-07 | 4.187E-07 | 2.851E-07 | 1.453E-07 | 0.000E+00 | 6.552E-07 |
| Unigene426143 | oqxB | 0.000E+00 | 0.000E+00 | 0.000E+00 | 0.000E+00 | 0.000E+00 | 0.000E+00 | 0.000E+00 | 0.000E+00 | 1.060E-07 | 0.000E+00 | 0.000E+00 | 0.000E+00 |
| Unigene426168 | Staphylocc | 0.000E+00 | 0.000E+00 | 0.000E+00 | 0.000E+00 | 0.000E+00 | 0.000E+00 | 4.449E-07 | 0.000E+00 | 0.000E+00 | 0.000E+00 | 0.000E+00 | 0.000E+00 |
| Unigene426267 | tetA(58) | 0.000E+00 | 0.000E+00 | 0.000E+00 | 0.000E+00 | 0.000E+00 | 0.000E+00 | 2.418E-06 | 0.000E+00 | 0.000E+00 | 0.000E+00 | 0.000E+00 | 0.000E+00 |
| Unigene426295 | YojI | 0.000E+00 | 0.000E+00 | 0.000E+00 | 0.000E+00 | 0.000E+00 | 0.000E+00 | 0.000E+00 | 0.000E+00 | 0.000E+00 | 0.000E+00 | 0.000E+00 | 9.642E-07 |
| Unigene426299 | novA | 0.000E+00 | 0.000E+00 | 0.000E+00 | 0.000E+00 | 0.000E+00 | 0.000E+00 | 1.191E-06 | 0.000E+00 | 0.000E+00 | 0.000E+00 | 0.000E+00 | 0.000E+00 |
| Unigene426436 | tva(A) | 0.000E+00 | 0.000E+00 | 0.000E+00 | 3.622E-07 | 0.000E+00 | 0.000E+00 | 0.000E+00 | 1.675E-07 | 0.000E+00 | 0.000E+00 | 0.000E+00 | 5.087E-07 |
| Unigene426472 | NmcR | 0.000E+00 | 0.000E+00 | 0.000E+00 | 2.507E-07 | 0.000E+00 | 0.000E+00 | 6.412E-07 | 1.932E-07 | 0.000E+00 | 0.000E+00 | 0.000E+00 | 0.000E+00 |
| Unigene426540 | catB2 | 0.000E+00 | 0.000E+00 | 0.000E+00 | 3.134E-07 | 0.000E+00 | 0.000E+00 | 9.733E-07 | 0.000E+00 | 2.262E-07 | 0.000E+00 | 6.509E-07 | 1.957E-06 |
| Unigene426628 | carA | 0.000E+00 | 0.000E+00 | 0.000E+00 | 0.000E+00 | 0.000E+00 | 0.000E+00 | 1.008E-06 | 0.000E+00 | 0.000E+00 | 0.000E+00 | 0.000E+00 | 1.128E-06 |
| Unigene426648 | novA | 0.000E+00 | 0.000E+00 | 0.000E+00 | 0.000E+00 | 0.000E+00 | 0.000E+00 | 0.000E+00 | 0.000E+00 | 2.138E-07 | 0.000E+00 | 0.000E+00 | 0.000E+00 |
| Unigene426661 | Corynebac | 0.000E+00 | 0.000E+00 | 0.000E+00 | 0.000E+00 | 0.000E+00 | 0.000E+00 | 2.589E-07 | 0.000E+00 | 0.000E+00 | 0.000E+00 | 0.000E+00 | 0.000E+00 |
| Unigene426686 | vanRB | 0.000E+00 | 0.000E+00 | 0.000E+00 | 0.000E+00 | 0.000E+00 | 0.000E+00 | 3.842E-07 | 0.000E+00 | 0.000E+00 | 0.000E+00 | 0.000E+00 | 0.000E+00 |
| Unigene426699 | mdtA | 0.000E+00 | 0.000E+00 | 0.000E+00 | 8.578E-08 | 0.000E+00 | 3.979E-07 | 7.208E-07 | 1.653E-07 | 1.857E-07 | 6.309E-08 | 2.267E-07 | 1.004E-07 |
| Unigene426743 | macB | 0.000E+00 | 0.000E+00 | 0.000E+00 | 0.000E+00 | 0.000E+00 | 0.000E+00 | 1.128E-06 | 0.000E+00 | 0.000E+00 | 0.000E+00 | 0.000E+00 | 0.000E+00 |
| Unigene426879 | Acinetoba | 1.042E-07 | 5.719E-08 | 0.000E+00 | 0.000E+00 | 0.000E+00 | 0.000E+00 | 1.096E-06 | 0.000E+00 | 0.000E+00 | 0.000E+00 | 0.000E+00 | 5.851E-07 |
| Unigene426905 | PDC-9 | 1.369E-07 | 0.000E+00 | 1.075E-07 | 2.765E-07 | 8.517E-07 | 2.960E-07 | 1.000E-06 | 4.794E-07 | 0.000E+00 | 0.000E+00 | 6.264E-07 | 5.502E-07 |
| Unigene426947 | tetA(58) | 0.000E+00 | 0.000E+00 | 0.000E+00 | 0.000E+00 | 0.000E+00 | 0.000E+00 | 0.000E+00 | 5.421E-07 | 0.000E+00 | 0.000E+00 | 0.000E+00 | 0.000E+00 |
| Unigene427030 | bcrA | 0.000E+00 | 0.000E+00 | 0.000E+00 | 0.000E+00 | 0.000E+00 | 4.142E-07 | 7.421E-07 | 1.044E-06 | 0.000E+00 | 0.000E+00 | 6.209E-07 | 2.113E-06 |
| Unigene427031 | optrA | 0.000E+00 | 2.109E-07 | 4.085E-07 | 2.910E-07 | 4.625E-07 | 4.847E-07 | 1.099E-06 | 8.224E-07 | 7.000E-08 | 2.497E-07 | 1.429E-06 | 2.877E-06 |
| Unigene427040 | efmA | 0.000E+00 | 0.000E+00 | 0.000E+00 | 0.000E+00 | 0.000E+00 | 0.000E+00 | 7.796E-07 | 0.000E+00 | 0.000E+00 | 0.000E+00 | 0.000E+00 | 0.000E+00 |
| Unigene427064 | baeR | 4.287E-08 | 9.410E-08 | 2.103E-07 | 2.164E-07 | 7.620E-07 | 0.000E+00 | 1.139E-06 | 1.401E-06 | 0.000E+00 | 6.208E-07 | 1.177E-06 | 4.966E-06 |
| Unigene427152 | mtrA | 0.000E+00 | 1.031E-07 | 0.000E+00 | 3.321E-07 | 2.088E-07 | 0.000E+00 | 5.720E-07 | 9.324E-07 | 0.000E+00 | 0.000E+00 | 0.000E+00 | 0.000E+00 |
| Unigene427178 | msbA | 0.000E+00 | 4.326E-08 | 0.000E+00 | 0.000E+00 | 0.000E+00 | 3.196E-07 | 8.506E-07 | 0.000E+00 | 0.000E+00 | 0.000E+00 | 2.705E-07 | 3.960E-07 |
| Unigene427232 | Brucella su | 0.000E+00 | 0.000E+00 | 1.842E-07 | 1.895E-07 | 0.000E+00 | 0.000E+00 | 9.001E-07 | 0.000E+00 | 0.000E+00 | 0.000E+00 | 0.000E+00 | 9.613E-07 |
| Unigene427264 | vatE | 0.000E+00 | 6.672E-08 | 0.000E+00 | 0.000E+00 | 0.000E+00 | 1.314E-07 | 2.691E-07 | 0.000E+00 | 0.000E+00 | 0.000E+00 | 0.000E+00 | 7.186E-08 |
| Unigene427283 | cmeB | 0.000E+00 | 0.000E+00 | 0.000E+00 | 0.000E+00 | 0.000E+00 | 0.000E+00 | 1.017E-06 | 2.235E-07 | 0.000E+00 | 0.000E+00 | 0.000E+00 | 0.000E+00 |
| Unigene427330 | novA | 0.000E+00 | 0.000E+00 | 0.000E+00 | 0.000E+00 | 2.930E-07 | 0.000E+00 | 9.340E-07 | 0.000E+00 | 0.000E+00 | 0.000E+00 | 0.000E+00 | 0.000E+00 |
| Unigene427367 | tetA(58) | 0.000E+00 | 0.000E+00 | 0.000E+00 | 0.000E+00 | 2.568E-07 | 0.000E+00 | 5.815E-07 | 1.545E-06 | 0.000E+00 | 0.000E+00 | 3.846E-07 | 6.956E-07 |
| Unigene427378 | bcrA | 0.000E+00 | 0.000E+00 | 0.000E+00 | 0.000E+00 | 0.000E+00 | 0.000E+00 | 7.186E-07 | 0.000E+00 | 0.000E+00 | 0.000E+00 | 0.000E+00 | 0.000E+00 |
| Unigene427387 | vatB | 0.000E+00 | 0.000E+00 | 0.000E+00 | 0.000E+00 | 0.000E+00 | 0.000E+00 | 3.842E-07 | 0.000E+00 | 0.000E+00 | 0.000E+00 | 0.000E+00 | 0.000E+00 |
| Unigene427407 | MexW | 0.000E+00 | 0.000E+00 | 0.000E+00 | 0.000E+00 | 0.000E+00 | 0.000E+00 | 6.566E-07 | 1.259E-07 | 0.000E+00 | 0.000E+00 | 0.000E+00 | 0.000E+00 |

| | | | | | | | | | | | | | |
|---|---|---|---|---|---|---|---|---|---|---|---|---|---|
| Unigene427656 | evgS | 1.754E-07 | 0.000E+00 | 0.000E+00 | 0.000E+00 | 0.000E+00 | 0.000E+00 | 1.359E-06 | 0.000E+00 | 0.000E+00 | 0.000E+00 | 0.000E+00 | 0.000E+00 |
| Unigene427705 | cpxA | 2.708E-07 | 0.000E+00 | 3.916E-07 | 0.000E+00 | 0.000E+00 | 7.087E-07 | 3.281E-06 | 1.913E-06 | 3.427E-07 | 0.000E+00 | 0.000E+00 | 1.988E-06 |
| Unigene427774 | tetA(58) | 0.000E+00 | 0.000E+00 | 0.000E+00 | 0.000E+00 | 0.000E+00 | 0.000E+00 | 7.217E-07 | 4.757E-07 | 0.000E+00 | 0.000E+00 | 0.000E+00 | 9.635E-07 |
| Unigene427948 | kdpE | 0.000E+00 | 0.000E+00 | 0.000E+00 | 0.000E+00 | 0.000E+00 | 0.000E+00 | 5.595E-07 | 0.000E+00 | 0.000E+00 | 0.000E+00 | 0.000E+00 | 4.268E-07 |
| Unigene428006 | macB | 0.000E+00 | 0.000E+00 | 3.317E-07 | 8.533E-08 | 1.878E-07 | 1.523E-07 | 1.122E-06 | 0.000E+00 | 0.000E+00 | 0.000E+00 | 0.000E+00 | 0.000E+00 |
| Unigene428010 | patA | 0.000E+00 | 0.000E+00 | 1.913E-07 | 3.937E-08 | 6.497E-08 | 0.000E+00 | 1.143E-06 | 2.503E-07 | 0.000E+00 | 1.086E-07 | 2.453E-07 | 5.069E-07 |
| Unigene428019 | OprN | 0.000E+00 | 0.000E+00 | 0.000E+00 | 0.000E+00 | 0.000E+00 | 0.000E+00 | 5.509E-07 | 0.000E+00 | 0.000E+00 | 0.000E+00 | 0.000E+00 | 0.000E+00 |
| Unigene428219 | cpxA | 0.000E+00 | 0.000E+00 | 0.000E+00 | 0.000E+00 | 0.000E+00 | 0.000E+00 | 5.736E-07 | 0.000E+00 | 0.000E+00 | 0.000E+00 | 0.000E+00 | 0.000E+00 |
| Unigene428249 | tetA(58) | 0.000E+00 | 0.000E+00 | 0.000E+00 | 0.000E+00 | 3.367E-07 | 0.000E+00 | 5.032E-07 | 0.000E+00 | 0.000E+00 | 5.909E-07 | 0.000E+00 | 0.000E+00 |
| Unigene428300 | APH(6)-Id | 0.000E+00 | 0.000E+00 | 1.272E-07 | 8.728E-08 | 0.000E+00 | 0.000E+00 | 1.339E-06 | 1.513E-07 | 0.000E+00 | 0.000E+00 | 0.000E+00 | 3.065E-07 |
| Unigene428391 | TriA | 1.148E-07 | 0.000E+00 | 0.000E+00 | 0.000E+00 | 4.144E-07 | 0.000E+00 | 9.845E-07 | 1.139E-06 | 0.000E+00 | 0.000E+00 | 4.267E-07 | 1.051E-06 |
| Unigene428440 | Acinetoba | 0.000E+00 | 0.000E+00 | 0.000E+00 | 0.000E+00 | 0.000E+00 | 0.000E+00 | 2.224E-06 | 0.000E+00 | 0.000E+00 | 0.000E+00 | 0.000E+00 | 0.000E+00 |
| Unigene428470 | macB | 0.000E+00 | 0.000E+00 | 6.977E-08 | 7.180E-08 | 5.529E-07 | 0.000E+00 | 4.984E-07 | 4.150E-07 | 7.771E-08 | 3.168E-07 | 4.067E-07 | 1.961E-07 |
| Unigene428472 | Staphyloc | 0.000E+00 | 0.000E+00 | 0.000E+00 | 0.000E+00 | 0.000E+00 | 0.000E+00 | 5.539E-07 | 0.000E+00 | 0.000E+00 | 0.000E+00 | 0.000E+00 | 0.000E+00 |
| Unigene428553 | cmrA | 0.000E+00 | 0.000E+00 | 0.000E+00 | 0.000E+00 | 0.000E+00 | 0.000E+00 | 6.904E-07 | 0.000E+00 | 0.000E+00 | 0.000E+00 | 0.000E+00 | 0.000E+00 |
| Unigene428578 | MuxC | 0.000E+00 | 0.000E+00 | 0.000E+00 | 0.000E+00 | 0.000E+00 | 3.810E-07 | 5.200E-07 | 3.565E-07 | 1.541E-07 | 0.000E+00 | 0.000E+00 | 3.332E-07 |
| Unigene428591 | bcrA | 0.000E+00 | 0.000E+00 | 0.000E+00 | 0.000E+00 | 0.000E+00 | 0.000E+00 | 5.622E-07 | 2.695E-07 | 0.000E+00 | 0.000E+00 | 0.000E+00 | 4.913E-07 |
| Unigene428594 | vanSM | 0.000E+00 | 0.000E+00 | 0.000E+00 | 0.000E+00 | 0.000E+00 | 0.000E+00 | 4.138E-07 | 0.000E+00 | 0.000E+00 | 0.000E+00 | 0.000E+00 | 0.000E+00 |
| Unigene428668 | tlrC | 0.000E+00 | 0.000E+00 | 0.000E+00 | 0.000E+00 | 0.000E+00 | 0.000E+00 | 8.136E-07 | 0.000E+00 | 0.000E+00 | 0.000E+00 | 0.000E+00 | 3.160E-07 |
| Unigene428997 | msbA | 0.000E+00 | 0.000E+00 | 0.000E+00 | 0.000E+00 | 0.000E+00 | 0.000E+00 | 5.200E-07 | 0.000E+00 | 0.000E+00 | 0.000E+00 | 0.000E+00 | 0.000E+00 |
| Unigene429085 | AcrE | 0.000E+00 | 0.000E+00 | 0.000E+00 | 0.000E+00 | 0.000E+00 | 0.000E+00 | 7.593E-07 | 6.743E-07 | 0.000E+00 | 0.000E+00 | 0.000E+00 | 0.000E+00 |
| Unigene429224 | tetB(60) | 0.000E+00 | 0.000E+00 | 0.000E+00 | 0.000E+00 | 0.000E+00 | 0.000E+00 | 7.702E-07 | 0.000E+00 | 5.851E-08 | 0.000E+00 | 0.000E+00 | 0.000E+00 |
| Unigene429379 | oqxA | 0.000E+00 | 0.000E+00 | 0.000E+00 | 0.000E+00 | 0.000E+00 | 0.000E+00 | 0.000E+00 | 0.000E+00 | 0.000E+00 | 0.000E+00 | 0.000E+00 | 1.987E-07 |
| Unigene429403 | macB | 0.000E+00 | 0.000E+00 | 8.607E-08 | 2.067E-07 | 3.898E-07 | 2.845E-07 | 6.796E-07 | 8.874E-07 | 9.587E-08 | 3.583E-07 | 5.351E-07 | 1.728E-06 |
| Unigene429431 | tetB(60) | 8.967E-08 | 0.000E+00 | 2.200E-07 | 0.000E+00 | 1.992E-07 | 4.847E-07 | 8.435E-07 | 1.204E-06 | 0.000E+00 | 0.000E+00 | 1.026E-06 | 1.590E-06 |
| Unigene429526 | catB11 | 0.000E+00 | 0.000E+00 | 0.000E+00 | 0.000E+00 | 1.949E-07 | 0.000E+00 | 2.524E-06 | 0.000E+00 | 1.917E-07 | 0.000E+00 | 5.351E-07 | 1.382E-07 |
| Unigene429634 | arlR | 0.000E+00 | 0.000E+00 | 9.555E-08 | 1.967E-07 | 3.245E-07 | 0.000E+00 | 1.078E-06 | 0.000E+00 | 5.322E-08 | 0.000E+00 | 0.000E+00 | 7.482E-07 |
| Unigene429692 | OXA-18 | 0.000E+00 | 0.000E+00 | 7.134E-08 | 0.000E+00 | 0.000E+00 | 0.000E+00 | 5.633E-07 | 0.000E+00 | 0.000E+00 | 0.000E+00 | 8.316E-08 | 0.000E+00 |
| Unigene429703 | lmrD | 2.705E-07 | 0.000E+00 | 0.000E+00 | 0.000E+00 | 5.009E-07 | 0.000E+00 | 1.131E-06 | 8.420E-07 | 0.000E+00 | 5.357E-07 | 7.220E-07 | 1.741E-06 |
| Unigene429720 | tetA(58) | 0.000E+00 | 0.000E+00 | 1.473E-07 | 0.000E+00 | 7.784E-07 | 0.000E+00 | 4.431E-07 | 1.168E-07 | 0.000E+00 | 0.000E+00 | 5.725E-07 | 4.141E-07 |
| Unigene429877 | poxtA | 0.000E+00 | 0.000E+00 | 0.000E+00 | 0.000E+00 | 5.385E-07 | 0.000E+00 | 6.974E-07 | 0.000E+00 | 0.000E+00 | 0.000E+00 | 2.218E-07 | 8.021E-07 |
| Unigene429932 | pp-flo | 0.000E+00 | 0.000E+00 | 0.000E+00 | 0.000E+00 | 0.000E+00 | 0.000E+00 | 5.539E-07 | 0.000E+00 | 0.000E+00 | 0.000E+00 | 0.000E+00 | 3.697E-07 |
| Unigene429996 | rosA | 0.000E+00 | 0.000E+00 | 0.000E+00 | 0.000E+00 | 1.328E-07 | 0.000E+00 | 4.631E-07 | 3.489E-07 | 0.000E+00 | 0.000E+00 | 0.000E+00 | 0.000E+00 |
| Unigene430022 | baeS | 0.000E+00 | 0.000E+00 | 0.000E+00 | 0.000E+00 | 0.000E+00 | 0.000E+00 | 6.071E-07 | 0.000E+00 | 0.000E+00 | 0.000E+00 | 0.000E+00 | 0.000E+00 |
| Unigene430044 | macB | 0.000E+00 | 0.000E+00 | 0.000E+00 | 0.000E+00 | 0.000E+00 | 0.000E+00 | 7.566E-07 | 4.510E-07 | 0.000E+00 | 0.000E+00 | 0.000E+00 | 0.000E+00 |
| Unigene430078 | MexC | 0.000E+00 | 0.000E+00 | 0.000E+00 | 0.000E+00 | 0.000E+00 | 0.000E+00 | 5.912E-07 | 0.000E+00 | 0.000E+00 | 0.000E+00 | 0.000E+00 | 0.000E+00 |
| Unigene430121 | adeF | 0.000E+00 | 0.000E+00 | 1.684E-07 | 3.465E-07 | 0.000E+00 | 1.855E-07 | 1.465E-06 | 1.631E-06 | 0.000E+00 | 0.000E+00 | 4.206E-07 | 1.796E-06 |
| Unigene430200 | otr(B) | 0.000E+00 | 0.000E+00 | 0.000E+00 | 0.000E+00 | 0.000E+00 | 0.000E+00 | 8.267E-07 | 0.000E+00 | 0.000E+00 | 0.000E+00 | 0.000E+00 | 0.000E+00 |
| Unigene430229 | bcr-1 | 0.000E+00 | 0.000E+00 | 0.000E+00 | 0.000E+00 | 0.000E+00 | 0.000E+00 | 8.699E-07 | 0.000E+00 | 0.000E+00 | 1.812E-07 | 0.000E+00 | 1.602E-07 |
| Unigene430243 | NmcR | 0.000E+00 | 1.863E-07 | 2.331E-07 | 2.742E-07 | 1.508E-07 | 8.073E-07 | 2.029E-06 | 7.528E-07 | 2.226E-07 | 0.000E+00 | 5.824E-07 | 1.043E-06 |
| Unigene430285 | arlR | 0.000E+00 | 0.000E+00 | 0.000E+00 | 0.000E+00 | 0.000E+00 | 0.000E+00 | 3.761E-07 | 0.000E+00 | 0.000E+00 | 0.000E+00 | 0.000E+00 | 0.000E+00 |
| Unigene430478 | tetA(46) | 2.152E-07 | 0.000E+00 | 7.679E-08 | 9.878E-08 | 3.260E-07 | 1.269E-07 | 7.362E-07 | 8.449E-07 | 0.000E+00 | 4.359E-08 | 5.818E-07 | 2.127E-06 |
| Unigene430488 | macB | 0.000E+00 | 0.000E+00 | 0.000E+00 | 0.000E+00 | 0.000E+00 | 0.000E+00 | 5.598E-07 | 0.000E+00 | 0.000E+00 | 0.000E+00 | 0.000E+00 | 0.000E+00 |
| Unigene430579 | MexI | 0.000E+00 | 0.000E+00 | 0.000E+00 | 0.000E+00 | 0.000E+00 | 0.000E+00 | 6.650E-07 | 0.000E+00 | 0.000E+00 | 0.000E+00 | 0.000E+00 | 0.000E+00 |
| Unigene430640 | tetA(46) | 0.000E+00 | 0.000E+00 | 0.000E+00 | 0.000E+00 | 0.000E+00 | 0.000E+00 | 5.248E-07 | 0.000E+00 | 0.000E+00 | 0.000E+00 | 0.000E+00 | 7.761E-07 |
| Unigene430674 | norB | 0.000E+00 | 0.000E+00 | 0.000E+00 | 0.000E+00 | 0.000E+00 | 0.000E+00 | 1.228E-06 | 0.000E+00 | 0.000E+00 | 0.000E+00 | 0.000E+00 | 0.000E+00 |
| Unigene430710 | patA | 2.893E-07 | 0.000E+00 | 0.000E+00 | 0.000E+00 | 0.000E+00 | 0.000E+00 | 9.924E-07 | 0.000E+00 | 6.323E-08 | 0.000E+00 | 0.000E+00 | 0.000E+00 |
| Unigene430904 | macB | 0.000E+00 | 0.000E+00 | 2.901E-08 | 0.000E+00 | 4.269E-07 | 2.557E-07 | 1.374E-06 | 1.587E-06 | 0.000E+00 | 0.000E+00 | 0.000E+00 | 9.784E-07 |
| Unigene431263 | lmrC | 0.000E+00 | 0.000E+00 | 0.000E+00 | 0.000E+00 | 0.000E+00 | 0.000E+00 | 6.412E-07 | 0.000E+00 | 0.000E+00 | 0.000E+00 | 0.000E+00 | 0.000E+00 |
| Unigene431315 | vanRI | 0.000E+00 | 0.000E+00 | 0.000E+00 | 0.000E+00 | 0.000E+00 | 2.051E-07 | 2.430E-06 | 0.000E+00 | 0.000E+00 | 0.000E+00 | 0.000E+00 | 0.000E+00 |
| Unigene431363 | efrA | 0.000E+00 | 0.000E+00 | 0.000E+00 | 1.968E-07 | 0.000E+00 | 0.000E+00 | 7.119E-07 | 0.000E+00 | 0.000E+00 | 0.000E+00 | 0.000E+00 | 7.143E-07 |
| Unigene431474 | cpxA | 0.000E+00 | 0.000E+00 | 0.000E+00 | 0.000E+00 | 0.000E+00 | 0.000E+00 | 2.178E-06 | 0.000E+00 | 0.000E+00 | 0.000E+00 | 0.000E+00 | 0.000E+00 |
| Unigene431475 | mtrA | 0.000E+00 | 0.000E+00 | 0.000E+00 | 0.000E+00 | 0.000E+00 | 0.000E+00 | 1.495E-06 | 0.000E+00 | 0.000E+00 | 0.000E+00 | 0.000E+00 | 0.000E+00 |
| Unigene431583 | patA | 0.000E+00 | 0.000E+00 | 0.000E+00 | 0.000E+00 | 0.000E+00 | 0.000E+00 | 7.299E-07 | 0.000E+00 | 0.000E+00 | 0.000E+00 | 0.000E+00 | 0.000E+00 |
| Unigene431584 | tetA(60) | 1.635E-07 | 0.000E+00 | 0.000E+00 | 0.000E+00 | 0.000E+00 | 0.000E+00 | 7.601E-07 | 0.000E+00 | 0.000E+00 | 0.000E+00 | 0.000E+00 | 0.000E+00 |
| Unigene431807 | lmrD | 0.000E+00 | 0.000E+00 | 0.000E+00 | 0.000E+00 | 0.000E+00 | 0.000E+00 | 1.036E-06 | 0.000E+00 | 0.000E+00 | 0.000E+00 | 0.000E+00 | 0.000E+00 |
| Unigene431879 | Staphyloc | 0.000E+00 | 0.000E+00 | 0.000E+00 | 0.000E+00 | 0.000E+00 | 0.000E+00 | 8.032E-07 | 0.000E+00 | 0.000E+00 | 0.000E+00 | 0.000E+00 | 0.000E+00 |
| Unigene431945 | mtrA | 0.000E+00 | 0.000E+00 | 0.000E+00 | 0.000E+00 | 0.000E+00 | 0.000E+00 | 9.883E-07 | 0.000E+00 | 0.000E+00 | 0.000E+00 | 0.000E+00 | 0.000E+00 |

| | | | | | | | | | | | | | |
|---|---|---|---|---|---|---|---|---|---|---|---|---|---|
| Unigene431962 | smeS | 0.000E+00 | 0.000E+00 | 0.000E+00 | 0.000E+00 | 7.245E-08 | 0.000E+00 | 3.248E-07 | 2.284E-07 | 0.000E+00 | 0.000E+00 | 0.000E+00 | 2.312E-07 |
| Unigene432022 | srmB | 0.000E+00 | 0.000E+00 | 2.275E-07 | 0.000E+00 | 1.031E-07 | 0.000E+00 | 5.133E-07 | 3.790E-07 | 0.000E+00 | 0.000E+00 | 0.000E+00 | 0.000E+00 |
| Unigene432063 | lmrB | 0.000E+00 | 0.000E+00 | 0.000E+00 | 0.000E+00 | 0.000E+00 | 0.000E+00 | 4.427E-07 | 0.000E+00 | 0.000E+00 | 0.000E+00 | 0.000E+00 | 0.000E+00 |
| Unigene432085 | adeG | 0.000E+00 | 0.000E+00 | 0.000E+00 | 0.000E+00 | 2.403E-07 | 0.000E+00 | 0.000E+00 | 0.000E+00 | 0.000E+00 | 0.000E+00 | 0.000E+00 | 8.948E-07 |
| Unigene432205 | tetA(58) | 0.000E+00 | 0.000E+00 | 0.000E+00 | 0.000E+00 | 0.000E+00 | 0.000E+00 | 6.616E-07 | 0.000E+00 | 0.000E+00 | 0.000E+00 | 0.000E+00 | 0.000E+00 |
| Unigene432210 | novA | 0.000E+00 | 2.026E-06 | 0.000E+00 | 0.000E+00 | 6.762E-06 | 0.000E+00 | 1.869E-06 | 0.000E+00 | 0.000E+00 | 0.000E+00 | 0.000E+00 | 0.000E+00 |
| Unigene432443 | adeL | 0.000E+00 | 0.000E+00 | 0.000E+00 | 0.000E+00 | 0.000E+00 | 0.000E+00 | 4.279E-07 | 2.256E-07 | 0.000E+00 | 0.000E+00 | 0.000E+00 | 0.000E+00 |
| Unigene432532 | tet(51) | 0.000E+00 | 0.000E+00 | 0.000E+00 | 0.000E+00 | 0.000E+00 | 0.000E+00 | 3.934E-07 | 0.000E+00 | 0.000E+00 | 0.000E+00 | 0.000E+00 | 0.000E+00 |
| Unigene432606 | otr(B) | 0.000E+00 | 0.000E+00 | 0.000E+00 | 0.000E+00 | 0.000E+00 | 0.000E+00 | 0.000E+00 | 0.000E+00 | 0.000E+00 | 0.000E+00 | 0.000E+00 | 5.299E-07 |
| Unigene432627 | mefC | 5.173E-07 | 2.555E-07 | 4.315E-07 | 1.045E-07 | 5.460E-07 | 1.594E-06 | 1.918E-06 | 2.234E-06 | 3.392E-07 | 6.339E-07 | 1.302E-06 | 2.690E-06 |
| Unigene432707 | vanRI | 0.000E+00 | 0.000E+00 | 0.000E+00 | 0.000E+00 | 0.000E+00 | 0.000E+00 | 5.749E-07 | 0.000E+00 | 0.000E+00 | 0.000E+00 | 0.000E+00 | 0.000E+00 |
| Unigene432719 | macB | 8.277E-08 | 0.000E+00 | 0.000E+00 | 0.000E+00 | 0.000E+00 | 0.000E+00 | 6.412E-07 | 1.159E-06 | 0.000E+00 | 0.000E+00 | 3.787E-07 | 9.294E-07 |
| Unigene432861 | Bifidobacte | 0.000E+00 | 0.000E+00 | 0.000E+00 | 0.000E+00 | 0.000E+00 | 0.000E+00 | 1.352E-06 | 9.505E-07 | 0.000E+00 | 0.000E+00 | 0.000E+00 | 0.000E+00 |
| Unigene432870 | Klebsiella p | 0.000E+00 | 0.000E+00 | 0.000E+00 | 0.000E+00 | 0.000E+00 | 0.000E+00 | 2.707E-06 | 0.000E+00 | 0.000E+00 | 0.000E+00 | 0.000E+00 | 0.000E+00 |
| Unigene432904 | golS | 0.000E+00 | 0.000E+00 | 0.000E+00 | 0.000E+00 | 0.000E+00 | 0.000E+00 | 3.639E-06 | 0.000E+00 | 0.000E+00 | 0.000E+00 | 0.000E+00 | 0.000E+00 |
| Unigene432977 | oqxA | 7.507E-08 | 0.000E+00 | 7.366E-08 | 0.000E+00 | 0.000E+00 | 2.976E-07 | 9.416E-07 | 0.000E+00 | 0.000E+00 | 3.066E-07 | 0.000E+00 | 2.662E-07 |
| Unigene433062 | rosB | 0.000E+00 | 0.000E+00 | 0.000E+00 | 0.000E+00 | 7.546E-07 | 2.319E-07 | 2.374E-06 | 0.000E+00 | 0.000E+00 | 0.000E+00 | 6.543E-07 | 8.028E-07 |
| Unigene433063 | optrA | 0.000E+00 | 0.000E+00 | 0.000E+00 | 0.000E+00 | 0.000E+00 | 0.000E+00 | 3.057E-06 | 1.266E-06 | 0.000E+00 | 0.000E+00 | 0.000E+00 | 0.000E+00 |
| Unigene433105 | patA | 0.000E+00 | 0.000E+00 | 0.000E+00 | 0.000E+00 | 0.000E+00 | 0.000E+00 | 6.208E-07 | 0.000E+00 | 0.000E+00 | 0.000E+00 | 0.000E+00 | 0.000E+00 |
| Unigene433182 | LlmA 23S r | 0.000E+00 | 0.000E+00 | 0.000E+00 | 0.000E+00 | 0.000E+00 | 0.000E+00 | 0.000E+00 | 3.806E-07 | 0.000E+00 | 0.000E+00 | 0.000E+00 | 3.854E-07 |
| Unigene433223 | TaeA | 1.893E-07 | 7.619E-07 | 0.000E+00 | 0.000E+00 | 4.207E-07 | 1.706E-07 | 1.048E-06 | 4.052E-07 | 0.000E+00 | 0.000E+00 | 4.331E-07 | 0.000E+00 |
| Unigene433294 | cpxA | 0.000E+00 | 0.000E+00 | 0.000E+00 | 0.000E+00 | 0.000E+00 | 0.000E+00 | 8.661E-07 | 0.000E+00 | 0.000E+00 | 0.000E+00 | 0.000E+00 | 0.000E+00 |
| Unigene433443 | tetA(58) | 0.000E+00 | 0.000E+00 | 0.000E+00 | 0.000E+00 | 1.771E-07 | 1.292E-07 | 7.057E-07 | 1.489E-06 | 0.000E+00 | 0.000E+00 | 0.000E+00 | 8.008E-07 |
| Unigene433703 | emrY | 2.309E-08 | 7.603E-08 | 2.266E-08 | 2.332E-08 | 0.000E+00 | 1.997E-07 | 1.073E-06 | 1.887E-07 | 0.000E+00 | 0.000E+00 | 2.377E-07 | 2.456E-07 |
| Unigene433714 | macB | 0.000E+00 | 0.000E+00 | 0.000E+00 | 6.227E-08 | 0.000E+00 | 3.000E-07 | 1.092E-06 | 2.159E-07 | 3.370E-08 | 0.000E+00 | 0.000E+00 | 0.000E+00 |
| Unigene433796 | iri | 5.314E-08 | 0.000E+00 | 0.000E+00 | 0.000E+00 | 0.000E+00 | 0.000E+00 | 8.527E-07 | 0.000E+00 | 0.000E+00 | 0.000E+00 | 0.000E+00 | 0.000E+00 |
| Unigene433957 | MexB | 0.000E+00 | 0.000E+00 | 0.000E+00 | 0.000E+00 | 0.000E+00 | 0.000E+00 | 6.202E-07 | 0.000E+00 | 0.000E+00 | 0.000E+00 | 0.000E+00 | 0.000E+00 |
| Unigene434093 | MuxC | 0.000E+00 | 0.000E+00 | 0.000E+00 | 0.000E+00 | 0.000E+00 | 0.000E+00 | 8.066E-07 | 0.000E+00 | 0.000E+00 | 0.000E+00 | 0.000E+00 | 0.000E+00 |
| Unigene434133 | TaeA | 0.000E+00 | 0.000E+00 | 0.000E+00 | 8.693E-08 | 0.000E+00 | 0.000E+00 | 1.016E-06 | 0.000E+00 | 0.000E+00 | 0.000E+00 | 0.000E+00 | 0.000E+00 |
| Unigene434178 | macB | 0.000E+00 | 0.000E+00 | 0.000E+00 | 2.903E-07 | 0.000E+00 | 0.000E+00 | 2.000E-06 | 1.965E-06 | 0.000E+00 | 0.000E+00 | 0.000E+00 | 1.553E-06 |
| Unigene434236 | vanHD | 4.861E-07 | 0.000E+00 | 6.360E-08 | 0.000E+00 | 2.160E-07 | 0.000E+00 | 1.255E-06 | 4.161E-07 | 0.000E+00 | 0.000E+00 | 6.673E-07 | 8.811E-07 |
| Unigene434239 | NmcR | 0.000E+00 | 0.000E+00 | 0.000E+00 | 0.000E+00 | 0.000E+00 | 0.000E+00 | 5.109E-07 | 0.000E+00 | 0.000E+00 | 0.000E+00 | 0.000E+00 | 0.000E+00 |
| Unigene434246 | patA | 2.061E-07 | 9.049E-08 | 3.236E-07 | 2.914E-07 | 5.496E-07 | 0.000E+00 | 1.232E-06 | 5.775E-07 | 9.012E-08 | 0.000E+00 | 3.301E-07 | 1.608E-06 |
| Unigene434329 | iri | 0.000E+00 | 0.000E+00 | 0.000E+00 | 0.000E+00 | 0.000E+00 | 0.000E+00 | 6.787E-07 | 0.000E+00 | 0.000E+00 | 0.000E+00 | 0.000E+00 | 0.000E+00 |
| Unigene434334 | OXA-18 | 0.000E+00 | 0.000E+00 | 0.000E+00 | 0.000E+00 | 3.650E-07 | 1.332E-07 | 6.363E-07 | 5.273E-07 | 0.000E+00 | 2.745E-07 | 3.758E-07 | 5.340E-07 |
| Unigene434364 | patB | 2.374E-07 | 0.000E+00 | 1.165E-07 | 3.595E-07 | 4.834E-07 | 3.421E-07 | 1.226E-06 | 1.247E-06 | 8.647E-08 | 3.085E-07 | 9.503E-07 | 2.619E-06 |
| Unigene434517 | otr(B) | 0.000E+00 | 0.000E+00 | 0.000E+00 | 0.000E+00 | 0.000E+00 | 0.000E+00 | 1.161E-06 | 0.000E+00 | 0.000E+00 | 0.000E+00 | 0.000E+00 | 0.000E+00 |
| Unigene434534 | bcrA | 0.000E+00 | 0.000E+00 | 0.000E+00 | 0.000E+00 | 0.000E+00 | 0.000E+00 | 9.545E-07 | 0.000E+00 | 0.000E+00 | 0.000E+00 | 1.409E-07 | 0.000E+00 |
| Unigene434570 | AAC(6')-Ic | 0.000E+00 | 0.000E+00 | 0.000E+00 | 0.000E+00 | 0.000E+00 | 7.653E-08 | 1.880E-06 | 0.000E+00 | 0.000E+00 | 0.000E+00 | 0.000E+00 | 0.000E+00 |
| Unigene434582 | YojI | 0.000E+00 | 0.000E+00 | 0.000E+00 | 0.000E+00 | 0.000E+00 | 0.000E+00 | 1.715E-06 | 0.000E+00 | 0.000E+00 | 0.000E+00 | 0.000E+00 | 0.000E+00 |
| Unigene434638 | macB | 0.000E+00 | 0.000E+00 | 0.000E+00 | 0.000E+00 | 2.299E-07 | 0.000E+00 | 5.344E-07 | 8.856E-07 | 0.000E+00 | 2.305E-07 | 7.890E-08 | 4.892E-07 |
| Unigene434656 | YojI | 2.827E-07 | 0.000E+00 | 2.034E-07 | 0.000E+00 | 6.071E-07 | 2.648E-07 | 1.189E-06 | 6.599E-07 | 0.000E+00 | 3.569E-07 | 0.000E+00 | 1.314E-06 |
| Unigene434742 | rpoB2 | 0.000E+00 | 0.000E+00 | 0.000E+00 | 0.000E+00 | 0.000E+00 | 0.000E+00 | 4.820E-07 | 0.000E+00 | 0.000E+00 | 0.000E+00 | 0.000E+00 | 0.000E+00 |
| Unigene434764 | evgS | 0.000E+00 | 0.000E+00 | 0.000E+00 | 1.736E-07 | 0.000E+00 | 0.000E+00 | 9.131E-07 | 0.000E+00 | 0.000E+00 | 0.000E+00 | 0.000E+00 | 0.000E+00 |
| Unigene434815 | tlrC | 0.000E+00 | 0.000E+00 | 0.000E+00 | 0.000E+00 | 0.000E+00 | 0.000E+00 | 3.857E-07 | 7.119E-07 | 0.000E+00 | 0.000E+00 | 0.000E+00 | 0.000E+00 |
| Unigene434953 | Streptomy | 1.896E-07 | 0.000E+00 | 4.651E-07 | 2.074E-07 | 8.426E-07 | 0.000E+00 | 1.416E-06 | 1.660E-06 | 0.000E+00 | 3.520E-07 | 4.880E-07 | 1.849E-06 |
| Unigene435104 | tetA(58) | 0.000E+00 | 0.000E+00 | 0.000E+00 | 0.000E+00 | 0.000E+00 | 0.000E+00 | 1.211E-06 | 0.000E+00 | 0.000E+00 | 0.000E+00 | 0.000E+00 | 3.952E-07 |
| Unigene435131 | vanHD | 1.012E-07 | 0.000E+00 | 0.000E+00 | 0.000E+00 | 0.000E+00 | 0.000E+00 | 1.344E-06 | 0.000E+00 | 0.000E+00 | 0.000E+00 | 0.000E+00 | 0.000E+00 |
| Unigene435185 | evgS | 1.541E-07 | 0.000E+00 | 2.337E-07 | 9.904E-08 | 4.047E-07 | 3.332E-07 | 1.132E-06 | 7.032E-07 | 0.000E+00 | 3.590E-07 | 6.891E-07 | 8.280E-07 |
| Unigene435220 | macB | 0.000E+00 | 0.000E+00 | 0.000E+00 | 0.000E+00 | 0.000E+00 | 0.000E+00 | 3.503E-07 | 0.000E+00 | 0.000E+00 | 0.000E+00 | 0.000E+00 | 0.000E+00 |
| Unigene435335 | efrB | 0.000E+00 | 0.000E+00 | 0.000E+00 | 0.000E+00 | 0.000E+00 | 0.000E+00 | 0.000E+00 | 0.000E+00 | 0.000E+00 | 0.000E+00 | 0.000E+00 | 3.613E-07 |
| Unigene435345 | vanHF | 2.174E-07 | 0.000E+00 | 0.000E+00 | 0.000E+00 | 0.000E+00 | 0.000E+00 | 0.000E+00 | 0.000E+00 | 0.000E+00 | 0.000E+00 | 0.000E+00 | 0.000E+00 |
| Unigene435382 | Staphylocc | 0.000E+00 | 0.000E+00 | 0.000E+00 | 0.000E+00 | 0.000E+00 | 0.000E+00 | 3.707E-07 | 0.000E+00 | 0.000E+00 | 0.000E+00 | 0.000E+00 | 0.000E+00 |
| Unigene435430 | vatB | 0.000E+00 | 0.000E+00 | 0.000E+00 | 0.000E+00 | 0.000E+00 | 1.955E-07 | 4.503E-07 | 0.000E+00 | 0.000E+00 | 0.000E+00 | 0.000E+00 | 4.810E-07 |
| Unigene435475 | Bifidobacte | 0.000E+00 | 0.000E+00 | 0.000E+00 | 0.000E+00 | 0.000E+00 | 0.000E+00 | 4.783E-07 | 5.044E-08 | 0.000E+00 | 0.000E+00 | 1.977E-07 | 0.000E+00 |
| Unigene435481 | baeR | 0.000E+00 | 0.000E+00 | 0.000E+00 | 0.000E+00 | 0.000E+00 | 0.000E+00 | 4.566E-07 | 0.000E+00 | 0.000E+00 | 0.000E+00 | 0.000E+00 | 0.000E+00 |
| Unigene435659 | facT | 0.000E+00 | 0.000E+00 | 0.000E+00 | 0.000E+00 | 0.000E+00 | 0.000E+00 | 5.188E-07 | 0.000E+00 | 0.000E+00 | 0.000E+00 | 0.000E+00 | 7.916E-08 |
| Unigene435715 | Escherichia | 0.000E+00 | 0.000E+00 | 0.000E+00 | 0.000E+00 | 0.000E+00 | 0.000E+00 | 5.359E-07 | 0.000E+00 | 0.000E+00 | 0.000E+00 | 0.000E+00 | 0.000E+00 |

| | | | | | | | | | | | | | |
|---|---|---|---|---|---|---|---|---|---|---|---|---|---|
| Unigene435734 | hp1181 | 0.000E+00 | 0.000E+00 | 0.000E+00 | 1.877E-07 | 0.000E+00 | 0.000E+00 | 2.341E-06 | 1.112E-06 | 0.000E+00 | 0.000E+00 | 5.583E-07 | 0.000E+00 |
| Unigene435742 | smeB | 0.000E+00 | 0.000E+00 | 0.000E+00 | 0.000E+00 | 0.000E+00 | 0.000E+00 | 0.000E+00 | 1.782E-07 | 0.000E+00 | 0.000E+00 | 0.000E+00 | 0.000E+00 |
| Unigene435882 | cpxA | 5.723E-08 | 1.884E-07 | 0.000E+00 | 0.000E+00 | 0.000E+00 | 0.000E+00 | 5.701E-07 | 0.000E+00 | 0.000E+00 | 1.275E-07 | 0.000E+00 | 0.000E+00 |
| Unigene435972 | bcrA | 0.000E+00 | 0.000E+00 | 5.916E-07 | 0.000E+00 | 4.122E-07 | 6.518E-07 | 1.386E-06 | 0.000E+00 | 0.000E+00 | 0.000E+00 | 0.000E+00 | 2.467E-06 |
| Unigene435973 | basS | 0.000E+00 | 0.000E+00 | 0.000E+00 | 0.000E+00 | 0.000E+00 | 0.000E+00 | 9.575E-07 | 0.000E+00 | 0.000E+00 | 0.000E+00 | 0.000E+00 | 1.278E-07 |
| Unigene435996 | tetA(58) | 0.000E+00 | 0.000E+00 | 0.000E+00 | 0.000E+00 | 0.000E+00 | 0.000E+00 | 4.802E-07 | 0.000E+00 | 0.000E+00 | 0.000E+00 | 0.000E+00 | 0.000E+00 |
| Unigene436040 | macB | 0.000E+00 | 0.000E+00 | 0.000E+00 | 1.309E-07 | 0.000E+00 | 0.000E+00 | 0.000E+00 | 3.026E-07 | 0.000E+00 | 0.000E+00 | 0.000E+00 | 0.000E+00 |
| Unigene436180 | bcrA | 5.211E-08 | 0.000E+00 | 1.534E-07 | 0.000E+00 | 0.000E+00 | 1.690E-07 | 4.614E-07 | 4.866E-07 | 8.543E-08 | 0.000E+00 | 2.384E-07 | 1.201E-06 |
| Unigene436262 | adeR | 0.000E+00 | 0.000E+00 | 0.000E+00 | 0.000E+00 | 6.957E-07 | 0.000E+00 | 3.150E-07 | 0.000E+00 | 0.000E+00 | 0.000E+00 | 0.000E+00 | 4.711E-07 |
| Unigene436327 | Corynebac | 0.000E+00 | 0.000E+00 | 0.000E+00 | 0.000E+00 | 2.613E-07 | 6.356E-08 | 4.555E-07 | 0.000E+00 | 0.000E+00 | 0.000E+00 | 0.000E+00 | 6.950E-08 |
| Unigene436350 | Corynebac | 0.000E+00 | 0.000E+00 | 0.000E+00 | 0.000E+00 | 0.000E+00 | 0.000E+00 | 4.014E-07 | 0.000E+00 | 0.000E+00 | 0.000E+00 | 0.000E+00 | 0.000E+00 |
| Unigene436358 | mtrA | 0.000E+00 | 0.000E+00 | 0.000E+00 | 0.000E+00 | 0.000E+00 | 0.000E+00 | 4.315E-07 | 0.000E+00 | 0.000E+00 | 0.000E+00 | 0.000E+00 | 0.000E+00 |
| Unigene436464 | acrB | 0.000E+00 | 0.000E+00 | 0.000E+00 | 0.000E+00 | 0.000E+00 | 0.000E+00 | 6.915E-07 | 0.000E+00 | 0.000E+00 | 0.000E+00 | 0.000E+00 | 0.000E+00 |
| Unigene436592 | novA | 0.000E+00 | 0.000E+00 | 0.000E+00 | 0.000E+00 | 3.213E-07 | 0.000E+00 | 5.122E-07 | 0.000E+00 | 0.000E+00 | 0.000E+00 | 0.000E+00 | 0.000E+00 |
| Unigene436781 | patA | 0.000E+00 | 0.000E+00 | 0.000E+00 | 0.000E+00 | 0.000E+00 | 0.000E+00 | 7.767E-07 | 0.000E+00 | 0.000E+00 | 3.040E-07 | 0.000E+00 | 0.000E+00 |
| Unigene436853 | Streptomy | 0.000E+00 | 6.926E-08 | 6.192E-08 | 0.000E+00 | 6.310E-07 | 0.000E+00 | 7.683E-07 | 4.052E-07 | 0.000E+00 | 0.000E+00 | 0.000E+00 | 6.341E-07 |
| Unigene436857 | vmlR | 0.000E+00 | 0.000E+00 | 0.000E+00 | 0.000E+00 | 0.000E+00 | 0.000E+00 | 2.006E-06 | 0.000E+00 | 0.000E+00 | 0.000E+00 | 0.000E+00 | 0.000E+00 |
| Unigene436875 | Corynebac | 0.000E+00 | 0.000E+00 | 0.000E+00 | 0.000E+00 | 0.000E+00 | 0.000E+00 | 1.183E-06 | 0.000E+00 | 0.000E+00 | 0.000E+00 | 0.000E+00 | 0.000E+00 |
| Unigene436990 | lmrC | 0.000E+00 | 0.000E+00 | 0.000E+00 | 0.000E+00 | 0.000E+00 | 0.000E+00 | 5.725E-07 | 0.000E+00 | 0.000E+00 | 0.000E+00 | 0.000E+00 | 0.000E+00 |
| Unigene436992 | ugd | 0.000E+00 | 0.000E+00 | 0.000E+00 | 0.000E+00 | 0.000E+00 | 0.000E+00 | 1.102E-05 | 0.000E+00 | 0.000E+00 | 0.000E+00 | 0.000E+00 | 8.479E-06 |
| Unigene437064 | efrB | 2.104E-07 | 6.268E-07 | 8.258E-07 | 7.891E-07 | 1.336E-07 | 0.000E+00 | 1.331E-07 | 0.000E+00 | 0.000E+00 | 0.000E+00 | 0.000E+00 | 3.553E-08 |
| Unigene437087 | Acinetoba | 1.481E-07 | 0.000E+00 | 2.422E-07 | 5.234E-07 | 1.919E-07 | 0.000E+00 | 8.740E-07 | 1.556E-06 | 0.000E+00 | 2.749E-07 | 7.058E-07 | 8.751E-07 |
| Unigene437142 | efrB | 0.000E+00 | 0.000E+00 | 0.000E+00 | 0.000E+00 | 1.161E-07 | 0.000E+00 | 5.010E-07 | 0.000E+00 | 0.000E+00 | 0.000E+00 | 0.000E+00 | 5.351E-07 |
| Unigene437187 | mdtN | 0.000E+00 | 6.216E-08 | 8.335E-08 | 0.000E+00 | 0.000E+00 | 0.000E+00 | 7.208E-07 | 1.653E-07 | 0.000E+00 | 0.000E+00 | 1.620E-07 | 2.008E-07 |
| Unigene437282 | vanSA | 0.000E+00 | 0.000E+00 | 0.000E+00 | 0.000E+00 | 0.000E+00 | 0.000E+00 | 6.535E-07 | 3.063E-07 | 0.000E+00 | 0.000E+00 | 0.000E+00 | 0.000E+00 |
| Unigene437319 | oleC | 0.000E+00 | 0.000E+00 | 0.000E+00 | 0.000E+00 | 0.000E+00 | 0.000E+00 | 9.087E-07 | 0.000E+00 | 0.000E+00 | 0.000E+00 | 0.000E+00 | 0.000E+00 |
| Unigene437326 | vanTG | 0.000E+00 | 0.000E+00 | 0.000E+00 | 0.000E+00 | 0.000E+00 | 0.000E+00 | 7.741E-07 | 0.000E+00 | 0.000E+00 | 0.000E+00 | 0.000E+00 | 0.000E+00 |
| Unigene437371 | vanHF | 0.000E+00 | 1.393E-07 | 0.000E+00 | 0.000E+00 | 2.821E-07 | 1.716E-07 | 5.621E-07 | 8.521E-07 | 3.469E-08 | 1.414E-07 | 4.357E-07 | 1.351E-06 |
| Unigene437460 | patB | 0.000E+00 | 5.522E-07 | 0.000E+00 | 8.990E-07 | 1.161E-06 | 1.841E-06 | 6.297E-06 | 6.280E-06 | 4.653E-07 | 1.725E-06 | 0.000E+00 | 5.948E-06 |
| Unigene437484 | mefC | 1.296E-07 | 1.138E-07 | 1.018E-07 | 1.571E-07 | 4.033E-07 | 2.803E-07 | 1.636E-06 | 8.777E-07 | 2.267E-07 | 0.000E+00 | 1.394E-06 | 3.065E-06 |
| Unigene437531 | vanHD | 0.000E+00 | 0.000E+00 | 0.000E+00 | 0.000E+00 | 0.000E+00 | 0.000E+00 | 5.025E-07 | 0.000E+00 | 0.000E+00 | 0.000E+00 | 0.000E+00 | 0.000E+00 |
| Unigene437566 | YojI | 0.000E+00 | 0.000E+00 | 0.000E+00 | 0.000E+00 | 3.872E-07 | 0.000E+00 | 5.786E-07 | 0.000E+00 | 0.000E+00 | 0.000E+00 | 0.000E+00 | 0.000E+00 |
| Unigene437580 | macB | 0.000E+00 | 0.000E+00 | 0.000E+00 | 0.000E+00 | 0.000E+00 | 0.000E+00 | 6.236E-07 | 0.000E+00 | 0.000E+00 | 0.000E+00 | 0.000E+00 | 0.000E+00 |
| Unigene437587 | tetA(46) | 0.000E+00 | 0.000E+00 | 0.000E+00 | 0.000E+00 | 0.000E+00 | 0.000E+00 | 4.580E-07 | 0.000E+00 | 0.000E+00 | 0.000E+00 | 0.000E+00 | 0.000E+00 |
| Unigene437598 | smeS | 0.000E+00 | 0.000E+00 | 0.000E+00 | 0.000E+00 | 0.000E+00 | 0.000E+00 | 1.002E-06 | 0.000E+00 | 0.000E+00 | 0.000E+00 | 0.000E+00 | 0.000E+00 |
| Unigene437639 | vanSM | 0.000E+00 | 0.000E+00 | 0.000E+00 | 0.000E+00 | 0.000E+00 | 0.000E+00 | 6.485E-07 | 0.000E+00 | 0.000E+00 | 0.000E+00 | 0.000E+00 | 0.000E+00 |
| Unigene437734 | tlrC | 0.000E+00 | 0.000E+00 | 0.000E+00 | 0.000E+00 | 0.000E+00 | 0.000E+00 | 9.570E-07 | 0.000E+00 | 0.000E+00 | 0.000E+00 | 0.000E+00 | 0.000E+00 |
| Unigene437870 | msrC | 0.000E+00 | 0.000E+00 | 0.000E+00 | 0.000E+00 | 0.000E+00 | 2.705E-07 | 2.769E-07 | 3.894E-07 | 0.000E+00 | 0.000E+00 | 0.000E+00 | 0.000E+00 |
| Unigene437876 | farA | 0.000E+00 | 1.319E-07 | 0.000E+00 | 0.000E+00 | 0.000E+00 | 0.000E+00 | 0.000E+00 | 0.000E+00 | 0.000E+00 | 0.000E+00 | 0.000E+00 | 0.000E+00 |
| Unigene437895 | vanHO | 0.000E+00 | 0.000E+00 | 0.000E+00 | 0.000E+00 | 0.000E+00 | 0.000E+00 | 5.707E-07 | 0.000E+00 | 0.000E+00 | 0.000E+00 | 0.000E+00 | 2.032E-07 |
| Unigene437911 | vanHO | 0.000E+00 | 0.000E+00 | 0.000E+00 | 0.000E+00 | 0.000E+00 | 0.000E+00 | 4.732E-07 | 0.000E+00 | 0.000E+00 | 0.000E+00 | 0.000E+00 | 0.000E+00 |
| Unigene437926 | efrA | 0.000E+00 | 0.000E+00 | 0.000E+00 | 1.687E-08 | 0.000E+00 | 0.000E+00 | 1.017E-06 | 0.000E+00 | 0.000E+00 | 0.000E+00 | 0.000E+00 | 0.000E+00 |
| Unigene438026 | vatF | 0.000E+00 | 0.000E+00 | 8.986E-08 | 0.000E+00 | 3.561E-07 | 0.000E+00 | 0.000E+00 | 0.000E+00 | 0.000E+00 | 0.000E+00 | 9.428E-07 | 3.247E-07 |
| Unigene438027 | patA | 0.000E+00 | 0.000E+00 | 0.000E+00 | 3.196E-07 | 0.000E+00 | 0.000E+00 | 0.000E+00 | 5.277E-07 | 0.000E+00 | 4.029E-07 | 0.000E+00 | 0.000E+00 |
| Unigene438028 | macB | 0.000E+00 | 0.000E+00 | 0.000E+00 | 0.000E+00 | 0.000E+00 | 0.000E+00 | 6.462E-07 | 2.434E-07 | 0.000E+00 | 0.000E+00 | 2.862E-07 | 0.000E+00 |
| Unigene438088 | evgA | 0.000E+00 | 0.000E+00 | 0.000E+00 | 0.000E+00 | 0.000E+00 | 0.000E+00 | 5.641E-07 | 0.000E+00 | 0.000E+00 | 0.000E+00 | 0.000E+00 | 0.000E+00 |
| Unigene438185 | golS | 0.000E+00 | 0.000E+00 | 0.000E+00 | 0.000E+00 | 0.000E+00 | 0.000E+00 | 5.454E-07 | 0.000E+00 | 0.000E+00 | 0.000E+00 | 0.000E+00 | 0.000E+00 |
| Unigene438257 | macB | 0.000E+00 | 0.000E+00 | 0.000E+00 | 0.000E+00 | 1.476E-07 | 0.000E+00 | 0.000E+00 | 0.000E+00 | 0.000E+00 | 0.000E+00 | 0.000E+00 | 0.000E+00 |
| Unigene438281 | MuxC | 0.000E+00 | 0.000E+00 | 0.000E+00 | 0.000E+00 | 1.401E-07 | 0.000E+00 | 8.373E-07 | 5.887E-07 | 0.000E+00 | 0.000E+00 | 0.000E+00 | 3.975E-07 |
| Unigene438358 | tetA(58) | 0.000E+00 | 0.000E+00 | 0.000E+00 | 0.000E+00 | 0.000E+00 | 0.000E+00 | 4.894E-07 | 0.000E+00 | 0.000E+00 | 0.000E+00 | 0.000E+00 | 0.000E+00 |
| Unigene438599 | macB | 0.000E+00 | 6.988E-08 | 0.000E+00 | 1.607E-07 | 4.598E-07 | 0.000E+00 | 7.399E-07 | 3.344E-07 | 0.000E+00 | 0.000E+00 | 0.000E+00 | 9.407E-07 |
| Unigene438600 | Enterobact | 0.000E+00 | 0.000E+00 | 1.580E-07 | 0.000E+00 | 5.366E-07 | 0.000E+00 | 6.236E-07 | 3.445E-07 | 0.000E+00 | 0.000E+00 | 2.456E-07 | 0.000E+00 |
| Unigene438613 | tetA(58) | 0.000E+00 | 0.000E+00 | 0.000E+00 | 0.000E+00 | 0.000E+00 | 0.000E+00 | 5.809E-07 | 0.000E+00 | 0.000E+00 | 0.000E+00 | 0.000E+00 | 0.000E+00 |
| Unigene438639 | mphO | 0.000E+00 | 1.185E-07 | 1.766E-07 | 1.090E-07 | 5.997E-07 | 3.112E-07 | 1.354E-06 | 1.008E-06 | 3.933E-08 | 6.013E-07 | 4.116E-07 | 1.404E-06 |
| Unigene438789 | adeL | 0.000E+00 | 0.000E+00 | 0.000E+00 | 0.000E+00 | 0.000E+00 | 0.000E+00 | 1.372E-06 | 0.000E+00 | 0.000E+00 | 0.000E+00 | 0.000E+00 | 0.000E+00 |
| Unigene438792 | msbA | 1.289E-07 | 0.000E+00 | 0.000E+00 | 0.000E+00 | 0.000E+00 | 0.000E+00 | 9.983E-07 | 9.777E-07 | 0.000E+00 | 0.000E+00 | 5.159E-07 | 6.854E-07 |
| Unigene438895 | mgrA | 0.000E+00 | 0.000E+00 | 0.000E+00 | 0.000E+00 | 0.000E+00 | 0.000E+00 | 1.735E-06 | 0.000E+00 | 0.000E+00 | 0.000E+00 | 0.000E+00 | 0.000E+00 |
| Unigene439038 | tetT | 0.000E+00 | 0.000E+00 | 0.000E+00 | 0.000E+00 | 0.000E+00 | 0.000E+00 | 1.344E-06 | 0.000E+00 | 0.000E+00 | 0.000E+00 | 0.000E+00 | 0.000E+00 |

| | | | | | | | | | | | | | |
|---|---|---|---|---|---|---|---|---|---|---|---|---|---|
| Unigene439062 | lin | 0.000E+00 | 0.000E+00 | 7.542E-07 | 0.000E+00 | 0.000E+00 | 0.000E+00 | 1.701E-06 | 0.000E+00 | 0.000E+00 | 0.000E+00 | 3.077E-07 | 0.000E+00 |
| Unigene439080 | golS | 0.000E+00 | 0.000E+00 | 0.000E+00 | 0.000E+00 | 0.000E+00 | 0.000E+00 | 1.099E-06 | 0.000E+00 | 1.086E-06 | 2.674E-06 | 0.000E+00 | 0.000E+00 |
| Unigene439125 | Acinetoba | 0.000E+00 | 2.427E-07 | 9.642E-08 | 0.000E+00 | 0.000E+00 | 0.000E+00 | 1.359E-06 | 0.000E+00 | 0.000E+00 | 0.000E+00 | 0.000E+00 | 0.000E+00 |
| Unigene439127 | Klebsiella p | 0.000E+00 | 0.000E+00 | 0.000E+00 | 0.000E+00 | 0.000E+00 | 0.000E+00 | 1.584E-06 | 8.994E-07 | 0.000E+00 | 0.000E+00 | 0.000E+00 | 0.000E+00 |
| Unigene439131 | mtrA | 0.000E+00 | 0.000E+00 | 0.000E+00 | 0.000E+00 | 0.000E+00 | 0.000E+00 | 5.521E-07 | 0.000E+00 | 0.000E+00 | 0.000E+00 | 0.000E+00 | 2.527E-07 |
| Unigene439224 | smeR | 0.000E+00 | 0.000E+00 | 0.000E+00 | 0.000E+00 | 0.000E+00 | 0.000E+00 | 3.904E-07 | 0.000E+00 | 0.000E+00 | 0.000E+00 | 0.000E+00 | 0.000E+00 |
| Unigene439298 | arlR | 0.000E+00 | 0.000E+00 | 0.000E+00 | 0.000E+00 | 0.000E+00 | 0.000E+00 | 6.844E-07 | 0.000E+00 | 0.000E+00 | 0.000E+00 | 0.000E+00 | 0.000E+00 |
| Unigene439387 | bacA | 0.000E+00 | 1.317E-07 | 5.102E-07 | 0.000E+00 | 0.000E+00 | 0.000E+00 | 1.771E-06 | 7.470E-07 | 0.000E+00 | 0.000E+00 | 1.007E-06 | 2.222E-06 |
| Unigene439426 | baeR | 0.000E+00 | 0.000E+00 | 0.000E+00 | 0.000E+00 | 1.039E-07 | 0.000E+00 | 8.284E-07 | 2.403E-06 | 0.000E+00 | 0.000E+00 | 7.492E-07 | 8.848E-07 |
| Unigene439615 | oleB | 0.000E+00 | 0.000E+00 | 0.000E+00 | 0.000E+00 | 0.000E+00 | 0.000E+00 | 5.539E-07 | 0.000E+00 | 0.000E+00 | 0.000E+00 | 0.000E+00 | 0.000E+00 |
| Unigene439705 | evgS | 0.000E+00 | 0.000E+00 | 0.000E+00 | 0.000E+00 | 0.000E+00 | 0.000E+00 | 3.020E-07 | 0.000E+00 | 0.000E+00 | 0.000E+00 | 3.568E-07 | 4.147E-07 |
| Unigene439714 | Acinetoba | 0.000E+00 | 0.000E+00 | 0.000E+00 | 0.000E+00 | 0.000E+00 | 0.000E+00 | 7.702E-07 | 0.000E+00 | 0.000E+00 | 0.000E+00 | 0.000E+00 | 3.797E-07 |
| Unigene439718 | patA | 0.000E+00 | 0.000E+00 | 0.000E+00 | 0.000E+00 | 4.106E-07 | 0.000E+00 | 1.091E-06 | 0.000E+00 | 0.000E+00 | 0.000E+00 | 0.000E+00 | 6.311E-07 |
| Unigene439755 | efrA | 0.000E+00 | 7.290E-08 | 6.517E-08 | 3.354E-08 | 0.000E+00 | 3.590E-07 | 8.454E-07 | 5.427E-07 | 0.000E+00 | 0.000E+00 | 1.899E-07 | 2.748E-07 |
| Unigene440023 | adeL | 0.000E+00 | 0.000E+00 | 0.000E+00 | 2.203E-07 | 0.000E+00 | 0.000E+00 | 7.242E-07 | 0.000E+00 | 0.000E+00 | 1.620E-07 | 4.990E-07 | 8.164E-07 |
| Unigene440074 | ugd | 0.000E+00 | 0.000E+00 | 0.000E+00 | 0.000E+00 | 2.717E-07 | 0.000E+00 | 4.253E-07 | 0.000E+00 | 0.000E+00 | 0.000E+00 | 0.000E+00 | 6.194E-07 |
| Unigene440091 | tetA(58) | 0.000E+00 | 0.000E+00 | 0.000E+00 | 0.000E+00 | 3.032E-07 | 0.000E+00 | 1.079E-06 | 7.281E-07 | 0.000E+00 | 0.000E+00 | 4.905E-07 | 1.290E-06 |
| Unigene440100 | poxtA | 0.000E+00 | 9.049E-08 | 0.000E+00 | 0.000E+00 | 0.000E+00 | 0.000E+00 | 1.004E-06 | 3.369E-07 | 0.000E+00 | 0.000E+00 | 0.000E+00 | 0.000E+00 |
| Unigene440177 | bcrA | 0.000E+00 | 0.000E+00 | 0.000E+00 | 0.000E+00 | 0.000E+00 | 0.000E+00 | 1.237E-06 | 0.000E+00 | 0.000E+00 | 0.000E+00 | 0.000E+00 | 0.000E+00 |
| Unigene440219 | rphB | 0.000E+00 | 0.000E+00 | 0.000E+00 | 0.000E+00 | 0.000E+00 | 0.000E+00 | 1.813E-06 | 9.908E-07 | 2.613E-07 | 0.000E+00 | 0.000E+00 | 0.000E+00 |
| Unigene440243 | msbA | 0.000E+00 | 0.000E+00 | 5.452E-08 | 0.000E+00 | 4.321E-07 | 0.000E+00 | 1.045E-06 | 2.032E-06 | 0.000E+00 | 0.000E+00 | 0.000E+00 | 1.204E-06 |
| Unigene440281 | macB | 0.000E+00 | 0.000E+00 | 0.000E+00 | 0.000E+00 | 0.000E+00 | 0.000E+00 | 9.869E-07 | 0.000E+00 | 0.000E+00 | 0.000E+00 | 0.000E+00 | 0.000E+00 |
| Unigene440290 | NmcR | 7.222E-08 | 0.000E+00 | 0.000E+00 | 0.000E+00 | 3.209E-07 | 1.952E-07 | 1.039E-06 | 6.743E-07 | 0.000E+00 | 2.011E-07 | 2.478E-07 | 1.665E-06 |
| Unigene440299 | Escherichia | 0.000E+00 | 0.000E+00 | 0.000E+00 | 0.000E+00 | 0.000E+00 | 0.000E+00 | 1.012E-06 | 0.000E+00 | 0.000E+00 | 0.000E+00 | 0.000E+00 | 0.000E+00 |
| Unigene440323 | tva(A) | 0.000E+00 | 0.000E+00 | 0.000E+00 | 0.000E+00 | 0.000E+00 | 0.000E+00 | 9.129E-07 | 0.000E+00 | 0.000E+00 | 0.000E+00 | 0.000E+00 | 4.875E-07 |
| Unigene440328 | macB | 0.000E+00 | 0.000E+00 | 0.000E+00 | 0.000E+00 | 5.023E-08 | 0.000E+00 | 6.505E-07 | 5.805E-07 | 9.883E-08 | 0.000E+00 | 1.138E-06 | 2.031E-06 |
| Unigene440430 | lmrB | 8.643E-08 | 0.000E+00 | 0.000E+00 | 3.491E-07 | 0.000E+00 | 1.401E-07 | 3.826E-07 | 0.000E+00 | 1.889E-07 | 1.011E-06 | 0.000E+00 | 0.000E+00 |
| Unigene440521 | acrB | 0.000E+00 | 0.000E+00 | 0.000E+00 | 0.000E+00 | 0.000E+00 | 0.000E+00 | 7.618E-07 | 5.356E-08 | 0.000E+00 | 0.000E+00 | 0.000E+00 | 0.000E+00 |
| Unigene440522 | lmrD | 0.000E+00 | 0.000E+00 | 0.000E+00 | 0.000E+00 | 0.000E+00 | 0.000E+00 | 1.649E-06 | 0.000E+00 | 0.000E+00 | 0.000E+00 | 0.000E+00 | 0.000E+00 |
| Unigene440657 | poxtA | 0.000E+00 | 0.000E+00 | 0.000E+00 | 0.000E+00 | 0.000E+00 | 0.000E+00 | 1.075E-06 | 0.000E+00 | 0.000E+00 | 0.000E+00 | 0.000E+00 | 0.000E+00 |
| Unigene440712 | poxtA | 0.000E+00 | 4.577E-08 | 0.000E+00 | 0.000E+00 | 0.000E+00 | 3.156E-07 | 0.000E+00 | 0.000E+00 | 0.000E+00 | 0.000E+00 | 0.000E+00 | 4.930E-07 |
| Unigene440791 | lsaC | 0.000E+00 | 0.000E+00 | 0.000E+00 | 0.000E+00 | 0.000E+00 | 0.000E+00 | 8.245E-07 | 0.000E+00 | 0.000E+00 | 0.000E+00 | 0.000E+00 | 0.000E+00 |
| Unigene440795 | bacA | 0.000E+00 | 0.000E+00 | 0.000E+00 | 0.000E+00 | 0.000E+00 | 0.000E+00 | 0.000E+00 | 0.000E+00 | 0.000E+00 | 0.000E+00 | 4.058E-07 | 0.000E+00 |
| Unigene440830 | lmrD | 2.823E-07 | 0.000E+00 | 4.309E-07 | 0.000E+00 | 0.000E+00 | 5.765E-07 | 3.229E-06 | 2.307E-06 | 0.000E+00 | 3.844E-07 | 9.330E-07 | 1.594E-06 |
| Unigene440832 | vanHB | 0.000E+00 | 0.000E+00 | 1.949E-07 | 3.343E-07 | 8.828E-07 | 0.000E+00 | 7.695E-07 | 6.956E-07 | 0.000E+00 | 4.795E-07 | 4.923E-07 | 1.291E-06 |
| Unigene440958 | tetA(58) | 0.000E+00 | 0.000E+00 | 0.000E+00 | 0.000E+00 | 0.000E+00 | 0.000E+00 | 1.062E-06 | 0.000E+00 | 0.000E+00 | 0.000E+00 | 0.000E+00 | 0.000E+00 |
| Unigene441007 | oleB | 3.587E-07 | 1.072E-06 | 5.866E-07 | 0.000E+00 | 0.000E+00 | 0.000E+00 | 2.117E-06 | 0.000E+00 | 1.089E-07 | 0.000E+00 | 0.000E+00 | 0.000E+00 |
| Unigene441076 | Streptomy | 0.000E+00 | 0.000E+00 | 0.000E+00 | 0.000E+00 | 0.000E+00 | 0.000E+00 | 2.041E-07 | 0.000E+00 | 0.000E+00 | 0.000E+00 | 0.000E+00 | 3.634E-07 |
| Unigene441106 | oqxB | 0.000E+00 | 0.000E+00 | 0.000E+00 | 0.000E+00 | 0.000E+00 | 2.684E-07 | 1.832E-06 | 0.000E+00 | 0.000E+00 | 0.000E+00 | 0.000E+00 | 0.000E+00 |
| Unigene441183 | tetB(46) | 0.000E+00 | 0.000E+00 | 0.000E+00 | 0.000E+00 | 3.372E-07 | 0.000E+00 | 9.161E-07 | 1.031E-06 | 0.000E+00 | 0.000E+00 | 0.000E+00 | 2.022E-06 |
| Unigene441300 | lmrC | 0.000E+00 | 0.000E+00 | 0.000E+00 | 0.000E+00 | 0.000E+00 | 0.000E+00 | 6.041E-07 | 0.000E+00 | 0.000E+00 | 0.000E+00 | 0.000E+00 | 0.000E+00 |
| Unigene441424 | tetB(46) | 0.000E+00 | 0.000E+00 | 0.000E+00 | 0.000E+00 | 0.000E+00 | 1.790E-07 | 1.008E-06 | 0.000E+00 | 0.000E+00 | 0.000E+00 | 0.000E+00 | 0.000E+00 |
| Unigene441434 | Klebsiella p | 0.000E+00 | 5.701E-07 | 0.000E+00 | 0.000E+00 | 0.000E+00 | 4.011E-08 | 1.273E-06 | 0.000E+00 | 0.000E+00 | 0.000E+00 | 2.122E-07 | 0.000E+00 |
| Unigene441439 | cpxA | 0.000E+00 | 0.000E+00 | 0.000E+00 | 0.000E+00 | 0.000E+00 | 0.000E+00 | 1.796E-06 | 0.000E+00 | 0.000E+00 | 0.000E+00 | 4.367E-07 | 0.000E+00 |
| Unigene441442 | salA | 1.261E-07 | 1.384E-07 | 2.475E-07 | 1.273E-07 | 4.203E-07 | 1.818E-07 | 9.304E-07 | 6.133E-07 | 2.297E-07 | 0.000E+00 | 5.289E-07 | 7.204E-07 |
| Unigene441512 | iri | 0.000E+00 | 0.000E+00 | 0.000E+00 | 0.000E+00 | 0.000E+00 | 0.000E+00 | 5.465E-07 | 0.000E+00 | 0.000E+00 | 0.000E+00 | 0.000E+00 | 0.000E+00 |
| Unigene441580 | Klebsiella p | 0.000E+00 | 0.000E+00 | 0.000E+00 | 0.000E+00 | 0.000E+00 | 0.000E+00 | 1.281E-06 | 0.000E+00 | 0.000E+00 | 1.172E-07 | 1.204E-07 | 0.000E+00 |
| Unigene441610 | efrA | 0.000E+00 | 0.000E+00 | 0.000E+00 | 0.000E+00 | 0.000E+00 | 0.000E+00 | 3.402E-07 | 0.000E+00 | 0.000E+00 | 0.000E+00 | 0.000E+00 | 0.000E+00 |
| Unigene441639 | Corynebac | 1.030E-04 | 1.737E-04 | 1.661E-04 | 0.000E+00 | 8.632E-06 | 2.666E-07 | 3.071E-06 | 1.655E-06 | 5.314E-05 | 0.000E+00 | 2.716E-06 | 6.195E-07 |
| Unigene441667 | evgS | 0.000E+00 | 0.000E+00 | 0.000E+00 | 0.000E+00 | 1.891E-07 | 3.313E-07 | 9.045E-07 | 7.949E-07 | 0.000E+00 | 0.000E+00 | 3.506E-07 | 8.050E-07 |
| Unigene441684 | macB | 0.000E+00 | 0.000E+00 | 0.000E+00 | 0.000E+00 | 0.000E+00 | 0.000E+00 | 1.450E-06 | 0.000E+00 | 0.000E+00 | 0.000E+00 | 0.000E+00 | 0.000E+00 |
| Unigene441688 | patA | 0.000E+00 | 0.000E+00 | 0.000E+00 | 0.000E+00 | 0.000E+00 | 0.000E+00 | 2.107E-06 | 1.344E-06 | 0.000E+00 | 0.000E+00 | 0.000E+00 | 0.000E+00 |
| Unigene441732 | tetA(46) | 0.000E+00 | 0.000E+00 | 0.000E+00 | 0.000E+00 | 0.000E+00 | 0.000E+00 | 5.166E-07 | 0.000E+00 | 0.000E+00 | 0.000E+00 | 0.000E+00 | 0.000E+00 |
| Unigene441741 | mtrA | 1.483E-07 | 0.000E+00 | 0.000E+00 | 0.000E+00 | 4.471E-07 | 3.893E-07 | 9.846E-07 | 1.137E-06 | 0.000E+00 | 3.775E-07 | 6.057E-07 | 7.511E-07 |
| Unigene441803 | vanHF | 0.000E+00 | 0.000E+00 | 0.000E+00 | 0.000E+00 | 5.099E-07 | 2.756E-07 | 0.000E+00 | 0.000E+00 | 0.000E+00 | 0.000E+00 | 0.000E+00 | 0.000E+00 |
| Unigene441837 | TriC | 0.000E+00 | 0.000E+00 | 0.000E+00 | 0.000E+00 | 0.000E+00 | 0.000E+00 | 6.805E-07 | 0.000E+00 | 0.000E+00 | 0.000E+00 | 0.000E+00 | 1.090E-06 |
| Unigene441857 | efrB | 0.000E+00 | 1.728E-07 | 0.000E+00 | 0.000E+00 | 0.000E+00 | 0.000E+00 | 5.228E-07 | 0.000E+00 | 0.000E+00 | 0.000E+00 | 3.002E-07 | 0.000E+00 |
| Unigene441867 | Acinetoba | 0.000E+00 | 8.729E-07 | 0.000E+00 | 0.000E+00 | 5.197E-07 | 0.000E+00 | 1.269E-06 | 0.000E+00 | 0.000E+00 | 0.000E+00 | 0.000E+00 | 0.000E+00 |

| | | | | | | | | | | | | | |
|---|---|---|---|---|---|---|---|---|---|---|---|---|---|
| Unigene441926 | AcrS | 0.000E+00 | 0.000E+00 | 3.046E-07 | 0.000E+00 | 0.000E+00 | 0.000E+00 | 6.870E-07 | 0.000E+00 | 0.000E+00 | 0.000E+00 | 0.000E+00 | 0.000E+00 |
| Unigene441978 | smeR | 0.000E+00 | 0.000E+00 | 1.687E-07 | 0.000E+00 | 3.819E-07 | 4.831E-07 | 7.609E-07 | 8.828E-07 | 0.000E+00 | 0.000E+00 | 6.685E-07 | 2.235E-06 |
| Unigene441979 | vanSM | 1.899E-08 | 0.000E+00 | 1.025E-07 | 1.151E-07 | 4.326E-07 | 3.183E-07 | 1.020E-06 | 1.064E-06 | 7.266E-08 | 3.174E-07 | 8.256E-07 | 2.739E-06 |
| Unigene441986 | Staphylocc | 7.883E-08 | 0.000E+00 | 2.320E-07 | 0.000E+00 | 5.473E-07 | 2.130E-07 | 6.107E-07 | 2.070E-07 | 8.616E-08 | 2.415E-07 | 4.509E-07 | 7.687E-07 |
| Unigene441992 | oleB | 0.000E+00 | 0.000E+00 | 1.152E-07 | 0.000E+00 | 4.782E-07 | 0.000E+00 | 1.126E-06 | 8.678E-07 | 0.000E+00 | 0.000E+00 | 0.000E+00 | 1.017E-06 |
| Unigene442096 | adeR | 0.000E+00 | 0.000E+00 | 0.000E+00 | 0.000E+00 | 0.000E+00 | 0.000E+00 | 2.370E-07 | 0.000E+00 | 0.000E+00 | 0.000E+00 | 0.000E+00 | 0.000E+00 |
| Unigene442201 | evgS | 5.525E-08 | 0.000E+00 | 5.015E-07 | 0.000E+00 | 6.445E-07 | 0.000E+00 | 1.819E-06 | 0.000E+00 | 0.000E+00 | 0.000E+00 | 5.688E-07 | 2.253E-06 |
| Unigene442222 | lmrD | 0.000E+00 | 0.000E+00 | 0.000E+00 | 0.000E+00 | 0.000E+00 | 0.000E+00 | 2.416E-06 | 1.889E-06 | 0.000E+00 | 0.000E+00 | 1.494E-06 | 1.705E-06 |
| Unigene442230 | lsaC | 7.658E-08 | 0.000E+00 | 3.757E-08 | 0.000E+00 | 2.127E-07 | 0.000E+00 | 3.390E-07 | 6.258E-07 | 1.256E-07 | 0.000E+00 | 0.000E+00 | 0.000E+00 |
| Unigene442265 | basS | 0.000E+00 | 0.000E+00 | 0.000E+00 | 0.000E+00 | 0.000E+00 | 0.000E+00 | 0.000E+00 | 0.000E+00 | 0.000E+00 | 0.000E+00 | 0.000E+00 | 3.401E-07 |
| Unigene442367 | ceoB | 8.382E-08 | 6.899E-08 | 1.028E-07 | 1.693E-07 | 3.259E-07 | 2.039E-07 | 8.465E-07 | 8.438E-07 | 1.374E-07 | 1.517E-07 | 9.588E-07 | 2.700E-06 |
| Unigene442368 | adeG | 5.476E-08 | 3.005E-08 | 8.060E-08 | 2.488E-07 | 4.563E-07 | 2.960E-07 | 1.242E-06 | 7.350E-07 | 1.197E-07 | 0.000E+00 | 9.082E-07 | 2.039E-06 |
| Unigene442413 | eptA | 0.000E+00 | 0.000E+00 | 0.000E+00 | 0.000E+00 | 0.000E+00 | 0.000E+00 | 0.000E+00 | 3.646E-07 | 0.000E+00 | 0.000E+00 | 0.000E+00 | 0.000E+00 |
| Unigene442430 | Acinetoba | 0.000E+00 | 1.504E-07 | 5.884E-07 | 0.000E+00 | 4.759E-07 | 2.037E-07 | 1.252E-06 | 4.600E-07 | 0.000E+00 | 0.000E+00 | 4.116E-07 | 1.438E-06 |
| Unigene442471 | efrA | 6.920E-08 | 0.000E+00 | 0.000E+00 | 0.000E+00 | 3.844E-08 | 0.000E+00 | 6.893E-07 | 2.827E-07 | 0.000E+00 | 1.156E-07 | 3.958E-08 | 1.636E-07 |
| Unigene442498 | Acinetoba | 5.005E-08 | 8.239E-08 | 2.210E-07 | 1.516E-07 | 4.448E-07 | 2.435E-07 | 1.108E-06 | 9.347E-07 | 0.000E+00 | 0.000E+00 | 7.442E-07 | 1.923E-06 |
| Unigene442540 | msbA | 0.000E+00 | 0.000E+00 | 0.000E+00 | 0.000E+00 | 0.000E+00 | 0.000E+00 | 9.924E-07 | 0.000E+00 | 0.000E+00 | 0.000E+00 | 0.000E+00 | 0.000E+00 |
| Unigene442637 | adeL | 0.000E+00 | 0.000E+00 | 0.000E+00 | 0.000E+00 | 0.000E+00 | 0.000E+00 | 9.826E-07 | 0.000E+00 | 0.000E+00 | 0.000E+00 | 0.000E+00 | 0.000E+00 |
| Unigene442690 | novA | 0.000E+00 | 0.000E+00 | 0.000E+00 | 0.000E+00 | 0.000E+00 | 0.000E+00 | 7.622E-07 | 0.000E+00 | 0.000E+00 | 0.000E+00 | 0.000E+00 | 8.140E-07 |
| Unigene442726 | macB | 0.000E+00 | 0.000E+00 | 0.000E+00 | 0.000E+00 | 0.000E+00 | 0.000E+00 | 1.012E-06 | 0.000E+00 | 0.000E+00 | 0.000E+00 | 8.079E-07 | 0.000E+00 |
| Unigene442799 | oleC | 0.000E+00 | 0.000E+00 | 0.000E+00 | 7.585E-08 | 2.295E-07 | 1.218E-07 | 9.560E-07 | 1.754E-07 | 1.231E-07 | 0.000E+00 | 2.578E-07 | 5.771E-07 |
| Unigene442837 | tlrC | 0.000E+00 | 0.000E+00 | 0.000E+00 | 0.000E+00 | 0.000E+00 | 0.000E+00 | 1.102E-06 | 0.000E+00 | 0.000E+00 | 1.498E-07 | 0.000E+00 | 9.221E-07 |
| Unigene442883 | tetA(46) | 0.000E+00 | 0.000E+00 | 0.000E+00 | 0.000E+00 | 0.000E+00 | 0.000E+00 | 6.924E-07 | 0.000E+00 | 0.000E+00 | 0.000E+00 | 0.000E+00 | 0.000E+00 |
| Unigene442959 | tva(A) | 2.528E-07 | 1.585E-07 | 2.126E-07 | 1.458E-07 | 4.814E-07 | 0.000E+00 | 1.399E-06 | 9.272E-07 | 0.000E+00 | 0.000E+00 | 7.022E-07 | 2.646E-06 |
| Unigene443003 | vanHD | 3.973E-07 | 0.000E+00 | 0.000E+00 | 0.000E+00 | 0.000E+00 | 2.505E-07 | 8.428E-07 | 1.005E-06 | 3.619E-07 | 0.000E+00 | 5.681E-07 | 1.057E-06 |
| Unigene443005 | vgaB | 0.000E+00 | 0.000E+00 | 0.000E+00 | 0.000E+00 | 0.000E+00 | 0.000E+00 | 8.882E-07 | 0.000E+00 | 2.274E-07 | 4.305E-07 | 0.000E+00 | 7.378E-07 |
| Unigene443125 | mexM | 0.000E+00 | 0.000E+00 | 0.000E+00 | 0.000E+00 | 0.000E+00 | 0.000E+00 | 1.764E-07 | 0.000E+00 | 0.000E+00 | 0.000E+00 | 0.000E+00 | 0.000E+00 |
| Unigene443229 | Pseudomo | 0.000E+00 | 0.000E+00 | 0.000E+00 | 4.490E-08 | 2.964E-07 | 2.884E-07 | 1.033E-06 | 1.142E-06 | 0.000E+00 | 0.000E+00 | 8.138E-07 | 2.838E-06 |
| Unigene443371 | Corynebac | 0.000E+00 | 0.000E+00 | 0.000E+00 | 1.483E-07 | 0.000E+00 | 0.000E+00 | 9.982E-07 | 0.000E+00 | 0.000E+00 | 0.000E+00 | 0.000E+00 | 0.000E+00 |
| Unigene443408 | TaeA | 0.000E+00 | 0.000E+00 | 0.000E+00 | 0.000E+00 | 0.000E+00 | 0.000E+00 | 5.857E-07 | 3.088E-07 | 1.446E-07 | 0.000E+00 | 0.000E+00 | 1.564E-07 |
| Unigene443429 | Staphylocc | 1.649E-07 | 1.357E-07 | 1.214E-07 | 2.082E-07 | 2.519E-07 | 1.337E-07 | 8.669E-07 | 7.218E-07 | 0.000E+00 | 1.378E-07 | 5.659E-07 | 1.827E-06 |
| Unigene443521 | msbA | 9.276E-08 | 0.000E+00 | 2.275E-07 | 1.405E-07 | 4.122E-07 | 3.510E-07 | 4.620E-07 | 7.038E-07 | 0.000E+00 | 0.000E+00 | 5.305E-07 | 8.772E-07 |
| Unigene443616 | vatE | 2.924E-07 | 0.000E+00 | 0.000E+00 | 0.000E+00 | 0.000E+00 | 0.000E+00 | 4.789E-06 | 0.000E+00 | 8.309E-07 | 0.000E+00 | 0.000E+00 | 0.000E+00 |
| Unigene443659 | Agrobacte | 0.000E+00 | 0.000E+00 | 0.000E+00 | 0.000E+00 | 0.000E+00 | 0.000E+00 | 1.067E-06 | 0.000E+00 | 5.547E-08 | 0.000E+00 | 0.000E+00 | 0.000E+00 |
| Unigene443665 | vanRM | 0.000E+00 | 0.000E+00 | 0.000E+00 | 0.000E+00 | 0.000E+00 | 0.000E+00 | 8.308E-07 | 0.000E+00 | 0.000E+00 | 0.000E+00 | 0.000E+00 | 0.000E+00 |
| Unigene443776 | tet(42) | 0.000E+00 | 0.000E+00 | 0.000E+00 | 5.326E-08 | 5.860E-08 | 0.000E+00 | 1.313E-06 | 0.000E+00 | 0.000E+00 | 0.000E+00 | 0.000E+00 | 4.053E-07 |
| Unigene443990 | cmlv | 0.000E+00 | 0.000E+00 | 1.866E-07 | 3.018E-07 | 3.321E-07 | 2.937E-07 | 8.721E-07 | 5.075E-07 | 0.000E+00 | 0.000E+00 | 6.527E-07 | 2.248E-06 |
| Unigene443991 | adeL | 0.000E+00 | 2.029E-07 | 0.000E+00 | 0.000E+00 | 0.000E+00 | 0.000E+00 | 8.594E-07 | 7.769E-07 | 0.000E+00 | 2.471E-07 | 1.269E-06 | 2.666E-06 |
| Unigene444132 | vanHF | 0.000E+00 | 0.000E+00 | 0.000E+00 | 0.000E+00 | 0.000E+00 | 0.000E+00 | 5.688E-07 | 0.000E+00 | 0.000E+00 | 0.000E+00 | 0.000E+00 | 8.353E-07 |
| Unigene444140 | tetA(58) | 0.000E+00 | 0.000E+00 | 0.000E+00 | 0.000E+00 | 0.000E+00 | 0.000E+00 | 8.887E-07 | 5.624E-07 | 0.000E+00 | 0.000E+00 | 0.000E+00 | 0.000E+00 |
| Unigene444344 | dfrA9 | 0.000E+00 | 2.654E-07 | 0.000E+00 | 0.000E+00 | 0.000E+00 | 0.000E+00 | 9.366E-07 | 8.467E-07 | 0.000E+00 | 0.000E+00 | 0.000E+00 | 0.000E+00 |
| Unigene444466 | oleB | 0.000E+00 | 0.000E+00 | 0.000E+00 | 0.000E+00 | 0.000E+00 | 0.000E+00 | 3.627E-07 | 0.000E+00 | 0.000E+00 | 0.000E+00 | 0.000E+00 | 0.000E+00 |
| Unigene444556 | tetA(58) | 0.000E+00 | 0.000E+00 | 0.000E+00 | 0.000E+00 | 0.000E+00 | 0.000E+00 | 8.932E-07 | 0.000E+00 | 0.000E+00 | 0.000E+00 | 0.000E+00 | 0.000E+00 |
| Unigene444564 | mgrA | 0.000E+00 | 0.000E+00 | 0.000E+00 | 0.000E+00 | 0.000E+00 | 0.000E+00 | 1.111E-06 | 5.024E-07 | 0.000E+00 | 0.000E+00 | 7.385E-07 | 1.017E-06 |
| Unigene444570 | oleC | 0.000E+00 | 0.000E+00 | 1.874E-07 | 1.072E-07 | 9.431E-08 | 1.835E-07 | 1.362E-06 | 2.725E-07 | 0.000E+00 | 0.000E+00 | 2.913E-07 | 4.766E-07 |
| Unigene444648 | tetB(60) | 0.000E+00 | 8.813E-08 | 0.000E+00 | 0.000E+00 | 6.914E-07 | 0.000E+00 | 1.711E-06 | 0.000E+00 | 0.000E+00 | 0.000E+00 | 4.133E-07 | 0.000E+00 |
| Unigene444659 | optrA | 0.000E+00 | 4.334E-07 | 0.000E+00 | 0.000E+00 | 0.000E+00 | 2.028E-06 | 1.857E-06 | 0.000E+00 | 2.751E-06 | 0.000E+00 | 0.000E+00 | 4.667E-07 |
| Unigene444676 | oqxA | 0.000E+00 | 0.000E+00 | 0.000E+00 | 0.000E+00 | 0.000E+00 | 0.000E+00 | 5.001E-07 | 0.000E+00 | 0.000E+00 | 0.000E+00 | 0.000E+00 | 5.007E-07 |
| Unigene444718 | novA | 0.000E+00 | 0.000E+00 | 0.000E+00 | 0.000E+00 | 0.000E+00 | 0.000E+00 | 6.472E-07 | 5.006E-07 | 0.000E+00 | 0.000E+00 | 2.676E-07 | 0.000E+00 |
| Unigene444753 | tetA(60) | 3.074E-07 | 0.000E+00 | 5.746E-07 | 4.435E-07 | 8.295E-07 | 5.697E-07 | 1.410E-06 | 8.886E-07 | 2.400E-07 | 7.339E-07 | 1.122E-06 | 3.634E-06 |
| Unigene444856 | MexW | 0.000E+00 | 0.000E+00 | 0.000E+00 | 0.000E+00 | 2.451E-07 | 0.000E+00 | 1.194E-06 | 0.000E+00 | 0.000E+00 | 3.276E-07 | 0.000E+00 | 4.056E-07 |
| Unigene444884 | MdtK | 0.000E+00 | 0.000E+00 | 0.000E+00 | 0.000E+00 | 0.000E+00 | 0.000E+00 | 2.201E-06 | 0.000E+00 | 0.000E+00 | 0.000E+00 | 0.000E+00 | 0.000E+00 |
| Unigene444982 | tetT | 0.000E+00 | 0.000E+00 | 0.000E+00 | 0.000E+00 | 0.000E+00 | 0.000E+00 | 3.721E-07 | 0.000E+00 | 0.000E+00 | 0.000E+00 | 0.000E+00 | 0.000E+00 |
| Unigene445007 | evgS | 0.000E+00 | 0.000E+00 | 0.000E+00 | 0.000E+00 | 0.000E+00 | 0.000E+00 | 8.506E-07 | 0.000E+00 | 0.000E+00 | 0.000E+00 | 0.000E+00 | 0.000E+00 |
| Unigene445097 | TaeA | 0.000E+00 | 0.000E+00 | 0.000E+00 | 0.000E+00 | 0.000E+00 | 0.000E+00 | 0.000E+00 | 0.000E+00 | 0.000E+00 | 0.000E+00 | 0.000E+00 | 4.240E-07 |
| Unigene445232 | Brucella su | 0.000E+00 | 0.000E+00 | 1.705E-07 | 7.520E-08 | 1.103E-07 | 3.757E-07 | 1.181E-06 | 4.201E-07 | 0.000E+00 | 1.659E-07 | 1.846E-07 | 3.521E-07 |
| Unigene445282 | msbA | 0.000E+00 | 2.033E-08 | 0.000E+00 | 9.351E-08 | 0.000E+00 | 2.202E-07 | 1.066E-06 | 3.459E-07 | 0.000E+00 | 0.000E+00 | 1.695E-07 | 1.313E-07 |
| Unigene445335 | Enterobact | 0.000E+00 | 0.000E+00 | 0.000E+00 | 0.000E+00 | 0.000E+00 | 0.000E+00 | 7.821E-07 | 0.000E+00 | 0.000E+00 | 0.000E+00 | 0.000E+00 | 0.000E+00 |

| | | | | | | | | | | | | | |
|---|---|---|---|---|---|---|---|---|---|---|---|---|---|
| Unigene445348 | vanYB | 0.000E+00 | 0.000E+00 | 0.000E+00 | 0.000E+00 | 0.000E+00 | 0.000E+00 | 1.098E-06 | 0.000E+00 | 0.000E+00 | 0.000E+00 | 0.000E+00 | 0.000E+00 |
| Unigene445381 | efrA | 0.000E+00 | 4.934E-07 | 1.059E-06 | 0.000E+00 | 0.000E+00 | 0.000E+00 | 4.312E-06 | 3.953E-06 | 0.000E+00 | 0.000E+00 | 1.817E-06 | 3.259E-06 |
| Unigene445473 | efrA | 0.000E+00 | 0.000E+00 | 0.000E+00 | 0.000E+00 | 0.000E+00 | 0.000E+00 | 1.632E-06 | 0.000E+00 | 0.000E+00 | 0.000E+00 | 0.000E+00 | 4.711E-08 |
| Unigene445546 | novA | 0.000E+00 | 0.000E+00 | 0.000E+00 | 0.000E+00 | 0.000E+00 | 0.000E+00 | 1.002E-06 | 0.000E+00 | 0.000E+00 | 0.000E+00 | 0.000E+00 | 0.000E+00 |
| Unigene445561 | novA | 0.000E+00 | 0.000E+00 | 0.000E+00 | 0.000E+00 | 0.000E+00 | 0.000E+00 | 1.578E-06 | 0.000E+00 | 0.000E+00 | 0.000E+00 | 0.000E+00 | 0.000E+00 |
| Unigene445771 | oleC | 0.000E+00 | 0.000E+00 | 0.000E+00 | 3.364E-08 | 0.000E+00 | 1.801E-07 | 7.743E-07 | 2.333E-07 | 0.000E+00 | 0.000E+00 | 0.000E+00 | 0.000E+00 |
| Unigene445791 | smeR | 0.000E+00 | 0.000E+00 | 0.000E+00 | 0.000E+00 | 0.000E+00 | 0.000E+00 | 6.015E-07 | 0.000E+00 | 0.000E+00 | 0.000E+00 | 1.243E-07 | 0.000E+00 |
| Unigene445805 | catA4 | 0.000E+00 | 0.000E+00 | 0.000E+00 | 0.000E+00 | 0.000E+00 | 0.000E+00 | 3.741E-07 | 0.000E+00 | 0.000E+00 | 0.000E+00 | 0.000E+00 | 0.000E+00 |
| Unigene445847 | tetA(46) | 0.000E+00 | 0.000E+00 | 0.000E+00 | 0.000E+00 | 0.000E+00 | 0.000E+00 | 5.762E-07 | 2.026E-07 | 2.371E-07 | 0.000E+00 | 2.730E-07 | 8.462E-07 |
| Unigene445906 | kdpE | 0.000E+00 | 0.000E+00 | 0.000E+00 | 9.448E-08 | 0.000E+00 | 2.023E-07 | 6.731E-07 | 0.000E+00 | 0.000E+00 | 0.000E+00 | 0.000E+00 | 0.000E+00 |
| Unigene446076 | bcrA | 0.000E+00 | 0.000E+00 | 0.000E+00 | 0.000E+00 | 0.000E+00 | 0.000E+00 | 1.609E-06 | 0.000E+00 | 0.000E+00 | 0.000E+00 | 0.000E+00 | 0.000E+00 |
| Unigene446131 | Corynebac | 0.000E+00 | 0.000E+00 | 0.000E+00 | 0.000E+00 | 0.000E+00 | 0.000E+00 | 5.786E-07 | 0.000E+00 | 0.000E+00 | 0.000E+00 | 0.000E+00 | 0.000E+00 |
| Unigene446294 | bacA | 0.000E+00 | 0.000E+00 | 0.000E+00 | 0.000E+00 | 0.000E+00 | 0.000E+00 | 1.071E-06 | 8.132E-07 | 0.000E+00 | 0.000E+00 | 4.870E-07 | 2.882E-06 |
| Unigene446352 | macB | 0.000E+00 | 0.000E+00 | 0.000E+00 | 0.000E+00 | 0.000E+00 | 0.000E+00 | 8.749E-07 | 0.000E+00 | 0.000E+00 | 0.000E+00 | 0.000E+00 | 0.000E+00 |
| Unigene446375 | rphB | 0.000E+00 | 0.000E+00 | 0.000E+00 | 0.000E+00 | 4.221E-07 | 0.000E+00 | 1.547E-06 | 0.000E+00 | 0.000E+00 | 0.000E+00 | 0.000E+00 | 8.982E-07 |
| Unigene446473 | rphB | 0.000E+00 | 0.000E+00 | 0.000E+00 | 0.000E+00 | 0.000E+00 | 0.000E+00 | 7.074E-07 | 0.000E+00 | 0.000E+00 | 0.000E+00 | 0.000E+00 | 0.000E+00 |
| Unigene446530 | mdsA | 0.000E+00 | 0.000E+00 | 0.000E+00 | 0.000E+00 | 0.000E+00 | 0.000E+00 | 4.253E-07 | 0.000E+00 | 0.000E+00 | 0.000E+00 | 0.000E+00 | 0.000E+00 |
| Unigene446543 | vanHB | 0.000E+00 | 0.000E+00 | 0.000E+00 | 0.000E+00 | 1.833E-07 | 3.925E-07 | 5.114E-07 | 7.705E-07 | 0.000E+00 | 0.000E+00 | 1.510E-07 | 7.413E-07 |
| Unigene446572 | rosB | 0.000E+00 | 0.000E+00 | 0.000E+00 | 1.458E-07 | 0.000E+00 | 0.000E+00 | 0.000E+00 | 0.000E+00 | 0.000E+00 | 0.000E+00 | 0.000E+00 | 0.000E+00 |
| Unigene446664 | TaeA | 0.000E+00 | 2.320E-07 | 0.000E+00 | 0.000E+00 | 0.000E+00 | 0.000E+00 | 1.085E-06 | 2.916E-07 | 0.000E+00 | 0.000E+00 | 0.000E+00 | 0.000E+00 |
| Unigene446792 | oleC | 0.000E+00 | 5.047E-07 | 0.000E+00 | 1.857E-07 | 0.000E+00 | 0.000E+00 | 1.120E-06 | 0.000E+00 | 5.026E-08 | 0.000E+00 | 0.000E+00 | 0.000E+00 |
| Unigene446800 | APH(2'')-II | 0.000E+00 | 0.000E+00 | 0.000E+00 | 7.342E-08 | 0.000E+00 | 0.000E+00 | 6.839E-07 | 3.819E-07 | 0.000E+00 | 0.000E+00 | 2.911E-07 | 8.164E-07 |
| Unigene446865 | MCR-4.2 | 0.000E+00 | 0.000E+00 | 0.000E+00 | 0.000E+00 | 0.000E+00 | 0.000E+00 | 5.881E-07 | 0.000E+00 | 0.000E+00 | 0.000E+00 | 0.000E+00 | 0.000E+00 |
| Unigene446960 | farB | 1.543E-07 | 0.000E+00 | 0.000E+00 | 0.000E+00 | 0.000E+00 | 0.000E+00 | 0.000E+00 | 9.904E-07 | 0.000E+00 | 0.000E+00 | 0.000E+00 | 1.003E-06 |
| Unigene446978 | msbA | 7.209E-08 | 5.934E-08 | 7.074E-08 | 2.366E-07 | 2.803E-07 | 2.338E-07 | 1.137E-06 | 7.784E-07 | 7.880E-08 | 0.000E+00 | 5.360E-07 | 2.727E-06 |
| Unigene447030 | patA | 0.000E+00 | 0.000E+00 | 8.396E-08 | 0.000E+00 | 3.327E-07 | 1.850E-07 | 9.707E-07 | 1.099E-06 | 7.014E-08 | 2.383E-07 | 3.915E-07 | 9.609E-07 |
| Unigene447031 | Corynebac | 0.000E+00 | 0.000E+00 | 2.966E-07 | 1.221E-07 | 0.000E+00 | 0.000E+00 | 4.014E-07 | 0.000E+00 | 0.000E+00 | 0.000E+00 | 6.915E-08 | 0.000E+00 |
| Unigene447032 | AxyY | 0.000E+00 | 0.000E+00 | 0.000E+00 | 0.000E+00 | 4.798E-07 | 0.000E+00 | 0.000E+00 | 0.000E+00 | 0.000E+00 | 0.000E+00 | 0.000E+00 | 0.000E+00 |
| Unigene447035 | patB | 1.035E-07 | 0.000E+00 | 1.015E-07 | 1.045E-07 | 4.215E-07 | 0.000E+00 | 8.779E-07 | 1.208E-06 | 0.000E+00 | 0.000E+00 | 7.101E-07 | 1.468E-06 |
| Unigene447061 | vatH | 0.000E+00 | 0.000E+00 | 0.000E+00 | 0.000E+00 | 0.000E+00 | 0.000E+00 | 1.191E-05 | 0.000E+00 | 0.000E+00 | 0.000E+00 | 0.000E+00 | 1.386E-05 |
| Unigene447124 | H-NS | 0.000E+00 | 0.000E+00 | 0.000E+00 | 0.000E+00 | 0.000E+00 | 1.723E-07 | 1.588E-06 | 0.000E+00 | 0.000E+00 | 0.000E+00 | 0.000E+00 | 0.000E+00 |
| Unigene447139 | vanTN | 0.000E+00 | 0.000E+00 | 1.479E-07 | 0.000E+00 | 1.005E-06 | 0.000E+00 | 1.801E-06 | 7.036E-07 | 0.000E+00 | 0.000E+00 | 0.000E+00 | 0.000E+00 |
| Unigene447274 | Streptomy | 0.000E+00 | 0.000E+00 | 0.000E+00 | 0.000E+00 | 0.000E+00 | 0.000E+00 | 8.157E-07 | 0.000E+00 | 0.000E+00 | 0.000E+00 | 0.000E+00 | 0.000E+00 |
| Unigene447443 | macB | 0.000E+00 | 0.000E+00 | 0.000E+00 | 0.000E+00 | 0.000E+00 | 0.000E+00 | 9.022E-07 | 0.000E+00 | 0.000E+00 | 0.000E+00 | 0.000E+00 | 0.000E+00 |
| Unigene447444 | evgA | 0.000E+00 | 0.000E+00 | 0.000E+00 | 0.000E+00 | 0.000E+00 | 0.000E+00 | 4.785E-07 | 0.000E+00 | 0.000E+00 | 0.000E+00 | 0.000E+00 | 0.000E+00 |
| Unigene447450 | adeN | 0.000E+00 | 0.000E+00 | 0.000E+00 | 0.000E+00 | 0.000E+00 | 0.000E+00 | 4.600E-07 | 2.156E-07 | 0.000E+00 | 0.000E+00 | 0.000E+00 | 1.365E-06 |
| Unigene447503 | oleC | 0.000E+00 | 0.000E+00 | 0.000E+00 | 0.000E+00 | 0.000E+00 | 0.000E+00 | 6.570E-07 | 0.000E+00 | 0.000E+00 | 0.000E+00 | 0.000E+00 | 0.000E+00 |
| Unigene447505 | oleC | 0.000E+00 | 0.000E+00 | 0.000E+00 | 0.000E+00 | 0.000E+00 | 0.000E+00 | 6.250E-07 | 0.000E+00 | 0.000E+00 | 0.000E+00 | 0.000E+00 | 0.000E+00 |
| Unigene447534 | MuxC | 0.000E+00 | 0.000E+00 | 0.000E+00 | 0.000E+00 | 0.000E+00 | 0.000E+00 | 6.863E-07 | 4.258E-07 | 0.000E+00 | 0.000E+00 | 7.093E-07 | 4.743E-07 |
| Unigene447595 | TaeA | 0.000E+00 | 0.000E+00 | 9.302E-08 | 0.000E+00 | 0.000E+00 | 0.000E+00 | 1.102E-06 | 3.320E-07 | 0.000E+00 | 0.000E+00 | 0.000E+00 | 0.000E+00 |
| Unigene447610 | patA | 0.000E+00 | 0.000E+00 | 0.000E+00 | 0.000E+00 | 0.000E+00 | 0.000E+00 | 1.232E-06 | 0.000E+00 | 0.000E+00 | 0.000E+00 | 0.000E+00 | 0.000E+00 |
| Unigene447760 | macB | 0.000E+00 | 0.000E+00 | 0.000E+00 | 0.000E+00 | 0.000E+00 | 0.000E+00 | 6.590E-07 | 0.000E+00 | 0.000E+00 | 0.000E+00 | 0.000E+00 | 0.000E+00 |
| Unigene447828 | MexL | 2.040E-07 | 2.239E-07 | 0.000E+00 | 0.000E+00 | 1.700E-07 | 0.000E+00 | 5.080E-07 | 0.000E+00 | 0.000E+00 | 0.000E+00 | 0.000E+00 | 0.000E+00 |
| Unigene447848 | tetB(P) | 0.000E+00 | 0.000E+00 | 0.000E+00 | 0.000E+00 | 3.415E-07 | 0.000E+00 | 8.081E-07 | 5.383E-07 | 0.000E+00 | 0.000E+00 | 0.000E+00 | 0.000E+00 |
| Unigene447932 | dfrA3 | 0.000E+00 | 0.000E+00 | 0.000E+00 | 0.000E+00 | 0.000E+00 | 2.629E-07 | 0.000E+00 | 0.000E+00 | 0.000E+00 | 0.000E+00 | 0.000E+00 | 4.311E-07 |
| Unigene448048 | evgS | 0.000E+00 | 0.000E+00 | 0.000E+00 | 0.000E+00 | 0.000E+00 | 0.000E+00 | 7.939E-07 | 0.000E+00 | 0.000E+00 | 0.000E+00 | 0.000E+00 | 0.000E+00 |
| Unigene448069 | catB2 | 0.000E+00 | 0.000E+00 | 0.000E+00 | 0.000E+00 | 0.000E+00 | 0.000E+00 | 7.537E-07 | 7.949E-07 | 0.000E+00 | 0.000E+00 | 0.000E+00 | 1.020E-06 |
| Unigene448074 | Bifidobacte | 0.000E+00 | 0.000E+00 | 0.000E+00 | 0.000E+00 | 0.000E+00 | 0.000E+00 | 3.520E-07 | 0.000E+00 | 0.000E+00 | 0.000E+00 | 0.000E+00 | 0.000E+00 |
| Unigene448151 | TaeA | 0.000E+00 | 0.000E+00 | 0.000E+00 | 0.000E+00 | 0.000E+00 | 0.000E+00 | 4.378E-07 | 0.000E+00 | 0.000E+00 | 0.000E+00 | 0.000E+00 | 0.000E+00 |
| Unigene448191 | oleB | 0.000E+00 | 0.000E+00 | 0.000E+00 | 0.000E+00 | 0.000E+00 | 0.000E+00 | 1.319E-06 | 0.000E+00 | 0.000E+00 | 0.000E+00 | 0.000E+00 | 0.000E+00 |
| Unigene448212 | oleC | 0.000E+00 | 5.846E-08 | 0.000E+00 | 0.000E+00 | 5.918E-08 | 0.000E+00 | 5.306E-07 | 0.000E+00 | 5.822E-08 | 0.000E+00 | 0.000E+00 | 4.407E-07 |
| Unigene448302 | tetA(58) | 0.000E+00 | 0.000E+00 | 0.000E+00 | 0.000E+00 | 0.000E+00 | 0.000E+00 | 7.969E-07 | 3.735E-07 | 4.372E-08 | 0.000E+00 | 6.863E-07 | 1.040E-06 |
| Unigene448496 | mtrA | 0.000E+00 | 0.000E+00 | 0.000E+00 | 0.000E+00 | 3.579E-07 | 0.000E+00 | 4.279E-07 | 0.000E+00 | 0.000E+00 | 0.000E+00 | 1.474E-07 | 0.000E+00 |
| Unigene448518 | blt | 0.000E+00 | 0.000E+00 | 0.000E+00 | 0.000E+00 | 0.000E+00 | 0.000E+00 | 6.865E-07 | 0.000E+00 | 0.000E+00 | 0.000E+00 | 0.000E+00 | 0.000E+00 |
| Unigene448561 | cpxA | 0.000E+00 | 0.000E+00 | 0.000E+00 | 0.000E+00 | 0.000E+00 | 0.000E+00 | 4.220E-07 | 0.000E+00 | 0.000E+00 | 0.000E+00 | 0.000E+00 | 0.000E+00 |
| Unigene448573 | MuxB | 0.000E+00 | 0.000E+00 | 0.000E+00 | 0.000E+00 | 1.594E-07 | 0.000E+00 | 0.000E+00 | 3.349E-07 | 0.000E+00 | 0.000E+00 | 0.000E+00 | 0.000E+00 |
| Unigene448580 | evgS | 0.000E+00 | 5.954E-08 | 0.000E+00 | 0.000E+00 | 2.009E-07 | 2.150E-07 | 6.605E-07 | 0.000E+00 | 0.000E+00 | 1.813E-07 | 2.689E-07 | 1.026E-06 |
| Unigene448590 | sul4 | 0.000E+00 | 0.000E+00 | 0.000E+00 | 0.000E+00 | 0.000E+00 | 0.000E+00 | 6.854E-07 | 0.000E+00 | 0.000E+00 | 0.000E+00 | 0.000E+00 | 0.000E+00 |

| | | | | | | | | | | | | | |
|---|---|---|---|---|---|---|---|---|---|---|---|---|---|
| Unigene448591 | macB | 1.908E-07 | 0.000E+00 | 0.000E+00 | 1.156E-07 | 0.000E+00 | 1.650E-07 | 1.605E-06 | 1.113E-06 | 0.000E+00 | 0.000E+00 | 0.000E+00 | 3.247E-06 |
| Unigene448592 | vatF | 0.000E+00 | 0.000E+00 | 0.000E+00 | 0.000E+00 | 0.000E+00 | 4.496E-07 | 8.054E-07 | 9.708E-07 | 0.000E+00 | 0.000E+00 | 2.973E-07 | 2.273E-06 |
| Unigene448596 | clbB | 0.000E+00 | 0.000E+00 | 0.000E+00 | 0.000E+00 | 0.000E+00 | 0.000E+00 | 7.443E-07 | 4.859E-07 | 0.000E+00 | 0.000E+00 | 0.000E+00 | 1.893E-07 |
| Unigene448670 | Staphylocc | 0.000E+00 | 6.947E-08 | 4.658E-08 | 0.000E+00 | 3.340E-07 | 2.053E-07 | 1.103E-06 | 1.201E-06 | 0.000E+00 | 0.000E+00 | 4.163E-07 | 8.978E-07 |
| Unigene448675 | acrB | 0.000E+00 | 0.000E+00 | 0.000E+00 | 0.000E+00 | 0.000E+00 | 0.000E+00 | 7.392E-07 | 0.000E+00 | 0.000E+00 | 0.000E+00 | 0.000E+00 | 4.386E-07 |
| Unigene448679 | ugd | 0.000E+00 | 0.000E+00 | 0.000E+00 | 0.000E+00 | 0.000E+00 | 0.000E+00 | 1.164E-06 | 0.000E+00 | 0.000E+00 | 0.000E+00 | 0.000E+00 | 0.000E+00 |
| Unigene448725 | Corynebac | 0.000E+00 | 0.000E+00 | 0.000E+00 | 0.000E+00 | 0.000E+00 | 0.000E+00 | 6.535E-07 | 0.000E+00 | 0.000E+00 | 0.000E+00 | 0.000E+00 | 0.000E+00 |
| Unigene448726 | msbA | 0.000E+00 | 0.000E+00 | 0.000E+00 | 0.000E+00 | 0.000E+00 | 0.000E+00 | 7.964E-07 | 0.000E+00 | 0.000E+00 | 0.000E+00 | 0.000E+00 | 0.000E+00 |
| Unigene448802 | mgrA | 2.729E-07 | 0.000E+00 | 1.530E-07 | 0.000E+00 | 5.197E-07 | 1.686E-07 | 6.041E-07 | 4.551E-07 | 0.000E+00 | 0.000E+00 | 0.000E+00 | 4.608E-07 |
| Unigene448842 | Staphylocc | 0.000E+00 | 0.000E+00 | 0.000E+00 | 0.000E+00 | 0.000E+00 | 0.000E+00 | 9.577E-07 | 0.000E+00 | 0.000E+00 | 0.000E+00 | 0.000E+00 | 0.000E+00 |
| Unigene448944 | poxtA | 0.000E+00 | 0.000E+00 | 0.000E+00 | 0.000E+00 | 0.000E+00 | 0.000E+00 | 1.050E-06 | 0.000E+00 | 0.000E+00 | 0.000E+00 | 0.000E+00 | 0.000E+00 |
| Unigene448976 | MSI-1 | 0.000E+00 | 0.000E+00 | 0.000E+00 | 0.000E+00 | 0.000E+00 | 0.000E+00 | 6.437E-07 | 0.000E+00 | 0.000E+00 | 0.000E+00 | 0.000E+00 | 0.000E+00 |
| Unigene449049 | carA | 0.000E+00 | 4.490E-08 | 0.000E+00 | 0.000E+00 | 0.000E+00 | 4.423E-08 | 4.075E-07 | 1.910E-07 | 0.000E+00 | 2.735E-07 | 6.552E-07 | 9.188E-07 |
| Unigene449073 | msbA | 0.000E+00 | 0.000E+00 | 0.000E+00 | 0.000E+00 | 0.000E+00 | 0.000E+00 | 8.808E-07 | 0.000E+00 | 0.000E+00 | 0.000E+00 | 0.000E+00 | 0.000E+00 |
| Unigene449208 | carA | 3.913E-08 | 1.718E-07 | 2.496E-07 | 1.383E-07 | 5.434E-07 | 4.653E-07 | 7.578E-07 | 1.096E-06 | 0.000E+00 | 8.717E-08 | 6.490E-07 | 1.596E-06 |
| Unigene449273 | arlR | 0.000E+00 | 0.000E+00 | 2.754E-07 | 0.000E+00 | 8.836E-07 | 0.000E+00 | 1.087E-06 | 8.737E-07 | 0.000E+00 | 0.000E+00 | 4.281E-07 | 1.770E-06 |
| Unigene449274 | adeS | 1.930E-07 | 1.854E-07 | 4.024E-07 | 0.000E+00 | 6.969E-07 | 2.347E-07 | 8.811E-07 | 6.195E-07 | 0.000E+00 | 0.000E+00 | 3.588E-07 | 1.226E-06 |
| Unigene449416 | chrB | 0.000E+00 | 0.000E+00 | 0.000E+00 | 0.000E+00 | 0.000E+00 | 0.000E+00 | 1.664E-06 | 0.000E+00 | 0.000E+00 | 0.000E+00 | 0.000E+00 | 0.000E+00 |
| Unigene449461 | patA | 0.000E+00 | 0.000E+00 | 0.000E+00 | 0.000E+00 | 0.000E+00 | 0.000E+00 | 1.236E-06 | 0.000E+00 | 0.000E+00 | 0.000E+00 | 0.000E+00 | 0.000E+00 |
| Unigene449475 | vanHB | 0.000E+00 | 0.000E+00 | 0.000E+00 | 0.000E+00 | 0.000E+00 | 0.000E+00 | 4.447E-07 | 2.085E-07 | 0.000E+00 | 0.000E+00 | 4.596E-07 | 0.000E+00 |
| Unigene449501 | patA | 0.000E+00 | 0.000E+00 | 0.000E+00 | 0.000E+00 | 0.000E+00 | 0.000E+00 | 6.654E-07 | 5.566E-07 | 0.000E+00 | 0.000E+00 | 0.000E+00 | 0.000E+00 |
| Unigene449531 | efrA | 0.000E+00 | 0.000E+00 | 0.000E+00 | 0.000E+00 | 0.000E+00 | 0.000E+00 | 6.904E-07 | 0.000E+00 | 0.000E+00 | 0.000E+00 | 0.000E+00 | 0.000E+00 |
| Unigene449534 | tet(V) | 0.000E+00 | 0.000E+00 | 0.000E+00 | 0.000E+00 | 0.000E+00 | 0.000E+00 | 1.886E-06 | 0.000E+00 | 0.000E+00 | 0.000E+00 | 0.000E+00 | 0.000E+00 |
| Unigene449536 | mtrA | 0.000E+00 | 0.000E+00 | 0.000E+00 | 0.000E+00 | 0.000E+00 | 0.000E+00 | 9.721E-07 | 0.000E+00 | 0.000E+00 | 0.000E+00 | 0.000E+00 | 0.000E+00 |
| Unigene449551 | macB | 0.000E+00 | 0.000E+00 | 0.000E+00 | 0.000E+00 | 0.000E+00 | 0.000E+00 | 1.425E-06 | 0.000E+00 | 0.000E+00 | 0.000E+00 | 0.000E+00 | 0.000E+00 |
| Unigene449703 | oleB | 0.000E+00 | 0.000E+00 | 0.000E+00 | 0.000E+00 | 0.000E+00 | 1.586E-07 | 5.954E-07 | 0.000E+00 | 0.000E+00 | 0.000E+00 | 0.000E+00 | 0.000E+00 |
| Unigene449757 | novA | 0.000E+00 | 0.000E+00 | 0.000E+00 | 0.000E+00 | 0.000E+00 | 0.000E+00 | 1.301E-06 | 0.000E+00 | 0.000E+00 | 0.000E+00 | 4.137E-07 | 4.810E-07 |
| Unigene449792 | patB | 0.000E+00 | 0.000E+00 | 0.000E+00 | 0.000E+00 | 0.000E+00 | 0.000E+00 | 4.427E-07 | 0.000E+00 | 0.000E+00 | 0.000E+00 | 4.576E-08 | 0.000E+00 |
| Unigene449885 | patA | 0.000E+00 | 0.000E+00 | 0.000E+00 | 0.000E+00 | 0.000E+00 | 0.000E+00 | 0.000E+00 | 7.274E-07 | 0.000E+00 | 0.000E+00 | 0.000E+00 | 9.821E-07 |
| Unigene450024 | vanHF | 0.000E+00 | 0.000E+00 | 0.000E+00 | 0.000E+00 | 0.000E+00 | 0.000E+00 | 8.747E-07 | 0.000E+00 | 0.000E+00 | 0.000E+00 | 0.000E+00 | 0.000E+00 |
| Unigene450232 | oleC | 0.000E+00 | 0.000E+00 | 0.000E+00 | 0.000E+00 | 0.000E+00 | 0.000E+00 | 5.539E-07 | 0.000E+00 | 0.000E+00 | 0.000E+00 | 0.000E+00 | 0.000E+00 |
| Unigene450239 | bcrA | 0.000E+00 | 0.000E+00 | 0.000E+00 | 0.000E+00 | 0.000E+00 | 0.000E+00 | 4.210E-07 | 0.000E+00 | 0.000E+00 | 0.000E+00 | 0.000E+00 | 0.000E+00 |
| Unigene450258 | tetA(58) | 0.000E+00 | 0.000E+00 | 0.000E+00 | 0.000E+00 | 0.000E+00 | 9.561E-08 | 7.830E-07 | 1.067E-06 | 6.444E-08 | 0.000E+00 | 5.733E-07 | 1.045E-06 |
| Unigene450334 | MexB | 3.104E-08 | 3.179E-07 | 0.000E+00 | 0.000E+00 | 8.851E-07 | 0.000E+00 | 1.053E-06 | 0.000E+00 | 3.166E-07 | 0.000E+00 | 0.000E+00 | 2.201E-07 |
| Unigene450339 | macB | 0.000E+00 | 1.845E-07 | 2.887E-07 | 1.698E-07 | 8.872E-07 | 6.361E-07 | 1.349E-06 | 1.079E-06 | 0.000E+00 | 0.000E+00 | 4.327E-07 | 1.888E-06 |
| Unigene450354 | eptA | 0.000E+00 | 0.000E+00 | 0.000E+00 | 0.000E+00 | 8.949E-07 | 0.000E+00 | 2.335E-06 | 0.000E+00 | 0.000E+00 | 0.000E+00 | 0.000E+00 | 0.000E+00 |
| Unigene450522 | Streptomy | 4.678E-08 | 0.000E+00 | 0.000E+00 | 0.000E+00 | 1.039E-07 | 1.011E-07 | 4.142E-07 | 3.276E-07 | 1.023E-07 | 0.000E+00 | 2.676E-07 | 2.212E-07 |
| Unigene450586 | mdtB | 0.000E+00 | 0.000E+00 | 1.240E-07 | 1.276E-07 | 0.000E+00 | 6.829E-08 | 9.322E-07 | 3.441E-07 | 4.603E-08 | 0.000E+00 | 1.445E-07 | 4.231E-07 |
| Unigene450621 | CTX-M-16 | 0.000E+00 | 0.000E+00 | 4.140E-07 | 0.000E+00 | 0.000E+00 | 3.421E-07 | 5.838E-07 | 3.694E-07 | 0.000E+00 | 0.000E+00 | 0.000E+00 | 6.858E-07 |
| Unigene450658 | mdtG | 0.000E+00 | 0.000E+00 | 0.000E+00 | 0.000E+00 | 0.000E+00 | 0.000E+00 | 2.567E-06 | 1.291E-06 | 0.000E+00 | 0.000E+00 | 0.000E+00 | 0.000E+00 |
| Unigene450679 | mgrA | 0.000E+00 | 0.000E+00 | 0.000E+00 | 0.000E+00 | 0.000E+00 | 0.000E+00 | 6.412E-07 | 0.000E+00 | 0.000E+00 | 0.000E+00 | 0.000E+00 | 0.000E+00 |
| Unigene450688 | vanRM | 0.000E+00 | 0.000E+00 | 0.000E+00 | 0.000E+00 | 0.000E+00 | 0.000E+00 | 5.671E-07 | 0.000E+00 | 0.000E+00 | 0.000E+00 | 0.000E+00 | 0.000E+00 |
| Unigene450766 | bcrA | 0.000E+00 | 0.000E+00 | 0.000E+00 | 0.000E+00 | 0.000E+00 | 0.000E+00 | 7.767E-07 | 0.000E+00 | 0.000E+00 | 0.000E+00 | 0.000E+00 | 0.000E+00 |
| Unigene450846 | sdiA | 0.000E+00 | 0.000E+00 | 0.000E+00 | 0.000E+00 | 2.533E-07 | 0.000E+00 | 8.074E-07 | 0.000E+00 | 0.000E+00 | 0.000E+00 | 5.215E-07 | 1.240E-06 |
| Unigene450859 | oleC | 0.000E+00 | 0.000E+00 | 0.000E+00 | 0.000E+00 | 0.000E+00 | 1.268E-07 | 8.112E-07 | 8.556E-07 | 0.000E+00 | 0.000E+00 | 3.689E-07 | 0.000E+00 |
| Unigene450902 | AcrS | 2.059E-07 | 5.650E-08 | 3.031E-07 | 0.000E+00 | 8.579E-07 | 0.000E+00 | 0.000E+00 | 0.000E+00 | 0.000E+00 | 0.000E+00 | 2.356E-07 | 0.000E+00 |
| Unigene450952 | vanRE | 0.000E+00 | 0.000E+00 | 4.969E-08 | 6.392E-08 | 0.000E+00 | 1.916E-07 | 9.667E-07 | 1.921E-07 | 1.384E-08 | 0.000E+00 | 1.158E-07 | 1.796E-07 |
| Unigene450984 | tet(41) | 0.000E+00 | 0.000E+00 | 0.000E+00 | 0.000E+00 | 0.000E+00 | 0.000E+00 | 1.161E-06 | 0.000E+00 | 0.000E+00 | 0.000E+00 | 0.000E+00 | 0.000E+00 |
| Unigene451090 | optrA | 0.000E+00 | 0.000E+00 | 0.000E+00 | 0.000E+00 | 0.000E+00 | 0.000E+00 | 5.641E-07 | 0.000E+00 | 0.000E+00 | 0.000E+00 | 0.000E+00 | 0.000E+00 |
| Unigene451100 | bcrA | 0.000E+00 | 0.000E+00 | 0.000E+00 | 0.000E+00 | 0.000E+00 | 0.000E+00 | 7.401E-07 | 7.096E-07 | 0.000E+00 | 0.000E+00 | 0.000E+00 | 0.000E+00 |
| Unigene451116 | Acinetobac | 0.000E+00 | 0.000E+00 | 0.000E+00 | 0.000E+00 | 0.000E+00 | 0.000E+00 | 1.451E-06 | 0.000E+00 | 0.000E+00 | 0.000E+00 | 0.000E+00 | 0.000E+00 |
| Unigene451185 | Streptomy | 0.000E+00 | 3.192E-08 | 0.000E+00 | 0.000E+00 | 0.000E+00 | 3.144E-07 | 8.046E-07 | 0.000E+00 | 0.000E+00 | 0.000E+00 | 1.996E-07 | 1.719E-07 |
| Unigene451234 | mtrA | 0.000E+00 | 0.000E+00 | 0.000E+00 | 0.000E+00 | 1.017E-07 | 9.900E-08 | 1.419E-06 | 0.000E+00 | 0.000E+00 | 0.000E+00 | 4.714E-07 | 2.165E-07 |
| Unigene451236 | rosA | 0.000E+00 | 0.000E+00 | 0.000E+00 | 0.000E+00 | 1.784E-07 | 1.157E-07 | 6.221E-07 | 1.187E-06 | 0.000E+00 | 0.000E+00 | 0.000E+00 | 5.379E-07 |
| Unigene451265 | oleC | 0.000E+00 | 0.000E+00 | 0.000E+00 | 0.000E+00 | 0.000E+00 | 0.000E+00 | 8.031E-07 | 0.000E+00 | 0.000E+00 | 0.000E+00 | 0.000E+00 | 0.000E+00 |
| Unigene451408 | evgS | 0.000E+00 | 0.000E+00 | 0.000E+00 | 0.000E+00 | 0.000E+00 | 0.000E+00 | 7.282E-07 | 7.680E-07 | 0.000E+00 | 0.000E+00 | 0.000E+00 | 0.000E+00 |
| Unigene451437 | MexK | 0.000E+00 | 0.000E+00 | 0.000E+00 | 4.490E-08 | 0.000E+00 | 0.000E+00 | 3.937E-07 | 0.000E+00 | 0.000E+00 | 0.000E+00 | 0.000E+00 | 0.000E+00 |
| Unigene451454 | vanYB | 0.000E+00 | 0.000E+00 | 0.000E+00 | 0.000E+00 | 2.160E-07 | 0.000E+00 | 2.870E-07 | 1.513E-07 | 0.000E+00 | 0.000E+00 | 0.000E+00 | 0.000E+00 |

| | | | | | | | | | | | | | |
|---|---|---|---|---|---|---|---|---|---|---|---|---|---|
| Unigene451534 | patA | 3.961E-07 | 0.000E+00 | 0.000E+00 | 2.000E-07 | 0.000E+00 | 0.000E+00 | 8.402E-07 | 4.623E-07 | 0.000E+00 | 0.000E+00 | 0.000E+00 | 5.462E-07 |
| Unigene451580 | vanTrL | 0.000E+00 | 0.000E+00 | 0.000E+00 | 0.000E+00 | 0.000E+00 | 0.000E+00 | 0.000E+00 | 0.000E+00 | 0.000E+00 | 0.000E+00 | 0.000E+00 | 6.359E-07 |
| Unigene451610 | tetB(60) | 0.000E+00 | 0.000E+00 | 5.888E-07 | 1.045E-07 | 0.000E+00 | 0.000E+00 | 1.832E-06 | 6.038E-07 | 0.000E+00 | 0.000E+00 | 0.000E+00 | 1.394E-06 |
| Unigene451634 | novA | 0.000E+00 | 0.000E+00 | 0.000E+00 | 0.000E+00 | 1.087E-07 | 0.000E+00 | 6.225E-07 | 4.853E-07 | 0.000E+00 | 0.000E+00 | 3.077E-07 | 2.891E-07 |
| Unigene451668 | carA | 0.000E+00 | 0.000E+00 | 0.000E+00 | 0.000E+00 | 0.000E+00 | 0.000E+00 | 6.312E-07 | 0.000E+00 | 0.000E+00 | 0.000E+00 | 0.000E+00 | 0.000E+00 |
| Unigene451792 | MdtK | 0.000E+00 | 0.000E+00 | 0.000E+00 | 0.000E+00 | 0.000E+00 | 0.000E+00 | 3.290E-07 | 0.000E+00 | 0.000E+00 | 0.000E+00 | 0.000E+00 | 0.000E+00 |
| Unigene451823 | efrA | 0.000E+00 | 0.000E+00 | 0.000E+00 | 0.000E+00 | 0.000E+00 | 0.000E+00 | 2.328E-06 | 0.000E+00 | 0.000E+00 | 0.000E+00 | 9.763E-07 | 0.000E+00 |
| Unigene451849 | OpmD | 0.000E+00 | 0.000E+00 | 0.000E+00 | 0.000E+00 | 0.000E+00 | 0.000E+00 | 5.287E-07 | 0.000E+00 | 0.000E+00 | 0.000E+00 | 0.000E+00 | 0.000E+00 |
| Unigene451874 | adeH | 0.000E+00 | 0.000E+00 | 0.000E+00 | 0.000E+00 | 0.000E+00 | 0.000E+00 | 9.884E-07 | 0.000E+00 | 0.000E+00 | 0.000E+00 | 0.000E+00 | 0.000E+00 |
| Unigene451905 | OprA | 0.000E+00 | 6.902E-07 | 6.627E-07 | 0.000E+00 | 0.000E+00 | 0.000E+00 | 8.506E-07 | 0.000E+00 | 0.000E+00 | 0.000E+00 | 0.000E+00 | 0.000E+00 |
| Unigene451939 | vanC | 0.000E+00 | 0.000E+00 | 0.000E+00 | 0.000E+00 | 0.000E+00 | 0.000E+00 | 8.241E-07 | 0.000E+00 | 0.000E+00 | 0.000E+00 | 0.000E+00 | 0.000E+00 |
| Unigene451946 | AcrF | 0.000E+00 | 0.000E+00 | 0.000E+00 | 0.000E+00 | 0.000E+00 | 0.000E+00 | 4.894E-07 | 0.000E+00 | 0.000E+00 | 0.000E+00 | 0.000E+00 | 0.000E+00 |
| Unigene451952 | vanSM | 2.343E-07 | 4.550E-07 | 4.244E-07 | 3.458E-07 | 0.000E+00 | 0.000E+00 | 3.950E-06 | 3.282E-06 | 5.713E-07 | 1.104E-06 | 1.278E-06 | 3.238E-06 |
| Unigene452082 | MexC | 0.000E+00 | 0.000E+00 | 0.000E+00 | 0.000E+00 | 0.000E+00 | 0.000E+00 | 7.739E-07 | 0.000E+00 | 0.000E+00 | 0.000E+00 | 0.000E+00 | 0.000E+00 |
| Unigene452083 | evgS | 0.000E+00 | 0.000E+00 | 0.000E+00 | 0.000E+00 | 0.000E+00 | 0.000E+00 | 5.311E-07 | 0.000E+00 | 0.000E+00 | 0.000E+00 | 0.000E+00 | 0.000E+00 |
| Unigene452149 | farB | 0.000E+00 | 0.000E+00 | 0.000E+00 | 0.000E+00 | 0.000E+00 | 0.000E+00 | 7.208E-07 | 0.000E+00 | 0.000E+00 | 0.000E+00 | 0.000E+00 | 4.016E-07 |
| Unigene452158 | patB | 0.000E+00 | 0.000E+00 | 1.669E-07 | 3.436E-07 | 5.670E-07 | 0.000E+00 | 1.130E-06 | 7.446E-07 | 0.000E+00 | 0.000E+00 | 9.730E-07 | 1.408E-06 |
| Unigene452164 | Staphylocc | 0.000E+00 | 0.000E+00 | 0.000E+00 | 0.000E+00 | 0.000E+00 | 0.000E+00 | 9.161E-07 | 0.000E+00 | 0.000E+00 | 0.000E+00 | 0.000E+00 | 0.000E+00 |
| Unigene452239 | Acinetoba | 0.000E+00 | 0.000E+00 | 0.000E+00 | 0.000E+00 | 0.000E+00 | 0.000E+00 | 8.105E-07 | 0.000E+00 | 0.000E+00 | 0.000E+00 | 0.000E+00 | 0.000E+00 |
| Unigene452274 | adeL | 0.000E+00 | 0.000E+00 | 0.000E+00 | 0.000E+00 | 0.000E+00 | 0.000E+00 | 5.200E-07 | 0.000E+00 | 0.000E+00 | 0.000E+00 | 0.000E+00 | 0.000E+00 |
| Unigene452372 | macB | 0.000E+00 | 0.000E+00 | 0.000E+00 | 0.000E+00 | 0.000E+00 | 0.000E+00 | 2.132E-06 | 0.000E+00 | 0.000E+00 | 0.000E+00 | 0.000E+00 | 0.000E+00 |
| Unigene452518 | tetA(60) | 0.000E+00 | 0.000E+00 | 0.000E+00 | 0.000E+00 | 0.000E+00 | 0.000E+00 | 7.835E-07 | 0.000E+00 | 0.000E+00 | 0.000E+00 | 0.000E+00 | 0.000E+00 |
| Unigene452523 | vanSG | 0.000E+00 | 0.000E+00 | 0.000E+00 | 0.000E+00 | 0.000E+00 | 0.000E+00 | 3.424E-07 | 0.000E+00 | 0.000E+00 | 0.000E+00 | 0.000E+00 | 0.000E+00 |
| Unigene452535 | Pseudomo | 0.000E+00 | 0.000E+00 | 0.000E+00 | 0.000E+00 | 0.000E+00 | 0.000E+00 | 7.591E-07 | 0.000E+00 | 0.000E+00 | 0.000E+00 | 0.000E+00 | 0.000E+00 |
| Unigene452549 | adeJ | 0.000E+00 | 0.000E+00 | 0.000E+00 | 0.000E+00 | 0.000E+00 | 0.000E+00 | 3.435E-07 | 0.000E+00 | 0.000E+00 | 0.000E+00 | 0.000E+00 | 0.000E+00 |
| Unigene452567 | cmeB | 0.000E+00 | 0.000E+00 | 0.000E+00 | 0.000E+00 | 0.000E+00 | 0.000E+00 | 0.000E+00 | 0.000E+00 | 0.000E+00 | 0.000E+00 | 0.000E+00 | 3.401E-07 |
| Unigene452605 | macB | 0.000E+00 | 0.000E+00 | 0.000E+00 | 0.000E+00 | 0.000E+00 | 0.000E+00 | 1.014E-06 | 0.000E+00 | 2.002E-07 | 0.000E+00 | 0.000E+00 | 0.000E+00 |
| Unigene452641 | adeR | 0.000E+00 | 0.000E+00 | 0.000E+00 | 0.000E+00 | 0.000E+00 | 0.000E+00 | 5.378E-07 | 0.000E+00 | 0.000E+00 | 0.000E+00 | 0.000E+00 | 1.067E-06 |
| Unigene452703 | patA | 0.000E+00 | 0.000E+00 | 0.000E+00 | 0.000E+00 | 0.000E+00 | 0.000E+00 | 7.531E-07 | 0.000E+00 | 0.000E+00 | 0.000E+00 | 0.000E+00 | 0.000E+00 |
| Unigene452723 | lmrC | 0.000E+00 | 0.000E+00 | 0.000E+00 | 0.000E+00 | 0.000E+00 | 0.000E+00 | 1.052E-06 | 0.000E+00 | 0.000E+00 | 0.000E+00 | 0.000E+00 | 3.825E-07 |
| Unigene452725 | tva(A) | 0.000E+00 | 0.000E+00 | 0.000E+00 | 0.000E+00 | 0.000E+00 | 1.243E-07 | 9.161E-07 | 0.000E+00 | 0.000E+00 | 0.000E+00 | 0.000E+00 | 1.060E-06 |
| Unigene452976 | oleC | 0.000E+00 | 0.000E+00 | 0.000E+00 | 0.000E+00 | 0.000E+00 | 0.000E+00 | 9.619E-07 | 1.449E-07 | 0.000E+00 | 0.000E+00 | 0.000E+00 | 2.446E-07 |
| Unigene452983 | efrA | 1.600E-07 | 0.000E+00 | 1.177E-07 | 0.000E+00 | 0.000E+00 | 1.730E-07 | 0.000E+00 | 7.937E-07 | 0.000E+00 | 0.000E+00 | 0.000E+00 | 6.619E-07 |
| Unigene453026 | vmlR | 0.000E+00 | 0.000E+00 | 8.447E-08 | 0.000E+00 | 2.869E-07 | 1.861E-07 | 1.334E-06 | 4.019E-07 | 0.000E+00 | 2.877E-07 | 9.354E-07 | 1.272E-06 |
| Unigene453037 | adeL | 0.000E+00 | 0.000E+00 | 0.000E+00 | 0.000E+00 | 0.000E+00 | 8.163E-07 | 4.178E-07 | 0.000E+00 | 0.000E+00 | 0.000E+00 | 0.000E+00 | 0.000E+00 |
| Unigene453042 | oleC | 0.000E+00 | 0.000E+00 | 0.000E+00 | 0.000E+00 | 0.000E+00 | 0.000E+00 | 6.009E-07 | 0.000E+00 | 0.000E+00 | 0.000E+00 | 0.000E+00 | 0.000E+00 |
| Unigene453070 | adeR | 0.000E+00 | 0.000E+00 | 4.078E-07 | 0.000E+00 | 0.000E+00 | 9.985E-08 | 5.622E-07 | 0.000E+00 | 0.000E+00 | 1.543E-07 | 0.000E+00 | 0.000E+00 |
| Unigene453071 | adeS | 0.000E+00 | 0.000E+00 | 1.915E-07 | 0.000E+00 | 1.239E-07 | 0.000E+00 | 1.142E-06 | 9.761E-08 | 0.000E+00 | 0.000E+00 | 3.826E-07 | 5.602E-07 |
| Unigene453162 | macB | 0.000E+00 | 0.000E+00 | 0.000E+00 | 0.000E+00 | 0.000E+00 | 0.000E+00 | 0.000E+00 | 6.465E-07 | 0.000E+00 | 0.000E+00 | 0.000E+00 | 0.000E+00 |
| Unigene453252 | oleB | 0.000E+00 | 0.000E+00 | 6.138E-08 | 0.000E+00 | 9.267E-08 | 0.000E+00 | 8.770E-07 | 4.868E-08 | 0.000E+00 | 0.000E+00 | 7.156E-08 | 2.218E-07 |
| Unigene453255 | oleB | 0.000E+00 | 0.000E+00 | 0.000E+00 | 0.000E+00 | 9.907E-08 | 0.000E+00 | 1.020E-06 | 1.041E-07 | 1.624E-08 | 9.934E-08 | 0.000E+00 | 0.000E+00 |
| Unigene453289 | cpxA | 0.000E+00 | 0.000E+00 | 4.810E-07 | 0.000E+00 | 0.000E+00 | 0.000E+00 | 1.031E-06 | 0.000E+00 | 0.000E+00 | 0.000E+00 | 0.000E+00 | 0.000E+00 |
| Unigene453335 | Vibrio cho | 0.000E+00 | 0.000E+00 | 0.000E+00 | 1.483E-07 | 0.000E+00 | 0.000E+00 | 0.000E+00 | 2.143E-07 | 0.000E+00 | 0.000E+00 | 0.000E+00 | 0.000E+00 |
| Unigene453379 | optrA | 0.000E+00 | 0.000E+00 | 0.000E+00 | 0.000E+00 | 0.000E+00 | 0.000E+00 | 8.862E-07 | 0.000E+00 | 0.000E+00 | 0.000E+00 | 0.000E+00 | 0.000E+00 |
| Unigene453399 | kdpE | 0.000E+00 | 0.000E+00 | 0.000E+00 | 0.000E+00 | 0.000E+00 | 0.000E+00 | 5.954E-07 | 0.000E+00 | 0.000E+00 | 0.000E+00 | 0.000E+00 | 0.000E+00 |
| Unigene453400 | smeS | 0.000E+00 | 0.000E+00 | 0.000E+00 | 0.000E+00 | 0.000E+00 | 0.000E+00 | 6.986E-07 | 4.912E-07 | 0.000E+00 | 0.000E+00 | 0.000E+00 | 0.000E+00 |
| Unigene453412 | MuxB | 1.869E-07 | 0.000E+00 | 1.376E-07 | 0.000E+00 | 1.687E-07 | 1.894E-07 | 8.922E-07 | 6.955E-07 | 0.000E+00 | 3.384E-07 | 0.000E+00 | 5.938E-07 |
| Unigene453490 | tet(39) | 0.000E+00 | 0.000E+00 | 0.000E+00 | 0.000E+00 | 0.000E+00 | 0.000E+00 | 3.219E-07 | 0.000E+00 | 0.000E+00 | 0.000E+00 | 0.000E+00 | 0.000E+00 |
| Unigene453545 | Acinetoba | 0.000E+00 | 0.000E+00 | 0.000E+00 | 0.000E+00 | 0.000E+00 | 0.000E+00 | 2.513E-06 | 0.000E+00 | 0.000E+00 | 0.000E+00 | 0.000E+00 | 0.000E+00 |
| Unigene453559 | NmcR | 0.000E+00 | 0.000E+00 | 0.000E+00 | 0.000E+00 | 0.000E+00 | 0.000E+00 | 5.109E-07 | 0.000E+00 | 0.000E+00 | 0.000E+00 | 5.281E-07 | 5.877E-07 |
| Unigene453566 | acrB | 4.981E-07 | 1.401E-06 | 1.242E-06 | 2.410E-07 | 2.836E-06 | 0.000E+00 | 2.102E-06 | 0.000E+00 | 0.000E+00 | 0.000E+00 | 6.053E-07 | 0.000E+00 |
| Unigene453822 | TriC | 0.000E+00 | 0.000E+00 | 0.000E+00 | 0.000E+00 | 0.000E+00 | 3.071E-07 | 1.179E-06 | 6.218E-07 | 0.000E+00 | 0.000E+00 | 4.468E-07 | 7.975E-07 |
| Unigene453823 | mdtE | 0.000E+00 | 0.000E+00 | 2.047E-07 | 0.000E+00 | 0.000E+00 | 0.000E+00 | 6.928E-07 | 5.567E-07 | 0.000E+00 | 0.000E+00 | 5.455E-07 | 0.000E+00 |
| Unigene453848 | Acinetoba | 0.000E+00 | 0.000E+00 | 0.000E+00 | 0.000E+00 | 0.000E+00 | 0.000E+00 | 1.132E-06 | 0.000E+00 | 0.000E+00 | 0.000E+00 | 0.000E+00 | 0.000E+00 |
| Unigene453986 | vanRF | 0.000E+00 | 0.000E+00 | 0.000E+00 | 0.000E+00 | 0.000E+00 | 0.000E+00 | 8.127E-07 | 0.000E+00 | 0.000E+00 | 0.000E+00 | 0.000E+00 | 0.000E+00 |
| Unigene453987 | patB | 0.000E+00 | 0.000E+00 | 0.000E+00 | 0.000E+00 | 0.000E+00 | 0.000E+00 | 8.040E-07 | 0.000E+00 | 0.000E+00 | 0.000E+00 | 1.558E-07 | 5.367E-07 |
| Unigene454014 | adeL | 0.000E+00 | 0.000E+00 | 0.000E+00 | 0.000E+00 | 0.000E+00 | 3.568E-07 | 8.767E-07 | 0.000E+00 | 0.000E+00 | 0.000E+00 | 0.000E+00 | 8.973E-07 |
| Unigene454078 | clbB | 0.000E+00 | 0.000E+00 | 0.000E+00 | 0.000E+00 | 0.000E+00 | 0.000E+00 | 2.541E-06 | 0.000E+00 | 0.000E+00 | 0.000E+00 | 0.000E+00 | 0.000E+00 |

| | | | | | | | | | | | | | |
|---|---|---|---|---|---|---|---|---|---|---|---|---|---|
| Unigene454194 | bcrA | 0.000E+00 | 0.000E+00 | 0.000E+00 | 0.000E+00 | 0.000E+00 | 0.000E+00 | 6.570E-07 | 0.000E+00 | 0.000E+00 | 0.000E+00 | 0.000E+00 | 0.000E+00 |
| Unigene454212 | catB9 | 0.000E+00 | 0.000E+00 | 2.030E-07 | 0.000E+00 | 0.000E+00 | 1.118E-07 | 6.870E-07 | 0.000E+00 | 0.000E+00 | 0.000E+00 | 0.000E+00 | 0.000E+00 |
| Unigene454213 | OCH-3 | 0.000E+00 | 0.000E+00 | 0.000E+00 | 0.000E+00 | 6.194E-08 | 0.000E+00 | 7.096E-07 | 0.000E+00 | 0.000E+00 | 0.000E+00 | 0.000E+00 | 2.307E-07 |
| Unigene454263 | OpmB | 0.000E+00 | 0.000E+00 | 0.000E+00 | 0.000E+00 | 0.000E+00 | 0.000E+00 | 1.165E-06 | 0.000E+00 | 0.000E+00 | 0.000E+00 | 0.000E+00 | 0.000E+00 |
| Unigene454359 | macB | 0.000E+00 | 0.000E+00 | 0.000E+00 | 0.000E+00 | 2.312E-07 | 0.000E+00 | 8.224E-07 | 1.388E-07 | 0.000E+00 | 0.000E+00 | 0.000E+00 | 0.000E+00 |
| Unigene454378 | baeR | 0.000E+00 | 0.000E+00 | 2.475E-07 | 0.000E+00 | 0.000E+00 | 0.000E+00 | 1.814E-06 | 0.000E+00 | 0.000E+00 | 0.000E+00 | 0.000E+00 | 1.093E-06 |
| Unigene454678 | tetA(58) | 0.000E+00 | 0.000E+00 | 4.117E-07 | 1.513E-07 | 0.000E+00 | 0.000E+00 | 1.028E-06 | 0.000E+00 | 0.000E+00 | 0.000E+00 | 0.000E+00 | 0.000E+00 |
| Unigene454744 | MexW | 0.000E+00 | 0.000E+00 | 0.000E+00 | 0.000E+00 | 0.000E+00 | 0.000E+00 | 4.836E-07 | 0.000E+00 | 0.000E+00 | 0.000E+00 | 0.000E+00 | 0.000E+00 |
| Unigene454849 | efrA | 0.000E+00 | 0.000E+00 | 0.000E+00 | 0.000E+00 | 0.000E+00 | 0.000E+00 | 6.357E-07 | 0.000E+00 | 0.000E+00 | 0.000E+00 | 0.000E+00 | 0.000E+00 |
| Unigene454958 | efrB | 0.000E+00 | 0.000E+00 | 0.000E+00 | 1.457E-07 | 0.000E+00 | 0.000E+00 | 8.940E-07 | 0.000E+00 | 0.000E+00 | 0.000E+00 | 0.000E+00 | 0.000E+00 |
| Unigene454959 | efrA | 0.000E+00 | 0.000E+00 | 0.000E+00 | 0.000E+00 | 0.000E+00 | 0.000E+00 | 7.661E-07 | 0.000E+00 | 0.000E+00 | 0.000E+00 | 0.000E+00 | 0.000E+00 |
| Unigene454978 | mefC | 0.000E+00 | 0.000E+00 | 0.000E+00 | 0.000E+00 | 3.023E-07 | 0.000E+00 | 1.122E-06 | 2.887E-07 | 0.000E+00 | 0.000E+00 | 3.961E-07 | 6.725E-07 |
| Unigene455031 | tet(A) | 0.000E+00 | 0.000E+00 | 3.588E-07 | 1.846E-07 | 4.932E-07 | 1.129E-07 | 8.093E-07 | 6.707E-07 | 0.000E+00 | 0.000E+00 | 9.261E-07 | 9.261E-07 |
| Unigene455078 | tva(A) | 0.000E+00 | 0.000E+00 | 0.000E+00 | 0.000E+00 | 1.599E-07 | 0.000E+00 | 8.762E-07 | 6.721E-07 | 0.000E+00 | 0.000E+00 | 0.000E+00 | 5.955E-07 |
| Unigene455101 | evgS | 0.000E+00 | 0.000E+00 | 0.000E+00 | 0.000E+00 | 0.000E+00 | 0.000E+00 | 4.716E-07 | 0.000E+00 | 0.000E+00 | 0.000E+00 | 0.000E+00 | 1.259E-07 |
| Unigene455102 | oleB | 0.000E+00 | 0.000E+00 | 0.000E+00 | 2.822E-07 | 0.000E+00 | 0.000E+00 | 7.733E-07 | 0.000E+00 | 1.018E-07 | 0.000E+00 | 0.000E+00 | 0.000E+00 |
| Unigene455189 | vanHF | 0.000E+00 | 0.000E+00 | 0.000E+00 | 0.000E+00 | 0.000E+00 | 0.000E+00 | 7.515E-07 | 0.000E+00 | 0.000E+00 | 0.000E+00 | 0.000E+00 | 0.000E+00 |
| Unigene455202 | tcr3 | 0.000E+00 | 0.000E+00 | 1.050E-07 | 0.000E+00 | 2.377E-07 | 0.000E+00 | 6.392E-07 | 4.495E-07 | 0.000E+00 | 0.000E+00 | 2.447E-07 | 4.551E-07 |
| Unigene455217 | bcrA | 0.000E+00 | 0.000E+00 | 0.000E+00 | 0.000E+00 | 0.000E+00 | 0.000E+00 | 2.127E-06 | 0.000E+00 | 0.000E+00 | 0.000E+00 | 0.000E+00 | 0.000E+00 |
| Unigene455261 | arlR | 0.000E+00 | 0.000E+00 | 0.000E+00 | 0.000E+00 | 0.000E+00 | 0.000E+00 | 5.720E-07 | 9.324E-07 | 0.000E+00 | 0.000E+00 | 4.837E-07 | 4.999E-07 |
| Unigene455455 | efrB | 0.000E+00 | 0.000E+00 | 0.000E+00 | 0.000E+00 | 7.338E-07 | 0.000E+00 | 1.698E-06 | 1.069E-06 | 0.000E+00 | 0.000E+00 | 0.000E+00 | 0.000E+00 |
| Unigene455496 | bcrA | 0.000E+00 | 5.344E-08 | 0.000E+00 | 0.000E+00 | 0.000E+00 | 0.000E+00 | 0.000E+00 | 0.000E+00 | 0.000E+00 | 0.000E+00 | 2.228E-07 | 0.000E+00 |
| Unigene455530 | golS | 0.000E+00 | 0.000E+00 | 0.000E+00 | 0.000E+00 | 0.000E+00 | 0.000E+00 | 2.406E-07 | 0.000E+00 | 0.000E+00 | 0.000E+00 | 0.000E+00 | 0.000E+00 |
| Unigene455565 | AxyY | 0.000E+00 | 0.000E+00 | 0.000E+00 | 0.000E+00 | 0.000E+00 | 0.000E+00 | 3.664E-07 | 0.000E+00 | 0.000E+00 | 0.000E+00 | 0.000E+00 | 0.000E+00 |
| Unigene455576 | AxyY | 0.000E+00 | 0.000E+00 | 0.000E+00 | 0.000E+00 | 0.000E+00 | 0.000E+00 | 3.585E-07 | 2.100E-07 | 0.000E+00 | 0.000E+00 | 4.116E-07 | 0.000E+00 |
| Unigene455597 | Bifidobacte | 0.000E+00 | 0.000E+00 | 0.000E+00 | 0.000E+00 | 0.000E+00 | 0.000E+00 | 1.099E-06 | 0.000E+00 | 0.000E+00 | 0.000E+00 | 0.000E+00 | 0.000E+00 |
| Unigene455654 | cpxA | 0.000E+00 | 0.000E+00 | 0.000E+00 | 0.000E+00 | 0.000E+00 | 0.000E+00 | 6.380E-07 | 0.000E+00 | 0.000E+00 | 0.000E+00 | 2.198E-07 | 0.000E+00 |
| Unigene455660 | MuxB | 0.000E+00 | 0.000E+00 | 0.000E+00 | 0.000E+00 | 0.000E+00 | 0.000E+00 | 1.215E-06 | 0.000E+00 | 0.000E+00 | 0.000E+00 | 0.000E+00 | 0.000E+00 |
| Unigene455704 | patA | 0.000E+00 | 0.000E+00 | 1.820E-07 | 0.000E+00 | 0.000E+00 | 0.000E+00 | 5.646E-07 | 5.414E-07 | 0.000E+00 | 0.000E+00 | 0.000E+00 | 1.645E-07 |
| Unigene455789 | patA | 0.000E+00 | 0.000E+00 | 0.000E+00 | 0.000E+00 | 2.049E-07 | 0.000E+00 | 0.000E+00 | 0.000E+00 | 0.000E+00 | 0.000E+00 | 0.000E+00 | 0.000E+00 |
| Unigene455792 | evgS | 0.000E+00 | 0.000E+00 | 0.000E+00 | 0.000E+00 | 3.001E-07 | 0.000E+00 | 9.467E-07 | 0.000E+00 | 0.000E+00 | 0.000E+00 | 0.000E+00 | 0.000E+00 |
| Unigene455904 | cmrA | 0.000E+00 | 0.000E+00 | 0.000E+00 | 0.000E+00 | 0.000E+00 | 4.544E-07 | 6.978E-07 | 0.000E+00 | 0.000E+00 | 0.000E+00 | 0.000E+00 | 0.000E+00 |
| Unigene456083 | MuxB | 0.000E+00 | 0.000E+00 | 0.000E+00 | 0.000E+00 | 0.000E+00 | 0.000E+00 | 6.055E-07 | 0.000E+00 | 0.000E+00 | 0.000E+00 | 0.000E+00 | 0.000E+00 |
| Unigene456084 | mdtA | 0.000E+00 | 0.000E+00 | 0.000E+00 | 0.000E+00 | 0.000E+00 | 0.000E+00 | 3.069E-07 | 0.000E+00 | 0.000E+00 | 1.236E-07 | 0.000E+00 | 0.000E+00 |
| Unigene456114 | macB | 0.000E+00 | 0.000E+00 | 0.000E+00 | 0.000E+00 | 0.000E+00 | 0.000E+00 | 6.227E-07 | 5.746E-07 | 0.000E+00 | 0.000E+00 | 2.413E-07 | 0.000E+00 |
| Unigene456173 | oleC | 1.393E-07 | 0.000E+00 | 0.000E+00 | 1.055E-07 | 0.000E+00 | 4.141E-07 | 8.093E-07 | 4.065E-07 | 3.806E-08 | 0.000E+00 | 0.000E+00 | 0.000E+00 |
| Unigene456207 | patB | 0.000E+00 | 0.000E+00 | 0.000E+00 | 0.000E+00 | 0.000E+00 | 0.000E+00 | 7.589E-07 | 0.000E+00 | 0.000E+00 | 0.000E+00 | 0.000E+00 | 0.000E+00 |
| Unigene456253 | efrA | 0.000E+00 | 0.000E+00 | 0.000E+00 | 1.867E-07 | 0.000E+00 | 0.000E+00 | 9.003E-07 | 0.000E+00 | 0.000E+00 | 0.000E+00 | 0.000E+00 | 0.000E+00 |
| Unigene456269 | facT | 0.000E+00 | 0.000E+00 | 0.000E+00 | 0.000E+00 | 0.000E+00 | 0.000E+00 | 7.492E-07 | 0.000E+00 | 0.000E+00 | 0.000E+00 | 0.000E+00 | 0.000E+00 |
| Unigene456296 | lfrA | 0.000E+00 | 2.203E-08 | 0.000E+00 | 0.000E+00 | 0.000E+00 | 0.000E+00 | 7.776E-07 | 0.000E+00 | 0.000E+00 | 0.000E+00 | 0.000E+00 | 2.373E-07 |
| Unigene456365 | Streptomy | 0.000E+00 | 0.000E+00 | 2.687E-08 | 1.659E-07 | 3.042E-07 | 1.776E-07 | 1.121E-06 | 0.000E+00 | 0.000E+00 | 0.000E+00 | 0.000E+00 | 3.884E-07 |
| Unigene456405 | evgS | 5.978E-08 | 0.000E+00 | 7.332E-08 | 3.018E-08 | 1.162E-07 | 0.000E+00 | 8.766E-07 | 2.442E-07 | 0.000E+00 | 1.498E-07 | 0.000E+00 | 7.066E-08 |
| Unigene456438 | msbA | 0.000E+00 | 0.000E+00 | 0.000E+00 | 0.000E+00 | 0.000E+00 | 2.529E-07 | 9.924E-07 | 0.000E+00 | 0.000E+00 | 0.000E+00 | 0.000E+00 | 0.000E+00 |
| Unigene456481 | efrA | 0.000E+00 | 0.000E+00 | 1.473E-07 | 0.000E+00 | 3.892E-07 | 2.705E-07 | 1.717E-06 | 2.337E-07 | 0.000E+00 | 0.000E+00 | 0.000E+00 | 7.690E-07 |
| Unigene456499 | vatF | 0.000E+00 | 0.000E+00 | 0.000E+00 | 0.000E+00 | 0.000E+00 | 0.000E+00 | 0.000E+00 | 0.000E+00 | 0.000E+00 | 1.688E-07 | 0.000E+00 | 8.360E-07 |
| Unigene456537 | TaeA | 0.000E+00 | 0.000E+00 | 0.000E+00 | 0.000E+00 | 0.000E+00 | 0.000E+00 | 0.000E+00 | 0.000E+00 | 0.000E+00 | 0.000E+00 | 0.000E+00 | 4.025E-07 |
| Unigene456701 | baeR | 0.000E+00 | 0.000E+00 | 0.000E+00 | 0.000E+00 | 0.000E+00 | 0.000E+00 | 1.078E-06 | 0.000E+00 | 0.000E+00 | 0.000E+00 | 0.000E+00 | 0.000E+00 |
| Unigene456704 | arlR | 0.000E+00 | 0.000E+00 | 0.000E+00 | 0.000E+00 | 0.000E+00 | 0.000E+00 | 6.478E-07 | 0.000E+00 | 0.000E+00 | 0.000E+00 | 0.000E+00 | 0.000E+00 |
| Unigene456783 | arlR | 0.000E+00 | 0.000E+00 | 0.000E+00 | 0.000E+00 | 0.000E+00 | 0.000E+00 | 1.054E-06 | 0.000E+00 | 0.000E+00 | 0.000E+00 | 0.000E+00 | 0.000E+00 |
| Unigene456849 | basS | 0.000E+00 | 5.079E-08 | 1.816E-07 | 9.347E-08 | 2.571E-07 | 0.000E+00 | 5.378E-07 | 2.971E-07 | 5.058E-08 | 0.000E+00 | 5.823E-07 | 6.564E-07 |
| Unigene456886 | evgS | 0.000E+00 | 0.000E+00 | 0.000E+00 | 1.372E-07 | 3.924E-07 | 0.000E+00 | 6.616E-07 | 7.295E-07 | 5.940E-08 | 0.000E+00 | 0.000E+00 | 5.781E-07 |
| Unigene456894 | arnA | 0.000E+00 | 0.000E+00 | 0.000E+00 | 2.140E-07 | 0.000E+00 | 0.000E+00 | 2.508E-06 | 0.000E+00 | 0.000E+00 | 0.000E+00 | 0.000E+00 | 0.000E+00 |
| Unigene457022 | novA | 0.000E+00 | 0.000E+00 | 4.302E-07 | 3.690E-07 | 5.683E-07 | 0.000E+00 | 1.375E-06 | 9.169E-07 | 0.000E+00 | 3.053E-07 | 0.000E+00 | 2.116E-06 |
| Unigene457108 | smeR | 0.000E+00 | 2.181E-07 | 0.000E+00 | 0.000E+00 | 0.000E+00 | 0.000E+00 | 1.100E-06 | 1.657E-07 | 0.000E+00 | 0.000E+00 | 0.000E+00 | 0.000E+00 |
| Unigene457109 | kdpE | 0.000E+00 | 0.000E+00 | 0.000E+00 | 0.000E+00 | 0.000E+00 | 0.000E+00 | 1.200E-06 | 0.000E+00 | 0.000E+00 | 0.000E+00 | 0.000E+00 | 5.916E-07 |
| Unigene457117 | evgS | 0.000E+00 | 0.000E+00 | 0.000E+00 | 0.000E+00 | 0.000E+00 | 0.000E+00 | 1.998E-06 | 0.000E+00 | 0.000E+00 | 0.000E+00 | 0.000E+00 | 0.000E+00 |
| Unigene457166 | MuxC | 0.000E+00 | 0.000E+00 | 0.000E+00 | 0.000E+00 | 0.000E+00 | 0.000E+00 | 1.875E-06 | 0.000E+00 | 0.000E+00 | 0.000E+00 | 0.000E+00 | 0.000E+00 |
| Unigene457285 | adeH | 0.000E+00 | 0.000E+00 | 0.000E+00 | 0.000E+00 | 0.000E+00 | 0.000E+00 | 2.257E-06 | 0.000E+00 | 0.000E+00 | 0.000E+00 | 0.000E+00 | 0.000E+00 |

| | | | | | | | | | | | | | |
|---|---|---|---|---|---|---|---|---|---|---|---|---|---|
| Unigene457286 | emrA | 0.000E+00 | 0.000E+00 | 0.000E+00 | 0.000E+00 | 7.925E-07 | 0.000E+00 | 1.382E-06 | 0.000E+00 | 0.000E+00 | 0.000E+00 | 3.060E-07 | 0.000E+00 |
| Unigene457303 | efrA | 0.000E+00 | 0.000E+00 | 5.999E-08 | 9.261E-08 | 3.736E-07 | 1.652E-07 | 5.751E-07 | 2.498E-07 | 0.000E+00 | 0.000E+00 | 3.147E-07 | 5.059E-07 |
| Unigene457378 | msbA | 0.000E+00 | 0.000E+00 | 0.000E+00 | 0.000E+00 | 0.000E+00 | 0.000E+00 | 6.268E-07 | 1.322E-06 | 0.000E+00 | 0.000E+00 | 2.776E-07 | 7.650E-07 |
| Unigene457383 | adeG | 0.000E+00 | 0.000E+00 | 0.000E+00 | 0.000E+00 | 0.000E+00 | 0.000E+00 | 4.888E-07 | 0.000E+00 | 0.000E+00 | 0.000E+00 | 0.000E+00 | 0.000E+00 |
| Unigene457400 | msbA | 0.000E+00 | 0.000E+00 | 0.000E+00 | 0.000E+00 | 0.000E+00 | 0.000E+00 | 8.887E-07 | 0.000E+00 | 0.000E+00 | 0.000E+00 | 1.312E-07 | 4.068E-07 |
| Unigene457445 | lmrC | 0.000E+00 | 0.000E+00 | 0.000E+00 | 0.000E+00 | 0.000E+00 | 0.000E+00 | 9.190E-07 | 0.000E+00 | 0.000E+00 | 0.000E+00 | 0.000E+00 | 0.000E+00 |
| Unigene457469 | lmrD | 0.000E+00 | 0.000E+00 | 0.000E+00 | 0.000E+00 | 0.000E+00 | 0.000E+00 | 4.164E-07 | 0.000E+00 | 0.000E+00 | 0.000E+00 | 0.000E+00 | 0.000E+00 |
| Unigene457499 | efrA | 0.000E+00 | 0.000E+00 | 0.000E+00 | 2.615E-07 | 1.598E-07 | 1.866E-07 | 1.433E-06 | 5.373E-07 | 0.000E+00 | 0.000E+00 | 0.000E+00 | 4.421E-07 |
| Unigene457502 | tva(A) | 1.757E-07 | 0.000E+00 | 1.293E-07 | 0.000E+00 | 1.464E-07 | 0.000E+00 | 9.721E-07 | 4.101E-07 | 1.440E-07 | 0.000E+00 | 0.000E+00 | 0.000E+00 |
| Unigene457609 | mtrA | 0.000E+00 | 0.000E+00 | 0.000E+00 | 0.000E+00 | 0.000E+00 | 0.000E+00 | 0.000E+00 | 0.000E+00 | 0.000E+00 | 0.000E+00 | 1.592E-07 | 0.000E+00 |
| Unigene457812 | farB | 0.000E+00 | 0.000E+00 | 0.000E+00 | 0.000E+00 | 0.000E+00 | 0.000E+00 | 2.837E-06 | 0.000E+00 | 0.000E+00 | 0.000E+00 | 0.000E+00 | 0.000E+00 |
| Unigene457813 | adeL | 0.000E+00 | 0.000E+00 | 0.000E+00 | 0.000E+00 | 0.000E+00 | 0.000E+00 | 2.366E-06 | 0.000E+00 | 0.000E+00 | 0.000E+00 | 0.000E+00 | 0.000E+00 |
| Unigene457862 | novA | 0.000E+00 | 0.000E+00 | 0.000E+00 | 0.000E+00 | 0.000E+00 | 0.000E+00 | 7.911E-07 | 0.000E+00 | 0.000E+00 | 0.000E+00 | 0.000E+00 | 0.000E+00 |
| Unigene457914 | evgS | 0.000E+00 | 7.729E-08 | 0.000E+00 | 2.418E-07 | 2.660E-07 | 0.000E+00 | 5.456E-07 | 5.918E-07 | 0.000E+00 | 0.000E+00 | 5.961E-07 | 4.994E-07 |
| Unigene457923 | patB | 0.000E+00 | 0.000E+00 | 0.000E+00 | 0.000E+00 | 3.999E-07 | 0.000E+00 | 2.833E-06 | 6.537E-07 | 0.000E+00 | 4.902E-07 | 7.321E-07 | 8.983E-07 |
| Unigene458022 | Acinetoba | 0.000E+00 | 0.000E+00 | 0.000E+00 | 0.000E+00 | 0.000E+00 | 0.000E+00 | 7.005E-07 | 0.000E+00 | 0.000E+00 | 0.000E+00 | 0.000E+00 | 0.000E+00 |
| Unigene458077 | novA | 0.000E+00 | 2.143E-07 | 2.767E-07 | 0.000E+00 | 9.399E-07 | 1.173E-07 | 1.777E-06 | 6.330E-07 | 0.000E+00 | 0.000E+00 | 2.978E-07 | 1.231E-06 |
| Unigene458326 | arlR | 0.000E+00 | 0.000E+00 | 0.000E+00 | 0.000E+00 | 0.000E+00 | 0.000E+00 | 1.694E-06 | 0.000E+00 | 0.000E+00 | 0.000E+00 | 0.000E+00 | 0.000E+00 |
| Unigene458327 | eptA | 0.000E+00 | 0.000E+00 | 0.000E+00 | 0.000E+00 | 0.000E+00 | 0.000E+00 | 1.673E-06 | 3.438E-07 | 0.000E+00 | 0.000E+00 | 0.000E+00 | 0.000E+00 |
| Unigene458414 | catB8 | 0.000E+00 | 0.000E+00 | 0.000E+00 | 0.000E+00 | 1.696E-07 | 0.000E+00 | 4.223E-07 | 0.000E+00 | 0.000E+00 | 0.000E+00 | 3.492E-07 | 3.608E-07 |
| Unigene458463 | macB | 0.000E+00 | 0.000E+00 | 0.000E+00 | 0.000E+00 | 0.000E+00 | 9.207E-08 | 6.599E-07 | 1.657E-07 | 0.000E+00 | 0.000E+00 | 0.000E+00 | 0.000E+00 |
| Unigene458520 | ACC-2 | 0.000E+00 | 0.000E+00 | 2.906E-07 | 0.000E+00 | 0.000E+00 | 0.000E+00 | 5.463E-07 | 0.000E+00 | 0.000E+00 | 0.000E+00 | 0.000E+00 | 1.167E-07 |
| Unigene458616 | PEDO-1 | 0.000E+00 | 1.173E-07 | 2.447E-07 | 7.196E-08 | 0.000E+00 | 0.000E+00 | 9.070E-07 | 9.149E-07 | 2.337E-07 | 0.000E+00 | 1.019E-06 | 1.979E-06 |
| Unigene458686 | msrC | 2.729E-07 | 0.000E+00 | 0.000E+00 | 3.937E-08 | 0.000E+00 | 3.372E-07 | 7.335E-07 | 0.000E+00 | 2.557E-07 | 0.000E+00 | 0.000E+00 | 0.000E+00 |
| Unigene458744 | lmrC | 0.000E+00 | 0.000E+00 | 5.306E-08 | 0.000E+00 | 0.000E+00 | 0.000E+00 | 7.780E-07 | 4.418E-07 | 0.000E+00 | 1.205E-07 | 2.474E-07 | 4.474E-07 |
| Unigene458804 | vatB | 0.000E+00 | 0.000E+00 | 0.000E+00 | 0.000E+00 | 0.000E+00 | 0.000E+00 | 8.093E-07 | 0.000E+00 | 0.000E+00 | 0.000E+00 | 0.000E+00 | 0.000E+00 |
| Unigene458884 | bcrA | 0.000E+00 | 0.000E+00 | 9.746E-08 | 0.000E+00 | 0.000E+00 | 3.221E-07 | 6.596E-07 | 1.546E-07 | 0.000E+00 | 1.106E-07 | 0.000E+00 | 3.913E-07 |
| Unigene458893 | macA | 0.000E+00 | 0.000E+00 | 0.000E+00 | 2.337E-07 | 0.000E+00 | 0.000E+00 | 2.113E-06 | 0.000E+00 | 0.000E+00 | 0.000E+00 | 0.000E+00 | 0.000E+00 |
| Unigene458915 | macB | 0.000E+00 | 0.000E+00 | 0.000E+00 | 0.000E+00 | 0.000E+00 | 0.000E+00 | 6.823E-07 | 0.000E+00 | 0.000E+00 | 0.000E+00 | 0.000E+00 | 0.000E+00 |
| Unigene458919 | tetA(60) | 0.000E+00 | 0.000E+00 | 0.000E+00 | 0.000E+00 | 0.000E+00 | 0.000E+00 | 9.783E-07 | 0.000E+00 | 0.000E+00 | 0.000E+00 | 0.000E+00 | 1.261E-06 |
| Unigene458999 | vanSG | 7.157E-08 | 0.000E+00 | 7.023E-08 | 0.000E+00 | 2.651E-07 | 1.805E-07 | 1.109E-06 | 1.420E-06 | 0.000E+00 | 2.126E-07 | 2.456E-07 | 1.156E-06 |
| Unigene459008 | tetA(46) | 5.181E-07 | 9.478E-08 | 3.051E-07 | 0.000E+00 | 4.816E-06 | 1.046E-05 | 1.682E-06 | 8.891E-06 | 5.437E-06 | 3.636E-06 | 0.000E+00 | 9.799E-07 |
| Unigene459013 | patB | 0.000E+00 | 0.000E+00 | 0.000E+00 | 0.000E+00 | 1.047E-06 | 0.000E+00 | 2.521E-06 | 0.000E+00 | 0.000E+00 | 0.000E+00 | 0.000E+00 | 0.000E+00 |
| Unigene459056 | lmrC | 0.000E+00 | 0.000E+00 | 0.000E+00 | 0.000E+00 | 0.000E+00 | 0.000E+00 | 8.059E-07 | 0.000E+00 | 0.000E+00 | 0.000E+00 | 0.000E+00 | 0.000E+00 |
| Unigene459248 | macB | 0.000E+00 | 0.000E+00 | 0.000E+00 | 0.000E+00 | 9.602E-08 | 0.000E+00 | 4.304E-07 | 9.584E-07 | 4.723E-08 | 0.000E+00 | 3.954E-07 | 4.086E-07 |
| Unigene459255 | emrB | 0.000E+00 | 0.000E+00 | 0.000E+00 | 5.137E-08 | 2.826E-08 | 1.375E-07 | 5.349E-07 | 2.375E-07 | 0.000E+00 | 0.000E+00 | 0.000E+00 | 2.556E-06 |
| Unigene459318 | tetB(60) | 0.000E+00 | 0.000E+00 | 0.000E+00 | 0.000E+00 | 0.000E+00 | 0.000E+00 | 0.000E+00 | 2.546E-07 | 7.946E-08 | 0.000E+00 | 0.000E+00 | 5.156E-07 |
| Unigene459343 | Acinetoba | 0.000E+00 | 0.000E+00 | 0.000E+00 | 0.000E+00 | 0.000E+00 | 0.000E+00 | 8.062E-07 | 0.000E+00 | 0.000E+00 | 0.000E+00 | 0.000E+00 | 0.000E+00 |
| Unigene459463 | vanRF | 0.000E+00 | 3.918E-07 | 0.000E+00 | 0.000E+00 | 0.000E+00 | 3.859E-07 | 3.612E-06 | 0.000E+00 | 0.000E+00 | 0.000E+00 | 0.000E+00 | 0.000E+00 |
| Unigene459474 | arlR | 0.000E+00 | 0.000E+00 | 0.000E+00 | 1.176E-07 | 4.399E-07 | 1.511E-07 | 6.702E-07 | 6.253E-07 | 0.000E+00 | 0.000E+00 | 5.328E-08 | 2.753E-07 |
| Unigene459616 | bcrA | 1.356E-07 | 0.000E+00 | 7.542E-07 | 3.196E-07 | 0.000E+00 | 6.842E-07 | 2.302E-06 | 5.805E-07 | 0.000E+00 | 0.000E+00 | 7.240E-07 | 3.313E-06 |
| Unigene459666 | vgaB | 0.000E+00 | 6.248E-08 | 0.000E+00 | 0.000E+00 | 0.000E+00 | 0.000E+00 | 1.040E-06 | 2.990E-07 | 0.000E+00 | 0.000E+00 | 0.000E+00 | 6.729E-07 |
| Unigene459694 | mgrA | 0.000E+00 | 0.000E+00 | 0.000E+00 | 0.000E+00 | 0.000E+00 | 0.000E+00 | 2.835E-07 | 9.968E-08 | 0.000E+00 | 0.000E+00 | 0.000E+00 | 1.009E-07 |
| Unigene459705 | tva(A) | 0.000E+00 | 0.000E+00 | 0.000E+00 | 0.000E+00 | 0.000E+00 | 0.000E+00 | 7.136E-07 | 0.000E+00 | 0.000E+00 | 0.000E+00 | 0.000E+00 | 0.000E+00 |
| Unigene459768 | baeR | 0.000E+00 | 0.000E+00 | 0.000E+00 | 0.000E+00 | 0.000E+00 | 0.000E+00 | 2.556E-07 | 1.617E-07 | 0.000E+00 | 0.000E+00 | 0.000E+00 | 6.550E-07 |
| Unigene459808 | mgrA | 0.000E+00 | 0.000E+00 | 0.000E+00 | 0.000E+00 | 7.379E-07 | 0.000E+00 | 5.146E-07 | 0.000E+00 | 0.000E+00 | 0.000E+00 | 0.000E+00 | 1.570E-07 |
| Unigene459811 | sdiA | 0.000E+00 | 0.000E+00 | 0.000E+00 | 0.000E+00 | 0.000E+00 | 9.653E-08 | 3.162E-06 | 8.860E-07 | 0.000E+00 | 0.000E+00 | 0.000E+00 | 7.916E-07 |
| Unigene459812 | lmrD | 0.000E+00 | 0.000E+00 | 0.000E+00 | 0.000E+00 | 0.000E+00 | 0.000E+00 | 2.420E-06 | 0.000E+00 | 0.000E+00 | 0.000E+00 | 0.000E+00 | 0.000E+00 |
| Unigene459884 | tetA(58) | 0.000E+00 | 2.391E-08 | 4.275E-08 | 8.798E-08 | 0.000E+00 | 0.000E+00 | 4.821E-07 | 0.000E+00 | 0.000E+00 | 0.000E+00 | 0.000E+00 | 0.000E+00 |
| Unigene459885 | tlrC | 0.000E+00 | 2.523E-08 | 4.512E-08 | 4.643E-08 | 0.000E+00 | 0.000E+00 | 7.634E-07 | 4.831E-07 | 0.000E+00 | 0.000E+00 | 0.000E+00 | 0.000E+00 |
| Unigene459918 | baeS | 0.000E+00 | 0.000E+00 | 3.744E-07 | 0.000E+00 | 0.000E+00 | 0.000E+00 | 1.042E-06 | 0.000E+00 | 0.000E+00 | 0.000E+00 | 0.000E+00 | 4.811E-07 |
| Unigene460006 | oleC | 0.000E+00 | 0.000E+00 | 0.000E+00 | 0.000E+00 | 0.000E+00 | 0.000E+00 | 9.993E-07 | 0.000E+00 | 0.000E+00 | 0.000E+00 | 1.721E-07 | 0.000E+00 |
| Unigene460036 | Streptomy | 0.000E+00 | 0.000E+00 | 0.000E+00 | 0.000E+00 | 0.000E+00 | 0.000E+00 | 1.075E-06 | 0.000E+00 | 0.000E+00 | 0.000E+00 | 0.000E+00 | 0.000E+00 |
| Unigene460068 | vmlR | 0.000E+00 | 0.000E+00 | 6.016E-08 | 1.238E-07 | 3.065E-07 | 3.314E-07 | 8.821E-07 | 6.083E-07 | 1.340E-07 | 2.732E-07 | 7.364E-07 | 2.065E-06 |
| Unigene460160 | macB | 1.588E-07 | 0.000E+00 | 0.000E+00 | 2.005E-07 | 3.529E-07 | 7.297E-07 | 8.349E-07 | 2.595E-06 | 4.340E-07 | 0.000E+00 | 5.904E-07 | 1.314E-06 |
| Unigene460208 | carA | 0.000E+00 | 0.000E+00 | 2.262E-07 | 0.000E+00 | 0.000E+00 | 0.000E+00 | 1.446E-06 | 0.000E+00 | 0.000E+00 | 0.000E+00 | 0.000E+00 | 0.000E+00 |
| Unigene460224 | OXA-18 | 7.797E-08 | 1.284E-07 | 7.651E-08 | 1.181E-07 | 1.299E-07 | 1.264E-07 | 7.767E-07 | 6.371E-07 | 0.000E+00 | 0.000E+00 | 6.243E-07 | 1.843E-06 |
| Unigene460250 | srmB | 0.000E+00 | 0.000E+00 | 0.000E+00 | 0.000E+00 | 0.000E+00 | 0.000E+00 | 6.363E-07 | 0.000E+00 | 0.000E+00 | 0.000E+00 | 0.000E+00 | 0.000E+00 |

| | | | | | | | | | | | | | |
|---|---|---|---|---|---|---|---|---|---|---|---|---|---|
| Unigene460361 | lmrB | 0.000E+00 | 0.000E+00 | 0.000E+00 | 0.000E+00 | 0.000E+00 | 0.000E+00 | 4.706E-07 | 0.000E+00 | 0.000E+00 | 0.000E+00 | 0.000E+00 | 0.000E+00 |
| Unigene460482 | bcrA | 0.000E+00 | 0.000E+00 | 0.000E+00 | 0.000E+00 | 0.000E+00 | 0.000E+00 | 6.192E-07 | 0.000E+00 | 0.000E+00 | 0.000E+00 | 0.000E+00 | 0.000E+00 |
| Unigene460536 | cpxA | 0.000E+00 | 0.000E+00 | 0.000E+00 | 0.000E+00 | 0.000E+00 | 0.000E+00 | 7.028E-07 | 0.000E+00 | 0.000E+00 | 0.000E+00 | 0.000E+00 | 5.838E-07 |
| Unigene460553 | vanTG | 0.000E+00 | 0.000E+00 | 0.000E+00 | 0.000E+00 | 0.000E+00 | 0.000E+00 | 1.919E-06 | 1.151E-06 | 0.000E+00 | 0.000E+00 | 0.000E+00 | 6.359E-07 |
| Unigene460667 | Corynebac | 0.000E+00 | 0.000E+00 | 0.000E+00 | 0.000E+00 | 0.000E+00 | 3.965E-07 | 7.443E-07 | 0.000E+00 | 2.673E-07 | 0.000E+00 | 0.000E+00 | 6.504E-07 |
| Unigene460762 | MexH | 0.000E+00 | 0.000E+00 | 0.000E+00 | 0.000E+00 | 0.000E+00 | 0.000E+00 | 1.079E-06 | 0.000E+00 | 0.000E+00 | 0.000E+00 | 0.000E+00 | 0.000E+00 |
| Unigene460773 | carA | 1.139E-07 | 0.000E+00 | 1.955E-07 | 0.000E+00 | 0.000E+00 | 0.000E+00 | 5.986E-07 | 1.994E-07 | 0.000E+00 | 0.000E+00 | 2.605E-07 | 3.365E-07 |
| Unigene460901 | lsaC | 0.000E+00 | 0.000E+00 | 0.000E+00 | 0.000E+00 | 0.000E+00 | 0.000E+00 | 7.167E-07 | 0.000E+00 | 0.000E+00 | 0.000E+00 | 0.000E+00 | 0.000E+00 |
| Unigene460913 | tetB(60) | 0.000E+00 | 0.000E+00 | 1.336E-07 | 1.375E-07 | 3.026E-07 | 0.000E+00 | 8.040E-07 | 1.431E-06 | 0.000E+00 | 0.000E+00 | 0.000E+00 | 2.200E-06 |
| Unigene460985 | Corynebac | 8.473E-08 | 0.000E+00 | 0.000E+00 | 0.000E+00 | 0.000E+00 | 0.000E+00 | 0.000E+00 | 0.000E+00 | 0.000E+00 | 0.000E+00 | 0.000E+00 | 0.000E+00 |
| Unigene461136 | novA | 0.000E+00 | 0.000E+00 | 0.000E+00 | 0.000E+00 | 0.000E+00 | 0.000E+00 | 6.790E-07 | 0.000E+00 | 0.000E+00 | 0.000E+00 | 0.000E+00 | 5.021E-07 |
| Unigene461223 | MexA | 0.000E+00 | 0.000E+00 | 0.000E+00 | 0.000E+00 | 1.423E-07 | 0.000E+00 | 5.198E-07 | 0.000E+00 | 0.000E+00 | 0.000E+00 | 0.000E+00 | 0.000E+00 |
| Unigene461232 | basS | 0.000E+00 | 0.000E+00 | 0.000E+00 | 0.000E+00 | 0.000E+00 | 0.000E+00 | 6.359E-07 | 0.000E+00 | 0.000E+00 | 0.000E+00 | 0.000E+00 | 0.000E+00 |
| Unigene461408 | bcrA | 0.000E+00 | 1.540E-07 | 0.000E+00 | 0.000E+00 | 0.000E+00 | 0.000E+00 | 0.000E+00 | 6.553E-07 | 0.000E+00 | 0.000E+00 | 0.000E+00 | 6.083E-07 |
| Unigene461413 | tetT | 0.000E+00 | 0.000E+00 | 0.000E+00 | 0.000E+00 | 0.000E+00 | 0.000E+00 | 5.159E-07 | 0.000E+00 | 0.000E+00 | 0.000E+00 | 0.000E+00 | 0.000E+00 |
| Unigene461475 | bcrA | 0.000E+00 | 0.000E+00 | 0.000E+00 | 0.000E+00 | 0.000E+00 | 0.000E+00 | 3.321E-06 | 0.000E+00 | 0.000E+00 | 0.000E+00 | 0.000E+00 | 0.000E+00 |
| Unigene461538 | PmrF | 2.532E-07 | 0.000E+00 | 0.000E+00 | 3.835E-07 | 7.383E-07 | 0.000E+00 | 1.226E-06 | 1.256E-06 | 0.000E+00 | 0.000E+00 | 0.000E+00 | 1.197E-06 |
| Unigene461587 | cmlv | 0.000E+00 | 0.000E+00 | 0.000E+00 | 0.000E+00 | 0.000E+00 | 0.000E+00 | 1.924E-06 | 0.000E+00 | 0.000E+00 | 0.000E+00 | 0.000E+00 | 0.000E+00 |
| Unigene461599 | efrA | 0.000E+00 | 0.000E+00 | 0.000E+00 | 0.000E+00 | 0.000E+00 | 0.000E+00 | 1.201E-06 | 0.000E+00 | 0.000E+00 | 0.000E+00 | 4.137E-07 | 0.000E+00 |
| Unigene461667 | bcrA | 0.000E+00 | 0.000E+00 | 0.000E+00 | 0.000E+00 | 0.000E+00 | 0.000E+00 | 1.114E-06 | 0.000E+00 | 0.000E+00 | 0.000E+00 | 0.000E+00 | 0.000E+00 |
| Unigene461749 | lmrC | 8.060E-08 | 4.423E-08 | 3.954E-08 | 2.442E-07 | 1.343E-07 | 0.000E+00 | 6.690E-07 | 6.115E-07 | 0.000E+00 | 2.245E-07 | 4.610E-07 | 1.334E-06 |
| Unigene461862 | lmrD | 0.000E+00 | 0.000E+00 | 0.000E+00 | 0.000E+00 | 0.000E+00 | 0.000E+00 | 7.033E-07 | 1.483E-07 | 0.000E+00 | 0.000E+00 | 0.000E+00 | 0.000E+00 |
| Unigene461897 | Streptomy | 0.000E+00 | 0.000E+00 | 0.000E+00 | 0.000E+00 | 0.000E+00 | 0.000E+00 | 6.202E-07 | 0.000E+00 | 0.000E+00 | 0.000E+00 | 0.000E+00 | 0.000E+00 |
| Unigene462023 | optrA | 0.000E+00 | 0.000E+00 | 0.000E+00 | 0.000E+00 | 0.000E+00 | 0.000E+00 | 3.721E-07 | 0.000E+00 | 0.000E+00 | 0.000E+00 | 0.000E+00 | 4.968E-07 |
| Unigene462035 | efrB | 0.000E+00 | 0.000E+00 | 0.000E+00 | 6.003E-08 | 1.981E-07 | 0.000E+00 | 5.264E-07 | 0.000E+00 | 0.000E+00 | 0.000E+00 | 0.000E+00 | 0.000E+00 |
| Unigene462097 | macB | 0.000E+00 | 0.000E+00 | 0.000E+00 | 0.000E+00 | 8.726E-08 | 0.000E+00 | 1.043E-06 | 0.000E+00 | 0.000E+00 | 0.000E+00 | 0.000E+00 | 0.000E+00 |
| Unigene462221 | baeR | 0.000E+00 | 1.446E-07 | 0.000E+00 | 0.000E+00 | 0.000E+00 | 0.000E+00 | 8.263E-07 | 9.740E-07 | 0.000E+00 | 0.000E+00 | 0.000E+00 | 7.787E-07 |
| Unigene462389 | bcrA | 2.139E-08 | 0.000E+00 | 8.396E-08 | 0.000E+00 | 2.377E-08 | 9.250E-08 | 9.944E-07 | 9.988E-08 | 0.000E+00 | 7.149E-08 | 0.000E+00 | 5.057E-08 |
| Unigene462499 | mdtO | 0.000E+00 | 0.000E+00 | 0.000E+00 | 0.000E+00 | 0.000E+00 | 0.000E+00 | 8.456E-07 | 0.000E+00 | 0.000E+00 | 0.000E+00 | 0.000E+00 | 0.000E+00 |
| Unigene462504 | MexF | 0.000E+00 | 0.000E+00 | 0.000E+00 | 0.000E+00 | 0.000E+00 | 0.000E+00 | 4.203E-07 | 0.000E+00 | 0.000E+00 | 0.000E+00 | 0.000E+00 | 0.000E+00 |
| Unigene462511 | patA | 0.000E+00 | 0.000E+00 | 0.000E+00 | 0.000E+00 | 0.000E+00 | 0.000E+00 | 4.683E-07 | 0.000E+00 | 0.000E+00 | 0.000E+00 | 0.000E+00 | 0.000E+00 |
| Unigene462631 | adeN | 0.000E+00 | 0.000E+00 | 3.677E-07 | 0.000E+00 | 8.326E-07 | 1.563E-06 | 3.081E-06 | 0.000E+00 | 2.516E-06 | 0.000E+00 | 0.000E+00 | 6.328E-07 |
| Unigene462665 | oleC | 0.000E+00 | 0.000E+00 | 0.000E+00 | 0.000E+00 | 0.000E+00 | 0.000E+00 | 9.352E-07 | 0.000E+00 | 0.000E+00 | 0.000E+00 | 0.000E+00 | 4.661E-07 |
| Unigene462667 | vanSA | 0.000E+00 | 0.000E+00 | 3.579E-07 | 2.046E-07 | 0.000E+00 | 0.000E+00 | 1.256E-06 | 2.602E-07 | 0.000E+00 | 0.000E+00 | 4.868E-07 | 1.485E-06 |
| Unigene462686 | Pseudomo | 0.000E+00 | 0.000E+00 | 0.000E+00 | 0.000E+00 | 1.594E-07 | 0.000E+00 | 0.000E+00 | 0.000E+00 | 0.000E+00 | 0.000E+00 | 0.000E+00 | 0.000E+00 |
| Unigene462729 | efrA | 0.000E+00 | 0.000E+00 | 2.279E-07 | 0.000E+00 | 0.000E+00 | 0.000E+00 | 1.028E-06 | 0.000E+00 | 0.000E+00 | 0.000E+00 | 0.000E+00 | 0.000E+00 |
| Unigene462740 | mtrA | 0.000E+00 | 0.000E+00 | 0.000E+00 | 3.909E-07 | 0.000E+00 | 2.511E-07 | 0.000E+00 | 0.000E+00 | 1.100E-06 | 0.000E+00 | 0.000E+00 | 1.098E-06 |
| Unigene462810 | efrA | 0.000E+00 | 0.000E+00 | 0.000E+00 | 0.000E+00 | 0.000E+00 | 0.000E+00 | 2.247E-06 | 0.000E+00 | 0.000E+00 | 0.000E+00 | 0.000E+00 | 0.000E+00 |
| Unigene462849 | lmrC | 0.000E+00 | 5.180E-08 | 0.000E+00 | 0.000E+00 | 3.670E-07 | 1.020E-07 | 6.268E-07 | 4.958E-07 | 5.158E-08 | 0.000E+00 | 5.398E-07 | 1.729E-06 |
| Unigene462943 | basS | 0.000E+00 | 4.910E-08 | 1.756E-07 | 4.518E-08 | 1.491E-07 | 1.209E-07 | 9.161E-07 | 8.095E-07 | 0.000E+00 | 3.987E-07 | 6.141E-07 | 1.825E-06 |
| Unigene462962 | mdtN | 0.000E+00 | 0.000E+00 | 0.000E+00 | 0.000E+00 | 0.000E+00 | 0.000E+00 | 6.175E-07 | 0.000E+00 | 0.000E+00 | 0.000E+00 | 0.000E+00 | 0.000E+00 |
| Unigene463073 | emrY | 0.000E+00 | 0.000E+00 | 0.000E+00 | 0.000E+00 | 0.000E+00 | 0.000E+00 | 5.591E-07 | 0.000E+00 | 0.000E+00 | 0.000E+00 | 0.000E+00 | 0.000E+00 |
| Unigene463081 | patA | 1.273E-07 | 0.000E+00 | 0.000E+00 | 9.644E-08 | 1.768E-07 | 0.000E+00 | 1.233E-06 | 1.152E-06 | 0.000E+00 | 4.256E-07 | 4.006E-07 | 7.902E-07 |
| Unigene463103 | Acinetoba | 0.000E+00 | 0.000E+00 | 0.000E+00 | 0.000E+00 | 0.000E+00 | 0.000E+00 | 5.641E-07 | 1.983E-07 | 0.000E+00 | 0.000E+00 | 0.000E+00 | 1.339E-07 |
| Unigene463457 | vgaB | 0.000E+00 | 0.000E+00 | 0.000E+00 | 0.000E+00 | 5.116E-07 | 2.489E-07 | 1.506E-06 | 5.620E-07 | 2.288E-07 | 0.000E+00 | 0.000E+00 | 8.908E-07 |
| Unigene463491 | TaeA | 0.000E+00 | 0.000E+00 | 0.000E+00 | 0.000E+00 | 0.000E+00 | 0.000E+00 | 3.158E-07 | 0.000E+00 | 0.000E+00 | 1.816E-07 | 0.000E+00 | 0.000E+00 |
| Unigene463493 | tet(V) | 0.000E+00 | 0.000E+00 | 0.000E+00 | 0.000E+00 | 0.000E+00 | 0.000E+00 | 4.473E-07 | 0.000E+00 | 0.000E+00 | 0.000E+00 | 0.000E+00 | 0.000E+00 |
| Unigene463543 | MuxC | 0.000E+00 | 0.000E+00 | 0.000E+00 | 0.000E+00 | 0.000E+00 | 0.000E+00 | 6.838E-07 | 0.000E+00 | 0.000E+00 | 0.000E+00 | 0.000E+00 | 0.000E+00 |
| Unigene463557 | mtrA | 0.000E+00 | 0.000E+00 | 0.000E+00 | 0.000E+00 | 0.000E+00 | 5.360E-08 | 6.585E-07 | 0.000E+00 | 0.000E+00 | 0.000E+00 | 0.000E+00 | 2.344E-07 |
| Unigene463660 | patA | 0.000E+00 | 0.000E+00 | 0.000E+00 | 0.000E+00 | 0.000E+00 | 0.000E+00 | 0.000E+00 | 2.816E-07 | 0.000E+00 | 0.000E+00 | 0.000E+00 | 0.000E+00 |
| Unigene463682 | carA | 0.000E+00 | 0.000E+00 | 1.186E-07 | 0.000E+00 | 0.000E+00 | 0.000E+00 | 7.582E-07 | 0.000E+00 | 0.000E+00 | 0.000E+00 | 0.000E+00 | 0.000E+00 |
| Unigene463766 | evgS | 0.000E+00 | 1.342E-07 | 0.000E+00 | 0.000E+00 | 0.000E+00 | 0.000E+00 | 4.398E-07 | 1.070E-07 | 0.000E+00 | 0.000E+00 | 2.797E-07 | 1.445E-07 |
| Unigene463891 | tet(43) | 0.000E+00 | 0.000E+00 | 0.000E+00 | 9.616E-08 | 0.000E+00 | 0.000E+00 | 6.060E-07 | 0.000E+00 | 0.000E+00 | 0.000E+00 | 0.000E+00 | 0.000E+00 |
| Unigene464063 | YojI | 0.000E+00 | 0.000E+00 | 0.000E+00 | 0.000E+00 | 0.000E+00 | 9.331E-08 | 4.458E-07 | 0.000E+00 | 0.000E+00 | 0.000E+00 | 5.595E-07 | 5.101E-07 |
| Unigene464094 | evgS | 0.000E+00 | 0.000E+00 | 0.000E+00 | 0.000E+00 | 2.862E-07 | 0.000E+00 | 1.426E-06 | 3.932E-07 | 0.000E+00 | 0.000E+00 | 0.000E+00 | 0.000E+00 |
| Unigene464143 | kdpE | 0.000E+00 | 0.000E+00 | 0.000E+00 | 0.000E+00 | 1.559E-07 | 0.000E+00 | 9.320E-07 | 0.000E+00 | 0.000E+00 | 0.000E+00 | 0.000E+00 | 5.530E-07 |
| Unigene464150 | patB | 0.000E+00 | 0.000E+00 | 0.000E+00 | 0.000E+00 | 0.000E+00 | 0.000E+00 | 8.474E-07 | 4.234E-07 | 0.000E+00 | 0.000E+00 | 0.000E+00 | 0.000E+00 |
| Unigene464190 | vatE | 0.000E+00 | 0.000E+00 | 0.000E+00 | 0.000E+00 | 0.000E+00 | 0.000E+00 | 4.791E-07 | 0.000E+00 | 0.000E+00 | 0.000E+00 | 0.000E+00 | 0.000E+00 |

| | | | | | | | | | | | | | |
|---|---|---|---|---|---|---|---|---|---|---|---|---|---|
| Unigene464403 | basS | 0.000E+00 | 0.000E+00 | 0.000E+00 | 0.000E+00 | 0.000E+00 | 0.000E+00 | 1.051E-06 | 1.847E-07 | 1.384E-07 | 0.000E+00 | 0.000E+00 | 2.619E-07 |
| Unigene464489 | lmrC | 0.000E+00 | 0.000E+00 | 0.000E+00 | 0.000E+00 | 0.000E+00 | 0.000E+00 | 5.332E-07 | 0.000E+00 | 0.000E+00 | 0.000E+00 | 0.000E+00 | 0.000E+00 |
| Unigene464532 | novA | 0.000E+00 | 0.000E+00 | 0.000E+00 | 0.000E+00 | 0.000E+00 | 0.000E+00 | 6.334E-07 | 0.000E+00 | 0.000E+00 | 0.000E+00 | 0.000E+00 | 0.000E+00 |
| Unigene464589 | cpxA | 0.000E+00 | 0.000E+00 | 0.000E+00 | 0.000E+00 | 0.000E+00 | 0.000E+00 | 5.857E-07 | 0.000E+00 | 0.000E+00 | 0.000E+00 | 0.000E+00 | 2.085E-07 |
| Unigene464610 | mdtC | 0.000E+00 | 0.000E+00 | 0.000E+00 | 0.000E+00 | 0.000E+00 | 0.000E+00 | 9.833E-07 | 0.000E+00 | 0.000E+00 | 0.000E+00 | 0.000E+00 | 0.000E+00 |
| Unigene464939 | macB | 0.000E+00 | 0.000E+00 | 0.000E+00 | 0.000E+00 | 0.000E+00 | 0.000E+00 | 8.213E-07 | 4.812E-08 | 0.000E+00 | 0.000E+00 | 0.000E+00 | 0.000E+00 |
| Unigene465011 | baeS | 0.000E+00 | 0.000E+00 | 0.000E+00 | 0.000E+00 | 0.000E+00 | 0.000E+00 | 2.585E-06 | 0.000E+00 | 0.000E+00 | 0.000E+00 | 0.000E+00 | 0.000E+00 |
| Unigene465120 | efrA | 1.233E-07 | 0.000E+00 | 9.076E-08 | 1.868E-07 | 2.398E-07 | 3.666E-07 | 9.896E-07 | 3.959E-07 | 1.011E-07 | 2.404E-07 | 5.995E-07 | 1.093E-06 |
| Unigene465167 | bacA | 0.000E+00 | 0.000E+00 | 0.000E+00 | 0.000E+00 | 0.000E+00 | 0.000E+00 | 6.293E-07 | 0.000E+00 | 0.000E+00 | 0.000E+00 | 0.000E+00 | 0.000E+00 |
| Unigene465272 | tetB(60) | 1.268E-06 | 7.612E-07 | 1.128E-06 | 1.181E-06 | 1.862E-05 | 5.570E-06 | 1.489E-05 | 1.726E-05 | 1.124E-05 | 1.885E-05 | 1.879E-05 | 8.245E-06 |
| Unigene465324 | cpxA | 0.000E+00 | 0.000E+00 | 0.000E+00 | 0.000E+00 | 0.000E+00 | 0.000E+00 | 4.083E-07 | 0.000E+00 | 0.000E+00 | 0.000E+00 | 0.000E+00 | 0.000E+00 |
| Unigene465473 | oleB | 0.000E+00 | 0.000E+00 | 0.000E+00 | 0.000E+00 | 0.000E+00 | 0.000E+00 | 6.543E-07 | 0.000E+00 | 0.000E+00 | 0.000E+00 | 0.000E+00 | 0.000E+00 |
| Unigene465532 | adeG | 0.000E+00 | 0.000E+00 | 0.000E+00 | 0.000E+00 | 0.000E+00 | 0.000E+00 | 8.744E-07 | 0.000E+00 | 0.000E+00 | 0.000E+00 | 0.000E+00 | 0.000E+00 |
| Unigene465543 | catB10 | 0.000E+00 | 0.000E+00 | 0.000E+00 | 0.000E+00 | 0.000E+00 | 0.000E+00 | 5.771E-07 | 0.000E+00 | 0.000E+00 | 0.000E+00 | 0.000E+00 | 0.000E+00 |
| Unigene465748 | patB | 0.000E+00 | 0.000E+00 | 0.000E+00 | 0.000E+00 | 0.000E+00 | 0.000E+00 | 4.003E-07 | 0.000E+00 | 0.000E+00 | 0.000E+00 | 0.000E+00 | 0.000E+00 |
| Unigene465873 | poxtA | 0.000E+00 | 0.000E+00 | 0.000E+00 | 0.000E+00 | 0.000E+00 | 0.000E+00 | 1.023E-06 | 0.000E+00 | 0.000E+00 | 0.000E+00 | 0.000E+00 | 0.000E+00 |
| Unigene465927 | BJP-1 | 0.000E+00 | 0.000E+00 | 0.000E+00 | 0.000E+00 | 0.000E+00 | 0.000E+00 | 5.273E-07 | 4.171E-07 | 0.000E+00 | 0.000E+00 | 3.633E-07 | 0.000E+00 |
| Unigene465930 | tlrC | 0.000E+00 | 0.000E+00 | 0.000E+00 | 1.509E-07 | 0.000E+00 | 0.000E+00 | 0.000E+00 | 0.000E+00 | 8.167E-08 | 0.000E+00 | 8.547E-08 | 8.832E-08 |
| Unigene465932 | oleC | 0.000E+00 | 0.000E+00 | 0.000E+00 | 0.000E+00 | 0.000E+00 | 0.000E+00 | 4.027E-07 | 0.000E+00 | 0.000E+00 | 0.000E+00 | 0.000E+00 | 1.229E-07 |
| Unigene466030 | MexI | 0.000E+00 | 0.000E+00 | 0.000E+00 | 0.000E+00 | 0.000E+00 | 0.000E+00 | 6.633E-07 | 0.000E+00 | 0.000E+00 | 0.000E+00 | 0.000E+00 | 0.000E+00 |
| Unigene466031 | MexH | 0.000E+00 | 0.000E+00 | 0.000E+00 | 0.000E+00 | 0.000E+00 | 0.000E+00 | 7.711E-07 | 0.000E+00 | 0.000E+00 | 0.000E+00 | 0.000E+00 | 0.000E+00 |
| Unigene466094 | Staphylocc | 1.461E-07 | 0.000E+00 | 0.000E+00 | 0.000E+00 | 0.000E+00 | 0.000E+00 | 1.173E-06 | 0.000E+00 | 0.000E+00 | 0.000E+00 | 0.000E+00 | 1.036E-06 |
| Unigene466175 | macB | 0.000E+00 | 0.000E+00 | 0.000E+00 | 0.000E+00 | 0.000E+00 | 0.000E+00 | 6.181E-07 | 0.000E+00 | 0.000E+00 | 0.000E+00 | 0.000E+00 | 1.100E-06 |
| Unigene466197 | catB10 | 0.000E+00 | 0.000E+00 | 0.000E+00 | 0.000E+00 | 0.000E+00 | 0.000E+00 | 1.033E-06 | 0.000E+00 | 0.000E+00 | 0.000E+00 | 0.000E+00 | 0.000E+00 |
| Unigene466238 | patA | 0.000E+00 | 0.000E+00 | 0.000E+00 | 0.000E+00 | 0.000E+00 | 0.000E+00 | 8.622E-07 | 1.137E-07 | 0.000E+00 | 0.000E+00 | 0.000E+00 | 0.000E+00 |
| Unigene466271 | Brucella su | 0.000E+00 | 0.000E+00 | 0.000E+00 | 0.000E+00 | 0.000E+00 | 4.399E-07 | 4.503E-07 | 5.277E-07 | 0.000E+00 | 1.007E-07 | 2.069E-07 | 6.573E-06 |
| Unigene466289 | vatB | 0.000E+00 | 0.000E+00 | 0.000E+00 | 0.000E+00 | 0.000E+00 | 0.000E+00 | 1.015E-06 | 0.000E+00 | 0.000E+00 | 0.000E+00 | 0.000E+00 | 0.000E+00 |
| Unigene466290 | OXA-10 | 0.000E+00 | 0.000E+00 | 0.000E+00 | 0.000E+00 | 0.000E+00 | 0.000E+00 | 6.011E-07 | 0.000E+00 | 0.000E+00 | 0.000E+00 | 0.000E+00 | 0.000E+00 |
| Unigene466368 | tet(B) | 0.000E+00 | 0.000E+00 | 0.000E+00 | 0.000E+00 | 0.000E+00 | 0.000E+00 | 5.796E-07 | 0.000E+00 | 0.000E+00 | 0.000E+00 | 0.000E+00 | 0.000E+00 |
| Unigene466446 | AcrS | 0.000E+00 | 1.104E-07 | 0.000E+00 | 0.000E+00 | 0.000E+00 | 0.000E+00 | 8.347E-07 | 9.977E-07 | 1.649E-07 | 2.240E-07 | 6.902E-07 | 1.010E-06 |
| Unigene466492 | sdiA | 0.000E+00 | 4.781E-08 | 2.565E-07 | 0.000E+00 | 0.000E+00 | 0.000E+00 | 9.161E-07 | 3.051E-07 | 0.000E+00 | 0.000E+00 | 9.966E-08 | 3.604E-07 |
| Unigene466512 | tetA(58) | 0.000E+00 | 0.000E+00 | 0.000E+00 | 0.000E+00 | 0.000E+00 | 0.000E+00 | 6.213E-07 | 0.000E+00 | 0.000E+00 | 0.000E+00 | 0.000E+00 | 0.000E+00 |
| Unigene466524 | macB | 0.000E+00 | 0.000E+00 | 0.000E+00 | 0.000E+00 | 0.000E+00 | 4.979E-07 | 1.853E-06 | 2.248E-06 | 9.152E-08 | 0.000E+00 | 8.621E-07 | 1.089E-06 |
| Unigene466529 | Staphylocc | 0.000E+00 | 0.000E+00 | 0.000E+00 | 0.000E+00 | 0.000E+00 | 2.614E-07 | 1.026E-06 | 0.000E+00 | 0.000E+00 | 0.000E+00 | 0.000E+00 | 0.000E+00 |
| Unigene466564 | macB | 4.015E-08 | 4.407E-08 | 1.182E-07 | 0.000E+00 | 2.676E-07 | 2.170E-07 | 5.777E-07 | 4.686E-07 | 0.000E+00 | 2.236E-07 | 6.430E-07 | 1.898E-06 |
| Unigene466570 | oleB | 6.317E-08 | 4.622E-08 | 1.446E-07 | 1.701E-07 | 6.082E-07 | 1.821E-07 | 1.002E-06 | 8.111E-07 | 0.000E+00 | 0.000E+00 | 4.336E-07 | 1.394E-06 |
| Unigene466600 | abeS | 0.000E+00 | 0.000E+00 | 0.000E+00 | 0.000E+00 | 0.000E+00 | 0.000E+00 | 1.066E-06 | 0.000E+00 | 0.000E+00 | 0.000E+00 | 0.000E+00 | 0.000E+00 |
| Unigene466623 | sul4 | 0.000E+00 | 0.000E+00 | 0.000E+00 | 0.000E+00 | 0.000E+00 | 0.000E+00 | 9.597E-07 | 0.000E+00 | 0.000E+00 | 0.000E+00 | 0.000E+00 | 0.000E+00 |
| Unigene466714 | novA | 0.000E+00 | 0.000E+00 | 0.000E+00 | 0.000E+00 | 1.502E-06 | 0.000E+00 | 1.301E-06 | 1.510E-06 | 0.000E+00 | 0.000E+00 | 0.000E+00 | 0.000E+00 |
| Unigene466715 | msbA | 0.000E+00 | 9.891E-08 | 0.000E+00 | 0.000E+00 | 6.407E-07 | 3.702E-07 | 7.780E-07 | 6.311E-07 | 1.182E-07 | 1.807E-07 | 6.597E-07 | 8.309E-07 |
| Unigene466851 | lmrD | 0.000E+00 | 0.000E+00 | 0.000E+00 | 6.666E-08 | 0.000E+00 | 0.000E+00 | 2.192E-07 | 0.000E+00 | 0.000E+00 | 0.000E+00 | 0.000E+00 | 7.803E-08 |
| Unigene466910 | macB | 0.000E+00 | 0.000E+00 | 0.000E+00 | 4.509E-08 | 0.000E+00 | 3.379E-07 | 7.412E-07 | 0.000E+00 | 0.000E+00 | 0.000E+00 | 0.000E+00 | 0.000E+00 |
| Unigene466949 | efrB | 5.604E-08 | 2.050E-08 | 9.165E-08 | 5.659E-08 | 2.490E-07 | 1.818E-07 | 8.477E-07 | 9.812E-07 | 1.429E-07 | 0.000E+00 | 4.060E-07 | 9.716E-07 |
| Unigene466959 | adeL | 0.000E+00 | 0.000E+00 | 0.000E+00 | 0.000E+00 | 0.000E+00 | 0.000E+00 | 9.297E-07 | 0.000E+00 | 0.000E+00 | 0.000E+00 | 1.830E-07 | 8.038E-07 |
| Unigene466961 | cmx | 0.000E+00 | 0.000E+00 | 1.572E-07 | 0.000E+00 | 0.000E+00 | 0.000E+00 | 1.034E-06 | 1.558E-07 | 0.000E+00 | 1.487E-07 | 0.000E+00 | 7.259E-07 |
| Unigene466967 | Streptomy | 1.118E-07 | 6.658E-07 | 1.880E-07 | 0.000E+00 | 1.951E-07 | 1.553E-07 | 9.718E-07 | 2.124E-06 | 0.000E+00 | 0.000E+00 | 2.922E-07 | 1.321E-07 |
| Unigene466977 | APH(6)-Id | 0.000E+00 | 0.000E+00 | 0.000E+00 | 1.552E-07 | 0.000E+00 | 0.000E+00 | 8.506E-07 | 0.000E+00 | 0.000E+00 | 0.000E+00 | 0.000E+00 | 6.814E-07 |
| Unigene466998 | evgS | 0.000E+00 | 0.000E+00 | 0.000E+00 | 3.881E-08 | 0.000E+00 | 0.000E+00 | 5.529E-07 | 0.000E+00 | 1.680E-07 | 0.000E+00 | 0.000E+00 | 9.085E-08 |
| Unigene467045 | vmlR | 1.031E-07 | 9.049E-08 | 8.091E-08 | 1.041E-07 | 0.000E+00 | 2.006E-07 | 1.049E-06 | 3.369E-07 | 0.000E+00 | 0.000E+00 | 0.000E+00 | 0.000E+00 |
| Unigene467081 | ceoB | 0.000E+00 | 0.000E+00 | 0.000E+00 | 9.923E-08 | 0.000E+00 | 0.000E+00 | 7.613E-07 | 0.000E+00 | 0.000E+00 | 3.831E-07 | 0.000E+00 | 0.000E+00 |
| Unigene467134 | bcrA | 0.000E+00 | 0.000E+00 | 0.000E+00 | 0.000E+00 | 9.022E-08 | 1.317E-07 | 4.943E-07 | 3.318E-07 | 0.000E+00 | 0.000E+00 | 8.360E-07 | 4.320E-07 |
| Unigene467137 | mexP | 0.000E+00 | 0.000E+00 | 0.000E+00 | 0.000E+00 | 0.000E+00 | 1.572E-07 | 0.000E+00 | 0.000E+00 | 0.000E+00 | 0.000E+00 | 0.000E+00 | 0.000E+00 |
| Unigene467175 | TaeA | 0.000E+00 | 0.000E+00 | 0.000E+00 | 0.000E+00 | 0.000E+00 | 0.000E+00 | 3.015E-07 | 0.000E+00 | 0.000E+00 | 0.000E+00 | 0.000E+00 | 0.000E+00 |
| Unigene467333 | tetA(58) | 0.000E+00 | 0.000E+00 | 0.000E+00 | 0.000E+00 | 0.000E+00 | 0.000E+00 | 2.563E-06 | 0.000E+00 | 0.000E+00 | 0.000E+00 | 0.000E+00 | 0.000E+00 |
| Unigene467347 | smeF | 0.000E+00 | 0.000E+00 | 0.000E+00 | 0.000E+00 | 0.000E+00 | 0.000E+00 | 6.030E-07 | 0.000E+00 | 0.000E+00 | 0.000E+00 | 0.000E+00 | 0.000E+00 |
| Unigene467403 | tetA(58) | 0.000E+00 | 9.226E-08 | 3.712E-07 | 0.000E+00 | 0.000E+00 | 0.000E+00 | 1.279E-06 | 5.397E-07 | 0.000E+00 | 0.000E+00 | 0.000E+00 | 1.515E-06 |
| Unigene467423 | tetT | 0.000E+00 | 0.000E+00 | 0.000E+00 | 0.000E+00 | 2.866E-07 | 0.000E+00 | 0.000E+00 | 0.000E+00 | 0.000E+00 | 0.000E+00 | 3.794E-07 | 0.000E+00 |
| Unigene467557 | arlR | 1.832E-07 | 5.025E-08 | 3.145E-07 | 0.000E+00 | 1.017E-07 | 2.475E-07 | 4.054E-07 | 5.879E-07 | 0.000E+00 | 1.071E-06 | 2.095E-07 | 4.330E-07 |

| | | | | | | | | | | | | | |
|---|---|---|---|---|---|---|---|---|---|---|---|---|---|
| Unigene467665 | tva(A) | 5.595E-07 | 0.000E+00 | 0.000E+00 | 3.477E-07 | 0.000E+00 | 1.396E-07 | 8.574E-07 | 6.531E-07 | 4.704E-07 | 0.000E+00 | 0.000E+00 | 0.000E+00 |
| Unigene467666 | bcrA | 0.000E+00 | 0.000E+00 | 0.000E+00 | 0.000E+00 | 0.000E+00 | 0.000E+00 | 5.622E-07 | 0.000E+00 | 0.000E+00 | 0.000E+00 | 0.000E+00 | 0.000E+00 |
| Unigene467804 | macB | 1.740E-07 | 0.000E+00 | 0.000E+00 | 1.757E-07 | 0.000E+00 | 4.389E-07 | 1.477E-06 | 2.370E-07 | 1.268E-07 | 1.939E-07 | 0.000E+00 | 5.142E-07 |
| Unigene467843 | oleC | 0.000E+00 | 0.000E+00 | 0.000E+00 | 0.000E+00 | 0.000E+00 | 0.000E+00 | 2.382E-06 | 0.000E+00 | 0.000E+00 | 0.000E+00 | 0.000E+00 | 0.000E+00 |
| Unigene467854 | lmrC | 0.000E+00 | 0.000E+00 | 0.000E+00 | 0.000E+00 | 4.269E-07 | 0.000E+00 | 1.323E-06 | 2.542E-06 | 0.000E+00 | 0.000E+00 | 6.350E-07 | 1.161E-06 |
| Unigene467866 | macB | 0.000E+00 | 0.000E+00 | 3.438E-07 | 0.000E+00 | 2.977E-07 | 1.560E-07 | 1.095E-06 | 2.165E-07 | 0.000E+00 | 0.000E+00 | 2.594E-07 | 0.000E+00 |
| Unigene467904 | patA | 0.000E+00 | 3.623E-08 | 9.716E-08 | 0.000E+00 | 7.700E-07 | 1.784E-07 | 6.941E-07 | 1.926E-07 | 7.215E-08 | 0.000E+00 | 1.888E-07 | 7.803E-07 |
| Unigene467916 | msbA | 0.000E+00 | 0.000E+00 | 3.798E-07 | 0.000E+00 | 0.000E+00 | 0.000E+00 | 0.000E+00 | 0.000E+00 | 0.000E+00 | 0.000E+00 | 0.000E+00 | 0.000E+00 |
| Unigene467973 | mphB | 0.000E+00 | 0.000E+00 | 0.000E+00 | 0.000E+00 | 0.000E+00 | 0.000E+00 | 1.043E-06 | 0.000E+00 | 0.000E+00 | 0.000E+00 | 0.000E+00 | 0.000E+00 |
| Unigene468027 | tetS | 0.000E+00 | 0.000E+00 | 0.000E+00 | 0.000E+00 | 0.000E+00 | 0.000E+00 | 1.269E-06 | 3.825E-07 | 0.000E+00 | 0.000E+00 | 0.000E+00 | 0.000E+00 |
| Unigene468033 | macB | 0.000E+00 | 0.000E+00 | 0.000E+00 | 0.000E+00 | 0.000E+00 | 0.000E+00 | 2.394E-07 | 1.893E-07 | 0.000E+00 | 0.000E+00 | 0.000E+00 | 8.309E-07 |
| Unigene468323 | smeR | 0.000E+00 | 0.000E+00 | 0.000E+00 | 0.000E+00 | 0.000E+00 | 0.000E+00 | 3.383E-07 | 0.000E+00 | 0.000E+00 | 0.000E+00 | 0.000E+00 | 0.000E+00 |
| Unigene468361 | msbA | 0.000E+00 | 0.000E+00 | 5.518E-07 | 0.000E+00 | 4.790E-07 | 0.000E+00 | 1.390E-06 | 0.000E+00 | 0.000E+00 | 0.000E+00 | 4.289E-07 | 0.000E+00 |
| Unigene468389 | macB | 0.000E+00 | 0.000E+00 | 0.000E+00 | 0.000E+00 | 0.000E+00 | 0.000E+00 | 5.356E-07 | 0.000E+00 | 0.000E+00 | 0.000E+00 | 0.000E+00 | 0.000E+00 |
| Unigene468438 | TaeA | 6.571E-08 | 9.015E-08 | 1.612E-07 | 2.488E-07 | 9.308E-07 | 0.000E+00 | 1.073E-06 | 1.246E-06 | 0.000E+00 | 2.379E-07 | 4.510E-07 | 1.049E-06 |
| Unigene468562 | novA | 3.913E-08 | 0.000E+00 | 0.000E+00 | 0.000E+00 | 0.000E+00 | 0.000E+00 | 9.094E-07 | 1.827E-07 | 0.000E+00 | 0.000E+00 | 0.000E+00 | 1.387E-07 |
| Unigene468660 | tetA(58) | 0.000E+00 | 6.361E-08 | 2.843E-07 | 0.000E+00 | 6.438E-08 | 0.000E+00 | 8.124E-07 | 6.765E-08 | 0.000E+00 | 1.076E-07 | 2.873E-07 | 2.512E-07 |
| Unigene468689 | vanG | 0.000E+00 | 0.000E+00 | 0.000E+00 | 0.000E+00 | 0.000E+00 | 0.000E+00 | 5.413E-07 | 0.000E+00 | 0.000E+00 | 0.000E+00 | 0.000E+00 | 0.000E+00 |
| Unigene468849 | tetA(58) | 0.000E+00 | 0.000E+00 | 0.000E+00 | 0.000E+00 | 0.000E+00 | 0.000E+00 | 4.787E-07 | 4.418E-07 | 0.000E+00 | 0.000E+00 | 6.185E-08 | 1.917E-07 |
| Unigene468884 | tetB(60) | 0.000E+00 | 0.000E+00 | 0.000E+00 | 0.000E+00 | 0.000E+00 | 0.000E+00 | 8.894E-07 | 0.000E+00 | 0.000E+00 | 0.000E+00 | 0.000E+00 | 0.000E+00 |
| Unigene468986 | macB | 0.000E+00 | 0.000E+00 | 8.549E-08 | 0.000E+00 | 0.000E+00 | 0.000E+00 | 6.268E-07 | 0.000E+00 | 0.000E+00 | 0.000E+00 | 0.000E+00 | 0.000E+00 |
| Unigene469029 | AAC(6')-II | 0.000E+00 | 0.000E+00 | 0.000E+00 | 0.000E+00 | 0.000E+00 | 9.055E-07 | 0.000E+00 | 0.000E+00 | 0.000E+00 | 0.000E+00 | 1.474E-07 | 0.000E+00 |
| Unigene469032 | tetA(60) | 0.000E+00 | 0.000E+00 | 0.000E+00 | 0.000E+00 | 0.000E+00 | 0.000E+00 | 2.835E-07 | 0.000E+00 | 0.000E+00 | 0.000E+00 | 0.000E+00 | 0.000E+00 |
| Unigene469063 | patA | 2.242E-07 | 0.000E+00 | 6.285E-08 | 0.000E+00 | 4.625E-07 | 1.039E-07 | 9.924E-07 | 1.121E-06 | 1.400E-07 | 1.070E-07 | 4.396E-07 | 1.893E-06 |
| Unigene469127 | vanG | 0.000E+00 | 0.000E+00 | 0.000E+00 | 0.000E+00 | 0.000E+00 | 0.000E+00 | 6.924E-07 | 0.000E+00 | 0.000E+00 | 0.000E+00 | 0.000E+00 | 0.000E+00 |
| Unigene469142 | MexF | 0.000E+00 | 0.000E+00 | 0.000E+00 | 0.000E+00 | 0.000E+00 | 0.000E+00 | 6.852E-07 | 0.000E+00 | 6.444E-08 | 0.000E+00 | 2.529E-07 | 0.000E+00 |
| Unigene469152 | Acinetoba | 0.000E+00 | 0.000E+00 | 0.000E+00 | 0.000E+00 | 0.000E+00 | 0.000E+00 | 0.000E+00 | 0.000E+00 | 0.000E+00 | 0.000E+00 | 0.000E+00 | 2.650E-07 |
| Unigene469189 | macB | 0.000E+00 | 0.000E+00 | 0.000E+00 | 0.000E+00 | 0.000E+00 | 0.000E+00 | 4.872E-07 | 0.000E+00 | 0.000E+00 | 0.000E+00 | 2.797E-07 | 0.000E+00 |
| Unigene469201 | vanRO | 0.000E+00 | 0.000E+00 | 0.000E+00 | 0.000E+00 | 0.000E+00 | 0.000E+00 | 6.728E-07 | 0.000E+00 | 0.000E+00 | 0.000E+00 | 0.000E+00 | 0.000E+00 |
| Unigene469322 | lmrD | 1.834E-07 | 0.000E+00 | 2.400E-07 | 0.000E+00 | 4.075E-07 | 0.000E+00 | 7.443E-07 | 0.000E+00 | 0.000E+00 | 0.000E+00 | 8.858E-07 | 7.949E-07 |
| Unigene469329 | vatF | 0.000E+00 | 0.000E+00 | 0.000E+00 | 0.000E+00 | 0.000E+00 | 0.000E+00 | 1.098E-06 | 0.000E+00 | 0.000E+00 | 0.000E+00 | 0.000E+00 | 0.000E+00 |
| Unigene469330 | tetA(46) | 0.000E+00 | 0.000E+00 | 0.000E+00 | 0.000E+00 | 0.000E+00 | 0.000E+00 | 1.665E-06 | 3.725E-07 | 0.000E+00 | 0.000E+00 | 0.000E+00 | 0.000E+00 |
| Unigene469351 | Streptomy | 0.000E+00 | 0.000E+00 | 0.000E+00 | 0.000E+00 | 0.000E+00 | 0.000E+00 | 9.351E-07 | 0.000E+00 | 0.000E+00 | 1.107E-07 | 0.000E+00 | 0.000E+00 |
| Unigene469394 | macB | 1.668E-07 | 1.373E-07 | 2.046E-07 | 0.000E+00 | 4.170E-07 | 4.509E-07 | 6.924E-07 | 9.736E-07 | 0.000E+00 | 0.000E+00 | 9.541E-07 | 2.711E-06 |
| Unigene469423 | Staphyloc | 0.000E+00 | 5.442E-08 | 4.866E-08 | 0.000E+00 | 0.000E+00 | 0.000E+00 | 7.500E-07 | 3.859E-07 | 0.000E+00 | 0.000E+00 | 0.000E+00 | 0.000E+00 |
| Unigene469517 | Streptomy | 1.824E-07 | 0.000E+00 | 0.000E+00 | 0.000E+00 | 0.000E+00 | 0.000E+00 | 7.737E-07 | 6.031E-07 | 2.658E-07 | 0.000E+00 | 3.477E-07 | 6.467E-07 |
| Unigene469537 | novA | 0.000E+00 | 0.000E+00 | 4.092E-07 | 0.000E+00 | 3.243E-07 | 2.254E-07 | 8.770E-07 | 0.000E+00 | 0.000E+00 | 0.000E+00 | 0.000E+00 | 0.000E+00 |
| Unigene469663 | macB | 0.000E+00 | 0.000E+00 | 0.000E+00 | 0.000E+00 | 0.000E+00 | 0.000E+00 | 5.140E-07 | 0.000E+00 | 0.000E+00 | 0.000E+00 | 0.000E+00 | 2.288E-07 |
| Unigene469682 | macB | 0.000E+00 | 0.000E+00 | 7.952E-07 | 0.000E+00 | 0.000E+00 | 7.456E-08 | 9.161E-07 | 0.000E+00 | 0.000E+00 | 0.000E+00 | 2.170E-07 | 0.000E+00 |
| Unigene469726 | macB | 2.975E-07 | 0.000E+00 | 0.000E+00 | 1.502E-07 | 0.000E+00 | 0.000E+00 | 4.939E-07 | 0.000E+00 | 0.000E+00 | 0.000E+00 | 0.000E+00 | 0.000E+00 |
| Unigene469844 | novA | 0.000E+00 | 5.397E-07 | 0.000E+00 | 0.000E+00 | 8.676E-07 | 0.000E+00 | 2.369E-06 | 0.000E+00 | 0.000E+00 | 0.000E+00 | 0.000E+00 | 0.000E+00 |
| Unigene469883 | Chlamydia | 0.000E+00 | 0.000E+00 | 0.000E+00 | 0.000E+00 | 0.000E+00 | 0.000E+00 | 1.004E-06 | 0.000E+00 | 0.000E+00 | 0.000E+00 | 0.000E+00 | 0.000E+00 |
| Unigene469937 | tetA(58) | 0.000E+00 | 0.000E+00 | 0.000E+00 | 0.000E+00 | 0.000E+00 | 0.000E+00 | 8.456E-07 | 0.000E+00 | 0.000E+00 | 0.000E+00 | 0.000E+00 | 0.000E+00 |
| Unigene469949 | NmcR | 0.000E+00 | 0.000E+00 | 0.000E+00 | 0.000E+00 | 0.000E+00 | 0.000E+00 | 7.749E-07 | 0.000E+00 | 0.000E+00 | 0.000E+00 | 0.000E+00 | 0.000E+00 |
| Unigene469986 | MexL | 0.000E+00 | 0.000E+00 | 0.000E+00 | 0.000E+00 | 0.000E+00 | 0.000E+00 | 1.078E-06 | 0.000E+00 | 0.000E+00 | 0.000E+00 | 0.000E+00 | 7.675E-07 |
| Unigene470051 | vgaE | 0.000E+00 | 0.000E+00 | 0.000E+00 | 0.000E+00 | 0.000E+00 | 0.000E+00 | 5.032E-07 | 0.000E+00 | 0.000E+00 | 0.000E+00 | 0.000E+00 | 0.000E+00 |
| Unigene470149 | kdpE | 0.000E+00 | 0.000E+00 | 0.000E+00 | 0.000E+00 | 0.000E+00 | 0.000E+00 | 5.344E-07 | 0.000E+00 | 0.000E+00 | 0.000E+00 | 0.000E+00 | 0.000E+00 |
| Unigene470417 | vanHD | 0.000E+00 | 0.000E+00 | 0.000E+00 | 3.364E-07 | 0.000E+00 | 1.801E-07 | 8.849E-07 | 0.000E+00 | 1.456E-07 | 3.340E-07 | 5.335E-07 | 5.907E-07 |
| Unigene470433 | adeA | 0.000E+00 | 0.000E+00 | 0.000E+00 | 0.000E+00 | 0.000E+00 | 0.000E+00 | 7.217E-07 | 0.000E+00 | 0.000E+00 | 0.000E+00 | 0.000E+00 | 0.000E+00 |
| Unigene470592 | cpxA | 1.190E-07 | 1.045E-07 | 1.869E-07 | 0.000E+00 | 7.141E-07 | 2.573E-07 | 1.133E-06 | 6.669E-07 | 1.821E-07 | 2.122E-07 | 8.442E-07 | 1.351E-06 |
| Unigene470593 | MexH | 0.000E+00 | 0.000E+00 | 0.000E+00 | 1.630E-07 | 0.000E+00 | 0.000E+00 | 8.336E-07 | 0.000E+00 | 1.470E-07 | 0.000E+00 | 0.000E+00 | 1.272E-06 |
| Unigene470612 | oleB | 0.000E+00 | 0.000E+00 | 0.000E+00 | 0.000E+00 | 0.000E+00 | 0.000E+00 | 4.530E-07 | 0.000E+00 | 0.000E+00 | 0.000E+00 | 2.676E-07 | 0.000E+00 |
| Unigene470653 | tetA(46) | 0.000E+00 | 0.000E+00 | 2.295E-07 | 5.905E-08 | 1.516E-07 | 4.004E-07 | 1.747E-06 | 7.509E-07 | 0.000E+00 | 3.691E-07 | 2.676E-07 | 7.373E-07 |
| Unigene470659 | TaeA | 0.000E+00 | 0.000E+00 | 0.000E+00 | 0.000E+00 | 0.000E+00 | 0.000E+00 | 1.547E-06 | 0.000E+00 | 0.000E+00 | 0.000E+00 | 0.000E+00 | 3.004E-07 |
| Unigene470684 | tlrC | 0.000E+00 | 0.000E+00 | 0.000E+00 | 0.000E+00 | 0.000E+00 | 0.000E+00 | 8.143E-07 | 0.000E+00 | 0.000E+00 | 0.000E+00 | 0.000E+00 | 5.073E-07 |
| Unigene470688 | bcrA | 9.389E-08 | 1.443E-07 | 0.000E+00 | 0.000E+00 | 0.000E+00 | 1.421E-07 | 8.729E-07 | 2.038E-06 | 3.284E-07 | 7.531E-07 | 1.289E-06 | 1.953E-06 |
| Unigene470744 | arlR | 0.000E+00 | 0.000E+00 | 0.000E+00 | 0.000E+00 | 0.000E+00 | 0.000E+00 | 1.196E-06 | 0.000E+00 | 0.000E+00 | 0.000E+00 | 0.000E+00 | 0.000E+00 |
| Unigene470754 | patA | 0.000E+00 | 0.000E+00 | 0.000E+00 | 0.000E+00 | 4.427E-07 | 8.616E-08 | 7.939E-07 | 5.117E-07 | 0.000E+00 | 0.000E+00 | 0.000E+00 | 2.826E-07 |

| | | | | | | | | | | | | | |
|---|---|---|---|---|---|---|---|---|---|---|---|---|---|
| Unigene470787 | optrA | 0.000E+00 | 0.000E+00 | 0.000E+00 | 0.000E+00 | 0.000E+00 | 0.000E+00 | 6.870E-07 | 2.818E-07 | 3.769E-08 | 0.000E+00 | 0.000E+00 | 0.000E+00 |
| Unigene470811 | bcrA | 0.000E+00 | 0.000E+00 | 0.000E+00 | 0.000E+00 | 0.000E+00 | 0.000E+00 | 1.064E-06 | 6.948E-07 | 0.000E+00 | 0.000E+00 | 2.619E-07 | 1.570E-06 |
| Unigene470906 | cmlv | 7.990E-08 | 0.000E+00 | 2.091E-07 | 8.069E-08 | 1.775E-07 | 2.015E-07 | 1.739E-06 | 2.176E-06 | 5.822E-08 | 2.670E-07 | 0.000E+00 | 3.148E-07 |
| Unigene470982 | rosB | 0.000E+00 | 0.000E+00 | 1.508E-07 | 0.000E+00 | 0.000E+00 | 0.000E+00 | 3.828E-07 | 3.140E-07 | 0.000E+00 | 0.000E+00 | 2.198E-07 | 0.000E+00 |
| Unigene471104 | adeL | 0.000E+00 | 0.000E+00 | 0.000E+00 | 0.000E+00 | 0.000E+00 | 0.000E+00 | 5.261E-07 | 3.531E-07 | 0.000E+00 | 0.000E+00 | 0.000E+00 | 0.000E+00 |
| Unigene471125 | macB | 0.000E+00 | 0.000E+00 | 0.000E+00 | 0.000E+00 | 0.000E+00 | 0.000E+00 | 6.616E-07 | 0.000E+00 | 0.000E+00 | 0.000E+00 | 0.000E+00 | 0.000E+00 |
| Unigene471216 | adeL | 0.000E+00 | 0.000E+00 | 0.000E+00 | 0.000E+00 | 0.000E+00 | 0.000E+00 | 1.167E-06 | 0.000E+00 | 0.000E+00 | 0.000E+00 | 0.000E+00 | 0.000E+00 |
| Unigene471245 | oleC | 0.000E+00 | 0.000E+00 | 0.000E+00 | 0.000E+00 | 0.000E+00 | 0.000E+00 | 8.736E-07 | 0.000E+00 | 0.000E+00 | 0.000E+00 | 0.000E+00 | 0.000E+00 |
| Unigene471291 | macB | 0.000E+00 | 0.000E+00 | 0.000E+00 | 0.000E+00 | 0.000E+00 | 0.000E+00 | 5.671E-07 | 0.000E+00 | 0.000E+00 | 0.000E+00 | 0.000E+00 | 0.000E+00 |
| Unigene471364 | tetA(58) | 0.000E+00 | 0.000E+00 | 0.000E+00 | 0.000E+00 | 0.000E+00 | 0.000E+00 | 5.752E-07 | 0.000E+00 | 0.000E+00 | 0.000E+00 | 0.000E+00 | 0.000E+00 |
| Unigene471436 | MuxB | 0.000E+00 | 0.000E+00 | 0.000E+00 | 0.000E+00 | 0.000E+00 | 1.652E-07 | 8.696E-07 | 8.407E-07 | 0.000E+00 | 0.000E+00 | 0.000E+00 | 4.902E-07 |
| Unigene471459 | almG | 0.000E+00 | 0.000E+00 | 0.000E+00 | 0.000E+00 | 0.000E+00 | 0.000E+00 | 7.939E-07 | 0.000E+00 | 0.000E+00 | 0.000E+00 | 0.000E+00 | 0.000E+00 |
| Unigene471487 | mgrA | 0.000E+00 | 0.000E+00 | 0.000E+00 | 0.000E+00 | 0.000E+00 | 0.000E+00 | 8.046E-07 | 0.000E+00 | 0.000E+00 | 0.000E+00 | 0.000E+00 | 0.000E+00 |
| Unigene471531 | arlR | 0.000E+00 | 0.000E+00 | 0.000E+00 | 0.000E+00 | 0.000E+00 | 0.000E+00 | 1.045E-06 | 0.000E+00 | 0.000E+00 | 0.000E+00 | 0.000E+00 | 0.000E+00 |
| Unigene471609 | Acinetobac | 1.025E-07 | 1.125E-07 | 0.000E+00 | 7.761E-08 | 2.846E-07 | 1.939E-07 | 7.089E-07 | 1.495E-06 | 1.680E-07 | 3.995E-07 | 6.740E-07 | 2.544E-06 |
| Unigene471648 | evgS | 0.000E+00 | 1.071E-07 | 2.155E-07 | 6.160E-08 | 1.355E-07 | 1.451E-07 | 1.188E-06 | 2.421E-07 | 4.000E-08 | 0.000E+00 | 1.954E-07 | 5.624E-07 |
| Unigene471786 | efrA | 0.000E+00 | 0.000E+00 | 0.000E+00 | 0.000E+00 | 0.000E+00 | 0.000E+00 | 7.797E-07 | 0.000E+00 | 0.000E+00 | 0.000E+00 | 0.000E+00 | 0.000E+00 |
| Unigene471851 | otr(B) | 7.645E-08 | 0.000E+00 | 0.000E+00 | 0.000E+00 | 1.911E-07 | 0.000E+00 | 1.206E-06 | 2.231E-07 | 0.000E+00 | 0.000E+00 | 0.000E+00 | 0.000E+00 |
| Unigene471881 | mdtP | 0.000E+00 | 0.000E+00 | 0.000E+00 | 0.000E+00 | 0.000E+00 | 0.000E+00 | 9.721E-07 | 0.000E+00 | 0.000E+00 | 0.000E+00 | 0.000E+00 | 0.000E+00 |
| Unigene471914 | macB | 2.102E-07 | 0.000E+00 | 2.475E-07 | 0.000E+00 | 0.000E+00 | 1.818E-07 | 1.256E-06 | 0.000E+00 | 0.000E+00 | 4.214E-07 | 1.106E-06 | 9.936E-07 |
| Unigene471949 | tlrC | 0.000E+00 | 0.000E+00 | 3.616E-08 | 0.000E+00 | 0.000E+00 | 0.000E+00 | 7.749E-07 | 0.000E+00 | 0.000E+00 | 0.000E+00 | 0.000E+00 | 0.000E+00 |
| Unigene471978 | MuxB | 0.000E+00 | 0.000E+00 | 4.252E-07 | 2.292E-07 | 0.000E+00 | 6.580E-07 | 3.003E-06 | 1.927E-06 | 3.946E-07 | 7.470E-07 | 1.109E-06 | 1.512E-06 |
| Unigene472090 | vanHA | 0.000E+00 | 0.000E+00 | 0.000E+00 | 0.000E+00 | 0.000E+00 | 0.000E+00 | 7.618E-07 | 8.458E-08 | 0.000E+00 | 0.000E+00 | 2.487E-07 | 1.285E-07 |
| Unigene472176 | msbA | 6.247E-08 | 0.000E+00 | 7.662E-08 | 4.731E-08 | 2.776E-07 | 8.441E-08 | 8.124E-07 | 1.185E-06 | 1.707E-08 | 2.088E-07 | 3.216E-07 | 5.169E-07 |
| Unigene472230 | adeB | 0.000E+00 | 0.000E+00 | 3.672E-07 | 0.000E+00 | 0.000E+00 | 0.000E+00 | 8.284E-07 | 0.000E+00 | 0.000E+00 | 0.000E+00 | 1.070E-07 | 6.636E-07 |
| Unigene472363 | Streptomy | 0.000E+00 | 0.000E+00 | 0.000E+00 | 0.000E+00 | 0.000E+00 | 0.000E+00 | 5.352E-07 | 0.000E+00 | 0.000E+00 | 0.000E+00 | 0.000E+00 | 2.858E-07 |
| Unigene472417 | MexK | 1.489E-07 | 7.002E-08 | 1.252E-07 | 0.000E+00 | 1.181E-07 | 1.609E-07 | 4.001E-07 | 3.227E-07 | 0.000E+00 | 9.475E-08 | 5.108E-07 | 3.268E-07 |
| Unigene472593 | blt | 0.000E+00 | 0.000E+00 | 0.000E+00 | 0.000E+00 | 0.000E+00 | 0.000E+00 | 2.612E-07 | 5.509E-08 | 0.000E+00 | 0.000E+00 | 0.000E+00 | 3.459E-06 |
| Unigene472630 | kdpE | 0.000E+00 | 0.000E+00 | 6.033E-08 | 0.000E+00 | 0.000E+00 | 0.000E+00 | 1.191E-06 | 0.000E+00 | 0.000E+00 | 0.000E+00 | 0.000E+00 | 0.000E+00 |
| Unigene472689 | mtrA | 0.000E+00 | 0.000E+00 | 0.000E+00 | 0.000E+00 | 0.000E+00 | 1.544E-07 | 0.000E+00 | 0.000E+00 | 0.000E+00 | 0.000E+00 | 0.000E+00 | 0.000E+00 |
| Unigene472806 | mdtN | 0.000E+00 | 0.000E+00 | 0.000E+00 | 0.000E+00 | 0.000E+00 | 0.000E+00 | 3.943E-07 | 0.000E+00 | 0.000E+00 | 0.000E+00 | 0.000E+00 | 0.000E+00 |
| Unigene472835 | Pseudomo | 0.000E+00 | 0.000E+00 | 0.000E+00 | 0.000E+00 | 0.000E+00 | 0.000E+00 | 1.191E-06 | 0.000E+00 | 0.000E+00 | 0.000E+00 | 0.000E+00 | 0.000E+00 |
| Unigene472838 | MuxC | 0.000E+00 | 0.000E+00 | 0.000E+00 | 0.000E+00 | 0.000E+00 | 0.000E+00 | 0.000E+00 | 3.262E-07 | 0.000E+00 | 0.000E+00 | 0.000E+00 | 0.000E+00 |
| Unigene472851 | MexW | 0.000E+00 | 0.000E+00 | 0.000E+00 | 2.633E-07 | 0.000E+00 | 0.000E+00 | 7.869E-07 | 0.000E+00 | 1.813E-07 | 0.000E+00 | 0.000E+00 | 3.922E-07 |
| Unigene472914 | evgS | 0.000E+00 | 0.000E+00 | 0.000E+00 | 0.000E+00 | 0.000E+00 | 0.000E+00 | 6.351E-07 | 0.000E+00 | 0.000E+00 | 0.000E+00 | 0.000E+00 | 0.000E+00 |
| Unigene472933 | macB | 0.000E+00 | 0.000E+00 | 0.000E+00 | 0.000E+00 | 8.003E-07 | 3.407E-07 | 9.965E-07 | 0.000E+00 | 2.952E-07 | 0.000E+00 | 0.000E+00 | 1.224E-06 |
| Unigene472971 | MexD | 0.000E+00 | 0.000E+00 | 0.000E+00 | 0.000E+00 | 0.000E+00 | 0.000E+00 | 5.352E-07 | 0.000E+00 | 0.000E+00 | 0.000E+00 | 6.915E-08 | 0.000E+00 |
| Unigene472976 | farB | 0.000E+00 | 0.000E+00 | 2.590E-07 | 0.000E+00 | 5.188E-07 | 0.000E+00 | 1.079E-06 | 0.000E+00 | 0.000E+00 | 0.000E+00 | 0.000E+00 | 9.359E-07 |
| Unigene472977 | farA | 0.000E+00 | 0.000E+00 | 0.000E+00 | 0.000E+00 | 0.000E+00 | 0.000E+00 | 5.622E-07 | 3.234E-07 | 0.000E+00 | 0.000E+00 | 0.000E+00 | 3.275E-07 |
| Unigene473022 | TriA | 0.000E+00 | 0.000E+00 | 0.000E+00 | 0.000E+00 | 4.995E-07 | 0.000E+00 | 9.620E-07 | 0.000E+00 | 0.000E+00 | 0.000E+00 | 9.943E-07 | 3.047E-06 |
| Unigene473042 | mtrA | 0.000E+00 | 0.000E+00 | 0.000E+00 | 0.000E+00 | 0.000E+00 | 0.000E+00 | 0.000E+00 | 0.000E+00 | 0.000E+00 | 0.000E+00 | 4.716E-07 | 0.000E+00 |
| Unigene473046 | vanL | 0.000E+00 | 0.000E+00 | 0.000E+00 | 0.000E+00 | 0.000E+00 | 0.000E+00 | 3.028E-07 | 0.000E+00 | 0.000E+00 | 0.000E+00 | 0.000E+00 | 0.000E+00 |
| Unigene473075 | aadA10 | 2.427E-07 | 8.879E-08 | 0.000E+00 | 0.000E+00 | 5.842E-07 | 3.936E-07 | 7.163E-07 | 3.494E-06 | 0.000E+00 | 0.000E+00 | 8.329E-07 | 9.133E-06 |
| Unigene473089 | mtrA | 0.000E+00 | 0.000E+00 | 0.000E+00 | 0.000E+00 | 0.000E+00 | 0.000E+00 | 5.254E-07 | 0.000E+00 | 0.000E+00 | 0.000E+00 | 0.000E+00 | 0.000E+00 |
| Unigene473130 | mdtB | 0.000E+00 | 0.000E+00 | 0.000E+00 | 0.000E+00 | 0.000E+00 | 0.000E+00 | 3.009E-07 | 3.174E-07 | 0.000E+00 | 0.000E+00 | 0.000E+00 | 0.000E+00 |
| Unigene473146 | efrB | 0.000E+00 | 0.000E+00 | 0.000E+00 | 0.000E+00 | 0.000E+00 | 0.000E+00 | 6.030E-07 | 0.000E+00 | 0.000E+00 | 0.000E+00 | 0.000E+00 | 0.000E+00 |
| Unigene473273 | evgS | 0.000E+00 | 0.000E+00 | 2.621E-07 | 0.000E+00 | 1.696E-07 | 0.000E+00 | 1.056E-06 | 1.336E-07 | 0.000E+00 | 0.000E+00 | 2.619E-07 | 6.991E-07 |
| Unigene473438 | optrA | 0.000E+00 | 1.403E-07 | 1.464E-07 | 6.455E-08 | 1.420E-07 | 0.000E+00 | 1.014E-06 | 0.000E+00 | 0.000E+00 | 0.000E+00 | 1.950E-07 | 5.037E-07 |
| Unigene473450 | adeR | 0.000E+00 | 4.523E-07 | 0.000E+00 | 0.000E+00 | 0.000E+00 | 2.475E-07 | 2.078E-06 | 0.000E+00 | 0.000E+00 | 0.000E+00 | 0.000E+00 | 0.000E+00 |
| Unigene473479 | OXA-18 | 0.000E+00 | 0.000E+00 | 0.000E+00 | 0.000E+00 | 0.000E+00 | 0.000E+00 | 1.333E-06 | 1.224E-06 | 0.000E+00 | 0.000E+00 | 0.000E+00 | 9.183E-07 |
| Unigene473498 | ramA | 0.000E+00 | 0.000E+00 | 0.000E+00 | 0.000E+00 | 0.000E+00 | 0.000E+00 | 1.880E-06 | 6.610E-07 | 0.000E+00 | 0.000E+00 | 0.000E+00 | 0.000E+00 |
| Unigene473538 | LlmA 23S r | 0.000E+00 | 0.000E+00 | 0.000E+00 | 0.000E+00 | 5.794E-07 | 0.000E+00 | 1.367E-06 | 5.767E-07 | 0.000E+00 | 0.000E+00 | 3.768E-07 | 9.409E-07 |
| Unigene473601 | arlS | 2.756E-07 | 2.612E-07 | 4.425E-07 | 3.668E-07 | 7.376E-07 | 1.124E-06 | 3.050E-06 | 2.179E-06 | 0.000E+00 | 6.698E-07 | 1.132E-06 | 2.976E-06 |
| Unigene473682 | TaeA | 0.000E+00 | 0.000E+00 | 0.000E+00 | 0.000E+00 | 0.000E+00 | 3.286E-07 | 1.278E-06 | 0.000E+00 | 0.000E+00 | 0.000E+00 | 0.000E+00 | 1.437E-06 |
| Unigene473721 | Corynebac | 2.715E-07 | 7.823E-07 | 0.000E+00 | 0.000E+00 | 0.000E+00 | 0.000E+00 | 1.127E-06 | 0.000E+00 | 0.000E+00 | 0.000E+00 | 0.000E+00 | 0.000E+00 |
| Unigene473791 | vanHF | 0.000E+00 | 0.000E+00 | 6.768E-08 | 6.965E-08 | 0.000E+00 | 0.000E+00 | 4.962E-07 | 0.000E+00 | 0.000E+00 | 0.000E+00 | 0.000E+00 | 0.000E+00 |
| Unigene473817 | cpxA | 0.000E+00 | 0.000E+00 | 1.216E-07 | 0.000E+00 | 0.000E+00 | 2.680E-08 | 7.134E-07 | 0.000E+00 | 0.000E+00 | 0.000E+00 | 0.000E+00 | 0.000E+00 |
| Unigene473836 | oleC | 0.000E+00 | 0.000E+00 | 0.000E+00 | 0.000E+00 | 0.000E+00 | 0.000E+00 | 8.747E-07 | 0.000E+00 | 1.329E-07 | 0.000E+00 | 0.000E+00 | 0.000E+00 |

| | | | | | | | | | | | | | |
|---|---|---|---|---|---|---|---|---|---|---|---|---|---|
| Unigene473919 | TaeA | 5.309E-08 | 0.000E+00 | 0.000E+00 | 0.000E+00 | 2.163E-07 | 0.000E+00 | 9.597E-07 | 1.074E-06 | 0.000E+00 | 0.000E+00 | 4.454E-07 | 5.857E-07 |
| Unigene473931 | Acinetobac | 0.000E+00 | 1.957E-07 | 0.000E+00 | 6.003E-08 | 0.000E+00 | 1.928E-07 | 3.290E-07 | 0.000E+00 | 0.000E+00 | 0.000E+00 | 5.440E-07 | 3.513E-07 |
| Unigene473998 | mgrA | 1.328E-07 | 0.000E+00 | 0.000E+00 | 0.000E+00 | 0.000E+00 | 0.000E+00 | 0.000E+00 | 0.000E+00 | 0.000E+00 | 0.000E+00 | 0.000E+00 | 0.000E+00 |
| Unigene474041 | acrB | 0.000E+00 | 0.000E+00 | 0.000E+00 | 0.000E+00 | 0.000E+00 | 0.000E+00 | 2.556E-07 | 0.000E+00 | 0.000E+00 | 0.000E+00 | 0.000E+00 | 0.000E+00 |
| Unigene474298 | mdtC | 0.000E+00 | 0.000E+00 | 0.000E+00 | 0.000E+00 | 0.000E+00 | 0.000E+00 | 6.424E-07 | 5.733E-07 | 0.000E+00 | 0.000E+00 | 2.554E-07 | 0.000E+00 |
| Unigene474515 | acrB | 0.000E+00 | 0.000E+00 | 0.000E+00 | 0.000E+00 | 0.000E+00 | 0.000E+00 | 6.065E-07 | 0.000E+00 | 0.000E+00 | 0.000E+00 | 4.559E-07 | 0.000E+00 |
| Unigene474543 | patA | 0.000E+00 | 0.000E+00 | 0.000E+00 | 0.000E+00 | 0.000E+00 | 0.000E+00 | 9.857E-07 | 5.901E-07 | 0.000E+00 | 0.000E+00 | 0.000E+00 | 1.280E-06 |
| Unigene474639 | msbA | 0.000E+00 | 0.000E+00 | 0.000E+00 | 0.000E+00 | 3.355E-07 | 0.000E+00 | 1.170E-06 | 2.071E-06 | 0.000E+00 | 0.000E+00 | 0.000E+00 | 3.927E-06 |
| Unigene474704 | efrA | 8.090E-08 | 0.000E+00 | 1.588E-07 | 8.170E-08 | 1.798E-07 | 1.749E-07 | 8.954E-07 | 4.722E-07 | 1.769E-07 | 1.352E-07 | 4.627E-07 | 1.626E-06 |
| Unigene474756 | MexF | 0.000E+00 | 0.000E+00 | 0.000E+00 | 0.000E+00 | 0.000E+00 | 0.000E+00 | 4.135E-07 | 0.000E+00 | 0.000E+00 | 0.000E+00 | 0.000E+00 | 0.000E+00 |
| Unigene474762 | Corynebac | 0.000E+00 | 6.453E-08 | 0.000E+00 | 1.583E-07 | 3.484E-07 | 1.271E-07 | 6.291E-07 | 4.575E-07 | 8.569E-08 | 0.000E+00 | 3.363E-07 | 7.182E-07 |
| Unigene474794 | optrA | 2.143E-07 | 0.000E+00 | 0.000E+00 | 0.000E+00 | 6.668E-07 | 2.317E-07 | 6.642E-07 | 9.007E-07 | 0.000E+00 | 5.253E-07 | 0.000E+00 | 6.587E-07 |
| Unigene474810 | carA | 0.000E+00 | 0.000E+00 | 2.925E-08 | 0.000E+00 | 1.325E-07 | 1.611E-07 | 1.056E-06 | 1.287E-06 | 0.000E+00 | 0.000E+00 | 8.183E-07 | 7.046E-07 |
| Unigene474882 | Acinetobac | 0.000E+00 | 0.000E+00 | 0.000E+00 | 0.000E+00 | 2.959E-07 | 0.000E+00 | 7.369E-07 | 0.000E+00 | 0.000E+00 | 0.000E+00 | 0.000E+00 | 0.000E+00 |
| Unigene474909 | macB | 0.000E+00 | 0.000E+00 | 0.000E+00 | 0.000E+00 | 0.000E+00 | 0.000E+00 | 6.783E-07 | 0.000E+00 | 0.000E+00 | 0.000E+00 | 0.000E+00 | 0.000E+00 |
| Unigene474949 | efrB | 8.198E-08 | 8.998E-08 | 1.609E-07 | 0.000E+00 | 2.277E-07 | 2.216E-07 | 7.485E-07 | 1.053E-06 | 1.792E-07 | 5.251E-07 | 1.078E-06 | 2.931E-06 |
| Unigene475014 | PmpM | 0.000E+00 | 0.000E+00 | 0.000E+00 | 0.000E+00 | 0.000E+00 | 0.000E+00 | 5.431E-07 | 0.000E+00 | 0.000E+00 | 0.000E+00 | 0.000E+00 | 0.000E+00 |
| Unigene475053 | evgS | 0.000E+00 | 0.000E+00 | 0.000E+00 | 0.000E+00 | 0.000E+00 | 0.000E+00 | 1.031E-06 | 2.313E-08 | 0.000E+00 | 0.000E+00 | 0.000E+00 | 0.000E+00 |
| Unigene475092 | tetA(58) | 1.209E-07 | 0.000E+00 | 2.373E-07 | 1.628E-07 | 1.343E-07 | 1.743E-07 | 1.026E-06 | 1.082E-06 | 8.809E-08 | 0.000E+00 | 4.149E-07 | 2.525E-06 |
| Unigene475201 | tlrC | 0.000E+00 | 0.000E+00 | 2.320E-07 | 0.000E+00 | 0.000E+00 | 0.000E+00 | 0.000E+00 | 0.000E+00 | 0.000E+00 | 0.000E+00 | 0.000E+00 | 0.000E+00 |
| Unigene475347 | Bifidobacte | 0.000E+00 | 0.000E+00 | 1.621E-07 | 3.233E-07 | 4.589E-08 | 0.000E+00 | 1.040E-06 | 0.000E+00 | 0.000E+00 | 0.000E+00 | 0.000E+00 | 0.000E+00 |
| Unigene475423 | novA | 0.000E+00 | 0.000E+00 | 0.000E+00 | 0.000E+00 | 0.000E+00 | 0.000E+00 | 6.380E-07 | 0.000E+00 | 0.000E+00 | 0.000E+00 | 0.000E+00 | 0.000E+00 |
| Unigene475424 | sul4 | 0.000E+00 | 0.000E+00 | 2.455E-07 | 0.000E+00 | 0.000E+00 | 0.000E+00 | 3.231E-07 | 9.736E-08 | 0.000E+00 | 0.000E+00 | 0.000E+00 | 0.000E+00 |
| Unigene475439 | rpoB2 | 0.000E+00 | 0.000E+00 | 0.000E+00 | 0.000E+00 | 0.000E+00 | 0.000E+00 | 9.073E-07 | 6.978E-07 | 1.680E-07 | 4.186E-07 | 8.206E-07 | 7.066E-07 |
| Unigene475447 | rosB | 0.000E+00 | 0.000E+00 | 2.633E-07 | 0.000E+00 | 2.385E-07 | 0.000E+00 | 8.315E-07 | 2.819E-07 | 0.000E+00 | 0.000E+00 | 0.000E+00 | 3.806E-07 |
| Unigene475473 | evgS | 3.672E-08 | 0.000E+00 | 0.000E+00 | 3.708E-08 | 3.808E-07 | 1.720E-07 | 1.138E-06 | 2.343E-06 | 0.000E+00 | 2.318E-07 | 4.481E-07 | 1.244E-06 |
| Unigene475494 | tlrC | 0.000E+00 | 0.000E+00 | 0.000E+00 | 0.000E+00 | 0.000E+00 | 0.000E+00 | 1.176E-06 | 6.677E-07 | 0.000E+00 | 0.000E+00 | 0.000E+00 | 3.864E-07 |
| Unigene475508 | AcrE | 0.000E+00 | 0.000E+00 | 0.000E+00 | 4.131E-08 | 9.090E-08 | 0.000E+00 | 5.886E-07 | 1.433E-07 | 0.000E+00 | 0.000E+00 | 0.000E+00 | 3.385E-07 |
| Unigene475554 | vanRG | 0.000E+00 | 0.000E+00 | 0.000E+00 | 0.000E+00 | 0.000E+00 | 0.000E+00 | 8.714E-07 | 0.000E+00 | 0.000E+00 | 0.000E+00 | 0.000E+00 | 0.000E+00 |
| Unigene475828 | evgS | 0.000E+00 | 0.000E+00 | 0.000E+00 | 0.000E+00 | 0.000E+00 | 0.000E+00 | 7.844E-07 | 0.000E+00 | 0.000E+00 | 0.000E+00 | 0.000E+00 | 0.000E+00 |
| Unigene475876 | tetB(60) | 0.000E+00 | 0.000E+00 | 0.000E+00 | 0.000E+00 | 0.000E+00 | 0.000E+00 | 1.159E-06 | 2.073E-07 | 0.000E+00 | 0.000E+00 | 1.219E-07 | 0.000E+00 |
| Unigene475892 | TaeA | 0.000E+00 | 0.000E+00 | 0.000E+00 | 0.000E+00 | 0.000E+00 | 0.000E+00 | 1.083E-06 | 0.000E+00 | 0.000E+00 | 0.000E+00 | 0.000E+00 | 0.000E+00 |
| Unigene475978 | bcrA | 0.000E+00 | 0.000E+00 | 0.000E+00 | 0.000E+00 | 0.000E+00 | 0.000E+00 | 9.955E-07 | 0.000E+00 | 0.000E+00 | 0.000E+00 | 0.000E+00 | 0.000E+00 |
| Unigene475981 | evgA | 0.000E+00 | 0.000E+00 | 0.000E+00 | 0.000E+00 | 0.000E+00 | 0.000E+00 | 1.482E-06 | 0.000E+00 | 0.000E+00 | 0.000E+00 | 0.000E+00 | 0.000E+00 |
| Unigene476010 | MexK | 0.000E+00 | 0.000E+00 | 0.000E+00 | 0.000E+00 | 0.000E+00 | 0.000E+00 | 1.126E-06 | 0.000E+00 | 0.000E+00 | 0.000E+00 | 0.000E+00 | 0.000E+00 |
| Unigene476064 | cpxA | 0.000E+00 | 0.000E+00 | 1.160E-07 | 0.000E+00 | 0.000E+00 | 0.000E+00 | 0.000E+00 | 5.245E-07 | 0.000E+00 | 0.000E+00 | 0.000E+00 | 7.547E-07 |
| Unigene476376 | OXA-18 | 0.000E+00 | 1.737E-07 | 0.000E+00 | 0.000E+00 | 0.000E+00 | 0.000E+00 | 2.627E-06 | 0.000E+00 | 3.891E-07 | 0.000E+00 | 2.715E-07 | 9.820E-07 |
| Unigene476408 | efrA | 6.294E-07 | 7.799E-07 | 5.379E-07 | 0.000E+00 | 0.000E+00 | 2.634E-07 | 1.191E-06 | 0.000E+00 | 0.000E+00 | 0.000E+00 | 0.000E+00 | 0.000E+00 |
| Unigene476584 | cpxA | 0.000E+00 | 0.000E+00 | 0.000E+00 | 0.000E+00 | 0.000E+00 | 0.000E+00 | 4.029E-07 | 0.000E+00 | 0.000E+00 | 0.000E+00 | 0.000E+00 | 0.000E+00 |
| Unigene476710 | vmlR | 0.000E+00 | 0.000E+00 | 1.325E-07 | 1.819E-07 | 3.001E-07 | 0.000E+00 | 8.471E-07 | 9.985E-07 | 0.000E+00 | 0.000E+00 | 3.605E-07 | 1.543E-06 |
| Unigene476711 | srmB | 0.000E+00 | 0.000E+00 | 0.000E+00 | 0.000E+00 | 4.236E-07 | 0.000E+00 | 7.033E-07 | 3.956E-07 | 4.630E-08 | 0.000E+00 | 4.846E-07 | 2.654E-06 |
| Unigene476723 | otrC | 0.000E+00 | 0.000E+00 | 0.000E+00 | 0.000E+00 | 0.000E+00 | 0.000E+00 | 1.096E-06 | 0.000E+00 | 0.000E+00 | 0.000E+00 | 0.000E+00 | 0.000E+00 |
| Unigene476724 | carA | 0.000E+00 | 0.000E+00 | 0.000E+00 | 0.000E+00 | 0.000E+00 | 0.000E+00 | 9.565E-07 | 0.000E+00 | 0.000E+00 | 0.000E+00 | 0.000E+00 | 0.000E+00 |
| Unigene476804 | optrA | 0.000E+00 | 0.000E+00 | 0.000E+00 | 0.000E+00 | 0.000E+00 | 0.000E+00 | 5.988E-07 | 0.000E+00 | 0.000E+00 | 0.000E+00 | 0.000E+00 | 0.000E+00 |
| Unigene476858 | OpmD | 1.299E-07 | 0.000E+00 | 0.000E+00 | 1.530E-07 | 1.924E-07 | 0.000E+00 | 7.188E-07 | 3.033E-07 | 0.000E+00 | 0.000E+00 | 4.953E-08 | 5.118E-07 |
| Unigene476859 | MexD | 0.000E+00 | 5.613E-08 | 7.025E-08 | 1.033E-07 | 3.182E-07 | 8.846E-08 | 7.132E-07 | 6.089E-07 | 5.590E-08 | 0.000E+00 | 2.808E-07 | 6.045E-07 |
| Unigene477079 | evgA | 0.000E+00 | 0.000E+00 | 0.000E+00 | 0.000E+00 | 0.000E+00 | 0.000E+00 | 1.185E-06 | 0.000E+00 | 0.000E+00 | 0.000E+00 | 0.000E+00 | 0.000E+00 |
| Unigene477090 | ceoB | 0.000E+00 | 2.128E-07 | 0.000E+00 | 9.789E-08 | 0.000E+00 | 1.572E-07 | 7.510E-07 | 5.092E-07 | 0.000E+00 | 0.000E+00 | 0.000E+00 | 3.437E-07 |
| Unigene477096 | tetT | 0.000E+00 | 0.000E+00 | 0.000E+00 | 0.000E+00 | 0.000E+00 | 0.000E+00 | 1.223E-06 | 0.000E+00 | 0.000E+00 | 0.000E+00 | 0.000E+00 | 0.000E+00 |
| Unigene477103 | QepA1 | 0.000E+00 | 0.000E+00 | 0.000E+00 | 0.000E+00 | 4.922E-07 | 0.000E+00 | 2.802E-07 | 0.000E+00 | 0.000E+00 | 0.000E+00 | 0.000E+00 | 0.000E+00 |
| Unigene477159 | AAC(6')-Ia | 0.000E+00 | 0.000E+00 | 0.000E+00 | 0.000E+00 | 0.000E+00 | 0.000E+00 | 8.911E-07 | 0.000E+00 | 0.000E+00 | 0.000E+00 | 0.000E+00 | 0.000E+00 |
| Unigene477223 | APH(6)-Id | 0.000E+00 | 0.000E+00 | 1.123E-07 | 0.000E+00 | 0.000E+00 | 0.000E+00 | 9.290E-07 | 3.563E-07 | 0.000E+00 | 0.000E+00 | 0.000E+00 | 8.118E-07 |
| Unigene477389 | oleB | 0.000E+00 | 0.000E+00 | 0.000E+00 | 0.000E+00 | 0.000E+00 | 0.000E+00 | 8.308E-07 | 0.000E+00 | 0.000E+00 | 0.000E+00 | 0.000E+00 | 0.000E+00 |
| Unigene477460 | msbA | 0.000E+00 | 0.000E+00 | 0.000E+00 | 0.000E+00 | 0.000E+00 | 0.000E+00 | 2.469E-06 | 0.000E+00 | 0.000E+00 | 0.000E+00 | 0.000E+00 | 0.000E+00 |
| Unigene477461 | msbA | 0.000E+00 | 0.000E+00 | 0.000E+00 | 0.000E+00 | 0.000E+00 | 0.000E+00 | 1.698E-06 | 0.000E+00 | 0.000E+00 | 0.000E+00 | 0.000E+00 | 0.000E+00 |
| Unigene477542 | evgS | 0.000E+00 | 0.000E+00 | 0.000E+00 | 0.000E+00 | 0.000E+00 | 0.000E+00 | 6.107E-07 | 0.000E+00 | 0.000E+00 | 0.000E+00 | 0.000E+00 | 0.000E+00 |
| Unigene477563 | mdtN | 0.000E+00 | 0.000E+00 | 0.000E+00 | 0.000E+00 | 0.000E+00 | 0.000E+00 | 1.847E-06 | 0.000E+00 | 0.000E+00 | 0.000E+00 | 0.000E+00 | 0.000E+00 |
| Unigene477788 | novA | 0.000E+00 | 0.000E+00 | 0.000E+00 | 0.000E+00 | 0.000E+00 | 0.000E+00 | 5.352E-07 | 0.000E+00 | 0.000E+00 | 0.000E+00 | 0.000E+00 | 0.000E+00 |

| | | | | | | | | | | | | | |
|---|---|---|---|---|---|---|---|---|---|---|---|---|---|
| Unigene477829 | bcrB | 0.000E+00 | 1.298E-04 | 0.000E+00 | 4.295E-08 | 4.725E-08 | 0.000E+00 | 5.178E-07 | 0.000E+00 | 2.464E-06 | 0.000E+00 | 0.000E+00 | 0.000E+00 |
| Unigene477885 | evgS | 0.000E+00 | 1.657E-07 | 1.111E-07 | 7.625E-08 | 0.000E+00 | 4.898E-07 | 1.086E-06 | 1.234E-06 | 0.000E+00 | 0.000E+00 | 6.478E-07 | 2.678E-06 |
| Unigene477892 | tetA(46) | 0.000E+00 | 0.000E+00 | 0.000E+00 | 0.000E+00 | 0.000E+00 | 0.000E+00 | 8.150E-07 | 0.000E+00 | 0.000E+00 | 0.000E+00 | 0.000E+00 | 0.000E+00 |
| Unigene477959 | lmrC | 0.000E+00 | 0.000E+00 | 0.000E+00 | 0.000E+00 | 0.000E+00 | 0.000E+00 | 7.939E-07 | 0.000E+00 | 0.000E+00 | 0.000E+00 | 0.000E+00 | 0.000E+00 |
| Unigene478138 | mef(B) | 9.902E-08 | 0.000E+00 | 0.000E+00 | 0.000E+00 | 0.000E+00 | 0.000E+00 | 7.671E-07 | 4.238E-07 | 1.804E-07 | 0.000E+00 | 3.020E-07 | 3.121E-07 |
| Unigene478169 | carA | 0.000E+00 | 0.000E+00 | 0.000E+00 | 0.000E+00 | 0.000E+00 | 0.000E+00 | 2.104E-06 | 0.000E+00 | 0.000E+00 | 0.000E+00 | 0.000E+00 | 0.000E+00 |
| Unigene478302 | msbA | 0.000E+00 | 0.000E+00 | 0.000E+00 | 0.000E+00 | 0.000E+00 | 0.000E+00 | 1.179E-07 | 0.000E+00 | 0.000E+00 | 0.000E+00 | 0.000E+00 | 0.000E+00 |
| Unigene478319 | vmlR | 8.277E-08 | 0.000E+00 | 0.000E+00 | 1.254E-07 | 5.057E-07 | 0.000E+00 | 1.099E-06 | 1.304E-06 | 0.000E+00 | 0.000E+00 | 1.894E-07 | 1.027E-06 |
| Unigene478346 | tetB(46) | 0.000E+00 | 0.000E+00 | 0.000E+00 | 0.000E+00 | 0.000E+00 | 0.000E+00 | 2.214E-06 | 0.000E+00 | 0.000E+00 | 0.000E+00 | 0.000E+00 | 0.000E+00 |
| Unigene478427 | bcrA | 0.000E+00 | 0.000E+00 | 0.000E+00 | 0.000E+00 | 0.000E+00 | 0.000E+00 | 5.325E-07 | 0.000E+00 | 0.000E+00 | 0.000E+00 | 0.000E+00 | 0.000E+00 |
| Unigene478436 | novA | 0.000E+00 | 0.000E+00 | 0.000E+00 | 0.000E+00 | 0.000E+00 | 0.000E+00 | 4.904E-07 | 0.000E+00 | 0.000E+00 | 0.000E+00 | 0.000E+00 | 0.000E+00 |
| Unigene478460 | PmrF | 0.000E+00 | 0.000E+00 | 0.000E+00 | 0.000E+00 | 2.726E-07 | 2.652E-07 | 5.819E-07 | 4.909E-07 | 0.000E+00 | 0.000E+00 | 2.406E-07 | 5.800E-07 |
| Unigene478499 | bacA | 0.000E+00 | 0.000E+00 | 0.000E+00 | 0.000E+00 | 0.000E+00 | 0.000E+00 | 4.083E-07 | 0.000E+00 | 0.000E+00 | 0.000E+00 | 0.000E+00 | 0.000E+00 |
| Unigene478793 | bacA | 4.847E-08 | 0.000E+00 | 1.902E-07 | 0.000E+00 | 3.769E-07 | 0.000E+00 | 8.583E-07 | 4.526E-07 | 0.000E+00 | 0.000E+00 | 0.000E+00 | 0.000E+00 |
| Unigene478855 | carA | 0.000E+00 | 0.000E+00 | 0.000E+00 | 0.000E+00 | 0.000E+00 | 1.454E-07 | 1.290E-06 | 0.000E+00 | 0.000E+00 | 0.000E+00 | 0.000E+00 | 0.000E+00 |
| Unigene478889 | GOB-10 | 0.000E+00 | 0.000E+00 | 0.000E+00 | 0.000E+00 | 0.000E+00 | 0.000E+00 | 0.000E+00 | 1.302E-07 | 0.000E+00 | 0.000E+00 | 0.000E+00 | 0.000E+00 |
| Unigene478961 | facT | 0.000E+00 | 0.000E+00 | 4.629E-07 | 0.000E+00 | 2.745E-07 | 0.000E+00 | 1.765E-06 | 0.000E+00 | 0.000E+00 | 0.000E+00 | 0.000E+00 | 0.000E+00 |
| Unigene479003 | efrB | 1.824E-07 | 0.000E+00 | 4.474E-08 | 0.000E+00 | 1.520E-07 | 1.479E-07 | 6.560E-07 | 3.193E-07 | 0.000E+00 | 3.047E-07 | 2.608E-07 | 8.623E-07 |
| Unigene479027 | efrB | 0.000E+00 | 0.000E+00 | 0.000E+00 | 0.000E+00 | 0.000E+00 | 0.000E+00 | 7.578E-07 | 0.000E+00 | 0.000E+00 | 0.000E+00 | 0.000E+00 | 0.000E+00 |
| Unigene479061 | bcrA | 0.000E+00 | 0.000E+00 | 0.000E+00 | 0.000E+00 | 0.000E+00 | 0.000E+00 | 0.000E+00 | 0.000E+00 | 0.000E+00 | 0.000E+00 | 0.000E+00 | 3.325E-07 |
| Unigene479262 | arlR | 0.000E+00 | 0.000E+00 | 0.000E+00 | 0.000E+00 | 0.000E+00 | 0.000E+00 | 7.683E-07 | 0.000E+00 | 0.000E+00 | 0.000E+00 | 0.000E+00 | 0.000E+00 |
| Unigene479528 | tetB(60) | 0.000E+00 | 0.000E+00 | 2.258E-07 | 0.000E+00 | 0.000E+00 | 0.000E+00 | 8.279E-07 | 0.000E+00 | 0.000E+00 | 0.000E+00 | 0.000E+00 | 0.000E+00 |
| Unigene479572 | baeR | 0.000E+00 | 0.000E+00 | 0.000E+00 | 0.000E+00 | 0.000E+00 | 0.000E+00 | 0.000E+00 | 0.000E+00 | 0.000E+00 | 0.000E+00 | 0.000E+00 | 1.386E-06 |
| Unigene479578 | BCL-1 | 0.000E+00 | 0.000E+00 | 0.000E+00 | 0.000E+00 | 0.000E+00 | 0.000E+00 | 6.001E-07 | 0.000E+00 | 0.000E+00 | 0.000E+00 | 0.000E+00 | 0.000E+00 |
| Unigene479600 | oleC | 0.000E+00 | 0.000E+00 | 7.421E-07 | 4.166E-07 | 0.000E+00 | 0.000E+00 | 4.718E-06 | 0.000E+00 | 9.018E-07 | 1.417E-06 | 1.730E-06 | 3.698E-06 |
| Unigene479679 | eptA | 0.000E+00 | 0.000E+00 | 4.591E-08 | 0.000E+00 | 1.299E-07 | 0.000E+00 | 1.294E-06 | 1.365E-07 | 0.000E+00 | 0.000E+00 | 2.141E-07 | 4.147E-07 |
| Unigene479722 | tva(A) | 8.357E-08 | 9.172E-08 | 2.460E-07 | 0.000E+00 | 4.642E-07 | 2.710E-07 | 8.787E-07 | 1.390E-06 | 0.000E+00 | 3.026E-07 | 1.195E-06 | 3.235E-06 |
| Unigene479808 | MexK | 0.000E+00 | 6.820E-08 | 1.829E-07 | 1.255E-07 | 5.062E-07 | 2.575E-07 | 2.613E-06 | 2.442E-06 | 2.264E-07 | 8.422E-07 | 1.244E-06 | 1.897E-06 |
| Unigene479809 | MexJ | 0.000E+00 | 2.228E-07 | 0.000E+00 | 1.464E-07 | 0.000E+00 | 0.000E+00 | 1.958E-06 | 5.417E-07 | 0.000E+00 | 0.000E+00 | 0.000E+00 | 1.131E-06 |
| Unigene479956 | vanRG | 0.000E+00 | 0.000E+00 | 0.000E+00 | 0.000E+00 | 0.000E+00 | 0.000E+00 | 4.736E-07 | 0.000E+00 | 0.000E+00 | 0.000E+00 | 0.000E+00 | 0.000E+00 |
| Unigene479962 | baeR | 0.000E+00 | 0.000E+00 | 0.000E+00 | 3.079E-07 | 0.000E+00 | 0.000E+00 | 1.302E-06 | 0.000E+00 | 0.000E+00 | 0.000E+00 | 0.000E+00 | 0.000E+00 |
| Unigene479970 | otr(B) | 0.000E+00 | 0.000E+00 | 0.000E+00 | 0.000E+00 | 2.367E-07 | 0.000E+00 | 0.000E+00 | 0.000E+00 | 0.000E+00 | 0.000E+00 | 0.000E+00 | 0.000E+00 |
| Unigene480019 | novA | 0.000E+00 | 0.000E+00 | 0.000E+00 | 0.000E+00 | 0.000E+00 | 2.407E-07 | 0.000E+00 | 0.000E+00 | 0.000E+00 | 0.000E+00 | 0.000E+00 | 0.000E+00 |
| Unigene480024 | kdpE | 0.000E+00 | 0.000E+00 | 0.000E+00 | 0.000E+00 | 0.000E+00 | 0.000E+00 | 6.133E-07 | 0.000E+00 | 0.000E+00 | 0.000E+00 | 0.000E+00 | 0.000E+00 |
| Unigene480025 | smeS | 0.000E+00 | 0.000E+00 | 0.000E+00 | 0.000E+00 | 0.000E+00 | 0.000E+00 | 7.454E-07 | 9.141E-08 | 0.000E+00 | 0.000E+00 | 0.000E+00 | 0.000E+00 |
| Unigene480032 | efrA | 0.000E+00 | 0.000E+00 | 0.000E+00 | 0.000E+00 | 2.116E-08 | 0.000E+00 | 1.222E-06 | 1.334E-07 | 0.000E+00 | 0.000E+00 | 0.000E+00 | 2.251E-07 |
| Unigene480046 | lmrD | 0.000E+00 | 0.000E+00 | 0.000E+00 | 9.531E-08 | 8.738E-08 | 0.000E+00 | 1.428E-06 | 1.102E-07 | 0.000E+00 | 0.000E+00 | 1.440E-07 | 2.603E-07 |
| Unigene480178 | vanSM | 0.000E+00 | 0.000E+00 | 0.000E+00 | 0.000E+00 | 5.276E-06 | 0.000E+00 | 1.109E-06 | 0.000E+00 | 0.000E+00 | 0.000E+00 | 0.000E+00 | 0.000E+00 |
| Unigene480228 | evgS | 0.000E+00 | 9.640E-08 | 4.309E-08 | 0.000E+00 | 5.855E-07 | 0.000E+00 | 7.291E-07 | 3.588E-07 | 0.000E+00 | 1.468E-07 | 0.000E+00 | 4.672E-07 |
| Unigene480329 | optrA | 0.000E+00 | 0.000E+00 | 1.312E-07 | 0.000E+00 | 0.000E+00 | 1.084E-07 | 0.000E+00 | 0.000E+00 | 0.000E+00 | 0.000E+00 | 0.000E+00 | 0.000E+00 |
| Unigene480330 | TriC | 1.149E-07 | 1.949E-07 | 3.485E-07 | 1.793E-07 | 2.785E-07 | 7.002E-07 | 3.353E-06 | 1.829E-06 | 1.827E-07 | 4.422E-07 | 6.333E-07 | 1.482E-06 |
| Unigene480331 | TriB | 5.945E-08 | 5.220E-07 | 4.375E-07 | 0.000E+00 | 0.000E+00 | 6.105E-07 | 4.441E-06 | 2.463E-06 | 0.000E+00 | 0.000E+00 | 6.120E-07 | 1.546E-06 |
| Unigene480344 | Acinetobac | 0.000E+00 | 0.000E+00 | 0.000E+00 | 0.000E+00 | 6.831E-08 | 0.000E+00 | 4.083E-07 | 0.000E+00 | 0.000E+00 | 0.000E+00 | 0.000E+00 | 0.000E+00 |
| Unigene480365 | adeN | 0.000E+00 | 0.000E+00 | 0.000E+00 | 0.000E+00 | 0.000E+00 | 0.000E+00 | 0.000E+00 | 0.000E+00 | 0.000E+00 | 0.000E+00 | 0.000E+00 | 5.781E-07 |
| Unigene480458 | tetA(58) | 0.000E+00 | 0.000E+00 | 0.000E+00 | 0.000E+00 | 0.000E+00 | 0.000E+00 | 4.047E-07 | 8.536E-07 | 0.000E+00 | 0.000E+00 | 0.000E+00 | 0.000E+00 |
| Unigene480515 | evgS | 0.000E+00 | 0.000E+00 | 0.000E+00 | 0.000E+00 | 0.000E+00 | 1.466E-07 | 7.005E-07 | 0.000E+00 | 0.000E+00 | 0.000E+00 | 0.000E+00 | 0.000E+00 |
| Unigene480571 | adeL | 0.000E+00 | 0.000E+00 | 0.000E+00 | 0.000E+00 | 0.000E+00 | 0.000E+00 | 0.000E+00 | 4.454E-07 | 0.000E+00 | 0.000E+00 | 0.000E+00 | 0.000E+00 |
| Unigene480726 | evgS | 4.175E-07 | 3.399E-07 | 6.343E-07 | 5.440E-07 | 6.733E-07 | 8.298E-07 | 3.473E-06 | 3.097E-06 | 5.299E-07 | 1.125E-06 | 1.756E-06 | 3.279E-06 |
| Unigene480868 | msbA | 0.000E+00 | 0.000E+00 | 0.000E+00 | 0.000E+00 | 0.000E+00 | 5.879E-07 | 1.118E-06 | 0.000E+00 | 0.000E+00 | 0.000E+00 | 7.109E-07 | 9.183E-07 |
| Unigene480967 | msbA | 1.323E-07 | 4.840E-08 | 0.000E+00 | 0.000E+00 | 2.450E-07 | 0.000E+00 | 6.345E-07 | 0.000E+00 | 4.820E-08 | 0.000E+00 | 0.000E+00 | 1.043E-07 |
| Unigene480997 | bcrA | 0.000E+00 | 0.000E+00 | 0.000E+00 | 3.042E-07 | 5.260E-07 | 2.792E-07 | 6.193E-07 | 8.038E-07 | 4.704E-08 | 1.918E-07 | 3.446E-07 | 3.561E-07 |
| Unigene480999 | basS | 0.000E+00 | 0.000E+00 | 7.189E-07 | 0.000E+00 | 0.000E+00 | 0.000E+00 | 1.495E-06 | 0.000E+00 | 0.000E+00 | 0.000E+00 | 0.000E+00 | 0.000E+00 |
| Unigene481114 | Streptomy | 0.000E+00 | 0.000E+00 | 0.000E+00 | 0.000E+00 | 0.000E+00 | 0.000E+00 | 5.496E-07 | 0.000E+00 | 0.000E+00 | 0.000E+00 | 0.000E+00 | 0.000E+00 |
| Unigene481147 | macB | 0.000E+00 | 0.000E+00 | 0.000E+00 | 0.000E+00 | 0.000E+00 | 2.695E-07 | 1.609E-06 | 0.000E+00 | 0.000E+00 | 0.000E+00 | 0.000E+00 | 0.000E+00 |
| Unigene481234 | mgrA | 0.000E+00 | 0.000E+00 | 0.000E+00 | 0.000E+00 | 0.000E+00 | 2.281E-07 | 5.448E-07 | 0.000E+00 | 0.000E+00 | 0.000E+00 | 0.000E+00 | 0.000E+00 |
| Unigene481272 | evgS | 0.000E+00 | 0.000E+00 | 0.000E+00 | 0.000E+00 | 0.000E+00 | 0.000E+00 | 4.838E-07 | 3.925E-07 | 7.350E-08 | 0.000E+00 | 0.000E+00 | 2.385E-07 |
| Unigene481287 | TaeA | 0.000E+00 | 3.680E-07 | 0.000E+00 | 0.000E+00 | 0.000E+00 | 0.000E+00 | 2.429E-06 | 0.000E+00 | 0.000E+00 | 0.000E+00 | 0.000E+00 | 0.000E+00 |
| Unigene481380 | vanRI | 0.000E+00 | 0.000E+00 | 0.000E+00 | 0.000E+00 | 0.000E+00 | 0.000E+00 | 2.977E-07 | 0.000E+00 | 0.000E+00 | 0.000E+00 | 0.000E+00 | 0.000E+00 |

| | | | | | | | | | | | | | |
|---|---|---|---|---|---|---|---|---|---|---|---|---|---|
| Unigene481406 | patA | 0.000E+00 | 0.000E+00 | 0.000E+00 | 0.000E+00 | 0.000E+00 | 0.000E+00 | 7.899E-07 | 0.000E+00 | 0.000E+00 | 0.000E+00 | 0.000E+00 | 4.542E-07 |
| Unigene481498 | bcr-1 | 0.000E+00 | 0.000E+00 | 2.496E-07 | 0.000E+00 | 5.652E-07 | 0.000E+00 | 1.098E-06 | 5.938E-07 | 1.390E-07 | 0.000E+00 | 8.438E-07 | 8.720E-07 |
| Unigene481504 | tetA(58) | 0.000E+00 | 0.000E+00 | 0.000E+00 | 0.000E+00 | 0.000E+00 | 0.000E+00 | 8.163E-07 | 0.000E+00 | 0.000E+00 | 0.000E+00 | 0.000E+00 | 3.077E-07 |
| Unigene481557 | Acinetobac | 0.000E+00 | 0.000E+00 | 0.000E+00 | 0.000E+00 | 0.000E+00 | 0.000E+00 | 6.787E-07 | 1.302E-07 | 6.094E-08 | 0.000E+00 | 0.000E+00 | 0.000E+00 |
| Unigene481632 | mexN | 2.316E-07 | 1.040E-07 | 1.136E-07 | 1.382E-07 | 3.977E-07 | 2.845E-07 | 8.972E-07 | 9.217E-07 | 9.206E-08 | 3.284E-07 | 4.095E-07 | 7.467E-07 |
| Unigene481638 | Agrobacte | 0.000E+00 | 0.000E+00 | 0.000E+00 | 0.000E+00 | 8.726E-08 | 0.000E+00 | 1.738E-07 | 0.000E+00 | 0.000E+00 | 0.000E+00 | 8.984E-08 | 0.000E+00 |
| Unigene481647 | sul4 | 0.000E+00 | 0.000E+00 | 0.000E+00 | 0.000E+00 | 0.000E+00 | 0.000E+00 | 6.478E-07 | 0.000E+00 | 0.000E+00 | 0.000E+00 | 0.000E+00 | 0.000E+00 |
| Unigene481659 | mtrA | 0.000E+00 | 0.000E+00 | 0.000E+00 | 0.000E+00 | 0.000E+00 | 0.000E+00 | 0.000E+00 | 0.000E+00 | 0.000E+00 | 0.000E+00 | 6.478E-07 | 0.000E+00 |
| Unigene481696 | carA | 0.000E+00 | 0.000E+00 | 0.000E+00 | 0.000E+00 | 0.000E+00 | 0.000E+00 | 5.521E-07 | 0.000E+00 | 0.000E+00 | 0.000E+00 | 0.000E+00 | 0.000E+00 |
| Unigene481764 | vanRI | 0.000E+00 | 0.000E+00 | 0.000E+00 | 0.000E+00 | 0.000E+00 | 0.000E+00 | 3.904E-07 | 0.000E+00 | 0.000E+00 | 0.000E+00 | 0.000E+00 | 0.000E+00 |
| Unigene481809 | msbA | 0.000E+00 | 1.464E-07 | 0.000E+00 | 0.000E+00 | 0.000E+00 | 0.000E+00 | 8.366E-07 | 0.000E+00 | 0.000E+00 | 0.000E+00 | 2.034E-07 | 0.000E+00 |
| Unigene481835 | baeR | 0.000E+00 | 1.434E-07 | 2.137E-07 | 0.000E+00 | 4.356E-07 | 2.355E-07 | 1.109E-06 | 1.170E-06 | 0.000E+00 | 3.397E-07 | 1.545E-06 | 3.502E-06 |
| Unigene481884 | patB | 0.000E+00 | 0.000E+00 | 0.000E+00 | 0.000E+00 | 0.000E+00 | 0.000E+00 | 7.005E-07 | 0.000E+00 | 0.000E+00 | 0.000E+00 | 0.000E+00 | 0.000E+00 |
| Unigene481899 | tetA(60) | 0.000E+00 | 0.000E+00 | 0.000E+00 | 0.000E+00 | 0.000E+00 | 0.000E+00 | 1.323E-06 | 5.685E-07 | 0.000E+00 | 0.000E+00 | 0.000E+00 | 0.000E+00 |
| Unigene482010 | macB | 0.000E+00 | 0.000E+00 | 0.000E+00 | 0.000E+00 | 0.000E+00 | 0.000E+00 | 8.228E-07 | 0.000E+00 | 0.000E+00 | 0.000E+00 | 0.000E+00 | 0.000E+00 |
| Unigene482119 | TLA-3 | 0.000E+00 | 0.000E+00 | 0.000E+00 | 1.552E-07 | 0.000E+00 | 0.000E+00 | 1.089E-06 | 1.112E-06 | 2.352E-07 | 0.000E+00 | 5.275E-07 | 9.812E-07 |
| Unigene482124 | mdtB | 0.000E+00 | 0.000E+00 | 0.000E+00 | 0.000E+00 | 0.000E+00 | 0.000E+00 | 4.701E-07 | 0.000E+00 | 0.000E+00 | 0.000E+00 | 0.000E+00 | 0.000E+00 |
| Unigene482139 | APH(6)-Id | 0.000E+00 | 0.000E+00 | 0.000E+00 | 2.225E-07 | 1.632E-07 | 0.000E+00 | 2.073E-06 | 5.573E-07 | 2.007E-07 | 2.864E-07 | 5.041E-07 | 0.000E+00 |
| Unigene482147 | bcr-1 | 0.000E+00 | 0.000E+00 | 0.000E+00 | 0.000E+00 | 1.897E-07 | 0.000E+00 | 3.402E-07 | 0.000E+00 | 0.000E+00 | 0.000E+00 | 0.000E+00 | 4.441E-07 |
| Unigene482189 | PEDO-1 | 0.000E+00 | 0.000E+00 | 0.000E+00 | 0.000E+00 | 0.000E+00 | 0.000E+00 | 3.904E-07 | 0.000E+00 | 0.000E+00 | 0.000E+00 | 0.000E+00 | 0.000E+00 |
| Unigene482302 | vanRI | 0.000E+00 | 2.326E-07 | 6.700E-07 | 2.378E-07 | 7.847E-07 | 5.600E-07 | 1.121E-06 | 9.894E-07 | 1.544E-07 | 0.000E+00 | 7.811E-07 | 3.312E-06 |
| Unigene482308 | mtrA | 0.000E+00 | 0.000E+00 | 0.000E+00 | 0.000E+00 | 0.000E+00 | 0.000E+00 | 7.321E-07 | 0.000E+00 | 0.000E+00 | 0.000E+00 | 0.000E+00 | 0.000E+00 |
| Unigene482401 | tet(55) | 0.000E+00 | 0.000E+00 | 1.671E-07 | 0.000E+00 | 0.000E+00 | 0.000E+00 | 8.170E-07 | 0.000E+00 | 1.862E-07 | 1.581E-07 | 0.000E+00 | 0.000E+00 |
| Unigene482457 | vmlR | 0.000E+00 | 0.000E+00 | 0.000E+00 | 0.000E+00 | 0.000E+00 | 0.000E+00 | 1.016E-06 | 0.000E+00 | 0.000E+00 | 0.000E+00 | 0.000E+00 | 0.000E+00 |
| Unigene482466 | ugd | 0.000E+00 | 0.000E+00 | 0.000E+00 | 0.000E+00 | 0.000E+00 | 0.000E+00 | 6.139E-07 | 0.000E+00 | 1.212E-07 | 3.089E-07 | 0.000E+00 | 1.606E-06 |
| Unigene482467 | Acinetobac | 0.000E+00 | 1.193E-07 | 1.066E-07 | 0.000E+00 | 0.000E+00 | 0.000E+00 | 3.007E-07 | 0.000E+00 | 1.782E-07 | 0.000E+00 | 6.216E-08 | 0.000E+00 |
| Unigene482490 | basS | 0.000E+00 | 0.000E+00 | 0.000E+00 | 0.000E+00 | 0.000E+00 | 0.000E+00 | 0.000E+00 | 0.000E+00 | 0.000E+00 | 0.000E+00 | 0.000E+00 | 8.016E-07 |
| Unigene482491 | adeR | 0.000E+00 | 0.000E+00 | 0.000E+00 | 0.000E+00 | 0.000E+00 | 0.000E+00 | 0.000E+00 | 0.000E+00 | 0.000E+00 | 0.000E+00 | 0.000E+00 | 1.171E-06 |
| Unigene482519 | adeB | 0.000E+00 | 0.000E+00 | 0.000E+00 | 0.000E+00 | 0.000E+00 | 0.000E+00 | 4.279E-07 | 0.000E+00 | 0.000E+00 | 0.000E+00 | 0.000E+00 | 6.093E-07 |
| Unigene482536 | macB | 0.000E+00 | 0.000E+00 | 0.000E+00 | 0.000E+00 | 0.000E+00 | 0.000E+00 | 8.749E-07 | 0.000E+00 | 0.000E+00 | 0.000E+00 | 0.000E+00 | 0.000E+00 |
| Unigene482537 | macB | 0.000E+00 | 5.747E-08 | 0.000E+00 | 0.000E+00 | 0.000E+00 | 0.000E+00 | 8.403E-07 | 0.000E+00 | 0.000E+00 | 0.000E+00 | 0.000E+00 | 0.000E+00 |
| Unigene482544 | novA | 6.746E-08 | 0.000E+00 | 0.000E+00 | 0.000E+00 | 0.000E+00 | 0.000E+00 | 8.213E-07 | 0.000E+00 | 0.000E+00 | 0.000E+00 | 0.000E+00 | 0.000E+00 |
| Unigene482598 | basS | 0.000E+00 | 0.000E+00 | 0.000E+00 | 0.000E+00 | 6.329E-07 | 0.000E+00 | 4.904E-07 | 0.000E+00 | 2.075E-07 | 4.231E-07 | 0.000E+00 | 5.237E-07 |
| Unigene482723 | vanHB | 0.000E+00 | 0.000E+00 | 0.000E+00 | 0.000E+00 | 0.000E+00 | 0.000E+00 | 0.000E+00 | 3.130E-07 | 0.000E+00 | 0.000E+00 | 0.000E+00 | 4.358E-07 |
| Unigene482761 | evgS | 0.000E+00 | 0.000E+00 | 0.000E+00 | 0.000E+00 | 0.000E+00 | 0.000E+00 | 7.746E-07 | 0.000E+00 | 0.000E+00 | 0.000E+00 | 0.000E+00 | 0.000E+00 |
| Unigene482862 | Corynebac | 0.000E+00 | 0.000E+00 | 0.000E+00 | 0.000E+00 | 0.000E+00 | 0.000E+00 | 4.899E-07 | 0.000E+00 | 0.000E+00 | 0.000E+00 | 0.000E+00 | 0.000E+00 |
| Unigene482890 | bacA | 0.000E+00 | 1.756E-07 | 7.850E-08 | 0.000E+00 | 3.999E-07 | 2.595E-07 | 1.151E-06 | 2.101E-06 | 0.000E+00 | 0.000E+00 | 0.000E+00 | 1.087E-06 |
| Unigene483003 | msbA | 0.000E+00 | 2.513E-07 | 0.000E+00 | 0.000E+00 | 0.000E+00 | 0.000E+00 | 1.077E-06 | 6.013E-07 | 0.000E+00 | 0.000E+00 | 0.000E+00 | 6.765E-08 |
| Unigene483039 | lmrD | 1.582E-07 | 0.000E+00 | 3.105E-07 | 0.000E+00 | 3.867E-07 | 2.737E-07 | 1.086E-06 | 1.182E-06 | 3.113E-07 | 0.000E+00 | 1.014E-06 | 2.057E-06 |
| Unigene483184 | QepA4 | 0.000E+00 | 0.000E+00 | 0.000E+00 | 0.000E+00 | 0.000E+00 | 0.000E+00 | 8.525E-07 | 0.000E+00 | 0.000E+00 | 0.000E+00 | 0.000E+00 | 0.000E+00 |
| Unigene483364 | NmcR | 0.000E+00 | 0.000E+00 | 0.000E+00 | 0.000E+00 | 0.000E+00 | 0.000E+00 | 4.130E-07 | 0.000E+00 | 0.000E+00 | 0.000E+00 | 0.000E+00 | 0.000E+00 |
| Unigene483414 | vatB | 0.000E+00 | 0.000E+00 | 0.000E+00 | 0.000E+00 | 0.000E+00 | 0.000E+00 | 4.580E-07 | 0.000E+00 | 0.000E+00 | 0.000E+00 | 0.000E+00 | 0.000E+00 |
| Unigene483483 | patA | 0.000E+00 | 0.000E+00 | 0.000E+00 | 0.000E+00 | 0.000E+00 | 0.000E+00 | 2.372E-06 | 0.000E+00 | 0.000E+00 | 0.000E+00 | 0.000E+00 | 0.000E+00 |
| Unigene483506 | baeR | 0.000E+00 | 0.000E+00 | 0.000E+00 | 0.000E+00 | 0.000E+00 | 0.000E+00 | 5.753E-07 | 0.000E+00 | 0.000E+00 | 0.000E+00 | 0.000E+00 | 0.000E+00 |
| Unigene483528 | Chlamydia | 0.000E+00 | 0.000E+00 | 0.000E+00 | 0.000E+00 | 0.000E+00 | 0.000E+00 | 9.009E-07 | 0.000E+00 | 0.000E+00 | 0.000E+00 | 0.000E+00 | 0.000E+00 |
| Unigene483643 | novA | 9.738E-08 | 0.000E+00 | 0.000E+00 | 0.000E+00 | 3.786E-07 | 0.000E+00 | 1.024E-06 | 5.683E-07 | 0.000E+00 | 0.000E+00 | 0.000E+00 | 1.151E-07 |
| Unigene483765 | efrB | 0.000E+00 | 0.000E+00 | 0.000E+00 | 0.000E+00 | 0.000E+00 | 0.000E+00 | 1.858E-06 | 9.294E-07 | 0.000E+00 | 0.000E+00 | 6.400E-07 | 0.000E+00 |
| Unigene483828 | kdpE | 0.000E+00 | 0.000E+00 | 0.000E+00 | 0.000E+00 | 0.000E+00 | 0.000E+00 | 0.000E+00 | 1.522E-06 | 1.018E-07 | 0.000E+00 | 0.000E+00 | 8.259E-07 |
| Unigene483881 | patA | 0.000E+00 | 0.000E+00 | 0.000E+00 | 0.000E+00 | 0.000E+00 | 0.000E+00 | 8.756E-07 | 0.000E+00 | 0.000E+00 | 0.000E+00 | 0.000E+00 | 4.676E-08 |
| Unigene483954 | optrA | 0.000E+00 | 0.000E+00 | 0.000E+00 | 0.000E+00 | 0.000E+00 | 0.000E+00 | 5.646E-07 | 0.000E+00 | 0.000E+00 | 0.000E+00 | 0.000E+00 | 4.386E-07 |
| Unigene484097 | CTX-M-38 | 0.000E+00 | 0.000E+00 | 0.000E+00 | 0.000E+00 | 0.000E+00 | 0.000E+00 | 6.437E-07 | 0.000E+00 | 0.000E+00 | 0.000E+00 | 0.000E+00 | 0.000E+00 |
| Unigene484124 | catB10 | 0.000E+00 | 0.000E+00 | 0.000E+00 | 0.000E+00 | 1.049E-07 | 0.000E+00 | 7.835E-07 | 0.000E+00 | 0.000E+00 | 2.629E-07 | 0.000E+00 | 3.905E-07 |
| Unigene484162 | efrA | 0.000E+00 | 0.000E+00 | 0.000E+00 | 0.000E+00 | 0.000E+00 | 3.204E-07 | 9.842E-07 | 0.000E+00 | 0.000E+00 | 1.321E-07 | 0.000E+00 | 2.453E-07 |
| Unigene484284 | patA | 0.000E+00 | 0.000E+00 | 8.654E-08 | 1.781E-07 | 4.246E-07 | 2.860E-07 | 1.269E-06 | 8.922E-07 | 0.000E+00 | 5.240E-07 | 6.726E-07 | 3.162E-06 |
| Unigene484345 | poxtA | 4.847E-08 | 0.000E+00 | 0.000E+00 | 0.000E+00 | 2.154E-07 | 0.000E+00 | 1.127E-06 | 2.263E-07 | 0.000E+00 | 0.000E+00 | 0.000E+00 | 0.000E+00 |
| Unigene484397 | farB | 0.000E+00 | 0.000E+00 | 0.000E+00 | 7.476E-08 | 0.000E+00 | 0.000E+00 | 1.584E-06 | 1.008E-06 | 0.000E+00 | 4.124E-07 | 4.235E-07 | 1.138E-06 |
| Unigene484409 | msbA | 1.779E-08 | 0.000E+00 | 0.000E+00 | 0.000E+00 | 2.569E-07 | 1.730E-07 | 1.102E-06 | 1.453E-06 | 0.000E+00 | 2.377E-07 | 5.086E-07 | 1.156E-06 |
| Unigene484417 | macB | 0.000E+00 | 0.000E+00 | 0.000E+00 | 4.295E-08 | 5.197E-07 | 0.000E+00 | 1.365E-06 | 1.489E-06 | 0.000E+00 | 0.000E+00 | 5.351E-07 | 1.458E-06 |

| | | | | | | | | | | | | | |
|---|---|---|---|---|---|---|---|---|---|---|---|---|---|
| Unigene484421 | MexK | 1.695E-07 | 0.000E+00 | 1.663E-07 | 0.000E+00 | 0.000E+00 | 0.000E+00 | 9.377E-07 | 0.000E+00 | 0.000E+00 | 0.000E+00 | 0.000E+00 | 0.000E+00 |
| Unigene484456 | basS | 1.112E-07 | 1.220E-07 | 0.000E+00 | 8.980E-08 | 3.211E-07 | 3.124E-07 | 7.135E-07 | 6.487E-07 | 7.290E-08 | 4.705E-07 | 7.629E-07 | 7.621E-07 |
| Unigene484479 | patA | 1.882E-07 | 0.000E+00 | 3.694E-07 | 0.000E+00 | 0.000E+00 | 4.409E-07 | 1.493E-06 | 1.611E-06 | 0.000E+00 | 0.000E+00 | 0.000E+00 | 1.409E-06 |
| Unigene484560 | adeG | 0.000E+00 | 0.000E+00 | 0.000E+00 | 0.000E+00 | 3.819E-08 | 0.000E+00 | 3.424E-07 | 0.000E+00 | 0.000E+00 | 0.000E+00 | 0.000E+00 | 0.000E+00 |
| Unigene484571 | lmrC | 0.000E+00 | 0.000E+00 | 0.000E+00 | 0.000E+00 | 0.000E+00 | 0.000E+00 | 8.040E-07 | 0.000E+00 | 0.000E+00 | 0.000E+00 | 0.000E+00 | 0.000E+00 |
| Unigene484612 | MexW | 0.000E+00 | 0.000E+00 | 0.000E+00 | 0.000E+00 | 0.000E+00 | 0.000E+00 | 4.836E-07 | 0.000E+00 | 0.000E+00 | 0.000E+00 | 0.000E+00 | 0.000E+00 |
| Unigene484783 | Acinetoba | 0.000E+00 | 0.000E+00 | 0.000E+00 | 0.000E+00 | 6.099E-08 | 0.000E+00 | 7.899E-07 | 0.000E+00 | 0.000E+00 | 0.000E+00 | 0.000E+00 | 2.920E-07 |
| Unigene484894 | vanHF | 0.000E+00 | 0.000E+00 | 0.000E+00 | 0.000E+00 | 0.000E+00 | 0.000E+00 | 7.698E-07 | 0.000E+00 | 0.000E+00 | 0.000E+00 | 0.000E+00 | 0.000E+00 |
| Unigene484982 | bcrA | 0.000E+00 | 0.000E+00 | 0.000E+00 | 0.000E+00 | 0.000E+00 | 0.000E+00 | 1.339E-06 | 8.575E-07 | 0.000E+00 | 0.000E+00 | 1.236E-06 | 1.277E-06 |
| Unigene485011 | Acinetoba | 0.000E+00 | 0.000E+00 | 0.000E+00 | 0.000E+00 | 9.890E-07 | 0.000E+00 | 0.000E+00 | 6.325E-07 | 0.000E+00 | 0.000E+00 | 0.000E+00 | 1.510E-06 |
| Unigene485045 | evgS | 0.000E+00 | 0.000E+00 | 1.473E-07 | 0.000E+00 | 3.892E-07 | 0.000E+00 | 8.585E-07 | 0.000E+00 | 5.470E-08 | 0.000E+00 | 0.000E+00 | 1.213E-06 |
| Unigene485063 | bcrA | 2.360E-07 | 2.072E-07 | 0.000E+00 | 0.000E+00 | 0.000E+00 | 1.020E-07 | 9.924E-07 | 6.060E-07 | 0.000E+00 | 3.154E-07 | 3.239E-07 | 1.506E-06 |
| Unigene485064 | lmrD | 0.000E+00 | 0.000E+00 | 0.000E+00 | 0.000E+00 | 1.905E-07 | 1.390E-07 | 9.489E-07 | 6.505E-07 | 0.000E+00 | 0.000E+00 | 3.433E-07 | 1.571E-06 |
| Unigene485160 | vmlR | 0.000E+00 | 0.000E+00 | 0.000E+00 | 0.000E+00 | 0.000E+00 | 0.000E+00 | 1.095E-06 | 0.000E+00 | 4.506E-08 | 0.000E+00 | 3.301E-07 | 0.000E+00 |
| Unigene485222 | Staphyloc | 0.000E+00 | 0.000E+00 | 0.000E+00 | 0.000E+00 | 0.000E+00 | 0.000E+00 | 2.964E-06 | 7.130E-07 | 0.000E+00 | 0.000E+00 | 0.000E+00 | 0.000E+00 |
| Unigene485235 | patB | 1.182E-07 | 1.298E-07 | 1.160E-07 | 2.090E-07 | 2.627E-07 | 1.278E-07 | 1.014E-06 | 1.139E-06 | 6.462E-08 | 0.000E+00 | 5.072E-07 | 2.760E-06 |
| Unigene485249 | MexL | 0.000E+00 | 0.000E+00 | 0.000E+00 | 0.000E+00 | 0.000E+00 | 0.000E+00 | 1.014E-06 | 0.000E+00 | 0.000E+00 | 0.000E+00 | 0.000E+00 | 0.000E+00 |
| Unigene485370 | arnA | 0.000E+00 | 0.000E+00 | 0.000E+00 | 0.000E+00 | 0.000E+00 | 0.000E+00 | 4.936E-07 | 0.000E+00 | 0.000E+00 | 0.000E+00 | 0.000E+00 | 0.000E+00 |
| Unigene485426 | vanHB | 0.000E+00 | 0.000E+00 | 0.000E+00 | 0.000E+00 | 0.000E+00 | 0.000E+00 | 8.015E-07 | 0.000E+00 | 0.000E+00 | 0.000E+00 | 0.000E+00 | 0.000E+00 |
| Unigene485613 | adeH | 0.000E+00 | 0.000E+00 | 0.000E+00 | 0.000E+00 | 0.000E+00 | 3.409E-07 | 8.288E-07 | 2.760E-07 | 0.000E+00 | 0.000E+00 | 9.017E-07 | 5.125E-07 |
| Unigene485635 | efrA | 0.000E+00 | 6.729E-08 | 9.024E-08 | 0.000E+00 | 3.406E-08 | 2.983E-07 | 9.839E-07 | 1.431E-07 | 0.000E+00 | 1.707E-07 | 7.013E-08 | 7.972E-07 |
| Unigene485656 | emrA | 0.000E+00 | 0.000E+00 | 1.584E-07 | 0.000E+00 | 0.000E+00 | 6.398E-07 | 7.741E-07 | 4.396E-07 | 0.000E+00 | 0.000E+00 | 0.000E+00 | 3.180E-07 |
| Unigene485756 | oleD | 0.000E+00 | 0.000E+00 | 0.000E+00 | 0.000E+00 | 0.000E+00 | 0.000E+00 | 1.270E-06 | 0.000E+00 | 0.000E+00 | 0.000E+00 | 0.000E+00 | 7.233E-07 |
| Unigene485764 | macB | 0.000E+00 | 0.000E+00 | 0.000E+00 | 0.000E+00 | 0.000E+00 | 0.000E+00 | 2.586E-06 | 0.000E+00 | 0.000E+00 | 3.214E-07 | 0.000E+00 | 0.000E+00 |
| Unigene485833 | Acinetoba | 9.565E-08 | 0.000E+00 | 2.112E-07 | 0.000E+00 | 0.000E+00 | 2.843E-07 | 1.217E-06 | 4.187E-07 | 1.045E-07 | 0.000E+00 | 0.000E+00 | 0.000E+00 |
| Unigene485887 | Escherichia | 0.000E+00 | 0.000E+00 | 0.000E+00 | 0.000E+00 | 0.000E+00 | 0.000E+00 | 5.809E-07 | 0.000E+00 | 0.000E+00 | 0.000E+00 | 0.000E+00 | 0.000E+00 |
| Unigene485934 | Brucella su | 0.000E+00 | 0.000E+00 | 2.906E-07 | 0.000E+00 | 0.000E+00 | 0.000E+00 | 1.056E-06 | 0.000E+00 | 0.000E+00 | 0.000E+00 | 4.517E-07 | 0.000E+00 |
| Unigene486016 | bcrA | 0.000E+00 | 0.000E+00 | 0.000E+00 | 0.000E+00 | 0.000E+00 | 0.000E+00 | 9.240E-07 | 3.790E-07 | 0.000E+00 | 0.000E+00 | 0.000E+00 | 4.934E-07 |
| Unigene486017 | oleC | 0.000E+00 | 0.000E+00 | 0.000E+00 | 0.000E+00 | 0.000E+00 | 0.000E+00 | 4.670E-07 | 8.373E-07 | 0.000E+00 | 0.000E+00 | 9.171E-07 | 0.000E+00 |
| Unigene486098 | Acinetoba | 0.000E+00 | 0.000E+00 | 0.000E+00 | 0.000E+00 | 0.000E+00 | 0.000E+00 | 1.088E-06 | 0.000E+00 | 0.000E+00 | 0.000E+00 | 0.000E+00 | 0.000E+00 |
| Unigene486101 | Acinetoba | 1.121E-07 | 0.000E+00 | 0.000E+00 | 0.000E+00 | 0.000E+00 | 0.000E+00 | 3.721E-07 | 0.000E+00 | 0.000E+00 | 0.000E+00 | 1.282E-07 | 0.000E+00 |
| Unigene486139 | msbA | 0.000E+00 | 3.433E-08 | 0.000E+00 | 6.317E-08 | 6.950E-08 | 1.691E-07 | 2.769E-07 | 3.651E-07 | 0.000E+00 | 0.000E+00 | 3.220E-07 | 9.613E-07 |
| Unigene486147 | mtrA | 0.000E+00 | 0.000E+00 | 0.000E+00 | 0.000E+00 | 7.663E-08 | 0.000E+00 | 9.924E-07 | 0.000E+00 | 0.000E+00 | 0.000E+00 | 0.000E+00 | 0.000E+00 |
| Unigene486194 | vanTG | 0.000E+00 | 0.000E+00 | 0.000E+00 | 0.000E+00 | 0.000E+00 | 0.000E+00 | 6.107E-07 | 0.000E+00 | 0.000E+00 | 0.000E+00 | 0.000E+00 | 0.000E+00 |
| Unigene486357 | msbA | 0.000E+00 | 0.000E+00 | 0.000E+00 | 0.000E+00 | 1.434E-07 | 0.000E+00 | 7.145E-07 | 2.010E-07 | 0.000E+00 | 0.000E+00 | 0.000E+00 | 4.070E-07 |
| Unigene486413 | novA | 0.000E+00 | 0.000E+00 | 4.223E-08 | 0.000E+00 | 5.738E-07 | 0.000E+00 | 0.000E+00 | 7.033E-07 | 0.000E+00 | 0.000E+00 | 1.477E-07 | 3.052E-07 |
| Unigene486457 | evgA | 0.000E+00 | 0.000E+00 | 0.000E+00 | 0.000E+00 | 0.000E+00 | 0.000E+00 | 7.537E-07 | 0.000E+00 | 0.000E+00 | 0.000E+00 | 0.000E+00 | 0.000E+00 |
| Unigene486525 | bcrA | 0.000E+00 | 0.000E+00 | 0.000E+00 | 0.000E+00 | 0.000E+00 | 0.000E+00 | 7.201E-07 | 0.000E+00 | 0.000E+00 | 0.000E+00 | 0.000E+00 | 0.000E+00 |
| Unigene486561 | oleB | 0.000E+00 | 3.116E-08 | 0.000E+00 | 8.601E-08 | 2.523E-07 | 1.535E-07 | 7.541E-07 | 1.591E-06 | 0.000E+00 | 0.000E+00 | 5.846E-07 | 1.510E-06 |
| Unigene486715 | patA | 0.000E+00 | 0.000E+00 | 0.000E+00 | 0.000E+00 | 0.000E+00 | 2.384E-07 | 0.000E+00 | 0.000E+00 | 0.000E+00 | 0.000E+00 | 0.000E+00 | 0.000E+00 |
| Unigene486719 | vanSG | 0.000E+00 | 0.000E+00 | 0.000E+00 | 0.000E+00 | 0.000E+00 | 0.000E+00 | 7.874E-07 | 8.044E-07 | 1.215E-07 | 0.000E+00 | 0.000E+00 | 1.051E-06 |
| Unigene486772 | mdtB | 0.000E+00 | 0.000E+00 | 0.000E+00 | 0.000E+00 | 0.000E+00 | 1.852E-08 | 6.827E-07 | 1.200E-07 | 0.000E+00 | 0.000E+00 | 1.372E-07 | 6.076E-08 |
| Unigene486811 | mdtN | 0.000E+00 | 0.000E+00 | 0.000E+00 | 0.000E+00 | 0.000E+00 | 0.000E+00 | 2.233E-06 | 1.134E-06 | 0.000E+00 | 0.000E+00 | 0.000E+00 | 0.000E+00 |
| Unigene486872 | evgS | 0.000E+00 | 0.000E+00 | 0.000E+00 | 0.000E+00 | 0.000E+00 | 0.000E+00 | 4.147E-07 | 0.000E+00 | 0.000E+00 | 0.000E+00 | 0.000E+00 | 0.000E+00 |
| Unigene486878 | srmB | 0.000E+00 | 0.000E+00 | 0.000E+00 | 0.000E+00 | 0.000E+00 | 0.000E+00 | 8.858E-07 | 0.000E+00 | 0.000E+00 | 0.000E+00 | 0.000E+00 | 0.000E+00 |
| Unigene486911 | cmlA8 | 0.000E+00 | 8.258E-08 | 0.000E+00 | 0.000E+00 | 0.000E+00 | 0.000E+00 | 9.438E-07 | 0.000E+00 | 0.000E+00 | 0.000E+00 | 0.000E+00 | 5.633E-07 |
| Unigene486926 | TaeA | 0.000E+00 | 0.000E+00 | 0.000E+00 | 0.000E+00 | 0.000E+00 | 0.000E+00 | 1.641E-06 | 0.000E+00 | 0.000E+00 | 0.000E+00 | 0.000E+00 | 0.000E+00 |
| Unigene486928 | TriC | 0.000E+00 | 0.000E+00 | 0.000E+00 | 0.000E+00 | 9.330E-07 | 0.000E+00 | 2.056E-06 | 0.000E+00 | 0.000E+00 | 0.000E+00 | 0.000E+00 | 0.000E+00 |
| Unigene486929 | TriA | 0.000E+00 | 0.000E+00 | 0.000E+00 | 0.000E+00 | 0.000E+00 | 0.000E+00 | 2.107E-06 | 0.000E+00 | 0.000E+00 | 0.000E+00 | 0.000E+00 | 0.000E+00 |
| Unigene486930 | vanSF | 0.000E+00 | 4.440E-07 | 1.588E-07 | 0.000E+00 | 5.692E-07 | 0.000E+00 | 2.238E-06 | 0.000E+00 | 0.000E+00 | 0.000E+00 | 0.000E+00 | 8.925E-07 |
| Unigene486957 | vmlR | 1.860E-06 | 3.532E-06 | 2.418E-06 | 1.726E-06 | 0.000E+00 | 2.283E-06 | 2.838E-06 | 0.000E+00 | 1.044E-06 | 5.937E-06 | 4.716E-06 | 1.367E-06 |
| Unigene487080 | tetB(46) | 0.000E+00 | 8.879E-08 | 0.000E+00 | 0.000E+00 | 1.798E-07 | 4.373E-08 | 3.134E-07 | 0.000E+00 | 0.000E+00 | 0.000E+00 | 0.000E+00 | 3.825E-07 |
| Unigene487108 | efrA | 0.000E+00 | 0.000E+00 | 2.603E-07 | 0.000E+00 | 0.000E+00 | 0.000E+00 | 6.852E-07 | 0.000E+00 | 0.000E+00 | 0.000E+00 | 0.000E+00 | 0.000E+00 |
| Unigene487257 | PEDO-1 | 1.849E-07 | 8.117E-08 | 3.628E-07 | 1.494E-07 | 6.983E-07 | 0.000E+00 | 9.822E-07 | 1.165E-06 | 4.041E-08 | 8.238E-08 | 6.344E-07 | 1.311E-06 |
| Unigene487258 | adeL | 0.000E+00 | 1.697E-07 | 9.102E-08 | 2.498E-07 | 7.557E-07 | 2.674E-07 | 1.027E-06 | 6.135E-07 | 1.352E-07 | 0.000E+00 | 4.952E-07 | 5.482E-07 |
| Unigene487412 | mtrD | 1.643E-08 | 1.262E-07 | 0.000E+00 | 4.977E-08 | 2.738E-07 | 3.019E-07 | 4.727E-07 | 2.109E-07 | 2.155E-07 | 1.830E-07 | 8.456E-07 | 3.689E-07 |
| Unigene487418 | mecC | 0.000E+00 | 0.000E+00 | 0.000E+00 | 0.000E+00 | 5.656E-07 | 2.202E-07 | 2.029E-06 | 6.339E-07 | 0.000E+00 | 0.000E+00 | 0.000E+00 | 0.000E+00 |
| Unigene487559 | efrA | 1.248E-07 | 0.000E+00 | 2.142E-07 | 0.000E+00 | 2.079E-07 | 2.023E-07 | 1.070E-06 | 1.019E-06 | 0.000E+00 | 0.000E+00 | 3.568E-07 | 1.438E-06 |

| | | | | | | | | | | | | | |
|---|---|---|---|---|---|---|---|---|---|---|---|---|---|
| Unigene487586 | Bifidobact | 0.000E+00 | 0.000E+00 | 0.000E+00 | 0.000E+00 | 0.000E+00 | 0.000E+00 | 8.363E-07 | 0.000E+00 | 0.000E+00 | 0.000E+00 | 0.000E+00 | 0.000E+00 |
| Unigene487687 | tetA(58) | 0.000E+00 | 0.000E+00 | 0.000E+00 | 0.000E+00 | 0.000E+00 | 0.000E+00 | 1.152E-06 | 0.000E+00 | 0.000E+00 | 0.000E+00 | 0.000E+00 | 0.000E+00 |
| Unigene487753 | vanHD | 0.000E+00 | 0.000E+00 | 0.000E+00 | 0.000E+00 | 0.000E+00 | 0.000E+00 | 1.861E-07 | 0.000E+00 | 0.000E+00 | 0.000E+00 | 0.000E+00 | 0.000E+00 |
| Unigene487755 | baeS | 0.000E+00 | 0.000E+00 | 0.000E+00 | 0.000E+00 | 0.000E+00 | 0.000E+00 | 8.439E-07 | 0.000E+00 | 1.389E-07 | 0.000E+00 | 0.000E+00 | 3.505E-07 |
| Unigene487971 | Acinetoba | 0.000E+00 | 1.330E-07 | 4.756E-07 | 0.000E+00 | 7.000E-07 | 5.240E-07 | 1.958E-06 | 9.901E-07 | 2.649E-07 | 0.000E+00 | 9.148E-07 | 3.036E-06 |
| Unigene488177 | evgS | 0.000E+00 | 0.000E+00 | 1.351E-07 | 0.000E+00 | 4.078E-07 | 0.000E+00 | 7.871E-07 | 0.000E+00 | 0.000E+00 | 0.000E+00 | 4.986E-07 | 8.678E-07 |
| Unigene488228 | evgS | 0.000E+00 | 0.000E+00 | 0.000E+00 | 0.000E+00 | 0.000E+00 | 0.000E+00 | 5.565E-07 | 0.000E+00 | 0.000E+00 | 0.000E+00 | 0.000E+00 | 0.000E+00 |
| Unigene488299 | Staphyloc | 0.000E+00 | 0.000E+00 | 5.007E-07 | 0.000E+00 | 7.024E-07 | 2.878E-07 | 1.817E-06 | 8.805E-07 | 1.819E-07 | 7.291E-07 | 8.375E-07 | 2.740E-06 |
| Unigene488407 | adeR | 0.000E+00 | 0.000E+00 | 0.000E+00 | 0.000E+00 | 0.000E+00 | 0.000E+00 | 9.708E-07 | 0.000E+00 | 0.000E+00 | 0.000E+00 | 0.000E+00 | 4.147E-07 |
| Unigene488432 | tetB(60) | 0.000E+00 | 0.000E+00 | 0.000E+00 | 0.000E+00 | 0.000E+00 | 0.000E+00 | 1.055E-06 | 0.000E+00 | 0.000E+00 | 0.000E+00 | 0.000E+00 | 0.000E+00 |
| Unigene488481 | Erm(34) | 2.954E-06 | 2.562E-06 | 3.400E-06 | 1.842E-06 | 0.000E+00 | 0.000E+00 | 5.652E-07 | 1.277E-07 | 5.980E-07 | 8.126E-08 | 0.000E+00 | 0.000E+00 |
| Unigene488626 | patA | 0.000E+00 | 0.000E+00 | 0.000E+00 | 0.000E+00 | 7.907E-07 | 0.000E+00 | 1.297E-06 | 1.222E-06 | 0.000E+00 | 0.000E+00 | 4.310E-07 | 1.039E-06 |
| Unigene488635 | srmB | 0.000E+00 | 0.000E+00 | 0.000E+00 | 3.055E-07 | 5.281E-07 | 1.869E-07 | 6.217E-07 | 7.062E-07 | 0.000E+00 | 0.000E+00 | 0.000E+00 | 1.175E-06 |
| Unigene488706 | tetA(58) | 0.000E+00 | 0.000E+00 | 0.000E+00 | 0.000E+00 | 0.000E+00 | 0.000E+00 | 4.798E-07 | 0.000E+00 | 0.000E+00 | 0.000E+00 | 0.000E+00 | 0.000E+00 |
| Unigene488779 | otr(B) | 0.000E+00 | 0.000E+00 | 0.000E+00 | 0.000E+00 | 0.000E+00 | 0.000E+00 | 5.264E-07 | 0.000E+00 | 0.000E+00 | 0.000E+00 | 0.000E+00 | 0.000E+00 |
| Unigene488818 | novA | 0.000E+00 | 2.160E-07 | 0.000E+00 | 0.000E+00 | 7.289E-08 | 0.000E+00 | 0.000E+00 | 7.658E-08 | 0.000E+00 | 4.385E-07 | 0.000E+00 | 0.000E+00 |
| Unigene488863 | cmrA | 0.000E+00 | 0.000E+00 | 0.000E+00 | 0.000E+00 | 1.502E-07 | 0.000E+00 | 1.137E-06 | 0.000E+00 | 0.000E+00 | 0.000E+00 | 3.093E-07 | 9.907E-07 |
| Unigene488872 | PmrF | 0.000E+00 | 0.000E+00 | 0.000E+00 | 0.000E+00 | 0.000E+00 | 0.000E+00 | 1.021E-06 | 0.000E+00 | 0.000E+00 | 0.000E+00 | 0.000E+00 | 0.000E+00 |
| Unigene488880 | lmrC | 2.582E-07 | 0.000E+00 | 0.000E+00 | 0.000E+00 | 0.000E+00 | 0.000E+00 | 0.000E+00 | 0.000E+00 | 0.000E+00 | 0.000E+00 | 0.000E+00 | 0.000E+00 |
| Unigene489137 | efrA | 0.000E+00 | 0.000E+00 | 0.000E+00 | 0.000E+00 | 3.065E-07 | 0.000E+00 | 1.798E-06 | 0.000E+00 | 0.000E+00 | 0.000E+00 | 6.663E-07 | 1.486E-06 |
| Unigene489148 | tcr3 | 0.000E+00 | 0.000E+00 | 0.000E+00 | 1.502E-07 | 0.000E+00 | 0.000E+00 | 3.293E-07 | 2.315E-07 | 0.000E+00 | 0.000E+00 | 3.970E-07 | 1.758E-07 |
| Unigene489327 | Staphyloc | 1.469E-07 | 0.000E+00 | 1.802E-07 | 0.000E+00 | 4.488E-07 | 1.191E-07 | 6.097E-07 | 5.573E-07 | 4.014E-08 | 1.636E-07 | 2.941E-07 | 6.511E-07 |
| Unigene489423 | vanHB | 0.000E+00 | 0.000E+00 | 0.000E+00 | 0.000E+00 | 0.000E+00 | 0.000E+00 | 4.029E-07 | 0.000E+00 | 0.000E+00 | 0.000E+00 | 0.000E+00 | 0.000E+00 |
| Unigene489443 | msbA | 0.000E+00 | 0.000E+00 | 0.000E+00 | 0.000E+00 | 0.000E+00 | 0.000E+00 | 5.641E-07 | 0.000E+00 | 0.000E+00 | 0.000E+00 | 0.000E+00 | 0.000E+00 |
| Unigene489585 | Acinetoba | 0.000E+00 | 0.000E+00 | 0.000E+00 | 0.000E+00 | 0.000E+00 | 0.000E+00 | 2.188E-06 | 0.000E+00 | 0.000E+00 | 0.000E+00 | 0.000E+00 | 0.000E+00 |
| Unigene489599 | basS | 0.000E+00 | 0.000E+00 | 0.000E+00 | 0.000E+00 | 0.000E+00 | 0.000E+00 | 7.366E-07 | 0.000E+00 | 0.000E+00 | 0.000E+00 | 0.000E+00 | 0.000E+00 |
| Unigene489626 | mecD | 0.000E+00 | 0.000E+00 | 0.000E+00 | 1.857E-07 | 0.000E+00 | 0.000E+00 | 2.545E-07 | 5.367E-07 | 0.000E+00 | 0.000E+00 | 2.104E-07 | 0.000E+00 |
| Unigene489657 | evgS | 9.818E-08 | 1.078E-07 | 0.000E+00 | 0.000E+00 | 0.000E+00 | 1.698E-07 | 6.519E-07 | 5.959E-07 | 0.000E+00 | 0.000E+00 | 2.021E-07 | 5.338E-07 |
| Unigene489744 | patB | 0.000E+00 | 0.000E+00 | 0.000E+00 | 0.000E+00 | 0.000E+00 | 0.000E+00 | 7.335E-07 | 0.000E+00 | 0.000E+00 | 0.000E+00 | 0.000E+00 | 5.530E-07 |
| Unigene489842 | macB | 0.000E+00 | 0.000E+00 | 0.000E+00 | 1.341E-07 | 0.000E+00 | 0.000E+00 | 1.470E-07 | 0.000E+00 | 0.000E+00 | 0.000E+00 | 0.000E+00 | 0.000E+00 |
| Unigene489905 | basS | 0.000E+00 | 0.000E+00 | 0.000E+00 | 0.000E+00 | 0.000E+00 | 0.000E+00 | 9.853E-07 | 0.000E+00 | 0.000E+00 | 0.000E+00 | 0.000E+00 | 0.000E+00 |
| Unigene489926 | Streptomy | 0.000E+00 | 0.000E+00 | 0.000E+00 | 0.000E+00 | 0.000E+00 | 0.000E+00 | 8.756E-07 | 0.000E+00 | 0.000E+00 | 0.000E+00 | 0.000E+00 | 0.000E+00 |
| Unigene489980 | oleB | 0.000E+00 | 0.000E+00 | 0.000E+00 | 0.000E+00 | 0.000E+00 | 0.000E+00 | 1.272E-06 | 0.000E+00 | 0.000E+00 | 0.000E+00 | 0.000E+00 | 0.000E+00 |
| Unigene489994 | novA | 0.000E+00 | 0.000E+00 | 0.000E+00 | 0.000E+00 | 0.000E+00 | 0.000E+00 | 3.383E-07 | 0.000E+00 | 0.000E+00 | 0.000E+00 | 0.000E+00 | 0.000E+00 |
| Unigene490069 | Acinetoba | 0.000E+00 | 0.000E+00 | 0.000E+00 | 0.000E+00 | 0.000E+00 | 0.000E+00 | 6.135E-07 | 3.806E-07 | 0.000E+00 | 0.000E+00 | 0.000E+00 | 0.000E+00 |
| Unigene490213 | lmrD | 0.000E+00 | 2.912E-07 | 0.000E+00 | 1.786E-07 | 0.000E+00 | 0.000E+00 | 2.284E-06 | 0.000E+00 | 0.000E+00 | 0.000E+00 | 0.000E+00 | 9.408E-07 |
| Unigene490279 | MexD | 0.000E+00 | 0.000E+00 | 0.000E+00 | 1.982E-07 | 0.000E+00 | 5.657E-07 | 5.430E-07 | 6.490E-07 | 0.000E+00 | 0.000E+00 | 4.863E-07 | 1.005E-06 |
| Unigene490291 | vanRI | 0.000E+00 | 0.000E+00 | 3.249E-07 | 0.000E+00 | 4.992E-07 | 0.000E+00 | 1.125E-06 | 7.729E-07 | 2.068E-07 | 0.000E+00 | 0.000E+00 | 1.202E-06 |
| Unigene490317 | macB | 0.000E+00 | 0.000E+00 | 1.893E-07 | 0.000E+00 | 2.858E-07 | 1.159E-07 | 7.828E-07 | 1.076E-06 | 0.000E+00 | 0.000E+00 | 7.110E-07 | 1.064E-06 |
| Unigene490323 | adeL | 0.000E+00 | 0.000E+00 | 0.000E+00 | 0.000E+00 | 0.000E+00 | 0.000E+00 | 9.402E-07 | 0.000E+00 | 0.000E+00 | 0.000E+00 | 0.000E+00 | 0.000E+00 |
| Unigene490427 | mexY | 0.000E+00 | 0.000E+00 | 0.000E+00 | 0.000E+00 | 0.000E+00 | 0.000E+00 | 5.160E-07 | 3.768E-07 | 0.000E+00 | 0.000E+00 | 0.000E+00 | 0.000E+00 |
| Unigene490460 | efpA | 0.000E+00 | 0.000E+00 | 0.000E+00 | 2.656E-07 | 0.000E+00 | 0.000E+00 | 7.410E-07 | 0.000E+00 | 0.000E+00 | 0.000E+00 | 0.000E+00 | 0.000E+00 |
| Unigene490463 | msbA | 0.000E+00 | 0.000E+00 | 0.000E+00 | 0.000E+00 | 0.000E+00 | 0.000E+00 | 1.177E-06 | 0.000E+00 | 0.000E+00 | 0.000E+00 | 0.000E+00 | 3.697E-07 |
| Unigene490469 | efrA | 0.000E+00 | 4.941E-08 | 8.835E-08 | 0.000E+00 | 0.000E+00 | 0.000E+00 | 1.146E-06 | 0.000E+00 | 0.000E+00 | 0.000E+00 | 0.000E+00 | 0.000E+00 |
| Unigene490522 | CTX-M-8 | 0.000E+00 | 0.000E+00 | 0.000E+00 | 0.000E+00 | 0.000E+00 | 0.000E+00 | 5.203E-07 | 0.000E+00 | 0.000E+00 | 0.000E+00 | 0.000E+00 | 0.000E+00 |
| Unigene490637 | oleB | 0.000E+00 | 0.000E+00 | 0.000E+00 | 0.000E+00 | 2.833E-07 | 0.000E+00 | 4.515E-07 | 1.786E-07 | 0.000E+00 | 0.000E+00 | 0.000E+00 | 4.822E-07 |
| Unigene490670 | oleC | 0.000E+00 | 0.000E+00 | 0.000E+00 | 0.000E+00 | 0.000E+00 | 0.000E+00 | 1.137E-06 | 0.000E+00 | 0.000E+00 | 0.000E+00 | 0.000E+00 | 0.000E+00 |
| Unigene490733 | mtrA | 4.253E-08 | 0.000E+00 | 8.346E-08 | 0.000E+00 | 7.560E-07 | 4.138E-07 | 1.177E-06 | 1.440E-06 | 2.324E-07 | 5.685E-07 | 1.168E-06 | 4.223E-06 |
| Unigene490844 | Bifidobact | 0.000E+00 | 0.000E+00 | 0.000E+00 | 0.000E+00 | 0.000E+00 | 0.000E+00 | 1.221E-06 | 0.000E+00 | 0.000E+00 | 0.000E+00 | 3.410E-07 | 8.103E-07 |
| Unigene490849 | novA | 0.000E+00 | 0.000E+00 | 0.000E+00 | 6.487E-08 | 0.000E+00 | 0.000E+00 | 8.176E-07 | 0.000E+00 | 0.000E+00 | 0.000E+00 | 0.000E+00 | 0.000E+00 |
| Unigene490861 | lin | 0.000E+00 | 0.000E+00 | 0.000E+00 | 0.000E+00 | 0.000E+00 | 0.000E+00 | 2.233E-06 | 0.000E+00 | 0.000E+00 | 0.000E+00 | 0.000E+00 | 0.000E+00 |
| Unigene490862 | macB | 0.000E+00 | 0.000E+00 | 0.000E+00 | 0.000E+00 | 0.000E+00 | 0.000E+00 | 2.539E-06 | 0.000E+00 | 0.000E+00 | 0.000E+00 | 5.725E-07 | 0.000E+00 |
| Unigene490962 | adeB | 0.000E+00 | 0.000E+00 | 0.000E+00 | 4.137E-08 | 2.731E-07 | 1.181E-07 | 7.254E-07 | 1.020E-06 | 0.000E+00 | 1.217E-07 | 1.874E-07 | 6.618E-07 |
| Unigene491026 | tetA(60) | 0.000E+00 | 0.000E+00 | 0.000E+00 | 0.000E+00 | 0.000E+00 | 0.000E+00 | 5.979E-07 | 0.000E+00 | 0.000E+00 | 0.000E+00 | 0.000E+00 | 0.000E+00 |
| Unigene491089 | Staphyloc | 0.000E+00 | 0.000E+00 | 0.000E+00 | 0.000E+00 | 0.000E+00 | 0.000E+00 | 7.115E-07 | 0.000E+00 | 0.000E+00 | 0.000E+00 | 0.000E+00 | 0.000E+00 |
| Unigene491191 | oleB | 0.000E+00 | 0.000E+00 | 1.650E-07 | 0.000E+00 | 6.537E-07 | 2.726E-07 | 1.116E-06 | 6.378E-07 | 0.000E+00 | 0.000E+00 | 0.000E+00 | 6.956E-07 |
| Unigene491194 | efpA | 0.000E+00 | 0.000E+00 | 0.000E+00 | 0.000E+00 | 0.000E+00 | 0.000E+00 | 1.193E-06 | 0.000E+00 | 0.000E+00 | 0.000E+00 | 0.000E+00 | 0.000E+00 |
| Unigene491204 | MuxC | 0.000E+00 | 0.000E+00 | 0.000E+00 | 0.000E+00 | 0.000E+00 | 0.000E+00 | 7.939E-07 | 0.000E+00 | 0.000E+00 | 0.000E+00 | 0.000E+00 | 0.000E+00 |

| | | | | | | | | | | | | | |
|---|---|---|---|---|---|---|---|---|---|---|---|---|---|
| Unigene491236 | poxtA | 0.000E+00 | 0.000E+00 | 0.000E+00 | 0.000E+00 | 0.000E+00 | 0.000E+00 | 7.881E-07 | 0.000E+00 | 0.000E+00 | 0.000E+00 | 0.000E+00 | 0.000E+00 |
| Unigene491260 | vmlR | 0.000E+00 | 0.000E+00 | 0.000E+00 | 8.522E-08 | 0.000E+00 | 0.000E+00 | 1.541E-06 | 1.478E-06 | 0.000E+00 | 0.000E+00 | 0.000E+00 | 0.000E+00 |
| Unigene491339 | eptA | 1.787E-07 | 1.307E-07 | 0.000E+00 | 1.403E-07 | 5.955E-07 | 1.502E-07 | 6.811E-07 | 6.025E-07 | 0.000E+00 | 4.202E-07 | 6.131E-07 | 1.267E-06 |
| Unigene491343 | MexF | 0.000E+00 | 0.000E+00 | 0.000E+00 | 0.000E+00 | 0.000E+00 | 0.000E+00 | 7.337E-07 | 0.000E+00 | 0.000E+00 | 0.000E+00 | 0.000E+00 | 0.000E+00 |
| Unigene491367 | sdiA | 8.930E-08 | 0.000E+00 | 4.381E-08 | 2.254E-07 | 3.968E-07 | 2.896E-07 | 8.894E-07 | 7.817E-07 | 0.000E+00 | 0.000E+00 | 6.639E-07 | 2.111E-06 |
| Unigene491375 | mdtC | 0.000E+00 | 0.000E+00 | 0.000E+00 | 0.000E+00 | 0.000E+00 | 0.000E+00 | 4.279E-07 | 0.000E+00 | 0.000E+00 | 0.000E+00 | 0.000E+00 | 0.000E+00 |
| Unigene491412 | baeS | 0.000E+00 | 0.000E+00 | 0.000E+00 | 0.000E+00 | 0.000E+00 | 0.000E+00 | 1.907E-06 | 0.000E+00 | 0.000E+00 | 0.000E+00 | 0.000E+00 | 0.000E+00 |
| Unigene491413 | kdpE | 0.000E+00 | 0.000E+00 | 0.000E+00 | 0.000E+00 | 0.000E+00 | 0.000E+00 | 2.329E-06 | 0.000E+00 | 0.000E+00 | 0.000E+00 | 0.000E+00 | 0.000E+00 |
| Unigene491577 | lmrD | 0.000E+00 | 0.000E+00 | 2.030E-07 | 0.000E+00 | 2.299E-07 | 2.796E-07 | 1.260E-06 | 1.087E-06 | 0.000E+00 | 4.034E-07 | 8.284E-07 | 3.180E-06 |
| Unigene491597 | vanRD | 0.000E+00 | 0.000E+00 | 0.000E+00 | 0.000E+00 | 0.000E+00 | 0.000E+00 | 0.000E+00 | 1.850E-06 | 0.000E+00 | 0.000E+00 | 6.679E-07 | 1.183E-06 |
| Unigene491598 | vanSN | 5.912E-08 | 6.489E-08 | 1.740E-07 | 5.970E-08 | 2.627E-07 | 2.397E-07 | 9.815E-07 | 5.521E-07 | 1.131E-07 | 1.811E-07 | 8.115E-07 | 2.131E-06 |
| Unigene491599 | adeR | 0.000E+00 | 0.000E+00 | 0.000E+00 | 0.000E+00 | 2.359E-07 | 0.000E+00 | 7.051E-07 | 4.958E-07 | 0.000E+00 | 0.000E+00 | 6.478E-07 | 5.774E-06 |
| Unigene491691 | Acinetobac | 0.000E+00 | 1.142E-07 | 2.859E-07 | 0.000E+00 | 0.000E+00 | 0.000E+00 | 1.221E-06 | 0.000E+00 | 0.000E+00 | 0.000E+00 | 0.000E+00 | 7.380E-07 |
| Unigene491777 | lsaC | 2.118E-07 | 0.000E+00 | 0.000E+00 | 0.000E+00 | 0.000E+00 | 0.000E+00 | 1.125E-06 | 1.286E-06 | 0.000E+00 | 0.000E+00 | 6.784E-07 | 0.000E+00 |
| Unigene491782 | tetA(58) | 0.000E+00 | 0.000E+00 | 0.000E+00 | 0.000E+00 | 0.000E+00 | 0.000E+00 | 8.297E-07 | 0.000E+00 | 0.000E+00 | 0.000E+00 | 0.000E+00 | 0.000E+00 |
| Unigene491838 | vanRI | 0.000E+00 | 0.000E+00 | 0.000E+00 | 0.000E+00 | 0.000E+00 | 4.526E-08 | 4.634E-07 | 0.000E+00 | 0.000E+00 | 0.000E+00 | 0.000E+00 | 0.000E+00 |
| Unigene491844 | msbA | 0.000E+00 | 0.000E+00 | 0.000E+00 | 0.000E+00 | 0.000E+00 | 5.263E-08 | 8.083E-07 | 1.705E-07 | 0.000E+00 | 1.085E-07 | 2.785E-07 | 1.727E-07 |
| Unigene491867 | acrB | 0.000E+00 | 0.000E+00 | 0.000E+00 | 0.000E+00 | 5.831E-08 | 0.000E+00 | 6.390E-07 | 0.000E+00 | 0.000E+00 | 0.000E+00 | 0.000E+00 | 0.000E+00 |
| Unigene491964 | tetA(58) | 1.384E-07 | 0.000E+00 | 0.000E+00 | 0.000E+00 | 0.000E+00 | 0.000E+00 | 9.956E-07 | 4.442E-07 | 0.000E+00 | 0.000E+00 | 1.979E-07 | 0.000E+00 |
| Unigene492014 | optrA | 0.000E+00 | 0.000E+00 | 0.000E+00 | 0.000E+00 | 0.000E+00 | 0.000E+00 | 1.466E-06 | 0.000E+00 | 0.000E+00 | 0.000E+00 | 0.000E+00 | 0.000E+00 |
| Unigene492061 | cpxA | 0.000E+00 | 0.000E+00 | 0.000E+00 | 1.191E-07 | 3.932E-07 | 0.000E+00 | 1.045E-06 | 6.610E-07 | 0.000E+00 | 0.000E+00 | 5.668E-07 | 1.004E-06 |
| Unigene492062 | smeR | 0.000E+00 | 0.000E+00 | 0.000E+00 | 0.000E+00 | 0.000E+00 | 0.000E+00 | 8.894E-07 | 4.690E-07 | 0.000E+00 | 0.000E+00 | 0.000E+00 | 1.108E-06 |
| Unigene492079 | novA | 8.030E-08 | 0.000E+00 | 0.000E+00 | 0.000E+00 | 0.000E+00 | 0.000E+00 | 1.777E-07 | 9.373E-08 | 0.000E+00 | 0.000E+00 | 5.052E-07 | 0.000E+00 |
| Unigene492190 | MexK | 0.000E+00 | 0.000E+00 | 0.000E+00 | 0.000E+00 | 0.000E+00 | 0.000E+00 | 7.835E-07 | 0.000E+00 | 0.000E+00 | 0.000E+00 | 0.000E+00 | 0.000E+00 |
| Unigene492283 | efrB | 0.000E+00 | 0.000E+00 | 0.000E+00 | 0.000E+00 | 0.000E+00 | 0.000E+00 | 1.191E-06 | 0.000E+00 | 0.000E+00 | 0.000E+00 | 0.000E+00 | 0.000E+00 |
| Unigene492419 | lmrD | 0.000E+00 | 0.000E+00 | 1.745E-07 | 1.796E-07 | 4.940E-08 | 4.326E-07 | 8.366E-07 | 0.000E+00 | 0.000E+00 | 2.972E-07 | 0.000E+00 | 0.000E+00 |
| Unigene492535 | basS | 0.000E+00 | 1.039E-07 | 0.000E+00 | 0.000E+00 | 0.000E+00 | 0.000E+00 | 8.731E-07 | 0.000E+00 | 0.000E+00 | 0.000E+00 | 0.000E+00 | 0.000E+00 |
| Unigene492536 | arlR | 0.000E+00 | 0.000E+00 | 0.000E+00 | 0.000E+00 | 0.000E+00 | 0.000E+00 | 5.219E-07 | 0.000E+00 | 0.000E+00 | 0.000E+00 | 0.000E+00 | 3.040E-07 |
| Unigene492552 | adeS | 0.000E+00 | 0.000E+00 | 0.000E+00 | 0.000E+00 | 0.000E+00 | 0.000E+00 | 2.646E-07 | 3.907E-07 | 0.000E+00 | 0.000E+00 | 0.000E+00 | 0.000E+00 |
| Unigene492625 | tetA(60) | 0.000E+00 | 0.000E+00 | 0.000E+00 | 0.000E+00 | 0.000E+00 | 0.000E+00 | 5.507E-07 | 0.000E+00 | 0.000E+00 | 0.000E+00 | 0.000E+00 | 0.000E+00 |
| Unigene492631 | farA | 0.000E+00 | 0.000E+00 | 0.000E+00 | 0.000E+00 | 0.000E+00 | 0.000E+00 | 6.669E-07 | 0.000E+00 | 0.000E+00 | 0.000E+00 | 0.000E+00 | 0.000E+00 |
| Unigene492649 | mgrA | 0.000E+00 | 1.845E-07 | 2.750E-07 | 1.132E-07 | 1.868E-07 | 0.000E+00 | 1.178E-06 | 1.047E-06 | 0.000E+00 | 1.873E-07 | 5.770E-07 | 3.246E-06 |
| Unigene492664 | efrA | 0.000E+00 | 0.000E+00 | 0.000E+00 | 0.000E+00 | 2.754E-07 | 0.000E+00 | 7.134E-07 | 0.000E+00 | 2.168E-07 | 0.000E+00 | 3.403E-07 | 0.000E+00 |
| Unigene492695 | mtrA | 0.000E+00 | 0.000E+00 | 0.000E+00 | 0.000E+00 | 0.000E+00 | 3.861E-07 | 6.918E-07 | 6.775E-07 | 0.000E+00 | 0.000E+00 | 0.000E+00 | 0.000E+00 |
| Unigene492750 | evgS | 5.525E-08 | 3.638E-07 | 1.626E-07 | 1.953E-07 | 7.519E-07 | 0.000E+00 | 2.736E-06 | 0.000E+00 | 0.000E+00 | 0.000E+00 | 6.952E-07 | 8.163E-07 |
| Unigene492797 | lmrD | 0.000E+00 | 0.000E+00 | 0.000E+00 | 0.000E+00 | 0.000E+00 | 0.000E+00 | 6.065E-07 | 0.000E+00 | 0.000E+00 | 0.000E+00 | 0.000E+00 | 0.000E+00 |
| Unigene492913 | ceoB | 5.112E-08 | 0.000E+00 | 0.000E+00 | 0.000E+00 | 0.000E+00 | 0.000E+00 | 2.829E-07 | 2.685E-07 | 0.000E+00 | 0.000E+00 | 2.047E-07 | 3.021E-07 |
| Unigene492964 | macB | 0.000E+00 | 0.000E+00 | 8.122E-08 | 1.254E-07 | 5.977E-07 | 3.132E-07 | 9.619E-07 | 7.729E-07 | 1.809E-07 | 0.000E+00 | 1.278E-06 | 3.131E-06 |
| Unigene493105 | macB | 0.000E+00 | 0.000E+00 | 0.000E+00 | 0.000E+00 | 0.000E+00 | 0.000E+00 | 9.721E-07 | 0.000E+00 | 0.000E+00 | 0.000E+00 | 0.000E+00 | 0.000E+00 |
| Unigene493122 | Klebsiella p | 0.000E+00 | 0.000E+00 | 0.000E+00 | 0.000E+00 | 0.000E+00 | 0.000E+00 | 1.974E-06 | 0.000E+00 | 0.000E+00 | 0.000E+00 | 0.000E+00 | 1.004E-06 |
| Unigene493293 | TaeA | 0.000E+00 | 0.000E+00 | 0.000E+00 | 0.000E+00 | 2.999E-07 | 0.000E+00 | 7.256E-07 | 0.000E+00 | 0.000E+00 | 0.000E+00 | 4.853E-07 | 2.735E-07 |
| Unigene493475 | vanHO | 0.000E+00 | 0.000E+00 | 0.000E+00 | 0.000E+00 | 0.000E+00 | 0.000E+00 | 1.268E-06 | 0.000E+00 | 0.000E+00 | 0.000E+00 | 0.000E+00 | 7.795E-07 |
| Unigene493546 | APH(6)-Ic | 0.000E+00 | 0.000E+00 | 5.833E-08 | 0.000E+00 | 0.000E+00 | 0.000E+00 | 3.290E-07 | 0.000E+00 | 2.599E-07 | 0.000E+00 | 0.000E+00 | 0.000E+00 |
| Unigene493607 | vanTG | 0.000E+00 | 0.000E+00 | 0.000E+00 | 1.397E-07 | 6.146E-08 | 3.588E-07 | 7.960E-07 | 1.937E-07 | 0.000E+00 | 0.000E+00 | 0.000E+00 | 2.616E-07 |
| Unigene493767 | efrA | 0.000E+00 | 0.000E+00 | 0.000E+00 | 0.000E+00 | 0.000E+00 | 0.000E+00 | 7.217E-07 | 0.000E+00 | 0.000E+00 | 0.000E+00 | 0.000E+00 | 0.000E+00 |
| Unigene493802 | evgS | 0.000E+00 | 8.758E-07 | 5.304E-07 | 0.000E+00 | 7.007E-07 | 0.000E+00 | 1.496E-06 | 0.000E+00 | 0.000E+00 | 0.000E+00 | 2.356E-07 | 0.000E+00 |
| Unigene493876 | lmrC | 4.220E-08 | 0.000E+00 | 0.000E+00 | 0.000E+00 | 2.813E-07 | 0.000E+00 | 5.604E-07 | 7.388E-07 | 0.000E+00 | 0.000E+00 | 0.000E+00 | 0.000E+00 |
| Unigene493897 | smeC | 0.000E+00 | 0.000E+00 | 0.000E+00 | 1.433E-07 | 0.000E+00 | 7.670E-08 | 1.099E-06 | 0.000E+00 | 3.360E-07 | 0.000E+00 | 0.000E+00 | 0.000E+00 |
| Unigene494042 | macB | 0.000E+00 | 0.000E+00 | 0.000E+00 | 0.000E+00 | 0.000E+00 | 0.000E+00 | 6.004E-07 | 0.000E+00 | 0.000E+00 | 0.000E+00 | 0.000E+00 | 0.000E+00 |
| Unigene494098 | tlrC | 0.000E+00 | 0.000E+00 | 0.000E+00 | 0.000E+00 | 0.000E+00 | 0.000E+00 | 1.002E-06 | 0.000E+00 | 0.000E+00 | 0.000E+00 | 0.000E+00 | 0.000E+00 |
| Unigene494103 | Bifidobacte | 0.000E+00 | 0.000E+00 | 0.000E+00 | 0.000E+00 | 0.000E+00 | 0.000E+00 | 7.899E-07 | 0.000E+00 | 0.000E+00 | 0.000E+00 | 0.000E+00 | 0.000E+00 |
| Unigene494116 | patA | 0.000E+00 | 4.762E-08 | 0.000E+00 | 0.000E+00 | 0.000E+00 | 2.345E-07 | 9.604E-07 | 2.532E-07 | 0.000E+00 | 0.000E+00 | 1.241E-06 | 1.487E-06 |
| Unigene494131 | msbA | 0.000E+00 | 0.000E+00 | 0.000E+00 | 0.000E+00 | 0.000E+00 | 0.000E+00 | 2.520E-06 | 0.000E+00 | 0.000E+00 | 0.000E+00 | 0.000E+00 | 0.000E+00 |
| Unigene494155 | kdpE | 0.000E+00 | 0.000E+00 | 1.292E-07 | 9.969E-08 | 4.021E-07 | 0.000E+00 | 8.740E-07 | 8.834E-07 | 0.000E+00 | 0.000E+00 | 6.022E-07 | 1.672E-06 |
| Unigene494179 | smeS | 0.000E+00 | 0.000E+00 | 0.000E+00 | 0.000E+00 | 0.000E+00 | 2.737E-07 | 1.331E-06 | 7.388E-08 | 0.000E+00 | 0.000E+00 | 0.000E+00 | 6.733E-07 |
| Unigene494299 | macB | 0.000E+00 | 0.000E+00 | 0.000E+00 | 0.000E+00 | 0.000E+00 | 1.558E-07 | 6.911E-07 | 2.243E-07 | 0.000E+00 | 0.000E+00 | 3.297E-07 | 0.000E+00 |
| Unigene494315 | patA | 0.000E+00 | 0.000E+00 | 8.848E-08 | 0.000E+00 | 1.002E-07 | 0.000E+00 | 1.264E-06 | 1.052E-07 | 6.570E-08 | 0.000E+00 | 0.000E+00 | 4.974E-07 |
| Unigene494348 | vanRA | 0.000E+00 | 0.000E+00 | 0.000E+00 | 0.000E+00 | 0.000E+00 | 0.000E+00 | 8.368E-07 | 3.395E-07 | 0.000E+00 | 0.000E+00 | 0.000E+00 | 0.000E+00 |

| | | | | | | | | | | | | | |
|---|---|---|---|---|---|---|---|---|---|---|---|---|---|
| Unigene494366 | efrA | 0.000E+00 | 0.000E+00 | 0.000E+00 | 0.000E+00 | 0.000E+00 | 0.000E+00 | 1.098E-06 | 0.000E+00 | 0.000E+00 | 0.000E+00 | 0.000E+00 | 0.000E+00 |
| Unigene494425 | vmlR | 8.090E-08 | 0.000E+00 | 0.000E+00 | 1.225E-07 | 4.494E-07 | 1.312E-07 | 9.402E-07 | 5.194E-07 | 1.769E-07 | 2.704E-07 | 5.553E-07 | 1.578E-06 |
| Unigene494448 | msbA | 0.000E+00 | 0.000E+00 | 0.000E+00 | 0.000E+00 | 0.000E+00 | 0.000E+00 | 5.793E-07 | 0.000E+00 | 0.000E+00 | 0.000E+00 | 0.000E+00 | 0.000E+00 |
| Unigene494506 | msbA | 1.516E-07 | 0.000E+00 | 0.000E+00 | 0.000E+00 | 0.000E+00 | 5.461E-08 | 5.032E-07 | 0.000E+00 | 0.000E+00 | 0.000E+00 | 1.734E-07 | 1.194E-07 |
| Unigene494634 | Acinetobac | 0.000E+00 | 0.000E+00 | 0.000E+00 | 0.000E+00 | 0.000E+00 | 0.000E+00 | 2.155E-06 | 9.945E-07 | 0.000E+00 | 0.000E+00 | 0.000E+00 | 0.000E+00 |
| Unigene494666 | msbA | 0.000E+00 | 0.000E+00 | 0.000E+00 | 0.000E+00 | 0.000E+00 | 0.000E+00 | 0.000E+00 | 0.000E+00 | 0.000E+00 | 4.795E-07 | 0.000E+00 | 6.741E-06 |
| Unigene494693 | eptA | 0.000E+00 | 0.000E+00 | 3.500E-07 | 0.000E+00 | 0.000E+00 | 0.000E+00 | 8.334E-07 | 0.000E+00 | 0.000E+00 | 0.000E+00 | 0.000E+00 | 0.000E+00 |
| Unigene494753 | TaeA | 0.000E+00 | 0.000E+00 | 0.000E+00 | 0.000E+00 | 0.000E+00 | 0.000E+00 | 7.217E-07 | 0.000E+00 | 0.000E+00 | 0.000E+00 | 0.000E+00 | 0.000E+00 |
| Unigene494781 | vanTG | 0.000E+00 | 0.000E+00 | 1.890E-07 | 0.000E+00 | 0.000E+00 | 0.000E+00 | 1.218E-06 | 5.461E-07 | 0.000E+00 | 1.226E-07 | 0.000E+00 | 7.482E-07 |
| Unigene494806 | emrA | 0.000E+00 | 0.000E+00 | 0.000E+00 | 0.000E+00 | 1.332E-07 | 0.000E+00 | 0.000E+00 | 0.000E+00 | 0.000E+00 | 0.000E+00 | 0.000E+00 | 0.000E+00 |
| Unigene494850 | patA | 0.000E+00 | 0.000E+00 | 0.000E+00 | 0.000E+00 | 0.000E+00 | 0.000E+00 | 5.753E-07 | 0.000E+00 | 0.000E+00 | 0.000E+00 | 0.000E+00 | 0.000E+00 |
| Unigene494857 | patA | 0.000E+00 | 0.000E+00 | 2.326E-07 | 9.573E-08 | 1.580E-07 | 1.537E-07 | 1.102E-06 | 7.746E-07 | 0.000E+00 | 2.640E-07 | 1.084E-06 | 2.185E-06 |
| Unigene494968 | novA | 0.000E+00 | 0.000E+00 | 0.000E+00 | 0.000E+00 | 0.000E+00 | 0.000E+00 | 6.653E-07 | 0.000E+00 | 0.000E+00 | 0.000E+00 | 0.000E+00 | 9.948E-07 |
| Unigene494979 | Corynebac | 0.000E+00 | 0.000E+00 | 0.000E+00 | 0.000E+00 | 0.000E+00 | 0.000E+00 | 3.653E-07 | 0.000E+00 | 0.000E+00 | 2.206E-07 | 0.000E+00 | 6.242E-07 |
| Unigene495039 | carA | 0.000E+00 | 0.000E+00 | 0.000E+00 | 0.000E+00 | 0.000E+00 | 0.000E+00 | 5.982E-07 | 0.000E+00 | 0.000E+00 | 0.000E+00 | 0.000E+00 | 2.904E-07 |
| Unigene495312 | smeR | 0.000E+00 | 0.000E+00 | 0.000E+00 | 2.362E-07 | 0.000E+00 | 0.000E+00 | 5.178E-07 | 2.730E-07 | 0.000E+00 | 0.000E+00 | 0.000E+00 | 0.000E+00 |
| Unigene495318 | adeG | 0.000E+00 | 0.000E+00 | 0.000E+00 | 0.000E+00 | 0.000E+00 | 0.000E+00 | 0.000E+00 | 1.419E-07 | 0.000E+00 | 0.000E+00 | 0.000E+00 | 0.000E+00 |
| Unigene495325 | macB | 5.073E-07 | 2.344E-07 | 8.908E-07 | 2.966E-07 | 1.127E-06 | 5.195E-07 | 1.773E-06 | 1.434E-06 | 0.000E+00 | 0.000E+00 | 8.857E-07 | 3.819E-06 |
| Unigene495458 | oleB | 0.000E+00 | 0.000E+00 | 0.000E+00 | 0.000E+00 | 0.000E+00 | 0.000E+00 | 1.070E-06 | 0.000E+00 | 0.000E+00 | 0.000E+00 | 0.000E+00 | 0.000E+00 |
| Unigene495531 | novA | 0.000E+00 | 0.000E+00 | 0.000E+00 | 0.000E+00 | 1.355E-07 | 0.000E+00 | 4.723E-07 | 1.067E-06 | 0.000E+00 | 0.000E+00 | 0.000E+00 | 7.206E-07 |
| Unigene495657 | mdtC | 0.000E+00 | 0.000E+00 | 0.000E+00 | 6.330E-08 | 1.277E-07 | 1.016E-07 | 1.491E-06 | 0.000E+00 | 1.599E-07 | 5.819E-08 | 2.270E-07 | 4.322E-07 |
| Unigene495658 | TriA | 0.000E+00 | 0.000E+00 | 0.000E+00 | 0.000E+00 | 0.000E+00 | 9.331E-08 | 1.719E-06 | 0.000E+00 | 1.887E-07 | 0.000E+00 | 0.000E+00 | 2.721E-07 |
| Unigene495659 | smeF | 0.000E+00 | 1.243E-07 | 0.000E+00 | 0.000E+00 | 2.768E-07 | 0.000E+00 | 1.956E-06 | 0.000E+00 | 1.981E-07 | 0.000E+00 | 2.850E-07 | 4.016E-07 |
| Unigene495660 | Pseudomo | 0.000E+00 | 0.000E+00 | 1.767E-07 | 0.000E+00 | 0.000E+00 | 0.000E+00 | 1.794E-06 | 0.000E+00 | 0.000E+00 | 0.000E+00 | 0.000E+00 | 1.064E-07 |
| Unigene495661 | cpxA | 0.000E+00 | 0.000E+00 | 0.000E+00 | 0.000E+00 | 1.128E-07 | 1.372E-07 | 2.078E-06 | 1.185E-07 | 0.000E+00 | 8.481E-08 | 2.032E-07 | 1.500E-07 |
| Unigene495673 | tetA(46) | 0.000E+00 | 0.000E+00 | 0.000E+00 | 0.000E+00 | 0.000E+00 | 0.000E+00 | 1.701E-06 | 0.000E+00 | 0.000E+00 | 0.000E+00 | 0.000E+00 | 0.000E+00 |
| Unigene495773 | mdtH | 0.000E+00 | 0.000E+00 | 0.000E+00 | 1.348E-07 | 0.000E+00 | 0.000E+00 | 2.423E-06 | 1.091E-06 | 0.000E+00 | 0.000E+00 | 0.000E+00 | 0.000E+00 |
| Unigene495903 | arlR | 0.000E+00 | 0.000E+00 | 0.000E+00 | 0.000E+00 | 0.000E+00 | 0.000E+00 | 1.528E-06 | 0.000E+00 | 0.000E+00 | 0.000E+00 | 0.000E+00 | 0.000E+00 |
| Unigene495948 | smeS | 0.000E+00 | 0.000E+00 | 1.484E-07 | 0.000E+00 | 2.101E-07 | 0.000E+00 | 8.790E-07 | 0.000E+00 | 0.000E+00 | 1.053E-07 | 0.000E+00 | 2.906E-07 |
| Unigene496030 | macB | 0.000E+00 | 0.000E+00 | 0.000E+00 | 0.000E+00 | 0.000E+00 | 7.603E-08 | 3.892E-07 | 3.284E-07 | 0.000E+00 | 0.000E+00 | 0.000E+00 | 0.000E+00 |
| Unigene496078 | tlrC | 0.000E+00 | 1.002E-06 | 5.119E-07 | 0.000E+00 | 0.000E+00 | 0.000E+00 | 1.034E-06 | 0.000E+00 | 0.000E+00 | 0.000E+00 | 0.000E+00 | 0.000E+00 |
| Unigene496138 | dfrA26 | 0.000E+00 | 0.000E+00 | 0.000E+00 | 0.000E+00 | 0.000E+00 | 0.000E+00 | 4.841E-07 | 0.000E+00 | 0.000E+00 | 0.000E+00 | 0.000E+00 | 0.000E+00 |
| Unigene496182 | lmrC | 1.712E-07 | 1.342E-07 | 4.799E-08 | 7.408E-08 | 2.173E-07 | 1.586E-07 | 9.473E-07 | 7.136E-07 | 8.019E-08 | 0.000E+00 | 1.035E-06 | 2.197E-06 |
| Unigene496207 | LpeB | 0.000E+00 | 0.000E+00 | 0.000E+00 | 0.000E+00 | 0.000E+00 | 0.000E+00 | 1.303E-06 | 0.000E+00 | 0.000E+00 | 0.000E+00 | 0.000E+00 | 0.000E+00 |
| Unigene496234 | Streptomy | 0.000E+00 | 0.000E+00 | 0.000E+00 | 0.000E+00 | 0.000E+00 | 0.000E+00 | 7.874E-07 | 0.000E+00 | 0.000E+00 | 0.000E+00 | 0.000E+00 | 0.000E+00 |
| Unigene496287 | efrA | 0.000E+00 | 0.000E+00 | 0.000E+00 | 7.761E-08 | 3.415E-07 | 0.000E+00 | 6.380E-07 | 9.420E-07 | 0.000E+00 | 0.000E+00 | 4.396E-07 | 4.088E-07 |
| Unigene496300 | smeS | 0.000E+00 | 0.000E+00 | 0.000E+00 | 0.000E+00 | 0.000E+00 | 0.000E+00 | 4.978E-07 | 0.000E+00 | 0.000E+00 | 0.000E+00 | 0.000E+00 | 0.000E+00 |
| Unigene496325 | arnA | 0.000E+00 | 0.000E+00 | 0.000E+00 | 0.000E+00 | 0.000E+00 | 0.000E+00 | 9.829E-07 | 3.987E-08 | 0.000E+00 | 0.000E+00 | 0.000E+00 | 0.000E+00 |
| Unigene496351 | tlrC | 0.000E+00 | 0.000E+00 | 0.000E+00 | 0.000E+00 | 0.000E+00 | 0.000E+00 | 3.656E-07 | 0.000E+00 | 0.000E+00 | 0.000E+00 | 0.000E+00 | 0.000E+00 |
| Unigene496363 | vanHM | 0.000E+00 | 0.000E+00 | 0.000E+00 | 0.000E+00 | 0.000E+00 | 7.628E-08 | 4.295E-07 | 0.000E+00 | 0.000E+00 | 0.000E+00 | 0.000E+00 | 0.000E+00 |
| Unigene496516 | cipA | 3.871E-07 | 0.000E+00 | 0.000E+00 | 0.000E+00 | 0.000E+00 | 5.579E-08 | 9.710E-07 | 0.000E+00 | 0.000E+00 | 0.000E+00 | 0.000E+00 | 0.000E+00 |
| Unigene496527 | carA | 1.521E-07 | 0.000E+00 | 0.000E+00 | 2.304E-07 | 5.914E-07 | 0.000E+00 | 1.136E-06 | 1.953E-06 | 0.000E+00 | 4.236E-07 | 7.829E-07 | 2.876E-06 |
| Unigene496695 | mecB | 0.000E+00 | 0.000E+00 | 0.000E+00 | 0.000E+00 | 2.465E-08 | 0.000E+00 | 1.105E-06 | 0.000E+00 | 0.000E+00 | 0.000E+00 | 1.015E-07 | 0.000E+00 |
| Unigene496711 | novA | 0.000E+00 | 0.000E+00 | 0.000E+00 | 0.000E+00 | 0.000E+00 | 0.000E+00 | 9.117E-07 | 0.000E+00 | 0.000E+00 | 0.000E+00 | 0.000E+00 | 0.000E+00 |
| Unigene496733 | tetA(58) | 0.000E+00 | 0.000E+00 | 0.000E+00 | 0.000E+00 | 7.943E-08 | 0.000E+00 | 8.704E-07 | 0.000E+00 | 0.000E+00 | 0.000E+00 | 0.000E+00 | 0.000E+00 |
| Unigene496814 | QepA4 | 0.000E+00 | 0.000E+00 | 1.624E-07 | 0.000E+00 | 2.102E-07 | 6.136E-07 | 1.832E-06 | 1.215E-06 | 0.000E+00 | 0.000E+00 | 0.000E+00 | 7.268E-07 |
| Unigene496826 | patA | 0.000E+00 | 0.000E+00 | 0.000E+00 | 0.000E+00 | 0.000E+00 | 0.000E+00 | 3.897E-07 | 0.000E+00 | 0.000E+00 | 0.000E+00 | 0.000E+00 | 0.000E+00 |
| Unigene496851 | vanRD | 0.000E+00 | 0.000E+00 | 0.000E+00 | 0.000E+00 | 0.000E+00 | 0.000E+00 | 4.555E-07 | 0.000E+00 | 0.000E+00 | 0.000E+00 | 0.000E+00 | 0.000E+00 |
| Unigene496856 | efrB | 0.000E+00 | 0.000E+00 | 0.000E+00 | 0.000E+00 | 0.000E+00 | 0.000E+00 | 7.443E-07 | 0.000E+00 | 0.000E+00 | 0.000E+00 | 0.000E+00 | 0.000E+00 |
| Unigene496859 | efrB | 0.000E+00 | 0.000E+00 | 0.000E+00 | 0.000E+00 | 0.000E+00 | 0.000E+00 | 4.138E-07 | 0.000E+00 | 0.000E+00 | 0.000E+00 | 0.000E+00 | 0.000E+00 |
| Unigene496996 | PDC-6 | 0.000E+00 | 0.000E+00 | 0.000E+00 | 0.000E+00 | 0.000E+00 | 0.000E+00 | 5.254E-07 | 0.000E+00 | 0.000E+00 | 0.000E+00 | 0.000E+00 | 0.000E+00 |
| Unigene497044 | vanSM | 0.000E+00 | 0.000E+00 | 0.000E+00 | 0.000E+00 | 0.000E+00 | 0.000E+00 | 1.910E-07 | 0.000E+00 | 0.000E+00 | 0.000E+00 | 0.000E+00 | 0.000E+00 |
| Unigene497148 | mdtH | 0.000E+00 | 6.108E-07 | 0.000E+00 | 0.000E+00 | 0.000E+00 | 0.000E+00 | 1.701E-06 | 0.000E+00 | 0.000E+00 | 0.000E+00 | 0.000E+00 | 0.000E+00 |
| Unigene497163 | vatF | 0.000E+00 | 0.000E+00 | 0.000E+00 | 0.000E+00 | 0.000E+00 | 0.000E+00 | 0.000E+00 | 3.039E-07 | 0.000E+00 | 0.000E+00 | 0.000E+00 | 0.000E+00 |
| Unigene497225 | tetA(60) | 0.000E+00 | 0.000E+00 | 8.786E-08 | 1.507E-08 | 0.000E+00 | 0.000E+00 | 2.126E-05 | 0.000E+00 | 2.854E-06 | 0.000E+00 | 0.000E+00 | 0.000E+00 |
| Unigene497236 | Streptomy | 0.000E+00 | 0.000E+00 | 0.000E+00 | 0.000E+00 | 0.000E+00 | 0.000E+00 | 4.881E-07 | 0.000E+00 | 0.000E+00 | 0.000E+00 | 0.000E+00 | 0.000E+00 |
| Unigene497288 | facT | 0.000E+00 | 0.000E+00 | 4.763E-07 | 8.170E-08 | 0.000E+00 | 0.000E+00 | 1.313E-06 | 3.463E-07 | 0.000E+00 | 0.000E+00 | 3.702E-07 | 4.144E-07 |
| Unigene497304 | rosA | 0.000E+00 | 0.000E+00 | 0.000E+00 | 0.000E+00 | 2.415E-07 | 0.000E+00 | 4.812E-07 | 4.440E-07 | 0.000E+00 | 0.000E+00 | 0.000E+00 | 3.212E-07 |

| | | | | | | | | | | | | | |
|---|---|---|---|---|---|---|---|---|---|---|---|---|---|
| Unigene497319 | oqxB | 0.000E+00 | 0.000E+00 | 0.000E+00 | 0.000E+00 | 0.000E+00 | 0.000E+00 | 7.005E-07 | 0.000E+00 | 0.000E+00 | 0.000E+00 | 0.000E+00 | 0.000E+00 |
| Unigene497320 | mdtA | 0.000E+00 | 0.000E+00 | 0.000E+00 | 0.000E+00 | 0.000E+00 | 0.000E+00 | 4.337E-07 | 0.000E+00 | 0.000E+00 | 0.000E+00 | 0.000E+00 | 0.000E+00 |
| Unigene497340 | mtrA | 0.000E+00 | 0.000E+00 | 0.000E+00 | 0.000E+00 | 0.000E+00 | 0.000E+00 | 7.809E-07 | 0.000E+00 | 0.000E+00 | 0.000E+00 | 0.000E+00 | 0.000E+00 |
| Unigene497361 | evgS | 0.000E+00 | 0.000E+00 | 0.000E+00 | 0.000E+00 | 0.000E+00 | 0.000E+00 | 6.038E-07 | 3.184E-07 | 0.000E+00 | 0.000E+00 | 0.000E+00 | 0.000E+00 |
| Unigene497386 | patA | 0.000E+00 | 0.000E+00 | 0.000E+00 | 8.170E-08 | 3.595E-07 | 0.000E+00 | 1.209E-06 | 2.361E-06 | 4.421E-08 | 0.000E+00 | 5.553E-07 | 1.148E-06 |
| Unigene497457 | oleC | 0.000E+00 | 1.357E-07 | 2.427E-07 | 0.000E+00 | 0.000E+00 | 1.783E-07 | 7.757E-07 | 9.143E-07 | 0.000E+00 | 3.215E-07 | 0.000E+00 | 4.873E-07 |
| Unigene497459 | vmlR | 1.081E-07 | 0.000E+00 | 0.000E+00 | 0.000E+00 | 0.000E+00 | 1.169E-07 | 1.017E-06 | 5.049E-07 | 0.000E+00 | 0.000E+00 | 1.856E-07 | 1.470E-06 |
| Unigene497538 | MexF | 0.000E+00 | 0.000E+00 | 0.000E+00 | 0.000E+00 | 0.000E+00 | 0.000E+00 | 5.206E-07 | 0.000E+00 | 0.000E+00 | 0.000E+00 | 0.000E+00 | 0.000E+00 |
| Unigene497668 | msbA | 0.000E+00 | 0.000E+00 | 0.000E+00 | 0.000E+00 | 0.000E+00 | 0.000E+00 | 1.786E-06 | 0.000E+00 | 0.000E+00 | 0.000E+00 | 0.000E+00 | 0.000E+00 |
| Unigene497764 | AcrF | 0.000E+00 | 0.000E+00 | 0.000E+00 | 0.000E+00 | 0.000E+00 | 0.000E+00 | 3.366E-07 | 0.000E+00 | 0.000E+00 | 0.000E+00 | 0.000E+00 | 4.944E-07 |
| Unigene497800 | MexF | 0.000E+00 | 0.000E+00 | 0.000E+00 | 0.000E+00 | 0.000E+00 | 0.000E+00 | 6.149E-07 | 0.000E+00 | 0.000E+00 | 0.000E+00 | 0.000E+00 | 0.000E+00 |
| Unigene498113 | Streptomy | 0.000E+00 | 0.000E+00 | 0.000E+00 | 0.000E+00 | 0.000E+00 | 0.000E+00 | 1.208E-06 | 0.000E+00 | 0.000E+00 | 0.000E+00 | 5.946E-08 | 0.000E+00 |
| Unigene498188 | adeN | 0.000E+00 | 0.000E+00 | 0.000E+00 | 0.000E+00 | 2.717E-07 | 0.000E+00 | 8.120E-07 | 1.370E-06 | 0.000E+00 | 0.000E+00 | 1.287E-06 | 2.602E-06 |
| Unigene498251 | vanTE | 0.000E+00 | 0.000E+00 | 0.000E+00 | 0.000E+00 | 0.000E+00 | 0.000E+00 | 1.225E-06 | 0.000E+00 | 0.000E+00 | 0.000E+00 | 0.000E+00 | 0.000E+00 |
| Unigene498311 | msbA | 0.000E+00 | 0.000E+00 | 7.050E-07 | 0.000E+00 | 4.867E-07 | 1.004E-06 | 3.801E-06 | 2.393E-06 | 0.000E+00 | 0.000E+00 | 0.000E+00 | 3.004E-06 |
| Unigene498357 | bcrA | 0.000E+00 | 0.000E+00 | 0.000E+00 | 0.000E+00 | 0.000E+00 | 0.000E+00 | 5.228E-07 | 1.838E-07 | 0.000E+00 | 0.000E+00 | 0.000E+00 | 0.000E+00 |
| Unigene498413 | lfrA | 0.000E+00 | 0.000E+00 | 0.000E+00 | 0.000E+00 | 0.000E+00 | 0.000E+00 | 1.152E-06 | 0.000E+00 | 0.000E+00 | 0.000E+00 | 0.000E+00 | 0.000E+00 |
| Unigene498595 | evgS | 1.775E-07 | 0.000E+00 | 2.177E-07 | 0.000E+00 | 0.000E+00 | 9.594E-08 | 9.331E-07 | 2.072E-07 | 0.000E+00 | 0.000E+00 | 2.538E-07 | 2.360E-07 |
| Unigene498598 | adeR | 0.000E+00 | 0.000E+00 | 0.000E+00 | 0.000E+00 | 0.000E+00 | 0.000E+00 | 1.567E-06 | 0.000E+00 | 0.000E+00 | 0.000E+00 | 0.000E+00 | 0.000E+00 |
| Unigene498599 | vanSL | 1.435E-07 | 0.000E+00 | 3.238E-07 | 8.693E-08 | 1.434E-07 | 1.396E-07 | 1.111E-06 | 2.344E-07 | 7.840E-08 | 0.000E+00 | 2.790E-07 | 4.748E-07 |
| Unigene498627 | bcrA | 0.000E+00 | 0.000E+00 | 2.980E-07 | 1.314E-07 | 4.338E-07 | 2.814E-07 | 9.604E-07 | 9.116E-07 | 7.587E-07 | 0.000E+00 | 8.933E-07 | 1.949E-06 |
| Unigene498667 | oleB | 0.000E+00 | 0.000E+00 | 0.000E+00 | 0.000E+00 | 0.000E+00 | 0.000E+00 | 1.406E-06 | 0.000E+00 | 0.000E+00 | 0.000E+00 | 1.817E-07 | 0.000E+00 |
| Unigene498673 | Bifidobacte | 0.000E+00 | 0.000E+00 | 0.000E+00 | 0.000E+00 | 7.917E-08 | 0.000E+00 | 0.000E+00 | 0.000E+00 | 0.000E+00 | 0.000E+00 | 0.000E+00 | 0.000E+00 |
| Unigene498699 | Streptomy | 0.000E+00 | 0.000E+00 | 0.000E+00 | 7.392E-08 | 2.440E-07 | 1.187E-07 | 2.025E-07 | 0.000E+00 | 0.000E+00 | 0.000E+00 | 0.000E+00 | 0.000E+00 |
| Unigene498788 | QepA2 | 0.000E+00 | 0.000E+00 | 0.000E+00 | 0.000E+00 | 0.000E+00 | 0.000E+00 | 4.066E-07 | 0.000E+00 | 0.000E+00 | 0.000E+00 | 0.000E+00 | 0.000E+00 |
| Unigene498793 | vanHD | 0.000E+00 | 0.000E+00 | 5.753E-07 | 5.224E-07 | 1.073E-06 | 0.000E+00 | 1.336E-06 | 0.000E+00 | 0.000E+00 | 0.000E+00 | 9.862E-07 | 4.240E-06 |
| Unigene498807 | patA | 1.374E-07 | 5.025E-08 | 1.797E-07 | 0.000E+00 | 0.000E+00 | 0.000E+00 | 4.054E-07 | 0.000E+00 | 0.000E+00 | 3.060E-07 | 3.143E-07 | 7.577E-07 |
| Unigene498856 | Staphylocc | 6.839E-08 | 0.000E+00 | 0.000E+00 | 0.000E+00 | 0.000E+00 | 2.957E-07 | 7.569E-07 | 3.725E-07 | 0.000E+00 | 0.000E+00 | 4.433E-07 | 8.353E-07 |
| Unigene498858 | macB | 0.000E+00 | 0.000E+00 | 3.984E-08 | 0.000E+00 | 2.707E-07 | 0.000E+00 | 1.034E-06 | 5.213E-07 | 0.000E+00 | 0.000E+00 | 4.645E-07 | 3.360E-07 |
| Unigene498933 | bcrA | 0.000E+00 | 0.000E+00 | 0.000E+00 | 0.000E+00 | 0.000E+00 | 0.000E+00 | 7.273E-07 | 0.000E+00 | 0.000E+00 | 0.000E+00 | 0.000E+00 | 0.000E+00 |
| Unigene498945 | evgS | 5.453E-08 | 1.396E-07 | 7.134E-08 | 1.468E-07 | 0.000E+00 | 2.358E-07 | 6.437E-07 | 4.031E-07 | 1.192E-07 | 2.835E-07 | 0.000E+00 | 7.949E-07 |
| Unigene499012 | patA | 0.000E+00 | 0.000E+00 | 0.000E+00 | 0.000E+00 | 0.000E+00 | 0.000E+00 | 4.411E-07 | 0.000E+00 | 0.000E+00 | 0.000E+00 | 0.000E+00 | 0.000E+00 |
| Unigene499042 | evgA | 0.000E+00 | 0.000E+00 | 0.000E+00 | 0.000E+00 | 0.000E+00 | 0.000E+00 | 4.740E-07 | 0.000E+00 | 0.000E+00 | 0.000E+00 | 0.000E+00 | 0.000E+00 |
| Unigene499118 | mecA | 0.000E+00 | 0.000E+00 | 0.000E+00 | 0.000E+00 | 0.000E+00 | 0.000E+00 | 1.045E-06 | 0.000E+00 | 0.000E+00 | 0.000E+00 | 0.000E+00 | 0.000E+00 |
| Unigene499311 | efmA | 2.516E-06 | 5.012E-05 | 3.435E-05 | 9.190E-07 | 5.055E-06 | 1.869E-05 | 3.792E-06 | 0.000E+00 | 9.362E-06 | 0.000E+00 | 0.000E+00 | 4.176E-06 |
| Unigene499346 | emrB | 0.000E+00 | 0.000E+00 | 0.000E+00 | 0.000E+00 | 0.000E+00 | 0.000E+00 | 1.056E-06 | 0.000E+00 | 0.000E+00 | 0.000E+00 | 0.000E+00 | 0.000E+00 |
| Unigene499374 | carA | 0.000E+00 | 0.000E+00 | 2.305E-07 | 0.000E+00 | 7.830E-07 | 5.587E-07 | 1.196E-06 | 5.485E-07 | 0.000E+00 | 9.422E-07 | 7.525E-07 | 3.277E-06 |
| Unigene499416 | Escherichia | 0.000E+00 | 0.000E+00 | 0.000E+00 | 0.000E+00 | 0.000E+00 | 0.000E+00 | 1.912E-06 | 0.000E+00 | 0.000E+00 | 0.000E+00 | 0.000E+00 | 0.000E+00 |
| Unigene499505 | novA | 0.000E+00 | 0.000E+00 | 0.000E+00 | 0.000E+00 | 0.000E+00 | 0.000E+00 | 6.175E-07 | 0.000E+00 | 0.000E+00 | 0.000E+00 | 1.368E-07 | 0.000E+00 |
| Unigene499536 | Acinetobac | 0.000E+00 | 0.000E+00 | 0.000E+00 | 0.000E+00 | 0.000E+00 | 0.000E+00 | 7.179E-07 | 4.899E-07 | 0.000E+00 | 0.000E+00 | 0.000E+00 | 0.000E+00 |
| Unigene499582 | mdsB | 0.000E+00 | 0.000E+00 | 0.000E+00 | 0.000E+00 | 0.000E+00 | 0.000E+00 | 5.496E-07 | 0.000E+00 | 0.000E+00 | 0.000E+00 | 0.000E+00 | 0.000E+00 |
| Unigene499596 | oleC | 0.000E+00 | 0.000E+00 | 0.000E+00 | 0.000E+00 | 0.000E+00 | 0.000E+00 | 8.028E-07 | 0.000E+00 | 0.000E+00 | 0.000E+00 | 0.000E+00 | 0.000E+00 |
| Unigene499662 | smeS | 0.000E+00 | 0.000E+00 | 0.000E+00 | 0.000E+00 | 0.000E+00 | 0.000E+00 | 9.842E-07 | 0.000E+00 | 0.000E+00 | 0.000E+00 | 0.000E+00 | 0.000E+00 |
| Unigene499829 | vanRI | 0.000E+00 | 0.000E+00 | 0.000E+00 | 0.000E+00 | 0.000E+00 | 0.000E+00 | 9.340E-07 | 9.030E-07 | 0.000E+00 | 0.000E+00 | 0.000E+00 | 1.136E-06 |
| Unigene500076 | tetA(46) | 0.000E+00 | 0.000E+00 | 0.000E+00 | 0.000E+00 | 4.427E-07 | 0.000E+00 | 7.057E-07 | 6.512E-07 | 1.742E-07 | 3.108E-07 | 2.735E-07 | 1.083E-06 |
| Unigene500291 | vanRE | 0.000E+00 | 0.000E+00 | 0.000E+00 | 0.000E+00 | 0.000E+00 | 0.000E+00 | 7.746E-07 | 0.000E+00 | 0.000E+00 | 0.000E+00 | 0.000E+00 | 0.000E+00 |
| Unigene500414 | lmrC | 0.000E+00 | 0.000E+00 | 0.000E+00 | 0.000E+00 | 0.000E+00 | 0.000E+00 | 3.176E-06 | 0.000E+00 | 0.000E+00 | 0.000E+00 | 0.000E+00 | 0.000E+00 |
| Unigene500424 | MuxB | 0.000E+00 | 9.032E-08 | 1.413E-07 | 0.000E+00 | 2.286E-07 | 2.447E-07 | 7.059E-07 | 3.842E-07 | 3.823E-07 | 2.521E-07 | 6.825E-07 | 1.046E-06 |
| Unigene500779 | baeR | 0.000E+00 | 0.000E+00 | 0.000E+00 | 0.000E+00 | 0.000E+00 | 0.000E+00 | 6.904E-07 | 0.000E+00 | 0.000E+00 | 0.000E+00 | 0.000E+00 | 0.000E+00 |
| Unigene500928 | QepA4 | 9.197E-08 | 0.000E+00 | 0.000E+00 | 0.000E+00 | 3.065E-07 | 0.000E+00 | 8.652E-07 | 6.709E-07 | 0.000E+00 | 0.000E+00 | 1.052E-07 | 8.153E-07 |
| Unigene501044 | msbA | 0.000E+00 | 0.000E+00 | 0.000E+00 | 0.000E+00 | 0.000E+00 | 0.000E+00 | 1.728E-06 | 0.000E+00 | 0.000E+00 | 0.000E+00 | 5.309E-07 | 0.000E+00 |
| Unigene501056 | bcrA | 0.000E+00 | 0.000E+00 | 0.000E+00 | 0.000E+00 | 0.000E+00 | 0.000E+00 | 6.644E-07 | 0.000E+00 | 0.000E+00 | 0.000E+00 | 0.000E+00 | 0.000E+00 |
| Unigene501074 | mdsB | 0.000E+00 | 0.000E+00 | 0.000E+00 | 0.000E+00 | 0.000E+00 | 0.000E+00 | 9.101E-07 | 7.352E-07 | 0.000E+00 | 0.000E+00 | 3.602E-07 | 3.309E-07 |
| Unigene501093 | macB | 4.928E-07 | 0.000E+00 | 0.000E+00 | 0.000E+00 | 0.000E+00 | 0.000E+00 | 1.136E-06 | 0.000E+00 | 0.000E+00 | 0.000E+00 | 0.000E+00 | 5.825E-07 |
| Unigene501172 | vanRM | 0.000E+00 | 0.000E+00 | 0.000E+00 | 0.000E+00 | 0.000E+00 | 0.000E+00 | 3.862E-07 | 0.000E+00 | 0.000E+00 | 0.000E+00 | 0.000E+00 | 0.000E+00 |
| Unigene501186 | dfrA3 | 0.000E+00 | 0.000E+00 | 0.000E+00 | 0.000E+00 | 0.000E+00 | 0.000E+00 | 8.086E-07 | 0.000E+00 | 0.000E+00 | 0.000E+00 | 0.000E+00 | 0.000E+00 |
| Unigene501253 | macB | 1.258E-07 | 3.453E-08 | 9.262E-08 | 0.000E+00 | 3.845E-07 | 5.102E-07 | 7.661E-07 | 1.175E-06 | 0.000E+00 | 0.000E+00 | 1.116E-06 | 2.938E-06 |
| Unigene501268 | kdpE | 0.000E+00 | 0.000E+00 | 0.000E+00 | 0.000E+00 | 0.000E+00 | 0.000E+00 | 5.316E-07 | 0.000E+00 | 0.000E+00 | 0.000E+00 | 0.000E+00 | 0.000E+00 |

| | | | | | | | | | | | | | |
|---|---|---|---|---|---|---|---|---|---|---|---|---|---|
| Unigene501327 | efrB | 0.000E+00 | 0.000E+00 | 0.000E+00 | 0.000E+00 | 0.000E+00 | 0.000E+00 | 1.120E-06 | 0.000E+00 | 0.000E+00 | 0.000E+00 | 0.000E+00 | 0.000E+00 |
| Unigene501423 | rosB | 0.000E+00 | 0.000E+00 | 0.000E+00 | 0.000E+00 | 0.000E+00 | 0.000E+00 | 0.000E+00 | 0.000E+00 | 0.000E+00 | 0.000E+00 | 4.659E-07 | 0.000E+00 |
| Unigene501442 | mdtA | 0.000E+00 | 2.026E-07 | 0.000E+00 | 0.000E+00 | 0.000E+00 | 0.000E+00 | 1.751E-06 | 0.000E+00 | 0.000E+00 | 0.000E+00 | 0.000E+00 | 0.000E+00 |
| Unigene501443 | mdtB | 0.000E+00 | 0.000E+00 | 0.000E+00 | 0.000E+00 | 0.000E+00 | 0.000E+00 | 2.071E-06 | 7.360E-07 | 0.000E+00 | 0.000E+00 | 0.000E+00 | 0.000E+00 |
| Unigene501581 | PmrF | 0.000E+00 | 0.000E+00 | 0.000E+00 | 2.446E-07 | 0.000E+00 | 0.000E+00 | 2.374E-06 | 0.000E+00 | 0.000E+00 | 0.000E+00 | 0.000E+00 | 0.000E+00 |
| Unigene501637 | YojI | 2.281E-07 | 0.000E+00 | 1.424E-07 | 2.512E-07 | 8.292E-07 | 2.017E-07 | 1.193E-06 | 1.234E-06 | 0.000E+00 | 1.617E-07 | 5.217E-07 | 1.470E-06 |
| Unigene501800 | rosA | 0.000E+00 | 0.000E+00 | 0.000E+00 | 0.000E+00 | 0.000E+00 | 0.000E+00 | 6.592E-07 | 0.000E+00 | 0.000E+00 | 0.000E+00 | 0.000E+00 | 0.000E+00 |
| Unigene501888 | kdpE | 0.000E+00 | 3.041E-07 | 2.266E-07 | 0.000E+00 | 0.000E+00 | 0.000E+00 | 9.200E-07 | 1.132E-06 | 0.000E+00 | 5.144E-07 | 1.109E-06 | 9.280E-07 |
| Unigene501889 | msbA | 0.000E+00 | 3.515E-08 | 0.000E+00 | 0.000E+00 | 5.692E-07 | 3.808E-07 | 9.924E-07 | 5.607E-07 | 0.000E+00 | 0.000E+00 | 9.158E-07 | 9.842E-07 |
| Unigene501900 | oleC | 0.000E+00 | 2.164E-07 | 9.674E-07 | 1.138E-07 | 6.884E-07 | 5.481E-07 | 1.528E-06 | 8.220E-07 | 2.463E-07 | 0.000E+00 | 7.411E-07 | 3.463E-06 |
| Unigene501956 | patB | 0.000E+00 | 0.000E+00 | 3.882E-07 | 0.000E+00 | 0.000E+00 | 1.026E-06 | 9.632E-07 | 1.431E-06 | 0.000E+00 | 9.254E-07 | 0.000E+00 | 0.000E+00 |
| Unigene502057 | lmrD | 0.000E+00 | 0.000E+00 | 0.000E+00 | 0.000E+00 | 3.209E-07 | 0.000E+00 | 8.792E-07 | 0.000E+00 | 0.000E+00 | 0.000E+00 | 0.000E+00 | 7.682E-07 |
| Unigene502063 | oleB | 0.000E+00 | 0.000E+00 | 0.000E+00 | 0.000E+00 | 0.000E+00 | 0.000E+00 | 9.212E-07 | 0.000E+00 | 0.000E+00 | 0.000E+00 | 3.483E-07 | 3.840E-07 |
| Unigene502144 | efrB | 5.575E-08 | 0.000E+00 | 1.641E-07 | 0.000E+00 | 1.239E-07 | 0.000E+00 | 3.702E-07 | 0.000E+00 | 0.000E+00 | 0.000E+00 | 0.000E+00 | 0.000E+00 |
| Unigene502226 | Enterobact | 8.050E-08 | 0.000E+00 | 0.000E+00 | 1.626E-07 | 0.000E+00 | 1.450E-07 | 6.830E-07 | 1.065E-06 | 5.866E-08 | 2.092E-07 | 8.287E-07 | 1.269E-06 |
| Unigene502267 | evgS | 0.000E+00 | 0.000E+00 | 0.000E+00 | 0.000E+00 | 0.000E+00 | 0.000E+00 | 5.293E-07 | 9.303E-07 | 0.000E+00 | 0.000E+00 | 0.000E+00 | 1.696E-06 |
| Unigene502273 | vanO | 0.000E+00 | 0.000E+00 | 0.000E+00 | 0.000E+00 | 0.000E+00 | 0.000E+00 | 1.307E-06 | 0.000E+00 | 0.000E+00 | 0.000E+00 | 0.000E+00 | 0.000E+00 |
| Unigene502434 | tcr3 | 0.000E+00 | 0.000E+00 | 0.000E+00 | 3.215E-07 | 0.000E+00 | 0.000E+00 | 3.735E-06 | 2.824E-06 | 0.000E+00 | 0.000E+00 | 1.384E-06 | 2.408E-06 |
| Unigene502445 | macB | 0.000E+00 | 0.000E+00 | 0.000E+00 | 0.000E+00 | 0.000E+00 | 0.000E+00 | 1.207E-06 | 0.000E+00 | 0.000E+00 | 0.000E+00 | 0.000E+00 | 0.000E+00 |
| Unigene502650 | bacA | 0.000E+00 | 0.000E+00 | 0.000E+00 | 0.000E+00 | 0.000E+00 | 0.000E+00 | 0.000E+00 | 0.000E+00 | 0.000E+00 | 0.000E+00 | 2.429E-07 | 6.694E-07 |
| Unigene502689 | YojI | 4.483E-07 | 0.000E+00 | 0.000E+00 | 0.000E+00 | 3.113E-07 | 0.000E+00 | 4.342E-07 | 0.000E+00 | 0.000E+00 | 0.000E+00 | 5.770E-07 | 7.949E-07 |
| Unigene502751 | macB | 0.000E+00 | 0.000E+00 | 0.000E+00 | 0.000E+00 | 2.286E-07 | 2.860E-07 | 1.074E-06 | 6.520E-07 | 0.000E+00 | 0.000E+00 | 0.000E+00 | 7.298E-07 |
| Unigene502961 | patB | 6.274E-08 | 4.132E-07 | 0.000E+00 | 0.000E+00 | 0.000E+00 | 0.000E+00 | 2.291E-06 | 0.000E+00 | 0.000E+00 | 0.000E+00 | 0.000E+00 | 8.158E-07 |
| Unigene503077 | bcrC | 0.000E+00 | 0.000E+00 | 0.000E+00 | 0.000E+00 | 0.000E+00 | 0.000E+00 | 8.843E-07 | 0.000E+00 | 0.000E+00 | 0.000E+00 | 0.000E+00 | 0.000E+00 |
| Unigene503234 | Streptomy | 0.000E+00 | 0.000E+00 | 0.000E+00 | 0.000E+00 | 6.568E-08 | 0.000E+00 | 5.889E-07 | 2.070E-07 | 0.000E+00 | 0.000E+00 | 0.000E+00 | 0.000E+00 |
| Unigene503406 | vanHO | 0.000E+00 | 0.000E+00 | 0.000E+00 | 0.000E+00 | 0.000E+00 | 0.000E+00 | 3.633E-06 | 2.646E-06 | 0.000E+00 | 0.000E+00 | 0.000E+00 | 0.000E+00 |
| Unigene503458 | optrA | 0.000E+00 | 0.000E+00 | 0.000E+00 | 0.000E+00 | 0.000E+00 | 0.000E+00 | 4.438E-07 | 0.000E+00 | 0.000E+00 | 0.000E+00 | 0.000E+00 | 0.000E+00 |
| Unigene503474 | carA | 0.000E+00 | 0.000E+00 | 8.584E-08 | 0.000E+00 | 2.592E-07 | 3.152E-08 | 5.809E-07 | 5.446E-07 | 6.374E-08 | 2.274E-07 | 1.334E-07 | 7.238E-07 |
| Unigene503669 | lmrD | 0.000E+00 | 0.000E+00 | 0.000E+00 | 0.000E+00 | 0.000E+00 | 0.000E+00 | 6.937E-07 | 0.000E+00 | 0.000E+00 | 0.000E+00 | 0.000E+00 | 0.000E+00 |
| Unigene503682 | vmlR | 0.000E+00 | 0.000E+00 | 0.000E+00 | 0.000E+00 | 0.000E+00 | 0.000E+00 | 1.022E-06 | 0.000E+00 | 0.000E+00 | 0.000E+00 | 0.000E+00 | 0.000E+00 |
| Unigene503737 | msbA | 0.000E+00 | 4.210E-08 | 1.882E-07 | 2.131E-07 | 8.097E-07 | 1.451E-07 | 1.189E-06 | 1.231E-06 | 1.048E-07 | 2.778E-07 | 6.801E-07 | 1.247E-06 |
| Unigene503810 | MexK | 0.000E+00 | 0.000E+00 | 4.399E-08 | 9.055E-08 | 9.962E-08 | 1.939E-07 | 5.954E-07 | 3.140E-07 | 0.000E+00 | 0.000E+00 | 2.564E-07 | 3.180E-07 |
| Unigene503925 | Klebsiella p | 0.000E+00 | 0.000E+00 | 0.000E+00 | 0.000E+00 | 0.000E+00 | 0.000E+00 | 8.661E-07 | 0.000E+00 | 0.000E+00 | 0.000E+00 | 0.000E+00 | 0.000E+00 |
| Unigene503927 | oleB | 0.000E+00 | 2.142E-07 | 0.000E+00 | 0.000E+00 | 3.407E-07 | 0.000E+00 | 6.787E-07 | 0.000E+00 | 1.219E-07 | 0.000E+00 | 0.000E+00 | 4.284E-07 |
| Unigene503978 | salA | 9.276E-08 | 0.000E+00 | 9.102E-08 | 0.000E+00 | 1.546E-07 | 1.504E-07 | 8.213E-07 | 5.414E-07 | 1.014E-07 | 0.000E+00 | 5.305E-07 | 1.645E-06 |
| Unigene504059 | efrA | 0.000E+00 | 0.000E+00 | 0.000E+00 | 0.000E+00 | 0.000E+00 | 0.000E+00 | 6.525E-07 | 0.000E+00 | 0.000E+00 | 0.000E+00 | 0.000E+00 | 4.356E-08 |
| Unigene504288 | efrA | 0.000E+00 | 0.000E+00 | 0.000E+00 | 0.000E+00 | 5.220E-07 | 0.000E+00 | 0.000E+00 | 0.000E+00 | 0.000E+00 | 0.000E+00 | 0.000E+00 | 0.000E+00 |
| Unigene504310 | lmrC | 1.624E-07 | 1.337E-07 | 0.000E+00 | 0.000E+00 | 4.511E-07 | 0.000E+00 | 3.595E-07 | 1.469E-06 | 4.438E-08 | 0.000E+00 | 2.322E-07 | 6.239E-07 |
| Unigene504388 | tetA(58) | 0.000E+00 | 0.000E+00 | 0.000E+00 | 0.000E+00 | 0.000E+00 | 0.000E+00 | 2.802E-07 | 0.000E+00 | 0.000E+00 | 0.000E+00 | 0.000E+00 | 0.000E+00 |
| Unigene504392 | mgrA | 0.000E+00 | 0.000E+00 | 0.000E+00 | 0.000E+00 | 0.000E+00 | 0.000E+00 | 9.556E-07 | 0.000E+00 | 0.000E+00 | 1.480E-07 | 0.000E+00 | 0.000E+00 |
| Unigene504422 | mdtC | 0.000E+00 | 0.000E+00 | 0.000E+00 | 0.000E+00 | 0.000E+00 | 0.000E+00 | 6.918E-07 | 0.000E+00 | 0.000E+00 | 0.000E+00 | 0.000E+00 | 0.000E+00 |
| Unigene504473 | cmlB | 1.541E-07 | 5.637E-08 | 0.000E+00 | 0.000E+00 | 3.709E-07 | 0.000E+00 | 6.821E-07 | 7.794E-07 | 0.000E+00 | 2.861E-07 | 0.000E+00 | 8.499E-07 |
| Unigene504708 | kdpE | 0.000E+00 | 0.000E+00 | 0.000E+00 | 0.000E+00 | 0.000E+00 | 0.000E+00 | 5.155E-07 | 0.000E+00 | 0.000E+00 | 0.000E+00 | 0.000E+00 | 0.000E+00 |
| Unigene504776 | Corynebac | 0.000E+00 | 0.000E+00 | 0.000E+00 | 0.000E+00 | 1.670E-07 | 0.000E+00 | 5.322E-07 | 0.000E+00 | 0.000E+00 | 0.000E+00 | 0.000E+00 | 0.000E+00 |
| Unigene504818 | NmcR | 0.000E+00 | 0.000E+00 | 0.000E+00 | 0.000E+00 | 0.000E+00 | 0.000E+00 | 7.005E-07 | 0.000E+00 | 0.000E+00 | 0.000E+00 | 0.000E+00 | 0.000E+00 |
| Unigene504844 | efrA | 0.000E+00 | 0.000E+00 | 0.000E+00 | 1.144E-07 | 0.000E+00 | 0.000E+00 | 7.521E-07 | 0.000E+00 | 0.000E+00 | 0.000E+00 | 0.000E+00 | 0.000E+00 |
| Unigene504869 | MSI-1 | 0.000E+00 | 1.633E-07 | 0.000E+00 | 0.000E+00 | 0.000E+00 | 0.000E+00 | 7.683E-07 | 4.630E-07 | 0.000E+00 | 0.000E+00 | 0.000E+00 | 0.000E+00 |
| Unigene504952 | bacA | 0.000E+00 | 0.000E+00 | 0.000E+00 | 0.000E+00 | 0.000E+00 | 0.000E+00 | 8.996E-07 | 5.421E-07 | 0.000E+00 | 0.000E+00 | 0.000E+00 | 4.575E-07 |
| Unigene505107 | oleC | 0.000E+00 | 0.000E+00 | 0.000E+00 | 0.000E+00 | 0.000E+00 | 0.000E+00 | 1.578E-06 | 0.000E+00 | 0.000E+00 | 0.000E+00 | 0.000E+00 | 0.000E+00 |
| Unigene505149 | dfrA20 | 0.000E+00 | 0.000E+00 | 0.000E+00 | 0.000E+00 | 0.000E+00 | 0.000E+00 | 7.005E-07 | 0.000E+00 | 0.000E+00 | 0.000E+00 | 0.000E+00 | 0.000E+00 |
| Unigene505152 | macB | 0.000E+00 | 0.000E+00 | 0.000E+00 | 0.000E+00 | 0.000E+00 | 0.000E+00 | 0.000E+00 | 0.000E+00 | 0.000E+00 | 0.000E+00 | 0.000E+00 | 7.857E-07 |
| Unigene505233 | tet36 | 0.000E+00 | 0.000E+00 | 1.715E-07 | 0.000E+00 | 0.000E+00 | 6.299E-08 | 3.224E-07 | 2.721E-07 | 6.369E-08 | 1.298E-07 | 2.888E-07 | 0.000E+00 |
| Unigene505247 | lmrD | 1.442E-07 | 6.332E-08 | 1.415E-07 | 8.739E-08 | 1.602E-07 | 3.430E-07 | 1.469E-06 | 5.051E-07 | 1.576E-07 | 9.962E-07 | 5.280E-07 | 9.889E-07 |
| Unigene505371 | adeS | 0.000E+00 | 0.000E+00 | 1.635E-07 | 1.442E-07 | 7.934E-08 | 5.147E-08 | 1.159E-06 | 3.612E-07 | 1.301E-07 | 0.000E+00 | 3.812E-07 | 5.346E-07 |
| Unigene505372 | arlR | 0.000E+00 | 0.000E+00 | 0.000E+00 | 0.000E+00 | 0.000E+00 | 0.000E+00 | 1.588E-06 | 0.000E+00 | 0.000E+00 | 0.000E+00 | 0.000E+00 | 5.653E-07 |
| Unigene505376 | macB | 0.000E+00 | 0.000E+00 | 0.000E+00 | 0.000E+00 | 1.114E-06 | 0.000E+00 | 3.028E-06 | 9.047E-07 | 0.000E+00 | 0.000E+00 | 6.258E-07 | 0.000E+00 |
| Unigene505395 | efrA | 1.130E-07 | 0.000E+00 | 0.000E+00 | 0.000E+00 | 6.589E-07 | 0.000E+00 | 7.189E-07 | 0.000E+00 | 0.000E+00 | 0.000E+00 | 0.000E+00 | 0.000E+00 |
| Unigene505438 | tetA(58) | 1.033E-06 | 7.430E-07 | 3.566E-06 | 5.649E-06 | 0.000E+00 | 0.000E+00 | 4.338E-07 | 0.000E+00 | 0.000E+00 | 0.000E+00 | 0.000E+00 | 4.211E-08 |

| | | | | | | | | | | | | | |
|---|---|---|---|---|---|---|---|---|---|---|---|---|---|
| Unigene505493 | novA | 1.655E-08 | 0.000E+00 | 9.746E-08 | 6.687E-08 | 0.000E+00 | 2.505E-07 | 1.173E-06 | 1.739E-07 | 0.000E+00 | 0.000E+00 | 9.468E-08 | 1.565E-07 |
| Unigene505573 | Bifidobacte | 0.000E+00 | 0.000E+00 | 0.000E+00 | 0.000E+00 | 0.000E+00 | 0.000E+00 | 6.978E-07 | 0.000E+00 | 0.000E+00 | 0.000E+00 | 0.000E+00 | 0.000E+00 |
| Unigene505605 | msrA | 0.000E+00 | 0.000E+00 | 0.000E+00 | 0.000E+00 | 0.000E+00 | 0.000E+00 | 9.424E-07 | 7.229E-07 | 0.000E+00 | 0.000E+00 | 0.000E+00 | 1.510E-06 |
| Unigene505622 | tetA(46) | 0.000E+00 | 0.000E+00 | 0.000E+00 | 0.000E+00 | 0.000E+00 | 0.000E+00 | 5.019E-07 | 0.000E+00 | 0.000E+00 | 0.000E+00 | 0.000E+00 | 0.000E+00 |
| Unigene505991 | lin | 0.000E+00 | 0.000E+00 | 0.000E+00 | 0.000E+00 | 0.000E+00 | 0.000E+00 | 9.161E-07 | 0.000E+00 | 0.000E+00 | 0.000E+00 | 0.000E+00 | 0.000E+00 |
| Unigene506176 | Pseudomo | 0.000E+00 | 0.000E+00 | 0.000E+00 | 0.000E+00 | 0.000E+00 | 0.000E+00 | 8.506E-07 | 0.000E+00 | 0.000E+00 | 0.000E+00 | 0.000E+00 | 0.000E+00 |
| Unigene506267 | tetA(58) | 2.057E-08 | 0.000E+00 | 6.056E-08 | 1.039E-07 | 4.571E-08 | 2.002E-07 | 9.336E-07 | 2.882E-07 | 0.000E+00 | 0.000E+00 | 1.412E-07 | 0.000E+00 |
| Unigene506314 | tetA(58) | 4.107E-08 | 4.507E-08 | 0.000E+00 | 0.000E+00 | 1.141E-07 | 0.000E+00 | 6.818E-07 | 0.000E+00 | 0.000E+00 | 0.000E+00 | 0.000E+00 | 2.185E-07 |
| Unigene506481 | mtrA | 0.000E+00 | 0.000E+00 | 0.000E+00 | 0.000E+00 | 0.000E+00 | 0.000E+00 | 1.045E-06 | 8.814E-07 | 0.000E+00 | 0.000E+00 | 0.000E+00 | 0.000E+00 |
| Unigene506647 | baeR | 0.000E+00 | 0.000E+00 | 0.000E+00 | 0.000E+00 | 0.000E+00 | 0.000E+00 | 4.791E-07 | 0.000E+00 | 0.000E+00 | 0.000E+00 | 0.000E+00 | 0.000E+00 |
| Unigene506829 | macB | 0.000E+00 | 0.000E+00 | 0.000E+00 | 3.622E-08 | 0.000E+00 | 0.000E+00 | 5.160E-07 | 4.187E-07 | 1.176E-07 | 1.199E-07 | 0.000E+00 | 1.696E-07 |
| Unigene506878 | mtrA | 0.000E+00 | 0.000E+00 | 0.000E+00 | 0.000E+00 | 4.035E-07 | 2.945E-07 | 1.306E-06 | 1.590E-06 | 0.000E+00 | 0.000E+00 | 6.751E-07 | 6.440E-07 |
| Unigene506979 | TriA | 0.000E+00 | 0.000E+00 | 0.000E+00 | 0.000E+00 | 0.000E+00 | 0.000E+00 | 7.152E-07 | 0.000E+00 | 0.000E+00 | 0.000E+00 | 0.000E+00 | 0.000E+00 |
| Unigene506989 | evgS | 0.000E+00 | 0.000E+00 | 0.000E+00 | 0.000E+00 | 0.000E+00 | 0.000E+00 | 3.676E-07 | 0.000E+00 | 0.000E+00 | 0.000E+00 | 0.000E+00 | 0.000E+00 |
| Unigene506998 | OXA-45 | 0.000E+00 | 0.000E+00 | 1.077E-07 | 0.000E+00 | 0.000E+00 | 0.000E+00 | 4.253E-07 | 0.000E+00 | 0.000E+00 | 0.000E+00 | 0.000E+00 | 0.000E+00 |
| Unigene507010 | bcrA | 0.000E+00 | 0.000E+00 | 0.000E+00 | 0.000E+00 | 0.000E+00 | 0.000E+00 | 6.532E-07 | 0.000E+00 | 0.000E+00 | 0.000E+00 | 0.000E+00 | 0.000E+00 |
| Unigene507134 | dfrG | 0.000E+00 | 0.000E+00 | 0.000E+00 | 0.000E+00 | 0.000E+00 | 0.000E+00 | 6.041E-07 | 0.000E+00 | 0.000E+00 | 0.000E+00 | 0.000E+00 | 0.000E+00 |
| Unigene507286 | rpoB2 | 0.000E+00 | 0.000E+00 | 0.000E+00 | 0.000E+00 | 5.481E-07 | 0.000E+00 | 5.460E-07 | 1.645E-07 | 0.000E+00 | 0.000E+00 | 0.000E+00 | 4.999E-07 |
| Unigene507289 | tetA(58) | 0.000E+00 | 0.000E+00 | 0.000E+00 | 0.000E+00 | 0.000E+00 | 0.000E+00 | 2.107E-06 | 0.000E+00 | 0.000E+00 | 0.000E+00 | 0.000E+00 | 0.000E+00 |
| Unigene507434 | Streptomy | 0.000E+00 | 0.000E+00 | 0.000E+00 | 0.000E+00 | 0.000E+00 | 0.000E+00 | 1.134E-06 | 4.112E-07 | 0.000E+00 | 0.000E+00 | 4.030E-07 | 0.000E+00 |
| Unigene507508 | macB | 0.000E+00 | 0.000E+00 | 0.000E+00 | 0.000E+00 | 0.000E+00 | 0.000E+00 | 1.177E-06 | 0.000E+00 | 8.943E-08 | 0.000E+00 | 0.000E+00 | 0.000E+00 |
| Unigene507514 | iri | 0.000E+00 | 0.000E+00 | 0.000E+00 | 0.000E+00 | 0.000E+00 | 2.908E-07 | 6.848E-07 | 0.000E+00 | 0.000E+00 | 0.000E+00 | 0.000E+00 | 0.000E+00 |
| Unigene507525 | acrD | 0.000E+00 | 9.216E-07 | 6.001E-07 | 0.000E+00 | 0.000E+00 | 0.000E+00 | 1.331E-06 | 2.783E-07 | 0.000E+00 | 0.000E+00 | 3.439E-07 | 0.000E+00 |
| Unigene507634 | tva(A) | 1.600E-07 | 0.000E+00 | 0.000E+00 | 1.212E-07 | 0.000E+00 | 2.595E-07 | 1.284E-06 | 2.008E-06 | 0.000E+00 | 0.000E+00 | 0.000E+00 | 1.182E-06 |
| Unigene507644 | PmrF | 0.000E+00 | 0.000E+00 | 0.000E+00 | 0.000E+00 | 0.000E+00 | 0.000E+00 | 1.500E-06 | 0.000E+00 | 0.000E+00 | 0.000E+00 | 0.000E+00 | 0.000E+00 |
| Unigene507723 | oleC | 0.000E+00 | 0.000E+00 | 0.000E+00 | 0.000E+00 | 0.000E+00 | 0.000E+00 | 3.646E-07 | 2.563E-07 | 0.000E+00 | 0.000E+00 | 0.000E+00 | 0.000E+00 |
| Unigene507807 | efrA | 0.000E+00 | 0.000E+00 | 2.699E-07 | 0.000E+00 | 1.528E-07 | 0.000E+00 | 1.522E-06 | 0.000E+00 | 0.000E+00 | 0.000E+00 | 1.966E-07 | 2.032E-07 |
| Unigene507813 | cmlB | 0.000E+00 | 0.000E+00 | 7.488E-08 | 0.000E+00 | 0.000E+00 | 0.000E+00 | 5.912E-07 | 0.000E+00 | 0.000E+00 | 0.000E+00 | 0.000E+00 | 3.608E-07 |
| Unigene507817 | QepA2 | 0.000E+00 | 0.000E+00 | 0.000E+00 | 0.000E+00 | 0.000E+00 | 0.000E+00 | 8.021E-07 | 0.000E+00 | 0.000E+00 | 0.000E+00 | 0.000E+00 | 0.000E+00 |
| Unigene507819 | cmlv | 0.000E+00 | 0.000E+00 | 0.000E+00 | 0.000E+00 | 0.000E+00 | 0.000E+00 | 6.824E-07 | 0.000E+00 | 0.000E+00 | 0.000E+00 | 0.000E+00 | 0.000E+00 |
| Unigene507850 | efrA | 0.000E+00 | 0.000E+00 | 0.000E+00 | 0.000E+00 | 0.000E+00 | 0.000E+00 | 0.000E+00 | 9.079E-07 | 2.834E-07 | 0.000E+00 | 0.000E+00 | 1.379E-06 |
| Unigene507871 | evgA | 0.000E+00 | 1.093E-07 | 0.000E+00 | 0.000E+00 | 0.000E+00 | 0.000E+00 | 1.378E-06 | 5.815E-07 | 0.000E+00 | 0.000E+00 | 0.000E+00 | 8.832E-07 |
| Unigene507908 | lmrC | 1.505E-07 | 0.000E+00 | 0.000E+00 | 1.900E-07 | 3.344E-07 | 0.000E+00 | 9.161E-07 | 8.783E-07 | 0.000E+00 | 0.000E+00 | 3.658E-07 | 1.112E-06 |
| Unigene508001 | basS | 0.000E+00 | 0.000E+00 | 0.000E+00 | 0.000E+00 | 0.000E+00 | 0.000E+00 | 4.375E-07 | 0.000E+00 | 0.000E+00 | 0.000E+00 | 0.000E+00 | 0.000E+00 |
| Unigene508083 | NmcR | 0.000E+00 | 0.000E+00 | 0.000E+00 | 0.000E+00 | 2.006E-07 | 0.000E+00 | 9.191E-07 | 2.149E-06 | 0.000E+00 | 0.000E+00 | 0.000E+00 | 5.122E-07 |
| Unigene508085 | srmB | 0.000E+00 | 0.000E+00 | 0.000E+00 | 0.000E+00 | 0.000E+00 | 0.000E+00 | 6.380E-07 | 0.000E+00 | 0.000E+00 | 0.000E+00 | 0.000E+00 | 0.000E+00 |
| Unigene508141 | patA | 5.832E-08 | 9.601E-08 | 2.289E-07 | 1.178E-07 | 3.887E-07 | 3.152E-07 | 1.097E-06 | 8.850E-07 | 0.000E+00 | 2.599E-07 | 9.006E-07 | 2.757E-06 |
| Unigene508143 | PmrF | 0.000E+00 | 0.000E+00 | 0.000E+00 | 0.000E+00 | 0.000E+00 | 0.000E+00 | 1.004E-06 | 0.000E+00 | 0.000E+00 | 0.000E+00 | 0.000E+00 | 0.000E+00 |
| Unigene508156 | tetA(60) | 0.000E+00 | 0.000E+00 | 0.000E+00 | 0.000E+00 | 5.703E-07 | 4.440E-07 | 5.454E-07 | 8.149E-07 | 0.000E+00 | 2.059E-07 | 5.168E-07 | 5.583E-07 |
| Unigene508215 | macB | 0.000E+00 | 0.000E+00 | 0.000E+00 | 0.000E+00 | 0.000E+00 | 0.000E+00 | 4.875E-07 | 5.876E-07 | 0.000E+00 | 0.000E+00 | 0.000E+00 | 7.438E-08 |
| Unigene508241 | patA | 0.000E+00 | 0.000E+00 | 0.000E+00 | 0.000E+00 | 0.000E+00 | 0.000E+00 | 9.402E-07 | 8.499E-07 | 0.000E+00 | 0.000E+00 | 0.000E+00 | 5.260E-07 |
| Unigene508253 | tetA(60) | 9.676E-08 | 0.000E+00 | 1.139E-07 | 1.368E-07 | 0.000E+00 | 3.557E-07 | 1.264E-06 | 1.536E-06 | 0.000E+00 | 0.000E+00 | 9.740E-07 | 3.248E-06 |
| Unigene508268 | cpxA | 0.000E+00 | 0.000E+00 | 3.910E-07 | 0.000E+00 | 3.874E-07 | 0.000E+00 | 1.130E-06 | 0.000E+00 | 0.000E+00 | 0.000E+00 | 0.000E+00 | 0.000E+00 |
| Unigene508327 | floR | 0.000E+00 | 3.271E-08 | 0.000E+00 | 0.000E+00 | 0.000E+00 | 0.000E+00 | 9.237E-07 | 0.000E+00 | 0.000E+00 | 0.000E+00 | 1.364E-07 | 0.000E+00 |
| Unigene508413 | TriC | 0.000E+00 | 0.000E+00 | 2.155E-07 | 0.000E+00 | 0.000E+00 | 0.000E+00 | 6.076E-07 | 3.204E-07 | 0.000E+00 | 0.000E+00 | 0.000E+00 | 0.000E+00 |
| Unigene508513 | macB | 0.000E+00 | 0.000E+00 | 0.000E+00 | 0.000E+00 | 0.000E+00 | 0.000E+00 | 4.452E-07 | 2.935E-07 | 0.000E+00 | 0.000E+00 | 0.000E+00 | 0.000E+00 |
| Unigene508550 | vanSA | 0.000E+00 | 0.000E+00 | 0.000E+00 | 0.000E+00 | 0.000E+00 | 0.000E+00 | 8.460E-07 | 0.000E+00 | 0.000E+00 | 0.000E+00 | 0.000E+00 | 0.000E+00 |
| Unigene508590 | catB2 | 0.000E+00 | 0.000E+00 | 0.000E+00 | 0.000E+00 | 0.000E+00 | 0.000E+00 | 7.767E-07 | 0.000E+00 | 0.000E+00 | 0.000E+00 | 0.000E+00 | 0.000E+00 |
| Unigene508637 | efrA | 0.000E+00 | 0.000E+00 | 0.000E+00 | 0.000E+00 | 1.184E-07 | 0.000E+00 | 0.000E+00 | 0.000E+00 | 0.000E+00 | 0.000E+00 | 7.312E-07 | 0.000E+00 |
| Unigene508690 | OprJ | 0.000E+00 | 0.000E+00 | 0.000E+00 | 0.000E+00 | 0.000E+00 | 0.000E+00 | 6.065E-07 | 1.163E-07 | 0.000E+00 | 0.000E+00 | 0.000E+00 | 2.944E-07 |
| Unigene508757 | tlrC | 8.390E-08 | 1.842E-07 | 2.264E-07 | 4.236E-08 | 5.126E-07 | 7.256E-07 | 2.646E-06 | 8.079E-07 | 2.293E-07 | 5.374E-07 | 1.296E-06 | 3.421E-06 |
| Unigene508758 | vanHB | 0.000E+00 | 3.679E-08 | 2.960E-07 | 6.770E-08 | 4.096E-07 | 0.000E+00 | 1.929E-06 | 1.174E-06 | 6.595E-07 | 1.494E-06 | 8.436E-07 | 4.121E-06 |
| Unigene508786 | adeS | 0.000E+00 | 0.000E+00 | 0.000E+00 | 0.000E+00 | 0.000E+00 | 0.000E+00 | 7.780E-07 | 0.000E+00 | 0.000E+00 | 0.000E+00 | 0.000E+00 | 0.000E+00 |
| Unigene508936 | patA | 0.000E+00 | 0.000E+00 | 0.000E+00 | 0.000E+00 | 0.000E+00 | 0.000E+00 | 6.011E-07 | 0.000E+00 | 0.000E+00 | 0.000E+00 | 0.000E+00 | 0.000E+00 |
| Unigene508937 | iri | 0.000E+00 | 0.000E+00 | 0.000E+00 | 0.000E+00 | 1.960E-07 | 0.000E+00 | 4.295E-07 | 0.000E+00 | 0.000E+00 | 0.000E+00 | 0.000E+00 | 0.000E+00 |
| Unigene508949 | bcrA | 0.000E+00 | 0.000E+00 | 0.000E+00 | 0.000E+00 | 3.164E-07 | 0.000E+00 | 7.706E-07 | 0.000E+00 | 0.000E+00 | 0.000E+00 | 5.792E-07 | 3.741E-07 |
| Unigene508950 | macB | 1.312E-07 | 0.000E+00 | 0.000E+00 | 0.000E+00 | 6.560E-07 | 2.128E-07 | 1.343E-06 | 6.510E-07 | 1.076E-07 | 0.000E+00 | 5.629E-07 | 1.086E-06 |
| Unigene509026 | macB | 0.000E+00 | 0.000E+00 | 5.748E-07 | 0.000E+00 | 0.000E+00 | 7.999E-07 | 4.265E-06 | 3.419E-06 | 7.750E-07 | 1.649E-06 | 2.010E-06 | 3.972E-06 |

| | | | | | | | | | | | | | |
|---|---|---|---|---|---|---|---|---|---|---|---|---|---|
| Unigene509031 | cmlv | 0.000E+00 | 0.000E+00 | 0.000E+00 | 0.000E+00 | 0.000E+00 | 0.000E+00 | 3.824E-07 | 0.000E+00 | 0.000E+00 | 0.000E+00 | 0.000E+00 | 0.000E+00 |
| Unigene509277 | tetB(46) | 0.000E+00 | 0.000E+00 | 0.000E+00 | 0.000E+00 | 0.000E+00 | 0.000E+00 | 5.539E-07 | 0.000E+00 | 0.000E+00 | 0.000E+00 | 0.000E+00 | 0.000E+00 |
| Unigene509293 | MCR-1.7 | 0.000E+00 | 0.000E+00 | 0.000E+00 | 0.000E+00 | 0.000E+00 | 0.000E+00 | 2.666E-07 | 0.000E+00 | 0.000E+00 | 0.000E+00 | 0.000E+00 | 0.000E+00 |
| Unigene509296 | msbA | 0.000E+00 | 0.000E+00 | 0.000E+00 | 0.000E+00 | 0.000E+00 | 0.000E+00 | 4.511E-07 | 0.000E+00 | 0.000E+00 | 0.000E+00 | 0.000E+00 | 0.000E+00 |
| Unigene509299 | vanTC | 0.000E+00 | 0.000E+00 | 6.834E-08 | 0.000E+00 | 0.000E+00 | 0.000E+00 | 5.781E-07 | 0.000E+00 | 0.000E+00 | 0.000E+00 | 3.983E-07 | 3.293E-07 |
| Unigene509322 | vatB | 0.000E+00 | 0.000E+00 | 4.777E-08 | 0.000E+00 | 0.000E+00 | 0.000E+00 | 9.699E-07 | 0.000E+00 | 0.000E+00 | 0.000E+00 | 0.000E+00 | 0.000E+00 |
| Unigene509374 | macB | 0.000E+00 | 0.000E+00 | 0.000E+00 | 0.000E+00 | 0.000E+00 | 0.000E+00 | 4.387E-07 | 1.322E-07 | 0.000E+00 | 0.000E+00 | 0.000E+00 | 0.000E+00 |
| Unigene509567 | vmlR | 0.000E+00 | 0.000E+00 | 0.000E+00 | 5.905E-08 | 0.000E+00 | 0.000E+00 | 3.883E-07 | 0.000E+00 | 0.000E+00 | 0.000E+00 | 0.000E+00 | 0.000E+00 |
| Unigene509578 | salA | 0.000E+00 | 0.000E+00 | 0.000E+00 | 0.000E+00 | 0.000E+00 | 0.000E+00 | 7.719E-07 | 0.000E+00 | 0.000E+00 | 0.000E+00 | 0.000E+00 | 5.299E-07 |
| Unigene509655 | cpxA | 0.000E+00 | 0.000E+00 | 0.000E+00 | 0.000E+00 | 0.000E+00 | 0.000E+00 | 6.447E-07 | 0.000E+00 | 0.000E+00 | 0.000E+00 | 0.000E+00 | 0.000E+00 |
| Unigene509676 | mdtM | 0.000E+00 | 0.000E+00 | 8.405E-07 | 0.000E+00 | 0.000E+00 | 0.000E+00 | 1.777E-06 | 0.000E+00 | 0.000E+00 | 0.000E+00 | 0.000E+00 | 0.000E+00 |
| Unigene509738 | evgS | 8.678E-08 | 0.000E+00 | 0.000E+00 | 0.000E+00 | 2.892E-07 | 0.000E+00 | 8.323E-07 | 7.090E-07 | 0.000E+00 | 0.000E+00 | 0.000E+00 | 7.180E-07 |
| Unigene509804 | smeS | 0.000E+00 | 0.000E+00 | 0.000E+00 | 0.000E+00 | 0.000E+00 | 0.000E+00 | 9.185E-07 | 0.000E+00 | 0.000E+00 | 0.000E+00 | 0.000E+00 | 2.368E-07 |
| Unigene509808 | bcrA | 0.000E+00 | 0.000E+00 | 0.000E+00 | 0.000E+00 | 0.000E+00 | 0.000E+00 | 7.904E-07 | 0.000E+00 | 0.000E+00 | 0.000E+00 | 0.000E+00 | 0.000E+00 |
| Unigene509892 | MexA | 0.000E+00 | 0.000E+00 | 0.000E+00 | 0.000E+00 | 0.000E+00 | 0.000E+00 | 1.588E-06 | 0.000E+00 | 0.000E+00 | 0.000E+00 | 0.000E+00 | 0.000E+00 |
| Unigene509901 | tcr3 | 0.000E+00 | 0.000E+00 | 0.000E+00 | 0.000E+00 | 0.000E+00 | 0.000E+00 | 6.588E-07 | 0.000E+00 | 0.000E+00 | 0.000E+00 | 0.000E+00 | 0.000E+00 |
| Unigene509996 | bcrA | 0.000E+00 | 0.000E+00 | 0.000E+00 | 0.000E+00 | 0.000E+00 | 0.000E+00 | 1.946E-06 | 0.000E+00 | 0.000E+00 | 0.000E+00 | 0.000E+00 | 4.800E-06 |
| Unigene510050 | farA | 2.122E-07 | 0.000E+00 | 1.190E-07 | 2.755E-07 | 5.388E-07 | 9.830E-08 | 9.393E-07 | 7.430E-07 | 1.988E-07 | 0.000E+00 | 1.179E-06 | 2.866E-06 |
| Unigene510115 | bcrA | 0.000E+00 | 0.000E+00 | 0.000E+00 | 0.000E+00 | 0.000E+00 | 0.000E+00 | 7.975E-07 | 0.000E+00 | 0.000E+00 | 0.000E+00 | 0.000E+00 | 0.000E+00 |
| Unigene510167 | novA | 1.273E-07 | 0.000E+00 | 0.000E+00 | 0.000E+00 | 7.427E-07 | 0.000E+00 | 1.163E-06 | 3.010E-06 | 0.000E+00 | 0.000E+00 | 1.020E-06 | 1.656E-06 |
| Unigene510251 | golS | 0.000E+00 | 0.000E+00 | 0.000E+00 | 0.000E+00 | 0.000E+00 | 0.000E+00 | 4.315E-07 | 0.000E+00 | 0.000E+00 | 0.000E+00 | 0.000E+00 | 0.000E+00 |
| Unigene510291 | efrA | 0.000E+00 | 0.000E+00 | 0.000E+00 | 3.234E-08 | 3.202E-07 | 1.385E-07 | 7.089E-07 | 1.869E-06 | 0.000E+00 | 0.000E+00 | 5.128E-07 | 6.435E-07 |
| Unigene510346 | patA | 0.000E+00 | 0.000E+00 | 0.000E+00 | 0.000E+00 | 0.000E+00 | 0.000E+00 | 7.874E-07 | 0.000E+00 | 0.000E+00 | 0.000E+00 | 0.000E+00 | 0.000E+00 |
| Unigene510380 | lmrD | 0.000E+00 | 0.000E+00 | 0.000E+00 | 0.000E+00 | 0.000E+00 | 0.000E+00 | 4.740E-07 | 0.000E+00 | 0.000E+00 | 0.000E+00 | 0.000E+00 | 0.000E+00 |
| Unigene510390 | vanHA | 0.000E+00 | 0.000E+00 | 0.000E+00 | 0.000E+00 | 0.000E+00 | 0.000E+00 | 6.766E-07 | 0.000E+00 | 0.000E+00 | 0.000E+00 | 0.000E+00 | 0.000E+00 |
| Unigene510400 | arlR | 0.000E+00 | 0.000E+00 | 0.000E+00 | 0.000E+00 | 0.000E+00 | 0.000E+00 | 5.895E-07 | 6.218E-08 | 0.000E+00 | 0.000E+00 | 0.000E+00 | 0.000E+00 |
| Unigene510404 | vanSA | 2.089E-08 | 2.293E-08 | 1.230E-07 | 0.000E+00 | 4.642E-08 | 2.259E-07 | 1.041E-06 | 2.439E-07 | 0.000E+00 | 0.000E+00 | 0.000E+00 | 1.235E-07 |
| Unigene510755 | Pseudomo | 0.000E+00 | 0.000E+00 | 0.000E+00 | 0.000E+00 | 0.000E+00 | 0.000E+00 | 3.430E-07 | 0.000E+00 | 0.000E+00 | 0.000E+00 | 0.000E+00 | 0.000E+00 |
| Unigene510787 | lmrD | 0.000E+00 | 0.000E+00 | 0.000E+00 | 0.000E+00 | 0.000E+00 | 0.000E+00 | 1.718E-07 | 0.000E+00 | 0.000E+00 | 0.000E+00 | 0.000E+00 | 0.000E+00 |
| Unigene510848 | smeS | 2.065E-07 | 0.000E+00 | 0.000E+00 | 1.622E-07 | 9.431E-07 | 0.000E+00 | 7.364E-07 | 6.159E-07 | 0.000E+00 | 1.789E-07 | 6.299E-07 | 4.068E-07 |
| Unigene510849 | Pseudomo | 0.000E+00 | 0.000E+00 | 0.000E+00 | 0.000E+00 | 4.373E-07 | 0.000E+00 | 8.230E-07 | 6.127E-07 | 0.000E+00 | 4.873E-07 | 6.004E-07 | 3.619E-07 |
| Unigene510856 | tetA(60) | 0.000E+00 | 0.000E+00 | 0.000E+00 | 0.000E+00 | 0.000E+00 | 0.000E+00 | 0.000E+00 | 4.673E-07 | 0.000E+00 | 0.000E+00 | 0.000E+00 | 0.000E+00 |
| Unigene510992 | Acinetoba | 0.000E+00 | 6.216E-07 | 4.168E-07 | 0.000E+00 | 0.000E+00 | 0.000E+00 | 4.178E-07 | 1.102E-07 | 0.000E+00 | 0.000E+00 | 0.000E+00 | 0.000E+00 |
| Unigene511005 | iri | 0.000E+00 | 4.631E-08 | 1.656E-07 | 1.065E-07 | 3.516E-07 | 2.737E-07 | 6.538E-07 | 3.694E-07 | 0.000E+00 | 2.115E-07 | 3.379E-07 | 6.733E-07 |
| Unigene511131 | cmlv | 0.000E+00 | 0.000E+00 | 3.938E-07 | 0.000E+00 | 0.000E+00 | 2.983E-07 | 1.138E-06 | 1.025E-06 | 0.000E+00 | 0.000E+00 | 1.205E-06 | 3.736E-06 |
| Unigene511168 | TriC | 0.000E+00 | 0.000E+00 | 1.482E-07 | 0.000E+00 | 0.000E+00 | 0.000E+00 | 5.014E-07 | 0.000E+00 | 0.000E+00 | 0.000E+00 | 0.000E+00 | 0.000E+00 |
| Unigene511264 | tetA(58) | 0.000E+00 | 0.000E+00 | 0.000E+00 | 0.000E+00 | 0.000E+00 | 0.000E+00 | 4.881E-07 | 0.000E+00 | 0.000E+00 | 0.000E+00 | 0.000E+00 | 0.000E+00 |
| Unigene511303 | PER-7 | 0.000E+00 | 0.000E+00 | 0.000E+00 | 0.000E+00 | 0.000E+00 | 0.000E+00 | 8.480E-07 | 8.555E-07 | 0.000E+00 | 0.000E+00 | 4.573E-07 | 0.000E+00 |
| Unigene511341 | ugd | 0.000E+00 | 0.000E+00 | 0.000E+00 | 0.000E+00 | 0.000E+00 | 0.000E+00 | 2.560E-06 | 0.000E+00 | 0.000E+00 | 0.000E+00 | 0.000E+00 | 0.000E+00 |
| Unigene511661 | novA | 0.000E+00 | 1.414E-07 | 0.000E+00 | 0.000E+00 | 0.000E+00 | 2.090E-07 | 6.774E-07 | 7.521E-08 | 0.000E+00 | 0.000E+00 | 0.000E+00 | 0.000E+00 |
| Unigene511781 | MexW | 2.040E-08 | 2.239E-08 | 0.000E+00 | 6.180E-08 | 7.932E-08 | 2.426E-07 | 8.692E-07 | 2.024E-07 | 0.000E+00 | 0.000E+00 | 8.167E-08 | 1.808E-07 |
| Unigene511782 | mexM | 0.000E+00 | 0.000E+00 | 0.000E+00 | 0.000E+00 | 0.000E+00 | 0.000E+00 | 6.219E-07 | 3.279E-07 | 0.000E+00 | 9.389E-08 | 0.000E+00 | 0.000E+00 |
| Unigene511820 | vanG | 0.000E+00 | 4.072E-07 | 6.472E-07 | 0.000E+00 | 1.145E-06 | 0.000E+00 | 8.669E-07 | 0.000E+00 | 0.000E+00 | 1.837E-07 | 0.000E+00 | 0.000E+00 |
| Unigene511925 | novA | 0.000E+00 | 0.000E+00 | 3.764E-08 | 1.937E-07 | 6.606E-07 | 2.281E-07 | 1.019E-06 | 5.373E-07 | 1.677E-07 | 1.068E-07 | 7.240E-07 | 1.111E-06 |
| Unigene512034 | tlrC | 2.919E-07 | 1.831E-07 | 0.000E+00 | 0.000E+00 | 6.950E-07 | 0.000E+00 | 7.385E-07 | 8.276E-07 | 2.279E-07 | 1.858E-07 | 1.193E-06 | 4.930E-07 |
| Unigene512066 | vmlR | 0.000E+00 | 0.000E+00 | 0.000E+00 | 0.000E+00 | 0.000E+00 | 0.000E+00 | 4.763E-07 | 0.000E+00 | 0.000E+00 | 0.000E+00 | 0.000E+00 | 0.000E+00 |
| Unigene512200 | macB | 9.004E-08 | 0.000E+00 | 4.418E-08 | 0.000E+00 | 2.001E-07 | 0.000E+00 | 2.990E-07 | 3.679E-07 | 0.000E+00 | 0.000E+00 | 5.150E-07 | 1.118E-06 |
| Unigene512274 | cpxA | 0.000E+00 | 0.000E+00 | 0.000E+00 | 0.000E+00 | 0.000E+00 | 0.000E+00 | 9.016E-07 | 4.358E-07 | 0.000E+00 | 0.000E+00 | 0.000E+00 | 0.000E+00 |
| Unigene512279 | otr(B) | 0.000E+00 | 0.000E+00 | 0.000E+00 | 0.000E+00 | 0.000E+00 | 0.000E+00 | 4.178E-07 | 0.000E+00 | 0.000E+00 | 0.000E+00 | 0.000E+00 | 0.000E+00 |
| Unigene512299 | macB | 0.000E+00 | 0.000E+00 | 0.000E+00 | 0.000E+00 | 0.000E+00 | 0.000E+00 | 1.294E-06 | 0.000E+00 | 1.278E-07 | 0.000E+00 | 0.000E+00 | 8.756E-07 |
| Unigene512342 | mexM | 0.000E+00 | 0.000E+00 | 0.000E+00 | 0.000E+00 | 0.000E+00 | 0.000E+00 | 7.341E-07 | 5.592E-07 | 0.000E+00 | 0.000E+00 | 0.000E+00 | 0.000E+00 |
| Unigene512358 | smeS | 0.000E+00 | 0.000E+00 | 0.000E+00 | 0.000E+00 | 5.775E-08 | 0.000E+00 | 4.602E-07 | 0.000E+00 | 0.000E+00 | 0.000E+00 | 0.000E+00 | 5.530E-07 |
| Unigene512367 | Acinetoba | 0.000E+00 | 0.000E+00 | 0.000E+00 | 0.000E+00 | 0.000E+00 | 0.000E+00 | 6.107E-07 | 0.000E+00 | 0.000E+00 | 0.000E+00 | 0.000E+00 | 3.805E-07 |
| Unigene512375 | novA | 0.000E+00 | 0.000E+00 | 0.000E+00 | 0.000E+00 | 0.000E+00 | 0.000E+00 | 5.731E-07 | 0.000E+00 | 0.000E+00 | 0.000E+00 | 0.000E+00 | 0.000E+00 |
| Unigene512394 | efrA | 0.000E+00 | 0.000E+00 | 0.000E+00 | 0.000E+00 | 0.000E+00 | 2.515E-07 | 7.403E-07 | 0.000E+00 | 0.000E+00 | 0.000E+00 | 5.988E-07 | 5.156E-07 |
| Unigene512404 | efrB | 0.000E+00 | 0.000E+00 | 0.000E+00 | 0.000E+00 | 0.000E+00 | 0.000E+00 | 3.326E-07 | 0.000E+00 | 0.000E+00 | 0.000E+00 | 0.000E+00 | 0.000E+00 |
| Unigene512474 | basS | 0.000E+00 | 0.000E+00 | 0.000E+00 | 0.000E+00 | 0.000E+00 | 0.000E+00 | 3.254E-07 | 0.000E+00 | 0.000E+00 | 0.000E+00 | 0.000E+00 | 0.000E+00 |
| Unigene512507 | baeS | 0.000E+00 | 9.529E-07 | 0.000E+00 | 0.000E+00 | 0.000E+00 | 0.000E+00 | 1.030E-06 | 0.000E+00 | 0.000E+00 | 0.000E+00 | 0.000E+00 | 0.000E+00 |

| | | | | | | | | | | | | | |
|---|---|---|---|---|---|---|---|---|---|---|---|---|---|
| Unigene512612 | novA | 0.000E+00 | 0.000E+00 | 0.000E+00 | 0.000E+00 | 0.000E+00 | 0.000E+00 | 5.992E-07 | 0.000E+00 | 0.000E+00 | 0.000E+00 | 0.000E+00 | 0.000E+00 |
| Unigene512620 | tetA(58) | 0.000E+00 | 0.000E+00 | 0.000E+00 | 0.000E+00 | 1.332E-06 | 0.000E+00 | 1.327E-06 | 1.189E-06 | 3.931E-07 | 0.000E+00 | 1.063E-06 | 0.000E+00 |
| Unigene512693 | kdpE | 0.000E+00 | 0.000E+00 | 0.000E+00 | 0.000E+00 | 0.000E+00 | 0.000E+00 | 4.127E-07 | 0.000E+00 | 0.000E+00 | 0.000E+00 | 0.000E+00 | 0.000E+00 |
| Unigene512739 | macB | 0.000E+00 | 0.000E+00 | 0.000E+00 | 0.000E+00 | 0.000E+00 | 0.000E+00 | 6.395E-07 | 0.000E+00 | 0.000E+00 | 0.000E+00 | 0.000E+00 | 0.000E+00 |
| Unigene512766 | adeG | 0.000E+00 | 0.000E+00 | 0.000E+00 | 0.000E+00 | 0.000E+00 | 0.000E+00 | 9.012E-07 | 0.000E+00 | 0.000E+00 | 0.000E+00 | 0.000E+00 | 0.000E+00 |
| Unigene512800 | rosB | 0.000E+00 | 0.000E+00 | 0.000E+00 | 0.000E+00 | 0.000E+00 | 0.000E+00 | 1.000E-06 | 0.000E+00 | 0.000E+00 | 0.000E+00 | 1.879E-07 | 0.000E+00 |
| Unigene512993 | Enterobact | 0.000E+00 | 0.000E+00 | 0.000E+00 | 0.000E+00 | 0.000E+00 | 0.000E+00 | 0.000E+00 | 0.000E+00 | 0.000E+00 | 0.000E+00 | 0.000E+00 | 3.884E-07 |
| Unigene513005 | msbA | 0.000E+00 | 0.000E+00 | 1.765E-07 | 0.000E+00 | 6.660E-07 | 2.268E-07 | 8.956E-07 | 8.047E-07 | 0.000E+00 | 0.000E+00 | 5.143E-07 | 1.382E-06 |
| Unigene513011 | Staphylocc | 1.606E-07 | 0.000E+00 | 0.000E+00 | 1.802E-07 | 0.000E+00 | 0.000E+00 | 8.295E-07 | 0.000E+00 | 0.000E+00 | 0.000E+00 | 0.000E+00 | 4.008E-07 |
| Unigene513019 | cmlB | 0.000E+00 | 0.000E+00 | 0.000E+00 | 0.000E+00 | 0.000E+00 | 0.000E+00 | 8.202E-07 | 0.000E+00 | 0.000E+00 | 0.000E+00 | 0.000E+00 | 4.542E-07 |
| Unigene513058 | oleC | 0.000E+00 | 0.000E+00 | 0.000E+00 | 0.000E+00 | 0.000E+00 | 0.000E+00 | 1.146E-06 | 0.000E+00 | 0.000E+00 | 0.000E+00 | 0.000E+00 | 0.000E+00 |
| Unigene513170 | OXA-18 | 0.000E+00 | 0.000E+00 | 0.000E+00 | 0.000E+00 | 0.000E+00 | 0.000E+00 | 6.342E-07 | 0.000E+00 | 0.000E+00 | 0.000E+00 | 0.000E+00 | 0.000E+00 |
| Unigene513326 | oleC | 0.000E+00 | 0.000E+00 | 0.000E+00 | 0.000E+00 | 0.000E+00 | 0.000E+00 | 2.786E-07 | 5.141E-07 | 0.000E+00 | 0.000E+00 | 0.000E+00 | 2.975E-07 |
| Unigene513334 | macB | 0.000E+00 | 0.000E+00 | 0.000E+00 | 0.000E+00 | 0.000E+00 | 0.000E+00 | 2.308E-06 | 0.000E+00 | 0.000E+00 | 0.000E+00 | 0.000E+00 | 0.000E+00 |
| Unigene513402 | tet(C) | 0.000E+00 | 0.000E+00 | 0.000E+00 | 0.000E+00 | 0.000E+00 | 0.000E+00 | 5.539E-07 | 0.000E+00 | 0.000E+00 | 0.000E+00 | 0.000E+00 | 0.000E+00 |
| Unigene513425 | bcr-1 | 0.000E+00 | 0.000E+00 | 0.000E+00 | 0.000E+00 | 0.000E+00 | 0.000E+00 | 1.401E-06 | 0.000E+00 | 0.000E+00 | 0.000E+00 | 0.000E+00 | 0.000E+00 |
| Unigene513432 | macB | 0.000E+00 | 0.000E+00 | 0.000E+00 | 0.000E+00 | 0.000E+00 | 0.000E+00 | 1.003E-06 | 0.000E+00 | 0.000E+00 | 0.000E+00 | 0.000E+00 | 0.000E+00 |
| Unigene513439 | ramA | 0.000E+00 | 0.000E+00 | 0.000E+00 | 0.000E+00 | 0.000E+00 | 0.000E+00 | 4.106E-07 | 0.000E+00 | 0.000E+00 | 0.000E+00 | 0.000E+00 | 0.000E+00 |
| Unigene513534 | novA | 0.000E+00 | 0.000E+00 | 8.884E-07 | 3.341E-07 | 5.416E-07 | 9.599E-07 | 2.505E-06 | 2.236E-06 | 3.045E-07 | 1.183E-06 | 1.812E-06 | 2.840E-06 |
| Unigene513551 | cmlv | 0.000E+00 | 0.000E+00 | 0.000E+00 | 0.000E+00 | 0.000E+00 | 0.000E+00 | 1.075E-06 | 0.000E+00 | 0.000E+00 | 0.000E+00 | 0.000E+00 | 6.183E-07 |
| Unigene513608 | smeR | 0.000E+00 | 0.000E+00 | 0.000E+00 | 0.000E+00 | 0.000E+00 | 0.000E+00 | 2.490E-06 | 0.000E+00 | 0.000E+00 | 0.000E+00 | 0.000E+00 | 0.000E+00 |
| Unigene513862 | tva(A) | 0.000E+00 | 0.000E+00 | 2.026E-07 | 0.000E+00 | 0.000E+00 | 0.000E+00 | 3.998E-07 | 0.000E+00 | 0.000E+00 | 0.000E+00 | 8.855E-08 | 5.490E-07 |
| Unigene513962 | mecA | 0.000E+00 | 0.000E+00 | 0.000E+00 | 0.000E+00 | 0.000E+00 | 0.000E+00 | 3.240E-07 | 0.000E+00 | 0.000E+00 | 0.000E+00 | 0.000E+00 | 0.000E+00 |
| Unigene514007 | macB | 0.000E+00 | 0.000E+00 | 0.000E+00 | 0.000E+00 | 0.000E+00 | 0.000E+00 | 1.031E-06 | 0.000E+00 | 9.260E-08 | 0.000E+00 | 0.000E+00 | 1.352E-06 |
| Unigene514009 | tetA(58) | 0.000E+00 | 0.000E+00 | 0.000E+00 | 0.000E+00 | 0.000E+00 | 0.000E+00 | 3.992E-07 | 3.508E-07 | 0.000E+00 | 0.000E+00 | 0.000E+00 | 2.132E-07 |
| Unigene514030 | MCR-1.9 | 1.776E-07 | 0.000E+00 | 7.470E-08 | 2.563E-08 | 2.819E-07 | 1.372E-07 | 1.095E-06 | 1.155E-06 | 0.000E+00 | 1.979E-07 | 5.806E-07 | 1.890E-06 |
| Unigene514193 | Acinetobac | 0.000E+00 | 0.000E+00 | 2.550E-07 | 0.000E+00 | 0.000E+00 | 0.000E+00 | 7.479E-07 | 2.427E-07 | 0.000E+00 | 0.000E+00 | 0.000E+00 | 0.000E+00 |
| Unigene514310 | Staphylocc | 0.000E+00 | 0.000E+00 | 0.000E+00 | 0.000E+00 | 0.000E+00 | 1.955E-07 | 1.376E-06 | 8.180E-07 | 0.000E+00 | 0.000E+00 | 8.016E-07 | 6.947E-07 |
| Unigene514322 | msbA | 0.000E+00 | 0.000E+00 | 0.000E+00 | 0.000E+00 | 0.000E+00 | 0.000E+00 | 7.278E-07 | 0.000E+00 | 0.000E+00 | 0.000E+00 | 0.000E+00 | 0.000E+00 |
| Unigene514330 | tetA(58) | 0.000E+00 | 0.000E+00 | 0.000E+00 | 0.000E+00 | 0.000E+00 | 5.057E-07 | 1.036E-06 | 1.493E-06 | 0.000E+00 | 0.000E+00 | 0.000E+00 | 2.654E-06 |
| Unigene514348 | arnA | 0.000E+00 | 0.000E+00 | 0.000E+00 | 0.000E+00 | 0.000E+00 | 0.000E+00 | 6.505E-07 | 4.749E-07 | 1.482E-07 | 0.000E+00 | 0.000E+00 | 3.206E-07 |
| Unigene514512 | tetA(60) | 0.000E+00 | 0.000E+00 | 0.000E+00 | 0.000E+00 | 0.000E+00 | 0.000E+00 | 5.364E-07 | 0.000E+00 | 0.000E+00 | 0.000E+00 | 3.881E-07 | 0.000E+00 |
| Unigene514618 | mdtB | 0.000E+00 | 0.000E+00 | 0.000E+00 | 3.975E-07 | 0.000E+00 | 0.000E+00 | 4.357E-07 | 0.000E+00 | 0.000E+00 | 0.000E+00 | 0.000E+00 | 7.755E-07 |
| Unigene514640 | TolC | 0.000E+00 | 1.904E-06 | 1.283E-06 | 5.331E-07 | 0.000E+00 | 0.000E+00 | 1.753E-06 | 0.000E+00 | 0.000E+00 | 0.000E+00 | 0.000E+00 | 0.000E+00 |
| Unigene514651 | bcrA | 0.000E+00 | 0.000E+00 | 0.000E+00 | 0.000E+00 | 0.000E+00 | 0.000E+00 | 1.110E-06 | 0.000E+00 | 0.000E+00 | 0.000E+00 | 0.000E+00 | 0.000E+00 |
| Unigene514787 | YojI | 0.000E+00 | 1.514E-07 | 0.000E+00 | 0.000E+00 | 2.682E-07 | 0.000E+00 | 6.489E-07 | 1.892E-06 | 0.000E+00 | 0.000E+00 | 0.000E+00 | 1.386E-06 |
| Unigene514792 | tetA(58) | 0.000E+00 | 0.000E+00 | 0.000E+00 | 0.000E+00 | 0.000E+00 | 0.000E+00 | 9.782E-07 | 0.000E+00 | 0.000E+00 | 0.000E+00 | 0.000E+00 | 7.722E-07 |
| Unigene514802 | bcrA | 0.000E+00 | 0.000E+00 | 0.000E+00 | 0.000E+00 | 0.000E+00 | 1.762E-07 | 5.413E-07 | 0.000E+00 | 0.000E+00 | 0.000E+00 | 1.865E-07 | 4.625E-07 |
| Unigene514814 | tva(A) | 0.000E+00 | 0.000E+00 | 0.000E+00 | 0.000E+00 | 0.000E+00 | 0.000E+00 | 9.485E-07 | 0.000E+00 | 0.000E+00 | 0.000E+00 | 0.000E+00 | 0.000E+00 |
| Unigene514849 | PmpM | 0.000E+00 | 0.000E+00 | 0.000E+00 | 0.000E+00 | 0.000E+00 | 0.000E+00 | 4.458E-07 | 0.000E+00 | 0.000E+00 | 0.000E+00 | 0.000E+00 | 0.000E+00 |
| Unigene514884 | efrB | 0.000E+00 | 0.000E+00 | 0.000E+00 | 4.244E-08 | 0.000E+00 | 0.000E+00 | 4.652E-07 | 2.453E-07 | 0.000E+00 | 0.000E+00 | 0.000E+00 | 2.981E-07 |
| Unigene514907 | APH(6)-Ic | 0.000E+00 | 0.000E+00 | 0.000E+00 | 0.000E+00 | 0.000E+00 | 0.000E+00 | 7.291E-07 | 0.000E+00 | 0.000E+00 | 0.000E+00 | 6.029E-07 | 0.000E+00 |
| Unigene514921 | vanHB | 0.000E+00 | 0.000E+00 | 0.000E+00 | 0.000E+00 | 0.000E+00 | 7.385E-08 | 1.663E-06 | 0.000E+00 | 0.000E+00 | 0.000E+00 | 0.000E+00 | 0.000E+00 |
| Unigene515009 | patA | 0.000E+00 | 0.000E+00 | 0.000E+00 | 0.000E+00 | 0.000E+00 | 0.000E+00 | 4.680E-07 | 0.000E+00 | 0.000E+00 | 0.000E+00 | 0.000E+00 | 0.000E+00 |
| Unigene515048 | novA | 0.000E+00 | 0.000E+00 | 0.000E+00 | 0.000E+00 | 3.222E-07 | 6.271E-08 | 7.383E-07 | 1.727E-06 | 0.000E+00 | 0.000E+00 | 4.976E-07 | 1.303E-06 |
| Unigene515073 | msbA | 0.000E+00 | 0.000E+00 | 1.643E-07 | 0.000E+00 | 0.000E+00 | 0.000E+00 | 0.000E+00 | 0.000E+00 | 0.000E+00 | 0.000E+00 | 0.000E+00 | 3.959E-07 |
| Unigene515182 | PmrF | 0.000E+00 | 0.000E+00 | 0.000E+00 | 0.000E+00 | 0.000E+00 | 0.000E+00 | 5.671E-06 | 0.000E+00 | 0.000E+00 | 1.142E-06 | 0.000E+00 | 0.000E+00 |
| Unigene515203 | tetB(60) | 0.000E+00 | 0.000E+00 | 0.000E+00 | 9.367E-08 | 2.061E-07 | 3.677E-07 | 6.502E-07 | 9.023E-07 | 1.352E-07 | 1.378E-07 | 3.891E-07 | 1.279E-06 |
| Unigene515225 | vanHF | 0.000E+00 | 0.000E+00 | 0.000E+00 | 0.000E+00 | 0.000E+00 | 0.000E+00 | 1.575E-06 | 6.077E-07 | 3.414E-07 | 0.000E+00 | 5.162E-07 | 1.313E-06 |
| Unigene515273 | msbA | 1.548E-07 | 0.000E+00 | 3.798E-08 | 0.000E+00 | 3.870E-07 | 3.975E-07 | 1.242E-06 | 1.220E-06 | 2.115E-08 | 5.174E-07 | 9.962E-07 | 3.042E-06 |
| Unigene515462 | tcr3 | 0.000E+00 | 0.000E+00 | 0.000E+00 | 0.000E+00 | 2.481E-07 | 1.975E-07 | 8.988E-07 | 1.493E-06 | 0.000E+00 | 0.000E+00 | 0.000E+00 | 5.999E-07 |
| Unigene515505 | tlrC | 1.047E-07 | 9.190E-08 | 1.438E-07 | 2.748E-07 | 2.558E-07 | 3.621E-07 | 1.645E-06 | 1.320E-06 | 4.576E-08 | 3.265E-07 | 1.389E-06 | 3.415E-06 |
| Unigene515592 | mtrA | 0.000E+00 | 0.000E+00 | 0.000E+00 | 0.000E+00 | 0.000E+00 | 0.000E+00 | 0.000E+00 | 0.000E+00 | 0.000E+00 | 0.000E+00 | 0.000E+00 | 8.672E-07 |
| Unigene515715 | tetB(60) | 0.000E+00 | 0.000E+00 | 0.000E+00 | 0.000E+00 | 0.000E+00 | 0.000E+00 | 7.767E-07 | 0.000E+00 | 0.000E+00 | 2.171E-07 | 0.000E+00 | 0.000E+00 |
| Unigene515734 | Acinetobac | 0.000E+00 | 0.000E+00 | 0.000E+00 | 0.000E+00 | 0.000E+00 | 4.539E-07 | 2.905E-07 | 2.451E-07 | 0.000E+00 | 0.000E+00 | 6.004E-07 | 0.000E+00 |
| Unigene515755 | macB | 0.000E+00 | 0.000E+00 | 0.000E+00 | 1.204E-07 | 0.000E+00 | 0.000E+00 | 9.237E-07 | 8.698E-07 | 0.000E+00 | 0.000E+00 | 0.000E+00 | 5.989E-07 |
| Unigene515767 | carA | 2.118E-07 | 0.000E+00 | 0.000E+00 | 0.000E+00 | 0.000E+00 | 0.000E+00 | 6.564E-07 | 4.450E-07 | 0.000E+00 | 0.000E+00 | 0.000E+00 | 6.510E-07 |
| Unigene515777 | cmlv | 0.000E+00 | 3.192E-08 | 0.000E+00 | 0.000E+00 | 0.000E+00 | 0.000E+00 | 9.334E-07 | 6.450E-07 | 0.000E+00 | 0.000E+00 | 0.000E+00 | 0.000E+00 |

| | | | | | | | | | | | | | |
|---|---|---|---|---|---|---|---|---|---|---|---|---|---|
| Unigene515826 | hp1181 | 0.000E+00 | 0.000E+00 | 0.000E+00 | 0.000E+00 | 0.000E+00 | 0.000E+00 | 1.024E-06 | 0.000E+00 | 0.000E+00 | 0.000E+00 | 0.000E+00 | 0.000E+00 |
| Unigene515880 | novA | 0.000E+00 | 0.000E+00 | 1.487E-07 | 4.081E-07 | 8.979E-07 | 3.823E-07 | 9.505E-07 | 9.435E-07 | 1.104E-07 | 0.000E+00 | 8.668E-07 | 1.373E-06 |
| Unigene515948 | farB | 0.000E+00 | 0.000E+00 | 4.436E-08 | 0.000E+00 | 0.000E+00 | 0.000E+00 | 2.001E-07 | 0.000E+00 | 4.941E-08 | 0.000E+00 | 0.000E+00 | 6.947E-07 |
| Unigene515973 | basS | 7.048E-08 | 0.000E+00 | 1.614E-07 | 0.000E+00 | 3.393E-07 | 0.000E+00 | 6.760E-07 | 6.033E-07 | 0.000E+00 | 7.851E-08 | 4.300E-07 | 1.305E-06 |
| Unigene516232 | MexK | 0.000E+00 | 0.000E+00 | 0.000E+00 | 0.000E+00 | 0.000E+00 | 0.000E+00 | 6.844E-07 | 0.000E+00 | 0.000E+00 | 0.000E+00 | 0.000E+00 | 4.386E-07 |
| Unigene516276 | rosB | 0.000E+00 | 1.083E-07 | 1.937E-07 | 3.489E-07 | 0.000E+00 | 0.000E+00 | 7.102E-07 | 0.000E+00 | 0.000E+00 | 1.100E-07 | 0.000E+00 | 0.000E+00 |
| Unigene516347 | MexK | 0.000E+00 | 7.901E-07 | 0.000E+00 | 0.000E+00 | 0.000E+00 | 0.000E+00 | 1.163E-06 | 0.000E+00 | 0.000E+00 | 0.000E+00 | 0.000E+00 | 0.000E+00 |
| Unigene516411 | tetA(58) | 0.000E+00 | 0.000E+00 | 0.000E+00 | 0.000E+00 | 0.000E+00 | 0.000E+00 | 7.924E-07 | 0.000E+00 | 0.000E+00 | 0.000E+00 | 0.000E+00 | 0.000E+00 |
| Unigene516423 | fexA | 0.000E+00 | 0.000E+00 | 0.000E+00 | 1.128E-07 | 0.000E+00 | 2.012E-07 | 1.071E-06 | 4.781E-07 | 0.000E+00 | 0.000E+00 | 2.129E-07 | 3.521E-07 |
| Unigene516469 | Acinetoba | 0.000E+00 | 0.000E+00 | 0.000E+00 | 0.000E+00 | 0.000E+00 | 0.000E+00 | 7.061E-07 | 0.000E+00 | 0.000E+00 | 0.000E+00 | 2.432E-07 | 2.514E-07 |
| Unigene516538 | patA | 0.000E+00 | 0.000E+00 | 0.000E+00 | 3.663E-07 | 0.000E+00 | 2.178E-07 | 8.474E-07 | 8.467E-07 | 0.000E+00 | 3.143E-07 | 6.915E-07 | 4.287E-07 |
| Unigene516558 | evgS | 0.000E+00 | 0.000E+00 | 0.000E+00 | 0.000E+00 | 0.000E+00 | 0.000E+00 | 7.301E-07 | 0.000E+00 | 0.000E+00 | 0.000E+00 | 0.000E+00 | 0.000E+00 |
| Unigene516866 | LlmA 23S r | 3.576E-07 | 0.000E+00 | 0.000E+00 | 0.000E+00 | 9.029E-07 | 0.000E+00 | 8.995E-07 | 7.209E-07 | 0.000E+00 | 0.000E+00 | 9.296E-07 | 1.268E-06 |
| Unigene517021 | Klebsiella p | 0.000E+00 | 0.000E+00 | 0.000E+00 | 0.000E+00 | 0.000E+00 | 0.000E+00 | 4.083E-07 | 0.000E+00 | 0.000E+00 | 0.000E+00 | 0.000E+00 | 0.000E+00 |
| Unigene517042 | vanHO | 1.606E-07 | 0.000E+00 | 0.000E+00 | 0.000E+00 | 0.000E+00 | 0.000E+00 | 9.243E-07 | 0.000E+00 | 0.000E+00 | 0.000E+00 | 2.204E-07 | 1.443E-06 |
| Unigene517062 | vanSM | 0.000E+00 | 3.902E-08 | 1.047E-07 | 1.436E-07 | 3.950E-07 | 0.000E+00 | 8.394E-07 | 1.024E-06 | 0.000E+00 | 3.036E-07 | 5.829E-07 | 8.825E-07 |
| Unigene517108 | evgS | 0.000E+00 | 0.000E+00 | 0.000E+00 | 0.000E+00 | 0.000E+00 | 0.000E+00 | 8.839E-07 | 5.397E-07 | 0.000E+00 | 0.000E+00 | 0.000E+00 | 0.000E+00 |
| Unigene517158 | MexV | 0.000E+00 | 0.000E+00 | 0.000E+00 | 0.000E+00 | 0.000E+00 | 0.000E+00 | 3.422E-07 | 0.000E+00 | 0.000E+00 | 0.000E+00 | 0.000E+00 | 0.000E+00 |
| Unigene517180 | efrA | 0.000E+00 | 0.000E+00 | 1.143E-07 | 0.000E+00 | 1.510E-07 | 7.769E-06 | 2.365E-06 | 8.774E-06 | 4.543E-06 | 0.000E+00 | 0.000E+00 | 9.413E-07 |
| Unigene517230 | carA | 0.000E+00 | 0.000E+00 | 0.000E+00 | 0.000E+00 | 0.000E+00 | 0.000E+00 | 4.836E-07 | 3.188E-07 | 0.000E+00 | 0.000E+00 | 1.874E-07 | 5.165E-07 |
| Unigene517232 | kdpE | 0.000E+00 | 0.000E+00 | 0.000E+00 | 0.000E+00 | 0.000E+00 | 0.000E+00 | 8.796E-07 | 1.392E-06 | 0.000E+00 | 0.000E+00 | 6.644E-07 | 9.756E-07 |
| Unigene517363 | efrB | 0.000E+00 | 7.873E-08 | 0.000E+00 | 0.000E+00 | 0.000E+00 | 5.687E-07 | 1.111E-06 | 3.907E-07 | 0.000E+00 | 0.000E+00 | 0.000E+00 | 0.000E+00 |
| Unigene517386 | mdtO | 0.000E+00 | 0.000E+00 | 0.000E+00 | 0.000E+00 | 0.000E+00 | 0.000E+00 | 4.460E-07 | 0.000E+00 | 0.000E+00 | 0.000E+00 | 0.000E+00 | 0.000E+00 |
| Unigene517387 | mdtN | 0.000E+00 | 0.000E+00 | 0.000E+00 | 0.000E+00 | 0.000E+00 | 0.000E+00 | 6.662E-07 | 0.000E+00 | 0.000E+00 | 0.000E+00 | 0.000E+00 | 0.000E+00 |
| Unigene517443 | tetT | 0.000E+00 | 0.000E+00 | 2.618E-07 | 0.000E+00 | 3.754E-07 | 2.692E-07 | 1.732E-06 | 7.681E-07 | 0.000E+00 | 0.000E+00 | 0.000E+00 | 1.366E-06 |
| Unigene517468 | bcrA | 0.000E+00 | 4.595E-07 | 0.000E+00 | 0.000E+00 | 0.000E+00 | 0.000E+00 | 7.414E-07 | 0.000E+00 | 0.000E+00 | 0.000E+00 | 0.000E+00 | 0.000E+00 |
| Unigene517499 | tet36 | 1.234E-07 | 0.000E+00 | 1.817E-07 | 0.000E+00 | 2.286E-07 | 0.000E+00 | 8.653E-07 | 5.764E-07 | 0.000E+00 | 2.750E-07 | 4.236E-07 | 8.755E-07 |
| Unigene517600 | adeL | 0.000E+00 | 0.000E+00 | 0.000E+00 | 0.000E+00 | 1.513E-07 | 0.000E+00 | 4.522E-07 | 0.000E+00 | 0.000E+00 | 0.000E+00 | 0.000E+00 | 4.293E-07 |
| Unigene517667 | OprN | 0.000E+00 | 0.000E+00 | 0.000E+00 | 0.000E+00 | 0.000E+00 | 0.000E+00 | 8.431E-07 | 0.000E+00 | 0.000E+00 | 0.000E+00 | 3.268E-07 | 6.753E-07 |
| Unigene517770 | vatB | 0.000E+00 | 0.000E+00 | 1.473E-07 | 0.000E+00 | 0.000E+00 | 2.705E-07 | 8.862E-07 | 4.089E-07 | 0.000E+00 | 0.000E+00 | 2.862E-07 | 1.006E-06 |
| Unigene517936 | mtrA | 0.000E+00 | 0.000E+00 | 0.000E+00 | 0.000E+00 | 0.000E+00 | 0.000E+00 | 8.328E-07 | 0.000E+00 | 0.000E+00 | 0.000E+00 | 0.000E+00 | 0.000E+00 |
| Unigene518108 | cmeA | 0.000E+00 | 0.000E+00 | 0.000E+00 | 0.000E+00 | 0.000E+00 | 0.000E+00 | 7.751E-07 | 0.000E+00 | 0.000E+00 | 0.000E+00 | 0.000E+00 | 0.000E+00 |
| Unigene518130 | Klebsiella p | 0.000E+00 | 0.000E+00 | 0.000E+00 | 0.000E+00 | 0.000E+00 | 0.000E+00 | 7.780E-07 | 0.000E+00 | 0.000E+00 | 0.000E+00 | 0.000E+00 | 0.000E+00 |
| Unigene518140 | macB | 0.000E+00 | 0.000E+00 | 0.000E+00 | 0.000E+00 | 0.000E+00 | 0.000E+00 | 4.228E-07 | 0.000E+00 | 0.000E+00 | 0.000E+00 | 0.000E+00 | 0.000E+00 |
| Unigene518162 | otr(B) | 0.000E+00 | 0.000E+00 | 1.231E-07 | 0.000E+00 | 0.000E+00 | 0.000E+00 | 9.721E-07 | 0.000E+00 | 0.000E+00 | 0.000E+00 | 0.000E+00 | 0.000E+00 |
| Unigene518182 | MexK | 0.000E+00 | 0.000E+00 | 0.000E+00 | 0.000E+00 | 0.000E+00 | 0.000E+00 | 6.665E-07 | 4.686E-07 | 0.000E+00 | 0.000E+00 | 0.000E+00 | 5.220E-07 |
| Unigene518255 | Streptomy | 0.000E+00 | 4.962E-08 | 0.000E+00 | 0.000E+00 | 0.000E+00 | 0.000E+00 | 8.506E-07 | 0.000E+00 | 0.000E+00 | 0.000E+00 | 0.000E+00 | 0.000E+00 |
| Unigene518258 | LpeB | 0.000E+00 | 0.000E+00 | 0.000E+00 | 0.000E+00 | 0.000E+00 | 0.000E+00 | 7.291E-07 | 0.000E+00 | 0.000E+00 | 0.000E+00 | 0.000E+00 | 0.000E+00 |
| Unigene518543 | cmlB | 0.000E+00 | 0.000E+00 | 2.352E-07 | 1.345E-07 | 2.367E-07 | 2.591E-07 | 8.548E-07 | 9.016E-07 | 0.000E+00 | 1.483E-07 | 5.484E-07 | 2.330E-06 |
| Unigene518554 | tetA(58) | 0.000E+00 | 0.000E+00 | 0.000E+00 | 0.000E+00 | 0.000E+00 | 0.000E+00 | 3.308E-07 | 3.987E-07 | 0.000E+00 | 0.000E+00 | 0.000E+00 | 5.047E-07 |
| Unigene518556 | mdsA | 0.000E+00 | 0.000E+00 | 0.000E+00 | 0.000E+00 | 2.471E-07 | 0.000E+00 | 8.924E-07 | 1.201E-06 | 0.000E+00 | 0.000E+00 | 0.000E+00 | 3.944E-07 |
| Unigene518640 | MuxC | 0.000E+00 | 9.155E-08 | 0.000E+00 | 1.123E-07 | 2.780E-07 | 3.006E-08 | 4.000E-07 | 4.219E-07 | 6.078E-08 | 0.000E+00 | 2.544E-07 | 3.944E-07 |
| Unigene518758 | efrA | 9.738E-08 | 0.000E+00 | 0.000E+00 | 0.000E+00 | 0.000E+00 | 0.000E+00 | 7.544E-07 | 0.000E+00 | 0.000E+00 | 0.000E+00 | 0.000E+00 | 8.633E-07 |
| Unigene518767 | tetO | 0.000E+00 | 0.000E+00 | 0.000E+00 | 0.000E+00 | 0.000E+00 | 0.000E+00 | 1.089E-06 | 1.220E-06 | 0.000E+00 | 0.000E+00 | 0.000E+00 | 2.907E-07 |
| Unigene518772 | tlrC | 2.161E-08 | 0.000E+00 | 0.000E+00 | 0.000E+00 | 2.400E-08 | 4.672E-08 | 9.565E-07 | 1.513E-07 | 0.000E+00 | 0.000E+00 | 0.000E+00 | 5.108E-08 |
| Unigene518780 | oleB | 0.000E+00 | 0.000E+00 | 0.000E+00 | 0.000E+00 | 0.000E+00 | 0.000E+00 | 7.131E-07 | 2.632E-07 | 0.000E+00 | 0.000E+00 | 0.000E+00 | 0.000E+00 |
| Unigene518831 | oleC | 0.000E+00 | 0.000E+00 | 0.000E+00 | 0.000E+00 | 0.000E+00 | 0.000E+00 | 6.895E-07 | 0.000E+00 | 0.000E+00 | 0.000E+00 | 0.000E+00 | 0.000E+00 |
| Unigene518967 | oleC | 0.000E+00 | 0.000E+00 | 0.000E+00 | 0.000E+00 | 0.000E+00 | 0.000E+00 | 6.593E-07 | 0.000E+00 | 0.000E+00 | 0.000E+00 | 0.000E+00 | 0.000E+00 |
| Unigene519056 | bcrA | 0.000E+00 | 0.000E+00 | 0.000E+00 | 0.000E+00 | 0.000E+00 | 0.000E+00 | 5.513E-07 | 0.000E+00 | 0.000E+00 | 0.000E+00 | 0.000E+00 | 0.000E+00 |
| Unigene519257 | Acinetoba | 0.000E+00 | 0.000E+00 | 0.000E+00 | 0.000E+00 | 1.626E-07 | 5.275E-08 | 8.371E-07 | 0.000E+00 | 0.000E+00 | 0.000E+00 | 0.000E+00 | 0.000E+00 |
| Unigene519358 | macB | 0.000E+00 | 0.000E+00 | 2.727E-07 | 0.000E+00 | 0.000E+00 | 0.000E+00 | 1.143E-06 | 0.000E+00 | 0.000E+00 | 0.000E+00 | 0.000E+00 | 0.000E+00 |
| Unigene519372 | tetB(60) | 0.000E+00 | 0.000E+00 | 0.000E+00 | 0.000E+00 | 0.000E+00 | 0.000E+00 | 8.506E-07 | 0.000E+00 | 0.000E+00 | 0.000E+00 | 0.000E+00 | 0.000E+00 |
| Unigene519408 | adeL | 0.000E+00 | 0.000E+00 | 1.408E-07 | 0.000E+00 | 0.000E+00 | 3.102E-07 | 6.351E-07 | 4.605E-07 | 0.000E+00 | 0.000E+00 | 0.000E+00 | 0.000E+00 |
| Unigene519409 | cmlv | 0.000E+00 | 0.000E+00 | 0.000E+00 | 0.000E+00 | 0.000E+00 | 2.067E-07 | 9.370E-07 | 1.594E-07 | 0.000E+00 | 0.000E+00 | 0.000E+00 | 1.614E-07 |
| Unigene519531 | adeL | 1.109E-07 | 0.000E+00 | 0.000E+00 | 0.000E+00 | 6.573E-07 | 0.000E+00 | 6.139E-07 | 3.021E-07 | 0.000E+00 | 0.000E+00 | 4.230E-07 | 4.808E-07 |
| Unigene519562 | arnA | 0.000E+00 | 1.495E-07 | 0.000E+00 | 0.000E+00 | 1.059E-06 | 0.000E+00 | 2.073E-06 | 9.539E-07 | 0.000E+00 | 0.000E+00 | 3.895E-07 | 0.000E+00 |
| Unigene519620 | tcr3 | 0.000E+00 | 0.000E+00 | 0.000E+00 | 0.000E+00 | 0.000E+00 | 0.000E+00 | 1.074E-06 | 0.000E+00 | 0.000E+00 | 0.000E+00 | 0.000E+00 | 0.000E+00 |
| Unigene519688 | arnA | 0.000E+00 | 0.000E+00 | 0.000E+00 | 0.000E+00 | 0.000E+00 | 0.000E+00 | 6.489E-07 | 0.000E+00 | 0.000E+00 | 0.000E+00 | 0.000E+00 | 0.000E+00 |

| | | | | | | | | | | | | | |
|---|---|---|---|---|---|---|---|---|---|---|---|---|---|
| Unigene519707 | tetT | 0.000E+00 | 0.000E+00 | 0.000E+00 | 2.462E-07 | 0.000E+00 | 0.000E+00 | 1.130E-06 | 0.000E+00 | 0.000E+00 | 0.000E+00 | 0.000E+00 | 0.000E+00 |
| Unigene519753 | PEDO-1 | 0.000E+00 | 0.000E+00 | 0.000E+00 | 0.000E+00 | 0.000E+00 | 0.000E+00 | 9.003E-07 | 0.000E+00 | 0.000E+00 | 0.000E+00 | 0.000E+00 | 4.371E-07 |
| Unigene519754 | novA | 0.000E+00 | 0.000E+00 | 0.000E+00 | 0.000E+00 | 0.000E+00 | 0.000E+00 | 1.414E-06 | 0.000E+00 | 0.000E+00 | 0.000E+00 | 0.000E+00 | 0.000E+00 |
| Unigene519786 | TaeA | 0.000E+00 | 0.000E+00 | 0.000E+00 | 0.000E+00 | 0.000E+00 | 0.000E+00 | 5.789E-07 | 0.000E+00 | 0.000E+00 | 0.000E+00 | 0.000E+00 | 0.000E+00 |
| Unigene519798 | Acinetobac | 0.000E+00 | 0.000E+00 | 0.000E+00 | 0.000E+00 | 0.000E+00 | 0.000E+00 | 4.043E-07 | 0.000E+00 | 0.000E+00 | 0.000E+00 | 0.000E+00 | 0.000E+00 |
| Unigene519852 | Brucella su | 0.000E+00 | 0.000E+00 | 0.000E+00 | 0.000E+00 | 0.000E+00 | 0.000E+00 | 1.116E-06 | 6.066E-07 | 0.000E+00 | 0.000E+00 | 0.000E+00 | 0.000E+00 |
| Unigene519878 | patA | 1.209E-07 | 0.000E+00 | 2.076E-07 | 3.357E-07 | 3.358E-07 | 1.960E-07 | 1.104E-06 | 8.820E-07 | 9.911E-08 | 1.010E-07 | 2.420E-07 | 1.072E-06 |
| Unigene519958 | patA | 0.000E+00 | 0.000E+00 | 0.000E+00 | 0.000E+00 | 0.000E+00 | 0.000E+00 | 7.284E-07 | 0.000E+00 | 0.000E+00 | 0.000E+00 | 0.000E+00 | 0.000E+00 |
| Unigene519963 | rosB | 0.000E+00 | 0.000E+00 | 0.000E+00 | 0.000E+00 | 0.000E+00 | 0.000E+00 | 7.312E-07 | 0.000E+00 | 0.000E+00 | 0.000E+00 | 0.000E+00 | 0.000E+00 |
| Unigene520042 | evgS | 0.000E+00 | 0.000E+00 | 0.000E+00 | 0.000E+00 | 0.000E+00 | 0.000E+00 | 5.077E-07 | 0.000E+00 | 0.000E+00 | 0.000E+00 | 0.000E+00 | 0.000E+00 |
| Unigene520071 | optrA | 0.000E+00 | 0.000E+00 | 0.000E+00 | 0.000E+00 | 0.000E+00 | 0.000E+00 | 1.145E-06 | 0.000E+00 | 0.000E+00 | 0.000E+00 | 0.000E+00 | 6.359E-07 |
| Unigene520146 | bcrA | 0.000E+00 | 0.000E+00 | 0.000E+00 | 0.000E+00 | 0.000E+00 | 0.000E+00 | 6.583E-07 | 0.000E+00 | 0.000E+00 | 0.000E+00 | 0.000E+00 | 0.000E+00 |
| Unigene520149 | tetA(60) | 4.836E-08 | 0.000E+00 | 4.745E-08 | 4.883E-08 | 3.492E-07 | 1.568E-07 | 1.097E-06 | 6.209E-07 | 0.000E+00 | 1.347E-07 | 5.255E-07 | 1.801E-06 |
| Unigene520173 | ykkD | 0.000E+00 | 0.000E+00 | 0.000E+00 | 0.000E+00 | 0.000E+00 | 0.000E+00 | 8.583E-07 | 0.000E+00 | 0.000E+00 | 0.000E+00 | 0.000E+00 | 0.000E+00 |
| Unigene520234 | oleC | 0.000E+00 | 0.000E+00 | 0.000E+00 | 0.000E+00 | 0.000E+00 | 0.000E+00 | 4.997E-07 | 0.000E+00 | 0.000E+00 | 0.000E+00 | 0.000E+00 | 1.779E-07 |
| Unigene520318 | carA | 0.000E+00 | 0.000E+00 | 0.000E+00 | 0.000E+00 | 0.000E+00 | 0.000E+00 | 7.419E-07 | 4.118E-07 | 6.169E-07 | 0.000E+00 | 0.000E+00 | 1.084E-06 |
| Unigene520394 | oleC | 0.000E+00 | 0.000E+00 | 0.000E+00 | 0.000E+00 | 0.000E+00 | 0.000E+00 | 9.782E-07 | 1.749E-06 | 0.000E+00 | 0.000E+00 | 0.000E+00 | 7.268E-07 |
| Unigene520516 | MexW | 0.000E+00 | 0.000E+00 | 0.000E+00 | 0.000E+00 | 0.000E+00 | 0.000E+00 | 4.580E-07 | 0.000E+00 | 0.000E+00 | 0.000E+00 | 0.000E+00 | 0.000E+00 |
| Unigene520591 | tetB(46) | 0.000E+00 | 0.000E+00 | 0.000E+00 | 0.000E+00 | 0.000E+00 | 0.000E+00 | 6.683E-07 | 0.000E+00 | 0.000E+00 | 0.000E+00 | 0.000E+00 | 9.085E-07 |
| Unigene520677 | facT | 0.000E+00 | 0.000E+00 | 0.000E+00 | 0.000E+00 | 0.000E+00 | 0.000E+00 | 5.539E-07 | 0.000E+00 | 0.000E+00 | 0.000E+00 | 0.000E+00 | 2.218E-07 |
| Unigene520691 | MexL | 4.598E-08 | 0.000E+00 | 2.256E-07 | 0.000E+00 | 0.000E+00 | 1.491E-07 | 1.476E-06 | 1.288E-06 | 0.000E+00 | 3.073E-07 | 3.682E-07 | 1.957E-06 |
| Unigene520700 | TaeA | 0.000E+00 | 0.000E+00 | 0.000E+00 | 0.000E+00 | 0.000E+00 | 0.000E+00 | 0.000E+00 | 0.000E+00 | 0.000E+00 | 0.000E+00 | 5.152E-07 | 1.301E-06 |
| Unigene520705 | acrB | 0.000E+00 | 0.000E+00 | 0.000E+00 | 0.000E+00 | 0.000E+00 | 0.000E+00 | 0.000E+00 | 0.000E+00 | 3.564E-07 | 0.000E+00 | 0.000E+00 | 0.000E+00 |
| Unigene520736 | bacA | 0.000E+00 | 0.000E+00 | 0.000E+00 | 0.000E+00 | 0.000E+00 | 0.000E+00 | 5.261E-07 | 0.000E+00 | 0.000E+00 | 0.000E+00 | 0.000E+00 | 0.000E+00 |
| Unigene520765 | NmcR | 0.000E+00 | 0.000E+00 | 1.417E-07 | 3.646E-08 | 0.000E+00 | 0.000E+00 | 1.079E-06 | 1.264E-07 | 0.000E+00 | 0.000E+00 | 1.652E-07 | 3.414E-07 |
| Unigene520844 | Acinetobac | 0.000E+00 | 0.000E+00 | 9.642E-08 | 1.488E-07 | 3.002E-07 | 1.328E-07 | 6.525E-07 | 7.456E-07 | 0.000E+00 | 0.000E+00 | 8.430E-08 | 7.840E-07 |
| Unigene520861 | optrA | 0.000E+00 | 1.014E-06 | 0.000E+00 | 0.000E+00 | 0.000E+00 | 0.000E+00 | 1.909E-06 | 0.000E+00 | 4.489E-08 | 0.000E+00 | 0.000E+00 | 0.000E+00 |
| Unigene520872 | novA | 0.000E+00 | 0.000E+00 | 0.000E+00 | 4.873E-08 | 0.000E+00 | 0.000E+00 | 5.340E-07 | 3.379E-07 | 0.000E+00 | 0.000E+00 | 0.000E+00 | 0.000E+00 |
| Unigene520906 | PmrF | 0.000E+00 | 0.000E+00 | 0.000E+00 | 0.000E+00 | 0.000E+00 | 0.000E+00 | 3.781E-06 | 2.572E-06 | 0.000E+00 | 0.000E+00 | 0.000E+00 | 2.642E-06 |
| Unigene520913 | carA | 0.000E+00 | 0.000E+00 | 0.000E+00 | 0.000E+00 | 0.000E+00 | 4.634E-08 | 4.270E-07 | 2.002E-07 | 0.000E+00 | 0.000E+00 | 0.000E+00 | 5.574E-07 |
| Unigene520936 | macB | 0.000E+00 | 0.000E+00 | 0.000E+00 | 0.000E+00 | 0.000E+00 | 0.000E+00 | 7.960E-07 | 0.000E+00 | 0.000E+00 | 0.000E+00 | 0.000E+00 | 0.000E+00 |
| Unigene521032 | cpxA | 0.000E+00 | 0.000E+00 | 0.000E+00 | 1.721E-07 | 4.327E-07 | 0.000E+00 | 7.005E-07 | 7.956E-07 | 0.000E+00 | 2.441E-07 | 0.000E+00 | 9.784E-07 |
| Unigene521043 | vanTG | 0.000E+00 | 0.000E+00 | 0.000E+00 | 0.000E+00 | 0.000E+00 | 0.000E+00 | 3.842E-07 | 0.000E+00 | 0.000E+00 | 0.000E+00 | 0.000E+00 | 0.000E+00 |
| Unigene521046 | vatF | 0.000E+00 | 0.000E+00 | 0.000E+00 | 0.000E+00 | 0.000E+00 | 0.000E+00 | 7.069E-07 | 0.000E+00 | 0.000E+00 | 0.000E+00 | 0.000E+00 | 6.388E-07 |
| Unigene521050 | oleB | 0.000E+00 | 0.000E+00 | 0.000E+00 | 0.000E+00 | 0.000E+00 | 0.000E+00 | 0.000E+00 | 0.000E+00 | 0.000E+00 | 0.000E+00 | 0.000E+00 | 3.634E-07 |
| Unigene521079 | baeR | 0.000E+00 | 0.000E+00 | 0.000E+00 | 0.000E+00 | 0.000E+00 | 0.000E+00 | 6.731E-07 | 0.000E+00 | 0.000E+00 | 0.000E+00 | 0.000E+00 | 0.000E+00 |
| Unigene521201 | msbA | 0.000E+00 | 0.000E+00 | 2.195E-08 | 0.000E+00 | 1.740E-07 | 0.000E+00 | 5.199E-07 | 0.000E+00 | 0.000E+00 | 0.000E+00 | 0.000E+00 | 0.000E+00 |
| Unigene521269 | poxtA | 0.000E+00 | 0.000E+00 | 0.000E+00 | 0.000E+00 | 0.000E+00 | 3.116E-07 | 1.063E-06 | 0.000E+00 | 0.000E+00 | 0.000E+00 | 5.495E-07 | 0.000E+00 |
| Unigene521322 | OprN | 0.000E+00 | 0.000E+00 | 0.000E+00 | 0.000E+00 | 6.474E-07 | 6.300E-07 | 3.409E-06 | 2.211E-06 | 3.412E-07 | 8.810E-07 | 1.024E-06 | 2.263E-06 |
| Unigene521430 | macB | 2.832E-07 | 1.776E-07 | 1.191E-07 | 0.000E+00 | 0.000E+00 | 0.000E+00 | 9.402E-07 | 0.000E+00 | 0.000E+00 | 0.000E+00 | 0.000E+00 | 4.781E-07 |
| Unigene521480 | smeS | 0.000E+00 | 0.000E+00 | 2.005E-07 | 0.000E+00 | 0.000E+00 | 3.190E-07 | 6.281E-07 | 6.624E-07 | 0.000E+00 | 0.000E+00 | 4.155E-07 | 2.952E-07 |
| Unigene521711 | mtrA | 0.000E+00 | 0.000E+00 | 0.000E+00 | 0.000E+00 | 0.000E+00 | 0.000E+00 | 5.104E-07 | 0.000E+00 | 0.000E+00 | 0.000E+00 | 0.000E+00 | 0.000E+00 |
| Unigene521714 | mdtA | 1.284E-07 | 0.000E+00 | 5.040E-08 | 0.000E+00 | 6.562E-07 | 0.000E+00 | 9.379E-07 | 8.093E-07 | 0.000E+00 | 0.000E+00 | 6.463E-07 | 8.499E-07 |
| Unigene521727 | msrA | 0.000E+00 | 0.000E+00 | 2.062E-07 | 0.000E+00 | 6.537E-07 | 0.000E+00 | 1.489E-06 | 2.404E-06 | 0.000E+00 | 0.000E+00 | 0.000E+00 | 2.087E-06 |
| Unigene521730 | patA | 0.000E+00 | 3.181E-06 | 0.000E+00 | 0.000E+00 | 4.782E-06 | 0.000E+00 | 3.208E-06 | 0.000E+00 | 0.000E+00 | 0.000E+00 | 1.105E-06 | 0.000E+00 |
| Unigene521793 | tetA(58) | 8.406E-08 | 4.613E-08 | 4.124E-08 | 3.396E-07 | 3.736E-07 | 1.363E-07 | 1.070E-06 | 6.869E-07 | 0.000E+00 | 0.000E+00 | 7.693E-07 | 3.378E-06 |
| Unigene521814 | tlrC | 0.000E+00 | 0.000E+00 | 0.000E+00 | 0.000E+00 | 5.742E-07 | 1.016E-07 | 1.040E-06 | 1.316E-06 | 5.136E-08 | 0.000E+00 | 0.000E+00 | 1.222E-06 |
| Unigene521859 | vanSG | 0.000E+00 | 0.000E+00 | 0.000E+00 | 1.144E-07 | 2.517E-07 | 0.000E+00 | 9.778E-07 | 1.428E-06 | 0.000E+00 | 0.000E+00 | 0.000E+00 | 8.033E-07 |
| Unigene522001 | macB | 0.000E+00 | 1.429E-07 | 0.000E+00 | 0.000E+00 | 0.000E+00 | 0.000E+00 | 1.873E-06 | 2.026E-06 | 0.000E+00 | 0.000E+00 | 7.445E-07 | 4.564E-06 |
| Unigene522368 | cmlv | 0.000E+00 | 0.000E+00 | 0.000E+00 | 2.696E-08 | 3.856E-07 | 0.000E+00 | 8.570E-07 | 4.986E-07 | 0.000E+00 | 0.000E+00 | 2.443E-07 | 1.262E-06 |
| Unigene522389 | oleB | 2.720E-07 | 1.360E-06 | 0.000E+00 | 0.000E+00 | 0.000E+00 | 3.921E-07 | 1.706E-06 | 0.000E+00 | 0.000E+00 | 0.000E+00 | 0.000E+00 | 0.000E+00 |
| Unigene522432 | TaeA | 0.000E+00 | 0.000E+00 | 0.000E+00 | 0.000E+00 | 3.043E-07 | 0.000E+00 | 6.496E-07 | 7.307E-07 | 0.000E+00 | 0.000E+00 | 0.000E+00 | 4.162E-07 |
| Unigene522458 | rosA | 0.000E+00 | 0.000E+00 | 0.000E+00 | 0.000E+00 | 0.000E+00 | 0.000E+00 | 8.093E-07 | 2.439E-07 | 0.000E+00 | 0.000E+00 | 0.000E+00 | 4.939E-07 |
| Unigene522705 | cpxA | 0.000E+00 | 0.000E+00 | 0.000E+00 | 1.449E-07 | 0.000E+00 | 0.000E+00 | 1.111E-06 | 0.000E+00 | 0.000E+00 | 0.000E+00 | 0.000E+00 | 0.000E+00 |
| Unigene522718 | lmrC | 0.000E+00 | 0.000E+00 | 0.000E+00 | 2.114E-07 | 0.000E+00 | 0.000E+00 | 7.414E-07 | 0.000E+00 | 0.000E+00 | 0.000E+00 | 0.000E+00 | 0.000E+00 |
| Unigene522758 | tetA(58) | 0.000E+00 | 0.000E+00 | 3.641E-08 | 0.000E+00 | 0.000E+00 | 0.000E+00 | 2.033E-06 | 0.000E+00 | 0.000E+00 | 0.000E+00 | 0.000E+00 | 0.000E+00 |
| Unigene522762 | Brucella su | 0.000E+00 | 0.000E+00 | 0.000E+00 | 0.000E+00 | 0.000E+00 | 0.000E+00 | 1.960E-06 | 0.000E+00 | 0.000E+00 | 0.000E+00 | 0.000E+00 | 0.000E+00 |
| Unigene522803 | tetA(58) | 0.000E+00 | 0.000E+00 | 1.453E-07 | 0.000E+00 | 0.000E+00 | 0.000E+00 | 1.311E-06 | 0.000E+00 | 0.000E+00 | 0.000E+00 | 3.388E-07 | 0.000E+00 |

| | | | | | | | | | | | | | |
|---|---|---|---|---|---|---|---|---|---|---|---|---|---|
| Unigene522892 | mtrA | 5.249E-08 | 0.000E+00 | 0.000E+00 | 0.000E+00 | 0.000E+00 | 0.000E+00 | 4.647E-07 | 0.000E+00 | 0.000E+00 | 0.000E+00 | 0.000E+00 | 0.000E+00 |
| Unigene522909 | iri | 3.643E-07 | 0.000E+00 | 2.750E-07 | 1.981E-07 | 2.802E-07 | 5.755E-07 | 1.116E-06 | 2.813E-06 | 0.000E+00 | 6.867E-07 | 3.205E-07 | 1.457E-06 |
| Unigene523122 | Acinetoba | 1.145E-07 | 2.513E-08 | 1.348E-07 | 0.000E+00 | 2.289E-07 | 9.900E-08 | 9.122E-07 | 6.146E-07 | 5.004E-08 | 1.785E-07 | 8.118E-07 | 1.326E-06 |
| Unigene523170 | Streptomy | 0.000E+00 | 0.000E+00 | 0.000E+00 | 0.000E+00 | 0.000E+00 | 0.000E+00 | 4.530E-07 | 4.096E-07 | 0.000E+00 | 0.000E+00 | 0.000E+00 | 0.000E+00 |
| Unigene523288 | adeB | 0.000E+00 | 1.224E-07 | 0.000E+00 | 0.000E+00 | 0.000E+00 | 0.000E+00 | 3.702E-07 | 0.000E+00 | 0.000E+00 | 0.000E+00 | 0.000E+00 | 4.613E-07 |
| Unigene523387 | catB2 | 0.000E+00 | 0.000E+00 | 0.000E+00 | 0.000E+00 | 0.000E+00 | 0.000E+00 | 6.854E-07 | 0.000E+00 | 0.000E+00 | 0.000E+00 | 0.000E+00 | 0.000E+00 |
| Unigene523411 | Staphylocc | 0.000E+00 | 0.000E+00 | 0.000E+00 | 0.000E+00 | 1.905E-07 | 0.000E+00 | 0.000E+00 | 0.000E+00 | 0.000E+00 | 2.865E-07 | 0.000E+00 | 1.520E-07 |
| Unigene523416 | smeR | 0.000E+00 | 0.000E+00 | 0.000E+00 | 0.000E+00 | 0.000E+00 | 0.000E+00 | 4.253E-07 | 0.000E+00 | 0.000E+00 | 0.000E+00 | 0.000E+00 | 0.000E+00 |
| Unigene523446 | tet36 | 0.000E+00 | 0.000E+00 | 0.000E+00 | 0.000E+00 | 0.000E+00 | 0.000E+00 | 7.908E-07 | 0.000E+00 | 0.000E+00 | 0.000E+00 | 0.000E+00 | 0.000E+00 |
| Unigene523447 | Corynebac | 0.000E+00 | 0.000E+00 | 0.000E+00 | 0.000E+00 | 8.263E-07 | 0.000E+00 | 1.043E-06 | 0.000E+00 | 0.000E+00 | 0.000E+00 | 2.269E-07 | 9.378E-07 |
| Unigene523455 | AAC(6')-IIt | 0.000E+00 | 0.000E+00 | 0.000E+00 | 0.000E+00 | 0.000E+00 | 0.000E+00 | 7.051E-07 | 0.000E+00 | 0.000E+00 | 0.000E+00 | 0.000E+00 | 0.000E+00 |
| Unigene523465 | basS | 0.000E+00 | 0.000E+00 | 0.000E+00 | 5.659E-08 | 0.000E+00 | 0.000E+00 | 0.000E+00 | 0.000E+00 | 0.000E+00 | 0.000E+00 | 1.923E-07 | 1.325E-07 |
| Unigene523494 | Bifidobacte | 3.647E-07 | 0.000E+00 | 2.684E-07 | 0.000E+00 | 4.812E-07 | 0.000E+00 | 1.186E-06 | 6.120E-07 | 7.475E-07 | 0.000E+00 | 1.565E-06 | 1.671E-06 |
| Unigene523529 | tetB(60) | 0.000E+00 | 0.000E+00 | 0.000E+00 | 0.000E+00 | 0.000E+00 | 0.000E+00 | 3.587E-07 | 0.000E+00 | 0.000E+00 | 0.000E+00 | 0.000E+00 | 3.831E-07 |
| Unigene523555 | msbA | 0.000E+00 | 0.000E+00 | 8.060E-08 | 0.000E+00 | 1.141E-07 | 0.000E+00 | 7.045E-07 | 1.222E-06 | 0.000E+00 | 0.000E+00 | 0.000E+00 | 5.583E-07 |
| Unigene523608 | baeS | 0.000E+00 | 0.000E+00 | 0.000E+00 | 0.000E+00 | 0.000E+00 | 0.000E+00 | 4.625E-07 | 0.000E+00 | 0.000E+00 | 0.000E+00 | 0.000E+00 | 2.470E-07 |
| Unigene523629 | rpoB2 | 0.000E+00 | 0.000E+00 | 0.000E+00 | 0.000E+00 | 0.000E+00 | 0.000E+00 | 1.017E-06 | 0.000E+00 | 0.000E+00 | 0.000E+00 | 0.000E+00 | 0.000E+00 |
| Unigene523680 | MexK | 0.000E+00 | 0.000E+00 | 1.106E-07 | 0.000E+00 | 0.000E+00 | 0.000E+00 | 0.000E+00 | 4.603E-07 | 0.000E+00 | 0.000E+00 | 0.000E+00 | 0.000E+00 |
| Unigene523731 | tetB(58) | 0.000E+00 | 0.000E+00 | 0.000E+00 | 0.000E+00 | 0.000E+00 | 0.000E+00 | 6.081E-07 | 0.000E+00 | 0.000E+00 | 0.000E+00 | 0.000E+00 | 0.000E+00 |
| Unigene523880 | Acinetoba | 0.000E+00 | 0.000E+00 | 0.000E+00 | 0.000E+00 | 0.000E+00 | 0.000E+00 | 9.130E-07 | 0.000E+00 | 0.000E+00 | 0.000E+00 | 0.000E+00 | 0.000E+00 |
| Unigene523917 | TaeA | 0.000E+00 | 0.000E+00 | 0.000E+00 | 0.000E+00 | 0.000E+00 | 0.000E+00 | 2.586E-06 | 1.254E-06 | 0.000E+00 | 0.000E+00 | 6.632E-07 | 0.000E+00 |
| Unigene523918 | LlmA 23S r | 0.000E+00 | 0.000E+00 | 0.000E+00 | 0.000E+00 | 0.000E+00 | 2.978E-07 | 2.473E-06 | 0.000E+00 | 0.000E+00 | 0.000E+00 | 0.000E+00 | 6.875E-07 |
| Unigene523980 | oleB | 0.000E+00 | 0.000E+00 | 0.000E+00 | 0.000E+00 | 3.065E-07 | 0.000E+00 | 1.221E-06 | 3.220E-07 | 0.000E+00 | 0.000E+00 | 4.208E-07 | 0.000E+00 |
| Unigene523993 | adeK | 0.000E+00 | 0.000E+00 | 0.000E+00 | 0.000E+00 | 0.000E+00 | 0.000E+00 | 7.581E-07 | 2.579E-08 | 0.000E+00 | 0.000E+00 | 0.000E+00 | 0.000E+00 |
| Unigene524049 | adeL | 0.000E+00 | 0.000E+00 | 0.000E+00 | 0.000E+00 | 0.000E+00 | 0.000E+00 | 5.915E-07 | 0.000E+00 | 0.000E+00 | 0.000E+00 | 0.000E+00 | 0.000E+00 |
| Unigene524083 | bcrA | 0.000E+00 | 5.817E-08 | 1.820E-07 | 1.338E-07 | 0.000E+00 | 3.152E-07 | 1.467E-06 | 1.268E-06 | 0.000E+00 | 0.000E+00 | 1.213E-06 | 2.631E-06 |
| Unigene524093 | macB | 0.000E+00 | 0.000E+00 | 0.000E+00 | 0.000E+00 | 0.000E+00 | 0.000E+00 | 6.537E-07 | 0.000E+00 | 0.000E+00 | 0.000E+00 | 0.000E+00 | 0.000E+00 |
| Unigene524243 | tetA(60) | 0.000E+00 | 0.000E+00 | 0.000E+00 | 0.000E+00 | 0.000E+00 | 0.000E+00 | 6.007E-07 | 0.000E+00 | 0.000E+00 | 0.000E+00 | 0.000E+00 | 4.529E-07 |
| Unigene524541 | Klebsiella p | 0.000E+00 | 0.000E+00 | 0.000E+00 | 0.000E+00 | 0.000E+00 | 0.000E+00 | 5.316E-07 | 0.000E+00 | 0.000E+00 | 0.000E+00 | 0.000E+00 | 0.000E+00 |
| Unigene524572 | tetA(60) | 0.000E+00 | 0.000E+00 | 0.000E+00 | 0.000E+00 | 0.000E+00 | 0.000E+00 | 4.235E-06 | 1.997E-06 | 4.429E-07 | 0.000E+00 | 0.000E+00 | 2.395E-06 |
| Unigene524599 | SPG-1 | 0.000E+00 | 0.000E+00 | 0.000E+00 | 0.000E+00 | 0.000E+00 | 0.000E+00 | 1.197E-06 | 0.000E+00 | 0.000E+00 | 0.000E+00 | 0.000E+00 | 0.000E+00 |
| Unigene524652 | efrB | 0.000E+00 | 0.000E+00 | 0.000E+00 | 0.000E+00 | 0.000E+00 | 0.000E+00 | 4.625E-07 | 0.000E+00 | 0.000E+00 | 0.000E+00 | 0.000E+00 | 0.000E+00 |
| Unigene524658 | PmrF | 0.000E+00 | 0.000E+00 | 2.418E-07 | 0.000E+00 | 0.000E+00 | 1.332E-06 | 3.454E-06 | 0.000E+00 | 0.000E+00 | 0.000E+00 | 0.000E+00 | 0.000E+00 |
| Unigene524674 | Streptomy | 0.000E+00 | 0.000E+00 | 0.000E+00 | 0.000E+00 | 0.000E+00 | 0.000E+00 | 3.370E-07 | 2.370E-07 | 0.000E+00 | 0.000E+00 | 0.000E+00 | 1.200E-07 |
| Unigene524688 | tetB(P) | 0.000E+00 | 0.000E+00 | 0.000E+00 | 0.000E+00 | 1.292E-07 | 0.000E+00 | 1.223E-06 | 0.000E+00 | 2.331E-07 | 0.000E+00 | 0.000E+00 | 0.000E+00 |
| Unigene524761 | Klebsiella p | 6.987E-08 | 2.301E-07 | 1.714E-07 | 1.058E-07 | 2.717E-07 | 7.553E-08 | 5.413E-07 | 0.000E+00 | 0.000E+00 | 7.783E-08 | 3.597E-07 | 2.065E-07 |
| Unigene524820 | carA | 0.000E+00 | 0.000E+00 | 0.000E+00 | 0.000E+00 | 0.000E+00 | 0.000E+00 | 1.241E-06 | 0.000E+00 | 0.000E+00 | 0.000E+00 | 4.752E-08 | 0.000E+00 |
| Unigene524937 | MexK | 0.000E+00 | 0.000E+00 | 2.091E-07 | 0.000E+00 | 0.000E+00 | 0.000E+00 | 7.074E-07 | 6.839E-07 | 0.000E+00 | 0.000E+00 | 3.047E-07 | 4.407E-07 |
| Unigene524943 | aadA9 | 0.000E+00 | 0.000E+00 | 0.000E+00 | 0.000E+00 | 0.000E+00 | 0.000E+00 | 2.922E-07 | 0.000E+00 | 0.000E+00 | 0.000E+00 | 0.000E+00 | 0.000E+00 |
| Unigene525167 | baeR | 0.000E+00 | 0.000E+00 | 0.000E+00 | 0.000E+00 | 0.000E+00 | 1.939E-07 | 0.000E+00 | 0.000E+00 | 0.000E+00 | 0.000E+00 | 0.000E+00 | 1.166E-06 |
| Unigene525264 | vgaALC | 0.000E+00 | 0.000E+00 | 0.000E+00 | 0.000E+00 | 0.000E+00 | 0.000E+00 | 0.000E+00 | 4.454E-07 | 0.000E+00 | 0.000E+00 | 0.000E+00 | 0.000E+00 |
| Unigene525278 | macB | 0.000E+00 | 0.000E+00 | 0.000E+00 | 0.000E+00 | 2.939E-07 | 0.000E+00 | 5.857E-07 | 0.000E+00 | 0.000E+00 | 0.000E+00 | 0.000E+00 | 0.000E+00 |
| Unigene525356 | bcrA | 0.000E+00 | 0.000E+00 | 0.000E+00 | 0.000E+00 | 0.000E+00 | 0.000E+00 | 1.232E-06 | 1.462E-06 | 0.000E+00 | 0.000E+00 | 0.000E+00 | 2.193E-06 |
| Unigene525429 | Corynebac | 0.000E+00 | 0.000E+00 | 0.000E+00 | 0.000E+00 | 0.000E+00 | 0.000E+00 | 4.602E-07 | 0.000E+00 | 0.000E+00 | 0.000E+00 | 0.000E+00 | 0.000E+00 |
| Unigene525444 | vanTG | 0.000E+00 | 0.000E+00 | 0.000E+00 | 0.000E+00 | 0.000E+00 | 0.000E+00 | 6.462E-07 | 0.000E+00 | 0.000E+00 | 0.000E+00 | 0.000E+00 | 0.000E+00 |
| Unigene525506 | catB2 | 0.000E+00 | 0.000E+00 | 0.000E+00 | 0.000E+00 | 0.000E+00 | 0.000E+00 | 1.161E-06 | 0.000E+00 | 0.000E+00 | 0.000E+00 | 0.000E+00 | 0.000E+00 |
| Unigene525553 | evgS | 0.000E+00 | 0.000E+00 | 0.000E+00 | 0.000E+00 | 0.000E+00 | 0.000E+00 | 7.341E-07 | 0.000E+00 | 0.000E+00 | 0.000E+00 | 0.000E+00 | 0.000E+00 |
| Unigene525594 | fexA | 2.677E-08 | 0.000E+00 | 0.000E+00 | 0.000E+00 | 2.082E-07 | 2.025E-07 | 6.813E-07 | 5.936E-07 | 0.000E+00 | 0.000E+00 | 4.593E-07 | 9.808E-07 |
| Unigene525634 | efrA | 0.000E+00 | 0.000E+00 | 2.206E-07 | 9.731E-08 | 2.498E-07 | 6.250E-07 | 1.671E-06 | 3.374E-07 | 0.000E+00 | 0.000E+00 | 1.837E-07 | 2.658E-07 |
| Unigene525647 | ugd | 0.000E+00 | 0.000E+00 | 8.143E-08 | 0.000E+00 | 2.458E-07 | 0.000E+00 | 7.960E-07 | 6.780E-07 | 0.000E+00 | 0.000E+00 | 1.582E-07 | 6.212E-07 |
| Unigene525694 | bcrA | 1.060E-07 | 0.000E+00 | 0.000E+00 | 0.000E+00 | 0.000E+00 | 0.000E+00 | 6.453E-07 | 4.331E-07 | 0.000E+00 | 1.771E-07 | 3.638E-07 | 5.012E-07 |
| Unigene525707 | oleB | 0.000E+00 | 0.000E+00 | 1.602E-06 | 0.000E+00 | 0.000E+00 | 0.000E+00 | 3.105E-06 | 0.000E+00 | 0.000E+00 | 0.000E+00 | 0.000E+00 | 0.000E+00 |
| Unigene525841 | patA | 0.000E+00 | 0.000E+00 | 0.000E+00 | 8.358E-08 | 0.000E+00 | 0.000E+00 | 1.282E-06 | 7.246E-07 | 0.000E+00 | 2.766E-07 | 8.995E-07 | 2.837E-06 |
| Unigene525898 | efrA | 0.000E+00 | 0.000E+00 | 0.000E+00 | 0.000E+00 | 3.503E-07 | 0.000E+00 | 5.235E-07 | 0.000E+00 | 0.000E+00 | 0.000E+00 | 4.509E-07 | 0.000E+00 |
| Unigene525933 | Rm3 | 0.000E+00 | 0.000E+00 | 0.000E+00 | 0.000E+00 | 0.000E+00 | 0.000E+00 | 5.582E-07 | 0.000E+00 | 0.000E+00 | 0.000E+00 | 0.000E+00 | 0.000E+00 |
| Unigene525957 | vanHA | 0.000E+00 | 0.000E+00 | 0.000E+00 | 0.000E+00 | 0.000E+00 | 0.000E+00 | 7.609E-07 | 0.000E+00 | 0.000E+00 | 0.000E+00 | 0.000E+00 | 0.000E+00 |
| Unigene526023 | adeL | 0.000E+00 | 0.000E+00 | 0.000E+00 | 0.000E+00 | 1.599E-07 | 1.945E-07 | 3.983E-07 | 1.470E-06 | 0.000E+00 | 0.000E+00 | 5.351E-07 | 5.955E-07 |
| Unigene526090 | RlmA(II) | 0.000E+00 | 0.000E+00 | 0.000E+00 | 0.000E+00 | 0.000E+00 | 0.000E+00 | 5.954E-07 | 0.000E+00 | 0.000E+00 | 0.000E+00 | 0.000E+00 | 0.000E+00 |

| | | | | | | | | | | | | | |
|---|---|---|---|---|---|---|---|---|---|---|---|---|---|
| Unigene526100 | Enterobact | 0.000E+00 | 0.000E+00 | 0.000E+00 | 0.000E+00 | 0.000E+00 | 0.000E+00 | 4.444E-07 | 0.000E+00 | 0.000E+00 | 0.000E+00 | 0.000E+00 | 0.000E+00 |
| Unigene526103 | smeE | 0.000E+00 | 0.000E+00 | 0.000E+00 | 0.000E+00 | 0.000E+00 | 0.000E+00 | 9.996E-07 | 0.000E+00 | 0.000E+00 | 0.000E+00 | 0.000E+00 | 0.000E+00 |
| Unigene526206 | efrA | 0.000E+00 | 0.000E+00 | 0.000E+00 | 0.000E+00 | 0.000E+00 | 0.000E+00 | 0.000E+00 | 0.000E+00 | 0.000E+00 | 0.000E+00 | 1.388E-07 | 1.052E-06 |
| Unigene526235 | novA | 0.000E+00 | 0.000E+00 | 0.000E+00 | 0.000E+00 | 0.000E+00 | 0.000E+00 | 1.466E-06 | 0.000E+00 | 0.000E+00 | 0.000E+00 | 0.000E+00 | 0.000E+00 |
| Unigene526252 | adeG | 8.320E-08 | 0.000E+00 | 8.164E-08 | 1.400E-07 | 0.000E+00 | 0.000E+00 | 1.228E-06 | 1.457E-06 | 0.000E+00 | 0.000E+00 | 3.172E-07 | 7.539E-07 |
| Unigene526296 | cmlv | 0.000E+00 | 0.000E+00 | 0.000E+00 | 0.000E+00 | 0.000E+00 | 0.000E+00 | 6.517E-07 | 0.000E+00 | 0.000E+00 | 0.000E+00 | 0.000E+00 | 0.000E+00 |
| Unigene526365 | LlmA 23S r | 0.000E+00 | 0.000E+00 | 2.626E-07 | 1.351E-07 | 0.000E+00 | 2.894E-07 | 1.926E-06 | 1.281E-06 | 0.000E+00 | 4.174E-07 | 0.000E+00 | 1.266E-06 |
| Unigene526379 | bcrA | 0.000E+00 | 0.000E+00 | 0.000E+00 | 0.000E+00 | 0.000E+00 | 0.000E+00 | 7.733E-07 | 0.000E+00 | 0.000E+00 | 0.000E+00 | 0.000E+00 | 0.000E+00 |
| Unigene526380 | poxtA | 0.000E+00 | 0.000E+00 | 0.000E+00 | 3.169E-07 | 0.000E+00 | 0.000E+00 | 8.435E-07 | 0.000E+00 | 0.000E+00 | 0.000E+00 | 0.000E+00 | 5.299E-08 |
| Unigene526487 | efrA | 0.000E+00 | 0.000E+00 | 0.000E+00 | 0.000E+00 | 0.000E+00 | 0.000E+00 | 9.896E-07 | 1.148E-06 | 0.000E+00 | 3.984E-07 | 6.478E-07 | 1.127E-06 |
| Unigene526529 | macB | 0.000E+00 | 0.000E+00 | 5.650E-07 | 0.000E+00 | 1.119E-06 | 1.945E-07 | 1.673E-06 | 8.401E-07 | 0.000E+00 | 0.000E+00 | 1.111E-06 | 3.743E-06 |
| Unigene526541 | macB | 0.000E+00 | 0.000E+00 | 4.527E-07 | 2.496E-07 | 8.421E-07 | 2.850E-07 | 6.930E-07 | 0.000E+00 | 1.441E-07 | 0.000E+00 | 0.000E+00 | 1.831E-06 |
| Unigene526552 | MuxC | 8.222E-08 | 0.000E+00 | 1.513E-07 | 2.491E-07 | 8.677E-07 | 2.666E-07 | 1.092E-06 | 1.068E-06 | 0.000E+00 | 1.259E-07 | 4.467E-07 | 1.203E-06 |
| Unigene526582 | adeB | 0.000E+00 | 0.000E+00 | 0.000E+00 | 0.000E+00 | 0.000E+00 | 0.000E+00 | 5.954E-07 | 0.000E+00 | 0.000E+00 | 0.000E+00 | 0.000E+00 | 5.781E-07 |
| Unigene526587 | MexW | 0.000E+00 | 0.000E+00 | 0.000E+00 | 0.000E+00 | 0.000E+00 | 0.000E+00 | 5.931E-07 | 0.000E+00 | 0.000E+00 | 0.000E+00 | 0.000E+00 | 4.054E-07 |
| Unigene526667 | Acinetoba | 0.000E+00 | 0.000E+00 | 1.184E-07 | 2.436E-08 | 0.000E+00 | 3.651E-07 | 1.869E-06 | 8.448E-07 | 0.000E+00 | 1.613E-07 | 3.864E-07 | 0.000E+00 |
| Unigene526715 | macB | 0.000E+00 | 0.000E+00 | 0.000E+00 | 0.000E+00 | 0.000E+00 | 0.000E+00 | 7.939E-07 | 0.000E+00 | 0.000E+00 | 0.000E+00 | 0.000E+00 | 0.000E+00 |
| Unigene526759 | patA | 0.000E+00 | 0.000E+00 | 0.000E+00 | 0.000E+00 | 0.000E+00 | 0.000E+00 | 7.561E-07 | 0.000E+00 | 0.000E+00 | 0.000E+00 | 0.000E+00 | 4.711E-07 |
| Unigene526814 | vanG | 0.000E+00 | 0.000E+00 | 0.000E+00 | 0.000E+00 | 0.000E+00 | 0.000E+00 | 1.057E-06 | 0.000E+00 | 0.000E+00 | 0.000E+00 | 0.000E+00 | 0.000E+00 |
| Unigene526843 | oleC | 0.000E+00 | 0.000E+00 | 0.000E+00 | 2.369E-07 | 0.000E+00 | 0.000E+00 | 9.275E-07 | 0.000E+00 | 0.000E+00 | 0.000E+00 | 0.000E+00 | 3.566E-07 |
| Unigene526847 | carA | 0.000E+00 | 0.000E+00 | 0.000E+00 | 0.000E+00 | 0.000E+00 | 0.000E+00 | 0.000E+00 | 2.512E-07 | 0.000E+00 | 0.000E+00 | 0.000E+00 | 0.000E+00 |
| Unigene526865 | bcr-1 | 1.027E-07 | 2.819E-08 | 0.000E+00 | 7.780E-08 | 3.138E-07 | 8.328E-08 | 8.527E-07 | 4.496E-07 | 1.403E-07 | 4.005E-07 | 4.994E-07 | 5.160E-07 |
| Unigene527155 | bcrA | 0.000E+00 | 0.000E+00 | 0.000E+00 | 0.000E+00 | 0.000E+00 | 0.000E+00 | 4.787E-07 | 0.000E+00 | 0.000E+00 | 0.000E+00 | 0.000E+00 | 0.000E+00 |
| Unigene527342 | arlR | 3.101E-07 | 0.000E+00 | 2.738E-07 | 0.000E+00 | 4.823E-07 | 4.693E-07 | 1.030E-06 | 1.194E-06 | 2.711E-07 | 5.181E-07 | 7.804E-07 | 1.393E-06 |
| Unigene527383 | efrB | 0.000E+00 | 0.000E+00 | 0.000E+00 | 0.000E+00 | 0.000E+00 | 0.000E+00 | 5.178E-07 | 0.000E+00 | 0.000E+00 | 0.000E+00 | 0.000E+00 | 0.000E+00 |
| Unigene527405 | bcrA | 0.000E+00 | 0.000E+00 | 0.000E+00 | 0.000E+00 | 0.000E+00 | 0.000E+00 | 0.000E+00 | 0.000E+00 | 0.000E+00 | 0.000E+00 | 0.000E+00 | 9.442E-07 |
| Unigene527421 | tcr3 | 0.000E+00 | 0.000E+00 | 1.494E-07 | 0.000E+00 | 0.000E+00 | 0.000E+00 | 0.000E+00 | 0.000E+00 | 0.000E+00 | 0.000E+00 | 0.000E+00 | 0.000E+00 |
| Unigene527514 | cpxA | 0.000E+00 | 0.000E+00 | 0.000E+00 | 0.000E+00 | 0.000E+00 | 0.000E+00 | 1.114E-06 | 0.000E+00 | 0.000E+00 | 0.000E+00 | 0.000E+00 | 0.000E+00 |
| Unigene527659 | adeL | 0.000E+00 | 0.000E+00 | 0.000E+00 | 0.000E+00 | 0.000E+00 | 0.000E+00 | 2.335E-07 | 0.000E+00 | 0.000E+00 | 0.000E+00 | 0.000E+00 | 0.000E+00 |
| Unigene527683 | msbA | 0.000E+00 | 0.000E+00 | 0.000E+00 | 0.000E+00 | 2.533E-07 | 0.000E+00 | 5.046E-07 | 0.000E+00 | 0.000E+00 | 0.000E+00 | 0.000E+00 | 4.850E-07 |
| Unigene527748 | oleB | 0.000E+00 | 0.000E+00 | 0.000E+00 | 0.000E+00 | 0.000E+00 | 0.000E+00 | 5.413E-07 | 0.000E+00 | 0.000E+00 | 0.000E+00 | 0.000E+00 | 2.891E-07 |
| Unigene527799 | macB | 0.000E+00 | 0.000E+00 | 0.000E+00 | 0.000E+00 | 0.000E+00 | 0.000E+00 | 1.557E-06 | 1.594E-06 | 0.000E+00 | 0.000E+00 | 0.000E+00 | 5.870E-07 |
| Unigene527907 | novA | 0.000E+00 | 0.000E+00 | 2.053E-07 | 0.000E+00 | 2.325E-07 | 3.582E-07 | 1.448E-06 | 6.310E-07 | 1.144E-07 | 2.331E-07 | 0.000E+00 | 9.482E-07 |
| Unigene527959 | msbA | 8.820E-08 | 0.000E+00 | 0.000E+00 | 0.000E+00 | 0.000E+00 | 1.430E-07 | 1.074E-06 | 0.000E+00 | 0.000E+00 | 0.000E+00 | 0.000E+00 | 5.213E-07 |
| Unigene528029 | OprM | 0.000E+00 | 0.000E+00 | 0.000E+00 | 0.000E+00 | 0.000E+00 | 0.000E+00 | 4.701E-07 | 0.000E+00 | 0.000E+00 | 0.000E+00 | 0.000E+00 | 5.578E-08 |
| Unigene528049 | msbA | 0.000E+00 | 0.000E+00 | 0.000E+00 | 0.000E+00 | 0.000E+00 | 2.195E-07 | 2.996E-07 | 0.000E+00 | 7.397E-08 | 0.000E+00 | 0.000E+00 | 0.000E+00 |
| Unigene528116 | macB | 0.000E+00 | 0.000E+00 | 1.663E-07 | 0.000E+00 | 1.883E-07 | 0.000E+00 | 1.031E-06 | 1.483E-07 | 0.000E+00 | 0.000E+00 | 3.877E-07 | 3.505E-07 |
| Unigene528321 | Chlamydia | 2.568E-08 | 0.000E+00 | 0.000E+00 | 0.000E+00 | 2.853E-08 | 0.000E+00 | 1.052E-06 | 1.799E-07 | 0.000E+00 | 0.000E+00 | 5.875E-08 | 6.071E-08 |
| Unigene528388 | vmlR | 0.000E+00 | 0.000E+00 | 0.000E+00 | 0.000E+00 | 0.000E+00 | 0.000E+00 | 4.941E-07 | 0.000E+00 | 0.000E+00 | 0.000E+00 | 0.000E+00 | 0.000E+00 |
| Unigene528427 | efrA | 0.000E+00 | 0.000E+00 | 0.000E+00 | 0.000E+00 | 0.000E+00 | 0.000E+00 | 9.244E-07 | 0.000E+00 | 0.000E+00 | 0.000E+00 | 0.000E+00 | 0.000E+00 |
| Unigene528475 | adeL | 0.000E+00 | 0.000E+00 | 0.000E+00 | 1.805E-07 | 5.560E-07 | 5.024E-07 | 1.424E-06 | 1.460E-06 | 0.000E+00 | 7.964E-07 | 1.145E-06 | 4.183E-06 |
| Unigene528528 | MuxB | 0.000E+00 | 0.000E+00 | 0.000E+00 | 0.000E+00 | 0.000E+00 | 1.646E-07 | 2.247E-07 | 0.000E+00 | 0.000E+00 | 0.000E+00 | 3.483E-07 | 3.600E-07 |
| Unigene528574 | MexG | 0.000E+00 | 0.000E+00 | 0.000E+00 | 0.000E+00 | 0.000E+00 | 0.000E+00 | 4.284E-07 | 0.000E+00 | 0.000E+00 | 0.000E+00 | 0.000E+00 | 0.000E+00 |
| Unigene528578 | adeL | 0.000E+00 | 0.000E+00 | 0.000E+00 | 1.842E-07 | 2.431E-07 | 2.366E-07 | 1.171E-06 | 8.515E-07 | 0.000E+00 | 0.000E+00 | 7.510E-07 | 2.069E-06 |
| Unigene528640 | macB | 0.000E+00 | 0.000E+00 | 0.000E+00 | 0.000E+00 | 0.000E+00 | 0.000E+00 | 7.566E-07 | 0.000E+00 | 0.000E+00 | 0.000E+00 | 0.000E+00 | 0.000E+00 |
| Unigene528648 | Staphyloco | 0.000E+00 | 0.000E+00 | 0.000E+00 | 0.000E+00 | 0.000E+00 | 0.000E+00 | 9.140E-07 | 0.000E+00 | 0.000E+00 | 0.000E+00 | 0.000E+00 | 0.000E+00 |
| Unigene528745 | evgS | 0.000E+00 | 0.000E+00 | 0.000E+00 | 0.000E+00 | 5.134E-07 | 0.000E+00 | 9.498E-07 | 4.431E-07 | 0.000E+00 | 0.000E+00 | 5.286E-07 | 1.385E-06 |
| Unigene528760 | Acinetoba | 0.000E+00 | 0.000E+00 | 0.000E+00 | 0.000E+00 | 3.703E-07 | 0.000E+00 | 8.167E-07 | 1.417E-06 | 0.000E+00 | 0.000E+00 | 2.995E-07 | 9.004E-07 |
| Unigene528770 | macB | 0.000E+00 | 6.453E-08 | 0.000E+00 | 1.385E-07 | 0.000E+00 | 1.271E-07 | 6.074E-07 | 3.432E-07 | 1.500E-07 | 0.000E+00 | 4.260E-07 | 0.000E+00 |
| Unigene528779 | bcrA | 0.000E+00 | 0.000E+00 | 0.000E+00 | 0.000E+00 | 0.000E+00 | 0.000E+00 | 1.022E-06 | 0.000E+00 | 0.000E+00 | 0.000E+00 | 0.000E+00 | 0.000E+00 |
| Unigene528827 | patA | 0.000E+00 | 0.000E+00 | 0.000E+00 | 0.000E+00 | 0.000E+00 | 0.000E+00 | 1.897E-06 | 0.000E+00 | 0.000E+00 | 0.000E+00 | 0.000E+00 | 0.000E+00 |
| Unigene528856 | vanRB | 0.000E+00 | 0.000E+00 | 0.000E+00 | 0.000E+00 | 0.000E+00 | 0.000E+00 | 9.350E-07 | 0.000E+00 | 0.000E+00 | 0.000E+00 | 0.000E+00 | 0.000E+00 |
| Unigene528877 | macB | 0.000E+00 | 0.000E+00 | 0.000E+00 | 0.000E+00 | 0.000E+00 | 0.000E+00 | 3.171E-06 | 0.000E+00 | 0.000E+00 | 0.000E+00 | 0.000E+00 | 0.000E+00 |
| Unigene528878 | tetB(60) | 0.000E+00 | 0.000E+00 | 0.000E+00 | 0.000E+00 | 5.037E-07 | 0.000E+00 | 0.000E+00 | 0.000E+00 | 0.000E+00 | 0.000E+00 | 0.000E+00 | 0.000E+00 |
| Unigene529024 | Streptomy | 0.000E+00 | 0.000E+00 | 0.000E+00 | 0.000E+00 | 9.422E-07 | 0.000E+00 | 1.305E-06 | 1.036E-06 | 0.000E+00 | 5.314E-07 | 0.000E+00 | 1.582E-06 |
| Unigene529162 | Chlamydia | 0.000E+00 | 0.000E+00 | 0.000E+00 | 0.000E+00 | 1.916E-07 | 0.000E+00 | 6.489E-07 | 0.000E+00 | 0.000E+00 | 0.000E+00 | 0.000E+00 | 0.000E+00 |
| Unigene529186 | poxtA | 0.000E+00 | 0.000E+00 | 0.000E+00 | 0.000E+00 | 0.000E+00 | 0.000E+00 | 6.295E-07 | 0.000E+00 | 0.000E+00 | 0.000E+00 | 0.000E+00 | 0.000E+00 |
| Unigene529299 | novA | 0.000E+00 | 0.000E+00 | 3.017E-08 | 3.105E-08 | 3.415E-07 | 1.329E-07 | 4.423E-07 | 3.588E-07 | 1.344E-07 | 0.000E+00 | 2.110E-07 | 7.268E-07 |

| | | | | | | | | | | | | | |
|---|---|---|---|---|---|---|---|---|---|---|---|---|---|
| Unigene529391 | macB | 0.000E+00 | 0.000E+00 | 0.000E+00 | 2.272E-07 | 6.664E-07 | 2.837E-07 | 8.299E-07 | 3.063E-07 | 0.000E+00 | 2.924E-07 | 0.000E+00 | 8.863E-07 |
| Unigene529412 | evgA | 9.917E-08 | 0.000E+00 | 4.866E-08 | 1.001E-07 | 4.958E-07 | 4.288E-07 | 7.683E-07 | 1.158E-06 | 0.000E+00 | 0.000E+00 | 7.374E-07 | 1.231E-06 |
| Unigene529422 | LlmA 23S r | 0.000E+00 | 0.000E+00 | 0.000E+00 | 0.000E+00 | 0.000E+00 | 0.000E+00 | 0.000E+00 | 0.000E+00 | 0.000E+00 | 0.000E+00 | 0.000E+00 | 2.035E-07 |
| Unigene529426 | oleB | 0.000E+00 | 0.000E+00 | 0.000E+00 | 0.000E+00 | 0.000E+00 | 0.000E+00 | 2.322E-06 | 1.224E-06 | 0.000E+00 | 0.000E+00 | 0.000E+00 | 3.949E-06 |
| Unigene529492 | Erm(34) | 0.000E+00 | 0.000E+00 | 0.000E+00 | 0.000E+00 | 0.000E+00 | 0.000E+00 | 2.564E-06 | 0.000E+00 | 0.000E+00 | 0.000E+00 | 0.000E+00 | 0.000E+00 |
| Unigene529734 | mdtC | 0.000E+00 | 0.000E+00 | 0.000E+00 | 0.000E+00 | 2.550E-07 | 9.306E-08 | 6.034E-07 | 4.019E-07 | 0.000E+00 | 0.000E+00 | 1.969E-07 | 3.731E-07 |
| Unigene529823 | poxtA | 2.122E-07 | 0.000E+00 | 0.000E+00 | 1.836E-07 | 1.347E-07 | 1.966E-07 | 8.722E-07 | 4.246E-07 | 1.656E-07 | 0.000E+00 | 6.588E-07 | 7.524E-07 |
| Unigene529872 | bcrA | 1.729E-07 | 1.897E-07 | 0.000E+00 | 0.000E+00 | 0.000E+00 | 0.000E+00 | 1.530E-06 | 0.000E+00 | 0.000E+00 | 0.000E+00 | 0.000E+00 | 0.000E+00 |
| Unigene529913 | novA | 7.649E-08 | 5.597E-08 | 0.000E+00 | 2.575E-08 | 3.116E-07 | 1.378E-07 | 6.491E-07 | 3.571E-07 | 5.574E-08 | 0.000E+00 | 1.167E-07 | 5.425E-07 |
| Unigene529919 | otr(B) | 0.000E+00 | 0.000E+00 | 0.000E+00 | 0.000E+00 | 0.000E+00 | 0.000E+00 | 9.507E-07 | 0.000E+00 | 0.000E+00 | 0.000E+00 | 0.000E+00 | 0.000E+00 |
| Unigene530119 | bcrA | 0.000E+00 | 0.000E+00 | 0.000E+00 | 0.000E+00 | 0.000E+00 | 0.000E+00 | 4.304E-07 | 0.000E+00 | 0.000E+00 | 0.000E+00 | 0.000E+00 | 0.000E+00 |
| Unigene530191 | tetA(58) | 0.000E+00 | 0.000E+00 | 0.000E+00 | 0.000E+00 | 0.000E+00 | 0.000E+00 | 7.628E-07 | 0.000E+00 | 0.000E+00 | 0.000E+00 | 0.000E+00 | 0.000E+00 |
| Unigene530251 | MexC | 0.000E+00 | 0.000E+00 | 0.000E+00 | 0.000E+00 | 1.065E-07 | 1.554E-07 | 4.509E-07 | 4.755E-07 | 1.048E-07 | 2.937E-07 | 2.193E-07 | 3.116E-07 |
| Unigene530257 | Streptomy | 0.000E+00 | 0.000E+00 | 0.000E+00 | 0.000E+00 | 0.000E+00 | 0.000E+00 | 8.319E-07 | 0.000E+00 | 0.000E+00 | 0.000E+00 | 0.000E+00 | 0.000E+00 |
| Unigene530336 | novA | 0.000E+00 | 0.000E+00 | 0.000E+00 | 0.000E+00 | 1.382E-07 | 0.000E+00 | 0.000E+00 | 0.000E+00 | 0.000E+00 | 0.000E+00 | 0.000E+00 | 0.000E+00 |
| Unigene530416 | PER-2 | 0.000E+00 | 0.000E+00 | 0.000E+00 | 0.000E+00 | 0.000E+00 | 0.000E+00 | 6.288E-07 | 4.560E-07 | 0.000E+00 | 0.000E+00 | 8.124E-08 | 0.000E+00 |
| Unigene530418 | adeC | 0.000E+00 | 0.000E+00 | 7.488E-08 | 0.000E+00 | 0.000E+00 | 0.000E+00 | 9.290E-07 | 0.000E+00 | 0.000E+00 | 0.000E+00 | 0.000E+00 | 0.000E+00 |
| Unigene530421 | MuxB | 0.000E+00 | 0.000E+00 | 0.000E+00 | 0.000E+00 | 0.000E+00 | 0.000E+00 | 2.967E-07 | 0.000E+00 | 0.000E+00 | 2.133E-07 | 0.000E+00 | 0.000E+00 |
| Unigene530485 | MuxB | 0.000E+00 | 0.000E+00 | 4.345E-08 | 0.000E+00 | 0.000E+00 | 0.000E+00 | 6.126E-07 | 0.000E+00 | 0.000E+00 | 0.000E+00 | 0.000E+00 | 0.000E+00 |
| Unigene530547 | mtrA | 0.000E+00 | 0.000E+00 | 0.000E+00 | 0.000E+00 | 3.815E-07 | 1.237E-07 | 5.701E-07 | 2.004E-07 | 1.251E-07 | 0.000E+00 | 0.000E+00 | 0.000E+00 |
| Unigene530548 | QepA4 | 4.465E-08 | 0.000E+00 | 1.752E-07 | 0.000E+00 | 4.464E-07 | 0.000E+00 | 5.930E-07 | 1.042E-07 | 0.000E+00 | 0.000E+00 | 2.043E-07 | 3.166E-07 |
| Unigene530784 | kdpE | 0.000E+00 | 0.000E+00 | 0.000E+00 | 0.000E+00 | 0.000E+00 | 0.000E+00 | 1.814E-06 | 0.000E+00 | 0.000E+00 | 0.000E+00 | 0.000E+00 | 2.431E-06 |
| Unigene530821 | bcrA | 0.000E+00 | 0.000E+00 | 0.000E+00 | 0.000E+00 | 6.358E-08 | 0.000E+00 | 3.801E-07 | 0.000E+00 | 0.000E+00 | 0.000E+00 | 0.000E+00 | 4.059E-07 |
| Unigene530855 | Staphylocc | 0.000E+00 | 0.000E+00 | 1.731E-07 | 2.969E-08 | 9.798E-08 | 0.000E+00 | 2.928E-07 | 4.461E-07 | 0.000E+00 | 1.310E-07 | 1.009E-07 | 2.085E-07 |
| Unigene530903 | otrC | 0.000E+00 | 0.000E+00 | 0.000E+00 | 0.000E+00 | 1.928E-07 | 1.407E-07 | 5.762E-07 | 0.000E+00 | 0.000E+00 | 0.000E+00 | 1.985E-07 | 0.000E+00 |
| Unigene531009 | OXA-18 | 0.000E+00 | 0.000E+00 | 0.000E+00 | 6.354E-08 | 0.000E+00 | 0.000E+00 | 5.571E-07 | 0.000E+00 | 0.000E+00 | 0.000E+00 | 0.000E+00 | 2.231E-07 |
| Unigene531125 | novA | 0.000E+00 | 0.000E+00 | 0.000E+00 | 0.000E+00 | 0.000E+00 | 0.000E+00 | 1.123E-06 | 0.000E+00 | 0.000E+00 | 0.000E+00 | 0.000E+00 | 0.000E+00 |
| Unigene531176 | evgS | 0.000E+00 | 0.000E+00 | 1.050E-07 | 0.000E+00 | 3.089E-07 | 2.775E-07 | 8.050E-07 | 2.996E-07 | 0.000E+00 | 0.000E+00 | 5.628E-07 | 3.540E-07 |
| Unigene531232 | oleC | 0.000E+00 | 0.000E+00 | 0.000E+00 | 0.000E+00 | 0.000E+00 | 0.000E+00 | 6.823E-07 | 0.000E+00 | 0.000E+00 | 0.000E+00 | 0.000E+00 | 0.000E+00 |
| Unigene531329 | vanSF | 0.000E+00 | 0.000E+00 | 0.000E+00 | 0.000E+00 | 0.000E+00 | 0.000E+00 | 1.023E-06 | 0.000E+00 | 0.000E+00 | 0.000E+00 | 0.000E+00 | 0.000E+00 |
| Unigene531504 | baeS | 6.561E-08 | 7.201E-08 | 6.438E-08 | 6.625E-08 | 0.000E+00 | 1.419E-07 | 1.343E-06 | 2.680E-07 | 3.586E-08 | 1.096E-07 | 7.505E-08 | 0.000E+00 |
| Unigene531647 | tetA(58) | 0.000E+00 | 0.000E+00 | 0.000E+00 | 0.000E+00 | 0.000E+00 | 0.000E+00 | 6.854E-07 | 0.000E+00 | 0.000E+00 | 0.000E+00 | 1.771E-07 | 5.490E-07 |
| Unigene531688 | TaeA | 0.000E+00 | 0.000E+00 | 0.000E+00 | 0.000E+00 | 0.000E+00 | 0.000E+00 | 7.134E-07 | 0.000E+00 | 0.000E+00 | 5.524E-08 | 0.000E+00 | 0.000E+00 |
| Unigene531759 | evgS | 0.000E+00 | 0.000E+00 | 0.000E+00 | 0.000E+00 | 0.000E+00 | 0.000E+00 | 9.547E-07 | 0.000E+00 | 0.000E+00 | 0.000E+00 | 0.000E+00 | 0.000E+00 |
| Unigene531814 | lmrD | 0.000E+00 | 0.000E+00 | 0.000E+00 | 0.000E+00 | 2.696E-07 | 0.000E+00 | 0.000E+00 | 0.000E+00 | 0.000E+00 | 0.000E+00 | 0.000E+00 | 0.000E+00 |
| Unigene531892 | bcrA | 1.155E-07 | 0.000E+00 | 1.359E-07 | 9.327E-08 | 5.130E-07 | 0.000E+00 | 1.099E-06 | 5.660E-07 | 0.000E+00 | 0.000E+00 | 2.905E-07 | 4.367E-07 |
| Unigene531898 | macB | 0.000E+00 | 0.000E+00 | 0.000E+00 | 0.000E+00 | 0.000E+00 | 3.388E-07 | 4.047E-07 | 3.048E-07 | 0.000E+00 | 0.000E+00 | 0.000E+00 | 0.000E+00 |
| Unigene531922 | lmrD | 3.137E-08 | 0.000E+00 | 0.000E+00 | 0.000E+00 | 0.000E+00 | 0.000E+00 | 6.597E-07 | 1.465E-07 | 0.000E+00 | 0.000E+00 | 0.000E+00 | 0.000E+00 |
| Unigene531958 | adeS | 0.000E+00 | 0.000E+00 | 0.000E+00 | 0.000E+00 | 0.000E+00 | 0.000E+00 | 4.215E-07 | 0.000E+00 | 0.000E+00 | 0.000E+00 | 0.000E+00 | 0.000E+00 |
| Unigene532076 | adeL | 0.000E+00 | 0.000E+00 | 0.000E+00 | 0.000E+00 | 0.000E+00 | 0.000E+00 | 6.960E-07 | 0.000E+00 | 0.000E+00 | 0.000E+00 | 0.000E+00 | 0.000E+00 |
| Unigene532095 | vanHA | 0.000E+00 | 0.000E+00 | 0.000E+00 | 0.000E+00 | 0.000E+00 | 0.000E+00 | 4.933E-07 | 0.000E+00 | 0.000E+00 | 0.000E+00 | 0.000E+00 | 0.000E+00 |
| Unigene532110 | hmrM | 0.000E+00 | 2.523E-08 | 0.000E+00 | 0.000E+00 | 0.000E+00 | 0.000E+00 | 8.397E-07 | 0.000E+00 | 0.000E+00 | 0.000E+00 | 0.000E+00 | 0.000E+00 |
| Unigene532234 | evgS | 0.000E+00 | 0.000E+00 | 1.643E-07 | 0.000E+00 | 6.744E-07 | 3.621E-07 | 1.089E-06 | 1.368E-06 | 2.059E-07 | 0.000E+00 | 1.078E-06 | 3.613E-06 |
| Unigene532239 | catB2 | 1.624E-07 | 0.000E+00 | 0.000E+00 | 0.000E+00 | 0.000E+00 | 0.000E+00 | 9.437E-07 | 0.000E+00 | 0.000E+00 | 0.000E+00 | 0.000E+00 | 0.000E+00 |
| Unigene532310 | adeL | 0.000E+00 | 0.000E+00 | 0.000E+00 | 0.000E+00 | 0.000E+00 | 0.000E+00 | 9.556E-07 | 0.000E+00 | 0.000E+00 | 0.000E+00 | 0.000E+00 | 0.000E+00 |
| Unigene532344 | kdpE | 0.000E+00 | 0.000E+00 | 0.000E+00 | 0.000E+00 | 0.000E+00 | 0.000E+00 | 9.776E-07 | 0.000E+00 | 0.000E+00 | 0.000E+00 | 0.000E+00 | 0.000E+00 |
| Unigene532437 | efrA | 9.197E-08 | 0.000E+00 | 0.000E+00 | 1.857E-07 | 0.000E+00 | 3.977E-07 | 1.052E-06 | 0.000E+00 | 1.340E-07 | 0.000E+00 | 0.000E+00 | 0.000E+00 |
| Unigene532689 | TaeA | 0.000E+00 | 0.000E+00 | 0.000E+00 | 0.000E+00 | 0.000E+00 | 0.000E+00 | 6.298E-07 | 0.000E+00 | 0.000E+00 | 0.000E+00 | 2.367E-07 | 1.345E-06 |
| Unigene532776 | macB | 0.000E+00 | 0.000E+00 | 0.000E+00 | 0.000E+00 | 0.000E+00 | 3.050E-07 | 9.366E-07 | 6.115E-07 | 0.000E+00 | 0.000E+00 | 0.000E+00 | 3.811E-07 |
| Unigene532813 | patA | 0.000E+00 | 0.000E+00 | 0.000E+00 | 0.000E+00 | 0.000E+00 | 0.000E+00 | 2.431E-06 | 0.000E+00 | 0.000E+00 | 0.000E+00 | 0.000E+00 | 0.000E+00 |
| Unigene532875 | MexD | 0.000E+00 | 0.000E+00 | 0.000E+00 | 0.000E+00 | 0.000E+00 | 0.000E+00 | 4.555E-07 | 0.000E+00 | 0.000E+00 | 0.000E+00 | 0.000E+00 | 0.000E+00 |
| Unigene532950 | vanHF | 1.459E-07 | 0.000E+00 | 4.653E-07 | 1.473E-07 | 8.104E-08 | 7.886E-08 | 6.055E-07 | 2.980E-07 | 0.000E+00 | 0.000E+00 | 0.000E+00 | 6.467E-07 |
| Unigene533016 | kdpE | 0.000E+00 | 0.000E+00 | 0.000E+00 | 0.000E+00 | 0.000E+00 | 0.000E+00 | 7.899E-07 | 0.000E+00 | 0.000E+00 | 0.000E+00 | 0.000E+00 | 0.000E+00 |
| Unigene533032 | tet(H) | 0.000E+00 | 0.000E+00 | 0.000E+00 | 0.000E+00 | 0.000E+00 | 0.000E+00 | 5.622E-07 | 0.000E+00 | 0.000E+00 | 0.000E+00 | 0.000E+00 | 0.000E+00 |
| Unigene533043 | oleC | 0.000E+00 | 0.000E+00 | 0.000E+00 | 0.000E+00 | 0.000E+00 | 0.000E+00 | 4.540E-07 | 0.000E+00 | 0.000E+00 | 0.000E+00 | 0.000E+00 | 0.000E+00 |
| Unigene533047 | lmrD | 0.000E+00 | 0.000E+00 | 0.000E+00 | 4.329E-08 | 0.000E+00 | 0.000E+00 | 5.219E-07 | 0.000E+00 | 1.406E-07 | 0.000E+00 | 0.000E+00 | 0.000E+00 |
| Unigene533110 | msbA | 0.000E+00 | 0.000E+00 | 0.000E+00 | 0.000E+00 | 1.406E-07 | 0.000E+00 | 4.203E-07 | 2.216E-07 | 1.384E-07 | 0.000E+00 | 0.000E+00 | 0.000E+00 |
| Unigene533148 | YojI | 0.000E+00 | 0.000E+00 | 0.000E+00 | 0.000E+00 | 0.000E+00 | 5.539E-08 | 3.970E-07 | 1.196E-07 | 1.680E-07 | 0.000E+00 | 0.000E+00 | 4.240E-07 |

| | | | | | | | | | | | | | |
|---|---|---|---|---|---|---|---|---|---|---|---|---|---|
| Unigene533227 | arnA | 0.000E+00 | 0.000E+00 | 0.000E+00 | 0.000E+00 | 0.000E+00 | 0.000E+00 | 5.122E-07 | 0.000E+00 | 0.000E+00 | 0.000E+00 | 0.000E+00 | 0.000E+00 |
| Unigene533303 | emrB | 0.000E+00 | 0.000E+00 | 8.922E-08 | 0.000E+00 | 1.010E-07 | 3.604E-07 | 1.040E-06 | 1.769E-07 | 0.000E+00 | 0.000E+00 | 1.387E-07 | 2.508E-07 |
| Unigene533304 | farA | 0.000E+00 | 3.067E-08 | 8.227E-08 | 1.693E-07 | 0.000E+00 | 0.000E+00 | 7.733E-07 | 1.305E-07 | 0.000E+00 | 0.000E+00 | 0.000E+00 | 0.000E+00 |
| Unigene533527 | novA | 0.000E+00 | 0.000E+00 | 0.000E+00 | 0.000E+00 | 0.000E+00 | 0.000E+00 | 0.000E+00 | 2.842E-07 | 0.000E+00 | 0.000E+00 | 0.000E+00 | 0.000E+00 |
| Unigene533532 | arlR | 2.360E-07 | 0.000E+00 | 0.000E+00 | 0.000E+00 | 0.000E+00 | 0.000E+00 | 0.000E+00 | 0.000E+00 | 0.000E+00 | 0.000E+00 | 0.000E+00 | 0.000E+00 |
| Unigene533542 | Corynebac | 0.000E+00 | 0.000E+00 | 0.000E+00 | 0.000E+00 | 0.000E+00 | 0.000E+00 | 5.954E-07 | 2.093E-07 | 0.000E+00 | 0.000E+00 | 0.000E+00 | 0.000E+00 |
| Unigene533556 | tetA(58) | 1.649E-07 | 0.000E+00 | 0.000E+00 | 1.249E-07 | 9.160E-08 | 0.000E+00 | 8.669E-07 | 1.925E-07 | 0.000E+00 | 0.000E+00 | 0.000E+00 | 0.000E+00 |
| Unigene533709 | msbA | 0.000E+00 | 0.000E+00 | 0.000E+00 | 0.000E+00 | 0.000E+00 | 0.000E+00 | 2.301E-06 | 0.000E+00 | 0.000E+00 | 0.000E+00 | 0.000E+00 | 0.000E+00 |
| Unigene533741 | mtrA | 0.000E+00 | 0.000E+00 | 0.000E+00 | 0.000E+00 | 0.000E+00 | 0.000E+00 | 7.844E-07 | 0.000E+00 | 0.000E+00 | 0.000E+00 | 0.000E+00 | 0.000E+00 |
| Unigene533746 | mecC | 0.000E+00 | 0.000E+00 | 0.000E+00 | 0.000E+00 | 0.000E+00 | 0.000E+00 | 3.025E-06 | 0.000E+00 | 0.000E+00 | 0.000E+00 | 0.000E+00 | 0.000E+00 |
| Unigene533776 | Bifidobacte | 0.000E+00 | 0.000E+00 | 7.307E-08 | 0.000E+00 | 0.000E+00 | 0.000E+00 | 1.607E-06 | 6.084E-07 | 0.000E+00 | 0.000E+00 | 7.666E-07 | 6.161E-07 |
| Unigene533813 | bcrA | 0.000E+00 | 0.000E+00 | 0.000E+00 | 0.000E+00 | 0.000E+00 | 0.000E+00 | 7.566E-07 | 0.000E+00 | 0.000E+00 | 0.000E+00 | 0.000E+00 | 5.622E-07 |
| Unigene533855 | Listeria mc | 8.036E-06 | 0.000E+00 | 0.000E+00 | 2.306E-06 | 3.529E-05 | 1.768E-05 | 2.445E-05 | 2.657E-05 | 3.444E-06 | 0.000E+00 | 1.397E-08 | 0.000E+00 |
| Unigene533890 | tetA(46) | 0.000E+00 | 0.000E+00 | 0.000E+00 | 0.000E+00 | 0.000E+00 | 4.326E-07 | 6.397E-07 | 0.000E+00 | 0.000E+00 | 0.000E+00 | 0.000E+00 | 0.000E+00 |
| Unigene534051 | novA | 0.000E+00 | 0.000E+00 | 2.750E-07 | 0.000E+00 | 0.000E+00 | 0.000E+00 | 8.683E-07 | 0.000E+00 | 0.000E+00 | 8.116E-07 | 0.000E+00 | 0.000E+00 |
| Unigene534134 | vanSA | 0.000E+00 | 0.000E+00 | 0.000E+00 | 0.000E+00 | 7.843E-07 | 2.544E-07 | 1.184E-06 | 6.991E-07 | 2.104E-07 | 5.004E-07 | 5.139E-07 | 8.344E-07 |
| Unigene534204 | tlrC | 0.000E+00 | 0.000E+00 | 0.000E+00 | 1.011E-07 | 0.000E+00 | 5.410E-08 | 1.662E-07 | 2.337E-07 | 0.000E+00 | 0.000E+00 | 0.000E+00 | 0.000E+00 |
| Unigene534226 | cmlv | 0.000E+00 | 0.000E+00 | 0.000E+00 | 0.000E+00 | 5.030E-07 | 0.000E+00 | 1.150E-06 | 1.181E-06 | 2.620E-07 | 5.044E-07 | 7.921E-07 | 1.196E-06 |
| Unigene534311 | oleC | 0.000E+00 | 0.000E+00 | 7.802E-08 | 1.873E-07 | 0.000E+00 | 1.719E-07 | 8.213E-07 | 1.083E-06 | 0.000E+00 | 0.000E+00 | 3.032E-07 | 4.699E-07 |
| Unigene534325 | bcr-1 | 0.000E+00 | 0.000E+00 | 0.000E+00 | 0.000E+00 | 0.000E+00 | 0.000E+00 | 5.565E-07 | 0.000E+00 | 0.000E+00 | 0.000E+00 | 0.000E+00 | 2.377E-07 |
| Unigene534354 | patA | 0.000E+00 | 0.000E+00 | 0.000E+00 | 0.000E+00 | 0.000E+00 | 0.000E+00 | 8.038E-07 | 0.000E+00 | 0.000E+00 | 0.000E+00 | 0.000E+00 | 0.000E+00 |
| Unigene534385 | optrA | 0.000E+00 | 0.000E+00 | 0.000E+00 | 0.000E+00 | 0.000E+00 | 0.000E+00 | 8.437E-07 | 0.000E+00 | 0.000E+00 | 0.000E+00 | 0.000E+00 | 0.000E+00 |
| Unigene534404 | msbA | 0.000E+00 | 0.000E+00 | 0.000E+00 | 0.000E+00 | 0.000E+00 | 0.000E+00 | 0.000E+00 | 4.884E-07 | 0.000E+00 | 0.000E+00 | 7.522E-07 | 0.000E+00 |
| Unigene534443 | oleB | 0.000E+00 | 6.298E-08 | 2.252E-07 | 0.000E+00 | 3.506E-07 | 4.963E-07 | 2.763E-06 | 6.029E-07 | 2.509E-07 | 4.155E-07 | 6.564E-07 | 2.476E-06 |
| Unigene534482 | TriA | 1.412E-07 | 0.000E+00 | 0.000E+00 | 0.000E+00 | 4.079E-07 | 9.159E-08 | 8.752E-07 | 1.253E-06 | 1.235E-07 | 0.000E+00 | 4.523E-07 | 1.369E-06 |
| Unigene534540 | vanHD | 0.000E+00 | 0.000E+00 | 0.000E+00 | 0.000E+00 | 0.000E+00 | 0.000E+00 | 1.425E-06 | 0.000E+00 | 0.000E+00 | 0.000E+00 | 0.000E+00 | 0.000E+00 |
| Unigene534570 | OprN | 5.801E-08 | 6.366E-08 | 0.000E+00 | 5.858E-08 | 1.289E-07 | 1.254E-07 | 4.173E-07 | 3.724E-07 | 6.340E-08 | 0.000E+00 | 1.327E-07 | 4.114E-07 |
| Unigene534576 | Acinetobac | 0.000E+00 | 0.000E+00 | 0.000E+00 | 0.000E+00 | 0.000E+00 | 2.012E-07 | 5.357E-07 | 0.000E+00 | 0.000E+00 | 0.000E+00 | 0.000E+00 | 5.721E-07 |
| Unigene534637 | novA | 0.000E+00 | 0.000E+00 | 0.000E+00 | 0.000E+00 | 0.000E+00 | 0.000E+00 | 4.083E-07 | 0.000E+00 | 0.000E+00 | 0.000E+00 | 0.000E+00 | 0.000E+00 |
| Unigene534639 | emrB | 0.000E+00 | 0.000E+00 | 0.000E+00 | 0.000E+00 | 0.000E+00 | 0.000E+00 | 4.203E-07 | 0.000E+00 | 0.000E+00 | 0.000E+00 | 0.000E+00 | 0.000E+00 |
| Unigene534682 | TaeA | 0.000E+00 | 9.299E-08 | 0.000E+00 | 0.000E+00 | 0.000E+00 | 2.748E-07 | 1.078E-06 | 6.428E-07 | 0.000E+00 | 0.000E+00 | 4.846E-07 | 0.000E+00 |
| Unigene534697 | Acinetobac | 0.000E+00 | 0.000E+00 | 0.000E+00 | 0.000E+00 | 0.000E+00 | 0.000E+00 | 0.000E+00 | 0.000E+00 | 1.645E-07 | 0.000E+00 | 0.000E+00 | 0.000E+00 |
| Unigene534752 | macB | 0.000E+00 | 1.298E-07 | 0.000E+00 | 1.791E-07 | 6.240E-07 | 1.598E-07 | 6.870E-07 | 2.070E-07 | 0.000E+00 | 0.000E+00 | 4.058E-07 | 4.892E-07 |
| Unigene534794 | tetA(46) | 9.236E-08 | 0.000E+00 | 0.000E+00 | 0.000E+00 | 0.000E+00 | 0.000E+00 | 6.133E-07 | 0.000E+00 | 0.000E+00 | 0.000E+00 | 0.000E+00 | 0.000E+00 |
| Unigene534843 | Streptomy | 0.000E+00 | 0.000E+00 | 0.000E+00 | 0.000E+00 | 3.622E-07 | 0.000E+00 | 2.406E-07 | 2.537E-07 | 0.000E+00 | 0.000E+00 | 6.216E-08 | 0.000E+00 |
| Unigene534874 | vanRF | 0.000E+00 | 0.000E+00 | 0.000E+00 | 0.000E+00 | 0.000E+00 | 0.000E+00 | 3.811E-07 | 0.000E+00 | 0.000E+00 | 0.000E+00 | 0.000E+00 | 0.000E+00 |
| Unigene534957 | Acinetobac | 0.000E+00 | 0.000E+00 | 0.000E+00 | 0.000E+00 | 0.000E+00 | 0.000E+00 | 5.032E-07 | 0.000E+00 | 0.000E+00 | 0.000E+00 | 0.000E+00 | 0.000E+00 |
| Unigene535036 | vanHO | 0.000E+00 | 0.000E+00 | 0.000E+00 | 1.035E-07 | 0.000E+00 | 0.000E+00 | 7.183E-07 | 3.588E-07 | 0.000E+00 | 0.000E+00 | 0.000E+00 | 0.000E+00 |
| Unigene535076 | iri | 0.000E+00 | 0.000E+00 | 1.658E-07 | 8.533E-08 | 4.068E-07 | 0.000E+00 | 4.364E-07 | 4.932E-07 | 0.000E+00 | 0.000E+00 | 3.544E-07 | 7.325E-07 |
| Unigene535094 | tlrC | 0.000E+00 | 0.000E+00 | 0.000E+00 | 0.000E+00 | 0.000E+00 | 0.000E+00 | 8.506E-07 | 0.000E+00 | 0.000E+00 | 0.000E+00 | 0.000E+00 | 0.000E+00 |
| Unigene535109 | adeG | 0.000E+00 | 0.000E+00 | 0.000E+00 | 0.000E+00 | 3.011E-08 | 0.000E+00 | 7.199E-07 | 0.000E+00 | 0.000E+00 | 9.058E-08 | 0.000E+00 | 0.000E+00 |
| Unigene535119 | vanHO | 1.933E-07 | 0.000E+00 | 0.000E+00 | 0.000E+00 | 0.000E+00 | 0.000E+00 | 8.557E-07 | 0.000E+00 | 0.000E+00 | 0.000E+00 | 0.000E+00 | 0.000E+00 |
| Unigene535129 | evgS | 0.000E+00 | 0.000E+00 | 0.000E+00 | 0.000E+00 | 0.000E+00 | 0.000E+00 | 2.965E-07 | 0.000E+00 | 0.000E+00 | 0.000E+00 | 0.000E+00 | 0.000E+00 |
| Unigene535358 | efrA | 8.701E-08 | 0.000E+00 | 0.000E+00 | 0.000E+00 | 0.000E+00 | 0.000E+00 | 9.309E-07 | 2.302E-06 | 0.000E+00 | 2.908E-07 | 0.000E+00 | 1.337E-06 |
| Unigene535406 | macB | 0.000E+00 | 0.000E+00 | 0.000E+00 | 0.000E+00 | 0.000E+00 | 0.000E+00 | 7.051E-07 | 0.000E+00 | 0.000E+00 | 0.000E+00 | 0.000E+00 | 0.000E+00 |
| Unigene535427 | msbA | 7.525E-08 | 0.000E+00 | 1.477E-07 | 0.000E+00 | 1.045E-08 | 2.237E-07 | 9.369E-07 | 1.757E-07 | 0.000E+00 | 4.191E-08 | 1.721E-07 | 1.112E-07 |
| Unigene535461 | qacA | 0.000E+00 | 0.000E+00 | 0.000E+00 | 2.462E-07 | 0.000E+00 | 0.000E+00 | 6.166E-07 | 0.000E+00 | 0.000E+00 | 0.000E+00 | 0.000E+00 | 0.000E+00 |
| Unigene535639 | kdpE | 0.000E+00 | 0.000E+00 | 0.000E+00 | 0.000E+00 | 0.000E+00 | 0.000E+00 | 8.028E-07 | 0.000E+00 | 0.000E+00 | 0.000E+00 | 0.000E+00 | 0.000E+00 |
| Unigene535711 | novA | 0.000E+00 | 0.000E+00 | 0.000E+00 | 0.000E+00 | 0.000E+00 | 0.000E+00 | 1.017E-06 | 0.000E+00 | 0.000E+00 | 0.000E+00 | 0.000E+00 | 0.000E+00 |
| Unigene535830 | optrA | 0.000E+00 | 0.000E+00 | 0.000E+00 | 0.000E+00 | 5.734E-07 | 0.000E+00 | 7.031E-07 | 6.488E-07 | 0.000E+00 | 0.000E+00 | 0.000E+00 | 0.000E+00 |
| Unigene536006 | efrA | 0.000E+00 | 8.879E-08 | 3.175E-07 | 1.634E-07 | 3.146E-07 | 1.749E-07 | 9.849E-07 | 6.138E-07 | 0.000E+00 | 0.000E+00 | 6.941E-07 | 2.678E-06 |
| Unigene536023 | msbA | 0.000E+00 | 0.000E+00 | 0.000E+00 | 0.000E+00 | 0.000E+00 | 0.000E+00 | 3.515E-07 | 2.317E-07 | 0.000E+00 | 0.000E+00 | 0.000E+00 | 4.693E-07 |
| Unigene536142 | efrB | 0.000E+00 | 0.000E+00 | 0.000E+00 | 0.000E+00 | 0.000E+00 | 0.000E+00 | 8.386E-07 | 0.000E+00 | 0.000E+00 | 0.000E+00 | 0.000E+00 | 0.000E+00 |
| Unigene536186 | mdtC | 1.417E-07 | 0.000E+00 | 0.000E+00 | 1.041E-07 | 3.293E-07 | 2.090E-07 | 1.055E-06 | 4.964E-07 | 1.408E-07 | 3.015E-07 | 6.633E-07 | 6.245E-07 |
| Unigene536245 | evgS | 0.000E+00 | 0.000E+00 | 0.000E+00 | 0.000E+00 | 0.000E+00 | 0.000E+00 | 6.701E-07 | 6.664E-07 | 0.000E+00 | 0.000E+00 | 9.103E-07 | 0.000E+00 |
| Unigene536427 | Staphyloco | 0.000E+00 | 0.000E+00 | 0.000E+00 | 0.000E+00 | 1.540E-07 | 0.000E+00 | 8.287E-07 | 0.000E+00 | 0.000E+00 | 0.000E+00 | 0.000E+00 | 1.082E-06 |
| Unigene536473 | tetA(58) | 1.471E-07 | 0.000E+00 | 1.031E-07 | 1.061E-07 | 9.339E-07 | 6.816E-08 | 5.815E-07 | 6.623E-07 | 0.000E+00 | 0.000E+00 | 6.731E-07 | 9.688E-07 |
| Unigene536591 | Pseudomo | 0.000E+00 | 0.000E+00 | 0.000E+00 | 0.000E+00 | 0.000E+00 | 0.000E+00 | 8.802E-07 | 1.420E-06 | 0.000E+00 | 0.000E+00 | 0.000E+00 | 7.742E-07 |

| | | | | | | | | | | | | | |
|---|---|---|---|---|---|---|---|---|---|---|---|---|---|
| Unigene536639 | sdiA | 0.000E+00 | 1.976E-07 | 2.651E-07 | 0.000E+00 | 4.001E-07 | 0.000E+00 | 5.979E-07 | 5.781E-07 | 0.000E+00 | 0.000E+00 | 4.120E-07 | 1.064E-06 |
| Unigene536913 | Streptococ | 0.000E+00 | 0.000E+00 | 0.000E+00 | 0.000E+00 | 0.000E+00 | 0.000E+00 | 3.591E-07 | 0.000E+00 | 0.000E+00 | 0.000E+00 | 0.000E+00 | 0.000E+00 |
| Unigene537063 | tetA(46) | 0.000E+00 | 0.000E+00 | 0.000E+00 | 0.000E+00 | 0.000E+00 | 0.000E+00 | 2.120E-06 | 0.000E+00 | 0.000E+00 | 0.000E+00 | 0.000E+00 | 0.000E+00 |
| Unigene537096 | bcrA | 0.000E+00 | 0.000E+00 | 0.000E+00 | 0.000E+00 | 0.000E+00 | 0.000E+00 | 5.671E-07 | 0.000E+00 | 0.000E+00 | 0.000E+00 | 0.000E+00 | 0.000E+00 |
| Unigene537128 | evgS | 0.000E+00 | 2.307E-07 | 1.237E-07 | 0.000E+00 | 8.405E-07 | 4.317E-07 | 6.978E-07 | 1.055E-06 | 0.000E+00 | 0.000E+00 | 5.770E-07 | 6.707E-07 |
| Unigene537141 | lmrC | 0.000E+00 | 0.000E+00 | 0.000E+00 | 0.000E+00 | 0.000E+00 | 0.000E+00 | 4.763E-07 | 0.000E+00 | 0.000E+00 | 0.000E+00 | 0.000E+00 | 0.000E+00 |
| Unigene537143 | patA | 0.000E+00 | 0.000E+00 | 0.000E+00 | 4.663E-08 | 0.000E+00 | 0.000E+00 | 5.622E-07 | 0.000E+00 | 0.000E+00 | 0.000E+00 | 0.000E+00 | 0.000E+00 |
| Unigene537163 | vanSN | 0.000E+00 | 0.000E+00 | 6.725E-08 | 0.000E+00 | 0.000E+00 | 2.223E-07 | 3.034E-07 | 3.200E-07 | 0.000E+00 | 0.000E+00 | 0.000E+00 | 0.000E+00 |
| Unigene537170 | macB | 0.000E+00 | 0.000E+00 | 0.000E+00 | 0.000E+00 | 0.000E+00 | 0.000E+00 | 8.982E-07 | 0.000E+00 | 0.000E+00 | 0.000E+00 | 0.000E+00 | 6.499E-07 |
| Unigene537195 | macB | 0.000E+00 | 0.000E+00 | 0.000E+00 | 0.000E+00 | 0.000E+00 | 0.000E+00 | 1.806E-06 | 0.000E+00 | 0.000E+00 | 0.000E+00 | 0.000E+00 | 0.000E+00 |
| Unigene537268 | vanSA | 0.000E+00 | 0.000E+00 | 1.123E-07 | 1.541E-07 | 2.967E-07 | 2.475E-07 | 9.713E-07 | 7.126E-07 | 1.251E-07 | 0.000E+00 | 6.983E-07 | 1.218E-06 |
| Unigene537320 | efrA | 1.242E-07 | 0.000E+00 | 1.624E-07 | 0.000E+00 | 9.195E-08 | 0.000E+00 | 8.245E-07 | 0.000E+00 | 0.000E+00 | 0.000E+00 | 0.000E+00 | 2.935E-07 |
| Unigene537366 | tetA(58) | 0.000E+00 | 0.000E+00 | 1.432E-07 | 0.000E+00 | 0.000E+00 | 0.000E+00 | 8.477E-07 | 0.000E+00 | 0.000E+00 | 0.000E+00 | 0.000E+00 | 0.000E+00 |
| Unigene537399 | golS | 0.000E+00 | 0.000E+00 | 0.000E+00 | 0.000E+00 | 0.000E+00 | 0.000E+00 | 2.684E-06 | 0.000E+00 | 0.000E+00 | 0.000E+00 | 0.000E+00 | 0.000E+00 |
| Unigene537409 | Acinetobac | 0.000E+00 | 0.000E+00 | 0.000E+00 | 0.000E+00 | 0.000E+00 | 0.000E+00 | 3.811E-07 | 0.000E+00 | 0.000E+00 | 0.000E+00 | 0.000E+00 | 0.000E+00 |
| Unigene537511 | evgA | 8.820E-08 | 0.000E+00 | 0.000E+00 | 0.000E+00 | 0.000E+00 | 3.814E-07 | 9.761E-07 | 0.000E+00 | 0.000E+00 | 0.000E+00 | 0.000E+00 | 3.128E-07 |
| Unigene537515 | farB | 0.000E+00 | 0.000E+00 | 0.000E+00 | 0.000E+00 | 0.000E+00 | 0.000E+00 | 7.809E-07 | 6.863E-07 | 0.000E+00 | 1.637E-07 | 0.000E+00 | 6.603E-07 |
| Unigene537567 | baeS | 0.000E+00 | 0.000E+00 | 0.000E+00 | 0.000E+00 | 0.000E+00 | 0.000E+00 | 2.546E-06 | 1.004E-06 | 0.000E+00 | 0.000E+00 | 0.000E+00 | 0.000E+00 |
| Unigene537568 | arlR | 0.000E+00 | 0.000E+00 | 0.000E+00 | 0.000E+00 | 0.000E+00 | 0.000E+00 | 1.370E-06 | 8.336E-07 | 0.000E+00 | 0.000E+00 | 0.000E+00 | 0.000E+00 |
| Unigene537590 | carA | 0.000E+00 | 0.000E+00 | 0.000E+00 | 0.000E+00 | 0.000E+00 | 0.000E+00 | 4.311E-07 | 0.000E+00 | 0.000E+00 | 0.000E+00 | 0.000E+00 | 0.000E+00 |
| Unigene537732 | novA | 0.000E+00 | 0.000E+00 | 0.000E+00 | 0.000E+00 | 2.860E-08 | 0.000E+00 | 7.692E-07 | 0.000E+00 | 0.000E+00 | 0.000E+00 | 0.000E+00 | 2.434E-07 |
| Unigene537778 | evgS | 0.000E+00 | 8.769E-08 | 0.000E+00 | 1.883E-07 | 3.551E-07 | 2.015E-07 | 8.548E-07 | 4.663E-07 | 8.733E-08 | 0.000E+00 | 3.961E-07 | 1.700E-06 |
| Unigene537832 | lmrD | 0.000E+00 | 0.000E+00 | 0.000E+00 | 0.000E+00 | 0.000E+00 | 0.000E+00 | 7.145E-07 | 0.000E+00 | 0.000E+00 | 0.000E+00 | 0.000E+00 | 3.180E-07 |
| Unigene537871 | catB11 | 0.000E+00 | 0.000E+00 | 0.000E+00 | 0.000E+00 | 0.000E+00 | 0.000E+00 | 2.858E-07 | 2.010E-07 | 0.000E+00 | 0.000E+00 | 0.000E+00 | 4.579E-07 |
| Unigene537960 | patA | 0.000E+00 | 0.000E+00 | 0.000E+00 | 1.455E-07 | 0.000E+00 | 0.000E+00 | 8.506E-07 | 4.486E-07 | 0.000E+00 | 0.000E+00 | 0.000E+00 | 1.703E-07 |
| Unigene538051 | smeS | 0.000E+00 | 0.000E+00 | 0.000E+00 | 0.000E+00 | 0.000E+00 | 0.000E+00 | 9.100E-07 | 0.000E+00 | 0.000E+00 | 0.000E+00 | 0.000E+00 | 0.000E+00 |
| Unigene538087 | Corynebac | 0.000E+00 | 0.000E+00 | 0.000E+00 | 0.000E+00 | 0.000E+00 | 0.000E+00 | 8.344E-07 | 0.000E+00 | 0.000E+00 | 0.000E+00 | 0.000E+00 | 0.000E+00 |
| Unigene538107 | bcrA | 0.000E+00 | 0.000E+00 | 0.000E+00 | 0.000E+00 | 5.153E-07 | 3.008E-07 | 1.283E-06 | 1.516E-06 | 3.042E-07 | 3.617E-07 | 1.167E-06 | 3.454E-06 |
| Unigene538225 | adeL | 0.000E+00 | 0.000E+00 | 0.000E+00 | 0.000E+00 | 0.000E+00 | 5.109E-07 | 1.287E-06 | 0.000E+00 | 7.946E-08 | 0.000E+00 | 0.000E+00 | 0.000E+00 |
| Unigene538234 | MCR-5.2 | 0.000E+00 | 0.000E+00 | 0.000E+00 | 0.000E+00 | 0.000E+00 | 0.000E+00 | 7.585E-07 | 0.000E+00 | 0.000E+00 | 0.000E+00 | 0.000E+00 | 0.000E+00 |
| Unigene538305 | optrA | 0.000E+00 | 0.000E+00 | 0.000E+00 | 2.131E-07 | 0.000E+00 | 0.000E+00 | 2.179E-06 | 0.000E+00 | 0.000E+00 | 0.000E+00 | 0.000E+00 | 0.000E+00 |
| Unigene538306 | LlmA 23S r | 4.109E-07 | 1.654E-06 | 1.284E-06 | 0.000E+00 | 0.000E+00 | 0.000E+00 | 1.735E-06 | 0.000E+00 | 0.000E+00 | 0.000E+00 | 0.000E+00 | 0.000E+00 |
| Unigene538345 | macB | 5.693E-08 | 9.373E-08 | 5.586E-08 | 2.875E-08 | 4.744E-07 | 9.232E-08 | 5.041E-07 | 9.303E-07 | 0.000E+00 | 2.220E-07 | 6.187E-07 | 2.019E-06 |
| Unigene538349 | macB | 0.000E+00 | 8.947E-08 | 0.000E+00 | 0.000E+00 | 0.000E+00 | 0.000E+00 | 0.000E+00 | 0.000E+00 | 0.000E+00 | 0.000E+00 | 8.392E-07 | 0.000E+00 |
| Unigene538444 | OprA | 9.316E-08 | 7.669E-08 | 0.000E+00 | 0.000E+00 | 0.000E+00 | 7.553E-08 | 8.764E-07 | 0.000E+00 | 2.546E-08 | 0.000E+00 | 0.000E+00 | 0.000E+00 |
| Unigene538454 | evgS | 1.258E-07 | 0.000E+00 | 8.233E-08 | 0.000E+00 | 3.961E-07 | 1.814E-07 | 6.500E-07 | 6.855E-07 | 0.000E+00 | 0.000E+00 | 5.038E-07 | 1.785E-06 |
| Unigene538482 | Acinetobac | 0.000E+00 | 0.000E+00 | 7.632E-08 | 0.000E+00 | 0.000E+00 | 0.000E+00 | 3.730E-07 | 0.000E+00 | 0.000E+00 | 2.888E-07 | 0.000E+00 | 3.678E-07 |
| Unigene538777 | mexQ | 0.000E+00 | 0.000E+00 | 0.000E+00 | 0.000E+00 | 0.000E+00 | 0.000E+00 | 5.954E-07 | 2.691E-07 | 0.000E+00 | 0.000E+00 | 3.077E-07 | 0.000E+00 |
| Unigene538797 | bacA | 0.000E+00 | 0.000E+00 | 0.000E+00 | 0.000E+00 | 0.000E+00 | 0.000E+00 | 1.499E-06 | 0.000E+00 | 0.000E+00 | 0.000E+00 | 0.000E+00 | 1.418E-06 |
| Unigene538809 | vanRF | 0.000E+00 | 0.000E+00 | 0.000E+00 | 0.000E+00 | 0.000E+00 | 0.000E+00 | 7.178E-07 | 3.097E-07 | 0.000E+00 | 0.000E+00 | 0.000E+00 | 0.000E+00 |
| Unigene538849 | adeL | 0.000E+00 | 0.000E+00 | 0.000E+00 | 0.000E+00 | 2.299E-07 | 0.000E+00 | 6.870E-07 | 0.000E+00 | 0.000E+00 | 0.000E+00 | 0.000E+00 | 0.000E+00 |
| Unigene539001 | efrA | 0.000E+00 | 0.000E+00 | 0.000E+00 | 0.000E+00 | 0.000E+00 | 0.000E+00 | 6.342E-07 | 0.000E+00 | 0.000E+00 | 0.000E+00 | 0.000E+00 | 0.000E+00 |
| Unigene539038 | patA | 0.000E+00 | 0.000E+00 | 0.000E+00 | 0.000E+00 | 3.275E-07 | 0.000E+00 | 3.263E-07 | 0.000E+00 | 0.000E+00 | 0.000E+00 | 5.058E-07 | 7.840E-07 |
| Unigene539120 | bcr-1 | 1.819E-07 | 0.000E+00 | 0.000E+00 | 0.000E+00 | 4.331E-07 | 0.000E+00 | 1.093E-06 | 0.000E+00 | 0.000E+00 | 0.000E+00 | 0.000E+00 | 6.452E-07 |
| Unigene539195 | efrA | 0.000E+00 | 0.000E+00 | 2.554E-07 | 0.000E+00 | 1.607E-07 | 0.000E+00 | 8.964E-07 | 1.013E-07 | 0.000E+00 | 0.000E+00 | 5.294E-07 | 7.180E-07 |
| Unigene539333 | basS | 0.000E+00 | 0.000E+00 | 0.000E+00 | 0.000E+00 | 0.000E+00 | 0.000E+00 | 1.780E-06 | 0.000E+00 | 0.000E+00 | 0.000E+00 | 0.000E+00 | 0.000E+00 |
| Unigene539611 | efrA | 0.000E+00 | 0.000E+00 | 0.000E+00 | 0.000E+00 | 0.000E+00 | 0.000E+00 | 1.307E-06 | 0.000E+00 | 0.000E+00 | 0.000E+00 | 0.000E+00 | 0.000E+00 |
| Unigene539689 | cpxA | 0.000E+00 | 0.000E+00 | 0.000E+00 | 0.000E+00 | 0.000E+00 | 4.025E-08 | 7.005E-07 | 0.000E+00 | 0.000E+00 | 0.000E+00 | 2.981E-07 | 0.000E+00 |
| Unigene539758 | tetA(58) | 0.000E+00 | 0.000E+00 | 0.000E+00 | 0.000E+00 | 0.000E+00 | 0.000E+00 | 8.098E-07 | 0.000E+00 | 0.000E+00 | 0.000E+00 | 0.000E+00 | 0.000E+00 |
| Unigene539814 | tcr3 | 0.000E+00 | 0.000E+00 | 0.000E+00 | 0.000E+00 | 1.002E-07 | 0.000E+00 | 1.024E-06 | 1.053E-07 | 2.466E-08 | 0.000E+00 | 0.000E+00 | 3.466E-07 |
| Unigene539939 | Acinetobac | 0.000E+00 | 0.000E+00 | 0.000E+00 | 0.000E+00 | 0.000E+00 | 0.000E+00 | 1.364E-06 | 0.000E+00 | 0.000E+00 | 0.000E+00 | 0.000E+00 | 0.000E+00 |
| Unigene540015 | rosA | 2.127E-07 | 0.000E+00 | 0.000E+00 | 0.000E+00 | 2.835E-07 | 0.000E+00 | 7.531E-07 | 0.000E+00 | 0.000E+00 | 0.000E+00 | 0.000E+00 | 3.519E-07 |
| Unigene540051 | macB | 0.000E+00 | 0.000E+00 | 0.000E+00 | 0.000E+00 | 0.000E+00 | 0.000E+00 | 1.191E-06 | 0.000E+00 | 0.000E+00 | 0.000E+00 | 0.000E+00 | 1.007E-06 |
| Unigene540083 | tlrC | 0.000E+00 | 0.000E+00 | 2.195E-08 | 0.000E+00 | 2.734E-07 | 1.451E-07 | 7.427E-07 | 6.267E-07 | 0.000E+00 | 0.000E+00 | 3.327E-07 | 6.346E-07 |
| Unigene540088 | emrB | 0.000E+00 | 0.000E+00 | 0.000E+00 | 0.000E+00 | 0.000E+00 | 0.000E+00 | 2.548E-06 | 0.000E+00 | 0.000E+00 | 0.000E+00 | 5.476E-07 | 0.000E+00 |
| Unigene540089 | baeS | 0.000E+00 | 0.000E+00 | 0.000E+00 | 0.000E+00 | 0.000E+00 | 0.000E+00 | 2.280E-06 | 0.000E+00 | 0.000E+00 | 0.000E+00 | 0.000E+00 | 0.000E+00 |
| Unigene540090 | baeR | 0.000E+00 | 0.000E+00 | 0.000E+00 | 0.000E+00 | 0.000E+00 | 0.000E+00 | 2.718E-06 | 0.000E+00 | 0.000E+00 | 0.000E+00 | 0.000E+00 | 0.000E+00 |
| Unigene540142 | rosB | 0.000E+00 | 0.000E+00 | 0.000E+00 | 0.000E+00 | 0.000E+00 | 0.000E+00 | 2.041E-07 | 0.000E+00 | 0.000E+00 | 0.000E+00 | 0.000E+00 | 0.000E+00 |

| | | | | | | | | | | | | | |
|---|---|---|---|---|---|---|---|---|---|---|---|---|---|
| Unigene540421 | golS | 0.000E+00 | 0.000E+00 | 0.000E+00 | 0.000E+00 | 0.000E+00 | 0.000E+00 | 7.495E-07 | 0.000E+00 | 0.000E+00 | 0.000E+00 | 0.000E+00 | 0.000E+00 |
| Unigene540446 | adeL | 0.000E+00 | 0.000E+00 | 1.022E-07 | 0.000E+00 | 0.000E+00 | 0.000E+00 | 1.306E-06 | 0.000E+00 | 0.000E+00 | 0.000E+00 | 0.000E+00 | 0.000E+00 |
| Unigene540517 | vanRF | 0.000E+00 | 0.000E+00 | 0.000E+00 | 0.000E+00 | 6.447E-07 | 5.228E-08 | 6.690E-07 | 8.467E-07 | 0.000E+00 | 0.000E+00 | 3.319E-07 | 1.458E-06 |
| Unigene540592 | acrD | 0.000E+00 | 0.000E+00 | 0.000E+00 | 0.000E+00 | 0.000E+00 | 0.000E+00 | 8.323E-07 | 0.000E+00 | 0.000E+00 | 0.000E+00 | 0.000E+00 | 0.000E+00 |
| Unigene540639 | vanSM | 0.000E+00 | 0.000E+00 | 0.000E+00 | 1.242E-07 | 0.000E+00 | 0.000E+00 | 3.402E-07 | 0.000E+00 | 0.000E+00 | 0.000E+00 | 0.000E+00 | 0.000E+00 |
| Unigene540784 | macB | 0.000E+00 | 0.000E+00 | 8.346E-08 | 0.000E+00 | 4.016E-07 | 0.000E+00 | 6.825E-07 | 1.092E-06 | 4.648E-08 | 7.107E-08 | 0.000E+00 | 3.092E-06 |
| Unigene540849 | rosA | 0.000E+00 | 0.000E+00 | 0.000E+00 | 0.000E+00 | 0.000E+00 | 0.000E+00 | 7.498E-07 | 0.000E+00 | 0.000E+00 | 0.000E+00 | 0.000E+00 | 0.000E+00 |
| Unigene540863 | MuxB | 0.000E+00 | 0.000E+00 | 0.000E+00 | 0.000E+00 | 0.000E+00 | 0.000E+00 | 4.941E-07 | 0.000E+00 | 0.000E+00 | 9.947E-08 | 0.000E+00 | 3.694E-07 |
| Unigene540878 | patA | 0.000E+00 | 0.000E+00 | 0.000E+00 | 0.000E+00 | 0.000E+00 | 0.000E+00 | 7.328E-07 | 0.000E+00 | 0.000E+00 | 0.000E+00 | 0.000E+00 | 0.000E+00 |
| Unigene540880 | tet32 | 0.000E+00 | 0.000E+00 | 0.000E+00 | 0.000E+00 | 0.000E+00 | 0.000E+00 | 4.106E-07 | 0.000E+00 | 0.000E+00 | 0.000E+00 | 0.000E+00 | 0.000E+00 |
| Unigene541074 | tet(A) | 0.000E+00 | 0.000E+00 | 0.000E+00 | 0.000E+00 | 6.277E-07 | 0.000E+00 | 7.674E-07 | 0.000E+00 | 0.000E+00 | 0.000E+00 | 0.000E+00 | 0.000E+00 |
| Unigene541112 | msbA | 0.000E+00 | 0.000E+00 | 0.000E+00 | 0.000E+00 | 0.000E+00 | 0.000E+00 | 1.307E-06 | 0.000E+00 | 0.000E+00 | 0.000E+00 | 0.000E+00 | 0.000E+00 |
| Unigene541130 | baeS | 0.000E+00 | 0.000E+00 | 0.000E+00 | 0.000E+00 | 0.000E+00 | 0.000E+00 | 7.692E-07 | 0.000E+00 | 0.000E+00 | 0.000E+00 | 0.000E+00 | 5.477E-07 |
| Unigene541280 | oleC | 0.000E+00 | 0.000E+00 | 0.000E+00 | 0.000E+00 | 0.000E+00 | 0.000E+00 | 1.027E-06 | 0.000E+00 | 0.000E+00 | 0.000E+00 | 0.000E+00 | 0.000E+00 |
| Unigene541291 | novA | 0.000E+00 | 0.000E+00 | 0.000E+00 | 0.000E+00 | 0.000E+00 | 4.847E-08 | 1.092E-06 | 0.000E+00 | 0.000E+00 | 0.000E+00 | 2.564E-07 | 5.299E-07 |
| Unigene541373 | TaeA | 0.000E+00 | 5.202E-08 | 0.000E+00 | 0.000E+00 | 0.000E+00 | 0.000E+00 | 1.312E-06 | 0.000E+00 | 0.000E+00 | 0.000E+00 | 3.253E-07 | 2.241E-07 |
| Unigene541438 | baeR | 0.000E+00 | 0.000E+00 | 0.000E+00 | 0.000E+00 | 0.000E+00 | 0.000E+00 | 5.046E-07 | 0.000E+00 | 0.000E+00 | 0.000E+00 | 0.000E+00 | 0.000E+00 |
| Unigene541439 | baeS | 0.000E+00 | 0.000E+00 | 0.000E+00 | 0.000E+00 | 0.000E+00 | 0.000E+00 | 5.322E-07 | 0.000E+00 | 0.000E+00 | 0.000E+00 | 0.000E+00 | 0.000E+00 |
| Unigene541481 | ugd | 0.000E+00 | 2.429E-07 | 0.000E+00 | 0.000E+00 | 0.000E+00 | 0.000E+00 | 2.174E-06 | 0.000E+00 | 0.000E+00 | 0.000E+00 | 0.000E+00 | 0.000E+00 |
| Unigene541489 | vatB | 0.000E+00 | 0.000E+00 | 0.000E+00 | 0.000E+00 | 6.170E-07 | 0.000E+00 | 1.690E-06 | 1.540E-06 | 0.000E+00 | 0.000E+00 | 0.000E+00 | 0.000E+00 |
| Unigene541605 | TriB | 0.000E+00 | 0.000E+00 | 0.000E+00 | 0.000E+00 | 0.000E+00 | 0.000E+00 | 5.160E-07 | 0.000E+00 | 0.000E+00 | 0.000E+00 | 0.000E+00 | 0.000E+00 |
| Unigene541614 | tetA(58) | 0.000E+00 | 0.000E+00 | 0.000E+00 | 0.000E+00 | 2.676E-07 | 0.000E+00 | 9.776E-07 | 1.593E-06 | 0.000E+00 | 0.000E+00 | 0.000E+00 | 7.593E-07 |
| Unigene541815 | adeL | 0.000E+00 | 0.000E+00 | 0.000E+00 | 0.000E+00 | 0.000E+00 | 0.000E+00 | 1.289E-06 | 0.000E+00 | 0.000E+00 | 0.000E+00 | 0.000E+00 | 0.000E+00 |
| Unigene541825 | patA | 0.000E+00 | 0.000E+00 | 0.000E+00 | 0.000E+00 | 0.000E+00 | 0.000E+00 | 1.201E-06 | 0.000E+00 | 0.000E+00 | 0.000E+00 | 0.000E+00 | 0.000E+00 |
| Unigene541888 | optrA | 0.000E+00 | 0.000E+00 | 0.000E+00 | 0.000E+00 | 0.000E+00 | 0.000E+00 | 1.431E-06 | 0.000E+00 | 0.000E+00 | 0.000E+00 | 0.000E+00 | 0.000E+00 |
| Unigene541907 | lmrC | 0.000E+00 | 0.000E+00 | 0.000E+00 | 0.000E+00 | 0.000E+00 | 0.000E+00 | 9.091E-07 | 0.000E+00 | 0.000E+00 | 0.000E+00 | 0.000E+00 | 0.000E+00 |
| Unigene541960 | novA | 0.000E+00 | 0.000E+00 | 0.000E+00 | 0.000E+00 | 0.000E+00 | 0.000E+00 | 0.000E+00 | 0.000E+00 | 0.000E+00 | 0.000E+00 | 0.000E+00 | 8.335E-07 |
| Unigene541988 | bcrA | 0.000E+00 | 0.000E+00 | 0.000E+00 | 0.000E+00 | 0.000E+00 | 0.000E+00 | 1.129E-06 | 2.599E-06 | 0.000E+00 | 0.000E+00 | 0.000E+00 | 5.482E-07 |
| Unigene542013 | smeC | 0.000E+00 | 0.000E+00 | 0.000E+00 | 0.000E+00 | 0.000E+00 | 0.000E+00 | 8.054E-07 | 0.000E+00 | 0.000E+00 | 0.000E+00 | 0.000E+00 | 0.000E+00 |
| Unigene542090 | smeE | 0.000E+00 | 0.000E+00 | 0.000E+00 | 1.137E-07 | 3.001E-07 | 1.095E-07 | 9.343E-07 | 6.700E-07 | 1.722E-07 | 1.881E-07 | 5.922E-07 | 6.652E-07 |
| Unigene542091 | Enterobact | 0.000E+00 | 0.000E+00 | 0.000E+00 | 2.264E-07 | 4.151E-07 | 1.616E-07 | 8.546E-07 | 6.105E-07 | 2.178E-07 | 0.000E+00 | 4.274E-07 | 4.416E-07 |
| Unigene542114 | macB | 0.000E+00 | 1.346E-07 | 0.000E+00 | 9.287E-08 | 1.362E-07 | 0.000E+00 | 6.446E-07 | 0.000E+00 | 6.701E-08 | 0.000E+00 | 0.000E+00 | 6.885E-07 |
| Unigene542169 | patB | 0.000E+00 | 0.000E+00 | 0.000E+00 | 0.000E+00 | 0.000E+00 | 0.000E+00 | 1.962E-06 | 0.000E+00 | 0.000E+00 | 0.000E+00 | 0.000E+00 | 0.000E+00 |
| Unigene542260 | TaeA | 0.000E+00 | 0.000E+00 | 0.000E+00 | 0.000E+00 | 0.000E+00 | 0.000E+00 | 1.134E-06 | 0.000E+00 | 0.000E+00 | 0.000E+00 | 0.000E+00 | 0.000E+00 |
| Unigene542307 | adeF | 0.000E+00 | 0.000E+00 | 0.000E+00 | 0.000E+00 | 0.000E+00 | 1.271E-07 | 4.555E-07 | 1.716E-07 | 0.000E+00 | 0.000E+00 | 0.000E+00 | 0.000E+00 |
| Unigene542346 | mgrA | 0.000E+00 | 0.000E+00 | 0.000E+00 | 0.000E+00 | 0.000E+00 | 0.000E+00 | 9.340E-07 | 0.000E+00 | 0.000E+00 | 0.000E+00 | 0.000E+00 | 5.819E-07 |
| Unigene542422 | patA | 0.000E+00 | 0.000E+00 | 0.000E+00 | 0.000E+00 | 0.000E+00 | 0.000E+00 | 5.648E-07 | 0.000E+00 | 0.000E+00 | 0.000E+00 | 0.000E+00 | 0.000E+00 |
| Unigene542528 | Rhodococ | 0.000E+00 | 0.000E+00 | 0.000E+00 | 0.000E+00 | 0.000E+00 | 0.000E+00 | 6.870E-07 | 0.000E+00 | 0.000E+00 | 0.000E+00 | 0.000E+00 | 0.000E+00 |
| Unigene542617 | basS | 0.000E+00 | 0.000E+00 | 0.000E+00 | 0.000E+00 | 2.946E-07 | 0.000E+00 | 8.386E-07 | 0.000E+00 | 0.000E+00 | 0.000E+00 | 0.000E+00 | 0.000E+00 |
| Unigene542638 | adeS | 2.106E-08 | 0.000E+00 | 1.240E-07 | 1.276E-07 | 2.339E-08 | 2.504E-07 | 9.089E-07 | 3.195E-07 | 0.000E+00 | 0.000E+00 | 9.635E-08 | 0.000E+00 |
| Unigene542665 | efrA | 0.000E+00 | 0.000E+00 | 0.000E+00 | 0.000E+00 | 2.482E-07 | 0.000E+00 | 7.417E-07 | 0.000E+00 | 0.000E+00 | 0.000E+00 | 0.000E+00 | 0.000E+00 |
| Unigene542666 | msbA | 0.000E+00 | 0.000E+00 | 0.000E+00 | 0.000E+00 | 0.000E+00 | 0.000E+00 | 4.594E-07 | 0.000E+00 | 0.000E+00 | 0.000E+00 | 0.000E+00 | 0.000E+00 |
| Unigene542671 | tetB(60) | 0.000E+00 | 0.000E+00 | 0.000E+00 | 0.000E+00 | 0.000E+00 | 0.000E+00 | 2.045E-06 | 1.630E-06 | 2.693E-07 | 0.000E+00 | 1.081E-06 | 1.845E-06 |
| Unigene542721 | patA | 0.000E+00 | 0.000E+00 | 0.000E+00 | 0.000E+00 | 0.000E+00 | 0.000E+00 | 0.000E+00 | 2.010E-07 | 0.000E+00 | 0.000E+00 | 0.000E+00 | 0.000E+00 |
| Unigene542727 | adeL | 0.000E+00 | 0.000E+00 | 1.358E-07 | 0.000E+00 | 3.459E-07 | 2.244E-07 | 8.424E-07 | 4.846E-07 | 0.000E+00 | 0.000E+00 | 5.145E-07 | 8.997E-07 |
| Unigene542782 | tetB(60) | 0.000E+00 | 0.000E+00 | 1.100E-07 | 1.698E-07 | 3.424E-07 | 2.726E-07 | 7.753E-07 | 7.523E-07 | 0.000E+00 | 0.000E+00 | 4.808E-07 | 2.683E-06 |
| Unigene542783 | vanHA | 1.022E-07 | 7.474E-08 | 0.000E+00 | 0.000E+00 | 3.783E-07 | 4.049E-07 | 1.093E-06 | 8.744E-07 | 0.000E+00 | 3.035E-07 | 7.790E-07 | 2.495E-06 |
| Unigene542792 | macB | 0.000E+00 | 0.000E+00 | 9.102E-08 | 0.000E+00 | 3.607E-07 | 0.000E+00 | 4.620E-07 | 6.496E-07 | 3.042E-07 | 0.000E+00 | 5.836E-07 | 3.838E-07 |
| Unigene542811 | kdpE | 0.000E+00 | 0.000E+00 | 0.000E+00 | 0.000E+00 | 0.000E+00 | 0.000E+00 | 2.195E-07 | 0.000E+00 | 0.000E+00 | 0.000E+00 | 0.000E+00 | 0.000E+00 |
| Unigene542838 | MuxC | 2.961E-07 | 5.042E-07 | 4.808E-07 | 4.845E-07 | 7.259E-07 | 7.615E-07 | 4.260E-06 | 2.371E-06 | 3.682E-07 | 7.847E-07 | 1.063E-06 | 2.691E-06 |
| Unigene542847 | Streptomy | 0.000E+00 | 0.000E+00 | 0.000E+00 | 0.000E+00 | 0.000E+00 | 0.000E+00 | 1.135E-06 | 0.000E+00 | 0.000E+00 | 0.000E+00 | 0.000E+00 | 0.000E+00 |
| Unigene542971 | novA | 0.000E+00 | 0.000E+00 | 0.000E+00 | 0.000E+00 | 0.000E+00 | 0.000E+00 | 7.634E-07 | 0.000E+00 | 0.000E+00 | 0.000E+00 | 3.156E-07 | 0.000E+00 |
| Unigene543205 | macB | 0.000E+00 | 0.000E+00 | 0.000E+00 | 1.923E-07 | 0.000E+00 | 0.000E+00 | 0.000E+00 | 0.000E+00 | 0.000E+00 | 0.000E+00 | 0.000E+00 | 0.000E+00 |
| Unigene543267 | lmrC | 0.000E+00 | 0.000E+00 | 0.000E+00 | 0.000E+00 | 0.000E+00 | 0.000E+00 | 5.713E-07 | 0.000E+00 | 0.000E+00 | 0.000E+00 | 0.000E+00 | 0.000E+00 |
| Unigene543308 | kdpE | 0.000E+00 | 0.000E+00 | 0.000E+00 | 0.000E+00 | 0.000E+00 | 0.000E+00 | 8.567E-07 | 0.000E+00 | 0.000E+00 | 0.000E+00 | 0.000E+00 | 0.000E+00 |
| Unigene543371 | macB | 0.000E+00 | 0.000E+00 | 0.000E+00 | 0.000E+00 | 0.000E+00 | 0.000E+00 | 2.423E-06 | 0.000E+00 | 0.000E+00 | 0.000E+00 | 0.000E+00 | 0.000E+00 |
| Unigene543418 | macB | 0.000E+00 | 0.000E+00 | 0.000E+00 | 0.000E+00 | 0.000E+00 | 0.000E+00 | 7.634E-07 | 0.000E+00 | 0.000E+00 | 0.000E+00 | 0.000E+00 | 0.000E+00 |
| Unigene543456 | Brucella su | 9.752E-08 | 1.070E-07 | 9.569E-08 | 1.313E-07 | 3.250E-07 | 1.054E-07 | 7.555E-07 | 3.415E-07 | 1.421E-07 | 2.173E-07 | 2.975E-07 | 4.995E-07 |

| | | | | | | | | | | | | | |
|---|---|---|---|---|---|---|---|---|---|---|---|---|---|
| Unigene543529 | novA | 0.000E+00 | 0.000E+00 | 0.000E+00 | 0.000E+00 | 0.000E+00 | 0.000E+00 | 7.537E-07 | 0.000E+00 | 0.000E+00 | 0.000E+00 | 0.000E+00 | 0.000E+00 |
| Unigene543547 | lmrD | 0.000E+00 | 0.000E+00 | 0.000E+00 | 0.000E+00 | 3.689E-08 | 0.000E+00 | 6.616E-07 | 0.000E+00 | 0.000E+00 | 0.000E+00 | 0.000E+00 | 5.888E-07 |
| Unigene543585 | novA | 0.000E+00 | 0.000E+00 | 0.000E+00 | 0.000E+00 | 0.000E+00 | 2.748E-07 | 0.000E+00 | 0.000E+00 | 0.000E+00 | 0.000E+00 | 0.000E+00 | 0.000E+00 |
| Unigene543765 | vanHM | 0.000E+00 | 0.000E+00 | 0.000E+00 | 0.000E+00 | 0.000E+00 | 0.000E+00 | 6.665E-07 | 0.000E+00 | 0.000E+00 | 0.000E+00 | 0.000E+00 | 0.000E+00 |
| Unigene543819 | tetB(46) | 0.000E+00 | 0.000E+00 | 0.000E+00 | 0.000E+00 | 0.000E+00 | 0.000E+00 | 2.520E-07 | 4.652E-07 | 0.000E+00 | 0.000E+00 | 0.000E+00 | 0.000E+00 |
| Unigene543898 | AcrS | 0.000E+00 | 0.000E+00 | 0.000E+00 | 0.000E+00 | 0.000E+00 | 0.000E+00 | 8.630E-07 | 0.000E+00 | 0.000E+00 | 0.000E+00 | 0.000E+00 | 0.000E+00 |
| Unigene543955 | kdpE | 0.000E+00 | 0.000E+00 | 0.000E+00 | 0.000E+00 | 0.000E+00 | 0.000E+00 | 2.898E-06 | 0.000E+00 | 0.000E+00 | 0.000E+00 | 0.000E+00 | 0.000E+00 |
| Unigene544089 | MuxB | 0.000E+00 | 0.000E+00 | 0.000E+00 | 0.000E+00 | 0.000E+00 | 0.000E+00 | 4.726E-07 | 0.000E+00 | 0.000E+00 | 0.000E+00 | 0.000E+00 | 0.000E+00 |
| Unigene544122 | tlrC | 0.000E+00 | 0.000E+00 | 0.000E+00 | 0.000E+00 | 0.000E+00 | 0.000E+00 | 9.883E-07 | 0.000E+00 | 0.000E+00 | 0.000E+00 | 0.000E+00 | 0.000E+00 |
| Unigene544234 | TaeA | 0.000E+00 | 4.560E-08 | 0.000E+00 | 0.000E+00 | 0.000E+00 | 0.000E+00 | 0.000E+00 | 4.364E-07 | 0.000E+00 | 0.000E+00 | 0.000E+00 | 1.964E-07 |
| Unigene544372 | arlR | 0.000E+00 | 0.000E+00 | 6.570E-07 | 0.000E+00 | 1.063E-07 | 0.000E+00 | 8.998E-07 | 0.000E+00 | 0.000E+00 | 0.000E+00 | 0.000E+00 | 0.000E+00 |
| Unigene544459 | oleC | 0.000E+00 | 0.000E+00 | 0.000E+00 | 0.000E+00 | 0.000E+00 | 0.000E+00 | 8.756E-07 | 0.000E+00 | 0.000E+00 | 0.000E+00 | 0.000E+00 | 0.000E+00 |
| Unigene544496 | mdtG | 0.000E+00 | 0.000E+00 | 0.000E+00 | 0.000E+00 | 0.000E+00 | 0.000E+00 | 7.504E-07 | 0.000E+00 | 0.000E+00 | 0.000E+00 | 0.000E+00 | 0.000E+00 |
| Unigene544529 | Escherichia | 0.000E+00 | 0.000E+00 | 0.000E+00 | 0.000E+00 | 0.000E+00 | 0.000E+00 | 5.432E-07 | 0.000E+00 | 0.000E+00 | 0.000E+00 | 0.000E+00 | 4.463E-07 |
| Unigene544548 | lmrD | 0.000E+00 | 0.000E+00 | 0.000E+00 | 0.000E+00 | 0.000E+00 | 0.000E+00 | 7.537E-07 | 0.000E+00 | 0.000E+00 | 0.000E+00 | 0.000E+00 | 0.000E+00 |
| Unigene544681 | tetB(60) | 0.000E+00 | 0.000E+00 | 0.000E+00 | 0.000E+00 | 0.000E+00 | 0.000E+00 | 1.130E-06 | 0.000E+00 | 0.000E+00 | 0.000E+00 | 0.000E+00 | 0.000E+00 |
| Unigene544698 | bcrA | 0.000E+00 | 0.000E+00 | 0.000E+00 | 7.133E-08 | 1.831E-07 | 0.000E+00 | 3.909E-07 | 8.520E-07 | 7.720E-08 | 0.000E+00 | 0.000E+00 | 0.000E+00 |
| Unigene544709 | arlR | 0.000E+00 | 0.000E+00 | 0.000E+00 | 0.000E+00 | 0.000E+00 | 0.000E+00 | 3.326E-07 | 0.000E+00 | 0.000E+00 | 0.000E+00 | 0.000E+00 | 0.000E+00 |
| Unigene544771 | vgaE | 0.000E+00 | 1.352E-07 | 4.030E-08 | 0.000E+00 | 4.563E-08 | 0.000E+00 | 6.818E-07 | 5.273E-07 | 0.000E+00 | 0.000E+00 | 4.698E-07 | 1.408E-06 |
| Unigene544909 | efrA | 0.000E+00 | 0.000E+00 | 0.000E+00 | 0.000E+00 | 0.000E+00 | 0.000E+00 | 1.092E-06 | 0.000E+00 | 0.000E+00 | 0.000E+00 | 0.000E+00 | 0.000E+00 |
| Unigene544968 | Streptomy | 0.000E+00 | 0.000E+00 | 0.000E+00 | 0.000E+00 | 0.000E+00 | 0.000E+00 | 1.092E-06 | 0.000E+00 | 0.000E+00 | 0.000E+00 | 0.000E+00 | 0.000E+00 |
| Unigene544984 | lmrD | 0.000E+00 | 0.000E+00 | 0.000E+00 | 0.000E+00 | 0.000E+00 | 0.000E+00 | 7.594E-07 | 0.000E+00 | 0.000E+00 | 0.000E+00 | 0.000E+00 | 0.000E+00 |
| Unigene545097 | vgaB | 0.000E+00 | 0.000E+00 | 0.000E+00 | 0.000E+00 | 0.000E+00 | 0.000E+00 | 1.840E-06 | 0.000E+00 | 0.000E+00 | 0.000E+00 | 0.000E+00 | 1.850E-06 |
| Unigene545170 | carA | 1.204E-07 | 0.000E+00 | 0.000E+00 | 0.000E+00 | 0.000E+00 | 0.000E+00 | 7.110E-07 | 3.749E-07 | 0.000E+00 | 8.945E-08 | 1.378E-07 | 7.593E-07 |
| Unigene545174 | tetB(46) | 1.232E-07 | 0.000E+00 | 2.015E-07 | 2.488E-07 | 1.369E-07 | 0.000E+00 | 9.545E-07 | 6.711E-07 | 0.000E+00 | 3.660E-07 | 3.758E-07 | 1.214E-06 |
| Unigene545258 | carA | 0.000E+00 | 0.000E+00 | 0.000E+00 | 0.000E+00 | 0.000E+00 | 3.988E-07 | 0.000E+00 | 9.330E-07 | 0.000E+00 | 0.000E+00 | 0.000E+00 | 2.180E-07 |
| Unigene545281 | macB | 0.000E+00 | 0.000E+00 | 0.000E+00 | 0.000E+00 | 3.573E-07 | 0.000E+00 | 9.061E-07 | 7.850E-07 | 0.000E+00 | 2.931E-07 | 0.000E+00 | 7.258E-07 |
| Unigene545355 | patA | 0.000E+00 | 0.000E+00 | 0.000E+00 | 1.020E-07 | 0.000E+00 | 1.638E-07 | 3.914E-07 | 3.538E-07 | 0.000E+00 | 0.000E+00 | 4.045E-07 | 1.373E-06 |
| Unigene545799 | optrA | 0.000E+00 | 0.000E+00 | 1.756E-07 | 0.000E+00 | 0.000E+00 | 0.000E+00 | 1.189E-06 | 9.751E-07 | 0.000E+00 | 0.000E+00 | 4.323E-07 | 2.469E-06 |
| Unigene545852 | dfrB4 | 0.000E+00 | 0.000E+00 | 6.303E-08 | 0.000E+00 | 0.000E+00 | 0.000E+00 | 1.671E-06 | 0.000E+00 | 0.000E+00 | 0.000E+00 | 0.000E+00 | 0.000E+00 |
| Unigene545928 | tet(Z) | 0.000E+00 | 0.000E+00 | 4.005E-07 | 0.000E+00 | 0.000E+00 | 0.000E+00 | 1.109E-06 | 0.000E+00 | 0.000E+00 | 0.000E+00 | 2.547E-07 | 6.140E-07 |
| Unigene545990 | poxtA | 0.000E+00 | 0.000E+00 | 0.000E+00 | 0.000E+00 | 0.000E+00 | 0.000E+00 | 6.741E-07 | 0.000E+00 | 0.000E+00 | 0.000E+00 | 0.000E+00 | 0.000E+00 |
| Unigene546000 | vanRM | 0.000E+00 | 0.000E+00 | 0.000E+00 | 0.000E+00 | 0.000E+00 | 0.000E+00 | 6.844E-07 | 0.000E+00 | 0.000E+00 | 0.000E+00 | 0.000E+00 | 6.432E-07 |
| Unigene546023 | LRA-10 | 0.000E+00 | 0.000E+00 | 0.000E+00 | 0.000E+00 | 0.000E+00 | 0.000E+00 | 6.904E-07 | 3.276E-07 | 0.000E+00 | 0.000E+00 | 0.000E+00 | 4.793E-07 |
| Unigene546027 | Pseudomo | 0.000E+00 | 0.000E+00 | 0.000E+00 | 0.000E+00 | 0.000E+00 | 0.000E+00 | 1.667E-06 | 0.000E+00 | 0.000E+00 | 0.000E+00 | 0.000E+00 | 0.000E+00 |
| Unigene546074 | mtrA | 1.416E-07 | 4.662E-07 | 3.242E-07 | 0.000E+00 | 0.000E+00 | 2.551E-07 | 1.776E-06 | 1.432E-06 | 0.000E+00 | 0.000E+00 | 1.026E-06 | 3.738E-06 |
| Unigene546263 | vgaB | 0.000E+00 | 0.000E+00 | 0.000E+00 | 0.000E+00 | 0.000E+00 | 0.000E+00 | 1.373E-06 | 0.000E+00 | 0.000E+00 | 0.000E+00 | 0.000E+00 | 0.000E+00 |
| Unigene546384 | oleB | 0.000E+00 | 0.000E+00 | 0.000E+00 | 0.000E+00 | 0.000E+00 | 0.000E+00 | 7.819E-07 | 0.000E+00 | 0.000E+00 | 0.000E+00 | 0.000E+00 | 5.781E-07 |
| Unigene546390 | baeS | 0.000E+00 | 0.000E+00 | 0.000E+00 | 0.000E+00 | 0.000E+00 | 0.000E+00 | 5.988E-07 | 0.000E+00 | 0.000E+00 | 0.000E+00 | 0.000E+00 | 0.000E+00 |
[truncated: 1,753,905 more chars]
